# Development of SSR markers and identification of major quantitative trait loci controlling shelling percentage in cultivated peanut (*Arachis hypogaea* L.)

**DOI:** 10.1007/s00122-017-2915-3

**Published:** 2017-05-15

**Authors:** Huaiyong Luo, Zhijun Xu, Zhendong Li, Xinping Li, Jianwei Lv, Xiaoping Ren, Li Huang, Xiaojing Zhou, Yuning Chen, Jingyin Yu, Weigang Chen, Yong Lei, Boshou Liao, Huifang Jiang

**Affiliations:** Key Laboratory of Biology and Genetic Improvement of Oil Crops, Ministry of Agriculture, Oil Crops Research Institute of the Chinese Academy of Agricultural Sciences, Wuhan, 430062 China

## Abstract

**Key message:**

**A total of 204,439 SSR markers were developed in diploid genomes, and 25 QTLs for shelling percentage were identified in a RIL population across 4 years including five consistent QTLs.**

**Abstract:**

Cultivated peanut (*Arachis hypogaea* L.) is an important grain legume providing edible oil and protein for human nutrition. Genome sequences of its diploid ancestors, *Arachis duranensis* and *A. ipaensis*, were reported, but their SSRs have not been well exploited and utilized hitherto. Shelling percentage is an important economic trait and its improvement has been one of the major objectives in peanut breeding programs. In this study, the genome sequences of *A. duranensis* and *A. ipaensis* were used to develop SSR markers, and a mapping population (Yuanza 9102 × Xuzhou 68-4) with 195 recombinant inbred lines was used to map QTLs controlling shelling percentage. The numbers of newly developed SSR markers were 84,383 and 120,056 in the *A. duranensis* and *A. ipaensis* genomes, respectively. Genotyping of the mapping population was conducted with both newly developed and previously reported markers. QTL analysis using the phenotyping data generated in Wuhan across four consecutive years and genotyping data of 830 mapped loci identified 25 QTLs with 4.46–17.01% of phenotypic variance explained in the four environments. Meta-analysis revealed five consistent QTLs that could be detected in at least two environments. Notably, the consistent QTL *cqSPA09* was detected in all four environments and explained 10.47–17.01% of the phenotypic variance. The segregation in the progeny of a residual heterozygous line confirmed that the *cpSPA09* locus had additive effect in increasing shelling percentage. These consistent and major QTL regions provide opportunity not only for further gene discovery, but also for the development of functional markers for breeding.

**Electronic supplementary material:**

The online version of this article (doi:10.1007/s00122-017-2915-3) contains supplementary material, which is available to authorized users.

## Introduction

Cultivated peanut (*Arachis hypogaea* L.), also known as groundnut, is an allotetraploid (AABB, 2*n* = 4*x* = 40) grain legume native to South America, but now grown in diverse environments in six continents between latitudes 40°N and 40°S (Sharma and Bhatnagar-Mathur 2006). It provides edible oil and protein for human nutrition. In 2014, the annual production of peanut (pods without shelling) was around 42.32 million tones throughout the world (FAOSTAT 2014). Cultivated peanut was formed through the natural hybridization of its two diploid ancestors, *A. duranensis* (AA, 2*n* = 2*x* = 20) and *A. ipaensis* (BB, 2*n* = 2*x* = 20). Because the assembly of chromosomal pseudomolecules of cultivated peanut is very challenging, the genome sequences of its diploid ancestors were reported recently, providing a foundation in understanding the genome of cultivated peanut (Bertioli et al. 2016).

**Fig. 1:**
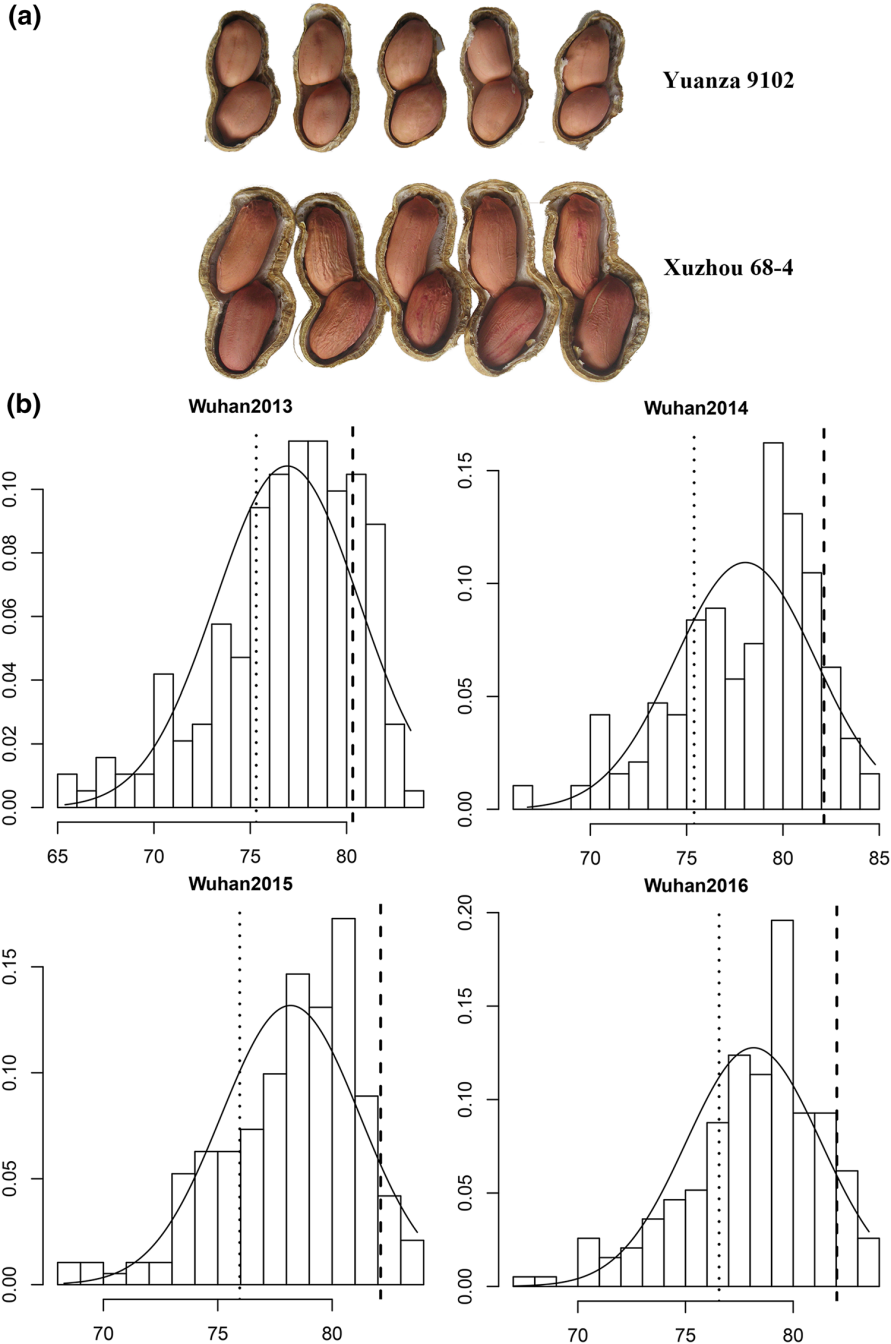
Phenotypic variation of shelling percentage in the RIL population. **a** Phenotypic difference between the parents. **b** Phenotypic distribution of shelling percentage in the RIL population across 4 years. The *y-axis* represented density, while the *x-axis* represented shelling percentage (%). The *dotted line* represented the shelling percentage of Xuzhou 68-4, and the *dashed line* represented the shelling percentage of Yuanza 9102

Shelling percentage (SP) is an important economic trait in peanut production. Peanut pod has two parts: kernel and hull (Fig. 1a). Kernels (seeds) contain rich edible oil, proteins, amino acids, and vitamin E, and are consumed worldwide as edible nut, peanut butter, or candy, and peanut oil extracted from the seeds (Bertioli et al. 2016; Ozudogru et al. 2013). The shelling of peanut hull is the first step needed to transform the peanut materials into a product (Guzel et al. 2005). Shelling percentage (weight of kernels/weight of pods) significantly varied among peanut varieties. For example, Jiang et al. (2013) reported that the shelling percentages of the Chinese core collection of peanut (574 accessions) ranged from 59.9 to 81.0%. Therefore, there is a great potential in the genetic improvement of shelling percentage in peanut breeding.

Quantitative trait locus (QTL) mapping has been widely conducted to identify the genomic regions associated with economically important traits. Molecular markers tightly linked to QTLs can be developed and further deployed in marker-assisted breeding (Janila et al. 2016; Sukruth et al. 2015; Varshney et al. 2014). Because of abundance, easy to use, and highly polymorphic, SSR markers were developed (Cuc et al. 2008; Ferguson et al. 2004; Gimenes et al. 2007; Guo et al. 2009; He et al. 2003; Hopkins et al. 1999; Moretzsohn et al. 2005; Shirasawa et al. 2012; Zhou et al. 2016) and widely used in the QTL mapping of disease resistance (Leal-Bertioli et al. 2015; Shoha et al. 2013), drought tolerance (Gautami et al. 2012; Ravi et al. 2011), quality traits (Mondal et al. 2015; Pandey et al. 2014), agronomic and yield traits (Faye et al. 2015; Huang et al. 2015) in cultivated peanut. However, SSR markers have not been screened on whole-genome level in the *Arachis* genus. Although genome sequences of *A. duranensis* and *A. ipaensis* were reported, their SSRs have not been well exploited and utilized hitherto.

Limited efforts were made in identifying QTLs controlling shelling percentage in cultivated peanut. Faye et al. (2015) detected two QTLs for shelling percentage with 5.74–6.97% phenotypic variant explained (PVE) under water stress condition in a RIL population. Huang et al. (2015) identified three QTLs for shelling percentage in an F_2:3_ population, including *qSPA5* (6.08% PVE), *qSPA7* (11.78% PVE), *qSPA9* (2.00% PVE). These QTLs were detected only in single environment. Jiang et al. (2014) reported five, two and two significant associated alleles for shelling percentage in three field trials, respectively, through association analysis in Chinese peanut mini-core collection of 298 accessions. Only one SSR allele, 9B4-260, was associated with shelling percentage in all three trials (1.49–2.98% PVE) (Jiang et al. 2014). No major QTLs were reported to be consistently expressed so far. Therefore, it is necessary to identify major and consistent QTLs of shelling percentage in order to accelerate the process of genetic improvement in peanut breeding programs.

In this study, SSRs markers were developed in the *A. duranensis* and *A. ipaensis* genomes, and a mapping population (Yuanza 9102 × Xuzhou 68-4) with 195 recombinant inbred lines (RILs) was used to map QTLs controlling shelling percentage in Wuhan, China, in four consecutive years. The peanut cultivar Yuanza 9102 showed significantly higher shelling percentage than Xuzhou 68-4 in previous screening of the Chinese peanut core collection.

## Materials and methods

### Plant materials and phenotyping

A mapping population comprising 195 recombinant inbred lines (RILs) was developed by crossing peanut cultivar Yuanza 9102 and Xuzhou 68-4 and advanced to the F_5_ generation by single seed descent method (Luo et al. 2017). The female parent, Yuanza 9102, belongs to *A. hypogaea* subsp. *hypogaea* var. *vulgaris* and is derived from interspecific hybridization between the cultivated peanut Baisha1016 and wild species *A. diogoi*. The male parent, Xuzhou 68-4, belongs to *A. hypogaea* subsp. *hypogaea* var. *hypogaea* and has larger pods but significantly lower shelling percentage than the female parent, Yuanza 9102 (Fig. 1a). The RIL population was used as mapping population to validate the quality of newly developed SSR markers, to construct a dense genetic map and to conduct QTL analysis for shelling percentage in this study. Generations F_5_–F_8_ of the RIL population were used in the present study for generating phenotyping data followed by QTL analysis.

The RIL population and the two parents were planted in the experimental field in OCRI-CAAS, Wuhan, China, in four consecutive years from 2013 to 2016. These experiments were treated as four environments and designated as Wuhan2013, Wuhan2014, Wuhan2015 and Wuhan2016 in this study. In each environment, the 195 RILs and the two parents were planted in a randomized complete block design with three replications. Each plot contained one row, with 12 plants in each row, 20 cm between plants and 30 cm between rows. Field management followed the standard agricultural practices. Eight representative plants in the middle of each row were harvested to investigate shelling percentage (${\text{SP}} = \frac{\text{Weight of kernels}}{\text{Weight of pods}} \times 100\%$), according to previously described standard procedures (Huang et al. 2015; Jiang et al. 2006).

Statistical analysis for the phenotypic data of shelling percentage was conducted using IBM SPSS Statistics Version 22 software. Treating the year as a single environment, the univariate variance analyses were performed with standard GLM method and variance components were estimated by restricted maximum likelihood (REML) method. The broad-sense heritability across the four environment trials was calculated based on the estimated variance components with the following formula: $H^{2} = \sigma_{\text{g}}^{2} /(\sigma_{\text{g}}^{2} + \sigma_{{{\text{g}} \times {\text{e}}}}^{2} + \sigma_{\text{e}}^{2} )$ based on plot mean and $H^{2} = \sigma_{\text{g}}^{2} /(\sigma_{\text{g}}^{2} + \sigma_{{{\text{g}} \times {\text{e}}}}^{2} /r + \sigma_{\text{e}}^{2} /rn)$ based on entry mean, where $\sigma_{\text{g}}^{2}$ is the genotypic variance component among RILs, $\sigma_{{{\text{g}} \times {\text{e}}}}^{2}$ is the RILs × environment interaction variance component, $\sigma_{\text{e}}^{2}$ is the residual (error) variance component, and r is the number of environment trials, n is the number of replications in each field trials (Holl and Nyquist 2010).

### Development of SSR marker in the genome sequences of *A. duranensis* and *A. ipaensis*

Genome sequences of *A. duranensis* and *A. ipaensis* were downloaded from the PeanutBase (Bertioli et al. 2016). SSR motifs were identified using the MISA script (Thiel 2014). For normal microsatellites, a minimum of 10, 6, 5, 5, 5 and 5 repeats were required for detecting mono-, di-, tri-, tetra-, penta- and hexa-nucleotide motifs, respectively. Compound microsatellites were interrupted by less than 100 base pairs. Primer3 software (https://sourceforge.net/projects/primer3/) was used to design SSR markers with the following parameters: minimum, maximum, and optimal sizes were 18, 27, and 20 nt, respectively; minimum and maximum GC content were 20 and 80%, respectively; minimum, maximum, and optimal Tm were 57, 63, and 60 °C, respectively; and product size range was from 100 to 300 bp. These markers were referred as newly developed markers in this context and designated with an initial letter ‘Ad’ and ‘Ai’ for *A. duranensis* and *A. ipaensis*, respectively, followed by the chromosome number, the corresponding subgenome character and an identifier.

### Genotyping of mapping population and construction of genetic map

A total of 2240 newly developed markers (Table S3) as well as 7200 previously reported markers (Bravo et al. 2006; Cuc et al. 2008; Ferguson et al. 2004; Gimenes et al. 2007; Guo et al. 2009, 2012; He et al. 2003; Hopkins et al. 1999; Hoshino et al. 2006; Huang et al. 2016b; Koilkonda et al. 2012; Leal-Bertioli et al. 2009; Macedo et al. 2012; Moretzsohn et al. 2005, 2009; Moretzsohn Mde et al. 2004; Nagy et al. 2010; Naito et al. 2008; Shirasawa et al. 2012; Wang et al. 2012a; Zhou et al. 2016) were used to screen polymorphism between the two parental genotypes. Polymorphic markers were used to genotype individual RILs. Based on known genomic positions, the 2240 newly developed markers were selected to validate the quality of newly developed SSR markers and to improve the quality of previous genetic map (Luo et al. 2017) for the identification of QTLs controlling shelling percentage. The polymorphism of the 2240 newly developed markers was compared to that of the 7200 previously reported markers. Genomic DNA was extracted from young leaves collected from RILs in F_5_ generations using a modified CTAB method (Doyle 1990). PCR amplification was conducted as described in Luo et al. (2017). The PCR products were separated on a 6% polyacrylamide gel and visualized by silver staining (Fountain et al. 2011).

Pearson’s Chi-square test was used to assess the goodness of fit to the expected segregation ratio 15:2:15 for co-dominant marker or 17:15 for dominant marker (*P* < 0.001). A genetic linkage map was constructed using the JoinMap 4.0 software (Van Ooijen 2006). The recombination ratio was converted to map distance using the Kosambi function (Kosambi 2011). The graphical presentation of genetic linkage map was generated with the MapChart 2.3 software (Voorrips 2002). The linkage groups (LGs) were designated as A1–A10 and B1–B10 by aligning the markers to the integrated consensus genetic map (Shirasawa et al. 2013) and the genome sequences of *A. duranensis* and *Arachis ipaensis* (Bertioli et al. 2016). This consensus genetic map was integrated based on 16 genetic maps and used as reference in other publications (Chen et al. 2016; Huang et al. 2015; Zhou et al. 2014).

### QTL and meta-analyses

Genome-wide QTL mapping was performed using the mean value of shelling percentage in each environment. The QTLs were scanned with the Windows QTL Cartographer 2.5 software (Wang et al. 2012b) through composite interval mapping (CIM). The threshold of LOD for declaring the presence of a QTL was determined by 1000 permutation tests at *P* < 0.05. When separated by a minimum distance of 20 cM, two peaks on one chromosome were considered as two different QTLs (Ravi et al. 2011). Otherwise, the higher peak was chosen to more closely approximate the position of the QTL. QTLs are designated with an initial letter ‘q’ followed by the abbreviation of trait name (SP), and the corresponding linkage group, similar to the previously described nomenclature (Udall et al. 2006). After the linkage group, the codes 1, 2, 3 and 4 were added for QTLs detected in 2013, 2014 2015 and 2016, respectively. Alphabetical letters were added if two or more QTLs were identified in the same linkage group in the same year. For example, if two QTLs for shelling percentage were detected on chromosome A09 in 2013, they were names as *qSPA09.1a* and *qSPA09.1b*, respectively. In addition, QTLs with more than 10% PVE were considered as major QTLs while other QTLs were considered as minor QTLs. If QTLs detected in different environments had overlapping 2-LOD support intervals, they were considered to be a consistent QTL and subjected to meta-analysis to estimate its position using the BioMercator software (Sosnowski et al. 2012). Consistent QTLs were designated with initial letters ‘cq’.

## Results

### Phenotypic variation of shelling percentage

Phenotypic evaluation of shelling percentage of two parental genotypes and RILs showed significant variation across four environments, i.e., Wuhan2013, Wuhan2014, Wuhan2015 and Wuhan2016 (Table 1). The shelling percentage of female parent, Yuanza 9102, varied from 80.32 to 82.13% while that of male parent, Xuzhou 68-4, varied from 75.31 to 76.56% in the four environments. The shelling percentage in the RIL population showed a continuous distribution skewed towards higher values in each environment (Table 1; Fig. 1b), indicating polygenic inheritance. The values of broad-sense heritability for shelling percentage was estimated to be 0.7769 based on plot mean and 0.9520 based on entry mean, indicating strong control by genetic factors. Variance analysis across the four trials also revealed that genetic, environmental effects and genotype × environment interactions significantly influenced shelling percentage (Table 2).

**Table 1:** The observed phenotypic performance of mean values of shelling percentage of two parents and RILs in four field trials

Year	P1 (%)	P2 (%)	RIL (%)	Min (%)	Max (%)	SD (%)	Skew	Kurt
2013	80.32	75.31	76.92	65.39	83.33	3.72	−0.833	0.447
2014	82.13	75.39	78.05	66.74	84.79	3.65	−0.742	0.174
2015	82.12	75.96	78.18	68.28	83.72	3.03	−0.827	0.582
2016	82.03	76.56	78.16	67.04	83.52	3.12	−0.803	0.568

*P1* female parent Yuanza 9102, *P2* male parent Xuzhou 68-4, *Min* minimum, *Max* maximum, *SD* standard deviation, *Skew* skewness, *Kurt* kurtosis

**Table 2:** Variance analysis for shelling percentage in the RIL population in four environments

Variables	*df*	Mean square	*F* value	*P* value
Environment	3	210.028	171.423	<0.001
Genotype	193	119.456	97.500	<0.001
Genotype × environment	570	5.784	4.721	<0.001
Error	1532	1.225		

### The abundance of SSRs in the genomes of *Arachis duranensis* and *A. ipaensis*

The availability of the pseudochromosomes of *A. duranensis* and *A. ipaensis*, the diploid ancestors of cultivated peanut, provides physical maps for genetic studies in the *Arachis* genus. A total of 264,135 and 392,107 SSR loci were identified by searching through the genome sequences of *A. duranensis* and *A. ipaensis*, respectively, with the MISA script. The average intervals of SSR loci were estimated as 4.10 and 3.45 kb in *A. duranensis* and *A. ipaensis*, respectively, indicating the high abundance of SRRs in their genomes. Of the repeat motifs observed, the mono-nucleotide motif was the most abundant, followed by di-, tri-, tetra-, penta- and hexa-nucleotide motifs (Table 3). The number of SSRs presented in compound formation in the *A. duranensis* and *A. ipaensis* genomes were 22,125 and 37,381, respectively.

**Table 3:** Numbers of the identified SSR loci and developed SSR markers in the *Arachis duranensis* and *A. ipaensis* genomes

Motifs	SSR loci		SSR markers	
	*A. duranensis*	*A. ipaensis*	*A. duranensis*	*A. ipaensis*
MNR	144,287	223,670	NA	NA
DNR	47,805	75,334	34,486	54,195
TNR	42,529	45,717	29,090	32,242
TTR	4988	6736	3761	5245
PNR	1657	2419	1379	2019
HNR	744	850	416	534
COM	22,125	37,381	15,251	25,822
Total	264,135	392,107	84,383	120,056

*MNR, DNR, TNR, TTR, PNR, and HNR* mono-, di-, tri-, tetra-, penta-, and hexa-nucleotide SSRs, respectively, *COM* compound microsatellites

The investigation of nucleotide composition characteristics revealed that some repeat types were dominant than others (Table S1). In the *A. duranensis* genome, the SSRs were found to be 232 repeat types and A/T (99.43%), AT/AT (48.97%) AAT/ATT (41.32%), AAAT/ATTT (55.41%), AAAAT/ATTTT (37.65%) and AAGAGG/CCTCTT (27.28%) were the most common repeat types corresponding to mono- to hexa-nucleotide repeats, respectively. There was similar tendency in the 231 repeat types found in the *A. ipaensis* genome, and the most common repeat types corresponding to mono- to penta-nucleotide repeats were A/T (98.81%), AT/AT (44.86%) AAT/ATT (35.00%), AAAT/ATTT (58.95%), and AAAAT/ATTTT (37.16%), respectively, while the most abundant hexa-nucleotide motif was AAAAAT/ATTTTT (18.00%) which was different from the *A. duranensis* genome.

### Development and validation of SSR markers

SSR markers were designed for di-, tri-, tetra-, penta- and hexa-nucleotide motifs as well as compound microsatellites using the Primer3 software. A total of 84,383 and 120,056 SSR markers were finally developed in the *A. duranensis* and *A. ipaensis* genome, respectively (Tables 3, S2). There were 15,251 and 25,822 SSR markers with motifs in compound formation in the *A. duranensis* and *A. ipaensis* genome, respectively, while the remaining markers amplified single motif. In the *A. duranensis* genome, SSR makers with di-, tri-, tetra-, penta- and hexa-nucleotide motifs accounted for 40.87, 34.47, 4.46, 1.63 and 0.49%, respectively (Table 3). Similarly, SSR makers with di-, tri-, tetra-, penta- and hexa-nucleotide motifs in the *A. ipaensis* genome accounted for 45.14, 26.86, 4.37, 1.68 and 0.44%, respectively (Table 3).

In order to validate the quality of the newly developed SSR markers, 2240 primer pairs were synthesized to screen polymorphic markers in both the parental genotypes and the RIL population. Among the 2240 newly developed markers, 365 markers amplified polymorphic bands while 1706 markers amplified same bands between the two parents (Table S2). A total of 180 newly developed SSR markers (accounted for 8.04%) amplified 185 polymorphic loci in the RIL population. In comparison, 682 previously reported markers amplified 693 polymorphic loci, which accounted for 9.47% of the 7200 previously reported markers screened.

### Construction of genetic map

**Fig. 2:**
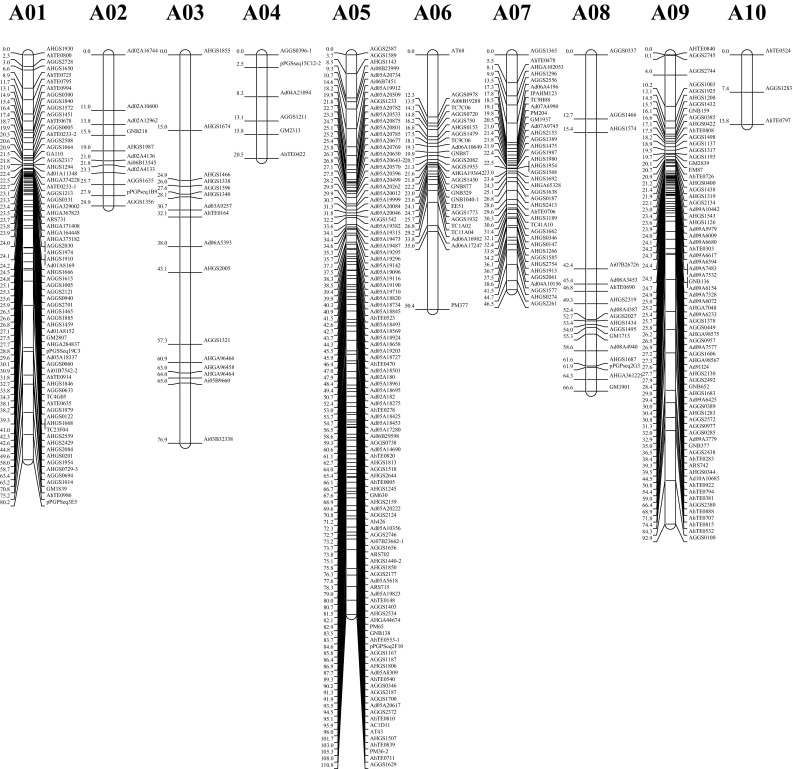

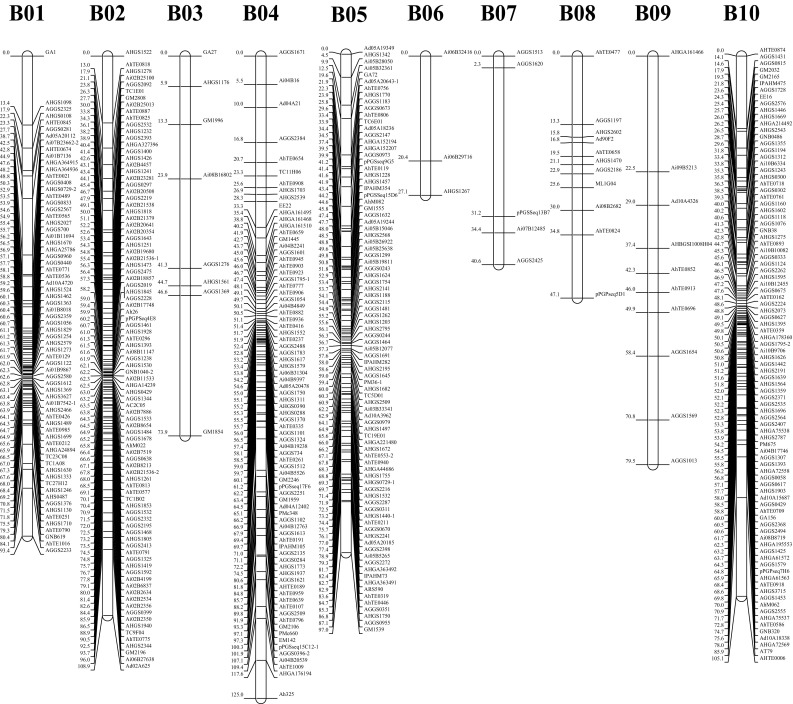
Graphical presentation of genetic linkage map constructed in the RIL population derived from a cross by Yuanza 9102 and Xuzhou 68-4

Polymorphic loci of both the 180 newly developed and the 682 previously reported markers were used to construct genetic linkage map with the JoinMap 4.0 software. Among the 862 polymorphic markers (Table S4), one maker (AHGS0729) amplified three genetic loci and 14 markers amplified two genetic loci, while the remaining 847 markers amplified single locus. Among these 878 genetic loci, 784 loci were co-dominant and 94 loci were dominant. Finally, a genetic linkage map containing the 830 loci was constructed spanning 1386.19 cM with an average inter-marker distance of 1.67 cM (Table 4; Fig. 2). The 830 loci were assigned to 20 LGs designated as A01–A10 for A subgenome and B01–B10 for B subgenome by aligning the markers to the integrated consensus genetic map (Shirasawa et al. 2013) and the genome sequences of *A. duranensis* and *A. ipaensis* (Bertioli et al. 2016). There were 371 loci for the A subgenome and 459 loci for the B subgenome with the map length of 588.48 and 797.71 cM, respectively. The length of LGs varied from 13.78 cM (A10) to 125.04 cM (B04) and the number of mapped loci ranged from 3 to 110 markers (Table 4; Fig. 2). The Chi-square analysis identified 258 loci (31.09%) that significantly deviated from expected ratios of 15:2:15 or 17:15 (*P* < 0.001), of which 26 and 232 loci skewed towards Yuanza 9102 and Xuzhou 68-4, respectively (Tables 4, S5). The skewed loci on LG A05 favored the female parent “Yuanza 9102” allele, while LG A01, A06, A07, A09, B01, B02, B04, B05 and B10 contained loci favoring the male parent “Xuzhou 68-4” allele. In addition, no more than five skewed loci were mapped on LG A02, A03, B03, B08 and B09. The most significant segregation distortion was observed on LG B05 whose percentage of skewed loci was 93.18% (Table 4).

**Table 4:** Description of the genetic linkage map constructed in this study

LG	Length	Loci	SDL	SDL %	P1	P2
A01	80.23	70	9	12.86	2	7
A02	29.92	11	5	45.45	0	5
A03	76.89	16	5	30.04	0	5
A04	20.47	6	0	0.00	0	0
A05	110.81	110	24	21.82	17	7
A06	50.35	26	17	65.38	0	17
A07	46.47	39	6	15.38	0	6
A08	66.63	17	0	0.00	0	0
A09	92.93	73	13	17.81	0	13
A10	13.78	3	0	0.00	0	0
A subgenome	588.48	371	79	21.24	19	60
B01	93.44	69	10	14.49	2	8
B02	108.91	92	13	14.13	1	12
B03	73.90	8	1	12.50	1	0
B04	125.04	81	57	70.37	1	56
B05	97.01	88	82	93.18	0	82
B06	27.10	3	0	0.00	0	0
B07	40.62	5	0	0.00	0	0
B08	47.08	11	4	38.91	0	4
B09	79.51	10	2	20.00	0	2
B10	105.11	92	10	10.87	2	8
B subgenome	797.71	459	179	39.06	7	172
Whole genome	1386.19	830	258	31.09	26	232

*LG* linkage group, *SDL* the number of segregation distortion loci in each linkage group (*P* < 0.001), *SDL* *%* the percentage of segregation distortion loci in each linkage group (*P* < 0.001), *P1* the number of SSR loci that segregated distortedly to the parent Yuanza 9102, *P2* the number of SSR loci that segregated distortedly to the parent Xuzhou 68-4

### Detection of QTLs for shelling percentage

**Fig. 3:**
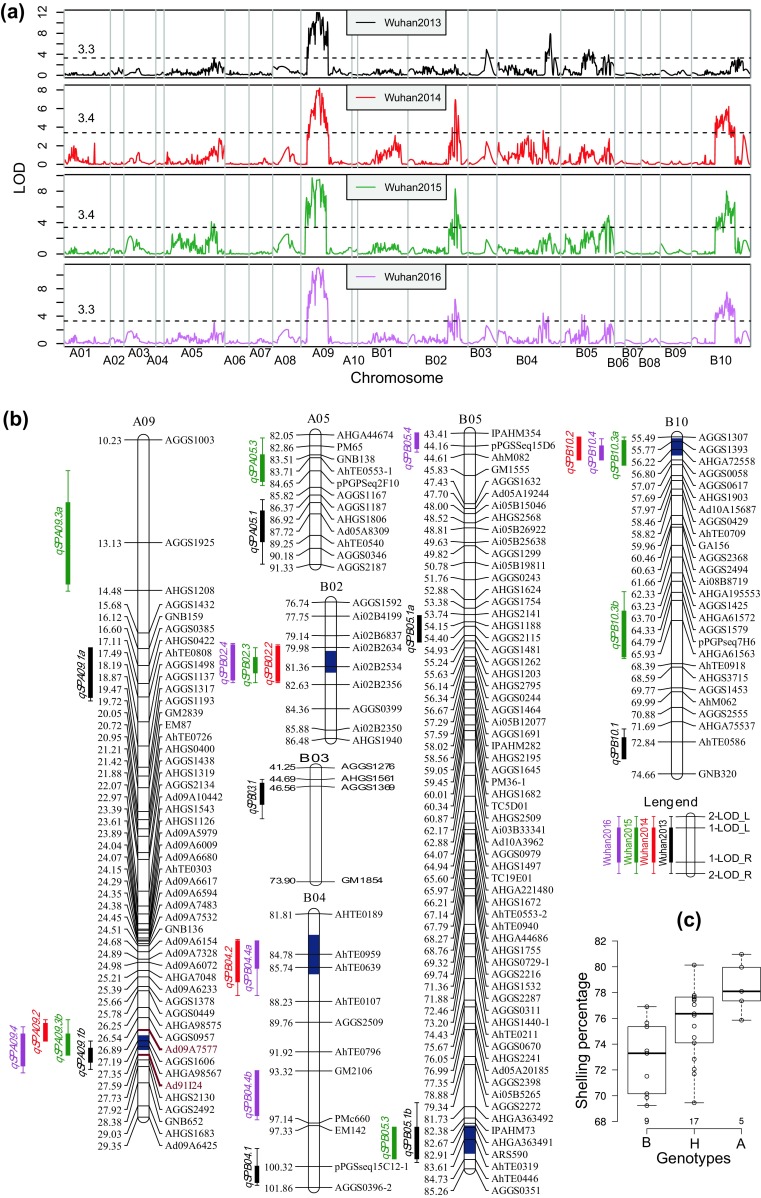
Overview of QTLs for shelling percentage in the RIL population. **a** Genome-wide overview of QTLs for shelling percentage across four environments. **b** QTLs location of shelling percentage in the corresponding linkage maps. Consistent QTLs obtained by meta-analysis in four environments are highlighted in *dark blue color* on the chromosome bars. **c** The boxplot of shelling percentage among three genotypic groups in the progeny of the residual heterozygous line RIL 15-71126. *Center lines* show the medians; *box limits* indicate the 25th and 75th percentiles as determined by R software; whiskers extend 1.5 times the interquartile range from the 25th and 75th percentiles; data points are plotted as *open circles*. *n* = 9, 17, 5 plants. *A*, *B*, *H* indicated homologous alleles from Yuanza 9102, homologous alleles from Xuzhou 68-4 and heterozygotes, respectively

Genome-wide QTL analysis was performed using the genetic map and phenotypic data of shelling percentage obtained from the RILs during 2013, 2014, 2015 and 2016 in Wuhan. Using composite interval mapping (CIM) analysis, 25 QTLs with 4.46–17.01% phenotypic variation explained (PVE) were identified to be associated with shelling percentage across four environments (Fig. 3; Table 5). Three major QTLs namely *qSPA09.1a*, *qSPA09.1b* and *qSPB04.1* and five minor QTLs namely *qSPA05.1*, *qSPB03.1*, *qSPB05.1a*, *qSPB05.1b* and *qSPB10.1* were detected in Wuhan2013 trial, which explained 4.46–17.01% phenotypic variation. In Wuhan2014 trial, one major QTL, *qSPA09.2*, and three minor QTLs namely *qSPB02.2*, *qSPB04.2* and *qSPB10.2*, were identified with 4.68–10.47% PVE. In Wuhan2015 trial, four QTLs namely *qSPA09.3a*, *qSPA09.3b*, *qSPB02.3* and *qSPB10.3a*, and three minor QTLs namely *qSPA05.3*, *qSPB05.3* and *qSPB10.3b* were detected with 5.28–12.20% PVE. In Wuhan 2016 trial, one major QTL, *qSPA09.4*, and five minor QTLs namely *qSPB02.4*, *qSPB04.4a*, *qSPB04.4b*, *qSPB05.4* and *qSPB10.4*, were identified with 5.32–14.39% PVE. A total of nine QTLs explaining more than 10% phenotypic variation were identified as major QTLs in four environments.

**Table 5:** QTL information of shelling percentage in peanut in four environments

Environment	LG	QTL	POS (cM)	CI (cM)	LOD	Additive	PVE (%)
Wuhan2013	A05	*qSPA05.1*	88.7	86.7–91.3	3.3	−0.81	4.46
	A09	*qSPA09.1a*	16.6	16.1–17.6	9.0	−1.47	14.67
	A09	*qSPA09.1b*	27.6	27.2–28	11.1	−1.55	17.01
	B03	*qSPB03.1*	46.6	44.6–55.9	4.9	−0.98	6.84
	B04	*qSPB04.1*	100.3	99–101.7	7.9	−1.26	11.45
	B05	*qSPB05.1a*	54.4	53.8–55.3	4.9	−1.04	6.91
	B05	*qSPB05.1b*	83.6	81.5–84.9	3.9	0.91	4.82
	B10	*qSPB10.1*	72.8	72.1–73.8	3.4	0.84	4.62
Wuhan2014	A09	*qSPA09.2*	26.9	26.6–27.2	7.6	−1.23	10.47
	B02	*qSPB02.2*	81.0	79.8–82.5	7.0	−1.21	9.72
	B04	*qSPB04.2*	84.8	83.7–87.8	3.6	−0.86	4.68
	B10	*qSPB10.2*	55.8	55.5–56.8	5.9	1.05	7.99
Wuhan2015	A05	*qSPA05.3*	83.7	82.3–85.7	4.1	−0.72	5.28
	A09	*qSPA09.3a*	13.1	11.1–14.5	7.2	−0.99	10.19
	A09	*qSPA09.3b*	27.4	26.6–27.6	8.9	−1.09	12.20
	B02	*qSPB02.3*	81.0	80–82.5	8.3	−1.08	11.20
	B05	*qSPB05.3*	83.6	82.9–84.7	4.9	0.79	5.59
	B10	*qSPB10.3a*	55.8	55.5–57.1	8.0	1.01	10.64
	B10	*qSPB10.3b*	67.9	64.3–68.1	6.6	0.95	9.27
Wuhan2016	A09	*qSPA09.4*	27.6	26.8–28.1	10.8	−1.26	14.39
	B02	*qSPB02.4*	81.0	79.7–82.5	6.5	−0.94	8.01
	B04	*qSPB04.4a*	84.8	83.8–87.8	4.5	−0.77	5.32
	B04	*qSPB04.4b*	96.3	93.3–96.9	3.9	−0.76	5.34
	B05	*qSPB05.4*	44.2	43.4–44.5	4.2	−0.75	5.45
	B10	*qSPB10.4*	56.2	55.6–56.8	7.5	0.97	9.28

*LG* linkage group, *POS* position, *CI* 2-LOD confidence interval, *LOD* logarithm of odds, *PVE* phenotypic variation explained

To further dissect the QTLs controlling shelling percentage, meta-analysis was conducted to integrate QTLs detected in multiple environments, whose confidence intervals were overlapped, into five consistent QTLs (Table 6; Fig. 3b) using the BioMercator software (Sosnowski et al. 2012). Specifically, chromosome A09 was associated with major and consistent QTL controlling shelling percentage in cultivated peanut. The consistent QTL *cpSPA09* was detected in all four environments and explained 17.01, 10.47, 12.20 and 14.39% of the phenotypic variance in 2013, 2014, 2015 and 2016, respectively. In addition, the consistent QTLs, *cqSPB02 and cqSPB10* were detected in three environments (2014, 2015 and 2016), and explained 8.01–11.20% and 7.99–10.64% of the phenotypic variance, respectively (Table 6; Fig. 3b). The consistent QTLs *cqSPB04* was detected in 2013 and 2016 (4.68–5.32% PVE), while *cqSPB05* was detected in 2013 and 2015 (4.82–5.59% PVE).

**Table 6:** Consistent QTLs of shelling percentage integrated by meta-analysis in four environments

Consistent QTL	LG	POS (cM)	CI (cM)	Consistent QTLs
*cqSPA09*	A09	27.24	27.04–27.45	*qSPA09.1b, qSPA09.2, qSPA09.3b, qSPA09.4*
*cqSPB02*	B02	81.01	80.24–81.77	*qSPB02.2, qSPB02.3, qSPB02.4*
*cqSPB04*	B04	84.81	83.37–86.24	*qSPB04.2, qSPB04.4a*
*cqSPB05*	B05	83.61	82.81–84.4	*cqSPB05.1b, cqSPB05.3*
*cqSPB10*	B10	56.06	55.58–56.54	*qSPB10.2, qSPB10.3a, qSPB10.4*

*LG* linkage group, *POS* position, *CI* confidence interval

In order to validate the consistent QTL *cpSPA09*, flanking marker Ad09A7577 and Ad91I24 (Fig. 3b) were used to screen the RIL population in F_7_ generation grown in Wuhan in 2015, and a plant named RIL 15-71126 was found to be heterozygous at the *cpSPA09* locus. Seeds of RIL 15-71126 were grown in Wuhan in 2016 and shelling percentages of 31 plants were measured. Among these residual heterozygous lines, five plants with homologous alleles from Yuanza 9102 had an average shelling percentage of 78.45%, while nine plants with homologous alleles from Xuzhou 68-4 had an average shelling percentage of 72.82% (Fig. 3c; Table S7). The shelling percentages of remaining 17 heterozygous plants averaged at 75.62%. Chi-square test revealed that the segregation of the *cqSPA09* locus among the 31 plants fitted the expected 1:2:1 segregation ratio (*P* = 0.52). Variance analysis and multiple comparisons revealed significant differences (*P* < 0.05) between each pair of the three genotypic groups (Fig. 3c; Table S7), which were congruent with the finding that the allele came from the parent Yuanza 9102 at the *cpSPA09* locus had additive effect in increasing shelling percentage.

## Discussion

SSRs were found to be abundant and dispersed throughout the *A. duranensis* and *A. ipaensis* genomes in this study. The recently completed genome sequences of the diploid ancestors of cultivated peanut provide physical maps of the highest resolution (Bertioli et al. 2016). A total of 264,135 and 392,107 SSR loci were identified from them, respectively. This number is much larger than the 375,180 loci found in another genome-derived SSR identification by genomic survey sequencing of the cultivated peanut Zhonghua 16 (Zhou et al. 2016) and other previous reports (Cuc et al. 2008; Ferguson et al. 2004; Gimenes et al. 2007; Guo et al. 2009; He et al. 2003; Hopkins et al. 1999; Huang et al. 2016b; Moretzsohn et al. 2005; Shirasawa et al. 2012). The average intervals of SSR loci were estimated as 4.10 and 3.45 kb in *A. duranensis* and *A. ipaensis*, respectively, which are neither the highest nor the lowest in plant (Shi et al. 2014; Wang et al. 2015; Yu et al. 2016). The mono-, di- and tri-nucleotide SSR motifs were more abundant than tetra-, penta-, and hexa-nucleotide motifs. Moreover, we found that the A/T (99.43%), AT/AT (48.97%) and AAT/ATT (41.32%) repeats were the most abundant mono-, di- and tri-nucleotide SSRs, respectively, in both genomes, which were congruent with previous reports in peanut (Zhou et al. 2016) or other species such as *Brassica*, rice and *Arabidopsis* (Katti et al. 2001; Shi et al. 2014; Temnykh et al. 2001).

A total of 84,383 and 120,056 SSR markers were finally developed in the *A. duranensis* and *A. ipaensis* genomes, respectively. Among the 2240 newly developed markers used in the genotyping of the RIL population in this study, 1706 markers amplified same bands and 365 markers amplified polymorphism bands in the two parents (Table S2). The quality of the newly developed markers was similar to recent reported SSR markers (Huang et al. 2016b; Zhou et al. 2016). Subsequently, around 8.04% of the 2240 newly developed markers were polymorphic in the RIL population, which was similar to percentage of the previously reported markers (9.47%). These results verified the validity and reliability of the newly developed SSR markers. Compared to the previous genetic map of the same RIL population (Luo et al. 2017), the mapped loci of the genetic map constructed in this study were improved from 743 to 830, and more importantly, the designated chromosomes were improved from 16 to 20. With known genomic positions, the newly developed SSR markers could be easily selected to improve the quality of genetic map by filling uncovered chromosomes or increasing the densities of covered chromosomes. Note that some of the newly developed markers were not mapped back to their original chromosomes in the constructed linkage map (Table S6), owing to the fact that there might be some segment exchanges among peanut chromosomes (Huang et al. 2016a). Collectively, SSR markers identified in this study should be useful in a variety of applications, such as studying of population structures, genetic map construction and mapping genes for important traits.

The broad-sense heritability estimated in this study was relatively high for shelling percentage in cultivated peanut, indicating that genetic factors play a major role in the determination of this trait. In this study, a RIL population was used to construct a dense genetic linkage map and conducting QTL analysis for shelling percentage. Because of a lack of polymorphism at the DNA level, the first SSR-based genetic linkage map for peanut only had 135 SSR loci (Varshney et al. 2009). However, a genetic linkage map containing 830 loci and covering a total length of 1386.19 cM with an average inter-marker distance of 1.67 cM was constructed in this study. The loci number and density of our map were relatively higher than that of previous reports (Chen et al. 2016; Huang et al. 2015; Qin et al. 2012; Ravi et al. 2011), except for the integrated consensus map (Shirasawa et al. 2013) and a recent report (Huang et al. 2016a), indicated a high quality of the linkage map constructed in this study. A total of 25 QTLs with 4.46–17.01% PVE were identified to be associated with shelling percentage across four environments. The LOD values of these QTLs ranged from 3.3 to 11.1 and were higher than the threshold of LOD for declaring the presence of a QTL that was determined by 1000 permutation tests. The 25 QTLs were mapped on chromosomes A05, A09, B02, B03, B04, B05 and B10. Because no QTL for shelling percentage had been reported on subgenome B, the 17 QTLs on chromosomes B02, B03, B04, B05 and B10 were novel. The chromosomes A05 and A09 that might harbor important genes for shelling percentage as two QTLs from this study and one QTL from an earlier study (Huang et al. 2015) were mapped on A05, and six QTLs from this study and one QTL from a previous study (Huang et al. 2015) were identified on A09. Collectively, shelling percentage was controlled by many QTLs on multiple chromosomes and environments often affected their effects. All the linked markers after validation can be deployed in marker-assisted selection (MAS) for the improvement of shelling percentage in peanut breeding.

Although parents had merely 5% difference in their trait means, this did not limit segregation variance and the power to detect QTLs in the RIL population. Kalih et al. (2014) successfully identified QTLs for plant height, heading stage, and Fusarium head blight in triticale even if parents had similar trait means. Miedaner et al. (2012) also identified QTLs for several agronomic traits in a segregating population whose parents did not differ much. In this study, significant variances and transgressive segregations were observed in the RIL population (Table 1; Fig. 1), indicating that the parents carry complementary alleles at several loci that were newly combined in the progeny (Tanksley 1993). This is in accordance with the fundamental rule of quantitative genetics for complex traits. The QTLs on chromosomes A05, A09, B02, B03 and B04 and two QTLs on chromosome B05 (*qSPB05.1a* and *qSPB05.4*) had negative additive genetic effects (Table 5), which revealed that maternal parent Yuanza 9102 as the source of alleles improving the shelling percentage. However, the QTL *qSPB05.3* and those on chromosome B10 had positive additive genetic effects (Table 5), suggesting that the alleles for increasing shelling percentage came from the parent Xuzhou 68-4. These QTLs explained the transgressive segregation of shelling percentage in the RIL population (Fig. 1).

Because of the fact that the identification of QTLs for shelling percentage was highly affected by environment, it is very important to assess their consistent performance across varied environments. Faye et al. (2015) detected two QTLs for shelling percentage with 5.74–6.97% PVE in a RIL population only under water stress condition. Huang et al. (2015) detected three QTLs for shelling percentage with 2.00–11.78% PVE in an F_2:3_ population in single environment. None of them were reported to be consistently expressed so far. Despite the significant G × E interactions (*P* < 0.001) present in the four trials conducted in this study, a consistent and major QTL, *cqSPA09*, has shown stable performance across all four environments. It was integrated from *qSPA09.1b, qSPA09.2, qSPA09.3b* and *qSPA09.4* by meta-analysis. The consistent QTL *cqSPA09* provided a significant level of consistent contribution to shelling percentage (13.75–26.82% PVE) in the four environments, and therefore may be an important interval for improving shelling percentage in peanut breeding. The segregation in the progeny of a residual heterozygous line, RIL 15-71126, confirmed that the allele came from the parent Yuanza 9102 at the *cpSPA09* locus had additive effect in increasing shelling percentage. Further studies, for example fine mapping, should be conducted to investigate its candidate genes. In addition, four consistent and minor QTLs namely *cqSPB02*, *cqSPB04*, *cqSPB05* and *cqSPB10*, were detected in two or three environments. Such QTLs with consistent performance for shelling percentage have been identified for the first time in peanut and will be very useful for further fine mapping of these QTL regions and development of diagnostic markers for peanut breeding.

### **Author contribution statement**

HL, XZ, YC, YL, BL and HJ conceived and designed the experiments. HL, JY, XR, LH and HJ performed SSR marker development. XR and HJ developed the RIL population. XR, XZ, YC and HJ conducted field trails. ZX, ZL, XL, JL, LH and WC performed genotyping. HL and HJ constructed the genetic linkage map and performed QTL analysis. HL, BL and HJ wrote the manuscript.

## Electronic supplementary material

Below is the link to the electronic supplementary material.

Supplementary material 1 (PDF 267 kb)

Supplementary material 2 (PDF 26672 kb)

Supplementary material 3 (PDF 1507 kb)

Supplementary material 4 (PDF 361 kb)

Supplementary material 5 (PDF 905 kb)

Supplementary material 6 (PDF 174 kb)

Supplementary material 7 (PDF 21 kb)

Supplementary material 8 (XLSX 627 kb)

Supplementary material 9 (XLS 215 kb)

## References

[CR1] Bertioli DJ, Cannon SB, Froenicke L, Huang G, Farmer AD, Cannon EK (2016). The genome sequences of *Arachis duranensis* and *Arachis ipaensis,* the diploid ancestors of cultivated peanut. Nat Genet.

[CR2] Bravo JP, Hoshino AA, Angelici CMLCD, Lopes CR, Gimenes MA (2006). Transferability and use of microsatellite markers for the genetic analysis of the germplasm of some *Arachis* section species of the genus *Arachis*. Genet Mol Biol.

[CR3] Chen W, Jiao Y, Cheng L, Huang L, Liao B, Tang M (2016). Quantitative trait locus analysis for pod- and kernel-related traits in the cultivated peanut (*Arachis hypogaea* L.). BMC Genet.

[CR4] Cuc LM, Mace ES, Crouch JH, Quang VD, Long TD, Varshney RK (2008). Isolation and characterization of novel microsatellite markers and their application for diversity assessment in cultivated groundnut (*Arachis hypogaea*). BMC Plant Biol.

[CR5] Doyle J (1990). Isolation of plant DNA from fresh tissue. Focus.

[CR6] Faye I, Pandey MK, Hamidou F, Rathore A, Ndoye O, Vadez V, Varshney RK (2015). Identification of quantitative trait loci for yield and yield related traits in groundnut (*Arachis hypogaea* L.) under different water regimes in Niger and Senegal. Euphytica.

[CR7] Ferguson ME, Burow MD, Schulze SR, Bramel PJ, Paterson AH, Kresovich S, Mitchell S (2004). Microsatellite identification and characterization in peanut (*A. hypogaea* L.). Theor Appl Genet.

[CR8] Fountain JC, Qin H, Chen C, Dang P, Wang ML, Guo B (2011). A note on development of a low-cost and high-throughput SSR-based genotyping method in peanut (*Arachis hypogaea* L.). Peanut Sci.

[CR9] Gautami B, Pandey MK, Vadez V, Nigam SN, Ratnakumar P, Krishnamurthy L (2012). Quantitative trait locus analysis and construction of consensus genetic map for drought tolerance traits based on three recombinant inbred line populations in cultivated groundnut (*Arachis hypogaea* L.). Mol Breed.

[CR10] Gimenes MA, Hoshino AA, Barbosa AV, Palmieri DA, Lopes CR (2007). Characterization and transferability of microsatellite markers of the cultivated peanut (*Arachis hypogaea*). BMC Plant Biol.

[CR11] Guo B, Chen X, Hong Y, Liang X, Dang P, Brenneman T (2009). Analysis of gene expression profiles in leaf tissues of cultivated peanuts and development of EST-SSR markers and gene discovery. Int J Plant Genom.

[CR12] Guo YF, Khanal S, Tang SX, Bowers JE, Heesacker AF, Khalilian N (2012). Comparative mapping in intraspecific populations uncovers a high degree of macrosynteny between A- and B-genome diploid species of peanut. BMC Genom.

[CR13] Guzel E, Akcali ID, Mutlu H, Ince A (2005). Research on the fatigue behavior for peanut shelling. J Food Eng.

[CR14] He G, Meng R, Newman M, Gao G, Pittman RN, Prakash C (2003). Microsatellites as DNA markers in cultivated peanut (*Arachis hypogaea* L.). BMC Plant Biol.

[CR15] Holland JB, Nyquist WE, Cervantes-Martínez CT, Janick J (2010). Estimating and Interpreting Heritability for Plant Breeding: An Update. Plant Breeding Reviews.

[CR16] Hopkins MS, Casa AM, Wang T, Mitchell SE, Dean RE, Kochert GD, Kresovich S (1999). Discovery and characterization of polymorphic simple sequence repeats (SSRs) in peanut. Crop Sci.

[CR17] Hoshino AA, Bravo JP, Angelici CMLCD, Barbosa AVG, Lopes CR, Gimenes MA (2006). Heterologous microsatellite primer pairs informative for the whole genus *Arachis*. Genet Mol Biol.

[CR18] Huang L, He HY, Chen WG, Ren XP, Chen YN, Zhou XJ (2015). Quantitative trait locus analysis of agronomic and quality-related traits in cultivated peanut (*Arachis hypogaea* L.). Theor Appl Genet.

[CR19] Huang L, Ren X, Wu B, Li X, Chen W, Zhou X (2016). Development and deployment of a high-density linkage map identified quantitative trait loci for plant height in peanut (*Arachis hypogaea* L.). Sci Rep.

[CR20] Huang L, Wu B, Zhao J, Li H, Chen W, Zheng Y (2016). Characterization and transferable utility of microsatellite markers in the wild and cultivated *Arachis* species. PLoS One.

[CR21] Janila P, Variath MT, Pandey MK, Desmae H, Motagi BN, Okori P (2016). Genomic tools in groundnut breeding program: status and perspectives. Front Plant Sci.

[CR22] Jiang H, Duan N, Ren X (2006). Descriptors and data standard for peanut (*Arachis* spp.).

[CR23] Jiang HF, Ren XP, Chen YN, Huang L, Zhou XJ, Huang JQ (2013). Phenotypic evaluation of the Chinese mini-mini core collection of peanut (*Arachis hypogaea* L.) and assessment for resistance to bacterial wilt disease caused by *Ralstonia solanacearum*. Plant Genet Resour C.

[CR24] Jiang HF, Huang L, Ren XP, Chen YN, Zhou XJ, Xia YL (2014). Diversity characterization and association analysis of agronomic traits in a Chinese peanut (*Arachis hypogaea* L.) mini-core collection. J Integr Plant Biol.

[CR25] Kalih R, Maurer HP, Hackauf B, Miedaner T (2014). Effect of a rye dwarfing gene on plant height, heading stage, and Fusarium head blight in triticale (× *Triticosecale* Wittmack). Theor Appl Genet.

[CR26] Katti MV, Ranjekar PK, Gupta VS (2001). Differential distribution of simple sequence repeats in eukaryotic genome sequences. Mol Biol Evol.

[CR27] Koilkonda P, Sato S, Tabata S, Shirasawa K, Hirakawa H, Sakai H (2012). Large-scale development of expressed sequence tag-derived simple sequence repeat markers and diversity analysis in *Arachis* spp.. Mol Breed.

[CR28] Kosambi DD (2011). The estimation of map distances from recombination values. Ann Hum Genet.

[CR29] Leal-Bertioli SC, Jose AC, Alves-Freitas DM, Moretzsohn MC, Guimaraes PM, Nielen S (2009). Identification of candidate genome regions controlling disease resistance in *Arachis*. BMC Plant Biol.

[CR30] Leal-Bertioli SC, Moretzsohn MC, Roberts PA, Ballen-Taborda C, Borba TC, Valdisser PA (2015). Genetic mapping of resistance to *Meloidogyne arenaria* in Arachis stenosperma: a new source of nematode resistance for peanut. G3.

[CR31] Luo H, Ren X, Li Z, Xu Z, Li X, Huang L (2017). Co-localization of major quantitative trait loci for pod size and weight to a 3.7 cM interval on chromosome A05 in cultivated peanut (*Arachis hypogaea* L.). BMC Genom.

[CR32] Macedo SE, Moretzsohn MC, Leal-Bertioli SC, Alves DM, Gouvea EG, Azevedo VC, Bertioli DJ (2012). Development and characterization of highly polymorphic long TC repeat microsatellite markers for genetic analysis of peanut. BMC Res Notes.

[CR33] Miedaner T, Hübner M, Korzun V, Schmiedchen B, Bauer E, Haseneyer G (2012). Genetic architecture of complex agronomic traits examined in two testcross populations of rye (*Secale cereale* L.). BMC Genom.

[CR34] Mondal S, Phadke RR, Badigannavar AM (2015). Genetic variability for total phenolics, flavonoids and antioxidant activity of testaless seeds of a peanut recombinant inbred line population and identification of their controlling QTLs. Euphytica.

[CR35] Moretzsohn Mde C, Hopkins MS, Mitchell SE, Kresovich S, Valls JF, Ferreira ME (2004). Genetic diversity of peanut (*Arachis hypogaea* L.) and its wild relatives based on the analysis of hypervariable regions of the genome. BMC Plant Biol.

[CR36] Moretzsohn MC, Leoi L, Proite K, Guimaraes PM, Leal-Bertioli SC, Gimenes MA (2005). A microsatellite-based, gene-rich linkage map for the AA genome of *Arachis* (Fabaceae). Theor Appl Genet.

[CR37] Moretzsohn MC, Barbosa AV, Alves-Freitas DM, Teixeira C, Leal-Bertioli SC, Guimaraes PM (2009). A linkage map for the B-genome of *Arachis* (Fabaceae) and its synteny to the A-genome. BMC Plant Biol.

[CR38] Nagy ED, Chu Y, Guo Y, Khanal S, Tang S, Li Y (2010). Recombination is suppressed in an alien introgression in peanut harboring *Rma*, a dominant root-knot nematode resistance gene. Mol Breed.

[CR39] Naito Y, Suzuki S, Iwata Y, Kuboyama T (2008). Genetic diversity and relationship analysis of peanut germplasm using SSR markers. Breed Sci.

[CR40] Ozudogru EA, Kaya E, Lambardi M (2013). *In vitro* propagation of peanut (*Arachis hypogaea* L.) by shoot tip culture. Methods Mol Biol.

[CR41] Pandey MK, Wang ML, Qiao L, Feng S, Khera P, Wang H et al (2014) Identification of QTLs associated with oil content and mapping *FAD2* genes and their relative contribution to oil quality in peanut (*Arachis hypogaea* L.). BMC Genet:133 10.1186/s12863-014-0133-4 PMC4278341 25491595

[CR42] Qin H, Feng S, Chen C, Guo Y, Knapp S, Culbreath A (2012). An integrated genetic linkage map of cultivated peanut (*Arachis hypogaea* L.) constructed from two RIL populations. Theor Appl Genet.

[CR43] Ravi K, Vadez V, Isobe S, Mir RR, Guo Y, Nigam SN (2011). Identification of several small main-effect QTLs and a large number of epistatic QTLs for drought tolerance related traits in groundnut (*Arachis hypogaea* L.). Theor Appl Genet.

[CR44] Sharma KK, Bhatnagar-Mathur P (2006). Peanut (*Arachis hypogaea* L.). Methods Mol Biol.

[CR45] Shi J, Huang S, Zhan J, Yu J, Wang X, Hua W (2014). Genome-wide microsatellite characterization and marker development in the sequenced *Brassica* crop species. DNA Res.

[CR46] Shirasawa K, Koilkonda P, Aoki K, Hirakawa H, Tabata S, Watanabe M (2012). In silico polymorphism analysis for the development of simple sequence repeat and transposon markers and construction of linkage map in cultivated peanut. BMC Plant Biol.

[CR47] Shirasawa K, Bertioli DJ, Varshney RK, Moretzsohn MC, Leal-Bertioli SC, Thudi M (2013). Integrated consensus map of cultivated peanut and wild relatives reveals structures of the A and B genomes of *Arachis* and divergence of the legume genomes. DNA Res.

[CR48] Shoha D, Manivannan N, Vindhiyavarman P, Nigam SN (2013). Identification of quantitative trait loci (QTL) for late leaf spot disease resistance in groundnut (*Arachis hypogaea* L.). Legume Res.

[CR49] Sosnowski O, Charcosset A, Joets J (2012). BioMercator V3: an upgrade of genetic map compilation and quantitative trait loci meta-analysis algorithms. Bioinformatics.

[CR50] Sukruth M, Paratwagh SA, Sujay V, Kumari V, Gowda MVC, Nadaf HL (2015). Validation of markers linked to late leaf spot and rust resistance, and selection of superior genotypes among diverse recombinant inbred lines and backcross lines in peanut (*Arachis hypogaea* L.). Euphytica.

[CR51] Tanksley SD (1993). Mapping polygenes. Annu Rev Genet.

[CR52] Temnykh S, DeClerck G, Lukashova A, Lipovich L, Cartinhour S, McCouch S (2001). Computational and experimental analysis of microsatellites in rice (*Oryza sativa* L.): frequency, length variation, transposon associations, and genetic marker potential. Genome Res.

[CR53] Thiel T (2014) MISA—MIcroSAtellite identification tool. http://pgrc.ipk-gatersleben.de/misa/misa.html. Accessed 20 Jan 2016

[CR54] Udall JA, Quijada PA, Lambert B, Osborn TC (2006). Quantitative trait analysis of seed yield and other complex traits in hybrid spring rapeseed (*Brassica napus* L.): 2. Identification of alleles from unadapted germplasm. Theor Appl Genet.

[CR55] Van Ooijen JW (2006). JoinMap 4. Software for the calculation of genetic linkage maps in experimental populations.

[CR56] Varshney RK, Bertioli DJ, Moretzsohn MC, Vadez V, Krishnamurthy L, Aruna R (2009). The first SSR-based genetic linkage map for cultivated groundnut (*Arachis hypogaea* L.). Theor Appl Genet.

[CR57] Varshney RK, Pandey MK, Janila P, Nigam SN, Sudini H, Gowda MV (2014). Marker-assisted introgression of a QTL region to improve rust resistance in three elite and popular varieties of peanut (*Arachis hypogaea* L.). Theor Appl Genet.

[CR58] Voorrips RE (2002). MapChart: software for the graphical presentation of linkage maps and QTLs. J Hered.

[CR59] Wang H, Penmetsa RV, Yuan M, Gong L, Zhao Y, Guo B (2012). Development and characterization of BAC-end sequence derived SSRs, and their incorporation into a new higher density genetic map for cultivated peanut (*Arachis hypogaea* L.). BMC Plant Biol.

[CR60] Wang S, Basten CJ, Zeng Z-B (2012b) Windows QTL Cartographer 2.5. Department of Statistics, North Carolina State University, Raleigh, NC. http://statgen.ncsu.edu/qtlcart/WQTLCart.htm. Accessed 01 Dec 2015

[CR61] Wang Q, Fang L, Chen JD, Hu Y, Si ZF, Wang S (2015). Genome-wide mining, characterization, and development of microsatellite markers in *Gossypium* species. Sci Rep.

[CR62] Yu J, Dossa K, Wang L, Zhang Y, Wei X, Liao B, Zhang X (2016). PMDBase: a database for studying microsatellite DNA and marker development in plants. Nucleic Acids Res.

[CR63] Zhou XJ, Xia YL, Ren XP, Chen YL, Huang L, Huang SM (2014). Construction of a SNP-based genetic linkage map in cultivated peanut based on large scale marker development using next-generation double-digest restriction-site-associated DNA sequencing (ddRADseq). BMC Genom.

[CR64] Zhou X, Dong Y, Zhao J, Huang L, Ren X, Chen Y (2016). Genomic survey sequencing for development and validation of single-locus SSR markers in peanut (*Arachis hypogaea* L.). BMC Genom.

