# Supplementary material for: Development of SSR markers and identification of major quantitative trait loci controlling shelling percentage in cultivated peanut (Arachis hypogaea L.)
Source: Theor Appl Genet. 2017 May 15;130(8):1635–48. doi: 10.1007/s00122-017-2915-3 (PMC5511596; doi:10.1007/s00122-017-2915-3)
Supplement: Supplementary file 1 — Supplementary material 1 (PDF 267 kb) [file 122_2017_2915_MOESM1_ESM.pdf]

Table S1 Numbers of SSR loci in different repeat types in the *Arachis duranensis* and *A. ipaensis* genomes.

a. Numbers of SSR loci in different repeat types in the *Arachis duranensis* genome

**Repeats**	**5**	**6**	**7**	**8**	**9**	**10**	**11**	**12**	**13**	**14**	**15**	**16**	**17**	**18**	**19**	**20**	**21**	**22**	**23**	**24**	**25**	**>25**	**Total**
A/T	-	-	-	-	-	83989	29988	13647	7540	4096	2096	926	374	217	136	103	62	42	51	34	22	135	143458
C/G	-	-	-	-	-	450	171	56	45	36	21	22	15	5	2				1	1		4	829
AC/GT	-	2199	1021	627	391	216	136	110	92	59	69	41	62	45	46	32	20	32	29	14	11	147	5399
AG/CT	-	7312	3261	2027	1282	767	475	348	332	271	254	243	254	240	232	195	158	153	94	93	77	129	18197
AT/AT	-	8892	4890	3484	2500	1466	1004	497	319	183	74	35	35	7	9	2	8		1	1	1	1	23409
CG/CG	-	759	29	5	4	2	1															0	800
AAC/GTT	1764	635	279	145	85	74	45	42	27	23	20	23	15	18	11	7	4	4	1	2	1	6	3231
AAG/CTT	6534	3406	1063	416	271	151	126	88	68	50	52	41	37	29	26	19	21	21	25	17	17	96	12574
AAT/ATT	6214	3054	1470	812	585	511	515	534	555	550	509	458	429	333	291	221	162	98	76	61	52	82	17572
ACC/GGT	925	434	166	60	46	4	12	4	6	1												0	1658
ACG/CGT	138	34	13	4	1	2	1															0	193
ACT/AGT	493	169	68	24	18	8	5	4	2	3	3	3	2	2	1	2	2	1			1	7	818
AGC/CTG	314	93	32	14	2	1																0	456
AGG/CCT	988	535	212	187	93	45	29	11		1												0	2101
ATC/ATG	1905	847	360	151	87	77	45	30	45	26	17	13	13	19	14	11	5	5	6	3	8	44	3731
CCG/CGG	138	35	16	4	2																	0	195
AAAC/GTTT	97	42	14	4	3		1	1		1									1			0	164
AAAG/CTTT	320	114	41	26	13	13	8	8	3	1	2		1									0	550
AAAT/ATTT	1881	549	187	70	35	17	11	10	2	1						1						0	2764
AACC/GGTT	10																					0	10
AACG/CGTT	1	1	1																			0	3
AACT/AGTT	146	48	27	3	2	1		2														0	229
AAGC/CTTG	3																					0	3
AAGG/CCTT	16	4																				0	20
AAGT/ACTT		2	1	1																		0	4
AATC/ATTG	20	5		1																		0	26
AATG/ATTC	75	15	6	3	2		1	1														0	103
AATT/AATT	304	84	31	14	7	4			1													0	445
ACAG/CTGT	1		1																			0	2
ACAT/ATGT	130	63	34	20	18	10	17	13	9	6	8	2	1		4			1				2	338
ACCT/AGGT	1	2																				0	3

ACGC/CGTG	9	6	2	1	1	2	1		1		1		4		1	29
ACGG/CCGT	2														0	2
ACTC/AGTG	28	15	9	2				1							0	55
AGAT/ATCT	87	40	22	6	6	6	7	3	1	3		3		1	1	186
AGCC/CTGG	2														0	2
AGCG/CGCT	1														0	1
AGCT/AGCT	5														0	5
AGGG/CCCT	16	3	1												0	20
ATCC/ATGG	7	1	2					1							0	11
ATGC/ATGC	11	1	1												0	13
AAAAC/GTTTT	21	4	2	1											0	28
AAAAG/CTTTT	91	19	4	2	2										0	118
AAAAT/ATTTT	505	77	26	11	3	1	1								0	624
AAACC/GGTTT	4	2													0	6
AAACG/CGTTT	1														0	1
AAACT/AGTTT	9	1	1												0	11
AAAGG/CCTTT	3	1													0	4
AAATC/ATTTG	23	1	2												0	26
AAATG/ATTTC	1														0	1
AAATT/AATTT	46	6	1		1										0	54
AACAC/GTGTT	5		1												0	6
AACAG/CTGTT	1	1													0	2
AACAT/ATGTT	1														0	1
AACCC/GGGTT	1														0	1
AACCG/CGGTT	18	1	1												0	20
AACCT/AGGTT	1														0	1
AACGC/CGTTG	1														0	1
AACTC/AGTTG	3	1	1												0	5
AACTG/AGTTC	4														0	4
AAGAG/CTCTT	34	8	4	1	2										0	49
AAGAT/ATCTT	401	58	16	6	2		1								2	486
AAGCC/CTTGG	1														0	1
AAGCT/AGCTT	1														0	1
AAGGG/CCCTT	5	1													0	6
AAGTC/ACTTG	1														0	1

AATAC/ATTGT	1			1				0	2
AATAG/ATTCT	4							0	4
AATAT/ATATT	29	13	7	3	2	2		0	56
AATCC/ATTGG	4	1						0	5
AATCT/AGATT	2	2		1				0	5
AATGC/ATTGC	1							0	1
AATGG/ATTCC	3							0	3
AATTC/AATTG	33	7		1				0	41
ACACC/GGTGT	2							0	2
ACACT/AGTGT	1							0	1
ACAGC/CTGTG	3							0	3
ACATC/ATGTG	1							0	1
ACCAT/ATGGT	3	1						0	4
ACCCC/GGGGT	2							0	2
ACCCG/CGGGT	2							0	2
ACCCT/AGGGT	13	3	1					0	17
ACCTC/AGGTG	2							0	2
ACGTC/ACGTG	1							0	1
ACTAT/AGTAT	2							0	2
ACTCT/AGAGT	1							0	1
AGAGG/CCTCT	2		1	1				0	4
AGATG/ATCTC	3	3						0	6
AGGCC/CCTGG		4						0	4
ATATC/ATATG	5	3	1					0	9
ATCCC/ATGGG		1						0	1
ATCCG/ATCGG	13							0	13
ATGCC/ATGGC	1							0	1
CCCCG/CGGGG	6							0	6
AAAAAC/GTTTTT	4	2						0	6
AAAAAG/CTTTTT	31	10	3		1		1	0	46
AAAAAT/ATTTTT	50	12	1	1		1		0	65
AAAACC/GGTTTT	2							0	2
AAAAGT/ACTTTT	1							0	1
AAAATC/ATTTTG	8	4	1	1				0	14
AAAATG/ATTTTC	3		1					0	4

AAAATT/AATTTT	11	3	1						0	15
AAACAC/GTGTTT	4								0	4
AAACAG/CTGTTT	1								0	1
AAACAT/ATGTTT		2							0	2
AAAGAG/CTCTTT	11	2	1				1		0	15
AAAGAT/ATCTTT	8	3	1						0	12
AAAGCC/CTTTGG	1								0	1
AAAGGG/CCCTTT	2	1							0	3
AAAGTG/ACTTTC	1								0	1
AAATAC/ATTTGT	1								0	1
AAATAT/ATATTT	4	1							0	5
AAATCC/ATTTGG	4								0	4
AAATGG/ATTTCC	1								0	1
AAATTC/AATTTG	2	1							0	3
AAATTG/AATTTC		2	1						0	3
AAATTT/AAATTT	3								0	3
AACAAG/CTTGTT	3	2	1						0	6
AACAAT/ATTGTT	13	3	3		2				0	21
AACACC/GGTGTT	18	6	2			1		1	0	28
AACACT/AGTGTT			1						0	1
AACAGC/CTGTTG				1					0	1
AACAGG/CCTGTT		1							0	1
AACAGT/ACTGTT	1								0	1
AACATC/ATGTTG	3								0	3
AACATG/ATGTTC	2								0	2
AACCAC/GGTTGT	1								0	1
AACCAG/CTGGTT	2								0	2
AACCAT/ATGGTT	1		1						0	2
AACCCT/AGGGTT	2	1							0	3
AACCGC/CGGTTG	1								0	1
AACGAG/CGTTCT	1								0	1
AACGCC/CGTTGG	1								0	1
AACGTC/ACGTTG	1								0	1
AACTAG/AGTTCT	1								0	1
AACTAT/AGTTAT	2		1						0	3

AAGAAT/ATTCTT	1											0	1
AAGACG/CGTCTT	2	1										0	3
AAGAGC/CTCTTG	1		1									0	2
AAGAGG/CCTCTT	84	60	32	17	4	1	1	1	1	1	1	0	203
AAGAGT/ACTCTT	1											0	1
AAGATG/ATCTTC	2											0	2
AAGATT/AATCTT		1										0	1
AAGCAG/CTGCTT	2	1										0	3
AAGCCC/CTTGGG		1										0	1
AAGGAC/CCTTGT			1									0	1
AAGGAG/CCTTCT	9	4	3	1	1		1					0	19
AAGGAT/ATCCTT	1											0	1
AAGGCT/AGCCTT	1											0	1
AAGGGG/CCCCTT	4											0	4
AAGGTG/ACCTTC	3											0	3
AAGTAG/ACTTCT	1											0	1
AAGTCC/ACTTGG	1											0	1
AAGTGG/ACTTCC	1											0	1
AATACT/AGTATT	1											0	1
AATAGG/ATTCCT	1											0	1
AATATC/ATATTG			1									0	1
AATATG/ATATTC	1											0	1
AATCAC/ATTGTG		1										0	1
AATCAG/ATTCTG	2	2										0	4
AATCAT/ATGATT	4	2										0	6
AATCCG/ATTCGG	2											0	2
AATCCT/AGGATT	19	1	1	1								0	22
AATCTC/AGATTG	1											0	1
AATCTG/AGATTC	1											0	1
AATGAG/ATTCTC		1										0	1
AATGAT/ATCATT	5		1									0	6
AATGCC/ATTGGC	1											0	1
AATGCT/AGCATT	1											0	1
AATGGG/ATTCCC	1											0	1
AATGTG/ACATTC	1			1								0	2

AATTAC/AATTGT	2									0	2
AATTCC/AATTGG	1									0	1
ACACAG/CTGTGT	1									0	1
ACACAT/ATGTGT	3									0	3
ACACCC/GGGTGT	1									0	1
ACACGC/CGTGTG	3	1								0	4
ACACGG/CCGTGT							1		1	0	2
ACACTC/AGTGTG	2									0	2
ACAGAG/CTCTGT	3	1								0	4
ACAGCC/CTGTGG	1									0	1
ACAGGG/CCCTGT		1								0	1
ACATAG/ATGTCT	1									0	1
ACATAT/ATATGT	4	2			1					0	7
ACATCG/ATGTCG	1									0	1
ACCACG/CGTGGT	3									0	3
ACCACT/AGTGGT	1	1								0	2
ACCAGC/CTGGTG	2			1						0	3
ACCATC/ATGGTG	1		1							0	2
ACCCCT/AGGGGT		1								0	1
ACCCTC/AGGGTG	2	1								0	3
ACCCTG/AGGGTC		1								0	1
ACCGAG/CGGTCT	1									0	1
ACCGCC/CGGTGG	5		1	1						0	7
ACCGCG/CGCGGT	1									0	1
ACCGGC/CCGGTG		2								0	2
ACCGTC/ACGGTG	2									0	2
ACCTCC/AGGTGG	5	4								0	9
ACCTCG/AGGTCG	1									0	1
ACCTGC/AGGTGC	3									0	3
ACGAGG/CCTCGT				1			1			0	2
ACGATG/ATCGTC	3	3								0	6
ACGGAG/CCGTCT		1		1						0	2
ACGGCG/CCGTCG			1							0	1
ACGGGG/CCCCGT	1									0	1
ACTATC/AGTGAT	2									0	2

ACTCAT/AGTATG	2																					0	2
ACTCCC/AGTGGG	1		1																			0	2
ACTCCT/AGGAGT	1	2		1																		0	4
ACTCGG/AGTCCG		1																				0	1
ACTCTC/AGAGTG	5	1	1																			0	7
ACTGAG/AGTCTC	1																					0	1
ACTGGG/AGTCCC	2																					0	2
AGAGAT/ATCTCT		1																				0	1
AGAGCT/AGCTCT	1																					0	1
AGAGGC/CCTCTG	1																					0	1
AGAGGG/CCCTCT	5	2	1																			0	8
AGATAT/ATATCT	1	3	1																			0	5
AGATGG/ATCTCC	1																					0	1
AGCATC/ATGCTG	1																					0	1
AGCATG/ATGCTC	1																					0	1
AGCCCC/CTGGGG	1																					0	1
AGCCCG/CGGGCT			1																			0	1
AGCCCT/AGGGCT			1																			0	1
AGCCGC/CGGCTG	1																					0	1
AGCCGG/CCGGCT	1																					0	1
AGCCTC/AGGCTG	3																					0	3
AGCTCC/AGCTGG	1	2																				0	3
AGGATC/ATCCTG	1																					0	1
AGGATG/ATCCTC	8	2	1	1																		0	12
ATCATG/ATCATG	1																					0	1
ATCCCC/ATGGGG	1																					0	1
Total	24366	29782	13398	8168	5475	87821	32603	15413	9049	5311	3126	1810	1243	916	773	596	442	357	286	227	191	657	242010

b. Numbers of SSR loci in different repeat types in the *Arachis ipaensis* genome

Repeats	5	6	7	8	9	10	11	12	13	14	15	16	17	18	19	20	21	22	23	24	25	>25	Total
A/T	-	-	-	-	-	117185	46947	24195	13661	8106	4766	2556	1303	705	421	236	212	135	118	68	43	341	220998
C/G	-	-	-	-	-	1305	644	267	161	90	67	34	26	23	16	11	5	2	6	2	1	12	2672
AC/GT	-	3262	1559	893	517	311	235	155	136	115	92	88	63	56	60	38	52	27	32	25	27	157	7900
AG/CT	-	14213	6106	3588	2121	1220	790	554	502	446	397	345	359	300	241	208	148	115	84	63	56	229	32085

AT/AT	-	11381	6399	4659	3332	2279	1395	958	650	461	336	314	234	212	159	127	112	109	102	72	58	444	33793
CG/CG	-	1452	84	16	3			1														0	1556
AAC/GTT	2153	704	338	159	79	58	60	43	42	42	21	20	18	23	7	7	6	7	6	2	3	15	3813
AAG/CTT	8837	2971	1017	407	257	145	106	98	63	80	59	43	44	35	39	34	19	29	35	29	28	154	14529
AAT/ATT	6526	2876	1427	759	547	399	366	375	348	307	273	283	224	197	166	121	110	82	67	56	42	448	15999
ACC/GGT	1492	598	219	98	50	30	19	7	5													1	2519
ACG/CGT	172	36	13	5	2	2	1	1														0	232
ACT/AGT	401	136	65	20	21	13	15	5	4	3	2	2	1	4		2	1	1	1	3		6	706
AGC/CTG	474	98	33	7	4																	0	616
AGG/CCT	1270	613	249	151	87	74	43	8	5	2												0	2502
ATC/ATG	2318	971	395	192	122	104	63	46	40	38	28	32	28	23	19	14	12	10	7	6	5	73	4546
CCG/CGG	188	55	7	3	2																	0	255
AAAC/GTTT	77	18	5	3	2	1	1															0	107
AAAG/CTTT	380	128	52	37	25	16	12	6	5	5	2	2										1	671
AAAT/ATTT	2353	1047	303	113	54	38	21	6	11	3	5	3	4	2			1		1		1	5	3971
AACC/GGTT	9		1																			0	10
AACT/AGTT	271	84	34	8	3	3	1		1		1							1				0	407
AAGC/CTTG	2	1																				0	3
AAGG/CCTT	20	4	2																			0	26
AAGT/ACTT	1	1	1																			0	3
AATC/ATTG	21	4	3	2																		0	30
AATG/ATTC	79	25	9	1				1														0	115
AATT/AATT	277	80	25	10	4	3		1													1	1	402
ACAG/CTGT	1	1	1																			0	3
ACAT/ATGT	183	66	43	34	28	29	27	23	12	21	19	13	9	6	10	4	1	2	1	2		6	539
ACCG/CGGT	1																					0	1
ACCT/AGGT	4		1																			0	5
ACGC/CGTG	17	10	7	2			2		1				1									0	40
ACGG/CCGT	2	1	1																			0	4
ACTC/AGTG	37	15	6	2	1	1			1	1												0	64
AGAT/ATCT	124	50	24	19	9	9	7	5	5	3	5	1	2			1	2					2	268
AGCG/CGCT	2	1																				0	3
AGCT/AGCT	1																					0	1
AGGG/CCCT	28	10	2																			0	40
ATCC/ATGG	4	5	1	1	1																	0	12

ATGC/ATGC	8	2	1							0	11
AAAAC/GTTTT	23	5	2	2						0	32
AAAAG/CTTTT	130	57	9	5	3		1			1	206
AAAAT/ATTTT	674	139	50	20	11	2	1	2		0	899
AAACC/GGTTT	7									0	7
AAACG/CGTTT	1									0	1
AAACT/AGTTT	4	4	1							0	9
AAAGC/CTTTG	1									0	1
AAAGG/CCTTT	2									0	2
AAAGT/ACTTT	1									0	1
AAATC/ATTTG	33	5								0	38
AAATG/ATTTC	5									0	5
AAATT/AATTT	38	15	2	1						0	56
AACAC/GTGTT	11	1								0	12
AACAG/CTGTT	1	3		1	1					0	6
AACAT/ATGTT		1		1	1					0	3
AACCC/GGGTT	4	1								0	5
AACCG/CGGTT	26	4								0	30
AACCT/AGGTT	1									0	1
AACGC/CGTTG	1									0	1
AACTC/AGTTG	3	1								0	4
AACTG/AGTTC	3									0	3
AAGAC/CTTGT	3									0	3
AAGAG/CTCTT	23	8	5	1		1	1			0	39
AAGAT/ATCTT	601	106	35	15	6	5	2		2	0	772
AAGCC/CTTGG		1								1	2
AAGGG/CCCTT	6		1							0	7
AAGGT/ACCTT	1									0	1
AAGTG/ACTTC	3									0	3
AATAC/ATTGT	3	1								0	4
AATAG/ATTCT	3									0	3
AATAT/ATATT	26	6	4	3	4					0	43
AATCT/AGATT	2									0	2
AATGC/ATTGC	3	1								0	4
AATGG/ATTCC	1									0	1

AATGT/ACATT	1								0	1
AATTC/AATTG	57	10	3						0	70
ACACC/GGTGT	1								0	1
ACACG/CGTGT	1								0	1
ACACT/AGTGT			1						0	1
ACATG/ATGTC	1								0	1
ACCAT/ATGGT	2	1							0	3
ACCCT/AGGGT	31	2	1			1			0	35
ACCGC/CGGTG	1								0	1
ACCTC/AGGTG	3								0	3
ACGCG/CGCGT	1								0	1
ACTCC/AGTGG	1								0	1
ACTCT/AGAGT	1								0	1
AGAGG/CCTCT	5	4							0	9
AGATG/ATCTC	5	4	2						0	11
AGCAT/ATGCT	1								0	1
AGCCT/AGGCT	1								0	1
AGGAT/ATCCT	3								0	3
AGGCC/CCTGG	2								0	2
AGGCG/CCTCG	1								0	1
AGGGG/CCCCT	3								0	3
ATATC/ATATG	8	1	1						0	10
ATCCG/ATCGG	41	2	1						0	44
ATGCC/ATGGC	1								0	1
CCCCG/CGGGG	6								0	6
CCCGG/CCGGG	1								0	1
AAAAAC/GTTTTT	5		1						0	6
AAAAAG/CTTTTT	42	18	2					1	0	63
AAAAAT/ATTTTT	113	31	6	2	1				0	153
AAAACC/GGTTTT	1								0	1
AAAACT/AGTTTT	1								0	1
AAAAGG/CCTTTT	1								0	1
AAAAGT/ACTTTT	1								0	1
AAAATC/ATTTTG	4	1	1						0	6
AAAATG/ATTTTC	3								0	3

AAAATT/AATTTT	14	7	1							0	22
AAACAC/GTGTTT	2									0	2
AAACAT/ATGTTT	1									0	1
AAACCC/GGGTTT	1									0	1
AAACTG/AGTTTC	1									0	1
AAAGAG/CTCTTT	10	2	1	1						0	14
AAAGAT/ATCTTT	4	4	2							0	10
AAAGGG/CCCTTT	3									0	3
AAATAC/ATTTGT	2									0	2
AAATAT/ATATTT		1								0	1
AAATCT/AGATTT	1									0	1
AAATGC/ATTTGC	1									0	1
AAATGT/ACATTT	1									0	1
AAATTC/AATTTG	1	2								0	3
AAATTG/AATTTC	2									0	2
AAATTT/AAATTT	1	1								0	2
AACAAG/CTTGTT	2	1	1							0	4
AACAAT/ATTGTT	10	12	6	4	1	2	1	1		0	37
AACACC/GGTGTT	19	3								0	22
AACAGT/ACTGTT	1									0	1
AACATC/ATGTTG	1		1							0	2
AACATT/AATGTT	1									0	1
AACCAC/GGTTGT	8	3	1							0	12
AACCAG/CTGGTT	1									0	1
AACCCT/AGGGTT	3	1		1						0	5
AACCTC/AGGTTG	2						1			0	3
AACGAC/CGTTGT	1			1						0	2
AACGAT/ATCGTT	1									0	1
AACGTC/ACGTTG	1									0	1
AACTAT/AGTTAT	1									0	1
AACTGG/AGTTCC		1								0	1
AACTTG/AAGTTC		1								0	1
AAGAAT/ATTCTT	2	3	1						1	0	7
AAGACG/CGTCTT	2	1								0	3
AAGAGC/CTCTTG	2									0	2

AAGAGG/CCTCTT	60	28	17	4	5	4		1			1	0	120
AAGATC/ATCTTG	1											0	1
AAGATG/ATCTTC	6		1			1						0	8
AAGCAG/CTGCTT	1	3										0	4
AAGCCC/CTTGGG	1											0	1
AAGGAG/CCTTCT	25	15	2	4	1	2				1		1	51
AAGGAT/ATCCTT	1											0	1
AAGGCT/AGCCTT	1											0	1
AAGGGC/CCCTTG	1											0	1
AAGGGG/CCCCTT	3											0	3
AAGGTG/ACCTTC	3	1										0	4
AAGTAG/ACTTCT		1	1									0	2
AATACT/AGTATT	1											0	1
AATAGC/ATTGCT		1										0	1
AATAGT/ACTATT		2										0	2
AATATC/ATATTG	1											0	1
AATCAC/ATTGTG	2											0	2
AATCAG/ATTCTG	3											0	3
AATCAT/ATGATT	5	1		1								0	7
AATCCT/AGGATT	10	1	1									0	12
AATCGG/ATTCCG	1											0	1
AATCTC/AGATTG	2											0	2
AATCTG/AGATTC		1										0	1
AATGAC/ATTGTC		1										0	1
AATGAG/ATTCTC	1											0	1
AATGAT/ATCATT	6	2	2	3	1	2		1	1			0	18
AATGCG/ATTCGC	1											0	1
AATGTG/ACATTC	2											0	2
AATTAT/AATTAT		1										0	1
AATTCC/AATTGG		1										0	1
ACACAT/ATGTGT	1	1										0	2
ACACCT/AGGTGT	1											0	1
ACACGC/CGTGTG	4	1		1		1	1					0	8
ACACGG/CCGTGT	2											0	2
ACACTC/AGTGTG	2											0	2

ACAGAT/ATCTGT	1								0	1
ACAGGG/CCCTGT		1							0	1
ACATAT/ATATGT	7	1		1					0	9
ACATCT/AGATGT	1			1					0	2
ACCACT/AGTGGT	4								0	4
ACCAGC/CTGGTG	1	1			1				0	3
ACCATC/ATGGTG	12	4				1			0	17
ACCCCT/AGGGGT		1							0	1
ACCCTC/AGGGTG	2								0	2
ACCGAG/CGGTCT		1							0	1
ACCGCC/CGGTGG	3	1	3						0	7
ACCGGC/CCGGTG	1								0	1
ACCGGG/CCCGGT			1						0	1
ACCGTC/ACGGTG			1						0	1
ACCTAG/AGGTCT		1							0	1
ACCTCC/AGGTGG	12	5							0	17
ACCTGC/AGGTGC	3	1							0	4
ACGAGG/CCTCGT	2		1						0	3
ACGATG/ATCGTC	1								0	1
ACGCGC/CGCGTG	2								0	2
ACGCGG/CCGCGT				1					0	1
ACGCGT/ACGCGT	1								0	1
ACGCTG/AGCGTC		1						1	0	2
ACGGCC/CCGTGG	2			1					0	3
ACGGCG/CCGTCG	1			1					0	2
ACGGGG/CCCCGT	1								0	1
ACTATC/AGTGAT	1			1					0	2
ACTCCC/AGTGGG	3								0	3
ACTCCT/AGGAGT	3								0	3
ACTCTC/AGAGTG	4	2							0	6
ACTGAG/AGTCTC	1								0	1
ACTGCC/AGTGGC	1								0	1
ACTGCT/AGCAGT	1	1	1			1	1		1	6
AGAGAT/ATCTCT	1								0	1
AGAGGC/CCTCTG	4	1							0	5

AGAGGG/CCCTCT	13	2	1																			1	17
AGATAT/ATATCT	1	2	1																			0	4
AGATGG/ATCTCC	1			1																		0	2
AGCAGG/CCTGCT	1																					0	1
AGCATG/ATGCTC	1																					0	1
AGCCAT/ATGGCT	1																					0	1
AGCCGG/CCGGCT	1																					0	1
AGCCTC/AGGCTG	2	1		1	1																	0	5
AGCCTG/AGGCTC		2																				0	2
AGCTCC/AGCTGG	2																					0	2
AGGATG/ATCCTC	7		3				1															0	11
AGGCGG/CCGCCT	1																					0	1
AGGGAT/ATCCCT	1																					0	1
AGGGCG/CCCTCG		1																				0	1
AGGGGC/CCCCTG	1																					0	1
ATCCCC/ATGGGG	2																					0	2
ATCGCC/ATGGCG	1																					0	1
Total	30090	41486	18611	11268	7308	123246	50763	26760	15655	9724	6075	3737	2317	1587	1138	804	682	520	462	328	265	###	354726
