# Supplementary material for: Development of SSR markers and identification of major quantitative trait loci controlling shelling percentage in cultivated peanut (Arachis hypogaea L.)
Source: Theor Appl Genet. 2017 May 15;130(8):1635–48. doi: 10.1007/s00122-017-2915-3 (PMC5511596; doi:10.1007/s00122-017-2915-3)
Supplement: Supplementary file 3 — Supplementary material 3 (PDF 1507 kb) [file 122_2017_2915_MOESM3_ESM.pdf]

Table S3 The 2,240 newly developed markers used in the genotyping of the RIL population.

ID	Chromosome	SSR_start	SSR_end	FORWARD PRIMER (5'-3')	REVERSE PRIMER (5'-3')	Bands in parents	Polymophism in RILs
Ad01A846	Aradu.A01	2175892	2176037	TCCATTCAAAGGACCAAAAGA	TCACTCAACTCAGCGTGTAGC	1	
Ad01A992	Aradu.A01	2520559	2520662	TCAAACGGTTCCTCTTCGTT	GGAAGAAGAACGTGCAACAA	1	
Ad01A1077	Aradu.A01	2771612	2771699	CGAAACCGTCCCTCATCTAA	TTGGTTGGTGTGATGCAGAT	1	
Ad01A1445	Aradu.A01	3687459	3687572	AACGTGTCACCAAAACTTATCAAA	AGGATGTCGCAAGATGAGAAA	1	
Ad01A2869	Aradu.A01	7389544	7389673	TTAGGAACTATTTCGATAGACAAAAA	CAGAAAGCTGGTTCTCCTTCA	1	
Ad01A3018	Aradu.A01	7770616	7770739	CAAATTCAGGACCAAAAATTCA	TCCCCAACCTCTCACCTAAA	1	
Ad01A3104	Aradu.A01	8005930	8006009	TTTGTCCCTCTTGGAGGTTG	AAAATTGAGGTGGCAAGGTG	2	
Ad01A3185	Aradu.A01	8318712	8318804	TGTGCTAAGCTGTCTCCTGC	AACAAGCGGCGAATAAAGAA	1	
Ad01A4177	Aradu.A01	11176754	11176839	CAAGTTAAGGATGCGTTTATTTTT	GCAGATCTCCCAAAAGAGTTG	1	
Ad01A4291	Aradu.A01	11452742	11452838	TAGGTTGTTGAGTTTGGGGG	GAAAATATTAGAGGTGAATTTGTCCC	1	
Ad01A5090	Aradu.A01	14569715	14569854	GAAGCTCTGCCACAATCACA	GCGAATGTAGATAGAATAGGGAATG	1	
Ad01A5132	Aradu.A01	14841047	14841224	AGGGCTGCACCAATAACAAC	GCTATGCATGCACCGAAAT	1	
Ad01A5465	Aradu.A01	16367057	16367180	TTCTCCATCAAGCCTTTTTGA	CGCTAGGCATCTGGAGTTTC	1	
Ad01A8152	Aradu.A01	33665408	33665488	GCTTCACGTGTGTAATTCCG	TGGTGCTTCATACGCCAATA	2	Yes
Ad01A8169	Aradu.A01	33746595	33746691	TGTCACGATTAAAAAGTTTCGG	TGGTAATTTTGCAGCATTATCTGT	2	Yes
Ad01A8429	Aradu.A01	35519808	35519887	TCATTCCCATTTGGGTTCAT	TCTCTCTTCTTTTTCGAAATTCAT	1	
Ad01A8451	Aradu.A01	35610051	35610149	CGAATACACACCCATCTCCC	CTAGAGGTTTCTCCGGCCTT	1	
Ad01A9058	Aradu.A01	39328275	39328378	GGGCGTGCTTTTTGTCATTA	CATGGGACGGTAACTGAGAC	2	
Ad01A9785	Aradu.A01	44728022	44728135	TTCAAAATACTTCCTCCTCCTCA	GAGTGTAGGTTCATCCTCTTCCA	1	
Ad01A9853	Aradu.A01	45218940	45219032	TGAGTGAGAGAGAGGAGGGAAG	CTCCTATCTTTCTTTTCCCCTT	1	
Ad01A9910	Aradu.A01	45652824	45652940	TCATTACAAGTATTCCGTCAAAAA	AAACATCAATATCGAGGAACCAA	2	
Ad01A11177	Aradu.A01	55686937	55687030	CTCCCATGTCATCGAGTCCT	AGCTTGGCGATGTTGGTATC	1	
Ad01A11348	Aradu.A01	56963494	56963574	CCAAAACGGTTTAGTCGTGA	CACAATTCAAAATTCTCCAGCA	2	Yes
Ad01A11768	Aradu.A01	60142926	60143143	TTGGGGGACATTTATGTTTTG	CAGATAACAGCGTACAGAAAGGA	0	
Ad01A11805	Aradu.A01	60442880	60442973	TTACCCACTCATCAGTCACCA	CGACGATAACGATAATGACGA	1	
Ad01A12246	Aradu.A01	63586308	63586444	TGATTCCGTTATCATTGTTGTTG	CAGAAGCACAAGCTGAACCA	1	
Ad01A12247	Aradu.A01	63587471	63587597	GGTGGTGGTGGTAGGTTCTG	ACCACCGCCATTATCATCA	1	
Ad01A12382	Aradu.A01	65046955	65047092	CTTTTCGTGATCCAGCCTTT	GGTGCCAGCTTAGGGACTTT	1	
Ad01A13053	Aradu.A01	70963487	70963665	ACTTCCTTAATGGCCTTCGG	GCAGGTAACTGGCATTGGAT	0	
Ad01A14236	Aradu.A01	80091650	80091748	TCTAATTTATTCTCTTCTTTCAATGGT	AAAACACTTTTTAAGATACGGTAATCA	0	
Ad01A15327	Aradu.A01	88794602	88794706	CAAAAATCAATAACAAGATTAGGGA	CCATTGTAAACACCCCTAGCA	1	
Ad01A15556	Aradu.A01	89591196	89591346	TCACAGACCTTTTGACACCG	AGCTCAATCTTCAGCTCGGA	1	

ID	Chromosome	SSR_start	SSR_end	FORWARD PRIMER (5'-3')	REVERSE PRIMER (5'-3')	Bands in parents	Polymophism in RILs
Ad01A15758	Aradu.A01	90281111	90281212	TTTTTGGTTGTAGCCCTTCTTC	GAGGGTTGTCCAATCCATCT	2	
Ad01A15941	Aradu.A01	90937449	90937560	GCCTCTTGTTCCTTGTCGTC	TGACCTCCTTTAGCCTCGAC	1	
Ad01A18005	Aradu.A01	96817750	96817832	TGCTATTTTCTTCCCTTGCG	CCACCTAGCCACTACCTCCA	1	
Ad01A18469	Aradu.A01	98713715	98713861	TGATGAAATTTGTTGTTGCTGA	ATCAACAGAAGCCCAAGCTG	1	
Ad01A18925	Aradu.A01	100161822	100161978	TCCATGTAACCATGTAACCAAGTT	AAGGAGGAGAAGGAGGAACG	1	
Ad01A18976	Aradu.A01	100304199	100304348	TTTTGCCTAAATTGCTTGGC	TGTTAGAATTGTTGGCATGGA	2	
Ad01A19145	Aradu.A01	100818178	100818262	GTACGCTTTTAATTTGCGGG	AATAAAGCTAGGGAGGCGCT	1	
Ad01A19585	Aradu.A01	102281317	102281474	ACCACCCGTTCTTCTTCCAT	CTTAAGAATTCCAATGGCGG	1	
Ad01A19715	Aradu.A01	102627202	102627305	ACCAGCACCTTCTTGCTTTC	GTTCTCCAAGGAACGGAGG	1	
Ad01A19990	Aradu.A01	103326847	103326931	GAGAAGAAGAGATGCGGACG	CGGTGCTGGTAACAAACAAA	1	
Ad01A20390	Aradu.A01	104545253	104545355	GGCGAGATTTCGAATTTTATTCT	CCTCAAGAATCACAACCACG	1	
Ad01A20947	Aradu.A01	106358335	106358501	CTAATCCCCAAAATTCGCTG	CACGTAAATGAAAAATGAAGAAGG	1	
Ad02A180	Aradu.A02	390520	390665	TTTATTAACACGCGCACCAA	GAAGGTGCAGCAAGAAGAAGA	2	Yes
Ad02A182	Aradu.A02	391137	391251	TTTATTAACACGCGCACCAA	CGTGCAGAAACGAAAGAAAA	2	Yes
Ad02A625	Aradu.A02	1484425	1484506	ATCCAATTCCCCAACATTCA	AAGAACAGCCATGGTAACGG	2	Yes
Ad02A725	Aradu.A02	1796747	1796867	CGTTCAGATTTCTGTCACGGT	TTTGTTAAGTTTGGTTGGGGA	0	
Ad02A1293	Aradu.A02	3486294	3486377	TTGTTCTTAGAGGCCTTTGTATGA	AAGCATGCTCATACCCATTG	1	
Ad02A1798	Aradu.A02	4715862	4716036	TTTCCGTTAAACATGACAGCC	AAATGATTTCAACCTTAAATTTGTTG	0	
Ad02A2779	Aradu.A02	7335579	7335686	TCCTCCTTTTCCTCCTCCTC	TTTCCATTCCTGAACCTCAAA	1	
Ad02A3952	Aradu.A02	10995133	10995239	CTGCCCCATGGAACCTATTA	TGAGCGACGTAGTGACGAAC	1	
Ad02A4008	Aradu.A02	11167140	11167239	TTCCAGAACCCCACCACTAC	TGCCATCAATAATGGTTCTTCTT	1	
Ad02A4133	Aradu.A02	11690140	11690244	GTTGGAGTCATTCGACCGTT	CGCTTTCAATCAAAAGACGA	2	Yes
Ad02A4136	Aradu.A02	11700071	11700169	TCCGTTCAATGTTGGAGTCA	GCGCTTTCAATCAAAAGACA	2	Yes
Ad02A4471	Aradu.A02	13020767	13020958	CAACTTTTCAAATCACGCTCC	GGAGGAGAAGGAGGAGAAGAA	1	
Ad02A5154	Aradu.A02	15853650	15853793	CAACATAGCAAGTACAAGATCGG	GTCAACGTCCCCAACATTTC	1	
Ad02A5473	Aradu.A02	17643624	17643776	AGTTTTGCTCATCCTGGAGC	CTGAATCCCTCTCTCCCTCC	1	
Ad02A5615	Aradu.A02	18485176	18485302	GTAGATCCGGAGGAGACGC	GTGAACCTGCCGGTTTTAGA	2	
Ad02A5633	Aradu.A02	18564280	18564388	GGCAGCACTAACACCCATTT	GATTCCGTTGTTTCGCTTTC	2	
Ad02A5661	Aradu.A02	18761806	18761898	TCAGATTGATGCGAGGACAG	TTATCGTTGCCTGCAGTCTC	2	
Ad02A5780	Aradu.A02	19721178	19721267	CGTGAGCTCCTTCTCTCTCC	GAGGAGCCTGTCCAACTCAC	2	
Ad02A5805	Aradu.A02	19875171	19875256	AGAAGGAGGAGAGGGAGACG	AACCCTCTTCACACAGCCAC	1	
Ad02A5861	Aradu.A02	20144959	20145087	GCCGGCAACTTCTTAAGTGA	TTTGGGATAACATTAGCAGGG	1	
Ad02A5972	Aradu.A02	20788408	20788550	GTCTGGATACCTGAGGCGAA	GCTCCTAACTCAGCCTGCAA	1	

ID	Chromosome	SSR_start	SSR_end	FORWARD PRIMER (5'-3')	REVERSE PRIMER (5'-3')	Bands in parents	Polymophism in RILs
Ad02A5993	Aradu.A02	20910626	20910731	GCTAAGCCATGTTTCTTTTCC	TCCCTCATAAACTTGTGATTGC	1	
Ad02A6022	Aradu.A02	21021831	21021978	TCCCTCACTTTATCACGTTGAA	ATCAAACAGAAGCAGCAGCA	2	
Ad02A6152	Aradu.A02	21993386	21993481	TTGTGCGGCCATATCATAAA	TGTTCATCCAATATAAGTGCCC	1	
Ad02A6275	Aradu.A02	22609420	22609522	TTTTGGGAACAACAATAACAACA	CCACAATTTCAGTTTTCAGATCC	1	
Ad02A6357	Aradu.A02	23185728	23185856	GGATGGGAAGTAGCCATTGA	CCTGACTCAGCCTACGAAGC	1	
Ad02A6382	Aradu.A02	23441584	23441705	GGTTGTTTTTGGAGAAACAGGT	CCAAAAGCGAAAACAGGAAA	1	
Ad02A6403	Aradu.A02	23581551	23581737	GGAAAACAAACCTTTATAAGGGAAA	TGATCTATCATCGTACCTGTTGG	1	
Ad02A6461	Aradu.A02	23984924	23985041	GACCTTGGCCTTATCGTTGA	TGAAAAATTAAAAACATTGGAAGGA	1	
Ad02A6531	Aradu.A02	24473990	24474109	AACGAATAAACCCCAAAAACG	CGTGCAAGAAGAAGAAGAACG	1	
Ad02A7015	Aradu.A02	28414221	28414322	CGACGACGATGATGATGATT	GCAGAAGAACAACAACAATGGA	1	
Ad02A7027	Aradu.A02	28465278	28465358	TACCTTGGCCGAACCTACAC	CTAGAGGATTCTCCGGCCTT	1	
Ad02A7118	Aradu.A02	29034904	29034984	CGAATTGCTCTGATGCCATA	AACCCGGTTTACTGAGCCTT	1	
Ad02A7541	Aradu.A02	32623347	32623484	CTAGTCGGCCTAAACTCCCC	TCTAGCCGTAAACCGAAGGA	1	
Ad02A8048	Aradu.A02	37363500	37363589	TGAAGCTTTGAAGTCCAAATTAAA	GGTTTGGGTTAGGATATGACCA	1	
Ad02A8360	Aradu.A02	40154423	40154557	GGCGATAGCTAAGACCTCCC	CCCCAAAAGAAAACCTAGCC	0	
Ad02A8999	Aradu.A02	45880197	45880310	TGAAAACATAAAACCCCACCTT	AGATCGACAGCTCAGGGTGA	1	
Ad02A9441	Aradu.A02	49451592	49451766	TTGTTGCTGCTGAATTTGTTG	TTCTCCCTTCACCCCAAAAT	1	
Ad02A10033	Aradu.A02	54389931	54390061	AAACGCAAAATCATTCTCCC	AAGGAAGGGGAGAGAGAGGG	0	
Ad02A10118	Aradu.A02	55064099	55064181	TCCCAGATGCTTACACGTCA	ATGGAGGATGTGAGTGGGAA	1	
Ad02A10228	Aradu.A02	56086726	56086855	TCCCTCTTCTCTTTCTCTTCCTC	TCCCTCTCTCCTTCTCTCCC	1	
Ad02A10600	Aradu.A02	58620569	58620691	TTGATGGTATTTCCTGTGAGTGA	GCAGTCGTTTTCAACCCCTA	2	Yes
Ad02A11078	Aradu.A02	62082831	62082975	AGAAAGAACGGGGGATTCAG	CGTCATCCTCATCAAAACCC	0	
Ad02A11079	Aradu.A02	62083122	62083240	GTGTGATGGATGGTGATGGA	GGACCAAAATGCCCCTATCT	1	
Ad02A11285	Aradu.A02	63609785	63609892	CCAACAATTGGATGACCACA	TTCTCGGGTTTCAATCAACC	1	
Ad02A11943	Aradu.A02	67828727	67828822	GAGGAAGAAGAACGTGCAGC	CGCGCACTAACTCATTTTGA	1	
Ad02A12178	Aradu.A02	69056490	69056672	TTTAAAATCAAGAAGCCATCCA	TCTTGGCCCAGTAAGCAATC	0	
Ad02A12699	Aradu.A02	72003226	72003345	CAGAAGCAGAAGCAACCAGA	GAGGAAAGAACAGGGGAAGG	1	
Ad02A12962	Aradu.A02	73499174	73499286	TCTTCTTCTCCCAACCAATGA	CGACGAGGACAATGATGATG	2	Yes
Ad02A13620	Aradu.A02	76565631	76565714	GGATTTTCACAGTTCGCCTT	GTTATGCGGAGAGAAAGCCA	1	
Ad02A14109	Aradu.A02	78566526	78566660	TCATCATCATCAAGAACCCAGA	TTTCGCTACAAAATGGTCCC	1	
Ad02A14679	Aradu.A02	80553852	80553969	TGCATTTCTAAGAGAGAGGACAT	GGTAGTTATGTTACAAATGCTGTCG	0	
Ad02A15088	Aradu.A02	82175600	82175689	AGAGAAGAGAAGGAGTGGGAAG	ACCCCAGTTATTTTCTCCCG	1	
Ad02A16352	Aradu.A02	85889136	85889285	CATTGTTTGTGACAGGTTGGA	TGAGTTCATAAGTTTCATTCAGAAAT	1	

ID	Chromosome	SSR_start	SSR_end	FORWARD PRIMER (5'-3')	REVERSE PRIMER (5'-3')	Bands in parents	Polymophism in RILs
Ad02A16537	Aradu.A02	86400202	86400325	ATTCGGGGTTGAGCAATATG	TGTGTGTAAAGTTTTAAGGGGAAA	1	
Ad02A16566	Aradu.A02	86509261	86509393	CCGTTTTCATTGATTGAATTG	GTGGTTGGTGTGCGAGTATG	1	
Ad02A16744	Aradu.A02	87131648	87131761	CGATAACATCTTTGTGTATTGACTCTC	TTAGCGGTAATCATGTTTAGAATGT	2	Yes
Ad02A17025	Aradu.A02	87868855	87869004	CGGTGTGCAGGTGTGAATTA	TTTGGGTTCCCACTCTTTTG	1	
Ad02A17359	Aradu.A02	88764798	88764881	TGTTGTTGATGTTGCCTTTGT	CAGTAGCAGCAAGAGCAAAAA	0	
Ad02A17360	Aradu.A02	88765427	88765540	TGCTGCTGTTATTATTGCTGCT	CAACACCACCACCACCAAC	2	
Ad02A17668	Aradu.A02	89490173	89490286	GGCCGTAATGTTTAGAAGTCG	AAAGCACATTCATGGAAGACG	1	
Ad02A18080	Aradu.A02	90360631	90360738	CCCATCACTCTGGATCTTCAA	CTGCCTTTCTCTTCATCGCT	1	
Ad02A18108	Aradu.A02	90435197	90435277	TTGCTGTCTACAACAGTGTGGAT	TTGTGTGGAGAATGTTTAAGGTTT	1	
Ad02A18256	Aradu.A02	90815378	90815547	TCGTTCGAACCAAAATACCA	GCTTCTGCTTCATTATTGTTGCT	2	
Ad02A19070	Aradu.A02	92685023	92685113	AGAGGAAAGTGGTGGAGGGT	AAACACCCAAAAGAAAGGGG	1	
Ad02A19283	Aradu.A02	93057512	93057611	AACAGCAACATCCTGCAATTT	ACATCGTGCTGAGTGGAATG	1	
Ad02A19370	Aradu.A02	93213837	93213969	GGGAATACTGAGAGATCATCAAGG	GGGAAGAACTGTTGGTGGAA	1	
Ad02A19420	Aradu.A02	93299024	93299111	AAGAACAACGACGGAGAACG	TGCAGTCCGTGAAAGTAACG	1	
Ad02A19426	Aradu.A02	93322702	93322784	ATAAATGGCCACGTTGCTTC	GATGACGGTGGAGAGACGAT	1	
Ad02A19543	Aradu.A02	93507836	93507929	TTTGACAGAAGTGATAATGTTTGGA	GTTTTTGCACAGAAAAATTGAA	1	
Ad03A41	Aradu.A03	118939	119063	ATTCGAGAAAATGGAAGGGG	TGAAAGATAGGTTTCGGTGGA	1	
Ad03A562	Aradu.A03	1328208	1328288	CAAATTCCCGACACCTCCTA	GCGTTTTTGTTGAATTGGGA	2	
Ad03A963	Aradu.A03	2149018	2149121	GGATCCAAGTGAGGCACATT	TCACTGAGTTCGTTCGTTCG	1	
Ad03A1878	Aradu.A03	4319448	4319542	TTGGTGTGAGCAAGAAGTGG	TTGTTGCCCCTTAAACAACC	0	
Ad03A1892	Aradu.A03	4347479	4347576	TCGCAAAATAAGGCTGTTGA	TTTTTCTTTCCGCCCTCTTT	1	
Ad03A2070	Aradu.A03	4765967	4766048	CTCCTCTCTCTCGCTGCTGT	TTATTTTCACCCTTCCACCG	1	
Ad03A2104	Aradu.A03	4819163	4819252	CAGTGGCGACTGAGTGTGTT	AATGCATCAATCTTTCCATGA	1	
Ad03A2259	Aradu.A03	5224171	5224279	ACGACGCAAGGAGTTTATGC	ATTCGTCTCCGAAATTGCC	2	
Ad03A3140	Aradu.A03	7565825	7565942	TCCTCATCAGAACCCAGAGC	GCCCTTGCCTCCTCTAGAAC	1	
Ad03A3492	Aradu.A03	8635500	8635600	CCAAACTCCAAATCCTCCAA	GGCTGATTTTGACAAGGGAA	1	
Ad03A3828	Aradu.A03	9690664	9690769	TGGCTTGAGCATGATTATCG	ATTTCCGTCCATCCATACCA	1	
Ad03A4482	Aradu.A03	10812434	10812525	CCAACAGGGGAACAGCTAGA	CCCCATTGGCACTACATTTT	1	
Ad03A4665	Aradu.A03	11649228	11649327	GACGATGAAAAAGACGCTCC	GCAATTTTAGATCACGCGTTT	1	
Ad03A5081	Aradu.A03	12864640	12864741	AATGCCCGAGTTAGGCTATG	TGGTTGGTGTTTAAGTGGTCA	1	
Ad03A5364	Aradu.A03	13766648	13766760	CTAAGCTCCATAACCGAGCG	CGCCAACCATCATTTTCAGT	1	
Ad03A5583	Aradu.A03	14443691	14443791	CGTACAGAGTCGACAGAGAGAGG	GCTGCATATCCAAGATCAAGC	1	
Ad03A5767	Aradu.A03	15600355	15600443	GGTTCTGTTCTGGTGGTCGT	TTTTTCGCTCCAAAATGGTC	1	

ID	Chromosome	SSR_start	SSR_end	FORWARD PRIMER (5'-3')	REVERSE PRIMER (5'-3')	Bands in parents	Polymophism in RILs
Ad03A5829	Aradu.A03	15815002	15815132	CTCTCCCCTTCCCCTCTT	GGAGGGAGAGAGAGCAAAGAA	1	
Ad03A5941	Aradu.A03	16446064	16446239	TTTCTCCTCATCTTCTGCTGC	CAAGTGCTACACTCAAAACGG	1	
Ad03A6363	Aradu.A03	17940338	17940429	CATCCATGATTCAAAACACACA	TTTGCCAACCTTCTTCTGCT	1	
Ad03A6716	Aradu.A03	19202841	19202941	TGCTCATGATGTTGAGGAGG	AACCGGAGATTCAGACATGC	1	
Ad03A7453	Aradu.A03	22155632	22155744	ACTCTGTTGGTGGGTTCCAA	GCCGAAACCTCTCTCTCACT	1	
Ad03A8741	Aradu.A03	27526946	27527055	AGAATGGAAAGGGGAAATGG	ACCACCCAAACCCTCTTCTT	1	
Ad03A9257	Aradu.A03	29737406	29737485	GCATGCATACATACGATACCAA	CAAGAAGTGGACGAGGAAGC	2	Yes
Ad03A9719	Aradu.A03	31631661	31631750	GATAACGCCAATTGGATAGTCA	TGTAGCCAACCCCACCTAGT	1	
Ad03A10896	Aradu.A03	37446991	37447101	CTAAAAAGGTCGAACGCTGC	GAACAGCCCAAATCTCCTGA	1	
Ad03A10902	Aradu.A03	37503013	37503095	CAACTCCCAACTTGATCTTTGA	TTGGGTCTGGCCATATTTTT	1	
Ad03A11035	Aradu.A03	38270609	38270701	CGTCGCTAGATCCCCAATAG	GGAATGCTTGTGTATATCATCCC	1	
Ad03A11523	Aradu.A03	40019588	40019680	TCAAGTTTCTGCGAGATAAAAGC	TTTTTAGTTAGGGGTAATTTGGTAAT	1	
Ad03A12321	Aradu.A03	45133345	45133424	AGAGGAGATGGTGGTGTTGG	TCTTCTTCTCTCGCGCTGTT	1	
Ad03A13940	Aradu.A03	59832600	59832687	CCAGTGGAGGACAATGAGGT	CCACACACTCGCCATAACAT	2	
Ad03A20541	Aradu.A03	112587165	112587268	TCAGATAAATACCAGCACCCTAGA	TTGTCAAAGAAAATAATCATATCACTC	1	
Ad03A20827	Aradu.A03	113819076	113819242	CTCCTAACTCAGCCTGCGAA	CGTCTGGACTCTTAAAGCGG	1	
Ad03A21320	Aradu.A03	116104456	116104575	GCATTACACAATGCATGAAAGAA	CCTCTTTTTAGATGTTATGTTTTCCC	1	
Ad03A23094	Aradu.A03	122308487	122308582	TTCGTTAGGTGAAAACCACC	TCGTACGTATATCCAATGCTCG	1	
Ad03A25225	Aradu.A03	128087042	128087132	AAACAACGAATGAAAAGGCG	ATGCGTTATTCAACCGCTTC	2	
Ad03A26250	Aradu.A03	130902805	130902907	CCTCTTGCAAGGAACCAGAC	GCAGATTCCACGTTCCACAT	1	
Ad03A27345	Aradu.A03	133757973	133758108	CCGGCTTGCGAAGATAACTA	CTGGAGACTTCTGAATTTGCG	1	
Ad03A27468	Aradu.A03	134042521	134042644	TGGTATCGTCGGCTACACAC	CAGAACGAAACCGAGTGAGA	1	
Ad03A27581	Aradu.A03	134359041	134359127	GAGCCACAAGTGCTTCTTCC	TTGAAAGAAGAGGGAACCGA	1	
Ad04A21	Aradu.A04	103018	103113	GGATGCACGTCATTCAGAAG	GCTCATCAAGCTGCTGAGAAA	2	Yes
Ad04A461	Aradu.A04	1341236	1341377	TTAGTGACAAGTTTGGCTTAAAAA	TGTGTGCTGATTCCAAAACTG	0	
Ad04A1557	Aradu.A04	4195842	4195974	ATAATGCAGGTAACGTGTAGACC	CCGGTAACATGGAGTTTAACAA	1	
Ad04A1757	Aradu.A04	4751607	4751729	ATGGATCTCCCCACCAGAG	CCTCCTATTCCACCACCGTA	1	
Ad04A2095	Aradu.A04	5761694	5761808	TCGAAATAGAAACAAAATAGCACG	TATTTATTTGCACGTCCCCC	1	
Ad04A2230	Aradu.A04	6187406	6187579	GGACTTTCAAAATGATCCACC	GTGCGCAAAGAAGAAAGAGG	1	
Ad04A2284	Aradu.A04	6339271	6339380	CCAACATACTTGGATTGTGGA	GAAGCCTCTACCCAAAATGG	1	
Ad04A2525	Aradu.A04	6975221	6975365	CCGTTAGACATAATAGCCCTCC	GCAGTCCTTCGTTATGAAATACAA	0	
Ad04A2797	Aradu.A04	7860995	7861152	CACCCCAATTACCCTAAGCC	TCCTTCATTGGGCTCCTAGA	1	
Ad04A2812	Aradu.A04	7914953	7915051	GGATGCATGTCCCTAAATCAG	TTTGGGGTAGAACTTAGGAACG	1	

ID	Chromosome	SSR_start	SSR_end	FORWARD PRIMER (5'-3')	REVERSE PRIMER (5'-3')	Bands in parents	Polymophism in RILs
Ad04A3067	Aradu.A04	8906195	8906274	CCCATCCCCTTACATGATTG	GGGCCATCACAACTCTGTTT	1	
Ad04A3269	Aradu.A04	9466735	9466861	TGAGACTGCACCGTTACCAA	CTTTGTATTGGGCCAGGGTA	1	
Ad04A4138	Aradu.A04	13332801	13333000	AGGTTGATGGATTCGGATGA	AGTGCGCAGGATAAGAATCG	1	
Ad04A4346	Aradu.A04	14450726	14450855	TCTCGCAAATTGGTCATTCC	CTGTTTAAAGAGAAATTTTTGGTGT	2	
Ad04A4579	Aradu.A04	15438141	15438225	TTTTAATTTCTCAAACTGAATTACCA	GCAGAACAAGTCATTTGCCA	0	
Ad04A5084	Aradu.A04	17620340	17620502	TCTCTCTCCTTTCCTCTTCCC	TTCTCCTCTTTCTTCCCTTCTTC	1	
Ad04A5923	Aradu.A04	22658627	22658741	GACCCAAATGTGGATGAAATG	GAACCTGCATTTCCTTTTTCC	1	
Ad04A6351	Aradu.A04	25728970	25729123	GCTCTCAAAGCCAACCTTCA	TGCACATATCTTCTCCACCG	1	
Ad04A6854	Aradu.A04	28408642	28408749	TGCCACTTCACCATCTTTGA	GGGTGAATCCATGCTTCAAT	1	
Ad04A6871	Aradu.A04	28541956	28542057	AAATCTCAAAGGCCACCAAA	AGAAAAAACGCGAACAGAGGA	1	
Ad04A6890	Aradu.A04	28581819	28581905	ATTTGGCCAATTTTAAGGGC	AGCTGTGACAGAAGCAGCAA	2	
Ad04A7863	Aradu.A04	35312406	35312495	CCATTTTCTTGAAAAACATAAATCC	TCCTTGGTTTTGAAAAGGTACA	1	
Ad04A8090	Aradu.A04	37588919	37588999	TGCCATTGATGGTAGTGGAA	CACAACACAACCCCCTCTTC	1	
Ad04A8117	Aradu.A04	37890425	37890613	TAGTGCTCACCCTCGAGCAA	TCTTCACCTTGGCTGAAACC	1	
Ad04A8903	Aradu.A04	43109821	43109900	GGGAAATTGTGGGAACAAAA	TTCTGAATTCAACATTGGGG	1	
Ad04A9456	Aradu.A04	48403744	48403859	AACCTTTGCTTTACGGCTAGA	TGATGCCAAAAAGCGTATGA	1	
Ad04A9900	Aradu.A04	53171122	53171203	ATCTCTGTACGTGCATCCCC	AATTCTCCAGATTCCCCCAT	1	
Ad04A10156	Aradu.A04	55706396	55706476	GCCAAGTTATGGTGTCTTCTCC	AGTTGATCCATTGATTCGGG	2	Yes
Ad04A10818	Aradu.A04	62667038	62667138	CCAACTCAAACCCACCCTAA	ATTACTCGCCCTCAAGCAAG	1	
Ad04A10872	Aradu.A04	63227250	63227380	TTCAAGCGAGCATTTGTTTTT	TTACATTTTTGGATTGAATTTTAGC	0	
Ad04A10897	Aradu.A04	63559185	63559305	AATATTGGTGGTGGCCGATA	TTAAATTTGAACCGCGAACC	0	
Ad04A11052	Aradu.A04	65071089	65071265	AAAACGTTAACGGGTTGGAT	AGCGGCCTTCTCTCTCTTTT	1	
Ad04A11387	Aradu.A04	68445087	68445168	CTGCGCCACACAACTACACT	CGAAGGGTCAACACCAAACT	1	
Ad04A11619	Aradu.A04	70474936	70475100	CCCTTTAGTGCCTTAACACGA	GGGGTGACAAATGTATTCCAA	1	
Ad04A12132	Aradu.A04	74597475	74597589	CCATCTCCATCACCCATTCT	AATCTAGAACCAAAGCCGCC	2	
Ad04A12402	Aradu.A04	76350205	76350330	CCTGCAACCTCACTTCCTTC	GCGCATCAAAGCAACATTTA	2	Yes
Ad04A12498	Aradu.A04	76926950	76927048	GGTTTGCACGTGATGCTAGA	TGCAAATATCTTGTAATCCCCA	1	
Ad04A12528	Aradu.A04	77258641	77258750	ATTTTCTTCCCCCTTGCACT	CGATGATAACGATAACGACGA	1	
Ad04A13343	Aradu.A04	84079616	84079707	GAGGTGGCGAGGTATTACGA	CCCACCTTGAAATTGTGACC	1	
Ad04A13748	Aradu.A04	87160039	87160118	GACAGAGGTTGGCAAGAAGC	CGTCGTCCTTGTCAGCAATA	2	
Ad04A14516	Aradu.A04	92251874	92252083	CCAATCCAAAAGCATAAAACTAGG	TTAGATGTAGTTTTTAGAGAGAGAGGC	1	
Ad04A14650	Aradu.A04	93079659	93079780	TGTTGGGAACAAAATTTAAAACG	GCAATATGTGATGTTGTTGGG	0	
Ad04A14906	Aradu.A04	94416671	94416761	CAAATTATTGGCTTGAGCATGA	CTGTCCATCCTTACCATCGG	2	

ID	Chromosome	SSR_start	SSR_end	FORWARD PRIMER (5'-3')	REVERSE PRIMER (5'-3')	Bands in parents	Polymophism in RILs
Ad04A15591	Aradu.A04	98395455	98395608	TCATCACTCCCCTCCCCT	GGGAGAGAGAGGGAAAGAAA	1	
Ad04A15698	Aradu.A04	98814538	98814659	CAGCCCCTTGATTTGATTCT	AACAACAAGCTCAACCAGCA	1	
Ad04A15908	Aradu.A04	100024809	100024914	GAGGGATAGGCACTGTATTGGA	CGCAATACCGATCCCAAAAT	1	
Ad04A16428	Aradu.A04	102588310	102588432	GTCCCCTGCACCCTTTCTAT	AGCGAAGTGACAACACCAAA	1	
Ad04A16643	Aradu.A04	103870932	103871017	GCAATGATAGAAAAACCAACCA	TTGAAACCTTCAAGTCACGTACA	1	
Ad04A16899	Aradu.A04	104884143	104884257	AAGGGGTGAGCGTAGAGTGA	TGAACTGAGAAGCAAATGGG	1	
Ad04A17826	Aradu.A04	108332214	108332331	TTGTTAAGAGGGTGCAACAAGA	TTTCGGTGCTATTTTCCTGA	0	
Ad04A17984	Aradu.A04	108787895	108787974	AAACTTCATTAACACGCGCA	CGTGCAGAAACGAAAGAAAA	1	
Ad04A18482	Aradu.A04	110619947	110620042	CATGTCGGAGGAGGTGTTTT	TTGAAAGAATAATCCAAGAAGCAA	1	
Ad04A19707	Aradu.A04	114711757	114711902	TGATTTGTTTGTGAAATTTTTGG	GTGTTTTCTCCCACCACCAC	2	
Ad04A21094	Aradu.A04	118753391	118753514	CTTCACTGTGAGGTGGAGGC	CCCTCTTAACGCACTGTTATCAA	2	Yes
Ad04A21449	Aradu.A04	119715357	119715476	AGATGGGCACGTGTTAAAGG	TGAGAACAAAATCCCATGCC	1	
Ad04A21670	Aradu.A04	120164301	120164442	TGCAAAGTGGGAGACAGAAA	CAAAATGACAGCCACCTCCT	0	
Ad04A21812	Aradu.A04	120575256	120575382	TGGTGATGATGATGGTGTGA	CACCATACCCTCCTTTCCCT	1	
Ad04A22108	Aradu.A04	121328083	121328215	TCTGCAATAAATTAGTCATTAGCCTG	GATTTTCTTTCCAAAAACAATAAAA	0	
Ad04A22784	Aradu.A04	123059422	123059555	CGAGATTAAACTTTGATTTGGATT	TTGCTGGTCCATTCTTCTGA	0	
Ad04A22916	Aradu.A04	123424617	123424716	AATGACATCTTCCGCTCTGG	CGTACCCCTTTTGGAGTCAA	1	
Ad05A880	Aradu.A05	1948296	1948386	AAATCTTGTAGTCTAAATTGCCTGC	ATGCTGTCCTGCTTTTGGAT	0	
Ad05A1015	Aradu.A05	2317513	2317642	ACTTCACCTGTTGCCATTCC	GACAAAAGGATGAGAAGCCAA	1	
Ad05A1766	Aradu.A05	4215732	4215847	TTCTTCCTTCTTTCTAAGTCAAATCC	TTTTGACCTCCCCAAGACAC	1	
Ad05A3091	Aradu.A05	7983899	7983979	CAACGACCAACGTGTCTATCA	TGCGTTACTAAAAGAAAGTCAGC	2	
Ad05A5040	Aradu.A05	14837039	14837244	GCCTCTCGCCAGATCCTAT	CTCTCATTGTCACGTCGTTCA	1	
Ad05A5506	Aradu.A05	16983491	16983592	CAAAGCCTTGGATCCTTTTG	TGAAGCAAGAAAAAGCAGTGAA	1	
Ad05A5618	Aradu.A05	17480986	17481078	GGGCTAAGATTCAACCTCCC	TCCAGAATGCACTGAACTCAA	2	Yes
Ad05A6083	Aradu.A05	19512930	19513010	CACTCAAATATCTTCGTGTTACACCT	AAAGAAGAAGAAGAAACACATAATGG	1	
Ad05A6457	Aradu.A05	21269703	21269789	CCGATGACAGCTGAAGCAT	CAACCAACCTGAAATCAAAATG	2	
Ad05A6813	Aradu.A05	22757512	22757654	AAAATGGTCCCCAAGGTTTC	CATGTTGCTTGATTTTGATTTTTC	1	
Ad05A7087	Aradu.A05	23898961	23899046	CCAGCAAAGCATTTAGACCA	TGTCGAGGAATAATCAGAAAAGAA	1	
Ad05A7404	Aradu.A05	25668161	25668242	TGCATTATTATTATTGGCTCCAC	GGAGTTGATGGGGATTCAGA	1	
Ad05A7725	Aradu.A05	27287440	27287550	TTTTGGAAAATGGGAGCACT	ATGCAACCTAACCTCCCATC	1	
Ad05A7920	Aradu.A05	28222833	28222968	GCCTTGCTTCTGCTTTCTTG	GCATAATTACCTCCTCCACCA	1	
Ad05A8309	Aradu.A05	31500819	31500972	TGCAAGAAGACGAAGAACGA	AAAAACGCGATGCAAAGTCT	2	Yes
Ad05A10356	Aradu.A05	46835750	46835935	TTTTTGGTCGAAAACCACG	GATGAGTGCCGGGAAGTAAA	2	Yes

ID	Chromosome	SSR_start	SSR_end	FORWARD PRIMER (5'-3')	REVERSE PRIMER (5'-3')	Bands in parents	Polymophism in RILs
Ad05A10410	Aradu.A05	47283518	47283603	TCACAATTTCGTCCACCAAG	AAAGCATGTAATTGACACCAAA	1	
Ad05A10428	Aradu.A05	47385002	47385132	GTAGGAAAAAGGATGCGACG	AGATTGGTGGTGACAGAGGC	1	
Ad05A11487	Aradu.A05	56111959	56112066	GAAACAACGGAGGATTTTGC	TCTTCGTCTTCATCCCCAAG	1	
Ad05A11997	Aradu.A05	60705572	60705697	AAGAGCAGGAGGAGGAGGAG	TCACGCGCTTTCTTCTTCTT	1	
Ad05A13419	Aradu.A05	74295694	74295773	TGCAATTGTGACATCGCTTT	AAGGAAGCATGGATTGTTGG	2	
Ad05A14690	Aradu.A05	81322053	81322133	AACACCAAGTCGGACGTTTT	GGGTTATTAAATTCGATGGACG	2	Yes
Ad05A16397	Aradu.A05	87518253	87518354	CAGAAGCACCAGCAAAAACA	GTCTCTGATCCCTCCACTGC	2	
Ad05A16591	Aradu.A05	88171732	88171840	TGGTAGCCAAGATCACTTTCG	TCAAAAGGGTGAGTTCGAGG	1	
Ad05A16836	Aradu.A05	89011034	89011157	GAAGAAGTGCACCCCATGAT	TGCTTGCTAAAACACAAGCATT	1	
Ad05A17280	Aradu.A05	90419102	90419206	TCCATCGAGGAATCACTCAAG	GCGCTTGATTTTTAGCATGA	2	Yes
Ad05A17484	Aradu.A05	90948599	90948685	ATGTTGAGAGAGAGGGAGCG	TCCCCTCTCTTTGTCTTCCT	2	
Ad05A17799	Aradu.A05	91857791	91857916	GCAGTGAGGCTCTGCTCTTT	CTATGCCTCAAGCACAAGGT	1	
Ad05A18148	Aradu.A05	93040040	93040055	TGAGGGCTACAGCATCACTTT	CCAAAGGCTACGCTTTTCAG	2	
Ad05A18149	Aradu.A05	93043329	93043344	CTTGTAGCCGTGGTGACAGA	TGCAACTATTGCCACCTTCA	1	
Ad05A18152	Aradu.A05	93055037	93055051	TGCTTGAAAGTTATGGCGAA	TGTTCAGCCCATGACAAAAA	1	
Ad05A18154	Aradu.A05	93071811	93071825	CGTTTGATTCATGTAGCCGA	GGAAGATGAAATGAACCGGA	1	
Ad05A18156	Aradu.A05	93077386	93077400	AAAGAAACCTCAGGGGAGGA	TGTTAGGTGGAGTTTTGGGG	1	
Ad05A18158	Aradu.A05	93080504	93080519	CACGTTAAATTTAGGGGCAGA	CAGTTTATCCAACGTTTAGTGGG	2	
Ad05A18162	Aradu.A05	93082508	93082519	TGTGCGTTAATTTATGCTCATT	TGCCTTGAGAATAAACACATGA	1	
Ad05A18164	Aradu.A05	93085460	93085471	TTACCGTCGAATTTATCCGC	GCTAACGTTGGGTTGCAAAT	1	
Ad05A18165	Aradu.A05	93098551	93098584	TGGATATCGATTTACAAACCCC	CAATTTCATACCCGGGCTTA	1	
Ad05A18166	Aradu.A05	93100670	93100681	CCAAATGCTAACCTTGGCTC	TTGGGCTTTTAGTCCCTTTG	1	
Ad05A18171	Aradu.A05	93115735	93115752	ATGGGCCTAATATGCGAATG	TGATCATGTTTGGGACGAGA	1	
Ad05A18173	Aradu.A05	93122484	93122591	CGCGCATTAATTCATTTTGA	GAGGAAGAAGAACGTGCAGC	1	
Ad05A18174	Aradu.A05	93122707	93122770	GCTGCACGTTCTTCTTCCTC	TTTCTCCTCCTCATCTTCTGC	1	
Ad05A18175	Aradu.A05	93123353	93123407	GAAGGAGAAAGAGAAGGCAAGA	ATCGTTTGGGTGAATTCCTG	1	
Ad05A18176	Aradu.A05	93123821	93123875	TTTCTCCTCCTCATCTTCTGC	CCACATACTGTTACGGCACG	1	
Ad05A18178	Aradu.A05	93128990	93129010	TTTGATATACGTGAATCGGGT	CGTTTTCCCTCCAAAATGAA	1	
Ad05A18180	Aradu.A05	93152882	93152899	TAATCCACCGATCAATTGCC	CCCATTCCAAATTAAACAGCA	2	
Ad05A18185	Aradu.A05	93175289	93175302	TTTTGACCTCATTTTGAGTTGC	AGCGAATTTGCAGAAGATGG	1	
Ad05A18194	Aradu.A05	93205487	93205512	CAAATGTGGTAGGGTGAGGG	AGGTGCATGAATGAAGGAGG	2	
Ad05A18199	Aradu.A05	93212809	93212840	TTTGATTTTGGCCATACAACTTT	TCTCTCTCCTTTAATTCTTCTCAAAA	0	
Ad05A18202	Aradu.A05	93222365	93222378	TGGCTCACGACTCAACTCAT	AAGGCTTGGACGAACTTCAA	0	

ID	Chromosome	SSR_start	SSR_end	FORWARD PRIMER (5'-3')	REVERSE PRIMER (5'-3')	Bands in parents	Polymophism in RILs
Ad05A18207	Aradu.A05	93231544	93231582	GTGAACTTGCACTATCCGCA	GCTTTGAAATTATTAAATGTGTCAAGT	1	
Ad05A18208	Aradu.A05	93232891	93232904	CGTGCTGATAACGTGTTGTG	TGAAAAAGAGAATTTTGGGCA	1	
Ad05A18209	Aradu.A05	93238523	93238542	AACGAGTTCGTTTTTCAAGGA	TCTCTCCTTGGCTTGTTTACG	0	
Ad05A18210	Aradu.A05	93245280	93245291	AGCCATTTTCGGATAGAGCA	AAGCAACGCTCTTCTTCCTTC	1	
Ad05A18211	Aradu.A05	93254222	93254233	ATGCATGCAAGATGAGATGG	CTAATACCCGATCCAAGCCC	1	
Ad05A18212	Aradu.A05	93256232	93256252	TTTCTTTTTGTCCATACGTTTCAA	TGTGTTGCTTTGCTTGTGAA	2	
Ad05A18213	Aradu.A05	93262456	93262467	CAAACGGCTTGTCAATCAGA	ATCACCCCACCCTTTTGTTT	1	
Ad05A18214	Aradu.A05	93266068	93266081	TCAGAGTATGCCCAGAAGCA	TCCCCATTTATTTTTGCGAC	1	
Ad05A18215	Aradu.A05	93269841	93269858	TGGCAGAGCAATAACAATAAACA	TTTTCTGCTAAAGCATCTCATTTG	1	
Ad05A18216	Aradu.A05	93270738	93270757	CCATATCCATTGCGACCTCT	TTCCATCCGCAAATTAGGTC	1	
Ad05A18217	Aradu.A05	93271409	93271420	ATCAGCCGCTAAAGTCCTCA	CTCCCTCTCTCTCAACGACG	1	
Ad05A18218	Aradu.A05	93271534	93271547	ATCAGCCGCTAAAGTCCTCA	CTCCCTCTCTCTCAACGACG	1	
Ad05A18220	Aradu.A05	93286586	93286601	CCTGTGCAGACCCGTTAAAT	TCAATCTTCCTTTCATCCCG	1	
Ad05A18221	Aradu.A05	93289241	93289252	GCTTCAGCTCACCATTTTCC	CACAGAGAATGCACAGAAGCA	2	
Ad05A18225	Aradu.A05	93298325	93298339	CAACAATATCTTTAAAAGCCAACG	TCGGATTGGATTTTGGATCT	1	
Ad05A18226	Aradu.A05	93300181	93300204	TTGGCAATTTTAGAGCAAGGA	TGCGATAAAAACTGTAAAAGCA	1	
Ad05A18228	Aradu.A05	93306164	93306184	GCCTAAAGACACAATTTGGCA	GCCACCCTTATTACCTGCAA	1	
Ad05A18229	Aradu.A05	93307326	93307346	CATGTTGAAAAGGTTGGGGT	GTGGGGTGAGGGGTTACTTT	1	
Ad05A18233	Aradu.A05	93318764	93318797	TTGGACCTCGTTGTTCTTCA	GCTCGGTGATCCAAAATAGC	1	
Ad05A18235	Aradu.A05	93327408	93327526	GTGCCCAAAATCATTGTTCC	ATGTACCCATGCACAACCAA	1	
Ad05A18236	Aradu.A05	93329194	93329223	TTTGTTTCGTGCGTTTGTTC	CAGGGCGGAAGACAATCTAA	2	Yes
Ad05A18245	Aradu.A05	93350071	93350088	GGTTTCTCCTCCACCAATGA	TTGCAGGGATCAGCAGTATG	1	
Ad05A18247	Aradu.A05	93351716	93351757	CACGTGCAATTGATCAGGAC	TTCCCTCAGCTAGGGTTCTTT	1	
Ad05A18249	Aradu.A05	93353834	93353847	AAAGGGGGTACCCGTACTGA	ATGAAAAGCAAGAGAGGGCA	1	
Ad05A18250	Aradu.A05	93358571	93358584	TTGCAAGTGCAAGCTGTCAT	TATGAGCCCATCACTTTCCC	2	
Ad05A18253	Aradu.A05	93367861	93367875	AACGTGAAAGGTTTTGGTGG	CGAGGGGGTTGAGTCTATGT	1	
Ad05A18255	Aradu.A05	93369624	93369639	CATGCATGGTGATCTTTTCAA	TGCACATTGGGAAATGTTGT	1	
Ad05A18257	Aradu.A05	93371311	93371325	TCATCCTCCCAACCTGAATC	ATCACTTGATGGTGGCCAAT	1	
Ad05A18258	Aradu.A05	93371771	93371785	AAGGCATTGCAGCTTAGGAA	CCAAAGGGAGTCCAAATTGA	1	
Ad05A18260	Aradu.A05	93378315	93378330	CAACATCTATTTTTACTTCCACCA	TGAAAAGAGAAGAAGAGGGTGA	1	
Ad05A18269	Aradu.A05	93424694	93424705	CAGATGAAGGCCCTTGGTAA	TCATGTTTTGGTGGGTTTCA	1	
Ad05A18270	Aradu.A05	93425757	93425771	GCCTAACCCATTCCCTTCTC	AGGGTCACCGAATCTCACAC	1	
Ad05A18271	Aradu.A05	93426310	93426344	GAGCGTTGAGGAGGAAGATG	CCTTATCGCCTTTTGTTGCT	1	

ID	Chromosome	SSR_start	SSR_end	FORWARD PRIMER (5'-3')	REVERSE PRIMER (5'-3')	Bands in parents	Polymophism in RILs
Ad05A18274	Aradu.A05	93437488	93437503	CCAATTGGGAAATGGAATTG	GCTCTTCCCACTTTTTGTGG	0	
Ad05A18275	Aradu.A05	93438851	93438874	CATCCAAATTTCTCACCTGCT	TTGTGGGTCAACTCGGGTAT	2	Yes
Ad05A18277	Aradu.A05	93445698	93445712	GCCGGTGAACTCTTACTTGG	CGATTGAAACCGGAGAAAAA	1	
Ad05A18287	Aradu.A05	93482168	93482182	CCACATTGCCCATTCTCTTT	ATGGAACCCACTTGGCATTA	1	
Ad05A18288	Aradu.A05	93484405	93484430	GGGAGACAGACAGGGATTTTT	TCTGCAAAAATGCAAAGGC	1	
Ad05A18289	Aradu.A05	93484691	93484702	CCACACCATTCCAATGAACA	GCTACAAGCACAAGTGGACAA	1	
Ad05A18290	Aradu.A05	93485843	93485872	GAACAATGTTTTGCAGGCAC	GCAAGAAGAGGTCAAGCCAC	1	
Ad05A18291	Aradu.A05	93486026	93486040	TCACTCTCTTACCTTTTCCACCA	TCAACTCCTGCAGCTTCTCA	1	
Ad05A18292	Aradu.A05	93486439	93486515	TGATGATGCATTGTTGTTGTTG	TCCAAGCATGGATCTCATCA	1	
Ad05A18294	Aradu.A05	93489992	93490007	AGGACGGAAAAGGACACAGA	TTTCTCCTCATCACCGAACC	1	
Ad05A18304	Aradu.A05	93506846	93506860	TCATGTCTCCGTTTTCCCTC	ACGTGAATGGGTAACAAAGATT	1	
Ad05A18305	Aradu.A05	93508481	93508492	TTTCCATTAAAAATTCATATCGTTTG	TTTCCACAACCACCTTGACC	2	
Ad05A18317	Aradu.A05	93527578	93527591	CAGTAAGTAGATTGCATCCGAAA	AGCGTGGAAGAAAATTGAGC	2	
Ad05A18320	Aradu.A05	93532341	93532360	ACCGTATAAACAAAACGAGATATACA	TGCATATCAATTAAAAACATTTCG	1	
Ad05A18322	Aradu.A05	93536529	93536595	AAATCATTTGAGAAAGTGAAAGGA	CCACCATAATTTAAGCCACCA	1	
Ad05A18329	Aradu.A05	93568056	93568106	TTCCAAATACACAATGATCGAAA	TCTCTGCTTGACTTGCTCTTT	1	
Ad05A18332	Aradu.A05	93572702	93572743	TGAGACATTGGTCCAAAAACA	TTATCATAATAGTTACACGGGAAAAA	0	
Ad05A18334	Aradu.A05	93577419	93577541	GTTGCTACACATACGGGCCT	TTTCTCCTCATCTTCTGCTGC	1	
Ad05A18335	Aradu.A05	93577983	93578097	TTCTTCAAAAACGATTTCACCA	GCGTTTCGAACCATTTCTCT	1	
Ad05A18337	Aradu.A05	93583591	93583605	CTCGAGATTGAACTCCGAGC	CACCACCCATCATTTGTCAC	2	Yes
Ad05A18344	Aradu.A05	93597000	93597052	TTCTTGGGCCACTTAACACA	TCAAAGATCAAAGAGCCACTCA	1	
Ad05A18356	Aradu.A05	93633418	93633437	GCACATGAACCGTGAGTGAC	CATGAACTGCATGTGTACAAAGA	1	
Ad05A18360	Aradu.A05	93640397	93640542	TGGCTCTTGAAAGAATGATGG	AGCACATGGTTAGGGACAGC	1	
Ad05A18372	Aradu.A05	93664301	93664324	TGCAATATCTGCTTGGTTGC	TCCCAGAATTACCTTCAACTCA	1	
Ad05A18373	Aradu.A05	93671943	93671962	GGGTTTTGGAACAAGCGTAT	GAACAATTTTGCTGCCATGA	1	
Ad05A18375	Aradu.A05	93679350	93679369	GGCCATGTAACCTTTTTGCT	GAGCGTCCAAGATGATGTGTT	1	
Ad05A18376	Aradu.A05	93684792	93684811	CAGATGCCATGACAAAACCA	TGAACCATCCATCAATTAGAGGT	1	
Ad05A18377	Aradu.A05	93686168	93686192	TGGTGCCTTTGTTATGGTGT	AAACAAAAGCCCAATGCAAC	1	
Ad05A18383	Aradu.A05	93704280	93704300	TTGAAATAAGGAGATGTGCCG	TCACAATGCAACACGCCTAT	1	
Ad05A18384	Aradu.A05	93704534	93704548	TTGCCAAATGACAATGATGAA	AGCCAAAGCCAAAGACAAGA	1	
Ad05A18385	Aradu.A05	93704715	93704729	TCTTGTCTTTGGCTTTGGCT	TTGGTGCCTTCAAGAACTCC	1	
Ad05A18386	Aradu.A05	93705657	93705695	TTGGCAGAAATGTTGTTGGT	ACGCCAAAGGAGTGCTAAAA	1	
Ad05A18387	Aradu.A05	93706229	93706244	CAGTTTCAGCCACATATTCACA	TTGTTGGCATTCATTTTGAAG	1	

ID	Chromosome	SSR_start	SSR_end	FORWARD PRIMER (5'-3')	REVERSE PRIMER (5'-3')	Bands in parents	Polymophism in RILs
Ad05A18388	Aradu.A05	93707223	93707346	CATTGAACACAAACCCAGCTT	TGGAGATGGGGAATAATGGA	1	
Ad05A18393	Aradu.A05	93719797	93719811	TGCTTCTAAAAGAAGAAATAAGAGCA	GGGGTAGAAGATGAGGAGGAA	1	
Ad05A18399	Aradu.A05	93751216	93751233	AATAGTATTTACCATGAATGACCAAA	CAATTACGATCACTCTCAACCAA	1	
Ad05A18403	Aradu.A05	93763724	93763738	CCTTCCATCCTGCCCTATTT	GCCACAGTACATGCTCAAATG	1	
Ad05A18404	Aradu.A05	93770428	93770439	CGTGCATCCTTTCTCCTCAT	ACACTGGGAGAGTGGGAGTG	1	
Ad05A18407	Aradu.A05	93778574	93778587	CACCAAGATAACACAAGGTGGA	CCATTACCTTCAACTTGCCA	0	
Ad05A18410	Aradu.A05	93788915	93788932	TGCTTGGCTGATGTCAAAAA	CCGATTCAAATTACACCGAAA	1	
Ad05A18411	Aradu.A05	93792886	93792906	AAAAGTTCGTCATTGGGCAC	GCCTGAGAAGAGCAGAAGCA	1	
Ad05A18414	Aradu.A05	93804598	93804617	TCAAATTTTAAATAACCAACAATGTC	TCAAAATCAATCCAATCCAATC	0	
Ad05A18415	Aradu.A05	93805426	93805451	TGTTCAAGGCTCTCCGAAGT	CGCAGGATAAAAGGTCAAGG	1	
Ad05A18417	Aradu.A05	93807466	93807479	TGATATTTCGAGTATTTCTTTCAAGG	TGCATATTCTTTTCGTCTTATTAATTC	0	
Ad05A18421	Aradu.A05	93854544	93854563	TCATCATCAAATTTTAAATAACCAACA	TCAAAATCAATCCAATCCAATC	1	
Ad05A18422	Aradu.A05	93856830	93856844	GGATTGCAGGATTCAGGTGT	CATATGCAGGGTAAGCCACA	0	
Ad05A18423	Aradu.A05	93857390	93857401	TTTTTCAAGGAAGAATTAACTTTTAGA	AAGTGCATTCCATATGCCGT	1	
Ad05A18425	Aradu.A05	93861994	93862031	AATGATGGCAACAACGTCAA	ACCAGTGGATTTAGCGATGG	2	Yes
Ad05A18426	Aradu.A05	93864049	93864068	CTTGGCGGTCATTCTCTCTC	CAACGAGGGAACCAAAAGAA	1	
Ad05A18430	Aradu.A05	93880668	93880699	AACGAGGGAACCGAAAGAAT	GGTCATTCTCTCTCGCGAAC	1	
Ad05A18433	Aradu.A05	93885755	93885766	TGTGATGATGATTAAAGGCATTG	CCATTTGGTTAACATTACACGC	1	
Ad05A18436	Aradu.A05	93903032	93903059	TTGCCATTTTCCAAATACGA	ATGTGGCGTTATGTGATTGG	2	
Ad05A18437	Aradu.A05	93909855	93909958	GGATTAGGGTTGGGAAAAGG	GCAATGTTTGGGTGATCAAA	2	
Ad05A18442	Aradu.A05	93949675	93949686	AAGTTGCCGCGTATTTTCAC	GAAAGCTTTTGACATGGTGATG	1	
Ad05A18443	Aradu.A05	93950554	93950571	TGGAACTGCTAGGCAAACCT	TCATGCGAAGCTAAACAAAAA	0	
Ad05A18447	Aradu.A05	93967449	93967460	GCTCGAAACGTACCCATGAT	GCAAGTGTCGAGGAAGAAGG	1	
Ad05A18450	Aradu.A05	93974133	93974160	AAAATCCTTTTACGGTGGGG	GGAAGACATGAGTTTGTAGCCA	1	
Ad05A18453	Aradu.A05	94001178	94001247	GACCATTATGATAAAATTTTGCACC	AAACAAATGCAATTTTCTCTCTTC	2	Yes
Ad05A18456	Aradu.A05	94009900	94009914	TCAGTGCTCACATCTGCTCC	GTCTGCAGGGTGGAAACAAC	1	
Ad05A18458	Aradu.A05	94014618	94014635	TTGGATTTGCTTGATTTTTGC	CGTTTGATTCATGTAGCCGA	1	
Ad05A18460	Aradu.A05	94019209	94019223	GGATACTCGGAAAGGGAAGC	ATGGGACAAGGTTTTGATGG	1	
Ad05A18463	Aradu.A05	94039485	94039500	GGTGAACCGAGCTTGAGATT	CTCCATCCGGTCTGATTGTC	0	
Ad05A18464	Aradu.A05	94044892	94044905	ACCATACAGAGTCAAGGCCG	GCCTCATAGCCCTTTTCCTC	2	
Ad05A18465	Aradu.A05	94046103	94046120	GTATGCGGTGCACAAATTCA	GTTGAATCCTCGGGACGTTA	0	
Ad05A18472	Aradu.A05	94082457	94082470	GGTTGGTTTATTATCGTGGTCA	CAAATCCAAAACACCATTCG	0	
Ad05A18474	Aradu.A05	94087326	94087377	CCTCCTCATCTTCTGCTGCT	GCTGCACGTTCTTCTTCCTC	1	

ID	Chromosome	SSR_start	SSR_end	FORWARD PRIMER (5'-3')	REVERSE PRIMER (5'-3')	Bands in parents	Polymophism in RILs
Ad05A18481	Aradu.A05	94133080	94133095	GGACTTCTGAAGCGAAGTGG	TGCAATGCTCAGAAAACGAG	1	
Ad05A18492	Aradu.A05	94157831	94157852	TTTTCCAACCCGTTCTCATC	TTTCTTGAGTTTGCGTGCAG	1	
Ad05A18493	Aradu.A05	94158618	94158639	TTACGGCTCTGCTCCTCAAT	AAGGGATGAGGAAGGAGGAA	2	Yes
Ad05A18499	Aradu.A05	94178425	94178519	TTGAACAAATGTAAAATGGGAGT	CCTTGATCTGCGATGGACTT	0	
Ad05A18500	Aradu.A05	94180230	94180269	GCAGACCTTCGGATTGAAAA	TCAGGCATGATTGGCTGTAA	0	
Ad05A18501	Aradu.A05	94183674	94183691	AAAAATTGGTCAAACCAGCG	CGACGTTGGCTTAGGAGAAG	2	Yes
Ad05A18503	Aradu.A05	94195949	94195964	AGCTTCAGGATGACCAGCAG	TGATAGTGCTCCAGCGAATG	1	
Ad05A18505	Aradu.A05	94200593	94200613	ATACCTGGCTTTCGGTGATG	TGCTCTCTCAATTGGCTTGA	1	
Ad05A18508	Aradu.A05	94209295	94209309	TGATATACGTGAATCGGGTAACA	CATTTTCCCTCCAAAACGAA	1	
Ad05A18509	Aradu.A05	94219656	94219667	CCCGATCAATTTTAATATGCC	AACAAATGCAATTCTCTCTCTCTTT	1	
Ad05A18510	Aradu.A05	94224061	94224072	TGTGCGAACTTGCACTATCC	TCAAATGCAATTCTCTCTCTCTTT	1	
Ad05A18511	Aradu.A05	94229645	94229660	TTGGCATGCACCTTATGTGT	TTTGGATGCAATGTTAAACCA	2	
Ad05A18513	Aradu.A05	94234866	94234877	GATGAAAAGAGGGGACATGC	TCATGCCAAGCAAAACTAAAAA	1	
Ad05A18521	Aradu.A05	94292134	94292178	TTTTTCAAAGAAAGTCCCGA	AAATTTCTTATGACGATGGCTT	0	
Ad05A18523	Aradu.A05	94292776	94292856	AGTGGGTGGGCACAGTAAAC	ATGACGCCCAATTTTTGGTA	1	
Ad05A18525	Aradu.A05	94306112	94306129	CAAGACATTCGTTGACCCCT	TGTGATGTATGCCTGCCCTA	1	
Ad05A18526	Aradu.A05	94323108	94323121	CGATTTTGGCTATTTTCTCTTATTC	TCAAAATCGTCCCTGAAAGA	1	
Ad05A18530	Aradu.A05	94326559	94326573	AAGAAGTTGGGCCTAACACG	ACAGAAGGAAATCGAAGGCA	1	
Ad05A18531	Aradu.A05	94327191	94327208	CCTCCTCATCTTCTGCTGCT	CAAGGTACGTTTCTTGCCTTC	1	
Ad05A18532	Aradu.A05	94327816	94327835	GGTCGATTTGGCTAGGGAAT	AAATGCTGTTGAAAGTAAACACCT	0	
Ad05A18535	Aradu.A05	94330387	94330398	CATTTTGCAAGTTGTGAAGAAAA	TTCAATAAAATTGCTGGCCC	0	
Ad05A18536	Aradu.A05	94330573	94330586	GGGCCAGCAATTTTATTGAA	ACGAGAAATGTTAGGGCCAA	1	
Ad05A18537	Aradu.A05	94330760	94330950	AAGTTATAAAAATTGTTGGCCCT	GCAACAAAGTCAATGTTTTAAATAAG	0	
Ad05A18538	Aradu.A05	94331930	94331953	CCCCTTCTCCTCTCTCGTGT	TCGAAGTAAAAACAAAATAGCACG	2	
Ad05A18543	Aradu.A05	94342428	94342487	TGTTCATTGCTTGTTGACTGC	GGAGGTTTGCGTTTGAGAAG	2	
Ad05A18544	Aradu.A05	94343589	94343609	CGAGTGTTCTAATTTTTGCTGTAGA	TCAAATCCGAATCCTTCAAA	1	
Ad05A18545	Aradu.A05	94365355	94365366	TGATGAGGATGCGGTGAGTA	CGGTGCCGTTTTTGAGTTAT	1	
Ad05A18546	Aradu.A05	94367109	94367166	GTCGATGGCCCACTGATACT	TCGTATATCGGCACCAATGA	1	
Ad05A18548	Aradu.A05	94372551	94372564	TGGTTTTCGACGAAGATAGGA	TTGGACAAAGTTGTGAAGCG	2	
Ad05A18552	Aradu.A05	94391055	94391069	AGAAAGGCATTACACCACGC	GCTCATCTCATCACTTTTCATCA	1	
Ad05A18553	Aradu.A05	94393514	94393531	CAAAACAAAAACAAAAAGACCAAA	TTTTATTGATGCGATTAAAATTCA	0	
Ad05A18554	Aradu.A05	94402815	94402829	TAGCAGCATTGAGGAAAGGC	CAGTTTGACCGGATTTTACACA	1	
Ad05A18556	Aradu.A05	94417562	94417573	TTGAATCATATGCAATACGTAACA	CATGCAACAATCAAAATATCAAAA	1	

ID	Chromosome	SSR_start	SSR_end	FORWARD PRIMER (5'-3')	REVERSE PRIMER (5'-3')	Bands in parents	Polymophism in RILs
Ad05A18558	Aradu.A05	94419684	94419790	TTCAAAGACGCGATGTAAGG	TGCGTGCAAGAAGAAGAAGA	1	
Ad05A18559	Aradu.A05	94419899	94419913	TGTTTCTTGCCTTCCCTTTC	GAGGAAGAAGAACGTGCAGC	1	
Ad05A18560	Aradu.A05	94421930	94421981	TCAAAATAACGTTTTCACATTTCA	GCATATAGCCCCATCATACCA	1	
Ad05A18562	Aradu.A05	94432630	94432722	TGTGAACAGCTTCTTTGGCA	CCTCAGCACCTTCTAGCACC	1	
Ad05A18563	Aradu.A05	94434950	94434965	TGTGAAAAGCCTTGAAGCAA	AAAACACGTACACGCACACC	1	
Ad05A18567	Aradu.A05	94440402	94440425	TCCCTCCTTTCCGGATATTT	TCAAAAAGATTTTAATGAGGCCA	1	
Ad05A18569	Aradu.A05	94449213	94449254	AAGAAGCGGATAACTTCGTTTG	CGAAATAATGCTCTGTTGTTGTG	2	Yes
Ad05A18570	Aradu.A05	94451471	94451542	GAAATACACGCGCCCAATAC	AACGCGAAGAAGAAGACGAA	1	
Ad05A18571	Aradu.A05	94452061	94452085	TCTGCAGAATGAACTGAACACA	TCGAATTGAGTGGAATGCAA	1	
Ad05A18573	Aradu.A05	94463865	94463892	CGGTAAATTTGACAGTAATCAACG	TCCAAAATTCATTGCGTTCTT	1	
Ad05A18575	Aradu.A05	94477122	94477133	ACTCGAAAGCGCAACACTCT	TTGTGTCCTTTTGGGTTTCC	1	
Ad05A18576	Aradu.A05	94481576	94481595	TTGGACAAAGTTGTGAAGCG	TGGTTTTCGACGAAGATAGGA	2	
Ad05A18579	Aradu.A05	94484564	94484575	CTTCGAGGCCGTAATACGAA	TTGATCTGGTAATGAAATATTAAACAA	1	
Ad05A18580	Aradu.A05	94486941	94486958	TCGAGTTTGGACAACTGACG	AAAAGATGCAATTGACACCAAA	1	
Ad05A18584	Aradu.A05	94507063	94507080	TGGAGGTTATGCTTGGAGTTG	TCTCGGTCATTCGTTCAACA	1	
Ad05A18585	Aradu.A05	94513668	94513683	CCGGTCCGAGCATAGAAGTA	TTTGTGATTTTCGGATGCAG	1	
Ad05A18587	Aradu.A05	94521727	94521738	AAAAATCGCAACTCGAAAGC	TATCTTCGCAACCGGATCTT	1	
Ad05A18590	Aradu.A05	94544979	94544996	TCGAGTTTGGACAACTGACG	TGACTCAAGGCTCAAACAAAAA	1	
Ad05A18591	Aradu.A05	94546099	94546116	TGAAAATTCCTCACCCTTTTTC	GGGTTCGAATTTTGAGATGAA	1	
Ad05A18592	Aradu.A05	94549421	94549435	ACCCCTCCATCCTTCTTCAT	TGACTCAGAGGTGGAAGCAA	1	
Ad05A18594	Aradu.A05	94577509	94577520	AGTCTTCACCATCGCGTCTT	CCCTGCGTTCATCATCTTCT	2	
Ad05A18595	Aradu.A05	94582402	94582419	GCTCTCACCACAGCAACGTA	TTTGAATGCCATTTTCGGAT	1	
Ad05A18596	Aradu.A05	94591807	94591824	CAAAGCAACGTTCCTCTCCT	TGAATGCGATATTGCAGAGC	2	
Ad05A18599	Aradu.A05	94599686	94599700	TGTGCATGTGGACGATAACA	TTAGAGATAACAGTCTTCTGTCAAAAA	1	
Ad05A18606	Aradu.A05	94610724	94610739	TCTGTAATTGCCGGGATCA	CTGCAAAAGTTTGGACAGAAT	1	
Ad05A18607	Aradu.A05	94611277	94611308	AAATATGAGGACCGGGGAAC	TACATGCAGATACCGCGAAG	1	
Ad05A18608	Aradu.A05	94612764	94612783	GGAGGATAAACATCATGCCAG	TTACAGGGTGCCAAAAGTCC	1	
Ad05A18610	Aradu.A05	94615841	94615856	CGCCAACGTGGTTAAGAATC	GAGGTACGTAAACTTTCACTTATCATT	1	
Ad05A18614	Aradu.A05	94626423	94626434	TGTGTTAAGTCTGTTGCACAAAT	TACCGGGAAGACTTTCGATG	0	
Ad05A18616	Aradu.A05	94634339	94634364	AAATCACCAATCCCTTTCCC	GCGGAAAGCAACAAAGAAAG	0	
Ad05A18617	Aradu.A05	94642960	94643007	TTTGGATGAGACTTTTCATAACCTT	TTGAGTAGAAAATAAAAATCCATAAGC	0	
Ad05A18618	Aradu.A05	94644675	94644689	CGTTTTCCCTCCAAAACAAA	CGTGAATCGGGTAACAAAGA	1	
Ad05A18619	Aradu.A05	94648054	94648065	TCACTGTTTTGTTATATGAAATTGAAA	TCCTTGTCTTTATTTTCCAAACC	1	

ID	Chromosome	SSR_start	SSR_end	FORWARD PRIMER (5'-3')	REVERSE PRIMER (5'-3')	Bands in parents	Polymophism in RILs
Ad05A18620	Aradu.A05	94648735	94648779	GATTGGAGAATCAAGCGGAA	TTTTCTTTGAAGCCTGACCG	1	
Ad05A18622	Aradu.A05	94668490	94668504	ACCACCTCCACCACTACCAG	CTAGGCCAAATACCCAACCA	1	
Ad05A18625	Aradu.A05	94675183	94675194	TTTAGAATGTGTCAATTCAATAATCA	TCTGTGGTTAAAATGGGTAAGGA	0	
Ad05A18631	Aradu.A05	94697984	94697995	TTTTGGAAATTAAACTATTTTCGTGA	TGTGCAAAACTTCCGTGAAT	0	
Ad05A18633	Aradu.A05	94703603	94703638	AAAAATGACAGGGCGAAATA	TTTGGGCTTAGTCCCAGTCT	0	
Ad05A18644	Aradu.A05	94729450	94729463	CTTGAATGCCATTTTCGGAT	CCCTCTCACTTGCTCTCACC	1	
Ad05A18648	Aradu.A05	94733670	94733684	GTGGACGGTAAGGAGTCCAA	TTCTTGTACATCGCGTCCAG	1	
Ad05A18650	Aradu.A05	94739949	94739984	AGTGGCCCAACAAAAACATT	CTTTGCACAATGTACAGCCC	0	
Ad05A18651	Aradu.A05	94740266	94740325	TTCACATGGAGTTAATCTTCAGAAA	CCGCTACCCATACAACACCT	0	
Ad05A18652	Aradu.A05	94740726	94740778	GAAACAAGGCGTTGACCATT	TCTGTGTTACTTTTGGGGTGT	1	
Ad05A18655	Aradu.A05	94747360	94747377	GTTATAAATATCCACAAACACAGTGAA	AAGAAAAATAACCCAAAAACACC	0	
Ad05A18656	Aradu.A05	94748986	94749003	CCCTCTCCTCCCTTGTTTTC	TCCGTTGGTAAATTCGAAGG	0	
Ad05A18658	Aradu.A05	94759044	94759089	TTGTCCATAAATCCGTCGGT	AGTCGACGCTGAAAGAGGAG	2	Yes
Ad05A18661	Aradu.A05	94762020	94762031	CCCTTTCCCTAATTCCCAAA	CCAACGCTAAAAAGCTAGAAAA	2	
Ad05A18670	Aradu.A05	94791708	94791743	CTCAGCAAGAAGTTTTGCCC	CACAGACGACAACCACCATC	1	
Ad05A18671	Aradu.A05	94792175	94792188	GCCCCTAGCATTCCTCGTAT	CGTGAAACCATACAAGATGGA	1	
Ad05A18672	Aradu.A05	94792455	94792466	AGGCATGCACGTACACTTGA	AATGACTTGATCTGATCTTGTAGCA	2	
Ad05A18689	Aradu.A05	94860454	94860465	TCATTCGGCAGATTGTTGAC	GGTGGGACTAAAAGAAAATAGGAA	1	
Ad05A18695	Aradu.A05	94870404	94870460	TCATTGTTGGTAATAAGTTGGCA	CAAATGCAATTCAACCGAGA	2	Yes
Ad05A18697	Aradu.A05	94875368	94875388	ACATGACAGAGCACAATGGC	AGCAAATTATCGGGTATGCG	2	
Ad05A18713	Aradu.A05	94905686	94905748	GCAATTCCTGGAGGATTCAA	TCATGCCAAGCAAAACTAAAAA	1	
Ad05A18715	Aradu.A05	94914201	94914212	TGAAGTTCTATACGAAAAGGATAAAAT	TTAAAATATAAAACGTCTCTCACCTTT	0	
Ad05A18716	Aradu.A05	94915169	94915188	TCTGCTCTGAATGGAAGCAA	TCACATTTCCAGCACCAAAA	1	
Ad05A18717	Aradu.A05	94918723	94918734	AAGCCTTGGTTTGCAGTGAT	TCTTCCAATTTTCCTGCCAT	1	
Ad05A18723	Aradu.A05	94940994	94941026	CCACCGGATACATAAATGCC	GCACCAACTTGGCGTTAAAT	2	
Ad05A18727	Aradu.A05	94954249	94954293	AGTTGTTGATGGCTTTTGCC	TGAATTGTTGACCATTTTTGGA	2	Yes
Ad05A18728	Aradu.A05	94954745	94954844	GAGGTTGGAACGGTTTACGA	TGGAGTCATGGAAGACGAAA	0	
Ad05A18734	Aradu.A05	94976871	94976896	TTGAGACTTCACGCAAGTGG	AAGGTTGTGGGCTTCTATGG	2	Yes
Ad05A18738	Aradu.A05	94987518	94987550	GGCATTTTTGTCCCCTTTTT	CTTGGGACATTGGTTGAGGT	1	
Ad05A18742	Aradu.A05	95001994	95002051	GCCCAACACGAGGAAAAACT	TGGAATGGTCGAAAGAAAGG	1	
Ad05A18747	Aradu.A05	95017604	95017625	GCAGGGTTGACAAACAGGAC	AACACACAAACCCCATATCCA	1	
Ad05A18751	Aradu.A05	95032207	95032317	AAAAATTGAGACGCGGGAAT	GAATGAAAAGTGTCCAATGTGC	1	
Ad05A18752	Aradu.A05	95034080	95034091	AAATTTGCCTCATGCACACA	GGCTCGAATGCATGTGATTA	1	

ID	Chromosome	SSR_start	SSR_end	FORWARD PRIMER (5'-3')	REVERSE PRIMER (5'-3')	Bands in parents	Polymophism in RILs
Ad05A18753	Aradu.A05	95034227	95034238	TAATCACATGCATTCGAGCC	CAAACTGCACCCAAAAACCT	1	
Ad05A18754	Aradu.A05	95042943	95042982	GCATTTTAGTGACCCGCAAT	GGCCGTATTGTTGCTCTGTT	1	
Ad05A18755	Aradu.A05	95044427	95044456	CACGACAGATAGAGTTGTTGCG	GACGCAGGGACGTAGTTAGG	1	
Ad05A18760	Aradu.A05	95050745	95050780	AATCAGCCACCAAAGTCAGC	TGCAAAATTATTTGGAAGAGGA	1	
Ad05A18761	Aradu.A05	95050973	95050987	TGCAGCTTTATGTGTTTCTTCTTC	CGAAGATATTTGGGTGCAAAA	1	
Ad05A18762	Aradu.A05	95051515	95051529	GCAAAACAAAAACAAACTTGAGAA	CCTTTTTCCCTTCCTCATCC	1	
Ad05A18763	Aradu.A05	95053244	95053255	CTGTACACACACTGGCCACC	GAAGGCTTTGGCTTTTACCC	1	
Ad05A18766	Aradu.A05	95057681	95057694	GAGCTCCTGACTCAACCTGC	GGATACCTGAAGCGAAGTGG	1	
Ad05A18770	Aradu.A05	95066018	95066032	CAATGTTAATGGTGTTAGTGTTAATGG	CAGCCACTTTGATTTCCAAC	1	
Ad05A18771	Aradu.A05	95077232	95077243	GGGGCTGAAAGAAGAAAAAGA	CCATCCAATTTGTTTGTTGGA	1	
Ad05A18773	Aradu.A05	95097301	95097312	TGCGAGAAAAATTCCATTCC	TTCGAAGAAGAGCAAATCGAA	1	
Ad05A18780	Aradu.A05	95103054	95103077	TGAAGAAGAGAGAAAAGGACCG	TCTATACCCCATATTCGCCG	1	
Ad05A18783	Aradu.A05	95120872	95120891	AAGCACATTCATCCACCACA	ATGACAATGGCTTCTTTGCC	1	
Ad05A18785	Aradu.A05	95121707	95121730	CATGAGATCCACGTCCTCCT	GTGGCTGGTAAACTTGGCAT	1	
Ad05A18789	Aradu.A05	95127105	95127128	CATCACCAAAGATTTAAACCCAA	TCATCAAAGCCCTTATTTCCA	1	
Ad05A18792	Aradu.A05	95145050	95145108	CATACATTTGCCAGCCTTCA	AGGTCAGCTGGACCAAACC	2	
Ad05A18797	Aradu.A05	95159897	95159911	TGTGGGTATGGCTATGGGTT	TGCATTCCCTGAACAATCTG	1	
Ad05A18799	Aradu.A05	95165426	95165491	ATGGCAAAAATTCGATGATG	TGGCTTCTACTTCTCCTCCTCA	2	
Ad05A18801	Aradu.A05	95173478	95173518	GTGCTGTTGGTGGAGGAAGT	AGTTTTTCGCCAGAATCGAA	1	
Ad05A18814	Aradu.A05	95204294	95204311	CAATGGTGTGGTGGTGAGAG	GAGGGAGGAAATGGTAGAGACC	1	
Ad05A18820	Aradu.A05	95212728	95212751	CAGCAATTAGTTTTAGAAGATGATGG	TCTAACAGTGAGAATAAACGATTGG	2	Yes
Ad05A18825	Aradu.A05	95230536	95230550	GCTACCGACCCTAACCCTTC	CCTGGTGCAACCTCATTCTT	1	
Ad05A18830	Aradu.A05	95236825	95236839	TTGCAGCACTCCAAGAATTG	GGCAGGTTAAACTGGTGGAA	1	
Ad05A18831	Aradu.A05	95237351	95237386	CCCTCCCACTTTCTTTCTCC	CCATGCCATAAAATAAATTGGA	1	
Ad05A18835	Aradu.A05	95256554	95256568	GGGGTTAAGTACGATTTTGGTC	ACTCCTACGAAGGGGGAAGG	1	
Ad05A18837	Aradu.A05	95268629	95268649	TGCATATGATCCCAAACCAA	GACCATTGGTTTTCATTGGC	1	
Ad05A18843	Aradu.A05	95276067	95276090	TCTGCATATGTGTGCGGATT	TGTCGTGCATTTGATTTGTG	2	
Ad05A18845	Aradu.A05	95284691	95284714	CAATTGTGGTCCCCTCCTTA	AGAATCAGTGAGCACACCCC	2	Yes
Ad05A18846	Aradu.A05	95284891	95284905	GGGGTGTGCTCACTGATTCT	GCATGTGCAGCCTCAAACTA	1	
Ad05A18852	Aradu.A05	95309631	95309642	TCTCGTGCTGATAACGTGTTG	CAAATGCAATTCTCTCTCTTTTTG	1	
Ad05A18854	Aradu.A05	95315833	95315844	ATGGGTATGATGGTTGTGGC	CAAGGCCTCAATACCTCCAA	1	
Ad05A18860	Aradu.A05	95336974	95336988	GAGCAATCGTGCTGCATTTA	GCTCTTACAATCAGGTCCGC	1	
Ad05A18861	Aradu.A05	95337125	95337139	GCGGACCTGATTGTAAGAGC	AATGGTTAGGGATTGGGGAG	0	

ID	Chromosome	SSR_start	SSR_end	FORWARD PRIMER (5'-3')	REVERSE PRIMER (5'-3')	Bands in parents	Polymophism in RILs
Ad05A18862	Aradu.A05	95340551	95340564	TACCACATGTCACTCTTTTATTGTAA	ATTAAGTGCCGAATTGCCTG	1	
Ad05A18867	Aradu.A05	95367014	95367025	ATGGCCAATTAGCTCTCCCT	CCAGCTGGCTGATAGGTTGT	1	
Ad05A18870	Aradu.A05	95388390	95388401	TTTGCCCAGTCAAAAACCTC	ATCAGAATAGCGTCGGCTTG	1	
Ad05A18877	Aradu.A05	95413587	95413604	GCTCATAGCGAAGGCGACTA	ATCGTTGCTCATCCCGTTAC	1	
Ad05A18880	Aradu.A05	95422441	95422458	TCTCAGCTGCTCGTGAAATC	GGGGCCCATACTGCTATGTA	1	
Ad05A18883	Aradu.A05	95429648	95429671	TTGTGCTGTTTATAAAACTTGAGC	TTGGATATGAATAAATGTGTAATTGAA	2	
Ad05A18885	Aradu.A05	95448626	95448643	TGCCAACGACGATAAACAAA	TCTTTCCACCAACTGCTCCT	1	
Ad05A18890	Aradu.A05	95459277	95459291	CCACATACTGTTACGGCACG	TTCTCCTCCTCGTCTTCTGC	1	
Ad05A18896	Aradu.A05	95466139	95466186	ATTTTGTCCCACGTGAAAGG	TTGGATATGAATAAATGTGTAATTGAA	2	
Ad05A18898	Aradu.A05	95480989	95481000	AAGCCGATCCAACAGTGAAC	GGGGGAGATTTGTTGGATTT	1	
Ad05A18904	Aradu.A05	95517385	95517400	TCGTGGTTTGACTGTGATTGA	CGCATCATGTGGTAAACAGG	1	
Ad05A18908	Aradu.A05	95539114	95539131	TGGCCAGAATGAATAGGGAC	GGACTCTTGTTTGCTTTTCCA	1	
Ad05A18909	Aradu.A05	95543441	95543452	TCCCTTTCAGGATATGAGTGC	CCGCTCCCAAAATACTTTCA	1	
Ad05A18912	Aradu.A05	95558610	95558627	GAGTCCATTTTGGAAGGCAA	GTGGTGTTGGTGGAGGTTGT	1	
Ad05A18914	Aradu.A05	95572872	95572889	TGTAGCACCTTCCTCTGCCT	CACCCACAGTCACCATCATC	1	
Ad05A18916	Aradu.A05	95589949	95589970	TGCCGAATTGAGGAGTAACA	GAACATAAAGGGCCACCAGA	1	
Ad05A18918	Aradu.A05	95595344	95595357	CTGTGGATCTCCCTCAGAGC	ACTCAACTCTAAGACGCCGC	1	
Ad05A18923	Aradu.A05	95613561	95613575	TCATCATTGCTGCTGCTACC	CAAGAAGACGCTGAAGAAGGA	1	
Ad05A18924	Aradu.A05	95613890	95613917	TTGACAAATTAATGTGAGTTACCAAA	CATAGCACACAAATTCGCACA	2	Yes
Ad05A18932	Aradu.A05	95630418	95630431	ACGAAGTCTTCAATGCCCAC	TCCGTGGGTACGGTAATGAT	0	
Ad05A18937	Aradu.A05	95641272	95641299	AATCCGTAGCAAACACCTTG	TATTGGTAAAAATTCGGGCG	1	
Ad05A18938	Aradu.A05	95641442	95641457	CACATTAAGTCGACTGCGCC	GGAAGTTTTATGTGCGACCG	1	
Ad05A18944	Aradu.A05	95656166	95656193	TGGGATTGCTTTTACTTCCC	ATCATTTGCCTTGTGCCTTC	1	
Ad05A18947	Aradu.A05	95676378	95676391	GTTGCTGGGTAAAGACCGAA	ATCATTTGCCTTGTGCCTTC	1	
Ad05A18949	Aradu.A05	95680313	95680326	TTGTCAAAAAGGTAGTGTGACAAG	TGGGGAGAGAAAGTGGACAT	1	
Ad05A18961	Aradu.A05	95710113	95710139	TGCTGTTGGACTCAAAGGTG	TGGTAGGAGGGGACAGAAGA	2	Yes
Ad05A18964	Aradu.A05	95712335	95712346	GTTCATGTCGCACACACACA	TGGCAGAGAGAATGATGCAC	1	
Ad05A18965	Aradu.A05	95713521	95713535	AACGACAATGACGACGAACA	AAGCTTCCATCAACGACACC	1	
Ad05A18968	Aradu.A05	95718808	95718821	CACTTGAGAAAATGCAATGTGA	ACTTGATGAGCTGGACGGTT	1	
Ad05A18970	Aradu.A05	95725698	95725719	GAGAAGAAGTTAGAAAGGGGTGG	CATCTCCTGGGGTCACACTT	1	
Ad05A18971	Aradu.A05	95726986	95726999	GCGTCTGGAGTTTTCTGCAT	TCGTAAAACAAAGATGGTGTGG	1	
Ad05A18976	Aradu.A05	95742950	95742973	ACTACGGTCTTGTCCGATGA	CCATCCAGAGGTGGTCAAAT	0	
Ad05A18978	Aradu.A05	95745016	95745029	AAGAATATGCGTTTGGGGAA	TTTGCGTGCATGCTTTATGT	0	

ID	Chromosome	SSR_start	SSR_end	FORWARD PRIMER (5'-3')	REVERSE PRIMER (5'-3')	Bands in parents	Polymophism in RILs
Ad05A18982	Aradu.A05	95747665	95747679	TGCGTATTTGCTGTAACTTTCAA	TCCTCTTTCAAGTAATTTTGTAGCA	1	
Ad05A18983	Aradu.A05	95748070	95748084	CCTCTTCATCTTCTGCTGCTT	TCATCTTTCAAATAATTTTGCAGC	1	
Ad05A18987	Aradu.A05	95752916	95752927	TGAAAAGGGATACTTCATTTTATTTTT	CAAATGGTAGAACCAAGGGC	0	
Ad05A18989	Aradu.A05	95762848	95762953	CGATCACCGCTTCTTTTTCT	CATCCATCACACCAACCAAA	1	
Ad05A18990	Aradu.A05	95763098	95763150	TGGATGGTGATAGAGTGGCA	GGTCCCTAAAGTAGGGGCTG	1	
Ad05A18991	Aradu.A05	95763616	95763635	TAGCAACTCGAATGGCAGTG	GCAAAATTGGTCCGGTTAGA	1	
Ad05A18992	Aradu.A05	95764361	95764378	TGTGCGAAGAAAATACGCAG	TAGGGCAATAGAAACGTGGG	2	
Ad05A18993	Aradu.A05	95768400	95768415	AAACCAGTTGAACCCTGGTC	GATTGGCAGGGAGAGAGAGA	2	
Ad05A18996	Aradu.A05	95795679	95795758	TCCCGATTAATGCCAATTATAC	GGTCTCCTTCCAATTCTTTCG	1	
Ad05A19006	Aradu.A05	95829254	95829269	TCCCTCTAGTATTTCTCGTTCTGA	GAAACGGTGGAAGAAGCAAG	1	
Ad05A19010	Aradu.A05	95843377	95843403	CTTCCACCATGGCAGTACCT	GAAGGAGACGTAGCGACGAG	1	
Ad05A19016	Aradu.A05	95864894	95864911	TCACCTAGTGAATTGAACATCCA	TGCAAATCGAGAACTTTTAGGA	2	
Ad05A19019	Aradu.A05	95873437	95873458	TAATTCCTTGTTTGTGGCCG	GGAAAGAGCTTTGGCATTTG	1	
Ad05A19025	Aradu.A05	95896165	95896178	TTTGAAGCCCGTAAAACAGC	CCGGCCAAAAACTAAACAAA	2	
Ad05A19029	Aradu.A05	95900582	95900595	AACTGCACCCCTAGTTGGTT	ATGTGGCGTGACGTAATTGA	2	
Ad05A19034	Aradu.A05	95903069	95903083	ATTGCATTCAGAGCTCCGTC	CAAGAAAATCTCCGAGCGAC	1	
Ad05A19037	Aradu.A05	95912486	95912518	CCTAATTGGCATGAACAAACAA	TCGAAGCATGGTTATTGAGAAA	0	
Ad05A19041	Aradu.A05	95942193	95942214	AAAATCAATCACATGCTGGC	TTCGTCTGATGTTTTGGCTG	1	
Ad05A19047	Aradu.A05	95958163	95958198	AACACCCCCATCACAAACAC	TTGTTTTGTTGTTTGTTCTCTGC	1	
Ad05A19049	Aradu.A05	95966237	95966258	GGTCAACAGTGGTCAAACCC	TGATATTCAGGTGAATCCATGC	1	
Ad05A19053	Aradu.A05	95992235	95992252	CGTTTGATTCATGTAGCCGA	TAAAAGAAGAACATTCATTGACTCTAA	1	
Ad05A19058	Aradu.A05	96004485	96004496	TTCACATAAAATCTCTGATTTCTAACA	CGAACACAAATTAGTATTAGCGTGA	0	
Ad05A19062	Aradu.A05	96014907	96014926	ACTGCTGTAGGGATGGTTGG	TCAGCCAACTATTTCAGGACA	0	
Ad05A19063	Aradu.A05	96023147	96023161	CAACACAACAGATGGTACATGC	ATGAAGCCTTTTGTTCCCCT	2	
Ad05A19065	Aradu.A05	96028450	96028537	CGTCACTTTGTCCCATGTTG	GGAAGAGGGACCATGAAAAA	1	
Ad05A19068	Aradu.A05	96029103	96029122	TTGCATCACAACTACACAAAAGAA	CTTGGCATGAGAGGCCTTAG	1	
Ad05A19069	Aradu.A05	96029352	96029375	CTAAGGCCTCTCATGCCAAG	CCCTGATGATGAACAAGGCT	1	
Ad05A19070	Aradu.A05	96030136	96030149	GTGCAGCAGCAAGTAGGACA	TCCCAAGTTGAAAAGAACAAAA	1	
Ad05A19071	Aradu.A05	96030306	96030317	TTTTGTTCTTTTCAACTTGGGA	CAGAAAGGAAGGAATGCAGC	1	
Ad05A19072	Aradu.A05	96031100	96031134	AAATGTTCCCTCTCCCTACCA	GGACCCATCCATCTGCTCTA	1	
Ad05A19073	Aradu.A05	96031433	96031518	CACCACCTGTCACACACCTC	TCTTCATTGCTATGAGTGGTGG	1	
Ad05A19074	Aradu.A05	96031639	96031656	TTGTCACAAATGCCAATGCT	TGGAGTTGTTGGGACTAGGG	1	
Ad05A19075	Aradu.A05	96031896	96031913	CCCAAACCACCATCACTTCT	TCAACCAACATAGTCCAAATGC	1	

ID	Chromosome	SSR_start	SSR_end	FORWARD PRIMER (5'-3')	REVERSE PRIMER (5'-3')	Bands in parents	Polymophism in RILs
Ad05A19077	Aradu.A05	96036095	96036114	TGTGATTTCGAAAAATAAAAGAAACA	TATGTACAAAACCCCACCCG	1	
Ad05A19080	Aradu.A05	96041845	96041874	ACTGCTGGCCAAGAAAGGTA	GGCAAAAAGAAAACAAAAGGG	1	
Ad05A19083	Aradu.A05	96043771	96043790	AGGAACTGGGATCTCACTGG	CATATCTTGGACCCGGCTTA	1	
Ad05A19085	Aradu.A05	96049000	96049013	TGAGGAACTCTTTTAGCTCACCA	AGCAATCACTTTTGGCCTTG	1	
Ad05A19088	Aradu.A05	96060604	96060615	ACGAGAGAGAGGGGGAGAGA	TTTGATTAGACATCTAAATTTCACCA	0	
Ad05A19089	Aradu.A05	96069150	96069164	TAGCGGGGTTTACACAGGTC	AGGATGAAAACGCGAAGAAA	1	
Ad05A19091	Aradu.A05	96071802	96071813	TTGGAATCGTTTAAACAAAAGG	TTCAGCCTTCATAATTATTTTTCTCTC	0	
Ad05A19092	Aradu.A05	96074841	96074868	TGTTGTGCAACTTATTTTTCCG	CAGGCATTTTAGAAGGTGGG	1	
Ad05A19093	Aradu.A05	96075413	96075432	GGTGCTTGAGATGGGAATGT	ATGGCAACAGTGGATGGAGT	1	
Ad05A19094	Aradu.A05	96079528	96079548	TTCTCATCGAAGCAAGCAAG	GGCTCATTCGATTTGGTGTT	1	
Ad05A19096	Aradu.A05	96082262	96082324	TTTGGTTGCAATTGATGACG	GCCTCATTCGTTTTGGTGTT	2	Yes
Ad05A19100	Aradu.A05	96095957	96095968	GAGACAATTTCTCCGAGGCA	AGAAGGGAGAGTGGAGAGGG	1	
Ad05A19101	Aradu.A05	96097336	96097363	GTTGGGGGAAAAATCCAGTT	CGTTTTTGGTGATTAACCCCT	0	
Ad05A19111	Aradu.A05	96112362	96112373	GTTGGGTCAGAGGACTTTGG	TGCATTTTTGAAGGTGGGTT	1	
Ad05A19114	Aradu.A05	96117514	96117534	TTCTCATCGAAGCAAGCAAG	GGCTCATTCGATTTGGTGTT	1	
Ad05A19116	Aradu.A05	96119587	96119699	ACACCAAAAATTCGTTGCAT	CGGTTCCTCTTCCTCTTCCT	2	Yes
Ad05A19121	Aradu.A05	96130272	96130286	CGATTTTCAATTATCAACTTCACA	AAGGAAAGGAAAGGGACAAGA	1	
Ad05A19122	Aradu.A05	96130509	96130522	CAATTTGCAATGGAATCGTT	TCAATGTGTCAAGCCTTATTCTT	2	
Ad05A19124	Aradu.A05	96132749	96132766	ACGACACGAAATACGTCGAT	TTGGACATGACATTCATCCG	1	
Ad05A19128	Aradu.A05	96138913	96138924	CCATCCATTTTCTTTCTCAGC	TTGGATTTAGGGGATTCAGG	1	
Ad05A19129	Aradu.A05	96140655	96140699	AGGTAGAGTTAAATTGAAGAATAACGA	GGCTATTTGGTTTTTCAAGGC	1	
Ad05A19133	Aradu.A05	96151451	96151464	TTTGTTTCGTTGTTTGTTGTTTG	TTTACAACAATTCCCGGAGG	0	
Ad05A19136	Aradu.A05	96157266	96157279	GCAACTTATTTTTCCGCACC	TTTACAACAATTCCCGGAGG	1	
Ad05A19137	Aradu.A05	96159832	96159870	AATTGCACACACGTCGACAC	TGTGCGCTTTCTTCAACAAC	1	
Ad05A19138	Aradu.A05	96168242	96168253	TGTGAACCAGAACATAGCGG	AAAAATCGAGGAAATGCGTG	1	
Ad05A19142	Aradu.A05	96175495	96175542	ACGCCCGAGTTGATCTTCTA	CATCCATAAAGCAAGCCGAT	2	Yes
Ad05A19149	Aradu.A05	96197292	96197319	CCTTGAAGCAAAAGAAACAGC	ACGCACTTGGTCTCGACTCT	1	
Ad05A19150	Aradu.A05	96204163	96204200	ATTGTTGCTGGAAAATTGGC	AAATCAGTTTCCGTTCACCC	2	
Ad05A19151	Aradu.A05	96207345	96207395	TGTGAATTCCCCTTCCCTTA	GGATTCTCTAATTATCAAACCTCACC	2	
Ad05A19152	Aradu.A05	96208897	96208914	CGCTAATCCCCGAAATTCTA	AAGACAGGAGAATGGCGAGA	1	
Ad05A19154	Aradu.A05	96213939	96213952	ACCGAAATCATAGCCACCAG	TTGACAATGATACATTAGCAAAAAT	0	
Ad05A19155	Aradu.A05	96217772	96217785	TCAAGAAAACAAAAATTAATCATCACA	GCCACTTGTGATCCAACAAC	1	
Ad05A19157	Aradu.A05	96222850	96222864	ATGATTGGCACTCAAGGGAC	TGCGCCTTTTGATATGTTTG	1	

ID	Chromosome	SSR_start	SSR_end	FORWARD PRIMER (5'-3')	REVERSE PRIMER (5'-3')	Bands in parents	Polymophism in RILs
Ad05A19158	Aradu.A05	96223669	96223684	TCCCAAATCAATTCAAACCAA	TTGTTGTGATTTTTGAATAATTGGA	1	
Ad05A19163	Aradu.A05	96230842	96230855	GGGTAAAACAAAATCGCCAA	ACACCTGAAGCGAAGTGGTT	1	
Ad05A19165	Aradu.A05	96232268	96232279	AAGAAATCGAAGCCGGAAGT	ATAAAGAAAGCCTACGGCGG	0	
Ad05A19166	Aradu.A05	96232450	96232477	CCGCCGTAGGCTTTCTTTAT	CGAAAATTTTTGTAAGGTGGG	1	
Ad05A19167	Aradu.A05	96234388	96234399	AGGAGCCTTGAAAAATGGGT	TATGGAGAGGCAGGGTTTTG	2	
Ad05A19170	Aradu.A05	96241659	96241776	TGATTGATGATGGATTGTTGC	CTCTGATCTTCCACCCCAAA	1	
Ad05A19174	Aradu.A05	96245018	96245031	CGTCTTCTGCTTCTTCTGGG	TGCATCACACCAACCAAACT	1	
Ad05A19176	Aradu.A05	96252758	96252777	CAAAACGGGAGTACTCTTGGTAA	CCATGCTCACTGAGAGGTCA	1	
Ad05A19180	Aradu.A05	96268553	96268567	CCTTCCAGATCCGACAAGAA	CCTTCCTTCCCCTTAACTGC	1	
Ad05A19181	Aradu.A05	96269259	96269300	ACCTTCTAGGGAGCAGAGCC	CAATCCCAATGGAAAAGGAA	2	
Ad05A19182	Aradu.A05	96270265	96270284	TTTTCAAGGGGATCAAGTTCA	ATAAGTCCCAATCCCCAACC	1	
Ad05A19186	Aradu.A05	96284130	96284191	GAGCGTAGGTTGAGGAGTGC	ACCCAAACAAAACCAGCAAG	1	
Ad05A19188	Aradu.A05	96293571	96293585	TCAGCAAGCAACAGAAGCAT	TTATAATCTCGCTGGGGTGG	1	
Ad05A19189	Aradu.A05	96293805	96293899	TTCAAACGTATGCATATACTCATTAAA	GGAGAAGATGATGATGAAAACG	0	
Ad05A19190	Aradu.A05	96296154	96296169	GCATTGGCATTGAAGTGAGA	AATAACGTTAATGGGCGATCA	2	Yes
Ad05A19193	Aradu.A05	96302568	96302588	CCCAACATGCACTTTGAACA	CCCTGGCTCTCTCTTCCTCT	1	
Ad05A19194	Aradu.A05	96302728	96302739	AGAGGAAGAGAGAGCCAGGG	GGAGATCGTGGAAGCCATTA	1	
Ad05A19203	Aradu.A05	96325626	96325657	CGGACACTTTTGCCCTTTTA	ACGGATCTCTCACCATGTCC	2	Yes
Ad05A19204	Aradu.A05	96339213	96339224	TCGCAGCTCTGTTCCTTTTT	CCCTCAACATACTCAGCCGT	1	
Ad05A19208	Aradu.A05	96346426	96346441	TACTTCTTTGAATTTGGCGAC	TGGCTAATTTTAATGTGCGAA	2	
Ad05A19209	Aradu.A05	96348949	96348963	CGTCTCTGTCAGAACCACCA	GGTGAGACAGAGAAGGCAGG	1	
Ad05A19212	Aradu.A05	96356750	96356767	TGCAACGTTTCTCAACGTAAA	AACGCGAAAAAGGAAAACAA	0	
Ad05A19217	Aradu.A05	96376422	96376435	TTGGCCAATTGCAGACAATA	GGAGCTGGTGATGGATTTTC	1	
Ad05A19220	Aradu.A05	96386734	96386745	TGCCTCTAACTTTTTCGGGA	TACGAAACTAAGGCCGGCTA	1	
Ad05A19223	Aradu.A05	96399278	96399297	AACTTGTGTGGTTCGTGCTG	CCATTGCAGATTGCATAACG	1	
Ad05A19227	Aradu.A05	96417902	96417979	TGATTTTAGTCGCTTTACATGGG	TTTTGGTCTATTATCGGAATGG	1	
Ad05A19232	Aradu.A05	96447117	96447128	GAGAAAGGAGAAAGTGGGGG	TAGATGATGCGTGGGCATAG	0	
Ad05A19233	Aradu.A05	96447391	96447426	CGTGAGAAAATCAGTTACCAAAA	CCATATTTTTAAATTTTTCGAGAGTT	1	
Ad05A19234	Aradu.A05	96449889	96449900	TTTTAAAACCCACAATCTTAAATGTT	AGAGTGAGTTGTGCCAATCG	1	
Ad05A19239	Aradu.A05	96460701	96460716	CCATCTCCCCTCTTTGTTGA	TCGAATTATGTGCATGTAACGA	1	
Ad05A19244	Aradu.A05	96479128	96479145	CACAGGGCAAAAGAAAAAGG	GCCAACTCTCATTGACACCA	2	Yes
Ad05A19246	Aradu.A05	96479795	96479808	TGATCATCACTAGCTACAAGCCT	TCGTCCGTTTAAATTAATGTCG	2	
Ad05A19248	Aradu.A05	96484629	96484658	TCATCCTATCTACTTACACCAAAGTGA	TCCGATCCGTCCATTATCTT	1	

ID	Chromosome	SSR_start	SSR_end	FORWARD PRIMER (5'-3')	REVERSE PRIMER (5'-3')	Bands in parents	Polymophism in RILs
Ad05A19250	Aradu.A05	96497275	96497307	TGTGGTTTATTTTTGCTTTCCTC	CATCTAGACATCACCTTTGCAG	2	
Ad05A19255	Aradu.A05	96507317	96507328	CCCTATCTGCGGGTTGTAAA	TTAGGTTGAACCCGTCCCTA	1	
Ad05A19259	Aradu.A05	96509711	96509740	ATGATAACAATTGTGAGACAGAATG	ACATGACAGAGCACAATGGC	0	
Ad05A19260	Aradu.A05	96519056	96519067	AAGGAGTTTCTTCTCCGCATC	TCACTTTTCTTTGAAAAATCGAAA	0	
Ad05A19274	Aradu.A05	96536625	96536648	AAATCATCCTCACACGCACA	GAACCACACCTCAGCCACTT	2	
Ad05A19275	Aradu.A05	96537024	96537041	TGCAAATGTCGTAGCAGGAG	TCATCATCGTCCAAGTAATTTTG	1	
Ad05A19277	Aradu.A05	96540342	96540371	TGTATAAGACGGAATTCAAAAACC	TTTTTGCGAGGCGTTAGTTT	2	
Ad05A19284	Aradu.A05	96550586	96550597	TTGCTTCTTGTTAGTCAAGCCA	TTGAACCTTTCTCAACAGGAAA	1	
Ad05A19287	Aradu.A05	96555237	96555285	CCAACGGTGAACAACTGAGA	GGTGGGTGTTAGTGTTGGGT	0	
Ad05A19289	Aradu.A05	96555812	96555841	CTGGTGGGAAGGGTGACTAA	TGACACCAAAATATAACTTCGTTTAAT	1	
Ad05A19294	Aradu.A05	96563258	96563275	GATCAGAAAGAAGCCGTTGC	CAGATTTGACGGTGACGAGA	1	
Ad05A19295	Aradu.A05	96563511	96563552	AGAGGCAACGGTAGCAACAC	TGGAGCATCAACAACAACAA	2	Yes
Ad05A19296	Aradu.A05	96563713	96563727	AGAGGCAACGGTAGCAACAC	AAGAAGCAATTAATGGAGCATCA	2	Yes
Ad05A19297	Aradu.A05	96569116	96569127	CCTTTCATACCTTGACCCGA	TGGTGTTTGGAAGTGTTGGA	1	
Ad05A19301	Aradu.A05	96587124	96587135	AGGGAGAATCCCAAGCAGAT	CGAATCTCAATTATCAACTTCAGG	0	
Ad05A19302	Aradu.A05	96587407	96587418	CAACCTCCATTAGTTATTGATTCAT	CGGTCTGGTTGTCGCTCTAC	1	
Ad05A19305	Aradu.A05	96595537	96595548	ATCCGGTATGGGGTGAATTT	TCGCCTTATTTTTACCCATTTC	2	
Ad05A19306	Aradu.A05	96596063	96596206	ACTCCCGCTTGTTTTACACG	CGTGAAAGAGGAGAACAACGA	1	
Ad05A19307	Aradu.A05	96596369	96596383	TCGTTGTTCTCCTCTTTCACG	TGCGTTGAATCAATCCAAAT	0	
Ad05A19308	Aradu.A05	96598237	96598317	CAGCAGCAATAAGCCAACAA	GCTTCTTTTCCCCTACGCTT	1	
Ad05A19312	Aradu.A05	96601570	96601589	CAGGTGAGGAAATAATGCGAA	CTCTGGAAAGCAAATCTCGG	1	
Ad05A19313	Aradu.A05	96603245	96603260	CGCTATTTATTTGAAAACTTTCGTG	TAAAGGGAATGACATTATTTTGGG	0	
Ad05A19314	Aradu.A05	96614008	96614022	GAATCAACCAAAATAAATAATCAAAAC	GCTTGTAGCCTAAGTCCCTTCA	1	
Ad05A19315	Aradu.A05	96617462	96617475	AACCGTCTTAACAGCTTGCG	TGACGTTACGAGCTACTGCG	2	Yes
Ad05A19322	Aradu.A05	96657438	96657453	GCGTTTTATCACCCCACTTC	CAGGGGTAAGCCATTTTGAC	2	
Ad05A19324	Aradu.A05	96661095	96661106	TGTGGAAAAGAGGGTGAACA	TTGGAGGAAGTTTCTAAAACGAA	1	
Ad05A19326	Aradu.A05	96663636	96663741	CACACACATGGTGCACAAAA	CTTTGAGGACAGAAGGCCAG	1	
Ad05A19327	Aradu.A05	96665737	96665754	TACACCCTTGGGGTGCTTAG	GGTCGTGACATTGTTGATGC	1	
Ad05A19345	Aradu.A05	96710235	96710254	CGCAGATCCGCACAATAAT	TCGCATAATTGAACCTTCTCTTT	1	
Ad05A19347	Aradu.A05	96724728	96724749	GGAGATGTGGACAGTGGGTT	TGCCACGTAAGCACATCTTT	1	
Ad05A19349	Aradu.A05	96732909	96732923	GGGTGGCGTATAGGTTGCTA	CGCCCTTCTCTTCCTTATCC	2	Yes
Ad05A19350	Aradu.A05	96736740	96736759	AGGTACGGCGACAATTTCTG	AGCCACCAAATCCCTTTTCT	1	
Ad05A19356	Aradu.A05	96758808	96758837	CAGCCATTCCAAAGCAAAAT	CCCAAGAAATGAGGATATTGATG	1	

ID	Chromosome	SSR_start	SSR_end	FORWARD PRIMER (5'-3')	REVERSE PRIMER (5'-3')	Bands in parents	Polymophism in RILs
Ad05A19357	Aradu.A05	96761429	96761446	AACATGTTTTATAAGGGAGTCATAGAT	TTCATGAACCAAATTTTAAACCAG	0	
Ad05A19362	Aradu.A05	96765145	96765162	TTTGTTGGTGTTGGTGTTGG	AGCAAATTCAACAGCACCCT	1	
Ad05A19363	Aradu.A05	96765309	96765385	TTGCTGTTGAATTTTGCTGC	AGTGCCAGAATCGAAATCGT	2	
Ad05A19364	Aradu.A05	96766877	96766897	GGTTTGTTCTCATCCCCTCA	TCCAAGTGAGCTTCAACCAA	1	
Ad05A19365	Aradu.A05	96767005	96767020	TTGGTTGAAGCTCACTTGGA	TCATGGGAAAGATTTGGTCC	1	
Ad05A19366	Aradu.A05	96767246	96767259	GGACCAAATCTTTCCCATGA	CCCAAAGTGAGATGGGTTTT	1	
Ad05A19369	Aradu.A05	96773799	96773825	GCAGGCGATAACCCAATAAA	ATTGGCCTGTTTGTTTTTGC	2	
Ad05A19378	Aradu.A05	96818086	96818206	TGATGCAATGTTTAGGCCTTT	GCTAATGGATCCGGTGATTT	1	
Ad05A19381	Aradu.A05	96821767	96821782	CCGACGATAACGAACCCTAA	GAGCGACGGAAAGACAAGAG	2	
Ad05A19382	Aradu.A05	96821939	96821950	CCGACGATAACGAACCCTAA	GATGGCGAGACACAAACAGA	2	Yes
Ad05A19383	Aradu.A05	96822302	96822315	TAGTTTTGACGTGCGAAGCA	CAGATGTAGGACGTGAGGCA	0	
Ad05A19385	Aradu.A05	96841733	96841759	TGCGTCCAAAACAGCTACAC	GATTGTTCGGGACATCGAAT	1	
Ad05A19386	Aradu.A05	96845216	96845230	TGAAACCGCTTTTACCCAAC	TGCGAAAAAGAAGGAGGAGA	1	
Ad05A19393	Aradu.A05	96858314	96858328	CCAAAATGATTCCAGAGATGG	ATATGGAAGGATGAAGGCCC	1	
Ad05A19394	Aradu.A05	96861594	96861611	CATGAGGTGGTCAGACGAGA	TCATCTTTCAGGTTTGGTGATG	2	
Ad05A19398	Aradu.A05	96868392	96868403	AACGACAAAGAGAAGCCTCC	TACATGCATTCTTGGTGGGA	1	
Ad05A19399	Aradu.A05	96869203	96869235	AGCTCAGAGGCAACCAACTC	TGAACTATTCTGCTGCCTGG	1	
Ad05A19402	Aradu.A05	96885233	96885256	GCAGTAATCGTGCCCAAAAT	TTAGAGAAGGTGGCGTTTCG	1	
Ad05A19405	Aradu.A05	96892314	96892328	CTCTCTTCTCCTCCAACCCC	GGAGCTCCAGACGATTAACA	1	
Ad05A19406	Aradu.A05	96892663	96892701	GAAGGAAGGATTATGGTACATTATGA	CGTTGGTATCTATTAGTGCCG	1	
Ad05A19407	Aradu.A05	96896450	96896467	ATGGATTGCTCAACTTGGGT	TTTTTGGGCGGACTGTTTAG	1	
Ad05A19412	Aradu.A05	96908738	96908779	GTTTGCATGGCTCGGTATTT	AAAAGTCTAGGAGGCCAGCA	1	
Ad05A19413	Aradu.A05	96908953	96908967	TAACAAATTTGCTGGCCTCC	CATTCAAACACATTTAACATTTCCA	1	
Ad05A19414	Aradu.A05	96909604	96909615	CGAGGACGTTTCTTCATTGC	TGGTGGTTACCATGGATCAG	1	
Ad05A19415	Aradu.A05	96914484	96914495	TCAAATTGAAGAAGAGTAAGACACA	TTCAATGGTCAATATTCAAAATCA	1	
Ad05A19422	Aradu.A05	96939528	96939542	GACCGGCTCATTCTTACTGC	AAACCACGAGGAAGGAGGAT	1	
Ad05A19424	Aradu.A05	96948208	96948219	CAGAACCCTTGTTAGGGAGG	ATTGGTGGCATGAAAGAAGC	1	
Ad05A19426	Aradu.A05	96953859	96953927	GGTACACCCTCAAAGGGGAT	TGCCAACTGAAGCATCTGAC	1	
Ad05A19427	Aradu.A05	96959383	96959396	GCTGTTCGAATTACGAGGGA	TGAACTTCACACCAGAGCGT	1	
Ad05A19428	Aradu.A05	96965535	96965548	GGTTTTCGAAACATGGTGCT	CCAGGAGGACGTTTCTATGC	1	
Ad05A19435	Aradu.A05	96986490	96986509	CAGCAGCTCCAAGAAGACCT	AACCTTGCATAAACCCTCCA	1	
Ad05A19442	Aradu.A05	97035137	97035256	GAGCAGTTTGAGACAAGAAGCA	TGTGTGGTGTATTCACTTTCCA	1	
Ad05A19443	Aradu.A05	97036573	97036586	GCACAAGAATAAATTGGACCG	GGAGGGACCGAGAGAAGAAG	1	

ID	Chromosome	SSR_start	SSR_end	FORWARD PRIMER (5'-3')	REVERSE PRIMER (5'-3')	Bands in parents	Polymophism in RILs
Ad05A19445	Aradu.A05	97039445	97039465	GCCCCATTGAGATTCCTGTA	GTTGGGCGTATGCTTCTGTT	1	
Ad05A19446	Aradu.A05	97039598	97039686	CCAAAATCAACCAGAAGTCCA	CGCCAGCTTCTTCTTCTGTG	1	
Ad05A19447	Aradu.A05	97039929	97039961	CGGCGAAAAAGAAGAAGAAG	CTACGAAATGGTCCCCAAAA	1	
Ad05A19449	Aradu.A05	97041807	97041833	TTTGGACCCTACAACGGTTT	CAATTGCAGAGTACGCCAAA	1	
Ad05A19450	Aradu.A05	97042330	97042368	TCATGTACTCCTCATCATACTTGTAA	AAGAGAAGGAAGCTATTAGCACTTTT	1	
Ad05A19452	Aradu.A05	97046804	97046817	GGCACAGTGAGGTGTAACGA	ACCCCATCCCCACTATTCTC	1	
Ad05A19453	Aradu.A05	97052504	97052531	GCATTCAGAAAGTTCCTCGG	TGGCTACCTTGGTTGTACCC	1	
Ad05A19467	Aradu.A05	97103729	97103740	GGACCTTGGGTGTGGTAAGA	CAAGGCCTCAATACCTCCAA	1	
Ad05A19468	Aradu.A05	97107555	97107569	GCTTTGCACCTAGAGTTCCG	GTGTAGCACAAACCCCATCC	1	
Ad05A19470	Aradu.A05	97122459	97122486	GCGAGGCGTTACGAACTTTA	TGTTTGGGACACTTTTGGGT	1	
Ad05A19471	Aradu.A05	97123360	97123373	TGCCCTACCACCTTCTCTTG	ATCCATGTCCCTCCTTTTCC	1	
Ad05A19473	Aradu.A05	97131688	97131699	ATTCGCCTTGAGAAAAGCAT	CACAATGCAACAGAACGAAGA	2	Yes
Ad05A19476	Aradu.A05	97138534	97138554	TTTCCTGGGAATTTGTTTGA	TGCAGCAAATGAATGAAAGG	1	
Ad05A19478	Aradu.A05	97139267	97139287	CAAGGAGTTGAACAGCGTGA	CTTCTTCGCCACTTTCAAGG	1	
Ad05A19479	Aradu.A05	97139441	97139455	ACCCCCAATAACCCCAAATA	TTAGCAGCACCTACAACCCC	1	
Ad05A19487	Aradu.A05	97154625	97154662	ATGTTGCAAACATGCCAAAA	AACCACACTCTCTCTCCCCA	2	Yes
Ad05A19488	Aradu.A05	97159873	97159899	GATAAGCGCAACAAAGCGAT	TTGACAAATGAGAATTGCAACC	1	
Ad05A19491	Aradu.A05	97171590	97171646	TGTTTGACCAGATTTTACAGAGACA	CAGGAATATGGAAAAGAATTGG	1	
Ad05A19492	Aradu.A05	97172013	97172026	GCACATATCTAAATACGAGCACA	CTTGCGTCTTCCTAAAATTTAATC	1	
Ad05A19495	Aradu.A05	97177948	97177961	GTGTTGGTTGTGGGGAAAAA	CCCAATTTCTCTTTTCACCGT	1	
Ad05A19507	Aradu.A05	97198741	97198752	AAATTGGTGCAGGGAATCAA	TTGACATGGTTCTGCACCTC	1	
Ad05A19509	Aradu.A05	97202682	97202696	AAGCATAATGGGCAGGTCAC	CATGTTTGAAGCAAAAGGCA	1	
Ad05A19512	Aradu.A05	97208620	97208640	TGCAGCACACCACTTACTTGT	TTGTCCGGACCTAAGTGACTG	1	
Ad05A19514	Aradu.A05	97214643	97214666	GCATGCTAAGAAAAAGGAAGTGA	GGCTTGGATGATAGGTTGGA	1	
Ad05A19515	Aradu.A05	97221158	97221169	TCACCAAATATGATATAAGAACACAAA	TTTTAGGATCGGACCACGTT	1	
Ad05A19518	Aradu.A05	97234627	97234642	CTGCAAAATAAAGGTCGGCT	TTGTTTTCAGATGCAGGACG	1	
Ad05A19519	Aradu.A05	97239299	97239316	ACTTGCCGGTTAGTTTGTGG	GAGGGGGAACGAAAGAAAAA	1	
Ad05A19521	Aradu.A05	97247080	97247091	GCGCGGTGTGACTCTAAATA	AAATACCCACCGTCAAATGC	1	
Ad05A19523	Aradu.A05	97249075	97249086	GTGCGTGTGCTTGATTGATT	TCATTGCCATGGAGATCAGA	1	
Ad05A19525	Aradu.A05	97250068	97250088	TTCGACTTTTGTGCTCAACG	GCTTTTGGAGTATGTACCGTTTTT	1	
Ad05A19527	Aradu.A05	97258562	97258573	TTTGTCCTGCATCTGTCTGC	GTCCTAGGAAGCTGTGTCGC	1	
Ad05A19529	Aradu.A05	97262191	97262214	ATGTCTGCCCAACACACTCA	GGATAGATGGTGGTGCGTCT	1	
Ad05A19533	Aradu.A05	97270981	97270998	TGGAAAGCCAACACATTCAA	AAGCTGCAGCAACAACAATG	1	

ID	Chromosome	SSR_start	SSR_end	FORWARD PRIMER (5'-3')	REVERSE PRIMER (5'-3')	Bands in parents	Polymophism in RILs
Ad05A19534	Aradu.A05	97271519	97271530	AGGGGCAATTTTGATTTTGA	GGATTTGGATGGCAAAAAGA	1	
Ad05A19540	Aradu.A05	97283118	97283243	AAAGAGCCACACCACCAAAC	GAGCTTCTTGGCTTGGAATG	1	
Ad05A19542	Aradu.A05	97291496	97291513	GGGCAATTTAAATGAGAGGC	CCGCTTGATCGAACATAGAA	1	
Ad05A19545	Aradu.A05	97312846	97312859	CGATTCGTTTCTCCTGTCGT	GAGGAATGGGAAGTGACAGC	1	
Ad05A19548	Aradu.A05	97345800	97345815	TTTGTGACTAAATTTTAAGAGGTTTGA	AAAATTTGATGCGTAGATTCTTTT	1	
Ad05A19551	Aradu.A05	97355811	97355834	AAATGAGTTTGGTTGTCGCC	ATCTCGATGGACTCACCACC	1	
Ad05A19554	Aradu.A05	97357836	97357850	GAGTGAGAATGAAAATACAAAGAAAA	GGGATTAAGAAATTTGGTGTGC	1	
Ad05A19557	Aradu.A05	97358485	97358500	AACGTCGAATTTTTCCGTTG	GGAGATTTGATGCTGGGAGA	1	
Ad05A19568	Aradu.A05	97377645	97377656	TCCCTCTCTCTCCTTCCCTC	AAGGGGGAGAAAGAGCACAA	1	
Ad05A19569	Aradu.A05	97377849	97377860	TTCTCCCCCTTCCTTCTCTC	TCTCTCTCCCCCTTTCCTTC	1	
Ad05A19570	Aradu.A05	97378108	97378153	ACAAAAGGGGAAAGAAAGGG	TTTTCTTTTTCTCCCTCTCCC	1	
Ad05A19571	Aradu.A05	97378307	97378326	GAGGGGAGGAAAAGGAAAGA	CCCTCTCCCATCTCTCTTCG	1	
Ad05A19572	Aradu.A05	97378461	97378604	GAATGGGGAGAGAGAGGGAG	AAAACCAGTTTTTATGGTTCAGTTT	1	
Ad05A19573	Aradu.A05	97387616	97387635	AAAGGACTTGGACCCCATCT	TGTACGTTTCTCAAAAACAGCG	1	
Ad05A19574	Aradu.A05	97389390	97389419	CCTGCAAAGCTAAGACTGGC	TTTCTGTTGCAGCGAAGTTG	1	
Ad05A19577	Aradu.A05	97400014	97400028	TCCTGGTGTATGTTTTTCCTTG	CCCTATCAATTTCCAACCCA	1	
Ad05A19583	Aradu.A05	97409349	97409360	TCCTGATTGTCAGGTTGTTCC	GCGATTTGACAACTAGGAGTTT	1	
Ad05A19586	Aradu.A05	97415292	97415311	GCCATTGTCCAAGTCACCTT	TTTGACCTTTTTAACAAGAGAAAGT	1	
Ad05A19588	Aradu.A05	97417643	97417660	ACAAGCAACGATGCATGAAA	ATCGACGTGTCCTGTGTCAA	1	
Ad05A19592	Aradu.A05	97424630	97424649	TTTCGATGTCATTTCTTTTTCTTTC	TGTTGTCGTCGTTATTATCAGCA	1	
Ad05A19594	Aradu.A05	97426783	97426803	TTTTCAAAAACCAGTTTTAACTTTC	AAATTTCTTCGGTATGCATTTTA	1	
Ad05A19595	Aradu.A05	97434592	97434609	CTAATCTGCAGCAACCACCA	CATGAGGTGGTCAGACGAGA	1	
Ad05A19601	Aradu.A05	97449694	97449735	GCCTTTGTGAATACGAACCA	GGTGCGAAGACTTAGTACGGA	1	
Ad05A19606	Aradu.A05	97466616	97466633	TCCTTGGTGAGGTCCCTATAAA	TCTTTTCCAACACCAATGAATG	1	
Ad05A19609	Aradu.A05	97470262	97470276	GCTGGTTCTTGGTTCTGCTC	GCCACTGTTGAAAGGGGTTA	1	
Ad05A19611	Aradu.A05	97471291	97471305	TACTTTTCTCTCCCCCTCCC	TCGTCATCTTCATCCACATCA	1	
Ad05A19612	Aradu.A05	97471415	97471514	GGGTGGTAGTACATGGAAAGATG	GCTACAGCACCACCAGATGA	1	
Ad05A19612	Aradu.A05	97471415	97471514	CATCGCAATTGTTACGAGGTT	GCTCGTTGATAGCAAAAGTTACAA	1	
Ad05A19613	Aradu.A05	97472148	97472171	CTGTGTTGAATGCTGCTGGT	AGCAACAAACTCCATCACCC	1	
Ad05A19614	Aradu.A05	97472338	97472364	GGGTGATGGAGTTTGTTGCT	ACCAATTTCAGCACTGCTCC	1	
Ad05A19615	Aradu.A05	97472860	97472874	TGGGAAAAGGTATCGAGTGG	TCCGTTGGTTTCGCTTAACT	1	
Ad05A19617	Aradu.A05	97473222	97473239	AATAGAGCTGGGCAAGGTCA	CTGCTGGCTCCTTAGCACTT	1	
Ad05A19618	Aradu.A05	97474230	97474349	TTTCGGTTTCCTCAGCTTTC	GAAGATGAGGGTTGGAAGCA	1	

ID	Chromosome	SSR_start	SSR_end	FORWARD PRIMER (5'-3')	REVERSE PRIMER (5'-3')	Bands in parents	Polymophism in RILs
Ad05A19619	Aradu.A05	97475048	97475062	CCCGTACTTTCAATCTAATCAATG	GCGTGTGATGTTGGAGAGAG	1	
Ad05A19623	Aradu.A05	97478950	97478961	CCGTATATATATGAAGTGCTAATTGTG	TTGTGGTACAAACAAGACTAGAATAAC	1	
Ad05A19627	Aradu.A05	97484447	97484461	CAAAGTTAACAACCAACGCAA	TCACAACCTTGAGAGCAAGC	1	
Ad05A19629	Aradu.A05	97490639	97490650	TTTCCAAATCAAAACCCTAATTTT	ACCCCCTCCCCTCTTATCTT	1	
Ad05A19635	Aradu.A05	97497075	97497110	TTTACAATTGAATAATCATACTTTTCG	TTGACCTCAATTCACCTTTATTCA	1	
Ad05A19643	Aradu.A05	97519675	97519713	TCAACGCCAAATTTGATAAAGA	TCGATCCTTATTATAGACTCAATCTCA	1	
Ad05A19644	Aradu.A05	97520496	97520515	TTTTATGGTATTTTATGTAGAGGGAAG	GATTTCCATGTCGGTATCGC	1	
Ad05A19648	Aradu.A05	97534976	97535025	CCTCTGCTTCCTCCTCCTCT	TTTCTCTTCCTCCTCCCCAT	1	
Ad05A19649	Aradu.A05	97535419	97535433	GAGAAGAAGATGATGACGATGATG	TGTTCATCGTTGATGGTTTGA	1	
Ad05A19655	Aradu.A05	97547066	97547083	GCCATATTGAATTTTGCATCA	TTTCTTGGAAGCAATGACAGA	1	
Ad05A19658	Aradu.A05	97548337	97548348	AATCCCTTTTGGGGCACTAT	TTTTGAGTTGCGTTTCTCTAACA	1	
Ad05A19663	Aradu.A05	97550637	97550652	AATGAAATTCTTGTGCTGCCT	ATATTTGGGGTTACAGGCCC	1	
Ad05A19665	Aradu.A05	97559550	97559571	GATCAATGCATGGAGGACAA	TCCTCAGCCCAAGCATAACT	1	
Ad05A19667	Aradu.A05	97561522	97561536	TTTGGGTCCGTCACTGTGTA	GGATATGTCGTGGAGGGAGA	1	
Ad05A19668	Aradu.A05	97561761	97561774	TCTCCCTCCACGACATATCC	GATAACTCAAGGATTTAACCAAAAA	1	
Ad05A19671	Aradu.A05	97565357	97565396	AGTGGCACTCCCTCCCTTAT	AGGAAGCGAGTGTTGAGGAA	1	
Ad05A19672	Aradu.A05	97566381	97566465	GTCAGAGTGGGGTTTGGAGA	ATAGATGCCCTCCGTGTGAC	1	
Ad05A19673	Aradu.A05	97571178	97571198	TTTTGATTTTTATTAGACTTTTGCCA	TGTCAGAAAATAAACTGGAGACTGA	1	
Ad05A19674	Aradu.A05	97571558	97571608	AACAGTCCTCCGTCCAAAAA	CAAATCAATTTTCAACGCTTTTC	1	
Ad05A19675	Aradu.A05	97572870	97572887	TGTTTTTGCTCTTGGAAAGGA	AATTGTATGGAGGTGACGGC	1	
Ad05A19681	Aradu.A05	97588810	97588848	CATTTTTGTACTTGCAGGTTGAC	TATAAGTCGCCAAGTCGGGT	1	
Ad05A19682	Aradu.A05	97590422	97590467	CCCTCAGATTCCTGCTCAAG	TTTTCACTTTTCAAATGCATGA	1	
Ad05A19692	Aradu.A05	97620924	97620947	CGTAAGCAGCGGCTAGATTT	CACCCCAATTACCCTAAGCC	1	
Ad05A19694	Aradu.A05	97632792	97632806	CAGTCAATAGATGAAGAAGGGGA	AGCGACTGTTTTCAAGTGCT	1	
Ad05A19697	Aradu.A05	97643637	97643651	ACAGGCTGCAGAATACACCA	TTTATAGCAGACGCTCGGGT	1	
Ad05A19700	Aradu.A05	97647192	97647233	TGGCAATAATTTGGCCTGTT	ATTTGGTGACGTGCTTCCTT	1	
Ad05A19706	Aradu.A05	97653892	97653906	GTGATCACTGCAGCCTCTGA	CGTCCAGGAACAGAGAGGAG	1	
Ad05A19707	Aradu.A05	97659455	97659466	GAGAGAAGGAGTTGCGGTTG	CCAATGGTAACGAGCCCTAA	1	
Ad05A19710	Aradu.A05	97663702	97663719	GGTTTCAACCTCACTGGCAT	TCCAGCACTCAAGACCACAC	2	Yes
Ad05A19715	Aradu.A05	97674888	97674908	TTTTTCCAAACATTGATCAGGA	AAAGCCACATTTTACGAGCA	1	
Ad05A19717	Aradu.A05	97678323	97678342	AAGTTTCATTTTATGGATGTGTGG	GCCACAAATTGGAGGAAGAC	1	
Ad05A19722	Aradu.A05	97688451	97688465	TTCACAGATTTCAACAAAAATTTCA	ACCGCATCAACCCACCAC	1	
Ad05A19725	Aradu.A05	97693189	97693203	GAGCATCTTCATGCTTAGTTTTAATTC	TGGTGACAACAACGACAACA	1	

ID	Chromosome	SSR_start	SSR_end	FORWARD PRIMER (5'-3')	REVERSE PRIMER (5'-3')	Bands in parents	Polymophism in RILs
Ad05A19729	Aradu.A05	97698238	97698310	CCTTTAATTCTTCTCAAAATCTCTCTT	GGATTTTCTTGAATTTTGACTCTAAG	1	
Ad05A19730	Aradu.A05	97698494	97698535	TTGGTTCAATTTCAAACTTCACA	TGTGGAGTAGCCTGTGTTATGC	1	
Ad05A19731	Aradu.A05	97699933	97699948	ATCGATACGCCATGTTCTCC	GCCATGAACCCCTACTTCCT	1	
Ad05A19737	Aradu.A05	97720806	97720817	GATGACAAATGCTTAGGCGA	GCAACATGAAAGAGAGGAAGAGA	1	
Ad05A19738	Aradu.A05	97725185	97725196	TGACGATGGAGAGACATGGA	AATGTTGAGCCGAGCTGAGT	1	
Ad05A19739	Aradu.A05	97731096	97731125	ACCTACCGTTACGTCGTTCG	TTGGTTTTCTCGGGTAATGC	1	
Ad05A19741	Aradu.A05	97733828	97733841	TGTCCCATCTTTTTCCATCA	ATAAAAGCCCAGGAGGAGGA	1	
Ad05A19743	Aradu.A05	97738068	97738083	TTGCGAAGTTGGGATTTTTC	ACCATCACCAACGAATAGGC	1	
Ad05A19744	Aradu.A05	97738445	97738464	TGCTCTGGATCAAAGGTTTTC	TGGATTGCAACTACCTACCCA	1	
Ad05A19747	Aradu.A05	97755051	97755116	GTGTCACCTCACTCACCCCT	GCAACAGAAGAGGAAGCAGG	1	
Ad05A19748	Aradu.A05	97757731	97757758	TGAAATCACTGTCCTTCCCC	AGCGATGGCAAGAGAGAAAG	1	
Ad05A19752	Aradu.A05	97764809	97764823	TGAAGACGACGAAGACGATG	TCTTTTGCACGCCTCTTTCT	1	
Ad05A19753	Aradu.A05	97765965	97765976	TCCATCACACCTTCAGAAGAGA	GCTAGACTGCTGGAGGATGC	1	
Ad05A19755	Aradu.A05	97766743	97766763	TGGTTCTTTGGTTTTGGTGA	CACTCCATCACCATCTGCAC	1	
Ad05A19756	Aradu.A05	97766937	97766954	GTGCAGATGGTGATGGAGTG	CTGAGAGATATCGTCGGGCT	1	
Ad05A19758	Aradu.A05	97785818	97785832	GCATAAGTGAGACAAGCAGGG	GCTGTGAAGAATCATTGGCA	1	
Ad05A19760	Aradu.A05	97787299	97787310	TTCATTAATCTCCCCCTCCC	TCCTCCCACTCTCTAACGGA	1	
Ad05A19762	Aradu.A05	97789449	97789466	CGTAAATTTCGTCGCACTCA	CCCAAAACCTTTAGCTTCTTGTT	1	
Ad05A19764	Aradu.A05	97790160	97790189	GGGTGAGCAAAAATTGATTGA	TGCAACTGAATTTCCACCTTT	1	
Ad05A19765	Aradu.A05	97794933	97794944	TTTCTTTGTTTTCGTGTGAGTAAA	TGGAAAGAGAGGGTAATAATCAGA	1	
Ad05A19766	Aradu.A05	97796055	97796072	GCTACCAACTAACGCCACCA	AATCAAAACTCCACAACCGC	1	
Ad05A19769	Aradu.A05	97800229	97800258	GCGGAAGATAATAGTCTTTTCAGGT	TGTTAGGAGAGACTAACACAATTGAA	1	
Ad05A19770	Aradu.A05	97800566	97800577	CAAATGGGATAGCACACCCT	AAAGAATTCACCAAACGGTCA	1	
Ad05A19772	Aradu.A05	97801256	97801279	CTGCAATGTCTGATGGCCTA	TGTCCTGTACCTAATGCCCA	1	
Ad05A19774	Aradu.A05	97805071	97805088	GAAGAAATTCTCGCTGTCGC	ATGCTGAAAATGCGAGGAAC	1	
Ad05A19775	Aradu.A05	97805463	97805477	TTGAGGCTGAAGGAGGTCAT	CAGCATCCAAAAGCAATTCA	1	
Ad05A19778	Aradu.A05	97811727	97811741	GCTGGACGCTAAGAATTTGC	TGAACAGAAGCAAACGATGC	1	
Ad05A19780	Aradu.A05	97817976	97817991	TTCTTCCTCGTCGAAGCACT	CCAGGCCCAGGTCATTACTA	1	
Ad05A19782	Aradu.A05	97821926	97821937	TCAACGCATTGAACAGGAAG	AGTGGAGCTTCAAATGGTCG	1	
Ad05A19783	Aradu.A05	97824805	97824816	ACAGGTTGAACCGACTGACC	CTGTTGCTGCAGTGTGATTG	1	
Ad05A19786	Aradu.A05	97831713	97831728	TGAACCGGTAGGTCCAATTC	TGAATTTTCATAATGTGATTCCAC	1	
Ad05A19787	Aradu.A05	97832880	97832894	GTGTTTCCAATGTGGGCTTT	CCTCCCCTTTCGTTTCTTTC	1	
Ad05A19794	Aradu.A05	97861144	97861164	GCTCCTTCGTTTTCCTCCTC	TCAAGTTGATTGGATTCGGA	1	

ID	Chromosome	SSR_start	SSR_end	FORWARD PRIMER (5'-3')	REVERSE PRIMER (5'-3')	Bands in parents	Polymophism in RILs
Ad05A19795	Aradu.A05	97863035	97863049	TGAAGAAGAAGAAGCAGCAAAA	TTGGGTGTAATATGAAGACATTTGA	1	
Ad05A19798	Aradu.A05	97873466	97873483	TGCATTATTGTTTGCATGGAT	ATTTCTTGCAGGCATCTCGT	1	
Ad05A19805	Aradu.A05	97901270	97901281	AAATAAGGCTAGGCACTGCG	CAGCCCATCTCACTCTCTCC	1	
Ad05A19806	Aradu.A05	97901397	97901410	GGAGAGAGTGAGATGGGCTG	ACCCCCAACCTTCATCTTCT	1	
Ad05A19809	Aradu.A05	97913974	97913994	AATTTCGTGCTGCCTGATTC	TCTGAAACAAACACAACCCG	1	
Ad05A19812	Aradu.A05	97918635	97918698	TCAGCCTGACCAAACATCAA	TGTTCAGGCAGTCAATGGAT	1	
Ad05A19823	Aradu.A05	97947598	97947613	TGGAAGATGAAAATATCGGAAGA	TGCTTATGGTGGTTATAGTGGC	2	Yes
Ad05A19826	Aradu.A05	97958771	97958794	TTGGTTGAGAGGGAGTACCG	CAGGGAACACCGACTTTTGT	1	
Ad05A19828	Aradu.A05	97967790	97967804	CCCAAGGATTTTAAAAGCGAA	CATTGTCCACATGCACAAAAC	1	
Ad05A19829	Aradu.A05	97968043	97968057	TTTGTGCATGTGGACAATGA	AACAGTCCTTCGTAAAAACAAAGT	1	
Ad05A19834	Aradu.A05	97979665	97979680	TGCAGAGGAATCAGAAACTGAA	TTCTTGCTGCACCAATTCAA	1	
Ad05A19835	Aradu.A05	97979924	97979941	GGTGCAGCAAGAAAGCAAAT	TTCATGTTGCAGTTTAGGTTGG	1	
Ad05A19836	Aradu.A05	97980687	97980700	TGAGGATACGGAACCTGGAC	TGGTTCGGTTCTAATAATTGGG	1	
Ad05A19842	Aradu.A05	97986767	97986781	TTTCTCCTCCTCATCTTCTGC	CCACATATTGTTACGGCACG	1	
Ad05A19843	Aradu.A05	97987498	97987512	TTTCTGACCGCATCAAATCA	GGCCAAAACCACCACTTTTT	1	
Ad05A19846	Aradu.A05	97990662	97990688	TGTGCGTAAACTCAAGTTGCT	GTGCCAAGTGACGAGACAAA	1	
Ad05A19848	Aradu.A05	98002545	98002556	AAAAACAAATGGCCGAAGG	ATTTCACGGTTCTGCTCACC	1	
Ad05A19849	Aradu.A05	98004252	98004263	CCCTCTCTCTCCCACACACA	AAGACAAAGAGAGGGGAGGG	1	
Ad05A19850	Aradu.A05	98004387	98004402	CCCTCTCTCTCCCACACACA	AAGACAAAGAGAGGGGAGGG	1	
Ad05A19853	Aradu.A05	98007236	98007271	ACGATCATGACGTGCTGAGA	AAACACATCACACCTGCCAA	1	
Ad05A19857	Aradu.A05	98015577	98015588	GCTGTGCTATTCTTGTTCTTCC	TTTGTTCAAAAGGATTTGATTCAT	1	
Ad05A19865	Aradu.A05	98039154	98039169	TGTTGTCTGCTGTTGAAACAAA	TCGGACTACATACAACAGTGGA	1	
Ad05A19866	Aradu.A05	98039313	98039327	TCCACTGTTGTATGTAGTCCGA	GAAAACCAAAATCAGCAAAACA	1	
Ad05A19868	Aradu.A05	98046935	98046949	TGCCATACAATTGAGGACCA	TTTCTCCTGGACGGAATCAC	1	
Ad05A19874	Aradu.A05	98066856	98066875	CGAAAATTAAAGACAAAAATCAAATG	AAATAACAAAACAAACAAACGTTCA	1	
Ad05A19876	Aradu.A05	98074847	98074861	CAAAGGCAGTTGAAGGAAGC	CAACATCACAGCAACGAACC	1	
Ad05A19878	Aradu.A05	98079603	98079614	GCGAAGCCAATTTCTCTCAC	TGGGGATGTATTCTTGCACC	1	
Ad05A19880	Aradu.A05	98092581	98092592	ATTCTGCCACCATCCTCATC	GCAACGACGGAGTGAGAGAC	1	
Ad05A19886	Aradu.A05	98112620	98112633	CTTTGGACGCATAGGTGGTT	TGCCGAAATTATCGTTGGAT	1	
Ad05A19889	Aradu.A05	98114017	98114158	TGTTGTTGAAATTTGTTGTGGA	CTGTGTTTTCTCCCACCACC	1	
Ad05A19891	Aradu.A05	98116221	98116242	CAAAAATCAATAACAAGATTAGGGA	TCCCATCTGTAAACACCCCT	1	
Ad05A19893	Aradu.A05	98123083	98123096	TGAAGCGTAAATACCTCCACA	CTTCGGCGATGATGACAAG	1	
Ad05A19899	Aradu.A05	98169169	98169188	CTGCGCACTTATATGATTGGA	TCAGATATATCCACAAAACACAAAAA	1	

ID	Chromosome	SSR_start	SSR_end	FORWARD PRIMER (5'-3')	REVERSE PRIMER (5'-3')	Bands in parents	Polymophism in RILs
Ad05A19900	Aradu.A05	98169806	98169817	TTGAGCCAACTTTCCTCTTGA	GTTCAGAAGCGAAAAAGGGA	1	
Ad05A19902	Aradu.A05	98181339	98181386	ATCCGTCCATGCGAGTAAAC	TGTCACCGTGTTTCGTTGAT	1	
Ad05A19903	Aradu.A05	98181533	98181544	TCAACGAAACACGGTGACAT	CAAAAGAAAGGCCTGTTTGC	1	
Ad05A19905	Aradu.A05	98194837	98194856	CGAAAAGCTTGTGAAATTCTTG	AGAGTCAACGTTTGGTGCCT	1	
Ad05A19907	Aradu.A05	98201016	98201039	CGAATTTTGTGTAGGTATCCGC	AGATCGGATCGGATATTGG	1	
Ad05A19911	Aradu.A05	98204337	98204399	TACCGTGCTTAAATCCGTCC	AGAGCCACGTTCCTCTAACG	1	
Ad05A19912	Aradu.A05	98205970	98206095	CAGCCAAACATTGTGATATGC	GGATATTGCCTTGTTATGAACG	2	Yes
Ad05A19915	Aradu.A05	98209228	98209242	CAAGACTCATGCATGTCACAAA	GGCTTATTCCGATGTACGTG	1	
Ad05A19921	Aradu.A05	98228568	98228625	TCTTTCTCCTCCTCGTCTTCTG	CCACATACTGTTACGGCACG	1	
Ad05A19923	Aradu.A05	98236214	98236225	GAAATGGATCTTGGTTCCGA	TTGTTCAAAACCTTACCGCC	1	
Ad05A19927	Aradu.A05	98245235	98245252	GCCATTTGTGGGTTGTATCC	CGTGCTTTGGTGTTGTGTCT	1	
Ad05A19928	Aradu.A05	98247980	98247991	CCAGGCCTTGTCACAACATA	ACCCTCCTGCTCCCAAAA	1	
Ad05A19935	Aradu.A05	98260607	98260621	TCCTCTTCAAGTAATTTTGCAGC	TCCTTCTTCTCATCTTCTGTTGC	1	
Ad05A19936	Aradu.A05	98261067	98261081	TGAATGAATGTAGGTTCATCCTCT	CCTCCTCTTCTTCCTCCTCTTC	1	
Ad05A19937	Aradu.A05	98261935	98261950	TGTTTCACTTCGAGCAACAAA	CGTAGCATAGTCGATCTTTAACC	1	
Ad05A19938	Aradu.A05	98266442	98266453	TTGCTTCGTGGAAAGTTTGA	TCTGCCTAAAAGGCTAGCTCA	1	
Ad05A19940	Aradu.A05	98267473	98267493	AAGCTTCACATTTTGTATTGGATAAA	GCAACCATCACTTGCGTATG	1	
Ad05A19941	Aradu.A05	98268737	98268751	GCAACCTAGTGGAATCAATGG	GATGGACAAGGGGTGAAAGA	1	
Ad05A19944	Aradu.A05	98282660	98282679	GCGATGATATTTCCAGCGAT	CTCACACCACATTACCGTCG	1	
Ad05A19949	Aradu.A05	98289134	98289148	TTTTAGAAGAATTATGGGACATTATGA	TTCTTGAGAAAGTTCAAGACTCCA	1	
Ad05A19951	Aradu.A05	98298628	98298642	GTCGTGGTGCACAAAAGTTG	TCGACCTTCCATAGTGGGAC	1	
Ad05A19953	Aradu.A05	98307262	98307369	CACCACATACAACCTTTGGG	TCACGTCAATCCTTCTCAGTG	1	
Ad05A19958	Aradu.A05	98328316	98328335	TTTTGAAACCTTAATTGGCTCC	GTCGAGGGCACAGATTAGGA	1	
Ad05A19960	Aradu.A05	98340833	98340848	CGTCGAAAATATTCGCCAGT	GAGGTATAATGGCGGCAGAA	1	
Ad05A19962	Aradu.A05	98351058	98351072	GAATTAACCGGTTTGCCTCA	CCTTTGGTTCGTCATTCTCC	1	
Ad05A19973	Aradu.A05	98383225	98383249	CGCATTCTTTTCTTTCCTCG	TCTCTTTCTTGTTTCACCTTCTCA	1	
Ad05A19974	Aradu.A05	98383434	98383451	TGAGAAGGTGAAACAAGAAAGAGA	CCTCGTTGTTATCGTTGTCG	1	
Ad05A19975	Aradu.A05	98383651	98383668	CCATGACACCGAAAGTTTCTT	CGGGAACGACTTAGGGTACA	1	
Ad05A19976	Aradu.A05	98385913	98385936	TGCTCCTTTAAAGAGGTCTTGTG	GTCGTCATCGTCGTTGTCAT	1	
Ad05A19979	Aradu.A05	98392557	98392568	GGAATTCGTGCCGATTAAAA	CCCCGAAAAGAATATACGTGA	1	
Ad05A19981	Aradu.A05	98397882	98397896	ACGTGTGCGCGTCTATGTTA	ACTTGCCATGAAATCACCAA	1	
Ad05A19983	Aradu.A05	98400371	98400385	CGTAAGATGATGATAATAATGCCAA	TTCATTAAGCTCCTCTCAATTTTT	1	
Ad05A19984	Aradu.A05	98401133	98401150	CAGAAACCAGCGAGGTCATT	GCATTTAGAACGCGGAAAAG	1	

ID	Chromosome	SSR_start	SSR_end	FORWARD PRIMER (5'-3')	REVERSE PRIMER (5'-3')	Bands in parents	Polymophism in RILs
Ad05A19985	Aradu.A05	98401480	98401537	CAAGAGAGTATTACTGCCGGTG	AGGTTTTGTGGAGGCAAATG	1	
Ad05A19986	Aradu.A05	98401858	98401877	CATTTGCCTCCACAAAACCT	GCTGACACCAGAAACCCATT	1	
Ad05A19989	Aradu.A05	98405313	98405348	CTTCCTGATAGATGCACCCC	TCCCTCAACTAACCCCTCCT	1	
Ad05A19991	Aradu.A05	98405867	98405884	AAGAGGGAGTCAAGAACGCA	ATGCCCGAACCATACAACAT	1	
Ad05A19992	Aradu.A05	98407131	98407255	TTTGCCAAAAAGAAAGAATTG	TCCTTGCTAAAGCTATAAACCAAAA	1	
Ad05A19996	Aradu.A05	98419003	98419032	CAAGGAATTGCAGGGAGGTA	AGGATGGGATTTCCAGTTCTC	1	
Ad05A19999	Aradu.A05	98425589	98425606	CACCAACATTGGTCATTTGC	TCTCACACCAATTTCACATCA	2	Yes
Ad05A20000	Aradu.A05	98425763	98425782	CAAAATCACATTTCCACACCA	CTAAATAGTATTATTGATTGGGGAGAA	1	
Ad05A20001	Aradu.A05	98428053	98428152	TTTGGTCTAATATAAAAATGTGATTTG	AATTGACAATAGGCATCGGC	1	
Ad05A20002	Aradu.A05	98429522	98429539	CATCCATCCAGAGAGCCTTC	AATTTTTAATTGCCGCAAGC	1	
Ad05A20004	Aradu.A05	98456145	98456158	TCAAAAGTTCACAAATTAAATGGG	GCAGAGTGGAGGAATCAAGG	1	
Ad05A20009	Aradu.A05	98468858	98468875	AAAGAGGAACAACAGCGAGC	CGTACTGGGTTTGTCGTCG	1	
Ad05A20010	Aradu.A05	98470364	98470383	ATGGGGTTGTGTGTCCAAGT	GGATCTTGGATGTGCCCTTA	1	
Ad05A20012	Aradu.A05	98478371	98478386	GAGAGTCTGAGAGGGCATCG	TTATCCGTCCAAACCCAAAC	2	Yes
Ad05A20016	Aradu.A05	98498843	98498854	CGGAAAAGCACACCAAACTT	TTTTTCGAAATCTCCTCCCC	1	
Ad05A20018	Aradu.A05	98526409	98526511	GGAGGAGCAGCAGAAGAAGA	GTTGCAGCACAACTTCCTCA	1	
Ad05A20022	Aradu.A05	98541014	98541049	CCATTCAGAACCAAAACCAA	GGCTTACAGAGGAACCAACC	1	
Ad05A20026	Aradu.A05	98563783	98563794	TTTGAAGACACTCACCATAGCC	TCAGAGAACTATTAGACACAAACACTT	1	
Ad05A20027	Aradu.A05	98565420	98565434	AAGTGGGCCACCAGAGACTA	TGTCCTAACAATTTCCGGGT	1	
Ad05A20028	Aradu.A05	98567071	98567088	GGTTCTTGACATTGAAGAGCTG	TTTCATTTTTGATATCTTGCCAAA	1	
Ad05A20030	Aradu.A05	98568260	98568279	GAAAGCCAAGCTAGCCACTG	AGCACGCGACAGTGTAAAGA	1	
Ad05A20039	Aradu.A05	98590823	98590852	TCTCGCTGTCACAACTCACC	CAACGTAGGGAGGAGAGCAG	1	
Ad05A20042	Aradu.A05	98597044	98597058	TTCATCCCAAATTATCAAATGTTTA	TGCATGTAACAATGTTGAAATAAAA	1	
Ad05A20043	Aradu.A05	98599066	98599092	TCATGAGAAGGTAAAACAAGAAGAAA	TCAAAACAATCACAGGCCAA	1	
Ad05A20046	Aradu.A05	98606143	98606156	TGATAGAACTGCCGATCCAG	TCGGACGGTTCGATTTTTAT	2	Yes
Ad05A20052	Aradu.A05	98620364	98620378	AAAATTCTTGCCGAACTTCAA	CGGTCAATCTAACGACTCCTTT	1	
Ad05A20058	Aradu.A05	98634223	98634236	ATCAAGCTCCAGATGCAACC	TTGGGGTTGTCTGTCACGTA	1	
Ad05A20059	Aradu.A05	98635745	98635764	CCTCAGGTGAGTGAGGATCG	CATCCGGAAAGACAAAGCAT	1	
Ad05A20062	Aradu.A05	98641388	98641399	CATGAAACTCAGTAGAGCCCC	CTCAGCCCAAACACTCATCA	1	
Ad05A20066	Aradu.A05	98651978	98651995	GCCTCTTTCCTATCTTCACCC	TGCAAGTGAATTGTTGAATGC	1	
Ad05A20067	Aradu.A05	98671006	98671047	CTGAGTCAAGGCTTAGCGTG	CCAACCTAAGAACTTTGGATCTTG	1	
Ad05A20070	Aradu.A05	98686262	98686279	ACGTGGTGGTTATGGAGGAG	CACAGCCGCTTGATACTGAA	1	
Ad05A20071	Aradu.A05	98688603	98688614	CCTCCACCCTGGGTATTTTT	CATATTCCACTGGCCAACCT	1	

ID	Chromosome	SSR_start	SSR_end	FORWARD PRIMER (5'-3')	REVERSE PRIMER (5'-3')	Bands in parents	Polymophism in RILs
Ad05A20075	Aradu.A05	98705743	98705757	GCAAGTGGTGCAGCATAAAA	GATTTTTGGGTGACGGAAGA	1	
Ad05A20076	Aradu.A05	98709860	98709920	TTTTTGCACTCCTCGTTTGA	TTGTCCCTCTAAGGTAACGTTCT	1	
Ad05A20080	Aradu.A05	98722709	98722722	ACAACCGAGTGGAGTTGGAG	AGGGGGAAATCAGTTTTTGG	1	
Ad05A20083	Aradu.A05	98737532	98737553	AGTTGATTTATGAATTCACCTTTTAGT	CAAAATCTCAAACTCCAGATTCAA	1	
Ad05A20084	Aradu.A05	98738228	98738251	GGTGCATGGTAAAATGGCTT	CGACCCTATGTGTACGATGG	2	Yes
Ad05A20089	Aradu.A05	98795119	98795138	GGATGGTCCGAGTTGATTGT	CGAAAGGAGAGAAATACAAGAGC	1	
Ad05A20098	Aradu.A05	98836382	98836488	TAGGGCTGGGAAATGAATGA	TGCTTTAACTACCCAAAAACTGC	1	
Ad05A20099	Aradu.A05	98849715	98849730	GACACTTGTGGCAGCTTGAA	GAGGGAGAGGAAAATCGGAG	1	
Ad05A20101	Aradu.A05	98860700	98860711	CCAATTCATGCTCAAACCAA	GTGACATTCCGGGTTTTACG	1	
Ad05A20102	Aradu.A05	98864701	98864712	AAGCCCTTTCCAAGCTTTTC	TGTTCGAAATCTCAGCCCTT	1	
Ad05A20103	Aradu.A05	98868900	98868914	GCACCTTGTTTCTGCCTCTC	TGCACCTAGAGGTCCGTCTT	1	
Ad05A20106	Aradu.A05	98896363	98896374	CGAAGTTGGTTATTTCGGGA	TAAAGCTGAGGTGGCAAGGT	1	
Ad05A20107	Aradu.A05	98902122	98902136	TTTTCGTGACCCATGCATTA	GGCCGAATTACTCTTCTCCC	1	
Ad05A20109	Aradu.A05	98910711	98910728	CAGGGAAAAAGGCACTCTTG	CTTTGTGAAGTTGCCAAGCA	1	
Ad05A20110	Aradu.A05	98913829	98913846	CATCAACCTCCTCTTTCCCA	CTCCCAAGACTCAGAGGTGG	1	
Ad05A20112	Aradu.A05	98930390	98930436	GGTTTCGCGCATTTAACATT	CAACCATGTCTTTACATTGCCA	2	Yes
Ad05A20113	Aradu.A05	98958538	98958562	GTCACTGGAGGGTGGAGGTA	CCCAACAGAGGAACATGACC	1	
Ad05A20116	Aradu.A05	98993934	98993949	AGGAGCCTTGAAAAATGGGT	CTCCGGTTCTCATCCCATAC	1	
Ad05A20117	Aradu.A05	98995416	98995445	GATTGCAAGAAATCGAAGCC	AGCATGAAAGTAAAAGGCGG	1	
Ad05A20118	Aradu.A05	98995582	98995638	CCGCCTTTTACTTTCATGCT	GGTGGATAGAATGTAGCGATCC	1	
Ad05A20119	Aradu.A05	98997157	98997168	GGATCCACGAGACGACATTT	GCGTGAGGTTCTGTGTGGTA	1	
Ad05A20120	Aradu.A05	98999109	98999214	TGGTGGGGTTTAGGTTCCTT	AGCATGAAAGTAAAAGGCGG	1	
Ad05A20121	Aradu.A05	98999351	98999386	CCGCCTTTTACTTTCATGCT	AGTGCAACTTGTAGCGACCC	1	
Ad05A20122	Aradu.A05	99001485	99001496	TATGGTTTAGGCTTCACGCA	CATCATGAAATCCTTCAACCC	1	
Ad05A20123	Aradu.A05	99013978	99013992	CTACTACGCGAGTGATGCCA	AAACATCCGCAAAACGAAAG	1	
Ad05A20125	Aradu.A05	99015905	99015922	GAGTGAGAAATGGGCAAAGG	TCCAGCAACATAAATTCACAGC	1	
Ad05A20126	Aradu.A05	99017361	99017375	TTGAAACTTATTGAAAACGTTAAGGA	TCACATGCAATTGCCTTCAT	1	
Ad05A20130	Aradu.A05	99023872	99023911	TGAAGCACGACGATAGCAAC	ATCACGGCTACCATGACCTC	1	
Ad05A20133	Aradu.A05	99031843	99031854	ACTCCGGAACGAGTTTTCCT	CGGTTCGAATTAGTGGGAGA	1	
Ad05A20134	Aradu.A05	99035918	99035935	TACAGTGGAGAGGGAGTGGG	GTCTGGGGTCGAAATCAAAA	1	
Ad05A20135	Aradu.A05	99037422	99037436	GCTAATGTGGCATCCTCTCC	ACTGGTCGACTTCCACAACC	1	
Ad05A20137	Aradu.A05	99041013	99041105	GGAATGTTTTGGTTTGCTGC	TATTGACAAGCACAACCCCC	1	
Ad05A20139	Aradu.A05	99049493	99049507	GCTAATGTGGCATACTCTCCAA	ACTGGTCGACTTCCACAACC	1	

ID	Chromosome	SSR_start	SSR_end	FORWARD PRIMER (5'-3')	REVERSE PRIMER (5'-3')	Bands in parents	Polymophism in RILs
Ad05A20141	Aradu.A05	99053011	99053103	TTGTTTGGTGCCCTTGATAA	TCCAATACACCAGCAAACCA	1	
Ad05A20143	Aradu.A05	99103095	99103109	TTGATGACGGTGGAAAGATG	TCCGTCAACTTTTCAATCCA	1	
Ad05A20144	Aradu.A05	99107990	99108001	AGATGCTCACGCATCAAGAA	AGGAGCCTTGAAGAATGGGT	1	
Ad05A20147	Aradu.A05	99115381	99115398	GGATCATTCCCACATAACCG	CTTGATCATTCTGGGGCTGT	1	
Ad05A20149	Aradu.A05	99131221	99131235	CAGCCTTCCCCACATACCT	TGGCTTTTCTTCCCCCTAAT	1	
Ad05A20150	Aradu.A05	99131421	99131442	CACGTTGGTGTTTGGTGAAG	GTCATTTGCATCACCCTCCT	1	
Ad05A20155	Aradu.A05	99154376	99154393	AAATGGAGTTCTAATCCGCAA	AATGGAAATTCACCGCAAAA	1	
Ad05A20156	Aradu.A05	99154883	99154921	GCAAGGAGCTAGGTCAACGA	AAAATTTACGCATGAATCAATAAAA	1	
Ad05A20166	Aradu.A05	99188061	99188080	TCCACCCTCATTTGAGATCC	TGAAGTTCAGATTTGCGACG	1	
Ad05A20169	Aradu.A05	99198741	99198775	CTCTCTCAACTCCCGTGGAC	TGCATGCACAATATTCTTGAAA	1	
Ad05A20171	Aradu.A05	99202187	99202200	TGGATTGGTCTTGTTGTGGA	ATCTTGGGGAGAGGGAGAGA	1	
Ad05A20175	Aradu.A05	99215571	99215600	AGGGTTTGCACATACAAGGG	TTTTTGAATAAAAATTCGAGCTG	1	
Ad05A20182	Aradu.A05	99240371	99240382	GATCAGACATGCCGAAAGGT	TCCAACATCTAAACCCCAAAA	1	
Ad05A20185	Aradu.A05	99246452	99246504	CAGGGTATCCTTCACAGTTCAA	CATGATTTTGTTTCTGGCTCC	2	Yes
Ad05A20189	Aradu.A05	99259805	99259819	GCTTCATGGGAAGATTTGGA	AGATCACAAGCAGTTTCGGC	1	
Ad05A20190	Aradu.A05	99270374	99270385	TCAATAAATTCATCACGTGGAAA	TGATCGTGCAAGTTTTTCAGA	1	
Ad05A20201	Aradu.A05	99292503	99292516	CTCGAGGACGAGCAAAATTC	CTTGGAGGTGGAAGCTTTTG	1	
Ad05A20202	Aradu.A05	99292989	99293004	GTGCTAGGTGGGAGACAACC	CCATGCTTTCGCGTAACTTT	1	
Ad05A20204	Aradu.A05	99300676	99300747	AAGCCACACAACACAACACAA	CTCAGCGGTCAATAATGCAA	1	
Ad05A20210	Aradu.A05	99329290	99329303	TAAGACAACAGACCCCAGCC	TCTAACCCGACCCTAAACCC	1	
Ad05A20211	Aradu.A05	99330636	99330653	AATCTGCGTAACAAAATCTTTCA	GTGTGGAGCATTGCAACAAG	1	
Ad05A20212	Aradu.A05	99331118	99331141	TCATTGCCTCACATCTCATCA	GCACCACTCACCACATCATC	1	
Ad05A20213	Aradu.A05	99333529	99333543	CCACCACTGTTTCAGACCCT	CACTTTTCCAATGATGGGCT	1	
Ad05A20214	Aradu.A05	99334961	99334981	GCATTGGGCTTGTAACACCT	CACATCAAGTCCTGCCAATG	1	
Ad05A20222	Aradu.A05	99357818	99357868	TTGTCCAGCTTGTAGGAGTTGA	TTCAGATCCCCTCAGGTTTG	2	Yes
Ad05A20224	Aradu.A05	99371834	99371851	GCAAGAGCATCCGCTTTAAC	TCAGAGGGTCAAGGAGGCTA	1	
Ad05A20225	Aradu.A05	99373934	99373951	TACCACCCCCTCTCTCTCCT	CGTGCCTTACATTTCACAAGC	1	
Ad05A20243	Aradu.A05	99419066	99419083	ATGATTCATCAGGCTTTGGC	CGTTGGTGATGTGATGGTGT	1	
Ad05A20244	Aradu.A05	99427771	99427788	AGAGTGTGAGAGAGAGCGCC	CTCGCTTCACAAACACCAAA	1	
Ad05A20248	Aradu.A05	99441737	99441750	TTGCAGAAAAGGAAAAACGAA	CGCGGGTGTGATTTAAACTT	1	
Ad05A20253	Aradu.A05	99449036	99449086	GCCTCGGGAATTTAGATCAC	GAAATTTCACTTGAGAGTTGAGAGA	1	
Ad05A20254	Aradu.A05	99449645	99449664	CAAAGTCTTCTTAGCCGCCA	TGAGAGAGCGTGTGATGGAG	1	
Ad05A20257	Aradu.A05	99482029	99482040	CCTACCCACCTCCAACAATC	CCTCTTCCTCGTGTTGAAGC	1	

ID	Chromosome	SSR_start	SSR_end	FORWARD PRIMER (5'-3')	REVERSE PRIMER (5'-3')	Bands in parents	Polymophism in RILs
Ad05A20259	Aradu.A05	99492268	99492291	TGATTGAAAAACGGAATTTGG	CAACTCAATCCCAACCGAAC	1	
Ad05A20262	Aradu.A05	99503915	99503936	CTGCAAAGAGCCAAGGTTGT	GGGTTCGAACATTGGTGAAT	2	Yes
Ad05A20268	Aradu.A05	99517473	99517507	TGGCCAAATTCAAAGAGAAGA	ACAAAACAAGCCAAACGTGG	1	
Ad05A20270	Aradu.A05	99520772	99520786	TTTCACAATGAACACACACATCA	ATATTCAGGGGAAGCCAACC	1	
Ad05A20276	Aradu.A05	99533306	99533365	TTTGCTTGTGAATTTTGCATT	AAAGACAAATTTGGTATTATTCGTAAA	1	
Ad05A20281	Aradu.A05	99563742	99563755	AGGGCATATTTCCACTTCCC	GTCTAATGGCGAGTTAGCGG	1	
Ad05A20290	Aradu.A05	99581951	99581981	CGCATACAAGCGATTAAGGC	AGGAAGAAGAACGTGCACCA	1	
Ad05A20291	Aradu.A05	99582582	99582596	ATCGTTTGGGTGAATTCCTG	CCGTTTCCTGTTTTTAAAGCG	1	
Ad05A20293	Aradu.A05	99583660	99583675	TGATTAGCAGCAGGGAAAGC	CCAAACCTAGCCGCCTCTAT	1	
Ad05A20300	Aradu.A05	99599577	99599588	CCACCACTTCGAATGGATTT	ATGGTGCAGTGTTGGACAAA	1	
Ad05A20301	Aradu.A05	99601617	99601656	TCGGGTTAAGTACGATTTTGG	CCGACGACAACGAAGCAC	1	
Ad05A20302	Aradu.A05	99603920	99603934	TTCAAGTATATGCATCAAAACTCTTC	TAAGAGGGCCCTTGGACTTT	1	
Ad05A20304	Aradu.A05	99604814	99604828	TCAAATGATTGCAAACCAAAA	GCAGTAGCATCTCCCTTTCG	1	
Ad05A20307	Aradu.A05	99607772	99607783	ATCATTTGCCTTGTGCCTTC	GTTGCTGGGTAAAGACCGAA	1	
Ad05A20308	Aradu.A05	99613231	99613248	CACCACTCAAGCTCAAACCA	TGGGCATGTGTTGAGGAATA	1	
Ad05A20309	Aradu.A05	99613600	99613611	AGCCCCACAAGTTTCAAAAA	CCATTTTGACGGCCCTACTA	1	
Ad05A20312	Aradu.A05	99625358	99625381	ACAACCCACCATGCAAAAAT	ACGGACTTGAGAGGCAAGAA	1	
Ad05A20314	Aradu.A05	99639579	99639590	TGAATCATGGACATTTTTCTTTTT	TTGACATGTTTGTCGAATGAA	1	
Ad05A20316	Aradu.A05	99652354	99652367	CATGGCATGCATATTATCATCA	AGACTTAACTCATTATCTCGTGAGC	1	
Ad05A20317	Aradu.A05	99653939	99653998	TCTTTCTATCACAAAAACTAACATGC	TGTGATCATATGAATTTTTACTTGAA	1	
Ad05A20323	Aradu.A05	99659883	99659897	GCACAAGTCAGCATCGAAAA	TTTCCTTAGCTTCTTCTCCCA	1	
Ad05A20327	Aradu.A05	99698482	99698493	AAATTCGGTTCATCCCTTCC	GCCATCTTGCTCTTTGCTCT	1	
Ad05A20330	Aradu.A05	99705890	99705913	CGGTAACAAGGCCTCAAAAA	TGAGTTTTGAGTGTGCGTCC	1	
Ad05A20333	Aradu.A05	99717583	99717594	AACCGAAGCGAAGAAACAAA	TGGGAAAGAGAGAGTGTGGC	1	
Ad05A20336	Aradu.A05	99727541	99727567	TCTTAAGGGGAAAGGGGAGA	TGGCATTGATAGAAGAGTTGGTT	1	
Ad05A20339	Aradu.A05	99732867	99732888	TGGGTCAGAAAACACACGTC	GCAGTGTTTCCTTGTTGGGT	1	
Ad05A20340	Aradu.A05	99733332	99733355	CGGGTGTGTTGTGATTCTTG	GGAAGGGTCAATCTCCCATT	1	
Ad05A20342	Aradu.A05	99734569	99734599	TTGTTTGTTTCTGATATGCACC	GGCCATTACCATCCACAAGT	1	
Ad05A20343	Aradu.A05	99737313	99737330	CTATCGACACCTCGAAAGGC	GATGCAATGAGGAGAGCACA	1	
Ad05A20344	Aradu.A05	99738525	99738554	TTTTTGTCTGCTTTAGACCGA	CGGAAGTTAAAAGGCCCAAT	1	
Ad05A20349	Aradu.A05	99784298	99784319	TTTGGCTGATTCCTGAAACC	TCCTTCACAAATTTCGGAGC	1	
Ad05A20355	Aradu.A05	99796158	99796169	CGCTCACCATCCTTCTTCTT	TTCAATTTCAGTGGATGATCG	1	
Ad05A20362	Aradu.A05	99813266	99813307	TGCATGAAAGAGAAAGGAAAA	GGTTTGTTTCCCCATGAAAG	1	

ID	Chromosome	SSR_start	SSR_end	FORWARD PRIMER (5'-3')	REVERSE PRIMER (5'-3')	Bands in parents	Polymophism in RILs
Ad05A20363	Aradu.A05	99815350	99815363	GAGAGTTTTACGCCGGTGAG	GAGAAGATGGTAAGCGCTGC	1	
Ad05A20364	Aradu.A05	99822932	99822958	AATCACAATGGGCTGAGAGG	TTCAGGAGGCTAAGGCAAAA	1	
Ad05A20365	Aradu.A05	99831889	99831918	TGCCAGAAAGGAATTGATCC	TGCTTTGTGTTGCATAACTGG	1	
Ad05A20369	Aradu.A05	99838768	99838785	CGGATGGTACACAAATCGG	GACCGGATCGACTAGGTGAC	1	
Ad05A20371	Aradu.A05	99839795	99839809	CGCTCCTTCACCGTTACTTT	GAAACACGCGAAGAAAAAGG	1	
Ad05A20374	Aradu.A05	99854852	99854866	AGAAAATGAATTTAGGCATAGACTG	CAACAAAAAGCAAATTACGATCA	1	
Ad05A20381	Aradu.A05	99866568	99866579	GTAACCACACGTCCCCTGTC	GGACTCGTAGTGGCCATTTG	1	
Ad05A20387	Aradu.A05	99872349	99872363	GCGTGGGTTGAAAGTCCTAA	TGACTGAGGGAATACGGAGG	1	
Ad05A20394	Aradu.A05	99887781	99887794	CAGGAACGAACTGCTTTGGT	AATCACGTTTCCTGAGCGTT	1	
Ad05A20396	Aradu.A05	99898866	99898917	GCCGTGGCATTCATTTTATT	TGGTTATTTTGGATAGTATGAGCG	2	Yes
Ad05A20402	Aradu.A05	99904319	99904330	AAGTATAAAAATCCCTCCCCC	GATTAATTAGAAGAGTTTTGTTAGGCA	1	
Ad05A20403	Aradu.A05	99909880	99909900	TGAGTGTTTTGCAGAGAATGAAA	TCCGAATCACTATTGTCCCA	1	
Ad05A20405	Aradu.A05	99914008	99914025	TGCAAATCTAATATCTTCACCGA	TGGTGAAATATGGGCAAACA	1	
Ad05A20407	Aradu.A05	99915947	99915976	CGGTACTGCGTGTGAGAAAA	TCGAAGAGGAGGACGAGGTA	1	
Ad05A20408	Aradu.A05	99920942	99920956	CGGGATTAGCACACCTATCG	CTTGCCGGTAAGTTGTGGAT	1	
Ad05A20412	Aradu.A05	99942362	99942376	CCTCCCTCAACACCACAACT	AGAGAGAAAGGCTGGGAAGC	1	
Ad05A20413	Aradu.A05	99942591	99942606	ACACCATCAAGGACTACCGC	CCTAACCTTGCTTGTTCCTCC	1	
Ad05A20414	Aradu.A05	99947088	99947099	CTAGACTCGGCTTGCGAAAC	CTTATCGATGGAGGAGCCAA	1	
Ad05A20418	Aradu.A05	99951992	99952007	TTCAATTCACTAAGTATGCAGATGG	TTTGACATCCATTACCTAAACGG	1	
Ad05A20419	Aradu.A05	99953435	99953449	AGGTCTGATGGGTTCAATGG	TTGTGTTTCTTATTTTTCTTCCCTC	1	
Ad05A20421	Aradu.A05	99957978	99958072	AGGTTGCTTTGGACAACCAG	GGATCAAAGAAAAGCCTCCC	1	
Ad05A20422	Aradu.A05	99961750	99961770	GGTAAATGCCACTTCTCGCT	AGCTGACCTGCTAACCCAGA	1	
Ad05A20424	Aradu.A05	99973187	99973200	CCGGAATCTGTGAGTTCCAT	TGATACGGATACGGTCCCAT	1	
Ad05A20427	Aradu.A05	99979890	99979903	TCAACTCCTCTCGCACATGA	CGCCATTTTCATTCTCCTTC	1	
Ad05A20428	Aradu.A05	99994490	99994503	GAGATTCTTTCTTTGATTTTTCCAA	ATGCAATCCCCATTTCATGT	1	
Ad05A20431	Aradu.A05	99999972	100000019	TGAAATTGTTTGTTATGTTAAAAGTGT	TCCGAGAACCCTACAAAACG	1	
Ad05A20432	Aradu.A05	100003384	100003395	TATACGGCGTCGTTTAAGCC	AGAATTCAATGGCGTTGTCC	1	
Ad05A20433	Aradu.A05	100003518	100003535	TATACGGCGTCGTTTAAGCC	AGAATTCAATGGCGTTGTCC	1	
Ad05A20434	Aradu.A05	100007572	100007589	TTCATCTGATTGACCCACCA	TCTCAATATCCCGTGCTTGT	1	
Ad05A20436	Aradu.A05	100014533	100014574	TTTCGAAATGACTAAATGTGTCAAG	ACTATCCGCAAAGCAAGCAT	1	
Ad05A20437	Aradu.A05	100017589	100017600	CATTGGTCCCGAAGTACCAT	TGGATCAGACAAGTATGCCG	1	
Ad05A20438	Aradu.A05	100022317	100022331	ACCTGCTATTATTGGTGGCG	CGACTTGTTCCTCTGCTTCC	1	
Ad05A20440	Aradu.A05	100025799	100025818	TTAACCTTTGTGTAAAACTCCGA	GATGAAGCCCGGTTCAATTA	1	

ID	Chromosome	SSR_start	SSR_end	FORWARD PRIMER (5'-3')	REVERSE PRIMER (5'-3')	Bands in parents	Polymophism in RILs
Ad05A20441	Aradu.A05	100031680	100031704	GAAGATCCAGGCATTTAAGAGG	TTGGCATCAGCAAATACAGTG	1	
Ad05A20444	Aradu.A05	100036767	100036778	AAATGGGACCACAAATTCAGA	TGAAGGGATGGCTCAAATTC	1	
Ad05A20454	Aradu.A05	100069430	100069444	GAAAAAGGATCTTGCGTTGAA	TTGAGATCCTCACATGACGC	1	
Ad05A20456	Aradu.A05	100078247	100078290	CACACAAACACACCCACACA	GCTTTTTCTTCGTTTTCCCC	1	
Ad05A20457	Aradu.A05	100078652	100078679	GGAAAACGAAGAAAAAGCCC	ATTGTTGAGCGGCTACTCGT	1	
Ad05A20461	Aradu.A05	100083751	100083764	CATCCACCTCCATGAGATCC	AGCGTAGTGGCTACGAAGGA	1	
Ad05A20462	Aradu.A05	100085814	100085829	GCTCCTAACCCAACCTGTGA	AGTTCTGCAGCGAAGTGGAT	1	
Ad05A20464	Aradu.A05	100100060	100100085	ACCATTTTAAAAGCGCGACA	GCAAAGCAAAACAATGCAGA	1	
Ad05A20472	Aradu.A05	100132057	100132072	GGTTCCATCACACTCACACG	TGCCAAGTATGCAGGACAGA	1	
Ad05A20478	Aradu.A05	100146628	100146677	CCGGTGTGTGCTCTCTCTTT	ACAATTGCATTCAACCCCAT	2	Yes
Ad05A20495	Aradu.A05	100187040	100187060	GGGGATCCCAAGTACAGTCA	GGACTTGTGTCTCCCTTTCG	1	
Ad05A20498	Aradu.A05	100194421	100194479	CGTCATCTTTTTCTCTTCCTTTTT	TGATGATCATGATGACGAAGG	1	
Ad05A20499	Aradu.A05	100196163	100196186	ACCAAGGAGAGGAGGAGGAA	CGCGGGGCTTTACATTATT	2	Yes
Ad05A20506	Aradu.A05	100215948	100215962	TGAATCCATGCCATCTTCAA	AAAGCTGCAAGCTCGTTAGG	1	
Ad05A20508	Aradu.A05	100219511	100219528	TGACTCAAGATGGTGTGGGA	AGATGGCAATCCGGTAGATG	1	
Ad05A20509	Aradu.A05	100221309	100221356	ACGAGGAGCTGAGCAACAAT	CCCTACCTTAGCCAAACCCT	2	Yes
Ad05A20510	Aradu.A05	100227408	100227422	TCTGCGTGATGTTTGTTTGT	GCGTCATATTGGCAAGGACT	1	
Ad05A20511	Aradu.A05	100228169	100228198	AAAATACCTGTGCGATATTTGC	GAGTTATCCTTGCGCCCAT	1	
Ad05A20512	Aradu.A05	100231202	100231216	AAATATTCGCGTGATGTTTGC	GTTATCCCTGCGCTCACATT	1	
Ad05A20517	Aradu.A05	100236479	100236498	AACTGCTGGTTGCACTTTAGAA	CACATCACCATCAAAATCCA	1	
Ad05A20518	Aradu.A05	100237286	100237300	TTCCTCTTCCTCATCTTCTGC	CAAAAATGTTTCCTTTAATGCTG	1	
Ad05A20521	Aradu.A05	100254193	100254243	AAAACTGTCTTGAAATTGATTTTGTT	CCCAATTCATTGTTTTTCTGG	1	
Ad05A20526	Aradu.A05	100266792	100266803	CACACGCAATAGCTCCTTGA	GTTGCACCACGGTATCACAG	1	
Ad05A20527	Aradu.A05	100269727	100269744	TGCACTAAGTTTTAATTTCGCTTC	TCGCTGTAAGAATTATATCCATGA	1	
Ad05A20530	Aradu.A05	100276496	100276519	GCTCAAGTATCCCATCCAACC	GGTGCACATTCATGTATCCGT	1	
Ad05A20531	Aradu.A05	100277279	100277292	AGTGAGCAAGGGAAACAAATG	AAGCAGCATGATTTTGACACA	1	
Ad05A20532	Aradu.A05	100277892	100277905	GGCCTTCATCCTTTTTCACA	GGCCAAAAGGAGTGGTGATA	1	
Ad05A20533	Aradu.A05	100279312	100279326	CGGGTAATTTGTTGACTGGG	TACCGCCACTGTCGTTATCA	2	Yes
Ad05A20534	Aradu.A05	100280129	100280146	AAAATTTTTCTTAAGGGCCAAAA	CAAGTGAACCAAAATTAACACACA	1	
Ad05A20535	Aradu.A05	100280441	100280455	CCGAAATTATTTAATGATGGCA	TTCCAACGAAGCACACGTAG	1	
Ad05A20549	Aradu.A05	100299892	100299930	CAATTCAATTTCTCCAATAACCA	TTTCCCCTCTTGTTTGAGGT	1	
Ad05A20552	Aradu.A05	100308564	100308578	CCAGAACCATAATCAAGCCAA	TCATGTAGCCAACCCCATTT	1	
Ad05A20558	Aradu.A05	100316590	100316610	AAACATTAACTCCCTCTTCCTCC	GGTGCCAAATCCCTAACTTG	1	

ID	Chromosome	SSR_start	SSR_end	FORWARD PRIMER (5'-3')	REVERSE PRIMER (5'-3')	Bands in parents	Polymophism in RILs
Ad05A20560	Aradu.A05	100323417	100323444	AACACCCACTTTTCCTCACG	GGGTAACGAGGTGCTCAAAA	1	
Ad05A20568	Aradu.A05	100340859	100340894	CCACTCCTGGACCCAATAAA	CTTGGTGCCCCATTGATACT	1	
Ad05A20570	Aradu.A05	100343797	100343850	TGCCAAAAACTCCTTTCGTT	AAGCCAGGTAGCACCCTCTT	2	Yes
Ad05A20571	Aradu.A05	100352718	100352729	ATAGGGAGCTCTTGGGCATT	GGGGATGAGGTTAGGACGAT	1	
Ad05A20572	Aradu.A05	100352834	100352943	GCGCGCACATTGTACTTCT	AGAGAAAAGCAGGGTGCAAA	1	
Ad05A20577	Aradu.A05	100359388	100359403	GATTCGGTTCGGGTAATTGA	ATATCGATTCTGGAGGTGCG	1	
Ad05A20581	Aradu.A05	100362260	100362274	TTCTCACCCCCTAAATTCCC	AATCAGCGAATGAATCCAGG	1	
Ad05A20585	Aradu.A05	100378859	100378876	CATGTTATTTTAGAAGGAGTTTGGT	TCATCATTCGTCACGGAAAA	1	
Ad05A20586	Aradu.A05	100379167	100379182	TTTTCCGTGACGAATGATGA	CCGAACCGGACCATATACAC	1	
Ad05A20590	Aradu.A05	100385684	100385695	CTCTCCATCAAACGCTACCC	CGTATCCACGAGTTCCCACT	1	
Ad05A20591	Aradu.A05	100386444	100386492	TGTTCGTTGCGACACAAGAT	ATGGAGCTTGTTTGTTTGGG	1	
Ad05A20592	Aradu.A05	100386903	100386922	GGTCGAGAAATGATAGGATAACCA	TGAGTCTCAATCTCCAAATCTCT	1	
Ad05A20593	Aradu.A05	100387420	100387431	TCTACCAAACGCTACCCAATG	TGAAAAGTGGTGCTTTGCAG	1	
Ad05A20594	Aradu.A05	100390150	100390167	TGCATAGACCCTTGAAATCCA	ATGCACAATTGTTGCTGAGG	1	
Ad05A20598	Aradu.A05	100404014	100404029	CATACACTTCCAAGCCAGTCAA	CCGAGCCGAACCATATACAC	1	
Ad05A20601	Aradu.A05	100408938	100408949	TTCGTTGTTTGATTGCTTAGATT	GCAGAGAAGTCCCTGTGAGG	1	
Ad05A20602	Aradu.A05	100411036	100411084	TGTTCGTTGCGACACAAGAT	ATGGAGCTTGTTTGTTTGGG	1	
Ad05A20603	Aradu.A05	100411495	100411514	GGTCGAGAAATGATAGGATAACCA	TGAGTCTCAATCTCCAAATCTCT	1	
Ad05A20604	Aradu.A05	100412012	100412023	TCTACCAAACGCTACCCAATG	TGAAAAGTGGTGCTTTGCAG	1	
Ad05A20605	Aradu.A05	100413815	100413829	AACGTTTCGTTCACACTCCC	CATGCATGGCTAAAGGCTAA	1	
Ad05A20606	Aradu.A05	100419744	100419758	TCAAGGTTCCTGTGAGTCCC	TTGCCTGCAAAATTTCTTGA	1	
Ad05A20614	Aradu.A05	100447565	100447579	ACCAAGGAAAGGAGGAGGAA	GTTCCCAGTTAATCCGGTCA	1	
Ad05A20617	Aradu.A05	100451047	100451079	TTCCAACACCAATTCATCCA	GTGCCTCCTAATCCCCAGAT	2	Yes
Ad05A20619	Aradu.A05	100456887	100456928	ACTCCTGGCCCCAATAAATC	CTTGGTGCCCCATTGATACT	1	
Ad05A20622	Aradu.A05	100462687	100462701	GAGGTGAGGGAGAAAGAGGC	CCTTACCCTCTCTGCGTTGT	1	
Ad05A20623	Aradu.A05	100467379	100467436	ACAGAGAGCTCCGCATTGTT	TATTACCATCCTGCCTTCGG	1	
Ad05A20624	Aradu.A05	100470102	100470113	CGATGGCGTAACCGTATTCT	CCGGGATCTGAAGAAATGAA	1	
Ad05A20627	Aradu.A05	100476939	100476953	ATGGCCATAGCAGCTCAAAC	TGATTTGAATAACACAAATGCTGA	1	
Ad05A20630	Aradu.A05	100480052	100480083	TGATTTCTTGGGAAATGCGT	TTTCTCGCTTGCACTTCCTT	1	
Ad05A20637	Aradu.A05	100505886	100505919	TTTAATCGGAATAATCGGCG	AAGCGCATCGAGTCTCTCTC	1	
Ad05A20639	Aradu.A05	100507695	100507708	GGCTCAAGATCTCAGCAAGC	AATCGAATTCCGTCTTGTGC	1	
Ad05A20643	Aradu.A05	100516682	100516737	TGCATTCAACAGCAAGGAAC	ACAGGTTAGCCGTCATGGTC	2	Yes
Ad05A20646	Aradu.A05	100524382	100524439	CGACGATGAGAGAGAAAGGG	GAAAAAGCAAACGGTGTCGT	1	

ID	Chromosome	SSR_start	SSR_end	FORWARD PRIMER (5'-3')	REVERSE PRIMER (5'-3')	Bands in parents	Polymophism in RILs
Ad05A20648	Aradu.A05	100526481	100526498	TTCGTCGTTGATGGTTTGAA	ACGGAAGCGGGATAAGACTA	1	
Ad05A20649	Aradu.A05	100531727	100531750	CAAGATAAACCTCTTGGTCGG	TAGCGTCGTTTGATCCATGT	1	
Ad05A20650	Aradu.A05	100538453	100538530	TTGCCCCTATATTCCTGCTC	AAGGTGGTGTGGGATTACGA	2	Yes
Ad05A20653	Aradu.A05	100553322	100553336	CCGCCATCTCGATGTTAGAT	CCTCAGACCTTAGGGACGAA	1	
Ad05A20655	Aradu.A05	100560029	100560085	TCGTTAGAAACAAGCAAAATGAA	CACACACGAGTCGTAAGTCCA	1	
Ad05A20656	Aradu.A05	100560207	100560226	CTGATGGGTCTGCTTCCAAT	ATTCTTGGACGGTTCCCTCT	1	
Ad05A20659	Aradu.A05	100561762	100561798	AGCTCAGTACCGCAGCAGAT	GTCACAGCATTGCGTTTAGC	1	
Ad05A20661	Aradu.A05	100569631	100569644	TCTGTGAATTTGTCCCACCA	TTTCGCTTTCAAACACCTCC	1	
Ad05A20670	Aradu.A05	100595099	100595110	AGTGAAAATGGAAAGCCCCT	AAACGGTCAATCCCACTGAA	1	
Ad05A20675	Aradu.A05	100616059	100616092	TAATAACGACGACGGCTTCA	TCACTAACGACTAATAAAAGTGTGGG	1	
Ad05A20677	Aradu.A05	100625606	100625659	TTTGGTCATTGTCAAAATCCTT	TGCTGTCAGTCATTTTACAGGAA	2	Yes
Ad05A20694	Aradu.A05	100669515	100669532	AGTGCCCAGTGGCAAATAAC	GTTGTTGGTGTTGTTGCAGG	1	
Ad05A20699	Aradu.A05	100699839	100699850	TGACTCACAAATTATGATCCGAAC	TTTATTATTTTTATTCGAGTAATTGCC	1	
Ad05A20700	Aradu.A05	100700320	100700331	TCCGTCTTCTCTGGAAATGG	AATGCACAAGAACCAAGAACA	1	
Ad05A20701	Aradu.A05	100700649	100700678	TCCTGTGTGGTGTTAATACGCT	GCAAAGGAAATTCGGTGAAA	1	
Ad05A20704	Aradu.A05	100710658	100710673	GAGCACTGTGGGTACAGCAA	GGGAGACACACAAGGAAGGA	1	
Ad05A20709	Aradu.A05	100726088	100726101	CGTCGATTTGTGGCTCTTG	GTGAGGTGTTGGTGGTGATG	1	
Ad05A20710	Aradu.A05	100730679	100730693	TGACCCACTATCTCTCCCTCA	AGCATCAACCATGCACCATA	1	
Ad05A20711	Aradu.A05	100738429	100738443	TGGTTGAGAGATAAAAACTTGCT	GGCGCTAGATGAGGAGTACG	1	
Ad05A20715	Aradu.A05	100756211	100756231	CGATCCCTATCGTAATTATTCGT	TATGGGACATGGAAAAAGGG	1	
Ad05A20716	Aradu.A05	100758226	100758243	CGAATCTTCTGGTTGTCAGG	GGCATGGGATGCTATTATCAA	1	
Ad05A20717	Aradu.A05	100758750	100758761	TCCTTGCACTAGCAATTCTTCA	CCTATGGGGTCATACACTAACGA	1	
Ad05A20718	Aradu.A05	100759500	100759511	CCATAGGGTGTTAGGTGTCCA	TCGTAGAATCACTAAAAATGGGA	1	
Ad05A20719	Aradu.A05	100759687	100759704	TCCCATTTTTAGTGATTCTACGA	CCAACATCCATGGGAGTTTAGT	1	
Ad05A20721	Aradu.A05	100771286	100771300	AGAAGAGAGGAACGAAGCCC	CAGAAACAGCAGCAACCAAA	1	
Ad05A20723	Aradu.A05	100774678	100774697	TGCTGGAGGTGTAGATGCAG	TGGGAGGTTTGAGCCAATAG	1	
Ad05A20724	Aradu.A05	100776851	100776875	CGCCCAATTCCATTACTTGT	AGAGAGATGGGGAATGGAGC	1	
Ad05A20728	Aradu.A05	100792689	100792706	AAGAAATCACAATGGGCTGG	AGATGGCGAAGACAAATGCT	1	
Ad05A20729	Aradu.A05	100801819	100801833	TCCATGAGGTGGTCAAACAA	CCTGTCAAAAATAAAAACCTCG	1	
Ad05A20733	Aradu.A05	100810654	100810665	AAAGGTATGAGGCAAGAACTCAA	CACGCCTTAGTTTGAGGGAA	1	
Ad05A20734	Aradu.A05	100815130	100815187	GCAGTACTGAGTGATGCGGA	AAGGAGGTTAGCGTGTGGTG	2	Yes
Ad05A20744	Aradu.A05	100846337	100846408	GATCTGCTACAATCTTCTCAAGACA	CATGCAATCAAAACAGAGGC	1	
Ad05A20745	Aradu.A05	100850723	100850758	AATTTTGTTGAGTTGGCGCT	ATGGCCAATCAGAGAGAACG	1	

ID	Chromosome	SSR_start	SSR_end	FORWARD PRIMER (5'-3')	REVERSE PRIMER (5'-3')	Bands in parents	Polymophism in RILs
Ad05A20750	Aradu.A05	100853450	100853498	TCTGTCGTCCAAACGCTAAG	TGTTGATGAGGTGATTGATGC	1	
Ad05A20753	Aradu.A05	100860260	100860275	TTTTTCCTTCATCGGGTTTC	TGCAGTTTTGCAGCCACTTA	1	
Ad05A20754	Aradu.A05	100862102	100862115	TGTCATTCTTAAATTATGGGTTTTT	GCAACAAGGGTTGCAAACAA	1	
Ad05A20755	Aradu.A05	100868457	100868468	CGACCCTCTGTGATGTACCA	GGTTGAATGTCCACATAATTCG	1	
Ad05A20756	Aradu.A05	100869456	100869470	TTGAAAGTGAAGTACCAAAAATTGA	TATGTGTGTGATTGTGGGGG	1	
Ad05A20757	Aradu.A05	100871700	100871717	TGGTTGATTCTAAAATTGTGCC	TATTGATGCCGTGTGTCCAT	1	
Ad05A20763	Aradu.A05	100922406	100922417	GTCAGCAAACGAGAAGGAGG	AATTGATTTGGGTGGAGCAC	1	
Ad05A20767	Aradu.A05	100935607	100935645	GGGATCGTCTTCAACATTGC	TCATGAAAAGTGAAACCGGG	1	
Ad05A20769	Aradu.A05	100945292	100945327	AGTCCCTGTCACCAAAATCG	CCGCTCTCTTCACTCCTCAC	2	Yes
Ad05A20777	Aradu.A05	100976576	100976590	TGGCAACAATTAGTTGACCTTT	TGTCATTTCCCCCAACTATCA	1	
Ad05A20778	Aradu.A05	100978831	100978881	CCTTTTAAAATATAATGCATGTACCG	TGAAATCAATATTCAATGCGTTA	1	
Ad05A20781	Aradu.A05	100992474	100992488	CCTTCGGGCTCTTCTTTTCT	TGAAGAACGAAAACAATTCCTG	1	
Ad05A20782	Aradu.A05	100995011	100995022	CCATGTACTTATGTATGTCGGATCT	TGAGCAATTAAATCTCCACTGTT	2	Yes
Ad05A20785	Aradu.A05	101001213	101001278	CCACACAATGCGTGTTTTCT	GTTGTGAGTGTTGCAGTGGG	2	Yes
Ad05A20787	Aradu.A05	101005914	101005955	TTTCGTTAGAAACAAGTAATAAGGTGA	TTTCAACATTTTTCTTCATCAGACA	1	
Ad05A20789	Aradu.A05	101010707	101010720	AACCGAAAAATTAGTGAATCAAA	TTCTAACATTTTACTTCTCTGCCA	1	
Ad05A20793	Aradu.A05	101016116	101016127	GTCCCTCAATTTCAGCCAGA	TCATTAAAGAGTTTAGATAAAGGCTCC	1	
Ad05A20794	Aradu.A05	101016346	101016360	TTCCTCAAATTTTCGATTTGC	ATGGTGGTGATGGAGATGGT	1	
Ad05A20796	Aradu.A05	101022170	101022190	TGTGTTTGGATGGAAAGAAAA	TTTTATCAAATTAGAACACGCTAACT	1	
Ad05A20801	Aradu.A05	101039817	101039838	GCCCTCTTCCCCTTATTTTG	TTTGGCAAACAGTGATAGCG	2	Yes
Ad05A20806	Aradu.A05	101055740	101055806	TGTAGGTTCATCCTCTTCCAA	GAATTTTCGGTGCATGTGTG	1	
Ad05A20823	Aradu.A05	101118206	101118230	ATTGAAAAGGAAGCTGGTGC	AGTTCCAAGCGGTGCTTTTA	1	
Ad05A20830	Aradu.A05	101125151	101125183	TGGCACTCTTCTTTTCTTTTACG	AAATTTGAGTCTTCCTATCTTCGG	1	
Ad05A20835	Aradu.A05	101157814	101157827	CATGTAAGAGATCGCGGGTT	TCACCGTTTTCCTCTGCTCT	1	
Ad05A20839	Aradu.A05	101161001	101161015	TTTGCACTTTGCACCATTACA	GGCATGTTTGAGCTTTGTGA	1	
Ad05A20854	Aradu.A05	101192615	101192626	GGACCTTGGGTGTGGTAAGA	GGGAAACCGAAACCATACCT	1	
Ad05A20856	Aradu.A05	101219712	101219726	TACCGATGCAATGATGGAAA	TTATCATCAGGGCGAAGAGG	1	
Ad05A20858	Aradu.A05	101229012	101229092	GTGAGGGCAAATTTTGGAGA	TTATGCCAAAACATAAAAGATCAA	1	
Ad05A20863	Aradu.A05	101239357	101239413	CCTTTTTTAAAATATAATGCATGTACCG	TGCACACATTAATAGTGACCCAT	1	
Ad05A20869	Aradu.A05	101259696	101259722	ACAGGGAGTTTGCTGTAGCG	ATCCCTCCTCCTTCATTTCG	1	
Ad05A20873	Aradu.A05	101289456	101289469	AGCATGAACAGTTGGAAAGGA	CACAGAACGCCAGAAAACAA	1	
Ad05A20875	Aradu.A05	101290587	101290612	CAAGTTCATGCATATTCAAGGC	AATTTCATTGATGCCGTGGT	2	Yes
Ad05A20877	Aradu.A05	101294965	101294980	AGAGGTGGGGTGGAGTTAGG	AACCGTAGTCGCTGCAATTT	1	

ID	Chromosome	SSR_start	SSR_end	FORWARD PRIMER (5'-3')	REVERSE PRIMER (5'-3')	Bands in parents	Polymophism in RILs
Ad05A20879	Aradu.A05	101299223	101299236	TATTCTATCCATGGGGCCTG	GGATGATTATGAATTTAACACTCACTT	1	
Ad05A20881	Aradu.A05	101306840	101306857	AGCTTCGTGCTGATAACGTG	AAATAGAAGGGGTTTGAGGCA	1	
Ad05A20886	Aradu.A05	101321237	101321318	TCGAAAAATATCGACGGAAGA	TGCGTAGGATAATAATAATAAATCGTT	1	
Ad05A20887	Aradu.A05	101321472	101321525	TTTTTCAACAGACTTAATAAGCATCA	ATTTGGATCATCCTCAATGG	1	
Ad05A20891	Aradu.A05	101327367	101327381	CATCTTCCAAGTAATTTTGCAGG	TGCGTGCTGATAAGTTCTGC	1	
Ad05A20892	Aradu.A05	101327764	101327778	CAAGAAGAAACTTGAGAAGATAAAACA	TCGCAACAAATTTGGGTGTA	1	
Ad05A20893	Aradu.A05	101328311	101328388	GAGGAAGAAAAGCAGCAGGA	TATTCATGCACGCACGTTTT	1	
Ad05A20898	Aradu.A05	101358857	101358871	CCATGAGAAGCAAAGAGGTTG	TGATGGTGCATGTTTATTGTTT	1	
Ad05A20899	Aradu.A05	101359306	101359329	GAGCAATGTTAGGGGACAGC	GGCATTTATTGGGGTCAATG	1	
Ad05A20902	Aradu.A05	101367257	101367270	CAGCAAGTCTCCAGCACTCA	CCAAGTGTTTTCTTGTGGGG	1	
Ad05A20907	Aradu.A05	101386203	101386216	GCCAAAGGATACGTGCAAAT	AACGAGGATGCACTTCTGAG	1	
Ad05A20908	Aradu.A05	101392714	101392734	CTTCCCCAAAATCAACCAGA	TTGCTAAAAGTTGCTGCTGAA	1	
Ad05A20913	Aradu.A05	101416100	101416111	GGACAATTGGCACTCAATCA	TGCCGCAGAATGATACAAAA	1	
Ad05A20916	Aradu.A05	101423270	101423371	CAGCAACAATAGCAGAGATTGC	TTTTGCAACACAAAAATGGC	1	
Ad05A20921	Aradu.A05	101438644	101438658	ACCAATGTGTCAGGCTCCTC	TTCCAGCCATTTATTAGGCG	1	
Ad05A20924	Aradu.A05	101459824	101459841	CACACTCGCATGGTTAATCG	TGTTTGTCCTTCCTAGTGGTTG	1	
Ad05A20930	Aradu.A05	101481449	101481477	CCTTTTATTCGCAGGTCGAG	GAGGTGGGGAGGTATTACGA	1	
Ad05A20931	Aradu.A05	101483592	101483609	TCTGACAATCATAACATGCAACA	TGCTTGTTTCCTTTATTTCGTTT	1	
Ad05A20933	Aradu.A05	101504652	101504665	CGTCCCAGCATTTCTTCATT	CTCGGAGATTACTTCAGCCG	1	
Ad05A20935	Aradu.A05	101507071	101507088	AAGGGTCAATTTCCCACTCC	AAGCAGAAGCAAGGTGGAGA	1	
Ad05A20937	Aradu.A05	101514378	101514389	TCAACTCCACTCAATGCCAC	TATTGTTGGCTTGGGCCTTA	1	
Ad05A20941	Aradu.A05	101537006	101537043	ATGGCCATTATCTTTTCGGT	ACGATTTTGGGAACCTTGTG	1	
Ad05A21131	Aradu.A05	102133670	102133777	CGGAATGGAATTTGGCTCTA	GCGCGACGGTATTGTACTTT	1	
Ad05A22286	Aradu.A05	105703457	105703600	AAAATTCGAACTTCCGATACTGA	AAACTAATAAAACGCACTCTCGC	1	
Ad05A22322	Aradu.A05	105752345	105752469	CGTGCAGAAACGAAAGAAAA	TCCCACTCAAACGGTTCTTC	1	
Ad05A23198	Aradu.A05	107902859	107902943	CACGCATTGCTTATGCTTTC	CACCAGCACCACCATCAATA	1	
Ad05A23286	Aradu.A05	108099687	108099783	CGTTTCCTGTTTTCAAAGCG	TTTCTCCTCATCTTCTGCTGC	1	
Ad06A96	Aradu.A06	308951	309032	CGGTATTGGTGGTTATTCCG	AACAACACCGGTGCCAGTA	1	
Ad06A384	Aradu.A06	999235	999318	ACTGCCGTTACCAACACCTC	TGAATCGATAACGATAACAATGA	1	
Ad06A656	Aradu.A06	1720345	1720459	TCTGAAAAGTCCGCCAAATC	ATGGTGGAGAATAAGGCACG	1	
Ad06A1523	Aradu.A06	4283297	4283393	CGCGCATTAACTCATTTTGA	GAGGAAGAAGAACGTGCAGC	1	
Ad06A2118	Aradu.A06	6090935	6091106	TATGTTGGGCATTTTGGGTT	GAGGAGCCTTTGAAGGAAAGA	1	
Ad06A2553	Aradu.A06	7397403	7397482	TGATCCTATTCCCCAACAACA	ATCCGGTTTTGACGTAATGG	1	

ID	Chromosome	SSR_start	SSR_end	FORWARD PRIMER (5'-3')	REVERSE PRIMER (5'-3')	Bands in parents	Polymophism in RILs
Ad06A2830	Aradu.A06	8263616	8263717	ACCAGGAGTACATGCAAGGC	TGAAGGCAAAAGTTAAGGGG	1	
Ad06A3468	Aradu.A06	9968367	9968478	GAGGAAAAAGAACGTGCAGC	AAACGCGATGCAAGGTTTAC	1	
Ad06A3852	Aradu.A06	11089077	11089182	CTGGCTCCGAGGAGAGTATG	ACTCCATAACCCCCGTTCTC	1	
Ad06A3979	Aradu.A06	11414505	11414662	GAGCTTGGATTTGTGGGTTC	GCTTCACTCTTCATGTTCTTCG	1	
Ad06A4135	Aradu.A06	11915833	11915964	TTCTGGTGATTTTGATGAAGGA	TACACGCGCTATATTCACGC	1	
Ad06A4196	Aradu.A06	12101070	12101181	CCCAATATAAAGGCCCAGGT	AAAAACTCCGTTTGCTGAACA	2	Yes
Ad06A4451	Aradu.A06	12898974	12899109	CGTGCAGAAACGAAAGAAAA	AACGTAACTACACGTGCCCC	1	
Ad06A4890	Aradu.A06	14778673	14778849	GCTTCGAACCGTTCTTCTTC	TGGAAATCGAGAATAACAAAGAAA	1	
Ad06A5393	Aradu.A06	16534135	16534289	CCCAGCATAAAACCAATTCAA	TGTATGTATGCTGCTGTCTGAA	2	Yes
Ad06A6447	Aradu.A06	21364246	21364395	TTTGATCCTTTTCCATTCCTTT	GGGGGATACCATTTGGGTAA	2	
Ad06A7140	Aradu.A06	26595256	26595352	CCTACCCCTTCTTCCTCCAC	CTAGAGGTTTCTCCGGCCTT	1	
Ad06A7676	Aradu.A06	30867684	30867789	CACGCGTCCCTTCCTATTTA	GAGCGGAGGTGGTGGTTAT	2	
Ad06A7821	Aradu.A06	32246619	32246699	TTGGTTTGAGAATTAGGCATTGT	TTCATGCATCCCAAATTCAA	1	
Ad06A7936	Aradu.A06	33013262	33013377	CAGCACTAGCTTGTCCGAGA	CCCTCTGAGAACCACACACC	1	
Ad06A8185	Aradu.A06	35337353	35337476	CCTCATGCTCTACAGGAACGA	TCGCAGGCTTAGATAGGAGC	1	
Ad06A9949	Aradu.A06	54045073	54045204	CTCATCACCACCGTTGACAC	TCATTTCCTCCTCCCTCCTC	1	
Ad06A10214	Aradu.A06	56350866	56350949	GGAGAAAATGGGGGAGAGAG	GCTCCAGGTGGAGACTTGAT	1	
Ad06A10649	Aradu.A06	60452381	60452480	CCTTTTTCAATCAAAGCACGA	TGCCTTTATTTTGGTTGATGC	2	Yes
Ad06A10668	Aradu.A06	60685560	60685697	TGCTTTGACCACACCAAACT	ACACACTACCCCCTCTCCCT	1	
Ad06A11725	Aradu.A06	69462950	69463079	TGTGAGTACTCCAACAGGCTAGG	TGGAAAGGAAAAAGCGAAGA	1	
Ad06A11924	Aradu.A06	70849086	70849207	TCACCAAAGATTCCTGTGACAT	CCTCCCCTTCTTCTCATTTG	2	
Ad06A12584	Aradu.A06	76264355	76264532	AAACATACCCTCGAAAAATTGC	TCACCTTATGTGGTAAAAATGTGC	2	
Ad06A13507	Aradu.A06	82424464	82424547	TCCCCAATTAGAGGTGTCCA	GGCTCTTGAAAGAATGAGGG	1	
Ad06A13701	Aradu.A06	83382918	83383058	GCCGCTACTGCTGAGAGATAA	GCACACATGGAGGGAGAGTT	1	
Ad06A14432	Aradu.A06	87141029	87141143	TGTCGGAATTTTTCGATGGT	AGATGTTGCAGTGGTGGTCA	1	
Ad06A14770	Aradu.A06	88762128	88762228	TGATTTGCGTTTTGAACAGG	CGTACGTTCGAGTCTCACTCA	2	
Ad06A15098	Aradu.A06	90261859	90261947	ATTTCCCTCCAAAAGCCAAT	CTCCAACCACTCACTCCGTT	1	
Ad06A16982	Aradu.A06	98497851	98497939	GGAACTCGAGGTTGGTGACT	ATCCTTTCCTGAGTGTTGCG	2	Yes
Ad06A17247	Aradu.A06	99455061	99455198	TCTGAATTGAAAATTTCTGCATC	AAATGGTGGCTTGCAATACG	2	Yes
Ad06A17637	Aradu.A06	100886483	100886653	TTCTGCTGCTTCTTCTTCTTCA	CTCAAAATGCGCTTCAAACC	1	
Ad06A18300	Aradu.A06	103006436	103006516	TCCCCAACGTTGTATTTGGT	CTTCACTCTCTCTCCCCTTCC	1	
Ad06A18417	Aradu.A06	103390478	103390566	GGCCTAAACTCCACAAGCTG	CTTAAGTTTTATTCGCGCCG	1	
Ad06A19035	Aradu.A06	105118254	105118334	CATCCCATTGCTATCAACCC	GGGAATATTCAATTTAGGAATGG	1	

ID	Chromosome	SSR_start	SSR_end	FORWARD PRIMER (5'-3')	REVERSE PRIMER (5'-3')	Bands in parents	Polymophism in RILs
Ad06A20472	Aradu.A06	108679224	108679364	GGGGGTATGGCTAGAAATTGA	TGAGTTTGTGAGTGAGTGAGTGAG	1	
Ad06A20587	Aradu.A06	108927575	108927734	GAAGGAGAAAGAGAAGGCAAGA	TTTTTCTTTTTCCGTGCCTC	1	
Ad06A21007	Aradu.A06	109779119	109779206	TCAGCTAGCCCTCGCTTACT	TGCTTGCCTCTTTACCATTT	1	
Ad06A21851	Aradu.A06	111639669	111639786	TGTTAGGTTCCGAGCTTTGC	AAAATTTGATTGAGAAATAAAACTCCA	0	
Ad07A14	Aradu.A07	67161	67184	CCTTTATAAACACTAGCACCTGGA	GGCTCATTGGCAAGCAGTAT	1	
Ad07A16	Aradu.A07	68861	68875	TACCATTTTCTCTGGGGGAA	GCATCTCACATAGAAGGGGAA	2	
Ad07A17	Aradu.A07	76015	76053	ATTTCACAGGGGAGGGAAGT	GGCTAGGTGTGACTGAGAACG	2	
Ad07A19	Aradu.A07	85129	85156	TTCCTCCTTAATTTTTGTTTGTTTG	TGGTAAGTTCGCAATAAAGATTGA	0	
Ad07A28	Aradu.A07	97016	97048	TAGCTACGTCGGTGTCGTTG	TAGGACGACGACGATGATGA	1	
Ad07A32	Aradu.A07	106282	106386	AAAGGGAAAGCCCAGAAAAA	TGTGTTGGCGAAACAAGAAA	1	
Ad07A36	Aradu.A07	153452	153471	CACACACTTTCACCCTCCCT	TTCTGCCTAGCACCCATTCT	1	
Ad07A53	Aradu.A07	251568	251609	TGCACCGAATACAATGGAGA	TCGTTCTTAAAGTGAGCGGC	1	
Ad07A55	Aradu.A07	255271	255285	ATGGGAGTTGCTTTTTGGTG	CGTCACTAGCTTGACGGTTG	1	
Ad07A57	Aradu.A07	269375	269395	CTGGCTCAGAAAGAAGTCGG	TTCAGGAGGCTAAGGCAAGA	1	
Ad07A63	Aradu.A07	313709	313726	AGAGGGTCCAGGAGGCTAAA	ATCATCCATGCCTATACGGG	1	
Ad07A64	Aradu.A07	321907	321921	TCTTGTTGCCATAGCTGCTG	CATGGCCACGATTAGACCTT	1	
Ad07A69	Aradu.A07	346625	346642	TTTGTGGATGTGCTTTCTCG	TACAATCAGCCACGCGATAC	1	
Ad07A71	Aradu.A07	348306	348332	TCAGGCTCAAACTCAAGGGT	TGAATCCTACGTACCTCCGC	1	
Ad07A78	Aradu.A07	366825	366842	TTCATGCACCATTTCTTGGA	ACCAGGTTCAATCCTTGTGC	1	
Ad07A107	Aradu.A07	464220	464237	TGTTTAAAATTTTCGTAGTTCGC	TTCCATTTCCTGCTTTCACC	1	
Ad07A108	Aradu.A07	471440	471459	TCGTTGTGTTTTTGTCGAGC	TACCAAACACGCGGATAATG	0	
Ad07A115	Aradu.A07	484248	484274	CTTGCTTAGCTCCATCGCTT	TGGCACATGACACATGAAAA	1	
Ad07A120	Aradu.A07	509201	509218	TGCAAATGAAAAATTTCTGGA	AACACACAAATAGATCTTCAAAGCA	0	
Ad07A123	Aradu.A07	511555	511569	GAACCATTCCCATCCATGAC	GAAAAGCAAGAGCACGTAGAAA	1	
Ad07A133	Aradu.A07	553466	553480	TTTTGTCAACTCAAACCGTAATAA	ATCACAAATATTGCTGGCCC	0	
Ad07A141	Aradu.A07	573950	573964	AGAGGGAAGGGTGAAAGGAA	TGCAAGTAGACTGGCTAGGGA	1	
Ad07A150	Aradu.A07	601944	601958	TTTAAAGCACTGGAGGAAGATG	TGTTTCGCTTTCTTTATTATTGC	1	
Ad07A162	Aradu.A07	646025	646039	ACTGAGCAGGTAAAGCCACA	GGATTTGGCACTCAAAGCTC	1	
Ad07A165	Aradu.A07	647284	647319	TTGCTTTAATAAAACTGAATTGGA	AACACTCAATCTGTCAATCATACATTT	1	
Ad07A167	Aradu.A07	655534	655548	TGAGACCCAAATCCAAAAGG	TTGGTGCCATTGCTTTGTAA	1	
Ad07A169	Aradu.A07	657018	657032	CAATGGGTAGAGCCTTGCTT	TGAGGAAATTCGAAAAAGGAAA	1	
Ad07A194	Aradu.A07	900283	900297	GAGACTTACGCCCTAGCCAA	ACGACATTTGGGATCGCTAC	1	
Ad07A201	Aradu.A07	923304	923324	AGCTAAGATTGTGCGATGCC	TGGTATACGCGCTGTTATCG	1	

ID	Chromosome	SSR_start	SSR_end	FORWARD PRIMER (5'-3')	REVERSE PRIMER (5'-3')	Bands in parents	Polymophism in RILs
Ad07A206	Aradu.A07	934748	934762	ACGCTTTGGCAGCACTCTAT	TCTTTCCCCAACTTGCTCTT	1	
Ad07A207	Aradu.A07	935049	935072	TGGGGACCCTAGATGGGTAT	TCCCCTTCTTCTCTTTGTGG	1	
Ad07A216	Aradu.A07	949920	949934	TCCCTAAAATTGAATGCGCT	TGAGAGGATCCATTTCCAACA	1	
Ad07A222	Aradu.A07	960219	960233	TTGCTGCCTTTTACCAAACC	TCTAAACTAGGTCAAATAGCAGAGC	1	
Ad07A225	Aradu.A07	969763	969777	TGACCGTTTTTAAAGAAAACAAA	TTAGGGTTTTGTGCAATGGA	1	
Ad07A235	Aradu.A07	996024	996038	TGCAACTCAGTGACCAGACA	AGCTAGCAGGGGTGTTGCTA	1	
Ad07A241	Aradu.A07	1007653	1007667	TGCAACTCAGTGACCAGACA	AGCTAGCAGGGGTGTTGCTA	1	
Ad07A251	Aradu.A07	1041849	1041902	GTTGGGCCCAGTTATTCAGA	CATGTTCCAAATATCATGCCA	0	
Ad07A259	Aradu.A07	1064442	1064474	ACCCGTCAAATCCACAAGTC	AGGGATCAATATCCTTCGCA	0	
Ad07A266	Aradu.A07	1083980	1083994	GATGACGATGATGCACCTTG	CCAACAAGCATCACAGAGGA	1	
Ad07A267	Aradu.A07	1084200	1084217	TCCTCTGTGATGCTTGTTGG	GTCATTGCCAAATGGGTAGG	0	
Ad07A269	Aradu.A07	1086324	1086362	TTGGATATAGAATGGCAAGACAA	TCGCGTTAATCTCAATGACG	1	
Ad07A290	Aradu.A07	1133680	1133694	ATTCAAATGTTTGCCGGTGT	CGTCTAATGTGGCAGTCAGG	1	
Ad07A299	Aradu.A07	1158604	1158618	ACAGAGATTTGCGCTCCATT	GGCCTTGTTAATTAGGGGGA	0	
Ad07A300	Aradu.A07	1159043	1159072	TAGCCGGCACATTGATACAG	TAACAACAACGCTGCAGGAG	1	
Ad07A307	Aradu.A07	1166573	1166690	TCACTTAGAGATTGTAAGTTCGAGTC	AGTCACGTGCATCTGTCCAA	1	
Ad07A313	Aradu.A07	1191594	1191626	AAAGTTCCTAGCCGTCCGAT	CAAAACACCCTGTATGCTTGG	1	
Ad07A315	Aradu.A07	1194040	1194054	TTACAACCCGTGATGATGGA	AAGTTGCCATTGATGCTTCC	1	
Ad07A318	Aradu.A07	1205818	1205835	GAGAAGAAATCTTGTCGCCG	GCCACACACTCACTCACACA	1	
Ad07A335	Aradu.A07	1240063	1240080	TGAAAATGGCTCCTTCTGGA	GGGCATGTTGAAATCTCTGG	1	
Ad07A351	Aradu.A07	1285507	1285521	GCTGCTTACTTGAACCTCGG	CAAGAATATTGGCCCCTTGA	2	
Ad07A358	Aradu.A07	1292248	1292277	CGAATAAAAAGCGTCAAAACG	AATCACCACAAGAACCAGGC	1	
Ad07A371	Aradu.A07	1324882	1324896	TGCCATCGCATGAGAATAAA	CCATCATCGTCGTCATCATC	1	
Ad07A374	Aradu.A07	1333982	1333999	GCCGTCAAGTAAATAGGACCC	GCGAGAAATCCTCAATTCCA	1	
Ad07A391	Aradu.A07	1374616	1374635	TTTCTTCCAACCACATGCAA	TTGCATGCATGAGTTCCATT	1	
Ad07A394	Aradu.A07	1376431	1376448	CAATTTAATGGCGCCCAA	AGGGTTCGAATTTCGTCTTG	0	
Ad07A395	Aradu.A07	1376631	1376645	CAAGACGAAATTCGAACCCT	GTATCTCTCAGGGGAGGGGA	0	
Ad07A397	Aradu.A07	1377521	1377535	CTCAGGGGAGAGGAATGACA	CACCTGCCAGTTGAGTCACA	1	
Ad07A402	Aradu.A07	1384642	1384730	GCATGGCTAAGTGGTGGTTT	TTGGGTCCCTATATAATTAAGTCAA	0	
Ad07A403	Aradu.A07	1384865	1384942	TGCAGAAACTCAAATAAAAGAAAAA	AAATAAGGAGGGGCCACACT	0	
Ad07A418	Aradu.A07	1444007	1444069	CTTAAAGTTTTCCACGGCCA	AATCTTGTCGTTTTGGCACA	0	
Ad07A419	Aradu.A07	1446949	1446966	TGGAGAAGAAACGAAAGGGA	CCCCCAAGACCCAAAATAAT	1	
Ad07A427	Aradu.A07	1500143	1500157	AAGCAAGCCCAATCAACATC	AAAGGCAAAGAGAGAACGCA	1	

ID	Chromosome	SSR_start	SSR_end	FORWARD PRIMER (5'-3')	REVERSE PRIMER (5'-3')	Bands in parents	Polymophism in RILs
Ad07A429	Aradu.A07	1505996	1506096	TTGATTCATGTAGCCGACCC	TGAAACCCGAAAGAGATGGT	1	
Ad07A432	Aradu.A07	1510697	1510819	GATTTAGAACACCTGCGTCCA	TTCTTCTTCTTCGCTCCTGC	1	
Ad07A436	Aradu.A07	1511891	1512020	AAAGTGTTTGCTTTCATTATTATTCA	GAAAAAGTAAAATGATTATCCCAAAA	0	
Ad07A437	Aradu.A07	1512258	1512275	GGCGTGATGAGGACAAAAAT	CCCTCTCAACTCTCAAAAGATTGT	0	
Ad07A482	Aradu.A07	1619215	1619235	GCCTTTGTTGCCTCTTCTTG	CATCATCACTCCTCTTTACAACCA	1	
Ad07A484	Aradu.A07	1620427	1620458	TTGGAGGCTTCTCTCTTTGC	AAACTAATTTCACCATCCACCC	2	
Ad07A487	Aradu.A07	1628528	1628554	CGAAAGAACGAAGCGAAAAC	CTTCTGGGTTTTGAGCAAGC	2	
Ad07A491	Aradu.A07	1643150	1643170	ACCGCAACAGCAATACCAAT	TTCAAGAGCTTCGGTTTCGT	1	
Ad07A492	Aradu.A07	1644209	1644223	TGGGAACAGAAAACAGAGGG	GCTTTTTGGCCATGTTTGTT	1	
Ad07A495	Aradu.A07	1648874	1648888	GAAGGTGGTCAAGGTCAAGG	TTGGATCAAATTGGAGTGGG	1	
Ad07A497	Aradu.A07	1649857	1649877	TGCTAGTCATATCTGATTTTTGTTATC	GAAGCTCAGATCCAAGCCAG	0	
Ad07A500	Aradu.A07	1668886	1668933	CAGCACATGTCGTTGCTTCT	CTTTTTGGACCGCGAAATAA	1	
Ad07A507	Aradu.A07	1691549	1691581	GATGAATCCCTTTGGAAGCA	TCTCTTCCAAATTCGTTGGC	1	
Ad07A508	Aradu.A07	1691687	1691731	AAACCGCCTTTTATTTCCGT	ATGTTTTGGCTGATCCCTTG	1	
Ad07A515	Aradu.A07	1710389	1710403	ACACCCAAAATTCGTTGAAG	ACTGCACGTTTTCACTGCAC	1	
Ad07A516	Aradu.A07	1710524	1710538	CCTCCTCATCTTCTGCTGCT	ACTGCACGTTTTCACTGCAC	1	
Ad07A517	Aradu.A07	1710663	1710754	AAACGTGCAGTAACGGTTGA	CGCACGTTCTTCTTCCTCTT	1	
Ad07A520	Aradu.A07	1713552	1713569	TCCCGTGATTTGATTTGTCA	TGTTCCACCAGGCCACTAAT	1	
Ad07A524	Aradu.A07	1721119	1721147	AAGACACCATTGCATTCATCA	GGTCTTTGTTTTTGGGAAGGA	1	
Ad07A529	Aradu.A07	1734275	1734289	ACCATTATACCCTCCTCCGC	GTTGCCATTTTATTTGGCCT	1	
Ad07A537	Aradu.A07	1740782	1740802	TGCCTCCCTTGAAAAGAAAA	TGATCAATTTCTGAGTGAAGGAAA	1	
Ad07A538	Aradu.A07	1741002	1741133	CCACAATGATCTGCTCAACAA	TTTCTTCTTTGCAATGGGCT	1	
Ad07A541	Aradu.A07	1744332	1744385	CCACATACTGTTACGGCACG	TGAAGAAAGAGAAAGAGAAGGCA	1	
Ad07A543	Aradu.A07	1745212	1745403	CGTGCAGAAACGAAAGAAAAG	ACTCAAACGGTTCCTCTTCG	1	
Ad07A544	Aradu.A07	1746624	1746668	AAAAATACGGCCCAATACCC	TGAGTGTGTGTTGAAGTGTTGC	1	
Ad07A546	Aradu.A07	1748628	1748642	TCCAAACTCTCCTCACCCAC	TGATCATGGGGTTGTTTCCT	1	
Ad07A548	Aradu.A07	1752562	1752608	TTTTTCGCATATTCAAGGCA	TGACTTTATTTTTGGTGTAATGTCTCA	1	
Ad07A553	Aradu.A07	1766891	1766908	GCCTCTGCTTTCTGCTTCTG	CAAAAATACCCTCCACGAGC	1	
Ad07A554	Aradu.A07	1769990	1770013	AGGAAGCTAAGGCCAAGTCC	GTCGAGCCAAACTTGAGGAG	1	
Ad07A571	Aradu.A07	1813251	1813372	TGAAGTGAAAGAGATAGGGGAAA	GGCGTGCAAGAACAACTTAAT	1	
Ad07A572	Aradu.A07	1816274	1816303	TCCCATTCTCCCACGTACTC	GCATGTTTGTCTTCTTCGCA	1	
Ad07A579	Aradu.A07	1875262	1875306	TTCCATTAACTTCTCGTACTTTTCC	GGGGCAAATTGGTAATATCC	1	
Ad07A580	Aradu.A07	1875890	1875919	TTAGCACCAAAGCGTGTTCA	CAGTGGCTTCCCTAGGATTTT	1	

ID	Chromosome	SSR_start	SSR_end	FORWARD PRIMER (5'-3')	REVERSE PRIMER (5'-3')	Bands in parents	Polymophism in RILs
Ad07A582	Aradu.A07	1883995	1884009	TCATTTATTTGGGATTGATTAGCTT	ACAACCGCTGAGGATGTTTC	1	
Ad07A597	Aradu.A07	1935548	1935625	TGAAGTTGATAAATGAGAGCCG	CACCACAAAAATTAACTATTACGATGA	1	
Ad07A602	Aradu.A07	1941110	1941124	CAGCAAAGCGTGTTCAATCT	TCTCCTCCTATTTGCAATGCT	1	
Ad07A603	Aradu.A07	1942653	1942716	GCGTGAGAAAATTAAAGGAGTGA	CAACCTCAACGTTAGGAACCA	1	
Ad07A605	Aradu.A07	1943106	1943202	TTTTGTTACCGTCGTCACCA	CAGAAACACCTGCATCTCCA	1	
Ad07A606	Aradu.A07	1943832	1943864	TTTGGGTTCATTCAATCGCT	TTTACGCTGTGGGACTTTGTC	1	
Ad07A607	Aradu.A07	1944147	1944242	TAGGTCACGCGCTTCTTTTT	GGAGGAGAATGAGGAGGAGG	1	
Ad07A797	Aradu.A07	2425045	2425145	TTCGACATCGTTGTGAGAGC	ATTCTGGTTGTAGCTTGGGC	1	
Ad07A808	Aradu.A07	2444517	2444675	TTCGGTGACGTTCAACTCTG	ATGCAACACCTTCCAAATCC	1	
Ad07A926	Aradu.A07	2663561	2663693	TGTTTTGAGTTTTGGAGGGG	AATTCAAACCCCCGACACTT	0	
Ad07A978	Aradu.A07	2829348	2829515	GGTGAGCTCTTGTTCGAGGA	CATGGACACCTGAGGAGGAT	0	
Ad07A1112	Aradu.A07	3215858	3215993	CAGCGATAAACCAACAGCAA	TTCCCATTAATCCCTTCTGC	0	
Ad07A1818	Aradu.A07	4907309	4907414	GGCATGTAGCACATTAACTGAATC	TGGACATCAAATTTGTTCACATC	1	
Ad07A1926	Aradu.A07	5196305	5196424	TGGTTGATGGTTCTTCTGATTTT	GAAGCCTAAGCCCAGAATCC	1	
Ad07A2116	Aradu.A07	5698696	5698805	TGATCGATGTAGCTTCGCTG	TGGAGCATCCAAAAGAGGAC	2	
Ad07A2224	Aradu.A07	5927722	5927871	CCCCTGAATTCGCTACTATCC	CTGCTTCGTTGAACCCTAGC	1	
Ad07A2897	Aradu.A07	8039995	8040079	TAATGCCGTTGCAATCATTC	CATGTACGCCGCTAAATCAA	1	
Ad07A3267	Aradu.A07	9166276	9166493	TCATCAGTATTCACCACAAGTGC	CAATGTTGAATCCACCACGA	1	
Ad07A3763	Aradu.A07	10690300	10690380	GAACGCGACCAGAAGAGAAC	CTGCTGCAACGTAAACCTCA	1	
Ad07A4529	Aradu.A07	13136577	13136681	TCCCGATTCATCAACTCAAA	ACATGACAGAGCACAATGGC	1	
Ad07A4990	Aradu.A07	14716640	14716745	GACATGTACATTAAAGACCAAAATCG	ATGGCGTGAAAAGCAAGTTC	2	Yes
Ad07A5292	Aradu.A07	15913792	15913888	AACGAGAGAGAGCAACGAGC	GTTTGCGATTCCCCTCTACA	1	
Ad07A5319	Aradu.A07	16008745	16008833	TTCGTCCTTAACGTCTTGGG	GAGGGAGAAGTTGCAGGTTG	1	
Ad07A5481	Aradu.A07	16682319	16682437	GTGTGATGGATGGTGATGGA	CCCTTCTTCCCCAAAATCAT	1	
Ad07A5666	Aradu.A07	17279519	17279650	AAAAAGAAGACAACGATAATGACAA	TGCACCAAAACTAAACGCAA	1	
Ad07A6822	Aradu.A07	23065419	23065504	GCCTCTTCCACCAACTCAAC	CACAACTTTCCGTGACCCTT	1	
Ad07A7294	Aradu.A07	24886314	24886393	GAGGTCGTCATTTCCTCTCG	CGCATCACAATTTTCTCACG	2	
Ad07A7483	Aradu.A07	26237090	26237274	AGAGAGAAAGAACAAAAGAAAAGAAA	TCCTCCTCCTCTCTCTTCCC	2	
Ad07A7854	Aradu.A07	28173857	28173937	GATAATGCTGAGGGCACGTT	TAGAAGGATGGGGGACACAA	0	
Ad07A8335	Aradu.A07	30796007	30796157	TTTCACAATCTGTACCAGAAACTT	TTTTCCCCATCATTTTTATCC	0	
Ad07A8878	Aradu.A07	34916293	34916385	CCAGGTGCAGTATTTTATGGC	GAGTAAAGTGCAGCCATCGTT	0	
Ad07A9745	Aradu.A07	40477501	40477658	GGATCGGGTCCGTAACATTAT	CTCTCCAAATCATCATCCAACA	2	Yes
Ad07A10125	Aradu.A07	43528148	43528234	ATGATCAGGATTCGTGAGGC	CAAAACAGCACAAATGAGGC	1	

ID	Chromosome	SSR_start	SSR_end	FORWARD PRIMER (5'-3')	REVERSE PRIMER (5'-3')	Bands in parents	Polymophism in RILs
Ad07A11249	Aradu.A07	52304312	52304508	GGGAGAACCTGATTCCAACA	CATCTTCTGCTCCAACTTCATC	1	
Ad07A11412	Aradu.A07	53597871	53597960	GCTTACGCATGGATGAGCTT	ATAGGGAGCTCTTGGGCATT	1	
Ad07A12825	Aradu.A07	61786597	61786691	TCCTTGAGGTGTCAGCAGAA	CACTTTTTCACTTCCAATTTTCG	1	
Ad07A13140	Aradu.A07	63825387	63825583	TACCCTTCTCCTTTCACCCC	TGTTGCTGAAATTTGTTGTGG	1	
Ad07A14672	Aradu.A07	71109729	71109854	TCCTTCATTTCTTTCTTTTTGTTTTT	GAATATTGCTTGACTTGCTTTCG	0	
Ad07A15154	Aradu.A07	72380611	72380736	TGCTGCTTATTCGTTCATGC	TTTTGGACAAGAACCTCATCC	1	
Ad07A15468	Aradu.A07	73413555	73413688	TTGCCATCTCACCCTCTCTT	AACGGAACCAAATGCAAAAG	1	
Ad07A15701	Aradu.A07	74262398	74262493	TTGTTCTCTGCCTACCAGCA	TCGATGGATTTAGCTAGGACG	1	
Ad07A15861	Aradu.A07	74819096	74819197	TCTTCCCCAAAATCAAGCAC	TTGCTTCTGGTTGTTTCTGCT	2	
Ad08A811	Aradu.A08	3184171	3184253	CTGACTGCGATGCTGTTCAT	AAGGATAGCGGATGGTGTTG	1	
Ad08A1071	Aradu.A08	4248950	4249046	AAAAACACTTCCCCCTGCTT	TTCCTCTTCTCTTACCGCCA	1	
Ad08A1156	Aradu.A08	4507083	4507184	TGAACCAGAGAGAATGTGAAGG	AACTTAAGCAAGTCCCTCCTG	1	
Ad08A1796	Aradu.A08	6988638	6988720	TTACAGACCGACCCTCTTCG	CGAAGAGAACCAGGCAACTC	1	
Ad08A1820	Aradu.A08	7079004	7079119	GGTCGCAGGAATCACTACTGT	TGTATGACTCGGTGGTGATGA	1	
Ad08A2112	Aradu.A08	7891486	7891569	CAGGCTCACGAGGACAAACT	CGTTCCGATGCACAATTTTA	1	
Ad08A2362	Aradu.A08	8712586	8712690	TCCACAAAACCTAAGCCTCAA	AACCCCAAACCCATTCAAGT	2	
Ad08A2732	Aradu.A08	9848313	9848441	TGCTTCTTCACCAAAGAGAGAA	GAAATACACGCGCCCAATAC	1	
Ad08A3286	Aradu.A08	11497472	11497576	TCAGCAACAACAATCCTCCA	CTCCTTCTTCTTCTTCCCGC	1	
Ad08A3438	Aradu.A08	11919864	11919947	CGTGAAGAAGAAGGAACGTGA	CGTCCAACACACACGAAATC	1	
Ad08A3453	Aradu.A08	11970935	11971018	CGTGGATAAAGTCAATCACCA	AGAGGGATCAAGTGTGGACG	2	Yes
Ad08A3459	Aradu.A08	11990406	11990513	AAAGGATGGTTGGTTGGTTG	CCTCCCTTCTTCCATTCCAT	1	
Ad08A4387	Aradu.A08	15003893	15004027	CATGCCCATACATGAAATGC	AAGCTGCGGCTTCAGTAATC	2	Yes
Ad08A4940	Aradu.A08	16589752	16589892	TCCCTTCTCTTCTTCCACGA	AGTGTGTGTCTGCGTTGGAG	2	Yes
Ad08A5185	Aradu.A08	17385245	17385340	GCAGCACTCAACAAGGAACA	TCCTGTCTGAGAGGGAGCAT	1	
Ad08A6032	Aradu.A08	21593818	21593937	TCCTTTTCCGTTATTGCCTC	GAAAGGGACGAATGAACCAA	1	
Ad08A6126	Aradu.A08	22643076	22643164	TTGAATGCAAAAGCCTGATG	GTGCATGGACTTCACGTTTG	1	
Ad08A6679	Aradu.A08	24548345	24548460	ACATGACAGAGCACAATGGC	GGGTCATAGCCTCCCGTTAT	1	
Ad08A7006	Aradu.A08	25608666	25608829	TTTCAGAGCTTGATGTCAAAAA	GCTGCACGTTCTTCTTCCTC	1	
Ad08A7218	Aradu.A08	26258248	26258350	TGACACCAAGTCCAACTCCA	TCCTTAAGGTGAATTTTGGCA	1	
Ad08A7227	Aradu.A08	26265579	26265744	CCTTACTTCTCCCTTCATCCC	TTTCTGATGATGATGCCCTG	1	
Ad08A8078	Aradu.A08	28812263	28812355	AGGAACGCTTTTGACGAGAA	TGAGCATGATAGTAATCCGCAC	1	
Ad08A8326	Aradu.A08	29418097	29418222	CGTTGTCCTGCACAATTCAA	CAGTTCGAAAACAAGCACACA	1	
Ad08A8579	Aradu.A08	30059661	30059759	TGGGAGTATGACAAAACATAAAAG	TCCACGGTTATTCGTTACCC	1	

ID	Chromosome	SSR_start	SSR_end	FORWARD PRIMER (5'-3')	REVERSE PRIMER (5'-3')	Bands in parents	Polymophism in RILs
Ad08A8871	Aradu.A08	31065834	31065957	CTGATGATGACGACGACGAT	GCATCACACCAACCACAACT	1	
Ad08A9165	Aradu.A08	31859118	31859209	TTGCCTTTAGAATTGGACGG	CAAAAGAAGGTTTTGTCAGTCA	1	
Ad08A10782	Aradu.A08	36622804	36622946	TCAATGGCCAATAACAATAAACTC	CCGCAAATCATGCACTTTAG	1	
Ad08A10918	Aradu.A08	36978305	36978427	CCCTTGCACCATCATAGTAGAG	GCTGAGAGTGAGTGAGATTCCA	1	
Ad08A12248	Aradu.A08	40709572	40709672	TGACACTATTTGGGTGTACCG	CCCAAATTGCGTGCTTACAT	1	
Ad08A12438	Aradu.A08	41282047	41282136	AGCGCTACACTCAGAACGGT	TCAGAAACCATGAAAATCGAAA	1	
Ad08A13210	Aradu.A08	43528610	43528711	GGGGTCGAGTTGGTACTTCA	GAGGAGGAGGAGAAAGAGGAA	1	
Ad08A13316	Aradu.A08	43741970	43742088	GGTGACGACGGTAACGAAAT	TCCAAGTGATTTGAATTACGC	1	
Ad08A13889	Aradu.A08	45251884	45251980	GGCCAAATTAACACTCATTGG	CGCTGTTCGTGTGGTTCTTA	1	
Ad08A14487	Aradu.A08	46666959	46667080	TCGAAATAGAAACAAAATAGCACG	TATTTATTTGCACGTCCCCC	1	
Ad08A14984	Aradu.A08	47748305	47748433	ACATGACAGAGCACAATGGC	AAAAATGATCTAAACCTATCACACCC	1	
Ad08A15328	Aradu.A08	48352183	48352265	TCCGTTTAACCGAACGAAAC	CCCCGTGACTCACACATACA	1	
Ad08A15421	Aradu.A08	48524382	48524490	CAACTCCACCATGGATTCCT	CAACAACAAAGTCTTGTCCCA	1	
Ad09A239	Aradu.A09	620135	620301	GCATGCCCGTTATCATCATT	CTTTGAAATGTGGTGGATGCT	1	
Ad09A1202	Aradu.A09	2737299	2737499	TGACTCCACCTACGCACAAG	GCGCGACGGTATTGTACTTT	2	
Ad09A1309	Aradu.A09	3002896	3003030	AATGCAAAAATTGAAGCAAAA	TCATGACCACTCATGCCTTT	1	
Ad09A1515	Aradu.A09	3496158	3496298	CAATGGGTTGGTGACCGTAT	TTCTCTGTTGGTAAATGATTATCTCA	1	
Ad09A1716	Aradu.A09	4114902	4114982	TAACCACCTAAGCACAGGGC	CACGTTACCCCAACCAGAGT	1	
Ad09A2251	Aradu.A09	5767850	5767957	AAATGTGAAATACACGCGCC	CCTTCTGCTGCTTCTTCACC	1	
Ad09A2638	Aradu.A09	7040191	7040344	TGGTGATGACGATAACGGAA	CTCCTTCTCCTCCTCCTCGT	1	
Ad09A3077	Aradu.A09	8202950	8203084	TGAAAAAGTGAAAAGGGAGCA	CTATTTGGAGAGGTGGGCCT	1	
Ad09A3779	Aradu.A09	10766109	10766221	TATCAACCGTTAACCGGAGC	TGGCATGGAACCTAGAGTGA	2	Yes
Ad09A3850	Aradu.A09	11087170	11087308	CAGTGCACCACCCTTTCATT	ATATCACATAGCCATCGGGG	1	
Ad09A4409	Aradu.A09	12939523	12939610	TTCCATGTGTTGCAGGAGAG	ACACAGGCAACTCCTCCCTA	1	
Ad09A4517	Aradu.A09	13191823	13192038	CTCCTTTGTTATCCCCCTCA	AAGGAAAGAAAAGAGAGAGGAGC	1	
Ad09A4697	Aradu.A09	13912632	13912725	ACCTACCCAGCCCTCTCATT	CCACCTCCTCTATCATCGGT	1	
Ad09A5968	Aradu.A09	19633655	19633694	CGGACTAGCAGAGAGTTGGG	TCGTTCACTTTTGTGTGTTGG	1	
Ad09A5971	Aradu.A09	19654010	19654055	CGGACTAGCAGAGAGTTGGG	CACATCACAAGCAACGTTCA	1	
Ad09A5979	Aradu.A09	19680607	19680636	GGAGAAAGAGGGAAAAGGAAAA	CTCCCCTCTCTCCCCACTCT	2	Yes
Ad09A5985	Aradu.A09	19695540	19695571	CCACAATGGCAAAATTACCC	GAAAGCTTCGCAGAACCAAC	2	
Ad09A5990	Aradu.A09	19741858	19741875	AGGGTTTAATGCTGGTCAATG	CCCAAATTTCTTGGGCTTTT	1	
Ad09A5998	Aradu.A09	19767710	19767751	ATGCCATGCTTCTTTCACCT	CATCCCAGCACGAAAGAGTT	1	
Ad09A6009	Aradu.A09	19796659	19796706	ACGATGATGTGCGAACAATG	CAGGATCTTCAATACGAAGCAA	2	Yes

ID	Chromosome	SSR_start	SSR_end	FORWARD PRIMER (5'-3')	REVERSE PRIMER (5'-3')	Bands in parents	Polymophism in RILs
Ad09A6014	Aradu.A09	19811640	19811660	TCTTTTTGTTACCGTTGGGC	GAGGCATAGCAGAGCAAAGG	1	
Ad09A6026	Aradu.A09	19863205	19863218	ATTCAAAACCGTGGCAAGAC	TTTTCAAATGAGTTGCACACATAA	1	
Ad09A6037	Aradu.A09	19896695	19896724	CTTGCCTATAAATCCGCCAA	TAGATCTACATGCGCAACGC	1	
Ad09A6046	Aradu.A09	19934724	19934738	TTGGTTTACTTTTCCGTGCC	TGCTTAATGCTGCACGCTAC	1	
Ad09A6050	Aradu.A09	19981986	19982003	GGGTGTTCTACAAAATGCAGC	GGCCAAGCCTTCTTCAGTAA	1	
Ad09A6058	Aradu.A09	20012896	20012925	TCGCTCTTGCGTGTAGTAGTTT	GGTTGTGGGTTCGAGTCTCA	2	
Ad09A6069	Aradu.A09	20047838	20047861	CGTTCCACACACAACAAAGG	CCATTGTCGACTGCCTCTTT	1	
Ad09A6072	Aradu.A09	20052807	20052834	GGGGTCAAGTTCTGAAACCA	TTTTGTCGAAGGTGTTTCTTGA	2	Yes
Ad09A6083	Aradu.A09	20115210	20115227	GCGTCTGGAGTTTCCTATGC	ACTCGACCTGCGAAGCTAAG	1	
Ad09A6095	Aradu.A09	20184597	20184636	CATTTTAAAAAGTATGCGTACAAAAAG	CGATCCATAAGCATCATATCGT	1	
Ad09A6105	Aradu.A09	20207343	20207374	AGGGCTGCTTTGAGAAGGTT	GTGGGCTCCTACTCATTGGA	1	
Ad09A6114	Aradu.A09	20258806	20258826	AGCATGTACGCACACGTTTC	TGCGAAGAAGAAGAAGGAGAA	1	
Ad09A6128	Aradu.A09	20318641	20318652	TTGGGCTATTTTTGACCCAG	TGAGTAAAGAAACATTGAACCTGAA	1	
Ad09A6146	Aradu.A09	20357297	20357311	ATGTTCCCACACCCACAAGT	CGCAGTTGTGATGCGTTAGT	1	
Ad09A6154	Aradu.A09	20424204	20424219	ATGCTTGATGATGGTTGGGA	CAAACATCAACACCCACCAC	2	Yes
Ad09A6158	Aradu.A09	20440376	20440395	TCCCCTCCTAATAAAATTTTTGC	CAGCCGAAGGCTAGAGAGAA	1	
Ad09A6164	Aradu.A09	20474979	20474990	GCAGAATTTGCCCCTAGTGA	GGAACATTGAAACTGAAATTGAGA	1	
Ad09A6171	Aradu.A09	20515632	20515671	TTTGCAGCATTATGTGTTTCTTC	TTTTCGGTGTATGTGTGCTGA	1	
Ad09A6183	Aradu.A09	20551676	20551695	TTTTCGAACAATACGCAAATC	TGTGTTATCGCCGAAAATGA	1	
Ad09A6200	Aradu.A09	20645025	20645042	CAAGTTTGGTGTTATGGGGG	AATAAGCAAGTGCCTGCTGC	2	
Ad09A6209	Aradu.A09	20669115	20669130	GAATGTCCAAACTCGAGGGA	AATGCGTCTTGGGAATTTGT	1	
Ad09A6224	Aradu.A09	20750736	20750777	TCACGAAAAACATACAGTACCACC	AAACTCCTGCACGACAATTTTT	2	
Ad09A6233	Aradu.A09	20785957	20785968	AAGATGCGAGGGGTTGTATG	ATTGCAATGTTCGACTGACG	2	Yes
Ad09A6250	Aradu.A09	20840916	20840972	TGCATTCAGTGTTAAAAGAAATCA	CATTGAGCAAACCAAACTTCAA	1	
Ad09A6258	Aradu.A09	20862656	20862688	AGAGGGAGGAAGATGTGGTG	AGTCTTGAATCGCTTTCCGA	1	
Ad09A6265	Aradu.A09	20892616	20892651	GGCTCGCTGATGAAGATAGG	GGCGAGGCACTAAGGTATCA	1	
Ad09A6270	Aradu.A09	20910230	20910262	CATGAGGTGGTCAAACGAGA	AAATGTCACATGGTTGCTGC	1	
Ad09A6278	Aradu.A09	20924771	20924889	TCCCCTTCTCACACTCTCTCA	GCAGTCTGCAGGTGCTTTTT	1	
Ad09A6287	Aradu.A09	20946658	20946720	CGCTATCCTGAGGAGGACAC	GGACATTTACAACGACCCTGA	1	
Ad09A6297	Aradu.A09	20968814	20968829	CATCTTCCATCATTCCGCTT	TTATAAAATCCGCGTGAGGC	0	
Ad09A6306	Aradu.A09	20991898	20991915	TCGTCTCAAACTTTTCTTTTGATTT	AAAAAGAATTTGCGCATAAAATA	1	
Ad09A6323	Aradu.A09	21039589	21039604	TTAACACCAAATCCTAAACACTCA	TTTCTGTTCCAAATCCTGGG	0	
Ad09A6333	Aradu.A09	21082209	21082283	TGTTGCATTTATGACAGCCA	CAAAAATGATCGTGAACCACA	1	

ID	Chromosome	SSR_start	SSR_end	FORWARD PRIMER (5'-3')	REVERSE PRIMER (5'-3')	Bands in parents	Polymophism in RILs
Ad09A6338	Aradu.A09	21095181	21095207	TGGACGAAAATACCCTTCTCC	TTCTTGTTCAGCTTGGGCTT	1	
Ad09A6343	Aradu.A09	21098562	21098579	CTTTCGATCCAGTGGGAAAA	TTTTATGAAACCTCAATGCAAAAA	1	
Ad09A6351	Aradu.A09	21108054	21108083	ACCACATCCTTGACCACTCC	GGGACCAACTCGAACTGAAA	2	
Ad09A6355	Aradu.A09	21134975	21134996	TTGCCATTGCTTGAGAGTTG	CACCCAATTTTGCAAAGGAT	1	
Ad09A6363	Aradu.A09	21169082	21169114	GCACCCACTCCTGATCCTAA	AAACTTCTTAGTGCAAACCAACC	1	
Ad09A6371	Aradu.A09	21193566	21193593	TTTTCCCTCGCCATTTATTG	TTAGCACCTCATGATCGCAC	1	
Ad09A6379	Aradu.A09	21215385	21215447	TGCTCTTATTGGTCAAGGATTTT	AGGCTTATGCCTGTTCCAAT	1	
Ad09A6395	Aradu.A09	21265305	21265338	ATCCCTGGTGCTGGTTAGTG	ACTTGCTTGGCCAACACTCT	1	
Ad09A6407	Aradu.A09	21300978	21300989	TTCCATCCACTTGGCTTCTC	TGTTGGAATTGGTGTTGGGT	2	
Ad09A6411	Aradu.A09	21319286	21319305	TCCATCCAACACTTCCACAA	TCCCCATACCCTTGTGATTC	1	
Ad09A6416	Aradu.A09	21382945	21382998	GCTAGCCAAGTCAAGTGAGC	TTGCAAGCAGCAAACAAATC	1	
Ad09A6420	Aradu.A09	21419608	21419625	GTAGTCACTCGGCCACACCT	AAGACCAAATCCAAGGCTCA	1	
Ad09A6425	Aradu.A09	21472232	21472324	TTGGGTGCCTTGAATTGTAA	TATAACCCCCACTGACCGAG	2	Yes
Ad09A6431	Aradu.A09	21505097	21505114	CACGCAGGTTCTTCTTCTCC	TCGGTGATGACGATAACGAA	0	
Ad09A6444	Aradu.A09	21540874	21540887	AATCAAGAAAGGGAAGAGGGA	CGCCGTAAATTCAACGGTAA	1	
Ad09A6451	Aradu.A09	21585064	21585084	CCCTGCAGCTTTATTTGCTT	TCATCTCCTGTTTGGTCCTTC	1	
Ad09A6458	Aradu.A09	21606313	21606333	TGATTTTCTTATACTTTGAAATTTTGC	TTGAAAAACAAAACACACTCTCG	1	
Ad09A6463	Aradu.A09	21618354	21618429	CAATACAATGCCATCACACCA	CATGTAAGTGACGATTTGCCA	1	
Ad09A6470	Aradu.A09	21631549	21631584	GGAGAAGAGGGGGAGAGAAA	TCGTTGACAGAGATTCGACG	1	
Ad09A6477	Aradu.A09	21656683	21656696	AGTGCTTGGTGGGAGACAAC	ATTTTCTCCCCTCTCCTTGC	1	
Ad09A6492	Aradu.A09	21709342	21709367	CACATATGGGAATAAGCCGC	AAAAGATGCTCACGCATCAA	0	
Ad09A6499	Aradu.A09	21770206	21770220	AAGGAGTTTTTGTCGGAGAATTT	CTGCAGCGGAATTTTGACTT	1	
Ad09A6503	Aradu.A09	21782393	21782404	TCGTTGTTTTGAATAAAGAGAGAAAA	GAACAATTCAAAGCGGTCGT	1	
Ad09A6509	Aradu.A09	21819798	21819819	AGTGTGGCCTTTTGTCTCTCA	TGAAAGACGATGTATGGTTGATG	1	
Ad09A6514	Aradu.A09	21839408	21839440	AGCCCCTTTTCCTTTTTCAA	ATGTGCATGTCACCACCACT	1	
Ad09A6528	Aradu.A09	21877452	21877472	CGACATAACAATCTCCCTCCA	TGGTTTCAACCTTGAGTTTCTTG	1	
Ad09A6530	Aradu.A09	21878024	21878039	TTGTCAACAAGAATCCGACAA	TTTTTCCGTGTATCCCATTCA	1	
Ad09A6539	Aradu.A09	21892445	21892458	TGAGTTGGTTGATTATTGCTGC	GGAGCACGATAGACCTCCAG	1	
Ad09A6547	Aradu.A09	21932063	21932082	CATTTTCGAATTAGATTCGTGTCA	TGATAAGTGAAATGATAAGAGAGGGA	1	
Ad09A6551	Aradu.A09	21942070	21942081	ACAAGCAGGCAATTGATGAC	GGGAGATGGAGTCATGGAGA	1	
Ad09A6562	Aradu.A09	21974050	21974061	GAGTCTTCGCTTCGTACGCT	GCTTGAATGGAAGATGACTGAA	1	
Ad09A6578	Aradu.A09	22051236	22051274	AACACAGCAGACTTGCACCA	GGATCAGAGCGATCATGTCA	1	
Ad09A6584	Aradu.A09	22097325	22097338	TGGGTACACATGGAGAATGC	TTTGGTCCGAAGGACAATTT	1	

ID	Chromosome	SSR_start	SSR_end	FORWARD PRIMER (5'-3')	REVERSE PRIMER (5'-3')	Bands in parents	Polymophism in RILs
Ad09A6587	Aradu.A09	22106125	22106180	GCGAGAGATGAAGGGAGAGA	CCTCTTATTTGCTCATCCCG	1	
Ad09A6594	Aradu.A09	22157062	22157083	TTCCATGAGAAAGGAGACGG	CGACGACAGAGTTGTGGAAA	2	Yes
Ad09A6617	Aradu.A09	22289637	22289662	TCTTCTTCCTCTTCCCCCAT	AGAGAGAGACTGGCTGGCTG	2	Yes
Ad09A6624	Aradu.A09	22323816	22323829	TTGTTGGCTCTTGAAAGAATGA	AGGCCCTCCTATCCACTGAT	1	
Ad09A6630	Aradu.A09	22355713	22355728	CACACACACACAGTCACCCA	ATGGATGACGGAAACTCAGG	1	
Ad09A6639	Aradu.A09	22384597	22384608	TCCAGCCCCAGAAAACATAG	GCCAGTTTGGAAACATAAATCA	1	
Ad09A6642	Aradu.A09	22399906	22399921	AAAGTGGGGAAATCCATTCA	CATAAGCCAGTTTGGCAACA	1	
Ad09A6655	Aradu.A09	22436924	22436977	AGGAGGCACGATAGTCCACA	CAAAGAATTATTAGGCTTCGGA	1	
Ad09A6669	Aradu.A09	22459059	22459076	TCTCAAGCTTCATAACGTCCAA	AATCAAGTTTGTTCCCGCAC	1	
Ad09A6680	Aradu.A09	22547808	22547825	GGGTGGAATGTAAGACCCAG	CAAATTGCTATACTATTTTCACTCACA	2	Yes
Ad09A6690	Aradu.A09	22589655	22589674	TCCTGCCTCTCCATACACCT	GGGCATTCCCAGGAACTTAT	1	
Ad09A6707	Aradu.A09	22703347	22703358	GGGCGCAGAAGTTATGAAGA	TTAAATCGCAAAATCGCTCC	1	
Ad09A6713	Aradu.A09	22715018	22715029	TCCTCAACGTGTACGTAATAGAATG	TCGTTGGCAAAGAAAGAAAT	1	
Ad09A6734	Aradu.A09	22844209	22844268	GAGAAACGACAAAATAAACCTGC	GCTTGTTGCATCAATGAAGG	2	
Ad09A6745	Aradu.A09	22904553	22904570	TTCACAAACTCGTGAACCAAA	TTGTGTTGGATCGTTTTCCA	1	
Ad09A6752	Aradu.A09	22940957	22941025	CCTAATTATCACTTTACTCTTTGGGA	GATTTCAATTGACGAGTCGGA	1	
Ad09A6764	Aradu.A09	23021269	23021288	AAAATACAAAGCTTGCGGGA	AGCCACTTAGGCCAGGATTT	1	
Ad09A6775	Aradu.A09	23049925	23049978	GCATGAACGGCTTTAGGATT	CGTTTAATCACCCCCAAAAA	1	
Ad09A6778	Aradu.A09	23075173	23075186	GCGTGTTCTTCCTTCTGCTT	TGAAGTGCATGTTTTCTTTTGTG	1	
Ad09A6780	Aradu.A09	23090945	23090968	GGGCCGAAAATGATAACTGA	ACATCCAGTCAGGAAGGTGG	1	
Ad09A6793	Aradu.A09	23225193	23225204	AAGAACCTGGGATTGAGAGTCA	TCTCCAGCGATTTCTGGTCT	1	
Ad09A6804	Aradu.A09	23285838	23285855	TCACTCTCCTTTAATCTTCTCAAAA	TTTTGATTTTGAGCATATAACTTTGTT	1	
Ad09A6813	Aradu.A09	23332462	23332503	GCATCTGAGTTTCCACCGTT	CACCAAGCATCTAACCTACATCA	1	
Ad09A6819	Aradu.A09	23364001	23364014	TCTTATGGACCAGCATGCAA	TTTCAATGGTTAAACACTAAACGA	1	
Ad09A6843	Aradu.A09	23504638	23504652	CACGAACAACAATTTTGGTGAC	TGCCCAACGAATATTTCAAG	1	
Ad09A6851	Aradu.A09	23538848	23538873	AGCCTGCGAAGCTAAGACTG	GCTAGGCGTCTGGATCTTTG	1	
Ad09A6855	Aradu.A09	23564283	23564321	CGGTCACAAATCACAATACCA	ATGCAATTGGCTTGCTTCTT	1	
Ad09A6862	Aradu.A09	23628043	23628060	ACAACCCCCTCTTCCATACC	CTCGAAGGTGGAAGCTTTTG	1	
Ad09A6867	Aradu.A09	23648826	23648867	AATGTGTGCTGGACATTGGA	TGAGTCGGCTGCATCATAAA	2	
Ad09A6882	Aradu.A09	23724335	23724346	CCCAAAACCTATAAACATGACCA	TGCGTGTATTGGGTGAAATG	1	
Ad09A6891	Aradu.A09	23767555	23767569	AGTCCATCTCCCATGACTCG	TCCATCTCCCATCACTCTCC	1	
Ad09A6902	Aradu.A09	23819243	23819264	TGCAAAGGTGTCAGCACTTC	TGATTCATATGCATGACACTTCTCT	1	
Ad09A6911	Aradu.A09	23911515	23911526	AGAATCCTCCTCCAGGGAAA	TTTGTTGCAACTCGAACTGG	1	

ID	Chromosome	SSR_start	SSR_end	FORWARD PRIMER (5'-3')	REVERSE PRIMER (5'-3')	Bands in parents	Polymophism in RILs
Ad09A6921	Aradu.A09	24008759	24008774	GACGACGTTCCACCTCCTTA	TGCAAGATTGGAGTGTTTGG	1	
Ad09A6926	Aradu.A09	24016692	24016733	TCGAACAAATGGGCAAAAA	TGAGGGGGAAAATAGGGAAC	2	
Ad09A6940	Aradu.A09	24111258	24111273	TGCGTTTTGATGATCCGTAA	GATGGGAGAATGTTGCTGGT	1	
Ad09A6950	Aradu.A09	24157977	24157991	ATGCGTGGTTCTTTTTGTCC	CCATCTTCGACAAAGCTTCC	1	
Ad09A6958	Aradu.A09	24304315	24304329	TAAAATATTGCTCGCCCTCG	CCTACCTCGCCCTCTCTCTT	1	
Ad09A6964	Aradu.A09	24401879	24401896	GAAGAGGGAGGAGAAGAGCAA	CGAAAATCACCCACCACTTT	1	
Ad09A6973	Aradu.A09	24497646	24497714	AAGAGGCAACCGACTATGGA	TTCAACAATGAAACCATCGTG	1	
Ad09A6979	Aradu.A09	24588614	24588641	AGCCGTTGAGAGGAAGTGAA	CAGATCTACAGCGACGGTGA	1	
Ad09A6980	Aradu.A09	24590042	24590121	TTGAGACAAATTGTTGACCCTC	CTGACATGTCCTGGCAGCTA	1	
Ad09A6989	Aradu.A09	24668103	24668114	TCCGTTAAGGATGAGCTGTT	TCCCTTATGATCTGGCATCC	1	
Ad09A6998	Aradu.A09	24771364	24771381	TTTTGTTGTTTTTCTCTCTCCTCA	CGTTAAACGCCCAGAATGAT	1	
Ad09A7008	Aradu.A09	24908325	24908344	GATTCAGTTCTCAATTTGCTTTGA	TTGCTTGCTTTAATGGGAGG	1	
Ad09A7017	Aradu.A09	24984926	24984943	CACATACACTATGGCGGTGC	GAGTTTTGGCGGAAGATTTG	0	
Ad09A7019	Aradu.A09	24986152	24986179	CAAGCAGCAAACACGATGTC	ATCCAGGCCTAGCCACACTA	1	
Ad09A7027	Aradu.A09	25037125	25037172	ACTGAAGGATCATTCCCTCG	TGTTACAAATCCTTATATGCAAATCT	0	
Ad09A7037	Aradu.A09	25067809	25067859	GCTCAAATTCACACACCCAG	GAATGCACCCAAGTTTTCTGA	0	
Ad09A7045	Aradu.A09	25125015	25125028	GCTCTTGTTGGTGAGCATGA	CAACCCACCAATTCTTCCAT	0	
Ad09A7054	Aradu.A09	25168436	25168455	GCCATTCCCTTCAAGTCAAA	TCCCTCAGAAGCCCAACTTA	1	
Ad09A7068	Aradu.A09	25285677	25285697	CACCCTCGAGCAAAGAAAAG	AATTCCTTTCCCACCCAAAC	1	
Ad09A7077	Aradu.A09	25312281	25312292	GTGACCAAAGGGGAGTACGA	TCCCTTTCATTCCTTGTCCA	1	
Ad09A7080	Aradu.A09	25321480	25321499	CTCGACCTGCGAAGTTAAGG	GATGTAGGTCGAGAGGCACC	1	
Ad09A7086	Aradu.A09	25387653	25387668	CATGTGGTTTTAAGTGGGCA	TCCACTTCGAGAAGGATTGG	1	
Ad09A7091	Aradu.A09	25450225	25450273	GATGGGAAGGCATCATTCAT	CTCAGGTGCTAGAGTGATGGAA	1	
Ad09A7094	Aradu.A09	25499324	25499545	TTTCACAGCAACGCATTAGG	CGTCTTCTCAGTACGACGATCA	1	
Ad09A7096	Aradu.A09	25508864	25508890	ATTTGTGCGTGGGAAGACTC	TGTCAGACACCACCCTTTCA	1	
Ad09A7105	Aradu.A09	25529511	25529540	CCTTTTCCTTTCACACCCAA	AACGCAAGCAACAACAACAG	1	
Ad09A7111	Aradu.A09	25588352	25588363	CCCCGGATATCTCATGTTTG	GGATATTTGGTGAAGGGGCT	1	
Ad09A7115	Aradu.A09	25612095	25612108	ATGCCACAAGCACATGGTTA	TGAAGCAAGAAAAAGCAGTGAA	1	
Ad09A7117	Aradu.A09	25645361	25645372	TGAATGAGGGATTGGAGGAG	GCGCCACTCTCGAAATAAAC	1	
Ad09A7120	Aradu.A09	25665310	25665321	CGCTCCTATTGTCCCATTTT	TGTTGGTACTGGCACCATTG	1	
Ad09A7129	Aradu.A09	25690325	25690339	CAACATGGAAGAATAGAAAAGCC	CTGAATCCAAACATAGACGATCA	1	
Ad09A7135	Aradu.A09	25731162	25731173	CCGAGTTGCATGTATGAGGA	GGTTTCAGAAAAGCAAATCCC	1	
Ad09A7140	Aradu.A09	25752347	25752391	AAAGAGCAACAAAGAGACAAAAA	TGGGGTAGGATGTAAGACCTCA	1	

ID	Chromosome	SSR_start	SSR_end	FORWARD PRIMER (5'-3')	REVERSE PRIMER (5'-3')	Bands in parents	Polymophism in RILs
Ad09A7147	Aradu.A09	25807176	25807187	AACTGCATGTGAGTTTTTACCA	ATTTGGATGAATTGCCCTTT	1	
Ad09A7151	Aradu.A09	25831371	25831382	GAAATGCCTCGAATTGCATTA	TGCATCCATTCATCAGACAA	1	
Ad09A7164	Aradu.A09	25911386	25911397	ATGGGGCTTAGGTAAGGGTG	ATGGAAGCACCCCAATGATA	1	
Ad09A7171	Aradu.A09	25963160	25963177	CCCTTTCCCTTTTCTTGAGG	CTCCCATATAGCCGAAACCA	0	
Ad09A7179	Aradu.A09	26099260	26099271	TTATTTGGATTTGGGCCTTG	TTGGGTGTATAATTAAATCAGTTGTT	1	
Ad09A7186	Aradu.A09	26130617	26130628	GGACCATTGTTACATGGCAA	TGTGAAGTGATGGTGAGGGA	1	
Ad09A7191	Aradu.A09	26195633	26195650	GACGGGAACTCCTCAGATCA	GCGTCTTGGGGATTTGTAGA	1	
Ad09A7196	Aradu.A09	26233708	26233725	TAGAATATTGCTCGCCCTCG	CCCATAAACCCCACCTACCT	1	
Ad09A7201	Aradu.A09	26270504	26270523	TCCATCAAACACAGAGGCAA	GCCACTGCCTCACACAATTA	1	
Ad09A7211	Aradu.A09	26301886	26301948	CCGTTGATCATTTAGGACAGAA	TGATTTTTAAAGGAAGGCGG	1	
Ad09A7223	Aradu.A09	26374861	26374887	AAGTGCTGGCCAAAAATGAT	CATCTAAGGTGTAATGTTTCATTCAT	1	
Ad09A7227	Aradu.A09	26383583	26383608	AACAAAATAATCACGGCAGACA	GGCACCAAGTTATAGGCACC	1	
Ad09A7231	Aradu.A09	26398155	26398172	TACCTTGGCCGAACCTACAC	CTCGAAGGTGGAAGCTTTTG	1	
Ad09A7233	Aradu.A09	26442873	26443031	CCCTCTCGTAACGGAACCTA	CCTCTTGTGCGATCTCCTTC	1	
Ad09A7236	Aradu.A09	26459120	26459175	ATTTTGGGGACTGAAACCCT	AACTATGGACCCGAAAAGGC	1	
Ad09A7238	Aradu.A09	26460806	26460817	CGAGCATGCGTTATTCAACT	AAACCCATGTTTCTCCCTCC	1	
Ad09A7241	Aradu.A09	26503722	26503745	AACACAGAGGGAGAGAGAGGG	TCGAAAATCACCTTCCTCTTTT	1	
Ad09A7251	Aradu.A09	26605230	26605301	TTGGCCCTAGAATCAAAGTTC	TTGCAAAAGCTAAGTGCCAA	2	
Ad09A7259	Aradu.A09	26629284	26629301	TTCCGCAGAGTTGTCAAATG	CCTAATCAAAACACGCTATCCA	1	
Ad09A7269	Aradu.A09	26684941	26684958	AGGGAGACGATGAGAGACCA	ATTTCCAACCATTTTTCGCA	1	
Ad09A7274	Aradu.A09	26700682	26700696	CACTGTCAATGCCACACCTC	TTCCTTTGGTTAAATCGGCA	1	
Ad09A7280	Aradu.A09	26725106	26725120	TTGAACGAGACCCAATGTGA	TCTCATCTCCACCTCCATCC	1	
Ad09A7285	Aradu.A09	26741519	26741533	GGGGTTTTGGGTTCATCTTC	GCAACAACCAAATTCAGCAA	1	
Ad09A7289	Aradu.A09	26752628	26752693	TACCCTCCAGCTTCATGACC	ACTTCTGGGAAGCCAAGGTT	1	
Ad09A7300	Aradu.A09	26825212	26825251	AAGGGGTTCTCTTGTTAACCTGT	AATGTGAAGGGTATCGCACC	1	
Ad09A7306	Aradu.A09	26853022	26853035	TGATAATTAAATGATGGCCAAAAA	TGCCGATAGATGTGGAGTCA	2	
Ad09A7320	Aradu.A09	27137235	27137291	CGAACCTACCCTCTTCCCTC	TAAAATATTGCTCGCCCTCG	2	
Ad09A7328	Aradu.A09	27174947	27174991	ACAGTCATCCATGGCAAAGC	TGTAAACCCTCACGTTACATGG	2	Yes
Ad09A7337	Aradu.A09	27322637	27322656	TTGGAAAATTGGAAGCCAAC	CCAATCATTACCAGCTGCAC	1	
Ad09A7344	Aradu.A09	27342494	27342508	ACTAAAATTGGCATGCGGTC	ACATGAAAGCTGCTGTCGAA	1	
Ad09A7347	Aradu.A09	27348636	27348683	AACTGATTCCCCACTATTTTCTTG	ACATTAATTTATTTATCTTGAACGAGG	1	
Ad09A7355	Aradu.A09	27383290	27383303	ACCAAAAGCAACGTTCACCT	CGGACTAGCAGAGAGTTGGG	1	
Ad09A7367	Aradu.A09	27450316	27450329	AGACACCCCACCATGGTAAT	CGAGGCTAAAATGCAGTGTG	1	

ID	Chromosome	SSR_start	SSR_end	FORWARD PRIMER (5'-3')	REVERSE PRIMER (5'-3')	Bands in parents	Polymophism in RILs
Ad09A7373	Aradu.A09	27484786	27484800	GAGCAAAGCAAGCAGTGACA	GATGCCCTTCTTTTCCCTTC	1	
Ad09A7375	Aradu.A09	27488446	27488457	TTGGTGATCCAACCCTTTTT	CCACATCACCACATCTCCTG	1	
Ad09A7382	Aradu.A09	27587623	27587634	GAATGATAAAAACTTCACGGAGG	AGAGGCTGCATAGTGGAGGA	1	
Ad09A7385	Aradu.A09	27597330	27597343	CACTCTTTCTCCCCCTTCCT	CCCCTTCCCTTGTTTCTCTT	1	
Ad09A7394	Aradu.A09	27657627	27657642	CCAAGATCAGAATGCACAGG	GTGTTTGGAGGAATGGCAGT	1	
Ad09A7401	Aradu.A09	27690231	27690244	CGTATGGCATAATACATATGTTAAAAA	CCCACTTAGTTCATTCGCAA	0	
Ad09A7408	Aradu.A09	27763076	27763087	TTGAAGGGTGAGGTAGGTGG	ACGCCAGAAATGGCTAAAAA	1	
Ad09A7422	Aradu.A09	27894273	27894292	CATCGTGATTATCAGCGCAC	CATGGAACCACATGGTCGTA	1	
Ad09A7432	Aradu.A09	27920035	27920073	TGCATTGACTCAGGTGCTTC	CGAAACCAACTCCACCACTT	1	
Ad09A7437	Aradu.A09	27965327	27965341	CGTCCTCGAGCAAAAAGAAA	TAATTTCCCCATGCACCATT	0	
Ad09A7440	Aradu.A09	27984986	27985001	CATCAATCTGGCGTTGAATG	AAGGTAGGTGGGGTTTTTGG	1	
Ad09A7447	Aradu.A09	28037925	28037936	TCTTCCCCCATTTCCTCTCT	GGCCCTTGACCCAAAATAAC	1	
Ad09A7449	Aradu.A09	28040761	28040775	CCAACAAAAAGAGGTTCCGT	GGTCCTCTCCAACATAAGCC	1	
Ad09A7462	Aradu.A09	28140813	28140848	AAACCTCCATGTTTGTTGGC	CAGCGTCACCCTCATCTACA	1	
Ad09A7467	Aradu.A09	28176663	28176680	CAGGGAAAAAGGCACTCTTG	TGAAGTCACCAAGCATTCTCC	1	
Ad09A7472	Aradu.A09	28193318	28193329	CCAAGACTCACTTGTGACCG	TCCCTCCAATTTGCTCTCAC	1	
Ad09A7474	Aradu.A09	28199794	28199805	CCCAAACATAAGGGTGAGGA	TTTCTTATCTTTTTCAAAACCACC	1	
Ad09A7480	Aradu.A09	28240896	28240922	TGTTGGTGATGGTTAAGGCA	AGCCAAACAGGAACACAACA	1	
Ad09A7483	Aradu.A09	28246396	28246443	CAAACCAATCTAGCCCTAAGAGG	TGGGCATGCATGAAAATATC	2	Yes
Ad09A7494	Aradu.A09	28340150	28340169	AGGGCAGATGAAATCAGTGG	CTGCCGTTCGTCGTTATCTT	1	
Ad09A7505	Aradu.A09	28394420	28394437	TGTTGAGCCACCACTTTTCA	TGCTAGAAGGGAACCTGGAA	1	
Ad09A7517	Aradu.A09	28467585	28467604	GTGCGGGTTGAGCTTTAACT	TACACCCCTAGGCCAACATT	1	
Ad09A7520	Aradu.A09	28472047	28472060	CATTAGGCGTTAATGTGCCA	CCCTAGCGTAGTGCCATGTT	1	
Ad09A7529	Aradu.A09	28578763	28578818	ATGACAGCAGCAGCTCAGTG	CCCAACTCCCTTTCACACTC	1	
Ad09A7532	Aradu.A09	28598260	28598322	TGGTCATCACTTTTAACCATCAA	TGAAATTTAAAAAGTGGTTGGTACAC	2	Yes
Ad09A7543	Aradu.A09	28625052	28625063	GAAATGGAAAACCGGGAGTT	GCAAATTGGAGCACCGTAAC	1	
Ad09A7545	Aradu.A09	28628957	28628992	CACGTGCTAAATGAGCGAAA	CTTGCTTTGCACGTTTGTGT	1	
Ad09A7548	Aradu.A09	28631594	28631605	GAAACAAGCGAAAAACTCGC	CGTTTCTCACGTGTGTGCTT	1	
Ad09A7551	Aradu.A09	28632272	28632301	CACACACACCCACATACACG	GTATTTTTCGCTCGTTTCGC	1	
Ad09A7553	Aradu.A09	28635671	28635682	ACGAAACGAGCGAAAAACAT	CGTTTCTCACGTGTGTGCTT	1	
Ad09A7560	Aradu.A09	28652532	28652563	CTCTGTAAAAGCCTCGTCGG	AGGACCTGACATTGGTGGAG	2	
Ad09A7567	Aradu.A09	28711232	28711245	GGCTCACGTTTCTGAAGACC	CGATAAGCGAAAACATTCCAA	1	
Ad09A7577	Aradu.A09	28752604	28752619	CATCATGGAGGGCTTCCTTA	TTCAATCCCTTTTCAGGTCG	2	Yes

ID	Chromosome	SSR_start	SSR_end	FORWARD PRIMER (5'-3')	REVERSE PRIMER (5'-3')	Bands in parents	Polymophism in RILs
Ad09A7742	Aradu.A09	29926299	29926385	TGCTGTTTAAGTGGATTCATGC	CCCGAACTTGTACTTCGAGA	1	
Ad09A8429	Aradu.A09	35277638	35277727	CTCTTTCGGTGTTCTTGTCTCA	AAGGCGTTAAATTCCTTCCTG	1	
Ad09A9337	Aradu.A09	42264576	42264662	GGAGGAGAAGAAGCAGAAGGA	TACGTGCCTCCTCCAATCTC	1	
Ad09A9806	Aradu.A09	46912466	46912615	CAAGCGCCTTCTATTCTACTCC	AAGGGCATCAGTTTCATTTCA	1	
Ad09A10442	Aradu.A09	52956563	52956669	ACGCAAAAAGGGAGGAGAAT	ACATACGCGTGGAGCTTCTT	2	Yes
Ad09A11056	Aradu.A09	58273948	58274120	TTTTGCACGTGTCTGTGGTT	ATGCAAAACGAGCGAAAAAC	0	
Ad09A11275	Aradu.A09	59537796	59537882	GCTTTTGTCTTCCCTTTCCC	TTCTTCACCTTGGCCTAACC	1	
Ad09A12809	Aradu.A09	72781534	72781656	GAACTCTCTCCCATGGCTCC	CGAAGAAAGAAGAAAGGGGG	1	
Ad09A13461	Aradu.A09	78584028	78584167	ACTATGCCCATGGTGAAAAA	CGACGTGATGGAGGTTAGGA	2	
Ad09A16310	Aradu.A09	98066192	98066274	CCCAACACTTTTGCATTTCA	GCTCAAAAATTCATGTTCCACA	1	
Ad09A16382	Aradu.A09	98520771	98520850	CCCAACACTTTTGCATTTCA	TCTTAGCTCAAAAATCATGTTCCA	1	
Ad09A17943	Aradu.A09	106132860	106132953	GTTGGTTGGTGTTGGTGTTG	CGAATCCGTCCCTCATCTAA	2	
Ad09A18059	Aradu.A09	106694964	106695068	CGAGAAGATGGCACAAATCA	TCTTCGTCTTCATCCCCAAG	2	Yes
Ad09A19214	Aradu.A09	111804894	111804976	CCACAAAAGGGTTTCAGAGC	GGAATTGGAATATCCACTTTCGTA	1	
Ad09A20111	Aradu.A09	114307256	114307363	ACTGAAATGGTGACTTGCGA	CTTCATCGCTCACTTGCTCA	1	
Ad09A20520	Aradu.A09	115381631	115381730	CGAAGGAAGCTCATCCAGAC	CTTCATGCCATTGCAACATT	1	
Ad09A20810	Aradu.A09	116091910	116092046	TCGCTAGAAAGAAAATGTTGATGA	CAAGAAGGAGAAACACATTTTGG	1	
Ad09A21168	Aradu.A09	117134636	117134754	TCCCATCTCTAAGGAGGAAACA	AGCTGACCACTCGACCAACT	2	
Ad09A21954	Aradu.A09	118984140	118984273	TTGGAATTCAAATTGGTGAGTG	CCCACACCTTGCAAATTACC	1	
Ad09A22067	Aradu.A09	119216891	119217021	GGTGTTGAGAGGTGGCATTT	GTCTTTGGTCGGCAAGTGAG	1	
Ad09A22399	Aradu.A09	119991196	119991385	CTGCGAGGAGGAAGAGAGG	CGTCAATGGCTGAATGTCAA	1	
Ad09A22583	Aradu.A09	120376994	120377100	CTTTGGAGGAGGAAGAGGCT	TGGAAGATGACGATGATGGA	2	
Ad10A707	Aradu.A10	2188004	2188105	TTTAATTATGCGCTGCCACA	TCATGAACGCTGCCAAATAG	2	
Ad10A778	Aradu.A10	2344409	2344512	TTGTTTAGGTTGATTGGTACTTGC	CCTTGAGACATTTTCCGCTC	0	
Ad10A1454	Aradu.A10	4284971	4285051	GTTGTCTCTCGTTCCTTCGC	AGCGAACCCAACAAATTGAG	2	
Ad10A1537	Aradu.A10	4602523	4602638	CTATCCAGCTGTTCGGTCGT	TGCAAGATCTTTCTTCTTGGAA	1	
Ad10A1855	Aradu.A10	5601171	5601252	GCCCATTGTCTTGCTTCTTC	AAGAATCTGCGTGTACAAAGAGG	2	
Ad10A1964	Aradu.A10	5988399	5988527	TCCCCCATCCAAAAATAAAA	CTCTTCACCCAGTCACCACC	1	
Ad10A2278	Aradu.A10	7056672	7056831	TGTAAGCTTCCAAACGAACAAA	TTTATTGTGTTGAGCTCCTTTTT	0	
Ad10A2434	Aradu.A10	7621021	7621102	AGGGGACAAAATTACCCCAA	TTTCAAGGTTGTAATGGGGC	1	
Ad10A2722	Aradu.A10	8646182	8646329	CGTGCAAGAAGAAGAAGAACG	AAACGCGATGCAAGGTCTAC	1	
Ad10A3834	Aradu.A10	13208739	13208870	TGTTTTATCAAATATTTTTCACCCTT	TCTCTCGCTCCTCTCTCTCG	2	
Ad10A3961	Aradu.A10	13962788	13962936	GGTCCCCAAGGTTTCAATTT	TTCCACAACAAATTTCACAAAAA	1	

ID	Chromosome	SSR_start	SSR_end	FORWARD PRIMER (5'-3')	REVERSE PRIMER (5'-3')	Bands in parents	Polymophism in RILs
Ad10A3962	Aradu.A10	13963232	13963320	GTGATGATGGAGTGGCAGTG	TTTTTCGCTCCAAAATGGTC	2	Yes
Ad10A4326	Aradu.A10	15899716	15899850	CGCGAATTCGAATCTCCTAT	TTGATGCACAAAACAAAAGC	2	Yes
Ad10A4577	Aradu.A10	17134425	17134506	GCGAATAAAGGAGAGCAACG	ACCTGAGAGTCGTACCACCG	1	
Ad10A4720	Aradu.A10	17678478	17678599	AACGGGCTAAAACCGCTAAT	TCACGGAAGGTGATGATGAA	2	Yes
Ad10A4830	Aradu.A10	18187975	18188075	TCCCTCGTGATTTCGTTTTC	TCACTTCCATTAGTTGGGGG	1	
Ad10A5091	Aradu.A10	19975224	19975334	AAACTTTTTGTCACCGTCCTTC	TGACAATGAAACTTTATAACCGACA	0	
Ad10A5444	Aradu.A10	22122832	22122955	TGATGGACGAGAACGATGAA	CAAATCTCTACGCGTCCAAG	1	
Ad10A6122	Aradu.A10	26269988	26270068	TCTGGGTAAAGTACCGTGGC	CGCGTATCCCCAAGAGTCTA	1	
Ad10A6382	Aradu.A10	27850632	27850774	AAAATAAAAGCGCAACGCTC	TCTCGTTAGGCGTCTGGACT	1	
Ad10A6773	Aradu.A10	30648463	30648588	TCTCTCCTCCTGATCTTCTGCT	TGTTTTCTTCTTCTTCTTTGTTAATTT	0	
Ad10A8566	Aradu.A10	46053220	46053317	CCCCACAAAGGGAGAAAAAT	CAGCTTCCCCAGCTACCTC	1	
Ad10A9006	Aradu.A10	50018114	50018196	CCCAACACTTTTGCATTTCA	GCTCAAAAATTCATGTTCCACA	1	
Ad10A9098	Aradu.A10	50720163	50720249	GGGTTAGGAATCGTGATGGTT	CCTTTGTAAAACTCGAAACCG	1	
Ad10A10356	Aradu.A10	63732451	63732537	GCCATTGTTTCCGCTGTAGT	CGTGAGTCGCTACTGAATCAAA	1	
Ad10A10685	Aradu.A10	66003438	66003536	CTAAGTGTGCACGGTTTCCC	CATGCATCTTCTTTCCAATTCA	2	Yes
Ad10A10900	Aradu.A10	67344729	67344809	GAGGAAGAAGAACGTGCAGC	CGCGCATTAACTCATTTTGA	1	
Ad10A11120	Aradu.A10	68614671	68614752	CCGTACGATGGTACACCTGA	AAGTGAATAGCAAGGATGGATGA	1	
Ad10A11890	Aradu.A10	74375862	74375960	ATGCCTGTGTTGTGTGCTTC	AAGTGGGTGAAGACGGTGAC	1	
Ad10A12567	Aradu.A10	79393677	79393829	GTTGCTCACCCTCGAGAAAA	GCCAAAACCCACACATCTCT	1	
Ad10A12887	Aradu.A10	82018237	82018320	CCTTGGCCGAAACTACACAT	GCTTTTGTCTTCCCTTTCCC	1	
Ad10A13573	Aradu.A10	86395465	86395563	GCATGGGGAGAGAGAAAGAAA	TTCTCCCCTTCCCCTCTTT	2	
Ad10A14091	Aradu.A10	88840171	88840299	AGAATATCAAGCTCCTGGCG	TCCTATGGCAAGCGAAGTTT	2	
Ad10A14183	Aradu.A10	89243288	89243392	TCAATCCCCAACGTTTTTCT	TCCGTTTGTTAAAGTCGGAGA	1	
Ad10A14563	Aradu.A10	90987051	90987192	TTGTTGTTGCTGAATTTGTTGA	GAACATAAGCCTACGCCCAA	1	
Ad10A14670	Aradu.A10	91476961	91477042	CTCCTCCTCATCTCCTGCTG	ACTGCACATTTTCACTGCAC	1	
Ad10A15279	Aradu.A10	94486547	94486627	ATTCACGCCATTTTCACGTT	TCATTTTCGCGTGTTTCATC	1	
Ad10A15580	Aradu.A10	95536559	95536695	CGCGCACTAACTCATTTTGA	GAGGAAGAAGAACGTGCAGC	2	
Ad10A15608	Aradu.A10	95622983	95623091	GTTGGTGTTGGTTGGTGTTG	TTCGTCCCTAATGTCTTGGG	1	
Ad10A15687	Aradu.A10	95992824	95992910	TGCCCTATCCATGCTTCAAT	GTAGGACTGCCCGGTGTATC	2	Yes
Ad10A16640	Aradu.A10	99402880	99402959	TCAACGGTTGGATCAATGAA	CATAGTGCTGATGCTGCTGAA	1	
Ad10A18171	Aradu.A10	103886955	103887054	TGAGGGTTGAATCGAGTAAATTG	CGGAAAATTGTTAATCCGAAGA	1	
Ad10A18338	Aradu.A10	104320484	104320653	AAGGGGCCCACTTTGTATTT	GGAATTACAAGCTGAAAATCACTC	2	Yes
Ad10A18566	Aradu.A10	104823722	104823847	CACACGCTCAACACATACGA	CAAAAGAAGAAGACGCTCCG	1	

ID	Chromosome	SSR_start	SSR_end	FORWARD PRIMER (5'-3')	REVERSE PRIMER (5'-3')	Bands in parents	Polymophism in RILs
Ad10A19369	Aradu.A10	107107303	107107414	TGGAGAAAGAGAAGGCAAGAA	TTCTCCACGTACAAGCGTTTT	1	
Ad10A19546	Aradu.A10	107544391	107544504	CACCATCACCACCACCACTA	CAAAAATGAAGGATAGGTCAACAA	1	
Ad10A19710	Aradu.A10	107890608	107890722	GTGAAACGCACACCATCAAC	TCGACCCCATCTCATACACA	1	
Ad10A19712	Aradu.A10	107896034	107896138	CAGTTTGCAAGTTTCAGGCA	ACCAGTTTCGTCCAATCCTC	1	
Ad10A19801	Aradu.A10	108103731	108103821	GTGCAATTTGGGAAGCAAGT	TGATCAATCACCAATAAAGCCA	1	
Ad10A19968	Aradu.A10	108473506	108473613	GGCACTCAAGTTTGATTGAAAA	GGCACTCAAGTTTGATTGAAAA	0	
Ai01B3449	Araip.B01	8336815	8336955	TCCATTAGGTGACACTTTTCTTTG	CACAAATTTTGCTTCAATTCCA	0	
Ai01B3535	Araip.B01	8604177	8604393	CAGTCATAGCCCTATCTGTGAGA	CGCCTATTCACGTGCTGAAC	1	
Ai01B5245	Araip.B01	14534488	14534594	CCCTCTGATCACTGATGCTTT	TTTTTGTGTGACATAATTTAAGAGTGA	1	
Ai01B5367	Araip.B01	15002822	15002951	CCAATCATTGCCCATGTTTAC	AACAATAAGCCCAAAACCATGT	0	
Ai01B5607	Araip.B01	15889664	15889848	AAAAAGACGAATTAAAAGCATAAAA	ACGACGGTTGATGCTTGAG	0	
Ai01B5852	Araip.B01	16657378	16657500	TCTCCATTGAATGATGATAAATGAC	TCTTGCTCTACTCCTTCGCC	1	
Ai01B7136	Araip.B01	21940707	21940862	TCTTTGGAGGTTTTTGGTGG	TCCTCTTTATTTTGAAACATTCTCTTT	2	Yes
Ai01B7542	Araip.B01	23694767	23694881	CTTGCGGTTGTTGTTGTTGT	GGGTACAAGTCAAGGAAAGCC	2	Yes
Ai01B7608	Araip.B01	23948832	23948933	AGCCAGAAGCGTGACATTTT	GGGTGCGGTGAGTTTACAAG	2	
Ai01B7788	Araip.B01	24847018	24847195	TCTAGCTGCCCTGTTTCGAT	ATGCTCCACTTTATTGTGTTGA	1	
Ai01B7963	Araip.B01	25571278	25571400	TCAATCCCAAACGTTTCTCTC	TCCTCCGTCCAAAAATAAAA	1	
Ai01B8018	Araip.B01	25723222	25723357	TGTCCCTTAGCCAAAGATTCA	GGTATGGTCGGCAAGTCAGT	2	Yes
Ai01B8434	Araip.B01	27673941	27674050	AAACCGTATGCATTGATGCTC	ACTTTGCTTTGGGGCATTTT	1	
Ai01B9610	Araip.B01	33820698	33820804	TGCCGATCCATTTTCTTCTC	TGGGCTATTGAGACGAGCTT	1	
Ai01B9867	Araip.B01	34991624	34991748	GCTTCACCACCGCCATAC	CCCAGAATCAGCAACAACAA	2	Yes
Ai01B10318	Araip.B01	37301046	37301160	CTCACGGTGACAAAGCTTCA	GGGACGAGGGATACAAAAGG	1	
Ai01B10857	Araip.B01	40300802	40300929	ACTGGACGGAACGCATAAAC	TCAAAGCTCCTCCCTTCGTA	1	
Ai01B11045	Araip.B01	41439842	41440034	TCGCATTTCATTTTTAGGTCA	TCCTTGGAAAATTTCATTATTTTG	2	
Ai01B11162	Araip.B01	41926681	41926807	CAACCCAATATCCCAAAATACA	GGGATGCTGGACTTTTGATG	2	
Ai01B11694	Araip.B01	44474383	44474490	GACCCTGCATGAACAGGATT	AAAAACTGCTGAATGGTCCG	2	Yes
Ai01B15952	Araip.B01	69119283	69119465	TTAGAATGTTGCTCGCCCTC	CACACATCCCTCCATCTCCT	1	
Ai01B16294	Araip.B01	71122737	71122885	TGCTTTTGGAGAAACAAGTGC	GCCAGATAAACGCAATCAAC	1	
Ai01B16894	Araip.B01	74072797	74072897	TTCCTTTATGTGTTAGAGGAACAGA	TTTCAATTTGCTTTTATTCCTTTTC	0	
Ai01B18099	Araip.B01	81569866	81570026	CAAGAATTCTCTCTATAGGGCATCA	GGCTCAAATCTCACTTGCCT	1	
Ai01B19054	Araip.B01	87275020	87275190	AAAGGGGAGGTGGAGAAAGA	CTCTCCCCCTTGCTTCCC	1	
Ai01B19390	Araip.B01	89755614	89755784	TTGTAAGACCCCAAGTTTTCG	ACCCATACATGCCACGTTTT	1	
Ai01B20288	Araip.B01	95603358	95603498	TTTCAAGGTTGTAATGGGGC	TGGGTAGCTAGGCATGATTTC	1	

ID	Chromosome	SSR_start	SSR_end	FORWARD PRIMER (5'-3')	REVERSE PRIMER (5'-3')	Bands in parents	Polymophism in RILs
Ai01B21120	Araip.B01	101007835	101007994	CCATGCCTGGACCTTCATC	TGGAATTTTATGCAACGCAG	1	
Ai01B22102	Araip.B01	106506737	106506894	CCCTTTTACCCTTCTGCCTC	TCCAAAATTGTACACACACATTCA	2	
Ai01B22109	Araip.B01	106539177	106539330	AAAATTTGGAAATTGGGAACA	TTTCCACACACTCATGCATTC	1	
Ai01B22325	Araip.B01	107659075	107659212	TTGAGGAATTATTGACTTTGGACA	CGAAACCATGGGTTATTTCAA	2	
Ai01B23042	Araip.B01	111416789	111416924	TGAAGCAAAAGAAACAGCAAAA	AACCATTGATGCTCAAAGCC	1	
Ai01B23569	Araip.B01	113865036	113865141	TTCTTCCGAACAAAGATGGA	AAAAACAATGCCAAAAAGCG	1	
Ai01B23998	Araip.B01	115437488	115437602	AATTGGGAACCCCAAGAAAG	TGTAATGGGGCCAAGGTAAA	1	
Ai01B24617	Araip.B01	118165174	118165332	CGTATCGGAGAACAAGCACA	CACAAACACATCAACCTCCG	2	
Ai01B26086	Araip.B01	123957586	123957724	CCCTTTTGTTAATTTTCTCCGTT	CCAACTAAAAATTCTTTGAAAATTGA	2	
Ai01B26917	Araip.B01	126818368	126818471	AGACATAACAGTCTTTCGTCCAA	CCATTGTTTGGTGCCATCTT	2	
Ai01B28343	Araip.B01	131563616	131563733	TGACCTAGACTCGACTCACGA	AATTTTGAAGGATTAGAATTGAGTAAA	0	
Ai01B29003	Araip.B01	133754288	133754410	GAAGAAGAAGCTCGTGGTGG	TTTCAATTTGTTTTAAAATCGTCC	1	
Ai01B29350	Araip.B01	134632644	134632756	TTCTTCTTTTTCCGTGCCTC	CAATGGAAATCGAAAATAACGA	1	
Ai02B51	Araip.B02	150586	150770	GTTTTAAACGCCCAACCTGG	TCCATTGTTTCTTTTCAGTCACC	1	
Ai02B685	Araip.B02	1663450	1663552	GAAAGAGAGAGAAGCGAGAGAGA	CCCCCTTTTCCCTTTTTCTA	1	
Ai02B757	Araip.B02	1804282	1804384	TTTCACAATGTCAACCCCCT	AAATGGTATTTTGCTAACCATTAAG	1	
Ai02B2350	Araip.B02	5950694	5950822	GGTTACATGCTGCCGAAAAT	TTCTCGTCGTCATTGCTGAT	2	Yes
Ai02B2356	Araip.B02	5954408	5954543	CTCTAGGCTTGGGAGCTTCA	ATCTCTGGCGACGGAGGTAG	2	Yes
Ai02B2534	Araip.B02	6367106	6367218	GAGAGAGCATGGGGAGAGAA	TCCATTGCTTTCGTTCCTTT	2	Yes
Ai02B2634	Araip.B02	6596579	6596679	CTGCGAAGTTAAGGCAGACC	TGGTTCATTTTGAGGCCCTA	2	Yes
Ai02B4199	Araip.B02	11170675	11170798	GCTTATATAAAGGAAACGTGGCA	AGGGGTAAGCTTCGTTTCAA	2	Yes
Ai02B4457	Araip.B02	11995589	11995738	TTGAGGTAAATCTTCCCAACC	CAATTTGCACTAAGCCACAAA	2	Yes
Ai02B4975	Araip.B02	13491227	13491356	CAACTTTCTCCTGCCGAGTC	TGAAACTGAAACTGCAACGG	1	
Ai02B5334	Araip.B02	14444960	14445087	CACAAGCTTCTTTTCCTGCAC	CGAAAACAAGCACACAAACAA	1	
Ai02B5372	Araip.B02	14662693	14662802	TTCTTATCTTTTTCAAAACCACCT	GGGTTCGAATTTGGTGAGTG	1	
Ai02B6055	Araip.B02	17017965	17018117	GCAAGAGAAAAGGAAAGAGAAA	CCTTTCCCCCTCTCCCTCT	1	
Ai02B6072	Araip.B02	17044259	17044414	TGTGCGTGTGTGTTTGCAT	CACACAGTTTTAAAATATGAACAGAAA	2	
Ai02B6565	Araip.B02	18825907	18826026	GGAGGAAATGATGGTGTTGG	TATATTCACGCGCTTTCAGC	2	
Ai02B6837	Araip.B02	19749179	19749351	TGAAGAACAAGACGATCATTAACAA	GCTTGATTTCAAAAACCATGAA	2	Yes
Ai02B7219	Araip.B02	21625481	21625614	TCTCTCCTGAACACTTGTCATTTT	TGCAATACTAGATGTAACTAGAAGCAA	1	
Ai02B7519	Araip.B02	22997068	22997210	CTTCTCCGATGAACTAGCGG	GAGAAGCGATGCGAAGAGAG	2	Yes
Ai02B7671	Araip.B02	23681996	23682133	CGCCTCCTTCTCCATTTTCT	AAAAGAAGAAGACGCAATACTGG	2	
Ai02B7672	Araip.B02	23691457	23691624	TTGGTGATGACAATAACGAAAGA	GCCCACCATTTCATAACACC	2	

ID	Chromosome	SSR_start	SSR_end	FORWARD PRIMER (5'-3')	REVERSE PRIMER (5'-3')	Bands in parents	Polymophism in RILs
Ai02B7886	Araip.B02	24574199	24574310	GCAAGCCCGTATGCATTTAT	CATGCAACTCACCCATTCTC	2	Yes
Ai02B8092	Araip.B02	25636283	25636396	GGAAGCATGCTCATGTGAAA	TCCCATGTTCATTGATTCTTTTT	0	
Ai02B8213	Araip.B02	26193488	26193594	GGGGAGAGAGAGGGAGAGAA	CCAGTTAATCAAAACCAAAACCA	2	Yes
Ai02B8571	Araip.B02	27687240	27687344	AAAAGAACATTCAAAATTGCAAGTAA	CGATCCCTATCGTAATTATTCGT	0	
Ai02B8654	Araip.B02	28165852	28165953	TCTGAGCTACATCGCCACTG	AGCCAACCCCATCTAGTACG	2	Yes
Ai02B9273	Araip.B02	31316386	31316485	AAGAGAGAACGGGGAAGAGC	TTCGAGCTCTCCTTCTCAGC	1	
Ai02B10195	Araip.B02	36251743	36251855	GGGACGAAGAGTAATGAAAAAGG	GAAAAGGAACATGATCAAACCA	1	
Ai02B11229	Araip.B02	43346672	43346835	TCCCTTTTTATTTCTTTTTCTTTTT	GGGCATTTTTGTTCCCTTTT	1	
Ai02B11533	Araip.B02	45269405	45269565	AAACTCAGTTCGTCATCGCA	TCAGCAACAACAATATTCCACA	2	Yes
Ai02B11709	Araip.B02	46122296	46122428	TTGATTTTCAAATCTTTTTCAACTAAC	GGGTGAGGAGAGCAGAGATG	1	
Ai02B12974	Araip.B02	53818891	53818990	CAGTGGCAGAAGAGTCCACA	TGAACTCGTCGTTGAGCATC	2	
Ai02B13410	Araip.B02	56173911	56174086	AGAGGTTATTTTGGAGAAACAGG	GGTGGTAAGGTATTACGACCTCTAAA	1	
Ai02B13600	Araip.B02	57294505	57294621	GCCAGGTTCCAACATATCTCA	TCATCTTGTTGCATAGATTGGG	2	
Ai02B13762	Araip.B02	58325105	58325274	ATCGAGAGGGGGAGAGAGAG	GCACCAGTTTAAACCAGTTCAT	1	
Ai02B14963	Araip.B02	64856428	64856538	AATAAAACAATGCATGCCCC	TGGAAGACTATTTTGGACTTGC	1	
Ai02B15581	Araip.B02	68625059	68625210	TGAAACCAGGGTGTGTGCTA	CCCATCATCTTGCCCTATGT	1	
Ai02B16100	Araip.B02	71523300	71523447	AAGAGGGATAGGAAATTGATTATGT	CCTGTTTGAAACTTAAACCGAA	0	
Ai02B16596	Araip.B02	74236397	74236504	TTTTCCTACGTTCTCTTTCTGATT	CCATGAGCTCACGTGTCAGT	1	
Ai02B16698	Araip.B02	74698067	74698225	AAAGAAAGGGCAAAAAGAGGA	TTTTCTCCCCTCTCCCCTC	2	
Ai02B17331	Araip.B02	77422793	77422948	GAGGAGGAGGGAAGGGAAG	TCAGAAATTCTACAACAAAAATCCA	0	
Ai02B17702	Araip.B02	79096090	79096209	ACTTTTGGCACCAGAACCTG	GCCATTCACCATATACCATGC	1	
Ai02B17732	Araip.B02	79245272	79245478	CACTCTCTTATCATTCCACTTATCAT	TGATAAATGACTTAACAAAAACAGAAA	1	
Ai02B17748	Araip.B02	79307580	79307718	TTGCCATCGGATGTATTTGA	TCACAACGGCACCTGTTTTA	2	Yes
Ai02B18230	Araip.B02	81418267	81418422	AGGGGGAGCAAGATGAAAA	CCCATTCTCTCCCTCTCTTTC	1	
Ai02B18857	Araip.B02	83865502	83865622	TGCACCATTTTAACCAATTCA	TAGAGAGGGAGAAAAGGGGG	2	Yes
Ai02B19680	Araip.B02	86968158	86968312	ACTAACAACGAAAGGGCCAA	CATATTGAATTGAACATCCGACA	2	Yes
Ai02B20354	Araip.B02	89424776	89424887	TAGAGTTCCAAATCCGGACG	GAGGAACATAAAAAGTGCACGA	2	Yes
Ai02B20363	Araip.B02	89461067	89461278	TTTTTATGACGCCTGAAACAAG	TTTGTTATAATGAGTAAATATTGCCC	2	
Ai02B20508	Araip.B02	89993038	89993143	CCCGGATTTGATGAGTTAGC	CTGCTTCAACGAAGACGATG	2	Yes
Ai02B20641	Araip.B02	90529672	90529780	TTTTCCTCCATTTATTTCATTTACG	AGCGCGTGTACATGCTTTTT	2	Yes
Ai02B20920	Araip.B02	91536337	91536473	CCACAAACACCCTCTCAACC	CGATGTTGCCATGTCTTTTT	1	
Ai02B21252	Araip.B02	92635211	92635330	GAGAGAGAGGAAGGGGGAGA	CCACATTTTGGTTAACCTGTTT	0	
Ai02B21379	Araip.B02	93088601	93088741	AACGTCCAATCAATAAACCAAA	GGTTGGTCCAAGCTCAAGAA	2	Yes

ID	Chromosome	SSR_start	SSR_end	FORWARD PRIMER (5'-3')	REVERSE PRIMER (5'-3')	Bands in parents	Polymophism in RILs
Ai02B21536	Araip.B02	93571581	93571713	TGCGCATTAATGAGAGTGAAA	GAAGAAGAAGGAACGCGAAA	2	Yes
Ai02B21538	Araip.B02	93573643	93573775	TATCAGGCGCGTTACACATC	GAAGAAGAAGGAACGCGAAA	2	Yes
Ai02B21744	Araip.B02	94098826	94098941	GGATTACGCCCAAACGTAAG	TGTTGGCATACAGGTTGCAT	0	
Ai02B21859	Araip.B02	94600519	94600656	TGGGATCGAGGAAGAAAATG	TGGAAGGATGGAAAGACACC	1	
Ai02B22025	Araip.B02	95015394	95015525	TTCCTTCGCCTTTGTTATGG	AGATAGAAGTGTGTTAAATGTGGGT	2	
Ai02B22379	Araip.B02	95977247	95977360	CTCCGTACGATTTTGATCCC	GAAGAAGAAGATGAAAAAGATGTGAA	1	
Ai02B22681	Araip.B02	96841409	96841510	ATTCCCAACGTTTCTCTCCA	CACAGGAGCAAAAATGTCCA	2	
Ai02B22742	Araip.B02	96989358	96989483	ACCTTTCCATGCAACTGGAG	TATGAATGAACCACATGGCA	1	
Ai02B23003	Araip.B02	97717005	97717160	TCGTCCTCAGCACTTTCGTA	ACTTGTGCCTGCGACAAAAT	1	
Ai02B23178	Araip.B02	98309901	98310005	TTTTCTCTGCAAATTTAGGCAT	GCATTGTGTACGGCGAGTATT	1	
Ai02B23281	Araip.B02	98575352	98575468	TTGCACATGAGACAAAATGAAA	ATAGAGAAGCTCGGCCTCAA	2	Yes
Ai02B24043	Araip.B02	100653063	100653240	GTCGGGTTGAGAGATACCGT	TTGGCCAAAAACCTTAGCTTAC	1	
Ai02B24181	Araip.B02	101074300	101074456	TTTCTCACCTCTCCCTCACC	GAGAGAAAGAAGGGAAGGGG	0	
Ai02B24182	Araip.B02	101074760	101074903	AGATCGGAGGAGGAGGAGAG	TCATTCTCTCTCCCATATCCCT	0	
Ai02B24289	Araip.B02	101334387	101334493	GGATACACCCACCATGGTCTA	GGAGTGAGACCCACCCTTTT	1	
Ai02B24445	Araip.B02	101857948	101858074	GAGTGAGTCTTGGTGTAAGGCA	TGAACGAGAGAGAGCAGAACA	1	
Ai02B24516	Araip.B02	102037715	102037829	CAACAGCATGGTCATCCATT	TTCAACGTTTTTATCACGACAGA	1	
Ai02B24599	Araip.B02	102269863	102269993	TGCAGAAACATTGAGAAGCAG	CCATTGAAGAATCTCGCCAT	1	
Ai02B24624	Araip.B02	102317872	102317981	CCAAACTGGTTTGCTTGAAA	ATTCGGTGTTCCTCATCACA	1	
Ai02B25013	Araip.B02	103239963	103240075	GGGGAACTAATGATTGGTGTG	TCCCTATTTTCCCCAAAACC	2	Yes
Ai02B25046	Araip.B02	103280778	103280879	TGAGTGTGTGTGGAATGAAGG	TGTTGATCAATACAACTGTGTATCTTT	1	
Ai02B25100	Araip.B02	103451101	103451224	TGTTTTTCAGATGCAGGTCG	CAAGCTCCAGAAAGGTCGAG	2	Yes
Ai02B26210	Araip.B02	106171310	106171420	AGCGTAACTACACGCGCC	GAGGAAGAAGAACGTGCAGC	1	
Ai02B27181	Araip.B02	107928583	107928691	CCACCGCGTACTAATAGGGA	CTTGGTTTCGCAGGTTGAGT	2	
Ai02B27634	Araip.B02	108725072	108725179	CACGTTGAAATTGTTAGGTTTGA	GTTGTCAAATTTGCGAGCCT	2	
Ai03B1163	Araip.B03	2683684	2683807	TGAGGATGCAATTCCTTAGTG	GGCGGTGAAGATATTGGAAC	1	
Ai03B1991	Araip.B03	4155636	4155747	GGATCCAAGTGAGGCACATT	TCACTGAGTTCGTTCGTTCG	1	
Ai03B3140	Araip.B03	6587997	6588099	CACAGTTTCAACGGCAATGT	CCAAAAATTCGTCACATGTATCA	1	
Ai03B3342	Araip.B03	7020603	7020775	CAAATCAATCCCCAACTTTCTC	CATAATAGTCCCCCGTCCAA	1	
Ai03B4381	Araip.B03	9503066	9503248	AGATGGGTTGCTGCTGTAGG	CGCATCGCTTCTCTCTCTTT	1	
Ai03B6104	Araip.B03	14034441	14034625	GCTCTCCCTCTCTCTCACTCA	GGTTCGAGTCTCCTATCTTTCG	1	
Ai03B6119	Araip.B03	14091298	14091405	GCAACAGAGAGGAGAATGTGTG	GGCCAATTTTAAGGGCTAGG	1	
Ai03B6448	Araip.B03	15016298	15016398	CATTGAAACAAGAGATTGGATTT	TGGATTCACATTTGACACAGG	1	

ID	Chromosome	SSR_start	SSR_end	FORWARD PRIMER (5'-3')	REVERSE PRIMER (5'-3')	Bands in parents	Polymophism in RILs
Ai03B8833	Araip.B03	22185519	22185732	AGACAAGGGATAGAGAAAGAAAGAC	CAAAACCTAATTTCAAAACCAAA	1	
Ai03B9630	Araip.B03	25032569	25032680	TTTCTCCCCTCTCCCTCG	GAGAAAAGAGAAAAAGAAAGGGAA	1	
Ai03B10088	Araip.B03	26590789	26590914	TGACAATCGCAATTTCAAGG	AAATACAAAAACAATGCACAAAAA	0	
Ai03B10105	Araip.B03	26631151	26631352	GCACGTAAATGAAGAAGGAGAAA	ACTGCACGTTTTCACTGCAC	1	
Ai03B10865	Araip.B03	29429667	29429774	TTTCACTTTCATACACACAATCAA	TGGTTGGTTCTCGTAATAATTTCT	1	
Ai03B11430	Araip.B03	31731107	31731250	TCGGCCAAAGGGAAATAAAT	GGGTAGGATGTAAGACCCCAA	2	
Ai03B12091	Araip.B03	34651698	34651835	CGTGCAAGAAGAAGAAGAACG	ACGAATAAACCCCCAAAACG	1	
Ai03B15214	Araip.B03	48140478	48140617	CCTTTGTAAAACTCGAAACCG	TGTCTGTGTCTGTGTGTGTGC	1	
Ai03B18080	Araip.B03	56260118	56260263	GAGAGAGAATGGAAGGGGAGA	GGTTAACCAAAACCAAAACCAA	1	
Ai03B20724	Araip.B03	72473375	72473490	CTAAAATTCACGGCCTCCAG	TGGTATTTGAGGGTTTGGATG	1	
Ai03B20837	Araip.B03	73091893	73092025	TTTTTATCATCATCATCATTCATCA	AAAACACGTGCGCAACAAT	2	
Ai03B22384	Araip.B03	81966072	81966206	TTTGGTTAGATTGAACTTTATTTTGAA	GATACGGAGGAAGACGACGA	0	
Ai03B22494	Araip.B03	82599625	82599735	AGAGGGATCAAGTGTGGACG	TGCAAATATCTTGTAATCCCCA	1	
Ai03B23146	Araip.B03	86061602	86061712	TAACATGGATAGCCCAACGC	TGAACTATTAAAAATGGACAAAAGTG	1	
Ai03B23157	Araip.B03	86100397	86100499	CGAATCTGGATAGGCAGAGC	ATCCTTCGACGTCCTTACGA	1	
Ai03B26132	Araip.B03	103345661	103345791	GAAGTCGCGTCACACTCGTA	CGCATGTATATTTTGTATTTTTCCC	1	
Ai03B26555	Araip.B03	105401636	105401740	TGTCAATTTTTAAGGACGAAAAT	TGTTCTCAAATGTTTTCGTTGG	1	
Ai03B27046	Araip.B03	107790866	107791088	CGTCATGGAGGAGGGAGTCT	ATTGCCATGGCCAAGAAC	1	
Ai03B27999	Araip.B03	111679332	111679451	CATGCTAAATTGTGGCACATAG	GCGGACGAGCTACATACCAG	0	
Ai03B28258	Araip.B03	112775636	112775774	TATACACATGCAGGACCCCA	AGGAAAAGACACAACCACTGTCT	1	
Ai03B28386	Araip.B03	113419730	113419869	TTCTTTTTCTTCTTCCCGCC	CCAAAAAATTTCACAAACAAATCA	1	
Ai03B28927	Araip.B03	115761914	115762019	TTGTTGCAGACAACATAAGAGTAGC	TCTTTCTAAATGAAGTAGGTCGCA	1	
Ai03B29470	Araip.B03	117806690	117806791	TTTTTCGCTCCAAAATGGTC	CACAACAAATTTCAACAAAAGCA	0	
Ai03B30116	Araip.B03	120028313	120028414	GCCAGCTCTAAGCGAGGTAA	ACGAAATCAATTCTGGCACA	1	
Ai03B30551	Araip.B03	121403666	121403792	TGGTAAAGGAATGAGTTTGGGT	AAAGGTCATGAGTTCAAATTTCATT	1	
Ai03B30911	Araip.B03	122397341	122397455	CCGGAGTAGAGGAGCAGAAG	GCTTAACGCCTCACCCATAA	1	
Ai03B32338	Araip.B03	126266952	126267116	TGCATTGCATTATTTTGTGAA	CATCAGGGTCGATTTCGATT	2	Yes
Ai03B33186	Araip.B03	128224874	128225080	TTCATGCGGCTATTATTTTCTC	TAGCGACGATGACGACTGAC	2	
Ai03B33341	Araip.B03	128754974	128755099	CCCACCCAAATCCCCTATAC	TTTTATGGTAAAATTGTTAGAACGTCA	2	Yes
Ai03B34328	Araip.B03	131271231	131271353	TGTTGAAGATGATGATAACGAAAGA	TATACACGCGCACACGTTTT	1	
Ai03B34812	Araip.B03	132692883	132693020	GCTCTTGGTATTTATTGCTCGC	GCAGCATATTGCCCTATTCC	1	
Ai03B34889	Araip.B03	132856920	132857036	TGCTTTTAATTTGGTGCGTG	TGTGCAGAGAAGAGTAGACCCA	1	
Ai04B16	Araip.B04	66229	66445	ATGACCATCACTGCTCCACA	GGACTGGGGATTGCATAGAA	2	Yes

ID	Chromosome	SSR_start	SSR_end	FORWARD PRIMER (5'-3')	REVERSE PRIMER (5'-3')	Bands in parents	Polymophism in RILs
Ai04B1445	Araip.B04	3763755	3763872	AGCCAAACAACACAAGAGGG	TTCCTTTGAGCCTTTGCACT	0	
Ai04B2241	Araip.B04	5799828	5799928	TTCGACCATGTGAATGAAGC	GCCCGGGCTCTAACCTTAAT	2	Yes
Ai04B2635	Araip.B04	6905276	6905423	CGAAGGACTTCCTTTTGTGC	GCTTCAATTTCAGATTTCTGCTG	0	
Ai04B3132	Araip.B04	8194033	8194199	GCCTAATAGTCCAACACAGCC	CGAAAAAGTGGAACTTGGGA	1	
Ai04B3484	Araip.B04	9226523	9226692	AAACGATTTCAGAGCTTGATG	CCACATACTGTTACGGCACG	1	
Ai04B4849	Araip.B04	13502425	13502530	GTGCTTTTTGCAGGTTCTCC	TTGAGATGGCAAGGTATTACGA	2	Yes
Ai04B5273	Araip.B04	15012262	15012408	GCGAATGCAATAAAGGAAAA	GACGCTTATGTGTGGATTGC	2	
Ai04B5526	Araip.B04	15902137	15902251	CACGCGCCTCATATAACGTA	AACGCAAACAGAGGAGAACAA	2	Yes
Ai04B5942	Araip.B04	17349539	17349652	TTTTCTGCTGCGGAAGCTAT	CCTCACCCCCTGTATGTGAC	1	
Ai04B7769	Araip.B04	25638706	25638815	GCATGAACCTTTTTCATCTGG	TTTGTGCATACTGGGCTTCA	2	
Ai04B8073	Araip.B04	26841348	26841463	TCTGCCACTCCTTCTCTGGT	AACACAACTTTTATTCGGCCA	0	
Ai04B9397	Araip.B04	33208563	33208728	CCAAAGAAGTTGCAATGACG	CTCCGCTTAAGAGCAGAGC	2	Yes
Ai04B9499	Araip.B04	33748747	33748878	AAAACATGAAGATCAATCAAGAAGG	AAATATAATGCATGTATCGTGTAGACC	2	
Ai04B9972	Araip.B04	36640947	36641085	CCGGTTAACCAAAACCAAAA	ATGAGAGAGAGAGGGGAGGG	1	
Ai04B10462	Araip.B04	39325861	39325964	CGTGTGGGTTATGTTATTTTGAA	CCCCTCTGTTCCTAGAACCTT	1	
Ai04B12760	Araip.B04	52113951	52114105	TTTCTCTCCCCCTCCTCTTC	AGGGGAGAAATATGGGAAGG	1	
Ai04B12763	Araip.B04	52115149	52115273	AAGGGAGAGAGGGGGAAGA	CCTCCAATCTCTCTTTTTCCCTA	2	Yes
Ai04B13474	Araip.B04	57075528	57075645	ACAGAAGCTCTGGGTTGCAT	CAGAGAAAAGAAAAACCAGAACA	2	
Ai04B15745	Araip.B04	72870571	72870679	AAAATAATGGAGAATCCATGTGC	TGCCTAGCTATCCATAATTCCC	1	
Ai04B16305	Araip.B04	76412897	76413009	TGTTTCTTAACTTTTTCAAAACTACCT	GGGCAGAGATGTGAGATGAGA	1	
Ai04B17014	Araip.B04	80204142	80204288	GAAAGGAGGGGGAGAGAAAA	CCCTCCTTCCCCTCTCAC	0	
Ai04B17746	Araip.B04	83859814	83859925	GAAGAAAAACCTGCGTGTGC	TTAAATCACGCGCTCCTTCT	2	Yes
Ai04B18016	Araip.B04	85608832	85608933	TGGAAACTTTGGTTGATGAGG	AACATTGGGGTAATTTTGTCC	1	
Ai04B18107	Araip.B04	86386158	86386273	TGAAATATATGCGCCCAACA	GCAAAGAAGAAGGCGAAGAA	1	
Ai04B18767	Araip.B04	90378765	90378876	CTTTTTGCCCAAGTGGAGAG	TTTCGAAAATTACTAACCTTTTTCA	1	
Ai04B19238	Araip.B04	93246956	93247070	CATTTTGGGGTTTTACTTTTGG	ACTAGGGCTGTCCGGAGAAT	2	Yes
Ai04B19908	Araip.B04	96497885	96498012	AATGAAACTGCAACGCAATG	CCCGTATCCACCTTTCTCCT	2	
Ai04B20539	Araip.B04	99203646	99203783	AACAGCAAATCCTGCCTCAT	CCATTTCGTTACAAAAGTGCAA	2	Yes
Ai04B20695	Araip.B04	100015080	100015190	TTTGGGGTATTTTGTCCCCT	TGGTTGGGATAAAAGGGTGT	1	
Ai04B23475	Araip.B04	111621458	111621601	TGGGCTTTCAACCCAAATAA	GTCCCTTTTTCACTGGTTCG	2	
Ai04B23931	Araip.B04	113027725	113027843	TCTCTTCTTCCACTCTCTCTTTCA	AAAAAGGGGAAAAGCAAAGG	1	
Ai04B24958	Araip.B04	116693175	116693305	TTGTGCACGTAAAATGGAGC	TTTAATCAAATCCAACCAAATTGT	0	
Ai04B25623	Araip.B04	119169187	119169330	CTCCTAACTCAGCCCTCGAA	GGACTTCTGAAGCGAAGTGG	1	

ID	Chromosome	SSR_start	SSR_end	FORWARD PRIMER (5'-3')	REVERSE PRIMER (5'-3')	Bands in parents	Polymophism in RILs
Ai04B27696	Araip.B04	125353243	125353351	GAGGGCTAACACTACAAAAATCG	GTTTAGTGCGGATTGGATCG	1	
Ai04B28049	Araip.B04	126309018	126309165	AATTGGTGGTGTGGTGGTG	AAATGGTCCCCAAAGTTTCA	1	
Ai04B28641	Araip.B04	127883491	127883664	AAACTCATATTTTGAAATTTTGGACC	TCGATCTATTAACGAGTGATTGC	2	
Ai04B30470	Araip.B04	132244886	132244985	TTGAAACAATCGAAGAATCCG	TAAGTTTTAGCGCATGCCAG	1	
Ai04B30796	Araip.B04	132924612	132924764	GGATCAAGAGGAGACAGGGA	TTTCCTTCTTATCCTACGCACC	1	
Ai04B30953	Araip.B04	133269112	133269221	AAAAAGTGTGTCTTCAGTCATGTAAAA	ATCACAGGTTCAATCAGGGC	1	
Ai05B1430	Araip.B05	2961936	2962041	TTTTAATTGTCCTGTTTTAACAACTTT	TCTTGGACAGCCATCATACG	0	
Ai05B2659	Araip.B05	6021286	6021444	GCGTAAAACTCTATGTGGTAGGATG	TTTTGGATATAGCGTCACAAAAA	0	
Ai05B4675	Araip.B05	12019265	12019487	TTTTCAAAGCGCTACACTCA	TTCAGAAACTATGAAAATCGAAAAA	1	
Ai05B4933	Araip.B05	12754807	12754933	GGGAAGAGAAACAACAAAGCA	GAGGTGCGATTTATCGGAGA	1	
Ai05B5265	Araip.B05	13802774	13802995	CACGCACTAGATCTTCTTCTCC	TGTTGGTGATGATGATAATGGAA	2	Yes
Ai05B5809	Araip.B05	15475405	15475531	AAAGGAGCGAAAAATACGCA	CTCGTTTTGCACGTGTGTCT	1	
Ai05B6490	Araip.B05	17760885	17761014	AAATCCTTCAATCATATTCATTACCTT	TTGGACATTCAAATTATGTAAGTTTTT	0	
Ai05B8601	Araip.B05	25547746	25547851	TCGCAGAACTAAAACAGACGTT	ATGCCAAGAAGAAGAAGCGA	1	
Ai05B8834	Araip.B05	26466006	26466106	CTGATCGCCTGCCAAAAA	TTGGATCATCCTCAATGGGT	1	
Ai05B9660	Araip.B05	29550263	29550374	GACGAGAAACCTTCCCATGA	GACGAACTTGCCCAAATACC	2	Yes
Ai05B10202	Araip.B05	31763287	31763472	TTCTTCAAAAACGATTTCAGAGC	CCACATACTGTTACGGCACG	1	
Ai05B12077	Araip.B05	42226478	42226595	CAATGGATCGCCCTAAAAGA	TTTTTGCATCGTACCTTGTTT	2	Yes
Ai05B12424	Araip.B05	44197871	44198029	GAAGGAGAAAGAGAAGGCAAGA	TCACGATTCTTATCCCTCTTACAA	1	
Ai05B12488	Araip.B05	44473244	44473398	GGGACGATTTTTGGTGTGAT	TGCAAATAAAATGTGATCCACG	2	
Ai05B12998	Araip.B05	47302166	47302276	TCGAGTCCTCGCCTAAAATG	GGCGATGTTGGTATTCCACT	1	
Ai05B15046	Araip.B05	62061041	62061171	CGCCCACTGACTTCCAAATA	TATTGACGGCGATTACGGTT	2	Yes
Ai05B15820	Araip.B05	67083675	67083811	TCTCCCCCTCCTCTCTCTTC	AAGAGGGAGAGAAACAAAGACA	2	
Ai05B16037	Araip.B05	68458664	68458825	CCTTGTTCTCTTCTCGCATTTT	TCGTTCAACTGATTTGGAGTT	1	
Ai05B16421	Araip.B05	71469775	71469890	CCAAAAACCGTCATGAACAA	TGTTTGCATATATTTTGGTTTGC	0	
Ai05B16553	Araip.B05	72466510	72466618	TTTCATGCCCAATAAACGAG	TCTCTGTATTGTTAGTGACGTTGTT	0	
Ai05B17996	Araip.B05	83079063	83079236	AGAGGGATCAAGTGTGGACG	TGCAAATATCTTGTGATCCCC	1	
Ai05B18121	Araip.B05	83963084	83963196	TGTTTCCCTCGCCTTTATCTT	AACCGCATAATCTTTCAAAAACA	1	
Ai05B18201	Araip.B05	84625128	84625229	GCTTTATTTTGCATTTGGGG	TGGGAAACCTATTGGGAACA	1	
Ai05B19112	Araip.B05	91071913	91072027	GGATTTGGGAACGTATTCATGT	CGTCTTGGGGTTTCTTTGTC	1	
Ai05B19615	Araip.B05	94540034	94540213	TTCCACAACAAATTTCACAAAAA	TTTGCTACAAAATGGTCCCC	1	
Ai05B19811	Araip.B05	95636365	95636515	GGGGAAATGTAGGAGGGAGA	TACCTTACCCCAACCCCTTC	2	Yes
Ai05B20237	Araip.B05	98304893	98305098	GAATTTCAAAGGGGTAGGACTTG	CAAAACTAAGGCTGACCGGA	0	

ID	Chromosome	SSR_start	SSR_end	FORWARD PRIMER (5'-3')	REVERSE PRIMER (5'-3')	Bands in parents	Polymophism in RILs
Ai05B21629	Araip.B05	106899075	106899181	AGAGGAGGGGGAGAAGAATG	TCCCCTCACCTCTCTTTGTC	0	
Ai05B21706	Araip.B05	107241755	107241856	AAGAGGGATAGGAAATTGATTATGT	CTCAGCTATAGCACAAGCGG	1	
Ai05B22435	Araip.B05	111734740	111734882	CACAAATTCAGCCCAACAGA	TTTTATTGATTGTTGATTGATTGAA	1	
Ai05B22619	Araip.B05	112483731	112483874	CAAGCTGAAGGCTTCCAAAG	TGAAATGAGCAAGATGAACGA	2	
Ai05B25638	Araip.B05	125704397	125704513	GGATTGAAGGCTTGAAGCTG	CCGCTAGTTTCTCTGTCGCT	2	Yes
Ai05B25831	Araip.B05	126380630	126380734	CGCATGAGAGGGGAGTAAAA	TGATCAATTTCCATGGCTGA	0	
Ai05B26240	Araip.B05	127639989	127640177	TCAAGAATACTTTCATGATATGAGGTT	TGCTAATGTCTCCTTATCTAACGATTT	1	
Ai05B26922	Araip.B05	130005159	130005329	GTGTTGGTGCGATGATAACG	AAGTCCAACAAGTCATTTACACTCA	2	Yes
Ai05B27169	Araip.B05	130691293	130691424	CATGGAGGAAGAGGAAGTCG	CCAAAGCACTACACTCACCG	2	
Ai05B27759	Araip.B05	132542299	132542440	CACACTCAAAACACACTCTAAATCC	GAAAAAGAAAATCTGTTGCTAAAAA	0	
Ai05B28050	Araip.B05	133533188	133533290	AAATATCCGCCGGTAAATCC	AATGGTGGCAGAGGCAATAG	2	Yes
Ai05B28771	Araip.B05	136054612	136054722	ACTGCACGTTTTCACTGCAC	GAGAAAGAGAATGCAAGAAACGA	1	
Ai05B28787	Araip.B05	136106424	136106555	TCAAAAAGTCGAACAATTTGTGAT	CAAGAAAGAGGATTATGGATAGCAA	2	
Ai05B32361	Araip.B05	145894732	145894839	AGGGACGGAAGATGGGTATC	AGAGTGTGTGGGGAGTGAGG	2	Yes
Ai05B32370	Araip.B05	145909762	145909887	GCTGGCTCATTGTTGTTGAA	GGCCATTTGATTTGCAGAGT	1	
Ai06B357	Araip.B06	776402	776546	TCATTTTGTTTTCTTATTTTTGGGA	GATTGCCAGCGTGGATAAGT	1	
Ai06B632	Araip.B06	1247546	1247730	CCTCTTTCAAGTAATTTTGCAGC	CTCTTCTTCTCCCTCCTCCC	1	
Ai06B739	Araip.B06	1487910	1488013	TTTTTAATGTTTGTTGCGATAATAA	CCGACATGATGATTAAATTTTGAG	1	
Ai06B935	Araip.B06	1979928	1980089	AACCAGATCCAATTCGTGTTT	GTGCAACTTCACGAATCACC	1	
Ai06B3856	Araip.B06	9691939	9692045	CCTTTTTGTTTTACCTTTTCAAGTG	GGTGCCTGATTTTAGTGGTTG	1	
Ai06B4862	Araip.B06	13155036	13155236	AAAAAGCCGACACCACCG	TCAAACAATAATACTCAGAATCAGAAA	1	
Ai06B5104	Araip.B06	13912038	13912169	AGCTCAAAAACATGATCCACAA	ATCGGCATTTTTGGGTGAC	1	
Ai06B6024	Araip.B06	17022586	17022746	GCACGTTCTTCTTCATGCTG	GGAGGAAAAGGAAGGAGGAG	1	
Ai06B6479	Araip.B06	18613379	18613495	GGACTAACAGAGCGACAGGG	AATTTCGAAGCGCAAACACT	1	
Ai06B6657	Araip.B06	19283774	19283904	TTTCTTCTTCTTTGAGAGACGTG	TCAATCACCAACACCCACAC	1	
Ai06B6681	Araip.B06	19371494	19371594	GTCCGATGGTAAATTCGACG	GTAGTGAAGGCGACACCGAC	1	
Ai06B7451	Araip.B06	22278964	22279132	CATCAACATAGCAAAAATTCAACA	ATCGTTACTGTTGTCGCCAT	2	Yes
Ai06B8399	Araip.B06	26191497	26191601	TTCACTTTGGGGCATTTTGT	TGGGTAAACACTCATGCATTCT	0	
Ai06B8780	Araip.B06	27913230	27913333	TCATCCGTTTTGGAGGAAAG	TCGAAAATCTTCACCCATTTTT	1	
Ai06B9021	Araip.B06	28985373	28985532	GGGAGGCAATCTGATGTTTT	TTTTTAACATTCTGCATGACCAA	0	
Ai06B9106	Araip.B06	29417919	29418090	TTCCTCTTCAGGCTTCTTGTTT	AAGGTGGTACGACTCTCAGGT	1	
Ai06B9693	Araip.B06	32141773	32141877	TTGAAAATATTGGGAATTGGG	GGCATTTTTGTCCCCTTTTT	1	
Ai06B10201	Araip.B06	35317173	35317396	AATATCCTTCGGCCATTTTT	GGTTCTTTTTCCTCTTTCTTCAC	1	

ID	Chromosome	SSR_start	SSR_end	FORWARD PRIMER (5'-3')	REVERSE PRIMER (5'-3')	Bands in parents	Polymophism in RILs
Ai06B10907	Araip.B06	38741300	38741401	AGTCGATGTGGCGAGGTATT	GTGATGCGATTTCAATTTCG	1	
Ai06B11788	Araip.B06	43810546	43810687	CAGAAAGCTCAAACGCACAG	GGATTTTGAGCTCCTTTTGG	1	
Ai06B13545	Araip.B06	55862832	55862937	AGCCAATGCCAGTAGCTTGT	CGTAATTAGTCTGGCCCCAT	2	Yes
Ai06B15732	Araip.B06	65481970	65482069	ATTAAGCGGTGATGTGGAGG	CATGAAATGCAATGCAAACC	1	
Ai06B15782	Araip.B06	65855058	65855167	TCGAGAGGCACCTCACTAGG	TCCTAGACCTCCATCAAGCC	1	
Ai06B16693	Araip.B06	71767262	71767433	AAACCTAATGTGTGAATAAATGAATGA	AAAAGCAGGTGTTTACTTTGTGC	1	
Ai06B18381	Araip.B06	82405381	82405508	TTTGTCCCATGTGCATTGAT	TCCCAAGTTGAATCCTTGGT	1	
Ai06B18690	Araip.B06	84504597	84504731	GCTATTATGCTCCCCTCTGC	TGAAATTGGGAGTGGTTGGT	2	
Ai06B19288	Araip.B06	87663816	87663957	TTGAAAAATAAAAGGAAAGGAGGA	TTCCGACAGAACGAGAGGTT	2	Yes
Ai06B19579	Araip.B06	89103959	89104146	TTTATGAGTCAAATCAAATTTTCAA	TTGAAAAAGAGGGAGATATTTTCG	1	
Ai06B19719	Araip.B06	89761713	89761821	CGAATCTGGATAGGCAGAGC	ATCCTTCGACGTCCTTACGA	1	
Ai06B20094	Araip.B06	91720879	91721010	GTGCTTTGGGTGCTCATTG	CATTTTCTTTTCAATTCAACCACA	1	
Ai06B20411	Araip.B06	93371428	93371538	AGCCACCTTTGGCTTTTCTT	AGGCAATACTTGCAACGAGA	1	
Ai06B21393	Araip.B06	98898478	98898591	TGGAGTTATACAAAGGCTTGTGC	TTTCAATTATTATACATTTCTTGCCTT	1	
Ai06B21543	Araip.B06	99741561	99741692	TTTCTCCAACCTTTTGGTGC	ATGATGAATCATGGACGACG	1	
Ai06B21949	Araip.B06	101792114	101792253	TTCCACTTAGATCCATGACGG	TCCTTACCACTTTCTCTCCCC	1	
Ai06B22126	Araip.B06	102631128	102631259	CACCAACATCAACCACAACC	GTTTCCTGCTTTCTTGGTGG	1	
Ai06B22307	Araip.B06	103402903	103403003	CGAAAATCACTAACCATTTTTCAA	GCCCAGGTATAGAGGTTCCC	1	
Ai06B22668	Araip.B06	104907584	104907695	TGAAACCATAATTTTTATGAAACCA	GGAGGAGAAAGAGAGGAAGGG	1	
Ai06B22767	Araip.B06	105373994	105374108	AAATCGCGAAGACGTACCAC	GTACCCTAGCCGGCCTAAAC	1	
Ai06B23277	Araip.B06	107693694	107693806	CTCGCTATTGCTCAGTCCCT	TGAAATGGTCGCTTGCAATA	1	
Ai06B23527	Araip.B06	108822360	108822495	TGGAGATAGTTCAGCTTAGGAACA	TCGGGTATTTCATCGAGTCTTT	1	
Ai06B24038	Araip.B06	111051024	111051151	AAAAACCAATTTCAAAGCCC	ACCAAAATCACAAAGGCCAC	1	
Ai06B24686	Araip.B06	113419536	113419659	TCCCTTTACCACTAACCCCC	TAATGGCGATGGTGATGAGA	1	
Ai06B24907	Araip.B06	114347867	114347974	CCGATCACTTTAGATCGGCT	TTCCTCTGGTTCATTATCCTCC	1	
Ai06B25262	Araip.B06	115667509	115667608	AAAGGAAAACCAAAAATGATAAAAA	TGCCCATTCCAAAACATACA	1	
Ai06B25720	Araip.B06	117226047	117226191	TGAGTGTGAGAGGAAGAGCAC	AGAGAACCGGCGGATTTG	1	
Ai06B25796	Araip.B06	117500334	117500450	CATGCTGGAGCAACTAACGA	ATGGTTAAACCCAGCACAGC	1	
Ai06B25918	Araip.B06	117978633	117978764	TCAATTTTCTCGATCATGCTG	TCCTTTTTCGGTTAAGAGTCCA	1	
Ai06B25928	Araip.B06	117997856	117998062	CCCCTCTCCCTCCTATCAAC	CGAAAATTACAATGCAAATCG	1	
Ai06B26745	Araip.B06	120947923	120948048	CTGGAGGAGCTCACATCCCT	TGGAAGGAATCGGTTGAAAG	1	
Ai06B27039	Araip.B06	121912180	121912282	AATTGTGGGTTCGAGTCTCC	TTAAGAAAAATGTGCGTGCG	2	
Ai06B27348	Araip.B06	123012639	123012809	AAAAAGAGGAGAAACGCAAAGA	GTGTCAACACGTGCGCTACT	2	

ID	Chromosome	SSR_start	SSR_end	FORWARD PRIMER (5'-3')	REVERSE PRIMER (5'-3')	Bands in parents	Polymophism in RILs
Ai06B27638	Araip.B06	124107889	124108027	GGAGAAAGAGAGAGAAGAGGGC	TGAACCAAACTGCTTTGTCAT	2	Yes
Ai06B29285	Araip.B06	129091619	129091766	TCAACCACACAACCTTTCGAT	CCACCTACTTGTCATTAGGCAA	1	
Ai06B29452	Araip.B06	129552041	129552144	GGGTCGTACTCTCTTGCGTC	GAAGAGAGCTTTGACGGTGG	1	
Ai06B29531	Araip.B06	129751769	129751891	CATGGATGCCAATACCTTCC	AATCAAACATCTCTAAACCCCC	1	
Ai06B29598	Araip.B06	129899861	129899975	TGCTTTCTTGCTTTCCTGCT	CCACCAACATTACCACCACA	2	Yes
Ai06B29716	Araip.B06	130299166	130299324	ATGCACTAAGCCACGTTTCC	GTGAGATAAAATTGTAAGACTCCAAA	2	Yes
Ai06B30789	Araip.B06	132702877	132703002	GCATTTGTTTTGAATGAACAAAGA	AAGATCTGCCCCTCATTTTG	2	Yes
Ai06B31304	Araip.B06	133809775	133809948	CGCATACAAGCGATTAAGGC	GAGGAAGAAGAACGTGCAGC	2	Yes
Ai06B32416	Araip.B06	135916057	135916209	CAAGCCAAAAACTCAGTCCA	GCTCCATTTTGAAATCTCCG	2	Yes
Ai07B99	Araip.B07	258756	258855	GCCAGTTGCCACCAAAGTTA	AGATGGCATGCATAAACCGT	1	
Ai07B1340	Araip.B07	3236887	3237020	GGGGAGAGAGAGGGAGAGAA	TCCTTCCATTGCTTTCGTTC	1	
Ai07B1456	Araip.B07	3560903	3561033	ACCACACTCAAACACACCCA	TTGTGTATTCCCTGCAACCA	1	
Ai07B2047	Araip.B07	4886239	4886365	CTCCTTCTCCTTCCCCCTT	GAGGAGAGAGGACAGAGAAACAA	1	
Ai07B3006	Araip.B07	7501433	7501562	TTAATCGTGCCTCTGTGGTG	TGTTTCGAATTAGTTTGGGTTTG	2	
Ai07B3210	Araip.B07	8138095	8138214	ACAGAAGGAGATCGAAGGCA	CAAGTTGGGCCTAACACGC	1	
Ai07B3591	Araip.B07	9119264	9119368	CAGGTAATTAATGTGAAAATCAACCA	AACAATTTAGATGCCCGCAC	1	
Ai07B4496	Araip.B07	11665650	11665791	TTGTGATAGGGACCATAATATTGAGA	CAAACGTTATACTGACGGTAGGG	1	
Ai07B4966	Araip.B07	13247897	13248073	TCCTGCTGGATTTGAATAAGTATC	CAACCCCCTTTCTCTAAACCA	2	
Ai07B5643	Araip.B07	15893570	15893686	TGTGAATGTTCTTATCATAATTCTTGT	GGTCCAGTGAACTCAGAAAAAG	1	
Ai07B5769	Araip.B07	16216461	16216561	TGGTCCACAGAAACAAAATCA	TCATCCCTTCGGATCTGTTC	1	
Ai07B5845	Araip.B07	16475382	16475486	GCAGCAACAACAACAGCAAT	CGTTCCTTTCTTCCTCCTCC	1	
Ai07B6384	Araip.B07	18536622	18536781	GGGAGGGGAGAGAAATAAAGAA	CTCCCCTTCCCCTCTCTC	1	
Ai07B6696	Araip.B07	19731006	19731133	TCTTTCTCGGATCTCCCTCA	CTTTTACACCCTCACCCACG	1	
Ai07B6722	Araip.B07	19838656	19838784	ACTCCCATGTTGTCGAGTCC	TTGGTATTCCACTTGCACGA	0	
Ai07B6996	Araip.B07	20817518	20817628	TTTGGCTTGCTAGTTTTTCAA	TACCTTCCCTCTTTTGCTGC	1	
Ai07B7279	Araip.B07	21979677	21979842	AACAAGCAGACATAGATTCATATACAA	TGAGAACTTTTTGCCTTTTCTTT	0	
Ai07B8626	Araip.B07	27522975	27523082	TCAATGAGGCACAAGAGCTAAA	TGTAGGTCGAGAGACACCCC	1	
Ai07B8981	Araip.B07	29181073	29181224	GGAGGAGAGAGAGAAGGGAGA	TGAAACAATTTAAAAACCATATCCA	1	
Ai07B9112	Araip.B07	29687975	29688079	GACTTCCAACGCTTAATGGG	TCCACCATCAATGTTTTCTCC	1	
Ai07B9137	Araip.B07	29785081	29785198	TAATGCTGAGGGCACGTTTT	CATCATCAGAATAAAGGGTCTTGA	1	
Ai07B9654	Araip.B07	31927297	31927441	GGGAAGGAGAGAGAGGAATAGG	AAAACTGGTTTTATTTAAACTCCAAAA	1	
Ai07B10001	Araip.B07	33311850	33311957	CGATCCTTATCGTAATTATTCGTTT	TGCTTGTCTCACATCTGTTCG	1	
Ai07B10177	Araip.B07	34014395	34014546	GCCCCCATTACTATAGGATTTTT	TTTTGTGTACCTTATCCCAAATC	1	

ID	Chromosome	SSR_start	SSR_end	FORWARD PRIMER (5'-3')	REVERSE PRIMER (5'-3')	Bands in parents	Polymophism in RILs
Ai07B10449	Araip.B07	35228912	35229040	CCTCTTCCTGGTGATTTTGC	TGCAAAACTCAAAACTCCTCC	1	
Ai07B10890	Araip.B07	37212338	37212451	TTCCCTTTTCTTTGGGATGA	AAAAATGGGTGCACATTCA	0	
Ai07B11008	Araip.B07	37853656	37853781	ATTATTGCATGCACAGGGCT	TTGATTCTAATATTTTTGTGACGG	1	
Ai07B11610	Araip.B07	40844474	40844690	TGTTGGTGTTGTTGGTGATG	TTATCTTCTTCTCCTCCCCCA	1	
Ai07B11969	Araip.B07	42625920	42626120	TGCATTCTTTTCAATCACAACA	GGACAAAATGTCCCAAAAGAA	0	
Ai07B12303	Araip.B07	44567063	44567200	TGGAGGCCAGGTTAACACAT	CTTCTTCCTCCGTGAGCTTG	0	
Ai07B12343	Araip.B07	44711334	44711450	GAAAAGGGAGAAGACTGCCG	GCAACAACAATATTTCACAACAAA	2	
Ai07B12485	Araip.B07	45489746	45489899	TTTGTCATTGAAGGAAAAGGAGA	TAGGCAGATTGCACCTGTGT	2	Yes
Ai07B12703	Araip.B07	46754370	46754505	TTGTTTAACCACCTCTTGGCTT	CCCTTTCAAAACTTCCTAACCA	1	
Ai07B12808	Araip.B07	47252751	47252865	TCCCACGAATTGGGACTATC	TGAACGCCATTTTATGGAAC	1	
Ai07B12987	Araip.B07	48256804	48256904	CGTTCCCGAGCAAAAATAAA	ACCCCTCACCATAACCCTTC	1	
Ai07B13466	Araip.B07	51630231	51630419	TGGGGTGATTTTATTCCCCT	TGCATTGGTTTACTATGCTTTCA	1	
Ai07B14965	Araip.B07	61065047	61065188	AAATCAACAACATTCCATTTATGTG	AGGGGGTACACGCTTAAGGA	1	
Ai07B15156	Araip.B07	62463327	62463437	TTCTCCTATTGTGGAACGAAA	TTTGAATGGTTCTTTGTCCTTT	2	
Ai07B15170	Araip.B07	62516949	62517056	TGTAATGTCCTGGGAAAGCC	GAGGTTTGCAGGATTTGGAA	1	
Ai07B15442	Araip.B07	64172984	64173121	TACGGACAAAAGCAACACCA	GGGAATGGTATGCATGTAACG	1	
Ai07B16352	Araip.B07	69796613	69796734	TTTGTGTTTGTTTCTTATCTTTTTCAA	GTGAGTGGGGGAGAGATGTT	1	
Ai07B16615	Araip.B07	71526411	71526510	TTTTCAGGGTTGTAATGGGG	CAAAAGTGGGAAAAATTTGGG	1	
Ai07B17094	Araip.B07	74809105	74809235	TGCTCAGGTCCCTCAATTTC	TCACTTTCTCTTTCCCCTTCC	1	
Ai07B18406	Araip.B07	78615768	78615917	TCAAGCAATTTTTGGTCACG	TGCTTCTTCATCAGGGAGAGA	1	
Ai07B18410	Araip.B07	78628062	78628171	AATCCCCACTTTTGCATCAC	TGTGAATATTGGGAATTTGGG	1	
Ai07B20570	Araip.B07	93861652	93861783	TGGGGTAACTTTTGTCCCCT	TTTGGAAAATGGGTGCATTC	1	
Ai07B21222	Araip.B07	97450873	97451034	AAAACAGAAGTGGCGAGGTG	CGATTTTGTTTTGCCCATTC	1	
Ai07B21230	Araip.B07	97465991	97466104	GGCTAGTCGGCCTAAACTCC	CGTTCACGCACGGTAACTAA	1	
Ai07B21428	Araip.B07	98380128	98380316	GCTACATCACCATCGTCGG	CGAAAGGGTAGAAGAAGAAGGA	1	
Ai07B22560	Araip.B07	104067576	104067680	AACCCGTCTCTGGGCTTACT	TCGTGTTATGTTCCATGTTCG	1	
Ai07B22606	Araip.B07	104257260	104257396	TTATCACTCTCCCCTGGCTC	ACCATTTGGTTTCCCTTTCC	1	
Ai07B22805	Araip.B07	105160201	105160356	TCTCGTGCTGATAACGTGTTG	CAAATGCGATTCTCTCTCTCTTT	1	
Ai07B23662	Araip.B07	108883922	108884089	AGAGGGATCAAGTGTGGACG	TGCAAATATCTTGTAATCCCCA	2	Yes
Ai07B24086	Araip.B07	110897713	110897832	TGGGATTTATCGGAGGCTAA	TCCCCAATTTGAGTTCCATC	1	
Ai07B24806	Araip.B07	113469277	113469393	TTTGGGCCATTTTTATTTCAA	AAGAATTTTGAATTCGGGGTT	1	
Ai07B24856	Araip.B07	113664273	113664394	GGAAGGATCTGCCATGAAAA	TTTGGTTTTCCCTATCTGGC	1	
Ai07B26726	Araip.B07	119647662	119647813	ATGATTAACCCCCACACGAA	TCGCCATACATGTGTGTAAAGA	2	Yes

ID	Chromosome	SSR_start	SSR_end	FORWARD PRIMER (5'-3')	REVERSE PRIMER (5'-3')	Bands in parents	Polymophism in RILs
Ai07B26973	Araip.B07	120202957	120203093	AAGGATGGTTTGTTGGTTGG	CCTCCCTTCTTCCATTCCAT	1	
Ai07B28513	Araip.B07	124027904	124028045	CCGTTTTTCAAGACTCCTCG	CACCTGACCACACTGAATCTT	2	
Ai07B28858	Araip.B07	124906789	124906930	TGTGACCAGAATTCCCACAA	CCAGATCCCTAGCACTACCC	2	
Ai08B1164	Araip.B08	2684699	2684826	TGCTCCACCTCAGACCAAC	TTGACACGCACAATTATTTTATGA	1	
Ai08B1264	Araip.B08	2920573	2920707	GAGAAAGAGAAGGCAAGAAACG	CCTTCTTCTTCCTGCTGCAC	0	
Ai08B1584	Araip.B08	3780072	3780171	TGCGTGCTTTTGTACGAATC	CCTTCTTGCCTGGGTTATCA	2	
Ai08B2682	Araip.B08	6446355	6446482	TGGATGCCTATGCTGCTAAA	GCTCTCGTTTGGTTTCTTCG	2	Yes
Ai08B2811	Araip.B08	6889133	6889267	TCATTTTGCGATTTGTTCCA	AAGCGACAGGTCAAGCATAC	1	
Ai08B3313	Araip.B08	8407549	8407650	GAGCTAGGAACAGGGGCTCT	CAATAAATCCGACGGCAACT	1	
Ai08B3700	Araip.B08	9650167	9650305	TTACCAGCCTCTTTATGCCA	TGGGTAACGGATTCTACTTATGC	1	
Ai08B4590	Araip.B08	12912035	12912220	AGAGGGATCAAGTGTGGACG	TGCAAATATCTTGTGATCCCC	1	
Ai08B5046	Araip.B08	14508942	14509085	CGGGGACAATTTTGATTTTG	GGTGGTGGAGATGTGACTGA	1	
Ai08B5110	Araip.B08	14733283	14733397	CGAGATAACGAAACAACCGA	CGTTGGGATGTTCACCGT	1	
Ai08B5504	Araip.B08	16096508	16096612	CACATGTGGAAGAGGCACTG	TGCAGCTCGTTCTTCTTTGA	1	
Ai08B5650	Araip.B08	16535153	16535324	GGCTTCTTTCTCTGCGGATT	TGGAATTTTGGTGCATTTTG	2	
Ai08B5890	Araip.B08	17435942	17436087	CATTGTCGTCGTCACAGTCA	AAAAAGGATGATGAGAAAAGAAGAAA	1	
Ai08B6358	Araip.B08	19245667	19245811	TGTTAACGTTATGTTTTTGTAATGAAA	CGGAAGAGTACCAAGGCTCA	1	
Ai08B6392	Araip.B08	19354677	19354810	AGTGCAGCTTTGACATCCCT	GTCATCCTTGTCACGAAGCC	1	
Ai08B6402	Araip.B08	19374229	19374334	GCAGTCGCAACAACAAAAGA	AAGCAATTTTAGGTCACACGC	1	
Ai08B6600	Araip.B08	20008270	20008371	TGCCCTGTTTCTTAACTGGC	TAAGCAAATCCCCATCGAAC	1	
Ai08B7309	Araip.B08	22579223	22579334	GAGGTTGACTTGGAGAAACTGG	CTCGACCTGCGAAGTAAAGG	1	
Ai08B8719	Araip.B08	27913737	27913838	TGCAGAAATTAGGGGATTAGTG	CACGTTGATCTATTAGTGCCG	2	Yes
Ai08B9423	Araip.B08	31598363	31598539	TGAGCTTATTTCTTCATTGGGC	CATGCCGTAAAGCAAAGGAT	1	
Ai08B9636	Araip.B08	32536487	32536591	GAAAAACTGTGGAAACTTGGG	GCATTGGGGTAATTTTGTCC	1	
Ai08B9789	Araip.B08	33426382	33426507	CATTGGGGTGATTTTGTCCT	AAATCCATGTGCATTGATAAGC	1	
Ai08B9945	Araip.B08	34064180	34064320	TGCAAAACATGGTATGGAGG	TGAGTCCGGATATTTTCTATTGG	2	
Ai08B10055	Araip.B08	34548420	34548549	GATCTCTTTTGTGTATTTTGGGC	TGCTTGCGTACGAAGAAAAC	1	
Ai08B10317	Araip.B08	35840233	35840339	GAATGTCATGCAATGCAACC	TGCTTTTTCTCCCATCTCAA	1	
Ai08B10774	Araip.B08	38342993	38343101	TCCATATGTCACAGGCAGGA	TCAACAATAAACATCCATTTCCA	1	
Ai08B11147	Araip.B08	40464242	40464349	TTTGTTTTATCCAGCAGCCC	AGCATCCCACCGAATCCTAT	2	Yes
Ai08B11284	Araip.B08	41241868	41242032	AGGAGAGAGAGGAAATCACGC	GGGCTTAGCCAATGAACAAA	2	
Ai08B11983	Araip.B08	45108958	45109125	CTCCCTTCCCCCTTCACT	GGAAGAGGGAGAGAGAAATAGG	1	
Ai08B12206	Araip.B08	46272914	46273053	GCATCCAAACTAAATGCAGG	TTGAATTTTGGTATATTGAATTTTGC	1	

ID	Chromosome	SSR_start	SSR_end	FORWARD PRIMER (5'-3')	REVERSE PRIMER (5'-3')	Bands in parents	Polymophism in RILs
Ai08B12332	Araip.B08	47001609	47001722	GTGCACAATTTGGTGGAATG	TCCATCTAATTGACCCCACC	1	
Ai08B12334	Araip.B08	47008052	47008213	GCACTCTTCAAGGTCCTCCA	CAAAGACATCTTGATACCTTGTGTG	1	
Ai08B12408	Araip.B08	47444565	47444747	AGATGCCGTTACTAATGCCG	TGGTCAAAATTCAAATGCAAA	1	
Ai08B12844	Araip.B08	50320257	50320383	TGGGGTAATTTTGTCCCCTT	TTTTGAATATTGAGAAATTTGGG	2	
Ai08B12917	Araip.B08	50725214	50725323	AAATCACGACGTTTGGGTTC	GGCAGCTCCTCTCTTTTCTTC	1	
Ai08B13358	Araip.B08	53520207	53520314	TGCTAGCTCCAAGCAATCAG	GGTGGGTGAACACCAAACTT	1	
Ai08B15928	Araip.B08	70116821	70116930	CATGTAATGCACACAATGCAA	CCACCATTGCCTCATTCATA	1	
Ai08B16184	Araip.B08	71500431	71500544	TCACTCTGCACAACCAAACC	TGACGGTGAGAGTGAGTGATG	1	
Ai08B16530	Araip.B08	73595610	73595720	TTCAACCTGATCCTCAACCTTT	GGCTAGCATGGGTTCTAGCTT	1	
Ai08B16802	Araip.B08	75373888	75373991	ATGCATACATGCCACGTTTC	GAGCTTTCTCTGTAAGACTCCAAA	2	Yes
Ai08B16954	Araip.B08	76105544	76105645	CTGTCGTGGTTCTCCTCCTC	TTCATGCAAATGGGATGTTT	1	
Ai08B17086	Araip.B08	76913567	76913686	GCCATGTGTTGGCTTTTCTT	TCTCACCCACTTAATCATTGTTG	1	
Ai08B17435	Araip.B08	78028511	78028685	GCGATAAATGTAAACCAGACAGG	TGGCTGAATAGGAATTTTTCTTG	0	
Ai08B17761	Araip.B08	79738631	79738782	GGAGGGGGAGAGAGAGGG	TCCTTCCCCCTTCCTTCTT	1	
Ai08B18824	Araip.B08	87106785	87106887	GATCCTTGGTTCTCGAGACTTT	CACGAAGCACCAAAATAATTCA	1	
Ai08B18992	Araip.B08	88291825	88291957	GATGAGCGAGAGAGAGAAAGAGA	CTCTCCCCTTTCCTTCCCC	0	
Ai08B20545	Araip.B08	97265190	97265333	GCAGCGACATTCCTCATTTA	TCCCTCTAACCTAGCCACCA	1	
Ai08B21495	Araip.B08	102521374	102521477	GGGAGAAGAAAGAGAGAAAAGG	TTTTTCTCCCCTCTCCCTTG	0	
Ai08B22626	Araip.B08	107561821	107561970	CTCAAACCATCATAACGTTGAGT	TTTTTATATGTGCCCGTACAGTG	1	
Ai08B22632	Araip.B08	107574624	107574746	CAACACCAAATCTCTTGCTCTAA	GCAGAGTAAAGTGTAAAAGTGTGGA	2	
Ai08B22677	Araip.B08	107731797	107731911	TCTCCGCATGTATAACGGGT	TCGGAAAAAGGCTAAAGACG	1	
Ai08B22875	Araip.B08	108619059	108619214	CACGTGTGAAACCAAGGAAA	TTATGCGTGTTTTTCGCTCA	1	
Ai08B23399	Araip.B08	110817640	110817779	ATCCTACCCGGTTTACGTCC	TCATGAAACTGAGGTGGTAAGG	1	
Ai08B23571	Araip.B08	111465344	111465451	TACCATCACTCATCCGCATC	TCACTCATACTTGCACCTCTCG	1	
Ai08B23999	Araip.B08	113125436	113125645	TGTTCCACAACAAATTTCACAA	CTTCACCACCGCCATACC	2	Yes
Ai08B24261	Araip.B08	114076453	114076578	GGCCAGGTTCACACATTCAT	TGTGAGCTGGCCTCAATGTA	1	
Ai08B25768	Araip.B08	119564998	119565108	GAATTCGATTTGCGTGGACT	GCTTTCTCGCTCCACTGTTT	1	
Ai08B28815	Araip.B08	128128539	128128665	TTGGAAGAACCAACAAAAAGG	TTGTCCAAATTATCTTTCACCAA	0	
Ai08B29061	Araip.B08	128596411	128596556	TTTTTCCCTTTCTCTCCCAT	ACAATTGGGAAACGCTTCAA	1	
Ai08B29551	Araip.B08	129486910	129487019	GCCAATGATTGAAAGAGCGT	CCGTCAAATTTGTACCAGTCA	2	
Ai09B1095	Araip.B09	2476857	2477017	TTTCAACTCTGCGGCTAACA	TGGGTTAGGAAGTGAGAGAGC	1	
Ai09B1399	Araip.B09	3143308	3143439	GGAGTCAAACTCCAAACTCAAA	GAAGGGTGGCAGAAATTCAA	1	
Ai09B1486	Araip.B09	3428351	3428533	TTTTCTTTCTCCATGCCTCC	GAGGAAGAAGAACGTGCAGC	1	

ID	Chromosome	SSR_start	SSR_end	FORWARD PRIMER (5'-3')	REVERSE PRIMER (5'-3')	Bands in parents	Polymophism in RILs
Ai09B2631	Araip.B09	6358820	6358977	TCTACATAGGTTCCCACATTGA	ATCGGAGATGACAACGGATT	0	
Ai09B3105	Araip.B09	7755023	7755162	ATTGTTGTGTTTGGGTCCGT	AGAAGAGGAATTGGTGGTGC	2	
Ai09B3164	Araip.B09	7942108	7942236	CAGTGTAAACAAGATAAGAGAAGGG	CTCCTTCGATGAGGCTGTGT	1	
Ai09B3657	Araip.B09	9363457	9363573	GAAGGGGTTAGGAGGGTGAG	AGCTTTCGAAACGGACTTCA	1	
Ai09B4318	Araip.B09	11183697	11183798	AAAATGTTTGCTCGTCCTCG	CTCCCAAACCCAACCCTAAT	1	
Ai09B5213	Araip.B09	14080427	14080554	GCGATAATGATAAACTTGAGTGGA	TGCCAAACTTCAAAGACAGAAA	2	Yes
Ai09B5343	Araip.B09	14428068	14428204	GCAGGTATCACTCTATTCTTATCTTCC	GCATGATCCTCCAATTTAAGC	1	
Ai09B5522	Araip.B09	14864561	14864712	TCGTACCGTCCCATTCTACC	GGAATATTCATCATACACAGCCAA	2	
Ai09B5626	Araip.B09	15203036	15203152	AAGCTGCAGAATGGTTTTATTT	GAGAAGTCTCTGTGAGATAACCTGTG	1	
Ai09B6076	Araip.B09	16666782	16666925	CATGGTAAAAATTATCACATACACAA	TGGTCTTGATGGATAAGCAGG	1	
Ai09B6253	Araip.B09	17133876	17133992	TGGTTAAACCCAACACAGCA	TGTAACCCAATGTGGTCCCT	1	
Ai09B6579	Araip.B09	18319557	18319720	AATGGATGATAAGGTTGGCG	CAAAGATTCAATTCATCAATCACC	2	
Ai09B7201	Araip.B09	20709491	20709644	TTTGGCTCTTAGAAGGTCTAAACA	AATCCGCAACCTATTGGAAA	1	
Ai09B7988	Araip.B09	23979964	23980107	AAAGTGTTCACCAAGTGTTTGA	TCTTCAAGCCCAGAAAAATGA	1	
Ai09B8041	Araip.B09	24247941	24248042	ATTTTACACGCGCCACTTAT	CGTTGAATCAATCCAGATCCA	1	
Ai09B8102	Araip.B09	24404440	24404595	TGATTATGGGTGGGCGTAAT	TCCCAAAATTTTCACAGTCTCA	1	
Ai09B9921	Araip.B09	32112396	32112529	AGAGTTGCTAAAACTCCATCAAA	CCAAGAATGAAGCATGCAAT	0	
Ai09B10252	Araip.B09	33889491	33889638	CTGGCAAAAATCCTAACGTC	AATTAATTCACATTCTCAAGGAGTC	1	
Ai09B10389	Araip.B09	34693224	34693373	TCTTCTTCTTCTTTGTTTGATTTTT	TTCTTCTCCCTCCTCCTCATC	1	
Ai09B12502	Araip.B09	45887167	45887272	AAACTTCCAATCCCCAACAA	CTTCGAGAGAACACGGAAGG	1	
Ai09B13479	Araip.B09	51123184	51123307	GCTGAAGTCAATCCAGATCCA	CAACGTAAACCTGCTGTTGC	1	
Ai09B13628	Araip.B09	52186547	52186646	CCATGATGGAGAAAGGGAGA	CGGCATGAGTCTCTAAACCC	1	
Ai09B14054	Araip.B09	54610599	54610736	TGTTTCATTTAAGTGTGGGAGAA	GGCATATGCTATTCAAATCATCC	1	
Ai09B14094	Araip.B09	54831079	54831222	AACCATTTTCCTCCCAAACC	TAGTGTTGGTTTCGGCCAAG	1	
Ai09B16024	Araip.B09	69545785	69545893	AATCGGTGTTTTTGGGATGA	GCTCAAAAATCATGTTCCACAA	1	
Ai09B17576	Araip.B09	81368520	81368642	AAACCGTATAACCGCTCCAA	TTGTACCATCGCCTTTATCTTT	1	
Ai09B17640	Araip.B09	81769690	81769841	TCAGTGGCTTGTGTGTGTGTT	TGGTTCACAACTTTCGTCACA	1	
Ai09B19910	Araip.B09	96543683	96543792	TGGGATGCAACCTACCTACC	TGAAGGGTTTGAAGCTCGAT	1	
Ai09B20243	Araip.B09	98607203	98607336	CCAAACGTGTAAAGGCACAA	TAAACTCCGCGAGTTGCAG	1	
Ai09B20643	Araip.B09	101287236	101287400	GATGCAGCAGAGAGCTTCAA	TGTTTCAATGACAGGAAAAGCA	1	
Ai09B20935	Araip.B09	102708444	102708545	CAATCCAGATCCATAATGCAGA	TTTTTCCCACCCACACACTT	1	
Ai09B21904	Araip.B09	107865081	107865239	TCTTTGCCTATGTTATTCATCATTG	TTCACTTTTGGCTACACTTTTCA	1	
Ai09B22054	Araip.B09	108767606	108767763	AACCATATGGATTGATGGTGTAT	CCTAGCTACCCATTTCATTGC	1	

ID	Chromosome	SSR_start	SSR_end	FORWARD PRIMER (5'-3')	REVERSE PRIMER (5'-3')	Bands in parents	Polymophism in RILs
Ai09B23112	Araip.B09	113878319	113878474	CTGCAGCGAAGTGGATTTTT	TCAAGCTCCAAACTCAGCCT	1	
Ai09B23472	Araip.B09	115356176	115356303	TTTGGACAATTGACGGTTCA	AAAAAGATGCAATTGACACCAA	1	
Ai09B24635	Araip.B09	120829803	120829954	AACATCTTCGCCTCCTTCCT	CATCACCATTGAACCAACCA	1	
Ai09B24954	Araip.B09	122145891	122146024	CGCCCAGGCTTATAGTGAAG	CCTTTCTCCTGTCGAGTCCA	1	
Ai09B25734	Araip.B09	125214306	125214439	CGTTTGAACATTTTCCCAAGA	CCCTTCTCCGTAAGGGTTCT	1	
Ai09B26364	Araip.B09	127935132	127935239	CAATAATGTTTTCAGAGTAAAACACC	AAGCAACACACAAATCCACCT	1	
Ai09B27069	Araip.B09	130654843	130654961	CTACATCACGCAACCATTCG	CCCATATTATCATCTGCATCATC	1	
Ai09B27970	Araip.B09	133715662	133715855	CGAAGAGGGAGGAGAGAAAGA	GAGGAAGTAGAAGAAGCTTTAGGTTT	1	
Ai09B28003	Araip.B09	133820067	133820180	TTCTTTTTGGTACATAATATTTGGATT	CGCGGACAATTTGTATGAAA	1	
Ai09B28300	Araip.B09	135031749	135031901	AATCTTGCCAACTGCCTCAC	TTCTTAATTATGTTCCATGCCA	1	
Ai09B28487	Araip.B09	135580521	135580755	GGCAGTTTTGGGTGGCTC	AGAAGCAGCAGCAGGACG	1	
Ai09B28756	Araip.B09	136357001	136357150	AGCCGAAGATATCGGTTAGG	AGGTTTGTAATTAACAATCAATAGCA	1	
Ai09B28929	Araip.B09	136866584	136866721	TCACATCCCTCTCTCCCTCT	TGGATGAGGATGTATGGCAA	1	
Ai09B30090	Araip.B09	140254168	140254308	TGCTTCGTTTTATTCCTGTCC	TTGTGTGAATTGAAAGCAAAAAC	1	
Ai09B31536	Araip.B09	144130889	144130997	CCACTTCTTTCTTCTGCCTTTC	TTGCAGCAGAGTCACGGTAG	1	
Ai09B31714	Araip.B09	144540619	144540738	TCAGTTTCCAAAGCGCTACA	TTCCTGTAATCGTTTGGGTG	1	
Ai09B31870	Araip.B09	144835693	144835815	CGGTGGTGAGTGTTTGAGTG	AATTAATGGCACGGCAACTG	1	
Ai09B32075	Araip.B09	145325108	145325229	CATTTCCTCTCGCTCTCTCC	GAGAAAGAAGGGAAGGGGG	0	
Ai09B32212	Araip.B09	145606209	145606313	TGCCAACTCAAATCCAACAA	TGGAGGATGAAGAAGAAAAGGA	1	
Ai09B32544	Araip.B09	146330590	146330709	TCTGGGCTTCCTTCTTTTAGG	AAAAAGTGTGTCACTTCAAATCCA	1	
Ai09B32593	Araip.B09	146453284	146453402	GAAAGACATAGATTCAACCCCC	AAATGAAGGAGCGAGGGATT	1	
Ai10B47	Araip.B10	212039	212145	GCGAAATTGGGTTGTCAGAT	TCGGATCCTCTTGTCTTCCA	2	
Ai10B252	Araip.B10	593793	593943	AAAGCGCGTGCATTTACTG	AAACCATGACAAGAGGCAGAA	2	
Ai10B1190	Araip.B10	3035159	3035281	GCAACGCTGACTCACACACT	TGTTTTGGGTGATTCAAGTAGG	2	
Ai10B1259	Araip.B10	3228965	3229129	TTTCTTCTTTGTTCTGCCGC	GTCCCAGGAGCTTCCAGTTC	2	
Ai10B2613	Araip.B10	7077291	7077430	AACAAGTAGAAATTGGTCCATAAAA	GGATAGGATTATAGGAATTGTTCCA	0	
Ai10B2736	Araip.B10	7593230	7593334	TGCCAAACTTCAAGAACAAAAA	AAGTTGCACCCACTCGTGAT	1	
Ai10B3187	Araip.B10	9225984	9226085	GGCAGTAGGACGAAGACGAA	GAGTCACTGGCTCACTGCTG	1	
Ai10B3700	Araip.B10	10791144	10791246	TCTCGTGCTGATAACGTGTTG	GAGGCTTGATCAGAGGCAAT	2	
Ai10B6238	Araip.B10	20030716	20030846	TTTTTGGTGTTCATACTTCAAGG	TGTACATATATGCATTCAAGCCC	1	
Ai10B6334	Araip.B10	20437003	20437169	TGAAATGGTGGCTTGCAATA	CCTCTCTCTCCATCGCTCAC	2	Yes
Ai10B6684	Araip.B10	21879436	21879554	ATCACTCACGTCCGGGTAAG	CCCTCATAGAGGTTGACTTGG	1	
Ai10B7011	Araip.B10	23087409	23087563	GAGAAGCCATATTCAATTCACTCA	GAGAGGGAGGGAGAGAAGGA	1	

ID	Chromosome	SSR_start	SSR_end	FORWARD PRIMER (5'-3')	REVERSE PRIMER (5'-3')	Bands in parents	Polymophism in RILs
Ai10B7757	Araip.B10	26243290	26243409	GGTGGTAAGACGCTACGACCT	TTTCTGCATAAGCTTTCCACC	0	
Ai10B8533	Araip.B10	29838391	29838578	GAGAGGAGGGGGAGATGAAC	TCCCTCTTCTCTTTCTTTTCTTTT	2	
Ai10B9706	Araip.B10	35251915	35252052	TCCTTGTGGTTGATCGCATA	TCAGAAGTAGCGCAGCACAT	2	Yes
Ai10B9899	Araip.B10	36200952	36201058	TTCTTTCTCCCAACGCAATC	CTCACCAGTAATGCCAAGTAGA	1	
Ai10B10082	Araip.B10	37201979	37202080	GTGTGTTGGTGGGTTTTCAA	CCCTTTCTCCCCAATTTTTC	2	Yes
Ai10B12455	Araip.B10	50409494	50409606	TCAAGCCTCATGTCAAGCAC	CTGGTAAGAGCACTGGCACA	2	Yes
Ai10B13195	Araip.B10	54880258	54880397	GGAATCGGGTGCATACTCAT	TCACTTTTTCACACACTCATGC	1	
Ai10B13765	Araip.B10	58252643	58252804	CTGAACTTCGCAGGTTGTGA	CCCAAAATACATGTTTAAAACCC	1	
Ai10B14520	Araip.B10	64274968	64275089	TTAGAATGTTGCTCGCCCTC	CCACAGGATCACCTCAGGAT	1	
Ai10B15315	Araip.B10	69338032	69338143	GAGGAAGAGAGAAGGGAGGG	CCAGTTTTTATGGTTAAGTTTAGTTTT	1	
Ai10B16055	Araip.B10	73664183	73664293	CCCTCAGGCTTACAAGAACG	TTTTCGGGCTTCTCGTTAAA	2	
Ai10B16530	Araip.B10	77104218	77104391	GAATCCTCTTCCCCCATTTC	GGTTAGCGACAACAAGGGAG	2	
Ai10B16551	Araip.B10	77195047	77195160	GAAGGGTTGGCACTTTGTGT	TTTTGTCTTGATTATTTGCCTGAA	2	
Ai10B16637	Araip.B10	77745590	77745709	AGAAAGAGGGGGAAGGGG	TCCATTGCTTTCGTTCCTTT	1	
Ai10B16737	Araip.B10	78348753	78348866	CAAGAAGGCCACTAGGGTTG	AGTCAACATAAGAAGCCGATCA	1	
Ai10B17189	Araip.B10	81467722	81467839	TTTGGGGTATTTTTGTCTCCTT	TGGAAATTGGACATATTCATGC	1	
Ai10B19252	Araip.B10	94560678	94560847	GCCAAACAGGCTAAAACCGT	CTCCCGGAAGGTGATAATGA	1	
Ai10B20207	Araip.B10	99534666	99534803	CACGAAGAGTGCCGACATTA	GACTTCAGGCTTCAGGCAAC	2	
Ai10B21491	Araip.B10	106273350	106273463	CAGGGTGGAAGACTACCCAA	AAAGGGATACACCCAAAGGA	1	
Ai10B22066	Araip.B10	109090365	109090522	TCCCTCTCTCTTCCCCTTACTT	GGGAGAGCGAGGAAAAGG	1	
Ai10B22075	Araip.B10	109172169	109172273	CCAAAATCAAATTTCAGCAACA	TTTTCGCTCCAAAATGGTTC	2	
Ai10B22473	Araip.B10	110781404	110781528	AACCCGACAAGTCCAGTTTG	CTACGACGTAAGGGTGACCG	1	
Ai10B22816	Araip.B10	112008979	112009093	GTTGCAACGACGAATCTGTG	CACGATTATGGAGAGAGGCAG	2	
Ai10B23170	Araip.B10	113456227	113456346	CAGCCCCTTTTCATTTTCAA	GCTCCATCAAACATCTTTTAATTC	2	
Ai10B23974	Araip.B10	116959956	116960056	GTCGTGTGGAGCATCTCAAA	TGCATGTTTGATTGACTTGAA	1	
Ai10B24818	Araip.B10	120137746	120137889	TTGAGTCAATGTAAGGCTGAGAA	CCGGCTATTATCATCCACAA	1	
Ai10B25219	Araip.B10	121638716	121638817	CGAACCAACATTCTTTCCATC	TTTAAATCTTTTCCATTTAACTTGC	1	
Ai10B26233	Araip.B10	125007574	125007765	CCCTCGATATTATCATCTCTATCACA	CGGTGATGCTGAAAAACACA	1	
Ai10B26837	Araip.B10	126805715	126805814	TGAAGGAACACTTCTCCTTGG	AAATTTAGTGCGTGTGTGCG	2	
Ai10B29118	Araip.B10	132774127	132774274	TCTCTTCGATTTCGGCATCT	TAACCCTTCAACCCATGACC	1	
