# Supplementary material for: Development of SSR markers and identification of major quantitative trait loci controlling shelling percentage in cultivated peanut (Arachis hypogaea L.)
Source: Theor Appl Genet. 2017 May 15;130(8):1635–48. doi: 10.1007/s00122-017-2915-3 (PMC5511596; doi:10.1007/s00122-017-2915-3)
Supplement: Supplementary file 4 — Supplementary material 4 (PDF 361 kb) [file 122_2017_2915_MOESM4_ESM.pdf]

Table S4 Detailed information of the 862 polymorphism SSR markers genotyped in the RIL population.

ID	FORWARD PRIMER (5'-3')	REVERSE PRIMER (5'-3')	Source	Alias
Ad01A8152	GCTTCACGTGTGTAATTCCG	TGGTGCTTCATACGCCAATA	newly developed marker	A01A245
Ad01A8169	TGTCACGATTAAAAAGTTTCGG	TGGTAATTTTGCAGCATTATCTGT	newly developed marker	A01A246
Ad01A11348	CCAAAACGGTTTAGTCGTGA	CACAATTCAAAATTCTCCAGCA	newly developed marker	A01A313
Ad02A180	TTTATTAACACGCGCACCAA	GAAGGTGCAGCAAGAAGAAGA	newly developed marker	
Ad02A182	TTTATTAACACGCGCACCAA	CGTGCAGAAACGAAAGAAAA	newly developed marker	
Ad02A625	ATCCAATTCCCCAACATTCA	AAGAACAGCCATGGTAACGG	newly developed marker	
Ad02A4133	GTTGGAGTCATTCGACCGTT	CGCTTTCAATCAAAAGACGA	newly developed marker	
Ad02A4136	TCCGTTCAATGTTGGAGTCA	GCGCTTTCAATCAAAAGACA	newly developed marker	
Ad02A10600	TTGATGGTATTTCCTGTGAGTGA	GCAGTCGTTTTCAACCCCTA	newly developed marker	A02A291
Ad02A12962	TCTTCTTCTCCCAACCAATGA	CGACGAGGACAATGATGATG	newly developed marker	A02A348
Ad02A16744	CGATAACATCTTTGTGTATTGACTCTC	TTAGCGGTAATCATGTTTAGAATGT	newly developed marker	
Ad03A9257	GCATGCATACATACGATACCAA	CAAGAAGTGGACGAGGAAGC	newly developed marker	A03A292
Ad04A21	GGATGCACGTCATTCAGAAG	GCTCATCAAGCTGCTGAGAAA	newly developed marker	
Ad04A10156	GCCAAGTTATGGTGTCTTCTCC	AGTTGATCCATTGATTCGGG	newly developed marker	
Ad04A12402	CCTGCAACCTCACTTCCTTC	GCGCATCAAAGCAACATTTA	newly developed marker	
Ad04A21094	CTTCACTGTGAGGTGGAGGC	CCCTCTTAACGCACTGTTATCAA	newly developed marker	A04A552
Ad05A5618	GGGCTAAGATTCAACCTCCC	TCCAGAATGCACTGAACTCAA	newly developed marker	A05A179
Ad05A8309	TGCAAGAAGACGAAGAACGA	AAAAACGCGATGCAAAGTCT	newly developed marker	A05A247
Ad05A10356	TTTTTGGTCGAAAACCACG	GATGAGTGCCGGGAAGTAAA	newly developed marker	A05A295
Ad05A14690	AACACCAAGTCGGACGTTTT	GGGTTATTAAATTCGATGGACG	newly developed marker	A05A405
Ad05A17280	TCCATCGAGGAATCACTCAAG	GCGCTTGATTTTTAGCATGA	newly developed marker	A05A485
Ad05A18236	TTTGTTTCGTGCGTTTGTTC	CAGGGCGGAAGACAATCTAA	newly developed marker	A05A728
Ad05A18275	CATCCAAATTTCTCACCTGCT	TTGTGGGTCAACTCGGGTAT	newly developed marker	A05A742
Ad05A18337	CTCGAGATTGAACTCCGAGC	CACCACCCATCATTTGTCAC	newly developed marker	A05A760
Ad05A18425	AATGATGGCAACAACGTCAA	ACCAGTGGATTTAGCGATGG	newly developed marker	A05A788
Ad05A18453	GACCATTATGATAAAATTTTGCACC	AAACAAATGCAATTTTCTCTCTTC	newly developed marker	A05A798
Ad05A18493	TTACGGCTCTGCTCCTCAAT	AAGGGATGAGGAAGGAGGAA	newly developed marker	A05A809
Ad05A18501	AAAAATTGGTCAAACCAGCG	CGACGTTGGCTTAGGAGAAG	newly developed marker	A05A812
Ad05A18569	AAGAAGCGGATAACTTCGTTTG	CGAAATAATGCTCTGTTGTTGTG	newly developed marker	A05A845
Ad05A18658	TTGTCCATAAATCCGTCGGT	AGTCGACGCTGAAAGAGGAG	newly developed marker	A05A884
Ad05A18695	TCATTGTTGGTAATAAGTTGGCA	CAAATGCAATTCAACCGAGA	newly developed marker	A05A890
Ad05A18727	AGTTGTTGATGGCTTTTGCC	TGAATTGTTGACCATTTTTGGA	newly developed marker	A05A897
Ad05A18734	TTGAGACTTCACGCAAGTGG	AAGGTTGTGGGCTTCTATGG	newly developed marker	A05A899

ID	FORWARD PRIMER (5'-3')	REVERSE PRIMER (5'-3')	Source	Alias
Ad05A18820	CAGCAATTAGTTTTAGAAGATGATGG	TCTAACAGTGAGAATAAACGATTGG	newly developed marker	A05A925
Ad05A18845	CAATTGTGGTCCCCTCCTTA	AGAATCAGTGAGCACACCCC	newly developed marker	A05A932
Ad05A18924	TTGACAAATTAATGTGAGTTACCAAA	CATAGCACACAAATTCGCACA	newly developed marker	A05A956
Ad05A18961	TGCTGTTGGACTCAAAGGTG	TGGTAGGAGGGGACAGAAGA	newly developed marker	A05A963
Ad05A19096	TTTGGTTGCAATTGATGACG	GCCTCATTCGTTTTGGTGTT	newly developed marker	A05A1013
Ad05A19116	ACACCAAAAATTCGTTGCAT	CGGTTCCTCTTCCTCTTCCT	newly developed marker	A05A1018
Ad05A19142	ACGCCCGAGTTGATCTTCTA	CATCCATAAAGCAAGCCGAT	newly developed marker	A05A1028
Ad05A19190	GCATTGGCATTGAAGTGAGA	AATAACGTTAATGGGCGATCA	newly developed marker	A05A1050
Ad05A19203	CGGACACTTTTGCCCTTTTA	ACGGATCTCTCACCATGTCC	newly developed marker	A05A1053
Ad05A19244	CACAGGGCAAAAGAAAAAGG	GCCAACTCTCATTGACACCA	newly developed marker	A05A1066
Ad05A19295	AGAGGCAACGGTAGCAACAC	TGGAGCATCAACAACAACAA	newly developed marker	A05A1080
Ad05A19296	AGAGGCAACGGTAGCAACAC	AAGAAGCAATTAATGGAGCATCA	newly developed marker	A05A1081
Ad05A19315	AACCGTCTTAACAGCTTGCG	TGACGTTACGAGCTACTGCG	newly developed marker	A05A1092
Ad05A19349	GGGTGGCGTATAGGTTGCTA	CGCCCTTCTCTTCCTTATCC	newly developed marker	
Ad05A19382	CCGACGATAACGAACCCTAA	GATGGCGAGACACAAACAGA	newly developed marker	A05A1111
Ad05A19473	ATTCGCCTTGAGAAAAGCAT	CACAATGCAACAGAACGAAGA	newly developed marker	A05A1146
Ad05A19487	ATGTTGCAAACATGCCAAAA	AACCACACTCTCTCTCCCCA	newly developed marker	A05A1150
Ad05A19710	GGTTTCAACCTCACTGGCAT	TCCAGCACTCAAGACCACAC	newly developed marker	A05A1228
Ad05A19823	TGGAAGATGAAAATATCGGAAGA	TGCTTATGGTGGTTATAGTGGC	newly developed marker	
Ad05A19912	CAGCCAAACATTGTGATATGC	GGATATTGCCTTGTTATGAACG	newly developed marker	A05A1305
Ad05A19999	CACCAACATTGGTCATTTGC	TCTCACACCAATTTCACATCA	newly developed marker	A05A1337
Ad05A20012	GAGAGTCTGAGAGGGCATCG	TTATCCGTCCAAACCCAAAC	newly developed marker	A05A1344
Ad05A20046	TGATAGAACTGCCGATCCAG	TCGGACGGTTCGATTTTTAT	newly developed marker	A05A1355
Ad05A20084	GGTGCATGGTAAAATGGCTT	CGACCCTATGTGTACGATGG	newly developed marker	A05A1368
Ad05A20112	GGTTTCGCGCATTTAACATT	CAACCATGTCTTTACATTGCCA	newly developed marker	A05A1378
Ad05A20185	CAGGGTATCCTTCACAGTTCAA	CATGATTTTGTTTCTGGCTCC	newly developed marker	A05A1409
Ad05A20222	TTGTCCAGCTTGTAGGAGTTGA	TTCAGATCCCCTCAGGTTTG	newly developed marker	
Ad05A20262	CTGCAAAGAGCCAAGGTTGT	GGGTTCGAACATTGGTGAAT	newly developed marker	A05A1430
Ad05A20396	GCCGTGGCATTCATTTTATT	TGGTTATTTTGGATAGTATGAGCG	newly developed marker	A05A1471
Ad05A20478	CCGGTGTGTGCTCTCTCTTT	ACAATTGCATTCAACCCCAT	newly developed marker	A05A1504
Ad05A20499	ACCAAGGAGAGGAGGAGGAA	CGCGGGGCTTTACATTATT	newly developed marker	A05A1507
Ad05A20509	ACGAGGAGCTGAGCAACAAT	CCCTACCTTAGCCAAACCCT	newly developed marker	A05A1510
Ad05A20533	CGGGTAATTTGTTGACTGGG	TACCGCCACTGTCGTTATCA	newly developed marker	A05A1522
Ad05A20570	TGCCAAAAACTCCTTTCGTT	AAGCCAGGTAGCACCCTCTT	newly developed marker	A05A1530

ID	FORWARD PRIMER (5'-3')	REVERSE PRIMER (5'-3')	Source	Alias
Ad05A20617	TTCCAACACCAATTCATCCA	GTGCCTCCTAATCCCCAGAT	newly developed marker	A05A1549
Ad05A20643	TGCATTCAACAGCAAGGAAC	ACAGGTTAGCCGTCATGGTC	newly developed marker	A05A1558
Ad05A20650	TTGCCCCTATATTCCTGCTC	AAGGTGGTGTGGGATTACGA	newly developed marker	A05A1562
Ad05A20677	TTTGGTCATTGTCAAAATCCTT	TGCTGTCAGTCATTTTACAGGAA	newly developed marker	A05A1570
Ad05A20734	GCAGTACTGAGTGATGCGGA	AAGGAGGTTAGCGTGTGGTG	newly developed marker	A05A1590
Ad05A20769	AGTCCCTGTCACCAAAATCG	CCGCTCTCTTCACTCCTCAC	newly developed marker	A05A1601
Ad05A20782	CCATGTACTTATGTATGTCGGATCT	TGAGCAATTAAATCTCCACTGTT	newly developed marker	A05A1605
Ad05A20785	CCACACAATGCGTGTTTTCT	GTTGTGAGTGTTGCAGTGGG	newly developed marker	A05A1606
Ad05A20801	GCCCTCTTCCCCTTATTTTG	TTTGGCAAACAGTGATAGCG	newly developed marker	A05A1612
Ad05A20875	CAAGTTCATGCATATTCAAGGC	AATTTCATTGATGCCGTGGT	newly developed marker	A05A1624
Ad06A4196	CCCAATATAAAGGCCCAGGT	AAAAACTCCGTTTGCTGAACA	newly developed marker	A06A137
Ad06A5393	CCCAGCATAAAACCAATTCAA	TGTATGTATGCTGCTGTCTGAA	newly developed marker	A06A169
Ad06A10649	CCTTTTTCAATCAAAGCACGA	TGCCTTTATTTTGGTTGATGC	newly developed marker	A06A265
Ad06A16982	GGAACTCGAGGTTGGTGACT	ATCCTTTCCTGAGTGTTGCG	newly developed marker	A06A431
Ad06A17247	TCTGAATTGAAAATTTCTGCATC	AAATGGTGGCTTGCAATACG	newly developed marker	A06A442
Ad07A4990	GACATGTACATTAAAGACCAAAATCG	ATGGCGTGAAAAGCAAGTTC	newly developed marker	A07A169
Ad07A9745	GGATCGGGTCCGTAACATTAT	CTCTCCAAATCATCATCCAACA	newly developed marker	A07A292
Ad08A3453	CGTGGATAAAGTCAATCACCA	AGAGGGATCAAGTGTGGACG	newly developed marker	A08A90
Ad08A4387	CATGCCCATACATGAAATGC	AAGCTGCGGCTTCAGTAATC	newly developed marker	A08A119
Ad08A4940	TCCCTTCTCTTCTTCCACGA	AGTGTGTGTCTGCGTTGGAG	newly developed marker	A08A138
Ad09A3779	TATCAACCGTTAACCGGAGC	TGGCATGGAACCTAGAGTGA	newly developed marker	A09A119
Ad09A5979	GGAGAAAGAGGGAAAAGGAAAA	CTCCCCTCTCTCCCCACTCT	newly developed marker	
Ad09A6009	ACGATGATGTGCGAACAATG	CAGGATCTTCAATACGAAGCAA	newly developed marker	
Ad09A6072	GGGGTCAAGTTCTGAAACCA	TTTTGTCGAAGGTGTTTCTTGA	newly developed marker	
Ad09A6154	ATGCTTGATGATGGTTGGGA	CAAACATCAACACCCACCAC	newly developed marker	
Ad09A6233	AAGATGCGAGGGGTTGTATG	ATTGCAATGTTCGACTGACG	newly developed marker	
Ad09A6425	TTGGGTGCCTTGAATTGTAA	TATAACCCCCACTGACCGAG	newly developed marker	A09A195
Ad09A6594	TTCCATGAGAAAGGAGACGG	CGACGACAGAGTTGTGGAAA	newly developed marker	
Ad09A6617	TCTTCTTCCTCTTCCCCCAT	AGAGAGAGACTGGCTGGCTG	newly developed marker	
Ad09A6680	GGGTGGAATGTAAGACCCAG	CAAATTGCTATACTATTTTCACTCACA	newly developed marker	
Ad09A7328	ACAGTCATCCATGGCAAAGC	TGTAAACCCTCACGTTACATGG	newly developed marker	
Ad09A7483	CAAACCAATCTAGCCCTAAGAGG	TGGGCATGCATGAAAATATC	newly developed marker	
Ad09A7532	TGGTCATCACTTTTAACCATCAA	TGAAATTTAAAAAGTGGTTGGTACAC	newly developed marker	
Ad09A7577	CATCATGGAGGGCTTCCTTA	TTCAATCCCTTTTCAGGTCG	newly developed marker	

ID	FORWARD PRIMER (5'-3')	REVERSE PRIMER (5'-3')	Source	Alias
Ad09A10442	ACGCAAAAAGGGAGGAGAAT	ACATACGCGTGGAGCTTCTT	newly developed marker	A09A282
Ad09A18059	CGAGAAGATGGCACAAATCA	TCTTCGTCTTCATCCCCAAG	newly developed marker	
Ad10A3962	GTGATGATGGAGTGGCAGTG	TTTTTCGCTCCAAAATGGTC	newly developed marker	
Ad10A4326	CGCGAATTCGAATCTCCTAT	TTGATGCACAAAACAAAAGC	newly developed marker	
Ad10A4720	AACGGGCTAAAACCGCTAAT	TCACGGAAGGTGATGATGAA	newly developed marker	
Ad10A10685	CTAAGTGTGCACGGTTTCCC	CATGCATCTTCTTTCCAATTCA	newly developed marker	
Ad10A15687	TGCCCTATCCATGCTTCAAT	GTAGGACTGCCCGGTGTATC	newly developed marker	
Ad10A18338	AAGGGGCCCACTTTGTATTT	GGAATTACAAGCTGAAAATCACTC	newly developed marker	A10A449
Ai01B7136	TCTTTGGAGGTTTTTGGTGG	TCCTCTTTATTTTGAAACATTCTCTTT	newly developed marker	A01B163
Ai01B7542	CTTGCGGTTGTTGTTGTTGT	GGGTACAAGTCAAGGAAAGCC	newly developed marker	A01B172
Ai01B8018	TGTCCCTTAGCCAAAGATTCA	GGTATGGTCGGCAAGTCAGT	newly developed marker	A01B185
Ai01B9867	GCTTCACCACCGCCATAC	CCCAGAATCAGCAACAACAA	newly developed marker	A01B213
Ai01B11694	GACCCTGCATGAACAGGATT	AAAAACTGCTGAATGGTCCG	newly developed marker	A01B252
Ai02B2350	GGTTACATGCTGCCGAAAAT	TTCTCGTCGTCATTGCTGAT	newly developed marker	
Ai02B2356	CTCTAGGCTTGGGAGCTTCA	ATCTCTGGCGACGGAGGTAG	newly developed marker	
Ai02B2534	GAGAGAGCATGGGGAGAGAA	TCCATTGCTTTCGTTCCTTT	newly developed marker	
Ai02B2634	CTGCGAAGTTAAGGCAGACC	TGGTTCATTTTGAGGCCCTA	newly developed marker	
Ai02B4199	GCTTATATAAAGGAAACGTGGCA	AGGGGTAAGCTTCGTTTCAA	newly developed marker	
Ai02B4457	TTGAGGTAAATCTTCCCAACC	CAATTTGCACTAAGCCACAAA	newly developed marker	
Ai02B6837	TGAAGAACAAGACGATCATTAACAA	GCTTGATTTCAAAAACCATGAA	newly developed marker	
Ai02B7519	CTTCTCCGATGAACTAGCGG	GAGAAGCGATGCGAAGAGAG	newly developed marker	A02B174
Ai02B7886	GCAAGCCCGTATGCATTTAT	CATGCAACTCACCCATTCTC	newly developed marker	
Ai02B8213	GGGGAGAGAGAGGGAGAGAA	CCAGTTAATCAAAACCAAAACCA	newly developed marker	
Ai02B8654	TCTGAGCTACATCGCCACTG	AGCCAACCCCATCTAGTACG	newly developed marker	
Ai02B11533	AAACTCAGTTCGTCATCGCA	TCAGCAACAACAATATTCCACA	newly developed marker	A02B234
Ai02B17748	TTGCCATCGGATGTATTTGA	TCACAACGGCACCTGTTTTA	newly developed marker	
Ai02B18857	TGCACCATTTTAACCAATTCA	TAGAGAGGGAGAAAAGGGGG	newly developed marker	A02B349
Ai02B19680	ACTAACAACGAAAGGGCCAA	CATATTGAATTGAACATCCGACA	newly developed marker	
Ai02B20354	TAGAGTTCCAAATCCGGACG	GAGGAACATAAAAAGTGCACGA	newly developed marker	
Ai02B20508	CCCGGATTTGATGAGTTAGC	CTGCTTCAACGAAGACGATG	newly developed marker	A02B379
Ai02B20641	TTTTCCTCCATTTATTTCATTTACG	AGCGCGTGTACATGCTTTTT	newly developed marker	
Ai02B21379	AACGTCCAATCAATAAACCAAA	GGTTGGTCCAAGCTCAAGAA	newly developed marker	
Ai02B21536	TGCGCATTAATGAGAGTGAAA	GAAGAAGAAGGAACGCGAAA	newly developed marker	A02B406
Ai02B21538	TATCAGGCGCGTTACACATC	GAAGAAGAAGGAACGCGAAA	newly developed marker	A02B407

ID	FORWARD PRIMER (5'-3')	REVERSE PRIMER (5'-3')	Source	Alias
Ai02B23281	TTGCACATGAGACAAAATGAAA	ATAGAGAAGCTCGGCCTCAA	newly developed marker	
Ai02B25013	GGGGAACTAATGATTGGTGTG	TCCCTATTTTCCCCAAAACC	newly developed marker	
Ai02B25100	TGTTTTTCAGATGCAGGTCG	CAAGCTCCAGAAAGGTCGAG	newly developed marker	
Ai03B32338	TGCATTGCATTATTTTGTGAA	CATCAGGGTCGATTTCGATT	newly developed marker	A03B820
Ai03B33341	CCCACCCAAATCCCCTATAC	TTTTATGGTAAAATTGTTAGAACGTCA	newly developed marker	A03B842
Ai04B16	ATGACCATCACTGCTCCACA	GGACTGGGGATTGCATAGAA	newly developed marker	A04B01
Ai04B2241	TTCGACCATGTGAATGAAGC	GCCCGGGCTCTAACCTTAAT	newly developed marker	A04B56
Ai04B4849	GTGCTTTTTGCAGGTTCTCC	TTGAGATGGCAAGGTATTACGA	newly developed marker	A04B112
Ai04B5526	CACGCGCCTCATATAACGTA	AACGCAAACAGAGGAGAACAA	newly developed marker	A04B133
Ai04B9397	CCAAAGAAGTTGCAATGACG	CTCCGCTTAAGAGCAGAGC	newly developed marker	A04B207
Ai04B12763	AAGGGAGAGAGGGGGAAGA	CCTCCAATCTCTCTTTTTCCCTA	newly developed marker	A04B267
Ai04B17746	GAAGAAAAACCTGCGTGTGC	TTAAATCACGCGCTCCTTCT	newly developed marker	A04B345
Ai04B19238	CATTTTGGGGTTTTACTTTTGG	ACTAGGGCTGTCCGGAGAAT	newly developed marker	A04B363
Ai04B20539	AACAGCAAATCCTGCCTCAT	CCATTTCGTTACAAAAGTGCAA	newly developed marker	
Ai05B5265	CACGCACTAGATCTTCTTCTCC	TGTTGGTGATGATGATAATGGAA	newly developed marker	A05B133
Ai05B9660	GACGAGAAACCTTCCCATGA	GACGAACTTGCCCAAATACC	newly developed marker	A05B217
Ai05B12077	CAATGGATCGCCCTAAAAGA	TTTTTGCATCGTACCTTGTTT	newly developed marker	A05B258
Ai05B15046	CGCCCACTGACTTCCAAATA	TATTGACGGCGATTACGGTT	newly developed marker	A05B302
Ai05B19811	GGGGAAATGTAGGAGGGAGA	TACCTTACCCCAACCCCTTC	newly developed marker	A05B384
Ai05B25638	GGATTGAAGGCTTGAAGCTG	CCGCTAGTTTCTCTGTCGCT	newly developed marker	A05B485
Ai05B26922	GTGTTGGTGCGATGATAACG	AAGTCCAACAAGTCATTTACACTCA	newly developed marker	A05B517
Ai05B28050	AAATATCCGCCGGTAAATCC	AATGGTGGCAGAGGCAATAG	newly developed marker	
Ai05B32361	AGGGACGGAAGATGGGTATC	AGAGTGTGTGGGGAGTGAGG	newly developed marker	A05B645
Ai06B7451	CATCAACATAGCAAAAATTCAACA	ATCGTTACTGTTGTCGCCAT	newly developed marker	
Ai06B13545	AGCCAATGCCAGTAGCTTGT	CGTAATTAGTCTGGCCCCAT	newly developed marker	
Ai06B19288	TTGAAAAATAAAAGGAAAGGAGGA	TTCCGACAGAACGAGAGGTT	newly developed marker	
Ai06B27638	GGAGAAAGAGAGAGAAGAGGGC	TGAACCAAACTGCTTTGTCAT	newly developed marker	
Ai06B29598	TGCTTTCTTGCTTTCCTGCT	CCACCAACATTACCACCACA	newly developed marker	
Ai06B29716	ATGCACTAAGCCACGTTTCC	GTGAGATAAAATTGTAAGACTCCAAA	newly developed marker	
Ai06B30789	GCATTTGTTTTGAATGAACAAAGA	AAGATCTGCCCCTCATTTTG	newly developed marker	
Ai06B31304	CGCATACAAGCGATTAAGGC	GAGGAAGAAGAACGTGCAGC	newly developed marker	
Ai06B32416	CAAGCCAAAAACTCAGTCCA	GCTCCATTTTGAAATCTCCG	newly developed marker	
Ai07B12485	TTTGTCATTGAAGGAAAAGGAGA	TAGGCAGATTGCACCTGTGT	newly developed marker	A07B265
Ai07B23662	AGAGGGATCAAGTGTGGACG	TGCAAATATCTTGTAATCCCCA	newly developed marker	A07B505

ID	FORWARD PRIMER (5'-3')	REVERSE PRIMER (5'-3')	Source	Alias
Ai07B26726	ATGATTAACCCCCACACGAA	TCGCCATACATGTGTGTAAAGA	newly developed marker	
Ai08B2682	TGGATGCCTATGCTGCTAAA	GCTCTCGTTTGGTTTCTTCG	newly developed marker	A08B47
Ai08B8719	TGCAGAAATTAGGGGATTAGTG	CACGTTGATCTATTAGTGCCG	newly developed marker	
Ai08B11147	TTTGTTTTATCCAGCAGCCC	AGCATCCCACCGAATCCTAT	newly developed marker	A08B222
Ai08B16802	ATGCATACATGCCACGTTTC	GAGCTTTCTCTGTAAGACTCCAAA	newly developed marker	
Ai08B23999	TGTTCCACAACAAATTTCACAA	CTTCACCACCGCCATACC	newly developed marker	
Ai09B5213	GCGATAATGATAAACTTGAGTGGA	TGCCAAACTTCAAAGACAGAAA	newly developed marker	
Ai10B6334	TGAAATGGTGGCTTGCAATA	CCTCTCTCTCCATCGCTCAC	newly developed marker	A10B119
Ai10B9706	TCCTTGTGGTTGATCGCATA	TCAGAAGTAGCGCAGCACAT	newly developed marker	A10B181
Ai10B10082	GTGTGTTGGTGGGTTTTCAA	CCCTTTCTCCCCAATTTTTC	newly developed marker	A10B194
Ai10B12455	TCAAGCCTCATGTCAAGCAC	CTGGTAAGAGCACTGGCACA	newly developed marker	A10B227
AC1D11	TGCAGAGAGGTTGGATGGAGTA	CCCGCTAATCCCCGAAGT	Moretzsohn et al. (2005)	
AC2B03	CTCGCTATACTAGGTTTTGGGTGT	TGGTTTGCCTTTCTAGCCATTA	Moretzsohn et al. (2005)	
AC2C05	CAAGGAAGCGTGAATTGTTAG	TGTGGACTATGCTTGTCATGTT	Moretzsohn et al. (2005)	
Ad90F2	CTCAATGGCCGGTATGATTAGT	TTGAGATAACGCTTCCGAAAAT	Leal-Bertioli et al. (2009)	
Ad91I24	TGTCCGCCAAGTTTTACAGATA	CAGCTTACACTGGCATGTTCTC	Bertioli (unpublished)	
Ag39	TGTAGTCAGCTGCTCCAAAA	ATGAAAGTTCACTTGAGCAAA	Hoshino et al.(2006)	
Ag49	TGAAATGGTGGCTTGCAATA	CCTTTCTCTCCATCGCTCAC	Moretzsohn et al. (2009)	
AGGS0005	AATAGTATGATGGTGCGGTGGT	AGGGTAGAGGTTGGAGAGAAGG	Huang et al.(2016)	
AGGS0013	AATCCGACACAACGATAAGAGA	ATTGCCCATTAGCATTTACG	Huang et al.(2016)	
AGGS0022	ATTCGGTTCTTACATTCCCAAC	ATTCAAACGCAAACCCTTCTTC	Huang et al.(2016)	
AGGS0058	CTGGCCACCTAATCTTTCTGTT	GATTCCTATGCTCCTCCACCTC	Huang et al.(2016)	
AGGS0060	GTTGGGCCATCTTAAGAACAAG	TAGTCTAAACGTTCCGGGACCA	Huang et al.(2016)	
AGGS0100	CAGCACTAAATCTCCAAATCCA	CACTGGCAGAGATAGGAACCTT	Huang et al.(2016)	
AGGS0187	CCCTCATTTCGAATTCACTCAC	ATAGCAGCGACAGCAACATTTA	Huang et al.(2016)	
AGGS0243	CGCCGCATCCTACTACTACTG	CTTTCACGTGTCTTCCCTCTGT	Huang et al.(2016)	
AGGS0244	GGTGAGGCAGAGGGATATGTAG	TCGTTCTTCCCACTCTCAAGTT	Huang et al.(2016)	
AGGS0281	ATTAGACCGAACGAACCGAAGT	CTTTCACTTCTCTTCCCACCAC	Huang et al.(2016)	
AGGS0284	AGTGAGATCAGAAGCAGAGCAA	AGGCCAGGAGAGCTAAGTTTGT	Huang et al.(2016)	
AGGS0285	AACACACACGCACTAGCAACTT	ACCAAACCAAGAAGGTGAAAGA	Huang et al.(2016)	
AGGS0297	GATGGAAGAATCCGATGGTATC	TGAGAGCAAACAAGGAGAGAGA	Huang et al.(2016)	
AGGS0300	GCAAAGACGATTAGCTCAGAGA	ATACTCCTCCTTCACCCATCCT	Huang et al.(2016)	
AGGS0302	CTCTCTCTCTCTCTCGCCTGAC	TATCGTCGACGCTGTAAATGAG	Huang et al.(2016)	
AGGS0311	GGTGAAATACACAAGAGGAACG	CAAATACTCAAAGCCCAAAGGA	Huang et al.(2016)	

ID	FORWARD PRIMER (5'-3')	REVERSE PRIMER (5'-3')	Source	Alias
AGGS0329	CTGGAACCATAAGCTGTCATCA	TTCTCACACATGCACTCAGAAA	Huang et al.(2016)	
AGGS0331	AGGAGGAGAAATTCGTCATCAG	AAGCGTAAAGCTCTATGTGGTAGA	Huang et al.(2016)	
AGGS0333	TCAGCCAATAAATCTGACGGTA	GGAGGTTGACGGAGAGGAGATA	Huang et al.(2016)	
AGGS0337	GGAATTACAAGCTGAAAATCACTC	GGAATTACAAGCTGAAAATCACTC	Huang et al.(2016)	
AGGS0339	CCCTAATATTCGGCCATCCTTA	TGGTATGAGAATGAAGAGGAGGA	Huang et al.(2016)	
AGGS0346	GTGGTGCTGGTGCTATTGAGT	CAACTACCCTTCTCTCCACCAC	Huang et al.(2016)	
AGGS0351	GCCACTAGATTATAGCGACTCCA	TTGCACTCTTCTTAGGTCATGTG	Huang et al.(2016)	
AGGS0358	GTGCCATACTAGTCGAGGGATT	TGTAGTGCCTTGTCACCTCTGT	Huang et al.(2016)	
AGGS0385	GCCGATAACAAGAACCTCATCT	TGAGTGGTGCTTACATTTCTATGG	Huang et al.(2016)	
AGGS0389	CCGCTCATTTGATCATAAGCAT	TGGTGTTTGCTTTCAGAGTTGT	Huang et al.(2016)	
AGGS0396	CGTCGTCCATGTAATTTCAGTC	TCTATTGTTGCATGAGAACGTG	Huang et al.(2016)	
AGGS0399	TGAGGAGCCTTGAAAGATAGGT	CAGGTTGAGTTCGGAATTTGAT	Huang et al.(2016)	
AGGS0408	ATTTGCTCTTTGCTGAAGGAAG	CCCGTAAGGGCTTAACTTCAAT	Huang et al.(2016)	
AGGS0429	GTGTGGAATGTGGTGCATTAAG	CCACATTCATACCCATAAGCAT	Huang et al.(2016)	
AGGS0440	TCTTCACCCTATCACAGCCTCT	GGCTAATTGTCCCTAGGATAGGA	Huang et al.(2016)	
AGGS0449	CACAGTATTAGGGTTACAAGTTGC	ATTATGCGTCCAAACTCGAACT	Huang et al.(2016)	
AGGS0617	TGGAGTCCCTAGATCAAGATGC	ACCAAACCATCCCATCAGTTAC	Huang et al.(2016)	
AGGS0627	ATCATCACCTTCACCCTTTGTT	AAGCTAATGCACCAACAAATCC	Huang et al.(2016)	
AGGS0633	ATACCAACCCACCTAGCAGAAA	ATCGCCTTCAGAAGTATGTTCC	Huang et al.(2016)	
AGGS0638	GGTTGGTGGCTTCTATGATTTC	CGTTCTCTCATTCGTTCATTCA	Huang et al.(2016)	
AGGS0670	CTTCTCCTGGCAATGTATTTCA	GCCCTGCTTTCTGTATTGTCTT	Huang et al.(2016)	
AGGS0672	CTCAGTCGTGCTTCAAATCTCA	ATGGAGCTTCTAGGTGCAAATG	Huang et al.(2016)	
AGGS0673	AATGAAAGAAGGAAGGGAGGAG	GCGCGACAAGTATCCGTATT	Huang et al.(2016)	
AGGS0675	TCCCACATCACCGTCACTATTA	CGTGTCCTCCTCCTTGAAGATA	Huang et al.(2016)	
AGGS0694	AAGCGTGAAACCTACTCTGCTC	TCATCACTCTCACACCCATCTT	Huang et al.(2016)	
AGGS0720	CGTCAGTAACTGCGAATGAAAC	GGAGCACGCAATTAAGAGAAGA	Huang et al.(2016)	
AGGS0738	TTGAAGTAGTGGTGATGGTGGA	GAGGTTGACTTGGAGAAACAGG	Huang et al.(2016)	
AGGS0815	TCTCCAAGACGATCCATCTAAA	TCTGTTCTACAAGGACCAGCAA	Huang et al.(2016)	
AGGS0833	TCTGATTCCAAAGATGACGTGT	AGTCAAACAAGAAGCTCGTTCA	Huang et al.(2016)	
AGGS0940	TGCTCTGCTACTCTTCAAACCA	CCCAATGTGTGATCAATTACGA	Huang et al.(2016)	
AGGS0955	GACCCATCTTACCCAACAGAAG	GTGAAAGGCATCATCAGAAACC	Huang et al.(2016)	
AGGS0957	GAAGATTTATGGAAAGCGGATG	GTGGAGACAAAGGTGCACAATA	Huang et al.(2016)	
AGGS0960	GCGGCTTACTCTTGTTTGTAGG	TCCCTTCTACACAGACATCCAA	Huang et al.(2016)	
AGGS0973	GCCATATCGATCGAGGAATTAG	CCCACATTTCTTGTGATTGTGT	Huang et al.(2016)	

ID	FORWARD PRIMER (5'-3')	REVERSE PRIMER (5'-3')	Source	Alias
AGGS0977	GTGTTTGATTGAGCCTTTGTCA	ACACGCACGAGATACATAGTGG	Huang et al.(2016)	
AGGS0978	TTTCCTTCCTTCTCCTTTGTCT	TCTAGAAGAGGAGGATCAGTAGGC	Huang et al.(2016)	
AGGS0979	GTTGCCTGAAAGGATTTACCTG	CAGCAAGTTGCATTGAGAATGT	Huang et al.(2016)	
AGGS1003	GCTTGAAGAAGATCGAGCAAC	CTGAACCCGTCGTCTCTGTC	Huang et al.(2016)	
AGGS1005	CCAAGTTGGTATTCTCCAAAGC	GGGTTTGATTTGGGTGATTAGA	Huang et al.(2016)	
AGGS1013	AGGAAGGATAAGCAGGCAAAC	TGGCATAAGTCTTAGGGTTACAC	Huang et al.(2016)	
AGGS1014	GTGAAGTGGTGAGTGAGTGAGG	ATGTAGAGACGACACCCATGCT	Huang et al.(2016)	
AGGS1054	GACACTGCTCCTCTCCTCTAGC	TCCACACTCATGCGTACTAACC	Huang et al.(2016)	
AGGS1056	CGGCATGACCTACAATACAACT	TGTGCTCCTTCTTCTTCTTCTTC	Huang et al.(2016)	
AGGS1064	TGCTAGGTCGCCACTGTTACTA	TAGCAATTCTGCAACAACAACC	Huang et al.(2016)	
AGGS1076	AGAAGAAATGGCCTACTGGTGA	CTGTCATCTCCTTGCTGTAACG	Huang et al.(2016)	
AGGS1101	ATAATTTCTGGAAGCCCAAGGT	TGCTTCCAAGACTTCAAACTCA	Huang et al.(2016)	
AGGS1102	CATGAACAATTACACCCAGTCA	CTTGTCAAACTCTGTGACTTAGCA	Huang et al.(2016)	
AGGS1118	AGGTGTTCCTCTGTGGTCAGTT	TGGTTATGGTTCTCTCATCTGG	Huang et al.(2016)	
AGGS1122	CACTCCATCATCTAAGGGTCAA	ACCTATTTCCCAATCATCATGC	Huang et al.(2016)	
AGGS1124	CAAAGCAAAGAAGGGTAAGGAA	GCACAAGATAAACCACGAAATC	Huang et al.(2016)	
AGGS1137	TTCTACCTTTGTCCCAAGAAGG	AACTTTGGAGAATTGCTTGCTC	Huang et al.(2016)	
AGGS1160	GTGTCTTGTTGTGTCCGTATCG	AGGCAACAGAATGGAGAGAGAG	Huang et al.(2016)	
AGGS1167	TTCCATCTACCTCGCAGAAAGT	TTACCCTTTCCCTCTCTGCTTT	Huang et al.(2016)	
AGGS1183	GGGAGAGAGAAGAGCAGGAGA	CTCCCTCCCTTCCATCCTTT	Huang et al.(2016)	
AGGS1187	CTAGGGCTTGGAAGTGAGAGAA	GGTGCTGCTGTTACCGACTTAT	Huang et al.(2016)	
AGGS1193	GGGCTGCTCTTGTCTCTACTAGG	ACTCGTCCTCTTCTCCTCACC	Huang et al.(2016)	
AGGS1194	CCCTTAACACACACACAAGGAA	TTAGACCGAAAGGAGCGAGTAG	Huang et al.(2016)	
AGGS1197	CGTCCTTCTTCTTGGTGAACTC	AGTCATCATTGAGCATCGTGAG	Huang et al.(2016)	
AGGS1211	GAACAGCTTTGTGGTGTGACAT	ACAGACATAACAGTCAGTTTCACC	Huang et al.(2016)	
AGGS1213	CATGGCAGTAGTAGCAGCAGAG	GTGACGTTGAGGGAGAGAGAAG	Huang et al.(2016)	
AGGS1233	GTACCACCATGGCACCTTCT	TTCATCACTCACATCTACCTCACA	Huang et al.(2016)	
AGGS1238	CTTTCCTAATACGAGCAACACA	CGTAGGGAGAGAAAGTGAAAGA	Huang et al.(2016)	
AGGS1243	AGTGTTTGAACAGAAACGCAGA	TGGGCTAACTTACACCAGGTAGA	Huang et al.(2016)	
AGGS1254	ACCAAGAACGAAGTAGCTGCAA	AAGTCATTGCTAGAGCCCATGT	Huang et al.(2016)	
AGGS1262	AGAGGGAAGAGAACACTGACCA	AAGGATTGCACTACGGGTTAAA	Huang et al.(2016)	
AGGS1276	GCGAAATTGGAACAGTAAGAGG	GCAAGCCTACCAAGAGAAAGAA	Huang et al.(2016)	
AGGS1283	CGCAAAGTCCAAGTTCTGGTA	CTCATTGATAGCAAGGACCTGA	Huang et al.(2016)	
AGGS1299	GTTGCTCCGATTTCTACGTTTC	GCTTTGGTAAACATCCGATAGC	Huang et al.(2016)	

ID	FORWARD PRIMER (5'-3')	REVERSE PRIMER (5'-3')	Source	Alias
AGGS1307	TTGGTGAATGACTTAGGAACGA	ATGGGCAGAATGGTAAATGAAC	Huang et al.(2016)	
AGGS1312	CGGAATTAATTGTTAGGGACCA	CCACCACTACTACCATCGTCTC	Huang et al.(2016)	
AGGS1317	AATGCGTGCACCTTCAGTAAC	TTGTGGTTCTGCTCTCTCAGTC	Huang et al.(2016)	
AGGS1321	TCCTTCTCCCTCTTCACATCTC	AGAAGAGAGAGCCGCCGTAT	Huang et al.(2016)	
AGGS1324	TGTGTGTGTGTCAGTGTGTGTG	CATGTAGTGTACACAGGCAAATCA	Huang et al.(2016)	
AGGS1325	TTTGAAAGACCCACACTCTCTC	GGTTTGGAGAAGAGAGATGCAC	Huang et al.(2016)	
AGGS1344	TTTAGAGAGCCTTACCACCTTCC	GCACTAATCCCTCGATTCATTC	Huang et al.(2016)	
AGGS1355	TTAGACCGAAAGGAGCGAGTAG	TTACACCCTTTAACACACACACG	Huang et al.(2016)	
AGGS1356	TTGTTGAATGTCTCCAGAATGG	ATTCACACAACCACCCACATTA	Huang et al.(2016)	
AGGS1359	CACATTCTCTGCCATTGAACAT	TGGTAGACCAAAGAAAGCTGAA	Huang et al.(2016)	
AGGS1363	GACAACGTGTAGTAGCGACAGC	TTTCGCTGGTAAATCCGATAGT	Huang et al.(2016)	
AGGS1365	GATATTGAGGTATGGCGTCGTT	TTCAGATGGCAGTAAGTGTTGC	Huang et al.(2016)	
AGGS1369	AACTGGTGAAATGGAGTGGAGT	TTTAACCTCCCTTGTGGGTATG	Huang et al.(2016)	
AGGS1370	CAAGGAGAGCTTGGACACCTAT	CAGGAGGATACTTCCTGAGCAT	Huang et al.(2016)	
AGGS1376	GGGAAGAAGTGGAACAAACAAA	CGCTCTACACTCCTTCTCCTTC	Huang et al.(2016)	
AGGS1378	TGGTACTATCCCGCCATAATTT	GGGTTAATTCAACATGGGTCTC	Huang et al.(2016)	
AGGS1389	GCAGTGCAAGCGTTGATAACTA	CCTTATCTTCTGAAACGCCATT	Huang et al.(2016)	
AGGS1393	GAGAACATGGTTCGAAGAAACC	CACCGTGTTGTTATGTTGTTCC	Huang et al.(2016)	
AGGS1400	ATAACTCCCGGATCATGCAA	ATGTTGTCTTTCTCCCGATCTC	Huang et al.(2016)	
AGGS1403	CTAAGCTTGTTGCTTCCATTCC	GTTGTCTGCAAGAGGTTGTCTG	Huang et al.(2016)	
AGGS1425	CATCAGCGCAGGATAAATCAA	CTGAAGGAGTTTGCAGGAACTT	Huang et al.(2016)	
AGGS1431	TTCCTCTTCCTCTCTCCTTCTTC	AAGGGATAGAGTGGGAGCAAAG	Huang et al.(2016)	
AGGS1432	GCTTCTCTCCACCACTATTACCA	AAGAGAGGAAGGTGAGGAGGAT	Huang et al.(2016)	
AGGS1438	GTCGTCTCCTCTGTTCGAGTG	AGACAAGGAGGAGGAGGTGATA	Huang et al.(2016)	
AGGS1442	TTCTTGTTCTTCTCCATGCTCA	TGCACTACCTTCAATGTTACCG	Huang et al.(2016)	
AGGS1450	GGAATCAAACACAGGATTCACA	GTGAACTGAGCACAAACTCAGTAA	Huang et al.(2016)	
AGGS1451	AAATCCCTAGCCTCTCAAGACC	GGGAAGTGTGTGGATTAGGAAG	Huang et al.(2016)	
AGGS1453	CGGATGATTATGTCCTCCACTT	TCATTCACTTCCCTCATTCTGA	Huang et al.(2016)	
AGGS1461	CATGAGCGATGACAAAGATAGC	TCTGTCGCAAATTTGAGTTACC	Huang et al.(2016)	
AGGS1464	GGCAGAAAGGGATGTTCGTACT	TGTGTCTGAGCTCACTTCCTTG	Huang et al.(2016)	
AGGS1466	GGAAGGGAGAGAGATAACGAGA	CTTCTTTCACTGCATCCACCTT	Huang et al.(2016)	
AGGS1468	CTAACCAACGAAGCCCATAGAA	TGATGAAATGAGCAAGTGGAAC	Huang et al.(2016)	
AGGS1479	GTTTATCACACGGATGCGTCA	GGGAGACAACCCACCATAGTATT	Huang et al.(2016)	
AGGS1481	CCATCGCGAGTGTATAAGAAAG	TCTTTGACACTACCATTGTGGA	Huang et al.(2016)	

ID	FORWARD PRIMER (5'-3')	REVERSE PRIMER (5'-3')	Source	Alias
AGGS1483	TCTCTCTCTCTCTCGCCTTAAA	GAAATCCTTTGAAGGTTGTTGG	Huang et al.(2016)	
AGGS1484	TTTGCTCCAACTCCTCAATTTC	GCCGTAAAGCGAAGAATAACAA	Huang et al.(2016)	
AGGS1495	TTCTTCTTCCTCCCTCTCTGTC	CTTCACACACAATCAGCTCACA	Huang et al.(2016)	
AGGS1498	CTCTGTTCCCACCACTCTCACT	TCTTCTTTGAATACGCGAGAGA	Huang et al.(2016)	
AGGS1512	CTTGTTTAGTGAAACGCTGCAT	ATGAAAGAGAGACTGCGAGGTT	Huang et al.(2016)	
AGGS1513	ATGCTTCTGCTTCTGCTTCTCT	AAATTATTCTGCGGTCCTCGTA	Huang et al.(2016)	
AGGS1518	TGGTCTCACGTTGGACTTTGTA	TCCATTTCTCCCTCTCTTTCAA	Huang et al.(2016)	
AGGS1532	CATGTGATTGGTCGTCATCTTT	AGGATCTGCCAAGAGTTAATCG	Huang et al.(2016)	
AGGS1533	CACACACACACACACACACACA	AGGCAAAGTGGTTACTGGGTTA	Huang et al.(2016)	
AGGS1542	AGAGAAGGAACAGGTGTGAAGG	ATTGCCATCTCCTTGTGATCTT	Huang et al.(2016)	
AGGS1568	CTACAGCTATGCAAGCAACGAC	GGTGGCCTCTATCATCATCATC	Huang et al.(2016)	
AGGS1569	AGGCTGATCAATCTTCAACTCC	TCTCAAAGGTGCTTGATTTGC	Huang et al.(2016)	
AGGS1572	ATAACCCAAACACAACCACTCC	GTGATCGGAGTTTCGGATTAAA	Huang et al.(2016)	
AGGS1577	TGGGTTAGAGACAGAAGGGAAG	TCTCTCTATCACTCACGCACTC	Huang et al.(2016)	
AGGS1579	ACTTTAGAGCTGAGGTGGCATC	CTCTCCTCGGCTTTCTTTATCA	Huang et al.(2016)	
AGGS1585	TCTCAGTCCTGCTTCTTGTCTG	GACCTCCACCCTTTAATTTCCT	Huang et al.(2016)	
AGGS1589	TCAACACCAGCTCTTCTCTCAA	GCAAACCAGAAGGGAAGAATAG	Huang et al.(2016)	
AGGS1592	GGGAGAGCAGAGATTGAAGAAG	TAGTTGCAAGCTGTCATTAGGC	Huang et al.(2016)	
AGGS1596	TGTGGTGGATCTCTGAGCTAAA	CTTCACACAGGTTCTGCTTCTG	Huang et al.(2016)	
AGGS1600	CGCAATTCAACTGATTCCTTC	CCCTCCATTTCGTCTTCTACTA	Huang et al.(2016)	
AGGS1601	AAGAAGACCACGAAGATGTTGC	CTGCTTCTTTCTCGTCGTCAA	Huang et al.(2016)	
AGGS1606	CTCACACATACACAACCTGCTG	CATCGAGTAGTATGGATGCTTGA	Huang et al.(2016)	
AGGS1612	CCTCCATCGAAGTTCGTTCAC	TCTACAATGTTATGCCGATCCA	Huang et al.(2016)	
AGGS1613	ATGAAGAAAGACGACAGCATGA	CATGGATACCTGCGAGTAAAGA	Huang et al.(2016)	
AGGS1615	TGGCTTGTTGATAATGATGCTG	AATGACCATCCGTCATTCAACT	Huang et al.(2016)	
AGGS1620	AAATTATTCTGCGGTCCTCGTA	ATGCTTCTGCTTCTGCTTCTCT	Huang et al.(2016)	
AGGS1621	ATACTCTTTCCGAAGCTGCAAG	CTTCCAGCTCACCGTAACTACC	Huang et al.(2016)	
AGGS1629	CCCTAGGCTCTGATACCATGTAA	TCAGTGTTCACTCAATCACAGAGA	Huang et al.(2016)	
AGGS1632	GAAGTTGCTCGCGTGTATATTG	TCAGGTTTCAAGTCTCGATGAA	Huang et al.(2016)	
AGGS1635	GAAGATGTCATGCGCAATAAGT	GTCACGTGGTCTTCATGTGATT	Huang et al.(2016)	
AGGS1638	GATGGATGAGTGGTCATACACG	CCTCCTGATTGGATGCTCTATC	Huang et al.(2016)	
AGGS1639	GAATCCCTTTCTCTATCCACCA	CAATCTTTGGAGGAGATTGGAG	Huang et al.(2016)	
AGGS1643	CTTTGCTCATTGTTTCTTCACG	ACTCAGATGCCCTTTCTTCATC	Huang et al.(2016)	
AGGS1645	CCCTCTCCCTTATTTCTGCAA	AACGACAATGCGATAAGGATCT	Huang et al.(2016)	

ID	FORWARD PRIMER (5'-3')	REVERSE PRIMER (5'-3')	Source	Alias
AGGS1654	ACAACATCCAGCAACGGTAATA	TGATGCGTTTCACCGATTTA	Huang et al.(2016)	
AGGS1656	ATTGAAAGGGATGGAAGAAGGT	CTCCTCTGCTGCGTTAAGAAGT	Huang et al.(2016)	
AGGS1662	GTAACAAGCACGCATTCAATCA	CACCACCTGCAATCACTTCTAA	Huang et al.(2016)	
AGGS1671	GGAATTAGGAAAGCAGATTGGA	AGCCATCCAAGCATAGAAGAAA	Huang et al.(2016)	
AGGS1678	CTCCCATCTCTCTTCGTCTTTC	GAGAGGAGGGAAAGGAGTGA	Huang et al.(2016)	
AGGS1691	TGCTGCCCTCTCTCTCTCTTT	GGAGCACCATAAGAGAGAACTCA	Huang et al.(2016)	
AGGS1700	CCAACCATTCCCTTCTTCTCTT	TGGGAAATAATCCACAACACAG	Huang et al.(2016)	
AGGS1728	CGGAGAGTATGTGCTAATCACG	ATTACAGCACATGGTGCCTCTT	Huang et al.(2016)	
AGGS1750	TCGCAGATCTGATTAGAACCAA	AGATGAAGGTGTGCAAGATGG	Huang et al.(2016)	
AGGS1754	GGATCTTTGATCGGATTCCTAA	AAGAAAGAAGAGTGGTGCCAAA	Huang et al.(2016)	
AGGS1773	TGCAAGGTAAGGCTTAGGGTAA	CTGGTAGAGAACAGGGACACCT	Huang et al.(2016)	
AGGS1783	CACAAATCTCATAACGGTCCAA	TCTTCATTGGAGTAGCCACCTT	Huang et al.(2016)	
AGGS1795	GTCATCGCCGAACTCTTCTTAG	CCTTAATGGCAATGCTATCTCC	Huang et al.(2016)	
AGGS1840	TGAGTGAGGCTAACTGTGGTTT	CGAGTCTTTGACCAGATGACAG	Huang et al.(2016)	
AGGS1885	GGAAGTGAGGTTGGTGATCTTC	AAGGATATGGAAAGTGGTGGTG	Huang et al.(2016)	
AGGS1925	TAGTTCTCTCCCTCACCCTCTG	CTCGTAGCTAAATGCCCAAGTC	Huang et al.(2016)	
AGGS1932	AATTCCGATAGCAAACTTGACC	CATCAGTAGCAGCGACGATAGA	Huang et al.(2016)	
AGGS1953	TTTCACCTGAGAAACCAACCTT	TCAATTCACTCTGCTTTCTAGGG	Huang et al.(2016)	
AGGS1954	TCACCCGTATAACGGTTTAGTG	CGGTAGCTTTAACGGATGAGAT	Huang et al.(2016)	
AGGS1979	CATGGCACACTCTTCTACGTAAC	GAAGGTGGTGGCAGACATTATT	Huang et al.(2016)	
AGGS1987	TAGTGTACGGAACCACAAACGA	AGCAACTGGAGTTCGTTACCAT	Huang et al.(2016)	
AGGS2019	CTCCCTTCAAGTTCAAGAGCAC	GCAGCATCTGAACAAGAACAAA	Huang et al.(2016)	
AGGS2023	AAACGTAGAGCTCGACAAGAGC	CCCAAGACCCAACATAGAAATG	Huang et al.(2016)	
AGGS2027	CCTAAATTCGAACTGCAAACCT	AACAGTGGTAGAAGCGACGAA	Huang et al.(2016)	
AGGS2030	TCCTCGAAGAAGTGAAGTAGCC	GACTGGTGAGTAGTGACGGACA	Huang et al.(2016)	
AGGS2061	ACCAGGCAAGAATTATGGAAGA	CACCTTTCACCACCATTAATCC	Huang et al.(2016)	
AGGS2082	CCAACAGTGGTAGTTTGGTGAA	TTGAACTGTGAACCCTCTTTCC	Huang et al.(2016)	
AGGS2092	CTCCCTCCCTCTCTCTCTAACC	GTGTTTCTAGCAGCAACGACAG	Huang et al.(2016)	
AGGS2115	GCAAAGACGTTGAAATGATGAG	GAGAGGCAGATAGCAGAGCTTC	Huang et al.(2016)	
AGGS2117	ATGATGACAATCGGACAATGTG	AATTAGAGATAGTGGTGCGTGGA	Huang et al.(2016)	
AGGS2121	GACACTCTCTCACCCTTCCTTC	AAATCGACGTGGAATCGAAA	Huang et al.(2016)	
AGGS2124	TGAGTCAGTAAATACGCCGAAA	GGACACTTTGTAGTTCACCGTTT	Huang et al.(2016)	
AGGS2134	AGCACCACTTGAGGGACATTAT	TTTGCAGAGAACTCACAAAGG	Huang et al.(2016)	
AGGS2135	ACTGGAGGTCCTGCTGTTAGTC	ATACCAGTTCTCCAGCAAGCTC	Huang et al.(2016)	

ID	FORWARD PRIMER (5'-3')	REVERSE PRIMER (5'-3')	Source	Alias
AGGS2147	CCTGAGACCAGAGTTGCTTCTT	TTCTTGGAGCTCAACATAGTGG	Huang et al.(2016)	
AGGS2177	CTCTCAGTTTCTTTGTCGCTCA	GTTCTTGGGATGTGAGCAATTT	Huang et al.(2016)	
AGGS2186	CCACGACAGGGACTTCCTATAA	TGATGGAGTTGAATGAATGAGC	Huang et al.(2016)	
AGGS2187	GGTCTGTGAACAGAAGGGAGAC	ACTGTGGGTACAGCAAACAGTG	Huang et al.(2016)	
AGGS2195	TGTTTCATCTGCTCTTATGTGG	ATGCTCTTTCCGGTAGTGTGTT	Huang et al.(2016)	
AGGS2202	TATGCGCTCAAATCTCTCAAGT	ATGGGAGAATAGGTGAAGAAGC	Huang et al.(2016)	
AGGS2216	TGTGTGGGTTTAGGGTTTCTCT	AAATAGTGGTGCATCAGCCTAA	Huang et al.(2016)	
AGGS2219	ATCACAGCAAGGTTCACCTCAT	TGATGTAGAGGGAGAGGGCTTA	Huang et al.(2016)	
AGGS2224	TCTCTCTCTCTCTCTCCCTCTCC	CCTGCTTGGTTTGGGAGTAA	Huang et al.(2016)	
AGGS2228	TATAGGAGGCTATGGTGGTGGA	AGTCAGGTTTCACCGTTGATG	Huang et al.(2016)	
AGGS2233	AGACATTGCAACGACGAGTCTA	AGCAGAGCTTCTTCTTCTTCCA	Huang et al.(2016)	
AGGS2251	ACGCACCACGTTACCATTAGAT	GATGGCACGAAGCTAGAGAGAG	Huang et al.(2016)	
AGGS2261	CTCCCATCTTCCTTCACAGTTC	ACGGCGACTAAGTGATGAGTTT	Huang et al.(2016)	
AGGS2262	TCTCTCTCTCTCTCTCCCTCTCC	CTGCCTGGTTTGGGAGTAAA	Huang et al.(2016)	
AGGS2272	CCTTCTTTCACCGACTAACCAC	GTGAGTGTTCTTAACGGTGTCG	Huang et al.(2016)	
AGGS2287	TTTCTTTCTTCTCTGGTGTGAGC	AGGCACAATGCACTCAAGTATG	Huang et al.(2016)	
AGGS2317	CTCCTACTCCCTCCATCTCCTT	AGGAGACACTACTGCTGCACAC	Huang et al.(2016)	
AGGS2325	GAGAGGCATATTCCATTAACGA	ATAGAGTGGTGGCTATGGCATC	Huang et al.(2016)	
AGGS2332	TGTCTCAGATCTGCCATAGCTT	TCTACAACAATCCAAACCACCA	Huang et al.(2016)	
AGGS2359	GAGAGGACAGTGTCGGAGAGAT	TACCTGGTAAATCCGACGGTAA	Huang et al.(2016)	
AGGS2368	GTTTACCTCACCTTCACCGAAC	AGCAGAAGTGAGCAACAGGAAG	Huang et al.(2016)	
AGGS2371	CCTCTCCCTCCTCTCTTTCTCT	GTTATCTCTTGGTGCCTGCTTG	Huang et al.(2016)	
AGGS2372	TACCGTTGGATTAATGGAAACC	GAGAGAGGCAGAGAATGGAAGA	Huang et al.(2016)	
AGGS2380	AGAAACCCTCTCTTCAATCACG	GATGCCAGAGTGAGACAGATGA	Huang et al.(2016)	
AGGS2384	TTTCTGCCAATGCAACAAAG	AACTAAAGATGTGGCTTGAGTGG	Huang et al.(2016)	
AGGS2387	GAGAGAGAGAGCAACGTTGAGG	CATTCCTCTCTTCACGATCACA	Huang et al.(2016)	
AGGS2393	TGCGTCTATCACAACAGCAAC	TTGATGTCTGTGCATGTGTTTC	Huang et al.(2016)	
AGGS2398	GAGGTGAGTGGGCACATAGAAT	CTTGGCACATTCAACAAATACG	Huang et al.(2016)	
AGGS2407	CCTCTCCCTCCTCTCTTTCTCT	CTGCCTGGTTTGGGAGTAAA	Huang et al.(2016)	
AGGS2413	GGACCAATATTGAGCCAAACG	GATTTGCATGTTCTTCCTCTCC	Huang et al.(2016)	
AGGS2425	TTTCCTTCGCCTTTGTCATT	AAGCTCACAAGTGCTCTTCGAT	Huang et al.(2016)	
AGGS2438	AGCGTAGGAACCCAATTGAAA	TCTCAGTTAGCCGAGTGATGAA	Huang et al.(2016)	
AGGS2475	TGCTACTTTCATCGCATCACTT	CTACCGATCTCCCTTACCGATT	Huang et al.(2016)	
AGGS2488	CCTGGATTTCTCTCTCTCCATC	CATAACGAACAAACCACAAAGG	Huang et al.(2016)	

ID	FORWARD PRIMER (5'-3')	REVERSE PRIMER (5'-3')	Source	Alias
AGGS2492	AACGGTTCGTCAATCATAGCTT	AAAGAGCCATTCAGCTCATTTC	Huang et al.(2016)	
AGGS2494	ATCTCCCTACTCCCTCTGCTG	GAGGAGGTTTGGAAATGGAGTT	Huang et al.(2016)	
AGGS2508	TCAGGATCACCTTCAACAAGAA	TCGAACCCTTTCTCTCTCTCTC	Huang et al.(2016)	
AGGS2509	CCTGTATAAGGGAACAGGTGGA	AGCATCACCATCATCCTCTCTC	Huang et al.(2016)	
AGGS2532	GAAAGGCAAGGGTTGTGTTAAG	GGGCGTGAATCACTCTCATAAT	Huang et al.(2016)	
AGGS2535	CCCTTTCTTTCTTTCTCTCTCTC	GTTATCTCTTGGTGCCTGCTTG	Huang et al.(2016)	
AGGS2555	CGTCTCGCTGTCGTCTCTAAAC	CTCTAACGGTTGACTTCGACAA	Huang et al.(2016)	
AGGS2556	TTTCCAGTTGAGTTCACCATGT	CCACCACACACTCTCTTCACTC	Huang et al.(2016)	
AGGS2564	CCCTTTCTTTCTTTCTCTCTCTC	CTGCCTGGTTTGGGAGTAAA	Huang et al.(2016)	
AGGS2567	GCCTTCCTCCATTCTTGAGTTA	CCGACTCCTCTCTTCTTCTTCA	Huang et al.(2016)	
AGGS2572	GAAGAAAGAAGCGAACCAGAGA	AAACACTTTGGGAGGTGTGG	Huang et al.(2016)	
AGGS2576	AGACTCATCACTTGATCGTTGC	GAGTCATTCATTGTGTGTTTGC	Huang et al.(2016)	
AGGS2580	CGTTCTGTTCATCCCATCAATA	TTCACCGCTACTGAAGAAGAGA	Huang et al.(2016)	
AGGS2701	GGGTGGGTTACCAACATTCTAA	CGAAATAGCGGTAATCCACATT	Huang et al.(unpublished)	
AGGS2710	CCAAGGAACCAAGCTGATTT	TGAATAGGGTAACATGGTCACG	Huang et al.(unpublished)	
AGGS2728	GACGGAAACTCTGACAAAGTCC	GTCGGCTTCTTATCACACTCCT	Huang et al.(unpublished)	
AGGS2744	AGGATCAATGCAATTGAAGAGG	GTTAGAACTATTTGCGGCAGTT	Huang et al.(unpublished)	
AGGS2745	AGGATCAATGCAATTGAAGAGG	GTTAGAACTATTTGCGGCAGTT	Huang et al.(unpublished)	
AGGS2746	TCCTAGGACCAAGTAAGTTGTGG	AACGGGCATAATCTTCATCATC	Huang et al.(unpublished)	
AGGS700	ACACACCGTAAGTGTCCTTCCT	TTAGGAACTCCAACGAATCCTC	Huang et al.(2016)	
AGGS734	AGGAAGACGTTGAAGAAGATGC	TCTCAACCAAAGAGATCATGGA	Huang et al.(2016)	
AGGS750	CGCCATCCTCTTCATGTAAGTT	TACAAGAAGCTACGACGACGAC	Huang et al.(2016)	
Ah26	GAAAATGATGCCATAAAGCGTA	AGTGTAACACCCCGTTAGCC	Gimenes et al. (2007)	
Ah3	TCGGAGAACCAAGCACACATC	TTGCGCTCTTTCTCACACTC	Bravo et al. (2006)	
Ah325	TGAGGTTGCTCTTCTCCTTCCTC	GAGCACCAGTGACAACACAAGG	Moretzsohn et al. (2005)	
Ah426	TGGAATCTATTGCTCATCGGCTCTG	CTCACCCATCATCATCGTCACATT	Hopkins et al. (1999)	
AHBGSI1008H04	TGCTCTCTGTGGGTTTCA	GTCGCCATTTGTGGAAAA	Moretzsohn et al. (2009)	
AHGS0108	GGTGAGGGAAAGAATCCACA	ACAAGGGTGACTTTGTTGGC	Shirasawa et al. (2012)	
AHGS0122	TGAGGATCTTGGATCTTGGC	CGAATCCAGTGCCCACTAAC	Shirasawa et al. (2012)	
AHGS0147	TAACAGCCGGATCAAACTCC	ACCACCACCTGCAATCACTT	Shirasawa et al. (2012)	
AHGS0153	CTCCATCGTGTTTTGCTCCT	GCACTTGCAACGCTGTTCTA	Shirasawa et al. (2012)	
AHGS0201	CACAATACACACACGCACCA	TTACAACGGCATGCTTAACG	Shirasawa et al. (2012)	
AHGS0274	TTGCTGAGAACATCACTGCC	ATAGCGACGGCGACTAAGTG	Shirasawa et al. (2012)	
AHGS0288	CAGTTCTGAGCAAGCACTCG	ATTAGAACCCCTCACCCCAC	Shirasawa et al. (2012)	

ID	FORWARD PRIMER (5'-3')	REVERSE PRIMER (5'-3')	Source	Alias
AHGS0300	AAGAAGCATGGAAATGGTGG	CAATCAAATTGGGGAAATGG	Shirasawa et al. (2012)	
AHGS0344	TCACTGAGATGCAGAATGCAC	CGTTGAGGGTTGTTCCACTT	Shirasawa et al. (2012)	
AHGS0346	TTATGCCTACCACCCCACTC	TTGGGAGAAAATGATGGAGC	Shirasawa et al. (2012)	
AHGS0390	CATCATCCAATCACAATCCA	GTGGGGGAAGATCTACGACA	Shirasawa et al. (2012)	
AHGS0400	GGGCAGAGATTCATGGAGAG	CTTTCGAAGGTCTGCTCGTC	Shirasawa et al. (2012)	
AHGS0422	TCCTAATTGGCCCTTCTCCT	AAAGCCTAAGAAGAATCGAATCAA	Shirasawa et al. (2012)	
AHGS0429	AGCACACGTGCATAGGTGAG	TGGGTAGCTATGCATGATTTT	Shirasawa et al. (2012)	
AHGS0729	TGGTTGTTCTAACCCTTCGG	TCACTATCCCATCCCTGCTC	Shirasawa et al. (2012)	
AHGS1098	CTGTTGACGCTGTGAAATGG	TTACCCCTTTCCTTTTCCTGA	Shirasawa et al. (2012)	
AHGS1126	GGACCGACATTTTCCTCGTA	TCCCAACGAAAAAGAACCAC	Shirasawa et al. (2012)	
AHGS1130	AGGATCTCTGCTCAAATGCC	TGACAGCAACATGATACGCA	Shirasawa et al. (2012)	
AHGS1143	AATGCAGAACGGAAGAATGG	CGTCGAATTTTTCCAATGCT	Shirasawa et al. (2012)	
AHGS1176	GCTGATCCAAGCCCAAATTA	AGCTTGCTTTGATTAGGGTGA	Shirasawa et al. (2012)	
AHGS1188	TAAGAGAATGAGAAGCGGGG	TTTGCCTTCACTGGAAGCTC	Shirasawa et al. (2012)	
AHGS1189	TCTCACCATAACAGTCCCCC	CTGCCAGCTAGGACAGATCA	Shirasawa et al. (2012)	
AHGS1203	CTCATGCAACGACGTAGGAA	CACTACCATTGTGGATAACATTATTT	Shirasawa et al. (2012)	
AHGS1208	TGTTGATGAACGAAATGGGA	TCCAGAGCAACTGGACAATG	Shirasawa et al. (2012)	
AHGS1228	CACTTTTAAGGCGTGCCAAT	TGTCAGGGGAAGCTTATGTCA	Shirasawa et al. (2012)	
AHGS1232	ATCCTAACTCCCCTAGCCCA	GATTCTGCTTTCCCCTTTCC	Shirasawa et al. (2012)	
AHGS1241	GCTAAGCTAAATTACCCATTTTGTG	GTTTGAGCTTGTGCAGTGGA	Shirasawa et al. (2012)	
AHGS1245	CGTGATTAGCGTAACTGCCA	TCTTAGCTGCGAAATCCACC	Shirasawa et al. (2012)	
AHGS1246	AAATGCCATTCACACTGCAA	TCACTTCTCTTCCCACCACC	Shirasawa et al. (2012)	
AHGS1251	GGATTCGGTGAAACGACACT	GATGCCCTTTCTTCATCCAA	Shirasawa et al. (2012)	
AHGS1261	GCAGAGGTAGCAGGGATGAG	CCCCTCTCAACGTCTCTCAA	Shirasawa et al. (2012)	
AHGS1266	TCGGATATTACGACGACTCTGA	TGAGAACCCTTTTGAGGACG	Shirasawa et al. (2012)	
AHGS1267	TGCAATATAGTCGGCTGCAC	TCTGGTGTGCTTCAAAAGGA	Shirasawa et al. (2012)	
AHGS1273	ACTGGAAATACCCCTGACCC	TGCCGATCCATTTTCTTCTC	Shirasawa et al. (2012)	
AHGS1275	GAGTGAGCTCGGAGGAACAC	ATCACGAACTGCTCCTGCTT	Shirasawa et al. (2012)	
AHGS1278	GGAGCAGAATTCAGTTCATTTACA	ACTGGAGCCTATTCTTCGCA	Shirasawa et al. (2012)	
AHGS1283	TGAACACACACGCACTAGCA	TGACAACTTCTGAAGCGATGA	Shirasawa et al. (2012)	
AHGS1288	GTGAGGTTCTGACGGCTCTC	CCATAACATTCACCCCATCC	Shirasawa et al. (2012)	
AHGS1294	CATCCATTCATTCACATGGC	TCCTAACATGGAGTTCATAAACCA	Shirasawa et al. (2012)	
AHGS1296	GAGGTATGGCGTCGTTTGAT	GTGTTGCAAACACCTCCCTT	Shirasawa et al. (2012)	
AHGS1311	CACTTGAAGCACCAAATTGAGA	TGGGTGCCATTGTTTCATTA	Shirasawa et al. (2012)	

ID	FORWARD PRIMER (5'-3')	REVERSE PRIMER (5'-3')	Source	Alias
AHGS1319	CAAACCATGTCAGCATCTCG	GGTCTCGAACCTCCAATGAA	Shirasawa et al. (2012)	
AHGS1333	ATGACTTACCCCCATTTCCC	CGAAGAGGATGACGAGATCA	Shirasawa et al. (2012)	
AHGS1338	ACTGTCTCAACCCCATCCTC	TCATCGTCACTACCAGCACC	Shirasawa et al. (2012)	
AHGS1340	TACGCTTGCCAACTTCACAC	AACGGTTGCTGCAAAGAACT	Shirasawa et al. (2012)	
AHGS1342	CTGCATAGTGGCGGTGATAA	CATCGGATTAATTCAACGGT	Shirasawa et al. (2012)	
AHGS1369	TCAAACCGTGAACCCTTTTC	ATGGAGTCCACGCAGTCTTT	Shirasawa et al. (2012)	
AHGS1393	TGACTCGCTAACTCAGCAGC	CGAACTCGAAAGATCACGGT	Shirasawa et al. (2012)	
AHGS1395	TCCGGCGACATAGGAAATAG	TAAACTCAAATCGCGAACCC	Shirasawa et al. (2012)	
AHGS1419	TCTCCATGGTGATTGAACGA	TGCAAGCTGTCATTAGGCTC	Shirasawa et al. (2012)	
AHGS1426	ACCACACCGCAAGAAAGAAG	CGTTGAGATTGTTGTGTCCG	Shirasawa et al. (2012)	
AHGS1434	CTGGTTAGATTCTCCGGCAC	CAGCTCAATCCTACGCATCA	Shirasawa et al. (2012)	
AHGS1440	ACGTTGGGGACAACTTTGAG	TCCTGGGAGAAGTAGAGCCA	Shirasawa et al. (2012)	
AHGS1446	CAGGAGAGAGAGAAAAGAGAGGAA	GGCGGTAAATTCGACGATAA	Shirasawa et al. (2012)	
AHGS1457	GAAGGAGGGGGAAAGAGAGA	CGGAAGAAGAAGCAACTTGG	Shirasawa et al. (2012)	
AHGS1459	TTACCGTGAGCCCTAACCTG	TCGTCTTTCGGTTCCGATTA	Shirasawa et al. (2012)	
AHGS1462	TTGCACTGCACGACTCTTTC	GAAGCACCTTTTATGGCACC	Shirasawa et al. (2012)	
AHGS1465	GGTTGGCGATGAAGAGAGAG	CCTCAATGCCCTCTCCAATA	Shirasawa et al. (2012)	
AHGS1466	CAACCAACTACCCTTTTCCG	AGGCATGAGGACAATGCTAA	Shirasawa et al. (2012)	
AHGS1470	GACACCGCTGGACAAGAAAG	ATCAAAGCAACAACGGGAAC	Shirasawa et al. (2012)	
AHGS1473	CCCTAACCCTAATTCCCATCA	ACCGATCTCCCTTACCGATT	Shirasawa et al. (2012)	
AHGS1475	TACTCGCCGCTCTCTTTGAT	ACAAGATCGGTGGCAAAAAC	Shirasawa et al. (2012)	
AHGS1489	CGATAATTGCCTAGGAAGTAGAGA	AACTTCCCCGAACTTCTCGT	Shirasawa et al. (2012)	
AHGS1497	TCTTCCTAAGAAGGCAAGAGC	TGGGGAAGAATAAACCATGAA	Shirasawa et al. (2012)	
AHGS1507	TATTTGTTTCTGGTTCCGCC	TCTGTTAGAGAAGTCAAGTCTGTTG	Shirasawa et al. (2012)	
AHGS1522	ACCAAGGTGCGACATTTTTC	TTGATTTTGCAGGGTTAGGG	Shirasawa et al. (2012)	
AHGS1524	GTGACGGAAGAGAGTAGCGG	GCAGTGATTGTCTCTGGCAA	Shirasawa et al. (2012)	
AHGS1530	CAAGTGAGAGAAAGGGAAAGAGA	AGGTAATACACCAGTGCGCC	Shirasawa et al. (2012)	
AHGS1532	CTGGTGTGAGCTCGAAGATG	AAACCAAGCCCAATGTCAAC	Shirasawa et al. (2012)	
AHGS1543	ACGTCAGGTTCACAACGACA	ATTTTTCTCCGAGTCAGCCA	Shirasawa et al. (2012)	
AHGS1552	CGGTGACTCATTCCACACAC	TGCTTCTTTCTCGTCGTCAA	Shirasawa et al. (2012)	
AHGS1561	GGCTCCCATTCATTCTCAAA	TCGTTTAACCTCCCTTGTGG	Shirasawa et al. (2012)	
AHGS1564	CTCTCTGCCATTGCATGTGT	AACTGCTTTTAAAATCTCCCCC	Shirasawa et al. (2012)	
AHGS1574	TTGCTGGGCAGAAGAGAGAT	CTCCACCACCGTCTTCTTTC	Shirasawa et al. (2012)	
AHGS1579	GCTGAGCTGAGCCAGAAACT	TTTCTGGAATCGCATCTCCT	Shirasawa et al. (2012)	

ID	FORWARD PRIMER (5'-3')	REVERSE PRIMER (5'-3')	Source	Alias
AHGS1595	CCTTCACCCTTTGTTTCAGC	ATGCACCAACAAATCCAACA	Shirasawa et al. (2012)	
AHGS1602	ATCACTCACGTCCGGGTAAG	TAGCTTCGCAGGTCGAGTTT	Shirasawa et al. (2012)	
AHGS1617	CACCCTCAACTCCTTCTCCA	GAGCTTCTTCATCTCCGCAC	Shirasawa et al. (2012)	
AHGS1624	AGCTTGCTTTGATTAGGGTGA	GCTGAAGACTTCCAAAGCCA	Shirasawa et al. (2012)	
AHGS1626	GAAGGTCAAACTTACGGGCA	CAGAGGGGTTGCCAAAGATA	Shirasawa et al. (2012)	
AHGS1630	GATTAGAATTGCGCACCCTC	TTGGTGGTGTTGACACTGGT	Shirasawa et al. (2012)	
AHGS1650	TGCTAGCTGTTTTGCACTCA	CATTCGAGAGGCTACTACTCTCC	Shirasawa et al. (2012)	
AHGS1666	ATGCTGCAGGATTGATGACA	TTGACAAAAATGACCATCCG	Shirasawa et al. (2012)	
AHGS1668	TCCCTCCATCTCCTTTGATG	TTCTACTCGGATTTGGGGTG	Shirasawa et al. (2012)	
AHGS1669	CTCCTTCTCTCCCCATCACA	CATTCATTGTGTGTTTGCCC	Shirasawa et al. (2012)	
AHGS1670	GTCGATTGTCGGTTTGGTTC	GTCTCTGGAAGAGCCGTGAA	Shirasawa et al. (2012)	
AHGS1672	CGCCCCTTCTTTTCCTAAAT	GGCAGAGAAGAGGAAGCAGA	Shirasawa et al. (2012)	
AHGS1674	GACACAATGGATTCAACCCC	CCCTTTCCCTCTCTGCTTTC	Shirasawa et al. (2012)	
AHGS1682	CCCCTCTCCCTTATTTCTGC	ATCTCAATGGAGGCAACGAC	Shirasawa et al. (2012)	
AHGS1683	AGTTGGCAACAACTTGGGAG	GCACGGATTCAGTGTTTCAA	Shirasawa et al. (2012)	
AHGS1687	TTCAGTTTCCTTCCACCACC	TATCACAGGAACTGGGAGGC	Shirasawa et al. (2012)	
AHGS1692	CCAAAACAAAGCAAAGGGAA	ATCCCAACGAGACCACTCAG	Shirasawa et al. (2012)	
AHGS1696	TCCTTGTCCTTTGCATACCC	ATATTTCTGCTTCGGCGCTA	Shirasawa et al. (2012)	
AHGS1699	TGGAAAACCGCTTCTCTTTC	CAACACCCTCACTCCTCCAT	Shirasawa et al. (2012)	
AHGS1703	TCTACCGTCGACACTGCTTG	TCAACATAACAAAACCAGTTCAAA	Shirasawa et al. (2012)	
AHGS1710	CAGCAGCATGGCAACAGTAG	CGGAAGTCATTGCTAGAGCC	Shirasawa et al. (2012)	
AHGS1750	ATTGAAGAACTCACGGCGAC	CTGTTCTCACCGTAAAGCCC	Shirasawa et al. (2012)	
AHGS1755	GAGGAAGAACTGGGCATGAA	CATCAGCCTAAGCCCACATA	Shirasawa et al. (2012)	
AHGS1770	TGGAGGTTTCGTGTTTTCCT	AAGCTGAGATTGGGATAAGAAAG	Shirasawa et al. (2012)	
AHGS1773	TGGAGGGGTTGTTAGAGGTG	TTTTCCGATGGTAAATTCGC	Shirasawa et al. (2012)	
AHGS1805	ACCAATATTGAGCCAAACGG	AGCCAAGGATTTGCATGTTC	Shirasawa et al. (2012)	
AHGS1806	GCAAAATTTTTCTTCCCATTGA	TCTATCCTGACCACGAAGCC	Shirasawa et al. (2012)	
AHGS1813	TCACAACCACACACCTTCGT	TATCCCTATGACGGCACACA	Shirasawa et al. (2012)	
AHGS1818	TCGTGCGACAGAGAAGTCAC	CCAACAGAAAATTCATTCGC	Shirasawa et al. (2012)	
AHGS1829	CCTTTCCCACATATCCCTCA	GATCTCGTTCTTCTCGGCAC	Shirasawa et al. (2012)	
AHGS1845	CTCCAACTCGCCTTCAACTC	GACTCCCAAATTGTTGACCG	Shirasawa et al. (2012)	
AHGS1846	CCCACCTAGCAGAAACCAAA	TCAGTCCCATTCAAGGTTCC	Shirasawa et al. (2012)	
AHGS1850	TTGCATACAAACCTTGCAGC	TTGACGTTAATCACATGCCC	Shirasawa et al. (2012)	
AHGS1853	TTTTAGATCCATTTGGTTTCTCTTT	GGACCAAAAATCTCTGAGCG	Shirasawa et al. (2012)	

ID	FORWARD PRIMER (5'-3')	REVERSE PRIMER (5'-3')	Source	Alias
AHGS1855	GATGCAGCTGCTAAAAGCCT	TTTATTGAGTGGGGTCCTGC	Shirasawa et al. (2012)	
AHGS1903	CCAGGTTCACACATTCATCC	TCTGTGAGCTTGCCTCAATG	Shirasawa et al. (2012)	
AHGS1910	CCCCTTCCCTAAAACGTCAT	TGGCACAACAAGGAGAGATG	Shirasawa et al. (2012)	
AHGS1913	CCATCATCCGCTTTTTCACT	GCAACGAAAGGCTATGAATGA	Shirasawa et al. (2012)	
AHGS1928	TAAACGTGTTGGCACGAAAA	ACTCCGCATGCTGTGATTAG	Shirasawa et al. (2012)	
AHGS1930	AACATCTGCCAAATGCAACA	CTGCCATGTGTGCTTGAACT	Shirasawa et al. (2012)	
AHGS1937	GGATAGAGCAAAGCAGTGGC	CCGATTTCAGAGTCCCCATA	Shirasawa et al. (2012)	
AHGS1940	AACTCCGCGCCTATGTTCTA	GGGAGAGGATGATGGTGGTA	Shirasawa et al. (2012)	
AHGS1954	AATTTTTATGGGGGCTACGG	ACATGCCTTCCCCACCTAAT	Shirasawa et al. (2012)	
AHGS1974	CCTTTGTAAAACTCGACACCG	AGAGACTAGGACTGGGATGGG	Shirasawa et al. (2012)	
AHGS1980	TTTCCATCGGATTTTTCGTC	GAGGTGGCAACGGTGATAGT	Shirasawa et al. (2012)	
AHGS1987	CATCGATGAGGGGTGTTGTA	AATGCAAAATGGTGCTTGTTT	Shirasawa et al. (2012)	
AHGS2005	AATTTGATTGGGTCTTCCCC	TGAAAAACTGGCTCGCTCTT	Shirasawa et al. (2012)	
AHGS2027	CGAGGAAAAGAAAGGGTAGG	TTTTCTTCTTCCGGGGTTTT	Shirasawa et al. (2012)	
AHGS2073	GAGTTCAAACCCCAATCGAG	TGTTGAAAATATTGGGTATTGGG	Shirasawa et al. (2012)	
AHGS2084	GGAGGGAAGGGAGAGAGAAA	CTCCCCTCCCATCTCTTTGT	Shirasawa et al. (2012)	
AHGS2130	GCAGAGAGTTGGGAAGAGGA	CCGATTCAAGCCCGTTAGTA	Shirasawa et al. (2012)	
AHGS2141	AAACAGCGACAGTGACATGC	AATTTCAATCGCGAAACCAC	Shirasawa et al. (2012)	
AHGS2153	AAAAGGCACCTGGAAAAGGT	CGCCATTTCTTGAGTTTGGT	Shirasawa et al. (2012)	
AHGS2159	ACTACGCATTGGGAGTGGTG	AACAGCCGCCACTACTCTGT	Shirasawa et al. (2012)	
AHGS2188	AGCACACGAACACACACACA	CTCTCTCTCTCTCCCCAGCC	Shirasawa et al. (2012)	
AHGS2191	TCTTCTCCATGCTCACATGC	ACCACAGCATAGATCTGCCC	Shirasawa et al. (2012)	
AHGS2195	CAGCAGCAGGAGTAGGCTTC	CAAAACCCTGCCTTGAAAAA	Shirasawa et al. (2012)	
AHGS2241	CACATGGAAATGCAAATGGA	AGCGTGTCGTGCTTTCTTCT	Shirasawa et al. (2012)	
AHGS2319	GTGGTGAGAAGAAGAAGCGG	CAACGGTAACTGTGCCGTAA	Shirasawa et al. (2012)	
AHGS2344	CTATCTCAACCCGCGAAGTC	GAGAGGCATCTCAATAGGCG	Shirasawa et al. (2012)	
AHGS2413	TTCTTCTCTCGCCCTCGTTA	CCGTATCGTTGTTTTCTTTGAA	Shirasawa et al. (2012)	
AHGS2429	GGGATTAAGGAAGGAGCAGG	TTGCAAAGGCAATTGCTAAC	Shirasawa et al. (2012)	
AHGS2466	TTGGATTGGAATGCTGACAA	GAAAGGGATTGCCTCTCAAG	Shirasawa et al. (2012)	
AHGS2509	GTTAATCCCGCCTACGTTCA	CACATTCCCTTAAGATGTCGC	Shirasawa et al. (2012)	
AHGS2534	AAGCTTGTTGCTTCCATTCC	TCTTTCCCGATTGATTTTGC	Shirasawa et al. (2012)	
AHGS2539	CGAAACCAAGTATGGTTCCTTC	TAAGGCAACACAAGAACCCC	Shirasawa et al. (2012)	
AHGS2543	CGTCTACCAGGGCATCAATA	TCGGCTTCATAAGGGAATTG	Shirasawa et al. (2012)	
AHGS2559	TTCTCCTCTGTTCCCACCAC	CCATTCCCTTCTCCTTCCTC	Shirasawa et al. (2012)	

ID	FORWARD PRIMER (5'-3')	REVERSE PRIMER (5'-3')	Source	Alias
AHGS2568	GGGTGTGGAAATCTGCTGTT	TTGACCCAAGGGCAAATAAG	Shirasawa et al. (2012)	
AHGS2579	ATTCTGTGAAAAACGGGGTG	GTGTAAACCGACACGTGAGC	Shirasawa et al. (2012)	
AHGS2602	GGATTCTCCCAACAAAGCAA	GCACATCGAGTTTCTCACCA	Shirasawa et al. (2012)	
AHGS2644	GCTAGGGCACGGATATTTGA	TTTTACCTACATTGGGTAGGGC	Shirasawa et al. (2012)	
AHGS2754	AACAACTTGCCTCAACCCAC	AACGCGAGGTTTTTGTTTTG	Shirasawa et al. (2012)	
AHGS2787	AGTCCCCAATCATCAACAGC	ACCCCTCCATTTCGTCTTCT	Shirasawa et al. (2012)	
AHGS2795	CAAACGACTCGTCAATCGAA	CAAAGTTGGGTCAACGGATT	Shirasawa et al. (2012)	
AHGS3627	AGCTCGGCACACTTTCACTT	CAATCGGAATACCGGAGAGA	Shirasawa et al. (2012)	
AHGS3715	CACCCAGTCAAAATCTTCAACA	CTTGTCAAACTCTGTGACTTAGCA	Shirasawa et al. (2012)	
AhM022	TGCATGCACAAGGATATGAA	GCTACATCCAAAGCCATCGT	Naito et al. (2008)	
AhM062	CTCAAGCCCATGAAGACTCC	GATGAAATGAGCAGGTGCAA	Naito et al. (2008)	
AhM082	GGTCACTCTCTCTCGCAAGC	GAGCAACAGTGAAACGACGA	Naito et al. (2008)	
AHS0487	TAAGCAACCGGAGAAGCCTA	CACATCCCAATTGGAACCAC	Koilkonda et al. (2012)	
AhTE0005	ACAGTACGAACGGTCCCAAG	TGGTGCATGTTGTTGTTGTG	Shirasawa et al. (2012)	
AhTE0006	AATACCCGAAGCACCTGAAA	CCAACAACCCAAGGATGAAG	Shirasawa et al. (2012)	
AhTE0021	AGTCCCAAGTGGACACCAAG	TTAGACCAATTCATGTAAGGTCAC	Shirasawa et al. (2012)	
AhTE0107	CACTCGGAAAACCAAACTCC	CAAGAAGCCTACAAAAATGGTG	Shirasawa et al. (2012)	
AhTE0119	AAGCTAGCGATGGCGATAAC	TTTATGCCCGCAAACTTTTT	Shirasawa et al. (2012)	
AhTE0129	GCATGCAATCGAAAGATGAC	TGGAAGCAATAAGGACTTCG	Shirasawa et al. (2012)	
AhTE0148	GAATCCAGAGCTGCCATTGT	ATTTTTGCCTCTCAGCCTCA	Shirasawa et al. (2012)	
AhTE0162	CTTGCGATCAATGAGGTTATCA	CCACCTTCCAAATTCATATTGC	Shirasawa et al. (2012)	
AhTE0164	AACCACCACCATTCTTTTAAGG	TCGTCTCCTCTAAGGTTTGCTT	Shirasawa et al. (2012)	
AhTE0189	CAAGCAAGTTGTGAAGTGGTGT	CGCTGACGAGAAAAAGTCTCAT	Shirasawa et al. (2012)	
AhTE0191	ACATGATGTTTCTGCCTTCCTT	CAAGTACAGTGGTGGTCTTCCA	Shirasawa et al. (2012)	
AhTE0211	TTTACCAAACATTTCCGTATTT	CTTTTTGGTGGCTACTCCAATG	Shirasawa et al. (2012)	
AhTE0212	TGGTAGCCTGAAAATGAGGTTT	GCATTGTGCCGATCAGTTAATA	Shirasawa et al. (2012)	
AhTE0233	CAGCATATTGGCTGATCAACTT	CTGATGCCATATGTGTGAAGGT	Shirasawa et al. (2012)	
AhTE0237	AAAAATAGTGATGAGCTTGATTGA	AATAAAAACCCCAAAGCCACATA	Shirasawa et al. (2012)	
AhTE0251	GCGTCGTCTTCAATTAGGTTACT	AAAGATCAACAAAGAGCAATGAAA	Shirasawa et al. (2012)	
AhTE0261	ATATAACAAACTCCCCTCCTCCA	TCACCACAAAACAACTCAAAGAA	Shirasawa et al. (2012)	
AhTE0278	CTTTTCTTTTGTAATGAATCTGTTTTT	GCAATGCTAATATGCTAAATCGTT	Shirasawa et al. (2012)	
AhTE0283	TCGGACACTTCATTATTTTCGAC	TGTGTTTGTGGTTACGTCTGTTC	Shirasawa et al. (2012)	
AhTE0296	ACTTCAAATCTGACGCACCTACT	CAATTCATGTTCTAGCAAGGGTTA	Shirasawa et al. (2012)	
AhTE0303	AAGATGCATACCTTTGGTTTTCT	CTGCACTTAAGCCAACTTCTCAT	Shirasawa et al. (2012)	

ID	FORWARD PRIMER (5'-3')	REVERSE PRIMER (5'-3')	Source	Alias
AhTE0319	ATTCGAAAACCCAAAATTTCATT	AAAGTTGGTGTGTCATTCTGAAAA	Shirasawa et al. (2012)	
AhTE0335	GACAAAGGTAGAGGTTAATCCCA	CCTAACATTGGGTTGGAGGA	Shirasawa et al. (2012)	
AhTE0359	ATCGTTGCTTGTTAGCACGG	AAAAGCGCAAAGAAATGGGT	Shirasawa et al. (2012)	
AhTE0381	TGCTTTTCCGGAGTCACAAT	TGCATGTAGCAGAAAGCCAG	Shirasawa et al. (2012)	
AhTE0416	GAGTTACATCGGTATTGAATATGTGA	CTCCGTGATTGAGGCGTTA	Shirasawa et al. (2012)	
AhTE0422	TGGCGTAATCTTTTAAGAACCAA	AGAATTAATGTCATCAAACGAATG	Shirasawa et al. (2012)	
AhTE0426	CAACCCATGATTTGTGAATTAAG	TGACTACAATGTTTGGTCATTTTG	Shirasawa et al. (2012)	
AhTE0446	GTGCCACGAGGTACACGATA	AGACACACACCACACGCATT	Shirasawa et al. (2012)	
AhTE0470	AACTGTTGGATGCAGTGTGC	CCCCACTCCCATTATTCCTT	Shirasawa et al. (2012)	
AhTE0477	GGTGGTGGCCATTTCTCTT	TTTGCCCATTTTTGGTTCTG	Shirasawa et al. (2012)	
AhTE0478	TGAAGCAGCCACACCATACT	GACGGTTGACTAAAAATGTTGG	Shirasawa et al. (2012)	
AhTE0489	TGACTACAATGTTTGGTCATTTTG	GAACCACCCATGATTTGTGA	Shirasawa et al. (2012)	
AhTE0523	AAAATTGAGCGGTATTAGACATCA	GTGAGCACCTATCATCCACAAATA	Shirasawa et al. (2012)	
AhTE0524	GTATGGGCCACTAATAAGCTATGG	GGAATTCGGATGCTCAAAGTATAA	Shirasawa et al. (2012)	
AhTE0532	AGGCAGTCCAAGCAAAGTGT	TGTCAAATTCATTCAAAGGGG	Shirasawa et al. (2012)	
AhTE0536	ATTTGGGGTTTGATGACAGG	TCACACATGGACATCTTGCTT	Shirasawa et al. (2012)	
AhTE0540	CCACTAGACTGAAGGTTGGTTG	AGTTCGATGGTAGTGACCCG	Shirasawa et al. (2012)	
AhTE0553	CATGCATGGACCTTACCTTG	ACAGGAGGAGAAGCAGCCTT	Shirasawa et al. (2012)	
AhTE0565	TATGGATTGAGGCCATGGAT	TGTGCCATCCTAAAACACTCA	Shirasawa et al. (2012)	
AhTE0577	GAGTTGGCTCTGTGATTTGTGA	GCAAGGTGTATTAAATGAAACCAA	Shirasawa et al. (2012)	
AhTE0586	CATTGTCTCGTTCGCGTTTA	GAACGTGGGGAAAGTCAAAA	Shirasawa et al. (2012)	
AhTE0635	GAAGTGGGGTAGGGAACCAT	CACAAACAACATAACTACCTTTCTTTT	Shirasawa et al. (2012)	
AhTE0639	GCTTTTCATAGAAAACATTGAACCT	CGCGGATTTAATTTGTTTGTT	Shirasawa et al. (2012)	
AhTE0654	TGTTTTTATGGCTTGATCCCA	TTACTTGCGTGCCACCATTA	Shirasawa et al. (2012)	
AhTE0658	TGTTTTGGATGGTCACCTCA	AAAGACCCTAAAGCGAAGGC	Shirasawa et al. (2012)	
AhTE0659	AGTGGGATTGGAGCATTTTG	CTATTTCGGATTGAAAAATTATAACA	Shirasawa et al. (2012)	
AhTE0674	AATCCACTTACTCCCTTGCG	CATCGTTATCTGCATGGTGG	Shirasawa et al. (2012)	
AhTE0678	CATTTTCTTTTTCTTTATGACCG	AGGGAGCATACCCACATACA	Shirasawa et al. (2012)	
AhTE0690	AAATTTTTGAAATGGGAGAAGC	TTGTAACACATCAAAATCAAACCA	Shirasawa et al. (2012)	
AhTE0696	CTTTGTTTGTTTAGCCACACTTT	CGGTATTTGCCGAATGCTTA	Shirasawa et al. (2012)	
AhTE0706	GCCCTACAATCTATCATCCAATG	TGAGGACTTTGATGTTGGCA	Shirasawa et al. (2012)	
AhTE0707	TGTGGCTAAACAAACAAAGTACA	TCCATGCTTGGTTCTGTGTC	Shirasawa et al. (2012)	
AhTE0709	GCCACTCCAACCGAATAAGA	TGTTTTTGGCATCTTCATGG	Shirasawa et al. (2012)	
AhTE0711	CACTGAAGGGATGCAGTGAA	TGTGACATTGGAAAAAGGCA	Shirasawa et al. (2012)	

ID	FORWARD PRIMER (5'-3')	REVERSE PRIMER (5'-3')	Source	Alias
AhTE0718	TTCCCATGCCAATAATGGTT	TGATTTGGCCACACTTCAAA	Shirasawa et al. (2012)	
AhTE0725	TCCATCATCCAATGGTCAAA	CCTGCCCACCACTGACTACT	Shirasawa et al. (2012)	
AhTE0726	ACCTTTCACATCCACAAGCC	TCCATCATCCAAAGGTCAAA	Shirasawa et al. (2012)	
AhTE0756	GCCCGAGTTTCTGTTTTCAA	GCAACACATCAAAATCAAACCA	Shirasawa et al. (2012)	
AhTE0761	GCAACACATCAAAATCAAACCA	AAGGAACTTCAAGTAAGACCCTTTC	Shirasawa et al. (2012)	
AhTE0771	TGAAGTGTGGCCAAATCAAT	GCTCGAAGATGGAAAATCAGA	Shirasawa et al. (2012)	
AhTE0775	ACACTCACACACATGGTTCCT	TCCATCATCCAAAGGTCAAA	Shirasawa et al. (2012)	
AhTE0777	CCATTCAATCCATCATCCAA	TTCATGTTTCCATGTTCCCC	Shirasawa et al. (2012)	
AhTE0790	CAAACAAAAACTAAAGAAAAGGACAA	GGAGTGTGGCCAAATCAATAA	Shirasawa et al. (2012)	
AhTE0791	GCTGACATAGCATAAAAATCTAGGA	TGCCATCTTCATTGTAGTATCCA	Shirasawa et al. (2012)	
AhTE0794	TGATTTGGCCACACTTCAAA	CAAACCCGCCATTATCAATC	Shirasawa et al. (2012)	
AhTE0795	ATCAAGCCCTACAATCTATCATCC	ACGTTGCACGGAGCTTCTAC	Shirasawa et al. (2012)	
AhTE0796	TGTTGTTTTTGTTTAAAGTGTGGC	AGATGTCCACTTTCGTGCAA	Shirasawa et al. (2012)	
AhTE0797	ACCCCTTCATACTGTGCCTG	TGTGGCCAAATTAATAAATCACA	Shirasawa et al. (2012)	
AhTE0800	TGAAGTGTGGCCAAATCAAT	TCCTCTACCTTTCACTTCTTATCCA	Shirasawa et al. (2012)	
AhTE0806	TCCATCATCCAAAGGTCAAA	TAAGAATCTGTCGCTTGCCC	Shirasawa et al. (2012)	
AhTE0808	TCAAATCAAATCAAGCCCTACA	TTTCTAGCCGCCATATGTGA	Shirasawa et al. (2012)	
AhTE0810	GGTCCTTTGAGTTGCCAAAA	CATCCAATGGTCAAAATGATG	Shirasawa et al. (2012)	
AhTE0813	TCATCCAATGGTCAAAATGA	TGAAGGGTTATGAACTGGACAA	Shirasawa et al. (2012)	
AhTE0815	TTGTAACACATCAAAATCAAACCA	AAAAATATACTTTACCCCCTCTTTTT	Shirasawa et al. (2012)	
AhTE0818	TGTCATGGATTAAGATTATGACTAAAC	GCAACACATCAAAATCAAACCA	Shirasawa et al. (2012)	
AhTE0820	TTTTGGTTGAACCGATGAAA	TTTATTGATTTGGCCACACTTT	Shirasawa et al. (2012)	
AhTE0824	TTGGCCACACTTCAAATAAAAA	CAGCCTCTTGAGTCAGGAATG	Shirasawa et al. (2012)	
AhTE0825	CCGACAAATTTTTAGCGGTC	TGTGGCCAAATTAATAAATCACA	Shirasawa et al. (2012)	
AhTE0839	GCATGAGACAGGGGCACT	TGAAGTGTGGCCAAATCAAT	Shirasawa et al. (2012)	
AhTE0840	GCAACACATCAAAATCAAACCA	TCCAATTTGTTTTGGAGGGA	Shirasawa et al. (2012)	
AhTE0845	GCAACACATCAAAATCAAACCA	TTTTCCAACTTGCAATGGCT	Shirasawa et al. (2012)	
AhTE0852	GAATGAAGAACCAACCAATGC	CACTCCATCATCTAAGGGTCAA	Shirasawa et al. (2012)	
AhTE0874	TCCATCATCCAAAGGTCAAA	ATTTTCACGGGAACAATGGA	Shirasawa et al. (2012)	
AhTE0882	TCTAACCCTACAATTTATCATCCAA	TCGTTGGAAGCTGCAGTTAAT	Shirasawa et al. (2012)	
AhTE0887	CCCTACACTCCATCATCTAAAGGT	GGGGAGACTATGCCATTTGA	Shirasawa et al. (2012)	
AhTE0888	AACAAGCGCCAAGTACAACC	GCAACACATCAAAATCAAACCA	Shirasawa et al. (2012)	
AhTE0893	CCATGAAGGAGAAGACGAGC	TCCATCATCCAAAGGTCAAA	Shirasawa et al. (2012)	
AhTE0903	TCGTTGGAAGCTGCAGTTAAT	TCTAACCCTACAATTTATCATCCAA	Shirasawa et al. (2012)	

ID	FORWARD PRIMER (5'-3')	REVERSE PRIMER (5'-3')	Source	Alias
AhTE0906	TTGGCCAAATTTTAAATAAAAACAA	GGTAATTTATTCGAGACTGCAATG	Shirasawa et al. (2012)	
AhTE0908	TGAAGTGTGGCCAAATCAAT	TCCACCCAATCTTTATAGGTTTTT	Shirasawa et al. (2012)	
AhTE0913	GGTAAAAGCTAAGACAGGCTCAA	TGAAGTGTGGCCAAATCAAT	Shirasawa et al. (2012)	
AhTE0914	CCCTACACTCCATCATCTAAAGG	GGCTATTGCACGTTGCTTTT	Shirasawa et al. (2012)	
AhTE0918	TCAAAACAAAATTTCACGAGAG	TGTGAAAATATATCACTTTGACCATT	Shirasawa et al. (2012)	
AhTE0922	TGAAGTGTGGCCAAATCAAT	CCAAACAGAGCTCCATCACA	Shirasawa et al. (2012)	
AhTE0923	TCAAAATCTAACCCTTCAATTCATC	CTCCCAACCAATGTGTGCTA	Shirasawa et al. (2012)	
AhTE0936	GGAAGTAGCTTTTATGCTTTTGTCA	CATCATTTTGACCATTGGATG	Shirasawa et al. (2012)	
AhTE0940	GCAACACATCAAAATCAAACCA	CCGCATCATCTTAGAACGAA	Shirasawa et al. (2012)	
AhTE0945	TGTGGAGGATTCAGTTCTGC	GCAACTACTCCTATGAAGATGCC	Shirasawa et al. (2012)	
AhTE0959	GCATCCTCTTCTGCATTCCT	GGCATCTTCATGGGAGTAGC	Shirasawa et al. (2012)	
AhTE0985	CAGAATTTTCCAGAGGCTCG	TCCATCATCCAAAGGTCAAA	Shirasawa et al. (2012)	
AhTE0986	CGGCCGAAGTATCTTAGTTGA	CAATCTATCATCCAATGGTCAAAA	Shirasawa et al. (2012)	
AhTE0994	GCCTAGAGGCAAAACTCTTGAA	TGGCTAAATAAATAAACCACACTTTT	Shirasawa et al. (2012)	
AhTE1009	TCCTTTTATATGAACTAAGGCGATT	TGGGAGTACCCACCATTTTT	Shirasawa et al. (2012)	
AhTE1016	CAGCCTTTTACTTTGTTAGCCA	TGATTTGGCCACACTTCAAA	Shirasawa et al. (2012)	
AHGA102053	TGAAGTCGATTGCACCTGAG	GCTAATAAGCCAGCCCACAC	Zhou et al. (2016)	
AHGA14239	CGCCGTAAAGCGAAGAATAA	GAAAATGATGCCATAAAGCG	Zhou et al. (2016)	
AHGA152194	AGTGAGGTTGGAGATGGGTG	AAGAGCGAAATCAATGGGAA	Zhou et al. (2016)	
AHGA152207	TCCGAAACCCAAAAAGAAGA	AATAATTTTGCACAAGGCCG	Zhou et al. (2016)	
AHGA161466	GACCAATATCCAATGTGCCC	GCCGAAGCAACAAATTGACT	Zhou et al. (2016)	
AHGA161468	CCCTCTTTTTCTCCCTCTGC	TCCGCCATACAAAGCTAACC	Zhou et al. (2016)	
AHGA161495	GGCATTAAATCAAATATTAAACTCCAA	TGGGTCTTTATAAAATTAAGCCA	Zhou et al. (2016)	
AHGA161510	GCAAAGATGGCGATGAAGTT	GAAATCCAGAAATCAAGCTGC	Zhou et al. (2016)	
AHGA164448	TGACAAATGACTGAAACCAACA	TGTCACGAAAAATATTGTGGCT	Zhou et al. (2016)	
AHGA171408	TTGCCAAAATTTAGGACTTCAAA	TCCTGTCTCTGTAAACAAACGC	Zhou et al. (2016)	
AHGA176194	TGGCATGCCTCTCTTTCTCT	CCTCTTCTTAGAGTTATCGGTTGC	Zhou et al. (2016)	
AHGA178360	CCCAATTTAGTCCCTGATTCC	TGATGGTTGCTTTCTGTTGTG	Zhou et al. (2016)	
AHGA193642	TGAACAATTGCGATTTGGAC	CAATGACGATTGACGACGAC	Zhou et al. (2016)	
AHGA195553	TCCCTACTCCATCTGCTGCT	AGGGTTTCGTTGAGGAGGTT	Zhou et al. (2016)	
AHGA214492	AGATCTCCCGACCCAATTCT	TCTCTCTAGCGGGACTCAATTT	Zhou et al. (2016)	
AHGA221480	TCAGAATTTCAGATTCGGGG	ATGCAAACAGTTCGGTCCTC	Zhou et al. (2016)	
AHGA24894	TGAAATTAAAGGGGTGTTAGGG	GCGGTTGAGATTGCAGTGTA	Zhou et al. (2016)	
AHGA25786	AATTGCAATCCACTGCATTAGT	ACCCTCATTGCTCCAGTCAG	Zhou et al. (2016)	

ID	FORWARD PRIMER (5'-3')	REVERSE PRIMER (5'-3')	Source	Alias
AHGA284837	CCGCTAAAAGTTTGTCGCTAA	TTGCACAGGCATTGACTCTC	Zhou et al. (2016)	
AHGA327396	TCTTAAATTCGAACCGCGAC	TGGAAACACAGCGACAGAAG	Zhou et al. (2016)	
AHGA329002	TTTTTAAGAGCATAAAACAAGAAGGA	TGTCACGATTAAAAAGTTTCGG	Zhou et al. (2016)	
AHGA361224	ATTCTGGGAAGCCTGGAGAT	AATTTTGTGTTCGCTTGCAT	Zhou et al. (2016)	
AHGA363491	ATTTATCCGCCCAGTCCAAT	TGCCGTTAACGATTCTGTGA	Zhou et al. (2016)	
AHGA363492	TAGAGGGGAAGGGGTGTCAT	TCATCATGTGGGTATTTCTTTTT	Zhou et al. (2016)	
AHGA364915	GGACAGCAGGTATTGGAGGA	CTCATCAGACTCAATGCCGA	Zhou et al. (2016)	
AHGA364936	TCATCTCTCAGCCCACATCA	TTGTCAAAATAAATCGGACAAAA	Zhou et al. (2016)	
AHGA367823	TCACGATCCCTTCTCCTTCA	AGGGGGAGTCAAAGGAAAGA	Zhou et al. (2016)	
AHGA374228	GAAGGGAGCATTTCGAACAA	GTGTCCTAAATTCGAACCGC	Zhou et al. (2016)	
AHGA375182	GATACCTGAAATTTGAATTAGTTAGGA	AAACGCATAACTATGAAAACTGG	Zhou et al. (2016)	
AHGA44674	CCGAATTACTCGCTTAACAACC	GAAAATGGGGATCCATTCAA	Zhou et al. (2016)	
AHGA44686	AAGTGGAAATACTTTCTCTTTCTTTTT	TTAGCTTAGTTATTACTTTTTCCTTGC	Zhou et al. (2016)	
AHGA61563	CCTGGTCACCATCTCTTTCA	TGGTCTTTTAAGGTTTTTGTGTCA	Zhou et al. (2016)	
AHGA61572	TAAACCTTCGCAAACCATCC	GCTCAGGAACGAAAATGTCC	Zhou et al. (2016)	
AHGA65328	TTACCTAAGGCCGACACCAC	ACAACAACAACGGGAGGTTT	Zhou et al. (2016)	
AHGA7048	TGCCATTTGAGCATTTAACG	GGGGATGAGGGGAAATAATG	Zhou et al. (2016)	
AHGA72558	ATTTCAGGGACCATTTTGGG	ACGCTGCGACACTAAATTCG	Zhou et al. (2016)	
AHGA72569	CCATATCATAGCCGCCAAGT	TACATCCACGATGCAGAAGG	Zhou et al. (2016)	
AHGA75537	AACCAACCTCAATCTACAGCA	GAAGAATAGGCTTATTTCTCGAAGG	Zhou et al. (2016)	
AHGA75538	ACAGAATGGTTTCGGTGAGG	CGTGAGATTTACCGTCGGAT	Zhou et al. (2016)	
AHGA96458	CGGACTTCTCCACTTCCAAA	GGCCTTTGTTTCTTTAGGGC	Zhou et al. (2016)	
AHGA96464	CCGTCACCTCCTCTTCTCTG	GTCGACTCCTGCCCAATCTA	Zhou et al. (2016)	
AHGA96466	GTGGCACATAGACATCACCG	CGCAAACAAACACAACCAAA	Zhou et al. (2016)	
AHGA98567	TTCAGAAGGCAGTGTCGATG	GATATAAGACCCCCGGGATG	Zhou et al. (2016)	
AHGA98575	AGCCTCTCAATTCCCTTGGT	CAAAACACATCCCCAAGGTC	Zhou et al. (2016)	
ARS590	ACTAACAGCGTAGCCGTCGT	ACCCCTCTCAGTGTTTTCCA	He et al. (unpublished)	
ARS702	ATGGCACATGAACAGCAAAA	TCCTCTTGCAAGCAAAATCC	He et al. (unpublished)	
ARS715	CGATCCTAGACCCGATGAAA	TCGCGAAACTATAAGACCAACA	He et al. (unpublished)	
ARS731	TGTCACGATTAAAAAGTTTCGG	TGGTAATTTTGCAGCATTATCTGT	He et al. (unpublished)	
ARS742	GATTTAATGGGGGATGAGGG	TCACCCACACCCTTTTGATT	He et al. (unpublished)	
AT43	ACAAAGCAGAGAACCACCGG	GGTGGCATTTTGCTGTTCCA	Jiang et al.(unpublished)	
AT68	ACTTTGCTTTGCTTTCAGCCT	AGTCACACTGATGATCACAGCA	Jiang et al.(unpublished)	
AT77	AGCACACTTGAAAGGTGAGAA	TGAATCGCCACTACATCCCG	Jiang et al.(unpublished)	

ID	FORWARD PRIMER (5'-3')	REVERSE PRIMER (5'-3')	Source	Alias
AT79	TCCATTCCTTTTCCCCTTTCTCT	TTGTCACTACACCAAGCTAAGT	Jiang et al.(unpublished)	
EE16	ATGGGACCACGAACATTTGA	GAGCACGTTCAAGCACTCATT	Guo et al. (2009)	
EE22	AAGGTAAAGAAGAAGCGGAAAG	TTTCCCACTCCAGAATCACC	Guo et al. (2009)	
EE51	GGGAGGTTTGGGATCAACT	GACCTGCTGGTTCGGATAA	Guo et al. (2009)	
EM142	GAACCTTCCAGGACAAACTCG	CGTCTTCCTGCTCGTAATCG	Guo et al. (2009)	
EM87	CATGCTCCTCCAATTTATTACG	CGAGACTTGAGTGCCTTGTTG	Guo et al. (2009)	
GA1	GCGTGAAATGAGTGTTGTGAG	CATAGCCACCATAGACACCAAA	Budiman et al. (unpublished)	
GA110	GGAGAACCAGTGACGTGACATA	GGATTAATTCTGATACCATGAAAGG	Budiman et al. (unpublished)	
GA156	CTACTCCCTCTGCTGCTTCCT	TAGGGTTTCGTTGAGGAGGTT	Budiman et al. (unpublished)	
GA27	CATCCAAAGCCAAAGTTCACA	GCTTAGCTTGCTTTGATTAGGG	Budiman et al. (unpublished)	
GA72	ACTTTGGTGGCTTTCCTTCAT	TCTCTGTGCCCTCTTTCTTCA	Budiman et al. (unpublished)	
gi832	GCCACTTTATTCTAAGCACTCC	AAGAGACCACACGCTCACA	Moretzsohn et al. (2005)	
GM1445	GTTGGCAGAGTGGAAGAACTG	CGCTTTTATAATAGGCCGAGGT	Guo et al. (2012)	
GM1539	GCTCTTGTTAATTCGATTCCA	CCTCATCAGAGTCAGGCAAGA	Guo et al. (2012)	
GM1555	CGTAGACGTGAACCACTACCAA	CGCCTAGTGTCTCAGAAAACG	Guo et al. (2012)	
GM1577	GCGGTGTTGAAGTTGAAGAAG	TAACGCATTAACCACACACCA	Guo et al. (2012)	
GM1713	TCTGCATGAACTGGACCATC	CACACACACAACACTCAACACA	Guo et al. (2012)	
GM1839	GAATCTGAGAGTGAAACAGAGCA	GAATTTGGGAAGACGAGGTTG	Guo et al. (2012)	
GM1854	CCCCAACCCTTTCTTTCTCTT	TGGTGGTGTTTTGTTGTTGTT	Guo et al. (2012)	
GM1901	GAAACACCGATATTTTCGATACA	TGACGAGCAAGTCATGTATGTG	Guo et al. (2012)	
GM1922	GGAGAGTCGGTGAGAGGAGAG	CGCTCGTTTTCCTCTTTCATT	Guo et al. (2012)	
GM1937	TTCATCCTCTGCTTCCTTTGA	TGACCAAACCCATCATCATCT	Guo et al. (2012)	
GM1954	GAGGAGTGTGAGGTTCTGACG	TGGTTCATTGCATTTGCATAC	Guo et al. (2012)	
GM1959	GTGTTCTCAGCCATCTTTTCG	GTGAAGGTGTTGTGAATGCAG	Guo et al. (2012)	
GM1996	CATCCCATCATTTTCCCTCTT	TACAGTGAAGGTGGGATCCTG	Guo et al. (2012)	
GM2032	GCCGATGATGTACGTTTCTTC	GAGACGGCATGTCAAAAGAAT	Guo et al. (2012)	
GM2106	TTTGACATCATTGTTGATTGTTG	GATGAGGCCATTAAGGAGTGA	Guo et al. (2012)	
GM2165	CTACGCGCATCGCATAATATC	GTGAGATGGGGTTGGAGATTT	Guo et al. (2012)	
GM2196	CCTTGCTTTTCGGCTTCTATT	GAGCTTTGGCTTTTGTTGTTG	Guo et al. (2012)	
GM2246	GCAATTTATGTGCACCCTTTT	CGCTTGACACCAATGAAGTCT	Guo et al. (2012)	
GM2284	ACACCCCAAATAGCTTCGTCT	TCCACAACACCAACCTTCTTC	Guo et al. (2012)	
GM2313	GATGCTGCTAAATCGAGATGC	GTTGTTTTGTTTCACGCCAGT	Guo et al. (2012)	
GM2807	TTGGAATCTGTTTGGATGAGC	TCGAGCCTTCTACTTGATTTGA	Guo et al. (2012)	
GM2808	TGTTGCTTTGTTCTGCCTAATG	GGATGAATAAGATTAAGATGGCTGT	Guo et al. (2012)	

ID	FORWARD PRIMER (5'-3')	REVERSE PRIMER (5'-3')	Source	Alias
GM2839	TCTCTTTCTTTTCCTCATTCTCTCA	CCACAGCCATAGCAGCAGAG	Guo et al. (2012)	
GM630	CAGCAATTCAGCAAACTAATGAA	TCCTCCCACGTCCTTTTATTT	Guo et al. (2012)	
GNB0486	CATTGCAGTTTCCCAGGAGT	TAGCACTCAGTCGGTCTCCA	Wang et al. (2011)	
GNB1040	TCACTTTTGAGTGTGCCTGC	TCCATTAGTGAGAATACCCTAACAAG	Wang et al. (2012)	
GNB136	GATTTAATGGGGGATGAGGG	TCACCCACACCCTTTTGATT	Wang et al. (2012)	
GNB138	CAGCCCGATGAAGGAATAAA	CGACCTAAACTCCGTAGGCA	Wang et al. (2012)	
GNB159	CGAATCGGAACACCGTACTT	TTTTAAATGTTTGGCCCAGG	Wang et al. (2012)	
GNB218	GCCATATTTCTGTCAAATCAAAA	TACCATCTGGTTTACCCCCA	Wang et al. (2012)	
GNB320	GAATTCCTGGCTCGAACTTG	ACCCCTCCATTTCGTCTTCT	Wang et al. (2012)	
GNB329	CCCTTTTTCGCTTTCTTCCT	GTTCTCGTTTGTGCCCTCTC	Wang et al. (2012)	
GNB377	TGAATACAAGCTATTTGGTGCAT	GGAGTGAGTGAAGAATTGTTGAAA	Wang et al. (2012)	
GNB38	TCCAGGGTCACTGTTCTTCC	CGTTGGTTTCATCAAAGGCT	Wang et al. (2012)	
GNB619	TAACCACAAGCAAGGCAACA	AATGGCTTCCAGAAGCTTGA	Wang et al. (2012)	
GNB652	CAAAGTCGCACAAAGTGGAA	AACTCCGCAGGCTGTGACTA	Wang et al. (2012)	
GNB87	GCCTGTAGCACTGCAAACAA	GGAATAGGGGCAAGAATGGT	Wang et al. (2012)	
GNB877	TCAGCGGCTACGATGAATAA	TGGGTATCCACAACCACAAA	Wang et al. (2012)	
IPAHM037	CGTATGCATTATAAGTGCTCGACAA	AATCCGATATCCGCTTCGAC	Cuc et al. (2008)	
IPAHM093	TCCATCGTTAGTGGCACTGT	GTCGACTCCTGCCCAATCTA	Cuc et al. (2008)	
IPAHM105	CAGAGTTTGGGAATTGATGCT	GCCAGATCTGAGCAAGAACC	Cuc et al. (2008)	
IPAHM108	CTTGTCAAACTCTGTGACTTAGCA	CATGAACAATTACACCCAGTCA	Cuc et al. (2008)	
IPAHM123	CGGAGACAGAACACAAACCA	TACCCTGAGCCTCTCTCTCG	Cuc et al. (2008)	
IPAHM282	AAGCCTTTGCGAATATAACCA	TGCAGGACTTGTATTTTGAGGA	Cuc et al. (2008)	
IPAHM290	CCACCGCTGATGTGTAATTGTA	GACGTGTAGTTGAAAACAACAGTATCA	Cuc et al. (2008)	
IPAHM354	TCCGAATCAAAATTGGCACT	ACCTCTCCCTCTCAAGTTTTTGT	Cuc et al. (2008)	
IPAHM468	GGCTTTTGAAGTTCCCTTCC	TATGCCTCTTCCCCTTCCTT	Cuc et al. (2008)	
IPAHM475	GTGATTTCCTGGTTGGTGCT	AGCCTCAGCTGGTTTTGCT	Cuc et al. (2008)	
IPAHM73	ACTAACAGCGTAGCCGTCGT	ACCCCTCTCAGTGTTTTCCA	Cuc et al. (2008)	
ML1G04	GAAGGACTGAAGACTACTTT	ATCCAGTAGATTACGTTACA	Moretzsohn et al. (2005)	
PM204	TGGGCCTAAACCCAACCTAT	CCACAAACAGTGCAGCAATC	He et al. (2003)	
PM36	ACTCGCCATAGCCAACAAAC	CATTCCCACAACTCCCACAT	He et al. (2003)	
PM377	ACGCTCACATGTTTGCTTTG	GCTCGATTTGATTTGGGTGA	He et al. (2003)	
PM65	GGACGTCTGGCTGCTAGAGA	TCGGCATCAAAACAGTGAGA	He et al. (2003)	
PM675	AATACCCTTCCCCAATCACC	TGCTTCTGCTCGATGTTCTG	He et al. (2003)	
PMc348	TGCCTGTAAGTGTGGACCAA	ACTCCAAAACGGGGAGTGTT	He et al. (2003)	

ID	FORWARD PRIMER (5'-3')	REVERSE PRIMER (5'-3')	Source	Alias
PMc660	CTATTCGACGAGTTGCGATG	AGGTCCGCTAGGGTTTTCAT	He et al. (2003)	
pPGPseq1B9	CGTTCTTTGCCGTTGATTCT	AGCACGCTCGTTCTCTCATT	Ferguson et al. (2004)	
pPGPSeq2F10	GTGGTGGCGGTGTAATCTTT	AAACGCAAAACTCTCCCTCA	Ferguson et al. (2004)	
pPGPseq2G3	ATTCACAAGGGGACAGTTGC	ATTCAAGCCTGGGAAACAGA	Ferguson et al. (2004)	
pPGPSeq3E5	CGATGAGGACAGAGACACGA	CGCTTGAACCCGACTATTTT	Ferguson et al. (2004)	
pPGPSeq4E8	ACCATTGCACTTTGAAGCTCT	GCTTGGTTTGGGTTAGTTTGA	Ferguson et al. (2004)	
pPGPseq5D1	TGGCCAAAACAACTGATTGA	TCCCAACTTTTCCGTTCTTG	Ferguson et al. (2004)	
pPGPseq7H6	CATCCTCACGGGAGTCAGAT	ATACCTACGCGTTGTGGAGC	Ferguson et al. (2004)	
pPGSSeq11C8	GTGATTGGTTAGCCTGATTAAAA	TGTTTGGAAAGGAAAATGGA	Ferguson et al. (2004)	
pPGSSeq13B7	TTGAGAACACAAATGGCACA	TGTCAAGTGGTCAAAATTCTTTC	Ferguson et al. (2004)	
pPGSseq15C12	ACAATGCAATGACCGTTGTT	TTGTTGCATGAGAACGTGAA	Ferguson et al. (2004)	
pPGSSeq15D6	CAACGCCGAGACTAGAGACC	GGTTCCCTGCTCCTTCTTCT	Ferguson et al. (2004)	
pPGSseq17F6	CGTCGGATTTATCTGCCAGT	AGTAGGGGCAAGGGTTGATG	Ferguson et al. (2004)	
pPGSseq18E7	CAACGCCGAGACTAGAGACC	GGTTCCCTGCTCCTTCTTCT	Ferguson et al. (2004)	
pPGSSeq19C3	TCATCGCCAAACTCTTCTCC	TCGAAGAGTGCATGTTGACC	Ferguson et al. (2004)	
pPGSseq9B4	CATTGTTAATGCACCTTTGGAA	GGCAGATTTGGTTATTGCCT	Ferguson et al. (2004)	
pPGSseq9G5	CAAATTGTGCAGCCAAGAGA	CATATGCCCAGGAAGAGGAA	Ferguson et al. (2004)	
TC11A02	AATCGGAATGGCAAGAGACA	AGAGCAAAGGGCGAATCTATG	Moretzsohn et al. (2005)	
TC11A04	ACTCTGCATGGATGGCTACAG	CATGTTCGGTTTCAAGTCTCAA	Moretzsohn et al. (2005)	
TC11H06	CCATGTGAGGTATCAGTAAAGAAAGG	CCACCAACAACATTGGATGAAT	Moretzsohn et al. (2005)	
TC19B11	ATCTCTTCCAACAGTTTGGGG	ATGCATCGCAAACATCACTCT	Macedo et al. (2012)	
TC19E01	ATCAGAAACAGAACCCTGGAGA	GGGGAAGAAGAAAGCGGA	Macedo et al. (2012)	
TC1A02	GCAATTTGCACATTATCCGA	CATGTTCGGTTTCAAGTCTCAA	Moretzsohn et al. (2005)	
TC1A08	AAGGGGTTAAGGGCATGACT	CCACAAATGGGTCGTCGAT	Moretzsohn et al. (2005)	
TC1B02	AACATGCATGCAAATGGAAA	GCCAAAGTCACTTGTTTGCTT	Moretzsohn et al. (2005)	
TC1E01	CAGCAAAGAGTCGTCAGTCG	GAAAGTTCACTTGAGCAAATTCA	Moretzsohn et al. (2005)	
TC1G04	TGCTGTGAGAGAAATGGCAG	GCGCATTCTTCGATTAAAGG	Moretzsohn et al. (2005)	
TC23C08	AGCAGAGTGGAAAACGAAGAAG	GTCAGTTTGTGAATCGGGTTTT	Macedo et al. (2012)	
TC23F04	CACGTGTAATAGTTGCTCAAAAT	TATATGCATCAGACTCTCCAGC	Macedo et al. (2012)	
TC27H12	TAACGAATGACATCAATCCCTG	TCTCACTTTGCACTCTCCTCAA	Macedo et al. (2012)	
TC2A02	CTCCCTTGTGGGTATGTGGT	GGCTCCCATTCATTCTCAAA	Moretzsohn et al. (2005)	
TC2D06	AGGGGGAGTCAAAGGAAAGA	TCACGATCCCTTCTCCTTCA	Moretzsohn et al. (2005)	
TC2E05	GAATTTATAAGGCGTGGCGA	CCATCCCTTCTTCCTTCACA	Moretzsohn et al. (2005)	
TC3H02	CTCTCCGCCATCCATGTAAT	ATGGTGAGCTCGACGCTAGT	Moretzsohn et al. (2005)	

ID	FORWARD PRIMER (5'-3')	REVERSE PRIMER (5'-3')	Source	Alias
TC41A10	GTTTTGCTTCCTAATAATAAAGG	ATTCCCAAACTCTCTTCTCTC	Macedo et al. (2012)	
TC4A02	ATTCAAATCGGAATGGCAAG	GAGCAAAGGGCGAATCTATG	Moretzsohn et al. (2005)	
TC4G05	AACCCACTACGGGACTACCC	ACGACGTGGAGGAGAAGAGA	Moretzsohn et al. (2005)	
TC4H02	ACCGCAAACTCATCCATCTC	GATAGCGTCAGAGGCAGAGG	Moretzsohn et al. (2005)	
TC5D01	CATTGACCACTCACATCCGT	GATGGGAGTGTGTATTCGGC	Moretzsohn et al. (2005)	
TC6E01	CTCCCTCGCTTCCTCTTTCT	ACGCATTAACCACACACCAA	Moretzsohn et al. (2005)	
TC7A02	CGAAAACGACACTATGAAACTGC	CCTTGGCTTACACGACTTCCT	Moretzsohn et al. (2005)	
TC7C06	GGCAGGGGAATAAAACTACTAACT	TTTTCCTTCCTTCTCCTTTGTC	Moretzsohn et al. (2005)	
TC9C06	CAAATGGCAGAGTGCGTCTA	CCCTCCTGACTGGGTCCT	Moretzsohn et al. (2005)	
TC9C08	ACTTTTGGGGCAGGATGAG	GCCTCTATTGCTGAGATTATTGC	Moretzsohn et al. (2005)	
TC9F04	CCTAAACAACGACAAACACTCA	AAGCACAACACAGAACCCTAAA	Moretzsohn et al. (2005)	
TC9F10	ATCACAATCACAGCTCCAACAA	GGCAAGTCTAATCTCCTTTCCA	Moretzsohn et al. (2005)	
TC9H08	GCCAAAGGGGACCATAAAC	TCCATCTTCCATCTCATCCAC	Moretzsohn et al. (2005)	
