# Supplementary material for: Development of SSR markers and identification of major quantitative trait loci controlling shelling percentage in cultivated peanut (Arachis hypogaea L.)
Source: Theor Appl Genet. 2017 May 15;130(8):1635–48. doi: 10.1007/s00122-017-2915-3 (PMC5511596; doi:10.1007/s00122-017-2915-3)
Supplement: Supplementary file 5 — Supplementary material 5 (PDF 905 kb) [file 122_2017_2915_MOESM5_ESM.pdf]

Table S5 Segregation of the genotypes of the 830 polymorphic loci in the RIL population.

Nr	Chromosome	Locus	a	h	b	c	d	-	X2	Df	Signif.	Classification
1	A01	AHGS1930	106	11	75	0	0	3	5.43	2	*	[a:h:b]
2	A01	AhTE0800	98	0	0	97	0	0	0.9	1	-	[a:h+b+c]
3	A01	AGGS2728	93	0	102	0	0	0	13.44	2	****	[a:h:b]
4	A01	AHGS1650	100	11	83	0	0	1	1.7	2	-	[a:h:b]
5	A01	AhTE0725	118	0	0	77	0	0	14.56	1	******	[a:h+b+c]
6	A01	AhTE0795	0	0	115	0	80	0	11.46	1	*****	[a+h+d:b]
7	A01	AhTE0994	111	0	0	84	0	0	7.91	1	****	[a:h+b+c]
8	A01	AGGS0300	97	3	86	0	0	9	7.52	2	**	[a:h:b]
9	A01	AGGS1840	0	0	98	0	95	2	1.18	1	-	[a+h+d:b]
10	A01	AGGS1572	80	2	103	0	0	10	11.49	2	****	[a:h:b]
11	A01	AGGS1451	88	6	86	0	0	15	2.64	2	-	[a:h:b]
12	A01	AhTE0678	95	8	91	0	0	1	1.58	2	-	[a:h:b]
13	A01	AGGS0005	95	14	79	0	0	7	1.91	2	-	[a:h:b]
14	A01	AhTE0233-2	0	0	91	0	96	8	0.24	1	-	[a+h+d:b]
15	A01	AGGS2508	108	0	83	0	0	4	16.22	2	******	[a:h:b]
16	A01	AGGS1064	102	5	77	0	0	11	7.54	2	**	[a:h:b]
17	A01	GA110	97	13	85	0	0	0	0.85	2	-	[a:h:b]
18	A01	AGGS2317	95	13	83	0	0	4	0.91	2	-	[a:h:b]
19	A01	AHGS1294	98	13	83	0	0	1	1.3	2	-	[a:h:b]
20	A01	Ad01A11348	98	12	83	0	0	2	1.24	2	-	[a:h:b]
21	A01	AHGA374228	94	12	86	0	0	3	0.36	2	-	[a:h:b]
22	A01	AhTE0233-1	101	0	0	94	0	0	1.9	1	-	[a:h+b+c]
23	A01	AGGS1213	96	12	86	0	0	1	0.55	2	-	[a:h:b]
24	A01	AGGS0331	94	12	73	0	0	16	2.69	2	-	[a:h:b]
25	A01	AHGA329002	92	13	82	0	0	8	0.73	2	-	[a:h:b]
26	A01	AHGA367823	96	13	85	0	0	1	0.73	2	-	[a:h:b]
27	A01	ARS731	94	14	76	0	0	11	2.46	2	-	[a:h:b]
28	A01	AHGA171408	94	14	80	0	0	7	1.57	2	-	[a:h:b]
29	A01	AHGA164448	95	13	81	0	0	6	1.23	2	-	[a:h:b]
30	A01	AHGA375182	92	13	76	0	0	14	1.78	2	-	[a:h:b]
31	A01	AGGS2030	95	13	84	0	0	3	0.76	2	-	[a:h:b]
32	A01	AHGS1974	91	13	85	0	0	6	0.33	2	-	[a:h:b]
33	A01	AHGS1910	94	12	84	0	0	5	0.56	2	-	[a:h:b]
34	A01	Ad01A8169	94	16	83	0	0	2	2.04	2	-	[a:h:b]
35	A01	AHGS1666	94	13	87	0	0	1	0.34	2	-	[a:h:b]
36	A01	AGGS1615	93	13	84	0	0	5	0.57	2	-	[a:h:b]
37	A01	AGGS1005	97	11	85	0	0	2	0.9	2	-	[a:h:b]
38	A01	AGGS2121	96	8	89	0	0	2	1.73	2	-	[a:h:b]
39	A01	AGGS0940	93	15	83	0	0	4	1.4	2	-	[a:h:b]
40	A01	AGGS2701	99	8	84	0	0	4	2.64	2	-	[a:h:b]
41	A01	AHGS1465	99	2	83	0	0	11	9.86	2	***	[a:h:b]
42	A01	AGGS1885	0	0	95	0	96	4	0.63	1	-	[a+h+d:b]
43	A01	AHGS1459	99	0	95	0	0	1	13.02	2	****	[a:h:b]
44	A01	Ad01A8152	97	2	95	0	0	1	9.04	2	**	[a:h:b]
45	A01	GM2807	105	0	81	0	0	9	15.7	2	******	[a:h:b]
46	A01	AHGA284837	104	4	84	0	0	3	7.91	2	**	[a:h:b]
47	A01	pPGSSeq19C3	100	0	81	0	0	14	14.19	2	*****	[a:h:b]
48	A01	Ad05A18337	92	5	89	0	0	9	4.08	2	-	[a:h:b]
49	A01	AGGS0060	84	13	91	0	0	7	0.42	2	-	[a:h:b]
50	A01	Ai01B7542-2	94	0	101	0	0	0	13.27	2	****	[a:h:b]
51	A01	AhTE0914	122	0	0	73	0	0	19.27	1	*******	[a:h+b+c]
52	A01	AHGS1846	89	11	86	0	0	9	0.09	2	-	[a:h:b]

Nr	Chromosome	Locus	a	h	b	c	d	-	X2	Df	Signif.	Classification
53	A01	AGGS0633	89	6	93	0	0	7	3.09	2	-	[a:h:b]
54	A01	TC4G05	95	10	83	0	0	7	1.1	2	-	[a:h:b]
55	A01	AhTE0635	77	12	96	0	0	10	2.1	2	-	[a:h:b]
56	A01	AGGS1979	106	1	86	0	0	2	13.03	2	****	[a:h:b]
57	A01	AHGS0122	88	5	98	0	0	4	4.86	2	*	[a:h:b]
58	A01	AHGS1668	97	18	78	0	0	2	5.11	2	*	[a:h:b]
59	A01	TC23F04	93	3	96	0	0	3	7.25	2	**	[a:h:b]
60	A01	AHGS2559	97	7	88	0	0	3	2.67	2	-	[a:h:b]
61	A01	AHGS2429	98	13	82	0	0	2	1.49	2	-	[a:h:b]
62	A01	AHGS2084	95	9	86	0	0	5	1.2	2	-	[a:h:b]
63	A01	AHGS0201	112	0	83	0	0	0	17.6	2	******	[a:h:b]
64	A01	AGGS1954	70	1	114	0	0	10	21.45	2	*******	[a:h:b]
65	A01	AHGS0729-3	106	4	81	0	0	4	9.12	2	**	[a:h:b]
66	A01	AGGS0694	88	2	104	0	0	1	10.43	2	***	[a:h:b]
67	A01	AGGS1014	84	3	106	0	0	2	9.94	2	***	[a:h:b]
68	A01	GM1839	81	4	95	0	0	15	6.15	2	**	[a:h:b]
69	A01	AhTE0986	130	0	0	64	0	1	31.58	1	*******	[a:h+b+c]
70	A01	pPGPSeq3E5	92	9	86	0	0	8	0.86	2	-	[a:h:b]
71	A02	Ad02A16744	100	4	83	0	0	8	7.04	2	**	[a:h:b]
72	A02	Ad02A10600	105	0	88	0	0	2	14.46	2	*****	[a:h:b]
73	A02	Ad02A12962	107	9	71	0	0	8	8.05	2	**	[a:h:b]
74	A02	GNB218	103	11	79	0	0	2	3.28	2	-	[a:h:b]
75	A02	AHGS1987	102	12	80	0	0	1	2.66	2	-	[a:h:b]
76	A02	Ad02A4136	93	13	77	0	0	12	1.72	2	-	[a:h:b]
77	A02	Ai06B13545	103	14	77	0	0	1	4.03	2	-	[a:h:b]
78	A02	Ad02A4133	96	24	72	0	0	3	16	2	******	[a:h:b]
79	A02	AGGS1635	111	2	80	0	0	2	14.26	2	*****	[a:h:b]
80	A02	pPGPseq1B9	106	0	77	0	0	12	17.1	2	******	[a:h:b]
81	A02	AGGS1356	112	0	83	0	0	0	17.6	2	******	[a:h:b]
82	A03	AHGS1855	99	9	86	0	0	1	1.79	2	-	[a:h:b]
83	A03	AHGS1674	133	12	50	0	0	0	37.69	2	*******	[a:h:b]
84	A03	AHGS1466	116	2	76	0	0	1	17.82	2	******	[a:h:b]
85	A03	AHGS1338	111	7	73	0	0	4	10.24	2	***	[a:h:b]
86	A03	AGGS1596	105	9	67	0	0	14	9.01	2	**	[a:h:b]
87	A03	AHGS1340	109	12	73	0	0	1	7.13	2	**	[a:h:b]
88	A03	Ad03A9257	114	4	75	0	0	2	14.15	2	*****	[a:h:b]
89	A03	AhTE0164	115	5	71	0	0	4	15.11	2	*****	[a:h:b]
90	A03	Ad06A5393	117	3	66	0	0	9	21.74	2	*******	[a:h:b]
91	A03	AHGS2005	98	1	87	0	0	9	11.05	2	****	[a:h:b]
92	A03	AGGS1321	88	1	103	0	0	3	12.01	2	****	[a:h:b]
93	A03	AHGA96466	93	10	76	0	0	16	1.86	2	-	[a:h:b]
94	A03	AHGA96458	93	12	89	0	0	1	0.09	2	-	[a:h:b]
95	A03	AHGA96464	89	12	93	0	0	1	0.09	2	-	[a:h:b]
96	A03	Ai05B9660	86	6	92	0	0	11	3.01	2	-	[a:h:b]
97	A03	Ai03B32338	83	4	90	0	0	18	5.1	2	*	[a:h:b]
98	A04	AGGS0396-1	101	6	76	0	0	12	6.4	2	**	[a:h:b]
99	A04	pPGSseq15C12-2	98	9	75	0	0	13	3.63	2	-	[a:h:b]
100	A04	Ad04A21094	97	10	82	0	0	6	1.57	2	-	[a:h:b]
101	A04	AGGS1211	95	15	82	0	0	3	1.74	2	-	[a:h:b]
102	A04	GM2313	98	6	82	0	0	9	4.37	2	-	[a:h:b]
103	A04	AhTE0422	100	13	78	0	0	4	2.8	2	-	[a:h:b]
104	A05	AGGS2387	104	4	83	0	0	4	8.09	2	**	[a:h:b]
105	A05	AGGS1589	106	3	86	0	0	0	9.58	2	***	[a:h:b]

Nr	Chromosome	Locus	a	h	b	c	d	-	X2	Df	Signif.	Classification
106	A05	AHGS1143	12	0	173	0	0	10	161.8	2	*******	[a:h:b]
107	A05	Ai08B23999	104	19	70	0	0	2	10.64	2	****	[a:h:b]
108	A05	Ad05A20734	109	0	76	0	0	10	18.61	2	*******	[a:h:b]
109	A05	Ai06B7451	85	14	88	0	0	8	0.54	2	-	[a:h:b]
110	A05	Ad05A19912	86	7	92	0	0	10	2.13	2	-	[a:h:b]
111	A05	Ad05A20509	88	8	91	0	0	8	1.29	2	-	[a:h:b]
112	A05	AGGS1233	85	5	95	0	0	10	4.55	2	-	[a:h:b]
113	A05	Ad05A20782	92	2	94	0	0	7	8.65	2	**	[a:h:b]
114	A05	Ad05A20533	91	4	90	0	0	10	5.28	2	*	[a:h:b]
115	A05	Ad05A20875	81	5	91	0	0	18	4.15	2	-	[a:h:b]
116	A05	Ad05A20801	87	10	90	0	0	8	0.31	2	-	[a:h:b]
117	A05	Ad05A20785	88	7	92	0	0	8	2.1	2	-	[a:h:b]
118	A05	Ad05A20677	90	6	83	0	0	16	2.86	2	-	[a:h:b]
119	A05	Ad05A20769	89	7	89	0	0	10	1.92	2	-	[a:h:b]
120	A05	Ad05A20650	88	9	85	0	0	13	0.58	2	-	[a:h:b]
121	A05	Ad05A20643-2	91	5	91	0	0	8	4.08	2	-	[a:h:b]
122	A05	Ad05A20570	91	6	89	0	0	9	2.93	2	-	[a:h:b]
123	A05	Ad05A20396	95	3	90	0	0	7	7.09	2	**	[a:h:b]
124	A05	Ad05A20499	83	3	98	0	0	11	8.01	2	**	[a:h:b]
125	A05	Ad05A20262	89	10	86	0	0	10	0.28	2	-	[a:h:b]
126	A05	Ad05A20012	92	1	91	0	0	11	10.23	2	***	[a:h:b]
127	A05	Ad05A19999	91	5	89	0	0	10	4	2	-	[a:h:b]
128	A05	Ad05A20084	83	10	93	0	0	9	0.82	2	-	[a:h:b]
129	A05	Ad05A20046	80	7	100	0	0	8	4.29	2	-	[a:h:b]
130	A05	AGGS1542	87	2	105	0	0	1	10.8	2	****	[a:h:b]
131	A05	Ad05A19382	79	11	96	0	0	9	1.69	2	-	[a:h:b]
132	A05	Ad05A19315	0	0	102	0	85	8	4.42	1	**	[a+h+d:b]
133	A05	Ad05A19473	77	0	0	108	0	10	2.05	1	-	[a:h+b+c]
134	A05	Ad05A19487	78	5	103	0	0	9	7.61	2	**	[a:h:b]
135	A05	Ad05A19295	77	13	95	0	0	10	2.06	2	-	[a:h:b]
136	A05	Ad05A19296	76	10	97	0	0	12	2.76	2	-	[a:h:b]
137	A05	Ad05A19142	79	5	100	0	0	11	6.48	2	**	[a:h:b]
138	A05	Ad05A19096	80	5	102	0	0	8	6.84	2	**	[a:h:b]
139	A05	Ad05A19116	73	13	99	0	0	10	4.09	2	-	[a:h:b]
140	A05	Ad05A19190	71	6	108	0	0	10	10.75	2	****	[a:h:b]
141	A05	Ad05A19710	72	8	105	0	0	10	7.45	2	**	[a:h:b]
142	A05	Ad05A18820	67	15	102	0	0	11	8.24	2	**	[a:h:b]
143	A05	Ad05A18734	69	16	102	0	0	8	7.91	2	**	[a:h:b]
144	A05	Ad05A18845	77	1	108	0	0	9	15.87	2	******	[a:h:b]
145	A05	AhTE0523	75	5	114	0	0	1	12.83	2	****	[a:h:b]
146	A05	Ad05A18493	66	0	119	0	0	10	28.53	2	*******	[a:h:b]
147	A05	Ad05A18569	68	6	112	0	0	9	14.01	2	*****	[a:h:b]
148	A05	Ad05A18924	66	12	108	0	0	9	10.13	2	***	[a:h:b]
149	A05	Ad05A18658	67	1	116	0	0	11	24.14	2	*******	[a:h:b]
150	A05	Ad05A19203	59	0	0	129	0	7	18.12	1	*******	[a:h+b+c]
151	A05	Ad05A18727	66	3	114	0	0	12	20.07	2	*******	[a:h:b]
152	A05	AhTE0470	70	4	120	0	0	1	19.55	2	*******	[a:h:b]
153	A05	Ad05A18501	0	0	107	0	80	8	8.04	1	****	[a+h+d:b]
154	A05	Ad02A180	65	15	106	0	0	9	10.69	2	****	[a:h:b]
155	A05	Ad05A18961	72	16	99	0	0	8	5.86	2	*	[a:h:b]
156	A05	Ad05A18695	70	11	105	0	0	9	7.06	2	**	[a:h:b]
157	A05	Ad02A182	63	5	123	0	0	4	24.41	2	*******	[a:h:b]
158	A05	Ad05A18275	72	9	107	0	0	7	7.64	2	**	[a:h:b]

Nr	Chromosome	Locus	a	h	b	c	d	-	X2	Df	Signif.	Classification
159	A05	AhTE0278	52	10	129	0	0	4	33.45	2	*******	[a:h:b]
160	A05	Ad05A18425	62	34	89	0	0	10	50.65	2	*******	[a:h:b]
161	A05	Ad05A18453	74	12	99	0	0	10	3.62	2	-	[a:h:b]
162	A05	Ad05A17280	63	8	123	0	0	1	21.29	2	*******	[a:h:b]
163	A05	Ai06B29598	56	12	119	0	0	8	22.65	2	*******	[a:h:b]
164	A05	AGGS0738	91	1	97	0	0	6	10.76	2	****	[a:h:b]
165	A05	Ad05A14690	85	7	102	0	0	1	3.9	2	-	[a:h:b]
166	A05	AhTE0820	82	0	0	113	0	0	1.82	1	-	[a:h+b+c]
167	A05	AHGS1813	18	4	170	0	0	3	134	2	*******	[a:h:b]
168	A05	AGGS1518	87	14	77	0	0	17	1.39	2	-	[a:h:b]
169	A05	AHGS2644	70	7	109	0	0	9	10.69	2	****	[a:h:b]
170	A05	AhTE0005	69	9	109	0	0	8	9.79	2	***	[a:h:b]
171	A05	AHGS1245	72	10	100	0	0	13	4.77	2	*	[a:h:b]
172	A05	GM630	69	11	97	0	0	18	4.73	2	*	[a:h:b]
173	A05	AHGS2159	71	9	110	0	0	5	9.28	2	***	[a:h:b]
174	A05	Ad05A20222	18	3	164	0	0	10	129.7	2	*******	[a:h:b]
175	A05	AGGS2124	76	4	103	0	0	12	9.41	2	***	[a:h:b]
176	A05	Ah426	77	5	103	0	0	10	7.87	2	**	[a:h:b]
177	A05	Ad05A10356	73	9	109	0	0	4	8.01	2	**	[a:h:b]
178	A05	AGGS2746	77	10	108	0	0	0	5.68	2	*	[a:h:b]
179	A05	Ai07B23662-1	78	5	110	0	0	2	10.07	2	***	[a:h:b]
180	A05	AGGS1656	72	1	112	0	0	10	19.52	2	*******	[a:h:b]
181	A05	ARS702	77	3	108	0	0	7	12.4	2	****	[a:h:b]
182	A05	AHGS1440-2	71	13	101	0	0	10	5.38	2	*	[a:h:b]
183	A05	AHGS1850	73	6	102	0	0	14	7.62	2	**	[a:h:b]
184	A05	AGGS2177	83	8	96	0	0	8	2.2	2	-	[a:h:b]
185	A05	Ad05A5618	80	10	103	0	0	2	3.3	2	-	[a:h:b]
186	A05	ARS715	80	10	96	0	0	9	1.71	2	-	[a:h:b]
187	A05	Ad05A19823	91	7	89	0	0	8	2.03	2	-	[a:h:b]
188	A05	AhTE0148	90	12	86	0	0	7	0.1	2	-	[a:h:b]
189	A05	AGGS1403	93	4	97	0	0	1	5.9	2	*	[a:h:b]
190	A05	AHGS2534	96	11	86	0	0	2	0.65	2	-	[a:h:b]
191	A05	AHGA44674	87	10	83	0	0	15	0.24	2	-	[a:h:b]
192	A05	PM65	100	1	83	0	0	11	11.9	2	****	[a:h:b]
193	A05	GNB138	85	3	98	0	0	9	7.79	2	**	[a:h:b]
194	A05	AhTE0553-1	87	0	0	101	0	7	0.03	1	-	[a:h+b+c]
195	A05	pPGPSeq2F10	82	0	110	0	0	3	17.16	2	******	[a:h:b]
196	A05	AGGS1167	89	8	96	0	0	2	1.73	2	-	[a:h:b]
197	A05	AGGS1187	87	10	94	0	0	4	0.61	2	-	[a:h:b]
198	A05	AHGS1806	93	7	90	0	0	5	2.19	2	-	[a:h:b]
199	A05	Ad05A8309	85	6	101	0	0	3	4.62	2	*	[a:h:b]
200	A05	AhTE0540	88	0	0	107	0	0	0.24	1	-	[a:h+b+c]
201	A05	AGGS0346	102	0	81	0	0	12	14.77	2	*****	[a:h:b]
202	A05	AGGS2187	83	10	97	0	0	5	1.42	2	-	[a:h:b]
203	A05	AGGS1700	86	10	96	0	0	3	0.91	2	-	[a:h:b]
204	A05	Ad05A20617	102	0	84	0	0	9	14.26	2	*****	[a:h:b]
205	A05	AGGS2372	107	0	83	0	0	5	15.9	2	******	[a:h:b]
206	A05	AhTE0810	121	0	0	73	0	1	18.71	1	*******	[a:h+b+c]
207	A05	AC1D11	99	0	82	0	0	14	13.77	2	****	[a:h:b]
208	A05	AT43	101	8	83	0	0	3	3.22	2	-	[a:h:b]
209	A05	AHGS1507	103	9	80	0	0	3	3.74	2	-	[a:h:b]
210	A05	AhTE0839	117	0	0	78	0	0	13.49	1	******	[a:h+b+c]
211	A05	PM36-2	93	3	92	0	0	7	6.96	2	**	[a:h:b]

Nr	Chromosome	Locus	a	h	b	c	d	-	X2	Df	Signif.	Classification
212	A05	AhTE0711	112	0	0	81	0	2	9.65	1	****	[a:h+b+c]
213	A05	AGGS1629	111	5	61	0	0	18	18.61	2	*******	[a:h:b]
214	A06	AT68	113	0	77	0	0	5	19.94	2	*******	[a:h:b]
215	A06	AGGS0978	105	0	88	0	0	2	14.46	2	*****	[a:h:b]
216	A06	Ai06B19288	102	11	70	0	0	12	5.99	2	*	[a:h:b]
217	A06	TC7C06	100	7	80	0	0	8	4.29	2	-	[a:h:b]
218	A06	AGGS0720	113	1	74	0	0	7	19.12	2	*******	[a:h:b]
219	A06	AGGS750	110	2	70	0	0	13	17.62	2	******	[a:h:b]
220	A06	AHGS0153	119	0	75	0	0	1	23.58	2	*******	[a:h:b]
221	A06	AGGS1479	117	2	70	0	0	6	21.16	2	*******	[a:h:b]
222	A06	TC9C06	114	2	70	0	0	9	19.6	2	*******	[a:h:b]
223	A06	Ad06A10649	111	0	82	0	0	2	17.51	2	******	[a:h:b]
224	A06	GNB87	108	5	79	0	0	3	9.03	2	**	[a:h:b]
225	A06	AGGS2082	111	9	68	0	0	7	11.18	2	****	[a:h:b]
226	A06	AGGS1953	107	9	69	0	0	10	8.93	2	**	[a:h:b]
227	A06	AHGA193642	108	10	66	0	0	11	10.43	2	***	[a:h:b]
228	A06	AGGS1450	108	6	63	0	0	18	14.67	2	*****	[a:h:b]
229	A06	GNB877	116	6	65	0	0	8	17.79	2	******	[a:h:b]
230	A06	GNB329	118	7	69	0	0	1	15.51	2	******	[a:h:b]
231	A06	GNB1040-1	113	6	69	0	0	7	13.99	2	*****	[a:h:b]
232	A06	EE51	120	0	0	67	0	8	22.46	1	*******	[a:h+b+c]
233	A06	AGGS1773	119	0	76	0	0	0	23.11	2	*******	[a:h:b]
234	A06	AGGS1932	110	7	73	0	0	5	9.82	2	***	[a:h:b]
235	A06	TC1A02	113	10	69	0	0	3	11.11	2	****	[a:h:b]
236	A06	TC11A04	109	11	68	0	0	7	9.59	2	***	[a:h:b]
237	A06	Ad06A16982	119	3	69	0	0	4	21.1	2	*******	[a:h:b]
238	A06	Ad06A17247	116	5	70	0	0	4	16.12	2	******	[a:h:b]
239	A06	PM377	115	7	65	0	0	8	16.27	2	******	[a:h:b]
240	A07	AGGS1365	109	9	73	0	0	4	8.01	2	**	[a:h:b]
241	A07	AhTE0478	128	1	66	0	0	0	31.98	2	*******	[a:h:b]
242	A07	AHGA102053	117	10	58	0	0	10	20.3	2	*******	[a:h:b]
243	A07	AHGS1296	118	7	68	0	0	2	16.08	2	******	[a:h:b]
244	A07	AGGS2556	110	6	75	0	0	4	9.99	2	***	[a:h:b]
245	A07	Ad06A4196	113	3	79	0	0	0	13.71	2	****	[a:h:b]
246	A07	IPAHM123	113	0	82	0	0	0	18.26	2	******	[a:h:b]
247	A07	TC9H08	106	2	86	0	0	1	11.22	2	****	[a:h:b]
248	A07	Ad07A4990	108	9	74	0	0	4	7.23	2	**	[a:h:b]
249	A07	PM204	108	6	72	0	0	9	10.34	2	***	[a:h:b]
250	A07	GM1937	106	5	75	0	0	9	9.54	2	***	[a:h:b]
251	A07	Ad07A9745	105	9	73	0	0	8	6.5	2	**	[a:h:b]
252	A07	AHGS2153	107	11	75	0	0	2	5.76	2	*	[a:h:b]
253	A07	AGGS1389	108	10	75	0	0	2	6.39	2	**	[a:h:b]
254	A07	AHGS1475	110	11	72	0	0	2	8.08	2	**	[a:h:b]
255	A07	AGGS1987	104	12	73	0	0	6	5.43	2	*	[a:h:b]
256	A07	AHGS1980	107	12	75	0	0	1	5.63	2	*	[a:h:b]
257	A07	AHGS1954	107	11	73	0	0	4	6.53	2	**	[a:h:b]
258	A07	AGGS1568	105	7	77	0	0	6	6.52	2	**	[a:h:b]
259	A07	AHGS1692	116	4	74	0	0	1	15.51	2	******	[a:h:b]
260	A07	AHGA65328	109	10	73	0	0	3	7.56	2	**	[a:h:b]
261	A07	AGGS1638	108	4	75	0	0	8	11.61	2	****	[a:h:b]
262	A07	AGGS0187	103	10	80	0	0	2	3.3	2	-	[a:h:b]
263	A07	AHGS2413	105	12	76	0	0	2	4.65	2	*	[a:h:b]
264	A07	AhTE0706	0	0	86	0	109	0	0.6	1	-	[a+h+d:b]

Nr	Chromosome	Locus	a	h	b	c	d	-	X2	Df	Signif.	Classification
265	A07	AHGS1189	107	5	78	0	0	5	8.97	2	**	[a:h:b]
266	A07	TC41A10	105	11	75	0	0	4	5.1	2	*	[a:h:b]
267	A07	AGGS1662	102	6	82	0	0	5	5.35	2	*	[a:h:b]
268	A07	AHGS0346	103	12	80	0	0	0	2.9	2	-	[a:h:b]
269	A07	AHGS0147	101	4	85	0	0	5	7.01	2	**	[a:h:b]
270	A07	AHGS1266	102	12	78	0	0	3	3.2	2	-	[a:h:b]
271	A07	AGGS1585	100	8	82	0	0	5	3.17	2	-	[a:h:b]
272	A07	AHGS2754	106	6	79	0	0	4	7.22	2	**	[a:h:b]
273	A07	AHGS1913	105	9	80	0	0	1	4.3	2	-	[a:h:b]
274	A07	AGGS2061	104	5	86	0	0	0	6.29	2	**	[a:h:b]
275	A07	Ad04A10156	90	8	86	0	0	11	1.23	2	-	[a:h:b]
276	A07	AGGS1577	115	2	76	0	0	2	17.36	2	******	[a:h:b]
277	A07	AHGS0274	104	10	81	0	0	0	3.31	2	-	[a:h:b]
278	A07	AGGS2261	101	8	76	0	0	10	4.77	2	*	[a:h:b]
279	A08	AGGS0337	113	4	77	0	0	1	12.93	2	****	[a:h:b]
280	A08	AGGS1466	95	15	75	0	0	10	3.4	2	-	[a:h:b]
281	A08	AHGS1574	95	17	80	0	0	3	3.47	2	-	[a:h:b]
282	A08	Ai07B26726	98	10	84	0	0	3	1.44	2	-	[a:h:b]
283	A08	Ad08A3453	108	8	78	0	0	1	6.45	2	**	[a:h:b]
284	A08	AhTE0690	0	0	93	0	102	0	0.05	1	-	[a+h+d:b]
285	A08	AHGS2319	107	1	86	0	0	1	13.31	2	****	[a:h:b]
286	A08	Ad08A4387	99	7	81	0	0	8	3.85	2	-	[a:h:b]
287	A08	AGGS2027	95	10	80	0	0	10	1.52	2	-	[a:h:b]
288	A08	AHGS1434	102	15	78	0	0	0	3.84	2	-	[a:h:b]
289	A08	AGGS1495	101	10	82	0	0	2	2.37	2	-	[a:h:b]
290	A08	GM1713	93	15	72	0	0	15	3.95	2	-	[a:h:b]
291	A08	Ad08A4940	106	7	82	0	0	0	5.51	2	*	[a:h:b]
292	A08	AHGS1687	103	12	77	0	0	3	3.76	2	-	[a:h:b]
293	A08	pPGPseq2G3	99	14	72	0	0	10	4.75	2	*	[a:h:b]
294	A08	AHGA361225	102	0	88	0	0	5	13.77	2	****	[a:h:b]
295	A08	GM1901	91	21	73	0	0	10	10.08	2	***	[a:h:b]
296	A09	AHTE0840	114	0	0	81	0	0	10.51	1	****	[a:h+b+c]
297	A09	AGGS2745	118	1	69	0	0	7	24.11	2	*******	[a:h:b]
298	A09	AGGS2744	112	0	72	0	0	11	21.54	2	*******	[a:h:b]
299	A09	AGGS1003	108	5	80	0	0	2	8.74	2	**	[a:h:b]
300	A09	AGGS1925	97	7	88	0	0	3	2.67	2	-	[a:h:b]
301	A09	AHGS1208	106	0	86	0	0	3	15.02	2	*****	[a:h:b]
302	A09	AGGS1432	98	9	79	0	0	9	2.7	2	-	[a:h:b]
303	A09	GNB159	112	0	74	0	0	9	20.68	2	*******	[a:h:b]
304	A09	AGGS0385	114	5	75	0	0	1	12.83	2	****	[a:h:b]
305	A09	AHGS0422	118	2	75	0	0	0	19.2	2	*******	[a:h:b]
306	A09	AhTE0808	123	0	0	72	0	0	20.56	1	*******	[a:h+b+c]
307	A09	AGGS1498	114	1	75	0	0	5	19.16	2	*******	[a:h:b]
308	A09	AGGS1137	110	4	73	0	0	8	13.2	2	****	[a:h:b]
309	A09	AGGS1317	108	6	71	0	0	10	10.75	2	****	[a:h:b]
310	A09	AGGS1193	107	9	74	0	0	5	6.86	2	**	[a:h:b]
311	A09	GM2839	109	10	73	0	0	3	7.56	2	**	[a:h:b]
312	A09	EM87	101	11	77	0	0	6	3.31	2	-	[a:h:b]
313	A09	AhTE0726	113	0	0	82	0	0	9.6	1	****	[a:h+b+c]
314	A09	AHGS0400	109	1	84	0	0	1	14.32	2	*****	[a:h:b]
315	A09	AGGS1438	111	5	78	0	0	1	10.45	2	***	[a:h:b]
316	A09	AHGS1319	102	10	78	0	0	5	3.55	2	-	[a:h:b]
317	A09	AGGS2134	99	13	64	0	0	19	7.81	2	**	[a:h:b]

Nr	Chromosome	Locus	a	h	b	c	d	-	X2	Df	Signif.	Classification
318	A09	Ad09A10442	102	12	74	0	0	7	4.45	2	-	[a:h:b]
319	A09	AHGS1543	101	14	79	0	0	1	2.97	2	-	[a:h:b]
320	A09	AHGS1126	104	10	81	0	0	0	3.31	2	-	[a:h:b]
321	A09	Ad09A5979	102	11	79	0	0	3	3.03	2	-	[a:h:b]
322	A09	Ad09A6009	99	12	82	0	0	2	1.6	2	-	[a:h:b]
323	A09	Ad09A6680	99	13	72	0	0	11	4.43	2	-	[a:h:b]
324	A09	AhTE0303	97	13	83	0	0	2	1.16	2	-	[a:h:b]
325	A09	Ad09A6617	101	11	80	0	0	3	2.54	2	-	[a:h:b]
326	A09	Ad09A6594	97	11	80	0	0	7	1.69	2	-	[a:h:b]
327	A09	Ad09A7483	98	10	76	0	0	11	3.01	2	-	[a:h:b]
328	A09	Ad09A7532	97	9	76	0	0	13	3.11	2	-	[a:h:b]
329	A09	GNB136	101	10	82	0	0	2	2.37	2	-	[a:h:b]
330	A09	Ad09A6154	98	12	81	0	0	4	1.61	2	-	[a:h:b]
331	A09	Ad09A7328	102	9	76	0	0	8	4.52	2	-	[a:h:b]
332	A09	Ad09A6072	92	8	83	0	0	12	1.57	2	-	[a:h:b]
333	A09	AHGA7048	103	6	85	0	0	1	5.08	2	*	[a:h:b]
334	A09	Ad09A6233	95	6	76	0	0	18	4.65	2	*	[a:h:b]
335	A09	AGGS1378	102	4	86	0	0	3	7.11	2	**	[a:h:b]
336	A09	AGGS0449	112	4	76	0	0	3	12.89	2	****	[a:h:b]
337	A09	AHGA98575	101	3	88	0	0	3	8.14	2	**	[a:h:b]
338	A09	AGGS0957	96	6	90	0	0	3	3.4	2	-	[a:h:b]
339	A09	Ad09A7577	103	9	81	0	0	2	3.5	2	-	[a:h:b]
340	A09	AGGS1606	101	0	92	0	0	2	13.31	2	****	[a:h:b]
341	A09	AHGA98567	98	0	84	0	0	13	13.28	2	****	[a:h:b]
342	A09	Ad91I24	106	0	89	0	0	0	14.58	2	*****	[a:h:b]
343	A09	AHGS2130	102	0	92	0	0	1	13.48	2	****	[a:h:b]
344	A09	AGGS2492	110	5	77	0	0	3	10.41	2	***	[a:h:b]
345	A09	GNB652	109	2	80	0	0	4	13.52	2	****	[a:h:b]
346	A09	AHGS1683	97	10	87	0	0	1	0.95	2	-	[a:h:b]
347	A09	Ad09A6425	101	11	82	0	0	1	2.1	2	-	[a:h:b]
348	A09	AGGS0389	103	3	80	0	0	9	9.86	2	***	[a:h:b]
349	A09	AHGS1283	99	13	81	0	0	2	1.87	2	-	[a:h:b]
350	A09	AGGS2572	103	7	83	0	0	2	4.48	2	-	[a:h:b]
351	A09	AGGS0977	103	0	91	0	0	1	13.73	2	****	[a:h:b]
352	A09	AGGS0285	101	11	83	0	0	0	1.9	2	-	[a:h:b]
353	A09	Ad09A3779	102	10	80	0	0	3	3.04	2	-	[a:h:b]
354	A09	GNB377	102	0	91	0	0	2	13.54	2	****	[a:h:b]
355	A09	AGGS2438	95	4	92	0	0	4	5.68	2	*	[a:h:b]
356	A09	AhTE0283	107	2	86	0	0	0	11.5	2	****	[a:h:b]
357	A09	ARS742	93	5	79	0	0	18	4.73	2	*	[a:h:b]
358	A09	AHGS0344	100	6	86	0	0	3	4.29	2	-	[a:h:b]
359	A09	Ad10A10685	108	6	79	0	0	2	7.9	2	**	[a:h:b]
360	A09	AhTE0922	0	0	82	0	113	0	1.82	1	-	[a+h+d:b]
361	A09	AhTE0794	0	0	92	0	101	2	0.05	1	-	[a+h+d:b]
362	A09	AhTE0381	109	0	85	0	0	1	16.1	2	******	[a:h:b]
363	A09	AGGS2380	101	11	75	0	0	8	3.9	2	-	[a:h:b]
364	A09	AhTE0888	132	0	0	63	0	0	33.93	1	*******	[a:h+b+c]
365	A09	AhTE0707	124	0	0	71	0	0	21.88	1	*******	[a:h+b+c]
366	A09	AhTE0815	109	0	0	73	0	13	12.38	1	******	[a:h+b+c]
367	A09	AhTE0532	105	2	81	0	0	7	11.9	2	****	[a:h:b]
368	A09	AGGS0100	105	20	69	0	0	1	12.58	2	****	[a:h:b]
369	A10	AhTE0524	98	0	97	0	0	0	13.01	2	****	[a:h:b]
370	A10	AGGS1283	97	3	94	0	0	1	7.37	2	**	[a:h:b]

Nr	Chromosome	Locus	a	h	b	c	d	-	X2	Df	Signif.	Classification
371	A10	AhTE0797	100	0	0	95	0	0	1.52	1	-	[a:h+b+c]
372	B01	GA1	83	20	76	0	0	16	7.7	2	**	[a:h:b]
373	B01	AHGS1098	100	8	82	0	0	5	3.17	2	-	[a:h:b]
374	B01	AGGS2325	0	0	76	0	112	7	3.14	1	*	[a+h+d:b]
375	B01	AHGS0108	94	9	83	0	0	9	1.33	2	-	[a:h:b]
376	B01	AHTE0845	122	0	0	73	0	0	19.27	1	*******	[a:h+b+c]
377	B01	AGGS0281	99	5	89	0	0	2	4.96	2	*	[a:h:b]
378	B01	Ad05A20112	87	11	82	0	0	15	0.15	2	-	[a:h:b]
379	B01	Ai07B23662-2	112	5	70	0	0	8	14.14	2	*****	[a:h:b]
380	B01	AHTE0674	102	0	93	0	0	0	13.44	2	****	[a:h:b]
381	B01	Ai01B7136	89	0	90	0	0	16	11.94	2	****	[a:h:b]
382	B01	AHGA364915	91	12	89	0	0	3	0.02	2	-	[a:h:b]
383	B01	AHGA364936	89	12	89	0	0	5	0	2	-	[a:h:b]
384	B01	AhTE0021	90	0	0	104	0	1	0.02	1	-	[a:h+b+c]
385	B01	AGGS0408	104	0	91	0	0	0	13.92	2	*****	[a:h:b]
386	B01	AHGS0729-2	102	12	76	0	0	5	3.8	2	-	[a:h:b]
387	B01	AhTE0489	79	29	85	0	0	2	25.57	2	*******	[a:h:b]
388	B01	AGGS0833	108	0	81	0	0	6	16.71	2	******	[a:h:b]
389	B01	AGGS2567	103	3	87	0	0	2	8.68	2	**	[a:h:b]
390	B01	AhTE0565	114	0	0	81	0	0	10.51	1	****	[a:h+b+c]
391	B01	AHGS2027	99	7	83	0	0	6	3.54	2	-	[a:h:b]
392	B01	AGGS700	104	0	83	0	0	8	14.98	2	*****	[a:h:b]
393	B01	Ai01B11694	106	7	82	0	0	0	5.51	2	*	[a:h:b]
394	B01	AHGS1670	97	0	95	0	0	3	12.82	2	****	[a:h:b]
395	B01	AHGA25786	99	10	82	0	0	4	1.95	2	-	[a:h:b]
396	B01	AGGS0960	104	2	84	0	0	5	11	2	****	[a:h:b]
397	B01	AGGS0440	114	2	79	0	0	0	15.78	2	******	[a:h:b]
398	B01	AhTE0771	120	0	0	75	0	0	16.84	1	*******	[a:h+b+c]
399	B01	AhTE0536	108	0	0	87	0	0	5.67	1	**	[a:h+b+c]
400	B01	Ad10A4720	98	13	81	0	0	3	1.69	2	-	[a:h:b]
401	B01	AHGS1524	105	13	75	0	0	2	5.05	2	*	[a:h:b]
402	B01	AHGS1462	101	19	74	0	0	1	8.17	2	**	[a:h:b]
403	B01	AGGS1363	96	18	73	0	0	8	6.65	2	**	[a:h:b]
404	B01	Ai01B8018	99	17	77	0	0	2	4.83	2	*	[a:h:b]
405	B01	AGGS2359	97	17	76	0	0	5	4.84	2	*	[a:h:b]
406	B01	AGGS1056	99	15	76	0	0	5	3.85	2	-	[a:h:b]
407	B01	AHGS1829	100	14	76	0	0	5	3.64	2	-	[a:h:b]
408	B01	AGGS1254	100	15	78	0	0	2	3.44	2	-	[a:h:b]
409	B01	AHGS2579	105	12	78	0	0	0	3.99	2	-	[a:h:b]
410	B01	AHGS1273	103	9	81	0	0	2	3.5	2	-	[a:h:b]
411	B01	AhTE0129	103	9	81	0	0	2	3.5	2	-	[a:h:b]
412	B01	AGGS1122	112	6	77	0	0	0	10.05	2	***	[a:h:b]
413	B01	Ai01B9867	107	4	77	0	0	7	10.56	2	***	[a:h:b]
414	B01	AGGS2580	91	6	80	0	0	18	3.2	2	-	[a:h:b]
415	B01	AGGS1612	102	7	82	0	0	4	4.41	2	-	[a:h:b]
416	B01	AHGS1369	111	0	80	0	0	4	18.1	2	******	[a:h:b]
417	B01	AHGS3627	99	7	86	0	0	3	3.16	2	-	[a:h:b]
418	B01	Ai01B7542-1	105	3	86	0	0	1	9.31	2	***	[a:h:b]
419	B01	AHGS2466	102	0	91	0	0	2	13.54	2	****	[a:h:b]
420	B01	AhTE0426	100	0	95	0	0	0	13.14	2	****	[a:h:b]
421	B01	AHGS1489	101	7	85	0	0	2	3.68	2	-	[a:h:b]
422	B01	AhTE0985	109	0	0	85	0	1	6.75	1	***	[a:h+b+c]
423	B01	AHGS1699	102	0	92	0	0	1	13.48	2	****	[a:h:b]

Nr	Chromosome	Locus	a	h	b	c	d	-	X2	Df	Signif.	Classification
424	B01	AhTE0212	109	0	0	86	0	0	6.37	1	**	[a:h+b+c]
425	B01	AHGA24894	94	11	83	0	0	7	0.74	2	-	[a:h:b]
426	B01	TC23C08	91	0	90	0	0	14	12.07	2	****	[a:h:b]
427	B01	TC1A08	101	5	89	0	0	0	5.31	2	*	[a:h:b]
428	B01	AHGS1630	96	1	94	0	0	4	10.71	2	****	[a:h:b]
429	B01	AHGS1333	91	1	96	0	0	7	10.63	2	****	[a:h:b]
430	B01	TC27H12	99	6	85	0	0	5	4.2	2	-	[a:h:b]
431	B01	AHGS1246	94	8	90	0	0	3	1.51	2	-	[a:h:b]
432	B01	AHS0487	101	3	88	0	0	3	8.14	2	**	[a:h:b]
433	B01	AGGS1376	96	0	99	0	0	0	13.05	2	****	[a:h:b]
434	B01	AHGS1130	92	7	96	0	0	0	2.44	2	-	[a:h:b]
435	B01	AhTE0251	94	2	91	0	0	8	8.62	2	**	[a:h:b]
436	B01	AHGS1710	108	10	76	0	0	1	6.03	2	**	[a:h:b]
437	B01	AhTE0790	103	0	0	92	0	0	2.77	1	*	[a:h+b+c]
438	B01	GNB619	99	10	73	0	0	13	4.14	2	-	[a:h:b]
439	B01	AhTE1016	0	0	103	0	92	0	2.77	1	*	[a+h+d:b]
440	B01	AGGS2233	51	3	133	0	0	8	45.24	2	*******	[a:h:b]
441	B02	AHGS1522	76	20	90	0	0	9	7.56	2	**	[a:h:b]
442	B02	AhTE0818	0	0	97	0	98	0	0.64	1	-	[a+h+d:b]
443	B02	AHGS1278	94	12	85	0	0	4	0.45	2	-	[a:h:b]
444	B02	Ai02B25100	106	3	79	0	0	7	11.09	2	****	[a:h:b]
445	B02	AGGS2092	90	10	90	0	0	5	0.32	2	-	[a:h:b]
446	B02	TC1E01	95	3	96	0	0	1	7.33	2	**	[a:h:b]
447	B02	GM2808	87	11	85	0	0	12	0.04	2	-	[a:h:b]
448	B02	Ai02B25013	102	12	74	0	0	7	4.45	2	-	[a:h:b]
449	B02	AhTE0887	73	5	110	0	0	7	11.9	2	****	[a:h:b]
450	B02	AhTE0825	0	0	119	0	76	0	15.68	1	*******	[a+h+d:b]
451	B02	AGGS2532	93	10	91	0	0	1	0.42	2	-	[a:h:b]
452	B02	AHGS1232	103	10	70	0	0	12	6.54	2	**	[a:h:b]
453	B02	AGGS2393	94	10	87	0	0	4	0.61	2	-	[a:h:b]
454	B02	AHGA327396	97	5	84	0	0	9	5	2	*	[a:h:b]
455	B02	AGGS1400	92	8	86	0	0	9	1.41	2	-	[a:h:b]
456	B02	AHGS1426	101	6	83	0	0	5	4.92	2	*	[a:h:b]
457	B02	Ai02B4457	77	11	61	0	0	46	2.16	2	-	[a:h:b]
458	B02	AHGS1241	98	7	79	0	0	11	3.97	2	-	[a:h:b]
459	B02	Ai02B23281	102	9	80	0	0	4	3.47	2	-	[a:h:b]
460	B02	AGGS0297	98	8	83	0	0	6	2.58	2	-	[a:h:b]
461	B02	Ai02B20508	113	0	77	0	0	5	19.94	2	*******	[a:h:b]
462	B02	AGGS2219	103	9	82	0	0	1	3.28	2	-	[a:h:b]
463	B02	Ai02B21538	95	9	78	0	0	13	2.22	2	-	[a:h:b]
464	B02	AHGS1818	108	7	79	0	0	1	6.93	2	**	[a:h:b]
465	B02	Ai02B21379	108	10	73	0	0	4	7.18	2	**	[a:h:b]
466	B02	Ai02B20641	111	10	66	0	0	8	11.81	2	****	[a:h:b]
467	B02	Ai02B20354	109	14	65	0	0	7	11.44	2	****	[a:h:b]
468	B02	AGGS1643	110	11	71	0	0	3	8.54	2	**	[a:h:b]
469	B02	AHGS1251	106	16	70	0	0	3	8.62	2	**	[a:h:b]
470	B02	Ai02B19680	101	17	65	0	0	12	10.44	2	***	[a:h:b]
471	B02	Ai02B21536-1	97	16	75	0	0	7	4.39	2	-	[a:h:b]
472	B02	AHGS1473	107	0	86	0	0	2	15.3	2	******	[a:h:b]
473	B02	AGGS2475	101	1	84	0	0	9	12.02	2	****	[a:h:b]
474	B02	Ai02B18857	103	7	72	0	0	13	7.43	2	**	[a:h:b]
475	B02	AGGS2019	103	17	70	0	0	5	8.47	2	**	[a:h:b]
476	B02	AHGS1845	106	18	69	0	0	2	10.68	2	****	[a:h:b]

Nr	Chromosome	Locus	a	h	b	c	d	-	X2	Df	Signif.	Classification
477	B02	AGGS2228	101	17	75	0	0	2	5.89	2	*	[a:h:b]
478	B02	Ai02B17748	99	17	72	0	0	7	6.64	2	**	[a:h:b]
479	B02	Ah26	103	14	73	0	0	5	5.46	2	*	[a:h:b]
480	B02	pPGPSeq4E8	99	11	80	0	0	5	2.1	2	-	[a:h:b]
481	B02	AGGS1461	101	20	70	0	0	4	11.18	2	****	[a:h:b]
482	B02	AHGS1928	101	15	71	0	0	8	6.14	2	**	[a:h:b]
483	B02	AhTE0296	104	12	72	0	0	7	5.82	2	*	[a:h:b]
484	B02	AHGS1393	102	14	72	0	0	7	5.57	2	*	[a:h:b]
485	B02	Ai08B11147	95	12	74	0	0	14	2.64	2	-	[a:h:b]
486	B02	AGGS1238	101	16	72	0	0	6	6.33	2	**	[a:h:b]
487	B02	AHGS1530	106	13	73	0	0	3	6.14	2	**	[a:h:b]
488	B02	GNB1040-2	103	13	74	0	0	5	4.84	2	*	[a:h:b]
489	B02	Ai02B11533	101	13	69	0	0	12	6.2	2	**	[a:h:b]
490	B02	AHGA14239	108	9	75	0	0	3	6.85	2	**	[a:h:b]
491	B02	AHGS0429	106	7	80	0	0	2	6	2	**	[a:h:b]
492	B02	AGGS1344	107	5	82	0	0	1	7.9	2	**	[a:h:b]
493	B02	AC2C05	104	14	77	0	0	0	4.28	2	-	[a:h:b]
494	B02	Ai02B7886	99	13	76	0	0	7	3.14	2	-	[a:h:b]
495	B02	AGGS1533	104	17	71	0	0	3	8.27	2	**	[a:h:b]
496	B02	Ai02B8654	100	9	80	0	0	6	2.97	2	-	[a:h:b]
497	B02	AGGS1484	111	4	79	0	0	1	11.44	2	****	[a:h:b]
498	B02	AGGS1678	99	10	75	0	0	11	3.55	2	-	[a:h:b]
499	B02	AhM022	101	8	81	0	0	5	3.59	2	-	[a:h:b]
500	B02	Ai02B7519	108	1	85	0	0	1	13.8	2	****	[a:h:b]
501	B02	AGGS0638	103	0	90	0	0	2	13.8	2	****	[a:h:b]
502	B02	Ai02B8213	106	7	75	0	0	7	7.5	2	**	[a:h:b]
503	B02	Ai02B21536-2	95	5	88	0	0	7	4.41	2	-	[a:h:b]
504	B02	AHGS1261	99	10	75	0	0	11	3.55	2	-	[a:h:b]
505	B02	AhTE0813	121	0	0	74	0	0	18.04	1	*******	[a:h+b+c]
506	B02	AhTE0577	90	17	79	0	0	9	3.34	2	-	[a:h:b]
507	B02	TC1B02	104	11	73	0	0	7	5.5	2	*	[a:h:b]
508	B02	AHGS1853	107	10	77	0	0	1	5.35	2	*	[a:h:b]
509	B02	AGGS1532	108	34	50	0	0	3	61.71	2	*******	[a:h:b]
510	B02	AGGS2332	91	13	85	0	0	6	0.33	2	-	[a:h:b]
511	B02	AGGS2195	104	2	87	0	0	2	10.55	2	***	[a:h:b]
512	B02	AGGS1468	108	0	86	0	0	1	15.59	2	******	[a:h:b]
513	B02	AHGS1805	105	0	83	0	0	7	15.28	2	******	[a:h:b]
514	B02	AGGS2413	106	0	87	0	0	2	14.86	2	*****	[a:h:b]
515	B02	AhTE0791	0	0	93	0	102	0	0.05	1	-	[a+h+d:b]
516	B02	AGGS1325	107	0	79	0	0	9	16.9	2	******	[a:h:b]
517	B02	AHGS1419	103	9	80	0	0	3	3.74	2	-	[a:h:b]
518	B02	AGGS1592	108	7	70	0	0	10	10.25	2	***	[a:h:b]
519	B02	Ai02B4199	87	30	63	0	0	15	36.75	2	*******	[a:h:b]
520	B02	Ai02B6837	95	8	82	0	0	10	2.15	2	-	[a:h:b]
521	B02	Ai02B2634	97	16	73	0	0	9	5.06	2	*	[a:h:b]
522	B02	Ai02B2534	95	9	74	0	0	17	3.08	2	-	[a:h:b]
523	B02	Ai02B2356	101	8	79	0	0	7	4.02	2	-	[a:h:b]
524	B02	AGGS0399	105	0	81	0	0	9	15.7	2	******	[a:h:b]
525	B02	Ai02B2350	108	2	73	0	0	12	15.45	2	******	[a:h:b]
526	B02	AHGS1940	96	9	88	0	0	2	1.18	2	-	[a:h:b]
527	B02	TC9F04	100	6	83	0	0	6	4.68	2	*	[a:h:b]
528	B02	AhTE0775	116	0	0	79	0	0	12.46	1	******	[a:h+b+c]
529	B02	AHGS2344	110	13	65	0	0	7	11.63	2	****	[a:h:b]

Nr	Chromosome	Locus	a	h	b	c	d	-	X2	Df	Signif.	Classification
530	B02	GM2196	108	5	73	0	0	9	11.05	2	****	[a:h:b]
531	B02	Ai06B27638	107	5	74	0	0	9	10.27	2	***	[a:h:b]
532	B02	Ad02A625	92	11	80	0	0	12	0.86	2	-	[a:h:b]
533	B03	GA27	90	7	93	0	0	5	2.19	2	-	[a:h:b]
534	B03	AHGS1176	96	4	91	0	0	4	5.77	2	*	[a:h:b]
535	B03	GM1996	93	8	83	0	0	11	1.72	2	-	[a:h:b]
536	B03	Ai08B16802	87	22	81	0	0	5	9.41	2	***	[a:h:b]
537	B03	AGGS1276	91	15	89	0	0	0	0.71	2	-	[a:h:b]
538	B03	AHGS1561	95	15	83	0	0	2	1.56	2	-	[a:h:b]
539	B03	AGGS1369	99	11	79	0	0	6	2.32	2	-	[a:h:b]
540	B03	GM1854	71	1	112	0	0	11	19.97	2	*******	[a:h:b]
541	B04	AGGS1671	112	5	70	0	0	8	14.14	2	*****	[a:h:b]
542	B04	Ai04B16	105	1	80	0	0	9	13.94	2	*****	[a:h:b]
543	B04	Ad04A21	108	13	71	0	0	3	7.69	2	**	[a:h:b]
544	B04	AGGS2384	0	0	78	0	104	13	1.18	1	-	[a+h+d:b]
545	B04	AhTE0654	118	0	0	76	0	1	15.16	1	*******	[a:h+b+c]
546	B04	TC11H06	94	13	74	0	0	14	2.63	2	-	[a:h:b]
547	B04	AhTE0908	0	0	89	0	99	7	0.02	1	-	[a+h+d:b]
548	B04	AHGS1703	108	9	77	0	0	1	6.14	2	**	[a:h:b]
549	B04	AHGS2539	117	5	71	0	0	2	16.11	2	******	[a:h:b]
550	B04	EE22	109	14	60	0	0	12	14.61	2	*****	[a:h:b]
551	B04	AHGA161495	120	5	67	0	0	3	19.96	2	*******	[a:h:b]
552	B04	AHGA161468	119	8	66	0	0	2	16.98	2	******	[a:h:b]
553	B04	AHGA161510	120	5	66	0	0	4	20.59	2	*******	[a:h:b]
554	B04	AhTE0659	125	0	0	70	0	0	23.24	1	*******	[a:h+b+c]
555	B04	GM1445	115	10	62	0	0	8	16.28	2	******	[a:h:b]
556	B04	Ai04B2241	123	4	68	0	0	0	22.41	2	*******	[a:h:b]
557	B04	AGGS1601	114	8	68	0	0	5	13.23	2	****	[a:h:b]
558	B04	AhTE0945	109	0	0	86	0	0	6.37	1	**	[a:h+b+c]
559	B04	AhTE0903	114	0	0	81	0	0	10.51	1	****	[a:h+b+c]
560	B04	AhTE0923	146	0	0	46	0	3	65.59	1	*******	[a:h+b+c]
561	B04	AGGS1795-1	126	7	60	0	0	2	26.34	2	*******	[a:h:b]
562	B04	AhTE0777	144	0	0	51	0	0	56.96	1	*******	[a:h+b+c]
563	B04	AhTE0906	137	0	0	58	0	0	42.81	1	*******	[a:h+b+c]
564	B04	AGGS1054	118	3	65	0	0	9	22.93	2	*******	[a:h:b]
565	B04	Ai04B4849	111	7	76	0	0	1	9.05	2	**	[a:h:b]
566	B04	AhTE0882	126	0	0	69	0	0	24.64	1	*******	[a:h+b+c]
567	B04	AhTE0936	133	0	0	62	0	0	35.63	1	*******	[a:h+b+c]
568	B04	AhTE0416	132	0	0	63	0	0	33.93	1	*******	[a:h+b+c]
569	B04	AHGS1552	126	1	65	0	0	3	31.43	2	*******	[a:h:b]
570	B04	AhTE0237	123	2	70	0	0	0	24.45	2	*******	[a:h:b]
571	B04	AGGS2488	130	0	0	61	0	4	34.43	1	*******	[a:h+b+c]
572	B04	AGGS1783	122	0	0	66	0	7	24.51	1	*******	[a:h+b+c]
573	B04	AHGS1617	124	4	66	0	0	1	24.3	2	*******	[a:h:b]
574	B04	AHGS1579	126	6	63	0	0	0	25.06	2	*******	[a:h:b]
575	B04	Ai06B31304	118	9	61	0	0	7	19.12	2	*******	[a:h:b]
576	B04	Ai04B9397	125	7	62	0	0	1	24.13	2	*******	[a:h:b]
577	B04	Ad05A20478	0	0	59	0	128	8	17.63	1	*******	[a+h+d:b]
578	B04	AGGS1750	121	9	65	0	0	0	18.04	2	******	[a:h:b]
579	B04	AHGS1311	123	9	62	0	0	1	21.32	2	*******	[a:h:b]
580	B04	AHGS0390	117	8	60	0	0	10	19.9	2	*******	[a:h:b]
581	B04	AHGS0288	122	6	64	0	0	3	21.89	2	*******	[a:h:b]
582	B04	AGGS1370	121	9	65	0	0	0	18.04	2	******	[a:h:b]

Nr	Chromosome	Locus	a	h	b	c	d	-	X2	Df	Signif.	Classification
583	B04	AhTE0335	121	7	66	0	0	1	18.94	2	*******	[a:h:b]
584	B04	AGGS1101	122	5	60	0	0	8	26.01	2	*******	[a:h:b]
585	B04	AGGS1324	117	1	59	0	0	18	30.04	2	*******	[a:h:b]
586	B04	Ai04B19238	123	0	72	0	0	0	27.23	2	*******	[a:h:b]
587	B04	AGGS734	118	2	64	0	0	11	25.28	2	*******	[a:h:b]
588	B04	AhTE0261	116	2	77	0	0	0	17.4	2	******	[a:h:b]
589	B04	AGGS1512	111	5	78	0	0	1	10.45	2	***	[a:h:b]
590	B04	Ai04B5526	106	2	76	0	0	11	13.59	2	****	[a:h:b]
591	B04	GM2246	111	3	74	0	0	7	14.72	2	*****	[a:h:b]
592	B04	pPGSseq17F6	125	3	60	0	0	7	30.92	2	*******	[a:h:b]
593	B04	AGGS2251	107	12	74	0	0	2	6.02	2	**	[a:h:b]
594	B04	GM1959	100	5	72	0	0	18	8.27	2	**	[a:h:b]
595	B04	Ad04A12402	85	45	59	0	0	6	103.3	2	*******	[a:h:b]
596	B04	PMc348	0	0	64	0	122	9	11.61	1	*****	[a+h+d:b]
597	B04	AGGS1102	114	2	71	0	0	8	19.11	2	*******	[a:h:b]
598	B04	Ai04B12763	123	1	69	0	0	2	26.94	2	*******	[a:h:b]
599	B04	AGGS1613	94	15	77	0	0	9	2.7	2	-	[a:h:b]
600	B04	AhTE0191	116	0	78	0	0	1	20.87	2	*******	[a:h:b]
601	B04	IPAHM105	116	3	75	0	0	1	16.57	2	******	[a:h:b]
602	B04	AGGS2135	114	3	71	0	0	7	17.44	2	******	[a:h:b]
603	B04	AGGS0284	111	0	83	0	0	1	17.24	2	******	[a:h:b]
604	B04	AHGS1773	128	1	66	0	0	0	31.98	2	*******	[a:h:b]
605	B04	AHGS1937	110	10	73	0	0	2	7.94	2	**	[a:h:b]
606	B04	AGGS1621	110	0	84	0	0	1	16.65	2	******	[a:h:b]
607	B04	AHTE0189	101	0	87	0	0	7	13.65	2	****	[a:h:b]
608	B04	AhTE0959	118	0	0	76	0	1	15.16	1	*******	[a:h+b+c]
609	B04	AhTE0639	122	0	0	72	0	1	19.97	1	*******	[a:h+b+c]
610	B04	AhTE0107	122	1	72	0	0	0	24.63	2	*******	[a:h:b]
611	B04	AGGS2509	110	8	72	0	0	5	9.46	2	***	[a:h:b]
612	B04	AhTE0796	119	0	0	76	0	0	15.68	1	*******	[a:h+b+c]
613	B04	GM2106	103	9	76	0	0	7	4.82	2	*	[a:h:b]
614	B04	PMc660	98	0	0	86	0	11	3.01	1	*	[a:h+b+c]
615	B04	EM142	0	0	75	0	105	15	1.96	1	-	[a+h+d:b]
616	B04	pPGSseq15C12-1	86	15	80	0	0	14	1.49	2	-	[a:h:b]
617	B04	AGGS0396-2	91	8	78	0	0	18	1.92	2	-	[a:h:b]
618	B04	Ai04B20539	82	4	98	0	0	11	6.7	2	**	[a:h:b]
619	B04	AhTE1009	0	0	111	0	82	2	8.77	1	****	[a+h+d:b]
620	B04	AHGA176194	99	44	40	0	0	12	119.2	2	*******	[a:h:b]
621	B04	Ah325	55	0	140	0	0	0	52.52	2	*******	[a:h:b]
622	B05	Ad05A19349	0	0	97	0	91	7	1.68	1	-	[a+h+d:b]
623	B05	AHGS1342	92	12	91	0	0	0	0.01	2	-	[a:h:b]
624	B05	Ai05B28050	136	8	50	0	0	1	42.16	2	*******	[a:h:b]
625	B05	Ai05B32361	103	2	89	0	0	1	10.1	2	***	[a:h:b]
626	B05	GA72	114	3	76	0	0	2	15.24	2	******	[a:h:b]
627	B05	Ad05A20643-1	120	8	59	0	0	8	22.47	2	*******	[a:h:b]
628	B05	AhTE0756	122	0	0	73	0	0	19.27	1	*******	[a:h+b+c]
629	B05	AHGS1770	118	2	68	0	0	7	22.81	2	*******	[a:h:b]
630	B05	AGGS1183	114	10	71	0	0	0	10.53	2	***	[a:h:b]
631	B05	AGGS0673	107	4	74	0	0	10	11.55	2	****	[a:h:b]
632	B05	AhTE0806	135	0	0	60	0	0	39.14	1	*******	[a:h+b+c]
633	B05	TC6E01	124	9	55	0	0	7	27.7	2	*******	[a:h:b]
634	B05	Ad05A18236	129	0	56	0	0	10	43.06	2	*******	[a:h:b]
635	B05	AGGS2147	80	38	75	0	0	2	59.63	2	*******	[a:h:b]

Nr	Chromosome	Locus	a	h	b	c	d	-	X2	Df	Signif.	Classification
636	B05	AHGA152194	129	0	64	0	0	2	36.22	2	*******	[a:h:b]
637	B05	AHGA152207	134	6	53	0	0	2	39.51	2	*******	[a:h:b]
638	B05	AGGS0973	122	3	65	0	0	5	25.32	2	*******	[a:h:b]
639	B05	pPGSseq9G5	122	3	59	0	0	11	29.71	2	*******	[a:h:b]
640	B05	AhTE0119	129	17	48	0	0	1	38.16	2	*******	[a:h:b]
641	B05	AHGS1228	127	15	46	0	0	7	38.18	2	*******	[a:h:b]
642	B05	AHGS1457	110	17	51	0	0	17	24.17	2	*******	[a:h:b]
643	B05	IPAHM354	129	17	45	0	0	4	41.7	2	*******	[a:h:b]
644	B05	pPGSSeq15D6	124	1	69	0	0	1	27.52	2	*******	[a:h:b]
645	B05	AhM082	128	15	47	0	0	5	37.71	2	*******	[a:h:b]
646	B05	GM1555	131	0	57	0	0	7	43.6	2	*******	[a:h:b]
647	B05	AGGS1632	137	1	54	0	0	3	49.03	2	*******	[a:h:b]
648	B05	Ad05A19244	134	1	52	0	0	8	48.78	2	*******	[a:h:b]
649	B05	Ai05B15046	115	4	65	0	0	11	19.71	2	*******	[a:h:b]
650	B05	AHGS2568	136	3	47	0	0	9	52.25	2	*******	[a:h:b]
651	B05	Ai05B26922	119	3	63	0	0	10	24.85	2	*******	[a:h:b]
652	B05	Ai05B25638	135	7	51	0	0	2	41.26	2	*******	[a:h:b]
653	B05	AGGS1299	121	2	67	0	0	5	25.13	2	*******	[a:h:b]
654	B05	Ai05B19811	127	1	63	0	0	4	33.56	2	*******	[a:h:b]
655	B05	AGGS0243	130	17	44	0	0	4	43.59	2	*******	[a:h:b]
656	B05	AHGS1624	133	5	54	0	0	3	39.03	2	*******	[a:h:b]
657	B05	AGGS1754	128	13	51	0	0	3	33.03	2	*******	[a:h:b]
658	B05	AHGS2141	125	9	48	0	0	13	35.28	2	*******	[a:h:b]
659	B05	AHGS1188	128	6	55	0	0	6	33.13	2	*******	[a:h:b]
660	B05	AGGS2115	125	10	48	0	0	12	34.75	2	*******	[a:h:b]
661	B05	AGGS1481	129	2	54	0	0	10	40.87	2	*******	[a:h:b]
662	B05	AGGS1262	134	5	54	0	0	2	39.78	2	*******	[a:h:b]
663	B05	AHGS1203	123	8	48	0	0	16	34.49	2	*******	[a:h:b]
664	B05	AHGS2795	132	13	41	0	0	9	47.66	2	*******	[a:h:b]
665	B05	AGGS0244	130	12	44	0	0	9	42.43	2	*******	[a:h:b]
666	B05	AGGS1464	135	3	46	0	0	11	52.62	2	*******	[a:h:b]
667	B05	Ai05B12077	134	8	48	0	0	5	42.87	2	*******	[a:h:b]
668	B05	AGGS1691	131	11	50	0	0	3	36.54	2	*******	[a:h:b]
669	B05	IPAHM282	125	10	50	0	0	10	32.66	2	*******	[a:h:b]
670	B05	AHGS2195	131	9	54	0	0	1	33.46	2	*******	[a:h:b]
671	B05	AGGS1645	128	8	56	0	0	3	30.22	2	*******	[a:h:b]
672	B05	PM36-1	123	6	58	0	0	8	27.05	2	*******	[a:h:b]
673	B05	AHGS1682	130	4	60	0	0	1	32.75	2	*******	[a:h:b]
674	B05	TC5D01	127	8	57	0	0	3	28.64	2	*******	[a:h:b]
675	B05	AHGS2509	123	8	49	0	0	15	33.45	2	*******	[a:h:b]
676	B05	Ai03B33341	125	0	58	0	0	12	38.37	2	*******	[a:h:b]
677	B05	Ad10A3962	129	13	51	0	0	2	33.7	2	*******	[a:h:b]
678	B05	AGGS0979	124	1	68	0	0	2	28.15	2	*******	[a:h:b]
679	B05	AHGS1497	125	2	56	0	0	12	36.06	2	*******	[a:h:b]
680	B05	TC19E01	129	2	61	0	0	3	34.58	2	*******	[a:h:b]
681	B05	AHGA221480	131	4	58	0	0	2	35.2	2	*******	[a:h:b]
682	B05	AHGS1672	129	0	55	0	0	11	44.01	2	*******	[a:h:b]
683	B05	AhTE0553-2	132	0	0	56	0	7	41.12	1	*******	[a:h+b+c]
684	B05	AhTE0940	166	0	0	29	0	0	114.6	1	*******	[a:h+b+c]
685	B05	AHGA44686	121	5	55	0	0	14	29.43	2	*******	[a:h:b]
686	B05	AHGS1755	126	6	60	0	0	3	27.4	2	*******	[a:h:b]
687	B05	AHGS0729-1	129	4	58	0	0	4	33.78	2	*******	[a:h:b]
688	B05	AGGS2216	124	3	62	0	0	6	28.71	2	*******	[a:h:b]

Nr	Chromosome	Locus	a	h	b	c	d	-	X2	Df	Signif.	Classification
689	B05	AHGS1532	126	7	61	0	0	1	25.54	2	*******	[a:h:b]
690	B05	AGGS2287	122	6	60	0	0	7	24.81	2	*******	[a:h:b]
691	B05	AGGS0311	126	1	59	0	0	9	36.1	2	*******	[a:h:b]
692	B05	AHGS1440-1	117	4	55	0	0	19	28.05	2	*******	[a:h:b]
693	B05	AhTE0211	145	0	0	50	0	0	59.15	1	*******	[a:h+b+c]
694	B05	AGGS0670	127	0	63	0	0	5	35.66	2	*******	[a:h:b]
695	B05	AHGS2241	127	0	65	0	0	3	34.16	2	*******	[a:h:b]
696	B05	Ad05A20185	120	6	56	0	0	13	26.72	2	*******	[a:h:b]
697	B05	AGGS2398	131	5	59	0	0	0	32.88	2	*******	[a:h:b]
698	B05	Ai05B5265	117	3	65	0	0	10	22.35	2	*******	[a:h:b]
699	B05	AGGS2272	121	2	68	0	0	4	24.51	2	*******	[a:h:b]
700	B05	AHGA363492	112	0	71	0	0	12	22	2	*******	[a:h:b]
701	B05	IPAHM73	120	6	64	0	0	5	20.71	2	*******	[a:h:b]
702	B05	AHGA363491	118	6	66	0	0	5	18.28	2	******	[a:h:b]
703	B05	ARS590	119	4	63	0	0	9	23.32	2	*******	[a:h:b]
704	B05	AhTE0319	120	4	71	0	0	0	19	2	*******	[a:h:b]
705	B05	AhTE0446	122	3	70	0	0	0	22.18	2	*******	[a:h:b]
706	B05	AGGS0351	106	4	80	0	0	5	9.37	2	***	[a:h:b]
707	B05	AHGS1750	119	10	66	0	0	0	15.78	2	******	[a:h:b]
708	B05	AGGS0955	120	2	69	0	0	4	23.35	2	*******	[a:h:b]
709	B05	GM1539	109	1	72	0	0	13	18.12	2	******	[a:h:b]
710	B06	Ai06B32416	95	11	76	0	0	13	2.13	2	-	[a:h:b]
711	B06	Ai06B29716	85	6	91	0	0	13	2.92	2	-	[a:h:b]
712	B06	AHGS1267	85	2	93	0	0	15	8.49	2	**	[a:h:b]
713	B07	AGGS1513	100	6	86	0	0	3	4.29	2	-	[a:h:b]
714	B07	AGGS1620	100	7	84	0	0	4	3.61	2	-	[a:h:b]
715	B07	pPGSSeq13B7	100	0	89	0	0	6	13.28	2	****	[a:h:b]
716	B07	Ai07B12485	97	0	98	0	0	0	13.01	2	****	[a:h:b]
717	B07	AGGS2425	98	3	91	0	0	3	7.47	2	**	[a:h:b]
718	B08	AhTE0477	112	2	81	0	0	0	14.34	2	*****	[a:h:b]
719	B08	AGGS1197	98	17	77	0	0	3	4.67	2	*	[a:h:b]
720	B08	AHGS2602	110	9	76	0	0	0	7.21	2	**	[a:h:b]
721	B08	Ad90F2	101	15	63	0	0	16	9.99	2	***	[a:h:b]
722	B08	AhTE0658	0	0	85	0	110	0	0.85	1	-	[a+h+d:b]
723	B08	AHGS1470	102	0	89	0	0	4	13.68	2	****	[a:h:b]
724	B08	AGGS2186	104	0	88	0	0	3	14.22	2	*****	[a:h:b]
725	B08	ML1G04	108	9	78	0	0	0	5.81	2	*	[a:h:b]
726	B08	Ai08B2682	104	0	85	0	0	6	14.64	2	*****	[a:h:b]
727	B08	AhTE0824	129	9	55	0	0	2	31.09	2	*******	[a:h:b]
728	B08	pPGPseq5D1	104	16	66	0	0	9	10.04	2	***	[a:h:b]
729	B09	AHGA161466	120	1	71	0	0	3	24.09	2	*******	[a:h:b]
730	B09	Ai09B5213	111	7	72	0	0	5	10.67	2	****	[a:h:b]
731	B09	Ad10A4326	99	11	80	0	0	5	2.1	2	-	[a:h:b]
732	B09	AHBGSI1008H04	100	2	87	0	0	6	9.65	2	***	[a:h:b]
733	B09	AhTE0852	111	0	0	83	0	1	8.33	1	****	[a:h+b+c]
734	B09	AhTE0913	102	0	0	92	0	1	2.53	1	-	[a:h+b+c]
735	B09	AhTE0696	110	0	0	85	0	0	7.12	1	***	[a:h+b+c]
736	B09	AGGS1654	94	2	91	0	0	8	8.62	2	**	[a:h:b]
737	B09	AGGS1569	84	0	93	0	0	18	12.29	2	****	[a:h:b]
738	B09	AGGS1013	107	0	87	0	0	1	15.13	2	*****	[a:h:b]
739	B10	AHTE0874	109	0	0	86	0	0	6.37	1	**	[a:h+b+c]
740	B10	AGGS1431	79	9	89	0	0	18	1.01	2	-	[a:h:b]
741	B10	AGGS0815	104	0	91	0	0	0	13.92	2	*****	[a:h:b]

Nr	Chromosome	Locus	a	h	b	c	d	-	X2	Df	Signif.	Classification
742	B10	GM2032	93	11	84	0	0	7	0.51	2	-	[a:h:b]
743	B10	GM2165	90	9	88	0	0	8	0.68	2	-	[a:h:b]
744	B10	IPAHM475	94	0	98	0	0	3	12.89	2	****	[a:h:b]
745	B10	AGGS1728	95	0	95	0	0	5	12.67	2	****	[a:h:b]
746	B10	EE16	101	5	85	0	0	4	5.73	2	*	[a:h:b]
747	B10	AGGS2576	98	8	86	0	0	3	2.22	2	-	[a:h:b]
748	B10	AHGS1446	90	10	89	0	0	6	0.3	2	-	[a:h:b]
749	B10	AHGS1669	97	8	88	0	0	2	1.91	2	-	[a:h:b]
750	B10	AHGA214492	95	5	82	0	0	13	4.8	2	*	[a:h:b]
751	B10	AHGS2543	98	8	87	0	0	2	2.13	2	-	[a:h:b]
752	B10	GNB0486	104	7	79	0	0	5	5.64	2	*	[a:h:b]
753	B10	AGGS1355	90	3	96	0	0	6	7.22	2	**	[a:h:b]
754	B10	AGGS1194	105	1	77	0	0	12	14.73	2	*****	[a:h:b]
755	B10	AGGS1312	99	13	80	0	0	3	2.09	2	-	[a:h:b]
756	B10	Ai10B6334	76	8	109	0	0	2	7.48	2	**	[a:h:b]
757	B10	AGGS1243	97	13	81	0	0	4	1.53	2	-	[a:h:b]
758	B10	AHGS0300	98	10	79	0	0	8	2.32	2	-	[a:h:b]
759	B10	AhTE0718	101	0	0	94	0	0	1.9	1	-	[a:h+b+c]
760	B10	AGGS0302	97	11	84	0	0	3	1.03	2	-	[a:h:b]
761	B10	AhTE0761	125	0	0	70	0	0	23.24	1	*******	[a:h+b+c]
762	B10	AGGS1160	102	2	82	0	0	9	10.79	2	****	[a:h:b]
763	B10	AHGS1602	107	4	75	0	0	9	11.21	2	****	[a:h:b]
764	B10	AGGS1118	112	3	79	0	0	1	13.31	2	****	[a:h:b]
765	B10	AGGS1076	103	6	83	0	0	3	5.42	2	*	[a:h:b]
766	B10	GNB38	111	4	76	0	0	4	12.47	2	****	[a:h:b]
767	B10	AHGS1275	93	19	81	0	0	2	5.05	2	*	[a:h:b]
768	B10	AhTE0893	112	0	0	83	0	0	8.73	1	****	[a:h+b+c]
769	B10	Ai10B10082	99	4	86	0	0	6	6.47	2	**	[a:h:b]
770	B10	AGGS0333	106	0	88	0	0	1	14.71	2	*****	[a:h:b]
771	B10	AGGS1124	105	0	89	0	0	1	14.34	2	*****	[a:h:b]
772	B10	AGGS2262	98	11	76	0	0	10	2.82	2	-	[a:h:b]
773	B10	AHGS1595	86	1	105	0	0	3	12.76	2	****	[a:h:b]
774	B10	Ai10B12455	88	0	104	0	0	3	14.22	2	*****	[a:h:b]
775	B10	AGGS0675	108	0	86	0	0	1	15.59	2	******	[a:h:b]
776	B10	AhTE0162	90	0	105	0	0	0	14.23	2	*****	[a:h:b]
777	B10	AGGS2224	92	7	77	0	0	19	2.92	2	-	[a:h:b]
778	B10	AHGS2073	96	5	94	0	0	0	4.54	2	-	[a:h:b]
779	B10	AGGS0627	96	3	92	0	0	4	7.23	2	**	[a:h:b]
780	B10	AHGS1395	95	4	95	0	0	1	5.81	2	*	[a:h:b]
781	B10	AhTE0359	108	3	84	0	0	0	10.54	2	***	[a:h:b]
782	B10	AHGA178360	90	5	96	0	0	4	4.5	2	-	[a:h:b]
783	B10	AGGS1795-2	105	5	84	0	0	1	6.89	2	**	[a:h:b]
784	B10	Ai10B9706	97	8	89	0	0	1	1.85	2	-	[a:h:b]
785	B10	AHGS1626	98	11	84	0	0	2	1.18	2	-	[a:h:b]
786	B10	AGGS1442	91	11	88	0	0	5	0.12	2	-	[a:h:b]
787	B10	AHGS2191	84	12	87	0	0	12	0.08	2	-	[a:h:b]
788	B10	AGGS1639	92	13	85	0	0	5	0.39	2	-	[a:h:b]
789	B10	AHGS1564	92	15	87	0	0	1	0.86	2	-	[a:h:b]
790	B10	AGGS1359	93	13	80	0	0	9	1.14	2	-	[a:h:b]
791	B10	AGGS2371	94	14	85	0	0	2	0.78	2	-	[a:h:b]
792	B10	AGGS2535	90	16	84	0	0	5	1.73	2	-	[a:h:b]
793	B10	AHGS1696	88	13	83	0	0	11	0.35	2	-	[a:h:b]
794	B10	AGGS2564	91	16	86	0	0	2	1.51	2	-	[a:h:b]

Nr	Chromosome	Locus	a	h	b	c	d	-	X2	Df	Signif.	Classification
795	B10	AGGS2407	87	16	79	0	0	13	2.38	2	-	[a:h:b]
796	B10	AHGA75538	100	12	80	0	0	3	2.22	2	-	[a:h:b]
797	B10	AHGS2787	96	14	81	0	0	4	1.64	2	-	[a:h:b]
798	B10	PM675	90	12	80	0	0	13	0.62	2	-	[a:h:b]
799	B10	Ai04B17746	100	7	75	0	0	13	5.46	2	*	[a:h:b]
800	B10	AGGS1307	102	3	87	0	0	3	8.45	2	**	[a:h:b]
801	B10	AGGS1393	93	16	84	0	0	2	1.82	2	-	[a:h:b]
802	B10	AHGA72558	77	14	87	0	0	17	1.39	2	-	[a:h:b]
803	B10	AGGS0058	87	5	90	0	0	13	3.86	2	-	[a:h:b]
804	B10	AGGS0617	97	8	86	0	0	4	2.06	2	-	[a:h:b]
805	B10	AHGS1903	96	4	87	0	0	8	5.86	2	*	[a:h:b]
806	B10	Ad10A15687	92	13	81	0	0	9	0.87	2	-	[a:h:b]
807	B10	AGGS0429	95	1	83	0	0	16	10.75	2	****	[a:h:b]
808	B10	AhTE0709	109	0	0	86	0	0	6.37	1	**	[a:h+b+c]
809	B10	GA156	97	11	85	0	0	2	0.9	2	-	[a:h:b]
810	B10	AGGS2368	89	13	84	0	0	9	0.32	2	-	[a:h:b]
811	B10	AGGS2494	95	16	81	0	0	3	2.51	2	-	[a:h:b]
812	B10	Ai08B8719	89	16	84	0	0	6	1.72	2	-	[a:h:b]
813	B10	AHGA195553	92	9	93	0	0	1	0.86	2	-	[a:h:b]
814	B10	AGGS1425	99	1	91	0	0	4	11.05	2	****	[a:h:b]
815	B10	AHGA61572	90	13	85	0	0	7	0.28	2	-	[a:h:b]
816	B10	AGGS1579	90	0	87	0	0	18	11.85	2	****	[a:h:b]
817	B10	pPGPseq7H6	85	12	80	0	0	18	0.24	2	-	[a:h:b]
818	B10	AHGA61563	97	0	95	0	0	3	12.82	2	****	[a:h:b]
819	B10	AhTE0918	113	0	0	81	0	1	10.08	1	****	[a:h+b+c]
820	B10	AHGS3715	96	12	81	0	0	6	1.27	2	-	[a:h:b]
821	B10	AGGS1453	98	8	78	0	0	11	3.46	2	-	[a:h:b]
822	B10	AhM062	109	13	72	0	0	1	7.59	2	**	[a:h:b]
823	B10	AGGS2555	115	0	0	77	0	3	13.07	1	******	[a:h+b+c]
824	B10	AHGA75537	98	11	81	0	0	5	1.69	2	-	[a:h:b]
825	B10	AhTE0586	87	9	98	0	0	1	1.52	2	-	[a:h:b]
826	B10	GNB320	104	11	78	0	0	2	3.84	2	-	[a:h:b]
827	B10	Ad10A18338	86	9	95	0	0	5	1.2	2	-	[a:h:b]
828	B10	AHGA72569	89	9	94	0	0	3	0.94	2	-	[a:h:b]
829	B10	AT79	95	10	88	0	0	2	0.65	2	-	[a:h:b]
830	B10	AHTE0006	126	1	68	0	0	0	29.36	2	*******	[a:h:b]
