# Supplementary material for: Development of SSR markers and identification of major quantitative trait loci controlling shelling percentage in cultivated peanut (Arachis hypogaea L.)
Source: Theor Appl Genet. 2017 May 15;130(8):1635–48. doi: 10.1007/s00122-017-2915-3 (PMC5511596; doi:10.1007/s00122-017-2915-3)
Supplement: Supplementary file 6 — Supplementary material 6 (PDF 174 kb) [file 122_2017_2915_MOESM6_ESM.pdf]

Table S6 Genetic linkage map constructed based on 830 polymorphic loci in the RIL population.

-Chromosome A01

AHGS1930	0
AhTE0800	2.349
AGGS2728	3.039
AHGS1650	6.647
AhTE0725	8.896
AhTE0795	11.716
AhTE0994	13.118
AGGS0300	14.867
AGGS1840	15.374
AGGS1572	16.366
AGGS1451	17.383
AhTE0678	18.694
AGGS0005	19.906
AhTE0233-2	20.291
AGGS2508	20.571
AGGS1064	20.866
GA110	21.468
AGGS2317	21.78
AHGS1294	21.901
Ad01A11348	22.379
AHGA374228	22.504
AhTE0233-1	22.655
AGGS1213	23.052
AGGS0331	23.266
AHGA329002	23.365
AHGA367823	23.541
ARS731	23.652
AHGA171408	23.786
AHGA164448	23.894
AHGA375182	23.977
AGGS2030	24.006
AHGS1974	24.07
AHGS1910	24.135
Ad01A8169	24.155
AHGS1666	24.5
AGGS1615	24.816
AGGS1005	25.113
AGGS2121	25.334
AGGS0940	25.639
AGGS2701	25.875
AHGS1465	26.303
AGGS1885	26.647
AHGS1459	26.838
Ad01A8152	27.112

GM2807	27.489
AHGA284837	27.723
pPGSSeq19C3	28.835
Ad05A18337	29.553
AGGS0060	30.068
Ai01B7542-2	30.865
AhTE0914	31.848
AHGS1846	32.701
AGGS0633	33.766
TC4G05	34.345
AhTE0635	38.074
AGGS1979	38.211
AHGS0122	39.279
AHGS1668	39.324
TC23F04	41.047
AHGS2559	42.346
AHGS2429	42.599
AHGS2084	44.834
AHGS0201	49.556
AGGS1954	58.046
AHGS0729-3	58.695
AGGS0694	63.414
AGGS1014	65.171
GM1839	70.829
AhTE0986	75.236
pPGPSeq3E5	80.23

-Chromosome A02

Ad02A16744	0
Ad02A10600	11.049
Ad02A12962	13.767
GNB218	15.949
AHGS1987	18.98
Ad02A4136	20.981
Ai06B13545	21.821
Ad02A4133	23.339
AGGS1635	25.674
pPGPseq1B9	27.859
AGGS1356	29.918

-Chromosome A03

AHGS1855	0
AHGS1674	15.02
AHGS1466	24.934
AHGS1338	26.017
AGGS1596	27.574

AHGS1340	28.137
Ad03A9257	30.709
AhTE0164	32.129
Ad06A5393	37.986
AHGS2005	43.109
AGGS1321	57.303
AHGA96466	60.893
AHGA96458	62.998
AHGA96464	63.98
Ai05B9660	65.024
Ai03B32338	76.891

-Chromosome A04

AGGS0396-1	0
pPGSseq15C12-2	2.503
Ad04A21094	8.275
AGGS1211	13.053
GM2313	15.794
AhTE0422	20.47

-Chromosome A05

AGGS2387	0
AGGS1589	3.67
AHGS1143	8.506
Ai08B23999	9.346
Ad05A20734	10.694
Ai06B7451	14.602
Ad05A19912	18.216
Ad05A20509	19.891
AGGS1233	21.799
Ad05A20782	22.665
Ad05A20533	23.273
Ad05A20875	24.744
Ad05A20801	25.111
Ad05A20785	25.195
Ad05A20677	25.439
Ad05A20769	25.824
Ad05A20650	26.348
Ad05A20643-2	26.791
Ad05A20570	27.087
Ad05A20396	27.48
Ad05A20499	28.212
Ad05A20262	29.469
Ad05A20012	29.872
Ad05A19999	30.734
Ad05A20084	31.343

Ad05A20046	31.798
AGGS1542	32.216
Ad05A19382	33.578
Ad05A19315	34.122
Ad05A19473	34.365
Ad05A19487	34.602
Ad05A19295	35.292
Ad05A19296	35.652
Ad05A19142	37.061
Ad05A19096	37.462
Ad05A19116	38.269
Ad05A19190	38.507
Ad05A19710	39.367
Ad05A18820	39.854
Ad05A18734	40.34
Ad05A18845	40.906
AhTE0523	41.531
Ad05A18493	42.577
Ad05A18569	42.749
Ad05A18924	43.693
Ad05A18658	44.343
Ad05A19203	45.549
Ad05A18727	45.941
AhTE0470	46.363
Ad05A18501	46.972
Ad02A180	47.911
Ad05A18961	48.408
Ad05A18695	49.409
Ad02A182	50.744
Ad05A18275	52.394
AhTE0278	52.95
Ad05A18425	53.747
Ad05A18453	54.661
Ad05A17280	56.484
Ai06B29598	58.556
AGGS0738	59.257
Ad05A14690	60.561
AhTE0820	61.142
AHGS1813	62.673
AGGS1518	64.004
AHGS2644	65.408
AhTE0005	66.122
AHGS1245	66.71
GM630	67.619
AHGS2159	68.908
Ad05A20222	69.628
AGGS2124	70.836
Ah426	71.21

Ad05A10356	72.252
AGGS2746	72.739
Ai07B23662-1	73.168
AGGS1656	73.664
ARS702	73.808
AHGS1440-2	75.056
AHGS1850	75.778
AGGS2177	76.308
Ad05A5618	77.615
ARS715	78.284
Ad05A19823	79.047
AhTE0148	79.967
AGGS1403	80.74
AHGS2534	81.545
AHGA44674	82.051
PM65	82.856
GNB138	83.507
AhTE0553-1	83.713
pPGPSeq2F10	84.645
AGGS1167	85.817
AGGS1187	86.366
AHGS1806	86.915
Ad05A8309	87.719
AhTE0540	89.251
AGGS0346	90.177
AGGS2187	91.33
AGGS1700	91.851
Ad05A20617	93.53
AGGS2372	94.542
AhTE0810	95.093
AC1D11	95.877
AT43	98.028
AHGS1507	101.67
AhTE0839	102.97
PM36-2	105.34
AhTE0711	107.97
AGGS1629	110.81

-Chromosome A06

AT68	0
AGGS0978	12.273
Ai06B19288	13.547
TC7C06	14.142
AGGS0720	14.829
AGGS750	16.187
AHGS0153	16.76
AGGS1479	17.531

TC9C06	18.077
Ad06A10649	19.328
GNB87	19.887
AGGS2082	20.652
AGGS1953	21.256
AHGA193642	21.579
AGGS1450	21.81
GNB877	22.159
GNB329	22.998
GNB1040-1	23.567
EE51	24.123
AGGS1773	24.675
AGGS1932	25.695
TC1A02	26.789
TC11A04	29.15
Ad06A16982	33.762
Ad06A17247	35.048
PM377	50.354

-Chromosome A07

AGGS1365	0
AhTE0478	5.459
AHGA102053	8.096
AHGS1296	9.863
AGGS2556	13.542
Ad06A4196	17.278
IPAHM123	17.843
TC9H08	18.527
Ad07A4990	19.137
PM204	19.828
GM1937	20.534
Ad07A9745	20.985
AHGS2153	21.412
AGGS1389	21.781
AHGS1475	21.934
AGGS1987	22.398
AHGS1980	22.52
AHGS1954	22.542
AGGS1568	22.985
AHGS1692	23.35
AHGA65328	24.288
AGGS1638	25.071
AGGS0187	26.765
AHGS2413	28.638
AhTE0706	29.57
AHGS1189	30.289
TC41A10	30.595

AGGS1662	31.355
AHGS0346	32.065
AHGS0147	32.389
AHGS1266	33.804
AGGS1585	34.191
AHGS2754	36.055
AHGS1913	36.67
AGGS2061	37.543
Ad04A10156	38.592
AGGS1577	41.502
AHGS0274	44.714
AGGS2261	46.473

-Chromosome A08

AGGS0337	0
AGGS1466	12.729
AHGS1574	15.397
Ai07B26726	42.385
Ad08A3453	45.354
AhTE0690	46.81
AHGS2319	49.275
Ad08A4387	52.376
AGGS2027	52.676
AHGS1434	53.426
AGGS1495	54.002
GM1713	55.3
Ad08A4940	58.584
AHGS1687	61.626
pPGPseq2G3	61.937
AHGA361225	64.304
GM1901	66.626

-Chromosome A09

AHTE0840	0
AGGS2745	0.14
AGGS2744	3.98
AGGS1003	10.228
AGGS1925	13.131
AHGS1208	14.485
AGGS1432	15.68
GNB159	16.121
AGGS0385	16.596
AHGS0422	17.107
AhTE0808	17.494
AGGS1498	18.19
AGGS1137	18.867

AGGS1317	19.474
AGGS1193	19.716
GM2839	20.053
EM87	20.724
AhTE0726	20.947
AHGS0400	21.205
AGGS1438	21.423
AHGS1319	21.884
AGGS2134	22.072
Ad09A10442	22.971
AHGS1543	23.39
AHGS1126	23.613
Ad09A5979	23.893
Ad09A6009	24.041
Ad09A6680	24.07
AhTE0303	24.152
Ad09A6617	24.286
Ad09A6594	24.353
Ad09A7483	24.38
Ad09A7532	24.453
GNB136	24.508
Ad09A6154	24.675
Ad09A7328	24.885
Ad09A6072	24.976
AHGA7048	25.214
Ad09A6233	25.387
AGGS1378	25.657
AGGS0449	25.78
AHGA98575	26.247
AGGS0957	26.538
Ad09A7577	26.887
AGGS1606	27.189
AHGA98567	27.348
Ad91I24	27.591
AHGS2130	27.732
AGGS2492	27.919
GNB652	28.379
AHGS1683	29.034
Ad09A6425	29.351
AGGS0389	29.952
AHGS1283	30.439
AGGS2572	30.751
AGGS0977	31.288
AGGS0285	31.991
Ad09A3779	32.899
GNB377	35.009
AGGS2438	36.495
AhTE0283	38.428

ARS742	39.331
AHGS0344	39.53
Ad10A10685	44.531
AhTE0922	50.811
AhTE0794	54.35
AhTE0381	58.997
AGGS2380	66.448
AhTE0888	68.87
AhTE0707	71.777
AhTE0815	74.433
AhTE0532	84.346
AGGS0100	92.928

-Chromosome A10

AhTE0524	0
AGGS1283	7.35
AhTE0797	13.782

-Chromosome B01

GA1	0
AHGS1098	13.423
AGGS2325	17.86
AHGS0108	22.278
AHTE0845	23.291
AGGS0281	27.658
Ad05A20112	38.661
Ai07B23662-2	42.451
AHTE0674	42.778
Ai01B7136	44.854
AHGA364915	47.57
AHGA364936	48.211
AhTE0021	48.768
AGGS0408	50.309
AHGS0729-2	52.852
AhTE0489	53.483
AGGS0833	53.913
AGGS2567	54.581
AhTE0565	54.866
AHGS2027	55.428
AGGS700	55.659
Ai01B11694	56.157
AHGS1670	56.521
AHGA25786	56.85
AGGS0960	57.067
AGGS0440	57.676
AhTE0771	58.108

AhTE0536	58.785
Ad10A4720	59.214
AHGS1524	59.643
AHGS1462	60.123
AGGS1363	60.298
Ai01B8018	60.393
AGGS2359	60.624
AGGS1056	60.739
AHGS1829	61.081
AGGS1254	61.162
AHGS2579	61.32
AHGS1273	61.672
AhTE0129	61.859
AGGS1122	62.001
Ai01B9867	62.339
AGGS2580	62.558
AGGS1612	62.788
AHGS1369	63.084
AHGS3627	63.422
Ai01B7542-1	63.771
AHGS2466	63.867
AhTE0426	64.117
AHGS1489	64.329
AhTE0985	64.667
AHGS1699	64.931
AhTE0212	65.599
AHGA24894	65.892
TC23C08	66.489
TC1A08	67.035
AHGS1630	67.342
AHGS1333	67.578
TC27H12	67.958
AHGS1246	68.377
AHS0487	69.192
AGGS1376	70.798
AHGS1130	71.469
AhTE0251	71.784
AHGS1710	75.549
AhTE0790	79.327
GNB619	80.361
AhTE1016	84.126
AGGS2233	93.435

-Chromosome B02

AHGS1522	0
AhTE0818	13.04
AHGS1278	17.898

Ai02B25100	21.108
AGGS2092	23.832
TC1E01	26.298
GM2808	27.662
Ai02B25013	30.021
AhTE0887	33.778
AhTE0825	34.307
AGGS2532	36.124
AHGS1232	38.193
AGGS2393	38.905
AHGA327396	40.393
AGGS1400	41.428
AHGS1426	42.625
Ai02B4457	43.081
AHGS1241	43.443
Ai02B23281	44.068
AGGS0297	45.439
Ai02B20508	46.735
AGGS2219	47.946
Ai02B21538	49.101
AHGS1818	49.975
Ai02B21379	51.1
Ai02B20641	52.152
Ai02B20354	52.594
AGGS1643	53.414
AHGS1251	54.267
Ai02B19680	54.809
Ai02B21536-1	55.394
AHGS1473	56.298
AGGS2475	56.442
Ai02B18857	57.325
AGGS2019	58.233
AHGS1845	58.242
AGGS2228	58.954
Ai02B17748	59.386
Ah26	59.887
pPGPSeq4E8	60.247
AGGS1461	60.673
AHGS1928	60.992
AhTE0296	61.288
AHGS1393	61.486
Ai08B11147	61.637
AGGS1238	61.889
AHGS1530	62.096
GNB1040-2	62.198
Ai02B11533	62.328
AHGA14239	62.498
AHGS0429	63.047

AGGS1344	63.214
AC2C05	63.539
Ai02B7886	63.801
AGGS1533	64.28
Ai02B8654	64.541
AGGS1484	64.904
AGGS1678	65.234
AhM022	65.842
Ai02B7519	66.404
AGGS0638	66.581
Ai02B8213	67.138
Ai02B21536-2	67.757
AHGS1261	68.019
AhTE0813	68.511
AhTE0577	69.127
TC1B02	70.094
AHGS1853	70.355
AGGS1532	70.896
AGGS2332	71.49
AGGS2195	71.994
AGGS1468	72.519
AHGS1805	73.201
AGGS2413	73.457
AhTE0791	74.508
AGGS1325	74.793
AHGS1419	75.669
AGGS1592	76.737
Ai02B4199	77.754
Ai02B6837	79.136
Ai02B2634	79.975
Ai02B2534	81.363
Ai02B2356	82.632
AGGS0399	84.363
Ai02B2350	85.876
AHGS1940	86.48
TC9F04	88.873
AhTE0775	90.523
AHGS2344	92.536
GM2196	93.672
Ai06B27638	96.022
Ad02A625	108.91

-Chromosome B03

GA27	0
AHGS1176	5.887
GM1996	13.305
Ai08B16802	23.918

AGGS1276	41.253
AHGS1561	44.686
AGGS1369	46.557
GM1854	73.896

-Chromosome B04

AGGS1671	0
Ai04B16	5.498
Ad04A21	9.973
AGGS2384	16.831
AhTE0654	20.661
TC11H06	23.313
AhTE0908	25.614
AHGS1703	26.903
AHGS2539	28.286
EE22	33.263
AHGA161495	35.378
AHGA161468	38.79
AHGA161510	40.161
AhTE0659	41.931
GM1445	42.712
Ai04B2241	43.748
AGGS1601	45.026
AhTE0945	45.582
AhTE0903	46.029
AhTE0923	46.663
AGGS1795-1	47.439
AhTE0777	48.132
AhTE0906	49.085
AGGS1054	49.65
Ai04B4849	50.119
AhTE0882	50.525
AhTE0936	51.077
AhTE0416	51.442
AHGS1552	51.691
AhTE0237	51.935
AGGS2488	52.384
AGGS1783	52.842
AHGS1617	53.189
AHGS1579	53.439
Ai06B31304	53.846
Ai04B9397	54.155
Ad05A20478	54.623
AGGS1750	54.974
AHGS1311	55.134
AHGS0390	55.181
AHGS0288	55.253

AGGS1370	55.477
AhTE0335	55.702
AGGS1101	55.971
AGGS1324	56.528
Ai04B19238	57.356
AGGS734	58.113
AhTE0261	58.469
AGGS1512	59.049
Ai04B5526	59.715
GM2246	60.118
pPGSseq17F6	61.239
AGGS2251	62.237
GM1959	63.369
Ad04A12402	64.499
PMc348	65.069
AGGS1102	66.162
Ai04B12763	66.891
AGGS1613	67.947
AhTE0191	69.438
IPAHM105	69.679
AGGS2135	70.975
AGGS0284	71.058
AHGS1773	72.241
AHGS1937	74.458
AGGS1621	80.584
AHTE0189	81.813
AhTE0959	84.784
AhTE0639	85.736
AhTE0107	88.229
AGGS2509	89.762
AhTE0796	91.916
GM2106	93.32
PMc660	97.142
EM142	97.325
pPGSseq15C12-1	100.32
AGGS0396-2	101.86
Ai04B20539	107.09
AhTE1009	109.37
AHGA176194	117.59
Ah325	125.04

-Chromosome B05

Ad05A19349	0
AHGS1342	4.507
Ai05B28050	9.856
Ai05B32361	12.532
GA72	19.59

Ad05A20643-1	21.869
AhTE0756	22.317
AHGS1770	23.936
AGGS1183	25.824
AGGS0673	29.648
AhTE0806	33.38
TC6E01	33.862
Ad05A18236	34.384
AGGS2147	34.46
AHGA152194	37.414
AHGA152207	39.473
AGGS0973	39.873
pPGSseq9G5	41.157
AhTE0119	41.378
AHGS1228	41.634
AHGS1457	41.865
IPAHM354	43.406
pPGSSeq15D6	44.161
AhM082	44.613
GM1555	45.833
AGGS1632	47.426
Ad05A19244	47.696
Ai05B15046	47.997
AHGS2568	48.515
Ai05B26922	48.806
Ai05B25638	49.634
AGGS1299	49.821
Ai05B19811	50.783
AGGS0243	51.764
AHGS1624	52.875
AGGS1754	53.376
AHGS2141	53.743
AHGS1188	54.145
AGGS2115	54.404
AGGS1481	54.933
AGGS1262	55.244
AHGS1203	55.63
AHGS2795	56.14
AGGS0244	56.341
AGGS1464	56.667
Ai05B12077	57.288
AGGS1691	57.59
IPAHM282	58.018
AHGS2195	58.559
AGGS1645	59.049
PM36-1	59.446
AHGS1682	60.01
TC5D01	60.344

AHGS2509	60.873
Ai03B33341	62.171
Ad10A3962	62.875
AGGS0979	64.074
AHGS1497	64.943
TC19E01	65.596
AHGA221480	65.969
AHGS1672	66.205
AhTE0553-2	67.144
AhTE0940	67.791
AHGA44686	68.269
AHGS1755	68.755
AHGS0729-1	69.315
AGGS2216	69.743
AHGS1532	71.362
AGGS2287	71.878
AGGS0311	72.465
AHGS1440-1	73.198
AhTE0211	74.432
AGGS0670	75.673
AHGS2241	76.054
Ad05A20185	76.992
AGGS2398	77.353
Ai05B5265	78.884
AGGS2272	79.343
AHGA363492	81.732
IPAHM73	82.379
AHGA363491	82.672
ARS590	82.905
AhTE0319	83.612
AhTE0446	84.725
AGGS0351	85.256
AHGS1750	86.808
AGGS0955	87.081
GM1539	97.01

-Chromosome B06

Ai06B32416	0
Ai06B29716	20.444
AHGS1267	27.104

-Chromosome B07

AGGS1513	0
AGGS1620	2.272
pPGSSeq13B7	31.179
Ai07B12485	34.373

AGGS2425	40.619

-Chromosome B08

AhTE0477	0
AGGS1197	13.349
AHGS2602	15.79
Ad90F2	16.766
AhTE0658	19.526
AHGS1470	21.101
AGGS2186	22.94
ML1G04	25.633
Ai08B2682	29.97
AhTE0824	34.779
pPGPseq5D1	47.078

-Chromosome B09

AHGA161466	0
Ai09B5213	22.538
Ad10A4326	28.973
AHBGSI1008H04	37.378
AhTE0852	42.25
AhTE0913	46.042
AhTE0696	49.872
AGGS1654	58.369
AGGS1569	70.794
AGGS1013	79.513

-Chromosome B10

AHTE0874	0
AGGS1431	14.105
AGGS0815	14.648
GM2032	17.945
GM2165	19.265
IPAHM475	21.772
AGGS1728	23.627
EE16	24.335
AGGS2576	25.394
AHGS1446	25.745
AHGS1669	25.958
AHGA214492	26.237
AHGS2543	26.485
GNB0486	28.654
AGGS1355	29.563
AGGS1194	31.849
AGGS1312	33.421

Ai10B6334	33.833
AGGS1243	35.321
AHGS0300	35.75
AhTE0718	36.275
AGGS0302	38.509
AhTE0761	39.274
AGGS1160	39.958
AHGS1602	40.169
AGGS1118	40.576
AGGS1076	41.331
GNB38	42.335
AHGS1275	42.796
AhTE0893	44.682
Ai10B10082	45.308
AGGS0333	45.577
AGGS1124	46.058
AGGS2262	46.476
AHGS1595	46.887
Ai10B12455	47.162
AGGS0675	47.636
AhTE0162	48.116
AGGS2224	48.572
AHGS2073	48.766
AGGS0627	49.097
AHGS1395	49.458
AhTE0359	49.802
AHGA178360	50.134
AGGS1795-2	50.504
Ai10B9706	50.642
AHGS1626	50.792
AGGS1442	50.981
AHGS2191	51.217
AGGS1639	51.641
AHGS1564	51.814
AGGS1359	51.956
AGGS2371	52.069
AGGS2535	52.238
AHGS1696	52.343
AGGS2564	52.771
AGGS2407	53.285
AHGA75538	53.784
AHGS2787	53.948
PM675	54.158
Ai04B17746	54.493
AGGS1307	55.491
AGGS1393	55.767
AHGA72558	56.221
AGGS0058	56.798

AGGS0617	57.069
AHGS1903	57.685
Ad10A15687	57.967
AGGS0429	58.459
AhTE0709	58.821
GA156	59.962
AGGS2368	60.455
AGGS2494	60.634
Ai08B8719	61.656
AHGA195553	62.327
AGGS1425	63.225
AHGA61572	63.696
AGGS1579	64.327
pPGPseq7H6	64.791
AHGA61563	65.928
AhTE0918	68.391
AHGS3715	68.585
AGGS1453	69.769
AhM062	69.99
AGGS2555	70.881
AHGA75537	71.692
AhTE0586	72.839
GNB320	74.658
Ad10A18338	75.591
AHGA72569	77.989
AT79	85.903
AHTE0006	105.11
