# Supplementary material for: Development of SSR markers and identification of major quantitative trait loci controlling shelling percentage in cultivated peanut (Arachis hypogaea L.)
Source: Theor Appl Genet. 2017 May 15;130(8):1635–48. doi: 10.1007/s00122-017-2915-3 (PMC5511596; doi:10.1007/s00122-017-2915-3)
Supplement: Supplementary file 7 — Supplementary material 7 (PDF 21 kb) [file 122_2017_2915_MOESM7_ESM.pdf]

Table S7 Statistics analysis for shelling percentage in the progeny of a residual heterozygous line RIL 15-71126.

a. Means of shelling percentage in the residual heterozygous lines

Genotype	Mean(%)	N	Std. Deviation
A	78.45	5	2.04
H	75.62	17	2.80
B	72.82	9	2.79
Total	75.27	31	3.22

b. ANOVA of shelling percentage in the residual heterozygous lines

	Sum of Squares	df	Mean Square	F	Sig.
Between Groups	106.50	2	53.25	7.302	0.003
Within Groups	204.19	28	7.29		
Total	310.69	30			

c. Multiple comparisons of shelling percentage in the residual heterozygous lines

Genotype	Mean(%)	0.05	0.01
A	78.45	a	A
H	75.62	b	AB
B	72.82	c	B

0.05 and 0.01 indicated the significance level
