# Supplementary material for: Development of SSR markers and identification of major quantitative trait loci controlling shelling percentage in cultivated peanut (Arachis hypogaea L.)
Source: Theor Appl Genet. 2017 May 15;130(8):1635–48. doi: 10.1007/s00122-017-2915-3 (PMC5511596; doi:10.1007/s00122-017-2915-3)
Supplement: Supplementary file 2 — Supplementary material 2 (PDF 26672 kb) [file 122_2017_2915_MOESM2_ESM.pdf]

Table S2 SSR markers developed in the genome sequences of *Arachis duranensis* and A. *ipaensis*

ID	Chromosome	SSR nr.	SSR type	SSR	Size	Start	End	FORWARD PRIMER (5'-3')	REVERSE PRIMER (5'-3')	Product (bp)
Ad01A6	Aradu.A01	6	p4	(ATTT)6	24	73570	73593	TGTACCAAAATTGGTGTGGC	CGGTATAATTGTTCACCAACAAAA	222
Ad01A10	Aradu.A01	10	p3	(ATC)5	15	97647	97661	TGAAGGCTCAAAATCCCATC	CCATGGCTCCAGCAATAGTT	131
Ad01A21	Aradu.A01	21	p3	(ATT)5	15	116476	116490	TTCAAACGCTGACATTTTGG	AAAAGGGGGCTACGATGACT	247
Ad01A22	Aradu.A01	22	p3	(AGA)5	15	116879	116893	AGTCATCGTAGCCCCCTTTT	TTTTGGGATGTTCAGGAATTG	269
Ad01A25	Aradu.A01	25	p3	(TGT)5	15	118917	118931	AGCTGAAGATCGTTGCAGTG	GAACAAAATTAGGGTACCCACC	197
Ad01A26	Aradu.A01	26	p2	(AT)8	16	121263	121278	GTCTCAGCCTCTACAACCGC	TGTTCTTCCAATGTTTTTAGGTTTT	237
Ad01A29	Aradu.A01	29	p2	(CT)28	56	126629	126684	CACCCCAAGGAAATAGCAGA	CCATGGACATAGACCAACCC	205
Ad01A31	Aradu.A01	31	c	(T)10atgtttaca	55	129445	129499	GCTGTGCTTCTTAAGCAATAATTT	TGAGAATTGTGGTGTTGGTGA	258
Ad01A32	Aradu.A01	32	c	(A)10g(A)10	21	129712	129732	CTCTCAAGCTTGTCAATGGC	CTAACCATGTTGGCTTGGCT	270
Ad01A35	Aradu.A01	35	p3	(CCA)7	21	133278	133298	CCATTGAGGGACGCTACCTA	GCCCCATTCCTAGTTGTGAA	215
Ad01A36	Aradu.A01	36	p3	(TAT)7	21	133429	133449	TTCACAACTAGGAATGGGGC	AGTGCAGGCCTAGAACCAAA	119
Ad01A39	Aradu.A01	39	c	(TAC)5(TAT)	54	151500	151553	CGAATGTACCATTGTCATCAGC	CCTCACAGTCCCCAAATACC	266
Ad01A43	Aradu.A01	43	p2	(AG)12	24	158062	158085	ATTCTTGAATCCCATGCCAA	CCAAAACGAATTCCACCACT	172
Ad01A49	Aradu.A01	49	p4	(TTAT)5	20	174660	174679	TTACTCCATGGCCACAAATG	TGGATAGTGGGCTAGTGGCT	234
Ad01A52	Aradu.A01	52	c	(A)15caaagga	143	179051	179193	GGTTGGACTAGATCTTATGAAGTATGA	TCCCAAATCTGCTTCGTTTT	231
Ad01A61	Aradu.A01	61	p2	(AT)8	16	197936	197951	GTCGCCCAAGGTTATGAAAG	TAAGTATTTGGTGAGGCGGG	260
Ad01A69	Aradu.A01	69	c	(T)12gatttggtg	53	207691	207743	CCATTTTCTTCATGTTGCCC	TGGTTCGAGTAAGGCACAAA	268
Ad01A71	Aradu.A01	71	p2	(AT)7	14	212046	212059	TCCCATTTAATTGCTTTAAAATTC	GCTTGCAAACGTGCTACAAA	260
Ad01A72	Aradu.A01	72	p3	(AGG)5	15	219179	219193	AAATGAAGGAGCGGCTATGA	TCTCTTGATGCTGTGCTGCT	135
Ad01A75	Aradu.A01	75	c	(T)10cag(T)11	24	227653	227676	GCCTTTATTTTAGGCAGCCA	AAAAATTGCAAGACAAAAACACA	148
Ad01A78	Aradu.A01	78	p2	(AC)7	14	230082	230095	TCATCCTCCAGCTTAGTCCG	CCAAAGTGACAACATTGTGGA	251
Ad01A79	Aradu.A01	79	p4	(CTAT)5	20	231690	231709	ACTCTGACAGGGAAAGCAGC	ACTCCGGTCGCAATAACTGT	257
Ad01A83	Aradu.A01	83	p2	(AT)7	14	242628	242641	TCGGTAATCACTATCCAACACG	ATGCCAAAGATGGCGATAAG	155
Ad01A87	Aradu.A01	87	p2	(TC)10	20	244699	244718	TGCCATATTGGCATATTTACACA	TTTTGTGCAATGTGACCTTTTC	156
Ad01A88	Aradu.A01	88	p2	(CT)17	34	248235	248268	CGTTTATCTCTCCCTCGCTG	GACACATAACGGGGACGACT	225
Ad01A90	Aradu.A01	90	p3	(AGG)5	15	252509	252523	AATGAAACACGCGAAGAAAAA	CCAACTTACATAATGGCACACG	101
Ad01A93	Aradu.A01	93	p3	(ATA)5	15	256247	256261	GAAAAGGAAATGATCCATTTGTG	AAATTTCGCTGCCTCCTAGC	279
Ad01A94	Aradu.A01	94	c	(A)11ggttac(T	121	256638	256758	TGGCTCTCTAGACTTTTTCATCA	AACATGTGAGTAGAGAAGGCAGC	181
Ad01A95	Aradu.A01	95	p2	(AT)6	12	257856	257867	TCATCATTCACGGTTTGTTTT	GCATTTGGAAGCATTTTTGTC	154
Ad01A96	Aradu.A01	96	p2	(TA)6	12	258153	258164	CCGCGCCTAATTTTTCAATA	CGTCTTTATCTGAGTGGTTCCC	176
Ad01A98	Aradu.A01	98	p2	(AT)12	24	259239	259262	TTCACGTGACTAACTCCTATAGTCTTG	GGAAGCTATTACCATGAGGTAGC	280
Ad01A99	Aradu.A01	99	p3	(GAT)7	21	261285	261305	TCTGTGATTGCACTTGAGGC	TCATCAGCAATCGTAGCAGG	243
Ad01A101	Aradu.A01	101	p2	(AT)8	16	271863	271878	GCGGATGTGGTGACAGAGTA	AAAACCTTGCAAAAAGCTTCA	265

ID	Chromosome	SSR nr.	SSR type	SSR	Size	Start	End	FORWARD PRIMER (5'-3')	REVERSE PRIMER (5'-3')	Product (bp)
Ad01A102	Aradu.A01	102	p2	(AT)6	12	274138	274149	TTCGATTCAACACCCTATCG	CCTTCCCTTGGCTTTTCTTT	280
Ad01A104	Aradu.A01	104	p2	(AT)11	22	283000	283021	GAATGCATTTCAGAATCGTTCA	TGTTCTCTCTTTTGGCAGCA	209
Ad01A107	Aradu.A01	107	p2	(GA)8	16	287711	287726	GGCTTTGTGTTTGGCTTGAT	TTAGTTGTTTCCGTTGCGTG	175
Ad01A110	Aradu.A01	110	p3	(AAG)5	15	292111	292125	CCTCCTCATCTTCTGCTGCT	GCTGCACGTTCTTCTTCCTC	190
Ad01A111	Aradu.A01	111	c	(GAAGAC)5g	70	292272	292341	GAGGAAGAAGAACGTGCAGC	CTCCACTCAAACGGTTCCTC	182
Ad01A115	Aradu.A01	115	p3	(AGA)5	15	305216	305230	TTCAAGCCAAATCCATAGGC	CATTTCGTCATTTTGTGCAAG	130
Ad01A122	Aradu.A01	122	p3	(GAA)5	15	329393	329407	GAACCGCCATGTTTTCTTGT	ACCTTCCTTCTCCCTTGCAT	255
Ad01A123	Aradu.A01	123	c	(CT)8ttctctcga	51	329773	329823	TCGTTAGGGCGGTGATCTTA	GCATTATCTGGCGGGAACTA	247
Ad01A125	Aradu.A01	125	p2	(AT)6	12	341598	341609	CCAAACTTGTCTTCAATTCCC	TGACCCAATTTCTCTTCCCA	210
Ad01A126	Aradu.A01	126	p2	(AT)13	26	344192	344217	CGTTGGCAGTTTTATGCGTT	GGCGAAGATGTTTGTCTTGG	280
Ad01A127	Aradu.A01	127	c	(AG)9ggttttgag	62	345146	345207	CGCCATGAGCGTCTTTTTAT	CCAAACCCAGAGGTGAGAAA	142
Ad01A132	Aradu.A01	132	p3	(CAG)8	24	351845	351868	AATGAATGCTAATGCCGACC	AACCCAAACAAGGTGTCAGC	278
Ad01A141	Aradu.A01	141	p3	(AGG)7	21	381586	381606	CTGGCTCGGAGAGTAGTTGG	GGCAAAGAAGAATGCTCAGG	206
Ad01A142	Aradu.A01	142	p2	(TA)6	12	385574	385585	CATAGTTGGTGGGGGCATAG	TCAGTCCCAACCCCAAATTA	179
Ad01A143	Aradu.A01	143	c	(T)10atgttttaaa	60	385874	385933	TGATGATTCTCTCATTGGCG	TCAAACACTTCAAACCACAAGC	263
Ad01A146	Aradu.A01	146	c*	(TTC)6(TCTT	61	399137	399197	TATAAACACGCCGTTTCACG	TTCTGGTGATTTTGATGAAGGA	197
Ad01A148	Aradu.A01	148	p3	(TAT)6	18	411168	411185	GGGTTGAGCACAATCCAGTG	ATTTGCATTTCGCAGATTCC	242
Ad01A149	Aradu.A01	149	c	(A)10gaaagaaa	118	413161	413278	TTGAGGAGTGTCCAATTCCC	TAGGGATCACCCATGTGCTT	189
Ad01A157	Aradu.A01	157	p3	(ACT)6	18	434167	434184	CATTTTCCCCCTCTTCCAAT	GATGCCAAATGTTGTTGTGC	106
Ad01A158	Aradu.A01	158	p2	(AT)8	16	437228	437243	TTATTCCATGAGGTGAGGGC	TGAGCAAAGATAACGGAGACC	148
Ad01A159	Aradu.A01	159	c	(A)10tctaaaact	100	439534	439633	CCATTCCTGTCCGTCACTAAA	GATAGAGGCAGTGGTTAGCG	280
Ad01A160	Aradu.A01	160	p2	(AT)6	12	440205	440216	TGGCACATGAAACCTAACGA	TGACAATATGCAATTATATGCTGA	190
Ad01A167	Aradu.A01	167	p3	(AGG)5	15	457092	457106	CCGCCATCAAAAGAAGAAGA	AAAGGGTCCAGGAGGCTAAG	220
Ad01A170	Aradu.A01	170	p2	(AG)7	14	465810	465823	GCCAATGGTGGGAAGAAGTA	TCCATCACCTCCCTCTTTTG	214
Ad01A171	Aradu.A01	171	p3	(TTA)5	15	468214	468228	TCATAACCTCCGCCTTCAAC	GCACTTGTGGGAAAATTGCT	263
Ad01A175	Aradu.A01	175	p3	(ATA)7	21	479268	479288	TGATTAGCCATTCAAATCTCACA	AATGCATGTACCATGTAGACCTT	271
Ad01A179	Aradu.A01	179	p2	(CT)6	12	488805	488816	TTTTGCTCAAAAATGGTCCC	GTGGTGGTGGGAGAAAACAC	263
Ad01A181	Aradu.A01	181	p3	(TCT)5	15	490629	490643	AAGCCCAACAAAATACACGC	AGCAGCAGAAGATGAGGAGG	279
Ad01A182	Aradu.A01	182	c	(AAG)5aggaag	55	490947	491001	CCTCCTCATCTTCTGCTGCT	TGCATTTCGAACCGTTACTG	241
Ad01A185	Aradu.A01	185	c	(T)12ctcgggtaa	46	499303	499348	TTCTTCTGCTGCATTGCCTA	TCCAATTCATTTCAACAGATGC	110
Ad01A186	Aradu.A01	186	p3	(ATA)16	48	499603	499650	AACGAAACCAAAATATCAGTGGA	CCACTCGACCAATCCAAGTT	252
Ad01A188	Aradu.A01	188	p3	(AAT)5	15	500834	500848	AACATGCGTATGTCTAATGTGTTT	AGCATGGACAATAAAATGCAA	208
Ad01A191	Aradu.A01	191	c	(TTA)13g(TA'	58	502026	502083	GCTGACCACTCGACCAATCT	TCTTTTTGTTGTGGTTGGACA	157
Ad01A196	Aradu.A01	196	p3	(AAC)7	21	514962	514982	CAAGCAGAAGCAGAATGCAG	CTTCTCCTTGTTCGGTCGTC	254

ID	Chromosome	SSR nr.	SSR type	SSR	Size	Start	End	FORWARD PRIMER (5'-3')	REVERSE PRIMER (5'-3')	Product (bp)
Ad01A199	Aradu.A01	199	p3	(AAT)13	39	521265	521303	AATTTCCTCACACCACCGAC	TAATTGGGGGATTGCTTTTG	140
Ad01A202	Aradu.A01	202	p2	(TA)6	12	521743	521754	TAGACACATTGTACCCCGGC	CTAGCTTCGCCTTGGAGAGA	156
Ad01A204	Aradu.A01	204	c	(T)11cttcctgata	89	524334	524422	GACGAAACAAAAATCATAAAACCC	TCCCAATACTCCATCTTGCC	196
Ad01A205	Aradu.A01	205	p3	(ATT)6	18	527591	527608	ATGGCCAAACACCTCCACTA	GCTCCTAAATGGGCCTCAA	279
Ad01A207	Aradu.A01	207	p5	(AAATA)5	25	532708	532732	TTTACTGTCAATTTACTGGTATGAGC	TCAAAATAATGGCGTCCAAA	199
Ad01A208	Aradu.A01	208	p3	(GTT)7	21	533852	533872	GCCACCTCAAGGAACTGGTA	TCGAGAAGCAAGAGTGAGCA	205
Ad01A209	Aradu.A01	209	p2	(CT)15	30	535369	535398	CGGGAGGTGCAGTAACTTGT	ACAGCACCCTCAAGATTTGG	260
Ad01A211	Aradu.A01	211	p2	(AC)6	12	543163	543174	TGCTTTTCCCACTTTTCACA	GAACTGTGACCTTGTGGCCT	233
Ad01A213	Aradu.A01	213	p2	(GA)10	20	551210	551229	AACTTTAACGGAAATGGGGC	CATTGGGCCTCGTGATTAGT	260
Ad01A215	Aradu.A01	215	p2	(AT)9	18	558672	558689	GGGTGCCTTTTGTACGTCTT	TAGGCGCCATGAAGAATAGG	241
Ad01A226	Aradu.A01	226	p3	(CTG)5	15	585227	585241	CAGTGGTGTTGGTGATCGTC	AGAAACATCTCTGAGGCGGA	276
Ad01A230	Aradu.A01	230	c	(T)14aaaaaaag	97	590487	590583	ACAAGATGTGAAGAGTTGTACGTT	TCCGACGGTTTAATGTAATAATCTAAT	280
Ad01A234	Aradu.A01	234	p3	(ACA)7	21	601782	601802	CAAATACCAAACCCGTGGAT	CGTTTGATTCATGTAGCCGA	263
Ad01A237	Aradu.A01	237	p2	(GT)6	12	604857	604868	AAGAATTTAGCATGTGATGCCTT	CATTGTGTTAACAACGGTTGAA	267
Ad01A240	Aradu.A01	240	c*	(ATT)5(ATTA	44	620713	620756	TGGAGAGGGTTTGGACTTTG	CAACAAAGGAGAAGGGGACA	280
Ad01A241	Aradu.A01	241	p2	(GA)17	34	620891	620924	TGTCCCCTTCTCCTTTGTTG	AGCATAAACCCCGTTCTGTG	122
Ad01A242	Aradu.A01	242	p2	(AG)11	22	621197	621218	TGTTTTTAGGCGCTTTGCTT	ATGCCGAAGATGGAGAGAGA	279
Ad01A256	Aradu.A01	256	p2	(GA)9	18	659457	659474	TGACTTACCGGTGGTGATGA	TTAGCGTCGGATTTTTCGTT	277
Ad01A257	Aradu.A01	257	c	(TAT)8tg(TTA	47	663062	663108	TGGACTTTGGGATTGGAGAG	TTTCTGATTTTGCCTCCGTT	271
Ad01A258	Aradu.A01	258	p2	(AG)11	22	663261	663282	ATGTCCCCTTCTCCCTTGTT	TTGAAATCCAAAAGGCGAAC	209
Ad01A259	Aradu.A01	259	p2	(GA)14	28	663555	663582	TGTTTTTAGGCGCTTTGCTT	TCAATTAGGAGGGAGAGGCA	105
Ad01A262	Aradu.A01	262	p3	(GAG)7	21	676194	676214	AAGAAATCACAATGGGCTGG	ACTTGAGGTTTGCCGACTTG	204
Ad01A267	Aradu.A01	267	p2	(TG)14	28	698644	698671	TTTAATTCGAGCAGCATCCC	TGCCATTATGGGTCACAACT	168
Ad01A269	Aradu.A01	269	c	(TCTT)5tatttct	43	701696	701738	GTTCGAACCAAAACCACACA	ACTTTACACTGCTGCTCCGC	233
Ad01A273	Aradu.A01	273	p6	(TTATTG)5	30	720401	720430	TGCCTTCTCTTGCTTATTTGC	TGCATTTGGTCTTCAATCCA	244
Ad01A276	Aradu.A01	276	c	(GT)8(AT)8	32	727474	727505	TTAAGATGGGGCAGGAAGTG	GGCATCATAAACTCCAGCGT	232
Ad01A278	Aradu.A01	278	p2	(AT)9	18	736451	736468	TCACTTCAAGAAGGAAACAGCA	CATTAAAGGTTTCACTGCCCA	266
Ad01A283	Aradu.A01	283	p3	(ATT)8	24	753271	753294	GTTGACGTGGCATGCTTCTA	ACGGTGTCCGAAAAGAAAGC	268
Ad01A286	Aradu.A01	286	p2	(AT)6	12	759076	759087	TTTTTGTGATGTGGGGATTT	GTCCAACCCCAGCTTGTAAA	122
Ad01A288	Aradu.A01	288	c	(T)11ggaaacttt	92	760801	760892	TGTGGAACCCAACTAAACTGTG	GCAATGCCAAAGATGACAAG	257
Ad01A292	Aradu.A01	292	p3	(TAA)5	15	769875	769889	CGACGCACATCCTGATAAAT	CCAGTTGCCAAAAACCACTT	119
Ad01A293	Aradu.A01	293	c	(AT)7(A)12	26	770132	770157	CCTGAAAGGAAGAGAGGGGT	CACACAGAAAGGAGCCATGA	142
Ad01A296	Aradu.A01	296	p3	(GAA)8	24	775581	775604	CAAGAAAAGACCCCAAGCAG	AGGTTGGTTGTTGAGCCAAG	174
Ad01A307	Aradu.A01	307	c	(GATGGG)5ga	73	797990	798062	GGGAAGGAGAAGGAGAAGGA	TTCCCAATCTGTTTCATCCC	156

ID	Chromosome	SSR nr.	SSR type	SSR	Size	Start	End	FORWARD PRIMER (5'-3')	REVERSE PRIMER (5'-3')	Product (bp)
Ad01A308	Aradu.A01	308	p2	(AT)7	14	803936	803949	CAAACAATAAGTACCACACACAAAAA	GCATTTGAGCTTTGGCCTAC	247
Ad01A318	Aradu.A01	318	c	(AAATA)5ttag	118	847422	847539	CTCTTCTTTGACTAACAATGAGCA	TTCCTTCTTTAGAGATATGTGAATTTG	201
Ad01A319	Aradu.A01	319	p3	(TTC)5	15	853374	853388	TGTAAACACGCGATTCCTCA	CCACAATGAGGATGAAAATGC	138
Ad01A320	Aradu.A01	320	p3	(AAG)5	15	853914	853928	TTGAGAAAGTAAAATAAAAAGGCAAA	TCCAATTAAGTGGCTGAGGTG	277
Ad01A321	Aradu.A01	321	p3	(TTA)10	30	855727	855756	TTTGAATGATGGTTTGGGGT	TCTGCACATACGGGTGAGAG	121
Ad01A326	Aradu.A01	326	p2	(TA)6	12	876807	876818	TTTGTGCACTATGGAATTGGA	CGTGTACACCAAAATTAATTACCAA	268
Ad01A327	Aradu.A01	327	p2	(TA)7	14	877725	877738	GTGGCACTGGACCTTTTTGT	AAAAATATAGCCGAACCCCC	140
Ad01A333	Aradu.A01	333	p3	(CTC)5	15	893065	893079	GCTGCACGTTCTTCTTCCTC	GAAGGAAGTGGTGGTGTTGG	100
Ad01A335	Aradu.A01	335	p2	(TA)10	20	900690	900709	TTGACTTTGATGACAATGATAACAAC	CGGGCTTTTGAACAAGTCTC	275
Ad01A336	Aradu.A01	336	p2	(AT)7	14	905693	905706	TCCCATGTCAAGTGGAATGA	AAAGATCCATTGTTTTGGCG	259
Ad01A337	Aradu.A01	337	p2	(AG)6	12	911279	911290	CACTGCAGCAGAATTCGGTA	CCGAGTCCCAACTTCATTGT	200
Ad01A348	Aradu.A01	348	p2	(TC)6	12	935862	935873	CTTCTTTTCCCATGTGGTCC	AGACCAATCATTGCCCATGT	134
Ad01A350	Aradu.A01	350	c	(TTTTA)5tatat	72	940700	940771	ATGGAGTGGCAACCCCTACTG	GAACTGCCTCCTTTGTCAGC	189
Ad01A351	Aradu.A01	351	p3	(ACC)5	15	941314	941328	ATCCTCAATTCCTCGGCTTT	ACCCACTCCTCCTTCGTCTT	213
Ad01A352	Aradu.A01	352	p3	(CTT)6	18	942039	942056	GCCAAGTGGTGTTGATGTTG	AGCAGCACCATTCTTTCACC	193
Ad01A353	Aradu.A01	353	p2	(TC)10	20	942720	942739	TTTTCCCTTCTGCGGTATTG	ATGAGTCTCCCCAAAAGCCT	260
Ad01A354	Aradu.A01	354	p2	(CA)6	12	942892	942903	TTTTCCCTTCTGCGGTATTG	ATGAGTCTCCCCAAAAGCCT	260
Ad01A358	Aradu.A01	358	p2	(TA)7	14	950531	950544	TTCATTCCCTCCTTTACTCTGC	GAACGAAAGACGACATTCCC	234
Ad01A370	Aradu.A01	370	p2	(AT)7	14	973939	973952	AAAACTCGTCATCGTATAGAATTATCC	GCGTTAATTTGAATATAAGGCAAAA	177
Ad01A371	Aradu.A01	371	p2	(TA)6	12	974820	974831	CTGTTTGTGCCAAAAGGTTG	TCGAATTTGTTTGTGTTGTCATC	206
Ad01A374	Aradu.A01	374	c	(TC)7cctctctcc	47	983905	983951	CTCTCGCTAACCCTCTCCCT	GGGCTGATGGAGTCGTAGAG	190
Ad01A375	Aradu.A01	375	p3	(TAT)5	15	984440	984454	TTGTGTTGTTGATTTTGAATTGG	TGTCTGCAAATAAGAAGTCATCC	246
Ad01A376	Aradu.A01	376	p3	(TTC)5	15	990419	990433	TAATATGCGAGACAGCGTCG	AATTCCTGAGCTTCGGTTCA	198
Ad01A377	Aradu.A01	377	p2	(TA)6	12	990944	990955	GTCCAACTGCCACTTAGGCT	TGTGCGGAATTGAGTCTTCA	194
Ad01A379	Aradu.A01	379	p2	(AG)6	12	993264	993275	AAGCCTCTTCTTTAAAGCCCAT	TTCTCGTGTCTCGTGGTGTC	220
Ad01A381	Aradu.A01	381	p3	(ATC)5	15	995963	995977	AGCCAACCTCATTGTCCTCA	GGGGTTGAAGGGAACAAAAG	193
Ad01A383	Aradu.A01	383	p2	(AT)7	14	1002334	1002347	AGCGTGATTTTCATTGTGGA	GAGAAGAATGAGTTGGAGTCAGG	277
Ad01A384	Aradu.A01	384	p3	(TTC)5	15	1003891	1003905	TCCCTTATGGGCTGTGAGTC	CACGAAATCAAACCAAACCA	136
Ad01A390	Aradu.A01	390	p2	(TC)6	12	1009815	1009826	CATGGAAGAAAGAAAGCTCCA	TTCGTCCTCCTCTTCTGGTG	265
Ad01A396	Aradu.A01	396	p3	(AGG)7	21	1030453	1030473	CTCGGAAAGTAGTTGGGCAG	TTATTTCCGAGTTGTTCCCG	272
Ad01A397	Aradu.A01	397	p2	(GA)6	12	1036533	1036544	AATTGACACCTGGGCTTTTG	TTGCAGCGACCAATTTACAG	223
Ad01A404	Aradu.A01	404	p3	(ATA)5	15	1052826	1052840	ATTTCGGATGCTTTTGTTGC	AATTTTCTAGCAAAAACGGTTG	270
Ad01A405	Aradu.A01	405	c	(TA)6ttgggatct	97	1054686	1054782	ATGAGGCTGCTAGATCCCAA	CAAATATCTTATTGGCTCTCTAACAAT	228
Ad01A406	Aradu.A01	406	p2	(TA)6	12	1055780	1055791	CAACAATTGGAATGCTAGAGAA	TTTTGTTTTGCCCGTTTTTC	253

ID	Chromosome	SSR nr.	SSR type	SSR	Size	Start	End	FORWARD PRIMER (5'-3')	REVERSE PRIMER (5'-3')	Product (bp)
Ad01A407	Aradu.A01	407	p2	(AT)7	14	1058251	1058264	TGCAATACCACCACAACACA	AAGTGATCGGCCAAGGTATG	277
Ad01A421	Aradu.A01	421	c	(TC)6aagtgaat	71	1083177	1083247	TGTGCAAACAAAGTAACAAACTCA	GTAGTGTTGCTCGTTGGGGT	270
Ad01A422	Aradu.A01	422	c	(T)11ccatttaaa	82	1083673	1083754	TGCTGCTTCTTCTTCTTCTGC	CAAGCTCCATGCCTGAAAAT	245
Ad01A428	Aradu.A01	428	p2	(AT)10	20	1090686	1090705	ACAATTGCAGGCAGAAGGAG	AGGGTGTTACATACCGCACC	226
Ad01A430	Aradu.A01	430	c	(T)11caattttgg	39	1094500	1094538	CAAAAGCAATTGCCCATTTT	TTCTTTTCAACGTCCAACCC	266
Ad01A431	Aradu.A01	431	p2	(AT)7	14	1095135	1095148	GGAGTTCATTTTGATGCCCA	TTTTGGTTACATAGCATTATTCGT	146
Ad01A434	Aradu.A01	434	p2	(AT)8	16	1105857	1105872	TTTTTCTTCTCTGTCGTCGTAGG	ATTAAAATTAGACCGGATAAATCG	203
Ad01A439	Aradu.A01	439	p2	(TC)19	38	1114169	1114206	CTGGAACTTCCTCCATTCCA	CTGCCAACAAAACAAAAGCA	264
Ad01A444	Aradu.A01	444	p3	(CAT)6	18	1139500	1139517	ACCACCTCATTCCCCTTCTT	TCAGTTCGAAAATAGTCACACAAA	233
Ad01A445	Aradu.A01	445	p3	(CTT)5	15	1139815	1139829	TTGCATTTGGACTCGTTGTC	TTCACTCAAAAATGAACCGAAA	251
Ad01A447	Aradu.A01	447	p2	(TA)8	16	1147927	1147942	GACAGCTGGGAATTGGTGTT	CTTTAATGGGCCTCTCCCTC	237
Ad01A449	Aradu.A01	449	c	(TA)6aaataaaa	149	1153677	1153825	TGCCTCTCTCCAATCTCAGG	TTGGAAGAATTTTGAATGTTAATAAGG	278
Ad01A451	Aradu.A01	451	c	(T)10cttttaaatt	96	1158582	1158677	CGTTATCATTGCCATCACCA	CACAGTCCTCTGGAACAGCA	216
Ad01A453	Aradu.A01	453	p3	(CAT)7	21	1159967	1159987	AAGCGATAACAACCCACCAC	GTGTCTGAAGGGACCCAGAA	234
Ad01A455	Aradu.A01	455	c	(AT)8tacgatttc	78	1162617	1162694	TTTGGTTCCCCAAATAGCAA	GGAGAGTCGAAATTCAATTAGGTT	244
Ad01A457	Aradu.A01	457	c	(T)16caaaaa(T	41	1166063	1166103	AGCTAGCATCTCAAAATCCACA	TCAATTTTGTATGCACGGGA	208
Ad01A467	Aradu.A01	467	p3	(AAT)7	21	1187212	1187232	CCTAAACTCCGACCAGCATC	TTGCACTTCAGCAATTGAGC	157
Ad01A469	Aradu.A01	469	p3	(CGG)5	15	1194726	1194740	GCAGCCGATTCAGTCTTTTC	GAAGTTAAAGATGGCAGCCG	186
Ad01A470	Aradu.A01	470	p2	(TA)6	12	1194944	1194955	CGGCTGCCATCTTTAACTTC	CTTCATGCACATGATTCGCT	243
Ad01A471	Aradu.A01	471	p2	(TA)11	22	1195235	1195256	ACCTAGCCATCCTACGCAAA	GCAAATCAAATCTTGAACTGGTC	200
Ad01A472	Aradu.A01	472	c	(T)11ctcttcaatt	102	1197477	1197578	CCAAAAATTCCTTTCTTCTGC	CACCAGGCTCTATCTTTTTGG	245
Ad01A479	Aradu.A01	479	p4	(AAAT)5	20	1210305	1210324	AAAACAACCCCCATGCATAC	AGTCTTGGTTGCCAGTTTGG	107
Ad01A486	Aradu.A01	486	p2	(TA)8	16	1232952	1232967	TTTTGCAGGGGAAAACTACG	TCCTTTTTCATCACTTCTTTTGC	276
Ad01A488	Aradu.A01	488	p2	(CT)6	12	1237415	1237426	TGCATCAATAACAGAATTAACCCA	GGCGGTGTCTTTGTTTTTGT	267
Ad01A489	Aradu.A01	489	p2	(CT)7	14	1238304	1238317	GTCAATGTTGTGGACATCGC	AGATGTGCAGAAATTTGGGG	162
Ad01A497	Aradu.A01	497	p2	(AG)16	32	1273062	1273093	AAAATGCGATGGAATAACCAA	TTGCTTAAACTAGCAAGGAGGAA	269
Ad01A498	Aradu.A01	498	p2	(GA)25	50	1273290	1273339	CCCTGTAAAATCCTCGGACA	ACCCATTTCTTTGGTTTTGC	249
Ad01A499	Aradu.A01	499	p2	(GA)7	14	1280565	1280578	AATGGCAAGTATCCGCAGTC	CCTGCCTCGTTGCTTATCTC	119
Ad01A500	Aradu.A01	500	p2	(AT)8	16	1283351	1283366	CTGTGCCACATGTTTCTCTCA	CAATTGAAAATTACATCAAAATCCA	214
Ad01A503	Aradu.A01	503	p2	(TA)6	12	1296628	1296639	TTGTTTGTGTGTCGTCATCATT	CCAAAAGGTTGGAATGACTTTT	265
Ad01A506	Aradu.A01	506	c	(A)10gtgtgtaag	71	1298579	1298649	TTCATTGCATTTGGCCTTTT	GCACATATTGGGTTGCTTTCT	216
Ad01A507	Aradu.A01	507	p2	(AT)6	12	1300756	1300767	CCAACTTGGTCCACCACTCT	TTGATTTTGTAATTAATTCTGTGGG	122
Ad01A511	Aradu.A01	511	p2	(AT)6	12	1305788	1305799	CCTTTAATTTTATTTGAGTTGTGGA	ATTTCTAAGGCTTAAATTCAAAACA	280
Ad01A517	Aradu.A01	517	c	(A)10gtgaaatg	59	1322051	1322109	TGTAATAAAACCACGTGTGAAAA	TGGATGGTTCAATTTCTCAATG	271

ID	Chromosome	SSR nr.	SSR type	SSR	Size	Start	End	FORWARD PRIMER (5'-3')	REVERSE PRIMER (5'-3')	Product (bp)
Ad01A519	Aradu.A01	519	p3	(ATT)6	18	1330183	1330200	CGACCTGCGAGTTAAAAAGC	AAGGGATTGACATGCTTTGC	194
Ad01A522	Aradu.A01	522	p2	(AT)7	14	1334363	1334376	AAATGAATGTTATTTGGCCG	CATCATGTTTATGTTCTTTTGTTTCA	143
Ad01A524	Aradu.A01	524	p3	(TAA)19	57	1336198	1336254	TTTGCAATGTATTAGAAGAGTCAAAA	AGGCAAAGCAAATGCAAAGT	198
Ad01A525	Aradu.A01	525	p2	(AT)7	14	1337409	1337422	AAAAATTTCACCCAAATAAATCC	GCGGCCACAAATTAGTAAAAA	129
Ad01A528	Aradu.A01	528	p3	(ATA)5	15	1342860	1342874	GCGAAAATTAAATCGGATGG	CATTCATTTCTCTCACTCGCA	251
Ad01A529	Aradu.A01	529	p2	(AT)6	12	1352989	1353000	TGCTTTAGCTTAGCCGCATT	CATCTTTATTTAAGTGATTCCTTCTTG	241
Ad01A530	Aradu.A01	530	p3	(AAT)8	24	1354414	1354437	AAGCCTCCAAACAATTTCCA	AGCCATGGTATTTGGGTTCA	232
Ad01A532	Aradu.A01	532	p2	(AT)7	14	1358200	1358213	CACTTCAACACCCTTTGCAG	GGTGCAGTCGACTTTACGTG	200
Ad01A537	Aradu.A01	537	c	(CTT)5ctg(CT	135	1366813	1366947	ACAGAAGGAGATCGAAGGCA	AACACGCGCGTTACAAAAA	198
Ad01A539	Aradu.A01	539	c	(ATA)6ac(AA'	47	1368862	1368908	TTGTGACTTATTAGAGGGTTTTTGAA	TCTTGCGGTAAACTTATGAAAACA	245
Ad01A546	Aradu.A01	546	p2	(TA)8	16	1393466	1393481	CTGCACTTGAATTTTCACCG	TTGGAGCTTAAATGGATTTTGAA	199
Ad01A548	Aradu.A01	548	p3	(TTG)5	15	1399498	1399512	GGTTTGATCGGATGATTCGT	GAAGGGACGTGGCAATAAGA	247
Ad01A551	Aradu.A01	551	p2	(AT)9	18	1405427	1405444	TTTGAAGGAAGAGAAGTGAAATTAAA	TTTTCCCCTATTCTCTCTCCC	227
Ad01A561	Aradu.A01	561	p2	(AT)6	12	1423668	1423679	TGCAACCACATACCCTTTCA	TTCAATTTCAGTAACCATGTATTTG	231
Ad01A563	Aradu.A01	563	c	(A)15gaaaaaga	52	1425175	1425226	TTCAACGCCTGTCCTTAACC	TGTGCGTGTGTGTCTCTTCA	270
Ad01A565	Aradu.A01	565	p3	(GGA)7	21	1425694	1425714	AATTTTCCTTTCATGCGTGC	GTGTTGCTGTTCCTGGTCCT	222
Ad01A566	Aradu.A01	566	p3	(TAT)10	30	1426608	1426637	AGTTTACCTGCAAGCATGGG	TTTTCTTCCCTGTCAATCCG	205
Ad01A567	Aradu.A01	567	c	(TC)9a(CT)8	35	1428140	1428174	GTATGGGGAGGTCCAACCAT	TGCCAAATTAAATGTGCCCT	280
Ad01A568	Aradu.A01	568	p2	(AT)7	14	1430783	1430796	CGAAGAAAATCGGGATCTCA	CGATACCCTACCCGACTGAA	114
Ad01A570	Aradu.A01	570	p2	(TA)8	16	1433885	1433900	GCCTCAGATGAATCATGAATGT	CGACTTAATCTATCTGCCACCA	249
Ad01A572	Aradu.A01	572	p2	(CT)6	12	1437382	1437393	TTATTCACAATTTTCGCCAGC	GACAGGGAGGAACAGCAGTC	130
Ad01A576	Aradu.A01	576	p4	(TTTA)5	20	1444465	1444484	TGCAAATGAAAAGACGATAGACA	CGGTTACCAAATCCAAAGACC	280
Ad01A577	Aradu.A01	577	p4	(TTAT)7	28	1445682	1445709	GAATCCGGTGTAAATAAACCAA	TGTCCAATCCACCTGACAAA	255
Ad01A578	Aradu.A01	578	p2	(CA)8	16	1448321	1448336	GAGGACTCAATTCAACGATGG	TAGTGCATGCTATGGTGGCT	264
Ad01A585	Aradu.A01	585	p2	(TC)7	14	1460757	1460770	CATGGATAACCACGCAAGACT	GATTTCTTATTACTTGCTTTTCACTG	252
Ad01A587	Aradu.A01	587	p2	(TC)7	14	1466311	1466324	ATAACCACGCAAGGCTTAGG	GATTTCTTATTACTTGCTTTTCACTG	247
Ad01A589	Aradu.A01	589	p2	(AT)7	14	1473785	1473798	GGCAACTGATTAATATTTCTGTGG	CAGCACTTTCTACGTGCTATCG	256
Ad01A591	Aradu.A01	591	p2	(GA)8	16	1476742	1476757	AAGCCAAGCAAAATGCAAAC	TGGCTGCTGTCACTCTCACT	245
Ad01A595	Aradu.A01	595	p2	(TC)6	12	1486141	1486152	CATAATTCAGGCAATTATTTTTGTT	TCAGCTGATGCAAAAGGTC	171
Ad01A603	Aradu.A01	603	c	(T)12gaaatagg	49	1494103	1494151	GTCGGTGAAAATGGAATGCT	TGGTGTATTTCTGTCATTTCCAA	170
Ad01A604	Aradu.A01	604	c	(TC)10(TG)8	36	1498791	1498826	TGTTCATGGATCGGATCGTA	TCCGCATAATTGAACCTTCTC	263
Ad01A605	Aradu.A01	605	p2	(AT)6	12	1508007	1508018	TTTTTCCCAGATGGTTTCCA	CCAATGCATTTCTTCTACGC	243
Ad01A606	Aradu.A01	606	p3	(TAA)6	18	1516714	1516731	TATTGGCTAGCCTCAGGGTG	GAGGTGAAGAAGAACAGCCG	273
Ad01A610	Aradu.A01	610	p2	(CA)8	16	1527690	1527705	GCTATTCAAAACCCTCGCCT	CGCCAAAGATAAGGTTGCAT	132

ID	Chromosome	SSR nr.	SSR type	SSR	Size	Start	End	FORWARD PRIMER (5'-3')	REVERSE PRIMER (5'-3')	Product (bp)
Ad01A613	Aradu.A01	613	p3	(AAG)6	18	1534349	1534366	AAGAAGAAGAAGCAACAGGAGG	ACCCAAACGTAAGGGATCAA	263
Ad01A618	Aradu.A01	618	p3	(TTA)8	24	1543655	1543678	GCAAAAGCTAGTGCACTCCA	GTCGGTTTGTTTGTTCACCC	142
Ad01A622	Aradu.A01	622	p2	(CT)6	12	1564216	1564227	CTCGGATCCCTCTCTCTCCT	GACCGTTCATGCATGCTTCT	175
Ad01A626	Aradu.A01	626	p3	(TTA)6	18	1571499	1571516	GGAAACAAGGACCGCAACTA	CTGAACCAGAATTCCCCTGA	160
Ad01A627	Aradu.A01	627	p2	(CT)6	12	1572174	1572185	TGCTTCTCCAATTCCTCCAT	GAAAACGAAATGGCTTTGGA	185
Ad01A631	Aradu.A01	631	p2	(GA)8	16	1577784	1577799	TCACAGTTGCCACAGTCACA	CCACCTCCAACAATCACACA	249
Ad01A633	Aradu.A01	633	p2	(TA)9	18	1582226	1582243	ACCCGTACCAACCACACATT	CGGAAAAAGGATTCCAGGAT	239
Ad01A642	Aradu.A01	642	c	(A)11tcaatttag	38	1604988	1605025	TTCACGCTTCACACACACAA	TTTGACAAAAATATGCACTTAAAAA	259
Ad01A645	Aradu.A01	645	p4	(ATAC)9	36	1610178	1610213	CCACTGATTCCATGCACAAG	ATCGCAAATCCAATTTAGCA	265
Ad01A646	Aradu.A01	646	p2	(CT)6	12	1616326	1616337	TAAACCGGGAAAAGGGAATC	CGATGGTGATTGGTGAAGTG	243
Ad01A647	Aradu.A01	647	p2	(CT)17	34	1616550	1616583	CACTTCACCAATCACCATCG	CAACGATGCCAATGCTCTAA	279
Ad01A649	Aradu.A01	649	p3	(GTT)5	15	1618996	1619010	TTTGCTGTATTGTATGGGCG	TGTTTGCAATCAAAGCGAAA	255
Ad01A654	Aradu.A01	654	p2	(AT)7	14	1629012	1629025	CACGGCATATGGGACAATTA	TTCAGTCCCATGTGTCCTCA	267
Ad01A657	Aradu.A01	657	p2	(GA)7	14	1634788	1634801	TCTTCCACCACTTCCTTTGG	TCCTCTTTCTTTTGCCCTCA	190
Ad01A660	Aradu.A01	660	p2	(AT)11	22	1648361	1648382	TCTTTGGAGCATTTTGTCCC	AAGTGTGGAGAAATCCGAAAAA	203
Ad01A667	Aradu.A01	667	p3	(ATA)7	21	1666502	1666522	CCAAAGATTGATTTGTTGGGA	AAGTTCAATCATTTTTGGTTGTGA	225
Ad01A671	Aradu.A01	671	p2	(AT)7	14	1676856	1676869	CGACACTTATTTAAGCGGGC	TGGAAGCCTGAACAACAACA	111
Ad01A672	Aradu.A01	672	p2	(TA)6	12	1678035	1678046	AAGATGACAATTGTTTGACAAATTAAA	AGCACAGAAATAACGGGGAA	204
Ad01A675	Aradu.A01	675	p4	(AAAT)9	36	1679891	1679926	CTGAAGCTCACTACCTCATCACA	CCCACATTCCATAATCCGAC	151
Ad01A691	Aradu.A01	691	p2	(TC)10	20	1722669	1722688	CCTCTCTCTGCACCTCAACC	CACGAAAAACACCCGCTTAT	246
Ad01A695	Aradu.A01	695	c	(GAT)5gaaa(A	34	1735359	1735392	AAAATCGAAAGGAAAAGACGAA	GCAAATTTTGGTGTAAAACTGAGA	193
Ad01A696	Aradu.A01	696	c	(T)10attctagtta	99	1736028	1736126	TCACAATTCGTTACAAATACACAAAA	TCTCATCTTTTGCTGCTTCTTC	280
Ad01A698	Aradu.A01	698	p2	(TA)9	18	1750707	1750724	GCATTTTCGGTTTTGTTTTGA	TGCATTGTCCATTTGCTAGG	215
Ad01A701	Aradu.A01	701	p3	(GTT)5	15	1757095	1757109	TGTTTTCTCAAGGGGGAATG	AGAGAGGTGGGTCACAATGG	269
Ad01A703	Aradu.A01	703	p3	(TCT)6	18	1767976	1767993	CGTCCCGAAAAATTGGTCTA	GGATTCAGTGCCACCAAAGT	232
Ad01A706	Aradu.A01	706	c	(A)10gaaaaacg	111	1791472	1791582	ATGCTTGCATGCACTCCTCT	AAACAACCTACAAAAGGGTATGG	269
Ad01A707	Aradu.A01	707	p5	(TTTTA)5	25	1796113	1796137	CCTTCTTCCTCTCCCTCTCC	GCCATAAGAAGTGTGCGTGA	268
Ad01A708	Aradu.A01	708	p3	(TTA)11	33	1799328	1799360	CCCTCCTCCTCTCGTGTATG	CACTCGAAATAAGAACAAAATAGTACG	182
Ad01A709	Aradu.A01	709	c	(CTT)7tt(TCA	38	1804030	1804067	CAAAACTCTTCTTCTCCTTTCTCG	TGCTGCATCTTTGTCTTCTTCT	272
Ad01A711	Aradu.A01	711	p2	(TC)8	16	1809819	1809834	CGACGTAGCGGCTTACTCTT	AGGGTTGCGGATCTCTTGTA	273
Ad01A716	Aradu.A01	716	p3	(AAG)6	18	1818296	1818313	TTGTGGTGAGAGAGAGTGCG	CTTTCCAACGCTGACAGTGA	168
Ad01A725	Aradu.A01	725	p2	(AG)11	22	1849831	1849852	CCAGCCTATCCTCATTCCAG	ATCCCTACCTGGTTCCTCGT	188
Ad01A732	Aradu.A01	732	p3	(TCT)5	15	1863759	1863773	TGAGGCTGATCAAAAGGGTT	CAAGTCGAAGTTGGAGGAGG	164
Ad01A733	Aradu.A01	733	p2	(TG)8	16	1863913	1863928	TCTTTTGCTTCCTCCTCCAA	TGTTCCCGACTCTAACCTGG	187

ID	Chromosome	SSR nr.	SSR type	SSR	Size	Start	End	FORWARD PRIMER (5'-3')	REVERSE PRIMER (5'-3')	Product (bp)
Ad01A742	Aradu.A01	742	p4	(AAAG)5	20	1886822	1886841	TGTTTTTAGCAAGAATTCAATCCA	TCTTGCCATACACAAAAATAGCA	259
Ad01A745	Aradu.A01	745	p4	(TTTA)9	36	1893570	1893605	GGGTGTGGATACCTCTCCCT	GGCGCACCAAATATTGATTA	278
Ad01A751	Aradu.A01	751	p3	(TTA)6	18	1913311	1913328	TACAAAACACAGCTGCCCCT	TGGATTCCTCCCTTTGTGTC	153
Ad01A752	Aradu.A01	752	p3	(AAT)8	24	1917110	1917133	TTCCATGGGATTCAAAGCTC	GCCCAGATTCATTCAGAACC	230
Ad01A754	Aradu.A01	754	p3	(GGT)5	15	1923206	1923220	GCTGGAAGATGGGTAAGCTG	CACTCTATTGCCACCACCCT	278
Ad01A756	Aradu.A01	756	p3	(GAA)6	18	1924998	1925015	AAGGACGAAGAACCTGAGGA	GTGGCCATTTCTGCCTCTAT	156
Ad01A757	Aradu.A01	757	c	(C)12ttc(A)12	27	1926111	1926137	ATACGACATGCGGCTTTTTC	TGACAAAGAAAAGGTTGGTCC	236
Ad01A760	Aradu.A01	760	p3	(ATG)5	15	1934205	1934219	ATTTGATTGCTGCTTTTGGG	ATGGTGGCAATATCCCTGAA	229
Ad01A761	Aradu.A01	761	p3	(TAA)5	15	1935092	1935106	GTTGCCAAGGGGAACTCATA	GGTGTGGCCAGTTATTGGAG	215
Ad01A762	Aradu.A01	762	p2	(AG)7	14	1936262	1936275	TTTGGGAGAATGCTTTCCTG	TGTGCCATGTGTTGTCACTG	188
Ad01A766	Aradu.A01	766	c	(T)10ctttttcatca	48	1948766	1948813	CTCTTCTTCTCCCTCCTTCTCA	TCATCATTTTTCAAGTAATTTTGTACC	220
Ad01A769	Aradu.A01	769	p3	(ATA)15	45	1962165	1962209	AACATTGGCAGCAACACAAG	ATCGGGAAGTTTTCTGGGAT	232
Ad01A770	Aradu.A01	770	p2	(GA)9	18	1962589	1962606	GGGCTTGATCCTAAACGACA	GCGCACACTCTACACTGCTC	270
Ad01A771	Aradu.A01	771	p4	(AAAT)5	20	1968584	1968603	TGCATTGGGCAGATCTTACTC	TCGGAAAAATTACTCTAAAGAATCG	182
Ad01A774	Aradu.A01	774	p3	(CAT)5	15	1986503	1986517	ATGCCTTGAGAAACCAGAGC	GCAAGAGCACGAAATGTGAA	253
Ad01A775	Aradu.A01	775	p2	(GA)18	36	1991472	1991507	TGCAAACCAACAACATCAGA	TGTTGATGTACCTCCCTCCC	163
Ad01A778	Aradu.A01	778	p2	(GA)6	12	1996948	1996959	AACCGACGTCTCTTTGCTACT	TCGATCGATTTTCAGAACCA	279
Ad01A779	Aradu.A01	779	c	(TTA)5(TTG)8	39	2001189	2001227	CGCAGAATATGGTTCGTGAC	CAATTCGGGGATCATACACA	261
Ad01A780	Aradu.A01	780	p3	(AAT)6	18	2005836	2005853	TGAATTCCTGCCAACAACAA	CCTTCTTTCATGCACGCTTT	196
Ad01A781	Aradu.A01	781	p2	(CT)7	14	2007279	2007292	TAGCCGAAACATTTAGCACG	TCTTGTGGGAGTTGTGTTGC	264
Ad01A782	Aradu.A01	782	c	(TC)24tgagact	110	2007426	2007535	TCAGGCAAGCTTCCTTCAAT	AGAACTTCATCGTTGGGTGC	267
Ad01A784	Aradu.A01	784	p4	(TTTA)5	20	2008754	2008773	GGAAGTCGCATGTTTCCAAT	GTGGGCTAAGTCTTGGGTGA	255
Ad01A787	Aradu.A01	787	p3	(ATA)5	15	2013952	2013966	TCAACAAGAGCAACAGGCAC	CCAATCAACCATGTTGACCA	224
Ad01A789	Aradu.A01	789	p3	(CCT)5	15	2022507	2022521	CCTTCTCATCTTCTGCTGCTT	TTGATGGTGGAGGAGAAGGA	212
Ad01A791	Aradu.A01	791	p3	(AAG)5	15	2023505	2023519	TTCCTCCTCCTCATCTTCCA	GCGTTTCTCCTCCTCTTCCT	258
Ad01A794	Aradu.A01	794	p3	(AGG)7	21	2034843	2034863	AGAAGGGAGTGGTCGAGACA	AGGAGGCTAAAGCGAAGTCC	149
Ad01A797	Aradu.A01	797	p3	(CTC)6	18	2048230	2048247	AGGCTAAAGCTAAGTCCCGC	CGCCTTAACCTCACCAGAAG	203
Ad01A801	Aradu.A01	801	p2	(AG)7	14	2060238	2060251	TCCCGAGAAGATAACATACATGC	GCCAAATCTTCAAATCGAGC	261
Ad01A807	Aradu.A01	807	c	(A)13tgg(ACA	73	2076566	2076638	CTAATGCCTTCGATTTCCCA	CCGTTCTTTATTGCCCTTCA	242
Ad01A808	Aradu.A01	808	p2	(GA)13	26	2076854	2076879	TGAAGGGCAATAAAGAACGG	TGAAGAAACGAGGGGTTACG	253
Ad01A809	Aradu.A01	809	p2	(AT)7	14	2084565	2084578	GCATGCTTCCAGCCTAACTC	TAAACCGCAATACCATTCCC	222
Ad01A810	Aradu.A01	810	p3	(CAC)5	15	2085645	2085659	ATTTCCCGAATTTTTGGACC	GCTGTTTGTGTTTGTGGTGG	150
Ad01A814	Aradu.A01	814	p2	(TA)7	14	2100591	2100604	AGCGGCTACAATCACATTCTC	TTAGCATGTTTGGCCATGAA	208
Ad01A818	Aradu.A01	818	c	(A)10ctgatgaa	116	2115538	2115653	TGGTGATGAGGTGAGTTTGC	CATGCAGAGAATCTGTTTCCA	246

ID	Chromosome	SSR nr.	SSR type	SSR	Size	Start	End	FORWARD PRIMER (5'-3')	REVERSE PRIMER (5'-3')	Product (bp)
Ad01A826	Aradu.A01	826	p3	(AAT)8	24	2130054	2130077	TCAACGTTCCTGATTGGTCA	GTCCTCCCAACACCTTCAAA	235
Ad01A827	Aradu.A01	827	p6	(TTTTTA)5	30	2130318	2130347	TTTGAAGGTGTTGGGAGGAC	TGAGCTGTGGTTTCATTCTTTC	161
Ad01A829	Aradu.A01	829	p2	(TA)6	12	2133410	2133421	AACTGGGAATTGGGAAGAGG	AAAATTCCCCCGAAACAGAT	205
Ad01A830	Aradu.A01	830	p3	(TGC)5	15	2139229	2139243	GCTGCAGCCAACATAGATGA	GCGGGTTTAGGTTGTGAAGA	245
Ad01A831	Aradu.A01	831	p2	(GA)12	24	2141503	2141526	TTGTTCAAGTTCAAAAACACGG	TTGTGACTTTTCCTCCTCGG	101
Ad01A836	Aradu.A01	836	c	(A)10ttgtaccac	104	2146685	2146788	TTTTTATTCCATTGAATCTCTGAA	CGTACACAACTTTCATCTGCG	260
Ad01A837	Aradu.A01	837	p3	(GTG)5	15	2147997	2148011	GTTCAAATTGGTGGTTTGGG	ACCCAGTACCTTGGACATGC	226
Ad01A838	Aradu.A01	838	p3	(TTA)5	15	2154729	2154743	AGCCCAGATAAATTGTCCCA	ACATTGCTCCCTGTAATGCC	227
Ad01A839	Aradu.A01	839	p3	(TAT)5	15	2155173	2155187	CGTATCAACCCCAAAGACCA	TGCCACATACGTGTGATAAGTG	133
Ad01A840	Aradu.A01	840	p3	(AAG)6	18	2156262	2156279	AGGATCCAACAAGGATGGGT	ACACACCACTCTCCACTCCC	248
Ad01A844	Aradu.A01	844	p3	(GCT)5	15	2171715	2171729	ATGCAGCTCTCTTGTCGGAT	AATACTCCGCCAGTGACACC	195
Ad01A845	Aradu.A01	845	p2	(GA)17	34	2175405	2175438	TGCCATCATACAGACCCAAA	GATTCCACTGCCGGATAAGA	161
Ad01A846	Aradu.A01	846	c	(AG)12aaatcag	146	2175892	2176037	TCCATTCAAAGGACCAAAAGA	TCACTCAACTCAGCGTGTAGC	255
Ad01A847	Aradu.A01	847	p3	(CTT)10	30	2176306	2176335	ATCTTCTTCAAGCGCGTGTT	GACGAGGATGAGGACGAGAG	186
Ad01A849	Aradu.A01	849	p2	(AT)11	22	2179451	2179472	ATTTACCCGATAAAAGGCGG	AGGAGAGAAGAGGGAGACCG	266
Ad01A852	Aradu.A01	852	p3	(CAA)5	15	2186200	2186214	CACCTCCACCTGAATTTCGT	TTATTTGGATGGCTGGGAAC	261
Ad01A854	Aradu.A01	854	p2	(TG)6	12	2190985	2190996	GTGCTCCAGGATGAAATGGT	AGACCACCACATTTGCCTTC	172
Ad01A858	Aradu.A01	858	p2	(TA)9	18	2209644	2209661	ATGTCTGTGGTGCAGTCAGG	CCTTCATGCGTAGTGTGGAG	246
Ad01A867	Aradu.A01	867	p2	(AC)6	12	2240971	2240982	AATCGTTATTTTAATGGTCTCGTT	TTTGGGAGTCATCCCATCAT	207
Ad01A869	Aradu.A01	869	p2	(AT)6	12	2243834	2243845	TGCTTCTCGGAAGCCAATTA	TTTTCCTAGTGTCCTAACTCCCA	277
Ad01A874	Aradu.A01	874	p4	(TTAT)5	20	2252409	2252428	TGCGAGATGCTAACTTACACA	AACAATTTCCCACAGCAAGC	280
Ad01A875	Aradu.A01	875	c	(A)12tag(AAC	35	2257170	2257204	TATTTTCACACTGCCACGGA	TTGGTTACCATGGGTTGCTT	256
Ad01A881	Aradu.A01	881	p3	(GCA)5	15	2271417	2271431	GCTGATAAACAATCGGGGAA	TTCCCTCCAACAGAATCACC	224
Ad01A884	Aradu.A01	884	c	(A)11c(A)10	22	2279385	2279406	TTCTCTTTTGGGAAAATTGAACA	AGCTCAGGACAGAGGTCCAA	228
Ad01A888	Aradu.A01	888	p2	(CT)6	12	2285593	2285604	GCCCTAAATTCGAACCACAA	GAAGGGATCGTTTTGAGCAG	172
Ad01A891	Aradu.A01	891	c	(TC)9caattcaac	126	2295011	2295136	TCATGAGCCAAGAATGCAAA	TGTTTCTTGCCTTGGTGAAA	250
Ad01A893	Aradu.A01	893	c	(A)10ctcatgcgt	76	2297776	2297851	CGTGAGAGTTAAACAGTCCAAA	TCAATTCTTCCATGAGACACTTAC	268
Ad01A896	Aradu.A01	896	p3	(AAT)5	15	2300456	2300470	GGAGCCTCTTGAGTACCCCT	TGCTCTTCAAGTGTATGCACAG	241
Ad01A897	Aradu.A01	897	p2	(TC)7	14	2301121	2301134	TCGACTGCACCTAAGTTTTCA	TTGGACGACTCATGATGGAA	197
Ad01A898	Aradu.A01	898	p2	(GA)6	12	2306053	2306064	GGTGTTTTGGGTTGTTGAGG	GGCGCTACCATTACCACTGT	166
Ad01A914	Aradu.A01	914	p2	(TA)6	12	2329310	2329321	TGATTTCATTGCATTCCCTTT	ACATGGTGCATGCTTAGGGT	273
Ad01A923	Aradu.A01	923	p2	(AT)6	12	2340635	2340646	TAAGCTCTTGTCGCAGGGAT	AACGGTTCGAGTGTGATGAA	229
Ad01A928	Aradu.A01	928	p2	(TC)6	12	2348824	2348835	GCATCTTCTCGTTGGGACAT	TGCACAAGGAAAAGAAAGGG	207
Ad01A929	Aradu.A01	929	c*	(CTT)6(T)10*	26	2352902	2352927	TGCTAACTTTGGTTGGTGGC	ATTGCAAAGATGGGGACTGA	180

ID	Chromosome	SSR nr.	SSR type	SSR	Size	Start	End	FORWARD PRIMER (5'-3')	REVERSE PRIMER (5'-3')	Product (bp)
Ad01A931	Aradu.A01	931	p2	(AG)12	24	2365459	2365482	GACGAGGGAAATTGCAATACA	TCCTCTCTGCCGTGTTCTTT	173
Ad01A935	Aradu.A01	935	p2	(TG)7	14	2391423	2391436	ATCCCCAAAATCCAAACCTC	AAATCAAAGGAAGGTCAGCG	213
Ad01A937	Aradu.A01	937	p2	(TA)6	12	2404875	2404886	TCATATCAAAAGTGGTTTCCAAAA	TTTCATTCCCAAAGTACACCAA	278
Ad01A947	Aradu.A01	947	p2	(TA)7	14	2429599	2429612	TGAATGTTCAGAAACCCTGTT	TTTTGTGCCGTCTTCTCTGA	277
Ad01A950	Aradu.A01	950	p2	(GA)14	28	2440407	2440434	CAAGGTACCGTCTCCCTCTG	AGTCACGCTTTCACTCCGAT	121
Ad01A956	Aradu.A01	956	c	(GCC)5tcagctt	103	2442618	2442720	TGAAGGAAAGCATGAAGCCT	GGGATTCGACAGGTGAAGAG	172
Ad01A965	Aradu.A01	965	c	(T)13atctctacc	91	2473095	2473185	TTTTTACCCCGTCCAAAACC	CTGCAACCAATTCCACAATG	247
Ad01A967	Aradu.A01	967	c	(T)11ggcacagg	70	2474029	2474098	AAAGATCAGGGGTCGAATTG	CCCTACCCGACTTCTGACAA	145
Ad01A970	Aradu.A01	970	p2	(TA)10	20	2478160	2478179	GGTGCACCCTCACTATCAAT	GGGATACCGTGAGAAACCAA	274
Ad01A973	Aradu.A01	973	p3	(TAA)7	21	2489039	2489059	GATATGCATCGATGTGCTGC	GACCCAAAAACTTTATTTCAATGC	277
Ad01A975	Aradu.A01	975	p2	(AT)7	14	2490321	2490334	GATCATGTCTACAAACTTTCATGTG	TGTTTTGGTGCCTCTTGAGTT	200
Ad01A976	Aradu.A01	976	p3	(AAT)11	33	2492533	2492565	ACTCGACAGGCCAAGGATTA	AATGATGTCACAGACGGCTC	280
Ad01A977	Aradu.A01	977	p2	(AT)6	12	2493433	2493444	GCTGTTTGCTGTCAAGGGTT	TCACATTGTAATCACGGAAGATG	226
Ad01A979	Aradu.A01	979	p3	(TTG)5	15	2498285	2498299	GGTGGAACTAGGTTCGGACA	GCAGAAATAGAAGCAAAAGCAGA	249
Ad01A987	Aradu.A01	987	c	(A)13gtcatagta	62	2508022	2508083	TGCAGACGAAATTTTCATTCTAA	TGAGCTGTAAACCAATGCAC	246
Ad01A991	Aradu.A01	991	p2	(TG)24	48	2516814	2516861	CCATGTATCTGGGAAGTTGTCA	ACGGAATCGCGTATGTTAGC	259
Ad01A992	Aradu.A01	992	c	(TCT)8(CTT)5	104	2520559	2520662	TCAAACGGTTCCTCTTCGTT	GGAAGAAGAACGTGCAACAA	245
Ad01A993	Aradu.A01	993	c	(TTC)6atttacg	55	2520790	2520844	TTTTCTTTCGTTTCTGCACG	CCTCCTCATCTTCTGCTGCT	147
Ad01A995	Aradu.A01	995	p3	(TTG)10	30	2542290	2542319	CGTTTGATTCATGTAGCCGA	GAAAGCAATGTATGGGGCTG	259
Ad01A997	Aradu.A01	997	c	(A)11gtaaaact(	68	2548090	2548157	TTGGTAAGTGGATTAGGCTCA	TTTTTGTTTTCGTTGTTCAAGTC	168
Ad01A1004	Aradu.A01	1004	p2	(AG)6	12	2577617	2577628	AATTCGGACACTCACCGTTT	AGTCCTTGTTTTGGGCTGTG	206
Ad01A1010	Aradu.A01	1010	c	(CTT)7acgctcg	76	2590123	2590198	AAACGTGAAATACAAGCGCC	GAAGAAGAAGGAACGCGAAA	198
Ad01A1011	Aradu.A01	1011	p3	(GAA)5	15	2591101	2591115	CACCTCCGTCCATTCAATTC	ACTCGCGTTCTTCGTTATGG	262
Ad01A1012	Aradu.A01	1012	p3	(GAA)25	75	2591234	2591308	AGCAGAGGAGAACGACGAAG	TCATCTGTTGCAGCCTAACG	132
Ad01A1015	Aradu.A01	1015	p3	(TTG)8	24	2617660	2617683	ACATGACAGAGCACAATGGC	GAAAGCAATGTATGGGGCTG	275
Ad01A1017	Aradu.A01	1017	c	(A)10gaaataatt	75	2638575	2638649	AGTGTCGTGATTTGAGAATAAAAA	CAAGTGCTTCTTTGGGGAGA	274
Ad01A1019	Aradu.A01	1019	p2	(TA)7	14	2642584	2642597	TGAAGGGATTCGTGCTGATT	GCTTGTGAAGCCTTGAAGGTA	280
Ad01A1021	Aradu.A01	1021	p2	(TA)7	14	2654154	2654167	TGAAGGGATTCGTGCTGATT	GCTTGTGAAGCCTTGAAGGTA	280
Ad01A1023	Aradu.A01	1023	p3	(AAG)5	15	2658685	2658699	AAGAGCGTGTTTCTGTTCCTG	GCGAAAGCAGAGACAGAGGT	104
Ad01A1031	Aradu.A01	1031	c	(T)10gaacaatca	79	2670322	2670400	CACCAAATTAATGGCACACA	CAATCTTTCAGCTCGGCAG	129
Ad01A1032	Aradu.A01	1032	p2	(TA)7	14	2674097	2674110	GTTCTTGGCCTTGGTTCAAA	TTCGGCTATCATCCCTATCG	255
Ad01A1034	Aradu.A01	1034	p2	(AG)28	56	2676916	2676971	GGCAGTGACACCTGTAAGCA	TGTAAGCATTCCATGATCGC	239
Ad01A1037	Aradu.A01	1037	c	(ATA)10ag(A	47	2684421	2684467	GGCACTCCATCTCCACCTAA	GGGGAGCACTGTAGCTTTGT	226
Ad01A1038	Aradu.A01	1038	c	(A)11caaagagg	87	2685930	2686016	GAAGCAGGTAAAGTTCAAAGCC	GTGGTTCTTAAAGCCCGACA	259

ID	Chromosome	SSR nr.	SSR type	SSR	Size	Start	End	FORWARD PRIMER (5'-3')	REVERSE PRIMER (5'-3')	Product (bp)
Ad01A1040	Aradu.A01	1040	p2	(TG)6	12	2688767	2688778	TTTCGTTTAGCAGCCTTGTG	CCCACAACCTCTGAATTTCG	190
Ad01A1043	Aradu.A01	1043	p2	(AT)8	16	2695517	2695532	CGATCCTATCAGACTCGATCC	TCCTACCGATCAACTCATATTCC	267
Ad01A1051	Aradu.A01	1051	p3	(TTA)5	15	2710106	2710120	CAAAGGTACTCCATTTCTCCCA	TTTTTGTGCATCTTTACTCAAACT	278
Ad01A1053	Aradu.A01	1053	p3	(TGG)5	15	2712079	2712093	CCTTTGTCATGGAGTCCAAA	TTCTTTGTGTGCAGGGATTG	153
Ad01A1054	Aradu.A01	1054	p4	(ATTT)8	32	2714760	2714791	ATCAATACCCGATTTGGCTG	TGGAGAATTTCTTTGGTTCCAT	230
Ad01A1055	Aradu.A01	1055	p2	(AT)8	16	2718269	2718284	GGTACAGTATGCGAGATGCG	ATGCCAGGTAAGCAGAATGG	264
Ad01A1056	Aradu.A01	1056	c	(A)10gtaacactt	77	2718892	2718968	TAAGCCCGTTTAAATTTCGG	GCCAGCCCGTTTATCATTT	280
Ad01A1061	Aradu.A01	1061	p3	(TTA)5	15	2726740	2726754	AAATTTAAGTCATTTACCTGTTTGTGA	TTTGGTACATTTCATATCACATTCG	208
Ad01A1063	Aradu.A01	1063	p2	(TA)6	12	2734510	2734521	TTGACATGCGAAAATTTGGA	CAACACAAGACACGGACTGG	271
Ad01A1068	Aradu.A01	1068	c	(AAT)5catttag	38	2750399	2750436	TGGTAAAATTAAAATGGCTTTCA	CTGAAGAGGACAGACGGCTC	219
Ad01A1072	Aradu.A01	1072	c	(T)10gagtgcac	107	2759859	2759965	TGAGACAAATCTTACGGTGTATGAA	TGGGACGTTTGGATAAATCA	217
Ad01A1077	Aradu.A01	1077	c	(CCA)6acacca	88	2771612	2771699	CGAAACCGTCCCTCATCTAA	TTGGTTGGTGTGATGCAGAT	263
Ad01A1078	Aradu.A01	1078	p2	(TC)6	12	2772111	2772122	AAGCAAGAAGCAGAAGCAGC	AGAGCTGAACGGAACGAAAA	260
Ad01A1090	Aradu.A01	1090	c	(T)12aaatctata	84	2797756	2797839	TTTTGCATTATCATTCGACAACTT	TGGAAAATTTGCTTGGGAAG	276
Ad01A1093	Aradu.A01	1093	p4	(AAAT)6	24	2803229	2803252	ACTGCCAACACAAGAAACCC	ATCTGCCAAAATCTGCCAAC	248
Ad01A1094	Aradu.A01	1094	c	(A)10ctctttttgg	175	2804237	2804411	TTAGGTGGAATACCCATCACG	TCATTAATGCTATGTTTGTTTGAAAT	280
Ad01A1097	Aradu.A01	1097	p2	(TC)8	16	2812025	2812040	TCCCATGTGCCATGTTATTG	ACTTGACACTCGACCAACCC	168
Ad01A1106	Aradu.A01	1106	c	(T)11c(T)10	22	2829771	2829792	TTTTTCCAAGACGAGGACATTT	TTGATTAATTTTGAAGAGGGGG	253
Ad01A1108	Aradu.A01	1108	p2	(AT)8	16	2831508	2831523	TGAAGCACTATTGTGTCGCC	GACAAATTCATATTCATCATTTCCA	246
Ad01A1112	Aradu.A01	1112	p2	(AT)6	12	2845348	2845359	TTTTGCGCGAAGAAAAGAAT	AACCCGCAAATAAGATTGGA	268
Ad01A1119	Aradu.A01	1119	p2	(TC)6	12	2868480	2868491	TGCCAATGCTCTGTGCTATC	AACCTTACAGGACCGAACCC	197
Ad01A1120	Aradu.A01	1120	c	(TTC)5ctcttcc(	126	2875658	2875783	CCCCAAAAACATGATACAAGG	CGTGCAAGAAGAAGAAGAACG	202
Ad01A1121	Aradu.A01	1121	p3	(CTT)5	15	2875890	2875904	CTACTCCCTTTCAACGAGCG	TCGCTTTACCATCATCACCA	253
Ad01A1122	Aradu.A01	1122	p3	(AGA)8	24	2877448	2877471	CGGTGGCCTCCTTTTACTTT	TTCTTTTCTCGCGTTTTCGT	242
Ad01A1139	Aradu.A01	1139	p3	(TGT)5	15	2931706	2931720	ACGCTTCTTTCAGTGGCTGT	AGCTGAAATCCATCACCACC	219
Ad01A1140	Aradu.A01	1140	p2	(CT)6	12	2932054	2932065	AATTGCATCACCAAAATCCC	AGCTTCAATCCACGCATCTT	267
Ad01A1151	Aradu.A01	1151	p2	(TA)6	12	2955146	2955157	GGCTACTGGTGTTCTTGTTGG	ACAATGAAGAGCGTTTTTGAA	194
Ad01A1152	Aradu.A01	1152	p2	(CT)12	24	2956036	2956059	TCCTTCTGCAAAATGGGTTC	TTGCAATGCTAGTGAATCCG	242
Ad01A1153	Aradu.A01	1153	p3	(TTA)5	15	2956516	2956530	ATCTTCGTTCAACGGCTCTC	CCTCTTCCGGGTCAGTTACA	226
Ad01A1156	Aradu.A01	1156	p4	(ATAA)8	32	2968829	2968860	GACAGCGGGGTAATTGTAGC	AAGGGTTCTTGTTTGCATGG	181
Ad01A1160	Aradu.A01	1160	p3	(TTA)8	24	2973895	2973918	CGCTTGACTTCTAAATACCGAA	AAAACTTCCTTAAAATATTCCATGAG	244
Ad01A1164	Aradu.A01	1164	p2	(GT)6	12	2977343	2977354	CTGAGGCTGGTTGTTGGTTT	ACCCAAAAGAAGGAGGGAAA	168
Ad01A1165	Aradu.A01	1165	p3	(ACA)9	27	2981347	2981373	TGGTGACCAATCTTCTGCAA	ATCTACGACGACCCCACCTA	165
Ad01A1168	Aradu.A01	1168	p2	(AT)6	12	2990473	2990484	GTCAGAATATCTGAGCCGCC	AATGTTCCTGGATTCAAGCG	160

ID	Chromosome	SSR nr.	SSR type	SSR	Size	Start	End	FORWARD PRIMER (5'-3')	REVERSE PRIMER (5'-3')	Product (bp)
Ad01A1174	Aradu.A01	1174	p2	(AT)6	12	3003299	3003310	TGAAAGCATTCCAGCATTGT	TCAGCATGACGCGATTTAAC	258
Ad01A1175	Aradu.A01	1175	p2	(TA)8	16	3010016	3010031	ACACTCGCACCAAAACCTTC	TCTTAACTGACCCATAAAATCGC	257
Ad01A1177	Aradu.A01	1177	p3	(GAA)5	15	3015046	3015060	CTACCGCTTCTCCACCAAAG	CGTTTACGCATGGAACATTG	180
Ad01A1180	Aradu.A01	1180	c	(A)11taccaactc	87	3027443	3027529	TGAACAATTTGGTGTTGGGA	CAACCCGACCTCTGATGTTT	252
Ad01A1184	Aradu.A01	1184	p3	(TAA)8	24	3053711	3053734	GAGAGGTTATCTTCGTCTCCGA	AAGTGCAAAATGCCAAGACC	267
Ad01A1186	Aradu.A01	1186	p5	(TCAAT)5	25	3062887	3062911	TCAAATTCAAACAGCTCTGCC	TCGAAACAATGAACGACTCAA	199
Ad01A1187	Aradu.A01	1187	p3	(ATT)6	18	3063724	3063741	TCTGGACTTTTCGTAGCGGT	CGGAATATGGAATTTGGGAG	264
Ad01A1189	Aradu.A01	1189	c	(A)10ttattataaa	40	3064855	3064894	GAAATGAGTACAGGGATCCAAA	AAATCATATCACAAGCAAAATTACTCA	240
Ad01A1190	Aradu.A01	1190	c	(A)10tatatctatc	49	3067236	3067284	AACAAAGAACGCCAGAATGG	GGCGGAATTATTTGACCTCT	191
Ad01A1191	Aradu.A01	1191	p2	(CT)13	26	3067469	3067494	AGAGGTCAAATAATTCCGCC	CAGTCTCCGATTAAGTCCCG	279
Ad01A1194	Aradu.A01	1194	p2	(TA)7	14	3082520	3082533	CGTGGGTTCGAGTTTTCCTA	TCAAGTGATTTAATTGTGACATTGC	143
Ad01A1196	Aradu.A01	1196	p4	(AAAT)6	24	3089439	3089462	TGAAAAAATTTGAATGGTGCAG	GAACCGTTCGTCAATTAGAGGT	256
Ad01A1200	Aradu.A01	1200	p2	(GA)7	14	3097129	3097142	ATTTGACCCGCTTTCAACAC	TGTTGGAGCGTGAAGAATTG	197
Ad01A1204	Aradu.A01	1204	p2	(AC)6	12	3110897	3110908	ATCAATGACTAACTCGCCGC	AACAATTTGGAAGTGGTCGC	235
Ad01A1205	Aradu.A01	1205	p3	(TCT)5	15	3111932	3111946	CCAATAACAAAGCCTCGCAT	TATGAGTGTGCTGACCCCAA	277
Ad01A1207	Aradu.A01	1207	p2	(AG)8	16	3112442	3112457	TGGAACGGGTTTCTCAAGTC	TGTGAATTTGGTGTGCAGGT	270
Ad01A1210	Aradu.A01	1210	p2	(AG)20	40	3125303	3125342	TGAACACCAGACAAAGCTGC	AGGATCCTTTGCAGAAGCTG	185
Ad01A1211	Aradu.A01	1211	p2	(GA)11	22	3125494	3125515	GATCCAGCTTCTGCAAAGGA	CATTGTTGGGCTCCATCTTT	177
Ad01A1214	Aradu.A01	1214	c	(T)10agatttgga	75	3134881	3134955	GGCTCTTCTTCTCCTCCTCC	TTGCCTCTTTTTATTTTAATTCACC	262
Ad01A1216	Aradu.A01	1216	p2	(TA)6	12	3140429	3140440	CGATTTGAATCGTTTTTATATCTGC	CTTGCAACAGATGCTGAGATT	186
Ad01A1223	Aradu.A01	1223	c	(T)10caattaggt	71	3150218	3150288	AAAGGTTTAATTATTCTATTGGTTCCT	TTTTTATTATTTAAATTCCTGCACCA	183
Ad01A1224	Aradu.A01	1224	p4	(AAAT)5	20	3153210	3153229	GCAAAAGCTCTTAATACCCCA	CGTTTGATTCATGTAGCCGA	274
Ad01A1225	Aradu.A01	1225	p3	(ACA)6	18	3153395	3153412	GCAAAAGCTCTTAATACCCCA	CGTTTGATTCATGTAGCCGA	274
Ad01A1227	Aradu.A01	1227	p3	(CCA)5	15	3161289	3161303	TCAGAATTGAAATCGTCCCC	AGTGGTGGTGGTAAAGGTGC	138
Ad01A1230	Aradu.A01	1230	p3	(TTA)7	21	3166699	3166719	CGATTCAGGAACTGGGTCAT	CCTTCCAGCACTACAATCCAA	234
Ad01A1232	Aradu.A01	1232	p4	(ATAA)5	20	3181447	3181466	CCTTGAATAATGGGTGAGCAG	TTCAAAAGGGGATAATCGCA	208
Ad01A1233	Aradu.A01	1233	c	(T)12aagactaaa	101	3184429	3184529	CACTTATAGTACCTTTGTCACCATCTC	AAACCAAACTTTTATTATGCAAGAA	279
Ad01A1234	Aradu.A01	1234	p3	(TAA)9	27	3184926	3184952	TGCGCTTCTTTAGCCAATCT	TTTCAAACCTCGGTTTTGTTG	274
Ad01A1236	Aradu.A01	1236	p2	(TA)7	14	3186987	3187000	AGAACAAAAAGGGAGGGGAA	TTCAGGAATTGCTGCTTAGGA	278
Ad01A1238	Aradu.A01	1238	c	(T)11c(T)11	23	3190615	3190637	AAGAATCATGGGCATGGAAA	GCGAGAGTGGGGATGATAAA	137
Ad01A1239	Aradu.A01	1239	c	(AG)6attgcaatg	102	3190849	3190950	TCTTCTTCTTGGTAATTCCACTGTC	TCCAATTAAGCAAACCCAAA	249
Ad01A1241	Aradu.A01	1241	p3	(AAC)5	15	3194201	3194215	TCAATCTGTTATCCAATGGAGC	GCCGATTCTACCTAATGGGA	208
Ad01A1243	Aradu.A01	1243	p2	(AT)6	12	3198706	3198717	GCGACAAAAATGTTAAAATAACAGAA	TTCTCTGTTGTCATAGGGTTAGGA	222
Ad01A1244	Aradu.A01	1244	c	(CTT)6tcaaata	125	3199950	3200074	TCCAACACCAACAAAAACCA	TGGTGATGACGACAACAAAAG	223

ID	Chromosome	SSR nr.	SSR type	SSR	Size	Start	End	FORWARD PRIMER (5'-3')	REVERSE PRIMER (5'-3')	Product (bp)
Ad01A1245	Aradu.A01	1245	p3	(AGA)7	21	3201012	3201032	GGAGAATGAGGAGGAGGAGG	CAATTTTGGCATTCAAGCAA	183
Ad01A1248	Aradu.A01	1248	p2	(AT)6	12	3210202	3210213	AAAGAAGGAGATGTATATGTTTTACGA	CACAAAATTGACATTTAACCATGT	270
Ad01A1251	Aradu.A01	1251	p3	(CTT)10	30	3244329	3244358	CAAGGTACGTTTCTTGCCTTC	CCTCCTCATCTTCTGCTGCT	215
Ad01A1252	Aradu.A01	1252	p3	(CTT)5	15	3245085	3245099	GGAGATCGAAGGCAAAGAGA	CCTCTGCTGCGGATTTTAAC	193
Ad01A1261	Aradu.A01	1261	p2	(TC)8	16	3256656	3256671	CCCAATGGTCGCTGAATAGT	CGTTGTGTCTTTGGTGATGG	110
Ad01A1264	Aradu.A01	1264	p2	(AC)10	20	3258893	3258912	ACACATGCATGCAGGGATAA	CTGCAAAGCACAAAGAAGCA	256
Ad01A1265	Aradu.A01	1265	p2	(CT)6	12	3260086	3260097	TTGCTACCCACGAAAGTTGA	GGGGTGGGAATGAGAAAAAT	217
Ad01A1267	Aradu.A01	1267	p4	(AAAT)5	20	3262887	3262906	CAAAGAACCGATGAATCGAA	ATTCGCAAGTTCCATGATGA	231
Ad01A1276	Aradu.A01	1276	p3	(ATG)5	15	3288764	3288778	TCGAATGGAACTTATTGCACA	GGAGAGAGAAATATCCATAACCC	186
Ad01A1278	Aradu.A01	1278	p3	(GGA)5	15	3291664	3291678	CTCACTCAAAGGGGAGCAAG	GCCAAGCCACAACAACAATA	256
Ad01A1279	Aradu.A01	1279	c	(T)12cag(A)1(	25	3293938	3293962	CAATCTTTTAAGGCCCACGA	ATTCATGTGCAATCCAAGCA	265
Ad01A1281	Aradu.A01	1281	p2	(TA)13	26	3297983	3298008	AAAGCTCCTGACTCAGCTCG	TTTTGCAGATGCAGGTTGAG	278
Ad01A1282	Aradu.A01	1282	p2	(AG)19	38	3302223	3302260	CAGTTTGACGGCAACAGAGA	GGAGAAATTCGTCGCCATAA	235
Ad01A1283	Aradu.A01	1283	p2	(TG)8	16	3303406	3303421	CGATGATCATAGCTCGGTGA	GGAAAAACTTGCAACGGAAA	262
Ad01A1287	Aradu.A01	1287	p2	(AT)6	12	3314150	3314161	CACCAAATTCATTTTTCCCG	AAGAAATGATGCACTCATGACAA	194
Ad01A1288	Aradu.A01	1288	p3	(ACC)6	18	3315181	3315198	TTTTCGTACCCAAGGTCTGG	TTGCTGTTGATGGTGGTTGT	265
Ad01A1290	Aradu.A01	1290	p3	(TAT)14	42	3325279	3325320	TGAAGGTGCATTTGAAAGGA	TCCTGCAAAACTGCAAAACA	248
Ad01A1291	Aradu.A01	1291	p2	(CT)16	32	3327863	3327894	GCCGGTTTGGTTCTCATAACT	TCGTGAAATACTGCCCTTCTT	280
Ad01A1292	Aradu.A01	1292	c	(T)10ctattttagt	77	3328201	3328277	CCCACTTTTCTTTCCACTTCA	GGGAACCAAAAGCACCATAA	263
Ad01A1295	Aradu.A01	1295	p2	(AT)8	16	3338391	3338406	CATGAACAACAGGAGCTGGA	CAACATTTGTTGATGTGGCA	192
Ad01A1297	Aradu.A01	1297	p3	(ACA)5	15	3341124	3341138	TTTGGTCATTGCCTAAACCTG	CCCATCTAGCGGGATAAGGT	259
Ad01A1298	Aradu.A01	1298	p4	(AGAA)6	24	3341855	3341878	CAAACCCATCTTCAAGCCTC	ATGCATGCCAAATTCTCATT	241
Ad01A1301	Aradu.A01	1301	c	(GAA)6cttacga	59	3347013	3347071	CCACCGAACCGAACAATGTA	GCCACCGAACTGAACAATG	280
Ad01A1302	Aradu.A01	1302	p3	(GAA)5	15	3347348	3347362	TTTGTGATTTCGGTTCACCA	CGCATTTTTCTTCTCATCTCG	278
Ad01A1303	Aradu.A01	1303	p3	(GAA)5	15	3347464	3347478	GCGAACAGAGGAAAACAACG	GCTACCGAACCGAACAACAT	213
Ad01A1304	Aradu.A01	1304	p3	(GAA)6	18	3347791	3347808	ATGTTGTTCGGTTCGGTAGC	TTCTCTGCTCGCATTCTTCA	246
Ad01A1305	Aradu.A01	1305	p3	(TTC)6	18	3348173	3348190	CGCGCATTATTGAGAGTGAA	GGTGAAGCCTATAACGCGAG	105
Ad01A1306	Aradu.A01	1306	p3	(GAG)5	15	3348703	3348717	AAGAAGAACCTACGAGCGCA	TGGTGAACCGAAATCACAAA	164
Ad01A1307	Aradu.A01	1307	p2	(TA)7	14	3349411	3349424	GACTTGATGGCAGGTGTCCT	AGGGTTAATGTCTCGCGTTT	264
Ad01A1309	Aradu.A01	1309	p2	(TA)6	12	3350742	3350753	TCCAAATTTGTGGCTCTTTG	TCTCCTCACTCTAATTCGGCA	224
Ad01A1310	Aradu.A01	1310	c	(T)11accaatga;	102	3356210	3356311	ACTCGTCACCGTGGACAATA	TCTGGGTGGAGATGACACTT	188
Ad01A1312	Aradu.A01	1312	p3	(GAA)5	15	3363653	3363667	AATCGATATCGGGCTTCCTT	GAACGAGTTTCCGGTGAAAA	232
Ad01A1315	Aradu.A01	1315	p3	(TAT)16	48	3365475	3365522	CCGATTACCAAAACAGCCAT	AAAAACATGGAGGAGAATATCATTG	261
Ad01A1317	Aradu.A01	1317	p3	(TTA)5	15	3369242	3369256	TGGTGGTGGTGATGATGATT	CAGCAGAAGTCGCACCAATA	192

ID	Chromosome	SSR nr.	SSR type	SSR	Size	Start	End	FORWARD PRIMER (5'-3')	REVERSE PRIMER (5'-3')	Product (bp)
Ad01A1319	Aradu.A01	1319	p2	(AT)8	16	3372018	3372033	CCACTCTTTTTAGCGGTTCG	CGTTTGCCACCCCTAGTTTA	224
Ad01A1322	Aradu.A01	1322	p3	(AAT)5	15	3380871	3380885	CCATTCTTCAGATGTTTGCG	GGGAGGGAAAGTAATCAATGC	115
Ad01A1323	Aradu.A01	1323	p4	(TCTT)5	20	3383911	3383930	GATGATCACGTTGTCGTTGC	ACCGTTTCCGTTGCTTCATA	270
Ad01A1325	Aradu.A01	1325	p4	(TATT)5	20	3387621	3387640	CAAATTTTAACGCCACCAAAA	CCCAATGGATACCAAAATCG	260
Ad01A1327	Aradu.A01	1327	p3	(CTT)5	15	3402004	3402018	GCAAATTTGGGTGTAACACGA	AAAAGAAGAAGATGGTGATGTTGA	168
Ad01A1329	Aradu.A01	1329	c	(GAA)8agaggt	198	3407808	3408005	AGAGGACACTCGCGCCTAT	GCCCTATCTGTGAGAGAGAGACA	265
Ad01A1330	Aradu.A01	1330	p3	(TTG)5	15	3415131	3415145	CATGAGGTGGTCAGACGAGA	CACAAGTCACAACACATCCCA	217
Ad01A1331	Aradu.A01	1331	p2	(TA)6	12	3417315	3417326	TTCTTGATTTGTCTCGGCTTC	GAGTATCCTTCGGTCGTGGA	167
Ad01A1332	Aradu.A01	1332	c	(T)11atgataggc	46	3419841	3419886	TGAAGGGCATTTGAATAGTTAGG	CGATTTTTGAATTGGATTTCG	266
Ad01A1333	Aradu.A01	1333	c	(AAG)8gagggg	107	3424966	3425072	TGTTCGTCCCCAATCTTTTC	AATAGCGACGAGCAACGAGT	243
Ad01A1335	Aradu.A01	1335	p3	(TTA)6	18	3432243	3432260	AATGGCACCTTGTCCAAAAA	GCAAAGGTGCCTCTCACGTA	244
Ad01A1337	Aradu.A01	1337	p2	(TA)6	12	3437382	3437393	TCATTTGACAAAACTACCCAACA	CCATTTGTCATTTATACATTGAGGG	221
Ad01A1339	Aradu.A01	1339	c	(CCA)5cctc(C	40	3447089	3447128	GAGCAACTTTCTCAGCACCC	AGGCATCGGTTGAGTATTGG	198
Ad01A1340	Aradu.A01	1340	p2	(GA)22	44	3447901	3447944	ACGGAAGGTCATGTGGAAAC	TCTCCACCACTACCACCTCC	181
Ad01A1342	Aradu.A01	1342	c	(CCA)5cctc(C	70	3451101	3451170	GCATCTTTCTCAACACCCGT	TTGAGGGAGGAGGAGAGACA	242
Ad01A1343	Aradu.A01	1343	p2	(AG)7	14	3451926	3451939	AAATCCCGAGTGAGGAAGGT	AACATTTCTCCACCACCACC	279
Ad01A1344	Aradu.A01	1344	c	(A)10taaaaggc	102	3457498	3457599	AAGGGAGGTTAAGGGTGGTG	AATTCGTGGCTTCCAGCTAA	276
Ad01A1349	Aradu.A01	1349	p3	(AAT)6	18	3468858	3468875	CGTTGGTGACATTTTGTCTTTT	CAAGTCTATCCCACTCCACCA	249
Ad01A1354	Aradu.A01	1354	p4	(ATTT)5	20	3475294	3475313	AGGGGGTGGTTGTAGAAAGC	TTGCAAATTCGAGTCTCCCT	218
Ad01A1371	Aradu.A01	1371	p2	(TA)7	14	3523822	3523835	ATGCAGGGACAGTAAGCCAG	CCTTCGAATTACTCAGCCCC	247
Ad01A1373	Aradu.A01	1373	p3	(CAA)6	18	3524895	3524912	AGAGGCTCTCCTCCTGCTCT	CTTTTCTTGCTGCTGCTCCT	241
Ad01A1374	Aradu.A01	1374	p3	(GGT)6	18	3525100	3525117	TTTGGAAGATTGGGAAGCTG	CCATTCACACCCGAACTTCT	274
Ad01A1375	Aradu.A01	1375	p3	(GGT)7	21	3525436	3525456	AGAAGTTCGGGTGTGAATGG	TGCCTTTGAAATCGCTCTCT	265
Ad01A1376	Aradu.A01	1376	p3	(GAA)7	21	3536700	3536720	GCTCCCACTTTCTGTCTCCA	GGCGACAAGTCCATAAGCTC	224
Ad01A1377	Aradu.A01	1377	p3	(TTA)6	18	3541049	3541066	TGGGCTAACATTATGATTTGCAT	AGGAGGCCTGTGTGGTTATG	260
Ad01A1383	Aradu.A01	1383	p3	(TTA)6	18	3546756	3546773	AATACCCAAAAGGCATGCAG	GCAGTTGGGAATCCTGCTAA	230
Ad01A1384	Aradu.A01	1384	p3	(ATC)7	21	3547578	3547598	CCAAGATCCCCATGAAGAGA	GCATACACGCACGGTTATTG	278
Ad01A1385	Aradu.A01	1385	p2	(TC)6	12	3549546	3549557	CAATCCTCTTGGTTCGTCGT	ACTGACTGAGCATCCAAGGC	237
Ad01A1388	Aradu.A01	1388	c	(T)10ctctcccca	63	3560387	3560449	ACGGAATCCATCTTTGATGA	ATTAGCGTTTTGTCCGCACT	272
Ad01A1398	Aradu.A01	1398	p3	(AAC)6	18	3585764	3585781	TTGGGAGCTAAGGTAAAAATGC	CATGCAAATAACATTCACCGTT	239
Ad01A1402	Aradu.A01	1402	p3	(TCC)5	15	3591569	3591583	TCGTCGTTACCAATACCACC	CCGAAATTCGCTGCAACTAT	267
Ad01A1403	Aradu.A01	1403	p2	(TA)6	12	3593050	3593061	AAGAACGCGAAAACAAAGGA	CTGCCTTGCCTGGTAACTTT	252
Ad01A1405	Aradu.A01	1405	p2	(GA)21	42	3595720	3595761	TTTGGGGATTTTGGATTTGA	TAAATCCTGCCCAGCGTAGT	169
Ad01A1415	Aradu.A01	1415	p2	(AT)8	16	3609150	3609165	ACATTTCCTTCCCAAAAACA	GGACCGTAGCTGATTTGCAT	261

ID	Chromosome	SSR nr.	SSR type	SSR	Size	Start	End	FORWARD PRIMER (5'-3')	REVERSE PRIMER (5'-3')	Product (bp)
Ad01A1418	Aradu.A01	1418	p3	(TCT)5	15	3616482	3616496	CCGTTGATTTCTCCTTGCAT	AACGATAGAACCAACGGCAC	234
Ad01A1419	Aradu.A01	1419	p2	(TA)6	12	3617784	3617795	CTGGAGACTCTTTGGAAGCG	CTCAACTTGCAAAGCAAAGG	212
Ad01A1420	Aradu.A01	1420	p3	(TTG)5	15	3621521	3621535	AAGATTGTTGGTGCAGGAGC	ATGGAGCAGCAACAGTAGCA	248
Ad01A1421	Aradu.A01	1421	p3	(TTC)5	15	3623877	3623891	TCCATCTCCTCACCTCCATC	AGTAACAATGGCGGATTTGG	209
Ad01A1430	Aradu.A01	1430	c	(T)10ctactctca	68	3647207	3647274	GCGCATGCTCTCTCTTCTTT	GTAGTCGTCGTCTTCCTCGC	274
Ad01A1431	Aradu.A01	1431	p3	(GGT)6	18	3647576	3647593	CGTTCAGGACAACAGCAAGA	CCCTGATTCTGAAACCCAGA	150
Ad01A1432	Aradu.A01	1432	p3	(TGG)5	15	3647812	3647826	TGGTGGATCTGGAATTAGGG	CCCATTCCCACCAATCCTAT	253
Ad01A1435	Aradu.A01	1435	p2	(AT)9	18	3655475	3655492	TTCCATTCACAAAAATGCTTCT	GCCGGTTAAAAGAAATAATCAGTG	260
Ad01A1439	Aradu.A01	1439	p2	(CT)6	12	3671095	3671106	AAATCCAAGCCCCATTTACC	TCTGCAGAGAGCAGTGGAAA	132
Ad01A1441	Aradu.A01	1441	p3	(TAT)16	48	3675209	3675256	AAGAAGTGGTGGTGGTGGAA	GCCCTTTGGTTACCATTCTC	179
Ad01A1442	Aradu.A01	1442	p2	(AT)8	16	3679410	3679425	CTATAGCAGCAACGGCAACA	AGGGAAAACATGGTGTTTGG	232
Ad01A1445	Aradu.A01	1445	c	(A)10caaaaata	114	3687459	3687572	AACGTGTCACCAAAACTTATCAAA	AGGATGTCGCAAGATGAGAAA	203
Ad01A1448	Aradu.A01	1448	p3	(AGT)5	15	3690973	3690987	CAGGGACGGATCCAATTTTT	CTTGGTCTGCGCCTTGTAAT	274
Ad01A1449	Aradu.A01	1449	c	(T)10agtgtcaaa	39	3691109	3691147	CAGGGACGGATCCAATTTTT	CTTGGTCTGCGCCTTGTAAT	274
Ad01A1452	Aradu.A01	1452	c	(T)13ggagtaac	86	3712182	3712267	TGGCAGCCATAAGTTGTGAG	TTTAATATTGTCCGTAATAAAAAGCA	265
Ad01A1455	Aradu.A01	1455	p3	(TAT)5	15	3714527	3714541	CAATCAAAACCGCACAATG	CAGTTTGGATTGGATTAGATTTG	270
Ad01A1457	Aradu.A01	1457	p2	(CA)6	12	3722526	3722537	CAGGGTTTGAGCCTTGGTAA	ATGCATCCAAACTTCCTTGG	209
Ad01A1463	Aradu.A01	1463	p2	(AT)10	20	3747556	3747575	CGTTCCTCCATCACCAATGT	TCGGGTTGGCTTAATAATCG	233
Ad01A1469	Aradu.A01	1469	p4	(TTTA)5	20	3757066	3757085	CGAGAAATTTCCCATTATTTGA	TCTGCCACTGACCAATTCAG	280
Ad01A1471	Aradu.A01	1471	p3	(TCT)5	15	3761226	3761240	AACGCGTTTTGAACCATTTT	AAGGCAAGCAACGAAAGAAA	210
Ad01A1472	Aradu.A01	1472	c	(GAA)18gag((	78	3761707	3761784	CAACGAATAAAAAGGCGAAGA	ATGCGTTATTCAACCGCTTC	182
Ad01A1473	Aradu.A01	1473	p3	(CAC)7	21	3763769	3763789	GTCAGCCTAGGGATGTGCTC	ATCCATCCTGAACCTCATGC	234
Ad01A1474	Aradu.A01	1474	p3	(CCA)6	18	3764264	3764281	TTGGCTCCTTCCTTGTCACT	ATGTGTGTGCGGTGGTAGAA	269
Ad01A1475	Aradu.A01	1475	p2	(AT)8	16	3772658	3772673	ACCAGCAAAGGCAAGATCAG	GTGGTTCACTTCGTTGAGCA	197
Ad01A1479	Aradu.A01	1479	c	(A)18cgaaccct	47	3777842	3777888	TTGGTTGGCTTTTCAAATCA	AGAGCTTGGCTAGAGAGGGC	209
Ad01A1482	Aradu.A01	1482	c	(A)12tagttgaaa	53	3792371	3792423	AATGTTTGTAATGGGGCAGG	GCTTAGCCATCGATTTGACC	203
Ad01A1483	Aradu.A01	1483	p2	(GA)19	38	3797820	3797857	ACAGCGACGTGTTATAGCGA	CACGATCACGGAAAGAGACA	176
Ad01A1485	Aradu.A01	1485	p2	(TC)24	48	3800875	3800922	GGAAATGAGGTGGGATTAAAGA	TCTGAGGGTTGCTGAGAACA	280
Ad01A1488	Aradu.A01	1488	p4	(TTTA)6	24	3806339	3806362	TCTTTCAAAAACATGGACAGGA	AACCAACTCTGCCACTGACC	278
Ad01A1496	Aradu.A01	1496	c*	(AAG)6atagtga	59	3829560	3829618	TGAGAAGGTAAAACAAACAAGGAA	GCAGCAAATTTTGGGGATAA	218
Ad01A1498	Aradu.A01	1498	c*	(A)10(AAG)6*	26	3830372	3830397	TGTGATTCTTCTTCTTCTTTGTTTG	CAGCAAATTTCGGGGTAAAA	260
Ad01A1500	Aradu.A01	1500	p3	(ATG)6	18	3832999	3833016	CAAGGATGTGTTTGGTAAAGCA	TTGGGTCCTTAATTGCTTGC	223
Ad01A1503	Aradu.A01	1503	c	(T)10aattatgttt	35	3841823	3841857	AGGTAAGAAATGCAGCACCC	CCTCCAACATTGCAACTAACA	277
Ad01A1504	Aradu.A01	1504	p4	(AATT)5	20	3848878	3848897	TGAAAGCCAACAAATTAAAGGAA	CCCACTAAAATTGACGGAGC	211

ID	Chromosome	SSR nr.	SSR type	SSR	Size	Start	End	FORWARD PRIMER (5'-3')	REVERSE PRIMER (5'-3')	Product (bp)
Ad01A1505	Aradu.A01	1505	c	(AAAG)5aaaa	57	3850148	3850204	GCCTCCTCAACAATACTAATGC	TCATATTCCTCCGAGTTCCG	214
Ad01A1508	Aradu.A01	1508	c	(T)10cttttatttg	44	3856367	3856410	TTGTTGGTCTTTATAGTTTCGCA	TCCCTTTTGGTAGTAATTGGC	191
Ad01A1510	Aradu.A01	1510	p3	(GAT)5	15	3859047	3859061	ATTTTGGTTGCATCTCCGTC	CCACCACCCTAGCTGGAATA	253
Ad01A1514	Aradu.A01	1514	p2	(TA)10	20	3885665	3885684	TGTTCAAATCCTCAAACGACA	TGTGGCACATTTTGGTTGTAA	235
Ad01A1516	Aradu.A01	1516	c	(T)12agactcag	59	3887266	3887324	TGCGGAACATTGAATTTTAGA	TACAATCATCTGGTGGCGG	240
Ad01A1523	Aradu.A01	1523	p3	(CTA)5	15	3898035	3898049	GGTTTTGGGATTCTGGGTTT	TGAAACGACAACCGACAAAG	145
Ad01A1528	Aradu.A01	1528	p3	(ATT)7	21	3915216	3915236	TGGCAATATCACCAAAACCA	GCTCAATGAAGAAAAATTGCC	261
Ad01A1529	Aradu.A01	1529	c	(A)14gaaaactg	81	3918658	3918738	AAATGCGAGGCAACAAAAGT	TGCAACTTGATTTGGTAAAGGA	165
Ad01A1531	Aradu.A01	1531	p4	(ATTT)5	20	3919016	3919035	TGCTGCTCATTCACTGGAAC	ACCCAGTTGCAGATTTGTCC	236
Ad01A1534	Aradu.A01	1534	c	(AAG)5acatac	45	3921131	3921175	GGAGGAGGAGGAAGAAGTGG	GATCTGATTCGATTCGCGTT	245
Ad01A1535	Aradu.A01	1535	p4	(TATT)5	20	3921609	3921628	TGTACATTTGCGGATCGAAT	GCATATCCGAAGATCCACAA	245
Ad01A1537	Aradu.A01	1537	p2	(CT)10	20	3930361	3930380	AACCCCAAAATCCTTCCAAC	GGAAGAAGAAAGAAAGGGCG	211
Ad01A1538	Aradu.A01	1538	p3	(GAG)5	15	3931720	3931734	CAAATTCCATCAACGCCTCT	CGTCGTCATCGCTGTTCTAA	213
Ad01A1540	Aradu.A01	1540	p3	(ATT)7	21	3933622	3933642	GTTCACACCAACAACATGCC	CCTGGGAACTCATTGAAAGC	232
Ad01A1545	Aradu.A01	1545	p3	(AAT)5	15	3944597	3944611	TTTGTTAAATTGCCCTGATCTTT	GCATCAAACACAAATTGAGAGAA	261
Ad01A1546	Aradu.A01	1546	p3	(TTA)6	18	3944718	3944735	TTTGTTAAATTGCCCTGATCTTT	GCATCAAACACAAATTGAGAGAA	261
Ad01A1551	Aradu.A01	1551	p2	(TA)6	12	3956409	3956420	CACTATCTCGCGTCCTGACA	CTGATTTCTCAATGCAGGGG	262
Ad01A1552	Aradu.A01	1552	p2	(TA)9	18	3956699	3956716	GGCCAACAATTTTGGTGTTT	TCAAGATTGTTCATTTGGCA	237
Ad01A1555	Aradu.A01	1555	p3	(GAA)5	15	3959409	3959423	AGGGAGAAAGAGGTGGTGGT	TAATTGCAGTTCGTGGAACG	143
Ad01A1562	Aradu.A01	1562	p2	(AT)10	20	3972572	3972591	TTTCACAGAAAATAAGAGGCCA	CGAATGCAATCTGTTTCAAAG	246
Ad01A1566	Aradu.A01	1566	p3	(AAG)5	15	3982750	3982764	ATGCGTTCGTGGGTAATCTC	ACCCTTCTTCTCCACTGCAA	210
Ad01A1567	Aradu.A01	1567	p3	(AGA)5	15	3982902	3982916	GGAGAAGAAGGGTTCATTGC	TGGTGGCTGATTTGTTGGT	280
Ad01A1568	Aradu.A01	1568	p3	(AAT)6	18	3986636	3986653	CCTTTTCCGAACTGCATAGC	ACCGGATCAAATACCGGTTC	245
Ad01A1574	Aradu.A01	1574	c	(TCTTCC)6(T	120	4006236	4006355	TCAAACGGTTCCTCTTCGTC	CGTGCAGAAACGAAAGAAAAG	253
Ad01A1575	Aradu.A01	1575	p3	(AAG)6	18	4007228	4007245	TTTCTCCTCCTCATCTTCTGC	CAAGGTACGTTTCTTGCCTTC	205
Ad01A1576	Aradu.A01	1576	p6	(GAAGAG)5	30	4007395	4007424	GAGAAAGAGAAGGCAAGAAACG	TACACGCGCTTTATTCATGC	265
Ad01A1580	Aradu.A01	1580	p6	(TCCTTC)5	30	4012501	4012530	GGACCAAAACCCAAAAGTCA	CCCAAAGAAGAAGGAGGAGAA	266
Ad01A1583	Aradu.A01	1583	p3	(TTA)14	42	4015242	4015283	TTTGAGCCATCTGTTGAGGA	TCGTTGTGACTAATTTTGGTGTTT	137
Ad01A1584	Aradu.A01	1584	p3	(ATG)5	15	4017392	4017406	CGACGAAATCGAAAAAGCTC	TTGCTTCACCTCCTCAATCA	253
Ad01A1585	Aradu.A01	1585	p3	(AAT)5	15	4017847	4017861	AGGAGATTCGGTTGTTGTGG	ACAAGTCAAACACCTTGCCC	200
Ad01A1586	Aradu.A01	1586	c	(A)10ttgagatac	55	4018396	4018450	TATGCCCCAATTGCATTCTT	TGTGTATGCAGCAACCCATT	230
Ad01A1588	Aradu.A01	1588	p3	(AAT)15	45	4024458	4024502	GTACATTGGAGCCACCCAAC	TTTTGCAATTTGAAGCATTACA	261
Ad01A1593	Aradu.A01	1593	p2	(TC)6	12	4034083	4034094	ACCAAGTACCCCAGCAACAG	ATTGGTTCATTTTGAGGCCA	184
Ad01A1596	Aradu.A01	1596	p2	(AT)8	16	4036661	4036676	TTCCAGGTAAGTGGCAAACA	CTGGCCAGGGATGCTATTTA	171

ID	Chromosome	SSR nr.	SSR type	SSR	Size	Start	End	FORWARD PRIMER (5'-3')	REVERSE PRIMER (5'-3')	Product (bp)
Ad01A1598	Aradu.A01	1598	p2	(TA)8	16	4041569	4041584	AAATGATTTTGATTGGAGAAATGTT	ATTTCATGTGTTAAATATGTGAATGAT	262
Ad01A1603	Aradu.A01	1603	p2	(AG)8	16	4051253	4051268	AATGGTCATCCAAATGCACA	GCTTGCCAAATTTTCTACTTCTC	273
Ad01A1605	Aradu.A01	1605	p4	(AATA)6	24	4058155	4058178	AGGCGTGGGTCATAAGTTTG	TGTTTAACCCAAAATCCCCA	181
Ad01A1611	Aradu.A01	1611	c	(T)10attttcctttt	88	4067134	4067221	CAAAAACTCTTTTTCTTTCTCTTTTT	TCACAAAATACATGTCTAAATCACCA	262
Ad01A1616	Aradu.A01	1616	p3	(TAA)10	30	4086884	4086913	CCATCAAATCCAACAGGGAG	GGTTGCAGATTCGAGTCTCTTT	223
Ad01A1617	Aradu.A01	1617	c	(T)11gttttaaaga	103	4087015	4087117	ACCTATATTGCTATTAGGGCATAGTT	TGATTTCCTTTTTAGTAGTTTTGATTT	278
Ad01A1620	Aradu.A01	1620	p3	(ATG)5	15	4098882	4098896	AATTGGCCCAACAATGAAAG	TTGAGGGAGGGACACTGAAG	121
Ad01A1622	Aradu.A01	1622	p3	(ATT)5	15	4100268	4100282	TAGGCACCACCATATCATGC	ATAACCACCAAACCCAACCA	255
Ad01A1623	Aradu.A01	1623	p2	(TA)11	22	4100693	4100714	CATTGGTGAGTCCAATGCTG	CCTTATCGCTTGCCCAAATA	250
Ad01A1630	Aradu.A01	1630	c	(A)10tgcatcatta	96	4107023	4107118	AATGACAACGCCACTTCACA	TTCAATGCAATGGTCGGTAA	176
Ad01A1635	Aradu.A01	1635	p2	(AT)9	18	4119366	4119383	ACATAAAGGCCCTCCTCCAT	CAAACATTGGACTTTCGGCT	206
Ad01A1638	Aradu.A01	1638	c	(A)10g(A)10	21	4131688	4131708	TTGCCAGATTTAATGCTAATGC	TGTTGTCGAGAATAAAATGGCA	278
Ad01A1641	Aradu.A01	1641	p4	(TCTT)5	20	4142594	4142613	GCACTTGACCAGGTTGTTGA	CCATAGTGGCCAAACAATCC	208
Ad01A1646	Aradu.A01	1646	c	(CTT)16cgagta	159	4150601	4150759	TGTAACAGCGGATCGAAATG	TTAAACACTAAAACCCTAAGTCACC	229
Ad01A1652	Aradu.A01	1652	p3	(AAT)5	15	4178988	4179002	GACGGAGTCACGGTCTCAAT	AGATGGTGGTGATGAAAGGC	277
Ad01A1654	Aradu.A01	1654	p3	(TAA)8	24	4187372	4187395	TGCTCAAATCTTCTTCCATTCA	ATTATGGGAGATTCGCGTGT	248
Ad01A1655	Aradu.A01	1655	p3	(TCT)5	15	4188356	4188370	TTCTTCAACCGTTACTGCACA	CCTCCTCATCTTCTGCTGCT	240
Ad01A1656	Aradu.A01	1656	c	(GAA)7gaggaa	82	4189217	4189298	TCAGAAACCATGAAAATCGAAA	TCGTGGATTATGAACGACTCC	244
Ad01A1657	Aradu.A01	1657	c	(AC)6caaacttta	104	4190565	4190668	TTGGTTACTAATAACATGGTTGGTC	TTCATGTCATATGCTGAGTTTCAA	260
Ad01A1659	Aradu.A01	1659	p2	(CT)8	16	4192565	4192580	AAGCTTAGGCGAAGATGCAA	TGGAGGGGACGTGAACTAAA	228
Ad01A1661	Aradu.A01	1661	p2	(GA)8	16	4202626	4202641	TGAACTAGCAGCAGGGACAA	AGTGGATGCCAAATCTTGCT	147
Ad01A1663	Aradu.A01	1663	p2	(AT)10	20	4235061	4235080	AGGAGCCTTGAAAAATGGGT	TTATGGAGAGGCAGGGTTTG	264
Ad01A1666	Aradu.A01	1666	p2	(AG)12	24	4239410	4239433	ATAATCAGTCCATCCCGCAG	ACGTAAATATTCTCCGGCCA	273
Ad01A1667	Aradu.A01	1667	p2	(TA)9	18	4241778	4241795	TCCAAGCTTCCAATTTCCAC	GCCGGATTTTAACCCAATTT	175
Ad01A1668	Aradu.A01	1668	c	(CTT)7ccaccgc	143	4244214	4244356	TATTTATTCCCGCTGCACCA	CCAGAAATTTCACAAACAAATCA	242
Ad01A1669	Aradu.A01	1669	c	(TGA)8ccatgat	176	4244480	4244655	TGTTGCTGAAATTTATTGTGGAA	TGTGTTAGAACCCACCACCA	246
Ad01A1671	Aradu.A01	1671	c	(A)11ttcttatata	98	4250499	4250596	TGGTTAATCTTGATTGCCTTTTG	ACATCACTTAAGGCTCTACGCT	227
Ad01A1677	Aradu.A01	1677	p3	(AAG)15	45	4265967	4266011	AACACGCGAAGAAGAAGGAA	ACGCGTCTAACACACACGAA	217
Ad01A1678	Aradu.A01	1678	p2	(AT)6	12	4266861	4266872	ATGGTAGCCAACCTATCCCC	TGAGAAGGGTGGAATCTTGC	248
Ad01A1682	Aradu.A01	1682	p2	(AG)6	12	4269377	4269388	TTAAAATCCTTGCGTGTCCG	TCCAACAACAACTCTCCGTG	280
Ad01A1685	Aradu.A01	1685	c	(GA)6gcgcgcc	37	4274650	4274686	CGTCCCCAGTAGTGATCGTT	TGCGTTTCACCAATTTACCA	256
Ad01A1686	Aradu.A01	1686	p2	(AT)8	16	4275359	4275374	CTTTGTCACGGCAGGAACTT	TCATTTTGAATTATCAGAACACTAACA	252
Ad01A1691	Aradu.A01	1691	p2	(AT)6	12	4286692	4286703	ATTGATGGCCAATTGATGGT	TCCTACTGATCAAACACCGAGA	103
Ad01A1694	Aradu.A01	1694	p3	(ACA)5	15	4295233	4295247	GGAGCATATCAAGCCCAAAA	TCGTTGGCAGGTAAAGTTCC	153

ID	Chromosome	SSR nr.	SSR type	SSR	Size	Start	End	FORWARD PRIMER (5'-3')	REVERSE PRIMER (5'-3')	Product (bp)
Ad01A1695	Aradu.A01	1695	p3	(AAT)6	18	4298640	4298657	AATGCCTTGACGTTTTCCAC	GGATGTCGATCCTCACGAAT	177
Ad01A1697	Aradu.A01	1697	p3	(CAA)6	18	4300907	4300924	CCCAAATCACCCTCAACAAC	TCGATCTCCCTCAACCAAAT	274
Ad01A1698	Aradu.A01	1698	p3	(CAA)6	18	4301190	4301207	TGGTTGAGGGAGATCGAAAA	AAGCTGAAAGCGGTTGAGAA	142
Ad01A1705	Aradu.A01	1705	p2	(TA)6	12	4326320	4326331	CATGCAATGACCCCCACTAT	AGGCACCTGAAGCACTTGTT	275
Ad01A1715	Aradu.A01	1715	p3	(AAT)12	36	4367547	4367582	TTTGTTTTTATCAACGTGGGA	TTTTAGAGGGGGTTGGGTAAA	187
Ad01A1716	Aradu.A01	1716	c	(A)12cgtaaacc	54	4370426	4370479	TCGCCATTTTTCATTCATCA	TTGAGAATGGGATGGAAAGG	204
Ad01A1717	Aradu.A01	1717	c	(T)10caaaattttt	53	4371060	4371112	AAGGGGATTGGTTCTACGCT	TCGCACTCGAACCTCAAAAT	271
Ad01A1719	Aradu.A01	1719	p3	(TTA)6	18	4387342	4387359	TCTCAATATTCCAAACCAAATACTACC	AGAGCTCAATGCCTCAATGG	254
Ad01A1721	Aradu.A01	1721	p2	(AC)10	20	4389478	4389497	GAAATTCGATAAACGGCTGC	CGAGATTCCATGTATTCCACAA	213
Ad01A1723	Aradu.A01	1723	p3	(TTA)8	24	4394006	4394029	TGCAAATTACTTCAAGGGGG	CCAAAAGGTGGAAAGAGGAA	273
Ad01A1725	Aradu.A01	1725	p3	(AGA)5	15	4399347	4399361	TCTTTTCTTTGGGCTCGCTA	CCATGACACGAGTCTCGCTA	106
Ad01A1727	Aradu.A01	1727	p4	(TAAA)5	20	4405407	4405426	GAGGTATTTTTGTTGCGGGA	TTCTTCTTGTGTGGCAAACG	273
Ad01A1730	Aradu.A01	1730	p3	(AAT)5	15	4414348	4414362	ATGATTTCCTTTGGGGTCCT	CAATCCTAGCCATGAAGGGA	280
Ad01A1734	Aradu.A01	1734	p2	(CT)8	16	4417229	4417244	AGCGGCAGATTCTTCCATTA	TGAACTTGACAAAATGCTAATGCT	182
Ad01A1736	Aradu.A01	1736	p2	(AT)6	12	4424363	4424374	CATCAACTAGCCATTGCATCA	GAATTGATTTGGTTTTTGTAACGA	275
Ad01A1739	Aradu.A01	1739	p3	(TTC)6	18	4429034	4429051	AACTTTTCAAATCACGCGCT	CGTGTGCAAATTTGGAAGAA	178
Ad01A1745	Aradu.A01	1745	p3	(ATT)6	18	4436385	4436402	ACGTATACAGCATGCCCCTC	TCTCAATTACTTTTGAATTTTGATGC	252
Ad01A1747	Aradu.A01	1747	p3	(TTA)5	15	4441455	4441469	GGCTACTTGATCAACGATGGA	TAGCACTATTCGCAAGGCAA	177
Ad01A1753	Aradu.A01	1753	p3	(AAT)7	21	4453933	4453953	CAATCGGAATAATGTTGTAAGGTG	CACCATGATGTCTTTCACTGC	255
Ad01A1754	Aradu.A01	1754	p2	(GT)6	12	4455920	4455931	CACCAAAAATCAGCCACCAT	TTGTCCTGAGACAAATGGGA	190
Ad01A1760	Aradu.A01	1760	p2	(GA)7	14	4477071	4477084	CAAAAACCCGAATTTGATTGA	CGAATCCGTCTTCTCCACTC	162
Ad01A1761	Aradu.A01	1761	p2	(AG)7	14	4477502	4477515	TGGAAGCCATGCATTGTAGA	CCTCTTTCTTGTTGAGAAAGGC	248
Ad01A1771	Aradu.A01	1771	p2	(AG)6	12	4504538	4504549	CGGTAATTGGGTCCTGTGAC	GCTCTCCATTTTGAGTGCAT	249
Ad01A1774	Aradu.A01	1774	c	(GA)7ct(A)14g	65	4507826	4507890	CACACCGAGAAACACAAAACA	ATTCGCAGTGACAGCAACAG	139
Ad01A1775	Aradu.A01	1775	c	(A)11ttaaaaacc	77	4509052	4509128	ACATTTGGAGTGAGCTTCGG	CCAAAGCATTAACAAAATCGG	196
Ad01A1779	Aradu.A01	1779	p2	(AT)6	12	4516737	4516748	GCCAACGACAAACCCTTAAA	CGTGATTACCAAACACACCC	235
Ad01A1782	Aradu.A01	1782	c	(A)10ctaaggag	73	4522284	4522356	GCCCGGTATTAAACCTCCAT	TGAGGAAACTTCGGAAGAACA	199
Ad01A1783	Aradu.A01	1783	p2	(TA)6	12	4525844	4525855	GAGAATAGGTAGAGTCAAGCGAGA	GGACGTTCAACGCTCATTTT	242
Ad01A1784	Aradu.A01	1784	p2	(AT)10	20	4533058	4533077	CAATCAATGTGTGAGCATGG	TCTCAACTAAGCTCCGCTCA	121
Ad01A1786	Aradu.A01	1786	p2	(TA)6	12	4535034	4535045	CTTGCTTTGGGTTCAAGTCC	TCTCTCCTCCCTCCCATTTT	222
Ad01A1791	Aradu.A01	1791	p2	(GA)9	18	4559895	4559912	GCCAGAAGCGAGTTTTGTTC	GATTCTTCGCTTCGCAGTTC	267
Ad01A1792	Aradu.A01	1792	p2	(CT)6	12	4562069	4562080	TGTGATTGTTTTGACTTTGAGGA	CTGGCTGAGGAGCAGAGTTC	253
Ad01A1793	Aradu.A01	1793	p2	(CT)6	12	4562327	4562338	CCGCCTCTGTCCTACTTGTC	GTCCAGCTGCTTTGGACTTC	229
Ad01A1795	Aradu.A01	1795	p2	(TA)6	12	4565192	4565203	CAATAGGAGAGCAACAAAACCTC	CCCTTCCAAGACTAGGCACA	163

ID	Chromosome	SSR nr.	SSR type	SSR	Size	Start	End	FORWARD PRIMER (5'-3')	REVERSE PRIMER (5'-3')	Product (bp)
Ad01A1797	Aradu.A01	1797	p2	(GT)6	12	4567647	4567658	CAATCTGGAAAGCTGGAAGG	CGAGTACGAGGACGAAGAGG	137
Ad01A1798	Aradu.A01	1798	p4	(TTAT)7	28	4574111	4574138	CCGTCTCTACAACGCAACAG	AAAGAGTAAGTTCGATCCGAAAA	280
Ad01A1801	Aradu.A01	1801	c	(A)11g(A)10	22	4579235	4579256	AGAGAGAAAAAGCGTTGGGG	GATAAACCCTAAGAGGGGCG	180
Ad01A1805	Aradu.A01	1805	c	(T)10agaattgct	58	4583905	4583962	TCTGCTGTGTCTATTGACATTCAT	GCCTGAATGGAAGAGGTTTTT	277
Ad01A1806	Aradu.A01	1806	c	(CT)7cacacac[illegible]	60	4589176	4589235	GGGAAGAACCACAACCAGAA	AAAAGACCAAGACTTTTTGAGGG	267
Ad01A1817	Aradu.A01	1817	p4	(ATTT)5	20	4607907	4607926	GCAAGCAACCAAGATTGTCA	TCAAAAGCTCTGGCCAAAAT	139
Ad01A1822	Aradu.A01	1822	p2	(AT)7	14	4622512	4622525	ACTTAGGGTCCACCGTCTCC	TTTGGATCAAAATAAGGGCAA	165
Ad01A1827	Aradu.A01	1827	c	(A)10taaaaata[illegible]	73	4627666	4627738	CAGGACAAAATATGCAAACACAA	TCCGAGTGATTTTAATGAATGTG	270
Ad01A1833	Aradu.A01	1833	p3	(ATT)7	21	4637357	4637377	TGCCTATATCTTATTTAACAAACACAA	AAAAGTTGATTTGGTAAATCTTTCAG	256
Ad01A1835	Aradu.A01	1835	p2	(AT)8	16	4641238	4641253	GAAACAAATCCAATCATCTTAGTCC	TGTATGACCAACTTGCTTTGC	257
Ad01A1836	Aradu.A01	1836	p2	(AT)7	14	4641546	4641559	AAAGCAAAGCAAGTTGGTCA	GCTGCTAGTGTGGGAATCTTG	161
Ad01A1838	Aradu.A01	1838	c	(T)10attttttttat[illegible]	46	4646910	4646955	TCATTCAGGCTGAGTTTGTTTC	ATGCATATGGCTAGAACGGC	163
Ad01A1840	Aradu.A01	1840	c	(TA)11atgctaa	111	4648755	4648865	TCTATTGTAGACCCAGGCGG	CGTGACGACACCTGAATATGA	190
Ad01A1848	Aradu.A01	1848	p2	(AT)8	16	4656120	4656135	CAGTTCCTGTCTGTGCGTGT	AATGCTCATCTTCAAAGTTTTCTT	280
Ad01A1853	Aradu.A01	1853	p3	(TAT)5	15	4659585	4659599	CCCAGAAATAGAGTTAAGATTGGG	TTCCAAAATCTTAGTTTGACAAAAA	277
Ad01A1855	Aradu.A01	1855	p3	(TAA)6	18	4662175	4662192	CCTTGCTAGTAGGTATTCTTGTAATCA	TCAAGTGCAACTAAATGTCTTAACG	243
Ad01A1859	Aradu.A01	1859	p2	(TA)11	22	4670205	4670226	GACAAAGATTCACACCCACG	ATATACGTGGTCTGTGGCCG	208
Ad01A1861	Aradu.A01	1861	p2	(AG)7	14	4682643	4682656	AGTTGGAAGTGGTGGCTCTG	GCTTCAAACATTATGCTGCG	220
Ad01A1864	Aradu.A01	1864	p2	(TC)17	34	4691640	4691673	AAACAATGATGCCAAAAGGG	CCCAAATGAAGAAAGTTGCG	255
Ad01A1865	Aradu.A01	1865	p3	(TTG)5	15	4695737	4695751	TGAAGCCATGGTGTCGTTTA	TCCGACACACTAACCTTCCC	248
Ad01A1868	Aradu.A01	1868	p2	(CA)6	12	4730934	4730945	AGGGTCAACTGAAAAACATGC	GCAGAGGATGCATGACCTATC	258
Ad01A1873	Aradu.A01	1873	p2	(TG)6	12	4743845	4743856	AAAATGCCTTCATGTTTGCC	ATTAGGTCTAGCCGTGTGGG	254
Ad01A1875	Aradu.A01	1875	p2	(TC)27	54	4747514	4747567	CGATGCCTCCTTTGTTTCCT	CCATGACCGGTATTGCTTCT	210
Ad01A1877	Aradu.A01	1877	p3	(AAT)7	21	4749477	4749497	ATGGTTTTACCCTGCCCCTA	TCAAATTCAAATATGCAGTAAAATCT	280
Ad01A1878	Aradu.A01	1878	c	(GT)6gg(T)10	24	4752631	4752654	GCCAAGGAATTAGGCGAACT	TGACACACAGACACACACGG	148
Ad01A1881	Aradu.A01	1881	p2	(CA)6	12	4755431	4755442	TGCGGTACGTTGAAGAGAGT	CGAAAATGTTTGAGGTGTGTG	236
Ad01A1888	Aradu.A01	1888	p3	(AAC)5	15	4768508	4768522	ATGCAGTAGCCCCACAAAAC	GATTGCTGCACTGTTGCTTC	247
Ad01A1890	Aradu.A01	1890	p3	(AGA)26	78	4774526	4774603	CGACGAAGCTAATTACGTCAAA	TGTTCTCTTGTCGTGTTTTCG	167
Ad01A1892	Aradu.A01	1892	c	(T)10attaattttg	91	4775019	4775109	ATTTCGGGAAACCCGACTAA	GGGCAGAAACTCAAACCGTA	279
Ad01A1893	Aradu.A01	1893	p5	(TCTCT)5	25	4776401	4776425	TTGGATAAGAAATCGGTGCC	TCTATTCCCAGCCTCACAGAA	270
Ad01A1894	Aradu.A01	1894	p4	(CATA)5	20	4777220	4777239	CAGCCACAAACATTCATCTCA	CAACCGGAGCTTCATATTGTT	280
Ad01A1898	Aradu.A01	1898	c	(ATAA)6(AT)	36	4786698	4786733	CAAAAGGACACACGCACAAC	ACCCCTCTCACCTTCCATTT	230
Ad01A1900	Aradu.A01	1900	p2	(TA)8	16	4789684	4789699	CTTTGCATGACTTGGCTTTT	TGCCAGGTTTAGATTTGATGG	144
Ad01A1902	Aradu.A01	1902	p2	(AT)7	14	4800382	4800395	TTCGATCTGACTGTTTAACCTTT	GAGGTAATTTGAGGTGGAATATTTTT	280

ID	Chromosome	SSR nr.	SSR type	SSR	Size	Start	End	FORWARD PRIMER (5'-3')	REVERSE PRIMER (5'-3')	Product (bp)
Ad01A1903	Aradu.A01	1903	p2	(AT)6	12	4802989	4803000	GCGGGCTCTTTTTCTCTTTT	AAAATTTGTAATTGGATGCTTTGA	153
Ad01A1910	Aradu.A01	1910	p2	(TA)9	18	4851432	4851449	CCTTTCACCTTACACAAAATGC	CTGCGTTCAGTATTTTCAGAGG	121
Ad01A1915	Aradu.A01	1915	p3	(CAA)5	15	4872543	4872557	TCACTCACCGAAAAGTTCCC	AATGAAATCTGGGAAGCGG	132
Ad01A1920	Aradu.A01	1920	p2	(AG)7	14	4888724	4888737	ACCGAAACCCCCAAGTTATC	CTTCCCGAACACTTGTCCAT	104
Ad01A1927	Aradu.A01	1927	p2	(TA)6	12	4900691	4900702	CGACCGACTCATCCGTTTAT	GCGGAGACTGTGAGACTGTG	280
Ad01A1935	Aradu.A01	1935	p3	(AGT)5	15	4925254	4925268	ACAGAATGCGGTCTCCAAAC	TATCATGCTTCCAAGGCACA	265
Ad01A1940	Aradu.A01	1940	p2	(GT)7	14	4954474	4954487	GGGTTAGATGGGATATTGGGA	GTCCGTGATATTCAACCGCT	267
Ad01A1942	Aradu.A01	1942	c	(T)10acctataac	43	4960890	4960932	TCGTGGAAAGCTAGACCCAC	TGGCGCAAAACAATATCAGA	255
Ad01A1943	Aradu.A01	1943	c	(A)11gtttagatt[illegible]	86	4965943	4966028	AAGGAATGAATCGAAAAAGTCAA	CACGTTTTCTTTGGCTAGCTG	228
Ad01A1946	Aradu.A01	1946	p2	(AT)6	12	4968834	4968845	TTGGACCCAAATAAGTGCCT	TGTTTCCCTCGACTTTCCAC	204
Ad01A1951	Aradu.A01	1951	p3	(AAT)8	24	4977612	4977635	AAACGGGTGATCTGGATCTAA	ATTCTCCCAATTTTCACGCA	202
Ad01A1969	Aradu.A01	1969	p2	(AT)8	16	5009324	5009339	ACAGACATTGATATGGATACGAGA	TTCATTCAGCGCACCATTTA	256
Ad01A1975	Aradu.A01	1975	p4	(TTAT)6	24	5016129	5016152	CGCTTTTTCTTTTTATCGTCG	TTCAATCTTCATCACCATGTCA	220
Ad01A1979	Aradu.A01	1979	p3	(AGG)6	18	5028131	5028148	TCATCCAATCAAAACCAGCA	CGATTTGGGGAGGTCAGATA	209
Ad01A1983	Aradu.A01	1983	c	(AT)10(AG)7a	52	5037225	5037276	GGAAAAGTCTAAGGGGCCAG	AGGAAAATCGCACAAGCACT	146
Ad01A1984	Aradu.A01	1984	p2	(TA)7	14	5038321	5038334	GGAACGCATTCGTTATCATTG	TGATGAGGTCCACAAAGCAC	255
Ad01A1993	Aradu.A01	1993	p2	(AT)6	12	5055614	5055625	TTGAAAGAAGCTAAAACCAAAACA	GGTAATCAAGTCCAACCCGA	203
Ad01A1994	Aradu.A01	1994	p2	(AT)6	12	5056395	5056406	TTGAAAGAAGCTAAAACCAAAACA	CGAACCTGAACCAAACCAAT	245
Ad01A1997	Aradu.A01	1997	p3	(GAA)6	18	5062434	5062451	CAATTGCTTTGACGGTGATG	TGTGATGATGATGGTGGCTT	124
Ad01A2000	Aradu.A01	2000	p4	(AAAT)6	24	5073206	5073229	ACCCTTGACCTTTCGGATCT	TCTCCCTCTCAATGTGGCTT	232
Ad01A2002	Aradu.A01	2002	p3	(CAA)5	15	5075632	5075646	AGCAGGCAGAGGCAGAAGTA	CCCTTCCCCTCTGTTTCAAT	168
Ad01A2004	Aradu.A01	2004	p2	(AT)7	14	5076636	5076649	CCTATAAGATATTAGCCCTTCCATTG	TTCCACAAATTGGTTCCTCT	143
Ad01A2007	Aradu.A01	2007	p3	(GAA)5	15	5083567	5083581	GGAAGAATAATCGAAATCGCA	TTTTATGAACGTTAGGCGGC	224
Ad01A2008	Aradu.A01	2008	p3	(GAA)5	15	5086473	5086487	GTTAGGGGGCGTGTAGTCAA	TTTTTCTTCTCGCGTTTTCG	133
Ad01A2012	Aradu.A01	2012	p2	(TG)7	14	5115198	5115211	ATGGCAATTTTCTCTGGTGG	AGGAAGAAAGACGGAGAGGG	211
Ad01A2015	Aradu.A01	2015	p3	(TTA)6	18	5138592	5138609	TGCCTTTGATTCTGCAACTG	TCAACGACCTATGCATGGAA	217
Ad01A2026	Aradu.A01	2026	p2	(AT)12	24	5154742	5154765	GCGAAATTTTGTCGGTGACT	TTGCCAATGACCGTTTATGT	174
Ad01A2032	Aradu.A01	2032	p3	(TTC)6	18	5172539	5172556	TATTCCAATGCCCCTACCCT	ATGATGATGGGGTTGGATTG	229
Ad01A2035	Aradu.A01	2035	p3	(ATG)5	15	5175887	5175901	CCTCAACGTGTTCCCAATCT	GGGGTAGAAGGGTAAATGGG	194
Ad01A2037	Aradu.A01	2037	p3	(GCT)6	18	5179730	5179747	TCATCTGGGTATGCTCCATTT	AGGGTCATGCAATTCACACA	278
Ad01A2039	Aradu.A01	2039	c	(T)11gaaatattc	81	5186079	5186159	TCACAGGAAATACCACCACATT	GGTGGTTGAGTTCCTTTCATTT	275
Ad01A2042	Aradu.A01	2042	p2	(AT)7	14	5199927	5199940	TTTGATTTCTCACAAAGAAAAACA	TACGCCCGAGAAAGAAGAAA	224
Ad01A2043	Aradu.A01	2043	p3	(ATT)5	15	5200421	5200435	CAGCTGAACAATGAAAATCCA	AATCCATGGAACATGCACAA	172
Ad01A2045	Aradu.A01	2045	p3	(AGA)5	15	5202333	5202347	CAGGAGTCCATTGTCCTTTGA	AGCTTGTGTGGCCTAATTGC	231

ID	Chromosome	SSR nr.	SSR type	SSR	Size	Start	End	FORWARD PRIMER (5'-3')	REVERSE PRIMER (5'-3')	Product (bp)
Ad01A2046	Aradu.A01	2046	p2	(AT)6	12	5203198	5203209	CCCATCATGGTGTGATGAAA	TTGTTTCTAAACGCACATCTAAGAG	122
Ad01A2052	Aradu.A01	2052	c	(AAT)15agaaa	123	5223905	5224027	GCATGTGAATTTCGACTTTACC	CCTCGTTGCACATGATTACG	196
Ad01A2053	Aradu.A01	2053	p3	(ATT)6	18	5224302	5224319	GGCGAAACGAACTGTTTCAT	TCGTCGCAGTCTCAGGTTTA	173
Ad01A2055	Aradu.A01	2055	p2	(AT)9	18	5227067	5227084	GTCGCTTGATGCTTGTGAAA	GTTATGAGTGCGTCATCCGA	219
Ad01A2056	Aradu.A01	2056	p2	(TA)9	18	5242788	5242805	TCCATAATTTTTGAAAACCACTGTT	TCGTATGCAAATTTCTGCTCA	249
Ad01A2066	Aradu.A01	2066	p2	(AT)9	18	5263096	5263113	GCAAGCTAAGGGACATGCAG	TCTTGTTGAGAGGGAAATGGA	265
Ad01A2069	Aradu.A01	2069	p2	(AG)6	12	5269241	5269252	TTTGAGGAGTCATAAAATTTGTAAAG	GGACTGTGGTGAGCTAGCACTAA	225
Ad01A2070	Aradu.A01	2070	p2	(GA)6	12	5274215	5274226	ATCACACTATTGCTGCTGCG	CTGGCACCAGCTAACAACAC	232
Ad01A2074	Aradu.A01	2074	p2	(TC)16	32	5278061	5278092	TGTCGGATTGGAAGATACCG	GAGAGAATGAGAGGCGTTGC	131
Ad01A2076	Aradu.A01	2076	p2	(TA)11	22	5284620	5284641	TCCGTCATCTTGTCATATCTCG	CTGCACGTGCCATTATCGTA	271
Ad01A2080	Aradu.A01	2080	p3	(TAA)5	15	5294476	5294490	GAAATCGATTGCACCTGAGT	TGCTGGGGTATAGTGCCTTT	276
Ad01A2081	Aradu.A01	2081	p3	(TAA)5	15	5294664	5294678	CGATTGCACCTGAGTTTTCA	TGCTGGGGTATAGTGCCTTT	271
Ad01A2087	Aradu.A01	2087	c	(T)11acatatattc	121	5315522	5315642	GGATAAACCGATAAACATCTTATTGA	TTGAAATTATGGACAACAATTCG	276
Ad01A2090	Aradu.A01	2090	c	(A)10cctttgaaa	64	5333159	5333222	GCGGAAAGGTTTGTTCTTCA	TTGTGATCTCCCTTATTTCTTCG	273
Ad01A2092	Aradu.A01	2092	c	(A)10ctaaagttt	58	5352653	5352710	GGAAACAAGAGGAAGGACGG	CTTGAAAAACAATTTCTTTCCTAAAT	226
Ad01A2102	Aradu.A01	2102	p2	(TA)8	16	5377955	5377970	CCCCTTCTCTTAGCCACTGA	TTCTTTTTGTCATATGTACTTCAATTC	219
Ad01A2109	Aradu.A01	2109	p2	(TA)6	12	5389182	5389193	CAGAAATTTTTGTCAATTTATGACTTT	GGAAGGAGGGGGATAATAATG	280
Ad01A2116	Aradu.A01	2116	p3	(TAT)20	60	5406027	5406086	AAAATCGCAAATAGAATAACTTCTCA	TTTGTCTTTTACACAAATACCAAATAA	262
Ad01A2123	Aradu.A01	2123	p3	(AAT)8	24	5434654	5434677	TAGCATCACCATCATTTGCC	AGCCCAGAAACACTCCTCAA	233
Ad01A2125	Aradu.A01	2125	c	(A)10tcctttttaa	43	5436406	5436448	CTAACATTGCCCAATTCCCA	TTTTTGAAAAGAAGAATATTGTATGC	242
Ad01A2131	Aradu.A01	2131	p2	(TA)7	14	5443515	5443528	CAGCTGATCCCAAATTGGTT	AGAAATGAACGGAGCATTGG	237
Ad01A2142	Aradu.A01	2142	p2	(AG)6	12	5476245	5476256	CTATTCGTGGGTGGGAAATG	CTGGATGGTGTGCAGTGTTT	153
Ad01A2143	Aradu.A01	2143	p2	(TA)6	12	5478569	5478580	AGGCAACAGAGTAGCAAATCG	TGCATTATCAATTACACAAGTCACA	264
Ad01A2144	Aradu.A01	2144	p3	(TTC)6	18	5481193	5481210	CTCCATTTTCACCACCACCT	GGCGAGACCAATTATCGAAG	196
Ad01A2153	Aradu.A01	2153	p3	(CAT)5	15	5505181	5505195	TCGGCTAGCTTCCCTTTGTA	CAGAGCAACCTCAAAGAAATGA	271
Ad01A2154	Aradu.A01	2154	p3	(TTA)7	21	5506316	5506336	GCGTACAACAGAACATTTGGA	GAAAAATGCACCAAAAACTAAGC	241
Ad01A2156	Aradu.A01	2156	p4	(TCAC)5	20	5515179	5515198	CTTTGTGGAGTTGCTGTTGG	TAGTGGTGGAGGCATGATGA	198
Ad01A2158	Aradu.A01	2158	p2	(GA)6	12	5515863	5515874	CCACGTCAAAAGAGTGTTGC	CGGATCCACTAGCGGTAAAA	273
Ad01A2160	Aradu.A01	2160	p3	(AAT)5	15	5516384	5516398	TTGCCGTTTAAATTGATTCAG	TGAATCCCTCTTGAGAATTGG	252
Ad01A2170	Aradu.A01	2170	p3	(TTA)16	48	5538258	5538305	CCTCTGGGCAAAAAGTTGTC	CATATTGAAAGCCACCATTGTC	166
Ad01A2172	Aradu.A01	2172	p2	(TC)6	12	5543620	5543631	ACCTCCACCACACCAAAAAG	GAAGAATGGAGGGAGGAAGG	107
Ad01A2173	Aradu.A01	2173	p3	(CCT)5	15	5544067	5544081	CCCAGACTACCTCCCTCCAC	AGTGGAAGTTTTCGGGGTTT	269
Ad01A2175	Aradu.A01	2175	p2	(AT)6	12	5567352	5567363	CGCGTATGCATAATAACCAAA	AACGACCATGACGATGACAA	263
Ad01A2178	Aradu.A01	2178	p3	(TGT)5	15	5590170	5590184	CCATTGAATCCAAACCCATC	CAAAAATGGCACTTCAGGGT	272

ID	Chromosome	SSR nr.	SSR type	SSR	Size	Start	End	FORWARD PRIMER (5'-3')	REVERSE PRIMER (5'-3')	Product (bp)
Ad01A2179	Aradu.A01	2179	p3	(TAG)7	21	5590845	5590865	TCATAAAGAAAAGGGTGGCG	CGTGGAGACAAACCACTGAA	192
Ad01A2188	Aradu.A01	2188	p3	(ATG)5	15	5600044	5600058	TGCAAGAGCCATAAATGCAG	CAAACATGGTTGCTTTGGAA	242
Ad01A2189	Aradu.A01	2189	p5	(ATTAT)7	35	5600396	5600430	TCCACATGGGATTCACACAC	TCCATAGCACAAGTCACCAAA	230
Ad01A2190	Aradu.A01	2190	p2	(TA)9	18	5601099	5601116	GCCGAGCTTGAGTTTGGATA	CAAGTTCGGCTCACGAAAAT	240
Ad01A2191	Aradu.A01	2191	p5	(TTATA)6	30	5601438	5601467	TTTGTGTTCTTAAGATAGAAATTAGCA	TCTAATTGCTCCATTTTAATGTGTT	240
Ad01A2197	Aradu.A01	2197	p2	(AT)7	14	5616749	5616762	GATCTTAAGAATCAAAGAAAAGAAAAA	AAAATGAGGTGGTGCGAGAC	262
Ad01A2203	Aradu.A01	2203	p3	(TAT)10	30	5634109	5634138	GGCCAAAGCAAGAAGAAAGA	CATTTGTTTGAGGGGACCAC	133
Ad01A2211	Aradu.A01	2211	p3	(TTA)5	15	5659179	5659193	GGTTATTATTTTTCGTCTTTCTTGTTG	CGTGTTGGCTGAAAAATCAA	134
Ad01A2212	Aradu.A01	2212	p3	(GAA)15	45	5676991	5677035	CGCCGTTTATACCGTCAACT	CCGTTTCTCATATCCATGTGC	215
Ad01A2214	Aradu.A01	2214	p2	(CT)22	44	5677850	5677893	AATTCAAACCGCGAACTGTC	CAATGGCAGTAGTGGCAGAA	252
Ad01A2220	Aradu.A01	2220	p3	(AAC)9	27	5688620	5688646	GGTCCAGGCAGTATGCTCTC	ATGAGCCTGGATGCATGATT	241
Ad01A2226	Aradu.A01	2226	p2	(TA)9	18	5702745	5702762	CTTCTTTCACCTTGGGAGGA	GACACAGACATGTCATAAAATAGACAA	280
Ad01A2231	Aradu.A01	2231	p2	(TA)6	12	5707088	5707099	TTTGTTTCGTCTTTATTTGTTTCTG	CGCGAATACCCATCCATATC	264
Ad01A2235	Aradu.A01	2235	p2	(TC)15	30	5713059	5713088	CAAGCCACTTCCAACCCTAA	CGGAATCGTCAACCTTCTCT	163
Ad01A2242	Aradu.A01	2242	p3	(AAG)5	15	5735664	5735678	TGAAGATGAAACACGCGAAG	ACACGCACGTTATTCAACCA	195
Ad01A2245	Aradu.A01	2245	p2	(AG)6	12	5739704	5739715	TTGGATCTGCAGTTGCTCTG	TTTACACCCTCATAATCCCTTG	193
Ad01A2246	Aradu.A01	2246	p2	(AT)7	14	5740359	5740372	GAGGGTGTGGAATTTGCAGT	TGCATATGCTTGAGTTCCATTC	186
Ad01A2247	Aradu.A01	2247	c	(T)12gtttgataa	65	5742405	5742469	TGGAAGAAATGAGCAAGGCT	GACCGGATTAATTGGGGATT	278
Ad01A2249	Aradu.A01	2249	p3	(TTA)5	15	5746571	5746585	CCGTCAATGAAAAGTCTTGTAAA	ATCATGTCGTCAATCGTCCA	200
Ad01A2251	Aradu.A01	2251	p3	(TTA)6	18	5750481	5750498	GCCCTCCTGTTATCGATTTG	TCATGGTTGCACTTCATGGT	158
Ad01A2252	Aradu.A01	2252	p2	(TA)11	22	5751878	5751899	CGACAGAAAGAAAAGGAACACA	CTTTCATGGATTGCCCCTAA	216
Ad01A2253	Aradu.A01	2253	p3	(CTT)5	15	5756876	5756890	GAAACAAAATAGCATGCGCC	AACGAACCACCGTCAAGTTC	163
Ad01A2255	Aradu.A01	2255	p2	(AG)6	12	5760949	5760960	TATCCTTATGCCTTGGGCAC	CGCAAAGGAGGCTTTTGTAG	183
Ad01A2256	Aradu.A01	2256	p2	(TC)6	12	5761144	5761155	CTACAAAAGCCTCCTTTGCG	AGGAAACCGAATTCACCTCC	188
Ad01A2260	Aradu.A01	2260	p2	(AG)7	14	5771094	5771107	TGATTTGAAAAGGGTAGTGCAA	TGGTGGTGGCGACATATCTA	103
Ad01A2261	Aradu.A01	2261	p2	(AG)8	16	5775674	5775689	TCGAGGAAGAAGTGAGGGAA	TAGTTTGGTGGAGGACTGCC	112
Ad01A2266	Aradu.A01	2266	p3	(GAT)5	15	5789030	5789044	GTGAAGCTCAAAGGCAAAGG	GGTTGTGACTTGGTCTCGGT	230
Ad01A2267	Aradu.A01	2267	p3	(TTC)5	15	5826805	5826819	ACACCACTTCTCCCCCTCTT	TCTCTCCATCTCCCATGACC	245
Ad01A2269	Aradu.A01	2269	p3	(TGT)5	15	5846578	5846592	TGCTATCCCTCTCTGAATGGTT	ATCTCGAGGGACAACATTGG	280
Ad01A2272	Aradu.A01	2272	p3	(TAT)7	21	5850282	5850302	TGAAAAGAAAGTTATGCACCGA	GCTCGAGAGAGACAGCCACT	110
Ad01A2274	Aradu.A01	2274	p2	(AT)7	14	5854184	5854197	CAGACGCAATCCAAACAGAA	GAGGGTTTTGTGGACATGCT	240
Ad01A2276	Aradu.A01	2276	p3	(AAT)5	15	5855523	5855537	TTACTTGGACCCCACACACC	TGGTGGTGTCTTCTTACCCC	270
Ad01A2280	Aradu.A01	2280	p2	(AT)11	22	5860470	5860491	CCAAACATAAACATACCCTCACTG	TTATGTGTCCCTCCACATCG	153
Ad01A2281	Aradu.A01	2281	p3	(ATT)6	18	5861939	5861956	ACCGTAAACAAACGAAGCCA	CGTTCATATAAGATCCAACAATCC	214

ID	Chromosome	SSR nr.	SSR type	SSR	Size	Start	End	FORWARD PRIMER (5'-3')	REVERSE PRIMER (5'-3')	Product (bp)
Ad01A2286	Aradu.A01	2286	p2	(AT)8	16	5871807	5871822	TCATTGTTTTCCTTATGGTGGA	AACAATTTTCAGTTTGGCGG	275
Ad01A2288	Aradu.A01	2288	p2	(TA)6	12	5879069	5879080	TCCACCAATTTGAAACCTAACA	TTGTCCCTATTCTCAATGTCCA	260
Ad01A2289	Aradu.A01	2289	p2	(AT)9	18	5882275	5882292	GACGCTTTTTAAGATGTGTTGC	TTGCTATTCAAAGTGTGAGTCCT	215
Ad01A2290	Aradu.A01	2290	p2	(TA)9	18	5882466	5882483	TTTCTTTCTCTATACCATTTCAATCTG	AAATTTTTAGCGTGCAAGTGA	276
Ad01A2293	Aradu.A01	2293	p2	(TA)6	12	5885701	5885712	AACAAGAATTAGGCAAAGAAGCA	CAAGCCCAAATTCAAAATTCA	272
Ad01A2295	Aradu.A01	2295	p3	(GAG)9	27	5896525	5896551	AATCACAATGGGCTGAGAGG	GCAAGGAAGAATGCTCAGGA	153
Ad01A2302	Aradu.A01	2302	p4	(TTAT)5	20	5912414	5912433	AAGCAAGTAGTCTTTGATCTTCCA	TGCAAATCAATAATATCACTATCACAA	238
Ad01A2305	Aradu.A01	2305	p2	(TC)6	12	5913856	5913867	CACACAACACTTGCATTGCC	AAAGTGGGGTACCTGCTGTG	222
Ad01A2312	Aradu.A01	2312	c	(TCC)5ctcttctt	104	5928005	5928108	TCTCCAATGTTTGTATCCCACA	CGGCTTTTTCTTCTTCATCTTC	261
Ad01A2313	Aradu.A01	2313	c	(AAG)5cagcaa	41	5929697	5929737	GAATGTAGGTTCATCCTCTTCCA	AGACATTTCAATGTATTTGTATTCTGA	263
Ad01A2314	Aradu.A01	2314	c	(TTC)6tttgtttga	124	5929971	5930094	TCATAATTTTTCTAGTAATTTTGCAGC	TCCTCCTCCTCATCTTTTGC	200
Ad01A2315	Aradu.A01	2315	c	(A)10gaagaacg	102	5930624	5930725	CCAATGAGAAAAGGAGGAGG	TTTCTGTCCCTTGCTCCACT	262
Ad01A2316	Aradu.A01	2316	p3	(TTA)5	15	5933943	5933957	GTTCCTCTCATCGGCTCTTG	GTGGGCTCGATTTACGAGAA	251
Ad01A2317	Aradu.A01	2317	p3	(ATT)6	18	5937383	5937400	TGTGGAAGCACGTAATGAGG	AAGCTATAGGTTTTACTGGCTCACA	109
Ad01A2320	Aradu.A01	2320	p6	(GGTTTT)5	30	5941736	5941765	GCAGATCTCGCAAGAAGGAC	CTCGTTGTCGCTCAAGCTC	234
Ad01A2324	Aradu.A01	2324	p2	(TA)8	16	5949919	5949934	GATTGGTCATCTGCATTTCG	CGGATATTATCATGGATACAAAACA	252
Ad01A2327	Aradu.A01	2327	p3	(AAT)7	21	5958110	5958130	ACACTTGCATGGTTTGGTCA	CCCATTAGAACAGGCCCAAT	274
Ad01A2333	Aradu.A01	2333	c	(A)15ttcaatccg	93	5978870	5978962	TTATCTCAAAAGACAACGAGGC	GTGAATACCACATCCGACCC	235
Ad01A2336	Aradu.A01	2336	p2	(TA)8	16	5993496	5993511	GCCTTTTGCACGTCTTCTGT	AAATTAAAAGCGCAACGCTC	212
Ad01A2338	Aradu.A01	2338	p2	(TA)7	14	5997766	5997779	TGCTACCTTCGACCATGTCA	AAAAAGACTTCAGCGATCAAATA	251
Ad01A2339	Aradu.A01	2339	p3	(TTG)5	15	5999504	5999518	CCCCTACTCCACCCCTTTTA	CCCACATTGGAAGTTGCTCT	227
Ad01A2341	Aradu.A01	2341	p4	(AGTG)7	28	6001494	6001521	GGAAAGGGAAAGGAGGAAAC	TCTCCTCCCACCTCTCCATA	198
Ad01A2343	Aradu.A01	2343	c	(A)14tgacaattta	67	6004396	6004462	CAGTGTGCAATAAAACTCAGTCG	TTTTAGTTCCGTCCCTCGTTT	222
Ad01A2349	Aradu.A01	2349	p2	(AT)6	12	6014742	6014753	ACTCATGTCCAGCCGACTTT	AACATCGAGTAAACTATCCAGCAA	270
Ad01A2350	Aradu.A01	2350	p2	(CA)6	12	6018941	6018952	GGGAAACCGAAACCATACCT	GGACCTTGGGTGTGGTAAGA	277
Ad01A2354	Aradu.A01	2354	p2	(AT)6	12	6028107	6028118	TGACGTGAATGTGGAGCTGT	CTTTGCTCAGACACAACAAAAA	258
Ad01A2361	Aradu.A01	2361	c	(AAG)5tgcagc	150	6037667	6037816	GAGAAAGAGAAGGCAAGAAACG	ACTGCACGTTTTCACTGCAC	201
Ad01A2362	Aradu.A01	2362	p3	(AAG)5	15	6037921	6037935	AGTGCAGTGAAAACGTGCAG	AATTCGTCAAAGATCGGAACA	261
Ad01A2365	Aradu.A01	2365	p3	(ACA)6	18	6044792	6044809	CGCCTTACAAATACAAATAAAAGGA	GTAGCGGAGCCCACCTAGTA	270
Ad01A2366	Aradu.A01	2366	p2	(TA)6	12	6055509	6055520	GTGCATGCAAACCAACCATA	AAAAACAAAAGCGTCACTGC	116
Ad01A2369	Aradu.A01	2369	p3	(TGA)7	21	6060816	6060836	ACACATTGGAGGGCTACCTG	CTCAGGCTTCCATATCAGCAG	258
Ad01A2370	Aradu.A01	2370	p2	(GA)6	12	6064185	6064196	TGGCCAAAAAGGTAACCAAA	CGCAGCTACACCCTCTTCTC	277
Ad01A2371	Aradu.A01	2371	c	(T)10(A)11	21	6064348	6064368	GGGGAATTCTGGGGTTAAAA	CCCTTTTCACCATTCTCACTTC	238
Ad01A2380	Aradu.A01	2380	p3	(AAG)5	15	6105355	6105369	AAGAGGGATCGTTTAGCGGT	CCCTCTTCTTTTTCAACCCC	224

ID	Chromosome	SSR nr.	SSR type	SSR	Size	Start	End	FORWARD PRIMER (5'-3')	REVERSE PRIMER (5'-3')	Product (bp)
Ad01A2388	Aradu.A01	2388	p3	(ATT)5	15	6115660	6115674	ACCACGTGATCAATTTGGAA	AACAGAAATTGCTGCTGCCT	274
Ad01A2391	Aradu.A01	2391	p4	(AAAT)5	20	6122909	6122928	TGGTGAAGAGTTGTTTTTGAATG	TGCAACATACACACTTGCATTG	233
Ad01A2399	Aradu.A01	2399	p3	(ATT)5	15	6143418	6143432	CAACACAAGTGATGTTGGTGTG	TGTTTGAAGGATTTTCTGAGCA	280
Ad01A2404	Aradu.A01	2404	p2	(TA)6	12	6159066	6159077	AGCATTGTCAATTAGTAGTATGCG	AAGAGAAAGAAAATGGAAGCTAAA	224
Ad01A2405	Aradu.A01	2405	c*	(TTA)5(TA)6*	25	6160543	6160567	TGGTAGGGACGAAATTGAAAA	AAAAAGCACATCCAAATCACTTT	264
Ad01A2407	Aradu.A01	2407	p2	(GT)6	12	6166116	6166127	AGTAAGGCCAGGTTTTGGGT	ACCAAGCAATAGGGCACAAC	204
Ad01A2411	Aradu.A01	2411	p3	(TTA)5	15	6183070	6183084	AAAAACGAATAAATGGTAATCGG	AAGGTTAGGAACCTATTTGTCCTTT	270
Ad01A2413	Aradu.A01	2413	c	(T)10ggtaaaaa	202	6189872	6190073	GATTTGGAATTAAAATCTAGGGTTC	TGCCTCCAAGGAATTGCTAA	270
Ad01A2419	Aradu.A01	2419	p2	(CT)18	36	6197579	6197614	AATTGCATCACCAAAATCCC	TTTCTTGAACCCCTTTTGGA	265
Ad01A2420	Aradu.A01	2420	p2	(AT)6	12	6205762	6205773	TTGTCGACATGCATCTTTCA	GCGGAATTCATTTTGTTCTCA	277
Ad01A2422	Aradu.A01	2422	p4	(TTTA)5	20	6208211	6208230	CACGAGATGGCAATATGGAA	TAAGAGGCCAAGTGTCAGCA	114
Ad01A2434	Aradu.A01	2434	p3	(ATG)5	15	6229624	6229638	CACAAATGACCCATATGCCC	ACCGAAATTACCGTGGGAGT	254
Ad01A2439	Aradu.A01	2439	p3	(AAT)5	15	6242414	6242428	CCGGTTTTGATATTTTTGCTT	TCTGTGCATATATACCGTGCTT	175
Ad01A2440	Aradu.A01	2440	c	(A)11ttttttattg	77	6242984	6243060	GATTGGTTAGCCCTCTGTCAA	CACAGCCTTTTCTCTCCATGT	280
Ad01A2442	Aradu.A01	2442	p2	(AT)11	22	6245657	6245678	ACTCGACAAATTCGGTTTGC	ATTGACCCTGCGATGTAAGG	168
Ad01A2443	Aradu.A01	2443	p3	(CTT)5	15	6247309	6247323	AAAGGTCTCTGCCAATGTCG	TGTCGCCATCTGAGAAACAG	230
Ad01A2449	Aradu.A01	2449	c	(TG)7(TA)11	36	6271855	6271890	CCCTTTTGCAACCAACATTT	TGCAATTCTCTAGTCAATGCAAA	256
Ad01A2450	Aradu.A01	2450	p4	(TAAA)5	20	6273288	6273307	GCACACCAAATGTATGGAAAAA	GGCGATTTTAAACTGCCGTA	248
Ad01A2451	Aradu.A01	2451	c	(TC)6ttgttttcct	78	6276517	6276594	CATATCCCTCCGCACATTTT	GCAAGGTGGATTATGCGACT	227
Ad01A2453	Aradu.A01	2453	p3	(TTA)5	15	6279778	6279792	CTGGCAAACAATTGGGAACT	TGACCTTGCTAAAGGATTGAAA	111
Ad01A2455	Aradu.A01	2455	p3	(CCA)6	18	6282293	6282310	TCACAATTCCGCTAACACCA	CCATCAGCATCACACACTTG	206
Ad01A2456	Aradu.A01	2456	c	(A)10tcctaaaat	34	6283984	6284017	GCCACAGGCAGAAAAGTCAT	AGTGAGTGGGACTGTGGGAC	248
Ad01A2459	Aradu.A01	2459	p3	(TCT)5	15	6290858	6290872	GTAGTTATCGGGAGGGGGAC	AGAAGCAGAAGCAGAGGCAG	193
Ad01A2468	Aradu.A01	2468	p2	(AT)9	18	6303393	6303410	CCTACCGTGCAAGCCATACT	CCATCTCATCCACCCTCCTA	206
Ad01A2469	Aradu.A01	2469	p3	(TTG)7	21	6303619	6303639	TGATCCATGTGTCCAACTCC	CAACGTTCCACCCTGTTCTT	243
Ad01A2481	Aradu.A01	2481	p2	(TC)6	12	6342941	6342952	TACAACTCATTTGCGTGCCT	AGGTTGAAGTGTGTTACGTGCT	220
Ad01A2483	Aradu.A01	2483	p3	(CAT)5	15	6350257	6350271	GCGGGGTCAGATTGAGATTA	CGCATACCTCTATTTCCCGA	219
Ad01A2487	Aradu.A01	2487	c	(T)17aaaaaaaa	188	6365506	6365693	AACATGAAATCGACCCTCCTT	CGGATCGAGCGATTTAGAAC	280
Ad01A2488	Aradu.A01	2488	p2	(AT)7	14	6366452	6366465	TTTCCGTGATGATAACACCTTC	TTCAGAATGGCAAAATCTTCCT	218
Ad01A2489	Aradu.A01	2489	p2	(TA)9	18	6366676	6366693	GAAGGAAGATTTTGCCATTCTG	TCGACGTATCGTGTCGTGTT	212
Ad01A2490	Aradu.A01	2490	p3	(TTC)5	15	6367092	6367106	CCATCATCTGCAAACACCAC	CCGTTCTTGATTCTCTGCGT	111
Ad01A2491	Aradu.A01	2491	c	(ATT)6aacaatt	51	6370587	6370637	GATCCGGTTCGATTTTCAGA	TTCAACTTACCATTTCTCTATCCA	269
Ad01A2493	Aradu.A01	2493	p2	(TC)6	12	6380573	6380584	CTTGGCAGCTGTACCAGGAT	TTGTGTGTCACTTTCTGCCC	204
Ad01A2495	Aradu.A01	2495	p3	(ATT)24	72	6386143	6386214	GCCATTTCCAAATGCACATA	TAGCGCACATGCTCAGAGTC	191

ID	Chromosome	SSR nr.	SSR type	SSR	Size	Start	End	FORWARD PRIMER (5'-3')	REVERSE PRIMER (5'-3')	Product (bp)
Ad01A2498	Aradu.A01	2498	p2	(TA)7	14	6400043	6400056	GATCCGGTTCGATTTTCAGA	TGTAGCATTGCTCATATTCTAATTCA	277
Ad01A2508	Aradu.A01	2508	p3	(GTG)5	15	6431290	6431304	TTAGGGAAGGACAAGCCAAA	CTAGCACTAGAACCACCGGC	205
Ad01A2509	Aradu.A01	2509	p2	(TA)6	12	6431600	6431611	TGGCAAATTTATTTTCTGACGA	TTAGATGAACACCCCCTCCC	193
Ad01A2510	Aradu.A01	2510	p2	(TA)6	12	6433574	6433585	TGGCATCTTGTTTTTCGTGA	AAAAATGGTATGTTCGTGAAGGA	272
Ad01A2511	Aradu.A01	2511	p2	(TA)7	14	6433752	6433765	CACGTGCACTCCATCATTTT	AAGAAGCAAAGCATGTGAATGA	211
Ad01A2512	Aradu.A01	2512	p3	(TTA)6	18	6434353	6434370	TATCATAATGCACCCGCAAA	TCTTCATCGAGAAATTAGGAAGAAA	252
Ad01A2514	Aradu.A01	2514	p3	(ATA)5	15	6444352	6444366	AGCTTTATAGGCTTGGCCCT	GCTGCATGCTTTGGACACTA	157
Ad01A2521	Aradu.A01	2521	p3	(ACA)7	21	6460072	6460092	TCCTGCAGCAAAATAAAATCG	GGGACAAATCCGTCCAGTTA	268
Ad01A2524	Aradu.A01	2524	p2	(TC)18	36	6469080	6469115	TGCCGAGTCCCTTTTCTCTA	TCATGCATGCCTATTTCCAA	248
Ad01A2525	Aradu.A01	2525	p2	(CT)6	12	6475078	6475089	TCGCTGCTTATCCCATTACC	CGTCATATTTACAATTACCGCC	198
Ad01A2527	Aradu.A01	2527	p2	(AT)8	16	6478457	6478472	AATTAATGTTTGTGGGCGTG	ATACGCGTGAACACAACGAG	226
Ad01A2530	Aradu.A01	2530	p2	(TA)6	12	6485144	6485155	CTCACGCAAACTGCCACTAA	GCCAAAAATCAGTCATTTGTTCA	203
Ad01A2531	Aradu.A01	2531	p3	(TTC)5	15	6485375	6485389	TGTTTGAAGGATTTTCTGAGCA	CAAGTGATGTTGGTGTGAATCC	278
Ad01A2532	Aradu.A01	2532	p3	(ATC)5	15	6488297	6488311	TTTTCCTTGTCAAGTTGGGC	ATGGTTATGGTGGCTGTGGT	163
Ad01A2533	Aradu.A01	2533	p3	(TGT)5	15	6490086	6490100	GGGTTTGAGGAATGTTTTGG	TCTACCACCATTTTCGGGTC	245
Ad01A2534	Aradu.A01	2534	p2	(TA)9	18	6490655	6490672	ATTTCCAGGAATGGTACCCC	CACACCAGGACAAACACAGG	211
Ad01A2535	Aradu.A01	2535	p3	(ATC)6	18	6492387	6492404	GCCAATAACCTGTTTGCATTC	CCCAGATTGGTTTCTAATTTCC	256
Ad01A2537	Aradu.A01	2537	p2	(AT)6	12	6501437	6501448	TGACTTTTAAGTTCATTTTGCATAGG	TAATGCGGCAGTGATAGCTC	275
Ad01A2539	Aradu.A01	2539	c	(A)10gcccaaaa	98	6504210	6504307	TGGTTATTTTCAACATATGAACTCAA	CATGCCATCTTTTTGCGAC	233
Ad01A2540	Aradu.A01	2540	p4	(AAAT)5	20	6506187	6506206	AAAATATGCATGAGGTGCCC	TCGTTTACGATCCGTTGAAA	209
Ad01A2541	Aradu.A01	2541	p2	(TA)7	14	6511238	6511251	GAGGGATTTGGTGTGCAACT	AGGTGTTTTGAGCCCCTTTT	187
Ad01A2543	Aradu.A01	2543	p2	(CT)7	14	6516232	6516245	TAGTATGCAAAGGGTTGGGC	AAGCACCGTAGCCTCCTGTA	257
Ad01A2544	Aradu.A01	2544	p3	(ATT)13	39	6516938	6516976	CCCTAACCCAAGGTTTGTGA	CTCTCCCCTGATTACCACCA	262
Ad01A2548	Aradu.A01	2548	p2	(CT)16	32	6522391	6522422	CTGAAGCCCATCTTTCTTCG	GTCGCTGAAACTGGGAGAAG	275
Ad01A2550	Aradu.A01	2550	p2	(TA)7	14	6522993	6523006	TCTGAAGAGTTGCTTGGCCT	ACACTTCAAACAAACGCACC	196
Ad01A2551	Aradu.A01	2551	p2	(AT)9	18	6525622	6525639	CAGTATTGGTAATGGTGAAAGGC	GAAACCTCATGAGAACTCCCC	278
Ad01A2552	Aradu.A01	2552	p2	(AT)6	12	6526157	6526168	CCACAATGGATAACGGGAAC	TTGGAGACATGATCTTTGGAGA	210
Ad01A2553	Aradu.A01	2553	p3	(TTA)14	42	6536296	6536337	GTCCAGAACCGAATCTTCCA	GTACCTCCAGCGATCGAAAG	273
Ad01A2555	Aradu.A01	2555	p4	(ATGT)5	20	6539969	6539988	TTTGCCAATTATTGATGGGA	TCACTCTTGCAGTTGCCTCA	248
Ad01A2556	Aradu.A01	2556	c	(CTT)7caaatgt	141	6540312	6540452	CAACTATGACTTTCTTCCCCAA	GGGCCCCATTTCCCTATAC	270
Ad01A2557	Aradu.A01	2557	p2	(TA)6	12	6540613	6540624	GCCCGAAAAGAAAGACAAAA	TTTTGGTTCAATACAATTTCCTTT	189
Ad01A2561	Aradu.A01	2561	p2	(CT)8	16	6549921	6549936	CAGAAAACACAACACCCCCT	AGGGAAGTGACGACGAGAGA	236
Ad01A2562	Aradu.A01	2562	p3	(AAT)5	15	6553368	6553382	CAAAAACCACATGAAATGCG	GAAGCCTTTGGGGACTGACT	128
Ad01A2564	Aradu.A01	2564	p3	(ATC)6	18	6559903	6559920	ATGGGTCCTAAAAACGGTCC	TTGCTTTTGGTGTAAAATTGC	240

ID	Chromosome	SSR nr.	SSR type	SSR	Size	Start	End	FORWARD PRIMER (5'-3')	REVERSE PRIMER (5'-3')	Product (bp)
Ad01A2570	Aradu.A01	2570	c	(A)12cacaaatta	47	6570329	6570375	GCTCCACAAAACATGGCATA	GGAGGGACAATGAGGTTTGA	249
Ad01A2581	Aradu.A01	2581	p2	(TC)6	12	6596814	6596825	TCCGAACCTCACATTCTTCC	TGGAGGGTTTGTAATCGGAG	216
Ad01A2586	Aradu.A01	2586	p2	(AT)8	16	6603201	6603216	GGTGACTATGAGTGTCTCGGC	GGTGTGTGTTGTGGTGTCACT	249
Ad01A2588	Aradu.A01	2588	p2	(TA)8	16	6605299	6605314	ACAAGCTCCAGGCTTCTTCA	GTGGAAAATGGTACAAGGGG	193
Ad01A2589	Aradu.A01	2589	p2	(AT)8	16	6606344	6606359	TTAGAGTGCGTTTGGAAGCA	CACGATCATCTGTGTTAAACTTTTT	121
Ad01A2590	Aradu.A01	2590	p3	(AAT)7	21	6606927	6606947	GATCACTTTACGAGGCTAAAAAGTT	TCAAATCAAATAACGCATGGA	277
Ad01A2591	Aradu.A01	2591	p3	(TTC)5	15	6611104	6611118	TGGCAGTGTGTGAAGGAGAG	CCATGTTTAAGAAAGGCCCA	263
Ad01A2592	Aradu.A01	2592	p2	(CT)6	12	6611771	6611782	TGGGGGAGTTCTTGCTATTG	GGAATTCACGTGGGAACAAC	269
Ad01A2595	Aradu.A01	2595	c	(T)10caatgaaat	44	6618998	6619041	TCCGCCATAAACGGATTTAG	GCTATAACAGATGCGCACAAA	190
Ad01A2596	Aradu.A01	2596	p3	(ATC)6	18	6619274	6619291	TGTGCGCATCTGTTATAGCC	CGACGATAACGATAACAACGA	232
Ad01A2600	Aradu.A01	2600	c	(A)11ttt(A)10	24	6626125	6626148	TCGATGTCAATATGTTGCTCAAT	AGGCAACACCTAGCCTTTCA	197
Ad01A2601	Aradu.A01	2601	c	(TCC)5tcgaatt	99	6627623	6627721	TCCTCCCTTTCTCTTCCGTT	TGGATCCAACAATGTCATCAA	264
Ad01A2602	Aradu.A01	2602	c	(CAA)5taattca	54	6628513	6628566	TCTCAACCATGGATGCATTT	AAAGCTGACCTTGAACTGCC	252
Ad01A2603	Aradu.A01	2603	p3	(AAT)16	48	6631191	6631238	TTTGCCCTCCACCTATTGAC	TGCATAGCATTTTTCCAATGA	274
Ad01A2604	Aradu.A01	2604	p3	(AGG)5	15	6633205	6633219	TTCTTTCATGGTGGTGGTGA	CCCGTTACACATTCAAACGA	155
Ad01A2609	Aradu.A01	2609	p2	(TA)8	16	6645182	6645197	TTGGAATTAAAGAATTGGTAGGTTG	TGGCTAAGTTTACTGCATTCCA	257
Ad01A2611	Aradu.A01	2611	c	(A)10cttaaaaac	85	6652556	6652640	TTGCCAATGATGGATGCTTA	TTCGAATTCTAAGGGTAGTGCTT	238
Ad01A2613	Aradu.A01	2613	p5	(TATTT)7	35	6653672	6653706	AAAATAAAGAGGGAACGGAAGG	CTGGGGAAGATTTCTGGTGA	199
Ad01A2615	Aradu.A01	2615	p3	(AAG)5	15	6670608	6670622	TGCAAGCCATCTTCACAAAG	TGCGGAATTGGTCTATAGGG	117
Ad01A2621	Aradu.A01	2621	c	(A)13cagttctga	122	6683705	6683826	TTTCTCTGAAAAACCCCCGT	GCACAGACGGAAAAGAGAGG	233
Ad01A2622	Aradu.A01	2622	p3	(TTA)5	15	6686252	6686266	GAAAACTCGGCAAGCAAAAG	GAGGTCCAACTGCTGCTCTC	246
Ad01A2624	Aradu.A01	2624	c	(AT)9gaatcatc	129	6693505	6693633	TTCTCTCTCTCTTTCTTTCTTCGC	CGTGCTGATAACGTGTTGTG	181
Ad01A2625	Aradu.A01	2625	c	(TA)7ttacaatat	123	6698550	6698672	GGGTCTCACACATATGATGCAC	TCCAAGGATCCTGTTGTGAA	214
Ad01A2629	Aradu.A01	2629	p3	(TAT)6	18	6712957	6712974	TCTTTTTCCACCAGAAACCG	GCTTTGCAACGCTAACACAA	238
Ad01A2631	Aradu.A01	2631	p3	(CTT)20	60	6716676	6716735	ATAAAATAACGCCGCACCAC	GAAGCTCCGGTTTGTTGAAG	235
Ad01A2641	Aradu.A01	2641	p3	(TAT)8	24	6740947	6740970	TTTTTCTCACTCACAAAATCTCC	GCCACAAATCATAATTTTTAAACC	164
Ad01A2646	Aradu.A01	2646	p3	(ACA)6	18	6755044	6755061	TTCATCTGTTATTTAGCGTTTTCAA	GGAAGGTGGATGAGATGGAA	246
Ad01A2650	Aradu.A01	2650	p2	(AC)6	12	6769368	6769379	GGAGGTAATATGCCGAAATCC	CCCCACCTCATTATGTTCTTT	280
Ad01A2651	Aradu.A01	2651	p2	(AT)7	14	6771996	6772009	CCATCGTTAACGTGATTTGG	TTTTTGACTGATCACTTCTTAGTTTCA	259
Ad01A2653	Aradu.A01	2653	p2	(AT)7	14	6773988	6774001	GCATGTGAACACACAAGTTGC	TTTTTCAAATTCGATCCGAG	211
Ad01A2657	Aradu.A01	2657	p2	(TA)9	18	6779482	6779499	TTGACGACGAAAGAAAACGA	CAGCGTTACACACTTACACACG	274
Ad01A2664	Aradu.A01	2664	p3	(ATT)7	21	6791793	6791813	TGGAATTTGGCTTGCTTTTC	GCAGATTAGCAGTGTGGCAA	119
Ad01A2665	Aradu.A01	2665	p2	(TA)7	14	6798045	6798058	CGTTGAGCTCAGGATTGTTG	AAAGTACATGCATTTGCACACA	250
Ad01A2668	Aradu.A01	2668	p3	(TTC)5	15	6806668	6806682	GAAGGACCACGGTAAGGTGA	ACCACCACCATAGCTCCAAG	125

ID	Chromosome	SSR nr.	SSR type	SSR	Size	Start	End	FORWARD PRIMER (5'-3')	REVERSE PRIMER (5'-3')	Product (bp)
Ad01A2673	Aradu.A01	2673	p3	(TTG)8	24	6824381	6824404	CATGCATGATGAACACACCA	TGCAACATGTTTGGGACAGT	225
Ad01A2676	Aradu.A01	2676	p2	(TA)6	12	6830482	6830493	CCAAAATTAGATCTTGGGAGTCA	CCCTTTTGACAACACAAACA	279
Ad01A2678	Aradu.A01	2678	p3	(TGG)9	27	6839037	6839063	TGGGGGTTACAGTGAGGG	TTTTCACCTTCCTCACCCAC	218
Ad01A2684	Aradu.A01	2684	p4	(ATTT)5	20	6849621	6849640	TTACTCGTCCCAAGTCCCAG	TCGGATTAAGGGTGTTTTGG	201
Ad01A2687	Aradu.A01	2687	p2	(AC)7	14	6854352	6854365	GCATAATCTATGTTGTGGGCAA	TAATGTCGCTGGCATGTTGT	163
Ad01A2691	Aradu.A01	2691	p3	(TAT)20	60	6885692	6885751	TGGATTCCTAATGGCAGGAC	TTGCTCAGATGCTTATTAGCTGAC	181
Ad01A2694	Aradu.A01	2694	p2	(TA)6	12	6899942	6899953	ATCAATACATGCCGCCCTAT	CCAAGACAAGGGAAAGATAAAGG	279
Ad01A2698	Aradu.A01	2698	p2	(TA)8	16	6904343	6904358	TCCGAAACAATAGCATCCTT	TGATGTTCAATTTTTCACTCAATTT	261
Ad01A2703	Aradu.A01	2703	p3	(ATA)5	15	6912213	6912227	TGCGACATGCATCGTGTATT	ACTCCTTGGCCACTGTCATC	245
Ad01A2706	Aradu.A01	2706	p2	(TA)6	12	6925756	6925767	GCCCAAAATGTGTAGATGGG	ACCCTTGACATTGATGAGGC	268
Ad01A2707	Aradu.A01	2707	p2	(AT)12	24	6933059	6933082	TTGTTATTTAACGTATCTTATGCCA	AAGAATGCATGTAACCTTTTCATC	279
Ad01A2711	Aradu.A01	2711	p3	(AAG)5	15	6946124	6946138	AAAACCCACCGTTTTCATCA	GTAGGTCTTTTTGGCGGTGA	220
Ad01A2714	Aradu.A01	2714	p2	(AT)7	14	6952512	6952525	CGGTTGAAACTAGCTGTGCT	ACGAAGATGATGACGCACAC	278
Ad01A2716	Aradu.A01	2716	p3	(GTT)6	18	6966895	6966912	AGGCTGCTTCCCATACCTTT	AGTGTGTGAAAAGGGCAACC	170
Ad01A2717	Aradu.A01	2717	c	(T)10aatattaatt	83	6980544	6980626	TTGGGATGGATTTTCTTTGTTT	CCGATGGGAATTTTGAGAGA	266
Ad01A2724	Aradu.A01	2724	p2	(TA)6	12	6985586	6985597	TCAAACACACACATGCTAGTGG	CAATGTTGGTCTTGGTCCCT	278
Ad01A2727	Aradu.A01	2727	p2	(AT)7	14	6993394	6993407	GCGCCCAATAGTTTTAGATTTT	TTTAAAAGTGTTGCTTAAAAGATTGA	278
Ad01A2729	Aradu.A01	2729	p3	(TTA)15	45	6996016	6996060	TTATGTTAACCCCACTGCCC	GCTCAGTCACAAAGACATTCCA	277
Ad01A2730	Aradu.A01	2730	p2	(AT)6	12	6996178	6996189	TGGAATGTCTTTGTGACTGAGC	AAATAAAACACCATGGTTGGATG	102
Ad01A2731	Aradu.A01	2731	p5	(AAAAT)5	25	6999625	6999649	TGCCCCATGAAAATATGGAT	CACATACATGTGATGAAGTTTTGG	279
Ad01A2733	Aradu.A01	2733	p3	(TAT)20	60	7006983	7007042	GAGAAAAACCAGAGCCATGC	TTTACACCCTCACCCTTCCC	222
Ad01A2734	Aradu.A01	2734	p3	(TCT)5	15	7022589	7022603	GGATCCTGTCTGTGTACGGC	CATCAAAGAAGGATCGCCTG	253
Ad01A2735	Aradu.A01	2735	c	(T)10agaatacct	97	7036307	7036403	TGGGGGTTAGGTCATGAGTC	GCGTATCCAGGGTTTAGGGT	258
Ad01A2738	Aradu.A01	2738	p2	(AT)9	18	7058280	7058297	AAGCCAAAAGAACCAGTCCA	TCTGGAGAGGACAATAGTGCAA	256
Ad01A2744	Aradu.A01	2744	p2	(AT)9	18	7069609	7069626	GAAAAACTCAACTCACGATAAAAGA	TGCATCACATCCCTACAACTTT	251
Ad01A2748	Aradu.A01	2748	c	(AT)7(GT)8	30	7081792	7081821	TTTTACCAAATGGCATCATGT	TTGGGCCAGATAAACAATCC	255
Ad01A2749	Aradu.A01	2749	p2	(TA)10	20	7083052	7083071	AAACATACATTGCCTCTTCATATTTTT	TGGACTTTGGAAATCATTGG	223
Ad01A2750	Aradu.A01	2750	c	(TC)7tt(TC)6	28	7083786	7083813	GACTAAGATTCCTCGCACGC	TCCTCCTTCGTCCTCTGCTA	256
Ad01A2758	Aradu.A01	2758	p3	(AAT)8	24	7094733	7094756	CATCACGAGTTTGGAATTTGA	CCATGCCTGAACCTTTCATT	258
Ad01A2759	Aradu.A01	2759	c	(C)13(A)16	29	7094974	7095002	AATGAAAGGTTCAGGCATGG	TTGATGGCAACAAAACGAAG	117
Ad01A2760	Aradu.A01	2760	p3	(CCA)7	21	7103391	7103411	CTCACAACCTTCGCAACTCA	GAAGTAGAAGCGTTGGTGGC	174
Ad01A2761	Aradu.A01	2761	p3	(GTG)5	15	7104074	7104088	TGGCTGAAGAAGTTGGCTTT	CTCACAACCTTCGCAACTCA	179
Ad01A2762	Aradu.A01	2762	p2	(AG)7	14	7111043	7111056	GAAATTTGCCAAACACGTTAAA	ATGGGTGGAGATGGATGATG	207
Ad01A2764	Aradu.A01	2764	c	(A)11ctccatgc	72	7119087	7119158	TTAGGACCCCATAGACCGTG	ATGCAAATTGTGGCAGAAGA	257

ID	Chromosome	SSR nr.	SSR type	SSR	Size	Start	End	FORWARD PRIMER (5'-3')	REVERSE PRIMER (5'-3')	Product (bp)
Ad01A2765	Aradu.A01	2765	p4	(AATA)6	24	7119297	7119320	ACCTCTTGACCTCGGCTGTA	CTATCCGGTTTACCTTTGCG	183
Ad01A2766	Aradu.A01	2766	p2	(TC)6	12	7132288	7132299	AAGTTGGCTAGTGTGTGCCC	TGCAACAAACAAGCTTAAAAGG	263
Ad01A2768	Aradu.A01	2768	c	(A)10gtagttttat	42	7137267	7137308	ATTCCGATCCGTAAATGTGC	GCCACACTTTCTGAAATCGTTA	266
Ad01A2771	Aradu.A01	2771	p3	(CTT)5	15	7141779	7141793	TGACACTGTTATTGCCATGC	AGGTTCCAGCAAGAAGAGCA	250
Ad01A2773	Aradu.A01	2773	p3	(AAT)6	18	7143342	7143359	CAAGAATTGAGAAATTTTTAGTTGAA	CCTCCAAGCTTAACAAATAGAGC	280
Ad01A2774	Aradu.A01	2774	c	(GT)23(GA)15	76	7149779	7149854	AGTGCACAACAAACACAAAGC	TCTTGTTGCCTTTTGCAGTG	228
Ad01A2776	Aradu.A01	2776	p3	(CAT)5	15	7154409	7154423	CGAAATCATCAATTTACCACCA	TTGGCTTAGGGAAGAAATCG	277
Ad01A2779	Aradu.A01	2779	p3	(ACA)5	15	7161462	7161476	TGCATGGTGTTTATGAATTGC	CACATTAGCTGACCCCACCT	185
Ad01A2780	Aradu.A01	2780	p2	(CT)7	14	7164907	7164920	AATAACGTCAACACCAGCCA	GAAGAGAAATGACGCTTTTTGA	177
Ad01A2781	Aradu.A01	2781	p3	(AAT)16	48	7170050	7170097	AGAAGCAGAAACAGCAGAAACA	GGGAGAGAGGGAGAGGAAGA	263
Ad01A2782	Aradu.A01	2782	p2	(TA)8	16	7170500	7170515	TCTCTGCATGAAAAAGACGA	TCGAAGTTAAATGACACAAACACA	276
Ad01A2783	Aradu.A01	2783	p2	(TA)8	16	7171642	7171657	AAACCTAAAATCCTAAGCTACAAAGTC	GAGAAAATAAAAAGATTTTTGGTGAAA	248
Ad01A2785	Aradu.A01	2785	p3	(TTA)17	51	7172681	7172731	TCAACCTCCCTCCTTTTTCC	TCCAATTGCAGGTTTTGGTT	237
Ad01A2788	Aradu.A01	2788	p3	(ATT)5	15	7180275	7180289	ATGGGGCACAAGAAACATTC	CGGAATGTGATCAACCACTG	140
Ad01A2791	Aradu.A01	2791	p2	(AT)7	14	7186987	7187000	ACCCGTCTATTAAATGGGGC	AGATGGCAGTGATGCTGTTG	256
Ad01A2792	Aradu.A01	2792	p2	(TA)7	14	7189498	7189511	TTTGTGATCCATATATAATTTTGCTTG	TCCAGCACTGAAATGCAAAG	208
Ad01A2793	Aradu.A01	2793	p4	(AGAA)7	28	7189988	7190015	GGGCATTCAGCATTGTATCA	AATGCATTCGCCGTTTTT	251
Ad01A2796	Aradu.A01	2796	c	(A)10tagtgatcc	37	7194689	7194725	GCTTCCTTTCAAAATTTGTATGCT	GAATTTAATAAGGAGAGAGAAAATGC	241
Ad01A2803	Aradu.A01	2803	p4	(AATA)5	20	7211460	7211479	AGCAAAGTTGGGGATGAATG	TTCAACAAACAAGAGAGACCCA	267
Ad01A2807	Aradu.A01	2807	p2	(AT)13	26	7216531	7216556	AAGCAATTTCAGGCCATGTT	GCAAATATTGTCGAGCAAAGG	248
Ad01A2809	Aradu.A01	2809	c	(AAG)5atgaaa	57	7223912	7223968	CGTTCCTCCTCCTCCTTCTT	TTGGTCTCATTGTTCCACACA	257
Ad01A2810	Aradu.A01	2810	p3	(AAG)5	15	7224827	7224841	CAAAAATGCATCCAAATCCA	TTTCTCCTCACATTCCATTGC	268
Ad01A2812	Aradu.A01	2812	p3	(AAG)5	15	7225237	7225251	CATGATGACGTGTTTTGAGGA	TTCATTGGGTCTCGTTGTTCT	191
Ad01A2813	Aradu.A01	2813	c	(ATG)9acgacg	69	7225446	7225514	AGAACAACGAGACCCAATGAA	TATCGTTGTCACCTCCACCA	248
Ad01A2817	Aradu.A01	2817	p3	(TCA)7	21	7236459	7236479	TCTTCAGCAACAACACCCAC	TTATTGCAGCAGCATTTTCG	272
Ad01A2829	Aradu.A01	2829	c	(A)10tatagaaat	122	7249816	7249937	GAAATGTTTCACGGAGATGCT	CAACAATATCTGCCCCATCC	270
Ad01A2832	Aradu.A01	2832	p4	(TTAT)5	20	7255175	7255194	ATTTGTTGGGTTTGTGCCAT	ACGAAGATGCTCGAGTTGGT	210
Ad01A2834	Aradu.A01	2834	p3	(ATA)6	18	7264218	7264235	TCTTGTGGAGGAATTTGATGC	GTGGGCCCTCAAACATAAGA	280
Ad01A2836	Aradu.A01	2836	c	(A)11c(A)10	22	7267689	7267710	AATGAAGTTGGCTGAGCTGG	GGGTCTTTCCAAAGCATCAA	211
Ad01A2837	Aradu.A01	2837	c	(TCT)5tcatcatc	57	7268899	7268955	TCTGAGCAGCAGCATCCTTA	TTCACGATCCCGAGATTTTC	198
Ad01A2842	Aradu.A01	2842	p3	(ATT)5	15	7284320	7284334	TTTGAATCGGGTGAAGTTAAAAA	CAAATAAAGCATATGAGTAACATGACA	243
Ad01A2845	Aradu.A01	2845	c	(A)10catgcataa	76	7292872	7292947	GCACTCATTAAACAGGAAAGTGAA	GTTTGTCCCATGTTGGTTGA	264
Ad01A2846	Aradu.A01	2846	c	(A)10gaaaagat	62	7297770	7297831	TGAAAAATACTAGGAGATCAACACTTC	TGCTCATCATCCTTACAAGCC	136
Ad01A2847	Aradu.A01	2847	p3	(TAA)21	63	7302561	7302623	CACTTGGGTTGTGCAAAAGA	GCTTAGTGGGGTGCAATGAT	207

ID	Chromosome	SSR nr.	SSR type	SSR	Size	Start	End	FORWARD PRIMER (5'-3')	REVERSE PRIMER (5'-3')	Product (bp)
Ad01A2849	Aradu.A01	2849	p2	(CT)7	14	7310059	7310072	TGGAGTGCAAGTCAAGATGG	CCACGGTTGAAACTGTTGAA	177
Ad01A2853	Aradu.A01	2853	p2	(AT)7	14	7320704	7320717	GGGTTTTGTTGCCGATGTTA	TGGATCAAAATCTTTTTAGTGGG	266
Ad01A2855	Aradu.A01	2855	p2	(TA)9	18	7328242	7328259	TGCTTTTGGGGCTTGTTTTA	TTTTGTTTTACTTTTGTGTGTGTG	243
Ad01A2862	Aradu.A01	2862	p2	(TA)6	12	7360129	7360140	CACATGCCAATTCCTTGTTG	GAAATAACTATTTGGTCGGCTCA	249
Ad01A2863	Aradu.A01	2863	p3	(AAC)5	15	7361839	7361853	GGCTTCATGAATCATCTTTCAAC	ACCTGAGAGTCGTACCACCG	205
Ad01A2864	Aradu.A01	2864	p3	(ACA)7	21	7368748	7368768	CCGGTACTGTCGGTCAAAAT	ACATGACAGAGCACAATGGC	274
Ad01A2865	Aradu.A01	2865	c*	(TCT)7tcctcttc	187	7378583	7378769	ACTCAAACGGTTCCTCTTCG	TGCTGCTTCTTCTTCTTCAAAA	280
Ad01A2866	Aradu.A01	2866	p3	(AAG)7	21	7379285	7379305	GCAGGCATTACTTCAAAAACG	CAAGGTACGTTTCTTGCCTTC	186
Ad01A2867	Aradu.A01	2867	c*	(AAG)6(GAA(	50	7379454	7379503	GAGAAAGAGAAGGCAAGAAACG	TCTTCTTTCTCCGTGCTTCC	229
Ad01A2868	Aradu.A01	2868	p3	(ATA)5	15	7383288	7383302	TGATGGGTGATTGATTGAAAA	CACAAATTCAGCCCAACAGA	165
Ad01A2869	Aradu.A01	2869	c	(TTA)15aagttt	130	7389544	7389673	TTAGGAACTATTTCGATAGACAAAAA	CAGAAAGCTGGTTCTCCTTCA	267
Ad01A2874	Aradu.A01	2874	c	(T)12cgctnnnn	115	7404157	7404271	CAGCTGGTTAGGCCACAGAT	TTGTTGTTGTTAGTGGATTAACGTA	244
Ad01A2878	Aradu.A01	2878	p2	(GA)7	14	7415605	7415618	CAATCTTAATCCTAATATCCGAAAACA	AGCAGCATGATGATGAGTGG	259
Ad01A2879	Aradu.A01	2879	p3	(GAT)5	15	7415914	7415928	AGGTCTCCTCCTTCCACCAC	AGCCTTGATTTCAGCAAAGG	279
Ad01A2884	Aradu.A01	2884	c	(T)13cattctttcc	102	7417585	7417686	CAATGTACGAACAATGGCCT	CGTTACCATGCTTGTTGAAAGA	225
Ad01A2886	Aradu.A01	2886	p3	(TTG)5	15	7427285	7427299	TCATGTAGCCGACCCTTTTT	ATTCAAGTGATGGGCCAAAG	217
Ad01A2891	Aradu.A01	2891	p2	(TG)6	12	7434115	7434126	TGCTGGTCGCTAAACTTTTTC	TTTGAGAAGAAGAAACATACACTTGAA	248
Ad01A2893	Aradu.A01	2893	p2	(AT)7	14	7436436	7436449	CAATTTTCTACACGAGATTTAAGTTGA	TGTTAGGACACATGTTAGTATTTTGAA	184
Ad01A2894	Aradu.A01	2894	p2	(AT)10	20	7439846	7439865	CAGCCACAACCTGTAAGCAA	GCAAGTTGGGATCAGTGGAT	220
Ad01A2895	Aradu.A01	2895	p3	(AGA)6	18	7441622	7441639	GGTTTCAGTGATGCGATTGTT	GGAGAGAGGCAGAGAGCAGA	262
Ad01A2897	Aradu.A01	2897	p2	(AT)7	14	7443187	7443200	ATATCTATTTTACATCTGCATATCCG	TCGGATATATTCGCATAATTAAAA	280
Ad01A2898	Aradu.A01	2898	c*	(A)10(AT)6*	21	7444926	7444946	TCATGAAATGTTTGTAGAATGGC	AAAGTTGGAAGGGAGGGAAA	169
Ad01A2906	Aradu.A01	2906	p2	(AT)6	12	7460141	7460152	ACCTCCCAATGAACCAATCA	TTCCAAATTTTCTCCTGGATG	270
Ad01A2907	Aradu.A01	2907	p2	(AC)6	12	7465980	7465991	TCAAAAGCATTTTTGGGTGA	GAAGCCGGTCTATTGTTGGA	177
Ad01A2908	Aradu.A01	2908	p2	(TA)8	16	7466883	7466898	GAGTGCTCGTGTGGCTTATG	CATGTTATTTTAGAGAGGATTGGG	156
Ad01A2909	Aradu.A01	2909	p2	(AT)12	24	7477084	7477107	ACAGCCAACTCGACAAGTCC	CGAGTTTTCCTGGGTAGACG	246
Ad01A2910	Aradu.A01	2910	p2	(TC)8	16	7478978	7478993	TCTCTGCCCTTACTCGCATT	AGAAGAGGGAGCTGACCACA	179
Ad01A2912	Aradu.A01	2912	c	(A)14gaaaaagt	45	7491434	7491478	GATTTTCTTCATGGTGCGGT	GAATGGCAAAATTGGATTCG	120
Ad01A2913	Aradu.A01	2913	p3	(ACT)6	18	7492565	7492582	TGATTGGCATTGCTGAGTGT	ACTGTGTGAAGGTGTGGTGG	146
Ad01A2915	Aradu.A01	2915	p3	(TAT)16	48	7500657	7500704	CCATTTTTGACTCTCCAGGAA	AAAATTAGCCATCAAAATCGG	271
Ad01A2918	Aradu.A01	2918	p3	(TAA)10	30	7505331	7505360	TTGTTTGTTTGCTGGTGAGG	GCTTTTTGAAAAGAAAAATATTACGG	244
Ad01A2926	Aradu.A01	2926	c	(AG)7ttagttttga	39	7553733	7553771	AAAGACAGAAGATTGTGCTCAAGTTA	TCGCAACAAAGTACCTGCAT	247
Ad01A2931	Aradu.A01	2931	p2	(CT)13	26	7563775	7563800	CGGACTAGCAGAGAGTTGGG	TCCCTCCAATTTGCTTTCAC	264
Ad01A2934	Aradu.A01	2934	p2	(TA)6	12	7567211	7567222	CACGCCAACACTACAACTTAACA	GCATTTGAGGGAGGTTACCA	278

ID	Chromosome	SSR nr.	SSR type	SSR	Size	Start	End	FORWARD PRIMER (5'-3')	REVERSE PRIMER (5'-3')	Product (bp)
Ad01A2935	Aradu.A01	2935	p3	(TAT)9	27	7568166	7568192	GGGACCAGGACACATCAAGA	TTGTCCATTGTTCCAACTGC	229
Ad01A2936	Aradu.A01	2936	p3	(ATT)5	15	7570275	7570289	TATGCCCGTCGAAGAAAAAG	CCCATGGAGAGTGGATATGG	278
Ad01A2937	Aradu.A01	2937	p2	(TA)6	12	7573476	7573487	TCCACTCTTATCCACCCACA	GGTGGGACCGAGTTTAGGTA	251
Ad01A2948	Aradu.A01	2948	p4	(ATTT)5	20	7597940	7597959	ATGCTTCCACCTTCGATGAC	CCTTCCGAAATGATGAATCC	228
Ad01A2950	Aradu.A01	2950	p3	(GAA)5	15	7599460	7599474	CCCAGTAACGGGTCATTCAC	CCCTTCCTTGCCTTCTCTCT	203
Ad01A2951	Aradu.A01	2951	p2	(AC)6	12	7599669	7599680	GCATAAAATGCATGCACGAC	CAATGCAACCCAACAAACAG	234
Ad01A2952	Aradu.A01	2952	p3	(ATT)7	21	7599991	7600011	CTGTTTGTTGGGTTGCATTG	CCAACCTCCTTCATATCCCA	247
Ad01A2953	Aradu.A01	2953	p4	(TTAA)5	20	7600840	7600859	GGCGGCGAAGTGGTTAAATA	CCATGCATTAACTAATTCAAAGTCC	279
Ad01A2955	Aradu.A01	2955	c	(T)11cttatgctg	49	7602415	7602463	AGGCGTTGTAATTCATGGCT	CGCCTAAACTTATTGATCATGTTG	280
Ad01A2958	Aradu.A01	2958	p3	(TTC)5	15	7604582	7604596	GGATCTGGATTGATTTTGACG	AGATCACCTCCGTTCATTCAA	242
Ad01A2962	Aradu.A01	2962	p2	(TA)6	12	7624493	7624504	TTGCAATAATATCTGCTAACAAAATC	GCTCTGCCGGTGTCATAAAT	263
Ad01A2966	Aradu.A01	2966	p2	(AG)7	14	7629281	7629294	TGAAGGGAGGTGGATTGAAG	AACCGTCCGAGCCTAAAACT	236
Ad01A2971	Aradu.A01	2971	p3	(TAC)5	15	7642676	7642690	ATGCCCAATTTTGTATCCGA	ACACGTGATGTTTTGGGACT	279
Ad01A2974	Aradu.A01	2974	p2	(AT)7	14	7648340	7648353	TGTAAACGGTGGAGATTCAATG	TTTTTGGATAGACACATTGCTTT	265
Ad01A2976	Aradu.A01	2976	p2	(TA)6	12	7655947	7655958	TGGGAACTCACAATTTTCTTTG	GCTTGAGCTTGGAATTGAGC	234
Ad01A2979	Aradu.A01	2979	p3	(AAT)8	24	7663434	7663457	GCCCGACTTCTTAGGAAAGG	TCTGTTGAAACGAAATGGGA	204
Ad01A2982	Aradu.A01	2982	p3	(ACC)5	15	7671593	7671607	CGAAACGATGTCGTATGTGC	CCATTGATTCCATTGGTTCC	228
Ad01A2983	Aradu.A01	2983	p2	(TC)17	34	7673165	7673198	CAGACTCTCCAGCTGCTCCT	TCCCTTCCACTGAGAGCAAT	163
Ad01A2985	Aradu.A01	2985	p2	(TA)6	12	7688108	7688119	AACTGTCCCTACCAACCACG	TTGCCTTTGCAGTCTTCCTT	197
Ad01A2988	Aradu.A01	2988	p3	(CTT)5	15	7700641	7700655	CCATCCAAATCTTCAACTGC	TAGTGGCTGAAGGGGTTGAG	201
Ad01A2989	Aradu.A01	2989	p3	(TTC)6	18	7700841	7700858	CTCAACCCCTTCAGCCACTA	CTGTGGCAGATTACCCCATT	214
Ad01A2991	Aradu.A01	2991	p2	(TC)7	14	7701676	7701689	GGAAATCGACGGAAGTGAAA	GATTCCATTAGCGCAGGAAA	207
Ad01A2992	Aradu.A01	2992	c	(T)10c(T)10	21	7702441	7702461	TCTTCTGCTAATATTTGGGGTCA	GAAAAATCAAATGATAAAAACGCC	273
Ad01A2993	Aradu.A01	2993	p3	(AAT)15	45	7703941	7703985	AACGACGACACACTCACCAG	GCCCCATTATTACACTAGCTTTG	277
Ad01A2994	Aradu.A01	2994	p3	(TTA)6	18	7705702	7705719	GTGCAAGTAAATGCTTTAACGG	CCTTACTATTAGGCATTCTTGTAATTG	222
Ad01A2998	Aradu.A01	2998	p2	(AT)6	12	7722333	7722344	CCTCTCAGTTTAATTTTTGGCG	CCACCAACCAATTTCTTCTCA	264
Ad01A3002	Aradu.A01	3002	p3	(AAT)5	15	7732758	7732772	GCATCTGAATTAAAAAGGGAACA	CCAAGCTGGTAAGGGACAAA	224
Ad01A3009	Aradu.A01	3009	p3	(ATA)12	36	7742664	7742699	AGAACTTGAATGCAGCCTGG	TATGTGACCGTGTGCCTCAT	183
Ad01A3010	Aradu.A01	3010	p4	(AATT)5	20	7745817	7745836	GCTCCTTCAAGATGCGTTTC	CCTTTCTTTCGCTCTCCCTT	144
Ad01A3017	Aradu.A01	3017	p3	(CAA)5	15	7770465	7770479	TCTCTCCATTTGCCATCCTC	TGAATTTTTGGTCCTGAATTTG	274
Ad01A3018	Aradu.A01	3018	c	(GA)8ggagaaa	124	7770616	7770739	CAAATTCAGGACCAAAAATTCA	TCCCCAACCTCTCACCTAAA	231
Ad01A3019	Aradu.A01	3019	p3	(TTA)6	18	7773195	7773212	TGCTGTTTATTTTGTCCAGGAA	TGTAACAAAATGAAAATGGCTTT	246
Ad01A3020	Aradu.A01	3020	p2	(AT)7	14	7776160	7776173	TTCTGTTCTTAACCTGTGAGTACTTGA	ATTTGACCAACTCGCACCTT	259
Ad01A3021	Aradu.A01	3021	c	(A)10ggactgttt	105	7776714	7776818	GCAGCAAATCTTTCAGTCCA	CCCTTGTAGGTCACATAAGAAGC	228

ID	Chromosome	SSR nr.	SSR type	SSR	Size	Start	End	FORWARD PRIMER (5'-3')	REVERSE PRIMER (5'-3')	Product (bp)
Ad01A3024	Aradu.A01	3024	p4	(AATA)5	20	7784156	7784175	GAAGTCAGAAGGCATGCACA	AAAAATCAATGAATGCACAACAA	197
Ad01A3025	Aradu.A01	3025	c	(A)11tttgtcatttt	64	7784756	7784819	TTGTGCAGGACAAGTGGATG	CACAGTAAACCCCTTTTCGG	188
Ad01A3026	Aradu.A01	3026	p4	(AAAT)5	20	7785987	7786006	GCACGATTGGCTATAACGCT	CCGTTCACTACAGCAAAAGGA	253
Ad01A3034	Aradu.A01	3034	c	(A)10tggagaaa	62	7799962	7800023	AAAATAGCGAGCACCAATGC	TCCAACAATGCTGCTAGTGC	173
Ad01A3035	Aradu.A01	3035	p3	(CCG)7	21	7801738	7801758	GAAGACGTCGTTTCCAGAGC	CTACGACCAACTGCGACTGA	177
Ad01A3036	Aradu.A01	3036	p4	(ATAA)9	36	7801958	7801993	AGCTTCAACGGATCCATCAC	ATCGACGACGATCTCTACCG	198
Ad01A3038	Aradu.A01	3038	p2	(AT)8	16	7806650	7806665	GCATATGATTGGCAACCACC	AGAGCTTGGCTCGACCATTA	277
Ad01A3043	Aradu.A01	3043	p3	(ATA)5	15	7812965	7812979	GGGTTGAGAACTAGAGTTGGAA	TTGCTGCCCCATTCTCTAAT	269
Ad01A3048	Aradu.A01	3048	p3	(GAG)8	24	7829220	7829243	AATCACAATGGGCTGAGAGG	TACCGACTTAAGGTTTGCCG	206
Ad01A3055	Aradu.A01	3055	p2	(TA)9	18	7847061	7847078	CTTGAGCCAAAACCTCTCCA	AAAGGCAAAAAGGGAGTGCT	179
Ad01A3057	Aradu.A01	3057	p3	(AGA)6	18	7868486	7868503	TGTGTTGCGTTGTCAGTGAA	TACTCAGGTCAAGTGCACGC	229
Ad01A3058	Aradu.A01	3058	p3	(AAT)6	18	7868755	7868772	GCGTGCACTTGACCTGAGTA	CACGATTGATTTTTGGTGTG	262
Ad01A3060	Aradu.A01	3060	p2	(TC)6	12	7870468	7870479	GATCGTAGATGCACAGCAGG	TCTGTTCCCTCTTGGTCAGAA	276
Ad01A3061	Aradu.A01	3061	c	(T)10aattttttatt	91	7871045	7871135	AAAAGAATTAAGAGAGATCAACTTTTG	CGTGTAAGGATAAAACATGCACC	245
Ad01A3069	Aradu.A01	3069	p2	(GA)6	12	7886901	7886912	TCACACGAAGTGAACCGAAA	TGCCTGGTCTATGGAAGGAC	274
Ad01A3070	Aradu.A01	3070	p3	(TTC)6	18	7887099	7887116	CTCGGAGACCTTATGGTGGA	CATAGCTAACCCCATCGCAT	266
Ad01A3071	Aradu.A01	3071	p3	(TCA)10	30	7891321	7891350	AAAGCAAAGGCTTAAAGGGG	GCTGTTAAAGGTGGGGTTCA	245
Ad01A3076	Aradu.A01	3076	p3	(TTA)6	18	7910795	7910812	GACCAACATGTTATCCTTGCC	TTTTAAAAATTGCACCAAACATT	256
Ad01A3077	Aradu.A01	3077	c	(CTT)5ctcattct	54	7912397	7912450	TTTTCTTCCACTCCACGCTC	CGCGAATAAAGGAGAGCAAC	232
Ad01A3079	Aradu.A01	3079	p3	(CAA)9	27	7929402	7929428	TGTGATTCCATGTTTCTTTTCTTT	CAAATCCGACTTTCACCGAT	215
Ad01A3081	Aradu.A01	3081	p2	(TA)14	28	7936860	7936887	TCAAACGCCCCAAAGTAAAC	ACGAGGTGCCTTGACAACTT	255
Ad01A3084	Aradu.A01	3084	p3	(ATA)12	36	7940714	7940749	AATGGGCTGAGTTCGGTGTA	ATTTAGCAACCACCAAACCG	266
Ad01A3086	Aradu.A01	3086	p3	(AAT)5	15	7950535	7950549	TCGATAATCAAAATCATGGCA	TGGACATCCACGTTACAACC	198
Ad01A3088	Aradu.A01	3088	p3	(GTG)5	15	7978094	7978108	CGTTCTTCATGTACCTGGCA	TTCTGCATTCCCTCCAAAAC	227
Ad01A3089	Aradu.A01	3089	p2	(TA)6	12	7979893	7979904	TTGTTTACCTGCGTCTTTCTGA	TCTTTTGGGTTCAACAATGAT	264
Ad01A3092	Aradu.A01	3092	c	(T)10aacctttca	35	7984501	7984535	CAACTCTATTAATGCTTTCAAGGCT	TTTCATGCTCCAAATCCACA	224
Ad01A3101	Aradu.A01	3101	p3	(TTA)5	15	7998673	7998687	CAATTTGATTGTTCATGTACTGC	CACTATTTGCAAGGCAAGCA	105
Ad01A3103	Aradu.A01	3103	p3	(GAT)5	15	8004609	8004623	GGTGTTGGCAATGTTGAATG	CCACCCATATACCCTCTCTTG	126
Ad01A3104	Aradu.A01	3104	c	(GT)34(AT)6	80	8005930	8006009	TTTGTCCCTCTTGGAGGTTG	AAAATTGAGGTGGCAAGGTG	279
Ad01A3108	Aradu.A01	3108	p2	(GT)9	18	8017396	8017413	CATGATTTTCTCCGCCAGAT	TGTTCTACGGCATAGCCAGA	267
Ad01A3109	Aradu.A01	3109	p2	(AG)7	14	8017538	8017551	AGAAGATTCTGGCTATGCCG	AGAGAATCATGATACAAGGGCA	236
Ad01A3110	Aradu.A01	3110	c	(A)10tgcccttgt	89	8017714	8017802	GGTCTTGGATTTTCTATTTTCCA	CACAAATCTGGCAAACCACA	160
Ad01A3112	Aradu.A01	3112	p3	(TAT)8	24	8018713	8018736	TCATTGAAATCGCACCATGT	CGAATCCCTTTATTCGTCCA	185
Ad01A3113	Aradu.A01	3113	p3	(TAT)5	15	8019902	8019916	GAGGGGAGAAAGGTGGAGAG	AAAAACCGCCATTTTTGGTT	218

ID	Chromosome	SSR nr.	SSR type	SSR	Size	Start	End	FORWARD PRIMER (5'-3')	REVERSE PRIMER (5'-3')	Product (bp)
Ad01A3114	Aradu.A01	3114	p4	(AAAT)5	20	8021509	8021528	TGCTGTTTTTGGCTTTTCCT	CGTTTTACACTGTCATTGCATC	267
Ad01A3120	Aradu.A01	3120	c	(T)10aattcgaat	39	8028468	8028506	TTTTTGAACATTTCGCTGTCA	CAACCTAGAACCCCACGAAA	278
Ad01A3123	Aradu.A01	3123	c	(A)10tcaacaaa	123	8032388	8032510	ACGTTCAACGCTCAAAACTG	TTTGGTTGTTCAATTTTGTTTGA	237
Ad01A3133	Aradu.A01	3133	p3	(ATT)12	36	8081236	8081271	TCTCTAAATCCTAAACACCCTTTTC	AACACTAAACTAATGCTACGTGGA	280
Ad01A3134	Aradu.A01	3134	p2	(AT)11	22	8086284	8086305	TTTCGCCTTTGTCAACCTTC	TGGAAAAATTCTGGCTATGGA	275
Ad01A3137	Aradu.A01	3137	p2	(AC)11	22	8118233	8118254	ATTCTCTGCAGAATTGCGCT	TTCAGGATATTGCGTCACGA	210
Ad01A3138	Aradu.A01	3138	p3	(GAA)6	18	8118599	8118616	GCAAGGATCTGCTTATCGGA	GCCCACCACTCTCATGATTT	218
Ad01A3140	Aradu.A01	3140	p3	(ATC)5	15	8120522	8120536	CGCCAATCTCAAAAACCATT	TTTTCACGCTCCGCTCTATT	162
Ad01A3141	Aradu.A01	3141	c	(T)10ctttttgaat	104	8153715	8153818	TTGAATCCTACAAAATCGCAGA	TCACAAAAAGTCACAAATATGAAACA	280
Ad01A3143	Aradu.A01	3143	p3	(AAT)6	18	8160835	8160852	TCCGAAATTCAATTCTACCAA	GAGACGTTGATCGTCCTGAAC	280
Ad01A3145	Aradu.A01	3145	p2	(TA)6	12	8163822	8163833	TTTCTGGCCCTATCTCCAAC	CATGGAATAATGGCAGGGTT	163
Ad01A3148	Aradu.A01	3148	p3	(GTG)8	24	8174841	8174864	CGGAGAAGGAATTGTTGGAA	CATCATCGGTGGTTCTTCCT	183
Ad01A3151	Aradu.A01	3151	p3	(TAA)7	21	8183985	8184005	CCCACTTTTGGTGGATTTGT	ACGGCGACGACAGAAATTAG	255
Ad01A3152	Aradu.A01	3152	p3	(TTA)5	15	8185891	8185905	AGCCGTGTTCTTTTTGCTTG	CCCATCACCTATTTCTTATTCTTCA	280
Ad01A3156	Aradu.A01	3156	p2	(TC)22	44	8196415	8196458	GGTAAGCCCTCCTCTGCTCT	AGTGAACTCACGCACACTGC	242
Ad01A3169	Aradu.A01	3169	p3	(CTT)6	18	8227153	8227170	TGATTAATAGGACGGCGGAG	GAGGAAGAAGAACGTGCAGC	271
Ad01A3170	Aradu.A01	3170	p3	(CTT)5	15	8227386	8227400	GCTGCACGTTCTTCTTCCTC	GAAGGCAAGAAACGAAAGAAAA	165
Ad01A3171	Aradu.A01	3171	c	(TAT)7tac(T)1	34	8230868	8230901	CCACATACATGTTCCCCACA	TTGGCACTTAGCTAGGTTTATCG	118
Ad01A3173	Aradu.A01	3173	p2	(TG)6	12	8232311	8232322	TCATGCACCCCATGTTCTTA	CAGCATGGAAGAGGAAGCAT	270
Ad01A3177	Aradu.A01	3177	c	(TTA)6tttttattt	45	8245068	8245112	TTCCCTGTGAGGTAACTTGTG	TCTTTTGTTCCTCCTCCCCT	263
Ad01A3178	Aradu.A01	3178	p2	(CT)6	12	8252397	8252408	GCCATCATTCCACATCACTG	AAGTTTGGAGGGGGTTTGAG	165
Ad01A3181	Aradu.A01	3181	c	(GA)11c(AG)[illegible]	39	8291659	8291697	TTGCAGGTTGAATTGGATTG	TCCCCCTTCTCATCTTTTCA	214
Ad01A3182	Aradu.A01	3182	p2	(TA)6	12	8297617	8297628	TTCAGCAGAACTGCATTTGG	TCCTTTCGACATTGGAGACC	210
Ad01A3183	Aradu.A01	3183	p3	(AAT)20	60	8300194	8300253	AGGCAAAACCCTAAATGCCT	GGAATTATCGTGCAACTTAGCC	252
Ad01A3185	Aradu.A01	3185	c	(CT)13aatttgtt	93	8318712	8318804	TGTGCTAAGCTGTCTCCTGC	AACAAGCGGCGAATAAAGAA	270
Ad01A3188	Aradu.A01	3188	c	(T)13nnnnng(	33	8333735	8333767	GCTGGAATCAGTCAGAAGGC	GCTTGGAACATGGCTTGATT	213
Ad01A3191	Aradu.A01	3191	p3	(TTA)5	15	8334594	8334608	TCGGTTGAAGAGAGTTTTCATTT	TCCTTGACTTTGTTGCTGACT	280
Ad01A3192	Aradu.A01	3192	p4	(ATTC)5	20	8336973	8336992	GGAGACCCCCATAATTGCTT	TTTTGATCCATGCGACTCTG	272
Ad01A3193	Aradu.A01	3193	p2	(TC)12	24	8340267	8340290	CACAACCTCAACCTCCACCT	CCAATCGAAGACAATCGGAG	260
Ad01A3196	Aradu.A01	3196	p2	(TA)8	16	8345087	8345102	CCCTCAATCTCCAACCTCAG	TAAGTTGGATTTGCCTCCCA	274
Ad01A3197	Aradu.A01	3197	p3	(TTA)16	48	8347151	8347198	CGTTTGGTTATCTTGCACCA	GTGTGAGCCATTACCCACCT	215
Ad01A3200	Aradu.A01	3200	p3	(AAT)10	30	8353474	8353503	ATTCCTGATAGCCTGCGAAA	GGACGAATTTGAGTATTAACCCC	276
Ad01A3201	Aradu.A01	3201	c	(AAC)12(AAT	54	8353606	8353659	ATTCCTGATAGCCTGCGAAA	GGACGAATTTGAGTATTAACCCC	276
Ad01A3202	Aradu.A01	3202	p3	(ATT)8	24	8356516	8356539	GTGTGCCACGCTGTCAGTTA	GGGTTGCTGGGGTTTTAAGT	270

ID	Chromosome	SSR nr.	SSR type	SSR	Size	Start	End	FORWARD PRIMER (5'-3')	REVERSE PRIMER (5'-3')	Product (bp)
Ad01A3203	Aradu.A01	3203	p2	(GA)6	12	8360544	8360555	TCCATACCTATTAAATTGAACATTCG	CCTTTTCCCTTGGGAGAGTC	209
Ad01A3204	Aradu.A01	3204	p2	(TA)6	12	8368629	8368640	TATGATGGTCGCAGGTCAAA	TTGATCATAGGATTATTAAAAAGGACA	251
Ad01A3205	Aradu.A01	3205	p3	(TTA)19	57	8369949	8370005	TGCTGTCAAGTGCCAATTTC	TGAAGAACAACTATCCTTTCAATTC	237
Ad01A3206	Aradu.A01	3206	p2	(TC)6	12	8380637	8380648	TCGTACGGACACTGACTTGC	CGTTACCTGATGTTCTCGGG	163
Ad01A3208	Aradu.A01	3208	p3	(TTA)5	15	8406307	8406321	TGGGATGTCAATGCCCTAAT	CTTTGAGTGCACCAATGACG	157
Ad01A3209	Aradu.A01	3209	p2	(TA)6	12	8418294	8418305	TTGCGAGAATTTCCCAACAT	GAAAGAGGACACATGCACGA	222
Ad01A3211	Aradu.A01	3211	p3	(TTA)5	15	8422742	8422756	TATGGCTGGTTTGACTGCAC	AAAAGCCGGAAGTTAGAGGC	137
Ad01A3212	Aradu.A01	3212	p3	(TAA)5	15	8445227	8445241	CCGCCCTAGCTTCCTAGTCT	ATTCACGTAGCCCTCCACAC	254
Ad01A3213	Aradu.A01	3213	p2	(AG)8	16	8445915	8445930	GAAAGGTCAATGGATGACAGC	CGCGGAAGAGGGTTATGTTA	266
Ad01A3216	Aradu.A01	3216	c	(AGA)5aatggt[illegible]	106	8460910	8461015	GCAGAACCACTACCACCACC	GTTCACAATTTCACGCACCA	260
Ad01A3218	Aradu.A01	3218	p2	(AT)14	28	8466624	8466651	CAACACTCCTCAATGACCCA	GGGCCAACTTGTATGCTTGT	105
Ad01A3219	Aradu.A01	3219	p2	(AT)6	12	8467461	8467472	AATCAATGGGTGTATCTTGTTGA	GTTTGGTTTCTTGCTGCCAT	240
Ad01A3223	Aradu.A01	3223	p2	(GC)6	12	8535473	8535484	CCCTCTTCATTCCTTGGGTT	CCAGTTGGGTTGGAAAGCTA	215
Ad01A3225	Aradu.A01	3225	c	(A)10gctatgctt	87	8541884	8541970	AGTGGTCTTGATGGGCTTTG	TACACCTCTCCCCCTCTCCT	245
Ad01A3227	Aradu.A01	3227	p3	(AAT)5	15	8546980	8546994	TATAAATCCCAGTCAGGCGG	GAGAGGATGGGATGTAGCCA	261
Ad01A3229	Aradu.A01	3229	p3	(ATC)5	15	8561109	8561123	TCTTCCTCCGGATCTGTCAC	TGGGATGAGTTCCCTACTGG	209
Ad01A3231	Aradu.A01	3231	p3	(TAT)6	18	8568359	8568376	ACGTGCGTGTTATGGTAATTT	AAGTAAAAGAAGGGCAAATAAAGA	279
Ad01A3233	Aradu.A01	3233	p2	(TA)6	12	8571222	8571233	GAACTTGGAACGTGTCCTCC	CCATGGCCACCTAAGCAATA	225
Ad01A3234	Aradu.A01	3234	p2	(TA)7	14	8572595	8572608	TGGATTGACTCGATCAGCAT	CTCAAGTTCTTGTTGGGCGT	204
Ad01A3236	Aradu.A01	3236	p3	(AAG)5	15	8575950	8575964	TAGCTTGAGGTTGGGAATGG	TGATGATTGAGAAGATGGCG	268
Ad01A3238	Aradu.A01	3238	p2	(AT)6	12	8578839	8578850	TCTCCTTTGGAAGAAAAGTATGTTG	TGTGCGTTAATTTTCTGTGTTG	249
Ad01A3242	Aradu.A01	3242	p3	(TTA)8	24	8587888	8587911	TGCTGTTCTGTTCTGTTTGTTG	TCAGGATCCAGAACCTTCAA	264
Ad01A3245	Aradu.A01	3245	p2	(CT)25	50	8590030	8590079	AAAATGGTTGAAGCAGGTGG	ATTGTGATTGGGGATGGAAA	230
Ad01A3248	Aradu.A01	3248	p4	(ATGC)5	20	8593863	8593882	TGAACAACAACCAATTCCCA	TTGCTGCCCCTACACTTTTC	267
Ad01A3249	Aradu.A01	3249	p2	(TA)8	16	8596390	8596405	TCGATATCTGATTCATGAAATGTT	CAATCCGAGTTTTGATGAGC	168
Ad01A3250	Aradu.A01	3250	p3	(ATT)19	57	8596725	8596781	TGATTCAAGAATGTCTGAGAACAC	CTGGCATAAATGCTTCGTCA	260
Ad01A3253	Aradu.A01	3253	p3	(TCA)8	24	8601258	8601281	TCATGGATGCATGCTTTTCT	TCTTTAAAAATGTTGACTCCCCA	235
Ad01A3256	Aradu.A01	3256	p2	(AT)10	20	8605027	8605046	AGGCCACACACTCTGGTAGG	AGAGGGTTGACCATTTTTGC	196
Ad01A3258	Aradu.A01	3258	p4	(AAAT)5	20	8624796	8624815	CCCCCAAGTGCTAGAGAACA	TGGTAACTGACTTTAGTGGCTGA	243
Ad01A3260	Aradu.A01	3260	p3	(AAT)5	15	8630056	8630070	AAGTCATCCACATGCCACAA	GCTACATATAACATGCAATTAACCC	120
Ad01A3268	Aradu.A01	3268	p2	(AT)6	12	8638400	8638411	CATTAAGTGCAGAAATTTGATTTTTA	AAACAAAATTATTAGATGGAAGAGAAA	280
Ad01A3269	Aradu.A01	3269	c	(AGA)5acgag[illegible]	48	8648935	8648982	TTCATAATCCACTTCGCCCT	CATTCTTCGTTCTTCTCCTCG	172
Ad01A3270	Aradu.A01	3270	p3	(TTA)8	24	8655647	8655670	CCTCATTCTTCCCTGATCCA	AGCGAGCAAGATATGGAAGC	196
Ad01A3271	Aradu.A01	3271	p4	(ATAC)37	148	8656253	8656400	GGGAATTTTGCCTGCAAGT	GCCTGCAGTACAAATTGTGG	272

ID	Chromosome	SSR nr.	SSR type	SSR	Size	Start	End	FORWARD PRIMER (5'-3')	REVERSE PRIMER (5'-3')	Product (bp)
Ad01A3274	Aradu.A01	3274	p2	(TA)7	14	8684192	8684205	ATCACGCACTGTTGGATGAA	ATTACCGTGTGTGCGTGTGT	257
Ad01A3277	Aradu.A01	3277	c	(TTA)7attttagc	79	8686943	8687021	AGTAACCGGGACCAACTTCA	CAAGTTGTTACGTTAATAGTGACCCA	160
Ad01A3278	Aradu.A01	3278	p2	(AT)10	20	8687554	8687573	TTTGGCGGGAATAGATTCAT	AGCTCTCCACGGTTGACCTA	280
Ad01A3280	Aradu.A01	3280	p3	(TCA)6	18	8698212	8698229	ATGCTGTTACATGGGCATCA	TTAAGCTGGGGAGGATGAGA	135
Ad01A3282	Aradu.A01	3282	c	(A)10gtgatcttc	117	8699445	8699561	AAAAACCCAACTTTGGATCA	GTGGGTTGGTGGTTCTGTTC	237
Ad01A3286	Aradu.A01	3286	p2	(TA)8	16	8710876	8710891	TTGATAAAGAGAGGGCATAGCA	AAAAACAAAATTTGACGCGTAT	280
Ad01A3292	Aradu.A01	3292	p3	(AAT)5	15	8725409	8725423	ACAACCCAGATGGCAAAAGA	CTTTTGCCGCCACACTATTT	216
Ad01A3293	Aradu.A01	3293	c	(T)23cgaaatctc	42	8726863	8726904	TTTTTGGTTGTGAATTTGTTTTC	GAAGCAAGAAAAAGCAGTGGA	116
Ad01A3295	Aradu.A01	3295	p2	(AT)7	14	8727831	8727844	ATGCCAACATTTCAGGGAGT	CCAATGAAGATGGGTGATCC	270
Ad01A3296	Aradu.A01	3296	p3	(ATT)9	27	8728615	8728641	AAAATGCAAATTTGGTGTACATAGA	AATAAGGGCAAGGTTTCGGT	205
Ad01A3297	Aradu.A01	3297	p2	(TA)8	16	8729987	8730002	TTTGTTTGTATCGTGTTGTTTTGA	GATGGATACGATGCAATCGAG	229
Ad01A3299	Aradu.A01	3299	p3	(TTA)6	18	8742297	8742314	TGGTTATCTACTTCCACCAAGACC	TCGAGTTTAAATAGTCGTCCCTG	242
Ad01A3302	Aradu.A01	3302	c*	(TGT)7(TTGT	37	8745098	8745134	GGTCAAAGGAAATTGCTAGTGG	ATGGCCTGTTTCATCCAAAG	102
Ad01A3303	Aradu.A01	3303	c	(AGA)5tgaaga	79	8746186	8746264	TCTGTAGGGGATCAGGTTGC	GCTTTTTCTTCTGCACGCTC	259
Ad01A3304	Aradu.A01	3304	p2	(TA)6	12	8747740	8747751	TGGGACTTGAAGCCAATTTT	GAAGTGCTAACAATGCTAATTTTTGA	266
Ad01A3308	Aradu.A01	3308	c	(T)10acttgagct	42	8769950	8769991	ATTTTGCTGCTGTCTCTGGC	AAGAAAAAGTCCAGAAAACTAACATT	206
Ad01A3313	Aradu.A01	3313	p2	(GA)7	14	8792376	8792389	GGGGAGATTTGTTGGGTTTT	ATTTCAAGCTGATCCAACGG	238
Ad01A3316	Aradu.A01	3316	p5	(TTATC)5	25	8818408	8818432	CCTGGGTGCTCCAGTGTATT	CCAAATCCTAAGGCAGTCCA	134
Ad01A3321	Aradu.A01	3321	p3	(TTA)5	15	8892956	8892970	GTCGCTATTGGATTCTGGCT	TTCCATTTGCATTTTTAAAGGTT	265
Ad01A3325	Aradu.A01	3325	p4	(TTTA)5	20	8904398	8904417	TTTTTGAATTCTCTCCCTCTCA	TTTCAGGTTCAGGATCTGCC	268
Ad01A3326	Aradu.A01	3326	p3	(TCT)5	15	8907609	8907623	CCCATCACCTCTTCACCACT	TTGCCTTCCCTTTCCTCTTT	244
Ad01A3328	Aradu.A01	3328	p2	(AT)9	18	8915083	8915100	TGCTTGCATATGATTGAGGC	TGGGGTAATTTTGTCCCCTT	225
Ad01A3329	Aradu.A01	3329	p3	(TTA)7	21	8918083	8918103	AAAAGGAACACAGCATCTCCA	TCGCACAACTAACCAGCAAG	260
Ad01A3330	Aradu.A01	3330	p2	(CT)11	22	8925304	8925325	TGATGTGACCGAAGCAAGAG	CGATGAAGGAAGGAGGACAA	253
Ad01A3338	Aradu.A01	3338	c	(AT)6ttttaattat	94	8945135	8945228	TGAACACAATGTGACCAACTGA	AAGCCCCTATTCAGGTGAGG	251
Ad01A3339	Aradu.A01	3339	p2	(AG)9	18	8945342	8945359	TGGTTGAACACATGCAATGA	CACCATCAAGCTAAGGCAAA	153
Ad01A3340	Aradu.A01	3340	p2	(TA)6	12	8945912	8945923	TGCACTATCTCCTGGTTCAAA	TGCATGTCGAAACTCATCTACT	249
Ad01A3342	Aradu.A01	3342	p2	(TA)9	18	8951802	8951819	AACAAATCAGACATAGTTGTCATGC	TTAAAGGAGTTTCGTCATACACAA	264
Ad01A3345	Aradu.A01	3345	c	(TTG)5(TTC)5	30	8954390	8954419	TCCATTGTTGTTGTTGATGC	CCAGAAGCAGAAGAAACCAGA	148
Ad01A3346	Aradu.A01	3346	p3	(GAA)5	15	8954555	8954569	TCTGGTTTCTTCTGCTTCTGG	CCTTTTCCCTTCATCTCCAA	178
Ad01A3348	Aradu.A01	3348	p2	(AT)6	12	8955279	8955290	AGCACGAAGAGGAGCATCAT	TCAAGCTCGAGATGGTGTTT	263
Ad01A3350	Aradu.A01	3350	c	(AT)10aattaatt	41	8956124	8956164	CTTTCCATATTTGCCCCCAT	GCGGTTTGTGATGAGAGTGA	251
Ad01A3353	Aradu.A01	3353	p3	(GGT)6	18	8961680	8961697	AGCACTGCATCAGATTGTGG	GACGCTTCCTACTGATTCGG	242
Ad01A3360	Aradu.A01	3360	c	(T)10c(T)12	23	8986284	8986306	TGAAGGCTGCAGCATAATGA	CAAGCAGAGACATCAAGGCA	256

ID	Chromosome	SSR nr.	SSR type	SSR	Size	Start	End	FORWARD PRIMER (5'-3')	REVERSE PRIMER (5'-3')	Product (bp)
Ad01A3361	Aradu.A01	3361	p3	(GAG)5	15	8987708	8987722	CAAAGTTTGTGGGGATGCTT	CACATTTACTCCTCTGCCGC	221
Ad01A3362	Aradu.A01	3362	p2	(TA)8	16	8988103	8988118	CACATGCAAGAAGAGGAAAGTG	ACCGAATTGTAAAGCCTTGG	266
Ad01A3365	Aradu.A01	3365	p2	(CT)24	48	8989444	8989491	GTTTGGTGACCCTGCTCAGT	AATGCTGAATCGCACCTTCT	196
Ad01A3367	Aradu.A01	3367	p2	(GA)28	56	8998876	8998931	TTCTCCCCACAATCTTCAGG	CAAGTCAAAATTCACCCAGG	181
Ad01A3373	Aradu.A01	3373	p2	(TA)7	14	9008664	9008677	GGGATTCGATTTCACCGATA	TGTCTCCTTACATCTCACTCCA	279
Ad01A3375	Aradu.A01	3375	p3	(TGA)8	24	9016161	9016184	TCCAATTAGCAGTGAGGCAA	GTGTGTGGCCATCTTCAATG	168
Ad01A3376	Aradu.A01	3376	p3	(TTC)5	15	9019711	9019725	AAACGCGCTGCAAGTTCTAC	CGATACAAAACGAAGCTGGA	259
Ad01A3380	Aradu.A01	3380	p3	(ATC)6	18	9034854	9034871	GGTAGCATCCTAGCGCTGTC	CTTGCAAATTGTCATGGTGG	184
Ad01A3383	Aradu.A01	3383	p3	(TAA)6	18	9039337	9039354	CAAATCCCATATGCACTCCA	AATGGGGGATAATCTCGGTC	232
Ad01A3392	Aradu.A01	3392	c	(T)10a(ATT)5	26	9057896	9057921	ATATCATGCATGCCATACCA	AACATCAAAATCATGCATTAGCA	215
Ad01A3399	Aradu.A01	3399	p2	(TA)6	12	9070212	9070223	GGGAAAAATCCATGTATGCG	GCCTCACACATCAAAAGTGG	239
Ad01A3401	Aradu.A01	3401	p2	(TA)6	12	9071711	9071722	TTAAAACCAACTTCCAAACACTC	GGGTGCTATTTGGCTTGAAA	262
Ad01A3403	Aradu.A01	3403	p2	(TA)7	14	9072730	9072743	TTTGACCTAGTTTTATACGCTGAG	AGCGATTGAATTGAACAAAAA	260
Ad01A3405	Aradu.A01	3405	p3	(ATT)6	18	9073506	9073523	TCCCTCCTTGTTCACTTTTCA	TGCACATGAGTTTTCATCTTTTT	195
Ad01A3406	Aradu.A01	3406	c	(GA)15agcaatg	139	9079724	9079862	AGAAGCACCAATGTTCCGAC	AAACCATGACAAGAGGCAGAA	243
Ad01A3410	Aradu.A01	3410	p2	(TA)6	12	9082913	9082924	GGGATACCACAAGGAGCAAG	TCCTAAAGAACAGGAATAGGAGC	279
Ad01A3417	Aradu.A01	3417	c	(C)14tc(T)13	29	9102003	9102031	GGTGGACATCAGTCAAAGGG	GGAAAGGCTTGCATTCTCTG	233
Ad01A3418	Aradu.A01	3418	p4	(AGTT)7	28	9106732	9106759	CCTCACTTGCTGCAATCAAA	CTTCTCTCTGTTCCGTTCCG	208
Ad01A3419	Aradu.A01	3419	c	(A)14tagagttta	98	9118814	9118911	TAATGGTGGTGGTGGTGATG	CACAAAGCTGTTGATGCCAG	184
Ad01A3420	Aradu.A01	3420	p2	(GA)10	20	9119138	9119157	TCAATGCATGACAAAACCTTAAA	CAAAAACCCCAAGACCAAAA	239
Ad01A3423	Aradu.A01	3423	p2	(AT)7	14	9129966	9129979	TGAGACAAGGATTGTGTTTTGAA	CTTGAGGGGGAGTGTTGAAT	232
Ad01A3425	Aradu.A01	3425	p3	(TTA)7	21	9142766	9142786	CCTAACCCCAGTTCCAATCA	TGATGCTTTGTTTTCTGGGG	271
Ad01A3429	Aradu.A01	3429	p3	(ATG)7	21	9146900	9146920	TGTCATTTCCAACATGCACAC	TTGGCATTGCATGTTTCATT	250
Ad01A3434	Aradu.A01	3434	p3	(ATG)16	48	9154139	9154186	CCAACATGCACACTGCTACC	TTGGCATTGCATGTTTCATT	265
Ad01A3435	Aradu.A01	3435	c	(T)11agtttgtatg	47	9155170	9155216	AACAAAAGAAATCAGTGTGTTTGA	TTTGGAGAAAAGTGAAATTATGG	276
Ad01A3437	Aradu.A01	3437	p3	(TTA)7	21	9158520	9158540	CCTAACCCCAGTTCCAATCA	TGATGCTTTGTTTTCTGGGG	271
Ad01A3444	Aradu.A01	3444	p3	(TAT)18	54	9163655	9163708	AATTAATTTCTGTAGTGATGATTTTCC	CAATGTTCCATTTTGAACCAA	196
Ad01A3452	Aradu.A01	3452	p2	(AT)6	12	9204854	9204865	AAACGGGATGGTAGTGCTTG	GGAGCTGCTAATGGTGGTGT	227
Ad01A3454	Aradu.A01	3454	c	(A)11gtcactcta	59	9208620	9208678	CATTAGGAGGTGGTTTTGCAC	TTTTTCGCCTTTACAAAATCAAT	266
Ad01A3457	Aradu.A01	3457	p4	(TAAA)5	20	9211065	9211084	CGTTTGCACGTAAAAAGCAA	CCAAATATCTCGCTTTACTCTCTT	262
Ad01A3460	Aradu.A01	3460	p5	(ATCCG)5	25	9214279	9214303	TGTTGTGCTCCGACTTAGAA	CGGATCGGATCGAATATAGC	260
Ad01A3462	Aradu.A01	3462	p2	(AT)6	12	9216608	9216619	GCATGCGTTACCGTTTACAA	GCTCTGAGCTTAATGGGCAC	138
Ad01A3464	Aradu.A01	3464	c*	(AT)7(ATA)12	48	9222216	9222263	TCGGTTAGTCATCAGAGTCGG	CGGTTAATGAATAATTTGATAACTGA	177
Ad01A3470	Aradu.A01	3470	c	(A)11ggacggct	118	9251314	9251431	AGCCATGCATGCACACTCT	TGTTTCACCCTTTTGCTTCA	269

ID	Chromosome	SSR nr.	SSR type	SSR	Size	Start	End	FORWARD PRIMER (5'-3')	REVERSE PRIMER (5'-3')	Product (bp)
Ad01A3471	Aradu.A01	3471	p3	(CAT)5	15	9251546	9251560	GGTGGTGATTGATGATGTCG	ATCATAAGCAAGCCAAACGG	188
Ad01A3474	Aradu.A01	3474	p4	(TTTA)5	20	9254922	9254941	CACCCTTGGATCAAGGACAT	TTGCATTTTTGCAAGTGAGC	191
Ad01A3477	Aradu.A01	3477	p2	(AT)8	16	9262254	9262269	GCACATGCAAGTGATGGAAA	TATACGTGGCACAACCGAAA	251
Ad01A3478	Aradu.A01	3478	p3	(CTT)5	15	9263804	9263818	CTGCCACAAACTACGCACAT	GGGAATGAATCGATGCTGAG	272
Ad01A3480	Aradu.A01	3480	p3	(TGG)6	18	9272450	9272467	AGGCAACGTTCTTGTAACCG	AACACTCTCCAACTCTGCCC	152
Ad01A3482	Aradu.A01	3482	p2	(TC)6	12	9284332	9284343	ATGCCCTATGCTCTCTGACG	AGGAAGGGAGAGCTGAGAGG	256
Ad01A3483	Aradu.A01	3483	c	(TTTC)5(T)11	31	9284531	9284561	CCTCTCAGCTCTCCCTTCCT	CAAATTAACCCTAAGCCCCC	208
Ad01A3484	Aradu.A01	3484	c	(T)10ggtattaaa	44	9289795	9289838	GGATTTTCAGCTTCCATCCA	GGATTCGCCTCCTAAAGAGC	274
Ad01A3486	Aradu.A01	3486	p2	(TC)6	12	9293365	9293376	TCGCGTATGCCCTCTACTCT	GAGAAAAAGGCTGCAACTGG	200
Ad01A3490	Aradu.A01	3490	p3	(AAT)13	39	9298075	9298113	GAAGAACGAGCTGCGAAATC	CGCACTCTCTCACGAAATCA	225
Ad01A3498	Aradu.A01	3498	c	(TAAA)6agatg	133	9308750	9308882	AACCAATTCAAATTCGGATCTA	TTTGTTTGCAGGTGCATGAT	242
Ad01A3502	Aradu.A01	3502	p2	(TC)10	20	9315312	9315331	CCAACCCCTCAGTCAAGAAA	CAAAGCATTGACCCATGTGA	269
Ad01A3504	Aradu.A01	3504	p3	(ATA)7	21	9317895	9317915	GGCATTGAGGCACATGAGAT	CTGCACATTGCCAAATCAAC	265
Ad01A3507	Aradu.A01	3507	p3	(ATA)6	18	9320771	9320788	TTAATCATAGTTTCACTGAGGAAAAA	AAGCGTCTAAGGTGGCAAGA	212
Ad01A3509	Aradu.A01	3509	p3	(ATT)5	15	9321858	9321872	AAATTTTGTGGTCCATTTCCT	CTATAGCCAGCGACCGATTC	234
Ad01A3512	Aradu.A01	3512	p2	(AT)8	16	9329470	9329485	AGCTCACGAGCTGACTCAAA	GCTTGGATAAGTTCGGCTCA	266
Ad01A3515	Aradu.A01	3515	p3	(TAT)18	54	9339678	9339731	CCTCATCATTAATGGCCCC	TGTGTGGTTTGAGTGTTGCC	254
Ad01A3516	Aradu.A01	3516	p3	(CTT)23	69	9348915	9348983	ATACAGAGAAGGGGTGGGGA	GGCGGTAATAAAGACCGATG	137
Ad01A3517	Aradu.A01	3517	p3	(TGA)5	15	9349575	9349589	CGATGATTTTGAGTTCTGGG	GGACGAAAATACCCCTCTCC	253
Ad01A3518	Aradu.A01	3518	p3	(CTT)11	33	9349983	9350015	GCCTCTACGTTGGGGACTAA	AAGAAGAAGGCGTGCGTAAA	241
Ad01A3519	Aradu.A01	3519	p3	(GAA)6	18	9352260	9352277	ACCTCGCTTCGACATACAGG	GTTTGCAAAACGCCAGAAAT	236
Ad01A3520	Aradu.A01	3520	p3	(CAT)5	15	9385077	9385091	AAGAGGCAAAGATTGGAGCA	CCGTGACTGTTCACCCTTTT	277
Ad01A3525	Aradu.A01	3525	p2	(TC)6	12	9409354	9409365	CAGCCGACTCTAATGCTGTTC	TCATTGTAAGACCCATCCCA	242
Ad01A3529	Aradu.A01	3529	p3	(GAA)5	15	9420435	9420449	TGGTGTGAAGGGAAGACACA	AGAAGGAGAAGCCTCCAAGG	150
Ad01A3535	Aradu.A01	3535	c	(GT)7cagcgccc	101	9433207	9433307	ATCCAACGGTCGTAAAGCAC	ATCTTTCCCCAAGAACCCAG	267
Ad01A3539	Aradu.A01	3539	p2	(AT)6	12	9439008	9439019	TGCCTGCTTAGAAGGGAAAA	AGTGAATTGCGGCTAAAGAA	202
Ad01A3540	Aradu.A01	3540	p2	(AT)6	12	9440701	9440712	TCATACTCATTTAAGAGGTCACGAA	CCTTTTAGTTGGACCTGGGC	269
Ad01A3541	Aradu.A01	3541	p3	(AGG)7	21	9448298	9448318	GTCGGGACAAACTTGAGGAA	GGAGGCTAAAGCAAAGTCCC	134
Ad01A3542	Aradu.A01	3542	p2	(TG)9	18	9451626	9451643	GTGCCTGGTGTTGTTGAAAG	CACCTTCATCATACCATTCGT	280
Ad01A3543	Aradu.A01	3543	c	(TA)7atgcaaaa	118	9452087	9452204	AGGAGTCATGCCTGGAGGT	TTTCCCGCTAACTACCATTACAA	280
Ad01A3546	Aradu.A01	3546	p3	(TGG)5	15	9456545	9456559	GAAGACAAGGGAGGAAGCAA	TGCTTACCCCATTGGAAAAA	239
Ad01A3549	Aradu.A01	3549	p2	(AT)6	12	9464371	9464382	TCTTGGCCAACCATAACCAT	TGTTTTGAAATTGTTGTGTACTTGTG	195
Ad01A3552	Aradu.A01	3552	p2	(TA)8	16	9469133	9469148	ATGAGTTCTAGGAAATTAACCATTTT	GTCTCGGACCTAAACAAATAGTG	280
Ad01A3555	Aradu.A01	3555	p3	(ATC)5	15	9482957	9482971	TGTTGAAATACTTCGTTAAAAAGACA	TGGTCATGTCGGTTATGTTG	276

ID	Chromosome	SSR nr.	SSR type	SSR	Size	Start	End	FORWARD PRIMER (5'-3')	REVERSE PRIMER (5'-3')	Product (bp)
Ad01A3556	Aradu.A01	3556	p3	(TCT)5	15	9483270	9483284	AGCAGCCCACGTTGAGAATA	GCTCCACCTGCTTGTCTACC	272
Ad01A3557	Aradu.A01	3557	p3	(TAT)5	15	9484419	9484433	CACAGTTTCATTCAACAATCACAA	AGCGTATGGTCCATCAGGAG	252
Ad01A3559	Aradu.A01	3559	p3	(TTG)16	48	9497414	9497461	GCTGCTGGATTGCAGGTTAC	AGCATCAGCCTTCACCACTT	250
Ad01A3561	Aradu.A01	3561	p3	(TAG)5	15	9498874	9498888	TAAGCCGAAATCACCCAAAC	GTGATGGCTTCAGTGGCTTT	212
Ad01A3564	Aradu.A01	3564	p4	(TATC)5	20	9501947	9501966	TGGCATGACCAAGAAATAAAAA	TTTTGCCGTGTTTTTAAGGG	269
Ad01A3567	Aradu.A01	3567	p3	(ATT)11	33	9503738	9503770	TTACCCCAAACGTTGAGGAC	GGTTAAAGTGGCATAAGGCG	272
Ad01A3568	Aradu.A01	3568	p2	(TA)6	12	9515654	9515665	CAAATGAAAATGTGCATTGGAA	TCGAGTCCGTTCTTTGAGAAA	269
Ad01A3570	Aradu.A01	3570	p2	(TA)6	12	9523218	9523229	AACTCAAGTTGGATGAAACCTTTT	GGCCATTGTGTAGAGGGTGT	245
Ad01A3571	Aradu.A01	3571	p2	(TA)10	20	9523758	9523777	TGAATTTTAGGTTATTTTAAAGTTGGG	CTAGCATTTTTGTCAGGCGA	195
Ad01A3572	Aradu.A01	3572	p3	(TTC)6	18	9531955	9531972	TTTCTGAATGCTCGGTTCTTC	CGGCAACAGTAGCATAACAAA	254
Ad01A3577	Aradu.A01	3577	p3	(CTC)12	36	9538547	9538582	TCTTTTGCAGACATGGCAAG	AATCACAATGGGCTGAGAGG	177
Ad01A3579	Aradu.A01	3579	p3	(TCT)5	15	9551257	9551271	GGATCCTGTCTGTGTACGGC	CATCAAAGAAGGATCGCCTG	265
Ad01A3582	Aradu.A01	3582	c	(A)10tacaaaac	106	9555245	9555350	TCCGGAATTATTGCAAGTCC	CACACACATAAATTGCGGTCA	188
Ad01A3587	Aradu.A01	3587	c	(TA)10cacaaag	127	9564978	9565104	TCATCAGCGTTAGTGGCTTG	TCCATCAAAAGGTAAGTTTTGTACC	243
Ad01A3591	Aradu.A01	3591	p2	(TA)6	12	9573825	9573836	GGAAGTGGTGAAATGCAGGT	TCAAGATCTGATACCGATTGAAAA	114
Ad01A3595	Aradu.A01	3595	c	(TTA)12ttg(TT	60	9576219	9576278	TTTGTTGGAGCACATGGAAA	TCCGGACCAAATTTTAGATGA	166
Ad01A3599	Aradu.A01	3599	p2	(TG)8	16	9585570	9585585	TATTGCTGGTGGAAGACGTG	TCAAAATCAATTAAAAATTGGACC	215
Ad01A3601	Aradu.A01	3601	p3	(TCA)6	18	9586180	9586197	GGCAACCATGGCTACCTTTA	GGGTCAATGGCAATTTCAAG	236
Ad01A3602	Aradu.A01	3602	p3	(AAT)5	15	9587218	9587232	TGCCATTAATGATGAAGCGA	TCAAGTAAAGTGAAAGGCGCT	184
Ad01A3603	Aradu.A01	3603	p3	(TTA)20	60	9587721	9587780	GTCATGGGCATTACCCAAAG	AACTCTCATCACTTTCATTCCAA	209
Ad01A3606	Aradu.A01	3606	c	(ACC)8attatca	105	9600509	9600613	TCCCCAAAATTATGCAAAGC	CAGTGGTGGTGGTTGTTCTG	200
Ad01A3607	Aradu.A01	3607	c	(GAA)6gtggcg	87	9600874	9600960	CGTCATCCTCATCAAAACCC	AAAAAGAAAGAAAGGAGGGGA	277
Ad01A3608	Aradu.A01	3608	p2	(AT)6	12	9602013	9602024	TCAAGGGTTTTATGTGTCAGGA	TTGTCCTTTTTCCAATAACACTC	262
Ad01A3612	Aradu.A01	3612	p4	(TATT)7	28	9612390	9612417	TGAGCCACAAGTACCTGCAC	AAAAGAAATGTGATCCAATTAAAAA	275
Ad01A3615	Aradu.A01	3615	p3	(AAT)5	15	9621170	9621184	TGGCACCATTATTTGAAGGA	TAGCCATTGCTGAACCTTCC	256
Ad01A3621	Aradu.A01	3621	p2	(AT)6	12	9626200	9626211	CATTATGCATCAGTGGCACA	TGCAAGGAGAAAATATATCAAAAAGA	227
Ad01A3622	Aradu.A01	3622	c	(AT)8gtattctac	106	9630151	9630256	TACGCACCAACAAATTCCTG	GCGCCTGAAGAACATATTATCAA	280
Ad01A3623	Aradu.A01	3623	p3	(TAA)5	15	9631348	9631362	TGAATATTCTATTGACCTTGCACTC	CCAACAACAGAGAAACGTGG	280
Ad01A3624	Aradu.A01	3624	p3	(CTT)5	15	9631578	9631592	CCACGTTTCTCTGTTGTTGG	GCTTTTTGGTTTGGTTCCTG	144
Ad01A3625	Aradu.A01	3625	p2	(TA)6	12	9633384	9633395	ATGGAAGCCACTTTTGCTTG	AGAAGTCCCAATTCCCCTTG	257
Ad01A3632	Aradu.A01	3632	p3	(GAA)6	18	9655690	9655707	AGGAGTGGGTAGAGCGACAA	TGTGTTGTCAATCCCCAAAA	229
Ad01A3636	Aradu.A01	3636	c*	(T)10gg(TAT)	60	9663171	9663230	TGCCTTTAATCTTTGAGCGAA	AGTTTCAACTTTCTTGCCAATG	275
Ad01A3637	Aradu.A01	3637	p2	(TA)8	16	9665242	9665257	GGAGGTCTAAGATGGCTGCT	CATGACACGGACAAATCAACA	276
Ad01A3641	Aradu.A01	3641	p2	(AT)6	12	9684514	9684525	CTTTGCATCTGGCAATCAGA	TGGTGGGAATAAAGGGTTTG	245

ID	Chromosome	SSR nr.	SSR type	SSR	Size	Start	End	FORWARD PRIMER (5'-3')	REVERSE PRIMER (5'-3')	Product (bp)
Ad01A3645	Aradu.A01	3645	p2	(CT)7	14	9690474	9690487	AACCCAAGGATGAAGCACAC	CCTTGGCTTCGCCATTATTA	260
Ad01A3646	Aradu.A01	3646	p2	(CT)10	20	9690617	9690636	AACCCAAGGATGAAGCACAC	CCTTGGCTTCGCCATTATTA	260
Ad01A3649	Aradu.A01	3649	p2	(TC)10	20	9697655	9697674	CGGACACCAATCTCCCTCTA	TGCTTCCATGTTTCCTGATG	260
Ad01A3650	Aradu.A01	3650	p2	(TC)8	16	9705070	9705085	TCTACGGACACCAATCTCCC	GCTTCCATGTTACCTGATGGA	259
Ad01A3651	Aradu.A01	3651	p3	(ACA)5	15	9707105	9707119	CAAAACTGTGGAAGACGGTG	CATGAGGTGGTCAAACGAGA	280
Ad01A3656	Aradu.A01	3656	c	(A)14gaaacgtt[illegible]	93	9716706	9716798	TGGCCACAAATTCTTAAATAAAGG	TGCATTGCTCTTTCAATGTG	252
Ad01A3658	Aradu.A01	3658	c	(AT)6aaataaag	93	9720776	9720868	TTCTCCCTCACCTTTCCCTT	CTGTCACAAACTCCATTGATGA	268
Ad01A3659	Aradu.A01	3659	p2	(TA)9	18	9722650	9722667	GAGAAAGGATTGGTCCAGCA	TTTTTGGAATTGGTGTCCATT	169
Ad01A3664	Aradu.A01	3664	p4	(AAAT)8	32	9727186	9727217	GCCATCAATTTTGTGAGGCT	TCTCATTCGCATGTTTTGGA	233
Ad01A3665	Aradu.A01	3665	p5	(TATTT)5	25	9727691	9727715	TGTTGTGGATCAAAATTTCGTTTA	TTAGCAGCTTTTGAGCGTGA	227
Ad01A3669	Aradu.A01	3669	p3	(CAT)6	18	9735306	9735323	GAAGGTTATCCGGCATGTCT	AAACAAACGGAAGAACCACAA	198
Ad01A3670	Aradu.A01	3670	p2	(TA)6	12	9735626	9735637	CTGCCGGACTGGATGATACT	GCCGATAATTGCAACTCCAT	166
Ad01A3671	Aradu.A01	3671	p4	(GTTA)6	24	9736600	9736623	GTCGATGAATTCCGAAATGG	AAGCAGCATTCTTTGGCAAC	268
Ad01A3674	Aradu.A01	3674	p3	(ACA)7	21	9741050	9741070	CCCTGCAAAGACAACCAAAT	TTGATCCATGTAGTTGATCCCA	270
Ad01A3675	Aradu.A01	3675	p2	(AC)6	12	9744695	9744706	CTTCCGAATGACCTCCACAG	AAAGTGAGAGATGGCGTGGT	214
Ad01A3676	Aradu.A01	3676	p2	(GA)9	18	9745747	9745764	CCAAGAAAGTGAAGGGTGTTG	TCCCTTCTCTTCCCTCCTTT	144
Ad01A3683	Aradu.A01	3683	c	(T)10ctttttctttt[illegible]	109	9758208	9758316	TTCTTTTCTCTTTTTGTGAAGGTG	ACCGGATCAGAGTTGACGAT	183
Ad01A3684	Aradu.A01	3684	p3	(GAA)5	15	9759425	9759439	AGCGCGCTACTCACCATTAT	TTCCTCCGCCTCATCTTCTA	253
Ad01A3685	Aradu.A01	3685	p4	(AAAT)6	24	9761747	9761770	CAGCTTATATTATTGCCAATTTTCAC	TTCACGTATGCCATATAACTCAGAA	260
Ad01A3686	Aradu.A01	3686	p3	(TTA)24	72	9762870	9762941	CTTAGCAGCAACCGTCATCA	TCACCTCAACTCAGCTCAACA	206
Ad01A3690	Aradu.A01	3690	p2	(TA)9	18	9769997	9770014	GGCGTGTATATATTTTGGATTGC	GTATGTATATACCTTCAATTCGAGTCA	251
Ad01A3692	Aradu.A01	3692	p2	(TC)14	28	9774808	9774835	CCAAAACACCGTTCTTTCTCA	ATATGATTTTGCTTTGCGGG	258
Ad01A3693	Aradu.A01	3693	p2	(AC)7	14	9774965	9774978	CGCCCTTCTTTGACTCTCTG	TTAACCTGATCCGAAAACCG	276
Ad01A3695	Aradu.A01	3695	p3	(CTT)10	30	9782065	9782094	ACTCCGATTCACTCCCCTCT	AAACACAAAACCAAGGTGGC	232
Ad01A3698	Aradu.A01	3698	p2	(GA)6	12	9792619	9792630	CCTCCTCTTCTTCCACCCTC	GTTCCAACGACGGTGAGATT	273
Ad01A3699	Aradu.A01	3699	p3	(CGC)6	18	9792878	9792895	ATTCACCGAATCTCACCGTC	GAAACAGCTGGAGCCTCAAC	121
Ad01A3705	Aradu.A01	3705	p3	(TAA)7	21	9809772	9809792	TTCATTCCTTCACAAAATCAAAAA	GGACAGTATCACAATAACAAGGCA	237
Ad01A3706	Aradu.A01	3706	p3	(CCT)5	15	9814003	9814017	ATAAATGGAACCACAGGGCA	TACTTGCCAGACAGTGGCAG	203
Ad01A3710	Aradu.A01	3710	p3	(AAT)5	15	9827761	9827775	AAAAAGTGGAAGAATGTAAGCCA	GGTGCTTGGTGCATCATAAA	258
Ad01A3713	Aradu.A01	3713	p2	(AT)7	14	9834181	9834194	TCACAATTTTTCTACCCTCGG	CCAACTTCGTTCCAACACCT	234
Ad01A3715	Aradu.A01	3715	p4	(AAGA)5	20	9836732	9836751	CGACGAGATGCAAAAGCATA	TCCGGATCTTCTTCCTTCCT	240
Ad01A3717	Aradu.A01	3717	p2	(TA)6	12	9843447	9843458	TTTCAGATCCAAGAAGGAAAAA	TCTCTCTCTGGATTTCATATGCTTC	279
Ad01A3719	Aradu.A01	3719	p3	(TTG)6	18	9849450	9849467	CTAACATGGGACATGGGGTC	ACCAACTTCCCAAACCTCAA	268
Ad01A3720	Aradu.A01	3720	p2	(CT)7	14	9849586	9849599	GAGGTTTGGGAAGTTGGTTG	TTGCGTCAGCACTTAGCAGT	115

ID	Chromosome	SSR nr.	SSR type	SSR	Size	Start	End	FORWARD PRIMER (5'-3')	REVERSE PRIMER (5'-3')	Product (bp)
Ad01A3722	Aradu.A01	3722	p2	(AT)10	20	9854576	9854595	TTTTCAAGTGATATTGGAATTTAGAA	TGCAACTAAATGACTAAGCAACAA	280
Ad01A3728	Aradu.A01	3728	p2	(AG)9	18	9874662	9874679	GCACTACTTTCGCCGACTTC	CCTTTTTCTTAATCCCATCGC	187
Ad01A3736	Aradu.A01	3736	c	(TTC)5ttttccaa	92	9900904	9900995	GGAAGGGAGAGAGCACCATC	GAACCCAGAATCAACAACAACA	280
Ad01A3737	Aradu.A01	3737	p3	(TGA)5	15	9901117	9901131	TAGCCGTTCCACGTTCTTCT	TCGTCATCCTCATCAAACCC	270
Ad01A3738	Aradu.A01	3738	c	(GAT)11ggtag	84	9901257	9901340	GGGTTTGATGAGGATGACGA	AATCATGCAAAGCACCATCA	192
Ad01A3741	Aradu.A01	3741	c	(T)10gttaaaattt	79	9905728	9905806	AGATAGCTAACCAATTTTCAAACTAAG	CAAATAGAACTCAGTACTTTATTCGC	280
Ad01A3744	Aradu.A01	3744	p3	(TTA)17	51	9909747	9909797	AATTTTTCCGCCAACATCTG	AAATGGCAGGTTTTGAGTATCAC	240
Ad01A3745	Aradu.A01	3745	p3	(ATA)6	18	9911115	9911132	AAAATCGAAAAGGGTGGTCC	TGGGAAGTCGAGAACAGCTT	261
Ad01A3748	Aradu.A01	3748	c	(A)10gcaactgc	68	9919714	9919781	TGAACACCCTCAACTACCCC	TTGTAAAAGCTACTTGGATCCTGTC	151
Ad01A3749	Aradu.A01	3749	p2	(AT)7	14	9925883	9925896	TGCAATACCACCACAACACA	AAGTGATCGGCCAAGGTATG	277
Ad01A3750	Aradu.A01	3750	p2	(AG)7	14	9931497	9931510	CGACGTAGTGACGGACTTGA	CGAGACAGGACGGACTTTTC	271
Ad01A3752	Aradu.A01	3752	p3	(ATA)7	21	9934874	9934894	TTAAATTCAAGGATAAGGATGTTTTT	GGCTATTTGGTTTTTCAAGGC	247
Ad01A3755	Aradu.A01	3755	p3	(CTT)5	15	9962186	9962200	CATAACCCTCCACCTCCTCA	GCATCAAGCTGTGAGACCCT	118
Ad01A3756	Aradu.A01	3756	p3	(TCA)5	15	9964883	9964897	TATCATCTTGTTCTCCCCGC	ATTTTGGAAACAGGGCACAG	220
Ad01A3758	Aradu.A01	3758	p3	(AAT)5	15	9975629	9975643	TGCTACACCCAAACAGTTGA	TGGAGTTCCTCGCACTTTCT	271
Ad01A3765	Aradu.A01	3765	c	(T)11aattgggtc	70	9994597	9994666	CGCAAAATTTTCAATTAGGTCC	TTGGTCCCTATAGTTCCGTGA	268
Ad01A3767	Aradu.A01	3767	p2	(AG)6	12	9996562	9996573	TCAGTGGCTAAGAGAAGGGG	GGAGACTGGATGTAGGCAGC	273
Ad01A3769	Aradu.A01	3769	p3	(TCA)5	15	10013189	10013203	TGCCTCCTCTCACTGTCCTT	ACTGCACCTGAGTTTCCACC	265
Ad01A3770	Aradu.A01	3770	p2	(AT)9	18	10014236	10014253	GGATCTTGCAACACATACATAGG	CCTAATTACAAAATTAAAAGCAACG	244
Ad01A3772	Aradu.A01	3772	c	(AAT)6aac(A	51	10020578	10020628	TTGTGAGGGCACACAACCTA	TGAAAATCCGTATTTGAGTATTCATC	255
Ad01A3774	Aradu.A01	3774	c	(T)10aactttttgt	118	10025385	10025502	GCCAAGAAATTTATTTTGCTTCA	CTGGCCTGAACCATTTTGAT	262
Ad01A3776	Aradu.A01	3776	p2	(GA)7	14	10026533	10026546	TTGGATGGTACAGACGGTGA	ACACAAACAACACCACCCCT	103
Ad01A3777	Aradu.A01	3777	p3	(GAA)5	15	10026655	10026669	AGGGGTGGTGTTGTTTGTGT	GAAATTACGGTGGGGAAGGT	217
Ad01A3780	Aradu.A01	3780	p4	(TTAG)5	20	10030735	10030754	TCGCTTTACCATCATCACCA	CTACTCCCTTTCAACGAGCG	253
Ad01A3781	Aradu.A01	3781	p3	(CAT)5	15	10036763	10036777	TGCACATTGAGTTTTTCAGCA	TGTGATGAAGCAGCAGAACC	256
Ad01A3783	Aradu.A01	3783	c	(T)10cctcttattt	30	10044448	10044477	CGAGTCATTTATCAAAGTCCACC	TCTTGTTGTTTTTGTTCCCCA	225
Ad01A3787	Aradu.A01	3787	c	(TTC)6tcgttact	103	10057572	10057674	TATATTCACGCGCTTCGTGT	GAACCACACCTCAGCCATTT	213
Ad01A3789	Aradu.A01	3789	p2	(AT)11	22	10063087	10063108	AGCCGAAAGATGGGATTCTT	GAAGGATTTAAATGTGTGATCCAA	259
Ad01A3793	Aradu.A01	3793	p2	(TA)6	12	10074928	10074939	TCATGCAAAACCACTTTGGA	CCAAAAACTTATGGTGATGGG	277
Ad01A3798	Aradu.A01	3798	c*	(A)12(AAT)11	43	10092381	10092423	CCCGTTTTACGGGAGAAACT	GTGCCCCTCAGTTACGACAT	232
Ad01A3802	Aradu.A01	3802	p2	(AT)6	12	10105518	10105529	TCCATATGTCACAGGCAGGA	GACCCGAATCAAACCATGAC	273
Ad01A3804	Aradu.A01	3804	p2	(AT)6	12	10107065	10107076	CACATGAATCAAACTTCAAGGA	TGCGAAACTATAGGAACCAACA	216
Ad01A3810	Aradu.A01	3810	p2	(AT)11	22	10144952	10144973	TCCATATGTCACAGGCAGGA	CCTCCATTTCCAAGACAACAA	223
Ad01A3812	Aradu.A01	3812	p2	(TA)6	12	10169233	10169244	CCTTTGTTAGCTGCAACGGT	TGGAAAAAATGGAAAATGGACA	194

ID	Chromosome	SSR nr.	SSR type	SSR	Size	Start	End	FORWARD PRIMER (5'-3')	REVERSE PRIMER (5'-3')	Product (bp)
Ad01A3820	Aradu.A01	3820	p2	(TA)9	18	10183106	10183123	GCCGAGGTTAAGCTTGGATA	TCACGAGCTGGCTCAAATAA	274
Ad01A3823	Aradu.A01	3823	p2	(TA)7	14	10197831	10197844	CGATAAATTGAGTTGATGAGGTTG	TTACGAAATTGCTCAACTAACAAA	232
Ad01A3824	Aradu.A01	3824	p3	(TAA)5	15	10201294	10201308	CACCATGCATGATTCACTCC	ATTGTGAACGCAGCATACGA	206
Ad01A3825	Aradu.A01	3825	p3	(TTA)20	60	10208751	10208810	ATTTGGTGACGTGCTTCCTT	TTATGTTATGGTAATAATTTGGCCT	149
Ad01A3827	Aradu.A01	3827	p4	(TTAT)5	20	10219152	10219171	TCAGAATTTCTCCTATCATTCACTTT	TCAATTTTTCATCTTAGTTGCGA	189
Ad01A3829	Aradu.A01	3829	p3	(AAT)5	15	10222615	10222629	TTTGATGAATTGAAAAATATCCTGA	TGGTAGGCAAAGTTTCCTTTCT	212
Ad01A3830	Aradu.A01	3830	p3	(TTA)8	24	10222993	10223016	GAAAGGAAACTTTGCCTACCA	TTTCTGACAAGGTCATTCATGC	275
Ad01A3832	Aradu.A01	3832	p3	(ATA)5	15	10227444	10227458	GCATCACCATGTTTCGTAGC	GCCAAACACAAAAGAAAAAGG	277
Ad01A3833	Aradu.A01	3833	p2	(TA)7	14	10238394	10238407	TGCACATGTATCTCTGCCATT	TCATGATGTAAGATTTTTCATTGTCT	276
Ad01A3834	Aradu.A01	3834	p3	(ATA)5	15	10238868	10238882	TACCACACCGAAAGATGCAA	CAAATTGTAAAATCAACCACCG	134
Ad01A3842	Aradu.A01	3842	c	(GAT)5aaat(A	33	10277416	10277448	ATCCCTTCCCCCAAAATCTA	TGACACGTGCCACATGAAAT	219
Ad01A3843	Aradu.A01	3843	p3	(TGC)7	21	10278815	10278835	CCCTTGATTCACTTGCTGCT	GATTCACGCTGTTGTGATGG	238
Ad01A3846	Aradu.A01	3846	c	(A)10gaaaatcc	57	10281839	10281895	CAATGAATTGAAATCGTGCG	TGCGATTTGCTTTTTCTTTTC	270
Ad01A3847	Aradu.A01	3847	p4	(ATAA)5	20	10286628	10286647	TCGCCTCTTTCAACTAAATTCC	GAAAGCAAGTAGTGTTTGGTCTTCT	278
Ad01A3849	Aradu.A01	3849	p3	(AAT)15	45	10288243	10288287	TGATGTATCATTGAGTCAGTTCAAAA	TGGTGGAATTGTCGTTCAGA	246
Ad01A3852	Aradu.A01	3852	p2	(AT)10	20	10322489	10322508	TGAATCCCAAGAGTTTTGTGTG	AGTGCTTGAAAGGGAAAGGC	229
Ad01A3855	Aradu.A01	3855	p2	(TA)6	12	10344825	10344836	TTGTTTTGCCCAAGGAAATC	TGCTTAAATTCAGTGGGTGC	231
Ad01A3857	Aradu.A01	3857	c	(AG)9attcattgc	121	10351587	10351707	TTTAATACTAACCAGAGCTTAGCAGA	ACCTTTAGCGGTTTTTCGGT	231
Ad01A3859	Aradu.A01	3859	c	(T)13aaaaaaatt	72	10362060	10362131	TTCATCCAATCCACGAACAA	GGGTCTGGAGGCAGGTTAGT	278
Ad01A3865	Aradu.A01	3865	p2	(TA)6	12	10376873	10376884	GGCGTATTGAAAATTAGTCGC	GTGATCCCTTGCAACAAAAA	239
Ad01A3869	Aradu.A01	3869	p3	(CAT)6	18	10395616	10395633	GGGAATGAGAATTGTCCCAA	TTTGGGAAGAAGCATAACTGG	139
Ad01A3871	Aradu.A01	3871	p3	(AAT)5	15	10399771	10399785	CAAGGAACTTTCATCTGCACC	ATGCCTGATCCTGGAGATTG	245
Ad01A3872	Aradu.A01	3872	c	(TGA)9cgatgat	84	10407380	10407463	GAGAAATCGTTGGATCGGAA	TATGATCGAAGCAAGACGGC	240
Ad01A3873	Aradu.A01	3873	p2	(GA)7	14	10412065	10412078	CTTGGAAATTGGAGGTTGGA	GATCGAAGCAAGAGAGCGTT	248
Ad01A3874	Aradu.A01	3874	p2	(AG)7	14	10414292	10414305	ACGCACTGCATGATCGAATA	CCTTGTTGGTTTGTTTGCCT	153
Ad01A3876	Aradu.A01	3876	p3	(AAT)5	15	10426517	10426531	GCAACAACAACAATAATGGCA	TCCATCAAACCTCATGCAAC	215
Ad01A3877	Aradu.A01	3877	p2	(AT)12	24	10427239	10427262	CGAGTACTTGCCTCCACTGA	CAGTTTTCAACGGTTAATTTCACA	277
Ad01A3879	Aradu.A01	3879	p3	(TAA)20	60	10428926	10428985	GCATCCCATGAATTTGTTCC	CAAATCAATCCCCAATGTTTC	268
Ad01A3881	Aradu.A01	3881	p3	(ATT)5	15	10433486	10433500	CCATGTGACTTAAATCTGGCAA	AACAGCCGGCTTGATAGAAA	224
Ad01A3884	Aradu.A01	3884	p3	(ACT)6	18	10436364	10436381	ATCAAACGGTTGCCACTACC	TTCCATTTTGGAGGCTGACT	170
Ad01A3886	Aradu.A01	3886	p3	(AAT)7	21	10438176	10438196	GCATGGAGTGAATATTAGCGA	TGTGTTATATTGGGGTTGTTCTTG	224
Ad01A3887	Aradu.A01	3887	p2	(CT)10	20	10440822	10440841	GCTAAGCAAGAGCATCGTACA	TCGCTACCCATGCTTACCTC	269
Ad01A3890	Aradu.A01	3890	p3	(TTC)5	15	10446331	10446345	GCCTGCACAACTTCCTCTTC	CACAGAAAACGATGGAGGGT	216
Ad01A3895	Aradu.A01	3895	c	(T)11agacac(A	27	10452586	10452612	GCAAAGCAAATCAATTGGTACA	TGCTAGAAGGCCAGCACTTT	224

ID	Chromosome	SSR nr.	SSR type	SSR	Size	Start	End	FORWARD PRIMER (5'-3')	REVERSE PRIMER (5'-3')	Product (bp)
Ad01A3901	Aradu.A01	3901	p3	(ATT)6	18	10475084	10475101	TGAATGTTTGATCCTCGGAA	TCTCTTTCATCTCTAACAATGGGA	236
Ad01A3905	Aradu.A01	3905	p2	(AC)7	14	10482313	10482326	GACCACTCCCCTCCTTCTTC	GATGCGGTGTTGGGAATAAT	219
Ad01A3909	Aradu.A01	3909	p2	(AG)6	12	10505155	10505166	GTTAAGAACACACCGGCCAC	AGAAGGATTCATAACCGGGG	245
Ad01A3911	Aradu.A01	3911	p2	(AT)7	14	10515079	10515092	TGAGGCTTTGCAGCTGTACT	CGCTGGTACACAACTGTCTCA	279
Ad01A3912	Aradu.A01	3912	c	(AGA)5tgacga	125	10516394	10516518	TTGCAGAGATGAAATTTGCG	TCAGAAATAATAAGGCCCAAAAA	247
Ad01A3914	Aradu.A01	3914	p2	(TA)9	18	10517191	10517208	TTTTTACAAACAACCAACTACCAA	CAAAGAAACGCACATCGAGA	203
Ad01A3916	Aradu.A01	3916	p3	(GAA)10	30	10519934	10519963	ACGAGGAGAAACACGACGAT	GAAGAATTCTCCACATATAAGCGTT	180
Ad01A3917	Aradu.A01	3917	p2	(TG)6	12	10521892	10521903	GAAAGAGGTGACCAATGGGA	AATGAAAAGCCTCCTGCTGA	239
Ad01A3918	Aradu.A01	3918	p2	(GA)9	18	10524437	10524454	GCAACATGCGTAACCACATC	GTTCGAGCAGGCTTACCTTG	194
Ad01A3920	Aradu.A01	3920	c	(T)10aaatc(T)1	25	10525923	10525947	TCACTATATCAAACGCGCCA	TGCTGCAATGGTGGTAGTGT	264
Ad01A3921	Aradu.A01	3921	p3	(GAA)6	18	10526740	10526757	ACCTCAAGTTCACGGGAATG	GCATTGCATTGACGAGTTGT	214
Ad01A3923	Aradu.A01	3923	p2	(AT)8	16	10532777	10532792	GGTTGGTAAGACCGATCCAA	GAATCATCAACCTACTCTTCCCC	271
Ad01A3925	Aradu.A01	3925	c	(T)10acttttaaat	58	10535395	10535452	GAGTTCGTTTGAGTAATGGCAG	CTTGCATGAAAATGGTGGTG	278
Ad01A3930	Aradu.A01	3930	p3	(TTG)6	18	10543899	10543916	AACACCACCACCTTCTTTGC	GGAGCAAGAGTTTTCTCCCA	120
Ad01A3932	Aradu.A01	3932	p3	(CAC)7	21	10548116	10548136	TACCTCCTCTTCCACCACCA	TTCGGCTTCTTCTTTTGCAT	199
Ad01A3934	Aradu.A01	3934	p4	(TAAA)5	20	10578283	10578302	CCAGAGTGCAGAGAAGTCCC	GAGTACCCCTCCCCTGTTTC	259
Ad01A3937	Aradu.A01	3937	c	(A)10gtaag(A)	27	10589452	10589478	GCCCTATGCTATCATCACCAA	CTTCACTGAACGCACGAAAA	274
Ad01A3939	Aradu.A01	3939	c	(A)13ccgaaaaa	101	10602829	10602929	CCAATTAACGGATTGCAAGG	ATAATTACGTCGACGACCGC	261
Ad01A3944	Aradu.A01	3944	p2	(CT)7	14	10605647	10605660	CCAAAAGCTTACCAGCCAAA	GGTTTCGTGGATTTTGAGGA	276
Ad01A3946	Aradu.A01	3946	c	(A)11tattgaata	72	10623708	10623779	AGTAATTCATCCAAGTCCTGGTC	TGGCAAAGCCTTTTGAAACT	258
Ad01A3947	Aradu.A01	3947	p2	(TG)6	12	10627519	10627530	CCATCAGAGAGCAGAGGGAA	ACAAAAGCTGCAATGCACAC	198
Ad01A3948	Aradu.A01	3948	p4	(ATTT)6	24	10628385	10628408	TGCCTGAATTCTACGGTTCC	AGGTTGAGAATGAATTGCCC	139
Ad01A3953	Aradu.A01	3953	p2	(GA)7	14	10643378	10643391	TTGGCCGTTCACCCTTAATA	AGCGAAAATGGAAAGCTTGA	272
Ad01A3959	Aradu.A01	3959	p2	(AT)6	12	10652817	10652828	TTGAAGAAACTCAATTAACCAGATTT	CACGTGAGATTTATTATTTACCAGAA	156
Ad01A3961	Aradu.A01	3961	p3	(TTA)11	33	10662839	10662871	GCAAATAAAATAAAACCGTCACA	TGCTATATTTTTGTTGGTTATTCTCTC	196
Ad01A3962	Aradu.A01	3962	p2	(TA)6	12	10664277	10664288	TGGATACCGAAAGTTGGTTTG	AAATTTGAAGAAATTAAAAAGTTGTCC	257
Ad01A3965	Aradu.A01	3965	p2	(TA)6	12	10665895	10665906	AAACTCGAATGCAGCAAATG	TCTTGGAGATTTTTGTTATTCGG	237
Ad01A3966	Aradu.A01	3966	p3	(AAT)16	48	10666411	10666458	TTTTACGTTTAATTTTTGCCTTTTC	AGGTGCAGTCAATTTCATGTG	279
Ad01A3969	Aradu.A01	3969	p2	(AT)7	14	10671367	10671380	GAAAGTTCAATTTTAAGCAGATTTTT	TTTTTAAGAACTAAAATTTCTTCGGT	247
Ad01A3970	Aradu.A01	3970	p2	(TA)7	14	10675499	10675512	AAGCGCAGTTTCTTGATGAAC	AAGACCAAATGTATTTGTGTGGAA	159
Ad01A3972	Aradu.A01	3972	p4	(ATAA)5	20	10685563	10685582	TGGACAAGTCTAAGATGGGGTC	GGAAACCCACTTCATCACCA	267
Ad01A3973	Aradu.A01	3973	c	(TAT)6taaaaaa	49	10686257	10686305	CTCCCGTGCATCTTGCTTAG	CCCTAATTTTTCATGTGTTATCATTT	253
Ad01A3974	Aradu.A01	3974	c	(T)11acttttgga	118	10687522	10687639	TTTTGGGTTTATTTATTCTTTGCAT	CAATGCAAATTCTCAAGTTTGG	227
Ad01A3977	Aradu.A01	3977	p3	(TAT)6	18	10693045	10693062	TCCAAATATTTTCGGCATTTG	TTATGTGCCATTTTGATACCCT	252

ID	Chromosome	SSR nr.	SSR type	SSR	Size	Start	End	FORWARD PRIMER (5'-3')	REVERSE PRIMER (5'-3')	Product (bp)
Ad01A3987	Aradu.A01	3987	p2	(TC)7	14	10728992	10729005	ACCCAGCTTCTACCTCCGTT	CCTTGAATGGGACTGAAGGA	224
Ad01A3988	Aradu.A01	3988	p2	(CT)20	40	10729174	10729213	TCAGTCCCATTCAAGGTTCC	CCCACCTAGCAGAAACCAAA	254
Ad01A3990	Aradu.A01	3990	p3	(TTA)6	18	10734907	10734924	TTTTTGAGGCATTCATCTTGG	GGCTACATGAATCAAACAACG	278
Ad01A3991	Aradu.A01	3991	p3	(AAT)15	45	10735710	10735754	ATGTGGCAATTTTTCGTTCA	AAGCTGGAGATGGTACACCG	243
Ad01A3993	Aradu.A01	3993	p2	(AT)6	12	10740263	10740274	TCCAATGCATCATCCAAAAA	TGGCCAAGACAAACTCAAAA	154
Ad01A3994	Aradu.A01	3994	p2	(AT)6	12	10741393	10741404	TCGGCATATTGTCTCAGCAG	TCAGAAGTGCAGGTTTGTGG	195
Ad01A3999	Aradu.A01	3999	p3	(CAA)5	15	10745735	10745749	GAGAAAATGACGGTGAACGG	GCTCAGTTTTCAGTTTTGTGGA	255
Ad01A4000	Aradu.A01	4000	p3	(AAG)8	24	10746103	10746126	TTCTTCCATTCGTTGGGAAC	AGAAGCTCCAAAACCTGCAA	234
Ad01A4001	Aradu.A01	4001	c	(T)12caaattcaa	106	10746229	10746334	CGTTAACCAAATCAAAAGCG	GGCATGATCGTAACAACTTCA	276
Ad01A4003	Aradu.A01	4003	p3	(ATC)5	15	10752600	10752614	TTTGAAATTTGGCACGTGAT	CTTGATCGTGCTGACCATGT	188
Ad01A4004	Aradu.A01	4004	p3	(ATA)19	57	10754875	10754931	TGACCTCTCAAATAATCGAGCTT	ACTCGAACCTGCGACTCCTA	207
Ad01A4005	Aradu.A01	4005	c	(AG)7tgatagac	37	10755108	10755144	CTAGGAGTCGCAGGTTCGAG	TTCATGGATGAAGATGATGGA	257
Ad01A4006	Aradu.A01	4006	p3	(ATA)6	18	10755727	10755744	GGAGGACAAAAATGGTGGAA	ATGGAAAGCAACCACACACA	186
Ad01A4011	Aradu.A01	4011	p3	(AAT)5	15	10772510	10772524	TCATTTTCAACCATAAATGCACA	TCCATGTTAGATGCATTTCG	264
Ad01A4016	Aradu.A01	4016	p2	(AT)9	18	10780004	10780021	AGCAGAAGCAGAGCCTGAAC	TGCGCATTTAAGTAATCCTCAAT	280
Ad01A4024	Aradu.A01	4024	p2	(TA)7	14	10818473	10818486	CCATGGTTTCCCTCAAATGT	TCTTGTTTGAAAATGCTGGG	213
Ad01A4030	Aradu.A01	4030	p2	(AT)6	12	10839706	10839717	AACTGGTTTTGGACCATTGC	CAATTCCTTGCACTTGGTTG	272
Ad01A4031	Aradu.A01	4031	c	(ACC)6gcgacg	85	10840876	10840960	TATGCCAGCATCGTCAAGAA	ACAACGGTGGTCAGAAGAGG	244
Ad01A4032	Aradu.A01	4032	c	(AAG)5atgctga	122	10841094	10841215	TCTCCTCTTCTGACCACCGT	TCAATTCCAAGTCGCACTCA	246
Ad01A4033	Aradu.A01	4033	p3	(AAT)7	21	10841409	10841429	TTGCACCCCTCTTGTTCTTC	TCCCAACCTCCTCTTATCCC	225
Ad01A4034	Aradu.A01	4034	p2	(TC)14	28	10844773	10844800	TTTTGGAAGCTAGGTTTCCCT	GCCAACAACAAAACTCCGTT	125
Ad01A4037	Aradu.A01	4037	p2	(TA)6	12	10854918	10854929	GTGGGGCAACTTGTTCATTT	GGTGCAATTAAAGCCAGTCC	181
Ad01A4038	Aradu.A01	4038	p2	(AT)7	14	10855088	10855101	GGACTGGCTTTAATTGCACC	TTGCACAAATTATGGGATTGA	256
Ad01A4039	Aradu.A01	4039	p4	(TTAT)6	24	10856707	10856730	TTTGCTGACTCTGGGTTTCA	TTTCGATGTGCCTGATTCAC	150
Ad01A4044	Aradu.A01	4044	c	(A)10gaaaagaa	42	10861757	10861798	GCTTTGTTACAGATACTCGTTTGG	TTTGATAGAAACATTGAGGACCA	218
Ad01A4045	Aradu.A01	4045	p2	(AT)6	12	10862475	10862486	CATACCTAAAGAAATCCTCACATTTT	TGTTTCAACATAGTTTGGCCC	245
Ad01A4046	Aradu.A01	4046	c	(T)10gtgtttctat	34	10862887	10862920	TAGGGCGCACCAAATATTCA	TTGTCAGTTCGAATTTCATTCC	174
Ad01A4047	Aradu.A01	4047	c	(AAT)5(TAT)7	36	10864147	10864182	CAAATCAAAATGTATCACCGGA	AAAAGATATGCGACAAAAATTTAAAAC	245
Ad01A4050	Aradu.A01	4050	p3	(TTA)5	15	10865934	10865948	AGAAATGGCGAAACATGACA	ATCCCACTACTCCATTGCCA	182
Ad01A4051	Aradu.A01	4051	p3	(ATT)13	39	10866104	10866142	TGGCAATGGAGTAGTGGGAT	CTACCCCCAACCAACTTTGA	199
Ad01A4053	Aradu.A01	4053	p3	(AAT)12	36	10867503	10867538	TTTTTGTTTAGTTGGGTCCCT	TAGCCCAACCTGATTTCCAC	278
Ad01A4054	Aradu.A01	4054	p3	(AGT)7	21	10868090	10868110	AGTGATCATAATCCGGCAGC	TGTTGTTGTTGCTTCTTGGTG	264
Ad01A4055	Aradu.A01	4055	p3	(CAA)5	15	10868277	10868291	TGTCATCTTGGTGTTCCATTG	GAGTTGGCCTTCAAATTGCT	152
Ad01A4056	Aradu.A01	4056	p3	(TAA)6	18	10870006	10870023	GAAGCAGCTGAAAAACCGTC	CAGCATGGTGCAACTTTGTT	256

ID	Chromosome	SSR nr.	SSR type	SSR	Size	Start	End	FORWARD PRIMER (5'-3')	REVERSE PRIMER (5'-3')	Product (bp)
Ad01A4060	Aradu.A01	4060	c	(T)10aatgtggat	48	10876250	10876297	CCAATTGGCATATTTTGGATT	GCAATTGCACCTTTTTCTTTTC	258
Ad01A4061	Aradu.A01	4061	p2	(TG)6	12	10879100	10879111	GATGGGGATGATGCTACCAC	ATGCCAAAGCTACCACCCTA	234
Ad01A4065	Aradu.A01	4065	c	(T)10gggtcttta	55	10881142	10881196	GGAATTCGAACATCCGACAC	GACAAAACAAGCATAGGCCC	257
Ad01A4068	Aradu.A01	4068	p3	(ATA)5	15	10888331	10888345	TGGCTCCACATTGCTGTAAC	ATGCTGTCCTGCTTTTGGAC	268
Ad01A4071	Aradu.A01	4071	c	(A)10ttaaagcct	110	10891875	10891984	GAGTGGCAAACAACACCAAA	CCTCTTAGGAGGTTGGACGA	224
Ad01A4072	Aradu.A01	4072	p2	(TA)6	12	10893904	10893915	TGGTGAGGACCTCCAAGTTT	CCACAAAATAATAAAGCTCGAGAAA	158
Ad01A4073	Aradu.A01	4073	p2	(AT)7	14	10894161	10894174	TTGTGGGGATAAGTGCTCCT	GAAGTGTTGGGAAAGAACGG	266
Ad01A4074	Aradu.A01	4074	p3	(TCC)5	15	10894456	10894470	TGCTTTTTCTCACCCTCGTT	TTCACCCGAGAGAAGGAGAA	193
Ad01A4079	Aradu.A01	4079	p2	(CT)7	14	10901493	10901506	AAGCGAGCTCGAACAGAGAG	ACCGACTTCACCGATTTCAC	235
Ad01A4088	Aradu.A01	4088	p2	(AG)6	12	10935162	10935173	TTTCTGACCTGTTTTTACGGTTT	TGCCAGGAAGCTCAAAGAGT	225
Ad01A4092	Aradu.A01	4092	c	(T)10atggagtag	87	10943699	10943785	TCAAATAAGTGATAGTTTTCTTGTTGA	AGGCCCAATTAGAAAAAGCC	220
Ad01A4096	Aradu.A01	4096	p4	(TCTT)6	24	10949191	10949214	TCCAAATTCCAAATGGTGAAA	ATTTGTTGATTCTGGCCCAT	222
Ad01A4097	Aradu.A01	4097	p3	(CAA)6	18	10949785	10949802	TTCAAGCACCTCCTCTGCTT	AGCACCCCATTTTCAAGAAC	220
Ad01A4098	Aradu.A01	4098	c	(A)10tcaaataaa	56	10952827	10952882	TTCAGCTTCTGAAAGAATGAAAC	AGGAAGCTTGATGTTTTGGG	209
Ad01A4099	Aradu.A01	4099	p3	(AAT)12	36	10955810	10955845	CAATTCACACTGGCGTATGG	TGTTCCGTTTTATGGTGCAA	260
Ad01A4100	Aradu.A01	4100	p2	(TC)8	16	10958258	10958273	ATGGTGCTGAAAACTCACCC	TCACGTGAAGCCTATTTCACC	278
Ad01A4101	Aradu.A01	4101	p3	(AAG)5	15	10959600	10959614	CCTCCTCATCTTCTGCTGCT	GCACGTTCTTCTTCCTCTTCC	213
Ad01A4102	Aradu.A01	4102	c	(AAG)6aggaag	64	10959738	10959801	CGTGCAAGAAGAAGAAGAACG	AAAAATGCGATGCAAGGTCT	207
Ad01A4105	Aradu.A01	4105	p3	(CTA)5	15	10965118	10965132	TCCGGTTTAAAGGGAGGAAT	GCTGCTGCATTGTTGTGATT	231
Ad01A4106	Aradu.A01	4106	c	(A)10gatacaga	108	10965483	10965590	CCGAGTCACAGTGATAGCTGA	CCGTAAAATCAAAGCCCAAA	280
Ad01A4109	Aradu.A01	4109	p3	(TAT)15	45	10978045	10978089	CCAACATAGAAATGGAATGAGAAA	GAAGTGTACTTCACAGATAGCAGCA	278
Ad01A4113	Aradu.A01	4113	p2	(TG)6	12	10981621	10981632	GCTGTTACGTAATTAATTGCAGATG	TCAATTCTAAATGGCATTGGC	168
Ad01A4115	Aradu.A01	4115	p2	(AT)8	16	10985995	10986010	TGCTTGTTCTCATCTCCTCA	TTAAAAAGATGCCGGAAAAA	207
Ad01A4117	Aradu.A01	4117	p3	(AGA)5	15	10987689	10987703	CCTGCCCAAGTTAAACCAAA	GGAAAAAGAGAATCCGGAGG	262
Ad01A4119	Aradu.A01	4119	c	(T)10atctaacta	82	10993787	10993868	CTTTGTGTGGAAGGGTGGTT	CAAAAATACTTCAAATGCTAACCCA	173
Ad01A4130	Aradu.A01	4130	c	(A)13gaaccaaa	97	11026939	11027035	CTTTTTCCCAATACCCAGCA	GCTTCCCTCAGTCACGTGTT	253
Ad01A4132	Aradu.A01	4132	p2	(CT)6	12	11057470	11057481	CTTAGCCTGGACTACGGTGG	GAGGGGTAGTGGAGGGTTGT	252
Ad01A4137	Aradu.A01	4137	p2	(CT)9	18	11076203	11076220	GATTCCCCCTTTTTCGCTAC	CTCACTTAGAAATCGCGGAA	139
Ad01A4138	Aradu.A01	4138	p2	(AT)6	12	11077805	11077816	TTTCATTGTCAAAATGGGTTG	AGGCCAATTTCGTCAACATC	102
Ad01A4139	Aradu.A01	4139	p3	(TAA)5	15	11079707	11079721	GCATAAATTTTAGGGCTTTTTCA	CCACCTATATTCTCCAAAGTGTCA	274
Ad01A4141	Aradu.A01	4141	p2	(AT)7	14	11086943	11086956	AATGTTGGTGATTCCCCACT	TGCATTTAATTTTGGTTTGTGG	195
Ad01A4147	Aradu.A01	4147	p2	(AT)7	14	11102953	11102966	GCTTAGTGCATGAGTGGCAG	GGAGGAGAAGGGAAAGAAATG	175
Ad01A4148	Aradu.A01	4148	p3	(TAT)17	51	11103145	11103195	TGACCTCAACAAATGGGTTT	TGATGTGGGATTGCTGAACT	273
Ad01A4149	Aradu.A01	4149	p2	(TA)6	12	11104207	11104218	TGGTATGATCATGACGTGCTAAA	GGGCTATCTTGTAGATAATGCCA	277

ID	Chromosome	SSR nr.	SSR type	SSR	Size	Start	End	FORWARD PRIMER (5'-3')	REVERSE PRIMER (5'-3')	Product (bp)
Ad01A4162	Aradu.A01	4162	p2	(GA)25	50	11136204	11136253	AAAAAGAACGTAGCCAGCATT	GCAACGTGACTCTGACTCCA	209
Ad01A4164	Aradu.A01	4164	c	(TA)10ttaggat(	79	11146326	11146404	AACAGAACACTTTTCTAATTCAATTTT	GTTTTAAACCGTCGCAAAAG	279
Ad01A4167	Aradu.A01	4167	p3	(AAT)26	78	11160931	11161008	AATGATTGGTTGGAGAGTGGA	TGGGAAATCCTTGACACACA	268
Ad01A4168	Aradu.A01	4168	c	(AATA)5atattt	124	11161334	11161457	GGCCTTGTTTTTGTTCTTGTTC	AATCGGACCCTAGGTTTGGT	273
Ad01A4169	Aradu.A01	4169	p2	(AT)12	24	11164817	11164840	CGGTAAAGGAAAATAGGATTTCA	CAACCCTTTTTGGATGTGTG	166
Ad01A4172	Aradu.A01	4172	p2	(TA)9	18	11168956	11168973	CTCCTGGGGATTTTTGACAC	CGATGGTAATGGATGGGTTC	202
Ad01A4177	Aradu.A01	4177	c	(AAT)11aaaaa	86	11176754	11176839	CAAGTTAAGGATGCGTTTATTTTT	GCAGATCTCCCAAAAGAGTTG	279
Ad01A4178	Aradu.A01	4178	c	(A)10t(A)10	21	11177314	11177334	GGGGTATGTTCATTGACATGG	GTCAAGGCCCCCAAATCTA	268
Ad01A4180	Aradu.A01	4180	p2	(AG)7	14	11178257	11178270	TGGTACAGAGTATTGGGCGG	CACATGTCCAAACCACCTGA	199
Ad01A4181	Aradu.A01	4181	p2	(CT)9	18	11180785	11180802	GCACAAAGAGGGAGTGGGTA	TTAACAAACCCCTGCCATTC	273
Ad01A4184	Aradu.A01	4184	p2	(GA)16	32	11194129	11194160	TTTGAACACGAGACACACGA	GGGGTGTTGTTTTGCAGTGT	184
Ad01A4185	Aradu.A01	4185	p2	(GA)6	12	11197325	11197336	TTGCCCCTTGAATAAATGAA	CAGTTTAGGAATCAAATTGAAAAGAA	255
Ad01A4187	Aradu.A01	4187	c	(AAAT)5tagca	100	11202321	11202420	TTCACGTTGCTGTTTTGCTC	GCTGTTCCAATGTCATAGCC	221
Ad01A4188	Aradu.A01	4188	p2	(AT)6	12	11205800	11205811	CGGTATTCTCCGATGCACTT	TCAATTCCTCTCAATTTAGCCTTT	217
Ad01A4191	Aradu.A01	4191	p2	(AT)6	12	11209183	11209194	CTGCGAAACTAAGGCTGGTC	GCAATACTCAATTCACCCCC	231
Ad01A4196	Aradu.A01	4196	p3	(AAT)18	54	11230839	11230892	GTTCTTGACCAATATCCCGC	TTTATTTCAATTGCCGAGCC	274
Ad01A4197	Aradu.A01	4197	c	(T)11aacgtttac	145	11232939	11233083	TCCATCATCTCCAAAAGCAA	CAAAATGTTTTCAAATCCCCA	273
Ad01A4198	Aradu.A01	4198	p2	(AT)9	18	11235604	11235621	TTCGCTCATCGATTTATGACT	AGGCTTGTTAAGTCGAGCCA	277
Ad01A4199	Aradu.A01	4199	p3	(TAA)6	18	11236610	11236627	TTGCAAGAATTTTCAAGGGG	CAATGCAAGGTGGTAAAGGG	215
Ad01A4200	Aradu.A01	4200	p2	(AT)11	22	11238849	11238870	AGCGGTTCAAAATCGTTAGA	AACTTCTGACAATGGCCCAC	271
Ad01A4202	Aradu.A01	4202	p3	(TCT)6	18	11239296	11239313	CCCACAATCAACACAACACA	TTGGGAAGAAACTTTGGCTC	213
Ad01A4203	Aradu.A01	4203	p3	(ATA)5	15	11239421	11239435	CCCACAATCAACACAACACA	TTGGGAAGAAACTTTGGCTC	213
Ad01A4205	Aradu.A01	4205	p3	(TAT)7	21	11241768	11241788	AGCATTGTGTGGATTGCTCA	TCCTTTTCCTCCATTTTTGC	249
Ad01A4210	Aradu.A01	4210	c	(TA)6ttagtctct	96	11247370	11247465	AAAACCATGAGACTTCTAACCG	TGAAACTTGTCTTCTTGTCCCC	204
Ad01A4211	Aradu.A01	4211	c	(CTC)5t(TCC)	34	11249580	11249613	TCTAGGACGCCAAGAGGAGA	TGCGCTTCTTCTCAGACTCA	204
Ad01A4212	Aradu.A01	4212	c	(A)11ggagaga	37	11263756	11263792	TGTTGTTCATGAATTGTTGGC	TCCCCAATTAGAGGTGTCCA	222
Ad01A4213	Aradu.A01	4213	p3	(ATT)13	39	11277159	11277197	CCCACCTATATTGGGGCTTT	AAATGAACTCAGCCTGTTCTGTT	271
Ad01A4216	Aradu.A01	4216	c	(GAA)9ggagaa	50	11281215	11281264	TCGAAGAAATCGAAAGGGAA	CAAACCCTTCCTTCTCCTCC	219
Ad01A4218	Aradu.A01	4218	p3	(TCT)5	15	11287274	11287288	TCAGATGGTGCGAGTTGAAG	TGCACCTAGAAAAAGAAGTTGC	253
Ad01A4219	Aradu.A01	4219	p3	(TAT)5	15	11287527	11287541	TTTCTGCCATTCTGATTATGTAAG	AAGCCTACCACAACCTTCAA	277
Ad01A4230	Aradu.A01	4230	p3	(GAA)8	24	11308495	11308518	AGTGGTGGTGGTGCTTCACT	CCGTGTGCTACCACCTTTTT	143
Ad01A4231	Aradu.A01	4231	p3	(TCT)6	18	11310526	11310543	TCAGTTTCCAAAGCGCTACA	TGGAGGAAGAGGAAGAGGAA	134
Ad01A4232	Aradu.A01	4232	p3	(AAG)5	15	11311379	11311393	ACCCAAAGGACACCTTATGC	CACAATTTCACTGCACCGTC	269
Ad01A4233	Aradu.A01	4233	p3	(AAG)6	18	11311499	11311516	GACGGTGCAGTGAAATTGTG	GCTGCATTGTCCAATTGTTG	270

ID	Chromosome	SSR nr.	SSR type	SSR	Size	Start	End	FORWARD PRIMER (5'-3')	REVERSE PRIMER (5'-3')	Product (bp)
Ad01A4235	Aradu.A01	4235	p2	(TC)24	48	11315489	11315536	CCCCAAAACCCCAAATCTAT	GAAAGGAGCCTTCATTGCTG	244
Ad01A4240	Aradu.A01	4240	p2	(TA)7	14	11322260	11322273	TGCTGGCTAATGAATTGCTG	ACTTCTTGCGACCTTATCCTT	203
Ad01A4242	Aradu.A01	4242	p3	(TAT)7	21	11323624	11323644	TGCTGTATGAACAAGGCGAG	AACTCACAAGCAAAAGTGTCCA	142
Ad01A4244	Aradu.A01	4244	c	(T)10actttttttat	124	11327710	11327833	TCTGACTCCGTTTCTGGCTT	CAAGCCTCAAGAAAGGAAAAA	233
Ad01A4252	Aradu.A01	4252	p4	(TTTA)5	20	11371652	11371671	TTTTCCATTGTGCAACCAAA	GGAACGGATCACAGAAAACAA	222
Ad01A4254	Aradu.A01	4254	p3	(TTC)5	15	11373682	11373696	TCCATCACCCACTTCTTTCA	TAAAGGAAACAGGCCACAGG	165
Ad01A4255	Aradu.A01	4255	p2	(TA)9	18	11374816	11374833	GAATGTATTTGCTGTCTTTTGCT	GCCTTAGGTCCCTCGTTTTT	258
Ad01A4257	Aradu.A01	4257	p2	(TA)13	26	11376081	11376106	TCGAGTACTCTTGGCACACG	GCAGCTTGAGTTTGCATTGA	225
Ad01A4258	Aradu.A01	4258	p3	(AAT)8	24	11378780	11378803	GAGTGAATTCAAATTCCTGATGC	CCATGTGCATGCTGTAACCT	221
Ad01A4259	Aradu.A01	4259	p2	(TA)8	16	11382908	11382923	TTTCGTTGTCCGATCCATTT	GTTTTTGACGGGATCGCTAA	225
Ad01A4260	Aradu.A01	4260	p2	(AT)7	14	11383087	11383100	TTAGCGATCCCGTCAAAAAC	TTTTCGATTTCATTTATTCCCA	169
Ad01A4263	Aradu.A01	4263	p6	(ATTTTT)6	36	11396745	11396780	AAACACATGTTCATAAACGATAACTAA	TCTGCTAAAGTGCGCAGGTA	248
Ad01A4267	Aradu.A01	4267	c	(AGA)6gaagaa	144	11399893	11400036	ACGAGCACGTGAAATTACGA	TGTTGTTGCAGCAAATTTCAA	269
Ad01A4268	Aradu.A01	4268	p3	(ATT)12	36	11400195	11400230	CGCTCTTTTAATGAGAGTGGTTT	GGAGCAAAAATTCATGCGAG	273
Ad01A4272	Aradu.A01	4272	c	(T)11gttacggta	131	11414578	11414708	TGAGGTTGGATTGAAGGAGAA	GCTATTTGGTGCATGCTATGT	269
Ad01A4276	Aradu.A01	4276	p2	(TA)7	14	11423518	11423531	TTTTGGTGAGTTGCATTGGA	TATTCCCAGCCACAAGATCC	279
Ad01A4286	Aradu.A01	4286	p3	(TTA)7	21	11443653	11443673	CGTGAAAACAGCTTAACCATTG	AAGGGATCAAAAGACCATGC	208
Ad01A4288	Aradu.A01	4288	p3	(TTA)6	18	11445459	11445476	TTCAACAAATTCGATACGAAACA	TGTTCATCGGACTCAAAAGAAA	122
Ad01A4291	Aradu.A01	4291	c	(GA)10gtagtgt	97	11452742	11452838	TAGGTTGTTGAGTTTGGGGG	GAAAATATTAGAGGTGAATTTGTCCC	214
Ad01A4295	Aradu.A01	4295	c	(TTAT)7tttctaa	95	11461681	11461775	TGAACCATCCATCAATTAGAGGT	TTGTTTTTGTTTACTCATGCACA	256
Ad01A4305	Aradu.A01	4305	c	(CTA)5(TTA)(	33	11472565	11472597	ACGGAATACACTGACACGCA	ATAACATGCACGTCAAATGC	277
Ad01A4308	Aradu.A01	4308	p3	(TAA)7	21	11481449	11481469	TTACTCATGCAGTCGAACTAAAAA	CTGAGATGAAGGCGTGAACA	265
Ad01A4310	Aradu.A01	4310	c	(A)12catgagcc	36	11482898	11482933	AGGAAGGTTCGCATACATGG	TGTTGCTCCTTCTAGTCTCTACAAAA	214
Ad01A4311	Aradu.A01	4311	p3	(AGA)5	15	11493319	11493333	CAGCTGAGAATCTGACGTTGA	TACGTCCAATACACGCGCTA	280
Ad01A4312	Aradu.A01	4312	c	(T)10ctttggattt	48	11494026	11494073	ACTTGTCCCCCATGTTTGAA	TTGGAGATTCAAATTTCGCC	199
Ad01A4317	Aradu.A01	4317	c	(T)11gg(A)10	23	11500586	11500608	AGACTGCTCTTGCCTGCTCT	CATGGGATCCAATAACCCAC	151
Ad01A4318	Aradu.A01	4318	c	(CTC)5t(TTC)	31	11504366	11504396	TTTCAAATCAGGCGCTTCTT	AGAAGAAAATTTTGCGTGCG	155
Ad01A4323	Aradu.A01	4323	p2	(TA)8	16	11518008	11518023	AGCGAAAACACACGCCTATT	AGAAGCTCGATCTCAGCAGC	251
Ad01A4325	Aradu.A01	4325	p2	(TA)6	12	11523243	11523254	ATCCTTATGCTTTTGGGGCT	CTAAACCGGCCAGAACACAT	201
Ad01A4326	Aradu.A01	4326	p3	(TCC)8	24	11523617	11523640	CGTGAGTGTTGCACTTTTCG	GGAAGAAAAACGCAAAGAAGA	273
Ad01A4327	Aradu.A01	4327	p3	(GAT)8	24	11524232	11524255	TTCCCATATTTCTTTCTTTCTTTCA	TCCTTTTCATCTTCTGCTGCT	251
Ad01A4328	Aradu.A01	4328	p3	(AAG)5	15	11524618	11524632	CCTCTTTCAAGTAGTTTTGCAGC	CTTCTGCTCCCTCTTCCTCA	174
Ad01A4329	Aradu.A01	4329	p2	(AT)7	14	11525133	11525146	TTGGTGATGACGATGACGAT	TTTTGTCCTCCGTTGGAGTT	214
Ad01A4330	Aradu.A01	4330	c*	(AT)6(T)10*	21	11526471	11526491	CAGGGATCATTTTGTCTTCCA	AAAGGGCAATTCCCACTACC	202

ID	Chromosome	SSR nr.	SSR type	SSR	Size	Start	End	FORWARD PRIMER (5'-3')	REVERSE PRIMER (5'-3')	Product (bp)
Ad01A4332	Aradu.A01	4332	p2	(AT)9	18	11529080	11529097	GAAGGGAGGAAGAGATGGAGA	GCATGAATGCAAAATGGATG	262
Ad01A4337	Aradu.A01	4337	p3	(GGA)5	15	11536246	11536260	GCAACGATGATGATGACCAC	GAACATCCTCGCAAGAAAGC	241
Ad01A4338	Aradu.A01	4338	p4	(TTAT)5	20	11538689	11538708	AGCATCCTCACCCCCTAAAT	AGGAGGTGGGATAGGTGGTC	262
Ad01A4341	Aradu.A01	4341	p3	(CCA)5	15	11543809	11543823	TCAAGGGTGGTATAATCCGC	TGGGTTTAATGGAAACGGAG	199
Ad01A4349	Aradu.A01	4349	p2	(TA)11	22	11562908	11562929	CCCAAAATCCTAATTTGATCG	AAAACCGTCACTATCTGTCAAAA	280
Ad01A4351	Aradu.A01	4351	c	(TCT)8tgatcct	70	11568804	11568873	ATCGGTTGCCATTTTGACAT	GTGGTGGTTTAGGGCAAGAA	273
Ad01A4352	Aradu.A01	4352	p2	(AT)6	12	11570708	11570719	TTGGTTATTCATGGTAACAGGG	TTAAAGGCGGCCATATCAGT	280
Ad01A4360	Aradu.A01	4360	c	(AG)7t(GA)8	31	11585518	11585548	GGTTGCGTGATTTGTGTTTG	TTTTTCCCTTGTGTGTTGCC	192
Ad01A4361	Aradu.A01	4361	c	(ACA)5ataaat	205	11589836	11590040	CAGAACCAAAAGCAACAACAA	TTCTGCTTCTGCTTCCATCA	276
Ad01A4362	Aradu.A01	4362	c	(AAG)5cgacg	105	11590441	11590545	CCCCATTCTTTCTCTCTCCC	TCCTCCTCCTCACCCTCAC	245
Ad01A4367	Aradu.A01	4367	p3	(ACC)5	15	11601119	11601133	GGACAAAAATGCCATCCACT	CTGCCTCTGCTTTTGCTTCT	195
Ad01A4369	Aradu.A01	4369	p2	(GT)6	12	11604516	11604527	GGGATGGTGCATGCTTTTAC	TTTGAGCCACTTGCTTCTCTC	158
Ad01A4370	Aradu.A01	4370	p3	(AAT)5	15	11608973	11608987	CCCTGCATGTGGATTGTGTA	TGTGTGGATGTGTAATCGGG	270
Ad01A4371	Aradu.A01	4371	p2	(AT)10	20	11611108	11611127	TGGGGATAACTTGGAGGTGA	CGGTTTGCTAAACCTTTGGA	276
Ad01A4374	Aradu.A01	4374	p2	(TC)19	38	11613895	11613932	GAAACACGTTGACCGTGAATA	AACGGAGGGTTTTGTGAGTG	155
Ad01A4375	Aradu.A01	4375	p3	(CTT)6	18	11614086	11614103	CACTCACAAAACCCTCCGTT	AAGGAGAGAGCAACACGGAA	276
Ad01A4377	Aradu.A01	4377	p3	(CCA)5	15	11626007	11626021	GCCAAGAAATTAGGGCTTCC	AATGGTGCTGGAGTTGGTTC	221
Ad01A4378	Aradu.A01	4378	p3	(TTG)5	15	11626493	11626507	CGAGAGCTTGGAAATGGAAG	CCACCCTCCAAAAACTCTGA	265
Ad01A4379	Aradu.A01	4379	p3	(TCT)9	27	11627563	11627589	TTTGTCATTAAACCCGACCC	TCGGATCCTGGTTCTGATTC	268
Ad01A4380	Aradu.A01	4380	c	(AGA)5cgaac	59	11627760	11627818	GAACGAAAGTACCGAACCCA	GTCCCACGGAGACTGTGTTT	234
Ad01A4383	Aradu.A01	4383	p4	(ATAA)5	20	11635958	11635977	TTTGAAAATTAACCTCGCCG	GTGTGCTTAGGGAAGTTGGC	262
Ad01A4385	Aradu.A01	4385	c	(T)10ctgcttagt	91	11636568	11636658	CCCTTTTCTTTTTCACTTTGGA	TAGGGTCGGCTACGCTATTG	275
Ad01A4386	Aradu.A01	4386	p3	(ACA)5	15	11636890	11636904	TAGCCCTTTTTCCCCCTAAA	AAGATCGCCAGAGCACCTAA	257
Ad01A4394	Aradu.A01	4394	p2	(TA)6	12	11662716	11662727	TTGAAAACATAATGCCAATTTTT	GCATTGCTCATATTCTAATTCAACTT	176
Ad01A4403	Aradu.A01	4403	c	(CTT)6tcaaata	154	11715467	11715620	GCGCATTAATGACGAGTGAA	TTGGTGATATTGGTGATGACG	244
Ad01A4404	Aradu.A01	4404	p3	(AAG)5	15	11716050	11716064	GCAACTCCACTTAACCGAGC	CACAAAGACCAGCGAACTGA	216
Ad01A4408	Aradu.A01	4408	p2	(GT)9	18	11735705	11735722	AGGTGGTGAAGAAGGTGACG	ACACTTAGCCCAACATTGCC	266
Ad01A4411	Aradu.A01	4411	p4	(AATA)5	20	11749486	11749505	CAGAGTCTCGTGCTGATAACG	TCTCTGAGAAAACACGAGCAA	225
Ad01A4412	Aradu.A01	4412	p2	(GA)6	12	11753965	11753976	TTTCTGCCAATTTGAAAGGG	GCCAGCTCTTTCTGCAATTT	217
Ad01A4413	Aradu.A01	4413	p4	(AAAC)5	20	11761211	11761230	TCAGCTGCATGTTGTGAATG	ATGTTGATCTCTTGGGCCAC	246
Ad01A4417	Aradu.A01	4417	p2	(TA)8	16	11763826	11763841	CCCCCATTTCTGTAAACAACA	TCAATCGGGTATTGTAGCCC	279
Ad01A4428	Aradu.A01	4428	p2	(AT)7	14	11785028	11785041	TCTCGTGCTGATAACGTGTTG	TGCCACACATTCTTCTCTTTCT	188
Ad01A4429	Aradu.A01	4429	c	(AT)6agaaagg	49	11785677	11785725	TCAAAACAATGCATAATGTTGTAAA	GAGGTTAAGCCTCCGTCAATC	277
Ad01A4430	Aradu.A01	4430	c*	(AT)7(ATA)6	60	11791059	11791118	TCTTGTGCTGATAACGTGTTG	GAGGTTAAGCCTCCGTTAATTTT	277

ID	Chromosome	SSR nr.	SSR type	SSR	Size	Start	End	FORWARD PRIMER (5'-3')	REVERSE PRIMER (5'-3')	Product (bp)
Ad01A4431	Aradu.A01	4431	c	(T)10catgttgac	124	11792194	11792317	TGATTATTCGTGGAGAATGATTG	TTATTTACCCACGCATTGCC	277
Ad01A4432	Aradu.A01	4432	p3	(GAT)5	15	11799452	11799466	TCCGAGTCTCGCTTCTTGTT	GCAACAACTGCTATTTGCGA	263
Ad01A4433	Aradu.A01	4433	p3	(ATT)14	42	11801036	11801077	TCCTTCTTGGCGAGCTTTTA	GGTGGATTCACAATTCCCTG	190
Ad01A4435	Aradu.A01	4435	p2	(CT)14	28	11803197	11803224	GGCATGACAAAAATGATCCA	ATGATTCGGAGGTGAAGGTG	215
Ad01A4439	Aradu.A01	4439	p2	(GA)6	12	11812703	11812714	GCGGGTAGTCCAGCTAGGTT	CCATGTATGCAGGAGACATCA	279
Ad01A4440	Aradu.A01	4440	p4	(AAAT)5	20	11813240	11813259	AAGGAGCTGCTGGTGAAATC	TTTGGTCCATTTCCCATTGT	265
Ad01A4441	Aradu.A01	4441	p3	(GAG)6	18	11822201	11822218	GAAGGGAAGCATCGTCCATA	CTGATGCTGCGAAGGCTACT	273
Ad01A4443	Aradu.A01	4443	p2	(AC)6	12	11824293	11824304	TCCAACGGTGGTTATTGGAT	CCTGAGATAGGCGGATTGAG	255
Ad01A4447	Aradu.A01	4447	p2	(AT)9	18	11828118	11828135	TTGGACACCAAAATCATAGTCAA	AAATGTGGCAAGATAAGAACTATGG	196
Ad01A4449	Aradu.A01	4449	p3	(AGA)7	21	11832428	11832448	GAAGAAGAAGACGCTCCGTG	AATGGGAGGCTACACATCCA	192
Ad01A4455	Aradu.A01	4455	c	(T)10aatcttaaa	51	11845353	11845403	GGGCCACCATATCTTCTTTTC	CATTCTTGGCATGCATTTTC	204
Ad01A4458	Aradu.A01	4458	p2	(AG)10	20	11851473	11851492	GCATAAGAAAATGGCGGAGA	TTAGTTTTTGCGTTTTGCCC	280
Ad01A4459	Aradu.A01	4459	p2	(AT)11	22	11853197	11853218	TGCACCAATAACACATCGAAA	AATTGATTTTGAAAAAGTAACCCTT	230
Ad01A4463	Aradu.A01	4463	p3	(AAT)6	18	11867210	11867227	AAATTCCACCAGCCTCACAC	TGCAGCTATCTAAGGGTGCC	240
Ad01A4465	Aradu.A01	4465	c	(GA)8gtcagtga	122	11867574	11867695	AAGGTGCGGAAGGTAAGGTT	GGTGGACAGTGTGGAACAGA	229
Ad01A4473	Aradu.A01	4473	c	(TA)6gcttttcac	96	11878388	11878483	TGAAGAAAGACAGAAAGAAACAGC	AATTTCAAGAGGATAGTGGAAGAG	238
Ad01A4474	Aradu.A01	4474	p3	(TAT)17	51	11879144	11879194	TTGCATCAAATTCAATTCTCG	GAACATGAATTGCCAACCCT	272
Ad01A4477	Aradu.A01	4477	c	(TC)10(TA)11	42	11882838	11882879	TGGAGTTTAACGCTCAAAACAA	TGGAAGACAAGATTCAGGCG	239
Ad01A4478	Aradu.A01	4478	p3	(ATT)5	15	11886610	11886624	AATCACCCATGAGCATCCAT	TCGATGATGGAAGAGGCAGT	254
Ad01A4480	Aradu.A01	4480	p4	(TTTC)5	20	11889119	11889138	GCTTCATTTTGGCAACAGGT	ATACGGTTACTCCCACGCCT	218
Ad01A4481	Aradu.A01	4481	p3	(TAA)12	36	11890770	11890805	TTTGGTGACCTGTATGAGTTATCA	CAAGCAGCTTTCTCTGTTCCT	263
Ad01A4482	Aradu.A01	4482	p3	(TTA)15	45	11891338	11891382	TTCTTAGGTATGTCTGTGGGTCTG	TTCCCATTAGTTATTCATGGTTTTT	159
Ad01A4486	Aradu.A01	4486	p2	(TA)9	18	11906959	11906976	TGCAATATCATGTTAGAGAATTGAAA	TGTCGTTTTTGTTAGGATAGAGACC	238
Ad01A4493	Aradu.A01	4493	p2	(AT)7	14	11912527	11912540	CAATACCAATTTATTTGACAATACGTG	TTTATGCAATAATAAGTTTCAAAAGTG	152
Ad01A4494	Aradu.A01	4494	c	(CT)6aatccaaa	99	11913530	11913628	CGACTTTACTCACACAGCGG	GCTCGAGACATGATCCAGTG	172
Ad01A4495	Aradu.A01	4495	c	(A)11cgcttcag	36	11918092	11918127	CACCTGCACCTGAGTTTTCA	TGATAACCCGGACTAGTTAACCTC	256
Ad01A4496	Aradu.A01	4496	p3	(TAT)8	24	11918963	11918986	AGGGAATAAGAATAGTTCAGTTTAACA	TGGCCCCTAGACTTTTTCTTC	280
Ad01A4498	Aradu.A01	4498	p2	(TA)8	16	11924854	11924869	TCGTATCGCAAAGGATTATCG	TCGACTTCACCTAAATTTTTACCA	259
Ad01A4499	Aradu.A01	4499	c	(TA)12ctctgac	59	11928489	11928547	TTTCCTCTGCAAGCGAAGTT	TTGAGGTGGCAAGGTATTACG	259
Ad01A4505	Aradu.A01	4505	c	(ATAA)5aaaga	52	11943502	11943553	GGAAAATTATTTGGTGGGGG	GCACCGAATTGAACTTACCAA	266
Ad01A4511	Aradu.A01	4511	p3	(AAT)15	45	11951483	11951527	AACCCAGTTAAACTCCAGTCG	AGTTGAACTGCCGTCATCG	231
Ad01A4512	Aradu.A01	4512	p2	(TC)8	16	11954652	11954667	CCCTCTCCTTTTCCCCATAC	CCGATTCGTCGACAACTAATTT	235
Ad01A4514	Aradu.A01	4514	p2	(TC)7	14	11959982	11959995	TGCCCTTTGCACACAAAATA	CTGAAACTTCCTCTCGGGTG	239
Ad01A4515	Aradu.A01	4515	p3	(ATT)5	15	11960213	11960227	CACCCGAGAGGAAGTTTCAG	TCCACCACTTTGATGTGGAA	167

ID	Chromosome	SSR nr.	SSR type	SSR	Size	Start	End	FORWARD PRIMER (5'-3')	REVERSE PRIMER (5'-3')	Product (bp)
Ad01A4518	Aradu.A01	4518	p4	(AATT)7	28	11964511	11964538	TTCCAAGTTTGGGAGATTAAAAA	TCAATGCAGCTCAGAACTTTGT	259
Ad01A4519	Aradu.A01	4519	p3	(TAA)12	36	11965096	11965131	GCGCAGAATCTGTGTCTGAA	CGCATAATTTAATGAACTCTTCCA	137
Ad01A4522	Aradu.A01	4522	p3	(TAT)13	39	11967190	11967228	GAATTCATGCAAAAACGGGT	TTGTTGTGGTTTTGCAGCTC	220
Ad01A4524	Aradu.A01	4524	c	(AT)7gttggtgca	60	11968210	11968269	TGTGCTGAAAAAGTGAATAATTTTG	CCACCAGGTGCCATTAAAAA	262
Ad01A4532	Aradu.A01	4532	p2	(AT)6	12	11991662	11991673	AATAGAATTTTGGTTAGCGCC	ACCTATTGAAGGTGCGCAAG	193
Ad01A4535	Aradu.A01	4535	p3	(ATT)5	15	11994077	11994091	CACACTAAGAAACAATGCAAAGC	TGAATTGCTTTTTCAATCACTCA	268
Ad01A4538	Aradu.A01	4538	c	(A)10tatgaccct	120	11997105	11997224	AAAATGGAGAGAAAAATGTTGTAAAAA	TCATCAACCACCACCTTTGA	249
Ad01A4539	Aradu.A01	4539	p3	(AGA)6	18	11997412	11997429	TCAAAGGTGGTGGTTGATGA	TTCCATGCTTTTGTTCCCTT	168
Ad01A4540	Aradu.A01	4540	p3	(AAT)8	24	11997616	11997639	TGATATTGGTGTACGCTGCAT	CAAGCACAGCCTTAGTGACTG	269
Ad01A4545	Aradu.A01	4545	p2	(AT)7	14	12006606	12006619	AAGGGAAATGGGAATTTTGG	TCGAATATGATCCTCTTTTGATGA	126
Ad01A4546	Aradu.A01	4546	c	(A)10ctcggcgt	52	12008077	12008128	AGCATCGTCGATCTCGTTCT	ACATTACAGTTTCCTCCGTCA	223
Ad01A4548	Aradu.A01	4548	c	(TTA)6tccttctc	87	12013483	12013569	ATCAGCCACATTTATCCCCA	GGTGTTTGTGGGATTTGGAG	183
Ad01A4550	Aradu.A01	4550	c	(A)10cacatcaa	80	12016812	12016891	TCATGATACCAATCAATATCACCTT	TCCCGTGGATAATGAAACTTG	209
Ad01A4551	Aradu.A01	4551	p3	(ATA)6	18	12017031	12017048	CAAGTTTCATTATCCACGGG	TGGGGTTGGAACGTTATCAT	208
Ad01A4556	Aradu.A01	4556	p3	(TAT)15	45	12027757	12027801	TCTAATACTTGACTTCTAAACACCAAA	TCGAATCACCCTCATTCAAA	222
Ad01A4563	Aradu.A01	4563	p2	(AG)6	12	12108411	12108422	ATGTATTTGTGTGGCGACGA	CTTCTCACGGTCTTTGGGAG	280
Ad01A4565	Aradu.A01	4565	p2	(GA)6	12	12110105	12110116	GCAGAATCGAGCAGACACAA	CTGGCTCTGAAGCTACCCAC	220
Ad01A4567	Aradu.A01	4567	p3	(ATA)19	57	12133238	12133294	AGCCCATCCACAACTTCAAC	GCATCCCGTTTTACATGCTT	225
Ad01A4568	Aradu.A01	4568	c	(A)15ggatggca	67	12134677	12134743	TACGAAAAAGGGAATGTGGG	TGCAATGCACCTGAACTTGT	247
Ad01A4569	Aradu.A01	4569	c	(GT)8(GA)9	34	12135301	12135334	CTGAAGGTTGGGGGACAGTA	CCACACACTCGCCATAACAT	272
Ad01A4572	Aradu.A01	4572	p3	(TTC)5	15	12142136	12142150	TGTCTCAATTGTTCCTCCCC	TTTCAAAGACTGGTGGGGTC	200
Ad01A4573	Aradu.A01	4573	p3	(TAT)13	39	12142491	12142529	TTGAACTGAAGAAGATATATGTGGC	AACTCAAAACATTACCATTCATTAAAA	280
Ad01A4578	Aradu.A01	4578	p3	(AAT)5	15	12165649	12165663	CAACCAACCAAAGCAACAAA	GTAATGAGCGGAGGACCAAA	271
Ad01A4579	Aradu.A01	4579	p2	(GA)8	16	12166199	12166214	ATTTCATCCCTCGCAATACG	AGAATAGCTCCCCTGCCATT	207
Ad01A4585	Aradu.A01	4585	c	(A)11gaatacaa	44	12201681	12201724	GCAACATAGCCCAACAAAAA	CACACACAAAAATATTCATTTGAGAA	134
Ad01A4590	Aradu.A01	4590	p2	(AT)6	12	12227473	12227484	TTTTCACTTTCTGCCGACCT	AGAAAAGGCAACGGATCTCA	244
Ad01A4593	Aradu.A01	4593	p2	(AT)6	12	12256987	12256998	TTGCACAACCTTCATGCAAT	TTCTTAATAGCTAAGTCCTTAAACGG	160
Ad01A4594	Aradu.A01	4594	p2	(AT)6	12	12257848	12257859	CCGATTTTAGGCCTCATTCA	CTCAGCTGCTTAGCCTGAGA	199
Ad01A4595	Aradu.A01	4595	p3	(CTT)5	15	12268401	12268415	AAACTCATCACTTGGCTGGG	TGTTGATGCTGCAACTCCTC	242
Ad01A4597	Aradu.A01	4597	p3	(ATT)5	15	12279178	12279192	TATGTTCTCCAGCCAGCCTT	TTGAGGTGGCAAGGTATTACG	270
Ad01A4598	Aradu.A01	4598	p2	(AT)8	16	12280506	12280521	CGGAGGGTACGAGTTATCCA	CCTCCGATTAAAGTTGGCTG	165
Ad01A4599	Aradu.A01	4599	c	(AAT)5taacttc	77	12284423	12284499	GAAACCTAACATAATCATGTCAAAAA	TGTTGTTAGAATTGAATTTAGGAATGT	204
Ad01A4600	Aradu.A01	4600	p3	(ATA)14	42	12287797	12287838	GGAATAATAACAATACCAACAGCAA	AACTCGCTTGTACCGGCTAA	270
Ad01A4603	Aradu.A01	4603	p2	(TA)7	14	12322489	12322502	GCTCTTCCCGGTCTTCACTA	CTGTGAAAATTCAGGTACAGTTCA	229

ID	Chromosome	SSR nr.	SSR type	SSR	Size	Start	End	FORWARD PRIMER (5'-3')	REVERSE PRIMER (5'-3')	Product (bp)
Ad01A4606	Aradu.A01	4606	c	(TC)9ctcttcccc	65	12324932	12324996	ATCTCAATCTCATGCTCGCC	AGTCCGATCCAAACATGAGC	190
Ad01A4616	Aradu.A01	4616	p2	(AT)7	14	12374405	12374418	ATTCCCCATTCAAACCAACA	GGGACATAAAAAGACATACGGA	273
Ad01A4620	Aradu.A01	4620	c	(TTC)5gccgcc	156	12380917	12381072	AAAAAGAAAGAAAGCGATACAGAG	GCAACAACAATATTCCACAACAA	265
Ad01A4621	Aradu.A01	4621	c	(TTC)6tgaactg	136	12381266	12381401	GTTGACTGCCATTCTCAGCA	TTCTGTTTTAGTCCCCAACG	254
Ad01A4624	Aradu.A01	4624	c	(AG)12(AC)7	38	12392826	12392863	GGTTTAATCGACCTTAGGGTTCT	TGACGAATGGTTTTGAGGGT	239
Ad01A4626	Aradu.A01	4626	p2	(GA)21	42	12408473	12408514	GCTGCAGCTCCTTCTTTCTC	CCACTATCATCTCCTCTCCCTC	194
Ad01A4631	Aradu.A01	4631	p3	(GGA)6	18	12424830	12424847	CTGGCTCAGAAAGAAGTCGG	TCTTTTGCAGACATGGCAAG	223
Ad01A4641	Aradu.A01	4641	p4	(ATTT)7	28	12480023	12480050	TTCGGTTAAGCCCATGTCTC	AAAGTCACGGCTAGGCTCAA	253
Ad01A4650	Aradu.A01	4650	c	(AG)6attatgtgc	33	12544675	12544707	TTGTTGTTCATGAATGTTGGC	TCCCCAATTAGAGGTGTCCA	237
Ad01A4651	Aradu.A01	4651	p2	(CA)8	16	12548624	12548639	TTTTCGTCCTCGAAAATTGA	TACCAGCCTGCCTTGTAACC	179
Ad01A4652	Aradu.A01	4652	p2	(AG)14	28	12560736	12560763	ACGGCACTCAACGTTTTTCT	CGTCCGATTTATCCGTCAGT	199
Ad01A4657	Aradu.A01	4657	c	(CT)7ca(T)10	26	12583739	12583764	TGGACAGACTTTTTCAACCAG	TGCTTTGACACTCAATCTAGTCG	272
Ad01A4659	Aradu.A01	4659	p2	(AT)6	12	12584970	12584981	CCGAAGATACAACGCTTACGA	TGTGTGCGTGTGTGTGTACC	276
Ad01A4660	Aradu.A01	4660	p3	(TCA)5	15	12586468	12586482	TATATCCGGAGCAGGTGGAC	CCCATTCCTCTGATTTTCCA	223
Ad01A4662	Aradu.A01	4662	p3	(GGA)5	15	12594055	12594069	GAACCCGCACTCAAGAGAAG	TGGAGGATCCGTTATTGCTC	211
Ad01A4669	Aradu.A01	4669	p3	(TGG)5	15	12605953	12605967	TCCGTGAATTTCAATTTCTGC	TCCTCATTATCCACTGCGAG	255
Ad01A4670	Aradu.A01	4670	c	(AG)11ggaagg	73	12611016	12611088	GAAGCACCTTTTATGGCACC	CTTGCACTGCACGACTCTTT	268
Ad01A4671	Aradu.A01	4671	p2	(TC)20	40	12621910	12621949	TGCAAATGGTGTTAACTGGAA	GTTGTGATGGTGTGGTGAGG	157
Ad01A4672	Aradu.A01	4672	p2	(GA)23	46	12622473	12622518	GGGGTTCAAGTGGAAAGACA	CTCCCCCACTCTCACCACTA	193
Ad01A4674	Aradu.A01	4674	p2	(TA)6	12	12626280	12626291	GAGTCACTTGACTATTTTTCTTTTTGG	AAGCTATGCTGCACTTTTTGAA	235
Ad01A4675	Aradu.A01	4675	p3	(TGG)5	15	12627874	12627888	TGCTTCGCTGTCTCATCTTG	ACACTAACATCACCGCCACA	218
Ad01A4678	Aradu.A01	4678	p2	(AG)10	20	12634612	12634631	GGGCATAGCGAAGGTGACTA	ATTGCTGCTCATCCCGTTAC	187
Ad01A4680	Aradu.A01	4680	p3	(TAA)5	15	12636047	12636061	CCCGTAGAAAATACGGAAACA	ATAACAACGGGGACCAAACA	214
Ad01A4682	Aradu.A01	4682	c	(A)10tacttctttta	44	12647709	12647752	TCCATGCATTGATGTTATGACT	AGCCTGGGGTCTGCAATTAT	279
Ad01A4684	Aradu.A01	4684	p2	(TA)7	14	12648617	12648630	CCTAATTGTAGCAAGTATCTTCATTG	TTTTTGCCTTTATTTTTCAAACTG	264
Ad01A4686	Aradu.A01	4686	p2	(AT)6	12	12671299	12671310	ATTTGGCTCCTGTTCACACC	TTCGTTCCCGTAGAGTTTGG	206
Ad01A4687	Aradu.A01	4687	c*	(TA)7(TATG)8	44	12672972	12673015	AGGAGGAACAAGGAAAGGGA	GGAAGGGGTGATAACTTGGA	241
Ad01A4693	Aradu.A01	4693	p2	(TA)8	16	12692574	12692589	GGATGGCAATACTACCCGAA	AACCCTAACCCGTCCCTATG	233
Ad01A4695	Aradu.A01	4695	p2	(CT)14	28	12697952	12697979	AGCCATGCACGTTATGGTTA	AGGGCATACGGATTTTTGTG	222
Ad01A4700	Aradu.A01	4700	p2	(AG)6	12	12728759	12728770	GAAGGGTTTTTGGGGAAGAG	ATCCCCACCTACCCTACCAC	107
Ad01A4702	Aradu.A01	4702	p2	(CT)6	12	12751421	12751432	TGAATTAAAGCGCAATCACG	GAAAAGGAAGGAGGAAAGAAGAA	229
Ad01A4703	Aradu.A01	4703	p2	(TA)7	14	12760982	12760995	TGCTCATGTGAGACCAGAAAA	GCCTAGCTACCCATAATTCCC	184
Ad01A4704	Aradu.A01	4704	p3	(TTA)6	18	12773545	12773562	CAGATGCACTGTTTCTGCGT	TTGTCCAGTGTTGTGCATCAT	119
Ad01A4705	Aradu.A01	4705	c	(AAT)8(ATT)1	57	12776000	12776056	TCATAATACCAAACCACAAAGGAA	CAAATGGTTTTAACCTTGAATTTTT	215

ID	Chromosome	SSR nr.	SSR type	SSR	Size	Start	End	FORWARD PRIMER (5'-3')	REVERSE PRIMER (5'-3')	Product (bp)
Ad01A4707	Aradu.A01	4707	p3	(TAA)12	36	12779027	12779062	AGCCTCTCCCATGCCTACTT	GAGCCAAATCAAAAGCCTTG	273
Ad01A4708	Aradu.A01	4708	c	(A)11gaaagact	115	12788401	12788515	ATCAGAAGGTGAGGCCATTG	TGTGTGAGTGTATTCGATCTCTCTT	196
Ad01A4712	Aradu.A01	4712	p2	(TA)6	12	12793451	12793462	GAGCTTATGAGCTTGTGGGC	CTGGAAAGATGAGGGTGGAA	266
Ad01A4715	Aradu.A01	4715	c	(A)12(CA)7	26	12796913	12796938	TCAAACGATCATGCCAACAG	GGCAAATGTCTCTTGCAATC	260
Ad01A4717	Aradu.A01	4717	p2	(TA)6	12	12798973	12798984	AAATTTGGCCTTACTCGTAGAA	TCATATTCTATCCTACCTGCACTCC	263
Ad01A4720	Aradu.A01	4720	p3	(ATA)23	69	12823312	12823380	CATCACCACTAACGCATTCAA	TGTTCATGATGGTTATTATTAGGACAA	276
Ad01A4722	Aradu.A01	4722	p5	(TTATC)5	25	12850771	12850795	TGTGGAACTGAGAATGCTGC	AGGCGGTTCCCCTAAAGATA	244
Ad01A4724	Aradu.A01	4724	p2	(CT)6	12	12872889	12872900	GGGCGTTTAACACCAGAAAA	TTTTTGGGGAAGAGGTGTTG	240
Ad01A4730	Aradu.A01	4730	p2	(TC)7	14	12915887	12915900	GAACAAAAGAAGCGTGGTCC	ATGTAAGAGAGAGCGAGGCG	205
Ad01A4731	Aradu.A01	4731	p2	(CT)8	16	12922106	12922121	TGCTCCCTCTTCTTCTTCCT	TGTGTAAGCGGCATGACCTA	198
Ad01A4732	Aradu.A01	4732	p3	(TTA)17	51	12924560	12924610	GGAACTGCCTCACTAGGCG	AGAGGGCTGCCATAAATGTG	280
Ad01A4736	Aradu.A01	4736	c	(TC)9tt(TC)15	50	12935285	12935334	AAGTCCACAATACCCGGTCA	TCCTTGGAACCTATTCCCCT	278
Ad01A4739	Aradu.A01	4739	p2	(TA)6	12	12944604	12944615	TTCACAAGAGAATTTATGAATTGGA	CGGTTTAAAACGTCACGAAA	267
Ad01A4748	Aradu.A01	4748	p3	(GTG)5	15	12972174	12972188	AAAAGGATCATCCCGAACCT	TCAGGTAACCCTCGGAACAG	111
Ad01A4752	Aradu.A01	4752	c	(A)10gaaaaaaa	32	13014755	13014786	GGAGACATGTTACTGAGGATCTGA	CCGGATACATACATGCCACA	264
Ad01A4753	Aradu.A01	4753	p3	(ATT)5	15	13061283	13061297	TGCTCCCATATGAAGCAGTG	TTCAATTTCAAAATATTTCCACTTTCT	260
Ad01A4755	Aradu.A01	4755	c	(CT)9t(TC)10	39	13063896	13063934	GTTCTGGCCGCTATCATTGT	AAAATCAAGAATTGCACGGC	225
Ad01A4756	Aradu.A01	4756	c	(TAT)14(TGT)	60	13068113	13068172	TCGATTTTGACAACTATGCAAT	GTTGGCAAGATGGGTCTGTT	280
Ad01A4760	Aradu.A01	4760	c	(T)10attatttgga	51	13083127	13083177	TCACCAGGTGGAAACTCTAACTC	TTGTTCGCTTTGCTGTTACG	279
Ad01A4766	Aradu.A01	4766	p3	(CTT)6	18	13096972	13096989	AATGGGACGAAGCTGAGAGA	AGAACGGTGAACCAACAACC	249
Ad01A4767	Aradu.A01	4767	p2	(TG)10	20	13103062	13103081	GCAGCATATCCGTTACAATCC	AAAACACAATCAATTCCATCCA	268
Ad01A4771	Aradu.A01	4771	p3	(TAA)13	39	13133553	13133591	AAATTTTGCACACATAATGCATAGA	CGGTGTAAGCTTTACAATTCTTTTT	231
Ad01A4772	Aradu.A01	4772	p2	(CT)7	14	13135982	13135995	CGCAAAGTCTTTTTCCCTCA	GGTGATAGAGGCGACGGTAA	234
Ad01A4780	Aradu.A01	4780	p3	(TAA)10	30	13153454	13153483	CCATCTGCACGAAAACACAC	CAAATCAATTCAATGGGTATTTCA	214
Ad01A4782	Aradu.A01	4782	p2	(TA)7	14	13164134	13164147	CTGGCCATTCAGCTTTGTTT	CACGTTTTCTTTTTGTTCTACGC	257
Ad01A4786	Aradu.A01	4786	p3	(GAA)5	15	13190949	13190963	TTTTCCCCTCCTGTCTTCCT	CAATTTAAAACTCTTCCTTTTCCG	190
Ad01A4787	Aradu.A01	4787	c	(TTC)6tctgttcg	52	13192340	13192391	TCAAGCAAACTACGTGACGG	AATGCGAACAGAGGAGAACG	268
Ad01A4788	Aradu.A01	4788	p3	(TTC)5	15	13192499	13192513	CGTTCTCCTCTGTTCGCATT	CATGCAGCAATCATTCCCTA	207
Ad01A4789	Aradu.A01	4789	p3	(TCC)5	15	13192789	13192803	AGCTAGGGAATGATTGCTGC	AGAAGAAAAACGCGACCAGA	258
Ad01A4790	Aradu.A01	4790	c	(AGA)5acaaga	116	13193628	13193743	CATTGAAGTCAATCCAGATCCA	ACCAAGTTGATCCAATCCCA	241
Ad01A4793	Aradu.A01	4793	p3	(TAT)19	57	13203016	13203072	TGATACATTCCGATACTTGATTTTG	TGGGTTATTTAGCCACGGTT	278
Ad01A4794	Aradu.A01	4794	p2	(TG)10	20	13204354	13204373	CCCACGTTTATAACCAATAGCA	TTTGACTTAATGTGAGAGCCG	248
Ad01A4796	Aradu.A01	4796	p3	(AAG)5	15	13218052	13218066	GAAGATGGTGATGAGGATGATG	ACTAGAAGCGCGGAAGATGA	241
Ad01A4797	Aradu.A01	4797	p3	(AAG)5	15	13221965	13221979	GAAGATGGTGATGAGGATGATG	ACTAGAAGCGCGGAAGATGA	241

ID	Chromosome	SSR nr.	SSR type	SSR	Size	Start	End	FORWARD PRIMER (5'-3')	REVERSE PRIMER (5'-3')	Product (bp)
Ad01A4798	Aradu.A01	4798	p3	(AAG)5	15	13226244	13226258	TGCTGCATTTTCTTTCTCTTCTT	TCCTCCTCCTAATCTTCTGCTG	277
Ad01A4802	Aradu.A01	4802	p2	(TC)8	16	13238651	13238666	GTAAATTCGGCAGCTGTGGT	ACGGCAAGGAACACCAATAG	270
Ad01A4804	Aradu.A01	4804	p3	(CAC)5	15	13242397	13242411	CAAACCCACCACCAAAATTC	AAGGGGCGAGAGAGAAAAAG	277
Ad01A4805	Aradu.A01	4805	p2	(AG)6	12	13242982	13242993	TCCAGTCCCTATCCAGTTCG	CATCTTTTTCCCACCACCAC	252
Ad01A4806	Aradu.A01	4806	p2	(TC)7	14	13245913	13245926	CTGAGGGTCGTCTCTTCCAA	AACGCAAGGCATTAAAATCG	179
Ad01A4810	Aradu.A01	4810	p2	(TA)6	12	13250147	13250158	AAATGGCAAATTCACATCCC	TGCAATTCTCTTTCTCTTTACTTTT	243
Ad01A4813	Aradu.A01	4813	p3	(TAA)12	36	13258778	13258813	TGTATCAAACTCGCAGTAGTAATGA	CGCTTTCTAAAACAAATCCTGAA	280
Ad01A4814	Aradu.A01	4814	p2	(TA)6	12	13261951	13261962	CGGAAGTACATGAATGGAGG	CGAGCTCACGAGCTGACTTA	262
Ad01A4816	Aradu.A01	4816	c	(T)10gtaatttttg	107	13263256	13263362	TTGTGATTGTTGCTATTTAGGTTGA	AAAACTAATCCCCAATACGACG	263
Ad01A4825	Aradu.A01	4825	p2	(AT)6	12	13286529	13286540	TGTCGATGTTATTGGCGAAA	TCTGTCAACTTAATCTGCATTTACA	279
Ad01A4828	Aradu.A01	4828	p4	(AAAG)5	20	13305770	13305789	CAACCTCGGAAAAATATGGG	GATTATGTTGACGCGTCCCT	235
Ad01A4834	Aradu.A01	4834	p3	(ATG)5	15	13327865	13327879	CGCACACACAACATCAATCA	TTTATTACCGCGATGCCAAT	257
Ad01A4835	Aradu.A01	4835	p4	(AAAG)5	20	13328932	13328951	AGCATTTCGTCATGTTAACCAC	TGCACCCTCCAAAAAGAGTT	104
Ad01A4836	Aradu.A01	4836	p2	(GA)6	12	13332402	13332413	TCCACATAGTCGTAAGGGGC	TCTGCAGCGACCAACTTATG	236
Ad01A4847	Aradu.A01	4847	p3	(AAC)5	15	13375055	13375069	GATGTCTTTTGCTTTTGCTTTTT	TCGTTTGATTCATGTAGCCG	273
Ad01A4856	Aradu.A01	4856	c	(T)11ggtcttgag	119	13408777	13408895	AACAACATATCAACAAAAGCTCAA	TAGGCTCCAAATAGCATGGG	241
Ad01A4858	Aradu.A01	4858	p3	(AAG)5	15	13414739	13414753	TGTTGGTGTTGTTGCTGATG	CCGAACCGAACAACATTCAT	196
Ad01A4859	Aradu.A01	4859	p2	(TA)6	12	13423814	13423825	GCTCAAAAACCCGATTTGAA	CACCTCGGTGAGTTGTCTGA	231
Ad01A4860	Aradu.A01	4860	p3	(GGA)6	18	13427158	13427175	ATCGGGGTAGAGGAGAAGGA	GATGCCAAGAAGAGGTCTCG	166
Ad01A4861	Aradu.A01	4861	c	(T)10cttttttc(T	29	13430081	13430109	TCCCCAATTAGAGGTGTCCA	TGGTTCTTGAAAGAATGAAGGAA	230
Ad01A4862	Aradu.A01	4862	c	(A)10ttaaaatcc	31	13440537	13440567	CTGGCACCAACGTCATACAA	CAAGTGGGCTTGTTTCTGGT	271
Ad01A4865	Aradu.A01	4865	p3	(TTA)6	18	13443661	13443678	TTATGCACGCCTTGGATGTA	TACCGTCCGAAACAACAACA	158
Ad01A4866	Aradu.A01	4866	c	(AAC)6aataag	54	13445396	13445449	CGATTTCAACTCGGAGATCAT	TGACATTACGCGTCCTGAAG	234
Ad01A4869	Aradu.A01	4869	p2	(CT)10	20	13455556	13455575	ATTCGTCTGCGACAACCTTC	AAGAAGACCTGGGAAGCACA	149
Ad01A4871	Aradu.A01	4871	p2	(TA)9	18	13470175	13470192	TTTAGGGATGCCAATAGATAGAA	AATTGAATCAAATTAAATGTTAGGGA	273
Ad01A4873	Aradu.A01	4873	p4	(AAAG)6	24	13474322	13474345	CGTCGGAGCAGGTTGATAAT	CCGGTCATCGAACCATTTTA	273
Ad01A4878	Aradu.A01	4878	p2	(TA)6	12	13487062	13487073	TTGTTTGAATTCATTAACTGAGGG	GTAATTCGATTTCCCGAGCA	245
Ad01A4881	Aradu.A01	4881	p2	(TA)7	14	13498365	13498378	TTTGAAGAGTGGCAAATTAAATGA	TTGCTACCATGCATAGGATGAC	276
Ad01A4882	Aradu.A01	4882	p3	(TAT)16	48	13498841	13498888	AAACCAATGTGAGTCCCCTG	TGCAAGTCAGGTTTGGTCAG	211
Ad01A4883	Aradu.A01	4883	p4	(AAAG)5	20	13502625	13502644	GCGTTAATATTCAAATAAGCAATG	TCAAAACTCATTCATCATTATTGTCA	183
Ad01A4884	Aradu.A01	4884	p3	(TAA)9	27	13502812	13502838	TCGAAATAGAAATAAAATAGCACGA	CTCCCCTCTCTCTCTCGGTT	174
Ad01A4887	Aradu.A01	4887	p3	(GAG)6	18	13510753	13510770	CGCGAAGAAAAACAAGAAGG	TCACCGAGAGTTTTTCACCC	146
Ad01A4888	Aradu.A01	4888	c	(T)12atcctaaag	112	13511274	13511385	ATGGTCCATTGTAAAGGGTCT	AAGTACAGTGCGGAGAGCGT	263
Ad01A4889	Aradu.A01	4889	c	(A)10ttattactcc	71	13511576	13511646	ACGCTCTCCGCACTGTACTT	TTGGGCAAAGAGGATTTCTG	202

ID	Chromosome	SSR nr.	SSR type	SSR	Size	Start	End	FORWARD PRIMER (5'-3')	REVERSE PRIMER (5'-3')	Product (bp)
Ad01A4890	Aradu.A01	4890	p3	(ATA)8	24	13520330	13520353	TCTGGAAATGCCAGTGCATA	TGCACGGGCAATATACTCTTC	241
Ad01A4895	Aradu.A01	4895	p2	(TC)11	22	13552033	13552054	CCTTCTCCCTTCTTGGAACC	AACCACACGGAGGCATAGAC	209
Ad01A4905	Aradu.A01	4905	p3	(TTC)6	18	13576614	13576631	TCCCTCATTTCGCTCAATTC	CCTGCAGACACTCTTGGTCA	260
Ad01A4907	Aradu.A01	4907	p2	(AG)17	34	13582033	13582066	CACTCCTTGCTCCTTGCTTC	CCTTCTTGGTGTGCAAGACA	262
Ad01A4909	Aradu.A01	4909	p3	(TTG)5	15	13610144	13610158	TGGGCTTGATTTCTTGAGGT	CCATCAACAACACAATTAGTCACA	212
Ad01A4912	Aradu.A01	4912	p2	(CT)6	12	13625643	13625654	TGTTTGTGCCATTCCTGTGT	GGGAGGCAACCCAATAATTT	249
Ad01A4913	Aradu.A01	4913	p2	(AT)12	24	13628936	13628959	TTAGAACGAAACAGGGGGAG	ACTTGCCGGTTAGTTTGTGG	216
Ad01A4914	Aradu.A01	4914	p2	(AT)8	16	13632426	13632441	CGTTTAACACCCAAAATGCC	CGAAATTTTGAGTTAACTTGGTCA	219
Ad01A4917	Aradu.A01	4917	c	(TCA)5(TCT)(	50	13654392	13654441	TTCGGTATTATTGTTAAGAAATTTTGG	TGACAAAGTAAACCAAAAAGGTGA	156
Ad01A4921	Aradu.A01	4921	p2	(AT)6	12	13672227	13672238	CTCCCACTTGAGTTTGAGGG	GCCACACTAACACGCACTACA	226
Ad01A4922	Aradu.A01	4922	c	(TA)10attaataa	46	13675098	13675143	GGAGTTGCATGCTCATCTCA	TGCGTGCTTGTCTTTTAGGA	274
Ad01A4925	Aradu.A01	4925	p2	(AG)8	16	13692567	13692582	GGGGAATCATGCAGACACTT	CATGAAGCGAAGAGTTCACAA	251
Ad01A4927	Aradu.A01	4927	p5	(TTTAT)5	25	13697743	13697767	TTTGAAAATCCTCACCCAAAA	CCATGATGGAGAAAGGGAGA	155
Ad01A4928	Aradu.A01	4928	p2	(TA)7	14	13709098	13709111	TTCGCTTGCTTCCAACTTTT	CTCCAACACGAAGACAAGCA	205
Ad01A4930	Aradu.A01	4930	p5	(TTATC)5	25	13717578	13717602	GGACGACCCAGTACACTTGC	AACACGACCCAAATCCAAAG	278
Ad01A4931	Aradu.A01	4931	p5	(TTATC)6	30	13727427	13727456	CCTGGGTGCTCCAGTGTATT	GAGACGACCGACCTCTTCAG	245
Ad01A4932	Aradu.A01	4932	p3	(AAG)5	15	13735989	13736003	TGAGGCATTTGTTTGGTTGA	CCACCAACATTGCTTCCTTT	207
Ad01A4933	Aradu.A01	4933	p2	(AT)8	16	13760830	13760845	ATAAAGGGGTGAAGCGGAAT	GTGAGGGTTCGATGCTCAAT	263
Ad01A4934	Aradu.A01	4934	p4	(TATG)7	28	13761344	13761371	TGCCACATATTCATGCATCC	TTGGGTTTGGGAATTGGATA	222
Ad01A4944	Aradu.A01	4944	p2	(AT)7	14	13791549	13791562	CATCGCTGTTGGAGAAGTCA	CGCTTTAGAGCCGTGATGAT	224
Ad01A4946	Aradu.A01	4946	p3	(TTA)16	48	13794046	13794093	AAACGCAAAGAAGACGATGG	CATGAATGTTCCCTCTTGCAT	279
Ad01A4947	Aradu.A01	4947	p3	(AAC)9	27	13810161	13810187	TTGGCACAAGATGGGTAAAA	CGTTTGATTCATGTAGCCGA	261
Ad01A4948	Aradu.A01	4948	p3	(TAT)5	15	13810475	13810489	TTCTTCTCAATGTCCAAATTTTC	GGTCAATGATGGAATAGTTTAATACG	279
Ad01A4950	Aradu.A01	4950	p3	(CTT)5	15	13822127	13822141	ATTATGTGGGCTGCATCCAT	TCCCATTTCATTTTAGTTGCC	262
Ad01A4952	Aradu.A01	4952	p3	(TAA)5	15	13829003	13829017	ACGGTTATTATAAAAATTTTGGATGT	CACTTACAGCACACACATTGC	185
Ad01A4953	Aradu.A01	4953	p2	(TA)7	14	13833727	13833740	GCAACGAAGGAGAAGAATGAA	TTTGTGGGAGTTGATGGTGA	280
Ad01A4955	Aradu.A01	4955	p3	(ACC)6	18	13845318	13845335	ATGCGAGACAAGAGGACCAT	TACATGTGCGAAACTGCTCC	274
Ad01A4956	Aradu.A01	4956	p2	(AT)10	20	13847345	13847364	CTTGCATGACTGCCTCTCTG	TAGCTGACCTTGTCTTCGCA	132
Ad01A4958	Aradu.A01	4958	p2	(AG)6	12	13872639	13872650	TCATCCTTCCCTTCATTTTGA	CCTAGAATTTCAATTGGCCG	242
Ad01A4959	Aradu.A01	4959	p2	(TG)10	20	13873885	13873904	CAAGAGGACGATGTCCGAGT	ATGGCCAATAGCAACAAACC	235
Ad01A4960	Aradu.A01	4960	p3	(CAG)5	15	13879824	13879838	ACTATCAAACCCAGGGGAGG	GAATGTGACAATGTGTATTGCTGA	260
Ad01A4961	Aradu.A01	4961	c	(AACCG)5cat	69	13895011	13895079	GATTCAAACCGAAAACCGAA	TCAAAATTTAGGAATCGGCG	252
Ad01A4962	Aradu.A01	4962	p2	(CT)6	12	13895207	13895218	GCAACGCCGATTCCTAAAT	GCGTCACTGAGAAGAGGAGG	262
Ad01A4964	Aradu.A01	4964	p2	(CT)6	12	13906856	13906867	GGATTGCACAATAAAGGCGT	ATTAGGGTTGGGATTGGGAG	246

ID	Chromosome	SSR nr.	SSR type	SSR	Size	Start	End	FORWARD PRIMER (5'-3')	REVERSE PRIMER (5'-3')	Product (bp)
Ad01A4965	Aradu.A01	4965	p2	(AT)9	18	13928789	13928806	GAACCCGAAGCAACCTGTAA	TCGATTAAAAATGGATTAGTTCAA	275
Ad01A4966	Aradu.A01	4966	p2	(AT)9	18	13930717	13930734	ATTGATCCACTTGCTCCTGG	GCGCATCCCTAAACACAAAA	156
Ad01A4968	Aradu.A01	4968	p3	(TTC)6	18	13949437	13949454	CCTCTCCACTTATTTGCACCA	TGATGGAGGAGAAGGAGGAA	264
Ad01A4969	Aradu.A01	4969	c	(CTT)5ttt(TCA	42	13949909	13949950	TTCTTCTCCCTCCTCCTCATC	CATCATCTTTCAAGTAATTTTGCAG	220
Ad01A4970	Aradu.A01	4970	c	(CTT)5ttcatcat	46	13950334	13950379	TTCCTCTTCCTCCTCCTCATC	CAAGCAGTTTTGCAGCATGT	202
Ad01A4975	Aradu.A01	4975	p3	(ATG)5	15	14019552	14019566	GTGAACCTAGCCCAGGTGAG	TAGCCCAAGGTGGGTGATTA	248
Ad01A4976	Aradu.A01	4976	p2	(AT)8	16	14025134	14025149	TGATAGTCACAACCACCAAGC	TGAATCAATTGACCCGATCC	265
Ad01A4978	Aradu.A01	4978	p3	(TTC)6	18	14030880	14030897	CCTTCTCCTTTTCCTCCTCC	TCAGCAGCATCGAGTGAATC	245
Ad01A4979	Aradu.A01	4979	p3	(AGA)5	15	14031539	14031553	AAATGAACCGAACATTGCCT	TCCATCAAGTGCTTTTGCAG	214
Ad01A4980	Aradu.A01	4980	p3	(CAA)5	15	14031860	14031874	TCTACTGCTTCTTCGTCGGG	CGTGATTACACGCACGTCTT	238
Ad01A4981	Aradu.A01	4981	p3	(AGA)5	15	14032018	14032032	GACGCTCTATGCGTAAACGA	GACAAGATTTTGCTAATCCGGTA	276
Ad01A4983	Aradu.A01	4983	c	(T)10ctttttaactt	62	14036757	14036818	TGCTAGAATTTTTGCTGTACCC	CATGCCTTCAGTCCTTTAGAGA	272
Ad01A4985	Aradu.A01	4985	p3	(TTA)9	27	14041019	14041045	TCCCACAAGAAACAACACCA	GCAACAGTGCAACTCAGTGAA	237
Ad01A4987	Aradu.A01	4987	p3	(TTA)15	45	14047665	14047709	AACACCACTCCAACCTCTGG	AAGGACGCATTCAAACTCTCA	129
Ad01A4989	Aradu.A01	4989	c	(TC)17cttgtcct	146	14072677	14072822	GAGATGGACGGTCAGGATGT	GCGAGAGAAAGAAAGAGGGAA	273
Ad01A4992	Aradu.A01	4992	p2	(TA)8	16	14081236	14081251	TTTAGGTTAACAAATACATTCATTTCA	TTTCTTTAATTCTTCTCAAAATCTCTC	134
Ad01A4994	Aradu.A01	4994	c	(GA)6tttgggga	120	14085962	14086081	AGAAGGGAGATCGGGAAGG	GCTCAAACTCTCTCCCATGC	219
Ad01A4996	Aradu.A01	4996	p4	(ATTA)5	20	14099015	14099034	GCAATTATTGATGTGTTGTGTGC	TTCTGCACATTCAAGGGTTG	202
Ad01A4998	Aradu.A01	4998	p3	(GAC)6	18	14101849	14101866	CATAGTCCACTGCCTCAGCA	CAATAGGTGGTCAAATTCTTTGTTT	248
Ad01A5000	Aradu.A01	5000	p3	(TTG)8	24	14106027	14106050	ACATGACAGAGCACAATGGC	ATTTTGCAGGCATCAAAGGA	237
Ad01A5004	Aradu.A01	5004	p3	(TTC)10	30	14142055	14142084	TCACCTAGGCCGAACCTACA	CTAGAGGATTCTCCGGCCTT	187
Ad01A5008	Aradu.A01	5008	p2	(CT)6	12	14157012	14157023	GAACCCTAAAACCTAGAGAGAGGA	CGTGCCTCATTAAAGGGAAA	199
Ad01A5011	Aradu.A01	5011	p2	(AC)6	12	14214108	14214119	AAAGTCCCAAATGCATGCTC	TTCCTGTAGAGCCTGAGGGA	129
Ad01A5014	Aradu.A01	5014	p2	(AC)6	12	14226696	14226707	TTAGAGCCACCAATCCCCTA	GCAAGCTCTAGGATGGAACG	251
Ad01A5016	Aradu.A01	5016	p2	(TA)6	12	14240574	14240585	TGGGGGACCAAATTGAAGTA	TATGGGCAACTAGGCAAAGC	277
Ad01A5017	Aradu.A01	5017	p3	(CCT)5	15	14244862	14244876	CGTCAGATTTATCCGCCAAT	AAGGAAAGGGAGGAGGATGA	279
Ad01A5018	Aradu.A01	5018	p2	(AT)9	18	14251803	14251820	TGTACGTGGCACCTTTACCA	CAAGCTCCTAACCCATCCTG	228
Ad01A5019	Aradu.A01	5019	p2	(TC)21	42	14259140	14259181	GCACCTTCTGTCCATCATCA	CACCCTTACCCATCACCATC	139
Ad01A5020	Aradu.A01	5020	p2	(AT)8	16	14260713	14260728	ATGCCTGTCCTACTGGCTGT	GGTAGGGTACTCTCCCTCGG	218
Ad01A5021	Aradu.A01	5021	p2	(TC)19	38	14266604	14266641	ACACCCTTATCTGCAGCACC	GGCTGGGCTATGTTGATGTT	151
Ad01A5022	Aradu.A01	5022	p3	(GAA)15	45	14267052	14267096	AATTTGATTTTGGCGACAGTG	CCGTCGCGAGTTCTATCTTC	264
Ad01A5023	Aradu.A01	5023	p2	(AG)8	16	14267274	14267289	GAAGATAGAACTCGCGACGG	GACTATTTGCAGCACCCTCC	216
Ad01A5026	Aradu.A01	5026	p3	(TTC)6	18	14298091	14298108	CGTATGCGTAACTGATGCGT	GCAATGAATGGAAGTATGCAA	237
Ad01A5031	Aradu.A01	5031	p2	(TA)6	12	14324305	14324316	ATCACCCACGAACCAAAGAG	ATGGGTCTGGGTCAGTCAAC	241

ID	Chromosome	SSR nr.	SSR type	SSR	Size	Start	End	FORWARD PRIMER (5'-3')	REVERSE PRIMER (5'-3')	Product (bp)
Ad01A5035	Aradu.A01	5035	p3	(TAT)6	18	14327008	14327025	CATGAGCTGCTGGTGATGAT	CATGTTCACATGAGAGAATGTGG	272
Ad01A5038	Aradu.A01	5038	p3	(TTG)5	15	14329016	14329030	ACCTGGCTGTGGTTCTCACT	CCATAGACGGAGACGACGAT	200
Ad01A5039	Aradu.A01	5039	c	(CT)7tggttaata	35	14329300	14329334	TCCCCTTCAGCTGCAGTTAC	AAAAAGCACAATACGCACCC	270
Ad01A5040	Aradu.A01	5040	p2	(AG)8	16	14329573	14329588	GGGTGCGTATTGTGCTTTTT	TTTTGCAACACCTACCCCTC	201
Ad01A5041	Aradu.A01	5041	p2	(TC)9	18	14330594	14330611	AATCGAAAAGCATCTGAGGG	CGCCCACAGGGTAAAACTTA	278
Ad01A5042	Aradu.A01	5042	p2	(TC)9	18	14331195	14331212	ACCTCACTCTCACCACCCTG	CATTCCAGCTCAGCGAATTT	262
Ad01A5046	Aradu.A01	5046	p2	(TG)20	40	14350333	14350372	GCGAGACTCTTTGGTGAAGC	CGATAACCGTCATTACACTCG	261
Ad01A5047	Aradu.A01	5047	p2	(TA)9	18	14357095	14357112	AATTTCAGGCTCTGGACACG	CTTTAACTCCCCGGGTTCTC	176
Ad01A5048	Aradu.A01	5048	p2	(TG)16	32	14358630	14358661	AACGGCAACAGACGACTACC	CACACCCCTCATTTCCCTAA	157
Ad01A5049	Aradu.A01	5049	p4	(ATTT)5	20	14377129	14377148	GTTTGGAGTTTTGCCCACAT	TTTTGGTTTGGATTGCTTCA	246
Ad01A5056	Aradu.A01	5056	p3	(TTA)12	36	14402891	14402926	TGGTGATGAAGTGCCTAGTTT	TTGCGTCTTCGTATTCAGAAA	279
Ad01A5057	Aradu.A01	5057	p2	(TA)7	14	14405806	14405819	TTGATGATTTTCGGTGTGGA	CCACAAATTCAACCCAACAG	272
Ad01A5058	Aradu.A01	5058	p2	(GA)6	12	14411178	14411189	GAAAAGGAAGGAGGGAAGAAGA	GCCAGAAGCATAACATGGGT	277
Ad01A5059	Aradu.A01	5059	p2	(TA)6	12	14426511	14426522	CAAGAGGAGCTTTGATACCATGA	CACGATCTACAAATTCAACCCA	262
Ad01A5060	Aradu.A01	5060	c	(T)11gattttgtag	88	14428424	14428511	TTGGATACCTTTTAATTTTTGGATG	TCAGGAAGGGTACAATGCAA	280
Ad01A5061	Aradu.A01	5061	p2	(CT)9	18	14433123	14433140	GCCAGTAAATCCGACGGTAA	GGAGGTTGACGAAGGTGAAA	174
Ad01A5062	Aradu.A01	5062	p3	(CTT)5	15	14455128	14455142	GATTGAGAAAACCCGCTGTC	CAACAACGGCACAGAGAGAC	272
Ad01A5066	Aradu.A01	5066	p2	(AC)7	14	14470612	14470625	AAGCTGCGAAGTAAAGACGG	TCACTGAGCGTGTTGGAGAC	225
Ad01A5067	Aradu.A01	5067	p2	(TA)7	14	14471832	14471845	TCGTCGCACTTGAAACTTTG	ATTTCTAGCCGGCTTTTGCT	100
Ad01A5068	Aradu.A01	5068	p2	(AT)8	16	14473287	14473302	CAAATCTCCTCGCACATTGA	GCCTTTACTTCGCAGGTTGA	125
Ad01A5069	Aradu.A01	5069	p3	(AAT)21	63	14475677	14475739	TCACATGCCGATGATAAAGC	TCCTGGCACCTATTCCTCAC	198
Ad01A5072	Aradu.A01	5072	c	(T)10cttttggacg	91	14480978	14481068	CGAATTCGTGCCCTTGTTAT	TGTTCGACTTAATTTTGGCAAT	219
Ad01A5076	Aradu.A01	5076	p3	(TTA)14	42	14500164	14500205	CCACAAAATTTATAATTGTGCTTGTTA	CAAATCGCTTAAACCATCAACA	275
Ad01A5077	Aradu.A01	5077	p4	(TAAA)7	28	14501583	14501610	ACGGATGAACTGGATGAACC	AAAATGGAGGCGATGATGAG	222
Ad01A5082	Aradu.A01	5082	p3	(AAC)5	15	14532146	14532160	GCAAGTTAAAGAGCGCCATC	AAGGCTAGGGTTTGCACTGA	116
Ad01A5083	Aradu.A01	5083	c	(TTA)9(TTG)1	78	14537368	14537445	TTGGTATTCCACTTGCACGA	CTCCCATGTCATCGAGTCCT	219
Ad01A5084	Aradu.A01	5084	p3	(AAT)6	18	14549139	14549156	CCCTATGTATTTAAATTTCGAGTATGA	AATGCACATGCGCAATAAAA	203
Ad01A5089	Aradu.A01	5089	p6	(GATGAG)5	30	14567186	14567215	AGACCATCCGGAGAGTGATG	TAGCTGTTGGATTGTCAGCG	170
Ad01A5090	Aradu.A01	5090	c	(AC)30aattaaa	140	14569715	14569854	GAAGCTCTGCCACAATCACA	GCGAATGTAGATAGAATAGGGAATG	263
Ad01A5091	Aradu.A01	5091	p3	(GAA)6	18	14570688	14570705	CCAATGACAATGTGAACGGA	GATTGTACCCAGAACCCCAA	272
Ad01A5096	Aradu.A01	5096	p2	(AT)7	14	14616005	14616018	TGGACTGAGCAACAATGGTC	TTTGTCATGAAATGAATCTTGC	231
Ad01A5097	Aradu.A01	5097	p2	(TA)6	12	14620872	14620883	AGGGAACATAACGCTCATCG	TATGCGGTTGTGTTGATGGT	188
Ad01A5104	Aradu.A01	5104	c	(A)14taaaagtat	57	14643666	14643722	CGTGGTGGGAGAGAGAAAAG	GCAATCACAACAACCACCAC	270
Ad01A5105	Aradu.A01	5105	p2	(TC)12	24	14643993	14644016	CGCTCTCTCTCTCCTGCTGT	AGGTTCCAGGGGAAGAAAAA	136

ID	Chromosome	SSR nr.	SSR type	SSR	Size	Start	End	FORWARD PRIMER (5'-3')	REVERSE PRIMER (5'-3')	Product (bp)
Ad01A5106	Aradu.A01	5106	p3	(TTA)23	69	14644664	14644732	TTGATTGCTTACCATGTTGAATG	TCGCTTGAGTGGGAGTAGTTT	278
Ad01A5108	Aradu.A01	5108	p2	(AT)7	14	14657974	14657987	GCTTTCCTTCTTCCCACATC	TTATCAGCCAAATGGAAGCC	216
Ad01A5114	Aradu.A01	5114	p3	(AAG)5	15	14683607	14683621	TAAAATATTGCTCGCCCTCG	AAATCTTTCCCACCCAAACC	208
Ad01A5119	Aradu.A01	5119	p3	(TTG)6	18	14693889	14693906	ACTGATGAAGAATTTGGCGG	TTCTTAAAGGCGAAAAGGCA	232
Ad01A5120	Aradu.A01	5120	p3	(ATC)6	18	14694326	14694343	TCTTCCTCCTCCTCTCCCAT	AACGACGATGATGACAACGA	265
Ad01A5124	Aradu.A01	5124	p2	(TG)6	12	14740250	14740261	CGTCTCCCAAGGAACTAGCA	CTCATTCCAAGCCAAGAAGC	232
Ad01A5125	Aradu.A01	5125	p2	(AG)6	12	14752618	14752629	TCCTGCAAACACAGATAGCG	GTTCGTCTGCCTTCTTCCAG	164
Ad01A5126	Aradu.A01	5126	p2	(GC)6	12	14768067	14768078	GGATGAAGGGTAGGAGAGGG	AACTCTGGGCCAACATTGAG	260
Ad01A5128	Aradu.A01	5128	p2	(AT)9	18	14773381	14773398	TTCCATCTCAATTGTAAAAGTGCT	ATGGGTTTATCAACCTCCCC	263
Ad01A5131	Aradu.A01	5131	p2	(TC)7	14	14826221	14826234	TCACCTCCTTCTTTTTCAAAATC	CGAACACAGAGAGAAAGAGATGG	238
Ad01A5132	Aradu.A01	5132	c	(ACA)17ataata	178	14841047	14841224	AGGGCTGCACCAATAACAAC	GCTATGCATGCACCGAAAT	258
Ad01A5140	Aradu.A01	5140	p3	(AAG)5	15	14876116	14876130	CACCAGGTGAATGTTGTTCG	TCCTCTGTTCGCGTTTTTCT	275
Ad01A5143	Aradu.A01	5143	p3	(AAC)6	18	14876793	14876810	TTTTGCAACCCCAAGTTTTC	AGGGTGGAGGAGGATGAGTT	212
Ad01A5145	Aradu.A01	5145	p2	(AG)6	12	14886245	14886256	CCAACTGAACAAAGTAAAAAGAACAA	ACCCCAAGTCTACCCCAAAT	276
Ad01A5146	Aradu.A01	5146	p2	(TA)6	12	14895708	14895719	AAAATTGATTTTCATCCGGAGTT	CCAACGATGTACCGGTGTTT	277
Ad01A5147	Aradu.A01	5147	p3	(TTG)5	15	14895922	14895936	AAACACCGGTACATCGTTGG	TTTCTCAAACATCTCATCCATTG	241
Ad01A5148	Aradu.A01	5148	c	(A)11gcattaaag	73	14898248	14898320	CCATAATATCCTCGCCTTGC	CTTCCGTTGGTGGTCATTCT	277
Ad01A5149	Aradu.A01	5149	c	(A)14ttggtgcgg	41	14900445	14900485	CAGTGCAAAGAATCCCGAAT	CCTATGCATGTTTGCTTGGA	221
Ad01A5154	Aradu.A01	5154	p2	(TC)10	20	14919610	14919629	TCATCTTCTCCGATCCTTCG	CGCTAGTGACGACAAAGCAG	125
Ad01A5155	Aradu.A01	5155	p2	(TG)6	12	14920643	14920654	TCATCCCTGGTGTTTTGGAT	TGTGTCACCATCAATCCCTG	272
Ad01A5164	Aradu.A01	5164	p3	(TTG)5	15	14945230	14945244	AGCCCTTCCTCTACCACCAT	AGCAACCAAAGCATCAACAA	172
Ad01A5165	Aradu.A01	5165	p3	(TTG)5	15	14945390	14945404	TGTTGATGCTTTGGTTGCTT	CGAAAATACTCCTCCCTCCC	240
Ad01A5167	Aradu.A01	5167	c	(A)10gttattttta	56	14949727	14949782	AAACAATTTAAAAGCAGAGAAATGC	CCGGTAAGTAATAATGATGATGAAGA	262
Ad01A5169	Aradu.A01	5169	p2	(TC)6	12	14953238	14953249	CCCCACCAAACCTTTATCCT	TTTCAAGTTGGTTGGTTGGA	219
Ad01A5174	Aradu.A01	5174	c	(A)10gtgagaac	93	14977349	14977441	AGACGCAAAACCCAATGAAC	TAGCCTGCGCTAGGAATAGG	277
Ad01A5176	Aradu.A01	5176	c	(TC)6tt(TC)12	38	14986754	14986791	CTCACTTCCTCTGCGACACA	ATGGAAAGGCAAGGAGAGGT	278
Ad01A5177	Aradu.A01	5177	p3	(TTG)8	24	14987190	14987213	ATGGAGCAAGTTGGTGACCT	TCCCTGCAAACATGAACAAA	263
Ad01A5178	Aradu.A01	5178	c	(CT)20t(TC)6	53	14987455	14987507	TTTGTTCATGTTTGCAGGGA	GAAAATGTCGGGAAGAGAGG	203
Ad01A5181	Aradu.A01	5181	p4	(TATT)5	20	15001024	15001043	CGATTCGATCCGCACATT	CATATCCGATCTGCGTGTCC	275
Ad01A5184	Aradu.A01	5184	p4	(GAGG)5	20	15005914	15005933	CCTCACTCCACCATTCACCT	TCTTTTTCTCAACCAACGGC	210
Ad01A5185	Aradu.A01	5185	p2	(AG)22	44	15006065	15006108	GCCGTTGGTTGAGAAAAAGA	TGCTACCATTTTCATTGCCA	230
Ad01A5190	Aradu.A01	5190	c	(AC)6tatgatcat	83	15042702	15042784	GCTTTGAAAATCTCGGCAAC	GAAAGAAACATCAGGAAGAGAATCA	257
Ad01A5194	Aradu.A01	5194	p2	(TC)8	16	15057433	15057448	GACCTGAGGGGAAAGAAACC	TTGGACATGCCCACTTTGTA	229
Ad01A5195	Aradu.A01	5195	c	(A)10tatatttata	84	15066226	15066309	TGTTTGGATGCTCTAAGCCA	TCCAGCCAAACAAAATAATCC	263

ID	Chromosome	SSR nr.	SSR type	SSR	Size	Start	End	FORWARD PRIMER (5'-3')	REVERSE PRIMER (5'-3')	Product (bp)
Ad01A5198	Aradu.A01	5198	p5	(TTTTA)6	30	15073288	15073317	ATTGATGCTCAGAGCCTTGG	AAACAAACCGTCAAATTGGG	171
Ad01A5204	Aradu.A01	5204	p2	(CT)6	12	15105356	15105367	CTTTTTCTCTCCCAATTTTCG	AGGGAGGGGGTTCGAATTAT	137
Ad01A5206	Aradu.A01	5206	p3	(TCT)6	18	15123343	15123360	CACGGTCATCCCTTCTGTTT	TTAGAATGTTGCTCGCCCTT	119
Ad01A5210	Aradu.A01	5210	p3	(ACA)5	15	15132010	15132024	GCACGATGTTGCATCACTTC	ATGCCAAAACAAAGACCGAG	138
Ad01A5211	Aradu.A01	5211	p3	(AGA)5	15	15132589	15132603	AAACGGTCACGTTGAAAAAGA	CCGGTTTGTTTTGTTCCTTC	119
Ad01A5213	Aradu.A01	5213	p2	(TC)6	12	15140007	15140018	TTGGCAGAGTCTACATTGCG	GAGCACGATGCTGAATTGAA	204
Ad01A5215	Aradu.A01	5215	p2	(TG)6	12	15142367	15142378	AAAGGGGAAAAAGCAACCAC	TGGACCAAGCATGTCCTATG	185
Ad01A5219	Aradu.A01	5219	p3	(GTT)6	18	15158459	15158476	GATGGTCAACGACGGATTCT	AAACCACACCACTTCTTGCC	159
Ad01A5220	Aradu.A01	5220	p3	(AGC)5	15	15159806	15159820	ATCCTGTGATACAGCTGGGG	TGAAGAATCAGCTGCTGTGG	124
Ad01A5222	Aradu.A01	5222	c	(TAA)7aatcatt	106	15164725	15164830	AATATACACGGTGCCAGTTCG	AATTTCAAAGGTTCCCACCC	257
Ad01A5228	Aradu.A01	5228	p3	(CTT)6	18	15196598	15196615	GGAAAGGACATGCGTGAAGT	CAGAGGAGGGTGTCGAGAAG	273
Ad01A5233	Aradu.A01	5233	p3	(GAG)6	18	15226314	15226331	TGGGTTTTGTGTGTGTTGTT	CCCTATGTCTCCATCAAGCC	116
Ad01A5236	Aradu.A01	5236	p3	(CTC)5	15	15233220	15233234	TACCACCACCACACCATCAC	TTGGTGCATTCTGGTCTTACC	246
Ad01A5240	Aradu.A01	5240	p2	(AC)6	12	15245973	15245984	AAAGTCCCAAATGCATGCTC	GCCTGAAGGATGGACTGATT	119
Ad01A5248	Aradu.A01	5248	p2	(TA)9	18	15268989	15269006	TTCGGGTGTCACCTACCTCT	AGGGGCATGATTTGTTTTTG	149
Ad01A5249	Aradu.A01	5249	c	(A)10gtga(AA	39	15275314	15275352	TGGTTTGAGTAATTGGGTGTTG	TACTTTGGGGCATTTTGTCC	211
Ad01A5250	Aradu.A01	5250	p2	(CT)18	36	15277583	15277618	ACCATCCATCATTCGACTCC	ATGCTGCAGGATTGATGACA	197
Ad01A5251	Aradu.A01	5251	c	(AGA)5ataaga	79	15277983	15278061	AAAAATGAAAACAGCGCCAC	TGATTCGTTGTGAAGGACCA	230
Ad01A5252	Aradu.A01	5252	p2	(CT)18	36	15278675	15278710	ACCATCCATCATTCGACTCC	ATGCTGCAGGATTGATGACA	197
Ad01A5253	Aradu.A01	5253	c	(AGA)5ataaga	79	15279075	15279153	AAAAATGAAAACAGCGCCAC	TGATTCGTTGTGAAGGACCA	230
Ad01A5254	Aradu.A01	5254	p2	(CT)18	36	15279767	15279802	ACCATCCATCATTCGACTCC	ATGCTGCAGGATTGATGACA	197
Ad01A5255	Aradu.A01	5255	c	(AGA)5ataaga	79	15280167	15280245	AAAAATGAAAACAGCGCCAC	TGATTCGTTGTGAAGGACCA	230
Ad01A5256	Aradu.A01	5256	p2	(CT)18	36	15280859	15280894	ACCATCCATCATTCGACTCC	ATGCTGCAGGATTGATGACA	197
Ad01A5257	Aradu.A01	5257	c	(AGA)5ataaga	79	15281259	15281337	AAAAATGAAAACAGCGCCAC	TGATTCGTTGTGAAGGACCA	230
Ad01A5258	Aradu.A01	5258	p2	(CT)18	36	15281951	15281986	ACCATCCATCATTCGACTCC	ATGCTGCAGGATTGATGACA	197
Ad01A5259	Aradu.A01	5259	c	(AGA)5ataaga	79	15282351	15282429	AAAAATGAAAACAGCGCCAC	TGATTCGTTGTGAAGGACCA	230
Ad01A5260	Aradu.A01	5260	p2	(CT)18	36	15283043	15283078	ACCATCCATCATTCGACTCC	ATGCTGCAGGATTGATGACA	197
Ad01A5261	Aradu.A01	5261	c	(AGA)5ataaga	79	15283443	15283521	AAAAATGAAAACAGCGCCAC	TGATTCGTTGTGAAGGACCA	230
Ad01A5262	Aradu.A01	5262	p2	(CT)18	36	15284135	15284170	ACCATCCATCATTCGACTCC	ATGCTGCAGGATTGATGACA	197
Ad01A5263	Aradu.A01	5263	c	(AGA)5ataaga	79	15284535	15284613	AAAAATGAAAACAGCGCCAC	TGATTCGTTGTGAAGGACCA	230
Ad01A5264	Aradu.A01	5264	p2	(CT)18	36	15285227	15285262	GTTAGCGCTGACAAAACGGT	ATGCTGCAGGATTGATGACA	276
Ad01A5265	Aradu.A01	5265	c	(AGA)5ataaga	79	15285627	15285705	AAAAATGAAAACAGCGCCAC	TGATTCGTTGTGAAGGACCA	230
Ad01A5266	Aradu.A01	5266	c	(TC)8tt(TC)22	99	15286318	15286416	ACCATCCATCATTCGACTCC	TTGATAGTGATGCTGCAGGG	245
Ad01A5267	Aradu.A01	5267	p3	(ATG)16	48	15287090	15287137	CGGTGAAGCCTCTGAAAAAC	TCCACCCCTAACTCTCATCG	270

ID	Chromosome	SSR nr.	SSR type	SSR	Size	Start	End	FORWARD PRIMER (5'-3')	REVERSE PRIMER (5'-3')	Product (bp)
Ad01A5272	Aradu.A01	5272	c	(TA)8gaatctcc	137	15309076	15309212	AACGTCTGGAGACCCTCCTT	GACCTGCGAAGTTAAGGCTG	234
Ad01A5273	Aradu.A01	5273	p2	(CA)9	18	15309773	15309790	GCTGGCCGGAGATTATTACA	ATGCAGGTCGAGAGACACCT	248
Ad01A5274	Aradu.A01	5274	p2	(TA)6	12	15314303	15314314	AGCCGGATCTTGAGTCTTGA	CACTCCGATCGACAACGTAA	103
Ad01A5278	Aradu.A01	5278	p3	(AAG)6	18	15331127	15331144	TAGGAAGGTTTATTCCGGGC	TGGCGGACAAACTTTAAACC	265
Ad01A5279	Aradu.A01	5279	p2	(TC)6	12	15334153	15334164	TTGGCAGCAAGAGTTTCCTT	CCCCAAAATAGCAGCAAAAA	197
Ad01A5281	Aradu.A01	5281	p3	(AAG)5	15	15345994	15346008	CTGGGCGATAAAGCCAATAA	TATTTAAGTGGCGGGCAAAC	147
Ad01A5284	Aradu.A01	5284	p3	(TAA)7	21	15350081	15350101	CTTGCATTTTCCTTCTTCCG	AATTTTTCCAGGAGCAAGCA	279
Ad01A5285	Aradu.A01	5285	p3	(GAA)5	15	15350350	15350364	CAAATCCAGTGTGGCAAATG	GCCACCAATGAAAGAGGTGT	113
Ad01A5286	Aradu.A01	5286	p3	(GAT)5	15	15353275	15353289	TTTCAACCACCGTGAAGTGA	ATTTCACATGGCCCTCTGTC	193
Ad01A5287	Aradu.A01	5287	p2	(AG)6	12	15362174	15362185	TTGAAGGGTGAGGTAGGTGG	CGGTGTTTAACGCCAGAAAT	257
Ad01A5289	Aradu.A01	5289	c	(TC)8ta(TC)2(	58	15368926	15368983	CAAAATTGTGATGGCAGTGG	AAGGAGAAAAAGGCGAAAGG	241
Ad01A5294	Aradu.A01	5294	p4	(TTAG)5	20	15384413	15384432	ATACGCACGACCCTGTTTTC	CCAGCAGCAACGTCTCAATA	272
Ad01A5295	Aradu.A01	5295	p5	(AAAAT)5	25	15386185	15386209	TTTGGGAACTTTGGTTGAGG	TACTTTGGGGCATTTTGTCC	197
Ad01A5296	Aradu.A01	5296	p2	(AT)6	12	15392552	15392563	AACTCACGAGCTGCCTCAAT	ACTCGTGAGCTGGTGTGTCA	268
Ad01A5297	Aradu.A01	5297	p2	(TA)7	14	15399162	15399175	CTCACTTCCAGCCCTAATCG	CAACACCACACCCTGTCAAG	270
Ad01A5303	Aradu.A01	5303	p2	(AG)12	24	15420869	15420892	AAAGCGAATTCGGACAGAGA	GTTTGTCATCGCTGAACCCT	258
Ad01A5306	Aradu.A01	5306	p2	(TC)8	16	15435537	15435552	TTTTTGATGAGCAGGGGAAC	CTGGCAGTGATGCAAAGAAA	134
Ad01A5311	Aradu.A01	5311	p3	(AAT)5	15	15458782	15458796	CACGTCATGGTCGTACCAAA	CCCCGCCTTTGTATGTAAGA	276
Ad01A5314	Aradu.A01	5314	p3	(TAA)14	42	15491828	15491869	TTTCCTTTTAAAGCTACCCCAA	CATCCCAAAAGGGAACAAAG	225
Ad01A5315	Aradu.A01	5315	c	(AG)18aa(AG)	52	15519301	15519352	GGCTCATAGCGATGGTGACT	TGTCTCAATTGGAGCCACTG	238
Ad01A5316	Aradu.A01	5316	p2	(GT)6	12	15529737	15529748	AACAGAAAGGAGGTCTGGGG	TGTAGACAACCCATGGCAAA	254
Ad01A5318	Aradu.A01	5318	p3	(GTG)8	24	15536387	15536410	TGCCACGAATAAAGCTGTTG	ACTCCAACGCTAACCACCAC	251
Ad01A5320	Aradu.A01	5320	p3	(ACA)8	24	15537602	15537625	TTTATTTAAATGCCCCAACCC	AACCGACCCTATTTAGTGGGA	208
Ad01A5321	Aradu.A01	5321	p2	(AT)7	14	15545967	15545980	ACTTGGCGGTTAGTTTGTGG	AACGCACCATGTGGTAAACA	272
Ad01A5324	Aradu.A01	5324	p3	(AAT)13	39	15575251	15575289	CAGGCTGTCATAGCCCTCTT	TGCAAAAGTGGATAAAGAAGACA	272
Ad01A5328	Aradu.A01	5328	p2	(AG)11	22	15579174	15579195	TCGAAGTCGCTGCTGTTAGA	GGCAGTCAAAGTTAGAGCCG	242
Ad01A5330	Aradu.A01	5330	p2	(CA)6	12	15583379	15583390	ATGGAAACAATGGATGGTGG	TTCTGAACTTGATGGGCTCC	271
Ad01A5333	Aradu.A01	5333	c	(AG)9ccgtgag	124	15591950	15592073	CTAATTAAACCACGGGAGCC	AGAACTGGCTTCTCCCTTCC	259
Ad01A5336	Aradu.A01	5336	p6	(TTATGA)6	36	15616323	15616358	TTCCCTGGGTAAAAGCAGTG	ATCCCCAACCCGAGTAATTC	215
Ad01A5338	Aradu.A01	5338	p2	(AT)9	18	15630607	15630624	TAGGGCACCAATTGATTTGA	CAATGTCCCAAGGTTTCCAC	230
Ad01A5344	Aradu.A01	5344	p2	(TA)7	14	15660216	15660229	GTTTGAATGTGAATGTAATAGTGTTGT	GGGACCTAGGGCAAAAACTC	213
Ad01A5345	Aradu.A01	5345	p3	(TTA)6	18	15677727	15677744	AAGCAGAGATCCAACAGGGA	TCGCACAACTAACCAGCAAG	273
Ad01A5347	Aradu.A01	5347	p3	(TTA)5	15	15686787	15686801	CAGGGACAAAGCATCTCCAT	AAAAACAATGCCAAAAAGCG	276
Ad01A5348	Aradu.A01	5348	p4	(AAAT)5	20	15692741	15692760	TGATTTAGTTTTCAATTTGCTTTGA	AGCTGCTTGCTTTAATGGGA	274

ID	Chromosome	SSR nr.	SSR type	SSR	Size	Start	End	FORWARD PRIMER (5'-3')	REVERSE PRIMER (5'-3')	Product (bp)
Ad01A5353	Aradu.A01	5353	p2	(AT)7	14	15729048	15729061	TGGAGGTTTCTTGGTTGTCC	GAAGAGAAATTGAAACGTGGC	186
Ad01A5356	Aradu.A01	5356	c	(CA)8cgcacaca	43	15736257	15736299	TGGAAAGAAACTTTGATCTCGAC	TCATGCAAGCTATCCCACAA	266
Ad01A5357	Aradu.A01	5357	p2	(AC)6	12	15747734	15747745	GAAAACCGGGCGTTAATTTT	AAGGAATGCGTAACCGCTAA	270
Ad01A5358	Aradu.A01	5358	p2	(AC)10	20	15753550	15753569	GGACCAAACGGGCTAAAAAT	TTTCACAAGGAATGCGTAGC	245
Ad01A5359	Aradu.A01	5359	p2	(AT)6	12	15775112	15775123	ATCCGCACGAAAGACCTTAG	AACACCTTTTGAAGGCATCC	246
Ad01A5360	Aradu.A01	5360	p4	(ATAC)8	32	15776219	15776250	CTAAGCCTTACCTCATGCCG	CGTGTGAATGCTTTGCACTT	180
Ad01A5365	Aradu.A01	5365	p2	(AT)6	12	15797608	15797619	AGCAGCTCTCCCAGTATCCA	GCCCTCCTCGTGTCTTTTCT	247
Ad01A5366	Aradu.A01	5366	c	(TA)6ttttgaaca	61	15799927	15799987	CGGAGAAAGCAAATCAGACC	CGGATTTTCACAAGACGAAGA	212
Ad01A5369	Aradu.A01	5369	p2	(CT)9	18	15805524	15805541	CTTCTCTCTCGCACAGCTCC	ACACACACATGGCTACCGAA	277
Ad01A5370	Aradu.A01	5370	p3	(GAT)6	18	15811712	15811729	TGTTCAGGCTGATTACACCG	CCCAGCAGTAGAAGTCGGAG	247
Ad01A5371	Aradu.A01	5371	p3	(CAA)5	15	15815760	15815774	TGCACCTAGAGGTCCGTCTT	CCGGAAAACTTGTGGCTTTA	212
Ad01A5373	Aradu.A01	5373	p2	(GT)15	30	15851130	15851159	GACAACTTCAGAACGGCGAT	CCCTTTCCTCACTCTCCCTC	233
Ad01A5377	Aradu.A01	5377	p3	(AAT)7	21	15881662	15881682	AATTTCTAACAACAACAACAATAACAA	TCTTCTTCCCACAACTCTTTGA	278
Ad01A5378	Aradu.A01	5378	p3	(GAA)5	15	15881898	15881912	TCTTTAGATTTTTATTTCTTTGGCA	TGCTTTCCCTCAACGGTACT	190
Ad01A5381	Aradu.A01	5381	p3	(TCT)5	15	15901130	15901144	TGCGTTTGGACAGAACACAT	ATGAAAACCAAATGGGTGGA	255
Ad01A5382	Aradu.A01	5382	p3	(TTA)6	18	15905956	15905973	GTTTGTCACATCGGACACGA	ACTTGAAGTTTCGGATGCGT	224
Ad01A5385	Aradu.A01	5385	p2	(AC)10	20	15917609	15917628	TAAGCCTCTGGGATGAGTGG	TGTTCGACAACAACCGTGAT	247
Ad01A5388	Aradu.A01	5388	p2	(GA)6	12	15931453	15931464	GCGTCAACACCAAACTTCAA	TAAAGCGCAATCATGCAAAC	198
Ad01A5389	Aradu.A01	5389	p2	(TA)7	14	15935033	15935046	GCAGAGAATTCCCAGTGAGG	CTTTTCCGTCCAGTACCCCT	225
Ad01A5391	Aradu.A01	5391	p2	(AT)7	14	15938910	15938923	TCATTTTCCCCAAGTCAAGG	CCTTCATTTTCCTTTTATTGCAC	216
Ad01A5392	Aradu.A01	5392	p3	(CAG)5	15	15954572	15954586	TCAGAACCTTGCCAAAAACC	AGAGATCCATCAGGAGCCAA	196
Ad01A5394	Aradu.A01	5394	p2	(AT)11	22	15961952	15961973	AACTCGCTTGCGTGAATACC	TGCACAACACCAATCAGACA	230
Ad01A5398	Aradu.A01	5398	p2	(AT)7	14	15966241	15966254	TGGCATTCCAATTCAAGGTT	GCGGCGGCTAGATTTCTTAT	229
Ad01A5399	Aradu.A01	5399	p2	(GT)6	12	15979366	15979377	TGAATTGGGTTCAGGGACTT	GCGTGCAAAGAAAGCTAAGG	277
Ad01A5400	Aradu.A01	5400	p3	(ATT)8	24	15979689	15979712	GGCCCTCTATTCCCAGTTTC	AGACAAGGGCGGAATTAGGT	278
Ad01A5403	Aradu.A01	5403	p3	(GAA)5	15	15989528	15989542	GGATTTTGGATGCTAGGAGTTG	TGAAGCGCATCATCATTCTT	215
Ad01A5404	Aradu.A01	5404	p3	(GAA)5	15	15989663	15989677	AGACAAGAATGATGATGCGCT	CATCGACCTTTTTCGGTGAT	238
Ad01A5406	Aradu.A01	5406	c	(T)12gggtattac	73	15996328	15996400	CATAGGAAACTGCATGCGTAA	CGATTTAGTATTTGACCATTGAAA	273
Ad01A5407	Aradu.A01	5407	c	(TCC)5ttctattg	103	15998689	15998791	CCCCTTTCGTTACTGTCATCA	CTTGCGTCTTGAATGAACCA	272
Ad01A5412	Aradu.A01	5412	p2	(TA)7	14	16055094	16055107	TGATGGTGGAAATGTTCGTG	CATTGCAAAACCCTCATTCA	250
Ad01A5413	Aradu.A01	5413	p2	(CT)6	12	16064960	16064971	TTGCCTAATAGGAGCAGGGA	ATTAGGGTTGGGTTTGGGAG	222
Ad01A5416	Aradu.A01	5416	p2	(TA)7	14	16096126	16096139	TGATGGTGGAAATGATCGTG	CCTCATTCAATCATCGACCC	212
Ad01A5418	Aradu.A01	5418	p2	(GA)9	18	16100640	16100657	GTAGAAGCGGCAACAGAACC	GCGAATCCTTTTTCCATTCA	185
Ad01A5419	Aradu.A01	5419	p3	(AAT)17	51	16101037	16101087	AATTCACCGATAAAACCGACA	AACATTTGGACTGGACTTTTATTT	262

ID	Chromosome	SSR nr.	SSR type	SSR	Size	Start	End	FORWARD PRIMER (5'-3')	REVERSE PRIMER (5'-3')	Product (bp)
Ad01A5422	Aradu.A01	5422	p5	(AAATA)5	25	16125695	16125719	GGCAATTTTTGTTGCGAGAT	GCTCAAATAGCATAGTCTCCACA	162
Ad01A5423	Aradu.A01	5423	p3	(AAG)5	15	16140132	16140146	ACAGTGGATCGATTTCAGGC	TCTTGACTTTCCCATGGAGG	269
Ad01A5425	Aradu.A01	5425	p3	(CTC)5	15	16172929	16172943	CTCCGAGTTTCGTGAAGACC	CAAGATTGGACGAGAGGAGG	269
Ad01A5426	Aradu.A01	5426	p3	(ATA)18	54	16190823	16190876	CAATGTCATTCTAATCCGAGC	AATTTCTGCCGCTATCTGTCA	279
Ad01A5427	Aradu.A01	5427	p3	(TAA)15	45	16192879	16192923	CATCTTCCGGCATTTCCTTA	TTTTTGCATTAGTTACGTTTTAATTCT	201
Ad01A5429	Aradu.A01	5429	p3	(AGA)5	15	16217448	16217462	TAAAATATTGCTCGCCCTCG	TCTTCTCCCACTTAGCCGAA	104
Ad01A5431	Aradu.A01	5431	p3	(AGA)20	60	16221502	16221561	CTCGGAAGTGGAAGCTTTTG	CTTCACCTAGGCCGAACGTA	246
Ad01A5432	Aradu.A01	5432	c	(A)12cttgagag	90	16228138	16228227	TGCATAGAGACCGAAAATACTGA	GTTTTCTGTTTTCGGGGAGG	264
Ad01A5433	Aradu.A01	5433	p2	(AT)6	12	16228347	16228358	AATAAATCCCTCCCCGAAAA	CGCAGTCATCACTAACATGC	270
Ad01A5434	Aradu.A01	5434	p3	(AAT)5	15	16229438	16229452	CGCTCATTTTGTCCTTCCAT	TGGCCTTGTGACACTTACCA	254
Ad01A5442	Aradu.A01	5442	c	(TA)8aaactttag	86	16245751	16245836	ATGTATGCGCGACTGTTCAT	TGGCCTACAGAATTACATCCG	248
Ad01A5444	Aradu.A01	5444	p2	(AG)6	12	16264174	16264185	ATAACGGACGGCGACTAAAA	GTGGTGGAACGTGGAAGTCT	247
Ad01A5446	Aradu.A01	5446	c	(TGG)5tgctgct	44	16277924	16277967	TTGGTTGGTGTGATGCAGAT	TTACCTCCTCCACCATCACTG	143
Ad01A5448	Aradu.A01	5448	p3	(CTT)5	15	16282180	16282194	TCGAATCGTTACTGCACGTT	CCTCCTCATCATCTGCTGCT	235
Ad01A5449	Aradu.A01	5449	c	(AT)8tattcacta	94	16317708	16317801	TAATACTTCACCAAATATCTTTCACC	ATGCACTTTATGAAAGGGAATTAT	277
Ad01A5451	Aradu.A01	5451	p2	(AT)9	18	16318787	16318804	TTTAACTCCCCAGATTCCCC	TATGGCTAATGAGCTCGCCT	212
Ad01A5452	Aradu.A01	5452	p4	(TTAT)5	20	16321536	16321555	TAACATGTGGTGGCAATGCT	TGCTTGCTTTTTCTTTTTCTTTC	266
Ad01A5454	Aradu.A01	5454	p3	(CTT)5	15	16323802	16323816	CGCATTTCAATAACGCTTCT	TGAGAGAAAACACGCGAAGA	193
Ad01A5455	Aradu.A01	5455	p3	(ATT)8	24	16326574	16326597	TGCTGCCAAGCCTAATTCTT	TGGTTATTAAACGGTAACTAATCCATC	235
Ad01A5457	Aradu.A01	5457	p2	(AG)8	16	16328097	16328112	CTGCCGTAAAATCCCAAAAA	ACGTTTCGCGCTTTTGTAAC	219
Ad01A5458	Aradu.A01	5458	p2	(TC)7	14	16328350	16328363	AAAGTACTAGCCGCAATCCG	TGTTTTCCAGATGCAGTTCG	249
Ad01A5462	Aradu.A01	5462	p3	(TCT)7	21	16342983	16343003	ACGAACCTCCATCTCTCCCT	CTAGAGGATTCTCCGGCCTT	169
Ad01A5464	Aradu.A01	5464	p2	(CT)8	16	16365945	16365960	TCATCATTGTTTCTGCATTTCC	CATGGCATGCATATACGGAG	226
Ad01A5465	Aradu.A01	5465	c	(AC)30agatata	124	16367057	16367180	TTCTCCATCAAGCCTTTTTGA	CGCTAGGCATCTGGAGTTTC	250
Ad01A5466	Aradu.A01	5466	c	(CT)8aatgccttt	44	16382276	16382319	CGACGGTAATCTTGCCCTAA	TATTAAGGGAGCAACGACGC	275
Ad01A5470	Aradu.A01	5470	p3	(GAA)5	15	16417793	16417807	AGCATCTTCGAGTTCGTCGT	AGGAGGAAGGAAGTGACGGT	244
Ad01A5472	Aradu.A01	5472	p2	(CT)6	12	16436817	16436828	ATTTTTGCATGCCATTTGGT	TGAAGGGTGAAGTAGGTGGG	221
Ad01A5475	Aradu.A01	5475	p2	(AG)6	12	16453454	16453465	TTTGCAGAACTCACACTCGAA	CAAAGTCATTAGTGGCCTCAGA	246
Ad01A5476	Aradu.A01	5476	p2	(AG)6	12	16457847	16457858	TTTGCAGAACTCACACTCGAA	CAAAGTCATTAGTGGCCTCAGA	245
Ad01A5483	Aradu.A01	5483	p6	(TAGTTA)7	42	16468014	16468055	GGCCTTGCAAACTGGAATAA	TGGAAAAGAATTTCCTAATCAAA	278
Ad01A5485	Aradu.A01	5485	p2	(TA)11	22	16478583	16478604	CTACGGCTACGCTTTTCAGG	TGCTTGTGAATCTTTAGAATGTATTT	242
Ad01A5486	Aradu.A01	5486	p2	(GC)6	12	16484134	16484145	GCAAAGATTGGATCAAGGGA	TTAAAGTGGGCCACGGATAG	276
Ad01A5488	Aradu.A01	5488	p2	(TA)8	16	16493271	16493286	TGTTAGGTTAGGCTGGTGGC	CCATATAGCGAGCACTGCAA	166
Ad01A5496	Aradu.A01	5496	p5	(TTGTT)5	25	16514299	16514323	AATGTTGGAACCTGGATGGA	CGCAATGAGAACAGCAAAAA	206

ID	Chromosome	SSR nr.	SSR type	SSR	Size	Start	End	FORWARD PRIMER (5'-3')	REVERSE PRIMER (5'-3')	Product (bp)
Ad01A5497	Aradu.A01	5497	p2	(TA)7	14	16515526	16515539	CCTTTGGTTGCAGCTCTTTC	AGCTGCCTAAACTGCAGGAC	184
Ad01A5498	Aradu.A01	5498	p4	(TTAT)5	20	16515828	16515847	TGATGAAACTGACGGAAGCA	CAACTGCGGGATTATCCAAT	206
Ad01A5499	Aradu.A01	5499	p3	(GGT)5	15	16518965	16518979	CCTTATCCTGCTTACCGGTGT	GCAAGTGGTGCAGCATAAAA	150
Ad01A5501	Aradu.A01	5501	p2	(CT)6	12	16530525	16530536	CATCCTTGACCCTTCACGAT	ATTAGGGTTGGGTTTGGGAG	198
Ad01A5503	Aradu.A01	5503	c	(T)11acttttatttc	34	16560924	16560957	GGGCATTTTGTCCCCTTTAT	GACTTTGGGACATTGGTTGG	242
Ad01A5505	Aradu.A01	5505	c	(A)13gagtttttt	116	16568555	16568670	CCTTCGACCAATACACCCAT	TCATCACCCAACAATATCAAACA	271
Ad01A5508	Aradu.A01	5508	p3	(TTG)5	15	16580439	16580453	TGGTGTCCGATACATTAGCAG	AAACCATCAAACAACAACAACAA	213
Ad01A5509	Aradu.A01	5509	p2	(TA)6	12	16586281	16586292	CTTTGTTTGAACAATGTGCG	CAACCCCCTTTGAAAATTCC	272
Ad01A5513	Aradu.A01	5513	p3	(ATA)16	48	16612270	16612317	ACCAACCAGCCAACAAAGAC	TTGAATTTGGGGCAGAAAAA	273
Ad01A5517	Aradu.A01	5517	p2	(TA)6	12	16627262	16627273	CAACCAAGTAAAACAGCCAAA	TCATCTGACAATCACGACCC	221
Ad01A5521	Aradu.A01	5521	p4	(TATT)5	20	16636054	16636073	TTCGAAATTCTCTCCCTCTCA	TTCTTGTCCTTGCTCCTGCT	239
Ad01A5522	Aradu.A01	5522	p3	(TCT)8	24	16639217	16639240	GGATCCCCACCTACCTCAAT	CTAGAATGTTGCTCGCCCTC	185
Ad01A5523	Aradu.A01	5523	c	(A)10ttgattcttg	62	16640050	16640111	GCTTGAACAGCGGCATATTT	GGGCCCTCCTATTCATTGAT	280
Ad01A5527	Aradu.A01	5527	c	(TAA)11tgaaa	141	16661517	16661657	TTTTATTATTCTGATTCTTCTAGCACC	TCCGTTATGCTCACATCATAATA	261
Ad01A5530	Aradu.A01	5530	p2	(GA)7	14	16669381	16669394	GAAGGAGGTGAGTCTGTGCC	GCTGACTTCTCTTCCCTCCA	263
Ad01A5532	Aradu.A01	5532	p2	(AG)6	12	16673139	16673150	ATGCATGGGAGTGTGAGTGA	TATAAGATGCCTGCGTCCCT	237
Ad01A5536	Aradu.A01	5536	p2	(AT)8	16	16683185	16683200	TGGGTCCCTGTCACTAAAGG	GGCTAAATCCGTCCCTATCC	139
Ad01A5538	Aradu.A01	5538	c	(T)10cgtttttac	30	16701681	16701710	GCACACTCTTTTTGCCTTGG	TTGTTGTTCATGAATGTTGGC	197
Ad01A5542	Aradu.A01	5542	c	(TC)16(TG)10	108	16725256	16725363	TTTTGTTCATAACCCGAGCA	ACAGCTTTTGCAGCTGGTTT	189
Ad01A5543	Aradu.A01	5543	p3	(CTT)7	21	16726056	16726076	TGCCCAGTGATGAATGCTTA	GCAACAACACCAACAACTGC	156
Ad01A5544	Aradu.A01	5544	c	(TC)7aaatgcata	84	16726443	16726526	GTTCATGATGATGCCCAATG	GTGACGTTCATCAAAACCCC	226
Ad01A5547	Aradu.A01	5547	p2	(TA)10	20	16728278	16728297	ACTGGAGCCTGCATCACTCT	TTCATAGCCTTCAAGCACCA	146
Ad01A5549	Aradu.A01	5549	p2	(AT)9	18	16736671	16736688	ACGCACTTCAATTCTTTCGG	TGCATCCAGACGAAAAGAGA	160
Ad01A5556	Aradu.A01	5556	p3	(TGA)5	15	16787771	16787785	TGGAAGAGTGACAGGGATGA	GCAATGGGTAGTACCAAATATGAA	199
Ad01A5557	Aradu.A01	5557	p4	(TGTA)9	36	16789160	16789195	TGAATGCATCCCTTCATTGT	TTGGTTGCTGCTGTTGTTTC	226
Ad01A5558	Aradu.A01	5558	p2	(TA)7	14	16792393	16792406	TGGGGTATTTTTGTCCCCTT	CTTGGGACATTGGTTGAGGT	218
Ad01A5560	Aradu.A01	5560	p2	(TC)6	12	16836817	16836828	CCCAATGATGCACAATTCAA	CCCACCGGTTTCATTCATAG	271
Ad01A5561	Aradu.A01	5561	c	(TCT)5ccttctat	95	16838085	16838179	TTTGATGTGCCCAGTGATGT	ATCCATCAAGGTGGGTGAAC	275
Ad01A5565	Aradu.A01	5565	p2	(AT)8	16	16847635	16847650	CGAGATATGGCAAGTCAGGC	CATCAAACGAAGGGCATCTT	152
Ad01A5568	Aradu.A01	5568	p2	(GT)6	12	16899027	16899038	ATTGGTCACCCCAATTTCTG	TGCTTTTGGAACCGTCTTTT	141
Ad01A5569	Aradu.A01	5569	c	(GT)10(GA)10	40	16900310	16900349	CCTTCCTTGCCTAGAATCCA	TGTCGCATGCTATGTTGTTG	280
Ad01A5572	Aradu.A01	5572	c	(T)10caaacaaa	77	16909536	16909612	AGACGGAGAAAGGAGGAAGG	CATTAGTCTCCACTCTCCAATCA	227
Ad01A5573	Aradu.A01	5573	p3	(TTC)5	15	16911449	16911463	GCTACTGTCGCATCTCCTCC	CGAATTTTACAGGCATCATCAA	151
Ad01A5574	Aradu.A01	5574	p2	(TG)6	12	16914494	16914505	TAATGGCTCATCCCAAAACC	CCGAGTTGGATCTTTTCTGC	226

ID	Chromosome	SSR nr.	SSR type	SSR	Size	Start	End	FORWARD PRIMER (5'-3')	REVERSE PRIMER (5'-3')	Product (bp)
Ad01A5575	Aradu.A01	5575	p2	(AT)7	14	16916097	16916110	TTTTCCTTTGTATTTCTTTTTGGTT	CACCGACTAATTTTTGGTATGC	277
Ad01A5576	Aradu.A01	5576	p3	(AAT)11	33	16917157	16917189	AGCGACCGCTTGGATAACTA	TTCTCATGCAATACATCACTTCTG	265
Ad01A5580	Aradu.A01	5580	p2	(AT)7	14	16940213	16940226	CGTGAAAGCATCTCAAGCAC	TCCGGTTTACGTTTATCGCT	226
Ad01A5581	Aradu.A01	5581	p3	(AAC)5	15	16950393	16950407	AGGAGGCAGAGACAATGGTG	TTGAGGGGATTGTGATTGGT	172
Ad01A5582	Aradu.A01	5582	p3	(TAT)8	24	16968638	16968661	GATCTTGCCTATGTCACGGG	ATGCCATGCAATGAACTTGA	190
Ad01A5583	Aradu.A01	5583	p2	(GA)7	14	16970321	16970334	AACGAACATGATGGCAGACA	CATCCTCCTAGCTCCTTCCC	169
Ad01A5586	Aradu.A01	5586	c	(A)14ccatatgta	116	16976069	16976184	ATGGGAGTAATTTATCCTTTCTTTT	TGGTCCATTTATACAAATCCATAAG	276
Ad01A5588	Aradu.A01	5588	p2	(CT)6	12	16997353	16997364	ACCATCTTCTTCTCCCCCAT	AAGAAGCTGGCAGTGGTGAT	245
Ad01A5597	Aradu.A01	5597	p2	(TA)7	14	17069415	17069428	CCGCCAAGAAAGAAGAACTG	TCAAGGCATACCAAAAAGGC	139
Ad01A5598	Aradu.A01	5598	p3	(AAG)5	15	17070364	17070378	GAGTAGCAACCCCAGATGGA	ACATAGCCTTGGATTGCCAC	210
Ad01A5599	Aradu.A01	5599	p2	(AT)7	14	17070864	17070877	AAGTTTTCAGGCGCGTTTTA	ACAGCAAAGCGAAAGCAAAT	250
Ad01A5603	Aradu.A01	5603	p3	(ATA)15	45	17073075	17073119	GGTTTGGCCAGTTCACAGAT	TGTCTTTGGTTGGATGATGC	221
Ad01A5608	Aradu.A01	5608	p2	(GA)6	12	17094787	17094798	GGGATGGAGGTGATTAGCAA	CAGGCAGCAGTCTGGTGTTA	240
Ad01A5614	Aradu.A01	5614	p2	(TG)11	22	17126158	17126179	CGCTCTCTGCTCTCTCGAAT	CGCCACTCACCTACTCCTTC	128
Ad01A5620	Aradu.A01	5620	p2	(GA)14	28	17167257	17167284	CCTGTCTTCGATTCGACCAT	CCTACGATTATGCCTCCCTG	263
Ad01A5621	Aradu.A01	5621	p2	(GA)20	40	17169018	17169057	GGTGATACTTCAAGGGGAAGG	GCCTAGGCCTTTTCAAATCTT	277
Ad01A5623	Aradu.A01	5623	p2	(TA)7	14	17207763	17207776	TTCATGACGAAAGTGCCAAG	CTATCTGAAATGGGGCAAGC	264
Ad01A5626	Aradu.A01	5626	p4	(GAGG)5	20	17214464	17214483	AAGGAGAAAATGGGAGGACG	AAGATGGTCGTGGCTTCATC	228
Ad01A5627	Aradu.A01	5627	p2	(CT)6	12	17217456	17217467	TCAACCAAAGCCAAGAATGT	TGCATAGACTCAGGAGAGCAGA	268
Ad01A5628	Aradu.A01	5628	p3	(ACA)6	18	17221444	17221461	CGGCTCATTTAGCTTGTGCT	TGGCATCGTTTGATTCATGT	244
Ad01A5633	Aradu.A01	5633	c	(TTG)5ttatttac	161	17246993	17247153	TTTGAATCTTGATGCCATGC	CAGTTACAACAGCTGGGGCT	269
Ad01A5638	Aradu.A01	5638	p3	(GAT)10	30	17278147	17278176	GCCCCACAGCATTAACATTC	ACAGGGAAAAGAAGCAGCAA	199
Ad01A5639	Aradu.A01	5639	p3	(CTG)5	15	17278885	17278899	TGCTGCTTGATGAGTTCTGG	CAATTTCAATTCACATGGCG	275
Ad01A5640	Aradu.A01	5640	p2	(TA)10	20	17279956	17279975	GACTGGACAAATCAGTTTAACAAAAA	GGGATGTATCTATAAAGTGGCAGAA	247
Ad01A5642	Aradu.A01	5642	p2	(CT)16	32	17283680	17283711	AACCAGTTTGATCCGACCAG	GCAACGAGACAGATGAACGA	272
Ad01A5643	Aradu.A01	5643	p2	(CT)6	12	17285578	17285589	CGGATCCCCTTTCTCCTAAG	CAAATTCACAACTTCACCAGG	144
Ad01A5646	Aradu.A01	5646	p2	(TA)6	12	17290590	17290601	CACCAATCGTAATACATGCCA	TGGTCTCCAGATTTGCTGC	129
Ad01A5650	Aradu.A01	5650	p2	(TA)8	16	17300186	17300201	CCGAAAATTTTAATATGGAGGG	TGTGATAATTTTTAACTAAACACGCT	252
Ad01A5653	Aradu.A01	5653	p3	(TAT)8	24	17312832	17312855	AAGTGGAAAGTGGGAACGAA	TCATGGGTGAATCCAAACCT	225
Ad01A5658	Aradu.A01	5658	p2	(AC)74	148	17366815	17366962	AAAACCATATAACCCACAAAGAAAA	GAGGGATAGAATTTATGATATGCAA	276
Ad01A5660	Aradu.A01	5660	p3	(AAT)20	60	17370573	17370632	TTTGGAAAAATGTACACACAAACA	TGAGGAAATGGACAAAGAAGC	279
Ad01A5664	Aradu.A01	5664	p2	(TC)6	12	17431797	17431808	TGGCAACATAAAGGAATCAACA	GGAGCTAGAAAGGGGGATTG	229
Ad01A5665	Aradu.A01	5665	p2	(AT)11	22	17434698	17434719	CTACGACGTAAGGGTGACCG	TCCCTTCTCAATCTCAGCGT	266
Ad01A5666	Aradu.A01	5666	p3	(ATC)5	15	17439272	17439286	CTTCGAATCTTCTATCGGCG	CATTGATGCCTTTGCCTTTT	157

ID	Chromosome	SSR nr.	SSR type	SSR	Size	Start	End	FORWARD PRIMER (5'-3')	REVERSE PRIMER (5'-3')	Product (bp)
Ad01A5667	Aradu.A01	5667	c	(CAC)8(CTC)	39	17442748	17442786	CGTTTTTCTCCGTGTCTCTTC	TGAATTGAACCACACCTAGCC	266
Ad01A5670	Aradu.A01	5670	p3	(AAT)13	39	17445250	17445288	TCTTCTTCTTCCTCGCTGGA	TCCGTCTCCTCCTTCGTAGA	211
Ad01A5673	Aradu.A01	5673	p2	(GC)6	12	17460247	17460258	GGGTAGTGGAGGTGGTGAGA	TAACCCTGCAGCCAACTTCT	241
Ad01A5674	Aradu.A01	5674	p2	(GC)6	12	17460486	17460497	GTTATGCCCCAGGAATGAGA	GTCCATATGCTTGCAACACG	133
Ad01A5675	Aradu.A01	5675	p2	(GC)6	12	17460635	17460646	CGTGTTGCAAGCATATGGAC	TACGCACGCTCTCACAAATC	191
Ad01A5676	Aradu.A01	5676	p3	(TCT)5	15	17488597	17488611	TTCCTTCCATTTTTGCAAGC	CTAGAGGTTTCTCCGGCCTT	278
Ad01A5678	Aradu.A01	5678	p3	(AAC)5	15	17494470	17494484	TGGAAATTGAAATGGAGCACT	TGTTCCGTACTTGCTGCTTG	264
Ad01A5679	Aradu.A01	5679	p2	(TA)14	28	17495270	17495297	GATTTGGAATCGACGCCTAC	GTGCTAGGTGGGAGACAACC	219
Ad01A5680	Aradu.A01	5680	c	(A)11gcagtgaa	32	17522585	17522616	TGGCTCTTGAAAGAATGATGG	TCCCCAATTAGAGGTGTCCA	197
Ad01A5685	Aradu.A01	5685	c	(CT)11tt(ACC	39	17554370	17554408	GCCTCCACCTCTTTCTCCAT	GGTAGTGGGTGGTATGGTGG	110
Ad01A5686	Aradu.A01	5686	p2	(GA)10	20	17555535	17555554	TGAGGATCTTGGATCTTGGC	CACCAGACCACCAGTAACCC	197
Ad01A5688	Aradu.A01	5688	p2	(AT)11	22	17562818	17562839	GGTACCAAATTAAACATAATTCAAACA	TCGCAATTAAATGGAGACAGAA	266
Ad01A5689	Aradu.A01	5689	c	(T)11agaattttg	32	17564951	17564982	GCATGGCCCTGTGTTACTTT	CAGGGTTCGATTTGCATTTT	147
Ad01A5695	Aradu.A01	5695	p2	(AG)11	22	17587352	17587373	CCAAACATAGTGCAAAGCGA	TTTGGCTTGATTGGGAAGTC	238
Ad01A5696	Aradu.A01	5696	p3	(CTT)5	15	17588533	17588547	GTGCCCCGTGTCTACTTTGT	AACGCGAAGAAGAAAACGAA	246
Ad01A5697	Aradu.A01	5697	p3	(GAA)5	15	17590230	17590244	AACGCGAAAGAGGAGAACAA	CGGGAAAAGCTCTGCATCTA	223
Ad01A5699	Aradu.A01	5699	p2	(CT)6	12	17596124	17596135	AAATCAAAGGGGCAAGTGTG	GAGTGGGGGATTCAAGGATT	271
Ad01A5701	Aradu.A01	5701	p2	(AT)11	22	17624867	17624888	TGGTCTTATCTTCGCAGGTT	TCGCCACATGTAACACCCTA	280
Ad01A5702	Aradu.A01	5702	p2	(TA)6	12	17625586	17625597	CATGGAACTAATCGGACCGT	GAGGAGAGTTCTTGAGGGGG	211
Ad01A5703	Aradu.A01	5703	c	(A)13ctaattatc	114	17634564	17634677	TTTGTGTGTGTATTTTTCTCCTGA	CCTTGTAAATGTTTCTTTCCCC	254
Ad01A5705	Aradu.A01	5705	c	(AT)7aaatgact	51	17645773	17645823	TGATGGACTTTTAACCCGTTTT	TTATGGCAGGGGCTAAATTG	113
Ad01A5710	Aradu.A01	5710	p3	(ATA)5	15	17652082	17652096	AAAGACCCAATTGAAAAGAAAAA	TACGCAAAAGTGATTGCCAG	166
Ad01A5711	Aradu.A01	5711	p2	(TA)6	12	17652867	17652878	CTTGTCGAGGTTGTTGCTCA	GGATGTCGCATTTGTGTCAG	272
Ad01A5716	Aradu.A01	5716	p5	(TTATC)5	25	17706447	17706471	TGATGGTGCTGATCCTGTGT	AACACGACCCAAATCCAGAG	280
Ad01A5718	Aradu.A01	5718	p3	(TTC)6	18	17717917	17717934	TGAGTTGACTCTCAGATTTAGGC	AAGTTGGCCCCGACTACTCT	227
Ad01A5719	Aradu.A01	5719	p3	(ATA)5	15	17731497	17731511	GAGTTAAGACCTTTTGAGTAGCTTTTT	AGCTTTAAGTATGCGTTAAATCAAT	254
Ad01A5720	Aradu.A01	5720	p3	(TTA)7	21	17733409	17733429	CATGGGTACAAATGTCAGCG	GTAAGTGGAAAAACCGCACC	255
Ad01A5721	Aradu.A01	5721	p6	(AAAAAG)5	30	17754730	17754759	GGCAACCCGATAAATTCGTA	ATTCCTGCGCAACTCTCTGT	241
Ad01A5722	Aradu.A01	5722	p2	(TC)6	12	17755292	17755303	ATTATGAAGAATGCGCGACC	AGATGATTCGCCTCCCTTTT	244
Ad01A5723	Aradu.A01	5723	p2	(GA)18	36	17762173	17762208	TTGAACACAGAGAGAGAAGAGAAGA	CACCTTCCTCTTTTTCAAAATTC	241
Ad01A5725	Aradu.A01	5725	p3	(TTA)5	15	17800119	17800133	TGATAGGAGCAAGGATTGCC	GGAAGCATCAAAAGAAGAAACC	238
Ad01A5726	Aradu.A01	5726	p2	(AT)7	14	17812251	17812264	ACAAAGAGTCCTGGTGGTGG	TCTTTCACAGCTCTTTGCGA	246
Ad01A5727	Aradu.A01	5727	c	(A)10caactaag	49	17819912	17819960	TCAAGATAAGAAAAGATAGCAAAGATT	TTTAAATTTGGTGTCCCCCTC	262
Ad01A5729	Aradu.A01	5729	p3	(TGT)5	15	17841298	17841312	TTTTGGATATATTTGGTTGTGGA	GATGCAAAGGTTTGCTGTCA	231

ID	Chromosome	SSR nr.	SSR type	SSR	Size	Start	End	FORWARD PRIMER (5'-3')	REVERSE PRIMER (5'-3')	Product (bp)
Ad01A5730	Aradu.A01	5730	c	(A)13ttggcaatt	81	17843931	17844011	TTGGTGTTTCTTCTTGATGAGC	TGCAGTTGTAGAAATGTTGTATGC	249
Ad01A5732	Aradu.A01	5732	p2	(AT)10	20	17845653	17845672	CACAAAATATTGCATCAAAATATGAC	AATTCATCTCGCATGATTTTATTT	173
Ad01A5734	Aradu.A01	5734	p4	(ATTT)5	20	17848753	17848772	GGCAACCTTTTTCGACTGAG	TGTTGATAAGCTGGATTTTAGCTG	276
Ad01A5735	Aradu.A01	5735	p3	(CTT)5	15	17849342	17849356	AGGCTCCATTTCTCCCTCTC	CCACCGAATGAAGAGCTGAT	171
Ad01A5736	Aradu.A01	5736	c*	(GAA)5(AAG.	30	17861223	17861252	GATCACCTTTTTCTTGGGCA	GATTGCATTGTAAGGGCGTT	270
Ad01A5741	Aradu.A01	5741	c	(ATT)16ttctga	101	17911950	17912050	ATGCCCTGAATCCCAAGTTA	GGGTACAAGTCAAGAAAAGCCA	280
Ad01A5744	Aradu.A01	5744	p3	(AAT)24	72	17926930	17927001	TCTTAGAAGAGGATTGGCGG	GTGATGCAGCAAGTGAGGAG	242
Ad01A5745	Aradu.A01	5745	p3	(AGA)5	15	17935269	17935283	AGAAGGTTTGCTCGTCCTCA	AAAACCCCACCTACCTCACC	245
Ad01A5746	Aradu.A01	5746	p2	(AG)6	12	17935569	17935580	ATAGGTGAGGGGTTTTTGGG	GCCAGAAATGGCAGAAAGAG	176
Ad01A5750	Aradu.A01	5750	p3	(CTT)9	27	17972204	17972230	TCAGTTCCTGCTGTGATTCG	ATTCTTCCTTGCCACCACAC	225
Ad01A5751	Aradu.A01	5751	p2	(CT)6	12	17979730	17979741	TTCCAAAATCGAAGGTATCCA	GGAATCAGGGGGACACTTCT	241
Ad01A5753	Aradu.A01	5753	p2	(GA)6	12	17984725	17984736	AGTTGGGCCAGCTTTACTCA	TGAGTGTCATGCAGCACAAA	245
Ad01A5754	Aradu.A01	5754	c	(T)14gattttgaa	56	18001803	18001858	AACAATGCATGCAAGACACC	TTCTTTGAAAGCTTGGCTGG	266
Ad01A5755	Aradu.A01	5755	p3	(GAA)5	15	18006548	18006562	CGCAAGGTCATAGCACAGAA	TTCTTTGGTGGAGAAGGTGG	128
Ad01A5756	Aradu.A01	5756	p2	(GA)6	12	18009058	18009069	TCCAATGCATGATTCGAAAA	CTCTTGCTTGAGTGTCGTGC	207
Ad01A5763	Aradu.A01	5763	p2	(GA)6	12	18021201	18021212	GAGCCGTCACCGTCACTATT	GGACAGAACTCCCCTTCCTC	117
Ad01A5767	Aradu.A01	5767	c	(GA)23ggaact(	108	18038736	18038843	ACCGGTAAAGACATTGCAGC	TTTTCTTTTTCTCCGCCTGA	246
Ad01A5772	Aradu.A01	5772	p3	(AGA)5	15	18044138	18044152	CCTGCATCCTGAATATGCGT	TGGAACATCCCCAAGGTCTA	280
Ad01A5773	Aradu.A01	5773	p2	(CT)18	36	18048818	18048853	CATCTCCCTCTCATTTCGGA	AGAGGGCAACCTACCCAGAT	250
Ad01A5774	Aradu.A01	5774	p3	(CAC)7	21	18054097	18054117	CCCATTTTCCCTCTTCACAA	CCTCTCAACGATGCCATTTT	198
Ad01A5779	Aradu.A01	5779	c	(TCA)5(TCT)'	36	18071713	18071748	CAATTAGCCAAGGGTGGAGA	CTGAAAGGCGGTGGTGTAGT	134
Ad01A5780	Aradu.A01	5780	p2	(AC)6	12	18076646	18076657	AAAGTCCCAAATGCATGCTC	TTCCTGTAGAGCCTGAGGGA	131
Ad01A5781	Aradu.A01	5781	p3	(TCT)6	18	18084737	18084754	TCCATCATCTCTTTCCCACC	TCTCTCCATCTCCCGTGACT	267
Ad01A5785	Aradu.A01	5785	p3	(AGA)19	57	18106102	18106158	AGAAGGAGGAAGAGGAACGC	AGCTTGAGAGTACATCACGGG	165
Ad01A5794	Aradu.A01	5794	p2	(AT)6	12	18141351	18141362	TCTTCTTTTCCAAAACCTTCCA	ACGGAGGTTGAGACAGGAGA	212
Ad01A5796	Aradu.A01	5796	p3	(ATG)5	15	18161337	18161351	CCCATGCATCGATACCATTT	CGACGACGATGCATCAAG	274
Ad01A5797	Aradu.A01	5797	p2	(TA)7	14	18164151	18164164	GTGGTGGTGAGGTGATGATG	CCACAGATTCGCACCTCATA	202
Ad01A5799	Aradu.A01	5799	p2	(AC)6	12	18176020	18176031	TGGAGTGCAAGTGCATATGAG	AAAGAGAAAACTCGACCCAAAA	276
Ad01A5800	Aradu.A01	5800	c	(TC)8ccctctct(	38	18178088	18178125	GAAATTTTGGTGTGCTCCGT	ACTGTTGCCGTCTGAGGAGT	271
Ad01A5801	Aradu.A01	5801	p3	(AAT)7	21	18181980	18182000	GGTGCTCTAGAAATGGACGG	GGTAACATTTTGCGCCACTT	194
Ad01A5802	Aradu.A01	5802	p2	(GA)9	18	18184742	18184759	CGAAAAAGTGGCAAGAGAGC	CAATGGGTTACCGCCATATT	254
Ad01A5803	Aradu.A01	5803	p2	(AG)6	12	18191543	18191554	TTCTTTTGGTGTTTTAAATCTCTGG	AGCTCCCAAGCAAAACTGAA	228
Ad01A5804	Aradu.A01	5804	p3	(TAT)10	30	18195561	18195590	CATGCACAGTCATATTAAATTCTTGA	TGTTAAGTTTTATTTTTATGCATCTGA	236
Ad01A5805	Aradu.A01	5805	p2	(AG)6	12	18196811	18196822	TGATCAGAGTCTCGTACTGATAACG	TCATTATATGGGCAGCTGATTTT	209

ID	Chromosome	SSR nr.	SSR type	SSR	Size	Start	End	FORWARD PRIMER (5'-3')	REVERSE PRIMER (5'-3')	Product (bp)
Ad01A5807	Aradu.A01	5807	p3	(ATA)6	18	18205150	18205167	TTAAAGATTCAGAAATTATTCACTGG	TCAGTATATTTAGTCACGTTATGTTCC	237
Ad01A5808	Aradu.A01	5808	p2	(AT)13	26	18213133	18213158	TTTTGACCAAGTCAACCCAA	CCTTGGTGTTTTTCCTCCAA	185
Ad01A5810	Aradu.A01	5810	p3	(AAT)5	15	18223309	18223323	GCAGGAACAAGGATAAAGGC	AAACTTGGACGTTGGTTTCG	206
Ad01A5818	Aradu.A01	5818	p3	(GAT)5	15	18255885	18255899	TGCAACAATGGTGTGCTGTA	GCGAGGAGAAGCACCACTAC	276
Ad01A5819	Aradu.A01	5819	p3	(TTG)6	18	18256433	18256450	GGTGTGTCGTCTCACATTGG	TCCTTCACCAACCTACAGCC	198
Ad01A5822	Aradu.A01	5822	p4	(AAAG)5	20	18264460	18264479	TTTACTGGGTTGGGCCATTA	CACAACCGAACCAAATTCCT	209
Ad01A5825	Aradu.A01	5825	p2	(CT)6	12	18290228	18290239	GGAACATTGAACCTGGAATGA	TTTGACCCAGTTCTCGATCC	211
Ad01A5827	Aradu.A01	5827	p2	(TA)8	16	18294029	18294044	TCAGTGACCTGTTGCTAAATGC	GGACAGCTGATTTCGTGGTT	158
Ad01A5828	Aradu.A01	5828	p2	(AT)6	12	18297612	18297623	CCTCATAGTCCAAGAGCCTCA	TTGCTGAAACTTCATTGGGT	276
Ad01A5832	Aradu.A01	5832	c	(TCT)6ct(TC)2	66	18312143	18312208	GGAGAGAGGCAGAGAGCAGA	CGACTGGCAAAGACGTTACA	196
Ad01A5835	Aradu.A01	5835	p2	(TA)7	14	18346981	18346994	ATTTGTTGACCCTCTGTCGC	AGTTCCTCTGTACGCGCATC	274
Ad01A5836	Aradu.A01	5836	c	(GC)7(GT)9	32	18347842	18347873	CTATGTGTGTGTGCGTGTGC	AAAAGGAAAAGCGCCACTCT	222
Ad01A5841	Aradu.A01	5841	p2	(AT)7	14	18359004	18359017	TCAGAGAATTATGTGAGATCAGGA	TTGCCAAAAGGACCCACTTA	269
Ad01A5852	Aradu.A01	5852	p3	(TCT)10	30	18388808	18388837	CGCAAACACCATAGCTTCAA	GGCCCGATTAACCAATTTTT	238
Ad01A5853	Aradu.A01	5853	p3	(TTC)5	15	18397448	18397462	ATTGGGCATCAATTCGAAAG	CTGAGAAAGAACAGGGCAGG	236
Ad01A5857	Aradu.A01	5857	p3	(TCT)7	21	18461787	18461807	CCCATAAACCCTACCCACCT	TTTAGAATGTTGCTCGCCCT	245
Ad01A5860	Aradu.A01	5860	p2	(GT)7	14	18468711	18468724	GGGGACAAAATTACCCCAAT	TAGGTTGTAATGGGGCCAAG	257
Ad01A5861	Aradu.A01	5861	p3	(ACA)9	27	18474900	18474926	AGAATAATAGCGCGTTTGGC	ACATGACAGAGCACAATGGC	214
Ad01A5862	Aradu.A01	5862	c	(TC)16tt(TC)7	48	18489582	18489629	TTCACGTTCTGTTCATCCCA	TTTCTGCTCAGTGTTGTCGC	199
Ad01A5863	Aradu.A01	5863	p2	(TC)8	16	18489893	18489908	GAGCTCGACAGTAACCAGGC	ACTACAGTCCCGTCTCACCG	168
Ad01A5864	Aradu.A01	5864	p2	(TC)25	50	18492618	18492667	TCTCCTCCTTCTCCGTCTCA	AAGGAAGGATGCACGAACAG	215
Ad01A5865	Aradu.A01	5865	p2	(TC)6	12	18492929	18492940	TCGTGCATCCTTCCTTTTTC	GGAGCCGTCATAAACGTCAT	243
Ad01A5866	Aradu.A01	5866	p2	(TA)6	12	18493150	18493161	ATGACGTTTATGACGGCTCC	CCTCCTTGGAGAAGAGCTGA	271
Ad01A5870	Aradu.A01	5870	p3	(TAC)6	18	18520922	18520939	CAAAAAGTTTTGCCACCACA	TCAAAACACAGGTTGTTGAGTTC	268
Ad01A5879	Aradu.A01	5879	p3	(TAT)5	15	18568366	18568380	CAGATAACGGAGGGCCATT	CCTCAAATCTTCTCTTTTGGTTTC	177
Ad01A5882	Aradu.A01	5882	p4	(AAAT)7	28	18583267	18583294	CGAAGTGACTTGCGTCAAAA	CAGAGTGCAAGTGCATGTGA	227
Ad01A5883	Aradu.A01	5883	p4	(AAAT)5	20	18611977	18611996	AAAAGTGAACTCGACCCAGAAA	AACGGAAGAAGATGGAGTGC	279
Ad01A5886	Aradu.A01	5886	p2	(AT)6	12	18620755	18620766	CCAAATCTGGGCTTAGCCTT	TGCTTTCACGAATTGACCCT	273
Ad01A5888	Aradu.A01	5888	p3	(ACA)7	21	18624033	18624053	CATCTCCATATTTAAAGTTGATTTGA	ACATGACAGAGCACAATGGC	231
Ad01A5891	Aradu.A01	5891	p3	(ATT)12	36	18636887	18636922	AGCAACTGTAGAAGCAGGGC	AGAGGAGTGGGAGGAAGCTC	241
Ad01A5894	Aradu.A01	5894	p2	(TC)7	14	18642232	18642245	TTGACAGCCACAGAAGCAAC	GGGTGCTGATTCAAAAGCTC	126
Ad01A5896	Aradu.A01	5896	p2	(GA)19	38	18675744	18675781	CGGTGACAGACTCAGACGAA	TGCCCAATCCCTTTTCTCTA	177
Ad01A5899	Aradu.A01	5899	p3	(TCT)5	15	18689464	18689478	ATTTCTTTCCCACCCAAACC	TAAAATATTGCTCGCCCTCG	207
Ad01A5902	Aradu.A01	5902	p2	(AG)6	12	18725026	18725037	CCTAACACTCACTCCACCGC	GGATGGTGGCAATAGCGTAG	157

ID	Chromosome	SSR nr.	SSR type	SSR	Size	Start	End	FORWARD PRIMER (5'-3')	REVERSE PRIMER (5'-3')	Product (bp)
Ad01A5905	Aradu.A01	5905	p2	(CT)7	14	18741510	18741523	AACCAAATTGGCAACATCAA	GGAAACATGCAGAGACTCGAT	252
Ad01A5908	Aradu.A01	5908	p2	(AT)6	12	18744242	18744253	TCAACAATTTCGTGAGTCTTTTT	CGTGAAGCGGACATCAGTAA	249
Ad01A5910	Aradu.A01	5910	p2	(AT)6	12	18755450	18755461	GAGCTTGGAAAATCGGCTAA	AACATGGTCCAACCCACAAT	256
Ad01A5912	Aradu.A01	5912	p3	(TTA)5	15	18759649	18759663	GGAGTCGACAATCATTAAACTGG	CCAATCATCATGTTTAAGAAATCC	275
Ad01A5916	Aradu.A01	5916	c	(A)10gaagaaaa	57	18789353	18789409	GCCAATTTCTTCGTCATCGT	TTCCTGATAAATTTTGCATATCTCA	262
Ad01A5917	Aradu.A01	5917	p3	(ATG)5	15	18790188	18790202	AAGGTGCACTCGGTCTATTTT	TGACCACATCACCACTTCCA	275
Ad01A5919	Aradu.A01	5919	c	(T)10gtcaagtgg	70	18794388	18794457	ATGCTGGAAGACGAAATGCT	GTTGAGGCTGGTTGTTGGTT	229
Ad01A5923	Aradu.A01	5923	p3	(ACC)5	15	18799490	18799504	CGTCTCCAACGTTTTATTTGG	TGTTTCTGCTTTGCTTCTGC	275
Ad01A5924	Aradu.A01	5924	c	(A)11tttaaattct	113	18808256	18808368	CCTGTCAAATCACTTGCCAAA	TCATGGAGGTGCATGTTGTT	256
Ad01A5926	Aradu.A01	5926	p2	(AC)8	16	18812048	18812063	TTAGGTGTAGCTTTAATAGTGGTTTTT	AGAAAAGATCGGGCATGCTT	162
Ad01A5929	Aradu.A01	5929	p6	(ACTCTC)5	30	18816075	18816104	GTGGTGCTACCGTGTGTCTG	AGAATGGTAGGGTGACGGTG	257
Ad01A5930	Aradu.A01	5930	p3	(AAT)13	39	18819886	18819924	CCACACAATGCGTGTTTTCT	GTTGTGAGTGTTGCAGTGGG	186
Ad01A5933	Aradu.A01	5933	p3	(AAC)5	15	18852944	18852958	GAACTTGTTTGGAAACTCTTGGA	TCCATATAGCCGACCTCACC	227
Ad01A5934	Aradu.A01	5934	p3	(TTA)6	18	18861671	18861688	TTCCTGAGCGTTGCTCTTTT	TCCTCAGGTAGAACGAACTGC	218
Ad01A5935	Aradu.A01	5935	p3	(TTA)7	21	18868775	18868795	TCCGTTTTTCAAGGCTCCTA	AGCTTGATTCCCTGCATCAC	198
Ad01A5937	Aradu.A01	5937	c	(AG)20acagaca	76	18887367	18887442	AGGCAGCATTGCTTACTGGT	CCTTCTTGAACTGCTCCTGC	279
Ad01A5943	Aradu.A01	5943	p3	(ATT)6	18	18937262	18937279	GCCTAAATTATCAACACCGCA	GAACAATTACATGGAGGCCAA	240
Ad01A5949	Aradu.A01	5949	p3	(TAT)11	33	18958883	18958915	GGTAAGATTTTACAAAACCAAGCCT	CATTTAAATTTAGCCCTCACTAGC	194
Ad01A5951	Aradu.A01	5951	p2	(TA)6	12	18965646	18965657	TCAACCAAAGTCTCACAATCTCA	TGGCAAATTTCTTAAAATACCAAA	264
Ad01A5953	Aradu.A01	5953	p4	(AAAT)6	24	18966129	18966152	CGCATAGGGCTTATTTAAGTTTG	GGCTAAAGCCTCAAAAGATACA	238
Ad01A5955	Aradu.A01	5955	c	(A)10gaagaccg	83	18982788	18982870	TCACCAAAGAAGGACCGAAG	AGGTTGCTCGCTTTGTCTGT	204
Ad01A5956	Aradu.A01	5956	p3	(TTC)5	15	18985970	18985984	CCATTTTCAACAATTGCCCT	TTGGATCAGCGAATGTTTCA	246
Ad01A5959	Aradu.A01	5959	p2	(TA)6	12	18989664	18989675	AACCGTCTTTTAGAATATGTTTTTCA	ACTTCCAATTTCCTTGGCCT	176
Ad01A5962	Aradu.A01	5962	p3	(GAG)5	15	19034934	19034948	TTGACAAAAATATGCACGCAC	TTGCAAACAAAGGAAGGGTT	271
Ad01A5964	Aradu.A01	5964	p2	(CT)19	38	19035598	19035635	CCCCTTCCCTAAAACGTCAT	AACCACGTGTTGCTGCTATG	264
Ad01A5966	Aradu.A01	5966	c	(TA)7ataatatta	49	19040670	19040718	GGTCTTTCGAACGGAATCAA	TCAAATGCAAATCTCTCTCTCTTT	208
Ad01A5967	Aradu.A01	5967	p2	(TA)6	12	19045988	19045999	TCTCGTGCTGATAACGTGTTG	TCAAATGCAAATCTCTCTCTCTTT	191
Ad01A5968	Aradu.A01	5968	p3	(ATT)6	18	19050966	19050983	TCAACACCACATTTGGTATATTTG	GAACAATTACATGGAGGCCAA	251
Ad01A5969	Aradu.A01	5969	c	(T)10gaaa(T)1	24	19064179	19064202	TCCCCAATTAGAGGTGTCCA	TGTGGTTCATGAATGTTGGC	237
Ad01A5972	Aradu.A01	5972	c	(TCC)5ttcttatt	46	19075410	19075455	AATCACAAAGGCCACCAAAC	AGAAAAACGCGAACAGAGGA	176
Ad01A5973	Aradu.A01	5973	c	(TCC)6ttcttcttt	60	19075844	19075903	TGGTGAACCGAAATCACAAA	AACAACCAATTTCAAAGCCC	204
Ad01A5974	Aradu.A01	5974	p3	(GAA)5	15	19076228	19076242	TCCAAATCGCACTCATTCAA	GCTCGCGTTATAGGCTTCAC	218
Ad01A5975	Aradu.A01	5975	p3	(AGA)5	15	19076367	19076381	AAGAAAGAAAACGCGAGCAG	TGAGAGTGAAAACACGTGCC	121
Ad01A5983	Aradu.A01	5983	p3	(TAA)5	15	19101521	19101535	GTAAACGCCCCTAGCAATAA	ACGATCATGACGTGCTAAAAT	280

ID	Chromosome	SSR nr.	SSR type	SSR	Size	Start	End	FORWARD PRIMER (5'-3')	REVERSE PRIMER (5'-3')	Product (bp)
Ad01A5987	Aradu.A01	5987	p2	(TA)6	12	19130798	19130809	AAGGGGTAAACAAAAACCGC	AGCCGGATCTTGAGTCTTGA	135
Ad01A5989	Aradu.A01	5989	p3	(AAT)5	15	19146660	19146674	ACGTTTGGTTTGGGAATTGA	CCATACATTCATGCATCCCA	276
Ad01A5990	Aradu.A01	5990	p2	(AG)6	12	19148016	19148027	TCGGCCCAAAAACACATATT	CAACATGGAAGTTCATAAGCCA	273
Ad01A5991	Aradu.A01	5991	p2	(TA)10	20	19156505	19156524	GGTCGCGTGGATTAATTTGT	CCCACCATGGTATGATCGTT	271
Ad01A5992	Aradu.A01	5992	p3	(TAT)8	24	19163945	19163968	TCTCCATTAAAATTGACATATCACAG	ACCTGCAAGTTCATGAGTCG	275
Ad01A5993	Aradu.A01	5993	c	(GGT)6ggcgga	90	19165935	19166024	ATATGTGGCGCACCTTATCC	CAACCACCACTATCACCACG	269
Ad01A5995	Aradu.A01	5995	p3	(TAA)5	15	19187260	19187274	TTCACTTTGGGGCATTTTGT	TAGGAACCTTGGGACATTGG	245
Ad01A5997	Aradu.A01	5997	p2	(TA)8	16	19193105	19193120	CATGAAACACGGATATTGCG	CAAAATAAACCAACAAAAACATGC	164
Ad01A5998	Aradu.A01	5998	c	(TTC)5ctcttcct	126	19200357	19200482	GAATAAACCCAAAAACGCGA	AACGTGCAAGAAGAAGAACGA	207
Ad01A5999	Aradu.A01	5999	p3	(CTT)5	15	19200586	19200600	TTTCGTTTCTTGCTTTCTCTTTC	GAGGAAGAAGAACGTGCAGC	134
Ad01A6005	Aradu.A01	6005	p2	(AT)6	12	19230451	19230462	ATAATCGCGAAGGCGTATCA	GTCTGAGAGACGGCGAAGAT	275
Ad01A6006	Aradu.A01	6006	c	(CT)8tctttgagt	49	19234894	19234942	TGAATCACCTTTAGCCTCCG	ATGCCAATCCCTCAAAAGTG	207
Ad01A6008	Aradu.A01	6008	c	(T)11ggctattttg	100	19238265	19238364	TTTCAAATGTATTTGGCGGC	TCGTAATACCTTGCCACTTCA	271
Ad01A6010	Aradu.A01	6010	p3	(TAT)15	45	19249206	19249250	TGTTTGAGGGAACGACAATCT	GCGCCAGGTTTGTATGTTTT	167
Ad01A6011	Aradu.A01	6011	p2	(TA)8	16	19251280	19251295	AATTCACCGGCCTTTTTCTC	GCAGCGGTCAAAAGTTTCTC	251
Ad01A6012	Aradu.A01	6012	c	(AT)6tcatgcata	117	19251922	19252038	TCGGCCAAGTGATGATGATA	CAGAAACTCTCTACTGCAACGG	270
Ad01A6015	Aradu.A01	6015	c	(TAA)18ttgatt	144	19253883	19254026	TTGCAGAACTACATTATGTCATTGTAT	TTCATCTGAGTTTCGTGGCTT	242
Ad01A6016	Aradu.A01	6016	p2	(AT)8	16	19260025	19260040	TTCCCTCGTGTTGTTGTTGA	CGGATATTACGACGACTTCCA	173
Ad01A6017	Aradu.A01	6017	p2	(TG)9	18	19260180	19260197	TGGAAGTCGTCGTAATATCCG	GGAAGAGCACGAAAAGCACT	197
Ad01A6019	Aradu.A01	6019	p2	(AT)7	14	19262793	19262806	GGCCACGTGTCACTCATTTA	TCGGTACTCTTCGGTGAACTT	219
Ad01A6022	Aradu.A01	6022	p3	(AAC)7	21	19265783	19265803	TGCTTTTGGCTACGTCACTC	TACTTGAGCTTAGCCGACCC	100
Ad01A6023	Aradu.A01	6023	p2	(AC)8	16	19274260	19274275	TGTGCCACTCTGATAGCTCG	TTCGTTTCTATTTAAATGGTGTGC	274
Ad01A6025	Aradu.A01	6025	p2	(TC)6	12	19280499	19280510	ATACCGCACCAACTCCATTC	CCATCGCATAGGACCAATCT	280
Ad01A6026	Aradu.A01	6026	p2	(AT)7	14	19283138	19283151	GCCTTTTCAATGGTGTTTCC	GACAGTCCGGTGTACAAGCA	114
Ad01A6027	Aradu.A01	6027	p3	(GCT)5	15	19287061	19287075	TCCTCAACTCCTTCCACCAC	TTGCTTCATTCATTCGCTTG	277
Ad01A6031	Aradu.A01	6031	p2	(TA)6	12	19299887	19299898	CACTTGCCCCACTCTCATTT	GTGACGGCTGGGATTTAAGA	112
Ad01A6032	Aradu.A01	6032	p2	(GA)6	12	19306998	19307009	TCATGTTGCTGAAATCCCG	TTGATGATGGAGGAAAAGGAA	193
Ad01A6033	Aradu.A01	6033	p2	(TA)6	12	19313339	19313350	TGATTATTTTCCCTCGCCTTT	CTGCGCCACTCTGATAGCTT	263
Ad01A6034	Aradu.A01	6034	p3	(TGA)5	15	19321657	19321671	TCACATTTTTGGGCTTTCAA	TAGCTTCAACTGCCGAGGTT	193
Ad01A6036	Aradu.A01	6036	p3	(AGA)5	15	19328650	19328664	TTACTTGAGATTGAGCAGGGG	ACCTAGCCTCGAAGTCACGA	191
Ad01A6040	Aradu.A01	6040	p2	(AT)6	12	19336844	19336855	TGTTCGATCACGTGCTTTTT	CCACCAAACGCTACCTTAAGAA	279
Ad01A6041	Aradu.A01	6041	p3	(TTA)6	18	19340791	19340808	ACTCCCAGACAATGGTGCTC	CCTTGAAGCAAAAGAAACAGC	247
Ad01A6045	Aradu.A01	6045	p2	(CT)6	12	19387967	19387978	TAATCCACCCGGTGTTTTTC	CTACATGTGGCTTGTGGGTG	260
Ad01A6046	Aradu.A01	6046	c*	(AT)8ttctcccaa	144	19388607	19388750	GAATCAAATCGCTTGACCAAA	TCATTGACTTTCATTTACCTCCTTC	241

ID	Chromosome	SSR nr.	SSR type	SSR	Size	Start	End	FORWARD PRIMER (5'-3')	REVERSE PRIMER (5'-3')	Product (bp)
Ad01A6048	Aradu.A01	6048	p2	(GA)8	16	19398945	19398960	GGAGAGGAGAGGGAGGTGAC	CAGTGGCATCTTTTCATCCC	130
Ad01A6049	Aradu.A01	6049	p2	(AC)9	18	19401400	19401417	AATCCGCTTCCTCCAAGTTT	CCCTTTTCCACCAATTTTCA	205
Ad01A6052	Aradu.A01	6052	p3	(TTA)7	21	19425162	19425182	AATTTTCGGACAAAAGTGTCA	GCCCCTTATACCTTAGGGTCG	276
Ad01A6053	Aradu.A01	6053	p3	(TCT)5	15	19425693	19425707	GCCGAGAAGAGAAACAGTGC	TTCCAAATTTAAGCCAAATCA	221
Ad01A6055	Aradu.A01	6055	p3	(GAT)5	15	19429516	19429530	TGGAATTCGGTCATTGAAGA	CTCGTATTTGATGATGCCCA	204
Ad01A6057	Aradu.A01	6057	p2	(TC)8	16	19430833	19430848	GCTGGTGCTGTGAGAAATGA	GAGCATGATGAAGCTGTGGA	243
Ad01A6058	Aradu.A01	6058	p2	(AG)7	14	19439970	19439983	CCAAGACCCAAAAGCAAAAG	AATAAAGGGGGTTGGGTGTC	195
Ad01A6059	Aradu.A01	6059	p2	(TG)37	74	19442583	19442656	GTTTTGAACGCCAGCTTAGG	CACAAAAAGGGTCACAGGAAA	227
Ad01A6062	Aradu.A01	6062	p3	(ATT)5	15	19447363	19447377	CCACAACAACATCATCAGCC	GGTGGAGTGGGTGGTAAGAA	256
Ad01A6063	Aradu.A01	6063	p2	(AG)8	16	19449356	19449371	AATGGTCGCAGAAAATGAGG	ACGTGCCAAAAACACAACAA	265
Ad01A6064	Aradu.A01	6064	c	(TA)6ttagcagg	55	19450499	19450553	CCCTACTTGCTCCATTTCCA	AAACAAAACCAAAATGCAATGA	249
Ad01A6065	Aradu.A01	6065	c	(A)10gaaagaag	94	19454309	19454402	TGGAGGAGAAGGATGAAACG	AGAATTGTTGTTCACATTCAAATAAT	200
Ad01A6069	Aradu.A01	6069	p2	(AT)9	18	19461372	19461389	TCAAGGCATGCAACCAAATA	GTGAACAGCTGCAGCAACAT	184
Ad01A6070	Aradu.A01	6070	p3	(TTA)5	15	19464792	19464806	TGCTTTTCTCACGTTGAATGA	ATCTGCTGTTTCCCTCGGTA	279
Ad01A6071	Aradu.A01	6071	p3	(TAA)12	36	19467507	19467542	CCTTCTCCGAAACCCAAAAT	TGCAAATCCACTCTCAGCTC	212
Ad01A6072	Aradu.A01	6072	p2	(AC)7	14	19469994	19470007	GGGCTAAGGTAAGGGTGAAGA	AAAAACAATGCCAAAAAGCG	160
Ad01A6075	Aradu.A01	6075	p3	(GGT)5	15	19484586	19484600	AAGAGTCAGAGAGAGGGGGC	AGGTCCTTCTTCTTCCTCCG	206
Ad01A6081	Aradu.A01	6081	p4	(AAAT)9	36	19503933	19503968	CCTCAAAATCAAATGGCTACC	CAACTCAACCCTCCGGATAA	177
Ad01A6085	Aradu.A01	6085	c	(TA)9tttgggtgt	72	19512336	19512407	AAACGAAGGTCAGAGGCAGA	CCAATTAACCATGATATTAGATCACA	225
Ad01A6087	Aradu.A01	6087	p2	(TG)6	12	19514984	19514995	ATAGTAAGCGCGGTGGAGAA	TACCATCACGCCCTAAGCAT	245
Ad01A6090	Aradu.A01	6090	p2	(AT)7	14	19540226	19540239	TGGCAATATGCTAGTCTCCTTG	GGGTCACTGGTTGAATTGGT	270
Ad01A6091	Aradu.A01	6091	c	(T)12cagaatttta	146	19543610	19543755	TCGAGGTATTCGGAGTTTCG	TTGGCGTGTGACTTTTGTTC	274
Ad01A6092	Aradu.A01	6092	p2	(CT)8	16	19563335	19563350	CGGGGAGGGTATGATTCTAAG	AAGTGAAGTGTCCCACCCTC	160
Ad01A6093	Aradu.A01	6093	c	(TC)11ta(TC)6	36	19573201	19573236	AGCAAGCAAGCAGACAGACA	ACACCCATTGGATTCTCGAC	249
Ad01A6094	Aradu.A01	6094	p2	(TA)6	12	19574369	19574380	GCGATCTCCGTCTCTACCAA	TGGCAGTTGATGAAGACTAATTG	236
Ad01A6095	Aradu.A01	6095	c*	(AT)11(T)10*	31	19583109	19583139	AGCTTAAGGGGGAGTGTTGG	CCCGAATAGACAAAGAAGGG	208
Ad01A6096	Aradu.A01	6096	c	(A)11gagcaccc	122	19583864	19583985	CAGCAGCTTCTTGCCTGTAA	TGCTCTTGAGGCACTTGTTC	257
Ad01A6098	Aradu.A01	6098	p2	(AT)6	12	19590348	19590359	AGTCACGGCTAGGTTCAAGG	TTGAACAGTTTTGGCATCTCA	185
Ad01A6101	Aradu.A01	6101	p3	(TTA)6	18	19598297	19598314	TTTTTGACACAGTGGTATAATCAAA	TTTTTGTTGAATTTAAGTTGACTTGT	265
Ad01A6102	Aradu.A01	6102	p3	(TTC)5	15	19598891	19598905	TGAGAAGGAGGAAGAGGAAGAG	TGCTGCAAAATTACTTGGAAGA	213
Ad01A6104	Aradu.A01	6104	p3	(TGA)5	15	19599489	19599503	GGTAAAACAAAAAGAAAACGATGAA	TGGCTGATGTGTGGTTCAAT	280
Ad01A6109	Aradu.A01	6109	p2	(AT)6	12	19613900	19613911	AAATCCGGTTGCGAAGATAA	TTTCAGATGCAGGACGTGAG	197
Ad01A6111	Aradu.A01	6111	c*	(A)10(AT)6*	21	19628190	19628210	GGGGAAACTGTGGGAAACTT	CATTGGGGTAATTTTGTTCCC	233
Ad01A6116	Aradu.A01	6116	p3	(GCT)5	15	19654632	19654646	ATGATGGAGGGGATAAAGGG	TGTGTCAGGTGGAACAGGAG	195

ID	Chromosome	SSR nr.	SSR type	SSR	Size	Start	End	FORWARD PRIMER (5'-3')	REVERSE PRIMER (5'-3')	Product (bp)
Ad01A6118	Aradu.A01	6118	p2	(TA)6	12	19662313	19662324	CATTTGAATTGATATTAGGCTATTTTT	ACCACATGACAGCGAAACAA	254
Ad01A6121	Aradu.A01	6121	p2	(AG)7	14	19667730	19667743	GACATGCGCGATCTACAGAA	TGAACCTGGAATGAAGAAATCA	261
Ad01A6124	Aradu.A01	6124	p2	(AT)6	12	19699815	19699826	TTTGGACAGCATGAATGTGAA	TGGGCTAGACTGAAGGCACT	223
Ad01A6128	Aradu.A01	6128	p2	(CT)6	12	19738957	19738968	CATCAATCTGGCGTTGAATG	AGGTAGGTGGGGTTTATGGG	272
Ad01A6132	Aradu.A01	6132	p2	(TA)8	16	19789876	19789891	AACATTTTCGTTCATCCGCT	CCGGGTTTTACATGTGTGTTT	228
Ad01A6133	Aradu.A01	6133	p2	(TC)17	34	19810090	19810123	ATTGGCCCGTGATCATAGAG	TGGTCCTTTTCATTGACCCT	170
Ad01A6135	Aradu.A01	6135	p2	(TA)6	12	19819588	19819599	ATGCACACGAGCCAATGTTA	GACAGACATGGCGACTGAGA	265
Ad01A6138	Aradu.A01	6138	p3	(TGA)5	15	19823826	19823840	GGTGGTTGAACCCACTCCTA	GTCTTCCTCCTCTGCTGCTG	231
Ad01A6140	Aradu.A01	6140	p2	(AT)6	12	19830186	19830197	CCGTCCAATTGGCTTAGTGT	TGGAAAAGGAAGTTATAGGGCA	212
Ad01A6142	Aradu.A01	6142	p4	(AATT)5	20	19833223	19833242	AGCTGATTTTGGCGTACACA	CAAACAAAAATTAAATAAAACAGTTCC	273
Ad01A6145	Aradu.A01	6145	p2	(TA)6	12	19849294	19849305	AGGTTGAGCTTGGAGCTTGA	TGGGTGTCACATTGTAAGGC	236
Ad01A6146	Aradu.A01	6146	p2	(GC)6	12	19853262	19853273	ACAAGGCTCCTAAAACCGCT	TGCCATATGTTGTTTTGCGT	113
Ad01A6148	Aradu.A01	6148	p3	(ATC)6	18	19861227	19861244	GTAGGATCGACCCTGACTCG	TGGCATACAACCCCAATTTT	268
Ad01A6156	Aradu.A01	6156	p3	(TAA)6	18	19890799	19890816	AGGTCCAAAAATGTTGCCAG	CTTAGCTTCGCAGGCTGAGT	247
Ad01A6157	Aradu.A01	6157	p2	(TA)6	12	19891022	19891033	GACAACCAAGGAAGCAGCAT	CTGGATCCTGAAAAGCGAAG	263
Ad01A6159	Aradu.A01	6159	p3	(TAA)7	21	19899153	19899173	CCTAAATGCATGCTCACGAA	GCACGCAAGTAATCCTTTCC	233
Ad01A6160	Aradu.A01	6160	p3	(TTA)24	72	19902656	19902727	GAGTACTTCACGAAGTTCAAAAGTAAA	TGTATGACAAATGAAAACACAAAA	258
Ad01A6162	Aradu.A01	6162	p4	(TATG)6	24	19938579	19938602	AAACTCAAGCTTGGCTCACC	AACTCGTAAATGGGCTCGAT	272
Ad01A6164	Aradu.A01	6164	p2	(TA)9	18	19968433	19968450	CCCGAAGAACCGAACAGTTA	AAAGATGTATGTACGCTTTGAATGA	159
Ad01A6165	Aradu.A01	6165	p4	(TTTC)5	20	19991458	19991477	CTCTGCTTCACGCTTCTCCT	AACACATCCCTCAGCAGCTT	189
Ad01A6168	Aradu.A01	6168	p2	(TA)7	14	19995277	19995290	TCGACAATGCCATTTGCTAT	GTTTTCGGTAAGCTGCGTGT	271
Ad01A6170	Aradu.A01	6170	p4	(ATAC)5	20	20005026	20005045	GCGTCTGAAGGTTTTGTGGT	GGAAAGGATTACTTGCGTGC	256
Ad01A6171	Aradu.A01	6171	p5	(TTATT)5	25	20009836	20009860	TGAAAAGAAGGCGAAGAAAGA	CGTTAAACGCCAGAATGGAT	219
Ad01A6172	Aradu.A01	6172	p3	(TAT)14	42	20024686	20024727	AAGCCTTGGATGTCCATTGT	TGTACCTGATGCTGTTCTGCTT	228
Ad01A6173	Aradu.A01	6173	p2	(AT)9	18	20052141	20052158	TCCCAAATTCCCAACTATGG	CTTTCCGACCCCTCTTTCTC	146
Ad01A6176	Aradu.A01	6176	p2	(TA)6	12	20054861	20054872	ATACAAAAATGATGATTCCTAGACTTT	AACGTTTGAATTAAAATTCGGG	201
Ad01A6178	Aradu.A01	6178	p3	(ATG)5	15	20056809	20056823	AAATGTAAGTTCATCCTCTTCCAA	GCTCCTCATCTTCTGCTGCT	207
Ad01A6180	Aradu.A01	6180	c	(AG)6aa(AG)1	48	20063031	20063078	GCTAAAGGGGAACACGAACA	ACTTGCCGGTTAGTTTGTGG	278
Ad01A6181	Aradu.A01	6181	p2	(AT)6	12	20067538	20067549	GGAGTGAAAAGGTGTGACCC	TGTGTAGCAAAATTCAAGGCA	246
Ad01A6183	Aradu.A01	6183	p3	(ATA)5	15	20082961	20082975	GAAGAAGAGGAAGAAGGAAAAAGAA	TTAGCAATTGGGAAACAGGG	280
Ad01A6185	Aradu.A01	6185	p3	(TTA)9	27	20085559	20085585	CCCCTTAACGTAAGTGGCAA	GACTAAGTAAACGGCGCGTG	245
Ad01A6191	Aradu.A01	6191	p3	(GAA)5	15	20114653	20114667	CATCCGTATTCTAAACCCCAA	CTCCGCAGCCACTGTTATTT	170
Ad01A6195	Aradu.A01	6195	c	(AGAA)5aa(A	37	20121984	20122020	TCTCTCTACCACTGCCACCC	AATGACAGCAACCCTCCAAC	108
Ad01A6197	Aradu.A01	6197	p3	(ATC)8	24	20125428	20125451	CCACCATCATCTACAACCATCA	TGCGTTACCCCATCTCTTTC	206

ID	Chromosome	SSR nr.	SSR type	SSR	Size	Start	End	FORWARD PRIMER (5'-3')	REVERSE PRIMER (5'-3')	Product (bp)
Ad01A6201	Aradu.A01	6201	p2	(TA)6	12	20130226	20130237	GGGGTAAAACAAAATCGCAA	TATCTTCGCAACCGGATTTT	144
Ad01A6206	Aradu.A01	6206	p3	(AGA)6	18	20206520	20206537	CCAAGCAACCGAAAATCAAT	TGGCCGAACCTACACTTACC	263
Ad01A6207	Aradu.A01	6207	p3	(GAT)5	15	20213644	20213658	TGGAATCTGTTCAGGCCAAT	GAGGCTTTGGCAGAAGTGAC	223
Ad01A6208	Aradu.A01	6208	p3	(TAC)6	18	20234627	20234644	AACAAAAAGTATTGCCACCACA	AAAACACAGGTTGTTGAGTTCG	269
Ad01A6212	Aradu.A01	6212	p2	(CT)6	12	20252375	20252386	GAAAGGCTAGCTCCATTAGGG	CCAACAGAGAAACGGTTTGAA	231
Ad01A6213	Aradu.A01	6213	p2	(CT)6	12	20257121	20257132	GAAAGGCTAGCTCCATTAGGG	TTCCTTCCTTGATCCAACAGA	244
Ad01A6218	Aradu.A01	6218	p3	(CGA)6	18	20274957	20274974	AAAAGTTAAGTGGCAGCCCA	TGTGGTTTTGCTGTTGTGGT	185
Ad01A6228	Aradu.A01	6228	p2	(AT)9	18	20290493	20290510	GCAAGTTATGGCTAGTTTTAGCTG	CGGTATGATCTTTGAACGCA	229
Ad01A6229	Aradu.A01	6229	c	(TTAT)6tatttta	125	20295867	20295991	AAGATCTTAAACTAAAATAAATGCGAA	TCTGATCCCTGCTTCAAACAT	221
Ad01A6233	Aradu.A01	6233	c	(T)10attttttcttt	37	20317691	20317727	GGGGTAATTTTATCCCCTATCC	AAGTGTGGGAAAATTGGGTTT	208
Ad01A6236	Aradu.A01	6236	p2	(TA)12	24	20329168	20329191	ATCATTGACGCTTTCGCATC	AAGGAGCTCACCACATGTCC	240
Ad01A6238	Aradu.A01	6238	p2	(TC)21	42	20345254	20345295	TAGTGGTATTTTCCGGCGTC	GGAAGGATGCGCAAATAGAA	272
Ad01A6241	Aradu.A01	6241	p5	(AAGAA)5	25	20385550	20385574	TCCTTTCAGGTTCAGGATTCA	TTCGAAATTCCTCTTCTTCTTTTC	260
Ad01A6248	Aradu.A01	6248	p5	(TTATC)5	25	20424376	20424400	CCTGGGTGCTCCAGTGTATT	AACACGACCCAAATCCAGAG	166
Ad01A6251	Aradu.A01	6251	p2	(TA)11	22	20441092	20441113	TTCACTAAAAATTTGGTTTCTTCTG	GACTAGCATTTTTGGGCTGG	260
Ad01A6257	Aradu.A01	6257	p2	(CT)6	12	20485494	20485505	AAATTGGGAAAATAAGTAAAGGGAA	CGCATTTCAACCCAGTTTTT	251
Ad01A6258	Aradu.A01	6258	p2	(AG)23	46	20489738	20489783	AGTGGAGGCATGAAGACTGC	GTCCAACTCCACCTCGTTGT	192
Ad01A6259	Aradu.A01	6259	p3	(GGA)5	15	20493487	20493501	TTATGGTGGGATGTGGACAA	TCATCAAACATATCATCAGCCA	254
Ad01A6260	Aradu.A01	6260	p5	(AGAAG)5	25	20494307	20494331	TTTAAAGGGTGATGGGGTGT	TGCATGCACCCATCTCTAAC	110
Ad01A6262	Aradu.A01	6262	p3	(TTA)15	45	20499476	20499520	ACAAACTTACCAACTGGAAGAACT	GGATTTCAGCAAAGTGATGGA	204
Ad01A6266	Aradu.A01	6266	c	(A)12cacttatta	100	20505752	20505851	TTTCTGACATTTCACACGACAA	TGCCGATTCAGTTGTGGATA	260
Ad01A6268	Aradu.A01	6268	p3	(CAA)6	18	20540820	20540837	TTCTCCCACCCACACTCTTC	AAACGTTATGGCTTCGATGG	242
Ad01A6269	Aradu.A01	6269	c	(AT)7tcattctttc	45	20544327	20544371	CCAATTACCTCCCCATCCTT	TTGATGATGATAATGGGTTGTGA	213
Ad01A6271	Aradu.A01	6271	p6	(TTTTTC)5	30	20553585	20553614	AGCCTTGGATCCTTGCTCTT	GTGGTGGCCTTGCATTTATC	236
Ad01A6272	Aradu.A01	6272	p2	(CT)8	16	20557613	20557628	CTGTGATGAAGAAACAATCCCA	CGCATTTCAACCCAGTTTTT	206
Ad01A6275	Aradu.A01	6275	p2	(AT)6	12	20584423	20584434	AAATTCCCCCGAAGCAAAT	CTCAGAGGTAGGAGGTTGCG	245
Ad01A6276	Aradu.A01	6276	p3	(TCA)5	15	20586368	20586382	TTTTGACTATGCCACCTGTTT	CCAGCAGCTCAAAATCAACTC	280
Ad01A6278	Aradu.A01	6278	c*	(CGTG)10(GT	53	20613690	20613742	AAGGGATAAAATTACCCCAATGA	GTTGTAATGGGGCTAAGGCA	186
Ad01A6281	Aradu.A01	6281	p2	(CT)6	12	20628156	20628167	AGCTCCCTCTCTCTCCTTCG	CTTATTCCTGCGTCCACCAT	209
Ad01A6293	Aradu.A01	6293	p2	(AT)8	16	20779137	20779152	GTCACCATTCGCTCCAAGTT	TTGGACAGTCTCTCCGTGTG	153
Ad01A6298	Aradu.A01	6298	p2	(AC)7	14	20809589	20809602	TAATCGCCTTGCAACCTCTC	AATAGTGACGGTGACGGCTC	280
Ad01A6299	Aradu.A01	6299	p2	(TA)7	14	20813502	20813515	CCCTTTTCAGAATATGGACGA	CCGCGTAACCTGTCAAAAAT	259
Ad01A6302	Aradu.A01	6302	p3	(CTT)5	15	20820972	20820986	TCATGCGTACGCTATATGCTG	CACCATTCGAGATCAACCCT	273
Ad01A6303	Aradu.A01	6303	p2	(AT)12	24	20824355	20824378	ACGGTGGTACGACTCTCAGG	CTAGCCGACCTAAACTTCGC	255

ID	Chromosome	SSR nr.	SSR type	SSR	Size	Start	End	FORWARD PRIMER (5'-3')	REVERSE PRIMER (5'-3')	Product (bp)
Ad01A6305	Aradu.A01	6305	c*	(TA)8(TATG)!	50	20835738	20835787	GGTTGCACTCCAATCATCAA	TAAATCACAGCCCCTCCAAG	260
Ad01A6306	Aradu.A01	6306	p2	(TA)6	12	20839749	20839760	GCACGCAAGTAATCCTTTCC	TATTCAGTCCGTCCTCAGGC	244
Ad01A6308	Aradu.A01	6308	p2	(AT)6	12	20893787	20893798	CGTTTGGATCCTAGAAAGCG	GCCAAGGAAACAGCATAACC	222
Ad01A6309	Aradu.A01	6309	c	(TTA)9ctatttttt	127	20894002	20894128	TTTTGGTCATTCACAAAGCG	AACGAAAATGGCAGCGATAG	258
Ad01A6315	Aradu.A01	6315	p2	(TC)7	14	20916436	20916449	GCTTCTCTTTGACGCAGCTT	TCCCATCACTGCTACCAACA	193
Ad01A6316	Aradu.A01	6316	p3	(ACA)5	15	20917144	20917158	AACGACAAAAACTTGGTCACAA	TGGTCGGAGGTGATGTGTAA	153
Ad01A6317	Aradu.A01	6317	c	(AG)12aatgagt	130	20917624	20917753	CGATCCCTGAAGTGATGGTT	GGTCGTAATACCTTGCCATCTC	229
Ad01A6319	Aradu.A01	6319	p3	(TAA)5	15	20923096	20923110	TGATCTCCACTAAAAGTGAAGCAA	TGCAGATGAAAGAAACAAGCA	249
Ad01A6320	Aradu.A01	6320	c	(T)10ccttcgaga	31	20930806	20930836	AACAAATTTTTGTGTTTAGCTTCA	AACAAAAGCCATCGACATGA	269
Ad01A6323	Aradu.A01	6323	p4	(ATTT)5	20	20937970	20937989	GAGCCGCTTAAGCTAGGGTT	TGCATGCGAAAAAGAAAGAA	202
Ad01A6324	Aradu.A01	6324	p2	(TA)11	22	20946241	20946262	TTTCATCATTTTGACAACGCTT	GCACCTTGAATCTTTGGTTTG	265
Ad01A6325	Aradu.A01	6325	p3	(GGT)5	15	20946800	20946814	TCTGAGTCACACGCCAAAAG	TCTGGCCCATTATCTTCCAC	181
Ad01A6327	Aradu.A01	6327	p3	(AGC)5	15	20969226	20969240	AGAGCTGAGGATGGAAACGA	TTATGTGCTCTCTGGACCCC	150
Ad01A6328	Aradu.A01	6328	p2	(AT)9	18	20969748	20969765	TCATAACGGGATAGAGAGAAGAGAA	AACCAAATTTTGAACCAGCC	160
Ad01A6329	Aradu.A01	6329	p2	(AG)7	14	20971377	20971390	CTGGGGGTTGGAACTACTTG	GAGAGTGAAAACCCCAACCA	233
Ad01A6330	Aradu.A01	6330	p4	(TTGT)5	20	20972930	20972949	AGAAGGCCAAGTCGGTGATA	GGCCTTCTTTCTTGCAGCTA	208
Ad01A6331	Aradu.A01	6331	p4	(TTAT)5	20	20973080	20973099	GGGACAATGTTCCCATTCAC	TAGAGTTGAATTCCCCGGC	202
Ad01A6339	Aradu.A01	6339	p2	(TA)12	24	20991137	20991160	TGCAAAACAAATTACACGTAAAGA	TGCCCTTTCTCTTACCCTTTC	190
Ad01A6340	Aradu.A01	6340	c	(A)12tcaatggta	67	20996126	20996192	CAATTTTCACTCCAGGGTGG	CTTGTCCCCAAGCAAAGAAA	243
Ad01A6341	Aradu.A01	6341	p2	(AG)7	14	20999522	20999535	TGTGGTGGTTGCAATGAGTT	ATCCTTCTCTCCACCACTGC	143
Ad01A6343	Aradu.A01	6343	p3	(TCA)6	18	21006705	21006722	TTGTTGGCTTGTGGAAATGA	GGTGCAGTGAAACAGCAAAA	206
Ad01A6350	Aradu.A01	6350	p3	(TTG)5	15	21061325	21061339	GCACCCACTCTTGACCCTAA	ACGCAAACCAACCTGAAATC	185
Ad01A6351	Aradu.A01	6351	p2	(GA)6	12	21068376	21068387	GAAAAACTGTGAAGGGCAGC	CATCACCACCCAACCTCTCT	267
Ad01A6352	Aradu.A01	6352	p2	(GA)6	12	21068502	21068513	TTTGTTTCTCACCAGTCACCA	GTGGGTGGATATATTATTATTGGAA	241
Ad01A6354	Aradu.A01	6354	p3	(AGA)6	18	21102195	21102212	CTAGAGGTTTCTCCGGCCTT	GCCAAAACCCACACATCTCT	168
Ad01A6357	Aradu.A01	6357	p2	(CT)6	12	21147266	21147277	CCAATTTGGCAACATGAGTC	TTTCGGCCCAAAAATACATAA	275
Ad01A6358	Aradu.A01	6358	p2	(AT)8	16	21154121	21154136	GGAAAATTCCCGCCTTTTAT	TTGATTCTGCGCTGTGATTC	272
Ad01A6363	Aradu.A01	6363	p2	(CT)11	22	21193365	21193386	GCACATCGCTAGCGTCATTA	GGGAGAGGAGACGACAACAA	220
Ad01A6367	Aradu.A01	6367	p2	(AT)6	12	21210643	21210654	TCGCTCATTCTATCCTCTCAAA	AATAGGCCATTTCCCACAAA	269
Ad01A6375	Aradu.A01	6375	p2	(AG)6	12	21244359	21244370	GCAGAAGCTCTAGGTGGTGG	TAGCGCCAACTTTACACACG	255
Ad01A6380	Aradu.A01	6380	p2	(AG)7	14	21258755	21258768	TGACGATAGGGGTTTGCAGT	AGGATTAATGGAATGCTGCG	228
Ad01A6382	Aradu.A01	6382	c	(A)10g(T)10	21	21274619	21274639	TTTTACCCGAGAGTTGTGCC	ATGCGAATGCAGTGAACAAA	206
Ad01A6385	Aradu.A01	6385	p2	(GA)6	12	21282055	21282066	AGATGATTCGCCTCCCTTTT	ATTATGGAAATTTCGCGACC	278
Ad01A6386	Aradu.A01	6386	p2	(AT)6	12	21284179	21284190	CAAAAAGGATTAAACGCACCA	TCGTGAGTACCTTTCCCCTG	217

ID	Chromosome	SSR nr.	SSR type	SSR	Size	Start	End	FORWARD PRIMER (5'-3')	REVERSE PRIMER (5'-3')	Product (bp)
Ad01A6388	Aradu.A01	6388	p2	(TC)9	18	21286954	21286971	CCTAATCTAGAGAGAGGAGAGAGCC	AATGCTGGGGACAATTTCTG	176
Ad01A6393	Aradu.A01	6393	c	(A)10gagaatttc	98	21295257	21295354	AATTTTGGAATAAGTGATTTTATGAGA	TTTGGTCATTTGCTTGAAAAA	266
Ad01A6394	Aradu.A01	6394	p2	(TA)9	18	21299251	21299268	AAGTGATGAATCAAATTTTAAACCG	TTGTGAAATTGTCTCCGCTG	235
Ad01A6395	Aradu.A01	6395	p3	(GTT)10	30	21303883	21303912	TGTTTTCCCCTCACGTTTTC	TCCTCCGTCCAAAAATAAAA	272
Ad01A6396	Aradu.A01	6396	p3	(TAA)5	15	21304036	21304050	TGTTTTCCCCTCACGTTTTC	TCCTCCGTCCAAAAATAAAA	272
Ad01A6397	Aradu.A01	6397	p2	(TA)8	16	21304329	21304344	TGCTGGAGTAAAACCTTGGG	ACGCCAATAGTGCATCAAAA	275
Ad01A6399	Aradu.A01	6399	p2	(AC)10	20	21310935	21310954	AGCCTTTCCTGTTCTTGCTG	CATGAATGGTGCAACTCACA	190
Ad01A6400	Aradu.A01	6400	p4	(TAAA)5	20	21313522	21313541	TTCCTACTTTGGTCGATGGAA	TCACATGTCCTTTACGGGATT	232
Ad01A6401	Aradu.A01	6401	p3	(TCC)6	18	21315648	21315665	TTCTCGTTATCGTCGTCATCA	CACCCAAATTTGTTGCAGAA	250
Ad01A6404	Aradu.A01	6404	c	(T)11aaatttcaa	72	21326358	21326429	CCACTCCACCCATTTGAATC	GCTAATTCAAGCCAATTTATTTTT	276
Ad01A6409	Aradu.A01	6409	p3	(ATT)15	45	21399869	21399913	TTTTCTTGTCGTACCCGACC	GTGTTGCCGCACTAAGGATT	186
Ad01A6410	Aradu.A01	6410	p2	(TA)6	12	21400326	21400337	CCCAAACTAGCGTGGGAGTA	GTTTAAAACCCAAAAGCGCA	277
Ad01A6412	Aradu.A01	6412	c	(TTC)5tctactcg	87	21402413	21402499	GCTCGCGTTATAGGCTTCAC	TCCAAATCGCACTCATTCAA	218
Ad01A6415	Aradu.A01	6415	p3	(GAA)5	15	21440195	21440209	GGTTCAGTGGCCTTTGTGAT	TTTTCTGCTCGCATTCTTCA	280
Ad01A6416	Aradu.A01	6416	p3	(AGA)14	42	21441825	21441866	CTTCAACTTCCTCACCCCAC	GCTGGTTCATCGGAGAAGAG	257
Ad01A6417	Aradu.A01	6417	p2	(CT)6	12	21442156	21442167	CCTCCTTCGCATCACTTCTC	TAGGATCGTTGTTGCTGCTG	107
Ad01A6420	Aradu.A01	6420	c	(ATA)5(TTA)5	30	21465421	21465450	GATGGGGGACACAAAGTGAG	GCATGTGCTGCATCATTCTT	190
Ad01A6423	Aradu.A01	6423	p2	(AG)6	12	21471155	21471166	TACAGCGATTTTTGGGCTGT	CAAAGAACATTGAACCTAGAATCG	254
Ad01A6424	Aradu.A01	6424	p3	(TTA)7	21	21472298	21472318	TAACTCAACATTGGGGCAAA	AGCCTTAAGCGGAAAAACAA	262
Ad01A6430	Aradu.A01	6430	p2	(TC)20	40	21544782	21544821	CGGTCATCTCAGTTGCTTGA	CGACAATGATAGCAGAGGAGG	259
Ad01A6434	Aradu.A01	6434	c	(GAA)6a(AAC	34	21563657	21563690	AGCCATTCTTGGTTCTCTGTG	ACAAACATCACGCACCACAC	125
Ad01A6435	Aradu.A01	6435	p2	(AT)6	12	21565696	21565707	AAATTTGACTTGTGGTGAGATAAAT	GGGTTGTAATGGTATTTTTCAATATG	280
Ad01A6437	Aradu.A01	6437	p3	(AGA)7	21	21575791	21575811	TTGCCTTCCCTTTCCTCTTT	AAACCCACACCTCTCTCCCT	186
Ad01A6441	Aradu.A01	6441	p3	(ACA)14	42	21624419	21624460	CCAACCTGAAATCAAAAGGC	CATCCACTCCTGATTCTAATGAAA	221
Ad01A6443	Aradu.A01	6443	p3	(ATG)10	30	21631870	21631899	GAGTGATTGAGGGTGGGAGA	TTGTTCCAATTTTCTCCCTAATG	217
Ad01A6444	Aradu.A01	6444	p2	(TC)14	28	21633889	21633916	GCTCCCTTCTGTCGACACTC	AACGAGCAATCTCAACCACC	273
Ad01A6445	Aradu.A01	6445	p3	(TTA)5	15	21634243	21634257	TCGTTGCTATTGTTGCTAGGG	TGGTCATCTTCTCCACCCTC	244
Ad01A6446	Aradu.A01	6446	c	(T)10cgaaaata	28	21637880	21637907	GATTGTGACCCAGCTTTGCT	TTGGCTCTTGAAAGAATGGG	244
Ad01A6455	Aradu.A01	6455	p2	(AC)7	14	21687871	21687884	TTTCTTACAAATTCCACCCACA	CCATATGGCCAACCCTTACA	187
Ad01A6457	Aradu.A01	6457	c	(A)12gaaaagag	55	21697683	21697737	TTGAGAGCTTGCTACCATCG	TCTTCTCCCAATTTTCTGCAA	155
Ad01A6461	Aradu.A01	6461	p2	(AT)6	12	21736711	21736722	AAAAAGTGGGCGATTTTAGTT	TGATACATGCACTTGTAACATCAT	255
Ad01A6462	Aradu.A01	6462	p2	(TC)10	20	21737180	21737199	TCATTCCCAATTCCAATTCC	CAAGATGAAGGCCTGAGTTG	267
Ad01A6481	Aradu.A01	6481	p3	(ATT)5	15	21861017	21861031	TCGCCCTTGTTGCTTTAACT	GCACGTCATGATCGTACCAA	273
Ad01A6482	Aradu.A01	6482	p3	(AGA)23	69	21872915	21872983	ATGGAGGATGTGAGTGGGAA	TGCAATTGAGCGTACCATGT	280

ID	Chromosome	SSR nr.	SSR type	SSR	Size	Start	End	FORWARD PRIMER (5'-3')	REVERSE PRIMER (5'-3')	Product (bp)
Ad01A6486	Aradu.A01	6486	p3	(CTT)6	18	21887988	21888005	TCCTCACTCACCAAAAATTCG	GTTCTTGTCCTTGCTCCTGC	187
Ad01A6489	Aradu.A01	6489	p2	(AT)6	12	21908944	21908955	TATCAAAACTCACGCTTCCG	GGTTTGACGCAGAAGTCACA	273
Ad01A6490	Aradu.A01	6490	p4	(TACA)11	44	21916144	21916187	AAACCGCAATACCACTCCAA	TTGTTCCCTTGCCTTTATCTTT	216
Ad01A6492	Aradu.A01	6492	p3	(AGA)6	18	21944966	21944983	TAAAATATTGCTCGCCCTCG	CCCCCTCTTCTCTACCTTGG	110
Ad01A6493	Aradu.A01	6493	p3	(TAC)5	15	21948334	21948348	CATGCCTGATGATGATGAGAA	GAGCAAGGGCGTGATCTAAG	260
Ad01A6496	Aradu.A01	6496	p2	(AT)7	14	21985843	21985856	CCGGTCCGAGCATAGAAGTA	CTCCTCGTTGATGCATGTTG	244
Ad01A6498	Aradu.A01	6498	c	(A)10gcatataa	105	21990597	21990701	TCAATAAAAATGTCTTATCGGTTGA	ATACAGTCCGCTACGCGTTT	262
Ad01A6502	Aradu.A01	6502	p3	(ATA)5	15	22009654	22009668	TTTTCCTTTTGGACCAAGAAAT	GAACACCCTTGCAGCTTCTC	216
Ad01A6507	Aradu.A01	6507	p3	(AAT)9	27	22024686	22024712	CTTGGAGACGGCTTCAGTTC	GCAATTTGCGAATTTGCTTT	242
Ad01A6511	Aradu.A01	6511	p3	(AGA)6	18	22043697	22043714	TAAAATATTGCTCGCCCTCG	AAATCTTTCCCACCCAAACC	209
Ad01A6513	Aradu.A01	6513	p2	(AG)6	12	22057093	22057104	AGAGGTGGTGAGGTGATTGG	ATTTTTGCATGCCATTTGGT	133
Ad01A6516	Aradu.A01	6516	p3	(GTT)5	15	22077251	22077265	TCTTAGGACAACTTGCGGCT	TGCACCTAGAGGTCCGTCTT	215
Ad01A6517	Aradu.A01	6517	c	(A)11caaaacca	89	22081963	22082051	TGGCCCTCAATCTACCAAAA	TGAAGCACTATGGGAGAGGAA	254
Ad01A6518	Aradu.A01	6518	c	(T)13cattcgtta	84	22082742	22082825	TGATTTGATTTTGTGGTGTTGG	GGATTGTTATTGGGTGTTGGA	277
Ad01A6519	Aradu.A01	6519	c	(TAA)13tag(T	75	22085406	22085480	CGGTTCAATCACCAATTTGA	TTACCCGTTTTTCAAGGCTC	267
Ad01A6522	Aradu.A01	6522	p4	(TCTT)6	24	22113868	22113891	CTCATCCTCCTAGGGAAGGC	AAACACCAAAGCCATGCACT	222
Ad01A6523	Aradu.A01	6523	p3	(GAA)6	18	22116059	22116076	TCATCAACACCAGAACCCAC	TCTGCCACTGTTCCTCCTCT	272
Ad01A6525	Aradu.A01	6525	c	(AGG)9atg(A(	72	22129724	22129795	TCACCCCTTTCTCCATCAAC	CTTCTTCTCCGATGCCTGAG	257
Ad01A6526	Aradu.A01	6526	p3	(GAT)15	45	22130676	22130720	TAATCCACATTGAACCGACG	TCAGGGTGGGTTACCAACAT	108
Ad01A6527	Aradu.A01	6527	c	(T)10cactctcct	71	22130972	22131042	TGATGATAATTTCTTTTCCTTTCTTC	ATGCGTGTCCCAGTGTCATA	248
Ad01A6528	Aradu.A01	6528	p2	(TA)8	16	22134582	22134597	TTCTCTAGACATATTTGGACACACC	TGATGTACGGACATTACACACAGA	279
Ad01A6529	Aradu.A01	6529	c	(GA)9c(AG)1	45	22134935	22134979	TCGTCTTTCGGTTCCGATTA	TTACCGTGAGCCCTAACCTG	201
Ad01A6532	Aradu.A01	6532	p2	(TC)17	34	22143650	22143683	CACTTGAAAACGCAATTACATACA	GGCTACCATGACTCACGACA	266
Ad01A6533	Aradu.A01	6533	p3	(TAA)15	45	22149545	22149589	CTTGAACGGCTAGGAAACCA	GCCCCTACCTTGTGGTATTG	130
Ad01A6537	Aradu.A01	6537	p3	(AAT)7	21	22157226	22157246	CGAGAAACAAACTTGTGGCA	TGAGTGTCTTGCTGGCATTT	273
Ad01A6542	Aradu.A01	6542	p3	(TAC)7	21	22177103	22177123	TTTGACAAGTGAAAGCAGGAA	TCAAGTGAAAGATAGCGAGGC	280
Ad01A6543	Aradu.A01	6543	p3	(CTT)11	33	22177862	22177894	TCAGTTCCTGCTGTGATTCG	ATTCTTCCTTGCCACCACAC	231
Ad01A6545	Aradu.A01	6545	p5	(GTGAG)5	25	22189755	22189779	AAGAAGACGATAAAGCCGCA	TATTCCCCCAACCCTAGTCC	232
Ad01A6546	Aradu.A01	6546	p2	(GA)18	36	22190859	22190894	GGATGAGTATCATACGTGATTGGA	TTCACCCCTTGTCCATCTTC	209
Ad01A6548	Aradu.A01	6548	p3	(AAC)10	30	22205560	22205589	GAGAAGCTCTTCTGAGCAAACC	AAGCCTTTCTTTTTCCGCTT	202
Ad01A6549	Aradu.A01	6549	p2	(TG)7	14	22214868	22214881	TGACCAGCAGTGAGATTTGG	TAATAAATGGGGTGGGAGCA	255
Ad01A6551	Aradu.A01	6551	p2	(CT)6	12	22229354	22229365	TCAACCGCACTGTCAACTTC	ATGGAAGAGCACCAACATCC	172
Ad01A6555	Aradu.A01	6555	p3	(AAT)6	18	22241990	22242007	CAGGAACAAGGAAAAAGGCA	TTGGATGTTGGCTTTGTGAA	204
Ad01A6558	Aradu.A01	6558	p2	(TA)7	14	22268745	22268758	GCCTTCGCAAAATACGAACT	TCCCATATTTTCACCCCTTTC	278

ID	Chromosome	SSR nr.	SSR type	SSR	Size	Start	End	FORWARD PRIMER (5'-3')	REVERSE PRIMER (5'-3')	Product (bp)
Ad01A6559	Aradu.A01	6559	p3	(AAT)5	15	22269629	22269643	TTCCCTCGTCTTTTCCTAAGTG	AAGAGTGTAAGAATAAAAGCTTTACGA	259
Ad01A6563	Aradu.A01	6563	c	(T)10aaacatcaa	57	22274623	22274679	TATAACAGGCCAGGCCAAAC	AAAAATTGATGAATTGCCCG	222
Ad01A6564	Aradu.A01	6564	p2	(AC)8	16	22278083	22278098	CCCTGAAACCCTAACCCATT	CTTCCTCTCCCTCTCTGCCT	159
Ad01A6565	Aradu.A01	6565	p2	(TA)7	14	22281822	22281835	ACCCTCGCCTTTACCTTGAT	TAAACCGCAATACTGCTCCC	198
Ad01A6566	Aradu.A01	6566	p2	(AC)11	22	22285598	22285619	CAACCCACCCACTTAGAAGC	CTTCCTCTCCCTCTTTGCCT	128
Ad01A6567	Aradu.A01	6567	p2	(TA)6	12	22290810	22290821	ACCCTCGCCTTTACCTTGAT	CGCCCCTATAATTATTAAAATCCA	280
Ad01A6569	Aradu.A01	6569	p2	(GA)15	30	22299075	22299104	AGCTCATAGCAATGGCGACT	ACCCCTTCACAGCTCACATC	203
Ad01A6572	Aradu.A01	6572	c	(AG)6atttaagg	88	22310563	22310650	GTAAAGAGCGGTAGAGCCGA	GGGGATCACATCGTTATCGT	269
Ad01A6574	Aradu.A01	6574	p2	(TG)8	16	22322526	22322541	TATATGGGCCAGATCCCAAA	CCAACTTGAAACTACACCGACA	227
Ad01A6579	Aradu.A01	6579	p2	(CT)29	58	22335312	22335369	CTTCTTCTTCTCCTGTGCCG	TTACAACAAGCTGCGACGAC	233
Ad01A6580	Aradu.A01	6580	c	(TA)8gacacaca	41	22347358	22347398	AATCGCAACCCATGAGAATC	AGGCTAATCTTGGCGTCTGA	236
Ad01A6581	Aradu.A01	6581	c	(TA)8atgtttactg	113	22349507	22349619	TTTCAGCAACAAATATCAACAGA	TTGGGAAGAGAAAACTGAGGA	275
Ad01A6586	Aradu.A01	6586	p2	(TC)6	12	22363730	22363741	AACACATTGCTCCGATCCTT	TGGTCAAGCCCCATTTCTAA	268
Ad01A6588	Aradu.A01	6588	p2	(TA)6	12	22364692	22364703	TTGTGTACTCATGGTTGGAATTG	ATATGAAAAAGGCAAGGGCA	278
Ad01A6591	Aradu.A01	6591	c	(A)11g(A)10	22	22372007	22372028	TTCCCTATCACAATGGAGCC	CATGGCAAATTGAGATGCAC	214
Ad01A6593	Aradu.A01	6593	p2	(AT)6	12	22410373	22410384	CCCAACACTTTTGCATTTCA	CCACACCATTAGGCTCAAAA	242
Ad01A6596	Aradu.A01	6596	p2	(AG)6	12	22445855	22445866	GCAATGGAAGTCCCAACAGT	GCACCTTCTTCCTCACCAAG	152
Ad01A6598	Aradu.A01	6598	p2	(GA)6	12	22448683	22448694	AGAAAACCAGTAAGCGCAGC	ATTATGAAGAATGCGCGACC	213
Ad01A6599	Aradu.A01	6599	p3	(CAA)8	24	22457518	22457541	GGCACGGATGAAGATGAGAT	AAACGGGACATTCAAGCAAC	181
Ad01A6602	Aradu.A01	6602	p2	(TA)6	12	22464558	22464569	GCTTTAGGCATATAGATGTTATGGTTT	AATTTCTCTGTCAGAAACCTGTAGA	267
Ad01A6604	Aradu.A01	6604	p3	(TAT)6	18	22468354	22468371	GACGATCTTGTGAGCTGCAT	ACCACAAGCACCAACATTCA	263
Ad01A6607	Aradu.A01	6607	p2	(CT)6	12	22487547	22487558	TGGGTGATTAGACCTTTGCC	GGTACTCTCCAAAGCCAACG	139
Ad01A6608	Aradu.A01	6608	p3	(TGA)6	18	22514358	22514375	AGCAATTTCGAGTTCTTGCG	GGACGAGTCTGAAGAGGCAC	258
Ad01A6612	Aradu.A01	6612	p2	(TC)24	48	22566233	22566280	TGGGTGATTAGACCTTTGCC	CCAAGTTGATCCCTCCAAGA	194
Ad01A6613	Aradu.A01	6613	p3	(TTA)5	15	22568405	22568419	ACCTTGGCAAGAAGATCGTG	CTTCAATTAAGTCTGCGTTCTCT	277
Ad01A6617	Aradu.A01	6617	p2	(AT)6	12	22596418	22596429	AGTTGACGGACAAAAATCGG	ATGGACTGCTCAACTTGGGT	250
Ad01A6619	Aradu.A01	6619	p3	(TTC)5	15	22600466	22600480	GAGCTCCGGACGATTAACAT	TTTCTTCTTCTCCAACCCCA	136
Ad01A6620	Aradu.A01	6620	p3	(CCT)5	15	22604794	22604808	AGGCCCAATTAGGTAGCGTT	GATAATAGGTTGGGTGGGGG	253
Ad01A6621	Aradu.A01	6621	c	(A)11gcttatgct	89	22619926	22620014	CTAGAGGATTCTCCGGCCTT	TTCTACACCTTGGCCGAAAC	177
Ad01A6623	Aradu.A01	6623	p5	(AAGAT)5	25	22644088	22644112	AGGACAACCCGACCTCTTTT	CCTGGGTGCTCCAGTGTATT	237
Ad01A6625	Aradu.A01	6625	p3	(CAT)5	15	22664227	22664241	CTGTCGAAGTTGAGGAAGGC	GAGCAGGTTCAGGTTATCGC	171
Ad01A6627	Aradu.A01	6627	p2	(AG)6	12	22692818	22692829	GATCACGAAAAAGCACACCA	CATTTGGATCCCTCTTTCCA	118
Ad01A6630	Aradu.A01	6630	p2	(TC)6	12	22732058	22732069	AACATGAAAGGTTCATAAGCCAA	GGAATGCATCCACACATCAT	254
Ad01A6634	Aradu.A01	6634	p3	(TTC)6	18	22747129	22747146	CACCTTGACCGAAACACCTT	CATGACTCGGAGGTGGAAGT	260

ID	Chromosome	SSR nr.	SSR type	SSR	Size	Start	End	FORWARD PRIMER (5'-3')	REVERSE PRIMER (5'-3')	Product (bp)
Ad01A6635	Aradu.A01	6635	c	(T)10caatctc(T	31	22752144	22752174	TCCCATGTGCATTGATTCTT	GGGAACATGGGATGATAGGA	243
Ad01A6641	Aradu.A01	6641	p2	(AT)8	16	22811700	22811715	AGCGTCTCCTAAAACTGCCA	TGTGTCATTGGAAACTACTGTGTC	201
Ad01A6647	Aradu.A01	6647	p3	(TAA)12	36	22821549	22821584	TCATTCTTGGCTGATTTGGA	CTGGCACCACTCGGTTTATT	201
Ad01A6653	Aradu.A01	6653	p3	(TTC)6	18	22871395	22871412	AAATCTTTCCCACCCAAACC	TAAAATATTGCTCGCCCTCG	209
Ad01A6656	Aradu.A01	6656	p2	(AG)7	14	22910511	22910524	GAACACTTGGAGCAGCACAA	TTGCATTTCAAACTTAGTACCACA	280
Ad01A6657	Aradu.A01	6657	c	(TG)9ttctccact	117	22918803	22918919	ATATGTTTTGGGGGCCTTTT	AACTAGGAGCCTTGACGGAA	251
Ad01A6659	Aradu.A01	6659	p2	(CT)14	28	22932918	22932945	TGAAAATCACTAACCACTTTTTCG	TTGATGTGTCAGAGGAAAGGAA	203
Ad01A6662	Aradu.A01	6662	p2	(CT)7	14	22968161	22968174	GGAACATTGAACCTGGAATGA	GCAATCAGCGATTTCTAGGC	242
Ad01A6663	Aradu.A01	6663	p5	(TTTTA)6	30	22970964	22970993	TTTGGAGCATTTTGTCCCTT	TAAGTGTGGGGGAAAAGTGG	244
Ad01A6664	Aradu.A01	6664	p2	(TC)8	16	22976033	22976048	GGAACATTGAACCTGGAATGA	TGACAGAATTCGCAATCAGC	271
Ad01A6665	Aradu.A01	6665	p2	(CT)17	34	22979874	22979907	ATCCTAGGCACTTCCCAACC	TAGGAAGAGAGGCAAGACGC	227
Ad01A6669	Aradu.A01	6669	c	(TC)9tt(TC)6	32	22983861	22983892	ATAGAAGCGGAGGGGATGAA	TTGCTAGCTGTTTTGCACTCA	277
Ad01A6670	Aradu.A01	6670	p3	(TTA)12	36	22990390	22990425	TAGTGACCGGCATTCATGTG	GCCCTTGTTGATGGCTATGT	207
Ad01A6671	Aradu.A01	6671	p4	(TTTA)5	20	23000265	23000284	GAGCTTGTTTGAATGCCGTT	CCGAGTATGGTCGATTTTGG	224
Ad01A6675	Aradu.A01	6675	p3	(AGA)5	15	23004833	23004847	AGCAACCACACACAAAACGA	ATCCCTCCACTTCCCAATTC	208
Ad01A6677	Aradu.A01	6677	c	(T)10atctattttg	42	23008024	23008065	ACTTAAACCGCAACTCCCCT	CTCAAACATCAATCCAAGGTCA	227
Ad01A6678	Aradu.A01	6678	p2	(AT)12	24	23008469	23008492	TTTGTATATGATGTTTTGGAAGCA	ACCCCTACCAGCTCAACCTT	271
Ad01A6681	Aradu.A01	6681	p4	(TAAA)5	20	23043403	23043422	GTTCTTGTCCTTGCTCCTGC	TTCGAAATTCTCTCCCTCTCA	243
Ad01A6682	Aradu.A01	6682	p2	(TA)7	14	23082340	23082353	TTTGGTAACAGGAACGGAGG	GCCGGCTAGCCATATACAGA	178
Ad01A6683	Aradu.A01	6683	p2	(AT)8	16	23091684	23091699	GCCGCAAACGACACATAAAT	TCCAATCCAAAACAAACCAA	240
Ad01A6684	Aradu.A01	6684	p2	(AT)7	14	23091922	23091935	TTGGTTTGTTTTGGATTGGA	TTTACTCATATCGACTGATATCGAAC	280
Ad01A6685	Aradu.A01	6685	p3	(AGA)6	18	23113013	23113030	TTCTTTGGGTTTGGGTTCAC	ATGGCTGAACACACTCTCCC	265
Ad01A6687	Aradu.A01	6687	p2	(CT)9	18	23119308	23119325	AAAAACAATGCCAAAAAGCG	AGTTTGGCCCAGAAACACAG	192
Ad01A6688	Aradu.A01	6688	p2	(TC)6	12	23121533	23121544	AGAATACGCCCCCAGCTATT	TCAAAACAAGAAGAAATCCTAAATCC	196
Ad01A6689	Aradu.A01	6689	p2	(TA)6	12	23124511	23124522	TCTTTTGGGCTGTCTTTTGG	GAGGTGGCGAGGTATTACGA	226
Ad01A6691	Aradu.A01	6691	p2	(CT)6	12	23135381	23135392	AAGAGAAATTTTGCTCGCCA	TTGAATTTGAATGGTGGGGT	235
Ad01A6693	Aradu.A01	6693	p4	(TTAT)5	20	23140438	23140457	TTCAGCTTTGGGGTGATTTT	TATGGGAATTTGTGGGAACA	185
Ad01A6695	Aradu.A01	6695	p2	(GA)11	22	23143099	23143120	TTGCATCAATCCACAAGCAT	CCCCTTCTTCCTTCCATCTC	246
Ad01A6697	Aradu.A01	6697	p3	(GAG)6	18	23178608	23178625	TCCCAGAAGAAGAGCCAGAA	CGCCAAAAAGAGATCTCAGG	212
Ad01A6698	Aradu.A01	6698	p2	(AG)8	16	23179894	23179909	GTTCAGCCCACTAATCTCCG	GAAGCAACTGAATAACACTACACACA	276
Ad01A6699	Aradu.A01	6699	p2	(TG)8	16	23183088	23183103	ATGCATCATGTTTGTTTGCG	TCGCACATGTAGCTGAAAGG	236
Ad01A6702	Aradu.A01	6702	p2	(AT)10	20	23189644	23189663	AAAAAGTTGGCTCCTTAATATTACTC	TTGTGTATGTGTAGTGAGACGACG	113
Ad01A6707	Aradu.A01	6707	p3	(TTA)13	39	23219274	23219312	TCTACCGTATCAATCCCCTTT	CATGAAACGTCAAAGAAATGGA	274
Ad01A6708	Aradu.A01	6708	c	(AT)7tgaataata	95	23220820	23220914	AAATGGTCTAGGAAATGGGGA	AAGGATCGAAAACAGGAGCA	279

ID	Chromosome	SSR nr.	SSR type	SSR	Size	Start	End	FORWARD PRIMER (5'-3')	REVERSE PRIMER (5'-3')	Product (bp)
Ad01A6709	Aradu.A01	6709	p2	(CT)6	12	23241412	23241423	CAAGTTACGCGAACCCTAGC	GGTGCAGAAGGGGTTGAATA	226
Ad01A6711	Aradu.A01	6711	p2	(AT)6	12	23247426	23247437	CCCAACACTTTTGCATTTCA	AACCACACCATTAGGCTCAAA	253
Ad01A6712	Aradu.A01	6712	p2	(AT)9	18	23252741	23252758	TTTTGGGAACATGGGATGAT	TTGTCCCATGTGCATTGATT	203
Ad01A6714	Aradu.A01	6714	p2	(GC)6	12	23269522	23269533	ATAGGGAGCTCTTGGGCATT	GAGGAAGGAGAAAAGCAGGG	241
Ad01A6717	Aradu.A01	6717	p2	(TA)6	12	23280961	23280972	TCCCGCTTTTATTGGTCAAA	AAAAATACTGGCCACCTAAAAA	223
Ad01A6718	Aradu.A01	6718	p2	(TC)6	12	23281998	23282009	CCGACAGAAAATCCGTCTACA	GTGGAGGGAGCTCGTATGAC	267
Ad01A6721	Aradu.A01	6721	p3	(TAT)7	21	23327313	23327333	GATTCTTGAAACCTGATTTGGA	GATCCTCGATGTGGCTAAGG	280
Ad01A6722	Aradu.A01	6722	p3	(TTA)6	18	23327685	23327702	AGCCACATCGAGGATCATTC	CGAAGTAGCTGCTGCAAAAGT	273
Ad01A6725	Aradu.A01	6725	p2	(AC)6	12	23329573	23329584	AATGGCATAAGGCAAGTTGG	ATTGTCCATACCCTTTGGCA	246
Ad01A6726	Aradu.A01	6726	p2	(TA)10	20	23330432	23330451	TGTGCATCAATTCCCTTTCA	TTTTTGACCTCCAGACTATTTCAA	163
Ad01A6727	Aradu.A01	6727	p4	(AGTG)6	24	23335776	23335799	GTGAGATGGAGGCAGAGGAG	TTTTCCCCCTTTTCCTGAAG	265
Ad01A6734	Aradu.A01	6734	p4	(TATT)5	20	23355838	23355857	TTTCGAAAACTCCTCTCTCTCA	CAGAGTCCTTTCAGGTTCCG	274
Ad01A6736	Aradu.A01	6736	p5	(AATAA)5	25	23372053	23372077	GGGTTAGCATATGACCCCAG	TTTCTCTCTCCTCACTTTTTCG	243
Ad01A6739	Aradu.A01	6739	p3	(ATT)6	18	23397498	23397515	CGAAAAAGCTCGAAAAATGG	CCCCGATCTTTTATCAAACTACA	147
Ad01A6746	Aradu.A01	6746	p2	(AT)9	18	23405783	23405800	TTTGAGCTAAAAACCCTTAAAATC	TTCAAAAATTATTCCCGCCA	271
Ad01A6747	Aradu.A01	6747	p2	(GC)6	12	23419983	23419994	TTTTCAAGGAAGCACCATCC	ACAATTCGGGCACAACTCTC	104
Ad01A6748	Aradu.A01	6748	p3	(TTA)5	15	23425457	23425471	CCTTTTTAAAATATAATGCATGTACCG	GTTCTAATTCTCATTCAAGTGCAA	149
Ad01A6750	Aradu.A01	6750	c	(CT)20tattaacc	137	23444903	23445039	GCCATATATCGGGTGAGTCG	GACCAAGAATCTTCTCTCAAAACA	260
Ad01A6756	Aradu.A01	6756	p2	(AC)7	14	23480781	23480794	CCCTTGGCCCAACTAAACTA	TCAACATCAAGCCTCCTCAA	249
Ad01A6758	Aradu.A01	6758	p3	(CAA)5	15	23484303	23484317	TGCACCTAGAGGTCCGTCTT	CCCAGCACCTTGTTTCTGTT	235
Ad01A6759	Aradu.A01	6759	p3	(CTT)12	36	23492696	23492731	CGAAGCGCTAGTGGGTCTAC	CAGCAAAACCAGATGCAAAA	228
Ad01A6761	Aradu.A01	6761	c	(A)11cgcaggca	66	23506070	23506135	CAAGAACGGTTGTGGGTTTT	AACAGGAGTGTGGCCCATTA	267
Ad01A6762	Aradu.A01	6762	c	(A)10tttttttatg	58	23506341	23506398	TTAGTCATTGGGCTTCCGAC	AATAGTCGTGCCCCCTTTCT	188
Ad01A6764	Aradu.A01	6764	p3	(TTC)6	18	23541524	23541541	ATTTTCCCACCCTAAATGGC	CTAGAGGATTCTCCGGCCTT	274
Ad01A6767	Aradu.A01	6767	p2	(TA)6	12	23577497	23577508	TCGTTAGGCGTCTGGACTCT	AAGTCATGCGGAGAGAAAGC	102
Ad01A6768	Aradu.A01	6768	p2	(GA)7	14	23587634	23587647	CTAACTTCGTTTCTTTGTCATTTTT	TGCACTGGTTAGTGGCTTTG	272
Ad01A6774	Aradu.A01	6774	p2	(TA)7	14	23621903	23621916	TCCCCAATTAGAGGTGTCCA	TGGCTCTTGAAAGAATGATGG	210
Ad01A6777	Aradu.A01	6777	p2	(CT)10	20	23668887	23668906	CATTGAACCTGGAATTGGAAA	TTTCTGGGCTGTTTTTGACC	219
Ad01A6778	Aradu.A01	6778	p2	(TG)10	20	23672477	23672496	TGTTTTTCAGATGCAGGTCG	GACTGGCTGGAGAACACACA	255
Ad01A6779	Aradu.A01	6779	p3	(GTT)5	15	23679835	23679849	ACTTCTAGCCGACCCCACTT	AAATTTGACCGGCAGTATCG	115
Ad01A6780	Aradu.A01	6780	c	(T)10catgtgttg	90	23688373	23688462	AGAACGATTCTTAACTAAATGAAAATG	TTGATTCATGTAGCCGACCC	279
Ad01A6782	Aradu.A01	6782	p2	(GA)6	12	23706976	23706987	TTGCAACAATCCACAAGCAT	TCCCTTCTTCCTTCTATCTCACC	236
Ad01A6784	Aradu.A01	6784	p2	(CT)21	42	23725722	23725763	TCGAGAGCATGATGAGATGG	GGACCAAAAATCACAAACGG	247
Ad01A6788	Aradu.A01	6788	c	(T)12aaatacaaa	107	23736753	23736859	TGTCAATTTTCGGGTGCTAA	TGTCTACTTTTAAAATTTTTGGAGATT	259

ID	Chromosome	SSR nr.	SSR type	SSR	Size	Start	End	FORWARD PRIMER (5'-3')	REVERSE PRIMER (5'-3')	Product (bp)
Ad01A6794	Aradu.A01	6794	p3	(AGA)12	36	23785117	23785152	GCTTTTGTCTTCCCTTTCCC	TACACCTTTCCCCCTCTCCT	203
Ad01A6799	Aradu.A01	6799	p2	(TA)6	12	23813421	23813432	CCACGTGCATTTGCTTCTTA	CCATAAATGCCCTTGTTAGAAAA	122
Ad01A6801	Aradu.A01	6801	c	(A)13gataaagt	123	23818852	23818974	ATTCACAACGTGGAAGAGGG	TTCAACAGAGATAAAGGCCAA	219
Ad01A6802	Aradu.A01	6802	p2	(AT)8	16	23821423	23821438	TTGGTGGAGGAGATTTGCAT	TTGGGATCTATTGAAACCGC	136
Ad01A6803	Aradu.A01	6803	p2	(TA)6	12	23836227	23836238	TTATGCTCGGACCGGTTATC	AAGGGGTAAACAAAAACCGC	160
Ad01A6804	Aradu.A01	6804	p2	(TG)9	18	23836526	23836543	TGTAGAGCCTGAGGGATGGA	AAAGTCCCAAATGCATGCTC	133
Ad01A6805	Aradu.A01	6805	p3	(CAA)5	15	23837371	23837385	TGCACCTAGAGGTCCGTCTT	GCACCTTGTTTCTGCCTCTC	231
Ad01A6806	Aradu.A01	6806	p2	(TA)6	12	23843971	23843982	AGCCGGATCTTGAGTCTTGA	AAGGGGTAAACAAAAACCGC	135
Ad01A6807	Aradu.A01	6807	p3	(TGG)5	15	23857445	23857459	GCGAGGGAGAGAAGAATGTG	GCACGCGTAGGCTCTTAATC	272
Ad01A6811	Aradu.A01	6811	p3	(AGA)11	33	23883078	23883110	TGAACGCGATCAGAAGAGAA	CCATCCCTTCACTCGTTTGT	216
Ad01A6814	Aradu.A01	6814	p2	(CT)7	14	23909504	23909517	CCAAGTCCAAAAAGCATAGACC	AGGCCAAATAGGCAACAGAA	277
Ad01A6817	Aradu.A01	6817	p2	(AT)6	12	23945921	23945932	TTTTATCGCTGAAAAATTTTAGTTG	TCCACTTGAGGTTGGAGACC	258
Ad01A6818	Aradu.A01	6818	p4	(AAAT)5	20	23952258	23952277	GAATTGGTGAGGGGGAGATT	CGGATTAAAATGGCATTGCT	238
Ad01A6819	Aradu.A01	6819	p2	(TC)7	14	23953605	23953618	AGCCTCAGTGTCCCACTCAT	AGGGACACAATGCTTATCGG	265
Ad01A6822	Aradu.A01	6822	p2	(AC)7	14	23960199	23960212	CCTCAGACTGAATAATACACAAAACA	CAGAAGCGTTGAAATCACGA	234
Ad01A6824	Aradu.A01	6824	p2	(GA)8	16	23963755	23963770	CTGAGAGAGCTCGTGTGACG	TCACGTGAATTACTCCCATACC	175
Ad01A6833	Aradu.A01	6833	p4	(TTTA)5	20	24024250	24024269	TTCTCCGTTCTTGCTTGCTT	TTGAATCCCAGAAAAATGCC	218
Ad01A6834	Aradu.A01	6834	p3	(CTC)6	18	24027498	24027515	GTTTGTGGCCAATCGAAGAT	AATACAAGGCCCTGCAGATG	126
Ad01A6835	Aradu.A01	6835	p3	(AAT)8	24	24028106	24028129	GGAATGCTTGCAACAACCTT	AAATCTCAATTATTTGTGCCAACA	231
Ad01A6837	Aradu.A01	6837	p3	(TCT)7	21	24080329	24080349	CCTACCTCGCCCTCTCTCTT	CTCCCAAGACTCAGAGGTGG	268
Ad01A6838	Aradu.A01	6838	c*	(A)11(AT)7*	24	24082103	24082126	GGTGGCAATACTTTTTGTGC	GCCTTTTGGTTTTAAGGGCT	244
Ad01A6843	Aradu.A01	6843	p2	(AC)6	12	24099790	24099801	TTCCAATTAATGTAACAGTCTTACCAA	AATGTATGCATGTCGACCAAA	210
Ad01A6844	Aradu.A01	6844	p2	(TG)6	12	24099929	24099940	TTTGGTCGACATGCATACATTA	TGTTTAGTTGTTCTTTATTGATTTCCA	269
Ad01A6845	Aradu.A01	6845	p2	(TA)8	16	24100281	24100296	CAATCAAAGCCAACGGTCTT	AGCAGCAAAGGCTGTGTTTT	168
Ad01A6848	Aradu.A01	6848	p3	(GAA)21	63	24107087	24107149	GAGGACAAACACGACGATGA	CACCTCCATTTAAAACAAGTCCA	236
Ad01A6851	Aradu.A01	6851	p3	(ATA)13	39	24112645	24112683	TGTGTTGTGCATTAAAATCTTTTGT	TTTTTGCCTTTTCATCTTCCTT	143
Ad01A6853	Aradu.A01	6853	p2	(TA)8	16	24117459	24117474	CGAAACCGTATGCTGTTTTCTT	TTCTTTTTCAGGATGCACGA	279
Ad01A6854	Aradu.A01	6854	c	(TC)17tttgagc	64	24120994	24121057	AACAACGGAGGCAATAGTGG	GCCATCGACATAGTGGGTTT	280
Ad01A6855	Aradu.A01	6855	p2	(GT)13	26	24121292	24121317	ATGGCTCCATCATCGATAGC	ACGCCATCACTCTTCCTCAC	240
Ad01A6859	Aradu.A01	6859	c	(A)10ttaagtaat	44	24145191	24145234	GGGGAACCTTATGGGAACAT	TTTGTCCCATGTGCATTGAT	252
Ad01A6860	Aradu.A01	6860	p2	(CG)6	12	24150523	24150534	CCTATGCGTACGTGTGATGG	TACGCGTGGGAATGTACAAA	127
Ad01A6861	Aradu.A01	6861	c	(CTC)8tgcattt	70	24158092	24158161	GCTACACATCCAAGTCTTTTTGG	CCACTAGGAAGAGGAGGAGGA	267
Ad01A6862	Aradu.A01	6862	p3	(AAG)5	15	24158817	24158831	TTTTGTTGCATTTTGAGTTTGAA	TGAAGTTTCGGTGCATTTTG	234
Ad01A6863	Aradu.A01	6863	p2	(AT)6	12	24186419	24186430	TCTTGGGAAAAGATAGCAAATCA	ATGCCAATGGGATTCAAAAT	145

ID	Chromosome	SSR nr.	SSR type	SSR	Size	Start	End	FORWARD PRIMER (5'-3')	REVERSE PRIMER (5'-3')	Product (bp)
Ad01A6864	Aradu.A01	6864	p2	(AT)6	12	24200541	24200552	TTTTTATTTCACGAGTAAATGCG	TGAGCTAATTGATCCCCTTTG	248
Ad01A6865	Aradu.A01	6865	p3	(GAA)5	15	24214909	24214923	CACTCAAAACAAGGGGCATT	GCATGGCAACACCAAACTTA	225
Ad01A6866	Aradu.A01	6866	p4	(AATA)5	20	24238032	24238051	TCGTGCCCTGTTTCTTATCC	TGCAAATGCAATAAACAAAAGAA	213
Ad01A6869	Aradu.A01	6869	p2	(AT)7	14	24257696	24257709	GGAAACTGTGGAAACATGGG	TGGGGTAATTTTGTCCCCTT	232
Ad01A6871	Aradu.A01	6871	p2	(AT)7	14	24274143	24274156	TTTGGGAACATGGGTAGAGG	ATTATTCGCTTCACCTTGGG	243
Ad01A6872	Aradu.A01	6872	p3	(AAT)5	15	24307127	24307141	TGAAGCAAGAAAAAGCAGTGAA	ATGCCACAAGCACATGGTTA	218
Ad01A6879	Aradu.A01	6879	p3	(TCT)54	162	24397864	24398025	TATCCACCTCCTCCAACGAC	GAAGGAGAGAGGGGAATTGG	263
Ad01A6882	Aradu.A01	6882	p2	(AT)6	12	24443544	24443555	CAGGGTTGTAATGGGGCTAA	TAAAGCAATGCACATGGGAC	211
Ad01A6885	Aradu.A01	6885	c	(AATCCT)5aa	59	24448674	24448732	CAGTCCAGACATGGAACATTG	TTTTGACCCAGTTTTCGACC	260
Ad01A6886	Aradu.A01	6886	p3	(AGA)6	18	24461291	24461308	TGAAAATATTGCTCGCCCTC	CCCATAAACCCCACCTACCT	233
Ad01A6887	Aradu.A01	6887	p3	(TAT)13	39	24480141	24480179	TGAGTTTAGAATTGCTAATGCTTCAT	TGCTCGGTCAAGAGAAAGAA	268
Ad01A6888	Aradu.A01	6888	p2	(TA)6	12	24490863	24490874	TGCCATGTTTCTAATCTATGCG	TCAGCTTCATGACAGGGTTG	127
Ad01A6891	Aradu.A01	6891	c	(TAA)6tatgtac	101	24496598	24496698	AGCGATCCAACCCACTTATG	ATTCGGGAGTCAGGTTCGTA	238
Ad01A6896	Aradu.A01	6896	p2	(GT)7	14	24540594	24540607	TTGGAATTGGGGTTCACATT	TATCCCTTGAACTTCGTGCC	143
Ad01A6897	Aradu.A01	6897	p2	(TA)7	14	24541084	24541097	CAACCAGATGCACAGAAGAAA	TTGATCATGTTTTGGTCGGA	268
Ad01A6900	Aradu.A01	6900	p2	(AG)6	12	24553588	24553599	CCTTGGTAAATCAACCTCTCTTG	TCCATTGGAAAATGGTTTGAA	265
Ad01A6901	Aradu.A01	6901	p2	(TA)8	16	24563022	24563037	AGGATGCATATGGTCAAGTGG	GAGTTGGGAACATTGGTTGG	280
Ad01A6904	Aradu.A01	6904	p2	(GA)6	12	24615787	24615798	CATCCTTCCCCTCATCTTGA	TCTCTCGATCTCCACCCTTG	194
Ad01A6906	Aradu.A01	6906	p3	(TTG)7	21	24622343	24622363	GGAAATTGTGGGTATGGTGG	CCTCTTCAGAGAGCAAGGGA	128
Ad01A6907	Aradu.A01	6907	p2	(TA)10	20	24637388	24637407	ACTTCATTCTAGTACTTTTGGATCATT	GGTGTAGCCTTAGGTTTCATTTTT	263
Ad01A6908	Aradu.A01	6908	p2	(TA)8	16	24660836	24660851	GGCGTCTAGAGAGAGAGCGA	GCCTTAGGTCCCTCATTTTT	244
Ad01A6909	Aradu.A01	6909	p2	(AT)6	12	24675732	24675743	AGGACCGTGCACCATTTAAG	CACATCATCCATTCACAACCA	190
Ad01A6911	Aradu.A01	6911	c	(AC)6(AT)6	24	24704939	24704962	GGAACCTTCGATAACCATACGA	GCCTTACAATGAATGTTTGTCAC	256
Ad01A6913	Aradu.A01	6913	p2	(AT)9	18	24730725	24730742	GCCTAAGCTTTACCTCACGTC	CAGCTTAAACTCCGTGAGCC	275
Ad01A6914	Aradu.A01	6914	p2	(AC)9	18	24738193	24738210	TTTCAAGGTTGTAATGGGGC	GGGACAAAATTACCCCAATG	263
Ad01A6916	Aradu.A01	6916	p2	(GT)13	26	24746236	24746261	CGCTTCCCTGGGATACTTAG	GAGTCCTTTGACTTGCGAGG	199
Ad01A6917	Aradu.A01	6917	p2	(TA)6	12	24748518	24748529	CTGCGGGCTATGGATAGTGT	TCATGCCATAAAGCAAAGGA	252
Ad01A6918	Aradu.A01	6918	p3	(AGA)6	18	24761130	24761147	ATGGTCTTGATGGCTTTTGG	ACAACCCCCTCTTCTTCACC	272
Ad01A6920	Aradu.A01	6920	p4	(GTTA)5	20	24793417	24793436	TCAAGATCTTCTTTTGACTTTCCA	TTGAAGGAGAGAAAAGTGGGA	267
Ad01A6921	Aradu.A01	6921	p2	(TA)9	18	24811034	24811051	ATAAGTGTGTGGAGGTCGCC	TTCAATCAAACCAACTGCTCA	212
Ad01A6925	Aradu.A01	6925	p3	(TCT)6	18	24870790	24870807	AAACCCCTCTCCCTCCACTA	CTAGAGGTTTCTCCGGCCTT	246
Ad01A6927	Aradu.A01	6927	p3	(AAC)5	15	24876420	24876434	TGCCTATGGCAAAAAGATGA	AACATCCAGTCAGGAGGGTG	248
Ad01A6928	Aradu.A01	6928	p3	(GAA)5	15	24888441	24888455	ACATAGAATGTTGCTCGCCC	CAAAATCTCTTTCCCACCCA	125
Ad01A6930	Aradu.A01	6930	p2	(TC)7	14	24900942	24900955	CGAAAATCACTAACCATTTTTCAA	GTTCTTGTCCTTGCTCCTGC	260

ID	Chromosome	SSR nr.	SSR type	SSR	Size	Start	End	FORWARD PRIMER (5'-3')	REVERSE PRIMER (5'-3')	Product (bp)
Ad01A6932	Aradu.A01	6932	p3	(TTC)6	18	24931046	24931063	TACACCTCTCCCCCTCTCCT	ACTCGGAGGTGGAAGCTTTT	211
Ad01A6934	Aradu.A01	6934	c	(T)10ctttttctttc	48	24936451	24936498	GCCCAAAGCACTCTGTCTTC	TGAAGCAAGAAAAAGCAGTGAA	232
Ad01A6935	Aradu.A01	6935	p3	(AGA)7	21	24947318	24947338	AGGTTTCTCCGGTCTTTGGT	AAACCCACACCTCTCTCCCT	161
Ad01A6939	Aradu.A01	6939	c	(CT)8caccacca	101	24996008	24996108	CCCCCTTCTTCCTTCTTCCT	CCCAAATAAATGTCCAACACC	209
Ad01A6944	Aradu.A01	6944	p2	(CT)7	14	25023145	25023158	CATGGAAAAGTTCATAAGCCAA	GGAAATCCAACAATTCGCTC	244
Ad01A6945	Aradu.A01	6945	p3	(AGA)7	21	25031616	25031636	TCTTGAGGTTTGTCTGGCCT	AAACCCACACCTCTCTCCCT	167
Ad01A6946	Aradu.A01	6946	p2	(AG)10	20	25043947	25043966	AAACCTGGGATCGAGAGTCA	TTTCTGGGCTGTTTCTGACC	244
Ad01A6947	Aradu.A01	6947	p3	(AAG)8	24	25049266	25049289	CTCGAAGGTGGAAGCTTTTG	TTTTTGGCCGAATACACCTC	213
Ad01A6948	Aradu.A01	6948	p2	(GA)6	12	25060769	25060780	GAGAGAGAAGAGAAGAGGGTTCG	AATTTTTGAAAATCACCCCC	249
Ad01A6951	Aradu.A01	6951	p3	(TTC)6	18	25074385	25074402	AAATCTTTCCCACCCAAACC	TCCTCGAGCAAAGGAAGAAA	234
Ad01A6953	Aradu.A01	6953	p3	(TGA)6	18	25121754	25121771	CGACCTTTCCCTTTTTAGCC	AGGACGAGTTTGAAGAGGCA	232
Ad01A6954	Aradu.A01	6954	p2	(AG)6	12	25185753	25185764	TTTTTGGGGAAGAGGTGTTG	CTTGGTGCAGGGATGAATTT	128
Ad01A6955	Aradu.A01	6955	p2	(TA)6	12	25197828	25197839	ACCAGTTTTACCGTTTGGGG	TGGTGGGGAAGCAATTTTAG	235
Ad01A6957	Aradu.A01	6957	c	(T)10gtatttctct	41	25204747	25204787	GATCGAACCGGACTAGCTCA	TATGACCCATTCATTTGCCA	188
Ad01A6958	Aradu.A01	6958	p3	(CAA)5	15	25221473	25221487	CTCGTATGTGCCAAGGGACT	AGGCTGCTTCCCATACCTTT	230
Ad01A6959	Aradu.A01	6959	p3	(CAA)5	15	25229293	25229307	AGTGTGTGAAGAGGGCAACC	GTGTAGCACAAACCCCATCC	246
Ad01A6960	Aradu.A01	6960	c	(A)10gagagaaa	32	25237742	25237773	TGTGGTTCATGAATGGTTGG	TCAGCTTTGCTAGAGTCCCC	248
Ad01A6964	Aradu.A01	6964	p4	(AAAT)5	20	25287402	25287421	TGTTTGCTGAATGAATGCTTG	TCCCCAATTAGAGGTGTCCA	243
Ad01A6965	Aradu.A01	6965	p2	(TC)7	14	25297124	25297137	GAAGGAGGTCTCCTATGGGC	GGATGTGATTGGTTGAGGGT	244
Ad01A6966	Aradu.A01	6966	p2	(TC)12	24	25307241	25307264	AATCATTCTCAATCGCGACC	ACACGCACACGTAGAACCAG	266
Ad01A6967	Aradu.A01	6967	c*	(CA)7(CACG)	97	25316553	25316649	TTGTTCCTTTTGCTACATTTTCAA	AACAGCATTTGTTTGCCACA	269
Ad01A6968	Aradu.A01	6968	p2	(AT)7	14	25322081	25322094	TCCACTCCATTACATTTGTTTTT	GCTGATGAGCATCTTCCTTTTT	252
Ad01A6972	Aradu.A01	6972	p3	(ATT)17	51	25326640	25326690	GGAAACACAGGTGACCCTTC	GCGCTGAAGAAATAAAAGGAAA	215
Ad01A6974	Aradu.A01	6974	p2	(AT)8	16	25354015	25354030	TGGCAACGACAACATCTAACA	CCGAAGGACAAGATAGCCAA	189
Ad01A6975	Aradu.A01	6975	c	(T)10gtatggact	64	25356477	25356540	CCCTTTCCCTCAAAACCTTC	TGTAAGGCTTTCAACTCAATCAA	121
Ad01A6977	Aradu.A01	6977	c	(GAA)5gacgct	150	25374039	25374188	CTATTGGTTAAACACTTGTTGGC	TTTTTACTCCCACAATCTCTCTTTTT	207
Ad01A6979	Aradu.A01	6979	c	(A)10gaaaaaaa	142	25375789	25375930	TTGGACACTAGAATTTGAGGTTGA	GAATTTTGCAATCTTTTGTTTCA	210
Ad01A6982	Aradu.A01	6982	p2	(AG)7	14	25380352	25380365	TGCAATAAAAGAAAATGGAAGGA	TTTGCACCCTGTTTTTCTCC	233
Ad01A6986	Aradu.A01	6986	p2	(AG)6	12	25411269	25411280	CGGAAAAGCACACCAAACTT	TTTTCGAAATCTCTTCCCCC	147
Ad01A6989	Aradu.A01	6989	p3	(GAA)5	15	25421079	25421093	CTCCCATGACTCAGAGGTGG	CCCATATGGCCGAAACATAC	223
Ad01A6990	Aradu.A01	6990	p2	(CT)7	14	25429714	25429727	CGACGTGGAATCGAAAAACT	CAGCTACCAAACCCTTTCCA	185
Ad01A6991	Aradu.A01	6991	p2	(TA)6	12	25432069	25432080	AGGAATGTCATTTGGAACATCA	TTTTTCACATGTGCCACTTCA	263
Ad01A6999	Aradu.A01	6999	p2	(TA)9	18	25462513	25462530	CTTACCAAACCCAGGAGCAG	TCCTTTGAACCCGACGTAAC	230
Ad01A7001	Aradu.A01	7001	p3	(GAT)7	21	25511646	25511666	CTGTTCAGGCCGATTACACC	GGTACCCCTTTTGGACGATT	274

ID	Chromosome	SSR nr.	SSR type	SSR	Size	Start	End	FORWARD PRIMER (5'-3')	REVERSE PRIMER (5'-3')	Product (bp)
Ad01A7004	Aradu.A01	7004	p2	(AG)7	14	25560562	25560575	CAATTGGGCAACATCAATCA	CCCAAATCCAAGTCCAGAAA	274
Ad01A7005	Aradu.A01	7005	p2	(AG)6	12	25563953	25563964	CAATTGGGCAACATCAATCA	CCCAAATCCAAGTCCAGAAA	272
Ad01A7008	Aradu.A01	7008	p3	(ATA)7	21	25586221	25586241	TTACTGAGTGCAAGCAACCG	TCCAATAACAAAAGAAAGTTGGC	269
Ad01A7009	Aradu.A01	7009	p2	(AT)6	12	25587108	25587119	CCCGCATCATTCCTACAGAT	AAAAGCAAAGAAAATTAAAGAAGCA	241
Ad01A7011	Aradu.A01	7011	p2	(AT)24	48	25601093	25601140	CCTGCTTAAAACTTAAAACCGC	TTGTAAGAGGGATAGGAAAGTACGA	246
Ad01A7013	Aradu.A01	7013	p3	(TTC)6	18	25613402	25613419	CATGACCGAAACTTCACCCT	CCTCGAGCAATGGAAGAAAG	167
Ad01A7014	Aradu.A01	7014	c	(AT)8gaaactaa	53	25618774	25618826	AACCGCATAACCTTTCATAAACA	TTTTTCCCTCGCCTTTATCTT	220
Ad01A7015	Aradu.A01	7015	p2	(TA)6	12	25622314	25622325	CCATGTTGGAGAAAGGGAGA	TTTTTCAAAACTTCCTAACCGC	170
Ad01A7016	Aradu.A01	7016	p5	(AAGAT)5	25	25633121	25633145	AACACGACCCAAATCCAAAG	TAATCCTTTGTCACCGGAGG	270
Ad01A7019	Aradu.A01	7019	p4	(ATTT)5	20	25662641	25662660	AGCCAGCCAAGCTTCAACT	TCGAGTTTGGACAACTGACG	185
Ad01A7020	Aradu.A01	7020	p2	(AT)6	12	25696958	25696969	AGGCACATGCTATTCACAACA	ACACCAAGAATGGAGATGCC	254
Ad01A7021	Aradu.A01	7021	p2	(AG)21	42	25707316	25707357	CCCAAGCTCGAGTTTCTCAC	TGGGTTTGATTTGGGTGATT	224
Ad01A7023	Aradu.A01	7023	p3	(ACA)6	18	25721715	25721732	AAAATCCACAATGGCTCTCG	TGGCGTTGTTTGATTCATGT	149
Ad01A7026	Aradu.A01	7026	p2	(GA)10	20	25725458	25725477	CGATCTACCAATTGACCATGAA	CGCCGTCTACTTTCTCAACC	258
Ad01A7027	Aradu.A01	7027	p3	(AGA)5	15	25725704	25725718	GGTTGAGAAAGTAGACGGCG	GCATTCATTTCCGCATTCTT	264
Ad01A7030	Aradu.A01	7030	c	(CAC)7caacac	81	25733968	25734048	ACGTTTTCACACTTGAGCCC	TGGTGGGTTTTGGTTCTGAT	279
Ad01A7031	Aradu.A01	7031	p2	(AT)6	12	25737107	25737118	TGGTCATAGGATTATTAAAAAGGACA	TGACAGTCGCAGGTCAAAGA	250
Ad01A7034	Aradu.A01	7034	p2	(AG)9	18	25766684	25766701	CATGGAAGGGAGAAGCAGAG	ACAGTGACAGTGATGGCTCG	201
Ad01A7036	Aradu.A01	7036	p2	(CT)6	12	25803646	25803657	ACAGCTCAACCAAAATTGCC	CACCATGCAAAAAGGAAAGAA	270
Ad01A7037	Aradu.A01	7037	p4	(AAAT)5	20	25806322	25806341	GGAATTTGTGGGAGCAAAAG	TGGGTGATTTTGTCCCCTT	182
Ad01A7040	Aradu.A01	7040	p2	(TA)7	14	25843080	25843093	CAGGATATGGATGACGGAGC	GGCAGCACCACTCTGATAGC	256
Ad01A7041	Aradu.A01	7041	p3	(GAA)5	15	25850617	25850631	GGCTCTATTTGGGCAGAACA	AGCCCTAGCCATCTAGCCTC	258
Ad01A7050	Aradu.A01	7050	p3	(TCT)5	15	25900820	25900834	CCCATAAACCCCACCTACCT	TTTCTCGCCCTCAAGAAAAA	230
Ad01A7055	Aradu.A01	7055	p2	(CT)6	12	25999576	25999587	CTTCTCACCTTCCAAAACGG	ACATAGTGAAGGCGAGCCAC	213
Ad01A7057	Aradu.A01	7057	p3	(AAG)5	15	26004490	26004504	TCACCCTCGAGCAAGAGAAG	ATTTCTTTCCCACCCAAACC	195
Ad01A7058	Aradu.A01	7058	p3	(AGA)5	15	26013267	26013281	TTAGAATGTTGCTCGCCCTC	CACGGTCATCCCTTCTGTTT	161
Ad01A7063	Aradu.A01	7063	p3	(AGA)5	15	26064311	26064325	TCTCCAGCCTTAAGTGCCAT	TTCCCCATCAACTCTTCACC	217
Ad01A7067	Aradu.A01	7067	p2	(GT)6	12	26104471	26104482	CCAAAAGCGGAAAACCATAA	ACCTCAACCAATGTCCCAAG	105
Ad01A7075	Aradu.A01	7075	p2	(TG)7	14	26169752	26169765	AGATGGAGGGTGGAAGTGTG	AAATCCCCATTTTGTGCGTA	273
Ad01A7076	Aradu.A01	7076	p2	(AT)10	20	26172486	26172505	CATGGTGGGCATAACCATAA	TTCAAGTTTTGATCTTTGATTCTAGC	279
Ad01A7078	Aradu.A01	7078	p2	(AT)7	14	26182959	26182972	TCAAAATTCCTCGGACAACA	TGCACCGAGAGTTACATGCT	182
Ad01A7080	Aradu.A01	7080	p2	(GA)7	14	26191531	26191544	TTAGCTTCGTCGCCAAGATT	TCAGGCAAGCTCTAGCAGTG	228
Ad01A7081	Aradu.A01	7081	p2	(TA)7	14	26201229	26201242	TTACTTGGGGCATTTTGTCC	TGAAAATCTTGGGAATTGGG	179
Ad01A7082	Aradu.A01	7082	p2	(GA)7	14	26204953	26204966	AGGACAGTGGGAGTGACCAG	CCTCCATTTCCAACCTTCAA	233

ID	Chromosome	SSR nr.	SSR type	SSR	Size	Start	End	FORWARD PRIMER (5'-3')	REVERSE PRIMER (5'-3')	Product (bp)
Ad01A7085	Aradu.A01	7085	p3	(ACA)8	24	26212415	26212438	CTAGAGGTTTCTCCGGCCTT	CAGGATCTCCACCTATCCCA	211
Ad01A7086	Aradu.A01	7086	p3	(TGA)5	15	26226181	26226195	CCTGAAAGAGCAATTCCGAG	AAACCGGCTGAGTAGCTTCA	200
Ad01A7087	Aradu.A01	7087	p3	(CAT)5	15	26239267	26239281	TTGGCAAAAGTAAACGGAGG	CGGAAGAGTTTGGATTCTGC	227
Ad01A7089	Aradu.A01	7089	p3	(TAC)5	15	26272499	26272513	AACAAAAAGTATTGCCACCACA	CCACTTCCCAAAACACAGGT	278
Ad01A7091	Aradu.A01	7091	c	(TTCTC)7ttatc	139	26304436	26304574	CGTGACCACCTGCCATATTT	CTCAGCACAATCAGCAAATCA	267
Ad01A7100	Aradu.A01	7100	p2	(AG)7	14	26339087	26339100	TGAAAACAAAAGAACATTAAACTTGG	ATGCATCCATTCAGGGAGAG	152
Ad01A7101	Aradu.A01	7101	p3	(TTC)6	18	26372427	26372444	CCCATAAACCCCACCTACCT	TAAAATATTGCTCGCCCTCG	239
Ad01A7104	Aradu.A01	7104	p2	(TA)8	16	26388890	26388905	GGTTTCCTCACACGCAAGTT	CTCAGAGGCATAGCCAGTCC	266
Ad01A7105	Aradu.A01	7105	p2	(AT)13	26	26392758	26392783	GCGAACTGACTTCTTTTGGG	CTCCTAACTCAGCCTGCGAA	213
Ad01A7107	Aradu.A01	7107	c	(GA)13t(AG)8	43	26397363	26397405	GAGGGAGCGGATAAAAGAGG	TCCTTTTCTTCTTCGCGGTA	227
Ad01A7108	Aradu.A01	7108	p2	(GA)7	14	26406629	26406642	TGAATGGAATTTGGTGGGTT	GCACGCCTAAATAGAGCAGG	266
Ad01A7110	Aradu.A01	7110	p2	(GA)8	16	26414084	26414099	AATTTTGGAGGTAGGTGGGG	TAACGCCAGAATAGGGCATC	276
Ad01A7112	Aradu.A01	7112	p2	(TG)7	14	26458609	26458622	TGCGATATGGATTTGTTGAGA	AACTAGGGACAAGGTGAGGATG	202
Ad01A7114	Aradu.A01	7114	p3	(CTT)5	15	26472618	26472632	TTTCTCCTTCCATGGTTTGG	CAGGGAGAAGGAGAGCTGAA	144
Ad01A7115	Aradu.A01	7115	p2	(GC)6	12	26500138	26500149	ACTAATTGCTCCCCCTTGCT	GAGGATTTACCAATCGACGC	177
Ad01A7117	Aradu.A01	7117	p2	(GA)6	12	26505150	26505161	ATTTCAATCCAGTTTTCGGC	GGTTCATAAGCCATTTGGGA	277
Ad01A7118	Aradu.A01	7118	p2	(AT)10	20	26534192	26534211	TGAAGAACAAAAGCTTGCGA	ATGCCACAAGCACATGGTTA	228
Ad01A7120	Aradu.A01	7120	p3	(TTC)5	15	26566023	26566037	CAAACCCGACCTACCTTCAA	TCCTCGAGCAAAAGAGGAAA	221
Ad01A7123	Aradu.A01	7123	p2	(CT)7	14	26579642	26579655	TATCGCTGGTAATCTTGCCC	AGGAAGGAGGCAGTTCCAGT	237
Ad01A7126	Aradu.A01	7126	p2	(AT)12	24	26584488	26584511	GGTTGATGGTTGAATGATGG	GGCTCATTTGGTTTGGTCTG	234
Ad01A7127	Aradu.A01	7127	c	(T)10cattttatttt	38	26585862	26585899	GTGATTGAGGCTGTGATTGC	GTTGGTAAGGCTTGGGAACA	232
Ad01A7130	Aradu.A01	7130	p5	(AAAAT)6	30	26615767	26615796	TGACCCCAGAGTCTTTCTCG	CCTAACCAATTTCTCTCTCCTCA	259
Ad01A7133	Aradu.A01	7133	p3	(AGA)5	15	26661273	26661287	TCCTCGAGCAAAAGAGGAAA	CCGCCATATTCTTCACCACT	266
Ad01A7134	Aradu.A01	7134	p2	(AG)7	14	26661535	26661548	TTGGAAGGGAAAGTGGTTTG	CAAGATCCCCACCTACCTCA	145
Ad01A7137	Aradu.A01	7137	p2	(TA)6	12	26686150	26686161	TGAAAGGCCTGCTTTTTCAC	TTTTTGGTGTTGATTGCTTGA	220
Ad01A7138	Aradu.A01	7138	p2	(TG)12	24	26696062	26696085	CTGCCTGAAGCTATTCCCTG	TCGCAACAAAGTAAATTGCAT	256
Ad01A7142	Aradu.A01	7142	p3	(TTG)5	15	26730832	26730846	AGGTATGGTTTCGGTTTCCC	TCATACCATCAAGGCATCCA	197
Ad01A7143	Aradu.A01	7143	p3	(CAA)5	15	26734692	26734706	AGTGTGTGAAAAGGGCAACC	GTGTAGCACAAACCCCATCC	247
Ad01A7145	Aradu.A01	7145	p4	(AATT)5	20	26768334	26768353	CATCCGATATCCAACCAACC	TTCGATTCTGGCTTTGCTTC	272
Ad01A7146	Aradu.A01	7146	p4	(AAAT)6	24	26770745	26770768	GTGGCAGTGACCGGTAGATT	AAAAATCATTTGTTTCCTTCGC	241
Ad01A7148	Aradu.A01	7148	p2	(CT)6	12	26793088	26793099	CTGAACGCCAGTAACAAGCA	TGGGGAAGAGTGGATGGATA	277
Ad01A7149	Aradu.A01	7149	p3	(TTC)7	21	26793253	26793273	TCTCCATTCATGGTCATCCA	CGTCCTCGAGCAAGAGAAGA	204
Ad01A7152	Aradu.A01	7152	p3	(AGT)5	15	26824484	26824498	CCGTCCAATTATGCATCTCA	TATGAGGTTTGGGCAAGGAG	217
Ad01A7153	Aradu.A01	7153	p3	(AAG)5	15	26845069	26845083	TCATTGGAATTCTTCCTCGC	GAGGATCTGGATGAGGTGGA	234

ID	Chromosome	SSR nr.	SSR type	SSR	Size	Start	End	FORWARD PRIMER (5'-3')	REVERSE PRIMER (5'-3')	Product (bp)
Ad01A7155	Aradu.A01	7155	p2	(AG)7	14	26905327	26905340	TGCGGTGACGATACTGTAGG	AGCCATTTCATTTCGGCTTA	250
Ad01A7158	Aradu.A01	7158	c	(T)14caaatagtt	40	26923898	26923937	TCAGACCCAGATCAGCATCA	TCGGTTTTTACACCGGAAAG	218
Ad01A7176	Aradu.A01	7176	p2	(CT)6	12	27088800	27088811	AGATTTCGCAAACAGCGAAT	CAAAGAACATTGAACCTGGGA	251
Ad01A7178	Aradu.A01	7178	p3	(CAA)7	21	27129799	27129819	TCCATAGCTGAGTCTGGCCT	GGGTTCGTGTTCTGGGTAGA	104
Ad01A7180	Aradu.A01	7180	p2	(CT)7	14	27137343	27137356	TGTCAGCAACTTGAGCCTTG	TGGATCTCCTTCATCCTTGC	218
Ad01A7185	Aradu.A01	7185	p2	(AC)6	12	27166916	27166927	TCAACTCAAACCCACACTAACC	CCGTCCCTCTTTCTCTTTCC	123
Ad01A7187	Aradu.A01	7187	c	(T)10c(T)10	21	27177111	27177131	GTTGGTTTGAATGGGGAATG	GCCTATCTCCAAATTCAGCG	174
Ad01A7189	Aradu.A01	7189	c	(GT)8ttgggttct	53	27192030	27192082	TTTGAAGAAGGCAAGGCAAT	TGCGTAGCAGACAACTTTGA	199
Ad01A7190	Aradu.A01	7190	p2	(TA)6	12	27197955	27197966	TTGCCCTTAGGCTAGTTTGTG	TACACCTGGTCAACGCACAT	260
Ad01A7200	Aradu.A01	7200	p3	(TAT)5	15	27245377	27245391	TTTGAGTGAAAAACAAAACTCCA	CACATGACTTCATACGTAATAATTTGA	212
Ad01A7201	Aradu.A01	7201	c*	(TAT)7(T)10*	30	27246187	27246216	GAATCATATATTCTTATCAGGTGCCA	TTTCCCTCCAAAATGAATGC	276
Ad01A7207	Aradu.A01	7207	p2	(AC)6	12	27265635	27265646	GCAAGGGTGAGGCTATGTGT	AGGGGTAGCTAGGCGTGATT	121
Ad01A7210	Aradu.A01	7210	p2	(CT)6	12	27291081	27291092	TCTTCGATGAATGGCATGTG	GCAAGCAAACCTCTCTCACC	114
Ad01A7211	Aradu.A01	7211	p2	(TA)6	12	27293401	27293412	TTGGATTAATTGAGGGCTGG	AGTTACATTTGCCCCATCAA	272
Ad01A7212	Aradu.A01	7212	c	(AT)9gggtctgt	118	27294573	27294690	GCACGAAAGCGTTAACACAC	TATGGTAAGTTTGGGGGTGG	276
Ad01A7219	Aradu.A01	7219	c	(TA)6tcataagg	72	27316119	27316190	TTTTGCCAAACGTGTTAGAGA	ATGCCAGCATCATGGAATCT	250
Ad01A7220	Aradu.A01	7220	p3	(TTA)7	21	27321054	27321074	GCTGTTTACCCCATGCTCTT	AAAAGATAAAAATGATGAAAAACGA	275
Ad01A7223	Aradu.A01	7223	p2	(AT)15	30	27394485	27394514	GAGTTCGCACACCATTCTCA	ACATTGCCCAAGCCAATAAT	113
Ad01A7224	Aradu.A01	7224	p3	(AAG)6	18	27406859	27406876	AGTGGTTTTGATGGGCTTTG	CCGAATACACCTATCCCCCT	249
Ad01A7226	Aradu.A01	7226	p2	(AG)7	14	27420591	27420604	TGACCTGTACGAACCAGCAG	CATTGAACCTGTGATGCAGAA	260
Ad01A7227	Aradu.A01	7227	p2	(CT)6	12	27428323	27428334	ATCCAAGGGGAAGTGTTGTG	AATAATGGCGCCCAAACAT	218
Ad01A7235	Aradu.A01	7235	p2	(CT)6	12	27482634	27482645	ACAGCAGTGGGAGCAGAGAT	AAGCAACGTTCATGTTTCCC	278
Ad01A7236	Aradu.A01	7236	p3	(TCA)5	15	27485416	27485430	ATGCTTGCTTGCTAGCTTCC	GGTGCAGTGAAACAGCAAAA	236
Ad01A7238	Aradu.A01	7238	p3	(GAA)7	21	27540214	27540234	AGGGCATCATCATACCTTGG	GGTTTTGGTTTTGGTTAACTGG	260
Ad01A7239	Aradu.A01	7239	p2	(TC)10	20	27540456	27540475	CCAGTTAACCAAAACCAAAACC	TGAGTGAGAGAGAGGGGGAA	188
Ad01A7240	Aradu.A01	7240	c	(TC)6tgct(TC)	28	27540615	27540642	TCTTTATCTCCTCTCCCCTTCA	GACGAGGTGGAGAAAGAGGG	268
Ad01A7241	Aradu.A01	7241	p2	(CT)9	18	27540806	27540823	TCTTTATCTCCTCTCCCCTTCA	GACGAGGTGGAGAAAGAGGG	268
Ad01A7243	Aradu.A01	7243	c	(TC)7ccctttcct	39	27541128	27541166	TCTCTCTGTCTCTCTCTCCGC	AAAGAGAGGGGAGGAAAAGG	263
Ad01A7244	Aradu.A01	7244	p2	(GA)19	38	27541582	27541619	AGGGAGAGGAGGGGAAAGA	CCTCCCCTTTCTTGCTCTCTA	121
Ad01A7245	Aradu.A01	7245	c	(A)11gagtaattt	35	27544918	27544952	TCCAATTGCAGGTTTTGGTT	ACTGCTGCTGCTGATGAAAA	261
Ad01A7246	Aradu.A01	7246	c	(AG)10at(AG)	74	27550725	27550798	AATTTCATGAACCAGTCGGC	GAGGCCTGACTTTTTCCCTC	189
Ad01A7248	Aradu.A01	7248	p2	(TC)21	42	27561100	27561141	CGGCGAGGTTGTTTTCATAA	TCAGAAATGGATACAAATGCAAA	212
Ad01A7249	Aradu.A01	7249	p2	(TC)9	18	27581122	27581139	TTTAATGCATTTTTGCACGC	CGTGTATTTTGGCTTGGGTT	207
Ad01A7250	Aradu.A01	7250	p2	(CG)6	12	27585258	27585269	TCCATACCTTGACCCGAGAG	TGGTGTTTGGAAGTGTTGGA	252

ID	Chromosome	SSR nr.	SSR type	SSR	Size	Start	End	FORWARD PRIMER (5'-3')	REVERSE PRIMER (5'-3')	Product (bp)
Ad01A7252	Aradu.A01	7252	p3	(AGA)7	21	27592550	27592570	TATCTCCCATGACTCGGAGG	TCTTCTCCCACTTAGCCGAA	222
Ad01A7255	Aradu.A01	7255	p2	(TC)6	12	27606199	27606210	ATTTTTGCACGCCTAAATGG	AATTTTGGAGGTAGGTGGGG	153
Ad01A7256	Aradu.A01	7256	p2	(AT)6	12	27617957	27617968	GTTGGATTTTCCTTGGGGAT	TTCGTGGTTTTCCAATCCAT	263
Ad01A7257	Aradu.A01	7257	p3	(ATT)6	18	27621910	27621927	CGTTGCCCAAAGTTCTTAGC	TGGAGGATCCTAATTGCAGG	192
Ad01A7258	Aradu.A01	7258	p4	(ATTT)6	24	27623037	27623060	ACATAAGGGCTGGTCCAGAA	TGGGATCAGACCAGAAAAGC	277
Ad01A7259	Aradu.A01	7259	p5	(TTTTC)5	25	27625913	27625937	TTGTGTTCCCTGATGTCAACT	TCCAAACTCTTTTGGTGATGG	272
Ad01A7260	Aradu.A01	7260	p3	(GGA)5	15	27628049	27628063	GGTGCCAGGAATGAAGAAAG	CATGGTCACGGAGAGAGACA	133
Ad01A7266	Aradu.A01	7266	p3	(TTC)6	18	27661953	27661970	TCTTCTCCCACTTAGCCGAA	TGCCATTGATGGTAGTGGAA	159
Ad01A7273	Aradu.A01	7273	p4	(TTAT)5	20	27726618	27726637	ACAACAAGCCACCATGACAA	GCGTCATTGATGCTTACGTG	236
Ad01A7280	Aradu.A01	7280	c	(T)12cctttgtga[illegible]	109	27753226	27753334	CACTTCACCTTGATTGCTTGAG	TCCAACGGTGAACAACTGAA	242
Ad01A7281	Aradu.A01	7281	p2	(CT)7	14	27756605	27756618	CTGCCAGTAAATCCGATGGT	GAAGGAGCTGGTTTTGGTGA	223
Ad01A7283	Aradu.A01	7283	p3	(TAT)5	15	27766678	27766692	TGAGATTCGTGCCAAATGTT	TCAACAACGTGTGTGGTGAT	277
Ad01A7285	Aradu.A01	7285	p2	(CT)22	44	27767473	27767516	TTCTGGAAGCTTCTGGTAGTTTA	TCATTGAAATCAGTGTCGCA	265
Ad01A7293	Aradu.A01	7293	p3	(ATC)5	15	27804967	27804981	CCTCTTCTTCCACCGACTTG	GTCAAACCAAAGCCACCAGT	184
Ad01A7297	Aradu.A01	7297	p2	(TA)7	14	27816789	27816802	CTATCCGGCCTTCCTTTACC	CCACACAAAGACTTACGCGA	249
Ad01A7303	Aradu.A01	7303	p2	(GA)6	12	27860718	27860729	AAGGAAATTGGGATTGGGAC	CTCTCTCACCATCAGCACCA	181
Ad01A7306	Aradu.A01	7306	p2	(TA)6	12	27905314	27905325	GTGTCCCTTTTGGGTTTCCT	AAAAATCGCAACTCGAAAGC	209
Ad01A7307	Aradu.A01	7307	c	(A)11gtgttatag	41	27906159	27906199	TTGGTGTCCTAGGTTTTGTGA	TTGGATAGTCAAATTAAAGCCCA	273
Ad01A7310	Aradu.A01	7310	p2	(TA)10	20	27907514	27907533	TGTCCTTACACTTGTTTTATTCGC	CTCCTGATGCCAAAGTCTCA	277
Ad01A7311	Aradu.A01	7311	p3	(GAA)5	15	27908839	27908853	TGTTTCTGCTCCTACAAAAGG	TTGGCTGTAAAAACAGAAGTAGAGG	100
Ad01A7312	Aradu.A01	7312	p3	(ATT)7	21	27909052	27909072	TTGGCCTATATACCTTAGAAATCAAAA	TTCCCTTCATGGAGCTGATT	223
Ad01A7314	Aradu.A01	7314	c	(GAA)6tctacg[illegible]	110	27910714	27910823	GCAAAATGTTGGTGTTATTGG	TTTCAAATCACGTGCTTCCTT	228
Ad01A7317	Aradu.A01	7317	p2	(TA)6	12	27919765	27919776	TTGCATGTGCAGTAGCTGTT	CCATTTTATGCTTTTTAATTGCC	253
Ad01A7322	Aradu.A01	7322	p3	(TGA)6	18	27927010	27927027	GTCAGTGGGAGGGTCTTCAG	AACAACCAACACCAACAGCA	280
Ad01A7323	Aradu.A01	7323	p2	(AG)6	12	27927241	27927252	AAAACCCAAAAAGGGAGTGG	TGGAAAGAAAAATGGGAGCA	199
Ad01A7325	Aradu.A01	7325	p2	(AT)8	16	27934930	27934945	AGCGGAAGAAGAAGGGATTT	GAATGCAGCAGGTGAAGAAA	279
Ad01A7330	Aradu.A01	7330	p3	(ATC)24	72	28022889	28022960	TGATCAAATGTCTCTTTGGTAAACA	TTGGACACAACATAGCTTTGAA	164
Ad01A7331	Aradu.A01	7331	c	(AAT)22gaagt	118	28023529	28023646	TTTAATTTCGAACAAATGTCTTTCA	AACAATTTGATAAAAGTATCGTCATTT	266
Ad01A7333	Aradu.A01	7333	p3	(AAT)5	15	28027863	28027877	TGACAATTGAGCTTTCACCG	CCTCCACCATAAGTACCTGC	209
Ad01A7336	Aradu.A01	7336	p2	(TC)6	12	28060818	28060829	CCCAAATTTCAAACACCTCC	CATCTTGTGCAGTGGGATTG	263
Ad01A7337	Aradu.A01	7337	p3	(TAT)7	21	28061074	28061094	CTCAACCTCCCTCCTTTTCC	CTCGAGTTTGGGTTGTTGGT	275
Ad01A7338	Aradu.A01	7338	p4	(GTGC)5	20	28067843	28067862	GTACGCCTAAACTTCGCAGG	AATGCTCGAGCCATAAGTCAA	265
Ad01A7340	Aradu.A01	7340	c	(TTTTC)6ttca[illegible]	132	28078663	28078794	AGGCTTCCCTGTTGAGCAT	ATTCACGGCAGTTAGCTTCG	262
Ad01A7343	Aradu.A01	7343	p2	(AT)9	18	28099183	28099200	TTTTTCTTGTCCTGACTTTTGGA	TGTTTGTAGCATAAACGAATTAAAATG	272

ID	Chromosome	SSR nr.	SSR type	SSR	Size	Start	End	FORWARD PRIMER (5'-3')	REVERSE PRIMER (5'-3')	Product (bp)
Ad01A7344	Aradu.A01	7344	p3	(TAT)6	18	28105864	28105881	CAATGTAAGCATGCGCAAGTA	TGGTCCATCAGAGAGAAACAAA	147
Ad01A7345	Aradu.A01	7345	p2	(AT)7	14	28107417	28107430	ACCCTCACACGTACCCCATA	GGCGACAAACATTTTTGAACA	265
Ad01A7346	Aradu.A01	7346	p2	(CT)6	12	28123902	28123913	CTTGAGCCTTGAGCTTCACC	CCCTCCATAATGCTTCAACC	267
Ad01A7347	Aradu.A01	7347	p2	(CT)6	12	28133316	28133327	CTTCAAAGCCATTTTTCGGA	AAGCCATTAACCAAAGAAGCA	174
Ad01A7348	Aradu.A01	7348	c	(CGC)5cctactt	53	28135388	28135440	TAGGGTTTTGCATTTGGAGG	TCACAGAAGAAGAAGCAGCG	269
Ad01A7350	Aradu.A01	7350	p2	(TG)6	12	28146393	28146404	CCACAAAGGTTTCCAAAGGA	ACGTCATGCAAGATTCTCCC	122
Ad01A7351	Aradu.A01	7351	p2	(AT)9	18	28146552	28146569	AGTGCATGCATGACCAATGT	GAAGCAACAATTACAACCCTCC	153
Ad01A7353	Aradu.A01	7353	p2	(AT)7	14	28153068	28153081	CACCAGGTTGTAATGATTTTCG	CTGGTTGAACCGGTTGACTT	272
Ad01A7354	Aradu.A01	7354	p2	(AG)6	12	28155014	28155025	GGGGGAATACATCCATTCAA	AACCAAATTGGCAACATCAA	233
Ad01A7355	Aradu.A01	7355	p3	(AAG)5	15	28157171	28157185	CCTCTCCCATTGGACTTGAC	ATGGCCTAGGGAGAGGTGTT	216
Ad01A7356	Aradu.A01	7356	p3	(CAC)7	21	28160157	28160177	ACCAACCAACACCAACACC	CGCCTCTACTTCTTGCTTCG	252
Ad01A7360	Aradu.A01	7360	c	(AC)6(AT)6	24	28192709	28192732	CCAAACAGAAAAGGACGGAA	ACTGGTTGGCTCAATGGTGT	272
Ad01A7367	Aradu.A01	7367	p3	(CAA)5	15	28238819	28238833	TCACTGGAAATGCTCTGCAC	GTTGCCCATTTGCATTTTG	278
Ad01A7368	Aradu.A01	7368	c	(TA)10tg(TA)(	34	28247976	28248009	CGTGAGTGTTACGACTTCGC	TATCCCTCAGGCTCACAAGG	241
Ad01A7370	Aradu.A01	7370	p6	(ATTTTA)7	42	28254795	28254836	AAGAGAGTGTCATGTGACATATTTTAG	TTCCCCATCTCTCTTCCTTTC	251
Ad01A7373	Aradu.A01	7373	c	(TAA)6aacaaa	64	28255986	28256049	ATCTTGCAGGAGAGGGGAGT	TTTTCCCTTTCGGTTTCCTT	277
Ad01A7374	Aradu.A01	7374	p3	(AAT)24	72	28256250	28256321	AAGGAAACCGAAAGGGAAAA	AACTAGACGCCTGTGGCAAT	261
Ad01A7375	Aradu.A01	7375	p3	(TCA)6	18	28275675	28275692	TCCTATGCCATCGTAGGGAG	ACATTCCGATCCAACACACA	165
Ad01A7376	Aradu.A01	7376	p3	(TAA)20	60	28276667	28276726	TGAGTCAGGAATAGTGAGCTTCC	AGGACAACTTGGTATCTCTTCG	177
Ad01A7380	Aradu.A01	7380	p3	(AAC)5	15	28281109	28281123	CATTGTGGTGAATGCAGACC	ACATGACAGAGCACAATGGC	215
Ad01A7389	Aradu.A01	7389	p3	(AAT)17	51	28339121	28339171	TGTGTCATATCAATCCACACAAG	TGAATCTCATTCACATTCGGA	279
Ad01A7392	Aradu.A01	7392	p3	(AAG)6	18	28385856	28385873	TAAGGCTTCATTCTCGTCGC	AGGTTGGCCACCATTTCTAA	249
Ad01A7393	Aradu.A01	7393	p2	(GA)9	18	28387069	28387086	GAATGCTCTTTGTGAAGCCA	TCACAAAGTCACAGGCATCC	227
Ad01A7394	Aradu.A01	7394	p2	(TA)6	12	28393981	28393992	TCCGATTTGTGTGAAAACGA	CAGGAGCTGAGGGAAGAAGA	264
Ad01A7396	Aradu.A01	7396	p2	(TA)8	16	28400661	28400676	TTTTGCATTTATGCATGTTGG	AAGAGGGCCTCGAATAGACA	250
Ad01A7402	Aradu.A01	7402	p2	(AG)6	12	28509852	28509863	TTGAAGGGTGAGGTAGGTGG	TGCAGGGATGACTTTTCCTT	146
Ad01A7404	Aradu.A01	7404	p3	(GAA)5	15	28519037	28519051	CCAGCAAGCAAGATCAATCA	TTGGGTTACCTCCCAACAAG	192
Ad01A7407	Aradu.A01	7407	p3	(TTC)6	18	28551038	28551055	TTTTCTTTTGGGCTAGTGCC	TTGCTTAATGTTCCAAGCCC	231
Ad01A7408	Aradu.A01	7408	p3	(GTT)16	48	28562639	28562686	TCCTAACCAGCACATCTCCA	CCAAATCATCCCATCTTGCT	206
Ad01A7409	Aradu.A01	7409	p3	(ATC)6	18	28564190	28564207	CAAACCCTAACCTGGAGCAA	TGAGTCGAGGCAATGAAGTG	243
Ad01A7413	Aradu.A01	7413	p2	(CT)7	14	28651357	28651370	AAGGCACATTGAACCTGGAA	TTTTTGACCCAGTTTTTGGC	219
Ad01A7414	Aradu.A01	7414	p2	(CT)8	16	28657724	28657739	AAGGCACATTGAACCTGGAA	ATCCATTCATGGGGACGATA	161
Ad01A7418	Aradu.A01	7418	p3	(TCT)6	18	28666608	28666625	TTTCACGCGCTTCCTAATTT	TCAATTCAAACCATCAGCGA	178
Ad01A7419	Aradu.A01	7419	p3	(TCA)5	15	28666896	28666910	CCTCCTCATCTTCTGCTGCT	CGAATGTAAGTCCATCATCTTCC	211

ID	Chromosome	SSR nr.	SSR type	SSR	Size	Start	End	FORWARD PRIMER (5'-3')	REVERSE PRIMER (5'-3')	Product (bp)
Ad01A7420	Aradu.A01	7420	p3	(CTT)5	15	28667849	28667863	TTCTTCTCCCTCCTCCTCATC	CGAATGTAAGTTCATCCTCTTCC	215
Ad01A7421	Aradu.A01	7421	p3	(CTT)6	18	28668232	28668249	TCTTCCTCCTTCTCATCTCCTG	TTCTCCTCCTCCTCCTCCTC	262
Ad01A7422	Aradu.A01	7422	c	(AGA)5aa(GA	35	28668429	28668463	GAAAAAGGAGGAGAAACGCA	TCAAATTGCCTAAAAGGGGA	240
Ad01A7423	Aradu.A01	7423	c	(AAT)5atgaaa	96	28692639	28692734	AAGACGGAAACAATATAAAAGCAA	TCAACAAGACAACAATTAGTCATCAA	254
Ad01A7426	Aradu.A01	7426	c	(TA)6ataaatata	40	28716860	28716899	TAATTTCCGACAGGGCTTTT	TTGAGGTGTCAATTTCTTATCAATTC	267
Ad01A7431	Aradu.A01	7431	p3	(TTA)14	42	28726855	28726896	TTTTTACGATTACCCGTGATAAGTT	AAAATTGGGTTTTCCCCAAC	279
Ad01A7432	Aradu.A01	7432	p2	(AG)10	20	28736713	28736732	TTTGGGCTATTTTTGACCCA	TGAATCTAGCATCAAAAGTTCACA	249
Ad01A7435	Aradu.A01	7435	p2	(GA)19	38	28749841	28749878	CGGGATCCTTCAGCAGTAAC	TACACACCTTGCTGCTCCAC	273
Ad01A7437	Aradu.A01	7437	p3	(TCT)7	21	28750777	28750797	ATGGCGTGCTCCATATTCTC	GTAGCGATGCCAAGAGAAGG	182
Ad01A7438	Aradu.A01	7438	p2	(AG)7	14	28751211	28751224	TGAAGAGGATAGTGGCTGGG	CTGCAGCACCTAAATCACCA	130
Ad01A7442	Aradu.A01	7442	p3	(TCT)5	15	28772998	28773012	GCCGAATACACCTTTCTCCA	GCTTTTGTCTTCCCTTTCCC	191
Ad01A7443	Aradu.A01	7443	p3	(TTA)5	15	28776771	28776785	AAGCAGAGATCCAACAGGGA	CAACTCCGCACAACTAACCA	270
Ad01A7444	Aradu.A01	7444	p2	(AC)6	12	28785442	28785453	CGGGAGTTGAGACTAGCACC	TGTAAATCAATGGCAGCGAG	221
Ad01A7446	Aradu.A01	7446	p3	(AAG)5	15	28791649	28791663	GAGGAAGAAGAACGTGCAGC	CATAAAACAGCGCCAAAACA	262
Ad01A7448	Aradu.A01	7448	c	(TA)7c(AT)7	29	28796258	28796286	AGCCCCTACAGCAAAGTCAA	AATAAAATGAGATCGACAAAGCA	177
Ad01A7449	Aradu.A01	7449	p3	(ATT)6	18	28797001	28797018	TCATCAGCTTGACAACAGCA	CACGTGAGACTGATAGTTAACCAAA	242
Ad01A7450	Aradu.A01	7450	p3	(CAA)5	15	28797554	28797568	TATACTTCGACAAAGGCGGC	GGAGGAAACTTTTGGTTTGG	170
Ad01A7452	Aradu.A01	7452	c	(T)12gaattttgtt	82	28800183	28800264	ACAAATAGGCAATGGAGCGA	TGAATTTACAATGATTTTGGATGTTT	277
Ad01A7456	Aradu.A01	7456	c	(T)11gtttttatatt	72	28803156	28803227	TTGAGTAAACCATCAGTTCAAACA	ACAACTCATCCAATTTGCCA	242
Ad01A7457	Aradu.A01	7457	p4	(AAAT)6	24	28824066	28824089	TCACGTTTGAGAGCACCAAG	TTCGAAAACAAAGGGGAAAA	224
Ad01A7459	Aradu.A01	7459	p2	(CT)9	18	28857539	28857556	CTAATGCACTTGTGATGCGG	TCAAGCACAAGCCACCATAG	260
Ad01A7460	Aradu.A01	7460	p3	(GTT)5	15	28880821	28880835	AGTGGTGAGTTGGGTTGGAG	AAGCCACCCAAAACTTTCCT	254
Ad01A7461	Aradu.A01	7461	p2	(TA)7	14	28882235	28882248	GAGGACATAAAAAGACATACGAAAGA	TGACGAGGCATTATGACACC	260
Ad01A7462	Aradu.A01	7462	c	(A)10gagttttttt	115	28885498	28885612	ATATGCACATCGACGCGTAA	GGCACCAAAATAATGCAAGAA	234
Ad01A7465	Aradu.A01	7465	p2	(TA)12	24	28894995	28895018	AAATTGGGCCTTCTAAACAGTC	AAGAACAATTTATTTGTCAGATTGAA	263
Ad01A7469	Aradu.A01	7469	p2	(AC)17	34	28925160	28925193	CGTCGACCTTCTGAATTTCC	AAGCTCCCAATCATCTTCCA	259
Ad01A7471	Aradu.A01	7471	p2	(AT)8	16	28937401	28937416	ACGGCTAGGGTTTCCACTTT	GGCAAAAATCTCCAGCAGAG	171
Ad01A7472	Aradu.A01	7472	p4	(TAAC)5	20	28941927	28941946	AACGCTTTGAAGGCAATGTT	CCACCACAACCGTTTACCAT	238
Ad01A7475	Aradu.A01	7475	c	(A)10gcaatgctt	67	28958376	28958442	GGAAATTTTAATTTTGCAATGTTTCT	CCTCCGAGTCACAATCCCTA	262
Ad01A7479	Aradu.A01	7479	p3	(TTG)6	18	28968126	28968143	CGTTTGATTCATGTAGCCGA	TAACCAAACGAAGCCCAAAG	280
Ad01A7480	Aradu.A01	7480	p3	(GAG)5	15	28968914	28968928	AGAGAGGAAGAGGGAGCCAC	TGTCCTTTCTTGTCGCCTCT	179
Ad01A7483	Aradu.A01	7483	p2	(TA)7	14	28971163	28971176	CCAATAAGTGTCGGCTACCA	CATGTCCCATACATGGTGGA	278
Ad01A7486	Aradu.A01	7486	p3	(TGG)5	15	28978776	28978790	GTACCCACGGGGTTTGTATG	GGAATCGGTCGAAGTCCTCT	227
Ad01A7487	Aradu.A01	7487	p3	(TCT)5	15	28979500	28979514	AGGAGAACCTCAAGTCCGCT	TGGTGGAAGGCCAGTATTTT	249

ID	Chromosome	SSR nr.	SSR type	SSR	Size	Start	End	FORWARD PRIMER (5'-3')	REVERSE PRIMER (5'-3')	Product (bp)
Ad01A7488	Aradu.A01	7488	p3	(AAT)17	51	28984751	28984801	TTGTTAGTTAAATATTAATGCTCGTGG	AAGTAACAACATGCTTCTTCAGACA	262
Ad01A7492	Aradu.A01	7492	p3	(GAT)5	15	28989506	28989520	TGTGCTGCTAGCCTGCTACT	GAACCACCGTCTTCTGCATC	259
Ad01A7494	Aradu.A01	7494	p2	(CT)10	20	29006838	29006857	CATTGAACATGGAATTGAGAAGA	AATTTGCATCCAGCGATTTC	221
Ad01A7495	Aradu.A01	7495	p2	(CT)9	18	29013108	29013125	CATTGAACATGGAATTGAGAAGA	TTTCTGGGCTGTTTTTGACC	203
Ad01A7497	Aradu.A01	7497	p3	(TTC)8	24	29037212	29037235	ATCCAAGCAATTTTGGCATC	AGGAAGAACGCGAAGAACAA	181
Ad01A7498	Aradu.A01	7498	c	(AGA)6gaaa((	191	29037972	29038162	TCGTTGGTGATGACGATAACA	GGTCCAACACCAACAAAAATC	270
Ad01A7499	Aradu.A01	7499	p3	(TTA)6	18	29038336	29038353	TTTGGATGTGAAACATAGCCC	TCCTGATTGCCTGAAAAAGAA	239
Ad01A7500	Aradu.A01	7500	p3	(TTG)8	24	29039216	29039239	CGATGTTGGTGTTCCACTTG	TATCCTGTATGCCGACACCA	143
Ad01A7501	Aradu.A01	7501	p3	(GAA)5	15	29044600	29044614	CACAATGAAAGAAGCAGCGA	GGTTTGATTGGTAGGCAAGG	240
Ad01A7502	Aradu.A01	7502	p2	(TC)12	24	29057439	29057462	TCTACCTTGAAGGCACAGCA	CAACATCAGAGTAGCAGCGG	280
Ad01A7505	Aradu.A01	7505	c	(A)10ggtaaaga	73	29062863	29062935	TTTCAATTGGAAAAAGGCAT	TCACTGAGTGCTCACCCAAC	178
Ad01A7507	Aradu.A01	7507	c	(T)12(A)11gaa	142	29067460	29067601	TTTCCCTCAACCTTCTCTGC	CCCGATTCATGTACGACAAA	280
Ad01A7513	Aradu.A01	7513	p3	(CAT)6	18	29094160	29094177	AAGCCTGAGCCTAAGGGAAG	TGCGTATGATCAGAATGGGA	182
Ad01A7514	Aradu.A01	7514	p2	(TG)7	14	29094658	29094671	CGAATTTGGTCTTCAGAGGC	GGAGCTCCCGTGAAATACAA	246
Ad01A7516	Aradu.A01	7516	p2	(TA)6	12	29095960	29095971	TGGTTATGCAAGTAAAAAGTGTGAA	GGAGACCACACTCCTCCAAC	273
Ad01A7517	Aradu.A01	7517	p2	(TC)6	12	29103260	29103271	CATTAGCGGGAATTTTTCCA	TCCTCACCATCGATCAATCA	167
Ad01A7518	Aradu.A01	7518	c	(T)10aattgagtc	68	29120575	29120642	TGCGAAATTTTCAATTAGGTCC	TTAGTTGGGTCCATGCATCA	209
Ad01A7527	Aradu.A01	7527	p4	(AAAT)5	20	29156287	29156306	TGGTAGAGCATAGACTTTGTTGTTG	TGTATCGATCAGAGATTGTCGAA	263
Ad01A7531	Aradu.A01	7531	c	(T)10cttttatttga	104	29159377	29159480	TCAGATTCCTGCATCAATTTTT	TTTTAATTGGGTCCCTGCAC	169
Ad01A7537	Aradu.A01	7537	p2	(GA)7	14	29184037	29184050	ATTTAGACTCCCGGGTCGTT	TCCACATGATTGCCATGTTT	273
Ad01A7538	Aradu.A01	7538	p4	(CTAA)5	20	29188252	29188271	AAGCATTCCCAAAGGCAATA	TCATCATCCATGAAACTCATCT	280
Ad01A7539	Aradu.A01	7539	p2	(GA)6	12	29189629	29189640	ATTTAGACTCCCGGGTCGTT	CAATTGCCATGTTTGTGGTC	265
Ad01A7541	Aradu.A01	7541	p2	(AG)6	12	29205278	29205289	TTTGGAAGGGAGATGGTTTG	GCCAGGATTGCACTCTAAGG	246
Ad01A7543	Aradu.A01	7543	p2	(AC)8	16	29220012	29220027	ATGCACACATGCAAACATCA	ATGGAAGCACCCCAATGATA	280
Ad01A7544	Aradu.A01	7544	p3	(GAA)6	18	29222904	29222921	TGACATCGAGAAAATATGATGGA	TCGTACGCGAAACACCATTA	172
Ad01A7548	Aradu.A01	7548	c	(AT)6tttttttcaa	66	29254647	29254712	ACAACCGTCCCTTAGCTTTT	CTTTGAAAATAGGCAACGGC	263
Ad01A7549	Aradu.A01	7549	p2	(TA)6	12	29255069	29255080	AGGATGCATTTTTCAGGTGAT	TGTTTTGATCAATCCAATATTTTTGT	275
Ad01A7551	Aradu.A01	7551	c	(A)11cttgtttcac	88	29265053	29265140	TCCATCATGCAACTCAATGC	TGCTTTGATTAGGGCTAACACTT	224
Ad01A7552	Aradu.A01	7552	p2	(CA)7	14	29316937	29316950	TTAGCTCATCCCAAGCCAAG	TGAATTCTCGGGTCATCTCC	253
Ad01A7553	Aradu.A01	7553	p3	(TTG)5	15	29343682	29343696	GTGTAGCACAAACCCCATCC	AGTGTGTGAAAAGGGCAACC	247
Ad01A7554	Aradu.A01	7554	p2	(CA)6	12	29347475	29347486	GGGAAACCGAAACCATACCT	GGACCTTGGGTGTGGTAAGA	278
Ad01A7557	Aradu.A01	7557	p2	(CT)6	12	29374681	29374692	ACATTGAACCTGGGATCGAG	TTTTTGACCCAGTTTTTGGC	231
Ad01A7558	Aradu.A01	7558	p2	(AG)9	18	29404606	29404623	AAAATGGCCAAACAACCAAA	GGTCCCATTCCTTGATCCTT	272
Ad01A7559	Aradu.A01	7559	p2	(TC)8	16	29407137	29407152	TGATTACGCCTCCCTGATTC	GCCTTTCCTTCCTTTCACCT	265

ID	Chromosome	SSR nr.	SSR type	SSR	Size	Start	End	FORWARD PRIMER (5'-3')	REVERSE PRIMER (5'-3')	Product (bp)
Ad01A7560	Aradu.A01	7560	p2	(TC)8	16	29411773	29411788	AGCTCCTCCTTTTGTTGCCT	ATATGCAGATTGGGTTGGGA	189
Ad01A7565	Aradu.A01	7565	p2	(AT)7	14	29451139	29451152	GGCTCTTGAAAGAACGACGA	CCACCGGATACATAAATGCC	260
Ad01A7569	Aradu.A01	7569	p3	(AAT)22	66	29477147	29477212	TTTGTTGGCTAGTTCCAAGTGA	GCCCTTAGGTTGATGTTATCTGA	248
Ad01A7573	Aradu.A01	7573	c	(AT)9gaacaaaa	62	29488611	29488672	AAAACACTATCCCAATTCCAAA	AGCAACAGCAGAGGGAGTGT	280
Ad01A7576	Aradu.A01	7576	p2	(GA)6	12	29496653	29496664	GGGGAGATTTGTTGGGTTTT	CCAACGGTGAACAACTGAGA	223
Ad01A7578	Aradu.A01	7578	p2	(CA)7	14	29540857	29540870	ACGGTGCTCAATGCTTTTCT	AAGAGCGCGAATTGAAATGT	227
Ad01A7580	Aradu.A01	7580	p3	(CAA)5	15	29562625	29562639	GGTTCGAAGAACAAGACCCA	TCCCAGTTGGAATTGGAAAA	257
Ad01A7581	Aradu.A01	7581	p2	(AG)6	12	29566250	29566261	GCATCCATTCATGGAGACAA	TGGAGTTCATAAATCAAATTGGG	236
Ad01A7582	Aradu.A01	7582	p3	(TCT)6	18	29590780	29590797	CAAACAACACAACCCCCTCT	CTCGGAAGTGGAAGCTTTTG	235
Ad01A7586	Aradu.A01	7586	p4	(TATT)6	24	29613872	29613895	TTGAAGCTTCCCAAACGAAC	TCCATGCATCCAACAAACAT	218
Ad01A7587	Aradu.A01	7587	p3	(ACA)5	15	29616119	29616133	TGTTCAGCACTGAAATCCCA	ACAATACTGATGGAAGGGCG	255
Ad01A7589	Aradu.A01	7589	p2	(CT)9	18	29627956	29627973	ATTCGAACGAAACTACGCCA	CCCACACATACGATGATTGC	222
Ad01A7592	Aradu.A01	7592	p3	(AAT)7	21	29628960	29628980	TTCAACACAAAAGTGAAGCAAAA	GTCGGGTTGAAAAGGAACAG	246
Ad01A7593	Aradu.A01	7593	p2	(GT)6	12	29634334	29634345	GGAGCACCGTAGCTCAAGAT	AATGGAAAATCGGGGGTTAG	237
Ad01A7594	Aradu.A01	7594	p5	(TTTTA)6	30	29679851	29679880	AATTTTCGAATCCCTTGGGT	CCATGATGGAGAAAGGGAGA	250
Ad01A7597	Aradu.A01	7597	p2	(CT)11	22	29734786	29734807	GTTCCACGCAGCTACATTCA	ACAACGACCACCACCACTTT	272
Ad01A7599	Aradu.A01	7599	p2	(AT)6	12	29749298	29749309	CCTACAAATCGCAAGCAACA	GCATTAGCAAAATTTGGTCACA	278
Ad01A7600	Aradu.A01	7600	p2	(TG)6	12	29752652	29752663	GCGAAAGCTTTTGTTCAAGG	CATACACAATCATGGCCCAA	204
Ad01A7610	Aradu.A01	7610	p2	(TA)7	14	29798904	29798917	AAATGTGGCAAGATAAGAACTATGG	ATACTAGCGGCTCAACAGGC	267
Ad01A7611	Aradu.A01	7611	p2	(TA)11	22	29800809	29800830	TGAGCCATGTAAGTTTTAATTTATTGT	AAACGGAAGATCATTAGACCCTT	159
Ad01A7613	Aradu.A01	7613	c	(GA)9gggggga	66	29816032	29816097	GGAGAAGGAATGGGGAAGAG	CTGGCAATTTGCATTTTCCT	269
Ad01A7614	Aradu.A01	7614	p2	(AC)20	40	29816994	29817033	ACATGTCGCACTCGAAACAG	TCTTTTGGGTATGGACTGGG	205
Ad01A7615	Aradu.A01	7615	p2	(AC)7	14	29817862	29817875	ACATGTGCATTCCCAAACAA	TGATCGGGCATTTTAACTCC	226
Ad01A7616	Aradu.A01	7616	p3	(GTT)5	15	29827186	29827200	CTGCATGGAGTGTTGGAATG	GCGGGAATTTTACACCAAGA	200
Ad01A7618	Aradu.A01	7618	p2	(TG)8	16	29847664	29847679	GGCGTCTGAATTTTCTTTGC	TATCACGCTCCAAGCTCGAC	240
Ad01A7623	Aradu.A01	7623	p2	(AG)10	20	29862197	29862216	GCAAAATTTGATAAAGCGCC	CCCTCCTTCTTCGTTCACAC	260
Ad01A7624	Aradu.A01	7624	p3	(TTA)9	27	29862915	29862941	AGGAACACGAAATTTCAGCAG	CCCACTTTTTCCTTTACCTTCA	251
Ad01A7627	Aradu.A01	7627	p2	(CT)10	20	29866048	29866067	TGTCTTAAATTCGAACCGCA	AAAGAAGAGCAGAAGCAGCG	217
Ad01A7628	Aradu.A01	7628	c	(TC)6ccttcactc	47	29872647	29872693	AACTGGTTTCACTGAACTGAATTG	AAAAGCGGAGGGAGAGAAAG	270
Ad01A7630	Aradu.A01	7630	p2	(CT)6	12	29873404	29873415	TCTCTCTCCCCCTTCCCTTA	CGCTCTTTCTTCCCCTTTTT	266
Ad01A7631	Aradu.A01	7631	c	(GA)8aaaagaaa	151	29875247	29875397	AGGGGGAGAAAGAGGGGC	TCTCTCCTTCTTTTCCTTTCTCTC	249
Ad01A7633	Aradu.A01	7633	p2	(AG)13	26	29875964	29875989	GGGAGAGAGAAAAAGGAGGG	GCACCAGTTTAAACCAGTTCAT	234
Ad01A7636	Aradu.A01	7636	p4	(ATTT)5	20	29879526	29879545	TGCTGCTTTGTTTCCTCGTA	TTTTTAAGTATTGGTCACACGAGA	198
Ad01A7642	Aradu.A01	7642	p3	(TTA)17	51	29894152	29894202	TCTTCTTGCCCATCACATCA	TGCACTCCATTAATCCCTCA	146

ID	Chromosome	SSR nr.	SSR type	SSR	Size	Start	End	FORWARD PRIMER (5'-3')	REVERSE PRIMER (5'-3')	Product (bp)
Ad01A7645	Aradu.A01	7645	p2	(TA)6	12	29898453	29898464	AGATTCCCATCGCCCTTATT	GGCAAAATGGACACACATCA	273
Ad01A7646	Aradu.A01	7646	c	(A)10gaaaaaga	81	29910028	29910108	TCATGCATTGAATGTTGAGC	GCTTAACCTCCCACCTAACC	267
Ad01A7647	Aradu.A01	7647	p3	(ATA)20	60	29926175	29926234	ACATCGCACTCTCCCTGTCT	TTTGTTTTGATGTTGTCAAATGAT	215
Ad01A7655	Aradu.A01	7655	p2	(GT)10	20	29968071	29968090	AAAGAACCCTAGCCCCTCAA	AAGTCACAATGCTTACCGGC	160
Ad01A7656	Aradu.A01	7656	p2	(AT)6	12	29997081	29997092	CGGTTATATAGCCAACCCGA	CTTGGGTAGATGCTGTGGGT	151
Ad01A7658	Aradu.A01	7658	p2	(GA)6	12	30009425	30009436	TGGAGAATCCAAACACAATGG	CAAAACCACCTAACTACTTTTCCC	265
Ad01A7660	Aradu.A01	7660	p2	(TA)6	12	30036093	30036104	CATAATGTTTTGTGTTTTTGAGCA	CGAAGTTGGACCTTGGGATA	124
Ad01A7663	Aradu.A01	7663	p3	(GAA)5	15	30055321	30055335	ATCAACCGAAGAGAGGAGCA	TGCTTCGTGTGGAAGTGTTC	185
Ad01A7664	Aradu.A01	7664	p3	(TTC)5	15	30057012	30057026	TTGCACCCTGTTTTTCTCCT	CAAACAAGTCAAGAAGCACCA	280
Ad01A7665	Aradu.A01	7665	p2	(AG)6	12	30060987	30060998	AGGCTACAGAGTGGGGGAAT	CAACATCAGATAATCAACAAGGGA	194
Ad01A7667	Aradu.A01	7667	p2	(AG)15	30	30083064	30083093	TGGCTCTTGAAAGAATGATGG	TCCCCAATTAGAGGTGTCCA	250
Ad01A7669	Aradu.A01	7669	p3	(TTG)10	30	30092823	30092852	CTCTCCCTCGAGGTCCCTAC	AACAGGCAGAAGCAGAAAGC	278
Ad01A7672	Aradu.A01	7672	c*	(A)12(AAG)6*	28	30126355	30126382	AGGGGAGAAACCAGCAATTT	CTGGTGTTTGGTTTGTGGTG	125
Ad01A7673	Aradu.A01	7673	p2	(TC)6	12	30127227	30127238	GCCCCAACTGCTGTCATAGT	GGGAAAGAGAGAGATGGGCT	163
Ad01A7676	Aradu.A01	7676	p2	(CT)7	14	30168627	30168640	CAAATCTGCCAACCTGCTTT	AAAACCTTGTTAATGGGGGTG	130
Ad01A7677	Aradu.A01	7677	c	(T)10cattggatc	30	30188517	30188546	AATCGCTATTGGTTTGACGG	CCCACACGTAAGAACCGACT	238
Ad01A7679	Aradu.A01	7679	p2	(AT)7	14	30235509	30235522	CCAAACATTGTGGTAGGGATG	GAATCTATCATGTTGTGGCGG	279
Ad01A7684	Aradu.A01	7684	p2	(AG)6	12	30251632	30251643	TAAAGTGGGGAATCCATCCA	GGAACATTGAACCTGAAAATTGA	187
Ad01A7686	Aradu.A01	7686	p2	(TA)9	18	30258365	30258382	TGGACCTCACATCCAGACTT	TCAAAAAGGAAGCAAGCAAA	280
Ad01A7687	Aradu.A01	7687	p3	(ATA)6	18	30259187	30259204	ATTCCATGGAGCTTCTGCTG	TCATGAATCCACTCATCCCA	209
Ad01A7692	Aradu.A01	7692	p4	(TTTA)5	20	30282231	30282250	CAGCACAGGAAACACCAAGA	TCCTCCTGCAAAACAAAAGG	203
Ad01A7696	Aradu.A01	7696	p2	(AT)6	12	30293828	30293839	TTCCAACGGATTCAGAAACA	GAACCTGCAACAAAACAGCA	189
Ad01A7698	Aradu.A01	7698	p4	(TATG)7	28	30304391	30304418	TGTTTTTCAGATGCAGGTCG	CTCAACTTGGGAAGCAAAGG	192
Ad01A7699	Aradu.A01	7699	p2	(TG)31	62	30305245	30305306	ATTTTTCAGATGCAGGACGC	TCGACCTCTAGGAGGGACAA	243
Ad01A7700	Aradu.A01	7700	p2	(TA)6	12	30305483	30305494	ATTTCTAACCGGCCTTTGCT	GGAGCCTTGAACAAAAGGGT	176
Ad01A7701	Aradu.A01	7701	p2	(GA)6	12	30306918	30306929	AAAGGGGGTTGGACTGTTTT	TAGCGAGATTCATTGGCACA	222
Ad01A7702	Aradu.A01	7702	p2	(GA)17	34	30309719	30309752	TGATTGAATTCTGGGGACAAG	TGATTTGATAAGGGTTTAGGGC	279
Ad01A7703	Aradu.A01	7703	p2	(GA)10	20	30310970	30310989	GCAGCAAAAGAGGAAACCAA	TCGTGTCTCTGTCCTCATCG	134
Ad01A7711	Aradu.A01	7711	p3	(AAT)8	24	30351728	30351751	TTGTGATTTTTGTGTATTATGTTTGTT	TGAAACAAATCTAAAAGTTCTTGATGA	205
Ad01A7720	Aradu.A01	7720	p2	(TA)6	12	30384905	30384916	TCCATCCTCCAACCACTCTC	GGGAAGGAAGGGAGCATAAA	143
Ad01A7721	Aradu.A01	7721	p3	(TAT)18	54	30385045	30385098	TCCATCCTCCAACCACTCTC	AAAAAGCGACGAGGGATTTC	279
Ad01A7724	Aradu.A01	7724	p3	(TTG)6	18	30395681	30395698	AACATCCAGTGAGGAGGGTG	AAGCCCAACCTTCTACAACAAA	234
Ad01A7725	Aradu.A01	7725	p3	(TAT)18	54	30396831	30396884	TCGGTTGAAAACGTACACCA	AGACAATTAGACCCCAGGGC	204
Ad01A7730	Aradu.A01	7730	c	(T)10a(AT)11	33	30405195	30405227	GGCATGTGATGATTTCTGGA	GCCAAATCTCATCCACCAGT	208

ID	Chromosome	SSR nr.	SSR type	SSR	Size	Start	End	FORWARD PRIMER (5'-3')	REVERSE PRIMER (5'-3')	Product (bp)
Ad01A7733	Aradu.A01	7733	p3	(AAT)5	15	30420897	30420911	CGGAGTGAAACAAGTCGGTT	TTGGCGTTACACGTTACGAA	147
Ad01A7735	Aradu.A01	7735	p3	(ATT)16	48	30429284	30429331	TGCTGTTAAAAGTGTTTCCATTTC	CGAGAATTTAGTGGCTTAGCTG	227
Ad01A7736	Aradu.A01	7736	c	(TCT)6tcgc(CT	55	30430621	30430675	TTTTTCGCTCCAAAATGGTC	GCAGTGGTGAAGTAGGGGAG	246
Ad01A7740	Aradu.A01	7740	p2	(CT)6	12	30457324	30457335	GGAACATTGAACCTGTGATGAA	ACGCATTGCAGTGACAAAAA	260
Ad01A7745	Aradu.A01	7745	c	(CAC)5catcatc	66	30482124	30482189	TAACCACAAGCAAGGCAACA	AATGGCTTCCAGAAGCTTGA	242
Ad01A7747	Aradu.A01	7747	p2	(AT)6	12	30496623	30496634	AGGAGCTCGCCAAATGTCTA	GCAACTCGCTGCCTATTTTC	266
Ad01A7748	Aradu.A01	7748	p2	(CA)7	14	30503039	30503052	AAGCACAGCAGAAAATGCCT	TCAACTGATGGATGGCTTCA	266
Ad01A7749	Aradu.A01	7749	p3	(CCA)6	18	30503230	30503247	AGCCATCCATCAGTTGAAGC	CTGCTGCTGCAAGAAGAGAA	175
Ad01A7750	Aradu.A01	7750	p2	(GA)6	12	30541076	30541087	TTCGAACACAGAGAGGGAAGA	GAAAACTCCTCACCCTTTTTCA	252
Ad01A7751	Aradu.A01	7751	p2	(AG)7	14	30564315	30564328	AATAAAACAGGGCAGGGTCA	CGCGCTTCATTTTCCATACT	234
Ad01A7752	Aradu.A01	7752	c	(GAT)12(GAC	69	30596109	30596177	CCTTGACACAAGAAGTTTCTTAATTTT	CTCCATGTCCTCTTCTTCTTCA	245
Ad01A7756	Aradu.A01	7756	c	(A)10ccatgcatg	66	30621746	30621811	ATCACAAGTTCACGCACCAC	GAGGGCAACAAGGACAAAGA	261
Ad01A7758	Aradu.A01	7758	c	(A)10tcagttagt	39	30629158	30629196	GAACGATTAGACATTTTTGGATTG	GCTTTCCTCTACGATTTGGG	180
Ad01A7760	Aradu.A01	7760	c	(TC)16tgtt(TC	56	30632708	30632763	AATTGCTTTGACACTCGCCT	GGAACAGAGGGAAGGCTGAT	280
Ad01A7762	Aradu.A01	7762	c	(AG)8cgtgccaa	120	30637074	30637193	ATCTCTTTGTTGCTGCTGCG	TGCATGATTTATCCAAACATAAAA	226
Ad01A7764	Aradu.A01	7764	p2	(TA)6	12	30673603	30673614	TCCTAACGTCAGCCTTACACC	CCAAGGATTGGTCCTTCATC	271
Ad01A7766	Aradu.A01	7766	p3	(ATT)17	51	30693783	30693833	TGATTTGAGTTCAATACCATTCG	TCTCCAGTCCATTCTTTGGG	239
Ad01A7768	Aradu.A01	7768	c	(CTT)39cactttg	197	30698739	30698935	TGATTTATAGCCTGATTATGTTCCC	CATAGTGATGGAGAAGTGACGA	263
Ad01A7769	Aradu.A01	7769	c	(T)10gaaaatata	60	30714803	30714862	TCACATATGCATTGACCTTTCA	TGGAGAAACTGTGGGAAACC	276
Ad01A7771	Aradu.A01	7771	p2	(AT)6	12	30737898	30737909	TTAGTTTTAATTTTTCCTGTAGCAACT	CTGACTGCTTGTTGGGACCT	242
Ad01A7773	Aradu.A01	7773	p3	(TCT)8	24	30754941	30754964	AAACCATTTTCCCACCCATT	TGCTTTGACCACACCAAACT	268
Ad01A7774	Aradu.A01	7774	p2	(TA)6	12	30767399	30767410	CAATTTTGGCCCATCAATCT	CCTCAGACCTTAGGAACGGA	212
Ad01A7776	Aradu.A01	7776	p5	(TTTAT)5	25	30772065	30772089	TGAATCTTCAAGGTGTTCCTGA	CCATAACGGAGAAAGGGAGA	277
Ad01A7778	Aradu.A01	7778	p2	(TA)8	16	30800141	30800156	CCAAAGAGGATTCCCCAGAG	TGCCACCACATCAAGGTAAC	273
Ad01A7780	Aradu.A01	7780	c	(TC)8tgtccaa(T	35	30805390	30805424	CTTCCCTCGACAACGACG	AGAGAGGGAAGAGTGCACCA	201
Ad01A7781	Aradu.A01	7781	p2	(TA)6	12	30810573	30810584	TTTGTGTCCCTTTGGGTTTC	ACTCAAAAGCGCAACACTCC	197
Ad01A7782	Aradu.A01	7782	p2	(CT)7	14	30819832	30819845	TTTTTCGAAATCTCCCCTCC	CGGAAAAGCACACCAAACTT	152
Ad01A7784	Aradu.A01	7784	c	(TA)6atcacgcg	85	30828668	30828752	AGCCGGATCTTGAGTCTTGA	TGCAATTTGTAACACCCTACCA	280
Ad01A7785	Aradu.A01	7785	p3	(TTG)5	15	30829966	30829980	GGAAGATGAACAAGGGGTGA	TCATTCTCATTGTCAACGGC	254
Ad01A7790	Aradu.A01	7790	p2	(GA)6	12	30919149	30919160	CCTCTCACTCACCCAACCAT	CCTTTCACCCCCTTTCTCTC	271
Ad01A7791	Aradu.A01	7791	p2	(TA)9	18	30963885	30963902	GACGAGGCTTTTACCGTCTG	GCTCTCTCTCTTACCCTTTCAA	280
Ad01A7792	Aradu.A01	7792	p3	(GAG)5	15	30983288	30983302	AATCACAATGGGCTGAGAGG	TTCAGGAGGCTAAGGCAAAA	110
Ad01A7794	Aradu.A01	7794	c	(ATG)8acgac(	56	30990599	30990654	AGCAAGAGGTGGGATTGAGA	TCGTCCTTGTCCTCATCTCC	219
Ad01A7795	Aradu.A01	7795	p3	(GAG)8	24	30999691	30999714	AATCACAATGGGCTGAGAGG	GCAAGGAAGAATGCTCAGGA	153

ID	Chromosome	SSR nr.	SSR type	SSR	Size	Start	End	FORWARD PRIMER (5'-3')	REVERSE PRIMER (5'-3')	Product (bp)
Ad01A7799	Aradu.A01	7799	p2	(TC)17	34	31031104	31031137	TCCCCAATTAGAGGTGTCCA	TGGCTCTTGAAAGAATGATGG	228
Ad01A7802	Aradu.A01	7802	p2	(AT)7	14	31068389	31068402	GAGGGTTCAAATTCTGTCTCG	TTTAATTGTGGGGGCAAAAA	174
Ad01A7804	Aradu.A01	7804	p3	(TGA)5	15	31073081	31073095	TGGTGATGGCAGTGTTCATT	AAACCCATCAGTTCGCAATC	219
Ad01A7806	Aradu.A01	7806	p2	(GC)6	12	31096091	31096102	GGCTGTTTGGGGGTTAGAAT	CTTGGTGTGGGCGTAACTCT	206
Ad01A7808	Aradu.A01	7808	c	(TC)6ttctcttaca	48	31104044	31104091	TGGTTGCAATCAAAGAAAGG	TCGGTGCTTGTAGGAGAACA	278
Ad01A7810	Aradu.A01	7810	p3	(TTA)5	15	31118173	31118187	TTTGACATACGTGAATCGGG	TCTGTTTTCCCTCCAAAACG	275
Ad01A7811	Aradu.A01	7811	p2	(TC)24	48	31118696	31118743	TCTCTCTCCCTTTGTCGCTC	AGCAGAAGCAAAGCAAGCAT	197
Ad01A7813	Aradu.A01	7813	p2	(TC)9	18	31121753	31121770	ATATTCCTCCCTCCGCTGTT	ACGGTATCCACTTGCACACA	267
Ad01A7815	Aradu.A01	7815	p2	(TA)10	20	31125866	31125885	TGAATTCTCATAAGCAACGGA	TTGCACTTTTGGGATTCTTG	221
Ad01A7820	Aradu.A01	7820	p2	(TA)9	18	31218498	31218515	AGCTTTCAATCAACGCCAGT	TGAGATCCCCATATGTCCTCT	226
Ad01A7822	Aradu.A01	7822	p2	(AT)16	32	31227789	31227820	AGCTGCTTGCTTTAATGGGA	TTTGCAATCATATCTTCTCAATCA	270
Ad01A7827	Aradu.A01	7827	p2	(AG)18	36	31291398	31291433	AGTCTTCGACCCAACACCAC	CCGATTCATAACATGAGAACGA	208
Ad01A7828	Aradu.A01	7828	p3	(TTG)11	33	31301212	31301244	TATGACAGAGCTCAATGGCG	TTGTCCCATTCGAACACGTA	199
Ad01A7829	Aradu.A01	7829	p4	(ATTT)5	20	31313788	31313807	AACTATTTTTAAGCCACTTTTTCG	TCCATGTTGGAGTAAAGGGG	252
Ad01A7831	Aradu.A01	7831	p2	(TC)16	32	31373301	31373332	TTCGAAAATTCCTCACCCTTT	TCGAACTTAGAGAGGAAAAGAAGG	272
Ad01A7834	Aradu.A01	7834	p5	(GATAA)5	25	31446295	31446319	ATATCCTCCCGATGTAGGGG	GAGCCCCTGGCTCTTTTTAT	235
Ad01A7836	Aradu.A01	7836	p3	(AAT)7	21	31451781	31451801	GAAGCTTCCAAAATTCAATTCAT	TCTTCGTTTTGCTTTAGACGC	279
Ad01A7839	Aradu.A01	7839	p5	(AAGAT)5	25	31466414	31466438	AGGCGGTTCCCCTAAAGATA	ATGGTGCTGATCCTTTGTCC	232
Ad01A7840	Aradu.A01	7840	p2	(TC)8	16	31471127	31471142	TCTTCTCCTTTCCTCACCCA	CCAGAACTGAGGGCGATAAA	208
Ad01A7842	Aradu.A01	7842	c	(T)10g(T)11cta	72	31480821	31480892	ACTACCAATCACTGTCCGCC	GGAGCTGTGTGACCGATTTT	211
Ad01A7843	Aradu.A01	7843	p3	(AAC)5	15	31482162	31482176	CGGAGGCAGTAACAACCATT	TACCTTTGGGTGAGGTGGAG	134
Ad01A7844	Aradu.A01	7844	p2	(TC)10	20	31482356	31482375	CTCCACCTCACCCAAAGGTA	CACATGCCATAGCTGCTTGT	261
Ad01A7845	Aradu.A01	7845	p3	(TTG)5	15	31484550	31484564	GAAGATGGACAAAGGGCAAA	ATTCGCCACCACTATTCCTG	243
Ad01A7846	Aradu.A01	7846	p3	(TTC)5	15	31493056	31493070	GCGCGAACTAGAATAGTGCC	CAGAGAAGTGTAAAAGTGCGGA	210
Ad01A7848	Aradu.A01	7848	p3	(AGA)8	24	31517854	31517877	TTGCCTTCCCTTTCCTCTTT	AACCCTCTCTTCTCCCCCTT	215
Ad01A7851	Aradu.A01	7851	p2	(TC)9	18	31553055	31553072	GATTAGACATTCAAATCTCATCATTTA	AGGAAAGGGAGGGAGGGAG	280
Ad01A7853	Aradu.A01	7853	p4	(TAAC)7	28	31559016	31559043	CCTGCATCACTCAACCAAAA	CCTTTCAACCATCACCCAAC	230
Ad01A7854	Aradu.A01	7854	p2	(AG)6	12	31560287	31560298	TTCATTGATCATTCACTTCTCCTT	CCAAACAACCAATTGCAAAC	280
Ad01A7858	Aradu.A01	7858	p2	(AT)6	12	31566474	31566485	GAGCGCTACCGACTCATCTT	TTTTTCGCTTTGGTAACTGC	214
Ad01A7861	Aradu.A01	7861	p3	(TCA)5	15	31611178	31611192	TGCTTGCTGGACTCATCATC	AGCAACAGCCCAACAGAGAT	139
Ad01A7862	Aradu.A01	7862	p2	(AG)8	16	31614122	31614137	CGTGCCAAGTGAAAAATCCT	AGTTTTGAGGCTCTGGGAGG	263
Ad01A7864	Aradu.A01	7864	p3	(TTG)10	30	31623761	31623790	CCGTGCCATATCGTGTTTTT	TATCCAACGACGACGACGA	171
Ad01A7865	Aradu.A01	7865	p3	(TAT)14	42	31623951	31623992	ACGACCGGTGTAATTATCATATC	CCATTTTTCCTCCAAAATGAA	279
Ad01A7869	Aradu.A01	7869	c	(TA)8(TG)8	32	31651825	31651856	AGCATGTCCTCATGCTAACTCA	GGGTTCAATTGCTCATTTCC	280

ID	Chromosome	SSR nr.	SSR type	SSR	Size	Start	End	FORWARD PRIMER (5'-3')	REVERSE PRIMER (5'-3')	Product (bp)
Ad01A7870	Aradu.A01	7870	c	(AT)7nnnnnnn	91	31652669	31652759	CACGGGTTGTGTGTTCTTTG	TGCATTGATGTTGTTTTGGG	251
Ad01A7871	Aradu.A01	7871	p2	(GA)12	24	31681342	31681365	AGAGGGAGAGAGAGGGTTCG	CCTAACCACTTTTCCACTTCCA	240
Ad01A7874	Aradu.A01	7874	p2	(AG)6	12	31697499	31697510	TGCATAAAAAGGGGAATCAAA	GCTGGGAGTCGAACAGAATC	247
Ad01A7879	Aradu.A01	7879	p2	(TG)6	12	31727316	31727327	TCGATTATTTTGTGGGAGGC	CACCGTTATTCAAGCCTTTTT	171
Ad01A7880	Aradu.A01	7880	p3	(TTC)5	15	31728342	31728356	CATCACGTTTTCAAAGCCCT	GGAAGAAGAAAAGGAGGAGGA	111
Ad01A7890	Aradu.A01	7890	p3	(TTC)6	18	31814997	31815014	ATATGGCCGAACCTTAACCC	CTAGAGGTTTCTCCGGCCTT	270
Ad01A7891	Aradu.A01	7891	p3	(AGA)5	15	31835464	31835478	GCTTCTATGCCAGAAGGCAG	TCAATTTTTGAAATTCTCTTCTTCTC	251
Ad01A7893	Aradu.A01	7893	p3	(AGA)6	18	31864637	31864654	TAAAATATTGCTCGCCCTCG	ACAATTCAATTTCCCACCCA	212
Ad01A7894	Aradu.A01	7894	p3	(AGA)5	15	31867523	31867537	CTAGAGGAGACTCCGGCCTT	TCCCACATAGCCGAACCTAC	175
Ad01A7897	Aradu.A01	7897	p3	(AGA)6	18	31897858	31897875	ATGCTTTTACCGCACCAAAC	AACCGCTCTCCCTCCACTAT	189
Ad01A7899	Aradu.A01	7899	c	(TG)8(TA)6	28	31913902	31913929	CTCCCGGTTCTCATTCCATA	AAGCTCCAGACTTGACCTGC	262
Ad01A7900	Aradu.A01	7900	p5	(TCTTT)5	25	31938571	31938595	TTCAAGGATCAAATCAAAGCC	AACGCATCACAACACCAAAA	269
Ad01A7906	Aradu.A01	7906	p2	(AT)10	20	31951132	31951151	TTTGGATTTATTCTTGAACACTATTTT	TGTGTTTTTGCCGCTATTGA	233
Ad01A7915	Aradu.A01	7915	p3	(TAA)5	15	31990580	31990594	CAAGGAAATGAAGTGGGGTC	AGGGCTGAATTTGGAATGTG	193
Ad01A7916	Aradu.A01	7916	p2	(TA)6	12	31999529	31999540	TTTGTTAGCCCAGGAACCAG	CCCATTAGCCACAATCAAAAA	243
Ad01A7919	Aradu.A01	7919	p2	(AT)7	14	32007025	32007038	TCCTCAAGTTGTGGGAACTAAAA	ATTGCCTTATGGCCCTCTCT	221
Ad01A7920	Aradu.A01	7920	p3	(AGT)6	18	32007547	32007564	TCGTTTTCAAACTCCACTAGCA	TTCCCAGCAGTGGCTCTAAT	237
Ad01A7921	Aradu.A01	7921	c	(T)10attatttttaa	113	32009925	32010037	TTTTTGCTGTATGCTATAGGGATTT	TTGCATCTTCGTCATCATTTG	248
Ad01A7926	Aradu.A01	7926	p5	(TTATC)5	25	32050077	32050101	CCTGGGTGCTCCAGTGTATT	AGATAAAGGCGGTTCCCCTA	127
Ad01A7927	Aradu.A01	7927	p6	(AGAAAA)6	36	32066084	32066119	GGGAAAGTTGAATTTGGAAACA	TACTTTGGGGCATTTTGTCC	231
Ad01A7930	Aradu.A01	7930	p3	(ATG)5	15	32071659	32071673	TGAATTCTGAAGAGGGACGG	TGCATGGACCGAAGCTATAA	252
Ad01A7931	Aradu.A01	7931	p2	(CT)9	18	32077307	32077324	AGTGAAACTGGCCAAAATGC	CCCCTCTTCACCGTCAAATA	205
Ad01A7932	Aradu.A01	7932	p2	(AG)6	12	32081292	32081303	TGTGCCCAACTTTGATTGAA	TGCAAAAACATAAAAGATTGCC	159
Ad01A7934	Aradu.A01	7934	c	(CT)11(AT)11	44	32085807	32085850	GGCTTCTAAACCAATTCGCA	TTTTCAGAAGCACCCTCACC	245
Ad01A7936	Aradu.A01	7936	c	(A)11tatcttttcta	57	32106517	32106573	CCTTGGTGTTTTTCCTCCAA	TGAGTTGAAAAGATTTTAAGAAGGAA	261
Ad01A7938	Aradu.A01	7938	p2	(TA)6	12	32157010	32157021	TCCGGAGACTAACTCGGAAA	TTTCTACCGTATCTTCTAAACTCAACA	278
Ad01A7943	Aradu.A01	7943	p2	(AT)11	22	32172925	32172946	CCTTTCCAAAAAGAGTGAAGTTG	CATGTTGGTACATGACATTTCG	268
Ad01A7944	Aradu.A01	7944	p3	(ATG)9	27	32175171	32175197	AATCAAAACTGGTTGTGGGC	CATGTCTCCATGAGCATTTCA	259
Ad01A7945	Aradu.A01	7945	p3	(GAT)5	15	32179901	32179915	GGGAGTAGTTTGTGGGTGGA	AAAACTCCTCACCCTCACCA	201
Ad01A7946	Aradu.A01	7946	c	(A)12g(A)11	24	32180738	32180761	CCCCAACTATAAACCATGCTC	TTTGCTTCAGTGCAATTTCG	265
Ad01A7947	Aradu.A01	7947	c	(T)12gtgatgtga	69	32185095	32185163	GGATTCAAGAACCAATCAATCA	TTCTCATGGTAGCCAAGTTTTC	250
Ad01A7948	Aradu.A01	7948	p2	(GA)16	32	32185279	32185310	GCAACAAAACACAACTAAAGCAA	TGCTAAAATCCTCGTCTCCC	227
Ad01A7949	Aradu.A01	7949	p2	(TA)10	20	32192345	32192364	CGAAATGGGAGCATGGTTAT	ATGGGGTTTTTCTGGGGATA	279
Ad01A7950	Aradu.A01	7950	p2	(TA)6	12	32199374	32199385	AGATGCAAGTCGAGAGGCAC	CTCGACCTGCGAAGTTAAGG	256

ID	Chromosome	SSR nr.	SSR type	SSR	Size	Start	End	FORWARD PRIMER (5'-3')	REVERSE PRIMER (5'-3')	Product (bp)
Ad01A7951	Aradu.A01	7951	c	(TAT)5tactact	130	32202045	32202174	TGCTTCACTAAATATTTTTCATCCTT	TTTTAATTCTCTTTAAGACCCCAA	272
Ad01A7953	Aradu.A01	7953	p2	(CA)10	20	32203365	32203384	CCTATTCGATTGTATTTCCTTGG	CGGATTGGAAGAAATCGAAA	158
Ad01A7954	Aradu.A01	7954	p2	(TA)6	12	32205085	32205096	TTGTTTTCAGATGCAGGTCG	AAGCTAAGGCCAGCCAGAGT	253
Ad01A7957	Aradu.A01	7957	p2	(TA)7	14	32233806	32233819	TGAGGCCATTGTTTCATTAGC	TTAGTTGGGGCATTTTGTCC	273
Ad01A7958	Aradu.A01	7958	p2	(AG)6	12	32239556	32239567	CGCGATCTGCAGAATTAACA	CATTGAACCTGGAAATTGAGAA	260
Ad01A7959	Aradu.A01	7959	p3	(CAT)11	33	32249535	32249567	CTACCACCTCCTCCTCCCTC	CGGTCCAAGTGCACCTTATT	227
Ad01A7960	Aradu.A01	7960	p3	(CTT)5	15	32249823	32249837	TGCATTTGGTCTCGTTGTTC	TGATGAGGAGTTTTGAGAAGTTTTT	231
Ad01A7963	Aradu.A01	7963	p3	(AGA)5	15	32262067	32262081	TCTCCCTCGAGCAAAGAAAA	CTTCCCCATAAATCCCACCT	230
Ad01A7968	Aradu.A01	7968	p3	(TTC)7	21	32304788	32304808	GAGTCGCGTTCTGTGCAATA	CTTGTGATAGCCACTGCGAG	276
Ad01A7970	Aradu.A01	7970	c	(A)10gaagaagt	120	32319266	32319385	TTCAATTCCAAATTTCCAACAA	AGGGTTTATATATACGAAACACATCA	273
Ad01A7972	Aradu.A01	7972	p2	(AT)7	14	32325570	32325583	GGAGGGTCCTCTTTTGTGAA	AGCGTGCGCTTTTCATTTT	278
Ad01A7973	Aradu.A01	7973	p2	(AT)7	14	32325707	32325720	ACGCTCGAGGAATCGAATTA	GCTCCTTGCTAGGTGTCTGG	249
Ad01A7974	Aradu.A01	7974	p2	(CT)12	24	32330183	32330206	ACGGACACATAACTGGGCTC	TGGTTATCCATGACCGTGTG	270
Ad01A7976	Aradu.A01	7976	p2	(GC)6	12	32341919	32341930	GTCCTCTCCCATTTTGCTCA	TACACCGATAGGCTCTGGCT	237
Ad01A7979	Aradu.A01	7979	p3	(TTA)5	15	32366797	32366811	GAAAATTTTTAAGTTTGGTGTTTTCA	CCATGATGGAGAAAGGGAGA	234
Ad01A7980	Aradu.A01	7980	p2	(TA)12	24	32375434	32375457	TGGACCTATGAAGCAAAGGC	GTGCAGGTATGGAGGCATCT	252
Ad01A7984	Aradu.A01	7984	p3	(TGG)7	21	32383077	32383097	TTGACCAGCAATCTAGAGCCT	TACTCCAACCCCAATCTCCA	116
Ad01A7985	Aradu.A01	7985	p4	(CTCC)7	28	32385327	32385354	CCATAGTGACAGCCATCGTG	GCCTGCAATTAGTGACGGTT	266
Ad01A7986	Aradu.A01	7986	p2	(AC)6	12	32392470	32392481	TTGTGGGTCTTCACCAAGTTT	ATCCCTTCTCAAGCTTTCCG	216
Ad01A7990	Aradu.A01	7990	p3	(AAT)5	15	32410347	32410361	GCACCAGAACACTGTGAACC	CAACGCAGTCACAAAGAACC	202
Ad01A7993	Aradu.A01	7993	p3	(CAA)6	18	32437903	32437920	CAATCAAATCGGGAGAGCAT	CATGAGGTGGTCAAACGAGA	210
Ad01A7997	Aradu.A01	7997	c	(ATT)5gtagtta	99	32448106	32448204	TTTGGGAGTGTGGAAAAGAA	GACAAAAATACCCTTTTAAATTAATCA	280
Ad01A7999	Aradu.A01	7999	p2	(AT)7	14	32459222	32459235	ACTTAAATGCCATTGGGTGC	AGCGTTCATTGCTGACAGTG	162
Ad01A8000	Aradu.A01	8000	p2	(AC)8	16	32459898	32459913	TGGAAATTTTCGCTGGTTTT	CCATTGAGGCACATCCCTAC	249
Ad01A8004	Aradu.A01	8004	c	(A)22tcttaaattc	56	32464921	32464976	AAAAGGAAACCATGCACACC	CAACGTTGCAAATTCCATTG	145
Ad01A8008	Aradu.A01	8008	p2	(TA)8	16	32486267	32486282	TATTTGGTTGCATGCCTTGA	TTGATGCTACATGGAGTAACCC	277
Ad01A8009	Aradu.A01	8009	p2	(GA)9	18	32502266	32502283	AGAAGGAGGGGAAAAATTCG	CGAAAACCACTTTCCCCTTT	273
Ad01A8010	Aradu.A01	8010	p2	(GA)6	12	32506231	32506242	GTTCTTGTCCTTGCTCCTGC	TTCTTTTCCTTTTAATTATTTTCGGA	268
Ad01A8014	Aradu.A01	8014	c	(ATC)9attattac	53	32562639	32562691	TGATATTCAGCGCCCAAAGT	CGCAAAGTAAATGACACCCC	216
Ad01A8017	Aradu.A01	8017	p3	(CAT)7	21	32588663	32588683	TACCACCACCACCTCCATTT	AACGATGACGACGATGATGA	128
Ad01A8020	Aradu.A01	8020	p2	(CT)20	40	32610550	32610589	AGAGCTCGACAAGAGCTTCG	GCCCAAGACCCAACATAGAA	230
Ad01A8021	Aradu.A01	8021	p3	(TTA)10	30	32620333	32620362	CCTTGTAGGCCCTCCTAACC	CTTGTTCTCTCCTTGCCGAC	258
Ad01A8022	Aradu.A01	8022	p3	(TTA)15	45	32622416	32622460	TCGAATTTGATGAGAGAAAAAGG	TGCATGCAGTCATTGTTTTG	130
Ad01A8023	Aradu.A01	8023	p2	(GA)6	12	32625917	32625928	AAATGAAGCAGTGTGGAGGG	AATAAGGACGTTTTGCACGC	195

ID	Chromosome	SSR nr.	SSR type	SSR	Size	Start	End	FORWARD PRIMER (5'-3')	REVERSE PRIMER (5'-3')	Product (bp)
Ad01A8024	Aradu.A01	8024	p2	(AT)11	22	32639636	32639657	ACTAGCCCCTGACTCAGCCT	GGATCTTGGGAAGCGAAGTA	249
Ad01A8026	Aradu.A01	8026	p3	(TTA)13	39	32652365	32652403	GCATGTAAATCGAACCAAATGA	TGAATTGTTGATTCTTTTTGGA	260
Ad01A8032	Aradu.A01	8032	p4	(AAAT)5	20	32685445	32685464	AATGAGGAGGGTTTATGGGG	TTGATCCTGGCATCTCAACA	253
Ad01A8035	Aradu.A01	8035	p2	(CT)6	12	32692934	32692945	GGAAAGAACTTGGCCAAAAA	GACTTTCACCGGAAGATCCA	217
Ad01A8038	Aradu.A01	8038	p4	(AATT)5	20	32799426	32799445	TTGGAAGTGTATTTGAATGATAATCTG	GATTGATAATATACCGTGCGAGC	248
Ad01A8040	Aradu.A01	8040	p3	(AGA)6	18	32812270	32812287	ACAAAGAAGCTTTGCGTTGAA	TTCTCCTCTGTTCGCGTTTT	100
Ad01A8041	Aradu.A01	8041	p2	(AT)6	12	32814185	32814196	GGTCACCACGGTACCTCAAT	AAACAACCCCAAGTTTGTGC	257
Ad01A8042	Aradu.A01	8042	p2	(CT)14	28	32817696	32817723	TCACTGCTTAAATAGCTTCTGCC	ACATACATGTGGGGAGGGAG	174
Ad01A8045	Aradu.A01	8045	p2	(TC)10	20	32857251	32857270	CGTTGTGCCTTCACATCATC	AATCAACCCTAAACCCCGAT	253
Ad01A8046	Aradu.A01	8046	p2	(TC)7	14	32870414	32870427	TGAGAATTCAAAAACCAAATAGCA	TTTTTGACCCAATTTTTGGC	277
Ad01A8047	Aradu.A01	8047	p2	(CT)11	22	32876713	32876734	GCAACATAAAGTAATGAACAAGGG	TTTTTGGGCAGTTTTTGACC	274
Ad01A8048	Aradu.A01	8048	p2	(CT)6	12	32883053	32883064	GCAACATAAAGTAATCAACAAGGG	TTTTTGACCCAGTTTTTGGC	245
Ad01A8049	Aradu.A01	8049	p5	(AAGAT)6	30	32884424	32884453	AAGATAAAGGCGGTTCCGTT	CCTGGGTGCTCCAGTGTATT	117
Ad01A8050	Aradu.A01	8050	p3	(TTC)22	66	32894498	32894563	CACGACAGTTCCCTTGGAAT	GAAGCAAGTGGAGCGTTTTC	181
Ad01A8052	Aradu.A01	8052	p4	(CTTT)6	24	32926102	32926125	CTCTGTGGTGGTGGTGTGTC	ATCACTCCCACCTTACACGC	184
Ad01A8054	Aradu.A01	8054	p2	(CT)6	12	32936623	32936634	CAAATTCCATTAACAGGAAAATCA	GGAGGAATAAGGGGGATTCA	273
Ad01A8056	Aradu.A01	8056	p2	(AT)9	18	32950550	32950567	CCATCCATCCTTGATTGGTC	GACCACGCAAAAATCCTCTC	268
Ad01A8057	Aradu.A01	8057	p2	(AT)10	20	32965752	32965771	AGAAGGGACTTTCCATAACCTT	AATTGTAGTAAGTATCTCCATCGAAAA	273
Ad01A8058	Aradu.A01	8058	p3	(TTA)18	54	32966480	32966533	CCTTTTTAAAATATAATGCATGTACCG	GTTCTAATTCTCATTCAAGTGCAA	157
Ad01A8060	Aradu.A01	8060	p4	(ATAC)6	24	32973965	32973988	AATAGGGTCCCATCCAAACC	TGGGTGGTTAAAATGCATGA	139
Ad01A8062	Aradu.A01	8062	p2	(CT)7	14	32990863	32990876	AGCCCATCTCTCTCTTTCCC	GCAGGAACCTAGGAGCAGTG	225
Ad01A8063	Aradu.A01	8063	p3	(GAA)7	21	33031942	33031962	ACCACTGAGCTATGACGGCT	CAACTTCTCTTTCCCACCCA	280
Ad01A8065	Aradu.A01	8065	p2	(CT)7	14	33041007	33041020	AATAAGCAGCAGTCTGGCGT	TGAAAGTGAGGTAGGTGGGG	276
Ad01A8066	Aradu.A01	8066	p3	(ATC)11	33	33049153	33049185	GCAATCCTGAATATGTTTGGC	CCTTGAAGCAAAAGAAATAGCAA	196
Ad01A8067	Aradu.A01	8067	p3	(ATC)5	15	33053897	33053911	TGCTTCATTGCTGTCCTTTG	TCTTCATGCACTGCCATTTT	230
Ad01A8068	Aradu.A01	8068	p3	(TTG)5	15	33054540	33054554	TCAAGTGTTTTGGGTTGTCTT	ATTCCAGCTGCAGTTCTGGT	217
Ad01A8070	Aradu.A01	8070	c	(T)11aaataaaag	50	33107437	33107486	GGATTTAGGGTTGTGCATGG	CTGTGTGGCTTTACGAGCTG	235
Ad01A8071	Aradu.A01	8071	p2	(AC)6	12	33107658	33107669	CAGCTCGTAAAGCCACACAG	ATGAGAAAGCTCTTTGCCCC	117
Ad01A8080	Aradu.A01	8080	p2	(TA)10	20	33143585	33143604	GGTTGAGGTGTGTGTGTCATTT	TGGGTAATCTTTGCCCCTTT	280
Ad01A8082	Aradu.A01	8082	p3	(AGA)5	15	33167515	33167529	AGAATATTGCTCGCCCTCAA	CGAACCTACTCCCTCTCCCT	173
Ad01A8087	Aradu.A01	8087	p3	(GAA)5	15	33221312	33221326	GTCTTGGCGGTTGTTTTCAT	TAACCTGGGCTCAAAATTGG	246
Ad01A8092	Aradu.A01	8092	p3	(TCT)5	15	33260967	33260981	TTTTACCCAGATTGGTCCCC	GCTAGTGTTCAACAACGGCA	262
Ad01A8093	Aradu.A01	8093	p2	(CT)6	12	33271188	33271199	CCTCGAACATTGATCCTGCT	GAGAATTTTGCGGAAATGGA	251
Ad01A8094	Aradu.A01	8094	p2	(CT)6	12	33282282	33282293	GGCTGCCATGAGCTTTAGAT	TTATTGAAACGGGTGTGCCT	100

ID	Chromosome	SSR nr.	SSR type	SSR	Size	Start	End	FORWARD PRIMER (5'-3')	REVERSE PRIMER (5'-3')	Product (bp)
Ad01A8095	Aradu.A01	8095	c	(ATA)5atttttttt	87	33282501	33282587	CCCCGTACTGGGTATTTCAA	CACCAAGCAACCCCATTTAT	238
Ad01A8096	Aradu.A01	8096	p3	(TGC)5	15	33283292	33283306	AAAATTTCGTTGTTTTGCCG	CTTGCCATGACACGATCAAC	265
Ad01A8097	Aradu.A01	8097	p2	(TA)6	12	33304929	33304940	GGACATTATAGTCCGGGAAGA	TCAAATTGAGTCAGTTCAATGG	114
Ad01A8098	Aradu.A01	8098	p3	(ATA)5	15	33305895	33305909	CCTATGGGGTCACATATTAACGA	TCAGTGAGGATCTCATCCAAA	239
Ad01A8099	Aradu.A01	8099	c	(T)11agttgggtc	72	33307698	33307769	TGAGTCCTTGCACCAATTTTT	TGGTTGTCAAAATAACCCATGT	271
Ad01A8100	Aradu.A01	8100	p3	(TCT)5	15	33334747	33334761	CCCATCACCTCTTCACCACT	CTGGCCTTAGGTGCCATAAA	267
Ad01A8102	Aradu.A01	8102	p2	(AC)7	14	33349357	33349370	TTCAATGAACACCCAAAGCA	GACAACCCTTACATCTGCCC	216
Ad01A8103	Aradu.A01	8103	p2	(AG)6	12	33349982	33349993	CAAGCAAACCAAACAAAGCA	CCACCAACTGAAGGTGGAAT	222
Ad01A8105	Aradu.A01	8105	p3	(ATT)5	15	33357528	33357542	ATTTGGACGTTGGCTTTGTC	CAGGGAAAAAGGCACTCTTG	194
Ad01A8112	Aradu.A01	8112	p2	(GT)6	12	33445736	33445747	TGGGAATTTGGATGCTACTCA	TAAGGATGAGGATGGCCAAG	243
Ad01A8113	Aradu.A01	8113	p2	(TC)6	12	33454170	33454181	CCTAATTGGAGCAGGGATGA	GGTTTGGGAGGGAAAAGTGT	125
Ad01A8115	Aradu.A01	8115	p3	(AGA)5	15	33468999	33469013	TCCTCGAGCAAAAGAGGAAA	AAAACCCCACCTACCTCACC	227
Ad01A8116	Aradu.A01	8116	p3	(TCT)6	18	33471801	33471818	CTCTCTTCTCCCCCTTGACC	CCAGCCTTAGGTGCCATAGA	173
Ad01A8118	Aradu.A01	8118	p3	(AGG)6	18	33480379	33480396	AGCATCGCGTCTGCTAAAAT	TTGTGCTGTTGCTACTTGGG	254
Ad01A8120	Aradu.A01	8120	p2	(GC)6	12	33504390	33504401	CTCAATCCCCCTTCAATCCT	CCAGTTGGGTTGGAAAGCTA	221
Ad01A8121	Aradu.A01	8121	p3	(GGT)6	18	33510007	33510024	AAAAGGAGGGTGTGAACGTG	TCTTCTCCTTGTCGCCCTAA	140
Ad01A8123	Aradu.A01	8123	p2	(AT)10	20	33510638	33510657	TTCGTCGACGCATAGTGAAG	TTCGGTTTGGTTTGGTTTGT	216
Ad01A8124	Aradu.A01	8124	p3	(TTA)9	27	33512271	33512297	AGCACCAATGCCAATTCAAC	TCTTATGCAAACCAACCTGAAA	138
Ad01A8129	Aradu.A01	8129	p4	(AATA)5	20	33524337	33524356	TTAGTCCCCATGTCCGTCTC	ACTCCATCCCTCCAGAGGTT	179
Ad01A8130	Aradu.A01	8130	p3	(TAA)5	15	33524529	33524543	ACCTCTGGAGGGATGGAGTT	GTTTGTGCACCTCACCTGAA	220
Ad01A8133	Aradu.A01	8133	p4	(TATT)6	24	33580459	33580482	GAAAAGTTAAACAAGAAAAGCAAGA	TTCGAAATTTATGTTCTTTGATCTTA	204
Ad01A8135	Aradu.A01	8135	p3	(GAT)5	15	33584731	33584745	TTGACCCAATTTCCATTTCC	GAAAAGTGGGCCAAACACAG	260
Ad01A8136	Aradu.A01	8136	p2	(AG)19	38	33585133	33585170	CACTGCAGCCGTTTATAGCA	CTCCAAAACCCTAGCCCTTC	176
Ad01A8137	Aradu.A01	8137	p2	(GA)10	20	33585609	33585628	GGCAAAGATACGGTGCAAAT	GACCTTTCGCTGGTGTGTTT	203
Ad01A8138	Aradu.A01	8138	p2	(CT)8	16	33590614	33590629	CGATAGTGAGACGCTGGTGA	TACGCCACGTTGTCGTACAT	232
Ad01A8139	Aradu.A01	8139	c	(AG)8gtgagaga	45	33592743	33592787	GCGACGGTATTTCTAGCGAC	TCCGACAATAATCATGCCCT	255
Ad01A8140	Aradu.A01	8140	c	(ATA)5at(TTA	86	33594082	33594167	CCTGAGATGAAGTATGAAGGAAGG	GGCTCGATACCCAGGTCATA	193
Ad01A8143	Aradu.A01	8143	p3	(ATA)18	54	33598525	33598578	TGTCATTTCTCCGTTATTTCGTT	TTTCGTTGTGGTTTTTATCCAA	125
Ad01A8144	Aradu.A01	8144	p2	(AT)8	16	33604138	33604153	TTTTACCGTGGCCATAGACC	GGTTCGAAGGAGGTTGAGAA	207
Ad01A8145	Aradu.A01	8145	p2	(AT)8	16	33608493	33608508	GGCTATTCGAGTGGCGTTAG	CCATAGTTGTGGCTGGTGTG	171
Ad01A8146	Aradu.A01	8146	p2	(AG)8	16	33613977	33613992	TGCTCATCTCCGAGCAAGTA	CTGACTCCACCCAATCCACT	232
Ad01A8150	Aradu.A01	8150	p3	(AGA)7	21	33646293	33646313	CTAGAGGATTCTCCGGCCTT	CTTCACTCTCCCCTCTCCCT	250
Ad01A8151	Aradu.A01	8151	c	(AG)6c(CT)7c	48	33662262	33662309	GGCAAGATGGGAGACAACTT	TCTGAACAGGGTAAATGGGAA	237
Ad01A8152	Aradu.A01	8152	c	(TG)22t(TG)1	81	33665408	33665488	GCTTCACGTGTGTAATTCCG	TGGTGCTTCATACGCCAATA	279

ID	Chromosome	SSR nr.	SSR type	SSR	Size	Start	End	FORWARD PRIMER (5'-3')	REVERSE PRIMER (5'-3')	Product (bp)
Ad01A8154	Aradu.A01	8154	p3	(ATT)17	51	33669194	33669244	CAAAGAAACTGCTGAAGGGG	ATTGGGAGAGGGGGACTATG	207
Ad01A8155	Aradu.A01	8155	p4	(AAAT)5	20	33672366	33672385	CAATTGGTCATTTATTAAAGTGGAGA	AAACAAAATCTTTCTGGTGGGA	206
Ad01A8158	Aradu.A01	8158	c	(AATAA)7aga	77	33688130	33688206	GGGTTAGCATATGACCCCAG	CACTTTCTCTATCCTCACTTTTTCG	258
Ad01A8160	Aradu.A01	8160	p2	(CT)6	12	33693107	33693118	GCAGAGGGCAGATAGAGCAC	AGGGTGGGAATAAAGGATGG	166
Ad01A8161	Aradu.A01	8161	p2	(AT)8	16	33700502	33700517	AGGAGCCTTGGAAGAAAGGA	ATCTTGGGACTCGGTGTTTG	235
Ad01A8167	Aradu.A01	8167	p2	(AG)7	14	33743908	33743921	CAATTGGGCAACATCAATCA	GCTATAAAGTGGGGGAAATGC	240
Ad01A8168	Aradu.A01	8168	p2	(TA)6	12	33746283	33746294	TACCCTAGCCGGCCTAAACT	CGACTCTCGAGTGGGATTTT	202
Ad01A8169	Aradu.A01	8169	c	(CTC)6atcc(T(	97	33746595	33746691	TGTCACGATTAAAAAGTTTCGG	TGGTAATTTTGCAGCATTATCTGT	276
Ad01A8170	Aradu.A01	8170	p3	(GAA)5	15	33746910	33746924	TGCTGCAAAATTACCAGAAAGA	CATAAACGTTCGTATCCCCC	149
Ad01A8171	Aradu.A01	8171	p2	(TA)8	16	33747175	33747190	CGAACGTTTATGTTAATGGAGAG	AACAATGTTTTTCAATGAAGGGA	276
Ad01A8174	Aradu.A01	8174	p2	(AT)6	12	33754476	33754487	GAAACCATTTTCTGTTTGGCTT	TCATGTTAAAGGAAAGACATTTTCA	275
Ad01A8175	Aradu.A01	8175	p3	(AAT)20	60	33754865	33754924	TCAAAATAATTGGAATTTGGAGAA	CTCTTGTTCCTGGCCTCAAG	243
Ad01A8176	Aradu.A01	8176	p3	(ATT)18	54	33755527	33755580	CCATGTCATGTGCGAAAAAT	TGGAATCGTCATCACCTAGC	280
Ad01A8177	Aradu.A01	8177	p2	(AT)10	20	33756219	33756238	TTCTATGATTCGTGCGTTCG	TTGAGGGAAATCTGATTCGG	176
Ad01A8179	Aradu.A01	8179	c*	(A)10(AAT)5*	23	33775373	33775395	TGAAGCAAGAAAAAGCAGTGAA	ATCCATTGATGCTCAAAGCC	168
Ad01A8180	Aradu.A01	8180	p2	(AT)6	12	33780429	33780440	GCTACCCATAATTCCCACTTTTT	TGCATCAACTAAGTGTGGGG	279
Ad01A8188	Aradu.A01	8188	p2	(TA)6	12	33834602	33834613	CATTGCTTGTCGTGCAAGTT	GCATGTCCTCATGCTAAGCTC	276
Ad01A8189	Aradu.A01	8189	p2	(AG)6	12	33835302	33835313	ATCAAGGAGGGGGAATCATC	TTGAACCTGGAATTGGGAAG	138
Ad01A8193	Aradu.A01	8193	p3	(AAG)5	15	33858640	33858654	AGTGGTCTTGATGGGCTTTG	GCCGAAACCAAACACTTCTC	246
Ad01A8196	Aradu.A01	8196	p3	(AGA)12	36	33917985	33918020	TGGAAGCTTTTGTCTTGCCT	CCCCCATCCTTCTTCATTTT	265
Ad01A8197	Aradu.A01	8197	p2	(CT)6	12	33947186	33947197	TTGGAAGAAGAAACCCTAAACC	CACGTGCCTCAGTTACGAGA	215
Ad01A8199	Aradu.A01	8199	p2	(AT)6	12	33966494	33966505	TGCTTCTCTGATAGAGCGGA	TTTCCCTCGCCTTGTTATTG	273
Ad01A8200	Aradu.A01	8200	p2	(CA)6	12	33966609	33966620	GCGGATATACGATGACTTCCA	TTTCCCTCGCCTTGTTATTG	258
Ad01A8203	Aradu.A01	8203	p2	(TA)12	24	34005170	34005193	GTACCCTAGCCGGCCTAAAC	AGCGTGAAGTCAAAAGCGAT	136
Ad01A8204	Aradu.A01	8204	p2	(AT)7	14	34025150	34025163	CAAAACACCCTAACTTCAACCA	CTAACCCCCAAATTCTGCAA	273
Ad01A8209	Aradu.A01	8209	p2	(AT)7	14	34032192	34032205	TTCCCACTTCACCAAAAAGG	CATATCTTTTCCCTCATCATGC	259
Ad01A8211	Aradu.A01	8211	c	(T)10ag(T)10	22	34055110	34055131	GCAAGCAAATCATCAAGAACA	CTAACATTGCATGATTCCTGG	250
Ad01A8212	Aradu.A01	8212	p2	(AT)9	18	34085238	34085255	GATGTGCAATCCATACGACG	CCATGCGATCGTGTCATATT	253
Ad01A8213	Aradu.A01	8213	p2	(AG)6	12	34087758	34087769	CAATTGGGCAACATCAATCA	AAATCCAAGTCCAGAAGGCA	269
Ad01A8214	Aradu.A01	8214	p3	(GAA)6	18	34094801	34094818	CCAGCCTTAGGTGCCATAAA	ATTTCTTTCCCACCCAAACC	188
Ad01A8215	Aradu.A01	8215	p2	(TG)7	14	34096317	34096330	TTTGAATTGAAAGCTTCGGG	TTTCAAGCAAGGGGAATCAC	191
Ad01A8217	Aradu.A01	8217	p3	(AGA)6	18	34135800	34135817	CTTCCCTTTTCCTTTTTCGG	ACACATTACCCCCTCTCCCT	253
Ad01A8221	Aradu.A01	8221	p3	(AAT)6	18	34150078	34150095	GGCACTCTTGTTGAAGCTGA	AAACTTGGACGTTGGCTTTG	192
Ad01A8222	Aradu.A01	8222	p3	(TCT)6	18	34152545	34152562	CCTACCTCGCCCTCTCTCTT	TAAAATATTGCTCGCCCTCG	225

ID	Chromosome	SSR nr.	SSR type	SSR	Size	Start	End	FORWARD PRIMER (5'-3')	REVERSE PRIMER (5'-3')	Product (bp)
Ad01A8223	Aradu.A01	8223	p2	(TG)24	48	34155857	34155904	TTTCTCTCAATTTCGTTAGGGTTC	TGATCAAAAGCAATAAAGGCAA	268
Ad01A8227	Aradu.A01	8227	p3	(AGA)5	15	34212223	34212237	TCGCTCATGACTCAGAGGTG	CCCACCCTAAATAGCCGAAT	226
Ad01A8228	Aradu.A01	8228	p2	(AG)6	12	34212393	34212404	GGTGGGAAAGAGATGATGGA	CTCAGGATCTGTGGACCCAT	163
Ad01A8234	Aradu.A01	8234	p3	(TCT)7	21	34260433	34260453	CCCATATGGCCGAAACATAC	CTATGGACCCCTGAGATGGA	236
Ad01A8235	Aradu.A01	8235	p3	(ACA)9	27	34263745	34263771	CACTTCAAAGGGCCAGAGAG	GAAGTGCCAGGAATTGTGGT	105
Ad01A8237	Aradu.A01	8237	p2	(AT)10	20	34290280	34290299	ACGTTTTGCGCTTTTCAAAC	TGGGGAAGGTAAATGTGGAG	261
Ad01A8238	Aradu.A01	8238	p3	(TTA)7	21	34298040	34298060	TAGGGCATTTTGTCCCCTTT	TTAAGTGTGGGGAAGTTGGG	223
Ad01A8239	Aradu.A01	8239	p3	(GAA)6	18	34299550	34299567	TGGAAGTGAAGAGGGGTGAG	GCATGGCTCGTTCAGTTTTT	130
Ad01A8240	Aradu.A01	8240	p3	(ATC)6	18	34362852	34362869	GAAGTCGAAGAAGTCGGCAC	GAAAAGGCACAGGCTGTAGG	265
Ad01A8243	Aradu.A01	8243	p3	(AGA)5	15	34385837	34385851	TAAACACCCATTTTGGCACC	CACTCCGGTCTATCTCTCGG	251
Ad01A8244	Aradu.A01	8244	p2	(AT)7	14	34422892	34422905	AAGGATAACAAGGTGCCACG	AACTCCGCAGGTAGTGGCTA	233
Ad01A8245	Aradu.A01	8245	p2	(AT)8	16	34423024	34423039	AAGGATAACAAGGTGCCACG	AACTCCGCAGGTAGTGGCTA	233
Ad01A8246	Aradu.A01	8246	c	(TA)6tgtatatat	34	34427342	34427375	AAATCCCAACGTCAAAATGG	AAAGCGAGAGGGGTGAGAAT	201
Ad01A8247	Aradu.A01	8247	p3	(TTA)6	18	34431060	34431077	GCAAATGCACTAAAAGAAAATGG	CCTGCTTTTCTCCTTCCTCC	227
Ad01A8253	Aradu.A01	8253	p2	(TA)6	12	34519042	34519053	CAAATCCAGTGAGGGTTCAA	TGGTGGAATCTTGTGCAATG	272
Ad01A8255	Aradu.A01	8255	p3	(TCT)6	18	34553253	34553270	ACACAACCCTATCTTCCCCC	TTGCCTTCCCTTTCCTCTTT	211
Ad01A8261	Aradu.A01	8261	p3	(ATC)6	18	34610352	34610369	GAAGTCGAAGAAGTCGGCAC	CAATTTCAAGTTCTTGCGCC	161
Ad01A8262	Aradu.A01	8262	c	(T)13ggggaaga	49	34636352	34636400	GGTGAGGTAGGTGGGGTTTT	TGGCTCAGAACGTGATTTTG	222
Ad01A8263	Aradu.A01	8263	c	(A)14gaattccca	65	34646134	34646198	CCTTCCCTAAATCGTTAACAAAAA	TGGTGGCAAAGAGATGAATG	225
Ad01A8273	Aradu.A01	8273	p2	(AT)9	18	34735134	34735151	TGATTTTTAAAGGCGTTGGC	TCCATAAGTATTGTCTTAGTTGGGTTT	273
Ad01A8277	Aradu.A01	8277	p2	(AG)7	14	34748095	34748108	ACAATGCTTGGATGGTTGGT	TTTATCGGTTTGGTTCTCGC	115
Ad01A8278	Aradu.A01	8278	p2	(AG)6	12	34750158	34750169	CGTGCAGAAAATTGCAAAAA	AAAGGAACATTGAACCTGGAA	273
Ad01A8279	Aradu.A01	8279	p3	(TTG)5	15	34752949	34752963	CCCCACTTAATGGGACAAGA	GGTGCTGTGGGATAGTGCTT	270
Ad01A8283	Aradu.A01	8283	p2	(TA)6	12	34767721	34767732	TTGCCTTTAGTTTTCAAATTGTTTC	TGGCATAAATTTTACCTAGTTGTAGC	252
Ad01A8285	Aradu.A01	8285	p2	(TA)7	14	34768939	34768952	AATCATGTATTCTTCATGAGTTAATTG	GCCGGAGTTAGCATGTGATT	261
Ad01A8287	Aradu.A01	8287	p3	(ATT)16	48	34775230	34775277	GGGTAATTGAAATAGTTTATGTACGGA	AATGGCACCGGTCTAATCAT	243
Ad01A8288	Aradu.A01	8288	c	(TAT)5tac(TA	33	34775455	34775487	ATCATGATTAGACCGGTGCC	GGTGAGGTGTCGATCCTTGT	243
Ad01A8289	Aradu.A01	8289	p2	(AT)8	16	34781804	34781819	GTACCCTAGCCGGCCTAAAC	GCGATATACGGACAAGGCAT	117
Ad01A8292	Aradu.A01	8292	p4	(TCTT)5	20	34783493	34783512	CGTCACAAAATCCACGTGTC	GTGGAGGTGGAGAGTGAGGA	181
Ad01A8304	Aradu.A01	8304	p2	(CT)6	12	34860480	34860491	ATGAGTCCCGCAAAGATGAC	GCGGAAGAGAGGGAGACAG	244
Ad01A8305	Aradu.A01	8305	p4	(CTTT)5	20	34860608	34860627	CTCTGCTCCCTCAACTCCAC	ATCTGCCAAGCCAATGTTTC	275
Ad01A8307	Aradu.A01	8307	p2	(CT)6	12	34869352	34869363	ATGAGTCCCGCAAAGATGAC	GCGGAAGAGAGGGAGACAG	244
Ad01A8308	Aradu.A01	8308	p4	(CTTT)5	20	34869480	34869499	AAGCAAAGTGCTAGGTGGGA	CGCGTGACTCTCTGCTTCTT	267
Ad01A8309	Aradu.A01	8309	p2	(TA)6	12	34872881	34872892	TTCACTAAAATAGCCCAAAACG	CCATAAATTTGTCATTGAATTCTCA	268

ID	Chromosome	SSR nr.	SSR type	SSR	Size	Start	End	FORWARD PRIMER (5'-3')	REVERSE PRIMER (5'-3')	Product (bp)
Ad01A8321	Aradu.A01	8321	p2	(TC)19	38	34909121	34909158	TCATCGTCATTGAAGGCTTG	CGACATGATCGAACTCGAAA	224
Ad01A8326	Aradu.A01	8326	p2	(TA)9	18	34940958	34940975	GTGACCTGATGACAGGGGAG	TACGACCGGGTTGGTAGTTC	269
Ad01A8327	Aradu.A01	8327	p2	(TA)10	20	34944097	34944116	AAAAATGCCAATAGCAAAAATAA	GGCCGTACGTAGGAAGTTGA	268
Ad01A8331	Aradu.A01	8331	p2	(AG)6	12	34946786	34946797	TGAACAAGGCTTTGACCACA	TTTGTATCTCTGACGTGGCA	200
Ad01A8332	Aradu.A01	8332	p2	(TA)12	24	34946911	34946934	TGCCACGTCAGAGATACAAA	GCAAATTGATAAAAACGCTCC	194
Ad01A8334	Aradu.A01	8334	p2	(AG)6	12	34963751	34963762	GAGAGAGGGCTGCTAGGGTT	AACCGGTGAACTGCTTGAAC	166
Ad01A8337	Aradu.A01	8337	p2	(GC)7	14	34981987	34982000	CAGTACCAGCGATTGCTTCA	CCTTTGGTTCTGCACTCCTC	146
Ad01A8339	Aradu.A01	8339	p2	(CT)6	12	34992808	34992819	GCACGCCTAAATAGAGCAGG	TTGGGAGGGAAAAGTGTTTG	248
Ad01A8340	Aradu.A01	8340	p2	(TA)7	14	35000954	35000967	GGGGTTTAAGACCCTGGAAA	CCAAATAACATAAAACCCAATTTCT	275
Ad01A8341	Aradu.A01	8341	c	(AT)10act(TA)	35	35023246	35023280	GTCTTGGCCTGCGAAGATAA	GAGCATGCCAGCTTCTCTCT	226
Ad01A8342	Aradu.A01	8342	p2	(AT)11	22	35041151	35041172	TGCATGTGCCTTATCCATAACT	GCATCACGGTAACCAACCTT	211
Ad01A8344	Aradu.A01	8344	p4	(TTAT)6	24	35049233	35049256	CGTTTGAAATCCCCAAAGAA	CATCGAATGATCTTCTACAACCA	237
Ad01A8347	Aradu.A01	8347	p2	(GT)6	12	35056077	35056088	GCTCGAACACACAAAACCTG	CAACATTGGTTTGCACAGGA	245
Ad01A8348	Aradu.A01	8348	p2	(GT)7	14	35058142	35058155	GCTCGAACACACAAAACCTG	TAGGCTCAACATTGGTTTGC	252
Ad01A8352	Aradu.A01	8352	c	(A)10caaaaatc	97	35093412	35093508	CGGTCTTAGGTGCCATTGAT	GAACTCCCACCTTCCCTCTC	243
Ad01A8357	Aradu.A01	8357	p3	(AAT)5	15	35136001	35136015	CAGGAACAAGGAAAAAGGCA	GGACGTTGGCTTTGTGAAGT	199
Ad01A8358	Aradu.A01	8358	p3	(TTC)6	18	35139037	35139054	CGAACCTAACCTCTCCCCTC	TGGAAGCTTTTGTCTTCCGT	280
Ad01A8361	Aradu.A01	8361	p2	(TG)6	12	35194866	35194877	TGGGATCGACCCTTACTCAC	GCTTAGTGTGCCACGACAGA	210
Ad01A8362	Aradu.A01	8362	p3	(GAT)13	39	35199385	35199423	TTATGGCAGTGCAGTGAAGG	TCTTCCATTAACCCATCCCA	211
Ad01A8365	Aradu.A01	8365	p3	(AAG)5	15	35206315	35206329	CAACAGCAGCAATAAAAGAACG	CGTATACACACGCGCACTTC	145
Ad01A8368	Aradu.A01	8368	p3	(ACA)6	18	35222092	35222109	AACCACCATTTACCAAAGCA	TTGATGGGATTTTTATTGTTGTTG	184
Ad01A8369	Aradu.A01	8369	p2	(TG)8	16	35223952	35223967	CCCTCACAAGCTCGTCTTTC	CACCCACTCCCCAAACTCTA	267
Ad01A8370	Aradu.A01	8370	p2	(CT)20	40	35227257	35227296	TCAAATGTGAAACCCTTCCC	AAGGTGACGACCTTTGATGG	117
Ad01A8371	Aradu.A01	8371	p2	(TA)12	24	35229962	35229985	GGTGGTAGGGGTGGAAGAAT	TGCTTCGTGAGTGTTGTGTTT	246
Ad01A8372	Aradu.A01	8372	p2	(AC)21	42	35239236	35239277	TTTGGACAACTGACGGTTCA	GGAAGCCCCTATCCAAAAGA	221
Ad01A8373	Aradu.A01	8373	p2	(CT)7	14	35252951	35252964	GAGGCAACTTTACCAGCAGC	CGAAAGAGGAGGGAGGTTTC	257
Ad01A8374	Aradu.A01	8374	p2	(TA)6	12	35254273	35254284	TCATGATAACATTGGTTGGGA	TGATGGGCTATCACCTACCC	169
Ad01A8375	Aradu.A01	8375	c	(TA)9gtgcttgg	62	35254436	35254497	TGAGGGTAGGTGATAGCCCA	AGCACGCTAATTGGGACTCA	233
Ad01A8376	Aradu.A01	8376	p3	(TAT)9	27	35254838	35254864	GAATCATGACCCGGAAGCTA	GGGAAAAGTCCCAAACATCA	205
Ad01A8377	Aradu.A01	8377	c	(A)14gagc(AG	36	35263131	35263166	CGAACCAGGAGGAACTTGAG	TATTGGTGTGTTCCCCGTCT	264
Ad01A8378	Aradu.A01	8378	p2	(AT)8	16	35265438	35265453	TCCCACTTCAACACTTAATTTTGTT	TGATTGATTAGGGTTTATGGAAAA	278
Ad01A8380	Aradu.A01	8380	p3	(GGT)5	15	35272348	35272362	TATTGGGTTGGATTGCTGGT	TGCAGATTTGCCTCATTGAT	232
Ad01A8381	Aradu.A01	8381	p3	(TAA)5	15	35274659	35274673	AACCATGAATTTGCAGTGGC	ACCCGGTTCACTTTTGAATG	257
Ad01A8382	Aradu.A01	8382	p2	(TA)6	12	35297005	35297016	TTTAGCCGCTTAGGCCTGTA	TGGCTCTTGAAAGAATGATGAA	249

ID	Chromosome	SSR nr.	SSR type	SSR	Size	Start	End	FORWARD PRIMER (5'-3')	REVERSE PRIMER (5'-3')	Product (bp)
Ad01A8383	Aradu.A01	8383	p3	(AGA)6	18	35298682	35298699	ACTCACCCTCGAGCAAAGAA	CCCATCACCTCTTCACCACT	270
Ad01A8385	Aradu.A01	8385	p3	(AGA)6	18	35332540	35332557	GCTTTTGTCTTCCCTTTCCC	TACACCTCTCCCCCTCTCCT	185
Ad01A8389	Aradu.A01	8389	c	(TCA)9(TCT)2	108	35359341	35359448	CACCCACACACAAAATTGAA	TTCTTCGCAGAGATTGAGAGA	280
Ad01A8393	Aradu.A01	8393	p2	(TC)6	12	35364740	35364751	CAGGAAATGGCTACCTTGGA	GCTGGGAACCATTTGAAGAA	254
Ad01A8398	Aradu.A01	8398	p2	(TA)11	22	35374771	35374792	GAGACATCGTGAGAGGGAGC	TCAGGTTGAACACGAGCAAG	207
Ad01A8401	Aradu.A01	8401	p3	(TAA)12	36	35376399	35376434	TGTTGGTTAATTACTGGCCAAA	TCAAACCGCAAATCAACAAA	278
Ad01A8403	Aradu.A01	8403	p3	(AAG)7	21	35379054	35379074	TGAACCACGCTATCAAGGTG	CCAAAGCTGAAGAGAATGCC	269
Ad01A8408	Aradu.A01	8408	p3	(TCT)5	15	35409726	35409740	TACTCGTGAGGGCGATTAGG	AAACTACCTCGGAAGAGGGG	251
Ad01A8409	Aradu.A01	8409	p2	(AG)19	38	35414106	35414143	TGGAGTTCTGACGAGTTCCC	CCTCCCCTTCACTTGTCTGA	160
Ad01A8410	Aradu.A01	8410	p3	(AGA)5	15	35426862	35426876	CGACAAAACATGACGAATGG	TCACCGGGGATTATTTGTGT	256
Ad01A8412	Aradu.A01	8412	p3	(TTA)5	15	35439536	35439550	GAGTGAGTGTGGTTCGGGTT	TCAAACATTTTTGCTGCTAATGA	216
Ad01A8414	Aradu.A01	8414	p3	(ATA)7	21	35442602	35442622	TTGGACTTGCCTTTGTCCTT	TGGCAACTGCCTTACCACTT	231
Ad01A8415	Aradu.A01	8415	p2	(AG)6	12	35445119	35445130	CGGACAAAGTTGGCTCATAAA	AACCATGAACGAGGCAAAAG	280
Ad01A8417	Aradu.A01	8417	p2	(GA)16	32	35450751	35450782	AAAGGTAAAGTGATACCGCGAA	GTGTGCCCATACACACAAGC	159
Ad01A8420	Aradu.A01	8420	c	(A)10gatg(A)1	24	35478399	35478422	TCACCATAATACGTACTACAAATTTCA	CATGTCGTCATCCGCATATT	247
Ad01A8421	Aradu.A01	8421	c	(CAG)5(CAA)	30	35479369	35479398	CTGCCTCAAAGTAATCCCCA	CACTCTGGACCCTCGTTTGT	279
Ad01A8422	Aradu.A01	8422	p3	(CTC)6	18	35485144	35485161	TTTATCCAGGCGCAACTAGG	ATCACAATGGGCTGAGAAGG	256
Ad01A8427	Aradu.A01	8427	p3	(ACC)5	15	35505401	35505415	GCAAGTGGTGCAGCATAAAA	GGTGACGGAAGAGGTTGTTG	219
Ad01A8429	Aradu.A01	8429	c	(AGA)17at(A/	80	35519808	35519887	TCATTCCCATTTGGGTTCAT	TCTCTCTTCTTTTTCGAAATTCAT	239
Ad01A8431	Aradu.A01	8431	p3	(TTC)13	39	35538900	35538938	GGTGGTGAGTGAGGAAGGAA	GAGTAGCCGAACCCCCTTAC	273
Ad01A8432	Aradu.A01	8432	p2	(AG)15	30	35548586	35548615	TTCAAGCCTTGGCTGTTCTT	GGGCTAGGAATCCTTCTTGG	254
Ad01A8433	Aradu.A01	8433	p3	(TTA)6	18	35553765	35553782	GACTATGACCGCCTATCCCA	CGCAGTCGTACATTGCAAAA	178
Ad01A8436	Aradu.A01	8436	p2	(TC)6	12	35568242	35568253	CGTGCACTTCCAAATCCATA	GGAAAGGAGGTGGTGCTAAA	202
Ad01A8437	Aradu.A01	8437	p2	(TA)6	12	35568946	35568957	ATAGGGGAGGCGCTTGATTA	AGCCAAGCACCTCAAAAGTC	264
Ad01A8439	Aradu.A01	8439	p2	(GA)13	26	35570966	35570991	AGCTTCCTTTTCAAGCGACA	TTCGTTGGCACTACAGTTCG	256
Ad01A8441	Aradu.A01	8441	p3	(ATA)5	15	35572463	35572477	ATGCAATCCACGTCACAAAA	GCTTGTCGTTGCAATTGTTG	100
Ad01A8442	Aradu.A01	8442	p2	(AG)6	12	35572643	35572654	CCTAGCAACAATTGCAACGA	ACTCCCTTCCATCACAATGC	259
Ad01A8450	Aradu.A01	8450	p3	(GTA)5	15	35600687	35600701	GTGAAGTAGCTGTCGGAGGC	CCCCTATTGCCCTAACACAA	247
Ad01A8451	Aradu.A01	8451	p3	(TCT)33	99	35610051	35610149	CGAATACACACCCATCTCCC	CTAGAGGTTTCTCCGGCCTT	246
Ad01A8453	Aradu.A01	8453	c	(T)10cgtacattc	29	35616901	35616929	CCATTGATTCAGTTTTTAATTTGC	CTTGCTTTGATGGGAGGTGT	263
Ad01A8456	Aradu.A01	8456	p3	(AAT)6	18	35632266	35632283	AGGGAAAAAGGCACTCCTGT	AAACTTGGACGTTGGCTTTG	201
Ad01A8457	Aradu.A01	8457	p3	(TTC)6	18	35634628	35634645	CAACTTCTCTTTCCCACCCA	CCCAAGATTCAGAGGTGGAA	233
Ad01A8459	Aradu.A01	8459	p2	(AT)10	20	35643463	35643482	ACCTAACTGCAAACATGCCC	TGGCCTTTGTTCTCTGTTGA	272
Ad01A8460	Aradu.A01	8460	p2	(GA)17	34	35644437	35644470	CTCCATTTCCTTCCACCTCA	TTGGCAATTTGGGACAGAAT	271

ID	Chromosome	SSR nr.	SSR type	SSR	Size	Start	End	FORWARD PRIMER (5'-3')	REVERSE PRIMER (5'-3')	Product (bp)
Ad01A8461	Aradu.A01	8461	p2	(AG)16	32	35644696	35644727	TTTGCCAGGAAATTGAGACC	CCCCAAATCCACTTTCTCCT	194
Ad01A8468	Aradu.A01	8468	p2	(TG)11	22	35658865	35658886	ACGTGTCGGTTACGTTGACA	CTGATCATAAGGGTGGTCGG	197
Ad01A8469	Aradu.A01	8469	p2	(TC)7	14	35663473	35663486	TCAAAGTTCTGACGGTGGTG	CCATGTATCTCACCGAGGCT	267
Ad01A8471	Aradu.A01	8471	p3	(TTA)5	15	35667351	35667365	TTGTTGGAGCTTTTGCCTCT	CGGTTCTTCATTCAGTGGAA	272
Ad01A8472	Aradu.A01	8472	p3	(GTG)5	15	35668384	35668398	TTCTCGAGCCTTCACCACTT	TTCGAGGACGAAGCTGAAAT	232
Ad01A8473	Aradu.A01	8473	p3	(AAT)16	48	35674860	35674907	TTGCGTTATTTGGGCAGAGT	TCACGTTATTTTTAACATTTCTTTTCA	134
Ad01A8474	Aradu.A01	8474	p3	(TTA)5	15	35675304	35675318	TGTCAAAAATTGGAAAGAGTTGAA	TCCGAAAAATAAAGATGTCCAAA	161
Ad01A8475	Aradu.A01	8475	p3	(TTA)5	15	35675669	35675683	ATTCCACCCAATCCAATTCA	ACCTTTTGTCTGGTGTCCCA	271
Ad01A8476	Aradu.A01	8476	p3	(TAT)5	15	35677363	35677377	TTCGACGAAAATTTTGACAACT	AGACTTGCGTTATTTGGACTGA	246
Ad01A8477	Aradu.A01	8477	p3	(TTA)7	21	35685430	35685450	CAGTGACGTGCAAGCCTAAA	GCGGCCACTGCTCATAGTA	217
Ad01A8479	Aradu.A01	8479	p3	(TTA)5	15	35686200	35686214	TTGTCAAAAATTGGAAAGAGTTGA	TCCGAAAAATAAAGATGTCCAAA	162
Ad01A8480	Aradu.A01	8480	c*	(A)12(AAT)5*	25	35687342	35687366	TGGTCTATGGCATTTGGGTT	CCACACACTTGCTTCTGGTG	187
Ad01A8481	Aradu.A01	8481	p3	(ATT)10	30	35687896	35687925	TATAAACGGTGCCAGTTCGC	AATTTCAAAGGTTCCCACCC	244
Ad01A8482	Aradu.A01	8482	c	(A)10tttcgttaaa	43	35693942	35693984	TTTGATGCAAGAATCCTCGT	TGTGTTTTGATAAATGGCAACAT	278
Ad01A8483	Aradu.A01	8483	c	(GA)25(GT)16	82	35704053	35704134	GGGGAGAAGAAGAAAAGGAAA	CGCGTCAATCAGTATAAGCG	277
Ad01A8484	Aradu.A01	8484	p3	(ATT)5	15	35704787	35704801	GGTCGTGTCGCGTAATTTTT	CTTCCTCTTGGAAGATCCCC	240
Ad01A8486	Aradu.A01	8486	p3	(TAA)5	15	35739838	35739852	CTGGGTTTTCTCCCAGTGTT	CGTGAATCGGGTAACAAAGA	166
Ad01A8490	Aradu.A01	8490	p3	(AAT)5	15	35768755	35768769	CTCGTCCTTGCAAAATCACA	GGTTATGGCTGGTAGGGGTT	189
Ad01A8492	Aradu.A01	8492	p2	(TG)6	12	35844408	35844419	TCCAAACTCAGCCCAAAATC	AAGGAGCTGACCAGTGAGGA	205
Ad01A8493	Aradu.A01	8493	p2	(AC)7	14	35845247	35845260	GGCTAAGGTATGGGTGAGGA	AAAGGGGAACAAAATCACCC	247
Ad01A8494	Aradu.A01	8494	c	(A)10tttagatttta	94	35851780	35851873	GACCACGCAAAGGTTCTCTC	CTCATAGCCAAAGGGGTGAG	277
Ad01A8498	Aradu.A01	8498	p2	(AG)6	12	35911242	35911253	TTGTTGGCTCTTGAAAGAATGA	TTTGGTTAGGGACAGCTTGG	269
Ad01A8500	Aradu.A01	8500	p5	(AAGAT)5	25	35966720	35966744	AGGGCGGTACCCTTAAAAGA	TGTCCGACTTGTTGGACTTG	255
Ad01A8501	Aradu.A01	8501	p3	(AGA)6	18	35979737	35979754	TAAAATATTGCTCGCCCTCG	AAAACCTTTCCCACCCAAAC	210
Ad01A8517	Aradu.A01	8517	p3	(TCT)8	24	36067336	36067359	AAATCTTTCCCACCCAAACC	TGCCATTGATGGTAGTGGAA	264
Ad01A8519	Aradu.A01	8519	p2	(GA)8	16	36076244	36076259	ATTGGGTAGATTTGGGAGGG	ACAAGATCCCCACCTATCCC	203
Ad01A8525	Aradu.A01	8525	c	(TCTTCC)5(T	48	36124029	36124076	AGCGCTACACTCAGAACGGT	ACACCCAAAATTCGTTGCAT	199
Ad01A8526	Aradu.A01	8526	c	(CTT)6caaaaat	56	36124498	36124553	ACAGGAATTCACCCAAACGA	CTGCACGTTCTTCTTCATCG	273
Ad01A8527	Aradu.A01	8527	p3	(AAG)7	21	36124670	36124690	CGATGAAGAAGAACGTGCAG	AAACACGCTTCCAACCATTC	169
Ad01A8528	Aradu.A01	8528	p2	(AC)6	12	36125738	36125749	CGCACACAACACACAACTCA	CGGAGACCCAAGAAAATGAA	224
Ad01A8530	Aradu.A01	8530	c	(A)10tgagagga	72	36128739	36128810	CCGTCCTTGCTTGTTTGGTA	TGGCACTACAGCAACCTCAG	272
Ad01A8531	Aradu.A01	8531	c	(A)10gtagttgaa	109	36131837	36131945	CCACCGTTATAAGTTGGTTTG	GCGAGTGGGTCATAGAAAGG	236
Ad01A8532	Aradu.A01	8532	p3	(TTG)5	15	36135277	36135291	CGTTTGATTCATGTAGCCGA	GGGTTTTACTAAATATGGAGGCA	229
Ad01A8533	Aradu.A01	8533	p2	(CT)16	32	36136579	36136610	TCAATTCCGTACAACATAACTATCAA	AAAGGTTGCGATGTAGGTGG	226

ID	Chromosome	SSR nr.	SSR type	SSR	Size	Start	End	FORWARD PRIMER (5'-3')	REVERSE PRIMER (5'-3')	Product (bp)
Ad01A8535	Aradu.A01	8535	p2	(TC)7	14	36141006	36141019	CATTGCACTTGGTTTGGTTG	GCCCAGACGATCTTCATCTC	215
Ad01A8536	Aradu.A01	8536	p2	(TC)12	24	36141225	36141248	CGACGTCGATGAGATGAAGA	CGAGGGATCAGCAGAAAATC	255
Ad01A8538	Aradu.A01	8538	p4	(TATT)6	24	36148329	36148352	AAGGACTTACAATTCGTTTGCAC	GCCATTTCATCATTTTCCGT	221
Ad01A8539	Aradu.A01	8539	p3	(GTG)7	21	36155804	36155824	CGATGATGGTTTTGGTGATG	TGACAAAACTACCCCTTCTCTCTC	157
Ad01A8540	Aradu.A01	8540	p2	(CA)9	18	36157421	36157438	GCTCCACCCACCTCAACTTA	GAGGAGGAGGAAGAGGAGGA	226
Ad01A8541	Aradu.A01	8541	p3	(AAG)5	15	36157576	36157590	ACGTCCTTCTCTTTCCCCAT	ATTGTTAGAGCCGCCACATC	248
Ad01A8545	Aradu.A01	8545	p3	(ATA)5	15	36161961	36161975	ACTTCACCACCATCTGGCTT	ACACACGATTATTTTATCAGATTCAT	271
Ad01A8547	Aradu.A01	8547	p2	(AT)7	14	36166626	36166639	ACAAGCCACGCAGTCTCTTT	GGAACAAAGTCTCTGGCTGC	257
Ad01A8548	Aradu.A01	8548	c	(TAT)5taataca	71	36169917	36169987	GTAGGGAAAAAGGGCAAAAA	AAGGGACTTCTCTAGTCAGTTTTGTT	262
Ad01A8549	Aradu.A01	8549	c	(A)13ctatggctt	45	36170464	36170508	AAGGAAAGAAAAAGTGCAGGC	AAAAACACCAATAACAACCCAA	234
Ad01A8551	Aradu.A01	8551	p4	(AGAT)7	28	36173837	36173864	GCTCTGGTCAACGTCCCTAA	AGAGAATTGCGAAGCCAAAA	260
Ad01A8552	Aradu.A01	8552	p3	(TAG)5	15	36184579	36184593	GAGCAAGGGCGTGATCTAAG	CATGTCTGATGATGATGAGAAAAAT	257
Ad01A8554	Aradu.A01	8554	c	(CT)6cctcattaa	33	36210490	36210522	GGCCCAATTGAACTAAACCA	ACTCGAAATCAAGGCTGGAA	277
Ad01A8555	Aradu.A01	8555	p2	(AC)6	12	36218788	36218799	GGTCCTTGGCCCAACTAAAC	CAAGGCTGGAAATTCGAAGA	273
Ad01A8559	Aradu.A01	8559	p4	(TTAT)5	20	36231954	36231973	GAAGAAAACGAAAATAAGAAAATGG	AAAAGGGAAAAGTAAATAAGAATGAG	275
Ad01A8562	Aradu.A01	8562	p2	(TA)6	12	36236353	36236364	GGGATTTGGTAATCATCCCA	TTTTGGTGAAACATTTTGACACTT	203
Ad01A8564	Aradu.A01	8564	p2	(AT)9	18	36314728	36314745	AAAGCCTTCAAAGCCCTGTT	CAATCAGCCAAATTAAGGGG	206
Ad01A8565	Aradu.A01	8565	p3	(TCT)6	18	36329763	36329780	GCCGAAACACAACCATCTCT	CTGAGCTATGGACCCCTTGA	248
Ad01A8567	Aradu.A01	8567	p2	(TA)9	18	36341404	36341421	TCGAATATGCAATTGACACCA	CTGGCAATTAAGCCATGTCC	247
Ad01A8574	Aradu.A01	8574	p2	(AT)6	12	36407222	36407233	CATTTCGGTTCATTTGTGACT	TGCTGCCCTCTCAAGTCTTT	230
Ad01A8578	Aradu.A01	8578	c	(T)10atttaaaaa	75	36417829	36417903	GGATCGAGATTTTCGCCATA	TATTAAAATCGTCTTTTGCTTCTG	178
Ad01A8583	Aradu.A01	8583	p3	(TTA)18	54	36454530	36454583	TCAACATCTATCTGGAGAGCCA	ATTGTCTGCGACAACAGCAG	181
Ad01A8586	Aradu.A01	8586	p2	(TC)7	14	36465446	36465459	GCGACGGGAAAAACATTAAA	CTAAAACGTCACGAGGCCAT	248
Ad01A8588	Aradu.A01	8588	c	(CAA)5catattta	38	36476226	36476263	TGCCCTTTTTCCACTAGTTCAC	TCTGGATTGCTTTTGAATGTTG	229
Ad01A8592	Aradu.A01	8592	p2	(TC)6	12	36495371	36495382	AGTAACAGCAGCGTTCCTCC	GAAGAGAAAAACTCGTGCCG	236
Ad01A8598	Aradu.A01	8598	p4	(AAAT)5	20	36553047	36553066	TTGCTTGTTGTAAACGTCGC	TTTTTGAATTTAAGTTTGGTGTTACC	246
Ad01A8606	Aradu.A01	8606	p4	(AATA)5	20	36628758	36628777	TTTGAATCAAGGGATGTTGATG	ATTGATGCTCAGAGCCTTGG	172
Ad01A8607	Aradu.A01	8607	p3	(AGG)5	15	36630984	36630998	CGGTTCGGTTTGTCAGTTTT	TGAAAAGTGGATCGAAGGCT	243
Ad01A8613	Aradu.A01	8613	p2	(AT)10	20	36655526	36655545	CAATTGAACCTTCACCGGAT	AGCTCACTTAGCCATTTAGCC	147
Ad01A8614	Aradu.A01	8614	c	(AC)6tcatgtatt	110	36655675	36655784	GGCTAAATGGCTAAGTGAGCTAAA	CCATATGCATTGATGTCTTGGT	276
Ad01A8616	Aradu.A01	8616	p3	(TTA)8	24	36666751	36666774	TTTCAGAGAAGCAACTCTTACTGC	TCAAGACCTAATTGTATAGGATGTGAA	277
Ad01A8618	Aradu.A01	8618	p3	(TAT)8	24	36670655	36670678	AGAACTTCAATGCCAGCACC	GTGGAGGTGGGTGTTTGAGA	277
Ad01A8622	Aradu.A01	8622	p3	(GAG)5	15	36688152	36688166	AATCACAATGGGCTGAGAGG	TCTTTTGCAGACATGGCAAG	165
Ad01A8628	Aradu.A01	8628	p2	(AT)9	18	36721683	36721700	ACGTCTTATGTGTTATTTTGGGA	ACTACTGCGCCTCGTTGTTT	216

ID	Chromosome	SSR nr.	SSR type	SSR	Size	Start	End	FORWARD PRIMER (5'-3')	REVERSE PRIMER (5'-3')	Product (bp)
Ad01A8630	Aradu.A01	8630	p3	(GAG)6	18	36732257	36732274	CCTCACCAGAAGAGCCAGAC	GTGGCGTCAAGGTGGTTATC	174
Ad01A8635	Aradu.A01	8635	p3	(ATT)5	15	36749518	36749532	CCTTGACCAAACCTAAATGAGA	TGACGGTGAAGATGGATCAC	169
Ad01A8636	Aradu.A01	8636	p4	(AATA)8	32	36750293	36750324	GACTTTTCTTGGTGGTGGGA	TTTTCTTCCTCTTTTTCACATGC	211
Ad01A8640	Aradu.A01	8640	p2	(AT)7	14	36756703	36756716	ATTGGGGACCAGTTGACAAG	GGACCGCCAACATCATTAAG	226
Ad01A8642	Aradu.A01	8642	p3	(CAT)5	15	36765894	36765908	TAGCCATCCCCACGTAAATG	TGGAATTTATGTCGTGCCAA	220
Ad01A8644	Aradu.A01	8644	p2	(AG)6	12	36766430	36766441	TGGAAGTGAATGTTTTGGTTTC	ACAGTACCATTTTGCGGCTC	207
Ad01A8646	Aradu.A01	8646	p3	(ATT)6	18	36769357	36769374	AACTCCATTCCGACGACAAA	TCATGTGCTAAAGAGAACGACAA	253
Ad01A8649	Aradu.A01	8649	c	(A)10gacattgtt	86	36780653	36780738	CCGAAAACACCCTCTCTCAC	TGCTCGTGAGTTCTCACTCATT	276
Ad01A8651	Aradu.A01	8651	p2	(TA)6	12	36782356	36782367	GAACCGGTCAATTCAATCGT	GGTAGGGGTAGGGATGGGTA	227
Ad01A8652	Aradu.A01	8652	p2	(CT)20	40	36782787	36782826	ACCAATAACCACCGCTGAAG	GCAGAGAGGAAGCAGAGGAA	248
Ad01A8655	Aradu.A01	8655	p2	(TA)8	16	36819163	36819178	CCCAAAAGCATAATCAGTCCA	TCTTCCCCTTTTCCCTCCTA	250
Ad01A8656	Aradu.A01	8656	p2	(AG)9	18	36842234	36842251	ACCGTCACTACTGGTCTGCC	CCGGAACCCAAAAGAACATA	259
Ad01A8660	Aradu.A01	8660	c	(A)11cagttacat	75	36890998	36891072	AGCTCAACAGTCAACACAACTGA	CACTCGTGATCCCAATGAAG	279
Ad01A8662	Aradu.A01	8662	p3	(CAA)5	15	36895879	36895893	TTTCTTTGACCACACTGAATCAC	TGGAATATAGACAAGGGGCG	272
Ad01A8665	Aradu.A01	8665	p3	(GAT)6	18	36897437	36897454	TCGTTTTCAACATGGTTTGACT	ACCAATCAACCCTCCAATGA	192
Ad01A8668	Aradu.A01	8668	p2	(TA)6	12	36900742	36900753	TGCTCTCAAAAATAACCATGTAGTG	ATATGGGTGCCGGAAAATCT	262
Ad01A8672	Aradu.A01	8672	c	(A)10g(A)10ga	59	36927939	36927997	TGTGAGGAAGTTGGATTTGAA	TACTTTGGGGCATTTTGTCC	219
Ad01A8673	Aradu.A01	8673	p3	(TCA)5	15	36930866	36930880	GGTTGCAAACCTTGTCCCTA	AACAGCAGGTCAATTCTGGG	239
Ad01A8674	Aradu.A01	8674	p3	(AAT)7	21	36944711	36944731	GGACTGGACCTGTGATGGTC	GAATCAAGTGCAAGTAAGTGCC	261
Ad01A8675	Aradu.A01	8675	p3	(TCT)5	15	36952643	36952657	AAATCTTTCCCACCCAAACC	TAAAATATTGCTCGCCCTCG	208
Ad01A8680	Aradu.A01	8680	p2	(TA)7	14	36983115	36983128	ACCCAACTTCCATATGACGC	ACCATGACAAGGAGCTCACC	270
Ad01A8681	Aradu.A01	8681	p3	(AGG)5	15	36987274	36987288	TTGACGCTTGGATTCAACAA	AATCGCGCTATATGGTTTGG	117
Ad01A8683	Aradu.A01	8683	p2	(AG)7	14	36995962	36995975	CGTGTTGTGCATTTTGATTG	CATTTTTAGTTTCTAAGATGTTTTCCA	252
Ad01A8684	Aradu.A01	8684	p2	(TG)18	36	37006223	37006258	TGCACCTCTGGTTGTGTTGT	AAACAAATTCAATCACAATCTTCA	198
Ad01A8685	Aradu.A01	8685	p3	(ATT)5	15	37012232	37012246	TCACAACCATGCAAAATTCAA	TCATATGCATTCCGATGTTAGC	257
Ad01A8688	Aradu.A01	8688	p2	(CT)6	12	37014873	37014884	GACAACTGAGGAGCCACCAT	AATAAAGCCACCACCACTGC	229
Ad01A8693	Aradu.A01	8693	c	(AAT)5(ATA)	30	37016808	37016837	ATTAATCGGTTTCGCAGTGG	AAGGGCACAGAAAAAGGTCA	238
Ad01A8694	Aradu.A01	8694	p2	(TC)7	14	37022924	37022937	ACACCATTTTAGTCACCGCC	AAGGGGTAGTGGCACTGATG	194
Ad01A8695	Aradu.A01	8695	p2	(TA)7	14	37027872	37027885	TGCCTCTAGCTTTCCCAAGA	GGCCTCCATGAGGTACAAAA	244
Ad01A8700	Aradu.A01	8700	c	(AAAAG)5ata	40	37077890	37077929	CAATGGTTGTGTTCCATTGAT	TCGCCTGTTCTTTAATCCAGA	275
Ad01A8701	Aradu.A01	8701	c	(T)10cttaattaa	102	37087410	37087511	CGTCTTGTACAACGTTTCTTTCC	TCTCTCTTCTCACCCAGGGA	236
Ad01A8704	Aradu.A01	8704	c	(A)10gaagagcg	94	37100885	37100978	TCTTTTTGGTGGAGGAATGC	TTCTTGAGCAGAGCTTGCAG	279
Ad01A8705	Aradu.A01	8705	p3	(TCT)5	15	37103550	37103564	ACGAAATCGCTCTTACCCAA	AACGCGAAGAAGAAGACGAA	136
Ad01A8706	Aradu.A01	8706	p2	(TA)7	14	37124241	37124254	GAGGCTCATCACTAGGCGTC	AATCCGTTCTTCAGAAGCCA	236

ID	Chromosome	SSR nr.	SSR type	SSR	Size	Start	End	FORWARD PRIMER (5'-3')	REVERSE PRIMER (5'-3')	Product (bp)
Ad01A8708	Aradu.A01	8708	p2	(CT)6	12	37131539	37131550	GGAACATTAAACCTGGAATTTGA	TTTCGGCCCAGAAGACATAG	205
Ad01A8710	Aradu.A01	8710	p4	(AATA)5	20	37136039	37136058	TCCATACGAGATTGACGACG	TTAGCACGCGATTGATTTTC	163
Ad01A8711	Aradu.A01	8711	c	(T)12cctttttctt	187	37136836	37137022	CAATCTGGCACCACAAGAAA	TTGCCATGGTGTTAGAATAAACA	252
Ad01A8712	Aradu.A01	8712	c	(CT)6aacct(TC	59	37138107	37138165	GTGGAACGCTACGTTTTGGT	ATGGTGACGATAAAGCGACC	255
Ad01A8715	Aradu.A01	8715	p2	(CA)6	12	37179075	37179086	CATCACATCTTAAGCAGCCTT	GCATGGATTGTACCCGAACT	258
Ad01A8716	Aradu.A01	8716	p2	(GA)6	12	37179697	37179708	TACTTGCATGGTGAATCCGA	TTCTCACTCCCTTCCTTCCA	177
Ad01A8718	Aradu.A01	8718	c	(CA)8t(AC)8	33	37191400	37191432	TTGTGCAAGCAAAGTTGGAG	TGCAAGGTACGAAGATCGAA	269
Ad01A8719	Aradu.A01	8719	p2	(TA)15	30	37195173	37195202	GGGTCGAGTTCGTATGTTGG	CACCACACTCAATGCTCTCAA	156
Ad01A8724	Aradu.A01	8724	p2	(AT)6	12	37211911	37211922	ACATGTGCAAGGTGGAGTTG	TCGCACCTCACCACCTACTA	261
Ad01A8727	Aradu.A01	8727	p4	(ATTT)6	24	37231828	37231851	ATTGCATGTGAATCTGGCAC	CCTCACGAAACAGTTTTGGAAT	199
Ad01A8732	Aradu.A01	8732	c	(T)10caattaggt	71	37260942	37261012	TTTTTCCTTTTAATTGGTCCC	TGTTGGTCCCTATAGTTTCGC	239
Ad01A8734	Aradu.A01	8734	c	(CAT)15(CTT	84	37264241	37264324	GTCCTTTGCTTCTGCTACGG	ACACCACTCCGACACCTTTC	260
Ad01A8737	Aradu.A01	8737	p3	(TAT)17	51	37282134	37282184	TGATGATCCGTGACATTCGT	CAAGACACACGGATGGTGTT	209
Ad01A8738	Aradu.A01	8738	p2	(TA)9	18	37290405	37290422	CTTGATGCAACCCAGAGCTA	CTATGCTCTGGGGAAGTGGA	197
Ad01A8740	Aradu.A01	8740	p5	(AAAAG)5	25	37297880	37297904	TGAATGCTTGAACATTGCATA	GCCTTGGATCCTTTTTACCC	260
Ad01A8745	Aradu.A01	8745	p3	(AAG)9	27	37336039	37336065	TAGAATATTGGTCGCCCTCG	CCCATCACCTCTTCACCACT	194
Ad01A8748	Aradu.A01	8748	p2	(TA)7	14	37404704	37404717	ACGTTAGTTCGTTTTGCGCT	TCGAGCAATGATTTCTGTGG	252
Ad01A8750	Aradu.A01	8750	p2	(GC)6	12	37432263	37432274	AAGAACCGGAAAGCTGTCAA	CACGTTTTGCTTCAAGTGGA	221
Ad01A8752	Aradu.A01	8752	p3	(TCT)6	18	37453401	37453418	ACACAACACAACCCCCTCTC	TGCCATTGATGGTAGTGGAA	174
Ad01A8755	Aradu.A01	8755	p3	(TGA)5	15	37513103	37513117	GAGCTCGACCTCTCCCTTTT	GCTGTTGGAGTTGAGGAAGG	149
Ad01A8761	Aradu.A01	8761	p3	(TAA)5	15	37534353	37534367	GGGTAGACGTGGACAGTGGT	CCAGACAGTTACAGCAGCCA	190
Ad01A8762	Aradu.A01	8762	p3	(GGT)7	21	37538666	37538686	CATGGTTGGGATCTGAGACA	AAGATGCGAGGTTGGAGATG	278
Ad01A8764	Aradu.A01	8764	c	(T)10gtttttaaga	81	37539782	37539862	TCTTTTGTTTGTTTATTACATGGCA	AGCGTGTTTGGCAAATTTCT	163
Ad01A8765	Aradu.A01	8765	p3	(ATA)13	39	37542063	37542101	TCAGCCAATGGAGAATTATGA	CTCCGCACTGCTATACACACA	273
Ad01A8766	Aradu.A01	8766	p3	(ATT)6	18	37542240	37542257	GCGGAGAGTTTATTTGTGGC	CAAGCACAAAAAGACGGAGA	165
Ad01A8770	Aradu.A01	8770	p3	(ATT)5	15	37560704	37560718	ATGCCACAAGCACATGGTTA	AAGCAAAAGCTTGCGAAAAA	230
Ad01A8771	Aradu.A01	8771	p3	(AGA)6	18	37562340	37562357	TTTTCTTGGCCACAACATCA	CCACACACTTCTCCCTTTCC	265
Ad01A8772	Aradu.A01	8772	p4	(TAAA)5	20	37565547	37565566	CAGGTTCAGGATCTGCTTCA	CGAATTTCTCCTTCTCTCATCC	266
Ad01A8776	Aradu.A01	8776	p2	(AT)9	18	37617193	37617210	TGCTGACACCTACCAGAGGA	TCAGCCACCAAAATCAGTCA	123
Ad01A8778	Aradu.A01	8778	p3	(ATC)10	30	37623936	37623965	TGATTGTCATCACCATGAAGAA	ATGGGATTCATGTGGAAGGA	154
Ad01A8780	Aradu.A01	8780	p2	(CT)6	12	37645643	37645654	TCATGAACCTAATTGGGGAAA	TTGGATCAAGTTTCAAGCCC	278
Ad01A8782	Aradu.A01	8782	p2	(AT)11	22	37661885	37661906	TATTGAACCTTGCTTTGGGG	CACAGCCACTATAGGCACCA	145
Ad01A8783	Aradu.A01	8783	p2	(TA)6	12	37662653	37662664	CCACCATAGGCACCAAAACT	GTTTGGGAGAGTTGGGAACA	153
Ad01A8784	Aradu.A01	8784	p2	(CT)6	12	37671345	37671356	GGATTGGTGAAGCTTGGTGT	TTGAACCGAGCCAACTTCTT	131

ID	Chromosome	SSR nr.	SSR type	SSR	Size	Start	End	FORWARD PRIMER (5'-3')	REVERSE PRIMER (5'-3')	Product (bp)
Ad01A8787	Aradu.A01	8787	c	(A)11tttctaatttt	44	37692151	37692194	TCGAGTTTGGACAACTGACG	AAAAACATGCAATTGAAATCAAAC	231
Ad01A8788	Aradu.A01	8788	c	(AT)6tttttttatt	76	37714189	37714264	CTTGCTTTGATGGGAGGTGT	CAACTTGCTTTGATCTTATTCCC	259
Ad01A8789	Aradu.A01	8789	p3	(TTA)13	39	37717513	37717551	TTTTGAGAATTTTCGATTCGG	TTGGTATGCAATTGATTTTTGC	196
Ad01A8790	Aradu.A01	8790	c	(T)11acgatgaa	59	37717728	37717786	TGTTGTTAATTGCCATCTTGC	CCTATGATTTATGTTAAAAGTTCGCA	259
Ad01A8797	Aradu.A01	8797	c	(A)10taaacggt	30	37745075	37745104	TTGAAGACCAAAAAGTCACGAA	TTTATAAGTTGCGTAGCATGGG	164
Ad01A8798	Aradu.A01	8798	c	(T)10aatttttcgg	36	37745587	37745622	AGGGTTGACCTCCAAATTCC	AGTGACGACGACGATGATGA	261
Ad01A8799	Aradu.A01	8799	p3	(TCC)5	15	37751967	37751981	TGAGAGACGTCCCATGAGGT	ATTTGGAGACACAAGTGGGC	186
Ad01A8800	Aradu.A01	8800	p2	(AG)6	12	37756141	37756152	TTGAATTTGAATGGTGGGGT	CAGAATTGGCACAAAGAGCA	241
Ad01A8801	Aradu.A01	8801	p3	(TCC)5	15	37760135	37760149	TGAGAGACGTCCCATGAGGT	ATTTGGAGACACAAGTGGGC	186
Ad01A8803	Aradu.A01	8803	p2	(GA)23	46	37768960	37769005	TCAAGAAGCAACAATCGCAG	ACTCCTCCTTCACCCATCCT	210
Ad01A8808	Aradu.A01	8808	p3	(TTC)5	15	37780985	37780999	CCGGAGAGCTATTTTGGGTT	CCCCTAATGAAAGTGGGGTT	172
Ad01A8810	Aradu.A01	8810	p3	(AAT)6	18	37782095	37782112	TGCCGGTTATACGTGAAATCT	GCCATAAATCAAAAGACTCAGCA	273
Ad01A8812	Aradu.A01	8812	c	(A)10cggccatc	48	37799202	37799249	ATAGGGAGCTCTTGGGCATT	AGCACAATGCTAGCACAACG	154
Ad01A8813	Aradu.A01	8813	p2	(TA)7	14	37806884	37806897	AGTTGGAGAATTAGCGGTGC	CAATTGTGGCCATAACAGCA	102
Ad01A8815	Aradu.A01	8815	c	(T)10cagtttaaa	107	37817232	37817338	TCAAGCACAAGCCACCATAG	TCTTCCTAACGCACTGGTGA	267
Ad01A8827	Aradu.A01	8827	p2	(TA)8	16	37935479	37935494	TACTATGCATGCCACCTCCA	CGATACAGCAACAACCCAGA	160
Ad01A8828	Aradu.A01	8828	p2	(TA)6	12	37936769	37936780	TTCATCATTCATCCCTGCAA	CCCCCTAAAACTTGATTTCAT	195
Ad01A8838	Aradu.A01	8838	p3	(TTC)5	15	37961933	37961947	CAAGGCATCTTCACACCCTT	GGCAGAGGAAGAGAAGGAGG	276
Ad01A8839	Aradu.A01	8839	c	(T)10(A)13	23	37963560	37963582	AGAGTTTGTCGGGTCTGGTG	GCCGGTTGGACTAATTGAACT	264
Ad01A8842	Aradu.A01	8842	p3	(AAC)5	15	37969340	37969354	GAGGTACTCAAGGAGGAGGGA	TCTTTATTTAGCCGACCCCA	207
Ad01A8846	Aradu.A01	8846	c	(T)11gtcatgaat	96	37971990	37972085	GGCTTGTTTGGTGAGCCTAC	CGAACAGCTCTTCTGCAAGTT	277
Ad01A8847	Aradu.A01	8847	p2	(TC)6	12	37974586	37974597	CAAGCCCTTTTGGCTTTACA	AAAGGGAAGAAGGAAAAGAGGA	203
Ad01A8848	Aradu.A01	8848	p6	(TTATCT)5	30	37982385	37982414	AGGTCGGAGTTGTTGTGTCC	GATAAGGGCAGTTCCCATGA	262
Ad01A8849	Aradu.A01	8849	p2	(CT)9	18	37998503	37998520	TTTCTTTTCCCTCTTTTAAAATTCA	TCCTTGAGGTGTCAGAAGAAAA	190
Ad01A8851	Aradu.A01	8851	p3	(TCT)6	18	38016024	38016041	GGCGTTTTACATGCCTCATT	GTTTCTCCGGCCTTAGGTTC	268
Ad01A8852	Aradu.A01	8852	p3	(TTA)6	18	38028357	38028374	TGAAATTCCCACCATTGGAT	TCGCACAACTAACCAGCAAG	229
Ad01A8853	Aradu.A01	8853	p2	(GA)7	14	38030045	38030058	GGGTTTGGGAAGGAAAATGT	GATTGCACAGTAAGGGCGTT	275
Ad01A8854	Aradu.A01	8854	c	(A)12cacaagaa	131	38039673	38039803	TTTTTGCTTTTACTCTCACAAAAGA	CACCAGTCAAAATCGGACCT	255
Ad01A8857	Aradu.A01	8857	p2	(CT)13	26	38078740	38078765	TCCTAAATCCTAAGAGAGGGGA	GAAAACGTGCTCTACGGGAC	249
Ad01A8858	Aradu.A01	8858	p2	(AG)8	16	38083672	38083687	TTTGGGCTATTTTTGACCCA	CATTGAACCTGGATTGGAAGA	214
Ad01A8865	Aradu.A01	8865	p6	(AATAAA)5	30	38106731	38106760	TGTTTAGTGATGATGAGAAGAACAAA	CAGATTGAAGGAGCACCCAT	199
Ad01A8867	Aradu.A01	8867	p3	(ACA)6	18	38120572	38120589	AATTTAGCAAGATTGACTTAGAATCAT	CCGACTCCACCTAGTAGGACA	274
Ad01A8869	Aradu.A01	8869	p3	(TGA)7	21	38125109	38125129	TCGTCTTTAATGCTACATTTCTTTTG	GTCGTTGTCGTCTTCACCAC	280
Ad01A8874	Aradu.A01	8874	p2	(GA)9	18	38138972	38138989	CGCTTATTGAAGGCGAACAT	CACGACAGTCTGTAACCGGA	237

ID	Chromosome	SSR nr.	SSR type	SSR	Size	Start	End	FORWARD PRIMER (5'-3')	REVERSE PRIMER (5'-3')	Product (bp)
Ad01A8879	Aradu.A01	8879	c	(T)13ggtgactag	115	38145697	38145811	TTCATGCAAATGTGAAAGATGA	TGGCACCTTTATTACAAAACTGAA	276
Ad01A8880	Aradu.A01	8880	p2	(AT)7	14	38150210	38150223	ACGGGTTTCCTTGGGTAGAC	GCCAACCTGACAAGTCCAAT	234
Ad01A8886	Aradu.A01	8886	c	(A)10ttctcgcat	112	38197880	38197991	CACACTCAACGAACTGACGAA	GATGTGGATGGCCTTCTGTT	227
Ad01A8887	Aradu.A01	8887	p3	(AAT)6	18	38198733	38198750	CATTTTTGTTCTAAAACTTTGGCAT	CCTCCTTCACTGTCACTCTCAA	240
Ad01A8888	Aradu.A01	8888	p2	(AG)15	30	38202000	38202029	TCGTCCAAACTCTGGGTAGG	CGGTTACGATGAGGTTCCAC	237
Ad01A8889	Aradu.A01	8889	p2	(AG)7	14	38210317	38210330	GCGCTTCCAAATAAGTCGTC	CGGTTACGATGAGGTTCCAC	191
Ad01A8893	Aradu.A01	8893	p2	(GA)10	20	38215062	38215081	CTCCGGACGGTGAAATAGAA	GTTTTCAAGGTGGTGGCTGT	220
Ad01A8897	Aradu.A01	8897	p2	(TA)8	16	38223493	38223508	TTGAACGGCAGAAACCTCTT	TGCCCATCAAGACAATTGAA	240
Ad01A8902	Aradu.A01	8902	p2	(AC)6	12	38242868	38242879	AGAATGTCACGCTTCCGACT	TTTTTCCCTTGCCTTTATTTTT	266
Ad01A8903	Aradu.A01	8903	p2	(AT)6	12	38243737	38243748	GAAATGTTCAAGCGCACTCA	CTGGGAATGTTACCCCATTG	260
Ad01A8904	Aradu.A01	8904	p2	(CT)7	14	38245745	38245758	CGGAATAAAGCGGTGGTAAA	TTGCCATCATTGGAACAGAG	163
Ad01A8905	Aradu.A01	8905	p2	(GA)10	20	38247545	38247564	TCATAATGACGATGGGACGA	CCACCTCCAACAATCACACA	269
Ad01A8906	Aradu.A01	8906	p3	(TTA)18	54	38249907	38249960	AAGGGAATTGATCCCTTCAT	TTCTCATTGATCACAAAATTTCA	193
Ad01A8907	Aradu.A01	8907	p2	(CT)10	20	38251563	38251582	AAAGGAACATTGAACCTGGAA	ACGAACCAGCAGCGATTTAT	280
Ad01A8908	Aradu.A01	8908	p3	(AAG)11	33	38256333	38256365	CTAGAGGTTTCTCCGGCCTT	TAGCCAAACCTACTCCCCCT	265
Ad01A8909	Aradu.A01	8909	p3	(AAT)5	15	38261623	38261637	CTTCGCACAACTAACCAGCA	CGCCTATCTGAAATTCCCAC	233
Ad01A8910	Aradu.A01	8910	p2	(CT)6	12	38267886	38267897	TGAACCTGGAACTTAGAAGAAATTG	CAGCGATTTATGGGCTGTTT	253
Ad01A8911	Aradu.A01	8911	p3	(TGT)5	15	38281482	38281496	TTCTAGGATTGTTTTCGGCG	TCACCATATCCGCAATCTCA	156
Ad01A8914	Aradu.A01	8914	p2	(GC)6	12	38294406	38294417	CCTCTGGGTCACTTTCCAGA	AGGAAGCAGGATGGGGTAGT	222
Ad01A8916	Aradu.A01	8916	p2	(AG)7	14	38298916	38298929	GGGTTAGGGGACTTTTGCTC	TCACCAAGGAATGGTTGAGA	148
Ad01A8917	Aradu.A01	8917	p3	(ATC)6	18	38318803	38318820	GGACGAGTCTGAAGAGGCAC	GCAGGAGCAATTTCGAGTTC	229
Ad01A8918	Aradu.A01	8918	p5	(AAGAT)5	25	38319801	38319825	AGAGATAAGGGCGGTTCCAT	TGTGGAACTGAGAATGCTGC	257
Ad01A8919	Aradu.A01	8919	c	(AT)6(A)10	22	38355014	38355035	TTCTTTGCAGGTTCCTCTTTTT	CGATGAGAATTCCCAACCAA	247
Ad01A8920	Aradu.A01	8920	p2	(TA)7	14	38357016	38357029	TAGGGCAGAGAGGGACAAAA	GCAATATTCCGCTTCTCTCG	198
Ad01A8921	Aradu.A01	8921	p2	(AT)7	14	38364855	38364868	TGACTGATATTCTCTTTCCCAGG	GGTCAACGGATCACGGTACT	227
Ad01A8923	Aradu.A01	8923	p3	(TCT)24	72	38376737	38376808	CAGGCACTCAAGCTTTCACA	ACGAGGAGAAACACGACGAT	267
Ad01A8924	Aradu.A01	8924	p3	(GAA)6	18	38376946	38376963	ATCGTCGTGTTTCTCCTCGT	CTCTTCCTCTTTCTCCTCCTCA	161
Ad01A8929	Aradu.A01	8929	p2	(TA)6	12	38424251	38424262	TTGTACACCAACTCTTTTTAATTTTCA	TTTGGTGGGTTTGTACGAGT	183
Ad01A8933	Aradu.A01	8933	p2	(TG)6	12	38436950	38436961	ATCTTGCCTCCTTGGACCTT	CCAAGCTTTTCAAAATCACCA	167
Ad01A8934	Aradu.A01	8934	p2	(TG)9	18	38440481	38440498	TAATTCCATCCCGTGACTCC	CCAACCATCAAAATGCAAAA	171
Ad01A8935	Aradu.A01	8935	p3	(TTA)5	15	38440675	38440689	AGTGTGGCATCACTGGTATGTT	ACCCTTTTTAGTCGGGCCT	211
Ad01A8938	Aradu.A01	8938	p2	(TA)6	12	38444484	38444495	AAGTGAAGATATGGTTGTGTAGAACG	GAACGAGAAATACCGCGAAA	262
Ad01A8940	Aradu.A01	8940	p2	(TC)8	16	38453737	38453752	CTCAAATCAATTGGTGCCCT	CCCTCCGTCCATCTTTTAGG	157
Ad01A8941	Aradu.A01	8941	p2	(AG)7	14	38457861	38457874	AAAAACCTGGATCGAGAGTCA	GGCCCAGATCCAAGTACAGA	201

ID	Chromosome	SSR nr.	SSR type	SSR	Size	Start	End	FORWARD PRIMER (5'-3')	REVERSE PRIMER (5'-3')	Product (bp)
Ad01A8950	Aradu.A01	8950	p3	(TCT)6	18	38492687	38492704	TCTCTTTCCCACACAAACCC	CCGGTCTTAGGTGCCATAAA	273
Ad01A8952	Aradu.A01	8952	p3	(AAT)6	18	38503596	38503613	TCCTAGGATTAAGGACAAAGAGAGAA	CTCTCACTCTCACTCTCACTCTCA	213
Ad01A8954	Aradu.A01	8954	p2	(AT)7	14	38507757	38507770	TCTCTGTGTGACAAGGGCAG	AGCTGGCCCAATCTAGAAAA	253
Ad01A8958	Aradu.A01	8958	p4	(AAAT)5	20	38511865	38511884	TGCAAAATTGATGGTCCAAT	TTGGCACTTATGCATTTAAGG	238
Ad01A8959	Aradu.A01	8959	p2	(AT)11	22	38512031	38512052	TGCATAAGTGCCAATTTTACCTT	ACATAATCACTCACTTTGGGCT	266
Ad01A8960	Aradu.A01	8960	p3	(ATC)5	15	38512259	38512273	CAATGCCATCTAAAAATCCCA	TGTCCAGGCAAAAACGATTA	140
Ad01A8962	Aradu.A01	8962	p2	(AT)15	30	38515294	38515323	CCATACTTGTCTGTCTCATTAGTGG	CATTAGCTCATGAACTGACTCGAT	267
Ad01A8964	Aradu.A01	8964	p2	(GA)10	20	38516676	38516695	GGAGAAGAGATCGCGAGAGA	TTTGAGCAATCTGCACCAGT	146
Ad01A8965	Aradu.A01	8965	p3	(ATT)5	15	38526965	38526979	CAAGGGAAGTGGTTTTGGAA	AAAGTCCCTATTCCGATCTTTAATTT	239
Ad01A8968	Aradu.A01	8968	p3	(ATT)13	39	38540973	38541011	TGATGCTCAGAGTCTTGGACC	TCTGTGATTTTTGAATGCATGA	214
Ad01A8969	Aradu.A01	8969	p2	(AT)7	14	38545324	38545337	TTAGAACGAAACAGGGGGAG	GGGATCGACCCTTACTCACA	240
Ad01A8982	Aradu.A01	8982	p2	(TC)13	26	38593141	38593166	CTCTTTCCTCCCCTTCCTTC	GAAGGGGAAAGAGACAGATAAGG	263
Ad01A8983	Aradu.A01	8983	c	(CT)17ccctcct	85	38593302	38593386	CTCCTCTCCCATTCTCTCTCC	AAAGAGGGAAGGAGATACACACA	225
Ad01A8985	Aradu.A01	8985	p2	(GA)8	16	38598239	38598254	ATGGGGAGAGAGAGGGAGAG	TCCACTTTCTTTTTCCGCTC	219
Ad01A8991	Aradu.A01	8991	p3	(TTA)5	15	38666228	38666242	TCAAGTGCAATTAAATGTCTTAACG	TTCCTTACCAGTAGGCATTCTT	242
Ad01A9003	Aradu.A01	9003	p2	(TC)9	18	38770944	38770961	AATCCATTCCATTGCTTTCG	GAGAGGAGGGGGAGAGAGAA	239
Ad01A9004	Aradu.A01	9004	p2	(CT)7	14	38771095	38771108	TCTCTCTCCCCCTCCTCTCT	GGGGACAAAAAGAGAGAAAAAGA	270
Ad01A9005	Aradu.A01	9005	c	(GA)6aagagag	130	38771518	38771647	GAGAAAGAAGAAAAGGAAAGAGG	TCCCTCTCTATCTCGCTCTCC	207
Ad01A9006	Aradu.A01	9006	c	(GA)18aagaaa	118	38771814	38771931	TGAGGTGGAGAAAGAGGGAG	CCGGTTAACCAAAACCAAAA	234
Ad01A9009	Aradu.A01	9009	p5	(TATTT)5	25	38809075	38809099	TGGCTTTTTCTGCTTGCTTT	TTATTTTGCTGTGGTGGCAA	258
Ad01A9011	Aradu.A01	9011	p2	(CT)6	12	38844796	38844807	GATTTTGCTTGCCATTTGGT	GTGAGGGTTCGGCTATTTGA	259
Ad01A9012	Aradu.A01	9012	p3	(AAG)5	15	38866125	38866139	CACCACCCTTCTCATTCCAT	ACATTGTCGTGTGGTCGCTA	273
Ad01A9015	Aradu.A01	9015	p3	(ATA)11	33	38893578	38893610	CGCATCATCGTGATAAATGG	AATACTGATAATACTTTTAGGTTGCCA	131
Ad01A9017	Aradu.A01	9017	p3	(AGA)5	15	38921471	38921485	CCCTGTGAGATGTGAAGAAAGA	ATTCTCTCTCCCACTTTTTGAA	275
Ad01A9018	Aradu.A01	9018	p2	(CT)6	12	38923412	38923423	TGAACCTGGGATTGAGAGTCA	CGGCCCAGAGAACACATATT	208
Ad01A9019	Aradu.A01	9019	p2	(GT)8	16	38931662	38931677	TTTCTTCGCAGGTCGAGACT	TCGCACTCGAAAACGTTAAG	127
Ad01A9020	Aradu.A01	9020	p2	(AT)9	18	38932280	38932297	AAATCAGGAGCCTTCCAACT	AATTTGAAGCACCAAGCACC	153
Ad01A9021	Aradu.A01	9021	p2	(TC)6	12	38934923	38934934	AGCTGTTGAGCTATTCTTGGAA	TTGCTTTGAGCCAACAACAC	187
Ad01A9023	Aradu.A01	9023	p4	(ATAA)7	28	38950302	38950329	CAAGTTTTGAACAAATCAAACG	GATCCACGTAACCCGCTAAA	222
Ad01A9024	Aradu.A01	9024	p2	(AT)8	16	38974580	38974595	AGTTCGTTCCTTGCTACCCA	ACAACCCACCACGGTATGAT	276
Ad01A9027	Aradu.A01	9027	p2	(TA)6	12	39007052	39007063	CCATGATGGAGAAAGGGAGA	GGTGTTCCTTGGTGTTTTCC	201
Ad01A9028	Aradu.A01	9028	p2	(CT)6	12	39031868	39031879	CAAATCCACCCAAGTCCATT	CTAGAGGTTTCTCCGGCCTT	269
Ad01A9029	Aradu.A01	9029	p2	(AG)8	16	39093160	39093175	AGAATCAGTCGCCGTCACTT	GAAAGATGGTGGCAGCAAAT	276
Ad01A9030	Aradu.A01	9030	p2	(CT)7	14	39098926	39098939	CCATGGCCAAATTTTAAAGG	TCTCCATAAATTCATGCATCTCC	216

ID	Chromosome	SSR nr.	SSR type	SSR	Size	Start	End	FORWARD PRIMER (5'-3')	REVERSE PRIMER (5'-3')	Product (bp)
Ad01A9031	Aradu.A01	9031	p2	(AT)6	12	39099099	39099110	TCCATGGACCTCTCTCTCTCTC	TCGGGTCCAAAATTCAACTG	270
Ad01A9033	Aradu.A01	9033	p2	(AG)12	24	39101109	39101132	AAAGCCAACCACCACAGATT	TGGGTATATGATGCCTTGTCA	212
Ad01A9037	Aradu.A01	9037	p2	(AG)6	12	39135978	39135989	TTGGATCTTGCGGAAGCTAT	CACTGCTCAATCTCACCCCT	231
Ad01A9038	Aradu.A01	9038	c	(T)10cgtttttcttc	39	39143049	39143087	TCAGCTTTGCTAGAGTCCCC	TGTGGTTCATGAATGTTGGC	242
Ad01A9042	Aradu.A01	9042	c	(TTA)5tctttattt	48	39185137	39185184	ATCCAACAGGGACAAGCATC	GATCCCATGGAGATTGTTGG	226
Ad01A9044	Aradu.A01	9044	p3	(ATT)6	18	39210065	39210082	ATTGGCCCCCTAAAATTGAT	CTTGCTTGAACCTCGGAAAC	234
Ad01A9046	Aradu.A01	9046	p2	(AG)6	12	39213648	39213659	AAAGAAACTGGGATTGAGAGTCA	AAGGGACTTTTGGGCTGTTT	225
Ad01A9048	Aradu.A01	9048	p3	(TAC)5	15	39217926	39217940	AACAAAAAGTATTGCCACCACA	TCAAAACACAGGTTGTTGAGTTC	270
Ad01A9053	Aradu.A01	9053	p2	(TA)8	16	39252513	39252528	TATGAAGCAGTAGCGGGGTT	ACATCGAATCCTTTGGGTTG	262
Ad01A9056	Aradu.A01	9056	p3	(TAT)5	15	39327545	39327559	TGGTGTTGAGATTTTGGCTCT	CAATCAAGCTCCAATCCACA	208
Ad01A9058	Aradu.A01	9058	c	(AAT)18aaacg	104	39328275	39328378	GGGCGTGCTTTTTGTCATTA	CATGGGACGGTAACTGAGAC	278
Ad01A9059	Aradu.A01	9059	p2	(TC)9	18	39328913	39328930	TTATGCCCTTTTCTTATGGCT	AATTCCACAGGCTGGAGAGA	166
Ad01A9060	Aradu.A01	9060	c	(TTA)6ttg(TTA	36	39329078	39329113	TTTCTCTCTCCAGCCTGTGG	AGCCACCAAAACAGTCTCCA	156
Ad01A9063	Aradu.A01	9063	p3	(AGA)5	15	39345652	39345666	AAAAGAATGGAAGAAGAATGAATG	ATTGAAGGGGGAATGGAGAT	212
Ad01A9066	Aradu.A01	9066	p2	(TA)6	12	39374805	39374816	GAAACCACCGTGCACATTTT	CGCATCCAATGCATAACAAA	261
Ad01A9067	Aradu.A01	9067	p3	(TGA)6	18	39412702	39412719	AACATGCGACATTTTTGTGC	GCTTACGCAATCAAAATAAGCA	209
Ad01A9068	Aradu.A01	9068	c	(T)10cttttcggtt	76	39415652	39415727	GAAATGTTTAAGTGGGGGCA	TGAAGCAACCTACCAAAATAATGA	150
Ad01A9070	Aradu.A01	9070	p2	(AT)7	14	39476323	39476336	GAGAACCATGTCGGGTGAAC	GCTATGGCATCCTCAAGCTC	205
Ad01A9072	Aradu.A01	9072	p3	(ATG)5	15	39479964	39479978	GCCATTTATTGAATTGAGGCTT	GGAATTTGTGCAGCTTCCTC	274
Ad01A9074	Aradu.A01	9074	p2	(AT)9	18	39480878	39480895	CTAACAAACAGCATCCGCAA	CGCGTTTGAGAATAGGGTGT	255
Ad01A9075	Aradu.A01	9075	p2	(AT)6	12	39481257	39481268	CCTTTGCCAAAAGCATCATT	AAGTTCCTCCTTGGCAATCA	266
Ad01A9079	Aradu.A01	9079	p2	(TA)6	12	39486446	39486457	AACACTCATCTCTTTTATCGGATT	CCATTTACCTATAGTGATCTTCAGCA	277
Ad01A9081	Aradu.A01	9081	p2	(AT)8	16	39495577	39495592	TTGCCTTTTGGTTTAAAGAGC	GGTGGCATTGCTTTTGTTTT	279
Ad01A9082	Aradu.A01	9082	p2	(TA)6	12	39504406	39504417	CCACACTCAAACCAACCAAA	TATTTGCTTTGTCTTGGCCC	191
Ad01A9083	Aradu.A01	9083	c	(AAT)6aac(AA	75	39512403	39512477	TTCCCTATCTTGAGTAGATAGTAGCG	CAGAGGTCAAATATATTCCATAAGCA	280
Ad01A9084	Aradu.A01	9084	p2	(TA)9	18	39513847	39513864	CCATGTACTTCAAGGGCGTT	TCGACGTCTCATAATTTTGGTTT	185
Ad01A9085	Aradu.A01	9085	p2	(AT)6	12	39517293	39517304	GTCGCGACTGAACATCAGAG	TGAGCCTTCACGAATACGAA	268
Ad01A9086	Aradu.A01	9086	p2	(TG)8	16	39537490	39537505	CACCTGTTGCAGAAGCAGAA	GAGTGCATGCATGATTTTCAA	219
Ad01A9087	Aradu.A01	9087	p2	(AT)6	12	39541322	39541333	AATGGGGCTAAGGTAAGGGT	GCATGGAATGTGAAGGTGAA	156
Ad01A9089	Aradu.A01	9089	p3	(ATA)5	15	39570771	39570785	TGAACCTATGGGGTCACACA	TGGATCTCATCCAAATTTACAA	245
Ad01A9094	Aradu.A01	9094	p3	(TGT)6	18	39622293	39622310	GCTCATCTCCTTGCCATCTC	TTTTGGACAAATGATGCAGA	275
Ad01A9095	Aradu.A01	9095	p5	(AATAA)5	25	39644615	39644639	GGTCCATGATGAAGTAAGGGAG	AATTTTCGAATCCCTTGGGT	273
Ad01A9097	Aradu.A01	9097	p3	(TCT)5	15	39667328	39667342	CCTACCTCACCCTCTCCCTC	TAAAATATTGCTCGCCCTCG	225
Ad01A9100	Aradu.A01	9100	p3	(TGG)5	15	39724563	39724577	TACAACCGCAATCACAATCC	ATTTCCCTCCTTCAACCACC	217

ID	Chromosome	SSR nr.	SSR type	SSR	Size	Start	End	FORWARD PRIMER (5'-3')	REVERSE PRIMER (5'-3')	Product (bp)
Ad01A9103	Aradu.A01	9103	p5	(AATAA)5	25	39761948	39761972	GCAGCAGTAGTGGGGTTAGC	CCTAACCACTTTCTCTCTCCTCA	260
Ad01A9106	Aradu.A01	9106	p3	(TTC)5	15	39808059	39808073	GTTATTGTTGGCCCCTCCTT	TGTGTGTTTCCTGTGCTGTG	199
Ad01A9107	Aradu.A01	9107	p2	(TC)8	16	39812228	39812243	TGGCGTTCTCTTGACACTTG	ATCCTAAATCGGAACCCCAA	245
Ad01A9108	Aradu.A01	9108	p2	(AT)6	12	39814818	39814829	TGGTTTATTTGCCAATGGAAT	TCCAGTTCAGCTTCATCCCT	222
Ad01A9109	Aradu.A01	9109	p2	(AT)6	12	39831855	39831866	ACCAACAAGAACAAAAGCCAA	GATTTGGTTTTGAACCATAGGA	239
Ad01A9112	Aradu.A01	9112	c	(T)10aggttgatg	63	39866715	39866777	TAAGTTTGGTGTTGGGAGGC	TTTCTCTTCCAGGGTGTGCT	238
Ad01A9113	Aradu.A01	9113	p2	(CT)15	30	39867158	39867187	TCTTGACCCCTCAGGATTTG	TTGGGAGGGAAAAGTGTTTG	198
Ad01A9114	Aradu.A01	9114	p3	(AAT)19	57	39887504	39887560	TGGCATGCATTCAAATCAA	TCACATTCACATTGCTTGTGTT	190
Ad01A9115	Aradu.A01	9115	p2	(AC)6	12	39890098	39890109	TTGTGGGTCTTCACCAAGTTT	CGGAGTGGGTTTTATTTGGA	198
Ad01A9118	Aradu.A01	9118	p2	(GA)6	12	39940215	39940226	CGATTTTGGATCCATGGTG	TTCTCTTTCTTCTTTTTCAAAACTTTC	251
Ad01A9122	Aradu.A01	9122	p3	(TCT)6	18	40037251	40037268	ACCCCTTCACCACTCACATC	CTGGCCTTAGGTGCCATAAA	208
Ad01A9123	Aradu.A01	9123	p3	(TTC)5	15	40050195	40050209	ACCCCTTTCATTGGGTTAGG	TGTGGTCCAGCATGAGAAAG	222
Ad01A9125	Aradu.A01	9125	p2	(TA)6	12	40077432	40077443	CCGAAAGTGAGGATTTGGAA	TGGGAAGTGTTACCATGCAA	217
Ad01A9128	Aradu.A01	9128	p2	(AG)6	12	40101032	40101043	CGGAAAAGCACACCAAACTT	CCTCTTTCCACCACACCATT	103
Ad01A9129	Aradu.A01	9129	p2	(AT)7	14	40114107	40114120	AGTGAAACAAGGCCTCAATCA	AGCTCGAACACACAAAACCA	185
Ad01A9131	Aradu.A01	9131	p2	(AG)9	18	40133934	40133951	GGTGTGCTTTGAACACCAAA	AAGAGGGGAGGACTTCAAGC	247
Ad01A9135	Aradu.A01	9135	p5	(TTATC)5	25	40195467	40195491	CACCCTTGCCTTTTGGTTTA	TGGCTCTTGAAAGAATGAACG	171
Ad01A9148	Aradu.A01	9148	p3	(TTC)6	18	40332534	40332551	AAATCTTTCCCACCCAAACC	TAAAATATTGCTCGCCCTCG	210
Ad01A9153	Aradu.A01	9153	p3	(AGA)8	24	40451248	40451271	TGCAAGCTTTTGTCTTCCCT	ACTCCCTCTCCCCTCCCTAT	280
Ad01A9154	Aradu.A01	9154	p3	(TCC)5	15	40453064	40453078	ATAGGGAACTCCGTCCTGCT	CCGTTAGAAATTTGGAGGCA	217
Ad01A9157	Aradu.A01	9157	c	(CTT)5tgtcatca	47	40516487	40516533	TGCAACAAATTTCGGCATAA	CAAGAAGTAAATTTGAGAAGGAAAAA	217
Ad01A9160	Aradu.A01	9160	p3	(ATT)5	15	40520375	40520389	TTGATTTTCACAATTTTAAACGTAGA	ACATGCTCCATGAGCTGCTA	186
Ad01A9163	Aradu.A01	9163	c	(TA)9(GA)8	34	40526827	40526860	TTGGTGAGAAGGGGGATGTA	CCATGAGTGGAATCATCCAA	245
Ad01A9164	Aradu.A01	9164	p3	(TGT)5	15	40528799	40528813	TGTTTTCTCAAGGGGGAATG	AGAGAGGTGGGTCACAATGG	271
Ad01A9165	Aradu.A01	9165	p3	(CAT)5	15	40534925	40534939	AAATACCGGGGATCAGAAGG	TTGTGGAAAAGGGTTATGCC	196
Ad01A9166	Aradu.A01	9166	c	(T)10aaattatcaa	82	40536538	40536619	TGTCTTTTATACCCGAATTTTTAATTG	GGACCGTTAAGCCTGAAACA	236
Ad01A9167	Aradu.A01	9167	p2	(GA)10	20	40538340	40538359	TTTGCCCCAATAAGCCATAC	AGCGGTACGCTTTTGAATGT	241
Ad01A9168	Aradu.A01	9168	c	(AT)10nnnnnnn	102	40539641	40539742	CACGCGGTGATAAGTGTTTG	GCTTGATACAATAAGGCAGTTGG	266
Ad01A9169	Aradu.A01	9169	p2	(GA)24	48	40540433	40540480	CATGCTCGCGTGATAAGTGT	GCACCCAGTAGTGAGGCAAT	126
Ad01A9170	Aradu.A01	9170	p3	(TGA)7	21	40540597	40540617	CTGGGTGCTATGATGGGAAT	CCGGATAAAAGGTAAAGGCA	172
Ad01A9174	Aradu.A01	9174	p2	(TG)6	12	40543043	40543054	GAAAAGAGAATGGCACGAGG	TATCACTCCCCCAAAGCCAAC	103
Ad01A9177	Aradu.A01	9177	p3	(AGA)5	15	40565881	40565895	CGACAGCAACGACAACTGAT	TTGACTTGAATGCCACCAAA	262
Ad01A9178	Aradu.A01	9178	p2	(CT)7	14	40568358	40568371	ATGTTCACTACCCAACCCCA	GTCTTTGTCGGTGACCCCTA	219
Ad01A9179	Aradu.A01	9179	p2	(TA)8	16	40569941	40569956	TGCCTACGATCGAATTCATATTT	CCGATGCCAAAAGGATAAAA	175

ID	Chromosome	SSR nr.	SSR type	SSR	Size	Start	End	FORWARD PRIMER (5'-3')	REVERSE PRIMER (5'-3')	Product (bp)
Ad01A9183	Aradu.A01	9183	c	(A)10gagtgtatc	37	40579355	40579391	GAGCAAAATTCCATTCAGGG	CCATCCTTTCTTGCTCTCCA	272
Ad01A9186	Aradu.A01	9186	p3	(TTA)17	51	40593940	40593990	TCATTATTTCATTAGACAGCCTCTTC	CATCAAGTCCAATATCGCCA	177
Ad01A9189	Aradu.A01	9189	p3	(AGA)6	18	40603139	40603156	TTGCCTTCCCTTTCCTCTTT	AAACCCCTCTCCCTCCACTA	267
Ad01A9190	Aradu.A01	9190	p4	(AATT)5	20	40615484	40615503	TCAATCTTTCTTTTGCCTTAATGA	AGACACCACTGAGGTTTCCAA	277
Ad01A9191	Aradu.A01	9191	p3	(AAC)6	18	40619012	40619029	TCCTCAAAATAGCCACGGTT	TTGCTGAAATGACCAATGGA	271
Ad01A9193	Aradu.A01	9193	p2	(AG)6	12	40633594	40633605	ATAGGTGAGGGGTTTTTGGG	TTAATGCCAGAATTGGCACA	183
Ad01A9195	Aradu.A01	9195	c	(TA)8cacacata	43	40649814	40649856	GCGCTGAAGTCACGAAAAGT	CAAGACCGCTCCCAAAAATA	278
Ad01A9196	Aradu.A01	9196	c	(TC)9cc(TA)9	38	40651468	40651505	TGAGCTTAATCCTTCGTCGG	ATGCGTATGCACAGGATGAA	207
Ad01A9198	Aradu.A01	9198	p2	(TA)13	26	40669345	40669370	TTTTCGAATCAAGATTCAATTGTT	AAAAATAATACACTCTCTCTTTCTCCC	128
Ad01A9200	Aradu.A01	9200	p2	(AG)6	12	40678129	40678140	CCGGATACTTCATACAACAGACA	CATGAATGGTCCATAAGCCA	219
Ad01A9202	Aradu.A01	9202	c	(A)10gctatgctt	92	40727677	40727768	TTGCCTTCCCTTTCCTCTTT	CCACAGGATCACCTCAGGAT	253
Ad01A9207	Aradu.A01	9207	p3	(TTC)6	18	40769723	40769740	TCCCTAGCCTCTCAGGATCA	GTGCCTTTCCCTTCCTCTTC	264
Ad01A9208	Aradu.A01	9208	p4	(TTTA)6	24	40772455	40772478	GCTGACCCGTCGTTAATCAT	TTGTTGGTTTGAGCAAGCAG	259
Ad01A9209	Aradu.A01	9209	p2	(AT)6	12	40778950	40778961	TTGCTAAATTATAAACGCACACA	GGCCTCTTCAGATTCAGATCC	233
Ad01A9210	Aradu.A01	9210	p2	(AT)6	12	40801160	40801171	TTGCTAAATTATAAACGCACACA	GGCCTCTTCAGATTCAGATCC	233
Ad01A9213	Aradu.A01	9213	c	(T)11ggttaactg	69	40823651	40823719	GGAACCCAAGGAAAATTTGA	ACGTTCCATGGGTCTTGTTT	215
Ad01A9214	Aradu.A01	9214	p2	(TA)7	14	40825935	40825948	CACTCCGATCGACAACGTAA	AGCCGGATCTTGAGTCTTGA	103
Ad01A9217	Aradu.A01	9217	p3	(ATT)13	39	40878038	40878076	TGGGAGCGTAAGCGTTATTT	CGAGGCCTCACCTACTACCA	161
Ad01A9218	Aradu.A01	9218	p2	(TC)6	12	40887298	40887309	AGTAGCCCTGTTGAGAGGCA	AACTCACCGGTGGTGAAAAG	202
Ad01A9224	Aradu.A01	9224	p2	(CA)6	12	40964704	40964715	TGTATGAGCGCATAGGCTTG	CACAAGAGGCGAAAAAGAGG	232
Ad01A9228	Aradu.A01	9228	c	(A)10gagaaatg	94	40997620	40997713	TCTGAATGAATGCTTGAACAATG	CACCCTTGCCTTTTGGTTTA	236
Ad01A9229	Aradu.A01	9229	p2	(TA)7	14	41003261	41003274	TGAAAACATGCTTCTTTGGC	ACTAAAACCGCGTCATTTGG	263
Ad01A9230	Aradu.A01	9230	p2	(AG)7	14	41003726	41003739	TGCATCAAGGCAATTTCTGA	AAGAACATTGAACCTGGAATTGA	225
Ad01A9232	Aradu.A01	9232	p4	(AACT)7	28	41018151	41018178	AACCGCAACAATGAATAGCA	CCAAACACCATCCATAACCA	173
Ad01A9238	Aradu.A01	9238	p2	(AG)6	12	41076323	41076334	GGTTCAGGATCAGCTTCAACA	GGTAGAAGAAGATGAAAGCATGAA	278
Ad01A9239	Aradu.A01	9239	p2	(GA)6	12	41129926	41129937	TCGAACACAGAGGGAGAGAGA	TCTTTTTCAAAACCACCTAACCA	198
Ad01A9240	Aradu.A01	9240	p2	(CT)6	12	41144399	41144410	GTACTCACGAGGCGCTTTTC	TAGGAAGGAAGGGGAAGGAA	217
Ad01A9244	Aradu.A01	9244	p3	(AAG)21	63	41166664	41166726	CTAGAGGATTCTCCGGCCTT	ACCTCCCCTTCTCCTCCATA	201
Ad01A9246	Aradu.A01	9246	p2	(GA)14	28	41178948	41178975	AGCAGTGACCCAAGGCTCTA	AATGACGCCAACTTTATCCG	253
Ad01A9247	Aradu.A01	9247	p2	(AG)6	12	41190769	41190780	CATAGCCCCATACTGCCAAT	CTTTGGCTTTTGATGGGAAC	215
Ad01A9249	Aradu.A01	9249	p4	(AATA)5	20	41194090	41194109	AATCATGGCCTGTAAGTTGGA	AATATGGAGTTCGTCGGGAA	261
Ad01A9252	Aradu.A01	9252	p3	(TGG)6	18	41205901	41205918	AGTTTTGATGCATTGGAGGG	TAGCATTCGTCGTGGAGTTG	247
Ad01A9253	Aradu.A01	9253	p3	(AAC)6	18	41209494	41209511	CATCTGCCTTCACCACCTTT	GCTGCTGGATTGCAGGTTAC	213
Ad01A9256	Aradu.A01	9256	p3	(ATG)8	24	41223788	41223811	GATGCCCGATTCTCCTGTAA	TCTCTAGCACCGAAGCCAAT	268

ID	Chromosome	SSR nr.	SSR type	SSR	Size	Start	End	FORWARD PRIMER (5'-3')	REVERSE PRIMER (5'-3')	Product (bp)
Ad01A9258	Aradu.A01	9258	p2	(CT)10	20	41226625	41226644	TCACTACCCACCCAACAACA	ATGGTTGATCCTGGTTGGTC	100
Ad01A9259	Aradu.A01	9259	p2	(AT)9	18	41229794	41229811	GATGGCGAAGATCATTGGAT	TTTAGGCCGGCTAGGGTAAT	191
Ad01A9261	Aradu.A01	9261	p2	(AT)9	18	41267919	41267936	TGTGGTTTTCAGATGCAGGA	ACCCCAAAAAGACCCAAAAT	191
Ad01A9265	Aradu.A01	9265	p3	(ATC)6	18	41279007	41279024	CAGCAAACAACTTCGGGTTT	TGGTGGAAAGTGTGGTTTGA	148
Ad01A9266	Aradu.A01	9266	p2	(CT)10	20	41279836	41279855	GCAAGCAAAATTATCTAAACTCCAA	CAGAGTCGAAGAGGTGGAGG	243
Ad01A9275	Aradu.A01	9275	p3	(AGA)6	18	41347282	41347299	CTCTGGCCTTATGTGCCATT	CAGGATCCCCACTTACCTCA	280
Ad01A9277	Aradu.A01	9277	p2	(AT)7	14	41382006	41382019	GCGCTACCAACTTGTCTTCC	GTCAGGGCCTGGGATGTTAT	234
Ad01A9278	Aradu.A01	9278	c	(ATC)6gaaata	87	41382973	41383059	CCCCCATGTGTCACCTAATC	CTCCCAAACAAAAGTGGCAT	260
Ad01A9280	Aradu.A01	9280	p2	(TA)6	12	41389187	41389198	CACCACGAAAGAATGTCGAA	AGCAGGTTGAGTGGTTTGCT	157
Ad01A9284	Aradu.A01	9284	p2	(TA)6	12	41422201	41422212	GGGGTAAACAAAAATCGCAA	AGCCGGATTTTGAGTCTTGA	133
Ad01A9285	Aradu.A01	9285	p2	(TA)6	12	41428649	41428660	AACTCGAAAGCGCAACACTC	TTTGTGTCCCTTTGGGTTTC	199
Ad01A9288	Aradu.A01	9288	p2	(AT)10	20	41450725	41450744	CGAGACACAACAGGTACACACA	GACATGATTGGACATATCTAAATACCA	273
Ad01A9289	Aradu.A01	9289	p2	(AT)6	12	41454830	41454841	AAGGGAAAAAGGGGAAACAG	CGATGCTGTTTGTGTTTGCT	134
Ad01A9290	Aradu.A01	9290	p3	(AAC)5	15	41458055	41458069	CAGGCTGCATTATAGCCCTT	CTGCCCCAATTTTGAACACT	158
Ad01A9292	Aradu.A01	9292	p3	(TAA)13	39	41465266	41465304	TAAAGGCTTCGGCCATTTTT	CAAGTCCCTCCGCACAATA	276
Ad01A9296	Aradu.A01	9296	p2	(TA)7	14	41476899	41476912	TTGCGTTGAACTTGCTTACG	TTGTTGGATGTTTTCTTGGATG	229
Ad01A9298	Aradu.A01	9298	p2	(TA)6	12	41480383	41480394	TGCAAACACAAGGTTTCAACT	CGGATGAACACAACATAGAGC	280
Ad01A9299	Aradu.A01	9299	p3	(TCT)7	21	41497981	41498001	CTCATCAAGAGCATCATCGC	CTGCCCATACATCCACCTCT	156
Ad01A9300	Aradu.A01	9300	p3	(ATT)15	45	41499025	41499069	TTCCAATGAAAAGCGAAACA	CCAATCCACGTCATATTTGTCA	264
Ad01A9301	Aradu.A01	9301	p3	(ATT)5	15	41499186	41499200	TCAATGGATGGAGGTTTCATT	TGCCCACCGTAAACTTGATT	258
Ad01A9309	Aradu.A01	9309	p2	(GA)14	28	41570237	41570264	CATGCATGCCAATTTTCAAC	TTCCTTCACAGCTCATGTCG	276
Ad01A9310	Aradu.A01	9310	p3	(TGT)5	15	41571701	41571715	CTGAAAACTAAAATGCCTCTTCA	TTGCATGGAGTTATGGCTGA	186
Ad01A9314	Aradu.A01	9314	c	(AC)6(TC)8	28	41575854	41575881	TCACACCCTCTATCCCAAGC	GAAGAGCGAGAGAGAGGCAA	184
Ad01A9327	Aradu.A01	9327	p6	(TTTTGA)6	36	41622176	41622211	CCATTCAAAACATGCAACCA	CAGGTGGTGGGCAATCTACT	269
Ad01A9330	Aradu.A01	9330	p2	(GA)6	12	41630687	41630698	TAACCCTAGCCGCCTTACCT	CTCTGCTCCCTCAACTCCAC	254
Ad01A9334	Aradu.A01	9334	p3	(TTA)24	72	41639614	41639685	CACCCATTTTAATGAGGCAT	TTGCTGCTCCTGTTATTCCA	280
Ad01A9336	Aradu.A01	9336	p3	(AGA)5	15	41648509	41648523	ACCTTCTTCATGGCCACAAC	AAGCCACCTCCATCTCCTCT	269
Ad01A9337	Aradu.A01	9337	p3	(AGA)7	21	41660199	41660219	TAAAATATTGCTCGCCCTCG	CACCTTGACCGAAACACCTT	100
Ad01A9339	Aradu.A01	9339	p2	(GA)6	12	41686302	41686313	TTTCGAACACAGAGGGAGAGA	TTGACCACTTTTCAAGTTCCAA	188
Ad01A9340	Aradu.A01	9340	p2	(AT)9	18	41691388	41691405	CATCAAGCCCGATAAAGGAA	TGGTGACAGAGCAAAGGATG	280
Ad01A9341	Aradu.A01	9341	p4	(TTTA)5	20	41700081	41700100	CGGTTCATCTTGTTGCTCAG	ACCATGCATGAGTTTCCGA	275
Ad01A9342	Aradu.A01	9342	p4	(TTTA)5	20	41701379	41701398	CGAAAACCACTAACAACTTTTTCA	AGAGAGGGAGAGAGGGTTCG	244
Ad01A9345	Aradu.A01	9345	c	(GT)6atgtgtga(	34	41761592	41761625	GCACGAAATGTTCACACACA	CTAGTCGGCCTAAACTTCGC	208
Ad01A9347	Aradu.A01	9347	p3	(TTC)6	18	41798324	41798341	TACACCTCTCCCCCTCTCCT	GCTTTTGTCTTCCCTTTCCC	185

ID	Chromosome	SSR nr.	SSR type	SSR	Size	Start	End	FORWARD PRIMER (5'-3')	REVERSE PRIMER (5'-3')	Product (bp)
Ad01A9349	Aradu.A01	9349	p6	(ATTTTT)5	30	41803307	41803336	TTCTCTCTCCTCATCTTTTTCG	ATGATGGAGCAAGGGAGATG	200
Ad01A9351	Aradu.A01	9351	p3	(TCT)6	18	41819066	41819083	CCTAACGTACATGGCCGAAT	AAATGTTGCTTGCCCTTGAG	103
Ad01A9353	Aradu.A01	9353	p2	(TA)9	18	41829148	41829165	CATGCTGGAAGCTTCTGATTT	TACTTTCGCGAACCGAGTCT	113
Ad01A9360	Aradu.A01	9360	c	(TA)7ataatatta	48	41854353	41854400	AAAAATGCATTAAGGAAGAGCG	TGCAATTTTCTCTCTCTTTACTTTT	259
Ad01A9361	Aradu.A01	9361	p2	(TA)7	14	41859856	41859869	TTTGATCAGAGTCTCGTGCTG	TGCAATTTTCTCTCTCTTTACTTTT	195
Ad01A9366	Aradu.A01	9366	p2	(TA)6	12	41878304	41878315	TCTTCAACACTTGGCTCCCT	GTGGTGGAAGCTGAAGAAGC	185
Ad01A9367	Aradu.A01	9367	p2	(GA)21	42	41884397	41884438	CAAATGGTGGTGACGATGAC	CGACGGGTTTACTGTCGAAT	275
Ad01A9369	Aradu.A01	9369	p2	(AT)6	12	41893482	41893493	TGAACACGAACCGGTAATCA	TGCTTGTGCACATCAAATCA	280
Ad01A9372	Aradu.A01	9372	p2	(AC)6	12	41908562	41908573	CATGACCCCATATAAGGCAAA	TGATTGGAATGGTGTGTTGC	238
Ad01A9373	Aradu.A01	9373	p2	(TC)28	56	41910077	41910132	CGGTTGGGGTGATACTGTTC	CTCGGCAGATAAACGAGTGC	252
Ad01A9374	Aradu.A01	9374	p2	(TA)9	18	41911147	41911164	TCATGGTATTTAAGGCATCCAA	CCACTCCTTTGGGAAGTTCA	237
Ad01A9376	Aradu.A01	9376	c	(TA)8cacgcact	75	41920456	41920530	TGGAGGATGAGAAGGAATGC	TTTCATTCAATACAGGCACACA	266
Ad01A9381	Aradu.A01	9381	p3	(ATA)17	51	41933339	41933389	AAAAGAAGTGGCACATGAATTG	CCCATATGCATGCCATAACT	266
Ad01A9383	Aradu.A01	9383	p3	(GAA)5	15	41939132	41939146	TCAATTTAGCAACAAATTCCACA	TACTGGGTTCCCTTTCATGC	275
Ad01A9384	Aradu.A01	9384	p3	(TTA)9	27	41940517	41940543	GGTGGGGCTTCAATAATGCT	AGTGTGAGGAAGGCAGAGGA	260
Ad01A9385	Aradu.A01	9385	p3	(TGA)5	15	41940687	41940701	TCCCATGGACAATGTGAGTG	AGTGTGAGGAAGGCAGAGGA	174
Ad01A9387	Aradu.A01	9387	p3	(TTA)6	18	41952032	41952049	TTCGGAAAAGAGTTGAGCAAA	AGGTAGTTGGGCTTTTTGGG	180
Ad01A9388	Aradu.A01	9388	c	(TA)9ttattaattc	80	41960853	41960932	CCAGCTTAAACTCTGCAGGC	AGGCACGGACAGATCAAAAC	156
Ad01A9394	Aradu.A01	9394	c	(TCC)5tttttatct	83	41998309	41998391	CGTCACTGTTACCACCACCA	TTGAGTGAGTCGATTTTAGATTTTG	277
Ad01A9395	Aradu.A01	9395	p3	(CAC)6	18	41998758	41998775	CCACAAATTCAATGATAACAAAACA	AAATTTGGCCTTGTGATTCTTT	237
Ad01A9396	Aradu.A01	9396	p3	(CAT)10	30	41999156	41999185	TTTCGGTGTCAAAATTACGAA	CAGGTTCTTGTTAAGTGGGTGA	189
Ad01A9398	Aradu.A01	9398	p2	(TC)6	12	42002870	42002881	TCCCCTCTCTCATCCTCTTTC	TCCTCCTCTCTCTCCTCCTTC	280
Ad01A9400	Aradu.A01	9400	c	(AG)7aaagaga	143	42003493	42003635	GAGAGAGAGGGAGCAAAGAAG	TCTCTCCCCTATTCCTCTTCC	224
Ad01A9401	Aradu.A01	9401	c	(AG)8caaaaga	116	42003952	42004067	AGAGAAAGAAAGAGAGAAAGGAGAG	CCCACTCTGTCTCTCCCCA	270
Ad01A9402	Aradu.A01	9402	p2	(TA)8	16	42010011	42010026	GCTAGCCTAGGTGCCAGCTT	TGAGCCTGATTCGTTGTTAAA	236
Ad01A9404	Aradu.A01	9404	p2	(TA)7	14	42030162	42030175	CTGACAACCATTCGCAAAGA	TGCACAATCATACATTGCCA	145
Ad01A9405	Aradu.A01	9405	c	(TA)7(TG)23	60	42030412	42030471	GGAACTTCTTGAACAGGCCA	CAGAATCAATCCAAAACAATTCG	254
Ad01A9407	Aradu.A01	9407	p5	(TTTAT)5	25	42064814	42064838	CCTAACCACTTTCTCTCTCCTCA	GGGTTAGCATATGACCCCAG	213
Ad01A9408	Aradu.A01	9408	p3	(AAC)5	15	42082052	42082066	CGCCCGAATCAAGGATACTA	GAGGTGCTCTGTAAGGCTGG	244
Ad01A9414	Aradu.A01	9414	p3	(AAT)6	18	42139981	42139998	TAAAGGTCGATTCCACGGAG	AGAAGGATTGGATGAAGGCA	239
Ad01A9415	Aradu.A01	9415	c	(A)10t(A)10	21	42155595	42155615	TTGCAAACGTGTGCCTTATT	CCGTCCATCTTTGATTGCTT	230
Ad01A9416	Aradu.A01	9416	p3	(TCT)5	15	42169854	42169868	AACCTACTCCCTTCCCCTCA	GCTTTTGTCTTCCCTTTCCC	272
Ad01A9418	Aradu.A01	9418	c	(TTC)5tttttctat	47	42182736	42182782	CATCTGTGTCCCTTCCCTTT	TGGAGGATGTGAGTGAGAAAGA	244
Ad01A9420	Aradu.A01	9420	p3	(CTT)6	18	42209799	42209816	TTCAAAAATTGGACCGGACT	GGAAGAGGAACAGGAGGAGG	279

ID	Chromosome	SSR nr.	SSR type	SSR	Size	Start	End	FORWARD PRIMER (5'-3')	REVERSE PRIMER (5'-3')	Product (bp)
Ad01A9421	Aradu.A01	9421	c	(CTT)5tt(TCA	182	42210412	42210593	TTTTTCTCCCTCCTTCTCATC	AAGAGTAAAACAAGAAGAAACTTGAGA	242
Ad01A9422	Aradu.A01	9422	p3	(GAG)7	21	42211567	42211587	AAAAGAGGAGAAACACAAAGAAGAA	TTGCTCATGTCCCTCAATTTC	280
Ad01A9423	Aradu.A01	9423	p2	(CT)24	48	42212947	42212994	CCCATCATCCATTTCCACTC	ATTGGCGTGAGATTCAGAGC	236
Ad01A9424	Aradu.A01	9424	p3	(TCT)5	15	42213151	42213165	GCTCTGAATCTCACGCCAAT	TGGATCCTCAGCGTTAGTGA	108
Ad01A9425	Aradu.A01	9425	c	(T)10cgtaggaa	126	42219712	42219837	CCTCGGTTGGTATATGTCGG	TCATGATCTTCTCCTCACACAAA	276
Ad01A9426	Aradu.A01	9426	c	(TA)7acgttaga	73	42235153	42235225	TGTGATTAGGGAAGTATTTCATTTTG	TGAGAGAGTGTGTGGCAACC	271
Ad01A9427	Aradu.A01	9427	p3	(GAG)6	18	42243919	42243936	TTCTTTCAAGCGGTTGTTTTT	CGTTGATCACTGGAGCATGT	250
Ad01A9431	Aradu.A01	9431	c	(A)10ctcgtgcta	69	42256343	42256411	TCAAACGTAAGTAAATCTGGTTTTTG	CCTACTGTGGCTGTCGGTTT	255
Ad01A9436	Aradu.A01	9436	p5	(TTATC)5	25	42323507	42323531	CCTGGGTGCTCCAGTGTATT	CTAGACGATCGCCAAAGGAG	206
Ad01A9437	Aradu.A01	9437	p3	(AGA)6	18	42336523	42336540	TAGAGAATGTTGCTCGCCCT	CCCCCTCACCAATCACATAC	273
Ad01A9440	Aradu.A01	9440	p2	(TA)6	12	42379491	42379502	GCAAACCAAAACAATGCACA	AACTCTCCAGGTTCCCCAAT	233
Ad01A9441	Aradu.A01	9441	p2	(TG)19	38	42382521	42382558	TTTCAGATGCAGGACGTGAG	TCAACCTCTAGGAGGGGAAGA	190
Ad01A9442	Aradu.A01	9442	p2	(TA)6	12	42382871	42382882	AGCTAGCCTTTGCTTTGCAG	GCAAGATGTGATCGGGTGTA	260
Ad01A9443	Aradu.A01	9443	p3	(TCT)6	18	42442309	42442326	TGAATACACACCCCTCTCCC	ATGTTTTTCCGGCCTTAGGT	161
Ad01A9444	Aradu.A01	9444	c	(A)10ttaaaaatc	83	42460120	42460202	AGAAACTGTGGGAACATGGG	TGGGGTAATTTTGTCCCCTT	209
Ad01A9445	Aradu.A01	9445	p2	(GT)7	14	42460363	42460376	AGGGGACAAAATTACCCCAA	TTTCAAGGTTGTAATGGGGC	254
Ad01A9446	Aradu.A01	9446	p2	(TA)6	12	42472135	42472146	AAACCGTCAAAAATCCAATCA	GAGCTCAGGACTGAGGATGG	227
Ad01A9449	Aradu.A01	9449	p2	(AT)7	14	42499863	42499876	CCTTGAATTGTGCTTTGCTTT	ACCAAAAACAGAGGCTGTCA	248
Ad01A9450	Aradu.A01	9450	p3	(CAT)6	18	42503812	42503829	CAGGCATGAAACAATTCCAA	AGTTCATTGGCGGAACTTTG	156
Ad01A9452	Aradu.A01	9452	p2	(TC)11	22	42505829	42505850	AGCAACTATAATTCCGGGGG	CCAAGCACCGACAGAGATAA	191
Ad01A9453	Aradu.A01	9453	p4	(TTGA)6	24	42507090	42507113	AACAAAGCTGTTCGGAAGGA	TATCCAAGGAGACGAGGGTG	263
Ad01A9454	Aradu.A01	9454	p4	(ATGG)5	20	42507344	42507363	GGGCAATTCTGTTCCTGTGT	TCATCACTTCATTCTCCCCC	146
Ad01A9455	Aradu.A01	9455	p2	(TC)6	12	42507688	42507699	TCTCAACAGTCATACCATAACTCCTC	TAGCTTTTGCAATAGACATTTTTC	280
Ad01A9457	Aradu.A01	9457	p3	(CAT)5	15	42508979	42508993	CCCTCATTGAAGCTACAGCC	GTATAGGCCAGCGAGATGGA	186
Ad01A9458	Aradu.A01	9458	p4	(TCAT)5	20	42510210	42510229	ATAGTATATCCGGGGGCAGG	ATGGCAATTTGATGAGAGGG	267
Ad01A9459	Aradu.A01	9459	p2	(AT)7	14	42510482	42510495	CATGCATACACGCAAAACAA	GCTGTGTGAGTATAAAGGGGC	245
Ad01A9462	Aradu.A01	9462	c	(CT)6cctccata(	35	42536332	42536366	CAAACCTAAACCCCTCTCCC	TTGCCTTCCCTTTCCTCTTT	273
Ad01A9466	Aradu.A01	9466	p2	(TC)6	12	42547095	42547106	TGTTGCTGTTTGTGGGGATA	GCATGAGAGAAAGCACAGCA	163
Ad01A9472	Aradu.A01	9472	p5	(AAAAT)5	25	42571202	42571226	TGTTTGATTGCTTAGATTAGGCG	TTCCCAGTGAGGTAACCTGTG	250
Ad01A9477	Aradu.A01	9477	p2	(CT)8	16	42620924	42620939	CACGCCAGAATTGCACTCTA	TGAAAGTGAGGTAGGTGGGG	258
Ad01A9478	Aradu.A01	9478	p2	(AT)8	16	42631000	42631015	ATTCCTTCGGCTTTCCAAAT	AACTTTGGCGAAACAAGGTG	209
Ad01A9479	Aradu.A01	9479	p2	(AG)19	38	42634275	42634312	CGTAATACCTCACCACCGCT	GCGCAAAAATGTAACTCACC	215
Ad01A9480	Aradu.A01	9480	p2	(TA)8	16	42638337	42638352	TTTGTTAAAGATGTTTTCATGTTGTG	AAAAATTTTAAGAGGCTTGAGTTTG	255
Ad01A9481	Aradu.A01	9481	p2	(AT)10	20	42645043	42645062	TGGTCTCTGGCAATTGATGA	TGGGCTGTACAGAAGAATGC	103

ID	Chromosome	SSR nr.	SSR type	SSR	Size	Start	End	FORWARD PRIMER (5'-3')	REVERSE PRIMER (5'-3')	Product (bp)
Ad01A9482	Aradu.A01	9482	p2	(TC)8	16	42646647	42646662	TGGACAAGCTTCAGCTGCTA	CGCCTTGAAACCAATGATTC	272
Ad01A9483	Aradu.A01	9483	p2	(TA)6	12	42647587	42647598	CCAACCACATTAACAAGGCTC	TCCAGGCTTTCGTTTTCATC	178
Ad01A9485	Aradu.A01	9485	p2	(TA)6	12	42649094	42649105	CCACTGGATCCATGAAATGA	AAGCAGTGTGCCTTCTCCAT	258
Ad01A9486	Aradu.A01	9486	c	(A)10ggaaaaca	54	42649552	42649605	CAAGCAAGCACATAAGGCAA	GGTGTTTCAGTTGGTCGGAG	258
Ad01A9489	Aradu.A01	9489	c	(TAA)5tctttact	115	42658599	42658713	AAAAACGGTGGTCCTGATTT	TTTCATCTTATCCTAAATTCTGTTTTG	201
Ad01A9491	Aradu.A01	9491	p3	(AGA)5	15	42673596	42673610	CGTCCTCGAGCAAAAGAAGA	ACATAACCGAACCCTACCCC	174
Ad01A9492	Aradu.A01	9492	p3	(TAA)6	18	42681265	42681282	GAAAACGTAACAGGGGAGGG	TACTAGGTACGACCCGGTGC	267
Ad01A9494	Aradu.A01	9494	p2	(GT)9	18	42695234	42695251	CGACGTTCCCAACCTTTCTA	TCCCTCACTTGTCCGAAATC	161
Ad01A9498	Aradu.A01	9498	p2	(AT)8	16	42710019	42710034	CGACTGTTAACCCAAGGGAG	TCGTTCCTCCCTGCAAATTA	271
Ad01A9503	Aradu.A01	9503	p2	(GA)6	12	42725705	42725716	GCAATTGAAGGAAGAGAGAAGG	TGCTTATTAACGTCGTCCTCG	128
Ad01A9505	Aradu.A01	9505	p3	(AAC)5	15	42726765	42726779	GAGGAGGAGGAAAAGAAACAAA	TTGTTTGGAAAAATTCTTTTGAG	280
Ad01A9507	Aradu.A01	9507	p2	(TG)8	16	42740866	42740881	GCACACCCATGTGTTACGAG	GCAGTGACGAGCAAACAAAA	242
Ad01A9509	Aradu.A01	9509	c	(AG)6aa(AG)8	55	42743431	42743485	TGCCCCTTCCCTTTAAATCT	CATGCTGAGTGGAATAGCTCA	227
Ad01A9510	Aradu.A01	9510	p2	(AG)7	14	42759233	42759246	GGGTTTGGAGCAGAATCAGA	AGCCAATTGGGCAACATAAA	227
Ad01A9511	Aradu.A01	9511	p4	(TTTA)5	20	42763007	42763026	TGGGGTAATTTTGTCCCCTT	AAAAACTGTGGGAACATGGG	252
Ad01A9513	Aradu.A01	9513	p3	(TTC)6	18	42777011	42777028	ACACAACCCCCTCTTCTCCT	GCTTTTGTCTTCCCTTTCCC	212
Ad01A9514	Aradu.A01	9514	c	(T)10(A)10	20	42783059	42783078	TTGTCTGGTTGCTTAGATTTGG	GCAAACATTAAACTAGAAATTTTGAA	247
Ad01A9518	Aradu.A01	9518	c	(AAT)7ataat(A	54	42840729	42840782	CTGGGAGCACAATCTCATCC	TGCAATAAAAGAAAATGGAGGAA	278
Ad01A9519	Aradu.A01	9519	p2	(AT)10	20	42892247	42892266	CCAACAATCCAGCTACCGTT	GTAACGACCCAACTTCTGGC	217
Ad01A9521	Aradu.A01	9521	p3	(TTC)6	18	42895814	42895831	GCCAAAGTTGGCAAAGTAGC	TCGGGAATTTGGTTAATGGA	162
Ad01A9522	Aradu.A01	9522	c	(T)10a(T)10	21	42897693	42897713	TCAAATCATAACATGGGAAGGA	AGAGAGCTCGCCAATTTTGA	214
Ad01A9523	Aradu.A01	9523	p3	(ATT)8	24	42910099	42910122	GATCCGCATGTGGTGAGTTA	TGAACGTGTCTTTATTAAATGTGTCA	230
Ad01A9528	Aradu.A01	9528	c	(GAA)5gaggg	85	42943256	42943340	TCCAAATTTAAGCCAAATCACA	CTGTGATCCGCCACTGTTC	214
Ad01A9529	Aradu.A01	9529	p3	(GAT)5	15	42952190	42952204	AGAGGTGATGGAGCTCAGGA	GGGACAACAGAGGCTTTGAC	258
Ad01A9530	Aradu.A01	9530	p3	(CAT)5	15	42960583	42960597	TCCGGCTGAGTAGCTTCAAT	TCCTCTTCCTCAGCACCAAC	269
Ad01A9531	Aradu.A01	9531	p3	(TTC)7	21	42985483	42985503	TCCATCTCCCATCACTCTCC	CTAGAGGATTCTCCGGCCTT	162
Ad01A9532	Aradu.A01	9532	p2	(GC)6	12	42988499	42988510	ATGCGGTTCTAGTTGGGTTG	TGTCCCATCCCTCTTCAATC	231
Ad01A9534	Aradu.A01	9534	p3	(TTC)15	45	43000164	43000208	TAGGATCACCACCTACCCCA	CTAGAGGTTTCTCCGGCCTT	262
Ad01A9540	Aradu.A01	9540	p4	(AAAG)5	20	43085550	43085569	CCTAACCCAAAACCTAGCCC	CGGGTCAGACGTGTATCTCC	253
Ad01A9541	Aradu.A01	9541	p2	(AG)11	22	43085859	43085880	TCGCTGTCACCTCTAGTCCA	AACCAAGTGATGATCCTGGC	275
Ad01A9542	Aradu.A01	9542	p2	(AT)6	12	43089090	43089101	TGTCCTTGCATGCATTACTTG	TTTAATGCCCAAATTGCCTC	217
Ad01A9545	Aradu.A01	9545	p3	(AAT)5	15	43104130	43104144	TCTTCACAACTCCGCACAAC	CAGGGACAAAGCATCTCCAT	263
Ad01A9548	Aradu.A01	9548	p2	(AG)10	20	43134228	43134247	AATAATGCCAGGGAACATGC	TGGAAATTGAGAAGAAGAAATCC	179
Ad01A9550	Aradu.A01	9550	p3	(TTC)6	18	43171441	43171458	AAATCTTTCCCACCCAAACC	TAAAATATTGCTCGCCCTCG	210

ID	Chromosome	SSR nr.	SSR type	SSR	Size	Start	End	FORWARD PRIMER (5'-3')	REVERSE PRIMER (5'-3')	Product (bp)
Ad01A9553	Aradu.A01	9553	p3	(TAA)15	45	43184823	43184867	AAGGCAAAAAGACGCCAAC	TTGTTTTCCATTGACCCCAT	277
Ad01A9554	Aradu.A01	9554	p2	(AT)6	12	43189471	43189482	TCAGTGGCCATTCGAAATAA	GCCGATGAAACTTGTCTTTTT	280
Ad01A9555	Aradu.A01	9555	p3	(ATA)5	15	43190569	43190583	AATCGCAATATTCGTATTCTTTCT	TTCCTTCTCTTATTCTTTGGCTTT	258
Ad01A9565	Aradu.A01	9565	p2	(AT)7	14	43250419	43250432	GGTCTATGAAGCAAAGGCCA	AATCCCTCATGTTGGCAGAG	244
Ad01A9567	Aradu.A01	9567	p3	(TTC)5	15	43266730	43266744	CCTCCCCTCACACACTTTTC	ACCCAATTGGATGAAGATCG	258
Ad01A9568	Aradu.A01	9568	p3	(AAG)5	15	43272584	43272598	TAGAATATTGCTCGCCCTCG	CCTAATTGGTGCAGGGATGT	276
Ad01A9570	Aradu.A01	9570	c	(TTC)5(T)10	25	43300054	43300078	TGACTCAGAGGTGGAAGCAA	CCACCCTACGTAGCCGAATA	233
Ad01A9571	Aradu.A01	9571	p3	(TCC)5	15	43304567	43304581	CAGAGCTCATGAATGGCAGA	AGTGGGCCAGCTGAGTAAGA	275
Ad01A9575	Aradu.A01	9575	p3	(TCT)7	21	43322848	43322868	AATCCATCTCCCCTCACTCA	CTCCCAAGACTCAGAGGTGG	201
Ad01A9580	Aradu.A01	9580	p2	(GA)17	34	43349564	43349597	ACCGCAACCGAGAGTGTTAG	CAAATGCTTGCTCACCTGAA	176
Ad01A9582	Aradu.A01	9582	p3	(TAT)18	54	43401445	43401498	TTGAGCCTAGCCGTGACTTT	AAGGCTTTGAGCACCACTGT	180
Ad01A9583	Aradu.A01	9583	p2	(CT)9	18	43405396	43405413	GGAACATTAACCCTAGAAATTGAGA	AGACCCTTTTCGGTCCAGAT	241
Ad01A9586	Aradu.A01	9586	p3	(TAA)5	15	43409497	43409511	GGGGATGGAAAACTTGCTAA	AAGTTTTTGGCGCTGTTGTC	174
Ad01A9595	Aradu.A01	9595	p3	(AAG)6	18	43528908	43528925	AGTGGTCTTGATGGGCTTTG	CCCCCACTCTCCTCCATATT	238
Ad01A9601	Aradu.A01	9601	p2	(GT)6	12	43567095	43567106	TGCTTCCGTTATTGCTTGTG	TGCATGCTTCTTTTGTGACAT	234
Ad01A9604	Aradu.A01	9604	p3	(TTC)26	78	43596747	43596824	TAGACCACGCGTATTCGTCA	CCTTGAGAAGAAGGTGAGAAAGA	273
Ad01A9607	Aradu.A01	9607	p3	(ATT)5	15	43621911	43621925	TTTCCTGGTTTTTGTCCCTG	GGAACTGCTTACCTGGTGGA	261
Ad01A9611	Aradu.A01	9611	p3	(AAT)5	15	43624062	43624076	ATTGGTCATCAAATAGGCCG	TGGTAAGTTGGTGAGAGACAAAA	201
Ad01A9613	Aradu.A01	9613	p6	(AGAGAC)5	30	43630844	43630873	AAGGTCGATGCAAAATGCTT	GAATTGCCTGCTGGAATGTT	271
Ad01A9615	Aradu.A01	9615	p3	(TTG)8	24	43682279	43682302	TGATAGAGCACAATGGCGTC	TGGCATAGAGTTGATGGCAG	248
Ad01A9624	Aradu.A01	9624	p3	(TAA)12	36	43712691	43712726	TTTTGTAGCAGATGTGGGCA	TATCCGAATAATTGCAGGGC	240
Ad01A9626	Aradu.A01	9626	c	(T)10caagtcact	93	43723473	43723565	GTCCTCCACACCTCAACCTT	TCTCTTCTGTGTAATAATTCATTGGC	261
Ad01A9628	Aradu.A01	9628	p2	(CA)6	12	43734134	43734145	GTGTTCACACACCAAGCCAA	TGAGGTATGCTCATCCCCTG	136
Ad01A9629	Aradu.A01	9629	c	(TTAT)5tga(A	39	43739867	43739905	CCCAAAATTTCTCACTTTGCAT	GGAATTTGGGTGCCTACTGA	253
Ad01A9635	Aradu.A01	9635	p2	(TA)6	12	43800602	43800613	AGTGTCTCCTAATTTATCAATAGCATC	GCAAAAGAAAGGAATTGCCA	204
Ad01A9640	Aradu.A01	9640	p3	(TAA)17	51	43825140	43825190	TTAATTATGGGGCTGATGGC	TGTTGGATCGCCATTATTCA	231
Ad01A9649	Aradu.A01	9649	c	(AAC)8(AAT)	96	43854539	43854634	AATACAGGTGGGGGTTGATCT	TCCGACGAAGCTTTTACAGAA	211
Ad01A9650	Aradu.A01	9650	p2	(GA)13	26	43855622	43855647	TGCTATCAATTCACGTGGGA	TTTTGGATGGGTTCTCCAAG	173
Ad01A9651	Aradu.A01	9651	p3	(AAT)5	15	43857184	43857198	GAAACAAAATGCGGGAAAAA	GCATGCAACTTGGTTTTTGA	274
Ad01A9652	Aradu.A01	9652	p2	(GA)9	18	43859936	43859953	AGTAACTCGTGCCAGGGAGA	TGGTTACAGACGAAAAATCCG	239
Ad01A9655	Aradu.A01	9655	p2	(AT)11	22	43935184	43935205	GATTCACTATTTTTGGGGCG	TTTCAACAACCAAAAACTAACATGA	263
Ad01A9657	Aradu.A01	9657	c	(TA)6tttttttttct	39	43948548	43948586	GGGGCATTTTTGTCTCCTTT	CTTGGGACATTGGTTGAGGT	238
Ad01A9660	Aradu.A01	9660	p3	(ATT)5	15	43966777	43966791	GACGTCGGCTTTGTGAAGTC	CAGGGAAAAAGGCACTCTTG	192
Ad01A9663	Aradu.A01	9663	p3	(TTA)7	21	44000229	44000249	TCCCGACTTTCGATGGTTAG	AGCCCTATGCTTGCCTTTTT	240

ID	Chromosome	SSR nr.	SSR type	SSR	Size	Start	End	FORWARD PRIMER (5'-3')	REVERSE PRIMER (5'-3')	Product (bp)
Ad01A9664	Aradu.A01	9664	p3	(TTA)6	18	44003162	44003179	GGATGTAAGACCCAAGTTTTCG	TGAATGCCTTAAGCCAAAAGA	270
Ad01A9666	Aradu.A01	9666	p2	(AG)6	12	44008198	44008209	GCGTCACTTTTTCGTGACCT	AAAGAACCCGGGATTGAGAG	262
Ad01A9669	Aradu.A01	9669	p2	(GA)6	12	44013073	44013084	TGACTCAGAGGTGGAAGCAA	ACCTCCATCCATCTCTTCCC	252
Ad01A9670	Aradu.A01	9670	p2	(TA)6	12	44020772	44020783	TGCTTGTCCTCAAGCAAAGA	TAGGCACCAGGTGTGAGTTG	246
Ad01A9671	Aradu.A01	9671	p3	(AGG)5	15	44034717	44034731	CCAAAGAGAGAGTGCAAGGC	CATGAATGGCAGAAGGAGGT	198
Ad01A9674	Aradu.A01	9674	p3	(AAT)5	15	44043937	44043951	TAAGGGTCGATCCCACAGAG	ACAACGGTGATGCCCTACAT	279
Ad01A9675	Aradu.A01	9675	p3	(TAA)6	18	44047624	44047641	GAAAACCGAAAGCTCTTTTTCA	TGGTTAGGTTCTCACCACCC	275
Ad01A9676	Aradu.A01	9676	p2	(AT)7	14	44052574	44052587	TTGCTTAATGCAACCCAGAG	GAATGATGCGCAGACAAGAA	218
Ad01A9677	Aradu.A01	9677	p2	(CT)14	28	44061984	44062011	CAGACCCTCTTTTCACGAGC	TCCAGACTCGTCATCACTGC	204
Ad01A9678	Aradu.A01	9678	c	(TC)6(T)10	22	44066984	44067005	ATCCATTGATGCTCAAAGCC	TGGCTCCTGAAAGAATGAGG	190
Ad01A9681	Aradu.A01	9681	p2	(AT)9	18	44095019	44095036	GCCATTCGATAGCGAGGATA	TGGTGAGACCGTGTGGTTAG	239
Ad01A9682	Aradu.A01	9682	p3	(CAG)5	15	44097760	44097774	CCAGAGGCTAACTTCGATGG	GCCAGACGAGATGGAAAGAG	198
Ad01A9683	Aradu.A01	9683	p3	(CAA)5	15	44098844	44098858	CAGAAGCAGAAGCATGCAAA	TTTGTAGCCTGCCAACACTG	269
Ad01A9684	Aradu.A01	9684	p2	(AC)9	18	44099347	44099364	TCCTGGACCGAAGCTTTAGA	CACATGCAATTGACACCAAA	183
Ad01A9685	Aradu.A01	9685	p2	(TA)6	12	44100667	44100678	GCCACTCGATAGCGAGAATA	TCAGAGAGATGGGAGTCGTG	269
Ad01A9687	Aradu.A01	9687	p2	(CT)9	18	44103300	44103317	AACAACTGGAAGGATGACGG	AGAGACGAACGCAAGGAGAA	243
Ad01A9688	Aradu.A01	9688	p6	(GTGTGA)5	30	44103553	44103582	TTCTCCTTCTGGATCGCACT	ACCAATAACCCAGTGACCCA	241
Ad01A9692	Aradu.A01	9692	c	(T)10agttttttta	107	44115569	44115675	CTTCTCGCATTTGGCAATTT	CATCTCTCCCCAATCGTGTT	206
Ad01A9695	Aradu.A01	9695	p2	(AT)6	12	44137678	44137689	GAGCAAGGGCGTGATCTAAG	GCCTGATGATGATGAGAGCA	257
Ad01A9696	Aradu.A01	9696	c*	(A)11(AG)7*	24	44138016	44138039	TTGGCTCCTGAAAGGATGAG	ATGCCACAAGCACATGGTTA	269
Ad01A9700	Aradu.A01	9700	p3	(AAT)6	18	44174928	44174945	TTGCTTTGTTTTTATTTTCTTCTGG	AGCTGCTTGCTTTAATGGGA	261
Ad01A9701	Aradu.A01	9701	p2	(TA)9	18	44203133	44203150	CTTTACTTTGGTTTGGGGCA	CAGATTTTTGCTCAGCTCCC	258
Ad01A9706	Aradu.A01	9706	p2	(AT)8	16	44229224	44229239	TGTCCTTGCATGCATTACTTG	GATAGCCATAAACGCCCAAA	207
Ad01A9708	Aradu.A01	9708	p3	(CTT)8	24	44262190	44262213	GTGAAATCGAAGAAGCGAGG	ACAACTCGCTGACACACAGC	243
Ad01A9712	Aradu.A01	9712	p3	(ATG)9	27	44266269	44266295	ACTGGACCTTGAGGGAGGTT	TCCCCTTGCTGTACCATAGC	276
Ad01A9713	Aradu.A01	9713	c	(A)10ttattttcga	67	44273598	44273664	TCGAAAATACCTCCCTCTTTTTC	CAGAAGGGTGAGGATATGGGT	231
Ad01A9714	Aradu.A01	9714	p3	(TCT)5	15	44277113	44277127	CCTACTCCCTCTCCCCTCAC	TAAAATATTGCTCGCCCTCG	171
Ad01A9715	Aradu.A01	9715	p3	(TTC)5	15	44283723	44283737	AAACCCCTCATCCATCCTTC	AGCAATGCTTTTTCCACACC	171
Ad01A9718	Aradu.A01	9718	p2	(AG)8	16	44293355	44293370	GAGCTCCCAGATCAAGATGC	TCCCGTCAGTTACATCCACA	167
Ad01A9720	Aradu.A01	9720	p2	(TA)6	12	44301875	44301886	ACAAAATCAACCAAAAGCTCA	TCCAAAAGAAAAATTCAACCAAA	259
Ad01A9721	Aradu.A01	9721	c*	(CT)25(TGCG	73	44303405	44303477	CACCCCTCTCCCTTCTCTCT	AGGGAGCTTGAGCTTGAACA	275
Ad01A9722	Aradu.A01	9722	p3	(GAA)5	15	44305761	44305775	CATTGGAGAGCATGCTGAGA	GAGGCACAAACACAGAAGCA	255
Ad01A9725	Aradu.A01	9725	p2	(AG)6	12	44327505	44327516	GAGGAGATGAAAAGGGAGGG	TCACTCTCCTTCGCCAAGAT	234
Ad01A9727	Aradu.A01	9727	p2	(AG)7	14	44342016	44342029	CAATTCAAATGCCGGAATAAA	AATTACAGAACACGCCCCAG	278

ID	Chromosome	SSR nr.	SSR type	SSR	Size	Start	End	FORWARD PRIMER (5'-3')	REVERSE PRIMER (5'-3')	Product (bp)
Ad01A9728	Aradu.A01	9728	p2	(TA)6	12	44342892	44342903	ATGGGTTTATCAACCTCCCC	TTTCTTTCCCATCTCATTTGC	270
Ad01A9730	Aradu.A01	9730	p2	(AT)7	14	44346661	44346674	TATTGTGCAAATGACGCGAT	TGCATTCTGCATTTTTCGTT	261
Ad01A9731	Aradu.A01	9731	p2	(AG)9	18	44367307	44367324	GATAGTGCGCAGACAAGCAA	TGATAAAGACTTCAACGGAGGT	261
Ad01A9733	Aradu.A01	9733	p2	(GA)7	14	44390500	44390513	GAAAAGGAAGGAGGGAAGAAGA	ACAGCTCAACCAAAATTGCC	173
Ad01A9738	Aradu.A01	9738	c	(T)10cgttttctttt	48	44463507	44463554	AATTAGAGGTGCCCAGGGTT	TGTGGTTCATGAATGTTGGC	231
Ad01A9740	Aradu.A01	9740	p2	(TA)7	14	44476354	44476367	TAAAGGTGGTGAGGCGCTAC	GCTGGAGACTCTTTGGCAAG	197
Ad01A9742	Aradu.A01	9742	p3	(ATA)6	18	44497180	44497197	TTCAAAATTGGTTTTCAAAAGTTG	AATGAGATGGGGGAAGGAAA	276
Ad01A9743	Aradu.A01	9743	p2	(GA)6	12	44507985	44507996	TCAAAATAGAGATCTGAAACATGGA	TTCAGAGAGAGCTCGCCAAT	222
Ad01A9746	Aradu.A01	9746	p3	(GTC)5	15	44509909	44509923	CCTATGGTAATTCCGACGGT	CAACTCAGGGCCACGAGAC	268
Ad01A9747	Aradu.A01	9747	p2	(AT)11	22	44510595	44510616	GCGAAAACAAACCGAAACTT	ACGACTGAACATCGGAGCTT	231
Ad01A9748	Aradu.A01	9748	p2	(AT)11	22	44515489	44515510	ATTGAGCCTTCGCAAATACG	ACAGGTTCAGGAATCCTTGG	270
Ad01A9750	Aradu.A01	9750	p2	(TA)8	16	44518131	44518146	GGGTTTGGGATTTTTCCATC	TGTGGAAAGATCAGATGGCTC	189
Ad01A9751	Aradu.A01	9751	p2	(TA)8	16	44518390	44518405	CCACCAACTTTTGAGTTGACC	TGTTTTTCAGATGCAGGACG	160
Ad01A9752	Aradu.A01	9752	p4	(TATT)5	20	44531735	44531754	TGCTTATTTTAGACATACCCCACA	CGTAATTGAGGGGAGACACC	168
Ad01A9753	Aradu.A01	9753	p2	(AT)8	16	44544088	44544103	GGCAATTTGGGTAAGTGAGC	TCGAGAAATTATCTATAGGGCACC	236
Ad01A9758	Aradu.A01	9758	p3	(ATT)5	15	44571079	44571093	CAAACTTGGGTGTCGAACAA	CCCATCAGAGACATGAAGTCAG	269
Ad01A9762	Aradu.A01	9762	p2	(TA)10	20	44583387	44583406	AATTGCAATAAAAGCCACCG	AATCCTTTTTGCGAGCTTCA	197
Ad01A9764	Aradu.A01	9764	c	(AT)11taatgttc	79	44595631	44595709	TGGCACACTTCAAAAGCGTA	AGCATGAGATTGCGTGAGTG	230
Ad01A9766	Aradu.A01	9766	p2	(AT)8	16	44611713	44611728	TGAAATTCCCTTCCTTTGAA	TTAGCAATGGTTTGCCACAA	228
Ad01A9767	Aradu.A01	9767	p4	(TTAT)5	20	44618360	44618379	TGAGCTTGACCTAGGACTTATCAA	TGCCAAACCTCATTATTTTCG	268
Ad01A9768	Aradu.A01	9768	p4	(GAAA)8	32	44618986	44619017	TCTCCTCAGCAACACACAGG	CTCTCTTATCGGAACAGCGG	265
Ad01A9769	Aradu.A01	9769	p5	(AATAA)6	30	44631107	44631136	GGGTTAGCATATGACCCCAG	TTTCTTTCTCCTCACTTTTTCG	241
Ad01A9771	Aradu.A01	9771	p2	(TC)6	12	44675694	44675705	TTGTGGTGGGCAAAAGAAAT	TTAAACTGGCAGAACCCGAC	238
Ad01A9774	Aradu.A01	9774	p2	(AG)6	12	44700223	44700234	TACACTGTGGGCGATTTCTG	AGAACCTGGGATTGAGAGCC	227
Ad01A9776	Aradu.A01	9776	p3	(AGG)5	15	44713568	44713582	TTTCGGTTCATTCGATCACA	TTATTATCGTTATCGCCGCC	218
Ad01A9777	Aradu.A01	9777	p3	(GAA)5	15	44715249	44715263	GGCCCGATTAACCAATTTTT	CCATCATCTGCAAACACCAC	262
Ad01A9779	Aradu.A01	9779	p2	(AT)6	12	44716392	44716403	TCCACCAATGTGACTCAAACA	ACGAGGTGTGCAATTTGGTT	159
Ad01A9781	Aradu.A01	9781	p3	(CAT)5	15	44722882	44722896	TCCTTGGAGAGTTGGCTTGT	GATGAAATTCTGGCCCTTGA	141
Ad01A9782	Aradu.A01	9782	p3	(TAT)6	18	44724160	44724177	GGCTTCCTACATAGGCATTCA	CGAGATCTGTCCCTCAATCC	136
Ad01A9784	Aradu.A01	9784	p3	(ATC)5	15	44727551	44727565	CACCAACACCACCTCTTCCT	AAGAAAAAGACGACGATGGC	104
Ad01A9785	Aradu.A01	9785	c	(TCA)5ttatcatc	114	44728022	44728135	TTCAAAATACTTCCTCCTCCTCA	GAGTGTAGGTTCATCCTCTTCCA	234
Ad01A9786	Aradu.A01	9786	p3	(CTT)6	18	44728407	44728424	TTATTCTTCTTTCTCCTCTTCATCTT	TGAATGAAGGTTCGTCTTCTTCT	220
Ad01A9787	Aradu.A01	9787	p3	(CTT)5	15	44728792	44728806	TTCCTCTTCCTTCTCCTCATCTT	TGCTAAAGTTCTCAAAATAATGCTG	280
Ad01A9790	Aradu.A01	9790	p2	(AG)16	32	44738101	44738132	TGATATGAGATGGTTGTAGGTTGT	AGTTTGGGTATTACGATGACTTTT	280

ID	Chromosome	SSR nr.	SSR type	SSR	Size	Start	End	FORWARD PRIMER (5'-3')	REVERSE PRIMER (5'-3')	Product (bp)
Ad01A9793	Aradu.A01	9793	p2	(AT)9	18	44769447	44769464	ATTGAATCCTCACCGGATGT	AAATGCAAGCTCACTTAGCCA	231
Ad01A9794	Aradu.A01	9794	p3	(AAT)5	15	44788277	44788291	GTCGATCCCACGAAGATTGT	ACAACGGTGATGCCCTACAT	274
Ad01A9801	Aradu.A01	9801	p3	(AAT)5	15	44813883	44813897	TCAAAATTATTGCACAAGCCA	TTTTGAGATGTGTGTTGTGTGTAGA	239
Ad01A9802	Aradu.A01	9802	p2	(CT)24	48	44815140	44815187	TTATCCACCGGTAAATCCGA	GAAGGGAGCATTTCGAACAA	238
Ad01A9804	Aradu.A01	9804	p3	(AGA)6	18	44823530	44823547	TGCCATTGATGGTAGTGGAA	TACCCTCTCCCCTCTCCCTA	222
Ad01A9805	Aradu.A01	9805	p5	(AATAA)5	25	44824286	44824310	CAGATGCTCCTTACTGGCGT	GATCACCTTTGTGGCTCCAT	247
Ad01A9806	Aradu.A01	9806	p2	(AG)9	18	44868612	44868629	TGAAAGTGAGGTAGGTGGGG	CCTAATTGGAGCAGGGATGA	222
Ad01A9807	Aradu.A01	9807	p2	(AG)6	12	44896205	44896216	AAAAGTGGGGAAATCCATCC	GGAACATTGAACCTGAAAATTGA	178
Ad01A9808	Aradu.A01	9808	p2	(CA)6	12	44897755	44897766	AGCAGGAGCTATGTTTTCGC	CCGCTCATCAAGGGTAAAAA	279
Ad01A9809	Aradu.A01	9809	p3	(TTC)6	18	44915957	44915974	AAATCTTTCCCACCCAAACC	CCCTCGAGCAAGAGAAGAAA	195
Ad01A9811	Aradu.A01	9811	p2	(TA)8	16	44945472	44945487	GGTCTCATGCATATTTTGATACGA	CAAAGGCTACGCTTTTTAGCA	280
Ad01A9812	Aradu.A01	9812	p2	(TA)6	12	44952237	44952248	AAGCTCCAAGAAACAAGGCA	CCTCCGGTTCTCAAACCATA	270
Ad01A9815	Aradu.A01	9815	p2	(AT)8	16	44988002	44988017	TCTCATCTCGCTCCAAACCT	TCTTTCAATTTTGATGCCCA	152
Ad01A9817	Aradu.A01	9817	p2	(TA)6	12	44991121	44991132	TCAGACTCACATACGTTTGCC	GTTTGCTGCACATGGATCAG	265
Ad01A9818	Aradu.A01	9818	p3	(ATC)5	15	44992261	44992275	TCGCCTGGAGCTAGTGAAAT	TCCCTTATGATGGTGTCGGT	179
Ad01A9819	Aradu.A01	9819	p3	(AAG)5	15	44995465	44995479	AGTTGGCTTTGTGGACCATC	TCAATTCGAGCACCAAAACA	177
Ad01A9823	Aradu.A01	9823	p3	(AAG)6	18	45017822	45017839	AAAATATTGCTCGCCCTCAA	AACACAACCCCCTCTTCCTT	115
Ad01A9828	Aradu.A01	9828	p3	(CTT)5	15	45043782	45043796	ATGAGCTTCATCACCAAGGG	TGGAAAAGGAGGAATGTTGC	248
Ad01A9829	Aradu.A01	9829	p2	(AT)11	22	45056559	45056580	TGGGGTAATTTTGTCCCCTT	TGAGTATGGGAAAATTGTGGG	212
Ad01A9830	Aradu.A01	9830	p3	(AGA)6	18	45066445	45066462	TACCTTTTCCGGCCTTAGGT	TACCTTGGCCGAACCTACAC	170
Ad01A9833	Aradu.A01	9833	p2	(AT)14	28	45116654	45116681	GGGTTCAATAGCTCATTCCC	TGGTGGAATCTTGTGCAATG	276
Ad01A9834	Aradu.A01	9834	p5	(AAGAT)5	25	45118321	45118345	AAAGGCGGTTTCCCTAAAGA	CCTGGGTGCTCCAGTGTATT	122
Ad01A9835	Aradu.A01	9835	p5	(AAGAT)6	30	45134202	45134231	AAAGGCGGTTTCCCTAAAGA	CCTGGGTGCTCCAGTGTATT	127
Ad01A9836	Aradu.A01	9836	p2	(TC)7	14	45137857	45137870	CCATCAAGGCATCTCATTCA	ATCCTCCAACATCGTCAAGC	162
Ad01A9837	Aradu.A01	9837	p2	(AT)9	18	45145821	45145838	GAAACTGTGGGAACATGGGT	TCGCATGTGCATTGATCTTT	246
Ad01A9840	Aradu.A01	9840	p2	(GA)20	40	45166454	45166493	GGAAAGAGAGAGGAACAAGAGAGA	TAAACCCTAAAAGCGGGGTT	142
Ad01A9841	Aradu.A01	9841	p2	(GA)6	12	45167170	45167181	AAGACCAAGGGTTGAAGGGT	TGAGGATTGAACAATTAGAAGCA	249
Ad01A9843	Aradu.A01	9843	p3	(TCT)6	18	45174716	45174733	TCACCTTGACCGAACCTACC	TCTCCCATTACTCGGAGGTG	223
Ad01A9846	Aradu.A01	9846	c	(A)10gaaaaaac	117	45203967	45204083	CCTTATGCATTGGTGCTCCT	CCCATTTTTCACTTTTTCACA	255
Ad01A9850	Aradu.A01	9850	p2	(GA)11	22	45217502	45217523	AAGACCAAGGGTTGAAGGGT	TCACCAAGGAATGGTTGAGA	164
Ad01A9851	Aradu.A01	9851	c	(GA)7aagagaa	71	45218387	45218457	TCGGCATGCTAAATTTCGTT	CCCTCCCTTCTCTCTCTTCC	207
Ad01A9852	Aradu.A01	9852	c	(GA)6aagtgaga	197	45218581	45218777	GAGAAGGGAGGGGGAGAAA	CCCCTTCACTCTCTCTTTGC	273
Ad01A9853	Aradu.A01	9853	c	(GA)6aagagag	93	45218940	45219032	TGAGTGAGAGAGAGGAGGGAAG	CTCCTATCTTTCTTTTCCCCTT	231
Ad01A9854	Aradu.A01	9854	c	(GA)6aagtatgta	99	45219153	45219251	TGAGAAAGAAGGAAGGGAGAGA	TCAAAGTAGGGGTGTTCATAAAA	279

ID	Chromosome	SSR nr.	SSR type	SSR	Size	Start	End	FORWARD PRIMER (5'-3')	REVERSE PRIMER (5'-3')	Product (bp)
Ad01A9855	Aradu.A01	9855	p3	(GTC)6	18	45242527	45242544	CACCGCACAGAAAGAACTCA	AAACCCCAAGAACTCACCG	184
Ad01A9856	Aradu.A01	9856	p2	(AC)20	40	45248746	45248785	CGGCTGCGGTATTCTGATAG	GGATTTCGGTTATGAAATGCTT	242
Ad01A9858	Aradu.A01	9858	p4	(TTAG)5	20	45252488	45252507	TATTTTCTGCTTCCCATGCC	TCCAACACTTATCCCCTAAAACA	244
Ad01A9859	Aradu.A01	9859	p2	(GA)8	16	45257016	45257031	TCTCTTCCTCCCTCTCGGAT	ACCACCCCAAACCCTAACAC	196
Ad01A9861	Aradu.A01	9861	p4	(TTTA)5	20	45269627	45269646	CAAACAAGTCAAGAAGCACCA	ATGGCGTATATGCACGGATT	248
Ad01A9862	Aradu.A01	9862	c	(CG)6ccatgtgc	62	45269830	45269891	TATACGCCATGCGTGCTTAG	AGTTGTGCCCACTTTATGCC	162
Ad01A9863	Aradu.A01	9863	p3	(ATT)5	15	45279809	45279823	AAACTTGGACGTTGGCTTTG	CAGGGAAAAAGGCACTCTTG	232
Ad01A9865	Aradu.A01	9865	p3	(AAG)6	18	45310754	45310771	TCTAAAGGATTCTCCGGCCT	TACCCTCTCCCCTTTCCCTA	248
Ad01A9867	Aradu.A01	9867	p3	(TTC)5	15	45352009	45352023	GTTATTGTTGGCCCCTCCTT	GGGAAGGCTATTGTTGGAGA	108
Ad01A9874	Aradu.A01	9874	p2	(CT)6	12	45434644	45434655	CAGAATTGGCACAAAGAGCA	TTTTTGGGGAAGAGGTGTTG	162
Ad01A9878	Aradu.A01	9878	c	(A)11gagaggag	92	45457973	45458064	TGAAAACTCTGGGATTTGGG	TTTTTGTCCCCTTTCATTGC	221
Ad01A9880	Aradu.A01	9880	p2	(GC)6	12	45459210	45459221	GAAGATAGCTGGAAGTGGCG	TGAAATTGGGCCTAATGAGC	201
Ad01A9881	Aradu.A01	9881	p2	(GA)6	12	45471967	45471978	TTGAGGTTGAGGCTCTTGGT	TCCCACTTCAGAACCCATTC	240
Ad01A9883	Aradu.A01	9883	p2	(GA)7	14	45492076	45492089	TACAGAGGGAGAGAGGGGGT	TTTCAAAATCCTTTTTCTAACTTCTT	253
Ad01A9887	Aradu.A01	9887	p3	(ATG)5	15	45514480	45514494	CAGGTTCGAGTTCTTGCTCC	GAAGGCGGGATAATAGAGGC	155
Ad01A9891	Aradu.A01	9891	p2	(TC)6	12	45546684	45546695	GGCTATAAACGGCTCGTCAA	CCAAATGGACCAAAAATGCT	227
Ad01A9892	Aradu.A01	9892	p3	(AAG)9	27	45547271	45547297	TAGTGGCGGATAATGGAAGG	GTGTTCGCACCCTTATGCTT	182
Ad01A9895	Aradu.A01	9895	p3	(AAC)5	15	45578579	45578593	ATGGATTAAACAATGGCCGA	AACATCCAGTCAGGAGGGTG	260
Ad01A9897	Aradu.A01	9897	p2	(TA)6	12	45585024	45585035	GTCGCTTCCTTCATTTGGTC	AACACTCAAAAGGCAAAGCC	173
Ad01A9901	Aradu.A01	9901	p3	(ATT)5	15	45611010	45611024	CAATGCCCGACTCCAATAAT	GGATCGACCTCACTTTCGTG	280
Ad01A9903	Aradu.A01	9903	p2	(CT)7	14	45633069	45633082	GCCGGTAAATCTGACGGTAA	CATCAGTCACGGAGAAGGGT	216
Ad01A9904	Aradu.A01	9904	c	(TTA)7taatgga	120	45645978	45646097	TGTCTGCTAATGATGCTTGGA	TTGAGAAATATTAAGTGTCATGGTTT	280
Ad01A9905	Aradu.A01	9905	p2	(GA)6	12	45646218	45646229	GGAACCAAAGAAAGGAACCA	GTGGGTCACGATTTCCTCAT	233
Ad01A9910	Aradu.A01	9910	c	(AAT)13ttttgg	117	45652824	45652940	TCATTACAAGTATTCCGTCAAAAA	AAACATCAATATCGAGGAACCAA	191
Ad01A9914	Aradu.A01	9914	p3	(ATA)14	42	45665455	45665496	GGGGTGGTTATGGTATTTTCAA	AATACCTACCCAATCTTCCAAAA	273
Ad01A9915	Aradu.A01	9915	p2	(TA)6	12	45666286	45666297	AGCTCCGACTCGACCTACAA	TGTTGCTTTTCAGATGCAGG	251
Ad01A9917	Aradu.A01	9917	p2	(CA)21	42	45674811	45674852	ATCAAGCCAACCTGACAAGC	ACAATTCTCGTGGGATCGAC	255
Ad01A9918	Aradu.A01	9918	c	(T)10cgtttttctc	45	45688430	45688474	CCGGATACATACATGCCACA	TGAAGCAAGAAAAAGCAGTGAA	252
Ad01A9919	Aradu.A01	9919	p2	(GA)6	12	45705638	45705649	AAGACCAAAGGAGCAAGGGT	TTGCAATGGATCTCACCAAA	177
Ad01A9921	Aradu.A01	9921	p2	(AT)8	16	45739119	45739134	TTAAACCCTAACCTGCCCCT	TTAGGGGTGGCAACACTACC	203
Ad01A9922	Aradu.A01	9922	p3	(ACC)5	15	45765615	45765629	GTCGAAAATTCAAACGGAGC	CAGATGGAACCAGTCAAGCA	118
Ad01A9925	Aradu.A01	9925	p2	(TA)7	14	45772439	45772452	CAAGGGAAGGAACCTCCATA	TTGTGGGGTTTGATGAGGAT	255
Ad01A9930	Aradu.A01	9930	p3	(AAT)7	21	45788745	45788765	TCCTTTCTCACTCTCTTCTTTTCC	GGCACCAAAATAATGCAAGAA	265
Ad01A9933	Aradu.A01	9933	p2	(GA)7	14	45828335	45828348	TATGCGTCATTGTCGAGCTT	ACATTGGTTTCAGTAGCGGC	172

ID	Chromosome	SSR nr.	SSR type	SSR	Size	Start	End	FORWARD PRIMER (5'-3')	REVERSE PRIMER (5'-3')	Product (bp)
Ad01A9934	Aradu.A01	9934	p2	(TA)11	22	45846850	45846871	TGAACGTTTCACGCTTTTGT	GGCTTCTATTGTTGTCCCCA	213
Ad01A9935	Aradu.A01	9935	p2	(AT)9	18	45852988	45853005	AACAATTCAGGGCCTTTGC	CAACCTTGGCAAAGATTCAA	210
Ad01A9936	Aradu.A01	9936	p2	(CT)8	16	45856410	45856425	CAAGTTATGGCTCCCGAAAA	TTGAAAACCGGGCACTAATC	238
Ad01A9939	Aradu.A01	9939	p3	(AAC)5	15	45888987	45889001	CGCGATACACAAACAAGGAG	AACATCCAGTCAGGAGGGTG	266
Ad01A9941	Aradu.A01	9941	p3	(AGA)6	18	45905617	45905634	TAGAATATTGCTCGCCCTCG	TTAACCCCTCTCCCTCCACT	173
Ad01A9942	Aradu.A01	9942	p3	(AGA)6	18	45910928	45910945	TAAAATATTGCTCGCCCTCG	AAATCTTTCCCACCCAAACC	121
Ad01A9945	Aradu.A01	9945	p3	(TAT)14	42	45932047	45932088	GGTTCCTTGTCCCTGTTCCT	TTCATTAGTACCCCCTCCCC	234
Ad01A9946	Aradu.A01	9946	p2	(GA)6	12	45935038	45935049	TCGCAGAGTTAGCACTCAACA	GCCCAAACACCAAAAAGGTA	270
Ad01A9948	Aradu.A01	9948	p3	(AGA)5	15	45942071	45942085	GCCAAAGTGGGAATGAAAAG	GCACAAAATTCGGAAGGAAA	244
Ad01A9949	Aradu.A01	9949	p2	(TC)6	12	45953233	45953244	CGGCTTGAAGCAAACTATGG	CTGAGAGGACGGATGGTAGC	265
Ad01A9951	Aradu.A01	9951	p2	(AG)6	12	46028841	46028852	GACCCTGTTTTTGACCCAGA	AACATTGAACCTGGAATGAAGAA	205
Ad01A9952	Aradu.A01	9952	p4	(AAAT)6	24	46033721	46033744	AAAAACTGTGGGAACATGGG	AACTTTGCATTGGGGTAATTTT	254
Ad01A9953	Aradu.A01	9953	p2	(AG)6	12	46035136	46035147	GAAATTCCAACCACCTGCAC	AACATTGAACCTGGAATGAAGAA	262
Ad01A9954	Aradu.A01	9954	p4	(TTTA)5	20	46066163	46066182	TGCAATGAATGAAAATAAGCAAA	CGTGGATTTGCACCCTATTT	230
Ad01A9955	Aradu.A01	9955	p3	(TCT)6	18	46093388	46093405	CCGAACTTTAACCCCTCTCC	GCTTTTGTCTTCCCTTTCCC	277
Ad01A9956	Aradu.A01	9956	p3	(TTA)5	15	46096179	46096193	ATTCAGGAGGAGCACAGCAT	TCGCACAACTAACCAGCAAG	258
Ad01A9957	Aradu.A01	9957	c	(AC)9aacac(C.	37	46111746	46111782	CACACACTACCCATCCCCTC	GTGATGGTGGGACTCTTCGT	159
Ad01A9958	Aradu.A01	9958	c	(T)10ctgaaacc	56	46117474	46117529	TTTTGCTACATGCTAGTGGTCC	TATTAGTGTGCGGACCGGAT	254
Ad01A9960	Aradu.A01	9960	p4	(TTTA)5	20	46120589	46120608	CGCTTCTATTGTGCAAGCTG	GCATCAAAATAAATCAAGCAGA	272
Ad01A9961	Aradu.A01	9961	p3	(ACC)5	15	46122355	46122369	GCAAATGGTGCAGCATAAAA	GGAGCAAGGAGTTGAAGCAG	264
Ad01A9963	Aradu.A01	9963	p4	(ATAA)7	28	46134699	46134726	GCAAGTAGCAAGCCAAGACC	TTTGGTGGTTGGTTATTTTGC	148
Ad01A9964	Aradu.A01	9964	c	(A)10g(AGA)5	26	46137523	46137548	GCTTTTGTCTTCCCTTTCCC	CGAAACCACACACCTCTCCT	189
Ad01A9970	Aradu.A01	9970	p3	(GAA)5	15	46200568	46200582	CTCCAGCCTTAGATGCCATT	ACTCCCCCTTCTCCTCCATA	146
Ad01A9971	Aradu.A01	9971	p3	(AAG)5	15	46215897	46215911	GCTTTTGTCTTCCCTTTCCC	TACACCTCTCCCCCTCTCCT	193
Ad01A9974	Aradu.A01	9974	p2	(AG)6	12	46262472	46262483	GGTTGAATGGTTGAGGGTTG	CCATTCCCCTTCTTCTTCCT	278
Ad01A9975	Aradu.A01	9975	p5	(TTTTA)5	25	46277068	46277092	CCTCACTTTTTCGAAATTCTTCA	GGGTTAGCATATGACCCCAG	254
Ad01A9980	Aradu.A01	9980	p2	(AT)9	18	46321419	46321436	GCCACGTCTAATTCCTGGAC	CACCATGCATGAGTTTTCAA	260
Ad01A9982	Aradu.A01	9982	p4	(CTTT)7	28	46335571	46335598	CAGCTCAAAGAAGCAGCCTT	CACAGTAGTTGCTGCTGGGA	189
Ad01A9984	Aradu.A01	9984	p4	(AAAG)11	44	46355236	46355279	CAATTTTTCCCTTTACCGCA	CCTCCAAGGCCTGACAATAA	250
Ad01A9985	Aradu.A01	9985	p2	(GA)6	12	46355645	46355656	GGAAGACCAGAAACGATGGA	TACCGGTGACATGAATGGTG	255
Ad01A9987	Aradu.A01	9987	p2	(AG)6	12	46370673	46370684	TTGGAAGAAGAATGGATCGG	CAACACGGCTAGTGTGCAGT	262
Ad01A9990	Aradu.A01	9990	p5	(AAGAA)5	25	46407286	46407310	TAGCCTGAGCTTGTTGCAGA	CCTTTTCTCTCTCTTCTTTTTCG	279
Ad01A9991	Aradu.A01	9991	c*	(AT)6(TTATT	36	46408257	46408292	AAATGAAAACCAAAGTGACGC	GTTCTAAATTCGCGCACCAT	232
Ad01A9993	Aradu.A01	9993	p2	(AT)6	12	46416879	46416890	TGATCGGTCAATCTTGGATG	GCTTAAAAGCCATGTTCCACA	257

ID	Chromosome	SSR nr.	SSR type	SSR	Size	Start	End	FORWARD PRIMER (5'-3')	REVERSE PRIMER (5'-3')	Product (bp)
Ad01A9995	Aradu.A01	9995	p3	(TCT)6	18	46437256	46437273	ATCCATCCCAGCGCTAACTA	TGCAGGAGATATGCAACAGA	233
Ad01A9999	Aradu.A01	9999	p2	(AT)6	12	46461703	46461714	CAAGACCGCTCCCAAAAATA	GATATGGGCGCAGAAGTCAT	259
Ad01A10004	Aradu.A01	10004	p2	(AT)8	16	46474742	46474757	GCCAAGCTCATGAGGAATGT	TCCTTTACTGCTATGTTCTTTCAA	184
Ad01A10006	Aradu.A01	10006	p2	(AT)6	12	46496419	46496430	TTCCACCAAGTCCACACAAA	GCGGAGAATCCTTACCATGA	215
Ad01A10007	Aradu.A01	10007	p3	(TAG)5	15	46497097	46497111	AGACGTGGCTTATTGGGTTG	AGCAAAGCAATAGGAAGGCA	276
Ad01A10013	Aradu.A01	10013	p2	(AT)6	12	46527460	46527471	AATGGGGCTGAGGTAAAGGT	GGAAGCATGCTCATGTGAAA	254
Ad01A10015	Aradu.A01	10015	c	(CT)13tcccaac	74	46617750	46617823	GATGAATAGTGGCAATGGGG	TAGCCCAAAGTGGGTCAAAC	197
Ad01A10016	Aradu.A01	10016	p3	(AGG)7	21	46621145	46621165	GTTTCGGTTGCAACATCCTT	CCAACAAGGCATAGGACGAT	159
Ad01A10018	Aradu.A01	10018	p2	(TA)7	14	46622424	46622437	TGAAGAGATTTCTGGGTCGG	CATGAAATGGAGGAACCCAC	120
Ad01A10019	Aradu.A01	10019	p2	(CT)8	16	46624757	46624772	CGCTGACTCAGTGTAACATGC	TGAGTAGCAGCCCAAACAAA	274
Ad01A10025	Aradu.A01	10025	c	(T)11ctttttcctct	51	46670789	46670839	TCCCCAATTAGAGGTGTCCA	TTGTTGTTCATGAATGTTGGC	240
Ad01A10028	Aradu.A01	10028	p2	(AT)10	20	46695245	46695264	CCTTCGTGTGTACTTTGTTTCC	GAAAATTTATTTGTGGAATTTTCG	278
Ad01A10029	Aradu.A01	10029	p3	(AAG)11	33	46728590	46728622	CTAGAGGTTTCTCCGGCCTT	CCAAACCTACTCCCTCTCCC	266
Ad01A10031	Aradu.A01	10031	p2	(AT)10	20	46737982	46738001	TGGGTACCTACTCATGTAAGACCA	TGTAATGGGGCCAAGGTAAA	185
Ad01A10032	Aradu.A01	10032	p3	(TCT)5	15	46746918	46746932	ACTTGAACTTCATGCGACCC	ATGGAGGATGTGAGTGGGAA	235
Ad01A10033	Aradu.A01	10033	p2	(TA)6	12	46762890	46762901	GGGGTAAACAAAAATCGCAA	TTTGTGTCCCTTTGGGTTTC	217
Ad01A10034	Aradu.A01	10034	p2	(TA)6	12	46770985	46770996	CACAACTCGAAAGCGCAATA	AGCCGGATTTTGAGTCTTGA	120
Ad01A10035	Aradu.A01	10035	p2	(GT)6	12	46797982	46797993	TCTTCTCCTCCCTCACTCCA	GACCTAGATTGGACCGAACG	151
Ad01A10036	Aradu.A01	10036	c	(AT)7c(TG)9	33	46804612	46804644	TTTCCAGAAAGGTGCATGTG	GACTATGGCTGCAATTGCCTA	265
Ad01A10037	Aradu.A01	10037	p3	(AGA)6	18	46811640	46811657	TAAAATATTGCTCGCCCTCG	CCAAAAACCCCACCTACCTT	241
Ad01A10038	Aradu.A01	10038	p2	(TA)7	14	46834850	46834863	AACTTCATTTTCTGAGTGGTTGC	GTATACCTCAGCCGCAGCAT	249
Ad01A10040	Aradu.A01	10040	p3	(TGA)7	21	46856324	46856344	TTGATCAGCATCAGGCTCAG	TCGATTCCTCCTTTTGGATG	205
Ad01A10042	Aradu.A01	10042	p3	(TAT)16	48	46862997	46863044	TGCAACAAGTGAGAAGAGCG	TTTTTACCCGACGGTTTGAG	210
Ad01A10043	Aradu.A01	10043	p2	(AG)6	12	46898777	46898788	CACCTCGAGCTCCTTGTTTC	CTCGTGTCATCGTGAACAAA	167
Ad01A10044	Aradu.A01	10044	c	(TC)7cccctcttt	158	46901243	46901400	AACCAGTTTTTATGGTTCAGTTCA	GAAAGAGAAGGGGAAATGAGG	277
Ad01A10045	Aradu.A01	10045	p2	(TC)6	12	46901529	46901540	CCCTCTCTCTCCCCTTTTGT	CCTTCCTTCTCTCTCTTATTTTGG	277
Ad01A10046	Aradu.A01	10046	p2	(GA)7	14	46902280	46902293	GGAGAGAGAAAAAGGAAAAGGG	ATTCTCTCCCTCTCCCCCTC	277
Ad01A10049	Aradu.A01	10049	p3	(TAA)6	18	46919952	46919969	TCTAAAATTGCATAATTCCTAATCCA	TCAAAAAGGCGTCTAAAGATCA	112
Ad01A10052	Aradu.A01	10052	c	(AACCG)5cat	81	46927490	46927570	GATTCAAACCGAAAACCGAA	TTTTTGTGTCGTCTTTTAACCG	263
Ad01A10054	Aradu.A01	10054	p2	(GA)7	14	46935927	46935940	TGGTAAGAGTGATGGTGGCA	TGGTGACTCGCAATGGTTTA	160
Ad01A10056	Aradu.A01	10056	p3	(AAG)5	15	46937236	46937250	TGCTGCGAAAAATCAACAAA	GCAACCAAGTCCAACCAAGT	235
Ad01A10061	Aradu.A01	10061	c	(GA)9(GT)8	34	46970715	46970748	AGCAGAGGGGGAAGAAGAAG	CCATGGCAGTAGTAGCAGCA	266
Ad01A10062	Aradu.A01	10062	c	(TA)6(TG)10	32	46972092	46972123	ATAAAGGCGATTCATGGCG	GACCTTGTCCGGAACCAATA	274
Ad01A10063	Aradu.A01	10063	p3	(ACA)9	27	46991143	46991169	TGTCGCCGTGTTGGTAAGTA	ACATGACAGAGCACAATGGC	259

ID	Chromosome	SSR nr.	SSR type	SSR	Size	Start	End	FORWARD PRIMER (5'-3')	REVERSE PRIMER (5'-3')	Product (bp)
Ad01A10064	Aradu.A01	10064	p2	(AT)8	16	46994537	46994552	TCGTTTTCCTTCTCGCACTT	TTTTTCCTCTCGCATACGCT	168
Ad01A10066	Aradu.A01	10066	c	(T)10ctaatagaa	84	47015072	47015155	GGACAATTCGATTTGCTCAGT	TCCGAATTTTCAGGCTCATT	261
Ad01A10070	Aradu.A01	10070	p5	(TATTT)8	40	47036580	47036619	CGTTGGATCTTTATGCCGAT	TGCATTCTGCATTTTGCATT	143
Ad01A10071	Aradu.A01	10071	p2	(TA)6	12	47046797	47046808	TGGGAACTCACAATTTTCTTTG	GCTTGAGCTTGGAATTGAGC	234
Ad01A10075	Aradu.A01	10075	p2	(GT)20	40	47076827	47076866	GCCTGCCACCATAGTTGTTT	TCTGTGTTGTTGTTTGGCGT	156
Ad01A10078	Aradu.A01	10078	c*	(A)10(AAT)5*	23	47098320	47098342	AAACAGATTTCAGCCATTCTAAAAA	ACCCAGCGAAAGTTTCTCAA	237
Ad01A10085	Aradu.A01	10085	p2	(AC)7	14	47108108	47108121	TCCCTCACTCTCACATTCCC	CTGCTCTGACCTCCTCTGCT	136
Ad01A10086	Aradu.A01	10086	p2	(AT)6	12	47128950	47128961	AGTTGCCGGTTAGTTTGTGG	CGAGGACACCAAACCTAATTTC	232
Ad01A10088	Aradu.A01	10088	p3	(TGA)5	15	47166922	47166936	ATCTATCCGGGCCGATTATT	CCCGGTTCAAGATTTAGCAA	258
Ad01A10090	Aradu.A01	10090	p3	(CTT)5	15	47192710	47192724	ACTTCCGTTGGGAAGCATAG	CCCCTAATGAAGATGGGGTT	183
Ad01A10092	Aradu.A01	10092	p2	(CA)6	12	47209976	47209987	CCCAGTTGGTTCAAGATGCT	GGGCATCAATTTCCAAAGAA	124
Ad01A10093	Aradu.A01	10093	p2	(AG)7	14	47241129	47241142	CCATGATGGAGAAAGGGAGA	TTTTTCAAAATTCCCTAACCACA	247
Ad01A10094	Aradu.A01	10094	p5	(AAAAT)5	25	47249726	47249750	TTCCCTGTGAAGTAACCTGTG	TTGTTTCACCTGCTTCTTGG	239
Ad01A10099	Aradu.A01	10099	p2	(AG)6	12	47258332	47258343	TCTGACACATCTAATTTCACATGG	TCGAGATATCCCCTGAAAGG	269
Ad01A10103	Aradu.A01	10103	p3	(TCT)5	15	47296339	47296353	TCCTGGGTTATTCCGTTCAG	CCCCCATGGCTTTATGTAGA	247
Ad01A10104	Aradu.A01	10104	c	(A)13gtgaaaac	49	47300461	47300509	GCTGGGACTTCACTTACGGA	CCTTCCTTTAAGCAGCCAGA	225
Ad01A10105	Aradu.A01	10105	p2	(TA)7	14	47303104	47303117	TGAACCCTTTTGGTTTACGC	GATGCAAACATTGCTACCCA	191
Ad01A10107	Aradu.A01	10107	p2	(TA)6	12	47318832	47318843	CGCTCTCGTGTGACTCTCTG	GTGCTAGGTGGGAGACAACC	261
Ad01A10108	Aradu.A01	10108	p3	(CTT)5	15	47319859	47319873	TTGGAAGGAGGTTCGATGTC	ACCCCCAGTGAACAAGACAG	144
Ad01A10109	Aradu.A01	10109	p5	(TTATC)5	25	47341733	47341757	CCTGGGTGCTCCAGTGTATT	GCCCAATAAAGGGTCCAAAT	192
Ad01A10110	Aradu.A01	10110	p2	(GA)7	14	47343235	47343248	TTGGTGGTGGAAAAAGTGGT	TAAAATCCAAACCGCAGACC	245
Ad01A10115	Aradu.A01	10115	p5	(AAGAT)6	30	47411374	47411403	CGGTTCCCTTGAAGATACGA	GTCAAAGCTGATGTGTCCGA	272
Ad01A10116	Aradu.A01	10116	c	(A)10tatttttgttg	102	47418000	47418101	ATGGATGAATTGAAATGCCA	TACGCTTACACATGGCGTG	280
Ad01A10117	Aradu.A01	10117	p2	(GT)6	12	47424053	47424064	CACAAACTTATATGGCCAACCC	TTCAATGAACACCCAAAGCA	226
Ad01A10120	Aradu.A01	10120	p2	(GA)6	12	47435910	47435921	TTGAACACAGAGGGGGAGAG	TTTCGAAAATCACTAACCACTTTTT	256
Ad01A10125	Aradu.A01	10125	c	(TTC)6tta(TTC	36	47452562	47452597	CATGGGTCGTATGCACAGTC	GGAGGGGTCCTCTTGTAAGG	165
Ad01A10128	Aradu.A01	10128	p2	(CT)6	12	47463747	47463758	GCAGTTGTCTGATGTCTCGG	TACTGAACATTGCAGCAGCC	209
Ad01A10131	Aradu.A01	10131	p2	(AT)8	16	47474321	47474336	CCAAGGCTTGCCAGAGAATA	TCTGGATCTTTGAAGCGGAG	280
Ad01A10132	Aradu.A01	10132	p3	(TTG)5	15	47480574	47480588	CGTACGGAGCGTCTTCTTCT	CACCATGAGTACGTGTTCGG	279
Ad01A10139	Aradu.A01	10139	p3	(TTA)6	18	47517981	47517998	CACATTCATGCATCCCAAAT	GTGTGGGAAAAGTGGGTTTG	266
Ad01A10142	Aradu.A01	10142	p2	(AT)7	14	47529989	47530002	TTGTTCACGCAAAGCTAACG	TGCGGCTAGTTCCCTATGAT	102
Ad01A10143	Aradu.A01	10143	c	(TC)26ttttccctt	112	47538315	47538426	AGTTATGGCTCCCGAAAGGT	GCCCAAGGTCCAACATACAT	208
Ad01A10144	Aradu.A01	10144	p2	(AG)6	12	47539872	47539883	GATGTCATTGATGCAGGACG	CTTGTCTCTCCCATCTTCGG	180
Ad01A10150	Aradu.A01	10150	p3	(AAG)6	18	47588677	47588694	TTTCCTTCTCCCATGACTCG	ACACAACACAACCCCCTCTC	254

ID	Chromosome	SSR nr.	SSR type	SSR	Size	Start	End	FORWARD PRIMER (5'-3')	REVERSE PRIMER (5'-3')	Product (bp)
Ad01A10151	Aradu.A01	10151	c	(GT)6tttgga(A	30	47597827	47597856	GCAATTAGCCAAAGGAAGTTTT	AACACATTTTTGTCTTGTCCCA	271
Ad01A10153	Aradu.A01	10153	p2	(AT)8	16	47608127	47608142	TCCGAGACACGAGTTTTCCT	GTCCGGTTTGCTAAACCCTT	250
Ad01A10154	Aradu.A01	10154	p2	(CT)32	64	47613733	47613796	GGCCTTGGCCCTACATAACT	AATAGTGCGGCAATCAAAGC	236
Ad01A10159	Aradu.A01	10159	p2	(CT)8	16	47667739	47667754	TTTCCTTGTTAAGAAAGTTCCATTC	TTTGAAAAATTGTGGGTTAGGA	180
Ad01A10160	Aradu.A01	10160	p4	(AATA)5	20	47667896	47667915	TTTTCAAAAATCCTAACCCACAA	TGACCCTAGAGTCCTTCTGGA	251
Ad01A10161	Aradu.A01	10161	p2	(TC)6	12	47677695	47677706	CTTCTCTCTCCCATCCCTTTC	TCAGCTTCAACAAGAATGCC	235
Ad01A10162	Aradu.A01	10162	p3	(TTA)8	24	47687593	47687616	TCAACTTGTAAAATGAGCCACA	TTGTTGACTATTGACTCATGCAA	262
Ad01A10163	Aradu.A01	10163	p2	(TA)11	22	47692018	47692039	CCTTGGTGTTTTCCCTCAAA	TGGTTAGGAAGTTTTGAAAAGGA	231
Ad01A10164	Aradu.A01	10164	p3	(GGT)5	15	47694577	47694591	TGCTTTGCTTCTGCTTTTGA	CCTTTACCATCACCACCACC	208
Ad01A10165	Aradu.A01	10165	c	(T)12aaatttcttt	40	47699595	47699634	GGCAAAATTTAAGCAGGGGT	TGCTGTCAGTAACAACCAACG	271
Ad01A10166	Aradu.A01	10166	p3	(GGA)5	15	47710879	47710893	ATGGAGAATGCAGATCAGGG	TCCCAATCCAACGCTCTTAC	255
Ad01A10173	Aradu.A01	10173	p2	(CT)6	12	47814817	47814828	AAAGGAATAGAAAAACCATGCAA	CACGACAAGAAAAATGGCCT	217
Ad01A10174	Aradu.A01	10174	c	(A)10gtataagg	59	47815337	47815395	TGCTGCCTCCTCTTATCAGC	GGAGGCAACATGAATGGTTA	232
Ad01A10175	Aradu.A01	10175	p2	(AG)11	22	47816702	47816723	TCCTCTGCAAGGAGTGATGA	TCATTGCTTCTTCACTTTCACAA	215
Ad01A10177	Aradu.A01	10177	p2	(AT)6	12	47818201	47818212	GCTGCTAACCAAGTAGTGCCTT	TGGCTATTGCAAGTTAAACAACA	244
Ad01A10184	Aradu.A01	10184	p3	(AAT)6	18	47885898	47885915	TTCGTGGATTACAAGCCAAAT	TGCGAGTGTAAAATTGATCTTTTG	215
Ad01A10187	Aradu.A01	10187	p2	(TG)6	12	47887402	47887413	TATCAAACAAGGCCGTGACA	ATGCGTTTGCCTCTGTAAAT	269
Ad01A10188	Aradu.A01	10188	p3	(CAA)6	18	47887790	47887807	AAATGCCAATGGAAGAACCA	TCTCATCCTGAAAACGGTGA	129
Ad01A10190	Aradu.A01	10190	c*	(AT)8(T)10*	25	47899080	47899104	CAAAGCTGCTTGCTTTGATG	GATTCAGTTTTCAATTTGCTTTGA	269
Ad01A10191	Aradu.A01	10191	c	(ACA)6atatcaa	130	47900226	47900355	CCCAAATAATTAAGCCCAAAA	CCGGTCCAGTTTTAGAACCTT	264
Ad01A10193	Aradu.A01	10193	p4	(ATTA)6	24	47903027	47903050	CAGCCAATTTTAATGCTGGT	CCCAGTGCTATAGGAAAAGGA	271
Ad01A10197	Aradu.A01	10197	p3	(GAA)17	51	47929929	47929979	TGATTGAGAATGTGAGAATGGG	ACATCACCTGAACCCTACGC	194
Ad01A10202	Aradu.A01	10202	p3	(ATT)8	24	47957470	47957493	GGGGTTAGCATATGATCCCA	TCCTAATCACTTTCTCTCTCCTCA	225
Ad01A10211	Aradu.A01	10211	p3	(TTC)5	15	48018972	48018986	CAAACACTTTTCCCTCCCAA	GCAATGCTTTTACCACACCA	238
Ad01A10212	Aradu.A01	10212	p3	(TAT)8	24	48027694	48027717	GAGCGTTGCGCTTTTTATTT	CACCTAGCTTAACTCATGCTTTT	236
Ad01A10213	Aradu.A01	10213	p4	(TTAT)6	24	48034704	48034727	AACGCCTGAAAAATCACTCG	ACCGGAAGGTCATTCCCTAT	237
Ad01A10219	Aradu.A01	10219	p4	(TTAT)5	20	48128315	48128334	CAAAAGCCTGAAAAATCACTCA	GATGGATTTTATGGTGCTACTCG	173
Ad01A10220	Aradu.A01	10220	p2	(TA)8	16	48133021	48133036	TCGCTAGGCAACTGGATCTT	GCTAAGGTTGGCCAGAGGAT	233
Ad01A10221	Aradu.A01	10221	p2	(TA)8	16	48133157	48133172	TGTTTTTCAGATGCAGGTCG	AAGCTAAGGTTGGCCAGAGG	264
Ad01A10222	Aradu.A01	10222	p3	(AAG)6	18	48143246	48143263	TAAAATATTGCTCGCCCTCG	AAATCTTTCCCACCCAAACC	199
Ad01A10225	Aradu.A01	10225	p2	(CT)11	22	48169259	48169280	GGGTCACGACGATGCTAAAT	TCGGAGAGAGAAAGATGCGT	252
Ad01A10226	Aradu.A01	10226	p4	(ATAA)5	20	48169573	48169592	GGTTTGAATACGGTTTTAATTTGG	CCACAAAAATTAAACTAGGGGTC	259
Ad01A10228	Aradu.A01	10228	p2	(CT)12	24	48186684	48186707	TTCGAAAATCACCTTCCTCTTT	GAATGGAGGGGTTCGAATTT	241
Ad01A10229	Aradu.A01	10229	p3	(GAG)5	15	48188286	48188300	CCATGCAATGCAGAAGAGAA	CCCATGAGGTCTTCCACACT	119

ID	Chromosome	SSR nr.	SSR type	SSR	Size	Start	End	FORWARD PRIMER (5'-3')	REVERSE PRIMER (5'-3')	Product (bp)
Ad01A10230	Aradu.A01	10230	p2	(CT)7	14	48189759	48189772	GATTGCACAATAAGGGCGTT	AAAATGTTTGCTCGTCCTCG	241
Ad01A10231	Aradu.A01	10231	p2	(TA)7	14	48216742	48216755	GTACCCTAGCCGGCCTAAAC	CGCGAAGACGTATCACTCAC	177
Ad01A10232	Aradu.A01	10232	p2	(TA)6	12	48217801	48217812	CAACGCTGAGAAGAGAGGAGA	AACTTTGCATTTTGATTGATTGA	244
Ad01A10233	Aradu.A01	10233	p4	(TTTA)5	20	48218436	48218455	TTGATCTCCTTTCTTTTCCAACA	GATCATTTCCTATGCCGGTT	197
Ad01A10235	Aradu.A01	10235	c	(TAAA)5agaag	39	48238255	48238293	TGCCTTTTTCCTTGTTCCTG	TTTCCGAAAACCATTAACTCC	272
Ad01A10237	Aradu.A01	10237	p2	(TA)6	12	48239459	48239470	TTCGAAAAATGCAAGAACAAGA	TTAAGCCATGTCCAACCCTT	251
Ad01A10238	Aradu.A01	10238	p4	(TTAT)6	24	48239744	48239767	CAAAGGGATTTGGATATGGC	TCGAGTTTGGACAACTGACG	213
Ad01A10240	Aradu.A01	10240	p3	(TTG)5	15	48271106	48271120	AAGTTTTGAATCCGCTCTGC	TTTTCTTCAAAGGCTCCTCG	265
Ad01A10241	Aradu.A01	10241	p2	(AT)6	12	48272080	48272091	GCATATGATGCATGCCTGTC	TGTAGCCGGACATTGTGAAA	222
Ad01A10243	Aradu.A01	10243	p2	(AT)8	16	48290702	48290717	ATATCAAGGCTCCGGACTCA	TCATCACTGAGGCATGCTGT	272
Ad01A10244	Aradu.A01	10244	p2	(AT)10	20	48291438	48291457	CCATTGGACTGTCCCTCTGT	CACGTCCAACCGTGTTTATG	207
Ad01A10249	Aradu.A01	10249	p3	(TCT)5	15	48333721	48333735	GAGCTCTTCACAAGGAACGC	AAGTCTGCCCGATACAGCAC	274
Ad01A10250	Aradu.A01	10250	p3	(TCT)5	15	48341040	48341054	AATCGGAGCTCTGTAGCTCAA	GCCGAAGCCCAACATATAAA	164
Ad01A10251	Aradu.A01	10251	p3	(GAA)5	15	48345148	48345162	GCACCTCCGGAGTATTTTGA	CTGGCCTCCTCCTTCTTCTT	262
Ad01A10253	Aradu.A01	10253	p3	(AGA)6	18	48351327	48351344	CTTCCCTTTTCCTTTTTCGG	CCTTGGCCGAACATACATCT	177
Ad01A10254	Aradu.A01	10254	p3	(ATT)5	15	48353994	48354008	AAATTTGGACGTTGGCTTTG	TCATGCTTTTCCCTTTTGCT	179
Ad01A10256	Aradu.A01	10256	p2	(AT)6	12	48365626	48365637	ATGGGTTTATCAACCTCCCC	ACCCATCTCTCCCATCTCAA	278
Ad01A10257	Aradu.A01	10257	p3	(ATT)5	15	48373715	48373729	TCCTTCCTAACTCTCCCTACCA	TCCTTTCAAGTTTTAGCGTGC	252
Ad01A10259	Aradu.A01	10259	p2	(AT)10	20	48378537	48378556	TCCAAGACAAATAAACACCCAA	TACTGATGCACCATTCGAGC	261
Ad01A10261	Aradu.A01	10261	p3	(AGA)5	15	48385862	48385876	ATGGTCTTGATGGGCTTTTG	AAACCCACACCTCTCTCCCT	242
Ad01A10264	Aradu.A01	10264	p2	(TG)8	16	48408212	48408227	TTTCTCTCGGAATCGGTAATG	TTCCAGTCTAGAGCACATGGT	274
Ad01A10265	Aradu.A01	10265	p2	(TA)6	12	48410755	48410766	CAGAAATTTTCAACCCGTGC	TCCAAATTGATGTTGTCGGA	125
Ad01A10266	Aradu.A01	10266	p2	(AT)7	14	48411498	48411511	TGAAATCCAAGAATAAGATGAGTACC	GCAAGGCCTCTAATATTTCACA	280
Ad01A10267	Aradu.A01	10267	p3	(AAT)22	66	48414814	48414879	CTTGCACTATCCGCAAGACA	TGGAAATGTATAATATTTGAATTCGTT	272
Ad01A10270	Aradu.A01	10270	p3	(TAA)13	39	48442953	48442991	CTTCATCCACCTTGGGTTGT	GAGCGAAGCTACCAGCATCT	231
Ad01A10275	Aradu.A01	10275	p3	(TTC)6	18	48500464	48500481	AAATCTTTCCCACCCAAACC	GCCCTCAAGCAAGAGAAGAA	108
Ad01A10276	Aradu.A01	10276	p2	(TC)6	12	48511587	48511598	GGTGATTCCTACGGTGGAGA	AAAGCTGCCACTGCTTCATT	179
Ad01A10277	Aradu.A01	10277	p3	(AAG)5	15	48519158	48519172	CTAGAGGTTTCTCCGGCCTT	CGAACCTACTTCCTCTCCCC	251
Ad01A10278	Aradu.A01	10278	p2	(GA)6	12	48522451	48522462	GGAGAATCCAAACACAAGGG	CAAAACCACCTAACTACTTCTCCC	255
Ad01A10280	Aradu.A01	10280	p3	(AAT)5	15	48524360	48524374	CAACTCCGCACAACTAACCA	ATCCAACAGGGACAAGCATC	259
Ad01A10284	Aradu.A01	10284	p2	(TG)7	14	48562506	48562519	GCATCCAAACTCATCAACTTCA	TGGGATATTTTGGGTGTTCC	215
Ad01A10292	Aradu.A01	10292	p3	(TTC)5	15	48607590	48607604	CCGAAACCACACACCTCTCT	ACTCGGAGGTGGAAGCTTTT	198
Ad01A10293	Aradu.A01	10293	p5	(TTTTA)5	25	48612939	48612963	TTGTGTCTTTTATGTGTTTCCTCC	ATGATGGAGCAAGGGAGATG	271
Ad01A10294	Aradu.A01	10294	p3	(TTC)7	21	48628355	48628375	CACCGTAAATGGCCAAAACT	CTAGAGGTTTCTCCGGCCTT	265

ID	Chromosome	SSR nr.	SSR type	SSR	Size	Start	End	FORWARD PRIMER (5'-3')	REVERSE PRIMER (5'-3')	Product (bp)
Ad01A10295	Aradu.A01	10295	p3	(TTC)6	18	48642937	48642954	TACACCTCTCCCCCTCTCCT	CCTATGACTCGGAGGTGGAA	205
Ad01A10296	Aradu.A01	10296	p2	(AT)10	20	48662513	48662532	TCATCCAAGGAAGAAGCACC	TTGGTAAATAGTTTCCGGCG	250
Ad01A10297	Aradu.A01	10297	p2	(GA)8	16	48665186	48665201	GACGGCGGAAAACAAATAGA	CCCCAAAATTCATAGCGAGA	242
Ad01A10303	Aradu.A01	10303	p3	(CTC)5	15	48683794	48683808	GGGTCATTTGGCAAGCTACT	TGCCTGAGCCATTACCTATG	278
Ad01A10305	Aradu.A01	10305	p3	(AAC)5	15	48690863	48690877	TAGCCTCAACCTCAGCATCA	AGTCATATGCAAGTGGGTGC	280
Ad01A10309	Aradu.A01	10309	p3	(ATC)5	15	48724820	48724834	CAAATCGTCCCTCTCCAAAA	AACGAAGAGCCCAACAGAGA	205
Ad01A10311	Aradu.A01	10311	p3	(TCT)6	18	48733540	48733557	CCTAATTGGTGCAGGGATGT	CGCCCTCAATCAAAGAAAAG	197
Ad01A10314	Aradu.A01	10314	p3	(GTT)5	15	48758329	48758343	GCAGTTTGGTGCAAAAGGTT	AGGGAAATGTATCGATTCCG	210
Ad01A10316	Aradu.A01	10316	p2	(TA)7	14	48795424	48795437	TTTGATCAAGTGGGCAAGAA	GGTTTATCAACCTCCCCACA	160
Ad01A10318	Aradu.A01	10318	p2	(CT)6	12	48807977	48807988	CCCAAATCCAAGTCCAGAAA	ATAAGCCAATTGGGCAACAG	168
Ad01A10319	Aradu.A01	10319	p2	(CT)7	14	48813388	48813401	TGAGCATGCTTTTCATAATTCA	ATAAGCCAATTGGGCAACAG	267
Ad01A10321	Aradu.A01	10321	c	(T)10gttttcttttc	39	48834636	48834674	TCAGCTTTGCTAGAGTCCCC	TGGTTCATGAATGTTGGCTC	247
Ad01A10325	Aradu.A01	10325	p2	(CT)8	16	48867975	48867990	AGATCTGTGAACCCCCACAGG	GAGGAGATGAAGGGGGAGAG	264
Ad01A10326	Aradu.A01	10326	c	(TA)6ag(GA)1	48	48882153	48882200	GGCTGATTCCCTGTACATTTG	CTCTCCAGCTTCTCCTGCAC	143
Ad01A10328	Aradu.A01	10328	p2	(AC)13	26	48882855	48882880	TCGAAGAGTGCATGTTGACC	ACCATGGGTGCCATCATACT	201
Ad01A10333	Aradu.A01	10333	p3	(AGA)6	18	48929608	48929625	TGACTCAGAGCTGGAAGCAA	CCACCTACCTTTCCCCTCTC	275
Ad01A10334	Aradu.A01	10334	p3	(AAT)5	15	48934596	48934610	CCCACGGAGATTGTTGGTAT	TTCCAACCATATTCGCTCCT	275
Ad01A10343	Aradu.A01	10343	p2	(AG)8	16	48988677	48988692	GAGAAAGGAGTTGCAGTGGC	CCTATCGTCGTCTGCCTCTC	232
Ad01A10344	Aradu.A01	10344	p2	(GA)7	14	48988795	48988808	GAGAGGCAGACGACGATAGG	GCATTTTGTTCACTCGCTGA	245
Ad01A10346	Aradu.A01	10346	p4	(AGAT)13	52	49004884	49004935	CTTCTTTGGTGGTGTTGCCT	AGGCAAACCAAAGCTCCATA	265
Ad01A10347	Aradu.A01	10347	p3	(TTA)50	150	49005436	49005585	CGGTGTGCAGGTGTGAATTA	TTTGGGTTCCCACTCTTTTG	248
Ad01A10348	Aradu.A01	10348	p3	(TAA)11	33	49009822	49009854	GTTAACCGCCGGAATTTGTT	ATCGAGTTCGATATCCGCC	279
Ad01A10349	Aradu.A01	10349	p2	(AT)6	12	49017288	49017299	TCTAACCTCCCACCATCTCG	TTAGGTATGGGGAGGCAGG	211
Ad01A10351	Aradu.A01	10351	p3	(TTC)5	15	49034733	49034747	CTCCAAACCCAACCCCTAAT	AAAGGTTTGCTCGTCCTCAA	198
Ad01A10352	Aradu.A01	10352	p3	(AGA)5	15	49041952	49041966	CTAGAGGATTCTCCGGCCTT	TCACCTAGGCCGAACCTACA	172
Ad01A10356	Aradu.A01	10356	c	(T)10c(T)10	21	49063422	49063442	TCCCCAATTAGAGGTGTCCA	TGGCTCTTGAAAGAATGAAGG	232
Ad01A10358	Aradu.A01	10358	p2	(TA)7	14	49095836	49095849	GCACGATTACAGCCCAACTT	GTGCTAGGTGGGAGACAACC	255
Ad01A10359	Aradu.A01	10359	p2	(CT)9	18	49098603	49098620	GGAACATTGAACCTGGAATGA	AATGCATCCATTCAAGAGGG	195
Ad01A10360	Aradu.A01	10360	p2	(TA)6	12	49102626	49102637	TTTTCATTTCCTTCGACCCT	CCATATTTTGGGATCGCACT	237
Ad01A10361	Aradu.A01	10361	c	(A)11caaaaaaa	57	49115246	49115302	CACCAAACTTGAATTCTGTGTCA	ATTGCTCACATTAGGAGCGG	217
Ad01A10363	Aradu.A01	10363	p3	(AAT)12	36	49116897	49116932	ACTTGCATCACCAACCAACA	ATATCGACCAGCAAACTCGG	207
Ad01A10371	Aradu.A01	10371	p2	(TA)10	20	49189295	49189314	GCCCGAAGAAATTTGAATGA	CCAGTTAGTGCACCTAGAAAAACA	272
Ad01A10374	Aradu.A01	10374	p2	(TC)7	14	49215606	49215619	AAATGACCGAACCCTAACCC	CTAGAGGTTTCTCCGGCCTT	261
Ad01A10377	Aradu.A01	10377	p3	(TTC)5	15	49243027	49243041	TACCTTGGCCGAACCTACAC	TGCCATTGATGGTAGTGGAA	151

ID	Chromosome	SSR nr.	SSR type	SSR	Size	Start	End	FORWARD PRIMER (5'-3')	REVERSE PRIMER (5'-3')	Product (bp)
Ad01A10379	Aradu.A01	10379	p2	(TA)6	12	49249487	49249498	TTTTCAGGGTTGTAATGGGG	GGGACAAAATTACCCCAATGT	256
Ad01A10381	Aradu.A01	10381	c	(A)10ctagtttgt(	55	49290502	49290556	TGACCAAATATAAATCACCTTAATACA	ATCCCGTATTTGTGCATCAT	268
Ad01A10384	Aradu.A01	10384	p2	(TA)6	12	49321267	49321278	CGAATAAACTGACGTACGTGTCTC	TTCTTTCTCGTTTCATCATTTTCA	155
Ad01A10387	Aradu.A01	10387	p3	(AGA)5	15	49358590	49358604	GCTTTTGTCTTCCCTTTCCC	CCTTGGCCGAATACACATCT	195
Ad01A10393	Aradu.A01	10393	p2	(TC)6	12	49397023	49397034	ATTTTCCTCAATGTACGCGG	GAGAGACTGAGGCGGAGAGA	263
Ad01A10394	Aradu.A01	10394	p3	(AAG)5	15	49404791	49404805	ATGGATCGATTTCAGGCAAC	TCTTGACTTTCCCATGGAGG	266
Ad01A10395	Aradu.A01	10395	p2	(GA)6	12	49419728	49419739	CGTGCTCAATCGTGCAATAG	CGCCGGATAATTACACTTCC	243
Ad01A10396	Aradu.A01	10396	c	(CT)7(GT)27	68	49422122	49422189	CCCCTCTCTCTCCTCTGCTC	AAGTCATCGGGTCACACACA	114
Ad01A10397	Aradu.A01	10397	p2	(TG)16	32	49423209	49423240	CTGGGTCTTACATTCCACCC	CTGCTTTGTAGCCTTTTGCC	274
Ad01A10398	Aradu.A01	10398	p3	(TTC)7	21	49445528	49445548	ACTTTCACCTCACGTCCCAC	CGAAGAAGAAGATGAACGACG	259
Ad01A10401	Aradu.A01	10401	p4	(TATG)5	20	49478514	49478533	AAGGCTCAACAACTCACAAGG	GATTTTGAAATTGCATCTATCGC	211
Ad01A10402	Aradu.A01	10402	c	(AAC)11(AAT	57	49497797	49497853	TAATGGTGGGTTTGGCATTT	TGGCTCAATGTTGGTTCAAA	170
Ad01A10406	Aradu.A01	10406	p3	(TCC)5	15	49520946	49520960	ATGGTTCGAAGGCATACCAG	AAGGGAGGGATAAGAGGGGT	196
Ad01A10407	Aradu.A01	10407	p3	(TCT)6	18	49528125	49528142	CAAAATCTCTTTCCCACCCA	TCGTCGAGCAACAGAAGAGA	200
Ad01A10408	Aradu.A01	10408	p2	(AT)6	12	49542941	49542952	TTGAGGAATGTTTGGATGGG	ATACATGCATCCCCAAGCTC	271
Ad01A10412	Aradu.A01	10412	p3	(GTT)5	15	49581507	49581521	CACGCTGATGTGGACAGATA	TCAACCAAAGGGTGATGCTA	280
Ad01A10413	Aradu.A01	10413	c	(AGG)5tcgagg	71	49586691	49586761	GCGGAGAAAGATCTCAAACG	GTGTGGCAGTGACTTGATGG	159
Ad01A10414	Aradu.A01	10414	p3	(TCT)19	57	49587066	49587122	AAGGACGAGGAAGGAGGAAG	AAGAAGCAGCGCTTGAAGAC	271
Ad01A10420	Aradu.A01	10420	p2	(AT)6	12	49599267	49599278	TCCCAAGCACCAGGTAAAAC	ACCAAGACATGTCGGATTGG	275
Ad01A10421	Aradu.A01	10421	p3	(ACT)5	15	49599610	49599624	GTTCAACCCAAAGCAAGTGG	ATTTGCTCAGATTGGCTCGT	225
Ad01A10423	Aradu.A01	10423	p3	(AAT)10	30	49615038	49615067	CCAAGCCCAACTCCAATAGA	TACACGGCACGCTTACAAAA	280
Ad01A10424	Aradu.A01	10424	p3	(GAT)5	15	49615946	49615960	TGAAATTCCTGCACATGACC	TTGGTAAAGGTTTGGTGTTTGA	268
Ad01A10425	Aradu.A01	10425	p3	(GGA)10	30	49617140	49617169	TTCGTTGCATTCTAGGTTTGAA	AGACCAGAATGCGCTGAAAG	267
Ad01A10426	Aradu.A01	10426	p3	(GAA)7	21	49617620	49617640	AGGAGCAAAAGGAAGGAGGA	ATTCACGCGCCACTAAACAC	275
Ad01A10434	Aradu.A01	10434	p3	(AAG)5	15	49636020	49636034	ATTTCAGGCAACCACTCACC	TCTTGACTTTCCCATGGAGG	258
Ad01A10438	Aradu.A01	10438	c	(AAAT)5aatat(	36	49654772	49654807	GGGCGAACCTTAAGGAGTTT	TTTCTCTTTGCACTCCTTCG	106
Ad01A10440	Aradu.A01	10440	p2	(AT)6	12	49657080	49657091	ATGCAAACCCTTCACGAATC	TTCCGAGGGCATAAAAGATG	263
Ad01A10442	Aradu.A01	10442	c	(AC)9ataatagt(	37	49662635	49662671	TGGCACGGATAAGAACAACA	TAGCAGGCCTAAACTCTGCG	145
Ad01A10444	Aradu.A01	10444	p2	(TA)8	16	49676974	49676989	AGGCTTAGTGCGGAGCTACA	TTAAAAGTTTGGGCGTCACC	242
Ad01A10446	Aradu.A01	10446	p3	(GAA)5	15	49686017	49686031	TGGGATGGGTGAGAGAGAAG	AAGGGGATAACCCTAATGCG	198
Ad01A10449	Aradu.A01	10449	p3	(AAT)21	63	49702909	49702971	TTTCGTACTTCAAAACGTGAAAA	CGCAAGAATTCATTCGTCAT	262
Ad01A10453	Aradu.A01	10453	p2	(GC)6	12	49711633	49711644	ATAGGGAGCTCTTGGGCATT	AAAAAGCAGGGAGCAAATCC	272
Ad01A10455	Aradu.A01	10455	p2	(CT)6	12	49724673	49724684	TCACGTGTACGCAGAAGGTC	TGAACTTTGATTTTGGATTATGGA	247
Ad01A10456	Aradu.A01	10456	p3	(ATC)6	18	49750192	49750209	GACGAGTCTGAAGAGGCACC	GAGCAATTCCGAGTTCTTGC	259

ID	Chromosome	SSR nr.	SSR type	SSR	Size	Start	End	FORWARD PRIMER (5'-3')	REVERSE PRIMER (5'-3')	Product (bp)
Ad01A10459	Aradu.A01	10459	p3	(ATA)5	15	49785362	49785376	CGTTATGTTTTATTTTCTATGCATTTG	TGTCCTTCCCATTTTCTTCAA	253
Ad01A10465	Aradu.A01	10465	p2	(CA)8	16	49816167	49816182	CGAAGAACAAAGGGACCAAG	AACCTACAGGTACCCGACCC	274
Ad01A10467	Aradu.A01	10467	p3	(TTA)11	33	49819270	49819302	CATTCATGGCTTTGTTGTGG	ACGCCATCCACCTCTTCTTA	202
Ad01A10468	Aradu.A01	10468	c*	(AT)9(TATG)9	51	49820542	49820592	AAAACCTGAGGGCATGACTG	TTTGGAATCTCCTCATCGAAA	252
Ad01A10470	Aradu.A01	10470	p4	(TTAG)5	20	49832472	49832491	TTTCCCATCCACCATCATCT	TCAACAAAGAATCCAACACTGAA	208
Ad01A10472	Aradu.A01	10472	p2	(GA)6	12	49839626	49839637	AAAGGGAGAGAGAGGGTTCG	CACTTTTCAACTTCCAATTTTCG	172
Ad01A10473	Aradu.A01	10473	p3	(AGA)6	18	49848440	49848457	AGAATATTGCTCGCCCTCAA	ATTTCTTTCCCACCCAAACC	207
Ad01A10483	Aradu.A01	10483	p4	(AAAT)7	28	49928847	49928874	TTGCTCCAATTATGAGACTACAGTT	TTCACCGTTGATGTTAGATAGATTT	279
Ad01A10486	Aradu.A01	10486	p3	(TTC)6	18	49969342	49969359	CCCATAAACCCCACCTACCT	TAAAATATTGCTCGCCCTCG	241
Ad01A10487	Aradu.A01	10487	p2	(AT)7	14	49970946	49970959	GAAAAAGCAAAAAGCTTGCG	ATGCCACAAGCACATGGTTA	228
Ad01A10488	Aradu.A01	10488	p3	(ATA)5	15	49977054	49977068	TTTACTTCGCAACAACGAATTT	TGCGAGTAACATTAGAGTTGAAAGA	226
Ad01A10489	Aradu.A01	10489	p2	(CT)12	24	49977804	49977827	TACTTCCCATTCCATCGCTC	AATGTCAACGGCAAGGATTC	155
Ad01A10491	Aradu.A01	10491	p2	(AT)15	30	49980954	49980983	GGACATTGCTGCAAAACTGA	TCTTAGGCCTTAGCTGTGGG	179
Ad01A10493	Aradu.A01	10493	c	(T)10gtgt(TC)	44	49986037	49986080	TCATGGGTGTTTGTTAGGGG	CTCACCCGGATTCTTATCCA	248
Ad01A10494	Aradu.A01	10494	p4	(AAAT)5	20	49987352	49987371	AAGTGTGGGGAAAACTGTGG	TATTGACTTGGCATTGGGGT	208
Ad01A10500	Aradu.A01	10500	p4	(AAAT)6	24	50033206	50033229	TTTGAATCAAGGGATGTTGATG	ATTGATGCTCAGAGCCTTGG	173
Ad01A10501	Aradu.A01	10501	p3	(TTC)6	18	50041669	50041686	TCTTCTCCCACTTAGCCGAA	GGTCTTGATGGCTCTTGGAG	260
Ad01A10503	Aradu.A01	10503	p3	(ATT)11	33	50049565	50049597	ATGTCTTATGCCTAAAAATGTAAAACT	ATGCATGCAAGTCACATTTC	279
Ad01A10507	Aradu.A01	10507	p3	(TTG)9	27	50068312	50068338	TGTGTCAAGTTTTAATTATTCAGGA	TTGCACTATTCACAAGGCAAA	151
Ad01A10509	Aradu.A01	10509	p3	(ATA)5	15	50070366	50070380	TGTGACCACTCGTGATGATTC	AAAAATGATGGGTTGTATAACGAAA	230
Ad01A10510	Aradu.A01	10510	p2	(AG)6	12	50077303	50077314	TTTGGAGGGAAAACACCAAG	CCCAATTGGTTAGAGCGTTG	238
Ad01A10511	Aradu.A01	10511	c	(AG)7ggttcgaa	78	50091719	50091796	CCTTGATGTGTCAGAAGAAAAGAA	CTTTGCAATTCCAATTTTCG	209
Ad01A10512	Aradu.A01	10512	p2	(TA)6	12	50098442	50098453	TGGATGAATGATGGTGGAAA	CCAAAAACTCAACCATCCCA	256
Ad01A10516	Aradu.A01	10516	p2	(TA)6	12	50134047	50134058	GGGGTAAACAAAAATCGCAA	ATGTCCCTTGGGTTTCCTTT	214
Ad01A10517	Aradu.A01	10517	p2	(AG)6	12	50138385	50138396	TTTTTGGCCCAATTTCAAAC	TTGGCAAAACAAGGAAATCA	271
Ad01A10521	Aradu.A01	10521	p2	(AT)10	20	50173044	50173063	GCCTTGAAAACGGGTAAACA	CAGGAGAGTTAGGGGCTTGA	257
Ad01A10522	Aradu.A01	10522	p3	(CTT)8	24	50183574	50183597	GCGATTTGCACAAACTGAGA	AGGAAAGGAGGAATGGGAAA	218
Ad01A10524	Aradu.A01	10524	c	(CT)7ccctcccc	62	50192809	50192870	TCTCTCCCCTCTCTCTTCCC	CAGGGTTCCATGCGTAAAGT	152
Ad01A10525	Aradu.A01	10525	c	(CT)6cccatt(T	36	50193296	50193331	ACGCAAGCGAAAAATGCTAT	GAGCGCAAGGAAAGAGAGAA	163
Ad01A10526	Aradu.A01	10526	c	(CTC)5ttctctct	42	50193433	50193474	GCGCTCTTTTTCCCTCTCTT	TCCCTCTTCTCTTTCTCTCCC	129
Ad01A10527	Aradu.A01	10527	c	(GA)6ggaatag	63	50193664	50193726	GGAGAGAGAAAGAAGGGAAGGA	CTCTCCGCCCTCTCTCCA	141
Ad01A10529	Aradu.A01	10529	p2	(AG)7	14	50195635	50195648	TTTTGGCCCAGAAAACACAT	GGGACATTGAACTTGAAAATTG	210
Ad01A10530	Aradu.A01	10530	c	(T)12ggttaattc	32	50227136	50227167	TTGGTGTCATTTTGTTGTTTTTC	TGGATAAAGGGAAATGATATGGA	280
Ad01A10537	Aradu.A01	10537	p3	(TCT)5	15	50264111	50264125	ACTGCACGTTTTCACTGCAC	CCTCCTCATCTTCTGCTGCT	225

ID	Chromosome	SSR nr.	SSR type	SSR	Size	Start	End	FORWARD PRIMER (5'-3')	REVERSE PRIMER (5'-3')	Product (bp)
Ad01A10539	Aradu.A01	10539	p2	(AT)10	20	50291261	50291280	AACTTGGCTACCCATGCAAA	CGAATTTGTTGACATTGGAGA	152
Ad01A10542	Aradu.A01	10542	p2	(CT)19	38	50297290	50297327	GTCGCATTCCTCGATCTCAT	GGGAGTGAGCTTGGACAGAG	135
Ad01A10543	Aradu.A01	10543	p2	(AT)6	12	50309707	50309718	CACACGCATAGAAGCGGTTA	TGAAAGAATTGTGTTTGAAAATCC	208
Ad01A10549	Aradu.A01	10549	p3	(TTG)9	27	50374340	50374366	AGGTCCGAGAGTTTATGCGA	TCATGTCTTTGTCTTCTTGCTCA	221
Ad01A10550	Aradu.A01	10550	p3	(TTC)5	15	50377386	50377400	TTATGATTTTGGTTGCCGCT	GGACTTGAAGAGAGGCATGG	165
Ad01A10552	Aradu.A01	10552	p4	(AATA)5	20	50387899	50387918	AGGTTCAATTTGTTCACGGT	CGCCCCGTATTATCCTTATT	278
Ad01A10554	Aradu.A01	10554	p2	(AG)6	12	50415782	50415793	AGGGAACATGCAGAGACTCAA	CGGGTCATCTTCTCAAGGAA	257
Ad01A10555	Aradu.A01	10555	p2	(AT)6	12	50426601	50426612	TGCCACGACAGTTCTAAAGC	TCCGCAAGTTGTGACTGGT	280
Ad01A10556	Aradu.A01	10556	p2	(CT)18	36	50435178	50435213	CAAAAATTCCTCTCCCTCTCC	TTCATTCCCATTTGGATTCAT	253
Ad01A10557	Aradu.A01	10557	p2	(AG)6	12	50441428	50441439	CAAAGCTTTTTCCCAGACCA	ATTTTCCTCCCAAACCCAAC	228
Ad01A10558	Aradu.A01	10558	p3	(AAG)5	15	50472726	50472740	TCCTCATCATCTTCTGCTGC	TCTTGCACGTTCTTCTTCCTC	229
Ad01A10559	Aradu.A01	10559	c	(AGA)6ggaaa	40	50472905	50472944	CAACGTGCAGCAAGAAGAAG	CCCAAAAATACGATGCAAGG	256
Ad01A10560	Aradu.A01	10560	p2	(AT)9	18	50479718	50479735	CAAAAATAAAAGCATTCTTTACATCG	TTTTAAATACATAAAAATCAAACGACG	275
Ad01A10563	Aradu.A01	10563	p3	(AAG)6	18	50495922	50495939	TTAAAATGTTGCTCGCCCTC	GTTTTTCACCACCCACATCC	148
Ad01A10564	Aradu.A01	10564	p2	(GA)10	20	50517787	50517806	GAAAAGAGAGAAGGGGGTGG	GCTCACGATCTCTAACCCCA	192
Ad01A10566	Aradu.A01	10566	c	(AT)7ag(AC)9	34	50549241	50549274	CCGATGAAGGAATGAAGCTC	GACAGAGTGGAGGAGGCTTG	273
Ad01A10573	Aradu.A01	10573	p3	(AAT)5	15	50644491	50644505	TTAGATGCAAAATTAACGATGTTT	GGGTTTTGGGAGTGAGATCAT	275
Ad01A10579	Aradu.A01	10579	p4	(TATT)5	20	50730073	50730092	TTTCGAATTCTCTCCCTCTCA	TTCTTGTCCTTGCTCCTGCT	222
Ad01A10581	Aradu.A01	10581	p2	(GC)6	12	50756493	50756504	CCACCTTATGCCCACTTCAT	CAGGCACAGACTAGGCACAA	181
Ad01A10582	Aradu.A01	10582	p5	(CTTCT)5	25	50758455	50758479	CTAACTCTGTGCCAGGAGCC	CGGAGACGGTGGTGAAGTAT	250
Ad01A10583	Aradu.A01	10583	p3	(TTC)6	18	50766000	50766017	CAAACCCACCCTACATGGTC	TAAAATATTGCTCGCCCTCG	195
Ad01A10589	Aradu.A01	10589	p3	(ACA)5	15	50818929	50818943	ATCACCTCATCTTTGGCACC	AAGCGACTTGGAGAGGTTGA	120
Ad01A10590	Aradu.A01	10590	p2	(CT)6	12	50819160	50819171	ACACAGACTCGGATCCTTGG	CTTGACCAAGGGGAGTTTGA	216
Ad01A10592	Aradu.A01	10592	p2	(GA)9	18	50836181	50836198	GTCACTGTCGAACCCTAGCC	TTCTCCGTCAGAGCATCCTT	205
Ad01A10594	Aradu.A01	10594	p3	(TCT)5	15	50842228	50842242	TCGCATACAAACGATTAAGGC	GAGGAAGAAGAACGTGCAGC	173
Ad01A10595	Aradu.A01	10595	p3	(CTT)5	15	50842358	50842372	GCTGCACGTTCTTCTTCCTC	TCCTCTTCATCTTCTGCTGCT	240
Ad01A10596	Aradu.A01	10596	c	(A)10cgaa(GA	35	50842825	50842859	TCGTTTGGGTGTATTTCTGAAG	CGAGCCTTTCAATGCGTTAT	197
Ad01A10599	Aradu.A01	10599	p2	(AT)7	14	50868045	50868058	TCATTTGTTTTCTGGGGCTC	CCTATTCCTGTTAGCCTGAGATG	134
Ad01A10602	Aradu.A01	10602	p2	(CT)6	12	50870280	50870291	CTCACCTCCAACAATCACACA	GCTCTGCGGTAGAGATTTGG	141
Ad01A10604	Aradu.A01	10604	p2	(TC)6	12	50904731	50904742	CTCTTTCCGCCTTCCTCTCT	AAATCCTAATCTCCACCGCC	242
Ad01A10609	Aradu.A01	10609	p2	(GT)7	14	50926055	50926068	TTGACTGTGAGTGAGGTGGC	TTCCCATTGTTGGATTTTGG	266
Ad01A10610	Aradu.A01	10610	p2	(GT)8	16	50932938	50932953	TTGACTGTGAGTGAGGTGGC	TTCCCATTGTTGGATTTTGG	268
Ad01A10611	Aradu.A01	10611	p2	(GT)11	22	50940368	50940389	TTGACTGTGAGTGAGGTGGC	TTCCCATTGTTGGATTTTGG	267
Ad01A10612	Aradu.A01	10612	p3	(AAT)7	21	50942843	50942863	TTGAAGTCCTTATTGCTTCCATC	GCCTGTCTCTACTCGTCCCA	255

ID	Chromosome	SSR nr.	SSR type	SSR	Size	Start	End	FORWARD PRIMER (5'-3')	REVERSE PRIMER (5'-3')	Product (bp)
Ad01A10613	Aradu.A01	10613	p3	(TTA)14	42	50943934	50943975	TGGAGGAAGAAGGTGCTGT	AAAACTCGCTTCGAGCAAAA	123
Ad01A10614	Aradu.A01	10614	p2	(AG)10	20	50950766	50950785	TTTCCATTAAGCTGGCCTGT	AAATTGCATCCGCAACCTAA	233
Ad01A10617	Aradu.A01	10617	p3	(TAA)8	24	50959657	50959680	CTATCAAATAAGCAGAAATTCAAAAA	ACTACCATTATCGCCTACACAAA	205
Ad01A10621	Aradu.A01	10621	p3	(TTA)5	15	50981766	50981780	TACCAACAAGGAACGGGAAG	TGGCTCACATGCATTAGGTT	208
Ad01A10622	Aradu.A01	10622	p2	(CT)10	20	50983033	50983052	TCGGCAAGTTAGAAAGTTACCA	TCCATGGATCAGAACGTGAG	279
Ad01A10623	Aradu.A01	10623	p2	(CT)6	12	50984190	50984201	GGAACATTGAACTTGGAATGAA	AGGCTGGAGAATGAGGGAAT	198
Ad01A10624	Aradu.A01	10624	p2	(CT)7	14	50989492	50989505	AAGGGGGCATGTTAGGCTAT	TGAGAAAATGAGCCTTATCAAAGA	271
Ad01A10626	Aradu.A01	10626	p2	(CT)6	12	50995647	50995658	CTCATTTGCCACCCCTAATG	TTTGGAACGATACTGAGCGA	266
Ad01A10628	Aradu.A01	10628	p3	(TAA)6	18	50998638	50998655	GGTCAATTTGTAATTGTTGTTGACTT	CTCTCACTTGACAACAATGGAAT	275
Ad01A10629	Aradu.A01	10629	p2	(CT)6	12	51007273	51007284	AAAAAGAACATTAAACCTGGAGC	CCAGTTTCAAGCCCAGAAAA	221
Ad01A10631	Aradu.A01	10631	p2	(CT)6	12	51024410	51024421	AACATGGAGCTCAGAAGAAATTG	CCAATTTCAAGCCCAGAAAA	208
Ad01A10634	Aradu.A01	10634	p2	(TA)7	14	51038153	51038166	AAGTTGCAAGTAATTCGGCG	AATCTTATGAATCAACCAGTAATTTTT	279
Ad01A10640	Aradu.A01	10640	p2	(TC)9	18	51072734	51072751	CCCTCTCCTTCACACCTCTG	CGACGCACAGTGAGACAGAT	109
Ad01A10641	Aradu.A01	10641	p3	(ATA)5	15	51076958	51076972	CCCTTTGTACTGGGTTTGTCA	TTCACATACAACTTCAACCATCAA	186
Ad01A10643	Aradu.A01	10643	p2	(AG)8	16	51082700	51082715	GATCCGACGAGTGCTTCTGT	CAGAAACCTCTCCTTCACGC	233
Ad01A10647	Aradu.A01	10647	p3	(TGG)5	15	51085944	51085958	CTTGGTGTTGGTGTTGGTTG	TTCATCTTTCTCACCCCACC	258
Ad01A10648	Aradu.A01	10648	p3	(GTG)11	33	51086063	51086095	CTTGGTGTTGGTGTTGGTTG	TTCATCTTTCTCACCCCACC	258
Ad01A10649	Aradu.A01	10649	p3	(GAA)6	18	51086679	51086696	GGAAAATGAGGAGGAGGAGG	GCTTCTCTTCTCCATCACGC	168
Ad01A10651	Aradu.A01	10651	p2	(AC)9	18	51088840	51088857	GCGAAACTAAGTCTGGTCGG	GCTAGGCATTTGGAGCTTTG	219
Ad01A10655	Aradu.A01	10655	p2	(AG)16	32	51119645	51119676	AAAAACAATGCCAAAAAGCG	GCAAATATCTGCCGGTGAAT	242
Ad01A10657	Aradu.A01	10657	p4	(AATA)5	20	51122534	51122553	TCTTTTAGGGACGAATTTAACCA	TGGTTCCTGAAAATTCATTCG	267
Ad01A10658	Aradu.A01	10658	c	(TA)6at(TA)8	30	51123661	51123690	AAATATGCTATGAACTGTGTTGGA	ACAAGCCAAACTAAGCCAGG	255
Ad01A10661	Aradu.A01	10661	p2	(AC)20	40	51130557	51130596	TCGGCACAAACCTAACAACA	GAAGGCTCTGCTGAAGCAAT	239
Ad01A10663	Aradu.A01	10663	p2	(TG)6	12	51170562	51170573	TGAGGGATGGACTGACTATGC	TCACCGGGTCATTAGGGTTA	270
Ad01A10666	Aradu.A01	10666	p2	(CT)6	12	51197260	51197271	CTTGGTGCAGGGATGACTTT	TTGAAGGGTGAGGTAGGTGG	208
Ad01A10668	Aradu.A01	10668	p3	(TCA)13	39	51208462	51208500	TCACCAGTGAAGGTGATGGA	TGGTTTTTATTTCAAAATTTAACCAAG	233
Ad01A10669	Aradu.A01	10669	c	(A)10tctttcaca	38	51210311	51210348	TTGATCCATCAATCCTCGTG	GGGGAGGGGAGAATATGAAA	270
Ad01A10672	Aradu.A01	10672	p2	(GA)6	12	51242403	51242414	GGGGAAGAAGAGGAAAATGG	CACACAGGATCCCCACCTAC	205
Ad01A10677	Aradu.A01	10677	c	(CT)8ttctttctt(	55	51281592	51281646	CTCATCCCGTTACCGTCACT	AACGTCCGAGCTTATAGCGA	209
Ad01A10679	Aradu.A01	10679	p2	(AT)9	18	51292217	51292234	CGGTGGTACGACTTTCAGGT	CCCTAACCGGCCTAAACTTC	273
Ad01A10681	Aradu.A01	10681	p2	(CT)22	44	51297894	51297937	CCCCTCTCCCTCAAAATCAT	GCATGCAGTCATGCTTACAG	276
Ad01A10682	Aradu.A01	10682	p2	(AT)13	26	51300197	51300222	CGTAAAACAGAGGTGGTGTGG	AGCTTCGCAGGCTGAGTTAG	272
Ad01A10684	Aradu.A01	10684	p2	(CT)7	14	51311132	51311145	GCTCATAAGCCAAATAGGCAA	AGGGAACATGCAGAGACTCG	246
Ad01A10686	Aradu.A01	10686	p2	(AT)10	20	51317049	51317068	GGAAGAGCAACTCTAATCAACCA	CATGGCTGTATGCCACTTTG	158

ID	Chromosome	SSR nr.	SSR type	SSR	Size	Start	End	FORWARD PRIMER (5'-3')	REVERSE PRIMER (5'-3')	Product (bp)
Ad01A10688	Aradu.A01	10688	p3	(ATG)5	15	51334898	51334912	ACGGATCTCAAGAAAGCGAA	TCTATAACGCGACACCCACA	182
Ad01A10691	Aradu.A01	10691	p4	(TTAA)6	24	51358386	51358409	AGGGCAACTATCGCGTAAGA	TCGAGGGGTTTAATTTCACG	203
Ad01A10692	Aradu.A01	10692	p3	(TAT)20	60	51359274	51359333	AAGACAATTAACCGGTAGCGA	GCATGAATGGTGAAGGTGATT	280
Ad01A10694	Aradu.A01	10694	p3	(AGA)5	15	51363389	51363403	TCGTAGCAGCTTTGTGGTTG	GGGCAATGGCTTGATAAAAA	280
Ad01A10697	Aradu.A01	10697	p2	(GA)15	30	51372660	51372689	TACCTCCCTGCAATTCCTTG	AATTCAAAGCGCGAACTCTC	254
Ad01A10698	Aradu.A01	10698	p2	(AG)7	14	51377571	51377584	ATTGTGATTGGGGATGGAAA	TGGCCAAAACCCATATACCT	275
Ad01A10702	Aradu.A01	10702	p3	(TTA)6	18	51402525	51402542	GAACAAAGGTTGCTACATTCCC	TCATAGTGGGGATGTAAGTGGA	263
Ad01A10705	Aradu.A01	10705	p2	(AG)6	12	51414118	51414129	AGAATGGGACAAAAGGGAGG	AAATGGCGAAAAAGGGAACT	240
Ad01A10706	Aradu.A01	10706	p3	(ATC)5	15	51421686	51421700	GCATCTACCGTCCCATCATC	GAGCAATTCCGAGTTCTTGC	239
Ad01A10707	Aradu.A01	10707	p3	(TCT)7	21	51435088	51435108	CCCATAAACCCCACCTACCT	TAGAATGTTGCTCGCCCTCT	244
Ad01A10710	Aradu.A01	10710	p3	(TCT)5	15	51453316	51453330	CAGGATCCCCAACTACCTCA	TTAGAATGTTGCTCGCCCTC	214
Ad01A10720	Aradu.A01	10720	p3	(TTA)5	15	51529326	51529340	CCTTGCTTTCTTAGTTTTGCTTG	CTCCCTAACCACAAGGGTGA	266
Ad01A10722	Aradu.A01	10722	p3	(TTC)6	18	51551318	51551335	TACACCTCTCCCCCTCTCCT	GCTTTTGTCTTCCCTTTCCC	185
Ad01A10723	Aradu.A01	10723	p3	(TTA)6	18	51583776	51583793	TTCCTTGGGTTGACTTGGTC	GGAAGATGATGTGGATTGGAG	267
Ad01A10727	Aradu.A01	10727	p3	(ATT)6	18	51610802	51610819	AAAATTTTTGCACATGAATGAGA	GATTGAGAATATGTAGCAATTATAGCA	257
Ad01A10730	Aradu.A01	10730	p3	(ATG)7	21	51619805	51619825	TTTGGACCAACTCCATTATCA	GGAAAATTCAACGAATCAGCA	273
Ad01A10731	Aradu.A01	10731	p3	(ATT)6	18	51624503	51624520	AAATAGGAGGATGAGCACGC	CATATGGTGCCATGCAAAAG	113
Ad01A10737	Aradu.A01	10737	p3	(AGA)6	18	51670707	51670724	ACCACTGAGCTATGACGGCT	CAACTTCTCTTTCCCACCCA	272
Ad01A10740	Aradu.A01	10740	p2	(AT)13	26	51711115	51711140	TGCATGATTCATACGCCTTC	TGATTTTACAAAAGTGAGGCATGT	225
Ad01A10743	Aradu.A01	10743	p2	(TA)6	12	51723273	51723284	GACCAAATTGGAAAGAGGGA	TCACCAACATTTGAAAGCCA	259
Ad01A10744	Aradu.A01	10744	p2	(TA)9	18	51729372	51729389	GACTTGCGTCTTTATCCCCA	CAAACAAGCTTCCAAGACCC	212
Ad01A10745	Aradu.A01	10745	p3	(AAT)5	15	51731940	51731954	GCTTGGTGTCATTTTGTTGTTT	CCATGATGGAGAAAGGGAAA	257
Ad01A10746	Aradu.A01	10746	p3	(TTG)6	18	51739662	51739679	TGGCGTCATTTGATTCATGT	GAGGTGATGGATTCATAGGACA	182
Ad01A10747	Aradu.A01	10747	p4	(TTAT)5	20	51747201	51747220	AGGTTAGGGTGGTGAAGGCT	AAGATGTAAGGCTCCCGGAC	192
Ad01A10748	Aradu.A01	10748	c	(AC)7(AT)6	26	51761542	51761567	TTTTTCAGGCTCCTCGTTTA	TGGCGAAGTAGTTTTGGTGA	219
Ad01A10759	Aradu.A01	10759	p2	(AG)6	12	51851732	51851743	GAGTGAAGGAGGATGGGACA	TTGCATTGAAAGGAAATTGAA	278
Ad01A10760	Aradu.A01	10760	c	(CCA)8acacca	85	51857891	51857975	CCCTCACAACTACCAGCACA	GTTGGTGGTGGTCTTGCTTT	205
Ad01A10761	Aradu.A01	10761	p3	(CAC)5	15	51858077	51858091	CCAGCACAATTACCTCCTCC	GGTGTGTTGCTGTTGTTTGG	250
Ad01A10762	Aradu.A01	10762	p2	(AT)8	16	51859220	51859235	CCTGGGAGCATTAGCAAAAC	GCCAATGAAATGAAAGCCAT	193
Ad01A10763	Aradu.A01	10763	p3	(AAT)5	15	51859824	51859838	CCTTGCTTTTGCTTCTCTGG	AGAGGGGAAGCTCTTTCAGG	205
Ad01A10764	Aradu.A01	10764	p2	(AC)6	12	51861940	51861951	CACGTAACCGTAGCCTCCAT	CTCCAATCGGAAAACTCTGC	239
Ad01A10765	Aradu.A01	10765	p2	(TA)8	16	51863315	51863330	CTGACTTTGGTGGCGAATTT	GTTATACCGCAAGCAAGGGA	241
Ad01A10766	Aradu.A01	10766	p3	(AAT)5	15	51891049	51891063	GTTCGATCCCACGAAGATTG	CATTGACAACAGTGATGCCC	280
Ad01A10768	Aradu.A01	10768	p3	(AGA)6	18	51896434	51896451	GCCGGTTCCTTACCTTTTCT	CACACCACACACCTCACCTC	210

ID	Chromosome	SSR nr.	SSR type	SSR	Size	Start	End	FORWARD PRIMER (5'-3')	REVERSE PRIMER (5'-3')	Product (bp)
Ad01A10771	Aradu.A01	10771	p2	(AG)6	12	51907591	51907602	TGCCTCTTTACTGGCGTTTT	TTTTTCAAAACATCCTAACCACC	272
Ad01A10778	Aradu.A01	10778	p4	(AATA)5	20	51978955	51978974	TTCTTGTCCTTGCTCCTGCT	TCTCTCCCTCTCATCTCCTTC	228
Ad01A10779	Aradu.A01	10779	p2	(TA)8	16	51987252	51987267	TCAACTAGGATGTCAATGCCC	CGTACCCCTTTGCACATCTT	178
Ad01A10780	Aradu.A01	10780	p3	(CTT)6	18	51991410	51991427	CTTCACCTTTTTGGTTCCCA	GAGAAGAAAGAGGAGCCCGT	243
Ad01A10783	Aradu.A01	10783	p2	(TG)20	40	52001215	52001254	TTGGCTTCGTAGGACGAACT	CGAGGAGCCTTAAGGAAAGC	173
Ad01A10785	Aradu.A01	10785	p2	(CT)20	40	52008631	52008670	CTTCTTCTCACCTCAACCGC	GAATTATGAGAGGGGCAGCA	268
Ad01A10788	Aradu.A01	10788	p3	(TCT)7	21	52045453	52045473	CACTCAACACAACCCCCTCT	CTAGAGGATTCTCCGGCCTT	201
Ad01A10790	Aradu.A01	10790	p3	(AGA)6	18	52090466	52090483	TCCAACTTGTTGAATGGGGT	CCTTGGCCGAACTTACACAT	234
Ad01A10791	Aradu.A01	10791	p2	(TA)8	16	52100940	52100955	GAGGGAAGCTTGGGAACATT	AGGAGCATTTTTGTCCCCTT	246
Ad01A10792	Aradu.A01	10792	p2	(TA)6	12	52106429	52106440	TTTTTCATCAATTAGGGTGCC	TTCTAAGTGTGGGGAGGTCG	204
Ad01A10794	Aradu.A01	10794	p2	(CT)6	12	52186905	52186916	TTTTGACCCAGTTTTCAGCC	ACGTTGAACCTGGGATTGAG	206
Ad01A10795	Aradu.A01	10795	p3	(AGA)5	15	52194062	52194076	TTGGTCCTTAGCAAAATGGA	TATAGCATGCACAAACCCCA	120
Ad01A10796	Aradu.A01	10796	p2	(TA)6	12	52194517	52194528	TTGTGTTTAAAGCAACTGGGC	TTTTATGCTCTAGCAATTCATTTGTC	274
Ad01A10803	Aradu.A01	10803	p2	(AC)6	12	52229755	52229766	AGGCAAAAGAGGAAGGAAGC	AAAAATTGGGCTCTGGAGGT	277
Ad01A10809	Aradu.A01	10809	p2	(TA)6	12	52233809	52233820	TTTGGAAACTTTGGTTGGGA	CCTAAGCTTCATATGGCATCC	239
Ad01A10814	Aradu.A01	10814	p3	(GGT)7	21	52244756	52244776	GGCGTCAATCTCGTCGTATT	ACCCTCCATAATCCTCCACC	256
Ad01A10815	Aradu.A01	10815	p3	(AGA)6	18	52251155	52251172	GTTAACCGTCCTCGAGCAAA	AATGATCGAACCCTAACCCC	175
Ad01A10816	Aradu.A01	10816	p5	(AAAAC)5	25	52271198	52271222	CCATGATGGTTGCTTTGTTG	ATCCCCAAATTAGACCAGGG	265
Ad01A10817	Aradu.A01	10817	c	(ATT)5atcatt(A	48	52273839	52273886	GTAGGGAGGAGAACGGGAAC	CCTCATCGATTTCTCACCAA	115
Ad01A10822	Aradu.A01	10822	p4	(TAAT)5	20	52375342	52375361	TTCCCTCTCTTCTCTCCCTTG	TGGACAAAAGAGCTTCCAGTG	262
Ad01A10823	Aradu.A01	10823	p4	(TCTA)5	20	52378420	52378439	TCGAAAGAGGAAGAGGAGAAGA	ACCAAGGATGAAAGGGGAAG	223
Ad01A10825	Aradu.A01	10825	p2	(GA)6	12	52383140	52383151	ATAGCTGAGTGGTGGTTGGG	CAACCCCCTCTTCTTCTTCC	146
Ad01A10832	Aradu.A01	10832	p3	(AGA)6	18	52477161	52477178	TGGTGCCATTGATGGTAATG	CACCTTTGCCGAACCTACAT	154
Ad01A10834	Aradu.A01	10834	p2	(AG)6	12	52531484	52531495	GGTGCGAACATAGACTGGGT	CGCTTCACAAACACCTACCA	253
Ad01A10835	Aradu.A01	10835	p2	(AG)11	22	52535728	52535749	ATGTCGCTGTTAGGACTGGC	GACACATGCAAGGAAGGGTT	274
Ad01A10836	Aradu.A01	10836	p2	(CT)10	20	52574018	52574037	TTCGAATTCCCTCTCTCCTTA	TGAAGGGGGAAGAAGAAGAA	172
Ad01A10837	Aradu.A01	10837	p3	(TTC)5	15	52587765	52587779	CCTTGGCCGAATACACAACT	GCTTTTGTCTTCCCTTTCCC	186
Ad01A10840	Aradu.A01	10840	c	(A)10ttgatacat	84	52601690	52601773	GGGGACAAAATTACCCCAAT	GGGGCCAAGATAAGGATAGG	160
Ad01A10841	Aradu.A01	10841	p2	(TA)6	12	52608858	52608869	TGAAATTTACCATACCAAACCTGA	AACATAGAAAAACTGGGCCG	206
Ad01A10842	Aradu.A01	10842	p3	(GAA)5	15	52612148	52612162	AAAAGGAGCAAGAGGAAGCC	TCTCAAGCTTGGAATCCACC	273
Ad01A10845	Aradu.A01	10845	p3	(TTA)9	27	52652309	52652335	CCCAAAGCACTCTGTCTTCC	GGCTCTTGAAGGAATGATGG	280
Ad01A10846	Aradu.A01	10846	p2	(AG)6	12	52684255	52684266	TTGGGAAGCAATGGTAGAGG	CTTCTTTCCACGCACATTCA	225
Ad01A10847	Aradu.A01	10847	p2	(TA)8	16	52692258	52692273	TGTTTTTCAGATGCAGGTCG	AAGAATCTAACCACGGCTCG	259
Ad01A10852	Aradu.A01	10852	p2	(AG)6	12	52775842	52775853	CAAATCCAAGCCCAGAAAAA	TGGGAAAATAAACGAACCAAA	266

ID	Chromosome	SSR nr.	SSR type	SSR	Size	Start	End	FORWARD PRIMER (5'-3')	REVERSE PRIMER (5'-3')	Product (bp)
Ad01A10854	Aradu.A01	10854	p2	(TC)6	12	52785123	52785134	TCTTCTTTCTCCCTTTCCCC	TTTGCAATAATCCACAAGCA	252
Ad01A10856	Aradu.A01	10856	p2	(AG)6	12	52833126	52833137	CCCAAATCCAATGCAACTCT	TGCATTGAAAGGAAATGGAA	274
Ad01A10864	Aradu.A01	10864	p2	(TA)7	14	52900105	52900118	CGCCCAAACAAGCACTTTAT	ATCCTTACCTGACCAACCCG	148
Ad01A10865	Aradu.A01	10865	p2	(GC)6	12	52933250	52933261	TTTATTCCATTCGATGCCGT	TCGATTGTGCTTTTTGCTTG	188
Ad01A10869	Aradu.A01	10869	p3	(AGA)7	21	53020576	53020596	TGCCATTGATGGTAGTGGAA	TCCCATCACTCTCCTCCAAC	134
Ad01A10870	Aradu.A01	10870	p2	(AT)6	12	53038840	53038851	TGACGCACTATATGCTGGCT	TCAAATGGTTAGACACGCGA	210
Ad01A10876	Aradu.A01	10876	p2	(AG)6	12	53065461	53065472	AAAAACAATGCCAAAAAGCG	GAGAATGTGGAGAATGAAACCC	187
Ad01A10878	Aradu.A01	10878	p2	(AT)7	14	53077531	53077544	CCCAAAGCAATGTGAAGCTC	GGTTGTAATGGGGCTAGGGT	225
Ad01A10879	Aradu.A01	10879	c	(A)10tttctctttg	44	53080262	53080305	TGTTGCCAGGAATTGTTTGA	AAAAACATGCAATTGACACCA	249
Ad01A10880	Aradu.A01	10880	p2	(TA)8	16	53108769	53108784	AACACGAAGCGTTCACACAC	GAGTTGCGAGATGGTGACAA	273
Ad01A10881	Aradu.A01	10881	p2	(TC)22	44	53111764	53111807	GCTCAGAAAGAGCTTCGCAT	TACTTACGGGTTGGACTGGG	237
Ad01A10882	Aradu.A01	10882	p3	(AGA)6	18	53120634	53120651	TCTAGAGGATTCTCCGGCCT	ATGGCCGAACAAACACTACC	257
Ad01A10884	Aradu.A01	10884	p3	(AGA)6	18	53128643	53128660	CAGCCTTAGGTGCCATTGAT	ATGGCCGAACAAACACTACC	243
Ad01A10885	Aradu.A01	10885	p2	(AG)8	16	53148175	53148190	TGGAATAATCCATAAATCAATTCAAA	GGAGAGCTCGCCAATTTCTA	198
Ad01A10887	Aradu.A01	10887	p2	(AC)6	12	53156650	53156661	CCAACCAATGTCCCAAGTTT	AGCCAACTTTGAGCCTACGA	159
Ad01A10889	Aradu.A01	10889	p3	(ATT)5	15	53164131	53164145	CGACAAATTTTTCTTCCATTTCA	TGATCTTTGCATGTAGTACGCTT	118
Ad01A10890	Aradu.A01	10890	p2	(TA)7	14	53164320	53164333	AAGCGTACTACATGCAAAGATCA	TCTCTTTCCTTTAATTCTTCTCAAAA	248
Ad01A10893	Aradu.A01	10893	c	(AT)6(GT)6	24	53184304	53184327	TCTCAGGGACCACTTTAGGG	AAAAGTAAATAATTTCACATCATTGGA	266
Ad01A10898	Aradu.A01	10898	p2	(CT)6	12	53199430	53199441	TTTCTCCTCATCACCGAACC	GAAAAGGGTGTCGTGGAGAA	254
Ad01A10901	Aradu.A01	10901	p3	(CAC)6	18	53205730	53205747	TCACCCCTTTTCAACGTTTC	AAGGGAGAATGGAGGTGCTT	258
Ad01A10902	Aradu.A01	10902	p2	(AT)6	12	53209220	53209231	AACGGGCATTTAAGATTCACA	AGCTCAAAACTCACGGGTTG	120
Ad01A10903	Aradu.A01	10903	c*	(C)10(CA)9*	27	53210276	53210302	TGGTTGTCTTGCAAGTTTGTG	GCATGTCCTCATGCTAACCTC	263
Ad01A10906	Aradu.A01	10906	p2	(AT)8	16	53242519	53242534	GGAAGCATGCTCATGTGAAA	TTTTCAGGGTTGTAATGGGG	229
Ad01A10907	Aradu.A01	10907	p2	(TA)6	12	53247338	53247349	TGTTTGAGGTATGAATTTTCTAAATGA	GCTCTTTCCTAAAAATATTCGCA	266
Ad01A10908	Aradu.A01	10908	p3	(TTA)5	15	53247940	53247954	AATCACAATTGATTATTGATACCCT	CACGCATTAATAGTGACCCA	143
Ad01A10911	Aradu.A01	10911	p2	(AT)11	22	53251781	53251802	TTTTTCATGCCTTGCAATAAT	TTAAAGGTGCCATCACTATTTCT	211
Ad01A10912	Aradu.A01	10912	c	(A)10taataaaga	115	53252419	53252533	TCACCTGGATCGCAACATAC	TTTTAAAAGAGATTGGTTAGCATTCA	253
Ad01A10913	Aradu.A01	10913	c	(ATA)6agatgc	81	53255198	53255278	CCGAGATTTATTTGGGGTCA	TGGGTACCAGCAACTGAATG	253
Ad01A10920	Aradu.A01	10920	p4	(AACT)5	20	53306404	53306423	AAGGGACTCCAACAATGATGA	CATCCAACACCACCAATCAC	205
Ad01A10921	Aradu.A01	10921	p2	(TA)6	12	53326759	53326770	TGCTTGGATGGTGACAGAAG	ATCTAGCCACGGCTCGTAGA	216
Ad01A10925	Aradu.A01	10925	p2	(TA)9	18	53372591	53372608	AGGCTCAAAACTCCCAAGGT	CGGTGATTTTGGGTGACAAG	255
Ad01A10926	Aradu.A01	10926	p2	(GA)17	34	53383233	53383266	GTGGGTATGAAGGGGTGATG	GGAGAAAAAGGTGGCATTGA	262
Ad01A10929	Aradu.A01	10929	c	(T)10gtgtttttatt	34	53410309	53410342	ATGAATGCATGGGATTTTCG	TGGACAACTGACGGTTCATC	274
Ad01A10932	Aradu.A01	10932	p3	(AGA)6	18	53467820	53467837	CTAGAGGATTCTCCGGCCTT	ACAACCCCCTCTTCCATACC	191

ID	Chromosome	SSR nr.	SSR type	SSR	Size	Start	End	FORWARD PRIMER (5'-3')	REVERSE PRIMER (5'-3')	Product (bp)
Ad01A10934	Aradu.A01	10934	c	(CT)8(AT)9	34	53474948	53474981	TACGGAGCCATTCCGATAAT	CAACTGGCACCAATTTTTCA	155
Ad01A10935	Aradu.A01	10935	p2	(AG)7	14	53477677	53477690	GACCAAAAACACTTAGCGGG	CTCCCTTCTCTCACAAACCG	135
Ad01A10936	Aradu.A01	10936	p2	(AC)7	14	53484760	53484773	AACCAAACCAGCCACAACTC	CCCAAAGCCCTACTAAACCC	242
Ad01A10937	Aradu.A01	10937	p4	(AAAT)5	20	53509929	53509948	CCTAACGTCAAAATGGCACC	GGGAAGGGGGAAAACAAATA	133
Ad01A10944	Aradu.A01	10944	c	(T)11acaacgat	54	53589567	53589620	TGGGAAGATAAGAGGGGGAT	GAGGAGGTTCTTGTTCCCAA	278
Ad01A10945	Aradu.A01	10945	p2	(AT)9	18	53603873	53603890	GGAGAAAGCAAGATGATGTGG	TTGGTGTTCCTTGGTATTTTCC	249
Ad01A10946	Aradu.A01	10946	p2	(AT)6	12	53608920	53608931	AGGGATTCCTATCGAGGAGC	TTTTGGGTTATTTGGGATGC	234
Ad01A10947	Aradu.A01	10947	c	(TTC)5tccttttc	77	53626066	53626142	GTAGAGACCGGAACGCAGTC	GCAACGACGAGTCTGTGAAA	194
Ad01A10948	Aradu.A01	10948	p2	(TC)8	16	53635543	53635558	AATAAAGGCATTTTGCACGC	CTAGAGGTTTCTCCGGCCTT	263
Ad01A10950	Aradu.A01	10950	p3	(CAT)5	15	53700324	53700338	CATCAGTACGGGGAGAAGGA	TGATGCCGACTTGACTCTTG	207
Ad01A10951	Aradu.A01	10951	p5	(AAGAT)6	30	53706983	53707012	CGACAGGAAAGCCCAAATAA	CCTGGGTGCTCCAGTGTATT	207
Ad01A10953	Aradu.A01	10953	p5	(AAGAT)5	25	53726842	53726866	AAGGCGGTTCCGTTAAAGAT	CTGGGTGGCTCCAGTGTATT	124
Ad01A10955	Aradu.A01	10955	c	(T)10gattttatga	38	53728279	53728316	TGCAATGCATGAATTTTTCG	CGGTTCATCTTGTTGCTCAG	273
Ad01A10957	Aradu.A01	10957	p5	(TTATC)5	25	53738421	53738445	GTGTTGGACTGCCAATGATG	TGGAGATACAAGGACCCCAG	237
Ad01A10959	Aradu.A01	10959	p3	(TCT)5	15	53759922	53759936	ACCCCTTTCATTGGGTTAGG	TTTCCAAGGCTCAGAGGAGA	175
Ad01A10960	Aradu.A01	10960	p2	(AT)6	12	53764098	53764109	GCACATCAAGATGGCATGAA	TTTGAAATTTTTGGTGGGAAA	222
Ad01A10961	Aradu.A01	10961	p3	(TCT)5	15	53768501	53768515	AGCTTGGCACTTTGGAGAAC	TTTCCAAGGCTCAGAGGAGA	280
Ad01A10962	Aradu.A01	10962	p4	(TTAG)6	24	53770355	53770378	TTTCTGCCACTCACGCATAC	GCAAACAAGATTGATAATCCAACA	272
Ad01A10964	Aradu.A01	10964	p4	(TTAG)6	24	53781379	53781402	ATCATCCACCACCACCATTT	GCAAATCAGCAAATCAAAACAA	121
Ad01A10968	Aradu.A01	10968	p3	(AAG)5	15	53798160	53798174	AGGAGTCTCCAACCTTGGGT	ATGGCCGAAACCTAACCTTC	255
Ad01A10969	Aradu.A01	10969	p2	(AG)7	14	53801912	53801925	TTTCGAAAATAGAGGATGGAAG	TTCGAAAATCATCTCTCTTCTTCTC	124
Ad01A10975	Aradu.A01	10975	p3	(GAA)5	15	53902140	53902154	CCCCTAATGAAAAATGGGGT	TAGAGGAAAGGGGGCCTTAG	268
Ad01A10976	Aradu.A01	10976	p3	(GAA)5	15	53910276	53910290	AGCTCTTCCCCCTAACGAAA	TAGAGGAAAGGGGGCCTTAG	277
Ad01A10977	Aradu.A01	10977	p2	(AT)6	12	53963137	53963148	AACCACATCAATAGGCTCAAGTC	GGCGATCTTGGCTGAAAAAT	275
Ad01A10978	Aradu.A01	10978	p3	(TTC)6	18	53969649	53969666	TACCCTCTCCCCTTTCCCTA	ACTCGGAGGTGGAAGCTTTT	279
Ad01A10979	Aradu.A01	10979	p2	(CT)6	12	53988181	53988192	CCATTTAATGCCAGCAAGGT	TGAGTAGGTGGGGGTAGGTG	243
Ad01A10984	Aradu.A01	10984	p2	(GA)6	12	54044538	54044549	TTTGCAATAATCCACAAGCA	TCTTCTTTCTCCCTTTCCCC	262
Ad01A10987	Aradu.A01	10987	p2	(AT)11	22	54071829	54071850	TCGTTTTCAAATCATCCTTTCC	TGACCACTCACCAACTAAGGA	233
Ad01A10988	Aradu.A01	10988	p2	(AG)7	14	54072195	54072208	TTCCCATTTATTTTCACTATTGTTTTT	AAAGGAGATTTGCTCACAATTTTT	276
Ad01A10990	Aradu.A01	10990	p3	(ATC)6	18	54077641	54077658	TGAAGGTGTTGAAGAAGGTTCA	CTGGGTACAGCAAGGACCAT	195
Ad01A10993	Aradu.A01	10993	p2	(TA)8	16	54091360	54091375	TAGACCAACGATAGCAGGGG	TGTGGCATAAATTGATTGGAG	238
Ad01A10994	Aradu.A01	10994	p3	(ATA)5	15	54091889	54091903	TCTTCAAGCCCAGAAAAATGA	CTTTACCTGCAGTTGGGAGC	130
Ad01A10996	Aradu.A01	10996	p2	(TA)10	20	54098366	54098385	ATGGGTTTATCAACCTCCCC	TCTCCCATCTCAATTGTAAAAGC	279
Ad01A10997	Aradu.A01	10997	p4	(CTAA)7	28	54105811	54105838	TTCGAAAGGTAAGCATGAAGAA	TCCAACACTTCCAAACACCA	278

ID	Chromosome	SSR nr.	SSR type	SSR	Size	Start	End	FORWARD PRIMER (5'-3')	REVERSE PRIMER (5'-3')	Product (bp)
Ad01A10998	Aradu.A01	10998	p2	(AG)6	12	54124352	54124363	TGTCCTTGCTCCTGCTCATA	TTTCCTTCTAATTTTCGAATTTCC	237
Ad01A10999	Aradu.A01	10999	p2	(AT)9	18	54142933	54142950	CACAATCAAACCATCCCTCC	CGTGAGAACATTTCACGCTT	134
Ad01A11000	Aradu.A01	11000	p2	(AT)9	18	54145511	54145528	CTCGTGAAAGCAGGGAACAT	AGGTTGGGTTAAAGCTGGGT	275
Ad01A11001	Aradu.A01	11001	c	(A)10ttttactcaa	101	54160325	54160425	GCTCAAAAACATGTTCCACAA	TTTTAAGTGTGGGGAGGTCG	266
Ad01A11006	Aradu.A01	11006	p2	(CT)6	12	54182029	54182040	CAGGCAGCAGTCTGGTGTTA	TTTAGGCGTATGTAAGGGGG	161
Ad01A11008	Aradu.A01	11008	p2	(TA)11	22	54199210	54199231	GACGTAAGCCGTTCTCATCC	TTCTGAGCATATAACTTTGTTTTGG	267
Ad01A11011	Aradu.A01	11011	p2	(AT)10	20	54236339	54236358	CATCATTCAGTCATCCAGCC	CTCCTTGGACAGGTCAGAGC	139
Ad01A11012	Aradu.A01	11012	p2	(CT)6	12	54241364	54241375	CCAATTTGGCAACATGAGTC	AAAGTGGGGGAATACATCCAT	240
Ad01A11013	Aradu.A01	11013	p2	(TA)9	18	54242725	54242742	TACTTTGGGGCATTTTGTCC	GTGGAGAAATTGGGTTTGGA	236
Ad01A11015	Aradu.A01	11015	p3	(TCT)6	18	54265285	54265302	TCACACAACACAACCCCTTC	AGGATTCTCCGGCCTTATGT	194
Ad01A11017	Aradu.A01	11017	p2	(TA)6	12	54270816	54270827	CAAAATCCAATGAGGGTTCAA	GCCATGCAATGCAATTCTAA	264
Ad01A11018	Aradu.A01	11018	p4	(AATA)6	24	54288143	54288166	TTTGAATCAAGGGATGTTGATG	AAGCCTTGGACCTTGCTTTT	158
Ad01A11023	Aradu.A01	11023	p4	(TATG)5	20	54325991	54326010	AAGCCCAAAATTGTTGGAAA	TGCCTAGCTCATTTTGATCCT	176
Ad01A11024	Aradu.A01	11024	p2	(TA)13	26	54326185	54326210	CAACATCCAATCACAATCCA	TTAAGTGTGGGGAAGGATCTG	277
Ad01A11027	Aradu.A01	11027	p3	(AAG)5	15	54342721	54342735	ATGGATCGATTTCAGGCAAC	TCTTGACTTTCCCATGGAGG	265
Ad01A11029	Aradu.A01	11029	p2	(AT)7	14	54345728	54345741	TAAGTGTGGGGAAGAGTGGG	TTCACCACTTACTTTGGGGC	208
Ad01A11032	Aradu.A01	11032	c	(T)10ctcctttccc	42	54419183	54419224	TACTTTGGGGCATTTTGTCC	ACATTGGGTGCATAAAAGGG	228
Ad01A11033	Aradu.A01	11033	p2	(AT)10	20	54433611	54433630	TGTTCCCTACTTTCATGAGCTG	TCATGTAATGCAATGCAACCT	182
Ad01A11039	Aradu.A01	11039	p3	(TCT)6	18	54480086	54480103	CATTTTCACACAACGCAACC	CTAGAGGATTCTCCGGCCTT	203
Ad01A11045	Aradu.A01	11045	p2	(GT)7	14	54514100	54514113	ATAGGGGACAAAATCACCCC	GGGCTAAGGTAAGGGTGAGG	234
Ad01A11047	Aradu.A01	11047	p3	(ACA)5	15	54541361	54541375	GCTGACCCTGAACCTGAAAG	AATGGCAAGGATTGAGATGC	201
Ad01A11048	Aradu.A01	11048	p2	(TC)7	14	54544082	54544095	CCTAAATGGAGCAGGGATGA	GTTTGGAGGTAGGTGGGGTT	179
Ad01A11049	Aradu.A01	11049	p3	(TCT)5	15	54558944	54558958	CGTCTTCTTCCTCCATTCTTCT	ATGGCAAAAGAGTCACCCAA	168
Ad01A11050	Aradu.A01	11050	p2	(AG)14	28	54566034	54566061	TAAAAACGCTAATGGGTCGG	GTGACAATGGGATTTCACCC	162
Ad01A11051	Aradu.A01	11051	p2	(AC)10	20	54569324	54569343	TTCTTGAAAATTTCCTTGTTGTTTC	TAAGTGTGGGGGAGGATCTG	275
Ad01A11052	Aradu.A01	11052	c	(GA)7gg(GA)9	34	54577495	54577528	CATGCAAGCAACCCTAAAAA	CCCCTGATTTGCCAACTATC	280
Ad01A11053	Aradu.A01	11053	p2	(AT)6	12	54584303	54584314	TTTCTTGATCTTTTCCAAAGCA	CCACCCATTCTCTGGTGTTT	258
Ad01A11054	Aradu.A01	11054	p2	(AT)6	12	54604372	54604383	TTGCAAACGTGTGCCTTATT	AGGGGCTTGTGAGTATGGTG	245
Ad01A11059	Aradu.A01	11059	p2	(CT)6	12	54698983	54698994	TTTCTTCCTTCTCCCTTTTTCC	TCTTTCAAGCCTTCCAACTCA	263
Ad01A11061	Aradu.A01	11061	p3	(TAC)5	15	54739863	54739877	AGAACAAAAAGTGTTGCCACC	CCCAAAACACAGGTTGTTGA	275
Ad01A11072	Aradu.A01	11072	c	(AC)6(AT)11	34	54788296	54788329	GCCTAATGGGCTATATAAATGGTG	CCATTCTCAAGGTCACAAGAAA	280
Ad01A11073	Aradu.A01	11073	p3	(AAT)8	24	54792240	54792263	TCTCCACTGGCTGTTCACAC	ATCATGGAGGATGAGGATCG	230
Ad01A11074	Aradu.A01	11074	c	(CT)7cccttcccc	64	54794403	54794466	CTTTCCCTCTCTCTCCCCAC	AGAGAGGAAGAGGAATAGGGG	270
Ad01A11075	Aradu.A01	11075	c	(CT)6ttctctccc	121	54794580	54794700	TCTCTCCCCTATTCCTCTTCCT	GGAGAGAGAGGGAGCAAAGAA	233

ID	Chromosome	SSR nr.	SSR type	SSR	Size	Start	End	FORWARD PRIMER (5'-3')	REVERSE PRIMER (5'-3')	Product (bp)
Ad01A11076	Aradu.A01	11076	p2	(CT)8	16	54794888	54794903	CCCCTCCCTCTCTTTGTCTT	GAAAGGGGAGGGGAAGAAAG	140
Ad01A11077	Aradu.A01	11077	p3	(CTC)6	18	54795107	54795124	TTGTGCTCTTTTTCCCTTTTTC	TCCTTTTCTCTCTCTCATTCCC	250
Ad01A11080	Aradu.A01	11080	p2	(GA)14	28	54796085	54796112	GAGGGGGAAAGGAGAGAGAG	AACTGGTTTCACTGAACTGAATTG	274
Ad01A11084	Aradu.A01	11084	p2	(AC)9	18	54842054	54842071	TACCCATGCAAACATCCTGA	GGGGACAAAATTACCCCAAT	278
Ad01A11085	Aradu.A01	11085	p2	(AT)6	12	54842224	54842235	TGGGGTAATTTTGTCCCCTT	TATTGGGAATTTGGGTGCAT	126
Ad01A11086	Aradu.A01	11086	p3	(AGA)7	21	54846948	54846968	GGCCTTAGGTGCCATTGATA	AAATCTTTCCCACCCAAACC	272
Ad01A11089	Aradu.A01	11089	p3	(TTG)6	18	54905863	54905880	CAGCAATAGGGGGTACAACAA	GATTGAAAGGACCTTGAGGC	257
Ad01A11092	Aradu.A01	11092	p3	(AGA)6	18	54916620	54916637	GGTCTTGATGGCTCTTGGAG	TTCCATACCTTGGCCGAATA	279
Ad01A11094	Aradu.A01	11094	p2	(TA)6	12	54968718	54968729	AGGCGTCTGGAGCTTCTACA	CCTTTGCTTTACGACGTGAG	223
Ad01A11095	Aradu.A01	11095	p5	(TTTTC)5	25	54972364	54972388	CACTCACTTTGGGGCATTTT	TGTTTGGAAACATTTGGTTTGA	270
Ad01A11100	Aradu.A01	11100	c	(T)10caaaata(T	27	55073322	55073348	ATCCATTGATGCTCAAAGCC	AAAACAGTGAATGGCGAAAAA	158
Ad01A11105	Aradu.A01	11105	p3	(TTC)5	15	55095538	55095552	CGCGTCATTTGGAAGTTTTT	TCTGGCCTTAGGTGCCATAG	236
Ad01A11106	Aradu.A01	11106	p3	(TCT)6	18	55109179	55109196	CACCACTCACATCCATCCAC	TTTAGAATGTTGCTCGCCCT	268
Ad01A11108	Aradu.A01	11108	p3	(GAT)6	18	55139973	55139990	AGTGGTGCATCAAATCTCCC	GGTTTGGCTTGGTTGTCAGT	230
Ad01A11111	Aradu.A01	11111	c	(A)13ttatcaaaa	107	55165432	55165538	TGCAAAGTCACATTGTTGGTT	TGAGCGAAAGAATGAGAAAGA	245
Ad01A11114	Aradu.A01	11114	p2	(TA)9	18	55187099	55187116	CATTTAGGAACAATGGCACTGA	CCCCCTCTTTCAGATCAACA	185
Ad01A11115	Aradu.A01	11115	p3	(GAA)5	15	55187243	55187257	TGTTGATCTGAAAGAGGGGG	CACGTTGGCGTCTACAATGA	265
Ad01A11116	Aradu.A01	11116	p3	(ATG)5	15	55190242	55190256	CAAGTGTCCACCAGAAGGGT	ACCATGCCTTGATGAGCTTT	260
Ad01A11119	Aradu.A01	11119	p5	(AAAAT)5	25	55192473	55192497	TGAGGAGAATTTCCAGTGAGA	TGACAGCTACCCATTGTTTGA	257
Ad01A11122	Aradu.A01	11122	p3	(TTC)5	15	55211473	55211487	CAATCACCTCCACCCACTCT	TCCTCGAGCAAAGGAAGAAA	253
Ad01A11125	Aradu.A01	11125	p2	(TA)6	12	55244160	55244171	CCTCTGGTTCTCACCCCATA	AAGGCCGGCCAGAGTATATTA	276
Ad01A11126	Aradu.A01	11126	p3	(TAT)5	15	55246817	55246831	ATCGAGTTCAATATCTGCCG	ACTCACGACAATTATACTCAATGTTT	203
Ad01A11127	Aradu.A01	11127	p3	(TTA)12	36	55246963	55246998	AAACATTGAGTATAATTGTCGTGAGT	AAACCGCCTTTTATTTCCGT	241
Ad01A11128	Aradu.A01	11128	p2	(AT)6	12	55247195	55247206	CGGAAATAAAAGGCGGTTTC	TTCTCTGTCGCCGTTTTCTT	200
Ad01A11130	Aradu.A01	11130	p2	(GA)6	12	55256024	55256035	AAGGAAGGAGGGAAGAAGAAAA	GCCAGAAGCATAACATGGGT	280
Ad01A11132	Aradu.A01	11132	p2	(AT)8	16	55280809	55280824	CCTTGGTGTTTTTCCTCCAA	TGAAGAATTTCGAAAAAGTGAGG	272
Ad01A11133	Aradu.A01	11133	c	(TA)10ttcga(A	40	55295120	55295159	GCAGTAGCGGGGTTAGCATA	TTCCTAACCACTTTCTCTTTCTTCA	248
Ad01A11137	Aradu.A01	11137	c	(ACA)6aaagaa	94	55346857	55346950	CATGATTGGTGTTCCGTTCA	GCACGGAGCGTCTTTTTCTA	239
Ad01A11144	Aradu.A01	11144	p2	(AG)7	14	55401142	55401155	AAATGAATCAAAGCCAACCG	GGGTGATTAGACCTCAACCCT	200
Ad01A11145	Aradu.A01	11145	p2	(AG)6	12	55413660	55413671	TGGCTCTTGAAAGAATGATGG	TGCATGCCACAGACACATAA	253
Ad01A11146	Aradu.A01	11146	p4	(TTAT)6	24	55423458	55423481	ACCATGTGGGAAACAGGAAA	AAATTAAGTTTGGTGTCCCCG	222
Ad01A11152	Aradu.A01	11152	p3	(TTC)7	21	55502989	55503009	TTCTTGGCCGAATACACCTC	AGTGGTCTTGATGGGCTTTG	259
Ad01A11153	Aradu.A01	11153	c	(AC)9g(CA)1(	51	55512619	55512669	CAATCCCAGCTAAAGGCTGA	TCCCTCGCACTAGTTGAGTCT	280
Ad01A11154	Aradu.A01	11154	p3	(AGA)5	15	55514453	55514467	GTTTGGCTGCAGAGACATGA	CCTACTAGTCACCTCCCTTCCC	219

ID	Chromosome	SSR nr.	SSR type	SSR	Size	Start	End	FORWARD PRIMER (5'-3')	REVERSE PRIMER (5'-3')	Product (bp)
Ad01A11156	Aradu.A01	11156	p3	(TCT)5	15	55516284	55516298	TTCCCCAAGGTTCTCCTCTT	CAACAGCAACAGAACCGAAA	279
Ad01A11167	Aradu.A01	11167	p2	(TG)10	20	55581234	55581253	TTGATGGCAGTTGGTGAATG	TGGGTCCCAACTAATCCACT	141
Ad01A11168	Aradu.A01	11168	p2	(TA)6	12	55583206	55583217	TTGGTTGGAGAAGTGTTTGG	CCACATACAACCATCACCCA	229
Ad01A11169	Aradu.A01	11169	p2	(CT)10	20	55600528	55600547	AGAATGCAATCACACGAACG	GGGAGGAGGGAAGAAGAAAA	226
Ad01A11171	Aradu.A01	11171	p2	(GC)6	12	55615118	55615129	ACAAGGCTCCTAAAACCGCT	ATGTTACTCGCATTCAGGGG	106
Ad01A11172	Aradu.A01	11172	p2	(AT)12	24	55620358	55620381	TTGTTGGCTCTTGAAAGAATGA	TTTTTACCCATGCCTTTTGG	206
Ad01A11177	Aradu.A01	11177	c	(AAT)9(GAT)	94	55686937	55687030	CTCCCATGTCATCGAGTCCT	AGCTTGGCGATGTTGGTATC	221
Ad01A11182	Aradu.A01	11182	c	(AC)13(AT)6	38	55710050	55710087	TTACATGCTTTGCTTTCGCA	GAGGGACCCCTCAGAAAATG	274
Ad01A11184	Aradu.A01	11184	p2	(AT)7	14	55712050	55712063	TGAACTTCATGTTGGCGGTA	GGAGACAGGGACCCGTTTAT	272
Ad01A11186	Aradu.A01	11186	p3	(ATT)5	15	55736385	55736399	GCCCAAATGGGTGTATCTGT	GCACGTGGGCAGATATAGTG	254
Ad01A11188	Aradu.A01	11188	p2	(AT)8	16	55752646	55752661	GTACACCTGCCTGATGCGTA	TGCGTATGCATGAGTGGAAT	182
Ad01A11190	Aradu.A01	11190	p2	(TG)6	12	55757098	55757109	TGGAAAAAATTTGGGAACATTG	CTTCCACACACTCATGCATTC	170
Ad01A11192	Aradu.A01	11192	p2	(TA)8	16	55773764	55773779	GATAAAGAAGCGCTTGTCGG	CACTCTTGCGCAACTCTCTG	260
Ad01A11193	Aradu.A01	11193	p2	(TC)6	12	55774336	55774347	AAATTCAAGAACACGCGGAC	TTGCATGTCCTCAAGCAAAG	226
Ad01A11195	Aradu.A01	11195	p3	(GTG)5	15	55779104	55779118	TAGCAGGGGCTAGTTCTGGA	TCCATCTCTAACGACCACGA	170
Ad01A11196	Aradu.A01	11196	p3	(TAG)5	15	55791961	55791975	GCACCCCACAGATTAGAGGA	TGCCTGATGATGATGAGAGAAA	197
Ad01A11198	Aradu.A01	11198	c	(CT)7cacacacg	34	55837963	55837996	GCCTGGATTGACGTTCCATA	GTGAAGGTTGCGGTAGGTGT	205
Ad01A11199	Aradu.A01	11199	c	(ACC)8(AAC)	42	55838157	55838198	CCGTGACATCTTCTCCCAAT	GACCTACCGCTACTGCTCGT	239
Ad01A11200	Aradu.A01	11200	p3	(TAT)5	15	55839764	55839778	TGCATGTGTTTGTTTCATTTAATA	AAAAATAATAGGACTGGTACACCAAA	280
Ad01A11201	Aradu.A01	11201	c	(A)10taatgcgta	41	55842511	55842551	TTTCTTTTGAGAAATGATGTTTATGC	TCTCCACCACAAGGTTGGAT	164
Ad01A11203	Aradu.A01	11203	p3	(ACA)5	15	55845027	55845041	GAACCTCTTTTCCACCACCA	ACTGGACCTCACAAGATGGG	158
Ad01A11204	Aradu.A01	11204	p2	(TA)6	12	55860776	55860787	TGAGTTGTGTGGAAATCGGA	TTCAAACATCAATACCCAGCA	272
Ad01A11210	Aradu.A01	11210	p2	(GA)6	12	55920354	55920365	AATGGGAGAGAAAAGGGGAA	TGATTAGACATTCAAATCTCACCAT	270
Ad01A11216	Aradu.A01	11216	p2	(TA)6	12	55952268	55952279	ACACCCCATTACCCTAAGCC	GGATGCTTCGCGTTTTGTAT	178
Ad01A11218	Aradu.A01	11218	p2	(TA)11	22	55967421	55967442	AAGTGTCACAACAGTTCTAAGGC	GCAGGCTGTGACTAGTTCTCTTT	280
Ad01A11219	Aradu.A01	11219	p2	(AT)6	12	55967620	55967631	TGTCACAACAGTTCTAAGGCTCA	CGCAGGCTGTGACTAGTTCT	278
Ad01A11227	Aradu.A01	11227	p3	(TTG)8	24	56046990	56047013	TAGCATAGACCCCAACTGGC	AGAGGGGATCACCTTCCACT	251
Ad01A11229	Aradu.A01	11229	p3	(TTG)7	21	56060849	56060869	CAGGAAATGTGGTTGCTCCT	TGTGATGCGCTGGAGTTAAG	194
Ad01A11230	Aradu.A01	11230	p4	(AAAT)7	28	56065222	56065249	TTCTTTCTTTCGTTCCCCCT	GCAGAGAATTCCCAGTGAGG	221
Ad01A11232	Aradu.A01	11232	c	(AT)7agc(TA)	33	56077388	56077420	GATAAAGAAGCGCTTGTCGG	CTAGTGCCACTCCTGTGCAA	273
Ad01A11233	Aradu.A01	11233	c	(TA)9ttctttatttt	45	56085785	56085829	TTACTTGGGGCATTTTGTCC	GTTTGGGAGAGTTGGGAACA	238
Ad01A11239	Aradu.A01	11239	p2	(TA)7	14	56111707	56111720	CATTGGTCTTCTTAGTTAGAGACTTTT	AACGAGTTTTTCCATGGGC	207
Ad01A11242	Aradu.A01	11242	c	(TA)6cattacaa	45	56123533	56123577	TGAGATGGTAACAATAAATAAATGCAA	TCTCGTGCTGATAACGTGTTG	213
Ad01A11245	Aradu.A01	11245	p2	(TC)10	20	56160515	56160534	AGGAGCAGGGATGAAATCCT	ATTAGGGTTGGGTTTGGGAG	220

ID	Chromosome	SSR nr.	SSR type	SSR	Size	Start	End	FORWARD PRIMER (5'-3')	REVERSE PRIMER (5'-3')	Product (bp)
Ad01A11248	Aradu.A01	11248	p2	(AT)9	18	56227336	56227353	CGCCAGTGAGCTCTTTTTCT	TGGTTGTTTTTCTCTCTCCTCA	274
Ad01A11249	Aradu.A01	11249	p2	(AT)12	24	56237303	56237326	GGCCTCAGAGATGCTTGTTC	TCTTCATCAAGGCTCCTCGT	241
Ad01A11252	Aradu.A01	11252	p5	(AATAA)5	25	56278713	56278737	GGGTTAGCATATGACCCCAG	CCACTTTCTCTCTCCTTACTTTTTG	250
Ad01A11253	Aradu.A01	11253	p2	(CT)21	42	56287828	56287869	CAACAGCAGCAGAAATCCAA	ATGAATGAGAGGGATGCGAG	133
Ad01A11254	Aradu.A01	11254	p2	(GT)7	14	56288198	56288211	CCCTATCTCTTCCTCTGGGG	CTTTACCCAAACCGTAGCCA	206
Ad01A11257	Aradu.A01	11257	p2	(CT)6	12	56311662	56311673	CCCAAATCCTACTTTTCCCC	TTCTCAACAATCCACAAGCAA	280
Ad01A11259	Aradu.A01	11259	p2	(CT)9	18	56327541	56327558	TGAAAGAACATTGAACCTGGG	CGCGATCTGCAGAATTACAA	276
Ad01A11260	Aradu.A01	11260	p2	(AT)8	16	56347858	56347873	CTTACGCATCACCTGCACTT	CAAAAGAAAATGAAAGATGAGAAAAA	259
Ad01A11263	Aradu.A01	11263	p2	(GA)7	14	56369218	56369231	TTGCAACAATCCACAAGCAT	TTCCTTCTCCCTTCTTCCTTC	246
Ad01A11266	Aradu.A01	11266	p3	(ATA)12	36	56383913	56383948	TTGACTTAATGTCACTCTTCATGG	CACACAATTCGATCTTTGACG	225
Ad01A11269	Aradu.A01	11269	p3	(ATA)15	45	56388152	56388196	TGTTATGGCAATAATTTAGCCTG	TATTTGGGGACGTGCTTCAT	126
Ad01A11270	Aradu.A01	11270	c	(TA)6(TG)6ttt	47	56388406	56388452	GTTAGGGACTTACAAAACCTTTCTT	CGAATACCCCTAACAAGCCA	280
Ad01A11274	Aradu.A01	11274	p2	(TA)6	12	56395984	56395995	AAGGGTTAGGTTAATAAAAATGGAAA	ACAATTCAGATTGGAAAGTGCT	180
Ad01A11275	Aradu.A01	11275	p2	(AT)12	24	56397887	56397910	TTCATTCATCGACTTGCTTCA	TTAAAGCTGCAGCAACATGC	241
Ad01A11277	Aradu.A01	11277	p3	(TGA)6	18	56404598	56404615	GATGTCCCTCTTCCGACAAA	AATTAGCCATTCGTGGGTTG	269
Ad01A11282	Aradu.A01	11282	p3	(AAT)9	27	56417435	56417461	TCATAGTTGGAGATAGAGACACCG	ATGAATTTGCATTGGATCGG	217
Ad01A11283	Aradu.A01	11283	p4	(TTAT)5	20	56417802	56417821	TTGTCAGAGTTCGGCTGTTG	TTCATTTTTACTCTTCTCCTGCAC	266
Ad01A11284	Aradu.A01	11284	p3	(CAA)5	15	56426502	56426516	GTCAAGAATGCAGCACGAAA	TTGTGTTGTCTTGTCCCGAA	277
Ad01A11285	Aradu.A01	11285	p2	(AT)6	12	56428060	56428071	GGCATATTCACAAAACATGGC	TTCAATTTTGATCTTGGATTCTAGC	265
Ad01A11287	Aradu.A01	11287	p2	(AT)6	12	56431478	56431489	TCTGTTGAAAATGCTTTTATTCATCT	CGACTAGTTTCGGGTTCGAG	262
Ad01A11288	Aradu.A01	11288	p2	(GC)6	12	56439814	56439825	CCTAGTCCTGCAGCAACCTC	AGGAAAGCGGGATAGGGTAA	254
Ad01A11289	Aradu.A01	11289	p2	(TA)6	12	56445047	56445058	TCATGGCATCCAAGTTGAAA	GGGGAGGATCCGAAATTTTA	204
Ad01A11294	Aradu.A01	11294	p3	(TCA)8	24	56502765	56502788	TCGTCCTCATCCTCATCCTC	AAAAACAATGCCAAAAAGCG	161
Ad01A11296	Aradu.A01	11296	p2	(AT)10	20	56528683	56528702	ATGGCGGATTCCACTATGAC	TTTGCCCTGAATTGAATCTTG	279
Ad01A11299	Aradu.A01	11299	p2	(AT)6	12	56550168	56550179	CCTCAAGTGAATGAGGGTCG	TTTGCCATGAATTGAATCTTGT	263
Ad01A11300	Aradu.A01	11300	p3	(AAG)6	18	56566729	56566746	TAAAATATTGCTCGCCCTCG	AAATCTTTCCCACCCAAACC	208
Ad01A11301	Aradu.A01	11301	p4	(TAAA)5	20	56570300	56570319	AGGGGCAGTGATTTGAGATG	CCTATCCCTCCCTAATTTTCG	223
Ad01A11302	Aradu.A01	11302	p3	(TTC)12	36	56577338	56577373	CCAAAAACCCCACCTACCTT	TTAGAATGTTGCTCGCCCTC	256
Ad01A11304	Aradu.A01	11304	p3	(AGA)5	15	56642551	56642565	ATTGCCTTCCCTTTCGTCTT	CCCATCACCTCTTCACCACT	248
Ad01A11305	Aradu.A01	11305	p3	(TCA)6	18	56666040	56666057	GGAAGTCAGAGAGGTCAGCG	AGTCACCTTGTAGGCAGCGT	210
Ad01A11306	Aradu.A01	11306	p2	(AT)6	12	56674102	56674113	TTGGGAATTGGGTACATGCT	CCCACCTAACCTATGGCAAC	280
Ad01A11307	Aradu.A01	11307	p2	(AT)10	20	56687634	56687653	AATCGACCCTCACTCACCTG	TTATCTCCTGGAGTTCCCCC	100
Ad01A11308	Aradu.A01	11308	p2	(AT)11	22	56688756	56688777	CTCACCAAGCACCTACCCAT	TGGTTTATCAGGGTTCTCGG	261
Ad01A11311	Aradu.A01	11311	p2	(GA)20	40	56712468	56712507	GGGAGAGAGAGTTCGTGTGC	TATTTGGTGGGTTGTTGGGT	197

ID	Chromosome	SSR nr.	SSR type	SSR	Size	Start	End	FORWARD PRIMER (5'-3')	REVERSE PRIMER (5'-3')	Product (bp)
Ad01A11312	Aradu.A01	11312	p3	(TCC)6	18	56713798	56713815	GCCAACTCCATCAACACCTT	CTCGACGTCCTCTGGAACTT	228
Ad01A11314	Aradu.A01	11314	p3	(AGA)7	21	56720444	56720464	GTTTCTCCGGCCTTAGGTTC	TAAACCCCTCTCCCTCCACT	245
Ad01A11318	Aradu.A01	11318	p2	(AT)8	16	56775745	56775760	TTCAATGATGATACCCGCAC	GTCGCCTTGTCCCATATTGT	258
Ad01A11322	Aradu.A01	11322	p3	(AGA)5	15	56814650	56814664	TATCTCCCATGACTCGGAGG	CCTCTCTCCCTCTCCTCCAT	206
Ad01A11325	Aradu.A01	11325	p2	(TA)8	16	56821804	56821819	GTGCAACAGATGTCACCAGC	GCAAGCCACCTTCATTCATT	241
Ad01A11326	Aradu.A01	11326	c	(AC)18(AT)6	48	56841104	56841151	CCCCACCAGGGTAAATTCTT	ACCATTTTTGCGCAATTCTC	272
Ad01A11327	Aradu.A01	11327	p2	(TA)7	14	56844905	56844918	CATTTTGCACTTTGCCTTGT	CAATGTGAATCCTAAATGCCC	271
Ad01A11333	Aradu.A01	11333	p2	(TA)9	18	56891815	56891832	CAAAAGTGGGAAAATTTTGGG	TTTCAAGGTTGTAATGGGGC	170
Ad01A11334	Aradu.A01	11334	p3	(TCC)9	27	56899284	56899310	AGGAAGCCTCACACCTAGCC	CCCATCGACTTAGATTGGGA	269
Ad01A11336	Aradu.A01	11336	p2	(TC)18	36	56902699	56902734	CCTTTCTCCTGAGCGTATCG	CAGAAACTCACAACTTCACCAAG	159
Ad01A11341	Aradu.A01	11341	p2	(GC)6	12	56932745	56932756	GCAAAGATTGGATCAAGGGA	TTTCATTAAATTGGGCCACG	280
Ad01A11344	Aradu.A01	11344	p2	(TA)12	24	56949595	56949618	GGTGTTCTATAATTTTTCTGGATTTGA	TCAAGATGAAAAGATTGTTCAATAAA	268
Ad01A11345	Aradu.A01	11345	p3	(ATA)15	45	56953552	56953596	TTCTGACATCTTATGCTTGTTTCC	TTTACGTTAATGGAGAATTAGAACG	251
Ad01A11347	Aradu.A01	11347	p3	(AGG)7	21	56963275	56963295	CTGCCTGTAAATCCGATGGT	CCGTTTTGGTGCTATTTTCTG	170
Ad01A11348	Aradu.A01	11348	c	(GAA)18(GAT	81	56963494	56963574	CCAAAACGGTTTAGTCGTGA	CACAATTCAAAATTCTCCAGCA	270
Ad01A11349	Aradu.A01	11349	p3	(GGT)5	15	56964600	56964614	TCCATAATGACGATGATGGTG	TCCTATTGTTGCTGCTGCTG	255
Ad01A11350	Aradu.A01	11350	p2	(AC)15	30	56965843	56965872	TCAGACTTTGCCACCCTTCT	TTGGTTGAGTTGGTTGTAGGAA	271
Ad01A11351	Aradu.A01	11351	p2	(TA)6	12	56971737	56971748	AGAAATGCAACGAATCGAGC	CATCACTGAAGGCCACCTTT	268
Ad01A11356	Aradu.A01	11356	p4	(TTTA)5	20	57007312	57007331	CGAAAGTTTCTCCCTCTCTCA	CAGGTTCTGGATCAGCTTCA	267
Ad01A11358	Aradu.A01	11358	p2	(GA)9	18	57047085	57047102	GAGAGGGAGATGGGGAAAAG	AGTCCTTGACCCCTCAGGAT	233
Ad01A11359	Aradu.A01	11359	p2	(TA)7	14	57052116	57052129	GCGAACACGCGAATTAAGA	TGATGGGAGAGGTAGTAAAGGG	277
Ad01A11360	Aradu.A01	11360	p3	(TGT)5	15	57073552	57073566	TACCGTTGGTTCAGGTAGGC	CCATGATGCCATCTGCTATG	272
Ad01A11362	Aradu.A01	11362	p2	(CT)9	18	57076200	57076217	AGAGGTGGGGAAGATGCTTT	GCTGGTTAGTTGTGCGGAAT	270
Ad01A11363	Aradu.A01	11363	p3	(TTA)5	15	57088722	57088736	AGGATGTGGATCCTTCGTTG	TGCAATGAGAGAGAGAGCGA	171
Ad01A11365	Aradu.A01	11365	p2	(CT)6	12	57109657	57109668	ACCCCAACCAACGTAACTCA	TACAGTTTCCACCGCACATT	211
Ad01A11368	Aradu.A01	11368	c	(T)10ggaatttgg	41	57131208	57131248	TGCCAAAATGGGTTTATTCC	TGTCTTTCTGATTGATTGGGC	217
Ad01A11373	Aradu.A01	11373	c	(T)12aattttccta	104	57144721	57144824	TTGGACTTCCATGGACAATTT	CAAACAATGAAATCGCTCAGA	217
Ad01A11374	Aradu.A01	11374	p2	(AT)6	12	57152936	57152947	GGCATCGAATGGTACAAAGG	TTGGATGAGATCCTAACCAATTC	267
Ad01A11375	Aradu.A01	11375	p3	(TAT)5	15	57153458	57153472	CGAACCCGAGAAGAACAAAG	ATGAATCCGTCAACTTTCGG	232
Ad01A11376	Aradu.A01	11376	p2	(AG)7	14	57159089	57159102	GGACAAATGGGTAGAGGGGT	AGTGGTAACGGCATCGAATC	278
Ad01A11377	Aradu.A01	11377	p5	(TTTTA)5	25	57165456	57165480	AAAAACAATGCCAAAAAGCG	TTGAGGAGAGGGAAAGTGGTT	232
Ad01A11379	Aradu.A01	11379	c	(T)10cttcttcaag	63	57180241	57180303	TTCGAGTGTGAGGCGACTTT	TTGAAAACACGATGCTTTGG	145
Ad01A11380	Aradu.A01	11380	p3	(CAA)6	18	57182243	57182260	TTGGGGGATACCCCTTCTAC	TAGTGGGACAAAGCGTGGTT	276
Ad01A11383	Aradu.A01	11383	p3	(TCT)6	18	57243023	57243040	CACGGTCATCCCTTCTGTTT	CCTTAGGTGGCATCAATGGT	262

ID	Chromosome	SSR nr.	SSR type	SSR	Size	Start	End	FORWARD PRIMER (5'-3')	REVERSE PRIMER (5'-3')	Product (bp)
Ad01A11384	Aradu.A01	11384	p2	(TA)6	12	57250265	57250276	AGGGGACAAAATTACCCCAA	TTTCAAGGTTGTAATGGGGC	260
Ad01A11385	Aradu.A01	11385	c	(AT)7ac(AT)9	88	57251814	57251901	GGCCTAAACTCTGCAAGCTG	GCTCAAAGTCGCATACAGCA	280
Ad01A11386	Aradu.A01	11386	c	(AT)6tgcattttca	125	57259929	57260053	GATTCGTCATTTTGGGGAGT	TCCTATTCCTAATTTTCAACATTGC	277
Ad01A11387	Aradu.A01	11387	p3	(AGA)5	15	57272952	57272966	CTAGAGGTTTCTCCGGCCTT	TTCATTTTCACCCACCACAA	205
Ad01A11390	Aradu.A01	11390	p3	(TCA)5	15	57316869	57316883	CAAGCTTCTGAACTCGACCC	AGTTGGTGTGGTTTCCTTGC	227
Ad01A11391	Aradu.A01	11391	p2	(TA)8	16	57317492	57317507	TAATGCAGCTCCACTTGCTC	CGCATTAGCACTGATCCAAA	220
Ad01A11395	Aradu.A01	11395	p2	(TC)6	12	57339499	57339510	AAATTGCACAAAAACCAGGG	AAACCAATAAGCGTTGCGTC	275
Ad01A11398	Aradu.A01	11398	p2	(TC)7	14	57350706	57350719	CTCATCCCGTTACCATCGTT	GCATCATATTTATAATTACCGCCA	205
Ad01A11404	Aradu.A01	11404	p2	(AT)6	12	57356890	57356901	TGTGCTGGAGATGAAAATGG	AGGTGCCAAATCAAATACGC	231
Ad01A11405	Aradu.A01	11405	p3	(TTG)8	24	57362196	57362219	CGTTTGATTCATGTAGCCGA	GAGCAATACCAGGAGGACGA	239
Ad01A11413	Aradu.A01	11413	p3	(GAG)5	15	57400389	57400403	ATTCTGAACGAACTGAGGCG	GTCCAGGAGGTCAAGTCGAG	261
Ad01A11414	Aradu.A01	11414	p2	(TA)8	16	57410577	57410592	AAGTCGGACGAAGGTCTCAA	CCATTTCAACCCACAACTACA	187
Ad01A11416	Aradu.A01	11416	p3	(TAT)11	33	57413681	57413713	CATGTCATGGAGAACATGCAG	CACACTCATGCACAACAATTT	279
Ad01A11418	Aradu.A01	11418	p2	(AG)6	12	57423163	57423174	GGACCAGAAAGCACTGGAAG	CAAAGAACATTGAACCTGGGA	254
Ad01A11421	Aradu.A01	11421	c	(A)15gaaaaaaa	45	57455128	57455172	GACCTGTGCACAAATTCATCC	GTTAAGGGGTTCGTTCGGTT	216
Ad01A11427	Aradu.A01	11427	p2	(AT)6	12	57506063	57506074	GGTGGGTGGATTGTTGTGAT	CATTGTGCATTTTGGGTTGT	196
Ad01A11429	Aradu.A01	11429	p3	(ATT)5	15	57533317	57533331	AGGAGCAACAAAGGCAAAGA	AAGAGAGGTGAAGGATTTATGGAA	235
Ad01A11431	Aradu.A01	11431	p2	(CT)8	16	57540623	57540638	TTTCTGGGCTGTTTCTGACC	AAACCTGGGATCGAGAGTCA	242
Ad01A11437	Aradu.A01	11437	p2	(TA)6	12	57557895	57557906	TGTCCAAACAAGCACTAAGATTG	GACGTGCAATAAGCTCCTCC	118
Ad01A11439	Aradu.A01	11439	p3	(ATA)5	15	57561653	57561667	ATTGCCACTGCCTTCTGAAC	TGGCAGTTGAACCGATAATG	233
Ad01A11440	Aradu.A01	11440	p2	(TG)6	12	57563729	57563740	GTTATGGCCACCGGAAGTTA	CCTAAGTAGGGTCCAAGCCC	153
Ad01A11441	Aradu.A01	11441	p3	(TCT)10	30	57576333	57576362	CGTGTAAGCCTTTTTCTCGC	GACTTTTCCTTTGCGTTTCG	111
Ad01A11445	Aradu.A01	11445	p2	(GC)6	12	57602270	57602281	CGGTTCTAGTTGGGTTGGAA	ACAAGGCTCCTAAAACCGCT	168
Ad01A11446	Aradu.A01	11446	p2	(GC)6	12	57603491	57603502	GAGTTGGATTGGGCCTACAA	TGGAAAGCTATCATCTGGGG	169
Ad01A11448	Aradu.A01	11448	p2	(GC)6	12	57609814	57609825	GAGTTGGATTGGGCCTACAA	CATCTATTGCTTCGCGTTCA	126
Ad01A11450	Aradu.A01	11450	p2	(TC)6	12	57640127	57640138	TGAATAATTGGCTCCCAAGC	AACTAATAAGCGCAGCGTCC	267
Ad01A11452	Aradu.A01	11452	p2	(CT)9	18	57652478	57652495	GAAAATCACCTTCCTCTTTTTCAA	AGAGGGAGAGAGAGGGTTCG	249
Ad01A11456	Aradu.A01	11456	c	(AG)21aacgtaa	65	57792034	57792098	GGAAGAAGGAGGTGTCAAGAA	AACTTTCTGCCAGGAACACC	280
Ad01A11459	Aradu.A01	11459	c	(AT)6(AC)7	26	57813353	57813378	ACCTTACTCGCGTCCAGAGA	GCTTTGCAGGCTGTAATTGG	232
Ad01A11461	Aradu.A01	11461	p2	(TA)6	12	57821122	57821133	TGTCCCTTGTTTGGATCTTTG	AGCCTTACTTGGGGCATTTT	276
Ad01A11465	Aradu.A01	11465	p2	(AG)8	16	57855724	57855739	AAACCTGGGAACGAGAGTCA	TTTTGACCCAGTTTTCAGCC	237
Ad01A11466	Aradu.A01	11466	p2	(CT)7	14	57873090	57873103	GCTATAAAGTGGGGGAATGC	CAATTGGGCAACATCAATCA	272
Ad01A11467	Aradu.A01	11467	p2	(AT)14	28	57877634	57877661	TAAGTGTGGGGAAGAGTGGG	GCCGCTTTCACTTACTTTGG	223
Ad01A11468	Aradu.A01	11468	p2	(CT)8	16	57878708	57878723	GCTATAAAGTGGGGGAATGC	CAATTGGGCAACATCAATCA	274

ID	Chromosome	SSR nr.	SSR type	SSR	Size	Start	End	FORWARD PRIMER (5'-3')	REVERSE PRIMER (5'-3')	Product (bp)
Ad01A11469	Aradu.A01	11469	p2	(TC)16	32	57888866	57888897	TCCCCAATTAGAGGTGTCCA	TCATGAATGTTGGCTCTTGAA	266
Ad01A11471	Aradu.A01	11471	p4	(AACT)5	20	57900324	57900343	AAGAACGGCGCAATTATCAG	TCGAACACCGATTCTTTTCC	163
Ad01A11472	Aradu.A01	11472	p2	(CT)6	12	57904054	57904065	GGAACATTGAACCTAGAATGAAGA	CCAGTTTCAAGCCCAGAAAA	216
Ad01A11475	Aradu.A01	11475	p2	(GA)7	14	57919381	57919394	TTGTGAAAGCAGCGGTGTAG	CTTCCTTATCCGTCGCTGAG	165
Ad01A11476	Aradu.A01	11476	p2	(CT)9	18	57932277	57932294	TACACGCCTAAATGGTGCAG	TGGGAGGAAAAAGTGTTTGG	254
Ad01A11478	Aradu.A01	11478	p3	(TCT)6	18	57946801	57946818	TCTTCTCCCACTTAGCCGAA	GCTTTTGTCTTCCCTTTCCC	205
Ad01A11480	Aradu.A01	11480	p2	(AC)6	12	57961660	57961671	GCAGAGTGCACAAAGACGAG	TCTTGACTGCTTCAACCCCT	255
Ad01A11481	Aradu.A01	11481	p3	(AAT)6	18	57981118	57981135	TGTGTCAATGTCTGTGCATCAT	GGAAGCAGAATTTGTCGGTAG	280
Ad01A11487	Aradu.A01	11487	p4	(CATA)11	44	57994488	57994531	TATCCTCTCATGAGGCCACC	TTTCCATCTTTGGCTTTTTGTT	106
Ad01A11494	Aradu.A01	11494	p3	(ACA)5	15	58083418	58083432	TGACAAACCAACAAACGACA	CCATTTGGCTAGCATCGAAT	272
Ad01A11495	Aradu.A01	11495	p2	(AT)8	16	58118129	58118144	CCCTGCTTTCACAAGCTGTC	ATCACGGCATCGAATGGTAT	278
Ad01A11497	Aradu.A01	11497	p2	(TC)7	14	58153840	58153853	ATTCGATCGTCAATGAACCC	CAGTTCGCACGTGATTCTGT	224
Ad01A11499	Aradu.A01	11499	p2	(AG)9	18	58162403	58162420	GGTGCACACAACTGAATTGG	GCTTGGCTCGATTGCTATTC	236
Ad01A11500	Aradu.A01	11500	p2	(CT)13	26	58162790	58162815	AGGGGGAAGGAAGAAACAAA	CTGGGCATGAAAAATGTGTG	185
Ad01A11502	Aradu.A01	11502	p2	(TA)6	12	58196693	58196704	CTTTGCTTTGGGGCATTTT	GTGAGGATTGGGAACATTGG	275
Ad01A11503	Aradu.A01	11503	p2	(AG)6	12	58202722	58202733	TCAAAACAAGAAGAAATCCGAAA	AGAAATGCATCCATTCAGGG	114
Ad01A11505	Aradu.A01	11505	p2	(AT)6	12	58222097	58222108	GCCTTGAGCCTCCTCTTACA	GCACGGGGTGCTATTAGAAA	200
Ad01A11507	Aradu.A01	11507	p2	(TA)8	16	58227609	58227624	TCGCAGGTCAAAGGAAACTC	AAAAGGACATTAATAATTCGGAAAGA	231
Ad01A11508	Aradu.A01	11508	p3	(ACC)5	15	58237050	58237064	TCTTGGCGCTCAACATAGTG	CGCCGTTCTAAGGAATTTCA	206
Ad01A11509	Aradu.A01	11509	p2	(AC)13	26	58238885	58238910	AACACAAAACGGTATCGCTG	GGAACAGGAGCCATGATTGT	197
Ad01A11510	Aradu.A01	11510	p4	(CAAA)5	20	58240204	58240223	GTTTCAAATATCGGCCTCCA	ACACGTGAGCTCATGGTCAG	253
Ad01A11513	Aradu.A01	11513	p3	(TTA)7	21	58251729	58251749	TTTAAGTCATTTACCTGTTTGTGG	CATTTCATATCACATCCGTTTCA	204
Ad01A11515	Aradu.A01	11515	c	(TAA)6t(ACA	34	58253408	58253441	TCTCATGAATTAATTTTTCCTCCA	CGCCCATCCTTTGTAGACAT	273
Ad01A11516	Aradu.A01	11516	p3	(CTC)6	18	58258784	58258801	AGAGGGTCCAGGAGGCTAAA	ACGGCTGAGAAGGAGTTGAA	138
Ad01A11519	Aradu.A01	11519	p2	(TG)36	72	58273768	58273839	CGACTTTGCGATTTTGTTTTC	AGGCTCACGAGGACGAACTA	256
Ad01A11520	Aradu.A01	11520	p5	(AATAA)5	25	58278466	58278490	TGGAGAAAGGGAGATGATGTG	TTTTCGAGAACAAGGAGCATT	271
Ad01A11521	Aradu.A01	11521	p2	(TA)8	16	58285871	58285886	TGTAGCCTTTTGTCTCTCATTTTT	TCATCCCAATATTTTTGGTTCAT	259
Ad01A11525	Aradu.A01	11525	c	(A)10gaaaagga	36	58370671	58370706	TCTTGAAGCAAGAAAAAGCAG	CCACCGGATACATAAATGCC	250
Ad01A11529	Aradu.A01	11529	p3	(AAG)10	30	58398104	58398133	TAGAATATTGCTCGCCCTCG	AAATCCTTTTCCCACCCAAC	220
Ad01A11531	Aradu.A01	11531	c	(TAAA)5agga	41	58411331	58411371	TTCTTGTCCTTGCTCCTGCT	CGAAAATCATTAACCCTTTTTCA	273
Ad01A11534	Aradu.A01	11534	p2	(GA)9	18	58444469	58444486	GGAAAAGGTAAACGCCAACA	GCTCCTTTGACCTCAGCATT	227
Ad01A11538	Aradu.A01	11538	p2	(GA)8	16	58456212	58456227	TCCAAACACAAGGGTAAGGAT	TGAAAATTTCTCCTTCCTTTTCA	215
Ad01A11540	Aradu.A01	11540	p3	(TTC)11	33	58483799	58483831	ACCCTACCCCCTCTCCCTAT	CTTCCCTTTTCCTTTTTCGG	266
Ad01A11543	Aradu.A01	11543	c	(ATA)6g(TAA	34	58533333	58533366	TGCTTCGGTCCAGGAAAATA	AATTTGACAAGACAAAAACTAAATTG	191

ID	Chromosome	SSR nr.	SSR type	SSR	Size	Start	End	FORWARD PRIMER (5'-3')	REVERSE PRIMER (5'-3')	Product (bp)
Ad01A11544	Aradu.A01	11544	p2	(AC)8	16	58535129	58535144	CCTCCATTCACGCCTTCTAA	GTGTGATTGCAATTTCGTGC	276
Ad01A11546	Aradu.A01	11546	c	(TAA)5tgatgat	103	58551228	58551330	TTCCTAGAGAGAACGGCTCG	AAATTTGGTAAGACAAAAACTAAATTG	223
Ad01A11547	Aradu.A01	11547	p2	(AC)7	14	58554334	58554347	GGTAAGGGTAAGGGTGAGGG	AATGAAAGCACCCCAAATGA	238
Ad01A11548	Aradu.A01	11548	p3	(TTA)7	21	58559822	58559842	GCCTTAGGTGAGGTGCTCTG	GACTGCCCGAATCAAGGATA	222
Ad01A11549	Aradu.A01	11549	p3	(TTG)5	15	58565589	58565603	TATGTGATGTGTGGGGCTTG	CCATAACCGGCACATCTTCT	208
Ad01A11550	Aradu.A01	11550	c	(AT)8ag(AC)8	34	58567941	58567974	CGCGTAAACGATAAACGTGA	TTTATCTTCGCAAGCCGAGT	140
Ad01A11561	Aradu.A01	11561	p3	(ATT)6	18	58653866	58653883	CCAAACATGATGAAATTGTTGG	TTCTTAGAGTGAGTCAGTTCAATGG	235
Ad01A11566	Aradu.A01	11566	p2	(AG)7	14	58671817	58671830	GAGTGGGGAAATCATGGAGA	AAAGGAACATTGAACCTGGA	156
Ad01A11567	Aradu.A01	11567	p5	(AATAA)5	25	58675302	58675326	TTGGCGCTATCATCTTGTTG	TGAATATGCAATTGACACCAAA	258
Ad01A11568	Aradu.A01	11568	p3	(AAG)5	15	58680624	58680638	GCTTTTGTCTTCCCTTTCCC	CCTACTCCCTTTCCCCTCAC	272
Ad01A11570	Aradu.A01	11570	p2	(TA)6	12	58706137	58706148	AAAAAGTAAGGAGAGAGAAAGTGGTT	CAATTGGCTAGAGCGTTGGT	219
Ad01A11572	Aradu.A01	11572	p3	(AGA)6	18	58729053	58729070	CTAGAGGATTCTCCGGCCTT	TGGCCGAACCTACATCTCTC	170
Ad01A11576	Aradu.A01	11576	p2	(CT)7	14	58755560	58755573	CCTAAATGGAGCAGGGATGA	GGTTTTTGGGGAAGATGGAT	202
Ad01A11581	Aradu.A01	11581	p2	(TC)6	12	58787074	58787085	ACCTCCCTCTTTTTCAAAATTC	AGGGTGAAGATAGGGGTTCG	207
Ad01A11583	Aradu.A01	11583	c	(CT)6cctccata(	38	58834047	58834084	CCCCTTTTTCAATCAAAACAC	TTTTGGTTGTTGCACACTCTT	256
Ad01A11585	Aradu.A01	11585	p3	(TTC)9	27	58847093	58847119	TGCAGCCCTGTTTTCTTTTC	CCATGAATGCGAGTATGCAA	209
Ad01A11586	Aradu.A01	11586	p2	(AT)9	18	58862301	58862318	ACTTTGGGGCATTTTATCCC	TCAAATACGGGAAACTTGGG	271
Ad01A11590	Aradu.A01	11590	p2	(TC)8	16	58867108	58867123	TCACCAAGGAATGGTTGAGA	GATCCCATTTTGGGGAAGTT	206
Ad01A11591	Aradu.A01	11591	p2	(GC)6	12	58867312	58867323	GAACTTCCCCAAAATGGGAT	CCAGAAGCCAAAGTCAGGAG	228
Ad01A11592	Aradu.A01	11592	p2	(TC)8	16	58868583	58868598	ATATGGCCGAACCTTAACCC	CTAGAGGTTTCTCCGGCCTT	257
Ad01A11593	Aradu.A01	11593	p3	(CAT)5	15	58883389	58883403	GAGGAAGGTGGGACAACAGA	AGAGGTGATGGAGCTCAGGA	266
Ad01A11595	Aradu.A01	11595	p2	(TA)10	20	58915666	58915685	GGCCGATCCTAGAGCTTCTT	AGTGCAACACTCACAATCGG	124
Ad01A11597	Aradu.A01	11597	p2	(TA)7	14	58923102	58923115	GACTCCCAAACCCTTGTGAA	CTCTAGGATCGACCTGCGAA	162
Ad01A11604	Aradu.A01	11604	c	(TTA)13ttcaaa	107	58969171	58969277	TTCCCAGCATAATACCAATTCA	AGGTGTAAAGCCCTGTGTGG	188
Ad01A11606	Aradu.A01	11606	p2	(AT)7	14	58986679	58986692	ATGGACAGTCCCTCTATGCG	GTTCCCGATTTGCGATTTTA	276
Ad01A11607	Aradu.A01	11607	p2	(CT)8	16	58990753	58990768	TGAAGAAACAAACGGAAGCA	TTCCAAGCCCGAAAACATAG	267
Ad01A11608	Aradu.A01	11608	p2	(TG)27	54	58991431	58991484	TCATGCCGAGTTCAAGAGAA	ATTTGAGCCCTTGAATTTGC	228
Ad01A11615	Aradu.A01	11615	p3	(TAC)5	15	59042729	59042743	GAAAATTGTGGGAACATGGG	TGGGGTAATTTTGTCCCCTT	229
Ad01A11616	Aradu.A01	11616	p2	(AT)12	24	59051965	59051988	TTTGAGGAGAGAGAAAGGGG	CTTGGTGTTTTCCCTCCAAA	276
Ad01A11619	Aradu.A01	11619	p3	(ATA)5	15	59062540	59062554	GAACAAGAGAAGGAGATGGCA	TGGAAGCCCATAAAACTTGC	195
Ad01A11620	Aradu.A01	11620	p3	(TCT)5	15	59065687	59065701	CCCCACCTACCTTCCAAAAT	TAAAATATTGCTCGTCCCCG	230
Ad01A11621	Aradu.A01	11621	c	(CTT)58atttctt	204	59070916	59071119	CTTCTTCACGGTGCTCTTCC	TGTACATGCTGTCGCAACAAT	276
Ad01A11626	Aradu.A01	11626	p3	(AGA)6	18	59131396	59131413	GCTTTTGTCTTCCCTTTCCC	CACCTTGACCGAAACACCTT	198
Ad01A11627	Aradu.A01	11627	p2	(TC)7	14	59147934	59147947	TTTTTCCACTTCCAATTTTCG	TCGAACACAAAGGGAGAGAGA	177

ID	Chromosome	SSR nr.	SSR type	SSR	Size	Start	End	FORWARD PRIMER (5'-3')	REVERSE PRIMER (5'-3')	Product (bp)
Ad01A11628	Aradu.A01	11628	p3	(AGA)6	18	59182503	59182520	AGTGGTCTTGATGGGCTTTG	TACACCTCTCCCCCTCTCCT	235
Ad01A11630	Aradu.A01	11630	p3	(ATT)5	15	59189783	59189797	CCATGTGCTTCACTTTTTGC	GGAAGCATGCTCATGTGAAA	118
Ad01A11631	Aradu.A01	11631	p2	(AT)8	16	59197671	59197686	GATGCCAAAGGCAATCCTAA	GATCGCATGGAGGATCAGTT	221
Ad01A11632	Aradu.A01	11632	p3	(AGA)5	15	59226429	59226443	CTAGAGGTTTCTCCGGCCTT	ACCACAAAGCCATCTCCATC	164
Ad01A11634	Aradu.A01	11634	p2	(GT)9	18	59239909	59239926	GAATCGAAGCACCGTAGCTC	TTAAAAACGCTAACGGGGTG	208
Ad01A11635	Aradu.A01	11635	p3	(TTA)17	51	59243471	59243521	TCAAGTGTCCAACTCAACCAA	CCATATGCATTTCTCCTGGAA	252
Ad01A11636	Aradu.A01	11636	p3	(AGA)6	18	59253348	59253365	TGCCATTGATGGTAGTGGAA	ACACACTACCCCCTCTCCCT	225
Ad01A11637	Aradu.A01	11637	c	(A)10gataaa(T	27	59257198	59257224	CAAACAAAAAGTCAAAGAGTTAGTTG	CAATTAATGTGATTGGTTCAAAAA	278
Ad01A11640	Aradu.A01	11640	p2	(CT)16	32	59273513	59273544	GCACACTCTTTTTGCCTTGG	TTGTTGGCTCTTGAAAGAATGA	215
Ad01A11646	Aradu.A01	11646	p2	(GT)7	14	59349409	59349422	GATTTGGGAACCAGGGTTTT	TTAAAGAAATCGGCCGAAGA	186
Ad01A11650	Aradu.A01	11650	p2	(AT)8	16	59367957	59367972	TTTGTCTGCGCTTGAATTTTT	TCACTATTGTGCCATGTGTGTG	106
Ad01A11652	Aradu.A01	11652	p3	(TTA)8	24	59370765	59370788	TTTTCCGTATCTCCATTCATTG	TTGTCCCATTCTCTTATCATTCC	280
Ad01A11654	Aradu.A01	11654	p2	(TA)6	12	59378528	59378539	GTGAGTTTCAAGGGTTGGGA	TGAATTCCTGTTGCACATCC	192
Ad01A11656	Aradu.A01	11656	p3	(AAG)5	15	59388677	59388691	CCAGTTGAAGCATCAGCAAA	TCCACTGCATCACCATTTGT	125
Ad01A11657	Aradu.A01	11657	p3	(AAG)5	15	59388890	59388904	ACAAATGGTGATGCAGTGGA	TTTCAGCTTATTCGGCATCC	256
Ad01A11659	Aradu.A01	11659	p3	(AAT)5	15	59390787	59390801	GTGAACTTCCATCCTTCCGA	GATAACCCAGGAAACGGTCA	149
Ad01A11660	Aradu.A01	11660	p3	(AAG)5	15	59405429	59405443	GCCCTTTTCGTATCCATTCA	CGACAATGAAAATGACGGTG	190
Ad01A11661	Aradu.A01	11661	p3	(AAG)5	15	59417427	59417441	GCCCTTTTCGTATCCATTCA	CGACAATGAAAATGACGGTG	190
Ad01A11662	Aradu.A01	11662	p3	(GGA)5	15	59418552	59418566	CCTCCATCAATTGCTCCTTG	TCAAGGTTCCTGTGAGTCCC	247
Ad01A11663	Aradu.A01	11663	p3	(TCT)5	15	59424887	59424901	TTCCTCTACTGGCAATTCCG	GTTTCCGCTGTGGAGAGAAG	137
Ad01A11664	Aradu.A01	11664	p2	(AT)11	22	59428145	59428166	GCAGTGTATTATAGATGTGCTAAGGG	GCCTTAGGTCCCTCTTTTCTTT	280
Ad01A11672	Aradu.A01	11672	p2	(AG)16	32	59461947	59461978	AACAAAACGACATTGCCCTC	CAGAAACACGCGTAGTGGAA	101
Ad01A11673	Aradu.A01	11673	p2	(GA)14	28	59462109	59462136	TTCCACTACGCGTGTTTCTG	AGGCTTAGCGGAACACTTGA	240
Ad01A11679	Aradu.A01	11679	p3	(TCT)6	18	59481171	59481188	AAACCCACACCTCTCTCCCT	GAATTGCCTTCCCTTTCCTC	186
Ad01A11680	Aradu.A01	11680	c	(A)11gaaaacat	90	59485765	59485854	CCCAAATAAATATCCAACACCAA	CCCCACTTCTTCCTTCTTCC	200
Ad01A11683	Aradu.A01	11683	p3	(ACG)5	15	59509104	59509118	CAGAAACGACGATCAAGCAA	ACTGTCATCGCCGAACTCTT	228
Ad01A11684	Aradu.A01	11684	c	(GCT)5ggct(G	150	59509263	59509412	AAGAGTTCGGCGATGACAGT	TAGCAGAGCCTTCCCACTCT	224
Ad01A11689	Aradu.A01	11689	p3	(TAT)16	48	59521839	59521886	CTTGGCAATTTGTCCATCACT	TCCTTGCCTTCTCTTTTCCA	280
Ad01A11690	Aradu.A01	11690	p2	(CT)6	12	59522337	59522348	GAAGCACTTTAGGCAATCCG	TTTTTCCTTCAACATAGTTTTCCTG	258
Ad01A11692	Aradu.A01	11692	p3	(AGA)9	27	59538865	59538891	TTGCCTTCCCTTTCCTCTTT	GAAACCCTCACCTCTCTCCC	193
Ad01A11694	Aradu.A01	11694	p2	(CT)6	12	59548422	59548433	AAGAAGAAATTGAAAGAAGAACCC	TGCCTGATGTTTAAGGACCC	269
Ad01A11697	Aradu.A01	11697	p2	(AG)6	12	59556848	59556859	GCTATAAAGTGGGGGAATGC	TCATAAGCCAATTGGGAAACA	258
Ad01A11698	Aradu.A01	11698	p4	(ATTT)6	24	59558519	59558542	TGCAAATGCAATAAACAAAAGAA	TATGTGTGGGCAACGCTTAC	278
Ad01A11700	Aradu.A01	11700	p2	(AT)6	12	59569030	59569041	GCATTTGTTGGTTCGTGATG	TTTTTAGAGTTGTCCAAACTTATCAAT	245

ID	Chromosome	SSR nr.	SSR type	SSR	Size	Start	End	FORWARD PRIMER (5'-3')	REVERSE PRIMER (5'-3')	Product (bp)
Ad01A11705	Aradu.A01	11705	p5	(TTGAT)5	25	59574180	59574204	CCAGGCGCTTCTATTTTCAG	TTGCCTAGCATCATATTCATTCA	274
Ad01A11708	Aradu.A01	11708	p2	(AG)6	12	59590216	59590227	ATTTCTGTCTGCAACACCTACA	CCACCCCTAGTGATTGGTTG	275
Ad01A11710	Aradu.A01	11710	p3	(TCC)5	15	59600362	59600376	TGCTGGTTAGTTGTGCGAAG	AAACACGCGAAGAAAAATGG	156
Ad01A11714	Aradu.A01	11714	p2	(TC)25	50	59609211	59609260	CCTTTTTAAGCTGCCTTTGC	TCCTCTTCATCTTCTTCTGCTTC	257
Ad01A11715	Aradu.A01	11715	c	(AGG)6aagagg	67	59610136	59610202	TGATGGACGAGAAGGAGAAAA	CATTTACATTCATGCGACCG	201
Ad01A11718	Aradu.A01	11718	p2	(AG)6	12	59649099	59649110	GGGAAGCTTAAAAACAAAGGAA	TTTTGACCCAGTTTTCAGCC	230
Ad01A11719	Aradu.A01	11719	p2	(AC)6	12	59650584	59650595	GCTTTTTCTCCTGCTTCCCT	TGTGACCTAGCTTTCCATGCT	231
Ad01A11720	Aradu.A01	11720	p2	(AG)6	12	59653343	59653354	GGGAGTGGAGCAGAATCAGA	AGCCAATTGGGCAACATAAA	230
Ad01A11722	Aradu.A01	11722	p2	(TA)6	12	59691791	59691802	GAAGTGCGAAACACACAGGA	CAAAACAATCCACACACCTTG	262
Ad01A11723	Aradu.A01	11723	c	(GA)6gtgagaga	121	59723791	59723911	GGCCAGGAGAAAGAGGAGAG	CAACTGCCGTCGTAGTAGCA	250
Ad01A11731	Aradu.A01	11731	p2	(TA)8	16	59816310	59816325	CCGGTTAGTTTGTGGGTTGT	ACTACGGGGACACCAAACTT	274
Ad01A11733	Aradu.A01	11733	p3	(GAA)5	15	59831646	59831660	CCCAAGACTCAGAGGTGGAA	ATCTCCCCTCACTCACCTCC	192
Ad01A11738	Aradu.A01	11738	p5	(AAGAT)5	25	59896813	59896837	GCGGTTCCCTTGAAGATACA	GCTGATTCCTTCGTCTCTGG	229
Ad01A11743	Aradu.A01	11743	p3	(AGA)6	18	59959402	59959419	TAAAATATTGCTCGCCCTCG	TCTTCTCCCACTTAGCCGAA	104
Ad01A11744	Aradu.A01	11744	p3	(TCT)5	15	59972104	59972118	CCCATCACCTCTTCACCACT	TTAGAATGTTGCTCGCCCTT	280
Ad01A11747	Aradu.A01	11747	p2	(CT)8	16	59985834	59985849	AATTACAGAACACGCCCCAG	TCCTAATCAGAACCAAAAGGGA	267
Ad01A11750	Aradu.A01	11750	p6	(CACACG)6	36	60029278	60029313	ACACACACATTTATACACACACATTT	TGATATTATGTGCGTGTGCG	126
Ad01A11751	Aradu.A01	11751	p3	(GTT)5	15	60046707	60046721	TGTAGCACAAATCCCATCCA	CTTTGCACCTAGAGGTTCGC	176
Ad01A11754	Aradu.A01	11754	c	(A)11gagaaaga	74	60090555	60090628	TGTGGTTCATGAATGTTGGC	TCCCCAATTAGAGGTGTCCA	265
Ad01A11756	Aradu.A01	11756	p2	(AT)6	12	60115311	60115322	TTGAGATTGCATGTGTTCTGTG	TCTCATGTCAAAAACAAAATTACTGA	216
Ad01A11758	Aradu.A01	11758	c	(CTT)5ctcctcc	78	60117330	60117407	CAACGCCCGAGAATAGAGAC	ACATAGTGGTTCCGTGCTCC	280
Ad01A11760	Aradu.A01	11760	p3	(GAG)5	15	60128709	60128723	CGGGTATGGGGATAGATGTG	GCAGTGCCAGGGTAACTAGC	237
Ad01A11761	Aradu.A01	11761	p5	(TAAAA)5	25	60134217	60134241	AAAAGCTGATCGGCTAATTAAA	GACCCTTATTTGTTGCACCC	154
Ad01A11763	Aradu.A01	11763	p2	(TC)19	38	60136758	60136795	CCGGTTTTCAGAACATTGGA	CACTGCTCAATGGCAAAAGA	236
Ad01A11764	Aradu.A01	11764	p2	(TC)21	42	60137138	60137179	CCGGTTTTCAGAACATTGGA	CACTGCTCAATGGCAAAAGA	240
Ad01A11766	Aradu.A01	11766	p3	(ATA)25	75	60139163	60139237	ATATGCCATGCCCTGAAACT	CAGGCACCAAACTATGAGCA	213
Ad01A11767	Aradu.A01	11767	c	(AAC)6agcag(	67	60140004	60140070	TAAAGTGCGCCGTAAACCTT	TGAGCTCGCTAATGTTGGTG	210
Ad01A11768	Aradu.A01	11768	c	(TA)7(TG)10t	218	60142926	60143143	TTGGGGGACATTTATGTTTTG	CAGATAACAGCGTACAGAAAGGA	280
Ad01A11769	Aradu.A01	11769	p2	(CT)9	18	60157210	60157227	TCTTTTTCCCGGAGTACCCT	ATAGCTCGGCGCTAGAATGA	246
Ad01A11771	Aradu.A01	11771	p2	(AG)9	18	60167854	60167871	ATCCAAGCCCAAAGAACACA	GCCAATCTGGCAACATAAATC	280
Ad01A11772	Aradu.A01	11772	p2	(TA)6	12	60181378	60181389	TTCTGTATTTTACACTTTGGGCTG	GGTGGCCCTTAAAATCTTAGTGT	271
Ad01A11773	Aradu.A01	11773	p2	(AG)6	12	60183432	60183443	CAGGCCCAGAGAACACAGAT	CATAAGCCAATCTGGCAACA	241
Ad01A11774	Aradu.A01	11774	c	(T)10acataaaat	59	60186631	60186689	CTTGGTGTTTTCCCTCCAAA	TGTTGATTTAAAGAAAATGGAATAAG	272
Ad01A11776	Aradu.A01	11776	p3	(TAG)5	15	60221996	60222010	AGCTGAAGCGCCAGAAATTA	AACTTAATTGGGAATAAAACCAGAA	205

ID	Chromosome	SSR nr.	SSR type	SSR	Size	Start	End	FORWARD PRIMER (5'-3')	REVERSE PRIMER (5'-3')	Product (bp)
Ad01A11788	Aradu.A01	11788	p4	(AAAG)5	20	60316713	60316732	AATCCTAGCCACCACCCTTT	TCCACCTTCCTTCTTTCCCT	127
Ad01A11789	Aradu.A01	11789	p2	(GA)8	16	60316862	60316877	AGGGAAAGAAGGAAGGTGGA	TTTGAGGTTCTATCGGTGGC	258
Ad01A11790	Aradu.A01	11790	p2	(AG)6	12	60317014	60317025	GCCACCGATAGAACCTCAAA	CATCTTCAGCAGCAGCGATA	143
Ad01A11795	Aradu.A01	11795	p3	(TTC)6	18	60357164	60357181	AAACTTTAGAGGATCCAAATCCAA	GGTGAAGACTTATCTGGTGATGG	197
Ad01A11796	Aradu.A01	11796	p3	(TAC)5	15	60371426	60371440	CAACAAGTATTGCCACCACA	TCTCAAAACACAGGTTGCTGA	270
Ad01A11800	Aradu.A01	11800	p3	(TAT)15	45	60397738	60397782	GGGCAAGAAAAGTTAGGTGAC	TTTAAATTCAAGAATAACGGTGTTT	280
Ad01A11801	Aradu.A01	11801	p2	(TA)6	12	60399345	60399356	TTGGATTTAGGGGATTCAGG	CATTTTCTTCCCCAGCATGT	169
Ad01A11802	Aradu.A01	11802	p3	(TTA)6	18	60403079	60403096	GGATGACAAATTGAAGGAAACA	AATTTGCACTATCCGCAAGG	278
Ad01A11803	Aradu.A01	11803	c	(T)10aaatatcgt	64	60408465	60408528	CGGGTGAAATTGACATCTGA	ATGAAGCAACAATGGCACAA	271
Ad01A11804	Aradu.A01	11804	p3	(TAT)12	36	60431103	60431138	TGAGTTGTTGATCCCAACCAT	CCATCCGCTCTTAGTGAAGC	216
Ad01A11806	Aradu.A01	11806	p3	(GAG)6	18	60443698	60443715	GAGAGGAGGGGGAAAAGAAA	GTGATTATATGCGTGGGGCT	175
Ad01A11808	Aradu.A01	11808	p2	(AT)9	18	60460631	60460648	CCCTTTCAACTACAGGTGCG	TTCATGAATATGAAACGGAAGTT	270
Ad01A11809	Aradu.A01	11809	c	(AAT)15attnnı	95	60461839	60461933	TCTGCTTTGCAATCACACTTC	TCGGTAAAAATGAATGGATGAA	260
Ad01A11810	Aradu.A01	11810	c	(T)12gaataactc	95	60470534	60470628	GAAACCATAAATCTCTTCTGCCA	TTCACAAATCAATCCGCTCTT	269
Ad01A11814	Aradu.A01	11814	p3	(AAT)7	21	60481110	60481130	AAGAGGTGAAAGCACTTCTGG	TTTGGCAAGGTTACGTTCAT	206
Ad01A11815	Aradu.A01	11815	p2	(TA)6	12	60487075	60487086	CCTAACTCAGCCTGCAAAGC	CGATAGGCACCTCGCTAGAC	258
Ad01A11816	Aradu.A01	11816	p2	(TA)7	14	60489818	60489831	GGAGCATTTTTGTCCCCTTT	CTTGGGACATTGGTTGAGGT	240
Ad01A11821	Aradu.A01	11821	p2	(TA)7	14	60522572	60522585	TTCACCACTTACTTTGGGGC	TAAGTGTGGGGAAAAGTGGG	214
Ad01A11822	Aradu.A01	11822	p2	(AG)6	12	60527232	60527243	CGGAAAATATCAACAGCCAA	GCATCCATTCATAACGAGGC	267
Ad01A11823	Aradu.A01	11823	p2	(AG)6	12	60528937	60528948	AAACTGAAAATGCCTCAATACCA	GGGCGGGCATAATTTTAGTT	256
Ad01A11824	Aradu.A01	11824	p2	(TA)6	12	60539504	60539515	GGAAGGCAATGCAACCTAAA	TTTCTTTCCCATCTCATCTGC	184
Ad01A11826	Aradu.A01	11826	p2	(AG)6	12	60546722	60546733	CAATTGGGCAACATCAATCA	GGGCGGGCATAATTTTAGTT	240
Ad01A11830	Aradu.A01	11830	p2	(TA)6	12	60634892	60634903	TTACGTGATGTGGTGGCATT	TGCTCTTGCCTTTTTGGTTT	265
Ad01A11831	Aradu.A01	11831	p2	(AC)6	12	60651781	60651792	AGGTTGTAATGGAGCCAAGG	AAATAAGGGGACAAAATTACGC	260
Ad01A11834	Aradu.A01	11834	c	(T)10cgaaatttta	66	60670303	60670368	TTGGTGTCATTTTGTTGTTTTTC	CCATGATGGAGAAAGGGAAT	260
Ad01A11846	Aradu.A01	11846	p3	(GAT)5	15	60768134	60768148	GGTCATCTTGTGGGTAGCGT	ACACGAAAGACCACGACCTC	244
Ad01A11847	Aradu.A01	11847	p2	(TG)7	14	60804887	60804900	GGAAACACAGTTGCAAGCATT	GCAGGTAGGATGACCAACCA	266
Ad01A11850	Aradu.A01	11850	p2	(TA)8	16	60817595	60817610	TTGGATAGTCGGATCCATTG	GCCTTAGGTCCCTCATTGTTT	273
Ad01A11851	Aradu.A01	11851	p3	(TTA)6	18	60821293	60821310	GCGACCCTCAGTTTCAAAAA	ATAAACCGCCTTTGGTTTCC	232
Ad01A11853	Aradu.A01	11853	p2	(AT)6	12	60835240	60835251	AACACCCGCATTTGAATAAGA	CCACAATGGTGAAGCACAAA	222
Ad01A11855	Aradu.A01	11855	p2	(CG)6	12	60844490	60844501	GAGGAAGGAGAAAAGCAGGG	ATAGGGAGCTCTTGGGCATT	279
Ad01A11856	Aradu.A01	11856	c	(GC)6acagcgtg	50	60844607	60844656	GAGGAAGGAGAAAAGCAGGG	ATAGGGAGCTCTTGGGCATT	279
Ad01A11858	Aradu.A01	11858	p2	(GC)6	12	60857432	60857443	ACAAGGCTCCTAAAACCGCT	CGGTTCTAGTTGGGTTGGAA	168
Ad01A11859	Aradu.A01	11859	p2	(TG)6	12	60862197	60862208	TTCCTGTAGAGCCTGAGGGA	TAGTACGGCCTCTACCACGG	267

ID	Chromosome	SSR nr.	SSR type	SSR	Size	Start	End	FORWARD PRIMER (5'-3')	REVERSE PRIMER (5'-3')	Product (bp)
Ad01A11860	Aradu.A01	11860	p3	(AGA)5	15	60875449	60875463	TAAAATATTGCTCGCCCTCG	CCCATAAACCCCACCTACCT	241
Ad01A11863	Aradu.A01	11863	p3	(TCT)5	15	60893291	60893305	TCGAGTTTGGACAACTGACG	ACACGCCTGACATTCCCTAC	246
Ad01A11866	Aradu.A01	11866	c	(AT)7agaaaaaa	123	60911507	60911629	AAACCATATGCATTGATACTCTTG	CCTAACTACCCATGATTCCCTC	268
Ad01A11873	Aradu.A01	11873	c	(AT)7ag(A)10	26	60959271	60959296	TGAAGATCTTGGGAATTGGG	ACCCATGATTCCCACTTTTT	226
Ad01A11876	Aradu.A01	11876	p5	(CAGTT)5	25	61007349	61007373	AGAGGACCAAACACAGCCAC	TGAACTGAACTGAACTGAACTGC	246
Ad01A11877	Aradu.A01	11877	p5	(CAGTT)5	25	61007711	61007735	TGACAACACAACACAATGCAG	TGTTCGGTGAACTTGCTCTG	239
Ad01A11880	Aradu.A01	11880	p2	(CT)6	12	61053563	61053574	CCTCTCACTCCATCCCACTC	GTGGAAGAGGAAAAGGGGAG	248
Ad01A11882	Aradu.A01	11882	c	(T)10ctttttctttt	47	61076398	61076444	TCCCCAATTAGAGGTGTCCA	AAGCAAGAAAAAGCAGTGAGC	130
Ad01A11889	Aradu.A01	11889	p3	(TAA)21	63	61149536	61149598	GCAAGATTGGAAATTCTATTATGTGT	GCAACATGCTCTGAATTTTGAC	253
Ad01A11895	Aradu.A01	11895	p3	(AAT)5	15	61171626	61171640	TTGTGCAACCCAATTCAATAA	GCAACATGCTCTGATTTTGG	275
Ad01A11896	Aradu.A01	11896	p2	(AG)13	26	61173296	61173321	GCGCGAGAGAGAAAGAGAGA	TGCATGAAGTAAAATGGCTGTT	229
Ad01A11897	Aradu.A01	11897	p2	(AT)6	12	61173978	61173989	TTGTACTTTTGCATCACCCG	AAACATGTATTTTGTTTGAGTTAATGA	280
Ad01A11900	Aradu.A01	11900	p2	(TA)6	12	61187761	61187772	GGTTGTGTATGTGTTGGGTGA	TTGAACCCTTACCGGATGTG	251
Ad01A11901	Aradu.A01	11901	p3	(TTC)6	18	61194453	61194470	TCTTCTCCCACTTAGCCGAA	GCTTTTGTCTTCCCTTTCCC	207
Ad01A11903	Aradu.A01	11903	p3	(TCT)6	18	61210179	61210196	AATCTTTCCCATCCAAACCC	CCTTAGGTGCCATGAGTGGT	272
Ad01A11904	Aradu.A01	11904	c	(AT)8aaatatata	126	61231330	61231455	GAAGATTTTTGGGAAATTGGG	TCCATGATTCTCACTTTTTCACAT	268
Ad01A11905	Aradu.A01	11905	p2	(AT)6	12	61235326	61235337	GATCATACCAAGAGCTCAGGC	TTCCCTTGTCGTTTATTGTTTTT	250
Ad01A11906	Aradu.A01	11906	p4	(TTCT)5	20	61236009	61236028	CTCGTTCTCAATGCCAAACA	CCTCTGCCATAGGAGAGCAG	192
Ad01A11909	Aradu.A01	11909	p2	(AT)6	12	61248290	61248301	CACCATGCATGAGTTTTTCG	GGGGATTGTTTGAGTTTGGA	221
Ad01A11912	Aradu.A01	11912	p3	(AGA)6	18	61303157	61303174	TGCCTTCCCTTTCCTCTTTT	CTTGGCCGAAACATACACCT	190
Ad01A11913	Aradu.A01	11913	p2	(GA)6	12	61306373	61306384	AGAGGAGAAGAGAGGGTTCG	TCGAAAATTCCTCACCCTTTT	209
Ad01A11914	Aradu.A01	11914	c	(T)10gaaaattaa	206	61307405	61307610	TGAAGATCATGAAGAACACATGC	CGGTTCATCTTGTTGCTCAG	271
Ad01A11917	Aradu.A01	11917	p4	(TATT)5	20	61320860	61320879	TTCGAATTCTCCCTCTCTTCTC	TCCAATCACAATGGTGAGGA	115
Ad01A11919	Aradu.A01	11919	c	(TA)7ttgtatccc	54	61356611	61356664	TTGTCCCATGTGCATTGATT	GTGCTAGGTGGGAGACAACC	239
Ad01A11920	Aradu.A01	11920	c	(T)12agaatcact	33	61373363	61373395	TCCCCAATTAGAGGTGTCCA	TGTGGTTCATGAATGTTGGC	241
Ad01A11924	Aradu.A01	11924	p3	(ATA)5	15	61414239	61414253	TCGTGAGTTTCAGAACATTGC	CGGGTCGGGGAGTAAATAAG	262
Ad01A11925	Aradu.A01	11925	p2	(CT)6	12	61423533	61423544	ACCTCTCCGTATGCTGGATG	CACCAGTTGCATGCTTGATT	259
Ad01A11927	Aradu.A01	11927	p2	(CT)6	12	61429014	61429025	CCCTTGCTTCAAAACCTCAG	CAAGCTGCTAAAGGGTCAGC	215
Ad01A11929	Aradu.A01	11929	p2	(TA)9	18	61435212	61435229	AAACTGTCGATAAAAATTCAACCA	AGACGGAGACGGAGAACAAA	273
Ad01A11930	Aradu.A01	11930	p2	(TA)8	16	61437653	61437668	GCATTATGCAAGCCCACTAA	GCAACCAAACTCCTCCAAGT	116
Ad01A11932	Aradu.A01	11932	p2	(TC)11	22	61438695	61438716	TTGCTGAATGGCTATTTCCC	TCCAAGCTGGAACTGCTTCT	266
Ad01A11933	Aradu.A01	11933	c	(GAA)6agagaa	100	61442265	61442364	GAACAACGTGCAGCAAGAAA	TAAACCCCAAAAATGCGATG	224
Ad01A11934	Aradu.A01	11934	p3	(TAA)5	15	61446077	61446091	AGCCCATGGAGTTTTGTTTG	CCGAGATGTTATAGTTTGAGTGTCA	215
Ad01A11936	Aradu.A01	11936	p2	(TA)6	12	61448619	61448630	GTCCGGAGTGTTGGGTTGTA	AGTCCATATCCCCAGGCTTT	261

ID	Chromosome	SSR nr.	SSR type	SSR	Size	Start	End	FORWARD PRIMER (5'-3')	REVERSE PRIMER (5'-3')	Product (bp)
Ad01A11937	Aradu.A01	11937	c	(TTA)17gttact	84	61450659	61450742	AAACTATTTTGATAGGCAAAAATTAAG	CAAGCCACGTTTCCTTTCTT	254
Ad01A11938	Aradu.A01	11938	p2	(AT)6	12	61452870	61452881	AGGGCTAGGAGTGTTGGGTT	GTGTAACGACCCAATTCCCA	222
Ad01A11939	Aradu.A01	11939	p2	(AT)14	28	61482612	61482639	CAAATCCTTTGCACACCAGA	TGGATTGGATGATGTTGCAT	191
Ad01A11950	Aradu.A01	11950	p2	(CT)8	16	61603837	61603852	CTCAACCACAACTGCCAAAA	GCACCATGTGAAAAGGAAAGA	266
Ad01A11953	Aradu.A01	11953	p2	(AT)9	18	61627876	61627893	GAATTCTGAGCAAAAATCTAACTCAA	TTGTGCAATGGTTGCATCTT	103
Ad01A11954	Aradu.A01	11954	p2	(TA)7	14	61629874	61629887	TTGCACGAAAATTGTTGAGG	TTAGTTTCACCCAAGGCAGC	271
Ad01A11955	Aradu.A01	11955	p2	(TA)7	14	61632218	61632231	TTTTCAGATGCAGGTCGAGA	AAGTGCAACGCTCAGGAAAG	269
Ad01A11956	Aradu.A01	11956	p2	(TA)6	12	61632461	61632472	CTTTCCTGAGCGTTGCACTT	TGTCTGCATCGTCAAATAGTGTT	280
Ad01A11957	Aradu.A01	11957	p2	(TC)6	12	61637971	61637982	CGAAAATCGTCTGTGTCCCT	CATCTTCAATGCGATTCATTTC	272
Ad01A11958	Aradu.A01	11958	c	(T)11aatggttcg	205	61638094	61638298	TGTCATGTGTTAGTTTGATCCC	TCCTACTTTGTTCCTACATCTTGC	280
Ad01A11959	Aradu.A01	11959	p2	(CT)6	12	61641438	61641449	AGTCAGGGAACTGGCAGAGA	GAACCTGGGATCGAGAGTCA	149
Ad01A11962	Aradu.A01	11962	p2	(CT)6	12	61673361	61673372	AGTCAGGGAACTGGCAGAGA	GAACCTGGGATCGAGAGTCA	155
Ad01A11963	Aradu.A01	11963	p3	(AAT)15	45	61681702	61681746	CCAAACATTGACTCTTGAAATGAC	AGGGCCAAATATGTTTGATTT	248
Ad01A11965	Aradu.A01	11965	p2	(AT)7	14	61683982	61683995	TATTGGGTTGAAGATTGGGC	GCTGGATCATCTTTGCCTGT	134
Ad01A11968	Aradu.A01	11968	p4	(AATA)5	20	61707332	61707351	GCCGAGTAGCATGCATAACA	TGTGATCCAACTTGCCAAAA	198
Ad01A11971	Aradu.A01	11971	p2	(CT)6	12	61724221	61724232	AGCCAATTGGGCATCATAAA	GTTTTCGGCCCAGAAACATA	265
Ad01A11973	Aradu.A01	11973	p3	(AGA)6	18	61740065	61740082	TTAGGTGCCATTGATGGTGA	TCTTCTCCCACTTAGCCGAA	163
Ad01A11978	Aradu.A01	11978	p3	(TCT)6	18	61807591	61807608	AATGGCCGAAACACAAACAT	CCGGTCTTAGGTGCCATAAA	158
Ad01A11979	Aradu.A01	11979	p2	(AT)6	12	61819624	61819635	CATAGAGCCTGAGGGATGGA	CCACGAGGTGCTATTTGGTT	210
Ad01A11983	Aradu.A01	11983	p3	(TTC)6	18	61901705	61901722	CAACTTCTCTTTCCCACCCA	ACCACTGAGCTATGACGGCT	276
Ad01A11987	Aradu.A01	11987	p4	(AGTT)7	28	61922772	61922799	CCCTTCAACCATCATCCAAC	TCAACCTCAACAAAGAAATCCA	236
Ad01A11991	Aradu.A01	11991	p3	(ATA)8	24	61938530	61938553	TGTTGTTTTGGCCTCTGATG	ACCCCAGGAAATGTTGCATA	236
Ad01A11996	Aradu.A01	11996	p3	(TTC)6	18	61976253	61976270	AACATACACCCCCTCTCCCT	TGCCATTGATGGTAGTGGAA	224
Ad01A11999	Aradu.A01	11999	p2	(AT)11	22	62013413	62013434	TAAAGTGAAATTCAAAATTTAGAGGA	TTCTTTGCAAAAACATATGACAA	213
Ad01A12002	Aradu.A01	12002	p3	(GCC)5	15	62014394	62014408	CCAATGCCTTCTCTCTCCAT	GAGCTCGTGTGACGATGAAA	136
Ad01A12003	Aradu.A01	12003	p3	(AAT)8	24	62019620	62019643	GGAACAACAAGGGTTTTAGCA	AATCTCCGTGTGATTCGACC	261
Ad01A12006	Aradu.A01	12006	p2	(GA)20	40	62040286	62040325	TGCTCACAATGGGACAAAAA	TGGAAGTTGAAAGGGTGAGAA	170
Ad01A12009	Aradu.A01	12009	p2	(AT)6	12	62043862	62043873	AAACAGAACTCATCGCAGCA	TTCCCCCTTGAAAAATAGCA	228
Ad01A12012	Aradu.A01	12012	p3	(TAA)5	15	62053976	62053990	AAATGAGTGTGAGAGTTTTTCTTTTT	GACCATGGTGTTATCATGTATGC	279
Ad01A12013	Aradu.A01	12013	p3	(TTA)19	57	62054468	62054524	AACGACACCTCGTAGGAAAAA	GTGGCAAGTTGTTACTTTAATGG	258
Ad01A12014	Aradu.A01	12014	p2	(CA)6	12	62055423	62055434	CCCTAACCCTAACACACCCA	ACCCCCTCTCACTCTCCATT	176
Ad01A12015	Aradu.A01	12015	p3	(TCT)5	15	62059210	62059224	TCAAAACCACAGAACCACCA	GCGAGCTAGAGAGCCACAGT	204
Ad01A12017	Aradu.A01	12017	p2	(TC)13	26	62087004	62087029	TGTCGAAAATATCTCATCCCTTT	CAGAAGGGTGAGGATAGGGG	243
Ad01A12018	Aradu.A01	12018	p2	(CT)7	14	62091249	62091262	TTCATCATGGACCTCACAGG	AGGAGATGGAGGGATGTGTG	161

ID	Chromosome	SSR nr.	SSR type	SSR	Size	Start	End	FORWARD PRIMER (5'-3')	REVERSE PRIMER (5'-3')	Product (bp)
Ad01A12019	Aradu.A01	12019	c	(TCT)5attctttc	87	62091375	62091461	ATCTCTTTCCCACCCAATCC	CCTTGAGCTGGAAGCTTTTG	234
Ad01A12020	Aradu.A01	12020	p2	(TA)9	18	62096224	62096241	TGAGGAAGGTGGAAGAGGTG	TTCATTTTGGACTTCACCTTGA	253
Ad01A12028	Aradu.A01	12028	p2	(GC)6	12	62177551	62177562	GGTCCAGCATGAGAAAGCAT	CCAGTTGGGTTGGAAAGCTA	220
Ad01A12030	Aradu.A01	12030	p3	(TCT)6	18	62188396	62188413	TACCTTGGCCGAACCTACAC	ACTCGGAGGTGGAAGCTTTT	253
Ad01A12032	Aradu.A01	12032	p2	(AT)8	16	62202393	62202408	GTGTGGGGAACTGTGGAAAC	CTGGGGTAATTTTGTCCCCT	237
Ad01A12033	Aradu.A01	12033	p2	(AG)6	12	62203721	62203732	AGTTCTCGGCTCAGGAAACA	GGAATTGAGAAGAAGCAACCC	188
Ad01A12034	Aradu.A01	12034	p3	(AGA)11	33	62208957	62208989	GCTTTTGTCTTCCCTTTCCC	CCTCTCTCCCTCTCCTCCAT	197
Ad01A12040	Aradu.A01	12040	p4	(TTTA)5	20	62240385	62240404	AATGGTGCTTTTTCACCCAA	TCATTCTAACAAAACTCCAAACACA	272
Ad01A12041	Aradu.A01	12041	p2	(CT)6	12	62241809	62241820	CATTTTGGTATCCTTGATTTCATC	GCAAGTGTACGAAATCGCAT	246
Ad01A12043	Aradu.A01	12043	c	(T)10gtgtttttcg	41	62256194	62256234	GCATGCAATTGACACCAAAC	AATTCCTGGACCGGAGTCTT	188
Ad01A12045	Aradu.A01	12045	c	(AT)7(GT)23	60	62277490	62277549	TGATAAGTCAAGCGGACATTTG	CGGCCATTCAGCTATGTTTT	244
Ad01A12050	Aradu.A01	12050	p3	(ATT)6	18	62284050	62284067	TTGATTTTTCTTGGCATTTTCTT	AAGGAAAAACATGGTAAATTGCAT	236
Ad01A12051	Aradu.A01	12051	p3	(TAC)5	15	62291267	62291281	TCATGTCTGGTGATGATGAGAA	AGCTGAAGCGCCAGAAATTA	274
Ad01A12053	Aradu.A01	12053	c	(T)11aaaaaattc	97	62324880	62324976	TTTCGAAAACAAGGAGCATT	TGAGGAGAGAGAAAGTGGTTAGG	275
Ad01A12056	Aradu.A01	12056	p2	(TC)7	14	62345948	62345961	TTTCGAAAATATCTCATCCCTTC	GAGGCAGTGATTGGAGATGAA	217
Ad01A12057	Aradu.A01	12057	p2	(TA)6	12	62361338	62361349	TGTTTTTCAGATGCAGGTCG	TAAAGCTGAGGTGGCAAGGT	247
Ad01A12060	Aradu.A01	12060	c	(CT)9t(TC)19	57	62366778	62366834	TGTTGGTTGGGTCATTTGAA	GAAAAGGGATGACGCTGGTA	276
Ad01A12061	Aradu.A01	12061	p3	(AAT)5	15	62367278	62367292	CCCACGGAGATTGTTGGTAT	ACAACGGTGATGCCCTACAT	271
Ad01A12062	Aradu.A01	12062	p3	(TCT)5	15	62377549	62377563	ACCTCCCCTACTCTCCTCCA	TTTCCATCTCCCATGACTCC	210
Ad01A12065	Aradu.A01	12065	c	(A)10gaagaaaa	56	62439697	62439752	GGTGGTATGCATGCTTGATT	AAGTTCAAACCATTGGTGCC	221
Ad01A12066	Aradu.A01	12066	p3	(AAG)5	15	62458449	62458463	TGCCATTGATGGTTATGGAA	ACCGAACCCACACATCTCTC	144
Ad01A12068	Aradu.A01	12068	p3	(TCT)5	15	62482364	62482378	TAGCGTGGCTTTTGTTGTTG	AGGAAATGCGAAAAGGTTGA	244
Ad01A12070	Aradu.A01	12070	p3	(TTG)7	21	62501109	62501129	GAAGTGCCAGGAAATGTGGT	GGCATTCCCCAACATCATAC	227
Ad01A12074	Aradu.A01	12074	p3	(AGT)5	15	62541910	62541924	AATTCATTAGGGGGCTCTGG	GTGGGGTTAATGGATCATCG	259
Ad01A12075	Aradu.A01	12075	c	(CA)7gacactca	66	62554363	62554428	ATCTGCCCAACACACTCACA	GACTTGTCATCAATGCTCCTG	160
Ad01A12076	Aradu.A01	12076	p3	(AAG)5	15	62562276	62562290	GGATCGTAGGTGCCCTATTG	TTGATGCAAAAAGGGAAACA	279
Ad01A12079	Aradu.A01	12079	c	(AT)13gtatgtat	54	62591360	62591413	CATGCCGTAAAGCAAAGGTT	TTCACGCTTTGTAATTCCGT	173
Ad01A12080	Aradu.A01	12080	p3	(GAA)5	15	62601842	62601856	ACGGAAGGGAGGAGGAGATA	ATAACCCTAATGCGCGACAC	204
Ad01A12081	Aradu.A01	12081	p2	(TA)6	12	62608696	62608707	ACGAACCGCTCTGATACCAC	GTGAATGTTCGCGCTTTGTA	133
Ad01A12085	Aradu.A01	12085	p2	(TA)6	12	62632829	62632840	ACAGAGTTTGGAAGCCAGGA	CTCCTTTGTCCTTTTCGTGC	265
Ad01A12087	Aradu.A01	12087	p3	(AAT)7	21	62637666	62637686	CCTTGAAGGAAAAGAAACAGCA	AGAAAGTGGTGCTCAAAGCC	253
Ad01A12091	Aradu.A01	12091	p2	(AG)6	12	62653585	62653596	CCTGGTAAAAAGATCATCCTTGA	TAGCCCAAATCCAAGTCCAG	199
Ad01A12095	Aradu.A01	12095	c	(AAT)5aaagaa	61	62664420	62664480	AAAAGAATACCCTCCAAGGCA	TCACTAGGATTTTGGGTGGC	230
Ad01A12096	Aradu.A01	12096	p3	(TGC)5	15	62667061	62667075	TCACCATCCCCTCTATGCTC	GAGAGGCATAAACCCTCGTG	233

ID	Chromosome	SSR nr.	SSR type	SSR	Size	Start	End	FORWARD PRIMER (5'-3')	REVERSE PRIMER (5'-3')	Product (bp)
Ad01A12099	Aradu.A01	12099	p2	(TA)6	12	62673932	62673943	CATATGCAATTCTCCCTCTCTTT	TCTCGTGCTGATAACGTGTTG	176
Ad01A12100	Aradu.A01	12100	p3	(AAT)9	27	62684127	62684153	CACGAAGAAGATGGCACAAA	CAACATCAACACCCTAATAATCACTC	274
Ad01A12106	Aradu.A01	12106	p2	(TG)12	24	62728219	62728242	TCCCTCCGCATAACTTGTTC	TCCTAACTCAGCCCGTGAAG	157
Ad01A12110	Aradu.A01	12110	p3	(GGT)5	15	62739355	62739369	ATTACCCCGCCTTTTCTTGA	CCGTTTTCCAAATTTTGCTC	257
Ad01A12118	Aradu.A01	12118	p2	(TA)6	12	62776778	62776789	CACGATTTCGCTTGAAAATG	GGGTAGCAAAACCATCTAGGC	223
Ad01A12119	Aradu.A01	12119	p3	(TAT)5	15	62782927	62782941	GTACAACCTCCGTCCCTGAA	ACCACACCCACAATCTACCC	277
Ad01A12123	Aradu.A01	12123	p2	(AG)6	12	62810254	62810265	CGGAAAAGCACACCAAACTT	TTACAAAAACACGTTCGGCA	126
Ad01A12127	Aradu.A01	12127	p3	(TAT)18	54	62837349	62837402	AGTGAGTTATGAATTATGGACCAGT	TTTTGTTTTTCAATCATTTCTACGA	238
Ad01A12128	Aradu.A01	12128	p3	(GAA)5	15	62863999	62864013	AAGGGCGAGTGTCAAAAATG	TTGGTGCATCTTCCTCCTTT	224
Ad01A12129	Aradu.A01	12129	p3	(ATC)7	21	62880956	62880976	AGGCTTCTCGGCAGTATCAA	CCTTTCCCTTTTTAGCCTGG	264
Ad01A12130	Aradu.A01	12130	p2	(AT)9	18	62889362	62889379	CTTGGGACATTGGTTGAGGT	GGGCATTTTTGTCCCCTATT	226
Ad01A12135	Aradu.A01	12135	p2	(TA)6	12	62934449	62934460	TTTTGCCCCTAATCTCGTTG	TCATACCTTGACCGATTCCC	143
Ad01A12136	Aradu.A01	12136	p2	(AG)8	16	62939027	62939042	GAATTGAAAATTGGGCGCTA	CCACCCTCTCCTCTCTCTCA	199
Ad01A12137	Aradu.A01	12137	p2	(AT)13	26	62939401	62939426	CTCAAGCTCCATTTTCAGCC	CAAGGCAACTTCAACAAGCA	262
Ad01A12140	Aradu.A01	12140	c	(T)10gtttagata	50	62955327	62955376	ATGGCCACGGTACAATTCAC	GGATTGTTCACGTCCCACTC	256
Ad01A12143	Aradu.A01	12143	p3	(CTT)6	18	62957143	62957160	TTCCTCTTTCTCCCCCTCAT	ATCGTCGTGTTTCTCCTCGT	156
Ad01A12146	Aradu.A01	12146	p3	(GTG)6	18	62961555	62961572	AGAGATCTGACGGCTGAGGA	CAATTCCATTCCCCATTCAC	272
Ad01A12149	Aradu.A01	12149	p3	(TTC)5	15	63005893	63005907	ACCCCGACAGTTCCAACTTA	GGAAAAGAGGCTCACAATGG	279
Ad01A12150	Aradu.A01	12150	p3	(TTA)17	51	63029731	63029781	AGCGTAAATTTGCAGCCAAC	AGCCAATATCCACCGTCTTG	270
Ad01A12152	Aradu.A01	12152	p5	(ATTCA)5	25	63031370	63031394	TGCGAGTTTCAAAACCAAAA	GAGGTTTGGAGCATTGGTTC	101
Ad01A12156	Aradu.A01	12156	p3	(CCT)5	15	63046139	63046153	TTTCTTCTCCATCTGCGCTT	TGTGACACACAAACTCAATTCG	219
Ad01A12158	Aradu.A01	12158	p3	(AGA)5	15	63047050	63047064	AGAAGGAGGAACCCGAAAAA	TTCATTCAAATAGCCGGTCC	176
Ad01A12159	Aradu.A01	12159	p3	(AAT)20	60	63047171	63047230	GGACCGGCTATTTGAATGAA	GCAAAAACATCTGAATGGCA	265
Ad01A12160	Aradu.A01	12160	p2	(TC)11	22	63048453	63048474	TCTCTCAAACCACGTTGGC	GCAACTGCCACAAATTCAAA	280
Ad01A12161	Aradu.A01	12161	p2	(AC)8	16	63048993	63049008	TACCAAAATGTCCCGCTTTC	TTTGTAAGGAGGGATAGGAGTCA	274
Ad01A12162	Aradu.A01	12162	p2	(CT)6	12	63064384	63064395	TGACGACAACTCCTTCGACA	TCAGTCCTCTCCTTTCCCAA	269
Ad01A12165	Aradu.A01	12165	p2	(GC)6	12	63091731	63091742	TACTGGCACAACCACTTCCA	GGGTGGTATAGAGGAGGGGA	186
Ad01A12167	Aradu.A01	12167	p2	(GC)6	12	63108568	63108579	CAAGCAAGTGCAAGAAAAGAAA	GTTGTGCGAGCTTTATGCTG	261
Ad01A12169	Aradu.A01	12169	p2	(CA)7	14	63117428	63117441	AAAACAAGAACTTGGCCTTGA	ACCATCGCATGTTTTGAACC	277
Ad01A12174	Aradu.A01	12174	p3	(GGT)5	15	63130084	63130098	GCTCGACAGTCCATCACTCA	CGAGACCACGACACAAGAAG	277
Ad01A12180	Aradu.A01	12180	p3	(AGA)6	18	63150868	63150885	CTGGCCTTAGGTGCCATAAA	ACCCTAAATGAACGAACCCC	253
Ad01A12181	Aradu.A01	12181	p3	(AGA)6	18	63166479	63166496	AGCGATGCTTTTACCACACC	ACCCACACCTCTCTCCCTCT	109
Ad01A12182	Aradu.A01	12182	c	(TC)9c(CT)9	37	63190618	63190654	GCAGCTAAACCAGGATACTTCTTG	GCCCATTTTTGCTGAGTGTT	232
Ad01A12184	Aradu.A01	12184	p2	(CT)9	18	63238791	63238808	TCTTTTTCAAAACCACCTAACCA	TGAGAGGAGAGAGGGGATGA	237

ID	Chromosome	SSR nr.	SSR type	SSR	Size	Start	End	FORWARD PRIMER (5'-3')	REVERSE PRIMER (5'-3')	Product (bp)
Ad01A12186	Aradu.A01	12186	p5	(AAGAT)6	30	63257806	63257835	CTAAAGGGCCCGAATAGAGG	GGTGGTACCTGCAAAGGACA	274
Ad01A12187	Aradu.A01	12187	p5	(AAGAT)5	25	63260328	63260352	GCCCGAATAGAAAAACACGA	CCTACAAGGGACTCCAATGC	255
Ad01A12188	Aradu.A01	12188	c	(TTC)5atttgctg	120	63290857	63290976	GCAGTGACCAGTGTGCGTAT	CGCCGAAGAGAATACTGGAG	267
Ad01A12189	Aradu.A01	12189	p3	(TTC)6	18	63321509	63321526	TCTTCCCAATTACCCCCTTC	TAAAATATTGCTCGCCCTCG	274
Ad01A12191	Aradu.A01	12191	p2	(AG)14	28	63343111	63343138	TCCGCTCATCTGCTGACTTA	AGCACAATTCCTGCGCTACT	270
Ad01A12192	Aradu.A01	12192	p2	(AT)8	16	63344708	63344723	GCTTGAGCTTGGATTTGAGC	CAAAAATAATGCCAAAAAGCG	269
Ad01A12200	Aradu.A01	12200	p2	(TC)7	14	63412067	63412080	TCGAAAATCACATTCCTCTTTTT	GAGAGAGAAGAGAAGAGGGTTCG	246
Ad01A12205	Aradu.A01	12205	p3	(ATG)6	18	63438801	63438818	CGTCTGTTATTTGGACCAACTCT	GGAAAATTCAACGAATCAGCA	279
Ad01A12206	Aradu.A01	12206	c	(GT)6(AT)6	24	63440048	63440071	CCTAAAACATGTCAGTTTGTTGC	ACCAAAGGAGCATGCAATTC	266
Ad01A12207	Aradu.A01	12207	p3	(ATA)6	18	63440977	63440994	CGAATTCATTTTCGCAGTTG	GCCCATGCCACAACTTATCT	228
Ad01A12210	Aradu.A01	12210	p2	(GT)6	12	63444257	63444268	AAACTAACGTGGCTTGCTTGA	TGCACCAAATGGAAAACAAA	239
Ad01A12211	Aradu.A01	12211	p2	(TC)10	20	63445088	63445107	GTTTTCATGGTGTGGCACTG	GTGTGAATGGAAGCTGCAAA	259
Ad01A12213	Aradu.A01	12213	c	(T)10attttcaata	179	63446345	63446523	CCCTAGGTTCTTCACTTATTGGA	AAGGTGCATTTAGTTTGTGTTTTT	278
Ad01A12215	Aradu.A01	12215	p3	(TCT)6	18	63449163	63449180	GGACGGGTGAAGAGTGATGT	ACGAGAGAGGAAGAAAGGGC	192
Ad01A12216	Aradu.A01	12216	p3	(AAG)11	33	63450237	63450269	ACCTCGATTGGTTCTGTTCG	GGACATTTTCGTCCCTCAAA	133
Ad01A12217	Aradu.A01	12217	p2	(AT)7	14	63450458	63450471	TTCACATCAATAGTATTTGGAACAGT	TTTTATTCCTCTAATTTTGGGTGA	265
Ad01A12219	Aradu.A01	12219	p2	(GA)6	12	63471975	63471986	GTACAGTAGGGGGTAGCGCA	TCTTCTCTCACCATCACCCC	222
Ad01A12221	Aradu.A01	12221	p2	(GT)6	12	63486504	63486515	TTTTGGAGCATTTCAATCACA	AAAATGAGGTGTGTGGGTCC	131
Ad01A12223	Aradu.A01	12223	p3	(GAA)5	15	63490331	63490345	CCGGTCTTAGGTGCCATAAA	CCTCCATTTTTCTTCCCCAT	270
Ad01A12224	Aradu.A01	12224	p2	(GA)7	14	63493550	63493563	GAGAGGGGGTCCGAATTATC	CACCTAACCACTTTTCCACTCC	149
Ad01A12230	Aradu.A01	12230	p3	(AGA)5	15	63508817	63508831	CCTTAGGTGCCATCAATCGT	TCCATCATCTCTTTCCCACC	187
Ad01A12231	Aradu.A01	12231	p2	(GA)6	12	63512136	63512147	CAAGGGTGAAGATAGGAGCAG	TTTTATTCCCCCTTTAATTTTCG	134
Ad01A12233	Aradu.A01	12233	p3	(TTC)7	21	63533040	63533060	TCACATCCTCCCACTCTTCC	AGAATATTGCTCGCCCTTGA	259
Ad01A12235	Aradu.A01	12235	p2	(TG)31	62	63543643	63543704	TATGCTCGGACCGGTTATCT	GCTTTTGAAGAAAAAGGGGG	242
Ad01A12241	Aradu.A01	12241	p3	(GAA)5	15	63578053	63578067	CCACATTATGCAGAAAGCGAT	TCTCCTCCTCATTCTCCTCG	276
Ad01A12243	Aradu.A01	12243	c	(T)10gtttcttcat	106	63578885	63578990	TTTGCAATATATTTTTAGGGATGAA	GTTCTCCATGCAACCGAAGT	206
Ad01A12244	Aradu.A01	12244	c	(T)10gggtgttta	80	63580919	63580998	TAAGCAGCGCCAGAGATTTT	CATTTGATTGATTTCGGGCT	269
Ad01A12245	Aradu.A01	12245	c	(CCA)5ttatcatc	147	63585093	63585239	ATCAACAGAAGCCCAAGCTG	TGGGCGCTAAGTTTTGTTGT	278
Ad01A12246	Aradu.A01	12246	c	(TGG)7cggattc	137	63586308	63586444	TGATTCCGTTATCATTGTTGTTG	CAGAAGCACAAGCTGAACCA	267
Ad01A12247	Aradu.A01	12247	c	(ATG)5accataa	127	63587471	63587597	GGTGGTGGTGGTAGGTTCTG	ACCACCGCCATTATCATCA	264
Ad01A12248	Aradu.A01	12248	c	(T)10cattttttttc	35	63599959	63599993	TCCCCAATTAGAGGTGTCCA	TGTGGTTCATGAATGTTGGC	252
Ad01A12250	Aradu.A01	12250	p3	(TTG)6	18	63613934	63613951	ATGTAGCCGACCCCACATAG	CCATGGAGGCATGCTATTTT	252
Ad01A12256	Aradu.A01	12256	c	(T)10ggttaatatt	117	63657073	63657189	CCTTTCTGGTAAGCTCGTCG	TTCTGCCAAAGACCATCTCA	210
Ad01A12257	Aradu.A01	12257	p3	(ATG)6	18	63658261	63658278	ATTTTCGGAGAGGGTGGAAT	CACTCGTTCCCCAACACTTT	249

ID	Chromosome	SSR nr.	SSR type	SSR	Size	Start	End	FORWARD PRIMER (5'-3')	REVERSE PRIMER (5'-3')	Product (bp)
Ad01A12258	Aradu.A01	12258	p2	(TC)9	18	63667124	63667141	AACGCGTCATTACGAAAAATG	AAGATGATTCGCCTCCCTTT	269
Ad01A12259	Aradu.A01	12259	p3	(CTT)5	15	63678693	63678707	TGACTTTCCCATGGAGGTTC	ACCCCCAGTGAACAAGACAG	123
Ad01A12260	Aradu.A01	12260	p2	(AG)8	16	63680326	63680341	TGAACCTGGATCGAAAGTCAC	AGGAACGCAACCCAGTTTTC	230
Ad01A12261	Aradu.A01	12261	p3	(AGA)6	18	63791187	63791204	TTAGAATGTTGCTCGCCCTC	CCCTATCCATTCTTCCCCAT	256
Ad01A12264	Aradu.A01	12264	p2	(TC)7	14	63833232	63833245	TTTGGTGTTTCTTGTTAGTGAATTTT	GAAAAAGTCAAAGAAAGATTTTGAA	279
Ad01A12266	Aradu.A01	12266	p2	(GA)6	12	63864142	63864153	GTTAGTGGCTGTGGATGGCT	TCCCTCAACTCACAACACCA	220
Ad01A12271	Aradu.A01	12271	p3	(TTC)6	18	63927610	63927627	ATGGCCGAACCTTAACTCCT	GCTTTTGTCTTCCCTTTCCC	217
Ad01A12272	Aradu.A01	12272	p2	(AG)7	14	63945641	63945654	TTTTTGGGGAAGAGGTGTTG	CAGAATTGGCACAAAGAGCA	164
Ad01A12273	Aradu.A01	12273	p3	(AAG)5	15	63953126	63953140	AGAGTCCAAGCCAAGGCTAA	TGGTTGGTAATTCCCCAATG	248
Ad01A12275	Aradu.A01	12275	c	(T)12gaggtttct	66	63958736	63958801	GAGGAAAGTGCATCCCTTGA	TCTTCTTTTGCTCGAGGACG	265
Ad01A12280	Aradu.A01	12280	p2	(AT)9	18	63989927	63989944	TGCTTAGTGGGAGACAACCC	CGTGGAACTTTCTGGGTGTT	251
Ad01A12281	Aradu.A01	12281	p2	(TA)6	12	64009237	64009248	TTTCGGTACTTGCACATTTTTC	TTGTTAACTAGCTCACATTTGTTGC	273
Ad01A12282	Aradu.A01	12282	p3	(AAC)5	15	64012719	64012733	CGAACCCTCAGGAGATCAAG	GCTTCCACAATGTCTGGGAT	195
Ad01A12286	Aradu.A01	12286	p3	(AAT)13	39	64068826	64068864	ATTGTTGGTCAGCGACATGA	TATGGACGGGTAATCGGGTA	277
Ad01A12288	Aradu.A01	12288	p3	(AAT)5	15	64091712	64091726	GCAGGAACAAGGATAAAGGC	TTGGACGTTGGTTTTGTGAA	236
Ad01A12289	Aradu.A01	12289	p3	(TTC)6	18	64095413	64095430	CACAACACAACCCCCTCTTC	CAGCCTTAGGTGCCATTGAT	184
Ad01A12291	Aradu.A01	12291	p2	(CT)9	18	64160070	64160087	TCAAAACCACCTAACCACCA	AGAGGGAGAGAGAGGGTTCG	179
Ad01A12295	Aradu.A01	12295	p2	(AG)9	18	64246256	64246273	CGACGTGAAGAACAACGAGA	AAGTGTGAACCCTCTCCCCT	263
Ad01A12296	Aradu.A01	12296	p3	(AAG)5	15	64266645	64266659	GCTTTTGTCTTCCCTTTCCC	CCTTGGCCGAATACACATCT	195
Ad01A12298	Aradu.A01	12298	p3	(TTG)5	15	64282513	64282527	GGGGCTAAGATTCAACTCCC	CATCCAAAATTTATGCTTCGG	251
Ad01A12299	Aradu.A01	12299	p4	(TTCT)5	20	64289066	64289085	TGGGAGACATAGGCACATTG	GGCAATCGAGAGCATTGAGT	134
Ad01A12303	Aradu.A01	12303	p3	(GTA)6	18	64292145	64292162	TGATGATGAGGAAGGTGTCAG	TTCAGTTCTTTCCACCAGGC	214
Ad01A12307	Aradu.A01	12307	p2	(TA)9	18	64319569	64319586	ACCAATCTTTTTGGGGGTTC	TTAGGATTTGGGTCGTGCTC	275
Ad01A12309	Aradu.A01	12309	p2	(AT)9	18	64368111	64368128	CGTAGGCATGGACAGAACAA	TTGACGGACTTAGAGGCTTGA	249
Ad01A12310	Aradu.A01	12310	p2	(TC)8	16	64374047	64374062	GTGAATAATGCCACCATCCC	CCAAAGTTGCGTCAAACAGA	173
Ad01A12311	Aradu.A01	12311	p2	(TC)6	12	64422101	64422112	GTAAACGGCTCATCAATCGG	AGTTGGGCCAAACAGACAAA	223
Ad01A12315	Aradu.A01	12315	p2	(AG)6	12	64450106	64450117	AGATCCAAGCCCAAAGAACA	CATGAATGGTCCATAGCCAA	233
Ad01A12316	Aradu.A01	12316	p4	(AAAT)6	24	64475957	64475980	GTGGTGGCAACACTTTTTGT	TGGCTTTTTCTGCTTGCTCT	251
Ad01A12321	Aradu.A01	12321	p2	(AT)6	12	64516727	64516738	CACAACATCCAATCACAACCA	GTTGAAGGTGGAGGACCGTA	274
Ad01A12330	Aradu.A01	12330	p2	(AT)6	12	64558284	64558295	TTGAATGAAGTCGCCCCTAC	ATTCAACTCGTTCGGATCGT	208
Ad01A12331	Aradu.A01	12331	p2	(AC)7	14	64558613	64558626	TGCTTCTTGGTGGATTTTCC	CGCTGATGAGCTTCATTGAT	279
Ad01A12333	Aradu.A01	12333	p3	(TTA)12	36	64560708	64560743	GACATCCATTGCAAATACTCAAA	GGTTATGGTTACAAAGGAAAAAGAA	219
Ad01A12334	Aradu.A01	12334	p5	(TTATC)5	25	64633072	64633096	CCTGGGTGCTCCAGTGTATT	GCCCAATAAAGGGTCCAAAT	192
Ad01A12335	Aradu.A01	12335	p5	(TTATC)5	25	64646257	64646281	CCTGGGTGCTCCAGTGTATT	GCCCAATAAAGGGTCCAAAT	192

ID	Chromosome	SSR nr.	SSR type	SSR	Size	Start	End	FORWARD PRIMER (5'-3')	REVERSE PRIMER (5'-3')	Product (bp)
Ad01A12336	Aradu.A01	12336	p2	(TC)6	12	64648204	64648215	GGAACATTGAACTTGTGATTGAA	AAACGCTGGAAACGATTTTG	223
Ad01A12337	Aradu.A01	12337	p2	(CT)6	12	64654379	64654390	CCAATTAATCAACCAAAGCCA	GCAGAGACTCAAGGGAGGAG	280
Ad01A12338	Aradu.A01	12338	p2	(CT)6	12	64675917	64675928	TCAACCAAAGCCAAGAATGT	CAAGGGAGGAGATCAGACACA	278
Ad01A12339	Aradu.A01	12339	p2	(CT)6	12	64683439	64683450	CCAATTAATCAACCAAAGCCA	TGCAGAGACTCAAGGGATGA	271
Ad01A12341	Aradu.A01	12341	p2	(AG)8	16	64702514	64702529	AGCGCGGAGCAGTAGAATAA	CATTCGTGCATCCTAACCCT	218
Ad01A12342	Aradu.A01	12342	p4	(AAGA)8	32	64723463	64723494	TTACGCCTCCGTGATTCTCT	TTTAGATTGGGGAGTGGTGC	245
Ad01A12344	Aradu.A01	12344	p3	(GAA)5	15	64759140	64759154	TCAAGCAACCAGAAACAACAA	TCTTCTTACCGCCGTACCAC	236
Ad01A12345	Aradu.A01	12345	p3	(AAG)21	63	64759316	64759378	ACAGATGGCAGGGCAATAGT	CACCTGCCGCTTCTTTTT	134
Ad01A12347	Aradu.A01	12347	p2	(AT)6	12	64760199	64760210	GGGACGCCAAGACAATTCTA	GATCGCATGGAGGATCAGTT	189
Ad01A12354	Aradu.A01	12354	p2	(AT)7	14	64866211	64866224	CGAAAAATTTTGAAATTGAAATCTG	TCCTTGGTATTTTCCCTCCA	205
Ad01A12356	Aradu.A01	12356	p3	(TTC)5	15	64876064	64876078	GAACCCTACCCCTCTCTCCA	AAGCTTTTGCCTTCCCTTTC	277
Ad01A12357	Aradu.A01	12357	p2	(AT)11	22	64894618	64894639	TTTTGTGTGTTTTTCTCTTTCATCA	CCAGAGTCCTCCTGGACTGA	262
Ad01A12358	Aradu.A01	12358	p3	(TTC)5	15	64905833	64905847	GATGGATTGGGGGAATTCTT	CAAGGTTCCCATAAAACCCA	270
Ad01A12360	Aradu.A01	12360	p2	(CT)8	16	64909378	64909393	CTCTCCCTTTCTCCCTCCAT	AAAAGGAAAAGGCGAAGAGG	176
Ad01A12361	Aradu.A01	12361	p3	(CTC)7	21	64909755	64909775	CCCATTTCTCTCTCTCCCCT	CCGATCGAGTCTCGTAGAGC	275
Ad01A12364	Aradu.A01	12364	p5	(TTATC)5	25	64918426	64918450	TGTGGAGTGACCTTTCTGGA	CAGAGGAACACGACCCAAAT	142
Ad01A12365	Aradu.A01	12365	p5	(TTATC)5	25	64924341	64924365	CCTGGGTGCTCCAGTGTATT	CAATGCTCGACCTCTTCACA	235
Ad01A12367	Aradu.A01	12367	p5	(TTATC)5	25	64932885	64932909	CCTGGGTGCTCCAGTGTATT	TAAAGGGACACAGCCCAATC	171
Ad01A12368	Aradu.A01	12368	p3	(CAT)5	15	64941752	64941766	GAGGCTTTGGCAGAAGTGAC	AGAGGTGATGGAGCTCAGGA	248
Ad01A12369	Aradu.A01	12369	p2	(CT)6	12	64946294	64946305	CCAGTTTTCACCCCAGAGAA	AAACCTGGGATCGAGAGTCA	218
Ad01A12370	Aradu.A01	12370	p2	(CT)6	12	64953581	64953592	TTCTGACCCAGTTTTCGACC	AAACCTGGGATCGAGAGTCA	225
Ad01A12372	Aradu.A01	12372	p5	(TTATC)5	25	64969156	64969180	CCTGGGTGCTCCAGTGTATT	GACCGACCTCTTCGAGTCAG	229
Ad01A12382	Aradu.A01	12382	p2	(CA)69	138	65046955	65047092	CTTTTCGTGATCCAGCCTTT	GGTGCCAGCTTAGGGACTTT	279
Ad01A12383	Aradu.A01	12383	c	(AT)9agctctgg	98	65055369	65055466	CTCTGAGAAGCGGAAAATGG	TTGATTGATGCAACCCAGAG	245
Ad01A12384	Aradu.A01	12384	c	(AT)7(GT)27	68	65059649	65059716	ACCTTCTTGTGCTTGCCTTT	AAGCTCCTAACTTAGCCCGC	261
Ad01A12387	Aradu.A01	12387	p5	(TTTAT)6	30	65147788	65147817	CCTAACCACTTTCTCTCTCCTCA	GGGTTAGCATATGACCCCAG	259
Ad01A12393	Aradu.A01	12393	p2	(GC)7	14	65213029	65213042	GCCATTAGCCTAGTGTTGGC	GGGAGTTTTTGGGCATTTCT	143
Ad01A12396	Aradu.A01	12396	p2	(TA)6	12	65255043	65255054	ATGTCCCTTCGGGTTTTCTT	AGATGGCAAGGCATTACGAC	236
Ad01A12398	Aradu.A01	12398	p4	(TTAT)5	20	65260850	65260869	GGCTAAACCTCAGTACGTAAAACAA	TTTGATCAGAGTCTCGTGCTG	254
Ad01A12404	Aradu.A01	12404	p3	(ACA)17	51	65293587	65293637	GTGTTCGGTTCATCCCTCAC	TGATGTTCTACTTGTGCGCC	204
Ad01A12405	Aradu.A01	12405	p3	(AAT)7	21	65294040	65294060	AAAGGTGAAACGTGCGAAAG	AGTGAAAATGGTACGGCTGTT	280
Ad01A12410	Aradu.A01	12410	p3	(AAT)5	15	65306470	65306484	GCCTCCTCTGAAGGAAAAGC	AAAGGCTTGCTATTGCCAAA	203
Ad01A12411	Aradu.A01	12411	p3	(AGA)6	18	65331396	65331413	GCTTTTGTCTTCCCTTTCCC	CCTACCCTCTCTCACCTCCC	271
Ad01A12413	Aradu.A01	12413	p3	(CAT)6	18	65342622	65342639	TTGTTGGAGTTGAGGAAGGC	TTCGAGCTGACTATGCCAAA	246

ID	Chromosome	SSR nr.	SSR type	SSR	Size	Start	End	FORWARD PRIMER (5'-3')	REVERSE PRIMER (5'-3')	Product (bp)
Ad01A12415	Aradu.A01	12415	p2	(CT)21	42	65370520	65370561	GGAACATTGAACCTGGAATGA	AACACTGGGGATGATTTTGG	279
Ad01A12417	Aradu.A01	12417	p2	(CT)7	14	65378139	65378152	TGGAATGGAAAAATCATAGCC	GACATGCGCGATCTGTAGAA	267
Ad01A12419	Aradu.A01	12419	p3	(TCT)5	15	65390118	65390132	CTTCTTTCTTGTGGTGGGGA	ACGATTTTGAGCCGAAGAGA	174
Ad01A12420	Aradu.A01	12420	p2	(AG)15	30	65398134	65398163	GTGCTAGGTGGGAGACAACC	GAACCCTTCTTGCACAGCTC	276
Ad01A12426	Aradu.A01	12426	c*	(ATA)5(AATA	52	65461619	65461670	CCTGCTTTTCTCCTTCCTCC	GATTGCATTGAGGCTCGTCT	239
Ad01A12432	Aradu.A01	12432	p2	(AG)7	14	65528580	65528593	GTTCTTGTCCTTGCTCCTGC	CCTTTTCCTTTTAATTATTTCCGA	269
Ad01A12433	Aradu.A01	12433	p3	(AGA)5	15	65535024	65535038	AGTGGTCTTGATGGGCTTTG	TACACCTCTCCCCCTCTCCT	246
Ad01A12435	Aradu.A01	12435	p3	(CAT)6	18	65579775	65579792	TCAATTGTTCCATCTGGACG	CCGAGCTTGACCTGACTCTC	217
Ad01A12437	Aradu.A01	12437	p3	(AGA)9	27	65663563	65663589	TGCCATTGATGGTAGTGGAA	TCAAAAACATTTTCCCACCC	277
Ad01A12438	Aradu.A01	12438	p2	(CT)6	12	65679749	65679760	TTGGCAGAAGGAGCATTTTT	CTTAGACGGTTTGCTCGTCC	152
Ad01A12439	Aradu.A01	12439	p2	(TC)6	12	65691904	65691915	AAGACATATCTTTTTCAAAACCTCC	CCATGATGGAGAAAGGGAGA	263
Ad01A12443	Aradu.A01	12443	p2	(TA)6	12	65710902	65710913	ATGCAGTTTGTGCATCTTGC	GTTGTGTCTTGCAACATCGG	200
Ad01A12444	Aradu.A01	12444	p3	(ATA)10	30	65711776	65711805	TGACTGAATTGCTTTTGCTGA	TTCAAGAAGTTCACCAATCCC	277
Ad01A12445	Aradu.A01	12445	p2	(AT)7	14	65713936	65713949	TGGCCCATAGGATAGGTTTG	TCCTCATCCACAACATCAACA	210
Ad01A12446	Aradu.A01	12446	p2	(AC)6	12	65715033	65715044	GGCTTAGGTAAGGGTGAGGG	AATGAAAGCACCCCAAATGA	236
Ad01A12448	Aradu.A01	12448	p2	(AG)7	14	65743774	65743787	TGAAGAATTCGAATGAAAAGGG	TTTTCAAAACTACCTAACTAATTCCTC	248
Ad01A12449	Aradu.A01	12449	p3	(TCC)5	15	65752228	65752242	TGTTAGGCAAGTTCCCATGA	GTTTCCGATCGCGATTTTTA	271
Ad01A12450	Aradu.A01	12450	c	(GC)6gaattgtc	61	65761911	65761971	ATTGCACTTCCGCAACTTCT	GAGGAAGGAGAAAAGCAGGG	239
Ad01A12451	Aradu.A01	12451	p2	(TC)8	16	65770880	65770895	ACAGGCAGCAATCTAGCGTT	AATTTTGGAGGTAGGTGGGG	276
Ad01A12453	Aradu.A01	12453	p2	(TA)7	14	65794375	65794388	CCGTAGCTCGATTTATTGCC	TAAAGTTGAAAACCGGGCAC	250
Ad01A12454	Aradu.A01	12454	p5	(AATAA)5	25	65797119	65797143	TGAGGCACATGAAAAATGGA	TTTGGTGCCATTATCAAGGA	243
Ad01A12455	Aradu.A01	12455	p3	(TAC)5	15	65798690	65798704	TGGGACCCGTTCAACATTAT	ACTGGGGAAGAGGACTCCAT	238
Ad01A12457	Aradu.A01	12457	p2	(AT)6	12	65806110	65806121	AGGAGCACAACATCATATGCC	TTCTTAAACCATAAGGAGGGACA	165
Ad01A12459	Aradu.A01	12459	p3	(GAG)5	15	65839101	65839115	AAGTGTAAACCAAAGGGGCA	TTGTCGGGAATTGTTTGTGA	219
Ad01A12463	Aradu.A01	12463	p2	(AG)7	14	65859616	65859629	AGAGGGAAGGTAGTGGCGAT	CAACCACCACCCCTCTTCTA	161
Ad01A12464	Aradu.A01	12464	p3	(AAG)5	15	65867299	65867313	TAAAATATTGCTCGCCCTCG	ATTTCTTTCCCACCCAAACC	207
Ad01A12467	Aradu.A01	12467	p5	(ATTTT)5	25	65871593	65871617	CCTAACCAATTTCTCTCTCCTCA	GGGTTAGCATATGACCCCAG	272
Ad01A12468	Aradu.A01	12468	c	(TC)6cctc(CT)	32	65879592	65879623	CTGAACCAAACTGCTTTGTCAT	GAAGAGAGAGAAAGGAAGGGG	235
Ad01A12469	Aradu.A01	12469	c	(TC)6tgtctcttt	62	65879793	65879854	TCCCTCTCCCCTCTCTTTGT	TGAGAGAGATGGAGCGAAAAA	192
Ad01A12470	Aradu.A01	12470	c	(C)10tctctctcc	165	65880199	65880363	CGCCTCCTCATTCTGCTATC	TGAATGGGAAAAGTCAACACC	271
Ad01A12471	Aradu.A01	12471	p2	(CT)6	12	65880542	65880553	CCTTTTCTCCCATCCCTTTT	GAAGAAAAGAGTGCAAGGGG	213
Ad01A12472	Aradu.A01	12472	c	(AG)6aagggga	102	65881019	65881120	GGAGGGAGAGGATAGAAACAAAA	CTCCCTTCCCTCCCTTCTTT	212
Ad01A12473	Aradu.A01	12473	c	(GA)6aaggg(G	62	65881653	65881714	GGGAAAAAGAGGAGAAAGAGAGA	TGAATTGAAACTGAACTGAAACTG	280
Ad01A12475	Aradu.A01	12475	p3	(TCT)7	21	65912511	65912531	ACACACTACCCCCTCTCCCT	TGCCATTGATGGTAGTGGAA	228

ID	Chromosome	SSR nr.	SSR type	SSR	Size	Start	End	FORWARD PRIMER (5'-3')	REVERSE PRIMER (5'-3')	Product (bp)
Ad01A12477	Aradu.A01	12477	p3	(TCT)6	18	65926264	65926281	AAACCCACACCTCTCTCCCT	CCCCTTCCTCTTTCTTGAGG	176
Ad01A12480	Aradu.A01	12480	p2	(AC)6	12	65951489	65951500	AGGACAGAAACCGTGGAAGA	GCAAAAGAGAAGTGTTAGAAGGTCA	280
Ad01A12482	Aradu.A01	12482	p2	(TA)6	12	65982526	65982537	GTCATTTTCCGTTTCCCAGA	TGACATTTATCCGGTCACGA	157
Ad01A12483	Aradu.A01	12483	p2	(AT)7	14	65987301	65987314	CGAAGCGTTCACACGAACTA	TTTGAAATAGCATCATTTGTGATT	170
Ad01A12485	Aradu.A01	12485	c	(T)10ctttatcatt	46	65997934	65997979	GGCATTTTTGTCCCCTTTTT	TCAAAATCTTGGGAATTGGG	164
Ad01A12487	Aradu.A01	12487	c	(A)10gcagtgaa	34	66017843	66017876	TGTGGTTCATGAATGTTGGC	GCACACTCTTTTTGCCTTGG	203
Ad01A12491	Aradu.A01	12491	p2	(AG)6	12	66056150	66056161	AAACTGAAAATGCCTCAATACCA	CATTCAGAGGGAGGGCATAA	264
Ad01A12492	Aradu.A01	12492	p2	(TA)6	12	66056972	66056983	ATGGGTTTATCAACCTCCCC	TTTCTTTCCCATCTCATTTGC	270
Ad01A12496	Aradu.A01	12496	p2	(TC)8	16	66075638	66075653	GGCTGGCTCAACTAAGGAGA	CACAGCAAGCTCAATTGGAA	262
Ad01A12497	Aradu.A01	12497	p2	(GT)6	12	66077131	66077142	TCTTCCCTCTCCTACCCCAT	TCAACTCATCCCCTTGTTCC	124
Ad01A12498	Aradu.A01	12498	p2	(AG)8	16	66083717	66083732	GATTGGTGGGGATTGATGAG	CAACCACCATCCCTCTTCTC	163
Ad01A12502	Aradu.A01	12502	p2	(TC)10	20	66092044	66092063	TGAAAATTTGCAACATCCGA	TACTTTTGGGTCGGGATGAG	185
Ad01A12507	Aradu.A01	12507	p3	(ACC)5	15	66120051	66120065	AACCAGCAACAACCAGAAGC	TCTAGCCCTCTCCCTTGTCA	279
Ad01A12510	Aradu.A01	12510	p2	(TA)11	22	66121925	66121946	TTTTAGAGATGACATACACAAACAATG	TGCAACAAAGAGACACATAAAAA	198
Ad01A12512	Aradu.A01	12512	p3	(ATT)11	33	66132914	66132946	ATTATTGAGCACGGTTTCGG	CATGCCTCACACCATCTTTG	232
Ad01A12513	Aradu.A01	12513	p3	(ATC)6	18	66146800	66146817	GACGAGTCTGAAGAGGCACC	GCACCAGATCTCGACCTTTC	241
Ad01A12516	Aradu.A01	12516	p4	(AACT)5	20	66223633	66223652	CAGCAGCAATTAATGGTAAACG	TCAAACACTCCCAAACACCA	157
Ad01A12518	Aradu.A01	12518	p3	(TTA)5	15	66269838	66269852	TGCAATGAATGAAAATAAGCAAA	TCTCCTTCATCCTTTCCCCT	247
Ad01A12527	Aradu.A01	12527	p3	(CTT)5	15	66368481	66368495	TGGGATTTAGCCTCCTTTGA	CAAATATGCATTCCTTGGCA	230
Ad01A12533	Aradu.A01	12533	p5	(TTATT)6	30	66442018	66442047	TCCCCAATTAGAGGTGTCCA	TGTGGTGGCAATACTTTTGG	268
Ad01A12538	Aradu.A01	12538	p3	(CAT)5	15	66484211	66484225	CTTAAAGAGGTCGGCACAGC	AAAGAGCAGGTTCGGGTTCT	167
Ad01A12539	Aradu.A01	12539	p4	(ATTT)6	24	66510416	66510439	TCGCTTATCAGAGTGCAAGTACA	TGTCGAAATAACTTGCGTTGA	244
Ad01A12540	Aradu.A01	12540	p2	(AT)8	16	66511987	66512002	TGGCCCATTTAGTTGTTTCA	TTTGGGCTAACTTTTTACCTTTT	161
Ad01A12542	Aradu.A01	12542	p3	(AGA)6	18	66527005	66527022	TAAAATATTGCTCGCCCTCG	CAATCACCTCCCTCACCACT	279
Ad01A12545	Aradu.A01	12545	p2	(CT)6	12	66591070	66591081	TCAAATCTGACATGAGGATTCA	TTTCTGGGCTGTTTTTGACC	279
Ad01A12548	Aradu.A01	12548	p4	(GATA)5	20	66604162	66604181	TTGCTTGGAATTCAATTGTTTG	GAATGCAACAACCAACATGC	110
Ad01A12549	Aradu.A01	12549	p2	(TG)6	12	66604654	66604665	AGGCGTGCATACACACAAAA	CATGCGCTGGTACCTATGTG	132
Ad01A12550	Aradu.A01	12550	p3	(TTC)6	18	66614880	66614897	CCTACCCTCTCTCACCTCCC	GCTTTTGTCTTCCCTTTCCC	270
Ad01A12552	Aradu.A01	12552	p3	(AGA)5	15	66643620	66643634	CTCCGACCTTAGGTGCCATA	TCACCACTCTCATCCATCCA	248
Ad01A12555	Aradu.A01	12555	p3	(GAA)5	15	66657395	66657409	AGCTTGCTGAAAAGAGGCAC	ACCTTTGGTTGGGTTTCCTC	165
Ad01A12564	Aradu.A01	12564	p3	(AGA)5	15	66724828	66724842	CTAGAGGTTTCTCCGGCCTT	CTCTTCCCCAAAAACCCTTC	235
Ad01A12568	Aradu.A01	12568	p3	(AGA)6	18	66784405	66784422	CTGGCCTTAGGTGCCATAAA	CAAAATCTCTTTCCCACCCA	278
Ad01A12570	Aradu.A01	12570	p3	(TCT)6	18	66818617	66818634	ACCCATTCCCACCCTAAAAG	TGTCTTTGTTGCTCCTCCCT	210
Ad01A12571	Aradu.A01	12571	c	(AAT)14aagca	145	66828014	66828158	CAAAACAAAGCATAAAACAAGAAAAA	TGAATCCTAAAACATGCCCC	279

ID	Chromosome	SSR nr.	SSR type	SSR	Size	Start	End	FORWARD PRIMER (5'-3')	REVERSE PRIMER (5'-3')	Product (bp)
Ad01A12573	Aradu.A01	12573	p5	(ATTTT)5	25	66831841	66831865	TTCAATGACATCTTTTTCCTATCG	GGGGAGATGACGTGGATTTA	262
Ad01A12576	Aradu.A01	12576	p3	(AAT)6	18	66840411	66840428	CTGCCGGTTCTAGTTCTTCG	TGGGATTTGAAGAAGGTTGC	267
Ad01A12578	Aradu.A01	12578	p2	(CA)6	12	66847103	66847114	CCAAACGAACCAAAAATGCT	GTAGCCGCAAATAGCTCGTC	206
Ad01A12581	Aradu.A01	12581	p2	(AT)8	16	66884732	66884747	GGAATCAGAGGGAAAGCAGA	GGAAATGAAGGGGTCCAAGT	215
Ad01A12582	Aradu.A01	12582	p3	(GAA)5	15	66887520	66887534	CACAAATTGGGAGCACAAGA	CGCCACTCTACCTCACACAA	243
Ad01A12583	Aradu.A01	12583	p2	(CT)6	12	66893957	66893968	ACGCCAGAATTGGCATAAAG	ATAGGTGAGGGGTTTTTGGG	178
Ad01A12589	Aradu.A01	12589	p2	(CT)6	12	67006473	67006484	GGAACATTGAACCTGTGATGAA	CCGTCTCAGCGATTTCTAGG	262
Ad01A12590	Aradu.A01	12590	p3	(AGA)7	21	67022216	67022236	AGCAATGCTTTTTCCACACC	GACACGCCTCTCTTCCACTC	204
Ad01A12593	Aradu.A01	12593	c	(A)11tcttatc(T)	28	67046360	67046387	ATCCTAACGTCAAAATGGCG	CCAAAGGGGTGAGAGTTCAT	279
Ad01A12595	Aradu.A01	12595	p2	(CT)6	12	67055482	67055493	TTCATAAACCAAATTGGGAAAA	CAGATTATAGGCTGGGAGCG	266
Ad01A12596	Aradu.A01	12596	p2	(CT)10	20	67059130	67059149	TCATAAACCAAATTGGGGAAA	TTAGAGGCTGGAAGCAGAGC	259
Ad01A12597	Aradu.A01	12597	p4	(TTAA)5	20	67068328	67068347	TTGAATGAATAGAGGTGAGGGG	CAAAACCTCCTAACTAATTCTTCCC	217
Ad01A12599	Aradu.A01	12599	p3	(TGA)5	15	67079041	67079055	AGGAGCGATTCCGAGTTCTT	GGACGAGTCTGAAGAGGCAC	258
Ad01A12606	Aradu.A01	12606	p3	(TTA)5	15	67153414	67153428	AAATTTCGAATCCCTTGGGT	CCATGATGGAGAATGGGAGA	254
Ad01A12613	Aradu.A01	12613	p2	(GA)6	12	67224312	67224323	CGAACAGAGAGAAGAGAGAGGG	CAAAACCACCTAACTACTTTTCCA	273
Ad01A12615	Aradu.A01	12615	p3	(AGA)7	21	67254519	67254539	AAAATGTTGCTCGCCCTAGA	CACGGTCATCCCTTCTGTTT	162
Ad01A12616	Aradu.A01	12616	p2	(TA)6	12	67259275	67259286	TGCAACACTCCGATCGATAA	AGCCGGATCTTGAGTCTTGA	108
Ad01A12618	Aradu.A01	12618	p2	(CT)6	12	67314401	67314412	TTGGATGGCATAACATAAAAACA	AGGCTACAGAGTGGGGGAAT	266
Ad01A12620	Aradu.A01	12620	p3	(TTC)5	15	67319562	67319576	TGTTGCATCACAACACCAAA	CTTGCTGGGAAGAGACCAAG	244
Ad01A12622	Aradu.A01	12622	p2	(CT)6	12	67333866	67333877	CATTGAACCTGAGATGAAGAAGAA	AGAAGTCGCTGGCACAGATT	218
Ad01A12625	Aradu.A01	12625	p3	(TCT)6	18	67356699	67356716	ACACACTACCCCCTCTCCCT	CTAGAGGATTCTCCGCCCTT	246
Ad01A12629	Aradu.A01	12629	c	(T)11gtttttttttc	71	67394645	67394715	GATGATGTTGGACTCGCCTT	AGATGGCGGAGACAAGAGAA	144
Ad01A12630	Aradu.A01	12630	p3	(CTT)5	15	67394983	67394997	TTATCATCAGGGCGAAGAGG	TGCCAACGACGATAAACAAA	178
Ad01A12631	Aradu.A01	12631	p3	(AAC)5	15	67398874	67398888	TCTTCGAATGAGATTGTGAATGA	TTCGAGCTCTGAGATCTTCTCTC	259
Ad01A12632	Aradu.A01	12632	p3	(TAA)19	57	67403472	67403528	CCCGTGTTCCGTCATAGTTT	CGTTAGGTCTAAACGATAATAACAAAA	178
Ad01A12636	Aradu.A01	12636	p2	(CT)6	12	67486656	67486667	TCGAAAATCACTAACCTGTTTTCA	CTTGTCATTGCTCCTGCTCA	271
Ad01A12638	Aradu.A01	12638	p2	(AT)6	12	67500590	67500601	GCGCTTATGGGTGACAAGTT	AACCACACCATTAGGCTCAAA	271
Ad01A12640	Aradu.A01	12640	c	(A)10tcccatcaa	81	67513750	67513830	CAAGTGGTATATCACAAATAAACAAGA	TGCTCCTTGTCCTAAACTTGG	221
Ad01A12641	Aradu.A01	12641	p2	(CT)6	12	67518480	67518491	GGTGCAGAAAGGACAACGAT	CTATGGGAAGTTGCTGCCAT	127
Ad01A12642	Aradu.A01	12642	p3	(ATT)20	60	67521002	67521061	ATTGGAGGCCATTTGATTTG	CTCGTGTCGCGTCCTATGTA	169
Ad01A12647	Aradu.A01	12647	p3	(TTC)6	18	67624125	67624142	CTTCCCCATAAATCCCACCT	ATGCTTTTACCACGCCAAAC	264
Ad01A12651	Aradu.A01	12651	p3	(AAT)6	18	67632900	67632917	TCGATCCCACGAAAATTGAT	ACAACGGTGATGCCCTACAT	276
Ad01A12653	Aradu.A01	12653	c	(TTC)5tattctttc	87	67662401	67662487	CCTTGGCCGAATACACATCT	GCTTTTGTCTTCCCTTTCCC	198
Ad01A12656	Aradu.A01	12656	p2	(TA)9	18	67697849	67697866	CATTAGGGTAATTTTGTCCCCTT	AAGTGTGGGAAAACTGTGGG	231

ID	Chromosome	SSR nr.	SSR type	SSR	Size	Start	End	FORWARD PRIMER (5'-3')	REVERSE PRIMER (5'-3')	Product (bp)
Ad01A12657	Aradu.A01	12657	p3	(TTC)5	15	67702520	67702534	TAATGGCTTTGGGAGAGGTG	AGACGGTTGCCTTTGATGTT	267
Ad01A12662	Aradu.A01	12662	c*	(CT)6(T)14*	25	67753952	67753976	GGCAACACTTCCAAGGACAT	AAGTTTGGTGTGCTGACGTG	279
Ad01A12664	Aradu.A01	12664	p2	(CA)6	12	67773796	67773807	GCCCCCTCACCTTTACAAAT	TCCCTCCTTTCCTCCCTCTA	157
Ad01A12665	Aradu.A01	12665	p2	(TC)19	38	67774121	67774158	AGAGGAGGCTATTCCACCGT	GTTGCCTGCAGAGACATCAA	197
Ad01A12667	Aradu.A01	12667	p2	(AT)12	24	67800177	67800200	CACAAGATGTGATTGGGCAC	CCCGACATGTCCTGGTAGTT	221
Ad01A12668	Aradu.A01	12668	p2	(TA)7	14	67801048	67801061	GGAGTTTCCTGTGAAAGCGA	AGACTGGACCTGCGAAGCTA	219
Ad01A12669	Aradu.A01	12669	c*	(T)10gaactgtac	105	67806400	67806504	TAGCTACCCACGATTCCCAC	TTGAAAATCATGGGATTGGG	280
Ad01A12671	Aradu.A01	12671	p4	(TTTA)6	24	67807998	67808021	GCAAATGCAATAAAAGAAAATGG	TTTGCACCCTGTTTTTCTCC	217
Ad01A12672	Aradu.A01	12672	c	(CG)6ccatgcat	110	67808167	67808276	GGAAGGAGAAAAACAGGGTG	AGTTGTGCCCACTTTATGCC	194
Ad01A12673	Aradu.A01	12673	p2	(AT)8	16	67824792	67824807	TGGCTCTTGAAAGAATGATGG	TCCCCAATTAGAGGTGTCCA	212
Ad01A12674	Aradu.A01	12674	p3	(GTT)5	15	67851550	67851564	ACAATTGTAGCACAAGCCCC	CGAAGGAAGAACTTTGCACC	192
Ad01A12676	Aradu.A01	12676	p2	(GA)8	16	67874094	67874109	TGCTCTCACTTCTTGCTCCA	AAAGCAAAGATGCACGGTTC	239
Ad01A12677	Aradu.A01	12677	p2	(AG)20	40	67875155	67875194	CCAACAAACGGTGGTGTGTA	AGAACACACACACTCACTCACTCA	276
Ad01A12680	Aradu.A01	12680	c	(GA)9a(AG)8	35	67881081	67881115	CAGCTGCTGCTTCCTCCTAT	TGCAGCAACAAACAGAGGAT	279
Ad01A12681	Aradu.A01	12681	p2	(GA)6	12	67884446	67884457	TCTTCAGATTTTGGTTCGGG	CATAATTCTGCTCCGCCTTC	279
Ad01A12682	Aradu.A01	12682	p2	(GA)6	12	67902506	67902517	AGATTGGGCGGAGGAGAG	GCAACCTCTCTGGTTTCAGC	215
Ad01A12683	Aradu.A01	12683	p3	(AGA)7	21	67912255	67912275	GTGCTTTGACCACACACCAA	AAATGGCCGAACATTCACTC	208
Ad01A12684	Aradu.A01	12684	p2	(AT)8	16	67922949	67922964	AAGAGTAAACTAGAAACCCTTTTAGCA	TCAATGTGAATCTTTGAATGGC	203
Ad01A12687	Aradu.A01	12687	p2	(GC)6	12	68003683	68003694	CACAAGCATGCCACAACTCT	TTGTGCCCACTTTATGTCCA	175
Ad01A12700	Aradu.A01	12700	p2	(AT)6	12	68109749	68109760	TTTAAGGGCAAGTTAATCCATTTT	ATGCCAAGTGTCACAACCAA	278
Ad01A12703	Aradu.A01	12703	c	(T)11acttttaaaa	51	68117638	68117688	TTCAATCATTCTACGACATGAAA	TCATGAGTCCACACAAATGTAAAA	263
Ad01A12706	Aradu.A01	12706	p2	(TA)8	16	68135442	68135457	CCAGGGCATGAACAGTTTTT	AAGCCCAAAATTGTTGGAAA	259
Ad01A12708	Aradu.A01	12708	p3	(TTA)5	15	68142976	68142990	ACAACGGTGATGCCCTACAT	GTTGATCCCACGGAGATTGT	274
Ad01A12709	Aradu.A01	12709	c	(T)11c(A)10	22	68143331	68143352	ATTTCCCCCACCAAGTTTTT	GTTGGAAGCCTCAATCCAAA	270
Ad01A12713	Aradu.A01	12713	p3	(AGA)6	18	68218977	68218994	TCTCTTGCCTTAGGTGCCAT	CAGGATCCCCAACTACCTCA	264
Ad01A12716	Aradu.A01	12716	p3	(TTC)6	18	68299191	68299208	ACAACCAAATCTTCTCCCCC	TGCCATTGATGGTAGTGGAA	166
Ad01A12717	Aradu.A01	12717	p3	(AAG)6	18	68309193	68309210	CGGTCTTAGGTGCCATTGAT	TGGCCGAACCTACATCTCTC	159
Ad01A12718	Aradu.A01	12718	p3	(ATT)5	15	68312101	68312115	GCTCCAATGGCAGGAATAGA	CAAGAGAAGGAGATGGCAGC	122
Ad01A12723	Aradu.A01	12723	p3	(AGA)6	18	68413924	68413941	GTGCCATCAATGGTTATGGA	CCCAACCTACCTCACCCTTT	244
Ad01A12726	Aradu.A01	12726	p4	(TAAA)6	24	68436735	68436758	CTCCCCTCTTGGAGTGTTGA	GAGCCCATACTCACATCCGT	177
Ad01A12727	Aradu.A01	12727	c	(TTC)10ctcttc(	47	68441677	68441723	TTAACCTTGGCCGAAATCAC	CTATGCTTTTGCCACACCAA	137
Ad01A12731	Aradu.A01	12731	p3	(TTC)7	21	68450471	68450491	TTAACCTTGGCCGAAATCAC	GCTTTTGCCACACCAAACTT	118
Ad01A12732	Aradu.A01	12732	p2	(TA)7	14	68461077	68461090	CGAGTTTGATTGCCTCTCCT	GCAAGGAATGTAACACCCCA	280
Ad01A12737	Aradu.A01	12737	p2	(TA)10	20	68502209	68502228	GGGAGTGCAACCTTTGTCAT	AAGTGTAGCCTTAGATCTCGTTTTT	254

ID	Chromosome	SSR nr.	SSR type	SSR	Size	Start	End	FORWARD PRIMER (5'-3')	REVERSE PRIMER (5'-3')	Product (bp)
Ad01A12739	Aradu.A01	12739	p3	(TTA)6	18	68525483	68525500	CCTATCCGAAATTCCCATCA	CCCACGGAGATTGTTGGTAT	162
Ad01A12744	Aradu.A01	12744	p3	(TCT)6	18	68545819	68545836	TCAAAAACATTTTCCCAGCC	TGCCATTGATGGTAGTGGAA	257
Ad01A12746	Aradu.A01	12746	c*	(AT)7(T)10*	23	68562036	68562058	CGAAAAATTTTGAAATTGAAATCTG	CTTGGTGTTTTCCCTCCAAA	208
Ad01A12751	Aradu.A01	12751	c	(A)10ggaagaaa	52	68629557	68629608	TGTGGTTCATGAATGTTGGC	CAGGTTTGCTAGAGTCCCCA	267
Ad01A12753	Aradu.A01	12753	p2	(AT)9	18	68681710	68681727	TGAAAATTCTGGGATTTGGG	TTTTTGTCCCCTTTCATTGC	210
Ad01A12754	Aradu.A01	12754	p2	(AT)7	14	68702862	68702875	GGGAGGAGATCACCTTTCATT	TCGGGAATAATTTCGATTTTG	254
Ad01A12757	Aradu.A01	12757	p2	(GA)6	12	68703589	68703600	TAGAAGCCGATGGAGCACTT	TGCTCGTCGTCAGTATCGTC	251
Ad01A12758	Aradu.A01	12758	p2	(TA)6	12	68707751	68707762	TCCCCCAAAAAGTAAAGCAA	CCTCAATGCCTGATCTGTTG	235
Ad01A12762	Aradu.A01	12762	p2	(TC)16	32	68716395	68716426	CGACGAAGCTGGTAGTGACA	GAGGGAGGAGAGGAAGGAGA	182
Ad01A12767	Aradu.A01	12767	c	(TTA)5cttcgaa	55	68748368	68748422	CCCTAGCCACTTTCTCTCCC	GCAGTAGCGGGGTTAGCATA	261
Ad01A12768	Aradu.A01	12768	p3	(TTA)14	42	68758116	68758157	CCATGATGGAGAAAGGGAGA	TTGCTTTGATTTTATTCTCTTTTGG	174
Ad01A12772	Aradu.A01	12772	p3	(AGA)5	15	68786271	68786285	CTAGAGGTTTCTCCGGCCTT	GAAAACAAAAGCCATTCCCA	166
Ad01A12773	Aradu.A01	12773	p4	(TTAT)5	20	68847755	68847774	TTCACTTGACACCTTCACGC	TGCATGCGAAAAAGAAAGAA	261
Ad01A12779	Aradu.A01	12779	p2	(TA)6	12	68940087	68940098	CAGCTTTGGGGTGATTTTGT	TATGGGAATTTGTGGGAACA	189
Ad01A12782	Aradu.A01	12782	p2	(TA)6	12	68952422	68952433	ACACAAGTGGGAAAATTGGG	GGCTAAGGTGAGGGTAATGG	145
Ad01A12784	Aradu.A01	12784	p3	(GTT)5	15	68962318	68962332	GCTGGACTTAGTCAGGCGAA	TAATCACTAACGGCCTTGCC	210
Ad01A12787	Aradu.A01	12787	p3	(ATA)7	21	69010013	69010033	CACCAGTAGATTGTGAACCCG	TATTTTGGGGCAAGCTCAAC	261
Ad01A12791	Aradu.A01	12791	c	(A)10gaatttttta	78	69086525	69086602	CAGGTTTTGAGAAAAAGAGAAATCA	TCGAACATTCCTAAACTACCCA	267
Ad01A12792	Aradu.A01	12792	p3	(ATT)5	15	69093899	69093913	AAACTTGGACGTTGGCTTTG	GGAAAAGGGCACTCTTGTTG	193
Ad01A12794	Aradu.A01	12794	p3	(CTT)5	15	69099253	69099267	GAATTTTCGGTGTATATGTGCTGA	GCGAAGCTGAATTCGAAAAG	137
Ad01A12797	Aradu.A01	12797	p2	(GA)8	16	69104477	69104492	TGCTATCTCCTAACACTCCTCTTTT	TTGCACCATCCTAATATCATTACTGT	280
Ad01A12799	Aradu.A01	12799	p3	(AGA)7	21	69106619	69106639	TTTTGGATCAGACAACGTCA	TTAAGGGGCATTTGGACTTG	250
Ad01A12800	Aradu.A01	12800	p3	(GAA)5	15	69107039	69107053	CGAAAGCAGCACACAAACAT	GCACTTGAACTCGTTTCTGC	232
Ad01A12801	Aradu.A01	12801	p3	(GAG)5	15	69107457	69107471	GGAGGAGGAGAAATTCCAATG	GCTATGTCGCCGTCTTCTTC	147
Ad01A12802	Aradu.A01	12802	p4	(TTTA)6	24	69108683	69108706	TGGAATAAGACCTACTTTATGTGCC	GCAGTTTGGGTTTGACATCAT	265
Ad01A12809	Aradu.A01	12809	p3	(AGA)5	15	69171282	69171296	ACCTTCTTCATGGCCACAAC	CCCCCATCCATCTCCTATCT	267
Ad01A12811	Aradu.A01	12811	p3	(CTT)5	15	69218447	69218461	TCACACAACACAACCCCTTC	CTTCCCTTTTCCTTTTTCCG	215
Ad01A12815	Aradu.A01	12815	c	(T)10gaaaatag	109	69250795	69250903	CCTTTCTCAACAGAAAAGGCA	AAGGGTAGAGTTTTGGGCGT	276
Ad01A12819	Aradu.A01	12819	p3	(AGA)6	18	69275480	69275497	ACTCGGAGGTGGAAGCTTTT	CCACTCTCTCTTCTCCCCCT	226
Ad01A12820	Aradu.A01	12820	p2	(AG)8	16	69280300	69280315	TTGGTCAACCTTTACCAGGG	TGCATCCATTCATAAGCAGG	250
Ad01A12822	Aradu.A01	12822	p2	(GT)6	12	69292379	69292390	TTCAAAAGGAATGCGTAGCC	CGGGCTAAAAACGCTAACAG	227
Ad01A12823	Aradu.A01	12823	p3	(TTA)5	15	69314872	69314886	ACAACGGTGATGCCCTACAT	TAAGGGTCGATCCCACAGAG	279
Ad01A12824	Aradu.A01	12824	c	(AG)7atcgaa(A	34	69316702	69316735	CCCCTGTTTTCAGCATCAAT	CGTTTGGTCTCTCTTCTCCG	250
Ad01A12825	Aradu.A01	12825	p3	(TAT)8	24	69319191	69319214	CTGGGTTGTTGTGTAAGGGAA	CCCAAAACAACCATCACAAA	187

ID	Chromosome	SSR nr.	SSR type	SSR	Size	Start	End	FORWARD PRIMER (5'-3')	REVERSE PRIMER (5'-3')	Product (bp)
Ad01A12827	Aradu.A01	12827	p3	(TTC)6	18	69338851	69338868	CTATACCTTGGCCGAATCCA	CGGTCTTAGGTGCCATTGAT	163
Ad01A12830	Aradu.A01	12830	p2	(AT)11	22	69378669	69378690	CAAAAAGCCTGGTGGTTGTT	AGGATAGCCTAGGTGCCAGC	247
Ad01A12832	Aradu.A01	12832	p2	(TA)11	22	69398300	69398321	CTGGTGAGGGTTCAATTGCT	TGCAAGCTACCTACATGCGT	244
Ad01A12833	Aradu.A01	12833	p3	(AAC)5	15	69415895	69415909	CATAATTTTCATGACCGCCC	GAGGTGCTTTGTAAGGCTGG	233
Ad01A12837	Aradu.A01	12837	p3	(TCT)5	15	69517077	69517091	TAAAGGACCCTGTCTGCGTT	TATCTGAGGACGGGACTGCT	183
Ad01A12838	Aradu.A01	12838	p3	(ATA)12	36	69522863	69522898	ACATGGCCAAAATTCAACAA	TATGCATCCAAGCGATTTCA	188
Ad01A12839	Aradu.A01	12839	c	(CT)17(TC)7	48	69523351	69523398	GGTTGGCATGAGGAAGAAGA	GTGGAAGTTGCAGCAAGTGA	126
Ad01A12841	Aradu.A01	12841	p3	(TTA)6	18	69536639	69536656	TGGTTCTTTAATCAAACTCAAAACA	TGGATGGAACCCTTTAACGA	222
Ad01A12843	Aradu.A01	12843	p5	(TTTAT)7	35	69543166	69543200	GGGATTTGTGCACCAAGTTT	CAAAAACAAAAAGACCCAAATCA	248
Ad01A12845	Aradu.A01	12845	p2	(TA)9	18	69554809	69554826	TTGGTGTTTTCCCTCCAAAA	ATGATGGAGCAAGGGAGATG	280
Ad01A12852	Aradu.A01	12852	p3	(AGA)7	21	69623201	69623221	CTAGAGGATTCTCCGGCCTT	TACCCTCTCCCCTCTCCCTA	248
Ad01A12853	Aradu.A01	12853	p3	(ATT)5	15	69626173	69626187	AAATTTGGACGTTGGCTTTG	GAAACAGGGAAAAAGGCACA	203
Ad01A12854	Aradu.A01	12854	p3	(CTT)5	15	69638485	69638499	CCACTTCATTAGGGAAGGAGC	TCATGATTCCCCTTCTGAGG	176
Ad01A12855	Aradu.A01	12855	p2	(AT)7	14	69640508	69640521	TGGGATCGACCCTCACTTAC	GCGGCAACACCAAACTTAAT	274
Ad01A12856	Aradu.A01	12856	p3	(AAT)5	15	69661123	69661137	ATGGGTTAGCAAGATGCTCG	AGAAGTGATTGGGTTGCCTG	277
Ad01A12858	Aradu.A01	12858	c	(T)10gtgttcaaa	109	69663280	69663388	CATGGCCATAGTCCTTTCTTC	AAGGCTTTAAGCAACCATCA	280
Ad01A12867	Aradu.A01	12867	p5	(AATAA)5	25	69771194	69771218	GGGTTAGCATATGACCCCAG	CCTAACCACTTTCTCTCTCCTCA	216
Ad01A12869	Aradu.A01	12869	p2	(TA)8	16	69780286	69780301	CACCAGTTCATTGGAAGCAA	TTAGCGCAAGTTTAGGGGAA	237
Ad01A12870	Aradu.A01	12870	p2	(AG)6	12	69806176	69806187	GGCATAAGCCAAATAGGCAA	GGAGGAATGCATCCATTGAG	242
Ad01A12871	Aradu.A01	12871	p2	(GT)6	12	69843304	69843315	ATAGGGGAGTGTTGCAGGTG	CACGGTTAGCTTCCTGTCTTC	182
Ad01A12872	Aradu.A01	12872	p2	(AG)6	12	69846253	69846264	CGCGATCTGCAGAATTACAA	AAGAACCTGGGATTGAGAGTCA	244
Ad01A12875	Aradu.A01	12875	p2	(TC)7	14	69863576	69863589	GGGTCAGAGGCACACGTTAT	AAACACAATCACAAGCAGCG	160
Ad01A12881	Aradu.A01	12881	c	(AT)11ac(AT)	38	69886793	69886830	TGCCGAGATTCAGCGATTAT	GCCGATGAAATTACGAGAAAA	275
Ad01A12882	Aradu.A01	12882	p3	(TTA)5	15	69887007	69887021	AGAAGCATTGCCATGGAGTT	GATAAGGGCCCATTGGAGTT	103
Ad01A12884	Aradu.A01	12884	p2	(AT)9	18	69892490	69892507	TAGTGGGGCGTCCAACTTAC	TAAGGTTGTAATGGGGCCAA	237
Ad01A12886	Aradu.A01	12886	p2	(GT)6	12	69913455	69913466	TATTAGCCAGCCATGGTCGT	GAGAAGCACGAAAGTCAGGG	239
Ad01A12887	Aradu.A01	12887	p3	(TCT)6	18	69925322	69925339	CCCATTCCCTCTCCCTCTAC	CTAGAGGTTTCTCCGGCCTT	249
Ad01A12889	Aradu.A01	12889	p4	(TCAT)5	20	69940535	69940554	CAGTTGGGAGGGATTGACAT	TCTTGCTGATGATTTGCACC	273
Ad01A12890	Aradu.A01	12890	p2	(CT)7	14	69941035	69941048	GCATTCCATCGAACAGGTTT	GATTCTGTTCAGCTTCCGCT	242
Ad01A12894	Aradu.A01	12894	p2	(AC)6	12	69948089	69948100	CCTTCCATCCAAGCAAAAGA	TGAGAGGAAACAAAATGGAAAGA	111
Ad01A12897	Aradu.A01	12897	p2	(AT)7	14	69953686	69953699	CAAGAAATTATTTTGCACGTCATT	CAATTTGGATTGGTTGAATGG	227
Ad01A12898	Aradu.A01	12898	c	(A)10gcttctgct	91	69954728	69954818	TTTAAACAATACGAAACACACATATCA	CGGGGAGGGTTTGAAGAATA	244
Ad01A12899	Aradu.A01	12899	c	(T)10ctttgtttttt	117	69955249	69955365	CACAACGCAATTTTTGTTGAA	AACAAGTAAAGAAAGGTAGCTGTGA	276
Ad01A12901	Aradu.A01	12901	p3	(ATA)13	39	69966178	69966216	TTGATCCAAGCAATTTTCGC	TTTTTGGCCCACTTCGTAAA	163

ID	Chromosome	SSR nr.	SSR type	SSR	Size	Start	End	FORWARD PRIMER (5'-3')	REVERSE PRIMER (5'-3')	Product (bp)
Ad01A12903	Aradu.A01	12903	p2	(AT)11	22	69982323	69982344	GTTGGCCCACTTCGTAAAAA	TTTCAGTGAATAACACACATAGCAA	246
Ad01A12906	Aradu.A01	12906	p5	(TTATC)5	25	70003429	70003453	ACCTGCAAGGGACTCTGATG	AATTGCTAGGGAAGCCCAAT	259
Ad01A12907	Aradu.A01	12907	p2	(AG)8	16	70047255	70047270	TTCGTGCTGATAACGTGTTG	AAATAGGGCATGGATTTGTGA	251
Ad01A12909	Aradu.A01	12909	p2	(CT)7	14	70070163	70070176	CACTTTTATTCCTTTTGCATTGA	GCACGCACATTTATCAGTGG	195
Ad01A12918	Aradu.A01	12918	p3	(GAA)5	15	70152480	70152494	TCCTCGAGCAAAAGAGGAAA	CCCATAAACCCCACCTACCT	225
Ad01A12920	Aradu.A01	12920	p3	(AGA)7	21	70171198	70171218	TGCCATTGATGGTAGTGGAA	TCCTAAATGGCCGAACAAAC	243
Ad01A12921	Aradu.A01	12921	c	(AG)9aaagaga	60	70190293	70190352	CTGGGCTATTTCTGACCCAA	AAAGGAACATTGAACCTGGAAA	235
Ad01A12925	Aradu.A01	12925	p2	(AT)13	26	70202739	70202764	CAAGACCGCTCCCAAAAATA	TGGTTTATTGTACCCTCGCC	199
Ad01A12928	Aradu.A01	12928	p3	(ATT)17	51	70220756	70220806	TTATTCGCTCATCAGCACCC	GCAATTCATGATGGGTCACA	243
Ad01A12929	Aradu.A01	12929	p3	(GAT)7	21	70223265	70223285	GAGCTCTTCGGCGAGTTATG	GTAAAACACCATCTGCCGGT	202
Ad01A12930	Aradu.A01	12930	c	(CCT)6ctttctcc	97	70226793	70226889	CACTTTCGTTACCGTCGTCA	GAACAAAATGCATCCAAATCAA	263
Ad01A12931	Aradu.A01	12931	p3	(TGA)6	18	70235682	70235699	GGGCCAACAAGAACTGAAAA	TGTTGAGATTGGATGGTTGG	176
Ad01A12932	Aradu.A01	12932	p3	(TCT)6	18	70238103	70238120	GACCGGATGTAGGTTGCATT	GAACATGATGAGAAATTTTGAGGA	181
Ad01A12934	Aradu.A01	12934	p2	(TA)7	14	70254516	70254529	CCTGATTTATGGAAACCAATCAA	AAAAATGGGACACATATCTCAAG	143
Ad01A12936	Aradu.A01	12936	c	(T)10gcagcagc	31	70259057	70259087	ATGACCTTTGGCTAGACCCC	CACCAAATCAACCCCAATTC	278
Ad01A12939	Aradu.A01	12939	p2	(TA)6	12	70280387	70280398	TTAGTTTGGCAGATTTGCGT	AACAACAAAGTCTTGATCTCTAAACA	280
Ad01A12942	Aradu.A01	12942	p2	(AT)8	16	70283650	70283665	ACTCCTGGCCTTTTCTGGTT	GCCTCGTATAATGGACCACG	235
Ad01A12944	Aradu.A01	12944	p3	(TCT)5	15	70288434	70288448	CGGCCCATACACACTCCTAC	AGGAAACGGATCACGCAATA	278
Ad01A12945	Aradu.A01	12945	p3	(AAG)5	15	70289160	70289174	CCGAACACGTACTCATGGTG	TTATCTTCTTTTGCCCCCTG	221
Ad01A12946	Aradu.A01	12946	p2	(AT)6	12	70289667	70289678	GCTTTTCAGTTTCACCTCGG	TGCCTGGCCAAGAAGAGTAT	252
Ad01A12950	Aradu.A01	12950	p2	(TA)7	14	70301278	70301291	AGATGGCAAGGCATTACGAC	TATGCTCGGACCGGTTATCT	227
Ad01A12951	Aradu.A01	12951	p2	(AC)8	16	70306125	70306140	CCCTTGGCCCAACTAAACTA	TCGAAAACAAGGCTGGAAAT	280
Ad01A12952	Aradu.A01	12952	p3	(CAA)5	15	70309796	70309810	AGTGTGTGAAAAGGGCAACC	GCTTCCCAAACTTCTTCCCT	162
Ad01A12953	Aradu.A01	12953	p2	(AC)7	14	70313530	70313543	CCCTTGGCCCAACTAAACTA	ACTCGAAAACAAGGCTGGAA	278
Ad01A12954	Aradu.A01	12954	p2	(TA)6	12	70322737	70322748	AACTTGAAAACGCATCGCTC	TGTCCCTTTGGGTTTTCTTG	195
Ad01A12955	Aradu.A01	12955	p2	(GT)6	12	70374118	70374129	ATCAGAGCTCCGGATCAAAA	AAAGAAAACGGCCCAAGATT	229
Ad01A12956	Aradu.A01	12956	p2	(TA)7	14	70384619	70384632	TGGTTTATCAACCTCCCCAC	TGCTTGATCCTATGTTCCCTG	278
Ad01A12957	Aradu.A01	12957	p2	(TA)6	12	70384974	70384985	CGAGGTTGTAATGGGGCTAA	GGTGAGAGCATGCAAAGTGA	169
Ad01A12958	Aradu.A01	12958	p2	(AT)6	12	70392337	70392348	ATAACCGGTCCGAGCATACA	TTTCAGATGCAGGACGTGAG	263
Ad01A12959	Aradu.A01	12959	p2	(GT)6	12	70395252	70395263	CTCAAAAATCAAGGCTGGGA	TCAGCTCTCTTCTCCCCAAA	236
Ad01A12960	Aradu.A01	12960	p2	(TA)6	12	70400395	70400406	TCGTAACTTGAAAGCGCATC	ATCTTTGCAACCGGATTTTG	131
Ad01A12963	Aradu.A01	12963	p2	(GT)6	12	70418935	70418946	TGCAAAAGTGAGAATTTTGGG	GTTGTAATGGGACCAAGGCA	154
Ad01A12967	Aradu.A01	12967	p2	(TA)6	12	70434470	70434481	TATAGATGGGGCCGGAATTG	TGGATGGGACTTTCCATTTT	127
Ad01A12968	Aradu.A01	12968	p2	(TA)6	12	70440861	70440872	CCCTAAAGGTGCAAGGAATG	TTTGGACATGATTTGGGTCA	200

ID	Chromosome	SSR nr.	SSR type	SSR	Size	Start	End	FORWARD PRIMER (5'-3')	REVERSE PRIMER (5'-3')	Product (bp)
Ad01A12970	Aradu.A01	12970	p3	(TCT)23	69	70454493	70454561	TGATCTTGTTGGACCATAGCC	TATTGGGTTGGATGGGAGAA	266
Ad01A12971	Aradu.A01	12971	p3	(TAC)5	15	70454677	70454691	CCACTTCCACTTTCAGATTCG	GGTGGTGAAGGGAGTGAAGA	205
Ad01A12972	Aradu.A01	12972	p2	(AT)8	16	70456444	70456459	TTCCTCCAAACCTTAAACGAA	CCTTGGAGAAAGTGTTTGTGC	205
Ad01A12974	Aradu.A01	12974	p4	(AAAT)5	20	70465603	70465622	CCGATCTACAAAACTGTCAAATCA	CATTTTTACTCTTAAGTCACCAAAAA	240
Ad01A12983	Aradu.A01	12983	p2	(GC)6	12	70526386	70526397	CTCTCAGGCCTGCACTAAGC	AGAGGAGGGTGCATGATGAG	141
Ad01A12985	Aradu.A01	12985	p2	(AT)7	14	70534031	70534044	TCTCGTAATTATTAGCTTTCTTTATGG	TTTCAGCCACTTGACTGGATT	261
Ad01A12986	Aradu.A01	12986	p2	(GA)6	12	70539129	70539140	CCCACGGTTTGAAAGAAAGA	GCAGAGCAATGTGTGACGAT	249
Ad01A12987	Aradu.A01	12987	c	(TTA)8(TTG)7	45	70539464	70539508	GCCATTGAAGAAGAGAAGGAGA	CCAACTTGCAACCACCTGTA	280
Ad01A12988	Aradu.A01	12988	p2	(GA)15	30	70544885	70544914	GGGGGAGAAGAAGAAAAGGA	GCCAGTAGCATCAAATGGGT	251
Ad01A12990	Aradu.A01	12990	p2	(AT)6	12	70557840	70557851	CCTGTATGGGATCAAATTATAAATCA	TGAATTTGGTAGTTAATTTTTGTTGT	266
Ad01A12994	Aradu.A01	12994	p2	(TA)10	20	70583348	70583367	TGGCAGCTAAGAAGTTTGGG	TTTTGCATTGGATACTTTCCC	230
Ad01A12998	Aradu.A01	12998	p4	(GAAT)5	20	70632145	70632164	TTGTGGACCCAAATATGACG	TGGACGAGTTCAAGCACAAG	267
Ad01A13002	Aradu.A01	13002	p3	(TTA)11	33	70655902	70655934	GAGTTATCCATGATGACGTGAAA	AGATAACACCAAACCTCCCAA	269
Ad01A13005	Aradu.A01	13005	c	(TA)6ttttttc(T)	29	70676005	70676033	GCAGCTGTAATGGGGTTAGC	TCGTATCTTTTCAATTTGCTTTGA	246
Ad01A13006	Aradu.A01	13006	p2	(TA)6	12	70680683	70680694	GGGCATTTTTGTCCCCTTAT	TTTGGGTATGGGAAACTTGG	230
Ad01A13007	Aradu.A01	13007	p2	(GC)6	12	70682336	70682347	TTAGGCACGGATGCAGAAG	AGTTGTGCCCACTTTATGCC	146
Ad01A13008	Aradu.A01	13008	p3	(GAG)5	15	70691488	70691502	GATCCCTGACTTTCCACAGC	GATCGTCATAAGGCTCGCTC	221
Ad01A13009	Aradu.A01	13009	c	(AT)7tagttgctt[illegible]	97	70697020	70697116	TTGATTGATGCAACCCAGAG	GCGGAAAATGGCTGTAGGTA	236
Ad01A13010	Aradu.A01	13010	p3	(GAG)5	15	70698231	70698245	CCCTGACTTTCCACAGCCTA	CGCCTATCTCCTCATCTTGC	203
Ad01A13014	Aradu.A01	13014	c	(CA)7(AC)6	26	70739665	70739690	TGGCATACCCCAATCTTTTT	CGGCTAGAGGTAAGGCTTAGG	252
Ad01A13015	Aradu.A01	13015	p2	(TG)6	12	70741658	70741669	AAACGCCCTCAACAGAGTGT	GCACACCCCTGTGAGTTTTT	193
Ad01A13016	Aradu.A01	13016	p2	(CT)22	44	70743102	70743145	GGAGCTTTGAAGGAGGAGGT	TTGAAAACCGGGCACTAATC	257
Ad01A13018	Aradu.A01	13018	p2	(CT)6	12	70757717	70757728	CAAGAAATCGAGGCAGAAGG	AGCCATTGTGGGAAACAAAA	175
Ad01A13019	Aradu.A01	13019	c	(T)10gaactatat	41	70757877	70757917	TTTTGTTTCCCACAATGGCT	CCGCTATTTTACGCCTGTCT	243
Ad01A13020	Aradu.A01	13020	c	(TC)10ttct(TC	40	70758243	70758282	GCATCTGTTTTGGAGAAGGC	CGTACAGAGAATGCCGTTGA	203
Ad01A13023	Aradu.A01	13023	p2	(AG)6	12	70783319	70783330	TTTTTGACCCAGTTTTTGGC	GGAACATTGAACCTGTGATTGA	209
Ad01A13025	Aradu.A01	13025	p2	(AG)6	12	70785989	70786000	AAACCTGGGATCGAGAGTCA	CGGCCCAGAGAACACATATT	212
Ad01A13027	Aradu.A01	13027	c	(TA)7(CA)7	28	70802663	70802690	CCAGCCTTAACTTCGCAGAC	ACAGCGAAGATGGGAGTTTG	273
Ad01A13029	Aradu.A01	13029	p2	(AT)6	12	70814271	70814282	AAGGAAAAGGGGACCAAAAA	TTGCACACTCATGCATTCCT	154
Ad01A13033	Aradu.A01	13033	p2	(AC)8	16	70845152	70845167	ACTTCATGCAGTCATTATGGATT	GCTGAAACGATTGAGCTTCC	268
Ad01A13035	Aradu.A01	13035	p4	(TATT)5	20	70861023	70861042	CCTTCCCCCTTCTTTTTCAA	ATGGAGCAAGGGAGATGATG	164
Ad01A13036	Aradu.A01	13036	p3	(TTA)5	15	70865274	70865288	ATTCAACAGGGACAAGCACC	TAAGGGTCGATCCCACAGAG	201
Ad01A13037	Aradu.A01	13037	p3	(TCT)5	15	70866018	70866032	CCTACTCCCTCTCCCCTCAC	GCTTTTGTCTTCCCTTTCCC	271
Ad01A13038	Aradu.A01	13038	p3	(AGA)6	18	70870388	70870405	CTAGAGGATTCTCCGGCCTT	TACACCTCTCCCCCTCTCCT	161

ID	Chromosome	SSR nr.	SSR type	SSR	Size	Start	End	FORWARD PRIMER (5'-3')	REVERSE PRIMER (5'-3')	Product (bp)
Ad01A13040	Aradu.A01	13040	p2	(AC)8	16	70880072	70880087	AAACTAAAAGCGCAACGCTC	CTTAGCTTCGCAGGCTGAGT	150
Ad01A13041	Aradu.A01	13041	p2	(TA)8	16	70885139	70885154	TGATGCTGCTGGATTTTCTG	CCGGTGAGTCTTTTGGTGAT	114
Ad01A13046	Aradu.A01	13046	p2	(TA)7	14	70900666	70900679	CCTGTAAGCGTATTAGCACACG	ACCAGGGCCTAGAGTGTTGA	214
Ad01A13047	Aradu.A01	13047	p2	(TA)11	22	70904042	70904063	GGAAGTAGCCATGGCAAGAA	TTCTTCGTGCGCAAGTAATC	274
Ad01A13048	Aradu.A01	13048	p2	(TA)10	20	70907651	70907670	CAAGTTAAACCCCTGGTGCT	ATTGGAGAGAAGGATTGGGG	118
Ad01A13050	Aradu.A01	13050	c	(AC)21at(AC)	70	70916452	70916521	CGGCAGATATGAAACACCCT	ACCCCCTTTTTACAAGGCTC	204
Ad01A13051	Aradu.A01	13051	p2	(CT)6	12	70920835	70920846	TTTCGAAAATCGCTAACCAC	TTCGAACACAGAGGGAAACA	170
Ad01A13052	Aradu.A01	13052	p3	(TAA)5	15	70961121	70961135	TGATATCGCGTTTCCATTTTT	AGCTGCGGGCAATTATTAGA	264
Ad01A13053	Aradu.A01	13053	c	(GAA)23gagta	179	70963487	70963665	ACTTCCTTAATGGCCTTCGG	GCAGGTAACTGGCATTGGAT	241
Ad01A13054	Aradu.A01	13054	c	(CA)12t(AC)1	67	70965547	70965613	TTGCTTGTTTCTCAAGCCCT	CAATTTTTGCCACGTCAATG	241
Ad01A13055	Aradu.A01	13055	p3	(TTC)6	18	70984400	70984417	TCTTCTCCCACTTAGCCGAA	ACTCGGAGGTGGAAGCTTTT	219
Ad01A13056	Aradu.A01	13056	p2	(TA)15	30	70992669	70992698	TTAAACCTTGAACCTTGGGG	TTGATTTTGGGAAAGTTGGG	253
Ad01A13059	Aradu.A01	13059	p2	(TC)7	14	71018978	71018991	GGCCACCTCCCTATTCTCTC	TGTTGACCTACCTCGAAGCC	240
Ad01A13061	Aradu.A01	13061	p2	(CT)7	14	71029898	71029911	TGACAAATCCTTGACCCCTC	CCTCGAGCAAACGAAGAAAG	258
Ad01A13063	Aradu.A01	13063	p2	(CT)8	16	71036477	71036492	ATCAATCCTCGTGGGATCAA	GCCATTACCCTCCCAAAGAG	261
Ad01A13065	Aradu.A01	13065	p4	(TAAT)6	24	71039710	71039733	TGCTTTCATAGCCGACAATTC	GCCATCATTTTCCATTGTCC	149
Ad01A13067	Aradu.A01	13067	c	(TC)8tattgatca	104	71040160	71040263	GCAAACAAGTAACAAAAGGCAA	AGGAATGCTCCATCACCAAC	272
Ad01A13077	Aradu.A01	13077	p5	(TTTTA)5	25	71063267	71063291	CCTAACCACTTTTTCTCTCCTCA	GAAGCAGTAGTTGGGTTGGC	242
Ad01A13082	Aradu.A01	13082	p2	(GA)21	42	71091429	71091470	TGAGAGGATCTAATTTCTTTTTCTACC	GCAAGCAAGTAAGCTGTAAGCA	255
Ad01A13083	Aradu.A01	13083	p4	(ATTT)5	20	71092020	71092039	ATTCCCCCTTTTTCCACACT	ACTTTACAGAAACCCACTTTTGA	258
Ad01A13090	Aradu.A01	13090	p2	(AG)7	14	71129656	71129669	TTTTTGACCCAGTTTCAGGC	TTTGGAGCCATTGTTGATGA	183
Ad01A13092	Aradu.A01	13092	p4	(CTTT)5	20	71149349	71149368	ACTTCACCTTGGGGCATTTT	GGGAACATTGGATGGGATAA	242
Ad01A13093	Aradu.A01	13093	p3	(AGA)5	15	71150848	71150862	CTCAGGGACTGAAGGTGGAG	ATTTTCCTCAATGTACGCGG	277
Ad01A13094	Aradu.A01	13094	p3	(TTC)5	15	71166407	71166421	ATTTTCCTCAATGTACGCGG	GGAGTGGGCAAGTGAGAGAG	244
Ad01A13095	Aradu.A01	13095	p2	(TC)8	16	71176928	71176943	CCACACAGCTCAACCACAAC	GAAAAGGAAGGAGGGAAGAAGA	181
Ad01A13097	Aradu.A01	13097	p3	(ATT)17	51	71192159	71192209	TCGAAAATTTTTGCATTAAATCAG	TGGAAGACCGAAATACTGGC	272
Ad01A13099	Aradu.A01	13099	p3	(AGA)6	18	71205058	71205075	AGCAATTGCCTTCCCTTTCT	AAACCCACACCTCTCTCCCT	189
Ad01A13101	Aradu.A01	13101	p3	(CAT)7	21	71254273	71254293	CAGTATCTCGGGGTGAAGGA	GAGCTCGACCTCTCCCTTTT	265
Ad01A13102	Aradu.A01	13102	p3	(TTA)5	15	71271091	71271105	GCCACGCTTTAGCGTAACTC	GTGCTAGGTGGGAGACAACC	268
Ad01A13104	Aradu.A01	13104	p2	(GA)7	14	71284835	71284848	CCCCTTTGGTGCTAAACAGA	ACTATGATAATCGCGTGCCC	275
Ad01A13109	Aradu.A01	13109	p2	(AG)6	12	71362219	71362230	AAATCCATTCATTCATCGGG	TCCTTTAAGAGGAATCCTAATCC	138
Ad01A13110	Aradu.A01	13110	p3	(TAG)5	15	71393720	71393734	GAAGAGCAAGGGCATCAAAA	CATGCCTGATGATGATGAGAA	184
Ad01A13116	Aradu.A01	13116	p2	(AG)7	14	71458253	71458266	ATGGGGAGAAGAAGGAGGAA	AGTCCTCCAAGATCACCACG	161
Ad01A13118	Aradu.A01	13118	p2	(CT)6	12	71470229	71470240	AACATGAATGTCATTAAGCCAAA	AGATCCAAATCCAAGAGGCA	278

ID	Chromosome	SSR nr.	SSR type	SSR	Size	Start	End	FORWARD PRIMER (5'-3')	REVERSE PRIMER (5'-3')	Product (bp)
Ad01A13119	Aradu.A01	13119	p4	(AGAA)5	20	71479463	71479482	TGAAGCAAGAAAAAGCAGCA	TTTAGCTGCTTAGGCCAGGA	267
Ad01A13122	Aradu.A01	13122	p3	(AAG)5	15	71484583	71484597	CAGCCTTAGGTGCCATCAAT	AAATCTTTCCCACCCAAACC	273
Ad01A13124	Aradu.A01	13124	p3	(TCT)6	18	71508408	71508425	CCCATCACCTCTTCACCACT	CTGGCCTTAGGTGCCATAAA	214
Ad01A13125	Aradu.A01	13125	c	(T)15gnnntcca	41	71514853	71514893	CCTTCGTAAGTGGCCCAATA	ATTACATTGATGGGAGGGCA	211
Ad01A13130	Aradu.A01	13130	p3	(TTC)5	15	71533228	71533242	CTTTCAGTTTGTCGAGTTCTGG	TCATCACTCATGGAGAAACCA	213
Ad01A13132	Aradu.A01	13132	p2	(AT)7	14	71568435	71568448	TGTATATTCCTCCCCTGCCA	GAAAGGGACTTCTTGGCGAT	108
Ad01A13133	Aradu.A01	13133	p3	(TAA)5	15	71570827	71570841	AAGGCTTTGAGCACCACTGT	TTGAGCCTAGCCGTGACTTT	184
Ad01A13134	Aradu.A01	13134	p2	(TC)6	12	71574543	71574554	TGAGGAGATGAATGGCAGTG	AAAAGGCCAAACCAAACAAA	124
Ad01A13135	Aradu.A01	13135	p3	(TTC)25	75	71575944	71576018	TTAAAAAGCACCACCAACCC	AACCACGTGAAGAGTACGGC	279
Ad01A13139	Aradu.A01	13139	p3	(CTT)5	15	71650685	71650699	TCTTCTCCCACTTAGCCGAA	GCTTTTGTCTTCCCTTTCCC	207
Ad01A13140	Aradu.A01	13140	p2	(AG)26	52	71663591	71663642	AGTGGCGAGGGAAAGAAAAT	AATTTGAAGCCGAACCCTTT	131
Ad01A13142	Aradu.A01	13142	p2	(TA)8	16	71701977	71701992	TCCTTTTTACCCCTGCCTTT	TGGCTCTTGAAAGAATGATGG	212
Ad01A13144	Aradu.A01	13144	p5	(AAAAT)5	25	71703479	71703503	GCTCTGTTCTGGCGTTGAAT	GGGAGGCAACCCAATTTTAT	222
Ad01A13146	Aradu.A01	13146	p2	(GA)7	14	71739609	71739622	TTGGGAATGAGAAGGACTGG	ATTCATTTCCCTCAAAGCCC	237
Ad01A13147	Aradu.A01	13147	p2	(AT)13	26	71747342	71747367	TTCCTGTTCCTCAAGAACCAA	TCTCATGTATTGCCCACTGAA	211
Ad01A13152	Aradu.A01	13152	c	(GT)17t(TG)7	49	71783665	71783713	GAATAATGCGCACAAGAAGG	TTTCTATCTATAGGGCACCTCAA	213
Ad01A13154	Aradu.A01	13154	p3	(AAT)5	15	71850032	71850046	GGGAGGTCAGAATCCAACAA	TGGTGTTGATGAGCGGATAA	251
Ad01A13155	Aradu.A01	13155	p3	(GAG)5	15	71884844	71884858	AAAAAGAGAGTGCAAGGCCA	TCAGAGGATGTCCCAAGAGG	107
Ad01A13157	Aradu.A01	13157	p2	(AT)7	14	71891160	71891173	TACGAATCTGAGGCAACACG	TCATTTTGCGCCTTTCAAAT	277
Ad01A13158	Aradu.A01	13158	p2	(GT)6	12	71894284	71894295	AAATTGGAGCACCGTAGCAC	TTGAACCGGGTCTAAGTTGG	167
Ad01A13160	Aradu.A01	13160	p3	(AAT)5	15	71915913	71915927	CAGGGAAAAAGGCACTCTTG	TTTGGATGTTGGCTTTGTGA	196
Ad01A13161	Aradu.A01	13161	p3	(TCT)6	18	71918732	71918749	CCCCTCTTCTTTACCTTGGC	CGACCTTAGGTGCCATTGAT	172
Ad01A13164	Aradu.A01	13164	p2	(TA)6	12	71931123	71931134	AGATTCTTGGGAGCAATTCG	ACCAAATCAATTGGCACAGC	274
Ad01A13167	Aradu.A01	13167	p2	(TA)7	14	71943609	71943622	CTCAGCTGCAACAAGGATGA	ACATTTGGAGGCACAACACA	226
Ad01A13168	Aradu.A01	13168	p3	(ATA)5	15	71945796	71945810	ACTGCCATTACTATTGCCGC	CCTGGTCAAAACTTAAATGCAA	143
Ad01A13170	Aradu.A01	13170	p2	(AG)6	12	71950200	71950211	AAATTCCAGCGACCTACACG	CAAAAGGAACATTAAACATGGAA	276
Ad01A13171	Aradu.A01	13171	c	(T)10caaaaaaa	57	71951072	71951128	AAATCCGATCGTCAAAATGG	AAGCCAAAGGGGTGAGAGTT	243
Ad01A13173	Aradu.A01	13173	p2	(AG)9	18	71974278	71974295	AAAATTCCAGCAACCTGCAC	TTGGAAAAATAAACAAAAGGAACA	262
Ad01A13174	Aradu.A01	13174	p3	(TAT)6	18	71975991	71976008	CGATAATGTAGTGTTGTTGCGG	TGCAAAAGCTACTTTGATCTTTGA	260
Ad01A13179	Aradu.A01	13179	p4	(AATA)6	24	71994705	71994728	AACAGGGAGCTCACCAAATG	AACGCATATATGACGCGGAT	264
Ad01A13184	Aradu.A01	13184	p3	(TTC)5	15	72043578	72043592	ACCCCACAGGATCATCTCAG	CCCTCGAGCAAGAGAAGAAA	263
Ad01A13188	Aradu.A01	13188	c	(AT)12(GT)28	80	72058843	72058922	GAAGCGAAGTGGTTACTGGG	TACGAGCTCCTGACTCAGCC	279
Ad01A13191	Aradu.A01	13191	p3	(CTT)6	18	72069160	72069177	TCTTCTTCTCCTTCCTCATCATC	CCATTATTTGTTTCTTCTTCTTCTTT	186
Ad01A13192	Aradu.A01	13192	p3	(AAG)5	15	72070461	72070475	AAAATTGGTGCAGAGACCCA	CGTGTTCTTCCTTCTCCTCC	197

ID	Chromosome	SSR nr.	SSR type	SSR	Size	Start	End	FORWARD PRIMER (5'-3')	REVERSE PRIMER (5'-3')	Product (bp)
Ad01A13204	Aradu.A01	13204	p3	(TTA)5	15	72109164	72109178	TGATATACCTGAATGGGGTAACAA	CGTTTTCCCTCCAAAATGAA	159
Ad01A13205	Aradu.A01	13205	c	(AGG)11aagaa	90	72109992	72110081	CTTGCGTGCGTGAATTTAGA	GCTTTACCAAACTAAGCCTTAATCA	182
Ad01A13209	Aradu.A01	13209	p2	(AT)6	12	72117433	72117444	TGAATGATGGTTTGTGATGTGA	AGCCAAACGTGGCTCATGTA	151
Ad01A13210	Aradu.A01	13210	p3	(TTG)5	15	72124310	72124324	GTGGTGGTGGTTGAAGTTCC	GCACCAATGACAACACAAGG	261
Ad01A13212	Aradu.A01	13212	p2	(GT)6	12	72143918	72143929	ATCGCTTGTTTGGTGGTCTT	ATTCTAAGTTGCGCGTGGTT	145
Ad01A13213	Aradu.A01	13213	p4	(AAAT)6	24	72144893	72144916	TCATTCAAGTGTGGGGAAACT	TTGGGGAATTTTGTCTCTTTTT	258
Ad01A13215	Aradu.A01	13215	p3	(AAG)5	15	72149743	72149757	TGTGTGTGTGAATTTTGAAAGC	CATCAATCGAAGCCTCATCA	136
Ad01A13218	Aradu.A01	13218	p3	(AAG)5	15	72165548	72165562	AGTGGTCTTGATGGGCTTTG	TACCTTGGCCGAATACACCT	257
Ad01A13220	Aradu.A01	13220	p2	(TA)7	14	72178562	72178575	TGGGGTAATTTTATCCCCTTA	TTTGCATATGATTGAGGCCA	277
Ad01A13221	Aradu.A01	13221	p2	(GA)24	48	72195715	72195762	GCAGATCTTGCTACTGCTGCT	CCAGGTTTTGATCTATGACTGC	223
Ad01A13222	Aradu.A01	13222	p2	(GA)11	22	72195868	72195889	GCAGTCATAGATCAAAACCTGG	TTAGTTGGAGAACCAGTGACG	269
Ad01A13225	Aradu.A01	13225	p3	(ATT)5	15	72197240	72197254	ATGCGTTTTCAAGTGGCTTT	TGGTTTGCATGAATAATGCTG	244
Ad01A13228	Aradu.A01	13228	p2	(CT)28	56	72204082	72204137	CAAACCCCTCTTAGCCTTCC	GTTGAGCGAGACAGAAAGGG	245
Ad01A13231	Aradu.A01	13231	p3	(TCT)5	15	72227894	72227908	ATTTCTTTCCCACCCAAACC	TAAAATATTGCTCGCCCTCG	197
Ad01A13232	Aradu.A01	13232	p3	(AGA)5	15	72258938	72258952	CGATGTGGAAGGTGACACAG	GGTGCTAGAGGTGCTGAAGG	229
Ad01A13234	Aradu.A01	13234	p3	(ACC)6	18	72265252	72265269	CCAGAAAAATCAAGTGGTGCT	CGGAAGAGGTTGGTGAAAAA	227
Ad01A13236	Aradu.A01	13236	p3	(TAA)5	15	72284692	72284706	GGGGTTTATTTGTCCCTATTCTTT	CCGGTAAATTATTATTATTGTGAAAA	280
Ad01A13237	Aradu.A01	13237	c	(A)10tttataacat	116	72288992	72289107	TTTGGACAACTGACGGTTCA	TGAAGATCATGAAGAACACATGC	279
Ad01A13238	Aradu.A01	13238	p2	(TC)9	18	72290143	72290160	TCGAAAATTCCTTACCCTTTTT	AGAGGAGAAGAGAGGGTTCAAA	205
Ad01A13239	Aradu.A01	13239	p3	(TCT)6	18	72293470	72293487	ACCCTAAAATGACCGAACCC	TTAGAATGTTGCTCGCCCTC	190
Ad01A13243	Aradu.A01	13243	p3	(CTC)5	15	72350746	72350760	CGCATGTTTCTCCTCCTCTC	TGGCATAATAACACCATGAGGA	105
Ad01A13245	Aradu.A01	13245	p2	(TC)8	16	72352135	72352150	TTTTCTCCCTCACTTGTCCG	GGCGTGGTGGTACCAGTAGT	244
Ad01A13246	Aradu.A01	13246	p2	(AT)7	14	72355110	72355123	GAGGCTCATGACAATGCAGA	GCCCTGTTGGCTCCTATGTA	244
Ad01A13247	Aradu.A01	13247	p2	(GA)6	12	72359812	72359823	AGAGAGAGGGATCTGGGGAA	GTCACTCTCTCCCATGGCTC	142
Ad01A13248	Aradu.A01	13248	p3	(TTC)5	15	72361606	72361620	TCAAAATCCACCAGATGCAA	CAAGAAGGTAGGGTTGGGGT	191
Ad01A13250	Aradu.A01	13250	p3	(ATA)5	15	72373869	72373883	GCCACCTATTCAAAATGAGTGA	TGTGTAAAGCTGTGATCTTTCAG	266
Ad01A13256	Aradu.A01	13256	c*	(A)10(AG)8*	25	72412933	72412957	TGTGGTGGCAATACTTTTGG	GCTCAAAGCCTTGGATCCTT	273
Ad01A13258	Aradu.A01	13258	c	(TTC)5tttttctta	59	72451613	72451671	CAGACGCCTACGGATCATTT	AATGGAGGATGTGAGTGGGA	259
Ad01A13261	Aradu.A01	13261	p3	(TCT)5	15	72464741	72464755	ATTTCTTTCCCACCCAAACC	CGACCTTAGGTGCCATCAAT	271
Ad01A13263	Aradu.A01	13263	p3	(AGA)6	18	72501016	72501033	TTAGAATGTTGCTCGCCCTC	CTTCCCCATAAACCCAACCT	244
Ad01A13265	Aradu.A01	13265	p4	(AAAT)5	20	72529667	72529686	CGGAAAACAATTGAAAATCCC	TCATTGGGGTGATTTTGTCC	267
Ad01A13270	Aradu.A01	13270	p3	(AAT)5	15	72629085	72629099	GGGGATTGTTCGAGTATGGA	AAACATGCAATTGACACCAAA	256
Ad01A13273	Aradu.A01	13273	c*	(A)10(AT)11*	31	72644570	72644600	TCATTGGCTAAAAGCATGGA	GTTTGTCAATTGCTTGGGCT	121
Ad01A13277	Aradu.A01	13277	p3	(TCT)10	30	72654106	72654135	TTCTTTCTCCGTGCCTCCTA	GGAGGAAGAGGTGGTGTTGA	155

ID	Chromosome	SSR nr.	SSR type	SSR	Size	Start	End	FORWARD PRIMER (5'-3')	REVERSE PRIMER (5'-3')	Product (bp)
Ad01A13282	Aradu.A01	13282	p3	(CTT)5	15	72673513	72673527	TCTTGACTTTCCCATGGAGG	ACAGTGGATCGATTTCAGGC	269
Ad01A13284	Aradu.A01	13284	p2	(GC)6	12	72685891	72685902	ATAGGGAGCTCTTGGGCATT	GCGCACAACTCTCTGGTTTT	126
Ad01A13285	Aradu.A01	13285	p4	(AATA)6	24	72686221	72686244	GCGGATTTGCTCCCTACTTT	TGCAATGAATGAAAAGAAGCA	262
Ad01A13286	Aradu.A01	13286	p2	(AT)8	16	72691177	72691192	TCAAAGTCTCACGGGTTGTG	TCCGGAGGTAATTCTTTCCC	251
Ad01A13289	Aradu.A01	13289	c	(TCA)44(TCT	150	72723008	72723157	TATCTTCGTCTTCATCCCCA	TCAAGAAGATGGCACAAATCA	269
Ad01A13292	Aradu.A01	13292	p3	(ATA)5	15	72748562	72748576	ACTCGCACTATTTGCAAGGC	TTGGCTATTTGATCAATGATGG	179
Ad01A13293	Aradu.A01	13293	p2	(GA)6	12	72760072	72760083	AGCGGGAAGAACTGAGAATG	CCAACCTCCTTCTCTCACCA	207
Ad01A13296	Aradu.A01	13296	c	(GACCAC)5((	60	72783488	72783547	TTGCTGCTCAGAATGGTTTG	GCAAACAACCCAAGGGAGTA	165
Ad01A13298	Aradu.A01	13298	p3	(CAG)5	15	72786750	72786764	AAACAGCAACAGCAGCGTAA	GGTTCTCAGGACCTTGGTGA	235
Ad01A13304	Aradu.A01	13304	p2	(TG)6	12	72848683	72848694	GCTGCTTTTGCAATTTTGGT	CATTAGCAGGGTTGAGCAGC	129
Ad01A13306	Aradu.A01	13306	p3	(TTA)15	45	72849606	72849650	TGGGTCCAGTGTGAAAATGA	TATGGGCGCTTTAACCATTC	261
Ad01A13308	Aradu.A01	13308	p2	(CG)6	12	72853325	72853336	AGTTGTGCCCACTTTATGCC	AAAAACAAGGTGCAAATCCG	135
Ad01A13312	Aradu.A01	13312	p3	(AGA)6	18	72885501	72885518	TTTCCATCTCCCAAGACTCG	TTGGCCGAATCCTTACACTC	228
Ad01A13313	Aradu.A01	13313	p2	(AG)18	36	72904180	72904215	TTGGAGAGGAAAAATGGCAC	CTTCTTCTTCCCATTGCAGC	257
Ad01A13318	Aradu.A01	13318	p2	(CT)8	16	72968555	72968570	CCCTCTTCTCTCTCTTCCCC	CTTTCTTCGCCCTCCTCTCT	244
Ad01A13321	Aradu.A01	13321	p2	(TC)6	12	72977413	72977424	TTGCATGCATTGGTTATTGG	TGTTTGAAGTGACCGAATGG	155
Ad01A13322	Aradu.A01	13322	p2	(AT)6	12	72982732	72982743	AACTCGAAAGCGCAACACTC	TATTTGTGTCCCGTTGGGTT	201
Ad01A13324	Aradu.A01	13324	p2	(CT)17	34	73011583	73011616	TCCGACGAGATGCATAACAA	AGGCCTCAAAAGAAAACGGT	262
Ad01A13326	Aradu.A01	13326	p2	(AT)8	16	73017595	73017610	TGCGAAGATAAGGCCGACTA	TCTCTCATGCGAAGCGTAAA	195
Ad01A13327	Aradu.A01	13327	c	(TC)25tt(TC)6	86	73022061	73022146	AACTTCACATGGCCGTAAGC	CACAACACCCACATTTCAGC	190
Ad01A13333	Aradu.A01	13333	c*	(CT)6(T)12*	23	73125441	73125463	AGGCCCTCCTATCCACTGAT	TTGGCTCTTGAAAGAATGATGA	255
Ad01A13335	Aradu.A01	13335	p3	(TTC)6	18	73177876	73177893	AAATGGCCGAACCTACCTCT	TAAAATATTGCTCGCCCTCG	182
Ad01A13336	Aradu.A01	13336	p3	(TCT)6	18	73211169	73211186	CCCATCACCTCTTCACCACT	ATTGCTTACCCTCGAGCAAA	144
Ad01A13337	Aradu.A01	13337	p2	(TC)7	14	73213918	73213931	CGTTTGGTCTCTCTTCTCCG	TAGCCCCTGTTTCCAACATC	245
Ad01A13338	Aradu.A01	13338	p3	(AAT)15	45	73215514	73215558	GCCAGTCCCTTTGCTTTGTA	GGCCAAGACACAATCCTTTC	260
Ad01A13339	Aradu.A01	13339	p4	(ATAG)5	20	73215765	73215784	TGTGATCATGACCTTGCACC	TCATCAAATGCATCAGCCAT	272
Ad01A13342	Aradu.A01	13342	p2	(AG)8	16	73237048	73237063	ACCAAAGCCATACGATGGAA	TGTGCTTGTTGTTCTTTCCG	279
Ad01A13343	Aradu.A01	13343	p2	(TA)8	16	73238228	73238243	CCCAACATTTCAAAATTATCTCAA	TCCGGTAAGCCAACTAAGAA	280
Ad01A13346	Aradu.A01	13346	p3	(GTT)5	15	73241049	73241063	AAACCTCAGTGGTGCTCAGG	AAGCTACCATGCCAACCAAG	177
Ad01A13347	Aradu.A01	13347	p3	(ATT)7	21	73257435	73257455	CCACTTGAGTTTGAGGGGAG	ACCAAGGTTGCGAAAATCAT	265
Ad01A13348	Aradu.A01	13348	c	(AAT)14aagat	104	73257615	73257718	CTTGCACTATCTGCAAGACAAA	CAACCGTTAACCTCCTTGGT	240
Ad01A13351	Aradu.A01	13351	p3	(TCT)7	21	73294069	73294089	AAACCCACACCTCTCTCCCT	ATGCTTTTACCGCACCAAAC	110
Ad01A13353	Aradu.A01	13353	p2	(CT)6	12	73303728	73303739	AGCCAATTGGGCAACATAAG	GGCTGCAAAGTAGAGGAATGA	243
Ad01A13354	Aradu.A01	13354	p3	(CTT)6	18	73310747	73310764	GCGTCACCGTCTCTATGGAT	CGTGTTCTTTGTCTCCTCCC	134

ID	Chromosome	SSR nr.	SSR type	SSR	Size	Start	End	FORWARD PRIMER (5'-3')	REVERSE PRIMER (5'-3')	Product (bp)
Ad01A13355	Aradu.A01	13355	p3	(GAA)5	15	73328187	73328201	CTAGAGGTTTCTCCCGCCTT	AAATGACCGTCCCTAATCCC	248
Ad01A13356	Aradu.A01	13356	p2	(AG)6	12	73331586	73331597	AGAGGAGAAGAGAGGGTTCG	TTTTTGAAAATTACCTCCCTCA	219
Ad01A13358	Aradu.A01	13358	p3	(AAG)5	15	73349556	73349570	TATCTCCCATGACTCGGAGG	CTTCTTCTACACCTTGGCCG	231
Ad01A13361	Aradu.A01	13361	p2	(AG)7	14	73395255	73395268	GCAAAAGAACATTGAACCTGG	AAATCCAAGTCCAGAAGGCA	208
Ad01A13362	Aradu.A01	13362	c	(TA)7ttgtatccc	54	73396494	73396547	CCCAAATTTTTCCCACTTTTG	CCCACCATGGTATGATCGTT	278
Ad01A13364	Aradu.A01	13364	p5	(TTATC)5	25	73436598	73436622	ATGGTGCTGATCCTTTGTCC	GGAACACGACCCAAATCCTA	277
Ad01A13365	Aradu.A01	13365	p3	(TGA)6	18	73439478	73439495	GTCCGAGCCGATTATACCAA	CTGGGTGATTGATCTCAGCA	259
Ad01A13366	Aradu.A01	13366	p5	(TTATC)5	25	73446628	73446652	CCTGGGTGCTCCAGTGTATT	CAGGACGACCTGACCTCTTC	238
Ad01A13367	Aradu.A01	13367	p2	(TA)7	14	73455085	73455098	GAAGTTTTTGGGGGATTCGT	CCACCTTGGTCGAGATGTTT	214
Ad01A13369	Aradu.A01	13369	p6	(TTTTAT)5	30	73468829	73468858	CCTAACCAATTTCTCTCTCCTCA	GCAGTAGCGGGGTTAGCATA	273
Ad01A13374	Aradu.A01	13374	p5	(AATAA)5	25	73551994	73552018	CGGCGGGTATTGTTTATGTT	CAGGGCACAACTCCAATTTT	206
Ad01A13375	Aradu.A01	13375	p2	(TA)8	16	73552568	73552583	CATAGACCAACGCTCTAGCCA	TCCAAAGCATTAGTCTTCCAAAA	100
Ad01A13376	Aradu.A01	13376	p2	(AT)6	12	73559635	73559646	AAAGCGCAACACTCCTATCG	AGCCGGATCTTGAGTCTTGA	109
Ad01A13377	Aradu.A01	13377	p2	(AC)6	12	73565473	73565484	CGCCATCAAGTTTCTGTTGA	AGAGCCTGAGAGACGGACTG	198
Ad01A13379	Aradu.A01	13379	p6	(TTTCTT)5	30	73596835	73596864	CATGGAAATGTCGATTCAGC	CTTGAACCCTGATTCCCTGA	226
Ad01A13383	Aradu.A01	13383	p2	(TC)6	12	73611718	73611729	TGAACAGAGAGTCGCGTGAG	TGCTTGTCCTCAAGCAAAGA	272
Ad01A13385	Aradu.A01	13385	p2	(AC)6	12	73619378	73619389	AACCCACATCACCCTAAATCC	TGAGGCAGCAGTCTAGAGCA	238
Ad01A13386	Aradu.A01	13386	p2	(GA)7	14	73627441	73627454	GGAGAATCCAAACACAAGGG	TCGAAAATATCTCATCCCTTTTTC	230
Ad01A13389	Aradu.A01	13389	p2	(TA)6	12	73682680	73682691	CGATTTTGCTCTGGGTAGTCTT	GGGACAACTCAGTGGTGCTT	176
Ad01A13393	Aradu.A01	13393	p2	(TA)11	22	73693901	73693922	AAATCAATTTTGGAATGCCC	AAGTGTGGGAAAGTCATCCG	219
Ad01A13395	Aradu.A01	13395	p3	(TCT)6	18	73701978	73701995	CCCCTCTTCTTTACCTTGGC	CTAGAGGATTCTCCGGCCTT	185
Ad01A13397	Aradu.A01	13397	p3	(AGA)11	33	73736847	73736879	TGCCATTGATGGTAGTGGAA	TCACACAACACAACCCCTTC	193
Ad01A13403	Aradu.A01	13403	c	(AT)8ag(AC)3	92	73780008	73780099	TCAAAATGAAAGTGCAATGCT	TCATCTAAGTGTGTGGGAGGA	279
Ad01A13409	Aradu.A01	13409	p2	(TA)8	16	73863908	73863923	CCAGCAAGTCCTTTCCTGAG	TGATACCAGATGGCCCAAAT	159
Ad01A13410	Aradu.A01	13410	p2	(AG)7	14	73886262	73886275	TGGTGAAGAGGTGATGGTGA	GATTTGCACTCAAAGGGCAT	202
Ad01A13411	Aradu.A01	13411	p2	(AT)7	14	73898593	73898606	AGACTCAAACAAGAAACACAAAAA	TTCGAGTTTGGACAACCGAC	280
Ad01A13413	Aradu.A01	13413	c	(A)10gatatgata	102	73910745	73910846	CATAGCCAAAGGGGTGAGAG	GGAATTGCAAACGTGTCCTT	240
Ad01A13415	Aradu.A01	13415	p2	(GA)10	20	73934767	73934786	TGAAAGAAAAGGAAGGAGGGA	TGAATTAAAGCGCAATCACG	240
Ad01A13418	Aradu.A01	13418	c	(A)10gaaatatta	57	73951076	73951132	AAACGCACCATGTGACAAAC	GCACTTGCCGGTGATTTATT	238
Ad01A13420	Aradu.A01	13420	c	(T)12acttttatttg	106	73960374	73960479	TCTGTTGGTCCCTATAGTTTTGC	CTTTTAATTGGGTCCCTCCG	236
Ad01A13423	Aradu.A01	13423	p3	(GAA)6	18	74027263	74027280	AGGCCGAGAAGGAAGAGAAG	CAATACTCCTTCGGCAGAGC	103
Ad01A13425	Aradu.A01	13425	p2	(TA)6	12	74086238	74086249	TGCCAGCTTAAGGACTTCTTG	GCGCTACCAATTTGTCATCC	280
Ad01A13427	Aradu.A01	13427	p2	(AG)6	12	74103124	74103135	GGGATAATGCAACGAGAGGA	CAAAGTGCGAACTCTCTCCC	148
Ad01A13430	Aradu.A01	13430	p2	(AG)6	12	74130120	74130131	GCCGCATTTCAACCTAATTT	TTGAGAAAGCAAATAAAAGGAACA	243

ID	Chromosome	SSR nr.	SSR type	SSR	Size	Start	End	FORWARD PRIMER (5'-3')	REVERSE PRIMER (5'-3')	Product (bp)
Ad01A13434	Aradu.A01	13434	p2	(AG)6	12	74150874	74150885	CGGAAAAGCACACCAAACTT	TATAAAAACACGTTCGGCCC	124
Ad01A13435	Aradu.A01	13435	p4	(AACT)5	20	74190234	74190253	TCGAAACAATTCCTGAAGGC	TCTTCAACCATCATCCACCA	272
Ad01A13436	Aradu.A01	13436	p3	(TTA)5	15	74200716	74200730	CCAACAGGGACAAAGCATCT	CTTCGCACAACTAACCAGCA	256
Ad01A13437	Aradu.A01	13437	p4	(TATT)5	20	74204126	74204145	TTTTCGAATTCTCTCCCCCT	GTTCTTGTCCTTGCTCCTGC	241
Ad01A13439	Aradu.A01	13439	p2	(TC)6	12	74222860	74222871	GCTCGCCAATTTCTAGTTTTCA	TGACCTTAGATGGAAGGCAAT	280
Ad01A13440	Aradu.A01	13440	p2	(TC)6	12	74230544	74230555	TGACAGAGGCCACGTATCAG	TGAAAATAAAAGAACATTAAACCTGG	267
Ad01A13441	Aradu.A01	13441	p4	(TTTA)5	20	74232756	74232775	TTTTCGAATTCTCTCCCCCT	GTTCTTGTCCTTGCTCCTGC	237
Ad01A13443	Aradu.A01	13443	p3	(TTG)5	15	74258113	74258127	GCACCTTGTTTCTGCCTCTC	AGAGGGGAAGCTCTTTCAGG	245
Ad01A13445	Aradu.A01	13445	p2	(TC)7	14	74315933	74315946	TGAACCTGGAGCTCAGAAGAA	TTGGAGGAATGAAGGGAGGT	171
Ad01A13461	Aradu.A01	13461	p4	(AAAT)7	28	74437310	74437337	TGAGAATGACGAACTCGGAA	AATATTTTCAAATTCACAAAGGTTAAG	231
Ad01A13463	Aradu.A01	13463	c	(T)10atttgtttga	49	74439998	74440046	TCCTGCGTCTTTTTAATCGC	TGTTTTTGGGGTTTGATTTG	160
Ad01A13465	Aradu.A01	13465	p2	(AT)8	16	74461447	74461462	GCGTTTTCACCTTTATCGGA	TTTTTAATGAGGCACCAGGC	252
Ad01A13466	Aradu.A01	13466	c	(TCTA)10tctc	78	74471754	74471831	GATGAACATGCTGCTCGAAA	GCATCAAGCCAACATCCTCT	179
Ad01A13468	Aradu.A01	13468	p2	(AG)6	12	74495824	74495835	TTTACAGAAAATGCTGGGGC	CAACACAGGGTGGACATCTG	236
Ad01A13469	Aradu.A01	13469	p3	(TTC)5	15	74499378	74499392	CCCCCATAACCACCCTCTAT	GAGGGGCAAAGCTCTTCTCT	265
Ad01A13470	Aradu.A01	13470	p2	(TA)6	12	74503681	74503692	TGGGTAGGTAAGCATGATTTGA	GGAGCTAACGTAAGGGTGAGG	131
Ad01A13471	Aradu.A01	13471	p2	(AG)8	16	74504803	74504818	GAGCTGGATGGAGGAATCAA	CCAAAACAACACAAGGTGGA	162
Ad01A13472	Aradu.A01	13472	c	(A)10cctaataat	32	74508924	74508955	TGAGACAAGGGAAAAGGTGG	TTGGTCACGGTCAGAGTCAG	102
Ad01A13473	Aradu.A01	13473	p3	(CCT)6	18	74515967	74515984	TTCCAAGTTTTTCCGACCTG	GAAGAGGGAGGAATTGGAGG	276
Ad01A13475	Aradu.A01	13475	p2	(GC)6	12	74523796	74523807	GCCAGCACTATGCCTTTAGC	GCACAGTGTTAGCCCAAGGT	231
Ad01A13478	Aradu.A01	13478	p5	(TTATC)5	25	74557741	74557765	CCTGGGTGCTCCAGTGTATT	GACCGACCTCTTCAGCTCAG	253
Ad01A13479	Aradu.A01	13479	c	(T)10acaaaatct	115	74562437	74562551	GCATGTAAGGATGCGATTTG	TGGGATAAGAAAATGATGAAAAA	280
Ad01A13481	Aradu.A01	13481	p3	(GAT)9	27	74565801	74565827	TTTTCCCATTTTCTCCATCG	GCACAAACAAACTCGGTTCA	260
Ad01A13486	Aradu.A01	13486	c	(A)10gacatgaa	103	74664436	74664538	CATAGCCAAAGGGGTGAGAG	TTGCAAACGTGTGCCTTATT	238
Ad01A13487	Aradu.A01	13487	p2	(AT)6	12	74689527	74689538	GGAATTACAAAACGCGAAGC	GCCTAAACTCCGCAGGCTAT	129
Ad01A13490	Aradu.A01	13490	p2	(TG)9	18	74725784	74725801	AAGTGATTGGGAGGAGCAAA	CAAAAACAATCCTCATGCCC	224
Ad01A13495	Aradu.A01	13495	p2	(CT)6	12	74746577	74746588	CCAATTTGGCAACATAAATCAA	TGGAGACTACAAAGTGGGGG	254
Ad01A13496	Aradu.A01	13496	p3	(AGA)11	33	74752593	74752625	CCTCCTCATCTTCTGCTGCT	TGCACATTCTTCTTCTTCTTGC	216
Ad01A13498	Aradu.A01	13498	p3	(ACC)6	18	74757393	74757410	TGGAAACACAAGGAAGGCAT	TTTTCCATGTTGGCTTTTCC	244
Ad01A13500	Aradu.A01	13500	p4	(AAAT)8	32	74781961	74781992	CCCCAGTTAAGTGCTTGTGG	TTGGCATCTTACTCTCCTTTGA	254
Ad01A13502	Aradu.A01	13502	c	(T)11aaaataaat	88	74788085	74788172	TAAAGTGCTTGTCTGGCACG	TTTGCCAAACACGCTAAAAA	266
Ad01A13506	Aradu.A01	13506	p2	(TA)6	12	74796603	74796614	AATTCCCCGATTGTTCACAC	TCTGGAAGACAGCGAAGAAGA	263
Ad01A13508	Aradu.A01	13508	p2	(GA)8	16	74801880	74801895	GGAGAGTGGTATTTTGGGCA	GCATTCTAAGGGCATTTTGC	160
Ad01A13509	Aradu.A01	13509	p2	(AT)6	12	74804390	74804401	GGGTATATATGGCCAAGTGAGC	GGGGACAAAATTATCCCAATG	217

ID	Chromosome	SSR nr.	SSR type	SSR	Size	Start	End	FORWARD PRIMER (5'-3')	REVERSE PRIMER (5'-3')	Product (bp)
Ad01A13511	Aradu.A01	13511	c	(AT)7(GT)14	42	74823638	74823679	TGTTGCTTCTTGAAAGAATGATG	TTTGCTCTTGCCTTTTGGTT	246
Ad01A13512	Aradu.A01	13512	p2	(TA)7	14	74829985	74829998	TGGGCTTAAATCCTTTATCGG	ATCGAGTATGAACGATCGGC	259
Ad01A13513	Aradu.A01	13513	c	(AT)12g(TA)6	51	74830329	74830379	TCTGCAAAGATTAGACCGGC	GCTCCTCAGTGAGCAAAAGG	256
Ad01A13515	Aradu.A01	13515	p2	(AT)8	16	74848847	74848862	AAAAAGTGAGGAGAAAGAAAGTGG	CCTTGGTGTTTTCCTTCCAA	256
Ad01A13519	Aradu.A01	13519	p3	(AAG)7	21	74868765	74868785	TAGAATATTGCTCGCCCTCG	CCAAAAACCCCACCTACCTT	239
Ad01A13520	Aradu.A01	13520	p2	(GA)6	12	74872226	74872237	TCCAAACACAAGGGTGAAGA	TCGAAAATTACCTCCCTCTTTTT	222
Ad01A13533	Aradu.A01	13533	c	(TA)6caaacaaa	75	75006532	75006606	ACGAGCGAGGCCCTTTTAT	CTTGAGACTTAAGACCGTGGC	280
Ad01A13536	Aradu.A01	13536	p2	(AT)8	16	75013679	75013694	ATTGGGCTGTTCATTTCTCG	CGGCTGTTTATTTTGCGATT	280
Ad01A13537	Aradu.A01	13537	c	(CACG)6cacac	94	75013917	75014010	CCAAGGCTAGCCGGAGAA	CCCTGGGATATTTTGGTTCA	270
Ad01A13539	Aradu.A01	13539	p2	(AT)8	16	75040925	75040940	CTTGCTGGTTAGTTGTGCGA	CGGCTGTTTATTTTGCGATT	265
Ad01A13540	Aradu.A01	13540	p2	(AT)6	12	75041164	75041175	ACTCAGTCTGCGAAGCCAAG	TGTTTCAGATGCAGGTCGAG	257
Ad01A13543	Aradu.A01	13543	p2	(AT)10	20	75055426	75055445	CCTCAGGCTCTATAGGAACGAA	TCACCCATTTTTCAAGGCTC	196
Ad01A13544	Aradu.A01	13544	p2	(TA)14	28	75064443	75064470	ATGCTGCTTCCACAACCTTT	AATCTTCCATCGGTTTTCCC	269
Ad01A13551	Aradu.A01	13551	p2	(AT)7	14	75112877	75112890	TTTTTAAATGATTCTTGGGTTCA	TCAACTCATTTTCTGGAGTTGTG	220
Ad01A13553	Aradu.A01	13553	c	(GT)16gaaaaag	89	75140742	75140830	CCCCAATCTCATTACTACGCA	TCCATCGCTCACTCACTCAC	241
Ad01A13554	Aradu.A01	13554	p2	(AC)13	26	75142014	75142039	TGGACCTTCTCGTGCTACAA	TGACCATTTGGGAAGATGCT	278
Ad01A13555	Aradu.A01	13555	p2	(TC)6	12	75145355	75145366	CACCCTTGCCTTTTGGTTTA	TGTTGGCTCCTGAAAGAATG	162
Ad01A13560	Aradu.A01	13560	p2	(TA)7	14	75199021	75199034	GCCTATGCATTATCTCATGCTG	CTTGGCGGTAAGGTATTGGA	172
Ad01A13564	Aradu.A01	13564	p3	(TAA)8	24	75224923	75224946	TCATTTGTCCACTTTATCAAATGTC	TATTAAGCAAGGAGCGAGGC	167
Ad01A13566	Aradu.A01	13566	p2	(TA)10	20	75246009	75246028	GAATGGCATAAGGTGATGGG	GCAGGCCCTTATCATTCAAA	226
Ad01A13568	Aradu.A01	13568	p3	(TTG)9	27	75250666	75250692	TATGGGAGGTGAGGAAGCTG	AGACTGGAACGCAAAGTGGT	240
Ad01A13569	Aradu.A01	13569	p2	(CT)6	12	75266313	75266324	CCTAATTGGAGCAGGGATGA	TGAAAGTGAGGTAGGTGGGG	217
Ad01A13575	Aradu.A01	13575	p6	(AAAAAG)5	30	75353189	75353218	TGAAGCAAGAAAAAGCAGTGAA	AGCACATGGTTAGGGACAGC	229
Ad01A13577	Aradu.A01	13577	c	(TC)9tttctctcat	102	75363087	75363188	TTTTTCTTTCTTATCTCTTTTCAGCA	GAGAGGGGAGAGAGAGGATAGG	271
Ad01A13578	Aradu.A01	13578	c	(GA)9aagaagaa	140	75363500	75363639	AAAGAAAGAGAAGAAGGGGAGG	CCTCTTTCTAATCATCTCTCCCA	254
Ad01A13582	Aradu.A01	13582	p3	(TAT)5	15	75385337	75385351	AGCCGTGCTGAGTGCTAACT	CATGAACTTGTGAATAAAATTTGAGC	223
Ad01A13584	Aradu.A01	13584	p3	(ATC)5	15	75393532	75393546	TTAGGCCCAACTTTTGGTTG	AACCTCCACCTCCAGGGTAT	216
Ad01A13586	Aradu.A01	13586	p2	(CT)7	14	75404943	75404956	CTAAAGCGCAATCACACGAA	GAAAAGGAAGGAGGGAAGAAGA	226
Ad01A13588	Aradu.A01	13588	p2	(TA)7	14	75413799	75413812	TTTCCTTTGAGCCCATCATC	TCACAAAAAGCTGCGACAAC	247
Ad01A13590	Aradu.A01	13590	p5	(TTATC)5	25	75436544	75436568	CTTTTTGTTTGTTTCTTAACTTTTTCA	ATGAGATGGGGGAGGGATAA	237
Ad01A13594	Aradu.A01	13594	p5	(TTATC)5	25	75455334	75455358	CCTGGGTGCTCCAGTGTATT	ATGTTTAGCGACAAGCCGAC	269
Ad01A13595	Aradu.A01	13595	p3	(TAG)5	15	75471215	75471229	GAGCAAGGGCGTGATCTAAG	GCCTGATGATGATGAGAGCA	251
Ad01A13596	Aradu.A01	13596	p2	(AG)9	18	75476223	75476240	AAACCTGGGATCGAGAGTCA	GGACCAAAAAGCACTGGAAG	265
Ad01A13598	Aradu.A01	13598	p2	(AG)7	14	75503454	75503467	CAATTGGGCAACATCAATCA	GCTATAAAGTGGGGGAATGC	273

ID	Chromosome	SSR nr.	SSR type	SSR	Size	Start	End	FORWARD PRIMER (5'-3')	REVERSE PRIMER (5'-3')	Product (bp)
Ad01A13599	Aradu.A01	13599	p3	(ATA)6	18	75509756	75509773	TGTCGATAAAGATAAAACCGGAA	CGCCTTCTTTGTTGAAAAGG	214
Ad01A13608	Aradu.A01	13608	p3	(CCA)5	15	75548012	75548026	CATAACCGTCCCCAACATCT	GTACTTGCCTTTCTCCACCG	208
Ad01A13613	Aradu.A01	13613	p2	(CT)9	18	75571136	75571153	GGTCAATGTTGTGGATTCCC	GGAAGGGGAGAGAGAGAGTGA	278
Ad01A13614	Aradu.A01	13614	c	(TC)10ctcct(T	37	75571427	75571463	TCTTCCCCTTTCTTTCATTCTC	AAGGGGAGGAGAGAGGAGTG	187
Ad01A13615	Aradu.A01	13615	p2	(TC)7	14	75571572	75571585	CTCACTCCTCTCTCCTCCCC	AAAGGGGAGAGCATGGAAAA	185
Ad01A13616	Aradu.A01	13616	c	(TC)6ccacccttt	168	75571898	75572065	TCCTTTTTCTCCCATCTCCC	TGCTCTTTCTCCTCCTTCCTC	253
Ad01A13617	Aradu.A01	13617	p2	(AG)6	12	75572226	75572237	GGAAGGAGGAGAAAGAGCACA	TCTCTCCCTCCCCTTTCTTT	236
Ad01A13618	Aradu.A01	13618	p2	(GA)11	22	75572508	75572529	AGAGAGGGGAAGGGGAGAA	TCGCTCCCTCTCTCTCCTAA	274
Ad01A13619	Aradu.A01	13619	c	(GA)6gtgaggg	102	75572691	75572792	TTAGGAGAGAGAGGGAGCGA	ACGCCGGTTTTAGATCTCCT	249
Ad01A13620	Aradu.A01	13620	p2	(GA)9	18	75573074	75573091	AGGAGATCTAAAACCGGCGT	TCTCTCCCCCTCTCTCTCCT	220
Ad01A13622	Aradu.A01	13622	p2	(AG)9	18	75587214	75587231	CAGTGGTGGGGCTAGGTAGA	TAATACTTGGCGGGAAATGG	245
Ad01A13623	Aradu.A01	13623	p2	(GC)6	12	75592023	75592034	ACAAGGCTCCTAAAACCGCT	AGCCGACCTCTTTGCACTTA	215
Ad01A13627	Aradu.A01	13627	c	(T)10gtatatatt	41	75635210	75635250	TGAACATTTGAAGCACGTATCA	TGTCTGTCGCATCTTCCTTT	278
Ad01A13628	Aradu.A01	13628	p4	(TTTA)5	20	75640546	75640565	TTCGAAATTCTCTCCCTCTCA	TCAGGTTCGGGATCAACTTC	265
Ad01A13634	Aradu.A01	13634	p2	(AT)6	12	75663218	75663229	ATGGCTAGCCGGCTTAAACT	CTTACCTCGCGTCGTAAAGC	257
Ad01A13637	Aradu.A01	13637	p2	(TA)6	12	75668775	75668786	TTTTTCATTCACAGTTAAATCAGGA	CATGCCAAGATTTGATCCCT	265
Ad01A13642	Aradu.A01	13642	p3	(TTC)5	15	75676218	75676232	TCGCATTTTTCTTCTCTGCAT	AAATTGATGCGTTTCGGAAG	183
Ad01A13643	Aradu.A01	13643	p3	(AAG)5	15	75677692	75677706	AATGTTGTTCGATTCGGTGG	CTGCTCGCGTTTTCTTTCTT	264
Ad01A13644	Aradu.A01	13644	p3	(AAT)5	15	75678114	75678128	AAAATGTTTTCATTGGCATGTT	AAATTTTTGCCAAACTTCAAAGA	240
Ad01A13646	Aradu.A01	13646	p2	(AG)27	54	75691877	75691930	CATCCATTCATTCACATGGC	TCCTAACATGGAGTTCATAAACCA	276
Ad01A13647	Aradu.A01	13647	p3	(TTC)6	18	75693054	75693071	CAGGAATTCACCCAAAGGAT	CATCGTCGTGTTTCTTCTCG	280
Ad01A13648	Aradu.A01	13648	p3	(AAG)5	15	75693222	75693236	CGAGAAGAAACACGACGATG	GTATATGGGCCACGTGCTTT	273
Ad01A13655	Aradu.A01	13655	p2	(TC)24	48	75701677	75701724	CGTCATCGTCATTGCTGAAC	CGACGGCAACTAAGTGATGA	272
Ad01A13657	Aradu.A01	13657	p3	(TAA)8	24	75704849	75704872	TTCAATAGTTATCCTTTGCGTATCTT	GCAGCAATATCATTCTTCAACA	279
Ad01A13658	Aradu.A01	13658	p2	(TC)12	24	75706840	75706863	CACAGCACGCACCACTATCT	TTTTCCTCATGGAGTCCACC	256
Ad01A13660	Aradu.A01	13660	p3	(TTA)7	21	75711892	75711912	CAGTACCTTCAATGTGCCTCAA	AAAGAAACAAAAATGCCATGC	276
Ad01A13661	Aradu.A01	13661	p2	(TA)6	12	75735579	75735590	TCACATGTGCATCGATCTTT	GAAACTGTGGGAACATGGGT	254
Ad01A13667	Aradu.A01	13667	c	(AT)7gaattgaa	81	75803772	75803852	AAAGGCATTAACAAATAAATCACA	CTTCTTATTAGGCGCATACTTTTT	277
Ad01A13670	Aradu.A01	13670	p2	(CT)7	14	75808611	75808624	CCAAAATGGCCTAGCAAATG	GAAGGAAAAAGAGGGTTGGG	203
Ad01A13673	Aradu.A01	13673	p2	(TA)6	12	75813557	75813568	TGTCCGAGGTACGAATTTTT	TGAAGATATTTGCTAATTATTATCGTG	119
Ad01A13675	Aradu.A01	13675	p2	(AT)8	16	75818770	75818785	TTACATCCTGCTGGCTTCCT	TCCAAAGTTTGTCCATGCAA	203
Ad01A13676	Aradu.A01	13676	p2	(TA)7	14	75824765	75824778	ACTCGTGTCCTAGCTCGTCG	TTGACTTGCCAAATTGAGGA	201
Ad01A13690	Aradu.A01	13690	p3	(CTA)5	15	75903827	75903841	GCAGAGATGTTGCAGATGGA	CATCACTCAAAGGCACATGG	253
Ad01A13691	Aradu.A01	13691	p4	(TTAG)5	20	75914610	75914629	ATACGGGTAACCCCTGCTTC	TCATGCCAATAATCAGCAGC	249

ID	Chromosome	SSR nr.	SSR type	SSR	Size	Start	End	FORWARD PRIMER (5'-3')	REVERSE PRIMER (5'-3')	Product (bp)
Ad01A13692	Aradu.A01	13692	c	(A)10g(A)10	21	75916549	75916569	AAGTGTGGGAAGTTTGGGAA	TATTTTGGGCATTTTGTCCC	236
Ad01A13693	Aradu.A01	13693	p3	(TTC)5	15	75929667	75929681	TCTTCTCCCACTTAGCCGAA	GCTTTTGTCTTCCCTTTCCC	205
Ad01A13694	Aradu.A01	13694	p2	(TA)6	12	75940239	75940250	CATTCTCCCTGGATCGTGTT	CAAGCAAAAATGCAAGATGC	253
Ad01A13695	Aradu.A01	13695	p2	(AT)11	22	75940464	75940485	GCATCTTGCATTTTTGCTTG	TGGAATGCCCTAAAAGGAGA	262
Ad01A13696	Aradu.A01	13696	p2	(AG)6	12	75991478	75991489	GCTGCGTTTAGCCTTTCATC	CTCCCCTATGATGGACCAGA	207
Ad01A13699	Aradu.A01	13699	p3	(AAG)5	15	76020704	76020718	TTTTATCAAACCCTTCCCCC	TGTGGATTTATCAATTTTCCG	155
Ad01A13700	Aradu.A01	13700	p2	(TG)10	20	76022515	76022534	CTGAAAGGCGTCGTCAATTT	TACCTCAGTCATGCCAACCA	172
Ad01A13701	Aradu.A01	13701	c	(A)10ccccaact	149	76027227	76027375	CCCCTGACAATTACCTCGAA	CGGCCCTTGACAATTATCTC	261
Ad01A13702	Aradu.A01	13702	p4	(GTTT)6	24	76030972	76030995	AACCATCCTGAAGTTGTGGC	TCCATTGTTCTCATGTCCCA	233
Ad01A13703	Aradu.A01	13703	p2	(CT)28	56	76032471	76032526	TGATTGGGATCACGGTACAA	AGATGCCATCGTTAATTGGG	199
Ad01A13706	Aradu.A01	13706	c	(T)13atatataac	124	76038566	76038689	TCAACTCCGAAGAACTGCCT	TCCCTTCTTAATTTATGTCAGCA	272
Ad01A13714	Aradu.A01	13714	p3	(TGT)5	15	76069949	76069963	GGCACTTAGGACTGCCTTTG	GATCGCCCAAGTCAAGGATA	269
Ad01A13716	Aradu.A01	13716	p2	(TC)13	26	76088862	76088887	TCAGCTTTGCTAGAGTCCCC	TGGCTCTTGAAAGAATGATGC	203
Ad01A13718	Aradu.A01	13718	p3	(AGA)5	15	76137884	76137898	CCTATGACTCGGAGGTGGAA	CTCTTTCCCACCCAATCTCA	236
Ad01A13719	Aradu.A01	13719	c	(ATT)5agtatttt	177	76185130	76185306	GAGGGACATAAAATGTGTACGGA	ACACAATGTCCCCTCCCTTT	280
Ad01A13721	Aradu.A01	13721	p3	(TGA)6	18	76199659	76199676	CCCTTTTAGCCTGGACAACA	GGACGAGTCTGAAGAGGCAC	222
Ad01A13725	Aradu.A01	13725	p2	(AT)12	24	76224535	76224558	TTACAAAGCGCGAAATGTTG	TCACTAGCCGGCCTAAACTC	206
Ad01A13727	Aradu.A01	13727	c	(AC)9atatac(A	38	76254341	76254378	AGGAGCATGTGAGCCTGTTT	TTGGTGGTCTCTACTCATGACC	211
Ad01A13728	Aradu.A01	13728	p2	(TC)9	18	76259952	76259969	CTGCTACTGAAGGTCCTCCG	TGAAACATTAAACGGCGTCA	203
Ad01A13729	Aradu.A01	13729	p4	(AAAG)5	20	76263282	76263301	CTTGAAAATTTGGGGTGCAT	GGGGTAATTTTGTCCCCTTT	154
Ad01A13735	Aradu.A01	13735	c	(AG)9(TG)15	48	76296491	76296538	GCTGACACTAGCAGAAGGGG	CGGCAAATTTATCTGCTGGT	239
Ad01A13737	Aradu.A01	13737	p2	(AG)8	16	76299586	76299601	ACCCTTGACATCTAGCCCCT	GGTGGATGTTCATGGCTTCT	139
Ad01A13738	Aradu.A01	13738	p2	(AT)10	20	76311678	76311697	TTGTAATGGGGCCAAGGTAA	GGGGACAAAATTACCCCAAT	240
Ad01A13739	Aradu.A01	13739	p3	(TTC)6	18	76323842	76323859	TACCCTCTCCCCTCTCCCTA	CAGCCTTAGGTGCCATTGAT	233
Ad01A13740	Aradu.A01	13740	p3	(TCT)6	18	76335072	76335089	AACCGAACCTCATTCCCTCT	GTTTCTCTGGCTTTTGGTGC	251
Ad01A13741	Aradu.A01	13741	p3	(ATT)5	15	76351570	76351584	ATAAGAGTCCTTCGTCCAAAAA	GGTGAATAATGATATTTTGAAATCC	268
Ad01A13743	Aradu.A01	13743	p3	(ATA)5	15	76354707	76354721	CTTGCAAGCAACCAATGAAA	CCGCCACCTCTATCTTGTGT	266
Ad01A13745	Aradu.A01	13745	p3	(CGC)6	18	76358531	76358548	TCTCAGCGAGACTGTGGCTA	AGGATCCTTCCAGGAGGAAA	212
Ad01A13746	Aradu.A01	13746	p4	(TAAC)5	20	76358787	76358806	TGCCATACGAATCATTCTGC	CCAATCACTTGGTTTGCCTT	221
Ad01A13747	Aradu.A01	13747	p3	(CTC)6	18	76362013	76362030	AGAGATGGCGAAGAAGAACG	GCAAGAGCATCCGCTTTAAC	279
Ad01A13748	Aradu.A01	13748	c	(TA)6(AT)8	28	76378000	76378027	GCGTTTTTGGGTGACAAGTT	TTTTCAATCAATTGGGGTGC	194
Ad01A13749	Aradu.A01	13749	c	(TA)6(AT)8	28	76384874	76384901	GCGTTTTTGGGTGACAAGTT	TTTTCAATCAATTGGGGTGC	194
Ad01A13753	Aradu.A01	13753	p2	(GA)6	12	76412381	76412392	TCGTGCACCAGAGCTCTAAA	GGTGAGGTCGGAATGACTTC	248
Ad01A13758	Aradu.A01	13758	p3	(AAG)5	15	76436534	76436548	CCAACATGAAGAATGCCAAA	GCTGCCCTGTTCTGTTCTCT	249

ID	Chromosome	SSR nr.	SSR type	SSR	Size	Start	End	FORWARD PRIMER (5'-3')	REVERSE PRIMER (5'-3')	Product (bp)
Ad01A13759	Aradu.A01	13759	p2	(AG)6	12	76451392	76451403	TTCCAGCTAATCCTGGGCTA	GGAACATTGAACATGTGATGATG	223
Ad01A13760	Aradu.A01	13760	c	(A)10ttttcagatt	79	76452257	76452335	GCACCGTTGCTAGGGAATTA	TCAAATAAGATTTTGAAAAAGATACGA	208
Ad01A13761	Aradu.A01	13761	p2	(AT)7	14	76456808	76456821	ATCAATGCACATGGGACAAA	AATGGGGCTGAGGTAAAGGT	103
Ad01A13765	Aradu.A01	13765	p5	(TTTAT)5	25	76479376	76479400	AAGTTTTTGGCGCTGTTGTC	TCAAAGGAGCAACTATTCCCA	241
Ad01A13769	Aradu.A01	13769	p3	(ATA)21	63	76536549	76536611	TCAGTGCCTTGATACCACCTC	AGAAGCACGAAATCATGGGT	252
Ad01A13771	Aradu.A01	13771	p2	(TA)8	16	76545849	76545864	CACTCCATGATGAGTATCCCC	GATAAAGGGCAAGAAGGATCG	260
Ad01A13773	Aradu.A01	13773	p3	(TAT)5	15	76569317	76569331	TGATTTCGGTGTTGGACTCA	AGAGTGCCGGCATTACCTTA	174
Ad01A13780	Aradu.A01	13780	p2	(AT)9	18	76591298	76591315	GCGATATACGGACAAGGCAT	CAACCTAAACTTCGCGGGT	108
Ad01A13781	Aradu.A01	13781	p4	(TTAT)5	20	76602000	76602019	TTCCAATTGCCAAATCTTCC	GCACCAGCTTGTACCCATCT	202
Ad01A13782	Aradu.A01	13782	p2	(AT)6	12	76604269	76604280	TTTTGCCGAATTCACTATTGC	GGGTAAAACAAAAAGAACCTGATG	269
Ad01A13783	Aradu.A01	13783	p2	(GA)6	12	76625030	76625041	TGACGAGACACGTGAAGGAG	ATCACGCATCCAACCCTAAC	256
Ad01A13784	Aradu.A01	13784	p2	(AG)6	12	76627866	76627877	GACCTTAGATGGAAGGCAACA	TGCATCCATTTAGGGAGAGC	253
Ad01A13785	Aradu.A01	13785	p2	(TA)7	14	76629392	76629405	CGGGATTTGTTCAACTCGAT	TCTCAAACAAGAAGGGGTGG	234
Ad01A13787	Aradu.A01	13787	p3	(TAT)5	15	76641564	76641578	TCGATGAATCAATTTCGAGTTTT	CAGCGGTTCAAGTTATAAATAGGG	280
Ad01A13788	Aradu.A01	13788	c	(AGA)6tatgaag	37	76650541	76650577	CGTCCTCGAGCAAAGAAAAG	CCCATCACCTCTTCACCACT	268
Ad01A13790	Aradu.A01	13790	c	(A)10catgcaatt	125	76655046	76655170	TGCAATGCATGAATTTTTCG	TCGAGTTTGGACAACTGACG	270
Ad01A13791	Aradu.A01	13791	c	(GT)11(GC)6	34	76657398	76657431	CGGATTTTGGATTCGAGTGT	CACACACGCACATCTGCAC	229
Ad01A13794	Aradu.A01	13794	p4	(TGAG)5	20	76663583	76663602	AGCGAGTGAGAGACCGTCAT	CTGAACTCGATCCGAACCAT	172
Ad01A13799	Aradu.A01	13799	p3	(CTT)6	18	76673522	76673539	CATGAACGGTGGCATATCAG	AAACTTCACGGTTTGTTGGC	261
Ad01A13800	Aradu.A01	13800	c	(A)10ttgaagaag	96	76688752	76688847	CCATGATGGAACAAGGGAGA	TTTTTCAATTCTTATCCTTTTACGTTT	273
Ad01A13801	Aradu.A01	13801	p2	(AG)19	38	76697764	76697801	CCAAAAGGGAGCACAATCAT	TCCATCTCATCCACCTTGCT	238
Ad01A13803	Aradu.A01	13803	p3	(TCT)5	15	76703494	76703508	TTCACCTTGGCCAAACCTAC	TGCCATTGATGGTAGTGGAA	150
Ad01A13807	Aradu.A01	13807	p2	(AT)11	22	76719918	76719939	TTAATGTTCACCACAATTAATATCTCA	ATTTATGATGGGCGGTTCAG	277
Ad01A13812	Aradu.A01	13812	p2	(AG)6	12	76757559	76757570	GGGCCGTATTTTAACCCAGT	AAATTGGGAAGATAAGTAAAGGGAA	251
Ad01A13813	Aradu.A01	13813	p2	(CT)7	14	76758556	76758569	GCTTGCTTTGATTTGGATGA	ATCCAAGCCCAAGTTCTTCA	178
Ad01A13814	Aradu.A01	13814	p4	(CTTT)5	20	76771028	76771047	TTCACTTTGGGGCATTTTGT	GGGAACATTGGATGGGATAA	241
Ad01A13815	Aradu.A01	13815	p2	(GA)16	32	76774427	76774458	GGGAGAGAGAGAAGAGGGGA	ATTTTCCTCAATGTACGCGG	263
Ad01A13816	Aradu.A01	13816	p2	(TA)9	18	76815763	76815780	TCAAATCAATTGAGGTGCCC	TCATCTTTGAGTGCACCGAG	241
Ad01A13818	Aradu.A01	13818	p6	(AAAAAG)5	30	76834616	76834645	GATGTGGTGGCATTGCTTTT	TTTGGTTTAAAGAGCTTTTGGC	278
Ad01A13820	Aradu.A01	13820	p3	(TCT)5	15	76847987	76848001	ATGGCCGAACCTTAACTCCT	GCTTTTGTCTTCCCTTTCCC	279
Ad01A13825	Aradu.A01	13825	p3	(AGA)6	18	76989867	76989884	TTAGAATGTTGCTCGCCCTC	CCCATCACCTCTTCACCACT	278
Ad01A13828	Aradu.A01	13828	c	(T)10cttgatcca	44	77041091	77041134	ATGCCACAAGCACATGGTTA	TGGCTCCTGAAAGAATGAGG	270
Ad01A13832	Aradu.A01	13832	c*	(AAGA)15(AA	89	77053217	77053305	CATATTTGGGATCGGAGGTG	AACATGAAAGAATTGGAGGCA	246
Ad01A13838	Aradu.A01	13838	c	(T)10cggatttttt	37	77076549	77076585	CACCTAAATGGGAGCCGTAA	ACAAGGATCAGCACAACGTG	218

ID	Chromosome	SSR nr.	SSR type	SSR	Size	Start	End	FORWARD PRIMER (5'-3')	REVERSE PRIMER (5'-3')	Product (bp)
Ad01A13839	Aradu.A01	13839	c	(T)10ctggattttt	37	77077166	77077202	AGGAGGACACCTAAAGGGGA	AAGAAGAGGCCATTACGGCT	171
Ad01A13841	Aradu.A01	13841	c	(T)10acattgtgc	114	77077675	77077788	AATGACGCTATCCAGGGTTG	GCGTGAGCACAACGTAAAAA	244
Ad01A13844	Aradu.A01	13844	c	(T)10g(T)10	21	77100067	77100087	TACTTTGGGGCATTTTGTCC	TTGGGAATTTGGTACATGCTC	211
Ad01A13845	Aradu.A01	13845	p2	(AG)7	14	77112508	77112521	TTGTTGGCTCTTGAAAGAATGA	AGGCCCTCCTATCCACTGAT	222
Ad01A13846	Aradu.A01	13846	p3	(TTC)6	18	77166089	77166106	TCCCAACCCTAAATGACCAA	CAGCCTTAGGTGCCATTGAT	257
Ad01A13852	Aradu.A01	13852	p2	(AG)6	12	77234475	77234486	GTTGGGGTGGTTGAGAGAGA	TAGCACAAATACCAGCGACG	256
Ad01A13854	Aradu.A01	13854	p2	(CT)6	12	77243033	77243044	GACCCAAATCCAAGTCCAGA	AAGAACCTGGGATTGAGAGTCA	199
Ad01A13857	Aradu.A01	13857	p3	(AGA)6	18	77278194	77278211	ATGACTCGGAGGTCTTCCCT	TTCTACACCTTGGCCGAATC	208
Ad01A13860	Aradu.A01	13860	p3	(TCT)9	27	77317373	77317399	TGATATGGTGGTGGTGGTTG	ACACAAGCTTGGCATTACCC	265
Ad01A13863	Aradu.A01	13863	c	(T)10gtcgcttgt	120	77346021	77346140	AAAAGCATCCTTCCTGGTCA	GACCTCCCAATCTCACCTCA	225
Ad01A13864	Aradu.A01	13864	p2	(AT)6	12	77349855	77349866	CATCATTCAGTCATCCAGCC	GTCAGAGCCTGGGATGTTGT	119
Ad01A13865	Aradu.A01	13865	p2	(AT)8	16	77358040	77358055	TCAACTCATCTAAATAAAAATGCAGAA	CCAAAGGCTACGCTTTTCAG	272
Ad01A13866	Aradu.A01	13866	p3	(AGT)6	18	77361558	77361575	CCACTTCGAAATGCACTGAA	GGCAGAAAGGAAAGTTGCAG	257
Ad01A13868	Aradu.A01	13868	p3	(CAT)5	15	77381926	77381940	CCACCAGGAATGAAGACGTT	AGGTATGAGGTCGTGTTCCG	138
Ad01A13869	Aradu.A01	13869	p3	(TGA)5	15	77392717	77392731	GCCCAGAAGCACAAGGATAG	AAACGGGAGGAGAAATGACA	271
Ad01A13872	Aradu.A01	13872	c	(TCA)6gaagaa	123	77406856	77406978	ACCCACCACCACTACCACAT	TGTTGTGGATTTCTGATGATGA	183
Ad01A13875	Aradu.A01	13875	p5	(AAGAT)5	25	77450897	77450921	GAAGAGATAAGGGCGGTTCC	ATGTGTCCGACTTACTGGGC	269
Ad01A13882	Aradu.A01	13882	p3	(CTC)5	15	77563939	77563953	TTCATTCGAGCACTCCTCCT	CGAATATTGCTTCTGCGTGA	162
Ad01A13886	Aradu.A01	13886	p3	(ATA)12	36	77572429	77572464	GAGAAAAGACCATCTTTGTGTCG	GGTGAATTAATGTATGCACATCAG	243
Ad01A13887	Aradu.A01	13887	c	(ACT)13aa(TA	56	77581073	77581128	ATACTAACAGGCCCCGGATT	AGCAGCAGCAATATCACGG	261
Ad01A13891	Aradu.A01	13891	p4	(AAAG)6	24	77611856	77611879	AACCTAACCCTAACCACCGC	CCTTCCAGTCACAGCCTCTC	221
Ad01A13893	Aradu.A01	13893	p2	(TA)10	20	77627063	77627082	TGCGGGCTAAGGATAGTTCT	GCAAAAGTTAATTAAAGGTCACGA	277
Ad01A13894	Aradu.A01	13894	p2	(TG)7	14	77636918	77636931	TTCCTGTAGAGCCTGAGGGA	AAAGTCCCAAATGCATGCTC	130
Ad01A13896	Aradu.A01	13896	p3	(TCT)5	15	77645126	77645140	CACAATTTCACTGCACCGTC	TCCTCCTCATATTCTGCTGCT	225
Ad01A13899	Aradu.A01	13899	p3	(AGA)5	15	77648665	77648679	GGATCGATGAGTGTTTGTTCG	GTTCGAAAAGGCGATCATGT	123
Ad01A13901	Aradu.A01	13901	p3	(CAT)5	15	77650049	77650063	CTGACGCAGTGTTGGTGTTT	GGAATTGGAATGATTGGGTG	240
Ad01A13902	Aradu.A01	13902	p3	(GAG)6	18	77650545	77650562	CCCCATTGTATTTCCATCCA	AGAGCCTGAATGGTTCGTTG	216
Ad01A13904	Aradu.A01	13904	p2	(TA)6	12	77658952	77658963	TAAACCGCAATACCATTCCC	GGGCGCAGAAGTTATGAAGA	264
Ad01A13906	Aradu.A01	13906	p2	(TC)6	12	77672065	77672076	TCCCCAATTAGAGGTGTCCA	TGGCTCTTGAAAGAATGAGGA	200
Ad01A13910	Aradu.A01	13910	p3	(ATT)5	15	77701535	77701549	CGTGCTTATTATTCTTTGCATTC	GAACGTTTTTAATTTTCAGCCAA	279
Ad01A13911	Aradu.A01	13911	p2	(TC)9	18	77704278	77704295	TACTGCCGCTACCATCACAC	GTCATCGTTGCCTTTTTGGT	278
Ad01A13912	Aradu.A01	13912	p4	(TGTA)5	20	77709888	77709907	GATGTTGCTGACATGGCATT	ACCTTGCAAGTTCACATGGC	223
Ad01A13913	Aradu.A01	13913	c	(A)10caaaaac(	27	77714407	77714433	ATGTGACGCAACAATTGGAA	AGATTGGTGGCATTGGGTAA	169
Ad01A13914	Aradu.A01	13914	p3	(TCC)6	18	77716227	77716244	GCACTCCCCCAGTAATGAAA	GAAGTCCGGGAAGAAAGAGC	267

ID	Chromosome	SSR nr.	SSR type	SSR	Size	Start	End	FORWARD PRIMER (5'-3')	REVERSE PRIMER (5'-3')	Product (bp)
Ad01A13918	Aradu.A01	13918	p2	(TA)8	16	77729075	77729090	GGAGCACTTGGTTGAAAAGG	ACTACACCGCCCCACAATAA	257
Ad01A13921	Aradu.A01	13921	p3	(AAT)5	15	77735934	77735948	AAGAAATCTCAATTCTCGGCA	CCCACACCACTTCCTTTTGT	187
Ad01A13926	Aradu.A01	13926	p2	(AT)7	14	77757667	77757680	CACCGGAGAAGAAGAAAATGA	AAAAAGAGCACCAAATCGGA	212
Ad01A13929	Aradu.A01	13929	p2	(TC)9	18	77772735	77772752	AGGAAAAAGGGGAGCAACAT	AGGCTCCACAAATCCATGAG	256
Ad01A13930	Aradu.A01	13930	p2	(AT)11	22	77773361	77773382	TTGAAATTGTTGAGTTTTAATGCC	CTCCGGAACATTCTTCTGTTG	264
Ad01A13931	Aradu.A01	13931	p2	(CT)12	24	77775795	77775818	AGCCAAATGTTTGGAGTTGG	ACTGAAACAACCAGCATCCC	258
Ad01A13935	Aradu.A01	13935	p2	(AG)25	50	77781339	77781388	GAAATCCTGATTCCCCCAAC	ATGTCAGCTTGTTGCCACTG	256
Ad01A13939	Aradu.A01	13939	c	(T)10gagaaaac	50	77793096	77793145	TGTGCAAAGATTTAAGATTTTATTAGA	TGTTGCGACCATACTCAAGC	251
Ad01A13940	Aradu.A01	13940	p2	(AT)11	22	77794823	77794844	TGACCCGTATAATCCCCGTA	GACGTGCGAGAAGAGGGTAG	270
Ad01A13945	Aradu.A01	13945	p2	(AT)6	12	77805492	77805503	TCACTCACAGTTTTCGTCAATG	TTGTCCTCCAACCATTCCAT	235
Ad01A13947	Aradu.A01	13947	p2	(AC)7	14	77808778	77808791	AGTTGCTTCATGCTTCCGTT	CCTTCGTTTCTTCCAGCTTT	262
Ad01A13949	Aradu.A01	13949	c	(TC)11tt(TC)6	36	77812082	77812117	GATGTTGGTCCTTTGCCCTA	CCGTCACTATCACTGGCTGA	244
Ad01A13952	Aradu.A01	13952	p2	(TA)6	12	77815954	77815965	TGCCATCTCATGACTCTCTTCT	TTTTGAAAAAGACATTTGTACATCA	267
Ad01A13953	Aradu.A01	13953	p2	(AG)6	12	77821309	77821320	TTCAGTGCAAAATGCCAATC	GGGGCTACATCTCCTCATCA	265
Ad01A13955	Aradu.A01	13955	p2	(CT)7	14	77822970	77822983	TCTCTTATTCTCGCCGGAAA	TGCCCATGGAGGTTTCTTAG	152
Ad01A13960	Aradu.A01	13960	p2	(GA)6	12	77838874	77838885	TCTATCTTGAAGTCGCCGCT	ATAAACGGCAACTCCTCCCT	249
Ad01A13961	Aradu.A01	13961	p2	(AT)10	20	77840561	77840580	AATTCTGGGAATGGTACCCC	AAACCTTAGACTGTCCCCCG	106
Ad01A13964	Aradu.A01	13964	p2	(TA)7	14	77846103	77846116	CCATTTCCATCCATACCCAC	CTGTTAGTGAAGAATTGGAGCG	264
Ad01A13966	Aradu.A01	13966	c	(A)12gaagcaca	113	77852850	77852962	ATGGGTAATGTTGACGACGA	GATGTTATGATGATTAAGGCATTCTT	279
Ad01A13970	Aradu.A01	13970	p3	(CAT)9	27	77866327	77866353	TCCCAACAACTTTCTCCACA	TGGAGTACCGGATAGGGTTG	274
Ad01A13971	Aradu.A01	13971	c	(A)10tatattatat	106	77869716	77869821	TTTGAGTGAAAAATATAGGGTAATTGA	TGCCGTAACATGCCATACAC	271
Ad01A13973	Aradu.A01	13973	p2	(AC)6	12	77882016	77882027	CACCTTTCAAACCCTTTCCA	TGCGAATGAGTTGATGAATGA	252
Ad01A13977	Aradu.A01	13977	p3	(AGA)5	15	77904185	77904199	CCCGACTTAGGTGCCATAAA	ACCCACTCTTCCCCATTACC	223
Ad01A13980	Aradu.A01	13980	c	(TA)10(TG)11	58	77918922	77918979	CGCCACAAATAGAGAGCACA	CACCCTCTCGTCACTCTTCC	259
Ad01A13981	Aradu.A01	13981	p2	(TA)8	16	77919315	77919330	CTCGGAGAATTTTGCCATTC	TTTGATTTTGACCCCAGACC	258
Ad01A13987	Aradu.A01	13987	p3	(AGT)5	15	77926664	77926678	TCGCTTCAGCGTATTGTGAC	TTATTCAAGAGTGAGGGCCG	117
Ad01A13991	Aradu.A01	13991	c	(AT)6nnnnnnn	66	77978188	77978253	TTGGTCATGCATAATCATCAATAATA	TTCTCCAGCACTGCAAAACTT	273
Ad01A13994	Aradu.A01	13994	c	(A)13ccaaaaaa	44	77994648	77994691	CTAACATTGCATGATTCCTGG	AATGCAAGAAGAACGGAAACA	200
Ad01A13996	Aradu.A01	13996	p2	(TA)6	12	78015512	78015523	TCGACCTACAAAGCAAAGGC	TCAGATGCAGGACGTGAGAC	258
Ad01A13997	Aradu.A01	13997	p2	(AT)7	14	78018275	78018288	TGTTTTTCTTCTTAATTTTCGCA	AACATAAGCTTTTGAAAATTGGATT	209
Ad01A13998	Aradu.A01	13998	p2	(CA)7	14	78018830	78018843	CCAAGGTTCTGAAAACCGAA	TGCTGTGGTTGTTCTTTGATG	260
Ad01A14001	Aradu.A01	14001	p2	(CT)8	16	78063633	78063648	TACCTCGGTGACCTTATGGC	TAAGCGAATTGAGCACGATG	262
Ad01A14002	Aradu.A01	14002	p2	(TG)7	14	78064057	78064070	TGGCGAACATAGTCTCTGTGA	GGGCTCTTCTCTGCATTGAG	269
Ad01A14003	Aradu.A01	14003	p2	(TC)7	14	78067261	78067274	TGATTCATAGGCATACCGCA	CAACCTGTCAGAGGCACAAC	280

ID	Chromosome	SSR nr.	SSR type	SSR	Size	Start	End	FORWARD PRIMER (5'-3')	REVERSE PRIMER (5'-3')	Product (bp)
Ad01A14005	Aradu.A01	14005	p2	(GC)6	12	78076627	78076638	CCAGTTGGGTTGGAAAGCTA	ACAAGGCTCCTAAAACCGCT	141
Ad01A14006	Aradu.A01	14006	p2	(TA)8	16	78080055	78080070	CTCCACAGATGCATTGGTTTT	TTGATTTTGGGAAAGTTGGG	260
Ad01A14009	Aradu.A01	14009	p3	(AGA)5	15	78116816	78116830	GGTCTTGATGGGCTTTTGAG	CCCCCTTAGCCAAATACACA	252
Ad01A14011	Aradu.A01	14011	c	(T)11cgtttttctc	42	78134077	78134118	TCCCCAATTAGAGGTGTCCA	TTGTTGTTCATGAATGTTGGC	229
Ad01A14012	Aradu.A01	14012	c	(AGA)7tatgga	42	78152572	78152613	TTAGAATGTTGCTCGCCCTC	CCCCTTTAGCCGAATACACA	103
Ad01A14013	Aradu.A01	14013	p2	(TA)13	26	78164315	78164340	ATTTTTGGCATGGAGGACTG	CTATGCAATGCAAAATGCAA	264
Ad01A14014	Aradu.A01	14014	p2	(CT)6	12	78170845	78170856	TCCATAAGTGAAATTTGGGGA	CGATTTCTGGGCTATTTCCA	257
Ad01A14016	Aradu.A01	14016	p2	(TC)8	16	78204427	78204442	CATAGCGAGTTCTGAAGCCC	TCCTTGAAGCAAATAAAATCCT	205
Ad01A14018	Aradu.A01	14018	p2	(TC)10	20	78210488	78210507	CATAGCGAGTTTTGAAGCCC	TGAAAAAGGAATATTGAACCTGG	243
Ad01A14019	Aradu.A01	14019	p3	(GAA)5	15	78225060	78225074	TTGCAACAAGTGGAAGGGAT	TTGGTGTGGAATCGTCTTCA	280
Ad01A14023	Aradu.A01	14023	p2	(AC)7	14	78259843	78259856	CCAAATGCATGCTCACAAAC	CCTTCAAAGGCTCCTCGTTA	280
Ad01A14024	Aradu.A01	14024	p2	(TA)9	18	78260226	78260243	ACGAGGAGCCTTTGAAGGAA	GAGAAGCAGGTTCTGCAAGC	261
Ad01A14025	Aradu.A01	14025	p2	(CT)7	14	78265253	78265266	AGCGTCAGAAATCGTGTGTG	GCAAACGGTGAGGAAGAGAG	247
Ad01A14026	Aradu.A01	14026	p2	(TA)9	18	78265373	78265390	CTCTCTTCCTCACCGTTTGC	TCAAGCAACGAATCAAGCAC	148
Ad01A14029	Aradu.A01	14029	c	(T)11actttaattt	42	78286350	78286391	TTACTCCCTAATGGCTTCGG	TTGGTGTATGTTTGGGCTGA	185
Ad01A14033	Aradu.A01	14033	p3	(ATA)5	15	78298472	78298486	TTTCGGCCGATGTGAATAAT	TCATTTACTTGGCCAAAGCC	276
Ad01A14034	Aradu.A01	14034	p3	(TAA)7	21	78298741	78298761	TACTCCCTTAAGGCATTCGG	GAGCAATTTTTGAGGACGAAA	269
Ad01A14043	Aradu.A01	14043	c	(TG)8(TA)10	36	78341001	78341036	TTCTCCTAATTGTAGCAAGTATTTTCA	GAATCGTAGCTAGTAGCCCTTAGGT	214
Ad01A14045	Aradu.A01	14045	p2	(AT)8	16	78342804	78342819	AGCGGAAAGGACTCAGATCA	CTCCACCTCAGCAGCTTACC	159
Ad01A14046	Aradu.A01	14046	p2	(AT)8	16	78353021	78353036	GTGGTTGGTGAAGCTCAGG	TTCATCAACAGCCTCTGTGC	250
Ad01A14049	Aradu.A01	14049	p3	(TTC)5	15	78378371	78378385	GAACTTCCGGGTACTCTCCC	TCAACAATTGCCATGAACAAA	140
Ad01A14052	Aradu.A01	14052	p3	(GAC)6	18	78383213	78383230	GTGCAGCCCATCAACTACCT	GTTGTGGAGGAAAGGCAAAA	100
Ad01A14061	Aradu.A01	14061	p2	(CT)7	14	78457958	78457971	TGATTACGATTGTGATGGACCT	TCAGTCCAAAGACCCAAACA	268
Ad01A14063	Aradu.A01	14063	p2	(CG)6	12	78469839	78469850	TCCATACCTTGACCCGAGAG	TGGTGTTTGGAAGTGTTGGA	253
Ad01A14065	Aradu.A01	14065	p3	(AAG)6	18	78510407	78510424	GCTTTTGTCTTCCCTTTCCC	TACCCTCTCCCTCCTCCCTA	271
Ad01A14067	Aradu.A01	14067	c	(A)11gaagaaaa	95	78520387	78520481	GGGAACATTGGATGGGATAA	TATTGACGTTCACCTTGGGG	261
Ad01A14069	Aradu.A01	14069	p3	(TTC)5	15	78538831	78538845	ACTCTTTTCTTCCCGGTGCT	TGCTTTGCGAAATGAAAACA	216
Ad01A14070	Aradu.A01	14070	p2	(CT)8	16	78545427	78545442	AATCTCATACCCAACCCACG	ATTGATGAGAGATGGGGCAG	272
Ad01A14077	Aradu.A01	14077	p2	(TA)10	20	78676383	78676402	AGCATTGAGGCTCTTGTTGA	TCAAGGTGACAATTCCGACA	155
Ad01A14078	Aradu.A01	14078	p3	(CAA)13	39	78679928	78679966	AAGCCAGAGGGGATCACTTT	AGCCTTACATGGACACCAGG	252
Ad01A14079	Aradu.A01	14079	p3	(AAG)17	51	78686090	78686140	GCTTTTGTCTTCCCTTTCCC	CCTTGGCCGAATACACATCT	227
Ad01A14081	Aradu.A01	14081	p3	(AGA)5	15	78711136	78711150	CTCCGGCCTTAGGTGTCATA	AAACCCCTCTCCCTCCACTA	235
Ad01A14082	Aradu.A01	14082	p5	(TTATC)5	25	78712858	78712882	CCTGGGTGCTCCAGTGTATT	GCCCAATAAAGGGTCCAAAT	192
Ad01A14083	Aradu.A01	14083	p5	(TTATC)5	25	78721632	78721656	CCTGGGTGCTCCAGTGTATT	GCCCAATAAAGGGTCCAAAT	192

ID	Chromosome	SSR nr.	SSR type	SSR	Size	Start	End	FORWARD PRIMER (5'-3')	REVERSE PRIMER (5'-3')	Product (bp)
Ad01A14085	Aradu.A01	14085	c	(A)11gatagtaca	34	78729801	78729834	TGTGGTTCATGAATGTTGGC	TCCCCAATTAGAGGTGTCCA	210
Ad01A14089	Aradu.A01	14089	p2	(AC)6	12	78771371	78771382	TTTCAAGGTTGTAATGGGGC	TGGGAATTATGGGTAGTCAGG	154
Ad01A14094	Aradu.A01	14094	p2	(TC)11	22	78804792	78804813	ACTTCCGTCACCTCCTCCTT	CCACCCCTTACCCCTACCTA	280
Ad01A14095	Aradu.A01	14095	p2	(AT)8	16	78807561	78807576	GCTGTCCATTACGGCAAAAA	TGAAAATTTTGTAACGGGAAA	141
Ad01A14097	Aradu.A01	14097	p5	(AAGAT)5	25	78843213	78843237	GCCCAAATAGAGGAACACGA	CCTGGGTGCTCCAGTGTATT	180
Ad01A14103	Aradu.A01	14103	p2	(CT)6	12	78958223	78958234	CCAAGACCCATTCTCCTTCA	CCTCCAACCTCAATCCAAAA	279
Ad01A14104	Aradu.A01	14104	p2	(CT)6	12	78964430	78964441	CCAAGACCCATTCTCCTTCA	CCTCCAACCTCAATCCAAAA	279
Ad01A14107	Aradu.A01	14107	p3	(AGA)5	15	78989266	78989280	CCATTTTCTTGGCCACAACT	AAACCCACACCTCTCTCCCT	275
Ad01A14111	Aradu.A01	14111	p2	(TC)6	12	79036638	79036649	TTTCTGACCCAGTTTTTGGC	AAGAACCTGGGATTGAGAGTCA	204
Ad01A14112	Aradu.A01	14112	p2	(AG)6	12	79049974	79049985	TCTGTAACATGGAAGGGTTCA	TTCGGCCCAGAAAACTAAGA	240
Ad01A14114	Aradu.A01	14114	p2	(TA)9	18	79060718	79060735	CCAGCGTGACTCTCTGTTCA	GTGCTAGGTGGGAGACAACC	258
Ad01A14117	Aradu.A01	14117	p3	(TCT)5	15	79090554	79090568	AAATGGCCGAACATTCACTC	TGCCATTGATGGTAGTGGAA	236
Ad01A14119	Aradu.A01	14119	p3	(TCT)7	21	79099542	79099562	CAAAACCCTCTCTTCTCCCC	GGTCTTGATGGGCTTTTGAG	273
Ad01A14120	Aradu.A01	14120	p2	(TG)6	12	79112169	79112180	CCGGCAACTTGGGTTAACTA	GTGGCAAGGCAGCTTTTTAG	279
Ad01A14121	Aradu.A01	14121	p2	(AT)9	18	79165870	79165887	TGAATCTCATGCATGCTTCC	GCAGAGTGGAAGGAGTCGTC	143
Ad01A14128	Aradu.A01	14128	p3	(AAG)5	15	79258564	79258578	TAAAATATTGCTCGCCCTCG	CTTCCCCATAAATCCCACCT	245
Ad01A14132	Aradu.A01	14132	c	(TA)8(T)10	26	79290517	79290542	TACACTTTGCTTTGGGGCAT	TTGGGAATTGGGTACATGCT	211
Ad01A14133	Aradu.A01	14133	p3	(GTT)5	15	79298845	79298859	CTTGAATGATGGTCCGGAGT	AGGGACAATCACCAACAAGG	280
Ad01A14137	Aradu.A01	14137	p3	(AGA)5	15	79331140	79331154	CTAGAGGTTTCTCCGGCCTT	CAAACCCACCCATAATGGTC	276
Ad01A14138	Aradu.A01	14138	p3	(AGA)6	18	79344242	79344259	TGGAAGCTGTTGTCTTCCCT	CAAACCCACCCTACATGACC	214
Ad01A14139	Aradu.A01	14139	c	(AG)7gctctctc	64	79350214	79350277	CGCGATCTGCAGAATTAACA	AAGAACATTGAACCTGGAATTGA	243
Ad01A14140	Aradu.A01	14140	c	(CT)9a(TC)7	33	79370085	79370117	ATTCAACCCCACCAACGATA	ACAGGAGATGTGGGTGAAGG	219
Ad01A14141	Aradu.A01	14141	p2	(CT)6	12	79372070	79372081	ATGGGCAGTTGTAGCCAAAT	TTGGCCCAGAAACACAGATT	273
Ad01A14143	Aradu.A01	14143	p3	(CAT)6	18	79391137	79391154	CTGAAGGGGCACCGTATCTA	TGTTGGTTAGTTGTGCGGAA	236
Ad01A14144	Aradu.A01	14144	p4	(TATT)5	20	79416334	79416353	TGCACTATCAAGGCAATCATTC	CAGCAGGGTGACTTAATGGC	213
Ad01A14145	Aradu.A01	14145	p2	(AG)6	12	79441473	79441484	TTGTATTTGGTGAGGATTGCC	GTTTTGGGAATCGTGGAGAG	228
Ad01A14148	Aradu.A01	14148	p2	(TC)6	12	79493189	79493200	TCAGTCCTCCAGTGATGACG	GGAAAGAGAGAACGGGGAAG	243
Ad01A14156	Aradu.A01	14156	p3	(GTA)7	21	79518466	79518486	AAGAGCAAGGGCGTGATTTA	AACTTAATTGGGAATAAAACCAGAA	238
Ad01A14160	Aradu.A01	14160	p2	(TG)7	14	79531528	79531541	TTCCTGTAGAGCCTGAGGGA	AAAGTCCCAAATGCATGCTC	131
Ad01A14165	Aradu.A01	14165	p3	(ATA)5	15	79630272	79630286	GTGGCAGGAGTTGTGTTGAA	GAGGAACGGTTTCGATTCTG	234
Ad01A14166	Aradu.A01	14166	c	(TCT)6tttctatc	142	79632928	79633069	AATAAATCCCAAAAACGCGA	GAGGAAGAAGAACGTGCAGC	220
Ad01A14170	Aradu.A01	14170	p4	(TGTT)6	24	79642412	79642435	TTCGGAAGACACTGTTGCTG	ATGTACCAGACCGCTTCCAC	230
Ad01A14171	Aradu.A01	14171	c	(ATT)13agttttt	78	79645096	79645173	GGTTTCCTAAATGTTATGGGAGTTT	AGGGAACAAGGGAATCAACC	163
Ad01A14182	Aradu.A01	14182	p2	(AG)6	12	79670218	79670229	AGCTGTTTCTTCACCTTCCA	GCATGTACCTCACGTGCCT	236

ID	Chromosome	SSR nr.	SSR type	SSR	Size	Start	End	FORWARD PRIMER (5'-3')	REVERSE PRIMER (5'-3')	Product (bp)
Ad01A14186	Aradu.A01	14186	p3	(TTA)5	15	79673094	79673108	TTCCGTTAATAGAAGCCAGCA	ACGTGTGGTTGTTGCTTTGA	241
Ad01A14188	Aradu.A01	14188	p3	(AGA)7	21	79677957	79677977	GCGTCGAAAATTCCACTAGG	ATTGATGACGTGGTTGGACA	273
Ad01A14189	Aradu.A01	14189	p2	(TC)10	20	79682345	79682364	AACAAGAATTTGGGCTTGGA	TTAGCCCTTGACCACGAAAT	265
Ad01A14191	Aradu.A01	14191	p3	(TAC)5	15	79708068	79708082	TTGGGAATAAAGCCAGAATAGA	AGCTGAAGCGCCAGAAATTA	198
Ad01A14193	Aradu.A01	14193	p2	(AC)7	14	79743935	79743948	CTGCAGTCCTTTCTCAGCCT	TCATCGCATTAAGACGAACG	244
Ad01A14194	Aradu.A01	14194	p2	(AG)8	16	79746851	79746866	TCAGCGATTTCTGGGCTATT	GGAACATTGAACCTGTGATGAA	234
Ad01A14197	Aradu.A01	14197	p2	(TA)7	14	79758234	79758247	AGAACCAATGCACAAGGGAC	AAGCATTGAACCCTCACTGG	260
Ad01A14198	Aradu.A01	14198	p2	(GA)6	12	79800220	79800231	AAGGCAGAATTTTGACCCCT	GTCCACTCGTGCCAATTTCT	195
Ad01A14202	Aradu.A01	14202	p2	(TC)6	12	79847071	79847082	AGTCCAGAAGGCAGAGACCA	TCAGAACCAAAAGGGGAAAA	250
Ad01A14204	Aradu.A01	14204	p2	(GA)6	12	79857622	79857633	AAGAGGTGGTGGGGAAGAGT	ACACACCTAAAGGGTGCAGG	196
Ad01A14210	Aradu.A01	14210	c	(A)10tttgatgtat	38	79913631	79913668	TTGCAAACCGTTCTTCACAA	CATTCCCCAATCATGGACTC	100
Ad01A14213	Aradu.A01	14213	p3	(GAA)6	18	79937502	79937519	GAAACTTAAGTGCCACCGGA	CGCCCTTCCTCTCTCTCTTT	156
Ad01A14214	Aradu.A01	14214	c	(T)12gaaatataa	42	79943042	79943083	AAGTTTGGTGTCTGCATGTG	GGCCCCTCCTTGTATTTGTC	244
Ad01A14215	Aradu.A01	14215	c	(T)10cttagagct	78	79949955	79950032	TGTCTTTGGCACATTTTCCTT	TAACCCGGGATTATCAACCA	253
Ad01A14217	Aradu.A01	14217	p5	(AATAA)5	25	79965733	79965757	GCAATTTGTAAAAACTGTGCCA	CCTAACCAATTTTTCTCTCCTCA	170
Ad01A14221	Aradu.A01	14221	p2	(CT)6	12	79988606	79988617	TAGGATTGCATGAAAAGGGC	AGTGGTGAAGAGGTGATGGG	215
Ad01A14223	Aradu.A01	14223	p2	(TC)8	16	79995562	79995577	TTGGCTTTTTCTGCTTGCTT	CGAATTCATCCTTGAAACTGG	266
Ad01A14226	Aradu.A01	14226	p3	(AAG)6	18	80014794	80014811	TTAGAATGTTGCTCGCCCTT	CCCATAAACCCCACCTACCT	245
Ad01A14230	Aradu.A01	14230	p5	(TAAAA)7	35	80033724	80033758	TTGAGGAGAGAAAAATTGGTTAGG	TGCTCACAATTTTCGAATTCAT	232
Ad01A14236	Aradu.A01	14236	c	(ATT)13(AGT	99	80091650	80091748	TCTAATTTATTCTCTTCTTTCAATGGT	AAAACACTTTTTAAGATACGGTAATCA	209
Ad01A14241	Aradu.A01	14241	p2	(GC)6	12	80097312	80097323	CCATTCGATGCCTTGATGTA	GTACGCGTGCGTGTCTCTAC	195
Ad01A14243	Aradu.A01	14243	c*	(ATA)5(AAT/	65	80113808	80113872	TTACATCGTCAACTCCCACG	GGCGATGTTGGTATTCCACT	207
Ad01A14245	Aradu.A01	14245	p2	(AT)8	16	80129316	80129331	TGCTTCCATATTGGATTACAAGAG	TTTTCTCCTTCATCATAAAATTCAAA	279
Ad01A14246	Aradu.A01	14246	p2	(AT)7	14	80143289	80143302	TCATCAAGAACAACATGAAGATCA	TCGAGTTTGGACAACTGACG	248
Ad01A14247	Aradu.A01	14247	p3	(AGA)6	18	80149564	80149581	TGCCTTCCCATTCATCTTTC	GCCAAAACCCACACATCTCT	186
Ad01A14252	Aradu.A01	14252	p4	(TTAG)5	20	80195618	80195637	CGCGTACATGTCACCTCTTG	CCAACACTAATCCCCAAAACA	278
Ad01A14254	Aradu.A01	14254	p2	(CT)7	14	80200179	80200192	TTTTCCACTTCCAATTTTCG	AGAGGGAGAGAGAGGGTTCG	131
Ad01A14258	Aradu.A01	14258	p3	(AGA)6	18	80248570	80248587	GCTTTTGTCTTCCCTTTCCC	CAAAATCTCTTTCCCACCCA	224
Ad01A14259	Aradu.A01	14259	p2	(GA)6	12	80248712	80248723	TTTTGAAGGTAGGTGGGGTG	AAATAAGCATGGAACTGGCG	263
Ad01A14262	Aradu.A01	14262	p2	(TG)22	44	80280077	80280120	AGGAACATCGGTTACATGGC	GTGTGATTGCAATTTCGTGC	194
Ad01A14263	Aradu.A01	14263	p3	(GTA)5	15	80293658	80293672	AGCTGAAGCGCCAAAAATTA	CATGCCTGATGATGATGAGAA	236
Ad01A14264	Aradu.A01	14264	p2	(AT)9	18	80293988	80294005	TGTGGTGGCAATACTTTTTGTT	TGGTTTTAAGGGCTGTTTGG	222
Ad01A14276	Aradu.A01	14276	p2	(AG)25	50	80461421	80461470	TGACAATTTTAAAGGTGGTTTTTG	TCCTTTCTCTTTCCTCTTTTTCAA	151
Ad01A14277	Aradu.A01	14277	c	(T)10atttctcctt	85	80466294	80466378	CACGTGTTTTCACGCTTCAT	TTCGCTTGATCTGATTCTCCT	229

ID	Chromosome	SSR nr.	SSR type	SSR	Size	Start	End	FORWARD PRIMER (5'-3')	REVERSE PRIMER (5'-3')	Product (bp)
Ad01A14278	Aradu.A01	14278	p2	(GA)6	12	80470813	80470824	AATGAATAAACCTGCGACGG	AAGACGCCACCAAATCAAAC	246
Ad01A14279	Aradu.A01	14279	p2	(AT)8	16	80474250	80474265	CACGAGAGATCGACACCTCA	GAACTAACATGTGCAGCTCCC	140
Ad01A14284	Aradu.A01	14284	p4	(TAAA)5	20	80561512	80561531	GGATCAGCTTCAACGAGAATG	TCCCTCTCTCTCATCCCTTG	224
Ad01A14285	Aradu.A01	14285	p3	(TAT)18	54	80567814	80567867	TTCATGCATCTTTTTCATCCA	AAAAATTGGGAAATGGGTGC	231
Ad01A14286	Aradu.A01	14286	p4	(TATG)5	20	80568512	80568531	GAATGCCCTTGAAACAAAGTTC	TTGCATTTTGCATCTTTTGC	249
Ad01A14289	Aradu.A01	14289	p2	(GT)6	12	80585828	80585839	ATAGCCGCTGACAGCACAC	TAGAAATGGAAAACCGGGTG	269
Ad01A14293	Aradu.A01	14293	p2	(CT)6	12	80632396	80632407	CCCCCTTCTTCCTTTTTCCT	TTTCTCAACAATCCACAAGCA	238
Ad01A14294	Aradu.A01	14294	p3	(AAT)5	15	80639768	80639782	TCTTTTGTGGCAGAGATCCTT	CAAGAGTTTAAGAATAAAAGCTTTACG	271
Ad01A14295	Aradu.A01	14295	p3	(TAA)6	18	80640921	80640938	CATGTAAGCCACGTTTTCGT	AACAATTTTCGAGGGCAAAA	272
Ad01A14298	Aradu.A01	14298	p2	(GA)6	12	80665204	80665215	GAAAAGGGGGTGGGTAGGTA	TCCCTTCTTTCTCCCTCCAT	273
Ad01A14299	Aradu.A01	14299	p2	(CT)9	18	80665999	80666016	TACACAGTAAGTGCGCGTCC	ATAGGGAGATCCTGGGCATT	250
Ad01A14301	Aradu.A01	14301	p3	(TCT)6	18	80685136	80685153	CCCAAACACACCCTAAATGG	CTAGAGGTTTCTCCGGCCTT	273
Ad01A14302	Aradu.A01	14302	p5	(AAAAT)5	25	80690652	80690676	TCCTTGTGACTCCTCCCATC	TCAAATCTTTTTCAAATCTTTTCTC	214
Ad01A14304	Aradu.A01	14304	p2	(GT)7	14	80692561	80692574	CATGTGAATAAGTGGGCAAGAA	ATGCATGTATGGCCAAGTGA	279
Ad01A14305	Aradu.A01	14305	p2	(AG)6	12	80693690	80693701	TTTCAGGCGGTATTTTGACC	GGAACATTGAACCTGGAGTGA	224
Ad01A14309	Aradu.A01	14309	p2	(AT)6	12	80748006	80748017	TCAAGGCATGCAACCAAATA	ACCTATGCTGGAAGGGATGA	168
Ad01A14314	Aradu.A01	14314	p2	(AG)6	12	80801793	80801804	AAGAACCTGGGATTGAGAGTCA	GTGTCACTTTTCCGCGACTT	254
Ad01A14316	Aradu.A01	14316	p3	(CTT)5	15	80805942	80805956	TCTTGACTTTCCCATGGAGG	ATGGATCGATTTCAGGCAAC	266
Ad01A14320	Aradu.A01	14320	c	(AT)7tctaactat	40	80858058	80858097	TCGGTTTTTACACCGGAAAG	TCAGACCCAGATCAGCATCA	218
Ad01A14324	Aradu.A01	14324	p4	(TTAT)5	20	80868301	80868320	ACTCAGAGCCTTGGACCTTG	TTTGAATCAAGGGATGTTGATG	162
Ad01A14325	Aradu.A01	14325	p4	(TATT)6	24	80871089	80871112	AACGCACCATGTGGTAAACA	TTGAAATTAAGTTTGGTGTCCTCA	126
Ad01A14326	Aradu.A01	14326	c	(T)10gatcttgtg	98	80874872	80874969	TAATCCCTTCCACCTGATGC	GCCATATCAAATTGCAATCCA	263
Ad01A14327	Aradu.A01	14327	p2	(AG)16	32	80880333	80880364	CCGGTGGTGAAGAAAGAAAA	TCACTTGTTCGCTTCTCGTG	279
Ad01A14328	Aradu.A01	14328	p3	(CTT)5	15	80886503	80886517	GCATGCAAACTGCAGGACTA	CAGGAGACAATGGAAGCACA	277
Ad01A14330	Aradu.A01	14330	p3	(TCT)5	15	80921008	80921022	TACCTCCCCCTCACTCTCCT	AGTGGTCTTGATGGGCTTTG	242
Ad01A14333	Aradu.A01	14333	p2	(TG)7	14	80928419	80928432	TTTTGGTTGTGAAGGCAACA	TTCATTCCAAGCCAAGATCC	202
Ad01A14338	Aradu.A01	14338	p2	(AT)8	16	80942438	80942453	TTCGGTATTCAGGGATGGTC	GACCTCTAAAATGCAATGCG	240
Ad01A14339	Aradu.A01	14339	p3	(AAT)14	42	80944301	80944342	TCACTCTAAAATTTCTTTGGCTG	AAAACACAACAAATAAAACGAAAAA	231
Ad01A14344	Aradu.A01	14344	p2	(AG)9	18	80974891	80974908	CCGGTGGTGAAGAAAGAAAA	CGTACGCACATGACTCGTTT	233
Ad01A14353	Aradu.A01	14353	p2	(CT)6	12	81106449	81106460	TTTCGAAATCTTTCCTCCCC	ACCAGCTCTTTGGCAACTGT	214
Ad01A14359	Aradu.A01	14359	p2	(AT)7	14	81182653	81182666	GACAAAATTCTCCCAAGGCA	CTGAACACACAAATCACGGC	280
Ad01A14360	Aradu.A01	14360	p3	(AAC)5	15	81184866	81184880	ATTGGCAGGACAACTCCAAC	GAGGTGCTCTGTAAGGCTGG	156
Ad01A14365	Aradu.A01	14365	p2	(CT)8	16	81275152	81275167	GCTCAAGTTATGGCTCTCGG	CAAAGTGGACCAAACGGACT	206
Ad01A14368	Aradu.A01	14368	c	(T)10ctattaattt	58	81296304	81296361	TCCTCATCATTTTCTGCTGCT	CAGCATTATGTGTTTCTTCTCCTT	186

ID	Chromosome	SSR nr.	SSR type	SSR	Size	Start	End	FORWARD PRIMER (5'-3')	REVERSE PRIMER (5'-3')	Product (bp)
Ad01A14369	Aradu.A01	14369	p3	(CAT)5	15	81296626	81296640	TCTGCTTCTTCTGCTTCTTCTTC	TTTGCTACAAATGCACAACAA	278
Ad01A14370	Aradu.A01	14370	p5	(AAAAG)5	25	81298003	81298027	TTTTAATCGACCCCATTTGA	TATTGTTGAACCGGCAACTG	268
Ad01A14371	Aradu.A01	14371	p3	(ATT)18	54	81298400	81298453	TTGCTAAGATCGTTGTTCTTATTACC	ACGTCAGCAAATTGACTCCC	168
Ad01A14372	Aradu.A01	14372	p2	(TG)9	18	81350429	81350446	CTTCTTGAACAGGTCAGGGC	AAGCGTAGTTATTTTACGAGGCA	233
Ad01A14374	Aradu.A01	14374	p2	(TC)14	28	81355445	81355472	TTTCAATGCAAAAGCAGCAC	ATGTTATGCTTTTCGGGGC	225
Ad01A14376	Aradu.A01	14376	p4	(TAAT)6	24	81357328	81357351	GGAGCGAACAAGCAAGGTAG	ATTAGGGCCACACAAAACCA	219
Ad01A14377	Aradu.A01	14377	p2	(CT)10	20	81357473	81357492	TGGTTTTGTGTGGCCCTAAT	TTGACCAGATGACAGCCAAG	180
Ad01A14378	Aradu.A01	14378	p5	(AAAGA)5	25	81360931	81360955	CCGAAGCAACAACCAAAGTT	TTGATTTGTGGAATGGATTTGA	178
Ad01A14382	Aradu.A01	14382	p3	(ATT)7	21	81365885	81365905	GGATCATCCACAATGGGTAA	AATCAAACAACTCGCATGGTC	275
Ad01A14383	Aradu.A01	14383	c	(ATT)19gttgttg	91	81367347	81367437	CTGCTGGAAGCGTGGTTAAT	TGACTCCACCTACGCACAAG	241
Ad01A14393	Aradu.A01	14393	p2	(AG)6	12	81434351	81434362	CGTGACATGGGAGAAATTCA	CCCAAATCCAAGTCCAGAAA	280
Ad01A14394	Aradu.A01	14394	p2	(TA)6	12	81435247	81435258	GGAATGTCATGCAATGCAAC	CAAAATCCAGTGAGGGTTCAA	276
Ad01A14396	Aradu.A01	14396	p2	(AT)7	14	81450643	81450656	CTGACCTATCCAGGAACCGA	TGGATTTGACACTACTCACCAAA	280
Ad01A14397	Aradu.A01	14397	p5	(AAGAT)5	25	81451832	81451856	GCCCAAATAGAGGAACACGA	CCTGGGTGCTCCAGTGTATT	180
Ad01A14401	Aradu.A01	14401	c	(CTT)5gctgcac	54	81474507	81474560	CCACATACTGTTACGGCACG	TTTCTCCTCCTCATCTTCTGC	220
Ad01A14402	Aradu.A01	14402	p3	(GAA)9	27	81475020	81475046	ACACCCAAAATTCGTTGCAT	TCCTCTTCCTCCTCCTCCTC	259
Ad01A14403	Aradu.A01	14403	c	(A)10c(GAA)5	114	81475161	81475274	CGTGCAGAAACGAAAGAAAA	CGCGCATTAACTCATTTTGA	271
Ad01A14406	Aradu.A01	14406	p3	(ATA)18	54	81478746	81478799	TGCCTAAAACCAAACCAATGA	GAGATTTTGGCAGCTGAACC	203
Ad01A14409	Aradu.A01	14409	p2	(GT)8	16	81495478	81495493	ATAATGGAAACGGGATGCAG	TCATAACCCTAATACAACACACTCTCA	278
Ad01A14412	Aradu.A01	14412	p2	(GC)6	12	81523244	81523255	ATGCGGTTCTAGTTGGGTTG	AAGTGGCCATGGACAGAATC	134
Ad01A14413	Aradu.A01	14413	p2	(TA)7	14	81570739	81570752	GCCCAAAGCACTCTGTCTTC	TGGATCTTGAAAGAATGATGGA	261
Ad01A14419	Aradu.A01	14419	p2	(GA)6	12	81660736	81660747	TGAAAGTGAGGTAGGTGGGG	GGATTGCACAATAAGGGCAT	205
Ad01A14422	Aradu.A01	14422	p3	(GAA)5	15	81686980	81686994	CCACGATGGCAATGACAATA	AGGCGTCCCAGTATGTTTTG	200
Ad01A14423	Aradu.A01	14423	p3	(CAA)5	15	81687579	81687593	TAAGTCATCAGCATTGCCCA	CTTTGGCTCTTCCTCCTCCT	277
Ad01A14424	Aradu.A01	14424	p3	(AAG)10	30	81692530	81692559	AGCGATGCTTTTACCACACC	CCCATAAACCCCACCTACCT	274
Ad01A14426	Aradu.A01	14426	p2	(AT)11	22	81706513	81706534	CATTTGTTTTGAAAATCCTCATCA	CCATGATGGAGAAAGGGAGA	162
Ad01A14430	Aradu.A01	14430	p2	(AT)8	16	81734758	81734773	CAGCGTGGCATACGACATAC	ACAGAAAAGCCAAAAGGCAA	150
Ad01A14436	Aradu.A01	14436	p6	(AGAGAC)5	30	81799197	81799226	TTCCATACACAGCATGGGTT	TTGAATGCTTCTTGTGTTCCC	269
Ad01A14440	Aradu.A01	14440	p2	(CA)7	14	81802863	81802876	CACGTCGTAAAGCAAAGGCT	GAGCTGCGGCTAGATCGTTA	258
Ad01A14441	Aradu.A01	14441	p3	(AAT)13	39	81806501	81806539	CTCTGCACAATCAAACCGAA	GTGAGAGTGCGTGATGCTGT	216
Ad01A14443	Aradu.A01	14443	p2	(AT)7	14	81827521	81827534	GATTCCAGAGTGTTGGGCTC	TTTAAGGGCAAATTGAGTTTTT	274
Ad01A14444	Aradu.A01	14444	p2	(TA)6	12	81849314	81849325	CTAGACTCGACCTGCGAAGC	CGAAGATCTACCTTGGGGGT	186
Ad01A14447	Aradu.A01	14447	p2	(GA)6	12	81874898	81874909	ATAATGGCACCCAAACAAGC	CCAGCCTCTGTCGATCTCTC	147
Ad01A14448	Aradu.A01	14448	c	(T)10cttatataaa	95	81876384	81876478	CAACATAAACATTCGGTGATGG	CTGTGTGTACCAAATTCGTGG	250

ID	Chromosome	SSR nr.	SSR type	SSR	Size	Start	End	FORWARD PRIMER (5'-3')	REVERSE PRIMER (5'-3')	Product (bp)
Ad01A14457	Aradu.A01	14457	p4	(GATA)5	20	81964006	81964025	TCTATCGGTGCTTCCGGTAT	TGTAGATCACGGTAAGAGAAAGAGAA	226
Ad01A14460	Aradu.A01	14460	p4	(TTTA)5	20	81987074	81987093	TTGTCCCATGTGCATTGATT	TTTTATGGGAACATGGGATGA	221
Ad01A14464	Aradu.A01	14464	c	(ATT)18gagtg	212	82004832	82005043	GGGACCTATTTATCATTTTCCC	TTCCTTAGACATAATAGTCTTTCGTG	274
Ad01A14465	Aradu.A01	14465	p3	(AGC)6	18	82005417	82005434	AAACCCTAATTGCATCCCCT	AGTACCGCATACCGACCTTG	260
Ad01A14467	Aradu.A01	14467	c	(T)10cttctttttct	50	82008103	82008152	CGGTCGTTTCTTGTTCGATT	AGGTGATGTTGATGACGACG	276
Ad01A14468	Aradu.A01	14468	p3	(TCT)5	15	82008593	82008607	TGGGTGTAACACAGATATTTGGA	TGCAGCATTATGTGTTTCTTCTT	263
Ad01A14469	Aradu.A01	14469	c	(A)10caccacaa	45	82010139	82010183	TCCACCTGCAATTCTTCTCC	CCTCGTGGAATCAACATTCA	236
Ad01A14470	Aradu.A01	14470	p2	(GT)6	12	82016518	82016529	AGCCAAATTTGAGCCTACGA	TTCTCTCCCAATGTCCCAAG	162
Ad01A14471	Aradu.A01	14471	p2	(AG)7	14	82072718	82072731	TCAAAATCTCACAGGTTGTGTGT	TTAAGTGTGGGGAGGTTGGA	279
Ad01A14472	Aradu.A01	14472	p3	(AGA)5	15	82074671	82074685	TGGAGAATGTGAGTGAGAGAAAA	CACGTCCCTCACCTTTAAGC	251
Ad01A14475	Aradu.A01	14475	p4	(AGTG)6	24	82079034	82079057	CCCCTTTCTTTTCCTTGCTT	TTTTAAAAACAATCCCTAATTCTCAAA	220
Ad01A14476	Aradu.A01	14476	p2	(AG)8	16	82085153	82085168	CCGTGTTTTGACCCAGATTT	AAAAGGAACATTGAACCTGTGA	218
Ad01A14477	Aradu.A01	14477	p2	(AG)9	18	82088314	82088331	GTATGCGTGACATGCGTGAT	GGAACATTGAACCTGTGATGAA	274
Ad01A14479	Aradu.A01	14479	p2	(AG)6	12	82117852	82117863	TTGTCAAACACGTGATGGGT	CCTTTGCTTTCATCTTGTTCC	272
Ad01A14480	Aradu.A01	14480	p2	(TA)8	16	82119337	82119352	TTACTTGGGGCATTTTGTCC	GTTTGGGAGAGTTGGGAACA	121
Ad01A14482	Aradu.A01	14482	p2	(AG)6	12	82136275	82136286	TCCAAATCCATCAAGAGTTCG	ATTCAAGCTTGGGCAAAAGA	220
Ad01A14484	Aradu.A01	14484	p2	(CT)6	12	82162402	82162413	TCACCTTCCTCTGTTTCAAAAAT	CAGAGAGGAAGAGAGGGTTCG	234
Ad01A14485	Aradu.A01	14485	p2	(CT)7	14	82166801	82166814	ATTGTAAGGGCGTTTTGCAC	AGGTAGGTGGGATTTTTGGG	195
Ad01A14488	Aradu.A01	14488	p2	(TA)8	16	82185779	82185794	TGGGGTAATTTTATCCCCTTA	TTTGGGAAGATTGTGGGAAC	217
Ad01A14491	Aradu.A01	14491	p2	(TA)8	16	82208623	82208638	CTCAAAGCAAGAGGGGTGAG	TTGCAGACGTGTGCGTTATT	149
Ad01A14494	Aradu.A01	14494	p2	(GT)6	12	82227020	82227031	GATCGGCCCTTACTCACGTA	CCCATTTTCCCACACTTGAA	174
Ad01A14499	Aradu.A01	14499	p2	(AC)6	12	82283996	82284007	AGCAACACCATCAATCCACA	TATCAGCCATCCACCCAAAT	278
Ad01A14500	Aradu.A01	14500	p2	(TA)10	20	82297736	82297755	AGAGATCCCTTATGCCAACG	CACCCTACCATTCATTGCCT	206
Ad01A14501	Aradu.A01	14501	p3	(TAA)6	18	82299425	82299442	TGAATTTTGTTTGGTAATGAACTGA	AAAAACAGGATTTTAAAACACGAA	236
Ad01A14508	Aradu.A01	14508	p2	(TC)7	14	82311891	82311904	TTCCAACTTTTTGTTTGGCT	GAGAATCTTCACTTCCTCTCCA	280
Ad01A14515	Aradu.A01	14515	c	(GA)6gtggat(C	32	82331397	82331428	GCCGAAAGGAAGATGAGGTA	TTCTCTTCTGCCATAACCGC	261
Ad01A14519	Aradu.A01	14519	p2	(TC)8	16	82364247	82364262	TGAAACACGGTATAAAGAGAGTCAA	GCATGTATTTTATACTGGTAGCGAA	242
Ad01A14520	Aradu.A01	14520	p2	(TA)6	12	82370386	82370397	CCCTAACCGGCCTAAACTTC	GATAAACCGTGAAGCCGAAA	117
Ad01A14522	Aradu.A01	14522	p2	(TA)6	12	82397307	82397318	TACCCTAGCCGGCCTAAACT	CCATCTTGTTAGACGCACCC	271
Ad01A14526	Aradu.A01	14526	p3	(TCT)5	15	82440088	82440102	CCTACTCCCTCTCCCCTCAC	GCTTTTGTCTTCCCTTTCCC	271
Ad01A14527	Aradu.A01	14527	p2	(CT)8	16	82465524	82465539	CGTCCTTCTCCCTTTTTCCT	AATTTGCAACAATCCACAAGC	251
Ad01A14533	Aradu.A01	14533	p3	(ATT)8	24	82511106	82511129	TGGATTAAGAGCTGCTTGGAA	GTCCTCTCACCCTCAAGCAC	269
Ad01A14534	Aradu.A01	14534	p3	(AAT)5	15	82511429	82511443	CGAGAATGGCCCACAACTAC	TCTTGAACTTTCATGGAGCTGA	279
Ad01A14539	Aradu.A01	14539	p2	(AC)6	12	82547987	82547998	CGGTGCTGCGTATTCTTTTT	GGGAAGTGCATGTTCCTTTT	270

ID	Chromosome	SSR nr.	SSR type	SSR	Size	Start	End	FORWARD PRIMER (5'-3')	REVERSE PRIMER (5'-3')	Product (bp)
Ad01A14542	Aradu.A01	14542	c	(A)10taataataa	72	82566178	82566249	TTCTTCCTTTAATAAGTCTGACTATCC	TTGCGTCATTTTAAACCTTCTTT	178
Ad01A14546	Aradu.A01	14546	p2	(CT)8	16	82642485	82642500	GTGATAACAGGGCAACCTGC	CCAGAGGAGAGAATTCACCG	247
Ad01A14547	Aradu.A01	14547	p3	(TCT)5	15	82648698	82648712	CACCTCCCCTACTCTCCTCC	CTCGGAGTTGGAAGCTTTTG	195
Ad01A14551	Aradu.A01	14551	p3	(TGT)6	18	82669082	82669099	TTGGATGTGGATAGTGTGCC	ACATTCTTTTGGATGTCGCC	219
Ad01A14555	Aradu.A01	14555	p3	(AAT)5	15	82682818	82682832	TTGCCATTTTCTTCAAACACA	TTGCCATTTTCTTCAAACACA	253
Ad01A14557	Aradu.A01	14557	p3	(TCT)6	18	82705831	82705848	CACCCTAAATGGCCGACTAA	TCGTCCTCGAGCAATAGAAGA	177
Ad01A14558	Aradu.A01	14558	p2	(AT)10	20	82726775	82726794	CAAGCTGCGGCTAGTTCTCT	GTCCTTTGTAAGGCCCCAAT	265
Ad01A14560	Aradu.A01	14560	p2	(AT)8	16	82738586	82738601	TATGCAATGCAGCACAATCA	CTTCGTGTGGACGTTTTGTG	254
Ad01A14562	Aradu.A01	14562	p2	(AT)7	14	82744426	82744439	TTGTCCCCTTTCATTTCCTG	ATGAACCCCAAGAAAAAGGG	139
Ad01A14565	Aradu.A01	14565	p2	(AT)8	16	82783171	82783186	CATCATTCAGTCATCCAGCC	CTCCTTGGACAGGTCAGAGC	135
Ad01A14568	Aradu.A01	14568	p3	(TAC)5	15	82822573	82822587	CAACAAGTATTGCCACCACA	TGCTTCTCAAAACACAGGTTG	274
Ad01A14570	Aradu.A01	14570	c	(T)10aaaacgaa	60	82851708	82851767	AGCATGCAATTGACACCAAA	GTTAGTTGTGCGGACTGCAA	278
Ad01A14571	Aradu.A01	14571	p3	(TTC)6	18	82884455	82884472	TACCCTCTCCCCTTTCCCTA	CTAGAGGATTCTCCGGCCTT	247
Ad01A14576	Aradu.A01	14576	c	(A)10tgaaaaaa	97	82992223	82992319	TAGTTTTGGTGAGGCTTGGG	TTTTGTCCCCTTCATTGCTC	254
Ad01A14580	Aradu.A01	14580	p2	(TA)6	12	83051835	83051846	TTTCTTTCCCATCTCATTTGC	ATGGGTTTATCAACCTCCCC	270
Ad01A14583	Aradu.A01	14583	p2	(AT)13	26	83082595	83082620	TTTTCCCTCGCCTTTATCTG	ATGCGCCACTCAGATAGCTC	247
Ad01A14584	Aradu.A01	14584	p2	(TA)6	12	83088083	83088094	CGGTCTAGGTTTTGCCTCAA	CAAATGTAATTCTCTCTCTTTTTGTTT	236
Ad01A14585	Aradu.A01	14585	c	(TA)6ataatatta	47	83092982	83093028	TCGTTAGAGCTTCGTGCTGA	CAAATGCAATTCTCTCTCTTTTTG	203
Ad01A14586	Aradu.A01	14586	p2	(TC)6	12	83104455	83104466	TTCGAAAATCACTAACCCTTTTTC	TCCAAACACAAGGGTGAAGA	136
Ad01A14588	Aradu.A01	14588	p3	(AGA)6	18	83135466	83135483	CAGGACTCAGAGGTGGAAGC	CTCTCTTGACCGAAACCCAC	219
Ad01A14592	Aradu.A01	14592	p2	(CT)8	16	83174906	83174921	TGTCACAACGGCTGGACTAC	CCACCCCCTTTTCTTCCTAA	189
Ad01A14598	Aradu.A01	14598	p5	(ATTTT)6	30	83218582	83218611	TTGAATTTTGTCCCATGTGC	TGGGGAATTTTATGGGAACA	225
Ad01A14599	Aradu.A01	14599	p3	(ATA)5	15	83240143	83240157	AGCAAATTTACAAGCGCGAG	GTCTAAACTTCGCGGGCTAC	158
Ad01A14605	Aradu.A01	14605	c	(A)10gaaac(A)	25	83292508	83292532	GGGGATTGTAAAGATGGCTG	CTTTTTGGCTTTTCGAGTGG	215
Ad01A14607	Aradu.A01	14607	c	(A)11tttcagattt	83	83298469	83298551	TTGCAAACGTGTGCCTTATT	TTTAAGCCAGAGGGGTGAGA	214
Ad01A14609	Aradu.A01	14609	c	(T)10cttttatttga	103	83311117	83311219	TTGAGTTCTTGACTTCTTGCAC	AAGAGAAAGGTTTGATTATTATGTTGA	250
Ad01A14611	Aradu.A01	14611	c	(GAG)5a(AGC	34	83315178	83315211	ACGTGGCACAACTTAAGGCT	AAGATTTATCATCGTGGCCG	145
Ad01A14617	Aradu.A01	14617	p2	(GA)6	12	83371582	83371593	TGGGATGATCTCCCTCTCAC	TGGTGTTGGGTCAAAACCTT	246
Ad01A14619	Aradu.A01	14619	p3	(AGT)5	15	83398287	83398301	ACCCACTTCCCAAAACACAG	AACAAAAAGTATTGCCACCACA	279
Ad01A14623	Aradu.A01	14623	p3	(TTA)18	54	83407378	83407431	AAAAAGAAACAACTCCAGCATC	TATGCTCCATAAGCCAACCC	161
Ad01A14624	Aradu.A01	14624	p2	(GA)6	12	83409637	83409648	ACTGGTTTTGTGTGTGCGAG	AGCCTTAAGTGTGGGGAGGT	268
Ad01A14625	Aradu.A01	14625	p2	(TA)10	20	83413642	83413661	CGTCATCCAACTGACGAAAA	TTGGTATGTTTTTGTTTCCAGG	269
Ad01A14631	Aradu.A01	14631	p2	(AG)8	16	83457345	83457360	TGGACATTTGGTGAATTTGC	TTGTCACAAATTTCGTTGCG	271
Ad01A14632	Aradu.A01	14632	p2	(TC)6	12	83474669	83474680	AGGGGGCTCGCATAATTCTA	AAACTGAAAATGCCTCAATACCA	259

ID	Chromosome	SSR nr.	SSR type	SSR	Size	Start	End	FORWARD PRIMER (5'-3')	REVERSE PRIMER (5'-3')	Product (bp)
Ad01A14637	Aradu.A01	14637	p2	(TC)8	16	83514638	83514653	CTAAAACGCAATCACGCAAA	TCACCTCTGTTTTGTGCCAG	244
Ad01A14640	Aradu.A01	14640	c	(GA)6aaactgag	100	83569567	83569666	GACGGAATGGGTGAGAGAGA	TTCAAAACAAGAGGCAACCC	222
Ad01A14642	Aradu.A01	14642	p2	(TA)8	16	83619387	83619402	GGAAGGCAATGCAACCTAAA	TTTCTTTCCCATCTCATTTGC	192
Ad01A14644	Aradu.A01	14644	p2	(AG)6	12	83626486	83626497	AAACTGAAAATGCCTCAATACCA	CCATTCAGGGAAAAGGCATA	265
Ad01A14645	Aradu.A01	14645	p3	(TTC)6	18	83633077	83633094	TGATCGAAACTTCACCCTCC	CTAGAGGATTCTCCGGCCTT	256
Ad01A14652	Aradu.A01	14652	c	(GT)11(AT)6	34	83688891	83688924	GCTCGCAAAGATAAACCTGC	GATGTAGGACGTGAGGCTCC	278
Ad01A14654	Aradu.A01	14654	p2	(CT)6	12	83700281	83700292	GGTCGCGTGGAACAATTTAT	GTGCTAGGTGGGAGACAACC	272
Ad01A14655	Aradu.A01	14655	p2	(GA)9	18	83705035	83705052	TCCCTCTCACTCATCCAACC	TGGTGTTAGGGCAAAACCTT	242
Ad01A14658	Aradu.A01	14658	p3	(CAT)5	15	83751387	83751401	AACATCGGGATCAGACTTCG	CCTTTCCCTTTTTAGCCTGG	172
Ad01A14662	Aradu.A01	14662	p2	(AT)7	14	83771406	83771419	GTTCAGAACATTGGTTGGCA	TTGGGGTATTTTGTCCCCTT	277
Ad01A14663	Aradu.A01	14663	p2	(GT)6	12	83776587	83776598	GCTTTCATGCTTCTTCACCA	GCAAAAGCAAGGAAGCCTAA	256
Ad01A14666	Aradu.A01	14666	c	(T)11acctttgaa	40	83799171	83799210	TGAGTTGAGTGGCCATTGTT	GAAGCAGAGCACTCTCCTAACC	260
Ad01A14669	Aradu.A01	14669	p2	(GT)6	12	83806139	83806150	GCTTTCATGCTTCTTCACCA	GCAAAAGCAAGGAAGCCTAA	255
Ad01A14670	Aradu.A01	14670	c	(T)10gcatgcatg	40	83811143	83811182	TCCTTTTCCTGCATTTTTGG	CGAATTTTGGAATGTTGGCT	217
Ad01A14671	Aradu.A01	14671	p2	(TA)6	12	83818671	83818682	AACCGAATTTTCGGAACCAT	GTCCCAATCCCTTGGTGACT	138
Ad01A14672	Aradu.A01	14672	p3	(AGA)6	18	83825160	83825177	ATGCTTTTACCGCACCAAAC	CCCATAAACCCCACCTACCT	260
Ad01A14676	Aradu.A01	14676	p2	(TC)6	12	83871550	83871561	CCCTTTCCCCTTCTACCTCA	TTTGCAATAATCCACAAGCA	247
Ad01A14677	Aradu.A01	14677	p2	(AT)8	16	83875251	83875266	TTTTCAGGGTTGTAATGGGG	CCCAAATGCAAAATAAAGCAA	232
Ad01A14678	Aradu.A01	14678	p2	(GT)6	12	83881451	83881462	AACGCTTTCCTGGGGTACTT	GAGTCCTTTGACTTGCGAGG	279
Ad01A14679	Aradu.A01	14679	p2	(TA)6	12	83882447	83882458	TTGGATAGGTCAGAGCCTGG	TGTTGTTATTTTGCGAGGTGTT	272
Ad01A14686	Aradu.A01	14686	c	(A)10tatcttttcta	57	83984610	83984666	TGTTTTTCTCTTTCATCATAAAATTCA	ATGGAGCAAGGGAGATGGTA	280
Ad01A14690	Aradu.A01	14690	c	(A)11gaaaaaga	36	84018509	84018544	AAGTGTGGGAAAACTGTGGG	TTGGGGTAATTTTGTCCCCT	247
Ad01A14691	Aradu.A01	14691	p2	(TC)6	12	84026027	84026038	TGCATCCATTTAGGGAGAGC	TGGAATAATTCGTAAATCAATTCAAA	207
Ad01A14692	Aradu.A01	14692	p2	(CT)6	12	84043707	84043718	AAACCACCTAACCACTTTTCCA	TCCTTGAGGTGTCTGAAGAGAA	251
Ad01A14696	Aradu.A01	14696	c	(TA)7(TTAT)7	42	84102035	84102076	TTGAAATTTTGGTGGGAAGA	CAAGTTTGGTGTTCAAGGCA	275
Ad01A14703	Aradu.A01	14703	p3	(TTC)5	15	84185730	84185744	TTTTGGTAGAGCAATGAACAACT	ATCCAACCCCCAACCTAATC	264
Ad01A14707	Aradu.A01	14707	p3	(CAT)5	15	84233683	84233697	TAGCTTCAACTGCCGAGGTT	TAGGTTGGGGTCTGTTCAGG	266
Ad01A14708	Aradu.A01	14708	p2	(GA)7	14	84252105	84252118	AGGTTTGGAGGTGATTGGTG	TCCCCTCAAGATCTGTGGAC	157
Ad01A14710	Aradu.A01	14710	p2	(GT)6	12	84262136	84262147	GGACACCAAGGTCTCAAGGA	AACCAGTTGGTTCAAGGTGC	126
Ad01A14711	Aradu.A01	14711	p2	(TA)6	12	84269917	84269928	AGGATGTGCCTACCGTTGTC	ATAGGTTAGGCCCTGGGATG	156
Ad01A14712	Aradu.A01	14712	p2	(AT)10	20	84274166	84274185	GAATCGTTGTTAAAAGCGTCA	GACTTCTTGAACAGGTCAGGC	272
Ad01A14714	Aradu.A01	14714	p2	(AT)8	16	84295969	84295984	TTGCATTACTAAGCCGTCCC	CCCTCCTTGTTTTTGTCGAG	236
Ad01A14716	Aradu.A01	14716	p2	(AT)8	16	84346274	84346289	AATTTTCGAATCCCTTGGGT	GGGGTTAGCATATGATCCCA	271
Ad01A14718	Aradu.A01	14718	p2	(AT)6	12	84386721	84386732	CCTAACCACTTCCTCCCTCC	GGGGTTAGCATATGATCCCA	229

ID	Chromosome	SSR nr.	SSR type	SSR	Size	Start	End	FORWARD PRIMER (5'-3')	REVERSE PRIMER (5'-3')	Product (bp)
Ad01A14722	Aradu.A01	14722	p2	(GC)6	12	84403822	84403833	GGTAGCACAAGGGTGGCTTA	CGTTCCTCATCCTACACCGT	192
Ad01A14723	Aradu.A01	14723	p2	(GC)6	12	84403955	84403966	GTGAAGTGGCCTAGAGGACG	CCTTGTGCACTCCTGGTACA	193
Ad01A14724	Aradu.A01	14724	p2	(GC)6	12	84404104	84404115	TGTACCAGGAGTGCACAAGG	CAACAAAAGCAACCCTCCAT	188
Ad01A14726	Aradu.A01	14726	p3	(TTC)7	21	84447360	84447380	CCCCACCTACCTTCCAAAAT	TGCCATTGATGGTAGTGGAA	208
Ad01A14727	Aradu.A01	14727	p3	(TTC)6	18	84483479	84483496	TACCCTCTCCCCTTTCCCTA	TTAGGTGCCATTGATGGTGA	229
Ad01A14728	Aradu.A01	14728	p3	(AGA)6	18	84499750	84499767	TTAGAATGTTGCTCGCCCTC	CCACAGGATCACCTCAGGAT	231
Ad01A14729	Aradu.A01	14729	p2	(TC)13	26	84507129	84507154	CCTTTACTTGGGGGCATTTT	ACACCCCATTCCCATTTACA	249
Ad01A14733	Aradu.A01	14733	p2	(AT)6	12	84520875	84520886	GTAAAACTGGCCAAGATGCC	CAAGCCTTTAAGCGAAAAACA	229
Ad01A14734	Aradu.A01	14734	p2	(CT)8	16	84526382	84526397	GCTATAGAGTGGGGGAAATGC	CAATTGGGCAACATCAATCA	260
Ad01A14738	Aradu.A01	14738	c	(A)11gagaagga	68	84544260	84544327	TTCATGAATGTTGGCTCTTGA	AGCTTTGCTAGAATCGCCAA	266
Ad01A14739	Aradu.A01	14739	p2	(CT)6	12	84548545	84548556	AGCGCTGGAAGTGATTTTTG	AAACCTGGGATCGAGAGTCA	251
Ad01A14741	Aradu.A01	14741	p3	(CTT)5	15	84579702	84579716	TCACATCATGCGCCACTTAC	CGATGACGATGACAATGACC	263
Ad01A14743	Aradu.A01	14743	p3	(AAG)7	21	84614729	84614749	GCCCAAGCAAAATTGGACTA	TCCATTCTGCTGGATTCTCC	266
Ad01A14745	Aradu.A01	14745	p2	(AT)8	16	84627837	84627852	TGAAGATCATAGCGATGGTGA	ATGGCAACACTTCCAAGGAC	248
Ad01A14748	Aradu.A01	14748	p3	(AAG)5	15	84658636	84658650	TCTGGCCTTAGGTTCCATTG	CCTCTTCTTCACCTTGGCTG	172
Ad01A14749	Aradu.A01	14749	p2	(CT)8	16	84677244	84677259	GAACATTGAACCTGAGATGAAGAA	GTTTTCGGCCCAGAAACATA	207
Ad01A14755	Aradu.A01	14755	p2	(CT)6	12	84785193	84785204	CTTGGTGCAGGGATGACTTT	ATAGGTGAGGGGTTTTTGGG	141
Ad01A14758	Aradu.A01	14758	p3	(ACT)6	18	84827560	84827577	TGTCTGATGATGATGAGAAAAATG	AGCTGAAGCGCCAGAAATTA	228
Ad01A14763	Aradu.A01	14763	p2	(CT)10	20	84888429	84888448	CCTAACCACTTTTCCACTTCCA	AAACACAGATGGAGAAAGAGGG	188
Ad01A14768	Aradu.A01	14768	p2	(AG)6	12	85061042	85061053	TTTGTGAAGTGTTCTCTTGCAT	GGCACGTCGATTGCTAAGTT	258
Ad01A14769	Aradu.A01	14769	c	(A)11gagtgaga	41	85064510	85064550	TGAAAACTTTGGGATTTGGG	TTTTTGTCCCCTTTCATTGC	171
Ad01A14770	Aradu.A01	14770	p3	(GTA)5	15	85098374	85098388	AGCTGAAGCGCCAAAAATTA	TGCCTGATGATGATGAGAGG	236
Ad01A14771	Aradu.A01	14771	p3	(AAT)6	18	85111645	85111662	GCCTTGAATGTCCAGCCTTA	TTTTCGAATTTTTGGTCCTAATTT	273
Ad01A14780	Aradu.A01	14780	p2	(GA)6	12	85145283	85145294	TTAAGTTCAGCTCAATTAGCAAGATA	ATTCAGAGAGAGCTCGCCAA	278
Ad01A14781	Aradu.A01	14781	p2	(AG)8	16	85150773	85150788	TGGATCCAGAGCTACTCTCAAA	ATGCATCCATTCAGGGAGAG	131
Ad01A14782	Aradu.A01	14782	p3	(TTA)5	15	85151636	85151650	ATCCAACAGGGACAAAGCAC	CGATCCCACGGAGATTATTG	195
Ad01A14784	Aradu.A01	14784	p3	(TCT)5	15	85156538	85156552	CAAGATCCCCACCTACCTCA	TTAGAATGTTGCTCGCCCTC	211
Ad01A14786	Aradu.A01	14786	p3	(TTA)5	15	85159494	85159508	ATCCAACAGGGACAAAGCAC	CCCACGGAGATTGTTGGTAT	191
Ad01A14789	Aradu.A01	14789	p3	(AGA)5	15	85187270	85187284	CCAATTGCATTGAAGAAGAAA	CCCAATCCCAACCAACTTT	274
Ad01A14790	Aradu.A01	14790	p2	(AG)6	12	85188487	85188498	CAGAACTGCCACTCGTCTGA	AGTTTTTGCTCGTTTGCGTT	218
Ad01A14791	Aradu.A01	14791	c	(A)10tgatatttta	72	85189063	85189134	GCTAAGAGCAACTCCAACGG	CGGGTTATTTCCTCATTCTGG	264
Ad01A14794	Aradu.A01	14794	p2	(TA)6	12	85193704	85193715	TGATTTCCTCAGAAAAATTCAAA	AAGGTGGTTTGCTTGGTCAC	279
Ad01A14795	Aradu.A01	14795	p3	(AGA)7	21	85194195	85194215	AGCAACCACGAAGCAAAACT	TGAAGAGTGAGTGTGCGAGG	262
Ad01A14796	Aradu.A01	14796	p2	(TG)28	56	85202302	85202357	AGGCCTGGTAAGTGGGATTT	TCTCAATTTATTCCTGGCCG	246

ID	Chromosome	SSR nr.	SSR type	SSR	Size	Start	End	FORWARD PRIMER (5'-3')	REVERSE PRIMER (5'-3')	Product (bp)
Ad01A14797	Aradu.A01	14797	p2	(AT)6	12	85214756	85214767	CAAGGGATGTGGATTGTTTTG	TTGGTGAACCACGTTTTCTTT	256
Ad01A14798	Aradu.A01	14798	p2	(TC)16	32	85218143	85218174	ACGCTCTCGCATGACTCTCT	GTGCTAGGTGGGAGACAACC	268
Ad01A14799	Aradu.A01	14799	p2	(CT)8	16	85229019	85229034	ACCTAAATGGTGCAGGGATG	GGGGAAGAGGGATAGAGGTG	198
Ad01A14800	Aradu.A01	14800	p3	(TCT)6	18	85246697	85246714	TCGCGAAGAGAATAGATGGG	TAGAAATGGAAAACCGGACG	250
Ad01A14803	Aradu.A01	14803	p2	(AG)9	18	85275851	85275868	AACCAGCAGTGATTTTTGGG	GAACATTGAACCTGTGATGAAGA	248
Ad01A14805	Aradu.A01	14805	p2	(AG)6	12	85282491	85282502	TTTGCCGTGATCTGTACGAA	GAACATTGAACCTGTGATGAAGA	260
Ad01A14806	Aradu.A01	14806	c	(A)12gagagatt	37	85293264	85293300	TGATGTGGTGGCAATACTTTG	TATTGGCCTTTTCTGCTTGC	238
Ad01A14811	Aradu.A01	14811	p2	(AG)6	12	85355005	85355016	AAGAACCTGGGATTGAGAGTCA	CCCAAATCCAAGTCCAAAAA	193
Ad01A14815	Aradu.A01	14815	p2	(AG)7	14	85388708	85388721	CCCCCTCCACTACTTGTTCA	GTGGACCAGACATGAGCAGA	231
Ad01A14816	Aradu.A01	14816	p3	(TGA)5	15	85398365	85398379	TGTTCTTTCAAGGGTTGAGTGTT	AAACCAGCCTCCAGTGAAGA	233
Ad01A14818	Aradu.A01	14818	p2	(TA)8	16	85421206	85421221	TCATAACCAATGCTAGCCCC	CTTGTGAGTCGAGGCCTGTT	231
Ad01A14819	Aradu.A01	14819	p2	(TC)9	18	85423670	85423687	CGGACAAGAGTTTGTGCTTG	CAGCCCTTGACCATGAAAAT	271
Ad01A14820	Aradu.A01	14820	p2	(CT)6	12	85426412	85426423	TGGTCCGAAATTGAAGGAAA	CTTTTGCAAAGGCCATCATC	277
Ad01A14823	Aradu.A01	14823	p3	(AGA)9	27	85436196	85436222	CGGTCTTAGGTGCCATTGAT	ACACACTACCCCCTCTCCCT	244
Ad01A14826	Aradu.A01	14826	p2	(CT)24	48	85475409	85475456	ATTCTGGCACTCTGCCTGTT	TCATTGATCAAAACATTGGAGTC	252
Ad01A14827	Aradu.A01	14827	p2	(GA)6	12	85489214	85489225	CGTCATATTTACAATTACCGCC	TCCATTACCGTCGTTGTTGA	195
Ad01A14832	Aradu.A01	14832	p3	(AAT)5	15	85546734	85546748	CCAGAGCATGAAATCGAAGG	CAAGTGTTGGTTGCAATGATG	279
Ad01A14834	Aradu.A01	14834	p2	(AC)17	34	85552017	85552050	ATATTCCGAAGGATCCGCTT	AACGAGAATTCAGCTGGTGG	238
Ad01A14836	Aradu.A01	14836	p3	(AGA)6	18	85563424	85563441	CTAGAGGTTTCTCCGGCCTT	CCCATCACCTCTTCACCACT	262
Ad01A14837	Aradu.A01	14837	p2	(AG)10	20	85583283	85583302	GTTCTTGTCCTTGCTCCTGC	CCACCTAACTACTTTTCTCTCCC	271
Ad01A14841	Aradu.A01	14841	p3	(TTA)5	15	85618116	85618130	CAGCTCCACGTTTGAAGGAT	CCTTCACCCTGACTCCTGAA	272
Ad01A14842	Aradu.A01	14842	p3	(TAA)14	42	85620764	85620805	GTATTCGTCGGTCCCACACT	CTACAGGCCAGACTCGGAAA	245
Ad01A14847	Aradu.A01	14847	p3	(TAA)17	51	85646683	85646733	TATGCAATGCCGTTAACCAA	ATTCCTTATGGTTGCGTTGC	237
Ad01A14849	Aradu.A01	14849	p2	(TA)6	12	85648108	85648119	TGTAGCAATTTATGTATGCTTCCAA	TGTCTGAGGTACGAATTTTCTAGATG	248
Ad01A14852	Aradu.A01	14852	p2	(TA)6	12	85651916	85651927	GAAGCCTGTGACTTCACCGTA	TTGGGAATCGGATGAAGAAG	236
Ad01A14853	Aradu.A01	14853	c	(AAT)5aaatttta	61	85654064	85654124	CCTTCCCCTGTTTCGTTTTA	AACGCACCATGTGGTAAACA	267
Ad01A14854	Aradu.A01	14854	p2	(TC)6	12	85656221	85656232	CCCGTAGCATAACATGGGTC	ACACGCAGCATTAACACCAA	263
Ad01A14855	Aradu.A01	14855	p2	(AT)8	16	85664713	85664728	TGGCTGCACCACTCTGATAG	GAGTTGTACCGCGTTTGGTT	228
Ad01A14858	Aradu.A01	14858	p3	(GGT)5	15	85680441	85680455	TGGTCAATTCATGTCCTGCT	GGACTAGAAAATGCAAGCGG	187
Ad01A14860	Aradu.A01	14860	p3	(TCT)6	18	85685249	85685266	TTTGTGAGGGCACAACAGAG	CGCAACACTGAGGAAGATGA	279
Ad01A14861	Aradu.A01	14861	p3	(TCC)8	24	85693636	85693659	GGATTTCACTCTCGCGTCTC	TTTGAGAAAGCCGAGGAAGA	232
Ad01A14862	Aradu.A01	14862	p2	(GA)6	12	85695762	85695773	TCACATTTACAATTACCGCCA	TTTCTGGGTCCGTCACTGTT	239
Ad01A14863	Aradu.A01	14863	c	(GC)6acagcgtg	48	85709579	85709626	GCGCACAACTCTCTGGTTTT	TATAGGGTGCTCTTGGGCAT	134
Ad01A14867	Aradu.A01	14867	p2	(AG)7	14	85720120	85720133	GAGGGTGAGAAAAGGGGAAG	CCTATTCGCGATTCCTCTCA	231

ID	Chromosome	SSR nr.	SSR type	SSR	Size	Start	End	FORWARD PRIMER (5'-3')	REVERSE PRIMER (5'-3')	Product (bp)
Ad01A14874	Aradu.A01	14874	p2	(AT)7	14	85844148	85844161	TCCAACACTTTTGCATTCCA	GCTCAAATCTCACTTGCTCG	247
Ad01A14875	Aradu.A01	14875	p3	(TTA)7	21	85859811	85859831	AAATTAAGTTTGGTGTCCCCG	AACGCACCATGTGGTAAACA	234
Ad01A14879	Aradu.A01	14879	p2	(CA)7	14	85883827	85883840	CTATCCTCACCCCACCTTCA	GCTCCTTCCCTTGCTTCTCT	122
Ad01A14880	Aradu.A01	14880	p2	(GA)6	12	85883970	85883981	AGAGAAGCAAGGGAAGGAGC	ACCTACGTTGACAGCCCAAC	185
Ad01A14884	Aradu.A01	14884	p2	(TA)6	12	85901563	85901574	TTTTTGGGTGCAAACATTCTC	CATCATCCAATCACAATCCAG	237
Ad01A14887	Aradu.A01	14887	p2	(AC)6	12	85910138	85910149	CAAGTTAGGCCCAAGTCCAA	TCACAAGGAATGCGTAGCAG	195
Ad01A14889	Aradu.A01	14889	p3	(TTC)6	18	85944510	85944527	TCTTCTCCCACTTAGCCGAA	GGTCTTGATGGCTCTTGGAG	261
Ad01A14891	Aradu.A01	14891	p3	(TGA)5	15	85960910	85960924	TGAAGGAGCAGGTTCGAGTT	GGAGGGCGGAATATTAGAGG	164
Ad01A14893	Aradu.A01	14893	p2	(GA)12	24	85971799	85971822	AGAGAGGGAGAGAGGGTTCG	TGAAAATCACTTTCCTCTTTTTCAA	249
Ad01A14895	Aradu.A01	14895	c	(TG)8ta(TG)6	30	85977837	85977866	TTGCATTTTTCCTTATGCGA	TCACATTGCAATACCCTACCA	267
Ad01A14904	Aradu.A01	14904	c	(C)11tctctctnn	42	86029461	86029502	AATTGACATGCATGACGGAA	CCGTTTCGAAGGATATGGAA	218
Ad01A14905	Aradu.A01	14905	p2	(CT)12	24	86030286	86030309	CCAACCAGGGTTAGATTCCA	TGCTAAATGGGAAGGGTCTG	249
Ad01A14907	Aradu.A01	14907	c	(GT)8(AT)9	34	86039449	86039482	TGGGATGTCAATGCCCTATT	GCGTTTTTGGGTGACAAGTT	192
Ad01A14908	Aradu.A01	14908	p4	(TAAC)6	24	86040417	86040440	CCCTAATCAAAGAAACCAACAA	TCTTCCCTTCTCTCACCCAA	234
Ad01A14909	Aradu.A01	14909	p2	(AT)9	18	86054153	86054170	GCATGAGTGATCGTGGTTTG	ATTTTTGCCCTCGAAAATCC	224
Ad01A14910	Aradu.A01	14910	p2	(AC)6	12	86054630	86054641	CTGCATGAAAGGCAGTGAAA	CAAGGGTTTCTCTTCTCCCC	248
Ad01A14911	Aradu.A01	14911	p3	(TTC)6	18	86056760	86056777	CGCATATTTTCCAGCCAGAT	AGCAAAGCAATGGGTACGAG	261
Ad01A14914	Aradu.A01	14914	p2	(AT)7	14	86094836	86094849	AACTAAGAGAAAGTGCCGAAAAA	TGAATCAAATTGGTAATATGAAGGC	254
Ad01A14919	Aradu.A01	14919	p3	(TGA)5	15	86148930	86148944	ATTTGGCTTTGTCGCTCATT	GCCCTTGGACCCTTTTCTAT	250
Ad01A14923	Aradu.A01	14923	p2	(TC)14	28	86167355	86167382	TATCGTCGGAAGTACCCGTC	AGAAGCAGAAGCGACAGAGC	278
Ad01A14924	Aradu.A01	14924	p2	(AT)11	22	86170179	86170200	TGGTCACCAACAAACCTTCA	TAGCTTTCGGAACTTTTGGG	198
Ad01A14925	Aradu.A01	14925	p2	(AT)6	12	86183186	86183197	CAAAATTACAGCCCCTCCAA	TTAGCTTCCGGAACTTGTGG	242
Ad01A14926	Aradu.A01	14926	p2	(AT)6	12	86192868	86192879	TGGACTCATCAATTCTCCCA	TTGATTGTTGCTTTCACCAAA	183
Ad01A14927	Aradu.A01	14927	p3	(TAT)6	18	86193475	86193492	ATCAATCAACCCACGAAAAA	TGGGAAAAATAGGTGTGCATT	270
Ad01A14928	Aradu.A01	14928	p2	(TA)8	16	86194255	86194270	TGTATGTGATGGATGCAATTTTT	GGGGAGGATTCACCATTTTT	242
Ad01A14929	Aradu.A01	14929	p5	(AATAA)5	25	86219175	86219199	AGGCAGTAGTGGGGTTAGCA	CCTAACCACTTTCTCTCTCCTCA	246
Ad01A14931	Aradu.A01	14931	p3	(GTT)5	15	86223499	86223513	GGGTCTGCTGTTGGTTGTTT	TTAACCAAGGTTGGCAGGAC	243
Ad01A14933	Aradu.A01	14933	p2	(AT)7	14	86345430	86345443	CCCCTTGTCTTACCAAGTTCC	TATATTGGGGGTGGACGAAT	132
Ad01A14937	Aradu.A01	14937	c	(T)10attttttcg(	30	86355998	86356027	GCACACTCTTTTTCCCTTGG	TTGTTGGCTCTTGAAAGAATGA	177
Ad01A14941	Aradu.A01	14941	p5	(GCCTG)6	30	86454103	86454132	TTAACAAGCTTCAGGCCAGG	AAACCCTAAACCCAGAACCC	219
Ad01A14943	Aradu.A01	14943	p3	(TAT)6	18	86527446	86527463	TCTCTGCTGAAAGGATGCAA	CATGTTCCATGGGTCTTGTTT	227
Ad01A14944	Aradu.A01	14944	p2	(CT)6	12	86569018	86569029	ATTCAATGCCAGAAACAGGC	TGAGGTAGGTGGGGATTTTG	279
Ad01A14945	Aradu.A01	14945	p3	(TTA)5	15	86594457	86594471	TTGGAGTTATCGTTAAAAGGAGTTG	TATTAAGGGCCAAGCCCATC	231
Ad01A14948	Aradu.A01	14948	p3	(TAC)5	15	86620601	86620615	AAGGAGCTGAGTTTTCAAGCA	TTTGGAGCTTTTAAGGGTGC	187

ID	Chromosome	SSR nr.	SSR type	SSR	Size	Start	End	FORWARD PRIMER (5'-3')	REVERSE PRIMER (5'-3')	Product (bp)
Ad01A14949	Aradu.A01	14949	p2	(TG)9	18	86624773	86624790	TGCCTTAGGGCTAGACATGC	AGGGTCGGGGTTAAAAGTCA	248
Ad01A14953	Aradu.A01	14953	c	(TCT)5caccctc	108	86701049	86701156	CGTGGGTCCCTAGTTTCATC	TGGAAGGTACAACGGTAGGC	258
Ad01A14954	Aradu.A01	14954	p2	(AT)13	26	86711876	86711901	GGATGACCCAGTACACTTGC	AAGCATGCAATTGACATCAAAC	280
Ad01A14956	Aradu.A01	14956	p2	(AT)6	12	86723734	86723745	TTGATTTTGGGAAAGTTGGG	TTACTTGGGGCATTTTGTCC	269
Ad01A14957	Aradu.A01	14957	p2	(TA)7	14	86737771	86737784	GGACCCTAGAGCCATTCTCC	CGATGTGATGGATGCAACTT	252
Ad01A14958	Aradu.A01	14958	p3	(AAT)5	15	86738578	86738592	GTAGGGGGAAATTTTGGGAA	GCCCACATTCATGCCTTAGT	280
Ad01A14959	Aradu.A01	14959	c	(AAAT)5aataa	49	86832288	86832336	GGAAAACTGTGGGAACATGG	TTGGGGTAATTTTGTCCCCT	247
Ad01A14960	Aradu.A01	14960	c	(TAT)5taa(TA	63	86836743	86836805	ATCTGGAGTTCCCTCTGCAA	GCTAAGGTGGCAAGGTATTACG	258
Ad01A14961	Aradu.A01	14961	p3	(TAT)16	48	86838201	86838248	ATCTGGAGTTCCCTCTGCAA	TAAAGCTGAGGTGGCAAGGT	280
Ad01A14963	Aradu.A01	14963	p2	(GC)6	12	86866188	86866199	ATAGGGAGCTCTTGGGCATT	TGCTCCCAGCACAATGTTAG	160
Ad01A14964	Aradu.A01	14964	p3	(AAT)5	15	86866498	86866512	GTTTGGTCCCTGCTTTTCTG	GATACAGCGGTAGGGTGCAT	241
Ad01A14969	Aradu.A01	14969	p2	(TA)6	12	86904378	86904389	TGCCATTTTTATTTTGATAATGTCTT	TTTTGTATTTGCATTTTAGGTCACA	238
Ad01A14974	Aradu.A01	14974	p3	(TGG)6	18	86928798	86928815	GAGGTGCTGCATAAGTCGGT	TGGTCACAGAGATGGCTCAG	275
Ad01A14980	Aradu.A01	14980	p3	(ATT)6	18	87049213	87049230	TTGGATGTTGGCTTTGTGAA	GGATTCCTCTTTCTTTTCTTGTTTT	276
Ad01A14981	Aradu.A01	14981	p2	(AG)17	34	87067999	87068032	TCATGGATGTTGGCTCTTGA	TGGCTTTTTCTGCTTGCTTT	184
Ad01A14982	Aradu.A01	14982	p2	(TA)6	12	87069256	87069267	GCCTTTTGTCCCTCATTTTT	GAATACTCAAAATTATGATCATCCGA	274
Ad01A14984	Aradu.A01	14984	p3	(AGG)7	21	87092221	87092241	CGAGCAAAATGTTGGTGTTG	GCGCACGTAGGTTCTTCTTC	102
Ad01A14986	Aradu.A01	14986	p3	(TTC)6	18	87122129	87122146	CCCTTAGCCGAACCTACCTC	CTCGGAAGTGGAAGCTTTTG	202
Ad01A14987	Aradu.A01	14987	p2	(GA)6	12	87142427	87142438	GACCTTTCCGGCATAGAACA	CACAGCCTCCCTCTTCACTC	263
Ad01A14991	Aradu.A01	14991	c	(T)11attcttgtta	69	87155550	87155618	CTCCAGCAGCTCACGTACAG	TCAAAATTCTTCCTCTTCATTCA	248
Ad01A14994	Aradu.A01	14994	p3	(AAT)5	15	87161865	87161879	CGTGTACGCACAAGCTGTTT	CCTGCTTTTACCCGTTCACT	179
Ad01A14995	Aradu.A01	14995	p3	(AGA)6	18	87176894	87176911	AGTGGTCTTGATGGGCTTTG	TGGCTGAATCCTACACCTCC	253
Ad01A14996	Aradu.A01	14996	c	(T)10ggaaaaaa	66	87184724	87184789	GCATGCAATTGACACCAATC	GGGGATTGTTCGAGTATGGA	260
Ad01A14998	Aradu.A01	14998	c	(AT)11(AC)8	38	87193558	87193595	TGAGTTTTCTCTCTCTTTTCTGTTGA	TTTGCTTGGTTGAGCAAAGA	147
Ad01A15003	Aradu.A01	15003	p2	(CT)20	40	87223168	87223207	TATCTCCTTCACGCGCTCTT	CCACAAATCCAAACCGATTC	193
Ad01A15004	Aradu.A01	15004	p3	(TAT)9	27	87226788	87226814	TTTGTATGTTTGGGGGAAGG	CAAACCGCTCAATTTTGGAT	129
Ad01A15017	Aradu.A01	15017	p2	(GA)7	14	87250652	87250665	CGGTTTTCATGGTGAATCGT	CACTGCATTCTGCAACCCTA	219
Ad01A15022	Aradu.A01	15022	p4	(AATC)5	20	87279991	87280010	TCAACCACTGATGCTCAAAAG	CTCTTTGTAGCTGCCACCATC	211
Ad01A15028	Aradu.A01	15028	p3	(TTC)8	24	87343233	87343256	CACACAATTCAACCCCCTCT	CTAGAGGATTCTCCGGCCTT	204
Ad01A15030	Aradu.A01	15030	p5	(TTATC)5	25	87353150	87353174	CCTGGGTGCTCCAGTGTATT	TCTTCTCAAAAGAGCTCGGC	263
Ad01A15033	Aradu.A01	15033	p3	(TCT)5	15	87382628	87382642	CCAAACCTCTCTCCCTCTCC	CTAGAGGTTTCTCCGGCCTT	159
Ad01A15034	Aradu.A01	15034	c	(A)10g(A)10	21	87393942	87393962	TTGTTGGCTCTTGAAAGAATGA	TTGGCTTTTTCTGCTTGCTT	193
Ad01A15035	Aradu.A01	15035	p4	(AAAG)5	20	87402849	87402868	GCTTTTGCCTTCCTTCTCCT	CCCCTCATCCCTCTTTCAAT	247
Ad01A15036	Aradu.A01	15036	c	(A)10tctttttctta	39	87415953	87415991	TTAAACGCCAGGATGACACA	TCAAAACATTAATTGTTAGCAAGG	201

ID	Chromosome	SSR nr.	SSR type	SSR	Size	Start	End	FORWARD PRIMER (5'-3')	REVERSE PRIMER (5'-3')	Product (bp)
Ad01A15038	Aradu.A01	15038	p2	(AT)10	20	87420132	87420151	TGAAGAGAACTGGTCCGAGC	GAGGTCGTAATACCTTGCCATC	242
Ad01A15039	Aradu.A01	15039	p2	(AC)18	36	87426134	87426169	AGATTTCCAAGCACCAATCG	AACTTGAAGGGGAGGCTTGT	238
Ad01A15040	Aradu.A01	15040	p2	(TA)6	12	87451191	87451202	TGAGTTTTCCTGGGTGAAGG	CCAACTCGACAAATCCTGGT	250
Ad01A15041	Aradu.A01	15041	p3	(AAT)5	15	87488876	87488890	CAGGGAAAAAGGCACTCTTG	AAACTTGGACGTTGGCTTTG	199
Ad01A15042	Aradu.A01	15042	p4	(AAAG)5	20	87496756	87496775	GCTGGAAAAAGCACCATAGG	TAGGCATTAACAGGGCAACC	232
Ad01A15048	Aradu.A01	15048	c	(ATA)5gaaag(	30	87660333	87660362	AAAGCAGTGAAAAAGCAAAAGC	GCCTTGGATCCTTTTTACCC	127
Ad01A15049	Aradu.A01	15049	p2	(TC)6	12	87663814	87663825	GGAGGCTAAGTCTCAGGCAA	TCTCGTGCTGATAATGTGGTG	224
Ad01A15051	Aradu.A01	15051	p3	(TCA)6	18	87669746	87669763	ACATTGAAGCTGGAGGCAGT	TGAAGCAAAGGCTGAATGTG	229
Ad01A15052	Aradu.A01	15052	p2	(TA)9	18	87671380	87671397	TAAAGGAAGCAGGGCAGAAA	TGGGAGTTTCAATGGTCACA	242
Ad01A15055	Aradu.A01	15055	p2	(CT)7	14	87675773	87675786	ATTTCTGACCCAGTTTTCGG	CAATTGGGCAACATCAATCA	279
Ad01A15058	Aradu.A01	15058	p2	(GT)6	12	87700148	87700159	AGACAGAATCAGATTGGGCG	TTCATTTCGCCGTAACTTCC	205
Ad01A15059	Aradu.A01	15059	p2	(GA)23	46	87701242	87701287	AGCGACAGCGCAAGAATAAT	TACGCTTGCCAACTTCACAC	157
Ad01A15060	Aradu.A01	15060	p2	(GA)6	12	87703189	87703200	CTTCAGCGAACCTGTAACCC	TAGCAATGGCAAATGACGAG	229
Ad01A15061	Aradu.A01	15061	p2	(TA)10	20	87709584	87709603	GCCTATCGTGATTTGCACCT	TCAAAACTCGTCTCAAATCCAA	222
Ad01A15062	Aradu.A01	15062	p2	(GA)6	12	87710114	87710125	ACCTGCAACCCAACAGTTTC	TAGCAATGGCAAATGACGAG	220
Ad01A15069	Aradu.A01	15069	p3	(TCA)6	18	87737070	87737087	CCAATTACTCCTGTACCAGCAA	TTTTGGTAGGGTTTGATTTTTCA	214
Ad01A15071	Aradu.A01	15071	p3	(TAT)10	30	87738586	87738615	TCATCTCCAAGATTGTATTATCCAT	GAGGCAAATAGACGGAAGGA	278
Ad01A15074	Aradu.A01	15074	p4	(TTAG)6	24	87750748	87750771	TTTGCCTTTTCTCTTCCCCT	TCCTATCAGTAAGCAGCAGCA	205
Ad01A15075	Aradu.A01	15075	c	(AG)33aaagag	92	87752555	87752646	TGAAAATCTTGGGAATTGGG	TGGGACATTTTGTCTCCTTTTT	249
Ad01A15076	Aradu.A01	15076	p3	(TTA)5	15	87791329	87791343	CAGGTGCCGGGATATAAGAA	GCACCTGATACTGGTGCTCA	189
Ad01A15078	Aradu.A01	15078	p3	(AGA)6	18	87807126	87807143	AGAGGAGGAAGAAGGCGAAG	ACGAAATGACCGCTCTTACC	100
Ad01A15079	Aradu.A01	15079	p2	(AG)6	12	87810669	87810680	AGATAGGAGAGGGGGAGACG	CCCTAGCAACGACAGTGACA	231
Ad01A15080	Aradu.A01	15080	p2	(TC)6	12	87817756	87817767	TGAACCTGATGAAGAGTCGAAA	GACGTGCGCAATCTGTAGAA	260
Ad01A15084	Aradu.A01	15084	p2	(TA)7	14	87856696	87856709	GAAGCCGTGGAACTTTTGAC	TCTGTGCCTCCTAAAACTCCA	183
Ad01A15086	Aradu.A01	15086	c	(TTC)10ttttcttt	114	87858839	87858952	AACGAATAAACCCCAAAAACG	AGGAAGAAGAACGTGCGAGA	226
Ad01A15087	Aradu.A01	15087	p3	(GAA)5	15	87859205	87859219	TTGCTGCATGTTCTTCTTCC	ACAGGAATTCACCCAAAGGA	261
Ad01A15088	Aradu.A01	15088	c	(A)12ttcaacctg	92	87860328	87860419	TCCAAAATAGCCCCTGACAA	ACTTCGAAAGGACAACGAGG	228
Ad01A15089	Aradu.A01	15089	p3	(AAC)10	30	87869950	87869979	GGAACATGGGGTGAAAGAAA	ACATGACAGAGCACAATGGC	204
Ad01A15091	Aradu.A01	15091	c	(A)10gcgatgca	48	87891324	87891371	GGAAGCATGCTCATGTGAAG	AAGCAAAGCTTCTTCACCCA	179
Ad01A15093	Aradu.A01	15093	c	(AG)7gagtttca	64	87896102	87896165	TTTTCAATTCATCCTTCTTCACTTT	CTCACTCAATTCCTTGATGCTG	218
Ad01A15101	Aradu.A01	15101	c	(A)11gaaagaga	33	87924557	87924589	TGGCTCTTGAAAGGATGAGG	GATTGTGACCCAGCTTTGCT	258
Ad01A15110	Aradu.A01	15110	p3	(TAA)7	21	87970216	87970236	AATCCAAGGGAGGAAGGTTG	CATGTAGCGGGGCAGTAAGT	190
Ad01A15113	Aradu.A01	15113	p2	(AT)12	24	87978607	87978630	GAGCATCTCAAATCATAATTTTTCTG	AATGCTGGTGCATTTGGTTT	268
Ad01A15115	Aradu.A01	15115	p2	(TA)6	12	87982699	87982710	GGTCAACAAATTGAAAATCACG	TCATGGCTGTGGACAAGAAA	145

ID	Chromosome	SSR nr.	SSR type	SSR	Size	Start	End	FORWARD PRIMER (5'-3')	REVERSE PRIMER (5'-3')	Product (bp)
Ad01A15117	Aradu.A01	15117	p2	(TA)6	12	87983663	87983674	TCTCCACCACAAACCTGAAA	TCTGCGAATTAGGAAAAGGAA	251
Ad01A15119	Aradu.A01	15119	p2	(AG)8	16	87990681	87990696	TACACCAAAATCAGCCACCA	CCATGTGTTTCCACCCTCTC	236
Ad01A15121	Aradu.A01	15121	p3	(TTA)10	30	87998323	87998352	CAAATGCCCACATAACATGG	GACGAAAATGGAGATCAGGG	249
Ad01A15125	Aradu.A01	15125	p2	(CT)6	12	88010107	88010118	GGGTGGCAGTAGGTTGAGAA	TTTATGTGGAAAGTGACGGAA	273
Ad01A15128	Aradu.A01	15128	c	(TTC)6g(TCA	34	88025096	88025129	TAATCTTCACCGGCAACACA	GCAATTAGGGAAGCAGGTCA	170
Ad01A15130	Aradu.A01	15130	p3	(TAA)5	15	88030708	88030722	TGTGAAATAAACAAAGAACAATAAAAG	TCAAACGTCAAGACTAAGGAACC	128
Ad01A15131	Aradu.A01	15131	c	(T)11atacaagtg	69	88030849	88030917	GGTTCCTTAGTCTTGACGTTTGA	TATTTGGGAAGGACGACGAC	271
Ad01A15132	Aradu.A01	15132	p2	(AC)8	16	88038828	88038843	CCAAAATGTGATTTTTGTGGC	CTCATGGCCTCCATTTCAAC	223
Ad01A15133	Aradu.A01	15133	p2	(AC)8	16	88046662	88046677	GCCCTTGGCACAACTAAACT	CGAAAACAAGGCTGGAATTT	280
Ad01A15137	Aradu.A01	15137	p3	(TTC)6	18	88060803	88060820	CAAGTTCACGTGCTTCTTCG	GTCTTCACGCTTGGCTTTGT	117
Ad01A15143	Aradu.A01	15143	p3	(ATG)7	21	88071123	88071143	TTGTACTTCAAGCGGCAATG	GCAGTTCCTGCAATTAGGGA	234
Ad01A15144	Aradu.A01	15144	p3	(GAA)5	15	88071383	88071397	TTACTATGACGAGGGCGAGG	CTATGATCGGTCGAATCCGT	195
Ad01A15146	Aradu.A01	15146	p3	(GAA)6	18	88077379	88077396	TACCTGAGCTTGGGAACAGG	GTTGACGACGACACCATCAC	140
Ad01A15156	Aradu.A01	15156	p2	(TA)8	16	88099953	88099968	TCGACCAGTTCGATTTTCGT	CCACCTTACCGTAACCAACC	261
Ad01A15165	Aradu.A01	15165	c	(T)10ggtaaataa	55	88134697	88134751	CTTTCACTTTGACCGAGGGA	TGCTTTTTGTTTTCTTTGTCTCA	226
Ad01A15166	Aradu.A01	15166	p4	(AATA)5	20	88135194	88135213	GCCAAAAACAAAATAAGAAATGC	GCGATTTGAAGAAGGAGGAA	257
Ad01A15167	Aradu.A01	15167	p3	(AAT)5	15	88135528	88135542	TCAAGAGAAAATGGAGTGAGCA	TCAAGGTGTGGAACAATTAATAAAGA	193
Ad01A15169	Aradu.A01	15169	p3	(TAA)5	15	88140235	88140249	GTGACACCCTGCTTAAAGGC	GACCTGTCGGGTTAGGGAAT	269
Ad01A15170	Aradu.A01	15170	p2	(TA)6	12	88141435	88141446	GCACACCCAAACAATGTCAC	GCCACCATGTATTCCAGGTT	203
Ad01A15172	Aradu.A01	15172	p3	(AAT)5	15	88160707	88160721	TGCATACCTTTGTCGCTTGA	TGTTCACCTTAATAACACCCAGG	253
Ad01A15174	Aradu.A01	15174	p3	(TTC)7	21	88173430	88173450	AAATCTAGGTGCCGCATTTG	TCCTTCCAATTTTCAGGATCA	265
Ad01A15176	Aradu.A01	15176	p3	(ACA)5	15	88174297	88174311	TTTGTACCACTGGTGCTTGC	CTTCTTCACAGCCCCCAATA	229
Ad01A15180	Aradu.A01	15180	p3	(TAA)17	51	88186502	88186552	TTGGAATCTTGGATCTTGGA	AGTTGCGTTTGTTGCATTTG	197
Ad01A15184	Aradu.A01	15184	c	(T)12aaatttaaa	39	88193869	88193907	TTATGAGGCCACAAAGGGAC	CAAGGGAATTGTGTATTGGGTT	219
Ad01A15192	Aradu.A01	15192	p2	(AT)6	12	88209261	88209272	TCCGTTGTTTCAGTTTTCTTCT	AGTTCATTCATGTCCTTTCCG	208
Ad01A15195	Aradu.A01	15195	c	(A)10gagtaaag	194	88211736	88211929	CAAATAATTCATAGCAAATGCAAG	CAAAACATCATTCACCCTCCA	280
Ad01A15196	Aradu.A01	15196	c	(AAT)7agaaga	85	88213866	88213950	TCACTTTGAAGAGAGCGACA	GACGAATTTCGGCAATTTTTA	183
Ad01A15198	Aradu.A01	15198	p3	(AAT)5	15	88227754	88227768	TTTGGGGTAATTTTGTCTCCTT	GTGGGGAAATTTTGGGAACT	232
Ad01A15199	Aradu.A01	15199	p2	(GT)6	12	88231575	88231586	CAAAGGGTTATGAACGAATTTTT	GAAAAATTGAAGCCATATTAGACTTTT	279
Ad01A15200	Aradu.A01	15200	p2	(AT)7	14	88234958	88234971	CGCCATCGTCGAACTAAAAT	TTTGGTGTTTTGGGAGGTGT	200
Ad01A15201	Aradu.A01	15201	p3	(TAA)5	15	88235177	88235191	CTTCTTGCGTGAATCACCTG	GAGAAGACGCATAAGAAGAGGAA	256
Ad01A15202	Aradu.A01	15202	p2	(TA)7	14	88236290	88236303	CCGATATTTCAGTAATTTTAACCG	GCCCATTGGAGTTGCTCTTA	168
Ad01A15203	Aradu.A01	15203	p2	(AT)8	16	88240337	88240352	GGCCAGGACCTTGAGTAGTG	AAAAAGGCATTCTTCTAACAATTTA	273
Ad01A15204	Aradu.A01	15204	p2	(TA)6	12	88240721	88240732	TTGAGAATTGGATTTGGTCAAT	TGGAAGTTTCACCCCAACAT	231

ID	Chromosome	SSR nr.	SSR type	SSR	Size	Start	End	FORWARD PRIMER (5'-3')	REVERSE PRIMER (5'-3')	Product (bp)
Ad01A15211	Aradu.A01	15211	p2	(TA)6	12	88262746	88262757	GCACAAGATTTGCTGGAACA	TCCGATAATTGGCCATGAATA	220
Ad01A15223	Aradu.A01	15223	p2	(AG)6	12	88298358	88298369	AAACAAAGCACCACGACAGA	TACGCGGAGTTTGAGATCCT	276
Ad01A15224	Aradu.A01	15224	p2	(CT)15	30	88298535	88298564	AACGCAAACACCTTTCCAAC	TCATGTTTTGGGCCGATAGT	255
Ad01A15229	Aradu.A01	15229	p3	(CTG)5	15	88305775	88305789	ACTGCCCGAAACTGTTATGC	GGAGGGGTGGAAAGAGTAGC	265
Ad01A15231	Aradu.A01	15231	p2	(TA)8	16	88311753	88311768	GGAAAATGGGTTATTGTTGGAA	GCGTTCCTTTAGCAGCTACC	262
Ad01A15234	Aradu.A01	15234	p3	(ATT)5	15	88321762	88321776	TCCCTCCGTCAAAGTGAAAG	TGAGAAGGGTTGAGTAGCGG	236
Ad01A15235	Aradu.A01	15235	p3	(TTC)6	18	88321922	88321939	TCCTTCATCGATCTTCACAGG	TGCCGAAGAAAATCAAGAAAA	261
Ad01A15236	Aradu.A01	15236	p4	(TTAT)7	28	88322042	88322069	TCCTTCATCGATCTTCACAGG	TGCCGAAGAAAATCAAGAAAA	261
Ad01A15239	Aradu.A01	15239	c	(TTC)17taatca	162	88335826	88335987	CGGAGATAATGTGGGTTTGG	TTTCAAGTGCACCTAAATGGG	278
Ad01A15241	Aradu.A01	15241	p2	(TG)6	12	88389479	88389490	AATGAAAGCACCCCAAATGA	TAATGGGGCTTAGGTAGGGG	235
Ad01A15242	Aradu.A01	15242	c	(A)10gaacccag	42	88408830	88408871	CATTGGCAGAAAAGGGAGAA	GGTTAGCTCACCAACCTGCT	266
Ad01A15244	Aradu.A01	15244	p4	(AAAG)8	32	88412683	88412714	TAGCCACCCTTTCTCCTTCA	GATGGTGGCTTATGGCAGAT	266
Ad01A15246	Aradu.A01	15246	p2	(TC)6	12	88423313	88423324	TCTCTCATTTGCTTCACCCA	GCTGTTGTTAGGCCTTGGAG	275
Ad01A15247	Aradu.A01	15247	p4	(TCTT)5	20	88423576	88423595	CTCCAAGGCCTAACAACAGC	CACCCCCTTTCTCCTTCAGT	208
Ad01A15248	Aradu.A01	15248	p2	(TA)12	24	88427360	88427383	CATGTTCATACGAACCAAATACAA	GCGAGGACGATGTTCTCACT	254
Ad01A15249	Aradu.A01	15249	p2	(TC)6	12	88433544	88433555	CATGAACGGTGACTGGATTG	GGAAGACCAGAAACGATGGA	246
Ad01A15250	Aradu.A01	15250	p4	(TTCT)12	48	88433919	88433966	CCTCTCCGGGTCTTCTTCTC	CAATTTTTCCCTTTACCGCA	187
Ad01A15252	Aradu.A01	15252	p4	(AAAG)6	24	88438230	88438253	CCTAGCCACCCTTTCTCCTT	CGGCGATATCTCAAAGAAGC	232
Ad01A15253	Aradu.A01	15253	p2	(AT)8	16	88464350	88464365	TTTAACGAGGTGCCAAGACC	ATGTTTTGGAATAATGCGGC	151
Ad01A15254	Aradu.A01	15254	p5	(AAGAT)5	25	88468707	88468731	CGGTTCCCTTGAAGATACGA	TTCTGATTCTTTCATCCCCG	228
Ad01A15255	Aradu.A01	15255	p2	(GA)6	12	88470825	88470836	GTTTCGGTGGGATAGGGATT	GTTTAACGCCAGGATTGCAT	270
Ad01A15257	Aradu.A01	15257	p2	(CT)7	14	88476585	88476598	ACTGCATCCCCAACAGTAGC	TGTTCTGTCAAGAGGGGACC	269
Ad01A15258	Aradu.A01	15258	p2	(AG)7	14	88486261	88486274	AAGTGGGGGAATCCATTCAT	CATGAATGGTCCATAAGCCA	242
Ad01A15260	Aradu.A01	15260	p2	(CT)7	14	88541937	88541950	CCCATTAGCCTTAGCCCTTC	AAACGGCGATCAAAAAGAGA	244
Ad01A15261	Aradu.A01	15261	p2	(TG)7	14	88542360	88542373	TCAGAATTCGTTAGCAACTGGA	GCATAAGTCCGCTGAAAAGG	252
Ad01A15262	Aradu.A01	15262	p2	(TC)12	24	88545665	88545688	TCAGGCAAGGGTTCCTACTG	TGCCAGTCTCCTCTTCAACC	178
Ad01A15263	Aradu.A01	15263	p2	(TC)6	12	88548726	88548737	CTTCTTTCTCCCTTTGCCCT	TGGGGAATGATGACTCAAAAG	223
Ad01A15266	Aradu.A01	15266	p2	(AG)7	14	88578493	88578506	TAACAGAAATCACTGGGGGC	AAAGGAACATTGAACCTGGAA	247
Ad01A15279	Aradu.A01	15279	c	(T)14gcagattaa	107	88634955	88635061	GCAGCAGATTTTGTTTTGTGA	GGGATAGGACTGATGAGGAGTG	280
Ad01A15281	Aradu.A01	15281	p2	(CT)7	14	88643337	88643350	GCCTCTCGAAATGGAAATCA	AGAGCTAGCGATCGGACATC	269
Ad01A15285	Aradu.A01	15285	p3	(AAT)14	42	88671584	88671625	ATTTGGGTTTGGTGACTGGA	TGTTGCAGAGCTTATGGAGG	254
Ad01A15289	Aradu.A01	15289	p3	(CTT)23	69	88681454	88681522	GCTTGGTTTTCAGGCTCATC	ACGAAACGTGGTCAACACAA	134
Ad01A15290	Aradu.A01	15290	p2	(AG)12	24	88685744	88685767	ACTCTCCTTGCCTTCAACCA	GCAAAGTCAAGCTCAACGGT	246
Ad01A15293	Aradu.A01	15293	p2	(AT)8	16	88701998	88702013	AGCTTTGTCAAGGCAAGCAT	GGAAGTTATTGCGACACCAA	157

ID	Chromosome	SSR nr.	SSR type	SSR	Size	Start	End	FORWARD PRIMER (5'-3')	REVERSE PRIMER (5'-3')	Product (bp)
Ad01A15294	Aradu.A01	15294	c	(AC)7aa(AAT)	61	88703178	88703238	GTCCTAAGGCTGGTGGATGA	TGGATGGCATTCAGGTTTTT	216
Ad01A15295	Aradu.A01	15295	c	(GA)20tccacat	133	88704439	88704571	TTTCCCATAACAATGACCATCA	CCATTTGCCAATGATGTGAA	187
Ad01A15296	Aradu.A01	15296	p2	(AG)9	18	88704743	88704760	AGGGTTAAGGGATTGGGTTG	GCATTTGGTTTGCATTTTCA	274
Ad01A15301	Aradu.A01	15301	p3	(AAT)5	15	88713591	88713605	ATGTTTACCTGGCCGATTCA	TTGTGTTGTTCCTCCCCTTC	228
Ad01A15303	Aradu.A01	15303	p2	(TA)8	16	88714603	88714618	CGAAAGCATAGTGGTAAAAATCG	AGAACAAAAACCGATCATGC	225
Ad01A15306	Aradu.A01	15306	p2	(CT)14	28	88729481	88729508	TACCTCCCTTGGTGCATGA	CCACAGGGGAATTATAGGGG	279
Ad01A15312	Aradu.A01	15312	p3	(TAT)9	27	88750913	88750939	TGTCACGGAGAGAATATGCAG	AAAAATTGGATTGCGTATTAAAAA	117
Ad01A15313	Aradu.A01	15313	p2	(TA)7	14	88751214	88751227	AAAAATTGTTGTGCATGAGTGT	CAACAGTGGCTTTTAATTCACAAAT	161
Ad01A15321	Aradu.A01	15321	p3	(TTA)11	33	88770537	88770569	TGATGAAAATTCCACCACTCA	CTTGCACTATCCGCAAGACA	254
Ad01A15324	Aradu.A01	15324	p2	(CT)9	18	88776564	88776581	TTGATTGTGAAATGTGAACCA	AAAACCATCCCCAGACACAG	241
Ad01A15326	Aradu.A01	15326	p3	(TAA)16	48	88794316	88794363	TGGCAATAATTTGGCTTGTT	ATTTGGTGACGTGCTTCCTT	114
Ad01A15327	Aradu.A01	15327	c	(AT)11gagcaa	105	88794602	88794706	TGAGGAGAGGGAAAGTGGTT	CTTGGTGTTTTCCCTCCAAA	239
Ad01A15329	Aradu.A01	15329	p2	(CT)9	18	88804804	88804821	AGGAAATGGGAAGCAGAGGT	TCTGGTGGATTTGGGAAGAG	201
Ad01A15333	Aradu.A01	15333	p3	(AAT)15	45	88814366	88814410	GGACCATTAGAAGACGCCAA	GACTGAGGCTTCAACCCAAA	256
Ad01A15334	Aradu.A01	15334	p2	(AC)7	14	88821046	88821059	ACTAGCACCCCTGAGCAAGA	ATCATGGTGAAGGGAACCAA	184
Ad01A15335	Aradu.A01	15335	p3	(ATC)6	18	88823314	88823331	AGATCAAGATCCGAGGGTGA	TGCATCTGGTGTTTTATCCG	276
Ad01A15336	Aradu.A01	15336	p2	(CT)8	16	88823517	88823532	AAACACCAGATGCACGATGA	CCAAAATAGGGGAAAGAGAGG	160
Ad01A15337	Aradu.A01	15337	p3	(ATC)5	15	88825230	88825244	AAAGGGCAGAGGCCAATAGT	GTGAAGGGGTTGGGAATTTT	232
Ad01A15341	Aradu.A01	15341	p2	(AG)6	12	88830447	88830458	CAGAAGCTGCCATTCAACAA	TCCATAAACTCTTCGTGCCC	218
Ad01A15342	Aradu.A01	15342	p3	(CCT)5	15	88856126	88856140	TACTTTCGTCGTCGTCATCG	CATCGAAATTTCTTAGCCGTG	192
Ad01A15344	Aradu.A01	15344	c	(A)11g(T)11	23	88864130	88864152	GTTTTGTGTTCTGCAATGCG	GCTCATTCTCTGAAGACGCC	182
Ad01A15345	Aradu.A01	15345	p3	(CTC)7	21	88864286	88864306	GCGTCTTCAGAGAATGAGCC	ACGATGCGGTACAAATCACA	246
Ad01A15348	Aradu.A01	15348	c	(GCT)5ttggtgc	64	88875841	88875904	AGTTTTCGAGATCGGCAAGA	GGTTTTCGCATCACCTGTTT	208
Ad01A15351	Aradu.A01	15351	p4	(AAAT)7	28	88900653	88900680	AACTTCTGGAGTCAAGGGACA	AATGCCCGTGTGAATATGGT	255
Ad01A15353	Aradu.A01	15353	c	(AT)7gttaagttt	88	88915017	88915104	TCCTATTCCATCTTTGTTCAAACT	GAAACAAAATCACCTCCTTCTAGC	192
Ad01A15355	Aradu.A01	15355	p3	(ATT)6	18	88916755	88916772	TGAGTGGAAAAAGAGGTGGC	AATGTGAAGCGCATGTGAAG	191
Ad01A15360	Aradu.A01	15360	p3	(CAT)5	15	88927473	88927487	TGCAACACCACCAAGTGATT	CATGGACATTGACACGAAGG	270
Ad01A15364	Aradu.A01	15364	c	(ACA)5tactcca	76	88938383	88938458	CAGAAAAACGGACAGAATCTCC	GGATCCTCGTGTGGGTTATG	275
Ad01A15366	Aradu.A01	15366	p2	(AT)7	14	88944460	88944473	GCCAAACGACCAAATTTTCA	TGAGCCCTTCTTCTTTCCAA	270
Ad01A15370	Aradu.A01	15370	p2	(TA)6	12	88960470	88960481	AACAAATGCAATTCTCTTTCTTTTT	TCGTACTGATAACGTGTTGTGAAA	259
Ad01A15371	Aradu.A01	15371	c	(TA)6tgcaattat	69	88965856	88965924	AACAAATGCAATTCTCTCTCTTTTT	TCTCGTGCTGATAACGTGTTG	255
Ad01A15373	Aradu.A01	15373	p2	(AT)6	12	88966994	88967005	GTGGCAAAGCTGAGTGTTGA	TGACAAATTTGGAGGGCTTC	123
Ad01A15380	Aradu.A01	15380	p2	(AG)6	12	88981426	88981437	TTGTTGTGTTGCATGGCATA	GGTGTAGCGATTGGTAGGGA	241
Ad01A15382	Aradu.A01	15382	p2	(TA)10	20	88982637	88982656	CTAAGCAAGCTGTGCGTGAC	TCTGCTTCTGTAAACGCCAA	266

ID	Chromosome	SSR nr.	SSR type	SSR	Size	Start	End	FORWARD PRIMER (5'-3')	REVERSE PRIMER (5'-3')	Product (bp)
Ad01A15383	Aradu.A01	15383	p2	(AT)11	22	88984313	88984334	TGGTTCAAACCTCACGTCAA	TAATTGGGAATTGGAACCCA	241
Ad01A15385	Aradu.A01	15385	p2	(TA)6	12	88994269	88994280	CCTAACAATAAAAGACGAAGAAATACG	TTGCTTCACAGATTCCTGGAT	215
Ad01A15388	Aradu.A01	15388	p3	(TTA)12	36	89003035	89003070	TTTGACAATGGTTTCGCAGA	AACCGGAAAAATAAGACCCA	242
Ad01A15391	Aradu.A01	15391	p4	(AAAT)5	20	89005461	89005480	TGCACACTAATCGGATCGAA	GCACATTTGCGGATCGAAT	240
Ad01A15397	Aradu.A01	15397	c	(T)10agcttggtt	119	89038409	89038527	AAAGACAAAAGAACCGCGTG	ACTCACCACCACGAACATGA	276
Ad01A15400	Aradu.A01	15400	c	(A)10gaagaaag	37	89056234	89056270	TCAGGGGGTTGTAAAGATGG	GGGCCGGTTGTTTTCTTATT	227
Ad01A15401	Aradu.A01	15401	p3	(TCT)5	15	89072724	89072738	TCACCACTCAAATCCATCCA	CCCTCGAGCAAGAGAAGAAA	127
Ad01A15405	Aradu.A01	15405	c	(CA)9tccttcaga	88	89100028	89100115	GCAGGTAGAACAACCCTCCA	CCCCGTACTGGGTATTTCAA	268
Ad01A15407	Aradu.A01	15407	c	(ATGT)5atatac	42	89100651	89100692	ATTGGGCTCAAAAACCATGT	ACCGATCTCCATGGGTAACA	267
Ad01A15409	Aradu.A01	15409	p2	(TA)9	18	89103127	89103144	TTCCCCAAATATTGTGTAGGC	TCCTTGGGTTTGGACTCGTA	260
Ad01A15411	Aradu.A01	15411	p5	(TTATC)5	25	89119505	89119529	CCTGGGTGCTCCAGTGTATT	CAGAGGAACACGACCCAAAT	168
Ad01A15415	Aradu.A01	15415	p5	(TTATC)6	30	89183700	89183729	CCTGGGTGCTCCAGTGTATT	AAGGGCGGTGTTCATACTTG	239
Ad01A15416	Aradu.A01	15416	p3	(ATT)6	18	89189860	89189877	TTTCCTCCTCAGACGATTGG	TCCTCCTTCCTTTTTCGGTT	264
Ad01A15418	Aradu.A01	15418	p2	(TA)6	12	89190987	89190998	TGCAATTTTCTCTCTCTTTATTTTT	TCCTTCGGTTTATGTTGGAG	220
Ad01A15420	Aradu.A01	15420	c	(T)13cgtttaattc	33	89197859	89197891	GATTCAGTTCTCAATTTGCTTTGA	CTTGCTTTGATGGGAGGTGT	252
Ad01A15423	Aradu.A01	15423	c	(A)10gaaagag(	27	89228556	89228582	TGGATCCAATTGAACCACAAT	TTTCTCTTTCTCCTCTTTCTTCTCC	278
Ad01A15425	Aradu.A01	15425	p3	(AAT)7	21	89232032	89232052	TAGAGGCTGTTCCAGGAGGA	TGGATTTTGCTGTCAACTGG	255
Ad01A15427	Aradu.A01	15427	p2	(AT)6	12	89233485	89233496	AGGCCTCGGCAGTAACAATA	TCTCTCTCATTGTGTCTTTGGTTT	249
Ad01A15428	Aradu.A01	15428	c	(A)11gagagaaa	39	89235543	89235581	AGTTTAATATGCTCCCACCGA	TCAGAACTCCAAGATTCACGTT	149
Ad01A15429	Aradu.A01	15429	c	(T)12ctttttaaaa	88	89235855	89235942	TGAAAATGGAAAAGCATCCC	CAGGCCAGAAACAAGAGAGC	230
Ad01A15437	Aradu.A01	15437	p2	(TA)8	16	89247459	89247474	TTGCTATGGCTTATGGGTCC	AATTAAAATGTGGGGGAGGG	149
Ad01A15442	Aradu.A01	15442	c	(A)11ggaagaag	68	89256693	89256760	GCACATAGCAGCGAGTTCTG	CACAAATTTACCGGCCTTGT	276
Ad01A15443	Aradu.A01	15443	p2	(TA)6	12	89261569	89261580	GACGTATGTTCTCTTGCGGC	GCGATAATGTTGTTGAGGCA	278
Ad01A15444	Aradu.A01	15444	p2	(TA)12	24	89264544	89264567	ATCCTTCATTTGTTCTGCCG	GCCACGTAGATCACCGTTTT	274
Ad01A15445	Aradu.A01	15445	p5	(CCCCA)5	25	89266561	89266585	GGGGGAAAACAATGGAATTT	AACTCCACCACATCAGAGCC	139
Ad01A15450	Aradu.A01	15450	p2	(GT)9	18	89281723	89281740	CCCATTACGCAGAATCAGGT	TTGGGAATGTTTTGATGGGT	263
Ad01A15451	Aradu.A01	15451	p4	(CTTT)5	20	89286956	89286975	TCGTGTTCAAACTGCTCCAC	CAGAGAAAATTAGAGAACAAGAAGGAA	207
Ad01A15452	Aradu.A01	15452	p3	(GAT)7	21	89290597	89290617	TTTGATCTCGAAATCCCAGG	GATCAGTTGGAAGCCTCTGG	171
Ad01A15454	Aradu.A01	15454	p3	(TTC)9	27	89295789	89295815	AATTTACTGCACCCTGCCAC	AGGGAGGAAGAAGAAGCAGC	170
Ad01A15455	Aradu.A01	15455	p2	(CA)6	12	89298416	89298427	TCACTCAGAAAATGACCCCC	ATGCCTGTTTTTGTTCCGTC	264
Ad01A15457	Aradu.A01	15457	p2	(AT)7	14	89313470	89313483	TGCGTGCAGAGAGAACAATC	TGAGTTACTGTGGTGGCTGC	103
Ad01A15461	Aradu.A01	15461	p4	(TTTA)5	20	89320633	89320652	AAGCTCCCATGCAGTCTTTG	TGCACAATAACATTCTTTTATTCATTT	270
Ad01A15462	Aradu.A01	15462	p4	(AAAT)5	20	89320783	89320802	AAAATCAAATCTCCCCTCTTTT	CCTACCCTCCCAGGTGTTTT	267
Ad01A15465	Aradu.A01	15465	p4	(ATAA)5	20	89327341	89327360	GAAGGCAAAATGGGATGATG	TTTGGTGCCTCAATTTTTGC	279

ID	Chromosome	SSR nr.	SSR type	SSR	Size	Start	End	FORWARD PRIMER (5'-3')	REVERSE PRIMER (5'-3')	Product (bp)
Ad01A15468	Aradu.A01	15468	p2	(TA)6	12	89330262	89330273	TGCCTTTATCGTTTAGCACG	TCGCTAAAATCGGTCGCTAT	257
Ad01A15470	Aradu.A01	15470	c	(A)10taaatctta	45	89339047	89339091	AAGAGGCTACTTTGGCAGCA	TCAAGAATCCTTTTGGCACA	221
Ad01A15471	Aradu.A01	15471	c	(GA)7tctgggga	112	89340349	89340460	CGAAGAAAGAAGAAAGGGGG	GTCACTCTCTCCCATGGCTC	236
Ad01A15474	Aradu.A01	15474	p2	(TA)7	14	89355545	89355558	CCATTTAGCACGTCCGTTTT	GGGACTCCTTGTCGTTGAAA	188
Ad01A15475	Aradu.A01	15475	p3	(ATC)5	15	89369228	89369242	TAGGCATCTGTCGTAAGGGG	GAGGTTGAGGGCTGTTCTTG	276
Ad01A15481	Aradu.A01	15481	p2	(TA)6	12	89395539	89395550	TCAAACCATTTAGCACGTTTGT	GTCTTTGGGACTCCTGGTCA	225
Ad01A15482	Aradu.A01	15482	c	(T)10attaaaatc	72	89396877	89396948	TGGGGTGAAAATCAAAATCG	CGACAAATGGGTTAGGAGGA	173
Ad01A15489	Aradu.A01	15489	p2	(AT)6	12	89417766	89417777	AAACACAGAGACTTGGCGCT	ATTCTGTGCATCAATCATATCATAC	277
Ad01A15490	Aradu.A01	15490	p5	(AAATA)5	25	89417917	89417941	TGCATCCATTCATAAAACACAA	CATGGAAAGGTTCATAAGCCA	258
Ad01A15493	Aradu.A01	15493	p3	(CTC)9	27	89428871	89428897	TTTCTGCCGACTCGAGTTTT	CTGGCTCAGGAAGAAGTTGG	273
Ad01A15500	Aradu.A01	15500	p3	(GAA)5	15	89448332	89448346	TCCCAGAAGCAATCAGACAA	TTGAAGCTCCCCTGTACACC	265
Ad01A15501	Aradu.A01	15501	p3	(CAT)9	27	89449902	89449928	CAATTGGAGGCTCAGGGTTA	GCTTTTCATGACGAGGCATT	250
Ad01A15502	Aradu.A01	15502	p3	(GAA)11	33	89450906	89450938	ATGAGGAATGGGTTTAGGGG	TCGATTCTCCACAGTACGCA	252
Ad01A15503	Aradu.A01	15503	c	(T)10cttccc(T)	26	89451778	89451803	CATTGAAACAAAATATTTAACCAAGTC	TCAACAAAATCAAGTGAACATCAA	262
Ad01A15504	Aradu.A01	15504	c	(AGG)5taacgg	105	89452637	89452741	TTGTTGGTGATGACAATAACGA	TCAAGTTCAATTAAGTTGGCACA	250
Ad01A15508	Aradu.A01	15508	c	(A)11gaagaaga	178	89470324	89470501	TCATCACTAATTCCAAAACCAAA	CCCATCGTGTAAGGCTCTGT	255
Ad01A15512	Aradu.A01	15512	p2	(CT)7	14	89480438	89480451	GCTCATCCCGTTACTGTCGT	GTGGGACAAGGCTTTGTTGT	258
Ad01A15513	Aradu.A01	15513	p3	(ACA)7	21	89480584	89480604	CCCGTTACTGTCGTTGTTGA	TAATTCATGTAGCCGACCCC	277
Ad01A15515	Aradu.A01	15515	p3	(ATT)8	24	89486537	89486560	CCATGTAAGCTTCATTATACCACA	TCGGGTCAAACGTTGAGAAT	272
Ad01A15518	Aradu.A01	15518	p3	(TCT)5	15	89488675	89488689	CCGTTAAAGCCAAGACCTCA	TGCAGCTGAAACTGGAAACA	184
Ad01A15519	Aradu.A01	15519	c	(ATA)5agaaga	49	89488806	89488854	ATTTTCCAGCTTCCAGGTCA	GCGCATCGAATTAAACCAAA	234
Ad01A15520	Aradu.A01	15520	p4	(AGAA)5	20	89498451	89498470	CAGAAGTGGACCAAATGAAGC	CCCCTCCCCACTTACAATTT	223
Ad01A15521	Aradu.A01	15521	p4	(TATC)6	24	89499410	89499433	GGCAATAACCCCTCACACAC	CTCTTGCCACTGTGATGGTG	274
Ad01A15522	Aradu.A01	15522	p3	(TCT)5	15	89499543	89499557	GGCAATAACCCCTCACACAC	CTCTTGCCACTGTGATGGTG	274
Ad01A15523	Aradu.A01	15523	p3	(TAT)7	21	89501381	89501401	CCGTTTTTCCACCTGTGTTT	TTGAGGGAGCATGAAGAACA	245
Ad01A15527	Aradu.A01	15527	p2	(AT)8	16	89511213	89511228	TCAAATAATGCCCCAGGAAG	GATCCTCGGCCAGTTCACTA	280
Ad01A15529	Aradu.A01	15529	p5	(TCTTT)5	25	89514449	89514473	TCGTGATTCTCAGGCCTCTT	TGCAACTTGTCTTCCCTCCT	228
Ad01A15534	Aradu.A01	15534	p3	(CTG)6	18	89526534	89526551	AATGCACGACAATCAACACC	ATCATTTAAGCCGCCATCAG	261
Ad01A15535	Aradu.A01	15535	p3	(GTC)5	15	89530037	89530051	CATCTCCTTCGTCTCCTTCG	GCTAAGTCCGAGACGGTGAG	248
Ad01A15538	Aradu.A01	15538	p3	(ATA)5	15	89547649	89547663	GTTCTAATTCTCATTCAAGTGCAA	AATGCATGTAATACTGTGTAGACCTT	122
Ad01A15542	Aradu.A01	15542	p2	(TG)7	14	89554871	89554884	GTTGAGGCTGGTTGTTGGTT	TGCACTAGCTTTCACCCTGA	193
Ad01A15543	Aradu.A01	15543	p2	(TC)9	18	89561406	89561423	ATTCACAGGGCACACAAACA	TGATTCGGAAGGCTTATCGT	273
Ad01A15549	Aradu.A01	15549	p3	(AAG)6	18	89575347	89575364	CGAGGTTGTCGTGTCAAGAA	GCAAACAGGGCAATCAAAAT	277
Ad01A15550	Aradu.A01	15550	p3	(TAA)5	15	89575561	89575575	ATTTTGATTGCCCTGTTTGC	AGTTGTCAACGACTCGTCCC	176

ID	Chromosome	SSR nr.	SSR type	SSR	Size	Start	End	FORWARD PRIMER (5'-3')	REVERSE PRIMER (5'-3')	Product (bp)
Ad01A15551	Aradu.A01	15551	p2	(AT)7	14	89576618	89576631	AAGCTCTGATTGCGAAGTGG	TGACTTGTGCTGATTTTCGC	214
Ad01A15552	Aradu.A01	15552	c	(T)10gtcacgaa	30	89578603	89578632	TGGGGGTTCTTCAAGTTTTG	CGCTTACAACTTCGTGACCA	238
Ad01A15555	Aradu.A01	15555	p3	(CAT)10	30	89591005	89591034	ATTCCAGCAGCTGATGATCC	AAGAATGGCGGTGTCAAAAG	260
Ad01A15556	Aradu.A01	15556	c	(TTG)6ctggtttt	151	89591196	89591346	TCACAGACCTTTTGACACCG	AGCTCAATCTTCAGCTCGGA	262
Ad01A15557	Aradu.A01	15557	c	(TGG)5atctctg	80	89591932	89592011	TCTTTGTGGGAAGGATGAGG	GGATTCCCATATGACGCTTG	225
Ad01A15558	Aradu.A01	15558	p2	(TC)7	14	89592302	89592315	AGCTTCAAGCGTCATATGGG	CGGAACTATCCGACCCTACA	250
Ad01A15562	Aradu.A01	15562	p2	(AG)8	16	89596543	89596558	AGTAAAACGGCAACGAAAGC	ATGAACCAAAGGAGTGGGTG	157
Ad01A15565	Aradu.A01	15565	p3	(TTA)17	51	89604825	89604875	AGCGTCGGGAAAAAGTAACA	AACGTTCAAGCGCGTTAATA	237
Ad01A15566	Aradu.A01	15566	p3	(TCT)5	15	89605088	89605102	CACAATTTCACTGCACCGTC	ACCCAAAGGACACCTTATGC	266
Ad01A15567	Aradu.A01	15567	p6	(GAAGAG)6	36	89605936	89605971	TGGAGGAAGAGGAAGAGGAA	TCAGTTTCCAAAGCGCTACA	128
Ad01A15568	Aradu.A01	15568	p3	(TTA)7	21	89606095	89606115	TGTAGCGCTTTGGAAACTGA	TTGCTACCACAGTTACCGCTT	144
Ad01A15570	Aradu.A01	15570	p2	(TA)6	12	89609200	89609211	AAGCGAATACCTGATAAAAGTTCAA	GGGAATGCAGTATGCACAGA	279
Ad01A15572	Aradu.A01	15572	p2	(GA)8	16	89610018	89610033	GAGTGGGTTGTAGGTGGTGG	GGACATGTCTCCTCCCTTGA	184
Ad01A15574	Aradu.A01	15574	p2	(TA)6	12	89611802	89611813	ATGCAATAAAAATGCAGCCA	AAAAAGGGAAACTCAGGGGA	275
Ad01A15575	Aradu.A01	15575	p3	(AAT)7	21	89621717	89621737	CCATGATGTTAATGCTTTCCAA	GGACAATGTCAAGGGACGTTA	117
Ad01A15576	Aradu.A01	15576	p2	(TG)10	20	89625313	89625332	AGGCAAATTCAGTTGCCAAA	AGGCAACTCGCGAAATCTTA	268
Ad01A15579	Aradu.A01	15579	p3	(GAT)7	21	89633082	89633102	TCAAAGCTTTTCCAGTCGGT	TCGATCAACTTTTCCGATCC	271
Ad01A15580	Aradu.A01	15580	c	(AT)9(AC)9ata	68	89636554	89636621	CACAGAAAAGGCATTCTTGC	AACCGATTTGGTGTTCTTGC	149
Ad01A15583	Aradu.A01	15583	p2	(TA)7	14	89645873	89645886	CAAATTGATTTGAGCGAAAAA	TTGATTTGACCGAAAAATAGTGAA	194
Ad01A15584	Aradu.A01	15584	p3	(TAT)8	24	89647477	89647500	CTCGTCAGCCAGGTACAACA	TACAACCGTCCGGTTCAACT	211
Ad01A15591	Aradu.A01	15591	p3	(ATC)5	15	89659308	89659322	CTCCTTGTGTGTGTTCGTGG	CTTCAAGTCGTTGATTGCGA	208
Ad01A15593	Aradu.A01	15593	c	(AC)79(AT)6(	192	89661069	89661260	GCAACCTTCGGTAGCTGAAC	CATGCACATTTCTGATTTCAAG	280
Ad01A15596	Aradu.A01	15596	p2	(TC)23	46	89676354	89676399	CCCCAGTCTCAAGCTCTCTG	GTTGACAAGCAGATCCCCAT	239
Ad01A15598	Aradu.A01	15598	p2	(TC)6	12	89682826	89682837	ACAAGTGGTGCTCCAAAACC	TTTGTTTCCAATGACCCCAT	187
Ad01A15599	Aradu.A01	15599	p2	(TC)6	12	89684640	89684651	TGCATATAGGTTTGTGCCGA	GATATGGTCGGTTGGTCAGC	116
Ad01A15604	Aradu.A01	15604	p3	(CTT)5	15	89701921	89701935	ATTGACCTTGGAATTGCGAC	CGTCACAGGAACGAACAAGA	235
Ad01A15605	Aradu.A01	15605	p2	(AT)6	12	89703732	89703743	GTGCCCGTTTTCATTTTCAC	TTTAACCGTATTGATATTGATTGC	180
Ad01A15615	Aradu.A01	15615	p2	(AT)6	12	89736185	89736196	TCCAATATCCACCAGGCATT	TGCATCATTACACTTCCCCA	117
Ad01A15616	Aradu.A01	15616	c	(AT)7g(TAA)5	30	89741250	89741279	ATCCCATCTGTAAACACCCC	CAAAAATCGATAACAAGGTTAGGG	280
Ad01A15617	Aradu.A01	15617	p3	(TTA)6	18	89741470	89741487	TCGATTTTTGTAGTGTTAGCCC	TGGCAATAATTTGGCCTGTT	149
Ad01A15621	Aradu.A01	15621	p2	(AC)6	12	89747688	89747699	CAAAATCACTAATTTGTGCCC	GCTCTTTGCCCTCTCTTCCT	113
Ad01A15622	Aradu.A01	15622	p2	(AG)7	14	89753293	89753306	GCCCTCCATTCTTCATCAAA	CTCTTGTCTTCCCTTTCCCC	243
Ad01A15625	Aradu.A01	15625	p4	(TGTT)5	20	89759850	89759869	TTGCGTTCATGCATTTCATT	ACGATGAACGAGTCCTCACC	174
Ad01A15626	Aradu.A01	15626	p3	(AAG)5	15	89770294	89770308	ACAGCTACCCAACACTTCCG	CTGTTGTTGCATGGAGCTGT	275

ID	Chromosome	SSR nr.	SSR type	SSR	Size	Start	End	FORWARD PRIMER (5'-3')	REVERSE PRIMER (5'-3')	Product (bp)
Ad01A15628	Aradu.A01	15628	p4	(CCGT)5	20	89774064	89774083	TTCTGCAGCTCCGTTTCTTT	AAACCGTCAAACATCCGTTC	253
Ad01A15631	Aradu.A01	15631	c	(TTTA)5ttatta(	44	89784476	89784519	TGCAATAAAAGAAAATGGAGGAA	ATTGTCCCAACCTCATCCAC	272
Ad01A15632	Aradu.A01	15632	p2	(GC)6	12	89784849	89784860	GTGGATGAGGTTGGGACAAT	ATAGGGAGCTCTTGGGCATT	255
Ad01A15633	Aradu.A01	15633	c	(T)12attcctttc	58	89795268	89795325	ATAACCGATCCGGTTGAGTG	CCGGTAGTGAGACGAAGAGG	252
Ad01A15635	Aradu.A01	15635	p2	(AT)9	18	89817239	89817256	GTACCCGAAGAACCGAACAA	AGCTGTGTTTTGGAGATGGG	234
Ad01A15638	Aradu.A01	15638	p4	(CCGT)5	20	89829925	89829944	TTCTGCAGCTCCGTTTCTTT	CGACGAAGAACACGAACAAA	249
Ad01A15642	Aradu.A01	15642	p3	(TCT)5	15	89860033	89860047	GGATCCTGTCTGTGTACGGC	CATCAAAGAAGGATCGCCTG	265
Ad01A15643	Aradu.A01	15643	p3	(TCT)5	15	89867426	89867440	GGATCCTGTCTGTGTACGGC	CATCAAAGAAGGATCGCCTG	265
Ad01A15646	Aradu.A01	15646	p3	(AGA)6	18	89921530	89921547	TAAAATATTGCTCGCCCTCG	CCCTCAAACCCACCCTAAAT	198
Ad01A15647	Aradu.A01	15647	c	(AGA)5(AT)1	37	89924809	89924845	AGGAGTCTCCAACCTTGGGT	GGGTCATCCTGACCTGAAGA	207
Ad01A15652	Aradu.A01	15652	p4	(TTAA)5	20	89978285	89978304	CTTAAGAGTCCAACCCGGTG	AAAATCCATTTTACCGTGCAA	279
Ad01A15653	Aradu.A01	15653	c	(T)11caagattgg	110	89978475	89978584	TCAATTGAATTTTGCACGGT	CCATGCTCTTCATCTTTAATTCC	165
Ad01A15655	Aradu.A01	15655	c	(AT)6(GT)9	30	89981537	89981566	GTACGAATTTGTCTGCGGGT	TGAGTTCAACACTCACTTTCTTCA	128
Ad01A15658	Aradu.A01	15658	p2	(CT)6	12	89985328	89985339	AGCCGACACAAAATGATTCC	AGGTAAATGGGATTGGGAGG	237
Ad01A15660	Aradu.A01	15660	p3	(GAA)5	15	89990623	89990637	GGAAGGAGAGGAGGAGAAGG	GGACAAATCGACCCCTACAA	172
Ad01A15663	Aradu.A01	15663	p3	(ATA)9	27	89993827	89993853	GACGGTTGGGTGAAGAGAAG	CACATACATGTCATTGTCTCATCG	114
Ad01A15664	Aradu.A01	15664	p3	(TCT)5	15	89996896	89996910	TTGCTGCACGTTCTTCTTCA	CCTCCTCATCTTCTGCTGCT	184
Ad01A15665	Aradu.A01	15665	p6	(GAAGAG)8	48	89997454	89997501	ACACCCAAAATTCGTTGCAT	AGCGCTACACTCAAAACGGT	280
Ad01A15669	Aradu.A01	15669	c	(TTA)7(TTG)9	48	90007334	90007381	AATACACGGGTAATTGAAATAGTTTAT	TTGATTCTAATCGTAAAATGATCAAA	219
Ad01A15670	Aradu.A01	15670	p3	(TAT)9	27	90007560	90007586	TCATAAGATTTGATCATTTTACGATT	TTAGGGGAAAACCCCATTTT	280
Ad01A15671	Aradu.A01	15671	p3	(TTA)5	15	90008625	90008639	TTCGGAGCTCTATAAGATATGTTACCT	TTTCATATCACATTCCGTTTCAA	277
Ad01A15677	Aradu.A01	15677	p3	(TCT)5	15	90020504	90020518	GTGTTCAACCTCCCCCTTTT	TCATCCTCAACCCGAGTTCT	230
Ad01A15678	Aradu.A01	15678	p2	(TA)6	12	90023071	90023082	GAAGAAAATTTATATCCGAGGTACGA	CCATAAGCGATCCAGGCTAA	275
Ad01A15681	Aradu.A01	15681	p3	(ATT)22	66	90029308	90029373	GGGACCTATCTGTCTTTTTCCC	ATAATCATTCACCTGACTTCATATACA	234
Ad01A15682	Aradu.A01	15682	p3	(TTA)5	15	90031323	90031337	GACCATGGTGTTATCATGTATGC	CATTTCATATCACATCCGTTTCA	248
Ad01A15686	Aradu.A01	15686	p3	(GCG)5	15	90039977	90039991	CGGTGAGTGAGCTGAAATGA	GTACGCATTTTCGTCCCAGT	115
Ad01A15691	Aradu.A01	15691	p2	(TC)7	14	90058026	90058039	AAATGGTTTGGTTCAATCGG	CGGCATTATCAAATTGCACA	275
Ad01A15693	Aradu.A01	15693	p3	(AGA)9	27	90064238	90064264	CACGGATATTTCGAATTGACG	CGGTCCTTCGAAAATGAAAA	250
Ad01A15694	Aradu.A01	15694	p2	(TA)6	12	90067790	90067801	GCAAAAAGGGATTTGGTGAA	ACCGCCAGCAACTATGAACT	277
Ad01A15698	Aradu.A01	15698	p3	(CTC)8	24	90072273	90072296	AGGAGGCTAAAGCGAAGTCC	CCGAGACAAAGTCGAAGAGG	137
Ad01A15700	Aradu.A01	15700	p2	(AT)6	12	90081893	90081904	GGGAAAATTCTTTGATGGCT	AAATTTATTGTAGCGTGAGGAAAA	261
Ad01A15703	Aradu.A01	15703	p3	(TTA)5	15	90085260	90085274	AATAACACGCGGCCACTATC	GGACAAATTATGCTTCCCGA	198
Ad01A15704	Aradu.A01	15704	p2	(AT)9	18	90087219	90087236	TCATGCCGTCCATGTAAAAA	TCAACATTTGTTCGCCATGT	198
Ad01A15708	Aradu.A01	15708	p2	(AG)8	16	90106892	90106907	AGTGGAAAAGAAATGGGGGT	CCAACTCTCCTCCTCTCCCT	262

ID	Chromosome	SSR nr.	SSR type	SSR	Size	Start	End	FORWARD PRIMER (5'-3')	REVERSE PRIMER (5'-3')	Product (bp)
Ad01A15714	Aradu.A01	15714	p3	(GAA)7	21	90139374	90139394	AAGGCATCTTTGTCGTTGCT	TTTTTCTCCGTTCCCCTTCT	118
Ad01A15717	Aradu.A01	15717	p3	(ATA)7	21	90147127	90147147	TTTTCGTGCGAATAGTTATCTGA	TTCTGACATTTCGGTACAATTCA	278
Ad01A15718	Aradu.A01	15718	p3	(GAA)5	15	90149381	90149395	TGAAGGGTATGCCCTGAAAG	AGGGGGAGTATTGTTACCCG	148
Ad01A15720	Aradu.A01	15720	p2	(TA)6	12	90150783	90150794	TTCAGGGAGGTTGAATACGC	TCCAAGTCTCTCCCACGTTT	246
Ad01A15722	Aradu.A01	15722	p4	(TTAT)5	20	90152520	90152539	CAACGTAACTTTTCTTGTGGAGAA	CACGGTTTCCATCAGTTTCA	266
Ad01A15723	Aradu.A01	15723	p3	(ATT)11	33	90153277	90153309	TCATGAACCCACTCATTCCA	TCGTCATCATAGATTTGGAAGC	255
Ad01A15725	Aradu.A01	15725	p6	(TTTTTA)5	30	90160422	90160451	AGGTTGGGGCATCCTAGTGT	TGGAAGGAAAAATGAAGGGA	267
Ad01A15727	Aradu.A01	15727	c	(TCA)5(TCT)	187	90164199	90164385	TCCAATGAGTAACTCGCTGAAT	TTGGAAGCATCAAACAACACA	272
Ad01A15728	Aradu.A01	15728	p3	(AGA)5	15	90164531	90164545	GCAGTCTGATTGGAACAGCA	TAGTTTTGATTTGGGCCTGC	251
Ad01A15731	Aradu.A01	15731	p2	(AG)13	26	90167833	90167858	AGAGAGCTCGAAGAGGGACC	TAGCCACCACCTTCTTCACC	153
Ad01A15736	Aradu.A01	15736	p3	(GAA)5	15	90179382	90179396	AGCTGAAGGCTCGTCACAAT	TTCCACCCACTACTCCTCCA	187
Ad01A15741	Aradu.A01	15741	p2	(TA)10	20	90187473	90187492	TGTTGCCGTGCATTAAGTTC	AGTTAACGTCCACACACGGG	150
Ad01A15743	Aradu.A01	15743	c	(A)13gnnnnnn	46	90193947	90193992	GAAAACGGGGAACGTTGTTT	CATTACACGTTTACACTCACCAAA	263
Ad01A15746	Aradu.A01	15746	p2	(TC)10	20	90199742	90199761	AAATCGAAATCGTTCCTGATG	AAGGGGGACGATGAGCTAGT	202
Ad01A15756	Aradu.A01	15756	p2	(AT)6	12	90272489	90272500	AGCATTCAACCATCAACCAAG	TTCAATGGTCACGTTTATTTGTG	279
Ad01A15757	Aradu.A01	15757	c	(GT)10(GA)1	74	90280700	90280773	TTACCTTCAACCCTTGACCG	GGCCAAAGTCAAGTTCAACG	230
Ad01A15758	Aradu.A01	15758	c	(AG)14gagagt	102	90281111	90281212	TTTTTGGTTGTAGCCCTTCTTC	GAGGGTTGTCCAATCCATCT	256
Ad01A15761	Aradu.A01	15761	c	(AT)8aattatgta	67	90295760	90295826	GCCAAGAGTTTTCGCCTATT	TGGACAAGTTATTGCCATTCA	266
Ad01A15769	Aradu.A01	15769	p3	(TTA)6	18	90317720	90317737	ACCGTAATCGGTTGAGGCTA	CCGCAACATTTTCCTCATCT	275
Ad01A15773	Aradu.A01	15773	p2	(TC)9	18	90333495	90333512	ACGCGTCATGATGAAGAATG	AAACCCATAAGCACAGCGTC	238
Ad01A15775	Aradu.A01	15775	p4	(AATA)5	20	90337275	90337294	CCAAATCCTCTAATATTCCTCCTG	CACCCATGCAATGAGTGAA	219
Ad01A15777	Aradu.A01	15777	p4	(AAAT)5	20	90338038	90338057	GCCGATCACTACCAGACTTGA	ATTCCACAAGCGGAAACTTG	208
Ad01A15778	Aradu.A01	15778	p3	(CTC)6	18	90344184	90344201	TTTCAGAGAGTCCAGGAGGC	GCTGAGGGAGAGCTGAAGAA	139
Ad01A15780	Aradu.A01	15780	c	(TAT)5tacat(T	34	90370899	90370932	TCGATAACAAGGTTAAGGACTTACAA	TATCCCATCTGCAAACACCC	280
Ad01A15784	Aradu.A01	15784	p2	(CT)23	46	90390502	90390547	GGTGAGGGAAGTTTGTTCCA	TTCAACGCATGAACCAAAAG	279
Ad01A15787	Aradu.A01	15787	p3	(AAG)8	24	90397857	90397880	TTGCTTCAATGTGTGTGCAA	GATTCCTCTTCCTGCCTTCC	259
Ad01A15792	Aradu.A01	15792	p2	(TC)6	12	90409388	90409399	TCCATTGATGAGGTTGGTGA	CTCTTCTGTGACCCTACGGC	206
Ad01A15794	Aradu.A01	15794	p5	(TGTAT)8	40	90413071	90413110	ATAGACAGCAACCCTGTCCC	TGCAAGGATGACAAAGGTGA	247
Ad01A15798	Aradu.A01	15798	c	(ATT)6ttg(A)1	31	90436985	90437015	TGGCATTTTCTGTTGTTTGC	AAGCAAAATTCCAAGCCAAA	180
Ad01A15801	Aradu.A01	15801	p2	(AG)6	12	90455309	90455320	CGACGCACAGTGAGACAGAT	AGGCTCGAGTGCAACTTCAT	244
Ad01A15802	Aradu.A01	15802	p2	(GA)6	12	90457397	90457408	TTCTCCACGAATCAGATCCC	AGAAACCCTCTCGCTGAAGA	221
Ad01A15806	Aradu.A01	15806	p2	(TG)6	12	90462216	90462227	GGCACGCATCATATTTATTCC	TTTTGTGGGGGCATATGAAT	199
Ad01A15809	Aradu.A01	15809	p3	(TAA)13	39	90467530	90467568	GATGCACTCTGCCATTTTGA	TTATATCCTCGCCCACAAAAA	247
Ad01A15810	Aradu.A01	15810	c	(CTT)5ctctcat	119	90467818	90467936	GTGGACAAAAGGGAAGAACG	TGATGAGGTAGGGGGTTGAG	257

ID	Chromosome	SSR nr.	SSR type	SSR	Size	Start	End	FORWARD PRIMER (5'-3')	REVERSE PRIMER (5'-3')	Product (bp)
Ad01A15811	Aradu.A01	15811	p3	(AGA)9	27	90468186	90468212	TAATCACGGCAGTGGAATCA	TTGTTGTTGTCATCGTCCGT	216
Ad01A15812	Aradu.A01	15812	p3	(TCA)6	18	90468735	90468752	AGAAGCGCAATAAGCTTGGA	TTCTTTCTCGTCCTGAGGGA	155
Ad01A15813	Aradu.A01	15813	p2	(CT)6	12	90470682	90470693	GTGGTAGGAACCCAGCTCAG	AGGAGAGTACGAGCCAACGA	215
Ad01A15821	Aradu.A01	15821	p3	(CCT)8	24	90517613	90517636	TTCAGGAGGCTAAGGCAAAA	AATCACAATGGGCTGAGAGG	119
Ad01A15823	Aradu.A01	15823	p3	(AAG)5	15	90533802	90533816	CAATTTTGGTTCCCGTTTGT	CATTCAAATCCCCACCATTC	210
Ad01A15824	Aradu.A01	15824	p2	(TA)6	12	90537411	90537422	GGAAGCAAAAGAAGCCAAGA	CCATTCCATGAAAGCCAAGT	234
Ad01A15825	Aradu.A01	15825	p3	(CTT)6	18	90538281	90538298	TTACGCTGGTCAAATGTTGC	ACATGCACAGATGGGAAAAA	231
Ad01A15828	Aradu.A01	15828	c	(A)11ggttgtgac	57	90546823	90546879	ATGGCGTTGACTACAGAGCA	ACGGGTCCAAATCCATTACA	254
Ad01A15829	Aradu.A01	15829	p3	(ATT)5	15	90547080	90547094	TGTAATGGATTTGGACCCGT	GTTCTGGGCTTGTGTTGGTT	198
Ad01A15830	Aradu.A01	15830	p2	(AG)8	16	90547233	90547248	AACCAACACAAGCCCAGAAC	CTGCATATCCGAGGAGAAGG	214
Ad01A15832	Aradu.A01	15832	p3	(CTT)9	27	90548857	90548883	GAGTTCACGGAACGGAACAT	AAAGCGTGGAAACAGAAGGA	104
Ad01A15833	Aradu.A01	15833	p2	(CT)12	24	90549641	90549664	CATCTTGTGCATGCTCCGT	GTACAAAGGCTGAGTTGGCA	271
Ad01A15835	Aradu.A01	15835	p3	(TGG)5	15	90556232	90556246	GCATGGTTGATTGCTCCTTT	TGACTGGGCTTTCTCTTGCT	180
Ad01A15836	Aradu.A01	15836	p3	(TTC)5	15	90556369	90556383	GCATGGTTGATTGCTCCTTT	TGATGAAGATGGAAGATGATGC	222
Ad01A15838	Aradu.A01	15838	c	(C)18(A)10	28	90560797	90560824	CTTTTCTGGTTTTCACCCCA	TCCATACCCAATGATTTCAGG	271
Ad01A15839	Aradu.A01	15839	p3	(GGA)5	15	90571636	90571650	TCGAACCACTCGTTTCACAC	GCGTCTCCGACGGTAAGATA	267
Ad01A15840	Aradu.A01	15840	p3	(GAG)7	21	90571911	90571931	GAAAGCGACGTGCTACATGA	GCTTGACGACGACAAATTCA	162
Ad01A15841	Aradu.A01	15841	p3	(AGA)7	21	90572110	90572130	TCGGTGATTTCTGTGCAGTC	TCCCTCTCTTCACCACAACC	182
Ad01A15842	Aradu.A01	15842	p3	(ATT)5	15	90576502	90576516	CATGAACCACTACAATGCCG	AAGCAAACCAAACAAGTGGG	118
Ad01A15843	Aradu.A01	15843	p2	(TA)6	12	90578695	90578706	TCAATGCATGCAATTCCCTA	GCGAACACAAAGGGAACAAT	132
Ad01A15845	Aradu.A01	15845	p3	(TAC)5	15	90598377	90598391	CATGCCTGATGATGATGAGAA	AAGAGCAAGGGCGTGATTTA	256
Ad01A15847	Aradu.A01	15847	p2	(TA)7	14	90640299	90640312	CCATGGCTTCTTGCTACTGG	GCATGTCACGAGCCTTAGAA	247
Ad01A15849	Aradu.A01	15849	c*	(CT)10(ATCT	94	90645728	90645821	CATGCATTTTGATGCCCTAA	GGCAATGGTCTTATGCTCGT	169
Ad01A15852	Aradu.A01	15852	p2	(TA)6	12	90650743	90650754	ATGTGGCGAATTCTTTGGTG	GGTGCATGCTAATGCTATGG	178
Ad01A15862	Aradu.A01	15862	p2	(TA)6	12	90672650	90672661	CCACTGTTTTGTTATATGAAATTGAAA	CCCATATTACCGCTAACAGCA	261
Ad01A15864	Aradu.A01	15864	c	(T)10ct(A)11	23	90675645	90675667	TGTTGATTGCATGAGAACAAAAT	CAACAATTTTAGCTGTGCCTCA	261
Ad01A15866	Aradu.A01	15866	p2	(TC)6	12	90678341	90678352	CCCTCTCTCTCCCATATCTCTC	AAGAGGGGAGAGGACCATGA	139
Ad01A15867	Aradu.A01	15867	p2	(GA)9	18	90678867	90678884	GAGCGTGGTGGTGATGTATG	TTCTCTCCCCCTCCTCTCTC	230
Ad01A15868	Aradu.A01	15868	c	(GA)6ggaagac	74	90679088	90679161	AGACGAATGGCAAAAACTGG	TGCTTCTCTCTCTCCTTCCCT	260
Ad01A15869	Aradu.A01	15869	p2	(GA)9	18	90679266	90679283	AGGGAAGGAGAGAGAGAAGCA	CCCCTTTCCCCTCTTTTTCT	129
Ad01A15870	Aradu.A01	15870	c	(AG)6aagggga	101	90679513	90679613	AAAGGGAGAGAGAAGAAGAGAGA	ATTGCACCAGTTCAAACCAG	280
Ad01A15872	Aradu.A01	15872	p3	(AAC)5	15	90691383	90691397	TCAATTGAAAATGGCTAGTAAGAAGA	GTCACAGAGCCAATGCTCAC	257
Ad01A15873	Aradu.A01	15873	p3	(TGA)6	18	90692099	90692116	TGCTTTTCGTTGTAAAGGTGA	TGAGTTGAGTCAATCTTTTTCGAT	265
Ad01A15875	Aradu.A01	15875	p2	(TA)11	22	90693634	90693655	TCGAAAACAATAAAATTACTGAATGA	AACCCTCATCACCTTGCTTG	134

ID	Chromosome	SSR nr.	SSR type	SSR	Size	Start	End	FORWARD PRIMER (5'-3')	REVERSE PRIMER (5'-3')	Product (bp)
Ad01A15876	Aradu.A01	15876	c	(AAAAT)5tatt	125	90696335	90696459	CCAATAAGTTCGTAGTTAACATCCAG	CTTGTTCAGCAGCACACACA	187
Ad01A15879	Aradu.A01	15879	p3	(TAA)5	15	90705518	90705532	AAGGAAAGGCCTAGTGGAGTG	GGGAACTTGTTCCTTGTGGA	247
Ad01A15880	Aradu.A01	15880	p3	(TGG)7	21	90728409	90728429	CTCGATCTTAACGGCGAGAA	CAACCTTCTCTCTCCAACGC	138
Ad01A15882	Aradu.A01	15882	p2	(TA)7	14	90729610	90729623	GGTGTTCCTGTGGTAGGTGC	GGGTGGGAGTGCTGAGTAAA	241
Ad01A15884	Aradu.A01	15884	p3	(ATA)13	39	90732493	90732531	TTTGAAGCAAGGGATCTCTGA	CGGTAAAAATTCAGGTGCAA	251
Ad01A15886	Aradu.A01	15886	p3	(AAT)11	33	90734387	90734419	CACCATGATCTATGATGCACAA	CTTGTGGGTTCCACTGTCCT	198
Ad01A15888	Aradu.A01	15888	p2	(AT)8	16	90738788	90738803	CCCTCTTAAAACTGCTCTTTGC	TGGCTTTGCCTTATTGGATT	228
Ad01A15890	Aradu.A01	15890	p2	(CT)9	18	90739953	90739970	TTTTGGACCCAGCTTTATTCA	GCAATAACTCTCTGAGGGTGTG	207
Ad01A15891	Aradu.A01	15891	p2	(TC)10	20	90740378	90740397	CCTTCCCAAGCTGACACAA	TGATGAGGATGGCAAAGAGA	124
Ad01A15893	Aradu.A01	15893	p2	(TA)8	16	90746897	90746912	TTGCGTGTCCCACATATTTT	TCCCTAGTGTTATGATGAATGGA	261
Ad01A15896	Aradu.A01	15896	p3	(TAA)7	21	90757958	90757978	GGGGATGTTGGTGAACAAGT	AATGACACAGGACTTGCGTG	273
Ad01A15899	Aradu.A01	15899	p3	(CGG)5	15	90773508	90773522	CCAATTATGCTGCCGACTTT	CACCTCCAACAATGTTTCCC	234
Ad01A15901	Aradu.A01	15901	p2	(TA)6	12	90778733	90778744	CCACGTCACCCTCTTTTTGT	TTTTCAGGTCATTCCAATTGAT	280
Ad01A15903	Aradu.A01	15903	p3	(GAA)6	18	90782560	90782577	GGCGGTTGATATCCTTCAGA	AACTATCCCTGACACCGTCG	236
Ad01A15905	Aradu.A01	15905	p3	(TTA)19	57	90785634	90785690	TGATGCCATAAAAACGCAAA	CACATTTCATGTGGCTTGCT	235
Ad01A15907	Aradu.A01	15907	p3	(TTA)22	66	90790148	90790213	CATGTCAATGAAGGTATGGCA	GCATTTCATGTGGCTTGCTA	211
Ad01A15908	Aradu.A01	15908	p5	(AAGAT)5	25	90806485	90806509	TAAAGGCAGCTAAAGCCCAA	AAAGTCCTGCTGATCCTTCG	263
Ad01A15909	Aradu.A01	15909	c	(TTA)5(TTG)1	48	90810581	90810628	TGATGCCATAAAAACGCAAA	ATCTCTCCACTCAGCTTCGC	244
Ad01A15915	Aradu.A01	15915	p2	(AT)10	20	90842109	90842128	GGATGCGATAACATTGCGTT	CGTTCGTCCAGATTGTTTCA	239
Ad01A15918	Aradu.A01	15918	c	(A)11gattaattg	79	90848783	90848861	CATGTCAATGAAGGTATGGCA	ATCTCTCCACTCAGCTTCGC	263
Ad01A15921	Aradu.A01	15921	c	(CA)6tacatatgt	57	90869386	90869442	GCCCATTCATAACCCCACTT	GGTCCACCAAGCCGTAAATA	250
Ad01A15922	Aradu.A01	15922	c	(TA)6gac(AT)	29	90870491	90870519	TTGCCAAAAGTCCAAAGAAA	GCAACATACAAACCCCAAAAA	266
Ad01A15923	Aradu.A01	15923	c	(AT)12tttat(AC	43	90870984	90871026	TTGGTATCCCCAAAGTCTCG	ATGGAGAGGGCAGAGACAAA	171
Ad01A15925	Aradu.A01	15925	c	(T)10cgttttctttt	35	90887688	90887722	CCCCAATTAGAGGTGTCCAA	TGTGGTTCATGAATGTTGGC	226
Ad01A15932	Aradu.A01	15932	p4	(TAGG)5	20	90925400	90925419	GCAGTGGCAGAAAAATGGAT	GGGTGAGGAAACAGTTAAGGG	160
Ad01A15936	Aradu.A01	15936	p3	(TGT)6	18	90931190	90931207	GTTGATGTGTGGACCAGTCG	CAAGGAATCTGATTTCCCCA	204
Ad01A15939	Aradu.A01	15939	p2	(AT)6	12	90936076	90936087	CCCCTTATCCTGAGTCAAGC	TTCACATGACCTCCATATCACTAGA	273
Ad01A15940	Aradu.A01	15940	p2	(TA)6	12	90936463	90936474	ACGGTATGCTGCATCACAAA	TTTGCTTTACATGAATGTTGAACTC	258
Ad01A15941	Aradu.A01	15941	c	(GGT)5gatcatg	112	90937449	90937560	GCCTCTTGTTCCTTGTCGTC	TGACCTCCTTTAGCCTCGAC	276
Ad01A15942	Aradu.A01	15942	p3	(ACA)7	21	90943378	90943398	TGCAATTGTCATTGATGGCT	GGTGGGAACTGGTAAGGGAT	218
Ad01A15945	Aradu.A01	15945	p3	(CTC)5	15	90952279	90952293	GTGTGGGGAAAGAAATGGAA	GGCAGAAATGAGAGGAGTGG	263
Ad01A15946	Aradu.A01	15946	p4	(ATTA)5	20	90952638	90952657	CCACTCCTCTCATTTCTGCC	GCACCTGAATTTTCACCGTT	238
Ad01A15947	Aradu.A01	15947	p3	(TAT)16	48	90954467	90954514	TGTGTTGAGATGAAGCCCTC	TTAGGAAATTAGTATGCTGTGTTATGA	160
Ad01A15948	Aradu.A01	15948	p3	(ATA)5	15	90955744	90955758	TCCAATGATTTTATTAGTGGCAA	TTGCCTTCTTGTAACGACACC	254

ID	Chromosome	SSR nr.	SSR type	SSR	Size	Start	End	FORWARD PRIMER (5'-3')	REVERSE PRIMER (5'-3')	Product (bp)
Ad01A15953	Aradu.A01	15953	c	(T)14gatgatcta	44	90974141	90974184	CCTGATCTTCCCGACTGAAC	ACCCGTGGATCATACCAATC	221
Ad01A15955	Aradu.A01	15955	p2	(AT)6	12	90976415	90976426	TCTGTTGAAAATGCTTTTATTCATCT	ATCCGACTAGTTCCGGGTTC	265
Ad01A15957	Aradu.A01	15957	p4	(TTAT)6	24	90993736	90993759	TTGCCTTTTGTCACATTTGC	TTGTCCACCTCTTTCACCTTTT	172
Ad01A15961	Aradu.A01	15961	p3	(TAA)5	15	91005492	91005506	CAAAATGGTTGCGTAGCTTG	CACAAAAAGATGCTGCAGAAAA	168
Ad01A15962	Aradu.A01	15962	p3	(ATT)6	18	91010582	91010599	TCAGTGACACGAAAGGCTACC	CCCTTTTGGCAACAAGCTAA	278
Ad01A15963	Aradu.A01	15963	p3	(AAG)6	18	91011158	91011175	AAATTCCACACTTAATATGCAAAAA	AGTCAAAAGCCAAGTGGTGG	155
Ad01A15968	Aradu.A01	15968	p2	(TG)6	12	91028467	91028478	GTGGGATGAGGCTTTGTTGT	GGTTCCTGTCCTATGCCAAA	121
Ad01A15970	Aradu.A01	15970	p3	(TAA)5	15	91034093	91034107	GGTGCCAGAAAAACATTTCG	AGTGCCCTAAAGCAAGGTCA	183
Ad01A15977	Aradu.A01	15977	c	(A)11tagctactt	108	91041775	91041882	TAGACCAAGATTTCGGGGG	GCACCGCACAATAGTTTCCT	274
Ad01A15980	Aradu.A01	15980	p3	(GAA)6	18	91042835	91042852	AATGCAGGAAAAAGGTGACG	GATGTGATGGGATTGTGCTG	202
Ad01A15981	Aradu.A01	15981	p2	(TA)7	14	91043176	91043189	GGAAAAGGAGAGATCCAGGG	TGAAGGGTGCTTTCTGCTTT	235
Ad01A15986	Aradu.A01	15986	c	(A)10cctccatat	78	91064218	91064295	GAGTCAGTTAATCACAGTGGCAA	TTGAAATTGTTCAGAGTTCAAATAA	279
Ad01A15987	Aradu.A01	15987	p3	(ATT)5	15	91065156	91065170	TTTTTCACGTGCTTATGTGTG	GCATTGCACAAATTTTTAACTCA	193
Ad01A15988	Aradu.A01	15988	p2	(AT)7	14	91066194	91066207	TTCTGTCTCTCACTTTGCCTCA	TGGAATAGGTTGCTGTCTTTTATTT	260
Ad01A15996	Aradu.A01	15996	p2	(TA)6	12	91093272	91093283	ACAAGCTGCCATACAAGCCT	TGTTGACGATTGGACATGCT	266
Ad01A16002	Aradu.A01	16002	p2	(AT)11	22	91102370	91102391	TGCAATACGTCCCATGTAAAA	TGTGTATCTTAAAATCAGTCACCG	214
Ad01A16004	Aradu.A01	16004	p4	(ATTT)5	20	91106877	91106896	CCTCTGGTTCTTCTTGCATGT	ATTTTGCAGCCCATTGAAAG	244
Ad01A16005	Aradu.A01	16005	p3	(ACA)5	15	91118895	91118909	CAGAAAAACGGACAGAATCTCC	GTCACAGGCAAGATCCTCGT	280
Ad01A16010	Aradu.A01	16010	p3	(ATA)7	21	91127894	91127914	GCGCTCATGCTGTCAATTAG	TTTTGAATGGCAATTCGTGT	180
Ad01A16014	Aradu.A01	16014	p2	(GC)6	12	91147411	91147422	TTGCTCTTCAATCCTTGGCT	CAAAGAAGTTGAACTCGCCC	223
Ad01A16016	Aradu.A01	16016	p3	(CTA)6	18	91171214	91171231	GGGGTCATTGCATTCTCCTA	GATCTTGAAAGGCCTGCTTG	234
Ad01A16017	Aradu.A01	16017	p2	(TA)6	12	91171882	91171893	TTGACCCTATGGATTTCATGTG	AGATGCATGAGGCCATCTTC	139
Ad01A16018	Aradu.A01	16018	c	(TA)8(GA)15	46	91172481	91172526	AAAACAGGGCCTTCCACTTT	CTTTAGTGTCCCGGCAATGT	170
Ad01A16019	Aradu.A01	16019	p4	(TCTA)5	20	91173612	91173631	CCTCCTTCATGCCATCATTT	GGGTTGAATGAATGTTTATTCTTG	258
Ad01A16020	Aradu.A01	16020	c	(GA)10aaa(AC	35	91173821	91173855	TGACAGGCTGTGATGAGAAGA	CTTTCTGTGGTCCCCTTTCC	280
Ad01A16021	Aradu.A01	16021	p4	(AAAT)5	20	91175167	91175186	ATTTGGGTCACTCAAGGCAC	ACTGCACCTGAGTTTCCACC	208
Ad01A16025	Aradu.A01	16025	p3	(ATA)5	15	91189617	91189631	CTGGCGCAAAGAAAGAAAAC	AAGTCTGCTGCTCCTCGTTC	255
Ad01A16026	Aradu.A01	16026	p4	(AATA)7	28	91189935	91189962	ATGTTAAGATCCCGGCAGTG	CCTCTCAACTTCACCAAGCC	247
Ad01A16027	Aradu.A01	16027	p3	(TTC)5	15	91190861	91190875	TACGTCCAATACACGCGCTA	CGAGAAGAAGAAGAACGAGAAGA	187
Ad01A16028	Aradu.A01	16028	p4	(TTAA)8	32	91195228	91195259	TGGTGAATGCAGATTGTGCT	TTCCACTGCAACCAACAAGA	249
Ad01A16029	Aradu.A01	16029	p2	(TA)8	16	91195406	91195421	TTCTTGTTGGTTGCAGTGGA	TTATTTGCACATTGCTTCGC	120
Ad01A16033	Aradu.A01	16033	p3	(TAA)14	42	91203404	91203445	TAACCCCTCTTGTGGTGGAC	TTCCTGATGTACTCCACCATGT	190
Ad01A16035	Aradu.A01	16035	p4	(GTAT)6	24	91211161	91211184	TCAAAACATTTTTCGGCCTT	GATGCCAGCATTGAGGAACT	173
Ad01A16037	Aradu.A01	16037	p3	(ATT)6	18	91225460	91225477	ACAACTGGAGCATCACATGG	AACCTCTTGAAATTGAATCGGA	230

ID	Chromosome	SSR nr.	SSR type	SSR	Size	Start	End	FORWARD PRIMER (5'-3')	REVERSE PRIMER (5'-3')	Product (bp)
Ad01A16046	Aradu.A01	16046	c	(TTA)5ttg(TT/	33	91242687	91242719	GGCTCCAACCACTTTTTGAT	GTGGGCTTTTCTGGATTGAG	180
Ad01A16047	Aradu.A01	16047	p3	(GCA)5	15	91245798	91245812	ACCCATGGATCTTTCTGCAT	TCATATGATGCCAAGGCAAA	153
Ad01A16048	Aradu.A01	16048	c	(T)11ggacgaga	119	91247493	91247611	TCTGTCTTTGGACGTTGTCG	CCAGGGTCCAGGTACCATTT	181
Ad01A16050	Aradu.A01	16050	p3	(TTC)5	15	91260162	91260176	TCCTTCTCATCTTCTGCTGCT	TCACCTTTCAAGTAATTTTGCAG	216
Ad01A16053	Aradu.A01	16053	p3	(CTT)6	18	91261718	91261735	TGCTATTGTTAAGAAATTTCGGTG	TGGTGTTGTTGCTGCATTTT	198
Ad01A16059	Aradu.A01	16059	p3	(TTA)21	63	91274852	91274914	TTGCATGGTAGAGAGAATGGG	GAAGAAGCAAAACCGATTCAA	244
Ad01A16060	Aradu.A01	16060	p2	(CT)7	14	91281168	91281181	ATTCCCCCAACTTGGTTTGT	CTGCGTTGATAGGAGGCATT	197
Ad01A16063	Aradu.A01	16063	p5	(TTCTC)5	25	91284820	91284844	TAAGTGGCCCATAGTGACCC	GAAACGCTGAGTGGGAAGAG	125
Ad01A16064	Aradu.A01	16064	c	(T)10aacctatgt	106	91296666	91296771	TTGGATTAGTGAACACGCCA	TGACTATGTGATATGGTGATGGG	226
Ad01A16065	Aradu.A01	16065	p3	(TAA)7	21	91299211	91299231	CACATTTGCAATGTAATAGCCG	GAGGAAGAAGTCACGGAGCA	162
Ad01A16066	Aradu.A01	16066	p3	(TAT)18	54	91299935	91299988	CTCTCGATCTCAATCTTTCCG	TCATTGCATTTCGTTTTCCA	183
Ad01A16071	Aradu.A01	16071	p3	(GTG)5	15	91318542	91318556	CAGAAGGGATTCGAGGAGGT	CGCAATAGCAACACCAACAG	201
Ad01A16072	Aradu.A01	16072	p2	(AT)6	12	91321719	91321730	GCGACTCTCACTTAACCTTAAATTC	TGAAAGAAAAAGAAAAGATTTTTGA	279
Ad01A16073	Aradu.A01	16073	p4	(ATTT)6	24	91323301	91323324	CGGTTTATTTAGGTCCGATCC	CCCGAAAATTCGATTCATTG	241
Ad01A16084	Aradu.A01	16084	p3	(GGT)6	18	91337031	91337048	TTTGTACCCCTGGCACTTTC	AGCATTGCTAACAGCACCCT	212
Ad01A16085	Aradu.A01	16085	p3	(GCG)5	15	91339111	91339125	TATCACGCCGTTGTTGTTGT	TGCCCGATCAACTAACCTCT	175
Ad01A16086	Aradu.A01	16086	p2	(AG)6	12	91339433	91339444	TATTATGTTCCGGTTCCCCA	GGGACCTCGTTTTTCGTGTA	219
Ad01A16087	Aradu.A01	16087	p2	(GA)16	32	91340469	91340500	CAATGCAAGGTGGAAGGTCT	GGCAAGAAAGTCATTGACCC	162
Ad01A16092	Aradu.A01	16092	p3	(AGG)8	24	91350402	91350425	GTCGAGCCAAACTTGAGGAG	GGCAAAGAAGAATGCTCAGG	179
Ad01A16094	Aradu.A01	16094	p2	(AT)6	12	91355774	91355785	TGATACGATTTTTGCACACCA	CGTTCTTGTATGTTTGAACGGA	260
Ad01A16095	Aradu.A01	16095	p4	(TTAA)5	20	91356204	91356223	GCATGGTTGATTTTTCTTTTTGA	TTTCTGTTTGAGCGTGACTGA	115
Ad01A16098	Aradu.A01	16098	c	(TG)13(AG)16	58	91359474	91359531	ATTGGAACTCCGCACAAACT	TCGGAAGGGGGAGTCTCTAT	245
Ad01A16107	Aradu.A01	16107	p2	(AT)6	12	91385942	91385953	GGTTTAAATTCCCGCAACAA	AATTTCTCACGACCTGGCTG	274
Ad01A16108	Aradu.A01	16108	p2	(GT)7	14	91389391	91389404	TGGAGGCAGTAGCTGGACTT	CGCTACACACCCACTGAAGA	195
Ad01A16109	Aradu.A01	16109	p2	(CT)10	20	91390508	91390527	TTTAGCGTGGGACTTATCGG	GCAGATGGCTGGAGGTAGAG	240
Ad01A16111	Aradu.A01	16111	c	(T)12atgtattaga	75	91392947	91393021	TGAAACTTTGTTTGGTTGGAGA	GCCACTAATTATCGGCGAAT	265
Ad01A16113	Aradu.A01	16113	p2	(AC)6	12	91403570	91403581	GAAGAGACCGCCCAACAATA	GGAAACAAATTGAAAAAGGCA	101
Ad01A16114	Aradu.A01	16114	p2	(CA)6	12	91403798	91403809	AACACAAACACACCATCAACAA	CACCGACGATCTTCTTGACA	251
Ad01A16115	Aradu.A01	16115	p2	(AC)6	12	91404513	91404524	CACGTGGCTCAGTGTTGTCT	GGTGTGCACAATTTCATCCA	280
Ad01A16119	Aradu.A01	16119	p3	(AAT)6	18	91413575	91413592	AAGTCCGTGCAATTGAAACC	TTCCCTCGATCTCTTCGCTA	164
Ad01A16121	Aradu.A01	16121	p2	(TA)9	18	91428264	91428281	CAATCCAATTTCAAACCCCA	TTTATTCCCCAGCACCAGTC	160
Ad01A16123	Aradu.A01	16123	p2	(GA)8	16	91432393	91432408	TTGATGAATCTGTGGTGGGA	CACATGCACCCAAAATCAAG	161
Ad01A16124	Aradu.A01	16124	p2	(TC)6	12	91433047	91433058	ACAGTTCATCCTGCGAAAGG	GATTGAAATCCACGGCTTGT	248
Ad01A16125	Aradu.A01	16125	p3	(AAT)5	15	91441719	91441733	CAGAACGTGTGGAAATTAAGCA	TCAGTTGCTCACTTGCTTGC	222

ID	Chromosome	SSR nr.	SSR type	SSR	Size	Start	End	FORWARD PRIMER (5'-3')	REVERSE PRIMER (5'-3')	Product (bp)
Ad01A16126	Aradu.A01	16126	p3	(AAT)5	15	91442195	91442209	GAACGTGTGGAAATTGAGCA	GCAATCTTTTAGCTGCAGGG	203
Ad01A16130	Aradu.A01	16130	c	(CT)6tctctc(A/	33	91455892	91455924	GAGGTAGTGGGAGGGAGGAG	AACTGATGGAAGTGGATCGG	267
Ad01A16131	Aradu.A01	16131	p3	(ATA)7	21	91456589	91456609	AAGAATGGCAAATTGCAAGG	CAATAAGGTGACCGGGAATG	280
Ad01A16136	Aradu.A01	16136	c	(A)11caattc(CT	35	91482055	91482089	CAAAAGCAAAAGGGCAAACT	TTGAGGGAAATGTGAAAGGG	222
Ad01A16137	Aradu.A01	16137	p3	(CAG)5	15	91482397	91482411	CACCAAGATCCTTCGTCGAT	GTTTCCACGACGTCCTGATT	207
Ad01A16138	Aradu.A01	16138	p3	(TAT)11	33	91489710	91489742	TTTCTCAACTTATTGGGCCG	TCCCTAACTTATAAGGCGACGA	238
Ad01A16139	Aradu.A01	16139	p2	(TC)8	16	91493529	91493544	CAATCCACCCTCAGAAGGAA	GACAGTGCCCGGAATAAAAA	259
Ad01A16142	Aradu.A01	16142	p2	(AT)10	20	91496537	91496556	GCTTGAAACAGAACACGCAA	TTGACAACATGGGTGAGAGC	196
Ad01A16144	Aradu.A01	16144	p3	(AAT)7	21	91502210	91502230	TTATTGGGCCTGATGGACTC	TGTTCAGCCCATGACAAAAA	270
Ad01A16149	Aradu.A01	16149	p3	(ATT)6	18	91514071	91514088	CCAAGCATGTCAGGGTGTAA	TTGGGGCCTCTAATTCTAAACA	273
Ad01A16151	Aradu.A01	16151	p3	(CTT)6	18	91514430	91514447	GAAGAACAAGAACAAAGAACGG	TGCAACAAGGTGGAAGATGA	121
Ad01A16154	Aradu.A01	16154	p3	(ACC)5	15	91521063	91521077	TGCCTCTGATTCTGCAACTG	CTCATGACTGCGTCACCCTA	155
Ad01A16155	Aradu.A01	16155	p2	(GT)6	12	91521304	91521315	CCACCTTCCCCTTTCTTAGC	TGTCTTCACTAAAAACAAAGTTCATT	271
Ad01A16159	Aradu.A01	16159	p2	(AG)8	16	91526336	91526351	TGCAACAGCAAAGACCTCAG	CCATGTTCCACTTATTTTGGC	227
Ad01A16160	Aradu.A01	16160	p3	(TTC)8	24	91528324	91528347	GATCTCGTCGATGTCCGATT	AGCAAGGGAAAGAAAAAGGC	192
Ad01A16163	Aradu.A01	16163	p3	(GAT)5	15	91535910	91535924	TTGCTCTTCCTTCGTGAGGT	CTTCTTCCTCTGCATCCTCG	274
Ad01A16165	Aradu.A01	16165	c	(AAT)5c(ATA	55	91537579	91537633	TCTTCTTCTCCGGTTCCTCA	GATTTGTATTTCGTTTTGTGCAT	274
Ad01A16166	Aradu.A01	16166	c	(TCC)6tattctttt	104	91538531	91538634	CGAGTTTGAGATTCTTGAATTTTT	GGAGAAGGAGGAGGAAGAGAA	220
Ad01A16167	Aradu.A01	16167	c	(A)10gaggagaa	52	91539507	91539558	TCGGTTCATTTCTGTTCTGC	GCTCTTCCCGCTTTCTTCTT	235
Ad01A16170	Aradu.A01	16170	p5	(GGGTA)5	25	91549805	91549829	TGAGACTTGAACCCACAACCT	ACCCTACCCGCACCTAAAAT	233
Ad01A16171	Aradu.A01	16171	p2	(TC)6	12	91556942	91556953	CAGTCCACGTGTCTGTATTTGAG	AGGCAAGCCTAGAGCATACG	267
Ad01A16177	Aradu.A01	16177	p3	(ATA)6	18	91585261	91585278	TTGGATGGTGGTTATTCCTTTT	TGTGAAAAACAGTGGGATGA	256
Ad01A16178	Aradu.A01	16178	p3	(TCC)5	15	91585980	91585994	CGAACTAACCGCCAGAAAAG	TGGCCAGATCCTTTACCTTG	254
Ad01A16180	Aradu.A01	16180	p2	(TA)7	14	91588992	91589005	CCCTTGAGCACTTTACTTTTCC	TGTAAAACACAACCTGTCACAAAA	243
Ad01A16181	Aradu.A01	16181	p2	(AT)6	12	91590651	91590662	GAATTGAAAAATACCGTGAAGAA	TGAAACTTAGCATGTCATAAAATGTG	271
Ad01A16183	Aradu.A01	16183	p2	(AT)6	12	91608483	91608494	CTAGTTTGGGCCCACCTGT	TTTTCAAATTTATGATTGTTGGAAGT	254
Ad01A16184	Aradu.A01	16184	p2	(TA)8	16	91608857	91608872	TTGCCTGAATTTTGGCTAAT	CAAGCGATCAACGATGAAGA	261
Ad01A16185	Aradu.A01	16185	p3	(TGA)5	15	91609036	91609050	GGGTACCTGAATGACCGAGA	CAAGCGATCAACGATGAAGA	117
Ad01A16191	Aradu.A01	16191	p2	(TA)6	12	91616889	91616900	TCATCACAAGTCAGAGCCCA	CATCATCACCACATAGGCACA	267
Ad01A16194	Aradu.A01	16194	c	(TA)6gtacataca	34	91623140	91623173	ATGTCCCTCAACACCATCCT	CAGGTGAAGAGAAGTGTTTGGA	278
Ad01A16195	Aradu.A01	16195	p3	(TCA)6	18	91623287	91623304	TCCATCCAAACACTTCTCTTCA	GTCCTTTCGAGCTTGTGAGC	262
Ad01A16196	Aradu.A01	16196	p3	(CTA)5	15	91623586	91623600	GCTCACAAGCTCGAAAGGAC	ATTGTTTTGAACCGCCGTAG	196
Ad01A16199	Aradu.A01	16199	c	(A)12ctttccggc	38	91647754	91647791	GGTAACCGTACCCAAAAGCA	GGGCCCAATGTGAATGTTAC	277
Ad01A16203	Aradu.A01	16203	p2	(TA)9	18	91688707	91688724	AAGATCTTTGGTTAGAACACCCC	AACGTACTTCCCCTCGCTTT	180

ID	Chromosome	SSR nr.	SSR type	SSR	Size	Start	End	FORWARD PRIMER (5'-3')	REVERSE PRIMER (5'-3')	Product (bp)
Ad01A16204	Aradu.A01	16204	p3	(ATA)7	21	91693483	91693503	ATCGAGCTTGCCTCAGAAAA	GAAGAAGGATTCTTGCACGC	236
Ad01A16205	Aradu.A01	16205	p2	(AC)6	12	91696558	91696569	ATTTTCGAATTTTCGAGGGC	TCCTTCCCTACCCCTTCTTC	183
Ad01A16206	Aradu.A01	16206	c	(T)10atattgcat	40	91699267	91699306	CTGTGTGATTGCGTTTGCTT	CTGGTGCAGGCACTAACTCA	196
Ad01A16208	Aradu.A01	16208	p3	(TTA)6	18	91709603	91709620	TTGACTTTGATGGCCAATTTT	CTGCGTTCCACAGACTTGAA	224
Ad01A16210	Aradu.A01	16210	p3	(TAT)5	15	91712157	91712171	TGCATCCCCACAACTTGATA	GAAATGAGGAAAGAGCGTGC	227
Ad01A16211	Aradu.A01	16211	p2	(AC)14	28	91714918	91714945	TGCTATCAGTGGCTGGTTTG	TCCCTTCCAGTGTCTGTTCC	280
Ad01A16212	Aradu.A01	16212	c	(AG)7tgtttggt(	34	91718531	91718564	CTCACCAAGAAAGCCTCTGC	CATTTCAACAACGCCAACAC	193
Ad01A16213	Aradu.A01	16213	p2	(GA)27	54	91723507	91723560	CCTTTTCCCACTATCAGCCA	CTCCGCTATTTGATGCCTGT	223
Ad01A16216	Aradu.A01	16216	p3	(TAT)5	15	91733796	91733810	TTCGTTACCAACAAGCCCAC	TGTGACTCGTATGGCTTTCG	145
Ad01A16222	Aradu.A01	16222	p6	(TGTTCC)6	36	91743299	91743334	GGGCTCAGGAAGAGTGAATG	AGGGAAAAGTTCAGCCCCTA	181
Ad01A16229	Aradu.A01	16229	p3	(CTT)5	15	91754528	91754542	CCCCAACAACAAAACCCTTA	CATCTTCTTCAGGGGACCAA	142
Ad01A16230	Aradu.A01	16230	p3	(GCA)5	15	91755450	91755464	AGCCTCCAAAACCCTTTCTC	GCGGATTCTCCAAAAGATCA	262
Ad01A16231	Aradu.A01	16231	p3	(TTA)5	15	91757446	91757460	AACTTTTTGACAAACATAATGTCCA	CCCAAAACATCAGCCCATTA	277
Ad01A16238	Aradu.A01	16238	p3	(TAT)9	27	91783508	91783534	CTTTTCCGATATTCCAGGCA	CTCGGCGATTCCAAAATAAA	250
Ad01A16239	Aradu.A01	16239	c	(TA)6cctgcagg	91	91785654	91785744	ATTGATTGAACCCAAGCCAC	TGGCTGTGAATGAGTGATATTTG	222
Ad01A16245	Aradu.A01	16245	p2	(TA)6	12	91796661	91796672	CTCTGCCGTCTCTCTCATCC	CCAAAAACTTACCATAGAAAAAGCA	251
Ad01A16247	Aradu.A01	16247	p3	(CAA)6	18	91803973	91803990	TGATCCAAAAGATGCTCTCTCA	CATGGATCTGTGCCTAGTTGA	240
Ad01A16254	Aradu.A01	16254	p2	(AT)6	12	91849023	91849034	TCGTTGTTTTTCAGGAACCA	CCGACTTATAGGTCAAAGCAGC	164
Ad01A16255	Aradu.A01	16255	p3	(ATT)5	15	91851224	91851238	TTGAAAATGAAATATAGCGACTGG	TGCATGTGAGTCACGAGTTTT	255
Ad01A16256	Aradu.A01	16256	c	(T)10attttggaa	84	91868163	91868246	AGTTGTAGGTGATCGGACCAT	CACTCCTTGACATCACCCCT	256
Ad01A16258	Aradu.A01	16258	p3	(TCA)5	15	91884266	91884280	CTTCCGTCGTCTTGATTGGT	CATATGCAGATAAATTGTAACAAAAGC	237
Ad01A16261	Aradu.A01	16261	c	(CTG)5cttactg	41	91890640	91890680	CGCACTCCAACCCCTAGATA	AATCTTGGAATCGCAGGCTA	273
Ad01A16262	Aradu.A01	16262	p3	(AAC)8	24	91891230	91891253	CATTCCCAACCCAAACTACG	CTACCTTCGGCATGGTGATT	216
Ad01A16268	Aradu.A01	16268	p2	(CT)6	12	91905358	91905369	GAAGCGTTCCAATGGTTGAT	AATTCAAACTGGATGTGGGC	230
Ad01A16269	Aradu.A01	16269	p2	(TA)7	14	91905754	91905767	AACCTCCAAAAATGTGGAAAAA	TTGGAGGATCCCAAAATTCA	264
Ad01A16270	Aradu.A01	16270	p4	(TCAT)5	20	91906975	91906994	CTCTGGCTCATAATCAGGGC	GCACGGTAAGTGGAAGAGGA	255
Ad01A16271	Aradu.A01	16271	c	(CTTT)5tttttt(	36	91912656	91912691	TAATGGTATACGCCCCAAGC	TCAGGTGGCCACAATATTAAA	141
Ad01A16275	Aradu.A01	16275	p2	(AT)6	12	91926761	91926772	TTCCTCTCAAGAATGGAAGCA	TCAAGAAGGACACCAACCCT	150
Ad01A16280	Aradu.A01	16280	p2	(AG)6	12	91931464	91931475	CCCAAGCAAGTACCAAGAGC	TGGTGGAAAGTGACGACTGA	280
Ad01A16281	Aradu.A01	16281	p3	(ATT)7	21	91931860	91931880	CGACACCATACCTCCAAACA	TGGTTTGGAACCGTATGTCA	241
Ad01A16284	Aradu.A01	16284	p2	(AT)10	20	91935455	91935474	GACACCATCACCATGCCTAA	TGAATCCTGAAGCAACTCCA	237
Ad01A16285	Aradu.A01	16285	p3	(CAT)6	18	91938727	91938744	GGAGCATTTCGCTAGTGCTT	TGTCTCAGCAAATGTTACCCC	249
Ad01A16287	Aradu.A01	16287	p3	(TTG)6	18	91943283	91943300	GGATGATGCCAATGGCTAGT	ACCCCAAATTTTCTGCTCCT	273
Ad01A16289	Aradu.A01	16289	p3	(AAT)13	39	91950823	91950861	TGAATTCAGGTAGGCTTCCG	AGGTTGGGTGGCAAAGTTC	256

ID	Chromosome	SSR nr.	SSR type	SSR	Size	Start	End	FORWARD PRIMER (5'-3')	REVERSE PRIMER (5'-3')	Product (bp)
Ad01A16290	Aradu.A01	16290	p2	(TA)6	12	91951937	91951948	ATGAAAGTTCTGGAGCGACG	GCTCTTTTCGCAGAATCATTG	144
Ad01A16293	Aradu.A01	16293	p3	(GGA)5	15	91962705	91962719	ACAGAGGCAGAGGCTACCAA	GCCAAGAGAAAAACTCGTGC	122
Ad01A16296	Aradu.A01	16296	p3	(AAT)6	18	91968641	91968658	TTCTTAAAATCACACCCTCCTCA	GTCTTAACCCCTCTCAGCCC	263
Ad01A16297	Aradu.A01	16297	p4	(TTAT)5	20	91969236	91969255	GGTGCTCAAATTTAAAACGC	AAAGAATCAAATCTCCCCTCTTTT	275
Ad01A16298	Aradu.A01	16298	p3	(ATA)5	15	91971665	91971679	TGCAGAGTCAGGTGTGAGGA	GGAGAGCCTTGTGCAGATTC	210
Ad01A16302	Aradu.A01	16302	p2	(CA)6	12	91982664	91982675	AACCTGCACATTCAGAACCC	CCACCATCAGAACCACAACA	224
Ad01A16303	Aradu.A01	16303	p3	(TTG)8	24	91982886	91982909	TGCTGCGATGGAGAGATAGA	CTTAACGCACATTCGACCCT	138
Ad01A16304	Aradu.A01	16304	p3	(TAA)5	15	91984879	91984893	CCATTTTTCCTCCAAAATGAA	CGTGAATCGGGTAACAAAGA	278
Ad01A16306	Aradu.A01	16306	p6	(GAATAG)5	30	91992571	91992600	GGGGAGTGGCAGATAGGAAT	GGTTGGGTGGGAATAAGGTT	193
Ad01A16309	Aradu.A01	16309	p2	(AG)9	18	91996787	91996804	TTTGCTATTTGGGTCGGACT	TGCAACGTGTTACAGTGTCG	267
Ad01A16312	Aradu.A01	16312	c	(TC)8(TA)9	34	92003075	92003108	CGTCAATTGAGCATGCAAGT	GGAGGAGAGGAAGGGAACAC	205
Ad01A16315	Aradu.A01	16315	p2	(AT)11	22	92007615	92007636	TGCATTCAACAAGGGAGATG	AAGTGAAATTCTGGCCATGC	234
Ad01A16322	Aradu.A01	16322	p2	(AG)11	22	92025066	92025087	CCCTTCAAACCCCCTTTTT	GAGCTCTTGGATTGACTGCC	271
Ad01A16323	Aradu.A01	16323	p3	(ATC)5	15	92026495	92026509	TTGTTTAGCCAAAGGCAAGG	CTCAATCCGGCTCTCATTTC	250
Ad01A16327	Aradu.A01	16327	p4	(ATAC)5	20	92031196	92031215	CAACCACCACCTGAAGTCCT	AATGAGAACACGTGCAGCAG	179
Ad01A16328	Aradu.A01	16328	p2	(TC)6	12	92031557	92031568	AACGCATGGCCTACCTACAC	CCCACAACGAATAATAATGCAA	206
Ad01A16329	Aradu.A01	16329	c	(TA)9(CA)7	32	92041061	92041092	CAAAAGATGGGCTTTGGATG	GTCAGCCAGTTTCGTTTGGT	277
Ad01A16330	Aradu.A01	16330	p2	(AG)7	14	92043478	92043491	TAAAGTTGAAAACCGGGCAC	ACCACCCTCCCCTATCTCTG	201
Ad01A16331	Aradu.A01	16331	p3	(TAT)15	45	92064372	92064416	GCTGGTTAGTTGTGCGGAAT	GTGAAGCGTTCACACACGAT	197
Ad01A16337	Aradu.A01	16337	p4	(ACAT)5	20	92103241	92103260	TTTGAGTGTAACAGAAATGAACG	ATTGGTTGGTGCGTAAAAGC	263
Ad01A16338	Aradu.A01	16338	p2	(TA)7	14	92103726	92103739	ACCCCTAAAAAGGCAAAGGA	ACTTGTTCCACCTTCCATGC	233
Ad01A16340	Aradu.A01	16340	p2	(CA)6	12	92106508	92106519	TTGAGTCAACATTTTGGCTTTC	CCCCTCTGTTCATTGCACTT	262
Ad01A16341	Aradu.A01	16341	p3	(AGA)7	21	92111568	92111588	GTGGGTGTTGGAGAGGAAGA	AGAACAAATCGTGACCGTCC	277
Ad01A16342	Aradu.A01	16342	p3	(AAT)5	15	92113009	92113023	TCATCATCGTCACCACCACT	TTGGACTTGTGACCCTTTGA	274
Ad01A16347	Aradu.A01	16347	c	(TA)8(T)10	26	92121457	92121482	AATTCCGTTAGGTTTGGTAAAAA	TCCAAAAATCTTATCGGAGAAA	277
Ad01A16350	Aradu.A01	16350	c	(T)10catttaaatg	82	92126664	92126745	AAATGAAAGGACCAAGGGTT	CAAAATATGTCAAAAAGGTATCCAAA	207
Ad01A16354	Aradu.A01	16354	p2	(CT)7	14	92140699	92140712	TCACCATCAACTTTCACCGA	TGGCCATTCCTAGATCATTCA	230
Ad01A16355	Aradu.A01	16355	c	(T)13caattcctta	77	92140863	92140939	CCCTTTTCCTTTCTTTTGTGTG	CATTCATTTGGTGGAAAGCC	269
Ad01A16356	Aradu.A01	16356	p2	(GC)6	12	92142986	92142997	CCAGTTGGGTTGGAAAGCTA	AGTTGGATTGGTCCCACAAG	214
Ad01A16359	Aradu.A01	16359	p2	(CT)7	14	92157621	92157634	ACACCACGGTCATGGAAGTT	GGAAAGGCCAAACGTGTCTA	186
Ad01A16361	Aradu.A01	16361	c	(TA)9t(TG)8	35	92165105	92165139	AGGTGACGCTTTTCTTTGTGA	TGTGTACAAATAATGAGACTGGAAAA	278
Ad01A16363	Aradu.A01	16363	p3	(AAT)6	18	92167165	92167182	GAATAAGCTTTGCCGCTTTG	AGCCACAGAGAGACAGCACC	154
Ad01A16366	Aradu.A01	16366	p2	(TC)6	12	92171881	92171892	TGATTAGACATTCAAATCTCACCAT	GAGAGGAAGAGAGGGAGGGA	245
Ad01A16369	Aradu.A01	16369	p3	(CCA)5	15	92183145	92183159	ATGACCCCATTGAGAAGCAC	ACTGTGAAGCTGGGCAAGTT	109

ID	Chromosome	SSR nr.	SSR type	SSR	Size	Start	End	FORWARD PRIMER (5'-3')	REVERSE PRIMER (5'-3')	Product (bp)
Ad01A16370	Aradu.A01	16370	p3	(GGT)5	15	92183925	92183939	GAATATTTGGCACCCACGTT	TGGCTTCCAATGTATGGTCA	279
Ad01A16372	Aradu.A01	16372	p2	(TC)6	12	92189349	92189360	GGGGTTCCCTTCCAAAATAA	TTCATGAGAAAGAGAGGGGC	262
Ad01A16375	Aradu.A01	16375	p3	(TAA)11	33	92196225	92196257	TCGGAAGGTTCAATTTGTGT	GGCAATAGGCATCGTGAAGT	276
Ad01A16376	Aradu.A01	16376	p3	(TAA)6	18	92196494	92196511	ACTTCACGATGCCTATTGCC	TGTTGGGTGTGATGACAATG	270
Ad01A16382	Aradu.A01	16382	c	(TTC)7ctctcttc	87	92206083	92206169	AATGTGTGAGAGGCTGAGGG	TACAGACGTCGGTGGTTGAA	249
Ad01A16383	Aradu.A01	16383	p2	(AG)7	14	92211047	92211060	CTGAAGAGAAGAACGACGCC	TCCATAAGCCCATCTCGTTC	212
Ad01A16384	Aradu.A01	16384	c	(A)11ttaaacacc	105	92212240	92212344	TCACCGTTAGCAATTTCGTG	GCTCAAGATCTCCGCTCATC	253
Ad01A16385	Aradu.A01	16385	p3	(TCA)5	15	92213851	92213865	GGCCTCAGCTACTTTTGCAC	CGCCTTTGAAGAGTGTGTCA	275
Ad01A16386	Aradu.A01	16386	p3	(ATT)6	18	92214670	92214687	GCATCCGTAACCATGACTGA	CCCCCAACAAGGTCACTTTA	278
Ad01A16387	Aradu.A01	16387	p3	(AGA)5	15	92214958	92214972	TCCATTGTCTCAGGATGTGC	GGCTTGAAATGATTACAGGGA	269
Ad01A16390	Aradu.A01	16390	p3	(TGG)9	27	92220097	92220123	TTGTTTGGGGTTCTTGGTGT	AAAATCATCCTTCCCCGAAT	274
Ad01A16392	Aradu.A01	16392	p3	(ATT)6	18	92227884	92227901	TGTTTCCAAAGTGCAAGAGAA	TTTTTAATAGCTGTAGAAAGACTGTCA	251
Ad01A16394	Aradu.A01	16394	p3	(TTA)6	18	92229723	92229740	CAAATCAAACACCACCACCC	TTTTTACTTTACAAGAATGCGCC	258
Ad01A16396	Aradu.A01	16396	p4	(TTAT)5	20	92234233	92234252	AAACATATAACATCCATAATCAATGAA	AAAAATCCAAAATCTTAAATTCGAT	148
Ad01A16397	Aradu.A01	16397	p2	(AT)7	14	92234795	92234808	GCAAAAAGCAAGAAAAGATGA	CAGCCTAAGTTGAGGCCACT	149
Ad01A16398	Aradu.A01	16398	p5	(TCTCT)9	45	92235293	92235337	TTGGCATTGCCTTACACAAA	GTTTGAGTCGAGGGGATTGA	173
Ad01A16401	Aradu.A01	16401	p2	(CT)6	12	92246459	92246470	TGAGAAAACCCGAATCCATC	TGCATGAATGAAAGCTGGAG	124
Ad01A16402	Aradu.A01	16402	p3	(TTG)7	21	92250128	92250148	AAGAGAGGAACGAAGCCCTC	GCAGCAGAAACAGAAACAACC	270
Ad01A16403	Aradu.A01	16403	p2	(TC)9	18	92254016	92254033	CCATGTTAGCACTGATCTTCCA	CACCGTCCTAAGGTGCTTGT	259
Ad01A16404	Aradu.A01	16404	p4	(AAAG)5	20	92254431	92254450	CAAACACAACTTGGCCCTTT	GGAACATGAGTATGTATCCACCA	264
Ad01A16405	Aradu.A01	16405	p2	(AT)7	14	92255541	92255554	CGTCTTAATTCATTCGCGTG	TCTGTAAACAAACGCAGCCA	109
Ad01A16408	Aradu.A01	16408	p3	(ATA)7	21	92260741	92260761	GCATGCACTACTCCCCTTTC	CGGTGATCTTGCAGCTATGA	184
Ad01A16409	Aradu.A01	16409	p2	(TC)6	12	92264797	92264808	ACTAATCCACGCGTTCTTGG	GAACAGACCCCTAACCTACCC	155
Ad01A16412	Aradu.A01	16412	p2	(TA)6	12	92269589	92269600	TCGTGCTAAATCATGATCTTCTTC	CGAGCCACCCTAAATAAGCA	225
Ad01A16413	Aradu.A01	16413	p3	(AAT)5	15	92273802	92273816	TTTGGCACAAGATGGGTAAA	TGGCTATTTGATCAATAATGGAA	221
Ad01A16414	Aradu.A01	16414	p2	(TA)7	14	92276132	92276145	TCTCCCCTAAGCGGTAACAA	TGCAAGTGCAAAAATCAACC	269
Ad01A16417	Aradu.A01	16417	p3	(AAT)8	24	92280697	92280720	TTCCAAGCATCACAGTCAGG	TGCATCCCCTTGAAAAATTC	225
Ad01A16421	Aradu.A01	16421	p3	(AAG)5	15	92296454	92296468	GACACATTCTATCTTAAAAAGTGAAGC	TCATATCCTACGGGCCTCTG	159
Ad01A16422	Aradu.A01	16422	p2	(GC)6	12	92302416	92302427	AGGGGTGGCACAACTCTCTA	AAGCTACCAGCCCAAAACCT	271
Ad01A16423	Aradu.A01	16423	c	(TTAT)6tatcta	58	92318537	92318594	GCAGCACCAACAAAGACAGA	CAACATACAATCATCATCGACAAA	218
Ad01A16425	Aradu.A01	16425	p2	(AT)6	12	92328080	92328091	GCTTCATCAGGTCAGAGTTGC	CATCCTCGATCAAAGCCAAT	141
Ad01A16429	Aradu.A01	16429	c*	(T)10(TA)7*	23	92333236	92333258	GCTCCAGCCTAAGTCGTGTT	TGGAGTTTAATTTTGATGCACTG	217
Ad01A16430	Aradu.A01	16430	p2	(AG)17	34	92338581	92338614	TCTCTCCTGCACATGCTTTG	ACGCGGTTAAGGAAACAAGA	277
Ad01A16432	Aradu.A01	16432	p3	(ATA)7	21	92339588	92339608	AAAAATTCATCCAAAACATATCCT	GAGCCACACACAACACAACC	102

ID	Chromosome	SSR nr.	SSR type	SSR	Size	Start	End	FORWARD PRIMER (5'-3')	REVERSE PRIMER (5'-3')	Product (bp)
Ad01A16433	Aradu.A01	16433	p4	(AAAG)5	20	92339960	92339979	TATCACTTGTCCCCTTTCCG	GTAGACCTGGCGGTTCATGT	230
Ad01A16434	Aradu.A01	16434	c	(T)13ggaaaaat	125	92341344	92341468	ACTACGGCTACAATCACGGC	GGCCTCTCAAGCAAAGAATG	260
Ad01A16437	Aradu.A01	16437	p3	(ATA)5	15	92351954	92351968	TATCGCTCAACAAGTGGCAA	TTCCTCCTATCATCGGTTCG	268
Ad01A16443	Aradu.A01	16443	p3	(GAA)5	15	92363054	92363068	CGCGAAATGAAGGGTAAAAA	GGGAAGGAGCCTCTCGTATC	159
Ad01A16445	Aradu.A01	16445	c	(T)12accttgac(	32	92365387	92365418	TGAACTGGTTTTTGGCATCA	TCCCGATTTGTAATCCTCTCC	129
Ad01A16452	Aradu.A01	16452	p2	(CT)6	12	92376845	92376856	ACAGAAAATTCATGTGCCCC	TACAAAGTGGCGTGCTTTTG	255
Ad01A16453	Aradu.A01	16453	p3	(AAG)9	27	92380854	92380880	TGCTGCAGACAAAGTTCCAG	CAGCTGGGCCATGATTACTT	180
Ad01A16454	Aradu.A01	16454	p2	(AT)6	12	92382272	92382283	TTTGTCAAAATTAGGTCATACAAATTA	GACCAAACAAATCAGTTTAGCAA	202
Ad01A16456	Aradu.A01	16456	c	(GA)8ttgacgaa	80	92393424	92393503	TGCCTATACACATCAACAACCA	TTTGGGTTTGGGTCCAATAA	232
Ad01A16458	Aradu.A01	16458	p3	(ATA)19	57	92396310	92396366	TCAAGAAATTTTGATGGGGC	CGTGAAGTTGACTGCATCTGA	233
Ad01A16461	Aradu.A01	16461	c	(TAA)10tataga	119	92400224	92400342	GGCAATTCAGATCACAAGCA	TTCTGTTTCTCATCAACACCAA	270
Ad01A16462	Aradu.A01	16462	p3	(AAT)7	21	92400455	92400475	TCACCTTCATGAGCAGCATC	TGCTTTTGGCTTCCTCAGAT	230
Ad01A16463	Aradu.A01	16463	p3	(ATA)5	15	92400708	92400722	ATGGAGGACGTGGAGCTATG	GGTGGAAGCAGAAGCAGAAG	139
Ad01A16464	Aradu.A01	16464	p3	(TAT)6	18	92405878	92405895	GTGTTGCACGTGATGGATTC	AAAAAGTGTCCTTGTTTCATCC	277
Ad01A16465	Aradu.A01	16465	p2	(AT)9	18	92406160	92406177	TAGGATGAAACAAGGACACTTTT	AATACAGGATAAACAGACACACAAAT	262
Ad01A16466	Aradu.A01	16466	p3	(GAA)6	18	92412813	92412830	AGGTTGGCTGAGTGGAGAGA	TTCCCGCTCATTTTATTTGG	269
Ad01A16468	Aradu.A01	16468	p2	(TA)6	12	92415235	92415246	CGTATCAGCGTGTCTTGCAT	TGGACACGAAAAACGACAAA	149
Ad01A16469	Aradu.A01	16469	p2	(AT)9	18	92426706	92426723	GGGCCTTTTGGTATATTTGGA	TCACTAATCAGTCACCGTTATAAAAA	280
Ad01A16474	Aradu.A01	16474	p2	(AG)10	20	92444168	92444187	GGCTCATAGCGAAGGTGACT	CACCATTTTTCTGCCGAGTT	143
Ad01A16476	Aradu.A01	16476	p3	(AAT)25	75	92448458	92448532	CTTCCAAGCAAAAACCGTTC	TGCGATGACTTCGAGAATTG	216
Ad01A16477	Aradu.A01	16477	p3	(ATT)6	18	92448748	92448765	CAATTCTCGAAGTCATCGCA	GCCCTAGAGCTTCTGCTTCA	260
Ad01A16478	Aradu.A01	16478	p3	(ATT)5	15	92448871	92448885	GAAGCTCTAGGGCTGACGTG	CCTCTTCAACCACGAACCAT	113
Ad01A16479	Aradu.A01	16479	p3	(TTA)6	18	92449031	92449048	ATGGTTCGTGGTTGAAGAGG	GACTTAACGCCGTCACCAAT	219
Ad01A16482	Aradu.A01	16482	p3	(TAT)5	15	92451486	92451500	GAATTTTGAGTGTTCACCAAGTG	CATTGATAATGAACCTATCACACCA	161
Ad01A16485	Aradu.A01	16485	p2	(TA)7	14	92470054	92470067	GGGACTTGCCAAGAAATTGA	CGTGTCTCAAATGCAAATCC	221
Ad01A16488	Aradu.A01	16488	p2	(TC)6	12	92477351	92477362	ACCTCACCATTGTTTCTGCC	CCTCGACCTGTGACCCTAAA	212
Ad01A16489	Aradu.A01	16489	p2	(TA)6	12	92478777	92478788	TAACTTGTAATGCGGAGGGG	TTCAGATATGGCAAGGCTCC	275
Ad01A16492	Aradu.A01	16492	p2	(TC)9	18	92494723	92494740	ATCTCGGTTAAGCAGCGAAA	TTCACAATTCTCCGAAACGA	280
Ad01A16494	Aradu.A01	16494	p2	(TA)8	16	92500112	92500127	TTTTTCGAACGTAAATGCCC	ACCATGTGACAAAGCCCTTC	259
Ad01A16495	Aradu.A01	16495	p2	(TC)6	12	92504200	92504211	GGAATAGTCTCATTCAAAATCTGTT	CTTTACTACCCCTCCCAAAAA	200
Ad01A16497	Aradu.A01	16497	p2	(TA)8	16	92505818	92505833	CGCACCATGTGATAAATAGGAA	CCCCTGTTACGTTTTCCCTT	236
Ad01A16505	Aradu.A01	16505	p3	(AAG)5	15	92526490	92526504	GAGGTGTGAGTGGGAATTGG	TCCCAACATCAACAACCTCA	234
Ad01A16508	Aradu.A01	16508	p3	(AGA)7	21	92544157	92544177	GTGTTCCTCCAGCCACAAAT	CGTTTTGTTCATTCCATCACA	223
Ad01A16510	Aradu.A01	16510	p4	(TTTA)5	20	92549413	92549432	GTGAGCTTGTTTGTGCGAAA	CCGTGGTATGATCGTTCCTT	271

ID	Chromosome	SSR nr.	SSR type	SSR	Size	Start	End	FORWARD PRIMER (5'-3')	REVERSE PRIMER (5'-3')	Product (bp)
Ad01A16511	Aradu.A01	16511	p2	(AT)7	14	92551128	92551141	AAAGAGGGGGAGAGAGAGGA	CCTCTATCCCTATGCATCTTCC	248
Ad01A16517	Aradu.A01	16517	p3	(ATA)17	51	92559029	92559079	AGATGTTGTATTAACTCACATGAAATG	TGCCACCATAGACCTAGCCT	245
Ad01A16521	Aradu.A01	16521	c	(T)10ccacaaaa	42	92562836	92562877	CCGGTTTAATAAGAATGTCCAA	TTGCGTAGAAAAATAAACTAAATACGA	261
Ad01A16522	Aradu.A01	16522	p2	(TA)8	16	92567103	92567118	GCATTCAAATTCCAGCCAAT	TTCTTCCCCTTTCCCTCCTA	211
Ad01A16524	Aradu.A01	16524	p2	(AG)6	12	92582102	92582113	CCTGAATTTGACCTAAAATTTATTGA	CATGGCTTTCAATTCGGACT	239
Ad01A16525	Aradu.A01	16525	c	(CTT)5gctgcac	98	92583395	92583492	CGCATACAAGCGATTAAGGC	CGAGAAAGAAGAACGTGCAG	253
Ad01A16526	Aradu.A01	16526	c	(GAG)5gaagaa	90	92583953	92584042	GAAGAGGACGAGGAAGAAGGA	GTTGCTACACATGGGCCTTT	200
Ad01A16529	Aradu.A01	16529	p2	(CT)6	12	92592715	92592726	TCAATGAAATGGACTTGGCA	TGCTTTTGATCAGCTCATGG	246
Ad01A16530	Aradu.A01	16530	p2	(AC)7	14	92599791	92599804	AGGAGACAATGCAGGGTGAC	ACCCTGCAACCAATTAGCAC	146
Ad01A16533	Aradu.A01	16533	p2	(CT)13	26	92604972	92604997	GCTCCTCTTCGTTCACTGCT	GGAATAGGTTCAGCCCATGA	249
Ad01A16534	Aradu.A01	16534	p3	(ATA)5	15	92608169	92608183	TTCAATAATCCTATGACCAAGAACA	CTATCATGATTGGCCCTCGT	233
Ad01A16535	Aradu.A01	16535	p3	(TCC)5	15	92616607	92616621	TCAATGGTTTCCATAGTGCG	TCTTCCTCTTCCTTCTTCCCA	280
Ad01A16538	Aradu.A01	16538	p2	(TC)6	12	92623533	92623544	TGAACATGCAATTGACACCA	CCATTCTAAAGCTTGACTGGC	255
Ad01A16539	Aradu.A01	16539	p2	(CT)6	12	92631451	92631462	TGTCCAATTGAAGGTCACCA	AGAAACCGCTATAGGCAGCA	230
Ad01A16540	Aradu.A01	16540	p2	(CT)6	12	92639512	92639523	CGCACGCTCACTCGTAATAA	CGAATCTGAGGGCTCAAAAG	242
Ad01A16541	Aradu.A01	16541	c	(T)10cattctctttt	122	92640943	92641064	CACTGATCACTGGTGGTTGG	ATGCAATGACAAAAGGCAGG	202
Ad01A16543	Aradu.A01	16543	c	(TAA)10(GAA	85	92645281	92645365	GCAAAGAAGCTCGAAGGAAA	AATGTCCACGTTTTTCGGAG	279
Ad01A16544	Aradu.A01	16544	p2	(TC)6	12	92645918	92645929	CGACCTCCTCCTTCCTCTCT	GGAAAGCACAGGGGTGATAA	224
Ad01A16548	Aradu.A01	16548	p3	(GTA)5	15	92652026	92652040	TCTCTTTTGTTTGGCCCATT	GGATAAAGAGGAACCTTTCGG	268
Ad01A16550	Aradu.A01	16550	p3	(ATC)5	15	92660335	92660349	TGCAAGAATGAAAGGGGAAG	CCTATGCCATTCTTCCAAGC	265
Ad01A16553	Aradu.A01	16553	p3	(CGC)7	21	92662324	92662344	GGACTTCAACCACCGAGCTA	CAAACGCATAGAAAGGAGGC	187
Ad01A16554	Aradu.A01	16554	p2	(TA)6	12	92665136	92665147	TTGGCCTATTTGCAATGTGA	CAGGTGGCATTAAGGGTTTG	259
Ad01A16558	Aradu.A01	16558	c	(T)11aaatcaatt	61	92668893	92668953	AAGGTGCCTCTTGATGTTGG	GTCCCCATTAAGCAGATGGA	159
Ad01A16559	Aradu.A01	16559	c	(TCC)7aatgtta	43	92673062	92673104	TGCGTCTCTTTCTCCGTTTT	TGTTTGCAAAATGTTGGTGTT	165
Ad01A16560	Aradu.A01	16560	c	(T)16gtgtcatca	89	92674045	92674133	TGAAAAGGAGAAGGAGAACGA	TGAGTGACGGAGTGATATTGG	264
Ad01A16561	Aradu.A01	16561	c	(TC)10(AC)7	34	92677268	92677301	ATGGTTGTCCAGGTTGAAGC	GCAGTCTGTGTCTCTGTGTGG	254
Ad01A16562	Aradu.A01	16562	c	(A)13tctattctag	72	92677692	92677763	TTGGTAGGCTATGACCCAAA	TGCCTCAGTGACTAAAAGGGA	156
Ad01A16563	Aradu.A01	16563	p2	(TA)9	18	92679868	92679885	TCTTAGCCAACTGAGGCACA	TGGATGTCAAGATTCCCACA	180
Ad01A16565	Aradu.A01	16565	p4	(AATA)5	20	92681461	92681480	TGTTTGGACTTTAACGGAAATG	TGTTCCCTGCTAACATGCAC	147
Ad01A16566	Aradu.A01	16566	p2	(TA)8	16	92682335	92682350	ATTGCCATGGATGATGATGA	TCCCTCTCTCTCGCTCATGT	224
Ad01A16568	Aradu.A01	16568	p2	(TA)10	20	92685690	92685709	AAAGAGGAGAGAATAACATTCACC	GGCTCTCACTCAACCCATGT	186
Ad01A16569	Aradu.A01	16569	p2	(TA)9	18	92687505	92687522	GACCTGAAGAAGTCGGATCG	TTGAGTCAAAGAATTAAAGAACAAGC	278
Ad01A16570	Aradu.A01	16570	p3	(AAT)5	15	92687675	92687689	CAGCAATTCTTAACTCTCAATTGTATG	TTTAATTTTTCACGCGGAGG	234
Ad01A16573	Aradu.A01	16573	c	(A)11gaataaaa	108	92696770	92696877	TGTTTAATTATGAACTGGAGCTTG	TGCTAGTTTCCCAGATCCCTT	238

ID	Chromosome	SSR nr.	SSR type	SSR	Size	Start	End	FORWARD PRIMER (5'-3')	REVERSE PRIMER (5'-3')	Product (bp)
Ad01A16575	Aradu.A01	16575	p2	(AT)9	18	92700688	92700705	GGGACCAATTACAATATGACACA	TGTCTGTCTGTTATCGTGGAGAA	264
Ad01A16577	Aradu.A01	16577	p2	(TA)6	12	92703805	92703816	TTTGCATTTTGTGGTGGAAA	AAATTTGGTTGGATTTGACTCAT	261
Ad01A16578	Aradu.A01	16578	c	(T)10caaaaacc	49	92705046	92705094	TGTAAACCTCCCTCTTCCCC	CAGGAAAATCGAAACCTGGA	233
Ad01A16579	Aradu.A01	16579	p3	(GGT)5	15	92705725	92705739	CTACGACGAGAAGAGGACGG	CATCTCCATCACCACCACTG	227
Ad01A16585	Aradu.A01	16585	p2	(TC)29	58	92725830	92725887	AAGTCCCTCCTCTTCTCCCA	CAACCTGTCCTTTTGTGGGT	276
Ad01A16586	Aradu.A01	16586	c	(T)28cccacc(T	72	92726347	92726418	AAGCAACATGGTCCAAGGTC	AGCTGTTGCTGTAATGTGTGA	166
Ad01A16592	Aradu.A01	16592	p2	(TG)6	12	92769864	92769875	TTCAGACCCTCATCAAAGGG	TTGTGGACCCACTTGAACAT	226
Ad01A16596	Aradu.A01	16596	c	(A)10cccctaag	110	92774147	92774256	CAAAAATAGGATATTCTCGCAAAA	TTGGCTATATAACAGAAAATGCGA	221
Ad01A16597	Aradu.A01	16597	c	(A)25g(A)10	36	92775526	92775561	TTTCACTTTCAGCAGCCAAA	GGTTGAGACCTTCCCAAACA	222
Ad01A16599	Aradu.A01	16599	p2	(AG)8	16	92778635	92778650	AGAGAGAGCGAACAAGCGAG	TCTCGCAAACTCAAAAACCC	160
Ad01A16604	Aradu.A01	16604	p3	(TAT)17	51	92797076	92797126	GGGACCAACCAAACCATAGA	GATGTTGGAATTGGAGTGGG	167
Ad01A16610	Aradu.A01	16610	p3	(AAT)6	18	92805034	92805051	GGCTAAAGGGATGAGGGTTT	AATGAGTCCATGAAGTGGGC	231
Ad01A16612	Aradu.A01	16612	p2	(AT)10	20	92805645	92805664	CAAATAAGCATCTACTTGTTGAAGGA	TCATGGGATTGCATTGCTTA	157
Ad01A16618	Aradu.A01	16618	p2	(TA)6	12	92819690	92819701	CCCTCGTAAATCCGTTTCTTT	TGGTTTCCACCTATTTTAGGGA	238
Ad01A16619	Aradu.A01	16619	c	(AT)7gctatttttt	51	92820594	92820644	TTGTGCCAAGACAATACCCA	TACCCCACCTCCACGATAAG	202
Ad01A16621	Aradu.A01	16621	p2	(TC)10	20	92822333	92822352	TTTTCTCGCTTGTGATCCCT	CGTTGTATGGAAGAAGGGGA	171
Ad01A16628	Aradu.A01	16628	p2	(TC)18	36	92841712	92841747	GTCCCAGAAGCTCCACGTAA	CAGTGAGAAACCGATGAGGA	218
Ad01A16629	Aradu.A01	16629	p3	(CCT)6	18	92844693	92844710	CACCCCCTCTTCCTCCTACT	TGTGAATCATCCTTTGCAGG	211
Ad01A16632	Aradu.A01	16632	c	(T)12(CT)8gag	117	92853472	92853588	CACAATCCCCCAATTCTCTC	GGACATAGGGTGTGCTGGTT	183
Ad01A16638	Aradu.A01	16638	p2	(AG)6	12	92881751	92881762	TGGGAATTGAAGGGACCATA	AGCTATGTATGGCTGATCAGTGT	270
Ad01A16639	Aradu.A01	16639	c	(T)11gataaag(A	29	92882223	92882251	GGAACTCCTCTAACGGCGAT	TGAGAAGGCTAATCCAAGAAGA	249
Ad01A16641	Aradu.A01	16641	c	(TA)8aacggact	68	92890255	92890322	GCTATTGCGACATACACTGAGC	GGATCCTGATTCACGGCTACT	146
Ad01A16643	Aradu.A01	16643	c	(A)10cta(T)10	23	92900844	92900866	TGTATTGGTCACCCGAACCT	CAAGATGTGATATTAATGATTTAACGA	267
Ad01A16644	Aradu.A01	16644	p2	(TA)7	14	92901236	92901249	GCCACTTGCAACTTAATGACA	TCAATAATCGACTCCATGCATAA	253
Ad01A16647	Aradu.A01	16647	p2	(TA)6	12	92908676	92908687	CGAGATTCAACGAGCCAACT	AATCCAATCATTGACTCCACG	220
Ad01A16648	Aradu.A01	16648	p2	(AT)8	16	92913347	92913362	GCCTTTTGTCCCTCATTTTT	TCATCCCAATATTTTTGGTTCAT	276
Ad01A16652	Aradu.A01	16652	c	(CAA)5cacaata	46	92922784	92922829	AAGGGGGTTATTTCCCTCTTAAT	TCCCCCTCAAGAAGGATACC	142
Ad01A16653	Aradu.A01	16653	p2	(AT)6	12	92924071	92924082	CGTAAGTCATTTTCATCCCTCA	CATACATAAATTTTGATTTTGAGCA	253
Ad01A16656	Aradu.A01	16656	c	(A)15cttttttaga	83	92932589	92932671	TTGTCTTCAACCACCACACAA	TCGTTATGAAAATGAATGATCTGAA	255
Ad01A16661	Aradu.A01	16661	p2	(GT)7	14	92947926	92947939	ATCACTTTTTGTGGCTCGCT	TGAAATGATTTTTGGTGGCA	193
Ad01A16663	Aradu.A01	16663	p3	(TAC)5	15	92953437	92953451	CCCGAAAAATCAAGTGATGC	AATTTGGCTATTGGGTGACG	257
Ad01A16664	Aradu.A01	16664	p3	(TCT)8	24	92957342	92957365	CATCAAAATCCGATGGCTCT	GATCCCTTGATTGCTTGCAT	275
Ad01A16665	Aradu.A01	16665	p3	(TTC)6	18	92957558	92957575	ATCTATATCCTCCGGCCAGC	TGCAACGCTCAAGAGATACG	153
Ad01A16670	Aradu.A01	16670	p3	(AGG)5	15	92966027	92966041	CGTACAGAGCTTGAACGGAA	GCTACTTCCTCCTCCAAGGC	277

ID	Chromosome	SSR nr.	SSR type	SSR	Size	Start	End	FORWARD PRIMER (5'-3')	REVERSE PRIMER (5'-3')	Product (bp)
Ad01A16672	Aradu.A01	16672	c	(TA)7gttagtcat	119	92970068	92970186	CAACCACCTTCAGAAGCTCA	AGGATGCTGATCTTGATTTGG	264
Ad01A16673	Aradu.A01	16673	p2	(AT)6	12	92972381	92972392	TGTCCTTTGACACAGCAAGC	ATGGCAATGTAAGAGCCACC	271
Ad01A16679	Aradu.A01	16679	p2	(TC)6	12	92992780	92992791	GTTAGGGTTTTTCGATCCGC	GAGAGCCAGAAAGCGAAAGA	269
Ad01A16684	Aradu.A01	16684	p2	(TC)9	18	93004120	93004137	GGCCCTGTCTAGTTGATGCT	GGAAACAGAAAAGCGTAGCG	202
Ad01A16685	Aradu.A01	16685	p3	(TAA)5	15	93010411	93010425	CAAACCGCTTAAGGTTGTCC	GCGAAGAGAGGACTTTACCG	267
Ad01A16686	Aradu.A01	16686	p3	(TGA)5	15	93011625	93011639	GCCCTCAAAGTTCTCTTCCA	CCAACACACCATGCTTCAAC	252
Ad01A16687	Aradu.A01	16687	p3	(TAA)6	18	93012013	93012030	TTCAAATCACATTCCTGCCA	AATGGGGTGGGTTCATGTTA	279
Ad01A16691	Aradu.A01	16691	p3	(ATT)5	15	93017987	93018001	GAGAATCTAAATCTTATCCCAAAGC	GGGCCAATGCAAGAACTCTA	221
Ad01A16692	Aradu.A01	16692	p2	(CT)6	12	93023934	93023945	CGAACCGAACCGATAAATGT	GAGAACGACGATTGCTAGGC	152
Ad01A16693	Aradu.A01	16693	p3	(AAG)6	18	93024237	93024254	AATGCAACGGTGGTCTTAGG	CAAGTCCCACCCAACCTCTA	215
Ad01A16694	Aradu.A01	16694	p3	(TTG)7	21	93024578	93024598	GCCGTGGTCTCAGACAATTT	CTTTTCCAATTCCAAAGCCA	219
Ad01A16695	Aradu.A01	16695	p3	(CTT)10	30	93025493	93025522	TAGCCAAGCACCACAACTGA	TGTGTCTTGGAATTGGTGGA	200
Ad01A16698	Aradu.A01	16698	p4	(AATG)5	20	93033463	93033482	CTCCCCACAGATTGACCGTA	TGCCTTCTACGTGTTTGCAG	258
Ad01A16699	Aradu.A01	16699	p2	(TC)8	16	93034073	93034088	GCCTGAGCTGGAACTACCAA	CCCACCCAGCTTTTCTCTTT	269
Ad01A16704	Aradu.A01	16704	p2	(AT)13	26	93041765	93041790	TTGAACGGCAACATTTTTGA	TCCTCCTTTCACTGCAGCTT	230
Ad01A16705	Aradu.A01	16705	p3	(TAT)5	15	93043240	93043254	AGCACCTTGATTTCATTGCC	CTTGCTTACCACACCAACCA	279
Ad01A16708	Aradu.A01	16708	p2	(CT)17	34	93052754	93052787	TAGAGTTTTCGGCAGTGGCT	CACCGCATGTGAAAGAGAAA	276
Ad01A16713	Aradu.A01	16713	p3	(CTT)6	18	93060878	93060895	TTCGATTCGATTTTCGGTTC	GAGGAAGAAGAACGTGCAGC	247
Ad01A16715	Aradu.A01	16715	c	(T)10ag(T)10	22	93069585	93069606	TTTTCTTGGTTTTGTTTGACATTC	AATTCAATCAATTGTAACCGAAAT	264
Ad01A16719	Aradu.A01	16719	p3	(TAA)5	15	93075346	93075360	AATTCAACATTCCCACACGG	CACCGCAGCATTTTGACTTA	168
Ad01A16723	Aradu.A01	16723	p2	(GA)15	30	93081814	93081843	GACGAAACACCTGGGAAAAA	AGGGCTTTTTGGTCAACATA	243
Ad01A16727	Aradu.A01	16727	p3	(CTT)5	15	93096108	93096122	TCTCATTAAAATCACGCTCCC	GGAGGAGGAGGAAGAGAAGAA	119
Ad01A16728	Aradu.A01	16728	p3	(AGA)6	18	93096567	93096584	GGCACCAAAGAAAATCAGGA	TTTTCTTCTTCGCACACGC	118
Ad01A16730	Aradu.A01	16730	p3	(CTC)6	18	93108197	93108214	GGCAAAGAAAAACGCTCAAG	CAGAAGAGCCAGACTCCAGG	233
Ad01A16735	Aradu.A01	16735	p3	(ATA)6	18	93120291	93120308	AAAATTCGAAACACTTTAGATTAAACA	TGAATGAATGAGAATGAAAAACA	276
Ad01A16737	Aradu.A01	16737	p3	(TAA)16	48	93121836	93121883	TCTGTGCACAAAATCAGTCAA	TGTGAAGAAATGCTACGGCA	270
Ad01A16738	Aradu.A01	16738	p2	(TC)20	40	93125514	93125553	CTTCGTCCCAACCTGACATT	CAACCATCTGAAAAATTACAAGTGA	256
Ad01A16743	Aradu.A01	16743	p2	(AG)8	16	93139845	93139860	TGCAGCGAGAGAGAGAGTGA	GACCCAAGACCGAAGACAAA	226
Ad01A16746	Aradu.A01	16746	p3	(AAT)5	15	93155495	93155509	TGTCATCAAAATCCGTTGTTCT	GCATGCATTTTGCTTTTCAA	144
Ad01A16748	Aradu.A01	16748	p3	(ATA)11	33	93157993	93158025	TGCAGCAGGTTTAATTTGGA	TTTTCGGATTCTCATCAGCC	152
Ad01A16750	Aradu.A01	16750	p2	(AT)6	12	93161952	93161963	TGCTTTATTCACTCATAACTACTTGC	AAATTTGTGCAAATCAAAAGGTT	264
Ad01A16751	Aradu.A01	16751	p6	(CCTAAC)5	30	93162349	93162378	TCACGTGTATATTCGTATCAACCA	GGGTTATGGTTAGGGTTAGCG	175
Ad01A16752	Aradu.A01	16752	p3	(ATT)17	51	93163216	93163266	TTGTAACCAAAGTGGCAAAAA	TTTCACATTTCATATTTTAAATCATCC	259
Ad01A16754	Aradu.A01	16754	p3	(AAT)5	15	93163855	93163869	TCAGTCTTGGATTCAAAATAAACAA	TTCAAAGCATGCATTTTGCT	206

ID	Chromosome	SSR nr.	SSR type	SSR	Size	Start	End	FORWARD PRIMER (5'-3')	REVERSE PRIMER (5'-3')	Product (bp)
Ad01A16758	Aradu.A01	16758	p3	(ATA)15	45	93176012	93176056	TTTGCATTTTGTTGATTGGA	CTCGGATTCTCATCAGCCAT	151
Ad01A16760	Aradu.A01	16760	p6	(CTAACC)5	30	93180935	93180964	TTCCTAACCCTAACCCTAAATGA	GCGCTAGCTTTAGGGTCACAT	226
Ad01A16761	Aradu.A01	16761	p3	(ATT)10	30	93181532	93181561	CCAAAGTGGCAAGAAAAATG	CCAAAATAACCTTTTCACATTTCA	246
Ad01A16765	Aradu.A01	16765	p3	(ATC)5	15	93208010	93208024	GGCCCCTGACAAATTGAATA	ATGATGCAATTGCTGCTGAA	226
Ad01A16767	Aradu.A01	16767	p3	(AAT)6	18	93215649	93215666	CATTTGGACGGTGACTCCTT	TCATTTGAGTTATAGGCGAGTATTTTT	196
Ad01A16770	Aradu.A01	16770	p3	(AAT)5	15	93219365	93219379	TGTATGGAGCGAATCCAGTG	CCCAAATCCTACACAAACAAAA	166
Ad01A16771	Aradu.A01	16771	p2	(AT)13	26	93225515	93225540	TCGAGTTTGGACAACTGACG	TTCCAGGAATCATGCAATGT	239
Ad01A16773	Aradu.A01	16773	p3	(TAT)5	15	93227710	93227724	ATCCAATCCAATCCAAATCG	CAATGACATAACAAGAAAGCTAACG	221
Ad01A16777	Aradu.A01	16777	p2	(TA)6	12	93247119	93247130	ATTCAAAATGGCTGGCAATC	TTGGAAAGTGATCCAAAGCC	124
Ad01A16778	Aradu.A01	16778	p3	(TAA)5	15	93255392	93255406	TGCCTCTATTTTCCCTCCAA	CGTGAATCGGGTAACAAAGA	270
Ad01A16781	Aradu.A01	16781	c	(TA)12(TTTA	44	93269029	93269072	CACATTTTCTGATGGTTGCG	TGGGAGTGAGAGAGGGAAAA	277
Ad01A16782	Aradu.A01	16782	c	(TC)6tg(TC)6t	99	93276966	93277064	TTGTGATCTGCTTGGCTTTG	GCAACAAATCCGTGACCTTT	232
Ad01A16785	Aradu.A01	16785	p3	(ACC)7	21	93283015	93283035	ATCATCATCGACAGCAGCAG	GCGATTCTCAGGCAAGAAAC	208
Ad01A16786	Aradu.A01	16786	c	(TAA)5tcattctc	209	93283314	93283522	TGTCGTTGATGACAAGGAGC	TCACCGGATAAACCTCAAGC	278
Ad01A16787	Aradu.A01	16787	c*	(TA)6(A)10*	21	93285762	93285782	AGTAGCTGATTTTAGTGTGGGAA	CTCTCCGCTTGCTCATCTTC	224
Ad01A16788	Aradu.A01	16788	p2	(AT)7	14	93286680	93286693	CATTTGAGTTTTCACCGCAA	CCATATCAGTAGCCCTCGGA	271
Ad01A16790	Aradu.A01	16790	p2	(TA)7	14	93287458	93287471	TTGTTGATCCACTAGTGTTCTCCT	TCATAGTGCGACTTCGTTGC	256
Ad01A16797	Aradu.A01	16797	p2	(CT)6	12	93334717	93334728	ATGTTCCCTATGCCTACCCC	ATGGCAAGAGTGGCAAAATC	206
Ad01A16801	Aradu.A01	16801	p3	(CTC)5	15	93345706	93345720	ACTCCCGCTTGTTTTACACG	TGCAGAGAAGAAAAATGCGA	187
Ad01A16802	Aradu.A01	16802	p3	(AGA)11	33	93347430	93347462	GAAGAAGAAGACGCTCCGTG	TCAATACACACGAAATCGCC	110
Ad01A16806	Aradu.A01	16806	c	(A)10gag(T)1(	23	93358565	93358587	CAGACTTGGGCCGACATTAT	TTGAGTGGGAAGATGCAACA	279
Ad01A16808	Aradu.A01	16808	p3	(AGA)5	15	93366614	93366628	TGCGCTTCTTCTTTTCAGGT	CAAGACGCCAAGAAGAGGTC	184
Ad01A16815	Aradu.A01	16815	p2	(AT)10	20	93381297	93381316	TCAAGCAGCGCTATTGAAGA	AGCTGCTCCTCCCATATCCT	265
Ad01A16818	Aradu.A01	16818	p2	(TA)6	12	93392458	93392469	CATTTGTTTACATTTGTTGATTCG	TTTAAGCAAATTGCCCATGA	221
Ad01A16822	Aradu.A01	16822	p2	(TA)6	12	93399449	93399460	TTCCTTGATCCCCCAATGTA	TGAAGCAATTGTCAAGTAGCG	167
Ad01A16824	Aradu.A01	16824	p4	(AAAT)6	24	93405428	93405451	GCCGGGGATTGATAGTGATT	CGTCAGAGTGCAGAGACCAA	277
Ad01A16826	Aradu.A01	16826	p3	(AAT)5	15	93408833	93408847	TCCACGCTGAACTTGAAAAA	TGCTTGTCCTCAAGCAAAGA	255
Ad01A16827	Aradu.A01	16827	c	(AGG)5agaagg	39	93415001	93415039	TGCAGAGAAGAATAACGCGA	CACGCGCCTCATATAACGTA	180
Ad01A16828	Aradu.A01	16828	p4	(TTTC)5	20	93423258	93423277	TTTGAGACGTGAATGGTTGC	AGAAGGTTGGGGGAAATGAT	168
Ad01A16830	Aradu.A01	16830	p4	(ATTC)6	24	93434516	93434539	TCCCTCCATGTCCATCAAAT	CCGGCCTTACATGGAAGATA	214
Ad01A16831	Aradu.A01	16831	p2	(AG)6	12	93435231	93435242	AGGCATGGAACAAAATTGGA	AATTTGGGCCACACTTTTGA	273
Ad01A16832	Aradu.A01	16832	p3	(TTA)6	18	93435936	93435953	TTGCAATCATGCAAATCACA	AACTTCCATACATGCTCTCCAAA	272
Ad01A16833	Aradu.A01	16833	p2	(AT)6	12	93436275	93436286	CCACCTTCACGTATTAGTTGTTCA	CCATACTCACCTAAGAGATCACGA	170
Ad01A16835	Aradu.A01	16835	p2	(TA)6	12	93440212	93440223	TGACGCAAGAAAGTTTCACAA	TGGGTCATACAGCACCTGAG	270

ID	Chromosome	SSR nr.	SSR type	SSR	Size	Start	End	FORWARD PRIMER (5'-3')	REVERSE PRIMER (5'-3')	Product (bp)
Ad01A16840	Aradu.A01	16840	c	(T)10aacc(T)1	24	93442660	93442683	CCCCTATGTATAAAGGGACCA	AGAACAAGACCAAACCACCG	271
Ad01A16842	Aradu.A01	16842	p2	(AT)6	12	93444573	93444584	TGGAAAATAGTGCACCAGGA	GAATGGTTCATTGTGTTGCG	261
Ad01A16844	Aradu.A01	16844	p2	(TA)8	16	93454295	93454310	CAATCAATATCTTGGAATTTGGAA	AAAGGAGGAAAAACGCAAAAA	222
Ad01A16846	Aradu.A01	16846	c	(TTC)5gtttgatt	138	93455614	93455751	TGCAAGTTCATCCTCTTCCAA	TCTCCTCCTTATCTTCTGCTGC	221
Ad01A16851	Aradu.A01	16851	p3	(TAA)5	15	93488159	93488173	TTGCTAAGCCAAACCAGAGG	GTTTTGCCCCAAGAAATTCA	146
Ad01A16852	Aradu.A01	16852	p2	(TA)8	16	93488921	93488936	TGATAGGGGGTGGAAGAAGA	CAATGAGTACCAGAAGGCAACA	173
Ad01A16856	Aradu.A01	16856	p2	(TA)9	18	93496192	93496209	TCTCAAATGGATGCTGCTGA	TCATGACATTCCCATGCCTA	214
Ad01A16858	Aradu.A01	16858	p2	(TA)9	18	93502797	93502814	GCAGCCCCAAATTGAGTTTA	CTTAACCTTGGCGTGAGAGC	161
Ad01A16863	Aradu.A01	16863	p3	(CTC)6	18	93511773	93511790	TCTTTCCAGAGGGTCCAAGA	CGCCTTAACCTCACCAGAAG	222
Ad01A16866	Aradu.A01	16866	p2	(TC)6	12	93521983	93521994	CACAATAATAAAACACTCTCCTCTCTC	TTTGATCAGAGTGTCGTGTTGA	202
Ad01A16870	Aradu.A01	16870	p3	(ATA)12	36	93528861	93528896	CGGTTTCCTTTTACATTTACATTG	GGTAGGGATATTGCGAACCA	198
Ad01A16874	Aradu.A01	16874	p2	(AT)6	12	93535755	93535766	TGTTTGACATGGTGTGAGCC	ATGTGTTGATTCCCTAGCCG	167
Ad01A16875	Aradu.A01	16875	p4	(AATT)5	20	93537079	93537098	TGGAAGCTTACACGTGATGG	AGGGGGACAAAATTGGAGAT	165
Ad01A16882	Aradu.A01	16882	p2	(CT)10	20	93554569	93554588	CCTCCCTTCTCTTCCCTACG	ACCATAGCTGCAACACATGC	223
Ad01A16884	Aradu.A01	16884	p3	(TTA)9	27	93566628	93566654	AAGAGGTTAATGCGAAGCGA	GAGGAGTCACCCCATACTGC	273
Ad01A16885	Aradu.A01	16885	p3	(CAT)7	21	93567419	93567439	TCAGCATCAGCATCACACAA	AGGAGGAGCAGAGTCCAACA	267
Ad01A16887	Aradu.A01	16887	c	(ATT)5ttaaaata	71	93569673	93569743	TCGAACATGCAACTTCTTAATTG	AAACTCACTCCTCAATCTTTTTCC	278
Ad01A16889	Aradu.A01	16889	c	(T)13aaaaaagc	67	93576064	93576130	GCACCCTGAAATTAATCAGCA	CGAGCAAATGCTCTATGACC	193
Ad01A16890	Aradu.A01	16890	c	(CA)6ttactttcaa	58	93579862	93579919	TGACGTGAACTGCACACAGA	TGATGATCACACTGTGGCCT	167
Ad01A16891	Aradu.A01	16891	p2	(AG)10	20	93580389	93580408	CAGACCTCTTTGGTTCTCCG	TTCCCTTTTGATCCTTCACG	151
Ad01A16892	Aradu.A01	16892	c	(ATT)8gttgttgt	61	93590269	93590329	ACACACCCTCACCACTCTCC	TCATGGCATGTTCATCCAAC	245
Ad01A16898	Aradu.A01	16898	p3	(GAT)5	15	93626399	93626413	GGATCTCTGGTGGCTGACAT	ATCTCCTCCCTCGTCATCCT	269
Ad01A16900	Aradu.A01	16900	p3	(GAT)5	15	93630293	93630307	TTGTTGTACAGGGCAAACCA	ATCTTCCCCGTCTTCTCCAT	261
Ad01A16901	Aradu.A01	16901	p4	(TTCT)6	24	93635529	93635552	CAACTCTTGATTGGTGGCCT	GCAGTGAAAAATTTAAGCGGA	167
Ad01A16904	Aradu.A01	16904	p3	(GAT)6	18	93644466	93644483	TTGCTGTTCCTGTTGTCGTC	TCTTCTGCGCTTCCAATTCT	166
Ad01A16914	Aradu.A01	16914	p2	(AG)12	24	93663052	93663075	TATCCCTATGACGGCACACA	TCACAACCACACACCTTCGT	270
Ad01A16921	Aradu.A01	16921	c	(AC)6agtgtaaa	81	93689946	93690026	CCCTTCACGTGGTTTGTTCT	TTTGTTTTGTGAAGCCCCTC	239
Ad01A16925	Aradu.A01	16925	p2	(AT)7	14	93693093	93693106	TGGCCCTTTTAGTGGAAAGA	CCTTAAATGCAACAACCTTGG	280
Ad01A16928	Aradu.A01	16928	p3	(TGT)6	18	93702457	93702474	AGTCGATAGCGCTTGTGGTT	GCTTATCAGCAGGGCTCTTG	267
Ad01A16931	Aradu.A01	16931	p2	(TA)10	20	93709616	93709635	AGTAATGTTATGGGGCGCAG	GCCATTGCGATTGAGAATTT	175
Ad01A16936	Aradu.A01	16936	p3	(TTA)5	15	93721971	93721985	TACGCAAAAGAATCGCACAG	ATGAAGTAAACCCGCACGTC	266
Ad01A16938	Aradu.A01	16938	p2	(GA)7	14	93725755	93725768	GCACTTCTAGAACTTGCGGG	CAGCATCACGAAACTCGAAA	109
Ad01A16940	Aradu.A01	16940	p2	(AT)10	20	93727870	93727889	GAGATTTTGGCCATATCCGA	GGTAACTCATCAACACGACCC	203
Ad01A16941	Aradu.A01	16941	p2	(AT)14	28	93729916	93729943	AAATGATGGATGGAAATGGC	AATTGCTTTGGTTTCATCGG	226

ID	Chromosome	SSR nr.	SSR type	SSR	Size	Start	End	FORWARD PRIMER (5'-3')	REVERSE PRIMER (5'-3')	Product (bp)
Ad01A16945	Aradu.A01	16945	p3	(AAG)5	15	93755381	93755395	CCCTAATCTCTCGCTGACCA	AACCAAAAAGTGGGTCACCA	274
Ad01A16946	Aradu.A01	16946	p3	(TTA)9	27	93757299	93757325	GATGAGGACTGAGGGATGGA	TCAGCCACCTCCAAAACTGT	260
Ad01A16949	Aradu.A01	16949	p2	(AT)13	26	93764073	93764098	TGTTGTACGAGTGTGTGATGC	GCCATTTATGAGCTGACAAGG	230
Ad01A16954	Aradu.A01	16954	p2	(CT)24	48	93782533	93782580	TACGCTTTCCAGGCTCTTGT	GATGCACGCGAATTTTCATA	251
Ad01A16955	Aradu.A01	16955	c	(A)13caaaaaca	73	93782963	93783035	GGGTAAAAGGGAGAAGGGTG	AGGACCGGTAACACTGACATC	271
Ad01A16958	Aradu.A01	16958	p2	(AT)9	18	93789659	93789676	CACATGCATTGATCTTTGAACTT	ATGGGAAGATTGTGGGAACA	218
Ad01A16963	Aradu.A01	16963	p2	(GC)6	12	93799613	93799624	ACACAGCACAGGCAACAAAC	GGGATTGGTTTTCGATTGAC	259
Ad01A16968	Aradu.A01	16968	p2	(TC)6	12	93809982	93809993	CGCGATTCTCCTTTCTCATT	CAAAGAACATCGCACCTGAA	107
Ad01A16971	Aradu.A01	16971	p3	(AAG)5	15	93819904	93819918	GATCAGAAAACAAGGCCAGC	AACGGAAAACGCTGAAGAGA	239
Ad01A16974	Aradu.A01	16974	p3	(ATC)5	15	93821245	93821259	TGTAATCCAAAATGCATCGAA	AAGCAAGAAGGAAACGATGAA	203
Ad01A16975	Aradu.A01	16975	p3	(CTT)6	18	93821692	93821709	AGAAATTTCAGTGTTATTTGTTCTGA	TTTTTCCCTCCTTTTCTTCC	237
Ad01A16977	Aradu.A01	16977	p3	(GAG)6	18	93872686	93872703	CGCCTTAACCTCACCAGAAG	GGCCAAAGCTAAGTCTCGTG	202
Ad01A16982	Aradu.A01	16982	p3	(TTG)5	15	93880492	93880506	GTGGCAGAGTGAAGGAAAGC	AAGAAGGTGGGAAGGGAGAA	175
Ad01A16983	Aradu.A01	16983	c	(T)10aaaaaaaa	101	93881008	93881108	TGTGTTTGTTTGGATGTCATTATTT	TTTGGGTGAGATCTTTTGTCG	271
Ad01A16987	Aradu.A01	16987	p3	(TAA)5	15	93898790	93898804	TTGCTATTTCCTCCGATCCA	TACCAATTTGGAGAAACGCC	190
Ad01A16988	Aradu.A01	16988	p4	(ACAT)5	20	93899318	93899337	TTTCCTCCACAATCATCCCT	TTGATGGTGATGATGATGCC	240
Ad01A16989	Aradu.A01	16989	p3	(ACA)6	18	93899598	93899615	AGGTCTCCTTTTGCCACTCA	TGACTAGGGGATGAAGGTGG	117
Ad01A16990	Aradu.A01	16990	p2	(TA)6	12	93900413	93900424	TTTGCTCCCTTCCTATCCCT	AACACGTATCTCGCCATTCC	246
Ad01A16993	Aradu.A01	16993	p3	(TTC)5	15	93903483	93903497	GGGTCTATAATTGCGCACCA	GAGAAGAAGGAGGAGGAGGG	183
Ad01A16994	Aradu.A01	16994	p3	(AGA)6	18	93905140	93905157	AAAAACCTGGTCAATGCTTATCA	CGCCTTCCTCCTTCTTCTTC	278
Ad01A16996	Aradu.A01	16996	p3	(TGC)5	15	93910575	93910589	GATTACAGTTCCGCAGCTTCA	CCAGGGCAAATTGGTTAAAG	279
Ad01A17001	Aradu.A01	17001	p2	(CT)6	12	93921456	93921467	AATGTGTTACTCAACATGCACCT	TGTTGCTTGCATCAATGAGTT	236
Ad01A17002	Aradu.A01	17002	c	(T)10ggcacaga	75	93921755	93921829	GAATTAGCCCCTGCACAAAA	ATACCCCATTCGACCCCTAA	157
Ad01A17004	Aradu.A01	17004	p2	(AT)7	14	93924113	93924126	TAATTGGGGAGGGATGTCAA	GTTCAGGTTGGTCGGGTAGA	218
Ad01A17007	Aradu.A01	17007	c	(TC)18ttctggtc	117	93932451	93932567	ACCAACCAAACCAAGCTCAT	GATACGGCGTAGGAAAACGA	277
Ad01A17009	Aradu.A01	17009	p3	(CAG)7	21	93936678	93936698	CGAATACAGTTGCTGCTCCA	GAAGGCTGTTGTTGGGACAT	127
Ad01A17016	Aradu.A01	17016	p2	(TG)6	12	93951435	93951446	CGAGGATTAGGAGTTTTGGG	CAAAATTCTCCCGAGACAGG	169
Ad01A17018	Aradu.A01	17018	p2	(TC)7	14	93955593	93955606	ACGCACCTACCCTCACTCAC	TTGGGCTATGTTGATGTTCAAG	154
Ad01A17019	Aradu.A01	17019	p3	(AAG)5	15	93956032	93956046	AGGAGATGACGAAAACGCAT	GTCGCAAGCTCCATCTTCTC	218
Ad01A17020	Aradu.A01	17020	p2	(TC)7	14	93956957	93956970	TGGAAGAATGGAGAGGATGG	ACAGTTGGCAAGCATGTGAG	253
Ad01A17022	Aradu.A01	17022	p3	(ATG)5	15	93960900	93960914	TCTGCCCGTGAACTTTAACC	AATCTCTAGAGTGCGTGGGC	164
Ad01A17023	Aradu.A01	17023	p3	(ATA)5	15	93962421	93962435	CGGAGAAACCTGTAACTCCATT	CGTGGCCTACTATTTCCTGTG	273
Ad01A17024	Aradu.A01	17024	p2	(AG)13	26	93968042	93968067	AAGGCAAGAAATGGTTTCCC	TCTTCCGTGATGGATGTTCA	191
Ad01A17030	Aradu.A01	17030	p2	(AG)16	32	94006673	94006704	GAGGAAAACGAAACAACCGA	CCAACAAGCCCAATCTTCAT	279

ID	Chromosome	SSR nr.	SSR type	SSR	Size	Start	End	FORWARD PRIMER (5'-3')	REVERSE PRIMER (5'-3')	Product (bp)
Ad01A17032	Aradu.A01	17032	p2	(GA)6	12	94008528	94008539	TCGATTTTGTCCCCAATTTT	CCTCCTCCCCTCTTTTCTTG	208
Ad01A17033	Aradu.A01	17033	c	(TGG)5ttctgttt	56	94008963	94009018	ATGGTGGTGGGTTCTTGTTC	CAACGAAAATACCCTTCTCCC	200
Ad01A17035	Aradu.A01	17035	p2	(TC)7	14	94010214	94010227	GTGCTCTCTCTCATTTCGCA	TGTCATGATAAAGACGAAAAATGAA	256
Ad01A17039	Aradu.A01	17039	p3	(ATG)6	18	94030711	94030728	TGCGGTTCAATAAAGCTGTG	ACGGATCGAACGAATACCTG	265
Ad01A17040	Aradu.A01	17040	p2	(CT)9	18	94031711	94031728	GGGAATGGACCACAGAGAGA	TTTGCTTCAATTCACCACCA	228
Ad01A17041	Aradu.A01	17041	p3	(AAT)8	24	94034652	94034675	TAAGGTGGGTGTGGTAGGGA	GCGCCATGCAGTGTAAAAC	183
Ad01A17042	Aradu.A01	17042	p2	(AT)7	14	94035157	94035170	CAAAGCTGCTTCATGGGTTT	GCATGCCACTTCAATTCTGA	278
Ad01A17043	Aradu.A01	17043	c	(TA)6atgagaag	75	94035598	94035672	GCCCTTCCTTTCCCTTATGA	AACCCCTCTCTCTCTCTGCC	256
Ad01A17044	Aradu.A01	17044	p2	(AG)6	12	94035805	94035816	TCAGCACCAACATTGCTTTC	CCCTTCCTCCCTCCTACATT	174
Ad01A17045	Aradu.A01	17045	p4	(GAAG)5	20	94036613	94036632	CTCCCACAGCTTTCTTGAGC	TGGTCCATGCTCTTCTCCTT	224
Ad01A17046	Aradu.A01	17046	p4	(AGTA)6	24	94036777	94036800	AGCTGTGGAAAAGCATGACC	TGAATGAATGAAGCAGAACCC	269
Ad01A17047	Aradu.A01	17047	c	(T)11c(T)10	22	94037086	94037107	TTCACACCCAAATCACCCTT	GGGGACCATAAACAACCCAT	184
Ad01A17048	Aradu.A01	17048	p3	(AAT)7	21	94039843	94039863	TGACTGTTTTCCGTGGTCAA	GCGTCTAGGATGGTCCGTAA	225
Ad01A17049	Aradu.A01	17049	p2	(TA)8	16	94040630	94040645	TTGTCGGATTGAAAGATATCGTAA	TGAGTTAATTCAATGATCATGTCTC	195
Ad01A17051	Aradu.A01	17051	p2	(AT)7	14	94043956	94043969	CTTGCTGCTGCTCGTTGTAG	AATAATGGCGAGGCACAATC	162
Ad01A17053	Aradu.A01	17053	p4	(ATAC)6	24	94048941	94048964	CAGCAGACTGCACACAAACA	CCATGTGGGCCCTTCTTACT	263
Ad01A17054	Aradu.A01	17054	p4	(ACAT)7	28	94049317	94049344	TGGGTTTGACAAGTCAGGTG	TGCAAGAATGAATGTTTGGG	273
Ad01A17060	Aradu.A01	17060	p3	(CCT)7	21	94062740	94062760	GAGGAAGAGGCAGAAGCAGA	ACCTGATTGGTGATTGCTCC	261
Ad01A17061	Aradu.A01	17061	p3	(TTA)11	33	94066554	94066586	TTCACTTTGCCAAACACACC	TAGCTACCAAATCGGTCCGT	266
Ad01A17062	Aradu.A01	17062	c	(AAT)5gataa(A	34	94068054	94068087	TGGAGTAGATTGATATGTTGTTGTTG	GATTAACTTTTACTTAGTGCCAGCAT	271
Ad01A17063	Aradu.A01	17063	p4	(GAAT)9	36	94068259	94068294	CCGTTGAGTTGGAATTTTGTT	TGTATACATCTCATTCTCCTTCCTC	110
Ad01A17065	Aradu.A01	17065	p2	(AT)10	20	94082544	94082563	TCGAAAAGAAAATTGAGTGAGACA	TCATCTCCACCATTTTCAACA	192
Ad01A17066	Aradu.A01	17066	p2	(TA)6	12	94082748	94082759	TCAACCAATTATTCTCGATCACA	TTGACCCATGTGCACAAACT	195
Ad01A17068	Aradu.A01	17068	p3	(AAT)5	15	94089271	94089285	AACTCAGAAAACACACACAACG	GCGTTTGAGAGGAACGTAGC	280
Ad01A17069	Aradu.A01	17069	p4	(AAAG)5	20	94090805	94090824	AAGTGGGTTCTCCACACACC	GAGGCCACCTAAACCCTAGC	216
Ad01A17070	Aradu.A01	17070	p4	(GAAT)6	24	94091067	94091090	GGGTTTAGGTGGCCTCAAAT	CTTCACTCTTCACCATCCCG	203
Ad01A17079	Aradu.A01	17079	p2	(TA)9	18	94101386	94101403	GAACCAGTGGCAGGTTTCAT	GATGTGAGCCCATCCATGTT	218
Ad01A17080	Aradu.A01	17080	p2	(TC)18	36	94102436	94102471	TAATCGTGACCAAAATGGCA	GTCATTGGGGCAAATTGAGT	182
Ad01A17083	Aradu.A01	17083	p2	(TA)10	20	94109811	94109830	CACCGTGCATCACTATGTCC	AAAAGAAAGGTCGCTGAAAAA	141
Ad01A17085	Aradu.A01	17085	c	(A)10ttttatttgtt	118	94116584	94116701	ATGTAATGGACGCATGGACA	TCTGAGTTTTGCTTTTCCCC	200
Ad01A17086	Aradu.A01	17086	p3	(GAG)5	15	94128612	94128626	TGAACGAATGCAAGAGCATC	GTTCAGGACGCTAAGGCAAG	242
Ad01A17088	Aradu.A01	17088	p3	(TAA)20	60	94132651	94132710	TTTTCAGAATGATAATGCTTCTATTG	TTGTTATTTATTTCGCATTACTGTTT	235
Ad01A17090	Aradu.A01	17090	p3	(AAT)6	18	94141536	94141553	ACATTGAATTGGCGTGGACT	TTTGAAATTCATGGTGGAATAA	214
Ad01A17091	Aradu.A01	17091	p3	(TCT)5	15	94142272	94142286	CCTTGTCTCTCACCCTCACG	CCTCACAGAAACGAACGGAT	124

ID	Chromosome	SSR nr.	SSR type	SSR	Size	Start	End	FORWARD PRIMER (5'-3')	REVERSE PRIMER (5'-3')	Product (bp)
Ad01A17093	Aradu.A01	17093	p4	(TAAT)5	20	94145408	94145427	AACATAATAAAATTGTTGACGGATT	GCCTACTCACGCCAGTTTGT	209
Ad01A17094	Aradu.A01	17094	p2	(GA)7	14	94149134	94149147	TGCTTGTCCTCAAGCAAAGA	GGGCAGAGAGTTGTACTGGC	262
Ad01A17097	Aradu.A01	17097	p3	(AGA)5	15	94174471	94174485	ATACCGCATCAACGACAACA	CAAAGCAATTTTAAGTCATGCG	246
Ad01A17099	Aradu.A01	17099	p2	(TC)9	18	94177767	94177784	TAAGGAAGGCGATGATTCCA	AAAAAGGGGGCTAGGGATTT	171
Ad01A17100	Aradu.A01	17100	c	(T)10ggttttggtt	62	94178033	94178094	CGCAAGGAATCTGTCAAGGT	CTGAGCATCAAAACACCGAA	262
Ad01A17101	Aradu.A01	17101	p3	(TTA)15	45	94183186	94183230	GGCAACTGAAGTGGATGGAT	AAGCCAAAATAGCAACCCCT	249
Ad01A17102	Aradu.A01	17102	p2	(CT)9	18	94190536	94190553	TCAAATCGGATTCTCCGTTC	CAAGGACTTCGAGTTCAGGG	278
Ad01A17103	Aradu.A01	17103	p3	(GTG)6	18	94191067	94191084	TTGTGTATGCAACACGCAGA	TCCAGAACCAAACTCATCCC	221
Ad01A17106	Aradu.A01	17106	c*	(AG)22(A)69(	166	94199190	94199355	TTCAACTGCAGTGCCAATTT	CCCGTCATGGTAAGTTGGTT	271
Ad01A17109	Aradu.A01	17109	p3	(TAA)15	45	94206926	94206970	CCAATTACACCATGCATATCCT	AAGTGACACTATGGTTGCATTGA	255
Ad01A17110	Aradu.A01	17110	p3	(AAT)5	15	94208638	94208652	AGAGCAGCCTCACGGATAAA	CTCAGGTGAATATCCCCCAA	259
Ad01A17111	Aradu.A01	17111	p3	(ATA)14	42	94209031	94209072	TCGTTAAAATTATGGAGACCACTTT	GATCGGATCAAATCGGTTGT	236
Ad01A17112	Aradu.A01	17112	p2	(GA)8	16	94209972	94209987	TGGGGGAAGTGTGAAGAATC	TCACCATCCCCACTCTTCTT	210
Ad01A17115	Aradu.A01	17115	p3	(TTC)6	18	94212442	94212459	CCTCTCACTCACTCCCCAAC	TCTCACTGTGCCATGCTCTC	150
Ad01A17116	Aradu.A01	17116	p3	(CCT)6	18	94213292	94213309	TGGGTCCATTCATTCCTGTT	AGGAATTTGTGAAACGGCAG	225
Ad01A17119	Aradu.A01	17119	p2	(TA)8	16	94216972	94216987	AGCCATGTGCCTTCAAAATC	TTTCCCCGTGTGTCCTACTC	255
Ad01A17120	Aradu.A01	17120	p2	(AT)8	16	94217799	94217814	GGCTGGTTTTTACTAGAACCATCTT	GGAAAGATAAATAAAAATTCAACCTGA	275
Ad01A17122	Aradu.A01	17122	c	(TAA)6tgaattta	121	94218334	94218454	TTTGGTAGATTAGTCATCTCTCTCTCC	TGGGTTAATATCGTGTTTGCAT	280
Ad01A17124	Aradu.A01	17124	p3	(ATA)5	15	94222715	94222729	TGAATTGAAGATTGGGGCAT	CATCACCGCCATTATTTTGA	217
Ad01A17125	Aradu.A01	17125	p3	(TCA)9	27	94228588	94228614	CAGTGGTCCCAGGTGAAATC	TGGGTCTGTGCTTGGGTACT	277
Ad01A17126	Aradu.A01	17126	c	(TGA)5tggggtg	223	94229034	94229256	TGGCCATGAAGAAGGTGTAG	GGCATTCAGAGAGAGTAACTGTG	280
Ad01A17127	Aradu.A01	17127	p4	(TTAA)5	20	94230993	94231012	CGAAAATTATGAGCAATGAACTT	CGACGGATGTAAGACAATGAAA	264
Ad01A17130	Aradu.A01	17130	p2	(AT)7	14	94263917	94263930	TCACTCACAAAATTGCTGCC	GGAAGGGCTGCTTTCAAACT	110
Ad01A17133	Aradu.A01	17133	c	(AC)7caagcaat	90	94268870	94268959	TGTCATTCAAATCCAACCAAA	GGAGGAAAATGAGGAGGAGG	240
Ad01A17134	Aradu.A01	17134	p3	(AAG)5	15	94269537	94269551	CCCAAATCAATTTTGGATCAG	ATTTGGACTCGTTGTCCTGC	241
Ad01A17135	Aradu.A01	17135	p3	(AAG)5	15	94269934	94269948	TCGAAACAAGCACACAAACA	TCAAAACTCTTTCTCCTCTTCCTC	264
Ad01A17136	Aradu.A01	17136	p3	(GAA)8	24	94270504	94270527	TGACGACGGTAACGAAAGTG	CTGCTTTCTTCTCTCCTCGC	108
Ad01A17138	Aradu.A01	17138	p2	(AT)11	22	94291910	94291931	CATTGGTGAGCGACAAACTC	TTCTGATTAACTGCTAGCGAAAAA	273
Ad01A17144	Aradu.A01	17144	p3	(TAT)22	66	94298238	94298303	GAATGGGGATAGCAACCAAT	CGTTGTTAAATTGTGCGTGC	280
Ad01A17147	Aradu.A01	17147	p2	(TA)8	16	94305360	94305375	CAAACAATGCAATAAGCCAAAA	TCACCAAAAGTTGGTCGATG	234
Ad01A17151	Aradu.A01	17151	p4	(TTCT)6	24	94311167	94311190	CCTCTTCCTCTCCATCCAAA	GGGCAAAGGGCTTTTTATTC	155
Ad01A17152	Aradu.A01	17152	c	(CAT)7gatgtcg	119	94311394	94311512	CCTTAGCTTGCTTCATCTTCTTC	GGCAAATTAATGTGAGGATCAA	280
Ad01A17154	Aradu.A01	17154	p3	(ATT)5	15	94311793	94311807	TTTGCCTACCTTCTTCATTCA	CCATAACAATAGATGGAGACCTCA	276
Ad01A17155	Aradu.A01	17155	p3	(AAT)5	15	94313435	94313449	TTGGGGTCGTGATTATGATG	TCCTCTGGATCTCTTCCCTG	235

ID	Chromosome	SSR nr.	SSR type	SSR	Size	Start	End	FORWARD PRIMER (5'-3')	REVERSE PRIMER (5'-3')	Product (bp)
Ad01A17159	Aradu.A01	17159	p3	(ATG)10	30	94317328	94317357	TGTGAACTTAGCCCAGGTGA	TCAATGCCATCCAAAAGTCC	208
Ad01A17160	Aradu.A01	17160	p3	(ATT)7	21	94319366	94319386	ACCGTGGGAAACCAAAAA	CGTCTATACGAAGACGGAATATG	280
Ad01A17164	Aradu.A01	17164	p4	(ATGT)5	20	94323460	94323479	ACACTCAGCAGCAGCATCAT	GTGATGTGCAAGCCAACAAC	193
Ad01A17166	Aradu.A01	17166	p2	(TA)10	20	94324636	94324655	AATGCCAAAGTTTTCATGCC	CCTGCTTTAAGGTTTGAAGTCG	267
Ad01A17167	Aradu.A01	17167	p3	(TTA)5	15	94325640	94325654	GGCTACCCCTCTCTTTTTGG	TTGATGTACAAGCGTCTCGC	256
Ad01A17168	Aradu.A01	17168	p2	(TG)6	12	94328457	94328468	ATTCCAAGCCCATCAATCAC	TGCTAGTTTGGGTCTTTGGG	103
Ad01A17169	Aradu.A01	17169	p2	(TA)6	12	94329498	94329509	TGTCCATGCATAACACTGCC	CCCCCAAGAAAAGACTCTGA	235
Ad01A17174	Aradu.A01	17174	p4	(AATA)5	20	94343605	94343624	TCGGAAATTACACACAAAACTCC	ACCTTATCGCACCAGAGTCG	279
Ad01A17178	Aradu.A01	17178	p3	(CTT)5	15	94353917	94353931	GCAGGTCTTGCGATTCTTTC	CCTGAACTTGCTCATGCAAA	244
Ad01A17182	Aradu.A01	17182	p3	(ACA)5	15	94381624	94381638	GCACAGGCTACCAATCAGGT	CATGAGGTGGTCAAACGAGA	186
Ad01A17183	Aradu.A01	17183	p3	(GTG)5	15	94397099	94397113	GTGAGGACGAAGGTGGTGAT	CTACCATAGTGGCCCCGTAA	141
Ad01A17185	Aradu.A01	17185	p3	(AAG)6	18	94397790	94397807	TCACTGGAGCTGTTGAAACG	GAAGAGGACGAGGAGGCTCT	198
Ad01A17186	Aradu.A01	17186	p3	(GGA)9	27	94403737	94403763	CGATGACAATGACATGACGA	CATTTTTCACATCACGTGCC	270
Ad01A17187	Aradu.A01	17187	p3	(AAG)6	18	94403877	94403894	CATGACGATGATGATGGAGG	CATTTTTCACATCACGTGCC	258
Ad01A17190	Aradu.A01	17190	c	(T)10attttgttaa	110	94412489	94412598	GCCGCCGTAGAAAATTACAG	GAGGCAGCAACCAGATTTTC	242
Ad01A17192	Aradu.A01	17192	c	(T)10attttgttaa	185	94415967	94416151	TTGCTCTCCCTGTTTCTTGAG	TCATCATTCATCAACCATTAACAA	264
Ad01A17194	Aradu.A01	17194	c	(T)10attttgttaa	110	94421310	94421419	GCCGCCGTAGAAAATTACAG	GAGGCAGCAACCAGATTTTC	242
Ad01A17197	Aradu.A01	17197	c	(T)10attttgttaa	111	94431050	94431160	CTGCCGCCGTAGAAAATTAC	CGAACCAAAACTGTCGGTCT	243
Ad01A17200	Aradu.A01	17200	p4	(ATTT)7	28	94438677	94438704	CACCAGAGTCGGTCATTCATT	TCGGAAATTACACACAAAGCTC	278
Ad01A17202	Aradu.A01	17202	p3	(TCC)9	27	94440651	94440677	ACCACCTCCTCTACCTCCGT	TGACAATGACGTGACGATGA	102
Ad01A17208	Aradu.A01	17208	p3	(AAG)5	15	94457734	94457748	TCACTGGAGCTGTTGAAACG	GAAGAGGACGAGGAGGCTCT	212
Ad01A17211	Aradu.A01	17211	c	(GC)6agaaagg	76	94481337	94481412	ATGATTGTCAAGGGCCTCAA	GTGGGAGACACCCCACTATG	272
Ad01A17213	Aradu.A01	17213	p3	(TTG)7	21	94501705	94501725	TGGCATCGTTTGATTCATGT	CTACGATGCGCAACACAAGT	271
Ad01A17216	Aradu.A01	17216	p4	(ATAC)5	20	94505010	94505029	TCCCAGTTGAAACAAATTAAACG	GGGTTCACTGCTGCTATTATTG	267
Ad01A17217	Aradu.A01	17217	p5	(AAAAT)5	25	94506181	94506205	ATTGGACCAGACCAGACCAG	TTGCTTTCTTTTTCAATGATTTTG	265
Ad01A17218	Aradu.A01	17218	c	(A)12t(A)12	25	94506464	94506488	CATCAGCAGAATCAAGGTTCAA	CAGGGTCTTCTTCTGGTGTTG	276
Ad01A17220	Aradu.A01	17220	c	(AAG)5aggaag	49	94508315	94508363	CCTCCTCATCTTCTGCTGCT	ATCGTCGTGTTTCTCCTCGT	243
Ad01A17224	Aradu.A01	17224	p2	(CA)6	12	94518573	94518584	AAACTACAACGGCAACGTCC	CTGCCGAGTTTCGTTTCTCT	164
Ad01A17225	Aradu.A01	17225	p3	(CTT)5	15	94520651	94520665	CACATCACGCGCCACTTAC	CGATGACGATGACAATGACC	262
Ad01A17226	Aradu.A01	17226	c	(TC)7tt(TC)7	30	94522115	94522144	TTGAATATCGTGGGAAGCAA	TTTACAATTACCGCCAGTATGA	200
Ad01A17227	Aradu.A01	17227	p2	(TA)6	12	94523157	94523168	AACATGCCACGTCAGCTTC	GCCGGTAACATGAAGTTTAGC	279
Ad01A17229	Aradu.A01	17229	p3	(CTT)5	15	94529022	94529036	GAAGAGGACGAGGAGGCTCT	TCACTGGAGCTGTTGAAACG	212
Ad01A17232	Aradu.A01	17232	c	(AT)6gtttgcatg	200	94535464	94535663	GGTTAGGTGTCATAAGAAGATGGA	TGATAATTACCGTCTAAATTGATTTTT	279
Ad01A17234	Aradu.A01	17234	p3	(AAT)6	18	94541699	94541716	TTAGGAGATAATTGATGCACACA	AATGCATGTGCTGTGTAGATTT	106

ID	Chromosome	SSR nr.	SSR type	SSR	Size	Start	End	FORWARD PRIMER (5'-3')	REVERSE PRIMER (5'-3')	Product (bp)
Ad01A17236	Aradu.A01	17236	p3	(AGA)5	15	94553155	94553169	CTTAAAATTTGCTCGTCCCG	ATTCAATTCCCCACCCTTTC	207
Ad01A17237	Aradu.A01	17237	p3	(ATT)5	15	94556322	94556336	AATTATTGGCTCCAATGGCA	TTATTTTCGAAATCTCCCCC	274
Ad01A17241	Aradu.A01	17241	p3	(TCT)5	15	94577462	94577476	AAAATGGCCGAAACCTAACC	TTGCCTTCCCTTTTCCTTTT	280
Ad01A17246	Aradu.A01	17246	p2	(TA)11	22	94605262	94605283	AAATGTGGCAAAATAAAAACTATGT	AACGGAAGATCATTAGACCCC	245
Ad01A17256	Aradu.A01	17256	p2	(TA)7	14	94636072	94636085	TTACCCCTACGTTGCCAAAC	GCCATTGCGATTGAGAATTT	103
Ad01A17258	Aradu.A01	17258	p2	(TC)9	18	94638556	94638573	GGAATCCATGGCCATAAAAA	GCAGTGATGGTGATTGTTGG	212
Ad01A17261	Aradu.A01	17261	p2	(CT)13	26	94640011	94640036	CATCAGAGGCTCCACTCTCC	TGCTCCACTTGGGAAAAATC	178
Ad01A17262	Aradu.A01	17262	p2	(CT)8	16	94642862	94642877	ATTCCACGCATGGGTTATTC	TAATGCAGGACTTCACCGCT	231
Ad01A17263	Aradu.A01	17263	p3	(GTG)6	18	94651361	94651378	AAGGTGGTGATGACGGAGAC	TACCATAGTGGCCCCCATAA	135
Ad01A17267	Aradu.A01	17267	p3	(AAG)6	18	94656497	94656514	TCACTGGAGCTGTTGAAACG	GAAGAGGACGAGGAGGCTCT	215
Ad01A17268	Aradu.A01	17268	p3	(AAG)5	15	94658396	94658410	GCAAGCCCTGCTAAGATGAC	GGTCTTGCGATTCTTTCTGC	203
Ad01A17269	Aradu.A01	17269	p3	(GGA)5	15	94661002	94661016	CAGCGACATTAAGCTGAACAA	CTTCCTCTTCCGCCTTCTTT	153
Ad01A17271	Aradu.A01	17271	p3	(TTA)5	15	94664795	94664809	AATCTCGGTCAATCTCCAATG	CGAGGGCAAAACCTCTTAAA	263
Ad01A17273	Aradu.A01	17273	p3	(AAG)5	15	94665720	94665734	TGCAAAATCCACTGTCTTTCA	TGTTTTGTTGCTGCTTCTGC	129
Ad01A17279	Aradu.A01	17279	p2	(TA)10	20	94706395	94706414	TTACCCCTACGTTGCCAAAC	GCCATTGCGATTGAGAATTT	109
Ad01A17282	Aradu.A01	17282	p2	(TC)8	16	94708890	94708905	GGAATCCATGGCCATAAAAA	ATGGCGATGGTAGTGATGGT	243
Ad01A17285	Aradu.A01	17285	p2	(CT)9	18	94710336	94710353	CATCAGAGGCTCCACTCTCC	TGCTCCACTTGGGAAAAATC	170
Ad01A17286	Aradu.A01	17286	c	(CT)9ttcttcctag	51	94713183	94713233	ATTCCACGCATGGGTTATTC	GCTGCCAAGGTCAGAAGAAA	217
Ad01A17288	Aradu.A01	17288	p3	(GTG)5	15	94721659	94721673	AAGGTGGTGATGACGGAGAC	CACCACCGCAACCATATACA	255
Ad01A17294	Aradu.A01	17294	c	(GTTGGT)17(	213	94738775	94738987	AAGTTGCTGTTGTTTGGTTTTCT	CCCTCATCACTACCAACACAAT	280
Ad01A17295	Aradu.A01	17295	p2	(AT)13	26	94744118	94744143	TTCTTGGTCACAGAATTATTAAAAGA	AGTCGCAGGTCAAAGGAAAA	264
Ad01A17298	Aradu.A01	17298	p2	(CA)6	12	94774510	94774521	TCGAGGCTTTCATCTTGGTAA	TCTCTCTCTCTCTCTCTCTCTCTCTC	273
Ad01A17299	Aradu.A01	17299	c	(AT)11(AG)13	48	94774700	94774747	CTGTCTCATCACACACACACAC	AAAGTTTGTACTAAAGTGTCCAAATG	280
Ad01A17302	Aradu.A01	17302	p2	(AT)10	20	94784869	94784888	AGAAAATTTTACTTTAAAACAGTTGGA	AAGGAAGCACGTCACCAAAT	247
Ad01A17303	Aradu.A01	17303	p3	(TTA)16	48	94785106	94785153	ATTTGGTGACGTGCTTCCTT	ATGGCAATAATTTGGCTTGC	133
Ad01A17308	Aradu.A01	17308	p3	(CTT)6	18	94790488	94790505	GCAGTGGCGGTAATTGTTTT	CGGAAGCAAAGGAATTGAAG	266
Ad01A17311	Aradu.A01	17311	p3	(TTG)7	21	94798005	94798025	GGCCATCTCTATGGTGAGGA	TAGCAGGGTCACCTTCCAAT	183
Ad01A17314	Aradu.A01	17314	p3	(TGT)7	21	94800378	94800398	GTTATGCTTGGTGCTTGCCT	GAGGAGGATGATGGTGCTGT	277
Ad01A17315	Aradu.A01	17315	p5	(TGTAG)5	25	94801756	94801780	ACCACCAATCCACCATTGAT	TGGAACTGGAAATTGGAGGA	145
Ad01A17316	Aradu.A01	17316	p2	(TA)7	14	94804392	94804405	AGCAGCAGCAAAGACACAGA	TGGACGACACCTATCCTTCC	255
Ad01A17317	Aradu.A01	17317	p2	(CT)6	12	94804878	94804889	AAGTTGCCTTTGAAGGGGAT	CCAGACATTGAGAACAAAAACG	220
Ad01A17331	Aradu.A01	17331	p3	(TAT)5	15	94837148	94837162	AAAAAGGAGGGCTGACAAAT	CCCACATTGCAAGGGAATAC	210
Ad01A17334	Aradu.A01	17334	c	(AGA)11aagga	114	94840812	94840925	CCATGCAAGCAAATAGGTCA	AGTTGTGCAGCAGACACGAT	280
Ad01A17337	Aradu.A01	17337	c	(TAT)12(TAG	66	94844406	94844471	TGGCAGTGGTTTTCTCAACA	TTCGAATTGAGTCTCCAGGTT	270

ID	Chromosome	SSR nr.	SSR type	SSR	Size	Start	End	FORWARD PRIMER (5'-3')	REVERSE PRIMER (5'-3')	Product (bp)
Ad01A17338	Aradu.A01	17338	p2	(TA)6	12	94846645	94846656	CCGAACCAAACCAATCAAAT	TTGAAAGAAGCTAAAACCAAAACA	250
Ad01A17340	Aradu.A01	17340	p2	(TC)14	28	94848102	94848129	TTCCCCTTTTTCACACTTGC	ATTTGAACTCCCTGCCATTG	265
Ad01A17341	Aradu.A01	17341	p3	(TTA)13	39	94849037	94849075	TCGTGTTCTAGCGTTGAGGA	TGCAGTTCCTGACTCATTGAAA	275
Ad01A17343	Aradu.A01	17343	p2	(CT)9	18	94855798	94855815	TCATCCTCATCATCACCGAA	TACGTGAGCTTCGAACATGG	175
Ad01A17344	Aradu.A01	17344	p3	(TAT)5	15	94856731	94856745	TTATGATGCGTTTGGTTGGA	GCGACAAAGGTCACCTCAAT	266
Ad01A17345	Aradu.A01	17345	p2	(AC)14	28	94858938	94858965	TTCAAGTTCGGGCTAAGATCA	CCAGAAAGCATTGACAAAAGC	275
Ad01A17347	Aradu.A01	17347	p3	(TTG)7	21	94862778	94862798	GGTAGATAGGGGGAGGAACG	GCTATGCAAAGGGGAGCTAA	202
Ad01A17348	Aradu.A01	17348	p2	(GC)7	14	94874312	94874325	GCACACCCAAAGGATGTTCT	TTTCAATTCCCAACTCACTCG	245
Ad01A17349	Aradu.A01	17349	p3	(TTA)6	18	94879510	94879527	ATGGAGCTACCGCACTCAAG	CAGCTAAACACCAGAGTCCCA	230
Ad01A17350	Aradu.A01	17350	p2	(GT)9	18	94884890	94884907	CACTGGACCTATCCGAATCA	CCCAAAGACCTTCAATCTGC	260
Ad01A17351	Aradu.A01	17351	p2	(GT)6	12	94893839	94893850	CTGGAGCTTGATGATCTCCG	TCTTAAGGTTTTTGGTGCGG	267
Ad01A17354	Aradu.A01	17354	p3	(GAA)5	15	94897794	94897808	AGAGTGTTTCTGCTCCTGCG	TTGGTTTTCAATTTTTGGCG	100
Ad01A17355	Aradu.A01	17355	p3	(TAA)5	15	94898026	94898040	CGCCAAAAATTGAAAACCAA	TTGAACTCTGCCTCATGCAC	264
Ad01A17357	Aradu.A01	17357	p3	(CTT)5	15	94901839	94901853	CTCAACCAAGTCACAATGCG	TGACGGCAATATTGAAAGAGG	108
Ad01A17360	Aradu.A01	17360	p2	(AG)8	16	94910313	94910328	AACGGAAATTTCATCTTGCG	GCCTTCAAATTGGGTCTGAA	225
Ad01A17362	Aradu.A01	17362	p2	(AG)8	16	94918138	94918153	CTCACCCTACCTCAACACCC	CTCAACACAGCCAAATCCCT	276
Ad01A17365	Aradu.A01	17365	c	(TA)8aatatatag	46	94934187	94934232	GTCCTCCGTCCAAAAATGAA	CCCAAGGATTTTAAAAACGGA	272
Ad01A17368	Aradu.A01	17368	p2	(AT)6	12	94936037	94936048	TCACAAGCATGTGTACTAAACCC	TAAGCATGGTTTCCTCTGGG	162
Ad01A17370	Aradu.A01	17370	p3	(AAT)5	15	94940317	94940331	TTAGGTATGTGTATCATTTTCATTCAT	CCTATCTCAGCTCTCTGTGCTTT	272
Ad01A17373	Aradu.A01	17373	p3	(TAA)13	39	94944548	94944586	TCCGTAACAGCAGGACAACA	TTTGATTCGTGGTTCCAACA	206
Ad01A17374	Aradu.A01	17374	p4	(AAAG)5	20	94953540	94953559	CCAAAGTTTCAAAAGCCCAC	CCAAGGTTGGAAGGTGAAGA	218
Ad01A17375	Aradu.A01	17375	c	(GA)6gggggag	108	94957175	94957282	CTCTGTTTGTCACTGCTGGC	ACCGGTGAACCGATAAAACC	267
Ad01A17376	Aradu.A01	17376	p2	(TA)8	16	94957480	94957495	TATAGCTCTGGGTTGCGTCA	CAGCGAAAAGAGAAACCAGAA	150
Ad01A17377	Aradu.A01	17377	p2	(TC)6	12	94959539	94959550	CTTCTCCTTCTTCGGTCGTG	GTGCAACGAATCAAAGAGCA	251
Ad01A17380	Aradu.A01	17380	p2	(TA)6	12	94962245	94962256	TTTTGCGCAAGACATTTGTAA	CACCTCAAGCCTCTCTCGTC	261
Ad01A17383	Aradu.A01	17383	p3	(CTC)6	18	94967299	94967316	CAGAGATGGCGAAGAGGAAC	CCACCTTACCTCACCCTCAA	268
Ad01A17385	Aradu.A01	17385	c	(T)10ctaattggt	66	94975465	94975530	CGGAAGCATTTTGTGTTGGT	TTTTTAACCACTGTTTTGTGTTTTG	251
Ad01A17387	Aradu.A01	17387	p2	(AT)6	12	94983965	94983976	CAGCTATTGCATTTTGTTGTCA	GCTGTAGCTTTGTCTGGGCT	276
Ad01A17390	Aradu.A01	17390	p3	(TAT)5	15	94991152	94991166	TCTCAAATGTTGAGGGATACACC	AGTGAGCCCCCACTACAATTT	266
Ad01A17392	Aradu.A01	17392	c	(CAC)5ctctctt	51	94995295	94995345	TGTACGTTCTTTTTCACCCCTT	CGTCATCCATATGGCAAAAA	251
Ad01A17393	Aradu.A01	17393	p3	(AAG)5	15	94996705	94996719	TTCATCTTCTTTTTCTTCATCATCA	TGTTTTCTCCTCATCGTCGTT	245
Ad01A17396	Aradu.A01	17396	c	(T)10ctccatgat	52	95004768	95004819	TGTGAATGTGCATAATTGAGGA	TCAGGCTAATAGTACCAGGATCA	168
Ad01A17399	Aradu.A01	17399	c	(TGGTGT)5tg	78	95007265	95007342	GTTGGTGTTGGTTGGTGTTG	TGGTCTTGGTCCCAAAAGTT	272
Ad01A17400	Aradu.A01	17400	p2	(GA)8	16	95008876	95008891	TGACGGCAGGATAACATTGA	TTCTCGGTTCCAAATTCTCG	230

ID	Chromosome	SSR nr.	SSR type	SSR	Size	Start	End	FORWARD PRIMER (5'-3')	REVERSE PRIMER (5'-3')	Product (bp)
Ad01A17404	Aradu.A01	17404	p3	(TAA)5	15	95040496	95040510	TTGTCCCACCTATAAAATAAATGGT	CCCCCGAGATTTTTCCTTTA	154
Ad01A17406	Aradu.A01	17406	p3	(TCT)5	15	95045093	95045107	GCAAAATATTTAATCAAGTCCAACC	GGAAGAGGAGGAGAAATGAGG	128
Ad01A17407	Aradu.A01	17407	p3	(ATC)5	15	95045293	95045307	TCCTTTTTCTGTTTCTCTTCTTCC	TGCCTTCAATGACACCAGAA	274
Ad01A17408	Aradu.A01	17408	p4	(AAAG)5	20	95045661	95045680	TTGTTTTTGTACTTGTTTTCGAACT	ATCAGCAAAATAACACCGAAA	255
Ad01A17410	Aradu.A01	17410	p3	(GAA)5	15	95049185	95049199	CCAGTTATCCATATGAGCAGCA	GGGAACTTCGCTTCTGAGTG	217
Ad01A17412	Aradu.A01	17412	p3	(TAA)10	30	95052127	95052156	TGAACACTGGAGTGTGGAGTTC	GCATGGGAGACGACAAAATAA	268
Ad01A17413	Aradu.A01	17413	p3	(ACA)7	21	95063986	95064006	CAACGTTTTGATACGATTTGTCA	TTTGGTGAGTCAGCTGGGTA	272
Ad01A17415	Aradu.A01	17415	p2	(TA)6	12	95067963	95067974	TTCAATCCAAAACATTAATTCGT	GGGTTTTCTCAAACCGTCAA	211
Ad01A17422	Aradu.A01	17422	p2	(TA)6	12	95100770	95100781	CCTTGCTGCTTTACGGGATA	TCCCTTCTTTGTCGTTTTGG	158
Ad01A17425	Aradu.A01	17425	c	(CT)9(AT)6	30	95101808	95101837	CAGCAGATTCCACCCCATTA	TGCTCTTGTGCGTGCTTTAT	275
Ad01A17426	Aradu.A01	17426	p2	(AT)6	12	95102289	95102300	GGCGACATAAGTGTAATGGACA	TGAAACAAAAATGGATCCTCC	206
Ad01A17427	Aradu.A01	17427	c	(A)10c(A)10	21	95103344	95103364	CGGGCCAGAGGATAATAACA	TGATTTCTGAAGGAGTGGCA	206
Ad01A17428	Aradu.A01	17428	p3	(TAT)20	60	95103776	95103835	CCAATGCTGCCTAAGATGCT	TGGCAATTTCAGATTTCTGTTT	278
Ad01A17431	Aradu.A01	17431	p2	(TC)8	16	95105738	95105753	CCGATCCTCCCACATAACAC	GTGAGGTCTTCATCCACCGT	228
Ad01A17432	Aradu.A01	17432	p3	(TAA)5	15	95107367	95107381	GCAAGCAATTTAAAGACGCC	TTATTGGCCATAACCGAAGG	235
Ad01A17433	Aradu.A01	17433	c	(T)11gaaacctc[illegible]	86	95109792	95109877	CCTCGAAAAGATAACGAGGTTTT	ACCCTCACTATCTCACCCCA	278
Ad01A17440	Aradu.A01	17440	p2	(TA)7	14	95122984	95122997	TCAGACAAGCAAATAATAAAGGCA	TTAAACAACGTGTCCCATGC	238
Ad01A17442	Aradu.A01	17442	p3	(AAT)5	15	95126984	95126998	TGATACACACATTAATAGTAACCCATT	TTGTGTCCATACGATATAACTTTACAA	280
Ad01A17443	Aradu.A01	17443	p2	(AT)7	14	95127675	95127688	GCTCTTTCCTAAAAATATTGGCA	GCATCACTTGAAAGAATCATGG	236
Ad01A17444	Aradu.A01	17444	p3	(TAT)6	18	95131628	95131645	TCCTTCCTTTCTTTCTTCTTTTT	AGATAGAGTTAAATTCAAGGATAACGA	232
Ad01A17448	Aradu.A01	17448	p2	(CT)10	20	95136660	95136679	TGAGACAGGACGGACTTTCC	AGCGACGTAGTGACGGACTT	277
Ad01A17449	Aradu.A01	17449	p3	(TTA)6	18	95138817	95138834	GGGCAAATGAGATGACCATT	TGCTTGACCGATTGTCTCA	280
Ad01A17450	Aradu.A01	17450	p3	(TAA)5	15	95139083	95139097	TTGAACAAAAGAAAACACGGG	GACCACATCCTCGTGATTGA	270
Ad01A17453	Aradu.A01	17453	p3	(TTA)6	18	95153719	95153736	CTCCCCTCTTTGTTGGTCAG	GCGGTATAAGGGTTTTGACG	253
Ad01A17454	Aradu.A01	17454	p2	(GA)7	14	95154646	95154659	GAAGCTCAATGGTGGTGGAT	TTCTTTCCTATTCAATTCTTGCTG	207
Ad01A17455	Aradu.A01	17455	p3	(ATT)10	30	95158804	95158833	TTGACAAGTTCATGGGTGGA	TTCGTACCGTATGGGTTTGA	181
Ad01A17456	Aradu.A01	17456	p5	(AATAA)5	25	95159113	95159137	AATTTGGGTGGTTATGGTGG	TTGCGTATGCATGTTATGATGA	234
Ad01A17458	Aradu.A01	17458	c*	(TAA)15tttcaa	115	95161222	95161336	GACAGCCGAGAAGCTAAGGA	CGTGTTGTCCCAATCTTTCC	231
Ad01A17459	Aradu.A01	17459	p2	(TA)7	14	95161821	95161834	AGCAGAAACCAAACAAGCACT	CAATCCATTACGTTGACCCA	278
Ad01A17465	Aradu.A01	17465	p3	(CAT)5	15	95166343	95166357	GGGAACATTGTGCTCCAAAT	CCAAAAGAAGCCTTCACCAA	236
Ad01A17470	Aradu.A01	17470	c	(TC)21cccatca	90	95175372	95175461	TTTCTTAAATTCATCGCCGC	GAAAAGGGACTCCGATGACA	202
Ad01A17471	Aradu.A01	17471	p2	(CT)8	16	95175569	95175584	TGTCATCGGAGTCCCTTTTC	AACAAAATTGCAACACAAATTCA	197
Ad01A17472	Aradu.A01	17472	p3	(CAA)5	15	95179592	95179606	GGAAAGAGAGGTGGGACACA	TTTCTCAAGGGGAAATGGTG	269
Ad01A17473	Aradu.A01	17473	p2	(TC)6	12	95181032	95181043	CTCTTCTCGATTCCAAACCC	TGAATTGTGTTGAATTATATTGAAGTG	235

ID	Chromosome	SSR nr.	SSR type	SSR	Size	Start	End	FORWARD PRIMER (5'-3')	REVERSE PRIMER (5'-3')	Product (bp)
Ad01A17474	Aradu.A01	17474	p3	(TAT)6	18	95181403	95181420	TTTAATGTGATTTGCGAGAAAAA	TCACGGTTAAAGTTGAGGAAGA	180
Ad01A17475	Aradu.A01	17475	p2	(AT)6	12	95183383	95183394	CCACACATGTCCTTTGAGGTT	AAAAACAAATTATAGGTTCAGTTTGGA	254
Ad01A17476	Aradu.A01	17476	p2	(TC)6	12	95184331	95184342	GCCCATTGGTCTCACACTTT	TGAGAATGGTGATTGAGATGTG	174
Ad01A17477	Aradu.A01	17477	p3	(ATT)5	15	95187945	95187959	AACAGGGGCGAAGCTACATA	CTTCCTCCCACCTGCATTTA	246
Ad01A17486	Aradu.A01	17486	c	(TCA)7tctgaat	94	95208652	95208745	AACCCTATCGCTTGGAAGGT	CTGTAGGAACCGCTGAGGAG	240
Ad01A17487	Aradu.A01	17487	c	(A)11ttttctctatt	63	95213103	95213165	ACGTCCAGTGGGACAAGTTC	TTTCTTCTCGGATCATTGTGG	231
Ad01A17489	Aradu.A01	17489	p2	(GT)6	12	95218963	95218974	GGATTCTGTTCTCCCCCTTC	GTTGGGCCAAACGAGTTAAA	144
Ad01A17490	Aradu.A01	17490	p3	(TTG)8	24	95228860	95228883	CGTTTGATTCATGTAGCCGA	TGCCGCCAAAACCTATTTAC	268
Ad01A17493	Aradu.A01	17493	p3	(TTA)5	15	95235842	95235856	TGTTGTTAGAAAATGAATTTAGGCAT	CCCTTCAAATTGTTCATAACTTCC	200
Ad01A17496	Aradu.A01	17496	p3	(AAT)5	15	95276067	95276081	CCAATCAATGAAAGGTGGCT	GCAATGTTTTAGCTGCAGGG	271
Ad01A17497	Aradu.A01	17497	p3	(AAT)5	15	95276541	95276555	TCAATCAATGAAAGGTGGCTT	CAATCTTTTAGCTGCAGGGC	270
Ad01A17498	Aradu.A01	17498	c	(A)10gaaaatata	92	95278050	95278141	CAAAATGGAAGAAAAAGCGG	AAAAAGAAAACATTTTGATGGGTC	184
Ad01A17503	Aradu.A01	17503	p2	(AT)6	12	95295910	95295921	GCGACAAAGAGACACACACAA	CCCCCTCTCTCGTATTTAAGG	272
Ad01A17504	Aradu.A01	17504	c	(A)10ccacaaag	72	95296713	95296784	CCACAAAGGAGAGACCAAAAA	GCTCTCCCTGTGCATGTGTA	166
Ad01A17505	Aradu.A01	17505	p2	(TA)8	16	95297350	95297365	AATACCATCACGTGTCAGCG	TTTTATATGACATACGGACACACA	276
Ad01A17508	Aradu.A01	17508	p2	(TC)8	16	95304810	95304825	CACCTCAGTCACGACACGAT	GCCGTCGCATTATGAATGTA	241
Ad01A17510	Aradu.A01	17510	p4	(AAAT)6	24	95306705	95306728	TTGACCTAACCCGAAGTCTGTT	CCGCGGATTAAGAACGAATA	237
Ad01A17511	Aradu.A01	17511	p3	(TTA)18	54	95307588	95307641	TTCTGCTAAAGCATTCCATTTG	GCGGGCAGAACAATAACAAT	259
Ad01A17515	Aradu.A01	17515	c	(GTG)6gttgtgg	73	95312212	95312284	TGCTGTTTCTTGGTTGGTTG	ACCCTCACCACTACCAGCAC	128
Ad01A17519	Aradu.A01	17519	p3	(TCC)6	18	95356449	95356466	CATCATGCCTTGGTTAGAATG	TTCCTGGTTCCTAAACTGCAA	279
Ad01A17522	Aradu.A01	17522	p3	(TTA)5	15	95359072	95359086	CATGCGCGTCCATAGTACAC	TCCACCTCACACATACTCGTG	271
Ad01A17525	Aradu.A01	17525	p4	(ATTT)5	20	95364148	95364167	TCGGCTCTGTGAACTATCATTG	TTTGCTGACCACACGAATAAA	280
Ad01A17526	Aradu.A01	17526	p3	(GAA)5	15	95372160	95372174	CTTTCTTGGTGGGGACAAAA	CTCAAGCCTTAGCCGTTCAC	229
Ad01A17527	Aradu.A01	17527	p2	(TC)7	14	95372732	95372745	GTCAAGTTCAGGATTGGGGA	CGCAACAGAAACAGAAATGG	220
Ad01A17529	Aradu.A01	17529	p3	(TTA)17	51	95375693	95375743	CATGTCATCAAGCGACACAC	GCCACCAATTTTGTCATGTG	151
Ad01A17530	Aradu.A01	17530	p3	(AAT)5	15	95376180	95376194	ACACAATGAAATCACGGGAA	AATCCCAGCCAAACCTTCTT	253
Ad01A17532	Aradu.A01	17532	c	(AAT)14tatgtta	117	95382583	95382699	ATTGCAGCCACATCATTGAA	CAACTGTCGGCAGGTTCTCT	279
Ad01A17535	Aradu.A01	17535	p3	(TAA)6	18	95386102	95386119	TTTTCCAGGGTTAAAATGATCTG	TTTATTCGGAAGCCAAGAGG	250
Ad01A17536	Aradu.A01	17536	p3	(TAA)6	18	95389660	95389677	TAGTGGCCCCATTTGTGATT	TGTGCACTTACCCTGCTGAC	240
Ad01A17537	Aradu.A01	17537	c	(TA)11(GA)11	44	95393726	95393769	CAAGGCTCGAAAGTGTCCTC	TACGGCGGCTAAGAAAGAGA	280
Ad01A17539	Aradu.A01	17539	c	(TC)7taactttctc	117	95397428	95397544	CCGTTGCACACAGCTTCTAA	GTCATGGGAGAATCGGAGAA	262
Ad01A17541	Aradu.A01	17541	c	(GA)9aa(G)11	31	95401397	95401427	CCCTCAAAAGTTATTTGAACCC	GCAAAGAAAGTCCGATGACC	250
Ad01A17542	Aradu.A01	17542	p3	(CCT)5	15	95411401	95411415	ATGCTCAGGAGGCTTACCAA	CCGCCATCAAAAGAAGAAGA	235
Ad01A17543	Aradu.A01	17543	p3	(CAC)5	15	95422116	95422130	ACACCCCACTCCTTCCTCAT	CGATTTTCCTGCTCTGTTCC	272

ID	Chromosome	SSR nr.	SSR type	SSR	Size	Start	End	FORWARD PRIMER (5'-3')	REVERSE PRIMER (5'-3')	Product (bp)
Ad01A17545	Aradu.A01	17545	p2	(TA)9	18	95426681	95426698	ACGGTTATCCTTTGATGCGA	CATCACGGATGGATGCTCTA	228
Ad01A17546	Aradu.A01	17546	p2	(TA)6	12	95439702	95439713	TGGGAAGAACAAAAGTGGAGA	GGTGGTACACAATGGCACAC	275
Ad01A17548	Aradu.A01	17548	p3	(AAT)5	15	95440806	95440820	ATTAAAGCCCCCTGATCCAT	CAATCAACAAGAACGAGCGA	257
Ad01A17549	Aradu.A01	17549	p4	(AAAT)5	20	95441334	95441353	GTGTGCTGCCCTTCATAGGT	AGACGACGGTTACAGATGGC	266
Ad01A17550	Aradu.A01	17550	p3	(CTG)5	15	95441897	95441911	TGTTCAACACCTCCGAACAG	TGACATGATGCCACTTCCAT	233
Ad01A17552	Aradu.A01	17552	p2	(AT)13	26	95445011	95445036	TGTTCATTTCCACCACCAAC	ATACCTTGTGGGGTGCCATA	178
Ad01A17553	Aradu.A01	17553	p2	(AT)8	16	95445533	95445548	CAGTGTAAACGATATACGTGCATTC	TGTGCCATCTCACGTTCAAT	244
Ad01A17555	Aradu.A01	17555	p3	(CAC)8	24	95452812	95452835	GAAGCTTTGGAGGTGCTGAC	TGTGAAAGCATTGCCGTAAA	130
Ad01A17556	Aradu.A01	17556	p2	(TA)7	14	95453811	95453824	CCCACCTAGCCAGTGGAGTA	GGATGAACAAGGGAATTGGA	278
Ad01A17560	Aradu.A01	17560	p2	(TA)6	12	95456641	95456652	CGACTACACCTGAATTTTCACC	ACCCATACCATACACCATGAA	247
Ad01A17561	Aradu.A01	17561	p4	(AAAT)5	20	95457805	95457824	TCTTTTGTTTTTATTTTCTTCTTCACA	CAATTGCATTCGGAACCTCT	201
Ad01A17562	Aradu.A01	17562	p3	(TCA)7	21	95458440	95458460	CCAAACCCATTTGCATTTTT	GGGGACCTAATTTTTACTGGAA	264
Ad01A17571	Aradu.A01	17571	p3	(ATC)5	15	95481161	95481175	TTATGTAAGGGAGCATCGGC	TGTGAATCTAGCCCAGGTGA	205
Ad01A17578	Aradu.A01	17578	p3	(TAA)20	60	95496051	95496110	TAAGATGCATGGCAAGACCA	TGTTTCAGCTGCGCTCTTTA	223
Ad01A17581	Aradu.A01	17581	p3	(AAT)5	15	95514052	95514066	ACAAGTGAAAACTGCGCATA	TCGGTTGGTTTCGTCTTTTC	212
Ad01A17583	Aradu.A01	17583	p3	(TTC)12	36	95519317	95519352	GTCCAACGCTTTTTCTTCCA	AAAAGGAGAAGGAGGAACGC	125
Ad01A17584	Aradu.A01	17584	c	(AAT)5tgaaaa	63	95525886	95525948	TTGCTTGCAAGAAGAGACAGA	ATTACATACCGGCGCTTGAG	275
Ad01A17585	Aradu.A01	17585	p3	(TTC)5	15	95526526	95526540	TAAATTTGTTGCTGCGCTTG	CAACAACGGTGGTGTCAAAC	139
Ad01A17588	Aradu.A01	17588	c	(A)10gaagtcac	30	95536759	95536788	TGATCACCACGTCGGTATTG	TGGTACAAGGAAATTCGGGA	277
Ad01A17589	Aradu.A01	17589	p5	(GATTG)5	25	95537080	95537104	CACAATTCCAATAAGCGCAA	AATTTTGCGGGGTCTAGCTC	160
Ad01A17591	Aradu.A01	17591	c	(AT)8gaaataaa	82	95539595	95539676	TTCTGGGTCAAAACTACTAGATTACG	GCAGCGTTTGTTTCCAACTT	277
Ad01A17594	Aradu.A01	17594	p3	(TAA)10	30	95569240	95569269	TGTTAAGTTTTATTTTTATGCATCTGA	TCCTTACCATTTCCTTCCAAGA	279
Ad01A17595	Aradu.A01	17595	c*	(A)12(AAAG)	33	95580219	95580251	TGCCAATGATAGCACAAAGC	TGGTTTGCAAATTGAATGGA	214
Ad01A17598	Aradu.A01	17598	p2	(AT)10	20	95591865	95591884	TCTCAACCATGGCATAGACG	AGCCCGAACCTTAACTGCTT	209
Ad01A17600	Aradu.A01	17600	p2	(GA)8	16	95595151	95595166	TCAAACAGAGAGCGCGTAGA	CCCCATCTTTCCTTTCCATT	269
Ad01A17605	Aradu.A01	17605	p2	(TC)6	12	95608371	95608382	AGCACCGTTCACCCAATAAC	GTTGATGGCTACCGACGACT	176
Ad01A17608	Aradu.A01	17608	p3	(TGG)5	15	95613870	95613884	CCCACCTTCAACCTCAACTC	GGTGACAAGAATCCCCTCCT	198
Ad01A17610	Aradu.A01	17610	p3	(AAT)6	18	95622042	95622059	GGGAAACCTTAGCTTCAGGA	TGTACCGATTTGGAATGATATTAAA	278
Ad01A17612	Aradu.A01	17612	p3	(CTC)9	27	95629297	95629323	TCTTTTGCAGACATGGCAAG	AATCACAATGGGCTGAGAGG	203
Ad01A17615	Aradu.A01	17615	p3	(ACC)6	18	95641600	95641617	ATGTCCACCTCCAAAACCAC	CAGAATGCCGAATGTTCAAA	218
Ad01A17616	Aradu.A01	17616	p2	(AT)10	20	95646240	95646259	CGGTTGAGTCAATTTTTCCC	TTTTGGACAAGCAAATGAGC	253
Ad01A17617	Aradu.A01	17617	p2	(AT)9	18	95646960	95646977	GCAATAGCCACTACTAGCCTTGA	AAAGTTTTGCCAAGTAAGCGA	237
Ad01A17619	Aradu.A01	17619	p3	(AAT)5	15	95650787	95650801	ACAGATGAACCTACCGTGCC	TGGACTGTAAAGGTGTGCCA	272
Ad01A17620	Aradu.A01	17620	p2	(AT)13	26	95651662	95651687	ATACCTTGACCGCAACATCC	GCAGGTTCCTGTGGAACTGTA	277

ID	Chromosome	SSR nr.	SSR type	SSR	Size	Start	End	FORWARD PRIMER (5'-3')	REVERSE PRIMER (5'-3')	Product (bp)
Ad01A17621	Aradu.A01	17621	p3	(CTT)5	15	95653948	95653962	TCTAGAACGCTGGGGAGAGA	GGTGGGAGGCACATCTAAAA	245
Ad01A17622	Aradu.A01	17622	p2	(CT)8	16	95654224	95654239	TTTTAGATGTGCCTCCCACC	GTCTGGCTTTATTGACCGGA	272
Ad01A17625	Aradu.A01	17625	p2	(AG)6	12	95671549	95671560	ATGACAGGGAGGAGGGAGAT	CCACGTGACACACTCAATCC	182
Ad01A17628	Aradu.A01	17628	p3	(TTG)5	15	95676045	95676059	ATTTGTAGTCCGCCAACACC	CAGAAGCAACCAAAGCATGA	212
Ad01A17633	Aradu.A01	17633	p2	(AT)8	16	95693892	95693907	AAATGGATGGAAAAGGGGAC	TGTTTGAATTGGAGGCAAGA	137
Ad01A17636	Aradu.A01	17636	p2	(TA)7	14	95695657	95695670	GACTCGAACCTGCAACCTCT	AATTAACGAAAATTGGGTTAGAAA	151
Ad01A17637	Aradu.A01	17637	p2	(TA)7	14	95696001	95696014	TTGCCAAATAAATTGGTATAAAATGA	CGTTGAGTATTCATTTGTGTGGA	145
Ad01A17639	Aradu.A01	17639	p2	(GA)6	12	95709191	95709202	GGTAGCTTGCTAATGCTTCCA	CACCCATCTCCCACTAAGGA	266
Ad01A17640	Aradu.A01	17640	p2	(GA)9	18	95715085	95715102	TGCTTCCATGTTTCCTGATG	ATGTTTTCACGGACACCCAT	272
Ad01A17641	Aradu.A01	17641	c	(A)11taatcatttt	111	95717349	95717459	CTTCCATATTGGTCGCGAGT	TGAATTCAATGTATTTGGAGGTT	204
Ad01A17645	Aradu.A01	17645	p2	(AT)7	14	95725684	95725697	AACTGTGCAGAGCCAAAGGT	AAATTAAAATTATGCCGCCG	213
Ad01A17647	Aradu.A01	17647	c	(TTC)6(TTA)7	39	95727772	95727810	ACCGTTTCACGAAATTGTCT	GCAAAATCTCGATTCATAAACTCA	256
Ad01A17648	Aradu.A01	17648	c*	(A)10(AC)10*	29	95729645	95729673	AACGCTAATGCAGAAGCTCAA	GCTGGGGCTGAGAGATTTTA	275
Ad01A17649	Aradu.A01	17649	p2	(AG)8	16	95732832	95732847	CTAAGCATGCAGCGATTTGA	GGTTCCTTCCATCGATGTTG	210
Ad01A17652	Aradu.A01	17652	p2	(TC)27	54	95739801	95739854	GGTTGTACTTGTCCGCCATT	AACACCAATCATTTCCCCAA	161
Ad01A17660	Aradu.A01	17660	p3	(TCT)6	18	95753092	95753109	GCGGACTCTGGCACTTTTAG	CAGTGAACGGGTCTCCAAAT	218
Ad01A17661	Aradu.A01	17661	p2	(TC)6	12	95753333	95753344	ATTTGGAGACCCGTTCACTG	GAAATAAGCCTCACGGGACA	230
Ad01A17664	Aradu.A01	17664	p5	(GCCCC)5	25	95756770	95756794	TTGGATAAGATATGTTTGTGTGTTTG	TAGTTTGGCATGGTCTCCCT	164
Ad01A17665	Aradu.A01	17665	p3	(ATT)5	15	95759419	95759433	CTGCAGAAGGGGGTTAACAA	GGATATGGAAGCTTTTGCGA	259
Ad01A17667	Aradu.A01	17667	p3	(TCC)5	15	95761464	95761478	TCCTCCTCATCACTGCATCA	TAATGTGGTGGATCTGCGAA	168
Ad01A17673	Aradu.A01	17673	p2	(TC)14	28	95783971	95783998	GAAAAGTGGTTAATCCGCCA	TGTGATCTGGAAACTGCTGC	258
Ad01A17674	Aradu.A01	17674	p3	(TTG)7	21	95787582	95787602	AATGATGCTTGCTCATGCTG	AAGCAGCCGGAACACTTAAA	246
Ad01A17675	Aradu.A01	17675	p3	(GAA)5	15	95791089	95791103	GGTGCTTCTTTGGCTTCTTG	ACATCCCATTTGCTTCGTTC	256
Ad01A17677	Aradu.A01	17677	p3	(TTA)5	15	95796631	95796645	CCTTGCTTCACATGCTCAAA	TTCGATCGCAGAGAAAACAA	121
Ad01A17681	Aradu.A01	17681	p3	(CTC)8	24	95811275	95811298	TCTTGCCGACTTGTGACTTG	CTCGGAGAGTAGTTGGGCAG	245
Ad01A17685	Aradu.A01	17685	p4	(AAAT)6	24	95832607	95832630	TCGACCTTTGTGATTGTGTGA	AGAGGCTTATGCCTTTGATGA	277
Ad01A17687	Aradu.A01	17687	p2	(CA)9	18	95839633	95839650	CGCCGCCGATAATATAATAAA	CTGCTCTCTCCAGGAACCAC	263
Ad01A17688	Aradu.A01	17688	p3	(TAA)6	18	95842660	95842677	TCCTTGCTTTGTGTTTTCAAGA	CTGTGGGGTAGGGACAAGAG	270
Ad01A17689	Aradu.A01	17689	p3	(TCT)5	15	95843935	95843949	ATATTCACGCCGTTTTCACG	ACACGCGCGTTACTGAATC	162
Ad01A17691	Aradu.A01	17691	p3	(AAT)6	18	95848180	95848197	CATGACGCCAGAATATAAGCAA	AAAAGCAAATTCATAATTGGAGA	279
Ad01A17692	Aradu.A01	17692	p3	(TAA)13	39	95850716	95850754	TGAGATAAGGGGCCTCAAAA	CCAGCATGTAACCAGCAGAA	264
Ad01A17694	Aradu.A01	17694	p2	(TA)6	12	95852975	95852986	CCCTCCGAGTTGTTTGTTGT	GACGACCGTGAGAAAGAAGC	233
Ad01A17695	Aradu.A01	17695	p2	(AG)7	14	95853576	95853589	TGCTCGAAGACAATGACGAC	CTTCGGTGACGGTGATAAGG	250
Ad01A17698	Aradu.A01	17698	p2	(TA)9	18	95865836	95865853	GCTCACGCGCAGATATTAAA	TTAGCTACGCCTGGGAAGAA	240

ID	Chromosome	SSR nr.	SSR type	SSR	Size	Start	End	FORWARD PRIMER (5'-3')	REVERSE PRIMER (5'-3')	Product (bp)
Ad01A17700	Aradu.A01	17700	c	(ATC)6attactat	142	95882069	95882210	CACCACCACCATCTCCTTCT	AACGACGATGAGGAGAAAACA	265
Ad01A17701	Aradu.A01	17701	p3	(AGG)6	18	95882417	95882434	CACGTGTTTTCTCCTCATCG	ATAATGACGACGCACAAGCA	233
Ad01A17703	Aradu.A01	17703	p2	(GC)6	12	95900016	95900027	GGTGGCACAACTCTCTGGAC	ACACACTGCGAGCACAAGAC	216
Ad01A17704	Aradu.A01	17704	p2	(GC)6	12	95900150	95900161	GAGGAGTGCAGGATCAAAGG	GCTCTCAGGCTGCTACCAAG	140
Ad01A17711	Aradu.A01	17711	c	(ACC)6tccttctc	210	95927721	95927930	CCTTCTCCTTCTTCGTCATTTT	GGAGAAAACACGTGATAACGATAATA	280
Ad01A17713	Aradu.A01	17713	p2	(TG)6	12	95931455	95931466	TTCCTGTAGAGCCTGAGGGA	TCCCTGTTCACACCAAACAA	192
Ad01A17715	Aradu.A01	17715	p4	(TTAT)5	20	95940329	95940348	TCATTTAGATGTCGTGGATTCG	TATGATCGGATTGCGGATTT	252
Ad01A17719	Aradu.A01	17719	p3	(CAT)6	18	95956105	95956122	GCAACGTCCCTGTTTCTCTC	GCAAACACGCTGTGTCAGAT	231
Ad01A17720	Aradu.A01	17720	p3	(CAT)6	18	95956710	95956727	GATTGAAGCATCCAGTGCAA	GCAAACACGCTGTGTCAGAT	141
Ad01A17723	Aradu.A01	17723	p3	(TGA)5	15	95962435	95962449	TCTATGAAGTTTCGGGACGC	TTGGATCATGTCAAGCCTCA	267
Ad01A17724	Aradu.A01	17724	p3	(TCT)6	18	95963725	95963742	ACCTTTTGTTCGAAACGACG	GGAATCCAAGTCGATCCTCA	241
Ad01A17725	Aradu.A01	17725	p3	(TAT)6	18	95965399	95965416	GCAGATTTAGCGGATGGAAG	AGCAAAAGGTGCTTTGCACT	238
Ad01A17727	Aradu.A01	17727	c	(TAG)5taagtag	95	95970957	95971051	GTTGATCTTGTGGTGGCAAA	TGATAGAGTTTGTTCCAAGCAATG	151
Ad01A17728	Aradu.A01	17728	p2	(AT)6	12	95971235	95971246	CCCAATCCTCTCATGAAAATG	CATGCAAATGCAATGGAACT	280
Ad01A17732	Aradu.A01	17732	p2	(TA)6	12	95988934	95988945	TTGAACTAGCCATGGTTCCC	GGTTTCTGCACCCCAGATAA	220
Ad01A17733	Aradu.A01	17733	p3	(TGA)5	15	95989996	95990010	GCAAAAGGAGAAGAAAATGCT	TGCCAAATCGAATAAAAACAAA	280
Ad01A17735	Aradu.A01	17735	p3	(CTC)6	18	95996260	95996277	AGGCTAAAGCTAAGTCCCGC	CGCCTTAACCTCACCAGAAG	203
Ad01A17740	Aradu.A01	17740	p3	(GAA)6	18	96016013	96016030	GGTGTTGATCTGACATTGCG	GGGAGAGGTCCAGCACATTA	211
Ad01A17742	Aradu.A01	17742	p2	(CA)6	12	96017847	96017858	GCCGGAACCCATTATACCTT	CACCATTACCACCACCAACA	179
Ad01A17746	Aradu.A01	17746	p3	(TTA)6	18	96052980	96052997	TTGGCTATTTGATCAATGATGG	GCGAATTTGCACTATCCACA	185
Ad01A17747	Aradu.A01	17747	p3	(AAT)6	18	96057307	96057324	TTTTCCCCTCCCTCTCTTTT	ATGATGGATAGAACGGCGAC	188
Ad01A17749	Aradu.A01	17749	p3	(TGT)5	15	96062214	96062228	CCATATTTGTTGGGATTCCG	CGGACTTGGTAGGTCAGCTC	173
Ad01A17751	Aradu.A01	17751	p3	(TAA)5	15	96065398	96065412	GGTTCAATCACAGACAAAATGG	CAGGTGTCATCGACTTCACG	272
Ad01A17753	Aradu.A01	17753	p2	(CT)7	14	96067710	96067723	GCGACTTCAGTTCAATGCAA	AGCAAAGGAGTGAGTCGGAA	118
Ad01A17755	Aradu.A01	17755	c	(A)10g(A)10	21	96070635	96070655	TTCTAACAAGGGTTGCTCCAG	TCCATGCATTTATTTAGAGATCG	209
Ad01A17756	Aradu.A01	17756	c	(A)10taatttagg	75	96071070	96071144	TGTCGATGGTTTAATGAAGCAG	TTGCGGGAGGATGTTACCTA	278
Ad01A17757	Aradu.A01	17757	p2	(TA)7	14	96077270	96077283	TTCCATGCATACAAATTATTGAAA	CAATGAGTCCATTGTCGAACTT	252
Ad01A17762	Aradu.A01	17762	p3	(ATA)6	18	96086690	96086707	CTGTCGGACCGTATAAGTGGA	CGTGGGGTCTCAATCTCATT	256
Ad01A17765	Aradu.A01	17765	p4	(TTTC)9	36	96094072	96094107	ATGATGATTTGAGGCCGAAG	AATAACGGCAAAGGACATCG	216
Ad01A17772	Aradu.A01	17772	c	(A)11ttcacataa	58	96108683	96108740	TCGGGACTATGGCTATCTTTTT	CCATGTCATCTCCTTCCACA	256
Ad01A17773	Aradu.A01	17773	c	(A)10ttttttacaa	40	96112370	96112409	ATATAGTGCATGTTTGGGCG	AGAAATTTGCCAAACACGTTAAA	100
Ad01A17780	Aradu.A01	17780	c	(CCA)6ttatcatc	56	96135974	96136029	CCAAAATCAACCAGAAGCAGA	GTAGTGGTGGTGGTGGGTTC	212
Ad01A17786	Aradu.A01	17786	p2	(TA)10	20	96149995	96150014	CTAACCGACTGCTTTCTGGC	TAGCTACGGCAGCATCACAA	278
Ad01A17789	Aradu.A01	17789	p3	(AGA)5	15	96155582	96155596	ATGTTGTGTTGGTCAAAGCG	TGCTTCTTGTTCTGCTGCTG	147

ID	Chromosome	SSR nr.	SSR type	SSR	Size	Start	End	FORWARD PRIMER (5'-3')	REVERSE PRIMER (5'-3')	Product (bp)
Ad01A17796	Aradu.A01	17796	p5	(TAAAT)5	25	96199450	96199474	CCCAACCATCACATCAAACA	GCCAATATCAAAACTCAGGTCG	276
Ad01A17800	Aradu.A01	17800	p2	(TC)8	16	96204886	96204901	TGTAACTCAAGTCCCCCTCG	GAAGGCGAGTTCTATGCTGC	207
Ad01A17801	Aradu.A01	17801	p3	(ATA)6	18	96209835	96209852	CACCAGTAGAAAAGTGGTCAGG	TCCAAACATGCAGATTTTCG	265
Ad01A17803	Aradu.A01	17803	c	(GAG)6(GAA)	33	96219887	96219919	GCGGGAGACAAGGAAGTAGA	TCAATTTGGTCTCTCGACCC	239
Ad01A17804	Aradu.A01	17804	p2	(TA)6	12	96224983	96224994	AGATATGAATCCGGCAGCAC	CCTTTTGGAGTTAGGGCACA	182
Ad01A17806	Aradu.A01	17806	p2	(AT)7	14	96234590	96234603	GGGGTGAACATTTTGCATTC	TTCTTTATATCCTATGCACTCTTTTG	250
Ad01A17808	Aradu.A01	17808	p3	(AAG)5	15	96241463	96241477	TTTGGTACGATCATGAGGTGTT	TGAATGTAAGTTCATCTTCTTCCAA	199
Ad01A17810	Aradu.A01	17810	c	(T)12aaatagtga	51	96249970	96250020	ACCGCCGCTCTTCTAAAAAT	GGCGCCTCTCTAATGCTTCT	280
Ad01A17811	Aradu.A01	17811	p2	(AT)6	12	96250401	96250412	TTGCATTTTCGCTCAACTTC	AACCGCGACCTCTTTGAGTA	225
Ad01A17812	Aradu.A01	17812	c	(A)10tggttcatc	67	96253008	96253074	AGTAAGGAAGGGGAGGGTGA	ATTTTTAGAAGAGCGGCGGT	236
Ad01A17814	Aradu.A01	17814	p2	(CT)7	14	96261582	96261595	CTCTCTCTTCGAAACCCACG	GTCCCCAACGGTACAGAGAA	279
Ad01A17815	Aradu.A01	17815	p2	(TA)6	12	96263393	96263404	TCAATTCGATTTATTGTGTTTCA	GCCCCCTAAACATTTTCGTT	266
Ad01A17816	Aradu.A01	17816	p2	(TA)6	12	96268978	96268989	CTCCTCAAATATCCACCCGA	TTGCTTACTCGTAGGCTTCTCC	251
Ad01A17819	Aradu.A01	17819	c	(T)10catttaatta	116	96279379	96279494	AGGCAAAATTTAGGCACCAT	GAGGATCAACTGGAAAGAGGG	279
Ad01A17822	Aradu.A01	17822	p2	(AT)10	20	96297619	96297638	GTTGACGACCTTTCCTTTCG	TCGATTGCTCACACTCGATAA	170
Ad01A17824	Aradu.A01	17824	p3	(ATT)6	18	96301239	96301256	GCTTCGACCCTCAACTCAAC	GGAATGGAGTTCGTTTGGAA	203
Ad01A17826	Aradu.A01	17826	p3	(CTA)5	15	96304863	96304877	CCATCATGGCTTTCTGAACA	CCACAAACTTCACCGGAACT	258
Ad01A17827	Aradu.A01	17827	p3	(CTC)6	18	96305195	96305212	CCACTTCCTCCACCAAGAAA	TAAAACATGCGCATCCAGAG	243
Ad01A17829	Aradu.A01	17829	c	(T)10ctttttg(CT	32	96313917	96313948	GCTGCCTTGGTGACTGTGTA	CCTTTGAGCAAACACGAACA	244
Ad01A17832	Aradu.A01	17832	p2	(CT)23	46	96330262	96330307	GAGGATTTCGAGACAAACGC	GTAGCTCCGATTGACAAGCC	215
Ad01A17833	Aradu.A01	17833	p2	(TA)7	14	96337797	96337810	GTGCAAATGCTACATGCCAG	CTGACTTCCTTTGTTTCCGC	245
Ad01A17836	Aradu.A01	17836	c	(T)11gggacaat	101	96342472	96342572	TGCTTTCAAATTGTGTGTCCA	TGCGCTTGTCTTCAATTCTT	277
Ad01A17839	Aradu.A01	17839	p2	(AG)7	14	96359435	96359448	GGGTGAACTGGTGAAGTACGA	CTGTTTCCCTCACCAGCATT	271
Ad01A17841	Aradu.A01	17841	p3	(TTA)5	15	96361140	96361154	GCATTTGCAAGTTCCAAGAGA	CAAACCTTAACTTCAAAAGAACCAA	260
Ad01A17843	Aradu.A01	17843	p3	(TAT)5	15	96365658	96365672	TTCATTGCTCTTGTCGGTTG	TTCCCCAAATGGAATTTGAA	153
Ad01A17844	Aradu.A01	17844	c	(TA)6cacacact	105	96366307	96366411	AGGTTTGTGTTCTCCTTTCCTT	CGCTTTACCAGCTTTTACCG	265
Ad01A17846	Aradu.A01	17846	p3	(AGA)5	15	96370955	96370969	TTGAAGGTCCATGATACGCA	TTGTAGTAACGTGCAAGGCG	266
Ad01A17850	Aradu.A01	17850	p2	(AT)6	12	96372072	96372083	AAGGGTTTGATCCTTTTGGG	AACGGAATTATCCGCTGTTG	108
Ad01A17851	Aradu.A01	17851	p2	(TA)6	12	96372195	96372206	TCATAGCTCGTTGGAAACAAGT	CCACGTGTTTCTGTGAATGTC	280
Ad01A17853	Aradu.A01	17853	p2	(CT)10	20	96374655	96374674	TCACCACCCACCACTCACTA	AGAGAGAAAAGGGCAAAGGC	135
Ad01A17855	Aradu.A01	17855	p2	(TA)7	14	96382264	96382277	GAGACCGTAGGTCCTTTCCC	TCAAAATTTTTCGGGTACCAA	230
Ad01A17856	Aradu.A01	17856	p3	(CTT)5	15	96383310	96383324	GAGTGTGCAAAGATTGCGAA	TCCCTTCAATGCAATCACAA	210
Ad01A17859	Aradu.A01	17859	p3	(CAC)5	15	96420742	96420756	TGTAGGGGACCCACTCTCAC	ACCAACACTTTTCTGCGCTT	198
Ad01A17860	Aradu.A01	17860	p3	(CAC)5	15	96420874	96420888	AAGCGCAGAAAAGTGTTGGT	TTCCTGCACAGAAAGCATTG	211

ID	Chromosome	SSR nr.	SSR type	SSR	Size	Start	End	FORWARD PRIMER (5'-3')	REVERSE PRIMER (5'-3')	Product (bp)
Ad01A17861	Aradu.A01	17861	p3	(TCT)5	15	96421338	96421352	TTCTGTAGCGTGGATTGCAG	AGGAGGTCTGTTTGTGGTGG	144
Ad01A17862	Aradu.A01	17862	p2	(CT)8	16	96425168	96425183	TTCAAAGCGTGAACCCTCTC	AGATGGCGATGGTAGCAAAC	207
Ad01A17865	Aradu.A01	17865	p3	(TTC)6	18	96428930	96428947	CTCATCACCGCTACCACCTT	AAGGGAAGACATGGATGCTG	219
Ad01A17866	Aradu.A01	17866	c	(CCA)6ctacaa	116	96432042	96432157	ACCCTCACCACTACCAGCAC	AAGTGGTTTTGCTGTTTTTGC	277
Ad01A17868	Aradu.A01	17868	p3	(ATA)6	18	96436025	96436042	TCATGCAACTAACGCTCTGG	ACCAGAAGACATCGGACGTT	202
Ad01A17869	Aradu.A01	17869	p2	(AG)18	36	96439566	96439601	TTGCACTTCTCAAATGCCTG	ATCAAGAGTGCCGATATGCC	158
Ad01A17871	Aradu.A01	17871	p3	(TTA)5	15	96448364	96448378	TGCTTGCAGTTATAGTGTTGGG	GCTGACAGGTTGCTGACTGA	232
Ad01A17874	Aradu.A01	17874	p2	(TC)7	14	96463305	96463318	ACGCACAAAGGAGATCGAAT	AATTGGGGATTCAAGGGAAC	274
Ad01A17875	Aradu.A01	17875	p2	(AG)6	12	96465516	96465527	GGAACTTCTCATGGATCGGA	CACGGGTTTGTCTTGGGTAT	253
Ad01A17876	Aradu.A01	17876	p2	(TC)6	12	96465968	96465979	GAGGGCTCTCCCATTGTAAA	GGAACTTCTCATGGATCGGA	216
Ad01A17877	Aradu.A01	17877	p2	(AG)6	12	96491525	96491536	GGAACTTCTCATGGATCGGA	GAGGGCTCTCCCATTGTAAA	216
Ad01A17878	Aradu.A01	17878	p2	(TC)6	12	96492121	96492132	GGGCCAATTGTTTATTCAGC	GCAGGATCCTCAAGAACGAG	250
Ad01A17880	Aradu.A01	17880	p3	(TAA)6	18	96497728	96497745	CTTCAGGGGCTACATTGAGG	TGGGTTGGCAAGCTAAACTC	243
Ad01A17881	Aradu.A01	17881	p2	(AT)8	16	96498858	96498873	CGGCTATTAAATATCAATTTCACG	TTTTTAGAAACTCTTGAGTGCCA	268
Ad01A17883	Aradu.A01	17883	c	(A)11gaatttcta	110	96500592	96500701	CACGCAGATAAGACTTGGCA	CAAATAATTCTGTTATGAGGAGTAGGG	200
Ad01A17890	Aradu.A01	17890	p5	(GAATA)5	25	96536579	96536603	TTGTGCAATCATTATGTAATAGGAA	TGTGTTAATGTGGAGAAGACTTTGA	210
Ad01A17891	Aradu.A01	17891	p3	(AAG)6	18	96536916	96536933	CTTCTTTCCCTCTCCCCATC	CTGGGAGTGAAATCGTGGAT	264
Ad01A17894	Aradu.A01	17894	p3	(ATT)10	30	96544169	96544198	TCGTCCGAGTTGAATTCCTT	CACCGCACATTACACAGGTC	253
Ad01A17896	Aradu.A01	17896	p2	(AG)7	14	96551499	96551512	AAAAGTGGCCCTTCGAATTT	AAAAGGAGAGACGGGTGAGG	228
Ad01A17897	Aradu.A01	17897	p2	(TC)21	42	96553762	96553803	ATTGCTTTTGCTTGAAGCGT	TGAAAAACGCATTGTTTGGA	165
Ad01A17899	Aradu.A01	17899	c	(A)11caaacttac	37	96555367	96555403	TGTGATTGTTGATGGCAATG	CCATTTTTGCCAATTCGTTT	212
Ad01A17907	Aradu.A01	17907	c	(TCT)5tca(TT	36	96583831	96583866	AAGCGTGCACCAATCTCTCT	TGCTCAATGGAAATGGGAAT	268
Ad01A17909	Aradu.A01	17909	c	(A)12taaataaat	69	96585658	96585726	TGCAAGCATATAAATCCCCA	GTTGGCGACAACAACCTACC	239
Ad01A17910	Aradu.A01	17910	p3	(ATG)13	39	96586459	96586497	TTGCTGAAGCTCAAGATGACA	TCTAAGGGGCTTCCCTCATT	256
Ad01A17913	Aradu.A01	17913	c	(CT)10cgctact	117	96596692	96596808	CAATACGCATAGATGCAAACAG	AGGCGTTGAGAGAGTGGTGT	245
Ad01A17917	Aradu.A01	17917	p2	(CT)7	14	96608771	96608784	CGGCAGAAGATCCAATCTGT	ATCCTCCGTTTTTGACATGG	187
Ad01A17920	Aradu.A01	17920	c	(T)10gtcattcttt	113	96622769	96622881	GCGAGAGTGAATCTTGAAAAA	ATCTTCCGCCGATCAAACTT	280
Ad01A17921	Aradu.A01	17921	p2	(TA)9	18	96624708	96624725	TTAAAATCTTGACGTGGGGG	GGTTCATTGTTGCCGAACAT	218
Ad01A17922	Aradu.A01	17922	p3	(CTT)5	15	96626004	96626018	ATGGATTGTGATGCCACCTT	TGGATTTCTCCTTCAATGGC	185
Ad01A17923	Aradu.A01	17923	c	(T)13ggctggtat	76	96630044	96630119	TCCAACAAGGGAAAAAGATCC	AAATGGATCCCTCCACTTCC	253
Ad01A17925	Aradu.A01	17925	c	(A)12gagaaagt	40	96638380	96638419	ACGAGGAGAAACACGACGAT	CATGACGGCTCTTTCCAAAT	176
Ad01A17926	Aradu.A01	17926	p3	(TCT)5	15	96638715	96638729	CACTCACTCACACACACCCC	GGTGATTCCCGAAGAGTGAA	167
Ad01A17928	Aradu.A01	17928	p2	(TA)7	14	96642876	96642889	GCTTTGCTTTACCCTGTTGC	TTTTGGATGGAAAATGGAGG	144
Ad01A17929	Aradu.A01	17929	p3	(CAC)6	18	96643534	96643551	CTGAATCCCCTTCACCTTCA	AGAAGACCAGCGAGTCCAAA	144

ID	Chromosome	SSR nr.	SSR type	SSR	Size	Start	End	FORWARD PRIMER (5'-3')	REVERSE PRIMER (5'-3')	Product (bp)
Ad01A17933	Aradu.A01	17933	p3	(TCC)5	15	96647870	96647884	CGTTCTTCGTTATCGTCATCA	AATTGAACCACACCTCAGCC	230
Ad01A17934	Aradu.A01	17934	p3	(GAA)6	18	96649106	96649123	GGAGAAATAAAATGCAGCAACA	AGCGCGAAATGAGAATCTGT	205
Ad01A17935	Aradu.A01	17935	p3	(AAT)11	33	96649486	96649518	GAGTTGTTTTTGTTGAGTTTGAGC	TTGAACTTGGTCTACATATATGACATT	255
Ad01A17941	Aradu.A01	17941	p3	(CTT)17	51	96675996	96676046	GCCGTCGAGGGTATATTTGA	TCTAAGTCTAAATTCACGCAAAAA	278
Ad01A17942	Aradu.A01	17942	p3	(TCT)6	18	96683035	96683052	CACCTCATTGCTTGTGCTGT	CGCACACACTGTGATGAGAA	199
Ad01A17944	Aradu.A01	17944	p2	(TA)7	14	96685927	96685940	ATCGGATCGGATCGAAGTAT	CGCAGATCCGCACAATAATA	273
Ad01A17946	Aradu.A01	17946	p2	(AT)7	14	96691526	96691539	TCCAGCCCTTTTCTTCTCTG	GACAAATTCATTTGACCGAAAA	185
Ad01A17949	Aradu.A01	17949	p2	(AT)12	24	96694574	96694597	ACGGAAGCTACGTGATGGAG	GCCAAAAAGCAATTCCAAAA	258
Ad01A17951	Aradu.A01	17951	p2	(CT)6	12	96698945	96698956	TTGCAATTAGTTACATCAACACACA	TTGGAGTCGCTGTCAAGATG	164
Ad01A17954	Aradu.A01	17954	p2	(TA)7	14	96704984	96704997	GAGTTCATGGCGGGTAGGTA	CTAATTGGACCGTCCGTGTT	272
Ad01A17956	Aradu.A01	17956	p3	(TTC)8	24	96708489	96708512	CAAACCAGCAATTCATTAGCC	GGAAGAGATGATGGCGATGT	224
Ad01A17958	Aradu.A01	17958	p3	(ATA)16	48	96710805	96710852	TCTTTTGTGCAAGAAAGTTCG	TGCAGCAATAACAGCAACAA	220
Ad01A17964	Aradu.A01	17964	p3	(TTA)5	15	96729224	96729238	TCCCTGATCCTCTTGAGTTTTT	TTTACGCCTTCAAGGAAACA	146
Ad01A17967	Aradu.A01	17967	p3	(ATT)5	15	96742126	96742140	TTAACATACTCGCATACAAGACTGA	TTTAGGGTGGTTGCGGTAAA	274
Ad01A17969	Aradu.A01	17969	p3	(TAA)6	18	96743617	96743634	TGGTGTGTGCCATTAGTTTGA	TTTTCACAAAGAGGCTAATTTGG	280
Ad01A17972	Aradu.A01	17972	p2	(TC)11	22	96750495	96750516	CAAATAAGGCGGCAAATTGT	GCAAAATCGGAACGGTAAAA	193
Ad01A17976	Aradu.A01	17976	p3	(TAT)8	24	96760835	96760858	TGAAGAAACGAAGCTGCAAA	TGTTAACGCAGGTACCACCA	249
Ad01A17977	Aradu.A01	17977	p3	(CTT)6	18	96761005	96761022	ACGAAGTCGTGCACACAAAG	GTGCATATCAATCAGCGTGC	182
Ad01A17978	Aradu.A01	17978	p4	(AAAT)5	20	96762613	96762632	CCCTTACATGTAGCGCGTTT	CATCTCCCGATCGTATTCGT	184
Ad01A17981	Aradu.A01	17981	p3	(AAT)5	15	96765068	96765082	AAAAGTCACATGGTTGAACGC	TGCTTCTTTTTATTAGGGACGC	125
Ad01A17988	Aradu.A01	17988	c	(T)11gcattttgca	66	96785427	96785492	TCCAATCTTGTTCGCCTAGC	TCGGTTCAAATTATTACACAGATAA	229
Ad01A17994	Aradu.A01	17994	p3	(TGA)5	15	96789832	96789846	CCTCTCTTGGCTTCGATGATT	GGTATGGCATTTCAGAATTGAAG	276
Ad01A17995	Aradu.A01	17995	p5	(AATTG)5	25	96791559	96791583	AATCGAATTGAACGGAATGC	TACTCACGGTGAACCGAAAA	196
Ad01A17996	Aradu.A01	17996	p3	(AAG)5	15	96792141	96792155	AACGCGAAGAAGAAGGTGAA	TGGCCAGCATATAATCAGCA	232
Ad01A18000	Aradu.A01	18000	p2	(AC)7	14	96809162	96809175	CCGAATAGAAAGGGCACAAA	GTTGTCGGTGCAAAGTGCTA	267
Ad01A18004	Aradu.A01	18004	c	(CT)6catgcggt	114	96816384	96816497	GACTCTTTCTCTTCGCGGTG	ACACACTCCACACAAAAGCG	239
Ad01A18005	Aradu.A01	18005	c	(GTT)5aaattgtt	83	96817750	96817832	TGCTATTTTCTTCCCTTGCG	CCACCTAGCCACTACCTCCA	271
Ad01A18008	Aradu.A01	18008	p3	(TTC)9	27	96821610	96821636	GCAAAAGATGAGGAGGAGAAA	TTTGAAAGATGATGAACTTACATTTG	242
Ad01A18009	Aradu.A01	18009	p3	(AAG)6	18	96821830	96821847	TGCTGCATTTTTCTTCTTCTTC	TTCTTCCTCCTTCTCATCTTCTG	271
Ad01A18010	Aradu.A01	18010	p3	(GAT)5	15	96822216	96822230	GAATGTAAGTTCATCCTCTTCCAAA	CCTCCTCATTTTCTGCTGCT	220
Ad01A18011	Aradu.A01	18011	p2	(TA)6	12	96823895	96823906	TTTTTGACGGTTACAATTTTTATCA	GGGTGAGAGACTAATTTGGAAGAA	261
Ad01A18013	Aradu.A01	18013	p3	(TCA)8	24	96826092	96826115	GTGTGCTCCGTTCCGTTAAT	CGGGAGCATGAGAGAAAGAG	191
Ad01A18021	Aradu.A01	18021	c	(ATT)5caaaatt	129	96857225	96857353	TTTCTTTTGGGTTTAGCTATGACA	AGATATTTTTAATCTTGACTAACCACG	206
Ad01A18023	Aradu.A01	18023	p2	(TC)9	18	96858762	96858779	TATTTGGCGAGAATGCTCCT	TGTAGAAGCTCGGACCACCT	156

ID	Chromosome	SSR nr.	SSR type	SSR	Size	Start	End	FORWARD PRIMER (5'-3')	REVERSE PRIMER (5'-3')	Product (bp)
Ad01A18024	Aradu.A01	18024	p2	(TG)9	18	96858986	96859003	AGGTGGTCCGAGCTTCTACA	CTCTATGCGGAAGGTTTTGC	269
Ad01A18027	Aradu.A01	18027	c	(C)10aaa(AC)(	25	96869505	96869529	GGGAGAAAGGAACACATCCA	TCACCAGATAGCAAACGATCC	224
Ad01A18037	Aradu.A01	18037	p2	(TC)12	24	96899490	96899513	GGCGCGTACATAGATAACCAA	TCCCCTCTCTCACACACACA	277
Ad01A18041	Aradu.A01	18041	p3	(TAT)9	27	96905215	96905241	GCAGCTGCATATTTTGTTTTG	TGGAAGAGCGAATAGAGCTGA	196
Ad01A18042	Aradu.A01	18042	p3	(TGA)9	27	96905898	96905924	ATCCATGCATCCTCCTCCTT	GGTTCTCATCATGGGATTGG	113
Ad01A18043	Aradu.A01	18043	p3	(ATG)5	15	96906638	96906652	CTCAGCAGCATATCTTCCCC	CCCACATTTTCTGAAGCCAT	255
Ad01A18044	Aradu.A01	18044	p3	(AAT)5	15	96907380	96907394	TGCAACAGTCACTTTCTCCCT	CATGATTGTTTAATCATATCCAATTTT	251
Ad01A18049	Aradu.A01	18049	c	(T)11caataaaaa	66	96920040	96920105	TTGCTTTGGTCAAAGTGGTATG	TGTTGTGATAAACTGAAACACTAATTT	246
Ad01A18050	Aradu.A01	18050	p2	(TA)8	16	96922766	96922781	AGAGTTGGCAGAAATTGACGA	CGCATTTCACCTTGAGTTAGC	124
Ad01A18052	Aradu.A01	18052	p3	(TAA)6	18	96933935	96933952	AACTCCCATTCAGCAGTCTCT	TCGATGATGAGGAGTTTAAAAGG	277
Ad01A18054	Aradu.A01	18054	c	(A)10ctgaaaata	99	96939605	96939703	AGATGATTCGCCTCCCTTTT	ACACTAGTTCGTCGCCCAGT	210
Ad01A18055	Aradu.A01	18055	p3	(GTT)22	66	96945373	96945438	TTTTTCTGGGCTGGTGTTGT	TTTGCTATTTAATGTAGCAGCCC	266
Ad01A18060	Aradu.A01	18060	p2	(CT)6	12	96957862	96957873	CCCAAAATTCCCTTTCCAAC	GAGAGGGTGGTGGAATTGAG	261
Ad01A18061	Aradu.A01	18061	p5	(AATTG)6	30	96965720	96965749	CATTTAAGGGTTTGCCGCTA	GAGTTGGGCCCACAATCTAA	195
Ad01A18063	Aradu.A01	18063	c	(A)11tagaagag	95	96967979	96968073	TTTGATCTTGCTTTTATTTTCCAA	AAAGGTTAGGCATTATTTTCACC	280
Ad01A18064	Aradu.A01	18064	p3	(CTC)6	18	96970962	96970979	AGATGGCGAAGACAAATGCT	AGCCCGAAGAAGAAATCACGA	161
Ad01A18067	Aradu.A01	18067	p5	(AATTG)5	25	96985316	96985340	CATCCTGGCTCAAGACACCT	TATCGAGGGGCATTCTTGTC	280
Ad01A18068	Aradu.A01	18068	p3	(AAG)6	18	96990268	96990285	ATCTTGTTCCCAACCACAGC	TGCAGGGGATAAACCAACTC	156
Ad01A18072	Aradu.A01	18072	c	(CT)6(AT)7	26	96994806	96994831	CATAGGCTGCAAACTCCATGT	GGTGCTCTTTGGGTTTGAAT	273
Ad01A18080	Aradu.A01	18080	p3	(ATA)11	33	97003814	97003846	TTTTTGGTTGGCGAAGTACA	GTGCTTTGAGAGGGTTTTGG	216
Ad01A18081	Aradu.A01	18081	p3	(AAG)5	15	97004679	97004693	CCTAGCATTTCATTTTTCCGA	CGGATAATCCAATAATTCAACCA	272
Ad01A18082	Aradu.A01	18082	c	(GT)18(GA)12	60	97006339	97006398	AACGCGTTATCTATGGCTGC	ATCCGCGGGAATTATCTTTT	222
Ad01A18086	Aradu.A01	18086	p3	(TTA)7	21	97016185	97016205	TGCAGTAACAGGAGCAGGAA	AACAAGAGCAAGGAAGGTGG	192
Ad01A18087	Aradu.A01	18087	p3	(TCT)5	15	97027684	97027698	TCCATTCTTTGGGCAAGTTC	GAAGGGATGATTAGGCAGCA	208
Ad01A18088	Aradu.A01	18088	p2	(CT)6	12	97029241	97029252	TTTCGTGCACCAAGTTTTTG	CTTACAGCCAGCCAGGATTC	266
Ad01A18092	Aradu.A01	18092	p2	(TC)11	22	97054926	97054947	GGACGGTCCGATTTCAGTAA	GAGACGAGAATGATCGGGAA	250
Ad01A18093	Aradu.A01	18093	c	(ATT)5aattaac	99	97055435	97055533	TCCTTGTAAAGCTTGTACGAATAGTT	CAATTCTCCACGGTACGGTAA	256
Ad01A18094	Aradu.A01	18094	p3	(ATT)13	39	97055657	97055695	CCGTACCGTGGAGAATTGTAA	AACTACCTGCATACATACATTGTAAAC	244
Ad01A18095	Aradu.A01	18095	p3	(GAA)5	15	97059174	97059188	CCAATGACAACGTGAATGGA	AGATTGTACCCAGAACCCCC	268
Ad01A18096	Aradu.A01	18096	p2	(TC)8	16	97061359	97061374	CCATTTGCCATTTTCCTTTG	GAGGGATGGACATGGAAGAA	210
Ad01A18107	Aradu.A01	18107	p2	(GA)6	12	97089963	97089974	TCACCAAGGGAGGAGAGAGA	ACAGTGGCAAGTTCGATGGT	136
Ad01A18111	Aradu.A01	18111	p4	(AAGA)5	20	97134172	97134191	GGCACGAGCTTTGTTTCTTC	CAACCACCCGAACTCAGATT	164
Ad01A18112	Aradu.A01	18112	c	(GAA)9ttgaaga	76	97134454	97134529	AACCGTACGTGGAAATGGAA	AACAACTCACCACCACACCA	245
Ad01A18114	Aradu.A01	18114	p2	(AT)7	14	97136150	97136163	AACGCTTTGTCAATTGTCCC	AACGTAAAGATGCATTGGTGA	273

ID	Chromosome	SSR nr.	SSR type	SSR	Size	Start	End	FORWARD PRIMER (5'-3')	REVERSE PRIMER (5'-3')	Product (bp)
Ad01A18118	Aradu.A01	18118	c	(T)10atcgtcgtt	103	97140785	97140887	GTCTTCTTTTTCGCGTTCTTT	CCTCATCCTCTTCTTCTTCACC	264
Ad01A18119	Aradu.A01	18119	p3	(TCA)6	18	97141453	97141470	GGTTTAGCCATTTCGATGTCA	GTCGGAAAATCCAACGAAAA	180
Ad01A18120	Aradu.A01	18120	p2	(TC)7	14	97154170	97154183	TTCGAATACATTCTCTCTCTCATCTC	CAGGTTCAGGATCAGCTTCA	273
Ad01A18122	Aradu.A01	18122	c	(A)11tcaacttca	92	97167313	97167404	CATTGCTCAAATCTGACCCT	CGGTCTTAGAGGTGCGAACT	263
Ad01A18123	Aradu.A01	18123	p2	(TC)9	18	97167566	97167583	CCTTCATTTCTTCGCCAAAC	AAAACCTGATCGGACACTGC	152
Ad01A18125	Aradu.A01	18125	p2	(GA)6	12	97173261	97173272	ATGGGAATGGATCCTCTCAA	CCATTGGATCAACATCCTCC	237
Ad01A18126	Aradu.A01	18126	p4	(TAAA)5	20	97173467	97173486	GGGTAAGGTTCGGCCATTAT	GGCATTTTTACACGCCACTT	275
Ad01A18127	Aradu.A01	18127	p3	(ATT)20	60	97174361	97174420	GATCTTGTACGGGGTTGTGC	GCAAGGAAAAATACAATCCCA	183
Ad01A18128	Aradu.A01	18128	p2	(CT)10	20	97176048	97176067	AAGGCCACCGGATAAAGAAT	CAAAATTCATGAGTGCGTGC	170
Ad01A18130	Aradu.A01	18130	p2	(AG)8	16	97177891	97177906	CTCCTCTGCCCTTGCACTAC	TAGAAATGGCAAAGGGTTGG	277
Ad01A18134	Aradu.A01	18134	c	(TAA)13ttaata	69	97196842	97196910	GAGAGAACCGTTGCAAAAGC	TGGATCAATGCGACCTGTTA	244
Ad01A18138	Aradu.A01	18138	p2	(CT)12	24	97208900	97208923	TCCCTGTAGGACCTGCTGTC	TGCAATAGGTTGGGGTTAGG	258
Ad01A18140	Aradu.A01	18140	c	(ACA)5(ATA)	30	97217781	97217810	CTCTCAATCACTGGGGTGGT	TGTGCTGAGCAGAACCATTC	263
Ad01A18141	Aradu.A01	18141	p2	(AT)6	12	97217988	97217999	TAATGGGCTTCAGGATTTGC	TCCCACCTGCCAAAATAATC	272
Ad01A18142	Aradu.A01	18142	p2	(AT)6	12	97220035	97220046	GGCTGCTTCATCACTGGAAT	AACAAGCAGAGGCAAAAGGA	279
Ad01A18143	Aradu.A01	18143	p3	(TTC)7	21	97233737	97233757	ACTCACTGCGGGTTTAGACG	ACCATTGCTACGCGGAATAC	150
Ad01A18147	Aradu.A01	18147	p3	(AAG)5	15	97238123	97238137	TTGAACCACATTTCAGCCAC	TTCACGCGCTTTGTATTGTT	266
Ad01A18150	Aradu.A01	18150	p3	(AGA)5	15	97249128	97249142	GTATGGCAAAAGAAGACGGC	TGGAAAAGGCGATCATATCC	166
Ad01A18151	Aradu.A01	18151	p3	(CTT)6	18	97273290	97273307	ACAGGAATTCATCCAAAGGA	TCATCGTGTTTCTCCTCGTTC	280
Ad01A18152	Aradu.A01	18152	p3	(GAA)21	63	97273445	97273507	CGAGGAGAAACACGATGATG	TTTTTCTTTTTCCGTGCCTC	124
Ad01A18156	Aradu.A01	18156	p3	(ATC)5	15	97291337	97291351	TTGAAGTTAGGTAGGCAATGTTT	TGGCAAGTTGTTAGTTAACGG	115
Ad01A18162	Aradu.A01	18162	p2	(AT)21	42	97330859	97330900	TCTGACTCAACTTCATGTGAAAA	TTCCTTTTGGACTTGGCTTT	273
Ad01A18163	Aradu.A01	18163	p2	(CT)6	12	97331716	97331727	GTCGTCGTACCGGAGAACAT	CGACGGAATTGACAGACCTT	107
Ad01A18164	Aradu.A01	18164	p3	(TTG)5	15	97332537	97332551	GCAGTGGAGGTGTGATGGTT	GCTCTCCCTCATCATTTACCC	109
Ad01A18165	Aradu.A01	18165	p2	(AC)8	16	97334844	97334859	TGGGAATACCGTTTGGGTAA	GGAAGGTGGGTCTGCTCATA	241
Ad01A18166	Aradu.A01	18166	p3	(AAT)16	48	97338637	97338684	AACAAAAACGGTTCAATGCC	GGACCGGTTTTCTTTCAGTG	252
Ad01A18167	Aradu.A01	18167	p2	(AT)8	16	97339277	97339292	TACCTCACCCCGACGAATAG	ACTTTTCCATTGATGCTCCG	214
Ad01A18170	Aradu.A01	18170	p3	(AAG)7	21	97352201	97352221	TGAGAAGGTAAAACAAAAAGGAAAA	ATTTGGAGCAAATTTGGGTG	212
Ad01A18177	Aradu.A01	18177	p3	(TAA)13	39	97389631	97389669	TTACCGTCCGGAGCCTAAAT	GCTGGTTTGGGTTGAACG	280
Ad01A18178	Aradu.A01	18178	p3	(AAT)5	15	97389860	97389874	AGAACGTTCAACCCAAACCA	CAACCATTCTGATTATGTAAGCTTTT	218
Ad01A18180	Aradu.A01	18180	c	(AT)7tcccagca	105	97396134	97396238	AAACAAATTGCAATGGGGAA	GGGGGCATGTTAGCAATTAG	268
Ad01A18182	Aradu.A01	18182	p3	(TGA)9	27	97408316	97408342	TGTCCACGTATTGCAGGGTA	AGCACACCAGATGTTTGCAG	191
Ad01A18183	Aradu.A01	18183	p2	(AT)10	20	97409319	97409338	GAGACTGATCCGTCCATGTTT	GAGACGGACCAAGAAAGGGT	273
Ad01A18184	Aradu.A01	18184	p4	(ATAA)5	20	97411153	97411172	CCAAAAATCAACCTTGCCAT	GCACATCAGGTTGTTTGCAC	270

ID	Chromosome	SSR nr.	SSR type	SSR	Size	Start	End	FORWARD PRIMER (5'-3')	REVERSE PRIMER (5'-3')	Product (bp)
Ad01A18188	Aradu.A01	18188	p3	(ATA)6	18	97439493	97439510	ATGTTTGGTTTCAGCCGTTC	CGGGTTTGGAGGGTTTTTAT	190
Ad01A18190	Aradu.A01	18190	p2	(GA)7	14	97440262	97440275	CCGCCATAAAGTAGGACGAA	ACGTATGGCCGTCTAACGAG	228
Ad01A18191	Aradu.A01	18191	c	(CT)7t(TC)14	43	97441639	97441681	CCCTTCTCTCCACCACTACC	ATGGTGTCGTCCTCTTCAGG	184
Ad01A18192	Aradu.A01	18192	p3	(TAG)5	15	97442211	97442225	AACTCATCCATGCCCTTCAC	TGGTGTACCCCAAACCCTAA	270
Ad01A18194	Aradu.A01	18194	p3	(ATT)5	15	97457134	97457148	GATGTATTTGTGGTGCTGCAA	TTTCCATCATGCAATAGCCA	266
Ad01A18195	Aradu.A01	18195	c	(AAT)5ta(ATT	35	97457469	97457503	TGCCCAGAATGTCATCCATA	CACGCCTGATTCCTTTTGAT	122
Ad01A18197	Aradu.A01	18197	p3	(CTT)6	18	97459931	97459948	TGCGCTAGGCCATGATAAAT	CAAAAATATCTTCTTGAATGCTGC	240
Ad01A18198	Aradu.A01	18198	p3	(TCT)5	15	97460261	97460275	AAAATTTTTCCTCTTTTCCTTCTCA	TCCTCTTCCTAATAATTTTGCAGC	181
Ad01A18199	Aradu.A01	18199	p3	(AAT)5	15	97462198	97462212	AATTCCCGTTTACAGACCGA	GGATGTGGCATCCAGGTTAG	276
Ad01A18201	Aradu.A01	18201	c	(ACC)6actgac	71	97472264	97472334	GCGTCGAGGTTGTTGAGAAT	CCTCAAACATTTAGGCCACTT	221
Ad01A18203	Aradu.A01	18203	c	(T)11ggggggg	47	97477908	97477954	TTCGTCCTCTCAACATGGCT	AGTATGCCATGAAACTCCGC	164
Ad01A18211	Aradu.A01	18211	p2	(GA)8	16	97494486	97494501	TTTTCAGTAGAAAATGGAGCGA	TGATCATGAATGTCAGTCCCA	198
Ad01A18215	Aradu.A01	18215	p5	(ATTGA)6	30	97504275	97504304	TGCTAAATTCTAGACGCAGGTG	TCAAATTCGAACAACTATCTGCAT	228
Ad01A18218	Aradu.A01	18218	p3	(GAG)6	18	97524103	97524120	CTTGCGCTTCTTGGGATTAG	AAAGCTAAGTCTTGCGCTCG	162
Ad01A18220	Aradu.A01	18220	p3	(AGA)5	15	97552922	97552936	TTTTTCGTCCCCAACCTTTT	AGAGGCAAAAGTGGATCGTG	237
Ad01A18222	Aradu.A01	18222	p2	(CT)6	12	97557420	97557431	TAGCCACCACCTTCTCCATC	AGGAGTGGAGCAACGATGAC	190
Ad01A18224	Aradu.A01	18224	p2	(TA)7	14	97560575	97560588	AACAAATGCAATTCTCTCTCTCTTT	AAAATGGGCATCCACGTAAG	278
Ad01A18225	Aradu.A01	18225	c	(TA)6cattacaa	45	97561004	97561048	CAAAAGCAAATGCTATTCTATCTCTC	TCTCGTGCTGATAACGTGTTG	258
Ad01A18226	Aradu.A01	18226	p2	(TA)6	12	97565751	97565762	AACAAATGCAATTCTCTCTCTCTTT	CCGTTGCCGGAGAAGTATAA	158
Ad01A18230	Aradu.A01	18230	p3	(TTC)7	21	97611148	97611168	TGTAACCGTGCATCTCTTGC	TTGCATGTTGGGCTTAATGA	264
Ad01A18232	Aradu.A01	18232	p3	(AGG)5	15	97616156	97616170	GAAAAAGAGGAGGAGGAGGG	TTGCCTGTGCTTAATGCTTG	255
Ad01A18234	Aradu.A01	18234	p3	(AAG)6	18	97624245	97624262	TGTTGTTGCGTTTTGTTTTTG	TGGTGCATTTGGTCTCGTTA	267
Ad01A18235	Aradu.A01	18235	p2	(TG)6	12	97641273	97641284	AGAATCGCGTCTACGGTTGT	TTTTTGCGTTCACGATTTTA	211
Ad01A18236	Aradu.A01	18236	p2	(TG)6	12	97641435	97641446	TAAAATCGTGAACGCAAAAA	TGCGTTTTACCTTTCGTTTG	220
Ad01A18237	Aradu.A01	18237	p2	(AT)11	22	97641584	97641605	CAAACGAAAGGTAAAACGCA	TGGTCTTGAAAGTCTTCCTCG	138
Ad01A18242	Aradu.A01	18242	p2	(TA)8	16	97654137	97654152	TCTAAACTAAGCTCCGTCCTTGA	TCATCAAGCAATTGAAAAATGAT	131
Ad01A18247	Aradu.A01	18247	p3	(TGA)6	18	97669316	97669333	CAGCAGAAAAAGGGACCAAC	TCCCAGAGGTAGATGGAGGA	100
Ad01A18252	Aradu.A01	18252	p3	(TAA)7	21	97678913	97678933	TCATGGATCAAATTTAAGGCG	GTTTACGTTTGTCCTGGCCT	275
Ad01A18254	Aradu.A01	18254	p3	(GAG)5	15	97684257	97684271	TGGATTTGGAAGAGGAGGAA	TGAATAAAACGCTGTATCTAAACAAAA	187
Ad01A18255	Aradu.A01	18255	p2	(TA)8	16	97687979	97687994	TTGCAGGTCAAACGAAACTCT	AAGGACATTAATAATCCGGAAAGA	229
Ad01A18260	Aradu.A01	18260	p2	(AG)6	12	97717580	97717591	GAAATGCTTGGAATTTCGGA	CACCATAGAGCCTTCTTCAGC	198
Ad01A18261	Aradu.A01	18261	c	(T)11agaatgaa	49	97728942	97728990	TTTGGACATTAAATCCTTAAAAGTG	TAAAGGACCCGCAATGAAAG	181
Ad01A18264	Aradu.A01	18264	p3	(TAA)17	51	97735906	97735956	TCCTTGCCCAGTTTGTAACC	GAAAATGTGACGGGTGTGAA	217
Ad01A18269	Aradu.A01	18269	p2	(TA)6	12	97739756	97739767	TGTGTCAAAATGGTCGAAGTT	GGAAGACGAGAGGAGAAGAAGA	280

ID	Chromosome	SSR nr.	SSR type	SSR	Size	Start	End	FORWARD PRIMER (5'-3')	REVERSE PRIMER (5'-3')	Product (bp)
Ad01A18270	Aradu.A01	18270	p2	(AT)6	12	97743916	97743927	TTCAAACTTTCCAATCACGAGTT	AATACAAACATCGGCGATCC	241
Ad01A18272	Aradu.A01	18272	p4	(CATT)5	20	97768094	97768113	TTGCTGACACTATTCAGGCG	ATCAACCTTATGATTATTTTTGGAA	187
Ad01A18273	Aradu.A01	18273	p3	(TTA)5	15	97772223	97772237	CCAAAACAAAATTACGCCCA	TGTCCCATTTCATCGTTTGA	264
Ad01A18277	Aradu.A01	18277	p3	(AGA)5	15	97786689	97786703	CAGAAGTGGAAGCTTTTGCC	CTCCTACATAGCCGAACCCA	212
Ad01A18279	Aradu.A01	18279	p6	(AAATAA)5	30	97807251	97807280	ATGATGGAGCAAGGGAGATG	TTCTCTCTCCTCATCTTTTTCG	200
Ad01A18280	Aradu.A01	18280	c	(T)10atcttaaaa	123	97807561	97807683	TGAGTTGAAAAGATTTTAAGAAGGAA	TTTCGAAAACAAGGAGCATT	268
Ad01A18284	Aradu.A01	18284	c	(T)10c(T)10	21	97868165	97868185	TCCCCAATTAGAGGTGTCCA	TGAAGCAAGAAAAAGCAGTGAA	163
Ad01A18286	Aradu.A01	18286	c	(T)11ggcggcta	49	97904020	97904068	TATGTGTGTGGCATTGCTGA	TTGCAAAAATGGATGATCAAA	248
Ad01A18293	Aradu.A01	18293	p3	(TTA)5	15	97939445	97939459	GCAGAATAACAAATCTGCCCA	GCGAAAAGGGGCATTAGAA	276
Ad01A18294	Aradu.A01	18294	p2	(TA)6	12	97944062	97944073	TTGGCATTTCATCAAACACA	TTTGATTCTAGCCATTTCCCA	272
Ad01A18295	Aradu.A01	18295	p2	(TA)6	12	97945646	97945657	CTTTGCATGCTTTTCTTCCA	TTCCTGGCTTTCACGAGATT	208
Ad01A18296	Aradu.A01	18296	p3	(TTC)5	15	97949823	97949837	GAACTTCCGGGTACTCTCCC	GCTCCTCCCCCTAATGAAAG	191
Ad01A18300	Aradu.A01	18300	p2	(AG)8	16	97978533	97978548	GCTGAATTGAACCAATGTAACAA	AGACATCAATCTGTTTTATATTCCAA	260
Ad01A18302	Aradu.A01	18302	p3	(AAT)5	15	97994809	97994823	CCAAGGCCAAAATTCACCTA	TTGATCCTAGTGTGTTAATTGCC	182
Ad01A18305	Aradu.A01	18305	p2	(TA)7	14	98022373	98022386	CCAAATCAATTGAAAAATAAATCC	ATTTGTTGTTTTTCGAGAATGACT	244
Ad01A18313	Aradu.A01	18313	p2	(CT)6	12	98065883	98065894	TTGGACTTCAAAACCCTTCC	GGGTTGAGTGCATGGAACTT	236
Ad01A18319	Aradu.A01	18319	p2	(AT)11	22	98084844	98084865	AGCATACTTCGTCCATTGCC	ACGTGTGGACTTGCTTGTCA	280
Ad01A18320	Aradu.A01	18320	p3	(ATA)13	39	98085487	98085525	GCCCTGAAACTGGTGCTTTA	ACACACAGGCCACTCATTCA	261
Ad01A18321	Aradu.A01	18321	p2	(TA)6	12	98087530	98087541	GCAACAAAGGGGCAAGTTT	AGGGAGGAAGAGATCGAGGA	277
Ad01A18324	Aradu.A01	18324	p2	(TA)7	14	98099498	98099511	TCGCACCTTCATGTTTTCAC	GCAAATCTTGCGGGTCTATC	142
Ad01A18325	Aradu.A01	18325	p3	(TCA)6	18	98100727	98100744	CTGGTCAGTGGTCACCTTCA	CACACCGGCCTTGTATTTTT	130
Ad01A18327	Aradu.A01	18327	p3	(AGA)5	15	98105342	98105356	CGGAACAAATACCAACCCAG	TGCAGTTGCATCCACTCTTC	212
Ad01A18328	Aradu.A01	18328	p2	(TA)6	12	98110556	98110567	TGGTGCAAATCGTGAATCTC	CCTAAAAAGAATATTTCAACCTTCA	251
Ad01A18329	Aradu.A01	18329	p2	(TA)8	16	98117042	98117057	GGTGTGCGCACTTACAAAGA	TAATTGAAGCAGGAGGAGGC	257
Ad01A18330	Aradu.A01	18330	p3	(AAG)6	18	98117389	98117406	GCCTCCTCCTGCTTCAATTA	GCAAACTCGAACCGGTAAAG	216
Ad01A18331	Aradu.A01	18331	p3	(TCA)6	18	98118547	98118564	GAAGAGGGACTCCATGGTCA	AGCGCAATTGGTAGCAGAGT	157
Ad01A18332	Aradu.A01	18332	c	(AT)6aattataat	64	98119105	98119168	TATGCACCCTCACCTTCCTC	TTCATTTACCGGTACAACTTTCA	173
Ad01A18335	Aradu.A01	18335	p2	(AT)7	14	98129842	98129855	TGTGAGTAATTGCGAATCTACAA	TTCACTCGATCGTACACCCA	163
Ad01A18337	Aradu.A01	18337	p3	(AGG)5	15	98144753	98144767	GGAGGAGAAACGCAAAGAAA	TGGGTGGTTTAGGTGTTGGT	215
Ad01A18339	Aradu.A01	18339	p2	(AT)8	16	98146208	98146223	GGTGAAAGAGGAGAGGAGCA	CGTGTCGGAAAGTTTGTTGA	253
Ad01A18344	Aradu.A01	18344	p3	(ATT)19	57	98161216	98161272	TTTTCAAAATGTCATTCATCGTG	GACGCGTAATGTGAAAAACG	261
Ad01A18345	Aradu.A01	18345	p3	(CCT)5	15	98161510	98161524	CACATCTCTGCTGCTGGTTT	GATGGTGACGGTAACGAAGG	170
Ad01A18347	Aradu.A01	18347	c	(TCA)6tt(ATC	35	98167380	98167414	AAACTGTAAACGAGATAAGTCCTCC	GAAGAAGAAGAGGAGGATGAGG	273
Ad01A18348	Aradu.A01	18348	p3	(TCT)8	24	98175004	98175027	GATCCGGAGCTGAGATTCAT	GTAGGGAGTGGTGGAAGGGT	256

ID	Chromosome	SSR nr.	SSR type	SSR	Size	Start	End	FORWARD PRIMER (5'-3')	REVERSE PRIMER (5'-3')	Product (bp)
Ad01A18350	Aradu.A01	18350	p3	(ATT)22	66	98179888	98179953	TTCTTTGAGGGTGAATTGGG	CGAGCAATTGAAAGTTTTGG	199
Ad01A18351	Aradu.A01	18351	p3	(ATT)5	15	98180897	98180911	GTAGTCCCATATGCAGCGGT	TAAACATGGATTGTCGCTCG	261
Ad01A18352	Aradu.A01	18352	p3	(TGG)5	15	98186195	98186209	CCTCCTCCCAACAAGATGAA	ATTTCCCATTTTACCCTCCG	252
Ad01A18353	Aradu.A01	18353	p3	(ACA)9	27	98186648	98186674	CAGCAACAATACATGGCAGC	GGAGGCCTTGCATACATCAT	125
Ad01A18360	Aradu.A01	18360	p2	(TA)9	18	98208570	98208587	AGCAACGAAGGAGAAGAATGA	GATTTGTTTTGCCCAAGGAA	268
Ad01A18362	Aradu.A01	18362	c	(A)10g(A)10	21	98216760	98216780	TAGGCCCCATTGAATAACCA	GCCAGGCTGATTTTGTATTTT	249
Ad01A18363	Aradu.A01	18363	p4	(TAAA)5	20	98219941	98219960	GCCAACGGAACCTAAAAACA	CCTTGCAAAATTGACGAGGT	199
Ad01A18368	Aradu.A01	18368	p3	(GAA)6	18	98247081	98247098	TGCAAAGAAGACAAAAGCGA	CTTCAAGCGTTTTCTCCCAC	175
Ad01A18370	Aradu.A01	18370	p2	(TA)6	12	98252423	98252434	AACAAGTTAGGAGGCCGAGG	TGCGAACAAGTAGGACAATACA	275
Ad01A18377	Aradu.A01	18377	p3	(TAA)8	24	98272480	98272503	GAGAAATGGCTGAGGCTTGA	CACATGGTGAAAGGGTCACA	279
Ad01A18388	Aradu.A01	18388	p3	(TAT)19	57	98422110	98422166	TGAAGGGGCTTTCCACATAC	CACATCACATCAGAAACGGG	274
Ad01A18389	Aradu.A01	18389	p2	(GT)6	12	98423399	98423410	GACAGGAGGTGATTCTGGGA	TGCGTCCATATTCTTGGACA	239
Ad01A18394	Aradu.A01	18394	p3	(TTA)6	18	98453857	98453874	TTTGGTGATTTGCACCTTGA	TTGAGCACCACTATCTGGGA	165
Ad01A18395	Aradu.A01	18395	p3	(TTA)17	51	98454069	98454119	ATTGATGCTCAGAGCCTTGG	CCTTGAAGCAAAAGAAACAGC	107
Ad01A18396	Aradu.A01	18396	p2	(GA)6	12	98454724	98454735	ACGTTCATCTCCCTTTGGTG	GAAAATCTCGCGACTTACGC	272
Ad01A18400	Aradu.A01	18400	c	(A)10tgaacaac	83	98478959	98479041	TCCTTATCCAAATACTACGTGGC	TTAATGCCTCGAGCCGTATC	264
Ad01A18401	Aradu.A01	18401	p2	(TA)9	18	98482368	98482385	CTTTCAGCAATTCCACAGCA	ATGCCAATGAAATCCACTCC	271
Ad01A18403	Aradu.A01	18403	p3	(ATA)5	15	98484960	98484974	TGCATGCATTCATTGTTTTG	TTTTTGAATTTGATGAGAGAAAAA	134
Ad01A18411	Aradu.A01	18411	c	(A)12tcacactca	93	98503788	98503880	TTCATGCATGCCACATTTTT	TTTGTTTCTTTTCGCTTTTGTTT	254
Ad01A18413	Aradu.A01	18413	p3	(ATT)16	48	98506237	98506284	CACAAATTTTGCTTCAATTCCA	TCAAAATTCAAGAAGATCCACAAA	175
Ad01A18414	Aradu.A01	18414	p2	(AT)7	14	98506529	98506542	TGTGGATCTTCTTGAATTTTGACT	TCTCTCTCCTTTAATTCTTCTCAAA	280
Ad01A18424	Aradu.A01	18424	c	(A)10gttcaccg(	28	98526927	98526954	AACAAAAACAAGCGAGGAGG	AGCACCCAAACAAAAAGCTC	180
Ad01A18427	Aradu.A01	18427	p4	(TTAT)5	20	98535942	98535961	TTGTTCGGCCTCTTCTTGAT	AGCCCTGCCATATGTCAAAC	111
Ad01A18428	Aradu.A01	18428	p3	(ATT)14	42	98537664	98537705	CAGCAACTTGGAGGGAAAAG	ACAAAGCAAACAACTTCGGG	187
Ad01A18432	Aradu.A01	18432	p3	(CTC)5	15	98557450	98557464	TTCATCCTCACTGCTGAACG	CTTGACCTGAGCCTTTCTGG	259
Ad01A18433	Aradu.A01	18433	p2	(CT)13	26	98563756	98563781	CAGAGATTTGTTAGGGGGCA	TCAACAGGAATAGCTGCACG	216
Ad01A18435	Aradu.A01	18435	p3	(TCT)5	15	98574341	98574355	TTGCATAATCTGCCAATCCA	GGTTGATGGTGAGAGAGGGA	266
Ad01A18436	Aradu.A01	18436	p2	(CT)8	16	98589831	98589846	GACGGGGTTAGGGTTTGAGT	ATCATGTATGGCTGCTGCTG	240
Ad01A18438	Aradu.A01	18438	c	(A)10tgcgttata	38	98596646	98596683	CAATGGAGCTGCACTGAGAA	TCCTTCCCTTTCCCTTTCAT	272
Ad01A18439	Aradu.A01	18439	p3	(ATA)5	15	98598444	98598458	TCACAAGAACAGTATACCAAAAGCA	CGTATTGTTATTAAACGTACGAATG	269
Ad01A18442	Aradu.A01	18442	c	(T)10ataaagtct	94	98608288	98608381	GAACAGATCCCCAAAGCATC	CGGGAAGTCTATGAATCCGA	254
Ad01A18443	Aradu.A01	18443	p3	(TTC)5	15	98618537	98618551	AAATGGCTTGCCCTTTTCTC	TGGTGGTCCCCAACTAAGTG	192
Ad01A18444	Aradu.A01	18444	p3	(ATT)11	33	98618781	98618813	TTGGGGACCACCAACAAGTA	TTTGGGGACCAAAATAGGTAAA	213
Ad01A18446	Aradu.A01	18446	c	(CA)6c(TA)7	27	98636493	98636519	CGCTTGCCCCACTACACTAT	AGCAGTGCTTTGCATGTACG	102

ID	Chromosome	SSR nr.	SSR type	SSR	Size	Start	End	FORWARD PRIMER (5'-3')	REVERSE PRIMER (5'-3')	Product (bp)
Ad01A18447	Aradu.A01	18447	p3	(CCA)5	15	98641488	98641502	GCGATATTTCATTGGACAGC	GCTCGCCTTCACTCATTTTC	115
Ad01A18452	Aradu.A01	18452	p3	(TAA)5	15	98650094	98650108	GTACTGTTCCCTCCGATCCA	TTACTTTTGACACTCAGCATGTAAT	142
Ad01A18453	Aradu.A01	18453	p2	(AT)12	24	98652696	98652719	CCCACGTGAGGTTTCTTTCT	GCAATGAATGATTTCAGGGG	159
Ad01A18455	Aradu.A01	18455	p2	(AT)7	14	98658385	98658398	TTCCATGACATGCCACAAAT	TTAATTATCCCAGCCCGTTG	259
Ad01A18456	Aradu.A01	18456	p3	(ATC)5	15	98659374	98659388	AATAAGCATGAATGGGAGCG	TAAGCTGCTGTTGCTGCTGT	263
Ad01A18457	Aradu.A01	18457	p2	(TA)6	12	98662535	98662546	TGAAGCTGTTTTCATGCATTC	ATTCCTTGTCCCAAGATCCC	275
Ad01A18462	Aradu.A01	18462	c	(TA)7a(T)10	25	98680451	98680475	GCCTGTCTATGACAAAGGGG	GGCTTACATTGCTCAGGTTCA	222
Ad01A18469	Aradu.A01	18469	c	(TGA)7ccatgaa	147	98713715	98713861	TGATGAAATTTGTTGTTGCTGA	ATCAACAGAAGCCCAAGCTG	250
Ad01A18471	Aradu.A01	18471	p3	(TCA)5	15	98720456	98720470	CCACCTAGCCATTTGCATTT	ATTGTGCCATGCAGAACAAG	209
Ad01A18472	Aradu.A01	18472	p3	(ATG)5	15	98720676	98720690	GCAAGAAAAAGGATGATGGG	AATGGCATTCCTCACTTTGC	206
Ad01A18474	Aradu.A01	18474	c	(A)10gaaaaaaa	34	98722985	98723018	TCCCACTTCACATCCCAACT	TTTTTCAGCTCAACTCGGTG	234
Ad01A18476	Aradu.A01	18476	p3	(TTA)5	15	98727299	98727313	AGTAATGGCCGGAGGACTTT	GGAGTCTCTTTTCTTTTTCAGGG	256
Ad01A18478	Aradu.A01	18478	p3	(TTG)6	18	98732464	98732481	CATGAGGTGGTCAGACGAGA	CCAAAATGTAGGTACCTCAATGAA	205
Ad01A18479	Aradu.A01	18479	p3	(TTA)5	15	98733157	98733171	TCAGATTGTGTAATTTGATCGGA	TTTTTGATAAACTAGAACAAAATCGGT	114
Ad01A18480	Aradu.A01	18480	p2	(TA)7	14	98734787	98734800	TGGTATCCATCATCACCTGC	TTGGCACAAGTACGAGCAGT	106
Ad01A18482	Aradu.A01	18482	p2	(AT)6	12	98741215	98741226	CCCTAAGCCTTGTCTCTAGCC	CAAGCTGCGGCTAGTTCTCT	254
Ad01A18484	Aradu.A01	18484	p3	(AAG)6	18	98745139	98745156	TCTTCTTTCTAGTGGCTTTGCAG	TTCCGCCCTCTTAACAAGAA	275
Ad01A18485	Aradu.A01	18485	p2	(TA)6	12	98748703	98748714	TTTATCAGCACATCCAAAATTCA	TTTCTCTTCGCCATTCGTCT	180
Ad01A18486	Aradu.A01	18486	p2	(AG)8	16	98749856	98749871	ATGTTGGGCTTCGGTAACAG	CGGATTGGATCGCTTCTAAA	184
Ad01A18496	Aradu.A01	18496	p3	(CTC)5	15	98789849	98789863	AAATCCAAAATGCACCAAAA	AGCGTCAAGAGTTGACCAAAG	274
Ad01A18498	Aradu.A01	18498	p2	(GT)6	12	98793963	98793974	ACAAAGGCTCAACAAATGGC	ACCCGGCAATGTATGAAGAG	239
Ad01A18499	Aradu.A01	18499	p3	(CTT)5	15	98794350	98794364	TCTTAAGGCCTCTGCTTCCA	CTCTCTGAGCCACAAGACCC	167
Ad01A18501	Aradu.A01	18501	p3	(TAT)16	48	98800551	98800598	AGGCTTGTCAACCTTCCTCA	CTTCGTGTTGGTGACTTGGA	194
Ad01A18503	Aradu.A01	18503	p3	(CTC)6	18	98804232	98804249	AGGAGGCTAAAGCGAAGTCC	TAACCCCACCATCCAAAGAA	219
Ad01A18505	Aradu.A01	18505	p4	(AAAT)5	20	98823503	98823522	CGGTCTAACTGTTCACCAACAA	TTGGTGTGGCCAAACTTATG	207
Ad01A18508	Aradu.A01	18508	p3	(TCT)8	24	98829864	98829887	GAAGCAGTAGAGCACGAGGG	GTTGACTCCCACGTATGGCT	258
Ad01A18510	Aradu.A01	18510	p2	(AG)6	12	98833852	98833863	TCACCACGGGTAGAGTACGA	GAGGCCGACAAGCTATCAAG	272
Ad01A18511	Aradu.A01	18511	p2	(TG)15	30	98840521	98840550	GCTGCATTCCCCTGATTTTA	AGAAACCGAACAACGGTACG	225
Ad01A18512	Aradu.A01	18512	p2	(AC)6	12	98840989	98841000	TCCTAATGCCCCAATCGTAG	GCGCTAAATACGAAAACAAGAA	232
Ad01A18514	Aradu.A01	18514	p3	(CTT)6	18	98849672	98849689	GTCCTCATATTGACGCCGTT	GCATACATTCCCCTGCATCT	199
Ad01A18517	Aradu.A01	18517	p4	(TATT)5	20	98860204	98860223	AATATCCCGGATTTCTTGGC	TAAAAAGTTGCGGGTTCGAG	197
Ad01A18519	Aradu.A01	18519	p2	(AT)8	16	98867499	98867514	GGTATTACGTGTCCGGAATCA	TCTTCATTGGGTGAAGATTAATAGA	242
Ad01A18520	Aradu.A01	18520	p2	(TA)6	12	98871348	98871359	ACTTCTTCAATGCAGTGGCT	GCACAACATTAGTTTGGGCA	116
Ad01A18522	Aradu.A01	18522	p4	(TATT)5	20	98873155	98873174	TTGAATACATCTCAGTAGTTTGCTCA	TTCTTCGTCCTAAAAGCATAAACA	204

ID	Chromosome	SSR nr.	SSR type	SSR	Size	Start	End	FORWARD PRIMER (5'-3')	REVERSE PRIMER (5'-3')	Product (bp)
Ad01A18524	Aradu.A01	18524	p5	(ATAAA)5	25	98874095	98874119	TGGTTAAGTGTGATTTCGGTCTT	GATTCTCCACCTTCCCTTCC	223
Ad01A18525	Aradu.A01	18525	p3	(TTG)6	18	98874605	98874622	CCCTGCAGTGCTGTTTCAAT	AAGCAGGCAGAAGCAAAAAG	255
Ad01A18526	Aradu.A01	18526	p2	(TA)8	16	98881568	98881583	ATCCACCTCTTCCATGTTGG	TCAAAGTGATTCAGTTCAATGG	280
Ad01A18527	Aradu.A01	18527	p2	(TA)6	12	98883502	98883513	CCCGGTTCGAATTACTCTCA	AAGCCAATGCCATCTCAAAT	132
Ad01A18541	Aradu.A01	18541	c	(T)10cttttc(AT	30	98937702	98937731	GGACTTAGTAACAGATACCCCCA	CGGTGGTGCTCTTTTTCTTC	222
Ad01A18542	Aradu.A01	18542	p2	(TC)7	14	98938356	98938369	GCCGATAAATTCGACGGTAA	ACAAACTGGTTTCGGTGAGG	199
Ad01A18543	Aradu.A01	18543	p2	(CT)18	36	98938976	98939011	TCCTCCTCACTTTTGCGAGT	GTTTTCGACGACTCTAGCGG	163
Ad01A18545	Aradu.A01	18545	p2	(TC)6	12	98945166	98945177	AAGCATAACATGGGTCACGC	AGAAAACCAATAAGCGCAGC	274
Ad01A18547	Aradu.A01	18547	p2	(AG)8	16	98955024	98955039	TTCTAGTGGTGACGACGGTG	GAGAAATGCGTCGTTTTGGT	198
Ad01A18548	Aradu.A01	18548	p2	(TA)6	12	98958514	98958525	TTGAATGGCGACCATTTCTT	AGCAGCATGCATTGACTGTTA	175
Ad01A18549	Aradu.A01	18549	p2	(TA)6	12	98961257	98961268	GTGATCGGATGAATGCAATG	TGGTGGACACTCTTGTGGAA	216
Ad01A18552	Aradu.A01	18552	p3	(TAT)8	24	98964734	98964757	TTGGAAAAAGCCAAAAATGC	AGTCTTTGTTGGGTGATCCG	207
Ad01A18553	Aradu.A01	18553	p2	(GA)7	14	98967258	98967271	AGAAAGTGTCGGTGCTTCGT	TGAATAGTGTCACGCTGGGA	194
Ad01A18554	Aradu.A01	18554	p3	(TCC)6	18	98979553	98979570	CGTTATCACCACCACCATCA	TTCTGAGTTGTGTTCGTCGC	190
Ad01A18555	Aradu.A01	18555	c	(A)12ctattggtc	89	98982384	98982472	GGAATTTTGGCAAGCATCAT	CCCTTAAAATTTCCTTGCATGT	198
Ad01A18557	Aradu.A01	18557	p2	(AT)8	16	98984558	98984573	TCGCCATGCCAACATTACTA	TTGATGGGACAAAGAGGTCC	228
Ad01A18561	Aradu.A01	18561	p3	(CTT)5	15	98996869	98996883	AAGGCCAAGGAAACCAGTCT	TGTGGCTTCTATGCAGGTTG	236
Ad01A18562	Aradu.A01	18562	p2	(AG)14	28	98999008	98999035	GATGGCACTTTCCCAGGTTA	GGTAGCAACCAAAAGGAGCA	194
Ad01A18567	Aradu.A01	18567	c	(TA)7ttatataaa	56	99012920	99012975	TATCAACCCGATCTGCAACA	TCGCGGGTTACAGAAAAGAT	201
Ad01A18568	Aradu.A01	18568	p3	(GGC)5	15	99014247	99014261	GGCAAAATTTAGTCACAACTAACAG	ACGGTAGCACCTACTACGCC	271
Ad01A18569	Aradu.A01	18569	c	(T)10gtcaaagtg	102	99018321	99018422	CAAAGGGACGAACGTGATTT	CCTTCTGGTTGTAGGTTGGG	274
Ad01A18572	Aradu.A01	18572	p3	(ATA)9	27	99028642	99028668	CCTCTGCCTATGACACCCAT	AAATGTTCAAGCGACATGGA	202
Ad01A18573	Aradu.A01	18573	p3	(CTT)5	15	99028919	99028933	AATGCCAAACTTTTGACGAA	ATCCTGGGGTCTGATTCTCC	275
Ad01A18576	Aradu.A01	18576	p2	(AT)7	14	99040236	99040249	AAAAATTGCGGGTATGATCG	TCACACTTTTAAAACCTATTTATTCCA	276
Ad01A18577	Aradu.A01	18577	p3	(TAC)5	15	99044916	99044930	TTGGTGTGGAGCATGTTGTT	AAATGGAGGAGGGTAATGGC	256
Ad01A18580	Aradu.A01	18580	p4	(TTTC)6	24	99050096	99050119	CGCCAGTAGTCTCGAGTTTTG	GATCATGGAGCGTTCATGTG	226
Ad01A18584	Aradu.A01	18584	p2	(GA)6	12	99052384	99052395	CATGATAGAACGAGTGGCCC	TCGCTCAATCTTCTGCTCAA	265
Ad01A18585	Aradu.A01	18585	p2	(TG)99	198	99058596	99058793	TTTTAGGATATCTTCTTTTAATTTGGA	CAGCCTCTATAGGCAAATTTCA	274
Ad01A18587	Aradu.A01	18587	p3	(ATA)24	72	99061125	99061196	GTTTCACTCTGCACAACCGA	GTTTAGGGGAAGGGGAATGA	159
Ad01A18589	Aradu.A01	18589	c	(AAC)5(AAT)	33	99072179	99072211	ATGTGCCGAATACCCAAAAA	TCATCAAATCCCTCCTCCAG	235
Ad01A18594	Aradu.A01	18594	p3	(ATT)6	18	99081793	99081810	AAGCAACTCAGCCATTTCGT	ATGTGCTTTTGGGTATTGGG	228
Ad01A18595	Aradu.A01	18595	p3	(ATA)5	15	99082228	99082242	TCCAAAACACGGTGTGAAAA	TTCATTTTTAGCTGAGCCAATG	246
Ad01A18596	Aradu.A01	18596	p4	(TGTT)5	20	99083424	99083443	AACTTGCAGCAAAGGAACTTG	GGGAGGCTAGCTCCACTAGG	188
Ad01A18600	Aradu.A01	18600	p2	(AG)6	12	99100343	99100354	TTCATAAAATCCAAAGCGGG	CGTGTTTGAAAATGGGACCT	279

ID	Chromosome	SSR nr.	SSR type	SSR	Size	Start	End	FORWARD PRIMER (5'-3')	REVERSE PRIMER (5'-3')	Product (bp)
Ad01A18601	Aradu.A01	18601	p2	(AT)6	12	99100568	99100579	CCCGCTAGCCACATTTCTTA	CAAATGAGTGTCAAAATTGAGGA	279
Ad01A18608	Aradu.A01	18608	p2	(TA)6	12	99120285	99120296	CTCTACATGCAAGCAAGGCA	TGTCCATGCATCATAGTCACTAAC	234
Ad01A18613	Aradu.A01	18613	c	(AG)6aagcgatg	67	99139618	99139684	GCCGTTACAAGGTCATCGTT	GTCCCTTCCTCTCTTCGCAT	250
Ad01A18620	Aradu.A01	18620	c	(A)10tttagtttcg	47	99147458	99147504	TAAGTGGCGGAGCTACCTTG	GCAATTGGGCACTGACATAG	280
Ad01A18623	Aradu.A01	18623	p2	(TC)6	12	99150849	99150860	GGTTTGAGGCAAATTTTAGGG	CCATACATGCATGCTTCTCC	258
Ad01A18625	Aradu.A01	18625	p3	(TAT)15	45	99153858	99153902	CAAGTGTGTCGATAAGAATAAATCC	CAAACTCAGAAATTCGTTGGG	205
Ad01A18628	Aradu.A01	18628	p2	(CT)18	36	99164277	99164312	CACCTTCTTGATTGGGTGGT	GCAGCCAAAGAGGAGTACGA	242
Ad01A18629	Aradu.A01	18629	p2	(TC)9	18	99172247	99172264	TGTGTGATGTATGTGGTGGCT	CCCAGCAAAATAAACTTCCG	153
Ad01A18631	Aradu.A01	18631	p3	(TGG)5	15	99177972	99177986	TTGCTATGGAAAACGGCTCT	CTGTTGATAGTTCAGCGGCA	214
Ad01A18634	Aradu.A01	18634	p2	(AG)15	30	99182054	99182083	CCGTTGGAGTTGTTGGAGTT	TCCGTCTCTTTCGTACCCAC	271
Ad01A18635	Aradu.A01	18635	p2	(AC)15	30	99184828	99184857	AAAATCCGCTCGTAATGCAC	GCACGAGTTTCCTTTCTTGC	219
Ad01A18638	Aradu.A01	18638	p2	(AT)7	14	99204154	99204167	ATTCAGGTGCACACATTGGA	AACAGCCACGGTATTTCCAG	261
Ad01A18640	Aradu.A01	18640	c	(A)11gaaattttta	96	99206645	99206740	TTGAAACAACCATCACTGGC	CATCCCTACCTGAGACCCAA	259
Ad01A18642	Aradu.A01	18642	p3	(TTA)5	15	99214540	99214554	AATCAATTGGTGGGAGCTTG	CTTTTGGTTAAAATCAAATACAAATCA	234
Ad01A18643	Aradu.A01	18643	p3	(ATA)5	15	99225538	99225552	TGGTAAGAAAGATGGGCATTG	GAAGAGATGACGACGGAAGC	266
Ad01A18647	Aradu.A01	18647	p3	(CTT)6	18	99232290	99232307	TCCTGATTCCTGATCCATCC	GGAAGCAAATGCATGGAAGT	125
Ad01A18652	Aradu.A01	18652	p2	(AC)10	20	99243868	99243887	ATGGTGCCAAAATAGCCAAG	ATTAAAGCAAAAGGCGCAGA	244
Ad01A18653	Aradu.A01	18653	p4	(ATCA)5	20	99244783	99244802	ATTCTCGATTGAAGTGGCCT	CCCCCTTCGATGTACAAAAA	247
Ad01A18654	Aradu.A01	18654	p3	(TGG)6	18	99245569	99245586	ATGGTTACCCTCCACAACCA	CGGTAATATCGGACCACCAC	215
Ad01A18655	Aradu.A01	18655	p2	(AG)6	12	99245949	99245960	AGCCTCGACAGTTCCAAGAG	GAGCACTACCCCTTCCCAAT	274
Ad01A18662	Aradu.A01	18662	p3	(AAT)6	18	99283728	99283745	TTGCAAACAGTTAGTAAGTTGAGAA	TTTTTCTTCTCTGCATGTGTTTACTT	243
Ad01A18664	Aradu.A01	18664	p2	(AG)7	14	99286406	99286419	TTCGTCGCTGTGAAAGATTG	AAATTCGTCGGTAAAGCTCAA	252
Ad01A18667	Aradu.A01	18667	p2	(CA)10	20	99291120	99291139	CTGCCTACCCTTCACAGCTC	AGAAACTGCAACGGTAACGC	260
Ad01A18671	Aradu.A01	18671	p2	(TG)7	14	99301778	99301791	CCATGGCAAATACCCTAAGC	TCGAAGTACAGTGTCTTTTGAAGAA	276
Ad01A18672	Aradu.A01	18672	p2	(TA)6	12	99317271	99317282	GGATCTGCGATATTTTGGGT	TGTTGTGACGGATGCAAGAT	280
Ad01A18673	Aradu.A01	18673	c	(A)12tcttgcatc	48	99317431	99317478	TGGATTGTGATTGGATGATG	GGAAAGTCATGTTTTTCACCAA	193
Ad01A18677	Aradu.A01	18677	p2	(TA)6	12	99329726	99329737	CGAAAATTGAGTTTGGCTCATT	TCAATTCAACTTATTAATTCGTGAGC	264
Ad01A18682	Aradu.A01	18682	p2	(AG)17	34	99343600	99343633	CACATGAGTTGAGCGAAGGA	GCCATTCACTTTTTGCCATT	249
Ad01A18684	Aradu.A01	18684	p2	(TA)6	12	99349011	99349022	TGCATTTCGATATCCACTTTG	CAGTATCGCTTTAGGGCCAG	277
Ad01A18685	Aradu.A01	18685	p2	(TA)10	20	99350015	99350034	ACGAAAAATCGACCACTTCA	TTGGGTAGGATAGAGTGCGAG	275
Ad01A18687	Aradu.A01	18687	c	(AAG)5g(AGA	31	99353412	99353442	ACCTCCTCAAGCTCTCGTCA	CGAGAAGGACAAGGTTCTGC	134
Ad01A18690	Aradu.A01	18690	c	(A)10cggaggac	93	99367908	99368000	TGAGAGCGCATTGAATAACG	AAGGTTTCATTTGATCCAAAAAT	255
Ad01A18694	Aradu.A01	18694	p3	(ATT)5	15	99402146	99402160	CGCATTTTGGGTGACAATCT	GCAATCATCACATCATCCCA	278
Ad01A18703	Aradu.A01	18703	c	(A)10ggaaggga	41	99428751	99428791	TTCGAAGAGCCACTTCCACT	ACGTTCAGGCAAAAGGAAGA	280

ID	Chromosome	SSR nr.	SSR type	SSR	Size	Start	End	FORWARD PRIMER (5'-3')	REVERSE PRIMER (5'-3')	Product (bp)
Ad01A18706	Aradu.A01	18706	p3	(ATT)5	15	99441323	99441337	CAAATTTGGGCACACCTTCT	GAACACGCATGGGGATTTAT	218
Ad01A18711	Aradu.A01	18711	p2	(TC)7	14	99458444	99458457	GATGCATTTTCTTGGTGGCT	GCTGGTCCAACCGAATTTTA	199
Ad01A18712	Aradu.A01	18712	p2	(TA)6	12	99462281	99462292	GAGCTCTTCCCATCGTTGAT	TCCTACACACCACAGGGTGA	275
Ad01A18713	Aradu.A01	18713	p3	(CGA)5	15	99465434	99465448	AGTCTGAGACGGTGAGGAGC	CATCTCCTTCGTCTCCTTCG	260
Ad01A18718	Aradu.A01	18718	p2	(AT)12	24	99498629	99498652	TTTGTAATGACCCAATTTCTATTACG	CAGGGCCTGAAATGTTGTGT	275
Ad01A18720	Aradu.A01	18720	p3	(GCC)5	15	99503847	99503861	GACTCTGCGTCTCAAAACCC	AGGCTCTTCATCTTCGGTGA	115
Ad01A18721	Aradu.A01	18721	c	(A)10cactaatta	52	99508273	99508324	GGAAAGGAGGACCCCTATCAT	CTCCACTGGGTTGTGTTGAA	280
Ad01A18723	Aradu.A01	18723	p2	(CT)7	14	99514276	99514289	TTCTACCCCTCTCGGTGTTTT	GACAGCAACAGGACATGCAC	212
Ad01A18730	Aradu.A01	18730	c	(T)11ctttaaaaa	98	99532126	99532223	TGTGCAACATTGGATTGTCTT	GAGTCCCCAACTCAAGTGGA	214
Ad01A18734	Aradu.A01	18734	p3	(TCT)5	15	99538453	99538467	CCACATACTGTTACGGCACG	CCTCCTCATCTTCTGCTGCT	242
Ad01A18736	Aradu.A01	18736	p3	(TAA)7	21	99541785	99541805	TGTTCATTTAAAGAATATCCTCATCTT	TGTTATTCACATAATGGTTAATTTTGC	267
Ad01A18739	Aradu.A01	18739	p2	(GA)22	44	99559417	99559460	ATCTAATGCACGCCTTCCAC	GCACCATCATCATGCTTTGA	241
Ad01A18740	Aradu.A01	18740	p2	(AT)6	12	99561604	99561615	GCTTGCTTGGGAAGTTGGTA	TCACAAGAGCGCATAATTGC	140
Ad01A18749	Aradu.A01	18749	p2	(TA)6	12	99585971	99585982	CTTCCCTATGCGGTTCATGT	TATGCATGGTGCAGTGAGGT	231
Ad01A18756	Aradu.A01	18756	p3	(AAT)9	27	99595650	99595676	ACCAGGAATTATGGCACAGC	GCCACAACAACACTGGTTTC	172
Ad01A18757	Aradu.A01	18757	p3	(TTA)16	48	99599643	99599690	AAGGGTGTCAGCAATATCGG	GGCCAAGCCGGTTACTATTT	209
Ad01A18759	Aradu.A01	18759	p2	(TA)6	12	99621334	99621345	TCCGTGAGCTTTTAAGTGGC	AAAAACCAACACACAACATTGA	253
Ad01A18763	Aradu.A01	18763	p2	(AT)7	14	99638937	99638950	TGCTGTCTCATATTCGTACGTG	TTAATGGCTATCTCATGACAAAAA	270
Ad01A18768	Aradu.A01	18768	p2	(TC)8	16	99655109	99655124	GTCGGGTTCAGTACGTGGTT	TTTTACCCATGAAAGGCTGG	280
Ad01A18769	Aradu.A01	18769	p3	(AAT)21	63	99656897	99656959	TGTTATGACAATAATTTAGCCCTG	TTCTGTGAATCTAAGAAAGGTTTTG	255
Ad01A18770	Aradu.A01	18770	p2	(AT)9	18	99657617	99657634	TCAAATTCAATATCCGCACTTTT	TGCAGCAATGAACCTATAGCA	275
Ad01A18772	Aradu.A01	18772	c	(TTC)6attatcg	69	99665718	99665786	TCACGCTCCTCCTTAACACA	CAGCCAGCCACTTGATTAGA	277
Ad01A18785	Aradu.A01	18785	p3	(TTA)7	21	99699414	99699434	CAATCCTATCTTCAAATTGCTGG	CATCCATACCCCAAGACCTG	232
Ad01A18786	Aradu.A01	18786	p4	(CATG)5	20	99702478	99702497	ATTGCTTCTGTTTGGGGTTG	CAACTGTGCATCCATCCATT	239
Ad01A18790	Aradu.A01	18790	p3	(TGG)7	21	99711469	99711489	GCCTTGCTTCTGCTTTCTTG	TACCAGCACCACCACTACCA	227
Ad01A18791	Aradu.A01	18791	p3	(TTG)16	48	99713873	99713920	TGAAAAATTGACACCTCACTTCA	TTTCCCTCCAAAATGAATGC	241
Ad01A18794	Aradu.A01	18794	p3	(ATA)5	15	99715955	99715969	TTTTTGTACATTTCATATCACATCC	GTGCTCATCATTCGGAGCTT	279
Ad01A18800	Aradu.A01	18800	p2	(CA)6	12	99731628	99731639	AGCACGCCTGAACCTATCAT	TGATAGTGGCATCAAGGGAA	272
Ad01A18801	Aradu.A01	18801	p3	(AAT)7	21	99731989	99732009	TTCCCTTGATGCCACTATCAT	CTGATGAATCGCATTGGAGA	198
Ad01A18803	Aradu.A01	18803	p3	(ATG)5	15	99741953	99741967	GGTGACAAGACTCCCATCGT	TGCTGATCCATTATTGGCAG	162
Ad01A18804	Aradu.A01	18804	p2	(AT)6	12	99742622	99742633	GGGATGCATCTCTGCTTTGT	CATGACAAAATTGATCCCCC	255
Ad01A18809	Aradu.A01	18809	p4	(ATAG)6	24	99789195	99789218	TCATTAAGTGCAAAAACCTGATT	TTTGTTACCCGTGATATGATGAA	173
Ad01A18810	Aradu.A01	18810	p3	(TTA)5	15	99789338	99789352	TTTCATCATATCACGGGTAACAA	GGTGTCGATTCCTCATGAATTT	271
Ad01A18813	Aradu.A01	18813	p2	(AG)8	16	99811033	99811048	GATGGAGACGGGAGACAAAA	GATGGAGTATGGACGACGCT	273

ID	Chromosome	SSR nr.	SSR type	SSR	Size	Start	End	FORWARD PRIMER (5'-3')	REVERSE PRIMER (5'-3')	Product (bp)
Ad01A18816	Aradu.A01	18816	p3	(TTA)5	15	99814752	99814766	GATGTGACAAATGCACTGGC	AATTCACCGTCCGTAACAGC	216
Ad01A18817	Aradu.A01	18817	p2	(TA)7	14	99824209	99824222	CCGTTCGTTAATCGAAATGTT	GTTGCGAATTCGAGTCTCCT	176
Ad01A18821	Aradu.A01	18821	p3	(AAC)5	15	99835552	99835566	ATTTATTTCGTCCTCGGCCT	GCTTCTGCTTCTGGTTTTGC	276
Ad01A18822	Aradu.A01	18822	p2	(TA)7	14	99837696	99837709	TGTGGCAAAAATTGCAGGTA	GTGCATGCTTTCCAACCTTT	270
Ad01A18823	Aradu.A01	18823	p2	(TA)6	12	99840376	99840387	ACTTCCAGTTCACATTGCCC	AACAAATGCAATTCTCTCTCTTTTT	232
Ad01A18826	Aradu.A01	18826	p5	(TATTT)5	25	99856889	99856913	TCTCCTCTGCCTTCTTTTCG	TTTTTCTTTAAATCCAAACAAACCTT	100
Ad01A18829	Aradu.A01	18829	p3	(CTC)5	15	99859744	99859758	ATAGCATCTTTGGCGACGAC	CCTGGCCAAACACAAAACTT	222
Ad01A18831	Aradu.A01	18831	c	(AT)7taattgtac	87	99869799	99869885	TGTAAGTCGTCTTGTTCTTGTTAAA	GATCAGAGCATATGCATAGATATAAAA	233
Ad01A18835	Aradu.A01	18835	p3	(TTG)5	15	99882298	99882312	AACTTTTAACTCTCAAGTCAGGAGAA	AAAAGAATCCATCTGTGCATGTT	251
Ad01A18839	Aradu.A01	18839	p2	(TC)8	16	99898189	99898204	TAGAGTTGGGGGTGTTCAGG	AACAGGGACCCACACAGAAC	200
Ad01A18840	Aradu.A01	18840	p3	(TTC)5	15	99899856	99899870	ATTATCCCTCCCTTTTTGCG	ACCAGCCTTGGCTAACACAC	189
Ad01A18842	Aradu.A01	18842	p2	(TA)9	18	99909623	99909640	GCCAAGGTTATCAAACTCGC	TTGTAAGGGATTTGAACTTGTGTT	186
Ad01A18849	Aradu.A01	18849	c	(T)11cttttttatttt	101	99914728	99914828	TTGTGATGCCATGTGTATGC	CCTTGCCTTCCAAAAATCAA	234
Ad01A18851	Aradu.A01	18851	p2	(CT)8	16	99918930	99918945	CGTCGCTGCAGATTTATTCA	CAAGCTTTTCAAACGGCATT	257
Ad01A18852	Aradu.A01	18852	p2	(AT)9	18	99923902	99923919	GTTGTGAATCACGCACTGCT	TGTGTTTCAGTTGGTTGATTGTT	188
Ad01A18853	Aradu.A01	18853	p2	(CT)6	12	99925393	99925404	CCTAACCTAACCCCGCTCTC	CAACCATGCCACTAATCACG	151
Ad01A18857	Aradu.A01	18857	c	(TAC)6(TGC)5	33	99942821	99942853	ACTATTCAAGAGCGAGGGCA	ATGATGGCGGGTCTGTACTC	227
Ad01A18858	Aradu.A01	18858	p2	(TC)15	30	99946406	99946435	TCTCAAAATCTCAGTCTTTTCCAA	GGTAGATGCAATGGTGGCTT	269
Ad01A18859	Aradu.A01	18859	p2	(TC)27	54	99949171	99949224	CGGCTCGTTAATCAGAGCTT	CAATTGAACCGGATTTCACC	276
Ad01A18863	Aradu.A01	18863	p2	(AT)6	12	99953438	99953449	TCTTAAGGGTTCGTGGCATT	CTCGTATCGTGGTTGGTTCA	195
Ad01A18864	Aradu.A01	18864	p2	(AG)6	12	99954889	99954900	GAAGCCATCGTTGACCTTGT	TCCATGACTTCCCTTCCTTG	276
Ad01A18865	Aradu.A01	18865	p2	(TA)6	12	99956983	99956994	GCACGGACCCAAGGATAATA	ATAAACGAGTGATGGCTGGG	252
Ad01A18867	Aradu.A01	18867	c	(A)10gtagacac	29	99975578	99975606	TCCGGGTTACCAAAAATTCA	TCAATCCCTCAATAGCACCTG	250
Ad01A18872	Aradu.A01	18872	p2	(TG)7	14	99982836	99982849	GCAAGTTGGCCCATTTGTAT	ACCACCAACAACAATCACCA	224
Ad01A18881	Aradu.A01	18881	p2	(CT)6	12	100006230	100006241	GCCATTTTCGGATAGAGCAG	TCTTGAAGTGCAACGTCCCT	259
Ad01A18882	Aradu.A01	18882	p2	(CT)7	14	100015750	100015763	GCCATTTTCGGATAGAGCAG	TCTTGAAGTGCAACGTCCCT	253
Ad01A18887	Aradu.A01	18887	p3	(ATA)5	15	100036586	100036600	TTCGTGTTGGATACCCATAGC	CAGATGCATTCTTTGGGATG	220
Ad01A18889	Aradu.A01	18889	p3	(ATA)5	15	100040431	100040445	CATAAAGGATGACACGTTGGAA	GGACATTTTCGTCCAAAGGA	277
Ad01A18891	Aradu.A01	18891	p4	(AATA)5	20	100046453	100046472	GGAAAATCAAGCAGCTTCAAA	TTCGAATAATGTCACTGCTGC	200
Ad01A18892	Aradu.A01	18892	c	(T)10ctcaatgcc	72	100046841	100046912	AAGAAGCACTTGTTGGGAGG	GCCAGCTTGATGCTTCTTTC	234
Ad01A18894	Aradu.A01	18894	p6	(CGCGAC)5	30	100048456	100048485	AATCGACCTTCCTCCGATTC	AGCCCTACTTTCCTCTTCGC	237
Ad01A18897	Aradu.A01	18897	c	(T)10attcatc(T	27	100057005	100057031	GTGGCACACCAAACTTAGGG	CATGACAGATTCTTCACTTGGTG	196
Ad01A18898	Aradu.A01	18898	p3	(AAG)5	15	100057542	100057556	AGAAAGAGGAGGCGGAAAAA	GCGTCACTCAACCATTTTCA	209
Ad01A18899	Aradu.A01	18899	p2	(AT)8	16	100058785	100058800	AGTTGGCATTTTTGTCCCAG	CTTCCCCAACTGTAACCGAA	209

ID	Chromosome	SSR nr.	SSR type	SSR	Size	Start	End	FORWARD PRIMER (5'-3')	REVERSE PRIMER (5'-3')	Product (bp)
Ad01A18901	Aradu.A01	18901	p2	(TA)6	12	100067721	100067732	TTCTCGCTGCCATTTTCTCT	TGCTTGACCTTGTAATGTTGC	244
Ad01A18902	Aradu.A01	18902	p3	(AAG)5	15	100069962	100069976	AACACGAAGAAGAAGGCGAA	ACGCGTCCAATACACACAAA	160
Ad01A18904	Aradu.A01	18904	p2	(TA)8	16	100072048	100072063	CCTGGGGACAATGCAATAAC	TGATGTGTCGCTTTTTGAGC	191
Ad01A18905	Aradu.A01	18905	p2	(CT)6	12	100072815	100072826	TATGCATCTGATGGTTTCGG	CCTAAGAAGTCAGCGATTCGAG	142
Ad01A18906	Aradu.A01	18906	p2	(TA)6	12	100072931	100072942	TTACGCCTCATCCCTCTCTC	AGAGGAGAAGAGAATGTCATTTTAGTA	261
Ad01A18907	Aradu.A01	18907	p2	(CA)6	12	100075203	100075214	GCAAAATGATGGACACGTTG	CACAAAAAGCATGCAAGTGG	246
Ad01A18909	Aradu.A01	18909	p3	(TTC)9	27	100094974	100095000	CCAATGTTGTCCCTGATTCC	TGATCGCAAAGAACTTCCCT	212
Ad01A18912	Aradu.A01	18912	p3	(AAT)5	15	100109149	100109163	CAGTCAGTTGCCGTTTGCTA	TTTGTGTTTGTGGCCTGAGA	257
Ad01A18914	Aradu.A01	18914	p3	(AAT)7	21	100116308	100116328	TGCACTGAACCCTATCCCTC	ACAACGTCGGTTGACTCTCC	253
Ad01A18915	Aradu.A01	18915	p2	(AT)7	14	100116999	100117012	TCGCACCTGAACGATTAAGA	GCTTTTTGGTTACGTAAACGCT	206
Ad01A18919	Aradu.A01	18919	p3	(GTA)6	18	100129715	100129732	CCCGTCCAAAGATCGTACAC	TTATTTCAATCACGTGCCACA	272
Ad01A18925	Aradu.A01	18925	c	(A)13tccaatacc	157	100161822	100161978	TCCATGTAACCATGTAACCAAGTT	AAGGAGGAGAAGGAGGAACG	226
Ad01A18926	Aradu.A01	18926	c	(GC)7agcgtgct	48	100166818	100166865	CGCGCATAACTCTCTGGTTT	ATAGGGAGCTCTTGGGCATT	133
Ad01A18927	Aradu.A01	18927	p5	(TCAAT)5	25	100170863	100170887	CCCTACAAATGGGGTTTGTC	TGAATTAAATGGACGGAGGTG	166
Ad01A18930	Aradu.A01	18930	p3	(CTT)5	15	100177600	100177614	GTGGGGCTACATACAGCGTT	CTCGACGAGACACAGCAGAG	273
Ad01A18931	Aradu.A01	18931	p3	(TTC)5	15	100182926	100182940	CTCCCCTTGAGCTTCCTCTT	GCATGTCATCCTTCATGCAC	276
Ad01A18935	Aradu.A01	18935	p2	(AT)6	12	100191997	100192008	TCTGATAACACAAGTAAAACCAAAA	CTGGGTTTTCGAAAGGGACT	274
Ad01A18938	Aradu.A01	18938	p2	(AT)7	14	100196404	100196417	CCGTTGAAAATGAAGAAGGAA	AGCAGGAGCTCATCCAGTGT	271
Ad01A18940	Aradu.A01	18940	p2	(TA)8	16	100206026	100206041	CATAGAGCAAAGGCAAGGCT	GCCAGGGAGAGTAACACCAA	142
Ad01A18942	Aradu.A01	18942	p4	(TAAT)6	24	100207817	100207840	CAAAAGCAGCACCAACAAGA	TGATTGCAGAGGAACCATCA	219
Ad01A18943	Aradu.A01	18943	p3	(TTC)7	21	100215004	100215024	GTCACTGATCTCTGCACCCA	TGATGGTTTGGCATTTAGCA	163
Ad01A18948	Aradu.A01	18948	p2	(AT)6	12	100221957	100221968	GACACGCATTGGCTTTTACA	AAAAAGCTGTTGTTATGAGCTGAA	256
Ad01A18950	Aradu.A01	18950	p3	(AGA)5	15	100223775	100223789	GAATGCGACCAGAGGAGAAC	TTGTTTGTCATACACGCGCT	148
Ad01A18951	Aradu.A01	18951	p2	(TA)9	18	100224545	100224562	GGTTGTTGTGGAAAAGAGGAAC	TCCTCTTTCTCTCTCTTTCTCTTCC	103
Ad01A18953	Aradu.A01	18953	c	(T)10ctcttttatct	68	100227507	100227574	GATACCAACAAGTAATCGGTGC	TTTTTCCCTTAACACCCAAAAA	170
Ad01A18954	Aradu.A01	18954	p3	(TAT)5	15	100228241	100228255	GCACATATTCCCAAAACCGT	TCTTCTGTTTTCGTTGTCATTTTT	245
Ad01A18956	Aradu.A01	18956	p3	(GCA)5	15	100231184	100231198	CAGCACCACAACGATCAACT	ATGGTGGTGGTTCCTGAAGA	239
Ad01A18961	Aradu.A01	18961	p2	(CT)13	26	100264445	100264470	CGGGTTTCCTTTTCTTGTCA	CCCCCTTTTTGCTAATGCTA	278
Ad01A18962	Aradu.A01	18962	p3	(TGG)5	15	100266997	100267011	TTGTGTGCCCTGTTTTGCTA	AAGAGCAGCAAGACCAACTCA	190
Ad01A18963	Aradu.A01	18963	p3	(CTC)5	15	100270745	100270759	AAGGGGTATGAGCACAATGC	GCCAGAGAGATCAAACCCTG	228
Ad01A18967	Aradu.A01	18967	p3	(TAT)5	15	100283814	100283828	CCAATGTAAACATTATTAACAAAACCG	CGCTAAGAATACACTCGTTATGGA	269
Ad01A18968	Aradu.A01	18968	p3	(ATA)13	39	100283932	100283970	AACAAAACCGTTTACTGTTGATGA	CGCTAAGAATACACTCGTTATGGA	252
Ad01A18969	Aradu.A01	18969	p3	(TAA)13	39	100284167	100284205	TCTTCGTTTCTTTCCTAAACTCTTTC	TGTGAATCAGCTCGGTGAAG	255
Ad01A18970	Aradu.A01	18970	p3	(ATT)8	24	100296007	100296030	GCTACATATTTTTAATTTTGTTGTGTG	AAAATTTGGGAGATCATAAAACC	230

ID	Chromosome	SSR nr.	SSR type	SSR	Size	Start	End	FORWARD PRIMER (5'-3')	REVERSE PRIMER (5'-3')	Product (bp)
Ad01A18973	Aradu.A01	18973	p2	(AT)10	20	100300269	100300288	CTTACTGCTGTGGTGGTTCG	CCACAACACATAGCATAGACCA	188
Ad01A18974	Aradu.A01	18974	p2	(GT)6	12	100301816	100301827	CCATTACTATGGTGGATTCGG	TTTTCACTCTTGCACAGGCA	189
Ad01A18976	Aradu.A01	18976	c	(ATT)13aggatt	150	100304199	100304348	TTTTGCCTAAATTGCTTGGC	TGTTAGAATTGTTGGCATGGA	251
Ad01A18977	Aradu.A01	18977	p4	(TGAA)5	20	100306097	100306116	TTTCCCAAAAGAAAAAGCGA	GAAGAGTGGTGGTGGGAAGA	185
Ad01A18978	Aradu.A01	18978	p2	(TA)6	12	100307078	100307089	TCACCCAAAACAACCCTAGC	TGCTCATCCTTGAATGTGAAA	224
Ad01A18980	Aradu.A01	18980	p2	(GT)6	12	100307507	100307518	AAGAGGAGAAAATTGATTTGGTTG	TGCTTGCACTATCAGCCATC	155
Ad01A18981	Aradu.A01	18981	p2	(TC)6	12	100307623	100307634	TGATGGCTGATAGTGCAAGC	TGGATGGAGGTTGGTTTGAT	114
Ad01A18982	Aradu.A01	18982	p3	(TCT)5	15	100307787	100307801	CATGCATCATCGTTCATTCC	CATAATTGGCGGTGGTGTTT	262
Ad01A18983	Aradu.A01	18983	p3	(CTA)7	21	100308845	100308865	ATCATTCCCCCATCCTCTTC	GGGGCTTTGATGAGGACATA	234
Ad01A18984	Aradu.A01	18984	p4	(TTTA)5	20	100309606	100309625	CTGGTATGAGCGAGCCTCTT	TTTTGAGTTTGGACTTGTGTTGA	204
Ad01A18987	Aradu.A01	18987	c*	(A)11(AT)9*	28	100330407	100330434	GCTCAACTTGCGAAGTCAGG	AATCCAAGCGAAGTTGAGGA	209
Ad01A18989	Aradu.A01	18989	p3	(TAT)5	15	100332508	100332522	CCACAAACTCAAATTCCTCCA	TGGGGTTTGGAATTGAAGAT	274
Ad01A18990	Aradu.A01	18990	p2	(AG)21	42	100338204	100338245	GGAGTAACACTGTCGGGGAA	ATTCATTTCGCGCTCTCACT	207
Ad01A18995	Aradu.A01	18995	p3	(TAT)5	15	100372738	100372752	GCCGCTGTTTTCAAAGGTTA	CCCTACAACGAAGACCCTTG	225
Ad01A18997	Aradu.A01	18997	p2	(AT)6	12	100377181	100377192	TTTCCTCTGGGTTTGATTGC	TGTTTCGACCAAAATGCTTG	268
Ad01A18998	Aradu.A01	18998	p2	(TC)6	12	100378020	100378031	ACCATCTTTGGGCTGAGAAA	ATGTTGACACAATGAGGCCA	271
Ad01A19004	Aradu.A01	19004	c	(GGTTT)6gctc	81	100396528	100396608	ATATTCGTGTGAAGGAGCCC	AACCTCCAAGAGCTGCTCAA	193
Ad01A19005	Aradu.A01	19005	c	(A)14ctggttttg	164	100399339	100399502	GGTTTTTGCAAAGAAAAATTGG	TGCCTTGACTGATATGTTTGTTC	279
Ad01A19006	Aradu.A01	19006	p3	(ATA)5	15	100401031	100401045	AAAACCATGGAGAACCAACG	TTTAGATGGCATTTTAAGGTTTG	249
Ad01A19007	Aradu.A01	19007	c	(A)10gagatttgt	107	100401342	100401448	GCTTCCGAGAATCCCAGTTA	ACCGAAGGAGGGTAGGATTC	272
Ad01A19008	Aradu.A01	19008	p2	(GA)6	12	100402035	100402046	GCAAATGACCACAATGAACG	TGAATCCGGCCAGTTTATTT	221
Ad01A19009	Aradu.A01	19009	c	(CT)6agtctctcc	46	100404124	100404169	TGGAACTTCTAACCTCAGCCA	CCTTCAATTGCAAACCCAAT	199
Ad01A19010	Aradu.A01	19010	p3	(AAT)14	42	100404535	100404576	GTAATGTCAGCTCCGCGGTT	GCAGCAGTTTCAGTCAACCA	234
Ad01A19015	Aradu.A01	19015	p3	(AAT)5	15	100410533	100410547	TTCTCAAATGGTCAAGCAAAGA	TCACAATGCTATCGGGAGAA	238
Ad01A19017	Aradu.A01	19017	p4	(GAAA)6	24	100419005	100419028	CTTCTAGGCGGCTGTTTTTG	GTGCGAATACAAGCAAAGCA	262
Ad01A19019	Aradu.A01	19019	p2	(AT)8	16	100420848	100420863	GTTTCATGGGAAAAAGCAGC	CATGTGTGTTTGAGACAGTTATATGG	273
Ad01A19023	Aradu.A01	19023	p2	(AT)7	14	100439530	100439543	TCCTAAGCGGATCCCATAAA	TTTTTGACCGCGTTATTCATT	280
Ad01A19029	Aradu.A01	19029	p2	(AT)7	14	100453649	100453662	AATGACACAATAAACTCGTGACAA	AAAATGAAAAATGGATGACATGA	206
Ad01A19031	Aradu.A01	19031	p2	(AG)6	12	100455937	100455948	TAAGGGTATTCACCATCCGC	GGGCTTCATTGGAGTTTTCA	255
Ad01A19034	Aradu.A01	19034	p3	(GTT)10	30	100467209	100467238	TTAGTGCCTTCTGGCTCTGG	GGATTTGGCAGAAAAACCAA	104
Ad01A19035	Aradu.A01	19035	p2	(AT)6	12	100474702	100474713	TTCTCAATCCCAGAATCCCA	TATGGAGCGTGCATTTGTTC	247
Ad01A19039	Aradu.A01	19039	p2	(CT)6	12	100501011	100501022	GGGCTAGGAATCCTTCTTGG	GCCTCCATGGTTGAAACTGT	196
Ad01A19040	Aradu.A01	19040	p2	(TA)9	18	100505896	100505913	TTTGTGGATCTTCTTGAATTTTGA	TTGTTACATTACTACATTTTGCTGGA	241
Ad01A19043	Aradu.A01	19043	p2	(TA)6	12	100512628	100512639	GGCCTAAACATTCTTGCTGG	TGATAAATTGTGCCAACCCC	184

ID	Chromosome	SSR nr.	SSR type	SSR	Size	Start	End	FORWARD PRIMER (5'-3')	REVERSE PRIMER (5'-3')	Product (bp)
Ad01A19046	Aradu.A01	19046	p3	(TAT)5	15	100514840	100514854	TGACAGAAAGAATAAGATTCCGGT	GAACCTAGTTGGCGTTAGCC	277
Ad01A19050	Aradu.A01	19050	p3	(TAT)15	45	100533039	100533083	CAGATAGGTTAAAATTTGTTAATTTGG	AAATACAACTTTGTGGTATGCTTCA	217
Ad01A19052	Aradu.A01	19052	p4	(TTCA)7	28	100541949	100541976	CTATCATCAAAGCGGCCATT	ACAACACAGTCCGCCTATCC	265
Ad01A19054	Aradu.A01	19054	c	(AG)9ataccgtc	99	100565812	100565910	GCAGCTTTGAATACGTACATGG	CCCAAGCCAAACCCTAATTT	233
Ad01A19058	Aradu.A01	19058	p2	(AG)15	30	100580134	100580163	CCTCCCTGCTTGATCCAATA	TTTCATCCAAATACCCCTATTCA	280
Ad01A19059	Aradu.A01	19059	p5	(AAAAT)5	25	100583406	100583430	TACGGACAGGAAACCCTTTG	CATGTCACCATGCACATTTTC	202
Ad01A19060	Aradu.A01	19060	p3	(ATA)5	15	100593809	100593823	TCGTCTTTCGAACAATTATACCC	GATCTTGAACTTTGGGACGC	231
Ad01A19068	Aradu.A01	19068	p3	(TAA)5	15	100618012	100618026	TATGCAGGCACCTGAAACTG	GGGACAAAAGTCGAGGTGAA	231
Ad01A19069	Aradu.A01	19069	c	(AAT)6taaccac	107	100619556	100619662	TTTTTGGAAAATAAATTTCTGATAACC	GTTGCTGGTCCCTAGCATTC	264
Ad01A19071	Aradu.A01	19071	p3	(TGG)7	21	100621208	100621228	GTGATGGTGGGTTTTGGTTC	GTTTAGCGCCAAAATCGAAA	197
Ad01A19072	Aradu.A01	19072	p3	(TAT)6	18	100622944	100622961	AGGCTGCAGCTATCCACACT	CTCGAATCAATCGTCACCCT	254
Ad01A19073	Aradu.A01	19073	p3	(TAA)8	24	100623075	100623098	CCGTGTAACACACGCTAACA	TGCAAGGGAGAGAAAAGGAA	277
Ad01A19076	Aradu.A01	19076	c	(C)17(A)19	36	100628007	100628042	ACCTCCAAAGGAGCTCCAGA	TGCACTTCGTTTCATATGCC	229
Ad01A19077	Aradu.A01	19077	p3	(CCA)6	18	100628979	100628996	TCCTTGTTATCGTCGTTCCC	TTCGCTGTTTCACACTCCTG	262
Ad01A19078	Aradu.A01	19078	p2	(TC)9	18	100633197	100633214	TGCTGCTGTTACTGGATTCG	CGATAAGCTGGGTGGTTACG	187
Ad01A19079	Aradu.A01	19079	p3	(CTT)5	15	100634392	100634406	CTCCCCACGCTTCTTCTTTT	AGTGGAGTTGGTCATTTCGG	278
Ad01A19081	Aradu.A01	19081	p3	(TTG)5	15	100641607	100641621	CATGCACCAGAATTTCCTGA	AGCTGGGGCTGTACCAATAA	221
Ad01A19087	Aradu.A01	19087	c	(TGA)12ccatg	164	100658200	100658363	TTGCTGAAATTTATTGCGGA	TCATTCGGACGAAAATACCC	274
Ad01A19091	Aradu.A01	19091	c	(CCA)6ctaacc	158	100661501	100661658	CCCTTATCATCATCCGCATC	GAGGAAATCGGGTAAGGGAG	277
Ad01A19092	Aradu.A01	19092	c	(GA)7atggggtt	41	100662286	100662326	CACAGAGCTCCGATCTAGCC	TCTGCATCATCTTTTCCCATC	280
Ad01A19094	Aradu.A01	19094	c	(T)10ctttttc(G	31	100672852	100672882	CTCTGCTGGTGGTAATGGGT	AGCAAAAATTGCAGTTTCCA	224
Ad01A19096	Aradu.A01	19096	c	(TA)8tc(TA)6	30	100676312	100676341	TGTGCTTTTGATGGTCTCCA	TAAGAATGATTGCTTGGGGG	280
Ad01A19098	Aradu.A01	19098	p4	(TTAT)5	20	100682598	100682617	CCTCCTCCTTGTTATTGCCA	TAACGGAACAGAAACAGGGC	278
Ad01A19100	Aradu.A01	19100	p2	(AG)8	16	100692750	100692765	TCGATCATCCTAAATTGATTAAAAA	CCCATTGTTGCTTTAATAGGAC	280
Ad01A19101	Aradu.A01	19101	p3	(AAT)8	24	100693333	100693356	AACCACCAAGAAAGTTAGGCA	TGGGTTTAATTTCTGTAGCTCAA	219
Ad01A19103	Aradu.A01	19103	p3	(AAT)13	39	100697586	100697624	ATGGATCAAACTCTGGCTGG	ATGCATACCATCACCAACCC	252
Ad01A19105	Aradu.A01	19105	p3	(CTT)9	27	100700985	100701011	TGATGGCAACATTGGACCTA	GGTGCTCAAATCGGTTCTTC	167
Ad01A19108	Aradu.A01	19108	p2	(AT)7	14	100703337	100703350	TTCACCTGGCTCCCATTATC	GCGAAGCAACATCAATTTCA	273
Ad01A19110	Aradu.A01	19110	p4	(TCTA)6	24	100705588	100705611	AGACATGGCCATTGAAGACC	AGCACCAACACAGTTTTCCC	191
Ad01A19111	Aradu.A01	19111	c	(T)13gaaatttga	89	100708878	100708966	CAAACTCCCCACAGGAACAT	TTGAATAAATCAAGCATGAAAATCA	275
Ad01A19112	Aradu.A01	19112	c	(A)13ttat(GA)(	29	100709309	100709337	TGCAAATCAATCCAACAAACA	TGAACAAAAATCATGGCGAG	234
Ad01A19113	Aradu.A01	19113	p3	(TTG)7	21	100709915	100709935	TTCTCCATGCAGATCCATCA	ACCATAGCAACCCCATTCAA	224
Ad01A19114	Aradu.A01	19114	p3	(TTA)5	15	100710166	100710180	AGGAGCAAGATCCGTGAGAA	GAGTGTGAGTGAGAAGGGGC	202
Ad01A19118	Aradu.A01	19118	p2	(TA)6	12	100728122	100728133	GGGAGGCTCTGAAAAGAGAGA	TTGTTCAAATTTCAAACCCG	280

ID	Chromosome	SSR nr.	SSR type	SSR	Size	Start	End	FORWARD PRIMER (5'-3')	REVERSE PRIMER (5'-3')	Product (bp)
Ad01A19124	Aradu.A01	19124	p3	(ATA)18	54	100741014	100741067	TCTCTAGGTTTAATGTGTGCCTATAAA	AACATCTTCATGTAATAAAAATCCTTT	278
Ad01A19126	Aradu.A01	19126	p3	(GGT)5	15	100745572	100745586	TGCTTTCTGCTTCTGCTTGA	TTTTAGGGACAAAAATACCCTCC	258
Ad01A19129	Aradu.A01	19129	c	(A)10taacaaata	49	100756992	100757040	TGTAGGAGAATCCCCACAGG	TGGCCAATGATTGAAGTAAGG	279
Ad01A19133	Aradu.A01	19133	p5	(AAGAT)5	25	100783695	100783719	CAGAGGAACACGACCCAAAT	CCTGGGTGCTCCAGTGTATT	177
Ad01A19134	Aradu.A01	19134	c	(CCA)6gtatcat	112	100784831	100784942	AGAACAGAAGCCTACGCCAA	TTTGATTTTCTGAGGTGGGG	274
Ad01A19137	Aradu.A01	19137	c	(AAT)6aaaaga	88	100789651	100789738	AAACTCCAACACAAATGGGC	TGAATTTTACAATCTCAGCTTATTCC	223
Ad01A19138	Aradu.A01	19138	p3	(GAA)6	18	100790435	100790452	CAAGTCAACGCAATGGAGAA	TCTGGTAAAGGATATCGGCG	157
Ad01A19141	Aradu.A01	19141	p2	(TA)7	14	100804649	100804662	CGTACCAATTTTCCTTCACCA	TTGCCTTCCATTATTGGGTT	273
Ad01A19143	Aradu.A01	19143	p2	(AG)9	18	100807272	100807289	TGCAGCTTTTTCCTCTCTCA	CCCTTTGGAAATTGCATGAT	280
Ad01A19145	Aradu.A01	19145	c	(TCT)7ttttcttct	85	100818178	100818262	GTACGCTTTTAATTTGCGGG	AATAAAGCTAGGGAGGCGCT	191
Ad01A19149	Aradu.A01	19149	p2	(AC)7	14	100827644	100827657	TGTGTGCAACATCGAGTCTTC	GAAGTGGATTGCATGGTGTG	118
Ad01A19152	Aradu.A01	19152	p2	(CG)7	14	100846998	100847011	TTTTCTTCATTCCAGTGGGC	CGAAACAGCCAATCAACAGA	260
Ad01A19156	Aradu.A01	19156	p3	(AAT)6	18	100864272	100864289	GGTCATGTATGAGCAATGCAA	TCCCTTCAGCCCTAATCAAA	225
Ad01A19158	Aradu.A01	19158	p3	(TTA)20	60	100869162	100869221	TCGTTTGAATTTTGGTCTTGTG	CAAAATCAGTTACACCGTATCACAC	211
Ad01A19160	Aradu.A01	19160	p2	(AT)6	12	100873935	100873946	ACCATCTTAATTCATGACCTCTCT	TGGTTATTGGTAAGTGCAAAAGT	202
Ad01A19164	Aradu.A01	19164	p3	(AAC)5	15	100892178	100892192	TCAACCCAACCATGATTCAA	GGTCAAGGTATGGTTTCGGT	226
Ad01A19165	Aradu.A01	19165	p2	(GT)9	18	100892827	100892844	CGCGAAGAGAAGAGAGGTGT	CAAACGGGCTAAAAACGCTA	167
Ad01A19167	Aradu.A01	19167	p2	(AT)14	28	100899807	100899834	TCCTACTCCCCTCAGGTTCA	AAAATTCAATTGACATGGTTGTT	268
Ad01A19168	Aradu.A01	19168	p2	(AT)6	12	100900334	100900345	GCATGTCTTTCAAAACAAAATTCA	AAATTTCTCGCTCAAAGCAA	258
Ad01A19174	Aradu.A01	19174	p2	(TA)6	12	100916799	100916810	AGAGCTGGATGGGACAGAGA	TTAGAGTGGCGCAGTCAGAA	205
Ad01A19175	Aradu.A01	19175	p2	(CT)7	14	100919305	100919318	AGTTGAAACCCGTAAGCCAT	AGCTCAAGGGGGAAGAAGAG	229
Ad01A19177	Aradu.A01	19177	p3	(TTA)7	21	100923381	100923401	TTTCAAAATTACTAAATGTGGCAAG	CACTATCCGCAAAGCAAACA	214
Ad01A19179	Aradu.A01	19179	p2	(TC)7	14	100928038	100928051	GCACCTAAGTGTTGGGAAGC	TGTGTTGCGAAAGAGTGAGC	210
Ad01A19182	Aradu.A01	19182	c	(T)10aaaaaattt	68	100937233	100937300	TTCATCATGGATTATTGGTGTG	TGGATACCACAAATACGAGGG	265
Ad01A19185	Aradu.A01	19185	p2	(GA)8	16	100954649	100954664	TCGCTTTTTGCATGATTCAG	CTCCTCGCTATCAGTCCCAG	203
Ad01A19193	Aradu.A01	19193	p4	(AAAT)6	24	100978493	100978516	ATTGTCCCACCCTCCAAACT	TGAGCAATGTTCATTTGTAACCA	236
Ad01A19199	Aradu.A01	19199	p3	(AAT)7	21	101001626	101001646	CCTCGGCAAAATAACATTGAA	CCCCGTTTTAAAAGGTCAGG	276
Ad01A19205	Aradu.A01	19205	p3	(TGT)5	15	101023713	101023727	TATTGTGCCTGTGTTTGGGA	TCAATAAACATGAAGGCCCC	175
Ad01A19207	Aradu.A01	19207	p2	(AT)7	14	101030212	101030225	AACTGAATTAAACCGGGTCG	CCTGACCGGACTCGAAGTTA	267
Ad01A19212	Aradu.A01	19212	p2	(TA)7	14	101058144	101058157	GATCATACGTTTAATTCAGGCTTT	TCTCTCTAATTTCCCCTTCCC	232
Ad01A19217	Aradu.A01	19217	c	(T)10ctttctctca	162	101068540	101068701	GAACTCGCAAAGGTAAATGCT	TGCATGCAATGAAATCCTTT	279
Ad01A19218	Aradu.A01	19218	c	(A)11ctaaaaga	121	101089951	101090071	TTGCATTTTGCTTGAAGTGC	GCATACGCATAAAGCACCAA	269
Ad01A19220	Aradu.A01	19220	p3	(TCT)5	15	101098538	101098552	TCTGAAGGGGAAAAGGAAGG	AAATCCAGAATGCACCGAAA	260
Ad01A19221	Aradu.A01	19221	p2	(TG)6	12	101098764	101098775	TCATTTTCGGTGCATTCTGA	TTCCTCCTCTTCCTCCATCA	167

ID	Chromosome	SSR nr.	SSR type	SSR	Size	Start	End	FORWARD PRIMER (5'-3')	REVERSE PRIMER (5'-3')	Product (bp)
Ad01A19222	Aradu.A01	19222	c	(A)10ggaggag	104	101098919	101099022	TGGAGGAAGAGGAGGAAGAA	TTTCTACCATTGATGCTATTGCT	180
Ad01A19223	Aradu.A01	19223	p2	(AG)8	16	101102953	101102968	AGACCCAGAAAACGAAAGCA	CTCATCCTCTTGGGCTCTTG	273
Ad01A19224	Aradu.A01	19224	p3	(CTT)5	15	101103340	101103354	AAAAAGCATCATTACATCCGTG	GCAAAATATTGGTGCTGTTGG	245
Ad01A19225	Aradu.A01	19225	c	(A)10taccgcag	222	101106114	101106335	TTCACTAGGGAGAGAATGGGA	TTTTTCTTTTCTCACGCTTTTTC	272
Ad01A19228	Aradu.A01	19228	p2	(TA)6	12	101114014	101114025	GCAGCTCGGTCAAAAGTTTC	CCTTACGGGTTCCCTACTCC	257
Ad01A19233	Aradu.A01	19233	p2	(AT)8	16	101133669	101133684	TTGCATTTGACCATTGCTTC	GATTGGTTTGAAAGGGCAAA	274
Ad01A19234	Aradu.A01	19234	p3	(GAT)5	15	101136564	101136578	GTTTTGGTTGCGTCTTCGTT	CCCAGCTGGATTACTCCTCA	247
Ad01A19237	Aradu.A01	19237	p2	(TC)6	12	101159109	101159120	ACCCACACTTTCCTCTCCCT	AAAATGCTCCCAAAATGCAC	156
Ad01A19241	Aradu.A01	19241	p3	(AAG)7	21	101177499	101177519	AATGTTTTTAGGTGGCAGCG	GGGTGGGGAGAGAAAGAAAG	168
Ad01A19242	Aradu.A01	19242	p3	(AAT)10	30	101177858	101177887	GATTTTGATTTTCGCCCCTT	CAACGCTTATGACCTGAGCA	175
Ad01A19243	Aradu.A01	19243	c	(A)10tcattttttat	90	101178162	101178251	TTGCATCGAGGTCAAGTCAC	CGTTCGTTCTTATGCTGTTCA	223
Ad01A19246	Aradu.A01	19246	c	(A)11cgaaatata	151	101179455	101179605	CCACAATTTTTGTCGTGGGT	TGCGTTTAAATGTGCATTGTC	275
Ad01A19251	Aradu.A01	19251	p2	(AT)7	14	101195851	101195864	AACGATTCTCATCTATTAATTTCACA	ACGCACCCGTCTTAACTCAA	228
Ad01A19252	Aradu.A01	19252	p2	(AT)6	12	101196465	101196476	TTCGGTGATTCTTTTTGACTCAT	TCCTCTTTTCCTCTTTGTGGA	264
Ad01A19254	Aradu.A01	19254	p4	(TTAT)7	28	101200029	101200056	TTCCCACTTTCCAGCACTTT	TAGCTACGGATTGCCATGTG	190
Ad01A19255	Aradu.A01	19255	c	(T)10c(T)10	21	101200354	101200374	AAGCTAAAGCCGAAAGGGAG	TGAAAAAGTTGCCAGTAGCAATA	189
Ad01A19256	Aradu.A01	19256	p3	(TAT)6	18	101201533	101201550	GGAGAGAAAGGTCTCATCCCA	TTGGTACTGAGGAGGCAAAT	248
Ad01A19259	Aradu.A01	19259	p3	(AGG)5	15	101212400	101212414	CAACCCTGTTGAAGAAGGGA	CCTGACAAACTGCACGGTTA	245
Ad01A19266	Aradu.A01	19266	p3	(ATG)5	15	101237066	101237080	AAAATGACACTCCCTTATCCTCT	TGTCGTCACAAACACCACCT	259
Ad01A19270	Aradu.A01	19270	p3	(ATT)5	15	101250946	101250960	CTGCAGAAATCGAACTCGGA	GAACCAGGAGCTGGTGAACT	267
Ad01A19273	Aradu.A01	19273	p2	(TG)6	12	101259137	101259148	CAGGTGAAGCAGAGTTGGTG	CAAAGTCAACCCAAAACCAAA	249
Ad01A19274	Aradu.A01	19274	p4	(ATTT)6	24	101263438	101263461	TTGAACTGGATTTTCCAGGC	TTAGCGATTCATCATTGCCA	242
Ad01A19278	Aradu.A01	19278	p2	(AT)7	14	101279125	101279138	TTGCTACTGGCAACTTTTTCA	AATCTGAGTGAAGAATTGAATCG	216
Ad01A19284	Aradu.A01	19284	p2	(TC)14	28	101296724	101296751	TAACAAGAGGGGGTTGTTGC	TCAAGGATAATGACCTGGCA	214
Ad01A19286	Aradu.A01	19286	p3	(AAT)5	15	101297897	101297911	AAAGTCCTTGGCGGCTAGTT	TTGGGCCAAACACAGAAAAT	216
Ad01A19289	Aradu.A01	19289	p2	(TA)16	32	101305911	101305942	TTTTGAGGGCTGTGTTTGTG	GCACTGCTCACGAAAAATGA	187
Ad01A19291	Aradu.A01	19291	p2	(TC)9	18	101307647	101307664	TCTCAGCATCTTGTTTGGTCT	AGGTGAAGGTGATGAGGACG	190
Ad01A19293	Aradu.A01	19293	p3	(AAT)5	15	101314794	101314808	AGATTCGGCACCTTGAATTG	TTGATCGGACTATACACGTCG	264
Ad01A19295	Aradu.A01	19295	p3	(AAT)5	15	101316007	101316021	ACTGCCATCATGCTCCTTTT	CAGAAGTCAAGAGGTTAGGGGA	280
Ad01A19301	Aradu.A01	19301	c	(T)11gc(T)10	23	101336168	101336190	CGCCATTAATGTGCTAAACAGA	TGCATGAGCATGATTTGTCC	233
Ad01A19303	Aradu.A01	19303	c	(T)10catgttaaa	118	101337070	101337187	TCAAATCAATACTCACACTCTTCAAA	TCCAGGATGGTTTGTTCCTC	274
Ad01A19305	Aradu.A01	19305	p2	(AT)6	12	101351110	101351121	TGCTTGTACAAATTGCTGGG	TGCTGGAACATGAATTCCAA	102
Ad01A19306	Aradu.A01	19306	p2	(AT)9	18	101369033	101369050	CACCACAAGACACATTAAGCAAA	CTTCATGATTCACCAAGCGA	255
Ad01A19311	Aradu.A01	19311	p2	(TC)9	18	101384742	101384759	GGAGTTGTTGGTGCTTCCAT	GCTCAGCCAATAGGCTTTCA	235

ID	Chromosome	SSR nr.	SSR type	SSR	Size	Start	End	FORWARD PRIMER (5'-3')	REVERSE PRIMER (5'-3')	Product (bp)
Ad01A19314	Aradu.A01	19314	c	(CTA)5gtttctct	125	101392322	101392446	CTCTTGAGCACGTATCACTCTTG	GAGAAAACCTAAATTCAAATTGTGC	250
Ad01A19316	Aradu.A01	19316	p3	(ACT)5	15	101393165	101393179	TTCTCTACCACACAACTTGGGA	TTGTGTTGTTGTCAACTCTAAGCA	235
Ad01A19317	Aradu.A01	19317	p3	(ACT)6	18	101393747	101393764	GAGTTGACAACACCACAACCA	CCAGTTGTGCTCCAAGTTGT	182
Ad01A19319	Aradu.A01	19319	p3	(ACT)5	15	101394485	101394499	GCACAACTTGAATTTAGGTTTTCTCT	GCAGCGTTGTTGTCAACTCTAA	201
Ad01A19320	Aradu.A01	19320	p3	(ACT)6	18	101395148	101395165	GAGTTGACAACACCACAACCA	CCAGTTGTGCTCCAAGTTGT	176
Ad01A19322	Aradu.A01	19322	c	(TAC)5ttgtagta	112	101395857	101395968	TGTTTAGAGTTCACAACAACACTGC	AAACAAGAGGAAACCTAAATTCAA	257
Ad01A19323	Aradu.A01	19323	p3	(ACT)5	15	101396681	101396695	TTCCTTTTGCTTAGAGTTGACAAT	GCAAGAGGAAACCGAAATTCT	263
Ad01A19324	Aradu.A01	19324	p3	(ACT)7	21	101397182	101397202	TTGACAACGACACAACCACT	TGTTGTCAACTCTAAGCAAAAGAAA	234
Ad01A19325	Aradu.A01	19325	p2	(AT)6	12	101412463	101412474	TGGCAAAAATCAAGGTAAATGA	CATTTATAAGACCTAACATGCAAAAA	269
Ad01A19327	Aradu.A01	19327	c	(T)10ggcaga(T	27	101418552	101418578	ACACCACAAATGCATCGAAA	TTATTGGGCCTTACGCATTC	223
Ad01A19329	Aradu.A01	19329	p2	(TA)8	16	101432442	101432457	GACAGGGACCCGTGAAATAA	AGTAGTGGCGGCTGAGAGAA	164
Ad01A19330	Aradu.A01	19330	c	(ATA)8acaaaa	138	101442048	101442185	ACGGACCTTGTTTGGACTTG	ATGGTAGTGGCGGTGAGTTC	270
Ad01A19335	Aradu.A01	19335	c	(GA)12aaagag	135	101453126	101453260	CATCCCCTCTGCCCTTCTAT	AAGGTTGAAACTTGCGGAAA	268
Ad01A19336	Aradu.A01	19336	p3	(TTC)5	15	101453391	101453405	CCGTCTCCACAAGAATCCAT	CCAATTCTCCTTCCACGAAA	268
Ad01A19337	Aradu.A01	19337	p2	(TA)6	12	101461581	101461592	TGTGGTAGGGCACTTATCCC	TGCAAGCAAGGAAAACACAG	275
Ad01A19338	Aradu.A01	19338	c	(T)11aca(TTC)	29	101468501	101468529	GCGTATGTGAACACGCTATTT	TGCTACAAAAATCACCAAGAAAAA	190
Ad01A19339	Aradu.A01	19339	c	(T)10cttatttttat	86	101468669	101468754	TTTTTCTTGGTGATTTTTGTAGCA	GCATAACTCAAAACGTTCTCTCC	162
Ad01A19344	Aradu.A01	19344	p3	(ACC)5	15	101487546	101487560	TGGACTTCAAAACCGAAACC	CTTCCACGTCCACTTCCAAT	256
Ad01A19345	Aradu.A01	19345	c	(AGA)5atggtag	75	101487822	101487896	TGGTTCTTCTGCTTCCGACT	TGTCCAGCTGTTCCTCTGTG	168
Ad01A19347	Aradu.A01	19347	p2	(TC)8	16	101490701	101490716	TGCAGTTTGATGTGGGTGAT	GATGGGCCAAGAAAATCAAA	239
Ad01A19351	Aradu.A01	19351	c	(A)11c(A)10	22	101498549	101498570	CCTCTTTCTATGACACCCGC	TCCTCCACTCTGTTATATTTCTTCG	275
Ad01A19353	Aradu.A01	19353	p4	(GAAA)5	20	101502082	101502101	TTGAAATTTGGACCGCTAGG	TTTGTGCTTGACTTGGTTGC	227
Ad01A19354	Aradu.A01	19354	p2	(CT)9	18	101502401	101502418	ATCTTTGGCAACTCTGCACC	AACCACACCCTCCTCTTCCT	271
Ad01A19358	Aradu.A01	19358	p2	(CT)10	20	101524627	101524646	AAAGCGCAAATCTTCTCCCT	GGAACGACTGGTTTCGGTAA	180
Ad01A19359	Aradu.A01	19359	p3	(ATT)5	15	101526173	101526187	GCAATCTTTTAGCTGCAGGG	CAGAACCTGTGAAAATTGAGCA	205
Ad01A19360	Aradu.A01	19360	p3	(ATT)5	15	101526651	101526665	ATGTTTTAGCTGCAGGGCTC	CCAATCAATGAAAGGTGGCT	268
Ad01A19364	Aradu.A01	19364	p2	(AG)9	18	101547298	101547315	GTTCAGGCTCATAGCGAAGG	CTGCTCATCCCATTACCGTT	184
Ad01A19370	Aradu.A01	19370	p2	(TA)7	14	101558862	101558875	TATTTCCTTCGCCTTTTTGC	TGCCCAATAATCAGTCTCACA	197
Ad01A19371	Aradu.A01	19371	p3	(CTC)5	15	101562753	101562767	GGGAGGACAGTAGAGAGGGG	CGAATGCGATGATAGGGAAG	162
Ad01A19373	Aradu.A01	19373	p2	(AT)7	14	101567482	101567495	AAATTGGACTAAAAACCAAAAAGG	TTTGAGCTATCGATACAACTCG	158
Ad01A19375	Aradu.A01	19375	p3	(TAA)10	30	101570055	101570084	CGATTTGCGAATACCCCTAA	AATAGGAGGGCTAATACCAAAAA	230
Ad01A19376	Aradu.A01	19376	p3	(TAT)12	36	101570272	101570307	TTTTTGGTATTAGCCCTCCTATT	CTTTCATTATGTCACGGCGA	233
Ad01A19378	Aradu.A01	19378	p2	(AT)7	14	101576787	101576800	ACTATTTTGAGCTTGTATCATTTGAA	TTTTACATTTACTTTTTCTTCTTCTCC	271
Ad01A19382	Aradu.A01	19382	p3	(CGT)5	15	101603257	101603271	CCGTCATTTTTGAAGCTTGG	AGCAGCAGAAGCAAGAAAGC	216

ID	Chromosome	SSR nr.	SSR type	SSR	Size	Start	End	FORWARD PRIMER (5'-3')	REVERSE PRIMER (5'-3')	Product (bp)
Ad01A19383	Aradu.A01	19383	p3	(AGA)5	15	101604141	101604155	CAGCATTGGTCCAGTAGCCT	CTGAGCTGGGTTCCTACCAC	229
Ad01A19384	Aradu.A01	19384	p3	(TTG)7	21	101610173	101610193	TTGATTCATGTAGCCGACCC	CATTGCAACCGTTACTCTTGA	279
Ad01A19385	Aradu.A01	19385	p6	(GGACGG)5	30	101611976	101612005	CTTTATTTTGCGCAAGTCCC	AATGCGCCACAAATTCTTTC	203
Ad01A19386	Aradu.A01	19386	p3	(TGG)6	18	101618128	101618145	TGGGAGGGGAGTTACAGTGA	TTTTTCGTCCCCAAAGTCTG	241
Ad01A19387	Aradu.A01	19387	c	(T)12ggcattggg	121	101624537	101624657	TGTGTTGGTTTTCTTTCCCC	TAGCCAAAAGCTGACCCACT	264
Ad01A19390	Aradu.A01	19390	c	(AG)7aaaaaaat	48	101637086	101637133	GAAATTGTGGGAACATGGGT	TCACATATGCATTGATTTTTCATT	252
Ad01A19392	Aradu.A01	19392	p3	(ATG)6	18	101651680	101651697	TCTGGCTACATCCAATGCAA	CAAAAGCGTTGCTGTGAAAA	280
Ad01A19394	Aradu.A01	19394	p2	(TA)7	14	101660422	101660435	GGCACCATTCTAGGCGTTTA	GAGGTGATTGGTGAATGGGT	257
Ad01A19395	Aradu.A01	19395	p2	(AT)6	12	101666988	101666999	AGCCAACATAATCCACCAGC	TGCCTGGACATACCTTCACA	276
Ad01A19397	Aradu.A01	19397	p3	(AAG)5	15	101679931	101679945	TGACGATAACGAAAGGGGAG	CACGCGCTTTTTCTTCTTCT	104
Ad01A19404	Aradu.A01	19404	p3	(CTC)6	18	101693606	101693623	TTTCAGAGAGTCCAGGAGGC	GGAGTCTACGAACATGGGGA	271
Ad01A19407	Aradu.A01	19407	p2	(CA)7	14	101704621	101704634	TGGGATATTTTGGGTGTTCC	GCATCCAAACTCATCAACTTCA	215
Ad01A19408	Aradu.A01	19408	p2	(GC)6	12	101705766	101705777	AAGAAGCGGAAATGGAGGTT	GTCCTCTCCCATTTTGCTCA	186
Ad01A19413	Aradu.A01	19413	p4	(GAAA)5	20	101730078	101730097	AGGATATGTGACGTAGGCGG	TCTTTGTATCCCTGCGTTCC	131
Ad01A19414	Aradu.A01	19414	p2	(CT)7	14	101746477	101746490	AGATCCTTGAGGGATGGCTT	TTCAAGAAAACTGGTTCGGC	277
Ad01A19420	Aradu.A01	19420	p4	(ATAC)5	20	101766235	101766254	ATTCAATCTGCGAAGCCAAG	AGTCACATCACATGCGGAAA	277
Ad01A19426	Aradu.A01	19426	p3	(TAA)5	15	101795659	101795673	AACATGTACTCCTCATCATACTTGTAA	TTTGTTGGCGTTATCTTTAGGAA	260
Ad01A19427	Aradu.A01	19427	p2	(TC)7	14	101798325	101798338	CACCTTTGCTCCTCTCAACC	CAGAAGGTGCATTGGCAGTA	216
Ad01A19428	Aradu.A01	19428	p2	(CT)19	38	101807058	101807095	TTTTGGTCGGTTAAGGTTGC	CATCACCACCACCACTCAAG	192
Ad01A19430	Aradu.A01	19430	p3	(ACT)5	15	101808867	101808881	AACGCGAATTTCATGTCGTA	GGGAAAAGAGATTGGTGCAA	248
Ad01A19431	Aradu.A01	19431	p2	(TA)7	14	101809025	101809038	GTATCACACCAACCCCTTGC	CCCCTCCTTGCATTGTTAAG	258
Ad01A19432	Aradu.A01	19432	p3	(ATT)5	15	101809931	101809945	AGTTGTCACGACTCAGCACG	CTCTAACTTTCAGCCACGCC	269
Ad01A19435	Aradu.A01	19435	c	(A)10ttctattttct	42	101833169	101833210	GGATGTATTTGATTTGCATTTTCA	TCCAAGTCGGTGGCTAACTT	218
Ad01A19441	Aradu.A01	19441	p2	(AC)9	18	101847539	101847556	AAGGCTAGAGCTTTCCCAGG	CTTTGCTGGTACGGGTTGAT	164
Ad01A19442	Aradu.A01	19442	p2	(AT)7	14	101856042	101856055	AGTCTGCGAAGCTAAGGCTG	GAATCCCCGGTTCTCATTCT	275
Ad01A19447	Aradu.A01	19447	p3	(TAT)16	48	101876109	101876156	TTGGAGATGAACGAAATTTGAA	CCAGCCTGTTAGGCAAAAAT	280
Ad01A19453	Aradu.A01	19453	p3	(AAT)19	57	101902143	101902199	CCATGCCAACAACAAATTCA	AAATACACTGGCGAGCCAAC	263
Ad01A19458	Aradu.A01	19458	p3	(AGG)8	24	101923161	101923184	TTGGTTGAGAGGGAGTACCG	ACGCCGACTTTTGTTCAATC	261
Ad01A19460	Aradu.A01	19460	p2	(AG)6	12	101930587	101930598	TTCCTTTGGCTCTTGGATCA	TAGGAATGACCAGGGCTGAG	247
Ad01A19463	Aradu.A01	19463	p3	(TCT)5	15	101947568	101947582	CTTCTTGCGCACGTTCTTCT	GAAAACGTGCAGTAATGGTTGA	162
Ad01A19464	Aradu.A01	19464	p3	(CTT)5	15	101947708	101947722	CGCACGTTCTTCTTCCTTTT	GGTGCAGTGAAAACATGCAG	280
Ad01A19465	Aradu.A01	19465	c	(CTT)5gctgcac	66	101947825	101947890	GCTGCACGTTCTTCTTCCTC	TGCTCCTGTGTCACAATGAA	270
Ad01A19466	Aradu.A01	19466	p2	(AT)6	12	101950625	101950636	AACTGGATGGTGGTTGTGGT	TGGCATTCCAGATCTTCTTCA	280
Ad01A19467	Aradu.A01	19467	p2	(AG)9	18	101956458	101956475	GCACAACCTACCAAGCACAA	CTTCTTTTCTCCAATGCCCA	245

ID	Chromosome	SSR nr.	SSR type	SSR	Size	Start	End	FORWARD PRIMER (5'-3')	REVERSE PRIMER (5'-3')	Product (bp)
Ad01A19469	Aradu.A01	19469	c	(T)10ctgataattc	65	101964149	101964213	AAGACAAAATCAAGATTTCTCAAACA	GCCGAAACCCTAAACCCTAA	224
Ad01A19471	Aradu.A01	19471	p3	(AAG)8	24	101967446	101967469	CCCTGAGGTAACGGAATGAA	CGCACAAGGAACGAATCTCT	204
Ad01A19473	Aradu.A01	19473	c	(T)12ggtcatata	59	101972317	101972375	TGCTCCACCATTTTTAGGGT	AGGCATGTGATGTAGTGGCA	257
Ad01A19478	Aradu.A01	19478	p2	(AT)8	16	101977915	101977930	CCCAAGGACAACCACATTTC	CCACTGCTGCTATGCTTTCA	147
Ad01A19480	Aradu.A01	19480	p2	(AT)9	18	101994793	101994810	GGTCCTTGTATTTCCCACCA	CTCTGGGAAGATGGGTTTCA	240
Ad01A19482	Aradu.A01	19482	p3	(TCA)5	15	101995967	101995981	CATTTCATTATCCAGCACGG	CAAGTGGCATAGTAGAATGAAGC	263
Ad01A19486	Aradu.A01	19486	p3	(AGG)8	24	102011208	102011231	GGGTTTGTGGAAGCAAGAAA	CCTCCTCCAAAATCTCCCTC	212
Ad01A19492	Aradu.A01	19492	p3	(CAT)5	15	102024462	102024476	CAATGCCATGTGTCAATACCA	GATGGTGGTTCCGCAGTAAT	192
Ad01A19494	Aradu.A01	19494	p2	(TA)6	12	102027183	102027194	TGGATAAAAGTTTGTCATGTGGA	CGATGAAAGAAAGAAAGAAGAAAAA	236
Ad01A19495	Aradu.A01	19495	p4	(TTTA)5	20	102027407	102027426	TTTTTCTTCTTTCTTTCTTTCATCG	CATGCTGATACTGCATTGTGAA	175
Ad01A19496	Aradu.A01	19496	p2	(GA)7	14	102033245	102033258	AGAGTAGAAGACGGCCACGA	CAGATCCCCTCCCTCACATA	245
Ad01A19497	Aradu.A01	19497	p2	(AT)7	14	102033586	102033599	TTAACCCGGTTGAACCAGTC	AAAAATGTGGTTTGGCTACTTGA	280
Ad01A19498	Aradu.A01	19498	p3	(ATT)12	36	102034619	102034654	AAATGCACTTAAATGGGTGTTATT	ACAGCAACAGCAACAGCAAC	210
Ad01A19499	Aradu.A01	19499	p2	(AG)6	12	102037299	102037310	TTGCAATTAGAGAATATTATGTTGCAC	TTTGCCAAAAAGACTTGTATGTG	273
Ad01A19500	Aradu.A01	19500	c	(GAA)9a(AAC	73	102038674	102038746	TGATGGAGGAGAAGGAGGAA	CGCTCCTGCTACATGTTGTT	218
Ad01A19501	Aradu.A01	19501	p3	(AAC)12	36	102039414	102039449	GGCATGGGCTTGTGTTTTAG	CTGAATTCGGGTTGGAAAAA	227
Ad01A19502	Aradu.A01	19502	c	(A)10ggaatatat	38	102051609	102051646	TTGGTTGGCTGTTTGAGCTA	CAGCCATTGGGTGTTCAGTA	194
Ad01A19503	Aradu.A01	19503	p2	(AG)26	52	102054407	102054458	AAATAATTCTTGCATGGGCG	CCGGCATAGCATTAAAGGAA	183
Ad01A19506	Aradu.A01	19506	p2	(AC)8	16	102065863	102065878	GGGACAAGGATTAAGCACCA	AGCAATGAACGTCAGACACG	135
Ad01A19508	Aradu.A01	19508	p3	(TTA)6	18	102072461	102072478	TCACTACAGCTTGTACCGATTTTC	TTTCATAAAAACCACAATGCAG	255
Ad01A19509	Aradu.A01	19509	p3	(TAA)7	21	102083580	102083600	CTCTCATGCATCCGTTTTCC	CGTGAATCGGGTAACAAAGA	278
Ad01A19511	Aradu.A01	19511	p3	(TTA)6	18	102085351	102085368	AAATTTAAGTCATTTACCTGTTTGTGA	TTTGGTACATTTCATATCACATTCG	208
Ad01A19512	Aradu.A01	19512	p3	(TTA)5	15	102086442	102086456	GACCATGGTGTTATCATGTATGC	CATTTCATATCACATCCGTTTCA	248
Ad01A19517	Aradu.A01	19517	p2	(TA)8	16	102100345	102100360	CAAATGCAATTCTCTCTCTTTTTG	TCTCGTGCTGATAACGTGTTG	204
Ad01A19522	Aradu.A01	19522	p3	(CCT)5	15	102110843	102110857	GCACATTATCATGGGGTGAA	CGGCTATGTTTTTATCCATTG	280
Ad01A19523	Aradu.A01	19523	p3	(AAT)25	75	102113171	102113245	CCTTGTGCATGTTGCCTAGA	CACTTTGCATGCCTTTTGAA	169
Ad01A19529	Aradu.A01	19529	c	(AAG)7aggaag	120	102119534	102119653	CGTGCAAGAAGAAGAAGAACG	TGCTCCCATAGTTCCCAAAA	277
Ad01A19530	Aradu.A01	19530	p3	(TTA)20	60	102123539	102123598	CACACAAAACATATATTGCGCC	TCGAAAAACACTAATTTCCTTTCA	279
Ad01A19533	Aradu.A01	19533	p2	(GC)6	12	102135300	102135311	ATGACAAGCAAGAGCCAAGG	TTCTTTGCCTCGCTCATTTT	203
Ad01A19534	Aradu.A01	19534	p2	(TA)6	12	102137572	102137583	ATTGTAAACCAACCCGGTGA	CATTGAATAGAAACACGGCCT	216
Ad01A19536	Aradu.A01	19536	p3	(TTA)17	51	102147173	102147223	GAAAGGTTTTGTAAGCCCTTGTT	TGTTATAGCAATAATTTGGCATGTT	211
Ad01A19537	Aradu.A01	19537	p2	(TA)6	12	102150850	102150861	AGAATACAGGGGTTCAGATTGTT	TTTGTGCTATGAAGGGATTCAA	240
Ad01A19538	Aradu.A01	19538	p3	(GGT)5	15	102154780	102154794	GGCTTGCTTTGGAGAGAAGA	ACCATGGCTACCACCATCAT	239
Ad01A19544	Aradu.A01	19544	p3	(AAT)5	15	102167536	102167550	CCATCAAATGTGGCCTTCTT	CGTGGGGCCTGTAACTGTAT	170

ID	Chromosome	SSR nr.	SSR type	SSR	Size	Start	End	FORWARD PRIMER (5'-3')	REVERSE PRIMER (5'-3')	Product (bp)
Ad01A19550	Aradu.A01	19550	c	(CCT)5tctccttt	73	102184935	102185007	GCTTTTCAAATCACGCACCT	AAGAAAACGCAACATCAGGG	217
Ad01A19556	Aradu.A01	19556	p2	(AT)9	18	102200102	102200119	TCCGTTCCTGTCATAACAAAAT	TCCCCAAATAAAATTACTCCCA	238
Ad01A19557	Aradu.A01	19557	p3	(GAT)6	18	102202427	102202444	TTGAAATTTGGAGGTTTGGC	TCCAATCCATCAACCATGTG	153
Ad01A19558	Aradu.A01	19558	p4	(TATT)5	20	102203656	102203675	CACGAACTTGAAGGCCTCTT	TACTAACCCCACATGCCGAG	280
Ad01A19559	Aradu.A01	19559	c	(A)13caaaaaca	63	102205003	102205065	AAACCACAAAAGGATTGTTGAA	TCACTCCTTCACCTTCCACC	183
Ad01A19561	Aradu.A01	19561	p3	(CTC)6	18	102210482	102210499	GAGTCCAAAGCAAGGTCTCG	ACCTCACCAGAAGAGCCAGA	206
Ad01A19565	Aradu.A01	19565	p2	(AT)6	12	102223351	102223362	TTATTTCACACGCGTATTTGTT	CGAAACATATAAATACACAAATTCAAA	265
Ad01A19567	Aradu.A01	19567	p2	(AT)7	14	102229882	102229895	TGGAAACGTCGTCTCGTTTA	GCGACAATCGGGATAAAGAA	201
Ad01A19569	Aradu.A01	19569	p2	(TA)6	12	102238061	102238072	CAATAAAAGCCCTTCTCCCC	ATGACAAAAGTTAAAGATGCAAAA	223
Ad01A19573	Aradu.A01	19573	p3	(GAG)9	27	102264010	102264036	AATCACAATGGGCTGAGAGG	TCTTTTGCAGACATGGCAAG	168
Ad01A19574	Aradu.A01	19574	p3	(TAA)13	39	102266396	102266434	AACCACGGTTAATTGGTGAAA	CGATATTATGTTTTGGGATATTGAA	270
Ad01A19579	Aradu.A01	19579	c	(T)10caaaaaaa	82	102276352	102276433	TGCCGAAGATAACGAGAGGT	AGGTCGGTTGAAACACAAGAG	257
Ad01A19582	Aradu.A01	19582	p4	(TAAT)5	20	102279345	102279364	ATCAGCCTCACCAACGATTT	CTTCATTCCCGGTTACAGGT	257
Ad01A19583	Aradu.A01	19583	p3	(TAA)8	24	102280439	102280462	CTCTCCACAATGCCAACAAA	CTCAAAATCCAACCAGCCTC	225
Ad01A19584	Aradu.A01	19584	p3	(ACT)9	27	102280924	102280950	GGGGAACGCCAACTTTATTT	GACCCCCTTATCGCTTCTTC	267
Ad01A19585	Aradu.A01	19585	c	(GA)12ggagga	158	102281317	102281474	ACCACCCGTTCTTCTTCCAT	CTTAAGAATTCCAATGGCGG	241
Ad01A19588	Aradu.A01	19588	p2	(AT)9	18	102283665	102283682	GTGCATGACCCAAAATACGA	TCTAGGAGGGAAAGGGGAAA	125
Ad01A19589	Aradu.A01	19589	p3	(AAT)5	15	102288594	102288608	TGATGGAGTTGTGGATGATGA	AACCCCCTTTGTGCTCTTCT	251
Ad01A19597	Aradu.A01	19597	p3	(ATA)5	15	102293337	102293351	CAACTAGCGGCGGTTCTTAT	TGCTGTACCGTGAAGGTCTG	249
Ad01A19602	Aradu.A01	19602	c	(A)11cctaatttg	156	102305551	102305706	GAATATCCAACTCGGCCCTT	AAAAATTCTAAACGGCCTCTGA	262
Ad01A19603	Aradu.A01	19603	p3	(ATC)5	15	102309639	102309653	AGGCAGTGCTGAACACATCA	TACATGGTTTGCAGGAACGA	123
Ad01A19610	Aradu.A01	19610	p2	(TA)6	12	102341214	102341225	TTGTTGCTAATCCGCTGTTG	CGGAAAAGAAGCCATAGCAG	270
Ad01A19619	Aradu.A01	19619	p2	(GA)15	30	102365564	102365593	TGCGCAATAAGACACACACA	GCACCAAGAGAAGGCAAGAC	190
Ad01A19623	Aradu.A01	19623	p2	(TC)10	20	102382060	102382079	ATGACTGGGCAGCGTAATTC	GAAGAGAGAGAGCGCGAAGA	265
Ad01A19625	Aradu.A01	19625	p2	(CT)6	12	102389546	102389557	CAGAATTGGCATGAAGAGCA	TTTTTGGGGAAGAGGTGTTG	162
Ad01A19626	Aradu.A01	19626	p3	(TCT)5	15	102403143	102403157	TCTGGTGCTTCAAATTCACG	AGGAAGAGATGGTGGTGGTG	132
Ad01A19627	Aradu.A01	19627	p3	(ATC)7	21	102403272	102403292	TCTGGTGCTTCAAATTCACG	GACGACGATGATGATGACGA	233
Ad01A19628	Aradu.A01	19628	p3	(TCT)5	15	102404286	102404300	CCTTCCTTCTCCTCCTCCTC	CCGTGACACAAGTTCCTGTT	113
Ad01A19629	Aradu.A01	19629	p2	(AC)6	12	102405279	102405290	TAATATCCAAAGCAACCGCC	GCGGCTCAAAAGTCAAGTGT	208
Ad01A19630	Aradu.A01	19630	p2	(TA)8	16	102408949	102408964	GGAGGACGAGTAGTTGATTCAGTT	AAAGTACAAAGACTTGAGGGTACGA	220
Ad01A19631	Aradu.A01	19631	p2	(AT)6	12	102414618	102414629	GATCGGAGCGGGTAGTGTAG	ATGAGAGGCTGAGCTGAGGA	229
Ad01A19633	Aradu.A01	19633	p3	(TTC)5	15	102417838	102417852	CGTCCATCGAAAAGCTGAAC	TGTTGCTGGTTTTTATTTTTGTG	153
Ad01A19635	Aradu.A01	19635	p2	(AT)6	12	102436666	102436677	GGGAAGGAACGGGAGACTAC	GGGAACAGAACACCGTCATC	211
Ad01A19639	Aradu.A01	19639	p3	(GAT)5	15	102461206	102461220	CTCTTCCACCATCACCTTCC	AAACACAGGCAAAGCACACA	274

ID	Chromosome	SSR nr.	SSR type	SSR	Size	Start	End	FORWARD PRIMER (5'-3')	REVERSE PRIMER (5'-3')	Product (bp)
Ad01A19641	Aradu.A01	19641	p3	(CCG)5	15	102465167	102465181	GTCAGGACCTGGGATGTTGT	ATGAGCCTGATTCGTTGTCA	275
Ad01A19642	Aradu.A01	19642	p4	(AAAG)5	20	102481596	102481615	CCAAAGTTTCAAAAGCCCAC	CTAAGGCCATTGTGCAGGTT	148
Ad01A19644	Aradu.A01	19644	p2	(TA)6	12	102495628	102495639	ATTTCCGCTAAAAGGAACGG	ATTGGCCTGGAGAAGAACCT	136
Ad01A19645	Aradu.A01	19645	p3	(TGA)17	51	102496306	102496356	TTAACTGCTTGCGTTTCCGT	TGGTGTTTGCTGATGAGGTC	220
Ad01A19646	Aradu.A01	19646	p2	(AT)6	12	102498075	102498086	ATCGACAAAGTGTGTTTGGAT	CGGTTATCGAAAGATCGAGG	176
Ad01A19648	Aradu.A01	19648	p2	(TG)8	16	102498754	102498769	AGTGGCCCAACCTTACACTG	AGCCAGGCATGTGGATTTAG	214
Ad01A19652	Aradu.A01	19652	p3	(CTT)5	15	102506484	102506498	TCTTTCTCACCCTTTCGTTTG	TGTTGTTGCGACTTTCGTTT	255
Ad01A19653	Aradu.A01	19653	p2	(TG)6	12	102508042	102508053	TTAAGGGCCAATGCAAATCT	TTGGTTTCTGCATCCACGTA	211
Ad01A19655	Aradu.A01	19655	c	(T)10ccgtcttc(	99	102509715	102509813	AAGCAATTCTAGCATCCAAGTTT	TGATTGCACACAAACTCAATTAAA	239
Ad01A19656	Aradu.A01	19656	p3	(AGA)7	21	102511103	102511123	GGAGGGGGAAGAGTAGGATG	CACGCTAGCTCATTTACGCA	126
Ad01A19659	Aradu.A01	19659	c	(A)10gaaaagaa	103	102522896	102522998	TGATCACCAAAATTGACTTAACG	GAATAAATGTTCTATTTTAAAGGCAGG	275
Ad01A19661	Aradu.A01	19661	p3	(GAG)9	27	102537593	102537619	GCAGCATCAAGTGAACCTCA	AACCGCTTTTCAAATCATGC	260
Ad01A19663	Aradu.A01	19663	p2	(CT)6	12	102540315	102540326	AGCCATCTTTCCCCTCATTT	AGAACGTCAACGACATGCTG	168
Ad01A19667	Aradu.A01	19667	p2	(CT)11	22	102550190	102550211	TCATCCATTTTCCCATCACA	TTGGGAGTTTGGGACCATTA	100
Ad01A19668	Aradu.A01	19668	p3	(TAT)7	21	102550449	102550469	CCATTTACCATGCTCCCATT	TTCGAAAGCCCAATATGCTC	276
Ad01A19671	Aradu.A01	19671	p2	(CT)6	12	102553101	102553112	TTCCATTGCTCCCTCACTTC	CCTTCATCCACCACCATTTC	242
Ad01A19672	Aradu.A01	19672	p3	(ATG)6	18	102555841	102555858	TTTGGTTTCATCAAATGCCA	GCACCATAGGAAAAACACCAA	258
Ad01A19673	Aradu.A01	19673	c	(A)26gaagacag	125	102560952	102561076	TGTCCTACTCGGGATAGCGT	TTTTTGTTCCCATATTTTCAGTG	198
Ad01A19674	Aradu.A01	19674	p4	(AGAT)6	24	102561337	102561360	TTGAGAGAGCATAGGGGGAA	AAGCACAAGGGCATTACCAC	235
Ad01A19675	Aradu.A01	19675	p4	(TACA)5	20	102562612	102562631	CTCAGCCTTCACAGGACACA	AGCAGTCAGAAATGGTTCGG	257
Ad01A19676	Aradu.A01	19676	p4	(TTTC)5	20	102562870	102562889	CCGAACCATTTCTGACTGCT	ATAGGGTTGAAGGAAAGGGG	156
Ad01A19680	Aradu.A01	19680	p3	(TGT)18	54	102564250	102564303	TGCATGATGATCCATAAGTGA	AGTTTTCTGTTTATGCATTTGTCA	175
Ad01A19681	Aradu.A01	19681	p3	(ATA)6	18	102564447	102564464	TTGTTGTTGTTGTTAGATGGCA	GGGACGACATTGTCACCATA	280
Ad01A19682	Aradu.A01	19682	p2	(AT)8	16	102564788	102564803	TAAGCCCCACTTACATTGGC	TCAGTTGTGTAATGTGTACTATCAAAA	189
Ad01A19684	Aradu.A01	19684	p3	(TAT)5	15	102565834	102565848	AGATGGAAAGTGCAAACCTTG	TGCTTGCTAGAAGGTGCTGA	199
Ad01A19685	Aradu.A01	19685	p3	(AAG)7	21	102566107	102566127	GCAAGCACCACACAGAGAGA	CCACTGCTCCTTATGCATGT	263
Ad01A19686	Aradu.A01	19686	p4	(GCAC)5	20	102566962	102566981	AGAGGAGAGGAAGGGAGTGC	TTTCTTAGCTTGCTGGGCAT	131
Ad01A19687	Aradu.A01	19687	p2	(TA)8	16	102568437	102568452	CTCCATTGTTTGGGCATCTT	TGTTTCATATTAATGAACTCGGATG	245
Ad01A19688	Aradu.A01	19688	p2	(GA)6	12	102569492	102569503	CAGCTGTCACCCACAAAGAA	GGACTGAGCAACAATGAGCA	212
Ad01A19690	Aradu.A01	19690	p3	(TTA)5	15	102576718	102576732	GTGAATTGGGCGGTAAGAAA	TGCTGTCGTCTCATGCTACC	237
Ad01A19694	Aradu.A01	19694	p3	(TAT)13	39	102586934	102586972	CATGAAGCTCAGTGTGAAGAAGA	TGTCCCAATTGTAGTGATTCTACG	276
Ad01A19695	Aradu.A01	19695	p3	(ATT)7	21	102588549	102588569	CAAGTATGCCACGCAGTGAT	CCTGATAACCAGGTAATTATTTGAGA	218
Ad01A19696	Aradu.A01	19696	p2	(TA)7	14	102590380	102590393	CGGTAACAATAACCGTTAGGAA	GAAGCCAGGCTGCATAAAAT	228
Ad01A19698	Aradu.A01	19698	p4	(TATT)7	28	102594588	102594615	GCATCACCATCAATGAATGC	GCATGTTCCCTTTTCTTTCG	224

ID	Chromosome	SSR nr.	SSR type	SSR	Size	Start	End	FORWARD PRIMER (5'-3')	REVERSE PRIMER (5'-3')	Product (bp)
Ad01A19699	Aradu.A01	19699	p2	(AG)6	12	102601375	102601386	TGAAGACAAAAGATAAAGAAGAACTTT	TGTCCATTTATGGGCAACCT	198
Ad01A19703	Aradu.A01	19703	p3	(TAT)6	18	102608014	102608031	CGCGTCAGCTGGACATATAA	GGGTTTAAAGAGGCCGAAAC	261
Ad01A19704	Aradu.A01	19704	p3	(ATT)5	15	102613861	102613875	TGAGATAGCCCTGGAAAACA	CCCAAGAATTTTTCTTTACTTATTCC	193
Ad01A19707	Aradu.A01	19707	p6	(AAGAGA)5	30	102617427	102617456	TAAGAGCCGCTTCCAGAAAA	CGCAATTTGCTACTGGTGAA	263
Ad01A19711	Aradu.A01	19711	c	(T)10caaacaaa	119	102624726	102624844	CGGATGCACCGATTTCTTT	GTGTCTGTCAACTTCCGTGC	247
Ad01A19712	Aradu.A01	19712	p3	(ATT)21	63	102625376	102625438	CATGCAAAGAATATGTAAATTTGTGA	CTCACTGTACCATGGCGAAA	162
Ad01A19714	Aradu.A01	19714	p3	(TAA)22	66	102626102	102626167	CTTTTATGCCCTTTCCTCCC	TTTTTGCATGCTCCTATCCC	205
Ad01A19715	Aradu.A01	19715	c	(TTA)7tcatcat	104	102627202	102627305	ACCAGCACCTTCTTGCTTTC	GTTCTCCAAGGAACGGAGG	239
Ad01A19716	Aradu.A01	19716	p2	(AG)12	24	102628037	102628060	TGGAGTAGCTGTGTGGCTTG	CCCTCAGCCCTAAAAGGAAG	215
Ad01A19717	Aradu.A01	19717	c	(A)13ctaagtatt	116	102628577	102628692	ATTCGGAGGGTCCAAATTCT	TTTTGAGATCAGTTTGGTGTTTTC	279
Ad01A19721	Aradu.A01	19721	p2	(AT)11	22	102632083	102632104	AAAATACACAGAAAAATGTTAAAAGCA	CGCGTATAAGTTCAGGGACC	271
Ad01A19727	Aradu.A01	19727	p2	(AG)7	14	102647086	102647099	TTTAGGGTGAAAGCCCAAGTT	TCCTTTTTCACTAGCATTCGG	204
Ad01A19732	Aradu.A01	19732	p2	(GA)7	14	102656435	102656448	CAAAACCATGGAAGGGAAGA	ACTGACAGGGACTCGCATCT	139
Ad01A19735	Aradu.A01	19735	p2	(CT)7	14	102662911	102662924	ATGGCTCCAACTCATCAACC	CACAGCTAATGGCGGGTAAT	142
Ad01A19739	Aradu.A01	19739	p3	(TCT)5	15	102679571	102679585	AGAAGCATGCGGAGTTGATT	GAGTAGCCACACCCCACATT	259
Ad01A19740	Aradu.A01	19740	p3	(TTA)15	45	102688145	102688189	TACAACAAAAAGTGTGGCCG	CCAGATTAGTGCCGTTGGTT	209
Ad01A19742	Aradu.A01	19742	p2	(GC)6	12	102691336	102691347	ATTCAAACCCTCCCGGTTAC	TGCGTTACCCAAGTGAATGA	181
Ad01A19743	Aradu.A01	19743	p2	(CT)8	16	102694637	102694652	CGGAATGCCATCAGAAGAGT	CCAGCGAGAGTGGTTTCTGT	222
Ad01A19745	Aradu.A01	19745	p3	(TAA)18	54	102698597	102698650	CCTTGAAGTGCGAAGAAAGC	TGGCCCTAAACCTCACATCT	208
Ad01A19748	Aradu.A01	19748	p3	(CTT)6	18	102702446	102702463	CATGCTTTAACCACCACACG	CCAAGAAGAAGTTGAAGGCG	140
Ad01A19753	Aradu.A01	19753	p3	(AAT)5	15	102721216	102721230	GGGACAAACACACAATCAAATC	TTTTTCCCGCTATCACAAGG	220
Ad01A19754	Aradu.A01	19754	p2	(AC)6	12	102721371	102721382	TGTGTCCCCATTTTGTTTCA	GACGAAAGGTTTGGTGGGTA	120
Ad01A19756	Aradu.A01	19756	p2	(TA)6	12	102722586	102722597	ATTGATTCCCCCTGCTTCTT	GTTTTTGCAGTTCTCGAGCC	251
Ad01A19757	Aradu.A01	19757	c	(TCT)6tttttgcc	90	102729385	102729474	CCATCGTAAGAGAGCTCCGA	TATCTCACCGCGTTCTTGTG	273
Ad01A19758	Aradu.A01	19758	p3	(AGA)5	15	102729757	102729771	GATGCAATGGCATGTTGAAG	CTCCGACGATGGTAGAGAGC	251
Ad01A19759	Aradu.A01	19759	p2	(TC)14	28	102730960	102730987	CACCAATTTACCGTCGGATT	CGAGCAGTGAGAGAGGGAAC	122
Ad01A19765	Aradu.A01	19765	p4	(AATT)5	20	102749699	102749718	AGAAGCTAAATGCCGCTTGA	TGGCAGATTTTTATTTTTGGAGA	217
Ad01A19770	Aradu.A01	19770	p2	(GT)6	12	102779664	102779675	GGTACGAGCATGAAAGCACA	ACAACTATGGCCCCATCAAC	263
Ad01A19774	Aradu.A01	19774	p2	(TC)6	12	102788818	102788829	TGGTGCACTGCGATAAAGAG	TGAGGGTAGTTAGTTGGCGG	188
Ad01A19775	Aradu.A01	19775	p2	(AT)6	12	102790080	102790091	GCACACTTGCAATATACATGGG	TTATTCCCATAAAGAGAACTAATTTCA	265
Ad01A19776	Aradu.A01	19776	p2	(TA)7	14	102790946	102790959	TCCATGTTAAAAATTAATGCCA	TGTATCTATTCTCATCCTCTCTTGC	276
Ad01A19783	Aradu.A01	19783	c	(T)14cttttcaag	113	102811870	102811982	TGAGGCGGTAATCTTGTGTG	GGGAATAGTTCCTCAGCCAA	269
Ad01A19787	Aradu.A01	19787	p2	(AG)7	14	102815264	102815277	CCATTTCCCCTATTGTCCTTC	TTATTACCCTTATGGCCCCC	271
Ad01A19788	Aradu.A01	19788	c	(AGA)12gtcac	70	102818747	102818816	GGCTTGCATAGAAAAGCAGC	TGCTTGCTTCCTTCCAGTTT	247

ID	Chromosome	SSR nr.	SSR type	SSR	Size	Start	End	FORWARD PRIMER (5'-3')	REVERSE PRIMER (5'-3')	Product (bp)
Ad01A19791	Aradu.A01	19791	p2	(AT)13	26	102825451	102825476	GGAGTCTGGACCTCACATTCA	CCACCCTAAAATTCTACACCTGA	241
Ad01A19792	Aradu.A01	19792	p2	(CA)7	14	102826640	102826653	GGCCCGTTGACCTATTTTCT	TGTGAAACTGGGGATAAGCC	247
Ad01A19794	Aradu.A01	19794	c	(ATT)8t(TTA)	46	102832571	102832616	CTGCTCTCCGTTGAACTTCC	GTCCCGAAGGTTCCAGTTTT	205
Ad01A19799	Aradu.A01	19799	p3	(TTA)17	51	102843775	102843825	AGGGTTGGACGAATCAAAAG	TCCAGCTCCTACTGATGATGAA	263
Ad01A19806	Aradu.A01	19806	p2	(AC)7	14	102857546	102857559	GGCATGGGAGAGTGAGAGAG	AACCTCAGCCATTGTTGTCC	123
Ad01A19811	Aradu.A01	19811	c	(CG)7(CA)8	30	102878472	102878501	TGAAAATCAGAGGTGGTGGA	CCGTTTTGAGTGTGATGTGAA	194
Ad01A19812	Aradu.A01	19812	p3	(ATA)5	15	102890363	102890377	TCGAATAGTTGCAAATGAAAAA	CGGCATTCTATTTCTTCCGT	160
Ad01A19818	Aradu.A01	19818	c	(T)10aagaaaaa	46	102899091	102899136	TGCAAGTTTTTCTGAACCAAA	TTCAAAAACGGAAACGAAATC	279
Ad01A19819	Aradu.A01	19819	c	(TCA)6ccatctt	122	102900257	102900378	TCCTCTTTCTCTTCCTCCTCC	CTCTTCCAAGTAATTTTGCAGC	209
Ad01A19821	Aradu.A01	19821	p3	(TCA)11	33	102903511	102903543	CCATCCTCAAATCCAAGCAT	ATCCCCCAGGTCTTTAGAGG	179
Ad01A19827	Aradu.A01	19827	p2	(AT)11	22	102917600	102917621	CGCGAATATGCATTTTTAGTTG	TCTTATTCGCCAATATGTTTTAAATTC	136
Ad01A19828	Aradu.A01	19828	c	(T)10caggttcaa	30	102920930	102920959	TTTTGCTTGGCCTCAAAACT	AATTATGTGTGGGGTCCTGC	233
Ad01A19831	Aradu.A01	19831	c	(A)12g(A)10	23	102946263	102946285	TCCTCTACTATTTTAGCACTTGTTCTT	TGGAACTCACAACTACTCTCCTTC	206
Ad01A19834	Aradu.A01	19834	p3	(ATT)5	15	102947711	102947725	AACACGTCCGAAACTAAAAACAA	AAACACACTAATCAGCAATGTTAAGG	238
Ad01A19838	Aradu.A01	19838	p3	(GAA)5	15	102952065	102952079	GGAGAGTGTTTCTACTTCTGCGA	TTTTAAATTTTTGGCAGCGG	101
Ad01A19842	Aradu.A01	19842	p2	(AT)9	18	102966514	102966531	CAGTGCCCAGTGATGTAGGA	ATCGCATGGAGGATAGGTTG	227
Ad01A19843	Aradu.A01	19843	p3	(ATC)5	15	102967986	102968000	TCAACCACATTACCATTTCATCA	ATCGGCCAAGGTATGGTTTT	267
Ad01A19846	Aradu.A01	19846	p2	(TC)8	16	102973382	102973397	TCATTGAAAATTATGTGAATGGATTT	ATTGAGGATTTCAGAGGCCA	143
Ad01A19847	Aradu.A01	19847	p3	(AAG)25	75	102974959	102975033	CAACGTAACAACCCCATTCA	TTCGCTTGCGTTATAGGCTT	275
Ad01A19849	Aradu.A01	19849	p3	(TTA)11	33	102981434	102981466	CATCCAAACCAAAAGCCATC	TGACATTTGGTATCCTTGTGTTC	228
Ad01A19851	Aradu.A01	19851	p3	(AAT)19	57	102983950	102984006	AAGTTACATGCATAAGCTTTCGG	GGCTTTGGTTCCACAGAAGA	215
Ad01A19852	Aradu.A01	19852	p2	(TA)8	16	102984228	102984243	TCTTCTGTGGAACCAAAGCC	GTTGGTGCTAACGAAAAGGC	227
Ad01A19853	Aradu.A01	19853	p3	(AAT)5	15	102987186	102987200	TCATTTAAAATACGAATTGTGAGACA	TTGAAATTTGGATCATGGCA	275
Ad01A19855	Aradu.A01	19855	p2	(TC)8	16	102988390	102988405	TTTCTAAAATGCCGGTTTGC	TTGAAGCTGCGAGTTCCTTT	247
Ad01A19856	Aradu.A01	19856	p4	(TTAT)5	20	102991140	102991159	TTGTCCCATGTGCATTGATT	TGGGAACATGGGATGATATG	228
Ad01A19857	Aradu.A01	19857	p4	(TTAT)5	20	102995064	102995083	TGAACCATCCATCAATTAGAGGT	GGTGGTTAAAAGGAGAAAGCC	216
Ad01A19860	Aradu.A01	19860	p3	(TTA)18	54	103008556	103008609	TTGAGCATCCGACATATCAA	TCTCTATTTATGTGGGGGCG	272
Ad01A19861	Aradu.A01	19861	p3	(TAT)5	15	103008810	103008824	CTGTGTCCTTCCCCTCAAAA	GCATTTTGTTCTGATTTGGCT	278
Ad01A19864	Aradu.A01	19864	c	(TAT)5ttaatttg	87	103011189	103011275	TTGCAAGAATTTGTTTTTCGG	AATCTTCAAGAATTTATTTGTCACATT	273
Ad01A19866	Aradu.A01	19866	p2	(AT)8	16	103013234	103013249	CGAATTATTGCTTTGTGGGA	TCAGAGCATATGAAGTTGATGCT	252
Ad01A19869	Aradu.A01	19869	p2	(AT)7	14	103019526	103019539	CTGCCATTAATTTTGCCGTT	TTTGAGTAATTCGTTCCCCAG	134
Ad01A19870	Aradu.A01	19870	p3	(AAG)5	15	103020530	103020544	TCATCAACAAAAACACATCGAA	TACGTGTACGTTTGGTTGCC	271
Ad01A19875	Aradu.A01	19875	p2	(AG)6	12	103030560	103030571	AGGACTTGGAAGAAGAGAGGG	TAGTAGCTTTGTCTCCGGGC	242
Ad01A19876	Aradu.A01	19876	p3	(CTA)5	15	103030769	103030783	AGCTAAACATTCCTTGGGGC	GCTTTTGCCCTCAATTTTCA	222

ID	Chromosome	SSR nr.	SSR type	SSR	Size	Start	End	FORWARD PRIMER (5'-3')	REVERSE PRIMER (5'-3')	Product (bp)
Ad01A19881	Aradu.A01	19881	c	(A)13tgagtaga;	71	103048296	103048366	GTCTAGCAATCCCTCCCTCC	TGCAACCAAGCTCTCTTTCAT	233
Ad01A19883	Aradu.A01	19883	c	(AAAT)6tcaag	88	103057341	103057428	GGAGATTGAAGATAGTGGCACC	TCACCATCTTTGGTGGAAGA	274
Ad01A19884	Aradu.A01	19884	p3	(ACC)5	15	103058179	103058193	TGTCCTATCCAGGAGAACGG	GTAGATGGGGCTGTTCCTGA	201
Ad01A19886	Aradu.A01	19886	p2	(AG)6	12	103063353	103063364	GGCGAAAATTGAATCCAAGA	AAGTCGGTTTCGGTGTTGAC	169
Ad01A19890	Aradu.A01	19890	p2	(AC)17	34	103068489	103068522	CCATAGCCACTATAGGCACCA	CGTAACGCTCACAAAGCGAT	177
Ad01A19893	Aradu.A01	19893	p2	(TA)6	12	103084333	103084344	CCCCTCTTTGTTGAGAGCAA	GCAACAAATGAATGAAGTTCG	278
Ad01A19894	Aradu.A01	19894	p3	(TCT)5	15	103088183	103088197	CCCCTCTTCTATTCCTTGGC	GGGTTTTGGGGATGAATCTT	254
Ad01A19895	Aradu.A01	19895	c	(T)10cgaaa(T)	26	103094389	103094414	TCCCCAATTAGAGGTGTCCA	TCATGAATGTTGGCTCTTGAA	216
Ad01A19896	Aradu.A01	19896	p4	(CAAA)6	24	103104817	103104840	AGCCTCCTCCCATCTTTGAT	GCTGGGGAATTGCAATATGT	278
Ad01A19897	Aradu.A01	19897	c	(TC)7ccctttctc	49	103109636	103109684	AAAAACCAATTTTTATGGTTCAGTTT	GGGAAAGGAGAGAGAGGGAG	207
Ad01A19899	Aradu.A01	19899	c	(CT)7ccctttttt	119	103110159	103110277	CTCTCCCTCCCCTTCCTTCT	GGGCAGAGAGGAGAGAAACA	255
Ad01A19900	Aradu.A01	19900	p2	(TC)6	12	103110578	103110589	TCTCCTCTCTGCCCTCTCTG	GGATGGAGAAAAAGAGAGAAAAA	265
Ad01A19901	Aradu.A01	19901	c	(GA)6agaagag	164	103110760	103110923	TCCCTCTTCTCTTTCTCTTCCTC	CGCTCTCCTTTTCTCCCTCT	270
Ad01A19902	Aradu.A01	19902	p2	(AG)6	12	103111402	103111413	GGGAAAAGGAAAATAGAGAGGA	TCTCTCTCCCTCCCCCTC	242
Ad01A19903	Aradu.A01	19903	p3	(AAG)9	27	103112865	103112891	CATCGTCGTGTTTCTCCTCA	TCGTTGAAGTACGCCTTATGC	197
Ad01A19905	Aradu.A01	19905	p3	(CTT)6	18	103116147	103116164	TTGGAAGAACCCTGTTGGTC	GTATCAGGAGAGACGAGGCG	112
Ad01A19906	Aradu.A01	19906	p3	(CTT)6	18	103118011	103118028	GGTCTTGCGATTCTTTCTGC	CAATACATTTGCATGCCCTG	213
Ad01A19907	Aradu.A01	19907	p3	(CTT)6	18	103119991	103120008	GGTGGTGGCGTAGTAGTCGT	GTGGGAGGAGGGAGTAGGAG	270
Ad01A19910	Aradu.A01	19910	p4	(TTTG)5	20	103126500	103126519	GAATTGAATGGAAGATGAGCAA	GCCTAGGAGGCACTGAAACA	131
Ad01A19911	Aradu.A01	19911	p2	(TA)8	16	103132382	103132397	CATACATAAATTTTGATTTTGAGCA	TTTCTTACATTATTACATTTTGCTGGA	114
Ad01A19912	Aradu.A01	19912	c	(T)10aaaataatg	69	103138265	103138333	AAGTAAGTGGTAGAATGACTTCTCG	CGTTTGAGAAGCAGCAAAAA	183
Ad01A19913	Aradu.A01	19913	p3	(AAG)5	15	103149967	103149981	CCATGATCGTACCCTCTGCT	GTGCAGGGGGTATTGAAAGA	213
Ad01A19915	Aradu.A01	19915	p2	(TG)7	14	103154275	103154288	GGGGAGGTTATGATTCTTGG	GGCAATGTCTGGCACTTCTT	236
Ad01A19916	Aradu.A01	19916	p3	(AAT)6	18	103154468	103154485	AAGAAGTGCCAGACATTGCC	CCCATTTGAATACGTGAGCA	244
Ad01A19917	Aradu.A01	19917	c	(TG)6ta(AT)6a	125	103154840	103154964	AGAAGAGGGACAGGGTCGTC	AAATGGAGCCCCTACACTCC	242
Ad01A19919	Aradu.A01	19919	p2	(TG)6	12	103156552	103156563	CTGCCCAAACTTAAATCCCA	CCATTTCCTCTTTTCCCTCC	268
Ad01A19920	Aradu.A01	19920	p2	(AC)6	12	103160341	103160352	CTGCACAATCACAACCAACC	CTTGAAGCTTTGGGTGCTTC	190
Ad01A19923	Aradu.A01	19923	p2	(TA)6	12	103171655	103171666	ATTGCCTCTGTCATTCCCAG	CTGCGAAAACTAAAGCCGAC	267
Ad01A19925	Aradu.A01	19925	p2	(TA)7	14	103177642	103177655	CGATTTTAAATATTCAATTTGTGATTC	GGAAATGCTAAAAGCTAAATTGG	279
Ad01A19926	Aradu.A01	19926	c*	(TC)11ccgcaat	123	103178164	103178286	CGGTAACTGCTCTCACTCCC	TTGTCAGGGAGAAAAACAGGA	259
Ad01A19928	Aradu.A01	19928	p3	(ATG)5	15	103188934	103188948	GGTGCACCATACATGCCTAA	GCACCAATTACAGGACAGCA	137
Ad01A19930	Aradu.A01	19930	c	(T)10g(T)11	22	103189912	103189933	CATGGCTATGGCATTCTCAA	CTCCCTCCCCTTTCTGCTAC	246
Ad01A19931	Aradu.A01	19931	p3	(TTC)5	15	103193742	103193756	CCAAATATCTTCGTGTTACACCC	CCTTCCCAAGTTTCTTCTTGTTT	240
Ad01A19936	Aradu.A01	19936	p2	(AT)6	12	103200522	103200533	TCCCATTCCAGGAGAGTACG	CACTGGGGGCAATGTAAGTT	264

ID	Chromosome	SSR nr.	SSR type	SSR	Size	Start	End	FORWARD PRIMER (5'-3')	REVERSE PRIMER (5'-3')	Product (bp)
Ad01A19940	Aradu.A01	19940	p4	(TATG)19	76	103225585	103225660	GGGCTCTACTTGTTTGGGGT	CGACGTCGATAAAAGGGAAC	270
Ad01A19945	Aradu.A01	19945	c*	(CT)6(T)13*	24	103234107	103234130	AATTCAATTCCCCCAAGGAC	CTGGACGAGTTCAAGCACAA	264
Ad01A19947	Aradu.A01	19947	p2	(AT)7	14	103238783	103238796	TGGTCTGTCCTGTCGAGTTG	CACCTGGCCAAAAGTAAGTTC	127
Ad01A19948	Aradu.A01	19948	p2	(TA)8	16	103239414	103239429	AAAACAAAGGAGGATTGGGG	GGTGCAGTTTTGGCAGTGAT	272
Ad01A19951	Aradu.A01	19951	p2	(AC)7	14	103245815	103245828	AAAACCCAAATTCCCAAACC	ACTCCACAAACCCCTTTCCT	200
Ad01A19955	Aradu.A01	19955	c*	(AAT)6(ATA(	36	103254812	103254847	CCAGGAATCAGGCTTGACAT	TTCAATTGCTGAAAGGAGCC	204
Ad01A19959	Aradu.A01	19959	p3	(AAT)19	57	103261702	103261758	GCAATTGCAAGTTCAGTATGG	TGGATCTGTCCATTGCTAAAAA	231
Ad01A19960	Aradu.A01	19960	p3	(ATA)14	42	103265757	103265798	TGCCTCAACAGAAACAGCTC	TTTGTGCGATTCATATTGGTT	233
Ad01A19961	Aradu.A01	19961	p3	(AAT)15	45	103268241	103268285	CCCTATCCATCAATAGATTGCC	ACTGAGAAGGCAAAGACGGA	206
Ad01A19964	Aradu.A01	19964	p2	(AG)8	16	103280220	103280235	TCCCTAGGTACGAGACGGTG	CGTGTTCTTCTCTCCTCTTCG	178
Ad01A19969	Aradu.A01	19969	p2	(AT)6	12	103293221	103293232	CCTTTTGGTGGTGGAAAATG	TTCAGAATTTGCAATGCATCA	224
Ad01A19974	Aradu.A01	19974	p2	(TG)6	12	103308267	103308278	TATGGGAGATTGGGATTTGG	AAAATTCTTCCGAGGCAGGT	265
Ad01A19975	Aradu.A01	19975	c	(CCT)7cttcctt(	49	103309801	103309849	ACCCCTCATTCGTTTGTCAT	AATAAGGAAGAACGCGAGCA	172
Ad01A19976	Aradu.A01	19976	p3	(AAG)7	21	103310575	103310595	CGCGAAAAAGAAGGAAAATG	CGTCCAACACACACGAAATC	160
Ad01A19980	Aradu.A01	19980	c	(CTT)5tttcatca	44	103313932	103313975	CGTTGATGGTTTGAATTGAATG	AAAACAAGAAGAAACTTGAGAAGG	279
Ad01A19982	Aradu.A01	19982	p4	(GAGT)5	20	103318114	103318133	GGGGGAACCAAGAAAACATT	GGGATGCTCAGAACGTCACT	223
Ad01A19984	Aradu.A01	19984	p3	(AAT)5	15	103319663	103319677	TCAATTGAAAGCAGCCCTTC	GTGCCAGAGACTGGTGATGA	205
Ad01A19990	Aradu.A01	19990	c	(GTGGCG)5a	85	103326847	103326931	GAGAAGAAGAGATGCGGACG	CGGTGCTGGTAACAAACAAA	250
Ad01A19991	Aradu.A01	19991	p3	(ATT)5	15	103328366	103328380	CACAATGACATGACAGTTTTACG	TTTTTGTCAGCGCATGGATA	198
Ad01A19994	Aradu.A01	19994	p3	(ATG)5	15	103333494	103333508	TGAAAACCCTGCCACGTAAT	TCCATTGACCACTCTTTGTTG	219
Ad01A20001	Aradu.A01	20001	c	(AG)6tgtgtgtgt	39	103349475	103349513	AAGTGGAATTTTCAGATGCGA	GAATGACTTCTCTGCCCCTG	180
Ad01A20005	Aradu.A01	20005	p2	(AT)6	12	103357401	103357412	ATGGTTGCCAACTTCCTTGA	CCCACATCGATCAATAGCCT	270
Ad01A20006	Aradu.A01	20006	c	(T)12gtgatggca	51	103359078	103359128	TGACCAACAACTCTGCTTTGA	AAAATCTTTGGGAAATGGGC	224
Ad01A20008	Aradu.A01	20008	p2	(CT)10	20	103361771	103361790	TCGACAATTCTGCCTCCTCT	ACATGGGAGAGTGCTTGCTT	131
Ad01A20009	Aradu.A01	20009	p3	(TTA)6	18	103361952	103361969	AAGCAAGCACTCTCCCATGT	GCCCATGATGATGAAAAGGT	218
Ad01A20010	Aradu.A01	20010	c	(A)11tcaaatact	48	103363770	103363817	GCCCTAAACAGAGAATGAAGCA	TTTGTTGGCCCAGGTCTTAG	105
Ad01A20011	Aradu.A01	20011	p2	(TC)7	14	103370437	103370450	CGAACAGAGAGTTGTGCAGG	TGCTTGTCCTCAAGCAAAGA	272
Ad01A20015	Aradu.A01	20015	c	(A)11caactggc	43	103391511	103391553	GCTCAACACAACATGGTGGA	GCGAATCTTTTGGTTGTTGA	115
Ad01A20016	Aradu.A01	20016	c	(A)10gaaaataa	68	103392724	103392791	TTTTGGCTCGGGTTATTCTG	TTCTTTAATTTTTCTCCTCAACTTTTT	280
Ad01A20017	Aradu.A01	20017	p2	(AT)7	14	103400160	103400173	TCATGACTTTGAGCCACTTGA	TTCTTGTATTGTTTGAATCCTTGAA	276
Ad01A20018	Aradu.A01	20018	p4	(AAAT)7	28	103402306	103402333	CCCAAAGGTTAAGCAGACCA	CATGCAACTTGGAACACCAC	130
Ad01A20021	Aradu.A01	20021	p3	(GAG)11	33	103413633	103413665	CGGTGAGGAGGAGACAAAAA	CCTCAACTCCACCACCATTT	255
Ad01A20024	Aradu.A01	20024	p3	(ATA)5	15	103424544	103424558	GGGCCTAATTCTTTAAATGGG	GACTTCGAATGCCAGGGAG	240
Ad01A20027	Aradu.A01	20027	p3	(GAA)6	18	103446299	103446316	GAAGATGAAAGCTGGTTCCG	CCGTCCGATTTCTGGTTTTA	142

ID	Chromosome	SSR nr.	SSR type	SSR	Size	Start	End	FORWARD PRIMER (5'-3')	REVERSE PRIMER (5'-3')	Product (bp)
Ad01A20028	Aradu.A01	20028	p2	(AT)8	16	103448854	103448869	GGGATGAATTTGGAGAGCAA	AGAATGATGAAGACGGTGGC	279
Ad01A20030	Aradu.A01	20030	c	(A)12ttaaacata	110	103453004	103453113	TTCCACGCTTCCACTTATCA	ATTGCCAATCCATTATCCGA	213
Ad01A20031	Aradu.A01	20031	p2	(GA)6	12	103453917	103453928	ATAGCGGTGTTTGGAAGGTG	TCGAAGCAAGGAAACGAGAT	243
Ad01A20033	Aradu.A01	20033	p2	(GA)6	12	103459933	103459944	GCCTCCAAATTTCTGATGGA	AAGCCTCAACAAGGCTTCAA	203
Ad01A20034	Aradu.A01	20034	p3	(GTT)6	18	103462338	103462355	CGTTTGATTCATGTAGCCGA	GAACAACGTGATTTCATGCG	189
Ad01A20035	Aradu.A01	20035	p3	(TCC)8	24	103462505	103462528	CGCATGAAATCACGTTGTTC	GAGAAAGGAGGAGAAATGCAAA	112
Ad01A20037	Aradu.A01	20037	c	(CTT)5ctccatc	131	103465294	103465424	TTCTGCATCTGGTTTTGCTG	TTTTGGACAAAAATGGCCTC	218
Ad01A20041	Aradu.A01	20041	p3	(CCA)5	15	103469823	103469837	TGATGTTGTGAAGTGCCCAT	GCCTTGTGTTGTGGGCTAAT	271
Ad01A20042	Aradu.A01	20042	p3	(AAT)5	15	103471988	103472002	GGATTCGGTCCCCTCATATT	CCCAACTAATACCCCCGATT	249
Ad01A20044	Aradu.A01	20044	p3	(TGC)5	15	103480474	103480488	CCTCAGCTTCTTCTGAACCG	CCCGATTCTTCCTCTGAACA	279
Ad01A20045	Aradu.A01	20045	c	(A)10taaaccttta	107	103490078	103490184	CAAGTAGTTGCATCCGGGTT	TCTCTATCGCTCCCATCCTC	208
Ad01A20046	Aradu.A01	20046	p2	(AT)7	14	103491568	103491581	TTTGCGTTTTCTAGGGTTTTG	GGCTTTTTAGGGACTTTTAGGG	251
Ad01A20050	Aradu.A01	20050	p4	(TTAT)5	20	103499818	103499837	CGAATTTGATCTCGAATATATTTTTA	CGATCCACATACTAGTAAAATCGG	280
Ad01A20054	Aradu.A01	20054	p2	(TA)7	14	103508569	103508582	GGAAGACTATGCTATTTCAAAAGAAAC	GGTGTCTTCCTTTTTCAGCTATG	260
Ad01A20055	Aradu.A01	20055	p2	(AT)7	14	103511186	103511199	TTCCATCTTTTACTTCAACCTGA	CAATTTTCATGATTTTAAACCACTTG	276
Ad01A20056	Aradu.A01	20056	p3	(CTT)5	15	103511869	103511883	AATTTATTTCGTCCCCGACC	GAAGAAAAAGTGGCAGACGG	205
Ad01A20057	Aradu.A01	20057	p3	(GTG)7	21	103512291	103512311	GACAGTGGGTGGAGAGTGGT	CCCTTCTTCCCCAAAATCAT	166
Ad01A20058	Aradu.A01	20058	p2	(AT)7	14	103513029	103513042	AAAGTTTTGCTTCAACGTGATT	TGACTTTGAACCACTTCACTTCTT	221
Ad01A20064	Aradu.A01	20064	p2	(TA)7	14	103530643	103530656	AAAAATTGCATTCGGCCATA	ATAATGGCTGTCCCTTTCCC	186
Ad01A20068	Aradu.A01	20068	p3	(ATC)5	15	103535612	103535626	CAGATATCATGGCAAGGGCT	ACTCCTGGTCGTCTTCTGGA	265
Ad01A20069	Aradu.A01	20069	p3	(TTC)6	18	103536384	103536401	GAGAACCCCATATCCCGAAT	AATCCTTGGAAAACAATGCG	229
Ad01A20071	Aradu.A01	20071	p3	(AAT)6	18	103537238	103537255	GGAGTTTATATTCGCGGCAG	GTCGATGCCATGTTTCCTTT	266
Ad01A20072	Aradu.A01	20072	p3	(ACC)7	21	103541234	103541254	ACAAGAACCTTCGTGTTGCC	GTTGGAACCAGAATCCGAAA	198
Ad01A20077	Aradu.A01	20077	c	(A)13gagagaag	94	103549123	103549216	ACACCTTTCATGACCCCACT	TCAATCATGGAAATGGAGACC	162
Ad01A20078	Aradu.A01	20078	p2	(AG)6	12	103550600	103550611	AGGCAGTCCCACTAGCTGAT	TAGTCAAGCACAATTCCCCC	160
Ad01A20079	Aradu.A01	20079	c	(TA)6(GA)7t(A	39	103556627	103556665	GTCCCTCCTAATCCCCAAAT	ACGTGGTGAGGGTTATGGAA	272
Ad01A20081	Aradu.A01	20081	p2	(TA)8	16	103560858	103560873	GCAAAGTGGATCATTTTGGAA	TGCAACGCTCAGAAATAACG	276
Ad01A20083	Aradu.A01	20083	p3	(ATT)6	18	103577336	103577353	TTTTTGAACCACAAAATGGC	CAACGGTTGGACTTGTCCTT	114
Ad01A20084	Aradu.A01	20084	p2	(GC)6	12	103578545	103578556	CCAATTGGGTTGGAAAGCTA	AGTTGGATTGAGCCCACAAG	178
Ad01A20087	Aradu.A01	20087	p3	(ATT)23	69	103590565	103590633	TGCCTAACAAATTAAAAACAAATGA	TCACTGAAAGATCACATACATAATCAA	276
Ad01A20088	Aradu.A01	20088	p4	(ACTA)6	24	103599099	103599122	TTGGAATGCACAATTACTAAGCA	TCACCACCATTTACCATCCA	270
Ad01A20090	Aradu.A01	20090	c	(T)11ggtttttttt	77	103606248	103606324	GATGCGTCAGACATACACGC	TCACTTCATTCGGCAGCATA	196
Ad01A20094	Aradu.A01	20094	p2	(TA)9	18	103611339	103611356	GCTGGAGACTCTCTGGAAGC	AGCTCCAGACTCGACCTACG	270
Ad01A20098	Aradu.A01	20098	c	(AT)9cactcaaa	86	103634145	103634230	GGGTTGTGTGTTCTTTGGCT	TTTAGTTTACCCTTTTTAGCATATGAG	277

ID	Chromosome	SSR nr.	SSR type	SSR	Size	Start	End	FORWARD PRIMER (5'-3')	REVERSE PRIMER (5'-3')	Product (bp)
Ad01A20100	Aradu.A01	20100	p3	(TAA)5	15	103635528	103635542	TTATGGCATATAGTCCCACGC	TTGAATGTGTAGCATAGCCCA	189
Ad01A20101	Aradu.A01	20101	p3	(CTT)7	21	103635700	103635720	CACGCACGCTTTATCTTCTTC	AGCAATTCCATTCCATTCAA	271
Ad01A20102	Aradu.A01	20102	c	(AGA)6aacgcg	47	103636689	103636735	AGAAGACGCTCCAGCGTAAA	GGCATCCAAATTCATCCAAA	163
Ad01A20104	Aradu.A01	20104	p2	(TA)6	12	103640107	103640118	TCTGACAAAATTCAGATGATTAGG	GATTCATTGTGGGTTCGGTC	198
Ad01A20108	Aradu.A01	20108	p3	(GAA)5	15	103646063	103646077	TCCTCTTCCTACTGATTTTGCAG	TGATGACACACAAACAAATCCA	217
Ad01A20109	Aradu.A01	20109	c	(AGG)6agaaaa	83	103646376	103646458	AAGAAGACGACGATGATGACAA	CTCCTCCCATTGATGTTGCT	261
Ad01A20111	Aradu.A01	20111	p3	(TCT)5	15	103653562	103653576	CAAAATCTCTTTCCCACCCA	TCGAGCAAAAGGAGAAGGAA	103
Ad01A20114	Aradu.A01	20114	p2	(GA)26	52	103670072	103670123	GACAATGACGTGTAGCGGTG	CGTCGCAAGAGATTTTCCAT	269
Ad01A20115	Aradu.A01	20115	p2	(TA)6	12	103673641	103673652	CTCCTAAACTACGCCAACGC	CCACTTTTATACTAAAAATCAACCACA	211
Ad01A20117	Aradu.A01	20117	p3	(TAT)15	45	103689783	103689827	TCAAGCCATTTCAGCATCAG	GCATGCAAGCCACCTTATTT	238
Ad01A20118	Aradu.A01	20118	p2	(GT)7	14	103692044	103692057	GGCCTGAGCTTTAGTACGATCA	TCTTTGCGAATACAAACCGA	143
Ad01A20119	Aradu.A01	20119	p2	(AG)8	16	103695060	103695075	TGTGCCTTGCACTCTCACTC	AAAGATGAAGGGCTCGGATT	235
Ad01A20125	Aradu.A01	20125	p2	(TA)6	12	103702610	103702621	TGCATTGGTCAAAGTGTTGAT	TAGAGCCGGGTCACTTCAAT	262
Ad01A20126	Aradu.A01	20126	p4	(CACT)5	20	103703104	103703123	ATAGATGCGGCAATGGTGAC	TCCAATCAGAGTCGAGCAAA	212
Ad01A20128	Aradu.A01	20128	c	(T)10g(T)10	21	103703581	103703601	TGAATTTGGCTGTGGAATCA	AAGATTCGCTCTTCAATCGC	190
Ad01A20130	Aradu.A01	20130	p3	(ATA)10	30	103715013	103715042	GGCCAATTTTGGTTGAAAAA	TGTAACCACACAATTAGGCTCAA	254
Ad01A20132	Aradu.A01	20132	p2	(GC)6	12	103732533	103732544	GCTGGGGACGATTTCACTAA	CCAGTTGGGTTGGAAAGCTA	144
Ad01A20133	Aradu.A01	20133	p2	(TC)6	12	103734561	103734572	AGCTGCTGAAGAGAAAAGCG	GCATGCAAACAGTGAGGAAG	267
Ad01A20134	Aradu.A01	20134	p2	(AC)6	12	103738847	103738858	GAAAGGCGCTACGACCTCTA	GACAATGCTTTGTGGGGTTT	161
Ad01A20135	Aradu.A01	20135	p2	(TA)12	24	103740383	103740406	GGTTTTACAGGCCTAGTGCG	TTACTGCAAGCAAAAGGCAG	278
Ad01A20136	Aradu.A01	20136	p3	(AAT)6	18	103741244	103741261	TGGTCAGAAAAAGTGATTTTACG	GAGACACAAGAAAGGATGCAAA	191
Ad01A20137	Aradu.A01	20137	p3	(AAT)5	15	103741599	103741613	ACAGGAGCAATGGCAAAAAG	TGCTGGAATTTTGGTTCCTC	197
Ad01A20138	Aradu.A01	20138	p2	(TA)6	12	103741929	103741940	AGCTCATTCCCCTGGACTTT	TTGGGAAAAATGGTATCTGGA	130
Ad01A20139	Aradu.A01	20139	p2	(TC)6	12	103743283	103743294	TGCTCTCCCCTTTCATTTTG	AAAATTGGCTGGTTTTCGTG	162
Ad01A20141	Aradu.A01	20141	p2	(CA)10	20	103749812	103749831	CTCCATCCCTTGTGTATCATCA	GCCAATTCCGAAAAGGAGA	264
Ad01A20142	Aradu.A01	20142	p2	(GA)6	12	103766626	103766637	CGAGTGGAGTGGGAGAGAAG	GGAAATCATCTTTCTGGGCA	132
Ad01A20143	Aradu.A01	20143	p2	(TA)7	14	103783563	103783576	CGACACAAAAAGCGTCAAAA	CTGGTGCTGATACTGGAGCA	209
Ad01A20146	Aradu.A01	20146	p2	(TA)6	12	103788807	103788818	CACAAGATACACAAACACGTGAA	CAGCGTGTCTAGTCTTATTCTTAACAT	268
Ad01A20148	Aradu.A01	20148	p3	(TGT)5	15	103791244	103791258	TCATCTCTGATGAGGGGCTT	CCATCATCACCATCATGAGC	189
Ad01A20150	Aradu.A01	20150	p3	(ATA)11	33	103791965	103791997	TGAAAATTCACAAAGCAAAGTG	TATAGAGTTCGCCGAGGGTG	227
Ad01A20154	Aradu.A01	20154	p3	(TCT)6	18	103813628	103813645	GCTTCAGCAACTCCAAGACC	TTCAACAATGGCAACTTGGA	107
Ad01A20160	Aradu.A01	20160	p2	(TA)7	14	103820389	103820402	TGCTAGAAATTCTTCATCTTTCCA	TGGTTAAAAATTCAAAATGCCA	212
Ad01A20162	Aradu.A01	20162	c	(T)10ctcatatttc	209	103828241	103828449	CCTCAGTGATGTTGCTACTTCTTC	AAATAACAATGATAACAATGATGACAA	278
Ad01A20164	Aradu.A01	20164	p3	(TTA)5	15	103831995	103832009	TGTACTCTCGCATTTTCACTTTTT	AAGTTACCAAACAAAATTCATAACCA	254

ID	Chromosome	SSR nr.	SSR type	SSR	Size	Start	End	FORWARD PRIMER (5'-3')	REVERSE PRIMER (5'-3')	Product (bp)
Ad01A20166	Aradu.A01	20166	p3	(TTA)5	15	103838622	103838636	TGAGGAGAAGACAAAATACCCA	CAAAATGAGATAATTAAAAATAGGGGA	270
Ad01A20168	Aradu.A01	20168	p3	(GGT)6	18	103842767	103842784	GTTCCTGGGGCTGCTATGTA	TCACCAAGACCACAATTCCA	245
Ad01A20171	Aradu.A01	20171	p3	(GAA)5	15	103847200	103847214	ATGCGAGATGGAGGAAAAGA	AAGGGAAGAAAGCCACCACT	271
Ad01A20172	Aradu.A01	20172	p2	(AG)13	26	103850831	103850856	ATACCTGAATCTTGAGCGGC	TCGTCAGATCTACCGTCGAA	214
Ad01A20177	Aradu.A01	20177	c	(GT)6tttgttttgt	43	103877697	103877739	CATGAAAGAAGTTAATTTTTACAGCAA	GTACCCCAATGCCAAGTCCT	277
Ad01A20180	Aradu.A01	20180	p2	(AG)8	16	103881762	103881777	TATCCGGCCCATACTCAGAC	CCTTCTCCTTATGCGCTGAC	205
Ad01A20184	Aradu.A01	20184	p2	(AC)9	18	103888567	103888584	TGGAATCAAGGGGTCACATT	CCGGGTCAATTACTGGTTTG	276
Ad01A20187	Aradu.A01	20187	p2	(TA)6	12	103890322	103890333	AAAAATGCGTATTTCGTGTGTT	TCGGATAGATAATGACTTGCATTT	231
Ad01A20191	Aradu.A01	20191	p3	(GAA)7	21	103899751	103899771	GGTCACATGACGGAACACTG	CGAGATATGCCTCCTTCTTCA	192
Ad01A20193	Aradu.A01	20193	p3	(TTC)5	15	103913686	103913700	AAAAACCAGAAATCGGTCCC	AGGAAGAACATCACCGGGTT	269
Ad01A20194	Aradu.A01	20194	p2	(AT)8	16	103914327	103914342	TGACAACGAAGATGGAGCTG	GAAGCTCTGCCACAATCACA	159
Ad01A20197	Aradu.A01	20197	p3	(TTA)8	24	103930644	103930667	CATTGGGGCTATCTGCCA	GGCGTGCAGTTTATTCTATGC	271
Ad01A20202	Aradu.A01	20202	p2	(AG)6	12	103960747	103960758	CAGTGACCAAGGAGGAAGGA	AAGAAAAGAACGCGTTGGG	227
Ad01A20205	Aradu.A01	20205	c	(AT)8(AC)7	30	103968868	103968897	TTCCACGTATAACCCAAGGC	AAATTGAGGCACATCCAAGG	166
Ad01A20207	Aradu.A01	20207	c	(A)10tgaagaca	66	103979245	103979310	ACCAAATTTGAATGCCCAAG	TGAACCTTCGATTCCAACAA	224
Ad01A20211	Aradu.A01	20211	p3	(ATA)18	54	103994175	103994228	TCACCACTCTTGTATTTTACCACTC	GACAAACAAAATTACGACGCA	278
Ad01A20213	Aradu.A01	20213	p3	(TAA)18	54	103998653	103998706	CGCTATATCCCGCTTGTGTT	ATTTGAGCGACGTTGTGTTT	169
Ad01A20214	Aradu.A01	20214	p2	(TA)7	14	104000417	104000430	TCATCACGTATTAACATGTTCAGTTT	TGATGCACAGATATTGATACAAACA	217
Ad01A20216	Aradu.A01	20216	p2	(AT)8	16	104017237	104017252	TTCGTCTAGTATGTAAAAGCGAGAA	CACTGAGGACAATGACATAGCA	248
Ad01A20218	Aradu.A01	20218	c	(T)11ccttttccac	199	104019866	104020064	TTCACCACCAGCTATCATGC	TCAATAATAACAAAGTCGCGTG	258
Ad01A20221	Aradu.A01	20221	c	(ATG)5atcatgg	118	104022478	104022595	CAGCAGTGAATCAGGATGGA	CCCAGAGGAAGCATCAGAAG	190
Ad01A20222	Aradu.A01	20222	c	(AGA)7aagaga	73	104022772	104022844	CTTCTGATGCTTCCTCTGGG	ATTTCCCTTTTCCCATCCAC	276
Ad01A20223	Aradu.A01	20223	p2	(CT)7	14	104023284	104023297	AGCACACACCATGCAAGAAC	ATGCAGTGCAGCACAATCTT	220
Ad01A20224	Aradu.A01	20224	p3	(AAT)5	15	104023517	104023531	AGGGAAAGGGACAAAAATGC	TGTGAATGTTGATTGTTGAGGA	102
Ad01A20226	Aradu.A01	20226	p3	(AAT)8	24	104024643	104024666	GCACACACATGGTGTCCTTT	CATGTGATGATGAATCAATGTCC	242
Ad01A20231	Aradu.A01	20231	p2	(TA)6	12	104032722	104032733	TCGATGAAATTCGACGTCAC	AACGCCACAACAAATTTTCC	212
Ad01A20234	Aradu.A01	20234	p4	(AAAT)5	20	104040991	104041010	AGGATGATCTTGCTCGCACT	TTTCACAATGGAGGTTTGAGG	237
Ad01A20235	Aradu.A01	20235	p3	(TGG)8	24	104042382	104042405	CTCAACACGCTGCTGGAATA	CCACCCTACTGGTCTCCTCA	195
Ad01A20240	Aradu.A01	20240	c	(CTT)5cgctttct	117	104049144	104049260	CGCGCCTAATACACACAAAA	TGTTCGGTTCATTCTGCACT	279
Ad01A20241	Aradu.A01	20241	p3	(AGG)7	21	104049943	104049963	ACCATGGGAGAATGACGAAG	TAACGTGCTGCAACCTAACG	141
Ad01A20242	Aradu.A01	20242	p2	(TA)8	16	104064063	104064078	GCCTGGAGCCTTATCTACGA	CCCCTAATTGGGAATGACCT	253
Ad01A20248	Aradu.A01	20248	p2	(AG)10	20	104091786	104091805	CACTTGATGCATGATGAGAGAA	GTGGCCAACTCACCCTACAT	164
Ad01A20252	Aradu.A01	20252	p2	(AT)6	12	104123076	104123087	TGGGAAATGGGAAATGAGAA	GCCTCGTGAATACCCGTTTA	109
Ad01A20256	Aradu.A01	20256	p2	(TC)6	12	104136807	104136818	CGCAGTGCTTGTTTTCAGAG	TAGCACCTGAAGTGGAACCC	159

ID	Chromosome	SSR nr.	SSR type	SSR	Size	Start	End	FORWARD PRIMER (5'-3')	REVERSE PRIMER (5'-3')	Product (bp)
Ad01A20259	Aradu.A01	20259	c	(AAC)12(AAT	63	104139793	104139855	ATCATCGACGGTTCAAAAGC	TGCAGTTGGAGTTTTCATCG	275
Ad01A20260	Aradu.A01	20260	p3	(ATA)5	15	104142180	104142194	CCCTAATGAAGGTGGGGTTT	CATCTCACCCGTCTTCTATTTTG	171
Ad01A20265	Aradu.A01	20265	c	(AG)6aaagaga	112	104148946	104149057	AGTCACCGCTGCTCTCATTT	AAGGGTTGGCCTTCTTGTTT	268
Ad01A20266	Aradu.A01	20266	p3	(TGC)8	24	104149288	104149311	CGACGAACCGTTAGAAGAGC	AGGCATTGTGAGATGTGCAG	129
Ad01A20268	Aradu.A01	20268	p3	(ATA)20	60	104150891	104150950	TCAAGATAAAATTAGCCAAAATGG	GAGAAATTGAAAGTAAGGCTAACCC	235
Ad01A20270	Aradu.A01	20270	p2	(AT)13	26	104157356	104157381	TGGACGAGAATGAACTTTTGC	TGGATCAAATTAAGACGGCA	165
Ad01A20272	Aradu.A01	20272	c	(GC)6ggatggtc	87	104173591	104173677	CGGATGGCTTATTTCGAGAG	AGCTGTTTGGAGGTTTGGAA	260
Ad01A20274	Aradu.A01	20274	c	(T)11ctaatatca	87	104177975	104178061	TTTGACACGATGAGTGACCTAA	TGGAAGGACAAAATCATACTGAA	279
Ad01A20277	Aradu.A01	20277	p2	(AT)8	16	104195307	104195322	ATCCCAGCCAGAGAAGTGAA	ATTTTGGGGCTTTGCAATAA	156
Ad01A20279	Aradu.A01	20279	p2	(TA)6	12	104196739	104196750	TTTCAATCGCTTTGTTCTTTTT	CCACCCTTTCATTTCGTGTT	257
Ad01A20286	Aradu.A01	20286	p3	(TTA)7	21	104209601	104209621	TGTTATTGTTGTTGATGTTGTTGG	TGGAAATCAAGGAAACAATGAA	124
Ad01A20288	Aradu.A01	20288	p2	(AG)24	48	104222899	104222946	ACATGCCAATTGGAGTTTTT	ACACAAGATTCAAGGCCAAA	215
Ad01A20289	Aradu.A01	20289	c	(T)11gaaagaaa	117	104223121	104223237	TTTGGCCTTGAATCTTGTGT	GGTGCAGACCCCTTTTGTTA	259
Ad01A20291	Aradu.A01	20291	p3	(CTT)6	18	104234040	104234057	GGTTGTTGGGCTGCATCTAT	TAAGCCTAGGCCAAGTGACG	271
Ad01A20295	Aradu.A01	20295	p2	(TA)7	14	104237928	104237941	CATGCCAATCTGGTTTCTCA	AAAATGGAGTGGAACGTTTTC	269
Ad01A20296	Aradu.A01	20296	p2	(AC)7	14	104238249	104238262	TCGTGATTTTTAGGTGCGTG	TGCAATTTAACAGGGTCGTG	126
Ad01A20297	Aradu.A01	20297	p3	(ATA)5	15	104240881	104240895	CGATTAAAAAGACTATGAAAAGGATG	CGAGATCGTGGTCGTCATTA	214
Ad01A20299	Aradu.A01	20299	p2	(TA)6	12	104241366	104241377	ATTTTTGTTTCGACACAGCG	GCCACTAAAATTAATGCTACCATAG	275
Ad01A20306	Aradu.A01	20306	p3	(GCG)6	18	104266800	104266817	ATAATAATAGCGCCACCGCA	CTCGTCAGCGTCCCTATCTC	268
Ad01A20307	Aradu.A01	20307	p2	(AT)8	16	104267649	104267664	TAATTTGGGAGGTGATTGGG	CAAGCTCAAAACAAATGCCA	279
Ad01A20310	Aradu.A01	20310	p3	(ATT)5	15	104272614	104272628	CATCCGAGATTCGTTTCGAT	TGTGCCTTTAGGACACAAATTA	200
Ad01A20311	Aradu.A01	20311	p3	(TCA)5	15	104273290	104273304	TCATCCACACCTATTGCCAG	TGAGGCTCTGAACCAAGTCC	137
Ad01A20319	Aradu.A01	20319	p5	(TTTTA)5	25	104304676	104304700	TCCTCTGTCTGGGTTCCATC	TCCCTCAACACCACAATTCA	279
Ad01A20330	Aradu.A01	20330	p2	(AG)8	16	104334116	104334131	CAACTGCACTCTTCCCCTTC	AGCAAGCAAGAGGAGTGAGG	251
Ad01A20331	Aradu.A01	20331	c	(GAAAA)5gaa	122	104337072	104337193	GGAAAGATCTGGAACATGTGG	AGAATTGCGCAACATAATCTTT	273
Ad01A20332	Aradu.A01	20332	p2	(TA)7	14	104356590	104356603	TTACCAGCTGACATTGCTGC	CCAACCAAATAACATAACAAACCA	279
Ad01A20334	Aradu.A01	20334	p5	(AAGAA)6	30	104359365	104359394	CGGCAAAAGATTGGAGTTCT	CCTTGCATACGAGTTCACCA	216
Ad01A20337	Aradu.A01	20337	c	(T)12atctccaag	50	104371979	104372028	CGTGCACGGTAGTATGTGAAA	GATGCACCTTGCTTTCATTG	275
Ad01A20339	Aradu.A01	20339	p2	(AG)8	16	104374133	104374148	GGAGCCAAATTAGGGAATTGA	CCGCTTTCCTTCTCAATCAG	163
Ad01A20343	Aradu.A01	20343	p3	(TAG)5	15	104379374	104379388	AAGCCTTCTTGAGCAAACCA	AAAAGCAGGAGGAAACCCTT	259
Ad01A20349	Aradu.A01	20349	c	(A)12gaatcttag	68	104387708	104387775	TGATATGTCACCAGGAACTCAA	TCTTTTATTTCGAGGGTAATCG	269
Ad01A20350	Aradu.A01	20350	p2	(TA)6	12	104389258	104389269	GCAAACCAACCAAAACGACT	TGATGCCGTCACATTTTTGT	163
Ad01A20351	Aradu.A01	20351	p6	(TCCTCA)5	30	104393468	104393497	GCGTGGAGGATCAGTCCTAA	GACGATGATGACGCTGAAGA	255
Ad01A20358	Aradu.A01	20358	p2	(AT)6	12	104429602	104429613	TGTCCGAAATTCGATCTCAA	AGTGCACGTGGTGGTACAAA	277

ID	Chromosome	SSR nr.	SSR type	SSR	Size	Start	End	FORWARD PRIMER (5'-3')	REVERSE PRIMER (5'-3')	Product (bp)
Ad01A20359	Aradu.A01	20359	p3	(AAT)15	45	104429882	104429926	CAAATTCTTGGATTCGCACA	TCACCATTATGAAGATCGTTGTC	178
Ad01A20360	Aradu.A01	20360	p2	(AT)6	12	104430571	104430582	CAAAAGTTTGTTCCATTTTCCA	CCTAGCCATGCAGAAAGCAT	221
Ad01A20366	Aradu.A01	20366	p2	(TC)22	44	104452824	104452867	AGAGTGGCCGAAGTTCATGT	AAAACGTCAGAAATCGCCTG	231
Ad01A20367	Aradu.A01	20367	p3	(CCT)5	15	104453501	104453515	GGATTGTTTGCTGTGGTGTG	AAACAATGGTTTCCCCAACA	274
Ad01A20369	Aradu.A01	20369	p4	(AATA)5	20	104462013	104462032	CATTAATTTTGGAGAAAATGATTTGA	CAATCAAAATATGCAATATGACATTC	259
Ad01A20372	Aradu.A01	20372	c	(T)11gtgattcac	65	104471707	104471771	TGTCATGTGTGCTTCAATTCAT	TTCAGCATTTTGAACCTGGG	235
Ad01A20374	Aradu.A01	20374	p2	(TC)9	18	104477663	104477680	GGCAGTTAGGAGGTGCCATA	GAATTTCTTGGAGACCGCAA	203
Ad01A20377	Aradu.A01	20377	p3	(ACC)5	15	104479342	104479356	AAATTTGCTCAGGTTCACCG	CATTCGCTTCGTCAGAATCA	221
Ad01A20380	Aradu.A01	20380	p2	(AC)17	34	104497079	104497112	CCCAAATTCATTCAAAACCC	GCTCTTGTTGGTGAGCATGA	231
Ad01A20381	Aradu.A01	20381	p3	(AAT)5	15	104504064	104504078	GCAGAACCCGTTTGAGGTAA	TCACTCGGATTCTGGCTTCT	279
Ad01A20382	Aradu.A01	20382	p3	(AAC)6	18	104505655	104505672	GACAAAAGCGGTTTGGAAAA	ACATGACAGAGCACAATGGC	146
Ad01A20389	Aradu.A01	20389	p3	(AAT)5	15	104544879	104544893	TGGTTGGTTTTAGCACTCCTC	AATTCCGCCTCCTTGATTTT	156
Ad01A20390	Aradu.A01	20390	c	(AAT)14acctta	103	104545253	104545355	GGCGAGATTTCGAATTTTATTCT	CCTCAAGAATCACAACCACG	247
Ad01A20391	Aradu.A01	20391	c	(A)10cagtcaca	117	104546354	104546470	TGGGGTTAAAGTCGTCTAGGG	TGAGCTCGTTTATGAGTTAATTTGA	255
Ad01A20398	Aradu.A01	20398	c	(AGA)8gggaag	155	104570063	104570217	TCAGGAAGCAAAGTCAAGCA	AATTGCGAATCCTCTTCCCT	269
Ad01A20399	Aradu.A01	20399	p2	(TG)7	14	104573741	104573754	GCGATGGTTAAGCAACAACA	CACCAACAATGATGGCAAAG	231
Ad01A20401	Aradu.A01	20401	c	(GA)14tgaacgg	120	104592552	104592671	GGAGTGGCGATAGGAGCATA	TGCAAAATTGTTGGAGCTGA	272
Ad01A20405	Aradu.A01	20405	p2	(TA)12	24	104611599	104611622	TTTGGAGATTTATCATTGTGATAGTG	TCGCAGGTCAAAGAAAACTC	261
Ad01A20406	Aradu.A01	20406	p2	(TA)6	12	104618908	104618919	GAATGTGATTACGAAAGTCCG	TGGTTGAATCCCCGTTATGT	280
Ad01A20407	Aradu.A01	20407	p2	(TA)7	14	104625442	104625455	TCAAGAGGAGGGTCGAAAGA	ACTTCCCACATGCATTAGCC	221
Ad01A20408	Aradu.A01	20408	p2	(TA)7	14	104634285	104634298	CTTACCTCGCGCCATAAAAA	TGACGAGTCTCATCTGTCGG	174
Ad01A20409	Aradu.A01	20409	c	(TG)8gtgcgcgc	55	104635074	104635128	TGCTTTTGGAGAAACAGGTG	TGCGTAAACTGGATAAACGAGA	150
Ad01A20411	Aradu.A01	20411	p3	(AAC)7	21	104636977	104636997	TCGCGCTAATGAACAGTTTG	TGATAGAGCACAATGGCGTC	279
Ad01A20427	Aradu.A01	20427	p3	(ATT)5	15	104690954	104690968	ATGGCATTGGAATACGCCT	TGCCTCATCCCTTTATTCCA	111
Ad01A20428	Aradu.A01	20428	p3	(TTG)5	15	104691085	104691099	TGGAATAAAGGGATGAGGCA	TGGAAGCCATGCATTATTGT	226
Ad01A20429	Aradu.A01	20429	c	(AGA)7atctgag	102	104692138	104692239	TGAAAAGAGTAGAAGCATTTACCA	CCTCATTTTGGAGGAGACAAA	247
Ad01A20435	Aradu.A01	20435	p2	(TA)10	20	104697296	104697315	TGCGATGAATTGAAGCAATC	ATTGAAATTGTGCCATGCAA	251
Ad01A20436	Aradu.A01	20436	p2	(AT)7	14	104698331	104698344	TGGACATATCTGCCCAAAAA	GGAGATGAGAATGAGAGCCG	170
Ad01A20440	Aradu.A01	20440	p3	(TTA)5	15	104699644	104699658	CCAAGCAGAAGAAGCCAATC	TTGAAAAACGATAATGGTAGGGA	274
Ad01A20443	Aradu.A01	20443	p2	(TA)6	12	104700938	104700949	TTGAAAACATTGTCAACTAATAAAAGA	GGTCCAGAATAAAACCGTACCA	157
Ad01A20454	Aradu.A01	20454	p2	(CT)8	16	104728074	104728089	GCCGAATGATGGATCTGTCT	AGCACCACCATGAACTCCTC	162
Ad01A20455	Aradu.A01	20455	c	(A)10gacaaagt	116	104728932	104729047	AATTATTCCGCAAGCCCCTA	TGCTGAGCTTAAAATTTGGC	173
Ad01A20457	Aradu.A01	20457	p3	(AGA)5	15	104732826	104732840	AGGACCTGATGACGTCTTGG	TTCGGCTTCACTCGATTTTT	272
Ad01A20460	Aradu.A01	20460	c	(CT)7(CA)7ctc	174	104747513	104747686	CGAATCTTTGCGCTCTTTCT	CATTGTGATTTGTGAGGTGGA	271

ID	Chromosome	SSR nr.	SSR type	SSR	Size	Start	End	FORWARD PRIMER (5'-3')	REVERSE PRIMER (5'-3')	Product (bp)
Ad01A20462	Aradu.A01	20462	p3	(AAT)5	15	104754139	104754153	GCATTTTCATCTTGGATTAGCA	GACCGCAAAATGGCATCTAT	159
Ad01A20475	Aradu.A01	20475	c	(CTA)8(TTA)	72	104811432	104811503	AACCAATTTGTCAAACTCCTTTTT	GTGATAGGCAGGTTGCCATT	269
Ad01A20477	Aradu.A01	20477	p2	(AC)8	16	104819182	104819197	TGCAAGATCATTCCACAGAGA	TGAAAGTATTCCAAAGCGTGA	243
Ad01A20485	Aradu.A01	20485	p3	(GAC)5	15	104842495	104842509	GACATCACCACCTTTTGCG	GGTAATTCCGACGGTGATGT	280
Ad01A20492	Aradu.A01	20492	c	(ATC)6actcaga	92	104858825	104858916	TGGTTACTGCCGTGTACCAG	AGATGGAGGTTATGGAAGCG	278
Ad01A20495	Aradu.A01	20495	p3	(ATA)7	21	104869321	104869341	GATTTCCCACAAAGGGAAAAA	CACATTAAATGCATAAAACCTTAAAAA	178
Ad01A20496	Aradu.A01	20496	p3	(AAT)14	42	104871400	104871441	TTTTGTACATGACACACAAACTTATGA	TTTTTCTTCTCATCCTCCTTCA	250
Ad01A20503	Aradu.A01	20503	c	(ATT)7caagtga	132	104879963	104880094	TTATCCCCTTTCATGGGTCA	TTGTTGTTGGGTCGTTTTGA	242
Ad01A20504	Aradu.A01	20504	p3	(TCT)10	30	104880318	104880347	TCAAAACGACCCAACAACAA	GAGGAACATTATTGGACCCG	260
Ad01A20506	Aradu.A01	20506	c	(T)11aattacgct	104	104887313	104887416	GGGTTTTGCTATCTCAATTAGTCA	TGGAGACTTTTGTCAACTTACGA	253
Ad01A20509	Aradu.A01	20509	p2	(TA)7	14	104890778	104890791	TTTTTAGGTTTTTCTTTGACATCTATT	TCAATTGCAATGAGGGAGTG	266
Ad01A20511	Aradu.A01	20511	c	(A)10gaagaaga	39	104895226	104895264	TTTGCAGCATTATGTGTTTCTTC	CCTCCTCATCTTCTGCTGCT	188
Ad01A20513	Aradu.A01	20513	c	(TCT)5ttttcttct	63	104902014	104902076	TAACTAAATGCGCGTGCTTC	TGAAAACGACAAGAACGATGA	271
Ad01A20514	Aradu.A01	20514	p4	(AAAT)5	20	104903093	104903112	GTCGGATTGGGGATAATGTG	CCGGCCAATCTCTACAAGAA	249
Ad01A20517	Aradu.A01	20517	p3	(TGA)5	15	104916517	104916531	ACGTGGTTGAGGATTTGGAG	GGCTTTCTGCTCTTGTGACC	223
Ad01A20530	Aradu.A01	20530	p2	(AT)6	12	104970176	104970187	CCTTGACATTCCTAATAAGATGATACC	TCGAACCCTCCAATACTTGC	255
Ad01A20532	Aradu.A01	20532	p2	(TA)7	14	104978644	104978657	CGAAATCGAGGATGTTGTTC	ATGGTTAGGTTACACGGGCA	124
Ad01A20533	Aradu.A01	20533	p2	(TA)6	12	104981630	104981641	TTCTTTTGGATTAGTATTCAAGTGG	GCACTTGTCCGTAATAATTTTCAT	279
Ad01A20539	Aradu.A01	20539	p3	(TAT)17	51	104999658	104999708	GAAAAGTCTTGGATTTTGAAAGAAA	CCTTGGCTTATCAACGTGAA	280
Ad01A20540	Aradu.A01	20540	p3	(ATT)22	66	105001351	105001416	TTTCTTTCTCCCTTGACTTATCA	CTGCAAAATTTGATGACCGA	249
Ad01A20543	Aradu.A01	20543	c	(TTA)5ttgttgt(	37	105006633	105006669	GTTTGCATGGGAAAATGACC	CCGGTTTCTTCTGGACTTCA	228
Ad01A20545	Aradu.A01	20545	p2	(AG)6	12	105008975	105008986	CAAAGAGGGAGAGAGGGAGAA	AAGCCTGGATCTAGCCCATT	253
Ad01A20546	Aradu.A01	20546	p3	(GGT)5	15	105009649	105009663	GAGCAAAGCTTGACCTGACC	GAGGAAAGGAAAATGGAGGC	146
Ad01A20547	Aradu.A01	20547	p3	(TGA)5	15	105010026	105010040	CCCGAATTCATCACAAATCC	CACCACTCCAACAAACCAAA	235
Ad01A20551	Aradu.A01	20551	c	(T)10gccttggat	78	105021629	105021706	CCCCAATTAGAGGTGTCCAG	TCCGAATGAATGCTTGAACA	273
Ad01A20554	Aradu.A01	20554	p2	(TA)6	12	105035143	105035154	TTGCCTTTATTTTTCAAACTTGTTC	TGTCATAAATTTTACCTATTTGTAGCA	263
Ad01A20555	Aradu.A01	20555	p2	(AC)13	26	105039843	105039868	TGGATTTGAATCCTCTAAAGTTTG	ACGGCTGAGTGAGAGATGGT	236
Ad01A20556	Aradu.A01	20556	p2	(GA)14	28	105040021	105040048	TGTTGAGAGAGGGAGAGGGA	GGGACAAGCTCCACACATTT	280
Ad01A20558	Aradu.A01	20558	p3	(TGG)5	15	105053793	105053807	GACGCATTCTTGTTTTCGGT	CCTACGATCGTGTCTCGTCA	167
Ad01A20560	Aradu.A01	20560	c	(AT)7cccaaatt	102	105058622	105058723	TGTAATGTGAGTGGATGTGTGAA	TGTCTTTGTCTGAATGGAATAAGG	274
Ad01A20561	Aradu.A01	20561	c	(T)10cctttttggt	115	105062877	105062991	TTTTTCCAACAATTCCATCAAA	CGTGACACGTCATGATCTAACT	276
Ad01A20565	Aradu.A01	20565	p3	(CAT)7	21	105075443	105075463	AAACCAGCCTCCAGTGAAGA	GGATTGGGCTTTTCATAGTAAGG	266
Ad01A20567	Aradu.A01	20567	p3	(TGA)5	15	105078863	105078877	AATGGAAAGTGTTTGGGAAAA	TCCCTGTACCAGCAAAATCA	279
Ad01A20569	Aradu.A01	20569	p2	(TA)9	18	105084522	105084539	TAGACGCATAGGCATGGACA	TTGACTAGCCGTCCGAAACT	134

ID	Chromosome	SSR nr.	SSR type	SSR	Size	Start	End	FORWARD PRIMER (5'-3')	REVERSE PRIMER (5'-3')	Product (bp)
Ad01A20570	Aradu.A01	20570	p2	(TA)8	16	105086628	105086643	AAGGATAACAAGGTGCCACG	CGTTTAGTTACCGTGTGCGA	169
Ad01A20571	Aradu.A01	20571	p2	(AC)13	26	105086776	105086801	AGCTTTCGCACACGGTAACT	CGCAGGCAGTGGTTAGTCTT	105
Ad01A20572	Aradu.A01	20572	p2	(CT)8	16	105109841	105109856	ACGAGGGTTCCCTCTCTCTC	CCTAACCTTAACCCACGCAA	191
Ad01A20576	Aradu.A01	20576	p2	(TC)24	48	105122649	105122696	TTTGTTGCGCATGGTATCAC	AGCACGCAAAGGAGTGAGAT	208
Ad01A20577	Aradu.A01	20577	p2	(TA)10	20	105132512	105132531	TTTTAGCATCGCAATGAGGA	ATCCGAGGAGAACCGTTACC	244
Ad01A20578	Aradu.A01	20578	p2	(CT)6	12	105136015	105136026	CCATTCGTCCCATTCTCAAT	CAAACCAAAATCCACAAATTCA	269
Ad01A20581	Aradu.A01	20581	p2	(AC)6	12	105160183	105160194	AATTGGGAACCCTAATCCCA	TATTGGCTCGCGTTTCTCTT	238
Ad01A20582	Aradu.A01	20582	p5	(GAGAA)5	25	105160310	105160334	CACACACACGCACAGAGATG	TATTGGCTCGCGTTTCTCTT	173
Ad01A20584	Aradu.A01	20584	c	(TA)8ataatatta	107	105185151	105185257	TCTCGTGCTGATAACGTGTTG	AACAAATGCAATTCTCTCTCTTTTT	259
Ad01A20585	Aradu.A01	20585	p2	(TA)13	26	105191704	105191729	CCTTCTGTCGCAAGATGTGA	CGGTTATATAGGCAACCCGA	266
Ad01A20587	Aradu.A01	20587	c	(A)10tcaaaatat	78	105199670	105199747	TCCAAAAACCTAATAAAAGAAGCA	GGAGGGGGATCATGAATTTT	254
Ad01A20590	Aradu.A01	20590	c	(GAA)11a(AA	49	105203208	105203256	TTTCTTGTAGTGCATTGCCG	GCCTTCCCACATTCTTTCAC	244
Ad01A20593	Aradu.A01	20593	p2	(CA)10	20	105210663	105210682	TGAATGCATTTTTCTCATGCTT	TTGTGCCTGATTTGAACACC	278
Ad01A20599	Aradu.A01	20599	p3	(ATG)6	18	105250727	105250744	GGGAGGGTGGAGTATCGAAT	TCTCTGGTCCCAAAACCAAC	237
Ad01A20602	Aradu.A01	20602	p2	(AT)6	12	105257506	105257517	CAGCCAAAGCTGCTCAATTA	CCACATTTTTGTGGATAACTTTTG	279
Ad01A20604	Aradu.A01	20604	p3	(TTA)5	15	105258922	105258936	TTCTGACTTCTGATCCTTTTTGTG	AGGTTCATGCATCAGATCCTC	192
Ad01A20605	Aradu.A01	20605	p3	(ATA)14	42	105262871	105262912	CGATTTTGATTTTTACTTCGAACA	TGGATGAGTGGAAACTAAGGG	238
Ad01A20610	Aradu.A01	20610	p3	(ATT)6	18	105270725	105270742	GCCTCGTTCATCTCCAACTC	AGGCACAAAAAGTGGGAAAA	205
Ad01A20611	Aradu.A01	20611	p2	(TC)6	12	105270878	105270889	GCCTCGTTCATCTCCAACTC	GGACCCAATAACGTGCTTCA	275
Ad01A20612	Aradu.A01	20612	p3	(ACA)7	21	105271322	105271342	CAGCAGTGGCTATGGTTTGA	AAGCAGAAGACGAGTGGGAA	221
Ad01A20613	Aradu.A01	20613	p3	(ATG)7	21	105272192	105272212	CTTGGAGGGCATAGAGCAAG	TCAGGCAAATTGGACACTCA	244
Ad01A20615	Aradu.A01	20615	p4	(CTAT)6	24	105272920	105272943	AGTACCCATCCATCCATCCA	TCCCTATAAGTCTCATGGCCC	160
Ad01A20620	Aradu.A01	20620	p2	(AT)6	12	105284781	105284792	TTTTTGTTTTGGTTATGCATTTTT	TGCAAACTTTCAATAAATACCATTTT	261
Ad01A20625	Aradu.A01	20625	p2	(CT)18	36	105293977	105294012	AGAACACGTCCGCTCAGAAT	CAGCAAGATTGGATTGAGCA	196
Ad01A20627	Aradu.A01	20627	p3	(AGA)6	18	105298944	105298961	AAACGCGAAAGAGGAGAACA	ATTTTCACTCCACGCCTCAC	201
Ad01A20628	Aradu.A01	20628	p3	(TAA)18	54	105299143	105299196	GTGAGGCGTGGAGTGAAAAT	TGATTTTAGCCAAAAACTCGG	232
Ad01A20631	Aradu.A01	20631	p3	(ATT)5	15	105301474	105301488	TTGGCACCATCTTCTTTTGA	GTGCCTTTGCCACTTTCAAT	230
Ad01A20633	Aradu.A01	20633	p3	(ATA)5	15	105307113	105307127	ATCATCTTCCCCACCAACAA	CATATCCTCGTCCTCGTCGT	261
Ad01A20644	Aradu.A01	20644	c	(A)18c(A)11	30	105355154	105355183	TGCTTATCAAGAGGGGAAAAA	ATTCCATGGACTTTGGACGA	247
Ad01A20645	Aradu.A01	20645	c	(A)10caaaaaac	101	105359784	105359884	TGGGACTGAAGAAAACCAGTG	AAGTGTCCACGCACAATGAA	220
Ad01A20653	Aradu.A01	20653	p2	(TG)7	14	105385112	105385125	GCATTTGAGGTGGAAGCAAT	TGAAATTGGTCCAAAAAGCA	101
Ad01A20655	Aradu.A01	20655	p2	(CA)8	16	105391612	105391627	TGCGCATAGAGAACAAACCA	CTACTTCGGCCTCATCTTGG	264
Ad01A20658	Aradu.A01	20658	p3	(TAT)5	15	105400216	105400230	GGAGAAGCACTTGAGTGGTGA	GGGTAGTGATGGTGGAGAAAA	183
Ad01A20666	Aradu.A01	20666	p4	(TTTA)5	20	105417435	105417454	TAAAAGCCACGGGCTCATAG	CGAATCAGATTTTGCTTCCC	193

ID	Chromosome	SSR nr.	SSR type	SSR	Size	Start	End	FORWARD PRIMER (5'-3')	REVERSE PRIMER (5'-3')	Product (bp)
Ad01A20667	Aradu.A01	20667	c	(T)10gttttcaaa[illegible]	82	105418076	105418157	TCCATCCCTCAACCTAATCG	CCAGCAAAGTCCCCTAGACA	174
Ad01A20670	Aradu.A01	20670	c	(CG)6cacagcg[illegible]	61	105430309	105430369	CTTATGCGTGAAAAGCAGGG	ATAGGGAGCTCTTGGGCATT	280
Ad01A20675	Aradu.A01	20675	p4	(AAGA)6	24	105456222	105456245	CTCGGCAATTCATTGTCCTT	GCTAAGTTGAAGCAATCGGC	171
Ad01A20676	Aradu.A01	20676	p2	(AC)8	16	105458874	105458889	GTTGTAAAGGTTGGCCAGGA	GTTAAGCTGGGCGAGAGCTA	249
Ad01A20677	Aradu.A01	20677	c	(AT)8acgaggc[illegible]	121	105460706	105460826	GCATCCAACAATGTAGGGCT	AGACCTTGCATTAACACGGG	242
Ad01A20684	Aradu.A01	20684	c	(CCA)5ctaacc[illegible]	162	105494801	105494962	TCCTTAACATCATCTGCTTCATC	CATCACACAACCATCCGAAG	222
Ad01A20685	Aradu.A01	20685	p3	(TTG)6	18	105495467	105495484	GAAGGGAACATGGGTGAGAA	CTTTGAGTTCACAGGCACCA	231
Ad01A20689	Aradu.A01	20689	p3	(TTA)5	15	105519657	105519671	TCTTTTTCTGGTCCTACTTGCAC	ACGAATTCGATTCTGGCACT	234
Ad01A20691	Aradu.A01	20691	p3	(ATT)8	24	105553434	105553457	TTATTTGGACCTGGGCCTTT	TAGACCGCATGACTCACGTC	250
Ad01A20692	Aradu.A01	20692	p3	(TCC)6	18	105554783	105554800	AAGCCTCTTTCGCTTTCAGA	CTTCCGCTTCTTGGAATCAG	279
Ad01A20693	Aradu.A01	20693	p3	(AAG)6	18	105554936	105554953	GGGAAAGCCAAAGTTGATGA	CGAAAATTTCCACCGAAGAA	142
Ad01A20696	Aradu.A01	20696	p3	(AAT)6	18	105560239	105560256	CGCTTTCCAATTACTACAACCA	TCACCTCTTTCAATCTTAGCCA	269
Ad01A20697	Aradu.A01	20697	c	(TCA)5ctttc(C	35	105561047	105561081	GGGTTTTCTGTCTGGAACGA	AAGTTCCTTCGCTGCTTGAG	231
Ad01A20698	Aradu.A01	20698	c	(TTA)5atatttg[illegible]	73	105561693	105561765	TTGCTCATATGCTAATTCTAAATGC	CCATCACACCCAAAAACAAA	279
Ad01A20711	Aradu.A01	20711	p3	(ATA)5	15	105601624	105601638	CTGCACAGATAATGCATGAAAA	GGTGAAAACTCAAGTGCAGTCA	276
Ad01A20715	Aradu.A01	20715	p4	(AAAG)5	20	105604563	105604582	ACCCCTTCCCAGAATACCAG	CAGCCCATGAGGAGCTAAAC	277
Ad01A20718	Aradu.A01	20718	p3	(TGG)6	18	105615962	105615979	ACCCATGGAAAATGGGTTTC	TATCTGCTAGGAACCGGTCG	243
Ad01A20720	Aradu.A01	20720	c	(T)11aatacatg[illegible]	110	105622146	105622255	TGTTTTGTTTTGTCTTTTCCCC	TTTCCACAGCAGATTGAGACA	253
Ad01A20728	Aradu.A01	20728	c*	(TA)7(TATG)[illegible]	60	105638902	105638961	CCTTAAACCCTAAACCAACGAA	ACCAACAGCAGCGGATAACT	242
Ad01A20734	Aradu.A01	20734	c	(AT)7aatattag[illegible]	100	105668650	105668749	AAGCAAAATTGAGACATTTCAAGA	TTTTGAAAATATAAATTACAACGGACA	249
Ad01A20735	Aradu.A01	20735	p4	(AGAA)7	28	105670042	105670069	ACCTTCGCCTACCCAAAAAC	ACGGAGAAGAAGCGTAGTGG	234
Ad01A20737	Aradu.A01	20737	p3	(CCT)5	15	105673621	105673635	TAGGATTGGATGCTAACCGC	TTCCACCCAAAGAGGATGAG	252
Ad01A20738	Aradu.A01	20738	p3	(AAT)5	15	105679033	105679047	TTCTGCCGGAGCTAAAAAGA	TGACAATTTTAATTTGAAGGGG	114
Ad01A20739	Aradu.A01	20739	p2	(TA)7	14	105685856	105685869	TTAAGGTGGATTACTCAACATCATT	ATATAGCCTTTGGCACCACG	270
Ad01A20741	Aradu.A01	20741	p2	(AT)11	22	105688790	105688811	TTCTTGGTCATTGACAGAAAAAT	TTATTATGCAAACAAAAATAACCCT	219
Ad01A20745	Aradu.A01	20745	p3	(CAT)7	21	105717259	105717279	TTGAATAATGGCACGGTGAA	CATTGAGGGATTCGAAGGAA	145
Ad01A20746	Aradu.A01	20746	c	(CCT)6(CTT)[illegible]	45	105717413	105717457	TTCCTTCGAATCCCTCAATG	GCCGATGGAAACCAATCTAA	260
Ad01A20748	Aradu.A01	20748	p2	(AT)9	18	105718073	105718090	GAGAAATTTTGGTGCTTCCG	TTGCAACTCCAGATTGAAGC	198
Ad01A20751	Aradu.A01	20751	p3	(CAA)7	21	105726853	105726873	GCACGCCTATCCATTCTTGT	ACATGACAGAGCACAATGGC	235
Ad01A20754	Aradu.A01	20754	c	(A)29gaaaggtt[illegible]	102	105730333	105730434	TTCATGCTAGTTTGGAGATCTTG	CACAACTCGTGCTTTGCTTC	270
Ad01A20757	Aradu.A01	20757	p2	(GA)6	12	105736614	105736625	GTCTGACTGACCAATCCGGT	TAAAATCCGTGCCTCCAAAC	144
Ad01A20758	Aradu.A01	20758	p2	(AG)7	14	105737734	105737747	GGGGTTGCTGCTACTACTGC	AGAGGAAGAAGAAGGCGTCG	146
Ad01A20761	Aradu.A01	20761	p3	(TAT)15	45	105751633	105751677	TCATATTGCAATTTATGGTTGTTTTT	CCGACATCGAATCGCTCTAT	274
Ad01A20765	Aradu.A01	20765	p3	(AAC)6	18	105765022	105765039	ACAATGGCAAATGGAATGTG	TCATGTAGTCGACCCCACCT	258

ID	Chromosome	SSR nr.	SSR type	SSR	Size	Start	End	FORWARD PRIMER (5'-3')	REVERSE PRIMER (5'-3')	Product (bp)
Ad01A20767	Aradu.A01	20767	p3	(AAC)6	18	105774100	105774117	AGGTGTTTGGGCCTAGCATA	AGAGCCTCAGCAAATTCCAA	270
Ad01A20768	Aradu.A01	20768	c	(TCT)5tcctcctc	88	105777871	105777958	GCCTTTATCATCACCGTCGT	TTTTCCGTTCCAATGTCTCC	279
Ad01A20774	Aradu.A01	20774	c	(A)10caattctta	100	105784112	105784211	CCATTAAATTTTTGAAATGTTACACC	TTGTGTCCATTATAGTTGCTGTTG	275
Ad01A20777	Aradu.A01	20777	p2	(TC)7	14	105795046	105795059	CCACGTCACCCCTAAAAGAT	GGAACTGCACAAGGAAGGAC	163
Ad01A20785	Aradu.A01	20785	p3	(TTA)5	15	105834366	105834380	GCCTATGCACATTGGAAAATC	TGACCCGTTCTACTTTTGCTG	111
Ad01A20787	Aradu.A01	20787	p2	(AT)13	26	105839663	105839688	ACGAACCGCTTTGATACCAC	TTTTGCGCTTTTCAAATCCT	200
Ad01A20793	Aradu.A01	20793	c	(T)10a(T)11gg	83	105865361	105865443	TTCTTTTGGGTGCGATCAAT	ACCTGTCCGCCTCATACAAC	267
Ad01A20794	Aradu.A01	20794	p2	(TA)8	16	105866066	105866081	TCCATCGTATCAAATGCGAA	TGGATGGGAATATGAAAACACA	154
Ad01A20795	Aradu.A01	20795	p2	(AT)6	12	105866259	105866270	ACAAGACATCGATACATAGACATAAAA	CTGTGTTTGGTTTTGAGAATAGAA	280
Ad01A20800	Aradu.A01	20800	p3	(CTT)5	15	105874778	105874792	TGCACAATTCAAAATTTTCCC	TTTTTCCACGCGATGTTTTT	260
Ad01A20804	Aradu.A01	20804	p3	(GAT)5	15	105896607	105896621	ACAAAGAGCTACGGGCTTGA	TTGATTGGTGCAATTGAGGT	220
Ad01A20806	Aradu.A01	20806	p4	(ACAA)5	20	105899638	105899657	TGAGCACAATCTCTTTACACACG	TGAGATGAACGTGGAAACCA	203
Ad01A20807	Aradu.A01	20807	p3	(CTC)5	15	105899984	105899998	CATAGGCGGGAACAGAAGAG	ACACTTTCCGAACCGAATTG	216
Ad01A20814	Aradu.A01	20814	c	(GA)8tcatct(T	36	105910121	105910156	CATGGTTTGGGTGTTGACTG	TGAACAGGATCTAAAACTGAACAAA	278
Ad01A20817	Aradu.A01	20817	p2	(AC)17	34	105921423	105921456	TCCCAACCAAATCACCTTTC	TGATGGGAGTTGATGAATGTG	196
Ad01A20819	Aradu.A01	20819	p2	(GA)6	12	105931307	105931318	GCAGAATCCGAAGTGTTGGT	CCCTTCCTTCAACTCCATCA	253
Ad01A20820	Aradu.A01	20820	p3	(ATA)17	51	105940708	105940758	GCAATCCAAGTTATTTGAGACCA	GGCGGCACTGTCAACTTATT	203
Ad01A20821	Aradu.A01	20821	p3	(TAA)6	18	105943679	105943696	GGGGGAAAAATGTGTTCTTCT	TGTTTAACTTATTCAACACAATGAAAA	177
Ad01A20825	Aradu.A01	20825	p2	(GA)17	34	105953681	105953714	AAGGAAAATCCTTGACGGCT	ACCCAACCCAAGTTCCCTAC	191
Ad01A20827	Aradu.A01	20827	p4	(TAAC)6	24	105955671	105955694	ATCATGGCAATCATCCAACA	CCCCTCCCCTATTTCACATT	237
Ad01A20830	Aradu.A01	20830	p3	(GGA)7	21	105969465	105969485	CCAGAACTGGTGTCCGTTTT	GCAAACTTGACAGCTCTCCC	272
Ad01A20831	Aradu.A01	20831	p2	(GA)6	12	105972049	105972060	AAATTGGACAGCTGGAATGG	ACTCTCCTAAGGTCCTGCCC	165
Ad01A20832	Aradu.A01	20832	p2	(GA)17	34	105973409	105973442	CTGAAGAAGATGCAGGGAGG	GCAGCAGTGTGGAATCTGAC	262
Ad01A20836	Aradu.A01	20836	c	(A)10gaagatttt	99	105989858	105989956	CATCATCGGAAGAAATGGGT	GTCAGAAGATTCCCCTTCCC	243
Ad01A20844	Aradu.A01	20844	p3	(TAT)6	18	106009002	106009019	CAGAGAGAATGGATTTGGAACTG	GATGCAGGATTCAACACCAG	171
Ad01A20848	Aradu.A01	20848	p3	(AAG)7	21	106026952	106026972	TGAGGAGGGTTGAGATGTGA	CGAAAATATCTTCCCTCTTTTTCA	234
Ad01A20852	Aradu.A01	20852	p3	(AAT)15	45	106073972	106074016	TTGGCAGATTTTGAACTCGAT	TTGATTTTATGATAATTAGCGTTTAGA	201
Ad01A20853	Aradu.A01	20853	p2	(CA)9	18	106076487	106076504	CTCGCTGAGCATACTGGAGA	TCGAAAGTCGCATCACAGTC	216
Ad01A20854	Aradu.A01	20854	p2	(GA)7	14	106082938	106082951	CGACGCCATAGTGAGACAGA	GAAACCCTCTCTTCGATCACC	148
Ad01A20861	Aradu.A01	20861	p2	(TA)6	12	106105385	106105396	AGCATTTTTGTGGTTGGTGA	AGGCGTAAAACATGGAATCG	236
Ad01A20864	Aradu.A01	20864	p3	(TAA)5	15	106112293	106112307	TCTCCCCTCTTCCTTCCCTA	TTTTGATGTTGATACCATGTTTTTG	234
Ad01A20867	Aradu.A01	20867	p3	(TAT)5	15	106119508	106119522	TGTTCCTTGGTGTTTCCCTC	TTAAGAAGATTTGAAAAACTTTGGAT	247
Ad01A20870	Aradu.A01	20870	p3	(GGA)7	21	106124823	106124843	TCATCAACTTTGGCTTTCCC	AGAACCAGAACCCTCAACCA	258
Ad01A20875	Aradu.A01	20875	c	(AAT)7(A)13	34	106131709	106131742	CTACACATTGATGAGCCCCA	TGGCTGCCATAGAAACTTCC	260

ID	Chromosome	SSR nr.	SSR type	SSR	Size	Start	End	FORWARD PRIMER (5'-3')	REVERSE PRIMER (5'-3')	Product (bp)
Ad01A20876	Aradu.A01	20876	p3	(ATT)6	18	106132023	106132040	ATATCCACCGCTATTGGCAG	TTTTCAAGGCTCTGCCCTAA	197
Ad01A20877	Aradu.A01	20877	p2	(TG)7	14	106133333	106133346	TGGAAATGGACTTGCACAAA	TCTGGAAGACAATTGGAGGG	213
Ad01A20887	Aradu.A01	20887	p3	(GAA)6	18	106155318	106155335	TTCATCCAATTCTCCAGCAA	GGCGACTACCAAGATGGAAA	275
Ad01A20888	Aradu.A01	20888	p2	(AT)6	12	106157763	106157774	ATCAATCATTTGCTGGCTGC	CCATATGCTGCGGTAGGTTT	201
Ad01A20889	Aradu.A01	20889	p2	(AT)6	12	106157960	106157971	CCTACCGCAGCATATGGAAT	GACGGTTATCAATAAAAAGACGAAA	114
Ad01A20890	Aradu.A01	20890	p3	(ATT)17	51	106158920	106158970	TGGGCAGAGGTTACAACTAACTG	TTGCAGCCACTAGGTGTGTC	256
Ad01A20891	Aradu.A01	20891	p3	(CTT)7	21	106165600	106165620	AGCATCGGATACGGAAACTG	TTTAAATGTGGCGGGGATAA	243
Ad01A20900	Aradu.A01	20900	p2	(TC)14	28	106195811	106195838	ATTAATTGATCGTCGTCGCC	AAAATCCGGTTCTTACCGCT	241
Ad01A20907	Aradu.A01	20907	p3	(TTC)6	18	106235436	106235453	TTCTACACCTTGGCCGAATC	TGGGAAAGAGGAGGTTGATG	197
Ad01A20908	Aradu.A01	20908	p3	(TCT)5	15	106235635	106235649	CATCAACCTCCTCTTTCCCA	CCAACTGAGCTATTCCAGCC	151
Ad01A20914	Aradu.A01	20914	p3	(CAA)5	15	106257224	106257238	ACACGGCTGCGCTTATTAAC	TGTGCTCCTGTTACGCATTC	205
Ad01A20916	Aradu.A01	20916	p2	(TA)6	12	106278838	106278849	TTCACACAAATCAATACTCAACGA	TGAAGTCAAAACAATATGATCAACG	270
Ad01A20917	Aradu.A01	20917	p4	(AATA)5	20	106282709	106282728	TTGTTTCGAAAAATCTTCACCT	CCATGATGGAGAAAGGGAGA	137
Ad01A20918	Aradu.A01	20918	p3	(TCT)5	15	106283294	106283308	CGGATTCCGTATAATTACGTACC	AGGTGGTCTGCATGTAAGGG	259
Ad01A20919	Aradu.A01	20919	p2	(AT)7	14	106284241	106284254	CAAAAGTGGCACGTAGAGAGC	GCACCTACCGGATAGGAGTTT	199
Ad01A20922	Aradu.A01	20922	p2	(AG)6	12	106295238	106295249	AAGAGGTTGAGAGTCGTCGC	ATGTCACTCTTCCCAATGGC	245
Ad01A20923	Aradu.A01	20923	p2	(CT)8	16	106299947	106299962	CCAAACACAACTCACACCAA	ATCTCTGAGCCCTCCAGTCA	258
Ad01A20924	Aradu.A01	20924	p2	(CT)6	12	106308522	106308533	ATTCATGGGGAAGCACTCAC	AAGAACCTGGGATTGAGAGTCA	132
Ad01A20928	Aradu.A01	20928	p2	(TG)6	12	106330906	106330917	TTGTGGTCAATGGCAAGTTC	TTGGAGAGTTGAGGCTGACA	201
Ad01A20929	Aradu.A01	20929	p2	(AC)8	16	106331482	106331497	CAAGTTCAATTGAGAAAGCGG	CAAATTAACCACCCCTCCCT	255
Ad01A20930	Aradu.A01	20930	p2	(CT)7	14	106332230	106332243	GCTTAAAGCTCTTCCCCTCC	TGTGGTGGGTCTTTGTGAAG	159
Ad01A20931	Aradu.A01	20931	c	(T)11acttgtggt	32	106334191	106334222	TTTCGAAAAATGCAAGATGC	ATTGAAAGCTTGGCTGGCTA	256
Ad01A20932	Aradu.A01	20932	p3	(ATT)7	21	106336017	106336037	TGCTGAAAATGGTTCTCACCT	TCGTATGCATAAAACAACATTCAA	227
Ad01A20938	Aradu.A01	20938	c	(TTC)5gccttct	79	106341389	106341467	TTCTTCGTCTTCGCATTCCT	GCAGCATCATCAAGTGAACC	280
Ad01A20939	Aradu.A01	20939	p3	(AAG)5	15	106342425	106342439	TTGGTGATGACGATAACGAAA	TGCACATGTGTCTTCTTCCC	106
Ad01A20945	Aradu.A01	20945	c	(T)10gtatgttttc	87	106353859	106353945	AATCACACAACGAGCACGTAA	TCCTTTTTGTTTCACCTTCTCA	233
Ad01A20946	Aradu.A01	20946	p3	(AGG)5	15	106354742	106354756	AAATTTTGGCGTCATTCACC	ATCACCACCACCGTCTCCT	177
Ad01A20947	Aradu.A01	20947	c	(CTT)6tttcgatc	167	106358335	106358501	CTAATCCCCAAAATTCGCTG	CACGTAAATGAAAAATGAAGAAGG	249
Ad01A20949	Aradu.A01	20949	p4	(ATGT)9	36	106368804	106368839	GAATTGAATCAAGAACCATTAGTTTG	TGGATGTTGAATGGCTCTGA	252
Ad01A20950	Aradu.A01	20950	c	(AC)6at(AC)8	30	106370750	106370779	CCTTTCAGGCCGACTAATCA	TGTGATGCGAGTTTCGAGTT	280
Ad01A20951	Aradu.A01	20951	p2	(TA)7	14	106371556	106371569	ACATGTCCAGTAGCGAACCC	ACTGAAAGATCCACATGGCA	251
Ad01A20953	Aradu.A01	20953	p2	(TC)13	26	106379258	106379283	CCAAATTACAACCACCGCTT	GCTCCAGATCCCATCTTTGA	222
Ad01A20956	Aradu.A01	20956	p3	(TGG)6	18	106382999	106383016	ATTTGCATCGCTTTCTGCTT	CCACCAACATTACCACCACA	256
Ad01A20963	Aradu.A01	20963	p2	(AT)6	12	106403227	106403238	CCTCATGATCGCACTAAAAGTTC	TGTAATTTGATAAAAGATCGGGG	250

ID	Chromosome	SSR nr.	SSR type	SSR	Size	Start	End	FORWARD PRIMER (5'-3')	REVERSE PRIMER (5'-3')	Product (bp)
Ad01A20964	Aradu.A01	20964	p3	(GTC)5	15	106404173	106404187	CGGTTTCTCTCTCTCACCCA	CATCAGGTGTTCTAAAAGAAAAGTG	280
Ad01A20965	Aradu.A01	20965	p2	(GT)7	14	106416894	106416907	TGGGTAGCTAGGCATGAATTT	ACCCTCGCTGGATGTGTATC	259
Ad01A20967	Aradu.A01	20967	c	(TC)16(AC)8	48	106429980	106430027	GTTTGCCTCCGATGAAGAAG	TAATACCTCGCCACCTCTGC	238
Ad01A20972	Aradu.A01	20972	p3	(TAA)15	45	106452083	106452127	TGGCGATCCATCTATCCTTT	AGGGGGAGAACAACATAGCA	276
Ad01A20976	Aradu.A01	20976	p2	(TA)6	12	106456362	106456373	CCTTTAAACCTGCCGAACAT	GTTAATCGGCCGGGTTACTT	186
Ad01A20977	Aradu.A01	20977	p3	(ATT)6	18	106456475	106456492	GTAACCCGGCCGATTAACTT	CTTCGCAGATGACACCTGAA	171
Ad01A20978	Aradu.A01	20978	p3	(AGA)5	15	106457370	106457384	TTGTGCGCCTCTTCTTTCTT	TTTCCTTCTCCCCCTCCTTA	274
Ad01A20979	Aradu.A01	20979	p2	(TC)8	16	106461016	106461031	CTCAGTTGGTGGGAAAGTCC	GCAATCACAAGAAGATGGTTGA	224
Ad01A20981	Aradu.A01	20981	p3	(GAT)5	15	106491117	106491131	TCATACCTAAAAGGGCGTGG	TTGACCACATTCAGCAGAGC	176
Ad01A20985	Aradu.A01	20985	p2	(TA)8	16	106501395	106501410	AAGGGCAACAGAGGTAGGGT	CATAGCAGCGGAAAGGTAGC	212
Ad01A20988	Aradu.A01	20988	p4	(AATA)5	20	106506950	106506969	CGTCTGTTTTCGCAGATTTAAG	CGGATTTGATTTAATCGGATATT	280
Ad01A20989	Aradu.A01	20989	p2	(CT)12	24	106507946	106507969	TCCGCTAGTAAATTCGACGG	GACAGTAGCGGGGACACAAT	163
Ad01A20993	Aradu.A01	20993	p2	(TC)7	14	106529285	106529298	GATCCCACTAGGGGTGTTCA	GGAGAGGGGAGAGAAAGAGG	269
Ad01A20994	Aradu.A01	20994	p3	(ATG)7	21	106530441	106530461	CACAAGAAAGGGGCCTACAA	TGTGACCAGTTGAGGCTGAG	119
Ad01A20995	Aradu.A01	20995	p2	(TA)7	14	106531158	106531171	CAACAACCAAACACATCCTCA	CTCGACGGTGGAAAGAATGT	260
Ad01A21000	Aradu.A01	21000	c	(T)10catattc(T	27	106541979	106542005	AGCACATGGTTAGGGACAGC	ATTGATGGCACCTAAGACCG	257
Ad01A21010	Aradu.A01	21010	p3	(TCA)29	87	106564415	106564501	TTCGTGCATCAGCATTCTTC	TTTCGTTTTTGTTCTTATTCTTTTT	196
Ad01A21012	Aradu.A01	21012	p3	(TAA)13	39	106582096	106582134	TGGCCCTTATATCTTTCAAGTCA	TCCTGATATCTTTTTGAAGCCA	237
Ad01A21014	Aradu.A01	21014	p2	(AT)6	12	106584986	106584997	AAAAGGGAACTCGGACCCTA	TACGACTTGTTGATGGCTGC	117
Ad01A21015	Aradu.A01	21015	p3	(TAA)13	39	106603503	106603541	TGGCCCTTATATCTTTCAAGTCA	TCCTGATATCTTTTTGAAGCCA	237
Ad01A21016	Aradu.A01	21016	p3	(CAC)5	15	106609400	106609414	ACTTTCGAACTCGAAGCCAA	CGCTGCAACAAAAGCAATAA	222
Ad01A21017	Aradu.A01	21017	c	(TGA)11tgcgtt	102	106609557	106609658	TTATTGCTTTTGTTGCAGCG	CAACGATCCCAATCCACTTT	257
Ad01A21018	Aradu.A01	21018	p2	(TA)6	12	106614050	106614061	CCTTTTCCCTTTCAAAACACA	TGATCAGCATCATCATCGGT	262
Ad01A21021	Aradu.A01	21021	p2	(GA)6	12	106615144	106615155	TCACCTCCATCATCACCAGA	AAAGACGGAATAAAGGCTGAA	254
Ad01A21022	Aradu.A01	21022	p3	(TTA)7	21	106615529	106615549	TTTATTTGCATGTCCCCTCC	CGAAATAGAAACAAAATAGCACGA	177
Ad01A21023	Aradu.A01	21023	p2	(AG)9	18	106617591	106617608	CTGGCTTTGGTAACACGGAT	GCTTAGGGTATTTGCGACCA	242
Ad01A21032	Aradu.A01	21032	p2	(CG)6	12	106636957	106636968	TGGTTATCCATGGTGTGTGG	TCCATACCTTGACCCGAGAG	256
Ad01A21035	Aradu.A01	21035	p3	(TTG)5	15	106652767	106652781	GTGGATCCGCTAAATGGAGA	TGCAAGGGTGACTGAGACTG	266
Ad01A21037	Aradu.A01	21037	c	(ACT)6gctatta	93	106657826	106657918	AGCGTTTGCAATTGTGAATTA	GCCAGAAAAAGCCTAAACCA	233
Ad01A21041	Aradu.A01	21041	c	(TTC)5a(TCT)	43	106664067	106664109	AAGTCCCTTTTCCGTCGAAT	TGGTTAAACCCAACACAGCA	245
Ad01A21043	Aradu.A01	21043	p2	(CA)8	16	106667373	106667388	GAAAATGGTGGTGATGCTCC	CATGACCTTCTTTGTTGCCA	236
Ad01A21051	Aradu.A01	21051	p2	(TA)6	12	106695766	106695777	CAAGAAATGATGAAAAAGTTGGTG	GCATGGTTAGTTCTTCGAATTTT	266
Ad01A21053	Aradu.A01	21053	p2	(AT)7	14	106697400	106697413	AAAATCGTTCTTTGCCGTTG	TTCCAATTTCTTTTAGCCACG	193
Ad01A21056	Aradu.A01	21056	p3	(TTA)5	15	106713511	106713525	GGGATGAATTGATGGGTGAC	AAATTTCCGCACACTCTGCT	243

ID	Chromosome	SSR nr.	SSR type	SSR	Size	Start	End	FORWARD PRIMER (5'-3')	REVERSE PRIMER (5'-3')	Product (bp)
Ad01A21059	Aradu.A01	21059	c	(TA)7(T)11	25	106716483	106716507	GGAAGAAGGCCAGAGGTTTT	TGGTGATTGATGAGCATAGTCC	161
Ad01A21062	Aradu.A01	21062	p2	(TA)7	14	106722980	106722993	TGCTTGAGTTCTTCCCTGCT	GGTTGATAATAATTGATCCGGAAA	133
Ad01A21067	Aradu.A01	21067	c*	(A)13(AC)6*	24	106738830	106738853	CGAGAAAACATTTGCCTTTTG	GGTTCAAGGGACAGATGGAA	180
Ad01A21078	Aradu.A01	21078	p3	(TTA)5	15	106778940	106778954	AAATTTAAGTCATTTACCTGTTTGTGA	CATTTCATATCACATTCGTTTCAA	201
Ad01A21080	Aradu.A01	21080	p3	(TTA)5	15	106785134	106785148	ATACGTGGCTGAAAAATGCC	GCAATTTCCTTCCAATTCCA	131
Ad01A21082	Aradu.A01	21082	p4	(ATTA)5	20	106789831	106789850	CATGGAATGCAACATTCTTCA	ATGTGCCAATTGCGATGTTA	193
Ad01A21083	Aradu.A01	21083	p3	(ATA)15	45	106790878	106790922	CGAATTTTGACGGATAAAACA	GTAACCACGTTTGCTGAGGG	245
Ad01A21086	Aradu.A01	21086	c	(TC)8tt(TC)9	36	106799376	106799411	TCCCTTCCATTGCTTTTGTT	GAGGGGGAGAGAGAGGAAGA	259
Ad01A21087	Aradu.A01	21087	p2	(TC)6	12	106799641	106799652	TCCTTCTCTCTCCCCTCCTC	TCTCTCGCTCCTCTCTCTTTTC	265
Ad01A21088	Aradu.A01	21088	c	(GA)6aaagaga	164	106799801	106799964	TCCTCTGTCTTCTCCTTCTCTCTC	TCTCCTTTCTCCTCTCTTTTTCC	279
Ad01A21089	Aradu.A01	21089	p2	(GA)9	18	106800113	106800130	GAGGAGAGATAGAAAAAGGGGG	TCTCTCTCGCCTTCTCTTCG	149
Ad01A21090	Aradu.A01	21090	p2	(GA)7	14	106800292	106800305	AGAGAAAGAAAGATGAGGTGGAG	TCTTTTTCCCTTCTCTCTCCC	265
Ad01A21092	Aradu.A01	21092	p3	(AAT)5	15	106805005	106805019	TTGGGAATGCTTGTGATGAA	ACTGCACCACTTTTGGTTCC	242
Ad01A21096	Aradu.A01	21096	p2	(CT)7	14	106810689	106810702	TTCCAGAATCTTCAGCCAGC	CTCTTAATACGGCGGAGCAG	192
Ad01A21097	Aradu.A01	21097	p2	(TA)8	16	106812402	106812417	TGCATATAGCAAAATTAGTGATCAAA	TTAGTGATGGGGACTGCCTC	272
Ad01A21098	Aradu.A01	21098	p2	(TA)6	12	106813137	106813148	TGATAAACATATGCAATTCTCTCTCTC	TTTGATCAGAGTCTCGTGCTG	204
Ad01A21099	Aradu.A01	21099	p2	(TA)8	16	106818625	106818640	TGCAATTCTCTTTCTCTTTACTTTT	CGCCTCTGGGTTTATGATGT	257
Ad01A21102	Aradu.A01	21102	c	(T)10cttttcttctt	46	106823219	106823264	CCACACTGATACCGCAGATG	AGGAGGAGATGAGTGTGATGG	249
Ad01A21104	Aradu.A01	21104	p3	(TCC)5	15	106831030	106831044	CTAGAAGGTCCAGGATGCCA	GGAGATGGGAGGAGGAAAAG	166
Ad01A21105	Aradu.A01	21105	p2	(CT)6	12	106836931	106836942	GCAGTAATCGTCCCGACTTC	ACATGGAGAAAAATGCCAGG	248
Ad01A21107	Aradu.A01	21107	p4	(AGAA)6	24	106840746	106840769	TAAAACTGCGCTTCACACCT	TGCCAACAAGGTTCATGGAT	222
Ad01A21111	Aradu.A01	21111	p2	(TC)6	12	106855249	106855260	AATCTACACGGCAAAAACGG	TTGAAACCCGGAAATGAAAG	238
Ad01A21113	Aradu.A01	21113	p2	(AT)6	12	106866115	106866126	TTGCAAAAGCTGGAACTATCC	TGATCATGGTTGTAAGGGTTG	273
Ad01A21114	Aradu.A01	21114	p2	(GA)7	14	106867691	106867704	GGGGAAAAACATTCTTCGGT	GCCATGCTTCCTTTCTTCAC	149
Ad01A21124	Aradu.A01	21124	p3	(GAT)5	15	106901523	106901537	TCAATTCTCTTTTGCCCCAC	ACCTATGATGAAGGGCATGG	233
Ad01A21130	Aradu.A01	21130	p2	(CA)6	12	106917842	106917853	ACGCACCGTTTCAAAGAAAT	CCGATAACGCGGAAATTAAA	169
Ad01A21131	Aradu.A01	21131	p2	(AT)8	16	106918033	106918048	CAACAACTAATTGATCAACGTGAA	TGTGGAAGTGTGTAATGCGT	276
Ad01A21133	Aradu.A01	21133	p3	(AAG)5	15	106918791	106918805	TCCTCATCATCTTCTGCTGC	GCACGTTCTTCTTCCTCTTCC	237
Ad01A21138	Aradu.A01	21138	p2	(TA)10	20	106935999	106936018	GCATCACCTGTAAAGCGTAGC	TCATGCCTATTTTGATATGGCT	280
Ad01A21140	Aradu.A01	21140	p2	(AT)7	14	106944561	106944574	TCAATCTGCTTCATCCAGGAC	AATGGAGGTATGGAGGGCTT	175
Ad01A21141	Aradu.A01	21141	p3	(GAT)6	18	106948063	106948080	CTGTTCAGGCCGATTACACC	AGTCGGCACAGCAAAAGTCT	227
Ad01A21146	Aradu.A01	21146	p3	(TCT)5	15	106969078	106969092	TGGGCTTTATTAGCCCCATA	GAGAAGGCAAGAAACGAAAGAA	252
Ad01A21147	Aradu.A01	21147	c	(CTT)5gctgcac	41	106969282	106969322	ACCGTTACTGCACGTTTTCA	CCTCCTCATCTTCTGCTGCT	216
Ad01A21149	Aradu.A01	21149	p3	(ATA)5	15	106970771	106970785	TGCTCAAAGCAAAAGTCACAA	TTGATGCAGGTTGAGAGAGC	265

ID	Chromosome	SSR nr.	SSR type	SSR	Size	Start	End	FORWARD PRIMER (5'-3')	REVERSE PRIMER (5'-3')	Product (bp)
Ad01A21153	Aradu.A01	21153	c	(TCT)6atctacc	109	106976159	106976267	AACAGCCTCTAACTCCACGG	AATCCACGCACGCTATCTCT	201
Ad01A21154	Aradu.A01	21154	c	(AAG)6aaaaag	55	106976508	106976562	GGATTGCACCAACCAAAGAG	TGTCTACCCCTTTGTGTCCC	278
Ad01A21155	Aradu.A01	21155	p2	(TA)7	14	106985225	106985238	GCAACGCTCAAATGGATTCT	AGAAGCTGCTGGACCTTGAC	170
Ad01A21161	Aradu.A01	21161	p3	(CTT)5	15	107024630	107024644	AGAGGGAGCAAAACACGAGA	CAAGGCTCCTTCCTTATCCC	165
Ad02A1	Aradu.A02	1	p3	(TTA)7	21	11605	11625	GCTGTTTACCCCATGCTCTT	AAAAGATAAAAATGATGAAAAACGA	275
Ad02A4	Aradu.A02	4	p3	(ATA)8	24	22830	22853	CGTTTTTGGGTTTATTGTCCA	CCATAACAATTCCAGAAGGTTAAAA	215
Ad02A5	Aradu.A02	5	p3	(ATT)17	51	23957	24007	TTTTTCTACCTTTTTGTTTTACGAA	TTTTTAAACAATTGAATCTTGATGCT	256
Ad02A6	Aradu.A02	6	p2	(AG)6	12	27555	27566	TGCAATGCATATTTCCTTGA	CTGGGTGTTCTGACGGTCTT	272
Ad02A7	Aradu.A02	7	p2	(CA)6	12	32069	32080	TGCAGTTTGAGAAGAACAACAGA	TGCTGTTTTGGTTGATTGGA	234
Ad02A8	Aradu.A02	8	c	(A)10gaata(T)	25	32369	32393	GCATGAACTGCAACATGTCC	TTTTGGAACTGGGGTTTGTC	208
Ad02A9	Aradu.A02	9	p2	(GT)6	12	32957	32968	AACGGTTTCATGGAGCAAAA	GGACTTCACTTATGCGCCTC	240
Ad02A10	Aradu.A02	10	p3	(CAT)7	21	33114	33134	CGACCAACCTATGACTTGGA	CGTACCGCAAACATCCCTAA	244
Ad02A11	Aradu.A02	11	p5	(ATCCG)5	25	33405	33429	TTAGGGATGTTTGCGGTACG	CGCAAATCTAAATCGATCCAA	160
Ad02A12	Aradu.A02	12	c	(AAAT)5aacat	65	33598	33662	ATCTTAAAAAGCCAACGATAACA	AAACTGCTGAATTGGATCGG	277
Ad02A15	Aradu.A02	15	p3	(TAA)7	21	39476	39496	GGACCTGAAAGTCCGAAAGA	ATTTCGGTGTGTCGTGTGTG	203
Ad02A17	Aradu.A02	17	p3	(TTA)5	15	46940	46954	CAAACTTCGTTACTTCTTGCCA	GCAATCTGATCATTGTTATGCAC	280
Ad02A21	Aradu.A02	21	p2	(TA)6	12	54144	54155	TCAAATGCAAATCTCTCCCTC	TCCCAATCAGAATTTAATCTAGACA	231
Ad02A31	Aradu.A02	31	p3	(CCT)6	18	77476	77493	TCCTCCTCCTGATGATCGAC	ACTATGGCATACCTTTGGCG	219
Ad02A32	Aradu.A02	32	p2	(TA)6	12	79807	79818	CCTTTTGTCTTAGGGTTGCG	AACAACGAATTCATGCACCA	181
Ad02A33	Aradu.A02	33	p2	(TA)7	14	81043	81056	GCTACTTCTATTTGATGAGTTGGAT	TGCTCATCACTTGCTTCTGC	280
Ad02A35	Aradu.A02	35	p2	(TA)7	14	81968	81981	AACGGCGGTGGAAATACA	TGCACCTATGCCATTTGAATTA	280
Ad02A46	Aradu.A02	46	p3	(TAA)5	15	112162	112176	AGGGTCTGTCTTTAGTGGATCTG	GACGGCGAAGATCAGAGAAG	250
Ad02A53	Aradu.A02	53	p2	(AT)21	42	130910	130951	TGTGCTTTCCTTACCAGCCT	CCAACATGATTTGGTCATGG	156
Ad02A54	Aradu.A02	54	p2	(CA)6	12	131167	131178	AATCAGGTTGCTGGGAAAAA	CGACTGCACCTGAGTTTTCA	218
Ad02A55	Aradu.A02	55	p2	(TA)6	12	131506	131517	TTCAAAAAGCCATGCCTCA	GGTGCCCTTTGCGTATAGTC	141
Ad02A56	Aradu.A02	56	c	(AAT)6aaaccc	110	131684	131793	TCTTTGTTGGCACTTCTCCA	TGGGACAACTTTGATTTCCA	250
Ad02A57	Aradu.A02	57	p2	(TA)11	22	132602	132623	TCTTGTGATCCAAGCTTCTGAC	TTGCAAGCTTTGGAGAGAGA	279
Ad02A68	Aradu.A02	68	p2	(AT)8	16	159627	159642	TCTCTCTACATTCGCGTATCC	TCCGCATAATTGAATCTTCTCT	276
Ad02A73	Aradu.A02	73	c	(T)11(A)11	22	166783	166804	CTTGGAAAGGGGAAGAAAGG	CGACAAACCGGAAGAACTGT	205
Ad02A76	Aradu.A02	76	p3	(ATA)17	51	170156	170206	GCTAGGGACTAGTTTTCGATTTG	TTGGTCCCACGTTAAATTTTG	259
Ad02A78	Aradu.A02	78	p3	(ATA)5	15	171948	171962	TTCATCAAGCAGCATTGAGC	AGGATCCCAGTTGCTATTTTC	218
Ad02A83	Aradu.A02	83	p3	(AGC)7	21	182409	182429	GGGAGCAGCATCAGGTCTAA	GCAACCTGCCAAATTTCACT	226
Ad02A88	Aradu.A02	88	p2	(TA)6	12	200663	200674	GCGACTAGAGGAGGACGATG	TTGGAGCTCTTATCAACGAGG	271
Ad02A89	Aradu.A02	89	p2	(CA)7	14	214846	214859	TCAGCGAGACCCTAGAGCAT	TCCTTTATGCGTATATTTTGGC	266

ID	Chromosome	SSR nr.	SSR type	SSR	Size	Start	End	FORWARD PRIMER (5'-3')	REVERSE PRIMER (5'-3')	Product (bp)
Ad02A91	Aradu.A02	91	c	(A)12tttctctttt	33	217609	217641	GCTTCTTCGTGGTGAGAAGG	TTTTGAATTAGTGGGAGAGTTGTG	271
Ad02A93	Aradu.A02	93	p3	(GAA)5	15	224967	224981	TGAAAAGGCAAAGAGAATAACGA	TTTCCAAAGCGCTACACTCA	278
Ad02A94	Aradu.A02	94	p3	(GAA)6	18	225116	225133	TGGAGGAGGAGGAAGAAGAA	TTTCCAAAGCGCTACACTCA	246
Ad02A96	Aradu.A02	96	p2	(TA)8	16	227161	227176	CATGCTCTTCAATTCTTTTCATGT	CGGGTCAAAGAAAGCAATGT	279
Ad02A101	Aradu.A02	101	p3	(TTC)5	15	231602	231616	TTTAAACCTTGGCAGTTGGC	GTTTCCTGGAGTTGGAGCAG	196
Ad02A109	Aradu.A02	109	p2	(TA)6	12	251952	251963	TCGCATAGATGATGCTTTTGA	GGCTAAAAGCACTGGGAGAA	279
Ad02A110	Aradu.A02	110	c	(CA)8cgcacgca	43	260128	260170	CCAGCACACAACTCTGTTCC	TAGAAGGGTAAAGAGCGGCA	245
Ad02A111	Aradu.A02	111	c	(TA)6ataatatta	45	268758	268802	AAAGTGTGATTAAATTAAAAAGTTTCG	AAACAAATGCAATTCTCTCTCTCT	221
Ad02A113	Aradu.A02	113	p3	(CTT)5	15	270028	270042	TTGCGTGATCAACCTTTCTC	TCCAAATCGCACTCATTCAA	256
Ad02A114	Aradu.A02	114	c	(T)11gttcaagtt	72	272813	272884	GCACAATGCATGATGAAGGA	AGAAAGGTGTTCTAAGTGCCAA	178
Ad02A129	Aradu.A02	129	c	(A)10tacaggttt	111	303975	304085	TTGCTTCTAGTGTGAGAACCAAG	TGGACGCATGCAATAGAAAA	244
Ad02A130	Aradu.A02	130	p2	(AT)6	12	305542	305553	TCTGCGTCTGTGATCACTCC	GCATTTACGTGGGTGAACTG	197
Ad02A131	Aradu.A02	131	p2	(CA)6	12	305971	305982	CTGCCTGGCCTTAACACACT	GGTGGTGAGGGGTAGATGAA	230
Ad02A133	Aradu.A02	133	p3	(TTC)5	15	314665	314679	GAAGCTGCAACAGATGCTGA	TCCATTCTCTTCGTTGGGAC	209
Ad02A137	Aradu.A02	137	p2	(AT)6	12	320068	320079	GACCAAAAGACTTGATGAAGGA	TGGTCCTGCCTTATCAACCA	279
Ad02A138	Aradu.A02	138	p2	(AG)6	12	321983	321994	CCATTTTCTGTTACGCCGTT	AAACCCAACGTTCCCTCTCT	134
Ad02A141	Aradu.A02	141	p2	(AT)6	12	329517	329528	CGTTCCCGTGAAGATATATGG	TCTTGAGGAATCGCCTGTCT	202
Ad02A144	Aradu.A02	144	p2	(TA)8	16	333478	333493	TCGAGTTTGGACAACTGACG	GGAAGCCTCAGTCCAAATGA	255
Ad02A147	Aradu.A02	147	p3	(TAA)8	24	336825	336848	AACTCCATGCATATTTGGTGC	TTGTCAATCCTAAATGCACGAC	213
Ad02A149	Aradu.A02	149	c	(T)11gaaaatt(A	32	339030	339061	GAAAAATGCTTCTTCTCATAATCACA	TGCATCTACCGTACCAACCA	245
Ad02A150	Aradu.A02	150	p3	(AAT)5	15	339838	339852	GCATTGTTAATGAAGCAAGCA	GGTCAATGTCATCCTCATCCA	195
Ad02A154	Aradu.A02	154	p2	(TG)6	12	342285	342296	GTGAGTGAGGGGTGTGGTTT	CCTCCCCAAACCAACTGTTA	251
Ad02A155	Aradu.A02	155	p3	(CTG)5	15	345989	346003	TCAACAGGAAGTGGAGGAGG	CCAGAACCCTGACAACAACA	132
Ad02A156	Aradu.A02	156	p3	(AAC)5	15	350416	350430	ATGGGGCAACTCCCCTATAC	AACATCCAGTCAGGAGGGTG	249
Ad02A162	Aradu.A02	162	p2	(GA)11	22	358235	358256	CATAAGGTCCATCTCTCGCC	CTGCAATTGAGCTTGAACCA	193
Ad02A164	Aradu.A02	164	p3	(ATA)5	15	363750	363764	TTGAAAAATGGGTGGCTCTC	TGGACAAAAACAAGAAAACCTG	238
Ad02A165	Aradu.A02	165	c	(AT)7ctttaattat	34	367393	367426	CGAATCCAAACTTTTTCTTCCA	GGGCTAAAAATGTTGCGGTA	280
Ad02A166	Aradu.A02	166	p2	(AT)8	16	367986	368001	CGTTGTTGCCATTATCACCA	GGACCTATGAAATATTCAAAGATGAAA	236
Ad02A170	Aradu.A02	170	p2	(AT)6	12	374618	374629	CCCTACCCGCCCCTATAAT	TCTATCGCGCCTCTACCTGT	234
Ad02A172	Aradu.A02	172	p2	(AG)6	12	375310	375321	GAGGGAGAGACAAAGACATACCA	TCACGGAAGAGGTCAACTCA	267
Ad02A173	Aradu.A02	173	p2	(AT)7	14	375799	375812	CGATTCGACTCGTGGACATT	TCAGATTTAAGTAATATTCGCTCCG	280
Ad02A174	Aradu.A02	174	p2	(AG)6	12	376523	376534	GAGGGAGAGACAAAGACATACCA	TTCACTGAAGAGGTCAACTCAAA	280
Ad02A175	Aradu.A02	175	p2	(AT)11	22	383100	383121	TGAGAGCATCCTTTTAATGCTT	TAAATGGAGAGGGGGACAGG	183
Ad02A176	Aradu.A02	176	p2	(AT)7	14	383723	383736	TGGTCTATTCTGTTTTGTCCCA	CGAACGGAGTCGGATTTAAG	243

ID	Chromosome	SSR nr.	SSR type	SSR	Size	Start	End	FORWARD PRIMER (5'-3')	REVERSE PRIMER (5'-3')	Product (bp)
Ad02A177	Aradu.A02	177	p2	(AG)6	12	384322	384333	GAAGAGGAAAGAAAAGGGGC	AAAACACTCATCAAAGAGACCCA	269
Ad02A180	Aradu.A02	180	c	(TCT)6tcctcttc	146	390520	390665	TTTATTAACACGCGCACCAA	GAAGGTGCAGCAAGAAGAAGA	279
Ad02A182	Aradu.A02	182	c	(TCT)6tcctcttc	115	391137	391251	TTTATTAACACGCGCACCAA	CGTGCAGAAACGAAAGAAAA	246
Ad02A183	Aradu.A02	183	p3	(AAG)5	15	392029	392043	TCCTCTTCATCTTCTGCTGCT	CGTTTCTTGCCTTCTCTTTCTC	183
Ad02A184	Aradu.A02	184	c*	(AAG)9(GAA(	53	392191	392243	GAAGGCAAGAAACGTACATTGA	TACACGCGCTTTATTCATGC	277
Ad02A187	Aradu.A02	187	p3	(TGG)5	15	394431	394445	GAGCCATGGAGGTTGTTGTT	GTCGTCGTCATCATCACCTG	239
Ad02A188	Aradu.A02	188	c	(GAA)6gagagt	114	398424	398537	GAGGTTGCAATGGTGAAGGT	AAAACGACAAAATCAACGCC	230
Ad02A190	Aradu.A02	190	p4	(TTTA)5	20	400518	400537	TGAAAAATTCAATCGGAAATTG	AAAACCCTTGGTTGAGTGGA	230
Ad02A191	Aradu.A02	191	p3	(TGG)5	15	402683	402697	TGTTGTTTTTCTTGGTGGTGG	TCAGAATTGAAATCGTTCCCA	197
Ad02A192	Aradu.A02	192	p2	(TA)6	12	403058	403069	TGCCAACCAATTCTTTCTTCA	CAATGGCTGCTACCATGAGA	169
Ad02A197	Aradu.A02	197	p3	(TGA)10	30	409968	409997	CTGGGTTTCTTGACCAGCAT	CTTCAATTCCTCCGTTTGGA	234
Ad02A204	Aradu.A02	204	p3	(TTC)5	15	440932	440946	CCGCACGGTTTCATTCTAGT	TGCTTCACGCTGGTAAGAAG	278
Ad02A206	Aradu.A02	206	p3	(TCA)5	15	442538	442552	AAAGCTGCCACTCAAAAGGA	TCAGACATGTGGTTTCTCGC	217
Ad02A214	Aradu.A02	214	p2	(AG)9	18	474074	474091	TGCAGGTCTTCTTTTTCCAA	CTCTCAAATGCATCAATCGC	230
Ad02A216	Aradu.A02	216	p3	(TCA)8	24	475677	475700	GGGGTGGTTGCAAGAATAGA	GTTTTACCAACCCCTCCCAT	248
Ad02A224	Aradu.A02	224	p3	(TTC)5	15	486998	487012	TGGAAAGTGAAGCTTTCAAAAT	GCCATCCTCTTTAAGTCCCA	272
Ad02A226	Aradu.A02	226	p3	(TCA)6	18	492474	492491	TGATGACTTTCTGGACGCTG	TCAGACATGTGGTTTCTCGC	233
Ad02A228	Aradu.A02	228	p2	(TA)12	24	497659	497682	GGGGGACTATTTTCCTGAGC	GCTTCCCAACACATTAATCCA	174
Ad02A232	Aradu.A02	232	c	(A)10gaagcttat	89	500267	500355	TGATGCAATCTGGGACTTTG	CACTGCTTGACTGCTTCTGC	276
Ad02A241	Aradu.A02	241	p3	(AAT)7	21	516112	516132	AAAGGGAGAATGGCTCTGGT	AAGAATGAACCGTGCAGGAG	174
Ad02A242	Aradu.A02	242	p3	(TTA)6	18	523223	523240	ACCGACCTCATTTGAAAAGC	AGCCAAGAATATCCACGCC	237
Ad02A244	Aradu.A02	244	p2	(AG)6	12	526389	526400	GTGAGAAGGGTCGTCATCGT	CGCCTCTTTCCTTTCCTTCT	100
Ad02A245	Aradu.A02	245	c	(GC)9(AC)13(	74	529561	529634	AGGATGTTGCAGACGATAGGA	TGGAGTGTTGACAAAGCTGG	277
Ad02A247	Aradu.A02	247	p2	(AC)6	12	530244	530255	CACAAACTGAAGCAGTACCCTTT	GTTGATCTGCCAGGGTTGAC	276
Ad02A249	Aradu.A02	249	c*	(TAA)5(AAA7	38	531587	531624	TCGGAACAGGGGATATTTGT	ACCGAGCGGTCTTGTACTGT	255
Ad02A253	Aradu.A02	253	p3	(TGA)5	15	534687	534701	TTCCCTACGCTCTTCTTCCA	ATGCTCCAACATGACCCTTC	147
Ad02A258	Aradu.A02	258	p2	(TC)6	12	544080	544091	ATTGGATTCATTGCTTTGGG	CGTAGACACGCACACACAGA	220
Ad02A262	Aradu.A02	262	c	(AG)11ctctcct;	99	556509	556607	GGAAAACATATGGTTGATGTTAAGC	CAGCCATGAGTATTTATAATGACGA	252
Ad02A264	Aradu.A02	264	p3	(CTT)5	15	571518	571532	AACTTCAATCTACACGCCCG	TTTCACTCAATGTATCAAATTCACAA	151
Ad02A267	Aradu.A02	267	p3	(ACA)7	21	585481	585501	TGCCTTCTGTGAGATATTTGTTG	CACATGATAGGGCTGCAATG	218
Ad02A269	Aradu.A02	269	p2	(AT)7	14	605401	605414	TGGCACAACAAAGATGAACTCT	ATGGTCCACAAGCTTCCAAA	227
Ad02A270	Aradu.A02	270	p3	(CCA)6	18	613108	613125	TGCTTTCTTTGACCTCCACC	CAATTTACCACGCTAAGCAACA	218
Ad02A279	Aradu.A02	279	p2	(TA)8	16	630151	630166	GCAGGGTTACATCTTATCCTCTGT	GTCTACTCCTCCCTGGACCC	129
Ad02A281	Aradu.A02	281	p2	(AT)7	14	631515	631528	GTTGGGCTTGATGCAGTTTA	AAACTTTTGGTGACATTTACATTTTT	280

ID	Chromosome	SSR nr.	SSR type	SSR	Size	Start	End	FORWARD PRIMER (5'-3')	REVERSE PRIMER (5'-3')	Product (bp)
Ad02A282	Aradu.A02	282	p3	(AAT)5	15	632486	632500	CGCTTTCAATGATCTAAATGGA	TCATTCCAACCACCCTTTTC	154
Ad02A283	Aradu.A02	283	p3	(TAA)5	15	633629	633643	GACTCGAACTCGCAATCTGTT	TCACCATTCCTCCATTGTCA	264
Ad02A284	Aradu.A02	284	p2	(TA)7	14	634668	634681	TGCTCCTATTGAATGGTCATTTT	ACTTCACCAAGCCAAGGAAA	247
Ad02A293	Aradu.A02	293	p2	(TA)6	12	649303	649314	GTTCGAATTAGGGATGGCAA	GGACCTGTTAAATCCCCGTT	184
Ad02A294	Aradu.A02	294	p2	(TA)6	12	650598	650609	GCAGGGATAGGGATTAGGGA	GGACCTGTTAAATCCCCGTT	261
Ad02A295	Aradu.A02	295	p4	(TTAT)5	20	651576	651595	TGAACCATCCATCAATTAGAGGT	CAGATGCCATGACAAAACCA	280
Ad02A296	Aradu.A02	296	p4	(TTAT)5	20	656037	656056	TGAACCATCCATCAATTAGAGGT	TCGATTGGAATAATGAATTTTGTG	177
Ad02A299	Aradu.A02	299	p2	(TA)8	16	660424	660439	GGACGGACCCAGGTAGTGTA	GGCGTTAGAGCATTGAGAGG	177
Ad02A302	Aradu.A02	302	p3	(TAA)5	15	670928	670942	AGCATGCACATTCCTGTTTG	TCACTGCTGGCAGCTTTCTA	226
Ad02A308	Aradu.A02	308	p2	(TA)6	12	686473	686484	CTCCCTTACGTCAAAGTTTCG	AATTGCATGATTTTGCTGGG	200
Ad02A309	Aradu.A02	309	c	(ATA)6ctaaata	123	687907	688029	TTGGATGACTTCTCCAATCTGA	AAATGAGTTGGACATTCGCC	220
Ad02A311	Aradu.A02	311	p2	(GA)7	14	692204	692217	TGCGATCAAAGAGACAATGG	TGTGCCCTCCTCTTCTCACT	201
Ad02A314	Aradu.A02	314	p2	(TA)6	12	706449	706460	TGGCTGATTCAAAAGCACAG	GGATCAGAAAATGGGCTTCA	122
Ad02A320	Aradu.A02	320	p2	(TA)6	12	724994	725005	ATAGAAGATTCGAAGCCGCA	TGAGTGGCCATCATCATACC	134
Ad02A321	Aradu.A02	321	c	(T)11aattcatac	68	725429	725496	ACAATCATGGATGAGCAGGA	TGGTCACAACTTGAATTAGCAA	257
Ad02A322	Aradu.A02	322	p3	(ATT)5	15	725643	725657	GAAAAGTTGGGATTTTCCAGG	AAAAGACGATCTTCTCAATTAAATAAA	228
Ad02A325	Aradu.A02	325	c	(GC)6ac(AT)7	83	728664	728746	TTTAAACTCTATTTTCTTTTCCGTTG	GGAAGGCCAAAGTTACTGAAGA	173
Ad02A329	Aradu.A02	329	p2	(CT)7	14	738578	738591	TGTGGATACTCCTCAGTGGCT	CATTACGCGGGTCTCACTCT	254
Ad02A333	Aradu.A02	333	p3	(AAT)20	60	756138	756197	GACACGTGTGTTGAGCTCCT	GGGTTTTTGCACTTGAAAGC	272
Ad02A338	Aradu.A02	338	p2	(AT)11	22	788442	788463	GCTTTTAGCTATGGTTCCTTTGA	ACACCACCCCATAATTGCAT	211
Ad02A339	Aradu.A02	339	c	(ATA)5gtaaaa	126	793773	793898	TCCTATACATTTGGATTTTAAGTTTGA	TTCGCAAGACAAACATCTTCA	275
Ad02A343	Aradu.A02	343	p3	(AAT)13	39	812930	812968	AACCGACTCGTCAAGTCTCG	TGCATCTCCAGGTTGATTGA	163
Ad02A349	Aradu.A02	349	c	(A)10cacccaat	40	828639	828678	TGAAGCCAATTGTCCAAACA	TCAATTCGCCTCCTGAGATT	224
Ad02A359	Aradu.A02	359	p3	(TTA)15	45	846396	846440	AAGTTGTCTCCAATTTATGTGTTGA	TGAAGAATAATTAATTTACGGTGTTTG	238
Ad02A366	Aradu.A02	366	p3	(CTT)5	15	864617	864631	CGGTGCAACCTGTAGTGATG	TTACCGACAGGGAGGATGAG	182
Ad02A370	Aradu.A02	370	p2	(TA)9	18	871608	871625	TGTCATTGGAAGTGCTGGAA	AGTCAACGTCTGGGATTTGG	216
Ad02A371	Aradu.A02	371	c	(A)10tgttttttctt	77	873458	873534	AACAACATGGCTTTACCGAA	TGAAAGCAATAGTGGAATACACG	197
Ad02A376	Aradu.A02	376	p2	(TA)8	16	882767	882782	CACCGGTTGTTATGTCGTTG	GGTAAAAGGTTGGGGACTGA	243
Ad02A377	Aradu.A02	377	p3	(ATC)5	15	885508	885522	TTGCAGCAAATTTCAGTGTAAC	AAAACAAGATGAAACGTGAGAAGA	219
Ad02A378	Aradu.A02	378	c	(CTT)8ttcatcat	76	885882	885957	TCTTCATCTTCCTCCTCCTCA	TTTGCAGCATTATGTGTTTCTTC	202
Ad02A383	Aradu.A02	383	p3	(AAC)7	21	905363	905383	TGGGGAATTACAAAATGGGA	AACATCCAGTCAGGAGGGTG	264
Ad02A384	Aradu.A02	384	p5	(ATACC)5	25	906986	907010	GGAAACTTTTGAAGAATGTGGC	AATGGATTTCATTCATGGCAA	266
Ad02A396	Aradu.A02	396	p3	(AAT)16	48	931075	931122	TCCAACATATCAAACAATAAACGAA	TCGCATGAAAATCACCTCAA	257
Ad02A400	Aradu.A02	400	p3	(TTG)6	18	955126	955143	GGGCTTTGGCAGTGTGTATT	ATTCACAGCATGAGTTGGCA	188

ID	Chromosome	SSR nr.	SSR type	SSR	Size	Start	End	FORWARD PRIMER (5'-3')	REVERSE PRIMER (5'-3')	Product (bp)
Ad02A407	Aradu.A02	407	p3	(CAG)5	15	976605	976619	TATACAGGTGCCCTCCCAAG	AACATTTCTGATGCAGCCAA	275
Ad02A409	Aradu.A02	409	c	(T)10aatttcactc	80	978690	978769	CAAGCTTCCCAAATGGCTT	GGCTGAACCAGAATCCTTCA	247
Ad02A412	Aradu.A02	412	p2	(TA)9	18	983712	983729	CATTGGTACCTTCGCATTCA	CGTAGGGTAAAAATTGAAACACG	216
Ad02A413	Aradu.A02	413	p2	(AT)11	22	994490	994511	AAACATAAAGCACATGCCACC	TTTGGTGTCTCATTTTCTGAGC	212
Ad02A420	Aradu.A02	420	p2	(TA)13	26	1003729	1003754	TGGTCAGTCAAAACATGTTCAA	TCCTGAGTCACGATTTTAATTTTT	187
Ad02A425	Aradu.A02	425	p3	(AAT)5	15	1010518	1010532	CTTTTATCCGACCCTCCTCC	CGTAATCTCACATCGCGAAC	260
Ad02A426	Aradu.A02	426	p2	(AT)7	14	1011535	1011548	TTCGTGTGAGAGGTAAACATAATCA	CGGTGAAAGGATAGAATGGAA	279
Ad02A427	Aradu.A02	427	p2	(GA)7	14	1012075	1012088	TGACAGTGCAGGAAGAATCG	GCTCTTGCAGTGGCAGTTCT	271
Ad02A432	Aradu.A02	432	p3	(ATT)6	18	1029401	1029418	CAGCTCACGCCAAACATAAA	ACCGTGAAGTTTTCAGCACC	275
Ad02A439	Aradu.A02	439	c	(A)13ctaaaaac	62	1043447	1043508	ATCAGGAAGCTGAAATCGGA	AAAATCAAATCGGACCCTCC	258
Ad02A440	Aradu.A02	440	p2	(AT)8	16	1045095	1045110	TGGGTTTGACCGGTTTGTAT	AAATCGCCACCAAAATCATC	213
Ad02A442	Aradu.A02	442	p3	(GAA)7	21	1051626	1051646	GTGGTGGAGAGTGGGTGAAC	GCAGGTCATATACCGTGCCT	241
Ad02A445	Aradu.A02	445	c	(AT)7aagggtct	35	1055754	1055788	CAAGTACATGCACCCATTGC	GGTGAAAACTCATGCGAAGTC	149
Ad02A448	Aradu.A02	448	p3	(ATA)18	54	1057857	1057910	CAAAGTAGGGTTCCTCATCCC	CATTGGAAATGCAGATGTGG	157
Ad02A450	Aradu.A02	450	p3	(AAT)7	21	1060748	1060768	CATTGGTCATGGGACACAAA	TGTGACGTTATGTAATTGGATGT	233
Ad02A453	Aradu.A02	453	p3	(AAT)12	36	1070533	1070568	GGTTTTTCTTACTTTTTGTTTCCTT	GACTTGTAAGACCCCAAGTTTTT	280
Ad02A456	Aradu.A02	456	p6	(GTTTTT)5	30	1075281	1075310	AGCCACCCTTTCTTCAACCT	TGTGAAAATTCTTCGAGACGG	244
Ad02A460	Aradu.A02	460	c	(T)10ccttcgtcc	59	1079019	1079077	GCCTTTTCCTTCTGATGTGC	TCACCTTCATCATCCAATTCA	210
Ad02A462	Aradu.A02	462	p2	(AT)8	16	1082465	1082480	GCGGGAAGTTATTGGATTTTT	TTTTTCTATTTCATGAGCCGTG	227
Ad02A472	Aradu.A02	472	p2	(GA)9	18	1107639	1107656	AAGGGTGTGTGAGAGATCCG	TGCATGCCATTGTTTTGATT	152
Ad02A475	Aradu.A02	475	c	(T)20gataacca	68	1109176	1109243	TGGATACCACAAACATGAGGG	AGAGAGATCCGAGGGTCAAA	219
Ad02A476	Aradu.A02	476	c	(A)10taatccata	89	1109395	1109483	TATGAGGGCCCGATTTGTTA	TTGCACCACCAAGTCAACAT	191
Ad02A477	Aradu.A02	477	c	(TTC)6tccaaag	105	1114386	1114490	TGGGGTGTGTCTGAGTGTGT	GATTCTGATCAGCGTGTACCAA	177
Ad02A485	Aradu.A02	485	c	(T)11caaaaaaa	82	1144140	1144221	TTTGACTTTAAAAACGCATGG	TCATCTTTTGATTTTGCTTCCTT	183
Ad02A488	Aradu.A02	488	c	(CAA)6taatcat	117	1149081	1149197	CAAAAGAAAGAGGGAAGGGG	TTCCGTGATTGCAATTTTGA	189
Ad02A489	Aradu.A02	489	p4	(TTAA)6	24	1150548	1150571	AAACTGCATGAAATCAAACATTG	TTTGGATGTTACAGGCCAATC	269
Ad02A496	Aradu.A02	496	c	(TTTTA)5ttact	58	1155595	1155652	ACTCCGATGAAGCCAAATGA	AAAATTGTCGGAGACGATGC	211
Ad02A503	Aradu.A02	503	p3	(AAT)5	15	1167758	1167772	CAAACTTGCACTATCCGCAA	GAAAAATAATTCCGATCAAGTATTCAA	126
Ad02A505	Aradu.A02	505	p2	(GA)7	14	1170809	1170822	TCTTGTTAGTTTGGCGCAGA	TCGATGGTGATGGAGAATGA	168
Ad02A515	Aradu.A02	515	p2	(CT)10	20	1209997	1210016	TTGTGGCAAGAACCTAACCA	TATTCTCCCCAGAACCACCA	273
Ad02A516	Aradu.A02	516	p2	(AT)8	16	1216125	1216140	TTCACGTGTCGACCTGTCTC	ACGCCTCTTGCCTTTTCATA	196
Ad02A523	Aradu.A02	523	p3	(ATA)9	27	1239414	1239440	TGCGATATGAATATGCCTGC	TGCAACATTTGACACATTGAAG	190
Ad02A530	Aradu.A02	530	p4	(CTAT)10	40	1246917	1246956	ACTACCACGCTGCTCCAGTT	TCAAAAATATGCCACATCAGC	254
Ad02A534	Aradu.A02	534	c	(T)10catttttttc(	31	1258729	1258759	ATTCTCCAGGTACACCCACG	ATGGAGTTGGAATTTGAGCG	122

ID	Chromosome	SSR nr.	SSR type	SSR	Size	Start	End	FORWARD PRIMER (5'-3')	REVERSE PRIMER (5'-3')	Product (bp)
Ad02A535	Aradu.A02	535	p2	(TC)6	12	1263359	1263370	TTGGACGCAAGATCAACTTT	AAGCAGGCATCACCAGTTTT	278
Ad02A537	Aradu.A02	537	p3	(ATT)5	15	1271328	1271342	CCACTCCAAAGCCTTCTCAG	GCCTCAAGAATCAATTCCCA	277
Ad02A540	Aradu.A02	540	p3	(AAG)8	24	1281507	1281530	CACCTGATTGATCTGTGAAGGA	CGGAACGGAAAACGAAGTAA	276
Ad02A542	Aradu.A02	542	p2	(GA)8	16	1286788	1286803	ACGGGATAACTGGATTGCTG	CACAAGGCTCGTCCTCTTTC	253
Ad02A545	Aradu.A02	545	p2	(AT)7	14	1291113	1291126	TGTGAAGTCCATAGCCACCA	TTTTTAATTACACGTTGCACTTTTT	137
Ad02A546	Aradu.A02	546	p2	(AT)7	14	1292238	1292251	GGAAACGAAGATAAAGCAAAATG	AGTCATCACGAAACCACAAAA	251
Ad02A548	Aradu.A02	548	p2	(TG)6	12	1301946	1301957	GCCTTCTTTGCCTCACTCAT	TCTGGCAGTTATTTGGGTGT	258
Ad02A549	Aradu.A02	549	c	(A)11gt(A)11	24	1317081	1317104	AACCAGAAAAATCGGGAACC	GGATTGGGGCCTTTAGAGAG	276
Ad02A550	Aradu.A02	550	p2	(TC)7	14	1317207	1317220	CTCTCTAAAGGCCCCAATCC	GGTGGTGGCTATTCTTGGAA	143
Ad02A553	Aradu.A02	553	p3	(TTA)5	15	1330312	1330326	TTGATATACGTAAATGGGATAACAAA	CGTTTTCCCTCCAAAATGAA	199
Ad02A557	Aradu.A02	557	p3	(TCC)5	15	1342358	1342372	TCGTCATCGTCATCGTCTTC	AGAAGAAAGCACGCGAAAAA	122
Ad02A558	Aradu.A02	558	c	(AAG)5atgcaa	98	1343914	1344011	CAGAAACTCGTGCATTCTATTCA	TTTCGCACTCCTGTTACTGC	280
Ad02A559	Aradu.A02	559	p2	(TA)11	22	1345204	1345225	CAGAAATTTTGGTCTTTTTAGTCAA	TGCATGAGTGACGTTTACACC	267
Ad02A562	Aradu.A02	562	p2	(TC)6	12	1347085	1347096	TGATATTTCCGGGTACACTTGA	ATGAGAGGATGCCAAAAACG	185
Ad02A563	Aradu.A02	563	p4	(TTTA)6	24	1350572	1350595	CATTTCAAGTTTTGCCATAGGA	CAAAATGCAACAAACATGGC	280
Ad02A565	Aradu.A02	565	p3	(ATA)11	33	1354369	1354401	GGTGCTCAAAAGTCCCACAT	TTTCCCTTTTAAACCAGGGC	252
Ad02A566	Aradu.A02	566	c*	(GTGA)5(GA)	68	1355431	1355498	GATCGAATAGAAACAGGGGG	GGGGGAAAAGGCAATTTATC	280
Ad02A569	Aradu.A02	569	p3	(TAA)5	15	1368955	1368969	TGATGGATTAAATTCTCGTCTGTC	AAAAGATTAAGTTTGAATAGGTCCAT	279
Ad02A573	Aradu.A02	573	c	(T)10c(T)12	23	1377429	1377451	TGCAACAACAAAAACAAACCA	GCGGATTGGATGGCTAAATA	247
Ad02A577	Aradu.A02	577	p3	(TTA)5	15	1383153	1383167	TTGATTCCTAGCAAAATTGTATTAAA	GTGACAATATTTAAAAAGTGCATAAAA	218
Ad02A579	Aradu.A02	579	p2	(AT)6	12	1387421	1387432	TTTAACTCCCCAGATTCCCC	CCATTGGACAGTCCCTTTGT	269
Ad02A580	Aradu.A02	580	p2	(TG)7	14	1392004	1392017	TTTGGGACCGGCTATGTATC	CCACGCCATTTCTTTAGTCA	257
Ad02A582	Aradu.A02	582	p3	(ATT)8	24	1394066	1394089	GATTGGGACAATGGAATTGG	TTGATGTTTGCAGATGGGAA	244
Ad02A583	Aradu.A02	583	p3	(AAT)15	45	1394277	1394321	TTCCCATCTGCAAACATCAA	AGTGAGAGACCGGAAAGGAT	134
Ad02A584	Aradu.A02	584	p3	(ATA)5	15	1396345	1396359	TTTTTAATGAAAGTCTTGGGTGC	TGGTTGCTGCAGTTGGTATC	161
Ad02A585	Aradu.A02	585	c	(A)11ccatgtga	33	1397884	1397916	TGAGGAATCGTATGAAGTTGAAA	CACACATGCCGTATTTGTTTCT	226
Ad02A587	Aradu.A02	587	p2	(CT)13	26	1405032	1405057	AACAAGGGCAGAATGCAAAG	CCGCCATTGATATCGTTGTT	278
Ad02A588	Aradu.A02	588	p3	(TAT)7	21	1405768	1405788	TGCAAAAACTTGGATGTTACAA	AACGGAGAGCAGGGGATATT	252
Ad02A589	Aradu.A02	589	c	(TA)9cacgttctt	74	1406539	1406612	AAGGGTTACGTTGGAGGGTT	AAAGCAGACAATCAAATCAGCA	244
Ad02A592	Aradu.A02	592	p2	(TA)6	12	1423857	1423868	TCCCTTCTGTTTTTGCCATC	GGTTCCCATCTTTGAAGCAA	236
Ad02A596	Aradu.A02	596	p2	(AT)10	20	1426768	1426787	CCAATTTTCGGGAGATGAGA	AAGTGTTGGAGGGAAAAGGAA	198
Ad02A597	Aradu.A02	597	p2	(CT)6	12	1433453	1433464	CACATTGATTGGTGTCCGAG	GTTCTCCCAGCAAACCACAT	100
Ad02A598	Aradu.A02	598	c	(T)10cactcattg	99	1433738	1433836	TTGAATGAATGCCTGAACAGA	GCCTTGGATCCTTTTTACCC	247
Ad02A599	Aradu.A02	599	p2	(TA)12	24	1434563	1434586	TCCCTTCTGTTTTTGCCATC	GCTTCCCAACACATTAATCCA	131

ID	Chromosome	SSR nr.	SSR type	SSR	Size	Start	End	FORWARD PRIMER (5'-3')	REVERSE PRIMER (5'-3')	Product (bp)
Ad02A601	Aradu.A02	601	p3	(TTA)5	15	1438147	1438161	GCATTTGGGTGTAGAGTTCCA	CCAAACGCACAAAATAGCAA	225
Ad02A603	Aradu.A02	603	p3	(CAA)5	15	1449362	1449376	AGTCCAATTTCGACCCATCA	TCTGTGGTGGTGGTGTTGTT	204
Ad02A606	Aradu.A02	606	p2	(GA)6	12	1455816	1455827	TCATCGTCATCAGATCCGTC	CGGTGACGAGTCTCCTTCTC	213
Ad02A610	Aradu.A02	610	p2	(GA)6	12	1464598	1464609	CATTTTGCAGTGCCATCATC	CCCCTCTGTTCGTGTATGGT	246
Ad02A613	Aradu.A02	613	p2	(TA)9	18	1469456	1469473	AAGCTAAAATTCACTGACCGGTAT	AAATCGACTTCACCTAAGTTTTTACC	237
Ad02A624	Aradu.A02	624	c	(ATA)7ttccaaa	48	1484127	1484174	ACGTGGCAAACACACATCAT	ATAAGATCGAGGGTTGGGGT	258
Ad02A625	Aradu.A02	625	c	(TTA)6acactaa	82	1484425	1484506	ATCCAATTCCCCAACATTCA	AAGAACAGCCATGGTAACGG	261
Ad02A626	Aradu.A02	626	p3	(GGT)9	27	1485229	1485255	ACCTTTCAACAGGTCCAACG	CACGTGAAATCGTTCCCTTT	192
Ad02A632	Aradu.A02	632	p2	(TA)6	12	1493727	1493738	GGAAAGGTACCCAACAATATTCC	TCTCTCTCTCCTGGTGGGTG	115
Ad02A633	Aradu.A02	633	p3	(CTC)6	18	1498584	1498601	AGAGGGTCCAGGAGGCTAAA	TGAGAGGGAGCTGAAGAGGA	133
Ad02A637	Aradu.A02	637	p3	(ATA)12	36	1511429	1511464	GCGTAGTGCCTATGACTTAGTGG	AAAAATGATACTCGAATTCAAAACTT	213
Ad02A638	Aradu.A02	638	c	(A)10gaatgcct	75	1511578	1511652	AAAGTTTTGAATTCGAGTATCATTTTT	TCCGCATTATGAGGTCAGAT	244
Ad02A643	Aradu.A02	643	p3	(TAA)5	15	1520946	1520960	CATTAACACAGGGTTTGGACC	CACACGAAAATCAGCAGCAC	195
Ad02A644	Aradu.A02	644	p3	(AAT)18	54	1521645	1521698	GGAGGTAGATGCACCCAAGA	AGTCGATCCATCCCACCTAA	219
Ad02A645	Aradu.A02	645	p3	(AAT)7	21	1522158	1522178	GAGACCCAATTGAAAGGAAAAA	TGGTTACCTCATCTCAGAGCAA	216
Ad02A648	Aradu.A02	648	p2	(AT)6	12	1537314	1537325	TGACAATTGCCCCACACTAA	TTCATGAAATTCGTAATCTTTGTT	233
Ad02A650	Aradu.A02	650	p2	(AT)6	12	1545022	1545033	GCTACACATTTAGAGGTGGCAA	GCGAATATCTGTTCGCCTCT	270
Ad02A652	Aradu.A02	652	p2	(AT)11	22	1552574	1552595	CTCTACATCCGCGTATCCGA	TGCGCATAATTAAACCTTCTCTT	277
Ad02A655	Aradu.A02	655	p3	(TTG)5	15	1554263	1554277	CGAGGTCCCTGTAGTGCTGT	AAGCAGAAGCAGAAGCAACC	183
Ad02A656	Aradu.A02	656	p2	(TA)6	12	1556866	1556877	GCATTTGGTGTCAAGCACTG	TGAAATTCAAACTCAAATCCTTTTT	241
Ad02A660	Aradu.A02	660	p3	(TTG)6	18	1571530	1571547	AACATCCAGTCAGAGGGTGG	TTTGAGCAAGTTGAGCCTGA	247
Ad02A661	Aradu.A02	661	c	(T)11cgtatacta	69	1572500	1572568	CCAAAGGCTACGCTTTTCAG	TGGTTTTGATATGGCTATGCTT	272
Ad02A666	Aradu.A02	666	c	(TC)7tactcgca	120	1611059	1611178	CCACGGCATCACATACTCAC	CCTAGGATTGGGAATTGTTCA	181
Ad02A667	Aradu.A02	667	p2	(AC)6	12	1616178	1616189	CAACTCTTGATCCAAGCCCT	TCATGACCCCATCTTATTGTGA	266
Ad02A668	Aradu.A02	668	c	(A)10ttaatcaat	80	1616375	1616454	TTCTCACAATAAGATGGGGTCA	TTTGAGCCTTGAATGTGGTAA	265
Ad02A670	Aradu.A02	670	p2	(AT)6	12	1618913	1618924	CAAACTACAAAGTTGCATTGGG	ATTGGTTTCCATGATCTGCG	219
Ad02A674	Aradu.A02	674	p2	(AT)15	30	1634832	1634861	ATGTGACCGGGCACTTTAAC	ACCATTGGACAGTCCCTCTG	247
Ad02A675	Aradu.A02	675	p2	(TA)6	12	1635755	1635766	TCGACTTGGAGATACAGGGG	CTCAACCTGCGAAGCTAAGG	103
Ad02A681	Aradu.A02	681	p2	(AG)6	12	1653988	1653999	GAAGCCTCCACACTTGAAGC	CATCATGAAGCATCAATGGC	214
Ad02A688	Aradu.A02	688	p2	(TA)7	14	1672428	1672441	CCCTTACCTTCTCTCTGTCTCG	TCAGATAAATTGATTTGGTCAAAAA	185
Ad02A692	Aradu.A02	692	c	(T)10gtagtgtgt	87	1686270	1686356	TAAATCCGCCGCAAGTTATT	GAGAATTCGATTTGCTAGTGACAA	272
Ad02A694	Aradu.A02	694	p3	(ATA)18	54	1689752	1689805	AACACGGCATTTTTATTGAGATT	AAAAGATGCGTTTCATCAAAAAG	175
Ad02A706	Aradu.A02	706	p3	(AAT)5	15	1738687	1738701	ACTGCGTTCGTCCTGATTCT	TTTATCGCTATGTTGGCACC	253
Ad02A707	Aradu.A02	707	c	(TCTTCC)5tct	60	1739352	1739411	TCAGTTTCCAAAGCGCTACA	TCAGAAACCATGAAAATCGAAA	256

ID	Chromosome	SSR nr.	SSR type	SSR	Size	Start	End	FORWARD PRIMER (5'-3')	REVERSE PRIMER (5'-3')	Product (bp)
Ad02A708	Aradu.A02	708	p3	(AAG)5	15	1740177	1740191	ACCCAAAGGACACCTTATGC	TTGCTGCACATTCTTCTTCC	237
Ad02A709	Aradu.A02	709	p3	(AAG)8	24	1740297	1740320	GGAAGAAGAATGTGCAGCAA	TTAACGCGCTTGAACGTTACT	147
Ad02A711	Aradu.A02	711	p2	(AT)8	16	1743219	1743234	CGTGGACAGCATCACTCTTC	TTGTACTCTGCTACCTCTCCTGC	264
Ad02A712	Aradu.A02	712	c	(TGT)6(CGT)'	39	1743598	1743636	CGAAACAGAAGCAGCAGACA	GGCATGCCTCTTACTCAGGA	274
Ad02A714	Aradu.A02	714	p3	(AAT)6	18	1759936	1759953	GTCGAATAATGTGAAGCGCA	CCTTTGACTTGGGTGCAAAT	168
Ad02A715	Aradu.A02	715	p2	(TG)6	12	1760645	1760656	ACTGCAGAGAGGTTGGATGG	GGATGGGAACGAGAGACAAA	251
Ad02A716	Aradu.A02	716	p2	(TC)8	16	1766521	1766536	TCATGGAAGAAGAGGGGAAA	AAGAGACAATTGTGTGTGTGTAAGC	208
Ad02A718	Aradu.A02	718	p3	(ATT)5	15	1768256	1768270	TTATTGTCGCACGTAACCCA	GGTTTTCTACCTAAAGATGCCC	279
Ad02A719	Aradu.A02	719	p2	(AT)6	12	1769230	1769241	CCAACAACCAATCAAACTAAAACA	TGCTTGTGTATGGTGTAGCG	270
Ad02A720	Aradu.A02	720	p4	(AAAG)5	20	1776086	1776105	ATCTGCCAAGCCAATGTTTC	CTCTGCTCCCTCAACTCCAC	275
Ad02A721	Aradu.A02	721	p2	(GA)6	12	1776221	1776232	ATCTGCCAAGCCAATGTTTC	CTCTGCTCCCTCAACTCCAC	275
Ad02A722	Aradu.A02	722	p3	(ATC)19	57	1781398	1781454	TTTTTAACTTCCTACCCGCTTT	AAAAACAAAATAGCACGGCG	268
Ad02A724	Aradu.A02	724	p3	(TCC)11	33	1796015	1796047	ACCACTGCTTCAGACGGTTC	CACCAAGCTGCTGCATGTAT	163
Ad02A725	Aradu.A02	725	c	(A)13tagtaaga	121	1796747	1796867	CGTTCAGATTTCTGTCACGGT	TTTGTTAAGTTTGGTTGGGGA	172
Ad02A728	Aradu.A02	728	p2	(TA)8	16	1817603	1817618	AAGGTCTGTGATTTTTGTTCAGG	AGGATGTGATGCTTTTTCCC	248
Ad02A731	Aradu.A02	731	p2	(TA)6	12	1822195	1822206	CCAAGCACCCACGTCTTTTA	TGCAATGCAGAGTTTGGTATG	278
Ad02A739	Aradu.A02	739	p2	(AT)10	20	1834113	1834132	TTGATTTGACCGAAAAATGATG	TGGTGAATTAAATTTTGAACTGATCT	235
Ad02A740	Aradu.A02	740	c	(AAC)9(AAT)	42	1836094	1836135	GGCATGCCTCTTACTCAGGA	TTGAAGCAGAAGCTAAGCAGC	279
Ad02A741	Aradu.A02	741	p3	(TAT)7	21	1837697	1837717	CATTTTTGTACTTGCAGGTTGAC	TTTTTGGTGATTGTTTGCGT	144
Ad02A744	Aradu.A02	744	p2	(AT)6	12	1847283	1847294	CTCTGACTTGACGTGTCCCC	CCTTGCACTGTTACAGCATCA	143
Ad02A746	Aradu.A02	746	p3	(TCA)8	24	1861771	1861794	TGCTGATGACTTTCTGGACG	TCAGACATGTGGTTTCTCGC	242
Ad02A748	Aradu.A02	748	p2	(TA)7	14	1876069	1876082	GGTTGGATAGTCCTACACGGA	AATGGAATCAAATGGCCAAA	186
Ad02A749	Aradu.A02	749	p3	(AAG)5	15	1881326	1881340	TGACATGCCTCACAGACCTT	CTAGCTGAGCTGGCGAGTTT	251
Ad02A758	Aradu.A02	758	c	(CAC)8ttcaacc	74	1905797	1905870	GCCAAACACACCACATTTCA	GGCTCCAATCCCAAACACTA	203
Ad02A763	Aradu.A02	763	p3	(GAT)5	15	1916078	1916092	TCAGACATGTGGTTTCTCGC	TGCTGATGACTTTCTGGACG	242
Ad02A765	Aradu.A02	765	c	(A)10cataggttt	80	1921446	1921525	ATGCGCACAAACTCTAAGCA	TGCAACAGCTTTCATAATCCC	177
Ad02A771	Aradu.A02	771	p3	(GAT)5	15	1953773	1953787	TGTTGATCATCCTCCCTTCC	TGATGACTTTCTGGACGCTG	276
Ad02A777	Aradu.A02	777	p2	(TA)7	14	1982113	1982126	AAGCAAACGAGGAAAGAAAAGA	CTTTGAGGCTTCATCATCCA	275
Ad02A778	Aradu.A02	778	p2	(AT)8	16	1994680	1994695	GCTTCTGGGACCAGTTTCAA	CGTGACATATGCCAGCAAAG	234
Ad02A780	Aradu.A02	780	p3	(ATC)6	18	1998779	1998796	CAGAAACTAATCTGTCCAAAGCG	AATTTCGCCAACCAACTCAA	231
Ad02A781	Aradu.A02	781	p3	(TAA)19	57	1999201	1999257	CCCCAACACTTAGCATCCAA	GCTGGTTCCAAAAATTTCTCA	268
Ad02A784	Aradu.A02	784	p4	(CTAA)6	24	2007144	2007167	ATTTTGGCACCCAATCAAAA	CAAGAGTAAGACGGGCTGCT	280
Ad02A795	Aradu.A02	795	p2	(TA)6	12	2044278	2044289	TGCCACTTGATACTGAGAAAATG	ATTGGCAGAAGTTGGCAGAT	185
Ad02A800	Aradu.A02	800	p3	(TAA)5	15	2052780	2052794	TCATGGCATGTTCATCCAAC	TTGGAGATGTGACAGGAGCA	275

ID	Chromosome	SSR nr.	SSR type	SSR	Size	Start	End	FORWARD PRIMER (5'-3')	REVERSE PRIMER (5'-3')	Product (bp)
Ad02A803	Aradu.A02	803	p3	(AAC)5	15	2065368	2065382	CATAGTAGGCATGCAAGGCA	ACATGACAGAGCACAATGGC	263
Ad02A811	Aradu.A02	811	p2	(AT)7	14	2107166	2107179	GACAGGATCCTCGTGTGTGTT	CGGACAGAACCTCCTCTGTT	264
Ad02A814	Aradu.A02	814	p2	(CT)6	12	2136515	2136526	GAGACATTGGACTGCAACGA	ATGTGGGAAATTTTGGGTCA	197
Ad02A818	Aradu.A02	818	p3	(TAT)18	54	2155639	2155692	TCAAAAATTGACACTTCACTTCA	TTCTCGTGAACCCATTTTCC	275
Ad02A819	Aradu.A02	819	p3	(ATT)6	18	2156463	2156480	CGATTGTATTATCCTTCAAGCCA	TGGACGATTGTAGAAGCAACA	197
Ad02A821	Aradu.A02	821	p2	(TC)8	16	2198001	2198016	GCAAGTAAGCTGAACTGGCA	GCCATGGATCAGAACCTGAA	272
Ad02A822	Aradu.A02	822	p3	(AAT)6	18	2205396	2205413	CAGGAACAGGGATAAAGGCA	TTGGATGTTGGCTTTGTGAA	204
Ad02A823	Aradu.A02	823	p3	(TCT)5	15	2211408	2211422	TTCACCCATATAGCCGAACC	GAAGCTTTTGCCTTCCCTTT	208
Ad02A828	Aradu.A02	828	c	(AC)16(AT)7	46	2219012	2219057	CACACTCGCATGGATATTCG	TCGACTTGGAGATACAGGGG	235
Ad02A829	Aradu.A02	829	p2	(TA)8	16	2220617	2220632	CAAGAAATCGAAGCCGAAAG	AGGATGGAAATCAAAGACGG	266
Ad02A830	Aradu.A02	830	p3	(TAA)19	57	2220760	2220816	CCGTCTTTGATTTCCATCCT	CTGGAATGTAGCAACCCTCA	278
Ad02A835	Aradu.A02	835	p2	(CT)6	12	2243292	2243303	CAACAAAGGGAAGTGGGAAA	TAGCTTCCATGGCTTCCTGT	151
Ad02A836	Aradu.A02	836	p2	(CT)6	12	2252004	2252015	CAACAAAGGGAAGTGGGAAA	AAGATGGATTTCTGGCCCTT	236
Ad02A839	Aradu.A02	839	p2	(GA)7	14	2261974	2261987	TAGCCCCTTTTTGCTTGCTA	TGCACTTAGCAGCTGAACCA	211
Ad02A840	Aradu.A02	840	p2	(GA)7	14	2271280	2271293	TGCTTGCTAACACTTGCTGG	TGCACTTAGCAGCTGAACCA	200
Ad02A843	Aradu.A02	843	p2	(AC)9	18	2290047	2290064	CTGCAGCTTCATTTGCTCAG	CAGCCCTTCAAGAACCAAAG	270
Ad02A848	Aradu.A02	848	c	(AT)6aaaactatt	55	2313683	2313737	AGGGTTTTGAGGAGGCACTT	TTTTTCCACCAAATATATGTCCC	194
Ad02A850	Aradu.A02	850	p3	(ATG)5	15	2333062	2333076	GATTAGCCAGCCAGCAAGAC	GGCCTACCATCCAGCTATCTT	109
Ad02A851	Aradu.A02	851	p2	(AG)9	18	2335909	2335926	TGCAGAGTTTTGTGTCTGCAT	TCGTGTACCGTGGTGTTCTC	268
Ad02A852	Aradu.A02	852	c	(TCC)9(GCC)	45	2336136	2336180	GATACGCTTCCACTCCCAAA	CCACAAGCCTCTCACTCCTC	255
Ad02A853	Aradu.A02	853	p2	(AG)13	26	2336564	2336589	GAATGCTGCGTCGTAGACTG	TGGCGAATTCGAGTCTCTCT	275
Ad02A858	Aradu.A02	858	p4	(TAAA)5	20	2342610	2342629	GCCTATGCCACTACTGAGCTG	CGCTCAGGGAGAAAATTGAG	211
Ad02A860	Aradu.A02	860	c	(AGA)5ggagg(	35	2345574	2345608	AAGCTTCCCACAAGTCATTCA	TGGAAGCTGTTGTTGGTGAG	229
Ad02A863	Aradu.A02	863	c	(ATT)7g(TTA	51	2350013	2350063	TGGTTTTAACTTTGAATTTCTTGTAAA	CAATCCCAACGTTTCTCTCTG	249
Ad02A864	Aradu.A02	864	c	(A)10g(A)10	21	2360844	2360864	TGGCTCTTGAAAGAATGAGGA	TCCCCAATTAGAGGTGTCCA	192
Ad02A865	Aradu.A02	865	p3	(GAG)6	18	2368754	2368771	CCTCACCAGAAGAGCCAGAC	AATGGCGAAGACAAATGCTC	245
Ad02A866	Aradu.A02	866	c	(TCTTCC)6tct	96	2376195	2376290	TGCATATCTACGTATCCGCAA	GAACTCAGATTCGCAGTATTCG	264
Ad02A867	Aradu.A02	867	c	(AAG)5aggaag	55	2377185	2377239	AAAGGAAGAGGACGAAGAACG	AAATCAAATTACGCGCTGCT	237
Ad02A873	Aradu.A02	873	c	(CTT)5gctgcac	94	2386283	2386376	TGGTTTGTTCTTCGTCGTTG	GAAGGAGAAAGAGAAGGCAAGA	191
Ad02A877	Aradu.A02	877	p3	(GAA)6	18	2398441	2398458	CAGCAGCTCATTGAAAACGA	ATACTTCAGCCATGGCGTTC	163
Ad02A882	Aradu.A02	882	p3	(ATA)6	18	2410715	2410732	CCCTTTAAACCATTGTTTTGG	CAAGGCTTTTCAAGGGAAGTC	226
Ad02A884	Aradu.A02	884	p2	(GA)7	14	2413973	2413986	GGAATTGAATCGAGAGCGAA	GAACACGCGTCTCTTCCTTC	260
Ad02A885	Aradu.A02	885	p2	(CT)10	20	2414215	2414234	GGGGTGTGATCGGAGAAGT	TTTGAAGTTGAGCACAAGAAACA	109
Ad02A889	Aradu.A02	889	p2	(AT)6	12	2433820	2433831	TTATGATCAGGGGTGAAGCC	ATTTCCATTTCTTCGCCGAC	278

ID	Chromosome	SSR nr.	SSR type	SSR	Size	Start	End	FORWARD PRIMER (5'-3')	REVERSE PRIMER (5'-3')	Product (bp)
Ad02A890	Aradu.A02	890	p2	(AT)8	16	2434819	2434834	CATATGGTAAAGTAATAAACACCAACA	TCGACTATTTTAATTCTTAACCTTTGA	224
Ad02A892	Aradu.A02	892	c	(A)11ctgggtag	104	2436230	2436333	TGGGCCATACAATAAGGGAA	CCAAGTTATTCGTCAATGTGAAA	234
Ad02A893	Aradu.A02	893	c	(TA)6tgagaaca	37	2436436	2436472	CAAATTTAATATTTTCACATTGACGAA	GCTGCTTCTTATTATTATAGGGCTTC	266
Ad02A894	Aradu.A02	894	p2	(TA)6	12	2438136	2438147	TTTCTGTCAACATCTGTCAACTTTT	CAGATTTGGTGTTGTGAAAATCA	278
Ad02A896	Aradu.A02	896	c	(A)10caaaatca	123	2441670	2441792	GGACAACCCAATACTCCTCTCA	TCATCATCATGTGGTTCATCA	273
Ad02A898	Aradu.A02	898	p3	(ATT)6	18	2443733	2443750	TGTTGACTAATGAAACATGCTCG	GGTTATGATGTGTCAACCCTAAAA	240
Ad02A899	Aradu.A02	899	p3	(ATA)5	15	2444190	2444204	TCAAACGTGGCACCATAGAA	TCCAGCATATAGACCGGTACG	178
Ad02A901	Aradu.A02	901	p2	(AT)11	22	2445706	2445727	ATGGAAGCCTTGGGATTCTT	ATGGGTCTTGCTGAAAACCA	273
Ad02A902	Aradu.A02	902	p2	(TG)6	12	2446159	2446170	CTCCATGTTTCTCATTTGTTGC	TTCCATTTTCCTTACACTCTTGC	264
Ad02A903	Aradu.A02	903	p2	(TA)6	12	2449987	2449998	AACCACCATGAGAAGCATGA	TGAAAATTAATGCATGAAAATGG	246
Ad02A904	Aradu.A02	904	p3	(TAA)16	48	2450830	2450877	TCCTAAATTGGTTTTACTTTGTTTGA	CGATTCCGACCCTCAATATG	240
Ad02A905	Aradu.A02	905	p3	(GAA)5	15	2451846	2451860	CCGTTGTTGCTCTTGGAGAT	CACACAACCATCACACACCA	265
Ad02A907	Aradu.A02	907	p2	(AT)6	12	2452706	2452717	TGAATTGACCAACTTTTGTTCC	GCTTTTGAGACAAAATTAAAATAAAAC	185
Ad02A913	Aradu.A02	913	p2	(AC)7	14	2468394	2468407	TTGCATGTTGCTTGATTGTTT	TGTGGACAAACATGAGTGGG	268
Ad02A917	Aradu.A02	917	p4	(TAAA)6	24	2473193	2473216	AACGCAGCCTATATGTATGTGG	TCTTCGTCTTTCGCAACTCA	259
Ad02A918	Aradu.A02	918	p2	(AT)8	16	2473439	2473454	TGAGTTGCGAAAGACGAAGA	TTGTCCCCTAGCATTCTCCT	169
Ad02A919	Aradu.A02	919	p2	(GA)14	28	2474585	2474612	TTTCACAATGTCAACCCCCT	AATTTGGCATCTATGTGGGC	103
Ad02A920	Aradu.A02	920	p2	(TC)17	34	2478690	2478723	TGTGAAAATTGAAAAACTGTCCA	TGGAAGAGGTGGAGGAAATG	270
Ad02A921	Aradu.A02	921	p2	(TA)6	12	2480443	2480454	TTGCCTTGTTAATGGGAAGG	ACCAAAAGGCTGTTGAAACG	131
Ad02A925	Aradu.A02	925	p3	(AAG)5	15	2489559	2489573	CTAATGCAAGACATTGGGGG	TTCCCAAAATCAAACAACCC	213
Ad02A927	Aradu.A02	927	p3	(TAT)14	42	2490913	2490954	GGGCTAGTGGTAGATGTGCAA	AAAAGCATCCAACACCTCCA	209
Ad02A928	Aradu.A02	928	c	(CTT)6ctcccgc	182	2491482	2491663	AAAGACAAATTGACACCGCC	CCAAAAAATTTCACAAACAAATCA	254
Ad02A929	Aradu.A02	929	c	(TGA)9taatgtg	124	2491777	2491900	TGATTTGTTTGTGAAATTTTTGG	GTGTTTTCTCCCACCACCAC	262
Ad02A932	Aradu.A02	932	c	(TG)7(T)11	25	2498319	2498343	GCATAGCCTGGGACACAGTT	CCATGTATATCGCGCTTAGGA	263
Ad02A941	Aradu.A02	941	p3	(CAC)6	18	2534016	2534033	ATTCCCATCTTTCCATTCCC	TGGTGGCAAGAAGAACAATG	170
Ad02A942	Aradu.A02	942	p3	(TAA)14	42	2534543	2534584	CCGATGGAGAAAATATGGACA	CGACACGGAGACATGACAAC	276
Ad02A943	Aradu.A02	943	p4	(ATAA)5	20	2536800	2536819	TGGTGGATTTCGTTTTTCTTG	CCCCAATGAATTATGTTTCCA	236
Ad02A948	Aradu.A02	948	c	(T)10cagtgatca	88	2540157	2540244	CCCGCATCTCCAACTTAAAA	TCCACTAAGGTTGAAATGACGA	198
Ad02A951	Aradu.A02	951	p2	(TA)9	18	2547567	2547584	CATGTCCCTGTCCTAGGCTT	GATATCGTGCCACGTGCTTA	200
Ad02A955	Aradu.A02	955	p3	(CTC)8	24	2557054	2557077	TGCATCATCTAACGCTTCCA	TGTGGAGAGGAGGAAAAGGA	271
Ad02A958	Aradu.A02	958	p4	(TTAA)5	20	2560627	2560646	GAAACTAACTTTAGTCCCTATTGTGG	TCACAATGCTTGTCGTCAACT	264
Ad02A963	Aradu.A02	963	c	(TC)10tatctatc	53	2569919	2569971	CGGATTCCGAGAAAAACGTA	TCAGAATCAGCTCAAATCACAGA	211
Ad02A967	Aradu.A02	967	p2	(AG)12	24	2579663	2579686	CGCGTTCATTCATTCACATT	CTTAGCATCTACGCCGGAAG	235
Ad02A968	Aradu.A02	968	p3	(TCG)5	15	2579845	2579859	CCTCGGAAAGAGACGTATGC	TGAACAAGATCGCACTCGAC	189

ID	Chromosome	SSR nr.	SSR type	SSR	Size	Start	End	FORWARD PRIMER (5'-3')	REVERSE PRIMER (5'-3')	Product (bp)
Ad02A969	Aradu.A02	969	c	(T)10caaataaat	62	2580234	2580295	TGTGATGATTGTGGAATCGAA	CACACAACGTGGCTCAAAAT	280
Ad02A974	Aradu.A02	974	p3	(TGA)5	15	2595216	2595230	TCCTCAACGGATTGGAAGAG	AACCCCATTTCTCAAAACCC	228
Ad02A979	Aradu.A02	979	p2	(AT)9	18	2610202	2610219	GCACTGGCCAACACCTAATC	GGTGGGTCACCGGTAATATG	231
Ad02A981	Aradu.A02	981	p3	(TTC)5	15	2618113	2618127	TAGCGTGTGCATCCATCTTT	TGGGGTGTTAAGCAATAGCC	212
Ad02A990	Aradu.A02	990	p3	(ATA)6	18	2639316	2639333	TGGTATCTTTTATTTTAAAGGAGTTGA	CAAAGCGACATAATGAAAGTGTG	264
Ad02A991	Aradu.A02	991	c	(T)10aatatggat	100	2639579	2639678	CACACTTTCATTATGTCGCTTTG	CCCTCAGATTTGGGGAGTAA	260
Ad02A993	Aradu.A02	993	p3	(GAA)5	15	2641093	2641107	CGATCTAATCGTCGCACTCA	TCATCCACCGCTTAATCTCC	239
Ad02A1002	Aradu.A02	1002	p2	(AT)7	14	2691296	2691309	GAGCATTGCAGTTCTGGACA	GCTCCTTCCAATCATTCAGC	275
Ad02A1005	Aradu.A02	1005	p3	(CAC)5	15	2704703	2704717	CTGGAAAACCGTAGCTGCTC	GGAACAACCTGTGATGCTGA	245
Ad02A1006	Aradu.A02	1006	p3	(CAC)5	15	2709821	2709835	CCCATTGGAACACAACCTTC	GGAACAACCTGTGATGCTGA	104
Ad02A1013	Aradu.A02	1013	p3	(CAC)5	15	2722321	2722335	CCCATTGGAACACAACCTTC	GGAACAACCTGTGATGCTGA	104
Ad02A1016	Aradu.A02	1016	p3	(TAA)19	57	2725395	2725451	TTCCACCAATCATTATTCGTTTT	TTTGGTGACGTGCTTCTTTTT	141
Ad02A1017	Aradu.A02	1017	p2	(TC)9	18	2728514	2728531	CAGGACTCGGACAGAAGCTC	CTTGCTGTCGTCCATAGCC	262
Ad02A1018	Aradu.A02	1018	p3	(TAA)12	36	2728958	2728993	CCCTTCCTTCTTCTCCGTTC	GCCTTCTTGCACCATTGATT	242
Ad02A1022	Aradu.A02	1022	p2	(AT)6	12	2732260	2732271	TCAAGGATTATGGATTGCACC	GAGATTAGTTCCGATTCGACG	280
Ad02A1027	Aradu.A02	1027	p3	(CAC)5	15	2754664	2754678	CTGGAAAACCGTAGCTGCTC	AGCAACCTGTGATGCTGATG	243
Ad02A1029	Aradu.A02	1029	p2	(TA)8	16	2774539	2774554	TCTTTGCTACCTCATTGGTTTC	TTAACTAAGAGGAAGTGCCAAAAA	280
Ad02A1035	Aradu.A02	1035	p2	(TG)6	12	2789930	2789941	CCAACATTGTGTTCGTCAGG	CTGATGAACTTTGGACTGAACG	163
Ad02A1038	Aradu.A02	1038	p3	(TTA)6	18	2795987	2796004	TGAGGTTTGCATGATTGCAC	CGAATGGTGAGGAGAATGGT	257
Ad02A1041	Aradu.A02	1041	c	(ACA)5tactcca	74	2806000	2806073	GCCAAACGATCAAATTCTCAG	TGAATCTTGAACTGTTTAGGTAGTGA	266
Ad02A1046	Aradu.A02	1046	p2	(TG)19	38	2826491	2826528	TTCGATTCCGACCCTGTATC	GCTCATTGATCATTGCACAAG	243
Ad02A1048	Aradu.A02	1048	p3	(ATA)5	15	2829974	2829988	CACAGGTTGGCTAGGACCAT	ATTTTTGCTTACATCATTGACACT	275
Ad02A1049	Aradu.A02	1049	p2	(CT)7	14	2831612	2831625	TCCCTTCAACTAATCAACTTAATCC	GCCTTGTGCATAACCAAAAA	274
Ad02A1050	Aradu.A02	1050	p3	(AAT)5	15	2837091	2837105	TACTTGCATCCGAACCATCA	CTTCGAACCGGAAAATTTGA	205
Ad02A1051	Aradu.A02	1051	c	(ATT)5atcatg(	36	2839207	2839242	GGCAATGACTTTTGAAGGGT	TTCCCATAGCCATGTCCATTA	195
Ad02A1058	Aradu.A02	1058	p2	(AT)6	12	2845126	2845137	TGCAAGCATCTCTTTTGTTCTT	AGTTCTGTTTGGGAGCCTCA	187
Ad02A1059	Aradu.A02	1059	p2	(GA)7	14	2846488	2846501	GAACTGAAGTGGTGCGACAA	TTCGGATCCACTGTACCCTC	143
Ad02A1064	Aradu.A02	1064	p3	(GAA)6	18	2851823	2851840	CCATTGCTTCCATTATTTCCA	TTTGTTTCCATTTTTCAGCAA	109
Ad02A1065	Aradu.A02	1065	p3	(TAT)9	27	2852488	2852514	CCATTGAATGAATGTGAGAGTTT	TCTCTTGGGAAGAAACTATGTTG	280
Ad02A1066	Aradu.A02	1066	p4	(TTTA)5	20	2852820	2852839	CCCAAAAGTACATGCCTTCA	AAGGCCAATTACACACGTCA	262
Ad02A1068	Aradu.A02	1068	c	(T)10cccactag	74	2855506	2855579	CATCTAACTTGGTGCTGGGG	CATTTTTGGGAAACAACAGG	169
Ad02A1071	Aradu.A02	1071	p3	(GCG)5	15	2859214	2859228	CTTTACCCTCTGCAGCAACC	ATTGACGATGACTCGCTCCT	261
Ad02A1075	Aradu.A02	1075	p4	(TTTA)5	20	2876894	2876913	TCTGCCCTCCTTTTCCTACA	TGCTGGCTCTTGAAAGAATG	232
Ad02A1076	Aradu.A02	1076	p3	(AGA)5	15	2878641	2878655	CTTGATGCGATTTGGTGTTG	CAAGCCCTCTCTCTCTCCCT	255

ID	Chromosome	SSR nr.	SSR type	SSR	Size	Start	End	FORWARD PRIMER (5'-3')	REVERSE PRIMER (5'-3')	Product (bp)
Ad02A1077	Aradu.A02	1077	p2	(AG)6	12	2886510	2886521	TGAAGCAAGAAAAAGCAGTGAA	CCCAAAGCACTCTGTCTTCC	280
Ad02A1079	Aradu.A02	1079	p3	(AAG)5	15	2897752	2897766	TTGGATTTGATCTTTTTACCCC	ATGGACACGGAATTTGGAAG	252
Ad02A1080	Aradu.A02	1080	c	(CT)10gtgatt((	44	2901225	2901268	GGCTGAATCGGCCTAACATA	GAATCCCAAGCTCGACCATA	226
Ad02A1084	Aradu.A02	1084	p5	(TTATT)5	25	2911413	2911437	AATAAGACTGACACGTGATGATATTT	TTACCCAAATCAATCCCCAA	176
Ad02A1086	Aradu.A02	1086	p3	(TAA)25	75	2917628	2917702	CAGCCGTTTGATCAGAGATG	TCCTACATCTACCAACGGCA	276
Ad02A1091	Aradu.A02	1091	p3	(AAG)5	15	2928891	2928905	ACCAAAGGTGGAATTGGTGA	AGGAAGAGGAGGAACACTTGG	132
Ad02A1092	Aradu.A02	1092	p3	(TAT)14	42	2929789	2929830	GCAAGTTTAATCCCACCATTTT	TCAGCAAAGAATCAAGAGGACA	265
Ad02A1099	Aradu.A02	1099	p3	(ATA)5	15	2951146	2951160	ATTCTGTGCCCATTTGGGTT	AAAGATCAATTCTTCATATGACTATGC	176
Ad02A1100	Aradu.A02	1100	p3	(GAG)5	15	2990775	2990789	AAATCACAATGGGCTGAAGG	TCTTTTGCAGACATGGCAAG	157
Ad02A1102	Aradu.A02	1102	c	(T)10gttgtctaa	93	2993985	2994077	AAAACTCAACACGGAATCCTACA	AGTTCTTGGCCTACCGGATT	262
Ad02A1105	Aradu.A02	1105	c	(A)10tgattttttta	123	2995957	2996079	CCAAATCTCAATTGGCTTGC	GGTTGCTAAACTGCTCTCCAA	269
Ad02A1106	Aradu.A02	1106	c	(ATA)5attgcac	99	3001786	3001884	CCTCAGGCGAAATTTAAAAAGA	ATGCTTCTTTTGACGCGAAT	256
Ad02A1113	Aradu.A02	1113	p2	(AG)6	12	3027949	3027960	CAAAGGGGTAAAGGCAGTGA	CTGGATTTGTCCTTTTGGGA	266
Ad02A1115	Aradu.A02	1115	p2	(TC)7	14	3028493	3028506	CAAAAGTTTCAAAATGAACCACA	TTCGGTGACGAGAAGAATTG	269
Ad02A1119	Aradu.A02	1119	p2	(TA)8	16	3032562	3032577	TTGATCCATGTAGCTGATCCC	AAATTGAAATGATACCAAACATTAGA	266
Ad02A1130	Aradu.A02	1130	p2	(AT)6	12	3058670	3058681	CAAACCCAATACCCATGGTC	GGCGACTAAAAATGAAATAGGTTC	266
Ad02A1133	Aradu.A02	1133	p2	(TA)6	12	3062167	3062178	TTTCCAAGAGCTTGTTTCCC	AAAAATTTAAGTAGATCTTTGACCACA	265
Ad02A1134	Aradu.A02	1134	c	(T)10cacatgtta	86	3062848	3062933	GCATGGTAAGAAAGGGAAACA	TTGAAGTGCATATGCGAATAGAT	269
Ad02A1136	Aradu.A02	1136	p4	(ATTT)5	20	3064425	3064444	TTGTTCGACGATTTGGTTCA	GCGTTTGATATGAAGGGATTC	182
Ad02A1143	Aradu.A02	1143	c	(T)15actaatgca	38	3092650	3092687	AACCTTAAAAACGCATCTGGA	TGAGGCAAGTTGTTGCTCTG	275
Ad02A1144	Aradu.A02	1144	p2	(AC)6	12	3093159	3093170	CCCTTCTCTTCCTCCCAAAC	TTTCGTGTAATGCTTGGAGC	267
Ad02A1145	Aradu.A02	1145	p3	(TAA)5	15	3094157	3094171	CTCTGTTTCTCGCTCGCTCT	TCCACAAAATACATGCCCAA	251
Ad02A1146	Aradu.A02	1146	p2	(TA)6	12	3094820	3094831	TCGCAGATTCAAATCTCGTTC	ATGGTGTGGGATGGTAGTGG	264
Ad02A1148	Aradu.A02	1148	c	(AAG)5atgaag	49	3105884	3105932	CCATAACCGGGTCGAATAGA	CCTCTTTCAAACCAAACCCTT	177
Ad02A1149	Aradu.A02	1149	p2	(AT)7	14	3107514	3107527	TTCTCTAATGACATAGGGAGGG	TCACATTTCACATTGAACCAAA	123
Ad02A1154	Aradu.A02	1154	c	(A)10tcaaaactc	71	3116395	3116465	TCAACTTGTAATGGCTTCATTGAT	TTGGGTCAGAAACCTTAAACAAA	151
Ad02A1159	Aradu.A02	1159	p3	(AAT)9	27	3129053	3129079	CATCCTTTCGAGTTCTTCATCA	AAAAGCCCTTAAAAGCACAAG	194
Ad02A1161	Aradu.A02	1161	p2	(CA)6	12	3131597	3131608	TCTTTCAGAAAAGGAGAGTGAAA	TCGTTTGATTCATGTAGCCG	225
Ad02A1162	Aradu.A02	1162	p3	(AAT)9	27	3136568	3136594	TTTCCATTCACTTCAATTCATCA	TCTTCACGCATAATCTCTTTTTGA	272
Ad02A1163	Aradu.A02	1163	p2	(AT)6	12	3137463	3137474	GCATGATCCTGGTGATCAAA	TGCCCACATAAACACACAGC	205
Ad02A1164	Aradu.A02	1164	p3	(AAT)6	18	3137604	3137621	TGGGCAAATAGTCCCATGAT	GTCCACGTCAGCCCATTTTA	183
Ad02A1169	Aradu.A02	1169	p3	(CGG)5	15	3157208	3157222	TGGTCAAAAGGGGTATCTGG	CCACCACCTTCATGGAGACT	272
Ad02A1170	Aradu.A02	1170	p3	(TAT)11	33	3159445	3159477	ACCTTTAAGCATGCGGTTTC	GCATTGATATAGGACAGCACACA	275
Ad02A1171	Aradu.A02	1171	p3	(TAT)11	33	3159635	3159667	CCTTTAAGCATGCGGTTTCT	GCATTGATATAGGACAGCACACA	274

ID	Chromosome	SSR nr.	SSR type	SSR	Size	Start	End	FORWARD PRIMER (5'-3')	REVERSE PRIMER (5'-3')	Product (bp)
Ad02A1173	Aradu.A02	1173	p4	(TTTA)6	24	3164548	3164571	TGCGTAAATGGTCATCCAAA	GTGGGGTCAAAAATCAATGC	201
Ad02A1177	Aradu.A02	1177	p2	(GA)7	14	3169883	3169896	AACACGATCCTGAGGACACC	ACTGGTTCAAGAGCCTCACG	260
Ad02A1178	Aradu.A02	1178	p2	(GA)9	18	3170073	3170090	CGTGAGGCTCTTGAACCAGT	TGTTGCTCTTCCATTTTCCC	250
Ad02A1179	Aradu.A02	1179	p2	(AC)6	12	3170192	3170203	TGGGAAAATGGAAGAGCAAC	CTTCTCCTTTTGGCTTTCCC	102
Ad02A1180	Aradu.A02	1180	p2	(AT)6	12	3171409	3171420	GCTTTGTGAAAATTGATGAGTTG	CAAAGAAATGATTACGCTTTGATG	231
Ad02A1181	Aradu.A02	1181	p2	(CT)20	40	3172666	3172705	TGCAACCTCTGTCAAACTCG	AATAGGACGGCGACAGACAC	198
Ad02A1183	Aradu.A02	1183	c	(A)11gctaaaatg	76	3178477	3178552	GCATAGGAATCTTGTTTGGGG	GAATCCATGTTGTGATGGCA	257
Ad02A1185	Aradu.A02	1185	p2	(TA)6	12	3180389	3180400	GCATGATGTATGTGGGGCTA	CTGCAGACCAAAAAGTCAGAGA	280
Ad02A1186	Aradu.A02	1186	p3	(TAT)6	18	3181701	3181718	ACAAAAATTGGCTTGCTTGC	CCTTCCATCATTCACAACCA	228
Ad02A1187	Aradu.A02	1187	p3	(TAT)18	54	3182078	3182131	TTGCTTTCTGACCTCATGCTT	TGATGAGAAACTAAAAGCGAACA	268
Ad02A1190	Aradu.A02	1190	p3	(TCT)7	21	3184147	3184167	GCAATGTAGCTAACCTGGCTG	AGTTGCGGAAGCTGTTGATT	211
Ad02A1196	Aradu.A02	1196	p2	(TC)7	14	3193290	3193303	GCAGCCACACGAAAAATCTA	TCAGTGCAAAATCAAGCCAC	258
Ad02A1203	Aradu.A02	1203	p2	(TA)11	22	3235848	3235869	CTTCCAATCATTCAGCTCACA	GAGCATTGCAGTTCTGGACA	280
Ad02A1207	Aradu.A02	1207	p2	(TA)10	20	3250857	3250876	TCAGCTCACACTCATTGCTTG	GAGCATTGCAGTTCTGGACA	266
Ad02A1209	Aradu.A02	1209	c	(T)15aacattact	45	3265378	3265422	TGATTCCGAGAAATGCAACA	TACCGGTGAGGCTGTGAGTA	253
Ad02A1211	Aradu.A02	1211	p4	(TTTC)8	32	3270565	3270596	GCCATTGAAGAAATTGAAGCA	TGAGGCATTTACCAAATCAATG	274
Ad02A1217	Aradu.A02	1217	p2	(AG)9	18	3304044	3304061	GAGCCACACAATTTCAGGGT	CCAGGTGCCTAAACCCTCTT	149
Ad02A1218	Aradu.A02	1218	p3	(TTA)6	18	3305452	3305469	TGAACTTAAAAATTAGAGGAACGAAA	TCCAAAAAGAAAGAAAAAGAATGAA	195
Ad02A1222	Aradu.A02	1222	p3	(GAA)6	18	3316676	3316693	CGTACACGCCATTGATGAAG	AAGCTCAAGCCTTTTCCCTC	156
Ad02A1232	Aradu.A02	1232	c	(CAT)6caccaa	75	3340637	3340711	CTTTGACCCCCAAATCATGT	GAGGCCATCTCATCTCATCC	211
Ad02A1234	Aradu.A02	1234	p3	(GAG)9	27	3352871	3352897	AATCACAATGGGCTGAGAGG	TTCAGGAGGCTAAGGCAAAG	119
Ad02A1235	Aradu.A02	1235	c	(AT)8nnnnnnn	103	3355418	3355520	TGGGTATGAATTATGATGAACCTG	AGAGCGTGGACATTCTCAAA	270
Ad02A1237	Aradu.A02	1237	c	(T)10agttaatttt	69	3359363	3359431	TTAACCCCCTATTTGCTTCTTT	TGAAAATACAAACTACATGCATCC	269
Ad02A1241	Aradu.A02	1241	p3	(ATG)7	21	3365601	3365621	TGTTGTTGTAGGGCACCTGA	GGAAGTTAAGCTATGAAAAGGGC	257
Ad02A1242	Aradu.A02	1242	p3	(TAA)5	15	3368130	3368144	TGTCGGCAATGAAATGCTTA	GACGTGTATTGTCACATTGCC	189
Ad02A1243	Aradu.A02	1243	p2	(AT)6	12	3371619	3371630	AAAATAAGTTTCGATAATGAGAGCA	TGCAAATGCTGAAAATAACCA	276
Ad02A1244	Aradu.A02	1244	c	(A)15ccacaaaa	213	3372526	3372738	CATGCAAACTGGTAAGTCTTGG	GGTGGTGGTTGCTGCTACTT	275
Ad02A1245	Aradu.A02	1245	p3	(GAA)5	15	3373577	3373591	CCAACTTGAATCCTCGGAAA	AGTATGAGCCGGAGCTTGAA	264
Ad02A1249	Aradu.A02	1249	p2	(TC)13	26	3378781	3378806	TTGAAAGCATCTTTTGGAGG	TAACATAGCTGGTCGGCCTT	136
Ad02A1254	Aradu.A02	1254	c	(TGT)6tcctcag	128	3389980	3390107	TTTGAACACTTGGTTTGAGCA	TGAACCCCATTTGCAATTTT	265
Ad02A1262	Aradu.A02	1262	p3	(ATC)6	18	3408066	3408083	TAACATGGGAACGGTGGTTT	ACCCATGCCAACAATAGGAA	238
Ad02A1264	Aradu.A02	1264	p2	(AG)6	12	3422839	3422850	GCCTCCATGGTTGAAACTGT	GGGCTAGGAATCCTTCTTGG	196
Ad02A1268	Aradu.A02	1268	p2	(TC)8	16	3442416	3442431	ACTGCAGATTCACCGCTTTT	AAAGAGGATGATGACGTGGC	214
Ad02A1273	Aradu.A02	1273	p4	(ATTT)5	20	3462392	3462411	CCCCTTCTCTTAGCCACTGA	TCCCAAACGTTAGGACAAAAA	179

ID	Chromosome	SSR nr.	SSR type	SSR	Size	Start	End	FORWARD PRIMER (5'-3')	REVERSE PRIMER (5'-3')	Product (bp)
Ad02A1276	Aradu.A02	1276	p2	(TC)8	16	3473993	3474008	TTCCGATAATCTTATTTAATTTGTGC	TGCACATTTTTCCACTAAGCA	225
Ad02A1279	Aradu.A02	1279	p2	(TA)8	16	3476128	3476143	TGTAAATAACAACTTTTGCACTTCA	CAGTACAAAATCAAATAGAATTATCCG	203
Ad02A1280	Aradu.A02	1280	p2	(TA)6	12	3476517	3476528	TTGGTGGCTAATTTTAGTGTACTCAT	TGTTACTTGGCAGAGAGGCA	126
Ad02A1281	Aradu.A02	1281	p2	(TC)7	14	3477165	3477178	GAGTTCTGAATTTTCCAGCTGTC	GGAAAATCCAAGGTAGAAAGGAA	148
Ad02A1283	Aradu.A02	1283	p3	(TTA)12	36	3480433	3480468	ATTGATGTGGAACCTGAGCC	TTCCAATTACGCGTTGAAAA	270
Ad02A1286	Aradu.A02	1286	p3	(AAT)5	15	3482020	3482034	GGGTCTAACAATGCCAAAGTG	AAAAATTAAAAACGCGACAAAAA	239
Ad02A1287	Aradu.A02	1287	c	(AAAT)5aaag	81	3483205	3483285	TTTTATCATCACCGACCGATT	TTACCCAATTTTTGAGGGCA	239
Ad02A1288	Aradu.A02	1288	p2	(AT)7	14	3484175	3484188	GCGATTTACTGTTGTGGATGT	CCTGTGGCTCAATTAATGAAA	280
Ad02A1290	Aradu.A02	1290	p3	(TAA)6	18	3485426	3485443	TTTTGGGACAAACTTGAGCA	GTGTCACGGGACAAACAAAA	198
Ad02A1291	Aradu.A02	1291	p3	(GAA)5	15	3485690	3485704	CTTTGGTGTTGATTGGAGGAA	ACTTTCATCAAAACCGTGGC	244
Ad02A1292	Aradu.A02	1292	p3	(AAT)5	15	3486026	3486040	GCCACGGTTTTGATGAAAGT	CCATTTTTGGTTTTCTTGTGC	229
Ad02A1293	Aradu.A02	1293	p3	(ATT)28	84	3486294	3486377	TTGTTCTTAGAGGCCTTTGTATGA	AAGCATGCTCATACCCATTG	274
Ad02A1295	Aradu.A02	1295	p3	(TAT)6	18	3487840	3487857	AAAACTTGGTTAAAGCTGATTTTCA	ATGGGAAGAGGATTGATTGC	227
Ad02A1296	Aradu.A02	1296	p3	(ATC)7	21	3490513	3490533	TATGTGGGATTGTGGAAGCC	TGACGTTGAAAGATCCTTTGG	211
Ad02A1298	Aradu.A02	1298	p2	(TC)6	12	3497694	3497705	TCATCCGATTGATCCAGTCA	GTCACGGAGAGCATGGAAGT	252
Ad02A1301	Aradu.A02	1301	p3	(TTA)7	21	3502782	3502802	GCCATTATCTCCCTAACGGG	TCCGCGACGAATTAATCTTT	189
Ad02A1302	Aradu.A02	1302	p2	(AT)6	12	3503525	3503536	TGCAACCTTTTGTTGATACTCC	TCCACCATGAACACTCATCAA	135
Ad02A1308	Aradu.A02	1308	p3	(TCA)7	21	3514956	3514976	GCGGTGTCATTCCTCTGTTT	TTCATGCATGTTTCCAATCC	277
Ad02A1314	Aradu.A02	1314	c	(A)13cacacgcc	95	3523628	3523722	TGTCGGAGATTTGAATCGTG	GTTTACATAGTATTCCATGAGAAGTCA	249
Ad02A1316	Aradu.A02	1316	p3	(CAA)5	15	3526226	3526240	TCATTGAACCCCACACAAAA	AAGCCTTTTGGCAAACTCAA	235
Ad02A1318	Aradu.A02	1318	p3	(ATC)5	15	3533557	3533571	CTGTTGGGCTGCACATTAGA	AGCCCTACAAACCTCCACCT	259
Ad02A1322	Aradu.A02	1322	c	(T)10attacggta	95	3543409	3543503	TTTTGTGTATTCGGGTTCCA	TCAATGATCATATTAATTTACGAGGTC	276
Ad02A1325	Aradu.A02	1325	p2	(CA)6	12	3566369	3566380	ATGGCCAATAGCAACAAACC	ATTCGTCGTAATACCCGAGC	212
Ad02A1326	Aradu.A02	1326	p2	(AT)8	16	3566623	3566638	AGCTCGGGTATTACGACGAA	AAAAATGCATAAGGCGGTTA	230
Ad02A1328	Aradu.A02	1328	p3	(AAT)5	15	3572138	3572152	GGCTGAGGCAGAGGAGTTTA	TGCTGTTTTGGAAAATAGAGAAA	253
Ad02A1329	Aradu.A02	1329	p3	(TTA)9	27	3580671	3580697	GCTCATATCCCACACTGCAA	TCATTAGTTCACTCTACGATGCC	275
Ad02A1332	Aradu.A02	1332	p3	(TAA)5	15	3615095	3615109	CCTCTTCGTAATCACTACTTTTGG	GCATCAATCGATGTAAGGCT	278
Ad02A1335	Aradu.A02	1335	p3	(TTA)6	18	3624282	3624299	CCAAAATTGCTCATATCCCA	GGCTAATTAGTTCACTCTGCCA	280
Ad02A1336	Aradu.A02	1336	c	(GAT)5a(ATG	40	3625009	3625048	TGCATGGTTGAATTAAAGCTC	TCATCATCTTCACCATCCTCA	145
Ad02A1337	Aradu.A02	1337	p2	(TA)6	12	3626005	3626016	AGTCCCATTTTTCATGTGCC	GTTGGGTCATGGGTGAGTCT	263
Ad02A1341	Aradu.A02	1341	p2	(TA)6	12	3631690	3631701	CATTTTTCATGTGCCGAAAAT	GAGGTTGGGTCATAGGTGAGTC	280
Ad02A1346	Aradu.A02	1346	p2	(CT)9	18	3658271	3658288	CGCGCTCTGATTCCTTTATC	ATGCTTGGTGTTGAGCTGTG	276
Ad02A1347	Aradu.A02	1347	p3	(GAA)5	15	3660063	3660077	CAAGTGCTGTTGGTCGCTTA	TTGGAAAGCATGTGGGAAAT	251
Ad02A1348	Aradu.A02	1348	p3	(ATG)7	21	3660847	3660867	TTTTTGCAGATTGATGGGAA	AAGTCTGAGGTGGCCAAAGA	250

ID	Chromosome	SSR nr.	SSR type	SSR	Size	Start	End	FORWARD PRIMER (5'-3')	REVERSE PRIMER (5'-3')	Product (bp)
Ad02A1349	Aradu.A02	1349	p5	(AGAGA)5	25	3663332	3663356	TTTGAATGCAAATTGAAAGGT	TGCCATCTGTCCTCACTTCA	215
Ad02A1350	Aradu.A02	1350	p5	(AGAGA)5	25	3668109	3668133	GAGCTGAGACTGTTCTGGGG	TGCCATCTGTCCTCACTTCA	264
Ad02A1355	Aradu.A02	1355	p3	(GAA)7	21	3684566	3684586	TTTGCACAGAAGTGTCTCGG	CGGCCATGTATTGCTTCTTT	248
Ad02A1356	Aradu.A02	1356	p3	(AAT)6	18	3704269	3704286	CAGGGAAAAAGGCACTCTTG	AAACTTGGACGTTGGCTTTG	202
Ad02A1358	Aradu.A02	1358	p5	(TTATC)5	25	3715823	3715847	GTACTTGCAAGGGACTCCGA	CAGAGGAACACGACCCAAAT	247
Ad02A1359	Aradu.A02	1359	p3	(AAT)7	21	3718415	3718435	AACTTGCACTATCCGCAAGG	TGATGAAAATTCCACCACTCA	239
Ad02A1361	Aradu.A02	1361	p2	(AT)9	18	3723589	3723606	TCGCAGGTCAAAGGAAACTC	GGACATTAATAATCCGAAAAGATCA	142
Ad02A1370	Aradu.A02	1370	p3	(TAT)6	18	3764758	3764775	TGATTGCGGAAGTACAGTCAA	GAAAGAAGAAGAAAAATAAGACCCG	258
Ad02A1372	Aradu.A02	1372	p3	(TCT)5	15	3765686	3765700	GAAGCTCCGGACGATTAACA	CCAATTTGCGTGAATGAATG	197
Ad02A1375	Aradu.A02	1375	p3	(TGT)6	18	3770124	3770141	CCCACAATGCGAGGTTAGTT	AGTTCACTCTACCATGCCTTTT	244
Ad02A1376	Aradu.A02	1376	c	(A)10gtaaaaata	132	3771385	3771516	TGCTAACACCACCATTGCAT	CCGAGGGAAACAATGAACTT	269
Ad02A1379	Aradu.A02	1379	c	(CTT)5ctcctcc	103	3774435	3774537	GAATCTGAAGTGCAGGGGAG	GAAGAAGAATTGGTGGTGCG	210
Ad02A1380	Aradu.A02	1380	c	(TGA)20taatgt	181	3774708	3774888	TGTTGTTGTTAAAATTTGTTGTGGA	CTGTGTTTTCTCCCACCACC	271
Ad02A1381	Aradu.A02	1381	p3	(TCA)5	15	3776691	3776705	CCAGGGAACCTCGTATCTCA	TGGAAAGAAGGATGATGAGGA	197
Ad02A1383	Aradu.A02	1383	p3	(CAA)5	15	3784731	3784745	AGAGAGGTGGGTCACAATGG	GTGGCTTGAGGTTGAGCTTT	134
Ad02A1385	Aradu.A02	1385	p3	(TTA)5	15	3790470	3790484	CAGATTGGTCCAAAATTGTTCA	TGTTAGTTCACTCTACCATACCTTTTT	274
Ad02A1387	Aradu.A02	1387	p3	(TGA)5	15	3791658	3791672	TGGCCTTTATTGCAGATTGA	TCTGAGTAGCCAATTACAAAGCA	220
Ad02A1391	Aradu.A02	1391	c	(T)10aagtttactc	74	3807376	3807449	CCATGATGGAGAAAGGGAGA	CCTTCTGGCGTTGTAGACTTG	229
Ad02A1394	Aradu.A02	1394	p5	(TTATA)6	30	3809765	3809794	CCCTCTCATTCCTTCAACCA	TCTCAAATTTAATAAGAGTGTCATGTG	227
Ad02A1396	Aradu.A02	1396	p2	(TA)8	16	3814992	3815007	TGATGCATGAATCTTTATTTTTGG	GTAGCGTGATTCGTCGGATT	208
Ad02A1399	Aradu.A02	1399	p3	(ATC)5	15	3834802	3834816	TTTGAGTAGCCAATTACAAAGCA	TGGCCTTTATTGCAGATTGA	199
Ad02A1400	Aradu.A02	1400	p3	(AAT)6	18	3835831	3835848	AGTTCACTCTGCCATACCTTTT	CCAGATTGGTCCAAAATTGC	273
Ad02A1401	Aradu.A02	1401	p2	(AT)6	12	3838359	3838370	TGCAACGATGGTTCTCAAAG	TCTTAGCCAGCCAAAGCAAT	244
Ad02A1403	Aradu.A02	1403	c	(CTT)6tcctgctg	58	3844973	3845030	CTGCAGCTGCCCTTATAACC	TTGTTGGTGGTGATGACGAT	269
Ad02A1404	Aradu.A02	1404	p3	(AGG)5	15	3845704	3845718	ATCAAGAAGAAGGAACGCGA	AGTTGGCTCAAACCCAACAA	201
Ad02A1407	Aradu.A02	1407	p2	(TC)6	12	3857976	3857987	GAATCGCACAAAACTCAGGG	AAACCCATAAGTGCAGCGTC	279
Ad02A1412	Aradu.A02	1412	p3	(CAT)7	21	3870428	3870448	GATAGCCTCTGACGTGCCTC	TAAATCGGTCAGTTTTGCCC	236
Ad02A1413	Aradu.A02	1413	c	(A)10ttatttcttaa	78	3876755	3876832	GCAATATCGCGTCCAATTTT	TTTACGGCATTTTCATTCCC	277
Ad02A1415	Aradu.A02	1415	c	(A)11ccaaacgt	50	3889107	3889156	GAGAATCAGTTACTTAGCTTTCGG	GACGTTTGTCTCATGCAAGC	185
Ad02A1419	Aradu.A02	1419	p3	(GCC)5	15	3896355	3896369	CGCTGAAAATGGATGAGGAT	CAGCGACTTCAGCGGCTA	180
Ad02A1422	Aradu.A02	1422	p2	(CT)7	14	3910460	3910473	AGCAGAAGCAAAAGCAAAGC	CCGCAAACAGAGGAAGACAT	236
Ad02A1429	Aradu.A02	1429	p2	(AT)8	16	3930959	3930974	TCCTCTAATTCTTGTCAAAATCTCTC	CATACATAAATTTTGATTTTGAGCAT	165
Ad02A1431	Aradu.A02	1431	c	(TAA)5aaaatag	92	3935229	3935320	GGCTAAATAGCATTGTTCTCCC	GCACCACCCACTATGCTTTT	227
Ad02A1437	Aradu.A02	1437	p2	(AT)6	12	3944505	3944516	TTGGATTCCCATAAAATTAAGTCA	AAAAGGCTGAAGATCGACCA	279

ID	Chromosome	SSR nr.	SSR type	SSR	Size	Start	End	FORWARD PRIMER (5'-3')	REVERSE PRIMER (5'-3')	Product (bp)
Ad02A1440	Aradu.A02	1440	p2	(TG)9	18	3953721	3953738	TTCGGATATCGGTTCTGAGG	GGGTTACAAGTTGCAAAACTCA	113
Ad02A1445	Aradu.A02	1445	p3	(ACA)5	15	3970653	3970667	TGGCTAATGCCATGGTTATTTT	GACAAAGGGCGAAAGACAGA	224
Ad02A1446	Aradu.A02	1446	c	(A)10ttatgttaat	80	3973073	3973152	TGAGACCATTTTTGACGAACA	GCACCAGAGAACCCCTTTAG	264
Ad02A1448	Aradu.A02	1448	p3	(AGC)5	15	3978259	3978273	CTGCCACATATCCGATCCTT	AAGGTTGGTTTCACATGCACT	240
Ad02A1450	Aradu.A02	1450	p2	(GA)6	12	3985645	3985656	AGTCCCAGGTTCCCTCTCAT	GCCAGTACTTAGCCAGCAAAA	278
Ad02A1451	Aradu.A02	1451	p2	(AT)11	22	3986492	3986513	TGCCGAATTGAGGAGTAACA	GAACATAAAGGGCCACCAGA	104
Ad02A1452	Aradu.A02	1452	p2	(TA)7	14	3987762	3987775	TAGCCATCCTTAGCCACCAC	GGTCCAGGATATCCCACCTT	211
Ad02A1455	Aradu.A02	1455	p3	(TAT)11	33	3994080	3994112	AAATATTGGGCAAAAACCGA	TCTGCTGATTACATGCTGGC	224
Ad02A1466	Aradu.A02	1466	p2	(TC)7	14	4027743	4027756	TGTGGTAGACTGGGAGGGAC	CTTAGCCCCTTTTTGCTGTG	258
Ad02A1467	Aradu.A02	1467	p2	(TA)8	16	4028312	4028327	GTCTTATGTGTCACCCGCCT	CAACCAAACGCTGCCTTAGT	102
Ad02A1468	Aradu.A02	1468	c	(T)10cttcttataa	78	4029761	4029838	GAACCGCGACAAAAACAGAT	TTCATAGCATTAATGTTTCGGAAT	217
Ad02A1470	Aradu.A02	1470	p2	(AT)7	14	4032962	4032975	TCAGGACCAGAATTTTTCGG	TGTTCTCGATAAAAATGTTCTCAAA	259
Ad02A1471	Aradu.A02	1471	c	(T)10ggttaacat	31	4042888	4042918	AAGCATATCCGAGTAAATAATTCCA	TTATGGCCTGTGAAGAACCC	234
Ad02A1475	Aradu.A02	1475	c	(ATT)8tacggg	40	4054242	4054281	CGTTGGTCTATAAGCTCCGC	CACAACATACATTTTGCCGC	262
Ad02A1483	Aradu.A02	1483	p3	(AAT)5	15	4068930	4068944	AAAAATACATCATCAGCTAACCATT	GGCACAAAATATTTGACAAGACA	279
Ad02A1484	Aradu.A02	1484	c	(AT)7aggggac	95	4069429	4069523	AAAAATTTTCAACGAAAACAATCA	TGCATCTGAATTTTCACCCA	262
Ad02A1486	Aradu.A02	1486	p2	(TA)6	12	4073736	4073747	TCATATCATGAAACCACTCCTTT	CGGCCAATATCATTTATCAAAA	280
Ad02A1488	Aradu.A02	1488	p3	(GAA)9	27	4077538	4077564	TGGTTGGAAAGATGGGATTC	GCACCACCACCAACAAGAAC	276
Ad02A1489	Aradu.A02	1489	p3	(ATG)6	18	4077674	4077691	TGGTGGTGCAAACCAAATTA	CAACCAAGATTTTTGTGGGG	248
Ad02A1492	Aradu.A02	1492	p3	(TTA)5	15	4080276	4080290	GGCTGTTCGTTTAATCTAGGC	CCTTTGTTTATTTCCACCCTACA	280
Ad02A1498	Aradu.A02	1498	c	(AT)9(GT)6	30	4099602	4099631	AAGCATGGCAAAGAATTTGG	TGATTGTCCTCCACTCAGATG	261
Ad02A1504	Aradu.A02	1504	p2	(TA)6	12	4106422	4106433	AAAACAATTGGGTAAGCAAGTTA	ACAAGATAGGATCGGAATGGT	163
Ad02A1505	Aradu.A02	1505	p3	(TAT)5	15	4107973	4107987	CAAAAACCAATTAAAACCGCA	TTTCAAGTAGTTTAGATTTGAATGGA	270
Ad02A1506	Aradu.A02	1506	p2	(TA)6	12	4112724	4112735	TTGGTTCATGACTTGGCTGA	TGAGTCAAACTTGAATTTGGATAAAC	271
Ad02A1512	Aradu.A02	1512	p3	(ATT)16	48	4140231	4140278	TTGGTGGTTGATGAATCTTGAG	ATGCCTAGACCATTGCCATA	275
Ad02A1514	Aradu.A02	1514	p3	(ATA)7	21	4142923	4142943	CGTGTCGCATTATTATTGGATG	TTAGACCCGATTTGATCCGA	261
Ad02A1517	Aradu.A02	1517	c	(T)10ctaatattaa	38	4146069	4146106	TTGGTCAATTTTCCTGTCCA	CTCACATGGTTGACCTCCAA	233
Ad02A1518	Aradu.A02	1518	p3	(ATA)14	42	4148054	4148095	GCGAGTCGTAACCTTCTTGG	ATGGACCGAAACCAGTCAAC	258
Ad02A1520	Aradu.A02	1520	p3	(ATT)5	15	4148849	4148863	TTCTCAAAGGAGAATAGGTGATCC	GGTTCCTAGATCGCCCAAAT	159
Ad02A1523	Aradu.A02	1523	p2	(AT)6	12	4152429	4152440	GGTGCTCTTGTGTCCTACATGAT	GGCAAAGAACATAGAAAAATGGA	213
Ad02A1526	Aradu.A02	1526	p2	(TA)7	14	4154420	4154433	AAACATAATTAGATTCATTCCTTTTCG	TTTGGCTAAGGATGGAGAAAAA	114
Ad02A1529	Aradu.A02	1529	p3	(TTG)5	15	4159668	4159682	CATGAGGTGGTCAAACGAGA	GACATAGGATTGAGGAGCGAG	201
Ad02A1530	Aradu.A02	1530	p2	(AT)11	22	4160832	4160853	TTTTCCGAAAGCACATACCA	GCTTCAAGGTTTTGTCGTTGA	234
Ad02A1532	Aradu.A02	1532	p3	(GTG)7	21	4163219	4163239	CAATCATCAATGGCAGCAAG	CCCATCTTCCAAGTTTCCAA	123

ID	Chromosome	SSR nr.	SSR type	SSR	Size	Start	End	FORWARD PRIMER (5'-3')	REVERSE PRIMER (5'-3')	Product (bp)
Ad02A1544	Aradu.A02	1544	p3	(CCT)6	18	4190134	4190151	TGAAATTTTTGAACCGCCTC	TAACCCAACTTCAACGACCC	248
Ad02A1545	Aradu.A02	1545	p3	(AAT)9	27	4190954	4190980	TTTGGTCTTTATTGATGGCAAA	TCTCATGAATCTATAAGCCTATCGTAA	183
Ad02A1548	Aradu.A02	1548	p3	(ATT)5	15	4195515	4195529	AAAGCTTTCAAACGGATCTTAAA	TGAGTTTATGACATGCCCCA	268
Ad02A1549	Aradu.A02	1549	p3	(TAT)5	15	4195675	4195689	AAAGCTTTCAAACGGATCTTAAA	TGAGTTTATGACATGCCCCA	268
Ad02A1550	Aradu.A02	1550	p2	(CT)23	46	4199104	4199149	AAGCAGGTTGGTCAAAATGG	CTGGCAGAGAGATGAAAGGG	178
Ad02A1553	Aradu.A02	1553	p2	(CT)6	12	4210118	4210129	TCTGCTCATGGAAGTCATGG	CCTTTTGAGCCTCCAAGTGA	271
Ad02A1558	Aradu.A02	1558	p3	(ATT)5	15	4212636	4212650	CCAAGATAATTAAACAACTTTAAGGAA	AAAATCCCAGGGCTTCAACT	273
Ad02A1559	Aradu.A02	1559	c	(A)10cacataaa	49	4212834	4212882	CACCGAAATCTACCTCGCAT	CCCAAGCAGTAGAAAGTGGG	262
Ad02A1563	Aradu.A02	1563	p2	(TA)6	12	4223339	4223350	AGGGTTGATTTTGATTGCCA	TTGGCAAATACCCTTTTGGA	261
Ad02A1564	Aradu.A02	1564	c	(ATA)7g(TAA	37	4223723	4223759	TTTGCCAATCAAAGTAGGGC	GGTTGTGGTGATGGTGTTGA	254
Ad02A1565	Aradu.A02	1565	p2	(AT)6	12	4225866	4225877	CCCCAACTTGATGCAAGAAA	TTTGCTGTGTCATTTGTCTGG	278
Ad02A1566	Aradu.A02	1566	p2	(TA)6	12	4229417	4229428	ATCAGCAACCTTTGGCTCTG	TTCATTTCAATCAAGTCATCCAA	267
Ad02A1567	Aradu.A02	1567	p2	(AT)6	12	4230104	4230115	CGCAAATATTTCTTCGACCC	CCATAGAGATGCTGATCCAACT	149
Ad02A1568	Aradu.A02	1568	p3	(TAA)5	15	4231317	4231331	TACTAACTGGCCCGGATGAA	CACATCTTAGCTTGTCCCCAA	259
Ad02A1576	Aradu.A02	1576	p3	(CTT)5	15	4254499	4254513	CTCTTCCTCTTTCTCCTCCTCA	TTCCATGCCTCCTCTTTTTC	251
Ad02A1577	Aradu.A02	1577	c	(AAG)7aaaaaa	48	4254651	4254698	CCTCCTCATCTTCTGCTGCT	TTCTTTTTCCATGCCTCCTC	243
Ad02A1579	Aradu.A02	1579	p3	(AAT)11	33	4260336	4260368	GCATTTGTTTTGGTGCATTG	ACGAGGCACACCCTTTACAC	208
Ad02A1580	Aradu.A02	1580	c	(TTC)6caaattc	95	4261036	4261130	TCCTTCCCCTTCTCCTTCTC	GCATCATCAAGTGAACCTCAAA	247
Ad02A1583	Aradu.A02	1583	c	(A)12gacaacaa	42	4263527	4263568	AAATCACCCACCAAGATCCA	CTTGGGGCGTTGTTAGACAT	213
Ad02A1585	Aradu.A02	1585	p5	(TATTT)6	30	4264763	4264792	TGGAAAGGAGGAATTGATGG	GGGATGTGGCATTTATTTGG	206
Ad02A1587	Aradu.A02	1587	p3	(TTA)13	39	4266166	4266204	TGGAGAGGTTATTAAGTGTTTCAA	CACTGGCACTTCAGTTTGTCTC	280
Ad02A1588	Aradu.A02	1588	c	(AAT)7gaggca	63	4266401	4266463	GTGCATGACTTCCACGTTTG	TTCGAGTTTCAACATTCAGGG	198
Ad02A1589	Aradu.A02	1589	p2	(AT)9	18	4268174	4268191	GGATAGTTTTCTTTTCACTTTTGAAC	AGAATCGGCGTTTTTGTCAC	240
Ad02A1590	Aradu.A02	1590	p2	(AT)7	14	4270397	4270410	CCCAATCAAGTGGTACGGTC	TGTGTGAAATGTGACCAAATGA	211
Ad02A1602	Aradu.A02	1602	c	(AAG)5agggaa	59	4298142	4298200	CATCAAGAATGAAGAAGAATGTGC	GCCAATGGCCATGGTAAATA	271
Ad02A1605	Aradu.A02	1605	p3	(ATA)5	15	4302019	4302033	TTGTGTGCCAAATTTCGTGT	TTGCTTGAAAAAGATGCCCT	280
Ad02A1606	Aradu.A02	1606	p2	(TA)6	12	4302275	4302286	GGGCATCTTTTTCAAGCAAT	AGGTGCATGCTATGTTGCTG	158
Ad02A1607	Aradu.A02	1607	c	(TAA)6attcttaa	73	4303611	4303683	CCGTGTCTTTTCTAACATTGCC	CCACTGTTTTGAAGTGTGCAA	275
Ad02A1608	Aradu.A02	1608	p4	(TTTA)5	20	4306920	4306939	TTTTCCATTGTGCAACCAAA	GGAACGGATCACAGAAAACAA	222
Ad02A1610	Aradu.A02	1610	p3	(TTC)5	15	4308921	4308935	TCCATCACCCACTTCTTTCA	AAACAGGCCACATGGGTTTA	159
Ad02A1612	Aradu.A02	1612	c	(TA)6aaattaaaa	83	4310058	4310140	GAATGTATTTGCTGTGTTTTGC	ATAAGTGTAGCCTTAGGTCCCTC	260
Ad02A1618	Aradu.A02	1618	p3	(AAT)7	21	4318907	4318927	CGATTGCCGATCATTTGTAT	TTTCTTTCACATTATTCACGAGGA	271
Ad02A1619	Aradu.A02	1619	p3	(TAC)5	15	4319094	4319108	ACGGTAGTAGTGCCACGCTT	CGTCTCCGCGGAAATAATAA	180
Ad02A1623	Aradu.A02	1623	p3	(CGC)5	15	4321405	4321419	TATCCCTTCTCTCTCCCCGT	GGGATCTCATAGGACGCAAG	221

ID	Chromosome	SSR nr.	SSR type	SSR	Size	Start	End	FORWARD PRIMER (5'-3')	REVERSE PRIMER (5'-3')	Product (bp)
Ad02A1624	Aradu.A02	1624	c	(CT)6t(TC)9	31	4325422	4325452	CAGTTGCTGTGAGTGTTCCTG	TATGCAGTTCCCTGGTGTCA	221
Ad02A1625	Aradu.A02	1625	p2	(CT)10	20	4327431	4327450	CCAAAACAAGAATGCACGAC	TCTAATCCTTGGCCGTTCAC	235
Ad02A1626	Aradu.A02	1626	p3	(AAG)5	15	4329849	4329863	TGAAAAAGGCAGAAGCAAAGA	GGCAATTTTGTCGGGATCTA	154
Ad02A1628	Aradu.A02	1628	p3	(CAA)5	15	4341317	4341331	ACACCATCCCTCAGAACTGG	GCGTACGAATCCTTGCTTTC	274
Ad02A1631	Aradu.A02	1631	p3	(TTA)16	48	4344787	4344834	TTGGACCAAATGTGGATGTT	TTCTTAAGTTTGAGGCGGCA	186
Ad02A1632	Aradu.A02	1632	p2	(TA)6	12	4346918	4346929	GGTGTTTGCTTAGCTTGGCT	TTTGCATTTAAAAAGTTTCACCA	231
Ad02A1634	Aradu.A02	1634	p2	(AT)8	16	4348782	4348797	CATGCATTAGATCAAATTTTAAAAACA	TGCAAACAAAACATCAAATCC	162
Ad02A1640	Aradu.A02	1640	p2	(TA)6	12	4354723	4354734	TTCTCCAAAAGAATCACGCC	TCAGTCTGAGAATTGACTTTTGATT	226
Ad02A1646	Aradu.A02	1646	c	(T)11ggtagagta	86	4376093	4376178	TGGTAAATTATGCAAGTCAAAGACA	GCCCAAGAATTGAAGGCTATT	273
Ad02A1649	Aradu.A02	1649	c	(A)11ggcaggcc	113	4379085	4379197	TTTCTTTCGTTACGTATAGTTCATTTG	CCTTCGACTTGCCTCTTTCA	272
Ad02A1650	Aradu.A02	1650	p3	(TCC)6	18	4383461	4383478	ACCTCTTTCCCGACCAAGTT	GCCGGATTTAGAGGTCTTCC	159
Ad02A1653	Aradu.A02	1653	p4	(GTAT)7	28	4399148	4399175	ATGGATTTTGTTGAAGGCCA	GAAATTGCGTGCAGTTTTGA	229
Ad02A1656	Aradu.A02	1656	c	(T)12agttacaca	96	4404685	4404780	TGCTTGTGCATCATGTTTGA	TTTCTTGGTGAATGTTGTAGCC	201
Ad02A1664	Aradu.A02	1664	p3	(CTT)5	15	4415434	4415448	CTTCTTCTCATTCCTCCTCATCTT	TCGAGAGGACAAAACAAGAAAA	115
Ad02A1668	Aradu.A02	1668	p2	(TA)6	12	4417007	4417018	AAACCCTACACAGGTAACCCAA	TGCAATTCTCTCTCTCTTTACTTTTT	256
Ad02A1669	Aradu.A02	1669	p4	(TTTA)8	32	4422851	4422882	GCTGCCCATAATAATCATCCA	TTCTTGCATCCAATTGACCA	231
Ad02A1673	Aradu.A02	1673	c	(TTA)5taatggc	67	4429157	4429223	AAAATGTTGGTACTTTTGCGTTT	TGTCTTGCAACCCTGAAGAG	231
Ad02A1675	Aradu.A02	1675	p3	(TCA)7	21	4432945	4432965	ATGAGCCTCCACCTGAAAGA	TCGATGTGCCGTTACGTTTA	250
Ad02A1677	Aradu.A02	1677	p3	(TAA)6	18	4438262	4438279	ACAGAATGTGCCGGAAAGAC	TGCATTTGTTTCCAATTTTCA	266
Ad02A1679	Aradu.A02	1679	p2	(TA)7	14	4451681	4451694	AATTTCTGGTGCGGAAAATG	TGGTTGATCTTTGTCCATCATC	248
Ad02A1681	Aradu.A02	1681	p3	(ACA)7	21	4454578	4454598	CCTCCAATGCCAACGTTACT	GACCCCACTTACTGGGACAA	112
Ad02A1682	Aradu.A02	1682	p3	(TCA)7	21	4457274	4457294	ATGAGCCTGCACCTGAAAGT	ATTGCCGTTTCGTTTGAATC	250
Ad02A1685	Aradu.A02	1685	p3	(TAA)5	15	4467543	4467557	GAATTCACCCAGACGTTAAGAA	TTCTTGTACCAATGTTTTTGCTTT	228
Ad02A1687	Aradu.A02	1687	p2	(AG)6	12	4470119	4470130	AAGCAACGCTCTTCTTCCTTC	CTTCAAAGCCATTTTTCGGA	261
Ad02A1688	Aradu.A02	1688	p2	(AG)14	28	4480559	4480586	TTGGTCAAAATGGCAGAGTG	CCAAATCACTTGAGATGCGA	274
Ad02A1689	Aradu.A02	1689	p2	(GA)6	12	4485646	4485657	CAATGATAAGTGCCTGTTGGG	GCCATTTGAAATTTGCTGCT	204
Ad02A1693	Aradu.A02	1693	p3	(TTA)17	51	4491683	4491733	TCGTGTTAAAAACAAAAATTTGAGA	ATTTCCTTCTTCAGCTGCCA	129
Ad02A1698	Aradu.A02	1698	p3	(TAA)5	15	4500070	4500084	TCTTTAAAATTTGGTGATTTTTCGT	CACCATCGAATTATTCAAAATG	270
Ad02A1704	Aradu.A02	1704	c	(A)12ctcaacatt	63	4515167	4515229	TCCCCTTCTCTTTTTCTTTGG	AGTCGTTGGATTCTCACATTG	232
Ad02A1705	Aradu.A02	1705	c	(T)15aatcc(T)1	30	4520087	4520116	TCTCCCTCTCTTTGGAGCAA	CAACAAATTCATGAGCAACGA	269
Ad02A1706	Aradu.A02	1706	p3	(ATT)5	15	4522242	4522256	TTGGCTCCTCGTACTTTTCC	TAAAGCAGGGTGGAGTTTGG	120
Ad02A1711	Aradu.A02	1711	p3	(CAT)5	15	4530937	4530951	CCTCACCTTGTCCATCCACT	GCGTGTGGACTTTGATGATG	191
Ad02A1712	Aradu.A02	1712	c	(AGA)5t(GAA	31	4531768	4531798	TTTTGAGAGCTCCACAACCC	TCCCTCTCTCCCTCTTCACA	170
Ad02A1713	Aradu.A02	1713	p2	(GA)7	14	4531974	4531987	TGTGAAGAGGGAGAGAGGGA	CGAAATTGAAGGCACCACTT	164

ID	Chromosome	SSR nr.	SSR type	SSR	Size	Start	End	FORWARD PRIMER (5'-3')	REVERSE PRIMER (5'-3')	Product (bp)
Ad02A1714	Aradu.A02	1714	c	(CA)7aaccacta	33	4544939	4544971	AGAGCCAGATCGAGTATCCG	CATCGGTCAAGCCTGAAAAT	124
Ad02A1715	Aradu.A02	1715	p3	(TAA)5	15	4547725	4547739	TCCCAATACTTATTTAGGCGGA	TTCCCTTGGAGTTCTTGGTG	225
Ad02A1718	Aradu.A02	1718	p2	(AT)7	14	4554654	4554667	CGCGAAGACGTATCACTCAC	GTACCCTAGCCGGCCTAAAC	161
Ad02A1722	Aradu.A02	1722	c	(T)10ctttttttaat	156	4570096	4570251	TGCTTTCACAATTTCATTAGCC	TTGGTGATGGGAAAGAAAGG	229
Ad02A1731	Aradu.A02	1731	p3	(ATA)5	15	4579262	4579276	CACCAAATTCTTCTTGATCTCCA	AAAATTGAGGGTCAAAATGTCC	183
Ad02A1734	Aradu.A02	1734	p2	(AG)6	12	4586259	4586270	TCAAGAACCTGAACCAAGAAGA	TGGTTAGCCCATTAGTTGCTG	211
Ad02A1735	Aradu.A02	1735	p2	(AG)6	12	4586860	4586871	TTGAAATCGGTGTTGCTGTAA	TTTTGGTTCACAATACAATTCACA	229
Ad02A1738	Aradu.A02	1738	p3	(AGG)5	15	4588865	4588879	AAAGGGCTAGGGTTTGAGGA	CTGTCACTTGCATGCCCTAA	243
Ad02A1739	Aradu.A02	1739	c	(T)10gcctcttcc	67	4590659	4590725	CCACTGGACCATTCCAACTT	GTATGAGTGGGGTCAATGGG	258
Ad02A1742	Aradu.A02	1742	p4	(ATTA)5	20	4593052	4593071	CCCACCACTGTCCCTCTAAA	TGTCCCATAGACAATTATGCAA	145
Ad02A1744	Aradu.A02	1744	p3	(CTT)6	18	4594711	4594728	TGAGGCTATTACGGCTTGCT	TCTGCTGACCCTCGTTCTTT	231
Ad02A1746	Aradu.A02	1746	p3	(GAA)5	15	4599601	4599615	TTGACACACTCTATCTATCAAGTGAA	AACTGAAAGTGCGGTGGTTC	187
Ad02A1748	Aradu.A02	1748	p2	(AG)7	14	4604667	4604680	AGTACCTGCGGTAGACGGTG	CCACATGGCATCAGTATTGG	224
Ad02A1749	Aradu.A02	1749	p2	(TA)8	16	4608777	4608792	TAACCCCCTCTATCCTGGCT	CGTGGCATCCTTTTGTATCA	264
Ad02A1750	Aradu.A02	1750	p2	(AT)6	12	4609481	4609492	GCCATGCCGTTAAGTTTTCT	TGTTTGGATTCATTTGAATACTTTTG	278
Ad02A1755	Aradu.A02	1755	p2	(AT)11	22	4615835	4615856	TCAAGACCAATTATTTTTGTGAAA	AGTTGGATCCGGAAGTGAAA	188
Ad02A1760	Aradu.A02	1760	p2	(AG)10	20	4628961	4628980	ACGCATGCATGTTTTGAGAG	TGGCTGAGATTGTCCTGTTG	108
Ad02A1761	Aradu.A02	1761	c	(T)12aatgggaa	61	4629724	4629784	TGAGTTGTTGATCATTCAAAGATGT	TTGGGTTTGAATCTTGTTATTGG	210
Ad02A1762	Aradu.A02	1762	p3	(AAT)7	21	4629956	4629976	CCAATAACAAGATTCAAACCCAA	TTCCTCCCATGTTCGATTGT	207
Ad02A1764	Aradu.A02	1764	p3	(GTT)6	18	4630800	4630817	CTCTAGGCTTGGGAGCTTCA	CCACCACCTATTCGACTCGT	235
Ad02A1765	Aradu.A02	1765	p3	(GGT)5	15	4630928	4630942	CACCACCTCTTCCCATTTGT	AGGGTGGGTGGTGTGTAGAA	179
Ad02A1766	Aradu.A02	1766	p3	(AGA)5	15	4632718	4632732	CTCATGCTTTCTGGCATTCA	CACACAACACACCACCCTCT	231
Ad02A1767	Aradu.A02	1767	p3	(ATA)7	21	4633834	4633854	GGGGACACCAGACACCAATA	GCACTTCTTCCAGCATAGCC	154
Ad02A1769	Aradu.A02	1769	p2	(TA)8	16	4636696	4636711	TGACATTTATCGACACTCATCG	TGAAACACAACACGCAAACA	276
Ad02A1771	Aradu.A02	1771	p2	(AT)6	12	4637317	4637328	CAAAGGCCAGCAACTTCTTC	GAAAACCGGTGAGAAAATGTC	206
Ad02A1779	Aradu.A02	1779	p3	(AAT)5	15	4648649	4648663	CACAGAGCTGAGGGAGGTTC	TTTATGGGAGCATCCTCGTT	208
Ad02A1780	Aradu.A02	1780	p2	(AT)7	14	4650657	4650670	CCAAAGTTGAGCCAAAGAGC	CATCACACTCGAAACGTTGAA	280
Ad02A1781	Aradu.A02	1781	p2	(TC)17	34	4651041	4651074	TCATTCAAGCGAGGTAACCA	CCATGAAAGGTACCAGGCTC	262
Ad02A1786	Aradu.A02	1786	p3	(CAA)6	18	4662938	4662955	CAGGATTCAATTCTCCCGAA	CGTTTGATTCATGTAGCCGA	122
Ad02A1790	Aradu.A02	1790	p3	(CTT)6	18	4674638	4674655	CGGTATTGCCGGCTAATCTA	AAGAGGTGTCAATGGGTTGG	122
Ad02A1794	Aradu.A02	1794	c	(TGA)5taataat	120	4690647	4690766	TTTTTGAGGTAGGGGTGGTG	TCACCCCCAAAATTAACCAA	226
Ad02A1797	Aradu.A02	1797	c	(A)10caaaacac	59	4714296	4714354	TTCAAACCTCCACAATTTTACA	TTTGGTTGGTTGAATTGGTTC	273
Ad02A1798	Aradu.A02	1798	c	(ATT)12tgtaca	175	4715862	4716036	TTTCCGTTAAACATGACAGCC	AAATGATTTCAACCTTAAATTTGTTG	274
Ad02A1802	Aradu.A02	1802	p2	(TA)8	16	4728265	4728280	ACTCGACCTGCGAAGCTAAG	GCTGAACGTCTGGAGTTTCC	226

ID	Chromosome	SSR nr.	SSR type	SSR	Size	Start	End	FORWARD PRIMER (5'-3')	REVERSE PRIMER (5'-3')	Product (bp)
Ad02A1803	Aradu.A02	1803	p2	(AT)6	12	4729049	4729060	GTACGTGCATCCCCAAATTC	GGAGGTTTCGTTTGAAGTGG	271
Ad02A1804	Aradu.A02	1804	c	(A)10gatgaata	45	4733562	4733606	CGCAACTTAATAAAACATGCATAAA	TCTTCTCCTTATCTTTTGTTGCTTC	263
Ad02A1806	Aradu.A02	1806	p3	(AGG)5	15	4734987	4735001	AAAAAGGAGGAAGAAGGGGA	CACCACTACCACCACCTCCT	129
Ad02A1809	Aradu.A02	1809	p3	(TCA)5	15	4740027	4740041	TCCCTAACATCTTGGGTCAAA	TTTGTTATGGTGGCAGTGGA	197
Ad02A1810	Aradu.A02	1810	p2	(AG)6	12	4740309	4740320	TCGGAAAACAGAAAGAGGGA	CACTAACATTGCTGGCGAGA	238
Ad02A1813	Aradu.A02	1813	p2	(GT)6	12	4756676	4756687	GCGTACGCATGATACTGTGC	CATCCGGTTTAACCAATCGT	260
Ad02A1817	Aradu.A02	1817	p2	(CT)16	32	4764457	4764488	TCGGAAAATGAAAATGAAGGA	TTTGCAGAAGAAAGACAAAGGA	193
Ad02A1819	Aradu.A02	1819	p2	(AT)10	20	4768269	4768288	AATGAACCCAATGCGTTTTC	TTTCGGCTTCACGGTTTATC	274
Ad02A1822	Aradu.A02	1822	c	(A)13caagctag	50	4793713	4793762	TCATGGGAGAGGTTGATTGA	CAAAATCGGGAAAACATTGG	171
Ad02A1828	Aradu.A02	1828	c	(A)10tcttatattt	108	4829125	4829232	TCAAGCAATATTTTCTGTTAAATCAA	TGTTGCACTAACAGTTTCTGAATG	235
Ad02A1829	Aradu.A02	1829	p2	(CT)11	22	4831345	4831366	ATCGACCGAACCAGAAGTTG	TGACAAACCAGGTGGTGCTA	238
Ad02A1831	Aradu.A02	1831	p3	(TCA)6	18	4834946	4834963	TTCCAATGACAAAAGGTGGT	AGGCATATACGTCTTAATGGTGA	280
Ad02A1833	Aradu.A02	1833	p2	(TC)7	14	4840875	4840888	ACTCAAAATTCGAACCGCAA	CTGTCGGAGAGATGAGAGGG	108
Ad02A1835	Aradu.A02	1835	p2	(AT)9	18	4847536	4847553	TCATCAACACCCCTGTACCA	GCAGAAAAACTGTATGCCCAA	270
Ad02A1839	Aradu.A02	1839	c	(T)10cttttggga	38	4851749	4851786	TTCACCATCATGTCCTCCAA	CTCTCCCTCACCATTCCAAA	175
Ad02A1841	Aradu.A02	1841	p3	(TTA)5	15	4860947	4860961	TTTTGAAATGTATAATGAGGCCAA	TGGAAAATCAATACGGGCTC	266
Ad02A1842	Aradu.A02	1842	p3	(ATC)5	15	4861419	4861433	CCTGTCATGACCCCATTTTC	TCTTCAAAATGCTTGCTCCA	250
Ad02A1843	Aradu.A02	1843	p3	(TAT)6	18	4863118	4863135	AAATCCTCACATATCTTTACACGG	TTTCTTTGCTGCCTCTGGAT	152
Ad02A1847	Aradu.A02	1847	p3	(TAA)5	15	4866644	4866658	GCACTCAAAATTTAGCAGAGGAA	CAAGTCCTTGAGTACTAGCACAAAC	280
Ad02A1849	Aradu.A02	1849	p3	(TAT)15	45	4873088	4873132	ACCTTATTAATCAACTACAAACCCTT	TGGATGTTTACTCCCATCCAT	214
Ad02A1852	Aradu.A02	1852	c	(TTC)7actatttc	69	4875119	4875187	TTGGATACAAAGCTGAGGAGG	AGGGAGGAAGGAGTGGATGT	220
Ad02A1853	Aradu.A02	1853	p3	(ATG)5	15	4875990	4876004	TGAGGTTGATGGTGGTGCTA	ATCCGTCCTCAGTTATCCCC	239
Ad02A1856	Aradu.A02	1856	p3	(ATT)17	51	4889904	4889954	TCCCCCATAACCGTATTTCA	AACAAGCAGCAAATTTTGGAA	246
Ad02A1857	Aradu.A02	1857	p3	(TGA)5	15	4890981	4890995	TGTGCCCACCATTTGTCTAA	TGATTTCCTCTGACGAGCCT	274
Ad02A1863	Aradu.A02	1863	p2	(GA)8	16	4896778	4896793	GTCAGCATGTCCAGTAGGCA	TCTCTCTTCTGTGAACCGCA	112
Ad02A1864	Aradu.A02	1864	p3	(TGT)6	18	4897027	4897044	TCTGTGAGCGAGGTTCGTTA	TCACTTCCTCACTGTCACCG	264
Ad02A1870	Aradu.A02	1870	p3	(GAT)6	18	4911029	4911046	TTTGCAGCATTATGTGTTTCTTC	TCTTCCTCTTGCTCCTCTTCA	195
Ad02A1873	Aradu.A02	1873	p2	(GT)6	12	4925943	4925954	ATTCGAAGACCAAAACGTGG	CCCTTGGCCCAACTAAACTA	259
Ad02A1876	Aradu.A02	1876	p2	(AG)6	12	4935140	4935151	CAGCTAAGGGGGAGAGTGTG	CCCAGCTTCCTTCATTCATC	217
Ad02A1881	Aradu.A02	1881	p3	(CAT)5	15	4958969	4958983	GCCATGGATTCACCTTGTCT	GATTATGCCCATGATGTCCC	141
Ad02A1883	Aradu.A02	1883	c*	(T)10(TG)7*	23	4961347	4961369	CGGAGTAAGGTGTGTGCTTG	GGAGAGAAAAGGAGCAGGTTC	242
Ad02A1889	Aradu.A02	1889	p2	(TA)6	12	4975649	4975660	AAAGTGCGGCCAGAGAATAA	CGCGTTCATCCCTAAACTCT	189
Ad02A1890	Aradu.A02	1890	p4	(TTAT)5	20	4976276	4976295	ATCCGATCCATGAACACTCC	CGGGGTAAGAGATACCGTGA	137
Ad02A1892	Aradu.A02	1892	p2	(TA)9	18	4977488	4977505	TTTTGTAAATTCATGTGTTAGTTTGAA	TTCGACTTTTGTATTCAGCAAA	280

ID	Chromosome	SSR nr.	SSR type	SSR	Size	Start	End	FORWARD PRIMER (5'-3')	REVERSE PRIMER (5'-3')	Product (bp)
Ad02A1894	Aradu.A02	1894	c	(T)10c(A)10	21	4981438	4981458	TAAAATGCAGTCGTGGAGCA	TGACCGTAATCACCACTTTCC	157
Ad02A1895	Aradu.A02	1895	c	(A)10caaaaata	51	4983005	4983055	TGAAGCTGAATTTTGTTCATCATA	TTTGTGAGACTTGGAACAAAAA	259
Ad02A1900	Aradu.A02	1900	p2	(GA)7	14	4987144	4987157	GCCTTGCCGTACACTCTCTC	TCCGAAAACAAGAAAGAATAAAAGA	101
Ad02A1901	Aradu.A02	1901	p2	(TC)6	12	4988920	4988931	ACCCCATTTCTCAATTTCCC	TACACTCTCCCTCTCCCCCT	248
Ad02A1903	Aradu.A02	1903	c	(A)13ttaattacc	146	4989797	4989942	TGGGTGTGTGAGAACTGAAAA	TTGAAGGAATGGCATGTTATCTT	280
Ad02A1904	Aradu.A02	1904	c	(AAC)6aataata	102	4990108	4990209	TCCCTTTCAAAGATCCAACAA	CAACATGAAGCTGAGGCTGA	241
Ad02A1905	Aradu.A02	1905	p3	(TAT)13	39	4990776	4990814	TTTGGCATAAGTTTCTGAATGC	TGGGGTTGTTTTATTCAAAGAGA	226
Ad02A1906	Aradu.A02	1906	p3	(TGG)6	18	4991403	4991420	CAACAAATGGTGGTTATGATGG	CAATTGACCATGCCTCCTTT	212
Ad02A1909	Aradu.A02	1909	p2	(TG)10	20	4992706	4992725	GGGAACACATGGCACTCATT	AAAAACAAGAGCATTTCTCAAACA	255
Ad02A1911	Aradu.A02	1911	p2	(AT)6	12	4994306	4994317	TGCACCCTTTCTGCTTCTTT	TTTGAAATTTTGTGAATGGTCTC	245
Ad02A1918	Aradu.A02	1918	p3	(AAC)12	36	5012255	5012290	TCCCTCCCCTCTTCTTTCTC	TGATGCTTTGGTTGCTTCTG	235
Ad02A1920	Aradu.A02	1920	c	(GAT)8gac(G/	48	5039255	5039302	AAGGAGGATGATCGGAGGAG	CGATAGGGATGATAGCCGAA	239
Ad02A1925	Aradu.A02	1925	p3	(ACG)7	21	5051636	5051656	AGGAGGAGATGGTGATGACG	CGATAGGGATGATAGCCGAA	221
Ad02A1927	Aradu.A02	1927	p3	(TCC)5	15	5060376	5060390	TTGTAGCAAATTTCGGTGTAAAA	TCAAAACTGCAATGCTCAAAA	247
Ad02A1930	Aradu.A02	1930	p3	(AAT)21	63	5062929	5062991	CAATCATTTGGTGGTTGCTG	TAGGAGTTTCATGGTTGGGC	194
Ad02A1931	Aradu.A02	1931	c	(TAG)5tggact	85	5063315	5063399	TGGGAAAAGCATCTCCACAT	GTTGATGCCATTGTTGTTGC	245
Ad02A1935	Aradu.A02	1935	p2	(TC)6	12	5066644	5066655	ATCCAAACCACCTGAGATGC	GCTAAAGGGAAGCACGAAAA	173
Ad02A1936	Aradu.A02	1936	c	(T)10atttatttag	48	5070249	5070296	TGGAAATCCGGAAAGAGAGA	ATTCATTTGGCCACTGCTTC	252
Ad02A1937	Aradu.A02	1937	p2	(TA)6	12	5071292	5071303	GACCGATTCAACAACAATTTCA	GTGTTCCTAACGGAGATGCC	276
Ad02A1939	Aradu.A02	1939	p3	(TCT)5	15	5082618	5082632	CGAGGGTGCATTAGGGTTTA	GGCTCCATCATTAGAGAGCAA	253
Ad02A1941	Aradu.A02	1941	p2	(TA)8	16	5098071	5098086	TTCATGATCGTATATTCGGGG	ATTCATTTGGCCACTGCTTC	266
Ad02A1943	Aradu.A02	1943	p3	(TCT)5	15	5101691	5101705	TTGGAAACGCCACTAAAACC	GGCTCCATCATTAGAGAGCAA	128
Ad02A1946	Aradu.A02	1946	p3	(ATA)5	15	5119712	5119726	TCCACTGAAATTTTTGGTTTCA	TCAATTATTTTTGGAGCAATTAAAA	258
Ad02A1948	Aradu.A02	1948	p4	(ATTT)5	20	5121487	5121506	CGTGCACTACTCTCTTACCCTTT	TGGGCTTATTGCATCTCCTC	232
Ad02A1949	Aradu.A02	1949	c	(T)10atcaaattc	75	5121749	5121823	GAGGAGATGCAATAAGCCCA	TCAGGAGTTAAACTGTGTAGACCAA	233
Ad02A1950	Aradu.A02	1950	p3	(TCT)5	15	5122808	5122822	TTGGAAACGCCACTAAAACC	GGCTCCATCATTAGAGAGCAA	128
Ad02A1951	Aradu.A02	1951	p2	(TA)6	12	5123000	5123011	TGCAAAAAGCATTCCATCTTC	GCTAATGAAGACAAAATAAACCAGG	277
Ad02A1959	Aradu.A02	1959	p2	(AT)8	16	5133957	5133972	GAAGATAACCGATCCGAGCA	TTTCAGATGCAGGACGTGAG	194
Ad02A1963	Aradu.A02	1963	p2	(AT)6	12	5160800	5160811	AACCAACAAAATACATTCGCAA	CAAGCTAAGTAGTGTAATGTTGGC	275
Ad02A1966	Aradu.A02	1966	p2	(TA)6	12	5176903	5176914	CGCAGTTTTGCAGATTCTTG	TGCAATCTTTGTGGAGGTGT	255
Ad02A1971	Aradu.A02	1971	p2	(TC)14	28	5180309	5180336	TATTTCCATACCCCAACCCA	AACCCTGCTCTTCGATTTCA	246
Ad02A1973	Aradu.A02	1973	p3	(AAT)5	15	5191747	5191761	TTGCAGCAAACATTCAGCTC	AGCTGGTGTGTGGACACTAAGA	263
Ad02A1974	Aradu.A02	1974	c	(T)10aataggctt	57	5194187	5194243	TATACCGCTAGGCGCTAGGA	AATTATGCGGATGTACCCGA	265
Ad02A1977	Aradu.A02	1977	p2	(TA)10	20	5206043	5206062	TGTTGCTTTTCAGATGCAGG	AAATCGTGTTGCTCACGAAA	249

ID	Chromosome	SSR nr.	SSR type	SSR	Size	Start	End	FORWARD PRIMER (5'-3')	REVERSE PRIMER (5'-3')	Product (bp)
Ad02A1978	Aradu.A02	1978	p2	(TC)6	12	5210497	5210508	CCCTCTCCTCATCATCTTCC	GAATTTTGAGTCCCGTTTCG	109
Ad02A1988	Aradu.A02	1988	p3	(TAA)7	21	5243846	5243866	GCATACACAACACTTACTTAAATGGG	TGCGTACCTTACAAAATGAGAAAA	172
Ad02A1989	Aradu.A02	1989	c	(TAT)9c(ATT)	64	5244777	5244840	TTTAAATTTATCCGCATTAAGACTACT	TTTTTGCCAAATTTCAAGCA	141
Ad02A1990	Aradu.A02	1990	p3	(TTA)15	45	5245211	5245255	TTGGGACAAAATTGAAACAAAA	AGAGCAAGTTAGTGTGCAGTGTG	190
Ad02A1992	Aradu.A02	1992	p2	(AT)6	12	5250509	5250520	TCATGCACACCCCTACTTCA	TGAGAGATCGACACGTCACC	249
Ad02A1993	Aradu.A02	1993	p3	(TAA)5	15	5250847	5250861	TACTTCCGTTTTCCCTCCAA	ACGTGAATGGGTAACAAAGATT	270
Ad02A1999	Aradu.A02	1999	p2	(AT)7	14	5266190	5266203	AGAAGTTAGAACCACATCCATTTC	CGTGCTTGTATGCTGGATTC	271
Ad02A2000	Aradu.A02	2000	p3	(AAT)20	60	5273251	5273310	CGTGTCGTGTCTCATATCCG	GATCTTTGGCTGTTTTCTTTCAA	279
Ad02A2001	Aradu.A02	2001	p2	(AT)7	14	5273694	5273707	TTCTTCACCCTATTTTTGTTTTGA	GATGACTTGTTTTCAGGTGAATTG	274
Ad02A2004	Aradu.A02	2004	p2	(AT)6	12	5277858	5277869	CCTAGGACCAATTAGTTGTGGGT	TCTCACACGTATCTTTCACCAGA	169
Ad02A2008	Aradu.A02	2008	p2	(GA)9	18	5294205	5294222	TTTGTGTTTAAGAAGGGGTGC	GCGGTCCAACATCCTTTTT	176
Ad02A2009	Aradu.A02	2009	p2	(TA)6	12	5294748	5294759	AACTTTTGTTGGTTATGTAATGTTTTT	TCATATTAAGGTGGGAAGGGG	163
Ad02A2010	Aradu.A02	2010	p2	(GA)11	22	5298310	5298331	GAGATTCAGCGCTGGAAAAG	CGTGCAATACATTACCGTCG	261
Ad02A2012	Aradu.A02	2012	p2	(TA)7	14	5299189	5299202	TGTTGAGCCACATGGTGATT	TCTAATTGGTTCCCCAGCAC	236
Ad02A2014	Aradu.A02	2014	c	(T)10atcttttgaa	81	5300122	5300202	TTTCTGACGTTCTCTCTCACACA	GATCAATGGTGTAGGGTGGG	275
Ad02A2015	Aradu.A02	2015	p3	(TTG)9	27	5304870	5304896	AACATCCAGTCAGGAGGGTG	CCGAATTAATTAGATAAATCAATGTTC	260
Ad02A2018	Aradu.A02	2018	p2	(CT)7	14	5307510	5307523	AAAATGGAAGGACAATGTTTTTAGA	CACACACATGGTGAGGTCTTG	245
Ad02A2019	Aradu.A02	2019	p3	(ATA)17	51	5307630	5307680	GTTTGCAGATTAATGCCAGAA	CGGATGTTTATTATCCATAGTACTCG	280
Ad02A2020	Aradu.A02	2020	p3	(TAA)9	27	5307962	5307988	TTGTACAGATAATTTCATTGACTTCC	GCGATTGAAATTCCAAAAGAA	265
Ad02A2021	Aradu.A02	2021	p2	(TA)7	14	5311405	5311418	GCAGAGTCTGTCCAACCCAT	CAAACAAGGGCTGGTCTCTT	242
Ad02A2023	Aradu.A02	2023	p2	(TC)7	14	5315533	5315546	TGGGGGAAGGAAGAAGATTT	TGCTTCAATCATGATATTTATTAGACA	251
Ad02A2026	Aradu.A02	2026	c	(T)12aatttcccc	116	5318577	5318692	TTATCTCTTTTCCCAACACCC	GACCCACACACACCCTTTTC	233
Ad02A2027	Aradu.A02	2027	c	(CAT)6ataattg	66	5318941	5319006	GAGGAGGAGGAAGAAGCACC	GGTTGCGGTTGATTTTGATT	280
Ad02A2028	Aradu.A02	2028	p3	(CCA)5	15	5319246	5319260	TGAATCAAAATCAACCGCAA	TGGGAATTACCAGCCTGTTC	258
Ad02A2029	Aradu.A02	2029	p3	(CTC)5	15	5319954	5319968	GGCGGCTATAATCATCCTCA	TACCATTACCACCACCACCC	250
Ad02A2031	Aradu.A02	2031	c	(TA)6tccactgtt	83	5320921	5321003	AGACCTTTGTCATCAATCATCA	TCCACATCAACCCAACAATG	280
Ad02A2032	Aradu.A02	2032	p3	(TTC)6	18	5321183	5321200	TGGTGACGCAGAAAAGTTTG	GGAGAAGGAGAAGAAGAAGAATGA	257
Ad02A2035	Aradu.A02	2035	p2	(AT)7	14	5324986	5324999	TTAGCTAGGGGGCAGTAGCA	GTCCAAATATTTAGTCATTTTACAACA	276
Ad02A2037	Aradu.A02	2037	c	(TG)6(T)10	22	5326597	5326618	TGGATAGACATGTGAGGACGG	CGGTAACGTACGTGATCAAA	271
Ad02A2040	Aradu.A02	2040	p2	(AT)8	16	5329553	5329568	CCCCCACCCTATACACACAC	TTGGCGAATTTAAGTTGGTT	172
Ad02A2041	Aradu.A02	2041	p2	(AT)6	12	5330130	5330141	TGGATAACCAAAGTAGTTAAAAGATG	TCAATCAATCCAAACCAACAA	126
Ad02A2044	Aradu.A02	2044	c	(T)11agatttagg	71	5332226	5332296	TTCATATCAAGATGTTTTCATTGAAGT	TCGAAATGTCTAAAATTCTACCTTTGT	266
Ad02A2047	Aradu.A02	2047	p3	(ACC)8	24	5343704	5343727	TCAGAATTGAAACCATCCCC	GCTTTTTGCTTTTGCTGCTT	278
Ad02A2048	Aradu.A02	2048	p3	(ACA)5	15	5349655	5349669	AATTTCAGCATTCAGCACCC	TGAAGGGATGAAAAGATTGGA	243

ID	Chromosome	SSR nr.	SSR type	SSR	Size	Start	End	FORWARD PRIMER (5'-3')	REVERSE PRIMER (5'-3')	Product (bp)
Ad02A2052	Aradu.A02	2052	p2	(AT)9	18	5353424	5353441	CCAAAGCTCCTAACTCAGCC	TTTCAGATGCAGGTCGAGTG	276
Ad02A2053	Aradu.A02	2053	c	(T)10gaaaatatc	68	5356097	5356164	CAGGAAGAACAATAGGACCTGA	CGAGACCAACAAAATCAAACC	198
Ad02A2054	Aradu.A02	2054	p3	(TTA)5	15	5360501	5360515	GCTTCACAAGCAAAAAGCCT	TGATCTCCCAAGGGAAAATG	237
Ad02A2055	Aradu.A02	2055	c	(GT)9(AT)7	32	5361106	5361137	ATGGATGTCGTCTTTGGGAA	ATGATACACGGGACTCCGAA	277
Ad02A2061	Aradu.A02	2061	c	(A)10tttgtttaaa	43	5368542	5368584	TGTACGATATTTGGATGAAGAAAAA	TGGATGAGAAGAGAAAATAAAATGAA	278
Ad02A2066	Aradu.A02	2066	p3	(TTC)5	15	5399225	5399239	CCTGGGAAGACGGACATAAA	GCTTCCCCATCTGACACCTA	257
Ad02A2070	Aradu.A02	2070	c	(T)17aagatgaa	67	5423107	5423173	GGAAATGGAATTGTGGCACT	ACCAACACTCTCTGCTTCGC	201
Ad02A2072	Aradu.A02	2072	p2	(GA)7	14	5426357	5426370	TGCTTCCATGTTTCCTGATG	TTGCTACACAAAGCACGGAG	138
Ad02A2075	Aradu.A02	2075	c	(T)12ataaatttta	45	5435970	5436014	CGTGACACAGTGCTGCAATA	CCTCTCTCTTCGATCACATCC	172
Ad02A2082	Aradu.A02	2082	p2	(AG)11	22	5456086	5456107	GAGCTTTTGTCCTGGATTCG	TATTTGCATAGGCGTGTTGC	234
Ad02A2087	Aradu.A02	2087	p3	(TAA)5	15	5459695	5459709	GTTTGGAACACCTCCCAGAA	TGGCTTCATCAGGGATATGA	185
Ad02A2093	Aradu.A02	2093	c	(A)10tgaaaatca	142	5470187	5470328	TTTCCTTAGAAATGGATCCGAA	GCACAAATCAATGGTTGAGAAG	250
Ad02A2096	Aradu.A02	2096	p3	(ATA)11	33	5480028	5480060	TCCGAATATCCCAATTCCAA	GCACAAGTCAATGGCTGAGA	234
Ad02A2098	Aradu.A02	2098	p3	(TAT)17	51	5484595	5484645	CCCAAGTCTTTGCCTTGTTC	GGTTACAAACCAGGGTGTCTTT	162
Ad02A2100	Aradu.A02	2100	p2	(AG)6	12	5495569	5495580	AAAGGTTATGTTATTGTTAAAAGGTGA	TGCAGCAACACACAACAGAA	248
Ad02A2101	Aradu.A02	2101	p2	(TA)6	12	5500088	5500099	AACTATCGCTATGGCAACAAA	AGCGTTTTCATCTATCATGGG	272
Ad02A2102	Aradu.A02	2102	c	(CT)6cactcaca	36	5500376	5500411	CTGAACTTGGTTCGTGGGTT	AAGAAGGGGTCAGAGTGGGT	129
Ad02A2103	Aradu.A02	2103	c	(AG)6tgtgagt(	35	5501497	5501531	AGCGGAGTGTTGAGGAAGAA	TACGTGTCACTCCCTCCCTC	114
Ad02A2107	Aradu.A02	2107	p3	(TAC)5	15	5510595	5510609	CTGCTGGAGTGACCAGAACA	GCCGAAAATGAGCTGAAAAG	157
Ad02A2108	Aradu.A02	2108	c	(AAG)5aaagaa	104	5511638	5511741	ATCAGCTGAACCAACCAACC	GTGGACTTTAGGCCCAACAA	261
Ad02A2111	Aradu.A02	2111	p2	(AT)8	16	5520206	5520221	TGTTTTCATAACAGCTGGCAA	ATTTAGCAACAAATTCTACGAACA	226
Ad02A2115	Aradu.A02	2115	p2	(CT)10	20	5525619	5525638	TCACTCCTCACTCATCCCCT	TGAGAGATTTTTGGGTTGGC	256
Ad02A2121	Aradu.A02	2121	p3	(AGA)5	15	5533935	5533949	GATCCCCCTTCCCAAAATAA	AAGAAGTGCGGTGAAGATGG	276
Ad02A2122	Aradu.A02	2122	p3	(AAT)6	18	5534325	5534342	GGGTCACTCTCAATTTTCAACC	TCAATGTTCCACAATTCCACA	270
Ad02A2124	Aradu.A02	2124	p4	(TGAA)5	20	5537274	5537293	GGGATGTTGGAGGAATGATG	TGAAAACAATGTGGCCTTTTC	245
Ad02A2127	Aradu.A02	2127	p2	(AT)6	12	5542155	5542166	TGCGCACCATAATCTCGTAG	CAAGACGAGGCATGAAATGA	214
Ad02A2128	Aradu.A02	2128	c	(T)10ctcgagag	72	5543037	5543108	ATTTTGCCATAGCCTTTGAA	TTAAACATTTTAATTTAAGTGACTCCC	239
Ad02A2129	Aradu.A02	2129	p2	(AT)6	12	5546338	5546349	GTGCATTACTTTGGTTTGCC	TGCCTATATACAAAAGGTCAATCA	279
Ad02A2131	Aradu.A02	2131	p2	(TA)11	22	5546978	5546999	GGTCTAATCCTGTTCCGTCG	CAGAACACTTCAACAATTGCAT	117
Ad02A2136	Aradu.A02	2136	c	(AT)8ccatgcttt	102	5555042	5555143	CTTTTAATGCCATTGCCACC	CCGAACCAAACCTGAACACT	218
Ad02A2139	Aradu.A02	2139	p2	(TC)6	12	5566208	5566219	GGCAAAATTATCCGAACCCT	AAATGAGTCGGGCTATGGTG	258
Ad02A2140	Aradu.A02	2140	c	(A)10gaaaatata	88	5567857	5567944	TTTTCCGCTTAAATGATGATG	TTATGGCCATTACAAAATCCA	271
Ad02A2142	Aradu.A02	2142	c	(T)10aata(T)1(	24	5569759	5569782	TCCGACCGTTCCTTTTTATG	CGCCAAGCAATCCTTTCTTA	278
Ad02A2144	Aradu.A02	2144	p3	(ATA)7	21	5571024	5571044	TCTATTTGATAACTCATCCAGCTTGT	TGCATGCAAGCTTTTGTATGTAT	268

ID	Chromosome	SSR nr.	SSR type	SSR	Size	Start	End	FORWARD PRIMER (5'-3')	REVERSE PRIMER (5'-3')	Product (bp)
Ad02A2145	Aradu.A02	2145	p3	(TTA)5	15	5571576	5571590	CACTCCCACACAGAGCAACA	TCTCATCAATGATCAAACACCC	159
Ad02A2151	Aradu.A02	2151	p3	(ATT)9	27	5578970	5578996	AGACAAGCACACATCAACCG	CAATTGCAGAGTACGCCAAA	263
Ad02A2154	Aradu.A02	2154	p2	(CT)6	12	5582007	5582018	TTTGCTTTTTCTCGTTCTCTCT	AATGTTGACTTGGGCCTCAC	185
Ad02A2155	Aradu.A02	2155	p2	(GA)6	12	5582211	5582222	GTGAGGCCCAAGTCAACATT	AAGTCGATCCAACGGTGAAC	198
Ad02A2156	Aradu.A02	2156	p2	(AG)6	12	5586857	5586868	GGGGAGATTTGTTGGGTTTT	CCAACGGTGAACAACTGAGA	224
Ad02A2158	Aradu.A02	2158	p3	(TTA)9	27	5590568	5590594	TGTGGCAATTGATGCTGATT	AATGGAGAGTGGAGACCATGA	201
Ad02A2159	Aradu.A02	2159	p3	(ATA)20	60	5591831	5591890	CGTTGGCATGAAATTGAAAA	AAAGTTGGTAACATTTGAGTGAAAGA	248
Ad02A2162	Aradu.A02	2162	p3	(TAT)5	15	5600011	5600025	GATTGGATTTATATCTCCACGG	TTAAGATCGCGTGCTCAATG	185
Ad02A2163	Aradu.A02	2163	p2	(TA)8	16	5604691	5604706	CTTTAGGGCCTGTTCCATCA	AAGCAGTGATGAAGCCACCT	223
Ad02A2164	Aradu.A02	2164	p3	(GAA)5	15	5607009	5607023	AGAGAAGGAGGAGGAGGTGG	AAAATTGAGCATCGTAGCAGC	277
Ad02A2165	Aradu.A02	2165	p3	(ATA)5	15	5615490	5615504	CCTTCGATACCTCTGATGTTCC	TGGTAACAGTTTGCAATAAGGA	256
Ad02A2168	Aradu.A02	2168	p3	(TCT)7	21	5620895	5620915	TCCTTCCGTTAAGTGGGTGT	CAGTGACGAGCGATAGGGTT	198
Ad02A2169	Aradu.A02	2169	p2	(TA)6	12	5626689	5626700	TTGGGTTAGGGTATGAACAAAAA	GGCGTATTGGTCATGTGTTCT	266
Ad02A2170	Aradu.A02	2170	p2	(TA)6	12	5631792	5631803	AAAAGAAGAAAAAGGTGCTTTCAA	GAACAAGTCATGGATTATGCAA	280
Ad02A2171	Aradu.A02	2171	c	(T)10gtaatatttt	92	5642591	5642682	CGCGTGTTTCATCTTTTTCA	TTTTCGGTGTATGTGTGTTGA	216
Ad02A2174	Aradu.A02	2174	c	(GAT)5ataa(A	34	5643391	5643424	TGAATGTAAGTTCATTTTCTTCCAA	AACCTAGGTCACCCACTCCA	264
Ad02A2175	Aradu.A02	2175	p3	(AGG)11	33	5644291	5644323	CCTCAGCGATTTGATTGGAT	TCATGTGTCGACATTCTTTCTTC	242
Ad02A2176	Aradu.A02	2176	c	(CCA)5cattatc	146	5645438	5645583	CTGTGTTTTCTCCCACCACC	TGTTGTTGAAATTTGTTGTGGA	204
Ad02A2177	Aradu.A02	2177	p3	(AAG)5	15	5645685	5645699	CCACCACCACCACATTATCA	CAGAAGGGGCTTCTTCTTCC	189
Ad02A2180	Aradu.A02	2180	p3	(TAA)14	42	5667127	5667168	AAACCGAACTTTTTCGTAAGTCC	TCTAATGCGGGAATGAAAGG	199
Ad02A2185	Aradu.A02	2185	p3	(TAA)17	51	5673855	5673905	CACAAAAATTGCTGACCCCT	AAACTTGCATGTTGGAATGC	268
Ad02A2186	Aradu.A02	2186	p3	(TCT)9	27	5677749	5677775	GGGGTCTAGGGTTCTTCTCG	ACCCTCTTCATCACAGTCCG	129
Ad02A2187	Aradu.A02	2187	c	(GAA)5ggagaa	94	5680070	5680163	TGAAAAGGCAGAGAGAATAACG	TTTCCAAAGCGCTACACTCA	256
Ad02A2190	Aradu.A02	2190	p3	(TGT)5	15	5684744	5684758	CCAAAGCGGTCGATCTTAAA	TCAACCGAGGAAGGAAAATG	208
Ad02A2194	Aradu.A02	2194	p2	(AC)6	12	5716181	5716192	GGCCAAACAGGCTAAAAACA	ATCGCGGATGGAAGTAAGAA	176
Ad02A2199	Aradu.A02	2199	p3	(GAA)5	15	5737235	5737249	CAGAGAGTACACATAAAAGAATTCAAG	AAGGCATAGATTCAACCCCC	184
Ad02A2201	Aradu.A02	2201	p2	(CT)6	12	5738534	5738545	TTCCCTTCACAGCCATTTTC	AAGCAACGCTCTTCTTCCTTC	263
Ad02A2203	Aradu.A02	2203	p2	(CT)6	12	5747717	5747728	CCCTTCACAGCTATTTTCGG	ATGCTTCAACCAAAGCAACG	273
Ad02A2205	Aradu.A02	2205	p2	(AT)7	14	5751140	5751153	GTAGTCTCTCCAACGGTGCG	CGAAATGTTGCATTGACCTG	177
Ad02A2206	Aradu.A02	2206	p3	(TTC)5	15	5751477	5751491	TACGGGACACATGTGGAAAA	CGAGCAAAATGTTGGTGTTG	235
Ad02A2210	Aradu.A02	2210	p3	(AAT)5	15	5757745	5757759	GCCAAACACACTGCATCAAC	CGAAAGAGGCCTTTGAATTG	182
Ad02A2211	Aradu.A02	2211	p3	(GTT)7	21	5762017	5762037	CACCAGAAGCACAAAGCAAA	TGGTCTGAAGCCATGAAAAA	267
Ad02A2218	Aradu.A02	2218	p2	(AT)12	24	5773353	5773376	AGGGAAGCAGAGCATATTGG	GGATAAAACTGTTCCAATCAGAAT	245
Ad02A2221	Aradu.A02	2221	p3	(AGA)5	15	5780528	5780542	CTGCGTGTACGAATTTGGAG	TTTAAATCACGCGTTCCTTTTT	169

ID	Chromosome	SSR nr.	SSR type	SSR	Size	Start	End	FORWARD PRIMER (5'-3')	REVERSE PRIMER (5'-3')	Product (bp)
Ad02A2230	Aradu.A02	2230	p2	(TA)9	18	5802446	5802463	TGCAAAATTAAACCAAGAATGA	AAGTTGGCAACCATCAACCT	246
Ad02A2232	Aradu.A02	2232	p2	(AG)7	14	5809011	5809024	CACCTGAGAGGCTGAGATCC	ATGCAACAAAGCCCACTTTC	259
Ad02A2233	Aradu.A02	2233	c	(ATA)15(AGA	66	5809704	5809769	CTGAACATTTAAAACTAAACAAATTGA	AGAGTGTTAATCCAGTTTAGGAGC	225
Ad02A2236	Aradu.A02	2236	c	(TA)6tccaaaac	56	5812346	5812401	ATGGCTTTTTGCAAGAGAAA	CATTGGATTGGTGAAGTGGG	190
Ad02A2237	Aradu.A02	2237	p2	(AG)6	12	5816993	5817004	ATCAGCAACAACCCCAACTC	AAGCCTCTATCTTCGAGCCC	257
Ad02A2238	Aradu.A02	2238	c	(TG)6cgcgtgt(	43	5817240	5817282	CTGCAGCTCTTCTTGCCTCT	TCGGTGGTTAACCGAGAAAC	174
Ad02A2239	Aradu.A02	2239	p3	(TTA)17	51	5819560	5819610	TTCTTCACGACTTCATCCCC	TGGAAAAAGACTTTGCTGAAA	279
Ad02A2242	Aradu.A02	2242	p3	(TCA)5	15	5821065	5821079	TCCAAGCTGAACAAGAACCC	CATCTTCTTCTTCTTCGCCG	218
Ad02A2246	Aradu.A02	2246	p3	(ATT)6	18	5823625	5823642	GTTGCTGAGGCTGCTTCAAT	ACTTGAAACGGTTTTGGCAC	230
Ad02A2249	Aradu.A02	2249	p2	(AT)8	16	5837442	5837457	TGTGAACTGGCTCGATTATATG	AGCTCACGAGTTGGCTTAAA	280
Ad02A2257	Aradu.A02	2257	p2	(TA)6	12	5888322	5888333	TCTTTGCAGCAATTGTTTCATTAT	TCCTTCCTGGTGCATACCTC	153
Ad02A2263	Aradu.A02	2263	p2	(TA)9	18	5906343	5906360	GAATAACCATCCGCGAGAAA	ACTCGTTTCATTTAAAGGGATTT	269
Ad02A2264	Aradu.A02	2264	p2	(TA)7	14	5910077	5910090	CCGTCACTCTAATTCCACCC	AATATGCTGTGGCCCAAAAA	277
Ad02A2268	Aradu.A02	2268	p3	(ATA)9	27	5912020	5912046	TTAAGCCCGAACATTCCTTG	GCAAGTCTATGGGGCGTTTA	158
Ad02A2272	Aradu.A02	2272	c	(A)10ttcaacccg	51	5915735	5915785	CCTAAATCAGCCCCTGACAA	ATCCGGCTCCTGATGATTTT	221
Ad02A2273	Aradu.A02	2273	p2	(AT)7	14	5916427	5916440	AAAAACATAGGATTTGGTTTCAGTG	GGTCCGGTTTTCAGAACCTT	261
Ad02A2288	Aradu.A02	2288	p4	(TAAT)5	20	5940318	5940337	AACGCGCCTAACCTAAAATTC	TCATGGTGTGGTTTTTCTGG	256
Ad02A2289	Aradu.A02	2289	p2	(CT)7	14	5940726	5940739	TCCCGAAACTCTCTACCCAA	CCACTTGACCAACCCAAGTT	226
Ad02A2290	Aradu.A02	2290	p2	(AT)6	12	5940886	5940897	TCCCGAAACTCTCTACCCAA	CCACTTGACCAACCCAAGTT	226
Ad02A2291	Aradu.A02	2291	p3	(AAT)19	57	5943696	5943752	TGCATTTGGTCTTCAATCCA	GGCTCTTCCTCTCTGGTGTG	230
Ad02A2297	Aradu.A02	2297	c	(A)10cccaactc	39	5966306	5966344	AGAAAAACCCAAATCAGCCC	TTCGAGGTAATTGTCAGGGG	125
Ad02A2299	Aradu.A02	2299	p3	(TTA)5	15	5971488	5971502	TCAAACCACATCAATTGGGA	TGGGTTTGTCAAAGTTTCTGG	160
Ad02A2301	Aradu.A02	2301	p3	(ATC)5	15	5982904	5982918	ACCCAAACCAAAACCTCACA	GGTGTTGGGACCGAGTTAGA	280
Ad02A2312	Aradu.A02	2312	c	(CTT)5gctgcac	148	6015134	6015281	GCGCTTCAAACCATTCTTCT	TCCTCATCTTCTGCTGCTTCT	279
Ad02A2313	Aradu.A02	2313	p3	(GAA)5	15	6015502	6015516	AGCAGCAGAAGATGAGGAGG	GTTGCTACACATACGGGCCT	271
Ad02A2314	Aradu.A02	2314	p3	(ATG)5	15	6016806	6016820	ATGACAAGGGAAGCAACCAC	GCAACAACACTTGGAGCAAA	130
Ad02A2318	Aradu.A02	2318	p2	(TA)8	16	6023863	6023878	TTGTTCAAACAACACAAATCAAAA	TCATTTGGTAGCAGAAAGATATGA	203
Ad02A2327	Aradu.A02	2327	p3	(TTC)12	36	6031795	6031830	CAAAACCGGTGCTTATACCA	GCTGAAGAACAGTTAACAGCCA	224
Ad02A2330	Aradu.A02	2330	p3	(GAA)5	15	6047300	6047314	AATCGTCATGGCACAAAATG	TACAAAATTCCAAGCCCCAA	242
Ad02A2331	Aradu.A02	2331	p2	(TA)6	12	6048361	6048372	TTCCATTAATGCCCCTCTTG	AACACAGAACCTGCGTGTTG	262
Ad02A2334	Aradu.A02	2334	p2	(AT)8	16	6055723	6055738	GCATACGTAAAATTACTCAACTATGTG	AACCCAAACAAACCAATCCA	236
Ad02A2336	Aradu.A02	2336	p3	(AAT)13	39	6057934	6057972	AAAGTGGTTTGGTACATGTGAAA	TGGAATGATTTAAGGGCAAGA	276
Ad02A2340	Aradu.A02	2340	p3	(CAT)7	21	6068486	6068506	ACGAGCCTCCACTTCAAAGA	GCCCTTGCGTTTGAATATGT	236
Ad02A2344	Aradu.A02	2344	c	(A)10gtcacatat	119	6081237	6081355	CGTAAAAATGGGGGTGAATG	ATGTGACCAAGGCATTTTGG	265

ID	Chromosome	SSR nr.	SSR type	SSR	Size	Start	End	FORWARD PRIMER (5'-3')	REVERSE PRIMER (5'-3')	Product (bp)
Ad02A2350	Aradu.A02	2350	c	(ATA)5gaatatg	104	6092704	6092807	TGAAAGGTTGCAGTCACTTG	TTCCGCCAGTTGTTTAATTT	276
Ad02A2352	Aradu.A02	2352	c	(AG)6(A)10	22	6093142	6093163	AGGTCATTTGGCATCATTCA	TTCTTTTTCTTAATTGGGTGCC	238
Ad02A2354	Aradu.A02	2354	c	(ACC)5acacag	95	6101377	6101471	CGGTGACAGCAAGCAGAGTA	CTCTATAGCCGACTCACCGC	265
Ad02A2359	Aradu.A02	2359	p3	(CAT)6	18	6117842	6117859	ATAGTTGGAGTCGACCGTGG	GTTGGTGAGACTGGACCGTT	196
Ad02A2363	Aradu.A02	2363	p2	(AT)7	14	6122860	6122873	GCAATGATGCACGCTTAGAA	TCCAGCAATTAAAAGCAAAATG	240
Ad02A2364	Aradu.A02	2364	c	(AT)6aagatgcc	53	6123791	6123843	TTTTCCGTACCACACTTCCA	CCTACCAGTTCTAGTTCCCCA	262
Ad02A2369	Aradu.A02	2369	p3	(TAA)15	45	6137508	6137552	CATCCCTTTGGTCATTTTGG	TTTTTACTAGCCCTTGATGCTT	177
Ad02A2370	Aradu.A02	2370	p3	(TTC)5	15	6144399	6144413	ACGTTTCCTTGATTGCCATT	CATATTTTTCATCATTGATTGGGA	271
Ad02A2376	Aradu.A02	2376	p3	(AGA)5	15	6160332	6160346	CTGTTGTGGATGATGAACCG	TGTTCCAAGAATTGATGCCA	230
Ad02A2378	Aradu.A02	2378	p3	(CTT)5	15	6168520	6168534	AACCACCATGTAATATATCTGGC	ATGCTTCCCCAAATACACGA	101
Ad02A2382	Aradu.A02	2382	p3	(TTG)7	21	6173772	6173792	CCATCCAGAGGTGGTCAAAT	TCCCATTTAGCTCAAGGCAT	234
Ad02A2387	Aradu.A02	2387	p2	(TA)6	12	6201206	6201217	GCTCATTAGCGACTAACCCG	GAAGTGAAAGGGTGAAACCAA	260
Ad02A2389	Aradu.A02	2389	p2	(TA)9	18	6205151	6205168	TGGACTGGGTTAACCTGTTTG	ACCCCGCTCCATATTATTCC	183
Ad02A2395	Aradu.A02	2395	p2	(AT)7	14	6217630	6217643	AGAAATGTAATTGACAAACAAAACA	CATACATAAATTTTGATTTTGAGCA	240
Ad02A2396	Aradu.A02	2396	p3	(TAT)6	18	6217881	6217898	AAGATCCACAAATTTTGCTTCAA	CACGTATTATGCTTAAAAAGCAATTC	101
Ad02A2402	Aradu.A02	2402	c	(T)14accttttttt	120	6231577	6231696	TGGGATTGTTTTTGTTTATGGA	CCAATCATGAATCATCCCAA	276
Ad02A2403	Aradu.A02	2403	c	(A)11g(A)10	22	6231894	6231915	AGCGGTTCCAGTTAGCAAGA	GCACATGGAGTGAGTTGCTG	126
Ad02A2404	Aradu.A02	2404	c	(A)15tttaaaattg	80	6233813	6233892	TTTGGATTGCATCTTTTTGC	CATGCCTAATATGCTTCTCACA	228
Ad02A2407	Aradu.A02	2407	p2	(TA)6	12	6240776	6240787	CTCTTCGATCTCGCCTCAAC	GCATGCACCATCAAATTTCT	279
Ad02A2408	Aradu.A02	2408	p2	(TG)11	22	6245061	6245082	TAAAGCTGCAGATGGCAGTG	CCAAAGCCATTACTGGGAAA	176
Ad02A2409	Aradu.A02	2409	p2	(TC)6	12	6253292	6253303	TTAGCTCAATGTGGTGTTGTCT	TGAGTTCGAGAATGAAGAGTGAA	257
Ad02A2413	Aradu.A02	2413	p2	(AT)6	12	6256905	6256916	TGCAGTTCTTCATTAACTAGCGA	GGGGTCCACAGAATAGGTGA	272
Ad02A2419	Aradu.A02	2419	p3	(TAT)5	15	6265125	6265139	ATGCCACCAGCAACACATTA	CATGGCTTTGATGGGAAAAG	176
Ad02A2423	Aradu.A02	2423	c	(A)10taaaaaatt	79	6270785	6270863	GAGATTTGAATCCTTAATTTGCC	GCATGCAATATGATTTGTTTGTG	256
Ad02A2424	Aradu.A02	2424	p2	(TA)6	12	6272276	6272287	CAAAATTTGATATGATACATCGACA	TCCTTAACCAATTCTTCTTGATCC	209
Ad02A2426	Aradu.A02	2426	p3	(TTA)5	15	6278834	6278848	TGTGTTTAATTCCTTTTTCAAGTGAG	GCAAATTTAACAAGCCATGC	270
Ad02A2427	Aradu.A02	2427	p3	(TAT)6	18	6279549	6279566	TGCATTTTTAAATCCTTGTTCA	AACAGCAAATTCAACCAGCC	198
Ad02A2430	Aradu.A02	2430	p3	(TTA)14	42	6281359	6281400	AAATCCTTGTTCAAGAGAGTTGAAA	AACAGTAAATTCAACCAGCCA	279
Ad02A2432	Aradu.A02	2432	p2	(AT)12	24	6283943	6283966	ATTTCGACATGGCTTATGGC	AAGAGATTAGGAGATTGGTTGTGG	266
Ad02A2434	Aradu.A02	2434	c	(AT)9tgaaa(AT	41	6285075	6285115	TCAACCAAAAGACAAGAATAACAAA	CACGTGTGGTGGAAACAAAG	274
Ad02A2440	Aradu.A02	2440	p2	(CA)6	12	6302873	6302884	GGCAAGACCCAGAGTCAAAA	TGCTGCAGCTTTTCATTTGT	235
Ad02A2441	Aradu.A02	2441	p2	(TA)11	22	6303272	6303293	ATGGCTATGCTTCATTTTGC	GGGCATAACATTTTGAAGGC	247
Ad02A2442	Aradu.A02	2442	p2	(TC)6	12	6303538	6303549	GCCTTCAAAATGTTATGCCC	AACAGCATATTGGTCCACCTG	244
Ad02A2443	Aradu.A02	2443	p3	(ATT)24	72	6307277	6307348	TGTTTCATGCTCCATTCCAA	GCAAGGTGGTTTTCCACAAA	279

ID	Chromosome	SSR nr.	SSR type	SSR	Size	Start	End	FORWARD PRIMER (5'-3')	REVERSE PRIMER (5'-3')	Product (bp)
Ad02A2449	Aradu.A02	2449	p2	(TA)7	14	6320279	6320292	TGCAGCATTGTTCAAATTCA	GGCTTCGGACATAAGACTGG	209
Ad02A2450	Aradu.A02	2450	p2	(AT)6	12	6321060	6321071	CGCTTGATCCCAGATTTCAT	TTTCAATGTAGTTTTTGCACATTT	177
Ad02A2452	Aradu.A02	2452	c	(TCT)5tttctttcg	107	6324206	6324312	TCGAACCGTTCTTCTTCCTC	CCTCCTCATCTTCTGCTGCT	268
Ad02A2453	Aradu.A02	2453	c	(AAGAGG)5a	90	6324791	6324880	CCTCCTCATCTTCTGCTGCT	TGCTAACGGGCCTTTTAATG	249
Ad02A2456	Aradu.A02	2456	p3	(ATA)5	15	6332246	6332260	CAAGTCGTTTGAAGTGCAGG	TCTTGGCTGATCCAAATGAA	242
Ad02A2457	Aradu.A02	2457	p3	(AAG)8	24	6332442	6332465	GCAGGCCGAAAACAAAGTAT	GCAACCATGAAAAGATTCCAG	278
Ad02A2460	Aradu.A02	2460	p2	(TA)6	12	6333898	6333909	TCCGGAACAATAATGTTTCAAT	TCAAATAGGTTCTCGGAAAGGA	274
Ad02A2462	Aradu.A02	2462	p2	(TA)6	12	6335243	6335254	TGGAATTGTTCCTCAATTTCG	AGGTCATTGCTGGAATTGCT	237
Ad02A2464	Aradu.A02	2464	p3	(GAA)7	21	6342961	6342981	ATGGCAGGAGAATGTGAAGG	GTCGGAGAAGACAAGGCAAG	247
Ad02A2466	Aradu.A02	2466	p2	(CT)12	24	6346791	6346814	TGGTACAGGGTGATTGACGA	GGGCAGCAGAGCTAAACAAC	234
Ad02A2469	Aradu.A02	2469	p3	(GCA)5	15	6359179	6359193	CAGCGCCTTCATAGAGGAAC	GCTTAAAGTGGCAGTGAGGC	187
Ad02A2470	Aradu.A02	2470	c	(A)10ttatcaaac	114	6363202	6363315	TGGCTATGCAGATGCAAAAT	TGTCAAAAGGAACACAAACACA	279
Ad02A2473	Aradu.A02	2473	p3	(GCA)5	15	6369661	6369675	TGCCTTATGAGAGGAACCATTT	TATGGCCATTGTTTGCTGAG	248
Ad02A2478	Aradu.A02	2478	c	(T)14acttagaaa	102	6381536	6381637	TGTCGAAACAGACCCCAAAT	CACACTCCATACATACCATCCAA	266
Ad02A2480	Aradu.A02	2480	p3	(GAA)7	21	6399976	6399996	GTTCGGAGGAAAGGGAAGAG	GTAAGCTTTCGATCTTGCGG	201
Ad02A2481	Aradu.A02	2481	p2	(CT)6	12	6401647	6401658	ATTATGGGAACTGGGAAGGC	AGGTTGGATGCTGAGAATGG	236
Ad02A2484	Aradu.A02	2484	p3	(TAT)7	21	6405652	6405672	TGACAAGTTAGGACCACCGA	GAGGAATGTTAGGAGGCAACA	269
Ad02A2486	Aradu.A02	2486	p3	(TTA)6	18	6407289	6407306	GGAAAGCCTATGGTTTGGAA	ACCCCACTCCTCCATTCTCT	203
Ad02A2488	Aradu.A02	2488	p2	(AT)6	12	6408652	6408663	AGGCTAAACCCTCCCCTACC	CTTCCTTACCCCTACCTGCC	233
Ad02A2490	Aradu.A02	2490	p3	(AGT)5	15	6424625	6424639	CCGCTGCAATCTTCTGATTT	ACGAGTGCAATTATGGGACC	226
Ad02A2491	Aradu.A02	2491	p2	(TA)6	12	6425181	6425192	ATATGGGTGGGAGGCACATA	GATCTCGGAATCAGAACGGA	272
Ad02A2493	Aradu.A02	2493	p2	(TA)8	16	6426489	6426504	CTCCTCGTTTCTTTTGGCTG	TTTCCAAACCAACGAACCAT	170
Ad02A2501	Aradu.A02	2501	p3	(TTC)7	21	6435809	6435829	CTCCACGTCCAAGCAATTTT	AACGCGAAGAAGAAGGTGAA	149
Ad02A2502	Aradu.A02	2502	c	(AGA)5at(GA(	35	6436470	6436504	TCAGATTCAGAACACCTGCG	TCTCCTCTTCCTCCTCCTCC	187
Ad02A2503	Aradu.A02	2503	c	(AAG)6catagg	44	6436619	6436662	GGAGGAGGAGGAAGAGGAGA	TTCCCATGCTACACCCAAAT	247
Ad02A2506	Aradu.A02	2506	p3	(AAT)5	15	6447153	6447167	AAAATTAGTCGGAGTGAGCACAT	GATTGGTAAGTGCTTGAACACG	248
Ad02A2509	Aradu.A02	2509	p3	(TAA)16	48	6451375	6451422	TCTTCTTCCTTAATTCAAACATCAGA	AGTAGAGACCCCGCCTTGAT	163
Ad02A2514	Aradu.A02	2514	p2	(AT)7	14	6465801	6465814	TTTTTCCGTTTCATAACAGTGC	TGCAGATTGATGGAATGGTG	239
Ad02A2516	Aradu.A02	2516	p2	(AT)8	16	6480509	6480524	TTGATTGATTTAGGTATTGGTCTTTTT	CCTTGTACATGCTAGCTCACACT	268
Ad02A2518	Aradu.A02	2518	p2	(AT)8	16	6483532	6483547	TGCCCTTGAAAGTTTGTTCC	TGGCTAGTTAAACACAGCTTAAATG	255
Ad02A2519	Aradu.A02	2519	p2	(GA)6	12	6489132	6489143	GAGTGAGTTTGTTAATTTTGGAGGA	CATTCTTTCGTTAAAATTGAGATGA	225
Ad02A2521	Aradu.A02	2521	p3	(ATA)7	21	6490070	6490090	GAGAAATGAAAATTCGTTTAGAATCA	CTCTCCCAAACTCCCTCTCC	218
Ad02A2524	Aradu.A02	2524	p2	(AC)7	14	6492484	6492497	CACTGTGTTTTGGTGATCCG	AGAAGAATGCAGGTAGCCGA	184
Ad02A2527	Aradu.A02	2527	p3	(TTC)7	21	6497362	6497382	CCTGGTCTCAGCCATTTGAT	TTGGGGAAGAACAGTGAAGG	180

ID	Chromosome	SSR nr.	SSR type	SSR	Size	Start	End	FORWARD PRIMER (5'-3')	REVERSE PRIMER (5'-3')	Product (bp)
Ad02A2528	Aradu.A02	2528	p3	(TGC)5	15	6498616	6498630	GCACTCATGCCAGAGAAGGT	TGCCTTAGTAGATGAACCATTTTG	186
Ad02A2531	Aradu.A02	2531	p2	(TA)7	14	6502763	6502776	TCTCGTGCTGATAACGTGTTG	TTTTTCTTTCTCTCTCTTTACAGCAA	215
Ad02A2534	Aradu.A02	2534	p2	(GA)9	18	6516769	6516786	CAACATACCTGGAGGGAGGA	GCCAGTAAAACCGACGGTAA	275
Ad02A2538	Aradu.A02	2538	p2	(TA)6	12	6520873	6520884	GAATTGGAGGGTGTGCTTGT	AAAATGCGGTCAATATTTTTGT	280
Ad02A2539	Aradu.A02	2539	p3	(GCT)5	15	6524981	6524995	GAAAAACATGGAGGAGGCAA	TGTTGGTTCGTTCCATCAGA	278
Ad02A2541	Aradu.A02	2541	p2	(GA)6	12	6551777	6551788	CGTCAACCCCTTGTGTTTCT	CCTTTTCGTCTCCCTCCTCT	158
Ad02A2549	Aradu.A02	2549	c	(A)10catttttcg(	59	6588147	6588205	CCTTTTCAATCAGACGTTTCAA	AGATTGCAGATTCGAGTCTCTT	280
Ad02A2551	Aradu.A02	2551	p3	(GAA)5	15	6592529	6592543	AAACAAGCCATATTGGGAGC	GGAAAAGTGATGGTCCGCTA	251
Ad02A2557	Aradu.A02	2557	p3	(CAC)7	21	6632019	6632039	GACCCATCAGTCTCCCTTGA	GATAGCAACATGGCAGAGCA	245
Ad02A2558	Aradu.A02	2558	p2	(TC)17	34	6645954	6645987	AACCTGTATAGCCAGTAATCAGTCA	TTGATTCCAATCGGGTTGTT	252
Ad02A2562	Aradu.A02	2562	p3	(GCA)5	15	6655404	6655418	CATGCATGAAACTTTGGCAC	AAGAGAATCATGGACCACGG	184
Ad02A2563	Aradu.A02	2563	p3	(CTT)10	30	6658407	6658436	CCCCAAAGTTTCAGTTTGTTT	CCAGAAATTTCACAAAAAGAATTT	280
Ad02A2564	Aradu.A02	2564	p3	(GGT)5	15	6658711	6658725	TTTGGTTGGTGTGATGGAGA	TTTTTCGCTCCAAAATGGTC	179
Ad02A2570	Aradu.A02	2570	p3	(TAA)6	18	6690180	6690197	GCAAAGGCATTGATCCAAAT	TCAAGGTTTAAATCCGGCAA	259
Ad02A2574	Aradu.A02	2574	p3	(TGC)5	15	6705915	6705929	AAGAGAATCATGGACCACGG	CATGCATGAAACTTTGGCAC	184
Ad02A2575	Aradu.A02	2575	p4	(AAAT)5	20	6712501	6712520	CCAATGTTCTCCGAAGCCTA	GCATGGATTTGTTGTTGCAT	232
Ad02A2576	Aradu.A02	2576	p3	(ATG)6	18	6724066	6724083	TGGAGCTGGATGAGTGTGAG	ATGCAAGATGAAAAATCGGC	185
Ad02A2578	Aradu.A02	2578	p2	(TA)6	12	6733823	6733834	CCATCTCCCCTCTTTGTTGA	TCGAATTACTAGGAAGGAAACAAAA	217
Ad02A2581	Aradu.A02	2581	p3	(TAA)17	51	6737336	6737386	TGTTATAGCAATAATTTGGCATGTT	TTTGGTGACGTGCTTCTTTTT	121
Ad02A2582	Aradu.A02	2582	p3	(TAT)9	27	6737576	6737602	GGTTAGGGACTTACAAAACCTTTC	TCCCATTTGTAAACACCCCT	278
Ad02A2585	Aradu.A02	2585	p2	(AG)6	12	6742914	6742925	CCCTGGCTGTTCTCCCTTAT	AACGCATCAAGGGCTAGAAA	250
Ad02A2586	Aradu.A02	2586	p3	(GCA)5	15	6751867	6751881	CAATGATTGGCCCACAATAAG	TTCTAATCACGGTGCAGCAA	251
Ad02A2591	Aradu.A02	2591	p2	(TA)7	14	6767636	6767649	CAATAAAGTTCCGCAAATAGAAAAA	CGTATCATTTCGTTACGGACA	280
Ad02A2592	Aradu.A02	2592	c	(A)12gaaaagaa	117	6771387	6771503	ACCAATGCCAATACCCCTAA	CAAAATTGGCATAAGTTGTTGG	270
Ad02A2596	Aradu.A02	2596	p3	(CCA)6	18	6774170	6774187	CCTTTACCACCATCAGCACC	TTGCATTTGCTTTTGCTTTG	221
Ad02A2598	Aradu.A02	2598	p4	(TAAA)5	20	6775457	6775476	ATCAATGCTCTCGGCACTTT	ATTCGCAGCCGATATTTTTG	225
Ad02A2600	Aradu.A02	2600	p3	(TGA)5	15	6777411	6777425	CATCAACAATAATAAGGTAAACCCAA	TACCAGTTCTGGTCCGGTTC	247
Ad02A2602	Aradu.A02	2602	p2	(TA)6	12	6780424	6780435	ATTCCAGGATCACGTCCAAG	GGAATTCCCCTCTTCCAAAG	270
Ad02A2610	Aradu.A02	2610	c*	(GAA)5gaggaa	203	6809711	6809913	TGAAAAGGCAAAGAGAATAACGA	AAGCGCTACACTCACCGTAAA	280
Ad02A2611	Aradu.A02	2611	p3	(TAT)10	30	6810768	6810797	GAATGTGAAGGGGAGTTGGA	TGACGACCGATCCAATAACA	142
Ad02A2612	Aradu.A02	2612	p4	(TATT)5	20	6816474	6816493	TTTGCATATCTGTTCGCAAATC	GGGTATCAACAAATCCGATCC	280
Ad02A2614	Aradu.A02	2614	p4	(TTAG)5	20	6820379	6820398	GCGTGAATTGGAAGGGAATA	ATCTTGGGAAGATAACCCCG	256
Ad02A2615	Aradu.A02	2615	p2	(TA)7	14	6824607	6824620	TGACCAATGAACTAACAACGG	CATAACACTATGCTGGGGCA	270
Ad02A2617	Aradu.A02	2617	p3	(AAT)9	27	6827526	6827552	CGTAGCAACGGTTAAGCGAC	AGTGAAAACTCGTGTGTGCG	262

ID	Chromosome	SSR nr.	SSR type	SSR	Size	Start	End	FORWARD PRIMER (5'-3')	REVERSE PRIMER (5'-3')	Product (bp)
Ad02A2618	Aradu.A02	2618	p3	(ATA)6	18	6828000	6828017	CGATGAAACGTCATCAGTGG	GAAAGGAGAATGGAGCATCG	226
Ad02A2619	Aradu.A02	2619	p3	(TAA)9	27	6831175	6831201	TGGTTATATCAGTAAGCTTGCAAAA	TCCATAACCTTGGCAAAAGAA	212
Ad02A2620	Aradu.A02	2620	p2	(AT)8	16	6832018	6832033	GCACACATATGCGCACACAC	CGTGGATTTCAATCTCTACGC	228
Ad02A2621	Aradu.A02	2621	p4	(AAAT)5	20	6839151	6839170	AAAAAGCCAACGATAACATAACAA	CGATCCAATCCAATCCAAAC	222
Ad02A2627	Aradu.A02	2627	p2	(TA)6	12	6853594	6853605	GACATGGACACGAGACACATAAA	CGCGTGTCCAGCCTTATTAT	280
Ad02A2628	Aradu.A02	2628	p6	(ATTTTT)5	30	6854841	6854870	TGAGTTTGGTTTTCACTTTTCAGA	TCACCAAACGAAAAGCTAAAGAT	279
Ad02A2630	Aradu.A02	2630	p2	(TA)7	14	6862270	6862283	TCTGGATGGGCTCTTACGTT	TCCACGTCACTCATTCCATT	279
Ad02A2632	Aradu.A02	2632	p2	(CA)6	12	6864332	6864343	CAAGCCGCCTCACCTAATAA	CCCTTCCCCATCTCTCTTTC	114
Ad02A2635	Aradu.A02	2635	p3	(AAT)14	42	6868427	6868468	GGTTTGTTTCCCCATGAAAG	TTGATCCATGAGAGGAAGGG	217
Ad02A2636	Aradu.A02	2636	p2	(TC)8	16	6869569	6869584	TATTACAGCCATTGCCACCC	TTTTTGGTAGACCCAACCGT	161
Ad02A2637	Aradu.A02	2637	p3	(AAT)16	48	6871551	6871598	TGATGTCCATTTAGATCGATGTTT	GCATGCAAACACACGAAAGT	272
Ad02A2638	Aradu.A02	2638	p3	(CAA)5	15	6872573	6872587	CTGAATTCGCTTCATTTGTGA	TCTTCGCGTGTTTTCTCTCA	184
Ad02A2639	Aradu.A02	2639	c	(T)10atgtttatga	65	6876256	6876320	ATTTCCTTCACAAACCCCCT	TTGTGGAAACTCAGGTGCAG	248
Ad02A2640	Aradu.A02	2640	c	(A)10tttaattaaa	61	6877334	6877394	AAATTTTGATGCCGTTTCTG	CATCTTCTTCTTTGCTCCCC	224
Ad02A2641	Aradu.A02	2641	p2	(AT)12	24	6879784	6879807	AACCCTTACTTCTCTGCCGTC	TGGTGAATTAAATCTTGAACCG	172
Ad02A2646	Aradu.A02	2646	p3	(TAT)14	42	6917289	6917330	TCACTTATTTTTCTTAATAAACCATGC	GGCCTCTAATTGGAAATGCC	240
Ad02A2651	Aradu.A02	2651	c	(A)10ttaggagc	39	6930410	6930448	AAAACAACGAGATCTCTGACAAAA	CCCTATTCGGTCACTGACAA	212
Ad02A2654	Aradu.A02	2654	p2	(CT)9	18	6942914	6942931	GGTGACGACGGCTTTAGGTA	CCACACACGCAGAAAGAAAA	258
Ad02A2657	Aradu.A02	2657	c*	(TA)8(A)10*	25	6954670	6954694	TCTGAGAGTCTGAGCAGCCA	GCAACTTTCCATCGTTTCCT	149
Ad02A2658	Aradu.A02	2658	c	(CTT)5ttgttttct	110	6956997	6957106	TCAAATTGTTATAACCGCTTTTT	AGAGAATGAGGGGGAGGAAA	274
Ad02A2659	Aradu.A02	2659	c	(CTT)5ttccatcg	57	6957704	6957760	CATAACTACTTGCGTTAGGCCA	TGCTGCATTTTCTTCTTCTTCTT	233
Ad02A2661	Aradu.A02	2661	p2	(AT)6	12	6961613	6961624	TCTGCATAACCTTCTCTTTCTCG	TCCCGTTAATTTCAGGGTCA	195
Ad02A2662	Aradu.A02	2662	p2	(AT)10	20	6963499	6963518	CAATTCGAAAGATGACACCA	TCCAATAAAGCAATTTCACGG	229
Ad02A2663	Aradu.A02	2663	p2	(TA)6	12	6963960	6963971	CATCATGCATGCAGGTTTGT	GCAATAGAATGCGCAAGTGA	215
Ad02A2664	Aradu.A02	2664	p2	(CA)8	16	6964423	6964438	AGTCACTGGCAAGCTCGATT	GTGGATGGTGTATTCAGGGG	202
Ad02A2665	Aradu.A02	2665	p2	(AT)6	12	6966996	6967007	GCTGGCGTGTTATGAAACAA	TTGGATGTAAAGTGGTGCGA	213
Ad02A2666	Aradu.A02	2666	c	(GA)10aggaga	79	6970007	6970085	ACACCGTCTGAAGATCCACC	TCTTCTCTAGCCGTACCCCC	139
Ad02A2672	Aradu.A02	2672	p3	(TGA)13	39	7011036	7011074	GAGATAGGACCTCGTTGCCA	CTCTCCACTTCTGCCTCTGC	253
Ad02A2673	Aradu.A02	2673	p3	(AAT)5	15	7027030	7027044	CGAGTGGACCATTAAAAGTTCATA	AGCAACATTCATGGGTAGTTTG	280
Ad02A2674	Aradu.A02	2674	p2	(TA)6	12	7027440	7027451	AATTGAAGGTGGGAGAGGGT	TTGAGTTATGGTTCCCCTGG	216
Ad02A2677	Aradu.A02	2677	p5	(CAGCA)5	25	7033642	7033666	CCCAAGCCTCATAATCCAAA	GGCGGTCATCAATCAAAGAG	242
Ad02A2678	Aradu.A02	2678	p2	(TC)6	12	7034022	7034033	CTCTTCCATTCCCGATTCAA	ACCCCACATCAAAAAGCAAG	230
Ad02A2679	Aradu.A02	2679	c	(TTA)5(TCC)5	30	7035243	7035272	ATGGCCATTTTCTTGTGGTC	GGCATGCAATTCCTCAATCT	135
Ad02A2682	Aradu.A02	2682	p2	(AT)7	14	7050042	7050055	GTTATTCCGCCAAGTTGCAT	GTCACCAAAATGTTCCAGGG	164

ID	Chromosome	SSR nr.	SSR type	SSR	Size	Start	End	FORWARD PRIMER (5'-3')	REVERSE PRIMER (5'-3')	Product (bp)
Ad02A2683	Aradu.A02	2683	p3	(TCT)5	15	7052408	7052422	ATGAGAGGTGGGTCTGGTTG	CACGCCAATAAGTTCTGCAA	208
Ad02A2684	Aradu.A02	2684	p3	(ATA)5	15	7052688	7052702	ATCACATTCCGAAGCCAACT	CACTGAATTCTCCTCCCCAA	206
Ad02A2685	Aradu.A02	2685	p3	(GAT)5	15	7053078	7053092	CACGAACGTGAATGTGAAGG	AGGAATTGAAGCCAACGAAA	112
Ad02A2691	Aradu.A02	2691	p3	(GGA)6	18	7074450	7074467	TGGTGAAAGAAAGGGATTGG	CCTGCACCAACACAGACACT	143
Ad02A2692	Aradu.A02	2692	p4	(AATT)6	24	7075855	7075878	TCACAAGCTCCTCCTCGATT	GCAAGTTGCTCACTGTTCGT	277
Ad02A2693	Aradu.A02	2693	c	(TG)12(AG)12	48	7079054	7079101	AGGAGAAGAAGAGGATGGCG	AATGGAGGCACGGTAGTGAG	250
Ad02A2694	Aradu.A02	2694	p4	(ATTA)9	36	7085260	7085295	TCTGGCCTATCAATAAGTGCAT	TCCAAATGTGCTCTCATCCA	212
Ad02A2697	Aradu.A02	2697	p2	(TC)8	16	7086560	7086575	TTCTGGATCTGTCACTGCTTG	GCAAAGGTCGCTCTTTCACT	236
Ad02A2700	Aradu.A02	2700	p3	(GAT)7	21	7092307	7092327	TGGCTTCATGCACGTAAAAG	GAAGGAGGGACAAGTAGGGC	206
Ad02A2703	Aradu.A02	2703	p3	(ATA)5	15	7101317	7101331	ACTGCACCTGAGTTTCCACC	TGTTTCAATCCACACACCAAG	276
Ad02A2706	Aradu.A02	2706	p3	(GAT)6	18	7119897	7119914	GCTTGGAAACCCCAATTTTT	GACGTCATGATGAAACGCTC	140
Ad02A2707	Aradu.A02	2707	p2	(AT)6	12	7134802	7134813	TCTTTTAACGCCGCAATACC	TCAATTTTAGGATTGTGCTATTTGA	278
Ad02A2710	Aradu.A02	2710	p3	(TCT)6	18	7139176	7139193	ACGCTGATCTCTTTCACGGT	CAAGGAAACTTTGGAGCCAG	234
Ad02A2711	Aradu.A02	2711	p2	(TC)20	40	7139396	7139435	CTGGCTCCAAAGTTTCCTTG	GTCGAATAGGTGGAATGCGT	207
Ad02A2717	Aradu.A02	2717	p2	(TA)8	16	7145459	7145474	AAAAATGAGAATGAATTACGGAAA	CAGACAGGGGTTTATGTCGAA	211
Ad02A2718	Aradu.A02	2718	p2	(AT)7	14	7145941	7145954	AAAATTGAGGCACGAATTGC	GTTGCTCCTGCTACTCAGCC	180
Ad02A2723	Aradu.A02	2723	p3	(ATG)6	18	7184470	7184487	TGAGGTTGATGCTTGGACAC	GCAAGGTCGGTCATCATCTT	271
Ad02A2724	Aradu.A02	2724	p5	(AAATT)5	25	7185290	7185314	TTAGAGAAAAGTAAAACTCTGGAAAAA	TTCATGAGTCAGACGATTTTGTG	257
Ad02A2727	Aradu.A02	2727	p2	(AT)9	18	7195086	7195103	CCCTCTTTAGAGCCTCCTGC	GGCAAGACGAGAGAATAAGATCC	272
Ad02A2728	Aradu.A02	2728	p2	(TA)7	14	7195295	7195308	CCCTCTTTAGAGCCTCCTGC	GGCAAGACGAGAGAATAAGATCC	272
Ad02A2729	Aradu.A02	2729	p2	(GT)6	12	7195993	7196004	AGTCCTCAACCTTGCCACAA	TTTACAAAGGTGTTTGATTGGTG	280
Ad02A2731	Aradu.A02	2731	c	(T)11caaaagtta	118	7201291	7201408	TGGATTCAGTTAGGTTTATCCTTCA	TCTTCCTTTCTTGCCCTTCA	262
Ad02A2736	Aradu.A02	2736	p2	(TA)10	20	7214966	7214985	TGAACACATACTTTTGCGCC	AGGCAATGAAAAACTTGATGA	261
Ad02A2737	Aradu.A02	2737	p2	(TC)6	12	7221784	7221795	TGCAATCTCGGTTTCACTTG	TTTCAGCTCGAGCAAGCATA	280
Ad02A2738	Aradu.A02	2738	c	(T)11ggatgacta	125	7222761	7222885	TTTTATTGCTTTAATTCTTTTAACACC	GGGGAAATGCTTACAACCAA	280
Ad02A2739	Aradu.A02	2739	p3	(ATT)5	15	7224614	7224628	TCAGATCAACCTCCGATTCC	GCAACTAGCTCTGGCGATTC	262
Ad02A2741	Aradu.A02	2741	c	(CT)9t(TC)19a	130	7226566	7226695	GCAAGTCAGCGTTACTATGTGG	GCCATTTCTTCACCAGAACC	223
Ad02A2748	Aradu.A02	2748	c	(A)10gcgtgttat	39	7236443	7236481	TTTGCTCCAGTCACAACTGC	TGACTGATGAACTTGAATAACAACAA	263
Ad02A2751	Aradu.A02	2751	p2	(AG)6	12	7253196	7253207	TTCCCACCATCTTGCCTTAC	CATGCAAGAGGAAATGGGTT	221
Ad02A2754	Aradu.A02	2754	c	(A)10taattcactg	65	7263776	7263840	GGGTTTACAACACAAAACAATCA	CAAATTAGATTACAATGCTTTTCGC	133
Ad02A2757	Aradu.A02	2757	p4	(GTTA)5	20	7269398	7269417	AGAGAACAGGGAGATGGGGT	TATCCCGTGGAAGAGGACAG	253
Ad02A2760	Aradu.A02	2760	p3	(AAT)5	15	7274326	7274340	ACCCCTTTAGCCCATTCATT	ACGACCGTATAAAGTGTATCAAAAT	279
Ad02A2761	Aradu.A02	2761	p3	(AAT)5	15	7275548	7275562	TTTGGAGGGAAAACACCAAG	AGCGGTGTGCTTATGTTCCT	273
Ad02A2767	Aradu.A02	2767	p2	(AT)6	12	7282602	7282613	CCTCTTAGCATGCAGAGCTTC	TTGTTTGCATGAAAGGGGAT	271

ID	Chromosome	SSR nr.	SSR type	SSR	Size	Start	End	FORWARD PRIMER (5'-3')	REVERSE PRIMER (5'-3')	Product (bp)
Ad02A2769	Aradu.A02	2769	p2	(TA)7	14	7284858	7284871	GCCTTAAGTCCCTCATTGTTTTT	TTCATTCCAATACTTTTGGTTCA	241
Ad02A2772	Aradu.A02	2772	p4	(AAAG)5	20	7321866	7321885	CCAAAGTTTCAAAAGCCCAC	CCAAGGTTGGAAGGTGAAGA	218
Ad02A2777	Aradu.A02	2777	p2	(GA)8	16	7329363	7329378	CAAAAACACCAAACCAACACC	CATAGACCAGCCGTGACTGA	203
Ad02A2779	Aradu.A02	2779	c	(TTC)7ttgtcttt	108	7335579	7335686	TCCTCCTTTTCCTCCTCCTC	TTTCCATTCCTGAACCTCAAA	280
Ad02A2780	Aradu.A02	2780	p3	(AAT)8	24	7336594	7336617	AACCAAAGACATTTCATATCAAAATAA	GAAAGAGAACGTGATTTAAAAAGTTG	280
Ad02A2781	Aradu.A02	2781	p3	(AGA)5	15	7347178	7347192	GGGAATGGCATGAAGAAGAA	AAAAACGAAGCAGCGAAAAA	246
Ad02A2784	Aradu.A02	2784	p2	(CT)9	18	7356393	7356410	ACCCAATAAACACCGAGGGT	TGATGGACGAGATTGGATGA	192
Ad02A2788	Aradu.A02	2788	p3	(ATT)6	18	7363926	7363943	CATGCCTCGTGGTACACTTG	TGTTTCCATGAAAATGGTGG	174
Ad02A2790	Aradu.A02	2790	c	(T)12acaaaatca	95	7365805	7365899	TCTCACGGTTTTTGAAATAGGAA	CCTCTTCACCCCTCAACAAA	277
Ad02A2797	Aradu.A02	2797	p3	(ATT)5	15	7393089	7393103	TCTTCCTGCATAACCATTTTCA	TGATTTTTGGTTTTGGGTGA	263
Ad02A2799	Aradu.A02	2799	p3	(AAG)5	15	7395212	7395226	TGGGGACGATAGTGGAAGAG	AACGCACAGCACAACTCAAC	250
Ad02A2802	Aradu.A02	2802	p3	(TTA)5	15	7400007	7400021	GACGAAGTAGGGAGTGCAGC	CCTCTGTTGAACGAGCGAAT	157
Ad02A2808	Aradu.A02	2808	p2	(AG)29	58	7414361	7414418	ACATCATCAATGCCCTCACA	CAAGATTGTGCGCTTTTGAC	153
Ad02A2809	Aradu.A02	2809	p2	(TC)9	18	7417057	7417074	GCTCATCACTACGGTGAGCA	AGATGGCACCCAAGTCTGTC	222
Ad02A2810	Aradu.A02	2810	p3	(ATA)5	15	7417907	7417921	TGTTGGTGAAATTCTCAAGGG	TCAAACCACATCAATTGGGA	174
Ad02A2812	Aradu.A02	2812	c	(ATT)12aa(T)	49	7422439	7422487	AAAACATGCTCGCCAAGTCT	TTCCTTGCCTTGGATATGGA	221
Ad02A2814	Aradu.A02	2814	p2	(AT)6	12	7425544	7425555	AAAAAGGATATAGATACAGGGGTTAGG	GCCGGATGAATAATAACGGA	273
Ad02A2819	Aradu.A02	2819	p2	(TA)6	12	7475083	7475094	CAGCATGATGCAGACGTACA	ATTGTAGGGAGCAAACCCCT	130
Ad02A2820	Aradu.A02	2820	c	(A)10tatgttttcc	57	7475408	7475464	TTGTAAATGAAATTTAATGGCGA	TTGCGACAGTTTTCAGCAAT	261
Ad02A2821	Aradu.A02	2821	p3	(TAT)10	30	7476443	7476472	TCTTGGCAATTACCATGCAA	GCAAGATGTATGTTGGCCATT	210
Ad02A2822	Aradu.A02	2822	c	(T)10aaaatagt(	29	7485269	7485297	AAAAAGAGTCAAGCGGATTAGC	AGCATGTTCCGTTTATTGGC	157
Ad02A2824	Aradu.A02	2824	p3	(TTG)6	18	7487535	7487552	CGTCCAGGAGACAGTGAACA	GCAGGAGCAGAACAACAACA	240
Ad02A2832	Aradu.A02	2832	c	(A)10cagaaattg	55	7504882	7504936	GCCTCTCCACTTTTGAGTCG	CCTTGCCATTGGAAATGACT	275
Ad02A2835	Aradu.A02	2835	p3	(CTC)6	18	7528660	7528677	TGGCAAAGACAAATGCTCAG	CCTCACCAGAAGAGCCAGAC	237
Ad02A2836	Aradu.A02	2836	p2	(AT)6	12	7536162	7536173	AGATTTTAGGGGCGGTCACT	TTTGTTTCTTCCTGGGTTGC	254
Ad02A2838	Aradu.A02	2838	p2	(TA)6	12	7538572	7538583	AAATTGTATCTTGAGTTATGGTGCC	CAAAAAGTGCTTAAAGTACAACATTTC	270
Ad02A2839	Aradu.A02	2839	c	(TA)9gtacatgtg	70	7539657	7539726	GTTACCGCTACTTTGTGGCA	TGGGATGACAACTTAGCAACA	233
Ad02A2843	Aradu.A02	2843	p2	(TA)9	18	7555973	7555990	AAAGATTAGGGGTTAGAAAGGG	TTTCAATCGCAGTGATCGTC	272
Ad02A2844	Aradu.A02	2844	c	(ATC)5at(AA(	63	7556607	7556669	CAGCCGAAAACGTGACATTA	CATGCAGTTGCTGTGCTTTT	254
Ad02A2845	Aradu.A02	2845	p2	(TA)7	14	7559572	7559585	AACCCATCCCCTTCAATAGG	CCGTGGCAGGGTACTATTCT	248
Ad02A2846	Aradu.A02	2846	c	(A)10tgttacgcc	68	7566302	7566369	AGGGCAGCTGAAAATGATTG	GTTTGAGGCTTCAAGGGGAT	200
Ad02A2848	Aradu.A02	2848	c	(TC)7ttcctttctt	62	7566912	7566973	ATCCCAAATTTCCCAAAACC	AACACAAAAGGCTCACCTGC	264
Ad02A2850	Aradu.A02	2850	c	(GTG)5acagtaa	129	7568641	7568769	CCTGCGGTTGAGATCAGAGT	CCATTTTCTCCACCATTACTACC	247
Ad02A2852	Aradu.A02	2852	p2	(TA)6	12	7574393	7574404	TTTTCAGGAACCGGATGAAG	CGTTGATTGCTTTGATATCCG	206

ID	Chromosome	SSR nr.	SSR type	SSR	Size	Start	End	FORWARD PRIMER (5'-3')	REVERSE PRIMER (5'-3')	Product (bp)
Ad02A2853	Aradu.A02	2853	p3	(TTC)8	24	7575144	7575167	TGGAAGAGAGCTCCGATGAT	TTGCCATGGTAGAAAGGGAC	133
Ad02A2854	Aradu.A02	2854	p3	(TCT)6	18	7575824	7575841	ATCGGTCACTGTCACCACTG	TGTGCATATTTCGTTAGTTGCAT	271
Ad02A2859	Aradu.A02	2859	p3	(ATT)5	15	7599524	7599538	TGGTTAGAAAGGCCCATGAA	CTTTATGCTGGCCATATTTGA	132
Ad02A2861	Aradu.A02	2861	p2	(TC)12	24	7601101	7601124	CGTTCTTCCCCTTCATTCTG	AGGAGAATGTTGGTTGTGGC	204
Ad02A2863	Aradu.A02	2863	p2	(AT)6	12	7605134	7605145	CCGGTTCAACACTCACTTTTT	GGCACGTTCGACTCAATCTT	250
Ad02A2865	Aradu.A02	2865	p3	(CCA)5	15	7610722	7610736	GAATTTGATTGGCTGAGGGA	CAGAGGAACCACTCCCGATA	221
Ad02A2867	Aradu.A02	2867	p4	(ATAA)5	20	7615930	7615949	GCAAACCCAGAAGGCAATAA	GACTTTGCAGCGTGACAAGA	256
Ad02A2869	Aradu.A02	2869	p3	(TAT)13	39	7618649	7618687	TGGCTCAACGACAGACAAAG	TCACGAGACTGAAATGTGGC	254
Ad02A2871	Aradu.A02	2871	p3	(ATT)5	15	7620643	7620657	TGTTGCTTGCAAAATCGAAG	GAATCGATGGGCAGAGATGT	255
Ad02A2873	Aradu.A02	2873	c	(ATA)5aagaat[illegible]	52	7622067	7622118	CAGGATGGGGACACTCCTTA	TGCTGCCGACAACAATAATC	193
Ad02A2875	Aradu.A02	2875	p3	(GAT)6	18	7623014	7623031	GAAGCCACTTTTGCTTTGCT	TTGTTTGGAAATTCTGGGCT	183
Ad02A2876	Aradu.A02	2876	c	(A)10ggggtcttt	120	7623272	7623391	GAGGAGACAAAAGGATAAAGCTC	GAATATGAAGCCACCCCTGA	277
Ad02A2877	Aradu.A02	2877	c	(A)13gggggaa[illegible]	80	7623605	7623684	GGATGTGGGAATGAACCCTA	AGTGCAAGAGTCAACCGACC	277
Ad02A2879	Aradu.A02	2879	p2	(GC)6	12	7627856	7627867	GGCCCATAAGGCTCCTAAAA	CTGTTAAGCCGACCTCTTCG	235
Ad02A2880	Aradu.A02	2880	c	(A)13gaaaatgt[illegible]	39	7630695	7630733	AGGAGGAGGTGATGATGGTG	AGTAAAACAAAAACAATTCAATCAAG	238
Ad02A2881	Aradu.A02	2881	p2	(TA)6	12	7630933	7630944	GGTTCAAACTTTAGGAAGGAATTTT	CAGCACGGACTAGGCATTTT	199
Ad02A2884	Aradu.A02	2884	p2	(TA)12	24	7648917	7648940	AAATTTTAACCGTAATCACATCTCAA	TTGATCACCTGCCAGTGAAA	215
Ad02A2886	Aradu.A02	2886	p3	(GAA)5	15	7651371	7651385	AAAGGCAAAGAGAGAACGCA	CTTTGGTGGCTCAAATTGGT	227
Ad02A2888	Aradu.A02	2888	p2	(TA)9	18	7656153	7656170	CTCAACCCATTTCACGCTTT	CACACGTGAGTCATCCAAGC	269
Ad02A2891	Aradu.A02	2891	p2	(TA)7	14	7665420	7665433	TCGCAGGTCAAAGGAAACTC	TTCAATCAATCATGTGCCAAA	280
Ad02A2894	Aradu.A02	2894	c	(A)12gattagct[illegible]	196	7672428	7672623	GGCTTTGGCCCTTTATTGA	TAGGGGAAGCATAAGCCAGT	275
Ad02A2895	Aradu.A02	2895	p4	(AATA)6	24	7674524	7674547	ACCGAGGAGGTATAGCCGTT	AAACTATCCATCCGTCACGC	274
Ad02A2896	Aradu.A02	2896	p2	(AG)17	34	7679142	7679175	GAGCAGTGCAAATTCATCCA	TGACCATTGGGAGACATTGA	274
Ad02A2898	Aradu.A02	2898	p3	(TGG)5	15	7682938	7682952	TTTTGCTGCTGAATTTGCTG	CGTTGAAAACCGATTTGGAT	266
Ad02A2899	Aradu.A02	2899	p3	(TAT)8	24	7683545	7683568	AATATCAAACGGTTTCAACTTCA	AACAGTCCTCCGTCCAAAAA	236
Ad02A2904	Aradu.A02	2904	c	(T)12aattttttatt	104	7695075	7695178	TGGCTGAAGCACTTTCACTG	TTCTGCTGTTGTTGCTTTGC	204
Ad02A2908	Aradu.A02	2908	p2	(CT)6	12	7704530	7704541	TCTTTTTCCCACCACTACCG	CTACATCACGCAACCATTCG	185
Ad02A2909	Aradu.A02	2909	p3	(TTG)6	18	7705057	7705074	AAGAGTGGAAATCGGGTGTG	CACTACCATCACCACCACCA	152
Ad02A2910	Aradu.A02	2910	c	(A)10gttagatat	86	7706905	7706990	TTTGATGGTGAATCCTTCGG	TTTTGGTAAGGAGTTCAAAATTCAG	145
Ad02A2913	Aradu.A02	2913	p2	(TA)9	18	7710556	7710573	CAAGAGAAGCCCTGCTCAGA	TGTATGATGTGATGGATGCAAA	269
Ad02A2914	Aradu.A02	2914	p4	(TTAA)5	20	7714177	7714196	TCACAATGCTTGTCGTCAACT	TCTTTTCCATTAGGAGGAACTAACTT	280
Ad02A2915	Aradu.A02	2915	c	(T)11atattggtc[illegible]	154	7714607	7714760	TTTCGAAGTGAAAAGCATTTTAGA	AAAGAAATTGCTTCCAGCGA	271
Ad02A2916	Aradu.A02	2916	p2	(TC)6	12	7721143	7721154	GCGTACCGAAAGAGATGAGC	GAAGTGATGTTGGCAAGGGT	166
Ad02A2921	Aradu.A02	2921	p3	(AGG)7	21	7733047	7733067	CCCCTTTCTCCATCAACTCA	CTCTACCAGGGCAAGCTCAC	147

ID	Chromosome	SSR nr.	SSR type	SSR	Size	Start	End	FORWARD PRIMER (5'-3')	REVERSE PRIMER (5'-3')	Product (bp)
Ad02A2934	Aradu.A02	2934	p2	(TA)6	12	7763862	7763873	TTTTTGGGATGATAAGTATTTGG	CTTTTCTCGCTCCTCACCAC	172
Ad02A2935	Aradu.A02	2935	c	(A)10tgaagcat;	99	7767368	7767466	GGTAAAAATGACGACGGACG	GGATTTTAATTCCACTATTTTCTCC	261
Ad02A2939	Aradu.A02	2939	p3	(GAA)7	21	7785378	7785398	TCGAAGAACACCACACGAAG	ATGTTTTGCAGATGGGGTCT	219
Ad02A2943	Aradu.A02	2943	p3	(TCT)8	24	7806722	7806745	CACGCTGGAGTGTCTAACGA	GGTACGAGAATGACCAGGGA	179
Ad02A2945	Aradu.A02	2945	p2	(TA)6	12	7809338	7809349	TGAGAGATCGACACGTCACC	TTTGCATTAAAAATTCATACCGTTT	107
Ad02A2948	Aradu.A02	2948	p3	(CCT)5	15	7828339	7828353	AAAGGGTCCAGGAGGCTAAG	CCGCCATCAAAAGAAGAAGA	217
Ad02A2950	Aradu.A02	2950	p3	(TGG)5	15	7839719	7839733	TGTAGTCCAAGATCACATGCAA	ACTTGCACCAATCCCATTGT	271
Ad02A2955	Aradu.A02	2955	p2	(AT)6	12	7863589	7863600	CAATCTGGCAAAGGAGGTGT	TTCAAAAAGTGTGCAAATGACC	144
Ad02A2962	Aradu.A02	2962	c	(TA)6(T)10	22	7877729	7877750	TTGGTGTCTTGTGTGCGTTT	GGTAGGGGAGTTGGGGAGTA	229
Ad02A2963	Aradu.A02	2963	c	(A)11c(A)10	22	7879118	7879139	CTGTCCATCACTGGTCCCTT	CGAATTGGGGTGTTCTATGC	279
Ad02A2964	Aradu.A02	2964	p2	(AG)6	12	7886592	7886603	TTCGAGGGTTTGGGAATATG	GACCAACAGCCTCGTTCATT	262
Ad02A2966	Aradu.A02	2966	p3	(TAT)6	18	7895845	7895862	CGAATCGTGAAATGAAATAATAGTG	AATGATGTCACGTATTAACCGA	273
Ad02A2969	Aradu.A02	2969	p2	(TA)10	20	7902577	7902596	CCGTTCTTCTTTCATGGCTC	AGTGGTCTTTATGCCGCAAT	190
Ad02A2976	Aradu.A02	2976	c	(T)10cacactaa	43	7920034	7920076	TGTCTGAGCTGTTTTTCCTAACA	GGCTGTTTATTGTGTCCACTCTT	252
Ad02A2980	Aradu.A02	2980	c	(A)10gaaag(A)	25	7925397	7925421	AAAACATAGCATGGAACGCC	GGAGAAAATGAAGGGGAAGG	236
Ad02A2993	Aradu.A02	2993	p3	(CTT)5	15	8022159	8022173	AGACAAAACAAGAGCCACGG	GACGGGGCTGATACAATCAT	225
Ad02A2994	Aradu.A02	2994	c	(A)12gactgaga	69	8024097	8024165	GTTGGATCCTTACGCCGATA	CCATGGTATGGTCGCTTCTT	265
Ad02A2998	Aradu.A02	2998	p2	(TA)6	12	8030838	8030849	GCTTTTAATAAACATAAGCAACATCAA	GCAACGATTGAAACAATACACAA	224
Ad02A2999	Aradu.A02	2999	p2	(AT)6	12	8035506	8035517	CATGCTGATAACGTGTTGTGAA	TTTGCTCTGACACCTGCATT	241
Ad02A3001	Aradu.A02	3001	p3	(AAC)5	15	8044004	8044018	GGGGAGTGGTTTTAGCTTTCA	CTCACTTAGTGGGACAAGGCT	280
Ad02A3002	Aradu.A02	3002	c	(T)17nnnnnna;	96	8046059	8046154	TTTTTGGCCATATACTGCTATGA	TTGATGTAATTTGGTTTGAATTTTT	225
Ad02A3003	Aradu.A02	3003	p3	(TTG)6	18	8050098	8050115	AATTCATTCCGCTTAGTGCG	TAAGGAGCAAGGATCATGGG	130
Ad02A3005	Aradu.A02	3005	p3	(TTA)6	18	8052477	8052494	AACACGTTGCACTTGCTGAC	TCCATAAGACCACAACACCATC	218
Ad02A3006	Aradu.A02	3006	c	(A)26cgaaaaaa	70	8055894	8055963	AAGCCAACCAACTTGCTTCA	TTTCACTTTCACTTTCCAGATTTTC	245
Ad02A3008	Aradu.A02	3008	p2	(AC)12	24	8062179	8062202	ACGGGACACAACACAACAAA	ACGTGTTAGCGTGTCCAACC	247
Ad02A3011	Aradu.A02	3011	p3	(TCT)6	18	8065970	8065987	GTCTCCTACGTTTGGGGTGA	TTACCGTTTCGTGCTTCACA	252
Ad02A3020	Aradu.A02	3020	c	(T)10caaacaaa	212	8119163	8119374	TTGGTATGGGATGTACAAATCG	GGACCGTCCGATTAGTTTTTG	278
Ad02A3021	Aradu.A02	3021	c	(A)10ttagcaatg	54	8119518	8119571	CAAGCACAACTCACACACCC	ATTCTATATGCCATTCCGGC	167
Ad02A3026	Aradu.A02	3026	c	(CCA)6gtatcat	140	8125047	8125186	GAACATAAGCCTACGCCGAA	GTGGTGGTGGGTTCTGTTTT	204
Ad02A3027	Aradu.A02	3027	p3	(AGA)6	18	8125387	8125404	AAAACAGAACCCACCACCAC	CTCGTAATTCAGTCCTCGCC	278
Ad02A3028	Aradu.A02	3028	p3	(GAT)5	15	8126991	8127005	ATAGAGCGTTTCGTGGTGCT	ACATCCCTCTTCCCCAAATC	142
Ad02A3031	Aradu.A02	3031	c	(A)10cacaaagg	45	8131946	8131990	CCACCAAGAGGACGAAGAAA	TTCCTTTTGGGTAACCTTTTGA	251
Ad02A3032	Aradu.A02	3032	p3	(AAT)5	15	8132180	8132194	CAAAGGAAAAGAAAAACGCAA	GGTGAATTTGTGAGCGGTGT	258
Ad02A3033	Aradu.A02	3033	p3	(TCT)5	15	8137673	8137687	TGTTGTCTGATACCTTGATTCATTG	CTCAACCAAGCAGGAGGAAG	119

ID	Chromosome	SSR nr.	SSR type	SSR	Size	Start	End	FORWARD PRIMER (5'-3')	REVERSE PRIMER (5'-3')	Product (bp)
Ad02A3034	Aradu.A02	3034	p3	(CGT)10	30	8139033	8139062	TGACACCACACTGTCATCGTT	CAAATTCTAGATTGGCCCGA	267
Ad02A3037	Aradu.A02	3037	p2	(AT)7	14	8145061	8145074	TGCAACATGGGAGAGATTCA	GGAATGCACCCATTCAAAGT	242
Ad02A3039	Aradu.A02	3039	p3	(AGG)11	33	8146996	8147028	CAATCAACAGCATTCCTCCC	TTGCGTTAGGCTATGGATGA	272
Ad02A3043	Aradu.A02	3043	p2	(TA)7	14	8155364	8155377	CGATATCTCCCAACTCGACAA	TTGAAATGTAATTGACAATCAAAAA	204
Ad02A3047	Aradu.A02	3047	c	(A)11ggaaatag	52	8161230	8161281	TTGCTGGAGGAAGAGTATTTGTT	GCATGTCGCTTGAATCTTGA	266
Ad02A3050	Aradu.A02	3050	p3	(TCA)5	15	8177910	8177924	TCGGTGTATATGTGCCGATAA	CGAATGTAAGTTCATCCTCTTCC	264
Ad02A3051	Aradu.A02	3051	p3	(TCC)10	30	8178416	8178445	ACTCTCTCACCAACACCACC	GAACCACACCTCAGCCACTT	202
Ad02A3062	Aradu.A02	3062	p3	(AAC)5	15	8224348	8224362	GCAGGAACAGGAGCAGAAAC	TGAGTCGAGGGAGGAGAAAA	252
Ad02A3074	Aradu.A02	3074	p3	(AAT)5	15	8253583	8253597	TTGAAAAAGAGGGAGATATTTTCG	TTTTTCAAAACCAATTTCAATCA	236
Ad02A3078	Aradu.A02	3078	p2	(AT)10	20	8293004	8293023	AAAATGAATAAAAGAATGTTATTGTGC	AACGCATCCAATTAAAAGGC	279
Ad02A3084	Aradu.A02	3084	c	(AG)6gaaggag	84	8316203	8316286	CTTTTGCAATCCTAGAGCCG	CTCCACCACCACCACTAACC	272
Ad02A3086	Aradu.A02	3086	p4	(TTAA)5	20	8321764	8321783	AAGAACTAACTTTAATTTCTGTTGTGG	TTTCACAATGCTTGTCATCAA	264
Ad02A3092	Aradu.A02	3092	c	(A)10cagaagca	103	8339721	8339823	GCCATCAACCATCACTCAAA	CGCTGATGGAGGTTCTGTCT	182
Ad02A3098	Aradu.A02	3098	p3	(TAA)5	15	8354868	8354882	TGGGTCATAGGGTAAGTGGG	CCAATCACTTCAAAGCAAGC	240
Ad02A3101	Aradu.A02	3101	c	(T)10gttattttca	102	8377311	8377412	GCAAATATTGAAGGGTGTGTTTG	ACGCAAGGTCTAATGCGTTT	202
Ad02A3102	Aradu.A02	3102	p3	(TGT)5	15	8383607	8383621	GAGCTTGGATGCATGGTTTT	CGACGTGATTTTAGTGGGGT	240
Ad02A3105	Aradu.A02	3105	p3	(TGT)5	15	8388014	8388028	CGCCTTACTTGAGCTTGGAC	TTTTCTCCCACCAATTCGAC	183
Ad02A3106	Aradu.A02	3106	p3	(AAC)6	18	8393644	8393661	TTTTGCAAAGGATAAACGGC	TCCATATGGTGGTCAAACGA	173
Ad02A3108	Aradu.A02	3108	c	(TAC)12(TAT	78	8397429	8397506	CATGAAATGGACAAATTAAAGTCTTG	GCAAGTTGACATTTGTGTGTGA	262
Ad02A3109	Aradu.A02	3109	c	(T)10cttttttc(T	154	8398463	8398616	TTCTTTGATTGCCTTTGCCT	TGTCTGGCCAATTCAAAAGA	214
Ad02A3114	Aradu.A02	3114	c	(T)10gtagggtga	33	8406040	8406072	TTCCGATCCGTCACTTTCTC	CAAAATCCAACCGATTTTCC	277
Ad02A3115	Aradu.A02	3115	p2	(CA)6	12	8411395	8411406	CCAAAAGGTTATCAGCCAAAA	TCGCGGAGAGTATGGAAGTC	242
Ad02A3116	Aradu.A02	3116	c	(A)11gttaaatac	62	8412524	8412585	GTTGTCCACGAAAGACCCAC	TGTGGCAGGCATAGCAATAA	276
Ad02A3121	Aradu.A02	3121	c	(A)11gagtaaag	108	8447811	8447918	GCACGCTACCATAACAACGA	TGACGAAAAACAGCGTCTTG	276
Ad02A3123	Aradu.A02	3123	c	(A)12caaaacac	33	8448538	8448570	AGAAGCCTCTGTTGCCTTTG	GAAGCCCTGATTGTCTGGTC	242
Ad02A3124	Aradu.A02	3124	p3	(TAA)13	39	8451030	8451068	TGCAGTCAAACCCCACATAA	TGACTTTATGAATCTGTTGTGTTGT	227
Ad02A3125	Aradu.A02	3125	p3	(TAT)13	39	8454535	8454573	AAGAGGATTTAGGTTCAATCTCCA	TGCGAATATGATCGGGTGTA	193
Ad02A3129	Aradu.A02	3129	c	(A)11ggaaaaat	59	8458105	8458163	TGATCATATTCCTTGGTATGGAAA	TTTAGGTGGGTAATTTGGAGAGA	280
Ad02A3130	Aradu.A02	3130	p2	(TA)6	12	8458620	8458631	CCTAATTTTTCCAACATGGCA	CGGGTTGCTTGCATACTTCT	223
Ad02A3131	Aradu.A02	3131	p2	(TA)6	12	8462731	8462742	TCGTCGTCCATGTTGTATGA	AAAAATAAAAGCAACAAATAAAGAGG	266
Ad02A3135	Aradu.A02	3135	p3	(AAT)5	15	8469224	8469238	GCATGCCAGTTTCTACGACA	CGTCATGAACTCAAGGATCTAGC	222
Ad02A3144	Aradu.A02	3144	c	(A)11tacaaaata	50	8485815	8485864	CAGGCAGACTATGAATATGAATCAA	GAAATGAAAAAGACTGCAGAGGA	268
Ad02A3145	Aradu.A02	3145	p2	(GA)6	12	8490384	8490395	ATGATGAATGGCTAGTCCGC	TCTTCCGATACACCTGGGAC	104
Ad02A3146	Aradu.A02	3146	p3	(CTC)5	15	8493885	8493899	TCACCCAAAACATTTGGAGAC	TAGCGGGTGGAAGAAAGAGA	279

ID	Chromosome	SSR nr.	SSR type	SSR	Size	Start	End	FORWARD PRIMER (5'-3')	REVERSE PRIMER (5'-3')	Product (bp)
Ad02A3147	Aradu.A02	3147	p3	(ATA)6	18	8494551	8494568	GCGATACTCAAAATAACAAACAAAA	CGTCATCGATCGGTATTTGA	180
Ad02A3148	Aradu.A02	3148	p3	(GCA)5	15	8495337	8495351	AGGGCTTTACGGGAGAGAAG	CACCCATGAATCCAGAGAATG	263
Ad02A3153	Aradu.A02	3153	p3	(CAA)7	21	8503179	8503199	TTTCCAAGTCTAGCCAATAACCA	TGGCGCCAAGTCATACTTAAT	277
Ad02A3156	Aradu.A02	3156	p3	(ATA)5	15	8514155	8514169	GCTCGGCCAAGAGAAAAATA	CTTTGTCAAACATCTTTTAATTCCT	195
Ad02A3158	Aradu.A02	3158	p2	(CT)11	22	8520774	8520795	TGAAATCAAATCTTTTCCTCCAA	CGTGACACATTTTGGTTGCT	239
Ad02A3159	Aradu.A02	3159	p2	(AC)6	12	8522554	8522565	TCTGGGGTCGACTGAGAAAA	TGCCTGCAACATCAAGAATC	154
Ad02A3161	Aradu.A02	3161	p2	(AG)12	24	8527811	8527834	TGAAGGAGTGTGGTGAGTGC	CCCATAAACTCTTCCCCGAT	272
Ad02A3162	Aradu.A02	3162	p3	(TTC)5	15	8532837	8532851	TGAGGAACCAAGAAAGCAAAA	CCTGGATGATGATGAAGGGT	217
Ad02A3167	Aradu.A02	3167	c	(AG)8aagctgct	93	8556846	8556938	TGGACAATTTTACCCTCAGCA	GTTAGGTAAGGCAACGCGAG	173
Ad02A3168	Aradu.A02	3168	p3	(GGT)5	15	8557041	8557055	CTCGCGTTGCCTTACCTAAC	TGGATAGCGGAGAGTAGGGA	162
Ad02A3173	Aradu.A02	3173	p3	(TGA)6	18	8576126	8576143	CCAACTGAGTGAGATGGAAACA	TTGGAATTGGATCATTGGCT	184
Ad02A3176	Aradu.A02	3176	p3	(CAT)5	15	8581405	8581419	CGTCCCTCACCTGGATACAA	GATGAGGAAATGGACGAGGA	272
Ad02A3177	Aradu.A02	3177	p3	(AAT)5	15	8582881	8582895	CATTCTCATTGAAACGTTGAGC	AAAGATGTTGATCGGTCTGAA	271
Ad02A3178	Aradu.A02	3178	c	(AC)6aagtcacc	117	8583552	8583668	ACTACACTCACCGCACCTCA	GCAACAAGGACGAACAGTGA	272
Ad02A3181	Aradu.A02	3181	c*	(AT)7(ATAC)	32	8590092	8590123	CTAGCCCCTGACTGAACCTG	TCCCTGGGTGTATTTTGGTT	235
Ad02A3182	Aradu.A02	3182	c	(AC)9(AT)6	30	8600378	8600407	CAAGCCTTGTCACCAACAAA	CTTCTGGAACTTGTGGGCTC	208
Ad02A3185	Aradu.A02	3185	p3	(TGA)11	33	8611515	8611547	TGCTTGCTGCTTGTTTTGTC	GTGGGAACATGAACCTCCAC	276
Ad02A3187	Aradu.A02	3187	p3	(AAT)17	51	8620115	8620165	TTTTTAACCATCTCTCTCGAGTTC	TGTTCAAAAATTCTGGGTTTGTT	273
Ad02A3188	Aradu.A02	3188	c	(A)12gaaagcaa	74	8630616	8630689	TGTGGTTCCTAGTGATGGCT	GGAGTCCAAAGTCTTGAGTGAAA	280
Ad02A3196	Aradu.A02	3196	p2	(TC)6	12	8649735	8649746	ATGGAAATTTCGTGACCCAC	AGATGATTCGCCTCCCTTTT	275
Ad02A3198	Aradu.A02	3198	p3	(ATA)20	60	8650309	8650368	TCTGTGATTTTTGAATGCATGA	ACGCACTTGGTCTCGACTCT	259
Ad02A3200	Aradu.A02	3200	c*	(GAA)17cgaag	203	8671233	8671435	TGATTCATTCATTGTTTCAATTCAG	TCTTCTTTTCTGCTTGCGTTT	260
Ad02A3205	Aradu.A02	3205	p2	(AT)7	14	8677484	8677497	CAAATTGCATAATTTTCAAGATCC	TGATTTCTCTGTATTGATGCTCTTTT	278
Ad02A3206	Aradu.A02	3206	p2	(AT)7	14	8677842	8677855	GAGCAACAAAGAGACACACAAAA	TCTGATTAACAGAAAAGATGTGTCA	230
Ad02A3207	Aradu.A02	3207	p3	(AAT)12	36	8681505	8681540	CTTTCTTGTTCGTGGTGTGC	GGTCATCTTCATCGTCACCA	122
Ad02A3209	Aradu.A02	3209	c	(T)10aaatcacaa	65	8682959	8683023	CGAGTGACGAAACCAATTGTA	TTGCAACAAGATTTGCAATG	194
Ad02A3211	Aradu.A02	3211	c	(TTA)8tttggaa	59	8687396	8687454	ACCACTCGTTCCAAAAGCTC	GTTCGCCGTGTGCATAAAT	224
Ad02A3216	Aradu.A02	3216	p2	(AT)12	24	8712649	8712672	GGTCTGCACACCCTAAGTGAA	TCAGGGCCACAAACCTTATT	247
Ad02A3217	Aradu.A02	3217	p2	(TA)8	16	8713197	8713212	TCATCAAATCTCAATTAAATCCACA	TTTCCTTTGTTAACTGGAACCAA	203
Ad02A3220	Aradu.A02	3220	p3	(TAA)14	42	8720695	8720736	ATCGCTATCTCACCACCCAC	AGCTGCTGCTCCAGAGTTTC	276
Ad02A3228	Aradu.A02	3228	p3	(ATA)12	36	8734349	8734384	CAACAACAACAACAACAACAACA	AAATGGACATTTTAGTCCCCC	274
Ad02A3232	Aradu.A02	3232	p2	(GA)6	12	8752212	8752223	GATGAGAGCAGGAAAGCTGC	TCTCATCCCTGTCACTGCTG	154
Ad02A3234	Aradu.A02	3234	p3	(ATA)6	18	8753626	8753643	GCTTTTGTCTTCCCTTTCCC	TTCTTGGCCGAAACATAACC	197
Ad02A3236	Aradu.A02	3236	p2	(TC)17	34	8758361	8758394	ATCGTGAGGGCTGCAACTAT	ACGGACCCACATGGATAAAA	232

ID	Chromosome	SSR nr.	SSR type	SSR	Size	Start	End	FORWARD PRIMER (5'-3')	REVERSE PRIMER (5'-3')	Product (bp)
Ad02A3242	Aradu.A02	3242	p3	(ATT)5	15	8783635	8783649	TGAAACCCATTTACATTTTATTGAA	TGACCACATAACAAGTGGCAAT	278
Ad02A3244	Aradu.A02	3244	p3	(GCG)5	15	8794259	8794273	TCCAGAGAGTTATGCCACCC	CTCTTAAAGGTGGCGTCGAG	161
Ad02A3246	Aradu.A02	3246	p3	(TAT)5	15	8801425	8801439	TCAAACACAACCCCAAACAA	TTAGTTTGCCAAAGGATGGC	248
Ad02A3248	Aradu.A02	3248	p2	(TA)6	12	8804720	8804731	TTGCATAGTTATGGCAAATCAT	TGCTTGCTCATTGTTATTGTCA	262
Ad02A3250	Aradu.A02	3250	c	(A)10caaaaaaa	69	8810596	8810664	CCACCACTATCGTCAATAATAAATCA	TGGCAAGAAGTGGTGGGTAT	216
Ad02A3251	Aradu.A02	3251	p3	(AAT)20	60	8812943	8813002	ATAGTCCAACGCTGTGTCCC	AAAAGCTGTTTTACCAAACACAC	269
Ad02A3253	Aradu.A02	3253	p3	(TTC)5	15	8815605	8815619	ACAGCTGCTGAGAAATCGGT	AGAAGAAGAACATCACCGGG	176
Ad02A3254	Aradu.A02	3254	p2	(GT)10	20	8816222	8816241	GGTCCAGGTGAAAACGAAGA	GAAGCTCTGCCACAATCACA	146
Ad02A3257	Aradu.A02	3257	p2	(CT)11	22	8826280	8826301	AATGAAGATGGCCAAGATGC	GCACACACATGCAAACACAA	178
Ad02A3260	Aradu.A02	3260	p2	(AC)6	12	8834310	8834321	CCCTTGGCCCAACATAACTA	ACTCGAAAACAAGGCTGGAA	280
Ad02A3261	Aradu.A02	3261	p4	(AGTT)7	28	8841024	8841051	TGAGACCATTAGGGTTTGGG	CTCCAACCAACCAACCAACT	233
Ad02A3264	Aradu.A02	3264	p3	(ATG)5	15	8846264	8846278	CCAAGACCTTGCAACTGTGA	GCATAATTGGCTCTTTGGGA	171
Ad02A3265	Aradu.A02	3265	p3	(TTG)5	15	8847064	8847078	TCAAGCCTTGTCCCATTAGG	TTCGGTGAGAATGGTGTTCA	172
Ad02A3267	Aradu.A02	3267	p3	(TCA)6	18	8856968	8856985	TCAACATCTCTCTCCCACCC	CCCTCGAGCAAAAGAAGAAA	110
Ad02A3268	Aradu.A02	3268	p3	(CTT)7	21	8857768	8857788	TCTTCCTCTTCCTCGTTCTCA	CGTTCCTCCTCCTCCTCTTC	272
Ad02A3271	Aradu.A02	3271	p2	(AT)7	14	8864332	8864345	ATGTTTTTCACAAGGCTCGG	GGCCATATGCTCAGTAAGACG	280
Ad02A3272	Aradu.A02	3272	p3	(ATC)5	15	8869352	8869366	ACTCCACGCGAAAAAGAGAA	TAAGGTCAGAAACCGCCTGA	277
Ad02A3273	Aradu.A02	3273	c	(TA)6cttaattttc	116	8875495	8875610	CGAGAGGCATCTCATTAGGC	TGCAAAGTCAAGACTGACCG	216
Ad02A3277	Aradu.A02	3277	p4	(TTTA)5	20	8879696	8879715	TCTCCCATGTGATGAAAGCA	TGTAGGCAGCAAAGATAGCAAA	226
Ad02A3284	Aradu.A02	3284	p2	(CT)9	18	8890692	8890709	GACCGCCCTAAAAACCGTAT	ATGGCGTTTTGGAATTTGAG	191
Ad02A3285	Aradu.A02	3285	p3	(TCT)6	18	8890849	8890866	AACCTCAAATTCCAAAACGC	ATCGAAACGCAGTGAGAGGT	172
Ad02A3292	Aradu.A02	3292	p3	(ATG)6	18	8916180	8916197	ATCCGTTCGAAACCAGTCAG	GGATGCCTCTCGCAGATTAC	272
Ad02A3296	Aradu.A02	3296	p3	(ATC)20	60	8928024	8928083	AAATTATTAAGAGCCATGTGTAATTTG	TCGGTGGTTCCAGAAAACAA	275
Ad02A3307	Aradu.A02	3307	p2	(TA)6	12	8956826	8956837	CAGTGTAAACAAGGTTGGGC	TGTCTTTACCAACTGCGTGC	161
Ad02A3310	Aradu.A02	3310	p3	(AAG)7	21	8962516	8962536	TGGTGGCGGATCTTTGTTAT	CGAAACCTACCTCCCACAGA	173
Ad02A3313	Aradu.A02	3313	p3	(TCT)13	39	8979495	8979533	TGTCTTTTGGAATGTTCGTGA	TTTCACCGCTTCCTATCCAG	277
Ad02A3319	Aradu.A02	3319	p2	(TA)8	16	9002826	9002841	TGCCACAAATTAAAATAAAGCC	TTCCATTTGTGTTTGTGGTTT	263
Ad02A3320	Aradu.A02	3320	p3	(TAA)5	15	9009685	9009699	TTCTTTCTCCCCATTACCCC	TTTTCCCCTCAAGATTCCCT	239
Ad02A3321	Aradu.A02	3321	p3	(CAT)5	15	9009974	9009988	TGGTCCCCATCCACAATAAT	TTTGCTCATGGTTTTCTCCC	143
Ad02A3324	Aradu.A02	3324	p3	(GAA)5	15	9011089	9011103	TGCTTCTTGCAAGTGTAACAAAA	TGATAATTGCATTGTGGAAACC	111
Ad02A3325	Aradu.A02	3325	c	(TAT)7tggttttc	103	9011291	9011393	TTCCTCACTAAGCTCCATCTCA	TTGCTCTTGTTGGAACCCTC	236
Ad02A3326	Aradu.A02	3326	p3	(CTT)5	15	9016934	9016948	TGGCATCCAAACTCAACAAA	AAACACGCGAAGAAGAAGGA	189
Ad02A3339	Aradu.A02	3339	p2	(TA)8	16	9045648	9045663	GAGAGCTCGCCAATTTTCAC	CATGGAAAGGGTTCATAAGTCA	235
Ad02A3342	Aradu.A02	3342	p3	(TTG)6	18	9049097	9049114	CTTGCCCTGCATAATGTGTG	CATTCCCCTCCAAGAGATCA	268

ID	Chromosome	SSR nr.	SSR type	SSR	Size	Start	End	FORWARD PRIMER (5'-3')	REVERSE PRIMER (5'-3')	Product (bp)
Ad02A3353	Aradu.A02	3353	p2	(AT)6	12	9076173	9076184	TCACAACAATAGCACATTAGAATTGA	CGAGTTTGTTGTCAAAGCCA	238
Ad02A3355	Aradu.A02	3355	p3	(ACC)5	15	9080735	9080749	TCGAAAACTGGAGCTCACCT	CAGCCTCGTCTCTCTGCTCT	207
Ad02A3361	Aradu.A02	3361	p2	(AT)6	12	9100821	9100832	AAATGGTAACAGAACTAACAAATTCAA	GAAACAACTCACCCCTCCAA	244
Ad02A3362	Aradu.A02	3362	p2	(AT)7	14	9105905	9105918	TCAATGTTCAAACCTTTTAAAGAGAA	AGTGGTTTTGGCTACTGGGA	256
Ad02A3364	Aradu.A02	3364	p2	(AT)8	16	9111256	9111271	CATGATAACGAGCATAATGCAA	CAAATGCCCCCTCTTCCT	150
Ad02A3365	Aradu.A02	3365	p2	(AT)7	14	9123848	9123861	AACAGATCAGAACCGTGCATC	TCACATATACATATAAATGCCTGCG	280
Ad02A3375	Aradu.A02	3375	p3	(CTC)7	21	9161979	9161999	CTACCAGAGGGTGCAGGAAG	TTGGTTGAGAGGGAGTACCG	119
Ad02A3377	Aradu.A02	3377	p3	(TGA)6	18	9171379	9171396	ATTCCCAAATTGGAACCTCC	CAATGTGGAACCAAGAAGCA	211
Ad02A3379	Aradu.A02	3379	p3	(TAA)13	39	9174087	9174125	CATTGGCACCACTAGAAGCA	TCAAATGCCAAAGATGACCA	185
Ad02A3380	Aradu.A02	3380	p2	(TG)31	62	9183134	9183195	TGTTGAGATGCTTTGTGTTGC	TAAACCACAAAATCGCTCCC	259
Ad02A3381	Aradu.A02	3381	p2	(TG)9	18	9190265	9190282	TGGATGCAGAAGTCACGAAG	TTAAACCGCAAAATCGCTCT	264
Ad02A3385	Aradu.A02	3385	p2	(AT)10	20	9196238	9196257	TTGACCTCATAGATAGTCATCCAAA	AAACTGAAAACGGCAGTGATG	255
Ad02A3386	Aradu.A02	3386	p2	(CT)8	16	9205793	9205808	TGTAGTTAATCGGCGGAACC	GATGGACGAAGGGAAGTGAG	158
Ad02A3389	Aradu.A02	3389	p3	(ATT)20	60	9222825	9222884	CATTCTAGTTTGTCAAAAGGAGCA	GCTGCCATTAGTATTACCGC	221
Ad02A3390	Aradu.A02	3390	p3	(GAA)6	18	9226155	9226172	AAGAGGATGAACGACGAAGC	TTCCACGTATTTTTCCCCAC	240
Ad02A3394	Aradu.A02	3394	p2	(AT)6	12	9228193	9228204	TTATTCACCGAAACTGCGTG	AATGATGTGGCACAGGTGAA	236
Ad02A3395	Aradu.A02	3395	c	(TA)11gctacgt	65	9228331	9228395	TTCACCTGTGCCACATCATT	CCCTCCAATTTAGGTTGTGC	260
Ad02A3401	Aradu.A02	3401	p3	(AAC)5	15	9247098	9247112	TTTCAGCTTCTGGTGGCTCT	GAACCGGTTGAAAAACTTGG	156
Ad02A3402	Aradu.A02	3402	c	(AGA)5caatgta	61	9247321	9247381	CAACCGGTTCAGACAATGTG	AAAGGGCCTCGAGAGATAGC	243
Ad02A3403	Aradu.A02	3403	p2	(GA)11	22	9247507	9247528	CAGGCAAGGTTCGCTATCTC	TGTGAAAGTGGGTGACCAAA	148
Ad02A3404	Aradu.A02	3404	c	(TTGGTG)5gt	54	9248622	9248675	TGGTGTTGTTGCTGTTGTTTG	AACGTTGAGGGTCAGAATCG	223
Ad02A3406	Aradu.A02	3406	p2	(AG)7	14	9254919	9254932	ATCCGCCTCATCTAGTCACG	TGTTGGCTTCTACCACACCA	221
Ad02A3410	Aradu.A02	3410	p2	(TA)6	12	9262006	9262017	GAAAATTCAAATGGGGCTCA	TCAAATTCACAAGTGGGTCAA	265
Ad02A3411	Aradu.A02	3411	p3	(AAT)14	42	9265871	9265912	TAGACCGTCTTTGCAACCGT	TTCTTCATTTACATGATCGACTTGA	277
Ad02A3419	Aradu.A02	3419	c	(A)11tcaaaaag	89	9278779	9278867	AAATTTTACCGAATGATTAGCCA	CCTTGAAAGGGGAAAGGTTC	252
Ad02A3420	Aradu.A02	3420	p3	(TGA)5	15	9279524	9279538	ACCATGAAGAGGACAATGCC	TTTGATTTCTTCCCACTGGC	116
Ad02A3421	Aradu.A02	3421	p3	(GAA)6	18	9290516	9290533	TAAAATATTGCTCGCCCTCG	CAACTTCTCTTTCCCACCCA	126
Ad02A3426	Aradu.A02	3426	c	(T)10ccgtctctc	61	9304716	9304776	CAGCCTTAAAAATGCTTTCCTG	TGTCAATCAGTATCAGCCCA	227
Ad02A3427	Aradu.A02	3427	p3	(AAG)5	15	9312152	9312166	TGTTGTGGTGAGCTTTCTGC	CCAAAACAGAGTCCTATCATTGC	178
Ad02A3433	Aradu.A02	3433	c	(T)10atcttaactg	71	9321833	9321903	TCCTGAGAGAAATGAATTGAAAC	AAAGCAGATCCACCAACCAC	181
Ad02A3434	Aradu.A02	3434	p2	(AT)6	12	9322348	9322359	TTTACGGGTTAGCGAGAGATG	CCAAAAATACTAAATTTGTTGTCTCC	228
Ad02A3435	Aradu.A02	3435	p3	(ATG)11	33	9324380	9324412	CGAAGAGGGTGGAGTACCAA	CCACAACTGGAGTGGTCCTT	137
Ad02A3436	Aradu.A02	3436	p3	(AAT)20	60	9334735	9334794	ATTTCCCTCACACAATGCCT	ATAATCCAAAGGCATGCACC	249
Ad02A3437	Aradu.A02	3437	p3	(TAT)7	21	9335958	9335978	CCGGTCAATAATGTACAAAACA	TGCTTTAATTTTTGTGTTAACGG	192

ID	Chromosome	SSR nr.	SSR type	SSR	Size	Start	End	FORWARD PRIMER (5'-3')	REVERSE PRIMER (5'-3')	Product (bp)
Ad02A3441	Aradu.A02	3441	c	(TAT)5cattatg	117	9342367	9342483	AAATCATAGAAAACAAAAGGTGGTT	AAAAATCGAAGCCGGAAGTT	254
Ad02A3444	Aradu.A02	3444	p2	(AT)11	22	9348800	9348821	TTGGGCGGGCTATCTAGTAA	CAACTTGCGTGTGAGGAAAA	255
Ad02A3446	Aradu.A02	3446	p3	(AGA)5	15	9357679	9357693	GAGGAGTCTTGGAACAGGCA	ATGCCGAGAAAATGGTTTTG	276
Ad02A3447	Aradu.A02	3447	p2	(TA)8	16	9358628	9358643	GGATGCAAACTCTTGAGAAACA	AAGAAGCTGTTGGAGCTTGC	242
Ad02A3448	Aradu.A02	3448	p3	(TTA)19	57	9359707	9359763	ATTGCAAACTTGTCTCCCCA	TGCACCAATTTACTTTGCCA	244
Ad02A3452	Aradu.A02	3452	c	(T)11gcatttcttg	104	9361467	9361570	TTTTTCACGTTCACCCTTCTC	CCACAATATAAAGATTTTTGTCCCA	259
Ad02A3453	Aradu.A02	3453	p3	(AAT)7	21	9364344	9364364	AGTTGTAAGGACGAAATTGAACA	CAGCCACTGTGGTTTGCTTA	255
Ad02A3463	Aradu.A02	3463	c	(C)12ttttttccttc	66	9404132	9404197	CCAGAGGGGAAATCAACAGA	TGATTTGTTGGTTCGTGTGC	145
Ad02A3465	Aradu.A02	3465	p2	(TC)7	14	9423426	9423439	CATTTTCTCGTAGCTTCGCC	ACGCTCATGCACAGCTTAGA	226
Ad02A3466	Aradu.A02	3466	p3	(ATT)6	18	9432007	9432024	TCTAAATGGGTGGACAAGCG	GAGATGCGTCAAAGGGCTTA	234
Ad02A3469	Aradu.A02	3469	p3	(GTT)5	15	9449717	9449731	GCACCTTGTTTCTGCCTCTC	CACAGAACCGAAGGAAGAGC	253
Ad02A3470	Aradu.A02	3470	c	(A)10tactatacc	116	9455851	9455966	CCAAATCCTAATAGCAAACTTCA	CCATTCTGCAGCGACTATCA	241
Ad02A3476	Aradu.A02	3476	p3	(TTA)5	15	9470307	9470321	ACTTGGACCTTCACGACACC	AACACAAGGCTTGCGAAAAA	214
Ad02A3477	Aradu.A02	3477	p4	(TTTA)5	20	9476499	9476518	TTTCGAAAACTCCTCTCTCTCG	GCCTTTTTCCTTCTTCCTGC	234
Ad02A3478	Aradu.A02	3478	p3	(TCT)5	15	9479688	9479702	ATCTCTTTCCCACCCAAACC	GCAATTGCCTTTCCTTTCCT	278
Ad02A3480	Aradu.A02	3480	p2	(GA)7	14	9494949	9494962	AGAGAGAAGAGAGATGGGTTCG	TCATTTTTGAAAACAACTCACCA	247
Ad02A3482	Aradu.A02	3482	c	(AT)12ag(AC)	42	9517893	9517934	CCAAAAGACAGCCCAAAAGA	TGCGGATTTTGTGATTTTCA	261
Ad02A3483	Aradu.A02	3483	p2	(AT)8	16	9533661	9533676	AGGCCGTGGGAATAAAGATT	GCACCTTTCCAACAACATCC	227
Ad02A3487	Aradu.A02	3487	p2	(TA)6	12	9539890	9539901	CCTTTAATGGGATGGGACCT	CCTGTTTGGCAACTACACCA	171
Ad02A3489	Aradu.A02	3489	p2	(AT)6	12	9541803	9541814	AGTTCATAAAAAGATTTGGATGTGT	ATGTACATGAGTGTGGGGGC	272
Ad02A3491	Aradu.A02	3491	c	(AT)14nnnnnn	73	9544376	9544448	CACCTTGCCACATAGAGCTT	AGCGTTGCGCTTTTAATTTC	227
Ad02A3492	Aradu.A02	3492	p2	(TC)12	24	9550563	9550586	TTTTTGCTATCCCTTGTCGG	GATGCATCGACTTAGGGTGG	246
Ad02A3493	Aradu.A02	3493	c	(TGA)7ccatgat	133	9552267	9552399	TGGTGGTGGTGAGTTTTGAG	CCACCACCCCTATTATCATCA	264
Ad02A3497	Aradu.A02	3497	p3	(ATA)13	39	9575589	9575627	CATGGCCATTGACCAGAGAT	AACGAGCGTGCAAATAAACC	277
Ad02A3498	Aradu.A02	3498	p3	(ATC)5	15	9577733	9577747	AAAAACATTTACCTCCACTACAATG	TTCATTCCATAAAGTTAAAACATCAAA	132
Ad02A3500	Aradu.A02	3500	p2	(TG)7	14	9596778	9596791	TACGTGCAAACAGCCTCAAC	AGCAGGGACCATGTTTTGAC	249
Ad02A3502	Aradu.A02	3502	p2	(AT)6	12	9599124	9599135	TGTGGCAAACAAGGAATCAA	TGCCACAAACATGGGAACTA	248
Ad02A3503	Aradu.A02	3503	p3	(ATA)11	33	9599270	9599302	TAGTTCCCATGTTTGTGGCA	TGAGCGTGCAAATAAACCTT	204
Ad02A3504	Aradu.A02	3504	p2	(TA)7	14	9599844	9599857	TGATTGACCCCACCAAGATT	CCATTAGGTGACGTTTTTCTTTG	269
Ad02A3505	Aradu.A02	3505	p2	(AT)6	12	9607127	9607138	TCCGAGCCAAGTTAACGATT	TTTATATTTGGGTAAAACCCCC	203
Ad02A3507	Aradu.A02	3507	p3	(TTA)20	60	9610067	9610126	AATCACAATTGATTATTGATACCCT	GTTCTAATTCTCATTCAAGTGCAA	235
Ad02A3510	Aradu.A02	3510	c	(A)10gaagaaga	42	9621716	9621757	TTTGCAGCATTATGTGTTTCTTC	TCCTCCTCCTCATCTTCTGC	204
Ad02A3513	Aradu.A02	3513	c	(AAAT)5agaa(	48	9628267	9628314	GAGATCCACTTTGTTTCTTTTAGCA	TTCTCCCCTTCTCCTTTCGT	253
Ad02A3517	Aradu.A02	3517	c	(T)10aggatttta	67	9646306	9646372	ACCCATGCATTGCTTTCTTC	GAAAGCACAATCACAGCAGG	280

ID	Chromosome	SSR nr.	SSR type	SSR	Size	Start	End	FORWARD PRIMER (5'-3')	REVERSE PRIMER (5'-3')	Product (bp)
Ad02A3518	Aradu.A02	3518	p3	(GAC)5	15	9652440	9652454	TTTGCCACCTCAATAGGACC	TTTTTAAAATGTTGTGACGCAAT	259
Ad02A3520	Aradu.A02	3520	c	(TG)12tc(TA)!	44	9660423	9660466	AGCGAAGTGGATCATTTTGG	CTGCGAAGATAAGGCTGGTC	227
Ad02A3523	Aradu.A02	3523	p3	(ATA)13	39	9681938	9681976	TGGATATATCTGCATGTGCCA	CTCAGCTTCAGGACCAATCC	273
Ad02A3524	Aradu.A02	3524	c	(A)10gaaattga	99	9694082	9694180	CCCCATGTATATTGGGTTGC	AAAATCATACCCTCCTCTTGC	243
Ad02A3526	Aradu.A02	3526	p2	(TA)11	22	9699441	9699462	ATCTCTCTTAGGCGGCGTTT	TGCGAAAGTATAATTGCCACAC	270
Ad02A3532	Aradu.A02	3532	p3	(GAA)5	15	9733083	9733097	GACAAAGAATTGAAGGCCCA	ATGAGGTCGGAATGGTGAAG	213
Ad02A3533	Aradu.A02	3533	p3	(ATA)5	15	9736672	9736686	CATGGCAGGCCTTCAAGTAT	AGCTTTCATTTTCCCCAAGT	252
Ad02A3538	Aradu.A02	3538	p3	(ATT)6	18	9747763	9747780	ATATGTCGGGCATGGATGTT	AAGAAAAAGGAGGATGCAACAA	266
Ad02A3543	Aradu.A02	3543	c	(TCC)6tcaaga	73	9763918	9763990	TGGCAGTAGTTGTTCCATGC	GAAGCTGTTGTGAAGTGGCA	170
Ad02A3544	Aradu.A02	3544	p2	(TC)9	18	9764736	9764753	ACAATAATGGTGGTGGCTCC	CCAACCTGCTTGTTCCTCAT	158
Ad02A3547	Aradu.A02	3547	p3	(TCA)5	15	9769068	9769082	AATCCAAAAACCAATGACGC	CAACAGAGATGGATGCATGG	270
Ad02A3555	Aradu.A02	3555	c	(T)10attcgatttc	86	9789968	9790053	CGATGATTAATTAGCAGGTTGG	CGTAACCATGGTGGAGGAGT	277
Ad02A3565	Aradu.A02	3565	p2	(TA)7	14	9824637	9824650	TTTTCCATGCCCAAGGTTAG	TTTCCGGTCATATTAGGCCA	211
Ad02A3569	Aradu.A02	3569	p2	(GA)10	20	9838584	9838603	CCCGTCAAAGAAAAACAAAGA	TCTTGATTCCAAATCCTCGG	145
Ad02A3577	Aradu.A02	3577	p2	(AT)11	22	9864008	9864029	TGATTGGTGGAGGCAAAAAT	TTCACTACGCTGGCAAATTA	280
Ad02A3581	Aradu.A02	3581	p2	(TC)8	16	9890393	9890408	ACTCCACCAACCACCATCAT	AGAAACCTGGAGCAAAGCAA	278
Ad02A3582	Aradu.A02	3582	p2	(AT)6	12	9901797	9901808	CCATGATGGAGAAAGGGAAA	TCTTTCCTCATTTCAAAAATCAAA	261
Ad02A3583	Aradu.A02	3583	p3	(TCT)5	15	9910281	9910295	CCCATCACCTCTTCACCACT	TTAGAATGTTGCTCGCCCTC	278
Ad02A3584	Aradu.A02	3584	p5	(ATTAA)5	25	9917404	9917428	TGCACGTAGAAGATAGCCAA	ATGTGCGAATTGGTACCTCA	263
Ad02A3588	Aradu.A02	3588	c*	(TTAT)7(TTA	45	9931801	9931845	TCTGCTGCGGCATGTATAAG	TCCCATCGATCAATACACCA	172
Ad02A3589	Aradu.A02	3589	p2	(TC)9	18	9931969	9931986	TGGTGTATTGATCGATGGGA	GGGCTTAGCTTCTTGGGTTT	215
Ad02A3593	Aradu.A02	3593	p2	(TA)6	12	9939558	9939569	AATTTGAACTTTTACTTTACCCTACCT	GCCTTTTCATCCCTAATAAATACA	276
Ad02A3596	Aradu.A02	3596	c	(A)10tc(T)10	22	9941952	9941973	CCACCATCATCATCATCTCCT	TCATTTGCTTTTGTGATGCAA	107
Ad02A3598	Aradu.A02	3598	p3	(CAA)7	21	9942412	9942432	GGCATAATGTTCCCAGCAGT	CCAGGGAGGCTTCTCTTTTT	188
Ad02A3599	Aradu.A02	3599	p4	(TTTA)6	24	9942693	9942716	TCTTGTCTGCCTGAGAATTAAGC	ATCCCTTTGGAACCCTTTGT	256
Ad02A3600	Aradu.A02	3600	p2	(AT)6	12	9945786	9945797	TGAGGCATTTTTGGATCATTC	CGGCTCTCAAGTATTAACGTCAC	121
Ad02A3601	Aradu.A02	3601	p3	(TGC)5	15	9946642	9946656	CAACCACAATCCAACAGTGC	CCAACTCCAAGTGAGGCATT	137
Ad02A3604	Aradu.A02	3604	p4	(AAAG)5	20	9951686	9951705	ATGTCAACGGTTAAGGGGGT	GTGGATTTAGTTGCCAACCG	176
Ad02A3606	Aradu.A02	3606	p4	(TTTC)5	20	9952901	9952920	CCCTCTGCAGAAGAAGAACC	TGGAGTGGATTGCATTGTGT	257
Ad02A3620	Aradu.A02	3620	p3	(ATT)6	18	10002590	10002607	GGTTCGTTTAAGTGTTCGAATTTT	GGGAACTATTTTGTGAGCTAGACC	278
Ad02A3622	Aradu.A02	3622	c	(T)10ccaaaaaa	163	10003340	10003502	CAGCAAAACACAAAAGCGAA	AAGCGGAGGGAGAAGAGAAG	277
Ad02A3623	Aradu.A02	3623	p3	(ACG)5	15	10003607	10003621	CTTCTCTTCTCCCTCCGCTT	TTGCGATTCGAAATACCTCC	237
Ad02A3624	Aradu.A02	3624	p3	(TGG)5	15	10003766	10003780	AAGACCTCAACGACGACGAC	TGCGATTATTACAGCTCCGA	270
Ad02A3628	Aradu.A02	3628	p3	(CAT)5	15	10032337	10032351	GGTTTAGGTTCATGAGTGGCA	GGGTATCATACATGCACAGTGG	273

ID	Chromosome	SSR nr.	SSR type	SSR	Size	Start	End	FORWARD PRIMER (5'-3')	REVERSE PRIMER (5'-3')	Product (bp)
Ad02A3629	Aradu.A02	3629	p2	(TC)6	12	10032572	10032583	ACTGTTCCACAAGGTGGGTT	AAAGGGCTAAAAGGGCACAT	194
Ad02A3631	Aradu.A02	3631	p3	(TCT)5	15	10043379	10043393	TGGAGTTCTGCTACACATTTAAGC	AGGAGGAGATGGATGTGGTG	182
Ad02A3632	Aradu.A02	3632	p3	(AGG)8	24	10044134	10044157	TGGTGATGACGGTAACCAAA	TCTCCACATCCAAGCAATTTT	147
Ad02A3642	Aradu.A02	3642	p2	(AG)7	14	10052667	10052680	GCAGTGGCAAACAAAGTTGA	GGTTCATTCCGCCTAAAACA	268
Ad02A3643	Aradu.A02	3643	p5	(AATTA)5	25	10054479	10054503	TCGGTTCCACTGAACATTGA	ATCCACCATCTTTCTCACGC	191
Ad02A3652	Aradu.A02	3652	c	(TA)7tgtttaagta	100	10084570	10084669	TTTTGCTGAATATGCCGAGTT	TTGCTCAAATACCTCAAACAAAA	280
Ad02A3653	Aradu.A02	3653	p3	(GAG)6	18	10093732	10093749	GCAAGAGCATCCGCTTTAAC	GGCGAAGAAGAACACTCAGG	273
Ad02A3655	Aradu.A02	3655	p6	(GATGAC)6	36	10103157	10103192	ATCATGGTTTCTGCCTTTGG	AACACCAACCCTGATTGCTC	173
Ad02A3656	Aradu.A02	3656	p3	(ACC)5	15	10103764	10103778	TTCTTGGTGCTCAACACAGC	CGGCACCGTTCTAAGGAATA	219
Ad02A3658	Aradu.A02	3658	p2	(GA)6	12	10114058	10114069	GTCAAATTCTGGCCGGTTTA	GTCGCAGCTTCATCTTTTCC	215
Ad02A3659	Aradu.A02	3659	p3	(AAG)9	27	10114231	10114257	GGAAAAGATGAAGCTGCGAC	CAAGACTCCAACGCCAAAAT	225
Ad02A3660	Aradu.A02	3660	p2	(AT)6	12	10116482	10116493	GCCTCCTTCATTCAGCAAAA	CCAGTGGCAAGGTATGAGAAA	275
Ad02A3662	Aradu.A02	3662	p4	(AAAT)5	20	10125642	10125661	TTTCGTTAATACCCCCTCCC	GAAAACTTACTACGGGAACACCA	153
Ad02A3667	Aradu.A02	3667	p2	(TA)6	12	10144340	10144351	TTGTTGAGTGGGTAGAAAAACAA	TTCACAAATAAAAACATAACATATTCG	100
Ad02A3669	Aradu.A02	3669	p2	(AT)9	18	10147129	10147146	GACAGGTTCGACCCGTTTTA	CACGGAGATCAGATCCTTTTG	278
Ad02A3679	Aradu.A02	3679	c	(AT)6(AC)6	24	10169173	10169196	GCAACGCTCACGTAAACAAA	TCTGCAAGCGAAGTAGGGAT	204
Ad02A3680	Aradu.A02	3680	p3	(TTA)12	36	10177486	10177521	GACATTAGTTATTAGCAAGAATGACCC	GGGTTGTGATGACGATGATG	276
Ad02A3681	Aradu.A02	3681	p3	(TGA)5	15	10178619	10178633	GGCTTTGTTGTGTCTGGGTT	TCCAAATATACCCCTCCCTTG	248
Ad02A3685	Aradu.A02	3685	p2	(TA)6	12	10187214	10187225	AACATGTTTGTATAATGGACAAGATT	TGATTTCACCTAAAACAGCTCA	230
Ad02A3689	Aradu.A02	3689	p3	(GGA)5	15	10193148	10193162	AGACACCTCAAAGGCCTCAA	ACATGTCCTTCTCCGACCAC	194
Ad02A3690	Aradu.A02	3690	c	(A)10gaaaaaaa	38	10193375	10193412	TGGAGATATGGTGCTGGACA	CGTGCGACTCCTTTTCTTTT	186
Ad02A3691	Aradu.A02	3691	p2	(AG)6	12	10194822	10194833	CAAACAACTCCACCCGCTAT	CTTCAATCCAACCCCAAAAA	256
Ad02A3692	Aradu.A02	3692	p3	(ATC)6	18	10207777	10207794	TCATGGCATTCAAGTCCAAA	TAATCGACCCTTTGGTGAGC	258
Ad02A3695	Aradu.A02	3695	p3	(TCT)6	18	10221194	10221211	CACAAACCATGAATCCACACA	AATCCCTATAATTCCTTTTGTTTTT	253
Ad02A3697	Aradu.A02	3697	p3	(ATC)5	15	10228939	10228953	GGGATATAGTACCCGGCACA	GAGAGGGTGGTGACTTGGAA	258
Ad02A3699	Aradu.A02	3699	c	(T)11ga(T)10	23	10237096	10237118	CAGAAGTTTTGAAAATCAAACTGG	TCGATTGAACGGATACATTTTT	208
Ad02A3700	Aradu.A02	3700	p3	(ATG)8	24	10238484	10238507	CAAGCTTTCCCAAACCTTCA	GAAACGATCATTGCAGAGCA	263
Ad02A3704	Aradu.A02	3704	p2	(TA)6	12	10244188	10244199	TTGTGAATACCATGTGGAGCTT	TTTGCCAATTGAGACGTAGAG	278
Ad02A3707	Aradu.A02	3707	p2	(TC)24	48	10247321	10247368	ACTCCCGTCATCGTCGTTAC	ACGGCGACTAAGTGACGAGT	268
Ad02A3709	Aradu.A02	3709	p3	(TTA)5	15	10256659	10256673	AAACAAAGTTAACGCAAAACAAAA	TTTTTCGATTCTCTTAGCACG	275
Ad02A3711	Aradu.A02	3711	p2	(CT)8	16	10273974	10273989	AACCGGTGAACCAACTGAAC	GAGAAGAGTTCGGCGATGAC	189
Ad02A3712	Aradu.A02	3712	p2	(CT)10	20	10274109	10274128	GTCATCGCCGAACTCTTCTC	GACGAACAGAGACCAGAGGC	197
Ad02A3715	Aradu.A02	3715	p2	(TC)8	16	10275987	10276002	GGTGCTCTTGCCACTCTCTC	TCGTGGATTTTACCGTAGGC	250
Ad02A3718	Aradu.A02	3718	c	(TA)7gttgtatttt	105	10297067	10297171	CGTATTTGTTGTTGTTGTTAGAATGTT	TCTGATTCAATCCTCAATAAATAAAAA	244

ID	Chromosome	SSR nr.	SSR type	SSR	Size	Start	End	FORWARD PRIMER (5'-3')	REVERSE PRIMER (5'-3')	Product (bp)
Ad02A3720	Aradu.A02	3720	p2	(AT)10	20	10303731	10303750	TGCGTCAAGATTTTGTCATCTC	AATCGCGACTATTTGCATGA	258
Ad02A3722	Aradu.A02	3722	p3	(AAT)5	15	10306398	10306412	AACTTGGAAAAGTGGTGGTAAA	TTCTTCCATTTGAGGGCTTG	257
Ad02A3723	Aradu.A02	3723	c*	(AAT)5(T)10*	24	10310017	10310040	CTCCAATCCCTACCAAACCA	CACCGCAGCTAACAAAGACA	242
Ad02A3724	Aradu.A02	3724	p2	(AG)6	12	10310421	10310432	GAGGCTGAGAGAGCGAGAGA	TTATTTTCCCACGCACACAA	218
Ad02A3729	Aradu.A02	3729	p2	(GA)17	34	10314962	10314995	CGAGAGAGGGAGAGGTGTGA	TGACTCGCATGAATGCTTTT	111
Ad02A3730	Aradu.A02	3730	p3	(AAT)17	51	10315479	10315529	TTTTGGATACGCTGGAAACA	TCTTCGTTCTAGACCCAATCG	266
Ad02A3731	Aradu.A02	3731	p3	(TAA)12	36	10337870	10337905	TCCACAACACCTCTTTTCCTC	TTTCTTTCGATCTATCTATTTAACCAA	242
Ad02A3733	Aradu.A02	3733	p2	(CT)6	12	10339448	10339459	AAAATTGAAGTTTTACCCACAAAAA	CGACTGCAGTTGGAGATGAA	274
Ad02A3735	Aradu.A02	3735	p2	(TA)8	16	10353456	10353471	GAGCGTTGCGCTTTTAATTT	ACGCATGTTAGGCTAATGCC	253
Ad02A3736	Aradu.A02	3736	p2	(AT)13	26	10356622	10356647	TTTGGCCACACGTTCACTTA	TGCCGTCAATAAGGTACGAA	258
Ad02A3738	Aradu.A02	3738	p3	(GAT)6	18	10361650	10361667	TTTGTATTGGGCATCATCACA	TCATCCTCCATTTTCTTTCCC	248
Ad02A3742	Aradu.A02	3742	p3	(AGG)8	24	10380681	10380704	TGGTTCCGTTTCCAAAGAAG	AACCGCTGCTAAACCAGAAA	181
Ad02A3749	Aradu.A02	3749	p2	(AT)9	18	10413971	10413988	GGTGACCTTTTTGTTTTATCGC	TCAAAATATATTGGTCTCTAATGCTCA	257
Ad02A3755	Aradu.A02	3755	p3	(TGT)5	15	10426765	10426779	ATCTGCATCGCTTTCTGCTT	CCACCAACATTACCACCACA	251
Ad02A3760	Aradu.A02	3760	p4	(AAAG)5	20	10434333	10434352	CCAGTGGAGGAGGTAATTTCA	ATGCGCCTAACTTTTGCACT	176
Ad02A3761	Aradu.A02	3761	c	(TA)6attaacgg	47	10436704	10436750	TGACCGCATTCAAAAGGTAT	TGTTTGACCGAAAACTATACACTGA	280
Ad02A3768	Aradu.A02	3768	p3	(CGC)5	15	10442947	10442961	CGCTCGAGTCCAAAAGAAAC	GAGGCGTCGTCAGAGCTTAG	235
Ad02A3771	Aradu.A02	3771	p3	(ATC)7	21	10452704	10452724	TCTGCCCAATTTGGAGAATC	CGACGTTTCAGCGTCAATAA	216
Ad02A3772	Aradu.A02	3772	p3	(ATT)14	42	10453193	10453234	AAAAACCTTCATAACTTGGACGA	CGAAGAAAGGATACTATCAACGAA	270
Ad02A3775	Aradu.A02	3775	c	(A)11tcttatataa	89	10462221	10462309	CGTTATTGTGTTGAGCGAGC	AAAGTTGGATCTACGAGTTGTAACG	144
Ad02A3777	Aradu.A02	3777	p3	(TTG)5	15	10463135	10463149	TGCCATGCTCACTTTCACAT	ACAAAAATGTCCCACCCTCA	230
Ad02A3778	Aradu.A02	3778	p3	(TGA)5	15	10463429	10463443	TGAGGGTGGGACATTTTTGT	AGGTTTGAACCTTGAGGCAA	238
Ad02A3779	Aradu.A02	3779	p2	(GA)9	18	10463630	10463647	TCTGTTGCCTCAAGGTTCAA	AGGAGCCTACCATTGCATGT	278
Ad02A3780	Aradu.A02	3780	c	(T)10ccaaggttt	94	10464846	10464939	TGCTTGCTTGTATCACCTTATG	AAAAATTGCAGTGTCATAATTTTGTT	232
Ad02A3782	Aradu.A02	3782	p4	(ATAA)5	20	10468420	10468439	AAATTTGTTGCATGAGAAGACG	AGTGCTCTGTTGGCCATTCT	232
Ad02A3783	Aradu.A02	3783	c	(A)11ggaaagga	131	10472100	10472230	CGCTCACCTAATCTTCTCACA	CCTCTCTCTGAGTCGAACCG	263
Ad02A3787	Aradu.A02	3787	c	(A)10ttgatttttaa	63	10481762	10481824	TTGATGATTTGATATTCGCCA	GGTCGAGCCCATTCTTTTT	230
Ad02A3789	Aradu.A02	3789	p3	(TAT)10	30	10494137	10494166	ATCCCAATTTAAGCCTTCGC	AAGCTGAAGAGAAAGAGAGAAAACA	239
Ad02A3792	Aradu.A02	3792	p2	(AT)6	12	10499225	10499236	TGTGTAGAAAATCATAAGCTTAGGTCA	CGATTTTGTGAAGCAAAGCA	187
Ad02A3793	Aradu.A02	3793	p4	(TTAA)5	20	10499533	10499552	TCAACCGTGCCTGATGAATA	ACACACTCACCAATTTGCCA	156
Ad02A3794	Aradu.A02	3794	p3	(ATT)5	15	10499719	10499733	TGGCAAATTGGTGAGTGTGT	GGTCAACAGAATTCCGAACC	175
Ad02A3796	Aradu.A02	3796	p3	(AAT)5	15	10501055	10501069	AAAGCTTCTCCCTCCCCATA	TCTTCTTGTTGTTCCCTGGC	244
Ad02A3797	Aradu.A02	3797	p2	(TA)6	12	10502040	10502051	CGGGTATGAAATTGTTGGGT	CAAGGCATAAGCAAAATCAAA	222
Ad02A3800	Aradu.A02	3800	p2	(AT)8	16	10502766	10502781	TCGGTAGTTATATATCTCCACCAATG	TCTCAAGTAATGAAATGATCGGAA	264

ID	Chromosome	SSR nr.	SSR type	SSR	Size	Start	End	FORWARD PRIMER (5'-3')	REVERSE PRIMER (5'-3')	Product (bp)
Ad02A3802	Aradu.A02	3802	p3	(TAA)8	24	10506261	10506284	CTCATATGAACACCATTCTTTAACA	TCTTTTTAGGAGAAATAAAACCAACAA	273
Ad02A3803	Aradu.A02	3803	p2	(AT)9	18	10508344	10508361	GAAGAAGCTTGTTATCGAAAAGTT	CGAATCTGACTTCACCCGAT	230
Ad02A3808	Aradu.A02	3808	p3	(ACC)6	18	10529946	10529963	TGTGAAGGGATGTGGTGAAA	AAACCTTGCCATGGAGTCTG	148
Ad02A3810	Aradu.A02	3810	p2	(CT)19	38	10531940	10531977	TTAAAATGGTGAAGGAGGCG	CCGTATTTGTTTGAGTTTGAAGG	272
Ad02A3812	Aradu.A02	3812	p3	(AAT)15	45	10555443	10555487	AAAGAAAAGCAACGGAAGCA	TGCAGAACGTGATATGGAGG	256
Ad02A3814	Aradu.A02	3814	p3	(TAT)8	24	10570784	10570807	GTTACTTTCCTGCGCCACTT	ATTCGGAATTGAGCGAACAC	219
Ad02A3817	Aradu.A02	3817	p2	(AT)9	18	10585173	10585190	CTCAAAGCATGGAATGTGTCA	TGTCTTTGAATTTGGGGCTC	266
Ad02A3818	Aradu.A02	3818	p3	(GTG)5	15	10588309	10588323	CGGCACCGTTCTAAGGAATA	TTCTTGGTGCTCAACACAGC	222
Ad02A3821	Aradu.A02	3821	p3	(CTC)8	24	10595551	10595574	AGGAAGCTAAGGCCAAGTCC	CATCCGCTTTAACACCACCT	237
Ad02A3822	Aradu.A02	3822	p3	(CCT)5	15	10597768	10597782	AAAGGGTCCAGGAGGCTAAG	CCGCCATCAAAAGAAGAAGA	220
Ad02A3823	Aradu.A02	3823	p2	(TA)9	18	10617537	10617554	ATGGCTAGCCGGCTTAAACT	CGCGACATAAAGCAAAGGTT	258
Ad02A3825	Aradu.A02	3825	c	(T)11cgttttttttt	80	10619400	10619479	ACAAGTTGCTCACCCAGCTT	TGAGAGCAATTAATTTTGGGC	190
Ad02A3827	Aradu.A02	3827	p5	(TTATC)5	25	10622510	10622534	CAAATTCAGCCCAACAGGAT	CAGCAGAGCTCAAGAAGCG	257
Ad02A3829	Aradu.A02	3829	p2	(TA)7	14	10627939	10627952	TAATGGCCGAAGCCATAAAG	TGGAGGGTTTCACAGTTTCC	238
Ad02A3830	Aradu.A02	3830	p2	(AT)6	12	10628447	10628458	CGGTAGTTCGGTCAAAAAGC	ATGGCTAGCCGGCTTAAACT	264
Ad02A3831	Aradu.A02	3831	p3	(CAA)5	15	10637273	10637287	AAAGGGCAACTGCTGAGAAA	ACAATTGTAGCACAAGCCCC	242
Ad02A3832	Aradu.A02	3832	p3	(AGA)5	15	10645801	10645815	CACGTACCCTTCCTTTTCCA	TTCTTGGTGCTCAACACAGC	204
Ad02A3838	Aradu.A02	3838	p3	(CTC)5	15	10659060	10659074	TCTTTTGCAGACATGGCAAG	AGAAGAAATCACAATGGGCG	165
Ad02A3842	Aradu.A02	3842	p3	(AAT)15	45	10677363	10677407	TTTCTTCCACCCCTAACCTTC	TCACTTTTAGATGAAAAATTACAGAAA	256
Ad02A3844	Aradu.A02	3844	p3	(TAT)8	24	10678409	10678432	CCTGCGCATTTTCATCAAT	GGATCCGATTGATATTTTTGTT	252
Ad02A3845	Aradu.A02	3845	p3	(TCT)5	15	10679359	10679373	CAACACCGCAATGATCAAAC	ACAGTGGTTCGCAGCTTCTT	172
Ad02A3849	Aradu.A02	3849	p2	(TA)7	14	10685467	10685480	CCTCAAAATGATGTTCCTACAGC	CATTTTCATTGTGCATTGCC	257
Ad02A3850	Aradu.A02	3850	p3	(TGA)5	15	10686779	10686793	TCATGAAGTGATGGGTTGATTT	AAGCAGGGTCCCTACCAGTT	146
Ad02A3852	Aradu.A02	3852	p3	(TCA)5	15	10693785	10693799	TCCCTTCTTTTGCTGCTGTT	GAGGAAAACGATTCGGATGA	240
Ad02A3853	Aradu.A02	3853	p3	(AAT)25	75	10694258	10694332	ATTGATCATGGCCGTTTGTC	ATTTCATCTACGAGTGCATTTTT	275
Ad02A3855	Aradu.A02	3855	c	(CAA)15(TAA	63	10694893	10694955	AAGAATGATAACAACATCCAGCAA	TTTAAACATGTGAGTATCCTGTTTTT	251
Ad02A3856	Aradu.A02	3856	p2	(AT)10	20	10711262	10711281	TCGAATTTTATGTAAAACACATCTCA	GGAGGCTTGTACCAATGACAA	229
Ad02A3857	Aradu.A02	3857	p3	(TAA)11	33	10711939	10711971	ATTACTCTGGCCCAGCTCCT	GATGTTGTTCTTCAATTGTGGG	220
Ad02A3858	Aradu.A02	3858	p3	(AAG)6	18	10712951	10712968	GCTCCTCATCTTCTGCTGCT	CGTTTCAAACCGTTACTGCAT	240
Ad02A3859	Aradu.A02	3859	p3	(GAA)5	15	10716941	10716955	AGCCATAATCGCACCAATTC	TGCCATCATTCATTCCTTCA	151
Ad02A3861	Aradu.A02	3861	c	(T)11(A)10	21	10721963	10721983	GCACCCTCCTGAATCACAAT	TGCTGTAGAACAGGTAGTGCG	208
Ad02A3863	Aradu.A02	3863	p3	(TTA)11	33	10722321	10722353	TTCCACACCCACTATCTCCA	TGGATTTTGGTTCCCGTTTA	269
Ad02A3864	Aradu.A02	3864	p4	(AATA)6	24	10722523	10722546	CAGCTTAAACGGGAACCAAA	TGGCATTGTTTGTGAAGGAA	180
Ad02A3865	Aradu.A02	3865	p2	(AT)6	12	10731748	10731759	TGATGGGCATAGTTATGTAAAGAAA	AAGCAAATAACACACTTGCCA	280

ID	Chromosome	SSR nr.	SSR type	SSR	Size	Start	End	FORWARD PRIMER (5'-3')	REVERSE PRIMER (5'-3')	Product (bp)
Ad02A3866	Aradu.A02	3866	p2	(TA)10	20	10743573	10743592	GATGGTTTGGTGGTACTGGG	CCCATCTTCTTCCTTCCCTC	184
Ad02A3874	Aradu.A02	3874	p4	(AAAT)5	20	10758801	10758820	AAATCCCAATCTTGTTCCCC	GTGTCCCTTGCATATGGCTT	183
Ad02A3877	Aradu.A02	3877	p2	(TA)6	12	10762653	10762664	GTTTCCTCCCTTTGGAGCAT	TGGGTATTAATTTGAAGTTCTCTGC	153
Ad02A3882	Aradu.A02	3882	p4	(TAGA)5	20	10772038	10772057	GTTTTTGTGGCTCCCTTTGA	TCCCTTGGCTGAGACAAAAT	280
Ad02A3883	Aradu.A02	3883	p3	(AAC)5	15	10772327	10772341	ATGGTGGAAACTGGTGAAGC	CCGTACTTGATCCCAATTCC	238
Ad02A3885	Aradu.A02	3885	p2	(AT)7	14	10777506	10777519	GGATCTGGTGACAAGTCCAA	GCTTGTTCCTCATTTGACCC	279
Ad02A3890	Aradu.A02	3890	p2	(AT)10	20	10780649	10780668	AGCGGGCAAGATATTGAGTG	TGATCCATTTTGCTGATCCA	280
Ad02A3892	Aradu.A02	3892	c	(ATC)5tataatta	81	10791944	10792024	ACCAACACCACCCTCAACAT	AATTTGCCAATTTCTGCCAC	235
Ad02A3893	Aradu.A02	3893	p2	(TA)6	12	10794283	10794294	ATTCGGGTAATTTTGTCCCC	CGCATTCCATGAGCAACTAA	279
Ad02A3894	Aradu.A02	3894	p3	(TAT)17	51	10795999	10796049	AGCGGCCCACAAATAGTAGA	CGTAATATTGGCAATTAAGACAATG	224
Ad02A3895	Aradu.A02	3895	p2	(CT)6	12	10797704	10797715	TTCCATCTCATCCATCCTCC	AAAGGGGAGCACGAACATAA	221
Ad02A3897	Aradu.A02	3897	p3	(ATA)10	30	10811214	10811243	TGCACACATTAATAGTGACCCAT	CCTTTTTAAAATATAATGCATGTACCG	111
Ad02A3898	Aradu.A02	3898	p2	(TA)6	12	10811883	10811894	GCTCTTTCCTAAAAATATTGGCA	CAGGAGAAGTCCTATGTCTGAGG	195
Ad02A3901	Aradu.A02	3901	c	(TTA)5gatatca	54	10818139	10818192	TGCTTATTCAGATCAATGTCATGTT	TGACGGGGTCTTTTTCAAAT	274
Ad02A3902	Aradu.A02	3902	p2	(TA)6	12	10826645	10826656	TCTGCTCTCCTCACCCTTGT	TTCTTGTCCTTGCTCCTGCT	168
Ad02A3912	Aradu.A02	3912	p2	(AT)6	12	10851846	10851857	AAAAGGCAGCTAGAGTGGCA	GAGTGGGTTTTTGTTTGGGA	233
Ad02A3918	Aradu.A02	3918	p4	(AAAT)5	20	10870791	10870810	AATTATGTTGGTGGGTCTCCA	AACTCTCCCTCTCCTTTCGTG	100
Ad02A3919	Aradu.A02	3919	p4	(AAAT)6	24	10875523	10875546	AAGCTTGGTGGAGCAGCTTA	TCTCCATCGAAGCTCCTTGT	208
Ad02A3921	Aradu.A02	3921	p2	(AG)6	12	10879999	10880010	TGGAAGAGGGAGAGAGAGCA	GCGCTAGCAACAATAGTCTCA	149
Ad02A3932	Aradu.A02	3932	c	(TTC)5tgccttc	72	10912675	10912746	TTTCTTCACGCCTCTTTCAA	CAGTCATACAAACCCACCAAA	186
Ad02A3934	Aradu.A02	3934	p4	(ATTT)5	20	10934866	10934885	TCGAAAAATTAAGAACCAAGTTGTC	AACCAACTAGGGGTGAAGCTAA	278
Ad02A3935	Aradu.A02	3935	p2	(CA)6	12	10938760	10938771	TCCCTGAAGCATCGAAAGAT	TTCTCCACCCAACAAGGTTC	189
Ad02A3936	Aradu.A02	3936	p2	(AT)6	12	10939741	10939752	TCAATGACCAAATACCACCTGA	TTGAACTTAGCATTAAAACACTTGA	263
Ad02A3939	Aradu.A02	3939	c	(TA)6ataatatta	45	10940524	10940568	GAAATAAGAAGAATAAAAAGCCTGTTG	GGAAGTTAAGCCTCCAACTGAA	248
Ad02A3940	Aradu.A02	3940	p2	(AT)13	26	10945675	10945700	CCTTTATCCTTCTGGGATGC	GCCTCCGTCAATTTTACACA	268
Ad02A3941	Aradu.A02	3941	p2	(AT)10	20	10947006	10947025	GGACATAAAGTGGTTATCGGG	CTAACTCAACCCGCGAAGTC	213
Ad02A3943	Aradu.A02	3943	c	(A)11cgagtgtg	117	10954520	10954636	AAAGGAAAAGAGAAGTTTTGATTTG	CACAGGAACTGTAAATATGTAGGCA	273
Ad02A3949	Aradu.A02	3949	c	(TTA)13ttgtgc	110	10989134	10989243	CTATTTTGATAGGCAAAATTACGTT	TGGCATCAGTGGATGAAAGA	222
Ad02A3950	Aradu.A02	3950	p2	(AT)6	12	10989352	10989363	TCTTTCATCCACTGATGCCA	CGGAAACCTTATGGTCATGG	180
Ad02A3952	Aradu.A02	3952	c	(AT)9aaatccatt	107	10995133	10995239	CTGCCCCATGGAACCTATTA	TGAGCGACGTAGTGACGAAC	205
Ad02A3955	Aradu.A02	3955	p2	(AT)6	12	10999122	10999133	TCTGTTCGGCATAAGAACCC	TGCATCAAAAAGAAAAGTGGAA	212
Ad02A3960	Aradu.A02	3960	p3	(TTA)5	15	11019953	11019967	CAAATCAATCCCCAATATTTTTC	CAATCCCCAACGTTTCTCTC	236
Ad02A3967	Aradu.A02	3967	c	(T)10cgaaaaata	105	11039514	11039618	TTTCCTTGTTTCGCGTTAGC	GCTGAGAATTCCCAATGAGG	250
Ad02A3973	Aradu.A02	3973	c	(ACA)11acgaa	213	11060285	11060497	CAGTTATCAATTAAACAAAGCAGC	GTCTCTGATCCCCCACTGC	273

ID	Chromosome	SSR nr.	SSR type	SSR	Size	Start	End	FORWARD PRIMER (5'-3')	REVERSE PRIMER (5'-3')	Product (bp)
Ad02A3978	Aradu.A02	3978	c	(AT)9acatatata	81	11079009	11079089	ACCAGCAACAGCGGATAACT	AACCCTAAAACCCTGAACCC	255
Ad02A3980	Aradu.A02	3980	p3	(TTA)5	15	11086899	11086913	CTGATGTCGTCTCATTCAAAGG	ACGGAGAAAGACGACGATGA	279
Ad02A3981	Aradu.A02	3981	c	(TTAT)9taatta	57	11096057	11096113	CTTATTAATATGGTTAGCACCGC	CACATTGGTTGGCGTTCTTT	273
Ad02A3982	Aradu.A02	3982	c	(T)10gtatttttag	86	11098777	11098862	AAAATTTTGATCCCCTTGGTTT	AATTGCCATTGTCCTTGCTT	280
Ad02A3991	Aradu.A02	3991	c	(TTA)9ttg(TTA	57	11119590	11119646	AATTGTTAACGGAAATTTTTGG	TAACAAGGCCCAAGGTTCAT	224
Ad02A3996	Aradu.A02	3996	p2	(TA)10	20	11133574	11133593	CAGCTCATTAGTTTGTGAGTTGA	CATGCGTTTCCGAGCTATAA	279
Ad02A3999	Aradu.A02	3999	c	(TC)10atgtggtt	57	11145929	11145985	TTCGTTGATGTTCTTCTCCTTTC	CCGTTGACGGAAGGACATAC	253
Ad02A4001	Aradu.A02	4001	c	(TCT)5cacctttc	115	11152625	11152739	ACCGAACCAGGTCATGCTTA	TGAACTGGATGGGTGAGAGG	223
Ad02A4002	Aradu.A02	4002	p2	(CT)8	16	11152855	11152870	CCCAACCCCTCTTCTTTCTC	GCAGGGACAGCAGAATCAAT	273
Ad02A4006	Aradu.A02	4006	p2	(TA)6	12	11166472	11166483	TTGGAACCACTTCCCATCTC	CCTTCAGATTTTAAAAAGGGAAAAA	152
Ad02A4008	Aradu.A02	4008	c	(TGT)5tcctaaa	100	11167140	11167239	TTCCAGAACCCCACCACTAC	TGCCATCAATAATGGTTCTTCTT	261
Ad02A4010	Aradu.A02	4010	p2	(AC)7	14	11177060	11177073	CCCCCTTACTCCTAAAACGC	CGTTTGGCCTCCATAGTGAT	165
Ad02A4017	Aradu.A02	4017	p3	(AAG)5	15	11185873	11185887	GTGAGCAGGGGTACGGAATA	GAACCCTTTTTCACCACTGC	272
Ad02A4020	Aradu.A02	4020	p2	(AC)8	16	11193587	11193602	CTTATCCGGCGTCTCAATTC	TTGTGATGTGCCTCCGAGTA	212
Ad02A4022	Aradu.A02	4022	p3	(TAA)5	15	11204687	11204701	CTCCCATGTCATCCAGTCCT	GATGGCCCACTGATACTGCT	275
Ad02A4025	Aradu.A02	4025	c	(T)10ctttctc(T)	67	11210649	11210715	TTGTCATCGTGAGCGAAAAC	TTGTTGATGCAACCGAAAAT	158
Ad02A4028	Aradu.A02	4028	p2	(TA)6	12	11229287	11229298	TATTCGTTGGTGGATTGCTG	TGGTGGCATAAAAAGTGGAA	260
Ad02A4033	Aradu.A02	4033	p2	(TA)6	12	11242453	11242464	GAAAGTTGGCCATAAATCAGG	TTGAGGTATCAAGAAATTTTGCC	279
Ad02A4035	Aradu.A02	4035	p3	(AAT)5	15	11243420	11243434	GGTGCTCAAGGACCTGAGTT	AAAGATGAGTGTGGGAAAGCA	168
Ad02A4038	Aradu.A02	4038	p3	(TCG)7	21	11248114	11248134	GTCTCCTTCACTGGCTCGTC	TAAGAAGGTGAGCACCGGAC	130
Ad02A4044	Aradu.A02	4044	p3	(TTC)5	15	11303741	11303755	TTGCCATTTTCATTCAAGCA	TCTATTACTGCAAAGGAGGAACG	257
Ad02A4047	Aradu.A02	4047	p2	(AG)7	14	11313649	11313662	TGAAGAAGCAGCCAAAGACA	ACCCTTCTTGAACCTTCCGT	269
Ad02A4054	Aradu.A02	4054	p2	(TA)10	20	11328170	11328189	GCAATAAAACAAAAACAAATGCC	ACGTGTTGTGGTCTCGTGAT	228
Ad02A4056	Aradu.A02	4056	p2	(GT)6	12	11371186	11371197	GCTTGATTTTTGCTTGAATGC	GTTCTAAAATTGTGCGCGGT	184
Ad02A4057	Aradu.A02	4057	p2	(GC)6	12	11383810	11383821	AGTGGTTTTCCACAAGGACG	TCCCTTGCTTGGACTTCAAT	215
Ad02A4058	Aradu.A02	4058	p2	(GC)6	12	11386453	11386464	ACATGGCTCCTAAAACCGCT	CCAGTTGGGTTGGAAAGCTA	163
Ad02A4059	Aradu.A02	4059	p2	(TA)8	16	11402223	11402238	AAAATGTCACGCTTCTGGCT	TCTCTCGCCTTTAGCTTTGC	265
Ad02A4061	Aradu.A02	4061	p2	(CT)6	12	11406249	11406260	CAAGAGGAATAGATAAACCAAAGCA	GGCTATTTTTGGCCCAGTTT	264
Ad02A4063	Aradu.A02	4063	p2	(AT)9	18	11433126	11433143	TTGCGACATTTTCTTTTCCC	GCTTTGGCCTCATTTGCTTA	253
Ad02A4064	Aradu.A02	4064	p3	(TCA)5	15	11434425	11434439	AACTCTTCTTCTCCCGCCTC	CCCCTTCCAAGTAATTTTATAGCA	214
Ad02A4066	Aradu.A02	4066	c	(T)10ggacaaaa	192	11444140	11444331	ATTTAAAATCGTCCTTAACCTTTTT	TCCAGAAATTCACAGAATTCACA	266
Ad02A4067	Aradu.A02	4067	p3	(TGA)6	18	11444593	11444610	AGGGAATACGGGAAGAGGAA	ACCTCTTCACCACCACCATC	191
Ad02A4068	Aradu.A02	4068	p3	(TCT)5	15	11449061	11449075	TTCCTCATCTTCTGCTGCTTC	CATCCTGTTCCAAGTAATTTTGG	201
Ad02A4070	Aradu.A02	4070	p3	(GAG)5	15	11451550	11451564	CACGCAAAGAAAAAGGAGGA	GCATCACGTTTTCAATGCAC	159

ID	Chromosome	SSR nr.	SSR type	SSR	Size	Start	End	FORWARD PRIMER (5'-3')	REVERSE PRIMER (5'-3')	Product (bp)
Ad02A4072	Aradu.A02	4072	p3	(TCT)8	24	11460344	11460367	TGATTTGGTATGGTGCGTGT	TGGAAGGGAAATGGTTGAAG	185
Ad02A4074	Aradu.A02	4074	p2	(AT)10	20	11464047	11464066	GCACGATTTCATGATTGCAT	TGTGAAACATTTCTTTAATTTGGA	271
Ad02A4076	Aradu.A02	4076	p3	(CTT)5	15	11465312	11465326	GAAACCGTTCAAGGGTGAGA	TGAACAAGAAAAGAAGAAGACGA	278
Ad02A4078	Aradu.A02	4078	c	(A)10gagacgaa	73	11489910	11489982	CAACAAATATTATGAAATGGAAAATGA	TGGGATTTGGTGGGGTAGTA	256
Ad02A4079	Aradu.A02	4079	p2	(AT)6	12	11492906	11492917	TTGAGACACATAGACACATACACGA	TTGCATAAGACACAAAATGTAATCT	277
Ad02A4085	Aradu.A02	4085	p2	(TA)6	12	11514253	11514264	TAACATAAGCAATTTGCGGC	AGTTTTGGTCGGTTCTTCCC	238
Ad02A4086	Aradu.A02	4086	c	(T)18acattgtgc	77	11515825	11515901	CGCACATTGTTGAGGAGATT	GGAGCTCTTTGTTCCCATCA	247
Ad02A4091	Aradu.A02	4091	p2	(CT)6	12	11531499	11531510	TCCCTCTCACCTGCAACTCT	CCATCGCTAGTCAACGACG	212
Ad02A4092	Aradu.A02	4092	c	(T)10att(TTTA	33	11534146	11534178	TCCGACCGAAATAGAATAAAAGTC	TTGCAACGCACCACTTATTT	223
Ad02A4093	Aradu.A02	4093	p2	(AT)10	20	11539999	11540018	AAAATGACTACTTCCAGTTTACCATT	TTCAAGATCAAAATTTCAAAAACC	179
Ad02A4094	Aradu.A02	4094	p2	(TA)10	20	11540714	11540733	TTCGTTCCCCTGCATTACTC	GTGTGGGAAAATTGGGTTTG	271
Ad02A4098	Aradu.A02	4098	p3	(ACA)11	33	11557200	11557232	TGCAAACATTAATCGTGACCC	CCTTTTTAAAATATAATGCATGTACCA	100
Ad02A4099	Aradu.A02	4099	p2	(TA)6	12	11557780	11557791	TCTCCTCTTTTGGCTCTTTCC	AAAAGAAGATCTATGTATGAGGTACGA	273
Ad02A4101	Aradu.A02	4101	p3	(ATG)5	15	11565804	11565818	AAGATGGATTTCTGGCCCTT	CTGCTCAACCACCACAAGAA	115
Ad02A4102	Aradu.A02	4102	p6	(CGGAGA)6	36	11582500	11582535	GAGGAGGATGAGGGAAGGAC	CTTGTTCTCCCATCGTGGTT	181
Ad02A4103	Aradu.A02	4103	p2	(AT)9	18	11584507	11584524	CCTCCATGTATTATCCATTTGTCA	AACCCAACCAATATGTTTGTCA	163
Ad02A4104	Aradu.A02	4104	p2	(AT)6	12	11592842	11592853	GCGGCTATGTATAGTATTGCTAAAA	CGGTAACTATGGTAGATTTGGTG	254
Ad02A4111	Aradu.A02	4111	p3	(ATT)6	18	11614180	11614197	GCTAGCTTTTTGCCTTCCAA	TAATGGCCTCCATGCTTCTC	209
Ad02A4122	Aradu.A02	4122	c	(A)10gattgcttta	35	11624179	11624213	CCCGATACATGTCTTAGCTTGA	TCCTTTTGTCTTTTTACGTTTCTTTT	239
Ad02A4126	Aradu.A02	4126	p2	(CA)6	12	11661899	11661910	CGCTAATCCCCGAAATTCAT	GATGCGATGAGAAGGGAGAG	214
Ad02A4127	Aradu.A02	4127	p3	(AAT)21	63	11668677	11668739	TGTGCTTAGGACTCCCTTTG	TTATTTGCGAAAATCCCACA	150
Ad02A4128	Aradu.A02	4128	p2	(CT)7	14	11674663	11674676	TCGAGTTTGGACAACTGACG	AAGCCTCGGTCCAAAAGAAT	245
Ad02A4132	Aradu.A02	4132	p3	(ATT)12	36	11689501	11689536	AATTTGTCCTTTATCCCCGC	CGGAGAATCGAACCTTTCTT	275
Ad02A4133	Aradu.A02	4133	c	(T)11actaaaga	105	11690140	11690244	GTTGGAGTCATTCGACCGTT	CGCTTTCAATCAAAAGACGA	256
Ad02A4136	Aradu.A02	4136	c	(T)12ctccttttcta	99	11700071	11700169	TCCGTTCAATGTTGGAGTCA	GCGCTTTCAATCAAAAGACA	182
Ad02A4138	Aradu.A02	4138	p2	(AT)6	12	11702780	11702791	AATCAATGGTGGTGAGGCAT	GGTTGGTTATGGAGAAACAGGA	226
Ad02A4139	Aradu.A02	4139	p2	(TG)7	14	11707065	11707078	TCTTCTGTCGCCTCTTTGCT	TTGCAATGCCTTATTCTCCA	267
Ad02A4141	Aradu.A02	4141	c	(T)13acgttgtgc	118	11729123	11729240	GGAGGACACCTAAAGGGGAG	AGAACGAGGGTCAGCACAAC	228
Ad02A4145	Aradu.A02	4145	c	(TTA)8gaagag	65	11730554	11730618	ATCCTTGCGACAGACCAAGA	CCGTTCCTTAAAGACGAAAATG	278
Ad02A4146	Aradu.A02	4146	c	(A)10gttaggga	60	11733175	11733234	CCACTTTTGCAATTTTCCGT	TGATGGAGACAAAATGCATCA	200
Ad02A4152	Aradu.A02	4152	p3	(ACC)5	15	11745865	11745879	GAATTACAGAGGCAGAGCGG	TTTTACCAATAGCCAAGGCG	143
Ad02A4153	Aradu.A02	4153	c	(TTA)5gtgcctt	91	11746444	11746534	TGACAAGGACAAAATAATATGCAA	CGAAGTTGATCTATCCAAGTTATGC	256
Ad02A4157	Aradu.A02	4157	p3	(TTA)5	15	11750523	11750537	CTAGGTAGGTGAGGGGCGAT	GAAGGCAACGAAATTGGAAA	270
Ad02A4158	Aradu.A02	4158	p3	(TTA)10	30	11751238	11751267	CAATTGTAAGGATACCACAACACAA	AGGAACAAGCACATACACCAA	221

ID	Chromosome	SSR nr.	SSR type	SSR	Size	Start	End	FORWARD PRIMER (5'-3')	REVERSE PRIMER (5'-3')	Product (bp)
Ad02A4163	Aradu.A02	4163	p2	(TA)10	20	11756749	11756768	GGTTGCATATGGATCGGAGT	TCAAAGGAACAAGAAGAGCAA	269
Ad02A4164	Aradu.A02	4164	p2	(CT)8	16	11759655	11759670	TAACAGCAGTTTCGTGGCAG	CTCCACCCCCTAAACCTAGC	267
Ad02A4167	Aradu.A02	4167	c	(TCA)5tcg(TC	36	11764626	11764661	CACCATCGTCGCTACCTTCT	TTTTGGATCAAACAACGTCA	229
Ad02A4169	Aradu.A02	4169	p2	(CT)16	32	11783035	11783066	GGACAACAATGGCACTTCCT	GAGGCGAGAGAAGATTGACG	160
Ad02A4170	Aradu.A02	4170	p2	(CA)10	20	11786493	11786512	GGCAAGACCCAGAGTCAAAA	TGCTGCAGCTTTTCATTTGT	239
Ad02A4171	Aradu.A02	4171	p2	(TA)16	32	11786898	11786929	ATGGCTATGCTTCATTTTGC	GGGCATAACATTTTGAAGGC	264
Ad02A4172	Aradu.A02	4172	p2	(CT)13	26	11787184	11787209	CCAGGTGGAATCATTGCTCT	TGCATCGCACAGTCATGTAA	203
Ad02A4173	Aradu.A02	4173	p2	(AC)11	22	11789838	11789859	TTCGAGCAATGGATGCTAAG	TCTCCACGGAATCATTGACA	280
Ad02A4176	Aradu.A02	4176	p3	(ATA)13	39	11806308	11806346	TTTACCAAATTAACAGTGGCGA	CTACCTCCAAGCCAAAACCA	224
Ad02A4182	Aradu.A02	4182	p2	(TA)8	16	11829698	11829713	CCTTGATTGAATGTGTTGGG	GACAACCCACTTAGGCGAAA	142
Ad02A4183	Aradu.A02	4183	p2	(AT)7	14	11831498	11831511	TGCATGCATGTCCTATGGTT	CATAAGGGTGACCGAGCACT	171
Ad02A4189	Aradu.A02	4189	p2	(TA)6	12	11869720	11869731	TTTAAATTCTGGCTTAAACGAACA	GGGCCGTCAAAATAATGAAA	196
Ad02A4190	Aradu.A02	4190	p3	(ATG)5	15	11883110	11883124	ATGAGCCTCCACATGCTAGG	TCCGAGAATTCTGAACTTCCA	202
Ad02A4191	Aradu.A02	4191	p3	(TAT)17	51	11889060	11889110	AATATAGAGAGCGGCCCACA	TGCACAACACTTGAGATAAAGTCA	266
Ad02A4192	Aradu.A02	4192	p2	(AT)9	18	11891210	11891227	GGATGTCGCCAAAATTGGTA	TGCGTTGACCATGTATGCTT	181
Ad02A4193	Aradu.A02	4193	p4	(TCTT)6	24	11900828	11900851	GCTCTCTGCCACTGCTTGTT	TGATGATGTGTGATGAGGGG	231
Ad02A4195	Aradu.A02	4195	p3	(TCT)5	15	11901479	11901493	AGAAGGGACAGGGCAAGATT	GGAAGCTAAATTAGGGGAGGG	231
Ad02A4196	Aradu.A02	4196	p2	(TA)6	12	11901956	11901967	GCAGAAGTTGGATTTGGAGC	TGACCAAAAGAATCAACCCC	237
Ad02A4198	Aradu.A02	4198	p3	(AAT)24	72	11914114	11914185	TTTTTCGTCTCCAACGAACC	GAAAGCCATGGACGACTGAT	272
Ad02A4202	Aradu.A02	4202	c	(T)10gaagaatg	86	11926507	11926592	TTTCGCAGGAATGACATGAA	GGCAGAATAATATGTAAAAGGGG	198
Ad02A4205	Aradu.A02	4205	p2	(AT)9	18	11933403	11933420	CGTTTGAATAAGCGGGGTAA	TCGCAGGTTGTGACTAGCAG	132
Ad02A4206	Aradu.A02	4206	c	(TA)6g(AC)9	31	11933628	11933658	CTGCTAGTCACAACCTGCGA	TTTTCATTCCTAGGTTCTCATTCC	243
Ad02A4207	Aradu.A02	4207	p2	(AG)10	20	11944950	11944969	CAGAGAGGAAGAGGGGGTTC	CGAAAATACTTCCCTCTTTTTCAA	227
Ad02A4210	Aradu.A02	4210	p2	(TC)6	12	11996610	11996621	CACTCCTCTTTCTCTCGCGT	CCCTGAAGCTTCGCACTACT	255
Ad02A4211	Aradu.A02	4211	c	(TC)9nnnnnnn	101	12017234	12017334	CTCTCCCGTGTAGTGGAAGC	AACCAGAGGAGAGTGCGAGA	267
Ad02A4213	Aradu.A02	4213	c	(AC)8atgtatata	60	12042101	12042160	GGATGCTCAATTAGATGCACTTT	TCTCGTTAGGCATCTGGACC	243
Ad02A4215	Aradu.A02	4215	p2	(TA)8	16	12045295	12045310	GTAATGCACGCATATCGCAA	CTTGAGGTCACGTGGAGACA	252
Ad02A4216	Aradu.A02	4216	c	(CT)8ttcttgcaa	62	12048550	12048611	CGATCATCTCGTCCTCCATT	GCCCAAATAAATATTAGCCCA	225
Ad02A4218	Aradu.A02	4218	p2	(AT)8	16	12052782	12052797	TTGCAAAAGCATAATGATAAAGGA	ACAACTGGAATGCCACATCA	246
Ad02A4219	Aradu.A02	4219	p2	(TA)7	14	12054107	12054120	TGGAATGCCACATCATCAAT	GCTCACTTAGACTCGACTCGC	215
Ad02A4221	Aradu.A02	4221	p2	(GA)6	12	12057376	12057387	CAGTGGCAGAAGCGACATAA	CGAATTGCGAATCCTCTTTC	210
Ad02A4229	Aradu.A02	4229	c	(TA)6tcttggtttc	136	12065544	12065679	TGCCATGTTCTTCACCTTTG	GCTTGATGCGTATAGGCACTATTT	212
Ad02A4232	Aradu.A02	4232	p3	(AAT)5	15	12067133	12067147	AACAAAGCCAAAATTCCAGC	ATTTCCCATTTTCCCTTTGG	244
Ad02A4233	Aradu.A02	4233	p4	(AAAT)5	20	12069631	12069650	CCATAGCAAGCTGAAGTGTCC	TCTTGATTATGCTATGCAGCAA	235

ID	Chromosome	SSR nr.	SSR type	SSR	Size	Start	End	FORWARD PRIMER (5'-3')	REVERSE PRIMER (5'-3')	Product (bp)
Ad02A4235	Aradu.A02	4235	c	(T)10c(T)11cc	70	12072504	12072573	CGGGTGTGTGTTGTCATTGT	AAAACACTGGCTGAGGTTGG	280
Ad02A4241	Aradu.A02	4241	p3	(TTA)7	21	12110648	12110668	CAGCTTTTCACTCTGGTGATTG	CAGGCGTAGTCAACTTCACG	213
Ad02A4243	Aradu.A02	4243	p2	(TA)6	12	12130482	12130493	TTTTTCCACCAAATATATGTCCC	AGGGTTTTGAGGAGGCAGTT	183
Ad02A4246	Aradu.A02	4246	p2	(TG)7	14	12136006	12136019	TCACCTCAAAGAACAACACCA	TAGTGTTTCAGGGATTGCCA	277
Ad02A4248	Aradu.A02	4248	p3	(CAT)5	15	12141894	12141908	GCTAGGCCTACCATCCAGC	TGGCAAATTCTGGAGTGACA	268
Ad02A4249	Aradu.A02	4249	c	(TTA)6aattta(T	34	12142370	12142403	TCTTGCTGCCTTATTCAACG	AACAAGAATTTGGTTGGCTGA	257
Ad02A4251	Aradu.A02	4251	p3	(GTT)5	15	12205543	12205557	TCCATGTAGCTAACCCCACC	TGCATGGCATGACAATTACC	228
Ad02A4252	Aradu.A02	4252	p3	(GAA)6	18	12207506	12207523	TTGGGTTTTGCATGGGTTAT	ATCAAACCGTGGCTGTTCTC	142
Ad02A4253	Aradu.A02	4253	p2	(TC)6	12	12208010	12208021	GATTCTGTGGGAATCCCCTT	TTTTGTCTCGTCACTTGGCA	237
Ad02A4259	Aradu.A02	4259	c	(T)10ctttttttttc	35	12228144	12228178	CCGGATACATACATGCCACA	TGGCTCTTGAAAGAATGAGGA	280
Ad02A4260	Aradu.A02	4260	p3	(TGA)6	18	12236303	12236320	GATGTCCCTCTTCCGACAAA	AATTAGCCATTCGTGGGTTG	269
Ad02A4261	Aradu.A02	4261	p3	(ATT)6	18	12236872	12236889	AAGCGCTCTCAAGACACTCA	GCATGCTTCACATGCTTTCT	277
Ad02A4264	Aradu.A02	4264	c	(A)10gatgaatta	138	12246549	12246686	TCCTTTTCCGGTTAAGAGTCC	TCAATTTTCTCGATCATGCTG	251
Ad02A4269	Aradu.A02	4269	p3	(ATG)5	15	12252523	12252537	GATTAGCCAGCCAGCAAGAG	GCTAGGCCTACCATCCAGC	113
Ad02A4271	Aradu.A02	4271	p2	(AG)13	26	12255370	12255395	CAGTTTTGTGTCTGCATCCAA	TCGTGTACCGTGGTGTTCTC	272
Ad02A4272	Aradu.A02	4272	c	(TCC)7(GCC)	42	12255606	12255647	TTTCCCTCAGCGTAAACCAC	CACTCCTCTCGTCTCCAAGG	277
Ad02A4273	Aradu.A02	4273	p2	(AG)8	16	12256042	12256057	TCCGATGAGGACCCATAGAC	CACAATGCACACTCTCTCCG	219
Ad02A4274	Aradu.A02	4274	p3	(TTA)5	15	12271935	12271949	ATCAACCCCTTTCACTGTGC	AAGCCCACAACTGTGAAGAGA	270
Ad02A4275	Aradu.A02	4275	p3	(TTA)8	24	12275578	12275601	CTTCTAGCCGAACCGTCTTG	ATCGTCCAAACCCATTGAAA	255
Ad02A4276	Aradu.A02	4276	p4	(GATA)7	28	12288659	12288686	ATTGCTCTGGTCAACATCCC	CAAGAGCATTGTGAAGCCAA	261
Ad02A4278	Aradu.A02	4278	p3	(TTC)5	15	12323992	12324006	ATGCTACCTCGGCTTGAGAA	TGACGTACCATAAGAAGAGGGAA	260
Ad02A4279	Aradu.A02	4279	p5	(AAAAT)8	40	12333518	12333557	GCACAAGAAAAACAAGAAAAGACA	GATTTTGGCAATTGGGTCAG	279
Ad02A4280	Aradu.A02	4280	p3	(TTC)5	15	12335249	12335263	TTTCTTCTTTAGTCATTCCTGCG	CCGAATCTACTGCAACCTAACC	233
Ad02A4281	Aradu.A02	4281	p3	(TTC)5	15	12337243	12337257	TTCTCTTTAGCCATTCTTGCG	CCATAATCTACTGCAACCTAACCA	267
Ad02A4282	Aradu.A02	4282	p2	(AT)6	12	12337774	12337785	GCACTTGCCAATTCTCAAAGA	CTCCAAGGGAGAGTTATGCAA	202
Ad02A4283	Aradu.A02	4283	p3	(TTC)6	18	12341944	12341961	CCAAGGCAAGCAAATCTTTC	CAAAATGAAGCTTCCATAAATGC	275
Ad02A4290	Aradu.A02	4290	p4	(AATA)6	24	12360104	12360127	TTGCTGCCATACGTGTAAGTT	GCTGGCAAAGGTTCTTTTCTT	280
Ad02A4294	Aradu.A02	4294	p3	(GAA)5	15	12385825	12385839	ACCACTGAGCTATGACGGCT	CAACTTCTCTTTCCCACCCA	268
Ad02A4301	Aradu.A02	4301	c*	(TTTA)9(TTA	87	12414067	12414153	TTGCCTTTACAGGTGAAGAACAT	AACTGGCAACTGCACCTTCT	280
Ad02A4302	Aradu.A02	4302	p3	(TAT)12	36	12414367	12414402	AAAAAGAAGGTGCAGTTGCC	TGAACTCGAAGACATGCACA	208
Ad02A4303	Aradu.A02	4303	p2	(AG)21	42	12422429	12422470	TGGATGGCAAACAGACAGAG	GTTTGCTTTGGGTGTCCATT	270
Ad02A4304	Aradu.A02	4304	p3	(AAT)15	45	12426913	12426957	GAACTTTAATTCGTGATAAATTAGGC	GGGTGAGATTGAAATTGTGTAGTG	188
Ad02A4307	Aradu.A02	4307	c	(ACC)5(ATC)	33	12438659	12438691	TTTCGCTCCAAAATGGTTTC	GTGTGATGGATGGTGATGGA	238
Ad02A4308	Aradu.A02	4308	p3	(GAA)5	15	12438921	12438935	TCCATCACCATCCATCACAC	AAAGAAAGAAACGGATCGGG	264

ID	Chromosome	SSR nr.	SSR type	SSR	Size	Start	End	FORWARD PRIMER (5'-3')	REVERSE PRIMER (5'-3')	Product (bp)
Ad02A4313	Aradu.A02	4313	p2	(TC)8	16	12456330	12456345	TTCGCGCTAAGACAGAGTGA	CACCACCCTTATACATGCCC	206
Ad02A4314	Aradu.A02	4314	c	(A)11gttgacag	92	12473443	12473534	ACGCAACCTGCGTTTTTAG	CACAGCGTGCGTTTTATTTT	209
Ad02A4322	Aradu.A02	4322	p2	(AT)6	12	12486883	12486894	CCAGCATTTTTAGAATATTTATTAGCA	CCCTCTCTTTAAGGGGGAAA	269
Ad02A4323	Aradu.A02	4323	c	(T)10caactaag	62	12488707	12488768	TATTGGACCACACATGCCAC	CACTGCAATCCCTTAATTCCA	233
Ad02A4326	Aradu.A02	4326	c	(CAT)5ggaatta	43	12490390	12490432	AAGACCATTTTGTCCCACCA	CATCTTCAAACTTGGGTGGC	258
Ad02A4334	Aradu.A02	4334	c	(A)11tcctaatag	55	12504315	12504369	TGCAATGGCAAAGCTAACAG	TTGAAACCTTCTATATGCATGTCTAC	250
Ad02A4347	Aradu.A02	4347	p3	(AAT)8	24	12542572	12542595	GGATGCACTTATCCGTTGCT	CACTTTCGAAACCTCCCAAA	173
Ad02A4348	Aradu.A02	4348	p3	(AAG)8	24	12547766	12547789	TAACAGATGTTGCGGAGCAG	GCTTCAAGAACCCATTTCCA	153
Ad02A4350	Aradu.A02	4350	p2	(GA)12	24	12569579	12569602	GTTCCATTGGCTTGAAGGAA	CCGAAAGGTCCAAACTACCC	268
Ad02A4352	Aradu.A02	4352	p2	(TG)7	14	12576186	12576199	CCTCCACTGCAAGTGAAACA	AGCTGGTATGCATGTGCTTG	126
Ad02A4353	Aradu.A02	4353	p3	(CGG)5	15	12580438	12580452	AGCACAACAACTGGAAAGGG	CGTTCCTCCTCATCTCGAAG	183
Ad02A4359	Aradu.A02	4359	p2	(CT)8	16	12589165	12589180	TGAAACCCTGGATCTTCGAC	TTCATGTCTGTCGTCGGTGT	260
Ad02A4362	Aradu.A02	4362	p3	(TTG)5	15	12603256	12603270	TGATAGAGCACAATGGCGTC	AAAAATCGCGGGATAAAAGG	274
Ad02A4364	Aradu.A02	4364	p2	(TC)6	12	12616468	12616479	CTCTTTCTCCCCCTCCTCTG	AGAAGAGAGGATGTGCCACG	274
Ad02A4365	Aradu.A02	4365	p2	(GT)7	14	12670361	12670374	AGAGTGAGAGAGGTGGGGGT	GTCTCGTCTGGACGTTGGAT	251
Ad02A4366	Aradu.A02	4366	p2	(TA)6	12	12675499	12675510	TGACTCAAATTTTGTGACCACC	TCTTAGGCTTTACATAGCAGCAA	255
Ad02A4367	Aradu.A02	4367	p2	(TA)7	14	12680711	12680724	AAAAATTTTACCTGAGCCTTCC	GCGGTGCATATTTTTCAACC	205
Ad02A4370	Aradu.A02	4370	p2	(AC)8	16	12683915	12683930	ATGCTACACCGTCCAAAAGC	CAACATTCCCTGGTTTCCAA	181
Ad02A4373	Aradu.A02	4373	p3	(TCT)5	15	12696383	12696397	CTACCCATGGTTCGCAGACT	CAGGGACTAGAGGAGGAGGC	163
Ad02A4374	Aradu.A02	4374	p2	(GT)6	12	12701023	12701034	TATTGAAATGCCCGTTCCTC	ACAAATGTGAAACTGATCTTTGTAGT	238
Ad02A4381	Aradu.A02	4381	c	(T)10atc(T)11	24	12707497	12707520	TTTTAATGAATAGAGAGGTCTTGTGA	GCCTTTATGGCATTGCTGAT	119
Ad02A4383	Aradu.A02	4383	p3	(TTG)8	24	12714109	12714132	CGTTTGATTCATGTAGCCGA	TCGTCGTTGACGTCTTCTTG	231
Ad02A4386	Aradu.A02	4386	p3	(TTA)5	15	12741264	12741278	GACCATGGTGTTATCATGTATGC	CATTTCATATCACATCCGTTTCA	238
Ad02A4388	Aradu.A02	4388	p3	(AAT)13	39	12742030	12742068	TGGACGATTGTAGAAGCAACA	TCGATTATATTATCCTTCAAACCAAA	213
Ad02A4390	Aradu.A02	4390	c	(T)10agtatttttg	36	12745870	12745905	CACGTGTATGACACGGGTTAAT	ATTCACAAATTCCACGCACA	219
Ad02A4399	Aradu.A02	4399	p2	(AG)6	12	12762351	12762362	GAAAGATGCTCACGCATCAA	TTGGCCAACTAACCCACTTA	116
Ad02A4400	Aradu.A02	4400	p3	(CTT)5	15	12770002	12770016	AATACACGCGCTCAACACAC	AGGAGGAGAAGGAGAAACGC	136
Ad02A4401	Aradu.A02	4401	p3	(AGG)5	15	12770839	12770853	AACCAGAGGAGAACGACGAA	CCCCACTCCTCACTTGTTTG	154
Ad02A4404	Aradu.A02	4404	p2	(AC)7	14	12780216	12780229	GCAGCCCAACATGAATTTCT	TTGCAGACCATTGTCGTTGT	271
Ad02A4406	Aradu.A02	4406	p2	(GT)8	16	12783330	12783345	TGGGAACTTTGAATGGCTTC	TCGACACACCCCATTTTCTT	181
Ad02A4407	Aradu.A02	4407	c	(T)16acttttttttc	90	12783596	12783685	GCTTTTATGGGAACTGAACCA	CACACTCCTCCTCTTCTTCCA	270
Ad02A4409	Aradu.A02	4409	p2	(AT)6	12	12784836	12784847	TTGCCCAAAAAGTTGAGACA	TAAGATGGTGGTAGGGCGAG	267
Ad02A4413	Aradu.A02	4413	p2	(AG)6	12	12792529	12792540	CGTCGAGCTCACGAGTGATA	TTGTCTGCTCTGCTCCTGAA	235
Ad02A4414	Aradu.A02	4414	c	(TG)11(TA)8	38	12815348	12815385	CGGGATATTGACGCAGAAGT	CGCAATATCGCTCCAAAAAT	277

ID	Chromosome	SSR nr.	SSR type	SSR	Size	Start	End	FORWARD PRIMER (5'-3')	REVERSE PRIMER (5'-3')	Product (bp)
Ad02A4418	Aradu.A02	4418	c	(TGA)7ccatgat	136	12821856	12821991	TGGTGGTGGGTTTTGAGTTT	TAACCTTTCCCCTTCATCCC	269
Ad02A4420	Aradu.A02	4420	p2	(AG)7	14	12828016	12828029	CCTTGCTCCAAACTGTGGTT	GAAGCATCAAGGGCCATAAA	254
Ad02A4423	Aradu.A02	4423	p2	(CT)8	16	12843935	12843950	CGACCTCCTCCTTCCTCTCT	AAGACCTGGAAAGCACATGG	237
Ad02A4425	Aradu.A02	4425	p2	(AT)9	18	12847575	12847592	TTTGGTGTCTTGCATGTTTTTC	TTTCGAAATTTATGAGAGAAATAAGG	242
Ad02A4426	Aradu.A02	4426	p2	(TC)6	12	12848289	12848300	TTCGAAAATCACCTTCCACTTT	GAAGGATGAGAAAAATTCGAACA	207
Ad02A4427	Aradu.A02	4427	p3	(TCT)5	15	12851619	12851633	CCTTGGCCGAATACACATCT	TCGATGCTTTTACCACACCA	115
Ad02A4430	Aradu.A02	4430	p2	(GA)6	12	12860166	12860177	CTCAGATGAAACTGCAACGG	CATCGCTGCTCATTTCGTTA	226
Ad02A4434	Aradu.A02	4434	p3	(TGT)5	15	12885018	12885032	ACATGACGGGATGTATTGGC	TTGGACCATAACAGGCAACA	261
Ad02A4439	Aradu.A02	4439	p2	(TA)7	14	12901501	12901514	GCTTGAAGGAGACGCAAAAC	AACAATAGGGATGGCAGCAG	271
Ad02A4440	Aradu.A02	4440	p2	(TA)6	12	12904510	12904521	CAATGATCAGTGAACCAGCAA	CGTGCACCAGTATCCATCAG	204
Ad02A4441	Aradu.A02	4441	p2	(AT)8	16	12906051	12906066	GCAGTCCTTTTCAGCCTTTG	TTGGTGCCACAAATTACCCT	267
Ad02A4447	Aradu.A02	4447	p6	(TAGATA)5	30	12931015	12931044	TGCTGTGTTCTTGTTGTGCTT	TGGGGCCTGAACTTTGTTAC	175
Ad02A4453	Aradu.A02	4453	p2	(AT)7	14	12963283	12963296	CAAGACAAATCAGTTTAGTCGAAAA	TTCTTTGTCATCATAAGGTTAGAATTT	218
Ad02A4454	Aradu.A02	4454	p3	(ATT)6	18	12963791	12963808	GCTGGCTTGTAACCAAAGGA	CAGCTACAATTGACATGGTTCC	164
Ad02A4460	Aradu.A02	4460	p3	(ATT)5	15	12969713	12969727	CACGAATCTAACTTCACTTTCAGG	CATGCATGTATCCCAATGGA	156
Ad02A4461	Aradu.A02	4461	p3	(ATT)6	18	12971470	12971487	TGGTGAATACTGCTGGCTTG	CAGCTACAATTGACATGGTTCC	175
Ad02A4462	Aradu.A02	4462	p2	(TA)8	16	12973046	12973061	GGCATGGATAAATTTACATGTTTTT	GCCGACAAACTTAGCTCAACA	231
Ad02A4465	Aradu.A02	4465	p2	(AT)6	12	12983574	12983585	TTCTACATTTGCCATTGGGC	ACCACGTCACGATGGGTAA	262
Ad02A4467	Aradu.A02	4467	p3	(GAT)8	24	12986741	12986764	CATTGTACACGATCGCACCT	CATCATGGTCCTCAAAGGCT	276
Ad02A4469	Aradu.A02	4469	c	(A)11cgaaacaa	62	12988872	12988933	TTTCTGTAACCGTTTGGGTG	CGCTGTTATGTGCCTCGTTA	280
Ad02A4471	Aradu.A02	4471	c	(CCT)5ccatctc	192	13020767	13020958	CAACTTTTCAAATCACGCTCC	GGAGGAGAAGGAGGAGAAGAA	263
Ad02A4474	Aradu.A02	4474	p3	(ATA)16	48	13030207	13030254	TCCTCATGAATTTATTTTTCCTCC	TGAAGCTAGGTAGATAATATTTGGCA	230
Ad02A4475	Aradu.A02	4475	p3	(TAA)5	15	13032103	13032117	TTCCTCCAACATGAAAGCACT	TCGGAGGGAAGAAGAGTTCA	280
Ad02A4479	Aradu.A02	4479	c	(T)11ggtcttgctt	42	13068793	13068834	TTTTGTGTTTGCATTCAATTGTT	AGGAAAACTCGAACCTGCAA	248
Ad02A4486	Aradu.A02	4486	c	(TCA)8tctaagg	161	13083066	13083226	CATCATGGTCCTCAAAGGCT	CACCTGGCGAATGAGACATA	279
Ad02A4489	Aradu.A02	4489	c	(TA)7atacgaaa	82	13087581	13087662	CTCAGCTCACACACCGAAAA	CTCGGCAATAATGGTGGATT	218
Ad02A4491	Aradu.A02	4491	p2	(AT)9	18	13090227	13090244	CGTACGTGTGCGTATGCTAGA	TTCGATGTTTGAAACCCACA	206
Ad02A4494	Aradu.A02	4494	p3	(ATA)5	15	13096084	13096098	TGGAACCCACGGCTACTAAC	TGCACTCATGAAGGAGGAGA	213
Ad02A4495	Aradu.A02	4495	p3	(ATA)5	15	13097218	13097232	CGCTGGAAATTAAAACCCAA	ACTGAGGAGAGGAGTCGCTG	140
Ad02A4497	Aradu.A02	4497	p2	(TA)7	14	13110137	13110150	ACATGTCCAAACATCGCAAA	CAGCCCTTGACCAAATTCTC	176
Ad02A4498	Aradu.A02	4498	c	(A)11cttgtaata	41	13113806	13113846	GGTGCTGTCATAGTTGGAAGG	TTTTTAGAATCACAACAAAGAAAAACA	275
Ad02A4502	Aradu.A02	4502	p3	(TAT)14	42	13130285	13130326	CCAAAATGATTGCAACACATAAA	TTTGAACTCGTTGTCTTTCACAA	275
Ad02A4503	Aradu.A02	4503	p3	(ATA)9	27	13130470	13130496	TGATATTAAAAATAATTTGTGCCCG	TTTCCATTCCTTTAGGTATAACATCA	253
Ad02A4504	Aradu.A02	4504	p3	(TCC)5	15	13135018	13135032	GCTTTTGCAACTTTCCTTGC	TGAAGTTTCGTGTTTTGGTGTT	184

ID	Chromosome	SSR nr.	SSR type	SSR	Size	Start	End	FORWARD PRIMER (5'-3')	REVERSE PRIMER (5'-3')	Product (bp)
Ad02A4505	Aradu.A02	4505	p3	(AGA)5	15	13136468	13136482	ACATTGCAACGACGAGTCTG	CGTAGAGGGGCAGAGAACAG	201
Ad02A4508	Aradu.A02	4508	c	(A)11ttaagaatc	116	13149110	13149225	AATTTTGATGCAAGGCAAGG	TTTTAAAACGACTCATGACTTTTGA	183
Ad02A4509	Aradu.A02	4509	c	(T)10ggtaatg(A	49	13151607	13151655	TTCACACCAATTTAACTTAACATATCA	AGCATTTGGACATTTGGACA	265
Ad02A4516	Aradu.A02	4516	p3	(ATA)5	15	13161431	13161445	CCAATGATGCACCTTAATACACT	TCTTATTTAACTTATGTTTCATGGCAA	264
Ad02A4518	Aradu.A02	4518	p2	(TC)8	16	13163394	13163409	TTCGCTAATGGATGTATGTCAC	GCTTTGGCACTTTGGGTTTA	263
Ad02A4519	Aradu.A02	4519	p4	(TATT)6	24	13164062	13164085	CGAATCTCGAATATATCGACAAAA	TGTAAGTATCGCGAATCTGCAA	241
Ad02A4520	Aradu.A02	4520	p3	(CAC)5	15	13164574	13164588	TCATTTGGTATTAAAATCGTCCTTT	TGAAACCCAATGTACAACACAA	280
Ad02A4522	Aradu.A02	4522	c	(CAA)12tatcaa	69	13167886	13167954	AGAAGTCCCTGCAAACGCTA	GACAGCAGTGGCAGAACAAA	269
Ad02A4523	Aradu.A02	4523	p2	(TA)8	16	13169201	13169216	TGGCGAATCGTTACAAAACA	TGGAAAACGAAAAATCACGA	223
Ad02A4524	Aradu.A02	4524	c	(A)10ccagaattt	93	13175892	13175984	TCGGACAAAGAAAAATTATCAAGA	TCAAAGAAAATCAGCAAAAATCA	229
Ad02A4526	Aradu.A02	4526	c	(T)10atatataatt	106	13189875	13189980	TGTTTGTTCATCGTCAAAATTAAA	TCGTACATAAAGGATCATATCGG	243
Ad02A4530	Aradu.A02	4530	p3	(TGA)6	18	13194838	13194855	CCCAAATCCTCAAACACCAT	TTGCAAGGAATCATGATGGA	185
Ad02A4531	Aradu.A02	4531	p2	(TA)8	16	13196724	13196739	TGTTTCCATTGGACCATCTG	TCCAAAAATAGTGACGAATTCAAA	179
Ad02A4533	Aradu.A02	4533	c	(TA)6cacattaca	47	13205134	13205180	GAGGCCTTTGCCTCTGAGTA	GCATCTGTACCATAGATTGACCC	244
Ad02A4534	Aradu.A02	4534	c	(TA)6cacattaca	47	13209963	13210009	CTCCCAAGCCAAAATGAAAA	CGGGAGTGAGTGTTTCAGTG	247
Ad02A4535	Aradu.A02	4535	p2	(AT)9	18	13211452	13211469	ATGCCTCGAATCACGAATTT	TGAAAAACCACCGTCAACAA	280
Ad02A4538	Aradu.A02	4538	p3	(AAT)15	45	13263077	13263121	AAACAGCCGAATAATCTTAAAACTG	TGCGTCTAATCTCCTCCTCC	248
Ad02A4543	Aradu.A02	4543	p2	(GA)7	14	13301768	13301781	CGTGAAGAAGAAGGAGGAGG	ATGTCTTCCGGCAGATTGTC	276
Ad02A4546	Aradu.A02	4546	p3	(TCT)5	15	13311532	13311546	CCTTCTCTACATCTACGCGCT	CACACACATGATCAACCGAA	257
Ad02A4547	Aradu.A02	4547	p3	(CAA)5	15	13317261	13317275	GTTTGGCTTGGAACCTTTGA	TCTTTGAGTGCAAGTGCTGG	191
Ad02A4548	Aradu.A02	4548	c	(CAT)7caagtag	105	13317398	13317502	CCAGCACTTGCACTCAAAGA	GCTAGGACTTCCACCTTGATG	240
Ad02A4549	Aradu.A02	4549	p3	(AAT)7	21	13318306	13318326	ACTAAAAGTCCTTGCGCTGG	TGTGGGTTGCAATCAGAAAA	256
Ad02A4553	Aradu.A02	4553	p3	(TTC)6	18	13329263	13329280	GTTGAATCCTCGGGACGTTA	AGCCCTACTCCTCTTCCGAG	246
Ad02A4555	Aradu.A02	4555	c	(T)11ggtatacta	77	13337105	13337181	TTCAGGACCAAAGGCTATGC	TCAACTCAGTTAAATAAAAATGCAGAA	260
Ad02A4558	Aradu.A02	4558	c	(ATC)20aagtag	143	13345675	13345817	CAAAATGCAAAGTGTAAAGGGA	CAAACAAACAAAGAAGAAGAAAAGAA	264
Ad02A4559	Aradu.A02	4559	c*	(AT)11(T)10*	31	13345983	13346013	GGTGTGTGCTTGCGAGTTTA	TCTTCTTGGGTTGCATTTGG	104
Ad02A4561	Aradu.A02	4561	p2	(TA)6	12	13348329	13348340	CAGATGCAAATCCAACAATGA	AATTGAACTTGGGTTGGTGG	187
Ad02A4572	Aradu.A02	4572	p3	(TCA)6	18	13363464	13363481	GAGGAATTGCATCCAAAGGA	ATCCGAAGGAAGAGCAACAA	272
Ad02A4573	Aradu.A02	4573	p2	(AT)6	12	13364223	13364234	TTTTTGCCAGCATCAGCTAC	GCTTGAAAATATTGTTATTTTAGCCTT	125
Ad02A4578	Aradu.A02	4578	p3	(AAT)6	18	13370455	13370472	TCAAAACCCAAATCAATTCAAA	CACAAACTTAGAAAAAGAAAATGTTGA	248
Ad02A4580	Aradu.A02	4580	c	(CAA)6(CAT)	33	13375505	13375537	CTCCATGAGCATTCCATTTG	CAGCATATCGTCAAAGGGGT	229
Ad02A4582	Aradu.A02	4582	c	(TA)8tgcaattct	71	13390249	13390319	GGGATATGGTACGAGACCTTTTT	CGTCAAGATGACGATGAAGA	269
Ad02A4584	Aradu.A02	4584	p5	(TTATC)6	30	13408720	13408749	CCTGGGTGCTCCAGTGTATT	GTAGAGGGACACGACCCAAA	178
Ad02A4587	Aradu.A02	4587	c	(A)11tttcatacta	120	13438331	13438450	TGTCTAATTCATGGGTTTCGAG	TTTTCTAATATCATTTTGAGCATGAA	215

ID	Chromosome	SSR nr.	SSR type	SSR	Size	Start	End	FORWARD PRIMER (5'-3')	REVERSE PRIMER (5'-3')	Product (bp)
Ad02A4589	Aradu.A02	4589	p3	(GAA)5	15	13439166	13439180	CCCATCACCTCTTCACCACT	GCCCTCGAGCAATAAACAAA	278
Ad02A4590	Aradu.A02	4590	c	(T)10cgaaa(T)	25	13443855	13443879	CACACTCTTGTTGCCTTGGA	TGGCTTTTGAAAGAATGAGGA	187
Ad02A4592	Aradu.A02	4592	c	(T)10ggtgacta(	30	13459039	13459068	AACTCCCAAGTTTGCTAGTTTTT	TGCATCCATCTAATTTACCGC	234
Ad02A4593	Aradu.A02	4593	p2	(AC)7	14	13467155	13467168	TCACCGGGTCATTAGGGTTA	TTCCTGTAGAGCCTGAGGGA	280
Ad02A4594	Aradu.A02	4594	p2	(TA)6	12	13467455	13467466	AACTCGAAAGCGCAACACTC	TTGTGTCCTTTTGGGTTTCC	199
Ad02A4601	Aradu.A02	4601	p2	(TA)7	14	13488813	13488826	TCGCTGTGAATCTTAAATGCC	TGGTTTCATTGGAAACATGG	158
Ad02A4602	Aradu.A02	4602	p5	(TAAAA)6	30	13489079	13489108	ATACAGCCCAAAGTGCAAAA	TATAAGTGTCGGGAGGTCGC	210
Ad02A4607	Aradu.A02	4607	p2	(AT)6	12	13513100	13513111	TGGAATTTGGATGAATCGGT	TGTGTGGAAGTGTGAGGCAT	223
Ad02A4610	Aradu.A02	4610	p2	(TA)8	16	13552098	13552113	ACTAACGGCCTAAACTCCGC	AGTCGCGTAAAGCGGAAATA	209
Ad02A4614	Aradu.A02	4614	p2	(TC)6	12	13564312	13564323	CCCTCCTTCTCTTCTCCGTT	GTGATCACCAACACGGTCAG	120
Ad02A4615	Aradu.A02	4615	p2	(GT)6	12	13585482	13585493	AAGGAACTCGAAGCCACTGA	GTGTTAAAATTTTGGCCCGA	265
Ad02A4616	Aradu.A02	4616	p2	(CT)8	16	13585912	13585927	GCACCGAGCAAACTTAAACC	TCATGTCACTGACGAAGGGA	280
Ad02A4619	Aradu.A02	4619	c	(GA)6cagaaag	81	13589486	13589566	TAGAGAAGGGGTCGGTGATG	AAACCGAGAGAACTGTCCGA	280
Ad02A4623	Aradu.A02	4623	p5	(ACACA)5	25	13607384	13607408	CAGCCACAACCCCTATCCTA	TCTCTTCCCCTTCCCTGATT	137
Ad02A4630	Aradu.A02	4630	p3	(GAA)8	24	13631237	13631260	TCCTCATCTTCTGCTGCTTCT	TTAGCTTCACGTGGTTGCTG	248
Ad02A4631	Aradu.A02	4631	p2	(TA)7	14	13632344	13632357	GCGAATATAAAAATAAAAGCAACAAA	GCACGTTCTTCTTCCTCGTT	275
Ad02A4632	Aradu.A02	4632	c*	(AAG)5aatttca	194	13632524	13632717	CGTGCAGAAACGAAAGAAAA	CCCCACTCAAACGGTTCC	270
Ad02A4635	Aradu.A02	4635	c	(TG)6(TA)6	24	13640715	13640738	TTTGTGCGTTTTCTTTTGACA	AAGACATTGTTTGACACAGTCAGTT	235
Ad02A4636	Aradu.A02	4636	p4	(ATAG)5	20	13641466	13641485	GAGCTGACGTGGCATCATTA	CCTTCTCAAATCAAGCTTTCG	176
Ad02A4638	Aradu.A02	4638	p2	(TA)8	16	13646917	13646932	ATCGAGTCATTGCATGCGTA	TCTTTTGTCCCTTGGTTTCG	261
Ad02A4639	Aradu.A02	4639	p2	(TA)9	18	13647536	13647553	TCGGACGTTTTTCCTTTCAG	TCACATTCACAGCAATAATCCA	218
Ad02A4640	Aradu.A02	4640	p3	(TTA)5	15	13648775	13648789	CCCTCACCCTTGTTGCTTTA	GGTCGATATCGCGTTTCAGT	165
Ad02A4641	Aradu.A02	4641	p3	(ATT)19	57	13649658	13649714	GGTAGATAGGATATAACAACCCGAA	AATGGTTTCGTCCATTCTTTC	267
Ad02A4642	Aradu.A02	4642	p2	(TA)8	16	13649835	13649850	AGAAAGAATGGACGAAACCA	TTGGTTTCGGTGAAGGAAAG	149
Ad02A4643	Aradu.A02	4643	p2	(TA)6	12	13649953	13649964	CGAAACCAAGGGACAAAAGA	CACGGTTCTCCTCAGTCACA	198
Ad02A4644	Aradu.A02	4644	p2	(TA)9	18	13650454	13650471	TTCTACGAAGCACGGAGGAT	TCACATTCACAGCAATAATCCA	239
Ad02A4645	Aradu.A02	4645	p3	(TTA)5	15	13651693	13651707	CCCTCACCCTTGTTGCTTTA	GGTCGATATCGCGTTTCAGT	165
Ad02A4646	Aradu.A02	4646	p2	(CT)10	20	13656764	13656783	TATCGTCGCTGTTGAGTTCG	TGATTTCATGCATGCCTAGC	254
Ad02A4649	Aradu.A02	4649	p3	(TGC)6	18	13661165	13661182	TGCTGAATGCTGAATGCTGT	ACACCAGAACCAGAACCCAG	106
Ad02A4650	Aradu.A02	4650	p3	(GCA)5	15	13662827	13662841	GATCGGCTTCAGGTTCTCAG	TCCCCAACATCTGCAAAAAT	203
Ad02A4653	Aradu.A02	4653	p3	(AAC)5	15	13664039	13664053	GGCATTGGAATTCCTCAATTT	ACATGACAGAGCACAATGGC	258
Ad02A4654	Aradu.A02	4654	p2	(AT)8	16	13667245	13667260	AGGTACACAAATGGCTCCCA	AGGTCCCTGAAGTTGGGTTC	260
Ad02A4655	Aradu.A02	4655	p3	(TCA)5	15	13671865	13671879	TGATGACTTTCTGGACGCTG	TCAGACATGTGGTTTCTCGC	239
Ad02A4657	Aradu.A02	4657	c	(T)12gacagtata	191	13678261	13678451	TGGTTTTCCTTGTTTCCTTTTC	CCGAACATCTGTGAACTTTTACC	280

ID	Chromosome	SSR nr.	SSR type	SSR	Size	Start	End	FORWARD PRIMER (5'-3')	REVERSE PRIMER (5'-3')	Product (bp)
Ad02A4662	Aradu.A02	4662	c	(ACA)5cattcca	71	13681664	13681734	GCCAAACGACCAAATTTTCA	GGATCCTCGTGTGGGTTATG	268
Ad02A4663	Aradu.A02	4663	p2	(AG)7	14	13701685	13701698	CGAAAGAACATTGAACCTGGA	AGCAAATTCTGGAGCCCTTT	226
Ad02A4665	Aradu.A02	4665	p2	(CT)7	14	13711651	13711664	TGTTTGTTTCTTATCTTTTTCAAAACT	AGAGGGTTCGGATTTTTGGT	278
Ad02A4666	Aradu.A02	4666	p2	(CT)6	12	13711888	13711899	ATCCCTCACCCACCAAAAAT	CTTCTCTTCCCTCTCCGGTT	245
Ad02A4667	Aradu.A02	4667	p3	(TTC)5	15	13717231	13717245	AAAGCCCTCCATTCTTCCTC	CGGTCTTAGGTGCCATCAAT	208
Ad02A4670	Aradu.A02	4670	p3	(AAT)11	33	13735144	13735176	AGGCATTTGCGCTGATAGAA	CGATCGATAAAGTGACACTACAACA	268
Ad02A4681	Aradu.A02	4681	p2	(TA)9	18	13798204	13798221	GCGATGATCACTACGGTTGA	TGAGAGAAACGACGCAAATG	276
Ad02A4682	Aradu.A02	4682	p5	(AAGAT)6	30	13808907	13808936	CAGGGGCACTGTTCATACCT	CCTGGGTGCTCCAGTGTATT	199
Ad02A4685	Aradu.A02	4685	p2	(TG)6	12	13809761	13809772	CAGATTTGGAATTTTTAATTGTGTG	TGTCTGCACGTTCTCTTTTCTC	269
Ad02A4687	Aradu.A02	4687	p3	(TGA)5	15	13812474	13812488	TGCTTCGTCTTCTGCTTCTTC	TCATCATCCTCATCAGAACCC	234
Ad02A4688	Aradu.A02	4688	c	(TGG)7tgttggt	69	13812704	13812772	TGGTGGGTTTTGGTTCTGAT	CCCTAACGTTGAGGCTGAAA	254
Ad02A4689	Aradu.A02	4689	c	(AT)7aaatccatt	107	13813875	13813981	AAGGGACCCGTTAAATCCAC	TTCCCTTAGCGAATGCAGAT	262
Ad02A4693	Aradu.A02	4693	p2	(AT)7	14	13829996	13830009	TGGGAACTTGGGTTGATAGG	TGGGGTAATTTTGTCCCCTT	257
Ad02A4694	Aradu.A02	4694	p2	(GT)6	12	13830170	13830181	GGGGACAAAATTACCCCAAT	GTAATGGAGCCAAGGCAAGA	236
Ad02A4704	Aradu.A02	4704	p2	(TA)7	14	13863461	13863474	TTGTGCATACCAAATCAGCC	GTCGACATCAGCCAATCGTA	250
Ad02A4706	Aradu.A02	4706	p3	(AAC)5	15	13864855	13864869	GTCACCACTTAATTGTAAACATAAAAA	GAGTGTAACTTCAGGGGCCA	209
Ad02A4707	Aradu.A02	4707	p3	(GTT)5	15	13865485	13865499	GTGTAGGTCGAGGCCAATGT	TCTGCACCCAAGAAAGTGAA	154
Ad02A4713	Aradu.A02	4713	p2	(AT)10	20	13912942	13912961	TCTTGATGAGACGCAATGCT	GCCAAGTGCATTGTGTGATG	184
Ad02A4717	Aradu.A02	4717	c	(CGC)7cat(CA	39	13915677	13915715	CCCTTCAACGAAATCAAGGA	AAATTTACCCCATGGCCTTC	273
Ad02A4726	Aradu.A02	4726	p2	(TA)10	20	13929820	13929839	GTTGTTTCCAAATGCAGCCT	TGCTTCCCAAAATCACATCA	267
Ad02A4729	Aradu.A02	4729	p3	(TAT)6	18	13935556	13935573	GAGGTCACAGAATTGAGGGC	GAGTTAAATTCAAGGATAACGATGTTT	272
Ad02A4733	Aradu.A02	4733	c	(T)12aaatatcca	43	13939653	13939695	TGTTGGATTGTACTTGCCCA	CGTGCTCAACACGGAAAAAT	155
Ad02A4735	Aradu.A02	4735	c	(TTA)6c(TAT)	200	13946859	13947058	TCTTGATGCCATGCATGTTT	AATTCAAATAATTCTTGTAACGCA	276
Ad02A4736	Aradu.A02	4736	p3	(ATT)7	21	13947175	13947195	AGTCTTTTTATGAAACGGAGGTT	CAACAGTAGGAACTGCACGAA	256
Ad02A4737	Aradu.A02	4737	p2	(TA)7	14	13952619	13952632	TCCGATCTAAATATTTTGCCA	ATGTAGTGCGTGAAGCTTTTAAT	190
Ad02A4739	Aradu.A02	4739	p3	(TAT)7	21	13954029	13954049	ACGCCCAAACAAACAACAGT	TAATGGCTTCGGCCAAATAA	280
Ad02A4740	Aradu.A02	4740	p2	(AT)7	14	13954201	13954214	GGCCGAAGCCATTAAGATTT	TTGTGGTGGCTAATGCTTCA	221
Ad02A4741	Aradu.A02	4741	c	(TTA)5(TTG)6	39	13957529	13957567	GTTTTGAACCATTTGGGTGG	TCAACAATAACAATAATAACAACACCA	251
Ad02A4742	Aradu.A02	4742	p2	(AT)7	14	13961926	13961939	TCCACTTGCTCCCAAATACC	ATCCTTGTTGGATGTCACGG	249
Ad02A4746	Aradu.A02	4746	c	(T)13cttttggcaa	33	13970978	13971010	TTCACAAGATCCTTGGAGCA	TCTTGTGCTAATTTTGCCTGTG	278
Ad02A4752	Aradu.A02	4752	p2	(CT)10	20	13987936	13987955	CTGGTTTGAGACCACTGCCT	TCCGAAGCCAAAATTCAAAC	133
Ad02A4758	Aradu.A02	4758	p2	(CT)6	12	14050484	14050495	TTTTGTATAAAAAGACGTGTCAAAAA	CGTGAGTAGTGTGAATGGCG	280
Ad02A4760	Aradu.A02	4760	p3	(TAA)6	18	14053970	14053987	AGCCACGTCCAAAACAAAAG	TCGATTCACCACCACTTGAA	213
Ad02A4762	Aradu.A02	4762	c	(T)10aattgtaaa	72	14057593	14057664	TCATGGCATGTAGTGTGTCCT	AAATAGTTCGATTTCTGTATTTTTCAA	264

ID	Chromosome	SSR nr.	SSR type	SSR	Size	Start	End	FORWARD PRIMER (5'-3')	REVERSE PRIMER (5'-3')	Product (bp)
Ad02A4765	Aradu.A02	4765	p3	(TAT)10	30	14063407	14063436	TCTCCAAATAATGGTTTGCCA	TGGTTAAGTAAATGAAAGGATTGAGA	189
Ad02A4768	Aradu.A02	4768	p2	(GA)14	28	14068032	14068059	TCCACCGATTCAGAACAACA	TGAACTTTGCTCCATTGTCG	176
Ad02A4769	Aradu.A02	4769	p2	(AG)6	12	14069791	14069802	AGGACACAAACCAAATACAGCC	CATGTCCAAACATCATATCAATCC	100
Ad02A4772	Aradu.A02	4772	p3	(ATC)5	15	14074049	14074063	TTTTTCACTTTGCTACCATTATCA	TACCTGCGTCTCGAATGAAC	280
Ad02A4773	Aradu.A02	4773	p6	(GATCAG)5	30	14080347	14080376	GGAAGCTGTTCTGGGTCAAC	AGGCCACAAAGGATCACAAA	265
Ad02A4774	Aradu.A02	4774	c	(AAT)15(CAT	69	14083016	14083084	TTTTCCCATTTGGGTCACA	GCCGTTTGGACCATTAGAAA	276
Ad02A4778	Aradu.A02	4778	p2	(AC)10	20	14089007	14089026	CCACAGCAAAGAGAATTGCC	TGCCATTGCTTAAATTTGCC	274
Ad02A4779	Aradu.A02	4779	p3	(GCA)5	15	14089732	14089746	CCAAATAAAATCAATGCGCC	TTAGGTTGGTGCCTTCAAGC	262
Ad02A4780	Aradu.A02	4780	p2	(AG)9	18	14091182	14091199	TCCAGCTTTTTCCTTGCTGT	TAGTGTTGCGTGCATGTTGA	100
Ad02A4781	Aradu.A02	4781	p3	(AAC)8	24	14091324	14091347	TCCAGCTTTTTCCTTGCTGT	TGTGGGATAACGGATTAGGC	237
Ad02A4784	Aradu.A02	4784	p2	(CA)17	34	14101047	14101080	GAATGGGGATCCGACTATGA	TGAGGATTCGAAGTTTTGGG	245
Ad02A4785	Aradu.A02	4785	p2	(TA)13	26	14102443	14102468	TTCTCTCTCTTCTTTTAGTGAGCAA	TTGGTCAGAGTCTCGTGTTGA	229
Ad02A4787	Aradu.A02	4787	p2	(TG)6	12	14112175	14112186	GTCACGTAATGTGCAGGTGG	GCCAACAACAACAAGAACCTG	192
Ad02A4788	Aradu.A02	4788	c	(T)11cttttttgcc	73	14120143	14120215	TTACTAGTGCCTCCCCTCCC	GAAGAGACGTTGAAGCCCTG	259
Ad02A4796	Aradu.A02	4796	p2	(TG)6	12	14140093	14140104	TGTGTTCAGGCTTAGGGCTT	GGACCTTCCATCTGAAACCA	205
Ad02A4800	Aradu.A02	4800	p3	(GAA)6	18	14149079	14149096	TTTTTGTTTGTGTTTTGGGG	CCAGCTGAGAGAGACGGAGT	158
Ad02A4801	Aradu.A02	4801	p3	(ACA)5	15	14152864	14152878	AAATGGTTGCATTGTTTGGA	TCGTTTGATTCATGTAGCCG	236
Ad02A4802	Aradu.A02	4802	p3	(TAA)6	18	14156047	14156064	TTGGTTGCCGAAGACCTAAC	ACCTGCATGGCATTGTTTTT	272
Ad02A4806	Aradu.A02	4806	p2	(TA)9	18	14167735	14167752	TGTCACTTGAATTTGAGGTTGG	AGACCAGCATCTGAACCCTC	259
Ad02A4807	Aradu.A02	4807	p3	(TTA)5	15	14176044	14176058	AAATGGGACGGCAAATATGA	TCCATTGGAAAAGGAGGAAA	193
Ad02A4808	Aradu.A02	4808	p3	(TTG)5	15	14181952	14181966	TCATCATACCGTAGCAAACACC	GCAAGTGTTGTGCTTTCCAA	155
Ad02A4810	Aradu.A02	4810	p3	(ATA)8	24	14183212	14183235	CATGGTGAGAATTGAAGGCA	AGATCTCCCCCACAAGTCAA	276
Ad02A4811	Aradu.A02	4811	c	(T)10aataaaaag	74	14184170	14184243	TGATATTTCAAATTTATTGACAGCA	GGCATTTGTGGTTTGTGTGA	279
Ad02A4815	Aradu.A02	4815	c	(A)12gaagagaa	36	14191152	14191187	TCTCACGTCCTTCTTTGTGTTC	AAAACACGTATCATTCTCCTCCTC	228
Ad02A4820	Aradu.A02	4820	p3	(TTA)5	15	14201643	14201657	TTGAGATTAAAGTGTTCGAGCTAA	CACCCACCAATTTTATTGCC	217
Ad02A4826	Aradu.A02	4826	p2	(AT)14	28	14249505	14249532	GCCGCCGTTAAAGTTTGATA	AAGAATTTCAAAGCCATCCG	272
Ad02A4832	Aradu.A02	4832	p3	(CAA)5	15	14271425	14271439	TAGACCCTCCCTGTTGTTGG	ATACTGGCCAAACGTGTTCC	262
Ad02A4833	Aradu.A02	4833	p3	(ATC)6	18	14272723	14272740	CTTCAGTCCAAAGCCAGAGG	GCTGATGATGATGATGGTGG	187
Ad02A4835	Aradu.A02	4835	p2	(AG)6	12	14273322	14273333	TTCAAAATACGGTGTTTCGGT	TCATAGCCCCAAACACAAGC	237
Ad02A4836	Aradu.A02	4836	p2	(TA)7	14	14276210	14276223	TATATGTTTTCCCCCGCTCA	ATATAAGGGAGGGTCCAGCG	169
Ad02A4840	Aradu.A02	4840	p3	(TAT)18	54	14297507	14297560	TGGGAACTAAGAATGGGTGG	GCAAAATGCTTACTGCGACA	254
Ad02A4845	Aradu.A02	4845	p3	(GAT)7	21	14318830	14318850	TTGGAGACACATGGGATTCA	CATAGTCCTCATCATCCGAGAA	259
Ad02A4847	Aradu.A02	4847	p2	(TA)6	12	14325405	14325416	CATGCCAACACACGAATTTT	TCACATGTCAGCGTGTTCAA	268
Ad02A4848	Aradu.A02	4848	p3	(ATA)5	15	14326281	14326295	TTCAAAACTAATCCAACCCGA	GGTTCAAAGTGGTTGGGAGA	230

ID	Chromosome	SSR nr.	SSR type	SSR	Size	Start	End	FORWARD PRIMER (5'-3')	REVERSE PRIMER (5'-3')	Product (bp)
Ad02A4850	Aradu.A02	4850	p2	(TA)6	12	14329952	14329963	AAAGAAAATCTGGAATAGTGGTGG	CACTCATAATGGTAATTAGCGGA	145
Ad02A4851	Aradu.A02	4851	p3	(AAT)6	18	14330206	14330223	TGTCACTTGACGTGATTCGAT	CATGCACTTATGGGGAAGGT	209
Ad02A4856	Aradu.A02	4856	p2	(TA)6	12	14339610	14339621	TCCAGAGAGGTGAGCCAGAT	CCATTCACCCAACGTTATCA	238
Ad02A4857	Aradu.A02	4857	p2	(AT)9	18	14340259	14340276	AAAAACTCATTCATTCATCAAACTAA	TGGGGGTCTGGCATTTATTA	237
Ad02A4859	Aradu.A02	4859	p2	(TA)6	12	14358371	14358382	GAGCAACATGAGTTCACAGCA	GGAAGCCAACAATTGAATGAA	234
Ad02A4861	Aradu.A02	4861	p2	(TG)26	52	14371768	14371819	CGCTCCAACATATGAAAGAGTGA	TCCTTCAAGGCTCCTCAAAT	248
Ad02A4862	Aradu.A02	4862	p2	(AC)23	46	14371957	14372002	TTTGAGGAGCCTTGAAGGAA	AGCTTCGCAGGCTGAGTTAG	219
Ad02A4865	Aradu.A02	4865	c	(T)12cccttctgtt	83	14382921	14383003	CGTGCCCCAAAAGAAATAAA	GCAACCAAGCTGTGAATCAG	274
Ad02A4870	Aradu.A02	4870	p3	(TAT)19	57	14385730	14385786	TCATTTTTGCTAATTCTGGTTTT	TGCCTATGAGATCCACTCAACA	246
Ad02A4871	Aradu.A02	4871	p4	(TTAA)5	20	14386001	14386020	AATTGATTGGAGCACCTACG	GTAAGAGGATCGGAGTGGCA	224
Ad02A4873	Aradu.A02	4873	p2	(AT)6	12	14397469	14397480	TCTAACCAATCACGGCCTCT	AGGCCGAGGTGATTTGATTT	106
Ad02A4874	Aradu.A02	4874	p2	(GT)7	14	14399628	14399641	TCCTCCGCTAGCACTCTCTC	TGCCACTCATCCACTGACAT	147
Ad02A4876	Aradu.A02	4876	p3	(AAC)5	15	14404925	14404939	CAAACAGCAACAACAATAACAACA	GTGCAGGGACCCAATTAAAA	188
Ad02A4880	Aradu.A02	4880	p4	(CTTT)10	40	14421662	14421701	GTGACTAAGCTCCCTCACGC	GCTAAACCCTAACCCCCAAG	241
Ad02A4883	Aradu.A02	4883	p2	(TA)6	12	14425455	14425466	TAAAAGCCCAACCCAAACTG	TGTAATGATGGGTGTTTGGC	266
Ad02A4884	Aradu.A02	4884	c	(TGA)5tggtgat	56	14425866	14425921	GTCCCACATGAACCCATAGC	GCTTCATCATCATCATCCCC	270
Ad02A4885	Aradu.A02	4885	p4	(TTAA)5	20	14426132	14426151	TGAAGGTGTGGCCTCATACA	CTGGACCACAACTTAAAAACTCA	196
Ad02A4890	Aradu.A02	4890	c	(TA)7tgc(AAT	53	14429654	14429706	GCCATGTTCGAGAAAAGTAATTG	CATGTCATGCTTGTCACTTCAA	247
Ad02A4893	Aradu.A02	4893	c	(T)14gagaaaga	43	14431613	14431655	TGGATAGAACCGAAACCCAG	CACCAACCTTTCTGCCATTA	257
Ad02A4896	Aradu.A02	4896	p3	(AAT)6	18	14436661	14436678	CAAGAAAACGCAATCCATTCT	GGTTTGCGTGCCTTTACACT	232
Ad02A4897	Aradu.A02	4897	p3	(TAT)12	36	14436896	14436931	AGTGTAAAGGCACGCAAACC	GGCCTTAAGTTCGATCATCTTCT	263
Ad02A4901	Aradu.A02	4901	p2	(TA)6	12	14440482	14440493	AGTTGCCTCCAACGATTTTG	GGGCCTCAAAGTTTTCTGCT	201
Ad02A4902	Aradu.A02	4902	p3	(TAA)5	15	14441861	14441875	TCCCACACACATAAGTTTTCG	TTCCAAAGCTGGACAATCTTC	200
Ad02A4903	Aradu.A02	4903	p3	(ATT)5	15	14442005	14442019	GAAGATTGTCCAGCTTTGGAA	CGTCGATCGGTCTCATTCTC	168
Ad02A4904	Aradu.A02	4904	p3	(AAT)8	24	14442220	14442243	ACCGATCGACGGTTCTCTTA	AATTTCCTGCATTTCCAAGC	208
Ad02A4905	Aradu.A02	4905	p2	(TA)6	12	14449030	14449041	TCAAATTTCTAATAACAAGACTCGAA	AGCATTCATTAATTGTTGCGA	242
Ad02A4906	Aradu.A02	4906	p3	(TAT)6	18	14449510	14449527	TTTTGCCTTAAGACGCCATT	CGTTTCCGCTATAGCTCAGG	248
Ad02A4909	Aradu.A02	4909	c	(GA)7aaggagta	59	14455588	14455646	ATGGGTTATGGCTAACGCTG	CGTTGCGTTTTAGTCACTCG	228
Ad02A4910	Aradu.A02	4910	p2	(AT)11	22	14477848	14477869	CGTAAATCATTTTCATCCCTCA	TTTGATTTTGAGCATATAACTTTGTTT	251
Ad02A4913	Aradu.A02	4913	p2	(AG)22	44	14496984	14497027	GAGCCATTTGGAAAATCGAA	TTGCAAAGAATATGCAATGTACG	268
Ad02A4920	Aradu.A02	4920	p3	(AAT)5	15	14540997	14541011	GCCCAACCCACACATTTAAC	TTTGAGTTCAAGTTGGTTCCC	265
Ad02A4921	Aradu.A02	4921	p2	(AT)9	18	14551973	14551990	AGCCCAGGCGTTACTTACCT	TTTCGGATACAGGTGTGAAGG	267
Ad02A4922	Aradu.A02	4922	c	(AT)6ttttg(T)1	27	14557245	14557271	AAGATGATTCGCCTCCCTTT	CCCCAAATACATGATCCCAC	275
Ad02A4923	Aradu.A02	4923	p2	(TA)6	12	14560966	14560977	TCCTTGATTCTTGGGTGAGG	CATTCCAAGGCTTTCCAAAA	164

ID	Chromosome	SSR nr.	SSR type	SSR	Size	Start	End	FORWARD PRIMER (5'-3')	REVERSE PRIMER (5'-3')	Product (bp)
Ad02A4926	Aradu.A02	4926	p2	(TA)6	12	14587633	14587644	GACTGTTCGAACTCGAGGGA	GCGTCTTGGGGATTTGTAGA	241
Ad02A4929	Aradu.A02	4929	p2	(GT)6	12	14607408	14607419	TCACCGTGATCGTGTTGAAT	AGGATGTCGCATAAGAACGC	266
Ad02A4934	Aradu.A02	4934	p3	(TGA)10	30	14691824	14691853	ACGGGGTAGTGCAATGAAAC	TTGTTGGCTTGTGGAAATGA	229
Ad02A4940	Aradu.A02	4940	p3	(TTA)16	48	14717437	14717484	CCACAAGACAAGAACAAAATGG	AACGCAACATTTCTAAGGGAG	241
Ad02A4945	Aradu.A02	4945	p2	(GA)8	16	14764724	14764739	AGTCGACGGGAACTTTTGTC	ACGTACGTCTTGGTTTTGGG	191
Ad02A4946	Aradu.A02	4946	c	(TTG)5gagtctt	127	14765782	14765908	CCCGAACTATTGTCTCTGCC	GATGGTGAGAGCTTAGCGGT	280
Ad02A4948	Aradu.A02	4948	p3	(GAA)5	15	14772227	14772241	TCAGCAACTGCCATCAGAAC	CCCAGGTTAGTGGCTTCTCA	261
Ad02A4949	Aradu.A02	4949	p2	(GA)19	38	14774652	14774689	AGAGAGAAAGGGGGAAGCTG	CTTCCTCCATACTTGCGAGC	253
Ad02A4950	Aradu.A02	4950	p2	(TG)6	12	14787654	14787665	TTCGTTCCTGTAGAGCCTGG	ACACGACCTCTACCACGTCC	234
Ad02A4951	Aradu.A02	4951	p2	(TA)8	16	14795458	14795473	GACCGGTTATCTTCGCAGTC	ACAAGGATAACCAACGCGAG	100
Ad02A4954	Aradu.A02	4954	p2	(AT)7	14	14810332	14810345	TTTGAAAGCCACGAAAAACC	AAAGCTTGATTGTTGTTGATCC	246
Ad02A4956	Aradu.A02	4956	p2	(AT)9	18	14816747	14816764	TGTAATATTCTATTTTGTCATGTGTGC	TGGTGAGGAAAGTATAAAGTGGTTC	256
Ad02A4958	Aradu.A02	4958	p3	(ATT)5	15	14822243	14822257	TGAAAATATCGGGAATTGGG	CCACAAGGGAGTGGGACTTA	229
Ad02A4964	Aradu.A02	4964	p2	(AG)6	12	14840792	14840803	TGAAAGGTGAGGTAGGTGGG	ATTAAACGCCAGGATTGCAC	257
Ad02A4965	Aradu.A02	4965	p2	(TC)6	12	14850478	14850489	GGAGATTGCCATCTTAGCACA	AAAGCTGAGATGCGTTTGGT	258
Ad02A4967	Aradu.A02	4967	p2	(GA)8	16	14857933	14857948	AAAGGAAAGGGGAAGGTGGT	TGGTGGGTTCATATGCAGAA	271
Ad02A4969	Aradu.A02	4969	p2	(AT)10	20	14860628	14860647	CGCAGATCCGCACAATAAT	GGATCGGATCGAATCGAATA	260
Ad02A4970	Aradu.A02	4970	p2	(TA)12	24	14867442	14867465	ATTTACCGTGACAACGAGCC	AGCAAGAGTATTTTGGGTTAAGAA	214
Ad02A4971	Aradu.A02	4971	p3	(GAA)5	15	14875748	14875762	ATGGTAAAAATTTCCAGCGG	CTAGGCATCCAAGCGTTTTT	255
Ad02A4972	Aradu.A02	4972	p3	(AGA)5	15	14879676	14879690	GCTTTTGTCTTCCCTTTCCC	CCAACATAGCCGAACCAACT	198
Ad02A4975	Aradu.A02	4975	p5	(AAGAT)6	30	14890747	14890776	AGATAAAGGCGGTTCCCCTA	GTGTCCGACCTGTTGGATCT	266
Ad02A4976	Aradu.A02	4976	p3	(TTA)6	18	14898929	14898946	ATTGATGCTCAGAGCCTTGG	TGGTGGTATTTTCTGTGATTTTTG	229
Ad02A4980	Aradu.A02	4980	p2	(AG)6	12	14908596	14908607	TGAGACAGGACACGCAGACT	CTTCCTCCTTCTGCGAACAC	224
Ad02A4981	Aradu.A02	4981	c	(AG)11aaagag	65	14908775	14908839	GCGTATCGCTGAATGTGAGA	TCCTCTAAAGGCACCAAACG	209
Ad02A4984	Aradu.A02	4984	p3	(TAT)6	18	14924058	14924075	CTCTCGTGCAAATGGGAGAT	ATCCGTCTTTCCGGATTATT	253
Ad02A4989	Aradu.A02	4989	c	(GA)6aag(AG	40	14945113	14945152	AGGACGAGAAGAAACGGTGA	CTAAGTGGGAGCACTCCTCG	248
Ad02A4990	Aradu.A02	4990	c	(A)10gacgaact	175	14947483	14947657	CCCAAAAGCCAATTGGATAAA	TTCACGAAGAGGTTGGGATT	280
Ad02A4992	Aradu.A02	4992	p3	(GAA)6	18	14978680	14978697	TAAAATATTGCTCGCCCTCG	CACCTCAATTCCTCTTCCCA	210
Ad02A4997	Aradu.A02	4997	p3	(TTG)8	24	15015467	15015490	AATCCCTTGCAACACAAACC	GCAATGGCTCATGGAAAGAT	280
Ad02A4999	Aradu.A02	4999	p3	(CTT)5	15	15023586	15023600	CACTGGTTGTTGGCCTTAGTT	CCATTGATCGGCAAATTGTA	270
Ad02A5001	Aradu.A02	5001	p3	(TCT)5	15	15037306	15037320	CCGAATACACCTCTCCCTCA	GCTTTTGTCTTCCCTTTCCC	193
Ad02A5003	Aradu.A02	5003	p2	(TA)6	12	15053290	15053301	GCACAAAATCCAGTGAGGGT	GAATGTCATGCAATGCAATCTAA	245
Ad02A5004	Aradu.A02	5004	p2	(TG)6	12	15082106	15082117	GAAGTTGGGACGGAGCTACA	TGGGTGGATTCAATGGTTCT	265
Ad02A5009	Aradu.A02	5009	p2	(AT)9	18	15155011	15155028	AGTGGGTTTGAAATGTTCGG	TAAGCTGTCCAGCCTGACCT	266

ID	Chromosome	SSR nr.	SSR type	SSR	Size	Start	End	FORWARD PRIMER (5'-3')	REVERSE PRIMER (5'-3')	Product (bp)
Ad02A5010	Aradu.A02	5010	p2	(TA)9	18	15159257	15159274	CGCACCATGTGGTAAACAAG	TCACATATGGTTTGCCATGAA	211
Ad02A5012	Aradu.A02	5012	p3	(ATT)22	66	15163965	15164030	TTGATGACACCTCATTTTTATTAGTT	AAAATTCTTTTCAATGCACGA	186
Ad02A5013	Aradu.A02	5013	c*	(TAT)6(ATTA	40	15165389	15165428	AATGTGTGCATGTGCTGTTT	ACATTAATGGTGACAAATTGATATTGA	100
Ad02A5016	Aradu.A02	5016	p3	(AAT)5	15	15171977	15171991	TTTGGTACATTTCATATCACATTCG	GATTTAAGTCATTTACCTGTTTGTGA	252
Ad02A5018	Aradu.A02	5018	c	(ATA)5tcaataa	43	15175629	15175671	CGTTTTCCCTCCAAAACAAA	TTTGATATACGTGAATCGGGT	277
Ad02A5019	Aradu.A02	5019	p2	(TA)7	14	15177601	15177614	CGTATGGCATAATACATATGTCAAAAA	CCCACTTAGTTCATTCGCAA	240
Ad02A5021	Aradu.A02	5021	p2	(TA)7	14	15185360	15185373	CTTTGGCTGGAATGAACCAT	ACCATCCACGTTCACAACAA	261
Ad02A5026	Aradu.A02	5026	p5	(TTGTA)5	25	15195874	15195898	TCAAAGCCTTTAAGAAGGTACACA	AAGCGGAAAAAGTATGCCCT	262
Ad02A5027	Aradu.A02	5027	p3	(CAA)5	15	15198522	15198536	CGGCTTCTCTTCTTTCTCAAA	CGCATTTGTTGCCACTTTTT	274
Ad02A5028	Aradu.A02	5028	p3	(AGA)5	15	15220775	15220789	AAGCAGACCTTTCGGCAATA	CTGTACCTTGCTGGTGGGAT	178
Ad02A5031	Aradu.A02	5031	p3	(AAT)21	63	15227857	15227919	TGGCATGTTCCTGTTGAAGA	AGCAAATGCCCTAAAAGCTG	269
Ad02A5034	Aradu.A02	5034	p4	(TTTA)9	36	15258050	15258085	CTCGCCGGTCATCTCTTAAT	CCTAGCCATGTGGAAAACTGA	200
Ad02A5036	Aradu.A02	5036	p2	(TA)6	12	15262660	15262671	TTTAACATAAATAGTTGCCCCTCT	ATTATTGGTCCCACAACCCA	165
Ad02A5037	Aradu.A02	5037	p3	(ATA)7	21	15263487	15263507	AACACGTGAGAGCCACATTG	AGCTTTTGTAGTGGACACCCA	270
Ad02A5038	Aradu.A02	5038	p2	(AC)6	12	15264165	15264176	TTAGGTGGGTCGTCAATTCC	TGGGGATCTTTCAGAGATGG	272
Ad02A5039	Aradu.A02	5039	c	(A)10tattaacag	75	15268900	15268974	AAACAACGAAATTCTCAACAAAAA	TGACTGTCGGATTTGTATTTGG	270
Ad02A5050	Aradu.A02	5050	p3	(ATT)6	18	15294222	15294239	TGGTTGAAAAATGTTTTGTTGG	TCCGAATCACTATTGTCCCA	247
Ad02A5054	Aradu.A02	5054	p2	(AT)9	18	15297983	15298000	GCTGGTAACATGGAGTTTAACAAA	ACCTTATCATCCATGAGAACCA	205
Ad02A5055	Aradu.A02	5055	p3	(ATT)7	21	15304794	15304814	GGCTCTAGTGCAAGTGGGAG	TCCGAATCACTAATGTCCCAT	256
Ad02A5059	Aradu.A02	5059	p3	(TGT)5	15	15320083	15320097	TGATAGGGAACAATGGCGTT	AGCAGAACCATTCAACACCC	274
Ad02A5060	Aradu.A02	5060	c	(GA)7gg(GA)6	119	15324228	15324346	GAAGAAAGGAGTCGTGGTGC	TGCAATGTGCAACTTCTTCA	271
Ad02A5062	Aradu.A02	5062	p3	(GAA)5	15	15346187	15346201	AACCAATTAAGAAATGGCAACAA	TTGCATATTCGCATTGATGG	194
Ad02A5063	Aradu.A02	5063	c	(AAT)9aaaataa	75	15362485	15362559	GATAGGAAAGATGCTCACGCA	TGCAAATGCAATAAACAAAAGAA	268
Ad02A5064	Aradu.A02	5064	p2	(GC)6	12	15369517	15369528	TCAGAAACCCTTCCAAATCG	GGCTAGGCATGGATCAAAGA	211
Ad02A5067	Aradu.A02	5067	c	(A)10c(T)10	21	15388054	15388074	CCAAGGCACTTGGAAATGAT	TGGCAAATTCTTCATAGGATTC	246
Ad02A5069	Aradu.A02	5069	p3	(AAG)5	15	15398987	15399001	TTGCAAAAAGGTCCTTGTCA	GCGCTATCATGAATCCCAAC	270
Ad02A5070	Aradu.A02	5070	p3	(CAT)8	24	15407823	15407846	AGCTGAGAAGTGTGAGGGGA	TGGAGAGATTGGAGTACCCG	184
Ad02A5071	Aradu.A02	5071	p3	(TTC)8	24	15409830	15409853	AGGCAACGCCATTTAAACAC	TGATCCTCCGAAACCCTAGA	253
Ad02A5072	Aradu.A02	5072	p2	(TG)12	24	15410285	15410308	TGAAAAATTGGAAATTGCATGA	CAGTGTTGGGCACTTCACAC	255
Ad02A5073	Aradu.A02	5073	c	(T)10aattctaaa	60	15417532	15417591	GAGTGCAATGACTGGCATGT	ACAAGGGCAGGTGACGTTTA	221
Ad02A5074	Aradu.A02	5074	p2	(GT)6	12	15417795	15417806	ACGTCACCTGCCCTTGTTAC	TCCTTTCGCACTGGAAACTT	235
Ad02A5075	Aradu.A02	5075	p3	(GAA)14	42	15420314	15420355	TGAAGAATGCGAGCAGAGAA	AACCCCCTTACGCATTTGTT	172
Ad02A5078	Aradu.A02	5078	p5	(TCAAT)5	25	15425310	15425334	GAACGCCTCCTCATGGTAAA	TTGAATTGAATGGACAGAGGTG	152
Ad02A5079	Aradu.A02	5079	p2	(CG)6	12	15466282	15466293	GTTTGCCACCCCTACCTATG	TGGTGTTTGGAAGTGTTGGA	241

ID	Chromosome	SSR nr.	SSR type	SSR	Size	Start	End	FORWARD PRIMER (5'-3')	REVERSE PRIMER (5'-3')	Product (bp)
Ad02A5082	Aradu.A02	5082	p3	(TAA)5	15	15481085	15481099	CGTGTCATTTTGGCAGTGTT	GAGTGGTGGCCATGGATATT	209
Ad02A5085	Aradu.A02	5085	p3	(TAA)13	39	15485145	15485183	TGTGGGACAAATAATACCCCA	TTTTGCTGCTTCTCTTTCCTT	277
Ad02A5086	Aradu.A02	5086	p3	(TCT)9	27	15485567	15485593	CATGCTCCTCTTTTTCTTCTCC	CCTCCTCATCTTCTGCTGCT	231
Ad02A5087	Aradu.A02	5087	p3	(GAA)5	15	15486083	15486097	AAAGGCAAAGAGAGAACGCA	AATTTTTATGGCGTGCTTCG	153
Ad02A5090	Aradu.A02	5090	p3	(AAG)5	15	15509156	15509170	TAAAATATTGCTCGCCCTCG	CCTACCTCGCCCTCTCTCTT	225
Ad02A5091	Aradu.A02	5091	p5	(AAAAT)5	25	15509780	15509804	GGGTTGTTGCTTGTTTCTGG	GGGAGGCAACCCAATTTTAT	260
Ad02A5092	Aradu.A02	5092	p3	(ATA)20	60	15525748	15525807	TGACGTTGATGCAAATAACCA	GGAGTAAAGTTCAAATAATGGTGAAA	195
Ad02A5097	Aradu.A02	5097	p4	(TAAA)5	20	15543766	15543785	GGGGCACGACAACAGTAAAA	TCTTTTGTTTGGGGGTCTTG	246
Ad02A5100	Aradu.A02	5100	p2	(TA)6	12	15546620	15546631	TTTGCCAATCGTTCACACTC	TAGCCGGCTTAATTTTCACG	123
Ad02A5101	Aradu.A02	5101	p2	(AT)6	12	15549886	15549897	ATGAAACTCGCACGAAATCA	ACTCAAAATTGGACCAACGG	247
Ad02A5104	Aradu.A02	5104	p3	(ATA)8	24	15554008	15554031	TGTCGGATAAAATTCACCCTTT	ATATCGGGATAGCCGTACCC	169
Ad02A5105	Aradu.A02	5105	c	(T)13gtctcccaa	66	15556085	15556150	ACTAAATCGGACAATCCCGA	TTTCCATTTAATACATTCAACCTCA	180
Ad02A5107	Aradu.A02	5107	p4	(AAAG)5	20	15583796	15583815	AATCTACCCTAGCCGCCATT	TCCCTTCCCTGTTTCCTTCT	170
Ad02A5108	Aradu.A02	5108	p2	(GA)9	18	15584221	15584238	GAGATCGCGAAGAGAGATGG	TGTGCGGATCTCTCCTTTCT	247
Ad02A5113	Aradu.A02	5113	p2	(AC)6	12	15594655	15594666	TCTAGAATCATCAAATAAAATCCGAA	TCTCTCGTGCGATAACGATG	261
Ad02A5116	Aradu.A02	5116	p2	(TA)9	18	15626019	15626036	GGGTAGGGTACGGGTTGAGT	GCCTACCCGCAGATTACAAA	257
Ad02A5119	Aradu.A02	5119	p2	(TC)6	12	15658415	15658426	TGGGGGACGGAGATTCTACT	GGTGGCCGAACGTGTATTTA	270
Ad02A5121	Aradu.A02	5121	c	(T)10cgaaaa(T	27	15679464	15679490	CCGGATACATACATGCCACA	TGAGACATGTTACTGAGGATCTGA	272
Ad02A5123	Aradu.A02	5123	c	(T)10gaaaaaaa	102	15696703	15696804	CCAGAGTCCTCCTGGACTGA	TTTTGTGTGTTTTTCTCTTTCATCA	279
Ad02A5124	Aradu.A02	5124	p3	(ATT)16	48	15699450	15699497	TGGTGATTATGAAGATTGTGGTG	TGTTGGAGACAGCATAGCCA	278
Ad02A5127	Aradu.A02	5127	p2	(AG)7	14	15734480	15734493	TAAGGTCCTCACTATCGCCG	AGGGAGCATTGTGGGTACAG	279
Ad02A5128	Aradu.A02	5128	p2	(TA)6	12	15737614	15737625	GGATATGGGCGCAGAAGTTA	TTACCACAATAACCGCTCCC	269
Ad02A5129	Aradu.A02	5129	p3	(AAT)5	15	15741651	15741665	GGCACTCTTGTTGAAGCTGA	TTGGATGTTGGCTTTGTGAA	188
Ad02A5130	Aradu.A02	5130	p3	(TCT)5	15	15745228	15745242	CTCATATAGCCGAACCCCCT	AGGATACTCCGGTCTTGGGT	175
Ad02A5131	Aradu.A02	5131	p2	(AT)6	12	15750872	15750883	CACGTGGAGACCCGTTAAAT	GAAGGGGCTAAGGGTTTGAG	195
Ad02A5132	Aradu.A02	5132	p2	(TA)7	14	15751944	15751957	TGGTGATTATTGTCTTGGCAG	AAAGGAACTGGAAGCAGGGT	176
Ad02A5134	Aradu.A02	5134	p3	(ATT)5	15	15757983	15757997	AATTGGACATTTTAGCCCCC	GCCAAGTTATGGTGTCTTCTCC	264
Ad02A5136	Aradu.A02	5136	p4	(TATC)12	48	15771776	15771823	TGCGAAGCCAAAACACAATA	GTCAACGTCCCCAACATTTC	263
Ad02A5141	Aradu.A02	5141	p2	(TA)9	18	15787381	15787398	TCAACACAATGTCCAACCTGA	CTTGGAATGGCTCCAGTGAT	202
Ad02A5143	Aradu.A02	5143	c	(A)10gaatatgtt	140	15791426	15791565	TGGACAATTTCATGCTTCCA	TCCATTTTCTCTTTATCCAAACAA	263
Ad02A5146	Aradu.A02	5146	p3	(AAT)14	42	15797679	15797720	CCAAACCCATTGAAGCTGTT	AGCTTCTAGCCAAACCGTCA	269
Ad02A5150	Aradu.A02	5150	p2	(TA)6	12	15834796	15834807	AAAGGGATTGGAGTATTCGACA	AAAGGAACTGGAAGCAGGGT	243
Ad02A5151	Aradu.A02	5151	p3	(ATT)5	15	15840635	15840649	TGGACATTTTAGCCTCCCAA	GCCAAGTTATGGTGTCTTCTCC	261
Ad02A5154	Aradu.A02	5154	p4	(TATC)36	144	15853650	15853793	CAACATAGCAAGTACAAGATCGG	GTCAACGTCCCCAACATTTC	266

ID	Chromosome	SSR nr.	SSR type	SSR	Size	Start	End	FORWARD PRIMER (5'-3')	REVERSE PRIMER (5'-3')	Product (bp)
Ad02A5158	Aradu.A02	5158	p2	(TA)9	18	15866803	15866820	TCAACACAATGTCCAACCTGA	CTTGGAATGGCTCCAGTGAT	202
Ad02A5159	Aradu.A02	5159	p2	(GA)7	14	15868351	15868364	GAAAGCTTTGCATACCCCAA	CACCACTGCATTCTCTTCCA	248
Ad02A5160	Aradu.A02	5160	p2	(AG)8	16	15868469	15868484	TGGAAGAGAATGCAGTGGTG	TGAAGGGTTGAAGACAGTGC	213
Ad02A5161	Aradu.A02	5161	p2	(TC)7	14	15870584	15870597	CCCTCTCTCCACCACTACCA	AAAGATTATTGGGGCTCGCT	185
Ad02A5162	Aradu.A02	5162	p2	(CT)6	12	15872406	15872417	GCGTGCAGATCACGTGTAGT	TCCAACACTGCAAGTTGCTT	261
Ad02A5166	Aradu.A02	5166	p3	(CAC)6	18	15886913	15886930	CACAATTACCACCACCACCA	CCGTTCTGCTTTCTTGCTTT	272
Ad02A5167	Aradu.A02	5167	p2	(AG)6	12	15893925	15893936	GCAGAGAAAGGTGAGCCATT	GGCATATTGGTGCTGTGCTA	279
Ad02A5168	Aradu.A02	5168	p2	(AG)6	12	15896276	15896287	CGACGCACAGTGAGACAGAT	AACCTTCTCGTTCACGCCTA	188
Ad02A5170	Aradu.A02	5170	p2	(GA)6	12	15899216	15899227	TCCAACACTGCAAGTTGCTT	GCGTGCAGATCACGTGTAGT	258
Ad02A5171	Aradu.A02	5171	p2	(TA)7	14	15903533	15903546	AAAGGAACTGGAAGCAGGGT	TGGTGATTACTGTCTTGGCA	176
Ad02A5172	Aradu.A02	5172	p2	(AT)7	14	15904550	15904563	GGGGCTAAGGGTTTGAGAAG	GCAGGGATTTATCCGTCAGA	216
Ad02A5173	Aradu.A02	5173	p2	(TA)7	14	15911841	15911854	ATGACACCAATGCCCTCTTC	AAGAGCGAGGCACATAATACAA	265
Ad02A5175	Aradu.A02	5175	p2	(CT)7	14	15934004	15934017	AGAATGAGTGCCATTCTGGG	CGGAAAAGCACACCAAACTT	207
Ad02A5176	Aradu.A02	5176	c	(T)10gtttttctcc	32	15942776	15942807	CCAACTAGAGGTGTCCAGGG	TGTGGTTCATGAATGTTGGC	266
Ad02A5180	Aradu.A02	5180	p3	(ATA)5	15	15993076	15993090	TGCAACCACACAATTAGGCT	TTAAGTGTGGGGAGGTCGTC	262
Ad02A5182	Aradu.A02	5182	p5	(AATCA)5	25	15999906	15999930	TCTATGAATAAAATAGGCAACTGAGA	TCATAGCAAGCGTTCAAGTTT	271
Ad02A5184	Aradu.A02	5184	p2	(TG)7	14	16012436	16012449	TGGATCCCATTGTTGAGTGA	ACCCTTTTCCCTCCTTTTGA	177
Ad02A5188	Aradu.A02	5188	p2	(TA)9	18	16028480	16028497	TGCTGATCACTTTAACCCCA	TGCTTTCACTCATGTCCTGC	234
Ad02A5190	Aradu.A02	5190	p3	(TCC)5	15	16035422	16035436	AAGTTAGGCCAAACCACACG	TGCGAAGAAAAAGGAGGAGA	123
Ad02A5192	Aradu.A02	5192	p3	(GAA)9	27	16036350	16036376	AGGAGGAAGTGGTGGTGTTG	AGCCGTTACTGCACGTTTTT	120
Ad02A5193	Aradu.A02	5193	p3	(CAA)17	51	16036608	16036658	TTGGCTGGACATTATAGCCC	AAACGTATTGGTATTATCTTTGTCG	235
Ad02A5194	Aradu.A02	5194	p2	(AT)6	12	16038825	16038836	CGATCGAATCGCTTGAGTTT	TATTGGTTCAATCGGTGGGT	262
Ad02A5197	Aradu.A02	5197	p2	(AT)9	18	16062148	16062165	AACCCGACAAGTCCAGTTTG	GGTAGAGTACCGTGGCTTGC	224
Ad02A5198	Aradu.A02	5198	p2	(AT)6	12	16070172	16070183	TTCCAACACTTTTGCATTTCA	GGCTCAAATCTCACTTGCTTG	233
Ad02A5199	Aradu.A02	5199	c	(AAAAT)5aaa	84	16111623	16111706	TCTCAACCATTTTCTTTGTCTTTTC	GAAGATGATGATGCAGGCAA	230
Ad02A5201	Aradu.A02	5201	c	(AAAAT)5aaa	84	16134105	16134188	GCCTTCCTTACCCAATTAGTCA	GAAGATGATGATGCAGGCAA	268
Ad02A5204	Aradu.A02	5204	p3	(GTG)5	15	16179013	16179027	GAACCTGGTGGTTAGAGGCA	TTCTTCTCTGCAACCCATCC	233
Ad02A5208	Aradu.A02	5208	p3	(AAT)11	33	16203368	16203400	GGGAACATGGGTTGATAGGA	GGCGAATCTGTCCCCTTATT	198
Ad02A5209	Aradu.A02	5209	p2	(CT)8	16	16203982	16203997	GGCTTGGCTATATGATTCCTTG	TGGATTTATCAACCTCCCCA	234
Ad02A5211	Aradu.A02	5211	p4	(TTAT)5	20	16221173	16221192	AAATCCCTTCCCCCTTTTTC	TCGAACACCAAAAGGAAGAGA	228
Ad02A5212	Aradu.A02	5212	p3	(TTG)5	15	16237377	16237391	GACAGAGCTCAATGGCATCA	TGACATCCAAATAAACACCAAA	200
Ad02A5216	Aradu.A02	5216	p3	(TTA)6	18	16245467	16245484	CTTGCCCCTGAAGGTTACAA	GACCTCATGTCAACCGACAA	172
Ad02A5219	Aradu.A02	5219	p3	(AAT)6	18	16256280	16256297	CAGCAAGGCACTGATGACAC	TGGTTTACCATTGCTGCTTTT	181
Ad02A5223	Aradu.A02	5223	c	(A)10tatcaacac	76	16271262	16271337	TCGTAGTCCCGTATGGAAGG	TTGGACGTAGACTCTCAAAGCA	276

ID	Chromosome	SSR nr.	SSR type	SSR	Size	Start	End	FORWARD PRIMER (5'-3')	REVERSE PRIMER (5'-3')	Product (bp)
Ad02A5225	Aradu.A02	5225	p2	(TC)26	52	16287909	16287960	TGCCTTAGACATTGTTCTCTGTG	CCCTTTGATTGAAGGATGGA	222
Ad02A5226	Aradu.A02	5226	p2	(TC)7	14	16288719	16288732	AATGCAAAATGGTGCTTGTTT	CATCGATGAGGGGTGTTGTA	222
Ad02A5228	Aradu.A02	5228	p2	(TA)7	14	16297706	16297719	AGAATCGGAGGTTCTCAGCA	GAGGTTTGCAGGATTTGGAA	206
Ad02A5232	Aradu.A02	5232	p3	(GAA)6	18	16309286	16309303	CATTTTTCTTTGTTGGGATTGTT	ACACTCTTCTCCAAGGTCGG	165
Ad02A5236	Aradu.A02	5236	c	(AT)11gattggg	42	16315661	16315702	ATCCAGCAGGACCGTGTATC	GGTCACCTGGACTTGCTTGT	276
Ad02A5237	Aradu.A02	5237	p3	(CTG)6	18	16317867	16317884	ATTGAAATAACGAAACGGCG	AAAGAAATCCTAGGGTCCGC	143
Ad02A5239	Aradu.A02	5239	p2	(AG)12	24	16328401	16328424	AGCCTCCACGGTTGAAACTA	GGGCTAGGAATCCTTCTTGG	209
Ad02A5242	Aradu.A02	5242	p2	(TA)6	12	16336939	16336950	TGTTCCTTCCAATGAGCAAG	TTCAGCTGTGATTGTATTGAATTG	215
Ad02A5245	Aradu.A02	5245	p5	(TTATC)5	25	16376403	16376427	CCTGGGTGCTCCAGTGTATT	TTCTTCTCAAAGAGCTCGGC	222
Ad02A5246	Aradu.A02	5246	p3	(AAT)21	63	16383380	16383442	CCCCACATAATTGTGTTGAACTT	GGCCTCCTCTAAATTTGCCT	188
Ad02A5247	Aradu.A02	5247	p6	(AAAAGA)5	30	16386455	16386484	AATTAAGTCTTGCGCCGTGT	GATGAGGGGCCTCGTTATTT	225
Ad02A5248	Aradu.A02	5248	c	(TC)9ttcacctct	122	16386711	16386832	CCGTCAGTCCCACACATTCT	GGGACAGAACAGAGGGTCAA	224
Ad02A5251	Aradu.A02	5251	p2	(TA)9	18	16407080	16407097	GTTCATTTCCATTTCACGGG	TTTTGGACATTTCTGTAGTTTTGC	248
Ad02A5253	Aradu.A02	5253	c	(A)10gaacattca	60	16408133	16408192	AACTCCCACAACCCAATCAA	GCTCCACGATCTCCATTCAT	275
Ad02A5255	Aradu.A02	5255	p3	(AAT)13	39	16438957	16438995	ATCGGGCATGAATCTTGGTA	TGATCATGACAGTAGGATTGCAC	267
Ad02A5258	Aradu.A02	5258	p3	(TTG)8	24	16448050	16448073	TGTAGCCGACCCCATTTAGT	AAGGTTCTGAAAACCGAATCAA	273
Ad02A5261	Aradu.A02	5261	p2	(AT)6	12	16458758	16458769	GCTTTGTAATATCAAGTAAGCCCG	TGTGTTGAGGTGTTACTTAACGTG	225
Ad02A5262	Aradu.A02	5262	p2	(AG)7	14	16465120	16465133	AAGAAGGAAAGGGAGACCGA	CCACTCCATCGCGTTCTACT	226
Ad02A5264	Aradu.A02	5264	p3	(TAA)12	36	16473442	16473477	GACTCTCTGCGTCAAATACGG	GGCAAAATGTAATGTTGAGAGAA	154
Ad02A5265	Aradu.A02	5265	c	(AC)17(AT)9	52	16493284	16493335	CTGCCTCTGCTAGGGTTTTG	AATTGTTTCAGGAACCGACG	267
Ad02A5270	Aradu.A02	5270	p3	(TTG)16	48	16502859	16502906	AATGGCGTCGTTTGATTCAT	TGGCTTAACGGTAAGTGCTTG	244
Ad02A5271	Aradu.A02	5271	p3	(CCT)5	15	16508149	16508163	CGGTTAAATGAGCTGGCAAT	GCAAAACAGCAGTGGTGAGA	259
Ad02A5278	Aradu.A02	5278	p3	(CGG)5	15	16531745	16531759	AATTCTGTGCCTTTCCGTTG	AGCTTCGTTTCGCTGTTAGC	194
Ad02A5281	Aradu.A02	5281	p3	(ACT)5	15	16571146	16571160	ATGCGCGAAGGTTCATTTAG	TGTTTCTTCAGCTACCAGGGA	262
Ad02A5282	Aradu.A02	5282	p3	(TAT)5	15	16580071	16580085	GAAACCCAGTCAATGCCAGT	TTTGTTTACAACACTGGTTATTATGA	260
Ad02A5287	Aradu.A02	5287	c	(TA)7ctc(AAT	38	16600404	16600441	TGCGCACTGTCTTATCTCGT	TCAAATGCTTTTAACCTTGAATTTT	151
Ad02A5293	Aradu.A02	5293	c	(CT)6ggcgtcac	39	16621282	16621320	AAAACCCTCTTTTCCATCACC	CACAAGATGGTGACGCTCTC	159
Ad02A5295	Aradu.A02	5295	p2	(TA)6	12	16625509	16625520	CAATTTCGCAAGCTGAAACA	TCCTCCCAATGCATATGTTG	252
Ad02A5298	Aradu.A02	5298	p3	(TTA)6	18	16628656	16628673	CTGGGGACAGAGACTTTTCG	TTCCACTCCTGTCACTTCCTC	187
Ad02A5300	Aradu.A02	5300	p2	(TA)9	18	16636396	16636413	TTTACCCAACCTACCACCCC	TCCATTGTACACATTGGCTCTC	187
Ad02A5304	Aradu.A02	5304	p3	(TCT)5	15	16655383	16655397	ATTTCTTTCCCACCCAAACC	CTCCCTCGAGCAAGAGAAGA	197
Ad02A5305	Aradu.A02	5305	p2	(TG)7	14	16678874	16678887	CCGTAGCTCGAGATATTGCC	AAACCAGCCACAACTCCATT	116
Ad02A5309	Aradu.A02	5309	p2	(TA)6	12	16683329	16683340	GGAAGAAAATGGCCTGCATA	GCTCTTGGTTGTGGAGAAGC	180
Ad02A5311	Aradu.A02	5311	p3	(TGG)5	15	16687529	16687543	GCGATGTGTCCAAACAGAAA	ACCGTACCCCTTGCTAAACC	136

ID	Chromosome	SSR nr.	SSR type	SSR	Size	Start	End	FORWARD PRIMER (5'-3')	REVERSE PRIMER (5'-3')	Product (bp)
Ad02A5313	Aradu.A02	5313	p3	(AAG)6	18	16690914	16690931	CGGATCCTCAAGGAGGAGTA	AGCAAATGACACCACATCAAA	280
Ad02A5314	Aradu.A02	5314	p2	(AC)6	12	16697263	16697274	CAACACACCTCTCCCCCTTA	AGCCCCTCTCTCTCTGTTCC	141
Ad02A5317	Aradu.A02	5317	p3	(GAA)23	69	16726866	16726934	AGAGGGATCAAGTGTGGACG	TTTAAAGGCAATCACCATTCC	195
Ad02A5319	Aradu.A02	5319	p2	(AT)10	20	16739533	16739552	GCACATCTTTTGCCCTAAGC	ACAAATTCCCTCCACACTCG	113
Ad02A5320	Aradu.A02	5320	p3	(CAG)5	15	16756854	16756868	TTGGAAGTCCACCATCTTCC	TGTTGGCACTGGTTGTGTTT	278
Ad02A5323	Aradu.A02	5323	p2	(AC)6	12	16764817	16764828	CAAGGATTGAAGGGGATTGA	GGGAAATTGAATCAAAACATGAA	186
Ad02A5324	Aradu.A02	5324	p2	(TC)6	12	16764987	16764998	TTCATGTTTTGATTCAATTTCCC	CATCAAACACTTGGAAGGCA	269
Ad02A5328	Aradu.A02	5328	p3	(AAT)16	48	16805272	16805319	TGCTATATTGCTCTCTGGGCT	GCTTTGCAGTTGACTGAGGA	254
Ad02A5334	Aradu.A02	5334	p2	(TA)6	12	16825136	16825147	GAAAATTTATGTCCGAGGTACGA	AGCTCTTTTCTAAAAATATTGGCA	265
Ad02A5336	Aradu.A02	5336	p2	(TA)6	12	16826755	16826766	CGAGGAATCGACACCTCATT	CCACTGTTTTGTTATACGAAATTGA	183
Ad02A5341	Aradu.A02	5341	p2	(CA)8	16	16850117	16850132	AGATGAGGCATAGGAGGCAA	GGTGCTATCAGCATGCTTTG	169
Ad02A5345	Aradu.A02	5345	p2	(TC)6	12	16866832	16866843	TGTGTTCTTTGGGTTCTTCAAA	AATCGTATCCATGAAGCCGT	268
Ad02A5348	Aradu.A02	5348	p2	(CT)7	14	16871132	16871145	TCGGTGCAAAGAGAAAGGTT	TTGAATGTTCGTTGAAAGCG	156
Ad02A5350	Aradu.A02	5350	p2	(AT)6	12	16874639	16874650	AGATGGTGAGGCGCTATGAC	CAGGTGGTGGAGGAAAGCTA	279
Ad02A5353	Aradu.A02	5353	p2	(AT)7	14	16916290	16916303	TACAATCGACGGCATCGTAG	GCCACGAAAAGGTTCATCAT	152
Ad02A5354	Aradu.A02	5354	p2	(TA)6	12	16921379	16921390	TTTCGTGTCCAATTGTGTCTT	CAGACATAGAAATGTTGAATTGTTGA	280
Ad02A5356	Aradu.A02	5356	c	(T)10cattttctttt	62	16932683	16932744	AATTAGATGAGCGGGGCTTT	TGAAGCAAGAAAAAGCAGTGAA	176
Ad02A5357	Aradu.A02	5357	p4	(AAAT)5	20	16941108	16941127	CAGTTGCAGAGTACGCCAAA	TTGAGCCTAGCCGTGACTTT	198
Ad02A5358	Aradu.A02	5358	p2	(AT)9	18	16944578	16944595	TCCTGGGAAGCGAAGTAGTC	CAACGCTCGAGGATTCAAA	271
Ad02A5366	Aradu.A02	5366	p2	(TA)6	12	16954664	16954675	TCCACACGCTGTAGGTTGTT	GGATGACCTTTGAACCTGGA	273
Ad02A5368	Aradu.A02	5368	c	(T)10atagaatat	57	16960166	16960222	TTTTGTAACTTAATCCAATCAAACA	AAAGTGCATACATTTCTCTTCTACTG	137
Ad02A5370	Aradu.A02	5370	p2	(TA)10	20	16973763	16973782	TTCACTTTGGGGCATTTTGT	GAAAAGTTGGGAACATTGGG	239
Ad02A5372	Aradu.A02	5372	c	(AAG)12(ATC	63	16996633	16996695	TTGAATGAATGTAAGTTCATCCTCTT	CCTTCTCGTCTTCTGCTGCT	251
Ad02A5373	Aradu.A02	5373	c	(GAT)5gaaa(A	34	16997088	16997121	GCATTATGTGTTTCTTCTTCTTCTTT	TGCTGCAAATTTGGGTGTAA	273
Ad02A5374	Aradu.A02	5374	p3	(GAG)7	21	16997492	16997512	CAATTGGCAATCTGAACCTG	GCACGTTCTTCTTCATGCTG	214
Ad02A5380	Aradu.A02	5380	p3	(CAT)16	48	17005545	17005592	CATGTCTCCATGAGCATTTCA	TGGATGTGGACAGCATATCG	259
Ad02A5385	Aradu.A02	5385	p2	(TC)7	14	17020540	17020553	TCGAAAATTCCTCATCCTTTTT	CAGAGGAGAAGAGAGGGTTTGA	215
Ad02A5386	Aradu.A02	5386	p3	(TCT)6	18	17023709	17023726	CCCACACATCTCTCCCTCTC	TGGGATCACCTTCTTCTTGG	280
Ad02A5387	Aradu.A02	5387	p2	(AT)7	14	17033292	17033305	ATTGAGCCTTCGCGAATATG	TGAGAACCCTTTCGAGGATG	255
Ad02A5388	Aradu.A02	5388	p2	(GT)6	12	17037145	17037156	GGAGCACCGTAGCTCAAGAT	CGCTAGCAGGTTGGACTAGG	189
Ad02A5389	Aradu.A02	5389	p2	(CA)8	16	17065686	17065701	CATAATGGGAGCCCTCAAGA	TGTATTGGGTTGGCAGTTGA	198
Ad02A5398	Aradu.A02	5398	p3	(TTA)14	42	17088141	17088182	GTTGGAAAGACGAATCTCCG	AGCACCAATCGGGTTTGTAG	253
Ad02A5399	Aradu.A02	5399	c	(T)10cattctcatc	78	17089285	17089362	TGACGATTTTATTTATAATCCGTAGAG	AATTCTCCCTTCTCAACTCACTT	249
Ad02A5400	Aradu.A02	5400	p3	(AGA)6	18	17089557	17089574	TCAAGTGAGTTGAGAAGGGAGA	GGTTCGAGGCGTCACTATTC	181

ID	Chromosome	SSR nr.	SSR type	SSR	Size	Start	End	FORWARD PRIMER (5'-3')	REVERSE PRIMER (5'-3')	Product (bp)
Ad02A5401	Aradu.A02	5401	p2	(AT)9	18	17093477	17093494	CGCTAATTTTACGGGATTTACG	GCTGCCACCAAGATTTGAA	241
Ad02A5402	Aradu.A02	5402	c	(AT)8ag(AC)8	34	17095531	17095564	TCATCGTTCCAACATCAATCA	AGGCTCTTGTTGATGAGCATT	169
Ad02A5403	Aradu.A02	5403	p3	(AAG)5	15	17118499	17118513	GAAGCTTGCCCCTTGAGTCT	CTTCGTGACCGAACCAAGAT	235
Ad02A5407	Aradu.A02	5407	c	(GA)6a(AG)7	27	17124263	17124289	TGGCAGCTTCAACCTGAGTA	TTACCGTGACGAAAGAGCAA	201
Ad02A5409	Aradu.A02	5409	p3	(TAT)11	33	17125592	17125624	GCCAGTTACATTCCAGAAACG	GAATTTTTGGACGCACCTGT	276
Ad02A5412	Aradu.A02	5412	p3	(TTA)6	18	17141136	17141153	GAAAATTGGTGCAAGATCTCAA	TCAAAACATGCCTTGGTTCA	270
Ad02A5414	Aradu.A02	5414	p2	(AG)6	12	17164326	17164337	GCGGAGAGAGAGAGTGGATG	GATAACCCTAACACGCGACG	235
Ad02A5415	Aradu.A02	5415	p3	(AGA)6	18	17175752	17175769	TTAGAATGTTGCTCGCCCTC	TCTCAAGATCTGTGGACCCC	255
Ad02A5418	Aradu.A02	5418	p2	(TA)6	12	17198015	17198026	CCAATAAAATAGCTGGGTCATTAAA	CAAATGCAATTCTCGATCTCTTT	260
Ad02A5420	Aradu.A02	5420	p3	(TAT)8	24	17214540	17214563	GCGCTTTTCTGATCCTGTTT	TGCGTATCAAAGGGTCAACA	278
Ad02A5421	Aradu.A02	5421	p3	(TGA)6	18	17221642	17221659	CAGTAGTTCATCACAAGTACCCG	TCTCGTGTCACTATTACTGCTTCA	213
Ad02A5423	Aradu.A02	5423	c	(A)11ccaaaata	114	17239927	17240040	TGGACCTTCAATTCATAGCAAA	CCCACAGGATGCAGGATTA	212
Ad02A5425	Aradu.A02	5425	p2	(TC)7	14	17246822	17246835	CTTTTTGTTTGTTTCTTATCTTTTTCA	AGGGGCAGTGATTTGAGATG	279
Ad02A5426	Aradu.A02	5426	p4	(TATT)5	20	17246992	17247011	TCGAAATTCTTTCCCTCTCTTC	AGGGTAAGAACAGGGGCAGT	105
Ad02A5427	Aradu.A02	5427	p2	(AT)8	16	17266173	17266188	GCATTTGCTGCCATTATGAA	GCTATTGGTGTCAAATCAAAATTAAA	278
Ad02A5428	Aradu.A02	5428	p3	(TAA)20	60	17266546	17266605	ATCACACACACCCCACACAT	CTTGCGATCGACCTAGTTTGT	189
Ad02A5430	Aradu.A02	5430	p2	(TA)10	20	17273626	17273645	TTGAACCTCCCAAAACTTGA	TTCATCATTCATCCCTGCAA	221
Ad02A5433	Aradu.A02	5433	p2	(CT)6	12	17290683	17290694	CTTATTCCCCACACCATTCG	CGGAAAAGCACACCAAACTT	101
Ad02A5434	Aradu.A02	5434	p3	(AAT)5	15	17294923	17294937	TTTACTCCTCAAATTAAAATGGTGAT	CAGAGAGAGTGATGGCTGTTT	163
Ad02A5436	Aradu.A02	5436	p4	(AAAT)5	20	17298711	17298730	TGGTTTTGAGAATAAAATAAAACATGA	TCAGAGATACCGAACGAAAACA	253
Ad02A5438	Aradu.A02	5438	p5	(TTATT)5	25	17304141	17304165	TTGGGTTGACTTGGTCAAAA	CCCCAGGGTCCTTCTAGATT	261
Ad02A5439	Aradu.A02	5439	p2	(GA)8	16	17306239	17306254	GAGATCACGAGCGAGAGACC	AGAAGCCGAAAGTGAGGACA	267
Ad02A5440	Aradu.A02	5440	p2	(CT)7	14	17306441	17306454	TGTCCTCACTTTCGGCTTCT	TGCATCTGTACCATTGGCAT	164
Ad02A5442	Aradu.A02	5442	p3	(CTT)6	18	17319162	17319179	GGTCCTTTGCTGGTTTTGAA	GATGAGCGAAAGGAAACGAA	242
Ad02A5444	Aradu.A02	5444	p2	(AG)6	12	17337513	17337524	ACTTTTCCGCGACCTGTATG	TGAACCTGGGATTGAGAGTCA	256
Ad02A5447	Aradu.A02	5447	p3	(TCT)20	60	17381883	17381942	TCACACAACACAACCCCTTC	TATCTCCCATGACTCGGAGG	275
Ad02A5448	Aradu.A02	5448	p3	(GAA)5	15	17386010	17386024	TAAGAGAAGGTCGGCCAGAA	CCTCCCAACAAATGCTTCTT	224
Ad02A5451	Aradu.A02	5451	p2	(GA)6	12	17442560	17442571	GGCTTTGCATGCTTCATTTT	AGTCGCTTGTGCCTCCTAGA	261
Ad02A5453	Aradu.A02	5453	p3	(TAT)5	15	17445858	17445872	ATAGCAATTTTCGAGGACGA	ATTAATGAATGCAAACGGGG	264
Ad02A5454	Aradu.A02	5454	p2	(GA)6	12	17446185	17446196	GGCTTTGCATGCTTCATTTT	AGTCGCTTGTGCCTCCTAGA	261
Ad02A5456	Aradu.A02	5456	c	(CT)7gtctg(TC	87	17450016	17450102	GAGGCAAGGTGGTGGTGTAG	CTGGTCCGCTGTAGAGGAAG	263
Ad02A5457	Aradu.A02	5457	p2	(TC)9	18	17450296	17450313	CTTCCTCTACAGCGGACCAG	ACCTTCCATCACCACCACAT	203
Ad02A5458	Aradu.A02	5458	p2	(AC)6	12	17451107	17451118	CCCTTGGCCCAACATAACTA	TTCGAAAACAAGGCTGGAAT	280
Ad02A5460	Aradu.A02	5460	p2	(TC)9	18	17469138	17469155	GAAGCTGAACAGCGGTAAGG	CGAAACCCTAATCCCCAAAT	273

ID	Chromosome	SSR nr.	SSR type	SSR	Size	Start	End	FORWARD PRIMER (5'-3')	REVERSE PRIMER (5'-3')	Product (bp)
Ad02A5461	Aradu.A02	5461	p2	(AT)6	12	17469568	17469579	GGTTTCGTGTATGAAATTGGG	TTGAAAAACAATAATAATGCGGA	273
Ad02A5464	Aradu.A02	5464	c	(A)10gaagaaaa	34	17524531	17524564	TGTGGTTCATGAATGTTGGC	GCACACTCTTTTTGCCTTGG	194
Ad02A5469	Aradu.A02	5469	p3	(TTC)6	18	17582553	17582570	CTATACCTTGGCCGAATCCA	TCGGAGGTGAAAGCTTTTGT	215
Ad02A5470	Aradu.A02	5470	c	(ATT)5(GTT)1	72	17587220	17587291	AACTTTCTGTGCATTGGGCT	TCGACAAAAGGAAATTTCTGG	260
Ad02A5473	Aradu.A02	5473	p3	(AGA)51	153	17643624	17643776	AGTTTTGCTCATCCTGGAGC	CTGAATCCCTCTCTCCCTCC	225
Ad02A5474	Aradu.A02	5474	p3	(AAG)9	27	17657232	17657258	GAGGTAGGCAAGTGCTGAGG	TCTCTTCTTAACCTTGGCCG	243
Ad02A5475	Aradu.A02	5475	p3	(AGA)5	15	17660624	17660638	GGATAGGTGCGAATTCTTGG	TCGAAAATTCCTTTCCCCTT	217
Ad02A5476	Aradu.A02	5476	p2	(TC)6	12	17668226	17668237	TGACCCAGTTTTTAGCCCAG	GGGAAGCTTAAAAACAAAGGAA	237
Ad02A5478	Aradu.A02	5478	c	(TC)6tttttttttcg	44	17711295	17711338	TCCCCAATTAGAGGTGTCCA	TGTGGTTCATGAATGTTGGC	225
Ad02A5480	Aradu.A02	5480	p3	(TTC)8	24	17741293	17741316	CATCCACTCTTCCCCAAAAA	TAAAATATTGCTCGCCCTCG	261
Ad02A5482	Aradu.A02	5482	p3	(AAT)5	15	17746519	17746533	ATCGACTTAACCGCTGCAAT	TTGGTTTTGTTGTTGACTTGTT	266
Ad02A5490	Aradu.A02	5490	p3	(TCT)5	15	17775904	17775918	CTTCTTCTCCCACATAGCCG	CGGTCTTAGGTGCCATTGAT	174
Ad02A5491	Aradu.A02	5491	p3	(TTC)5	15	17802313	17802327	CCGCTCTGACTCTGCTTTCT	CACCAGTGAACAGCCAACAC	111
Ad02A5492	Aradu.A02	5492	p2	(CT)7	14	17805733	17805746	TGCATAACAAGCATTCTGCC	GTGAATAGCACTTCAGGGGG	235
Ad02A5494	Aradu.A02	5494	p3	(TTC)5	15	17810283	17810297	CCGCTTTCTGACTCTGCTTT	CATCATCATAATCGCCTCCA	196
Ad02A5496	Aradu.A02	5496	p3	(TTC)5	15	17813556	17813570	AAAGAGCGGCTTGACAAGAG	CCAAACGAACCAAAAATGCT	251
Ad02A5498	Aradu.A02	5498	p2	(GA)6	12	17816942	17816953	CCATGCTTTTACGTTTGGGT	AAAACAAAATGATGAGGAACATGA	260
Ad02A5499	Aradu.A02	5499	p3	(TTC)5	15	17850886	17850900	CCGCTCTCTGACTCTGCTTT	CACCAGTGAACAGCCAACAC	113
Ad02A5502	Aradu.A02	5502	p3	(AGA)7	21	17860860	17860880	CTGGCCTTAGGTGCCATTTA	TACCCTCTCCCCTCTCCCTA	236
Ad02A5505	Aradu.A02	5505	p3	(AGA)6	18	17873033	17873050	AGTGGTCTTGATGGGCTTTG	TACACCTCTCCCCCTCTCCT	244
Ad02A5506	Aradu.A02	5506	p3	(GAA)6	18	17877833	17877850	TAAAATATTGCTCGCCCTCG	CCTACCTCGCCCTCTCTCTT	231
Ad02A5507	Aradu.A02	5507	p2	(AT)8	16	17903459	17903474	CTCCCATATGCAGCCGTAAT	TGATTTAGTTTTCAATTTGCTTTGA	236
Ad02A5517	Aradu.A02	5517	p3	(CAG)5	15	17926298	17926312	GCTAAGGATCGTGGTTGAGC	TCCTTGGCATGCTCTTCTTT	101
Ad02A5519	Aradu.A02	5519	p3	(CAG)6	18	17934977	17934994	GCTAAGGATCGTGGTTGAGC	TCCTTGGCATGCTCTTCTTT	104
Ad02A5525	Aradu.A02	5525	p3	(CTC)6	18	17943729	17943746	AGAGGGTCCAGGAGGCTAAA	GGCTGAGAGGGAGTTGAAGA	136
Ad02A5527	Aradu.A02	5527	p3	(TCT)6	18	17973075	17973092	TCAACTTCAATTTCCCACCC	GTGCCATTGATGGTGAATGA	270
Ad02A5530	Aradu.A02	5530	p3	(TAA)5	15	17991594	17991608	TGGAGGATATCAATCGACCG	GTGCTGTCAAAATGAACCCA	223
Ad02A5531	Aradu.A02	5531	p2	(TA)6	12	17992847	17992858	CGCAACACTCAGGAACAAGA	AGCTTCTACAGCGGAATGGA	218
Ad02A5533	Aradu.A02	5533	p4	(TTTA)6	24	18013357	18013380	TCATATGTGCATTGATCCTTGA	GGGAAATTGTGGGAACAAAA	236
Ad02A5535	Aradu.A02	5535	p2	(AT)11	22	18027735	18027756	GTGAAGCTAAGGTTGGCTGG	GCGTCTGGAGCTTCTACACC	211
Ad02A5536	Aradu.A02	5536	p3	(ATA)13	39	18034270	18034308	AAAGAAAAGAAAGAAAGGATGAAAAA	TCCCAGACGTGCTATGAGAA	270
Ad02A5538	Aradu.A02	5538	c	(CA)6(CT)11t	63	18063646	18063708	TCGTTTTGGGCGTTAATAGG	CCGGTGGTGAAGAAAGAAAA	250
Ad02A5539	Aradu.A02	5539	p3	(TTG)5	15	18071865	18071879	CTTTCCACAAGCGTAGCACA	AGAAAGGGAGTCAAAGGGGA	233
Ad02A5544	Aradu.A02	5544	p3	(CAC)6	18	18099988	18100005	GCCATGTTTGCAAAACCTTC	TTGGTGATGGTGGTGGGTAT	280

ID	Chromosome	SSR nr.	SSR type	SSR	Size	Start	End	FORWARD PRIMER (5'-3')	REVERSE PRIMER (5'-3')	Product (bp)
Ad02A5545	Aradu.A02	5545	p2	(TC)7	14	18101455	18101468	CCATGTTTTAGCGGAGGAAA	GACCATGACCAGGAAAGGAA	278
Ad02A5549	Aradu.A02	5549	p2	(GA)10	20	18149823	18149842	GAAAGAAAGAAAGGGAGGGAG	TCAACCTTCTCTCACTCTATATCCC	279
Ad02A5550	Aradu.A02	5550	c	(AG)7gggaaga	99	18149997	18150095	GGGATATAGAGTGAGAGAAGGTTG	CTCTCCTTCGCTCCCCTTC	278
Ad02A5552	Aradu.A02	5552	p3	(AAT)15	45	18152489	18152533	GCATAAGAATCTGTCAGCGG	CTGAAGGATCATGTCCAGGG	263
Ad02A5553	Aradu.A02	5553	p2	(CT)8	16	18157005	18157020	GTCGTCCTATACCCAACCCA	TCAGAGGTGGTTGTTGGTGA	231
Ad02A5555	Aradu.A02	5555	p2	(CT)7	14	18165110	18165123	ATATATGTTCGGCCACACCC	TCACGGAAAAACACACCAAA	126
Ad02A5563	Aradu.A02	5563	p2	(AT)11	22	18197029	18197050	GAACAAAACATTTGGTAACTTTTTAAT	AATGATTTGAATTAATTTGGTAAGAGT	221
Ad02A5571	Aradu.A02	5571	p2	(AG)6	12	18243719	18243730	TGAAAATAAAAGAACATTAAACCTGG	TGACAGAGGCCACGTATCAG	263
Ad02A5573	Aradu.A02	5573	p3	(GAA)5	15	18250617	18250631	GGCCCGATTAACCAATTTTT	CCATCATCTGCAAACACCAC	270
Ad02A5577	Aradu.A02	5577	p3	(TCT)6	18	18262308	18262325	TGGAGAGTGGTTTCCTCCAC	GAACTGGGGCAAATCTTCAA	182
Ad02A5585	Aradu.A02	5585	p3	(TGA)5	15	18348922	18348936	ATGGAAGGTGTTTGGGAAAA	TCCCTGTACCAGCAAAATCA	278
Ad02A5586	Aradu.A02	5586	p2	(TA)9	18	18355270	18355287	GGGATTACAATCTGGGCTGA	TTCACAGGTTGTGTGTTCTTAGC	211
Ad02A5587	Aradu.A02	5587	p2	(AT)6	12	18355791	18355802	CACATCACTAGCCTTAAGCGAA	TTTTGCCCCCTATTTATCCA	268
Ad02A5592	Aradu.A02	5592	p3	(TTC)6	18	18411179	18411196	TACCCTCTCCCCTTTCCCTA	CTAGAGGATTCTCCGGCCTT	247
Ad02A5594	Aradu.A02	5594	p4	(CTTT)7	28	18421007	18421034	ACTCAAGGACCATGGCTACG	TACCAGTTGCCACTCCCTTC	260
Ad02A5595	Aradu.A02	5595	p5	(TTTTC)5	25	18440748	18440772	TTCTGCCTTCTCCTTTCTCG	TCCACAAGCAAATAATGAGGAA	239
Ad02A5599	Aradu.A02	5599	p2	(TA)9	18	18454525	18454542	TCCGATTCTCAGAACCTTGTTT	CGAGTGTAATTTTAACACGCTGA	275
Ad02A5603	Aradu.A02	5603	c	(A)13ccattaaat	121	18466815	18466935	TGCATTGATCCTTGAATTCTT	AGCCTTAAGCGGAAAAACAA	280
Ad02A5604	Aradu.A02	5604	p2	(CT)7	14	18471964	18471977	GATGTATCCGATCCAATCCG	GGCGACGAGCTCAGAAAATA	280
Ad02A5605	Aradu.A02	5605	c	(T)12a(TTG)6	31	18472477	18472507	CGGTATTGGTGCGGTAGTCT	TCCACACCACCACTTTTTCA	236
Ad02A5607	Aradu.A02	5607	p3	(GAG)6	18	18474586	18474603	TGCAACGTAACCAGATCGAA	CCCACTCCTCACTCGTTTGT	204
Ad02A5608	Aradu.A02	5608	p3	(ATG)14	42	18475474	18475515	TCGAGATTCCTTGTTGACCA	CAAGTCCCGGTAAATTCTCG	261
Ad02A5611	Aradu.A02	5611	p2	(CA)9	18	18479296	18479313	GTCCAGGTCCTGCAAATCAT	CCTTCACACCCTCCTGTCAT	267
Ad02A5615	Aradu.A02	5615	c	(GA)6gctgggg	127	18485176	18485302	GTAGATCCGGAGGAGACGC	GTGAACCTGCCGGTTTTAGA	267
Ad02A5618	Aradu.A02	5618	p2	(TA)9	18	18498320	18498337	CGTTTCTTCCAAGGCTCCTA	ACGTTCAAAGAATGGATGGC	186
Ad02A5619	Aradu.A02	5619	p4	(TTTA)7	28	18532896	18532923	TTGAGCCTAGCCGTGACTTT	AAGGCTTTGAGCACCACTGT	198
Ad02A5622	Aradu.A02	5622	p3	(ATA)5	15	18544981	18544995	TGAGACAACGTAGTCCTTTTGTG	TAAGTGCCGCATCAATTTTT	273
Ad02A5626	Aradu.A02	5626	p2	(TA)9	18	18550835	18550852	TCAGATACGGATAAATACTGTGGA	GATTTTCTTTCCAAAAACAATAAAA	279
Ad02A5630	Aradu.A02	5630	p3	(ATA)5	15	18557271	18557285	GCTTCATGTTTGGCACCTCT	TGCCTAACCTCTCCTCCAAA	236
Ad02A5631	Aradu.A02	5631	p3	(AAT)11	33	18560519	18560551	CAAACAAACATTAAACAGACATCG	AAGACATTGTCGGGGTTCAG	175
Ad02A5633	Aradu.A02	5633	c	(A)10gataatgtc	109	18564280	18564388	GGCAGCACTAACACCCATTT	GATTCCGTTGTTTCGCTTTC	228
Ad02A5636	Aradu.A02	5636	p2	(AT)8	16	18618647	18618662	TTAGCCGAGTTTGAGTTTGGA	ATTGAGCTTGGCTCACGACT	274
Ad02A5637	Aradu.A02	5637	p2	(AT)7	14	18623609	18623622	GACTTGATGTACGCTCCACG	AGAAAGAGAGCTCAGGTGCG	269
Ad02A5638	Aradu.A02	5638	p2	(TA)10	20	18624897	18624916	TGGGAACATGGGATGATAAAA	TTGTCCCATGTGCATTGATT	218

ID	Chromosome	SSR nr.	SSR type	SSR	Size	Start	End	FORWARD PRIMER (5'-3')	REVERSE PRIMER (5'-3')	Product (bp)
Ad02A5640	Aradu.A02	5640	p2	(TA)9	18	18631028	18631045	TGCAAAATTGGGTTTGACTG	TCCTCACCTTCCACCATCTC	243
Ad02A5641	Aradu.A02	5641	p2	(TA)10	20	18632222	18632241	TCGCTAGGCGTCTGGATACT	ACTAGCCCCTGACTCAGCCT	254
Ad02A5645	Aradu.A02	5645	c	(A)10ttttgaaatt	96	18679628	18679723	GGCAAATTCGAAATTCAAAAG	GTGGGGAGGAGGTAATTTCG	272
Ad02A5646	Aradu.A02	5646	p5	(ATAAA)5	25	18685204	18685228	GGGTTAGCATATGACCCCAG	TTTCTCTCTCCTCATCTTTTTCG	241
Ad02A5648	Aradu.A02	5648	p3	(ATA)7	21	18693667	18693687	GGCCACGTGTCATTTTCTTT	AAAAATGTCAGTTTAATTTCTTCCA	260
Ad02A5652	Aradu.A02	5652	p2	(CA)8	16	18741437	18741452	AAAACCCTCCCTACCCTCG	TTAGATGAAGGGGTGGTTGG	128
Ad02A5653	Aradu.A02	5653	p2	(AG)22	44	18742367	18742410	GGTTAGTGGGTTTCAGGAAGG	TGGGATCAAAATTAAAATCGTCA	227
Ad02A5656	Aradu.A02	5656	p2	(TA)7	14	18753825	18753838	TCAAAATCAGCAACATTAATCCA	TCAACGTTCCAACGAGAATG	278
Ad02A5659	Aradu.A02	5659	p2	(CT)8	16	18755610	18755625	CAAAGACATTGATGTTGGCG	GGAGAAGATGATGGGGTGTG	158
Ad02A5661	Aradu.A02	5661	c	(A)10taacactta	93	18761806	18761898	TCAGATTGATGCGAGGACAG	TTATCGTTGCCTGCAGTCTC	206
Ad02A5665	Aradu.A02	5665	p2	(TA)8	16	18767543	18767558	CACTTTGGGGCACTTTGTCT	TGTCCCTTGTTTGGATCTTTG	245
Ad02A5668	Aradu.A02	5668	c	(TAA)8aataata	61	18810903	18810963	GGGATCCGATTAAAAGGAGA	TGAGCATCTGAACAGTTCTCAAT	275
Ad02A5672	Aradu.A02	5672	c	(T)10gcaccatct	73	18835497	18835569	GCAATGCAGATGTTTCATCG	CATTGTTGTGTTCATCCCCA	269
Ad02A5675	Aradu.A02	5675	p3	(GAG)6	18	18838171	18838188	CGCGTGTTTTCTCTTCATCA	TCGGTGTTATTTTCTGATAAATTTTG	279
Ad02A5676	Aradu.A02	5676	c	(GAT)6gacgat	48	18839469	18839516	TTTCATGAAGACTTGAGTTGCAG	TTTGATTGAATTTTCTTCCCG	273
Ad02A5682	Aradu.A02	5682	p2	(AT)10	20	18857444	18857463	AACAATAATCGCTGCCTTGG	AAATGACAAATGACGAGGGG	160
Ad02A5683	Aradu.A02	5683	p3	(TTC)6	18	18864227	18864244	TACCCTCTCCCCTTTCCCTA	GCTTTTGTCTTCCCTTTCCC	270
Ad02A5684	Aradu.A02	5684	p3	(AAT)8	24	18871462	18871485	CCTAAACGTTGGGGACAAAA	TGTCACACTTGTCGAACATCTTT	207
Ad02A5686	Aradu.A02	5686	p3	(TCT)23	69	18874867	18874935	CACGCAGGCTCTTTTTCTTC	TGGCACCAGAGAGAAATTCA	185
Ad02A5694	Aradu.A02	5694	c	(T)13gttttcgttc	84	18968956	18969039	TCTTTTAACTTAATCCAAGCAATTTTT	TAGCCACTTCGTCGTCACAC	271
Ad02A5695	Aradu.A02	5695	c	(A)10caagtaatg	49	18970510	18970558	TGTTGAGACGTTGATGCTCC	TTACCCCTCACATTCACCGT	144
Ad02A5700	Aradu.A02	5700	p3	(AAT)7	21	18976915	18976935	CGGTTTAGCCGTCGATTTTA	TGTAACACCCATACCGTTAGTACATT	257
Ad02A5703	Aradu.A02	5703	p2	(TC)10	20	18982520	18982539	TGGCTGTCTTTGTTACACGC	TGTGCTTGAGCGTGAATTGT	190
Ad02A5706	Aradu.A02	5706	p2	(AC)6	12	18992344	18992355	TCACTCCAACAAACCACCAA	GACAGGGGTACCTGCAAAAA	182
Ad02A5707	Aradu.A02	5707	p5	(AAAAT)5	25	19018034	19018058	CTTACTGGCTTTTTCACGCC	CAACCCAGCCATTCACAAA	159
Ad02A5709	Aradu.A02	5709	p2	(AT)6	12	19044252	19044263	GGTGTTTTAGGTCCCAGCAA	GAAATGGATTACTCAGCTTGGG	237
Ad02A5710	Aradu.A02	5710	p3	(TTA)26	78	19046662	19046739	GCCGTATTATTCTATAAGGACCG	TGATGCAGAGATTAGAGATTAGAGATT	201
Ad02A5711	Aradu.A02	5711	p3	(TTC)5	15	19047036	19047050	GAAGCTCCGGACGATTAACA	AAAGGAACACAAATCGGACG	278
Ad02A5713	Aradu.A02	5713	p3	(CAA)7	21	19052372	19052392	TTGGTTGCGAGGGTAGAAAC	ATTTTCCCCAATTTTCCGAG	136
Ad02A5714	Aradu.A02	5714	p2	(GA)12	24	19052607	19052630	CTCGGAAAATTGGGGAAAAT	AAATTCGAAACCCACACTCG	248
Ad02A5717	Aradu.A02	5717	p3	(AAT)18	54	19108460	19108513	GGCTGTTTTTGAAGGTGACAA	TGCCATCAATAAAAGGACTCAA	189
Ad02A5719	Aradu.A02	5719	p2	(TA)9	18	19114523	19114540	AGTTCATTCGCAAGAGCATT	CGTATGGCATAATACATATGTTAAAAA	238
Ad02A5721	Aradu.A02	5721	p2	(TA)6	12	19119253	19119264	GCTCTTTCCTAAAAATATTGGCA	CAGAAGAAGTTCTATATCTGAGGTACG	189
Ad02A5726	Aradu.A02	5726	p2	(TA)6	12	19128172	19128183	ATCCATGCTGATTGGGAGAC	TTGGTTCTGGGAAATCAAGC	198

ID	Chromosome	SSR nr.	SSR type	SSR	Size	Start	End	FORWARD PRIMER (5'-3')	REVERSE PRIMER (5'-3')	Product (bp)
Ad02A5727	Aradu.A02	5727	p3	(AAG)6	18	19130416	19130433	CGCTGAGGAGAAAATGGAAG	CTGTTTCTGGGAACGGAAAT	248
Ad02A5729	Aradu.A02	5729	p2	(CT)7	14	19139423	19139436	CGATCTTGATGGCAGAACAA	CCCACGGTTTGAAAGAAAGA	264
Ad02A5736	Aradu.A02	5736	p2	(AT)15	30	19175787	19175816	GGATATGGGCGCAGAAGTTA	TTTAAACCGCAATACCGCTC	261
Ad02A5737	Aradu.A02	5737	p6	(AAATAA)5	30	19209955	19209984	TACTGGCAACTTTGCTGCAC	TTTCTCTCTCCTCATCTTTTTCG	157
Ad02A5739	Aradu.A02	5739	p2	(TA)12	24	19218164	19218187	GCGGTCACGTGGATCTATTT	CACCCACAGCAGAAGACTGA	245
Ad02A5743	Aradu.A02	5743	p3	(TTC)6	18	19240141	19240158	CCTACCTCGCCCTCTCTCTT	TAAAATATTGCTCGCCCTCG	231
Ad02A5747	Aradu.A02	5747	p2	(AC)9	18	19269610	19269627	ACCCTTGGCCCAATTAAACT	ATTCGAAGAGCAAAACGTGG	264
Ad02A5748	Aradu.A02	5748	p3	(TTC)5	15	19282856	19282870	CCCGAAGAGCTATTTTGGTTC	CCCCTAATGAAAGTGGGGTT	167
Ad02A5752	Aradu.A02	5752	p2	(TC)6	12	19310642	19310653	CAGCAGTCTGGCGTTAAACA	TAGGTGGTGTTTTTGGGGAA	257
Ad02A5753	Aradu.A02	5753	p2	(CT)6	12	19351190	19351201	AAAAACAATGCCAAAAAGCG	GGGAAGGGGAGACTAAGTGC	177
Ad02A5754	Aradu.A02	5754	p2	(TA)8	16	19362564	19362579	TTTGCGCTTTTCAAACATTG	CATTGCACGGGTTTAACACT	110
Ad02A5755	Aradu.A02	5755	p3	(TTC)6	18	19374273	19374290	AAACCCACACCTCTCTCCCT	CCAGCCTTAGGTGCCATAAA	157
Ad02A5759	Aradu.A02	5759	p2	(CT)6	12	19400638	19400649	GGAACATCGAACCTGGAATG	CGTGCAGAAAATTGCAGAAA	264
Ad02A5760	Aradu.A02	5760	p3	(AGA)5	15	19425209	19425223	CTCCCAAGACTCAGAGGTGG	CATCAACCTCCTCTTTCCCA	233
Ad02A5763	Aradu.A02	5763	p2	(TA)6	12	19461215	19461226	GGAAGCCAAGAATTGAATGAA	GAGCAACATGAGTTCACAGCA	234
Ad02A5764	Aradu.A02	5764	p2	(CT)6	12	19472247	19472258	GACGAACAGCCTCGTTCATTA	GAAATTTGAGGGTTTGGGAA	262
Ad02A5765	Aradu.A02	5765	p2	(TG)6	12	19508895	19508906	GGTTTTGTAGAGCTCGTCGC	GCCCAAGCCCAACATATAAA	212
Ad02A5767	Aradu.A02	5767	p2	(TA)8	16	19517150	19517165	TGTAGCCTTTTGTCTCTCATTTTT	TTGTATGGTATTATTTTGGGCG	187
Ad02A5768	Aradu.A02	5768	p3	(AAT)6	18	19528510	19528527	GGCACTCTTGTTGAAGCTGA	AAACTTGGACGTTGGCTTTG	192
Ad02A5769	Aradu.A02	5769	p2	(AC)6	12	19563193	19563204	AACCAAATTCAATCACAATTCTC	CGGTTTAAGCACCGGTACAC	245
Ad02A5770	Aradu.A02	5770	p2	(TA)6	12	19566838	19566849	AAATTCCGCCTTTTCCAAGT	ATCCCTCAAGCTCACGAAGA	209
Ad02A5772	Aradu.A02	5772	p3	(ATT)6	18	19608809	19608826	AAACTTGGACGTTGGCTTTG	CAGGGAAAAAGGCACTCTTG	202
Ad02A5774	Aradu.A02	5774	p3	(AGA)5	15	19627648	19627662	TTAGGTGCCATTGATGGTGA	TCAAATTCACTTTCCCACCC	274
Ad02A5776	Aradu.A02	5776	p3	(GAA)6	18	19668531	19668548	TAAAATATTGCTCGCCCTCG	CCTCTTCCCAATTACCCCAT	201
Ad02A5778	Aradu.A02	5778	p3	(TTC)5	15	19686725	19686739	TATGCATGCCCATTGTGAGT	TGCATTCCTGGGTACAACAA	111
Ad02A5780	Aradu.A02	5780	c	(TC)7ttctaggct	90	19721178	19721267	CGTGAGCTCCTTCTCTCTCC	GAGGAGCCTGTCCAACTCAC	279
Ad02A5781	Aradu.A02	5781	p2	(GT)6	12	19721407	19721418	CCCTTCCTCCTCTTCCATTC	TTCAACACACACAACTTTAACGC	131
Ad02A5783	Aradu.A02	5783	p4	(TGCG)5	20	19731673	19731692	CGAGAAGCTTGAAAAGGGAG	AGGTCGTAACATGCACCACA	162
Ad02A5786	Aradu.A02	5786	p2	(AT)8	16	19753101	19753116	GCAAAACCGCTCCCTAAAAT	TTTCCCCTTTCAAGATGTGG	279
Ad02A5787	Aradu.A02	5787	p2	(CT)7	14	19756572	19756585	ACTCCTCCTTCTCCATCGGT	GAAGGAGAGGGGGAAGAAGA	216
Ad02A5788	Aradu.A02	5788	p2	(AT)8	16	19758010	19758025	GCAAAACCGCTCCAAGAATA	TCCCTTTCAGGATATGAGCG	268
Ad02A5789	Aradu.A02	5789	p4	(ATTA)5	20	19765240	19765259	GCTAACAAATAGGATTTTGTTGGTG	TCACTAAAACACATATACTCCCAGC	147
Ad02A5797	Aradu.A02	5797	p2	(AT)8	16	19810610	19810625	AGTTTCGAGGGTTGGGAAGT	CCTTTGGAGGAATCACAGGA	227
Ad02A5798	Aradu.A02	5798	p3	(AAT)5	15	19816608	19816622	CCTGACTCATCAAGCAAGCA	TTGAAAGCTTCAAGGGCATC	234

ID	Chromosome	SSR nr.	SSR type	SSR	Size	Start	End	FORWARD PRIMER (5'-3')	REVERSE PRIMER (5'-3')	Product (bp)
Ad02A5801	Aradu.A02	5801	p2	(TG)6	12	19831204	19831215	GGACCTTGGGTGTGGTAAGA	CTCTCAAGGCCTCACACCTC	203
Ad02A5802	Aradu.A02	5802	p2	(AT)7	14	19852086	19852099	AATGAAACGGCTGGTTATGG	CAGGGAAACTCTCATCTCCG	196
Ad02A5803	Aradu.A02	5803	p2	(TA)14	28	19871615	19871642	ACACCGCCCGTCACACTAT	AACATCCAGTCAGGAGGGTG	270
Ad02A5805	Aradu.A02	5805	c	(TTG)6ctgatga	86	19875171	19875256	AGAAGGAGGAGAGGGAGACG	AACCCTCTTCACACAGCCAC	223
Ad02A5808	Aradu.A02	5808	p2	(TA)6	12	19891268	19891279	TTATGCTCGGACCGGTTATC	AACTCGAAAGCGCAACACTC	140
Ad02A5821	Aradu.A02	5821	p3	(TTA)6	18	19927944	19927961	TATTGTTTGTGCCTTGCAGC	GCATGTGCACTCTCTTGGAA	105
Ad02A5824	Aradu.A02	5824	p3	(TCC)7	21	19942989	19943009	AGATCTCGTGCCCGAGTAGA	CCAGAAGTCTTGCGCTTCTT	191
Ad02A5825	Aradu.A02	5825	p3	(TTA)15	45	19951394	19951438	CAAAACGTTATAAACTTTGGTGGA	AATGGTGGAAGGTGCATGTC	255
Ad02A5827	Aradu.A02	5827	p3	(AGG)7	21	19966205	19966225	CTCCAGCTCCTCAACCTTTG	GGAGTCCTACCAGAGGGTCC	245
Ad02A5828	Aradu.A02	5828	p2	(CT)6	12	19969080	19969091	TGCTGCTAAAGGAGCAGGTA	TCTCAGAAAACATTCCGAAGAA	276
Ad02A5832	Aradu.A02	5832	p2	(TA)11	22	19975288	19975309	ATTATTGCCAGAGACCGACC	TTTTGTAAATTTTTGTAGGGCAAA	128
Ad02A5838	Aradu.A02	5838	c	(T)10cattaatctt	73	20008908	20008980	GTCAGCCTGGTTATATCGGC	TGTTGATGGTGGAGAGTTCG	214
Ad02A5842	Aradu.A02	5842	p2	(TA)9	18	20028795	20028812	TTGAGCCTTTGAGAATACGAAA	TGCGGAACAACAAGAGCATA	233
Ad02A5844	Aradu.A02	5844	p2	(AT)6	12	20034220	20034231	ACCGTCAATTTTCACCGAAC	TAAGGAAAGCCAAAACGTGG	273
Ad02A5845	Aradu.A02	5845	p2	(CT)7	14	20040005	20040018	ACCAAGATTGGACTGAACGG	ACCATTTCAGCGTGTGTGAG	141
Ad02A5846	Aradu.A02	5846	p2	(AG)6	12	20070556	20070567	CGGAAAAGCACACCAAACTT	TAAAAACATGTTCGGCCTCC	123
Ad02A5849	Aradu.A02	5849	p2	(AT)7	14	20078564	20078577	AAAACTCAAGACTCGGCTCG	TTTCAGATGCAGGACAGGTG	244
Ad02A5855	Aradu.A02	5855	p3	(TTC)6	18	20116957	20116974	CCTAAATGGCCGAACCTACA	TGCCATTGATGGTAGTGGAA	239
Ad02A5861	Aradu.A02	5861	c	(TAA)16aatata	129	20144959	20145087	GCCGGCAACTTCTTAAGTGA	TTTGGGATAACATTAGCAGGG	276
Ad02A5862	Aradu.A02	5862	p3	(ATC)7	21	20145314	20145334	CACTGCCATCAAAACACCAC	TGTTCGTATGTGGGGTGAGA	243
Ad02A5864	Aradu.A02	5864	p6	(TATCTT)5	30	20150377	20150406	CGGCAATTCTCTCGTTAAAA	TATATGAGCGCCTCGTCACC	209
Ad02A5866	Aradu.A02	5866	p3	(TAA)5	15	20154948	20154962	TTCGTGCATCACACGAATTT	TTCGGTCTTTCACTCTTTTGG	238
Ad02A5868	Aradu.A02	5868	c	(TGC)5ttctttat[illegible]	64	20168596	20168659	AAAGGAGGAGAGGGCAAGAG	AATCTCAAATCCCACCACCA	203
Ad02A5869	Aradu.A02	5869	p2	(TC)6	12	20178136	20178147	GCCATGCAAACAGAGAGACA	TGCTTGTCCTCAAGCAAAGA	280
Ad02A5870	Aradu.A02	5870	p2	(AG)6	12	20182577	20182588	AAAGAGGGGGAGTTGTTGCT	GGCGACAACTAGGCAGTGAT	272
Ad02A5871	Aradu.A02	5871	p2	(TA)12	24	20185346	20185369	TTTTTCCCTCGCTTTTATTCTT	TACCCTCGCTATCCGAATGT	276
Ad02A5872	Aradu.A02	5872	c	(ATT)12gatga[illegible]	69	20193567	20193635	TGGCTGAAATTAATATTTTGCTGA	TTTTTGCAATTTGGGCTTTT	276
Ad02A5873	Aradu.A02	5873	p3	(TTC)7	21	20195976	20195996	TCTTCTCCCACTTAGCCGAA	CTTGGAGGTGGAAGCTTTTG	221
Ad02A5874	Aradu.A02	5874	p3	(TTC)6	18	20207968	20207985	ACACACTACCCCCTCTCCCT	TGCCATTGATGGTAGTGGAA	227
Ad02A5878	Aradu.A02	5878	c	(TC)8ttctcgtga	78	20233112	20233189	CTGCTTTACCCATGGACGAC	CCAGGACCGAGGTAGAGAGA	197
Ad02A5881	Aradu.A02	5881	p2	(TC)7	14	20263314	20263327	GCAACATGAATGGTGACTGG	TTGTCGTTGATGCAGCTCTC	142
Ad02A5882	Aradu.A02	5882	p2	(TC)8	16	20263440	20263455	GAGAGCTGCATCAACGACAA	GTCACGAGGAGGACCAAGAC	180
Ad02A5883	Aradu.A02	5883	p2	(GT)6	12	20263824	20263835	CATCTCCCTCTTCTCCCTCC	CCCAAAATTCCCAAATTGAA	189
Ad02A5884	Aradu.A02	5884	p2	(AT)6	12	20265654	20265665	AATCGGTTCAACCAGTGACC	CTCAAATTTTGCAGCCATGA	237

ID	Chromosome	SSR nr.	SSR type	SSR	Size	Start	End	FORWARD PRIMER (5'-3')	REVERSE PRIMER (5'-3')	Product (bp)
Ad02A5885	Aradu.A02	5885	p2	(AC)6	12	20268257	20268268	TCATTTTCTGCATGTGGCTC	TTGCCTCGTTGCTTTCTTCT	227
Ad02A5886	Aradu.A02	5886	p3	(CAT)6	18	20268516	20268533	GCATTTTCCGGACACTTCTC	GCAGGTCAAAGGCAAGAGTT	260
Ad02A5888	Aradu.A02	5888	p2	(CT)6	12	20278074	20278085	CTTTCTTCTCCGTCTGTGGC	TTGCTGTCGTGACCTAACCA	240
Ad02A5889	Aradu.A02	5889	p2	(TA)7	14	20283625	20283638	TGAATGTGGATTGGTAAAGTGG	ACATCAACTGAAGCCCCAAC	211
Ad02A5891	Aradu.A02	5891	p2	(TA)8	16	20310506	20310521	TCAGATGCAGGACGTGAGAC	TCGCGAAGATAAACTGGCTT	258
Ad02A5892	Aradu.A02	5892	p3	(TTA)19	57	20317346	20317402	CCTGAAACTAAATTCGGGCA	CGCCTTGGAATTCCTCAGTA	217
Ad02A5896	Aradu.A02	5896	c*	(A)10(AT)10*	29	20339569	20339597	TTGTTGGCTCTTGAAAGAATGA	TTGAGCCCTCCTATCCATTG	258
Ad02A5897	Aradu.A02	5897	p3	(CAA)6	18	20344576	20344593	ATCATTCCCACAGAACCGAG	GCACCTTGTTTCTGCCTCTC	264
Ad02A5898	Aradu.A02	5898	p2	(TA)6	12	20351317	20351328	TTATGCTCGGACCGGTTATC	ACGAACGAGGATAACCAACG	106
Ad02A5902	Aradu.A02	5902	p3	(ACC)5	15	20356195	20356209	GAAAAAGCAAATGGTGGAGC	GATTTTTGGGTGACGGAAGA	233
Ad02A5904	Aradu.A02	5904	p2	(AT)8	16	20388402	20388417	TTAAGTGTGGGGAGTCCGTC	TTAAATAAATTGGGGTGCCCT	205
Ad02A5906	Aradu.A02	5906	p3	(AGA)6	18	20410359	20410376	CAGCCTTAGGTGCCATTGAT	GCTGAAACTTCACTCTCCCG	241
Ad02A5913	Aradu.A02	5913	p3	(GAT)5	15	20477877	20477891	TCTTCGTGTTACACCCAAATACA	TGCATAATTCAAAACTCTTCTTCC	277
Ad02A5915	Aradu.A02	5915	p2	(CT)6	12	20483174	20483185	CAGAATGGTGGTGGTGACTG	ACCGGGTTATAGCAAGGGTT	252
Ad02A5918	Aradu.A02	5918	c	(T)11gccttggat	79	20492597	20492675	TCCCCAATTAGAGGTGTCCA	GGCTCTTGAAAGAATGAGGG	227
Ad02A5921	Aradu.A02	5921	p2	(TA)6	12	20504033	20504044	TTCATTCCAATACTTTTGGTTCA	ACCAAAGGCTACGCTTTTCA	244
Ad02A5924	Aradu.A02	5924	p2	(AC)9	18	20522866	20522883	TTTCTTTGTCTCCCCCTTGA	CGGAGTGCTCTCAAGCAATA	220
Ad02A5928	Aradu.A02	5928	p2	(AT)9	18	20549770	20549787	TTTAACTTCCCGGGTTTTCC	CCATTGGACAGTCCCTCTGT	149
Ad02A5929	Aradu.A02	5929	p3	(TTC)5	15	20553019	20553033	CAACTTGGCCGAACCTACAT	TTGCCTTCCCTTTTCCTTTT	192
Ad02A5930	Aradu.A02	5930	c	(A)10tttggc(A)	26	20554526	20554551	TGAAGCAAGAAAAAGCAGTGAA	GCTTGATGTAGCCGCTTAGG	211
Ad02A5931	Aradu.A02	5931	p2	(TA)6	12	20557468	20557479	GTTGATTTGGCGAAACAGGT	TCCAGACTCGACCTGTGAAA	102
Ad02A5934	Aradu.A02	5934	p2	(TA)6	12	20573343	20573354	TTCAAGCAAGTTCAATGAAAAA	TTAAGTGTGGGGAGGTCGTC	182
Ad02A5935	Aradu.A02	5935	p3	(AAT)5	15	20577067	20577081	CCCCTTTGGAATCTTTTTCA	TTTGGAGGGAAAACACCAAG	263
Ad02A5938	Aradu.A02	5938	p3	(AAT)8	24	20593159	20593182	GGGGCTCACACAAGATTCAT	TTGGATGTTGGCTTTGTGAA	144
Ad02A5939	Aradu.A02	5939	p3	(TCT)6	18	20595963	20595980	CACAAAACACAACCCCCTCT	CAGCCTTAGGTGCCATTGAT	186
Ad02A5940	Aradu.A02	5940	p2	(TA)7	14	20601467	20601480	ATGCCACAAGCACATGGTTA	TAGCAAAAGCTTGCGAAAAA	216
Ad02A5941	Aradu.A02	5941	p3	(AAG)5	15	20602337	20602351	TTGCCTTCCCTTTCCTCTTT	ATCTCTCTTCCCCGTCACCT	257
Ad02A5942	Aradu.A02	5942	p5	(AATAA)5	25	20606956	20606980	ATGATGGAGCAAGGGAGATG	TTTCTCTCTCCTCACTTTTTCG	163
Ad02A5945	Aradu.A02	5945	p3	(TCT)6	18	20636294	20636311	CTTGGCCGAACCTACACTTC	GGTCTTGATGGCTCTTGGAG	251
Ad02A5946	Aradu.A02	5946	p2	(AT)7	14	20647352	20647365	GGCATGCTGGAAGCTTATGT	AAAACTCAAGACTCGGCTCG	264
Ad02A5947	Aradu.A02	5947	p3	(GAA)13	39	20647855	20647893	ACGAAGTATCAGGGGTGACG	TCATTTAGACTCATGCGCGA	115
Ad02A5948	Aradu.A02	5948	c	(A)10taatattga	91	20648637	20648727	GACAACGAGATCCCCAAAAA	GGGGATTTGGGAGTAGGAAA	268
Ad02A5952	Aradu.A02	5952	p3	(TTC)6	18	20649960	20649977	TGAAGGCATATCCAAAAGGG	GCAAAATGAAAATTCGACCAA	253
Ad02A5953	Aradu.A02	5953	p3	(AAC)5	15	20650300	20650314	TTCCCTGAGTTCGTAGAGTGC	ATCCTTGAGGTGGTCAAACG	193

ID	Chromosome	SSR nr.	SSR type	SSR	Size	Start	End	FORWARD PRIMER (5'-3')	REVERSE PRIMER (5'-3')	Product (bp)
Ad02A5954	Aradu.A02	5954	p3	(AGA)6	18	20659911	20659928	GGTCTTGATGGCTCTTGGAG	TACCTTGGCCGAACCTACAC	254
Ad02A5955	Aradu.A02	5955	p2	(CT)7	14	20670157	20670170	GGGAAATTAGAAGAGCCAGGA	GTTTCGATCCAGTTTTTGGC	208
Ad02A5956	Aradu.A02	5956	p2	(AT)11	22	20678481	20678502	AAAGCGAGAGGGGTGAGAAT	GAGAAGACGACCCGAGGTTT	265
Ad02A5958	Aradu.A02	5958	p3	(CCA)5	15	20707071	20707085	AATGCGAAAGAGCTCACCAT	TTCAAACTATCCTCACCCGC	198
Ad02A5959	Aradu.A02	5959	p3	(ATT)5	15	20707373	20707387	CCCATCTTTCATGGGTACAA	GTAGCGTGTGGAGCTGATGA	272
Ad02A5966	Aradu.A02	5966	p3	(TTA)13	39	20723081	20723119	ATGAGGTGAAATGCGAGGTC	TTTAAAAGCATTTTTGGTGCTT	274
Ad02A5967	Aradu.A02	5967	p3	(CAT)5	15	20727451	20727465	TTGCCCTAAGTTCTTTCTTGG	TTTTGGGGGTTGTTGTCACT	203
Ad02A5968	Aradu.A02	5968	p4	(TAAT)5	20	20730433	20730452	GAGAGACCACCAAAAACTGTAGC	TGAAAGGTGGTTTAATGTCGG	236
Ad02A5970	Aradu.A02	5970	p3	(TTA)15	45	20753292	20753336	AGCATGGAAGATGAGGATCG	GCCTCACATTGGTTTTGTTG	278
Ad02A5972	Aradu.A02	5972	c	(AT)12acttagc	143	20788408	20788550	GTCTGGATACCTGAGGCGAA	GCTCCTAACTCAGCCTGCAA	238
Ad02A5981	Aradu.A02	5981	p3	(TAA)7	21	20843955	20843975	TTGTCCTATAAGTGTTTTTCTTTTTGA	TCTGGGAGAGGAAGACCGTA	263
Ad02A5982	Aradu.A02	5982	p2	(AT)6	12	20844439	20844450	TTTGGTGGAGTTTGTGTGGA	CCAGTCAAGTGTATCCGATTTTT	172
Ad02A5983	Aradu.A02	5983	p2	(GT)6	12	20846601	20846612	TTAAATATTTGATGAGGCAAACTCT	AACCTTGCATTGGTTATGTGTG	279
Ad02A5986	Aradu.A02	5986	p2	(TA)10	20	20856702	20856721	AAAAAGTTAGTCACCAATGTGTTTTT	GGAAAAAGTTACCTTAATTTCCACC	255
Ad02A5989	Aradu.A02	5989	p3	(CCT)6	18	20860486	20860503	ATGGCTGAATCTGATGGCTC	CGGTGAAGTGCAACTAGCAA	259
Ad02A5990	Aradu.A02	5990	p2	(TA)8	16	20862956	20862971	AAGCACGCATCTCCAAGAGT	TAGATGCAGTGGCGATGTTC	269
Ad02A5991	Aradu.A02	5991	p4	(CTTT)5	20	20872334	20872353	TTCACTTTGGGGCATTTTGT	TTGGGAATTGGGTACATGCT	229
Ad02A5992	Aradu.A02	5992	p2	(GA)6	12	20875648	20875659	AACCTGGAGCAGAGCAAAAA	ATTTTCCTCAATGTACGCGG	251
Ad02A5993	Aradu.A02	5993	c	(ACT)5attacta	106	20910626	20910731	GCTAAGCCATGTTTCTTTTCC	TCCCTCATAAACTTGTGATTGC	188
Ad02A5994	Aradu.A02	5994	c	(T)10caattaagt	131	20913118	20913248	TCTGTTGGTCCCTATAGTTTCG	AATTGAGTCCTTGCACCAAAA	233
Ad02A5996	Aradu.A02	5996	p3	(CTT)7	21	20931373	20931393	GTTTCGTGAGGGAAGGTTGA	TTCTTTGTAACACGGGGAGG	238
Ad02A5998	Aradu.A02	5998	p3	(TAT)6	18	20937828	20937845	AAGAGACGCCCTTGCAGATA	AGAAGCAACGGTAGCCAAGA	240
Ad02A6003	Aradu.A02	6003	p2	(AG)17	34	20948159	20948192	CGTTCTTTGCCGTTGATTCT	AGCACGCTCGTTCTCTCATT	277
Ad02A6009	Aradu.A02	6009	p4	(TATG)19	76	20988101	20988176	TTTGGCTGATTCCTGAAACC	CGGAACTGCTCCCAAAAATA	253
Ad02A6011	Aradu.A02	6011	p2	(TA)8	16	20990977	20990992	CGCCAACACTACGAGCACTA	CCCACAGCTTATGGTAAAATTGA	205
Ad02A6014	Aradu.A02	6014	p2	(CT)18	36	21013488	21013523	ACAGGATTCCCACAAGATGC	CGCCTCTGAATTGTGGTTTT	273
Ad02A6019	Aradu.A02	6019	p3	(TAA)5	15	21017280	21017294	TGTCGATCTCTCATATCTCCG	CGTGAATCGGGTAACAAAGA	278
Ad02A6020	Aradu.A02	6020	p2	(TA)8	16	21019573	21019588	TTGGGTAATAAAGATTTACACGGA	TCGAAGAAATAACATACTCATTCATT	280
Ad02A6022	Aradu.A02	6022	c	(TGT)20tctgtta	148	21021831	21021978	TCCCTCACTTTATCACGTTGAA	ATCAAACAGAAGCAGCAGCA	279
Ad02A6029	Aradu.A02	6029	p2	(AC)7	14	21030466	21030479	GGGTTGAACCGAGCCTAAGT	CATGCAAATTGGAGCACTGT	194
Ad02A6030	Aradu.A02	6030	p3	(GAA)5	15	21041969	21041983	CTAGAGGTTTCTCCGGCCTT	AAATGACCGAACCCTAACCC	259
Ad02A6032	Aradu.A02	6032	p3	(TTA)6	18	21066265	21066282	TTAAAGGCGTGGGTCTTTTG	CCAAAACTGATTCCCATCGT	141
Ad02A6033	Aradu.A02	6033	p2	(TG)8	16	21074535	21074550	TCCCCTTCCCTAATTCTGCT	CTAACTCCCCTAACCCACCC	158
Ad02A6040	Aradu.A02	6040	p3	(TTA)7	21	21103681	21103701	GTGCTGACCGGTTTTCTTTT	AACGGTCTGCACAAGGTAAA	218

ID	Chromosome	SSR nr.	SSR type	SSR	Size	Start	End	FORWARD PRIMER (5'-3')	REVERSE PRIMER (5'-3')	Product (bp)
Ad02A6042	Aradu.A02	6042	c	(T)11acgatgtg(	79	21105052	21105130	GTTGTGCTGACCGGAATTTT	AAACCGATCATCTCAACGAAA	201
Ad02A6043	Aradu.A02	6043	p3	(TCT)5	15	21116800	21116814	CCCAAAATGGCTGAAACCTA	GTGCCATTGATGGTGAATGA	241
Ad02A6046	Aradu.A02	6046	p3	(GGA)5	15	21177287	21177301	AAAAAGAGAGTGCAAGGCCA	CAGATTCCTTGGGTTCCTCA	143
Ad02A6047	Aradu.A02	6047	p3	(GAA)6	18	21182165	21182182	GCTTTTGTCTTCCCTTTCCC	CCTTGGCCGAAACTACACAT	199
Ad02A6050	Aradu.A02	6050	p2	(AT)6	12	21195902	21195913	TGAAAAGATTTTGATTTGATTTTTG	TGCATGCATCATGTGTTTTG	255
Ad02A6052	Aradu.A02	6052	c	(T)10atttttatga	60	21207426	21207485	GCATGCAATTGACACCAAAC	GGGGATTGTTCGAGTATGGA	239
Ad02A6054	Aradu.A02	6054	p2	(CA)6	12	21220251	21220262	AACGGCAAGCAAAACCTAGA	AGGTGCGCACTTCAATTCTT	151
Ad02A6055	Aradu.A02	6055	p2	(TC)6	12	21222543	21222554	GCAAACTTTTGGGCAAGGTA	TCCCTTGACTTTCCTTTGCT	128
Ad02A6059	Aradu.A02	6059	p2	(AG)6	12	21283590	21283601	CGGAAAAGCACACCAAACTT	CTTTTCGAAATCTCCCCTCC	149
Ad02A6062	Aradu.A02	6062	p3	(AGA)5	15	21330870	21330884	CTAGAGGTTTCTCCGGCCTT	CACCCATTCATAACCGAACC	270
Ad02A6070	Aradu.A02	6070	p3	(AGA)5	15	21394886	21394900	TGACTCAGAGGTGGAAGCAA	CCTCTCTCCCTCTCCTCCAT	191
Ad02A6073	Aradu.A02	6073	p3	(ATT)14	42	21438768	21438809	TGAGGTGATGTTTGAAAAGGAA	TACCTTTCAGCGGCTTGATT	274
Ad02A6076	Aradu.A02	6076	p4	(TTTA)5	20	21443945	21443964	ACGCAAAGATCAAGGGTCTG	GTAGACTCCCGAACCCAAAA	240
Ad02A6077	Aradu.A02	6077	c	(A)10cttattgtct	39	21444365	21444403	AGGAGTGTGTGGGTGTGTTG	GGCAGGTCAATGACTAATCG	280
Ad02A6079	Aradu.A02	6079	p2	(TA)8	16	21490385	21490400	TTCCACCCGACTTGAGTTTT	CCACACATAAATACATGGCGA	271
Ad02A6082	Aradu.A02	6082	p2	(AG)16	32	21507043	21507074	CTGTGGGATTCGACCTCACT	TGCTGCATTGTCCCAATTTA	275
Ad02A6083	Aradu.A02	6083	p2	(TC)6	12	21525201	21525212	TTGGCTTTTTCTGCTTGCTT	GAGGAACATCCACTTCCCAA	266
Ad02A6084	Aradu.A02	6084	c	(T)10catgtacta	33	21532896	21532928	TCCCCAATTAGAGGTGTCCA	TGATGTGGTGGCAATACTTTG	278
Ad02A6085	Aradu.A02	6085	p2	(GT)6	12	21538388	21538399	TTGGAGTGTGAAGATGCTGC	GGAAAAGGAGTAGCGTGTGC	236
Ad02A6088	Aradu.A02	6088	p3	(GAA)5	15	21572285	21572299	CCCCTAATGAAAGTGGGGTT	GGAGGATACCCGGAGAGCTA	179
Ad02A6089	Aradu.A02	6089	p3	(TTC)5	15	21590214	21590228	TCCATCTCCCATCACTCTCC	CGACCTTAGGTGCCATTGAT	148
Ad02A6090	Aradu.A02	6090	p3	(GAA)10	30	21600007	21600036	ATGCTTTTACCGCACCAAAC	AATCCCTTTTCCACCCAAAC	246
Ad02A6091	Aradu.A02	6091	p2	(AG)6	12	21600247	21600258	GTTTGGGTGGAAAAGGGATT	TAAATCCTTGACACCTCGGG	181
Ad02A6099	Aradu.A02	6099	p2	(AT)6	12	21755783	21755794	ATCCGACTAGTTCCGGGTTC	TCTGTTGAAAATGCTTTTATTCATCT	265
Ad02A6101	Aradu.A02	6101	p2	(AT)9	18	21757090	21757107	CGTTCGCCCATCAATCTAAT	CCCCGTTGCTACTTGGACTA	248
Ad02A6102	Aradu.A02	6102	p2	(AT)6	12	21760481	21760492	ATCCGACTAGTTCCGGGTTC	TCTGTTGAAAATGCTTTTATTCATCT	265
Ad02A6107	Aradu.A02	6107	p3	(AAT)17	51	21795091	21795141	TTCATCACACATTAAGGAGGAAAA	CTCTCTAACTGCCCCAGCAC	233
Ad02A6108	Aradu.A02	6108	p2	(GT)6	12	21796854	21796865	TCATGAAACCACTCATCCCA	AGGAAGGCCATGATCAAGAA	277
Ad02A6110	Aradu.A02	6110	p2	(TC)6	12	21807998	21808009	ACTTTGCATCTGCTTCTGCC	AAAGAGGTGGAGCAATGGTG	172
Ad02A6111	Aradu.A02	6111	p4	(TTTA)7	28	21809371	21809398	TTCTTCACTCTCTCTCTTCCGT	AAAATTTGATCAGAGTCTCGTGC	232
Ad02A6112	Aradu.A02	6112	p2	(GT)6	12	21814540	21814551	CAAGGGTTTCTCTTCTCCCC	AATTTTAAAGGTTTGGCCCG	173
Ad02A6113	Aradu.A02	6113	p4	(TTAT)5	20	21831127	21831146	AAAAGAAGCCTCCCATGTATAA	AAAAAGTTTGGAGTGTGTTGTAAA	280
Ad02A6114	Aradu.A02	6114	p2	(AT)8	16	21833000	21833015	CAGCGTTTTGGTGAAGAGGT	GATCTAGCTCCAATGGCTGC	202
Ad02A6116	Aradu.A02	6116	p2	(CT)12	24	21833510	21833533	AGCGACATTGTAGGGGTCAG	TGCGAATTTGAATTGAACCA	121

ID	Chromosome	SSR nr.	SSR type	SSR	Size	Start	End	FORWARD PRIMER (5'-3')	REVERSE PRIMER (5'-3')	Product (bp)
Ad02A6117	Aradu.A02	6117	p2	(AT)7	14	21834390	21834403	TTGCTGCTTGTTACCTGAGC	CAATGCAGAGTGTCACAACAAA	279
Ad02A6119	Aradu.A02	6119	p2	(CT)7	14	21844314	21844327	GGTTCTTCGGCAACAAACTC	AAGCTGGCGTAATACATGGG	263
Ad02A6123	Aradu.A02	6123	c	(A)10catatgcag	68	21872164	21872231	CGAGGCTGGAGAACTGAATC	TGCTTGAGAGTGATCAAAGGC	229
Ad02A6133	Aradu.A02	6133	c	(T)11gagatatct	49	21932726	21932774	TGTAAACATAGATACTTGGTGGTTGA	CAGCAACAAAACCAATGCAC	135
Ad02A6137	Aradu.A02	6137	c	(A)11catatgcag	69	21951029	21951097	CGAGGCTGGAGAACTGAATC	TGCTTGAGAGTGATCAAAGGC	229
Ad02A6140	Aradu.A02	6140	c	(T)10aatgtgagt	68	21959108	21959175	TGAAAATGGGTTCTTGATGCT	CGAGGCTGGAGAACTGAATC	274
Ad02A6146	Aradu.A02	6146	p2	(AC)10	20	21982630	21982649	TGAACCAGTCCGAGACAAAA	TTGGTCCTAAGAATGGCTGG	246
Ad02A6148	Aradu.A02	6148	p4	(CAAT)5	20	21985685	21985704	AAGCCTTAACTGTTTTCTCCCA	CGACAAGTGCATCAAGTCGT	223
Ad02A6155	Aradu.A02	6155	p3	(AGT)7	21	21996506	21996526	TGGGTTCTGACTTGTGACCC	TAGAACACTCGTAACCGCCC	166
Ad02A6160	Aradu.A02	6160	p3	(AAG)10	30	22007738	22007767	GAGGAGGAGGAACAGTGGC	CTTGAGGGAGAGAGTGACGG	267
Ad02A6163	Aradu.A02	6163	c	(A)10gaagaata	85	22016126	22016210	CATGAGACAGCTTCTCGTTAAAA	CACATTCCTCCTTCTTCTCTTCA	181
Ad02A6164	Aradu.A02	6164	c	(T)10ct(A)10	22	22016703	22016724	TCTCCCAAATCCAATTTCCA	CCTTCAAGCATTGACGTGTG	246
Ad02A6166	Aradu.A02	6166	p2	(AC)16	32	22019555	22019586	ACTCAGTCCGCGAAGTCAAG	GCTCTCTTTCTCTCTCCGCA	172
Ad02A6167	Aradu.A02	6167	p2	(AG)8	16	22021466	22021481	TTTTGACCCAGTTTTCGACC	GGAACATTGAACCTGTGATTGA	227
Ad02A6168	Aradu.A02	6168	p3	(AGA)6	18	22024264	22024281	CGCGAAAAAGAAGGAGAAGA	AAAACCGCTTTTGTCCAATG	164
Ad02A6170	Aradu.A02	6170	p2	(TA)6	12	22030992	22031003	CAATGGTGGGTGATTTTTGA	TCCTTCCCCCTTTTCTCAAT	247
Ad02A6175	Aradu.A02	6175	c	(TA)7(TG)15	44	22049272	22049315	AACGAGGTTTGAGAGGAGACC	CAACAACTAACACTAGCAAGCGA	173
Ad02A6176	Aradu.A02	6176	p2	(AT)7	14	22049721	22049734	TCGAGTTTGAATTTCGTGTTG	GCTTTGATGGGGCTGAATAG	180
Ad02A6178	Aradu.A02	6178	p2	(GA)8	16	22051568	22051583	TCAGAGAGACCCCTTTGTTGA	GAATTTGGGGCATCTCATTG	276
Ad02A6189	Aradu.A02	6189	p2	(CA)8	16	22076061	22076076	TGGCAGGTGTAACAAACCAA	CTTCTTCTTGGCCAGGTTGT	253
Ad02A6190	Aradu.A02	6190	p2	(CA)6	12	22076652	22076663	GGTGCATGGGAACACACATA	TTGGCCAAGTTGTTGATAAGG	265
Ad02A6191	Aradu.A02	6191	p2	(TC)18	36	22077199	22077234	AAATCAAAATCATCTTTAGCGTTTT	TGGTGGAGGTTTAGACGTGG	205
Ad02A6192	Aradu.A02	6192	p2	(TC)20	40	22077779	22077818	GGAGAGAGAGCAGCAGCAGT	TACCATCGCCGAGCAATAAT	238
Ad02A6196	Aradu.A02	6196	p4	(TTAT)6	24	22082090	22082113	TTAGTGAAAGAGCGGGGATG	CTGTTGTCTCCTTCGCTGTG	230
Ad02A6202	Aradu.A02	6202	p3	(GTG)5	15	22092239	22092253	GAGACGAGGGCATACTGACG	CCAGAAACATAGACGCAGTGG	276
Ad02A6203	Aradu.A02	6203	p2	(TA)8	16	22099159	22099174	GCGTTGCGCTTTTTAGTTTC	GTGGCGTGGTATTACGACCT	124
Ad02A6204	Aradu.A02	6204	p3	(TTA)5	15	22112424	22112438	TGTTGCTGAAAAATACCACCA	TGCAAACTTGCACTATCCGT	242
Ad02A6213	Aradu.A02	6213	p3	(ATT)11	33	22189958	22189990	TGAGTCATTATATTTGTTGAGATTTTG	GGGTTGTAAAATACCGCACC	272
Ad02A6215	Aradu.A02	6215	p2	(GA)6	12	22206929	22206940	TTAGAGGTTTCTCCGGCCTT	CCCTAAATGACCGAACGCTA	261
Ad02A6216	Aradu.A02	6216	p4	(AATA)5	20	22214337	22214356	AACGAACGAAAGTTTGGAAAAA	ATAACCCTTTCCCCCTGTTG	133
Ad02A6217	Aradu.A02	6217	p2	(TA)6	12	22215999	22216010	GCTGCCATCTCCTTTGATTC	CTATGCATGGAAGCCACCTT	279
Ad02A6219	Aradu.A02	6219	p3	(TTC)5	15	22244578	22244592	TTGTCATTTTCTCCCTTCGC	ACATGCAGTCATTGTGGTGC	176
Ad02A6223	Aradu.A02	6223	p2	(AT)8	16	22270996	22271011	GCAACAGACCCAATAAGTTCAA	TGAGCGTTACAACATCGTGA	280
Ad02A6224	Aradu.A02	6224	p2	(TA)6	12	22272023	22272034	TTGCGAAGTTAAAACCGGAC	TCTCGCTGAGGTATTCTGGG	190

ID	Chromosome	SSR nr.	SSR type	SSR	Size	Start	End	FORWARD PRIMER (5'-3')	REVERSE PRIMER (5'-3')	Product (bp)
Ad02A6225	Aradu.A02	6225	p2	(AG)6	12	22275562	22275573	TGGAGAAGGAGATCTTGGGA	AAATCGAACGGCATCAAATC	251
Ad02A6226	Aradu.A02	6226	p2	(CA)20	40	22278932	22278971	GAGCCTTTGCGAATACGAAC	TCACAATTTTGCGCTACCAA	161
Ad02A6227	Aradu.A02	6227	p2	(AG)6	12	22282093	22282104	GGGAAGAGGTGTTGAGGTGA	CAGAAAAGGGCAAGAAGCTG	218
Ad02A6228	Aradu.A02	6228	p2	(AT)6	12	22300019	22300030	TTTAAACCGCAATACCGCTC	GGATATGGGCGCAGAAGTTA	274
Ad02A6229	Aradu.A02	6229	p3	(CAT)5	15	22317697	22317711	TAGATTTCGGCTGACTGGCT	GATCAGGTCCGAGTTCTTGC	196
Ad02A6230	Aradu.A02	6230	p3	(GAT)7	21	22321876	22321896	GGCCATCTCATAGGCAGTGT	AAGAAGTCGGGACAGCAGAA	208
Ad02A6234	Aradu.A02	6234	p2	(TA)6	12	22370710	22370721	AAATGAAAGTGACATGTGTAACGA	ATTCTCTTTGGCCAGAGCCT	108
Ad02A6235	Aradu.A02	6235	p4	(TTAA)5	20	22380391	22380410	TTTCGAAAATCAACACCAAAA	CCATGATGGAGAAAGGGAGA	215
Ad02A6238	Aradu.A02	6238	p2	(CT)6	12	22421036	22421047	AAAGGAACATTGAACCTGGAAA	CGTGCAGAAATTGCAGAAAA	261
Ad02A6240	Aradu.A02	6240	p3	(CAT)5	15	22431192	22431206	GACTCCAGCTGACCGACTTC	TGTCTGAGCTGACTATGCCG	267
Ad02A6243	Aradu.A02	6243	p3	(TCT)5	15	22452329	22452343	CTTTCTCACCGCCATCTCTC	ATAAACGACGACAGGGTTGC	275
Ad02A6252	Aradu.A02	6252	c	(T)10attaaacct	73	22497750	22497822	TGCATCGTTTTACACGTTATCC	TCATCATTGTCATCCCGCTA	157
Ad02A6253	Aradu.A02	6253	p3	(TTA)6	18	22498467	22498484	AATCCAACCGTACTCGATGAA	CCTGATTGTTACCTAACGGGA	278
Ad02A6255	Aradu.A02	6255	p3	(TTC)5	15	22503759	22503773	GCAACGGTTAGGGTTTTTCA	ACCCCGAATCCTCACATTCT	280
Ad02A6259	Aradu.A02	6259	p2	(AG)6	12	22533384	22533395	AAGGGTGTTGAGGTGATTGG	CAGAAAAGGGCAAGAAGCTG	214
Ad02A6260	Aradu.A02	6260	p2	(GT)6	12	22542799	22542810	ATCTCCTTCCACTGGTGGTG	ACCCTAACCCATCAACCCTC	178
Ad02A6261	Aradu.A02	6261	p2	(CT)6	12	22560572	22560583	GCTTCTATGGCAGTTCTCGG	CTTATCACTGTCGGCAAGCA	127
Ad02A6262	Aradu.A02	6262	p2	(GT)7	14	22560993	22561006	ATCTCCTTCCACTGGTGGTG	ACCCTAACCCATCAACCCTC	176
Ad02A6263	Aradu.A02	6263	c	(CT)11(CA)7	36	22563000	22563035	CCCAAAACAAACCTTAACCC	CTTGTTCGACAGAGATGGCA	227
Ad02A6265	Aradu.A02	6265	p2	(TC)8	16	22579093	22579108	GAAGCAATAAAACACCCTTCTCTC	TTATGAGGGGATGTGAAGCC	221
Ad02A6267	Aradu.A02	6267	p3	(TTG)7	21	22580184	22580204	TAGATGGACAGGGCACAACA	CCTGCATCCTGCTTCAATTT	241
Ad02A6269	Aradu.A02	6269	p3	(ATT)7	21	22591926	22591946	GCAAGTGTAATTCACACCGCT	TGGGCCTTTATCTTCGTTTG	185
Ad02A6270	Aradu.A02	6270	p3	(AAT)20	60	22592393	22592452	TGGACCTCACCTTCATACCC	TGGAAACTTTGAGTTGCACG	229
Ad02A6271	Aradu.A02	6271	p3	(ACA)7	21	22594887	22594907	TGATTGAGACCCATGTGGAA	ACATGACAGAGCACAATGGC	223
Ad02A6273	Aradu.A02	6273	p2	(TA)6	12	22601459	22601470	CCCCTTGATTATTCTTTTTCCC	CAAGTTGTGGACACATTTTGAG	275
Ad02A6275	Aradu.A02	6275	c	(GGT)5taacag	103	22609420	22609522	TTTTGGGAACAACAATAACAACA	CCACAATTTCAGTTTTCAGATCC	274
Ad02A6277	Aradu.A02	6277	p2	(CT)15	30	22613867	22613896	AGTAACAGCAACCCCTCCCT	ACCACATCTCCATCCGCTAC	150
Ad02A6280	Aradu.A02	6280	p2	(TC)7	14	22620242	22620255	TCCTAAATATTTTCGATTGCATCA	AAACTGATGCACAAGTGGTCC	266
Ad02A6288	Aradu.A02	6288	c	(TA)11ttttgggt	69	22635866	22635934	CTCTCCAATGACAGCAGCAA	GGAAATTACATAATCCAAACCAGC	225
Ad02A6291	Aradu.A02	6291	p2	(GA)7	14	22646129	22646142	AGGTCGCTGCAAGGAAATAC	TGGTGGAAGAAATCGAAAGC	202
Ad02A6292	Aradu.A02	6292	p2	(CT)7	14	22646914	22646927	TGTGGTTCATGCCATTGTTT	CTCCGTTTTGGACATGGATA	223
Ad02A6296	Aradu.A02	6296	p5	(TTGGT)5	25	22658856	22658880	TCTTGGTTTGGGTTCTGCTC	TTTTCGATTCGGTTTGGTTC	181
Ad02A6300	Aradu.A02	6300	p3	(AAT)6	18	22697720	22697737	CCATTGCCACTGGTATCGTA	GCTGAGAAATCATCAGCTATCAAA	280
Ad02A6302	Aradu.A02	6302	p2	(AC)8	16	22714935	22714950	AATACGAATCCTCAACCCCC	TTAGTTCGATCGCCCTCACT	193

ID	Chromosome	SSR nr.	SSR type	SSR	Size	Start	End	FORWARD PRIMER (5'-3')	REVERSE PRIMER (5'-3')	Product (bp)
Ad02A6308	Aradu.A02	6308	p2	(TC)26	52	22744550	22744601	TTCTCGATTCTCCGATGCTT	ATCTCTTCAATCTCAGCGGG	145
Ad02A6316	Aradu.A02	6316	p2	(TA)8	16	22853221	22853236	TCTGTTCTCGCAAATCAGGA	TGGCCTCAGGACTTAGATGC	255
Ad02A6317	Aradu.A02	6317	p3	(TCA)6	18	22854726	22854743	CGCTCGATTCTCTCTTGCTT	ACCCAATTAAGGCCACAACA	261
Ad02A6318	Aradu.A02	6318	p3	(AGG)5	15	22861783	22861797	TAGCAATTGCCAACATGGAA	GCGACACTGTGTTTCAGCTT	265
Ad02A6319	Aradu.A02	6319	p2	(CA)7	14	22862301	22862314	GTGACCCGTGATGTGAAGTG	ATGGCGAGTGAGAACAGGAT	139
Ad02A6321	Aradu.A02	6321	p4	(TTTA)5	20	22881900	22881919	CGAAAATATCTCATCCTTTTTCAA	GAGTGAAGAAGAATGGGTAAGGA	235
Ad02A6322	Aradu.A02	6322	p3	(TTC)6	18	22885300	22885317	TCCTTCTTGGCCGAATACAC	GCTTTTGTCTTCCCTTTCCC	200
Ad02A6323	Aradu.A02	6323	c	(T)15atttttttata	99	22928293	22928391	ATCTTTCTGATCCACCTGCG	GCCGAAGACTGAGCTAGTGG	271
Ad02A6329	Aradu.A02	6329	p4	(AAAT)5	20	22977822	22977841	GCTTTAGTGATTTAAAGAATAATGAGG	GGGACACTAGAGTAATTAAAAACCAA	231
Ad02A6330	Aradu.A02	6330	p2	(AC)8	16	22978030	22978045	CTCACCATCACTCACCCTCA	CACAGCAAGACGCAGTGAAT	158
Ad02A6331	Aradu.A02	6331	p2	(GA)10	20	22978198	22978217	CATTCACTGCGTCTTGCTGT	CGAGCTCGCAGTAGTAGCCT	202
Ad02A6332	Aradu.A02	6332	p2	(AT)9	18	22987822	22987839	CAGCGCACAGTCGAATTTT	TTTCTGGGCACCAGATTAGC	237
Ad02A6333	Aradu.A02	6333	c	(AC)12(TC)15	54	22990439	22990492	AAAACTCATGGGAGGTCACG	GGCTGAAAGAGGTGTGTGGT	124
Ad02A6334	Aradu.A02	6334	p2	(AT)13	26	22992880	22992905	GGCTTGGACCAATGAGAATG	TTTCCTGATCAGCTTAGAGTTGC	267
Ad02A6335	Aradu.A02	6335	p3	(ATA)5	15	22993026	22993040	GCAACTCTAAGCTGATCAGGAA	ATTTATCCAGTTTGGCCCGT	278
Ad02A6336	Aradu.A02	6336	p2	(CA)20	40	23011911	23011950	AGCTACAAAACAGCCCCTCA	CTGGAGCTTATTGGGCTTGA	273
Ad02A6337	Aradu.A02	6337	p3	(TAA)15	45	23033176	23033220	GCAAAAGTCTATTATGGACAATTTTA	CCAAACACGGAAATAAGACGA	274
Ad02A6343	Aradu.A02	6343	p2	(TA)7	14	23042459	23042472	TTGATAGAATTGTAATTAGCAGCCA	TTTCCATTGAATTAATCATAAAGGTT	242
Ad02A6344	Aradu.A02	6344	p3	(TAT)9	27	23044112	23044138	CAATTTCGATGAAAAACGAAGA	TTGTTATGTTAATGGTGACCCA	250
Ad02A6348	Aradu.A02	6348	p2	(AT)12	24	23099246	23099269	CCCTAAAATTGATTCCCCGT	GGAATTTATTGACGAAGCGG	272
Ad02A6349	Aradu.A02	6349	p2	(TA)7	14	23099862	23099875	CATTCTCTTTGAAGAGGGATCA	CCTTTCCGTGTGGTGAGATT	191
Ad02A6352	Aradu.A02	6352	p2	(TA)10	20	23118082	23118101	AGTCCTTGGGTGTGTTTTGG	AAAATGAAAAGCGCAATGCT	258
Ad02A6353	Aradu.A02	6353	p5	(AAGAT)5	25	23125716	23125740	AGATAAAGGCGGTTCCCCTA	CCTGGGTGCTCCAGTGTATT	126
Ad02A6354	Aradu.A02	6354	p5	(AAGAT)5	25	23161206	23161230	AGGCGGTTCCCCTAAAGATA	CGACTTGTTGGACTTGGTGA	251
Ad02A6355	Aradu.A02	6355	p2	(TA)8	16	23163156	23163171	GCGTGACACTTTTACCATGC	TTTTGCTCAGCTCCCTCAAT	256
Ad02A6357	Aradu.A02	6357	c	(TA)14gcttactc	129	23185728	23185856	GGATGGGAAGTAGCCATTGA	CCTGACTCAGCCTACGAAGC	270
Ad02A6359	Aradu.A02	6359	p2	(GA)6	12	23193292	23193303	CCCTTCCCTCTCTTCCAGTC	AGAGAGAGGACGAAGATGCG	245
Ad02A6360	Aradu.A02	6360	p2	(GT)6	12	23193617	23193628	CGTCCTCTCTCTCCATCTCG	TTCCCTAACACACCCACACA	277
Ad02A6364	Aradu.A02	6364	p2	(GC)6	12	23236329	23236340	TGGCACAACTCTCTGGAAAA	GTTTGGAATGCTTGGATTGG	195
Ad02A6371	Aradu.A02	6371	p3	(ATA)6	18	23347154	23347171	GGCCCATTAAAAGGTGTCAA	TTCAGTCCAGGGAAAGACAGA	114
Ad02A6372	Aradu.A02	6372	p2	(TG)6	12	23357021	23357032	TTCCTGTAGAGCCTGAGGGA	AAAGTCCCAAATGCATGCTC	129
Ad02A6373	Aradu.A02	6373	p3	(CAA)5	15	23366911	23366925	TGCACCTAGAGGTCCGTCTT	ACAATTGTAGCACAAGCCCC	178
Ad02A6376	Aradu.A02	6376	p3	(AGA)7	21	23384838	23384858	ACTCGGAGGTGGAAGCTTTT	TGGCCGAACCTACATCTCTC	210
Ad02A6379	Aradu.A02	6379	c	(TCT)5tcccttac	42	23404522	23404563	GCGCTAGTAGCACAAACACG	AGAGAGGGATGATGGGGAAT	277

ID	Chromosome	SSR nr.	SSR type	SSR	Size	Start	End	FORWARD PRIMER (5'-3')	REVERSE PRIMER (5'-3')	Product (bp)
Ad02A6382	Aradu.A02	6382	c	(TA)11tgtatgta	122	23441584	23441705	GGTTGTTTTTGGAGAAACAGGT	CCAAAAGCGAAAACAGGAAA	254
Ad02A6385	Aradu.A02	6385	p2	(TA)9	18	23452668	23452685	AGCATCACTTATGGTGTGTTGA	TGAACTATAAGTTTGCTCCTGGAA	248
Ad02A6386	Aradu.A02	6386	p3	(TAA)8	24	23460928	23460951	TCAAAAATTTAATTCGCTGAAAA	AGGTTCAGGTTCGGATTCAA	280
Ad02A6388	Aradu.A02	6388	p2	(GA)6	12	23461459	23461470	TTTGCAATAAGCCAAGGAGG	ATTGTTGAGCTGGTCATCGT	267
Ad02A6389	Aradu.A02	6389	p3	(ATA)11	33	23462092	23462124	AAGTGGAGGATGATCGGTACA	GCATACAACTGAAGGCCTCTT	199
Ad02A6392	Aradu.A02	6392	p2	(TA)9	18	23484747	23484764	TCGAAAATATCTTTAATCAACGTCA	TCACCTTCGATGAGAAACGA	224
Ad02A6393	Aradu.A02	6393	p2	(AT)6	12	23486108	23486119	TTGCACGATTGTGGAATAAA	CCTCACTTTAATTAGTTGGGAGAAA	279
Ad02A6394	Aradu.A02	6394	p3	(ATA)10	30	23486906	23486935	TTCTCGTTCATTGTGCAATAATTT	TGGGTAGACAATGTTTGGCA	141
Ad02A6395	Aradu.A02	6395	p2	(GT)7	14	23490809	23490822	TGGAAATATTACCTAATTATTGTCGTG	AGACGTCTTGCTCTTGACAAA	279
Ad02A6399	Aradu.A02	6399	p3	(AGA)7	21	23541636	23541656	TGCCATTGATGGTAGTGGAA	TCACAAAACAAAACCCCCTC	179
Ad02A6400	Aradu.A02	6400	p2	(AT)9	18	23551525	23551542	CCTTGGTGTTTTTCCTCCAA	GAAAGGGAGATGATGTGGATTT	280
Ad02A6403	Aradu.A02	6403	c	(TG)13ta(TG)1	187	23581551	23581737	GGAAAACAAACCTTTATAAGGGAAA	TGATCTATCATCGTACCTGTTGG	268
Ad02A6405	Aradu.A02	6405	p3	(CTT)5	15	23588337	23588351	AAGAAGAAGAAAAAGAAGCAGCA	GAAAATGGTGATGATGATGGAA	212
Ad02A6408	Aradu.A02	6408	p2	(CG)6	12	23624240	23624251	AGAGTTATGCCTACGCCACG	TGGTGTTTGGAAGTGTTGGA	209
Ad02A6410	Aradu.A02	6410	p2	(TC)16	32	23636116	23636147	TTATCGGCAGGTTTACCGTC	GAGATGGGCTGTGACTTGGT	269
Ad02A6412	Aradu.A02	6412	p2	(GA)6	12	23658179	23658190	AGATGATTCGCCTCCCTTTT	TAAGAACTTTCGCGACCCAC	248
Ad02A6413	Aradu.A02	6413	p3	(ATT)5	15	23664723	23664737	CAAATTTGGACGTTGGCTTT	TGGATTCACAACCCAAGGAT	197
Ad02A6415	Aradu.A02	6415	p2	(TA)8	16	23671972	23671987	GCATGCAGAGCTCAAGAACA	TGAGCTTAACCCTTCATTAGGC	262
Ad02A6421	Aradu.A02	6421	p2	(AT)8	16	23756472	23756487	TCGTCGATAAATCTAACGATACTCA	AAAATGAAATGCAGAAACAAAGA	215
Ad02A6422	Aradu.A02	6422	p3	(ACC)5	15	23757037	23757051	TTTTTGCTCGAAAATGGTCC	ATGCTGGTGATGGAGTGACA	251
Ad02A6423	Aradu.A02	6423	p3	(TGG)5	15	23757406	23757420	TCTGAGTTTGGCAGTGGATG	TCGGACCAAAATGTCCCTAC	101
Ad02A6427	Aradu.A02	6427	p2	(AT)12	24	23764069	23764092	TGACAATAGAATAACATTGAATCATTT	CAAGACTTTAACACTTGTTCTATCACG	250
Ad02A6430	Aradu.A02	6430	c	(T)11aatcagtgt	76	23771333	23771408	CAATGGGTAATGGCATTTTCA	CTCCTTAATTTTCCAAACACTCA	269
Ad02A6435	Aradu.A02	6435	p3	(AAT)14	42	23775789	23775830	TGCGAAATTTCTGCGATAGA	CCTCCTCTGCATGCGATTAT	202
Ad02A6436	Aradu.A02	6436	p3	(TTA)12	36	23776073	23776108	ATAATCGCATGCAGAGGAGG	GGACCGAGTTTATTGGCTCA	265
Ad02A6437	Aradu.A02	6437	p2	(CT)10	20	23776416	23776435	AACGTGCCTAGCAAGCCTAA	AGAGGATACTCCGACGCTCA	235
Ad02A6441	Aradu.A02	6441	p2	(CT)7	14	23813613	23813626	TGACCCACATGAAAACGGTA	CTTCTGGGCAAGTCGTCAAT	243
Ad02A6442	Aradu.A02	6442	p3	(ATT)10	30	23813781	23813810	ATTGACGACTTGCCCAGAAG	CCGCTATTCGACGTGTCTCT	272
Ad02A6445	Aradu.A02	6445	p4	(GTGA)7	28	23876007	23876034	CCTCTCCGTTCTTCCCTTTC	CCTAACCCTATCCACACCCA	166
Ad02A6446	Aradu.A02	6446	p2	(TC)6	12	23901241	23901252	GAAAAGCTCCAGAGGTCACG	TCAAAACAAGAAGAAATCCTAAATCC	253
Ad02A6447	Aradu.A02	6447	p2	(TA)7	14	23911696	23911709	TGGAAAGCTTGTGGAGTTCA	TTGCACTAAGATCAAGGGGC	228
Ad02A6448	Aradu.A02	6448	p2	(CT)7	14	23915434	23915447	AGTGAAACCCTCACCCCTCT	TTAAACCGGTTGGACTGAGC	145
Ad02A6450	Aradu.A02	6450	p2	(GC)6	12	23927951	23927962	ACAAGGCTCCTAAAACCGCT	CGGTTCTAGTTGGGTTGGAA	168
Ad02A6453	Aradu.A02	6453	p2	(TA)7	14	23934931	23934944	TTGAGTGGTGAATTAGGCCA	AAAGGAACTGGAAGCAGGGT	152

ID	Chromosome	SSR nr.	SSR type	SSR	Size	Start	End	FORWARD PRIMER (5'-3')	REVERSE PRIMER (5'-3')	Product (bp)
Ad02A6454	Aradu.A02	6454	p2	(CT)6	12	23939125	23939136	TCCACTCCACCGACTATTCC	TCCAACACTGCAAGTTGCTT	235
Ad02A6461	Aradu.A02	6461	c	(AAT)19gtaca	118	23984924	23985041	GACCTTGGCCTTATCGTTGA	TGAAAAATTAAAAACATTGGAAGGA	216
Ad02A6463	Aradu.A02	6463	p2	(AT)7	14	24014184	24014197	CTTGCTTTGATGGGAGGTGT	TGATTCAGTCTTCAATTTTCTTTGA	265
Ad02A6468	Aradu.A02	6468	p2	(AG)6	12	24071508	24071519	CATTTCAACCCAGTTTTCGG	ACATGAATGTCATTAAGCCAAA	280
Ad02A6470	Aradu.A02	6470	p2	(AT)6	12	24102822	24102833	TCCGCATAATTAAACCTTCTCTTT	CCGCATATCCGCACAATAAT	261
Ad02A6474	Aradu.A02	6474	p2	(AT)10	20	24123512	24123531	TTCCTAGCCTCACTTTTGGG	CCAAACACAGCAAAGAATTTCA	272
Ad02A6475	Aradu.A02	6475	p2	(TC)6	12	24127121	24127132	TCCCCAATTAGAGGTGTCCA	TGTTGTTCATGAATGATTGGC	236
Ad02A6476	Aradu.A02	6476	p3	(GAA)6	18	24147947	24147964	GCTTTTGTCTTCCCTTTCCC	CACAACACAACCCCTCCTCT	225
Ad02A6477	Aradu.A02	6477	c	(T)10gtatttttatt	92	24152325	24152416	GCATGCAATTGACACCAAAC	GGGGATTGTTCGAGTATGGA	242
Ad02A6480	Aradu.A02	6480	p2	(TC)6	12	24158545	24158556	CACCCTCCTACCTTTCCCTC	TTTGGGGGAGTGAGAGACTG	223
Ad02A6486	Aradu.A02	6486	p3	(TCT)7	21	24195992	24196012	AAATGGCCTAAACCAAACCC	TAGAATATTGCTCGCCCTCG	185
Ad02A6488	Aradu.A02	6488	p2	(CT)7	14	24216907	24216920	CAAGCCCTTTTGGCTTTACA	GGATTCTAACCCCACTTCTCAA	187
Ad02A6489	Aradu.A02	6489	p2	(AT)6	12	24218668	24218679	TTTGAAATAGCCTGTTGGGC	AGAGACAAATGGCTGAGTGGA	149
Ad02A6492	Aradu.A02	6492	p2	(TG)9	18	24230208	24230225	TTTTGTTGGAGACAAGATTGGA	TCAATAACCCCTGGACAACC	239
Ad02A6494	Aradu.A02	6494	p4	(AAAG)5	20	24234429	24234448	GTTGGATTGGTTCATTGCCT	GGGACAGCCATCTAAGCAAA	101
Ad02A6497	Aradu.A02	6497	p2	(TC)17	34	24243212	24243245	AAAGAAAATTTGCGACCCAC	GAAAATCTTTACGCGAAGCG	265
Ad02A6511	Aradu.A02	6511	p4	(AAAG)5	20	24371240	24371259	TGATTACGGGACACTTCGTT	AGGGGTTGTTGAGTTCGTTG	136
Ad02A6512	Aradu.A02	6512	p2	(AT)6	12	24371533	24371544	ACCAATCACGTACCAAACGC	CATCTCCTGGCGAGAAACTC	269
Ad02A6513	Aradu.A02	6513	p2	(TG)6	12	24382217	24382228	ATTCGAAGAGCAAAACGTGG	CCCTTGGCCCAACATAACTA	261
Ad02A6518	Aradu.A02	6518	p3	(TAA)14	42	24439121	24439162	CACTAATGAAGTCAACGCCG	TTCAAAGCAGTCCCTTCACA	234
Ad02A6519	Aradu.A02	6519	p2	(AC)6	12	24444688	24444699	AAGGAAGCAACAAAGCCAGA	AGGGTTGGAACCAAGGAGTT	194
Ad02A6525	Aradu.A02	6525	p2	(AT)9	18	24451090	24451107	TTTGACCGAAAAATAGTAAATCAAAA	CCACTTTCTTTCATCATCTTGC	257
Ad02A6527	Aradu.A02	6527	c	(AAC)6(AAT)	36	24465258	24465293	CCGTTGGGCTTTTCTGTTAG	CGAAGTCCTAACACTCACCACA	280
Ad02A6528	Aradu.A02	6528	p3	(ATT)5	15	24472238	24472252	AAATTATTTTTCCTTTCTAATTCGTGA	TTTTTGTTGAGTATTTAATTTGTGACG	264
Ad02A6529	Aradu.A02	6529	p3	(TAT)12	36	24472466	24472501	TTTTTGTGTCGTTCAGATGAAAA	CTCTTCTCCCAAGACCCCAT	259
Ad02A6531	Aradu.A02	6531	c	(TTC)5ctcttcc(	120	24473990	24474109	AACGAATAAACCCCAAAAACG	CGTGCAAGAAGAAGAAGAACG	206
Ad02A6532	Aradu.A02	6532	p3	(CTT)5	15	24474213	24474227	AAAACGCGATGCAAGGTCTA	GAGGAAGAAGAATGTGCAGC	280
Ad02A6534	Aradu.A02	6534	p2	(TA)7	14	24481849	24481862	TCACATGGCCTTGCAAGATA	TTCCCAACAAGTAGGCATGG	194
Ad02A6536	Aradu.A02	6536	p3	(AAT)5	15	24486787	24486801	CGGATCGGATACCAAATTGT	TTTCCACATGGTGCGTAGAA	175
Ad02A6537	Aradu.A02	6537	p2	(AT)6	12	24508995	24509006	TGAAGAACAAAGCTTGCGAA	ATGCCACAAGCACATGGTTA	121
Ad02A6538	Aradu.A02	6538	p5	(TTATC)5	25	24514762	24514786	CCTGGGTGCTCCAGTGTATT	CAGCCCAAGCCTACAGAAAG	146
Ad02A6539	Aradu.A02	6539	c	(A)11ttcgaaaa	45	24539798	24539842	TGGCTCTTGAAAGAATGAGGA	CACACTCTTGTTGCCTTGGA	181
Ad02A6541	Aradu.A02	6541	p2	(TA)6	12	24563106	24563117	TCTCGTGCTGATAACGTGTTG	GGCTTAATCCTTTGAGTATTATACGG	186
Ad02A6544	Aradu.A02	6544	p2	(TA)6	12	24597809	24597820	CCCACAGTCTCCCTTTTGAA	AGCACACAACCTGTGGGATT	165

ID	Chromosome	SSR nr.	SSR type	SSR	Size	Start	End	FORWARD PRIMER (5'-3')	REVERSE PRIMER (5'-3')	Product (bp)
Ad02A6545	Aradu.A02	6545	p2	(CT)7	14	24605686	24605699	GGAACATTGAACCTGATATGAAGA	GAGTTCGGCCCAGAAATACA	200
Ad02A6548	Aradu.A02	6548	p2	(TA)9	18	24621884	24621901	CATCTAAGTGTGGGGGAGGA	CCATGCAATATGAAATGCAA	235
Ad02A6550	Aradu.A02	6550	p3	(GGC)5	15	24624167	24624181	GAAGAAGAAGCAGCACCGAC	AAATCGTCCCCGATCTTTTT	251
Ad02A6554	Aradu.A02	6554	p3	(CTC)5	15	24654144	24654158	GACGGCGATGTCTCTTTCTC	ACAGTCGCATCGTTGTCAAG	172
Ad02A6555	Aradu.A02	6555	p2	(TG)6	12	24654346	24654357	TCCTCTTGTGCTTGACAACG	TTTTCTACCCCAACTCCCCT	267
Ad02A6556	Aradu.A02	6556	p3	(AAT)6	18	24654723	24654740	GCGAACTTGCACTATCCACA	TTTCAAAATCACTAATTGTGTCAAG	239
Ad02A6559	Aradu.A02	6559	c	(A)11tattcaata	71	24674538	24674608	AAGTGTGGGAAAAGCTGTGG	TTGGGGTAATTTTGTCCCCT	253
Ad02A6561	Aradu.A02	6561	p2	(TA)6	12	24682595	24682606	GCCAGGGAGAATCAACAATG	TGAAAAGGTGTTGGATTGGG	240
Ad02A6562	Aradu.A02	6562	p2	(TC)6	12	24682733	24682744	TTAGTCGGTCGTCTCTTCGG	CCAACAGCTCAGACTCGACA	145
Ad02A6567	Aradu.A02	6567	p2	(TA)7	14	24693491	24693504	TCAGATGCAGGTCGTGAGAC	AGGTCCAAACTCGACCTGTG	264
Ad02A6569	Aradu.A02	6569	p2	(AT)7	14	24703447	24703460	TGAAGCAAGAAAAAGCAGTGAA	AAAAACAATGCCAAAAAGCG	237
Ad02A6571	Aradu.A02	6571	p2	(CT)6	12	24711520	24711531	CCTCTTTCCACCACACCATT	CGGAAAAGCACACCAAACTT	111
Ad02A6572	Aradu.A02	6572	c	(A)10tttgaaaaa	42	24717937	24717978	TTGTTGGCTCTTGAAAGAATGA	AGATTGTGACCCAGCTTTGC	249
Ad02A6573	Aradu.A02	6573	p2	(GC)6	12	24723954	24723965	CGGTTCTAGTTGGGTTGGAA	ACAAGGCTCCTAAAACCGCT	168
Ad02A6574	Aradu.A02	6574	p2	(CG)6	12	24727082	24727093	TCCATACCTTGACCCGAGAG	TGGTGTTTGGAAGTGTTGGA	252
Ad02A6576	Aradu.A02	6576	p2	(AT)7	14	24728558	24728571	TTTCCATTCTTGTGTGCATTG	TTTGATTTGGGGCATTTTGT	258
Ad02A6577	Aradu.A02	6577	p3	(TTC)5	15	24734444	24734458	CAAAGCATGTTTCCGTTGTG	CCTCGTCCTAACAAATGGGA	280
Ad02A6578	Aradu.A02	6578	p2	(AC)16	32	24735955	24735986	CGTATAACCGCCCCAAAATA	TATGGATGCAGAGGTCACGA	263
Ad02A6579	Aradu.A02	6579	p2	(TC)6	12	24739209	24739220	AGTTGGACCAGACAACACCC	CTGTGGCAGAGGAAAAGAGG	252
Ad02A6580	Aradu.A02	6580	p2	(TC)7	14	24739436	24739449	ACTGTACTTGTGGTGCGACG	CCCAGTTACCCTTTCCCCTA	182
Ad02A6582	Aradu.A02	6582	p2	(GC)6	12	24780192	24780203	GGATCGACAAAAGCGACACT	GGAATATGTGCCAGGAGTGC	198
Ad02A6583	Aradu.A02	6583	p2	(GC)6	12	24780474	24780485	GCCTGTGGCATAACCACTTT	CTGGTAGCACAAGGATGGCT	125
Ad02A6584	Aradu.A02	6584	p3	(ATC)5	15	24794159	24794173	CCAAACTCGCTGACTCTTCC	GTGGATGTTGAGGGCTGTTC	232
Ad02A6585	Aradu.A02	6585	p2	(CT)8	16	24797815	24797830	CGACCAACAGTCTCGTTCCT	TGATTAGAGGAATTGGCTTGG	214
Ad02A6586	Aradu.A02	6586	p3	(CTC)5	15	24811494	24811508	CAAAGCACTCCCTCACCATT	TGAAGATGAAACACGCGAAG	161
Ad02A6588	Aradu.A02	6588	p2	(AT)7	14	24830386	24830399	AGGCACGGACAAAACAAAAC	GGATGGTGACAGAGCAGAGG	260
Ad02A6595	Aradu.A02	6595	c	(T)10ctttgtttgt	64	24853635	24853698	AAAAGCAATATCAGATCAAAATTCG	GGAGGAAGAAAAAGAGGAGACA	205
Ad02A6597	Aradu.A02	6597	p2	(AG)10	20	24866821	24866840	ATTCCACCTCCTCCTTCCAT	GCCTTTCCACCATTTCACAT	184
Ad02A6598	Aradu.A02	6598	p2	(TC)9	18	24867312	24867329	TCGTTTCCGACGGTAAATTC	TAGAGAGAGAATGGGAGGCG	278
Ad02A6600	Aradu.A02	6600	p4	(TATT)6	24	24873220	24873243	GGGACTTAGCACAATCCGAA	TCACACCAAGTATTTGGCACA	256
Ad02A6609	Aradu.A02	6609	p2	(AT)9	18	24956417	24956434	TAAACCGCAATACCATTCCC	GGGCGCAGAAGTTATGAAGA	271
Ad02A6610	Aradu.A02	6610	p3	(TTA)5	15	24971319	24971333	GGAATTGACTAGGACCTGAGGA	CAAGGAATTGCAGGGAGGTA	269
Ad02A6611	Aradu.A02	6611	c*	(A)13(AT)6*	24	24975772	24975795	GGAAGCATGCTCATGTGAAA	AATGGGGCTAAGGTGAAGGT	228
Ad02A6613	Aradu.A02	6613	c	(A)10ttgttggct	116	25012865	25012980	TGCTTTCTGAATGAATGCTTG	CACACTCTTGTTGCCTTGGA	257

ID	Chromosome	SSR nr.	SSR type	SSR	Size	Start	End	FORWARD PRIMER (5'-3')	REVERSE PRIMER (5'-3')	Product (bp)
Ad02A6615	Aradu.A02	6615	p2	(TG)6	12	25023641	25023652	TTCCTGTAGAGCCTGAGGGA	AAAGTCCCAAATGCATGGTC	129
Ad02A6616	Aradu.A02	6616	p3	(CAA)5	15	25024627	25024641	ATCATTCCCACAGAACCGAG	TACAGCTTTATGCCCTGCCT	232
Ad02A6617	Aradu.A02	6617	p2	(TA)6	12	25034782	25034793	TTCTTTGCACTGGTGTCGTC	GGGATTGGAAGAGCAATCAA	269
Ad02A6621	Aradu.A02	6621	p5	(ATTTT)5	25	25072750	25072774	CTGGTTAGTTGTGCGGGATT	GATTCAGTTTTCAATTTGCTTTGA	272
Ad02A6625	Aradu.A02	6625	p3	(ACA)6	18	25123679	25123696	TTACCCGCTTTTCTTTGCAT	CAAAAACTTGGTGCGGATTT	229
Ad02A6627	Aradu.A02	6627	p3	(AAT)5	15	25128279	25128293	CCAACTTTTTCAGACATCATTCA	GGCGCCTAAAAGTAGCTCAA	222
Ad02A6633	Aradu.A02	6633	p2	(AG)6	12	25176034	25176045	TTTTTGGGGAAGAGGTGTTG	CAGAATTGGCACAAAGAGCA	162
Ad02A6637	Aradu.A02	6637	p3	(AAG)5	15	25201803	25201817	GCTTTTGTCTTCCCTTTCCC	GCCAAACCTACTCCCTCTCC	276
Ad02A6639	Aradu.A02	6639	p2	(GA)8	16	25208885	25208900	TGGAGAAATCCAAACACAAGG	TCAAAACCACCTAACTACTTTTCTCTC	277
Ad02A6643	Aradu.A02	6643	p3	(TTA)5	15	25245076	25245090	GGACTTTCACGCCAGGTTTA	TGCAAGAAGAAAGAGGAGGAA	168
Ad02A6644	Aradu.A02	6644	p3	(GAG)5	15	25245405	25245419	AAGAAAAAGGCGATGACGAC	TTCCTCTCCTCTCCCTTCCT	260
Ad02A6645	Aradu.A02	6645	c	(A)10gtgtatata	172	25247469	25247640	GCCTTGATCAGGCTCGTGTA	GGGATCTTGGTCATTTTAGCC	253
Ad02A6646	Aradu.A02	6646	p3	(ATA)5	15	25248147	25248161	TTTTTAAACAATTGAATCTTGATGCT	TCTTTCGCTCTCTTTTCTCTCA	269
Ad02A6648	Aradu.A02	6648	p2	(GA)6	12	25260290	25260301	TTGTGTTGGATCGTTTTCCA	CCTAGCACGTGGGAGACACT	261
Ad02A6650	Aradu.A02	6650	p2	(AT)9	18	25262448	25262465	TTAAGGCTGGCCGAAGAATA	AATTTGGAGCCATGCTTTTG	187
Ad02A6651	Aradu.A02	6651	c	(AGA)6aaagaa	45	25266468	25266512	TGCCATTGATGGTAGTGGAA	AATTCAAAACCATTTCCCCC	273
Ad02A6652	Aradu.A02	6652	p2	(TA)7	14	25288447	25288460	TCCACATTTTTCCAATCACAA	GGCGGGATTTCTATTTTGGT	256
Ad02A6656	Aradu.A02	6656	p2	(AT)10	20	25375646	25375665	TGTCAAGGTCGAATGTTCCTT	AACGTGAGTCGTGGGTTTTG	262
Ad02A6657	Aradu.A02	6657	p2	(TC)9	18	25378713	25378730	CAAAGACAGTTCGGCAGTGA	CCGCCATTCTTGTGCTTTAT	215
Ad02A6658	Aradu.A02	6658	p2	(CT)8	16	25378885	25378900	ATAAAGCACAAGAATGGCGG	AGTTGATGGAGCAGAAGGGA	110
Ad02A6659	Aradu.A02	6659	p2	(CT)6	12	25390975	25390986	TTTCTTACCCCTCATTCCCC	TCACGAAAAAGCACACCAAA	122
Ad02A6660	Aradu.A02	6660	p3	(AAT)5	15	25417234	25417248	TCGCACAACTAACCAGCAAG	ACGCCTATCTGAAATTCCCA	232
Ad02A6662	Aradu.A02	6662	p2	(AG)7	14	25420163	25420176	CCTCGAGCAATGGAAGAAAG	GGCATTTTACACGCCTCATT	258
Ad02A6665	Aradu.A02	6665	c	(A)13caagccat	50	25438498	25438547	TTGGCTCCTGAAAGGATGAG	ATGCCACAAGCACATGGTTA	239
Ad02A6669	Aradu.A02	6669	p2	(TA)8	16	25472429	25472444	AGCCGGATCTTGAGTCTTGA	GGGGTAAACAAAAATCGCAA	138
Ad02A6672	Aradu.A02	6672	p2	(TA)6	12	25484980	25484991	CCATCTGGTCTCGAAGGAAG	CACCCAAAACAAACCGTCTT	180
Ad02A6673	Aradu.A02	6673	p3	(ATG)12	36	25489891	25489926	AAGTTGTTTTGTTGCAATGTTGA	TGGTTTTGGGCAAAAGAAGA	274
Ad02A6675	Aradu.A02	6675	p2	(AG)13	26	25494643	25494668	GGTTTGGATTGGATTGGATG	GCAGGGCTCCTTTCTCTTCT	130
Ad02A6677	Aradu.A02	6677	p2	(TA)9	18	25497634	25497651	TTTCCTTTTCAGGACGAATGA	TTTCATGGTAAGTTTTGCCAG	265
Ad02A6678	Aradu.A02	6678	p4	(TAAA)5	20	25500724	25500743	AATTTTCGAATCCCTTGGGT	GGAGTAAGGGAGATGATGTGGA	251
Ad02A6679	Aradu.A02	6679	p2	(AT)8	16	25504144	25504159	TCCTTGGTATTTTCCCTCCA	TTTGGAATGTAGCAATGAAATAAA	249
Ad02A6683	Aradu.A02	6683	c	(CT)6cccatttca	65	25560037	25560101	ACCGAACTGAACCCGTATTG	GGTCTTGTAGAGCTCGTCGC	244
Ad02A6685	Aradu.A02	6685	p3	(TTA)5	15	25565352	25565366	CGTTTATGATTTGGTTTAATTTGG	CAAATGCAGTGAATATCGGAGA	263
Ad02A6690	Aradu.A02	6690	p3	(TAA)5	15	25600049	25600063	TCAAAGGAATTCAAAATTAAAGAGAAA	AAGCTCGCCATCATCTTTGT	238

ID	Chromosome	SSR nr.	SSR type	SSR	Size	Start	End	FORWARD PRIMER (5'-3')	REVERSE PRIMER (5'-3')	Product (bp)
Ad02A6693	Aradu.A02	6693	p3	(ATA)16	48	25606356	25606403	TCAAACCAACCTAAAATTAAAAAGC	TGCATATGATAGTTGTTAAATGTTTTC	247
Ad02A6696	Aradu.A02	6696	p2	(TA)7	14	25611320	25611333	CTCTGCAGGCTGTGGCTAGT	ACAGAGGTACAAAAGCGCGA	128
Ad02A6698	Aradu.A02	6698	c*	(A)10(AT)6*	21	25639225	25639245	TTGAAAACTCTGGGGTTTGG	TTTTTGTCCCCTTTCATTGC	214
Ad02A6702	Aradu.A02	6702	p3	(ATA)6	18	25698653	25698670	TGGTAACCATTTTGGCAGAT	AGAGGCATTTTGATGGAGAA	241
Ad02A6703	Aradu.A02	6703	p2	(AG)6	12	25700185	25700196	GTGGGCCAAATAGACCAAAA	ATGGCTCTTGGAATGTGGAG	190
Ad02A6708	Aradu.A02	6708	p2	(AT)11	22	25757397	25757418	GAAAAGCAATAAAAAGGGGACA	AATGGGGCTGAGGTAAAGGT	226
Ad02A6709	Aradu.A02	6709	p3	(AGA)6	18	25761954	25761971	TAAAATATTGCTCGCCCTCG	CATCCACTCTTCCCCAAAAA	249
Ad02A6710	Aradu.A02	6710	p2	(AG)6	12	25782511	25782522	TGGATCCAGAGTTACTCCCAA	AATTACAGAACACGCCCCAG	218
Ad02A6711	Aradu.A02	6711	c	(T)10cgttttctttt	47	25787652	25787698	TCCCCAATTAGAGGTGTCCA	TGTGGTTCATGAATGTTGGC	230
Ad02A6712	Aradu.A02	6712	p2	(AG)7	14	25803921	25803934	TGGATCCAGAGTTACTCCCAA	AATTACAGAACACGCCCCAG	220
Ad02A6713	Aradu.A02	6713	p3	(GAT)5	15	25804839	25804853	GAGTTGGAGTCTGTCCGAGC	GTTGAGGAAGGCAGAGCAAC	248
Ad02A6714	Aradu.A02	6714	p2	(AG)6	12	25832241	25832252	TGATCAATTCAAATGCTGGAA	CTCAAGGACTTCCATCGCAT	159
Ad02A6715	Aradu.A02	6715	p5	(AAAAT)5	25	25840824	25840848	GTGCTTCTTTTGGGCGTTTA	TCGAGCAAGAATGCTCAAGA	279
Ad02A6716	Aradu.A02	6716	p2	(GC)6	12	25849130	25849141	CTAGCGGAAAGTGCAGAACC	AATCTACAGATCGACGCGCT	232
Ad02A6718	Aradu.A02	6718	p3	(AGA)5	15	25908249	25908263	TGTGAAATACCTCAAATTGCAT	AGGCTACAGAGTGGGGGAAT	246
Ad02A6721	Aradu.A02	6721	p2	(TA)9	18	25940577	25940594	ACCAGCAGCAGCGAATAACT	TCCTAAAACCTTAAACCCTTACACC	267
Ad02A6723	Aradu.A02	6723	p3	(ATT)5	15	25945262	25945276	CAGTCAAGGCTGGTATGCAG	TCATCACCACAGCTCCACTC	142
Ad02A6727	Aradu.A02	6727	c	(TCC)5ccccaa	46	25957549	25957594	CATGTTGTTGATGGCACACA	CACACAAATGAACCGAAACAA	208
Ad02A6734	Aradu.A02	6734	p3	(AAG)5	15	26010280	26010294	GCTTTTGTCTTCCCTTTCCC	CTGAATACACCTCCCCCTCA	191
Ad02A6741	Aradu.A02	6741	p3	(TTA)6	18	26060519	26060536	TGAATAAAAATTTGCCCTCTTC	TGCTGAAAGTTCTATCCGCTAA	250
Ad02A6743	Aradu.A02	6743	p3	(ATT)16	48	26064665	26064712	GATCGTTTGTTATGTTGGAAGTG	CGAATCCGGCAGTATATGGT	279
Ad02A6744	Aradu.A02	6744	p3	(TTA)10	30	26065478	26065507	GCGAAAATCGACCGATTAAG	GTGGTCACATTGCAACATCC	280
Ad02A6747	Aradu.A02	6747	c	(T)12a(T)14	27	26086847	26086873	CGATTTCGAACGGTTTGATT	GCAGAAACAAGTGCGTTTCA	260
Ad02A6748	Aradu.A02	6748	p2	(TA)6	12	26090458	26090469	TGTACCAAATATGTCGGTATTTCAA	GGTGATTTAGTGTGACTCTATGTGC	233
Ad02A6752	Aradu.A02	6752	c	(T)10cag(A)10	23	26106688	26106710	CTCTGCCAGATCTGCCTTTC	ACCACCACCACCACTTCTCT	213
Ad02A6753	Aradu.A02	6753	c	(GTG)5gtttaga	41	26106821	26106861	CTCTGCCAGATCTGCCTTTC	CCTTATCACAAATACCATCACCC	266
Ad02A6755	Aradu.A02	6755	c*	(AT)9(ATAC)	52	26109089	26109140	GGCTACGCCACTCTGATAGC	TTTCTCTCGCCTTTAACTTTGC	266
Ad02A6757	Aradu.A02	6757	p2	(CT)7	14	26124946	26124959	CCCAAATCCAAGTCCAGAAA	CAATTGGGCAACATCAATCA	272
Ad02A6758	Aradu.A02	6758	p2	(TC)6	12	26132089	26132100	GGAAAAGCATCCATTCAGGA	CAATCCGTAACATGGGAGAAA	237
Ad02A6760	Aradu.A02	6760	p3	(TCT)5	15	26140927	26140941	GAAACCCAACCCTTCCTCTC	TTGCCTTCCCTTTTCCTTTT	276
Ad02A6761	Aradu.A02	6761	p2	(CT)18	36	26147081	26147116	AGCTCGTTTTGATTTGGGTG	CCAATCCCAAGAAGACTCCA	200
Ad02A6763	Aradu.A02	6763	c	(A)10tatttcg(A	27	26199292	26199318	TGTGGTTCATGAATGTTGGC	TCCCCAATTAGAGGTGTCCA	243
Ad02A6766	Aradu.A02	6766	c	(T)10cttttcttctt	37	26227195	26227231	CGGATACATTCATGCCACAG	TGTTGGCTCTTGAAAGAAGGA	269
Ad02A6769	Aradu.A02	6769	p2	(AG)6	12	26292125	26292136	GGTGAGGTAGGTGGGGTTTT	GATTTTGCTTGCCATTTGGT	216

ID	Chromosome	SSR nr.	SSR type	SSR	Size	Start	End	FORWARD PRIMER (5'-3')	REVERSE PRIMER (5'-3')	Product (bp)
Ad02A6771	Aradu.A02	6771	p3	(GAA)6	18	26354607	26354624	TAAAATATTGCTCGCCCTCG	TACCCCATACACCCCACCTA	157
Ad02A6772	Aradu.A02	6772	p3	(TTC)10	30	26371881	26371910	AGCCGAACCTACTCCCTCTC	ATGTTTCTCCGGCCTTAGGT	259
Ad02A6773	Aradu.A02	6773	p3	(ATC)6	18	26380417	26380434	AAAGTCGGCACAGCAGAAGT	CTGTTCAGGCCGATTACACC	229
Ad02A6774	Aradu.A02	6774	c	(TA)6(T)10	22	26390270	26390291	ATCCATTGATGCTCAAAGCC	AAAAGCAGTGAAAAGCAAAAGC	158
Ad02A6775	Aradu.A02	6775	p3	(AGA)7	21	26408849	26408869	GCTTTTGTCTTCCCTTTCCC	ATACCTTAGCCGAACCCACC	199
Ad02A6778	Aradu.A02	6778	p2	(GA)8	16	26452349	26452364	CGGAGAAGAGAGAGACCGTG	GAAAGGCAGTGGAGGATCTG	242
Ad02A6780	Aradu.A02	6780	c	(TAT)6tg(TTA	38	26456428	26456465	CCAACCAACACTTCATTACCAA	TGCAATAGTTTTATTAGTGGCATGT	137
Ad02A6795	Aradu.A02	6795	p3	(AGA)7	21	26577787	26577807	TAAAATATTGCTCGCCCTCG	TCTTCTCCCACTTAGCCGAA	108
Ad02A6798	Aradu.A02	6798	p2	(TA)7	14	26613949	26613962	AGGATCCCCTTCTTTGTTCC	ACCACAACCCTTGCGTCTAC	242
Ad02A6800	Aradu.A02	6800	p2	(TA)8	16	26621277	26621292	TCAAATGAAACTCTTGATGTTATTTAC	TCAGGAGATACTGAAACAGAACAAA	236
Ad02A6806	Aradu.A02	6806	p4	(TTTA)5	20	26644413	26644432	AGATAGTGGCACTCAGCATCC	GGTAATGGCAGGAAGCATGT	242
Ad02A6810	Aradu.A02	6810	p2	(TA)6	12	26646403	26646414	TTCGTTCATTTAGTTTAAAATGATGTC	TTTAGAAATAAAAGGGTACGAGTACAT	261
Ad02A6812	Aradu.A02	6812	c	(CCT)5tcttcttt	66	26654050	26654115	TTTTTCTTTTCCTACCCCGC	GCAAAATGTTGGTGTCGTTG	191
Ad02A6813	Aradu.A02	6813	c	(GAA)6agatga	91	26655474	26655564	GGAAAGAAGAAACACCTGCG	CTTCTCCGTCTCCTCCTCCT	209
Ad02A6815	Aradu.A02	6815	p2	(AG)6	12	26682930	26682941	TTGAATTTGAATGGTGGGGT	CTTGGTGCAGGGATGACTTT	207
Ad02A6817	Aradu.A02	6817	c	(ATAC)13aaat	103	26695073	26695175	GGGACTAATTTGATCATTAATCTGA	TTCCCTCGTCTTTGTTATCGTT	278
Ad02A6818	Aradu.A02	6818	p3	(TAC)5	15	26705459	26705473	CTCAATTGGAAATAAAACCAGAA	AAGCACCCCACAAATCAGAG	167
Ad02A6819	Aradu.A02	6819	p2	(TA)8	16	26723067	26723082	TTCACCACTTACTTTGGGGC	GGACTGGGTTGGTTTGAGAA	190
Ad02A6821	Aradu.A02	6821	c	(AGA)7at(AA	38	26730118	26730155	CTAGAGGATTCTCCGGCCTT	TTCTACACCTTGGCCGAAAC	197
Ad02A6823	Aradu.A02	6823	p2	(AC)7	14	26752716	26752729	GCATTTTTGTCCCCCTTTTT	GGGGACATTGGTTGAGGTAA	211
Ad02A6831	Aradu.A02	6831	c	(CA)11(TA)6	34	26849822	26849855	TTTGCATTTTTCCTATTTTTGC	TGGGCATAAAGAATCAAACAAA	230
Ad02A6833	Aradu.A02	6833	p5	(TTATC)5	25	26857858	26857882	CCTGGGTGCTCCAGTGTATT	GCCCAAATAGAGGAACACGA	179
Ad02A6834	Aradu.A02	6834	p2	(AG)13	26	26864677	26864702	TCTCGACCCAGGGTATGAAC	TCTTTTTCAAAACCACCTAACAA	181
Ad02A6836	Aradu.A02	6836	p3	(TCT)5	15	26906661	26906675	GCCGAACCTACTCCTTCTCC	GCTTTTGTCTTCCCTTTCCC	278
Ad02A6838	Aradu.A02	6838	p3	(ATA)5	15	26947630	26947644	TTGTCTATCAAAACATCAACCAAAA	GTTGAATTCACCTGGGTGCT	108
Ad02A6839	Aradu.A02	6839	p3	(ATA)5	15	26948965	26948979	AATTTTGACCTTGCTCAAATAAA	CGGACGGTGAAATTGTTGAG	261
Ad02A6840	Aradu.A02	6840	p3	(ATA)5	15	26968511	26968525	TTCCAATTGCCAAATCTTCC	AGAAGTTGGATTTTGGTGCG	258
Ad02A6843	Aradu.A02	6843	p3	(ATA)5	15	26972968	26972982	TGGAACTTGTCTATCAAAACATCAA	TGGGTACTACTGCGGATGGT	102
Ad02A6846	Aradu.A02	6846	c	(T)10ctttctc(T)	27	26997964	26997990	TCCCCAATTAGAGGTGTCCA	TGTGGTTCATGAATGTTGGC	243
Ad02A6847	Aradu.A02	6847	p3	(ATA)6	18	27014134	27014151	CCGTTTTTCCCTTAATGGCT	GGGAGTTGAACCCGAAAGTT	275
Ad02A6848	Aradu.A02	6848	c*	(TTTA)5(TTA	121	27067269	27067389	GCAAATGCATTAAAAGAAAATGG	ATTTGCACCCTGCTTTTCTC	246
Ad02A6851	Aradu.A02	6851	p3	(TTC)5	15	27107503	27107517	TACCCTCTCCCCTTTCCCTA	GCTTTTGTCTTCCCTTTCCC	270
Ad02A6852	Aradu.A02	6852	p3	(TTC)7	21	27123506	27123526	AAATCTTTCCCACCCAAACC	CCCTCGAGCAAGAGAAGAAA	197
Ad02A6854	Aradu.A02	6854	c	(TG)12(TA)6	36	27131601	27131636	GGATATGGTCGTCACCGATT	TGCCCTACTAAGAATGTTTTTCTTG	256

ID	Chromosome	SSR nr.	SSR type	SSR	Size	Start	End	FORWARD PRIMER (5'-3')	REVERSE PRIMER (5'-3')	Product (bp)
Ad02A6857	Aradu.A02	6857	p2	(AT)15	30	27141716	27141745	CTAAGCCTTACCTCATGCCG	GCTTAAACTTCGCAAGTGGC	277
Ad02A6863	Aradu.A02	6863	p2	(AT)6	12	27202014	27202025	AACAACTTTCGACCCCAAAA	TCTGAAGTTTAAATTTTGATCTCGTTT	251
Ad02A6864	Aradu.A02	6864	p2	(AC)15	30	27221289	27221318	GCCCTCAGAGCATAAATCCA	TTTTGTTGCCGTAGAGGCTT	276
Ad02A6866	Aradu.A02	6866	c	(T)10cgaaatata	60	27235478	27235537	TTGTGACTCAGCTTTGCTGG	TCATGAATGTTGGCTCTTGAA	271
Ad02A6869	Aradu.A02	6869	p3	(TAG)5	15	27263144	27263158	GCAAGGGCATGATTTAAGGA	CATGCCTGATGATGATGAGAA	257
Ad02A6872	Aradu.A02	6872	p2	(AT)7	14	27285469	27285482	GCTTTACATGGCGCAAAAGT	TCTCACAAATAATGAACCCCG	272
Ad02A6875	Aradu.A02	6875	p2	(TA)6	12	27295040	27295051	TCAAATGCAAATCTCTCTCTCTTT	TGCATGTAATTCCTTTGGCA	277
Ad02A6877	Aradu.A02	6877	p3	(TTC)5	15	27322880	27322894	GGAAAATTTGTTTTTCCGCA	CCCCTAATGAAAGTGGGGTT	212
Ad02A6879	Aradu.A02	6879	p3	(TTC)5	15	27347817	27347831	CTAGCATTTCCGGGAGGATA	ACAAGCAGAGCTCTACCCCC	215
Ad02A6881	Aradu.A02	6881	p2	(AG)6	12	27372033	27372044	GGGAAATGGAATTAGGGGAA	CAGCAGCTCCCTCTGACTCT	160
Ad02A6882	Aradu.A02	6882	p3	(TAT)6	18	27372413	27372430	ATGGTGCGAGGAAGAGAAGA	CATGGCTTCGGCCACTAATA	251
Ad02A6885	Aradu.A02	6885	p2	(TA)8	16	27395472	27395487	AGGACCAAAGGCTACGGTTT	TCAACTCAGTTAAATAAAAATGCAGAA	254
Ad02A6887	Aradu.A02	6887	p3	(ATA)5	15	27411036	27411050	TGGAACATGTCTATCAAAACATCA	GTTGGATTTTGGTGCGAAAC	179
Ad02A6888	Aradu.A02	6888	p3	(ATA)6	18	27415004	27415021	GGAACTAGTCTATCGATACATCAACC	CGCTAGGAAGTTGGGTTTTG	190
Ad02A6889	Aradu.A02	6889	p3	(ATA)5	15	27416522	27416536	TGGAACTTGTCTATCAAAACATCAA	AAATCGCCTGGGTGATACAG	110
Ad02A6890	Aradu.A02	6890	p3	(ATA)5	15	27428773	27428787	TGGAACTTGTCTATCAAAACAACAA	TGAACCCGGAAGTTCTCATC	131
Ad02A6891	Aradu.A02	6891	p2	(TG)6	12	27429766	27429777	GGACCTTGGGTGTGGTAAGA	CAAGGCCTCAATACCTCCAA	202
Ad02A6892	Aradu.A02	6892	p3	(CAA)5	15	27433300	27433314	ATCATTCCCACAGAACCGAG	GCACCTTGTTTCTGCCTCTC	261
Ad02A6893	Aradu.A02	6893	p2	(TA)6	12	27440095	27440106	TTATGCTCGGACCGGTTATC	AACTCAAAAGCGCACCACTC	141
Ad02A6894	Aradu.A02	6894	p3	(ATA)5	15	27441878	27441892	TGGAACTTGTCTATCAAAACAACAA	TGAACCCGGAAGTTCTCATC	131
Ad02A6895	Aradu.A02	6895	c	(T)10atttacgca	43	27447579	27447621	TCGTCACGTTACAGAGGCAA	AGTCGCACTTAAAAGCCGAG	274
Ad02A6896	Aradu.A02	6896	p2	(TA)10	20	27453366	27453385	CCTTTGGGTTTCCTTTAGGG	ACAAGGATAACCAACGCGAG	160
Ad02A6897	Aradu.A02	6897	p2	(TA)7	14	27470593	27470606	TGGGTTTCCTTAGGGGTTTT	ACAAGGATAACCAACGCGAG	150
Ad02A6898	Aradu.A02	6898	p2	(CT)7	14	27475675	27475688	CAAATTCGAAACCAGCGAAT	AAGAACCTGGGATTGAGAGTCA	228
Ad02A6899	Aradu.A02	6899	p2	(CT)8	16	27479217	27479232	TGACGGTGAAGATGGATCAC	TTGACCAAATCCGAATGAGA	174
Ad02A6900	Aradu.A02	6900	p2	(TA)6	12	27488371	27488382	TGTTTGTGTCCCTTTGGGTT	GGGGTAAACAAGAATCGCAA	218
Ad02A6903	Aradu.A02	6903	c	(A)11gcagtgaa	49	27542227	27542275	TGTGGTTCATGAATGTTGGC	CCAACTAGAGGTGTCCAGGG	244
Ad02A6907	Aradu.A02	6907	p2	(CT)6	12	27589082	27589093	CGTTAAACCTGGGATTGAGAA	GCATCACTTTTCCGTGACCT	262
Ad02A6909	Aradu.A02	6909	p3	(CTT)6	18	27595419	27595436	TGCACTCACCTACTGGAACG	TGAAGATGAAACACGCGAAG	187
Ad02A6911	Aradu.A02	6911	p3	(GGT)5	15	27619314	27619328	CGGCCTCTGCTTTCTACTTC	CACCACCACCAGCACCTT	274
Ad02A6913	Aradu.A02	6913	p3	(TTA)8	24	27619675	27619698	CATTTGCCAAACACACCCTA	TGTGGATACATTCAATTTGCG	150
Ad02A6914	Aradu.A02	6914	p3	(CTT)5	15	27621445	27621459	TTTCTCTTTCTCCTCCTCCTCA	TCGCGAAAAGTGAAGGAAGT	107
Ad02A6920	Aradu.A02	6920	p2	(TA)6	12	27628056	27628067	TCTCAAAAGGAGCTAGTTAAAAATAAA	TGCCCCTGATATTTCCTTCA	279
Ad02A6929	Aradu.A02	6929	p3	(GAT)6	18	27662040	27662057	GTGCCAGATCTCGACCTTTC	ATTCCGGCTGAGTAGCTTCA	178

ID	Chromosome	SSR nr.	SSR type	SSR	Size	Start	End	FORWARD PRIMER (5'-3')	REVERSE PRIMER (5'-3')	Product (bp)
Ad02A6930	Aradu.A02	6930	p3	(AGA)6	18	27678062	27678079	GCTTTTGTCTTCCCTTTCCC	CTTCTTCTCCCACATAGCCG	209
Ad02A6933	Aradu.A02	6933	p3	(AAT)6	18	27727749	27727766	TGGCCTTTTCGTCTTGAAAT	CCATCAACTATGATGAATCGGA	205
Ad02A6935	Aradu.A02	6935	p3	(ATT)5	15	27770179	27770193	TTTGTGAAGTCACCAAGCATTC	ATGAGCAGGAACAGGGAAAA	194
Ad02A6937	Aradu.A02	6937	p2	(AT)7	14	27796693	27796706	CCATGATGGAGAAAGGGAAA	TCTTTCCTCATTTCAAAAATCCA	244
Ad02A6938	Aradu.A02	6938	p2	(CT)13	26	27831888	27831913	TCTTCCTCCCACGAAAATTG	AAGAGCCTGAGCTTCTTCCC	186
Ad02A6939	Aradu.A02	6939	p3	(ATT)5	15	27832218	27832232	TTGTGTTTTCTTCGATTCGC	CACCCTTAACAACAATGCCA	235
Ad02A6940	Aradu.A02	6940	p2	(TC)9	18	27833020	27833037	CTCTCCCCTCTCTCCCATTT	AGAGGAGGGGGAGAAAAAG	169
Ad02A6941	Aradu.A02	6941	p2	(TC)8	16	27833224	27833239	TTTTCTCCCCCTCCTCTTTC	CCTTCTCTCAATCTCTATCCCC	264
Ad02A6942	Aradu.A02	6942	p2	(GA)6	12	27833592	27833603	AAAGAAAGAGAGGGGAGGGA	CTCCTTCTCTATGCTTTCCCA	265
Ad02A6943	Aradu.A02	6943	p2	(GA)6	12	27833809	27833820	GGGAGAAAGAGGGCAAAGAT	ACACGTGCTTGACGAATCTG	217
Ad02A6944	Aradu.A02	6944	c	(GA)8agagaaa	54	27834080	27834133	CGGGAACAATAGCAGCAGAT	CCGGTTAACGAAAACCAAAA	272
Ad02A6946	Aradu.A02	6946	p2	(CT)7	14	27838957	27838970	GCATTGGTAGGGGGCTCTAT	TCCTTCAATTCTCCACCCAA	114
Ad02A6947	Aradu.A02	6947	p2	(CT)7	14	27844045	27844058	TCTTCGTAAAGAGAGCCCCA	TCCTTCAATTCTCCACCCAA	194
Ad02A6953	Aradu.A02	6953	c	(AG)6gaagagta	165	27932899	27933063	TCACGAAAAAGCACACCAAA	CAAATCTCTCAAATCATTCCCA	257
Ad02A6954	Aradu.A02	6954	c	(A)10gagagaaa	32	27945685	27945716	GATTCAGTTTTCAATTTGCTTTGA	CTTGCTTTGATGGGAGGTGT	253
Ad02A6958	Aradu.A02	6958	p3	(AAT)5	15	27968021	27968035	TGGAGCATGATGTGTGATGA	CCAACAACAACCTCAATAACCA	276
Ad02A6965	Aradu.A02	6965	p2	(TA)10	20	28043530	28043549	TAGCCCCTGACTTAGCTTGC	GATCGAGAGGCTCCTTGCTA	271
Ad02A6966	Aradu.A02	6966	p2	(AT)8	16	28045759	28045774	TGCAATACCACCACAACACA	AAGTGATCGGCCAAGGTATG	278
Ad02A6967	Aradu.A02	6967	p2	(AG)6	12	28068247	28068258	CCACCGATCCATCAATGAGT	TGAGGGACGGCAGATCTATT	243
Ad02A6968	Aradu.A02	6968	p2	(TA)6	12	28073490	28073501	AAAATCGCAACTTGAAAGCG	TCCCTTTGTGTTTCCTTTGG	202
Ad02A6972	Aradu.A02	6972	p2	(TA)8	16	28132051	28132066	GCTTTTATTTTTCCCTCGCC	CCGGCTATGCCACTCTGATA	270
Ad02A6976	Aradu.A02	6976	p4	(AAAT)7	28	28166130	28166157	AAATTAAGTTTGGTGTCCCCG	CGCACCATGTGGTAAACAAG	136
Ad02A6981	Aradu.A02	6981	p2	(AG)6	12	28228676	28228687	ACGGAAAAGCACACCAAACT	CCACACCATTCGAATTTCCT	100
Ad02A6983	Aradu.A02	6983	p2	(TG)8	16	28251038	28251053	GAGCTCTTCACAAGGAACGC	GCCCAAGCCCAACATATAAA	209
Ad02A6984	Aradu.A02	6984	p2	(AG)7	14	28256595	28256608	CAACTGTACGTGAGTTTTTGCC	CGAACCGGTCAGATTATTGG	100
Ad02A6985	Aradu.A02	6985	p2	(AG)6	12	28264641	28264652	TTGAACCTGGGATCGAGAGT	TGGCCCAAATCTAAGTCCAG	202
Ad02A6988	Aradu.A02	6988	p2	(CT)7	14	28272897	28272910	ACCTTATTCCCCCACACCAT	TCATGGAAAAGCACACCAAA	111
Ad02A6991	Aradu.A02	6991	p2	(TA)10	20	28283975	28283994	GACCTGTCATCAAATTAAAAGCA	TTTCAATTCTTTAGTGTCAACTTTGT	236
Ad02A6998	Aradu.A02	6998	p3	(GAA)6	18	28333619	28333636	CTAGGTTGCCAGGTCCAAAA	GACCTGATTCAAGAGGCTCG	206
Ad02A7000	Aradu.A02	7000	p2	(TA)7	14	28342841	28342854	AGTCGCAGGTCAAAGGAAAA	TGGGACATTAGTGATTCGGA	230
Ad02A7001	Aradu.A02	7001	p3	(AAT)6	18	28343034	28343051	TCCGAATCACTAATGTCCCA	GAAAATTGTGTGGAGACTCACCT	263
Ad02A7005	Aradu.A02	7005	c	(TTTA)5tttgag	50	28370656	28370705	GTGTCTTGAATGGCTGGGTT	CAGCGATCAACAACGCTAGA	214
Ad02A7007	Aradu.A02	7007	p2	(AT)6	12	28375237	28375248	TTAAAATGGATCACACTTACAGAAA	AATTTAACAACAAAATGTGAGTGTTT	280
Ad02A7009	Aradu.A02	7009	p2	(TA)7	14	28384963	28384976	CTGGATTCTGGAAAGCGAAG	GGAAAGCCAAGGAAATAGCA	265

ID	Chromosome	SSR nr.	SSR type	SSR	Size	Start	End	FORWARD PRIMER (5'-3')	REVERSE PRIMER (5'-3')	Product (bp)
Ad02A7010	Aradu.A02	7010	p2	(TA)8	16	28399352	28399367	TTTGGATTTTGGCCTCTGAT	TGACCTAATTCGCATTTCCTG	269
Ad02A7011	Aradu.A02	7011	p2	(GT)6	12	28399988	28399999	CGCAGTTCACTCTCTTCCCT	AACGAATTGGGTGCCAGTAG	239
Ad02A7012	Aradu.A02	7012	p2	(AT)6	12	28403636	28403647	GGGTTTATTAACCTCCCCACA	CCCATCTCAATTGTAAAAGTGCT	266
Ad02A7013	Aradu.A02	7013	p2	(GT)6	12	28412031	28412042	AGACAAAGACGCTTGGCTTC	CCAAACCCTAGTCCCCAAAT	205
Ad02A7014	Aradu.A02	7014	c	(AAT)13g(AT	76	28413281	28413356	CAGGCAATGGCACAAAAATA	TGTGGCTTCAGCTCGTTTTA	152
Ad02A7015	Aradu.A02	7015	p3	(TGA)34	102	28414221	28414322	CGACGACGATGATGATGATT	GCAGAAGAACAACAACAATGGA	237
Ad02A7016	Aradu.A02	7016	p2	(TA)7	14	28414747	28414760	TGTGCTTCAAACATTTTCCA	TTCCGAATCAAAACTCCTTTG	276
Ad02A7017	Aradu.A02	7017	p2	(CT)9	18	28415513	28415530	TTTTATCTGCGGGCTTTGTC	CAACGAAGGTGACACAATGG	253
Ad02A7018	Aradu.A02	7018	c	(A)10gaattggta	44	28416007	28416050	TTTTCTTATTAATTGGGTCTATGCAC	TTTTTATTGAATTCTTGCGCC	182
Ad02A7019	Aradu.A02	7019	p2	(TA)13	26	28418228	28418253	AGACGCAACACTCACGTCAA	TGGGAGCTCCTTATAGCGAA	243
Ad02A7020	Aradu.A02	7020	p2	(AC)15	30	28419821	28419850	ACCACATCAAACCAAGTCCC	TTGTCCGAATAATCGGCTTC	246
Ad02A7021	Aradu.A02	7021	p2	(AC)22	44	28420164	28420207	CAGCAATTCAATTCCAACCC	AGAAAGTTGATGACGGTGGG	238
Ad02A7025	Aradu.A02	7025	p2	(CA)9	18	28444022	28444039	CTGGCTGGCGAAGTTAAGAC	GCTTTCTGCAAGCGAAGAAC	215
Ad02A7026	Aradu.A02	7026	p2	(AG)6	12	28456857	28456868	AAAATAAGTGAACCGGGCCT	AAACGACTCGTCAATCGGAG	209
Ad02A7027	Aradu.A02	7027	c	(TTC)7ttt(TTC	81	28465278	28465358	TACCTTGGCCGAACCTACAC	CTAGAGGATTCTCCGGCCTT	246
Ad02A7028	Aradu.A02	7028	p2	(TA)8	16	28488440	28488455	GCCATTGATGTTCAAAGCCT	CTTGGTGGCCTTGCATTTAT	270
Ad02A7030	Aradu.A02	7030	p2	(AG)6	12	28510045	28510056	GTTTGGGGAAGAGGTGTTGA	CAGAGAAGGGCAAGAAGCTG	223
Ad02A7034	Aradu.A02	7034	p3	(CTT)9	27	28569331	28569357	TTGTTTTTACGCCGTCAATG	CCAAATCTACAGCAGGGAGC	243
Ad02A7037	Aradu.A02	7037	p2	(TC)7	14	28575457	28575470	CTGAGTTGCAATCTTCCCGT	GCAGCAGCACATCTAGCATC	224
Ad02A7040	Aradu.A02	7040	p2	(TA)7	14	28581261	28581274	CCGACTGCACGACTCTGATA	TGGTCTATTGTACCCTCGCC	278
Ad02A7043	Aradu.A02	7043	c	(AGA)5atgttta	74	28607723	28607796	CAGCAGAGATTGCAGATCCA	TTTTTCGTTAACGCACCTCC	185
Ad02A7044	Aradu.A02	7044	p3	(TAT)5	15	28608090	28608104	TGGATGTGGAGTGTAAAACCC	ATACACAACCATTCCGGCTC	264
Ad02A7047	Aradu.A02	7047	p3	(AGA)8	24	28624932	28624955	ACCACTGAGCTATGACGGCT	ACTTCTCCTTCCCACCCAAT	275
Ad02A7049	Aradu.A02	7049	p3	(CTT)5	15	28646740	28646754	GCTGGACCTTTGCTTCAGAC	GTAATTCCCAGGAAATGCCA	192
Ad02A7051	Aradu.A02	7051	p3	(CAC)5	15	28650797	28650811	TGAAAGATGGGTTTTGTGTGA	GGTGACAGTGGAGAGGGGTA	189
Ad02A7054	Aradu.A02	7054	p2	(AT)17	34	28660591	28660624	GTTTTACGTGAAAATGCGGG	TAATCAGAGTGATGCAGCGG	175
Ad02A7056	Aradu.A02	7056	p2	(TA)6	12	28669028	28669039	GCCCTTGATTAGTAGTGATTGGA	GCTCAAAAAGCACATTTCACA	280
Ad02A7062	Aradu.A02	7062	p3	(AAT)6	18	28704608	28704625	CAGGAACAAGGAAAAAGGCA	TTGGATGTTGGCTTTGTGAA	204
Ad02A7063	Aradu.A02	7063	p3	(TCT)5	15	28707435	28707449	CAACTTCTCTTTCCCACCCA	ACCACTGAGCTATGACGGCT	274
Ad02A7064	Aradu.A02	7064	p2	(AG)13	26	28712120	28712145	CGACCACATCATTTTCGTTG	CAATTTTCCATCGCCATCTT	226
Ad02A7065	Aradu.A02	7065	p2	(AT)7	14	28714131	28714144	TGGCTGAACCAGTCACAGAG	TTGTCTGGTCTTGGTTTTTGC	204
Ad02A7075	Aradu.A02	7075	c	(AAC)6(AAT)	141	28784664	28784804	GGCTAGCATCAGGTTGGTTC	TATCCAAAAAGCCATGAGCG	237
Ad02A7076	Aradu.A02	7076	p2	(TA)8	16	28785323	28785338	CCATAGTATTTCCCAACACAACA	TTCTTGTCTTACCGAATTGAGAGA	272
Ad02A7077	Aradu.A02	7077	p2	(CA)36	72	28794324	28794395	TTGGTCTGATTCCGACTTCC	GAAGACAAACCCCTCTGCTG	244

ID	Chromosome	SSR nr.	SSR type	SSR	Size	Start	End	FORWARD PRIMER (5'-3')	REVERSE PRIMER (5'-3')	Product (bp)
Ad02A7080	Aradu.A02	7080	c	(A)12tttcg(A)1	27	28807260	28807286	TGGCTCTTGAAAGAATGATGG	CACACTCTTGTTGCCTTGGA	188
Ad02A7083	Aradu.A02	7083	p3	(TTC)6	18	28863428	28863445	TACATGGCCGAACCTACCTC	TAAAATATTGCTCGCCCTCG	184
Ad02A7084	Aradu.A02	7084	p2	(AT)6	12	28886216	28886227	AAGCGAAACAGAAATCGCAT	ACCTCGTTGAGCATTCTGGA	279
Ad02A7086	Aradu.A02	7086	p2	(AT)6	12	28892626	28892637	TTTTCATATATCTCGTTTACACGGT	AAAATCGTTCATCTTCCCTGTT	190
Ad02A7087	Aradu.A02	7087	p2	(TG)6	12	28895609	28895620	GGTGATGAGGTGTGACGATG	GGATTCGAGAGGAGATTCCC	265
Ad02A7094	Aradu.A02	7094	c	(AAT)5aaaaata	86	28930927	28931012	CCAGGTCCTACTCCTCCACA	CTCAAATATGGGACACCGCT	236
Ad02A7098	Aradu.A02	7098	p3	(GTG)5	15	28938647	28938661	TGCTTCTGATTCTAATCGTGCT	TTCGTCAAAATCATCCTCAATG	246
Ad02A7100	Aradu.A02	7100	p2	(CT)9	18	28946814	28946831	AAGGGTAAGTGTGGTGTGCC	CGTTAAGAGCGTGGGAGAAG	163
Ad02A7101	Aradu.A02	7101	p2	(TC)12	24	28946995	28947018	CTTCTCCCACGCTCTTAACG	TGATAGGCGCAGAAAAGAGG	209
Ad02A7102	Aradu.A02	7102	c	(T)10ctttcactat	115	28947150	28947264	TTCCCTTTCCTTTCCTTTCC	GTCATACACGAGAAACCGGG	266
Ad02A7103	Aradu.A02	7103	p2	(TC)6	12	28947420	28947431	ACCAACCGCACACTCTTGTT	TCGAGTCAAAAGGGGAAAAA	148
Ad02A7106	Aradu.A02	7106	p2	(CT)6	12	28964004	28964015	CCTCTCTCCACCACACCATT	CGGAAAAGCACACCAAACTT	103
Ad02A7111	Aradu.A02	7111	p2	(TA)10	20	28970724	28970743	TGCTGCTGATTTGAATTTGG	GCAGTTTTGCTTCCTTCCAC	265
Ad02A7113	Aradu.A02	7113	p3	(CAA)12	36	28973749	28973784	GGAGCATTTAAGAATGAGCCT	GATTAATACACGGGTTTTTCACA	171
Ad02A7115	Aradu.A02	7115	p3	(TAA)5	15	28976323	28976337	TTTTTCTGCCAAAATGAATGC	TCATCAATTTAATTTGGAGTTTTTCA	261
Ad02A7116	Aradu.A02	7116	p3	(AGA)7	21	29003933	29003953	TAAAATATTGCTCGCCCTCG	TACCCTCTCCCCTCTCCCTA	171
Ad02A7118	Aradu.A02	7118	c	(ATA)10(TGA	81	29034904	29034984	CGAATTGCTCTGATGCCATA	AACCCGGTTTACTGAGCCTT	272
Ad02A7125	Aradu.A02	7125	p3	(TCT)5	15	29117503	29117517	CCCATAAACCCCACCTACCT	TAAAATATTGCTCGCCCTCG	240
Ad02A7126	Aradu.A02	7126	c	(AC)20ataa(AT	58	29132912	29132969	CTCAACCCACGAAGTCAAGG	TGTTGCTTTTCAGATGCAGG	175
Ad02A7128	Aradu.A02	7128	p2	(TA)12	24	29134604	29134627	TCCACCAAGCCAAATCAAAT	TCGGCCAAGGTATGGTTTAG	236
Ad02A7129	Aradu.A02	7129	p2	(AT)10	20	29138344	29138363	CAGGGAAACTCTCATCTCCG	GGATTTAACCGTGAGCCAGA	153
Ad02A7131	Aradu.A02	7131	p2	(CA)6	12	29143345	29143356	TCACATTAACCTACCCACCTCC	AGGGATCGAGCTCAAGTTCA	217
Ad02A7136	Aradu.A02	7136	p2	(AT)11	22	29207415	29207436	CTGCATGGGGTTTCTACAGG	TGTCCATGTCTTATCTGCCAA	237
Ad02A7137	Aradu.A02	7137	p2	(GA)14	28	29211575	29211602	TAGAGTAACCAACGCACCCC	ATTTTGGTCATTCGCAAAGC	258
Ad02A7139	Aradu.A02	7139	c	(A)11ttgtgcaag	98	29222440	29222537	TCCCTCTTAGCAGTAATTGGC	TCAAAGTCCATCTATTATTTGGG	241
Ad02A7141	Aradu.A02	7141	p3	(GAG)9	27	29227084	29227110	GGTTGATTGGTGGGTGAGAA	TGTGCTTCACAAGCCTCAAC	193
Ad02A7142	Aradu.A02	7142	p2	(AT)6	12	29228933	29228944	TGAATAGACATGACGAACCAGTTT	CCCCTGTCTTAGAAACCAAAA	230
Ad02A7143	Aradu.A02	7143	p3	(AAC)5	15	29229414	29229428	CAACAGCAACAACAAAAGCAA	GCGTCACAGAGAGAGGGAGT	205
Ad02A7145	Aradu.A02	7145	p3	(TTA)7	21	29231056	29231076	GCAAAGTACTTGCATTGTCTAACTA	TGAGAAGAATGGATGTGACATTAAA	277
Ad02A7149	Aradu.A02	7149	p4	(AAAT)5	20	29261235	29261254	TCGAAAATTCACACCCACAA	CCATGATGGAGAAAGGGAGA	215
Ad02A7151	Aradu.A02	7151	p2	(AT)9	18	29273017	29273034	ATGGCTTTGACAGCATTTCG	GAAGTGTGGGGGCAAATAAA	236
Ad02A7152	Aradu.A02	7152	p4	(TACA)5	20	29275921	29275940	TGGATGACTGGAACGATGAA	GCAATCGGATCCTAAACGAA	165
Ad02A7153	Aradu.A02	7153	p4	(AATA)5	20	29281900	29281919	GTGAAAGGGAAAGGGAAAGG	CCCTTCCAACAACAATCACC	253
Ad02A7156	Aradu.A02	7156	p2	(TA)8	16	29289415	29289430	ATTAGATACACCGTCCGCGT	TGGTATCCACTGCTTCTCTTG	218

ID	Chromosome	SSR nr.	SSR type	SSR	Size	Start	End	FORWARD PRIMER (5'-3')	REVERSE PRIMER (5'-3')	Product (bp)
Ad02A7158	Aradu.A02	7158	p2	(TC)6	12	29297712	29297723	GCCTGCATTTTGGTCATTTT	TGACAAAAACGGTGGCAATA	271
Ad02A7159	Aradu.A02	7159	p2	(AT)6	12	29304402	29304413	GTTCGGTGATTTTGGGAGAC	GGCTCAAATCTCACTTGCTTG	266
Ad02A7160	Aradu.A02	7160	p2	(TA)7	14	29311225	29311238	TTTCAGATGCAGGACGTGAG	AGCCTTTGAACAAAAGGGGT	273
Ad02A7166	Aradu.A02	7166	p2	(GA)12	24	29322953	29322976	CGAAGCGGAAGAAGAGAAGA	CACAACAACAAACAACGGCT	241
Ad02A7169	Aradu.A02	7169	p3	(GTT)5	15	29344823	29344837	GTTGCTGAGGTTTTGTGGCT	CGAATAGTCGAAACCCGAAA	179
Ad02A7170	Aradu.A02	7170	p2	(AT)9	18	29351015	29351032	TTGGCATCTCTCCGGTTTAG	TCGCAACTAAGGAGAAATACGA	272
Ad02A7173	Aradu.A02	7173	c	(TTC)6tcatcgc	69	29361966	29362034	ATCATGTCACGTTCCCTTCC	AAAAGCAAACAGCATGAGGTC	243
Ad02A7174	Aradu.A02	7174	c	(AT)8gtg(TA)!	37	29366954	29366990	TTCCCTTCTCCTATTTTGTACCAT	CCAAAACCTACCCTAGACCCA	269
Ad02A7176	Aradu.A02	7176	p2	(AG)6	12	29388043	29388054	GGAAGGGTTCGAATTTTGAG	TCGAAAATCACCTTCCTCTTTT	165
Ad02A7178	Aradu.A02	7178	p3	(ATT)19	57	29391398	29391454	GCACCTGATTGAACCCTCTC	TTGCAGATTGAAGTATTGGCA	280
Ad02A7180	Aradu.A02	7180	p3	(TAA)6	18	29401183	29401200	TCATGGCATGTTCATCCAAC	CACTCTCTTTCACCGCCTTC	240
Ad02A7182	Aradu.A02	7182	c	(A)10gccatagc	36	29405190	29405225	CAAATCCTGATGGAGGGCTA	GGTGATGATTTTATTTGATGCC	160
Ad02A7183	Aradu.A02	7183	p2	(TA)7	14	29405816	29405829	TCGTAACTCAAAAAGCGCAA	TATGCTCGGACCGGTTATCT	202
Ad02A7184	Aradu.A02	7184	p3	(TTA)16	48	29411111	29411158	TTTCGGTGGAATGAATGAA	CTTCTAATACAATGCAATTAAAACAAA	131
Ad02A7185	Aradu.A02	7185	p3	(GAA)5	15	29434696	29434710	CGAGGGAATAATGCGAAGAC	ATTTCCTGGATTGGTCGATG	155
Ad02A7187	Aradu.A02	7187	p2	(AT)7	14	29448980	29448993	TGTAATGGGGCAAGGTAAGG	GGGGACAAAATTACCCCAAT	245
Ad02A7188	Aradu.A02	7188	p2	(TA)6	12	29449209	29449220	TGGGGTAATTTTGTCCCCTT	TGGGAACTTGGGTTGATAGG	245
Ad02A7189	Aradu.A02	7189	p3	(TCT)5	15	29467022	29467036	TCCCACATAGCCGAACCTAC	CGGTCTTAGGTGCCATTGAT	162
Ad02A7190	Aradu.A02	7190	c	(T)11aaatttttga	79	29491673	29491751	TTGAAATTTTGACTTGACTTGTAAGAA	TGCATGCATTCATTGTTTTG	204
Ad02A7192	Aradu.A02	7192	c	(AGTT)5at(TT	42	29525355	29525396	CACCATCCACCATCTTCTCC	CTCATGGGTAATCAGCAGCA	166
Ad02A7193	Aradu.A02	7193	p2	(AG)6	12	29539016	29539027	CCAGTTTCAAGCCCAGAAAA	CCAAAGAAAATCATAACATGAATGG	259
Ad02A7194	Aradu.A02	7194	p2	(AG)6	12	29567645	29567656	GCAAAAATCAGCGTGACAGA	CATTGAACCTGAGATGAAGAAGAA	250
Ad02A7195	Aradu.A02	7195	p3	(ATT)6	18	29574341	29574358	TGTAACGGGTAAAGCCCTCA	ATTGGGTTGTTGCCATTTTT	210
Ad02A7196	Aradu.A02	7196	p2	(GA)11	22	29580671	29580692	AAGGTCACCAGCAATTCGAT	CCTTATTCTCTCACCCACGC	256
Ad02A7202	Aradu.A02	7202	p2	(TG)10	20	29606086	29606105	AGGGAAGGGCTTGAAATTGT	CACCCCAAGTTTATCCCTCA	280
Ad02A7204	Aradu.A02	7204	p2	(TA)6	12	29619785	29619796	CTATACATGGGATGCCCGTC	CATGATTATTTTTAGGACTGCTCA	249
Ad02A7205	Aradu.A02	7205	p3	(TAA)20	60	29620007	29620066	AAGTGAGCAGTCCTAAAAATAATCA	TTCTTTAACTATTAGTGATCCGTCCA	252
Ad02A7208	Aradu.A02	7208	c	(A)11ttg(A)10	24	29640675	29640698	ACCAGCCGTTGAGACAGATT	TGCTCTACATCCCCAGGAAG	279
Ad02A7209	Aradu.A02	7209	p2	(AT)6	12	29645524	29645535	TGCGTGAATAGTCATCCAAAA	CCACTTGGACAATGGAACCT	229
Ad02A7211	Aradu.A02	7211	p3	(TTC)6	18	29674714	29674731	CTATACCTTGGCCGAATCCA	ATGCTTTTACCGCACCAAAC	120
Ad02A7223	Aradu.A02	7223	p2	(AT)9	18	29724846	29724863	CGTCTACCCAAGCGGTACAT	TGGTGTGCCTAGTAAGGGGA	246
Ad02A7230	Aradu.A02	7230	p3	(TAA)5	15	29737235	29737249	TTTCTCCAATATCTCCTTTCGG	TAAGGCAATTCAGTAGTATCCGT	251
Ad02A7231	Aradu.A02	7231	p2	(TA)7	14	29738578	29738591	CATAACTCGAAAGCGCAACA	TTCCTTTGGGTTTCCTTGTG	197
Ad02A7232	Aradu.A02	7232	p2	(TA)7	14	29753850	29753863	TGACTTGCAAAGCTAAGACTGC	ACCTCGTTGAGCATCTGGAG	234

ID	Chromosome	SSR nr.	SSR type	SSR	Size	Start	End	FORWARD PRIMER (5'-3')	REVERSE PRIMER (5'-3')	Product (bp)
Ad02A7233	Aradu.A02	7233	p3	(TTC)6	18	29763332	29763349	CATCAACCTCCTCTTTCCCA	CCCTCGAGCAAGAGAAGAAA	116
Ad02A7242	Aradu.A02	7242	p2	(AG)6	12	29923051	29923062	GGGGATTGGGAGAAATTCAT	CACCAAGGAAGGGTTGAAAA	146
Ad02A7243	Aradu.A02	7243	p5	(TTATC)5	25	29993027	29993051	GGTGCGGAGTGACCTTTTTA	CAGAGCAATCGACCTCTTCC	236
Ad02A7244	Aradu.A02	7244	p2	(TA)10	20	30003275	30003294	GCCTGGAATTGAGCTCATAAA	AGCTGGCTCAAGCTCACG	267
Ad02A7249	Aradu.A02	7249	c	(A)10cagagaaa	42	30038177	30038218	GAGGCAACCCAGTCATTAGC	GGTGCCCATTCTAGCGTTTA	182
Ad02A7250	Aradu.A02	7250	c	(AC)6(AT)7	26	30047519	30047544	ATTTCGTGCACCATGAAACA	GAATTCAAAATAATTTGTGTTATGCAA	263
Ad02A7251	Aradu.A02	7251	p3	(GGT)5	15	30048145	30048159	TCGTCTCGTGTTTCTTGTCG	TGTGTGGTAGTGGTGGCAGT	113
Ad02A7260	Aradu.A02	7260	p2	(AT)12	24	30115130	30115153	TGGTTGCTAACCATTGGACA	ATGCAGTAGGTTGGCTCCAC	200
Ad02A7261	Aradu.A02	7261	p4	(GTTA)6	24	30120586	30120609	CATTCACCATCCACCATCTTC	ACGCTTGAAAGGCAATTCTC	273
Ad02A7269	Aradu.A02	7269	p2	(CT)7	14	30156930	30156943	CACAAAATCTACTGGCCCCT	GACAAACACTTTATCGTTTGCAC	278
Ad02A7270	Aradu.A02	7270	p2	(AT)7	14	30160770	30160783	TCTCCATTGTTACTTCCTGCAA	AACCTTCCTGATCAAATGTGAAA	261
Ad02A7274	Aradu.A02	7274	p3	(AAG)6	18	30169131	30169148	TCATCCACAATGTCAGGGTC	AGAACACAACTATGCCCGGA	212
Ad02A7275	Aradu.A02	7275	p3	(TTC)5	15	30171255	30171269	GCGTCTGATAGAGCTTTGGC	CGATGAAAAGAGGAAAAAGCA	136
Ad02A7280	Aradu.A02	7280	p3	(AGA)5	15	30228911	30228925	GAGGCTCACACAAGCATTCA	TCAGGGGGAGGAGCTCTATT	155
Ad02A7282	Aradu.A02	7282	p3	(AGA)6	18	30277243	30277260	GAGGCTCACACAAGCATTCA	TCAGGGGGAGGAGCTCTATT	158
Ad02A7283	Aradu.A02	7283	p2	(TG)21	42	30282195	30282236	CCACCACGTGTTCCAAGTTA	CAAGTCCAGCTGCTAAACCC	223
Ad02A7285	Aradu.A02	7285	p2	(CT)8	16	30298578	30298593	TCCTCCCCTCTCTTTGTCTTC	AGAGGGAGAGAGGACGGAAA	214
Ad02A7286	Aradu.A02	7286	p3	(CTC)7	21	30298857	30298877	GTGCCCTTTTTCCTCCTCTC	TCCTTTTCTCTCTCTCATTCCC	236
Ad02A7289	Aradu.A02	7289	p3	(AAG)5	15	30305498	30305512	ACTCGGAGGTGGAAGCTTTT	TTCACCTTAGCCGAAACCAC	213
Ad02A7293	Aradu.A02	7293	p4	(AAAG)7	28	30322630	30322657	GGCCTAAGAAAATGATGGCA	GGCCTAGTTTGTGGTGCATT	220
Ad02A7294	Aradu.A02	7294	p2	(AC)10	20	30326267	30326286	GATCTTGGTCGGTTAGGCAA	TGAGCTGTGTCCAATTTTCC	250
Ad02A7297	Aradu.A02	7297	p5	(AAATA)5	25	30357634	30357658	TTTTTCCCTTTCGGTCACT	AGCTGCTTGCTTTAATGGGA	190
Ad02A7304	Aradu.A02	7304	p3	(AGA)6	18	30430051	30430068	CTCGGAAGTGGAAGCTTTTG	GCTTCTACCCACTCTTCCCC	272
Ad02A7305	Aradu.A02	7305	c	(A)10taaaaatag	56	30434796	30434851	GAGGTGATGGATTCATAGGACA	AATGCCAAAAGTTCTCCCCT	261
Ad02A7306	Aradu.A02	7306	c	(TTTA)6ttagaa	90	30446149	30446238	TTTCTCCCAGAGTGTGTTTGG	TCACATTAATATTCAAACCGAATCA	272
Ad02A7308	Aradu.A02	7308	p2	(AG)8	16	30490747	30490762	TCGCTATTCCGAACTCATTTG	AGCCATTATCCCACGTCACT	252
Ad02A7309	Aradu.A02	7309	p2	(TG)6	12	30503030	30503041	TCCGAGTCGCTAATGTCCTAA	TTCCAACTGAAGAGATAAGTGTGT	257
Ad02A7310	Aradu.A02	7310	p2	(TC)6	12	30508196	30508207	AGGGAAAATGAACATTGGGA	GGTTTGGGGTTGGTCAGATA	280
Ad02A7312	Aradu.A02	7312	c	(A)13caagggtg	93	30543912	30544004	GTGTTCCATGGTGTTTCCCT	TTCGAAAATTGTGAGAAATTAAAGG	159
Ad02A7313	Aradu.A02	7313	p3	(TAG)5	15	30545540	30545554	AGCTGAAGCGCCAAAAATTA	CATGCCTGATGATGATGAGAA	243
Ad02A7316	Aradu.A02	7316	p5	(AAGAT)5	25	30594646	30594670	CAGAGGAACACGACCCAAAT	TGCTGATCCTTCATCACCAG	277
Ad02A7317	Aradu.A02	7317	c	(T)10aagttgaaa	70	30603181	30603250	GTTTGATGATCCCTCTTGGC	TCTCATGGTTGACATTTTCCA	280
Ad02A7318	Aradu.A02	7318	p2	(AG)6	12	30632944	30632955	GATCACTGACCTCAGACGCA	TTCATTCCACACACCATTCG	268
Ad02A7324	Aradu.A02	7324	p3	(TTC)6	18	30773210	30773227	CAACTTCTCTTTCCCACCCA	ACCACTGAGCTATGACGGCT	273

ID	Chromosome	SSR nr.	SSR type	SSR	Size	Start	End	FORWARD PRIMER (5'-3')	REVERSE PRIMER (5'-3')	Product (bp)
Ad02A7328	Aradu.A02	7328	p2	(TC)7	14	30855731	30855744	CCAGATCCAAGCACAGACAA	TGGATCCAGAGTTACCCTCAA	177
Ad02A7329	Aradu.A02	7329	p2	(TA)8	16	30863381	30863396	TGAAGTTTGGGAAAGTTGGG	TTACTTGGGCCATTTTGTCC	266
Ad02A7330	Aradu.A02	7330	p3	(GAA)6	18	30867052	30867069	ACTAGCAGAGCTCTTCCCCC	GAACTTCCGGGTACTCTCCC	203
Ad02A7331	Aradu.A02	7331	p3	(GAA)5	15	30875424	30875438	GGCATGTAGAGCAAAGGAGG	GGGTTGTGAGCTTTCGTGTT	203
Ad02A7333	Aradu.A02	7333	p2	(TG)8	16	30897590	30897605	TCTTCCCCTTCCCTGATTCT	CCTAACCCACCTAACAGCCA	162
Ad02A7334	Aradu.A02	7334	p2	(TA)6	12	30910433	30910444	ATTTGGCAATGAAGGGTGAG	GGCATGGTTGGTTATTCACA	230
Ad02A7337	Aradu.A02	7337	p2	(CT)6	12	30982877	30982888	GATCGGAGCTATGGTTTGGA	TGGCCCATATTGACCTAAGC	143
Ad02A7340	Aradu.A02	7340	p2	(AG)18	36	30989170	30989205	CCAGCTCTCTCTGGACTTGG	CCACATCACATCCTCACCAC	248
Ad02A7343	Aradu.A02	7343	p2	(AT)9	18	31004779	31004796	CGTAGGCACGGACATAACAA	TTTATGGCCGTAGAGGCTTG	244
Ad02A7348	Aradu.A02	7348	p3	(TTC)6	18	31033307	31033324	TCTCTACAAGAGGGCTCCGA	GCCCTAAATTCAAAATGCGA	116
Ad02A7350	Aradu.A02	7350	c	(T)10ctatatttttt	37	31052696	31052732	TTTACTTTGGGGCATTTTGC	TGCATTGACGTTCTTGCATA	148
Ad02A7351	Aradu.A02	7351	p2	(AG)7	14	31094191	31094204	TGTCCCAACTTTTCTTTGCAT	CACTGGAGCAGAAACAACGA	204
Ad02A7352	Aradu.A02	7352	p3	(TTG)5	15	31094750	31094764	TTGTTGTGTCTTGTTGCTGCT	TGCTTTTGCTTCTACAGCCA	171
Ad02A7353	Aradu.A02	7353	p2	(AC)6	12	31103257	31103268	TTTCAAGGTTGTAATGGGGC	AGGGGACAAAATTACCCCAA	259
Ad02A7355	Aradu.A02	7355	p3	(AGA)6	18	31109638	31109655	TGCCATTGATGGTAGTGGAA	CCCACCCTAAATAGCCGAAT	243
Ad02A7359	Aradu.A02	7359	p3	(TTC)11	33	31136940	31136972	ACCCACATCTCTTCCCCTCT	TGCCATTGATGGTAGTGGAA	156
Ad02A7360	Aradu.A02	7360	p3	(TAT)8	24	31154768	31154791	TTGCAATGTTGCATAATAGTTAAAAA	GGGGACTAATTTGACCAACAAA	278
Ad02A7362	Aradu.A02	7362	p4	(AAAT)5	20	31166897	31166916	TCAATCCGCATACCAAACAA	GGCGTCATTAAGCCGTATGT	230
Ad02A7365	Aradu.A02	7365	c	(TTC)5atccaaa	130	31219731	31219860	TCATTCAGACCAAAATACCCTTC	TTGTTGTTTCTTGTTGCTGCT	279
Ad02A7366	Aradu.A02	7366	p2	(AG)6	12	31223027	31223038	GTCACTTTTCCGCAACCTGT	AAACCTGGGATCGAGAGTCA	259
Ad02A7371	Aradu.A02	7371	p3	(AGA)6	18	31271159	31271176	AGGTTTCTCCGGCCTTATGT	ACCCCTCCATCCTTCTTCAT	220
Ad02A7374	Aradu.A02	7374	p3	(AAC)13	39	31310849	31310887	TTTTGGAAAATGGATGCACA	TCCAATGCAAACCTAACTACCC	246
Ad02A7376	Aradu.A02	7376	p3	(AGA)7	21	31317527	31317547	GGCCTTAGGTGCCATCAATA	AAACCCACACCTCTCTCCCT	153
Ad02A7377	Aradu.A02	7377	p2	(CT)6	12	31344084	31344095	TACACGCCTAAATGGTGCAG	AGAGAGAGTTGCCGAATGGA	280
Ad02A7378	Aradu.A02	7378	p2	(CT)6	12	31344275	31344286	TCTTCCCCAAAAACCCTACC	GAGGGAGAGATAGGTTCGGC	179
Ad02A7381	Aradu.A02	7381	p3	(TGA)6	18	31383954	31383971	GCGTCAGATCTCGACCTTTC	GACGAGTCTGAAGAGGCACC	235
Ad02A7387	Aradu.A02	7387	p2	(AT)6	12	31452687	31452698	AATGTACCATTCCTTCCCCC	AAAATCGAAGCGACAACACC	207
Ad02A7389	Aradu.A02	7389	p2	(TA)11	22	31456039	31456060	GTCCTCCCAGAAGGTTCCTC	TGTTCATCTGCCATCTCCAA	237
Ad02A7390	Aradu.A02	7390	p3	(TTA)7	21	31458134	31458154	TTCAAGCACCTGATCATCCA	CCTTGCAATCTAACCACGCT	246
Ad02A7393	Aradu.A02	7393	c	(TCG)6(TCA)	151	31468848	31468998	TTGTTCTCATCCCCAAGTGTC	TCAGGAAGAAGCAACAAATCAA	264
Ad02A7394	Aradu.A02	7394	p3	(ATT)7	21	31470543	31470563	ATGATTTGAGTTTGGATTAATTAAGAT	CGGCAAAATATCGAACAAGAA	231
Ad02A7395	Aradu.A02	7395	p2	(CT)8	16	31473529	31473544	GGAACATTAAACCTGGAAATTGA	AGCGATTTCTGGACCCTTTT	248
Ad02A7397	Aradu.A02	7397	p2	(CT)6	12	31510689	31510700	GATTTTGCTTGCCATTTGGT	ATAGGTGAGGGGTTTTTGGG	155
Ad02A7398	Aradu.A02	7398	p3	(ATA)7	21	31534761	31534781	TGATGCTAGTTTGATGCCCA	AACCACCAGTCCACCATCAT	227

ID	Chromosome	SSR nr.	SSR type	SSR	Size	Start	End	FORWARD PRIMER (5'-3')	REVERSE PRIMER (5'-3')	Product (bp)
Ad02A7399	Aradu.A02	7399	p3	(GTT)5	15	31534952	31534966	CCTTCGACCCAGTTCTTTCA	CCCAATGATGTTATCCAGCA	204
Ad02A7401	Aradu.A02	7401	c	(T)10gaaaaaga	99	31565077	31565175	TTGATTTGAAAAAGATTTGAAAAAG	AGGGTACCTAGCTGGGCATT	265
Ad02A7403	Aradu.A02	7403	p2	(TA)8	16	31596612	31596627	CAATTGTGGCCATAACAGCA	TGTCTTTGATGGCATTGTGAA	149
Ad02A7404	Aradu.A02	7404	p2	(TA)7	14	31602038	31602051	GCATGTCTTCATGCTAAACCAA	TGCTTGGGATTTGATTGTTG	139
Ad02A7407	Aradu.A02	7407	p3	(AGA)5	15	31633098	31633112	TCAGCAGTAGATCAAAGCCAGA	ATCTCCTTCCCCTTTGCACT	212
Ad02A7410	Aradu.A02	7410	p3	(AGA)6	18	31642696	31642713	TTGCTCGTCCTTGAGCAATA	CTTGGCACCTCAGGATCTGT	215
Ad02A7412	Aradu.A02	7412	p3	(TAC)6	18	31650068	31650085	GACAAAAAGTATTGCCACCACA	TCCCAAAATAAAGGTCGTTGA	272
Ad02A7417	Aradu.A02	7417	p2	(TC)6	12	31693982	31693993	TATGAAAAATGTGCGACCCA	AAGATGATTCGCCTCCCTTT	250
Ad02A7418	Aradu.A02	7418	p2	(TA)6	12	31726093	31726104	CTCCCATCTCAATTGCAAAA	ATGGGTTTATCAACCTCCCC	264
Ad02A7419	Aradu.A02	7419	p3	(CTT)6	18	31733966	31733983	GTGCTCTTTTCCCTTGCTTG	ACAGTGGCCTGAGCTAGGAG	206
Ad02A7421	Aradu.A02	7421	p2	(CT)6	12	31783815	31783826	CAGGAAAGGGCAAGAAGTTG	TTGGGGAAGAAGTGTTGAGG	221
Ad02A7422	Aradu.A02	7422	c	(A)10cgaaaaaa	58	31785264	31785321	TTGTTGGCTCTTGAAAGAATGA	TCCCCAATTAGAGGTGTCCA	233
Ad02A7423	Aradu.A02	7423	p2	(GA)6	12	31805746	31805757	AGAGTGGGTGAAAGCGAGAA	CGCTCACGTGTGGATCTCTA	265
Ad02A7425	Aradu.A02	7425	p2	(AT)6	12	31821339	31821350	TGAAAATCTTGGGAATTGGG	TCCCTTTTGATCACCACACA	222
Ad02A7428	Aradu.A02	7428	p3	(TAA)5	15	31853677	31853691	TCTCATGCTTCCGTTTTTCC	TTGAGATACGTGAATCGAGTAACAA	277
Ad02A7430	Aradu.A02	7430	p2	(TA)6	12	31856544	31856555	TTCCATAATTTTGAAAACCACTGTT	CCCACTTAGTTCAATCGCAA	205
Ad02A7431	Aradu.A02	7431	p3	(TAT)21	63	31859235	31859297	TTCTTCATCTTTGTCTTTCTCTTTTT	CATTGGTATTCTCACCAAATTATATGT	280
Ad02A7433	Aradu.A02	7433	p2	(TA)7	14	31880219	31880232	AACCATGAGTTTTAAGGGTGG	TCATGATGGATAAAAGAATTTGGA	268
Ad02A7435	Aradu.A02	7435	c	(T)11gaaaaaag	39	31907044	31907082	AAGTGCACACTCCATTATTTTTC	ATTCAAGGGCAACGTTTTGA	280
Ad02A7438	Aradu.A02	7438	p3	(AGC)7	21	31913633	31913653	TTATCCACCATAACCTCCGC	CACCGTGCTTCCTTCTTCTC	146
Ad02A7440	Aradu.A02	7440	p2	(AT)8	16	31928795	31928810	TCTTTTGAATAGATCAATATTCGCA	TAAGCCTTTTGGTGTGGGTT	215
Ad02A7442	Aradu.A02	7442	p3	(CTT)5	15	31947526	31947540	CCAATGGAGGTTCAGCATCT	TCGACGTTCGAACACTCAAG	198
Ad02A7446	Aradu.A02	7446	p3	(TTG)5	15	31955211	31955225	AGAAGGAATCAGAGCCACCA	CCGGAAGTGGAAGAATAGCA	279
Ad02A7447	Aradu.A02	7447	p3	(GTG)7	21	31955607	31955627	ACATAATGGGCCGCAGACTA	GCGTTGCGCTTTTTATTTTC	205
Ad02A7449	Aradu.A02	7449	p3	(GAA)5	15	31966388	31966402	CCCTGTGCATCACAACATTC	CATTCATCGTTCGACTCCCT	269
Ad02A7452	Aradu.A02	7452	c	(T)10c(T)10	21	31984279	31984299	CACACTCTTGTTGCCTTGGA	GAAGCAAGAAAAAGCAGTGGA	125
Ad02A7455	Aradu.A02	7455	p2	(TC)6	12	32047770	32047781	ATTACAGAACATGCCCCAGC	CAATTCAAATGCCGGAATAAA	275
Ad02A7457	Aradu.A02	7457	p2	(TC)6	12	32055538	32055549	CACCCCAGCGAGTTCTAAAG	TGATCAATTCAAATGTCGGAA	269
Ad02A7458	Aradu.A02	7458	p2	(AT)8	16	32062411	32062426	ATTGACCAGAACTCCATGCC	TTGCCACGACAGAATTTCTTA	184
Ad02A7459	Aradu.A02	7459	p3	(AAG)5	15	32071483	32071497	TTCAGGCAACCATTCAACAA	TCTTGACTTTCCCATGGAGG	211
Ad02A7460	Aradu.A02	7460	p2	(AT)7	14	32090455	32090468	TTCTTTCTAAAATGAGAGTGCAACA	TTCCGGCGGACAGAATAATA	239
Ad02A7463	Aradu.A02	7463	p3	(CCT)5	15	32109342	32109356	ATGGTTTTTGCAGAGATGGC	GAAGTCTTGCGCTTCTTCGT	251
Ad02A7464	Aradu.A02	7464	c	(T)10ctttttctctt	50	32113730	32113779	TTCACCATTTACTTTGGGGC	TGGGTTTGGAAATGTTTGGT	209
Ad02A7474	Aradu.A02	7474	c	(AT)7(TA)7	28	32195809	32195836	GCGCCATTCTAGTAGCTCGT	TCGTCCTTATCTTTACAAGTTTTTG	236

ID	Chromosome	SSR nr.	SSR type	SSR	Size	Start	End	FORWARD PRIMER (5'-3')	REVERSE PRIMER (5'-3')	Product (bp)
Ad02A7480	Aradu.A02	7480	p3	(TAT)24	72	32232451	32232522	TGATACTTCGGGATGCACAG	TGAATCGAAAAGTCAGGACG	211
Ad02A7481	Aradu.A02	7481	p3	(TTA)15	45	32232891	32232935	CACGTTTGATTTTTGCATTGA	AAGATGTGGCAAAGGTGGAG	248
Ad02A7488	Aradu.A02	7488	p6	(AAGAAA)5	30	32259745	32259774	AACGACGGTAATCACAAGCC	AGAGTGGAGAGTGCTGCCAT	270
Ad02A7489	Aradu.A02	7489	p2	(AT)7	14	32261641	32261654	TGCCTTAGTAGAGGTGATGTCAA	CTCATGTGACGGAAGCTGAA	248
Ad02A7499	Aradu.A02	7499	p2	(TA)11	22	32303973	32303994	AAAAACAGGATTGCAGGGTG	TGTATGACTGCAAGGTTGGTG	244
Ad02A7501	Aradu.A02	7501	p2	(GA)7	14	32323109	32323122	TGGAGAAGCAGTGACGTTTG	AACCCTTTTCATTGCGTGTC	223
Ad02A7502	Aradu.A02	7502	p5	(TTATT)5	25	32324866	32324890	CAGTCCCCAGTTCATTTTCC	CAATCCCCAACGTTTCTCTC	233
Ad02A7507	Aradu.A02	7507	c	(A)10gaaaaaaa	99	32330578	32330676	AACCGTTTGGCAGAAAATTG	TGGCTAGGAGGGAGAATGAA	240
Ad02A7510	Aradu.A02	7510	p3	(CAT)5	15	32335768	32335782	TGTGGGTCATCATCATCTTCA	TGGTGTACCTGCTGCTTTCA	274
Ad02A7511	Aradu.A02	7511	p2	(AT)6	12	32336194	32336205	CACCAAATTTGAACATTAGAACCA	AGGGGTAGTTGGGGCTAACA	277
Ad02A7514	Aradu.A02	7514	c	(A)10taaaagaa	43	32376266	32376308	TTAAGTGTGGGGAAAGTGGG	TACTTTGGGGCATTTTGTCC	232
Ad02A7515	Aradu.A02	7515	p3	(AGT)5	15	32392848	32392862	GCCCACTCAACATGACTCAA	CATCCAATTTCCACCCAAGA	245
Ad02A7517	Aradu.A02	7517	p2	(AT)7	14	32418694	32418707	AACCCCTAAATCCCTAAACCC	TTGTCCAAATTCCAGCCAAT	248
Ad02A7518	Aradu.A02	7518	p2	(CT)7	14	32439754	32439767	TTTCTGGGCTGTTTTTGACC	GGAAAGCTTTGAAAACAAGGAA	263
Ad02A7520	Aradu.A02	7520	p3	(ATA)8	24	32446379	32446402	CAACAAACCACACAAGCCAA	TCCTTCCATGTTGGTGAATG	214
Ad02A7524	Aradu.A02	7524	p3	(ATC)7	21	32466725	32466745	CCTCAGGTCGTCTCTCAAGG	GCAAAAGGCAATTACCATCA	207
Ad02A7527	Aradu.A02	7527	p2	(GA)6	12	32492901	32492912	CGAAAATAGAGAAGGGAGAGGG	TCTCCTCTTTTTCAAAACCACA	157
Ad02A7529	Aradu.A02	7529	p2	(TA)6	12	32518293	32518304	TTGGCTAAGCCTTTTTGTCTTT	AACTGCGAAGATTTGGATGC	266
Ad02A7530	Aradu.A02	7530	p3	(AGC)5	15	32520946	32520960	GCTGCTTCCATTTTGTCCAT	TCACCATCATGAAACCCAGA	223
Ad02A7531	Aradu.A02	7531	p3	(AAT)11	33	32526690	32526722	ATGCCTTTGCATGGTCACTT	TGTGGAAAGATTACTCCCGC	270
Ad02A7532	Aradu.A02	7532	p3	(ATA)12	36	32529363	32529398	GGAAGCTTCACTAATCGCCA	TCTTTTTGCCCCTTTTCTTTC	215
Ad02A7535	Aradu.A02	7535	p3	(ATT)5	15	32531859	32531873	TTGCAAAAGCCTTTGATTGT	AAATTTTTGCTTTGCTAACCTCA	250
Ad02A7539	Aradu.A02	7539	p3	(TTC)6	18	32603144	32603161	TGGCCGAACCTACATCTTTC	CTAGAGGATTCTCCGGCCTT	169
Ad02A7540	Aradu.A02	7540	p2	(AT)11	22	32620516	32620537	GGCAATTTTGGAGAAACATGA	TTCGAGTTTTGATATCGGGC	269
Ad02A7541	Aradu.A02	7541	c	(CA)16g(AT)6	138	32623347	32623484	CTAGTCGGCCTAAACTCCCC	TCTAGCCGTAAACCGAAGGA	259
Ad02A7542	Aradu.A02	7542	p3	(TAT)5	15	32632742	32632756	TGGCAGGAGTTTGACTTGTT	GCAAAAAGGAATCTGCAGTAGC	126
Ad02A7543	Aradu.A02	7543	c	(AC)12(AT)6	36	32633062	32633097	TGAAACCTGTAACACCCCAA	CTAGTCGGCTTAAACTCCGC	213
Ad02A7546	Aradu.A02	7546	p3	(TCT)5	15	32644975	32644989	ATATGGCCGAACCTTAACCC	AGTTTTGATGCTTGCTTGGG	261
Ad02A7548	Aradu.A02	7548	p3	(AGA)6	18	32658254	32658271	CGTCCTCGAGCAAAAAGAAA	GATCCCCACCTACCCAATTC	179
Ad02A7552	Aradu.A02	7552	p3	(ATG)5	15	32713057	32713071	TGGTGCAGAGCTTTCAAACTT	TGCGTTGTTTTCAAAATTGC	279
Ad02A7553	Aradu.A02	7553	p2	(GA)9	18	32734060	32734077	TGTGTGCATATTTTATGATTCTTGC	TCTCGCTCCTCTCTCTTTCCT	278
Ad02A7554	Aradu.A02	7554	c	(GA)11ggggaa	48	32734221	32734268	GGGATAGAGATTGAGAAAAGGTTG	CAGTGCACCAGTTTAAACCAA	244
Ad02A7564	Aradu.A02	7564	p2	(CT)7	14	32761883	32761896	CATGCTTTACATGGGCATTG	AATTTTGAAGGGGAGTTGGG	249
Ad02A7568	Aradu.A02	7568	p3	(AGA)6	18	32836652	32836669	CTAGAGGATTCTCCGGCCTT	AAATCTTTCCCACCCAAACC	273

ID	Chromosome	SSR nr.	SSR type	SSR	Size	Start	End	FORWARD PRIMER (5'-3')	REVERSE PRIMER (5'-3')	Product (bp)
Ad02A7578	Aradu.A02	7578	p3	(TTC)12	36	32989606	32989641	CCGAACCTACCTCTCTCCCT	TGATGGTGGGTGAAAACAAA	157
Ad02A7579	Aradu.A02	7579	p3	(AAT)6	18	33005125	33005142	GGAAAAGGGCACTCTTGTTG	TTGGATGTTGGCTTTGTGAA	195
Ad02A7580	Aradu.A02	7580	p3	(TCT)5	15	33008171	33008185	CTCCTACATAGCCGAACCCA	CGGTCTTAGGTGCCATTGAT	166
Ad02A7582	Aradu.A02	7582	p5	(AAGAT)5	25	33042786	33042810	AAAGGGCCCAAATAGAGGAA	CCTGGGTGCTCCAGTGTATT	185
Ad02A7583	Aradu.A02	7583	p5	(AAGAT)5	25	33049159	33049183	GCCCAAATAGAGGAACACGA	CCTGGGTGCTCCAGTGTATT	179
Ad02A7584	Aradu.A02	7584	p2	(GA)6	12	33091848	33091859	GAAAAGGAAGGAGGGAAGAAGA	CGATCGCGTCAACTGAACTA	242
Ad02A7586	Aradu.A02	7586	c	(TC)6tttctctctc	48	33132481	33132528	GGTGAGGATGATGGCAAGTT	AAGGGAAGAGAAGAGAGCGG	224
Ad02A7587	Aradu.A02	7587	p4	(TTCT)5	20	33132648	33132667	CCTCCTCTCCTTCCTCGTCT	ACACATACTCCCCAACCCAA	262
Ad02A7588	Aradu.A02	7588	p2	(GC)6	12	33139289	33139300	AGAAGGAACGCAAGGAAACA	TCTGTGATTTCTGCAGGTCG	150
Ad02A7590	Aradu.A02	7590	p2	(AG)24	48	33183394	33183441	AGTGGTGAAGAGGTGATGGG	CGTTTAACGCTAGGATTGGC	247
Ad02A7594	Aradu.A02	7594	c	(T)11gtg(T)10	24	33201362	33201385	GCATGCAATTGACACCAAAC	AACATTGCACTGGTTCCTGG	167
Ad02A7595	Aradu.A02	7595	c	(GTAT)5taaag	58	33234183	33234240	CTGAGCATTTGATTGTGCTTT	GGAGCCTTTACCTGAAATTTTG	260
Ad02A7598	Aradu.A02	7598	p4	(AATA)5	20	33244614	33244633	ACCACTCGACCTGCTCAAAT	CCCAAAATCAAGCAATGACA	197
Ad02A7599	Aradu.A02	7599	p3	(AAT)22	66	33246456	33246521	GAAAAGCGCATCTGCTGAAT	TGTTGAGGTTCACCCACTCA	240
Ad02A7600	Aradu.A02	7600	p3	(TAA)8	24	33247053	33247076	TCAGGATCCAGAACCTTCAA	TTCCCCTAACCCATTACCAA	178
Ad02A7602	Aradu.A02	7602	p2	(TA)9	18	33249976	33249993	AGAGAAGGAGGACGAGGAGG	TACATGCACATCATGGCAAC	225
Ad02A7603	Aradu.A02	7603	p2	(CT)6	12	33254104	33254115	GATCTCGACCATCCCTTCAA	ATGCTTGGTGTTGAGCTGTG	246
Ad02A7604	Aradu.A02	7604	p2	(AC)7	14	33262142	33262155	CCCAACCAAGCCTCATTAAA	AAATGGTGCGTTAATCGGAG	161
Ad02A7605	Aradu.A02	7605	p2	(CT)7	14	33272556	33272569	CCTCTTTCCACCACACCATT	CGGAAAAGCACACCAAACTT	112
Ad02A7608	Aradu.A02	7608	p2	(TC)6	12	33308359	33308370	CCCCCTAACTACTTTTCCCTC	TCCAAACACAAGGGTAAGGAT	261
Ad02A7609	Aradu.A02	7609	p3	(TTC)6	18	33311763	33311780	TACACCTCTCCCCCTCTCCT	AGTGGTCTTGATGGGCTTTG	245
Ad02A7612	Aradu.A02	7612	p3	(AAT)16	48	33349423	33349470	TTTCTCTCTCCTCACTTTTTCG	ATGATGGAGCAAGGGAGATG	207
Ad02A7618	Aradu.A02	7618	p2	(AT)8	16	33476914	33476929	ACTTTTCCGGGTTGGGTATC	GGAGGAACAAAGAAGGGGAT	266
Ad02A7619	Aradu.A02	7619	p3	(AAT)5	15	33490413	33490427	ATGAGCAGGAACAGGGAAAA	AAACTTGGACGTTGGCTTTG	210
Ad02A7624	Aradu.A02	7624	p2	(GA)7	14	33511812	33511825	CACAGAGGGAAAGAGAGGGTT	CACTTTTTCACTTCCAATTTTCG	261
Ad02A7629	Aradu.A02	7629	p3	(AAT)11	33	33584210	33584242	TCTCGTTCAAATTCAAACCTCA	AAACTTTTGGCAGATAAAAACCTT	231
Ad02A7630	Aradu.A02	7630	p3	(ATT)5	15	33584615	33584629	AATTTTGAAAAGAAACTGACCG	CATCCTCTTTCTCTCTTGTCTGC	264
Ad02A7631	Aradu.A02	7631	p2	(TA)14	28	33585410	33585437	TGACCACAATCCAATCAATATG	CCCGTACAAGATCTAGAACCTCA	276
Ad02A7634	Aradu.A02	7634	p2	(TA)7	14	33610132	33610145	GGGATTACAATCTGGGCTGA	TCTCAAAGTAAGTCAGTTCAATGGAT	266
Ad02A7635	Aradu.A02	7635	p2	(AT)15	30	33611006	33611035	AGCTCTAACTCAGCCTGCGA	CTCGCTAGGCATCTGAATCC	260
Ad02A7636	Aradu.A02	7636	c	(T)13gaacagga	45	33628383	33628427	CCTAGGAGAGGCCCAACTCT	GGGAGTCTTTGGGCAAGTAG	180
Ad02A7637	Aradu.A02	7637	p3	(AGA)6	18	33655129	33655146	AGAATATTGCTCGCCCTCAA	AATCCCTTTCCCATCCAAAC	208
Ad02A7638	Aradu.A02	7638	p2	(AT)7	14	33687856	33687869	TTTTTGAAAATGTTTGGATTGA	GCCAGCGTAGGGACTTATTG	256
Ad02A7640	Aradu.A02	7640	p2	(AT)6	12	33705052	33705063	TTAGGAAAGGATTGCTTGCG	GGGGTTGAGGGAGAGATAGG	212

ID	Chromosome	SSR nr.	SSR type	SSR	Size	Start	End	FORWARD PRIMER (5'-3')	REVERSE PRIMER (5'-3')	Product (bp)
Ad02A7641	Aradu.A02	7641	p3	(TTA)5	15	33716051	33716065	TTCAAAACCAAATGCAGCTAA	GATTCACGGTTTGACCGATT	270
Ad02A7642	Aradu.A02	7642	p3	(TTG)10	30	33720365	33720394	AACATCCAGTCAGGAGGGTG	CACCACCACCACACTTTCAT	276
Ad02A7643	Aradu.A02	7643	p2	(AT)10	20	33722391	33722410	CGGTCTTAGGTGCCATCAAT	CCATATGGCCGAACCAATAC	249
Ad02A7645	Aradu.A02	7645	c	(AT)6actctactc	120	33734238	33734357	CCCGCAATCCTGACATTTAT	TTGTTTGATGCAACCCAGAG	269
Ad02A7648	Aradu.A02	7648	p4	(AAAG)8	32	33748767	33748798	AACCCAAAATTCCCAATTCC	CAACGCTCCACCTCTTTCTC	172
Ad02A7649	Aradu.A02	7649	p3	(AAG)8	24	33762835	33762858	GCTTTTGACTTCCCTTTCCC	TCTTCTCCCACTTAGCCGAA	206
Ad02A7651	Aradu.A02	7651	p2	(AG)6	12	33789055	33789066	GTCTGCCTTGTAGCCACCAT	ATTTTGACCACCCTGCACTC	277
Ad02A7656	Aradu.A02	7656	p2	(AT)8	16	33824436	33824451	AAATCAAGAGCACCGAGTCAA	TCACGGTTTTAGTCGTTCCC	158
Ad02A7665	Aradu.A02	7665	c	(A)10taagaaaa	77	33903626	33903702	CGGAACCTTGAAACGACATT	TTTTGTGATCTGTTGAAGAAGGA	166
Ad02A7666	Aradu.A02	7666	c	(CA)6tatcctctc	41	33905373	33905413	CCACGCCTTCTCAATTTGTT	CATAGCTGCTTCAAACACCG	278
Ad02A7667	Aradu.A02	7667	p3	(TGA)8	24	33906664	33906687	GATCACTTAGCGGCTCTTCG	CTGGTGAAAGGAACATCGGT	245
Ad02A7668	Aradu.A02	7668	p3	(CTC)9	27	33912309	33912335	TTTGCATACATGGCAAGGAA	AATCACAATGGGCTGAGAGG	165
Ad02A7669	Aradu.A02	7669	p2	(GC)6	12	33917089	33917100	CCAGTTGGGTTGGAAAGCTA	ACATGGCTCCTAAAACCGCT	162
Ad02A7673	Aradu.A02	7673	p3	(AAG)5	15	33946922	33946936	CTAGAGGTTTCTCCGGCCTT	CCTACTCCCTCTCCCCTCAC	249
Ad02A7676	Aradu.A02	7676	p2	(TC)18	36	33970322	33970357	TGGTTCAAAGGGTTATTGGC	GCTGACAATGCTCCTTGTTCT	272
Ad02A7678	Aradu.A02	7678	p2	(GC)6	12	34014747	34014758	TAATTGCTCCCCATTTGCTT	AGTGGTTTTCCACAAGGACG	225
Ad02A7679	Aradu.A02	7679	p5	(TTATC)5	25	34020247	34020271	CTGGGTGCTCCAACGTATTT	GAGTCCAGAAAGATGAGGCG	138
Ad02A7680	Aradu.A02	7680	p2	(CT)6	12	34026086	34026097	GCACCAGACTGGCGTTTAAT	TGGGATTTGATAGGTGAGGG	266
Ad02A7683	Aradu.A02	7683	p2	(AT)8	16	34107127	34107142	CCTTTTTCAATCAAAGCACGA	GTGACCGGTGTTTGCACTTA	279
Ad02A7685	Aradu.A02	7685	c	(A)10tttgatgag	64	34132605	34132668	GAATTTTGGGACATTGGGTG	TTCACTTTGGGGCATTTTGT	258
Ad02A7686	Aradu.A02	7686	p2	(TA)8	16	34146120	34146135	AATTCCCTTTATGCTCCAATTC	GACAAATGGCACGAAAGGTT	161
Ad02A7690	Aradu.A02	7690	p3	(AGA)6	18	34183694	34183711	CTAGAGGATTCTCCGGCCTT	CCTATTTGGCCGAAACTTCA	262
Ad02A7695	Aradu.A02	7695	p2	(CA)7	14	34234250	34234263	CCCTTAAAATGAGCACGGAA	ATTCTCCGCTTGATGCTTGT	100
Ad02A7697	Aradu.A02	7697	p2	(AT)9	18	34256726	34256743	AGTTGTTCAATGCCTTTGCC	CAGAACCCCAGCATGGTACT	131
Ad02A7705	Aradu.A02	7705	c	(GA)11gtgtgtta	91	34316500	34316590	CCAAGCAAAAAGTTGAAAGGA	ATTGGGCAGAAGAGGATGTG	260
Ad02A7706	Aradu.A02	7706	p2	(AG)9	18	34316842	34316859	CACATCCTCTTCTGCCCAAT	GGGAAGATGCTTTGCTTGTC	279
Ad02A7707	Aradu.A02	7707	p2	(GA)6	12	34323070	34323081	TCCCATACCCACAGTCAACA	GTGGCCTCTTCTCCTCCTCT	206
Ad02A7708	Aradu.A02	7708	p2	(GA)6	12	34323328	34323339	GCTGTTCCATCGCCACTATT	ATCGGCAGTGGTTATTGGAG	174
Ad02A7713	Aradu.A02	7713	p2	(GA)8	16	34394097	34394112	TGTTCGCAAGCAAAGAAGAA	GAATTGCACAAATCCAAGGC	234
Ad02A7715	Aradu.A02	7715	p2	(CT)19	38	34414371	34414408	CGCGTGACTCTCTGTTCCTT	GTGTTGGGTGGGAGACAATC	248
Ad02A7721	Aradu.A02	7721	p2	(GT)6	12	34436329	34436340	AGGTCAATGGTTCGAAAAGC	TTCAGTGATCCAAAATTTAAAAGC	139
Ad02A7724	Aradu.A02	7724	p2	(TA)7	14	34448678	34448691	GCTCACCGAATGTCCAACTT	CTGTTCAGGTGCGCATACAC	259
Ad02A7726	Aradu.A02	7726	p3	(ATT)5	15	34453214	34453228	TTGCGTTGGTTGTTAACTTTG	GGTGAAGCCCGGATCTTAAT	238
Ad02A7727	Aradu.A02	7727	p2	(TA)10	20	34455075	34455094	TCTTGTCTCTCGGTGTGGTG	CAAAGAGAACGCTTATGACACG	247

ID	Chromosome	SSR nr.	SSR type	SSR	Size	Start	End	FORWARD PRIMER (5'-3')	REVERSE PRIMER (5'-3')	Product (bp)
Ad02A7733	Aradu.A02	7733	p3	(TCT)5	15	34473296	34473310	CAGGGGACGCTCTCTCTATG	TGTGAGTGGAAGAGTGTGGC	217
Ad02A7734	Aradu.A02	7734	p3	(TTC)5	15	34474623	34474637	CGTGTAATGGTTCTCGCCTT	AATCGCAACCCTTGAGTGAC	126
Ad02A7737	Aradu.A02	7737	p2	(AG)9	18	34488037	34488054	TGCAAAGAAGGTTGCAAGTG	AAGGCTTGCTGCTTCACTCT	276
Ad02A7738	Aradu.A02	7738	p2	(AT)10	20	34491750	34491769	GGTCCGGAGTGTTGATTTGT	CCACAACACATAGCATAGACCA	128
Ad02A7739	Aradu.A02	7739	p2	(AT)6	12	34496516	34496527	TTCTGTATGAACCAAGGCCC	AAGCCTGTAATCGTGTTAGCG	222
Ad02A7741	Aradu.A02	7741	p3	(AGA)6	18	34571654	34571671	TAAAATATTGCTCGCCCTCG	CCCATAAACCCCACCTACCT	242
Ad02A7747	Aradu.A02	7747	c	(AT)8(AC)6	28	34599998	34600025	TGGGAAAACTTAGGACATTGG	TGGAGAATTTTGTCCCCTTTT	242
Ad02A7752	Aradu.A02	7752	p2	(TA)7	14	34665938	34665951	TGGGGAATTTTATGGGAACA	GGGTTGTAATGGGACTGAGG	248
Ad02A7754	Aradu.A02	7754	p3	(TTC)7	21	34675861	34675881	TACCCTCTCCCCTTTCCCTA	CTAGAGGATTCTCCGGCCTT	250
Ad02A7755	Aradu.A02	7755	c	(T)10cgattttca(	29	34684531	34684559	GCACACTCTTATTTGCCTTGG	TGTGGTTCATGAATGTTGGC	200
Ad02A7760	Aradu.A02	7760	c	(T)10acgttatgc	110	34756795	34756904	CGTTGTGCTAACCGGTTTTT	TTGGCTTTGTTTTTCTTTTTCC	249
Ad02A7763	Aradu.A02	7763	p3	(TTG)5	15	34781008	34781022	TTGTTGGCATTGACATTTGG	AGCTGACCAAAGAACCCTCA	246
Ad02A7764	Aradu.A02	7764	p2	(CT)7	14	34793724	34793737	GCCAGAATTGGCAGAAAGAG	ATAGGTGAGGGGTTTTTGGG	178
Ad02A7767	Aradu.A02	7767	p3	(TTC)6	18	34843814	34843831	CACCTCAATTCCTCTTCCCA	CCCTCGAGCAAGAAAGAAAA	261
Ad02A7770	Aradu.A02	7770	p5	(AATAA)5	25	34862210	34862234	AATTGGGAATCAACGCTCAC	AAGCCACCATGACAAGGAAC	240
Ad02A7772	Aradu.A02	7772	p2	(TA)8	16	34899173	34899188	GCAGTAGCGGGGTTAGCATA	TGGTGTTCCTTGGTGTTTCTC	260
Ad02A7773	Aradu.A02	7773	p2	(AC)6	12	34912553	34912564	ACCTCAACCAATGTCCCAAG	ATCGGTGATTTTTCACCACG	211
Ad02A7775	Aradu.A02	7775	p4	(AACG)7	28	34934945	34934972	GAGCTTGAGTCAGGGCAATC	TCTGTGGATCCAGCAAAGGT	232
Ad02A7776	Aradu.A02	7776	p3	(TTC)7	21	34948734	34948754	CCCATAAACCACACCCAAAC	TGAAAATATTGCTCGCCCTC	214
Ad02A7780	Aradu.A02	7780	p3	(GAG)5	15	34991463	34991477	TCACTCAAGAGGAGGAGGGA	TAGCATCAAAGAGGGGGATG	255
Ad02A7782	Aradu.A02	7782	c*	(AAAT)6(AAT	36	35009162	35009197	TGAGTATGGGAAAATTGTGGG	TCATTGGGGTGATTTTGTCC	216
Ad02A7784	Aradu.A02	7784	p2	(AC)7	14	35026126	35026139	CAATTTCCCAAGTTTTCCCA	AGCTCACTTACCCAAATGGC	201
Ad02A7785	Aradu.A02	7785	p3	(AGA)6	18	35035692	35035709	AGTGGTCTTGATGGGCTTTG	CGAAACCTACACCTCCCCTT	250
Ad02A7786	Aradu.A02	7786	p2	(AT)6	12	35084013	35084024	TCTCTGTGATTCCTCCGCTT	TGTTCTTGCATGCATTATTTGTT	279
Ad02A7788	Aradu.A02	7788	p2	(GA)6	12	35095219	35095230	TTTGCAATAATCCACAAGCA	CCTTCCCCTTCTACCTCACC	239
Ad02A7790	Aradu.A02	7790	p3	(AGA)6	18	35111195	35111212	TTAAAATGTTGCTCGCCCTC	CCAAAAACCCCACCTACCTT	241
Ad02A7793	Aradu.A02	7793	p3	(TTC)6	18	35184543	35184560	GAACTTCCGGGTACTCTCCC	ACTAGCAGAGCTCTTCCCCC	203
Ad02A7794	Aradu.A02	7794	p2	(CA)6	12	35186616	35186627	AATTTTAAAGGTTTGGCCCG	GCGAAGAGAAGAGATGGGTG	210
Ad02A7796	Aradu.A02	7796	p3	(GAA)10	30	35193677	35193706	TAAAGGTTGGAGCCATCAGG	TTTTCTCCTCCCTTCACTGATT	191
Ad02A7798	Aradu.A02	7798	p4	(AATA)5	20	35222754	35222773	CCTTGAAGCAAAAGAAACAACA	GTTACTCCCAGACAGTGGGG	250
Ad02A7802	Aradu.A02	7802	p2	(AT)8	16	35316240	35316255	GGGTGCATGCTCATGTATTG	TACTTTGGGGCGTTTTTGTC	196
Ad02A7803	Aradu.A02	7803	p2	(TC)6	12	35321353	35321364	CCTTCTACCTTCGCAAGCAC	ACGCGATGCTGCCTAGTAGT	211
Ad02A7804	Aradu.A02	7804	p2	(AT)8	16	35325199	35325214	GATTTGGGAACATTGGTTGG	TGGATTTACTTTGGGGCATT	259
Ad02A7805	Aradu.A02	7805	p2	(CT)6	12	35346929	35346940	TCATGGTGGTTGCTTGATTT	AGACCCTTTTCGGTCCAGAT	278

ID	Chromosome	SSR nr.	SSR type	SSR	Size	Start	End	FORWARD PRIMER (5'-3')	REVERSE PRIMER (5'-3')	Product (bp)
Ad02A7806	Aradu.A02	7806	p3	(TCC)5	15	35378996	35379010	GGCCTCACAACACCAAACTT	CCCAGTATTCTCCCAAGCAA	227
Ad02A7810	Aradu.A02	7810	p3	(TCT)7	21	35389444	35389464	CCACAGGATCACCTCAGGAT	GGAGAGAGGTAGGTTCGGCT	272
Ad02A7811	Aradu.A02	7811	p3	(TTC)7	21	35389604	35389624	AGCCGAACCTACCTCTCTCC	GCTTTTGTCTTCCCTTTCCC	194
Ad02A7816	Aradu.A02	7816	p4	(TTTA)7	28	35443963	35443990	TGCAATGAATGAAAATAAGCAAA	TTTGCACCCTGTTTTTCTCC	224
Ad02A7822	Aradu.A02	7822	p3	(TAT)7	21	35508286	35508306	TGCATTGGATCAGAGTCAGC	CCAGAGAATGGCAAACCAGT	125
Ad02A7824	Aradu.A02	7824	c	(T)10cgtttttctc	91	35523616	35523706	GCACACTCTTTTTGCCTTGG	TGTGGTTCATGAATGTTGGC	263
Ad02A7828	Aradu.A02	7828	p5	(TTATC)5	25	35653962	35653986	CTGATCCTGTGTCCCCAGAG	AACACGACCCAAATCCAGAG	257
Ad02A7829	Aradu.A02	7829	p3	(AGA)6	18	35673199	35673216	TCGTCCTCGAGCAATAGAAGA	ATTCCCCATGTCACACTTCC	246
Ad02A7831	Aradu.A02	7831	c	(T)11attttgtaaa	31	35697859	35697889	TGCGGATATGCAGAAATCTT	GTTCTGCTGAAGCTTGGCTA	280
Ad02A7832	Aradu.A02	7832	c	(TA)7(T)10	24	35709816	35709839	CCCATGATTCCCACTTTTTC	CTTGGGACATTGGTTGAGGT	208
Ad02A7834	Aradu.A02	7834	p5	(TTATT)5	25	35726281	35726305	CCTAACCACTTTCTCTCTCCTCA	TGGGGTTAGCATATGACCCT	260
Ad02A7835	Aradu.A02	7835	p2	(GT)6	12	35765654	35765665	GTAACCCAAAGAGGTGCTGG	CATTCGTCAGAATGTCCCCT	245
Ad02A7837	Aradu.A02	7837	p5	(TTATC)5	25	35774935	35774959	CCTGGGTGCTCCAGTGTATT	AAGATAAAGGCGGTTCCGTT	130
Ad02A7841	Aradu.A02	7841	p5	(TATTT)5	25	35826836	35826860	TGATGGCCATAGAACATGGA	TCTTCGTGCACTTGCTTGAC	243
Ad02A7842	Aradu.A02	7842	p4	(TATT)8	32	35829249	35829280	GGCCTCAGACCTTAATGACG	TCAAAGCCTTGGCTGATAAT	253
Ad02A7850	Aradu.A02	7850	p2	(AT)10	20	35849041	35849060	GCAAAATCAGAGCTATTGCAGA	CAAATACTTTTTGAATGGTAGGGA	264
Ad02A7854	Aradu.A02	7854	p2	(AT)7	14	35862352	35862365	CTTGGGGAGAACAGACTGGA	TCCGCGAGGCATATTCTACT	249
Ad02A7861	Aradu.A02	7861	p2	(TA)6	12	35898263	35898274	AAGCTAAAGTGGAACGCAGC	TGGAACTCGAGAGAAAGGTGA	259
Ad02A7862	Aradu.A02	7862	c	(TG)19(AG)16	70	35899432	35899501	GTTCTCAATCGCACAGCAAA	CACCAAACCATGTATGGCAG	242
Ad02A7863	Aradu.A02	7863	p2	(CT)19	38	35903865	35903902	TTGGTTTGATTGCGTTACCA	TTTCCCAAACACAGAGGGTC	238
Ad02A7864	Aradu.A02	7864	p3	(TGT)5	15	35905836	35905850	GGATTGCTCCTCAAATTTTCT	ATCAAGGTTGAGGAAGGGCT	272
Ad02A7865	Aradu.A02	7865	p3	(TTC)6	18	35905985	35906002	ACAAGCCCTTCCTCAACCTT	GGACTTTGGGTTGGTCAAGA	191
Ad02A7866	Aradu.A02	7866	p3	(CAT)5	15	35906662	35906676	ATGTGAATGGACAAGCACCA	GGTGCACTTGTACACCAAGC	275
Ad02A7867	Aradu.A02	7867	p2	(AT)10	20	35906958	35906977	GGATGCACCAATTGTGAGTG	GGTTTCGATCGGTTGACAAT	250
Ad02A7870	Aradu.A02	7870	p3	(TAT)5	15	35908970	35908984	AACATGGTGGAGGTTGAAGC	GCTTTGCCATCCCTAATTCA	233
Ad02A7873	Aradu.A02	7873	p3	(AAT)5	15	35917750	35917764	ATGGCCAAATCCAAAAACAA	TCCGTCGTAAATCTGAGTTTCAT	216
Ad02A7875	Aradu.A02	7875	c	(TTA)9aggtcga	124	35919764	35919887	GTCTTTTGCAACACCCCAGT	TCAAGGGAGTTGGAATTGGA	275
Ad02A7876	Aradu.A02	7876	p3	(GTG)7	21	35923114	35923134	GAAGCAGTGTTCCCCACAAT	GGACCAGAAGTACTGCGAGC	195
Ad02A7878	Aradu.A02	7878	c	(TTTA)5attcttg	163	35925048	35925210	TTTGCTTACATCCCTCACCC	TGAGTTTGATTTGGATGTTTGG	270
Ad02A7879	Aradu.A02	7879	p3	(ATT)18	54	35925840	35925893	TTTGAAGAGAGCAATGGCAA	GAAGAGGGGCAAAACTGTCA	239
Ad02A7881	Aradu.A02	7881	p2	(GA)10	20	35926322	35926341	CCACCACACCAAGATCCATA	AACAAAACCATTCCGGTTTC	274
Ad02A7885	Aradu.A02	7885	p3	(TAA)5	15	35938949	35938963	AATTCTGCATTTCCCAGCAC	GATCCTGCATCAACACGAGA	207
Ad02A7886	Aradu.A02	7886	p3	(CAA)5	15	35939128	35939142	AGCATCTCGTGTTGATGCAG	CCACTTCGGTCCACAATTTC	180
Ad02A7892	Aradu.A02	7892	p2	(GC)6	12	35951590	35951601	CCAGTTGGGTTGGAAAGCTA	ACAAGGCTCCTAAAACCGCT	162

ID	Chromosome	SSR nr.	SSR type	SSR	Size	Start	End	FORWARD PRIMER (5'-3')	REVERSE PRIMER (5'-3')	Product (bp)
Ad02A7893	Aradu.A02	7893	p3	(TTC)6	18	35969250	35969267	TAGGGAGCTCTGTTGCGATT	CACGATATCCATCCAAACCC	235
Ad02A7894	Aradu.A02	7894	p3	(ATA)8	24	35969918	35969941	GAATCGTTCGCGTTTCATTT	AGGGGCTGTGACAGAGAAGA	228
Ad02A7895	Aradu.A02	7895	p3	(TTC)6	18	35970087	35970104	GAATCGTTCGCGTTTCATTT	TAATGGAACAAGGGGCTGTG	238
Ad02A7897	Aradu.A02	7897	p2	(TC)20	40	35980790	35980829	CACGATATCACGGTGTGAAAA	CACACAAAGGAGCGAGAACA	190
Ad02A7899	Aradu.A02	7899	p3	(TCT)5	15	35996395	35996409	AAAATGGCCGAAACCTAACC	CGGTCTTAGGTGCCATTGAT	247
Ad02A7903	Aradu.A02	7903	p3	(AAT)5	15	36013170	36013184	GGCACTCTTGTTGAAGCTGA	TTGGATGTTGGCTTTGTGAA	182
Ad02A7904	Aradu.A02	7904	p3	(TCT)5	15	36016183	36016197	CGAACCCAATCTCTCCCTCT	GTTCTTTGGATTCGGGTTCA	244
Ad02A7909	Aradu.A02	7909	p2	(CA)6	12	36063573	36063584	ACATGAAAACCCTAGCACGC	CGGTAGTTATGGCAGAGGGA	216
Ad02A7910	Aradu.A02	7910	p3	(TCT)6	18	36070700	36070717	AAACCCACACCTCTCTCCCT	TTGCCTTCCCTTTCCTCTTT	183
Ad02A7918	Aradu.A02	7918	p2	(CT)6	12	36152468	36152479	CTCTTCCCACCACACCATTC	CGGAAAAGCACACCAAACTT	107
Ad02A7928	Aradu.A02	7928	p2	(TG)6	12	36208560	36208571	TCCTTAAAGCATTTCAAGCACA	TTGCTTATGATGACTCACCCA	271
Ad02A7934	Aradu.A02	7934	p2	(GA)6	12	36283552	36283563	AACTTGCAATAATCCACAAGCA	TCTTCTTTCTCCCTTTCCCC	254
Ad02A7936	Aradu.A02	7936	p3	(CTT)5	15	36313542	36313556	CCCATGGAGTTTCTGCATCT	CGATGAAGAGCGTAGCATGA	149
Ad02A7937	Aradu.A02	7937	p3	(TCT)6	18	36353471	36353488	AAACCCACACCTCTCTCCCT	TTGCCTTCCCTTTCCTCTTT	183
Ad02A7938	Aradu.A02	7938	p2	(AT)6	12	36356878	36356889	TGCGCTTATCTAAAATTCCCA	CACTCTCGCGTGACTCTCTG	279
Ad02A7939	Aradu.A02	7939	p2	(TG)33	66	36364977	36365042	TGTCTTCGCAGGTCGAGACT	TTGCAAGGTACCTGTGAGAAAA	160
Ad02A7942	Aradu.A02	7942	p3	(AAT)11	33	36404990	36405022	TTCTGGTTTTGATCTGCTACGA	TTCGTGGATGAATGGTTAAAA	268
Ad02A7946	Aradu.A02	7946	p3	(AAG)5	15	36446084	36446098	CAGCCTTAGGTGCCATCAAT	ATTTCTTTCCCACCCAAACC	271
Ad02A7948	Aradu.A02	7948	p2	(AT)7	14	36453833	36453846	AAATTGTTGCGAAAATACTTGGA	GAAAACTGACTCGATCAATCTATACG	260
Ad02A7949	Aradu.A02	7949	p4	(AATA)5	20	36459095	36459114	CAGCTACTGCTTCCTCTCGAA	TTTCCATGTCTTGGGAAACA	228
Ad02A7952	Aradu.A02	7952	p3	(AGA)5	15	36462539	36462553	CTCAACGCTTCCATCTCTCC	TCTCTCTCCCGCACTCGTAT	242
Ad02A7955	Aradu.A02	7955	p3	(CTT)7	21	36464267	36464287	CAAGGTACGTTTCTTGCCTTC	CCTCCTCATCTTCTGCTGCT	206
Ad02A7956	Aradu.A02	7956	c	(CTT)11ctg(C'	51	36465013	36465063	ACAGAAGGAGATCGAAGGCA	CCTAACACGCGCATTACAGA	181
Ad02A7960	Aradu.A02	7960	c	(CA)9t(AC)6	31	36477098	36477128	ACGCCAAAAAGCGTACAAAT	AACCACCATTCACCCACATT	218
Ad02A7961	Aradu.A02	7961	p3	(ATT)5	15	36479011	36479025	TCTTGTTAATTTGAGCTCCTTACTTTT	TCATACCGCCAGATTTTGAA	241
Ad02A7963	Aradu.A02	7963	p2	(AT)7	14	36502160	36502173	AGAACGCCCTAAAAGCAAAA	TCTTTTCCGAACACTTTGCAC	276
Ad02A7964	Aradu.A02	7964	p3	(AGA)6	18	36503560	36503577	CATGCATTTTATCGAGAACACA	TCCTCCCCTTCTTCCTTCTC	262
Ad02A7965	Aradu.A02	7965	p3	(AGA)6	18	36511625	36511642	TCGAAGGTGGAAGCTTCTGT	AAATCTTTCCCACCCAAACC	234
Ad02A7968	Aradu.A02	7968	p3	(GTG)6	18	36554735	36554752	TGGTTTTCTTGGAAGGGATG	CCTCTTACCTCCCCTTCACC	276
Ad02A7971	Aradu.A02	7971	p2	(AT)11	22	36562758	36562779	ACTCAGCCTGTGAAGCCAAG	GCGGAGTATTCCCTGGTGTA	212
Ad02A7973	Aradu.A02	7973	p2	(GA)16	32	36565991	36566022	GGGAGAACGGGAAAAAGAAG	GCAGAATAGCGACGTCAACA	226
Ad02A7976	Aradu.A02	7976	p2	(AC)22	44	36592381	36592424	CAAAGTGGGCCAAACAGATT	GGGCTGAGAAAGGAAGTGTG	181
Ad02A7977	Aradu.A02	7977	p2	(TA)8	16	36595227	36595242	TTACTCCTTCATCGGGCTTC	GAACCGTGGACTGTCCCTAA	215
Ad02A7978	Aradu.A02	7978	p2	(AG)18	36	36607494	36607529	AGAAATGCAACTGGACAGGG	AGAACTTGTGTGTCACCCCC	167

ID	Chromosome	SSR nr.	SSR type	SSR	Size	Start	End	FORWARD PRIMER (5'-3')	REVERSE PRIMER (5'-3')	Product (bp)
Ad02A7979	Aradu.A02	7979	p2	(AT)7	14	36617232	36617245	TTAAGTGTGGGGAGGTCGTC	TGAAACAATGGCCTCAATCA	249
Ad02A7980	Aradu.A02	7980	p2	(AT)8	16	36617683	36617698	GGAGAATTGGAAACATTGGG	TACTTTGGGCATTTTGACCC	237
Ad02A7984	Aradu.A02	7984	p3	(TTC)6	18	36672081	36672098	AAAACTTTCCCACCCAAACC	CCCTCGAGCAAGAGAAGAAA	232
Ad02A7989	Aradu.A02	7989	p2	(AG)8	16	36703807	36703822	AAGAGAAGAGCTCGGCAATG	TGCTGTGAAGAACCGTCTTG	250
Ad02A7990	Aradu.A02	7990	p2	(TG)20	40	36711058	36711097	GGAGTCAAACTCCATGCTCA	GTTTGCGGTCTTTCACGATT	253
Ad02A7991	Aradu.A02	7991	p4	(TAAA)7	28	36742632	36742659	TGGGGAATTTTATGGGAACA	TTACATGCTCATGCACCAAA	257
Ad02A7993	Aradu.A02	7993	c	(AT)6(GT)10	32	36755345	36755376	GCCTTGGCAACTTGTTTGAT	TTATGCGTGAAAAATCGGGT	167
Ad02A7995	Aradu.A02	7995	p2	(AT)8	16	36772441	36772456	GCCTTGGCAACTTGTTTGAT	TAAAAAGATTTTCGCCCTCG	121
Ad02A7997	Aradu.A02	7997	p3	(CAT)6	18	36802720	36802737	AGAAGAAGTCAGGGCAGCAG	AGTCTGAGTTCTTGCTCCCG	157
Ad02A7998	Aradu.A02	7998	p2	(AT)7	14	36823669	36823682	ACTTGCCGGTTAGTTTGTGG	GGGGACACCAAACTTAATTTCA	231
Ad02A7999	Aradu.A02	7999	p2	(GT)7	14	36833361	36833374	AAGAGAAAGTTTTGGGCACG	TCTGTCCAACAAAGTGTGGC	196
Ad02A8006	Aradu.A02	8006	p2	(TC)6	12	36861575	36861586	GGGTAGGATTGGTGGTGTCA	TTTGTGCATTCACTTTCAAAAA	263
Ad02A8009	Aradu.A02	8009	p2	(TA)6	12	36883174	36883185	TGACAAGGACTTTTGTCCCC	TACCTCGCTGAGTGTGTTGG	263
Ad02A8015	Aradu.A02	8015	p2	(CT)6	12	37004802	37004813	GGCATTTTACACGCCTCATT	AGTGGTGAAGAGGTGATGGG	218
Ad02A8016	Aradu.A02	8016	p2	(CT)6	12	37005077	37005088	TTAACCCCTCTCCCTCCACT	GCTTTTGTCTTCCCTTTCCC	270
Ad02A8017	Aradu.A02	8017	p3	(AGA)7	21	37016746	37016766	GGTCTTGATGGCTCTTGGAG	TCTTCTCCCACTTAGCCGAA	261
Ad02A8022	Aradu.A02	8022	p3	(ATT)5	15	37071626	37071640	AAACTTGGACGTTGGCTTTG	CAGGGAAAAAGGCACTCTTG	199
Ad02A8023	Aradu.A02	8023	p2	(AT)9	18	37110514	37110531	AGGCGTCTGAGCTTCTGAAA	AATGAGCTCCTGACTCAGCC	228
Ad02A8025	Aradu.A02	8025	p2	(TA)11	22	37127548	37127569	CCCTCCTCCATGTTGAAAAA	TCACTAAACTCCCCCTACTCCA	277
Ad02A8029	Aradu.A02	8029	p2	(AT)8	16	37132878	37132893	CCACAAAAACCTGCAAAACA	CCTTCCTTAGCTTCATAACGAAA	277
Ad02A8030	Aradu.A02	8030	p2	(TA)7	14	37134749	37134762	GCTGTCCCACATTTTGGTCT	TTGCCCAGGAAAAATCAGTC	239
Ad02A8036	Aradu.A02	8036	p2	(AG)11	22	37234281	37234302	TCAGCTTCAACAAGAATGCC	GAAAATTTCCCTCTCTCGCC	253
Ad02A8039	Aradu.A02	8039	c	(T)10cgtttccttc	55	37291814	37291868	CAACAAGAGGTGTCCAGGGT	TGTGGTTCATGAATGTTGGC	242
Ad02A8040	Aradu.A02	8040	c	(T)11aaaaatgtg	91	37296362	37296452	TCGAAAACCAAGAGTGGAAAA	TTGAGAAAGGTTTCATGTTTGA	280
Ad02A8041	Aradu.A02	8041	p2	(GA)6	12	37305211	37305222	TTTGCAATAAGCCACAAGCA	TCTTCTTTCTCCCTTTCCCC	257
Ad02A8045	Aradu.A02	8045	p4	(AAAT)5	20	37343737	37343756	TGTGTCTTTTATGTGTTTTTCTCCA	TTCCATGATAGTGCAAGGGA	277
Ad02A8046	Aradu.A02	8046	p3	(AAC)5	15	37360465	37360479	AAACACCACTTTTGCGATCC	ACTAACAGGTCGCCTCATGC	198
Ad02A8047	Aradu.A02	8047	p3	(TTA)6	18	37363275	37363292	TCCAAATCAGTCCCCAAAAA	CGTCTATATGCACAAAGCCAA	180
Ad02A8048	Aradu.A02	8048	c	(TAA)11tattga	90	37363500	37363589	TGAAGCTTTGAAGTCCAAATTAAA	GGTTTGGGTTAGGATATGACCA	277
Ad02A8050	Aradu.A02	8050	p2	(AT)6	12	37369302	37369313	AAACGATTATCCAACCGTCG	TCCCAACTACTCCAAAGGGA	272
Ad02A8053	Aradu.A02	8053	c	(TTC)5atcttcat	85	37371642	37371726	TGTAACCCAATGTGGTCCCT	TGGTTAAACCCAACACAGCA	194
Ad02A8056	Aradu.A02	8056	c	(AG)6gaagagg	66	37397507	37397572	CGGAAAAGCACACCAAACTT	TTTTCGAAATTCTCCTCCCC	203
Ad02A8057	Aradu.A02	8057	p3	(CAA)5	15	37419535	37419549	GGATCATTCCCACAAAATCG	CCCGCCTTTCCATAATTGTA	221
Ad02A8060	Aradu.A02	8060	p2	(TA)6	12	37432631	37432642	TCCCTTTGTGTTTCCTTTGC	AGCAGGGATAAACCAACACG	158

ID	Chromosome	SSR nr.	SSR type	SSR	Size	Start	End	FORWARD PRIMER (5'-3')	REVERSE PRIMER (5'-3')	Product (bp)
Ad02A8061	Aradu.A02	8061	p2	(AT)8	16	37445905	37445920	GGAAAAAGAGAAAGTGGTTAGGA	CCTTGGTGTTTTTCCTCCAA	198
Ad02A8064	Aradu.A02	8064	p4	(ATTT)5	20	37469038	37469057	TATTAGATGCAACCAGCGCA	TTCAGCTTGGCATGAAGCTA	233
Ad02A8069	Aradu.A02	8069	p3	(AAT)7	21	37476312	37476332	CCTTGAAGCAAAAGAAACAGC	ACTCCCAGACAGTGGTGCTC	244
Ad02A8072	Aradu.A02	8072	p2	(AC)6	12	37504432	37504443	ATTTCCCTACCAAACCCACC	CCCGAGCAAAAGAAGAAAGA	174
Ad02A8076	Aradu.A02	8076	c	(CAG)5catcag	40	37553387	37553426	CAGATGTGGATGGAAAAGGG	CCCTATGATGCTTTCCGTTC	102
Ad02A8077	Aradu.A02	8077	p2	(AC)6	12	37554362	37554373	AGGCCTTTCAGCCTCATTCT	GCGAATCTAAGCACCGTAGC	119
Ad02A8078	Aradu.A02	8078	p3	(GAA)6	18	37563493	37563510	ACCACTGAGCTATGACGGCT	CACTTCTCTTTCCCACCCAA	273
Ad02A8079	Aradu.A02	8079	p3	(ATT)7	21	37566011	37566031	AAACTTGGACGTTGGCTTTG	CAGGGAAAAAGGCACTCTTG	231
Ad02A8084	Aradu.A02	8084	p6	(ATTTTT)5	30	37613441	37613470	CCTAACCAATTTCTCTCTCCTCA	ATGATCGAGCAAGGGAGATG	212
Ad02A8086	Aradu.A02	8086	p2	(AT)8	16	37626705	37626720	TTCAAGTAATGGAGAAATAATGAAAA	GGAATGATGAAGCGTGGTTT	270
Ad02A8090	Aradu.A02	8090	p2	(AT)6	12	37686692	37686703	TTTCAAGGTTGTAATGGGGC	GCATGCAATACAAAAGTGGG	168
Ad02A8094	Aradu.A02	8094	p2	(AT)7	14	37711608	37711621	TTAAGTGTGGGGAGGTCGTC	TTTCTCAAATCAATTGGGGG	227
Ad02A8100	Aradu.A02	8100	p2	(AT)11	22	37764871	37764892	TCAAGAGGATAATCAATCAATTTTGT	TTTGGTTGAAAAAGATCGAAAAA	277
Ad02A8102	Aradu.A02	8102	p2	(TA)8	16	37770266	37770281	CCCTCAATTTCAGCCAAAAA	TGTTATCCAAAAGAAGTCAGCA	203
Ad02A8103	Aradu.A02	8103	p2	(CA)6	12	37771668	37771679	AAAATGGTCCCCAAGGTTTC	GGTGAAGCAAGTGAGCTCGT	205
Ad02A8104	Aradu.A02	8104	c	(TTC)5ttaccgc	76	37771782	37771857	AGCTCACTTGCTTCACCACC	CCCAGAATCAGCAACAACAA	280
Ad02A8105	Aradu.A02	8105	c	(ATG)5(GTG)	80	37772176	37772255	TTGGTTGGTGTGATGCAGAT	CCCTTCTTCCCCAAAATCAT	215
Ad02A8106	Aradu.A02	8106	p2	(AT)6	12	37775069	37775080	TGATTTAGTTCTCAATTTGCTTTGA	CTTGCTTTGATGGGAGGTGT	271
Ad02A8112	Aradu.A02	8112	p3	(TTC)5	15	37840867	37840881	CACAACACAAACCCCCTCTT	ACTCGGAGGTGGAAGCTTTT	235
Ad02A8115	Aradu.A02	8115	p2	(TA)9	18	37933445	37933462	ACTCGACCTGCGAAGCTAAG	CGTTGAGCGTCTAAAAACCC	280
Ad02A8119	Aradu.A02	8119	p2	(AG)7	14	38030460	38030473	TGGTATGGTTCGGCCATTAT	CAGGATTGGTAGAAAGGGCA	227
Ad02A8121	Aradu.A02	8121	p2	(CT)6	12	38041855	38041866	TGGTGTGCAAAGGCATTTTA	TTTGAAGGTGAGTGGGGAAG	183
Ad02A8122	Aradu.A02	8122	p3	(ATT)6	18	38108504	38108521	AAACTTGGACGTTGGCTTTG	CAGGGAAAAAGGCACTCTTG	202
Ad02A8123	Aradu.A02	8123	p2	(AG)6	12	38108950	38108961	AAGAAGAAGGATGGATTCGGA	CAAAACCACCTAACTACTTTTTCCTC	237
Ad02A8128	Aradu.A02	8128	p2	(AT)11	22	38153937	38153958	GCTTCGAGCTTTCAGCATTT	GAGCATTGATGGGAATGTCA	253
Ad02A8129	Aradu.A02	8129	p2	(TA)7	14	38154738	38154751	GCAACGGAATAAACTAAGCTCAA	TTGCTGCATTTTGCACTTTT	151
Ad02A8132	Aradu.A02	8132	c	(T)10cttttc(T)1	26	38176267	38176292	TCCCCAATTAGAGGTGTCCA	TGTGGTTCATGAATGTTGGC	243
Ad02A8137	Aradu.A02	8137	p3	(AGA)6	18	38226823	38226840	TAAAATATTGCTCGCCCTCG	TCTTCTCCCACTTAGCCGAA	103
Ad02A8139	Aradu.A02	8139	c	(TG)22t(TG)6	57	38239181	38239237	TCCTTTGCTGATAAGGGTGG	TCCCCTTCTTATCCTTCGCT	249
Ad02A8140	Aradu.A02	8140	p2	(GA)6	12	38239378	38239389	GGTGAGTGAGAAGGAAGCGA	CCTTTTATCTAGGGCACCCC	280
Ad02A8141	Aradu.A02	8141	p2	(CA)7	14	38243557	38243570	ATCAAGGCCACAAGAAGCAT	GTTAAACGGAGGCATCCAAG	199
Ad02A8142	Aradu.A02	8142	p3	(AAT)8	24	38259123	38259146	TTGCCAATTTTCTGTTGTTTG	CCATGATGGAGAAAGGGAGA	178
Ad02A8143	Aradu.A02	8143	p2	(TA)8	16	38288486	38288501	TGGATCCTTAGCTTTTGGGA	TCGGTTTATGAACGTAGCCG	216
Ad02A8145	Aradu.A02	8145	p3	(CTT)5	15	38317115	38317129	GTTTTTCGTCCCCAACCTTT	GCGAAGGGAAAAGGAAAGAA	193

ID	Chromosome	SSR nr.	SSR type	SSR	Size	Start	End	FORWARD PRIMER (5'-3')	REVERSE PRIMER (5'-3')	Product (bp)
Ad02A8146	Aradu.A02	8146	c	(T)10ggaaataat	48	38319369	38319416	TTGTACCAAAATGGGTGTGG	CGATATAACTGTTCACCAACAAAAA	219
Ad02A8147	Aradu.A02	8147	c	(T)10c(T)11	22	38323684	38323705	GCTTAAGCTGTGGGAAAGGA	CAGCAAACCTCAAAATGGGT	192
Ad02A8149	Aradu.A02	8149	c	(AT)6(AC)8gc	89	38340473	38340561	ACTTGCGCCACTCAAATAGC	TGGGCAACGAAGTTTTAGAAG	280
Ad02A8153	Aradu.A02	8153	p5	(TCAAA)5	25	38368679	38368703	TGGAAAACGGAAAGAAGTGG	GAACGACGTGGTGTTTTTGA	247
Ad02A8157	Aradu.A02	8157	p3	(TTC)6	18	38417023	38417040	GCTGGTTAGTTGTGCGGATT	CGTCCTCGAGCAAGAGAAGA	228
Ad02A8158	Aradu.A02	8158	p2	(AT)8	16	38418554	38418569	TGAAGCAAGAAAAAGCAGTGAA	ATCCATTGATGCTCAAAGCC	173
Ad02A8159	Aradu.A02	8159	p3	(AGA)5	15	38425787	38425801	CAGCCTTAGGTGCCATCAAT	CCTTGGCCGAATACACAACT	159
Ad02A8162	Aradu.A02	8162	p3	(TTA)24	72	38446253	38446324	ACCACCGTTTACTTTGGAGC	TGGAAAAATTGGGTTGGAAA	279
Ad02A8163	Aradu.A02	8163	p2	(GA)7	14	38449487	38449500	TCTTTAAAAAGAAGAAGGACGGA	CTCTCCTTTTTCTCCCCCTC	247
Ad02A8164	Aradu.A02	8164	p2	(AG)6	12	38449757	38449768	AGGAGGGGGAGAAAAAGGA	CCTCCCCTCCCTCTCTCTT	184
Ad02A8172	Aradu.A02	8172	c	(A)11ttgaaaat(	29	38504689	38504717	TTGTTGGCTCTTGAAAGAATGA	TTGGCTTTTTCTGCTTGCTT	151
Ad02A8177	Aradu.A02	8177	p2	(CT)6	12	38615217	38615228	TAACAAGCATGAAACTGGCG	GGTGTATGGGGAAGAGTGGA	270
Ad02A8180	Aradu.A02	8180	p5	(AAAAT)6	30	38620577	38620606	TGGTGTCTCTTTGTGTTTTTCC	GAAAAATTGCTTGTGATTTTTGAA	190
Ad02A8182	Aradu.A02	8182	p3	(AAT)7	21	38677039	38677059	AACAGGAGAAGGAGATGGCA	GGAGATGCTTCTTCCACCAA	110
Ad02A8184	Aradu.A02	8184	p3	(AAT)5	15	38695542	38695556	TCTGACTGGATTGCAAGCTG	GGTTTGCTATTGCCACACCT	240
Ad02A8185	Aradu.A02	8185	c	(T)10ctttcgttc(	32	38706226	38706257	TCCCCAATTAGAGGTGTCCA	TCATGAATGTTGGCTCTTGAA	229
Ad02A8191	Aradu.A02	8191	p4	(TTTA)5	20	38785137	38785156	TCATTTCAGAACATTGTCATGG	TCCAAAAGCCCTTCTCTCAA	194
Ad02A8192	Aradu.A02	8192	p2	(AT)8	16	38786410	38786425	CTGAAAAGCGTAGCCTTTGG	GCGTAGCCTTAGGTCCCTCT	209
Ad02A8194	Aradu.A02	8194	p3	(AGA)6	18	38850330	38850347	TAGAGGTTTCTCCGGCCATA	CACCAAAACGCACCCTAAAT	276
Ad02A8198	Aradu.A02	8198	p3	(AAG)5	15	38879887	38879901	GGCAACCCGATAATTTCGTA	TAAAAGTGCAAACGACGTGC	275
Ad02A8199	Aradu.A02	8199	p2	(TC)6	12	38880384	38880395	GCGATTCACACAAACCAAGA	TGCTTGTCCTCAAGCAAAGA	245
Ad02A8200	Aradu.A02	8200	p2	(CT)6	12	38904549	38904560	GCACGCCTAAATAGAGCAGG	GAGGGAGAGATAGGTTCGGC	259
Ad02A8201	Aradu.A02	8201	p3	(TCT)5	15	38915846	38915860	ACATACACTCCCCCTCTCCC	TGCCATTGATGGTAGTGGAA	225
Ad02A8202	Aradu.A02	8202	p3	(AGA)6	18	38933899	38933916	TAAAATATTGCTCGCCCTCG	TCTTCTCCCACTTAGCCGAA	104
Ad02A8203	Aradu.A02	8203	p2	(CG)6	12	38950263	38950274	TGTTCCAGAGAGTTAGGCCG	CATAAGAGGGGCCTAGAGGG	207
Ad02A8206	Aradu.A02	8206	c	(CT)9ccctccttc	61	38958560	38958620	TCTCCCCTCTCTCTCCACAC	GGGAAAAGGAACATAGAGAGGA	232
Ad02A8208	Aradu.A02	8208	c	(CTC)5ctttctct	40	38959388	38959427	TTCTCTCTTTCCTTGCGCTC	CCTTTTCTCTCTCTCTCATTCCC	279
Ad02A8209	Aradu.A02	8209	p2	(GA)6	12	38959598	38959609	TCTCTCCTCCTCCTCCTCCT	TCCTTCCCTTATTTCTCTCTCC	250
Ad02A8210	Aradu.A02	8210	c	(GA)6aggggga	136	38959783	38959918	AAGGGGAAAGAGGGAAAAGA	CTCCCTCCCCCTTCCTTC	274
Ad02A8211	Aradu.A02	8211	c	(GA)8a(AG)7	31	38960446	38960476	GGAAGGGAGAGGGAGAGAGA	TGAAGCCATAGTTTTTATGCAA	208
Ad02A8214	Aradu.A02	8214	p2	(AT)8	16	38968061	38968076	GGCACGTCACATACTAATTTCC	CCAAGTTCATCATCGTCCAA	244
Ad02A8218	Aradu.A02	8218	c	(T)10cgaaatttc	29	38996665	38996693	CACACTCTTGTTGCCTTGGA	TGGCTCTTGAAAGGATGAGG	184
Ad02A8219	Aradu.A02	8219	p3	(ACA)5	15	39012240	39012254	AGGTTGCGCCTAACTTGAGA	AGAGCTGCGCCTTACTTGAG	179
Ad02A8222	Aradu.A02	8222	p5	(AATAA)5	25	39062463	39062487	TGGGGTTAGCATATGACCCT	TCTCCCTCCTCACTTTTTCG	241

ID	Chromosome	SSR nr.	SSR type	SSR	Size	Start	End	FORWARD PRIMER (5'-3')	REVERSE PRIMER (5'-3')	Product (bp)
Ad02A8225	Aradu.A02	8225	p4	(ATAA)5	20	39068266	39068285	AGCCTAAAACGTCTCTCTATGC	GCTTCAAAAACCTGAGGCAG	171
Ad02A8228	Aradu.A02	8228	p3	(GAA)7	21	39088854	39088874	CCCAAAACTCAGAGGTGGAA	CAACTTCTCTTTCCCACCCA	240
Ad02A8231	Aradu.A02	8231	p3	(CAT)5	15	39116205	39116219	TGAAACCAAACCTTTGTGGC	GGTGATGAAGAAGGTGACCAA	237
Ad02A8232	Aradu.A02	8232	c	(TAAT)7tcaga	126	39116342	39116467	TTGCGTCACTCAATGGATTC	TATGCCTGCCTTCAGACTCA	190
Ad02A8233	Aradu.A02	8233	c*	(ATGT)5(TAT	58	39116996	39117053	TTTTGTTTTGATTTTCATTTTCCA	TGAGTTTTATTGGTTTTGAATTTTGA	244
Ad02A8236	Aradu.A02	8236	p3	(CTT)5	15	39119175	39119189	TTCCAAGTTGTGCCGAATCT	TGGCTTTATTCACACTTCATGG	279
Ad02A8237	Aradu.A02	8237	c	(A)10ttaagccc	88	39119414	39119501	GACTCGGCCCAAATCATAAA	GGGCCGAAACACAAAAAGTA	274
Ad02A8238	Aradu.A02	8238	p2	(TA)7	14	39120179	39120192	ATATGATGTGTGTGCCTGCG	TCCATGCAAAGGAAAAGCAT	187
Ad02A8239	Aradu.A02	8239	p3	(TAT)15	45	39121315	39121359	CCAAACCTAAACCCACCTCA	ATGAGGCCAAAGGTCACAAG	250
Ad02A8240	Aradu.A02	8240	p2	(AG)6	12	39129957	39129968	TCGCATCTGTGAAAATCAGC	TTGAAGAACATTGAGCCTGAGA	266
Ad02A8245	Aradu.A02	8245	p2	(GA)6	12	39174183	39174194	CAGAGAGAAGAGAGAGGGTTCG	ACCACCAAACCACTTTTCCA	181
Ad02A8250	Aradu.A02	8250	p3	(AGA)7	21	39232109	39232129	GGTGCCATCAATGGTTATGA	CCTACTCCCTCTCCCCTCAC	230
Ad02A8256	Aradu.A02	8256	p3	(TCC)6	18	39254669	39254686	AACCCCTTTCTTGTCCTCGT	GTTCACCCAAACAAGCCCTA	226
Ad02A8259	Aradu.A02	8259	p4	(ATTA)6	24	39261258	39261281	GGCATCTTGCATGGGTACTA	GCCTTTAGCGCATTCCATTA	278
Ad02A8262	Aradu.A02	8262	p3	(ACA)5	15	39267006	39267020	GGATGATCCCCTGTACATTTG	CCATGTAGCCGACTGCCTAT	193
Ad02A8267	Aradu.A02	8267	p5	(TTTTA)5	25	39292978	39293002	CTCGCGTGATTCTCTGTTCA	GTGCTAGGTGGGAGACAACC	272
Ad02A8272	Aradu.A02	8272	p2	(CT)6	12	39378489	39378500	GGTGTTCAGCAATCGGTCTT	CTTCCCCTTTTTAGCTGCCT	220
Ad02A8274	Aradu.A02	8274	p3	(AAT)15	45	39381769	39381813	ACCATAGTCAGGCCAGGTTG	TTGTACTCTTGGGCCATTCC	180
Ad02A8277	Aradu.A02	8277	p2	(TC)6	12	39387953	39387964	TACCTTTCCGTCTCCCTCCT	GCGCGTAGTTTCTCCATCTC	185
Ad02A8280	Aradu.A02	8280	p3	(TGG)5	15	39395660	39395674	CCCAAAAAGGATCAGGGTTT	TTTTCTTTGGCAAACGCTCT	267
Ad02A8284	Aradu.A02	8284	p2	(AT)7	14	39410807	39410820	TTTTTGAAAACGGAGATGGTT	GCCACTTGTCGCATAATGAA	261
Ad02A8289	Aradu.A02	8289	p2	(TA)7	14	39461103	39461116	CTTGGGACACTGGTTGAGGT	GGCATTTTTGTCCCCATTTT	216
Ad02A8292	Aradu.A02	8292	p4	(TTCT)6	24	39505166	39505189	TACACGTGGTTGACGCGTAT	AGGGGGAAGAAGAAGAACGA	184
Ad02A8294	Aradu.A02	8294	p3	(TTG)6	18	39513016	39513033	TTCATGAGGTGGTCAAACGA	AGATGTAAACAACTGATGCCCA	252
Ad02A8296	Aradu.A02	8296	p3	(TAA)5	15	39519977	39519991	TAGTTCGATGTCGCGTTTCA	GTCAGTTTGAAGCTACGGGC	277
Ad02A8298	Aradu.A02	8298	c	(T)10catttttctc	75	39521622	39521696	GGACAAAAGTAACGCTAACACG	AAAGCAAGCAAGCCAAATGT	279
Ad02A8299	Aradu.A02	8299	p3	(AAT)6	18	39521842	39521859	ACATTTGGCTTGCTTGCTTT	CCTCCTGATGAGTCTCGGAA	225
Ad02A8300	Aradu.A02	8300	p2	(GT)9	18	39522546	39522563	TTGGCCACTTTGAGCTTTTT	CAAAACACATTCTCATCAACCAA	186
Ad02A8303	Aradu.A02	8303	p3	(GAG)6	18	39539885	39539902	TCCATTAGAAACGTGGAGGC	CTGGTTAGTTGTGCGGGATT	268
Ad02A8304	Aradu.A02	8304	c	(T)12caaaa(T)	27	39541686	39541712	AGTCTTGGCTGTGGCCTAAA	TCGTGAAGCAAGAAAAAGCA	236
Ad02A8309	Aradu.A02	8309	p4	(AAAT)6	24	39598233	39598256	AGCCAGCCAGATTTCAACAA	CCAAGATCCGGCAATAACAT	179
Ad02A8311	Aradu.A02	8311	p2	(AT)9	18	39654042	39654059	GCTCCCAAAATCTCCATCAA	TTTCAGTTTCCCATCCCTTG	274
Ad02A8316	Aradu.A02	8316	p2	(TA)11	22	39686801	39686822	GCGCAGAAGTCACGAAGAAT	TAAACCGCAAAATCACTCCC	256
Ad02A8318	Aradu.A02	8318	c	(CT)13ccttttcc	55	39766841	39766895	GGGTGTGCATGGTTCAGTTT	GGAGAGAGAGGGGAGAAGGA	279

ID	Chromosome	SSR nr.	SSR type	SSR	Size	Start	End	FORWARD PRIMER (5'-3')	REVERSE PRIMER (5'-3')	Product (bp)
Ad02A8319	Aradu.A02	8319	c	(CT)6tcctctctc	185	39767106	39767290	TCTCCCCTTCCCCTCTTT	GAGAGGGAGAGAGGACGGAA	261
Ad02A8320	Aradu.A02	8320	p2	(CT)6	12	39767548	39767559	CCCTCTCTCTCCCTATCTCCA	GGAAAAGAGCGCAAGAAAAG	244
Ad02A8321	Aradu.A02	8321	p2	(GA)6	12	39767977	39767988	TCCTCCTCTCTCTCCTCCTTC	TCTGCTCTCTGCCCTCTCTC	279
Ad02A8322	Aradu.A02	8322	c	(AG)6aaggggga	132	39768102	39768233	GAGAGAGGGCAGAGAGCAGA	TGAGAGAGAAACTCCTCCCCT	212
Ad02A8323	Aradu.A02	8323	p3	(GAG)6	18	39768339	39768356	GGAGAGAGAAGGAGAGGGGA	TTTGTGCTCTTTTTCCCTTTTT	269
Ad02A8324	Aradu.A02	8324	p2	(GA)8	16	39768559	39768574	GAAAGGGGAGGGGAAGAAAG	CCCCTCCCTCTCTTTGTCTT	140
Ad02A8325	Aradu.A02	8325	p2	(AG)12	24	39768763	39768786	AAGACAAAGAGAGGGAGGGG	TTTCTTTCTCTTTCTCCCTTCA	160
Ad02A8328	Aradu.A02	8328	p3	(TCT)5	15	39782075	39782089	CTTCCCCATAAATCCCACCT	AGAATATTGCTCGCCCTTGA	238
Ad02A8332	Aradu.A02	8332	p2	(GA)6	12	39840999	39841010	GGTGTCAGAGGAAAAGCAGA	CACTTTTTCACTTCCAATTTTCG	280
Ad02A8334	Aradu.A02	8334	p4	(TTTA)5	20	39856464	39856483	CGAAAACCACTAACAACTTTTTCA	TTCAAACACAGAGAGGGAGAGA	248
Ad02A8338	Aradu.A02	8338	p6	(TGCAGG)5	30	39938101	39938130	GCAGCAGGAGAACCAACTTC	TCCCCTGCAAGTTCACCTAC	101
Ad02A8340	Aradu.A02	8340	p2	(GA)18	36	39942775	39942810	TTCGAAGGAAGAGGCAGAGA	GGCTACATCATCCAACCCTG	258
Ad02A8341	Aradu.A02	8341	p2	(AC)11	22	39944855	39944876	CTCAAAATTGGACCAAACGG	GAGAGCTTCGTGTAGCCACC	259
Ad02A8345	Aradu.A02	8345	c	(TA)8(T)11	27	40001923	40001949	ATTGGGGACACATGTGCATT	GAAAATTGTGGGAACATGGG	258
Ad02A8346	Aradu.A02	8346	p2	(AG)12	24	40020160	40020183	TTGCAGTGAAAGAATGGCAA	TGATGTGCATTGTTCGTGTTT	237
Ad02A8350	Aradu.A02	8350	p2	(TG)15	30	40100074	40100103	ATAGGTCATGCTAGCGCTCC	ATGATCGTTGAAAGTTGGGG	215
Ad02A8356	Aradu.A02	8356	p2	(AG)7	14	40115672	40115685	TGTTGATAGGAGTCGTTGGTG	CGAAGCATCCCATTCAAAGT	160
Ad02A8359	Aradu.A02	8359	p2	(CT)6	12	40154295	40154306	CCTAGGCTTAACGAACAGCG	CCATGGAGAAGAAGATCCGA	249
Ad02A8360	Aradu.A02	8360	c	(TC)12tatcta(T	135	40154423	40154557	GGCGATAGCTAAGACCTCCC	CCCCAAAAGAAAACCTAGCC	257
Ad02A8361	Aradu.A02	8361	c	(AG)8ggggg(C	33	40155313	40155345	AAGGTCGTGCCATACCAGTC	GGTGCTAATCCCTCGATTCA	199
Ad02A8362	Aradu.A02	8362	p2	(TC)14	28	40162409	40162436	TTGAAAATCACTTCCCTCTTTTTC	GGAAAGGGTTTGAATTTTTGG	230
Ad02A8364	Aradu.A02	8364	p3	(TTC)5	15	40175239	40175253	ACCCCTCCATCCTTCTTCAT	AAAAACAATGCCAAAAAGCG	254
Ad02A8366	Aradu.A02	8366	c	(TAT)26tcg(A	91	40233126	40233216	TCCCTAACCACTTTCTCTCCTC	AAGGGAGATGATGGGGATTT	240
Ad02A8368	Aradu.A02	8368	c	(A)10tttgg(A)1	26	40237564	40237589	TTTGGAATGCTTGGATTGGT	GTGGATGGTCGAAAATGCTT	126
Ad02A8371	Aradu.A02	8371	p3	(AGA)5	15	40275639	40275653	GGTCTTGATGGCTCTTGGAG	TCTTCTCCCACTTAGCCGAA	260
Ad02A8373	Aradu.A02	8373	p2	(AG)6	12	40319974	40319985	ATAGGTGAGGGGTTTTTGGG	GGATCTGTAGACCCCACAGG	107
Ad02A8374	Aradu.A02	8374	c	(AGA)6aaatatg	41	40326185	40326225	GGTCTTGATGGCTCTTGGAG	TCTTCTCCCACTTAGCCGAA	261
Ad02A8376	Aradu.A02	8376	p2	(CT)6	12	40333913	40333924	GGGCACTAGACTGGCGTTTA	TGAGGTGATTGGTGAATGGA	230
Ad02A8377	Aradu.A02	8377	p3	(AGA)5	15	40349459	40349473	TTAAAATTTTGCTCGCCCTC	TCCCACATAGCCGAACCTAC	100
Ad02A8379	Aradu.A02	8379	c	(T)10cac(T)10	23	40403948	40403970	TGGTTTTAAAGGGCTGTTGG	TGTTGGCTCCTGAAAGAATG	186
Ad02A8382	Aradu.A02	8382	p2	(AG)6	12	40437778	40437789	CGCGATCTGCAGAATTACAA	CAAAGAACATTGAACCTGGGA	248
Ad02A8383	Aradu.A02	8383	p3	(TAC)5	15	40445306	40445320	TTGGGAATAAAGCCAGAATAGA	AGCTGAAGCGCCAGAAATTA	198
Ad02A8386	Aradu.A02	8386	p3	(ACA)7	21	40485234	40485254	AAAGGCAACAAATGCAATCC	ATATGAGAAAGCCGGCAAGA	207
Ad02A8389	Aradu.A02	8389	p2	(AC)11	22	40490208	40490229	GGAGCCTATGAAGGAAGATGA	CGACCTCTGCTTTACAGAACG	180

ID	Chromosome	SSR nr.	SSR type	SSR	Size	Start	End	FORWARD PRIMER (5'-3')	REVERSE PRIMER (5'-3')	Product (bp)
Ad02A8390	Aradu.A02	8390	p3	(GAA)5	15	40496297	40496311	TGGACCAAAGAGCTTCCAGT	CGAAAACTCTTTCTTCTTCTCCC	254
Ad02A8393	Aradu.A02	8393	p3	(TAC)5	15	40532261	40532275	CATGTCTGATGATGATGAGAAAAAT	GAGCAAGGGCGTGATTTAAG	259
Ad02A8396	Aradu.A02	8396	c	(T)10c(T)11	22	40581001	40581022	GCCCAAAGCACTCTGTCTTC	GAAGCAAGAAAAAGCAGTGGA	245
Ad02A8398	Aradu.A02	8398	c	(A)10gaaaatca	73	40592282	40592354	AAAAACCGGTCAGCACAATG	CGCGTTCTAGAGGAGGACAC	275
Ad02A8399	Aradu.A02	8399	c	(A)11cagtcagc	90	40592939	40593028	CGTAGAAGAGGCCACTACGG	GTTGTGCTGACCGGATTTTT	266
Ad02A8401	Aradu.A02	8401	c	(A)10cggtcagc	102	40594929	40595030	AAAAATGGCCAGCACAAAAA	GCTGACCGGTTATTTTCCCT	248
Ad02A8404	Aradu.A02	8404	c	(A)15cagtcagc	99	40596353	40596451	GCGCCTTAAGGTGTCCTCTT	TTTTTACGTTGTGCTGACCG	206
Ad02A8406	Aradu.A02	8406	c	(A)10cgtttagca	68	40597484	40597551	ATTACGGCGCCTTTAGGTGT	TTTTTACGTTGTGCTGACCG	255
Ad02A8417	Aradu.A02	8417	c	(A)10cggtcatg	144	40604147	40604290	GCACAATGTCAAATAAAGGGG	CGTTGTGCTGACCTGTTTTT	256
Ad02A8431	Aradu.A02	8431	c	(A)10cgttcagca	35	40616441	40616475	GTGTCCTCCTCTGGATAGCG	CGTTGTGCTGACCGTTTTT	267
Ad02A8458	Aradu.A02	8458	p2	(AG)6	12	40644586	40644597	CGGAAAAGCACACCAAACTT	ATTGCAAGAGGCTGGCTAGA	275
Ad02A8478	Aradu.A02	8478	c	(A)10tcggagga	150	40656411	40656560	AGGTGTCCTCCTCTGCATTG	CCTAAAGGGGAGCCGTAATG	248
Ad02A8480	Aradu.A02	8480	c	(A)10caaaaaac	95	40657355	40657449	CCTCGGGATAGCGTCATTTA	ACCGGCTTATTTTCGCTTTT	208
Ad02A8494	Aradu.A02	8494	c	(A)10tcggagga	114	40662949	40663062	TGTCCTCCTCTGGATAGCGT	TAAAGGCGAGCTGTAATGGC	272
Ad02A8502	Aradu.A02	8502	c	(A)10gaaaaaaa	31	40665140	40665170	CTCCCCTTTAGGTGTCCTCC	TTACGTTGTGCTGACCCTTG	217
Ad02A8505	Aradu.A02	8505	c	(A)10gaaaaaaa	67	40665882	40665948	CTCCCCTTTAGGTGTCCTCC	TTACGTTGTGCTGACCCTTG	253
Ad02A8514	Aradu.A02	8514	c	(A)11gaaaaaaa	33	40673839	40673871	CCTCGGGATAGCGTCATTTA	ACCTAAAGGCGAGCCGTAAT	152
Ad02A8518	Aradu.A02	8518	p3	(GAA)5	15	40730540	40730554	GGAGACAGTGGTGGTGGAGT	GAACCCGAGAAGTATGCCAA	268
Ad02A8519	Aradu.A02	8519	p2	(GC)6	12	40730948	40730959	TTGGCATACTTCTCGGGTTC	TGCAACTCTTGCAACCTGAC	271
Ad02A8521	Aradu.A02	8521	p2	(TC)6	12	40757123	40757134	ACCCTCCACAGACCCTCTCT	AGCCCCAAATACAACACAGC	226
Ad02A8522	Aradu.A02	8522	p2	(TA)6	12	40767313	40767324	AAGGGGTAAACAAAAACCGC	TTATGCTCGGACCGGTTATC	160
Ad02A8523	Aradu.A02	8523	p3	(AGA)5	15	40778910	40778924	GTGATGATGGAGGAGGAGGA	CACCAAAAATTAAAGGCCCA	262
Ad02A8528	Aradu.A02	8528	p4	(CTTT)5	20	40826667	40826686	CAGCTTATCCAGATCAGCCAA	GGGCAATTTCACAAACACAA	280
Ad02A8532	Aradu.A02	8532	p2	(CT)6	12	40894044	40894055	TTTCGAAAATCTCTTCCCCC	TCACGAAAAAGCACACCAAA	152
Ad02A8533	Aradu.A02	8533	p3	(TTC)5	15	40921408	40921422	TTCCCCAAAAACCCATCATA	AGGTTTGCTCGTCCTTGAGA	237
Ad02A8534	Aradu.A02	8534	p4	(ATTT)6	24	40946411	40946434	AAAAATTTTCGAATTTATTAGGCA	CGTTGCCAGGGATTGATAGT	244
Ad02A8535	Aradu.A02	8535	p4	(AAAT)6	24	40971589	40971612	TTCATTTCCACTCAGGTCCA	ATCCCTTGGGTTGACTTGG	280
Ad02A8537	Aradu.A02	8537	p3	(CAT)5	15	41031880	41031894	TTTTTGCAGGGAAAGAATGG	TGCTCTTTCAAGGGTTGAGTG	256
Ad02A8538	Aradu.A02	8538	p2	(GA)6	12	41048540	41048551	TGCTTGTCCTCAAGCAAAGA	CGAACAGAGAGTTGTGCAGG	221
Ad02A8539	Aradu.A02	8539	p2	(AG)6	12	41055846	41055857	TTTTTGGGGAAGAGGTGTTG	TTTAACGCCAGAAAAGGCAC	170
Ad02A8541	Aradu.A02	8541	p3	(TTC)6	18	41108222	41108239	TTCTACACCTTGGCCGAAAC	CTAGAGGATTCTCCGGCCTT	179
Ad02A8543	Aradu.A02	8543	p3	(AGA)6	18	41144136	41144153	CTAGAGGATTCTCCGGCCTT	TGGTGTGCAAAGGCATTTTA	279
Ad02A8552	Aradu.A02	8552	p3	(ATT)14	42	41287449	41287490	TTTTGATGCACAATCACTAATCAA	CGACCCATTAGAAGAAAGAAGG	265
Ad02A8553	Aradu.A02	8553	p2	(AT)6	12	41288128	41288139	CTGAGAAATGTTTGGGCGTT	AACAAAAATTCAAAAGGTTTTCC	212

ID	Chromosome	SSR nr.	SSR type	SSR	Size	Start	End	FORWARD PRIMER (5'-3')	REVERSE PRIMER (5'-3')	Product (bp)
Ad02A8557	Aradu.A02	8557	p3	(AAT)6	18	41314289	41314306	GGAAAAGGGCACTCTTGTTG	TTGGATGTTGGCTTTGTGAA	195
Ad02A8560	Aradu.A02	8560	p3	(TTC)5	15	41359011	41359025	GTACTTCCGGGAGAGAACCC	GCTCCTCCCCCTAATGAAAG	194
Ad02A8561	Aradu.A02	8561	p3	(ATT)8	24	41369083	41369106	AAAAAGACAAAGCTGCTTAACCTTAC	ATGCTGCAGAGTGGTTGCTA	233
Ad02A8565	Aradu.A02	8565	p2	(CT)7	14	41442105	41442118	CAGCTCAACTTGAATTGCCA	GAAAAGGAAGGAGGGAAGAAGA	171
Ad02A8566	Aradu.A02	8566	p3	(AAT)5	15	41459905	41459919	CCAGAAGCAACCTTTCAGGA	CAAACTTGGACGTTGGCTTT	111
Ad02A8572	Aradu.A02	8572	p5	(AAGAT)5	25	41607498	41607522	CCAGCCTGCAGAGATAAAGG	GGTGTCCGACTTGTGGAACT	276
Ad02A8573	Aradu.A02	8573	p3	(AGA)6	18	41613252	41613269	TAGACGATTCTCCGGCCTTA	TTCTACACCTTGGCCGAAAC	178
Ad02A8578	Aradu.A02	8578	p3	(TTG)6	18	41681225	41681242	GCAGAAGGAATCAGAGCCAC	AGGAAACAACCACCAGATGC	245
Ad02A8583	Aradu.A02	8583	p2	(TA)7	14	41698413	41698426	ACCACAACCCTTGCGTCTAC	GGAAACGCTGGTAATTTGGA	263
Ad02A8589	Aradu.A02	8589	p2	(GA)8	16	41729884	41729899	CTTCGTTTCTCGTGCTTTCC	CCCTCTCTCCACCACTACCA	275
Ad02A8592	Aradu.A02	8592	p2	(CT)8	16	41747026	41747041	CATTGAACCTGGAATTGGAAA	TATTTTTGGCCCAGTTTTCG	205
Ad02A8594	Aradu.A02	8594	p2	(CT)9	18	41772024	41772041	TGGCCCAAATCTAAGTCCAG	AAACCTGGGATCGAGAGTCA	200
Ad02A8597	Aradu.A02	8597	p3	(TCT)16	48	41784380	41784427	TGGCCGAACCTACATCTCTC	ACTCGGAGGTGGAAGCTTTT	237
Ad02A8604	Aradu.A02	8604	p2	(TG)6	12	41831687	41831698	TTCCTGTAGAGCCTGAGGGA	AAAGTCCCAAATGCATGCTC	129
Ad02A8608	Aradu.A02	8608	p2	(TC)16	32	41861289	41861320	AAAGGAACATTGAACCTGGAAA	AGGGAACCTGCAGAGACTCA	198
Ad02A8609	Aradu.A02	8609	p2	(GA)6	12	41872776	41872787	TTCAGTTTGTTGGGGAGGAC	GAATACCAGGTAGCCGCTGA	137
Ad02A8613	Aradu.A02	8613	p2	(TA)14	28	41879339	41879366	TCACCATTCCGAGCCTAATC	GAATTGGTTGATTGACGTGG	210
Ad02A8615	Aradu.A02	8615	p4	(AAAT)5	20	41924446	41924465	TTTGCACCCTGTTTTTCTCC	GCAAATGCAATAAAAGAAAATGG	197
Ad02A8618	Aradu.A02	8618	c	(AAC)5ataaca	72	41960156	41960227	CCAGGACCAGTGGAGATTACA	ACTGGTTTGCGGTTGAATTT	226
Ad02A8619	Aradu.A02	8619	p2	(TC)10	20	41960633	41960652	AAACCCTAGTAGCAGCAGCG	GAAAGAGAGGAAGAGCCACG	279
Ad02A8620	Aradu.A02	8620	p2	(CT)6	12	41963084	41963095	TTTCAAGTTCAGCGTGGACA	GCTCATGGTTCGGAGAGAAA	274
Ad02A8622	Aradu.A02	8622	p2	(AG)6	12	41992870	41992881	TTTTTGGGGAAGAGGTGTTG	TGCAGGGATGACTTTTCCTT	124
Ad02A8623	Aradu.A02	8623	p2	(AC)6	12	42003748	42003759	TGGCACAAAACAGAGATTCG	GATCCTGAGGGATGGACTGA	198
Ad02A8624	Aradu.A02	8624	p2	(CT)6	12	42019778	42019789	GACCAAAGAAGCATGTTTTCAA	TGGGGAATTCATCCATTCAT	226
Ad02A8625	Aradu.A02	8625	p2	(AG)17	34	42029690	42029723	TGATTATGCAAAGGCAGTGG	TTTGCATTTTCCGATCTCCT	121
Ad02A8627	Aradu.A02	8627	p3	(TAA)5	15	42035693	42035707	ACGGATGTGGACCAGGTAAA	AATGGAGTGACTGGAAGACAAA	257
Ad02A8630	Aradu.A02	8630	p3	(TCT)6	18	42049485	42049502	CCCATAAACCCCACCTACCT	GTGGAAGCTTTTGTCTTCGC	251
Ad02A8631	Aradu.A02	8631	p2	(AC)7	14	42057086	42057099	TTTCAAGGTTGTAATGGGGC	GGGGAATAAAATCACCCCAA	256
Ad02A8633	Aradu.A02	8633	p4	(TATT)5	20	42098709	42098728	TCGAAAATATCTCATCCCTTTTTC	AGAACCCAAACACAAGGGTG	234
Ad02A8637	Aradu.A02	8637	c	(ATT)6ac(TA	38	42154949	42154986	TGACATTTTTCCCTTCTCCC	CCAAACGTTGGGGATAAAAA	179
Ad02A8638	Aradu.A02	8638	p3	(TTA)19	57	42155514	42155570	TGACGGCAGGATAACATTGA	GCGCATTACCGGTTGAATTA	214
Ad02A8645	Aradu.A02	8645	c	(A)10ttttgag(A	29	42170884	42170912	GCCTTAAATAATCGGAGGGG	GGGTCCAATTTGTATGTCGC	260
Ad02A8646	Aradu.A02	8646	p2	(TA)9	18	42171854	42171871	TCACAACCGATCCAATCAAA	TGGATTTGCGGTTTTGTAAAT	270
Ad02A8648	Aradu.A02	8648	p5	(AAGAT)5	25	42180225	42180249	CAGAGGAACACGACCCAAAT	CCTGGGTGCTCCAGTGTATT	174

ID	Chromosome	SSR nr.	SSR type	SSR	Size	Start	End	FORWARD PRIMER (5'-3')	REVERSE PRIMER (5'-3')	Product (bp)
Ad02A8650	Aradu.A02	8650	p3	(AGA)6	18	42237923	42237940	CTAGAGGATTCTCCGGCCTT	TACACCTCTCCCCCTCTCCT	161
Ad02A8651	Aradu.A02	8651	p2	(AG)6	12	42253977	42253988	GAAGGGTTTTTGGGGAAGAG	TTTAACGCCAGAAATGGCTC	176
Ad02A8653	Aradu.A02	8653	p2	(CT)6	12	42274101	42274112	TTTTTCGAAATCTCCCTCCC	CGGAAAAGCACACCAAACTT	147
Ad02A8659	Aradu.A02	8659	p3	(TTC)13	39	42334414	42334452	CCTTCTTCTTTCAAATTCGTGC	AAAAATGAACCAAAATGGCG	276
Ad02A8660	Aradu.A02	8660	c	(GAG)5gaaggt	88	42334820	42334907	CGTGAAGAAGAAGGAACACGA	TTTAGGTCACGCGCTTTTCT	229
Ad02A8662	Aradu.A02	8662	p3	(AAG)5	15	42362349	42362363	GCTTTTGTCTTCCCTTTCCC	AGCCGAACCTACTCCCTCTC	279
Ad02A8667	Aradu.A02	8667	c	(A)10gagggaga	104	42381984	42382087	CGATGACATGGTTATTGAAATGTT	TGTGAAGGAACAAAGGAGCA	195
Ad02A8668	Aradu.A02	8668	p3	(TTG)12	36	42383389	42383424	GGTGCCATTGGAATGTTTCT	TCCGCGCTTAGAAATAAAAA	250
Ad02A8669	Aradu.A02	8669	p3	(ATT)6	18	42389022	42389039	CAACTACCACCACTTTGCCA	GCGTAGTGCTAATCAAGCAGC	184
Ad02A8670	Aradu.A02	8670	p2	(TA)6	12	42390329	42390340	TAAATATGGTGGCGTTTTCG	GAAATGTGTGTCCACAGTCCA	106
Ad02A8678	Aradu.A02	8678	p2	(AT)7	14	42415996	42416009	TTCGAAAAAGTGAGGAGAGAGAA	TTGTGCCTTTTGTGTGTTTTTC	234
Ad02A8680	Aradu.A02	8680	p3	(AAT)5	15	42456585	42456599	CAGGGAAAAAGGCACTCTTG	AAACTTGGACGTTGGCTTTG	199
Ad02A8681	Aradu.A02	8681	p2	(AT)14	28	42465592	42465619	TCGTGTCTTGCATGTCTTTCTT	TTCTGCCTCATGTTTTGAACC	266
Ad02A8684	Aradu.A02	8684	c	(A)11gcaatcaa	41	42477394	42477434	TGTGGTTCATGAATGTTGGC	TCCCCAATTAGAGGTGTCCA	218
Ad02A8689	Aradu.A02	8689	p2	(TA)10	20	42550172	42550191	GGGTTAGCATATGACCCCAG	TTCAAAACTTCCTAACCACTTTCTC	233
Ad02A8690	Aradu.A02	8690	p2	(AG)7	14	42553108	42553121	ACAAATCAGGGGCACACTTC	TTTGCAACGTAAAGAAATCAACA	223
Ad02A8691	Aradu.A02	8691	p2	(CT)8	16	42559216	42559231	GGGGAAATGCATCCATTAAG	ATTGGGCAACAGGAAAATCC	279
Ad02A8692	Aradu.A02	8692	p3	(CAT)5	15	42561639	42561653	GAGGCTTTGGCAGAAGTGAC	AGAGGTGATGGAGCTCAGGA	249
Ad02A8693	Aradu.A02	8693	p2	(GA)12	24	42567107	42567130	ACCCACACCCACAACTCAAT	GCAACCTCCTCTCTCTCCCT	257
Ad02A8694	Aradu.A02	8694	p2	(GA)10	20	42604060	42604079	TCAGGTTGTGCTCCTCAATG	ATTAAGAACATTCGCGACCC	277
Ad02A8697	Aradu.A02	8697	p2	(GT)8	16	42631980	42631995	GCAAATAAACAAGGGGACAAA	AATGGGGCTGAGGTAAAGGT	259
Ad02A8698	Aradu.A02	8698	p3	(TTC)6	18	42644062	42644079	CCCATAAACCCCACCTACCT	TAAAATATTGCTCGCCCTCG	240
Ad02A8702	Aradu.A02	8702	p3	(GGT)7	21	42657724	42657744	ATTGCTGTGGAGTTTTTGGC	CCAACAAATGCTTCCTGCTT	239
Ad02A8703	Aradu.A02	8703	p2	(AT)7	14	42661615	42661628	AATCTTTGGGACCAAAACCA	GGGGCTGATAAATTGCTTCA	277
Ad02A8705	Aradu.A02	8705	p3	(TGG)6	18	42679030	42679047	ACTGCCTCTGCTTTTGCATT	TTGGACAAAAATGTCCTCCAC	270
Ad02A8706	Aradu.A02	8706	p2	(TA)8	16	42679236	42679251	TCAACGTGAGGGACCAAAAT	GGTGAGGCGTTACGACATCT	207
Ad02A8707	Aradu.A02	8707	p3	(TAA)7	21	42680416	42680436	CATAATACATGCGATATGTTTTTGTA	TGTGAGTTGGGCTTATTATGATTG	274
Ad02A8710	Aradu.A02	8710	c	(A)14ctcctaaaa	125	42684429	42684553	CATCTGATCCAATGTGTGCC	CATACGTTGTGCCATGCTTT	276
Ad02A8716	Aradu.A02	8716	p3	(TTA)6	18	42703942	42703959	CCCTATTATAATTTTTACGATTACCCG	TGGCATCGGTTGTAGTAATGA	263
Ad02A8717	Aradu.A02	8717	p2	(AT)6	12	42705584	42705595	TGGTCCATTGTGAGGACAAA	TGCTTGTGTCACCGTCAATTA	187
Ad02A8720	Aradu.A02	8720	p2	(TC)7	14	42742215	42742228	GGACCAGAAAGCACTGGAAG	AAGAACCTGGGATTCAGAGTCA	275
Ad02A8724	Aradu.A02	8724	p2	(CT)13	26	42793083	42793108	GGTTCATAGGACAATTTGGCA	GAGGAATCCATCCATTCATTG	276
Ad02A8726	Aradu.A02	8726	p5	(AAAAG)5	25	42839166	42839190	TGTGGGAAAATTGTGTTTGG	CTCACTTTGGGGCATTTTGT	219
Ad02A8727	Aradu.A02	8727	p4	(TTAG)6	24	42849568	42849591	CCCTGTTTAACCACCCCTTT	AACCTCAACAAAGAATCCAACAA	259

ID	Chromosome	SSR nr.	SSR type	SSR	Size	Start	End	FORWARD PRIMER (5'-3')	REVERSE PRIMER (5'-3')	Product (bp)
Ad02A8728	Aradu.A02	8728	c	(A)10gaaag(A)	25	42859754	42859778	AAGTGTGGGGAGATTTGTGG	CACGTTCACTCATGCATCCT	277
Ad02A8734	Aradu.A02	8734	p3	(TTA)6	18	42958888	42958905	TGACTCGAGACTCAAACAAGAAA	GCTGGCCATGAAGCTTTAGA	138
Ad02A8736	Aradu.A02	8736	p3	(TTC)6	18	42969709	42969726	ACCCCACAGGATCATCTCAG	TAAAATATTGCTCGCCCTCG	277
Ad02A8737	Aradu.A02	8737	c	(T)10gtttttctctt	73	42981469	42981541	TCTTTTTGCCCTTGGATCAC	TGTGGTTCATGAATGTTGGC	210
Ad02A8739	Aradu.A02	8739	p2	(TC)6	12	43033599	43033610	TGCATCCATTCAGACAGAGC	TCAAAACAAGAAGAAATCCTAAATCC	109
Ad02A8740	Aradu.A02	8740	p2	(TC)6	12	43041300	43041311	ATGCATCCATTCAGGGAGAG	TCAAAACAAGAAGAAATCCTAAATCC	110
Ad02A8741	Aradu.A02	8741	p2	(TA)9	18	43059989	43060006	TTACTTGGGACATTTTGCCC	GTTTGGGAGAGTTGGGAACA	208
Ad02A8742	Aradu.A02	8742	p3	(TGG)6	18	43061345	43061362	AGAGCAAGCGCTACACCAAT	CGTTCTCGGGCTAGAGTGAC	134
Ad02A8744	Aradu.A02	8744	c	(CT)6tcttcacct	47	43091886	43091932	CAGAAAAGGGCAAGAAGCTG	AGGCGAGTGGTGAAGAGAAA	257
Ad02A8747	Aradu.A02	8747	p2	(TA)8	16	43133205	43133220	GCATGTACCATAAACTCCCATGT	CCAGACGCTCGAGTAGACAA	236
Ad02A8764	Aradu.A02	8764	p3	(ATT)17	51	43316293	43316343	CTCATGAAAAGCAAGCCACA	AATCCGAATGGTCAAAGTGG	231
Ad02A8765	Aradu.A02	8765	p3	(TTC)6	18	43333036	43333053	CCTTGGCCGAACATACATCT	TGCCATTGATGGTAGTGGAA	149
Ad02A8770	Aradu.A02	8770	p2	(GA)9	18	43408811	43408828	TTTCGAAAATCCACACAAAAA	CCATGATGGAGAAAGGGAGA	235
Ad02A8771	Aradu.A02	8771	p4	(AATA)5	20	43423587	43423606	CCTTGAGCCTTTTAATCCCC	TTGGGGTAATTTTGTCCCCT	248
Ad02A8772	Aradu.A02	8772	p2	(AG)6	12	43424963	43424974	CGGACCAGAAAGTAATGGGA	GAACATTGAACCTGAGATGAAGAA	243
Ad02A8775	Aradu.A02	8775	p3	(TCT)6	18	43475574	43475591	TCTCATTTAGCCGAACCCAC	CTCGGAAGTGGAAGCTTTTG	215
Ad02A8777	Aradu.A02	8777	p2	(AT)6	12	43484405	43484416	TGGGGTAATTTTGTCCCCTT	AGGAAACTGTGGGAACATGG	206
Ad02A8779	Aradu.A02	8779	c	(AT)14ccatgc(	44	43490779	43490822	TTGGCTCTTGAAAGAATGATGA	TTTGCTCTTGCCTTTTGCTT	219
Ad02A8781	Aradu.A02	8781	p3	(AGC)5	15	43511673	43511687	TGATAGGAACAACGCAGCAG	GCGTGGAGAGAGAAGTGGTC	133
Ad02A8785	Aradu.A02	8785	p2	(AT)6	12	43526761	43526772	TGACAACAAATACTTTAATGTTTCAA	GGACCATGCTGTGAGTAGGC	276
Ad02A8786	Aradu.A02	8786	p3	(AGC)5	15	43527327	43527341	ATCCAAACGGTCACAGGAAC	GTGGAGAGAGAAGTGGTCGC	141
Ad02A8787	Aradu.A02	8787	p2	(GA)6	12	43556146	43556157	CTAGAGGTTTCTCCGGCCTT	CCCATCACCTCTTCACCACT	269
Ad02A8788	Aradu.A02	8788	p2	(AT)6	12	43578312	43578323	CCACAATATCGTTCCCAAGA	GATATGGGCGCTGAAGTCAC	262
Ad02A8790	Aradu.A02	8790	p2	(AT)6	12	43598971	43598982	GAGCCTTCGCGAATACAAAC	CCTTTCAGCTACAAGTGCGA	177
Ad02A8796	Aradu.A02	8796	p2	(AT)10	20	43627958	43627977	GTAGCAACTCGGAAAGTCGC	CACTCAAATCAATTGGGATGC	279
Ad02A8799	Aradu.A02	8799	p2	(AG)6	12	43650940	43650951	TTTGGAATTGAAAACTGGGC	CCTTCATCTCAAAACCGCAT	190
Ad02A8800	Aradu.A02	8800	p3	(TAT)21	63	43665503	43665565	CCGTCAAAAATCCAATCATCA	TAGGAAAAGCCTTTGCTGGA	201
Ad02A8801	Aradu.A02	8801	p2	(TA)14	28	43667675	43667702	GTGAACGTTTTGCGCTTTTT	CGCTTTTTGTAATACCCCGA	157
Ad02A8808	Aradu.A02	8808	p2	(AT)7	14	43760787	43760800	TGCCTTAATTTGCATTTTTGG	GCTTTATGGGAACATGGGTG	180
Ad02A8809	Aradu.A02	8809	c	(T)10cattttctttt	64	43768598	43768661	TCCCCAATTAGAGGTGTCCA	TGTGGTTCATGAATGTTGGC	249
Ad02A8811	Aradu.A02	8811	p2	(GC)6	12	43789125	43789136	CCTGCGACCTTGGTGTATTT	GTGCGGATTGCATAACATTG	234
Ad02A8812	Aradu.A02	8812	c	(TC)6(T)10	22	43791832	43791853	TCCCCAATTAGAGGTGTCCA	TGTGGTTCATGAATGTTGGC	235
Ad02A8814	Aradu.A02	8814	p2	(GT)10	20	43816106	43816125	TGTCATTGAAGTGTGGGGAA	TTACTTTGGGGGCATTTTTG	229
Ad02A8817	Aradu.A02	8817	c	(A)10gagaagaa	36	43855356	43855391	TGTGGTTCATGAATGTTGGC	TCCCCAATTAGAGGTGTCCA	253

ID	Chromosome	SSR nr.	SSR type	SSR	Size	Start	End	FORWARD PRIMER (5'-3')	REVERSE PRIMER (5'-3')	Product (bp)
Ad02A8818	Aradu.A02	8818	p3	(TTC)7	21	43881086	43881106	AATCTTTTCCCACCCAAACC	TAAAATATTGCTCGCCCTCG	212
Ad02A8822	Aradu.A02	8822	p3	(AGA)6	18	43909174	43909191	CTAGAGGTTTCTCCGGCCTT	CCTCTCTCCCTCTCCTCCAT	157
Ad02A8824	Aradu.A02	8824	p3	(TTC)6	18	43938041	43938058	TCCACTCTTGCCCATAAACC	CCCTCGAGCAAGAGAAGAAA	236
Ad02A8826	Aradu.A02	8826	c	(CT)6cacatcca	111	43977159	43977269	TCCCTCCTTATTTCTTCCTTCTC	TTGCAACAATCCACAAGCAT	257
Ad02A8833	Aradu.A02	8833	p2	(AT)11	22	44131850	44131871	TAAGTGTGGGGAAAAGTGGG	CATATGCATTGCTTTCACCG	221
Ad02A8836	Aradu.A02	8836	p3	(TTC)5	15	44188906	44188920	GTCTTCGGACTCTCCCTTCC	TCCTTTTCCAAGGCTCACAT	262
Ad02A8837	Aradu.A02	8837	p3	(ACA)5	15	44207475	44207489	TTTTTGGTTTGTCGAGCCAT	CATGGTCCTCAGCTATGGAAG	234
Ad02A8839	Aradu.A02	8839	p3	(AGA)5	15	44218351	44218365	AAGCTTTTGGCTTCCCTTTC	CGAACCTATACCCTCCCCTC	277
Ad02A8842	Aradu.A02	8842	p2	(AT)7	14	44236131	44236144	AACACTTGCATAGCCACCAT	TGTTAGCTTCAATGCAAACCAT	261
Ad02A8847	Aradu.A02	8847	p2	(CT)6	12	44289711	44289722	AACCACTTTTCCACTCCCAA	GTGGTTTATCAAGGGGGTCC	150
Ad02A8848	Aradu.A02	8848	p2	(AT)10	20	44297485	44297504	TTTGTGTGTTTTTCTCTTTCATCA	GGGTAAGCATATGACCCCAG	280
Ad02A8850	Aradu.A02	8850	p2	(AG)6	12	44319912	44319923	GAAGGGTTTTTGGGGAAGAG	CCCTCTTCATTCCTTGGGTT	267
Ad02A8853	Aradu.A02	8853	p2	(TC)6	12	44353274	44353285	TCCCTTTCCCTTTCTACCTCA	TTTGCAAGAATCCACAAGCA	241
Ad02A8854	Aradu.A02	8854	p3	(AAG)6	18	44379159	44379176	CTTCCCTTTCCCTTTTCTGG	TACCCCCTCTCCTCTCCCTA	262
Ad02A8858	Aradu.A02	8858	p2	(TC)14	28	44398332	44398359	GACTTCCTTCCATCGCCATA	TCGATCGATCAAGGAACACA	258
Ad02A8859	Aradu.A02	8859	c	(T)10ccacttaat	41	44403463	44403503	AATTCCTGGACCGGAGTCTT	GAAACATGCAATTGACACCAA	195
Ad02A8861	Aradu.A02	8861	p2	(TA)7	14	44422072	44422085	GGCTAAGGTAAGGGTAGGGG	ATGGGAAGATTGTGGGAACA	251
Ad02A8862	Aradu.A02	8862	p2	(AT)9	18	44427356	44427373	TAGGGTTGCTTGCATGTTCA	AGGGCAATTTTTGTATCCCC	230
Ad02A8863	Aradu.A02	8863	p2	(AG)8	16	44444807	44444822	TTTCGGCCCAGAAAACATAG	TGAACCTGTGATTGAAGAAGATG	225
Ad02A8865	Aradu.A02	8865	p2	(GC)6	12	44451003	44451014	GAGGTTGCTACAGGACGAGG	CCTTTTGACAGTCCGTGGAT	237
Ad02A8866	Aradu.A02	8866	p2	(CT)6	12	44491776	44491787	TGGAAGGTAAAAGTAGATGAAAGCA	TGGGATCGACCCTTACTCAC	252
Ad02A8867	Aradu.A02	8867	p2	(AG)8	16	44523214	44523229	AGCCAATTGGGCAATATCAA	GAGGAAGGCATCCATTCAAG	260
Ad02A8868	Aradu.A02	8868	p2	(AG)8	16	44529234	44529249	AGCCAATTGGGCAATATCAA	GAGGAAGGCATCCATTCAAG	260
Ad02A8872	Aradu.A02	8872	c	(AT)6(GT)34	80	44638942	44639021	GTTTTCAAGCGCCAGCTTAG	AGCACGAGATCGATTTTTCG	257
Ad02A8873	Aradu.A02	8873	p2	(GA)6	12	44639953	44639964	GTAGGGCCAACAGCGTAAAA	TAGCCCCAAATGGGTCATTA	231
Ad02A8875	Aradu.A02	8875	c	(TAG)7(AAG)	111	44649459	44649569	CTAGAATGTTGCTCGCCCTC	CTACCTTCTCTGCTCCCCCT	206
Ad02A8882	Aradu.A02	8882	p3	(TTA)5	15	44758597	44758611	AGAAGGATTGGATGAAGGCA	TAAGGGTCGATCCCACAGAG	236
Ad02A8885	Aradu.A02	8885	p2	(AG)6	12	44781554	44781565	GGAAGCAGTAAAGAGAGCCTTG	ATAAAGGAGGCTGGCTCCAC	197
Ad02A8886	Aradu.A02	8886	p4	(TTTA)6	24	44785827	44785850	CCCCAGTTAGTGGTGTCCAG	GAAAAAGCAGTGAATGGCAA	229
Ad02A8894	Aradu.A02	8894	p4	(TTAT)5	20	44866878	44866897	ACGCACTTGGTCTCGACTCT	CCTTAAAGCAAAAGAAACAGCAA	210
Ad02A8895	Aradu.A02	8895	c	(TA)7tgcg(T)1	29	44868095	44868123	TGAATGGCGAAAATCACAAA	CACAGGAACCAAACACCTCA	229
Ad02A8896	Aradu.A02	8896	p2	(AT)7	14	44871399	44871412	TTAGAACGAAACAGGGGGAG	GGGATCGACCCTTACTCACA	240
Ad02A8899	Aradu.A02	8899	p2	(TG)8	16	44881660	44881675	GTGTCTTTGGCGAACAAGGT	AGGATCTTGCGTCTTACCGA	216
Ad02A8900	Aradu.A02	8900	p3	(AGA)6	18	44890606	44890623	CCCATGACTAGGAGGTGGAA	CACCTTAGCCGAAACACCAT	218

ID	Chromosome	SSR nr.	SSR type	SSR	Size	Start	End	FORWARD PRIMER (5'-3')	REVERSE PRIMER (5'-3')	Product (bp)
Ad02A8901	Aradu.A02	8901	p2	(TA)6	12	44905102	44905113	TAAGTCTTGTTCCCCATGCC	GGTGTTCCAAAGCGACAGAT	267
Ad02A8902	Aradu.A02	8902	p2	(TC)6	12	44906750	44906761	GCACTACCTGATCCCTCTGC	GGGAGGGATGATGGAGAAAG	183
Ad02A8904	Aradu.A02	8904	p4	(AATA)6	24	44916199	44916222	TTTCGTGCACCACCTACTCA	CCCATAATTTCCCACTTTTGC	248
Ad02A8909	Aradu.A02	8909	p4	(ATTT)5	20	44940617	44940636	AAACCCTTTGGGAATGGCTA	TTCTTGGCTGAAGAAGTGGC	275
Ad02A8912	Aradu.A02	8912	p2	(AC)7	14	44959049	44959062	CCAATCTCTGCCGTGAAAAT	TCAGTTGTTTCGCTACCAACA	158
Ad02A8915	Aradu.A02	8915	p3	(AGA)7	21	44967942	44967962	TAAAATATTGCTCGCCCTCG	ATCAATTTCCCTCCCATTCC	211
Ad02A8918	Aradu.A02	8918	c	(TC)8(T)11	27	44994288	44994314	TCCCCAATTAGAGGTGTCCA	TGGCTCTTGAAAGAATGATGAA	225
Ad02A8921	Aradu.A02	8921	p4	(TAAA)5	20	45041541	45041560	TTCTTGTCCTTGCTCCTGCT	TTTTTGAATTCTCTCCCTCTCA	240
Ad02A8925	Aradu.A02	8925	p2	(AC)6	12	45099685	45099696	TGTATGGCCAAGTGACCTGA	AATGAAAGCACCCCAAATGA	211
Ad02A8926	Aradu.A02	8926	p2	(TC)6	12	45124146	45124157	TCCCCAATAAGAGGTGTCCA	TGTGGTTCATGAATGTTGGC	270
Ad02A8927	Aradu.A02	8927	p3	(TAC)5	15	45146166	45146180	CATGCCTGATGATGATGAGAA	AGCTGAAGCGCCAGAAATTA	238
Ad02A8929	Aradu.A02	8929	p2	(TC)6	12	45195982	45195993	ATTAAGAACATTCGCGACCC	TCAGGTTGTGCTCCTCAGTG	270
Ad02A8933	Aradu.A02	8933	p2	(AC)7	14	45245893	45245906	TGTTTTCCCACAAATTTCCC	TTTTGAGCCTTTAATTCCCC	154
Ad02A8934	Aradu.A02	8934	p2	(CT)6	12	45254687	45254698	TTTCGAAATCTCTCCTCCCC	GCTTGTCCTCAAGCAAAAGAA	119
Ad02A8936	Aradu.A02	8936	c	(TTTATT)5tat	77	45264264	45264340	CCTAACCACTTTCTCTCTCCTCA	GGGTTAGCATATGACCCCAG	260
Ad02A8939	Aradu.A02	8939	p2	(TC)6	12	45319958	45319969	TTCAGAGAGAGCTCGCCAAT	TCAAAATAGAGATCTGAAACATGGA	220
Ad02A8942	Aradu.A02	8942	p3	(TTA)14	42	45332489	45332530	ACAAACATAAGGCCTGGCAC	TCAAGCATCCCATAATACCCA	255
Ad02A8944	Aradu.A02	8944	p6	(AAAATT)5	30	45334107	45334136	CAGAAACATAGCAATAACTGCACA	TCAATGGTTGGATTGACGAA	271
Ad02A8948	Aradu.A02	8948	c	(A)10tcata(T)1	25	45369865	45369889	GAAGGCAGAATGGTGGAACT	CCAGTGAGGGTTCAATTGCT	278
Ad02A8951	Aradu.A02	8951	p3	(ATT)5	15	45453285	45453299	AAAGGCTTGCTATTGCCAAA	TTCGGCAGTGCTAAAGAAGC	221
Ad02A8952	Aradu.A02	8952	p2	(AG)6	12	45454534	45454545	TTGAGGTGTCAGAGGAAAAGAA	CGAAAGTCACTAACCACTTTTTCA	266
Ad02A8953	Aradu.A02	8953	p2	(TA)6	12	45471809	45471820	TTTTCAATCAATTGGGCTGC	CAAAAATGGAAGCGGAAGAA	257
Ad02A8958	Aradu.A02	8958	p2	(CT)8	16	45510573	45510588	TTCCTTCTCCCTTCTTCCTTC	TTGCAACAATCCACAAGCAT	250
Ad02A8959	Aradu.A02	8959	p2	(AT)7	14	45515272	45515285	GCTGTAGGGTCAACAGAGGG	TTTCCTGGTTGGGTATCTCG	268
Ad02A8963	Aradu.A02	8963	p3	(CAT)5	15	45586088	45586102	TGGAAAATGGATCAAGCCTC	GCGATGCTCACTTTGATTGA	187
Ad02A8964	Aradu.A02	8964	c	(T)10gccactctt	30	45595406	45595435	ATCCATTGATGCTCAAAGCC	AAAGCAGTGAAAAAGCAAAAGC	150
Ad02A8967	Aradu.A02	8967	p3	(AGA)5	15	45638359	45638373	AAAGGTTTGCTCGTCCTCG	AACCACTTTCCCTCCCAAAC	270
Ad02A8968	Aradu.A02	8968	p4	(AAAT)8	32	45653454	45653485	ATAAAGGGAATTTGGGCACC	TGGGGTAATTTTGTCCCCTT	146
Ad02A8971	Aradu.A02	8971	p2	(AT)8	16	45679537	45679552	CAATGCAACTGAACCTTCCA	ACTCCTCCCCACTCCTTTGT	272
Ad02A8974	Aradu.A02	8974	p3	(AGA)6	18	45685499	45685516	CTAGAGGTTTCTCCGGCCTT	CCTAAATGGCCGAACCTACA	262
Ad02A8978	Aradu.A02	8978	p2	(GC)6	12	45722096	45722107	ACAAGGCTCCTAAAACCGCT	CCAGTTGGGTTGGAAAGCTA	163
Ad02A8981	Aradu.A02	8981	p3	(AGA)6	18	45761539	45761556	GCTTTTGTCTTCCCTTTCCC	AGCCGAACCTACCTCTCTCC	192
Ad02A8984	Aradu.A02	8984	p2	(AG)8	16	45837947	45837962	GCTTCCTCCTCAGAGTCCTTT	CTCCCTCTCTCATCCCTTTCT	279
Ad02A8986	Aradu.A02	8986	p2	(TA)8	16	45847198	45847213	ATCCAATTCCCACACGTTGA	CATTATGCCAATTCGGGAAG	268

ID	Chromosome	SSR nr.	SSR type	SSR	Size	Start	End	FORWARD PRIMER (5'-3')	REVERSE PRIMER (5'-3')	Product (bp)
Ad02A8989	Aradu.A02	8989	c	(TTC)5cctgtga	42	45853875	45853916	GTCAACCCCTATTCCATCCC	AATGGGGGTAGGAGAGGAAA	270
Ad02A8992	Aradu.A02	8992	p3	(TGA)5	15	45864335	45864349	GTTAATAGTGGCCCATTTATATTGA	TTTCTTCCCCCATGTCATTC	267
Ad02A8993	Aradu.A02	8993	p2	(AC)6	12	45864681	45864692	AGATAGTGGGGGTGGGAAAG	AAAGGCTGTGGTAGTGGTGG	174
Ad02A8994	Aradu.A02	8994	p3	(TAA)11	33	45865178	45865210	AGCCCCTACCTTAGGAACCA	TTGAAGTTAGGTAGACAAAGTTTCACA	126
Ad02A8999	Aradu.A02	8999	p2	(GA)57	114	45880197	45880310	TGAAAACATAAAACCCCACCTT	AGATCGACAGCTCAGGGTGA	279
Ad02A9005	Aradu.A02	9005	p3	(AGA)6	18	45928253	45928270	TGCCATTGATGGTAGTGGAA	TGGCCGAACCTACATCTCTC	146
Ad02A9006	Aradu.A02	9006	p2	(CT)6	12	45949412	45949423	GCACTTGGAGGGCATAACAT	GAGGCTATAAAGTGGGGGAA	279
Ad02A9007	Aradu.A02	9007	p3	(AGA)11	33	45957549	45957581	TGCCATTGATGGTAGTGGAA	ACCCAAACCCACCCTAAAAT	278
Ad02A9008	Aradu.A02	9008	p2	(GA)7	14	45979467	45979480	GGGGGAGGGAAGAAGAAAAT	TAATATGGGTCACGCGACTG	269
Ad02A9010	Aradu.A02	9010	p2	(AG)6	12	46009960	46009971	GAGGTTCGGATTTGTGGATG	CAAAACCACCTAACTACTTTTCCC	235
Ad02A9011	Aradu.A02	9011	c	(T)13agcttc(A)	29	46014375	46014403	GACGAAAATCGGGAACAGAA	CCACCATTCCGAGGAGTAGA	266
Ad02A9012	Aradu.A02	9012	p2	(TA)6	12	46015686	46015697	CTGTGGTTTCCGAGGTCAAT	GGTGCATGCTATGACGAAGA	263
Ad02A9017	Aradu.A02	9017	p3	(TTG)6	18	46069329	46069346	GCAGAAGGAATCAGAGCCAC	AAGCGGAAGAACAGCAGAAG	274
Ad02A9018	Aradu.A02	9018	c	(GGT)11gctag	77	46069701	46069777	ATGCAGATGGTGATGGAGTG	TTGAGGGCCAGAATTGAAAC	242
Ad02A9019	Aradu.A02	9019	p3	(CTT)6	18	46077685	46077702	ATCAGGCATTTCCATTCTGC	GCTGGACTCTCATGCCAAGT	117
Ad02A9022	Aradu.A02	9022	p5	(TTATC)5	25	46106886	46106910	GCAAGGGACTCCGATACTGA	TTGACAGGGAAGCCCAATAG	253
Ad02A9025	Aradu.A02	9025	p3	(TAT)12	36	46150042	46150077	CGCAAGTTTCCGTTTACCTG	GAGGTGGTGAGGGATTACGA	162
Ad02A9026	Aradu.A02	9026	p2	(AT)8	16	46155931	46155946	TTGCTTATTGTTCCATTGCC	GCGACTCTGATAGCTCGGAC	275
Ad02A9027	Aradu.A02	9027	p2	(CT)6	12	46168953	46168964	CCACTTTTCTATCTCCAATTTTCG	ATAGAGAGGGAGGGGGTTCA	147
Ad02A9034	Aradu.A02	9034	p3	(TTC)6	18	46252115	46252132	CCTACCTCGCCCTCTCTCTT	TAAAATATTGCTCGCCCTCG	231
Ad02A9036	Aradu.A02	9036	p2	(GT)6	12	46273386	46273397	CAACCCTTGCAACCAACTTT	TTACCTCAACCAATGTCCCA	177
Ad02A9038	Aradu.A02	9038	p2	(CT)7	14	46281878	46281891	AATTACAGAACACGCCCCAG	TGGATCCAGAGTTACTCCTAAAACA	222
Ad02A9041	Aradu.A02	9041	p2	(AT)8	16	46286338	46286353	GTGGGTTTGGAAACATCTGG	GGACATTTTGTCCCCTTTTTC	162
Ad02A9042	Aradu.A02	9042	p2	(CT)7	14	46287536	46287549	AATTACAGAACACGCCCCAG	TGGATCCAGAGTTACTCCCAA	220
Ad02A9045	Aradu.A02	9045	p2	(AT)8	16	46307168	46307183	AAGCCAGCATCTGCCTTAAC	TTGAAAGCTTTTCATTCACCA	181
Ad02A9051	Aradu.A02	9051	p2	(AT)6	12	46347945	46347956	AGGAGCCTTGAAGGAGAAGC	GCCTTAGTTTCGCAGGTCAA	179
Ad02A9052	Aradu.A02	9052	p2	(AT)6	12	46348835	46348846	TGCAGATAGGCATGGATGAA	GATCGGACACTTGAACTCCC	254
Ad02A9053	Aradu.A02	9053	p2	(TA)7	14	46349506	46349519	TGTGCATATGTACGCACCAG	TTGTGGTATGGTTGTCGTTGA	249
Ad02A9055	Aradu.A02	9055	p2	(AG)7	14	46386418	46386431	GGTTCATGATCGGCTTCAAT	CCCTCCTTTGATAATTTGGACTC	210
Ad02A9056	Aradu.A02	9056	p3	(AGA)8	24	46397384	46397407	TTGAGGTTTCTTCGGCCTTA	AAATGACCGAACCCTAACCC	262
Ad02A9058	Aradu.A02	9058	p2	(AT)7	14	46484470	46484483	GTGTTGCGTGTCACGTTCTT	CGATAGAGTTTGTCTTTGCCC	273
Ad02A9062	Aradu.A02	9062	p2	(GC)6	12	46567381	46567392	TGGAAAGCTAACATCCAGGG	ACAAGGCTCCTAAAACCGCT	152
Ad02A9065	Aradu.A02	9065	p2	(TG)7	14	46578660	46578673	TTTAAGAACGAATATGTCCAATACAAA	TCAACCGGACCGATTCTAAG	236
Ad02A9067	Aradu.A02	9067	p3	(TTA)5	15	46596136	46596150	CGGCTACACTGCGTCTTGT	TTGATACACACATTAATAGTGACCCAT	258

ID	Chromosome	SSR nr.	SSR type	SSR	Size	Start	End	FORWARD PRIMER (5'-3')	REVERSE PRIMER (5'-3')	Product (bp)
Ad02A9068	Aradu.A02	9068	c	(TTC)6(T)13	31	46637503	46637533	GTGGAGGACCATTTATCCCC	GCAAATATGCGAATGCATCA	198
Ad02A9069	Aradu.A02	9069	p3	(TTC)5	15	46639879	46639893	CCCGGAGAGCTATTTTGGTT	CCCCTAATGAAAGTGGGGTT	169
Ad02A9071	Aradu.A02	9071	c	(AT)7gttatatttt	43	46668335	46668377	ACCAAATCGACTGCTAGCCT	CGGTATCTGGGTTGCTCAAT	216
Ad02A9080	Aradu.A02	9080	p2	(TC)7	14	46742081	46742094	GCCTCTGACAATACACATAGCAA	AAATTTGATCAGAATCTCGTGC	233
Ad02A9081	Aradu.A02	9081	p2	(GC)6	12	46743604	46743615	ACAAGGCTCCTAAAACCGCT	CGGTTCCAGTTGGGTTAGAA	168
Ad02A9083	Aradu.A02	9083	p2	(TC)6	12	46747822	46747833	TTCCTTATCTTTTTCAAAACCACC	GGAGGGGGTCCGAATTATTA	211
Ad02A9084	Aradu.A02	9084	c	(A)10ctaaaattt	105	46755401	46755505	ATCTGCACGTTCCAGGATTC	TCCTAATTGGAACCCAAAGAAA	248
Ad02A9089	Aradu.A02	9089	p3	(AAT)11	33	46803053	46803085	TGCATCTGAATTTCAAGTATGAGG	TGTCCTTGCCATTTTCTTCA	251
Ad02A9090	Aradu.A02	9090	p4	(TTAT)5	20	46807574	46807593	GCCTCTAACAATACACACAGCAA	TGAGTACCCGATTTCTGAACAA	276
Ad02A9094	Aradu.A02	9094	p3	(GAA)5	15	46821423	46821437	CGGTAACAGCAGCATAACAAA	TTTCTGAATGCTCGGTTCTTC	251
Ad02A9098	Aradu.A02	9098	p2	(AT)10	20	46832419	46832438	AATATGATTATTTGTGCAAGAGTTTTT	TGGATAACTTTGCAGAGATTTAGTT	270
Ad02A9101	Aradu.A02	9101	p3	(TAA)11	33	46834435	46834467	TGGTACAACAAGTTTGGGGC	TGACTGCTTTTCTAGGCCCTT	239
Ad02A9103	Aradu.A02	9103	p2	(TA)6	12	46836184	46836195	GTTTCGTACGCAAAGACGAG	CCCGGTAGAGAGCAACAAAA	117
Ad02A9105	Aradu.A02	9105	p6	(AATCCT)5	30	46843802	46843831	ACAGCAAAGGCTGTGATTGA	GGGAACATGCAGAGACTCAAG	272
Ad02A9106	Aradu.A02	9106	p3	(TCT)7	21	46845524	46845544	AAATGACCGAACCCTAACCC	CTAGAGGTTTCTCCGGCCTT	263
Ad02A9108	Aradu.A02	9108	p2	(AT)12	24	46855614	46855637	GCACTTTGCTGAGGCATTCT	CTAAACCGGCCAGAGAACAC	249
Ad02A9109	Aradu.A02	9109	c	(A)10gttaaattc	51	46856177	46856227	TTTAATGCGTCCCTTAACCA	TTCACCACTTAAACGTGTCACC	167
Ad02A9113	Aradu.A02	9113	p2	(GT)6	12	46863963	46863974	AGAGCTATTCGCAAGGATCG	GGAAAACCGGGCATTAATTT	276
Ad02A9115	Aradu.A02	9115	p2	(AG)6	12	46870471	46870482	TGCAGAAAACGTTCATAGCG	TTGATGAAGAGTCGAATTAAATCCT	229
Ad02A9116	Aradu.A02	9116	p2	(CT)8	16	46878220	46878235	TTTCAAAACCGCCTAACCAC	CAGAGGAGAAGAGAGGGTTCAA	260
Ad02A9117	Aradu.A02	9117	p3	(TCT)6	18	46881461	46881478	ATCTCTTTCCCACCCAAACC	CCAGCCTTAGGTGCCATAAA	279
Ad02A9119	Aradu.A02	9119	p3	(TAC)5	15	46895167	46895181	AACAAAAAGTATTGCCACCACA	CCACTTCCCAAAACACAGGT	277
Ad02A9128	Aradu.A02	9128	p3	(AGA)6	18	46973967	46973984	AGACGGTGGAGAACGAGAGA	GGCGACATTAGCAAGAAAGC	148
Ad02A9133	Aradu.A02	9133	p3	(AAG)8	24	47009430	47009453	TTGCCTTCCCTTTCCTCTTT	TCTTCTCCCACTTAGCCGAA	203
Ad02A9137	Aradu.A02	9137	p2	(AG)6	12	47066229	47066240	ATCTACCCATCCCATCACCA	TCGTATTCCTCCTTGATGGC	240
Ad02A9138	Aradu.A02	9138	p2	(AT)6	12	47071015	47071026	TTTGAATATTGGGAAATTTGGG	CCAAGGCAAGGGTGAAGATA	240
Ad02A9140	Aradu.A02	9140	p2	(GA)6	12	47089048	47089059	AGATGTTTCGCCTCCCTTTT	GGACGCTAACGCGTCATTAT	264
Ad02A9142	Aradu.A02	9142	p2	(TA)7	14	47105113	47105126	TTCTTTTGAACTCTTCATTCCCA	TTTTCAAAAATTCCCTTCTTCTTC	247
Ad02A9146	Aradu.A02	9146	p5	(AAAAT)5	25	47123925	47123949	CGCTCTCACGTGACTCTCTG	GTGCTAGGTGGGAGACAACC	280
Ad02A9147	Aradu.A02	9147	p3	(AGA)6	18	47145115	47145132	TCGTCCTCGAGCAAGAGAG	CACCTCAAATCCTCTTCCCA	199
Ad02A9149	Aradu.A02	9149	p2	(CT)6	12	47157933	47157944	AGCCAAAAGGGAAAAATCCA	CATGCAGAGACTCATCACGC	280
Ad02A9150	Aradu.A02	9150	p3	(TTA)5	15	47183146	47183160	TGCTGTGCTCAATTTTCAGA	CACAAAAAGGGAGAAACGGA	174
Ad02A9152	Aradu.A02	9152	p2	(CT)6	12	47192706	47192717	GAATCCATTGTTGCTTCTGCT	TGCTAACAATCAACTACTGTGCAA	272
Ad02A9162	Aradu.A02	9162	p3	(ATA)10	30	47216105	47216134	CAAACGACGTTGAGGACAAA	ATTCCCATATCCTGCACAAG	178

ID	Chromosome	SSR nr.	SSR type	SSR	Size	Start	End	FORWARD PRIMER (5'-3')	REVERSE PRIMER (5'-3')	Product (bp)
Ad02A9164	Aradu.A02	9164	p3	(TCT)7	21	47226508	47226528	AATGACGGAACCTAAACCCC	CTCGAGCACAAGAGGAAAGAA	168
Ad02A9165	Aradu.A02	9165	c	(T)13catttcaca	132	47229164	47229295	CCCATAATTCTCACTTTTTCCA	CCTTATGCATTGAGGTTCTTG	273
Ad02A9166	Aradu.A02	9166	p2	(GC)6	12	47232266	47232277	ATACCAGGAGTGCACAAGGG	TTCCAGAGACTTGAGCCCAC	162
Ad02A9169	Aradu.A02	9169	p2	(TA)9	18	47266805	47266822	CCAAGGCTAGCCGGAGAATA	GATGTATGTGCGAGAAGCCA	276
Ad02A9170	Aradu.A02	9170	p5	(TTCTT)5	25	47270352	47270376	TCAAGAATGATTCAACCCCC	AATGCAAACGCATCAGAACA	113
Ad02A9171	Aradu.A02	9171	p3	(CAT)6	18	47273175	47273192	CAGCCCCAAATTCTTCAAAA	TGCATTGGATAAGTGAATTGATG	167
Ad02A9173	Aradu.A02	9173	p2	(AT)8	16	47281109	47281124	GAATTTGTGTGTTGCTGTATCTTTT	TCTTTAAGAGAAAGGTTATTAGGGG	278
Ad02A9175	Aradu.A02	9175	p3	(CTT)5	15	47287165	47287179	CTGCAAAGGCCATTACCCTA	GGCATTAATTGTTCCACACG	222
Ad02A9177	Aradu.A02	9177	p2	(TA)9	18	47290740	47290757	TGTTCTGGATATGATCAATCTTGTG	TGGGATGGGATATATGGACC	278
Ad02A9178	Aradu.A02	9178	p3	(ATG)5	15	47296660	47296674	AATTTGAACAACCGAGGTGC	TCAGTCTCATCTCGTGGCAG	240
Ad02A9179	Aradu.A02	9179	p2	(CT)22	44	47296851	47296894	CTGCCACGAGATGAGACTGA	ACACGCCTACATGGCCTTAC	189
Ad02A9183	Aradu.A02	9183	c	(T)12ggtgacta	93	47301940	47302032	AAATTCGCCGTTAATGGAAG	AACTTTTGAAGAGAAGCATAGGAAA	279
Ad02A9185	Aradu.A02	9185	p3	(TCC)5	15	47307351	47307365	GTGGAATGCACGCTCAAGTA	CTCAATGTCATCCCTCCACC	189
Ad02A9187	Aradu.A02	9187	p3	(TCA)5	15	47344387	47344401	TGGTCATGAGGGATTGATGA	GGGATGAGAACTCCGTTGAA	151
Ad02A9188	Aradu.A02	9188	p2	(TG)6	12	47347128	47347139	TCAACATGCAAAAATCACGTT	CCTAGCTCATCCCAAACCAA	225
Ad02A9193	Aradu.A02	9193	p2	(TC)8	16	47420885	47420900	GCCTAATTGGTGCAGGATGT	GGTTTGGGTGGGAAAAAGAT	209
Ad02A9194	Aradu.A02	9194	p3	(TTC)6	18	47421053	47421070	ATCTTTTTCCCACCCAAACC	CTAGAGGTTTCTCCGGCCTT	195
Ad02A9195	Aradu.A02	9195	p2	(AG)7	14	47422837	47422850	CAATTGGGCAACATCAATCA	GGCTGTTTCTGACCCAGTTC	279
Ad02A9197	Aradu.A02	9197	p2	(AG)6	12	47428749	47428760	ACCAATTGGGCAACATCAAT	CCAGTTCTCGTCCCAGAAAA	266
Ad02A9198	Aradu.A02	9198	p3	(AGT)5	15	47435831	47435845	GAGGAACATCCACTTCCCAA	CATGTTTGATGATGATGAGAAAAA	184
Ad02A9199	Aradu.A02	9199	c	(AC)6agagaga	76	47449967	47450042	TCCTTTCAGGTTCAGGATCG	TTCCCTCCCCCTTATCTCTT	277
Ad02A9204	Aradu.A02	9204	p4	(ATAC)13	52	47463235	47463286	CGAGGATGGCAACTTTTACA	TTTTCTTCGCCTTTGTTATCG	278
Ad02A9205	Aradu.A02	9205	p2	(TA)10	20	47463498	47463517	CGATAACAAAGGCGAAGAAAA	ATGCCCAATGATCGAGACTT	213
Ad02A9206	Aradu.A02	9206	p2	(TC)6	12	47465991	47466002	TTATCCTTTGTCCTCGTCGC	CTAATTCACACTCTCCCGCC	239
Ad02A9207	Aradu.A02	9207	p4	(ATTT)7	28	47472323	47472350	GACCATCCCGAAGAGATCAA	CCTGTGAGCGAAGATTGTCA	258
Ad02A9209	Aradu.A02	9209	p2	(CT)8	16	47485712	47485727	TCCTACCAGAGCAAACACCC	AGAGAGCTCGAAGAGGGACC	171
Ad02A9211	Aradu.A02	9211	c	(T)10gggttaaa	101	47489017	47489117	TTCGAATCTATGGTGTGACAATTT	GCATATGCAACAAAACATAACATTT	251
Ad02A9217	Aradu.A02	9217	c	(T)10ctttcttttc	50	47502051	47502100	CACACTCTTGTTGCCTTGGA	TGGCTCTTGAAAGAATGAGGA	161
Ad02A9219	Aradu.A02	9219	c	(T)10cgaaa(T)	25	47528381	47528405	GGTATGGAGAGGTTTTCCCC	TCCGAATGAATGCTTGAACA	271
Ad02A9220	Aradu.A02	9220	p2	(TA)7	14	47545889	47545902	AGGTTGGAATTTTGAACCGA	ATTCCACAGCCTTCACCAAC	269
Ad02A9225	Aradu.A02	9225	c	(A)14gaaagact	71	47573789	47573859	GGTGTGTGTGGGGGTAATTC	AGTTGGCAGATTCACTTTTGG	257
Ad02A9228	Aradu.A02	9228	p3	(TTC)6	18	47588560	47588577	AATTTCCCACCCTATCCCAC	ACCACTGAGCTATGACGGCT	266
Ad02A9230	Aradu.A02	9230	p2	(TA)6	12	47631770	47631781	TGTGATACGAAAATGCTGATGA	TCTCCACGTTCAAAAAGCAA	226
Ad02A9232	Aradu.A02	9232	p2	(AG)30	60	47650021	47650080	TGCAAAGTAGAGTGCAAGGG	TACTCTTACGTGTCGCGTGC	160

ID	Chromosome	SSR nr.	SSR type	SSR	Size	Start	End	FORWARD PRIMER (5'-3')	REVERSE PRIMER (5'-3')	Product (bp)
Ad02A9235	Aradu.A02	9235	p3	(TCT)6	18	47663182	47663199	CAACACAACCCCCACTTCTT	CTAGAGGATTCTCCGGCCTT	194
Ad02A9236	Aradu.A02	9236	p3	(AAG)7	21	47671510	47671530	GCTGGACTCTCATGCCAAGT	ATCAGGCATTTCCATTCTGC	120
Ad02A9240	Aradu.A02	9240	p3	(AGA)6	18	47716988	47717005	GCTTTTGTCTTCCCTTTCCC	CACCTCTTCTTCACCTTGGC	246
Ad02A9244	Aradu.A02	9244	c	(CTT)5tgagccc	108	47762684	47762791	CCTGAGATGGAGAGAGGCAG	GGTGACAGGCTCATAGGGAC	268
Ad02A9247	Aradu.A02	9247	p5	(TTTAT)5	25	47787890	47787914	TTTCTCTCTCCTCACTTTTTCG	CCCCAAAGTCCTTCTGGATT	244
Ad02A9254	Aradu.A02	9254	p3	(AGA)14	42	47869034	47869075	ATGGAAGTGGTCTTGATGGC	TCACCTAGGCCGAACCTACA	279
Ad02A9256	Aradu.A02	9256	p2	(AT)10	20	47872969	47872988	TTTTTGACCAAGTCAACCCA	TGCATGCATTCATTGTTTTG	159
Ad02A9260	Aradu.A02	9260	p2	(TA)7	14	47904177	47904190	CACACGGGACAAAATTCAAA	TTTCAAGGTTGTAATGGGGC	215
Ad02A9264	Aradu.A02	9264	p2	(GA)20	40	47911916	47911955	TCGATGTTAATATTCGAAACCA	AGTGCCATGTCAATTTGTTTTT	205
Ad02A9267	Aradu.A02	9267	p2	(CT)8	16	47966918	47966933	AAATCACCCTCCTCTTTTTCAA	GGGAAGAGGGTTCGAATTTT	213
Ad02A9268	Aradu.A02	9268	p3	(TCT)5	15	47979113	47979127	GAAACCCTCACCTCTCTCCC	TTGCCTTCCCTTTCCTCTTT	184
Ad02A9269	Aradu.A02	9269	p3	(AAG)6	18	48046849	48046866	ACCCAATTGGATGAAGATCG	CCTCCCCTCACACACTTTTC	261
Ad02A9271	Aradu.A02	9271	p3	(TCT)6	18	48084937	48084954	CTCATATGGCCGAACCCTAA	CAGCCTTAGGTGCCATCAAT	249
Ad02A9272	Aradu.A02	9272	p5	(TTTAT)7	35	48088901	48088935	TGATGGATTGTCTTGCTCCA	TGCTTGAAAATAAACCGCTTG	260
Ad02A9276	Aradu.A02	9276	p3	(AGA)7	21	48120487	48120507	TAAAATATTGCTCGCCCTCG	ACAGGATCCCCACCTACCTC	279
Ad02A9277	Aradu.A02	9277	p3	(ATT)5	15	48123472	48123486	AAACTTGGACGTTGGCTTTG	CAGGGAAAAAGGCACTCTTG	199
Ad02A9279	Aradu.A02	9279	c	(CT)6aatgaattt	70	48130873	48130942	GGTCTTTTCGGCGACAGTAG	ATGACAAAAATTCCCCACCA	225
Ad02A9282	Aradu.A02	9282	c	(TA)6(TG)18	48	48152025	48152072	TCAGATGCAGGACGTGAGAC	GAAGCATAGGATGGCCAGAG	274
Ad02A9283	Aradu.A02	9283	p2	(CT)7	14	48154995	48155008	CGCTTGATTTAGCTTGGAGTTT	CCTCATTCCCTCTCACTTGC	200
Ad02A9287	Aradu.A02	9287	p3	(ATA)7	21	48175853	48175873	AAAGCAACAAACAACCACCC	TTTGGTCGTTAAAGATGAAATTAAGA	181
Ad02A9290	Aradu.A02	9290	p2	(CT)6	12	48200026	48200037	CCAGACTTGGCTAAAAACGC	AGAGGTGGTGAGGTGATTGG	153
Ad02A9293	Aradu.A02	9293	c	(T)11cgaaatctc	98	48286874	48286971	TCCTTTTCTCAATTTCCTTCCA	CGGAAAAGCACACCAAACTT	207
Ad02A9297	Aradu.A02	9297	p3	(TTC)5	15	48328715	48328729	CAACTTCTCTTTCCCACCCA	ACCACTGAGCTATGACGGCT	277
Ad02A9298	Aradu.A02	9298	c	(A)11cggccatc	49	48335027	48335075	ATAGGGAGCTCTTGGGCATT	GTGGATGAGGTTGGAACGAT	224
Ad02A9299	Aradu.A02	9299	p2	(AT)10	20	48349618	48349637	TCCGAACAAATTTTAGACAAATCA	TGGTCTTAATATAGAAAGCAAATCG	246
Ad02A9301	Aradu.A02	9301	p4	(TAAC)5	20	48375909	48375928	CAAAAGCATTCTCAAAGGCA	TACTTCACCGACGATGCTCA	280
Ad02A9302	Aradu.A02	9302	p2	(AT)6	12	48380304	48380315	TGTTATGCTCTATATACATGTGCGA	TTTTATTAAGGAACCTCGTTATCTTTT	130
Ad02A9304	Aradu.A02	9304	p3	(TTA)5	15	48386701	48386715	GGAGGTTGGGACTCAAAACA	TGTATTTCACTGCCTCAGCG	269
Ad02A9306	Aradu.A02	9306	p3	(TTC)5	15	48389761	48389775	TCACCTTGACCGAACCTACC	ACTCGGAGGTGGAAGCTTTT	176
Ad02A9318	Aradu.A02	9318	p3	(ATA)15	45	48441654	48441698	CGACGTATTCCGTATTGTGTTG	TTGGTCTTTTTAACATTCTGACCA	277
Ad02A9320	Aradu.A02	9320	p3	(TAA)6	18	48444515	48444532	GCTGCTAGCCTTATTTCCAAGA	TTCGAGATTGAAATTAGTTTTCCC	226
Ad02A9323	Aradu.A02	9323	p2	(AT)10	20	48450694	48450713	CGAGGCATATATTGGTGTCAA	GTCCCGAGCAACCCATATAA	262
Ad02A9325	Aradu.A02	9325	p4	(TTAA)5	20	48459651	48459670	TGTTCTATTTTCTTGTAGTGAGCCT	GCTAACAACAACACCACCAATG	155
Ad02A9327	Aradu.A02	9327	p2	(AT)8	16	48469990	48470005	TCGTCGACGGGGTTTATATT	TCCATATTTTTCCAATCACAGC	278

ID	Chromosome	SSR nr.	SSR type	SSR	Size	Start	End	FORWARD PRIMER (5'-3')	REVERSE PRIMER (5'-3')	Product (bp)
Ad02A9328	Aradu.A02	9328	p3	(TAA)9	27	48519238	48519264	CAATCTTGGCCCGAATTCTA	ACAACCGGTGCAATTACCAT	257
Ad02A9333	Aradu.A02	9333	p3	(ATA)7	21	48530143	48530163	TCGCCAAAATGAATGCACTA	TAGCCGGTGCCATTATCA	280
Ad02A9334	Aradu.A02	9334	p2	(TC)6	12	48530729	48530740	TGACTAGACATTCAAATCTCACCA	AGGAAAAGAGGGAGGGAGG	280
Ad02A9335	Aradu.A02	9335	p2	(CT)8	16	48530946	48530961	TCTATCTCTCCCTCTCCCCC	AGAGCAACAAAGAGACACACACA	196
Ad02A9336	Aradu.A02	9336	p2	(AT)6	12	48531715	48531726	TTCAAGGTGCTATTGCTATTTCTG	TCATTTATGCTTTCAATCATTGTT	233
Ad02A9342	Aradu.A02	9342	p2	(TA)6	12	48601083	48601094	TCTCATCATGCAGGGAGTTG	GGCATGAGCTGGAATTTGTT	279
Ad02A9343	Aradu.A02	9343	p3	(AGA)5	15	48604429	48604443	CCTTAAAGTTGGCTAATGTTGAC	GGAGGATGAGGAGGAGAAGG	213
Ad02A9344	Aradu.A02	9344	p3	(AAT)5	15	48608952	48608966	TCGCACAACTAACCAGCAAG	CAGGGACAAAGCATCTCCAT	252
Ad02A9346	Aradu.A02	9346	p3	(AGA)5	15	48611877	48611891	CTAGAGGTTTCTCCGGCCTT	CCTCTCTCCCTCTCCTCCAT	153
Ad02A9347	Aradu.A02	9347	p3	(AAT)5	15	48616497	48616511	CAAGTGCACTGGGTCGTCTA	CAGGGACAAAGCATCTCCAT	277
Ad02A9349	Aradu.A02	9349	p3	(AAT)17	51	48621946	48621996	TTTCTGATTTTGATTTGCTACGA	GGTTGAAAAATTTTGGTGTTGA	249
Ad02A9356	Aradu.A02	9356	p2	(GC)6	12	48750514	48750525	ACAAGGCTCCTAAAACCGCT	AGCCGACCTCTTTGCACTTA	225
Ad02A9359	Aradu.A02	9359	p2	(GA)6	12	48756068	48756079	GAAAAGGGGAGAGGGAAGTG	TCCTAACCCCATAACCACCA	271
Ad02A9362	Aradu.A02	9362	p4	(ATAC)5	20	48779743	48779762	AATATTACGCTTTCGGCTGC	AAATAGGGATAGGAGTTGCGA	267
Ad02A9363	Aradu.A02	9363	p2	(CT)6	12	48782277	48782288	CCTGACGGTGATGGAGCTAT	AAGGGGAGCCGTAGAAGAAG	256
Ad02A9364	Aradu.A02	9364	p5	(TTCTT)5	25	48782434	48782458	CGGTGACTCAGCAATAGCAA	ACGCACCTTCACATTTTTCC	217
Ad02A9365	Aradu.A02	9365	p3	(AAT)7	21	48789444	48789464	TTGCAAACGTGTGCCTTATT	GTTGTTGCCTAAAGGGGTGA	265
Ad02A9369	Aradu.A02	9369	p2	(GA)6	12	48842646	48842657	TGAGTCAATCCAAAAATTCAAAA	TTGATGTTGTTCTTGGTGTTCA	187
Ad02A9370	Aradu.A02	9370	c	(TTA)18tc(AT	74	48848019	48848092	TGCAAAACACATCACATGGT	AGAGGATGAAGGGTTCATGTTT	207
Ad02A9371	Aradu.A02	9371	p3	(TTA)5	15	48855041	48855055	TGTTTTTAGACGTTTTGGAAGGA	CAGCAACTCATTGACCACAGA	198
Ad02A9372	Aradu.A02	9372	p3	(TTG)11	33	48855775	48855807	GTCTCCCAATTCGTGCATTT	CGAATTCTTCCTTTCTTCTATTCG	188
Ad02A9374	Aradu.A02	9374	p3	(AAG)6	18	48882608	48882625	TGTGAGCGAACTCCACAAAG	GGCCAATTTTCCTACTGCAA	259
Ad02A9375	Aradu.A02	9375	p2	(TA)6	12	48885736	48885747	CACATTCCACATGCATTGCT	AAGTGGGAACATTGGGTGAG	272
Ad02A9376	Aradu.A02	9376	p2	(CA)22	44	48914155	48914198	AGCCGTTTAGGCAAGGATTT	TGAAGCAAGAAAAAGCATCAAA	273
Ad02A9379	Aradu.A02	9379	p3	(AGA)6	18	48924234	48924251	TAAAATATTGCTCGCCCTCG	CCTCTTCTTCACCTTGGCTG	106
Ad02A9382	Aradu.A02	9382	p2	(TA)7	14	48953419	48953432	GCTCAAAAACCATGTTTCACAA	AAGTTGTCCGACCAATCAGC	258
Ad02A9383	Aradu.A02	9383	p3	(AGA)6	18	48960144	48960161	TTTCTCCATCTCCCATGACC	CAAATCTCTTTCCCACCCAA	254
Ad02A9384	Aradu.A02	9384	p2	(GA)6	12	48960312	48960323	CGTATTAGGGACAGTTATTTTTGTCA	TGCTTGCTTAGGAGTGTGGA	236
Ad02A9391	Aradu.A02	9391	p3	(TAT)18	54	48991576	48991629	ATTTCATTCATCCACCGCTT	AATTGTTGGGACTGCATTGAG	221
Ad02A9392	Aradu.A02	9392	p2	(GT)6	12	49008904	49008915	GCTTGGGAAGATTGAGGTGA	GAGAACCGGGCGTTAATTTT	214
Ad02A9399	Aradu.A02	9399	p4	(AAAT)5	20	49034930	49034949	AGCAACCTCATGGTCTTTGAA	GGATTTTTACATCATACTTTGCCTC	240
Ad02A9400	Aradu.A02	9400	p2	(AG)6	12	49038784	49038795	GGGATATTTTTGGCCCAGTT	TTGAAGCAACAAATCTAAATTAGGG	211
Ad02A9403	Aradu.A02	9403	p5	(AATAA)5	25	49082054	49082078	AGGGTTAGCATATGACCCCA	CCTAACCAATTTCTCTCTCCTCA	257
Ad02A9410	Aradu.A02	9410	p3	(GAA)5	15	49179654	49179668	CTAGCAGCCTTCAATCCCAG	GCGACCCCTCTCTCTACCTT	246

ID	Chromosome	SSR nr.	SSR type	SSR	Size	Start	End	FORWARD PRIMER (5'-3')	REVERSE PRIMER (5'-3')	Product (bp)
Ad02A9411	Aradu.A02	9411	p3	(AGA)5	15	49179913	49179927	GAGTTGAGGGTTGGAGGTGA	CATACCCCTCGTGTGTGTGA	266
Ad02A9414	Aradu.A02	9414	p3	(AGA)5	15	49200105	49200119	CTAGAGGTTTCTCCGGCCTT	CCACCCTCATGACTGAACCT	265
Ad02A9416	Aradu.A02	9416	p2	(CA)6	12	49221617	49221628	AAGCAAAGTGGCAACAAACA	TGGTGGCAACGTATGAAGAA	214
Ad02A9417	Aradu.A02	9417	p2	(GA)7	14	49223350	49223363	GGAGAGGAAGCGATGATTGA	CTCGTAGCCCTCTATCGCAC	220
Ad02A9419	Aradu.A02	9419	p3	(GAA)5	15	49233438	49233452	TCAAACAGCAGCCAATCAAC	GCGACCCCTCTTTCTCTACC	221
Ad02A9427	Aradu.A02	9427	p3	(TGA)5	15	49316546	49316560	AAAGCCGATCTCTCCCTGTT	CCAAATTCGCCCCAGTAGTA	274
Ad02A9432	Aradu.A02	9432	p2	(AT)11	22	49355150	49355171	AAAGGTGATAAAATCCCCCG	CTGCGTACCAGCTTAGGGAG	147
Ad02A9433	Aradu.A02	9433	p2	(AC)6	12	49356334	49356345	CTAAGGCCCAGCCAAACTC	GGCTTTGTAAAGCTCAACGC	216
Ad02A9434	Aradu.A02	9434	c	(T)10cgaaaa(T	26	49362714	49362739	CACACTCTTGTTGCCTTGGA	TGGCTCTTGAAAGAAGGAGG	190
Ad02A9435	Aradu.A02	9435	p2	(AC)6	12	49413078	49413089	TCTCGTTTTAACGAATGGGC	AAGGGTTTTGTGTTTTCCCC	161
Ad02A9436	Aradu.A02	9436	c	(A)10taaaaagg	38	49446921	49446958	CACTTCATGCAGGGCCTAAT	TCATGATGAGAATATAGAAGAATCGAA	176
Ad02A9438	Aradu.A02	9438	p3	(ATT)5	15	49450299	49450313	TTTTAACCAGCCAATTTTAAACAA	TGTCTCTGAGCTTTTGAGATTACTG	231
Ad02A9440	Aradu.A02	9440	p3	(CTT)5	15	49451431	49451445	TCCTGCCGTCCTATCGTACT	TCCCCAAAATCAACCAGAAG	271
Ad02A9441	Aradu.A02	9441	c	(TGA)6tgtcctt	175	49451592	49451766	TTGTTGCTGCTGAATTTGTTG	TTCTCCCTTCACCCCAAAAT	254
Ad02A9442	Aradu.A02	9442	c	(CTT)8(ATT)1	60	49455525	49455584	GCAGCACCATAATAGTAGCCA	TGTACAAAATACCAATGGGGG	273
Ad02A9446	Aradu.A02	9446	p2	(CT)6	12	49466045	49466056	CAAAACCACCTAACTACTTTTCCC	GAAGGGGGTAGAAGAATCCAA	275
Ad02A9447	Aradu.A02	9447	p3	(AAT)6	18	49466506	49466523	GGAAAAGGGCACTCTTGTTG	AAACTTGGACGTTGGCTTTG	199
Ad02A9449	Aradu.A02	9449	p3	(TCT)5	15	49469675	49469689	CCAACATAGCCGAACCAACT	GCTTTTGTCTTCCCTTTCCC	199
Ad02A9455	Aradu.A02	9455	c	(A)12ttttacaaa	43	49535580	49535622	TCCTGGACCGGAGTTTTAGA	GAATGTATGCAATTGACACCAAA	203
Ad02A9456	Aradu.A02	9456	p3	(AGA)6	18	49574018	49574035	AATCGGAGGTGGAAGCTTTT	CTATACCTTGGCCGAACCAC	215
Ad02A9459	Aradu.A02	9459	p5	(TTATC)6	30	49620309	49620338	CTGTCGTGTCCGACCTGTT	AAAGATGAGGCGGTTCCTTT	277
Ad02A9465	Aradu.A02	9465	c	(TTC)6tcttctttt	99	49705447	49705545	TTGTGTTGGATCGTTTTCCA	GGATATTCCGGCCTTAGGTG	265
Ad02A9466	Aradu.A02	9466	p2	(TA)6	12	49712743	49712754	CTTTCCTAGGCGCATGTGAT	TCTCTAAGAGGCATGGTTGG	217
Ad02A9468	Aradu.A02	9468	p2	(CT)28	56	49745216	49745271	GAAAGGTCTGCTCAGCCATC	CCCAGCAACTCAAAATCACA	247
Ad02A9469	Aradu.A02	9469	p4	(AACT)5	20	49764463	49764482	AAGCAATAAGGGCAGCAATG	TCCAACACTTCCAAACACCA	154
Ad02A9470	Aradu.A02	9470	p2	(TG)6	12	49781949	49781960	TACCTCCCTGCAATTCCTTG	TCACCTTCCACTATCCCCTG	267
Ad02A9471	Aradu.A02	9471	p2	(TA)6	12	49783012	49783023	TCTGGAGCTTCTGCAATGAA	ACAAGGTCCTAACTCAGCCG	231
Ad02A9472	Aradu.A02	9472	p3	(AGA)5	15	49802117	49802131	CCCTTTCCCTTTTCTTGAGG	CCAACATAGCCGAACCAACT	188
Ad02A9473	Aradu.A02	9473	p3	(AAG)5	15	49815917	49815931	GGGGCATTCAAACTTGTCAT	CCAAATGGTTGCAATGAATG	265
Ad02A9475	Aradu.A02	9475	p3	(TAG)6	18	49833335	49833352	GAGCAAGGGCGTGATCTAAG	CATGCCTGATGATGATGAGAA	259
Ad02A9479	Aradu.A02	9479	c	(AGAA)10(A(	134	49856606	49856739	GAAAGAGAGGGCAGGGAAA	GCCCTCCTATCTCTCTCTCC	221
Ad02A9480	Aradu.A02	9480	c	(GA)19ggaaag	153	49857045	49857197	GAAAGAAAGACGAGGTGGAGAA	AAAACAATTTTAAAAACCATATCCA	242
Ad02A9483	Aradu.A02	9483	p3	(TCT)6	18	49866273	49866290	CCCCTCTTCTTTACCTTGGC	TGCCATTGATGGTAGTGGAA	162
Ad02A9485	Aradu.A02	9485	c	(A)10cgaaaaca	117	49903641	49903757	AAAGCGCCAAGATAAAGCAA	ATACCCATCGACGCGTAAAC	234

ID	Chromosome	SSR nr.	SSR type	SSR	Size	Start	End	FORWARD PRIMER (5'-3')	REVERSE PRIMER (5'-3')	Product (bp)
Ad02A9488	Aradu.A02	9488	p3	(TTA)12	36	49919284	49919319	TCCGACTTTTGCCGATATAA	CATGTCCGTTTTCAGAACCA	262
Ad02A9490	Aradu.A02	9490	p3	(ATT)5	15	49969721	49969735	AAACTTGGACGTTGGCTTTG	CAGGGAAAAAGGCACTCTTG	199
Ad02A9491	Aradu.A02	9491	p2	(AG)7	14	49978837	49978850	TGTGTGTGTATTGCGACCCT	CTCCCCACACAACTGGACTT	169
Ad02A9497	Aradu.A02	9497	p2	(CA)7	14	50010187	50010200	TTTCCCAAGTACCGTTTTCC	GCCTAACTTTGTTGATGGGG	143
Ad02A9500	Aradu.A02	9500	p2	(CT)8	16	50014191	50014206	TTTCTCACAACGACCCCTTC	TGTTTATGACGGCAGAGCAG	109
Ad02A9502	Aradu.A02	9502	p2	(TC)16	32	50033179	50033210	TCTTCTTCCTCTTCCCCCAT	GAGAGAGAAGAGGGGTTGGG	131
Ad02A9503	Aradu.A02	9503	p2	(TC)6	12	50033332	50033343	CCCAACCCCTCTTCTCTCTC	CCTGGAGCAGAGCAAAAATC	205
Ad02A9504	Aradu.A02	9504	p2	(TC)20	40	50076148	50076187	CCATTGGAACTCCAAAGCCT	GGGCAGCAGAGCTAAACAAC	275
Ad02A9511	Aradu.A02	9511	p2	(GA)6	12	50130052	50130063	ATCGCTTCTGCCACTGTCTT	CAGCGTAGACGAAGACCCTC	125
Ad02A9516	Aradu.A02	9516	p3	(AAC)5	15	50223898	50223912	GCAGGAGCAAAGACAAAAGC	AAGTTGTTTGCTCCAATGGC	253
Ad02A9518	Aradu.A02	9518	p3	(ATT)5	15	50247482	50247496	TTATTTCCTTTGGCCCTCCT	TGAAAAGCAAAAGCTTGTGAAA	168
Ad02A9519	Aradu.A02	9519	p3	(AAG)8	24	50249148	50249171	CTCCAGCCTTAGGTGCCATA	ATTTCTTTCCCACCCAAACC	277
Ad02A9522	Aradu.A02	9522	p2	(TC)8	16	50265510	50265525	CTTGCCGGTAAGTTGTGGAT	AGAGGGATATCGGGGAGAGA	153
Ad02A9524	Aradu.A02	9524	p3	(TTG)7	21	50268806	50268826	CCAATTTGCCACCAAATACC	ACACAGCATCTGCCATCAAG	250
Ad02A9527	Aradu.A02	9527	p3	(TCT)5	15	50278847	50278861	AATCCAAGGGTCCGATTTGT	AGGGCTGAGGGAAGAAGAAG	258
Ad02A9533	Aradu.A02	9533	p3	(TCT)6	18	50339335	50339352	TGTGGACCCCACAGGATTAT	CCTCGAGCAAACGAAGAAAT	218
Ad02A9534	Aradu.A02	9534	p3	(GAT)7	21	50341752	50341772	TAAAAGGGGGTGGTTCAGTG	TTGACCACATTCAGCAGAGC	175
Ad02A9536	Aradu.A02	9536	c	(T)10cgaaaaca	35	50358575	50358609	GCATGCAATTGACACCAAAC	GGGGATTGTTCGAGTATGGA	241
Ad02A9538	Aradu.A02	9538	p2	(AC)7	14	50373791	50373804	AAGGCAAAGGATGAAGCAAA	TGTGAATGCCATTTTAACCG	181
Ad02A9541	Aradu.A02	9541	p3	(TCA)5	15	50418391	50418405	GATTTCTGTTCTGCCCCAAA	AGGAGTTGAAGGTGCTGGTG	255
Ad02A9542	Aradu.A02	9542	c*	(AAT)6(AAT/	35	50423327	50423361	TGAGAAAATGCAAGCAATCA	CAAAAGAAAATGGAGGAGAAGAA	229
Ad02A9543	Aradu.A02	9543	c	(A)12cggccatc	50	50428882	50428931	ATAGGGAGCTCTTGGGCATT	AGAGAAAAGCAGGGTGCAAA	275
Ad02A9544	Aradu.A02	9544	p2	(GC)6	12	50429036	50429047	ATAGGGAGCTCTTGGGCATT	AGAGAAAAGCAGGGTGCAAA	275
Ad02A9545	Aradu.A02	9545	c	(A)10gaaaaaaa	91	50429239	50429329	GGATTTGCACCCTGCTTTT	GACAAAAGAAAATGGAAGAAGAGAA	265
Ad02A9546	Aradu.A02	9546	p2	(GT)7	14	50441477	50441490	CGCATACCAAGTGCTCATGT	CCTTGTCGCTTATGGTGGAT	264
Ad02A9548	Aradu.A02	9548	c	(T)10caaa(T)1	24	50454123	50454146	CCGGATACATACATGCCACA	TGAAGCAAGAAAAAGCAGTGAA	216
Ad02A9551	Aradu.A02	9551	p3	(AAC)5	15	50500185	50500199	ATCAAGGGTGGCAAGACAAC	GATTTTGTTGAGGGCTTGGA	171
Ad02A9556	Aradu.A02	9556	p3	(TCT)5	15	50553965	50553979	TCTACACCTCGGCCGAATAC	TATGACTCGGAGGTGGAAGC	221
Ad02A9558	Aradu.A02	9558	p2	(AC)6	12	50568990	50569001	TCTCTCTCTCACACGCTGGA	ATTCAATCCAAGATCCGTCG	238
Ad02A9564	Aradu.A02	9564	p3	(ATT)17	51	50634591	50634641	TGGTCTGAAAATTTTTGGGG	TTTTGAAAAATGGGCAATGT	167
Ad02A9565	Aradu.A02	9565	p2	(TA)8	16	50635489	50635504	CCTTTTAATAACTCAGATTGCAAAAC	GGCCAACTTGTACGGATTGT	222
Ad02A9566	Aradu.A02	9566	p2	(TA)7	14	50639845	50639858	AAAACCACAAGTACATGCAATATGA	TGCTCTATGCACATCCACAA	154
Ad02A9568	Aradu.A02	9568	p2	(AT)9	18	50682271	50682288	TGGTTTATCAACCTCCCCAC	TCCCTTCTTTCATGAGCTGTC	265
Ad02A9569	Aradu.A02	9569	p2	(AT)9	18	50682677	50682694	TGTAATGGGGCCAAGGTAAA	GGGGACAAAATTACCCCAAT	255

ID	Chromosome	SSR nr.	SSR type	SSR	Size	Start	End	FORWARD PRIMER (5'-3')	REVERSE PRIMER (5'-3')	Product (bp)
Ad02A9572	Aradu.A02	9572	p2	(AT)11	22	50724980	50725001	GCGTAGTTAATTTGCGAGGTG	GTCAGGACCTGGGATGTTGT	260
Ad02A9575	Aradu.A02	9575	p5	(AAAAT)5	25	50816484	50816508	TGGCTCCTTCCATCTCTGAT	TCAAATCCTTTTCAAACCTTTTC	210
Ad02A9583	Aradu.A02	9583	p2	(TA)6	12	50860465	50860476	TTTGTGTCCCTTTGGGTTTC	ACTCGAAAGCGCAACACTCT	200
Ad02A9585	Aradu.A02	9585	p2	(AG)7	14	50885465	50885478	AGGAAGAAGAACGGAGAGGC	TCCTTTGGTTGAATTCGCTT	266
Ad02A9588	Aradu.A02	9588	p3	(AGA)6	18	50894463	50894480	CTAGAGGATTCTCCGGCCTT	CCGAATCCATATCCCATCAC	165
Ad02A9589	Aradu.A02	9589	p4	(TAAA)5	20	50914395	50914414	CAGGTTCAGGATCAGCTTCA	TTTTCAAAATTTTCCACCTCTCA	266
Ad02A9590	Aradu.A02	9590	p4	(AATA)10	40	50924394	50924433	GGATTTGCACCCTGCTTTT	TGCAATGAATGAAAATAAGCAAA	278
Ad02A9595	Aradu.A02	9595	p2	(CT)6	12	51000555	51000566	TGGACAAACTCAAAGGGAGG	TCTTCCTCTCTTGGTCACCG	228
Ad02A9596	Aradu.A02	9596	p2	(TA)6	12	51000673	51000684	TCTAGAGCCGAAGTCACGGT	GCCGAGCCATTAAGAGAGAA	227
Ad02A9597	Aradu.A02	9597	p2	(AT)8	16	51001709	51001724	TATCAGGAACGAACTGCCCT	TTTTTCCCTCGTCGTTTATTG	237
Ad02A9598	Aradu.A02	9598	p2	(AT)6	12	51002894	51002905	TCGCCTTTCCCTAAGTGTCT	AAAAAGTTTGTGATTTGGAAAGTAA	279
Ad02A9599	Aradu.A02	9599	p3	(TCA)5	15	51005798	51005812	TTTCTTCCCAGTGGAGATGG	TAAAAGGGGGTGGTTCAGTG	146
Ad02A9600	Aradu.A02	9600	c	(TAA)5atag(A	43	51015283	51015325	CACATATATAAGCCACGTTTGG	AACACCTCGAATTTTTGAAGC	279
Ad02A9601	Aradu.A02	9601	c	(TTGAA)5tgca	80	51015851	51015930	CAAGTGGATGAGATTTGGAATG	GCTGTCTGCACAATGAATCG	231
Ad02A9602	Aradu.A02	9602	p2	(TC)6	12	51018925	51018936	CCAAGCTGTCAAAGGGAAAA	CAAATGGTGGGGAAGAGAGA	267
Ad02A9603	Aradu.A02	9603	p2	(AT)11	22	51025889	51025910	CCCCTATCCTCCAATTCGTT	CCGCTTTCAGCTACAGATACG	280
Ad02A9605	Aradu.A02	9605	p2	(CT)6	12	51027769	51027780	TCTAGAGCCGAAGTCACGGT	CGAGCCATTAAGACGGAAAA	237
Ad02A9606	Aradu.A02	9606	p2	(TA)7	14	51027896	51027909	TCTAGAGCCGAAGTCACGGT	CGAGCCATTAAGACGGAAAA	237
Ad02A9607	Aradu.A02	9607	p2	(AT)6	12	51029099	51029110	TTAGCACCTCATGATCGCAC	TTTGTGAAAATTATGGCTCGC	237
Ad02A9608	Aradu.A02	9608	c	(TAA)5atag(A	43	51032075	51032117	ATCCTTTTTCCTTGTGGCCT	TGTAACACCTCGAATTTTTGAAGA	278
Ad02A9609	Aradu.A02	9609	c	(AATTG)5aatg	71	51032641	51032711	ATCAAGCGGATGAGATTTGG	GCTGTCTGCACAATGAATCG	223
Ad02A9610	Aradu.A02	9610	p2	(AT)7	14	51033376	51033389	GACTCGGAAGGGAGACGAG	GGGGTATGAATTGTATCTGAGGA	197
Ad02A9611	Aradu.A02	9611	p2	(GA)8	16	51039863	51039878	AAACCAATAAGCGCAGCATC	GCCGCACCATTTTCACTATT	174
Ad02A9612	Aradu.A02	9612	p4	(TAAA)5	20	51046035	51046054	GCAGAGAATTCCCAGTGAGG	CCGTTGTCGGGAATTGATAG	266
Ad02A9615	Aradu.A02	9615	p4	(TAAA)6	24	51085558	51085581	TGGAAAAATGTGGGAACTCAG	GCTACCCGAAATCTCATTTTTG	277
Ad02A9616	Aradu.A02	9616	p2	(GT)8	16	51085731	51085746	CAAAAATGAGATTTCGGGTAGC	GGCTAAGGTAAGGGTGAGGG	143
Ad02A9621	Aradu.A02	9621	p2	(TC)6	12	51161305	51161316	AAGGGGATAACCCTAATGCG	GAATGGGGAAAGAGAGGGAG	268
Ad02A9622	Aradu.A02	9622	p2	(GT)6	12	51167454	51167465	GAGTGAAGTGCATGCTTCTTTG	TACGTGCTCTTCCTCCCACT	268
Ad02A9625	Aradu.A02	9625	c	(T)10ctttcttttct	74	51187175	51187248	TTATCGAACCTTGCTTTGGG	TTGGGAACATTGGTTGGAAT	245
Ad02A9628	Aradu.A02	9628	p3	(TAA)5	15	51201683	51201697	AAGGAATTGGTTGAATCCAAGA	TTGGAAGAAGACACAATTTATTTTT	278
Ad02A9629	Aradu.A02	9629	p2	(TA)8	16	51203266	51203281	CGGTAAAAGAAAAGCGCATC	ACTCCAACCGGCCTTAACTT	154
Ad02A9630	Aradu.A02	9630	c	(A)10gctgtacct	88	51212490	51212577	CAGCAAAACCAAAACAAACG	GGGCAACCAGTCAAGGACTA	251
Ad02A9631	Aradu.A02	9631	p2	(TA)6	12	51230385	51230396	AAAAGTGGGGTGTTTCAGTTG	TTCCACAATCTCAACCAACAA	194
Ad02A9634	Aradu.A02	9634	p3	(AAT)5	15	51268833	51268847	TTGACCCAGAACCTGAAAGG	AAACTTGGACGTTGGCTTTG	173

ID	Chromosome	SSR nr.	SSR type	SSR	Size	Start	End	FORWARD PRIMER (5'-3')	REVERSE PRIMER (5'-3')	Product (bp)
Ad02A9636	Aradu.A02	9636	p3	(ATT)8	24	51300065	51300088	TTGGATGTTGGCTTTGTGAA	CAGGGAAAAAGGCACTCTTG	235
Ad02A9637	Aradu.A02	9637	p2	(CT)14	28	51308879	51308906	TCGCACTTCACTTTCCACAC	AGCCTAGGGCTAGAAGTGGC	245
Ad02A9640	Aradu.A02	9640	p3	(AGA)6	18	51337816	51337833	CTAGAGGGTTCTTCGGCCTT	GGCCAAATACACATTCACCC	167
Ad02A9641	Aradu.A02	9641	p3	(TTC)5	15	51346032	51346046	TCCGGAGAGCTATTTTGGTTT	CCCCTAATGAAAGTGGGGTT	168
Ad02A9644	Aradu.A02	9644	p3	(GTT)5	15	51379293	51379307	GAGCCATCAAGAATACCCCA	GTCAACCTTTGAGGTGGGAA	231
Ad02A9647	Aradu.A02	9647	p2	(CT)8	16	51402439	51402454	TGAACCTGTGATTGAAGAAGATG	CGCGATCTGCAGAATTACAA	233
Ad02A9649	Aradu.A02	9649	p3	(GAA)6	18	51439368	51439385	ACCACTGAGCTATGACGGCT	CAACTTCTCTTTCCCACCCA	273
Ad02A9652	Aradu.A02	9652	p2	(CT)6	12	51467236	51467247	CACCGTCAGTTCTCAACCCT	TTTGCTCAGTTTCGGTTGAA	155
Ad02A9653	Aradu.A02	9653	p2	(AG)7	14	51473800	51473813	TGAAAATAAAAGAACATTAAACCTGG	TGACAGAGGCCACGTATCAG	264
Ad02A9662	Aradu.A02	9662	p2	(CT)6	12	51556183	51556194	TTTTTCGAAATCTCCCCTCC	CGGAAAAGCACACCAAACTT	150
Ad02A9667	Aradu.A02	9667	p3	(AAG)5	15	51632056	51632070	ACTCGGAGGTGGAAGCTTTT	ACAACCCCCTTTTCTTCACC	228
Ad02A9668	Aradu.A02	9668	p3	(TCT)6	18	51643173	51643190	TCTCTTTCCCACACAAACCC	TCTCCCATTACTCGGAGGTG	244
Ad02A9672	Aradu.A02	9672	p2	(CT)6	12	51722868	51722879	CAGAAAAGGGCAAGAAGCTG	TTTTTGGGGAAGAGGTGTTG	224
Ad02A9673	Aradu.A02	9673	p2	(TA)10	20	51750871	51750890	GGCAATTTGTCCCCTTTTTA	TGGGTATGGAAAACTTGGGA	231
Ad02A9681	Aradu.A02	9681	p2	(AT)7	14	51780761	51780774	AACCACTGCGCTACCTGTTT	TCAGAACCGAACCGGTAATC	263
Ad02A9686	Aradu.A02	9686	p3	(AGA)7	21	51809460	51809480	CTAGAGGTTTCTCCGGCCTT	CCCATAAACCCCACCTACCT	243
Ad02A9687	Aradu.A02	9687	p2	(AT)6	12	51840418	51840429	CTAATCCTTTCCGCCATTGA	CCATTGTACCATTTTTGCGA	171
Ad02A9690	Aradu.A02	9690	c	(T)12gcttttccat	67	51886025	51886091	CACACTCATCCCTTCGGTTT	TGACTCAGAGGTGGAAGCAA	254
Ad02A9692	Aradu.A02	9692	p4	(AAAT)6	24	51897621	51897644	AACAAGCAATGGCCTCAATC	ACCCGTGAAACTTTGAGCCT	181
Ad02A9696	Aradu.A02	9696	p3	(GAA)6	18	51953799	51953816	ACCACTGAGCTATGACGGCT	CAACTTCTCTTTCCCACCCA	276
Ad02A9697	Aradu.A02	9697	p3	(TCT)5	15	51961037	51961051	CACAACACCAACCCCTTCTT	TTGCCTTCCCTTTTCCTTTT	222
Ad02A9699	Aradu.A02	9699	p2	(AT)10	20	52003960	52003979	GATTGCAATTGAGCGTACCA	AGCAAAGTGCATCTTTTGGG	277
Ad02A9702	Aradu.A02	9702	p2	(GA)10	20	52046189	52046208	GAAAGTGGCTTGAATGGGAA	CATGCATGCAACATTGAACA	255
Ad02A9710	Aradu.A02	9710	p2	(AT)6	12	52149617	52149628	TTCACTTGATCACTTTACCAATTTTT	AGCGAGTTTGATAAAGAGCCA	271
Ad02A9712	Aradu.A02	9712	c	(A)11cggccatc	49	52169626	52169674	ATAGGGAGCTCTTGGGCATT	GCGCACAACTCTCTGGTTTT	134
Ad02A9713	Aradu.A02	9713	c	(GC)6ggattgtc	59	52169779	52169837	TGCACTTCTGCAACTTCTGG	AGAAAAGCAGGGTGCAAATC	278
Ad02A9723	Aradu.A02	9723	p2	(TG)10	20	52238957	52238976	CGGTGCTCAACTGCTCATAA	TTATATGCCATCCCCACCAT	115
Ad02A9725	Aradu.A02	9725	p4	(AAAT)5	20	52241106	52241125	GGCATGAAAGACTCAAACCC	CAAAGTTGGCAACCCCTAAA	247
Ad02A9732	Aradu.A02	9732	p3	(AAT)5	15	52264088	52264102	TTCAGTTTCGGAACAGGCAT	GCACGGTTTTGGAAAACATAA	161
Ad02A9737	Aradu.A02	9737	p2	(AC)8	16	52272431	52272446	GTTGCTTGGATGTTGTGGTG	TGGTCTTGATGGATAAGCAGG	196
Ad02A9738	Aradu.A02	9738	p4	(ATCT)5	20	52272961	52272980	GCAAGTTTGTAAGGCAAAGC	GGTATGGGACTTTCCAACACA	259
Ad02A9740	Aradu.A02	9740	p2	(AT)8	16	52309269	52309284	TGTTCATTCCTACTTGCTTCCA	TTTTTGTGTTTTTCTCTTTCATCA	238
Ad02A9742	Aradu.A02	9742	p3	(ATC)6	18	52330768	52330785	AGACTCACCAACAACCCCAG	CACAGGGACAGGACAAGGTT	248
Ad02A9743	Aradu.A02	9743	p2	(AT)7	14	52331633	52331646	AAACAAATTGGTTGTTCGCC	TTTTTCAATTTTCGAATGTCCA	169

ID	Chromosome	SSR nr.	SSR type	SSR	Size	Start	End	FORWARD PRIMER (5'-3')	REVERSE PRIMER (5'-3')	Product (bp)
Ad02A9745	Aradu.A02	9745	p3	(TTA)14	42	52332822	52332863	TTTGTGACGTTTTGTTGGGA	AACGAAACGCACGGTTTTAG	175
Ad02A9747	Aradu.A02	9747	p4	(AATT)6	24	52336196	52336219	TTTTGCCAATATAACCTTTCCTT	TTACATGCATAGCTTGCTCACT	219
Ad02A9751	Aradu.A02	9751	p3	(AAC)5	15	52350940	52350954	TTTTTCGGAATCAAAGCAGC	TTTTGTTCTAATCCCGCCAC	163
Ad02A9752	Aradu.A02	9752	p2	(TA)6	12	52364441	52364452	TTCCATTTTCCATTCCAAGC	CCAAATCTTTCGTCCTAAGCC	272
Ad02A9754	Aradu.A02	9754	p2	(AT)7	14	52389968	52389981	CCAACAGCCAGATCCAGATT	AGCAATGGCGATAAACATGA	157
Ad02A9759	Aradu.A02	9759	p2	(TG)6	12	52398868	52398879	GCTCATTTTGCACGTTTGTG	GGGCGAAATGAGTGAAAAAC	265
Ad02A9761	Aradu.A02	9761	p2	(TA)6	12	52406036	52406047	TTTGAATCTTGGTATTGAGCCA	CGTACATGCCTTGTCATTTTG	276
Ad02A9768	Aradu.A02	9768	p2	(GT)26	52	52441813	52441864	CGCACGTGTGTTTTTCTTTG	CACGTGCAAAACTAGCGAAA	252
Ad02A9769	Aradu.A02	9769	p2	(TG)8	16	52442210	52442225	CGCTAGTTTTGCACGTGTGT	CCGAAATGAGCGAAAAACAT	273
Ad02A9772	Aradu.A02	9772	p2	(GT)9	18	52443411	52443428	CGTATGCGTGTTTTTCGCTA	AGACACACGTGCAAAACGAG	165
Ad02A9773	Aradu.A02	9773	p2	(GT)6	12	52444961	52444972	TTGCGTTTTTCACTTGTTTCA	CACATACGTGCGAAATGAGC	234
Ad02A9775	Aradu.A02	9775	p2	(GT)6	12	52446206	52446217	TGCACGTGTTTGTGGTTTTT	TACGTGCAAAATGAGCGAAA	237
Ad02A9776	Aradu.A02	9776	p2	(GT)6	12	52447760	52447771	AGGGTGCGTGTCTTTTGTTC	CACACACGTACGAAACGAGC	280
Ad02A9778	Aradu.A02	9778	c	(GT)9ttg(T)10	31	52449230	52449260	CACATGTGGGTGTTTTGCAT	GTGCGAAAAGAGCGAAAAAG	212
Ad02A9779	Aradu.A02	9779	p2	(GT)7	14	52449918	52449931	GCTCGTTTCGCACGTATGTA	AGCGAAAAATGCTCCTGTGT	251
Ad02A9782	Aradu.A02	9782	p2	(GT)8	16	52452975	52452990	CGTTTCGCACGTATGAGTGT	GTGCGAAATGAGCGAAAAAT	140
Ad02A9784	Aradu.A02	9784	p2	(GT)6	12	52457389	52457400	GCACGTGTGTGTTTTTCGTT	ACACGAACACGTGCAAAAAG	230
Ad02A9785	Aradu.A02	9785	p4	(TTTA)5	20	52457552	52457571	TGTGTTTTTCGCTCATTTCG	AAAAATACGCACACGTGCAA	248
Ad02A9787	Aradu.A02	9787	p2	(AT)8	16	52460501	52460516	TTGTGTTGTTGCCAAATGCT	CTTCCTCCACAACCAACCAT	102
Ad02A9788	Aradu.A02	9788	p3	(AAG)5	15	52461925	52461939	GAAGCACTTCCATGCTGTTG	CTGCAACTTCTTCCAGCCAT	226
Ad02A9791	Aradu.A02	9791	p2	(TC)8	16	52467716	52467731	CCGAAAAAGGTTAGCATGGA	CCCCCACTTTTGACACCTAA	141
Ad02A9797	Aradu.A02	9797	p2	(TC)10	20	52497273	52497292	CTACTCCAGCGCAGCTTTCT	TGCTAAAGGTCCGCTCATCT	265
Ad02A9798	Aradu.A02	9798	p2	(TA)6	12	52507296	52507307	TTGACATTCAAAGTGTCCTTGC	AGCATTTAACGCCCAGAATG	228
Ad02A9799	Aradu.A02	9799	p3	(AGA)6	18	52518904	52518921	GCTTTTGTCTTCCCTTTCCC	ACCTCCCCTTCTCCTCCATA	180
Ad02A9800	Aradu.A02	9800	p3	(ATA)22	66	52552855	52552920	TTTGAATCCCTTGGGTTGAC	TGGAGAAAGGGAGGTGATTG	250
Ad02A9802	Aradu.A02	9802	p2	(CT)8	16	52599243	52599258	TCATGAAGGGCATAAGGAGG	TTTCTTAACTTTTTCAAAACCACC	236
Ad02A9804	Aradu.A02	9804	p2	(CT)6	12	52605541	52605552	CGAAAATCACCTTCCTCTTTTT	GAGAGAGAAGAGAAGAGGGTTCG	215
Ad02A9805	Aradu.A02	9805	p2	(CT)8	16	52608575	52608590	GCAGCAATCTGGCGTTAAAT	ATTAGGGTTGGGTTTGGGAG	277
Ad02A9807	Aradu.A02	9807	p2	(TG)8	16	52613410	52613425	TAGCCTGAGGGATGGACTGA	CGTTCGTACGACCTCTCACA	243
Ad02A9808	Aradu.A02	9808	p3	(CAA)5	15	52614400	52614414	AGAAAGGGAGTCAAAGGGGA	GCACCTTGTTTTTGCCTCTC	280
Ad02A9809	Aradu.A02	9809	p2	(AT)6	12	52648714	52648725	TTTGAAGTGGTTGAATGCCA	CAAAATCCAGTGAGGGTTCA	260
Ad02A9814	Aradu.A02	9814	p3	(AAT)6	18	52719931	52719948	GGCACTCTTGTTGAAGCTGA	TGAAGTCACCAAGCATTCTCC	172
Ad02A9818	Aradu.A02	9818	c	(T)11gtattcacta	54	52768709	52768762	CAGCTTTGCTAGAGTCCCCA	TTGTTGTTCATGAATGTTGGC	280
Ad02A9819	Aradu.A02	9819	p2	(GA)7	14	52817341	52817354	TTGCAACAATCCACAAGCAT	TTCCTTCTCCCTTCTTCCTTC	243

ID	Chromosome	SSR nr.	SSR type	SSR	Size	Start	End	FORWARD PRIMER (5'-3')	REVERSE PRIMER (5'-3')	Product (bp)
Ad02A9820	Aradu.A02	9820	p4	(TTTA)5	20	52825240	52825259	ATTTTTGAATTCTCTCCCTCTTC	GTGCCTTTTCCTTGTTCCTG	251
Ad02A9824	Aradu.A02	9824	c	(T)10caaaagaa	55	52841057	52841111	AAAAATTGTAAGGATAAATTTGGTGT	GAAGCAGGCAAGAATTGAGG	186
Ad02A9830	Aradu.A02	9830	p3	(TAT)5	15	52886253	52886267	TGACTTGGCAATTTGGAACA	GGTTTTCATTGGTGGAAATCA	108
Ad02A9832	Aradu.A02	9832	p2	(AT)13	26	52890197	52890222	AGAGGCAGCTTAGTTTTCATCA	TTCGATGGAGATTGTTAGTGAA	280
Ad02A9835	Aradu.A02	9835	p3	(TCT)7	21	52908280	52908300	TTCCCCATACACCCCACTTA	CTCTGGCCTTATGTGCCATT	225
Ad02A9838	Aradu.A02	9838	p2	(AG)7	14	52953792	52953805	AGAGGAGATGGAGATGGCCT	CATGATCCCCACCTACCCTA	230
Ad02A9839	Aradu.A02	9839	p2	(CT)6	12	52966800	52966811	TGCAATTTACTCTTTCTGCACTTT	GGTCGATCCCACAGAGATTG	173
Ad02A9841	Aradu.A02	9841	p2	(AT)8	16	52979736	52979751	GTGTGATTGCAATTTCGTGC	CTTCTGACTTCGATGGAGGG	271
Ad02A9848	Aradu.A02	9848	p2	(CA)24	48	53039882	53039929	CCAACCCTACCCTACCCACT	CAACCTTGGGTGCCATAAGT	277
Ad02A9851	Aradu.A02	9851	p3	(TTG)5	15	53070416	53070430	GTGGTTGAAGTGCCAGGAAT	TGACAACACAAGGTTGGCAT	248
Ad02A9853	Aradu.A02	9853	p2	(CT)6	12	53084715	53084726	TTACAGAATATGCCCCCAGC	GGGAAGCTTAAAACAAAGAAACA	256
Ad02A9855	Aradu.A02	9855	p2	(CG)6	12	53100607	53100618	TGGTGTTTGGAAGTGTTGGA	GCGCCACTCTCGAAATAAAC	160
Ad02A9857	Aradu.A02	9857	p2	(AT)9	18	53109876	53109893	CAAAATCCAGTGAGGGTTCAA	GCATGTCCTGATGCTAAATCC	258
Ad02A9858	Aradu.A02	9858	p2	(CT)6	12	53110763	53110774	GGCCCAGATCCAAGTACAGA	GGGAAGCTTAAAACAAAGAAACA	218
Ad02A9861	Aradu.A02	9861	p2	(TA)7	14	53156196	53156209	GGATTTGAAGAGTTTGGCGA	ATTGGGCTAAGGCTGAACAC	209
Ad02A9865	Aradu.A02	9865	p2	(AT)10	20	53163949	53163968	CCACGCGCATAATGTCTAAA	CGGAGGCTTAGGTTTCTGAG	271
Ad02A9866	Aradu.A02	9866	c	(T)10aagaaaata	50	53169432	53169481	TTTGTGAGACTTGGAACAAAAA	TGAAGCTGAATTTTGTTCATCATA	259
Ad02A9867	Aradu.A02	9867	p2	(GA)7	14	53170935	53170948	TTCAAGTCTACCCCTCGTTCA	TCCTAACACAACACATTTGCG	217
Ad02A9870	Aradu.A02	9870	p3	(GTA)6	18	53198968	53198985	AGGGCTTTTGACCCAACTTT	AAGCAAAATCGAAAATCAAAGC	240
Ad02A9871	Aradu.A02	9871	p3	(ATT)5	15	53204643	53204657	CAAACAGTCCCAATTAGGAATACA	TAGCGCATTCTCCTTTTGGT	209
Ad02A9872	Aradu.A02	9872	p2	(GC)6	12	53213130	53213141	AAGTGAAGGGGTGAGTGTGC	TAGGACCCTCTCATGCTTGG	258
Ad02A9873	Aradu.A02	9873	p2	(GC)6	12	53213263	53213274	CCAAGCATGAGAGGGTCCTA	TCAGGCCTATACCAAGCCAC	189
Ad02A9875	Aradu.A02	9875	p2	(AT)7	14	53234868	53234881	TTGAGGCCGTAATACCTTGC	GATTAATGAAAACACGGGACG	256
Ad02A9880	Aradu.A02	9880	p2	(AT)13	26	53267424	53267449	TTTCCCAAAATTGTTTTCGC	TTTGCTTTTTCCCTGTTTGC	120
Ad02A9883	Aradu.A02	9883	p2	(TC)17	34	53278403	53278436	CAGTGGTAGCAGTGGCAGAA	TCGCCATCGGAAATTAGAAC	237
Ad02A9888	Aradu.A02	9888	p3	(AAT)6	18	53280947	53280964	TCTTAAAAAGCCAACGGTAACA	CGATCCAATCCAATCCAAAC	229
Ad02A9890	Aradu.A02	9890	p2	(CA)6	12	53285560	53285571	ACCAAGCCCTTTCCAAACTT	GGACCTTGGGTGTGGTAAGA	164
Ad02A9891	Aradu.A02	9891	p3	(TTG)5	15	53290298	53290312	ACCCGACCAGTCTATTGTGC	CACAGAACCGAGGAAAGAGC	280
Ad02A9892	Aradu.A02	9892	p2	(CA)6	12	53293905	53293916	TGCCTATCTCAGGGCCTCTA	GGGTATGATGGTTGTGGCTT	254
Ad02A9896	Aradu.A02	9896	p3	(TTG)5	15	53336019	53336033	TGAGTTGTGGTTTCAGCTCG	GGAAAGGCATTGTTGGAGAA	228
Ad02A9897	Aradu.A02	9897	p2	(GC)6	12	53346231	53346242	ATAGGGAGCTCTTGGGCATT	TGAAGGAGAAAAGCAGGGTG	273
Ad02A9901	Aradu.A02	9901	p2	(TG)18	36	53359845	53359880	CGAAGTGGTTACCGGGTTTA	CAAGGCTGGCCAGAGAATAC	132
Ad02A9902	Aradu.A02	9902	p2	(AT)7	14	53360441	53360454	TTGGAATGCTTTCAAATTAATGA	TCTTTGTCTTTTACGATTTTCACA	228
Ad02A9903	Aradu.A02	9903	p3	(GAA)5	15	53366116	53366130	GGTCGTTGTGGAGTCCAGTT	ACACACTTTCCATTGGAGGC	271

ID	Chromosome	SSR nr.	SSR type	SSR	Size	Start	End	FORWARD PRIMER (5'-3')	REVERSE PRIMER (5'-3')	Product (bp)
Ad02A9904	Aradu.A02	9904	p2	(AT)6	12	53382920	53382931	CAACATGATGAGTATTATTCGTTTTT	TTCGACTTTTTAATTTGACATTGAG	142
Ad02A9909	Aradu.A02	9909	p3	(AGA)6	18	53404897	53404914	CTAGAGGATTCTCCGGCCTT	TGGCCGAACCTACATCTCTC	170
Ad02A9911	Aradu.A02	9911	c*	(AT)10(T)10*	29	53441888	53441916	CATGAACTGGACATGCCAAA	ATGGGAGTTTGTGGTAAGCG	256
Ad02A9912	Aradu.A02	9912	p2	(TG)30	60	53448728	53448787	CTACCCGACCTTGTCTTTGC	GTGCTTGAACCCCAAATCAT	151
Ad02A9913	Aradu.A02	9913	p2	(TA)6	12	53474368	53474379	AAAATCGCAACCAAAACGAT	ACGGACCAGACTCCTGAAAT	156
Ad02A9922	Aradu.A02	9922	p2	(TA)6	12	53548637	53548648	TTGACCGAAAAATAATGAATCAAA	TGGCCAATTGGGGATAGATA	265
Ad02A9928	Aradu.A02	9928	p2	(TA)6	12	53570965	53570976	AAAGAAGAAGGAGAAAATGTGTTTG	TCCTCTTATCTACCTCTCTCCCC	180
Ad02A9929	Aradu.A02	9929	p3	(AGA)6	18	53597081	53597098	CGGACTTAGGTGCCATTGAT	TTCAAAAGCACTTTCCCACC	278
Ad02A9932	Aradu.A02	9932	p3	(AAT)5	15	53609758	53609772	CAGGGAAAAAGGCACTCTTG	AAATTTGGACGTTGGCTTTG	199
Ad02A9934	Aradu.A02	9934	p2	(TC)6	12	53625457	53625468	ACACCCCAGCGAGTTCTAAA	AAGAACCTGGGATTGAGAGTCA	221
Ad02A9937	Aradu.A02	9937	p2	(GA)8	16	53643411	53643426	GGAGCTTTTCAGGTTCTCCC	CTCGGTTCTGTGGGAATGAT	233
Ad02A9938	Aradu.A02	9938	p2	(AG)6	12	53665849	53665860	CGGAAAAGCACACCAAACTT	CCTCTTTCCACCACACCATT	110
Ad02A9941	Aradu.A02	9941	p2	(AG)23	46	53703664	53703709	AGGTTTTGGCCTAAAGTGGC	ATGGCTCCCAGAAAGTGATG	231
Ad02A9942	Aradu.A02	9942	p2	(AG)7	14	53715069	53715082	CGGAAAAGCACACCAAACTT	AGCTTCCTCTTTCCACCACA	117
Ad02A9945	Aradu.A02	9945	p3	(TGA)5	15	53769306	53769320	GGGGATGAAGTAAGGGGAGA	ATCTCACCATGTCCCAGAGC	266
Ad02A9949	Aradu.A02	9949	p2	(AT)12	24	53785291	53785314	CATATGGGCACCACTGTTCA	CTCAATCCAAGTTGGCCATT	180
Ad02A9950	Aradu.A02	9950	p2	(TA)7	14	53801048	53801061	CCCAATTATTACAGCACCAATG	CAAGGGCATCATCCCTACAT	276
Ad02A9952	Aradu.A02	9952	p4	(AAAT)6	24	53803667	53803690	ATGGGAAAACTGTGGGAACA	TTGGGATAATTTTGTCCCCTT	204
Ad02A9953	Aradu.A02	9953	p3	(AGT)5	15	53812072	53812086	CCCGAATGCTCTAGTTCTGC	AGCGAGAGCAAGGACCACTA	266
Ad02A9954	Aradu.A02	9954	p2	(TA)8	16	53822037	53822052	TGCATGTTAAGATCGGAGAGA	CGGGACAACTTGTATCCAAAA	249
Ad02A9955	Aradu.A02	9955	p3	(GAA)5	15	53831879	53831893	CCGACCTTAGGTGCCATAAA	AAACCCCTCTCCCTCCACTA	235
Ad02A9956	Aradu.A02	9956	p2	(GA)6	12	53835186	53835197	ATCCTTGCTCCTGCTCATGT	TTTCGAAAATCACTAACCATTTCTT	258
Ad02A9961	Aradu.A02	9961	p3	(AGA)5	15	53855831	53855845	TTGCCTTCCCTTTTCCTTTT	CAAACCCCTTCTTCTCCCAT	211
Ad02A9963	Aradu.A02	9963	p2	(CT)7	14	53879577	53879590	AAAAACAATGCCAAAAAGCG	GAACATTGAACCTGGGATTGA	219
Ad02A9964	Aradu.A02	9964	p2	(TA)8	16	53890192	53890207	ATCCATTGATGCTCAAAGCC	AAAGCAGTGAAAAAGAAAACGC	135
Ad02A9966	Aradu.A02	9966	p3	(AGA)6	18	53917547	53917564	TAAAATATTGCTCGCCCTCG	CCCATAAACCCCACCTACCT	244
Ad02A9971	Aradu.A02	9971	p2	(AT)9	18	53978438	53978455	TTTTAGCATCTCATGATCGCA	GAGGTCTAGGAAGGCGTGAA	274
Ad02A9975	Aradu.A02	9975	p2	(TA)9	18	53994456	53994473	TGGCTAGTCGGCTTAAACTCC	ATCCCAAAAAGGCACTCACA	259
Ad02A9976	Aradu.A02	9976	p3	(AGA)6	18	54000203	54000220	TAAAATATTGCTCGCCCTCG	CCAAAAACCCCACCTACCTT	241
Ad02A9979	Aradu.A02	9979	p3	(TAC)6	18	54029090	54029107	AAAAATTATTGCCACCACGC	CAACAGGTTGTTGAGTTCCAA	273
Ad02A9985	Aradu.A02	9985	p2	(AT)10	20	54090591	54090610	CACACAGCACATGGTTAGGG	GGAAGCAAGAAAAAGCAGCA	220
Ad02A9986	Aradu.A02	9986	p3	(AAT)17	51	54101942	54101992	ATGGGCATCCATTTTGATCT	ACATTTGAGACGTTGGGAGG	270
Ad02A9987	Aradu.A02	9987	p2	(TC)9	18	54102094	54102111	TGGGCATCCATTTTGATCTT	CATTTGAGACGTTGGGAGGT	268
Ad02A9995	Aradu.A02	9995	p2	(AG)10	20	54118323	54118342	AAACCTGGGATCGAGAGTCA	CCCAATTTTTGACCCAGAGA	227

ID	Chromosome	SSR nr.	SSR type	SSR	Size	Start	End	FORWARD PRIMER (5'-3')	REVERSE PRIMER (5'-3')	Product (bp)
Ad02A9996	Aradu.A02	9996	p2	(AG)10	20	54123814	54123833	ACAGCCAATTGGGAAACATC	GGGAAATGCATCCATTCATAA	272
Ad02A9997	Aradu.A02	9997	c	(TA)8aaagaaag	52	54127837	54127888	TCACCACGATTTCAATATAAGAATG	CAGGTTTTCAAGGGGAATCA	227
Ad02A10000	Aradu.A02	10000	p3	(TTC)12	36	54140213	54140248	TCTATACGTTGGCCGAAACC	AATCGGAGGTGGAAGCTTTT	235
Ad02A10002	Aradu.A02	10002	p3	(TTC)6	18	54145465	54145482	TACACCTCTCCCCCTCTCCT	CTCGGAAGTGGAAGCTTTTG	199
Ad02A10004	Aradu.A02	10004	p2	(AT)8	16	54173488	54173503	TCCGATGAAGGAATGAAAGC	AACTCAGCGGCCTACGACTA	179
Ad02A10005	Aradu.A02	10005	p2	(AC)9	18	54176058	54176075	GTTGTAATGGGGCTAAGGCA	GAAAAAGTGGGAATTTTGGG	157
Ad02A10006	Aradu.A02	10006	p3	(AGA)6	18	54178918	54178935	GAAAGACGTGAGAGGAGAGGAA	ACAGGATGCATCTACGTCCC	253
Ad02A10007	Aradu.A02	10007	p2	(AT)6	12	54180552	54180563	TACGGGTGAGAATTGCTTCC	AGGGATGAGATACCCCCAAC	163
Ad02A10008	Aradu.A02	10008	p2	(TG)6	12	54188662	54188673	TTATCTCATAACCAGCGCCC	AAAAATGCTACGCAGTTGGG	130
Ad02A10009	Aradu.A02	10009	p2	(AT)10	20	54193276	54193295	GGTGTTCCTTGGTGTTTTCC	TGGACGGTTCATTTCCACTT	269
Ad02A10017	Aradu.A02	10017	p4	(ATCT)9	36	54216186	54216221	CCAAACTTCAGGGACAAAAA	TCACTAAAGAGCTTATGTGGCAA	174
Ad02A10019	Aradu.A02	10019	p2	(GT)7	14	54240595	54240608	ACATCCATATCCCCGTGTGT	TGATTTCTGCTTTGTGTGCG	116
Ad02A10020	Aradu.A02	10020	p2	(AT)6	12	54246091	54246102	AGCCTTCGTGAATACGAACC	GCTCATGCATCACGGAACTA	248
Ad02A10021	Aradu.A02	10021	c	(TTTA)5ttttggt	175	54253474	54253648	TGCCAATTTAATTAGTAAAGCTCA	TGCCAATTTAATTAGTAAAGCTCA	264
Ad02A10024	Aradu.A02	10024	p2	(CT)6	12	54276623	54276634	GAACTTGGAATTGGGAAGCA	GGGCATTTCTGGGCTATTTT	212
Ad02A10028	Aradu.A02	10028	p2	(CT)6	12	54319369	54319380	GAACTTGGAATTGGGAAGCA	ACTGCAGAAATCGTTGGGAG	230
Ad02A10029	Aradu.A02	10029	p3	(TTC)5	15	54340980	54340994	CAAAACTCTTTCCCACCCAA	ACCACTGAGCTATGACGGCT	280
Ad02A10031	Aradu.A02	10031	p3	(TCT)7	21	54372748	54372768	CACAAGCCCCTCTTCTTCAC	CGGTCTTAGGTGCCATTGAT	181
Ad02A10033	Aradu.A02	10033	c	(CT)11tcccttcc	131	54389931	54390061	AAACGCAAAATCATTCTCCC	AAGGAAGGGGAGAGAGAGGG	265
Ad02A10034	Aradu.A02	10034	p2	(CT)6	12	54390464	54390475	CTCCACTCTCTCTCTCCCCC	TTCATCTCTCTCTTGCTTTGGA	264
Ad02A10035	Aradu.A02	10035	c	(GA)10gcatgag	127	54391471	54391597	GATTGGATTGGATACCCACG	CGCCTCTCTCCCATTTCTCT	244
Ad02A10036	Aradu.A02	10036	p2	(AG)7	14	54392973	54392986	GTGCATCTTTGTTTTGGGCT	CCCTAAATTCTAGAGGGAAGAGAGA	177
Ad02A10039	Aradu.A02	10039	p3	(TCT)5	15	54414854	54414868	GCCGAATACACCTTTCTCCA	TGAATGGGGTTGGTCAGAAT	273
Ad02A10040	Aradu.A02	10040	p2	(AC)6	12	54425865	54425876	CAAGGATTGAAGGGGATTGA	GAGGATTGAGAATTGACAAAGGA	206
Ad02A10042	Aradu.A02	10042	p2	(TC)6	12	54429702	54429713	TCTCCCATTCTCACACCTCC	GGCTAAGCAAACACTGCCTC	261
Ad02A10044	Aradu.A02	10044	c	(AT)7ac(AT)9	34	54436720	54436753	TTCGATATTACCAGCGCCTT	AGGCTCTTGTCCTCCAACAA	228
Ad02A10046	Aradu.A02	10046	p2	(TA)10	20	54440562	54440581	GATGAACCTTGATGAATCTTGC	GCTGCTGCAACATTGTAGTATAAA	171
Ad02A10050	Aradu.A02	10050	p3	(AAG)5	15	54462417	54462431	GGTCTTGATGGCTCTTGGAG	TGGCCGAACCTACATCTCTC	258
Ad02A10051	Aradu.A02	10051	p2	(AC)9	18	54471031	54471048	GGCTTAGGTAAGGGTGAGGG	AATGAAAGCACCCCAAATGA	242
Ad02A10052	Aradu.A02	10052	p2	(GC)6	12	54481217	54481228	TCGATTGTGCTTTTTGCTTG	TTTGATGCCGTGATCTGTGT	231
Ad02A10053	Aradu.A02	10053	p3	(TTC)6	18	54493053	54493070	AAATCTTTCCCACCCAAACC	GCCCTTGAGCAAGAGAAGAA	193
Ad02A10055	Aradu.A02	10055	c	(A)10gagaaaaa	59	54516761	54516819	TTCATGAATGTTGGCTCTTGA	GCACACTCTTATTGCCTTGGA	215
Ad02A10057	Aradu.A02	10057	p6	(AAAATA)5	30	54547177	54547206	TACTAGAGCCCCCAAGCTCA	AGAGCAACAATTACACGCCC	204
Ad02A10058	Aradu.A02	10058	p2	(AT)10	20	54554682	54554701	TTTAAACCGCAAAATCGCTC	GGCGCAGAAGTTACGAAAAG	254

ID	Chromosome	SSR nr.	SSR type	SSR	Size	Start	End	FORWARD PRIMER (5'-3')	REVERSE PRIMER (5'-3')	Product (bp)
Ad02A10059	Aradu.A02	10059	p2	(CT)6	12	54558238	54558249	CTTCTCGCCATCGTGGTACT	GCAGAATTAGGGAAGAGGGG	276
Ad02A10061	Aradu.A02	10061	p2	(AT)10	20	54562019	54562038	TTTAAACCGCAAAATCGCTC	GGCGCAGAAGTTACGAAAAG	254
Ad02A10062	Aradu.A02	10062	p2	(GA)6	12	54574627	54574638	TGGGATGATCTCCCTCTCAC	TGTTGGGGCAAAACTTTGTT	246
Ad02A10066	Aradu.A02	10066	p3	(AAT)6	18	54625945	54625962	GATTCATTTCTCAATTTGCTTTGA	CTTGCTTTGATGGGAGGTGT	254
Ad02A10074	Aradu.A02	10074	p3	(TAC)5	15	54708724	54708738	CATGCATGATGATGATGAGAAA	AGCTGAAGCGCCAGAAATTA	237
Ad02A10076	Aradu.A02	10076	p6	(TTTATT)5	30	54729298	54729327	GGGACCAGACACCCTACAGA	TGTTGAAAACTTGAAATAATTGCC	269
Ad02A10079	Aradu.A02	10079	p3	(AGA)9	27	54751014	54751040	TTAAAAGATTGCTCGCCCTC	CCCATAAACCCCACCTACCT	249
Ad02A10081	Aradu.A02	10081	p3	(TTC)8	24	54813000	54813023	TGGCCGAACCTACATCTTTC	AGTATTTCCCCCGAACCATT	227
Ad02A10084	Aradu.A02	10084	p4	(TTAT)6	24	54829994	54830017	CATGATGTCAAGGCTGATCG	TCCTTCCATCTAGGACCGATT	279
Ad02A10085	Aradu.A02	10085	c	(T)11gttctaaag	70	54830922	54830991	TGGTTAAAGCCTTCTTTACAAAGTT	TCAATTCAAGATTTGAAACTACAACA	235
Ad02A10090	Aradu.A02	10090	p2	(AT)7	14	54842058	54842071	ATTCATCCATTGCCTCCAAG	ATGCAATGATTTGGTGAACG	201
Ad02A10095	Aradu.A02	10095	p2	(AT)10	20	54868490	54868509	TTTGTCCCATGTGCATTGAT	TCCCAAGTTGAATCCTTGGT	273
Ad02A10098	Aradu.A02	10098	p3	(AAT)6	18	54874390	54874407	ATGAGCAGGAACAGGGAAAA	TTGGATGTTGGCTTTGTGAA	209
Ad02A10100	Aradu.A02	10100	p3	(GAT)5	15	54928328	54928342	GGTCATCTTGTGGGTAGCGT	ATTCCGGCTGAGTAGCTTCA	237
Ad02A10102	Aradu.A02	10102	p3	(TGG)5	15	54959002	54959016	CGTGTCCCGATTATCCTTCT	CACCGCTACTTCCTCCAAAA	280
Ad02A10103	Aradu.A02	10103	p4	(TATT)7	28	54976164	54976191	TTTCGAAAATACCTCCCTCTTTT	TGGGTAAGGATAAAGGTGGTG	225
Ad02A10104	Aradu.A02	10104	p3	(TTC)6	18	54979533	54979550	CCCCCTCTTGTACACCTTGA	CAGCCTTAGGTGCCATTGAT	173
Ad02A10105	Aradu.A02	10105	p2	(TA)9	18	54983571	54983588	CCCTTAGCTCTCGGGACTTT	CTCGGTTTGCGAAGATAACC	208
Ad02A10108	Aradu.A02	10108	p3	(TTC)5	15	54999494	54999508	GGCTGAAACCACACACCTCT	CTAGAGGTTTCTCCGGCCTT	169
Ad02A10110	Aradu.A02	10110	p2	(TA)9	18	55010777	55010794	TGTCTAAGGTACGAATTTTCTAGATG	GCTCTTTCCTAAAAATATTGGCA	187
Ad02A10111	Aradu.A02	10111	p3	(TTA)5	15	55011433	55011447	CCTTTTTAAAATATAATGCATGTACCG	GTTCTAATTCTCATTCAAGTGCAA	149
Ad02A10113	Aradu.A02	10113	p5	(AATAA)5	25	55022360	55022384	GTTGGCATATGACCCCAGAG	TTTCTCTCTCCTCACTTTTTCG	245
Ad02A10114	Aradu.A02	10114	p4	(ATAA)5	20	55040980	55040999	GCAGTGAAGAACAAAGCTTGC	ATGCCACAAGCACATGGTTA	220
Ad02A10118	Aradu.A02	10118	c	(TTC)19tatcttt	83	55064099	55064181	TCCCAGATGCTTACACGTCA	ATGGAGGATGTGAGTGGGAA	273
Ad02A10119	Aradu.A02	10119	p2	(AT)7	14	55081023	55081036	TTGGGGTTTATCAAGGAGCA	CCAATGCTCAGGCGTTATTT	239
Ad02A10123	Aradu.A02	10123	p3	(CTT)7	21	55128261	55128281	GATCCATTGAACCCCAATCA	CCCCTTCTGGAGTGAATGAA	151
Ad02A10125	Aradu.A02	10125	p2	(TG)6	12	55194871	55194882	GAGGTGTTGGGCATCAATTT	CACCCCTATGGGCTTACTTG	100
Ad02A10129	Aradu.A02	10129	p2	(CT)6	12	55225796	55225807	AGCCGAACCTACTCCCTCTC	CTAGAGGTTTCTCCGGCCTT	257
Ad02A10133	Aradu.A02	10133	p2	(AT)8	16	55248548	55248563	TGTTGGCCTTCTAACCTTCG	TCAACTTAATGGGTAAAACGGAA	235
Ad02A10134	Aradu.A02	10134	p3	(CAA)6	18	55249427	55249444	CCAACCAAGAAGCCATGAAC	CTTCTGATTCTCGTAGCGGC	206
Ad02A10135	Aradu.A02	10135	p2	(CT)6	12	55249647	55249658	CATGACTTCGTCATCATCGG	ACTCAAGAACATGTGCACCG	173
Ad02A10136	Aradu.A02	10136	p3	(CAT)14	42	55252158	55252199	CGCGTCCTCCAATTTGTAAT	ATTTCGGATATGGGTGCAAA	270
Ad02A10137	Aradu.A02	10137	p2	(AT)7	14	55255056	55255069	GAGGAACTAACGAGGGAGGC	TATCCGCCGAGAGACAGATT	268
Ad02A10138	Aradu.A02	10138	p2	(AC)12	24	55267374	55267397	GGCTTAGGTAAGGGTGAGGG	AGAAAGCACCCCAATGCTAA	242

ID	Chromosome	SSR nr.	SSR type	SSR	Size	Start	End	FORWARD PRIMER (5'-3')	REVERSE PRIMER (5'-3')	Product (bp)
Ad02A10139	Aradu.A02	10139	p3	(AAG)7	21	55270275	55270295	TGAGGGAAGAGAAAAGAAGAAGAA	TGAACAGAGGATTGTGCGAG	279
Ad02A10144	Aradu.A02	10144	p2	(TA)6	12	55328556	55328567	CGGGGGCTTGTTATGCTAT	TCAGTCCGTCCTCAGGTTCT	217
Ad02A10145	Aradu.A02	10145	p3	(TCT)5	15	55335628	55335642	CCTACTCCCTCTCCCCTCAC	GCTTTTGTCTTCCCTTTCCC	273
Ad02A10149	Aradu.A02	10149	c	(A)11gg(A)10	23	55344473	55344495	TGTGTCTAGCGTGGCAAGTC	CATGCTCCGTTTTTCAGTTG	219
Ad02A10151	Aradu.A02	10151	p3	(AAT)6	18	55357693	55357710	AAATTGGTGCAAGGACTCAAA	TTCCTAGCTATCACACCAAGC	209
Ad02A10156	Aradu.A02	10156	p2	(CT)9	18	55392170	55392187	TTCTTCTCCTCCATGACCGT	GAGAAAGGGAGATCGCATCA	186
Ad02A10161	Aradu.A02	10161	c	(GAG)8gaa(G.	42	55401000	55401041	TGCAAGCGCAAATTTAGAAG	TGGTGTTCATATCATTCCCAAA	280
Ad02A10164	Aradu.A02	10164	c	(TC)7ttttcccttt	49	55428371	55428419	TCACAGCACGGCTAATCATC	GAAAGGGGAAGAGGGAGAGA	263
Ad02A10165	Aradu.A02	10165	p2	(TC)8	16	55428964	55428979	CCTTCTTCTCTTTCTCTCCTTCC	CCTCCTCGTTCTCCTCCTCT	188
Ad02A10167	Aradu.A02	10167	c	(GA)6tatatatat	100	55429355	55429454	GGGATAATTTGAGAATTTTGATTGA	GCATACAAAACTAATAACGTGAAAAT	280
Ad02A10168	Aradu.A02	10168	c	(GA)6ggaagac	64	55429623	55429686	GGGGAAGAGAGGGAGCAAA	CTCCCCCTCCCTTCTCTCT	271
Ad02A10169	Aradu.A02	10169	c*	(AT)6(TTAT)8	43	55444611	55444653	GGGGCATTTTTGTCCTTTTT	GAGTGGGAAAACTTGGGACA	273
Ad02A10170	Aradu.A02	10170	p3	(TCT)5	15	55453301	55453315	ACACCCACATTCTCCCACAT	GCTTTTGTCTTCCCTTTCCC	212
Ad02A10172	Aradu.A02	10172	p3	(TCT)5	15	55470140	55470154	TGCACCATTCACATCAACCT	CTTAAAATTTGCTCGTCCCG	272
Ad02A10175	Aradu.A02	10175	p2	(TC)6	12	55483117	55483128	CTCTTAACCCACCCACCAAA	CAGAAGGTCGCTGGAATAGG	219
Ad02A10177	Aradu.A02	10177	p4	(TTTA)5	20	55494845	55494864	CCGGGGATTGTATGTGATTC	AAAAGGAAACAAATTGGGAAAAA	160
Ad02A10180	Aradu.A02	10180	p3	(AGA)5	15	55520582	55520596	ACATAGAATGTTGCTCGCCC	CAAGATCCCCACCTACCTCA	216
Ad02A10184	Aradu.A02	10184	p2	(AG)8	16	55571920	55571935	TTTGAGCTCTAAAACCATGTGC	CCAAGCTTTAAGTGTGGGGA	263
Ad02A10186	Aradu.A02	10186	p2	(AT)6	12	55588880	55588891	ATTTCCTTGCCTTGCCCTAT	CTCATCAGTTCCCCCACACT	229
Ad02A10188	Aradu.A02	10188	p3	(AAG)5	15	55597286	55597300	ATGGATCGATTTCAGGCAAC	TCTTGACTTTCCCATGGAGG	266
Ad02A10191	Aradu.A02	10191	p4	(TATT)5	20	55632012	55632031	CCTATCCCTCCCTAATTTTCG	AGGGGCAGTGATTTGAGATG	229
Ad02A10192	Aradu.A02	10192	p3	(TTC)6	18	55635453	55635470	TTCTACACCTTGGCCGAAAC	ATGGTCTTGATGGCTTTTGG	261
Ad02A10193	Aradu.A02	10193	p2	(AT)6	12	55639551	55639562	CATAGCCAAAGGGGTGAGAA	TTGCAAACGTGTGCCTTATT	131
Ad02A10195	Aradu.A02	10195	p3	(ATT)6	18	55665190	55665207	TTGGATGTTGGCTTTGTGAA	ATGAGCAGGAACAGGGAAAA	208
Ad02A10201	Aradu.A02	10201	p2	(AG)6	12	55737035	55737046	AAGAACCTGGGATTGAGAGTCA	TTTTGACCCAGTTTTCAGCC	196
Ad02A10203	Aradu.A02	10203	c	(AT)7gttaattcc	50	55841739	55841788	TGTGGGAGAATTTTGGGAAG	GGGTAACTTTTGTCCCCTTTTT	243
Ad02A10206	Aradu.A02	10206	p5	(TTTAT)5	25	55876970	55876994	TTTCTCTCTCCTCACTTTTTCG	GGGTTAGCATATGACCCCAG	252
Ad02A10207	Aradu.A02	10207	p3	(AGA)6	18	55886910	55886927	GCCTTAGGTGCCATCAATGT	AAACCCACACCTCTCTCCCT	147
Ad02A10210	Aradu.A02	10210	p5	(TTTAT)5	25	55905214	55905238	CCTAACCACTTTCTCTCTCCTCA	GGGTTAGCATATGACCCCAG	260
Ad02A10211	Aradu.A02	10211	p3	(AGA)6	18	55915271	55915288	ATCACCTTTTTCTTGGCCCT	AAACCCACACCTCTCTCCCT	277
Ad02A10216	Aradu.A02	10216	p5	(AATAA)6	30	55996121	55996150	GGCCCTAGAGTCCTTCTGGA	TCACTTTCTCTCTACTCACTTTTTCG	239
Ad02A10218	Aradu.A02	10218	c	(A)10gtaaaaaa	38	56008299	56008336	TGGCTCTTGAAAGAATGAGGA	TTCAGCACACTCTTGTTGCC	185
Ad02A10219	Aradu.A02	10219	p2	(GC)6	12	56042667	56042678	GTAGGGCCCACATTCTCTCA	AGCAGAGGTGGTGGTTATGG	169
Ad02A10221	Aradu.A02	10221	p2	(GA)7	14	56056603	56056616	TCGAACACAGAGGGAGAGAGA	TTTTCAAAACCACCTAACCCC	133

ID	Chromosome	SSR nr.	SSR type	SSR	Size	Start	End	FORWARD PRIMER (5'-3')	REVERSE PRIMER (5'-3')	Product (bp)
Ad02A10224	Aradu.A02	10224	p3	(ATT)8	24	56083044	56083067	TCCAATTAAGGGTCAACCACA	TAGATTAGCCGCCCATTGTC	226
Ad02A10228	Aradu.A02	10228	c	(GAG)7agagag	130	56086726	56086855	TCCCTCTTCTCTTTCTCTTCCTC	TCCCTCTCTCCTTCTCTCCC	271
Ad02A10229	Aradu.A02	10229	c	(GA)7ggaagaa	45	56087008	56087052	GGTGGTGCAAGGTTTTGTCT	AGCCACACAAAATCATCGTG	277
Ad02A10230	Aradu.A02	10230	c	(GA)9gggaggg	131	56087349	56087479	TGAGCAAAAGAGAAAAATGAGAGA	CTTTCCCTCTCTCTCCCCAC	218
Ad02A10234	Aradu.A02	10234	p5	(TTTAT)5	25	56097976	56098000	TCGAAAATCCTCACCCAAAA	AGGGAGATGATGTGGATTGG	130
Ad02A10236	Aradu.A02	10236	p3	(TAC)5	15	56112936	56112950	AACAAAAAGTATTGCCACCACA	CCACTTCCCAAAACACAGGT	277
Ad02A10237	Aradu.A02	10237	p3	(TCT)5	15	56155042	56155056	TCCCACATAGCCGAACCTAC	GCTTTTGTCTTCCCTTTCCC	201
Ad02A10240	Aradu.A02	10240	p2	(CT)21	42	56160337	56160378	AAGACCTTCAACCGGTCTCC	TGCGACAGTGGTGATAGAGC	137
Ad02A10242	Aradu.A02	10242	c	(TCA)6ccatctt	53	56163354	56163406	TCCTACTCATCTTCTGTTGCTTTG	TGCTGCATTTGTTTTCTTCTTC	257
Ad02A10243	Aradu.A02	10243	c	(A)10tgacagttg	45	56164102	56164146	CACGCAAAGAAAAAGGAGGA	ACACTTTCCAAAGCGCTCAT	161
Ad02A10244	Aradu.A02	10244	p2	(AT)13	26	56164765	56164790	TAATATACTCGGCACCCGGA	GGCGAATTTGCCTTAAATGA	211
Ad02A10245	Aradu.A02	10245	p3	(AGA)6	18	56177176	56177193	GCTCGTCCTCGAGCAAAATA	CCCATAAACCCCACCTACCT	231
Ad02A10248	Aradu.A02	10248	p2	(TA)6	12	56198383	56198394	GTTAATCCCCGTGAGAACGA	GGACTTGTTGAAGTCGGAAAA	239
Ad02A10250	Aradu.A02	10250	p2	(TA)7	14	56214755	56214768	TTGGTAATCAAAACGCCCTC	GCACAATAATTCATTCAATTTCCA	168
Ad02A10254	Aradu.A02	10254	p2	(TA)7	14	56218670	56218683	TTGCCATCAAAACGAAGTCT	TTTGAAATTTCTTCATTCATCTACTC	280
Ad02A10255	Aradu.A02	10255	p2	(TA)6	12	56221748	56221759	AAATAAACGAGATAAGATTGAGAGACA	GCCAAAGATTGACCCACAAA	272
Ad02A10256	Aradu.A02	10256	p2	(AT)9	18	56221913	56221930	CTTTGGCCACCTTTAAGCAC	TGACCGAACTCAAGGGGTAG	237
Ad02A10259	Aradu.A02	10259	p3	(TCT)5	15	56263049	56263063	TACTCCCTCTCCCCTCCCTA	GCTTTTGTCTTCCCTTTCCC	270
Ad02A10262	Aradu.A02	10262	p2	(AT)9	18	56333437	56333454	TGACTGGTGCTTTTAGTGGC	CCAGCCTCATACACCCAAAT	277
Ad02A10263	Aradu.A02	10263	p2	(GC)6	12	56333745	56333756	ATGAGGCTGGTATTTGGGTG	CGCGTGGAACATTTCAAGT	258
Ad02A10268	Aradu.A02	10268	p2	(GT)7	14	56381917	56381930	GCAGTGAGCTCCTTGGATTC	TATTGGAACGATGGTGCAAG	162
Ad02A10269	Aradu.A02	10269	p2	(TC)6	12	56382315	56382326	AGGATTTTGTTGCACACGGT	CACCACTTCTGGACCGAGAT	201
Ad02A10275	Aradu.A02	10275	p2	(TA)8	16	56391320	56391335	AGCTCTTTTCTAAAAATATTGGCA	TCCGAAGTACGAATTTTCTAAATG	256
Ad02A10277	Aradu.A02	10277	p3	(GGC)5	15	56394081	56394095	GGAAAGCGAATTCCGACATA	TGCTGCTGTTGACTTTGCTC	276
Ad02A10281	Aradu.A02	10281	p2	(AT)6	12	56401197	56401208	CGACTGCACCTGAGTTTCAA	GCAAAAGCCTTAGGCAGAGA	240
Ad02A10282	Aradu.A02	10282	c	(A)10ggagaaga	109	56404175	56404283	TGAACCACACCTTAGCCACTT	TCACGCGTATTCACGTTTTT	270
Ad02A10284	Aradu.A02	10284	p2	(TA)14	28	56405025	56405052	GCGCCCAACAACTAGTGAAT	CCCTGTTTCTTGCTCAAAGC	253
Ad02A10286	Aradu.A02	10286	p3	(TTC)7	21	56432955	56432975	CATTCTTTGCGCCATCACTA	GTCCACCACACACGATTACG	240
Ad02A10288	Aradu.A02	10288	p2	(AG)19	38	56450407	56450444	GGGTTGGACTCGGTTCATTA	CATTGCACTTTGAAGCTCTCC	206
Ad02A10289	Aradu.A02	10289	p2	(CA)18	36	56452604	56452639	ACCGCATCTTACCCAGTGTC	TCATGCATCAATGGCTGATT	100
Ad02A10290	Aradu.A02	10290	p2	(GC)6	12	56467990	56468001	GAGGTTGCTGCAGGACTAGG	ACGCACTCTTTTGGCAACTC	243
Ad02A10291	Aradu.A02	10291	p3	(GTT)5	15	56483409	56483423	GGCCGATTTTGTGAGTTGTT	CAAGGCGGTAATCCTAACCA	172
Ad02A10294	Aradu.A02	10294	p2	(GC)6	12	56524988	56524999	CCAAGATTGGAAATGCATCA	CTTCAAAACCAATTGCTCCC	258
Ad02A10295	Aradu.A02	10295	p2	(AT)7	14	56529518	56529531	CTAGACTCGACCTGCGAAGC	TAGGATTGCCTCTGGCTTTC	278

ID	Chromosome	SSR nr.	SSR type	SSR	Size	Start	End	FORWARD PRIMER (5'-3')	REVERSE PRIMER (5'-3')	Product (bp)
Ad02A10296	Aradu.A02	10296	p2	(AC)6	12	56530546	56530557	ATCACTGACCCTGAGCGAGT	CGAAATCGGAGGTCTGAGAA	235
Ad02A10297	Aradu.A02	10297	c	(ATT)7(TTA)5	36	56540457	56540492	TTGGATGTTGGCTTTGTGAA	TGACCAATTAGAATGTAACACATCA	142
Ad02A10299	Aradu.A02	10299	c	(TCA)13(TCT	57	56566667	56566723	TGCACAATTCAAAACTCTCCA	TCCTCCTCCTTTTGGTGATG	240
Ad02A10301	Aradu.A02	10301	p3	(GCT)5	15	56580806	56580820	CGACATTGACGAGCATTCAC	CTTTTCCCATTCCCTCCATT	211
Ad02A10305	Aradu.A02	10305	p2	(AG)7	14	56592273	56592286	GCAGAGCTTTACGAAGACCG	TGTGAAAATCTGCAGCATCC	223
Ad02A10307	Aradu.A02	10307	p3	(TCA)5	15	56613369	56613383	TCCATTTTCTCCTTCTCCTCC	TGGAGAATTTTGAATTGTGCAG	214
Ad02A10308	Aradu.A02	10308	p3	(TCA)13	39	56613561	56613599	CATAGTTGAAAAGTTTCGGTGC	TCCTCCTCCTTTTGGTGATG	268
Ad02A10310	Aradu.A02	10310	p3	(GCT)5	15	56621972	56621986	TCTTCTTCCCACATCTTGGC	ATCGTTTTTGGGTTCTCCCT	164
Ad02A10314	Aradu.A02	10314	p2	(AG)9	18	56637932	56637949	ACGCGGAGCAACTCATTTAT	GCGGAATGCTTTGATCTTGT	241
Ad02A10317	Aradu.A02	10317	p5	(TTATC)5	25	56669774	56669798	TAATCCTTTGTCACCGGAGG	CAGAGGAACACGACCCAAAT	273
Ad02A10321	Aradu.A02	10321	p5	(TTAGT)5	25	56682765	56682789	GCTTTGGTGTTAGATACTTGGACA	TTCAGATTTCAGCAATCAATAAAC	259
Ad02A10324	Aradu.A02	10324	p3	(AGA)5	15	56712763	56712777	CTAGAGGTTTCTCCGGCCTT	CATCCATGACCGAACCCTAC	265
Ad02A10326	Aradu.A02	10326	p3	(TCT)7	21	56772523	56772543	AAACCCCTCTCCCTCCACTA	CCCTCGAGCAAAAGAAGAAA	158
Ad02A10335	Aradu.A02	10335	c	(CT)8ttctt(TC)	57	56861000	56861056	ATTCGAACTGGGAACCCTCT	GCCAGAGATGTCGAACCAAC	246
Ad02A10337	Aradu.A02	10337	p4	(TTTA)7	28	56864761	56864788	ATGGTTTGGATGGATGAGGA	CCAGAGAAAATTTCGGAACG	261
Ad02A10338	Aradu.A02	10338	p3	(AAC)6	18	56865661	56865678	GAACCCACCACCACCACTAC	TTGGGTGGGGTGATACAGAT	201
Ad02A10339	Aradu.A02	10339	p2	(CT)6	12	56873891	56873902	CTCATTCGCAGAACAAAGCA	ATGAACAACCACTGCCACAA	225
Ad02A10340	Aradu.A02	10340	c	(A)10gtgctgcta	104	56875132	56875235	TTTTCAAATTGGTAGTGCAGTTATG	CCAAATGCAACTCCACAAAA	276
Ad02A10341	Aradu.A02	10341	p3	(TTA)6	18	56879439	56879456	CATCGGTAACCAAAAATCCG	TTCATTTTCCTAGTTATATCCCATCA	261
Ad02A10343	Aradu.A02	10343	p3	(ATT)5	15	56922720	56922734	AAACTTGGACGTTGGCTTTG	CAAGAGAAGGAGATGGCAGC	132
Ad02A10344	Aradu.A02	10344	p2	(GT)6	12	56936499	56936510	TAACGAGAATCGTGTGGCTG	CGGCTAAAAATGCTAATGGG	233
Ad02A10345	Aradu.A02	10345	p3	(GTT)5	15	56938120	56938134	CGGCCATTAGTTCAGTTGGT	CCCAGTGATGTTATCCAGCA	275
Ad02A10347	Aradu.A02	10347	c	(CT)6ttccc(CT	172	56948421	56948592	AAAACTGGTTTTATTTAAACTCCAAAA	GAAAGGGAAGGAGAGGGAGA	261
Ad02A10348	Aradu.A02	10348	c	(CT)6tcttctcct	152	56948711	56948862	TCTCCTCTCCCTCCTCTCTCT	GGGGGAAAAGGAGAAAGAAA	212
Ad02A10349	Aradu.A02	10349	c	(CT)7ccttctcct	157	56949191	56949347	CTCTCCCTCCCCCTTCCT	GGGGAAAGAGGGAAAAGATG	277
Ad02A10350	Aradu.A02	10350	p2	(TC)6	12	56949496	56949507	TCCTATTCTTCTCTCTCCTTCCC	TTCTTGCGCTCTTTTTCTCC	276
Ad02A10351	Aradu.A02	10351	p3	(GAG)6	18	56949680	56949697	CCTTTTCTCTCTCTCTCATTCCC	TTCTTGCGCTCTTTTTCTCC	241
Ad02A10353	Aradu.A02	10353	p2	(GA)6	12	56950476	56950487	GGAGAGAGGGAGGGAGAGAG	TGAACTGAAACTGCACTAAACCA	267
Ad02A10356	Aradu.A02	10356	p2	(TC)6	12	56965602	56965613	CGGGGCTTTAATTTGTGAGA	AGGGTCAATGGAAGACCCTC	176
Ad02A10358	Aradu.A02	10358	c	(ATA)8gtaataa	86	56969130	56969215	AAAAATTGTGGAGCATTCAAA	GTTGTAAATTGGGCGGGTTA	266
Ad02A10359	Aradu.A02	10359	p2	(TA)6	12	56972328	56972339	TGCCTAAAACCAAACCAATGA	GAAGCTTGCTGGGTTGTTTC	161
Ad02A10361	Aradu.A02	10361	p3	(TAT)11	33	56978778	56978810	AACACGAAAATAAAAGGCGG	GAAGTGGCCATGTCGAAAAT	142
Ad02A10364	Aradu.A02	10364	p4	(ATAG)5	20	56997320	56997339	TCACCAGTCATACTTATTGGGAA	CGTTTCTCTTGGACTTCTTGG	254
Ad02A10365	Aradu.A02	10365	p2	(GT)36	72	56997873	56997944	TGCAGAAACTCTTACATGTGGTC	TTGCTTGAATCTTGTCGTGC	267

ID	Chromosome	SSR nr.	SSR type	SSR	Size	Start	End	FORWARD PRIMER (5'-3')	REVERSE PRIMER (5'-3')	Product (bp)
Ad02A10366	Aradu.A02	10366	p2	(TC)17	34	56999178	56999211	TTGCTGAGTCGCTCATTCTC	CCCCAATCTCATTACTACGCA	202
Ad02A10367	Aradu.A02	10367	p2	(AT)6	12	57002071	57002082	CGCTGTTCAAATGATGGATG	ACCAAACAAAGCCCAACTTT	239
Ad02A10371	Aradu.A02	10371	p2	(AT)6	12	57014520	57014531	CGGAAATAAAAGGTGCTTCG	CACCGTTAACTTCCACCGAT	156
Ad02A10373	Aradu.A02	10373	p3	(TTC)7	21	57029298	57029318	AATACACGCGCTCAACACAC	AACGCGAAGAAGAAGACGAA	148
Ad02A10376	Aradu.A02	10376	p2	(TC)7	14	57093489	57093502	AACCCCACTTTGCTCATCAC	GCAAGCAACTACTACCATGCAA	164
Ad02A10378	Aradu.A02	10378	p3	(CTT)5	15	57115269	57115283	TTTCAAACGTCTTGGAAGGG	CAATTGGGGCAAGCAATAAT	271
Ad02A10379	Aradu.A02	10379	p2	(CT)6	12	57116952	57116963	AAACCTGGGATCGAGAGTCA	TTTCTGGGCTGTTTCTGACC	214
Ad02A10380	Aradu.A02	10380	p3	(TTC)5	15	57123042	57123056	CTATACCTTGGCCGAATCCA	CTAGAGGATTCTCCGGCCTT	174
Ad02A10381	Aradu.A02	10381	c*	(A)11(AT)6*	22	57126873	57126894	GGGAAACATGGGTTGATAGG	AGCTTCCCATAATTTTCCCA	232
Ad02A10385	Aradu.A02	10385	p3	(AGA)6	18	57202299	57202316	TAAAATATTGCTCGCCCTCG	CCCATAAACCCCACCTACCT	239
Ad02A10387	Aradu.A02	10387	p3	(CTT)5	15	57230277	57230291	TCTTGACTTTCCCATGGAGG	AACCGTTCAACAACTGGAGC	249
Ad02A10388	Aradu.A02	10388	p2	(TA)10	20	57239527	57239546	TGGGGTATTTTTGTCCCCTT	GGAGAGTTGGAAACATTGGG	262
Ad02A10390	Aradu.A02	10390	p3	(ATA)5	15	57270303	57270317	GCGTTTGTTTCTGAGAACAGG	AGAGACACAAACAAAAGATACAACTTA	251
Ad02A10394	Aradu.A02	10394	p3	(ACC)5	15	57277315	57277329	TTCGTCCTTAACGTCTTGGG	TTGGTGTTGGTGTTGGTGTT	254
Ad02A10395	Aradu.A02	10395	c	(ACC)6aacacc	142	57277451	57277592	CCATACCACCACAACCACC	TTGGTATTGTTTTGTGTTGTTGC	248
Ad02A10396	Aradu.A02	10396	p2	(TA)9	18	57286599	57286616	TGTGACGAGAATTCCTGGGT	CCTTACTTCTTTCCGGCCTC	262
Ad02A10398	Aradu.A02	10398	p2	(AT)8	16	57296603	57296618	ACACGTTTGGCATCTTTGTG	CAGCCTGAATAACAAAGGCA	218
Ad02A10401	Aradu.A02	10401	c	(A)12cctaaaaa	159	57305403	57305561	GACGGTCCGATTAGAGGAGA	GGACCGTCCGATTAGTTTTTG	251
Ad02A10403	Aradu.A02	10403	p4	(GAAT)6	24	57309440	57309463	CAAAAAGAAAGGTTGGCATCA	TCCCTGGCATATTTTGAACC	170
Ad02A10404	Aradu.A02	10404	p4	(GAAT)5	20	57309669	57309688	GGTTCAAAATATGCCAGGGA	GTGCTGCATTTCTGATTCCA	234
Ad02A10405	Aradu.A02	10405	p3	(AAG)6	18	57312951	57312968	AGGTGGGACCATCCAAAGTA	GTCATCGCAGAGGACATCAA	273
Ad02A10406	Aradu.A02	10406	p3	(TCG)8	24	57313127	57313150	AGGTTGATGTCCTCTGCGAT	GCGAGTAACGACAACAACGA	207
Ad02A10408	Aradu.A02	10408	p2	(AT)9	18	57323684	57323701	CCCATGTGCACCCTAAGAAA	AATTCTAATTTGATGTTTCTTTGATCT	151
Ad02A10410	Aradu.A02	10410	p2	(TC)6	12	57328722	57328733	TGTATAAGTTGGGATTAGCGGA	CGGTGAGGAAGCAGAGAGAG	269
Ad02A10418	Aradu.A02	10418	c	(T)10acattttttc	34	57361471	57361504	TGACATTGTTGACCAAACTTTTTC	GGTGTTAGGCTTTGCTTTGC	242
Ad02A10421	Aradu.A02	10421	c	(TAC)8(TAA)	60	57376478	57376537	CCCCATGGTCTCCCTAATTT	TTTTGGACTTGCACTCATTCTT	159
Ad02A10422	Aradu.A02	10422	p2	(TC)9	18	57381319	57381336	ACTGATCTAGGCGACGTCCA	GATAACGCTGGCCAAGAGAG	202
Ad02A10424	Aradu.A02	10424	p3	(TTA)7	21	57382722	57382742	TTCTTTGATTGTGGGCACTG	ACCAAGTCATCAGCGACCTT	224
Ad02A10428	Aradu.A02	10428	p3	(TAT)8	24	57387680	57387703	ACGAACGTATGGGATTCTGC	TGGTTCCAAAAGACCAAAAA	122
Ad02A10429	Aradu.A02	10429	p3	(TCT)5	15	57392380	57392394	ATTTTCCAATCCTTGGGGAC	TTTTGAGACCATCGAATCCC	254
Ad02A10431	Aradu.A02	10431	p3	(ATA)7	21	57398572	57398592	ATGCACGTTCCTTAATGGTT	TGGTGGTGAATTATACTCCTGTT	255
Ad02A10433	Aradu.A02	10433	p2	(GA)7	14	57410175	57410188	CTTCAACTCACGCCTTTTCC	ATAACGCCATCACACGGATT	278
Ad02A10438	Aradu.A02	10438	p3	(AAT)7	21	57471474	57471494	CCTTGAAGCAAAAGAAACAGC	AGACAGTGGTGCTCAAAGCC	248
Ad02A10443	Aradu.A02	10443	p2	(CT)6	12	57527746	57527757	TTATGCAAGTGTGAGGCTGC	AAAACCTTGTTGATGGGGGT	245

ID	Chromosome	SSR nr.	SSR type	SSR	Size	Start	End	FORWARD PRIMER (5'-3')	REVERSE PRIMER (5'-3')	Product (bp)
Ad02A10445	Aradu.A02	10445	p2	(TA)12	24	57534083	57534106	TGCTGGAGACTTCCTTTTGG	TATCAAGACTCGGGACTCGG	264
Ad02A10446	Aradu.A02	10446	c	(TA)9gaataata	77	57535594	57535670	TTTTATCAACACATCCAAAACTCA	GCCATTGATTCTCTTTTCCC	206
Ad02A10447	Aradu.A02	10447	p2	(TA)7	14	57539172	57539185	GCTAGCACTACAAGAAAAATGGTG	TCAAAACCGTGACTCTAACCAA	155
Ad02A10448	Aradu.A02	10448	c	(TA)6gag(T)1(	77	57540640	57540716	GCCATTGATTCTCTTTTCCC	TCAACACATCCAAAACTCAGTAAAA	201
Ad02A10450	Aradu.A02	10450	p2	(TA)7	14	57551637	57551650	GCTCACGTCGTCAGAAGCAT	TGCATATTTTGTTGCCTCCT	273
Ad02A10451	Aradu.A02	10451	p2	(GA)6	12	57556571	57556582	CCATCGTTATTGGGTCCTTG	CTAGCGGCCAAAACAACTTC	250
Ad02A10452	Aradu.A02	10452	p2	(GA)7	14	57560845	57560858	TGTTGCTGTCATTAGAGCCG	GCCACAACAACTTCTTCGGT	175
Ad02A10453	Aradu.A02	10453	p2	(GC)6	12	57592733	57592744	ACAAGGCTCCTAAAACCGCT	AGCCGACCTCTTTGCACTTA	225
Ad02A10455	Aradu.A02	10455	c	(ATT)7gttattat	59	57595905	57595963	CGGTGGAGAGAGAAAGTCGT	TCAACCATTCCTTTTTCATTTG	270
Ad02A10456	Aradu.A02	10456	p3	(ATT)22	66	57620687	57620752	AAAAAGGATGTTATGTATCCATCTAAA	ACTCGATCCGTTCCTTACCC	274
Ad02A10457	Aradu.A02	10457	p6	(TTAGGA)5	30	57623671	57623700	TGACCCAGTTTTTAGCCCAG	CGTTGAACTTGAGATGCAGAA	214
Ad02A10459	Aradu.A02	10459	p2	(AT)6	12	57628892	57628903	TGAAAACATTTGGTTTGAGAATTG	TTCACCACTTACTTTGGGGC	168
Ad02A10461	Aradu.A02	10461	p3	(ATT)18	54	57632904	57632957	CTCTTTGTTTCGGCCATCTC	AAAAACAGAAAAGTTCACTGAGAAGA	197
Ad02A10462	Aradu.A02	10462	p4	(TCTA)8	32	57635188	57635219	TTCAAGTTTCAGTGTGGCATTT	TGGCTCCTGAAAGTTTGTTTC	190
Ad02A10465	Aradu.A02	10465	p3	(TTA)13	39	57643376	57643414	CCTGTGAATTGGCAATAAAGC	TTATTGGGACCAATTTGGGA	165
Ad02A10471	Aradu.A02	10471	p2	(TA)8	16	57686534	57686549	ACATGCCGCTGTGATTCTCT	CATCCTCCATAAACGCATCTT	273
Ad02A10472	Aradu.A02	10472	p3	(TAT)8	24	57686724	57686747	AGCTCCCAAATATGAAAACCA	CATGTCACTCCTTCCTTGTTCTT	184
Ad02A10475	Aradu.A02	10475	p2	(AT)11	22	57695547	57695568	TCCCATCTGTAAACACCCTAGC	CAAAAATCAATAACAAGATTAGGGA	262
Ad02A10480	Aradu.A02	10480	p2	(TC)6	12	57713564	57713575	CTCAGGATCTATGGACCCCA	GGGAGGGAAAGGATTTTGAA	166
Ad02A10484	Aradu.A02	10484	p3	(AAG)5	15	57728463	57728477	CCACCACTCCCACCATTATC	TTTTAAAATCGTTGCCCACC	267
Ad02A10485	Aradu.A02	10485	p2	(AT)8	16	57728748	57728763	AAAGGTCGGGCGAAATAAAT	AGCCTAAGCGATTTCACCAC	176
Ad02A10487	Aradu.A02	10487	p3	(AAG)5	15	57739092	57739106	CTAGAGGTTTCTCCGGCCTT	CTCTTCCCCATTATCCCTCC	249
Ad02A10491	Aradu.A02	10491	p2	(TC)8	16	57781589	57781604	AGCCTTAAGTGTGGGGAGGT	CTCACTGGTTTTGTGTGTTCG	276
Ad02A10493	Aradu.A02	10493	c	(AAT)7aaaattt	126	57797622	57797747	CTTTTGAGAATGGTGTGCGA	TTCAAAATTACTATATGTGCCAAGTTC	245
Ad02A10494	Aradu.A02	10494	c	(A)11tcttaaatca	70	57799548	57799617	TACTCCTAGCTCCAATGCCA	AACATGTTATGTAGGCTTATTCCATT	276
Ad02A10495	Aradu.A02	10495	p3	(ACC)8	24	57802478	57802501	TTTTCCGTCAAAATTCTCCG	GCGATAGAGATGGCAGAAGG	220
Ad02A10496	Aradu.A02	10496	c	(CT)15(CG)10	50	57803014	57803063	TCAGCAAAATCGTCTGACAAA	GAAATGTTCAGAAGGGAGCC	260
Ad02A10497	Aradu.A02	10497	p2	(TC)11	22	57803225	57803246	GCGCCCATATACACACATTG	GGCGGAGGAAGAAGAAAAAT	248
Ad02A10500	Aradu.A02	10500	p6	(TATTTT)6	36	57805802	57805837	TTGAAATTGGAACTCCGTCC	AATTTTGGGGTCAACAACCA	218
Ad02A10501	Aradu.A02	10501	p2	(TA)9	18	57806606	57806623	ATATGTACGGGGCAAACGAA	TCTCGTGCTGATAACGTGTTG	206
Ad02A10504	Aradu.A02	10504	p3	(ATA)5	15	57845731	57845745	TTGAATTTTAAACTGACCGTGC	ATCATCGAACCGAAACAACC	178
Ad02A10507	Aradu.A02	10507	p2	(AT)8	16	57858801	57858816	AGGCATCTTGTCCCCTTTTT	TTGTGCGAAGTTGTGGAGAG	204
Ad02A10508	Aradu.A02	10508	p3	(ATT)8	24	57876747	57876770	AAACTTGGACGTTGGCTTTG	GGAAAAGGGCACTCTTGTTG	205
Ad02A10511	Aradu.A02	10511	p3	(TTA)5	15	57933129	57933143	ACCCAATTTGGATTGGTGAT	TTCATGGCAAGGATTGAGAA	254

ID	Chromosome	SSR nr.	SSR type	SSR	Size	Start	End	FORWARD PRIMER (5'-3')	REVERSE PRIMER (5'-3')	Product (bp)
Ad02A10519	Aradu.A02	10519	p4	(AAAT)5	20	57982014	57982033	TATTCGCATATTCGATTCGC	TCGATATAGCCGCAAAATACAA	250
Ad02A10525	Aradu.A02	10525	p2	(CT)7	14	58012578	58012591	AAAAACAATGCCAAAAAGCG	TAGCAGAGGCAGGGAAGAAA	157
Ad02A10527	Aradu.A02	10527	p3	(TCT)5	15	58041552	58041566	CCGAAACACAAACATCTCTCC	TGACTCAGAGGTGGAAGCAA	221
Ad02A10529	Aradu.A02	10529	c	(A)10gag(A)1(	55	58042472	58042526	TGTGGTTCATGAATGTTGGC	GCCTTGGATCCCAACTCTTT	187
Ad02A10530	Aradu.A02	10530	p2	(CT)9	18	58050246	58050263	AGAAATCTTCTCCCACGCCT	GAGAGAAGAAGAGGCGCAGA	109
Ad02A10532	Aradu.A02	10532	p3	(CTT)5	15	58059469	58059483	TCTTGACTTTCCCATGGAGG	ACAGTGGATCGATTTCAGGC	269
Ad02A10533	Aradu.A02	10533	p3	(TAA)10	30	58066795	58066824	TCCCATCTTAAAATTTCCAACAA	GGTTGGGTGTTTACTCCACG	225
Ad02A10534	Aradu.A02	10534	p3	(ATT)5	15	58093677	58093691	GGCTCAATGGCAGGAATAGA	CAAGAGAAGGAGATGGCAGC	119
Ad02A10535	Aradu.A02	10535	p3	(TCT)5	15	58099297	58099311	ACAACCCCCTCTTCTTCACC	GGTCTTGATGGCTCTTGGAG	267
Ad02A10536	Aradu.A02	10536	p2	(CT)7	14	58120139	58120152	CTTCAGTTTCCTCCCACCAC	GGTTGCCAGACTGAAGGTGT	255
Ad02A10537	Aradu.A02	10537	c	(A)11gaaagaaa	44	58152888	58152931	TTGTTGTTCATGAATGTTGGC	TCAGCTTTGCTAGAGTCCCC	260
Ad02A10541	Aradu.A02	10541	p2	(AT)9	18	58206859	58206876	ACACCCTACCACGCAGAATC	GCGAGTGATGCGCTTTTATT	178
Ad02A10542	Aradu.A02	10542	p2	(CT)7	14	58209912	58209925	ACATGAGGGTTCTCTTCCCC	GTCAAACCGATTGAACCGAG	147
Ad02A10550	Aradu.A02	10550	p4	(TCTT)5	20	58276747	58276766	CTTCTGATTTGGAAGTGGGG	AGGGTTGAGTTTGACGATTCA	280
Ad02A10551	Aradu.A02	10551	c	(T)10cgaaaatat	28	58290502	58290529	TCCCCAATTAGAGGTGTCCA	TGTGGTTCATGAATGTTGGC	229
Ad02A10553	Aradu.A02	10553	c	(T)10gaaaatttt	34	58309393	58309426	TCCCCAATTAGAGGTGTCCA	TGAAGCAAGAAAAAGCAGTGAA	152
Ad02A10556	Aradu.A02	10556	p2	(AT)8	16	58325631	58325646	GGACTGAAAGGCCAAACAGA	ACGGCTTTGGAGAGCTTTTT	207
Ad02A10563	Aradu.A02	10563	p3	(TGA)6	18	58397491	58397508	ACGACAGAAAGGATGGGTTG	TTGAGAAGCAATGACAAGCG	158
Ad02A10564	Aradu.A02	10564	p3	(ATT)10	30	58398143	58398172	TGTTTGTTCTTATTGACTTTTTCTTTT	TCAATTCTTCTGTGGTCGCA	215
Ad02A10565	Aradu.A02	10565	p2	(CT)6	12	58401874	58401885	TGCAGATTTTGGCTGGTCTA	AAATTCATAAGAAGACACTATTGCCA	223
Ad02A10566	Aradu.A02	10566	c	(CTT)5gctgctc	68	58405509	58405576	TCTTGACCGAAAATTTCAAGG	TGCATTGCATCTTCTTCTTCC	210
Ad02A10568	Aradu.A02	10568	p2	(AT)6	12	58409704	58409715	AACGATGTTAATGCAACTTTGG	ACAAATGATACCCGATGCAA	278
Ad02A10569	Aradu.A02	10569	p3	(ACC)5	15	58420979	58420993	AAGTTTCAGAATCCCCACCA	TGGTGTAGTAACCGCCATCA	279
Ad02A10572	Aradu.A02	10572	c	(AAT)5aacttaa	42	58423101	58423142	CAGCTATGCATGTGTTGTGTTTT	AGCATCACTATTAGAGGATACGAGA	237
Ad02A10577	Aradu.A02	10577	p3	(AAT)5	15	58463976	58463990	CACCTCACTTTCATTAGTCGGA	GAACTGTATTACGATTTTATCTCATCA	273
Ad02A10578	Aradu.A02	10578	p3	(ATA)5	15	58466787	58466801	GGATAACATGCAAGCTCGGT	AAAATTTTTATAAGTGGGGTAGGAA	269
Ad02A10579	Aradu.A02	10579	p3	(ATA)15	45	58469046	58469090	TTCTTTCTTCCATGGCTTCG	AACACAGTCTCAATTCAAATCCAA	251
Ad02A10584	Aradu.A02	10584	p3	(TGT)5	15	58495544	58495558	TTCACTTTCAGGGTTTTGAGG	GCCATAACAACAACAGCAGC	250
Ad02A10588	Aradu.A02	10588	p2	(AT)9	18	58512057	58512074	GGATCCGATCTGCATCTTTC	GGAGTGAATGGTTGCTGGTT	207
Ad02A10589	Aradu.A02	10589	p5	(AAAAT)6	30	58519684	58519713	CTTGGTGTTTTCCCTCCAAA	GGACTGTCCATTCATACTTACTTCC	279
Ad02A10590	Aradu.A02	10590	p3	(GAA)6	18	58537378	58537395	TTGGGAAAAAGAGTTTCAAGC	GCTCTGTTGCATTTCATGGAT	199
Ad02A10592	Aradu.A02	10592	p2	(AT)7	14	58572756	58572769	TTGCACACTCATGCATTCCT	CATGAACCCCAAGAAAAAGG	138
Ad02A10593	Aradu.A02	10593	p3	(AAT)5	15	58574069	58574083	CCCACGGAGATTGTTGGTAT	TTGCCATGGAAAGGAGTAGG	243
Ad02A10596	Aradu.A02	10596	p2	(GT)6	12	58597198	58597209	CCGTTCAAATTGGAGCTCAT	CCGTCCACCATTGAGTTCTT	274

ID	Chromosome	SSR nr.	SSR type	SSR	Size	Start	End	FORWARD PRIMER (5'-3')	REVERSE PRIMER (5'-3')	Product (bp)
Ad02A10597	Aradu.A02	10597	p3	(TGG)5	15	58618009	58618023	GCATAGTGAAGGGCAAGAGC	GCCTCAAATCCTCCCTCTCT	217
Ad02A10600	Aradu.A02	10600	c	(GA)11ttgcaat	123	58620569	58620691	TTGATGGTATTTCCTGTGAGTGA	GCAGTCGTTTTCAACCCCTA	278
Ad02A10606	Aradu.A02	10606	p2	(AT)6	12	58633130	58633141	TGGGCATGTGAATTCATAAAA	AAGTTCAATTGATGAGTTGGTCTAA	259
Ad02A10607	Aradu.A02	10607	p4	(AAAT)5	20	58634042	58634061	GCAAATTACTTCGAAGAAGAAAAA	GATGAGGATGAGGAAGAAGAACA	227
Ad02A10611	Aradu.A02	10611	p3	(GAA)8	24	58649507	58649530	TGATCGCAAAGAACTTCCCT	CCAATGTTGTCCCTGATTCC	209
Ad02A10612	Aradu.A02	10612	p2	(AT)6	12	58652413	58652424	AAATTTTTCCTGCCTCCAAAA	TTGAGCTTTGCCTCTACCGT	234
Ad02A10615	Aradu.A02	10615	p3	(AGA)5	15	58678246	58678260	CGGTCTTAGGTGCCATTGAT	TAGCCGAACCCACTCTTCTC	158
Ad02A10616	Aradu.A02	10616	c	(T)10cgcca(T)	25	58689770	58689794	ATCCATTGATGCTCAAAGCC	TGAAGCATGAAAAAGCAGTGA	156
Ad02A10617	Aradu.A02	10617	p3	(TTA)23	69	58715159	58715227	TTTCTCTCTCTCTTCCCACCC	GGAGGTCTTCTTTTTGGATGAA	270
Ad02A10623	Aradu.A02	10623	p3	(TTA)5	15	58744688	58744702	CAGCAGCTTTTGTTGCTTTC	CTGCATGGGGCTTTTAACTC	254
Ad02A10624	Aradu.A02	10624	p2	(TA)6	12	58749288	58749299	AATAATCCGTGCTTCACAACG	TGAAATCAATCATCTGTATAGCG	234
Ad02A10627	Aradu.A02	10627	p2	(AT)9	18	58755051	58755068	TTAACTCCTCGGGTTTCCCT	GCACGCATCCCTAATCCTAA	189
Ad02A10628	Aradu.A02	10628	p2	(CT)6	12	58761075	58761086	GGCATTTTACGTGCCTCATT	CTCGTCCTCGAGCAAAGAAG	255
Ad02A10629	Aradu.A02	10629	p3	(TTG)5	15	58769506	58769520	TCGACTCCACCTAGTGAAATAAAA	TCGAATAGACTAACTTCCATTCTCAA	237
Ad02A10630	Aradu.A02	10630	p2	(AT)7	14	58769978	58769991	TTTTTGAGTTTGTCATTGGTCA	TGCTTGTAGCAAGGAACACAT	274
Ad02A10632	Aradu.A02	10632	c	(TGA)6tggtgat	72	58772611	58772682	GATGTTGGTGGTGGTGGAGT	AACGTTGAGGGTCAGAATCG	245
Ad02A10635	Aradu.A02	10635	p2	(GA)6	12	58804900	58804911	GCAGAAGGGGAGAGTGAGTG	TTTCCATTTCTGCAACTCCC	143
Ad02A10638	Aradu.A02	10638	p2	(AG)6	12	58817374	58817385	TTTTTGACCCAGTTCTTGGC	AGCCAATTGGGCAACATAAA	269
Ad02A10639	Aradu.A02	10639	p3	(TTG)5	15	58856680	58856694	AGTGGTAAACACCCGACCAG	AGAGGGGAAGCTCTTTCAGG	245
Ad02A10642	Aradu.A02	10642	p2	(TC)23	46	58889532	58889577	GGTTCCCTTCTTATTGCTCG	GGAAAACAACTAATGGGACACC	197
Ad02A10643	Aradu.A02	10643	p3	(TTA)5	15	58889750	58889764	GGTGTCCCATTAGTTGTTTTCC	GCAGTCTCACCAGATGCGTA	224
Ad02A10648	Aradu.A02	10648	p3	(AAG)5	15	58971411	58971425	TCATCCATTTTCCAAGGCTC	TGGGTTAGGGAGCTCTGTTG	173
Ad02A10649	Aradu.A02	10649	p2	(CT)7	14	59001422	59001435	CCTCTTTCCACCACACCATT	TCATGGAAAAGCACACCAAA	115
Ad02A10651	Aradu.A02	10651	p3	(TCT)5	15	59019263	59019277	TCCTTGACGGAATTCTCCAC	TTTCATGCAACCCTTCATCA	278
Ad02A10652	Aradu.A02	10652	c	(T)10cttttttctttt	52	59038404	59038455	GCCCAAAGCACTCTGTCTTC	GAAGCAAGAAAAAGCAGTGGA	249
Ad02A10653	Aradu.A02	10653	p2	(AG)6	12	59039978	59039989	CGGAAAAGCACACCAAACTT	CCACACCATTCGAATTTCCT	100
Ad02A10654	Aradu.A02	10654	p3	(TAA)5	15	59049072	59049086	TTCCTTTTTGCCTCTTGCAT	AAACATTGGGTATAATTGTCGTGA	279
Ad02A10657	Aradu.A02	10657	p2	(AT)6	12	59053653	59053664	AGGCTCAAAAACTGACAAGC	ATCTTTAAACGCACCGAGGA	233
Ad02A10658	Aradu.A02	10658	p2	(AC)6	12	59054099	59054110	TGCGTGAGTTTTAAGTTGCAT	TTGCACATCAACCAAATTCC	234
Ad02A10659	Aradu.A02	10659	p4	(AACT)5	20	59054994	59055013	CCTCAATTACAATCCAACAACG	TATCATTCCCCATCCACCAT	216
Ad02A10660	Aradu.A02	10660	p3	(AAT)5	15	59066189	59066203	GAACAGGAGAAGGACATCGC	TGGAGATGCTTCTTCCATCA	106
Ad02A10665	Aradu.A02	10665	p4	(TTTA)7	28	59131934	59131961	TCAGCTTTGCTAGAGTCCCC	TTCATGAATGTTGGCTCTTGA	279
Ad02A10666	Aradu.A02	10666	c	(A)10tttgctttga	37	59149786	59149822	GCTGGGGATTGATTGTGATT	AGAACAGAGGGGAGGGGTTA	187
Ad02A10670	Aradu.A02	10670	p2	(TC)6	12	59152343	59152354	GACACAACCGCGTCAACTAA	AGCGAAGTGTCAACACCAAA	242

ID	Chromosome	SSR nr.	SSR type	SSR	Size	Start	End	FORWARD PRIMER (5'-3')	REVERSE PRIMER (5'-3')	Product (bp)
Ad02A10671	Aradu.A02	10671	p2	(TC)7	14	59171087	59171100	GTCACCCTCTCTCATGGCTC	TGAGTTTCCCTTGTGTTCCC	140
Ad02A10672	Aradu.A02	10672	p2	(AT)9	18	59186859	59186876	CCGACTTAAACTCTGCGAGC	CTAAACCTTACCTCGCGCTG	189
Ad02A10676	Aradu.A02	10676	c	(T)10cgttctttttt	47	59196671	59196717	TCACATCCCATGTCACTTTTTC	AAACTTCATCCACTTGATTTTGA	260
Ad02A10677	Aradu.A02	10677	p3	(TGA)6	18	59197903	59197920	CTCTTCCAAGGTCTTGCTGG	AGCACAGTTTAACCCCACTATG	176
Ad02A10678	Aradu.A02	10678	p2	(TA)6	12	59206437	59206448	GAGAATTTGGGCCTGGTGTA	ATCGTCTCAATTTTGTCCCG	270
Ad02A10679	Aradu.A02	10679	p2	(AC)11	22	59206934	59206955	CTAGCCGCGATCTTTCTCCT	CATCCTCCTTCTCCCCTTTC	181
Ad02A10682	Aradu.A02	10682	p5	(AAGAT)5	25	59293570	59293594	CAGAGCCCAGAAAGATGAGG	CCTGGGTGCTCCAGTGTATT	141
Ad02A10683	Aradu.A02	10683	p5	(AAGAT)5	25	59299920	59299944	CAGAGCCCAGAAAGATGAGG	TGTGGAACTGAGAGTGCTGC	265
Ad02A10687	Aradu.A02	10687	p3	(TTA)9	27	59321132	59321158	TCGAATTTGGGAGACCACTT	CCAAAGGATTTTGAGGGTCA	158
Ad02A10696	Aradu.A02	10696	c	(A)10taatcc(A)	28	59399050	59399077	TGGCTCTTGAAAGAATGAGGA	CACACTCTTGTTGCCTTGGA	191
Ad02A10699	Aradu.A02	10699	p5	(TTATC)5	25	59425677	59425701	TCATGCTTTTCCCTTTTGCT	AGGAACATGACCCAAATCCA	271
Ad02A10700	Aradu.A02	10700	p3	(TTC)6	18	59428295	59428312	CCTCCATCTCTCCCTCTCCT	GGACTTGATGGCTTTTGGAG	241
Ad02A10704	Aradu.A02	10704	p2	(AC)6	12	59444521	59444532	GGAAAACCGGGCATTAATTT	CTTCACGTGGAACACGTAGC	275
Ad02A10707	Aradu.A02	10707	p4	(AAAT)5	20	59475928	59475947	GCCAGCAACCTGGAAGTTAG	TTGAAGTTGTTTGTTGAAGCAGA	237
Ad02A10708	Aradu.A02	10708	p3	(ATT)5	15	59476059	59476073	GGCTTTAAATACGCCATAGGA	GGATGTCCGATTGACTCGTT	219
Ad02A10712	Aradu.A02	10712	p3	(GAA)6	18	59526400	59526417	TAAAATATTGCTCGCCCTCG	CCTACCTCGCCCTCTCTCTT	228
Ad02A10713	Aradu.A02	10713	p3	(ATT)5	15	59528964	59528978	AAACTTGGACGTTGGCTTTG	CAGGGAAAAAGGCACTCTTG	199
Ad02A10717	Aradu.A02	10717	p2	(CT)7	14	59550469	59550482	TCCTTTAGGAGGAATCCTAATCC	TTCAGGAAATGCTAGGGACG	222
Ad02A10718	Aradu.A02	10718	p3	(ATT)5	15	59607930	59607944	AAACTTGGACGTTGGCTTTG	GGAAAAGGGCACTCTTGTTG	196
Ad02A10719	Aradu.A02	10719	p3	(ATA)5	15	59611953	59611967	TGAGCTAACTTGGTTGAACTTGA	ATCATGTGCCTGCACTAATTT	278
Ad02A10721	Aradu.A02	10721	p2	(AG)6	12	59628933	59628944	GCGTGAAAATTTCTGGGCTA	GCAAAATGGAAATCTGAATTTGA	224
Ad02A10722	Aradu.A02	10722	p5	(GCCTG)6	30	59635656	59635685	TATTGACAGCCAGGCCAGAT	GAGGTTCCCATGGACTCTCA	252
Ad02A10723	Aradu.A02	10723	p2	(AT)11	22	59643241	59643262	TTTTTGTTTTGTTTCCTTTCAAT	TTCCCAGTGAAGTAACCTGTG	278
Ad02A10724	Aradu.A02	10724	p4	(AAAT)5	20	59646416	59646435	GCCTATCTCCAAGTTCAGCG	GGGCAAAAGGGAAGAGAATC	132
Ad02A10725	Aradu.A02	10725	p2	(AT)6	12	59646625	59646636	ACCAACTCCCTTCCCAATTC	ACAGAAAAGCCAAAAAGCGA	279
Ad02A10726	Aradu.A02	10726	p3	(AGA)5	15	59654872	59654886	GGGTTCATGGTGAAGAAAGG	TTCTCTCTCCTCTTTATTAAAACTTCC	271
Ad02A10727	Aradu.A02	10727	c	(TCA)13(TTA	54	59664030	59664083	TCCTGGTCAAAATTCAAATGC	TTGCCATTACTGTTGCTGCT	276
Ad02A10731	Aradu.A02	10731	p4	(AGAA)8	32	59683472	59683503	AGGGGAAAAGGAAACCTTGA	CACGCCTCCCTGTTACTCTC	263
Ad02A10732	Aradu.A02	10732	p2	(TC)7	14	59695460	59695473	TGACCCCTCAGGATCTATGG	TTCAGCCAACGGGGTATAAG	275
Ad02A10733	Aradu.A02	10733	p2	(TA)7	14	59716029	59716042	GCAAACGAGATATCCATGTAACC	TTGATTACTATGACAGTCGCAGG	255
Ad02A10735	Aradu.A02	10735	p3	(TAA)25	75	59720145	59720219	AAAGTGAGGTGTTAATTTCTCATGAAT	TGAAGTTAGGTAGATAATTTTTGGCA	191
Ad02A10738	Aradu.A02	10738	p2	(TA)6	12	59724231	59724242	AAGATCCTAAATGATTCTGTTTCAA	TTTTGGTTCGCCTGTCCTAT	266
Ad02A10739	Aradu.A02	10739	p3	(TGT)5	15	59726154	59726168	GCACCAGAAGTGCTGATCTCT	TCTCATCCTTTCCGAACACC	124
Ad02A10746	Aradu.A02	10746	p2	(AT)7	14	59747773	59747786	GATACTTTTTATATTTGTATTGCTCGC	CAGATGGAAAATCTAATCCACCA	240

ID	Chromosome	SSR nr.	SSR type	SSR	Size	Start	End	FORWARD PRIMER (5'-3')	REVERSE PRIMER (5'-3')	Product (bp)
Ad02A10747	Aradu.A02	10747	c	(TCT)5gggtttg	128	59752779	59752906	TCTGCTTCTTGCTTCGATCTC	ACCAACATCAACACCACACC	272
Ad02A10748	Aradu.A02	10748	p3	(TGG)6	18	59753091	59753108	GGTTGTGGTTGTGGTGTTGA	TGGTTTGCTTTTGTTTCGTC	274
Ad02A10750	Aradu.A02	10750	p2	(AC)6	12	59759948	59759959	CACCCTTTTATCCCAACCAA	TGAGGCCATCATTTCATTAGC	235
Ad02A10751	Aradu.A02	10751	c	(TA)12tg(TA)(	38	59760430	59760467	TCAATTGAGGTGTCAATGCC	AGTTACCGTACCCCCTTGCT	272
Ad02A10756	Aradu.A02	10756	c	(T)10gaaaaaga	37	59790060	59790096	CATAGCCAAAGGGGTGAGAG	AACGAGGATTTATCCGCAAA	240
Ad02A10757	Aradu.A02	10757	p2	(TG)6	12	59792417	59792428	AGTGTCCCAAAGAGGTGCTG	CCCAGTTGGTTCAAGATGCT	142
Ad02A10759	Aradu.A02	10759	c	(A)10gaagaaaa	99	59799166	59799264	TGTGGGAGAACTTTTGGGAA	TGGGATGACTTTTGTCCCAT	272
Ad02A10761	Aradu.A02	10761	p3	(TCT)5	15	59811499	59811513	CCACAGGATCACCTCAGGAT	CGTCCTCGAGCAAGAGAAGA	259
Ad02A10763	Aradu.A02	10763	p2	(AT)6	12	59816365	59816376	GCCTTTAGCTACGCTGTTGG	TCGGTTGTTCAGGTATCCCA	184
Ad02A10764	Aradu.A02	10764	p2	(TA)6	12	59819103	59819114	GCAGCAGGCACTGAAAAATA	AGATGCAGCCTTGGGTTAAG	231
Ad02A10766	Aradu.A02	10766	p3	(GAA)5	15	59838048	59838062	AGACAAGGGGTGTTGACTGG	CCTCTAGTTGGGTTGCCTCC	142
Ad02A10768	Aradu.A02	10768	c*	(A)10(AAT)5*	23	59844446	59844468	CCTAACCACTTTTTCTCTCCTCA	CACATGACCCCAGAGTCCTT	277
Ad02A10770	Aradu.A02	10770	p2	(TC)6	12	59855477	59855488	CTTTCCTCCTCTGTTGCGTC	AGTCATCGTCGTCGTTTGTG	220
Ad02A10771	Aradu.A02	10771	c	(CT)8gcgtttcttt	47	59855781	59855827	CACAAACGACGACGATGACT	CCTTTCCTCAGCCACTGAAC	231
Ad02A10775	Aradu.A02	10775	p2	(AC)12	24	59899290	59899313	ATGTTTCCCACAAATTCCCC	ATGGCCAACCCTTACATCAA	199
Ad02A10776	Aradu.A02	10776	p2	(AG)6	12	59918293	59918304	TGCTTTTAGATGGGAAGCAA	GGCTATAAAGTGGGAGAATGC	277
Ad02A10778	Aradu.A02	10778	p2	(AT)8	16	59923251	59923266	CCAACCTTGATGCCTGTTCT	ATCCTAACGTCAAAATGGCG	268
Ad02A10779	Aradu.A02	10779	p3	(TCA)6	18	59928346	59928363	TCACTCACCATTCCCATCAA	GGTTGAATGATGTGGCGTAA	241
Ad02A10780	Aradu.A02	10780	p2	(TC)8	16	59928954	59928969	ATGGCTGTAAACGGTTCGTC	ACGGACCAAAAATGCTAACG	228
Ad02A10781	Aradu.A02	10781	p2	(AT)10	20	59932383	59932402	TATATTTGCTTTGGCCGGTT	TTGAGGTGGCAAGGTATTACG	233
Ad02A10782	Aradu.A02	10782	p2	(AT)8	16	59935557	59935572	GACCCTCTATTCCCGCTTTC	GGTGGTGTGGTATTACGACCTT	194
Ad02A10783	Aradu.A02	10783	p2	(TA)7	14	59938569	59938582	TTCATGCATCACCCACTAGA	AACTTTCCTTCAAAAGGCTCC	268
Ad02A10787	Aradu.A02	10787	p4	(AAAG)5	20	59992113	59992132	TGCTTAAACCATGTGCATTGA	TACTTTGGGGCATTTTGTCC	111
Ad02A10788	Aradu.A02	10788	p3	(AAG)5	15	60010024	60010038	CCAAGGTTCACATAAAACCCA	GGAGTCTTCGGACCCTCTCT	263
Ad02A10792	Aradu.A02	10792	p3	(TAT)12	36	60061693	60061728	CTTGGTTGAGCCACTTAGGC	TGAATCAAGAAAAAGCAGCAAA	280
Ad02A10793	Aradu.A02	10793	c	(A)12ttaacctttg	116	60070267	60070382	TGAATGCCAATAGCGTAGCTT	GATGGATGAGCATATGTCCAGTA	265
Ad02A10795	Aradu.A02	10795	c	(CT)6ttctctctct	43	60079696	60079738	CCGTGATGCATTACCATCAG	AGAGAGAGGGAATCGTGGCT	261
Ad02A10796	Aradu.A02	10796	c	(TG)24ta(TG)7	64	60079860	60079923	ACTGAAACCATCCTGCCCTT	ATTGCTGGTTAGTTGGACGC	180
Ad02A10798	Aradu.A02	10798	p2	(AG)7	14	60082925	60082938	TTGGAGGTTTGCTTGTCCTC	TACACGTTTGGCTCCCTCTT	152
Ad02A10800	Aradu.A02	10800	p2	(AT)8	16	60098106	60098121	TTGATCAGATCCTCTGGGCT	AGTTGCAAACGTGTGCCTTA	280
Ad02A10801	Aradu.A02	10801	p2	(CT)7	14	60098775	60098788	ATAAGCCAATTGGGCAACAT	TGACCTAGTTTTTGGCCCAG	279
Ad02A10802	Aradu.A02	10802	p2	(CT)6	12	60100520	60100531	TGGCCCAAATCCAAGTTTAG	CAATTGGGCAACATCAATCA	280
Ad02A10805	Aradu.A02	10805	c	(T)11gtaaaaata	57	60137320	60137376	TTGCAAATGTTAGAAACAAAAGAA	GTTGGCCGCAATGTTAATTT	278
Ad02A10806	Aradu.A02	10806	p2	(TA)6	12	60140262	60140273	AAACTACATTTGTTCAACTTCTCTTTT	AACAAATATTGCATTGGAGTGAA	241

ID	Chromosome	SSR nr.	SSR type	SSR	Size	Start	End	FORWARD PRIMER (5'-3')	REVERSE PRIMER (5'-3')	Product (bp)
Ad02A10808	Aradu.A02	10808	p2	(AT)11	22	60147079	60147100	TTTTATCTACGGAGATCCTGATTTTT	TTGTTGCAGAAATTCAATCCA	265
Ad02A10809	Aradu.A02	10809	p2	(AT)8	16	60147759	60147774	ACTTGGCATCATCGACAACA	TTTGGCAATTTTGACACCAA	183
Ad02A10812	Aradu.A02	10812	p3	(TAT)10	30	60155390	60155419	TCTATTTAAAAGGGGCGTGTT	GACGGCTTAAAAATTGTTGTGA	277
Ad02A10821	Aradu.A02	10821	c	(A)10tcat(AAT	34	60168279	60168312	TTTGTCTATATCCGCAACTCCT	GGAAAGTTTTGGGCCTCAGT	279
Ad02A10824	Aradu.A02	10824	p2	(AT)6	12	60177617	60177628	GTCAACCCAAGGGATTTGAA	AAGAGAGAGAATTATTTTGGCGT	270
Ad02A10830	Aradu.A02	10830	c	(TCT)7(CTT)5	74	60199157	60199230	CGCGCATTAACTCATTTTGA	CCTCCTCATCTTCTGCTGCT	194
Ad02A10831	Aradu.A02	10831	p3	(AAG)5	15	60199967	60199981	TTTCTCCTCCTCATCTTCTGC	CCACATACTGTTACGGCACG	246
Ad02A10832	Aradu.A02	10832	p3	(CAT)5	15	60200665	60200679	TCAGCTATAATCCTCAGTGTCATCTT	GCGTCAAATAGTTTCGTTGTTG	120
Ad02A10834	Aradu.A02	10834	p2	(GA)17	34	60205187	60205220	GGTGGAGGAAGGTTGTGCTA	CCTTCCTTTAAGCTTGGGCT	225
Ad02A10835	Aradu.A02	10835	p2	(AT)6	12	60216749	60216760	TGTGGTTCATGAATGTTGGC	TTATTTGCCGTGGATCACAA	207
Ad02A10837	Aradu.A02	10837	p4	(TAGT)5	20	60244357	60244376	TCATCCAACACTCCCAAACA	AAGCATGAAAAACTCAAAACCAA	253
Ad02A10839	Aradu.A02	10839	p3	(AGA)5	15	60275076	60275090	CTCGGAAGTGGAAGCTTTTG	CTCTCCTATAGCCGAACCCC	214
Ad02A10841	Aradu.A02	10841	p2	(TG)6	12	60295060	60295071	ATGCACTGCCCAGTAAAACC	AATACCCAAGCCGAAGACCT	147
Ad02A10842	Aradu.A02	10842	p3	(ATA)12	36	60299913	60299948	AGCATGCATTCTCTCCTGGT	TTTACCCATTTTTCAAGACTCC	280
Ad02A10843	Aradu.A02	10843	p2	(AT)8	16	60305869	60305884	AAGCATTGAACCCTCACTGG	TTCAATTCATAGCTCACTTAGCC	232
Ad02A10846	Aradu.A02	10846	c	(TTA)5tttcga(A	33	60324388	60324420	GGTGTTCCTTGGTGTTTTCC	CCATGATGGAGAAAGGGAGA	203
Ad02A10848	Aradu.A02	10848	c	(A)10cgaaaaaa	66	60342301	60342366	TGTGGTTCATGAATGTTGGC	GGATCCTTTTTACCCTTGCC	234
Ad02A10849	Aradu.A02	10849	p3	(TTC)5	15	60346419	60346433	CTATACCTTGGCCGAATCCA	GCTTTTGTCTTCCCTTTCCC	245
Ad02A10850	Aradu.A02	10850	p2	(TA)6	12	60362934	60362945	TTTGAGCCTTTTAATCCCCA	CCCCACTCTCTTAGCCACTG	252
Ad02A10852	Aradu.A02	10852	p2	(TA)7	14	60386264	60386277	TATGCTCAGAATGCCCAACA	TGTCAGAACACCCATCCAAA	163
Ad02A10854	Aradu.A02	10854	p2	(TA)10	20	60390604	60390623	AACCATGGCTTGAACACCTT	GGAAGCAAGTCCAGTTTCCA	206
Ad02A10863	Aradu.A02	10863	c	(A)11gagag(A)	27	60479064	60479090	TGTGGTTCATGAATGTTGGC	TGGTTCTTAAGCCCCCTCTT	231
Ad02A10868	Aradu.A02	10868	p2	(AT)6	12	60532660	60532671	TCGATGGACTGGCATAAAATC	TTGGCCATATTCATATCCTGA	177
Ad02A10871	Aradu.A02	10871	p2	(CT)7	14	60586937	60586950	AAAGGAATAAAAACCGGCGT	GGGTTTTTGGGGAAGAGGTA	253
Ad02A10873	Aradu.A02	10873	p4	(ATAC)7	28	60603378	60603405	GGGGAAGTGTCATTAAGGTGA	CTTTGCATTGCTTCACTGGA	236
Ad02A10876	Aradu.A02	10876	p3	(TTC)5	15	60646118	60646132	CCCCAATTTGAGAAAGGTCA	TCTCATGCTGAAACTGCTGC	229
Ad02A10878	Aradu.A02	10878	p2	(GT)6	12	60656913	60656924	CAAGAAATGCGTAGCTGCTG	CGGGCTAAAAACACTAACGG	214
Ad02A10882	Aradu.A02	10882	p2	(TA)6	12	60714982	60714993	CCTCTTGACAATTAGAACTCCTCA	TCACCAGAAAATCCGTTTTG	280
Ad02A10883	Aradu.A02	10883	p2	(AT)9	18	60722293	60722310	TGCTGAAACCTTGAGAAGGG	TAGGCATGGGACCCAAAATA	280
Ad02A10885	Aradu.A02	10885	c	(GA)6aggg(GA	28	60727188	60727215	AGCAAGAGGGGGAGAGAGAC	GAGCCCCAATAGAGACCACA	217
Ad02A10887	Aradu.A02	10887	p2	(TG)8	16	60752289	60752304	CAGCCTCACTCTCGGATCTC	TAACTTAGCCACCGCCTCAC	118
Ad02A10889	Aradu.A02	10889	p4	(AAAT)6	24	60790210	60790233	ACACATTGGGATGATTGGGT	TTTCATGCAGTGCAACTTGA	256
Ad02A10892	Aradu.A02	10892	c	(ATA)6aatcccc	80	60792639	60792718	CCATATCTGACACCACACCG	AGCTTTACAACGATGGCGAC	239
Ad02A10893	Aradu.A02	10893	p3	(AGA)6	18	60804166	60804183	TGCCATTTGATGGTAGTGGA	CAACTTCTCTTTCCCACCCA	250

ID	Chromosome	SSR nr.	SSR type	SSR	Size	Start	End	FORWARD PRIMER (5'-3')	REVERSE PRIMER (5'-3')	Product (bp)
Ad02A10895	Aradu.A02	10895	p2	(AT)8	16	60839378	60839393	AGCACGTTCATCTCGCTTTT	GAATTGCTAATGCTTCCTCAAA	253
Ad02A10896	Aradu.A02	10896	c	(AAT)6(ATT)(	36	60839538	60839573	AAGCATTAGCAATTCTAAGTTCATCA	CTTCGAACCCCGAATTAAAA	195
Ad02A10898	Aradu.A02	10898	p4	(AAAT)5	20	60862170	60862189	TGGAGTTTGAAATTGACTCATCA	CCTCTCTTTCTTTCTTTCATACGC	238
Ad02A10899	Aradu.A02	10899	p4	(AAAT)6	24	60867315	60867338	AGAGCTTCGTGCTGGTAACG	CCTCTCTTTCTTTCTTTCATACGC	185
Ad02A10902	Aradu.A02	10902	p3	(TTC)5	15	60886742	60886756	CCCATAAACCCCACCTACCT	CGTCCTCGAGCAAAGAAAAG	229
Ad02A10904	Aradu.A02	10904	p3	(AGA)6	18	60902819	60902836	GGTCTTGATGGCTCTTGGAG	CCAAACCTACATCTCCCCCT	246
Ad02A10905	Aradu.A02	10905	p3	(GAG)6	18	60912110	60912127	AGCTCAACCACGTGAGGACT	AAGCTCATCCAATCTGGTGC	233
Ad02A10907	Aradu.A02	10907	p2	(GA)9	18	60921262	60921279	TGAGAAAGAAGGGAACGGAA	ACCCACCAATACCTTTCCAA	120
Ad02A10913	Aradu.A02	10913	p2	(AT)11	22	60955359	60955380	TCAATTTCTCATGCAATACATCAC	GCCACCAGCATAACTGAGGT	222
Ad02A10914	Aradu.A02	10914	p3	(TTC)5	15	60956542	60956556	GTGGTCTTGCTTGTGTGCAA	AAAGAAGTGCTTGTCGGGAG	259
Ad02A10916	Aradu.A02	10916	p3	(TCT)5	15	60978062	60978076	CGAACCTACTTCCTCCCCTC	GGATCTCTTGATTTGCAGCC	272
Ad02A10923	Aradu.A02	10923	p3	(TTA)6	18	61051822	61051839	AAGCTGCTTGCTTTAATGGG	TTCTCTTTTGGTTTTCGAATTTTT	242
Ad02A10925	Aradu.A02	10925	p3	(TAT)6	18	61054166	61054183	TGTTGCTTGTGGATTGTTTAGG	CCATATGGGCATTTTCACAA	140
Ad02A10926	Aradu.A02	10926	p2	(GT)6	12	61059361	61059372	AAATAGTGCCACCACTTGGC	ACAACTTTCGTTGGGACCAC	278
Ad02A10927	Aradu.A02	10927	p2	(AG)6	12	61066642	61066653	TGGAGGAGAGGGAAATGAGA	CCTAAATGGAGCAGGGATGA	207
Ad02A10928	Aradu.A02	10928	p2	(CT)16	32	61074570	61074601	CCCCTTCTTCTTCCTCCTCA	ACTGCGTGACTGACTGGTTG	274
Ad02A10929	Aradu.A02	10929	p2	(AG)17	34	61075391	61075424	AGACGCAACTCGTAAGCACA	TCTCAAGGAACAAAGTGGGG	252
Ad02A10930	Aradu.A02	10930	p3	(TTC)5	15	61075861	61075875	CATGGCATAAAAGTGGGGTT	CCACTAGAGAACAGGGCAGC	239
Ad02A10933	Aradu.A02	10933	c*	(TATT)6(TTA	66	61085066	61085131	CCCCCGTCCAAAAATAAAAT	TGGGTTGTCAAACTTCCTCC	175
Ad02A10934	Aradu.A02	10934	p3	(AAT)10	30	61085400	61085429	CTCGGTTTCGGACTAATGGA	AGCCAGTCAAGCTTCCACAT	237
Ad02A10938	Aradu.A02	10938	c	(A)19gac(A)11	114	61089794	61089907	CCTTTATTCATCACCAATTGAGC	TGGCCAAACTCAAAGAGAAAA	275
Ad02A10939	Aradu.A02	10939	p2	(AC)14	28	61099049	61099076	CTTGGTGTTTTCCCTCCAAA	AGGAGAGAGAAAGTGGTTGGG	252
Ad02A10940	Aradu.A02	10940	p2	(CT)6	12	61109330	61109341	TGCAGGGATGACTTTTCCTT	TTTGGGGAAGAGGTGTTGAG	123
Ad02A10941	Aradu.A02	10941	p3	(ATT)6	18	61127852	61127869	ACTCCCAGACAGTGGTGCTC	CCTTGAAACAAAAGAAACAGCA	252
Ad02A10944	Aradu.A02	10944	p3	(CTT)8	24	61154456	61154479	TTTCTTCCACCCAATCCTTG	AGGAGAAGGTGGTGGTGTTG	270
Ad02A10946	Aradu.A02	10946	p3	(ACA)6	18	61163869	61163886	TGACAACTGGAGTTGCGATT	TACATCCTTTTTAACGCGCC	259
Ad02A10947	Aradu.A02	10947	c	(T)13attattaa(T	31	61165184	61165214	CAATCCCAGGTTCCCAACTA	GACACCATGACCAACAGTCG	177
Ad02A10949	Aradu.A02	10949	p2	(AT)7	14	61178738	61178751	TGGGGATTGCCTCATTAAAA	GTGCGAACGTTTCACGTATG	202
Ad02A10950	Aradu.A02	10950	p2	(AT)10	20	61187273	61187292	TTGACCAGAGTCTCGTGCTG	CACACAAAATCAATAACAATTCTTCC	243
Ad02A10954	Aradu.A02	10954	c	(AT)7(AG)24	62	61212147	61212208	TTGCTTGGAATTTGATTGTTTG	TGGACTCATCAATTCTCCCA	249
Ad02A10959	Aradu.A02	10959	p2	(CT)7	14	61265337	61265350	CATTGAACCTGGAATTGGAAA	TCTGCGAAATTTCAGACGTG	230
Ad02A10962	Aradu.A02	10962	c	(A)10ttagtgaat	70	61275020	61275089	TTTCTTTTTCAATTGGATCCCT	TCACCGAATTTTCAATTAGATCC	165
Ad02A10965	Aradu.A02	10965	p2	(AG)9	18	61283452	61283469	TGATGCTTTGGTTTCACATTTC	CTTTCTGGTGCTTTGGTGGT	153
Ad02A10966	Aradu.A02	10966	p2	(TA)6	12	61286930	61286941	TCGTGTTTACAGCTGTTCCG	GTGTGGAGGGTTCAAATGCT	258

ID	Chromosome	SSR nr.	SSR type	SSR	Size	Start	End	FORWARD PRIMER (5'-3')	REVERSE PRIMER (5'-3')	Product (bp)
Ad02A10969	Aradu.A02	10969	c	(A)14gaagagtt	55	61290040	61290094	TGGGTGTGTGACTGTGTACG	AGTGCCTCCAACAATCCAAC	254
Ad02A10971	Aradu.A02	10971	p2	(TG)7	14	61298899	61298912	TCTTTTCTCTTCCCAAGGCA	CAAAGTGTTGGGTGGGAGAC	230
Ad02A10975	Aradu.A02	10975	p3	(AAT)6	18	61323304	61323321	GGCGCAGATCCTGAAAATAA	TGCCAAAGAAAATGCTGATG	243
Ad02A10977	Aradu.A02	10977	p2	(TA)10	20	61331658	61331677	TGACGAAGATGAAAGAACACCTT	GGGTCATTGCTGTTGCTTTT	175
Ad02A10979	Aradu.A02	10979	c	(T)10gtgcctcct	71	61349973	61350043	AAGCAGATCGAAGGCAAAGA	TCATCTTCGCGTGTTTCATC	212
Ad02A10983	Aradu.A02	10983	p3	(AGC)5	15	61361650	61361664	TGAGAGAAAAAGTGTAATGAAGGG	ACTTTTGTGCCATTTTTCCG	269
Ad02A10990	Aradu.A02	10990	p3	(TTC)5	15	61389378	61389392	CCTTGGCCGAATACACATCT	ACTCGGAGGTGGAAGCTTTT	209
Ad02A10992	Aradu.A02	10992	p2	(TA)6	12	61406386	61406397	TTGTAACGTGTAATGGCCGA	GAAATGAAGCGCGAAATGTT	250
Ad02A10994	Aradu.A02	10994	p2	(TA)6	12	61408958	61408969	TCTCGTTCTTCCTCTGTTCCTC	CAGCCTTTTAGTCTCTCAGATCAA	248
Ad02A10998	Aradu.A02	10998	p5	(AATAA)5	25	61431665	61431689	CGCTATCTCCCATCTCCTTG	GTGCTAGGTGGGAGACAACC	233
Ad02A10999	Aradu.A02	10999	c	(A)10gagaagaa	54	61446271	61446324	TGTGGTTCATGAATGTTGGC	CAATTAGCGGTGTCCAAGGT	254
Ad02A11001	Aradu.A02	11001	p2	(TG)7	14	61453803	61453816	GAATTTGGTTTGCCCTTTGA	CCGATCTCAACTATTCGCGT	259
Ad02A11004	Aradu.A02	11004	p2	(AT)9	18	61467783	61467800	TGCTCAAAGCCTTGAATCCT	TGAAGCAAGAAAAAGCAGCA	193
Ad02A11006	Aradu.A02	11006	p2	(TA)6	12	61480850	61480861	GCTAGGCGTCTGGATCTTTG	AAATCGCAAAATGAAAAGCG	271
Ad02A11007	Aradu.A02	11007	p2	(AT)7	14	61481004	61481017	TTACCCGTTTTGTTCAAGGC	CTCAGAGTATGCCCAGAGGC	224
Ad02A11016	Aradu.A02	11016	p3	(CTC)5	15	61545304	61545318	AATAACCCCTTCCAACCACC	GCTGGGATCATTGTAGGGAA	240
Ad02A11021	Aradu.A02	11021	p3	(TTC)5	15	61575932	61575946	CACGGTCATCCCTTCTGTTT	GGCCTTAGGTGCCATCAATA	266
Ad02A11023	Aradu.A02	11023	c	(TA)6ct(TA)8	30	61599593	61599622	CCATACTTGTGTCATTGTGCATC	TCCCCATATCGTTAAACCCA	208
Ad02A11024	Aradu.A02	11024	p3	(CTT)5	15	61610714	61610728	GCATCTGCTTCTGATTCTGCT	CCACCTCCCTTACTTCACCA	259
Ad02A11026	Aradu.A02	11026	p3	(TTG)6	18	61627241	61627258	GAATTCAAAGCCGAGATTGC	TTGCTCCTTTTCGCGTAGTT	243
Ad02A11030	Aradu.A02	11030	p3	(CAA)5	15	61659491	61659505	AAAGGGCAACTGCTGAGAAA	ACAATTGTAGCACAAGCCCC	242
Ad02A11031	Aradu.A02	11031	p2	(AC)7	14	61663483	61663496	TTAAAGAAATCGGCCGAAGA	GATTTGGGAACCAGGGTTTT	186
Ad02A11032	Aradu.A02	11032	p2	(TA)6	12	61669678	61669689	GACTGGTTATCTTCGCAGCC	AAAATCGCAACTCAAAAGCG	140
Ad02A11034	Aradu.A02	11034	p2	(TA)6	12	61679567	61679578	TATTTGCTTTGTCTTGGCCC	GGATTCATCAATTTCCCCAC	235
Ad02A11036	Aradu.A02	11036	p3	(AGA)7	21	61690430	61690450	GCTTTTGTCCTCCCTTTTCC	ACAACCCCCTCTTCCATACC	217
Ad02A11037	Aradu.A02	11037	c	(AAG)6ataaga	83	61701928	61702010	TGATGGAAGATGTGAGTGGG	ACTTACGCATCGCTTGGAAT	272
Ad02A11038	Aradu.A02	11038	p2	(TA)9	18	61712961	61712978	ACTTGCATGCCATTATGCCT	CCTTCCCCTTTCCTTTCTTG	237
Ad02A11039	Aradu.A02	11039	p2	(TA)8	16	61714174	61714189	TCCTCCTACTTCCTTCCCGT	CCTTCCCCTTTCCTTTCTTG	269
Ad02A11042	Aradu.A02	11042	p2	(AG)10	20	61755056	61755075	TGGATCAGCCTATCCTCACC	TCTTCTCTTTGACGATGGGG	206
Ad02A11043	Aradu.A02	11043	p2	(TA)8	16	61757185	61757200	GTCAGTTGCCATGCTACCCT	TGCTTTGGCTTCTATGAACG	240
Ad02A11045	Aradu.A02	11045	p2	(AT)6	12	61761588	61761599	TGCATGTTAGTTTTCTGGAATTGTA	CAAGAAAATCAAATTTAAAATGCAC	248
Ad02A11046	Aradu.A02	11046	p4	(TAAT)9	36	61768192	61768227	TCGTAAAACATAGGTGGTGAGG	TGCTGGTCGAGCTAGGAGTT	179
Ad02A11047	Aradu.A02	11047	p3	(ATC)5	15	61773168	61773182	CCCACACAATTCAAAAACAAAA	TTTGAGGTTGTCGTTGATGC	264
Ad02A11057	Aradu.A02	11057	p3	(GAA)5	15	61818032	61818046	GCTCCTTTGGGATTTTCACA	CGAGATGATCCACAACATGC	280

ID	Chromosome	SSR nr.	SSR type	SSR	Size	Start	End	FORWARD PRIMER (5'-3')	REVERSE PRIMER (5'-3')	Product (bp)
Ad02A11060	Aradu.A02	11060	p3	(AGA)6	18	61869279	61869296	CTAGAGGATTCTCCGGCCTT	TTCTACACCTTGGCCGAAAC	179
Ad02A11061	Aradu.A02	11061	p3	(AGA)9	27	61877769	61877795	AAGTGGTCTTGATGGGCTTG	AAACCCACACCTCTCTCCCT	256
Ad02A11065	Aradu.A02	11065	p3	(CAA)5	15	61933314	61933328	TAGAGGTCCGCCTTTCAAGA	GCACCCTGTTTCTGCTTCTC	225
Ad02A11068	Aradu.A02	11068	p2	(TC)8	16	61952162	61952177	TTTGGCTTTTTCTGCTTGCT	TGGCTCTTGAAAGAATGATGG	161
Ad02A11069	Aradu.A02	11069	p3	(AAT)6	18	61977524	61977541	CTGTGGGATTCGACCTCACT	TTTCTGGTTTACTTTGATCTTTTGC	211
Ad02A11071	Aradu.A02	11071	c	(AT)11(AC)9	40	61995445	61995484	AGAACCCCATTTGGAGATGC	GGGTTAAGCATGCGTTGATT	128
Ad02A11075	Aradu.A02	11075	c	(T)10cgaaa(T)	25	62046720	62046744	AGCACACTCTTGTTGTCTTGGA	TGGCTCTTGAAAGAATGAGGA	189
Ad02A11077	Aradu.A02	11077	p3	(TCT)5	15	62078805	62078819	CACAATGCAGGCTAGCAAGA	GAGACAGTTGGACTCTCGGG	263
Ad02A11078	Aradu.A02	11078	c	(CTT)6tttgctga	145	62082831	62082975	AGAAAGAACGGGGGATTCAG	CGTCATCCTCATCAAAACCC	256
Ad02A11079	Aradu.A02	11079	c	(GTG)6atggg(	119	62083122	62083240	GTGTGATGGATGGTGATGGA	GGACCAAAATGCCCCTATCT	259
Ad02A11081	Aradu.A02	11081	p2	(CT)10	20	62088493	62088512	CTTCTTCCTGTCCGGCTCAT	GAAGAGAGGGAGATCCGAGG	271
Ad02A11085	Aradu.A02	11085	c	(T)10ataaaaaaa	55	62097493	62097547	GCAGTACCTTGGGAGGTCAA	TTTATCCCCACCTAGTGGCA	245
Ad02A11088	Aradu.A02	11088	p2	(AT)6	12	62101892	62101903	CACATATCCACACACACGCA	GCACGACTCAGGCTTATTGG	230
Ad02A11091	Aradu.A02	11091	p2	(AT)7	14	62108903	62108916	TTGTTCAGTTTGTGACTCCCC	CAACATCCAAGTTTTTGAACCA	273
Ad02A11092	Aradu.A02	11092	p2	(AT)11	22	62113758	62113779	ATTGCAAATAAGTGGAGCGG	GTAGCCCAAATCCTCACCAA	186
Ad02A11093	Aradu.A02	11093	p3	(AAT)5	15	62114613	62114627	AGTTCCGCCTAATTGGATCA	TTTTGGTGTGTTACTGAATGATTATG	181
Ad02A11094	Aradu.A02	11094	p3	(AGA)6	18	62119150	62119167	TGGCCTTTTAGGTGCCATTA	AATGGCCGAACATACACTCC	247
Ad02A11101	Aradu.A02	11101	c	(A)15cgagcctt	93	62204931	62205023	TGGGTTTGGGCCAACTTAT	CATTTCTCAAAATTAATACCAAATACG	265
Ad02A11105	Aradu.A02	11105	p2	(GA)6	12	62215187	62215198	CTTTCTTTCCAATGGGAGCA	GAAGGGAAATGTGATGTGGC	215
Ad02A11109	Aradu.A02	11109	p2	(TA)6	12	62219941	62219952	GCGAACGATGATGATGAATG	TGGTGCTTTTTAATGGCAGA	280
Ad02A11111	Aradu.A02	11111	c	(CAA)12t(AA	52	62225620	62225671	CGTTGGTCATTGTCGCATAG	TGCTCATTCGTGAGAAGGTG	244
Ad02A11112	Aradu.A02	11112	p3	(CCT)6	18	62226835	62226852	TTCTCCTCTTTTGTTCTTGCC	CTGGTAGAAAGGGGGATGGT	279
Ad02A11113	Aradu.A02	11113	p2	(AT)9	18	62246727	62246744	CGCATCGTAAAGCAAAGGTT	GGCTAGCCGGCTTAAACTCT	262
Ad02A11115	Aradu.A02	11115	p2	(AT)9	18	62256524	62256541	CGCATCGTAAAGCAAAGGTT	GGCTAGCCGGCTTAAACTCT	257
Ad02A11116	Aradu.A02	11116	p2	(CT)7	14	62276037	62276050	GAGGAATTTTCCGCAACTGA	CAGATCCAAGCCCAGAGAAC	280
Ad02A11117	Aradu.A02	11117	p3	(AGA)5	15	62286301	62286315	TACCTTTTCCGGCCTTAGGT	ACACACTACCCCCTCTCCCT	242
Ad02A11120	Aradu.A02	11120	p2	(CT)6	12	62357958	62357969	TGGAAATCTGAATTGGAGAAGAA	GCCAGTTTCAAACCCAAAAA	186
Ad02A11124	Aradu.A02	11124	p2	(TA)7	14	62390014	62390027	AATGCTTCGCGCTTTTGTAT	ACCAACCGCTCTGATACCAC	201
Ad02A11130	Aradu.A02	11130	p2	(TG)6	12	62403232	62403243	TTGTCTCCTTGGATCTTGGG	TTTCACCACCAAACCCTTTC	181
Ad02A11131	Aradu.A02	11131	p2	(TG)6	12	62416855	62416866	TTCCTGTAGAGCCTGAGGGA	AAAGTCCCAAATGCATGCTC	165
Ad02A11132	Aradu.A02	11132	p3	(AAG)7	21	62424859	62424879	ACCCAATTGGATGAAGATCG	CTGAGACTGGGAGGATTGGA	244
Ad02A11133	Aradu.A02	11133	p2	(CT)7	14	62426431	62426444	CCAATTTGGCAACATAAATCAA	GAATGAAAGGGGACGACTCA	237
Ad02A11134	Aradu.A02	11134	p2	(AT)7	14	62427530	62427543	CAGGGTTGTAATGGGGCTAA	GCAAAATGAAGGATTTTGGG	150
Ad02A11135	Aradu.A02	11135	c	(TTAT)7tttttat	67	62427735	62427801	CCAAAATCCTTCATTTTGCAT	TGAAGATTGGGAAGTTTGGG	265

ID	Chromosome	SSR nr.	SSR type	SSR	Size	Start	End	FORWARD PRIMER (5'-3')	REVERSE PRIMER (5'-3')	Product (bp)
Ad02A11136	Aradu.A02	11136	p2	(CT)8	16	62445034	62445049	CCAATTTGGCAACATACATCA	AAGGCTGCAAAGTGAAGGAA	255
Ad02A11137	Aradu.A02	11137	p2	(CT)8	16	62450445	62450460	GGCAACATAAATAAAACAAAAGCA	AAATCCAAGCCCAGAAAACA	267
Ad02A11138	Aradu.A02	11138	p2	(TA)6	12	62451242	62451253	TGGAATCTTATGCAATGCAATC	TGTCTTGCAAGGTTGTGACC	213
Ad02A11142	Aradu.A02	11142	p2	(AT)11	22	62514574	62514595	TCACCTTACAAAATTTTCACCC	CGCTTTGCGCTAGACTTTTT	195
Ad02A11148	Aradu.A02	11148	p3	(AAG)6	18	62537207	62537224	ACGAGGAGAAACACGACGAT	TTCTTTCTCCATGCCTCCTC	118
Ad02A11149	Aradu.A02	11149	p2	(GC)6	12	62556918	62556929	ACAAGGCTCCTAAAACCGCT	TTTTGCCATATGTTGCTTCG	116
Ad02A11151	Aradu.A02	11151	p2	(TA)7	14	62567821	62567834	TTACTTGGGCCATTTTGTCC	TTGGAAACATTGGTTGGGAT	124
Ad02A11152	Aradu.A02	11152	p2	(AT)8	16	62568510	62568525	TTTCAATCAATTGGGGTGGT	CCTTTATTTTGGTTGACGCA	244
Ad02A11153	Aradu.A02	11153	c	(CG)6cactatgc	69	62572124	62572192	TCCACGTGGGTTGAGATACA	CCTTGAAGGAGATTGAGAGGG	189
Ad02A11160	Aradu.A02	11160	p2	(AT)7	14	62646917	62646930	ATGACATACAGGTGCGTGGA	TTCAACCACGTAATAACGCA	205
Ad02A11161	Aradu.A02	11161	p3	(AGA)5	15	62679849	62679863	CTAGAGGTTTCTCCGGCCTT	GCCGAACCCTACTCCTCTCT	257
Ad02A11162	Aradu.A02	11162	p3	(AGA)5	15	62680200	62680214	GGTGAGGTAGGTGGGGTTTT	CTTCTTCCCCTTAGCCGAAC	232
Ad02A11164	Aradu.A02	11164	p2	(CA)9	18	62689894	62689911	AGGCAAACCACTGCAAGAGT	CGCTTCCCTGGGATACTTAG	277
Ad02A11165	Aradu.A02	11165	p2	(AC)7	14	62695404	62695417	GTCCTTTGACTTACAGGGCG	GGGCTTCCCTGGGATACTTA	259
Ad02A11169	Aradu.A02	11169	p3	(TCT)7	21	62722942	62722962	CGCCTCTAACCAGCTTCTTG	TGACCAGTTAGGGGTTCAGC	153
Ad02A11171	Aradu.A02	11171	p2	(TA)12	24	62725708	62725731	TGGTGCTCTTCAAATGCTTG	AGCTGACGAAAATGGAATGG	197
Ad02A11172	Aradu.A02	11172	c	(TAT)6tg(TTA	35	62729860	62729894	AAGCTGTTGAATAGGCTGCTG	TCATGTTTGGGAAACAAGGT	267
Ad02A11173	Aradu.A02	11173	p2	(GA)20	40	62733288	62733327	CTGCCCTCCACCAGTTCTAT	TCGATGCGATTAGACACGAG	274
Ad02A11174	Aradu.A02	11174	p3	(GCT)5	15	62733484	62733498	TCTGACGGATCCTCAATTCC	GGTTCTCTCTCTGGTGACGC	256
Ad02A11176	Aradu.A02	11176	p2	(CT)8	16	62734661	62734676	GGAATCAGAGCTTGACCTGC	ATACCCGCGAAATTTCAAAC	260
Ad02A11177	Aradu.A02	11177	c	(T)10atctatgca	128	62753882	62754009	TCCCATGTGCATTGATTCTT	TTTTATGGGAACATGGGACG	223
Ad02A11181	Aradu.A02	11181	p2	(TC)18	36	62791666	62791701	TGGTTTCGAAAATAATTCGTCA	TTTTGGATACATTTTGGTAAGTGG	269
Ad02A11182	Aradu.A02	11182	p4	(GTAT)5	20	62808314	62808333	GGTTCGAATTAGCTTCGTGG	TTTCCAATGGCTCCAAAGAC	190
Ad02A11183	Aradu.A02	11183	p2	(TA)7	14	62810913	62810926	TATTTGCTTTGGCTGGTTCC	TGTCCTCCACTCTCAGACGA	242
Ad02A11184	Aradu.A02	11184	c	(TC)6tttttttcgt	49	62817784	62817832	CCGGATACATACATGCCACA	TGAAGCAAGAAAAAGCAGTGAA	254
Ad02A11185	Aradu.A02	11185	p3	(AGA)7	21	62847526	62847546	TAAAATATTGCTCGCCCTCG	CCTACCTCGCCCTCTCTCTT	231
Ad02A11188	Aradu.A02	11188	c	(T)11cttttttttc(	33	62866270	62866302	ATCTTTTTGGTGTTTTGCCC	CGAAAGCAAAGGAAGTTTCG	243
Ad02A11191	Aradu.A02	11191	p3	(AAT)6	18	62906379	62906396	CCATTTTCCAACACCAAACC	CGGTTCCTTAGGCTTGAATG	150
Ad02A11201	Aradu.A02	11201	p3	(AAG)6	18	62955717	62955734	CCAATTGCAGCACATTTCAC	TCAACCAATTCCCTCTTTGC	280
Ad02A11205	Aradu.A02	11205	p2	(AG)15	30	62967105	62967134	GGGCCCTGTAAAGATTCTCC	ACGGATTTGTGGTGTTGGAT	216
Ad02A11208	Aradu.A02	11208	p2	(AT)6	12	62971415	62971426	GAAAAGGAAGGTGGTGGGAT	TCTGGACGTCAGATTAGGGG	276
Ad02A11211	Aradu.A02	11211	p2	(AT)7	14	62973637	62973650	TGGCTAGCTGAGTGCAGAGA	TTTTCGTCCTCGCATTTTTC	245
Ad02A11212	Aradu.A02	11212	c	(T)10cgaa(T)1	24	62985869	62985892	TCCCCAATTAGAGGTGTCCA	TGTGGTTCATGAATGTTGGC	234
Ad02A11215	Aradu.A02	11215	p2	(AT)9	18	63014532	63014549	TGTTTCACCATATTTAGGGTTGG	CACAAATAAGAACAATTATGTTTGATG	275

ID	Chromosome	SSR nr.	SSR type	SSR	Size	Start	End	FORWARD PRIMER (5'-3')	REVERSE PRIMER (5'-3')	Product (bp)
Ad02A11218	Aradu.A02	11218	p2	(AT)7	14	63049679	63049692	GCGATGATCACTGCTGTAAAA	CCGAGACGTGATTTGATCCT	132
Ad02A11221	Aradu.A02	11221	p2	(CA)9	18	63063962	63063979	ACGGAATCGGTTTGATGATT	GAGTAAAAACATGTGTGTGTATGTGTG	259
Ad02A11225	Aradu.A02	11225	c	(ATC)7tttctcat	133	63096450	63096582	TTCAAAATTCTTCTCCCTCCT	TTTTGAATCGTACAGAATTTATCAGAA	261
Ad02A11230	Aradu.A02	11230	p3	(TAT)9	27	63127553	63127579	CGCATCAGAAGTCTCTTCCA	GCAACCGTCCATTTTGAAAC	268
Ad02A11231	Aradu.A02	11231	p3	(AAG)5	15	63129160	63129174	GGAGGAGAGTATGACGAGAGGA	TTTATTTTGCCATGCACCAA	203
Ad02A11232	Aradu.A02	11232	c	(A)10tcaaggttt	37	63131418	63131454	CAAACCATCTCCAATTTTTGGT	GGCAATAGCTTTGAGCCTGT	182
Ad02A11234	Aradu.A02	11234	p2	(TA)6	12	63137598	63137609	CTGGCAGCCGAAGATACCTA	CAGTTTGGCCATTAAACCAA	230
Ad02A11235	Aradu.A02	11235	p2	(AT)10	20	63139757	63139776	CCGACTGCGTCACTCTGATA	AAAAGGGATAGGAGTCATGATTGT	237
Ad02A11236	Aradu.A02	11236	p3	(AAC)5	15	63143772	63143786	GCAACCCAATAATCTGAAGAAGA	TGTTGGGTTGTTGTGATTGAA	103
Ad02A11238	Aradu.A02	11238	p2	(AT)8	16	63159223	63159238	TTCTTTCCATTTTCAATCAACTCA	TTTTCAGCACTCGCATCTTT	224
Ad02A11241	Aradu.A02	11241	p4	(TAAA)5	20	63171210	63171229	CAGGTTCAGGATCTGCTTCA	TTTTCGAATTCTCTCCCCCT	265
Ad02A11242	Aradu.A02	11242	p3	(ATA)7	21	63178412	63178432	CCAATCACTCAACCCAAGGT	TGTCACTTTTATGTTCATGCACTC	247
Ad02A11245	Aradu.A02	11245	p3	(TAT)6	18	63187967	63187984	AGCCTCTCTTGTTGGTGTTG	CCCTGCTGAGTGCTGAAGTA	273
Ad02A11248	Aradu.A02	11248	p3	(AAG)7	21	63212599	63212619	GCTTTTGTCTTCCCTTTCCC	TACACCTCCCCCTCACACTC	190
Ad02A11249	Aradu.A02	11249	p3	(AGA)5	15	63228044	63228058	TTGCCTTCCCTTTTCCTTTT	CCCCCTCCTCTCCTTCTCTA	184
Ad02A11251	Aradu.A02	11251	p2	(TA)6	12	63259519	63259530	CAAACGTTGGGGACAAAAAC	TCGGCCAAGGTATGGTTTAG	278
Ad02A11254	Aradu.A02	11254	p3	(AGA)7	21	63270510	63270530	ACCACTGAGCTATGACGGCT	ACCCCATTCACCATTCACAT	260
Ad02A11255	Aradu.A02	11255	p3	(ATT)6	18	63273747	63273764	AAACTTGGACGTTGGCTTTG	GGAAAAGGGCACTCTTGTTG	199
Ad02A11257	Aradu.A02	11257	p3	(TCT)6	18	63321686	63321703	TCTTTCCCACTCAAACCCAC	CCCTCGAGCAAAAGAAGAAA	192
Ad02A11259	Aradu.A02	11259	p5	(AAGAT)6	30	63334197	63334226	AAAGGGCCCAAATAGAGGAA	TGTAAGGGACTCCGATGCTT	262
Ad02A11263	Aradu.A02	11263	p3	(TCT)5	15	63391900	63391914	CTTCCCCATAAACCCCACTT	TAAAATATTGCTCGCCCTCG	234
Ad02A11265	Aradu.A02	11265	p3	(AGA)5	15	63413269	63413283	GTGCCATTGATGGTGAATGA	TTCCCACAACACAAAACCCT	180
Ad02A11266	Aradu.A02	11266	p2	(AT)9	18	63421306	63421323	CGCCAGTGAGCTCTTTTTCT	TTGCATGCATTATTTGTTTTGA	255
Ad02A11267	Aradu.A02	11267	p3	(GAA)5	15	63452042	63452056	ATGAGGGTTGAAGGTGTTGG	TCCCTCTCTTCTTCTTCCCC	263
Ad02A11270	Aradu.A02	11270	c	(AT)7(AC)6	26	63490575	63490600	CACGCTTTCATTGTAAGGCA	TGTTGAGGCTCCTGTTGATG	206
Ad02A11271	Aradu.A02	11271	p2	(GT)6	12	63491411	63491422	GCTACAAACGGTCCGTCAAT	CTCAAGATTGGGCTGAATGG	227
Ad02A11274	Aradu.A02	11274	p3	(TTC)5	15	63549188	63549202	ACACCACCTCTCTCCCCTTT	CGGCGTTAGATGCCATAAAT	177
Ad02A11276	Aradu.A02	11276	p4	(AAAT)6	24	63579502	63579525	TGGACCATTCACCTCAAAAAG	AAGCCTCCGTTAATAACACCC	258
Ad02A11278	Aradu.A02	11278	p2	(AG)15	30	63596891	63596920	GAGAAGCGGCGTAGGTACTG	CCCTGCTTTTGTTGGTGAGT	206
Ad02A11280	Aradu.A02	11280	p3	(TTA)5	15	63598261	63598275	CCAGCATAATTGCATTTCAAAC	AAGAAATTATTATTATGATGCCGTG	246
Ad02A11285	Aradu.A02	11285	c	(AG)15aatgaaa	108	63609785	63609892	CCAACAATTGGATGACCACA	TTCTCGGGTTTCAATCAACC	278
Ad02A11286	Aradu.A02	11286	c	(CA)7g(AC)13	110	63611072	63611181	CAATTTAGTTAAGCAAAAACTGAGC	GCTAAGATCTTGACCATTTGGG	266
Ad02A11292	Aradu.A02	11292	p2	(TA)14	28	63658847	63658874	CTCTAGCCGACCATTGCTTC	AATTGCATCACACTCGTTCG	147
Ad02A11294	Aradu.A02	11294	p3	(TTA)7	21	63674952	63674972	CCTCTCCAGAAAGCAATTCG	TTAGAAACATTTGCCCTCCC	225

ID	Chromosome	SSR nr.	SSR type	SSR	Size	Start	End	FORWARD PRIMER (5'-3')	REVERSE PRIMER (5'-3')	Product (bp)
Ad02A11295	Aradu.A02	11295	p2	(CT)6	12	63680360	63680371	CCCAAGTCCACACTGACCTT	CCCCAACCCAACAGAGTAGA	135
Ad02A11297	Aradu.A02	11297	p2	(AT)10	20	63719443	63719462	ACCCTCCAACTACCAACGTG	GGAAGCAAAAAGTGTGCGA	264
Ad02A11300	Aradu.A02	11300	p3	(AGA)6	18	63741128	63741145	TAAAATATTGCTCGCCCTCG	AAATCTTTCCCACCCAAACC	208
Ad02A11303	Aradu.A02	11303	p3	(ATT)10	30	63764483	63764512	TGTAGCATGGTTGTTTGAAAATC	TTGGTTTCTCCTTTGAGACTGA	265
Ad02A11304	Aradu.A02	11304	p3	(TTC)5	15	63779190	63779204	CCCATCACCTCTTCACCACT	TTAGAATGTTGGTCGCCCTC	279
Ad02A11305	Aradu.A02	11305	p4	(TTAT)14	56	63784037	63784092	GCCTCCCCTGTTACGTTTTT	GCAGAGAATTCCCAGTGAGG	231
Ad02A11307	Aradu.A02	11307	p2	(CT)7	14	63786355	63786368	CGAATGCGTCATTCAAGAAA	AGATGATTCGCCTCCCTTTT	280
Ad02A11308	Aradu.A02	11308	p2	(AT)6	12	63793884	63793895	CACAAGACAACGTGGCCATA	ATGTGACAAGTCGCGTTCAG	280
Ad02A11310	Aradu.A02	11310	p2	(TA)12	24	63800307	63800330	AGTTTCCTGGGTGAATGCAG	AGACCCTCTGTTCACGCATC	124
Ad02A11311	Aradu.A02	11311	p2	(TA)7	14	63800748	63800761	TGAGCATTTTGGAGGCTCTT	TCACCAGTCGGCCTAAACTC	188
Ad02A11315	Aradu.A02	11315	p2	(CT)17	34	63822023	63822056	TGGCCATGTTTTGTTACTGC	TGTGCTAAAATCCAACACTCTGA	279
Ad02A11320	Aradu.A02	11320	p3	(GTT)7	21	63848055	63848075	AGGATGAAGATGACGTTGGG	GCTAGCTGAGCCCAAATACG	192
Ad02A11321	Aradu.A02	11321	p3	(ATT)6	18	63858499	63858516	TTGGATGTTGGCTTTGTGAA	AAAAGGCGCTCTTGTTGAAA	231
Ad02A11324	Aradu.A02	11324	p3	(AGA)6	18	63867390	63867407	ACCACTGAGCTATGACGGCT	CAACTTCTCTTTCCCACCCA	266
Ad02A11327	Aradu.A02	11327	p3	(GAT)5	15	63881356	63881370	GGGTCATCTTGTGGTAGCGT	GAAGTAACCGGAGGAGGGAC	176
Ad02A11328	Aradu.A02	11328	p2	(AG)7	14	63912039	63912052	GCCATCCCTAGTGAAAACCA	AGTTTCAGTGTTTGGCCACC	237
Ad02A11333	Aradu.A02	11333	p3	(AAC)5	15	63955626	63955640	GTCAACCTTTGAGGTGGGAA	GAGCCATCAAGAATACCCCA	231
Ad02A11334	Aradu.A02	11334	p2	(CA)10	20	63956112	63956131	TTTTTGTTTGGAAAGCGTACA	AAAACGGACGAAGAAGCTCA	255
Ad02A11336	Aradu.A02	11336	p2	(CT)8	16	63966145	63966160	GATGACTGGGCGTAACTCGT	CGCCACCCCTATTCTAACAA	266
Ad02A11337	Aradu.A02	11337	c	(ATA)23acaga	114	63967595	63967708	TTTTGCCTCCCAATCATTTC	TGTAACCGAAAAGTATACACAAAACC	251
Ad02A11338	Aradu.A02	11338	p3	(TTA)5	15	63971716	63971730	TCATTTGAATTTGGATAAATTAAGG	TTGGCAAAATATTGAACTTGATAA	236
Ad02A11345	Aradu.A02	11345	p2	(AG)7	14	63997153	63997166	TTATTCAATCCTTCACCCGC	TGAAGAACATTGAACCTGAAATTG	246
Ad02A11346	Aradu.A02	11346	c	(T)10cttgtttatc	67	64008913	64008979	TCTCTCTCCTTTAATTCTTGTCAAA	TTCATTACGGTATTACTTTCATTTAGG	199
Ad02A11352	Aradu.A02	11352	p4	(TAAT)5	20	64016686	64016705	GGTCCACAAGCAGGAATCAT	AAAAAGCTCTTAATTTGGTCTGG	209
Ad02A11354	Aradu.A02	11354	p3	(AAT)7	21	64019032	64019052	CAATGAGTTGTGCAAGTTGAGG	TGCAAATTCCAATTTCAAAAGA	238
Ad02A11356	Aradu.A02	11356	p4	(TTAT)5	20	64020979	64020998	TCAATTGCACTTTGTCATGAATC	TTTGGACTTGGTTGTTGTGTATG	280
Ad02A11357	Aradu.A02	11357	p3	(CAA)5	15	64021287	64021301	GCTGGAACTTCACTGGTGGT	GGAAGGGAAACCACAAAACA	202
Ad02A11360	Aradu.A02	11360	p2	(TA)7	14	64041042	64041055	GTACCCTAGCCGGCCTAAAC	CGCGAAGACGTATCACTCAC	176
Ad02A11369	Aradu.A02	11369	p2	(AT)11	22	64077300	64077321	TATTGTGCAAATGACGCGAT	CATTGCATGCTGCATTTTTC	274
Ad02A11372	Aradu.A02	11372	c	(TA)9tg(TA)6	32	64087932	64087963	CGCTTCGCTTTTCGTAATTC	AACACCCCGTTACCCTAAGC	241
Ad02A11373	Aradu.A02	11373	c	(TA)9tg(TA)6	32	64092508	64092539	CGCTTCGCTTTTCGTAATTC	AACACCCCGTTACCCTAAGC	241
Ad02A11376	Aradu.A02	11376	p2	(AT)6	12	64096963	64096974	GACTAGCCGGCCTAAACTCC	CGTGCCGTAAATCAAAGGAT	234
Ad02A11377	Aradu.A02	11377	p2	(CA)8	16	64098137	64098152	ACTTCACCCACCCTATCGAG	TGTGATGGAGAAGCTCGTTG	100
Ad02A11379	Aradu.A02	11379	p4	(TTAA)5	20	64126735	64126754	AATTGTTTTGTGAGTTAGATAATGATG	CAAACCAGATCAGACCATGC	177

ID	Chromosome	SSR nr.	SSR type	SSR	Size	Start	End	FORWARD PRIMER (5'-3')	REVERSE PRIMER (5'-3')	Product (bp)
Ad02A11380	Aradu.A02	11380	p5	(AAGAT)5	25	64133261	64133285	CCATAGGAGCTCAAAGCAGG	CCTGGGTGCTCCAGTGTATT	197
Ad02A11382	Aradu.A02	11382	p4	(TAAT)6	24	64141869	64141892	AATGGTGGCATGCCTAAGTC	GAAGTGGGGCTTCAAACAAA	146
Ad02A11383	Aradu.A02	11383	p5	(AAGAT)5	25	64158835	64158859	CCATAGGAGCTCAAAGCAGG	CCTGGGTGCTCCAGTGTATT	197
Ad02A11385	Aradu.A02	11385	p3	(TCA)5	15	64179575	64179589	TAACCCTCGGAACAGTCACC	TGAAGTGGAGATTGAATGTGATG	263
Ad02A11386	Aradu.A02	11386	p2	(TC)17	34	64189118	64189151	TTTTTACAATCCCTCTCTCTCCC	GGCCAAAATTGTGTTGGATT	221
Ad02A11387	Aradu.A02	11387	c	(A)11gagggag	48	64200505	64200552	TGTGGTTCATGAATGTTGGC	TCCCCAATTAGAGGTGTCCA	254
Ad02A11390	Aradu.A02	11390	p2	(AT)10	20	64227443	64227462	TCCTTCTGTGCATCTGGTTG	TCGCACATACATACAAATCATCA	205
Ad02A11391	Aradu.A02	11391	p3	(TGA)7	21	64230925	64230945	TGTCCAACTCCTTTATCGCC	AGAGGCAGCCACTTCTTCAG	200
Ad02A11394	Aradu.A02	11394	p2	(CT)6	12	64249613	64249624	GGTTCATAAACCAATTTGGCA	GGCTATAAAGTGGGAGAATGC	261
Ad02A11395	Aradu.A02	11395	c	(TAA)6(TTA)!	33	64259453	64259485	TGGATGTCTGATAAATAAATAAATGGA	CTTAACAGCACACACATTGCAC	184
Ad02A11396	Aradu.A02	11396	p2	(TA)6	12	64263986	64263997	TGACCTGCTTAAATTCAGTGG	AAGGATAATTTTCTGGGCGG	148
Ad02A11400	Aradu.A02	11400	p3	(CTT)6	18	64281709	64281726	CCAACTAAGTTCAACCAAGTCTTTT	ACCCAGGCTAAGTCGGAAAT	245
Ad02A11401	Aradu.A02	11401	p3	(CTC)9	27	64284062	64284088	AATAATTGCTCGTTGTCCCG	ATATCACCCCATCAAACCCA	253
Ad02A11402	Aradu.A02	11402	p2	(TA)6	12	64300702	64300713	ATCAAGCTTCAGACCGACCT	ATGCAGATCGAGAGGCACTT	254
Ad02A11403	Aradu.A02	11403	p3	(TCA)5	15	64301718	64301732	AGCAAGTAGAATGAACCGCC	GGGTAAGGGTGTTGATGGAG	277
Ad02A11408	Aradu.A02	11408	p3	(AAT)5	15	64319328	64319342	CAACTCGGCCAATCTCGTAT	TCTTCCTGGTCTTTTCGCTC	192
Ad02A11411	Aradu.A02	11411	p4	(CATA)5	20	64352691	64352710	ACCCTATCATGACCTCCGGT	GAGGTTTAAATCGCCGTCAA	229
Ad02A11412	Aradu.A02	11412	p2	(TA)6	12	64359768	64359779	GGGAGATGGATACCCGTG	CACCCCACGGAACCTAATAA	277
Ad02A11413	Aradu.A02	11413	p2	(AT)6	12	64367904	64367915	TCAGAGTATGCCCAGAACCA	TCGTACGCAAGCAATCCTTA	270
Ad02A11414	Aradu.A02	11414	c	(TA)6ataatatta	85	64376453	64376537	GACCGTGGTTAAACCGAAAA	TGAGATAGTAACAATAAACATATGCGA	276
Ad02A11420	Aradu.A02	11420	c	(AGA)5acaaca	54	64391845	64391898	CAAAGCACACAAGTTCACACA	TGGAGTTTCATTCATTAACCAAG	280
Ad02A11421	Aradu.A02	11421	p3	(ATT)7	21	64394118	64394138	TTCTTATGCCTGAAAATTTGGT	TGCAATGGCTAGAAACAATCA	144
Ad02A11424	Aradu.A02	11424	p3	(TTC)5	15	64401474	64401488	TCACGCGCTTCGTGTTATTA	GGAGGAAGAGGTGGTGTTGA	116
Ad02A11425	Aradu.A02	11425	p3	(ATC)5	15	64402165	64402179	GCGGCAAATTTGGATGTATT	TTTTCTTCTTCTTTGTTTGATTTTT	229
Ad02A11426	Aradu.A02	11426	p2	(GA)25	50	64405023	64405072	CCCTTGGTTGTTCACCCTTA	CACAGCCCAAAACCAAACTT	275
Ad02A11429	Aradu.A02	11429	p3	(TTC)5	15	64435841	64435855	ACCACTCCTCCTCAATGCAC	CTTTCAGGTTCAGGGTCAGC	209
Ad02A11430	Aradu.A02	11430	p3	(TTA)16	48	64452411	64452458	AAATTCAAAAGAAGGGCGGT	AAATAAATAAATGGCCATCAGTAAA	237
Ad02A11432	Aradu.A02	11432	p3	(AGA)6	18	64465647	64465664	TAAAATATTGCTCGCCCTCG	ACAACCCCTCTTCTCCCACT	112
Ad02A11435	Aradu.A02	11435	p2	(AG)7	14	64475285	64475298	CTGGGCACTCTCACTCATCA	CTAGGGGGTGGTAGCCTTTC	167
Ad02A11439	Aradu.A02	11439	p3	(TAC)5	15	64495793	64495807	GAGGTGAAGAAGAACAGCCG	CAAATTGGCTAACCTCAGGG	280
Ad02A11441	Aradu.A02	11441	p3	(TTA)7	21	64501756	64501776	GCTAAACGGATTGGAAGCAA	CCCACCATAGGCATGGATAC	261
Ad02A11443	Aradu.A02	11443	p2	(AG)7	14	64502430	64502443	CGATTCGTTTGTATTAAAATGATG	CACTTCTAAGACTTCGTCCGTC	250
Ad02A11444	Aradu.A02	11444	p2	(AT)8	16	64504055	64504070	TGGATGTGTTTCCTGAGCAC	AAGTTGGATGCATGGACAAT	207
Ad02A11446	Aradu.A02	11446	c	(AGG)5gaggg	76	64516755	64516830	TGGCAGCAGAGAAAATCAGA	AGGCTGCATTTAATTTGAAAAC	277

ID	Chromosome	SSR nr.	SSR type	SSR	Size	Start	End	FORWARD PRIMER (5'-3')	REVERSE PRIMER (5'-3')	Product (bp)
Ad02A11447	Aradu.A02	11447	c	(AGG)5gagggg	76	64525831	64525906	TGGCAGCAGAGAAAATCAGA	AGGCTGCATTTAATTTGAAAAC	277
Ad02A11453	Aradu.A02	11453	p2	(GC)6	12	64580595	64580606	GCTCTTTGACAAGTCCGCAG	TCGCGTTATGAGCATATGGA	187
Ad02A11455	Aradu.A02	11455	p2	(TA)11	22	64587943	64587964	TGGCTGGGTAAATGATTGAA	CACTGTGACATGGAATGTAATTTT	280
Ad02A11456	Aradu.A02	11456	c	(TA)9gtgactc(	52	64588390	64588441	CAAATCAATCCCCAACATTTC	TCAAATGGTTTTAACCTCGAA	219
Ad02A11457	Aradu.A02	11457	c	(TTA)6ttgttg(T	81	64588705	64588785	TTTTTGCATATGACATCTTTTGA	CCTCCGTCCAAAAATAAAATAAAA	280
Ad02A11462	Aradu.A02	11462	p3	(AGT)5	15	64595368	64595382	CCTTCCTTTCTTGTCAATTTTTG	GGAAGAGTTCCTCTAATGGATAAGG	266
Ad02A11463	Aradu.A02	11463	p2	(GA)7	14	64595785	64595798	GAGGAACTCCCATCCCATCT	TTGATAACGCGCAAGAGTTG	257
Ad02A11465	Aradu.A02	11465	p3	(AAT)7	21	64638550	64638570	TTTCGTGCACCAAGTTTTTG	AGCCAGTCAAGCTTCCACAT	273
Ad02A11478	Aradu.A02	11478	p2	(TA)6	12	64735257	64735268	TTTTCCTCCACTTTTGAGAACA	TTGCCATTGCTCCCTTTATC	137
Ad02A11480	Aradu.A02	11480	p3	(CAC)5	15	64736966	64736980	TCAGAGGCTTCTTCCACCAT	GGGAAGGGGGTGAAGAATAA	243
Ad02A11481	Aradu.A02	11481	p3	(CTT)7	21	64737087	64737107	CCACCACCACCATCCTTATC	GGGAAGGGGGTGAAGAATAA	164
Ad02A11486	Aradu.A02	11486	p2	(TA)6	12	64772958	64772969	TAGCATCTCGTAAGGGAGGG	CACAACTTAGCAACACCCGTT	205
Ad02A11491	Aradu.A02	11491	c*	(TA)7(TATG)'	40	64842828	64842867	AGGATTGAGAGACAGCTCGC	GAAGCAAAGGTCGGACAGAG	270
Ad02A11493	Aradu.A02	11493	p3	(ACA)7	21	64852085	64852105	TTTTTGTGCAGGAGAATGGG	AATGGTTCAGCCCAACAAAG	243
Ad02A11494	Aradu.A02	11494	p2	(TA)6	12	64852966	64852977	CATTAGTTTGTGAGCTGACTCGAT	AAAGCCCACGAGCTGACTC	278
Ad02A11495	Aradu.A02	11495	p2	(AG)8	16	64857439	64857454	TTTTCTCCTTGCATGGCTTC	AAAATTGTGACACGCAGCAC	178
Ad02A11497	Aradu.A02	11497	p2	(AT)7	14	64876057	64876070	ACACTTCGACTGTGTCACGC	ATGATGTTCTCACCCCCGTA	242
Ad02A11498	Aradu.A02	11498	p2	(AT)7	14	64880040	64880053	CAAAAATCAAAATTTCAACAACCA	TTTGGGTATGAAATCCGTCC	222
Ad02A11500	Aradu.A02	11500	p3	(CAA)5	15	64882928	64882942	TCGTCCTTCTTCACCAAAATG	TTCATGCTTCTGATTTTGCTTC	254
Ad02A11502	Aradu.A02	11502	p3	(AAT)5	15	64887220	64887234	ATTGTTCTCAATCTCATTAGTGTCA	TGTTCGGTTTCAATCTTTTAGGA	230
Ad02A11505	Aradu.A02	11505	p2	(TA)6	12	64908003	64908014	TGCAACGATTCTTGCTCATT	AGGATTGCGGAATTAGGTGA	270
Ad02A11506	Aradu.A02	11506	c	(CT)9(AT)8c(	79	64920025	64920103	AATCGCTCCTCTTCCTGTCA	CCGTCGCCGTAGAGAGATAG	143
Ad02A11507	Aradu.A02	11507	p3	(AGA)5	15	64936762	64936776	CTCGAAGGTGGAAGCTTTTG	CACCCATATAGCCGAACCAC	214
Ad02A11508	Aradu.A02	11508	p3	(ATT)5	15	64939506	64939520	TGGATGTTGGCTTTGTGAAG	CAGGAACAGGGATAAAGGCA	200
Ad02A11510	Aradu.A02	11510	p3	(TTC)6	18	64960281	64960298	TCTACACCTTGGCCGAAAAC	CGACCTTAGGTGCCATTGAT	167
Ad02A11514	Aradu.A02	11514	p4	(TTAT)5	20	64990374	64990393	TGAACCATCCATCAATTAGAGGT	CAGATGCCATGACAAAACCA	280
Ad02A11517	Aradu.A02	11517	p3	(ATA)5	15	64994702	64994716	AAAATCACTCAAATTTCAATCCAA	AATGGACATACAAGAGCGGG	240
Ad02A11518	Aradu.A02	11518	p3	(ATT)5	15	64998070	64998084	TTTGGTTTTAGTGGGCCATC	GGGATATGATCCTTCTCGGA	272
Ad02A11520	Aradu.A02	11520	p2	(AT)10	20	64999032	64999051	CCAATGAAAACAAGGCCATC	CGCAATATCTCCAACACACA	280
Ad02A11522	Aradu.A02	11522	p2	(CT)6	12	65002438	65002449	GGCTCAGGGGACACAGATTA	GCCAGTTGGGCAACATAAAT	270
Ad02A11527	Aradu.A02	11527	p3	(GAA)6	18	65072007	65072024	ATCTCCCAAGGCTCAGAGGT	CAACTTCTCTTTCCCACCCA	241
Ad02A11531	Aradu.A02	11531	p2	(AT)9	18	65090864	65090881	TCCCTGGGTAAAGTACCGTG	TGATGCTTGCCTGTCCTATG	198
Ad02A11532	Aradu.A02	11532	p2	(TG)10	20	65093122	65093141	TCCTTAGGCGTCTGAGCTTC	TCAAGCTCCTAACTCAGCCC	242
Ad02A11536	Aradu.A02	11536	p2	(AT)7	14	65116200	65116213	GAAATAAAAGCGCAACGCTC	CCCCTGAGGTGAAGTGGTTA	269

ID	Chromosome	SSR nr.	SSR type	SSR	Size	Start	End	FORWARD PRIMER (5'-3')	REVERSE PRIMER (5'-3')	Product (bp)
Ad02A11537	Aradu.A02	11537	p2	(TA)8	16	65117701	65117716	AAGTTATAGCCCGCCGAAGT	CTGGAAGGGCTGTGTTTGTT	274
Ad02A11542	Aradu.A02	11542	p3	(TCT)5	15	65161902	65161916	TTCTCTCATATCGCCGAACC	CTCGGAAGTGGAAGCTTTTG	218
Ad02A11543	Aradu.A02	11543	p2	(AG)6	12	65176841	65176852	GGTCTGTCTTGGAGCTACCG	CAACAAAACCTCAAGCGACA	276
Ad02A11546	Aradu.A02	11546	c	(T)11gtgtttta(T	145	65189010	65189154	ATTTAGGGAGAAGGTAGACTGAACT	CACACATAATTTCACGTTTCTTCC	270
Ad02A11547	Aradu.A02	11547	p2	(AT)6	12	65189704	65189715	AAAAACGGATCGATTCAACTAA	CCAAACACACTTTTGATATTCCA	273
Ad02A11549	Aradu.A02	11549	p2	(TA)6	12	65207902	65207913	TTGGTGGATAAAAATTGTGATGTT	TTATTTTACAACCATCCCAAAAA	273
Ad02A11553	Aradu.A02	11553	c	(CAC)6ttcctcc	110	65209645	65209754	GTTCACGCGCTTCTTCTTTC	GGTGATGACGACGATAACGA	280
Ad02A11554	Aradu.A02	11554	p3	(CAT)10	30	65209903	65209932	ACCACCTCCTGCTCCTCTTT	GAAAATGATAATGTTGACGACGA	133
Ad02A11556	Aradu.A02	11556	c	(ATC)5ataag(A	30	65210945	65210974	TGTTCATTCAAATAATTTTGGTGTT	GGTTCGATTAATTGCGGTTC	259
Ad02A11562	Aradu.A02	11562	p3	(AGA)5	15	65265716	65265730	CGGTCTTAGGTGCCATTGAT	CGAACCTACTTCTCCCCCTC	154
Ad02A11563	Aradu.A02	11563	p3	(ATT)5	15	65268900	65268914	TTGGACGTTGGTTTTGTGAA	CAGGAACAGGGATAAAGGCA	201
Ad02A11565	Aradu.A02	11565	p2	(AT)8	16	65293355	65293370	GCGGCGGCTAGATTCTTAT	GGCTTCGAACCCAAAGTAAA	280
Ad02A11566	Aradu.A02	11566	p3	(ATA)6	18	65301080	65301097	CCTGCAAATTAAATCTATTCCCTG	TTCACTAGAAAAACGTTAGTGTCAAAA	272
Ad02A11570	Aradu.A02	11570	p2	(TC)10	20	65334924	65334943	TTCGCATCTTCTTCCCTTTC	TGGATTTCAAGACATTGCCA	227
Ad02A11571	Aradu.A02	11571	p2	(TC)6	12	65342110	65342121	ACTCATCAGATTGCGTGGCT	GGTGAGGTCAAGGGACAAAA	274
Ad02A11575	Aradu.A02	11575	p3	(ATA)12	36	65352559	65352594	ACGCTCAGAAACGGTGCTAT	CATGAAATTGCAAATAAGACAGA	257
Ad02A11580	Aradu.A02	11580	p2	(CT)7	14	65381152	65381165	CTGTGGGGAATGGATCATCT	AATGCTGGAACCACCGTTAC	258
Ad02A11582	Aradu.A02	11582	p4	(AAAT)5	20	65383998	65384017	GTGCTCCTTTTGGTGTTGGT	TGAACCCATGCAAAATAGTTAATG	280
Ad02A11588	Aradu.A02	11588	c	(A)12tttttggtttt	113	65432743	65432855	CGGTGCAGTTTGTTCAACTT	CCCCTTTATGCACTTGATGG	279
Ad02A11594	Aradu.A02	11594	p2	(TA)6	12	65518658	65518669	CCAATCCGAACTGATTAAACTC	CGTAAAACCCAAACCAACTCA	280
Ad02A11601	Aradu.A02	11601	c	(T)10gtaattaatt	126	65553296	65553421	TTTGCACTTTCTTGCTTTCTTTC	CTTCAAGAGCGCTGACACTG	262
Ad02A11602	Aradu.A02	11602	p6	(TCTTTC)5	30	65573205	65573234	GAGGATGCTGCTACTTTGGC	TGCGAGCAGAAACAAGAAAA	259
Ad02A11604	Aradu.A02	11604	p3	(AAT)5	15	65586932	65586946	GCAGGAACAAGGATAAAGGC	TTGGACGTTGGTTTTGTGAA	202
Ad02A11605	Aradu.A02	11605	p3	(TTC)5	15	65589786	65589800	AACCAATTTCCCTCCCATTC	GCCTTAGGTGCCATTGGTAA	271
Ad02A11606	Aradu.A02	11606	p3	(TTC)5	15	65595823	65595837	AGCCGAAACTCCTTCTCTCC	GCTTTTGTCTTCCCTTTCCC	193
Ad02A11607	Aradu.A02	11607	p2	(TA)12	24	65602349	65602372	ATTTTCCTTGTCTTGGCCCT	TAAACCGACCAAGGGAGATG	186
Ad02A11608	Aradu.A02	11608	p2	(CT)7	14	65610747	65610760	CACAAAAGCATAAAAACGCAA	GGCTGTAGAGTGGAGGAATCA	274
Ad02A11609	Aradu.A02	11609	p2	(CT)8	16	65616682	65616697	CAAAATCCAAACAATCACTTGC	TGCAGAGTGGAGGAATCAGA	241
Ad02A11611	Aradu.A02	11611	p3	(CTT)5	15	65624539	65624553	CGTGGCAAGGAATAGGTGAT	TGAATCGCAAGCTTTCAGTG	176
Ad02A11615	Aradu.A02	11615	p2	(CT)10	20	65682389	65682408	GCAACATAAAGTAATCAACAAGGG	ACGTTTTGACCCAGTTTTCG	265
Ad02A11617	Aradu.A02	11617	p5	(TTTTA)5	25	65683765	65683789	GGGGCATTTTGTCTCCTTTT	GGGTTTGGAAACGTTTGGTT	218
Ad02A11619	Aradu.A02	11619	c	(A)13taaatttcg	32	65704652	65704683	GGCTCTTGAAAGAATGGAGG	CCGGATACATACATGCCACA	272
Ad02A11620	Aradu.A02	11620	p3	(TTC)5	15	65717757	65717771	GCGGATCTGAGGAGAGTGAG	CCCCTTCCGGAGTGAATAAT	266
Ad02A11621	Aradu.A02	11621	p2	(TA)6	12	65719971	65719982	CAAATCCGAAATCGTGTCACT	TCCACTTTTGCATTTCCCTC	218

ID	Chromosome	SSR nr.	SSR type	SSR	Size	Start	End	FORWARD PRIMER (5'-3')	REVERSE PRIMER (5'-3')	Product (bp)
Ad02A11623	Aradu.A02	11623	p3	(AGA)7	21	65756240	65756260	TAAAATATTGCTCGCCCTCG	TACCCTCTCCCCTTTCCCTA	173
Ad02A11627	Aradu.A02	11627	c	(A)10ttattaagta	76	65787295	65787370	TGTGGAAAATTTTATGGAACA	TTGTCCAATGTGCATTGATTC	244
Ad02A11628	Aradu.A02	11628	p2	(CT)6	12	65802852	65802863	TACATAGCGGACCACAACCA	AACTGCGAACAAGAGGGAGA	259
Ad02A11629	Aradu.A02	11629	p2	(AT)7	14	65807494	65807507	CCATTGGACTGTCCCTCTGT	ATATCGTAAGTGTGGCCGGA	153
Ad02A11630	Aradu.A02	11630	p2	(CT)6	12	65809825	65809836	CTTAGACCAGCGACGACTCC	GAGTTACCGTTGATCGGCAT	203
Ad02A11631	Aradu.A02	11631	p2	(AG)12	24	65812315	65812338	CAAGAACCAAGGAAATGGGA	TTCTCTCGCTTCTTTTCCGA	280
Ad02A11641	Aradu.A02	11641	p3	(CTT)8	24	65842995	65843018	CAAGGTACGTTTCTTGCCTTC	CCTCCTCATCTTCTGCTGCT	209
Ad02A11642	Aradu.A02	11642	p3	(CTT)5	15	65843635	65843649	ACAGAAGGAGATCGAAGGCA	TACGCGCGTTACAGAAGAAG	131
Ad02A11644	Aradu.A02	11644	p3	(GTT)7	21	65847342	65847362	TGACGGATTCTAATCCAGGG	AAGGGCTTGCTCTACCATCA	218
Ad02A11645	Aradu.A02	11645	p3	(TTG)5	15	65859601	65859615	GACCCCACCTAGTGGAACAA	AAATTACAAAGTAACGGTTAGACACAA	255
Ad02A11646	Aradu.A02	11646	p3	(ATT)18	54	65866445	65866498	GGAGCTTCTTGCTCCATTTG	CCCCAAACTCAACAGCAAGT	273
Ad02A11647	Aradu.A02	11647	p3	(AAT)7	21	65881987	65882007	AATTTTCATTAAACCTTGAAGACC	CATGGTTCAGTTGCGACTCT	222
Ad02A11648	Aradu.A02	11648	p3	(AAT)6	18	65897148	65897165	CAGGGAAAAAGGCACTCTTG	TTGGATGTTGGCTTTGTGAA	198
Ad02A11653	Aradu.A02	11653	p2	(AG)6	12	65926910	65926921	GGAAGAATGATGGGAGACGA	GGAACATTGAACCTGTGATGAA	155
Ad02A11654	Aradu.A02	11654	c	(T)10cgttcttttc	56	65927154	65927209	TTGTTCATCTTATTGCTCAGATTAGG	AAAAATGCAATTGACACCAAA	237
Ad02A11655	Aradu.A02	11655	c	(A)10gttttcaga	130	65947415	65947544	TTGCAAACGTGTGCCTTATT	CATAGCCAAAGGGGTGAGAG	265
Ad02A11660	Aradu.A02	11660	p2	(TC)6	12	65980953	65980964	CGTAGAAGCCGAAAGTGAGG	ACCAGAGAGTGAGAGGGGGT	251
Ad02A11661	Aradu.A02	11661	p2	(GT)6	12	65981111	65981122	ACCCCCTCTCACTCTCTGGT	CCTCATTCCCTCCAAAGTCA	178
Ad02A11663	Aradu.A02	11663	p2	(CT)6	12	66019330	66019341	TAAATACACGTTCAGCCCCC	CGGAAAAGCACACCAAACTT	121
Ad02A11665	Aradu.A02	11665	p2	(TA)6	12	66040252	66040263	GAGGCGCTACGACCTCTAAA	TCTTGGAGGTTGACCTGGAG	125
Ad02A11668	Aradu.A02	11668	c	(T)10gaaaaaac	84	66074079	66074162	CAAACTTGAAGCTAGAGGGGTG	TTGCAAACGTGTGCCTTATT	262
Ad02A11669	Aradu.A02	11669	p4	(TATT)5	20	66082752	66082771	CCATGATGGAGAAAGGGAGA	TCCCTTGGATTGACTTGGTC	266
Ad02A11671	Aradu.A02	11671	p2	(GT)7	14	66092603	66092616	TAGTGGCATGGTTCACAAGG	TTGAAAATTGGCAGCATGAC	226
Ad02A11675	Aradu.A02	11675	p2	(AT)6	12	66100469	66100480	GGATGAGTTTAAGGGAGGATCA	TAGGAAGCGTGCTACCTTGG	269
Ad02A11679	Aradu.A02	11679	c	(A)10gattggttt	120	66105298	66105417	TGTGGATTTACTTTAACACTTGTATCG	TCATTTGATCTAATCGAAGACATTTT	271
Ad02A11685	Aradu.A02	11685	p2	(AT)6	12	66159567	66159578	GCGATATACGGACAAGGCAT	GTACCCTAGCCGGCCTAAAC	117
Ad02A11686	Aradu.A02	11686	p2	(CT)7	14	66160624	66160637	TACCGGCTCATCAACTCACA	GATTAGAGGAATTGGCTTGGC	179
Ad02A11688	Aradu.A02	11688	c	(ATT)11g(TTA	55	66172658	66172712	TGGCTCCTCATACTTTCCCTT	CGATTTGCTAAAAATCAATAAAACA	280
Ad02A11689	Aradu.A02	11689	c	(TCT)7ta(TTC	41	66190766	66190806	CCTAAATGACCGAACCCTCA	CTGGCTTAGGTGCCATCAAT	274
Ad02A11691	Aradu.A02	11691	c	(TAA)14tag(T	66	66197738	66197803	GGAGAAAGAGGGGAAGGGG	CGAGTAACTTTTTCTTATCTCCTTCTT	252
Ad02A11693	Aradu.A02	11693	p3	(TAA)5	15	66200460	66200474	TTTTTCAGCAATACAAATTTTCAGA	TGAAACTCAATGATCAAAAAGCA	246
Ad02A11695	Aradu.A02	11695	c	(T)10caattaggt	71	66208555	66208625	TCCTTTTAATTGGGTCCCTG	TTAATTGGGTTCTTGCACCA	176
Ad02A11700	Aradu.A02	11700	p6	(GATTGA)5	30	66225236	66225265	GCTGGAAGGGAAACAAATGA	ACGCCAAATTCCATACTTCG	264
Ad02A11705	Aradu.A02	11705	c	(AATCCT)5aa	59	66302268	66302326	CCAAATTGGCAACATCAAAT	ATTCTGTGCCGGATTTCAAC	268

ID	Chromosome	SSR nr.	SSR type	SSR	Size	Start	End	FORWARD PRIMER (5'-3')	REVERSE PRIMER (5'-3')	Product (bp)
Ad02A11706	Aradu.A02	11706	p2	(CT)8	16	66304413	66304428	CCCAAATCCAAGTCCAGAAA	CAATTGGGCAACATCAATCA	274
Ad02A11707	Aradu.A02	11707	p3	(AAG)5	15	66305032	66305046	ATGGATCGATTTCAGGCAAC	TCTTGACTTTCCCATGGAGG	266
Ad02A11709	Aradu.A02	11709	p2	(CT)9	18	66308425	66308442	CCATTCATAAGGGGGCTTTT	CAAGGGCAAAGGAAAGTCAA	246
Ad02A11710	Aradu.A02	11710	p3	(GAG)6	18	66315859	66315876	CTCGGGCTGACTGAAAGAAC	GCTCAAGAAACAAGTTCGGC	213
Ad02A11711	Aradu.A02	11711	p2	(AT)11	22	66324204	66324225	TGAAGCAAGAAAAAGCAGCA	AACCATTGATGCTCAAAGCC	165
Ad02A11712	Aradu.A02	11712	p3	(AGG)5	15	66326618	66326632	TTCACTTCGTCAAATGCAGC	TCGCAACAGAGCAGAACATC	231
Ad02A11720	Aradu.A02	11720	p3	(CCA)7	21	66370734	66370754	CAGAATCGATTTCGTCCCTC	GTTATGGCGGAGGGGTTG	174
Ad02A11723	Aradu.A02	11723	p2	(TC)6	12	66374153	66374164	CCCTCATTTTGAAAGACCCA	GGGAGAATGAGAGTGGGGAT	183
Ad02A11728	Aradu.A02	11728	p2	(CT)10	20	66433080	66433099	AAAAACAATGCCAAAAAGCG	GCAGAATCCAACGGTACTCAA	269
Ad02A11730	Aradu.A02	11730	p2	(AC)7	14	66467969	66467982	TAGGGCTAAGGTGAGGGTGA	GGGGATAAAATTACCCCTAATGC	238
Ad02A11732	Aradu.A02	11732	p3	(AGA)6	18	66489848	66489865	CTAGAGGTTTCTCCGGCCTT	CGGACCTACTACCCTTTCCC	253
Ad02A11733	Aradu.A02	11733	p2	(AT)6	12	66500162	66500173	AGTCCCTGGGTTATTTTGGG	TTTCTTGGGGAGCAAGCTAA	189
Ad02A11734	Aradu.A02	11734	p2	(TA)7	14	66500282	66500295	TTAGCTTGCTCCCCAAGAAA	CGCAAAATAAAAAGCGCAAT	269
Ad02A11738	Aradu.A02	11738	p2	(TA)7	14	66562023	66562036	AGGTTGAGCTTGGAGCTTGA	TCATAAAACTGAGGTGGCGA	137
Ad02A11747	Aradu.A02	11747	p3	(ATT)7	21	66635938	66635958	TGTTGTTGTTGTTAGTGTTTTTGG	GGACAACAATTTTCTGATCACC	272
Ad02A11748	Aradu.A02	11748	p3	(ATT)6	18	66636815	66636832	TTTTTATTTTGGCGTGGGAG	CCCATGTCATCGAGTTGTTG	264
Ad02A11751	Aradu.A02	11751	c	(TCC)5ttcttctt	48	66641342	66641389	TTTGGCGTAGCTAATCGGAC	GGAGCTGTGGAAGAAGAAGAAA	253
Ad02A11755	Aradu.A02	11755	p2	(AT)6	12	66664976	66664987	AGTGTCGGATCGAATATCGG	ATTCGTAGATCCGCACAATAA	278
Ad02A11756	Aradu.A02	11756	c	(T)10cttgctgct	95	66666238	66666332	TATCAACTGCTCCGTCCTCC	CGACTAAGGCCAAACGAGAC	229
Ad02A11760	Aradu.A02	11760	p3	(CTT)5	15	66679358	66679372	ACCGATACCGTTTCGCATAG	AGAAACGCCAAACCCTTTTT	249
Ad02A11763	Aradu.A02	11763	p2	(AG)6	12	66739060	66739071	TCTCGCTCTAAAAACCGGAA	TTCCTCAACCTCCATAACCG	200
Ad02A11764	Aradu.A02	11764	p2	(GC)6	12	66743126	66743137	AGGATGGCTTAGAGTTGGCA	GCCTGTGGCATAACCACTTT	114
Ad02A11766	Aradu.A02	11766	p3	(TTC)6	18	66784694	66784711	CCCATAAACCCCACCTACCT	TAAAATATTGCTCGCCCTCG	240
Ad02A11767	Aradu.A02	11767	p4	(ATAC)11	44	66798676	66798719	TCCATCCAATTCTACCAAAACA	TTTACCCCAGTCAGTTTGGC	254
Ad02A11770	Aradu.A02	11770	c	(TTTCTC)5ttt	87	66804063	66804149	CTCAAATATGGGACACCGCT	CCAGGTCCTACTCCTCCACA	236
Ad02A11774	Aradu.A02	11774	p3	(CTC)5	15	66807192	66807206	AAGCCCAATAAAACACACGC	TGAAGATGAAACACGCGAAG	240
Ad02A11780	Aradu.A02	11780	p3	(TCC)8	24	66844328	66844351	TCACGCGACTTCTTCTTTGTT	TGTTGGTGATGACGATAACGA	137
Ad02A11781	Aradu.A02	11781	p3	(GAA)5	15	66845397	66845411	ATGCGAGCAAGAAGAAGGAA	TGACGTTCAACCAAGGTCAA	210
Ad02A11783	Aradu.A02	11783	p2	(AG)7	14	66892539	66892552	ATGACAGGCCTTCCCATACA	CCACACCATTCGAATTTCCT	278
Ad02A11787	Aradu.A02	11787	p3	(ATT)6	18	66925360	66925377	GCTCTAATGGCAGGAATGGA	CAAGAGAAGGACATGGCAGC	122
Ad02A11789	Aradu.A02	11789	p3	(TAT)13	39	66930976	66931014	TGTTTGGAAACTACCTGAAACTT	TCTTTCTGTAGATGGAGCCACTT	265
Ad02A11795	Aradu.A02	11795	c	(A)11gtttataaa	115	66972801	66972915	TCGTTTTAAATTTGGTTTGGC	TTTCTTTAGAAAAATGCTACAATCAT	261
Ad02A11799	Aradu.A02	11799	p3	(TAA)19	57	67020079	67020135	GCCCAAGATTCATGTGTGTG	CGAAAATTTTGGTAAAATCAGAA	210
Ad02A11804	Aradu.A02	11804	c	(A)11gagtttttt	116	67054261	67054376	GTACGCGTCCTTCAACCAAT	GCACCAAAATAATGCAAGGAA	250

ID	Chromosome	SSR nr.	SSR type	SSR	Size	Start	End	FORWARD PRIMER (5'-3')	REVERSE PRIMER (5'-3')	Product (bp)
Ad02A11806	Aradu.A02	11806	p2	(TA)6	12	67071811	67071822	TTTGTTCTAGCTGCATTGGTTC	CCTTAAGGAATATACACACACAGAAG	273
Ad02A11807	Aradu.A02	11807	p3	(TGA)5	15	67080205	67080219	TGAATGTAACTTCATCATCTTCCAA	CTCCTCCTCATCTTCTGATGC	213
Ad02A11817	Aradu.A02	11817	p4	(ATAA)5	20	67130474	67130493	TTTTGATCATTAACCCAATCACA	TGAAGCCAATCTCTTATTTGTGT	157
Ad02A11819	Aradu.A02	11819	p2	(TA)7	14	67136101	67136114	AGCTTAAACAAATCGCTGGG	TCCTTCAAAGCCAACAAGAGA	160
Ad02A11821	Aradu.A02	11821	p4	(TTAG)6	24	67145146	67145169	GATGTCCCTTCTTCCATCCA	TAGGCAATAAGGGCAGCAAT	252
Ad02A11822	Aradu.A02	11822	c	(A)10caaaaagc	96	67156953	67157048	TTTCCTTTTCCCATGACTCG	ACCCCTTTCTTCTCCCACAT	245
Ad02A11823	Aradu.A02	11823	p3	(ATT)5	15	67159974	67159988	AAACTTGGACGTTGGCTTTG	CAGGGAAAAAGGCACTCTTG	199
Ad02A11824	Aradu.A02	11824	p3	(AGA)10	30	67195980	67196009	TAAAATATTGCTCGCCCTCG	CCTACCTCGCCCTCTCTCTT	240
Ad02A11826	Aradu.A02	11826	c	(ATT)6atcattat	97	67200810	67200906	AAGAAGAAAATCTTGGGGGC	AAGGAGGTGCTTTGGGGTAT	227
Ad02A11828	Aradu.A02	11828	p4	(TATT)8	32	67206295	67206326	CCGGATACATACATGCCACA	TTCATGAATGTTGGCTCTTGA	274
Ad02A11829	Aradu.A02	11829	p3	(TTA)5	15	67218440	67218454	TCGATTCTGATGTTGAAACAATG	CGATGGTTATAGTGATTTGGGA	163
Ad02A11831	Aradu.A02	11831	p3	(AAT)5	15	67263025	67263039	GGAAAAGGGCACTCTTGTTG	TTGGATGTTGGCTTTGTGAA	192
Ad02A11833	Aradu.A02	11833	p3	(GTT)5	15	67280299	67280313	TACAGCTTTATGCCCTGCCT	AAAGGGCAACTGCTGAGAAA	266
Ad02A11834	Aradu.A02	11834	p3	(AAT)6	18	67283734	67283751	CCGAATCTGAGTGCAATACAA	TCACCCAAAAACGAAGTCAA	225
Ad02A11835	Aradu.A02	11835	p2	(AC)8	16	67284763	67284778	GAGGTGGCGAGGTATTACGA	GTTGAGCCTGGAGCTTGAAT	222
Ad02A11836	Aradu.A02	11836	p2	(AG)6	12	67285714	67285725	CTGTGAGGAAGAGAGGGACG	TCCTCCCCTTTCTGTTTGTG	237
Ad02A11838	Aradu.A02	11838	p2	(AG)7	14	67293427	67293440	AGCGAGTTAGTGTTAGATATTTTTGTT	ACTCAATCATTCCTGGCCC	196
Ad02A11840	Aradu.A02	11840	p3	(AGA)9	27	67303074	67303100	TAAAATATTGCTCGCCCTCG	TACCCTCTCCCCTCTCCCTA	177
Ad02A11846	Aradu.A02	11846	p3	(TTC)6	18	67356210	67356227	CACCTTGACCGAAACACCTT	CTAGAGGATTCTCCGGCCTT	174
Ad02A11848	Aradu.A02	11848	p3	(TTA)5	15	67367513	67367527	TCTTAGAATCGGCCCTTGAA	AACCATTTTGCGTGTGTGAA	152
Ad02A11851	Aradu.A02	11851	p2	(AT)11	22	67395541	67395562	TCCCCCGCCTTTATCTTTAT	TTTAAACCGCAATACCGCTC	183
Ad02A11855	Aradu.A02	11855	p3	(ATT)21	63	67415207	67415269	AATGCATGCCATTTTGAGTTT	GCAATGGGGTGCCTAACTAA	195
Ad02A11857	Aradu.A02	11857	p2	(AG)6	12	67421940	67421951	GCTGGATCTCTCCAAGTGCT	TCAAATTGGAATGGCAGAGAG	262
Ad02A11859	Aradu.A02	11859	p3	(CTT)5	15	67435061	67435075	TGTGTACAAGCGATTACGGC	GAGGAAGAAGAACGTGCAGC	247
Ad02A11860	Aradu.A02	11860	c	(AAG)5aatttca	108	67435987	67436094	TGAGGAAGAAGAACGTGCAG	CTGATCTCACGGAATGGTTG	265
Ad02A11861	Aradu.A02	11861	p2	(AT)12	24	67447516	67447539	TGTGACTTACGTTCTGGATTATGT	TGCAACTTGTTGCTGTTATCG	201
Ad02A11862	Aradu.A02	11862	p2	(TA)7	14	67455071	67455084	TGGAGAAAGGGAGATGATGTG	ATTTTCGAAAGTTTTGCATTAAA	280
Ad02A11868	Aradu.A02	11868	p3	(AGA)6	18	67486924	67486941	CTAGAGGATTCTCCGGCCTT	CCTAAATGGCCGAACCTACA	260
Ad02A11869	Aradu.A02	11869	c	(TG)15t(TG)1(	63	67516067	67516129	CTACCTCGTAAGGGTGACCG	AAGTCCGGTTGCTAAACCCT	204
Ad02A11870	Aradu.A02	11870	p2	(AT)6	12	67533905	67533916	TTTCCCTCATTAATTCAACTCACA	GCTTCAAAAATTCTCTTCATGTTTT	215
Ad02A11871	Aradu.A02	11871	p3	(TAT)8	24	67534063	67534086	AAAACATGAAGAGAATTTTTGAAGC	TAGCGGAGTACTTCCATCGC	203
Ad02A11872	Aradu.A02	11872	p3	(TAT)5	15	67535463	67535477	CCACTAAACTCCTAAAACTCCTTCA	GCTATTATTGTTGCCGCCAT	252
Ad02A11873	Aradu.A02	11873	p3	(TTA)5	15	67537765	67537779	TCTTGGCCGTACAATAAGCA	TTGGCAATGTTGATTTTTCTTG	229
Ad02A11876	Aradu.A02	11876	p2	(TA)7	14	67540121	67540134	TGACGATTGATGATGAAGATGA	GCAGATACAATTGCAAATTCAAA	223

ID	Chromosome	SSR nr.	SSR type	SSR	Size	Start	End	FORWARD PRIMER (5'-3')	REVERSE PRIMER (5'-3')	Product (bp)
Ad02A11877	Aradu.A02	11877	p3	(ATG)6	18	67544533	67544550	AAGATGGCATCATTCCGAAG	GATAGAAGGCGGTTTCACCA	243
Ad02A11878	Aradu.A02	11878	p3	(TTC)5	15	67547145	67547159	TCAGCTGTTTCAGTGTTGACG	TCACCAATTTCTGCCACATC	246
Ad02A11879	Aradu.A02	11879	p3	(TTC)7	21	67554862	67554882	CATCTACCCACTCTTCCCCA	CAAGCTAATATTGCTCGCCC	175
Ad02A11880	Aradu.A02	11880	p3	(CAA)5	15	67555922	67555936	TGGAAGCCATGCATTATTGT	TGGAATAAAGGGATGAGGCA	226
Ad02A11881	Aradu.A02	11881	p3	(TAA)5	15	67556052	67556066	TGCCTCATCCCTTTATTCCA	ATGGCATTGGAATACGCCT	111
Ad02A11883	Aradu.A02	11883	p2	(TA)6	12	67558186	67558197	CATTTGATCTGACGTTGAGTCC	TGAATATCCATGTTCCCGCT	270
Ad02A11886	Aradu.A02	11886	c	(A)10caacaaca	43	67577854	67577896	TCGATGATCCAAATTCCAAA	CCATATTCCATTGGCAAGGT	217
Ad02A11892	Aradu.A02	11892	p2	(GA)6	12	67607838	67607849	GCCGACAAGATTCGAAGAAA	GTGGAGACGGTGATGGATCT	111
Ad02A11894	Aradu.A02	11894	p2	(TA)13	26	67617278	67617303	CGTGGGCCTTTTTAATTTTTC	CAATCTGTGAGCAGCTATTCTTG	247
Ad02A11895	Aradu.A02	11895	p2	(AT)6	12	67625836	67625847	GCTTGTATGTGTAGCGGGGT	TTTCTGACCAAAAGGATTTTGAA	209
Ad02A11898	Aradu.A02	11898	p5	(GGGTA)5	25	67638526	67638550	GGGTGCAGGTAGGGTTACAG	TCCAATCACAATCTCAGCCA	254
Ad02A11904	Aradu.A02	11904	p3	(AAT)6	18	67669476	67669493	TAAGGGCAGAAACAAAGGGA	GGTTTGATGACGGTTTGGTT	256
Ad02A11906	Aradu.A02	11906	c	(AT)6aatcaata[illegible]	42	67700170	67700211	ATTAACAAAACGTGACCGGC	ACCATATCCGAAGTCGTTGC	275
Ad02A11910	Aradu.A02	11910	c	(A)10ttagcctaa	76	67708429	67708504	CGTGTTGACTTCCCACAAAA	TTACGGTGTCACGTTGGAAA	266
Ad02A11915	Aradu.A02	11915	p2	(GA)17	34	67716396	67716429	CCAACCTCCTTAAAACATATGAGG	TTGGATCTTCTTGGGATGCT	234
Ad02A11918	Aradu.A02	11918	p2	(AG)7	14	67722858	67722871	CGCACCTCATTGTTTTCCTT	CTAAACTCGAAATTTGCGCC	265
Ad02A11920	Aradu.A02	11920	p2	(AC)7	14	67723904	67723917	CTTGGATCTTGTGGTGCTGA	GGAAGCTTGGAGTATTGAGGC	277
Ad02A11924	Aradu.A02	11924	p3	(TTC)6	18	67764418	67764435	CTCCCCATCGACATCACTTT	CGACCAACCATTCTTCAACC	166
Ad02A11927	Aradu.A02	11927	p2	(GA)8	16	67774129	67774144	GGTGGGATACTTTGGCTCAG	TACCATCTGGTTTACCCCCA	191
Ad02A11929	Aradu.A02	11929	p4	(AAAT)5	20	67779357	67779376	TTGACCTCCATTCCAATATAAGC	AATGGGGTTTAATCTTGGGC	269
Ad02A11931	Aradu.A02	11931	p3	(ATA)5	15	67780241	67780255	CCCAACAACATAATTGGCGT	TGGTGAACGTGCAGTTTTTG	142
Ad02A11935	Aradu.A02	11935	c	(TG)7(TA)7	28	67793104	67793131	TTGGAAATCTGTGGTGAGACC	GCCCTTGTAAAACTCGAAACC	224
Ad02A11936	Aradu.A02	11936	p3	(AAT)18	54	67802859	67802912	ACGGTCAAATTTTTCGTCAA	CAAATGGTTTCAACCTTGAATTT	266
Ad02A11940	Aradu.A02	11940	p3	(TTG)5	15	67818000	67818014	TTGAGTGATTTATGTTTTGGGTTTT	TTGAACCTCTAACTTTCTTCATTTG	249
Ad02A11942	Aradu.A02	11942	p3	(CTT)8	24	67827957	67827980	CAAGGTACGTTTCTTGCCTTC	TTTCTCCTCCTCATCTTCTGC	211
Ad02A11943	Aradu.A02	11943	c	(GAA)18(GA[illegible]	96	67828727	67828822	GAGGAAGAAGAACGTGCAGC	CGCGCACTAACTCATTTTGA	246
Ad02A11949	Aradu.A02	11949	p4	(TATT)5	20	67844552	67844571	TTGGAAACCGGATTTTGAAG	ATATTTTGTTGCCGCCTCAC	263
Ad02A11950	Aradu.A02	11950	p2	(TA)8	16	67848469	67848484	ACTTAGGGCATTTTGTCCCC	AACTTGGGACGTTGGTTGAG	256
Ad02A11951	Aradu.A02	11951	c	(A)10ggaaatct[illegible]	117	67875082	67875198	TCAAACCCAAAACAAAACAAAA	CAAGTCAAGGTGAAGGGCAT	231
Ad02A11955	Aradu.A02	11955	c	(AAG)5aggaa[illegible]	55	67901707	67901761	TTTCTCCTCCTCATCTTCTGC	CTCCACGTACTGTTACGGCA	242
Ad02A11961	Aradu.A02	11961	p2	(TA)9	18	67909832	67909849	GATGGGAGTTCAGTGACGGT	AAATTCTTTGTAACCCCGCC	118
Ad02A11962	Aradu.A02	11962	p2	(CT)10	20	67912025	67912044	AAAATGGTTTGAGTTGGGTGA	TGATTAAGTGATGCAGGCCA	259
Ad02A11963	Aradu.A02	11963	p5	(AATAA)7	35	67942147	67942181	ATCATGTATGGCTGCTGCTG	GACGGGGTTAGGGTTTGAGT	234
Ad02A11964	Aradu.A02	11964	p3	(TTA)11	33	67961656	67961688	TAAAACGCAGATGAAGCTGG	TGGCCAGAATAAACAGGGAC	123

ID	Chromosome	SSR nr.	SSR type	SSR	Size	Start	End	FORWARD PRIMER (5'-3')	REVERSE PRIMER (5'-3')	Product (bp)
Ad02A11965	Aradu.A02	11965	p3	(AGT)5	15	67963159	67963173	TTTGTTGAGCTTCCATTCCC	TTGTTGCACTCAACCCACAT	172
Ad02A11967	Aradu.A02	11967	p3	(GTT)5	15	67976424	67976438	GCATGGTACTCCTGGATGGT	AGTGTGTGAAAAGGGCAACC	221
Ad02A11968	Aradu.A02	11968	p2	(AC)6	12	67977479	67977490	AAAGTCCCAAATGCATGCTC	TTCCTGTAGAGCCTGAGGGA	129
Ad02A11969	Aradu.A02	11969	p2	(CA)6	12	67980293	67980304	AGATACGGGAAACCGAAACC	ATGGGTATGATGGTTGTGGC	207
Ad02A11970	Aradu.A02	11970	p2	(AG)6	12	67991594	67991605	TTTTTGGGGAAGAGGTGTTG	AAAAACGTGTCTTTGCTCGC	151
Ad02A11972	Aradu.A02	11972	p3	(AAC)18	54	68000032	68000085	GCAAAGTTATGGTGTCTTCTCCA	TGGCAGGAATTGTTCTCCTT	174
Ad02A11975	Aradu.A02	11975	c	(AAG)11aacga	91	68017578	68017668	TTGGAAGAAATTCGAGCGAT	TTTTACTATGGGTGCCTCGC	226
Ad02A11976	Aradu.A02	11976	p2	(TA)6	12	68018048	68018059	GGAATTTGGGTTGTTTGTGG	TGTGGAGACTCACACTTGGG	169
Ad02A11979	Aradu.A02	11979	p2	(CT)6	12	68047802	68047813	GCAAGAAGCTGGCGTAAAAC	TTTTTGGGGAAGAGGTGTTG	215
Ad02A11982	Aradu.A02	11982	p2	(AT)6	12	68055752	68055763	GGTGGGCCCATCAATAATAA	GCCACCCAAGACATACATGG	174
Ad02A11983	Aradu.A02	11983	p2	(AG)6	12	68060520	68060531	TGTTGGGGATAATTTCTGGG	GGAACATTGAACCTGGAATGA	236
Ad02A11984	Aradu.A02	11984	p3	(GAA)8	24	68064909	68064932	CTCGGAGATGGAAGCTTTTG	ACCACATTCCCTCTCCCTCT	253
Ad02A11992	Aradu.A02	11992	p3	(ATC)7	21	68110234	68110254	GGTTTATCACCCAGCCTTGA	CCATGGAGTTTTCTCACGGT	132
Ad02A11993	Aradu.A02	11993	p3	(TAT)16	48	68113602	68113649	TATTTGAACGAGCATGTGGC	TCTTCTCGAAAGAAGCCAGC	184
Ad02A11994	Aradu.A02	11994	p3	(ATG)6	18	68114569	68114586	TTGAGCTTGTAAAGGTTGAACTTG	AAACCAGCCTCCAGTGAAGA	270
Ad02A11995	Aradu.A02	11995	p3	(TTA)22	66	68120695	68120760	TGCTTGCAGAGATGATACACTTC	TGTGCAGAGCCACTTTATGC	221
Ad02A11996	Aradu.A02	11996	c	(GA)9a(AG)1(	39	68121636	68121674	TCTCTGAGCGAACACACAAA	TGGACACGATTTCACACAAGA	273
Ad02A11999	Aradu.A02	11999	p2	(AT)6	12	68141672	68141683	TGCTTATTGTTTCCTCGCCT	ACCGTAATACCGCTCCAAAA	184
Ad02A12000	Aradu.A02	12000	p2	(AG)7	14	68142947	68142960	CTAGCTACGCTATCGCCACC	GTTTCTCCGACAAAAGCAGC	256
Ad02A12001	Aradu.A02	12001	p2	(AT)6	12	68146458	68146469	TGTTCCCTCGCCTGTATCTC	TCAAAAGTAACGCTTTCGGC	277
Ad02A12002	Aradu.A02	12002	p3	(AAG)5	15	68154510	68154524	GCTGAACCTCCATTGGAAAG	GAATTTGCAAGCTCCACTCC	263
Ad02A12007	Aradu.A02	12007	p2	(TC)10	20	68179821	68179840	ATGGATTGCGACAACAATGA	TAATGCACCAAACCAAAGCA	266
Ad02A12008	Aradu.A02	12008	c	(ATG)6tgggttc	119	68180181	68180299	TTGTGCCTTCTGATTTGCAC	GGATGGAATAACGGCAAAAG	204
Ad02A12009	Aradu.A02	12009	p3	(CAG)6	18	68182392	68182409	CGGCACCTTCAGAACAAAAT	AGGTTTGATGAAGGCGAATG	276
Ad02A12014	Aradu.A02	12014	c	(GA)6acaagctg	42	68191740	68191781	GAAATCAAAGCACGCACAAA	GAACAGCTGCAACTTAGCCC	274
Ad02A12017	Aradu.A02	12017	p5	(TATTT)5	25	68260431	68260455	GAAACCGGTCAAAAACCAAA	TCGGTCTGATTCTCACAACTTT	229
Ad02A12018	Aradu.A02	12018	p3	(AAT)5	15	68262829	68262843	TGAAGAAGCTTCAGCAGCAA	AACACTTGAAATAACTAACTAACAGCA	239
Ad02A12019	Aradu.A02	12019	p3	(TAT)16	48	68262959	68263006	TGCTGTTAGTTAGTTATTTCAAGTGTT	TGTACGGGCACATATGGAAA	268
Ad02A12020	Aradu.A02	12020	p3	(AAT)5	15	68263146	68263160	CATTAATTTATTTATCCTGAACGAGG	AACTGATTCCCCACTATTTTCTTG	255
Ad02A12021	Aradu.A02	12021	p3	(GAA)5	15	68265537	68265551	TAAACATGAAAGCTGCTGCC	AGTAAAATTGGCATGCGGTC	163
Ad02A12022	Aradu.A02	12022	p3	(TGT)5	15	68266901	68266915	AGTAGCAATGGCGACGTTTT	CAAGCTCGCACTCAATTCAA	196
Ad02A12027	Aradu.A02	12027	p3	(AAT)5	15	68279835	68279849	TAGAAAATTTGATTCGCGGG	TTTCACCAACGGGCTTTTAG	269
Ad02A12028	Aradu.A02	12028	c	(CT)8ccctcccc	46	68285284	68285329	ATAAGGCGCGACAAAGAGAA	ATTTCATTCGCGTCGATTTC	254
Ad02A12030	Aradu.A02	12030	c	(T)10caaatctta	86	68296877	68296962	ATGTGTTTTCCTCCTTCATCA	TTTTGAGGAAAAAGAGAAAGTGG	245

ID	Chromosome	SSR nr.	SSR type	SSR	Size	Start	End	FORWARD PRIMER (5'-3')	REVERSE PRIMER (5'-3')	Product (bp)
Ad02A12032	Aradu.A02	12032	p3	(TTC)7	21	68316906	68316926	AAGTTGTGGTGGACTGAGGC	GGTGAAGGGAGAGAGAGGAAG	280
Ad02A12034	Aradu.A02	12034	p3	(TTC)5	15	68330577	68330591	TTTCAAACCCCTTTTCTTTCC	CATAACAAAGCAATCACCCAAA	267
Ad02A12035	Aradu.A02	12035	c	(A)11ttataaggt	159	68354418	68354576	GACTCAAATCGTGGTCATCG	CAATAAACATATGCAATTCTCTCTCTT	265
Ad02A12036	Aradu.A02	12036	p2	(TA)6	12	68360103	68360114	TCTCGTGCTGATAACGTGTTG	TCTTAATGCAATTCTCTCTCTCTTT	183
Ad02A12037	Aradu.A02	12037	p2	(TC)12	24	68361845	68361868	TTCGCAGGGCTTTAACATTC	CCCTTAGGAATCAAAACGCA	178
Ad02A12040	Aradu.A02	12040	p2	(TA)6	12	68366035	68366046	TCTATCATGTCCGTTCTGTTCA	CGTGTTACGCGCACTAACTAA	273
Ad02A12044	Aradu.A02	12044	p2	(AT)7	14	68415686	68415699	GGGCTAGGAACACTGGAACA	TTGACATCTCAACAGAAAGAGAGTG	270
Ad02A12045	Aradu.A02	12045	c	(A)11caaaaaca	108	68418817	68418924	GTGGTGTGCGTACATCATAAA	ACCACAGTGCCACGCATA	280
Ad02A12049	Aradu.A02	12049	p2	(CT)6	12	68443598	68443609	GCACCAGACTGGCGTTAAAT	TGTTGAGGTGATTGGGTGAA	224
Ad02A12052	Aradu.A02	12052	p2	(TA)11	22	68523830	68523851	GAAAGTGGAGGTGCGGTTTA	TGACGAATTTAACCAAAAACGA	261
Ad02A12056	Aradu.A02	12056	p3	(AAT)9	27	68530969	68530995	CGCGAATTATTGGCCTCTAT	ACTGTTTGGTCACGGTAGGG	257
Ad02A12057	Aradu.A02	12057	p2	(AG)6	12	68537956	68537967	ACCAGCGAATTCTGAGCTGT	GGAACATTGAACCTCTAATCGAA	232
Ad02A12058	Aradu.A02	12058	c	(A)10taatcaaaa	77	68538791	68538867	TTGTTACTTGTGACAACCAAACG	CCATACGCAACCCTTCATGT	257
Ad02A12061	Aradu.A02	12061	p2	(AT)8	16	68542440	68542455	TCGAGCCGGATTTGATAACT	TGGGGGACCATTTTGAATTA	278
Ad02A12062	Aradu.A02	12062	p2	(TG)6	12	68543379	68543390	GGTGAATTTGTCGAAAAGAATG	CCATTACCTCCTTCACTTTTCC	128
Ad02A12063	Aradu.A02	12063	p2	(TA)14	28	68548958	68548985	ACCAGTTTTCTCGTGCTGCT	CCTGGCCTCTTCATATTCCA	200
Ad02A12064	Aradu.A02	12064	p3	(TAA)5	15	68549707	68549721	GATCTCTCATGCCTCCGTTT	ACGTGAATGGGTAACAAAGATT	279
Ad02A12065	Aradu.A02	12065	p4	(TAAA)5	20	68554423	68554442	TTGGCTCCCTAGCACTATCC	TGGACTTGTTGACCGTGGTA	171
Ad02A12069	Aradu.A02	12069	p2	(AG)11	22	68566931	68566952	CGATCCAAGCCTGAATTGAT	CTTCTTCGGTGAAAGGCTTG	162
Ad02A12070	Aradu.A02	12070	p2	(TA)10	20	68571499	68571518	GGCCTGACATGACCTGTTTT	AGCAACGTGGATGCCTATTC	247
Ad02A12073	Aradu.A02	12073	c	(ATA)5aaatgag	85	68577284	68577368	CCGCAAGGCAAATATCTTCA	CCTTGAAAAAGGATGACAAAGG	280
Ad02A12076	Aradu.A02	12076	p2	(TC)6	12	68579781	68579792	ACCGTGACCAACCTCTATCG	GGGACAATAGAACCGGTGAA	258
Ad02A12079	Aradu.A02	12079	p3	(TTC)5	15	68584824	68584838	TCCTGATTTGCTTCCGTTTC	GCTGGTCCCAAACTTGGATA	274
Ad02A12081	Aradu.A02	12081	p3	(GAT)5	15	68598976	68598990	AACTCAAAGGCTCATCATTGG	TTTGAGGAAGAAGAAGCTCCA	220
Ad02A12082	Aradu.A02	12082	p3	(GAA)5	15	68608624	68608638	ACAGTGGTTCGCAGCTTCTT	CAACACCGCAATGATCAAAC	172
Ad02A12085	Aradu.A02	12085	p3	(AGA)5	15	68629710	68629724	AAAAACGACGATGACATGAAAA	TGGTTCAAACCCAACAAAAA	148
Ad02A12086	Aradu.A02	12086	c	(CAC)6aattac(	81	68632544	68632624	CGCCTCTCAGTTTCCTTCAC	GAGGGATCGTGTTCCAAAAG	261
Ad02A12088	Aradu.A02	12088	p2	(AT)10	20	68635123	68635142	TGATTCTTTTGTCAACAATCAATAAT	TTCTGGAAGAATATTATCATCAACTC	265
Ad02A12091	Aradu.A02	12091	c	(A)11taggaatca	92	68656013	68656104	CAATATTGGGATGTGTGTGCAT	AAGTGATCAGGTGAAGTTTAGATTTTT	250
Ad02A12093	Aradu.A02	12093	p3	(ATA)5	15	68660478	68660492	CAAAATACACGCATGCACACT	TTGCCTTGGTGGGATATGAT	208
Ad02A12096	Aradu.A02	12096	p2	(CT)6	12	68670838	68670849	TTTTTGAAAATTCCTCACCTTTTT	CAGAGGAGAAGAGAGGGTTCG	251
Ad02A12097	Aradu.A02	12097	p2	(GA)6	12	68681619	68681630	ATGGTGATGCCAAAGAGGAG	AAAATTTGGCTTGAACACGG	273
Ad02A12099	Aradu.A02	12099	p3	(AAC)5	15	68697076	68697090	TTCAAGCCATTGCTTAGCCT	CGGTGGAAGTCCTCAAGAAG	154
Ad02A12100	Aradu.A02	12100	p2	(CT)20	40	68701906	68701945	CCAACCAACATCCTTGTTCC	TGCTTGGAATTGAACTGTGA	250

ID	Chromosome	SSR nr.	SSR type	SSR	Size	Start	End	FORWARD PRIMER (5'-3')	REVERSE PRIMER (5'-3')	Product (bp)
Ad02A12101	Aradu.A02	12101	c	(TA)7a(AG)6	27	68718058	68718084	GCGACACAACTCAAGCAATG	ACAATGCGTTACCGTTGGAT	168
Ad02A12105	Aradu.A02	12105	p2	(AT)11	22	68734028	68734049	GGATGTTTGAGTCCTCTTTAATGG	CGCCGTTAATGAAAAATTGG	259
Ad02A12106	Aradu.A02	12106	c	(T)10cgaaatttt	39	68738430	68738468	TAAGCACCCTCTTGTTGCCT	TGGCTCTTGAAAGAATGAGGA	196
Ad02A12107	Aradu.A02	12107	c	(T)10c(T)11	22	68748701	68748722	CCATGATGGACAAAAGGGAA	GATTCAGTTTTCAATTTGCTTTGA	170
Ad02A12110	Aradu.A02	12110	p2	(GA)7	14	68766342	68766355	TCATTCGGCTACCAACCTTC	TAGCGAGAAGGGACTTGCAT	264
Ad02A12112	Aradu.A02	12112	p3	(TAA)6	18	68770303	68770320	TGAAAAATTAGCCGTCAATGG	TGCTGATGCAACTCATGTCA	244
Ad02A12115	Aradu.A02	12115	p3	(TAT)5	15	68787371	68787385	GACACAATATAGCAAAACCTGAGAAA	GCTTTGATATTGGACCCGAA	153
Ad02A12121	Aradu.A02	12121	c	(A)10gtcactca	45	68805373	68805417	CAGATGAAGAACTTGGGCGT	TTTTCGATGTCTGATCTAATTTTGAC	264
Ad02A12124	Aradu.A02	12124	p2	(TC)21	42	68818193	68818234	CAGATCCGCATTTTTCCAAT	GAGGAATGTGCTTCTTGCCT	233
Ad02A12128	Aradu.A02	12128	p3	(CTT)5	15	68845026	68845040	GCACCTAACCACCACAACCT	CCACAGCTGCAGCAAAATAA	232
Ad02A12131	Aradu.A02	12131	p3	(AGA)5	15	68857708	68857722	TCATGGGAGAAAGATGAAAACA	GAGATGCAAGTTTGCTCAAGG	268
Ad02A12132	Aradu.A02	12132	p2	(AT)11	22	68870071	68870092	TCGTCGACATTTCGAGTGTAA	GGCTCTACGAGAATGAACTGC	218
Ad02A12133	Aradu.A02	12133	c	(ATAC)5(AT)	42	68893549	68893590	CGTTATTTTTCTGTGCGCTG	GCATAGTCAGTCCGTCCCTC	268
Ad02A12134	Aradu.A02	12134	p3	(TAA)5	15	68907194	68907208	TGTTAGCATCCGAATTTTTCA	TCATTTGAGTTTGGATCAATTAAGA	208
Ad02A12138	Aradu.A02	12138	c	(T)11(G)17	28	68927157	68927184	TTTGGTTTTTCCTTGATGGG	GTCCTCGATCCAAGTTTCCA	274
Ad02A12141	Aradu.A02	12141	p2	(AT)7	14	68932801	68932814	AACACTAAAATTGATACTTGTATGGC	TGGGGAATACCATTTGGGT	252
Ad02A12143	Aradu.A02	12143	p2	(AG)6	12	68934873	68934884	TCGTCCGAATACTGGGTAGG	TGACCCACATGAAAACGGTA	260
Ad02A12146	Aradu.A02	12146	p2	(TA)6	12	68946728	68946739	GGCACTGCAACACAAGGTTA	CGGGCCGAGACTAGTTGATA	232
Ad02A12148	Aradu.A02	12148	p2	(AT)6	12	68953641	68953652	TTCTCAACCATTCCAAGCCT	TGTGTAAATTGGGCCAGAGG	208
Ad02A12150	Aradu.A02	12150	c	(AC)70(AT)7	154	68962719	68962872	AGTCTGCAAAGTCAAGGTTGG	TATGGAGAGGCAGGGTTTTG	230
Ad02A12153	Aradu.A02	12153	p3	(TTG)8	24	68972990	68973013	TTGACTCCGTCCCAATTCTC	TATCTTGCCGCCACCATATT	260
Ad02A12154	Aradu.A02	12154	p2	(AG)6	12	68973269	68973280	GTGTGGTGGGCTTGAGAGAT	GCTGACAATAATGACCGCAA	205
Ad02A12155	Aradu.A02	12155	p2	(TA)7	14	68974109	68974122	TGTTTAAGCGAACGAGTAAGATGA	CCCTCTAAAGAAATGCTGAAACTC	280
Ad02A12163	Aradu.A02	12163	p2	(AT)6	12	68997750	68997761	CAACCAAAAGGAAGAAGCGA	TGCACATAAAAGATTAGGTTTCG	251
Ad02A12164	Aradu.A02	12164	p2	(CT)7	14	68999614	68999627	TCATCCCAAGCATTGTCTCA	GAGACTGTGAGCGACGAACA	153
Ad02A12165	Aradu.A02	12165	p2	(TA)7	14	69000189	69000202	CTCTTACTTTGCAAGGGCCA	AGTTGACGGAGTCAAGGACC	194
Ad02A12166	Aradu.A02	12166	p3	(CTT)5	15	69009891	69009905	GAGTGGTCCCCACATGATTC	AAAGCGTAAAAGTGCGGAGA	194
Ad02A12167	Aradu.A02	12167	p2	(TA)6	12	69011119	69011130	GCAGGTCACAGCACTCCTTT	ACAATTTGCCGATCGTTCAT	213
Ad02A12175	Aradu.A02	12175	p2	(GT)6	12	69041833	69041844	AACTCGCTTGGAATTGTGCT	AAAGTTGGGCTGGAATTGTG	222
Ad02A12177	Aradu.A02	12177	p3	(AAG)5	15	69051366	69051380	TGGCTCTTGAAAGAATGAGGA	TCCCCAATTAGAGGTGTCCA	214
Ad02A12178	Aradu.A02	12178	p3	(TAC)61	183	69056490	69056672	TTTAAAATCAAGAAGCCATCCA	TCTTGGCCCAGTAAGCAATC	268
Ad02A12190	Aradu.A02	12190	p2	(AT)7	14	69108022	69108035	CTCCAAACCCTATCCACGAA	AAAAGCAAGCGGATTTGTGA	277
Ad02A12196	Aradu.A02	12196	p2	(TA)8	16	69126794	69126809	CCTTCAAGGTTCCATCCAAA	CGGGTTTCAACTATTCGAGG	251
Ad02A12201	Aradu.A02	12201	p2	(AC)10	20	69177184	69177203	TTGGGTTAAAATAGGTTTTCAATTA	TTGGTGAGGGTGAGTAAGGG	162

ID	Chromosome	SSR nr.	SSR type	SSR	Size	Start	End	FORWARD PRIMER (5'-3')	REVERSE PRIMER (5'-3')	Product (bp)
Ad02A12203	Aradu.A02	12203	c	(T)10aaaaaaag	76	69188087	69188162	CAAATCTTAAGGATTAAAATCGTAACT	TTTGAATGCTAGCCCGAATC	242
Ad02A12206	Aradu.A02	12206	c	(A)10gagttttttt	113	69202201	69202313	ATACCCATCGACGCGTAAAC	CAGGTCACAAAGCACCAAGA	270
Ad02A12208	Aradu.A02	12208	p3	(CAA)5	15	69208490	69208504	ACCGCTGAGAAAGGAAGTCA	TCTTAGGACAATTTGCGGCT	271
Ad02A12209	Aradu.A02	12209	p3	(AAT)5	15	69258695	69258709	AATCGAACCCGTCTCTGATG	AGGTGCTGGTGATCCAAAAC	171
Ad02A12214	Aradu.A02	12214	c*	(AAT)9(T)10*	36	69282665	69282700	CCTAACCACTTTCTCTCTCCTCA	GGGTTAGCATATGACCCCAG	268
Ad02A12216	Aradu.A02	12216	p3	(TAT)7	21	69294155	69294175	AGGAGGCACTTTCTCCCTTT	GGTTTCACATTCAGGTGTGG	276
Ad02A12221	Aradu.A02	12221	p3	(TGA)5	15	69320424	69320438	TGGGTTTTCTGAGGTTGAGG	TCTTTCAAATTGCATGGCTG	210
Ad02A12233	Aradu.A02	12233	p2	(TC)13	26	69357881	69357906	GCTTCAAATTCTCAAACTTTCCA	TACTCGCCGGTTCAGTTCTT	275
Ad02A12234	Aradu.A02	12234	p2	(CT)7	14	69358201	69358214	AGAACTGAACCGGCGAGTAA	TACGCATCAGAGGTCCAACA	167
Ad02A12241	Aradu.A02	12241	p3	(AAT)19	57	69379122	69379178	TGATCAGAATGCCCATGAAA	TGAGCTTGGACCTCACAAAA	172
Ad02A12242	Aradu.A02	12242	p5	(AAGAT)5	25	69384184	69384208	AGGCGGTTCCCCTAAAGATA	TGTCCGACTTGTTGGACTTG	255
Ad02A12243	Aradu.A02	12243	p5	(AAGAT)5	25	69390221	69390245	AGATAAAGGCGGTTCCCCTA	CCTGGGTGCTCCAGTGTATT	126
Ad02A12244	Aradu.A02	12244	p2	(AG)7	14	69393246	69393259	TTTGGTTTTTCAACTAATCAAACA	CTCCGTCTCCAGTCGATGAT	210
Ad02A12254	Aradu.A02	12254	p2	(AT)6	12	69431640	69431651	TTTGTCCGAAGTACAAATTCTCAA	CACTGCCATCTTTGATCCCT	272
Ad02A12255	Aradu.A02	12255	p2	(TA)9	18	69445089	69445106	TGTTTCCCTTGCCTTTAACTTT	GCTGCGCCACTCTGATAACT	244
Ad02A12256	Aradu.A02	12256	p3	(TAC)5	15	69462009	69462023	GAACAAAAAGTATTTCCACCACA	CCACTTCCCAAAACACAGGT	278
Ad02A12257	Aradu.A02	12257	p3	(AAG)6	18	69472101	69472118	CTAGAGGATTCTCCGGCCTT	CACTTTGGCCGAACCTACAT	177
Ad02A12262	Aradu.A02	12262	p2	(AG)6	12	69512725	69512736	ATAGGTGAGGGGTTTTTGGG	GAAATAGGCATCAAGCTGGC	234
Ad02A12264	Aradu.A02	12264	p2	(TA)7	14	69520991	69521004	CGTGAAAGCCTCCAATGAAT	GGTCCATAAACCGAATGTTCA	223
Ad02A12268	Aradu.A02	12268	p3	(AGA)6	18	69554042	69554059	AGGATTCTCCGGCCTTATGT	ACAACCCCCTCTTCTTCACC	186
Ad02A12271	Aradu.A02	12271	c	(TC)6ctctctttttg	48	69565173	69565220	CATATACACCACTGCCACGC	TCTTTTTCCCTCTTCTCTCCAA	202
Ad02A12273	Aradu.A02	12273	c	(GA)7ggggagg	67	69566096	69566162	GGAGAGAGAGGAAAAGGAAAAA	CTCTCCGCTCTCCCTTCTTT	227
Ad02A12274	Aradu.A02	12274	p2	(TA)6	12	69575341	69575352	TCAACAATAGCAACCCAAACA	AGGGCTAGCAAAGGGAAAAG	256
Ad02A12275	Aradu.A02	12275	p2	(TA)8	16	69580176	69580191	CTACACTCCTTGTTGGGCGT	CTGAAGCAAGATGGCTAGGG	244
Ad02A12276	Aradu.A02	12276	p2	(TA)6	12	69581173	69581184	AAAGGGATTGGAGTATTCGACA	AAGGAACTGCAAGCAGGGTA	242
Ad02A12277	Aradu.A02	12277	p3	(AGA)26	78	69591093	69591170	GACGACCCCAGAGTTCTTCT	TTTTATTTCTCTCTCTCCTTTTTCG	277
Ad02A12279	Aradu.A02	12279	c	(ATT)6ttttctttc	60	69605046	69605105	TCCCCAATTAGAGGTGTCCA	TCATGAATGTGGGCTCTTGA	261
Ad02A12280	Aradu.A02	12280	p3	(TAC)5	15	69615872	69615886	CATGCCTGATGATGATGAGAA	AGCTGAAGCGCCAGAAATTA	237
Ad02A12282	Aradu.A02	12282	p2	(AT)11	22	69639306	69639327	CATCAAATTCGTCTCAATTTTCA	GAAGAATTATTACATGTTGAGGGACA	220
Ad02A12283	Aradu.A02	12283	p2	(TC)13	26	69641452	69641477	TTCCTTCACAGCTCATGTCG	CAGATAAAACTGCAACGGCA	208
Ad02A12287	Aradu.A02	12287	p2	(TG)19	38	69673484	69673521	ATCCCGCTTATCTGAACACG	GTCTATCCCTCAGGCTCACG	271
Ad02A12288	Aradu.A02	12288	p2	(AG)10	20	69675526	69675545	TAAACAAGTGGTGGTGCTGG	AAAATGCCAAAACCCATCAA	275
Ad02A12291	Aradu.A02	12291	p2	(AC)17	34	69692251	69692284	GCCGCAAAGGCTTCATAGTA	TTGGGTTTCCAGACTATGGG	172
Ad02A12296	Aradu.A02	12296	p3	(TTC)6	18	69743223	69743240	TTTTATTTCTCTCTCTCCTTTTTCG	CCACTGCTACTACCCAGGGA	263

ID	Chromosome	SSR nr.	SSR type	SSR	Size	Start	End	FORWARD PRIMER (5'-3')	REVERSE PRIMER (5'-3')	Product (bp)
Ad02A12300	Aradu.A02	12300	p2	(TA)7	14	69759711	69759724	GGATAAAAGTGGGGTTCCGT	TGGGCTAATATTTATTTGGGCT	269
Ad02A12303	Aradu.A02	12303	p2	(TA)6	12	69764560	69764571	ACAAATAAGCTCGTGGCACC	CCAATTTCTGACATCATATATAAACCA	264
Ad02A12309	Aradu.A02	12309	p2	(TA)6	12	69777727	69777738	TCGGTTGAACCCTCAATACC	ATTTTGGTTGTTGGAGCCAC	199
Ad02A12314	Aradu.A02	12314	p3	(TTG)5	15	69786893	69786907	GTTGAATACCTTGATCGCCC	TCGTAACTCGATTTCAATTTGG	220
Ad02A12315	Aradu.A02	12315	p3	(TAA)7	21	69789072	69789092	AATCCACTACCCTGCGAATG	CAACCTCCCATTCTCTAAGCC	166
Ad02A12316	Aradu.A02	12316	p2	(TG)15	30	69803859	69803888	CTGAGCTCCTGAAGCGAAGT	GAAGTGAAGGCTGGTCGAAG	207
Ad02A12317	Aradu.A02	12317	c	(T)11gcttttcttg	75	69829687	69829761	ACATGGCCAAAACACAAACA	CTCCCATGACTCAGAGGTGG	253
Ad02A12318	Aradu.A02	12318	p5	(AAAAT)7	35	69830495	69830529	TGAAGCAAGAAAAAGCAGTGAA	ATCCATTGATGCTCAAAGCC	170
Ad02A12319	Aradu.A02	12319	p2	(TG)17	34	69835451	69835484	CTGAGCTCCTGAAGCGAAGT	GGAATGCTCTGAAAACGGAA	272
Ad02A12325	Aradu.A02	12325	p2	(TA)6	12	69850497	69850508	GGATTTGTTCGGCTCGATAA	GGTGGTGTGTAAACTCTCCCA	219
Ad02A12331	Aradu.A02	12331	p4	(AAAT)6	24	69873895	69873918	TTTTCCAGGCATAGATCACCT	TTTACTGCTTAAACAAGACAAGACAA	277
Ad02A12336	Aradu.A02	12336	p2	(AG)8	16	69882966	69882981	ACCCATAAACTTTGCGAACG	GCAGAAAGGTGGTGAGGAAG	278
Ad02A12341	Aradu.A02	12341	p3	(CTG)5	15	69908437	69908451	TTCCCAAGAAAAATGCATCC	ATGGACGAAATTGATCCGAA	175
Ad02A12342	Aradu.A02	12342	p2	(AG)7	14	69909714	69909727	AGGAAGTGAGACCAGAGGCA	CGAAATACGGACACACTCCA	278
Ad02A12344	Aradu.A02	12344	p2	(TA)8	16	69928612	69928627	TTTGGACACACCTAAATACCGTT	GGGACACACGAACACACAAA	276
Ad02A12345	Aradu.A02	12345	p3	(CTC)5	15	69928814	69928828	CGTGTCAGTATCCGTGCATC	TTCATTATCCTCAACAACGAGA	210
Ad02A12346	Aradu.A02	12346	p3	(CTT)5	15	69929097	69929111	TCTTCTTCTCCTTTCTCCTCATC	TTGCGAAATTTTCAATTAGGTTC	229
Ad02A12349	Aradu.A02	12349	p2	(CT)6	12	69933260	69933271	GCCGTCACTCTTCTCCTCAC	GTACATTCGCACTTCGCAGA	163
Ad02A12351	Aradu.A02	12351	p2	(TC)13	26	69936171	69936196	CCCTTGACGCTGTCTAGTCC	CATCCTCTCTCACGCAAACA	271
Ad02A12356	Aradu.A02	12356	c	(A)14cagaaaat	92	69941522	69941613	TCTTGCAAATTCAGTGTGTCA	TTTTCTCCCGGAAACAAGTAAA	234
Ad02A12357	Aradu.A02	12357	c	(A)13ttacttgttt	103	69941808	69941910	TTTCCGGGAGAAAAGTTAATTG	TGAAATTCTCTGTCCGATCCA	273
Ad02A12359	Aradu.A02	12359	p2	(TG)6	12	69948803	69948814	GCTCTTCACAAGGAATGCGT	CTAAAACGGCTAACGGGTTG	240
Ad02A12361	Aradu.A02	12361	p2	(TA)6	12	69959034	69959045	CATAATTCGTACAAAGGATGGAA	GCTGGAAAAACTCTCCACCA	256
Ad02A12369	Aradu.A02	12369	p3	(TAC)5	15	70042370	70042384	AACAAAAAGTATTGCCACCACA	TCAAAACACAGGTTGTTGAGTTC	270
Ad02A12370	Aradu.A02	12370	p2	(AT)6	12	70049556	70049567	ATGATGGAGCAAGGGAGATG	TTTTTCAATTCTTATCCTTTTACGTTT	152
Ad02A12371	Aradu.A02	12371	c	(T)11cgaaaaaa	59	70049749	70049807	TGTTGATTTAAAGAAAATGGAATAAG	TCCTTGGTATTTTCCCTCCA	274
Ad02A12372	Aradu.A02	12372	p2	(AG)6	12	70065007	70065018	GTCCGGCTGAAAGTGGTTTA	TTTGAGGGTTTACCTGGTGC	129
Ad02A12374	Aradu.A02	12374	p3	(ATC)5	15	70092469	70092483	TCAAAATCGTCCCTGACCTT	GTGTAATGGTGGCAGTGGTG	205
Ad02A12375	Aradu.A02	12375	p3	(CAA)5	15	70092714	70092728	CACCACTGCCACCATTACAC	TTGGATCTGAAGTTGCGATG	275
Ad02A12376	Aradu.A02	12376	p2	(GA)9	18	70101092	70101109	GTTGCCCTAATGCATGATCC	CCTTGCTAGAGTGGCGTGTA	206
Ad02A12378	Aradu.A02	12378	p3	(TTC)6	18	70141120	70141137	TTCTTGGCCGAATACACCTC	AATGGTCTTGATGGGCTTTG	256
Ad02A12379	Aradu.A02	12379	p5	(TTATC)5	25	70146668	70146692	CCTGGGTGCTCCAGTGTATT	GCCCAATAAAGGGTCCAAAT	192
Ad02A12381	Aradu.A02	12381	p2	(CT)6	12	70157496	70157507	CAGAAAAGGGCAAGAAGCTG	ATAGGTGAAGGGTTTTGGGG	239
Ad02A12382	Aradu.A02	12382	p5	(TTATC)5	25	70176028	70176052	GGTGGTGCTGATTCCTTTGT	AGAGATAAAGGCGGTTCCGT	246

ID	Chromosome	SSR nr.	SSR type	SSR	Size	Start	End	FORWARD PRIMER (5'-3')	REVERSE PRIMER (5'-3')	Product (bp)
Ad02A12384	Aradu.A02	12384	p2	(TA)15	30	70194630	70194659	TGCCGTTAATTGCACATCAT	AAAGATTTATGATGCACGAACATT	266
Ad02A12390	Aradu.A02	12390	p2	(TA)7	14	70221887	70221900	CTTTGGGTTTCCTTTGGGAT	CGCAACTCAAAAGCGTAACA	196
Ad02A12391	Aradu.A02	12391	p2	(AC)6	12	70226964	70226975	GGCCCTTGGCCTAACTAAAC	AGCTGGAAATTCGAAGAGCA	270
Ad02A12394	Aradu.A02	12394	p3	(AAT)20	60	70260073	70260132	GACTTCAAAATAAGGTTACGGCA	CTCCTCTTCCCATGGTGTGT	204
Ad02A12397	Aradu.A02	12397	p3	(TAA)5	15	70266494	70266508	GCAACGTGGCTTGATTCTAA	CATTACATTCATTTGGCGGA	186
Ad02A12401	Aradu.A02	12401	p2	(AT)7	14	70283772	70283785	CTTGCTTTGATGGGAGGTGT	GATTCAGTTCTCAGTTTGCTTTGA	276
Ad02A12402	Aradu.A02	12402	p2	(AG)6	12	70297011	70297022	GCCCGGAGAACACAGATTAG	CCAATTGGGCAACATAAACC	269
Ad02A12405	Aradu.A02	12405	p2	(TC)7	14	70310268	70310281	TGGGCTATTTCTGACCCAGT	AAACCTGGGATCGAGAGTCA	231
Ad02A12406	Aradu.A02	12406	p2	(TA)14	28	70314158	70314185	AGTACGGCTCTCAAGGTGGA	TTGGAGGCTGAGAATTTTGG	273
Ad02A12407	Aradu.A02	12407	p2	(AT)6	12	70339508	70339519	CCATGATGGAGAAAGGGAAT	TTGGTGTCATTTTGTTGTTTTTC	275
Ad02A12408	Aradu.A02	12408	p2	(AT)8	16	70349286	70349301	GCGATATACGGACAAGGCAT	TACAGGTCCAGGTGCTCAGA	238
Ad02A12416	Aradu.A02	12416	p2	(AG)17	34	70375462	70375495	AAATGGTGGCTTGGGATATG	CTTCATCCCTCACATGCTCA	189
Ad02A12417	Aradu.A02	12417	p2	(AC)7	14	70376720	70376733	GACCTTGTCGTGCTGGAAAT	TGAGTATGTGTGTGTGGGGG	186
Ad02A12418	Aradu.A02	12418	p4	(TTGA)5	20	70377171	70377190	CAACAGAAGAGAATGCAAGGG	ATTTCATGCACCAGGACACA	215
Ad02A12424	Aradu.A02	12424	p2	(AT)6	12	70435565	70435576	GCGAAGATCCTTTGTTCTCG	GCGAAGATAACCGGTCTGAG	213
Ad02A12428	Aradu.A02	12428	p4	(TTAT)5	20	70485569	70485588	AAGGATGGTGAATATAGAAGGCTC	GTGTGATTGCAATTTCGTGC	223
Ad02A12429	Aradu.A02	12429	c	(T)10cgaaa(T)	25	70487556	70487580	CCGGATACATACATGCCACA	TGAAGCAAGAAAAAGCAGTGAA	211
Ad02A12431	Aradu.A02	12431	p3	(TTC)6	18	70502667	70502684	TTTTGCTATACGCGCCTCTT	CCAGATCGAAGAAGAAAACCA	273
Ad02A12436	Aradu.A02	12436	p3	(GGT)6	18	70550639	70550656	TACAGTGGAGAGGGAGTGGG	AAATCGTCCCCGATCTTTTT	184
Ad02A12437	Aradu.A02	12437	p3	(AAT)5	15	70554255	70554269	TTCCCAATTCCTAAAAGGCA	CTCCGAATGTAAGCCACCAT	218
Ad02A12438	Aradu.A02	12438	p3	(CAT)19	57	70557189	70557245	CGTCTCCTCCTTCCTTTTCA	CACATGCGTTTCGAATGAAC	223
Ad02A12440	Aradu.A02	12440	c	(T)13ctctatttgt	106	70557828	70557933	TGATGATTGTGACGATGACATA	CACAAAGGCGAAGGAATAATG	280
Ad02A12441	Aradu.A02	12441	p3	(ATA)16	48	70558360	70558407	TTCTCCTTCTTTAAATTTGCATCT	TTCCAATGAGCAATGATTCG	246
Ad02A12443	Aradu.A02	12443	p2	(AG)24	48	70569196	70569243	CATTGAGTGAGGTGGTCCCT	GGTTGAACAAACCCGACAAT	236
Ad02A12446	Aradu.A02	12446	p2	(AG)6	12	70588367	70588378	CGCAGTACTTAGAGCAGGGG	GGGTGAAAATTGGTGAAGGA	221
Ad02A12448	Aradu.A02	12448	p2	(AT)6	12	70601062	70601073	CTCGGAGGACTTATGCCAAG	AATTCATCCTGCACCACCTC	219
Ad02A12464	Aradu.A02	12464	p2	(GA)6	12	70643227	70643238	TAAAAACCCTACCCGGTTCC	GGTCTCCTCTCTCTGCCTGA	270
Ad02A12465	Aradu.A02	12465	p2	(AG)6	12	70648411	70648422	GGAGAATAAGAGAAAAGAGAGAAAAA	AAAACATTTGGTGCTAGGATTCA	259
Ad02A12469	Aradu.A02	12469	p2	(AT)8	16	70666882	70666897	TCACTCTATCAAAATTTAGCATCCAT	TTGACACAACGGTAGCTTGC	263
Ad02A12470	Aradu.A02	12470	p2	(TA)7	14	70669083	70669096	CCAAAAGAATTTCAAGCCGA	CATGGGTGCCACTAAATCAA	256
Ad02A12471	Aradu.A02	12471	p3	(AAT)20	60	70675856	70675915	CTCCCATGTCATCGAGTCCT	GGTTTGTTGACACGAAGTTGAA	278
Ad02A12474	Aradu.A02	12474	p2	(AC)6	12	70703770	70703781	ACACCAAAGCGACAATCCTT	GACGCTTGCATGCTTTACAA	130
Ad02A12475	Aradu.A02	12475	p3	(TAT)6	18	70713499	70713516	GATCCGCATGTGGTGAGTTA	CAGCCTTCTACGCTGGTTTC	208
Ad02A12476	Aradu.A02	12476	p3	(ATT)6	18	70721074	70721091	CACATCTGTACACTAGATGGACCC	TGCCAAAATTTAATTCTCATGC	279

ID	Chromosome	SSR nr.	SSR type	SSR	Size	Start	End	FORWARD PRIMER (5'-3')	REVERSE PRIMER (5'-3')	Product (bp)
Ad02A12479	Aradu.A02	12479	p3	(TTC)5	15	70723750	70723764	CAAACGCACCTCCTCAATCT	TGAAGAATGCGAGCAGAGAA	248
Ad02A12480	Aradu.A02	12480	p3	(TTC)5	15	70724180	70724194	TCGCGCAAATAGGTTCTTCT	GCAACTCCACTTAACCGAGC	115
Ad02A12483	Aradu.A02	12483	c	(AAT)6aac(A/	39	70750743	70750781	TCCAGGTTTTGTCCATGTCT	AATGAGAATATGAGAAATTTGATGC	268
Ad02A12486	Aradu.A02	12486	p4	(ATTT)5	20	70757192	70757211	GATATATCCGCAAAACACAAAATA	TTGGATTGCAAATTTTATGTAGG	260
Ad02A12487	Aradu.A02	12487	c	(T)11aaataaatc	82	70759413	70759494	AATCTATTTGATCAAGGAGGAGTAAG	ACCCTATCATCGTGCGACAG	249
Ad02A12492	Aradu.A02	12492	p2	(CA)6	12	70812992	70813003	TGTTTCTCCACTCTCACCCC	CCTCCTCCACTGACTCTTGC	245
Ad02A12493	Aradu.A02	12493	p3	(AGA)6	18	70829379	70829396	CCCTCGAGCAAAGAAATGAA	CCCATAAACCCCACCTACCT	228
Ad02A12494	Aradu.A02	12494	p3	(TGA)6	18	70841759	70841776	AGCCGAAAAAGGTACCCACT	GTGGCCAGTTTTCTTTGCTC	220
Ad02A12495	Aradu.A02	12495	p2	(AG)6	12	70851025	70851036	TGCTTGGAGTTGAACTGTGA	GTTGCCGCACTCTGATTTCT	275
Ad02A12500	Aradu.A02	12500	p2	(GT)6	12	70882633	70882644	GTGTGTTCTTCCCTTCCCCT	CTAAGATTGGACCGAACGGA	155
Ad02A12501	Aradu.A02	12501	p2	(AT)8	16	70888967	70888982	AAAGCCGATTGCGAAGATAA	AGGTTTCTCGCTGAGGCATA	270
Ad02A12502	Aradu.A02	12502	p2	(GT)7	14	70891904	70891917	GTGTGTTCTTCCCTTCCCCT	ACCAAGATTGGACTGAACGG	158
Ad02A12504	Aradu.A02	12504	p3	(TAA)22	66	70910283	70910348	TGATGGAGAAGAGGGATTGG	GCTATTTTTGAAACCGTCGC	264
Ad02A12509	Aradu.A02	12509	c	(CTC)5tctctcc	85	70947921	70948005	CCCCTCTCTCTCTCCCAATC	TTCTTGTAGTGGAGGGGAAGAA	245
Ad02A12510	Aradu.A02	12510	p2	(GA)6	12	70949364	70949375	TCGCCGAAAGAAAGAAGAAA	GCGTGGACTAACTCAGCCTC	173
Ad02A12512	Aradu.A02	12512	p2	(GA)8	16	70951041	70951056	ATGAGGGTGAGAAAGAGGGG	TCTCCCTACTGTCACCGACC	209
Ad02A12514	Aradu.A02	12514	p2	(TA)6	12	70956679	70956690	CCAAAATCGTATCGCTGTTTT	CAGACGAGGAAGCTTAGGAAG	280
Ad02A12515	Aradu.A02	12515	p2	(TC)16	32	70961453	70961484	AAAAGGGCTGAGACAGCAAA	GAAGGGCCAATAGAGGGAGA	279
Ad02A12516	Aradu.A02	12516	p2	(AT)6	12	70965535	70965546	TTGCCAACGAGGACTAGCTT	TCATTCCAAAATCCAAAAGGA	248
Ad02A12517	Aradu.A02	12517	c	(A)11caat(A)2	36	70965781	70965816	TCCTTTTGGATTTTGGAATGA	GAGAATGTCTGGTGCTGCAA	276
Ad02A12518	Aradu.A02	12518	p2	(TA)7	14	70972801	70972814	CCCTTAGCTCTCGGGACTTT	CCGGTCCGAGCATAGAAGTA	203
Ad02A12519	Aradu.A02	12519	p2	(TA)7	14	70985753	70985766	TCTAGTCAGCCTTGCTTCGC	CAATAATCACATCGCACTCAAAA	137
Ad02A12521	Aradu.A02	12521	c	(T)12gacaaattg	151	70992830	70992980	TGGTCAATTTTGAAAGAGAGGAA	AGTTCCTCTGATGCTTTCAATC	242
Ad02A12522	Aradu.A02	12522	p2	(TA)6	12	70996733	70996744	AAAGCCAATAAACCGCTCCT	GAGGACTGTCTCTGGGATGC	171
Ad02A12525	Aradu.A02	12525	p2	(AT)7	14	71002504	71002517	TTGGATAGATCCAGCGAGAGA	TTGTACAAACGACGGGTTTC	259
Ad02A12529	Aradu.A02	12529	c	(AT)16ttgtatttt	78	71009820	71009897	CGTTAAACGCCCAGAATGAT	AAAAATGGGTGTTTTCATTTTATG	247
Ad02A12532	Aradu.A02	12532	p2	(TA)6	12	71012608	71012619	TTCGTCCATAGACAGAGCCA	ATTGTAAGGGGCAAAGACCC	167
Ad02A12533	Aradu.A02	12533	p3	(TTG)12	36	71032337	71032372	AACATCCAGTCAGGAGGGTG	CGCATTTGCATAAACCTTACA	256
Ad02A12535	Aradu.A02	12535	p2	(TA)6	12	71039728	71039739	TTGAACCAAACCCCAATGTT	TCATTTTGATGCAGTTGGGA	268
Ad02A12536	Aradu.A02	12536	p2	(AT)10	20	71040781	71040800	CCAAATCTCAAATGGAGACCA	CACGGAAGGGTAGAATTGACA	277
Ad02A12538	Aradu.A02	12538	p2	(TA)9	18	71042052	71042069	ACCGTGTGAAAATCCGAGTC	TTCAAAGATTGCAGTGCTCG	171
Ad02A12551	Aradu.A02	12551	p2	(AT)8	16	71157282	71157297	TGCGGATCTTATCGCTTCTT	CTTTGATTTGTCACAGCCCA	260
Ad02A12552	Aradu.A02	12552	p2	(AT)7	14	71161023	71161036	CAAAAATCATTCATTACATGCAAAA	CACAGAACAGAGGCCAAAAA	221
Ad02A12553	Aradu.A02	12553	c	(T)12ggttttgctt	41	71161147	71161187	CAGTTGATTGATTGTGTTGAATTG	TTGTTCGGAAATTGGATACG	257

ID	Chromosome	SSR nr.	SSR type	SSR	Size	Start	End	FORWARD PRIMER (5'-3')	REVERSE PRIMER (5'-3')	Product (bp)
Ad02A12557	Aradu.A02	12557	c	(T)10catacaag	59	71170943	71171001	ATGGAGCAATCAATTTTGGG	CCGTTACAGAACATCCAATCA	274
Ad02A12560	Aradu.A02	12560	p2	(GA)7	14	71173856	71173869	GCATTGACGCGTCGTATAAA	GGATCATGTGGAAAGTGCAA	197
Ad02A12564	Aradu.A02	12564	c	(T)12accgaaag	62	71191546	71191607	TCAAACCCTCCAATTTGCAT	GCTTCCTTTCTCTCCTGCCT	139
Ad02A12567	Aradu.A02	12567	p3	(TAT)19	57	71199995	71200051	CTTCTCTTCACACGCACCAA	TTGTAGCAACGGAATTAAATGTG	181
Ad02A12568	Aradu.A02	12568	p2	(TA)8	16	71201205	71201220	AGTCAAAGCTGGCTGGAGAA	GAACTCCTGAAGCGAAGTGG	202
Ad02A12574	Aradu.A02	12574	p2	(CT)6	12	71244711	71244722	AGCATGGAGGGAGGAAAACT	GGCTGAACCATTTGGAAAAA	205
Ad02A12575	Aradu.A02	12575	p2	(TC)6	12	71254645	71254656	TGAATTAAAGCGCAATCACG	TCAGGTTGTGCTCCTCAGTG	280
Ad02A12583	Aradu.A02	12583	p2	(AT)6	12	71262700	71262711	TCATAAAAATAAACATGCACCATTC	GGGAACCGTATTCAGTGGAA	279
Ad02A12587	Aradu.A02	12587	p3	(ATT)7	21	71272381	71272401	AACTGGGCTCTTTGTTTTGC	ACAAATGGGGTCAAGAGCAC	264
Ad02A12590	Aradu.A02	12590	p3	(AAT)6	18	71288590	71288607	CCCCAAGTTGAATCCTTGGT	TTATCCCATGTGCATCGCT	277
Ad02A12593	Aradu.A02	12593	p3	(AGA)6	18	71341768	71341785	TCTAGAGGATTCTCCGGCCT	CCCTACATGGCCGAACATAC	266
Ad02A12598	Aradu.A02	12598	p3	(ATA)9	27	71368635	71368661	AACATCCTCAAAAATAAATTATCTCAA	TGAAGATTAGCGAGATCAAAAACA	280
Ad02A12599	Aradu.A02	12599	c	(CTT)5ttacc(T	32	71369505	71369536	GCTTTTGCTATTCCACAGGG	TTCACTCTTCAACGCATCCA	267
Ad02A12601	Aradu.A02	12601	p2	(TC)18	36	71385011	71385046	TTTCGAAAATTCCTCACCCTT	GAGAGAGAAGAGAAGAGGGTTCG	274
Ad02A12602	Aradu.A02	12602	c	(T)12cactttgttt	114	71388433	71388546	TTTCGCTCACGTCCCTTG	GCACCATGTAGTAAATAGGAAAGTAAA	280
Ad02A12603	Aradu.A02	12603	p3	(TAA)14	42	71411974	71412015	TGAGGTCAATTGCCAAATGTT	ACGCGGGAATGATTATAGCA	278
Ad02A12607	Aradu.A02	12607	p3	(TTA)5	15	71442610	71442624	TCTTGATGCATGTGCTGACA	TTGCTTGCTGATCCTATGAGAA	173
Ad02A12608	Aradu.A02	12608	p2	(TA)6	12	71448375	71448386	TTTCAAGGTTGTAATGGGGC	ACACAAGTGGGAAAATTGGG	162
Ad02A12613	Aradu.A02	12613	p2	(AT)6	12	71477333	71477344	GCATCCTTGGAAGTTTGCAT	CAAATACGTTTTGAACACAACTAAA	233
Ad02A12618	Aradu.A02	12618	p2	(AT)6	12	71494167	71494178	CCCAGTCGTACATCCCCTTA	AGAGGTGGCTTCATTTCCAA	109
Ad02A12624	Aradu.A02	12624	p2	(AT)6	12	71519230	71519241	TTGTAACTCTTTGCACGCCA	TGGTCATAGGATTATCAAAAAGGA	246
Ad02A12629	Aradu.A02	12629	p3	(TTC)5	15	71522492	71522506	CCTGGTTGTTTGAAAATGCC	TTTGGGAGAGAAGGCAGAGA	177
Ad02A12634	Aradu.A02	12634	p2	(GA)9	18	71539897	71539914	AACAAATCATCAGCCGGTTC	TCTCCTTCTCCCCCATATCC	260
Ad02A12636	Aradu.A02	12636	p3	(TTC)5	15	71554719	71554733	ACCCCTTTCATTGGGTTAGG	TTTCCAAGGCTCAAAGGAGA	180
Ad02A12639	Aradu.A02	12639	p3	(AAT)16	48	71589599	71589646	AAACGTAAGATTAATTTGTTGTCGAA	TGAGTGGACATAGTAAACCTTTGA	280
Ad02A12640	Aradu.A02	12640	p2	(TA)6	12	71598639	71598650	CGATTTGAGGGCCTATTTCA	GAGCTGCTCAGATTCGGTG	208
Ad02A12644	Aradu.A02	12644	p3	(ATA)5	15	71638981	71638995	GCAGTGAAAAGCTTGCAGAA	TTCACTTGACACCTTCACGC	259
Ad02A12647	Aradu.A02	12647	p2	(TC)7	14	71641826	71641839	ATGAACGTTCTCCCCCTTCT	CAAAGGTGGACGGGAGAATA	237
Ad02A12652	Aradu.A02	12652	p3	(AGA)5	15	71671393	71671407	TCTTCCATGGCCTTCCATAC	ATTAGCAATGTGGAGGTCCG	121
Ad02A12654	Aradu.A02	12654	p2	(TA)6	12	71677114	71677125	GAGCTGCTCAGATTCGGTG	CGATTTGAGGGCCTATTTCA	209
Ad02A12656	Aradu.A02	12656	p3	(AAT)7	21	71684602	71684622	ACCTTGCCAACTCCAACAAC	AACGGAATGGTATGGTGCTT	248
Ad02A12660	Aradu.A02	12660	c	(AAG)5aggaag	172	71698563	71698734	GAGAAAGAGAAGGCAAGAAACG	CAACAGTTACTGCACGTTTTCA	228
Ad02A12661	Aradu.A02	12661	p2	(TA)9	18	71703868	71703885	CAAAATTAGGCCAGACAAATCA	TGTCAAAATTAGGCCAAACAAA	251
Ad02A12666	Aradu.A02	12666	p3	(TTC)5	15	71725873	71725887	AAACCCACACCTCTCTCCCT	TTGCCTTCCCTTTCCTCTTT	186

ID	Chromosome	SSR nr.	SSR type	SSR	Size	Start	End	FORWARD PRIMER (5'-3')	REVERSE PRIMER (5'-3')	Product (bp)
Ad02A12668	Aradu.A02	12668	p2	(TA)8	16	71742714	71742729	TCATGCATTATTTATGAGCTAATTG	GCTGGTAACATGGAGTTTAACAAA	219
Ad02A12669	Aradu.A02	12669	c	(ATT)5tg(TTA	35	71743302	71743336	TTGATTAAAACTGCGAATACATACTAA	CACATGAATTATCATCATTTCTCAA	151
Ad02A12670	Aradu.A02	12670	p3	(AAT)5	15	71749976	71749990	CACTTTTGTTAGCGGGGAAA	ATCGCCAGCTTGTCATTCTT	219
Ad02A12673	Aradu.A02	12673	p2	(AT)9	18	71764195	71764212	GATTAATTACACGACCCGCC	TGGCGAAAAATGTAGGAAGG	154
Ad02A12674	Aradu.A02	12674	p2	(AT)7	14	71790711	71790724	CTGCGAAACTAAGGCTGGTC	GGAACAGAGGAAAGGCAGAG	195
Ad02A12677	Aradu.A02	12677	p3	(AAT)13	39	71825694	71825732	CTTGAGCGTTGCACTTTTCA	TCCGCCAATTTGTAAGTTATCC	266
Ad02A12678	Aradu.A02	12678	p3	(TAC)5	15	71872164	71872178	CATGCCTGATGATGATGAGAA	AGCTGAAGCGCCAAAAATTA	235
Ad02A12682	Aradu.A02	12682	p2	(TA)10	20	71894966	71894985	TTTCTTTCATTTTCAGCGGG	TATTGCTCGCTCTGTCCTCA	278
Ad02A12683	Aradu.A02	12683	p2	(TA)7	14	71898697	71898710	TGGGCATAGTGTTCCACACA	TTTCATAGGATTTGAGTTTAGGGC	252
Ad02A12686	Aradu.A02	12686	p2	(GT)7	14	71910511	71910524	GAGCTTTTCACAAGGAACGC	GGCTAAAAACGCTAACGGGT	247
Ad02A12692	Aradu.A02	12692	p2	(TA)6	12	71935946	71935957	CAGGTCAAAAGGCTCCTCAC	GCTAAGGCTAGCCGGAGAAT	229
Ad02A12697	Aradu.A02	12697	p2	(TA)6	12	71979768	71979779	CGCAACACTCCAATCGATAA	CTTCGCAACCGGATTTAGAG	114
Ad02A12698	Aradu.A02	12698	p3	(TTA)13	39	72000000	72000038	TTATTTTTCACGCACACCCA	TTTAGCTTGCTCAAAGGCGT	174
Ad02A12699	Aradu.A02	12699	p3	(GAA)40	120	72003226	72003345	CAGAAGCAGAAGCAACCAGA	GAGGAAAGAACAGGGGAAGG	278
Ad02A12701	Aradu.A02	12701	p2	(TC)24	48	72005975	72006022	AGGCTAAACTCAAACGCGAA	GCTCCGACGGTAGTCACATT	179
Ad02A12705	Aradu.A02	12705	p2	(TA)6	12	72038175	72038186	GGTTGTTTTGGAGTGCTCTTG	AAGCCTCACCAAAGTCCTCA	216
Ad02A12712	Aradu.A02	12712	c	(AC)69(AT)8	154	72140932	72141085	ATTGTGCACCAAAATCACCA	GGCTGAGGTAAGACGTGCTC	257
Ad02A12718	Aradu.A02	12718	p3	(GAT)5	15	72175405	72175419	GTCTTTTCTGAGAGCCGCAC	CCCTGATCTTCCATGCATCT	157
Ad02A12719	Aradu.A02	12719	p2	(AG)19	38	72175688	72175725	ATGGTTCCAAAACCTGCATC	ATAACCCACCACCCTTCACA	208
Ad02A12720	Aradu.A02	12720	p2	(AT)7	14	72177744	72177757	CTCCGGATTTTGTTGCGTAT	GAGGAGCATCTGCAAAGAGC	204
Ad02A12722	Aradu.A02	12722	p3	(TAT)5	15	72182221	72182235	GCTTAAAATTGCTAACGCTTCA	CGAGACCAGAAGGTTAGAGGG	280
Ad02A12724	Aradu.A02	12724	p2	(AT)8	16	72199600	72199615	AGGGGTTTTCTGTACGGACC	AGAAAACTTGCGAGGACAGC	200
Ad02A12725	Aradu.A02	12725	p3	(TAT)6	18	72202869	72202886	TTGATGAGCTTAAAATTGCTAACG	ACGACGTGGCTTTTGTTTCT	204
Ad02A12727	Aradu.A02	12727	p3	(TTA)9	27	72213716	72213742	TTACTGGGAATTGGGTCGTT	CATCTTCCATGGGTACATCG	229
Ad02A12731	Aradu.A02	12731	p3	(TAA)14	42	72233421	72233462	TGCTTTGTTTTGGAATGAAATG	CAATAACATGATTATGCTCACATATCC	264
Ad02A12735	Aradu.A02	12735	c	(AC)7at(AG)1	48	72251913	72251960	GGATCGACCCTGACTCACTC	TGAAGGCCCGATTCTGTAAT	242
Ad02A12736	Aradu.A02	12736	p3	(GAG)6	18	72293311	72293328	AATTCACCCCATCAAAACCA	TTCGGGCACTTCTTCTCAGT	191
Ad02A12740	Aradu.A02	12740	p2	(TA)11	22	72301366	72301387	AAGGGAAACTGATATGGGGG	GAGAGGATGAGAGTGACGGC	167
Ad02A12741	Aradu.A02	12741	p3	(ATG)5	15	72301703	72301717	GCTTGTCCATCAGCCTCTTC	CAGCAACAACACCCACAATC	142
Ad02A12746	Aradu.A02	12746	c	(A)10ttagtatag	39	72326683	72326721	TTTTAATTGGGTCCCTGCAC	TTGCAAAATTTTTAATTAGGTCCA	150
Ad02A12747	Aradu.A02	12747	p2	(TC)9	18	72327499	72327516	ACTGTCAAATTAATCCGGCG	CAAAGTTGTTGTGGTGGCAG	113
Ad02A12749	Aradu.A02	12749	p2	(AT)8	16	72334787	72334802	ACTTAAGCCCTGAAACTTGCT	GACGGGTTATTGTTATAGACCATCTT	259
Ad02A12756	Aradu.A02	12756	c	(AT)6aagattttt	45	72361703	72361747	TGGACGACAAGGCTTAATGA	AAACAAAAATTCGTGCCACA	254
Ad02A12759	Aradu.A02	12759	p3	(TTA)16	48	72376435	72376482	GGAAATTTAAAGCAGTAGCCGA	TGATTGTCATCAGTGTAAGCTGG	150

ID	Chromosome	SSR nr.	SSR type	SSR	Size	Start	End	FORWARD PRIMER (5'-3')	REVERSE PRIMER (5'-3')	Product (bp)
Ad02A12760	Aradu.A02	12760	c	(GA)8t(AG)15	47	72377778	72377824	TTTTGTTGTTATAGGGTTCTTTTGG	TCTTGCCACATGCCAGTAAA	270
Ad02A12761	Aradu.A02	12761	p3	(ATA)5	15	72380199	72380213	TGCTCTTGCTGGTTATTGATG	CCAAATTCCGTGTCTTATCCA	211
Ad02A12763	Aradu.A02	12763	p2	(TC)7	14	72393844	72393857	AACAGAAAGTTACGTGGGCG	TGCTTGTCCTCAAGCAAAGA	273
Ad02A12764	Aradu.A02	12764	p3	(TAG)7	21	72396349	72396369	CGGTGACCAACTAATCACGA	TGGGATCGACCCTCACTTAC	237
Ad02A12766	Aradu.A02	12766	p2	(TA)6	12	72415976	72415987	AGCCGGATCTTGAGTCTTGA	AAACCATCGCAACTCGAAAG	125
Ad02A12768	Aradu.A02	12768	c	(T)11gttttcttttc	63	72437213	72437275	TCCCCAATTAGAGGTGTCCA	TGTGGTTCATGAATGTTGGC	260
Ad02A12769	Aradu.A02	12769	p2	(TA)8	16	72448837	72448852	TAAATGGGACGAAGACGAGG	TGTGGTGACTGGTGAAAGGA	182
Ad02A12773	Aradu.A02	12773	p2	(AG)16	32	72497576	72497607	CTGGAAAAGGAAACCACCTG	CATTGTGGGAATCACAGCAG	215
Ad02A12774	Aradu.A02	12774	c*	(ATA)5(AATA	31	72498786	72498816	AACGCAAGTAGCAACAAGGG	ACCGGAGTTCTCGTCTCTCA	257
Ad02A12778	Aradu.A02	12778	p2	(CG)6	12	72526416	72526427	ACATTAAGCTCCTGGCATGG	TGTTGGATGAGGGGTAGGAG	201
Ad02A12779	Aradu.A02	12779	p3	(ATA)22	66	72539867	72539932	GCCCTTAAATAAATTTCATTAGCA	TTCGTTACACAATTTTTGCGA	241
Ad02A12780	Aradu.A02	12780	p5	(AAATA)5	25	72541998	72542022	GATCACGAATGATGTGGTGG	CTTTTGTTGGTCCGGTCTGT	145
Ad02A12782	Aradu.A02	12782	p2	(TC)7	14	72543244	72543257	CCCACCATCCATCTTTCTTG	TTCTTGTGTTGGTGGTTGGA	113
Ad02A12786	Aradu.A02	12786	c	(TA)7gtcttctttt	58	72551688	72551745	TTCTCAAAATGAGATGTCTCGAA	GCGCCTGAGAAATTGAGATG	212
Ad02A12787	Aradu.A02	12787	p3	(TTC)11	33	72561526	72561558	CCACTTCTTCCCTTCCCTTC	CACCAAGTTTTTGGAGCCAT	235
Ad02A12788	Aradu.A02	12788	p2	(GA)21	42	72565473	72565514	TTTTGAGGTAGGTGGGGTTG	CCTAAATGGAGCAGGGATGA	253
Ad02A12790	Aradu.A02	12790	p2	(CT)6	12	72577027	72577038	GTCCATAGAATATCGGGCCA	ATGGTGGAGTGAAGCAGGAT	221
Ad02A12791	Aradu.A02	12791	p3	(CTT)6	18	72577508	72577525	GAAAAAGTGCCGCTATCTGG	CTCGCGAAGATGGGTAGGTA	247
Ad02A12792	Aradu.A02	12792	c	(GAA)9gaggaa	98	72578126	72578223	ACACCCAAAATTCGTTGCAT	GTTTTCAAAGCGCTACACCC	245
Ad02A12794	Aradu.A02	12794	p2	(TC)6	12	72583204	72583215	ATTCCCATGCGCACTAGTTT	ATTTATGAGAAGGGCGCAAA	130
Ad02A12795	Aradu.A02	12795	c	(TCT)5tgctgca	64	72588796	72588859	TTCTTCAACCGTTACTGCACA	CCTCCTCATCTTCTGCTGCT	247
Ad02A12796	Aradu.A02	12796	p3	(GAA)19	57	72589459	72589515	TCAGAAACCATGAAAATCGAAA	TCACCGTGGATTATGAACGA	200
Ad02A12798	Aradu.A02	12798	p2	(AT)11	22	72590368	72590389	AAAGTGGACCCACACATATCC	ATTGGCGACATTTTAGACCG	100
Ad02A12800	Aradu.A02	12800	p3	(TTA)5	15	72608021	72608035	TGGAAATTTTTCCTGTTATCATCA	TCTGTTGTCTCCTCACACCG	253
Ad02A12806	Aradu.A02	12806	p2	(AT)6	12	72633364	72633375	CAATCCCTAGTTTCCAGCCC	CTCAACTCACCAACAAGCCA	246
Ad02A12815	Aradu.A02	12815	p2	(AT)9	18	72685041	72685058	AAATTAAAAGCGTGTCCCCA	TTGTTGAGTGTGTGTCTGCG	162
Ad02A12816	Aradu.A02	12816	p3	(TAA)7	21	72685810	72685830	TCCCTTAGCTGACTTTGCGT	GACGATGACACCACAACGAC	100
Ad02A12818	Aradu.A02	12818	p2	(CT)27	54	72695006	72695059	TCTTCTTCAATGGCAACGTG	CCGTCAATGTTTCCCTTTGT	239
Ad02A12820	Aradu.A02	12820	p2	(GA)6	12	72703639	72703650	CACCGCTGTTGTCAGAGAAA	GCGGTGAACGGTAGTAGCTC	209
Ad02A12821	Aradu.A02	12821	p2	(AC)6	12	72721338	72721349	TGACCTACTACCCGACCCAG	TTTCGCGAGCTCTCTCTCTC	262
Ad02A12822	Aradu.A02	12822	p2	(GA)6	12	72721507	72721518	TTAGGGGAGAGGGGAGAAGA	CTTTCTGTTTCCTGCGGTGT	205
Ad02A12823	Aradu.A02	12823	p2	(GA)6	12	72721766	72721777	ACACCGCAGGAAACAGAAAG	GGCAATGGAAGCTTTAGCAG	239
Ad02A12827	Aradu.A02	12827	c	(A)12gaaaactta	141	72730681	72730821	TTTGGCTATGGGTGCTTTTT	TGGATGACATCGTTATAACTGAAAAT	279
Ad02A12832	Aradu.A02	12832	p2	(TA)10	20	72742770	72742789	GTGCCAACCAAAATGACCTC	GGGTTTAGGTGTTTGGTGCT	221

ID	Chromosome	SSR nr.	SSR type	SSR	Size	Start	End	FORWARD PRIMER (5'-3')	REVERSE PRIMER (5'-3')	Product (bp)
Ad02A12833	Aradu.A02	12833	p2	(CT)8	16	72750250	72750265	GTTGCCGTGCTCTGATTTCT	GATTAGAGGAATTGGCTTGGC	261
Ad02A12834	Aradu.A02	12834	p3	(TCT)5	15	72803100	72803114	TACCTCCCCCTCACTCTCCT	CTCGGAAGTGGAAGCTTTTG	197
Ad02A12836	Aradu.A02	12836	p2	(GA)9	18	72826249	72826266	GGGAGAAGGAAAGGAGTTCG	GTTCCTCTTCTCCCCTCTGC	200
Ad02A12837	Aradu.A02	12837	p3	(TTA)5	15	72832362	72832376	TTTTCCTTGTTCAATTGCCTT	TGGTGTGCATAAAAAGAAAAGC	230
Ad02A12846	Aradu.A02	12846	p2	(CT)6	12	72885395	72885406	ATAACCCTAAGGACGCGAGG	ATAGCTGAGTGGTGGTTGGG	274
Ad02A12848	Aradu.A02	12848	p2	(TG)6	12	72896907	72896918	GGGGATAAAATTACCCCAATG	ATATGTATGGCCAAGCGAGC	226
Ad02A12849	Aradu.A02	12849	p2	(AG)6	12	72898040	72898051	CCAGGGAACATGCAGAGACT	CAAATTGGCAACATCAAGCA	249
Ad02A12851	Aradu.A02	12851	c	(A)11gtggcg(A	27	72918419	72918445	TGGCTCTTGAAAGAATGATGAA	ATGCCACAAGCACATGGTTA	280
Ad02A12852	Aradu.A02	12852	p2	(TA)13	26	72926361	72926386	TTTTGCGCTTTTCAAATCCT	TGTCGTGTTTGGGAGGTGTA	191
Ad02A12854	Aradu.A02	12854	c	(TTCT)7ttttcca	62	72935137	72935198	AAAAACAATGCCAAAAAGCG	CCCCTTTACCCCAAAAGAAA	176
Ad02A12855	Aradu.A02	12855	p3	(TCA)6	18	72935925	72935942	TGCGTGCTGATAAGTTCTGC	GAATGTAAGTTCATCGTCTTCCAA	254
Ad02A12856	Aradu.A02	12856	p3	(TCA)5	15	72936322	72936336	TTTTCGGTGTATGTGTGCTGA	TGTTTCTTCATCTTCTTTGTTTGA	219
Ad02A12857	Aradu.A02	12857	p3	(AAG)5	15	72936907	72936921	TCCTTTTCATCTTTTGTTGCTTC	TTCCGTGTTCCTCTTCTTCC	262
Ad02A12860	Aradu.A02	12860	p4	(AATT)5	20	72953429	72953448	TTGCCCGAGTTGATCTTTTT	GAACCATACATTTCGGTGGC	230
Ad02A12861	Aradu.A02	12861	p3	(TAT)5	15	72955258	72955272	CGTGTCATTCTGCGGATAAA	TTGCCAGCAATTCAGAAAGA	188
Ad02A12862	Aradu.A02	12862	p3	(TAA)12	36	72960061	72960096	CCCAAGTTCCCTCAGTGAATA	TTCAGTTGAGTCCTTGTGCAT	280
Ad02A12867	Aradu.A02	12867	p2	(AG)6	12	72977222	72977233	CCTCTTCCCACCAAACTCAA	GGCTGTTGTGGTGATGATTG	197
Ad02A12868	Aradu.A02	12868	p5	(TTTAT)5	25	72977997	72978021	CGATGAATGGAAGGACTGGT	AGATCACCACCACTCCAAGG	279
Ad02A12872	Aradu.A02	12872	p3	(GCC)7	21	73021936	73021956	ACAGGTATCTCCGTCGCAGT	GATTTCACCGGAGAGGACAA	276
Ad02A12876	Aradu.A02	12876	p3	(GAT)5	15	73041563	73041577	TGAAACAGCAAAAAGGACCA	CCACATTAAGCAAAGCGGAT	147
Ad02A12880	Aradu.A02	12880	p4	(ATTT)5	20	73070570	73070589	GTCATGACAAAAACCACATAATTT	GAGACGCATACAGCACCTGA	246
Ad02A12883	Aradu.A02	12883	p2	(CT)9	18	73085454	73085471	ACCCACACACTCATTCCCAT	ACGGCAGAACAAGCAAGAGT	177
Ad02A12884	Aradu.A02	12884	p3	(TTG)5	15	73085908	73085922	CGCTTCGAATTTCTCTCTGG	CCCCACCTTTCACTCTACCA	278
Ad02A12886	Aradu.A02	12886	p3	(ATT)5	15	73097037	73097051	CTTACCCACACAGCCACCAT	AACAATAATAACAATTACAGCCACAT	274
Ad02A12887	Aradu.A02	12887	c	(A)11cctatatca	63	73100369	73100431	GTCAACCGCCAATGAGTCTT	TGTGCTACTGAATTGTCTACGG	266
Ad02A12893	Aradu.A02	12893	p2	(GT)6	12	73121165	73121176	GGTGAGACAAAGCATGAAAATG	ACCCGGACTACACCACAAAC	162
Ad02A12895	Aradu.A02	12895	p2	(TC)6	12	73123261	73123272	AACGCTAAAGGTGGCGTAAA	GAAGGAGAAGAAGGAGCCGT	171
Ad02A12896	Aradu.A02	12896	p3	(CAA)5	15	73129511	73129525	ATCATTCCCACAGAACCGAG	GCACCTTGTTTCTGCCTCTC	261
Ad02A12900	Aradu.A02	12900	p3	(TTC)5	15	73146805	73146819	GTGGGCATTGCTTTGATTCT	CTTTCCAAGCAACGAGACCT	133
Ad02A12901	Aradu.A02	12901	c	(AGA)5aggagg	79	73146934	73147012	CCTCGAGGTAGCCTCCTTCT	ACTCAATTTCCTCCAAGAAACA	227
Ad02A12902	Aradu.A02	12902	p3	(TCC)9	27	73154116	73154142	CCTTCGATTTGCTATTTCCG	CGGAGGAGGAGGAAGAGACT	243
Ad02A12904	Aradu.A02	12904	c	(AAT)8ataataa	52	73174446	73174497	ACCCTGCTTTTCTCCATCCT	GCAAATGCAATAAAAGAAAATGG	242
Ad02A12905	Aradu.A02	12905	p3	(ATT)7	21	73183756	73183776	ATCCAACAGGGACAAAGCAC	CGCACAACTAACCAGCAAGA	260
Ad02A12906	Aradu.A02	12906	p3	(ATT)5	15	73189607	73189621	TCCAATAGGGACAAAGCACC	CTTCGCACAACTAACCAGCA	256

ID	Chromosome	SSR nr.	SSR type	SSR	Size	Start	End	FORWARD PRIMER (5'-3')	REVERSE PRIMER (5'-3')	Product (bp)
Ad02A12910	Aradu.A02	12910	p3	(ATT)5	15	73215166	73215180	TGGTTATATATTGCTGGCATAGC	CAATCTTTGAAACGGTGTCTTT	279
Ad02A12911	Aradu.A02	12911	c	(CT)12acccctc	57	73222910	73222966	GCAAAATGTAGCGTGGGATT	AGAGGCACGACTTGAGGAAA	272
Ad02A12916	Aradu.A02	12916	p3	(TGA)6	18	73260138	73260155	GAATCAATCGTCAATGGCCT	CTATTGCCACTTTCATCGCA	258
Ad02A12920	Aradu.A02	12920	p2	(TA)14	28	73287647	73287674	ATCAATGCACATGGGACAAA	AATGGGGCTGAGGTAAAGGT	224
Ad02A12921	Aradu.A02	12921	p2	(GT)6	12	73289802	73289813	CCCATATGGTCAACCCTCTC	CAATGTTTCCCACAAATCCC	192
Ad02A12925	Aradu.A02	12925	p3	(AGA)6	18	73317196	73317213	TGCCATTGATGGTAGTGGAA	TAAATGGCCGAACAAACACA	235
Ad02A12926	Aradu.A02	12926	p2	(AT)6	12	73337363	73337374	GGTGACCAAATATTCCAGTACCT	TTTTTGAATAATTCTGAACACTTTCTT	278
Ad02A12927	Aradu.A02	12927	p2	(AT)7	14	73342033	73342046	TGATTTTAGGGGAGGTTGGT	TGGGTGCAAAATCAAAAGTG	275
Ad02A12933	Aradu.A02	12933	p2	(AT)7	14	73396894	73396907	GATCTAAAGCTTTGTGTAACACCC	GGAGCTTTTGAAAGCGAAGA	263
Ad02A12943	Aradu.A02	12943	p3	(ACA)5	15	73449059	73449073	GGAAGGCCGTGTGAAATAAA	AACATCCAGTCAGGAGGGTG	250
Ad02A12944	Aradu.A02	12944	p3	(GGC)6	18	73453646	73453663	TCACTTGGGGATCCTGTCTC	TTGCATCTGTCGTCGTCTTC	205
Ad02A12945	Aradu.A02	12945	c	(TTTC)5tgctga	46	73454007	73454052	TCATCCGTGACGTTGTGTTT	TTCAGCCGCTACACCTCTTT	278
Ad02A12954	Aradu.A02	12954	p4	(TTTA)5	20	73490591	73490610	GGGCATCTTTAGTGGCATGT	CCAAAGCTCCGAATCCATTA	151
Ad02A12958	Aradu.A02	12958	p3	(TTA)5	15	73495606	73495620	GGACGGAGTATGAACAGCGT	AGAGATCCAATTGTGGGGAA	258
Ad02A12959	Aradu.A02	12959	p3	(TTA)16	48	73495764	73495811	TGAGTGACGTTGTTGTGATCATT	GAATGGCCTCTCATTTCTGC	260
Ad02A12961	Aradu.A02	12961	p2	(AT)9	18	73498703	73498720	TGTGCATTAAAATTAATCATCAAAA	TGGAGTCTCTTTTCTCAACACAA	238
Ad02A12962	Aradu.A02	12962	c	(TTC)16ttaacg	113	73499174	73499286	TCTTCTTCTCCCAACCAATGA	CGACGAGGACAATGATGATG	230
Ad02A12971	Aradu.A02	12971	c	(GGT)6(CGT)	36	73531245	73531280	CTCGCCTCTTGTTCTTCGTC	GGTAGCGACGGTGAAACTGT	176
Ad02A12972	Aradu.A02	12972	p3	(TTG)5	15	73531689	73531703	CTCCCTACGTCGCTGCTACT	TCCAACAACAACAACAACCAA	279
Ad02A12973	Aradu.A02	12973	p3	(GTT)5	15	73531807	73531821	TTGTTTCTGGATTGTTTTTGGA	TGTGCCCTTCTCTCCTTCTT	280
Ad02A12985	Aradu.A02	12985	p3	(ATC)5	15	73600118	73600132	ACTCGCTTACTCTCCCACGA	GTGAATTGGTGATGCGTTTG	199
Ad02A12987	Aradu.A02	12987	p2	(AT)6	12	73616684	73616695	CTTACCTCGCGTCGTAAAGC	ACTTAAACTCCGCGAGTCGT	246
Ad02A12988	Aradu.A02	12988	c	(T)12aatatccta	66	73629544	73629609	TGGAACTTGTGTGTGGCAAT	TTCCTACCGGAAATGTTTGG	267
Ad02A12989	Aradu.A02	12989	p3	(TTA)5	15	73634241	73634255	GGGAACACATTTGTTTTAGCA	TCGAATGTATTGCCCTACATAAA	279
Ad02A12993	Aradu.A02	12993	p2	(AG)10	20	73646402	73646421	AAAGAGAACCAAAGGGGCAT	GCTCCTTCTCGCACTGTCTC	274
Ad02A12995	Aradu.A02	12995	p2	(TA)9	18	73653483	73653500	TATTGTTGAGGGTTGGTGGG	TGGCCTTCCACATCTTTCTT	112
Ad02A12996	Aradu.A02	12996	p3	(ATA)6	18	73654219	73654236	TTGGCTTGCTTGTCACTGTAA	CATCTTTCAAAAACGTTGGACA	221
Ad02A12997	Aradu.A02	12997	p3	(TTA)24	72	73654978	73655049	ATTTAAGCGGTTGAGACCGA	GGCCAACCTAACAAGTCCAA	204
Ad02A12998	Aradu.A02	12998	p3	(CTC)6	18	73656872	73656889	AGAGGGTCCAGGAGGCTAAA	TTAACCTCCCCAGAAGAGCC	211
Ad02A12999	Aradu.A02	12999	p3	(ATT)17	51	73664159	73664209	ACCTGCAAAGACACTCCGAT	CTTGATTGCCTCTCTTTGGG	225
Ad02A13003	Aradu.A02	13003	c	(A)12gaaatt(T	33	73668503	73668535	GGCACAATTGAGAGTTGGCT	AACTCGATTGCCTCTCTGGA	140
Ad02A13006	Aradu.A02	13006	p3	(TAT)7	21	73669678	73669698	TCCAATCCAAATGGAAAATCA	TGCTGTCGCTAACAAAATCG	197
Ad02A13007	Aradu.A02	13007	p3	(ATT)19	57	73669952	73670008	CATCGTTTGATGGTGGTTTG	TCCTTTTGGATTCCATGAATTT	264
Ad02A13008	Aradu.A02	13008	c*	(CATA)6(AT)	35	73671009	73671043	TAATAAACGGGACGAGACGG	GAAATTCTTCAAGGGCCTCC	232

ID	Chromosome	SSR nr.	SSR type	SSR	Size	Start	End	FORWARD PRIMER (5'-3')	REVERSE PRIMER (5'-3')	Product (bp)
Ad02A13011	Aradu.A02	13011	p3	(TTA)6	18	73689937	73689954	TCACACAATTGAAGGTTGGC	TGGATTGCCTCTCTCTGGAT	140
Ad02A13014	Aradu.A02	13014	c	(AGA)5aa(AA	38	73700664	73700701	GATGGTGAAGGAAGGAATGG	AAAGAAAAGGGGGAAGGGTT	269
Ad02A13018	Aradu.A02	13018	c	(AG)6aaaatatg	79	73717553	73717631	CATTGCATGTTATGGTGGGA	CCAACGTACGCTCTCTGTCA	240
Ad02A13019	Aradu.A02	13019	p2	(TA)7	14	73723409	73723422	GGTACTTGTCTTTTATGGACGCA	TGAAAGTAATAAAATTCTCAGTTGGTT	253
Ad02A13023	Aradu.A02	13023	c	(A)14gaa(AC)	29	73730718	73730746	GGTTGACAAGTGGGGATGTT	GCAAGCTTCCTTGGATTCAC	272
Ad02A13024	Aradu.A02	13024	p2	(TA)9	18	73740523	73740540	GCTTTAAAGTTGTCATTCGAATATTTA	AAGTCACCAAAACGAAAGCAA	275
Ad02A13027	Aradu.A02	13027	p3	(GCA)6	18	73747681	73747698	CTGCGAAGGAGAAATGGAAG	AATCGTATGGAGATGACGGC	124
Ad02A13029	Aradu.A02	13029	p2	(TA)10	20	73749010	73749029	TGGAGATGGAATGAAGTGACC	AGCCTGCTATTGTCATGTCG	244
Ad02A13031	Aradu.A02	13031	p2	(TA)7	14	73775554	73775567	ATCTTCGCAGCTGGATTTTG	TCGTAACTCAAAAAGCGCAA	130
Ad02A13033	Aradu.A02	13033	p2	(TA)7	14	73792505	73792518	AAAGAGTTTGGCATTCAAAGATT	TTGCTACACAATCCAATCCG	114
Ad02A13035	Aradu.A02	13035	p2	(AT)6	12	73794747	73794758	CCTTTTTCTTTCCAAGGGCT	AATAGGCCGGCTTCCATTT	271
Ad02A13036	Aradu.A02	13036	p2	(AT)6	12	73801377	73801388	GGATCCTCGTGTGGGTTATG	TCCACAGACAACAACAACAGC	191
Ad02A13039	Aradu.A02	13039	p3	(TAA)5	15	73820367	73820381	TTAATCTTGCGCAACGACAC	TTGGATCATCCTCAATGGGT	239
Ad02A13042	Aradu.A02	13042	c	(AAC)21aataa	88	73828458	73828545	CTCCCATGTCATCGAGTCCT	CGATGTTGGTGTTCCACTTG	242
Ad02A13045	Aradu.A02	13045	p3	(ATT)6	18	73830774	73830791	GGTTCTCGGTCAGGTACAGC	TAGGGTTGGAGGATACCGTG	277
Ad02A13047	Aradu.A02	13047	p4	(TTAT)5	20	73831167	73831186	CTCCATTTAAGGGTCTGCCA	TGACTGTAGAGGGGCAACCT	210
Ad02A13050	Aradu.A02	13050	c	(A)11gaaaacaa	43	73847632	73847674	GAGAGGACGAACGAAAAGGA	CGTTGCCGGAGATTAACTGT	275
Ad02A13051	Aradu.A02	13051	p3	(ACA)7	21	73855864	73855884	TCAGGCAAAAATGGAACAACT	ACATGACAGAGCACAATGGC	157
Ad02A13055	Aradu.A02	13055	p2	(TA)7	14	73867307	73867320	CGACGGCAGACATGAGTAGA	GGTTCGATTTTCGGTTTTGA	206
Ad02A13060	Aradu.A02	13060	c	(T)12acttttggtc	37	73872892	73872928	CCAGCAGAGAAAGATGAGCC	GGTGGGGTTGAGGGTTAGAA	275
Ad02A13066	Aradu.A02	13066	p2	(AT)7	14	73884652	73884665	GCGCGTACTCGACTCTACCT	GAGCTTCAACCCTACCCGAC	258
Ad02A13068	Aradu.A02	13068	c	(CCA)5(TCA)	210	73891970	73892179	ACAACCACCACCACCCAC	TTGTTGTTGCTGATTCTGGG	263
Ad02A13069	Aradu.A02	13069	p2	(GA)6	12	73898190	73898201	AGCACTCGCACGAAAGAAAT	GTGTCATCCCTCGTCCTCAC	101
Ad02A13071	Aradu.A02	13071	p3	(ATA)10	30	73904422	73904451	GGAGACACGAAGGAAAAGCA	TACCCAGCAATGCTTGAACA	273
Ad02A13072	Aradu.A02	13072	p2	(TC)12	24	73908113	73908136	AATGGGGAGTGAGTGAGGTG	CTGTCCATGTGATGTTTCGTG	227
Ad02A13075	Aradu.A02	13075	c	(T)12gaagcagt	122	73911035	73911156	TGCAATTGATGCATAAGTTAGTTG	CCAAGCATCTTTTCATTTGGA	240
Ad02A13081	Aradu.A02	13081	c	(A)11tcaaatgta	83	73948272	73948354	GCGTTTAATGCCAGGATTGT	TGAAATGAATTTGAAAAGATAATTGAG	278
Ad02A13089	Aradu.A02	13089	p3	(TTG)6	18	73978339	73978356	ACCTGTTACCCTCCCTCCAG	AAGCTCAACCATAAGCGGAA	275
Ad02A13090	Aradu.A02	13090	p3	(TGG)6	18	73978769	73978786	ATGATGCAGATGGTGATGGA	TGAGAGACAGAATCGAAAGCG	222
Ad02A13092	Aradu.A02	13092	p4	(TTAT)7	28	73981065	73981092	AACGAAAAAGGGAACTGGCT	TAGCAATTGGGGCCTAACAA	238
Ad02A13093	Aradu.A02	13093	p3	(AGG)5	15	73990785	73990799	AGAGGAGGTCCCCAAGTTGT	GAGGTCTCAGGCTGGAGTTG	100
Ad02A13094	Aradu.A02	13094	p2	(AT)6	12	73991700	73991711	AGTTCTGCTGCGAAATGGTT	AAGTCGGACACAACACTCCC	272
Ad02A13096	Aradu.A02	13096	p3	(TGA)5	15	74002680	74002694	GTGAAGGATGGCAAAATCGT	AGGACGAGTTTGAAGAGGCA	204
Ad02A13097	Aradu.A02	13097	p2	(TC)15	30	74011408	74011437	AAGGTTCACCTCATGGCAAC	GGCAGCAGATGAGGTAGAGG	204

ID	Chromosome	SSR nr.	SSR type	SSR	Size	Start	End	FORWARD PRIMER (5'-3')	REVERSE PRIMER (5'-3')	Product (bp)
Ad02A13098	Aradu.A02	13098	p3	(GAA)12	36	74013142	74013177	GAAGTAGAAGCGGCGAACAC	ACGTTTCTGGCGATGTTTCT	159
Ad02A13102	Aradu.A02	13102	p3	(ATA)9	27	74016176	74016202	GTTGGTTGCTTGCACATACG	CCAATGTTTAAACTCGGATGC	198
Ad02A13103	Aradu.A02	13103	p3	(TAA)16	48	74020288	74020335	ATTCCATGTATGTCCCCCAA	TCAGCACCAGATTTCTGCAC	270
Ad02A13104	Aradu.A02	13104	p2	(AT)8	16	74020886	74020901	AAAAAGTTTCCGGGGACTGA	GGATGGTGAGTTACCCCCTT	264
Ad02A13107	Aradu.A02	13107	p2	(AC)6	12	74026307	74026318	CATTCAACAAACCCATGCTG	TGCCATCTTCTACCCTCCAA	253
Ad02A13110	Aradu.A02	13110	p3	(CTC)6	18	74034724	74034741	GGCAAAGAAAAACGCTCAAG	ACGGCTGAGAAGGAGTTGAA	167
Ad02A13111	Aradu.A02	13111	p2	(AG)6	12	74049293	74049304	AAGCCAAGGAAGGAGAGGAG	TCGGCGATAGAAGTTTGGTC	252
Ad02A13114	Aradu.A02	13114	p2	(AG)16	32	74091129	74091160	CATTCTCCAAGTGGGTCCAT	AACTGCGGTTGAGAATGCTT	169
Ad02A13118	Aradu.A02	13118	c	(A)10c(A)11	22	74100276	74100297	CAAAGGGGGAAATGAAGACA	AAGAGAATCCCCTCTTGGGA	133
Ad02A13119	Aradu.A02	13119	p3	(AAG)6	18	74100760	74100777	GTGCACCGTATTGTTGTTGC	GTTGGTGAGCTAAACCCGAA	261
Ad02A13120	Aradu.A02	13120	p2	(AT)8	16	74117884	74117899	AAAAAGGCCAAATATTGGAAA	GCACGATGGAAACAGCAAT	247
Ad02A13128	Aradu.A02	13128	c	(GA)14a(AG)6	41	74137814	74137854	ACCTCAACATGGAGAGAGCG	CAACTGTTCGTGCATTCGAC	202
Ad02A13129	Aradu.A02	13129	c	(GA)12aaa(AC	102	74140611	74140712	TCTCTTCCTCCATCCTTGAAA	CTTGTGATGAGGTGACACCAA	220
Ad02A13130	Aradu.A02	13130	p2	(GA)9	18	74145827	74145844	CCTGATGCTCTGATACCATGAA	GGGATTGTCAGGAGGAATCA	270
Ad02A13136	Aradu.A02	13136	p3	(ATT)5	15	74160048	74160062	TGCCTTTGTCAAATAAGAGCC	CTCCATCGTCGTCATGATTC	239
Ad02A13139	Aradu.A02	13139	p3	(AAT)6	18	74165472	74165489	CAAGCCCATAGTTAAAAGCCA	GCCAAGAAGATGGGAAACAA	251
Ad02A13142	Aradu.A02	13142	p3	(AAT)13	39	74169168	74169206	CATGCAGGTGAAGCCATTTA	AGGTGGCCTCCAATTGTTTT	270
Ad02A13148	Aradu.A02	13148	c	(TAT)7c(ATT)	40	74180042	74180081	AAACGAGTGCATACCAAAGTACAA	TTCCAATTATTTGCTCCCAA	280
Ad02A13154	Aradu.A02	13154	p2	(TA)10	20	74186483	74186502	CCATTAGCGTCAACAAAAATC	AAGGCTCTTTCTTTCTTTCTTGAG	280
Ad02A13157	Aradu.A02	13157	c	(A)10tattattgaa	64	74222977	74223040	TCTGTTAAACATAACAATTTCCCG	AAAACATGAAACTAAACAAACCCAA	277
Ad02A13159	Aradu.A02	13159	c	(T)14gtgtctcct	42	74226341	74226382	TGCAAAACATGATACATGTGAAA	CGAAGAGAAACACGACGATG	178
Ad02A13160	Aradu.A02	13160	p3	(GAA)6	18	74226514	74226531	CATCGTCGTGTTTCTCTTCG	TCCTCCTCATCTTCTGCTGC	148
Ad02A13161	Aradu.A02	13161	p4	(AAAT)10	40	74230265	74230304	AATTATTGAGCTCGTTTTAGACATATT	AAATCAAACCGAAATTTGATCTTC	274
Ad02A13168	Aradu.A02	13168	c	(A)10ttataattta	70	74237613	74237682	ATAAAGTGGGCGAGACTTGG	GGAAGCAGTTACTTCCGTGG	200
Ad02A13174	Aradu.A02	13174	p2	(GA)20	40	74271895	74271934	CAAAATTGCCATAGAAAGGAGAA	CATGAAGATTCTAGTCCATGCAA	257
Ad02A13175	Aradu.A02	13175	p6	(CATGAG)5	30	74276967	74276996	TCATCCTGCTGGAATTTGAA	GTAGCCGTTCAATAGCGGAA	278
Ad02A13178	Aradu.A02	13178	p3	(TTG)5	15	74283533	74283547	ACATGACAGAGCACAATGGC	GTGCAGTGCTTGAAGGTGAA	226
Ad02A13179	Aradu.A02	13179	c	(T)11actttcattt	70	74283724	74283793	TTCACCTTCAAGCACTGCAC	TGATGACATGGATAAGAGTGGG	203
Ad02A13180	Aradu.A02	13180	p2	(AG)6	12	74285638	74285649	GACGGTGGAGGCATAGATGT	CGGACACAGGTCATCATCAG	204
Ad02A13181	Aradu.A02	13181	p2	(TC)8	16	74288218	74288233	TTTATGATGGGCCGTTGAAT	TTTCGTTAGAGTTTCGTGCTGA	239
Ad02A13182	Aradu.A02	13182	p3	(TTA)5	15	74289268	74289282	AAGCATCCTTGAAAAGGGGT	TCCTTGCCATTTTTCTTCAAA	255
Ad02A13187	Aradu.A02	13187	p3	(ATG)5	15	74300558	74300572	TTTGCTACAAATGCACAAAAA	CTCCTCTTCATCTTCTGCGG	273
Ad02A13188	Aradu.A02	13188	p2	(AT)12	24	74318674	74318697	TGATCATACCACCAAGCTGC	AAAGACAATTGTATGTGTGTCTTTACC	256
Ad02A13189	Aradu.A02	13189	p2	(TA)8	16	74327204	74327219	TGATAGTCACAGGTCAAAGGAAAA	TGGGACATTAGTGATTCGGA	233

ID	Chromosome	SSR nr.	SSR type	SSR	Size	Start	End	FORWARD PRIMER (5'-3')	REVERSE PRIMER (5'-3')	Product (bp)
Ad02A13190	Aradu.A02	13190	p3	(AAT)6	18	74327399	74327416	CCGAATCACTAATGTCCCATTT	TGGGATCATGTATATGTTGGAGA	244
Ad02A13191	Aradu.A02	13191	p2	(AT)7	14	74328345	74328358	CAAAACCGCTCCCAAAAAT	TTTTTCCCCTTTCAGGCTTT	276
Ad02A13192	Aradu.A02	13192	p2	(TA)6	12	74356139	74356150	TCCCTTTTGATCACCACACA	TTGATTTTGGGAGAGTTGGG	273
Ad02A13195	Aradu.A02	13195	p4	(AATA)7	28	74405663	74405690	CATAACTAGCCCCCGACTCA	GAAGCGAAGTAGTGCTTGGG	276
Ad02A13199	Aradu.A02	13199	p3	(TAT)7	21	74420609	74420629	TTCTCCTTTTGCAAATTATTGAGA	GAGGCTTTGGACAACACCAT	280
Ad02A13200	Aradu.A02	13200	p3	(AAT)5	15	74422026	74422040	ACAATTTTGCTGGGTTTTGC	AAAAAGAGGTGCCACACTGC	264
Ad02A13203	Aradu.A02	13203	c	(T)11atttgacca	48	74424737	74424784	ACTACGAGCCAGCACAACCT	TGCGTGCTATGTCGATTACTG	195
Ad02A13206	Aradu.A02	13206	p2	(AG)8	16	74426773	74426788	GAGCAGAAGCTGTTGCCATA	CCAATTGAGATCATGGAGGG	233
Ad02A13210	Aradu.A02	13210	p2	(TC)16	32	74430871	74430902	CCTACCCCTTTACAGCTCCC	TGAAACTGCAAGGACAATGC	252
Ad02A13211	Aradu.A02	13211	p2	(TA)9	18	74436578	74436595	TGTCCTTTGGTTGATTGATGA	AGGCAGTTGCAGCCATAGTC	216
Ad02A13213	Aradu.A02	13213	p2	(GT)6	12	74460190	74460201	GTGTGTAATTCGAACCCAGC	TCATGAATCCACCAATGCAC	126
Ad02A13216	Aradu.A02	13216	c	(T)10gaattatca	77	74466196	74466272	TCGAGGGCAAAGAAAGAAGA	TTTTTAAGAGAACCATATGACGAAAA	277
Ad02A13217	Aradu.A02	13217	p3	(AAC)17	51	74475075	74475125	ATTGAAAGGCGGGAAAGATT	TTTCAGGATCCGCTACTGATT	186
Ad02A13218	Aradu.A02	13218	p3	(AGA)6	18	74503873	74503890	CTTGGGTGTCATTGATGGTG	ACCCAAAATAGCCGAAACCT	249
Ad02A13220	Aradu.A02	13220	p3	(ATT)8	24	74530015	74530038	TCGTGTATTCGACTCTCGTTTC	AAAAGCCATGCCTAACACAAA	202
Ad02A13225	Aradu.A02	13225	p3	(GAG)9	27	74538341	74538367	AGGGTTGAAATGTGAGTGTGG	AGGAAAACATCCAATGGCAG	229
Ad02A13229	Aradu.A02	13229	c	(TA)7tgaacaat	101	74545957	74546057	TCAACAACTCTCCCACTCAAGA	CGGAATGAGAATAAGAATGAATGA	187
Ad02A13238	Aradu.A02	13238	p4	(TATG)5	20	74567467	74567486	CGCGTCTGGAATTTCCTAAG	AAATTTTGACTCGACCTGCG	248
Ad02A13240	Aradu.A02	13240	p2	(AT)7	14	74572854	74572867	GACTAAACTGGGGCCATGTG	AGACATGGGTCAAGCTAGGG	185
Ad02A13244	Aradu.A02	13244	c	(T)10ggaaaaaa	110	74593853	74593962	GAGAGTCAAAATCTTGCCAGG	AAAGCCTTTTTGGTTATTGACA	274
Ad02A13248	Aradu.A02	13248	p2	(AT)9	18	74606679	74606696	GGAGACTATGTTCGTCACGG	ACAAGAGCCCAACGAACTTG	176
Ad02A13249	Aradu.A02	13249	p3	(TAT)5	15	74627553	74627567	CCTCCGTTGCAGTACCAAAT	GCAAGTTATTACATTAATGATACCCCA	241
Ad02A13250	Aradu.A02	13250	p2	(TA)9	18	74629336	74629353	TGCACCTTAAAAGATCAACTTCA	TTGAGGTTCACTCGATGCTG	279
Ad02A13251	Aradu.A02	13251	p2	(TA)6	12	74630609	74630620	GCCAATTCTCTCGTGCTGAT	CGTGGGATATAGCTGCTTTTG	232
Ad02A13259	Aradu.A02	13259	p3	(TAT)5	15	74690589	74690603	CCTAAATGGATCAGTTGGCCT	CGCGAAATAAATAACCTTCCA	276
Ad02A13265	Aradu.A02	13265	p3	(GAC)5	15	74715326	74715340	GGGGTTGTTGAGGACAAAGA	AAAAGAAAACACTAGACGCTACGA	246
Ad02A13267	Aradu.A02	13267	c	(CT)9ttc(T)12	33	74718941	74718973	GGGAGCTTCTGAGCCTTCTT	AGACCCTCTCTCCAACACGA	257
Ad02A13268	Aradu.A02	13268	c	(TTA)5cttctact	64	74719132	74719195	CTCGTGTTGGAGAGAGGGTC	GCAAGGGGAAACAAAAACAA	146
Ad02A13270	Aradu.A02	13270	c	(A)10gagaaaga	39	74742912	74742950	CTGCTCTCTCCAGGAACCAC	AACGATCAATTTGCCATTCA	198
Ad02A13271	Aradu.A02	13271	p2	(CT)6	12	74748463	74748474	ATGCATGCAAGATGAGATGG	ATAAAAACCGGATCCAAGCC	279
Ad02A13272	Aradu.A02	13272	p3	(CAC)5	15	74752896	74752910	AGGCTACACCTCCTTCGGTT	TGTGCCTGATTAGTCGCTTG	124
Ad02A13278	Aradu.A02	13278	p3	(AAG)5	15	74805994	74806008	AAAGTTTTGCATCGTCACCC	ATCCCAAATGTTTAGCGTGC	143
Ad02A13280	Aradu.A02	13280	p3	(AAG)5	15	74810765	74810779	TCATTGTTTTGCGTCATGGT	TGGACCCAATTTAATCGCTC	188
Ad02A13282	Aradu.A02	13282	p5	(AAGAT)7	35	74814694	74814728	AAAGGGCCCGAATAGAAGAA	CCTGGGTGCTCCACTGTATT	194

ID	Chromosome	SSR nr.	SSR type	SSR	Size	Start	End	FORWARD PRIMER (5'-3')	REVERSE PRIMER (5'-3')	Product (bp)
Ad02A13283	Aradu.A02	13283	p5	(AAGAT)6	30	74819779	74819808	TAGAGATAAGGGCGGTTCCC	GATGGTGCTGATCCTTCGTC	249
Ad02A13284	Aradu.A02	13284	p2	(AC)10	20	74820186	74820205	ATTAAACCCCAAAATCCCCA	TCCTCGATCCCCTTTTCTTT	179
Ad02A13300	Aradu.A02	13300	p3	(TAA)13	39	74867878	74867916	CATCATCTGAATCCCCCAAC	CGAACAATGCTTGGATCTGA	101
Ad02A13302	Aradu.A02	13302	p2	(GA)6	12	74899003	74899014	TGAAGCGATGCAAGTTTTTG	GGTGATGCCCCTCTTTTACA	210
Ad02A13304	Aradu.A02	13304	p2	(AT)6	12	74915261	74915272	TTTTTATTCAGGCAAATACTAAACAA	GGATCGGTATCTCTTTGTTGG	277
Ad02A13306	Aradu.A02	13306	p2	(AG)6	12	74929725	74929736	TCGAAAGCCTAAGTCCTTGC	TGCCACCCCTAGTCTCTTTG	189
Ad02A13307	Aradu.A02	13307	p2	(AG)6	12	74930177	74930188	ACACATATTATGGGGGTGCG	GGAGAGGTTTCCAGATGGTG	277
Ad02A13308	Aradu.A02	13308	p2	(AG)9	18	74930397	74930414	GGAGTTGCCAAAGGAGTGTC	GCAACTCCATCCCCCTTTAT	150
Ad02A13309	Aradu.A02	13309	p3	(TTC)5	15	74942177	74942191	CCTACCCCTCTCTCCACTCC	CTAGAGGTTTCTCCGGCCTT	247
Ad02A13312	Aradu.A02	13312	c	(A)12gatttaatta	116	74951481	74951596	AAGAGAAAAAATTCTAAATGGCTCC	CGTTTTTGTTACGGTAAGGACC	203
Ad02A13314	Aradu.A02	13314	p3	(TTC)7	21	74988098	74988118	CCCATAAACCCCACCTACCT	TAAAATATTGCTCGCCCTCG	243
Ad02A13316	Aradu.A02	13316	p2	(AT)8	16	74993941	74993956	GGGGCGATTTTACAATACCA	CTCACACGTACACGTGACCC	274
Ad02A13317	Aradu.A02	13317	p3	(TAA)5	15	74996811	74996825	ATTTTGTTGGAATTATGTATTTGTGT	AAAGACCTTTTTAATGATCGCAA	250
Ad02A13319	Aradu.A02	13319	c*	(AT)7(ATA)5*	27	75015573	75015599	CATGCTCACGAACATGTATGC	CCCATTTTTCAAGGCTCCTA	280
Ad02A13325	Aradu.A02	13325	p2	(TA)6	12	75066444	75066455	AGCCTTTGTAGAAAAGGGGG	AGCCGGATCTTGAGTCTTGA	153
Ad02A13326	Aradu.A02	13326	p2	(TA)8	16	75075123	75075138	CTGGTCCATGTGCTCAGAAG	GGTAAACCGTGAAGCCAAAA	255
Ad02A13329	Aradu.A02	13329	p2	(GC)6	12	75094168	75094179	TGGTGTTTGGAAGTGTTGGA	GCGCCACTCTCGAAATAAAC	160
Ad02A13330	Aradu.A02	13330	p2	(AG)8	16	75108202	75108217	TTCTTAGCAAAATTTAGTTGGGG	CACAGTGTTGATTCGCTGCT	145
Ad02A13333	Aradu.A02	13333	p3	(TTA)5	15	75141784	75141798	TGAAACCGGGTACGTTCATT	CATCATCTGAATCCCCCAAC	247
Ad02A13338	Aradu.A02	13338	p2	(AT)6	12	75150158	75150169	TGCAGTAATTTATTGACCAATGC	CCAAAAGAGACGTCACAAAAA	280
Ad02A13341	Aradu.A02	13341	p3	(TTC)7	21	75160271	75160291	CCCAACCCCTTATTACACCA	GGTTTCTCTCACGACTCGCT	103
Ad02A13342	Aradu.A02	13342	p3	(TCT)5	15	75162193	75162207	TTATTTTTCACGCACACCCA	AAGGAGCTCACCACATGTCC	269
Ad02A13344	Aradu.A02	13344	p2	(AC)6	12	75181744	75181755	CTGGAGCTTGGTGTTGGTTT	TCCCAGTGGTGTGCTAATGA	249
Ad02A13348	Aradu.A02	13348	p3	(TTA)11	33	75193255	75193287	TCATCATGCCCACCAATTTA	GGGGCTCGTTTCATTACAAA	154
Ad02A13351	Aradu.A02	13351	p4	(ATAA)5	20	75195238	75195257	TTCATCTTCAAAGGGGCAAC	GTAAGCCAAATCCGAGCTGA	129
Ad02A13352	Aradu.A02	13352	c	(A)10g(A)11	22	75195457	75195478	TGGTTCGTTTTGTGATCCTG	CTCAGTGAGCCACTGCAAAA	274
Ad02A13353	Aradu.A02	13353	c	(T)10cgaacaan	75	75196046	75196120	TGTGTGTTATATATCGGACGCAT	TGTGGGGTTGAAGACCTTTC	259
Ad02A13354	Aradu.A02	13354	c	(T)17gtgaaaaa	45	75196520	75196564	GGGGAAAAATTTGCTGAGTG	CACAGTGGTGGCATTACTTGA	251
Ad02A13360	Aradu.A02	13360	p2	(TA)8	16	75202683	75202698	ACACATGGGTTTTGGTGTCA	CCATTTGGGAGAAGGAAAGA	124
Ad02A13363	Aradu.A02	13363	p2	(TA)7	14	75214714	75214727	TGCAAGTCCGGCTTAGAACT	TTGCCGGAGACAAATTTCAT	162
Ad02A13366	Aradu.A02	13366	p4	(TTTA)10	40	75246083	75246122	CCATGATGGAGAAAGGGAGA	TCCTTTAGGTTAACTTGGTCAAAA	277
Ad02A13367	Aradu.A02	13367	p2	(TA)6	12	75266191	75266202	TCAAATCAATTGAGGTGCCC	TTAAGTGTGGGGAGGTCGTC	203
Ad02A13371	Aradu.A02	13371	p2	(TG)6	12	75302944	75302955	GTTGCTGTAGAGCCTGAGGG	CCGACGCACGAACATTAGTA	260
Ad02A13373	Aradu.A02	13373	p3	(ATT)7	21	75337927	75337947	TTGATTGAAAAATTTTAAGTTTGGTG	CCATGATGGAGAAAGGGAGA	267

ID	Chromosome	SSR nr.	SSR type	SSR	Size	Start	End	FORWARD PRIMER (5'-3')	REVERSE PRIMER (5'-3')	Product (bp)
Ad02A13376	Aradu.A02	13376	p3	(AAT)8	24	75361268	75361291	AAAAGAGAGAGGGGTTGGGA	GAAGAGTTTAAAAACGGTCCAA	271
Ad02A13381	Aradu.A02	13381	p2	(AT)7	14	75394652	75394665	CAAGCACACACATCCACCTC	TCCTGAGAACTGCAGGATGA	194
Ad02A13383	Aradu.A02	13383	p3	(AGA)5	15	75410086	75410100	GGTGCCTTCAATGGTTATGG	CACACATCCCTCCATCTCCT	137
Ad02A13387	Aradu.A02	13387	p3	(TTC)5	15	75459117	75459131	CCCCACCTACCTCCAAAAAT	TAAAATATTGCTCGCCCTCG	233
Ad02A13390	Aradu.A02	13390	c	(CT)8caaacattt	80	75493002	75493081	GAAGCACTTTAGGCAATCCG	TCAAGTTCGATTTCTCCTTCAA	280
Ad02A13400	Aradu.A02	13400	p2	(AC)6	12	75561829	75561840	CACCAAATCCTCCTCTTCCA	TTTATCAACCACTTGTATTTGATTTT	240
Ad02A13404	Aradu.A02	13404	p2	(TA)9	18	75577778	75577795	AGCCGGATTTTGAGTCTTGA	AGGGGGTAAACAAAAATCGC	145
Ad02A13405	Aradu.A02	13405	c	(A)10ttgaattttg	79	75586606	75586684	CATAGCCAAAGGGGTGAGAG	TTGCAAACGTGTGCCTTATT	212
Ad02A13407	Aradu.A02	13407	p2	(TA)8	16	75592838	75592853	GCACCATCATCTTTACGTGG	ACACACTACCTGCGCAACAA	272
Ad02A13409	Aradu.A02	13409	p4	(AATA)5	20	75600383	75600402	TTGTGCTTGTTTAGGTGCCA	TTACATGGCCACAACCTACAA	265
Ad02A13410	Aradu.A02	13410	p2	(AT)7	14	75601822	75601835	GCAAGCGTGAGGTTTTTCTT	GGCATTGTTGGATCGTTTCT	275
Ad02A13413	Aradu.A02	13413	c	(A)10gatattggg	67	75616059	75616125	TCCATTTCCAAAACTAAGGCA	TTCTTTGGGAGACAGAAACAGA	279
Ad02A13414	Aradu.A02	13414	p5	(TTATC)6	30	75624068	75624097	TGTGGAACTGAGAATGCTGC	CAGAGCCCAGAAAGATGAGG	272
Ad02A13418	Aradu.A02	13418	c	(T)11aatattgga	110	75644582	75644691	AAGTCTGGTGTAATATTCAGATATGGT	GAATGTTACAATTATTCGGGACC	270
Ad02A13421	Aradu.A02	13421	p2	(AT)11	22	75657292	75657313	ATCAAGAACGTGGAAATCCG	GGAGAAAATTGACATCTCCCTC	247
Ad02A13424	Aradu.A02	13424	p2	(TG)11	22	75664472	75664493	GCCGGGTTGCTATATTTTGA	TCGACTTGCGAAATCAAGG	185
Ad02A13427	Aradu.A02	13427	p3	(TAT)5	15	75667359	75667373	CCAAAGCAGTCACTAATGCG	CAGTCGATATGCCGGTATCC	231
Ad02A13428	Aradu.A02	13428	c	(AG)7aaaaaaga	95	75677644	75677738	GGTCAATTTATTGGACCTGATTA	AAGAAGGGATGAGAAGATGGA	280
Ad02A13429	Aradu.A02	13429	p2	(AT)6	12	75679632	75679643	GTCCCTTGAGCACCAGTCAT	AAAGCGATTGGATCACCAAC	263
Ad02A13430	Aradu.A02	13430	p3	(ACA)9	27	75681437	75681463	TTTTAGACGGCAGCAGAAAAA	CCTTCGACCCAGTTCTTTCA	275
Ad02A13432	Aradu.A02	13432	p3	(TAC)5	15	75692409	75692423	TGATGTCTGATGATGACGAGAA	AGCTGAAGCGCCAGAAATTA	238
Ad02A13440	Aradu.A02	13440	p2	(AT)6	12	75748883	75748894	AAATTACAAAACGCGAAGCG	ACTCCGCAGGCTGTGAATAG	181
Ad02A13441	Aradu.A02	13441	p2	(TC)25	50	75750658	75750707	CCCCAAACCCTTCTCTTCTC	TCGAAAATCGGGCACTAATC	227
Ad02A13443	Aradu.A02	13443	p2	(TA)6	12	75773155	75773166	CGCAATGAGAAAGCTGTGAG	ACAGGGGTGCTGTCTTTGAG	251
Ad02A13444	Aradu.A02	13444	p3	(TCT)5	15	75773300	75773314	GGATCCGCTCTCAAAGACAG	TCGAGGAAATCCTCATCACC	265
Ad02A13445	Aradu.A02	13445	p2	(GA)6	12	75773670	75773681	GACGAAGGTTGAGGAGCTTG	TTAAGCCAGGTGTCGGTCTC	239
Ad02A13446	Aradu.A02	13446	p2	(TC)9	18	75782505	75782522	TGAGACGTGCTAATGGCAAC	TTTGCCACATCATTCTTTTCTG	271
Ad02A13447	Aradu.A02	13447	c	(A)12ttactttggg	122	75784379	75784500	GTGCAAACCACATTCATTGC	AACCAAGGCTGAAGAAACCA	228
Ad02A13448	Aradu.A02	13448	p3	(CAA)5	15	75789646	75789660	ATCATTCCCACAGAACCGAG	GCACCTTGTTTCTGCCTCTC	260
Ad02A13452	Aradu.A02	13452	c	(AC)9atactcaa	99	75804373	75804471	AACAGAGACGCAAGGAGAAG	ACTCGTAGGGTCCGATTCCT	237
Ad02A13462	Aradu.A02	13462	p2	(TC)7	14	75839170	75839183	GCATGAGTCATCCAGTGTCA	CCGGGATGTTGCTTAATCTG	239
Ad02A13464	Aradu.A02	13464	p3	(ATT)5	15	75839832	75839846	TTAAGCAAGCAAGGTAGCCG	AGACCACCGTTTGTGTAGGTT	220
Ad02A13470	Aradu.A02	13470	p3	(CGA)5	15	75857786	75857800	CATGGTCAAACTTTGGTGGA	CCAAGTTCAAGCTCACACCC	171
Ad02A13471	Aradu.A02	13471	p3	(ATT)6	18	75858894	75858911	TTTCTTGAAAACCTCAATCTCTTTTT	CCACCCTTTTCACATTTGGT	195

ID	Chromosome	SSR nr.	SSR type	SSR	Size	Start	End	FORWARD PRIMER (5'-3')	REVERSE PRIMER (5'-3')	Product (bp)
Ad02A13474	Aradu.A02	13474	p2	(AC)6	12	75861300	75861311	CACGGCTCATATCACCACAG	ACCACCACCACCATTTCCTA	208
Ad02A13476	Aradu.A02	13476	p2	(TA)7	14	75870584	75870597	CGGTATATCTACCATAAGTGTACAACG	GTACGTGACTTGGGCCAGAC	263
Ad02A13479	Aradu.A02	13479	p4	(AATA)5	20	75880344	75880363	CCAGCATTTATGGATCGGAC	GTTGAGGGCAACAGTGGAAT	198
Ad02A13480	Aradu.A02	13480	p3	(TAT)5	15	75889346	75889360	TCTTCAAATTCCCACCTCAA	TGAATCTTGTCCTGGTGCTTT	225
Ad02A13481	Aradu.A02	13481	p2	(AT)8	16	75891000	75891015	AAAGAGGTTGCAACATTGGG	GCTTGAAGCCTCATGGAAAA	182
Ad02A13484	Aradu.A02	13484	c	(T)10gttaattttg	87	75897412	75897498	TCTCTTCTACCAACTTTACCCTTCA	CGCTTTACACTATCAGTGCATCA	273
Ad02A13488	Aradu.A02	13488	p2	(CT)9	18	75908708	75908725	AAAGGAACATTGAACCTGGAAA	GGCTGTTTTTGACCCAGTTC	241
Ad02A13490	Aradu.A02	13490	p3	(AGA)5	15	75920253	75920267	ATTGTTTGCGAAAGAGGGG	TCACCTGCTCAACCAATGAC	191
Ad02A13491	Aradu.A02	13491	p2	(TA)6	12	75922059	75922070	AGCAACCAAAACATTATCAGAGA	CCCGATGGTGAGAAGAATGT	279
Ad02A13494	Aradu.A02	13494	p3	(GAT)6	18	75933393	75933410	GATAGAGCCAGGCTTAGGCA	CACCCCAACAAGAAGAGGAA	216
Ad02A13498	Aradu.A02	13498	p2	(TA)8	16	75944950	75944965	CCGGGTTACTTGCATTGTTT	ATTGAAAGCCATTGTCAGCA	280
Ad02A13499	Aradu.A02	13499	c	(ACA)7(ATA)	51	75967399	75967449	TTTGCATGAGATACCCAAACA	TGGTGGTTGTTCATGTTGTTC	171
Ad02A13502	Aradu.A02	13502	p2	(TC)7	14	75969391	75969404	AGCGGTTTCAAATCTCCCTT	ATTGCTACCACCGTCGAAAG	158
Ad02A13503	Aradu.A02	13503	p2	(AT)6	12	75973045	75973056	CGAACCACCCTAACATATCCA	TTCGAAGTTGGTTTGTCTCTCTC	243
Ad02A13508	Aradu.A02	13508	p3	(GAA)5	15	75996188	75996202	ACTGCTGCTGCTCCTTCAAT	ACTCACACCGTCCTTCATCC	270
Ad02A13509	Aradu.A02	13509	p2	(CT)9	18	75996824	75996841	CCTGGTCATTAATATGACTCGC	CCTGCAAAATCATTGGGACT	136
Ad02A13514	Aradu.A02	13514	p3	(TGT)5	15	76023561	76023575	CCACCTTGTTGCCTCTTGTT	CGAACTCTATGCCTCCAAGG	278
Ad02A13515	Aradu.A02	13515	c	(T)10gctaaaatt	162	76027372	76027533	TGGATTTATTCCTTTGGGGA	TGTGCATCCTTCTCGAGTCA	278
Ad02A13516	Aradu.A02	13516	p3	(CTT)5	15	76042936	76042950	CAGGGACTATCCAGGGTTCA	GAAGACAACCACGATGACGA	225
Ad02A13519	Aradu.A02	13519	c	(A)10gattacagt	109	76045901	76046009	TTGAAAAAGTTAGCCGTCCA	AATGAGGCAATCCTCGTCAC	278
Ad02A13520	Aradu.A02	13520	p2	(AT)12	24	76050532	76050555	TTGTGATCAAAGTCTCATGCTG	TGGATGCCCATTTAATGACTT	253
Ad02A13526	Aradu.A02	13526	p2	(AT)7	14	76092146	76092159	TGTGGGGAATTTTATGGGAA	TTTTTGAATTTTGTCCCACG	229
Ad02A13529	Aradu.A02	13529	p2	(AT)6	12	76098103	76098114	AGCTCAAGTTCGGCTCATTG	GGATAAGTTTAGCTCATTAGCTCGT	273
Ad02A13535	Aradu.A02	13535	p2	(CT)8	16	76131281	76131296	CGGACGCCGTGTAATTATGT	TGTGAGTAGTGATGGAAATGGA	277
Ad02A13536	Aradu.A02	13536	p3	(CCA)8	24	76132807	76132830	TGAGGGTCAGAATCGAAACC	TGGTGGTTGTGGTAGTGGTG	187
Ad02A13537	Aradu.A02	13537	p3	(CAC)7	21	76132946	76132966	CACTACCACAACCACCACCA	CCTGCTTTCTTGCTTCTGCT	270
Ad02A13542	Aradu.A02	13542	p3	(TTA)5	15	76153332	76153346	CCATAGCTGAGTGATCCACCT	CTCCCCGAAAACAGAAAACT	280
Ad02A13543	Aradu.A02	13543	c	(ATTT)5tggttt	90	76153575	76153664	TTTATCTTTTAGTTTTCTGTTTTCGG	CCGAAAATACTGACCTTTTCTTTC	265
Ad02A13549	Aradu.A02	13549	p3	(AAG)5	15	76163463	76163477	TTTCTCCTCCTCATCTTCTGC	TGCACTGCACGTTCTTCTTC	198
Ad02A13550	Aradu.A02	13550	p3	(AAG)5	15	76163596	76163610	ACGTGCAGTGCAGTGAAAAC	CGATTTAAATTCACACATTCGTT	234
Ad02A13553	Aradu.A02	13553	c	(A)11gaagaaga	33	76164743	76164775	AAGGAGAAGGAAAAAGAGAAGCA	CTGGAACCCCAATCTTTTTG	251
Ad02A13556	Aradu.A02	13556	p3	(CCT)5	15	76180499	76180513	GAGTGAGTGCTGCTAGGGCT	CTGGTTCAGGAGAAGATCGG	153
Ad02A13559	Aradu.A02	13559	p2	(AT)6	12	76202516	76202527	TAAGCTTTTGAAGGGCTGGA	CGCCATAAAGTGGTCAGGTT	267
Ad02A13561	Aradu.A02	13561	c	(A)12(CA)9	30	76207714	76207743	TCCCAAAAGCGTAACCTGAC	GGAGCCAAAAGTCTTTGATGG	278

ID	Chromosome	SSR nr.	SSR type	SSR	Size	Start	End	FORWARD PRIMER (5'-3')	REVERSE PRIMER (5'-3')	Product (bp)
Ad02A13563	Aradu.A02	13563	c	(GAG)7ggagtg	134	76208332	76208465	TGAGAATTGAGAATAGGGATGG	TCCAACCAACAACCATCATC	274
Ad02A13564	Aradu.A02	13564	p2	(AT)10	20	76208651	76208670	GATGATGGTTGTTGGTTGGA	GCATCGAAGTTTCTTGACTCG	249
Ad02A13567	Aradu.A02	13567	p2	(AT)9	18	76219375	76219392	AAAAGCATAGATTAAATGCATATAGGA	TCACGAGCTGGCTCAAATAA	271
Ad02A13569	Aradu.A02	13569	p2	(CT)6	12	76237365	76237376	ACCTGTGACGCGATTCTACC	GTGTTGGAGGCCAAAAGAGA	183
Ad02A13570	Aradu.A02	13570	p2	(TA)6	12	76239316	76239327	CCTCCATCACCATTTTCACA	TCTTTTATTGAGGTCAAGAACTTGTTA	229
Ad02A13573	Aradu.A02	13573	p2	(CA)6	12	76264346	76264357	TTATTACCTTGCCTCGGTGG	ATGTTCCTCGCAAGGCTAAA	184
Ad02A13574	Aradu.A02	13574	p3	(TAT)16	48	76269968	76270015	CCTAAAGAAATGATTCAAACTCAAAA	CCTGCATAATTGGCTATCAAAC	194
Ad02A13575	Aradu.A02	13575	p2	(TA)7	14	76272516	76272529	CCCACCGTAAATTCACCATC	GATAGAATAGCGAATGTGTAGACTGTT	238
Ad02A13577	Aradu.A02	13577	p3	(TTC)7	21	76292166	76292186	CACTTCCCAAAAACCCTTCA	ATGCTTTTACCGCACCAAAC	256
Ad02A13581	Aradu.A02	13581	p2	(GT)7	14	76320370	76320383	TATGAGTTGCAAGCACTGCG	AATGGTGCGATGAACAGTTTT	280
Ad02A13583	Aradu.A02	13583	p5	(ATTTT)5	25	76350537	76350561	AGGGAGGTGATGTGAATTGTG	ATCCCTTGGGTTGAATTGGT	278
Ad02A13588	Aradu.A02	13588	p2	(AT)8	16	76378310	76378325	GAAACTGTGGGAACATGGGT	TGGGGTAATTTTGTCCCCTT	203
Ad02A13589	Aradu.A02	13589	p2	(TA)6	12	76380998	76381009	ATCAATGCACATGGGACAAA	GGGACTAAGGTAAGGGTAAGGG	205
Ad02A13590	Aradu.A02	13590	p2	(AG)10	20	76392782	76392801	AGTGCTTGGTGGGAGACAAC	ACTCCCACGCAACTCTCTGT	279
Ad02A13597	Aradu.A02	13597	p3	(TTA)16	48	76429829	76429876	TGCCTTAACGGAAACAAAGAA	CCTTACTATTAGGCATTCTTGTAATCA	243
Ad02A13599	Aradu.A02	13599	c	(GAA)5catcag	109	76441090	76441198	TGGTTCTGAAAATGTTGTTGTTG	GTCCATCCTTGCTACCGAAA	205
Ad02A13600	Aradu.A02	13600	c	(AT)7agag(AT	38	76442006	76442043	TCGGAAAAGATTTGCTGTGA	AATTGGCCCTGTGTTCTTTG	113
Ad02A13607	Aradu.A02	13607	c	(T)11ggttttattc	106	76533467	76533572	TGCACAAACAAGACTGAATCG	TTGGACTCGTTGTCTTGCAC	277
Ad02A13608	Aradu.A02	13608	p2	(AT)6	12	76535557	76535568	ACATCATTCGTTTGCACGAT	CACGTGAACTAGCCAAACCA	271
Ad02A13610	Aradu.A02	13610	p4	(TAAA)5	20	76538447	76538466	GGTGTGGATTGGGCTCTTTA	TGTCCTCATGTCAGACACACA	269
Ad02A13613	Aradu.A02	13613	c	(A)11ctaaggtca	74	76546779	76546852	TTAATGAATACAATCATACAATCACCT	AAATCAACATTCACTTTACACATTTTT	189
Ad02A13614	Aradu.A02	13614	p2	(TA)6	12	76550838	76550849	CAATTCCCGAAAGACTTCCA	TTTTGTGTGATGCAGGGTGT	236
Ad02A13617	Aradu.A02	13617	p3	(CTG)5	15	76552821	76552835	GAACGCTTCCTATCTGCGAC	ACACAATTCGCGGTAAAAGG	232
Ad02A13618	Aradu.A02	13618	p2	(AC)8	16	76562655	76562670	TCTCGTTAGGCGTCTGGACT	TGCTATGAGTCTCGCGTTAAA	217
Ad02A13619	Aradu.A02	13619	p2	(TA)6	12	76562782	76562793	TTTAACGCGAGACTCATAGCA	CCAAATGCATGCTCACAAAC	279
Ad02A13620	Aradu.A02	13620	p2	(TG)42	84	76565631	76565714	GGATTTTCACAGTTCGCCTT	GTTATGCGGAGAGAAAGCCA	195
Ad02A13623	Aradu.A02	13623	p3	(TTC)5	15	76575074	76575088	TTAACTTTCACTCCACGCCC	TGCAGAGAAGAAAAATGCGA	201
Ad02A13625	Aradu.A02	13625	p2	(CG)6	12	76584920	76584931	CCACCTGCACTCCTGGTACT	TAGTGAAAGAAGGGCATCCG	229
Ad02A13626	Aradu.A02	13626	p2	(AT)6	12	76596896	76596907	CTGCGCCACTCTGATAGCTC	CAGGATATGGACGACGAAGC	242
Ad02A13628	Aradu.A02	13628	p2	(CT)6	12	76624403	76624414	GTAGACCGGCGATGACTCTC	CTTCGTCGCCACTCACACTA	184
Ad02A13629	Aradu.A02	13629	p2	(TA)8	16	76630970	76630985	ACTCCCTAGATCATGGCACG	AATGCGTCTTGGGAATTTGT	212
Ad02A13632	Aradu.A02	13632	p2	(AT)7	14	76672852	76672865	GCCGTAAAACAGAGGTGGTG	CTGAGCGTCTGGAGTTTTCC	241
Ad02A13635	Aradu.A02	13635	p3	(ACT)5	15	76679647	76679661	GGCTACTGTGTCACCCACCT	AAGGGTTTCTGAGCCAAACA	133
Ad02A13637	Aradu.A02	13637	p3	(ATT)6	18	76680759	76680776	TGGAAACAAACATATAGCCCA	CAAAATTCGCAATCGTTAGAAA	271

ID	Chromosome	SSR nr.	SSR type	SSR	Size	Start	End	FORWARD PRIMER (5'-3')	REVERSE PRIMER (5'-3')	Product (bp)
Ad02A13638	Aradu.A02	13638	p3	(ATT)24	72	76686954	76687025	GCAAAACCCTAGCAGAGCAC	CCGTAACTCCACTTCAACTCG	240
Ad02A13640	Aradu.A02	13640	p2	(GA)6	12	76700264	76700275	TTGGGAGGGAAAAGTGTTTG	CCTAAATGGAGCAGGGATGA	242
Ad02A13647	Aradu.A02	13647	p2	(AG)7	14	76722745	76722758	AGGAAATACACATCTCCTCGAA	CCCACGCAATTTTGGAGTTA	276
Ad02A13648	Aradu.A02	13648	p2	(GA)10	20	76724296	76724315	GACAAGAAGAGTTCGGCGAC	AAAGCCCAATTTCCCAATTC	164
Ad02A13650	Aradu.A02	13650	c	(A)10ctagaacc	108	76725930	76726037	TCCATTTTGGAGTAATTTTTCAAT	GACATATGGTAAATGAGCAAAACA	233
Ad02A13653	Aradu.A02	13653	c	(TCA)6attcact	47	76734158	76734204	GACCCAGAACCCCATACCAT	TGAAGAAGAAGAAACGATGGG	273
Ad02A13654	Aradu.A02	13654	p3	(TCA)6	18	76734336	76734353	TCCATGTCATTGAATAATCCCA	GTTGTGGGACATGGTAACCC	209
Ad02A13655	Aradu.A02	13655	p3	(CTA)5	15	76734608	76734622	GGGTTACCATGTCCCACAAC	CGGAGGGAATGAATGAAGAA	176
Ad02A13656	Aradu.A02	13656	c	(AAC)5aataatc	93	76734982	76735074	TGCATTTGAATAACCCGTCA	TTCAGGCTTGAACACCATGA	173
Ad02A13657	Aradu.A02	13657	p3	(GGT)5	15	76735249	76735263	AGATTTCGATAACCATGGCG	TACCAGAACCCCCACCACTA	275
Ad02A13660	Aradu.A02	13660	p3	(GAA)5	15	76739864	76739878	AAGGGATGTTATTGGAGGGC	ACCCCTCGCCTAAAATGAGT	205
Ad02A13665	Aradu.A02	13665	p6	(ATTTTT)5	30	76778123	76778152	GCCGGTTAGTCTGTGGTTGT	CGAAACGAAAAGAATGGGAA	213
Ad02A13669	Aradu.A02	13669	p2	(AT)9	18	76796787	76796804	TTTTTAACCAATAAATAGCCAACAAA	TTTGTAGGACTTTTCACCATGC	238
Ad02A13672	Aradu.A02	13672	p3	(AAT)7	21	76797845	76797865	ATCATGTTTGCCTTGGCTTT	TGCTCAAATTAACTCGAGAATTG	268
Ad02A13674	Aradu.A02	13674	c	(T)13aacttttcaa	54	76799467	76799520	TTTCCCTCTGTACAATTCCCA	CGGCACGTGAGTGAGTTAAA	280
Ad02A13678	Aradu.A02	13678	p2	(TA)6	12	76818434	76818445	TTTCCAGTTCAGATTTATGGCA	CTTTGGAGCTTTTGGACCTG	229
Ad02A13679	Aradu.A02	13679	p2	(TC)22	44	76820012	76820055	TATGGGAGAGAGGATGTGGG	ATTGAGAGCCCCAGACTTGA	204
Ad02A13680	Aradu.A02	13680	c	(T)10ctttcccca	36	76822070	76822105	TGCAGTTCAAAACATGACGC	TTTCTGCCAACCAAAATTCG	276
Ad02A13681	Aradu.A02	13681	p3	(AAC)6	18	76822409	76822426	TTGGATGTGGAAGAATTTTGA	ACATGACAGAGCACAATGGC	278
Ad02A13683	Aradu.A02	13683	c	(TA)6cattacaa	45	76829375	76829419	AATGACCATGGGTGGCTAAC	TCTCGTGCTGATAACGTGTTG	274
Ad02A13686	Aradu.A02	13686	p2	(AG)7	14	76842776	76842789	GAACGACACCCTCCTCCATA	GACAACTGACACCATGACCG	235
Ad02A13690	Aradu.A02	13690	p2	(TA)7	14	76861766	76861779	TTTGGTGCATTAATGGTTAAAATG	TCATGCAACTTAATTTTCTTCAACA	245
Ad02A13698	Aradu.A02	13698	p3	(GTT)6	18	76895986	76896003	TGACGGATTCTAATCCAGGG	AAACCACACCACTTCTTGCC	150
Ad02A13699	Aradu.A02	13699	p3	(AGC)5	15	76897231	76897245	GTGAGGTCTTCATCCACCGT	CCGATCCTCCCACATAACAC	224
Ad02A13702	Aradu.A02	13702	p2	(AT)9	18	76910424	76910441	TGATTATAAGGAGCGGCCTG	TCCGTGATTACAAGTCTCCTGTT	261
Ad02A13703	Aradu.A02	13703	p2	(AT)6	12	76912594	76912605	CCACAATCCAATCAATCACG	AAATGGGATCTATTAATCTTTATCCAA	254
Ad02A13708	Aradu.A02	13708	p4	(AAAT)5	20	76917284	76917303	GGATGGTTGAGACTTGGGAG	TTTTCGTCTCACATGCAACTTT	187
Ad02A13709	Aradu.A02	13709	p3	(AGA)5	15	76919957	76919971	AAGATGCTCCGTGCGTAAAC	CCCTTGTTCAACGCTTCTTC	115
Ad02A13714	Aradu.A02	13714	p3	(ATC)5	15	76937994	76938008	GAATCATCCCACAACCCACT	CGTCCCATATAAGGTCACCG	181
Ad02A13715	Aradu.A02	13715	p3	(ATT)8	24	76938112	76938135	CGGTGACCTTATATGGGACG	TGGAATATGAAAAGCTTGGTG	217
Ad02A13719	Aradu.A02	13719	p3	(CCA)5	15	76958054	76958068	TCAGCACCTTTACCTCCACC	CTGCAGTTGCTTCTGCTTGA	263
Ad02A13721	Aradu.A02	13721	c	(A)10caaaaatta	42	76969155	76969196	TCTGGTTTGGTAATTGGATTTTT	TTATAGGGCTTTGACCACCG	262
Ad02A13723	Aradu.A02	13723	p4	(TTTC)11	44	76980462	76980505	CGTCCCTCTCTCTCAACGTC	CCTTTGCCACCTCACACTTT	193
Ad02A13726	Aradu.A02	13726	p3	(ATT)11	33	76986551	76986583	TGTTACATGGCTACCAATAAGGG	TGTAGTGAGCTGTGGCCCTC	276

ID	Chromosome	SSR nr.	SSR type	SSR	Size	Start	End	FORWARD PRIMER (5'-3')	REVERSE PRIMER (5'-3')	Product (bp)
Ad02A13732	Aradu.A02	13732	p2	(AG)14	28	76997679	76997706	TTCCCTTAGTGACGCTTGCT	TGCACTTAGCAGCTGAACCA	134
Ad02A13733	Aradu.A02	13733	p3	(CTT)5	15	76998758	76998772	CACCTTAGCCCTTTTCTTGC	ATGCTTGCCTTGGTAACTGG	270
Ad02A13734	Aradu.A02	13734	p2	(AG)6	12	77007189	77007200	ATTTTGTCGCCATCGAACAG	GAGCAACGCTTGTTAAGTGTCTT	257
Ad02A13735	Aradu.A02	13735	p2	(AT)7	14	77014709	77014722	CCTTTATGCACAAGGCTGAA	TCGGGAGAAATAAAATCGGA	279
Ad02A13743	Aradu.A02	13743	p3	(AGA)5	15	77056533	77056547	CCATCTGAGTCACCCGAAAT	TTCCCCTTCTTCTTCACCCT	192
Ad02A13744	Aradu.A02	13744	p2	(AT)9	18	77058005	77058022	ACGCTAATGAATTGGATCGG	AGATATGGCCACCGAAAGTG	159
Ad02A13747	Aradu.A02	13747	p2	(AT)8	16	77125933	77125948	TGGATTTCTATTCTTATTCGTTTTGA	AAATCCCGAGAACCTGAAAAA	259
Ad02A13753	Aradu.A02	13753	p2	(TA)7	14	77147300	77147313	TTGTGGCTTCGTGTGAATGT	ATGGGAACGCATGACTATCG	261
Ad02A13754	Aradu.A02	13754	p3	(CTC)6	18	77151685	77151702	TCTACCGACTTGGGTTTTGC	AGAGGGAGTCCCCACATTCT	198
Ad02A13755	Aradu.A02	13755	p2	(CT)7	14	77167103	77167116	TTTCGAAATCTCTCCTCCCC	TCACGGAAAAACACACCAAA	152
Ad02A13759	Aradu.A02	13759	p2	(GA)6	12	77187331	77187342	AAGAGACTTAACCTTGTTGTCTCG	TCAAAGCCATTCAACATCCA	266
Ad02A13760	Aradu.A02	13760	p4	(ATAA)5	20	77192415	77192434	CGGTTGATCCACCATCAAAT	TGTGTGTCTGTCAAAGTGGG	233
Ad02A13761	Aradu.A02	13761	c	(TA)6tcatagaa;	155	77193938	77194092	ACAAAATGCAATACCCTACCAT	ATTTTGGATATGTTTTGGGTTTAT	280
Ad02A13762	Aradu.A02	13762	p2	(AC)7	14	77195682	77195695	GGCCCAAAGTTGACCAAATA	GTAAATCGGAGCACCGTAGC	227
Ad02A13763	Aradu.A02	13763	p4	(TTAA)5	20	77201721	77201740	TGAAGTAATTTCATTATCCGTTCA	TGACTTGAATTAGATGAGATGACGA	218
Ad02A13765	Aradu.A02	13765	c	(A)10g(A)10	21	77215318	77215338	CGGACGAATTTCGGGTTAAT	TCAAAACGCGACAACTTCC	196
Ad02A13766	Aradu.A02	13766	p6	(AACAAG)6	36	77217445	77217480	CGAAAGCATGGGTCAAAACT	TGATGATGACCGAGTGGGTA	266
Ad02A13767	Aradu.A02	13767	p3	(TCA)7	21	77217646	77217666	TTACTGCACAGAAGGAGCCA	TGATGAAATTTGTTGTTGCTGA	228
Ad02A13768	Aradu.A02	13768	p3	(AGA)5	15	77217780	77217794	TACCCACTCGGTCATCATCA	CACCACCGCTTCTTCTTCTT	182
Ad02A13773	Aradu.A02	13773	p3	(ATA)6	18	77238311	77238328	CCCTTGTCTCTTGTCTCCCA	GGATGAAAAGATACACCGTGAAG	276
Ad02A13775	Aradu.A02	13775	p2	(GA)6	12	77238953	77238964	TCCAATTGCATTCTGCTCTG	TGCTGACTCATTGATACGCC	263
Ad02A13778	Aradu.A02	13778	p2	(TA)6	12	77240706	77240717	TTCAAGAGGAAATTGGTGGC	TGCCATTTGAGCATAGTTTCA	274
Ad02A13782	Aradu.A02	13782	p2	(TA)10	20	77242416	77242435	AGCCCGGAACAATAACTCCT	GACACCAGAAAACAGAGGAAAAA	237
Ad02A13792	Aradu.A02	13792	c	(CCT)5tcttcttt(	56	77258481	77258536	CGTGCATTTTAATAACGCAAAA	TGTTGGTGTTGTTGGTGATG	150
Ad02A13794	Aradu.A02	13794	p3	(AAG)5	15	77260007	77260021	CAATCAACACACAAATGAACCA	CTTCCAAATTCACGCACGTA	195
Ad02A13798	Aradu.A02	13798	p3	(TGA)5	15	77288088	77288102	AGAAAAAGGGACCCACTCGT	TGAGAAACAAATTCCTGCCC	166
Ad02A13801	Aradu.A02	13801	p5	(TTCGG)5	25	77303740	77303764	GTTTGGTTCGGTCGGTTTTT	TTGGTTTTCGGTTGGCTTAG	280
Ad02A13805	Aradu.A02	13805	p2	(AT)7	14	77325150	77325163	CGCGGATATCTGCTTTACATC	CCGCATAATTGAACCTTTTCTT	280
Ad02A13810	Aradu.A02	13810	p3	(TTC)5	15	77335738	77335752	AAAAATGTTTCCTCTAATGCTGC	CCTCCTCATCTTCGACTGCT	275
Ad02A13811	Aradu.A02	13811	p2	(TA)6	12	77340914	77340925	CACACACTGAGGGCATATCG	TGTACATCCGAGAGCTTGAAAA	257
Ad02A13813	Aradu.A02	13813	p2	(AT)8	16	77344272	77344287	GAGGAGTTGGAAGGGTGCTT	GCTTGAAGAAAATTATTACCCCA	277
Ad02A13816	Aradu.A02	13816	p3	(AAC)5	15	77350339	77350353	GCTACAACAGCTGGGGCTAC	CATGCACCAGAATTTCCTGA	224
Ad02A13817	Aradu.A02	13817	p2	(TA)9	18	77352817	77352834	CGTAAGTCATTTTCATCCCTCA	TTGATTTTGAGCATATAACTTTGTTT	280
Ad02A13821	Aradu.A02	13821	p3	(CTC)6	18	77364470	77364487	AGAGGGTCCAGGAGGCTAAA	ATGGGATTGGAAAGAGTCCC	106

ID	Chromosome	SSR nr.	SSR type	SSR	Size	Start	End	FORWARD PRIMER (5'-3')	REVERSE PRIMER (5'-3')	Product (bp)
Ad02A13823	Aradu.A02	13823	p3	(TAA)19	57	77380950	77381006	AGCTTTTTGGTGTTTCCCAA	GCACGCAAGTAATCCTTTCC	230
Ad02A13824	Aradu.A02	13824	p2	(TA)11	22	77381212	77381233	GACAACCAAGGAAGCAGCAT	GGATCCTGGAAAGCGAAGTA	277
Ad02A13825	Aradu.A02	13825	p2	(TG)13	26	77400067	77400092	TTCCCTGGCACTATGCTTCT	AAAGTCCCAAATGCATGCTC	123
Ad02A13828	Aradu.A02	13828	p2	(TA)6	12	77419554	77419565	AGCCGGATTTTGAGTCTTGA	GGGGTAAACAAAAATCGCAA	134
Ad02A13830	Aradu.A02	13830	p3	(TAA)7	21	77424099	77424119	CTTAGCTAGATGGCTTCGGC	TTGCTTGCAACCACACTTTT	269
Ad02A13831	Aradu.A02	13831	p3	(AAT)5	15	77427166	77427180	ACTTCCCTTATTGGAACCGC	GGCCCAACAATGTATACCAAA	272
Ad02A13834	Aradu.A02	13834	p3	(AAT)11	33	77429354	77429386	TTTTTCTTTTCTTTGTTCCATTTT	TCAATTTTTGAGGGCGAAAT	255
Ad02A13838	Aradu.A02	13838	p3	(AAT)9	27	77438565	77438591	TGGCAATAATTTGGCCTGTT	CGGTTCACTGGTTTTCTGGT	204
Ad02A13839	Aradu.A02	13839	p3	(AAG)6	18	77441093	77441110	GCCATGTGTTTCCTTCCACT	CATTTCCAGCCGTCTTCTTC	116
Ad02A13842	Aradu.A02	13842	p3	(ATT)6	18	77464701	77464718	TTGAATGAATCGCAACTTCG	ACGCAGCGAGAAATGAGAAT	255
Ad02A13857	Aradu.A02	13857	p2	(AC)6	12	77543089	77543100	GTTGAGCCCAAGTCCAACAT	GGGAGGATTTCGAGCTTACC	241
Ad02A13861	Aradu.A02	13861	p2	(GA)6	12	77562098	77562109	GAAGACTTGGGAAGCACAGG	CCACTGTTCATCCACGACAA	143
Ad02A13862	Aradu.A02	13862	p2	(AG)7	14	77562230	77562243	CGTGGATGAACAGTGGGTTA	GTCCCTGGAGACGAAAACAA	279
Ad02A13867	Aradu.A02	13867	p3	(ATC)5	15	77575857	77575871	ATCTGCATCACACCAACCAA	CTGGGTTTCCCTGAGTTTGA	224
Ad02A13868	Aradu.A02	13868	p3	(TAT)6	18	77576704	77576721	GGTCACGTGGCTGGATACAT	GGAATAATAACAATACCAACAGGAA	262
Ad02A13875	Aradu.A02	13875	c	(A)11taaagaga	120	77594693	77594812	AAGCAACAAGAGGAGAGGCA	AACCCTAATTTGAACTCATTGAGAA	221
Ad02A13876	Aradu.A02	13876	p3	(AGA)5	15	77599512	77599526	ACTCGGAGGTGGAAGCTTTT	CTTCCCCATAAACCCCATCT	268
Ad02A13883	Aradu.A02	13883	p3	(AGA)6	18	77633454	77633471	CTAGAGGATTCTCCGGCCTT	CCGAACCTACACTTCCCCTT	166
Ad02A13884	Aradu.A02	13884	p2	(AG)6	12	77651650	77651661	AGGGATGAGGCAAGAAACCT	CCTACCATTCCACCATCACC	252
Ad02A13885	Aradu.A02	13885	p4	(TTCT)9	36	77657702	77657737	CATGGGATCGACCTCACTCT	ATCAACACAGAGCAGCAGCA	231
Ad02A13888	Aradu.A02	13888	p2	(CT)6	12	77673398	77673409	GCATGCTTAAAAGGAGCAGG	ATTAGGGTTGGGTTTGGGAG	222
Ad02A13889	Aradu.A02	13889	p3	(TTG)5	15	77678538	77678552	TTCTCCTAGGTTTTGCAGGC	ATTGAAAGGACATTGAGGCG	181
Ad02A13890	Aradu.A02	13890	c	(AT)6nta(TAT	39	77679853	77679891	GCATCACCTGCCTCTACTCC	TCACCCCCTTTCAGGTACAGG	255
Ad02A13893	Aradu.A02	13893	p3	(TGA)6	18	77725385	77725402	TGGAGCTCAGGGGTAGCTTA	GAGGTCTTGGTAGCAGGCAG	238
Ad02A13902	Aradu.A02	13902	p3	(AGA)6	18	77817247	77817264	CTAGAGGATTCTCCGGCCTT	CACCCAAATCCACCCTAATG	276
Ad02A13904	Aradu.A02	13904	p3	(CAT)5	15	77847203	77847217	GAGGCTTTGGCAGAAGTGAC	GCGGCCTATGAGAATCTGAA	164
Ad02A13905	Aradu.A02	13905	p3	(TTA)6	18	77851485	77851502	TTTTGGCACAAGATCATCAAA	TGAAAAAGTTTAATATTTGGATTCG	258
Ad02A13906	Aradu.A02	13906	p2	(AC)8	16	77855852	77855867	CTTGGTCCATTCTTAACCCAA	ATGGAGTGCTTGCCTCAGTT	234
Ad02A13909	Aradu.A02	13909	p2	(AT)9	18	77859404	77859421	TGCAGCATGCACTACAACTT	TTAAAACTCTGCCCGCACTT	263
Ad02A13916	Aradu.A02	13916	c	(A)10tggcg(A)	64	77891805	77891868	AAAAGCAGTGAAAAGCAAAAGC	ATCCATTGATGCTCAAAGCC	159
Ad02A13917	Aradu.A02	13917	p2	(AT)6	12	77902217	77902228	AAATTGAGTGAGAAGTGACATCAAA	CCTCAAAGAAGGGCAACAAG	137
Ad02A13920	Aradu.A02	13920	p2	(TA)6	12	77903669	77903680	TTGAAATTTGTAACACTTGCACA	ATTGGTGCCAACTTTGCTTC	270
Ad02A13921	Aradu.A02	13921	p3	(GAA)5	15	77904571	77904585	GAAGAACGCTCTTGCTGCTT	TGGTAAGGGGAGGATCAACA	249
Ad02A13923	Aradu.A02	13923	p3	(AGG)6	18	77907314	77907331	GGTGCATTTGAACTCGTTGTT	AAGCATGATTACACGCACACA	205

ID	Chromosome	SSR nr.	SSR type	SSR	Size	Start	End	FORWARD PRIMER (5'-3')	REVERSE PRIMER (5'-3')	Product (bp)
Ad02A13924	Aradu.A02	13924	p2	(CA)6	12	77908984	77908995	AAAATTCTCCCGAGGCAAGT	GATGCGATGAGAAGGGAGAG	265
Ad02A13931	Aradu.A02	13931	p2	(AT)7	14	77930844	77930857	AACCTTTATTATTAATCCAATGGAGA	CCTCCAATAATATTTTTAGTTGTGTTT	236
Ad02A13935	Aradu.A02	13935	p2	(TA)8	16	77936845	77936860	TTGAATAAATAATTCTTGGAAGCC	TTTCAATCTTCAATAAGGGCTGA	272
Ad02A13939	Aradu.A02	13939	p2	(TA)11	22	77996076	77996097	CCATGATGGAGAAAGGGAGA	TTCTCTTTTGGTTTTCGAATTTTT	126
Ad02A13942	Aradu.A02	13942	p2	(TC)6	12	78037018	78037029	TGCAAATCCCTCTCTTTTGC	CCGAGTTGCATGTATGAGGA	237
Ad02A13944	Aradu.A02	13944	p2	(CT)8	16	78047971	78047986	GACGTGACTGGCAGAGACAA	AAAGGGAGGGGAGATCATTG	261
Ad02A13945	Aradu.A02	13945	p3	(TAT)8	24	78049972	78049995	AGTCCAGCATTTACAGCACTTT	AAAATTTCGGGTCGAAAAGG	229
Ad02A13947	Aradu.A02	13947	p3	(CTC)5	15	78054936	78054950	AACTCCGAAATTGGTTGCAG	TAGGATGACCCACCTCTTGC	214
Ad02A13948	Aradu.A02	13948	p2	(AG)6	12	78057405	78057416	TTTCGTCCCCAACCATTTT	ACAACATCGGTGGCAGTGT	280
Ad02A13954	Aradu.A02	13954	p3	(TAT)20	60	78081089	78081148	TTGTTGGTGTTGTTAGTAGCTCAAG	CATTCTCATTGGAACGTTGA	166
Ad02A13955	Aradu.A02	13955	c	(TAT)5ta(AT)9	35	78081346	78081380	AGCTCAACGTTCCAATGAGA	ACATTACCATTGAATTAAATTTTCTCT	223
Ad02A13956	Aradu.A02	13956	p4	(TATT)5	20	78086777	78086796	CAATGAAACAAGGCGAAATG	TGTGTTTGGTTGGCAGAAGA	201
Ad02A13957	Aradu.A02	13957	c	(T)11aaaaaaa(	30	78087357	78087386	ACACCACAAACTGCAGCAAG	TGACAAACAAGACCAGCGAG	245
Ad02A13958	Aradu.A02	13958	c	(T)10ctttaaattt	118	78087564	78087681	TCGCTGGTCTTGTTTGTCAG	TGGATACGTGACACAAAATCG	202
Ad02A13963	Aradu.A02	13963	c	(T)10cag(A)10	23	78093124	78093146	ATGCATGCAGGGAGCAAT	GCCATCCTGAATTGCATACAC	130
Ad02A13964	Aradu.A02	13964	p2	(TA)7	14	78093359	78093372	TCGTGTATGCAATTCAGGATG	CTAATATAGATGTTTCTGTCCTGGAA	204
Ad02A13965	Aradu.A02	13965	c	(A)13gtcatagta	62	78093936	78093997	TGCAGACGAAATTTTCATTCTAA	TGAGCTGTAAACCAATGCAC	246
Ad02A13968	Aradu.A02	13968	c	(T)11cttatgactt	62	78098172	78098233	TGAGCTGTAAACCAATGCAC	TGCAGACGAAATTTTCATTCTAA	246
Ad02A13969	Aradu.A02	13969	p3	(ATA)7	21	78106000	78106020	AAAAAGATAAGCAGTGAAAATTTAAGA	GAGAAATCAAAATGTGTAACTCGG	254
Ad02A13973	Aradu.A02	13973	p2	(AT)6	12	78115230	78115241	TCATATCGTGTCGTGTCCGT	TCGTGACATCTTTGGAGTGG	244
Ad02A13974	Aradu.A02	13974	p2	(GT)9	18	78119418	78119435	CAAAGGTTTCGGGCATAAAA	TCGACTTAGGTGGGGTGTTC	260
Ad02A13975	Aradu.A02	13975	p2	(TA)10	20	78128704	78128723	TGAGAAATGATTATTCTCCAAACAA	CCTTCCTACCCAGCATTCAC	214
Ad02A13976	Aradu.A02	13976	p3	(CCT)8	24	78129722	78129745	GAAACCTTTTTGAATCGCCA	ATGGTTTGAGGTTCGAGTGG	212
Ad02A13978	Aradu.A02	13978	p3	(TAA)6	18	78134928	78134945	TTGATTTTCAGTTAATTCGGTCAA	GCAAAAAGGGAGAGACAATATCA	229
Ad02A13984	Aradu.A02	13984	p2	(AT)11	22	78141253	78141274	GGATAGGTTTTATTGCATGACCA	AGGGACAAAGTGATGTGGTG	180
Ad02A13986	Aradu.A02	13986	c	(TC)6ttttctttag	69	78142881	78142949	TTTCTAGACATTGCCATTTTGC	GCACGGAATGTGTATTCAGC	146
Ad02A13988	Aradu.A02	13988	p3	(TGG)7	21	78146780	78146800	TCGTAGGATCCACCACCATT	TTGGGATTTCCCATTCTCAG	253
Ad02A13989	Aradu.A02	13989	p2	(AG)7	14	78147287	78147300	GCATGGTTCCAAAAGTGTGA	CCCACAAAGCCTCTTCTTCA	244
Ad02A13990	Aradu.A02	13990	p2	(AG)6	12	78149313	78149324	ATGAATGCATGCCACAAAAA	CCTGTGAAAATGTGTGCGAT	273
Ad02A13992	Aradu.A02	13992	p2	(TC)6	12	78167015	78167026	CCTAACTAGGTCGCCTCCCT	TTTATTTGCTCCCTTGGACC	255
Ad02A13994	Aradu.A02	13994	c	(AT)7gttcattaa	57	78171787	78171843	ATCGGACCCAAGTTAGACCC	AACGAAGAGCTCGACGAGAG	237
Ad02A13996	Aradu.A02	13996	c	(A)10tctttaaag	46	78178350	78178395	CTGAGCTTTGGGAGTTCGAT	TGATTTTGGGTGCTTGAATG	241
Ad02A14001	Aradu.A02	14001	p2	(AT)9	18	78191602	78191619	CGTAAGTCATTTTCATCCCTCA	TTTGATTTTGAGCATATAACTTTGTTT	243
Ad02A14007	Aradu.A02	14007	p4	(ATAG)5	20	78216027	78216046	AGCATGCTACGTCAGCTTCA	CCTTATGACAATCACTCTTTGTGG	237

ID	Chromosome	SSR nr.	SSR type	SSR	Size	Start	End	FORWARD PRIMER (5'-3')	REVERSE PRIMER (5'-3')	Product (bp)
Ad02A14010	Aradu.A02	14010	p3	(AAT)5	15	78224139	78224153	AAAACGTGATTGGTGTGGCT	GGGGACGGTGGTACTCACTA	222
Ad02A14012	Aradu.A02	14012	c	(ACA)5tactcca	81	78241558	78241638	CGGACAGAATTTCCTCTGTTTC	CAGGATCCTCGTGTGGGTTA	276
Ad02A14014	Aradu.A02	14014	c	(ACA)5tactcca	81	78248346	78248426	CGGACAGAATTTCCTCTGTTTC	CAGGATCCTCGTGTGGGTTA	276
Ad02A14018	Aradu.A02	14018	p2	(GA)7	14	78256966	78256979	TTTTCACCACTATAAAAAGCTCCA	TCCCCCATTAGTTTGTGCAT	274
Ad02A14020	Aradu.A02	14020	c	(A)10ggaaaaga	35	78258418	78258452	TTGGTTCAATCACTGGTTCG	TCTGCTATATTCTTCATCCGCA	278
Ad02A14025	Aradu.A02	14025	p3	(TTA)6	18	78275510	78275527	TGCCTCAACTCTCCTCATCA	AATAAGGATTCCGCTAGGGC	242
Ad02A14026	Aradu.A02	14026	p2	(CT)6	12	78277552	78277563	TCCTCTTTCTCGTTCCCTCA	GAAGTTCGCGCCTAAGACAG	196
Ad02A14027	Aradu.A02	14027	p2	(AC)8	16	78291300	78291315	TGTTGTGTGTGCCTTTCTTCA	ACCCGTGGGTACAGCAATAA	226
Ad02A14031	Aradu.A02	14031	p2	(TA)6	12	78309211	78309222	TTGCAAGGGTTGTTATTTACTTGA	TGAAAATAATTGCTTGTGCGTC	272
Ad02A14034	Aradu.A02	14034	p2	(AT)6	12	78315234	78315245	TTCAAACACCACCTTCCTTACA	TGCTCAATTAAACACATCCTAAAAA	251
Ad02A14036	Aradu.A02	14036	p4	(AAAT)6	24	78316815	78316838	GAGACAGCATGTGCAAGGAA	TCGCTTTCTAATTCAACGAAAA	259
Ad02A14040	Aradu.A02	14040	p2	(TC)7	14	78333366	78333379	CAGAAGGGTGGTGAGGAAGA	ACATGGTACGCACAATGCTT	143
Ad02A14042	Aradu.A02	14042	p4	(TTGT)6	24	78336075	78336098	GCATGGATGAAAAGGGAAGA	CACATGTAACCGCGTCCATA	277
Ad02A14044	Aradu.A02	14044	p3	(TCC)5	15	78338229	78338243	AAACACGCGCTTCTTCAACT	TGAAGAAGGAACGCAAAGAAA	159
Ad02A14045	Aradu.A02	14045	p2	(GA)6	12	78340345	78340356	TGAAATTTCGTGGTTGGAGA	GCCAAAATCCCTCTTTCTCA	215
Ad02A14051	Aradu.A02	14051	p3	(TAA)7	21	78381608	78381628	GGGATTTCAATGCAAGGTTC	TTCAAGATTTGAGTGTGTTTTCTTTT	218
Ad02A14053	Aradu.A02	14053	c	(T)12ccaatgtca	148	78391230	78391377	AACAACATTCAGGACAGACACA	TCACTTGATGGGCTTTGGAT	258
Ad02A14054	Aradu.A02	14054	p2	(TA)7	14	78391722	78391735	TAGTGAGCCATGCAGAATGC	ATATGTGGCCGTTTGGGTTA	138
Ad02A14058	Aradu.A02	14058	c	(CTCC)6tc(CT	64	78396444	78396507	AAGTTGTTCCCGTTGCACTC	TGCCAAATGTCACAATTCAAA	204
Ad02A14059	Aradu.A02	14059	p3	(AAC)10	30	78397456	78397485	TGAAGGGAGGGAGTATGCAG	CGTTTGATTCATGTAGCCGA	259
Ad02A14060	Aradu.A02	14060	p3	(TAA)5	15	78400778	78400792	TCCTTTATCCGAGTTTGGGA	TTGGAGCGACCAGTTTCTCT	280
Ad02A14063	Aradu.A02	14063	p3	(TAT)6	18	78412231	78412248	TGTGTGTGAATTGCTGAAATGA	CGACACAATTAAATTATAACCCCG	250
Ad02A14067	Aradu.A02	14067	p3	(TAT)5	15	78416896	78416910	ATCACGAAAAACATGCGGTA	ACTCCTGCACGACAATTTTT	243
Ad02A14068	Aradu.A02	14068	c*	(AT)9(ATAA)	40	78428657	78428696	AATTTTCGAATCCCTTGGGT	GGTCCATGATGAAGTAAGGGAG	279
Ad02A14070	Aradu.A02	14070	c	(T)11actttaaaa	46	78453803	78453848	CCAGATTTGCTATTTATTGTAAGTGC	CCCTTAATTACAAACATTAAACCC	277
Ad02A14073	Aradu.A02	14073	p2	(AT)8	16	78460982	78460997	GTGTTTGATAATGAAAAAGTAATTTGA	TTTCTATCCGATCATAAAGTCAACA	279
Ad02A14078	Aradu.A02	14078	p3	(AAG)5	15	78472236	78472250	TCATCACCCACTCCTTCTCC	TCAGCGAGGTTATCGAGGTT	200
Ad02A14079	Aradu.A02	14079	p3	(ATA)5	15	78476974	78476988	ACTAGATTGGGGTCGTGGTG	CGAATTGAAAGTGTAGGGCA	148
Ad02A14081	Aradu.A02	14081	p2	(TA)7	14	78480673	78480686	TTGGCTCACTTTTACAGATCACA	TGCGTTGTTTTTGTATTTTCCA	242
Ad02A14083	Aradu.A02	14083	p4	(ATAA)5	20	78482124	78482143	TTCTGGATAGTGTGGGTGATG	TGCAGTGTTGATTAGAGATAGCG	266
Ad02A14094	Aradu.A02	14094	p4	(AGAA)5	20	78507076	78507095	CGTACGGCCCCGTTATAGTA	CCCACACACACAACCAAAAA	268
Ad02A14097	Aradu.A02	14097	p2	(TA)7	14	78507938	78507951	AAATCGGATCGCGGATATTT	TCCACATAATTAAACCTTCTCTTTTT	275
Ad02A14098	Aradu.A02	14098	p3	(TTG)6	18	78511878	78511895	CGTTTGATTCATGTAGCCGA	CCTCACGTGAAGAGCTTCCT	266
Ad02A14099	Aradu.A02	14099	p2	(TC)13	26	78525177	78525202	CCTAAGGAGAAATTGGAAGAAAAA	ATCAGGGGATCCATCATTCA	138

ID	Chromosome	SSR nr.	SSR type	SSR	Size	Start	End	FORWARD PRIMER (5'-3')	REVERSE PRIMER (5'-3')	Product (bp)
Ad02A14100	Aradu.A02	14100	p3	(TGA)5	15	78534084	78534098	TCTCAATATTCGGGTCTGGC	ATCAAGCAAGCAAGCACCTT	194
Ad02A14103	Aradu.A02	14103	p2	(TA)8	16	78545378	78545393	GACCTAAACTTCGCAGGTCG	GAAAGATAAAGCGCATTCGG	113
Ad02A14107	Aradu.A02	14107	c	(T)10acttggtct	41	78559454	78559494	AAAATACCAGACTTTTTGAATTCTCAT	TTGTCATGGCAGAAACTAAATTG	193
Ad02A14108	Aradu.A02	14108	p3	(ACC)9	27	78566258	78566284	TTTTTCGCTCCAAAATGGTC	ATGGATGGTGATGGAGTGGT	190
Ad02A14109	Aradu.A02	14109	p3	(AAG)45	135	78566526	78566660	TCATCATCATCAAGAACCCAGA	TTTCGCTACAAAATGGTCCC	224
Ad02A14112	Aradu.A02	14112	p3	(AAT)7	21	78572154	78572174	GGGCTAGATTTACAAGAAAGTTATGTT	AAGGGAGGGGAATGTATTTCA	280
Ad02A14116	Aradu.A02	14116	p3	(TTC)7	21	78575688	78575708	GTTGGAAGGGCCAACAACTA	CAATGACAAAGGGTTTGGGT	276
Ad02A14121	Aradu.A02	14121	p2	(TA)7	14	78598635	78598648	CTTGAGAGCCGTAAGATGAAAA	GCCATAACGGAAGATACGGA	260
Ad02A14123	Aradu.A02	14123	c	(A)11taaatcacc	78	78603038	78603115	ATCAAATGCACCGAATCACC	TTTCCTTGTCTGGGTTGCTC	172
Ad02A14124	Aradu.A02	14124	c	(GTG)7cggaa(	41	78603788	78603828	CTATCTTCCTCCCGCTCCTC	TCTCCGATTTCTCCGACAAC	246
Ad02A14127	Aradu.A02	14127	p5	(AAGAT)5	25	78629868	78629892	AGGCGGTTCCCCTAAAGATA	GGAGTTGGTGTGTCCGACTT	156
Ad02A14133	Aradu.A02	14133	p3	(TTA)5	15	78649100	78649114	TCAATTTTTGTGATCTAGCATGATT	TGTTCAAAAATTATCAAAAGCGAA	274
Ad02A14135	Aradu.A02	14135	c	(A)10gactagtta	45	78651335	78651379	TTTTGGCTAAAAATCCAATTTC	TGATTAATCGGAACCAAACCA	149
Ad02A14137	Aradu.A02	14137	p3	(TTA)7	21	78653543	78653563	TCCCGTAGTCAAAGAAGCTAAA	TACCTAGGGCTCTCTGCTGC	264
Ad02A14140	Aradu.A02	14140	p5	(ACCCT)5	25	78673085	78673109	TGGGTAGGGTTAGGGTAGGG	TTGATATTTCTGCGGGTAGGA	178
Ad02A14145	Aradu.A02	14145	p2	(AT)6	12	78717748	78717759	GTTGGCTGGTGTTTCACCTT	CCATCATCGTTATACTGGCG	251
Ad02A14146	Aradu.A02	14146	p3	(TTA)6	18	78726740	78726757	CTTGTCTTCGCAGGCTGAGT	TAAAGCTGAGGTGGCAAGGT	258
Ad02A14147	Aradu.A02	14147	p2	(GA)6	12	78731053	78731064	TTAGAATGTTGCTCGCCCTC	CCCATAAACCCTCCATCCTT	150
Ad02A14148	Aradu.A02	14148	c	(T)10c(T)10	21	78738630	78738650	AAAAAGGAACAACAGCGGAG	GAAAATGGAGGTTGCCTTTC	176
Ad02A14149	Aradu.A02	14149	c	(T)10gcctctaat	37	78738855	78738891	GAAAGGCAACCTCCATTTTC	GGATGAAAAGATGTTTCACAAGC	268
Ad02A14150	Aradu.A02	14150	p2	(GA)6	12	78746329	78746340	GCATTTCATCAAACACTTGGAA	TCATGCTTTGATTCAATTTTCCT	274
Ad02A14151	Aradu.A02	14151	p2	(TC)6	12	78753021	78753032	CCTCAGCCTTTCTTGCCTAA	ACCCAAAAGCAACGTTCATC	128
Ad02A14152	Aradu.A02	14152	p2	(TC)6	12	78762599	78762610	CCTCAGCCTTTCTTGCCTAA	ACCCAAAAGCAACGTTCATC	124
Ad02A14155	Aradu.A02	14155	p3	(TGG)5	15	78773152	78773166	CCTTTGTCATGGAGTCCAAA	TTGCAGCACCGAAATTAACA	276
Ad02A14159	Aradu.A02	14159	p3	(TTC)6	18	78788597	78788614	CACCTTGACCGAAACACCTT	GAGGATTCTCCGGCTTTAGG	171
Ad02A14160	Aradu.A02	14160	p2	(CT)7	14	78800998	78801011	CTTCTCTCTCACTTTGGTGGC	ATGACTGTTGGGTTTTTCGC	150
Ad02A14161	Aradu.A02	14161	p3	(AAT)12	36	78803409	78803444	GCCTTCTGAATAAGATTTGACCA	CCATGGATGGATAAAGAAGGA	264
Ad02A14162	Aradu.A02	14162	c	(A)10gaaccaaa	47	78803741	78803787	TGCCAACACCACCAAGATTA	CAGAATCTCTTGCATCAAACACA	170
Ad02A14163	Aradu.A02	14163	c	(A)10gaaaaaac	71	78804397	78804467	TGCATTACCCATGCGAAGTA	GCAATTGATTTGGGTTATGTTG	250
Ad02A14167	Aradu.A02	14167	c	(A)10ttgcagag	60	78808487	78808546	TTGATGTGGAAATCACATTGC	CCAGTCATCATAACCGGGTC	259
Ad02A14173	Aradu.A02	14173	p3	(GAA)7	21	78830229	78830249	TAGGGTGAGGGCATAACAGG	CGTTGTACGATGAAGGAGGC	274
Ad02A14176	Aradu.A02	14176	p2	(TA)7	14	78850097	78850110	TACAAGACCGGTGTGATGGA	AGGAGGATTGGGGTAGCATT	178
Ad02A14177	Aradu.A02	14177	p2	(CT)8	16	78854697	78854712	CAAGCTAAGCCAAAGCCAAC	AGCGAAGGCAGTGAAGATGT	239
Ad02A14180	Aradu.A02	14180	p2	(AT)7	14	78892407	78892420	TGGATAAAATAAGGGACATTACGAA	CGCATGTATGCTGACTTTGC	169

ID	Chromosome	SSR nr.	SSR type	SSR	Size	Start	End	FORWARD PRIMER (5'-3')	REVERSE PRIMER (5'-3')	Product (bp)
Ad02A14184	Aradu.A02	14184	p2	(TC)23	46	78902048	78902093	CTCATGGACATAATCGTACTGGA	AGGGCGTGCATCAGAAAATA	254
Ad02A14185	Aradu.A02	14185	p3	(GAT)5	15	78902330	78902344	ATATTTTCTGATGCACGCCC	CCATACATTCAAATGGGGGA	204
Ad02A14187	Aradu.A02	14187	c	(AT)13(GT)7	40	78928905	78928944	TTTGTGGGATGTGCATGAGT	ATGACAACCCAAAACCAAGG	275
Ad02A14190	Aradu.A02	14190	p2	(AT)10	20	78939215	78939234	TTTTAGGAGAACCCCACCTC	TGGGTTTCAACATGTTTTACTCT	269
Ad02A14196	Aradu.A02	14196	p2	(AT)6	12	78958823	78958834	AGTTGCAGTGGTGGTCTCAA	ATGGTCCTCACTCCTGCTTG	275
Ad02A14198	Aradu.A02	14198	c	(TC)6accccctc	86	78959535	78959620	TCCTCTCTTTCCTTCTCTTCTTTT	GGAGGGGGAGAGAGAACAAA	207
Ad02A14200	Aradu.A02	14200	c	(GA)7aaaagag	40	78960342	78960381	GGGAGAAAGAGGAAGAGAGAGAA	ACATCTCCAATAGGGTGCG	280
Ad02A14201	Aradu.A02	14201	p3	(TTG)7	21	78963529	78963549	TGCGACTTTTGATATGTTTGGT	AGTCCTTCGACAAGCCAATC	197
Ad02A14202	Aradu.A02	14202	c	(A)10gcggttttt	61	78964546	78964606	CCAAAACCAAATTTAAAAACCC	TTTGGATAGAAAGTTAGAAACATTGG	148
Ad02A14205	Aradu.A02	14205	p3	(TTC)6	18	78980199	78980216	ACATACACCCTCCCCTCTCC	GCTTTTGTCTTCCCTTTCCC	266
Ad02A14207	Aradu.A02	14207	p3	(TTA)5	15	78990953	78990967	TGCGCAGCAAAATTGTATTC	TGGAAACGTTTAATATATGGATTTG	270
Ad02A14215	Aradu.A02	14215	p3	(AAT)14	42	79020525	79020566	CTCCTTTCTGGTCTAGGGGG	AGTCCATCTCAGGTAAGCAGAA	265
Ad02A14217	Aradu.A02	14217	p2	(AT)6	12	79026314	79026325	TTGTTTTTCTTTTTCTGGTGCAT	TGGAGTTTGCATCACTGGAG	213
Ad02A14223	Aradu.A02	14223	p4	(AAAG)6	24	79034416	79034439	AACCCAGGTAAGGGTGTGTG	TCAGGGACCACTTCCACAAT	185
Ad02A14224	Aradu.A02	14224	p3	(ATA)5	15	79035297	79035311	GCAAAACTTGAAAATCAACCAA	TGTAGCATGCATAAGTAGACCAAA	241
Ad02A14226	Aradu.A02	14226	p3	(ATA)6	18	79036476	79036493	TTATTTTTGCCGGGTTTTCA	CTGCCTTGATATTTTTGGGG	205
Ad02A14231	Aradu.A02	14231	p5	(AAAAT)5	25	79045128	79045152	TCCGTTCCTATCTTATTTTCATCA	TTTACATAACACCCTGATTCATTTTT	267
Ad02A14241	Aradu.A02	14241	c	(T)10c(TG)7(T	39	79094562	79094600	CCCTTGCTACCTTTCCTCCT	TTTTTATTGAAGTTACATTGAGTTTGA	253
Ad02A14242	Aradu.A02	14242	c	(T)10cttacttatt	58	79095028	79095085	CACTTTAATTCCTCAATTTCCTCAA	TTTTCCCCATCTGTTTCATTG	152
Ad02A14249	Aradu.A02	14249	c	(AGC)5aagtga	103	79105423	79105525	TGCTACAATTTCCGATTCCA	TACACCTCTTCCTCGCATCC	274
Ad02A14251	Aradu.A02	14251	p3	(TCT)6	18	79126557	79126574	ACAACCCCCTCTTCTTCACC	TGCCATTGATGGTAGTGGAA	167
Ad02A14252	Aradu.A02	14252	p4	(AAGA)9	36	79164864	79164899	TGAGGAGTTCTGGTCTGCAA	TTGGGCTCGATCAGTAGCTT	277
Ad02A14253	Aradu.A02	14253	c	(CAT)12(CA)8	98	79165897	79165994	CCCATAAAACATCGGTTTCAC	GAAAGATGCTCACGCATCAA	250
Ad02A14254	Aradu.A02	14254	p2	(GA)16	32	79167987	79168018	AAGGGAGAGAGATCGAAGGC	TACGCCGACACATCTATCCA	230
Ad02A14255	Aradu.A02	14255	p2	(AT)10	20	79168581	79168600	TTCAATTCAGCATTTATGGCA	CACCCCATTATTGCCATTTC	275
Ad02A14256	Aradu.A02	14256	p3	(TCC)5	15	79171200	79171214	ACCACCATACCACCACATCC	TTTCGGTTCATTCGATCACC	199
Ad02A14257	Aradu.A02	14257	c	(AGA)5catagta	72	79171656	79171727	CGGAGGAGAAAGAAAAAGAGG	TTTCTACTTCTCCAAACAATTTTACA	240
Ad02A14263	Aradu.A02	14263	p2	(TC)6	12	79195223	79195234	CGGCTAGTGGTGAGGAGAAG	TCATCATCGCCGTTTACCTT	219
Ad02A14265	Aradu.A02	14265	p2	(TA)8	16	79214357	79214372	TCACAAGGTTTTTCTTGCCA	TGTCCCAAACATTTAAAGAATGAA	208
Ad02A14268	Aradu.A02	14268	p3	(ATT)15	45	79215463	79215507	ATGCACGCATGTTGGACTTA	ACCTCCCGTTAGCATCAGAA	274
Ad02A14271	Aradu.A02	14271	p3	(TTA)7	21	79225460	79225480	CTTTGACACGGTTGGGACTT	ACAAAACCTCCTATTAGTATCCGA	243
Ad02A14278	Aradu.A02	14278	p2	(CT)6	12	79250320	79250331	TTCAAGGGCAAAGATATGGC	GAGAGAGAGGACGTGATGGC	213
Ad02A14279	Aradu.A02	14279	p3	(TTA)12	36	79251016	79251051	TTGTGTTGGTATGCATTATGTATGT	TCGCAAGTTACTGGCAAAAC	259
Ad02A14280	Aradu.A02	14280	p2	(AT)8	16	79260769	79260784	TGTGCACTAATAGGCCATGAT	CCAAATCAAATCCCTAACATCTC	280

ID	Chromosome	SSR nr.	SSR type	SSR	Size	Start	End	FORWARD PRIMER (5'-3')	REVERSE PRIMER (5'-3')	Product (bp)
Ad02A14281	Aradu.A02	14281	p2	(AT)6	12	79261768	79261779	ACCATAGCCATGGGACAAAG	ATCATTGCGCACCAAGAGAT	271
Ad02A14282	Aradu.A02	14282	p2	(AT)10	20	79264441	79264460	TGTTGTAAAAAGTTTGATTATTCAGC	TTTTTCTTTCTCTTTTTAAATGGTTG	264
Ad02A14287	Aradu.A02	14287	p4	(ATTT)8	32	79285257	79285288	TTGCTTCGCTTCCTTTCATT	TCAAGTGTTCAAGTGGGGTTC	134
Ad02A14288	Aradu.A02	14288	p2	(TA)6	12	79285610	79285621	CGAAGCGAATTTGAGTGAGG	CGGGATAGGGGTGATACAAG	248
Ad02A14289	Aradu.A02	14289	p3	(TAT)17	51	79286193	79286243	TTGCGTGCTTGAGTTTTCTTT	CATTTGCTACAACAACATCATCA	159
Ad02A14293	Aradu.A02	14293	p2	(AG)6	12	79295349	79295360	GAATTGTGAGAAGGGATAAATGC	TCCCAGATAGGATTGGGTTG	251
Ad02A14298	Aradu.A02	14298	p3	(GGT)5	15	79305156	79305170	TGCTTCTGCTTTGCTTCAAC	TGAAAATACCCTTCCTCCCC	228
Ad02A14299	Aradu.A02	14299	p3	(TTA)19	57	79305408	79305464	TTAAACCTTTGGGACCATTTT	GAAGGAAGATGCGTGGTGAT	273
Ad02A14303	Aradu.A02	14303	p2	(AT)11	22	79312483	79312504	CAGTCAATCCATTTCCCAGG	TGCATCCTTGGTCCTCTACC	238
Ad02A14304	Aradu.A02	14304	p2	(TA)12	24	79321684	79321707	CACGTGTTCCAGGTTGAGAC	GACAAGTCCGGCTGCTAAAC	216
Ad02A14306	Aradu.A02	14306	p2	(TC)7	14	79335322	79335335	TGGTGATGGAAATCTGGACA	GGCCATTTCAAGAGGGAACT	265
Ad02A14307	Aradu.A02	14307	p3	(TTA)6	18	79339251	79339268	AGTGGCGGAAAGAACAAGAA	CATACCGTGAAAAGGGCAGT	186
Ad02A14310	Aradu.A02	14310	c	(T)10aaattagct	81	79342227	79342307	AAAATTGTTTTTCTGGAATTATTGCT	AACACAAACCATTTGCACCA	276
Ad02A14314	Aradu.A02	14314	p2	(AT)8	16	79348970	79348985	GGATGTTGGTGGTGCAATTT	TCGCTAGATTGAACTGATCGAA	256
Ad02A14316	Aradu.A02	14316	p3	(AAG)6	18	79359803	79359820	CCTCCTCATCTTCTGCTGCT	TTCTTTCGTTTCTTGCCTTCTC	195
Ad02A14317	Aradu.A02	14317	p2	(AT)8	16	79360312	79360327	AATTAGGTTTGACTTTAATTGTGAGC	CCAAAAACAGGATTACATCCAAA	242
Ad02A14322	Aradu.A02	14322	p3	(TAT)5	15	79362489	79362503	GTGCCATATTTTCTGGCGTT	CGATTTTTAAATTTGAATAGACCCA	194
Ad02A14324	Aradu.A02	14324	p2	(CT)12	24	79363653	79363676	GGACGATGGCCTATTTCCTT	AGGAAGTGTTTGGTTCCGTG	152
Ad02A14325	Aradu.A02	14325	p3	(CAA)7	21	79363946	79363966	TTTCCGGTTATGAATCCCAC	AGGAAGGACGGAAGATTGCT	277
Ad02A14326	Aradu.A02	14326	p3	(GAA)5	15	79364429	79364443	GGGCTCGAAATAGCACACTC	CCTCTTCTTCCTCAACCACG	225
Ad02A14327	Aradu.A02	14327	p3	(GAT)5	15	79365605	79365619	TGCGGATACTGCTTCTGTTG	GAACCCCTCTTAGGTCCTGC	209
Ad02A14330	Aradu.A02	14330	p3	(AAT)5	15	79379353	79379367	TTGTGATTTTTGTGTATTATGTTTGTT	CAAATCTGAAATTTCTTGATGATCT	186
Ad02A14335	Aradu.A02	14335	c	(CAT)5cgtcgtt	182	79414816	79414997	CCACCTTCTCCTCTTCTTCCT	AAAAACGACAATGATGACGAT	275
Ad02A14341	Aradu.A02	14341	p3	(CTC)7	21	79432271	79432291	CGATCCATGAACACCCCTAC	TTCTAATTTGATGTTCTAATCTTGCT	254
Ad02A14342	Aradu.A02	14342	c	(CAT)5aaaaag	32	79432780	79432811	AACATGTTCACCAAGTAATTTTGG	AAAACACAACTTCTTTAATGCTGC	230
Ad02A14343	Aradu.A02	14343	c	(ATT)6t(TTA)	52	79438173	79438224	TTGGCAAATGTAAGTTCTCAAA	CCCTCTGAGCTTCTTTCCTTC	273
Ad02A14349	Aradu.A02	14349	p3	(AAT)12	36	79480335	79480370	CGAGTACACTTTCTCCGGTGT	TCACAATTTGAAGCGCTGTT	244
Ad02A14357	Aradu.A02	14357	p3	(AAG)6	18	79508509	79508526	AAGCAAGGGTTTCAAGCTCA	AAATTATTGACGCCGTGTCC	261
Ad02A14359	Aradu.A02	14359	p2	(TC)6	12	79512164	79512175	AATCAACATCTCCACCGACC	GGGTGAATTCTTGCACCCTA	236
Ad02A14362	Aradu.A02	14362	p2	(AT)11	22	79525550	79525571	TGGCTCTTGAAAGAATGATGG	GCCCAAAGCACTCTGTCTTC	276
Ad02A14366	Aradu.A02	14366	p2	(CG)6	12	79544638	79544649	CGCCACTCTCGAAATAAACC	TCGATGGTGTTTGGATGTGT	208
Ad02A14367	Aradu.A02	14367	p3	(TTA)5	15	79556093	79556107	CCTCCTCACCTTGGATTCTG	CAAGGCCAAAATTCACCTGT	278
Ad02A14378	Aradu.A02	14378	p2	(CT)8	16	79597589	79597604	TCGCGAGACTCTCCTCTCTC	GAAAGACGCGACACAGAACA	186
Ad02A14379	Aradu.A02	14379	p2	(TG)14	28	79598007	79598034	TTCTCCTTCTCCCATTCCCT	TCAACTCTCACCTCACACGC	199

ID	Chromosome	SSR nr.	SSR type	SSR	Size	Start	End	FORWARD PRIMER (5'-3')	REVERSE PRIMER (5'-3')	Product (bp)
Ad02A14381	Aradu.A02	14381	c	(AT)13tgaatcta	107	79599751	79599857	CCCAAGCACATAGCAAAAGA	CTCATTTTCGGGTTGCAAAT	264
Ad02A14383	Aradu.A02	14383	p2	(TG)19	38	79604365	79604402	GGATAGCACCACCTCTCTCG	TCTCAACTCTCACCTCACACG	238
Ad02A14385	Aradu.A02	14385	p2	(AT)7	14	79606600	79606613	AACCGGAAAATACTTTTTGTTGA	GAGGATGATGTTCTCACCCC	272
Ad02A14386	Aradu.A02	14386	p2	(AT)9	18	79607376	79607393	TAACCCTTAGCGTCACCACC	TTTGCGAAACTATAGGGACCA	186
Ad02A14388	Aradu.A02	14388	c	(TGA)7tttttttgt	121	79611449	79611569	ATTGAAGATGGATTTGGGGA	CACCCCACCCAGAACTTAGA	248
Ad02A14389	Aradu.A02	14389	p3	(TGA)5	15	79611714	79611728	TCTAAGTTCTGGGTGGGGTG	TATCATTCCCCCTTCTTCCC	234
Ad02A14390	Aradu.A02	14390	p2	(AT)13	26	79615530	79615555	AAGCAAGTGGGACAAACCAC	CTTGTTTGTGGCTGCAGTGT	265
Ad02A14391	Aradu.A02	14391	p3	(CCA)6	18	79626648	79626665	CGACAAGAGGAGCATGATGA	CACGCGACATTAAGTGGATG	119
Ad02A14394	Aradu.A02	14394	p4	(GAAG)5	20	79631885	79631904	TTTTGATGTAACAAGGAGCGG	TGAGGCAGAGGACAGTGATG	254
Ad02A14395	Aradu.A02	14395	p2	(AG)10	20	79632366	79632385	ACTCACCCACCCACTCACTC	TAGTCCAAATGAGGCGAACC	264
Ad02A14398	Aradu.A02	14398	c	(T)13aagcaagc	50	79646803	79646852	CGAGATATGGGGAGGGAAAT	TCAACTTCAAAGCCAATCCC	276
Ad02A14399	Aradu.A02	14399	p2	(TA)8	16	79648967	79648982	CATCCCCATCCATTTACTGC	CGGAATCATCCATGTAACAGAA	142
Ad02A14406	Aradu.A02	14406	p3	(TAT)7	21	79692974	79692994	AACGGAAAACGCTGAAGAGA	CCAGCTAGTGAAGAAGCGGA	233
Ad02A14410	Aradu.A02	14410	p3	(ATA)18	54	79707695	79707748	GGGGAAAATGCATATGGAAA	CCCCCACACACACATATACA	261
Ad02A14416	Aradu.A02	14416	p2	(AT)11	22	79734747	79734768	CTGCATGCTGAAGCAACAAT	GTGAACAGGCTCTTGGAACG	272
Ad02A14420	Aradu.A02	14420	p2	(AT)6	12	79743829	79743840	GTGCTTGGGTCTCAATCCAT	AAAACCGGTGGGAAATCTCT	223
Ad02A14428	Aradu.A02	14428	p2	(TA)6	12	79766367	79766378	TCAATCAATTCAATATGCAAGACA	TCCCATGAAAAGATGTCAAAAA	142
Ad02A14439	Aradu.A02	14439	p2	(TA)7	14	79794179	79794192	AGGAAGTGTTATCCCCCTCC	CGAACCGGACCATAAACACT	227
Ad02A14441	Aradu.A02	14441	p2	(TC)18	36	79798766	79798801	CTAAGCCGTGAACCAGCACT	GGGAGAAAAGTGCGAAGAGA	187
Ad02A14442	Aradu.A02	14442	p3	(TCT)6	18	79798985	79799002	CGTGAGCTCCATCTTCTCCT	CATCATCAACATAGCCTAGCCA	277
Ad02A14443	Aradu.A02	14443	p2	(GA)15	30	79799238	79799267	TGAAGAGGATAGTGGCTAGGCT	CATACACCCACCCTCACTCA	133
Ad02A14444	Aradu.A02	14444	p2	(TA)9	18	79813908	79813925	TATGGAACACAGAGCCGCTT	AGAAAAGGTTTAGACGGTACATACA	275
Ad02A14446	Aradu.A02	14446	c	(CT)21tccatata	160	79830131	79830290	CCCACAAACACCCTCTCAAC	CGATGTTGCCATGTCTTTTTA	280
Ad02A14447	Aradu.A02	14447	p3	(TAC)6	18	79830462	79830479	TCTGCACTGCAAAATCCAAG	TGAAGGGGAAGGTGAGAAAA	138
Ad02A14449	Aradu.A02	14449	p3	(AGA)5	15	79831059	79831073	TGGTTGTGGCTACGTGAAAA	GGTTCCTGCACTCTCTTGGA	278
Ad02A14451	Aradu.A02	14451	c	(A)10tctactccg	39	79837181	79837219	TGATGATAAAATCACGTTTCCA	CGAATTGAGATATAGCACCAAA	191
Ad02A14452	Aradu.A02	14452	p3	(TAT)5	15	79842122	79842136	TTTTGCAACAACTTCTTCTTTTG	GGCCCTTTTTGTCGAGTGTA	279
Ad02A14458	Aradu.A02	14458	p2	(CA)8	16	79857796	79857811	ACATTGAATCACAACAGCGG	TATGTGGGTGGGTGGATTCT	185
Ad02A14460	Aradu.A02	14460	c	(TAT)20gtgcg	77	79867864	79867940	AGCAAAGAAACAAAAAGCGG	TACGTATGCAATGGCTTTCC	173
Ad02A14461	Aradu.A02	14461	p2	(AT)6	12	79868403	79868414	ATGGAAAGCCATTGCATACG	TCTCTCGCCTCTCATTCCTC	241
Ad02A14464	Aradu.A02	14464	p2	(TA)9	18	79876171	79876188	AACAAAACGACCAAAAATTTCA	TCCATAATAACAAATAAACCACATGA	188
Ad02A14466	Aradu.A02	14466	p3	(TAA)5	15	79880569	79880583	CGCTTGCTTTGTTTTCATGT	AAAGCAAATAGCAACAGCAGAA	278
Ad02A14470	Aradu.A02	14470	p2	(AT)9	18	79885398	79885415	TGTCGGGTTGAAAAATACCG	TGTTCAATTTTGGCCCTTTT	256
Ad02A14480	Aradu.A02	14480	p3	(TCC)5	15	79910545	79910559	GATGGCGAAGAAGAATTCCA	CCACCTTAGCCTCACCTTCA	269

ID	Chromosome	SSR nr.	SSR type	SSR	Size	Start	End	FORWARD PRIMER (5'-3')	REVERSE PRIMER (5'-3')	Product (bp)
Ad02A14482	Aradu.A02	14482	p2	(TC)7	14	79928939	79928952	CGAACCTTCTTACTATTTTGTTTGTC	GATAAATGTAAGGTTGTGACAGTTTT	259
Ad02A14487	Aradu.A02	14487	p2	(TC)11	22	79936145	79936166	AATCACATGTGCCACGTTCT	ACCATATCGATCCCAAGCTG	239
Ad02A14492	Aradu.A02	14492	p3	(AAT)23	69	79938938	79939006	CCCAGCTATGAAAAGCCTGA	AGGTAAATAATCCCATTTTAGAATCTT	277
Ad02A14501	Aradu.A02	14501	c	(T)10gtta(T)12	26	79970786	79970811	GAAAAACCGGGGCTAGAGTT	CGTGGGCGATGGTCTTATAG	275
Ad02A14502	Aradu.A02	14502	p3	(TTA)8	24	79976106	79976129	TTTTCCGTATCTCCATTCATTG	TCCCATTCTCTTATCATTTCACTT	277
Ad02A14503	Aradu.A02	14503	p3	(TTC)6	18	79980664	79980681	GGGTCCTCAATTCAGCTTCA	CCAACCTGGATAAGCCCTTC	273
Ad02A14504	Aradu.A02	14504	p2	(AG)6	12	79983560	79983571	TCAAAATCTCACAGGTTGTGTGT	GTCGGAATGACTTCCAGAGG	264
Ad02A14505	Aradu.A02	14505	p2	(GA)7	14	79994330	79994343	GCCCAACTATGATCCATGCT	CTTGCTTGAGTGGCGTACTG	218
Ad02A14506	Aradu.A02	14506	p2	(GA)6	12	80005095	80005106	TCCAACGGATTCCATTTCTC	TTGTTTCCATGGTTTCCTGG	253
Ad02A14514	Aradu.A02	14514	p3	(ATT)6	18	80032411	80032428	AATAGAGATTTACAACATTTTGGTTG	CGACGACTATGAATAAAGAGACACC	276
Ad02A14525	Aradu.A02	14525	p3	(ATT)6	18	80073619	80073636	CCAAATGCAAGAAAAGGGAA	GGAGAAAAGTAGGGACCAGCA	200
Ad02A14527	Aradu.A02	14527	p2	(AT)7	14	80074569	80074582	CACACATAAATATTTGCACAGGG	CAAAAGCAAAAGGGAAAATCA	278
Ad02A14529	Aradu.A02	14529	c	(A)16caatgacc	82	80077602	80077683	GCGTCAAAATGCGTACAACA	AGGAAGCAGACTTGTCCCAA	280
Ad02A14531	Aradu.A02	14531	c	(A)12gatttttcaa	43	80079319	80079361	TGTGGAGTTCAAATTTTGCC	TTCATCAGTTCATGTTCCTATTTCA	226
Ad02A14534	Aradu.A02	14534	p2	(AT)6	12	80086481	80086492	CCTTAAGGGTTACTTTCTTCACTCA	ATTCCTCCATATCTGGTTGGT	204
Ad02A14537	Aradu.A02	14537	c	(T)10gtaaaaatt	79	80095680	80095758	AGATCAATCCACAACAAGGG	CTGCCAAATCGATCGAAAAT	209
Ad02A14540	Aradu.A02	14540	p5	(AAAAT)5	25	80099914	80099938	TTTGGTAGCCTGGCTCACTT	TTTTCACGGACCATGTAAGAAA	193
Ad02A14542	Aradu.A02	14542	p2	(AT)6	12	80101853	80101864	GTCCCCATATTCCCCACATT	GGTGCAAAGAGGACGAAGAG	234
Ad02A14543	Aradu.A02	14543	c	(TTA)7ttg(TTA	45	80106169	80106213	TGAGTCGTCCACAAAAAGAAGA	TTCCTTCGCATAAAACCTTGA	199
Ad02A14545	Aradu.A02	14545	p6	(TCATCC)7	42	80118525	80118566	CTAGGCGCTGTATCCTCGTC	AGCAGAAGTAATGAAGCGGC	251
Ad02A14546	Aradu.A02	14546	p3	(GGT)9	27	80119083	80119109	CAAAACTGTTGGTGTGACGG	GCATTGGTACCGAGAAGCAT	261
Ad02A14553	Aradu.A02	14553	p2	(AG)6	12	80137858	80137869	AGGAGATGGTGATGAGGTGG	AAAATCGTCCTTTGGGACAG	108
Ad02A14555	Aradu.A02	14555	p3	(TGA)5	15	80145856	80145870	AACGCACCTCACCTAAAACG	CCCCACAACAACACATTGAA	151
Ad02A14559	Aradu.A02	14559	c	(AT)6ttcacaaga	51	80149754	80149804	GGGAAACAATTGTCAAACCC	ACGTGCTTGTCCTCTAAGGC	226
Ad02A14560	Aradu.A02	14560	p3	(AAC)6	18	80152005	80152022	GGCATTGCATCAAACACCTT	CTAGCCCGTGGTTGTTCATT	262
Ad02A14562	Aradu.A02	14562	p2	(AT)6	12	80172344	80172355	AACTTAAAATTTGAAACCAAGATAGAA	TGGTGTCCGTCTAGTGGTTTT	107
Ad02A14564	Aradu.A02	14564	p3	(TTA)5	15	80182915	80182929	TCAAAGTTTCTGATTGTGCTTCA	TGTTCCCTTTTACAATTTGACA	240
Ad02A14565	Aradu.A02	14565	p3	(AAG)5	15	80184124	80184138	CTCAGCGTTGACATTGGCTA	GTCGTCTCGATCATCCCTTT	145
Ad02A14568	Aradu.A02	14568	p2	(TA)6	12	80191578	80191589	AAAAAGAGTAAATGATGAAAACAACA	TCAATTTGCAAGGTTGGTGA	274
Ad02A14569	Aradu.A02	14569	p2	(AT)7	14	80194343	80194356	TCTTCGAAACAAAGCACAAAAA	TGTTAAGTGCTGGCCAAAAA	224
Ad02A14575	Aradu.A02	14575	p4	(AAAT)6	24	80258269	80258292	CATCGCTTCCTGTTTTCCAT	TGAAAACAAAATTAAAATGCAATGA	280
Ad02A14577	Aradu.A02	14577	p2	(CT)10	20	80269508	80269527	CGTTGTTGTTGCTGCTCAAT	CTGAATCAACCTCTTTGAGGC	266
Ad02A14581	Aradu.A02	14581	p2	(GA)6	12	80278185	80278196	GGTTTCGGTGAGGAGACAGA	GCAAAGAACAGGGAAACCAC	269
Ad02A14584	Aradu.A02	14584	p2	(AG)22	44	80280433	80280476	TTGAAAGCGTATTTTAAACGAGTG	GATGCCTAGTTCATCATTTGG	280

ID	Chromosome	SSR nr.	SSR type	SSR	Size	Start	End	FORWARD PRIMER (5'-3')	REVERSE PRIMER (5'-3')	Product (bp)
Ad02A14588	Aradu.A02	14588	c	(A)10tgaattgca	104	80287455	80287558	AGAAAGCGTAGCCGTAGCCT	CAACAGCAACAAATTTACCAAAA	249
Ad02A14594	Aradu.A02	14594	p2	(AT)12	24	80296825	80296848	AACTTGGATTGAATGCACCA	GCCATTCCCTTCAAGTCAAA	273
Ad02A14595	Aradu.A02	14595	p2	(TA)6	12	80298249	80298260	GTTTATGTGACCCCCTGCAT	GACCATGCACGAAGTTCTGA	248
Ad02A14600	Aradu.A02	14600	p3	(TTA)5	15	80317212	80317226	TTGTTGAGAATTTTGATTAGGAATTT	CAAAAATTTGCACAACTAACTCAA	196
Ad02A14604	Aradu.A02	14604	c	(T)10aatccattg	55	80327035	80327089	TCATAACATCCCATGCCAAA	GACATGCTTTGTCTTCCCGT	152
Ad02A14605	Aradu.A02	14605	p2	(TA)6	12	80333793	80333804	CTGGATGGTGAAATTCAAGACA	TCGAAAGGAAAAATGGGAGA	249
Ad02A14609	Aradu.A02	14609	p3	(ATA)5	15	80340668	80340682	CATAAAATTGAGGTGGCAAGG	TTGCACTTTTTGATTTTGCG	128
Ad02A14610	Aradu.A02	14610	p2	(AT)8	16	80340938	80340953	CGCAAAATCAAAAAGTGCAA	AGTTTCTGCGACGAAGTGGT	276
Ad02A14612	Aradu.A02	14612	p3	(ATA)11	33	80350341	80350373	CAGTCCGTCCTCAGGCTCTA	TTGCGCTTTTTGATTTTGTG	228
Ad02A14614	Aradu.A02	14614	p2	(AT)7	14	80356177	80356190	GAATCTTGGGACATTGGGTG	TTTGATTTGGGGCATTTTGT	269
Ad02A14615	Aradu.A02	14615	c	(AGA)5tgatatg	178	80361882	80362059	GCTATGAACCAAAATTCTTATATTCAA	AGTCAAGCATATTCTTAATCAAGTCA	278
Ad02A14617	Aradu.A02	14617	p3	(AGG)5	15	80380355	80380369	TAGCAATTGCCAACATGGAA	GCGACACTGTGTTTCAGCTT	265
Ad02A14620	Aradu.A02	14620	p2	(TA)7	14	80394902	80394915	TTATTCATTTTTAGCGGGTTTT	CTTTCTCTGCCCCACCAATA	246
Ad02A14621	Aradu.A02	14621	p2	(CT)7	14	80395291	80395304	TCGAAACAGAAAACCCCATC	TGTTCAAATTGAGGGATCGAG	246
Ad02A14623	Aradu.A02	14623	p2	(AG)15	30	80397975	80398004	TATTTTCGCATCTCTGCTGC	TGTGATAGCTGGCACTTTCTT	197
Ad02A14624	Aradu.A02	14624	p2	(AT)6	12	80401792	80401803	GCCATGCAATGCAATTCTAA	CAAAATCCAGTGAGGGTTCAA	263
Ad02A14625	Aradu.A02	14625	p2	(AG)6	12	80412171	80412182	CATGAAGGATGTCAGGCTCA	TGACAGCTGGCACTCTCTTG	178
Ad02A14628	Aradu.A02	14628	p2	(AT)6	12	80421528	80421539	GAGCGGTTTAATTTGGATCG	CCTTGCTTGGTAGGCTGGTA	273
Ad02A14631	Aradu.A02	14631	p2	(TG)6	12	80437792	80437803	TCACAGCCAAGATCACAGGA	TTGTGGCAGTTCCCATGTAA	258
Ad02A14633	Aradu.A02	14633	p2	(GA)8	16	80445405	80445420	ACAACCGAGTGGAGTTGGAG	GGAAATCAACTTTCTGGGCA	140
Ad02A14636	Aradu.A02	14636	c	(AG)6cttcttcct	46	80457284	80457329	TTTCCAAAGCCTTCTTTCACA	TTGGTGTTGCGAAAATTTGA	231
Ad02A14638	Aradu.A02	14638	p3	(TGA)6	18	80464359	80464376	CCCTTCCATATGGCATGTTC	GTCTCTGAGGCAGATGGAGG	214
Ad02A14640	Aradu.A02	14640	p3	(AAT)12	36	80469182	80469217	TCATCGAAATAAAAAGTCAAAGATTTA	CAATCTCTCGTTCAATATTTTATCACA	280
Ad02A14641	Aradu.A02	14641	c	(TC)6taacctctc	138	80471149	80471286	TTGGTGACTCGCAATGTGTT	GCTTTGGTGGTTAGCGTGTT	271
Ad02A14642	Aradu.A02	14642	p2	(AT)12	24	80471496	80471519	CTGCAATACCGATCCAAAAA	GATATGGGCGCAGAAGTCAT	277
Ad02A14643	Aradu.A02	14643	p2	(CT)6	12	80474935	80474946	TCTTCCTCGCGTAGCTCTCT	GAAAGAAGGATCTGAGGGGG	252
Ad02A14644	Aradu.A02	14644	p2	(TC)8	16	80476152	80476167	TCTCTCATGCTCGGTGTGAC	TGCCATTAACTGCGAACAAA	215
Ad02A14645	Aradu.A02	14645	p2	(TC)8	16	80477372	80477387	TCTCTCATGCTCGGTGTGAC	GATGCCATTAACTGCGAACA	217
Ad02A14647	Aradu.A02	14647	p3	(TAA)6	18	80481590	80481607	CCCAAAACCTTTTAACTCGC	AGCATGTGTGGCTGGAGTATT	280
Ad02A14648	Aradu.A02	14648	p3	(TAA)5	15	80482510	80482524	TGTTGTAACTCTTCTCCCTGAGC	TTGGTGGAGAAAGCACTTTTTAG	229
Ad02A14650	Aradu.A02	14650	c	(A)11gaaagaag	86	80485543	80485628	TGTTGGTGGTTTCTAACTCAAA	AGCGCTATTCCCTTTTCCAT	188
Ad02A14651	Aradu.A02	14651	p2	(TA)6	12	80486366	80486377	CAGCAATATTATTTCGCCCA	ATGGTGGCAATCGGATGTAT	223
Ad02A14653	Aradu.A02	14653	c	(CTA)7(ATA)	54	80487142	80487195	CCTTAATTTTTGAATAACCCTTAAAA	AAACGTTAGGGACAAAAACGA	267
Ad02A14659	Aradu.A02	14659	p4	(TTAT)7	28	80493854	80493881	CACAAAATCTGCCAATTCTCTC	AGCCATGTGGGCATGTTTAT	150

ID	Chromosome	SSR nr.	SSR type	SSR	Size	Start	End	FORWARD PRIMER (5'-3')	REVERSE PRIMER (5'-3')	Product (bp)
Ad02A14660	Aradu.A02	14660	p3	(ATT)24	72	80494750	80494821	GAGGGAGATTGTGATCAACTTTT	GCAACATCCCAACCAACAC	261
Ad02A14662	Aradu.A02	14662	p3	(AAT)17	51	80505083	80505133	CACGTCCAGCAAGCTAATTACA	GGATTAGACTGGTGAACACACAA	241
Ad02A14665	Aradu.A02	14665	p2	(TA)11	22	80508111	80508132	TTGGGGGTCATTTTTCTTTG	CGTGATTGCCAAACATATCG	114
Ad02A14668	Aradu.A02	14668	p2	(AC)6	12	80522777	80522788	TATCATCAGATCCCGAAGCC	TGGCAGTGGTATTATGCTCG	173
Ad02A14670	Aradu.A02	14670	p3	(TTC)5	15	80530654	80530668	TCCCATTAAGGGAGTTCAACC	GCATGCATATGGCAAAACAC	148
Ad02A14671	Aradu.A02	14671	p2	(AT)6	12	80530785	80530796	ATGGTTTTTGTGCGTTGTGA	TGATTAATTTATTCTCGCGATTTTT	273
Ad02A14674	Aradu.A02	14674	p2	(TA)6	12	80543182	80543193	TGCTTATTTTAGTGCGAGATGTATG	TAAGTGTAGCCTTAGGTCTCATTTTT	279
Ad02A14676	Aradu.A02	14676	c	(AT)14ag(AC)	46	80546894	80546939	GTTCCACCTGCGAAGCTAAA	CATGCTGAAAACCTCCGATT	255
Ad02A14679	Aradu.A02	14679	p2	(GA)59	118	80553852	80553969	TGCATTTCTAAGAGAGAGGACAT	GGTAGTTATGTTACAAATGCTGTCG	193
Ad02A14680	Aradu.A02	14680	p4	(CTTT)5	20	80556253	80556272	TCTCCGAACTTTTTAACACCAAA	CACAGAAAATTGGCAGAGCA	145
Ad02A14681	Aradu.A02	14681	p3	(GGT)9	27	80557207	80557233	CCAACAGCTTCAACGAAACA	TTGAAGCACAGCTGAACCAT	177
Ad02A14684	Aradu.A02	14684	p2	(TA)7	14	80561471	80561484	GAATTGCTAACTGCCCTTGC	GTCAGATTTCACGCCGTCTT	248
Ad02A14691	Aradu.A02	14691	p3	(AAT)13	39	80578945	80578983	CAAAAGTCTTGACAGACGACAAA	TGCTCATGTTTTTCTACTGGTG	272
Ad02A14694	Aradu.A02	14694	p2	(AT)6	12	80603140	80603151	GTGTTGAGGCAATCCGATCT	AACAGGGCTACGTTCCCAAT	252
Ad02A14695	Aradu.A02	14695	p4	(TCTT)5	20	80604442	80604461	GGAAGGAATTCAAGCTTCCC	AACAACAAAACACCGAAGCC	251
Ad02A14699	Aradu.A02	14699	c	(T)10cagtcatgg	82	80621721	80621802	GGCCAAATTAGTAGGAGTGGAA	GCTGAAGAGGTCCAATCACC	249
Ad02A14711	Aradu.A02	14711	p2	(TA)6	12	80663917	80663928	CAGTGTAAACAAGGTTGGGC	TGTCTTTACCAACTGCGTGC	161
Ad02A14714	Aradu.A02	14714	p2	(TA)7	14	80674346	80674359	ATACCATGCATTGGTGGGTT	CGCTTTTGTGAGTGAGCGTA	224
Ad02A14715	Aradu.A02	14715	p3	(TTC)5	15	80674642	80674656	TACGCTCACTCACAAAAGCG	TGCAAAGAAAAAGGAGGAGAA	199
Ad02A14716	Aradu.A02	14716	p3	(TTC)5	15	80675262	80675276	CTCTTCCAAGTAATTTTGCAGC	TCCTCCTCATCTTCTGGTGG	198
Ad02A14721	Aradu.A02	14721	p2	(AT)8	16	80690241	80690256	TCTGGGTAAAGTACCGTGGC	TGCCTGTTCTATGCCTGATG	270
Ad02A14722	Aradu.A02	14722	p2	(AT)6	12	80691122	80691133	TCCCTCGCCTTTATTTCTACC	GGCTACGCCACTCTGATAGC	232
Ad02A14739	Aradu.A02	14739	p2	(GA)7	14	80785031	80785044	GGGGAGATTTGTTGGGTTTT	ATTTCAAGCCGATCCAACAG	237
Ad02A14744	Aradu.A02	14744	p2	(TG)6	12	80808873	80808884	GGATTGTGTGGGAGGAGGTA	CCTCAAAATACCGCTTCTTCC	176
Ad02A14748	Aradu.A02	14748	p3	(ATT)7	21	80847613	80847633	CAAAACGCCGTAGTCCATTT	CCACCTCCTCAAGTCCAAAA	172
Ad02A14750	Aradu.A02	14750	p2	(TA)7	14	80850177	80850190	CAAGTCCATTTCACAAGGATATATGA	GGATGCTGGCAAGGTATGTT	279
Ad02A14751	Aradu.A02	14751	p3	(TGG)6	18	80869462	80869479	CTGCAGTTGTGGTGACAAGG	GGACCAATAAGTGGAGCGAA	254
Ad02A14754	Aradu.A02	14754	p2	(TA)8	16	80897777	80897792	TCATTTCGGTATGAAAAATAATAAACA	GCTCTTTCCTAAAAATATTGGCA	222
Ad02A14755	Aradu.A02	14755	p3	(TTA)21	63	80898424	80898486	AAAATATCATGCATGTACCGTG	TGATGCACATATTAACAGTGACCTA	140
Ad02A14756	Aradu.A02	14756	p2	(AG)6	12	80899643	80899654	GAGAAGAACCCGATCCAAGC	ATGCATGCAAGATGAGATGG	268
Ad02A14761	Aradu.A02	14761	p3	(TAA)7	21	80919778	80919798	TTTTCCCTCCAAAACGAATAAA	GACTTTCAAATTTAGATTCATCGGA	280
Ad02A14764	Aradu.A02	14764	c	(A)12ggaac(A)	27	80937115	80937141	TGGGTTGAAGTCCAAGCTGT	GCATGCAATCGAGCGTATAG	247
Ad02A14765	Aradu.A02	14765	p3	(TTG)5	15	80940260	80940274	ACATGACAGAGCACAATGGC	TGATTGATGGATGTTATTTTCAA	187
Ad02A14766	Aradu.A02	14766	p2	(GA)14	28	80951305	80951332	GAAAGAGAAGAGAAGAGGGTTCG	TTCTTCACCCTTTTTCAAAATTC	258

ID	Chromosome	SSR nr.	SSR type	SSR	Size	Start	End	FORWARD PRIMER (5'-3')	REVERSE PRIMER (5'-3')	Product (bp)
Ad02A14767	Aradu.A02	14767	p2	(TA)6	12	80958291	80958302	CCCCTCCTCTGTTTCGTTTT	TTCCCAGTGAGGTAACCTGTG	238
Ad02A14768	Aradu.A02	14768	p2	(TC)13	26	80963174	80963199	TCATTATGAAGAATGCGCGA	AGATGATTCGCCTCCCTTTT	280
Ad02A14770	Aradu.A02	14770	p2	(GA)6	12	80967089	80967100	AGAGAGAAGAGAAGAGGGTTCG	TCCTCACCTTTTTCAAAATTCC	238
Ad02A14771	Aradu.A02	14771	p3	(GAA)5	15	80977680	80977694	GGAATCACAAGGCAAAGCAT	TGCTCTTGATGTTGCTCCTG	197
Ad02A14772	Aradu.A02	14772	p2	(GT)6	12	80986377	80986388	ACACCGTCTCCTCCTTCCTT	TTGCCCTAATTCCATCATCC	101
Ad02A14776	Aradu.A02	14776	p3	(TAA)6	18	81004855	81004872	GAAACATCAAGTACTAATTAAATGCAA	CGGCTTAAACTCATTGTGGA	210
Ad02A14780	Aradu.A02	14780	p2	(AT)10	20	81013856	81013875	AACTGGGATGTTGAACAAAGG	CTCCCCTTCTTTGACGATGA	279
Ad02A14782	Aradu.A02	14782	p3	(CCA)7	21	81015774	81015794	CTTTGAAGCTGCTCTGGCTT	TTGGTGTTCTTGCGCTACAG	232
Ad02A14783	Aradu.A02	14783	p3	(AAT)5	15	81016159	81016173	TTTTTGGTCCTTTCCTGTGG	AGAAACCGAGTAGTGAGTACGTTT	176
Ad02A14784	Aradu.A02	14784	c	(A)10ttggtttcat	142	81020861	81021002	TCCAAATTCCCAACTTTCATAA	GAACAACAACCACCGCTCTT	279
Ad02A14786	Aradu.A02	14786	p2	(TA)7	14	81028580	81028593	TCGTGTCGTGTCCTATGTCC	CCATCCTTTCTAACAAGAATAAATGA	191
Ad02A14787	Aradu.A02	14787	p3	(AAT)5	15	81040489	81040503	CCATCGTTCTACCAAGGCAT	TCCAAGTGCATTTCCATACAA	256
Ad02A14788	Aradu.A02	14788	p3	(TAA)14	42	81040829	81040870	AAACTCTTAAAAACAGGGTACTCACA	AAATATGAACATGGTTATTAGTCCAAA	252
Ad02A14791	Aradu.A02	14791	p2	(GC)6	12	81048243	81048254	ACAAGGCTCCTAAAACCGCT	GCTGTCAAGCCAACCTCTTC	231
Ad02A14792	Aradu.A02	14792	c	(CT)12t(TC)7	39	81050982	81051020	CCAACCAAAAAGGTGGAAAA	TGCATATGTTTAGGTCAGGCA	229
Ad02A14794	Aradu.A02	14794	p3	(CTT)6	18	81055669	81055686	AAACAAACGGATAACGCCAG	GGGTCTTCGTTGTTGAGGAA	198
Ad02A14799	Aradu.A02	14799	p2	(TA)7	14	81066220	81066233	GTTTCAGACTCCTGCAAGGC	AAAATCATCCAACTTCACCACC	269
Ad02A14800	Aradu.A02	14800	p2	(TC)6	12	81071819	81071830	ACTCTCTTCTCCCTAGGCCC	TCCTCTTTCTTCGGATTCCA	244
Ad02A14803	Aradu.A02	14803	p2	(AT)7	14	81076277	81076290	ATTCGGTGTTCCTCATCACA	AACATTTTAAGAGGCTTGAGCTTT	198
Ad02A14807	Aradu.A02	14807	p3	(AAT)12	36	81088105	81088140	CTGTATCCGTGTCTGGGCTT	TATAGAGTTTGCCGGGTTCG	279
Ad02A14809	Aradu.A02	14809	p3	(AAT)17	51	81089532	81089582	GGACTGGGGCAATTTCAGTA	AACAATGTGGGCAGTGAACA	149
Ad02A14810	Aradu.A02	14810	c	(AT)9gaaacgca	79	81095887	81095965	GGATCCTCGTGTGGGTTATG	TGAAAAACGGACAGAATCTCC	279
Ad02A14811	Aradu.A02	14811	c	(AT)9gaaacgca	79	81102795	81102873	GGATCCTCGTGTGGGTTATG	TGAAAAATGGACAGAATCTCCTC	279
Ad02A14812	Aradu.A02	14812	p4	(AAAT)6	24	81106841	81106864	CTCGGTTTCTGACGGAATTT	GGTCCATTTGCTTGCAGAGT	280
Ad02A14813	Aradu.A02	14813	p2	(GA)11	22	81110844	81110865	CGACCACCAAAGAAACTTCAG	CGGTGCATAGGTTGATGAGA	258
Ad02A14816	Aradu.A02	14816	p3	(ATT)7	21	81142031	81142051	CCACATGGCAAAACCATATC	TTTAAGCAAAAATCGCAACTC	189
Ad02A14819	Aradu.A02	14819	p2	(TA)7	14	81149126	81149139	AGCAATACCCCACTCCATTG	CATTTTGATGAGGGGCAACT	148
Ad02A14820	Aradu.A02	14820	p3	(TTG)5	15	81149790	81149804	CGTTTGATTCATGTAGCCGA	TTAGAACCCTGGAAGCACAGA	137
Ad02A14830	Aradu.A02	14830	p2	(AT)10	20	81177151	81177170	GGGTAAAACAAAACCGCAAA	GGTTTATGGAGAGGCAGGGT	246
Ad02A14831	Aradu.A02	14831	p2	(TG)6	12	81178025	81178036	TTTCTTTTGGGGAAGGTTTTT	CAACTGATCAACAAACAGAACACA	265
Ad02A14833	Aradu.A02	14833	p3	(AAT)5	15	81182198	81182212	TTCTGAGCTTCGTTGCAATA	GGCCTTTCAATTCTGAAGAAAA	205
Ad02A14837	Aradu.A02	14837	p3	(ATT)5	15	81219536	81219550	TGCAGCCTTTGATTTTCATC	CGCACAGAACTGGATCACAC	215
Ad02A14843	Aradu.A02	14843	p2	(GA)7	14	81229207	81229220	GCGACAGTCAACGATGAAGA	CAATCACACACACAAACCCC	202
Ad02A14844	Aradu.A02	14844	p3	(AAT)5	15	81231324	81231338	TCGCACAACTAACCAGCAAG	GATGCAAAAAGAGCACAGCA	259

ID	Chromosome	SSR nr.	SSR type	SSR	Size	Start	End	FORWARD PRIMER (5'-3')	REVERSE PRIMER (5'-3')	Product (bp)
Ad02A14846	Aradu.A02	14846	p3	(ATA)5	15	81243012	81243026	CCACTTATATCAATTTTTCCACCTT	GGTTTTAGGAATCAATATTGCACA	276
Ad02A14847	Aradu.A02	14847	p3	(AAT)10	30	81243492	81243521	TTGGATGAAGCAGCCTTTTT	CCGGTGCAAAGAAAGCTAAG	263
Ad02A14852	Aradu.A02	14852	p3	(GTT)6	18	81257435	81257452	TATCAGAGCTTCGGTTCGGT	AGAGAGGTGGGTCACAATGG	226
Ad02A14853	Aradu.A02	14853	p3	(TTA)7	21	81262988	81263008	TCCACCAAAAGAAATCAGGAA	TCCATACCCATGCAGATTCA	236
Ad02A14854	Aradu.A02	14854	p2	(CT)7	14	81263765	81263778	ACAGTTTGGGTCTGCTGCTT	CAATAGGGAGGGAGAGGGAG	257
Ad02A14855	Aradu.A02	14855	p2	(AT)7	14	81269170	81269183	TCAGTTTTTGAGGCTCAGAGG	TTCTGCCATCACAGAGCAAC	167
Ad02A14858	Aradu.A02	14858	p2	(TG)7	14	81275763	81275776	AGTGGTTGCTGAGAGGTTGG	TCATTTCACACAATGGACGG	263
Ad02A14863	Aradu.A02	14863	p3	(TAT)15	45	81281423	81281467	TTTTAAGTGATTATTGGTTCTTGCTT	TTGAAAGTTAATATTGAGACCAAAGAA	234
Ad02A14865	Aradu.A02	14865	p3	(TTC)7	21	81282034	81282054	GCTTTCCTTTCATTCCTTCG	CCTTGAGTTAAAGCGCGAAC	269
Ad02A14869	Aradu.A02	14869	p5	(TTATT)5	25	81294425	81294449	CTTGGCCTTTTTGCACACTT	TGCTAGCCTCTGAAATCTAATGC	280
Ad02A14870	Aradu.A02	14870	p3	(TGA)5	15	81299026	81299040	CAGAAAATCAAAACCAGCAAAA	CACAAAGCACCACCACAGTT	250
Ad02A14874	Aradu.A02	14874	p2	(CT)13	26	81308728	81308753	CACGCACCCTCTCCATTTAT	GTAGCAGGAGCGACGAAAAC	254
Ad02A14876	Aradu.A02	14876	c	(A)10ttagcctaa	76	81313224	81313299	TTCCCACAAAAGAATCCACC	ACGGTGTACAGGTGTCCGTT	274
Ad02A14877	Aradu.A02	14877	p3	(TTA)9	27	81313906	81313932	TTTGATTTGGCTGATTCACG	CCACCTATTCAACCCACCAT	222
Ad02A14879	Aradu.A02	14879	c	(A)10ttattattaa	56	81318095	81318150	TTTTTGCACTAAATGTATAAAATCGG	TGAGTCTGAAAATTCATGGACA	232
Ad02A14885	Aradu.A02	14885	p2	(GA)6	12	81353470	81353481	TGCACATTTTGATTGTTAAATTTGT	AAGTGTGCAACATGACATTTTCT	251
Ad02A14886	Aradu.A02	14886	c	(GA)7a(AG)6	27	81353712	81353738	AGAAAATGTCATGTTGCACACT	TCAAAATTAAAGAATTCTCTCTTCCTC	143
Ad02A14887	Aradu.A02	14887	p3	(CGA)5	15	81374202	81374216	GCAGCAAGAAACAGCAACAA	CCGTCACAGAGAGAGAAGGG	255
Ad02A14888	Aradu.A02	14888	p2	(TC)6	12	81374479	81374490	CTCTGCGTCACCCTTCTCTC	TTATCACCGCGACTGACAAG	183
Ad02A14889	Aradu.A02	14889	p2	(AC)7	14	81376887	81376900	CCTAACCCACCTAACAGCCA	TCTTCCCCTTCCCTGATTCT	161
Ad02A14890	Aradu.A02	14890	c	(A)10caacttaag	77	81385461	81385537	ACCGTCGCAAAATTTCTACC	AAGAACAAACGAAGAAGTATACCG	264
Ad02A14892	Aradu.A02	14892	p2	(AC)6	12	81387583	81387594	GGACACGGACACGTACACAC	GATTCCTTTGTTTGGCCTGA	185
Ad02A14893	Aradu.A02	14893	c	(T)11cgttttctct	32	81396313	81396344	TCAGCTTTGCTAGAGTCCCC	TGGTTCATGAATGTTGGCTC	235
Ad02A14894	Aradu.A02	14894	p2	(AG)6	12	81398297	81398308	AATTTGAAGGGTGGGGTAGG	CGAAATTTTGCTTGCCATTT	227
Ad02A14896	Aradu.A02	14896	p3	(AGA)7	21	81427929	81427949	TAGAATGTTGCTCGCCTTCA	CTTCCCCATAAATCCCACCT	247
Ad02A14897	Aradu.A02	14897	p2	(TA)6	12	81440746	81440757	TGAATTCGATTTGGAACGAAG	CCGGCCTATTCACAAATTCA	247
Ad02A14901	Aradu.A02	14901	p4	(TATT)6	24	81448265	81448288	TGTCCCAAAATTTTTCACTTG	TGTGCTTTCTAAGTGACGTTTCA	244
Ad02A14902	Aradu.A02	14902	p4	(TTTA)11	44	81448437	81448480	TGAAACGTCACTTAGAAAGCACA	AAATTTGGGAGAATGGGAGG	221
Ad02A14906	Aradu.A02	14906	c	(CT)9cattttccta	39	81470698	81470736	TTCATTCCAAAAGTTGAGAGACA	CCTCATCAGAGATCGTTAACTCAA	245
Ad02A14908	Aradu.A02	14908	c	(AG)8ta(TG)6	30	81477197	81477226	TGCCCATTTTTAAACTTCCG	AAAAGCATCCCAAAGAAGCC	275
Ad02A14910	Aradu.A02	14910	p3	(TTC)5	15	81478547	81478561	CGTCCAAACGTAAGAGACCAA	TGGTGATAACGAAAACGAAAGA	142
Ad02A14911	Aradu.A02	14911	p2	(AG)6	12	81491405	81491416	CTCTTGCGCTATTTCTTGGG	AAGACAAAGTCGCATCCACC	149
Ad02A14912	Aradu.A02	14912	p2	(AT)6	12	81496706	81496717	TGCCTACGTAACGTTTTGCT	TGAACTTGAGTTGATTGCTGC	194
Ad02A14913	Aradu.A02	14913	p3	(CTC)5	15	81497917	81497931	AGTGCCTCTTCCTCCTCCTC	GACACCGTGAAAGGTTGTGA	208

ID	Chromosome	SSR nr.	SSR type	SSR	Size	Start	End	FORWARD PRIMER (5'-3')	REVERSE PRIMER (5'-3')	Product (bp)
Ad02A14915	Aradu.A02	14915	p3	(AAG)5	15	81506542	81506556	GGAAGAAGAACGTGCAGCAA	ACTGCATGTTTCCACTGCAT	170
Ad02A14916	Aradu.A02	14916	p3	(ATT)6	18	81510506	81510523	ACATTGTTGCCCCTCTCATC	CAACCAAAGGAGAGCCAGAA	250
Ad02A14918	Aradu.A02	14918	p2	(AG)19	38	81523528	81523565	GTCAGGAAGCAACCCGATAA	TCATGCACACATGATTGACG	229
Ad02A14928	Aradu.A02	14928	p3	(GAA)6	18	81544682	81544699	GGGGTGAAGGTGAAGGAACT	TTGCCTCATGAACTCCTCAA	221
Ad02A14930	Aradu.A02	14930	p2	(TA)11	22	81547088	81547109	ATTGGGAATCATCCACCTCA	TGAATCAAAATATAAGCTGACACAG	265
Ad02A14933	Aradu.A02	14933	p2	(TC)16	32	81555436	81555467	GGTGGCACAGCTACAACAGA	CTTTATTGGCGCCATCATCT	218
Ad02A14934	Aradu.A02	14934	p2	(TC)6	12	81555652	81555663	GACCTCAACAGCGGTAGCTC	ATGGAGGGGGAAGAAGAAGA	228
Ad02A14935	Aradu.A02	14935	c	(A)13gattgaaat	39	81558951	81558989	TTCTCTTGTTGTTCCCTTATTGTG	CCATTGAAGCCATCCTCATT	141
Ad02A14937	Aradu.A02	14937	p3	(TTA)8	24	81573945	81573968	TTCCATCCTTGCAATTTCTTG	TTCATCAAACACCTAGAAGGCA	261
Ad02A14941	Aradu.A02	14941	c	(AAT)5agcgca	57	81579941	81579997	TTTGAGTTTGAAGGCAAGGG	GCAATGTAGAAACTTAATTGAAAGAAA	275
Ad02A14943	Aradu.A02	14943	p2	(TA)14	28	81582293	81582320	GGGTAGCTTGCAAAAAGAAAAA	AACCAGTGACATAATGACCTTAAAA	240
Ad02A14945	Aradu.A02	14945	p3	(ATA)5	15	81590811	81590825	TGGCTTAAAATTTCATATCAGTCAA	TCCTACTCCTCACACGGAAAA	102
Ad02A14955	Aradu.A02	14955	p3	(CTT)5	15	81609704	81609718	CCCAATAGAGAGGCCAAACA	TTTAAGGGTTGGCACCATTG	266
Ad02A14956	Aradu.A02	14956	p2	(AC)9	18	81609963	81609980	GGCCTTAACCCACATTCTGA	CTTTACCTTTGTGCCCTCCA	149
Ad02A14957	Aradu.A02	14957	p3	(ATA)12	36	81610520	81610555	CTTCCTTCCTTCCTTCACCC	ATGACCAAATTGCATCCTCC	254
Ad02A14959	Aradu.A02	14959	p2	(AT)6	12	81616981	81616992	AATGCTAACGGGTTGGACTG	CTTTCAGTGCCCCTCTCATC	115
Ad02A14960	Aradu.A02	14960	p2	(TA)6	12	81617725	81617736	TGACAGGAGTACCCGAACCT	TGGGGAGAACAGGAAGTGAC	265
Ad02A14961	Aradu.A02	14961	p2	(AT)9	18	81618185	81618202	ACCTCTCTTTCTCGCCGTCT	ATGCAATTTGTGAAGGAAAAA	242
Ad02A14967	Aradu.A02	14967	p3	(ATA)6	18	81641008	81641025	AATGCATTGCAAACCAACCT	TGCACCCACTCGTGATCTTA	192
Ad02A14975	Aradu.A02	14975	p2	(AT)8	16	81689833	81689848	TGAAGAACAATTTCGGAGACAA	ACTTTCTGGATTCGGTGGTG	258
Ad02A14978	Aradu.A02	14978	p3	(GAA)5	15	81700094	81700108	TGTACGGAGGTAGGGTTTGG	CACCACAACCTCCTCAACCT	127
Ad02A14979	Aradu.A02	14979	p3	(ATT)6	18	81703093	81703110	GGGCTTGATTTCGCATAGTC	CCCAGGTTGTTTTGAAGCAT	225
Ad02A14981	Aradu.A02	14981	p2	(TA)6	12	81716947	81716958	GACCTCCATCCAAGAAGTCG	AAAATTCATGTACTAAATCCAGAAAAA	269
Ad02A14987	Aradu.A02	14987	p3	(TTA)5	15	81734923	81734937	CACCACAGACAGCACCTTGT	GGCTGAGGCAGGTGTTGTAG	151
Ad02A14988	Aradu.A02	14988	p3	(CAC)5	15	81747760	81747774	TCCATTCCAACAGACCAACA	GCAATTGAGCCAAATGTGTG	219
Ad02A14989	Aradu.A02	14989	p3	(ACA)7	21	81752267	81752287	TGAACCGTACCAACCAATCA	CGTTTGATTCATGTAGCCGA	277
Ad02A14991	Aradu.A02	14991	p3	(TAA)5	15	81760518	81760532	CACAAAGTCAGGCGGGTATT	GGATGTGGAAGAAGGGGTTT	119
Ad02A14996	Aradu.A02	14996	p3	(TAA)11	33	81782222	81782254	CAATGGAAATCAGGGCAAAT	TCAAATCACGTTAGCTTCTCTGA	207
Ad02A14998	Aradu.A02	14998	p2	(GT)6	12	81792956	81792967	GAATCTGTGGTCCCAAGGTG	TGTGATGTACTCAATGACGCA	118
Ad02A15001	Aradu.A02	15001	p2	(TC)11	22	81803675	81803696	ATTTTAGCAGCCAGCACTCC	AGTGGGGATTGGAACATCAA	267
Ad02A15004	Aradu.A02	15004	p2	(AT)11	22	81809848	81809869	AAGCCTATAATCGGGTTAGCG	AGTTCGGGTGCCAGTTTATG	233
Ad02A15007	Aradu.A02	15007	c	(TA)9aaagaaag	84	81811997	81812080	CAAGCCACGTTTCCTTTCTT	AAACTATTTTGATAGGCAAAAATTAAG	254
Ad02A15008	Aradu.A02	15008	p2	(TA)8	16	81814172	81814187	CGACCCAATTCCCAGTAAGT	GGTCCAGAGTGTTGGGTTGT	276
Ad02A15009	Aradu.A02	15009	p2	(TA)9	18	81816311	81816328	CAAGCCACGTTTCCTTTCTT	TCTTAGAAATTGATTGACTGGCA	195

ID	Chromosome	SSR nr.	SSR type	SSR	Size	Start	End	FORWARD PRIMER (5'-3')	REVERSE PRIMER (5'-3')	Product (bp)
Ad02A15017	Aradu.A02	15017	p2	(GA)6	12	81834692	81834703	GTGAGGCTCAAACCCAACAT	ACGGGAATTCTGTTTTTCCC	138
Ad02A15018	Aradu.A02	15018	p2	(AT)6	12	81840370	81840381	GAGGTCGTGTTCATTCGGTT	CTTGTACAATTACGGAGACAAGTG	247
Ad02A15019	Aradu.A02	15019	c	(T)10atttaggtc	129	81840648	81840776	TTTTCCTTTTAATTGAGTTCTTGTACC	TTCCTATGCATGTCAACCACA	274
Ad02A15020	Aradu.A02	15020	p2	(CT)15	30	81842767	81842796	CCTCCCTCATTCGAATTTCA	AGGATGGAACAGAGGTGGTG	122
Ad02A15021	Aradu.A02	15021	p3	(TAA)5	15	81850683	81850697	TCTCTCATGCCTCCGTTTTC	CGTGAATCGGGTAACAAAGA	277
Ad02A15022	Aradu.A02	15022	p2	(AT)10	20	81874158	81874177	ATCCAAGATGGTTCCAGCAA	ATGATTTAAGCACGAGGGCA	241
Ad02A15026	Aradu.A02	15026	p3	(TTA)6	18	81890855	81890872	CCCCAAGGCACCTAGTTGAT	CCTTGCCATTTTTCCTCAAA	268
Ad02A15028	Aradu.A02	15028	p3	(CCA)5	15	81895308	81895322	TCTTCCGCTTCGTTTCTGTT	TTGATTGTCGGTCGTGGTAA	174
Ad02A15029	Aradu.A02	15029	p2	(CT)6	12	81895608	81895619	GACATGGCCGATCACTGTAA	TTGCTATCGCCTTTTCCTTC	251
Ad02A15031	Aradu.A02	15031	c	(T)11gctttttggt	57	81924307	81924363	AAGTTCAAACCATTGGTGCC	CAAATGTCTTTGGCCATGAA	122
Ad02A15033	Aradu.A02	15033	p3	(AAC)6	18	81936179	81936196	GGCATCTTCCCAAAATCTCA	TGGCGTTGTTTGATTCATGT	179
Ad02A15035	Aradu.A02	15035	c	(AT)9(AG)8	34	81946052	81946085	GGAAGTGTCCTGACTGTGAGC	ATTGGATTGGCAATGTCTCC	252
Ad02A15037	Aradu.A02	15037	p3	(ATC)5	15	81952197	81952211	CCAATCAAGGGAGAAGCAAA	GCGCTTTTGAAAGTGTAGACG	254
Ad02A15043	Aradu.A02	15043	p2	(CT)8	16	81963081	81963096	GTCATCGTCATCGTCGTCAT	GGTGGACGAAGACAAGGGT	276
Ad02A15044	Aradu.A02	15044	p2	(CT)7	14	81965253	81965266	GAGAGCGTAAAGCCACCTTG	CTGGTTCCACTCTCACAGCA	220
Ad02A15045	Aradu.A02	15045	p3	(ATT)7	21	81967351	81967371	TGACATTTTCATCCAGAGGC	AAACACAATTTGCATCCATCA	242
Ad02A15047	Aradu.A02	15047	p2	(AT)9	18	81971004	81971021	ACAAGTACTAGTTTGCTAGTTGCTACA	TTGCCGACAATAAAAATAATCG	244
Ad02A15049	Aradu.A02	15049	p2	(AT)9	18	81974601	81974618	GGTGAAACAAAATTTGAATTGG	TTTGACCGAAAAATGGTGAA	269
Ad02A15050	Aradu.A02	15050	p2	(TC)23	46	81975774	81975819	GCTTCGCATACGGTTCTCTC	TCCAGCATCAATAGCAACCA	169
Ad02A15052	Aradu.A02	15052	p5	(TATTT)5	25	81978725	81978749	TTCCTCTCCTCCCTCTTTCC	CTGTTTGGGATCCATTCTGC	123
Ad02A15058	Aradu.A02	15058	p3	(ACC)5	15	81987766	81987780	TGCAGATTTGCGTCATTGAT	GGTGACGGAAGAGGTTGTTG	276
Ad02A15064	Aradu.A02	15064	p2	(TC)16	32	82006098	82006129	TTGGTAGTTAACCCTTGGCA	TGTGGAAGTTGTGGTGGTGT	125
Ad02A15065	Aradu.A02	15065	p3	(TAT)9	27	82008171	82008197	AAATTCTTGCCATTGCTTGG	TTCAACTGGAACATGGGTGA	160
Ad02A15066	Aradu.A02	15066	p2	(TA)6	12	82008589	82008600	CAAATTTAGGATCACTGGCGA	TACGGTACTGCAGGCTGGAT	268
Ad02A15069	Aradu.A02	15069	p2	(GA)18	36	82034370	82034405	AGCAAACACATCAAGGCATCT	CCTCGACGAGACACAGAACA	265
Ad02A15071	Aradu.A02	15071	p2	(TA)9	18	82038072	82038089	TGCAATTTGAATATTTGTGACG	TTCAATTTTTGCCAAATCACC	259
Ad02A15073	Aradu.A02	15073	p3	(ACC)5	15	82060444	82060458	TTTGCTAGGAACTGGTCGAAG	TACCCATGGAAGATGGGTTT	238
Ad02A15074	Aradu.A02	15074	p5	(TTTCT)5	25	82063480	82063504	TACACTTTGCTTTGGGGCAT	TGGGATATTGGTTGGGAAAA	271
Ad02A15076	Aradu.A02	15076	p3	(CAA)5	15	82101234	82101248	AAAGGGCAACTGCTGAGAAA	GTGTAGCACAAACCCCATCC	238
Ad02A15078	Aradu.A02	15078	p2	(TA)7	14	82119054	82119067	TGAATCGAGTTTGAGCTTGG	GCGAACTTGTCCAAATGCTA	242
Ad02A15082	Aradu.A02	15082	p2	(TA)6	12	82136611	82136622	CGGACCGAGTTTTCCTCTTA	GGCTGAACGTGCACATAATTC	246
Ad02A15086	Aradu.A02	15086	p2	(AT)8	16	82160482	82160497	TGTTTTTCGGACCTTACCCA	ATCACAGGCAGGATTCTCGT	258
Ad02A15087	Aradu.A02	15087	c	(AAG)14(ATC	57	82161929	82161985	TCAAAGGTTGAATACCTTCCTCA	CCGAAACCCTTTCCTCTCTT	280
Ad02A15088	Aradu.A02	15088	p3	(AAG)30	90	82175600	82175689	AGAGAAGAGAAGGAGTGGGAAG	ACCCCAGTTATTTTCTCCCG	205

ID	Chromosome	SSR nr.	SSR type	SSR	Size	Start	End	FORWARD PRIMER (5'-3')	REVERSE PRIMER (5'-3')	Product (bp)
Ad02A15090	Aradu.A02	15090	p3	(ATA)13	39	82181776	82181814	CCCTCCCTTACTCCTTCCTG	AAGAGCTTGATGAAAGGGCA	229
Ad02A15096	Aradu.A02	15096	p3	(TTA)18	54	82247368	82247421	TGGAGCACTAGGTGCTGTTG	TTCCGTTTCATGCTTGTTCC	272
Ad02A15098	Aradu.A02	15098	p2	(AG)18	36	82264513	82264548	TTTGAACCTGGAATTTTCGC	TTTTTCACAACTGACGCGAG	259
Ad02A15105	Aradu.A02	15105	p2	(TA)6	12	82291375	82291386	AAATTCAACCATTCCAAATTTCA	TTCTATCCTATCCGCACCTTT	259
Ad02A15108	Aradu.A02	15108	p2	(AT)8	16	82297388	82297403	CGTGGTCACACAAATTCAACA	AAGAAAACGGCACTTTGGTG	161
Ad02A15109	Aradu.A02	15109	p3	(CTG)5	15	82298920	82298934	GGCAACCTCAATCTTCTTCG	TGCACTTGCCGATTAGTTTG	171
Ad02A15110	Aradu.A02	15110	p3	(AAG)5	15	82304056	82304070	TTTTTCTTCTTCTTTTTCCCCC	TCCTCCTCCTCATCTTCTGC	260
Ad02A15113	Aradu.A02	15113	p2	(TA)6	12	82307036	82307047	GCCCCGTTTTCTTCTTTTTC	GGTCAATTCAAACGTTAGGGA	270
Ad02A15117	Aradu.A02	15117	p2	(AT)7	14	82320825	82320838	CGTTCTTTTGGGTGGTCATT	CCCTTGAAATCCTCCCAAGT	176
Ad02A15120	Aradu.A02	15120	p3	(TAT)5	15	82332172	82332186	TCATGATTACCACGACTGGTGT	TCTTTCGCAACTCCGTTTTT	264
Ad02A15122	Aradu.A02	15122	p2	(TG)14	28	82339196	82339223	GTCGGAGGATGAGAGGGAAG	TGAAACAACTAACTGCAAAGCC	279
Ad02A15124	Aradu.A02	15124	c*	(TA)6(A)15*	26	82348317	82348342	AAGCTTTTGCTAGTATGGGTCC	AAGAAGAAACTGCCCAAGCA	216
Ad02A15126	Aradu.A02	15126	p3	(TAT)5	15	82350231	82350245	ACATGCATCAATTTGGGTCA	GCTCACATTTCAGCATCAAAGA	261
Ad02A15131	Aradu.A02	15131	p3	(AGT)5	15	82371929	82371943	TGCAAAATGACATGAATCACAA	AAGCAAGCTTCATTGGCAGT	189
Ad02A15138	Aradu.A02	15138	p3	(TTA)5	15	82390559	82390573	GGACCGTCCGATTTGAATAA	CATTTCATATCACATTCGTTTCAA	159
Ad02A15140	Aradu.A02	15140	p2	(GA)7	14	82395911	82395924	AGATGATTCGCCTCCCTTTT	GATCACATGCCTGACGCTAA	260
Ad02A15142	Aradu.A02	15142	c	(T)10aactttttag	117	82405572	82405688	TGTGGCAAACAGGAAAACAA	GAGTACCCCTCCCCTGTTTC	216
Ad02A15144	Aradu.A02	15144	p2	(TA)7	14	82431225	82431238	TCAGGCCATTAGACCCAATC	ATATGCCACTCAACCAAGCC	187
Ad02A15145	Aradu.A02	15145	c	(TTC)5caagttc	190	82445301	82445490	CCTCTTCTTCCTCCTCCTCG	TTTTGAATTGAATGGACGCA	249
Ad02A15146	Aradu.A02	15146	p5	(ATTCA)5	25	82445821	82445845	GGCTTTCTGCATAATGAACCA	AATGGATAGTGCTCCGTTCA	250
Ad02A15147	Aradu.A02	15147	c	(AGA)5atatgcg	99	82446289	82446387	ATTGCATTCCATTCAATTCG	GGTCAAACACCAACAAAACCA	237
Ad02A15150	Aradu.A02	15150	p3	(AAC)7	21	82461266	82461286	CATACGTCCTACCAACGGCT	GTATATGAACGTCGTGGGGG	206
Ad02A15158	Aradu.A02	15158	p2	(TA)10	20	82492277	82492296	GGACGTTGACGATGAGGACT	TAGAATTGGGTGCGGGATAG	165
Ad02A15159	Aradu.A02	15159	p5	(AAAAT)5	25	82496131	82496155	TGCTTCATCATTCTTCATAAATAGTTG	AAAGCCACAAAAGCATGTCA	278
Ad02A15160	Aradu.A02	15160	p2	(TC)6	12	82497479	82497490	AAGAGGCAGAGATTGGAGCA	CCTCCAAGGTTGAAACCAAA	246
Ad02A15161	Aradu.A02	15161	p4	(ATAA)5	20	82498231	82498250	TCGTGACTAGGGACGAGACA	GCGTGAAATAGCTTCCTCCA	269
Ad02A15167	Aradu.A02	15167	p4	(AAAG)5	20	82517038	82517057	AATGTAGCTTACCGATGGCG	AATGCAAAACCCTTCTCCCT	158
Ad02A15168	Aradu.A02	15168	p2	(AC)6	12	82519553	82519564	CGACTATATTGCCCCTAACCC	GCCATGTGTTTTGCATTCTG	178
Ad02A15169	Aradu.A02	15169	p2	(AG)7	14	82523900	82523913	TTGGAAGAAGGAATGAGAAAGA	GGGCTAAATTGAGTCGGAGG	163
Ad02A15171	Aradu.A02	15171	c	(A)10ctatgatct	87	82527942	82528028	TTAGACCCGATTTGATCCGA	TGTCGCATTATTATTGGACGTA	260
Ad02A15174	Aradu.A02	15174	p2	(AC)6	12	82547133	82547144	TGAACATTAGTGGATGCGATG	TTTCGCGCCAAAATTAGTCT	255
Ad02A15176	Aradu.A02	15176	p2	(TA)10	20	82554456	82554475	TGAGCTATATCTTTCATCGGGC	CTTACCTCGCGTCGTAAAGC	127
Ad02A15177	Aradu.A02	15177	p2	(AT)9	18	82556599	82556616	CCACAATCCAATCAATCACG	TCTTCGTCCAATGAAGACCA	243
Ad02A15181	Aradu.A02	15181	p3	(TGA)17	51	82573003	82573053	TTATATCCACCGGAGTTGGC	AGCCCCACTGTCTTCATCAT	260

ID	Chromosome	SSR nr.	SSR type	SSR	Size	Start	End	FORWARD PRIMER (5'-3')	REVERSE PRIMER (5'-3')	Product (bp)
Ad02A15183	Aradu.A02	15183	p3	(AAG)5	15	82579884	82579898	TTGTTTTCGGTTCACCATGA	ATACACACGAAATCGCCCA	275
Ad02A15184	Aradu.A02	15184	p2	(TA)6	12	82582023	82582034	TGTACATTGGTTTTGGCATTAAA	AAACTCAGTCTTTTGTATGTGAGTGAA	129
Ad02A15186	Aradu.A02	15186	p3	(TTG)5	15	82585509	82585523	TGGCTTCATCACCTTGAATG	AAGGCTCAGATTTGGCTTCA	238
Ad02A15187	Aradu.A02	15187	c	(T)13gt(A)10	25	82588170	82588194	TGTCTTCGAGACAAAAAGTCAAA	TTTTTCCCTTGAAAAGAAGTGC	243
Ad02A15190	Aradu.A02	15190	p2	(GT)6	12	82592882	82592893	GGTGTGAGTGGTGTTTGTGC	TCTCATGTTGACTCATTGCCTC	143
Ad02A15191	Aradu.A02	15191	c	(T)10aaacaaata	56	82596005	82596060	TGCAGTGCATGCAGTTATGT	TTTTGTGTTTCTCACTGAACCC	232
Ad02A15192	Aradu.A02	15192	c	(TTA)6tacttta	138	82596468	82596605	TTGTTGAATATTTCTGTTGTTATCG	ACCATGCATGAACAGACGTT	279
Ad02A15193	Aradu.A02	15193	p3	(TAA)21	63	82600955	82601017	AATTCACCGTCCGAAACAAC	GGCGAGCCAATATCCATCTA	250
Ad02A15194	Aradu.A02	15194	p3	(ATA)6	18	82607207	82607224	TGAAAAGTGAATCAAAGCATGAA	TTTCTGAACCCATCTTTGGC	236
Ad02A15201	Aradu.A02	15201	p3	(TAT)17	51	82640096	82640146	TCTTTGCCTATGTTATTCATCATTG	GCACACGACGTACCTGTTGA	262
Ad02A15202	Aradu.A02	15202	p4	(AAAT)6	24	82641758	82641781	GCGCACACACATGGTTCTAA	AATTTTTCGTGGCAGGCATA	221
Ad02A15204	Aradu.A02	15204	p3	(CTA)6	18	82647412	82647429	TGCTGGTCCACTGATGAAAA	GGCTTGCATACGTTAAAGCAG	159
Ad02A15205	Aradu.A02	15205	p2	(TA)6	12	82647791	82647802	ATCCATTTAGTATGAAAAAGAAACATT	AGCCAAATACCTCTGAGCGA	105
Ad02A15207	Aradu.A02	15207	p2	(TA)8	16	82649468	82649483	TTTTTGTTAAATAAACCCAACCAA	TTTTAGAAATCGACTCGAAAGAG	217
Ad02A15213	Aradu.A02	15213	p2	(AG)7	14	82669735	82669748	GGAATCGGGAAAGGAGAAAG	CCTCTCTCTGGCCATTGTGT	141
Ad02A15215	Aradu.A02	15215	p3	(TCA)5	15	82680141	82680155	AATGTCTTTGGCGATGAACC	TGAGTATGATCAGCCACATGC	188
Ad02A15220	Aradu.A02	15220	p3	(ATG)6	18	82684761	82684778	TGCAACAATCTTGTGCTTCC	ACACCATCATGCCATTGAAA	270
Ad02A15221	Aradu.A02	15221	p3	(GTT)6	18	82685016	82685033	TTTCAATGGCATGATGGTGT	ATCTTCCGGGTTGGTTCTTC	279
Ad02A15222	Aradu.A02	15222	p3	(CCT)5	15	82686831	82686845	TGAAGATCAGGTGTCCAACG	CAAGCCCTTCAAACCTTGAG	199
Ad02A15225	Aradu.A02	15225	p3	(CTT)5	15	82690814	82690828	TTTGTTGCAAATTTCGGTGT	TTATGATGATGACGATGATGAAA	165
Ad02A15229	Aradu.A02	15229	p3	(AAC)5	15	82706907	82706921	CCATGCCTAGTAACGTGCAA	ACATGACAGAGCACAATGGC	165
Ad02A15233	Aradu.A02	15233	p3	(GAC)5	15	82719390	82719404	CAGGTGCAGGAGTTCCATTT	TAACGCGCCTTCTCTGATTT	226
Ad02A15236	Aradu.A02	15236	p3	(TAC)7	21	82723870	82723890	ATCAATCATCAAAATCCGCC	TTGAAGACTTGCTTCGTGAGT	266
Ad02A15240	Aradu.A02	15240	p2	(AT)8	16	82734783	82734798	TCCAACGAATTTTCACACACA	GGATGGTCCGATTTGAATGT	258
Ad02A15247	Aradu.A02	15247	p2	(AT)7	14	82748762	82748775	ATGGCCCATGATTAGCGTAG	AAATGAGATAAGACACGCAGAGA	181
Ad02A15251	Aradu.A02	15251	c	(TAT)5aattatta	66	82765600	82765665	GCTTGTTCTGTCCTTGATCG	TCTTAATGCAAACCAACCTGAA	154
Ad02A15253	Aradu.A02	15253	p2	(AG)8	16	82773991	82774006	GGGAGAACCGTCTGGTTTTT	AGAGTATTACCGGTGGCGAG	250
Ad02A15256	Aradu.A02	15256	p2	(TA)11	22	82792312	82792333	ATCATGAACTGCCACAACCA	GAACAATGTGCAAGTGGATCA	237
Ad02A15257	Aradu.A02	15257	p4	(TTAT)5	20	82797129	82797148	GGTTGAGCCTCCGTTGATTA	AGCTTCGTGCTGATAACGTG	242
Ad02A15269	Aradu.A02	15269	p3	(TCT)6	18	82893998	82894015	CCCATCACCTCTTCACCACT	TAAAATATTGCTCGCCCTCG	276
Ad02A15271	Aradu.A02	15271	p3	(GGT)5	15	82936746	82936760	AAATGGGTTAAACGGGGAAG	TCATCATCGTCAAGAACCCA	247
Ad02A15272	Aradu.A02	15272	c	(TTG)5ttcttttga	84	82936926	82937009	TGGGTTCTTGACGATGATGA	TCCAGAAGTTGAAGCAGAAGC	225
Ad02A15274	Aradu.A02	15274	p3	(AAT)5	15	82941861	82941875	TGAAATTGTTGTTGTTAGTGAATTTG	GGGTCCTGCTTTTCCTCTTC	256
Ad02A15277	Aradu.A02	15277	c	(ATT)5aagtagt	51	82946610	82946660	CAAGGGACCAACTCCAGAAA	CAGACAGCACAAAAGTGTTGC	235

ID	Chromosome	SSR nr.	SSR type	SSR	Size	Start	End	FORWARD PRIMER (5'-3')	REVERSE PRIMER (5'-3')	Product (bp)
Ad02A15278	Aradu.A02	15278	p3	(CTT)5	15	82949516	82949530	CCAAACTCCTCCTCAACCAA	GAGGTTCAATACGCATGGCT	204
Ad02A15279	Aradu.A02	15279	p2	(TA)10	20	82950379	82950398	TTTAACAAAGTTGCTGACCCC	TACTAGCATTTCGGCAGCCT	157
Ad02A15282	Aradu.A02	15282	p3	(CTT)5	15	82966645	82966659	CCCCTCCATTATCGTCCTCT	ACGTGTCGTTGTAGCGTCAG	261
Ad02A15283	Aradu.A02	15283	p3	(GAC)5	15	82972618	82972632	CAGGTGCAGGAGTTCCATTT	TAACGCGCCTTCTCTGATTT	226
Ad02A15285	Aradu.A02	15285	p2	(AT)6	12	82975634	82975645	AAAAAGGGAGAATTTGTTAGCG	TTCACATGCAGCAGAGCAGT	138
Ad02A15286	Aradu.A02	15286	p2	(TA)6	12	82976107	82976118	CACGAACTGGCTCAAACTCA	CATTAGCTCGTGAGCTGGC	251
Ad02A15288	Aradu.A02	15288	p2	(GA)15	30	82978647	82978676	TCCAAAAGCAACCCAGTTTC	GGTTTGGGGTCAAAATCAAA	228
Ad02A15290	Aradu.A02	15290	p3	(CAA)5	15	82981847	82981861	TTCAGTTATGGAAGGTGCCC	GAATTTGCATTGATTTCGGC	234
Ad02A15292	Aradu.A02	15292	p2	(CT)6	12	82987704	82987715	TTTCAGAATTTTGATTACTGCCAA	GGTGGTGACTTCTCAGGCAT	267
Ad02A15294	Aradu.A02	15294	c	(T)10aagactata	43	82993884	82993926	TTTTTGTTTGTAAACGCCCTT	GAGGATCATTAACAGAGAAATTAACCA	197
Ad02A15296	Aradu.A02	15296	c	(GA)7aagagata	97	83016159	83016255	CAGGTTCAGCACGAAAAGAA	TGACAGTTGGCTCTCTCTTGC	244
Ad02A15298	Aradu.A02	15298	p3	(CCA)6	18	83025824	83025841	TGAGGGTGAGAATCGAAACC	TGTTTTTCTTGGTGGTGGAA	201
Ad02A15299	Aradu.A02	15299	p3	(ATT)5	15	83032082	83032096	GACATGTAATGCCGGATCAA	TTGGTTCGTGTGTGGTAACAT	146
Ad02A15303	Aradu.A02	15303	p3	(ATA)17	51	83042923	83042973	CAGCACCATGCATTTCATTC	GCAATTAGCAATTTCGTATGGTG	243
Ad02A15304	Aradu.A02	15304	p3	(TCA)5	15	83043792	83043806	CCACGTCCAGATTCATAGCA	ATGTGTTGAGGTTGGTGTGC	129
Ad02A15305	Aradu.A02	15305	p2	(TA)6	12	83044175	83044186	TCCTCTATTCCATTTCATCTGTCA	TGGCCATTGGTAAAGTCTTTTT	266
Ad02A15310	Aradu.A02	15310	p4	(TTAT)5	20	83051766	83051785	TGAACCATCCATCAATTAGAGGT	CAGATGCCATGACAAAACCA	280
Ad02A15315	Aradu.A02	15315	c	(A)10tatataggt	107	83059765	83059871	TTTCATTCAAAGGTATGCCG	TGTGTCACATGATTTTCAATTATTTC	277
Ad02A15318	Aradu.A02	15318	p2	(TA)6	12	83062371	83062382	AACTCGAAAAGCGCAACATT	TTTGTGTCCCTTTGGGTTTC	199
Ad02A15326	Aradu.A02	15326	p5	(TCTCT)5	25	83075821	83075845	CCAGGTAACGGCTAGCTGAC	GCCTTATTTCATCAGCCCAA	257
Ad02A15328	Aradu.A02	15328	p2	(TC)20	40	83081271	83081310	CTACTCCATAGGACAGCGGC	GAGGGAGAGGAAAATCGGAG	149
Ad02A15330	Aradu.A02	15330	p2	(TC)19	38	83091030	83091067	TCGGCTGAAATGATACTTTGG	TGACACCAACAGAAGGACCA	274
Ad02A15331	Aradu.A02	15331	c	(T)11gcttttttttc	88	83091616	83091703	AGGGCTTACTCTCAGCAAGG	CTCAAAAGCTCGAGCAAAGC	266
Ad02A15333	Aradu.A02	15333	c	(TAA)12(TAG	54	83108368	83108421	CAATAGTCACGCTAAAGGTATCCA	TGTAGATCTGGCATCTGGGG	226
Ad02A15334	Aradu.A02	15334	p2	(AT)7	14	83109916	83109929	GCTGCTTCAACAAGTGCTTC	TCAATCTCTCCTTAATTTTCTTGAAT	278
Ad02A15337	Aradu.A02	15337	p3	(TAT)5	15	83133495	83133509	AAAAGTAAGCGAGTGGGGGT	AATGAAGATAGGGCCAATGC	196
Ad02A15339	Aradu.A02	15339	p2	(AT)6	12	83134132	83134143	AACGAGTGTTTAATAATGAACCTAAAA	TGAATTTTGAAATCATTAACCTAATTG	165
Ad02A15341	Aradu.A02	15341	p2	(AT)9	18	83135104	83135121	GATCCCTGTAACCGACCTCA	TCTTCAGAACATTGTTGCATAGG	278
Ad02A15342	Aradu.A02	15342	p3	(TCT)6	18	83136133	83136150	CTTGGCCTTTTGATTGAACC	ATTTGGAGCGAAAAGTCGAA	191
Ad02A15344	Aradu.A02	15344	p2	(AT)7	14	83136902	83136915	CCAAATTGAGGAGTTTTGTGG	TTCGGAAATGTTAAAACAATCAA	202
Ad02A15345	Aradu.A02	15345	p2	(AC)7	14	83138311	83138324	AGCTCCTAACTCAGCTTGCG	TGTACGTGGCACCTTTACCA	238
Ad02A15347	Aradu.A02	15347	c	(TCA)5cca(TC	118	83156818	83156935	CCTTCCTCATCTTCTGCTGC	TTGAATAAATGCAAGTTCATCATC	228
Ad02A15350	Aradu.A02	15350	p3	(AAG)5	15	83157803	83157817	TCATTATCATCGCCATCTTCTTT	TCTTGCACCGAAATTTGCTA	280
Ad02A15352	Aradu.A02	15352	c	(AT)10tatattaa	81	83165692	83165772	CCGAAAGCTCACGAGATGAC	TGAGACAAGATTGAGCCTGG	280

ID	Chromosome	SSR nr.	SSR type	SSR	Size	Start	End	FORWARD PRIMER (5'-3')	REVERSE PRIMER (5'-3')	Product (bp)
Ad02A15356	Aradu.A02	15356	p2	(AT)10	20	83167774	83167793	TGTTGAGATTGGAGTTTTGATGA	GGGATGAGAACTTAATCATACGG	246
Ad02A15363	Aradu.A02	15363	p3	(TAT)14	42	83183003	83183044	AAAAACAAATGAAGCAAGGCA	GCAACTTGCATGTATGTATCGT	225
Ad02A15365	Aradu.A02	15365	p3	(AAT)5	15	83190117	83190131	TCGTTAAAAGCTAAAGTTTTAGTGACA	AATTGTCTAGAGAAGTTTGAATGCTT	226
Ad02A15369	Aradu.A02	15369	p2	(CT)9	18	83206776	83206793	ACCTTGAGGTTGTGATTGCC	TCGGCTGTCTGCATACAAAG	178
Ad02A15370	Aradu.A02	15370	p2	(TC)12	24	83208082	83208105	ATCAGATCAGATCAGCCGGT	AGGAGACGAGAGGGGCTAAG	208
Ad02A15372	Aradu.A02	15372	p3	(TTG)5	15	83216503	83216517	TGGCAACCTCATAACTGCAA	CTGGACTCAGTCGCACAAAA	273
Ad02A15373	Aradu.A02	15373	p3	(AAT)5	15	83217196	83217210	AATGCCAAAATGGGTTGCTA	CCATTGATATCCATTGCCCT	238
Ad02A15376	Aradu.A02	15376	c	(A)12gttcaatat	115	83221573	83221687	GTGGGTTCGAGTCTCACTCC	CGGATCAAGCACCCTATACC	267
Ad02A15377	Aradu.A02	15377	c	(A)10gagacact	46	83222427	83222472	TGAACCATTGGTATTTAGGCTTT	TATTTTCCCCATTCCCACAA	256
Ad02A15383	Aradu.A02	15383	p3	(ATA)7	21	83253307	83253327	TCCCATCTGTAAACACCCCT	CAAGGTTAGAGACTTACAAAACCTTTC	268
Ad02A15388	Aradu.A02	15388	p2	(TA)6	12	83259685	83259696	GGCAAATTATGTCTGGTTTTGA	CGGCATTTAAGTGGAATTGA	185
Ad02A15390	Aradu.A02	15390	p3	(AAT)19	57	83262611	83262667	TAAAACCCCCTTGCTTACCC	TTGTCCTTGGAATACCCCTG	249
Ad02A15391	Aradu.A02	15391	c	(TA)6caataaaa	103	83264259	83264361	CCCCACCATGTATCAGAAGC	TCACAAACAACGGATCATCG	258
Ad02A15393	Aradu.A02	15393	p3	(AAG)5	15	83265480	83265494	TCAAATTGCGCAAAGGATAG	AGATCGAATTGGCAACCCTA	208
Ad02A15395	Aradu.A02	15395	c	(TA)9c(AT)6	31	83269144	83269174	CCCTCTCAGGGACCTCAAAT	GCATTTTTGTTCAAGTAAGATTCTC	280
Ad02A15397	Aradu.A02	15397	p3	(TGT)5	15	83277153	83277167	GGAGGAGGTTGAGTATTGCC	GCGATGAGCAGCAACATTTA	215
Ad02A15398	Aradu.A02	15398	p3	(TAT)15	45	83278873	83278917	AAACACCCCAAAGTTTTCCA	TCTGACCCTTTTTATCTCCTTTTT	249
Ad02A15410	Aradu.A02	15410	c	(T)10aaataaaat	47	83315181	83315227	AAATGGAGTGAGGCAAGCTG	TACGTTTTGCTGTGGGCATA	251
Ad02A15412	Aradu.A02	15412	p2	(TA)11	22	83317515	83317536	CGAATTAAGCAAGGAAGCCA	CACGTCTGGGGCTTCATAAT	115
Ad02A15414	Aradu.A02	15414	p3	(ATG)6	18	83322358	83322375	CTGCACCAAACATCATCAGC	TCAATTTTGTTATGGCCCTTG	102
Ad02A15415	Aradu.A02	15415	p2	(GA)6	12	83324659	83324670	GTGGAGAAGCGCTGGATTAG	TTTGATTAGGACCAGGGTGG	276
Ad02A15419	Aradu.A02	15419	c	(A)10cttttaattta	56	83332199	83332254	TGAGAATCAACAAATTTCACCAA	TCAACACATTGAAGCTTGCC	269
Ad02A15420	Aradu.A02	15420	p2	(AT)6	12	83337370	83337381	CCACTCTCCTAGCACTCCTCC	TTTGATTCTGCATGCTCCAC	244
Ad02A15424	Aradu.A02	15424	c	(A)11ttctaattta	99	83346436	83346534	AAGACATTATGGCTCAATTGCT	GGCTCCACCCTGTATCAATG	276
Ad02A15427	Aradu.A02	15427	p2	(CT)6	12	83364321	83364332	CATGTTGGGTTTTGCATGAG	AATCCTGTGCCTTGCACTCT	189
Ad02A15432	Aradu.A02	15432	p2	(AT)7	14	83376085	83376098	CTTTGACATGCCTGACTCCA	TTTTCCCGTAGGGAAGTGG	272
Ad02A15433	Aradu.A02	15433	p2	(TA)8	16	83377093	83377108	GGAGATGTAAAGGCAAGGCA	TGGCATGATGAATTACCAAGA	153
Ad02A15435	Aradu.A02	15435	p2	(AT)6	12	83390802	83390813	CCTCTAAGCCCTTAAACCCG	CAAGGACTATGCCAATGCTG	271
Ad02A15437	Aradu.A02	15437	p3	(AGA)5	15	83406393	83406407	AAGAAGTAGCCGAACCCCAT	AGAACTGCGGTCAGAAGCAT	274
Ad02A15439	Aradu.A02	15439	p2	(TA)9	18	83410349	83410366	CCCCTCCTTCTCTTAGCCAC	TGGAACTCAACAGCAGCAAT	236
Ad02A15440	Aradu.A02	15440	p3	(AAG)6	18	83412068	83412085	CCATAACCAGGCAAATCCAC	GCCATATTAGGTCGCTCCAA	252
Ad02A15445	Aradu.A02	15445	p3	(TCT)5	15	83430649	83430663	AACGCTTTTTCGACACCATC	CTGCTCGTCAACGTATCCAA	280
Ad02A15447	Aradu.A02	15447	p3	(ATA)9	27	83434717	83434743	CCTCAGAGGAACCCATTTCA	TCATCAACAGCAAAAAGCCA	277
Ad02A15451	Aradu.A02	15451	p3	(TCC)7	21	83460035	83460055	ATTGCTTCCCAACTCCAAGA	AGCACCAAATCCATGAAACC	241

ID	Chromosome	SSR nr.	SSR type	SSR	Size	Start	End	FORWARD PRIMER (5'-3')	REVERSE PRIMER (5'-3')	Product (bp)
Ad02A15457	Aradu.A02	15457	c	(AC)8ccagctga	72	83486154	83486225	CAAACGGGCTAAAAACGCTA	GAGCTCTTCACAAGGAACGC	251
Ad02A15460	Aradu.A02	15460	p2	(AG)17	34	83499296	83499329	CAGGGGAGAGCAGAGATTGA	AAGCCAAGCTAAGTCAGCAAA	237
Ad02A15462	Aradu.A02	15462	p2	(AG)8	16	83506711	83506726	TTGAAGAGAAGAGCAGGGGA	GCACAAAATCAGGAAGGAAAA	227
Ad02A15463	Aradu.A02	15463	p2	(AT)10	20	83508389	83508408	ATCCTAACGTCAAAATGGCG	AGGGGGAAAAAGGAGAAACA	142
Ad02A15467	Aradu.A02	15467	p2	(AT)7	14	83531411	83531424	TTTGACAAAAATAATACAATTTGATCG	AGCCACCAGAGACCTAGCTT	145
Ad02A15476	Aradu.A02	15476	p2	(TA)6	12	83572753	83572764	GAGTCTCTGGGAAACACAAAGC	TAGGGCTTGAGGCATAGCTG	261
Ad02A15479	Aradu.A02	15479	p3	(CTT)5	15	83583873	83583887	TTTACGGCAAAAATGAAGGC	TGTTTCTGCTTCTGCAAATGA	103
Ad02A15480	Aradu.A02	15480	p2	(AG)17	34	83585505	83585538	GGGGAAGGGGTTGAATCTTA	AGCTGAACCAGGATACTTCTTG	268
Ad02A15482	Aradu.A02	15482	c	(CTC)5atcctca	40	83588659	83588698	TCCTCCTCCTCCTCATTCTTC	TCAGATTCAAAACACCTGCG	220
Ad02A15484	Aradu.A02	15484	p2	(AT)7	14	83591936	83591949	TTGTTGGGAAACCATCTTCA	GGGCAGCAACTTTTGTGATT	210
Ad02A15485	Aradu.A02	15485	p2	(TA)10	20	83592115	83592134	AATCACAAAAGTTGCTGCCC	TCATTATTGTGTCGTTAGGGGA	112
Ad02A15486	Aradu.A02	15486	p3	(ACT)5	15	83595296	83595310	ACTTGGCTTTGTTGTTGGCT	CAAACCCTTTGTTTTTCCCA	262
Ad02A15487	Aradu.A02	15487	p3	(AAT)9	27	83596024	83596050	TGGTTTATGTTGTTCGACGG	TGTCCAAATAAAAACAACAGGTG	277
Ad02A15489	Aradu.A02	15489	p2	(AT)7	14	83597440	83597453	TCTAAATCATTTGGGCCACC	CTCCGCATCTGTTGTAATGG	270
Ad02A15490	Aradu.A02	15490	c	(AAT)9gagtatt	89	83602530	83602618	CTGTTGTTGTCTTGTGCTCCA	AAAAATCGGTGAACTATACCTTGAA	223
Ad02A15491	Aradu.A02	15491	p2	(TA)6	12	83604694	83604705	ACAATATCGTGCTAGCGGGT	TGAGGGTGAGAACTCATGAAAA	208
Ad02A15496	Aradu.A02	15496	c	(GA)6tgttgaag	111	83627033	83627143	TCACACCTAATACAATCATTCATGG	CCTCCTAATTAGTTCCTAACGAAA	267
Ad02A15498	Aradu.A02	15498	p6	(CCTCAT)6	36	83632923	83632958	CCACTCCAATCTGAACGACA	ATCCAAGTGGGATGAGGTTG	199
Ad02A15500	Aradu.A02	15500	p3	(TTC)5	15	83635581	83635595	GGGCTGGCTATGTTGGTTTA	ATGCAGTGAGTTTTGGAGCC	118
Ad02A15501	Aradu.A02	15501	p4	(ATAC)7	28	83655935	83655962	TCAATTCATTAATTCACAAAATCCC	GTGGATCTGCCAAAACAGGT	263
Ad02A15509	Aradu.A02	15509	p2	(AG)7	14	83686922	83686935	TCGAGGAACAGAGCTCAGAGA	AAGCCAACAAGCAGCTCAGT	266
Ad02A15510	Aradu.A02	15510	p2	(AG)13	26	83688269	83688294	AGGTCGCACAGCTTGTTTCT	GCAACTTTCCCTTTCCTTTCA	179
Ad02A15512	Aradu.A02	15512	p2	(AT)6	12	83688905	83688916	TTGTTTTCCGCCACTAATCA	TGGGTTTTGCACTTTTTGAC	274
Ad02A15516	Aradu.A02	15516	p2	(CT)10	20	83710780	83710799	TTCTAACCAGGCCATTCAGG	GAAGGGAGAAGCGAGATGTG	269
Ad02A15517	Aradu.A02	15517	p3	(TCT)5	15	83710939	83710953	TTCCTCTCCTGCTTCCAAAA	CCCAGATGGAATCTCTGCAT	201
Ad02A15521	Aradu.A02	15521	c	(A)12gaaccaat	82	83715009	83715090	TTGGCCAACACAAATTAATCA	CATGCCATATCAGCAAATTTAGA	262
Ad02A15522	Aradu.A02	15522	p2	(TC)12	24	83718758	83718781	CTTCGACACCGACAACACTG	GTCGAGTCACCCATTCCTGT	280
Ad02A15523	Aradu.A02	15523	c	(TG)6tttgtcatct	37	83719107	83719143	CTCTTTCGGCCTTCCTTTCT	CATTCCCCTTTTTCCCAAAT	219
Ad02A15524	Aradu.A02	15524	p3	(CCA)5	15	83721148	83721162	CGGCAGCGGAGAGACTATAC	ATGTATAGGTGGCAGCGGAG	216
Ad02A15527	Aradu.A02	15527	p3	(AAT)5	15	83725263	83725277	CATTGGGTTGTCCTTTCAGG	CAGTGCTAACTCACCCCACA	255
Ad02A15529	Aradu.A02	15529	c*	(TTTC)5(TTC	137	83727120	83727256	TTTGCGTGATCAACCTTTTTC	TCCAAATCGCACTCATTCAA	265
Ad02A15530	Aradu.A02	15530	p5	(TGAAT)5	25	83727459	83727483	CTTCCGAAACGCATCAATTT	CCGAACCGAACACATAAACA	273
Ad02A15531	Aradu.A02	15531	p2	(CT)6	12	83739023	83739034	CAGAATTGGCACAAAGAGCA	GTTTCGGTTATGGGAGGGTT	280
Ad02A15534	Aradu.A02	15534	c	(T)10ctg(TA)6	25	83752448	83752472	ATTCCGATTGCAGAATGAGG	AGCACCAATAAAATCACGCC	259

ID	Chromosome	SSR nr.	SSR type	SSR	Size	Start	End	FORWARD PRIMER (5'-3')	REVERSE PRIMER (5'-3')	Product (bp)
Ad02A15535	Aradu.A02	15535	p3	(ATT)5	15	83755029	83755043	AAATTTGAATTGGTTGGACTTGA	GGGATAAGGAAGCAAGGACA	247
Ad02A15538	Aradu.A02	15538	p2	(AT)10	20	83756480	83756499	TAGGGATCAATTGAAAAGAAAAA	ATTTGTGGTTTTGGCCTCTG	262
Ad02A15543	Aradu.A02	15543	p3	(AAT)18	54	83768242	83768295	CCCCCTTTCCCCATTAAAA	CACCCAAAAACACTAAAAATGG	280
Ad02A15545	Aradu.A02	15545	p2	(AT)6	12	83772560	83772571	CATTTTGACAATCACGTGAGAA	TGCCAAAATGGGTTTATCTC	111
Ad02A15547	Aradu.A02	15547	c	(A)11caaaaaca	98	83779957	83780054	TTCCTATTAAACCCGGGCA	ATCAATTTAACGGCAGACGC	231
Ad02A15552	Aradu.A02	15552	p2	(TG)23	46	83785030	83785075	GTTGTTTTTCAGATGCGGGT	TCAAGACTGGCCGGAGAATA	274
Ad02A15555	Aradu.A02	15555	c	(A)11catttacat	108	83793822	83793929	CGATTCTGCATACACAACCAC	CTGAGTCATTAGAAGCCGCC	264
Ad02A15556	Aradu.A02	15556	p3	(TAA)5	15	83805894	83805908	TTCAAGAACAAAAATATCCAACACA	AGCGAGTGTTTCTGTTTGCC	280
Ad02A15558	Aradu.A02	15558	p2	(TA)6	12	83808230	83808241	TCTAGGCCTGACATGACTTGAC	CGTTAACGAAAATTGTGAATAAAAA	225
Ad02A15563	Aradu.A02	15563	c	(T)15gaaataag	67	83816825	83816891	TGAGGGTAAACCGCATTTTT	TTTCCCACCTGGTTTCAATC	220
Ad02A15564	Aradu.A02	15564	p2	(TA)7	14	83817598	83817611	CGTGGCTACCATTTTGATTTG	TGTGTGGTTTCGTTTTGCAT	278
Ad02A15565	Aradu.A02	15565	p2	(AG)6	12	83822115	83822126	TTTTTGATTGTGGGCTTGTG	TTGTTCTACACTTTTCTTCAACGG	278
Ad02A15566	Aradu.A02	15566	p2	(TC)8	16	83825526	83825541	TCAATTGATAATTCCTGTTTCACAA	TTGCCATTTGTATTTGCTTGA	270
Ad02A15568	Aradu.A02	15568	p2	(TA)13	26	83833527	83833552	ATCACATAAACCGCGGTAGG	CACATCACAGGCTCCAAAAA	254
Ad02A15569	Aradu.A02	15569	c	(TC)16(TA)8	48	83838535	83838582	TGCTGACGAAAATTGTGAATG	GACAAATCTGTCCAAACGTAAAT	236
Ad02A15570	Aradu.A02	15570	p3	(ATG)7	21	83839607	83839627	GGTCGTGTTCTGTGTTTTGTGT	CAATGCCATTCAGAAATCCC	272
Ad02A15573	Aradu.A02	15573	p3	(CCG)5	15	83843473	83843487	AGCTTACACCTAGCCAGCCA	AACCTCTCCAACCTCGACCT	269
Ad02A15580	Aradu.A02	15580	p3	(TCT)5	15	83855767	83855781	TTCACCCGCTTTTACATTTTT	GGAGGAAGAGAAGGAGGAGG	111
Ad02A15581	Aradu.A02	15581	p3	(AAG)5	15	83856314	83856328	TTCATCATCGCCATCTTTTTC	TGCTGCATTTTTCTTCTTCTTTT	253
Ad02A15589	Aradu.A02	15589	p3	(TCA)5	15	83870240	83870254	TCAAGTCACAACGCTCGTCT	AGATGATGACGACAACGACG	272
Ad02A15590	Aradu.A02	15590	p2	(TA)6	12	83876357	83876368	AACAAATGCAATTCTCTCTCTCTTT	TGATCAGAGTCTCGTGCCAA	208
Ad02A15596	Aradu.A02	15596	c	(CAG)5(CAT)	33	83886929	83886961	AGGAGGATCAACATGGAGGA	ATGACTTCAGGGACGGACAC	263
Ad02A15598	Aradu.A02	15598	c	(T)11cttttcttctc	102	83890641	83890742	ACTTGCTGCTCCTTGTTGCT	TCCATACTAATTGAAGGTGAACAAA	277
Ad02A15599	Aradu.A02	15599	p2	(TA)7	14	83890941	83890954	TGGAAAATCACTCTTCCTCTTT	AAAGGGTGGGTATGTGACATTT	158
Ad02A15600	Aradu.A02	15600	p2	(AG)19	38	83894148	83894185	TTGGATTGTTCAATGCATCC	CTCCTTCTCTCCCTCTCCGT	261
Ad02A15602	Aradu.A02	15602	c	(CT)21t(TC)6	55	83913725	83913779	TGGGTCCGTCACTATTCACA	CGGACATAGCGAAGGTGACT	239
Ad02A15606	Aradu.A02	15606	p3	(ATC)5	15	83921841	83921855	AACCACCGTCTTTCGCATAA	TTGTTATTCTGCTGCTGCTGTT	267
Ad02A15613	Aradu.A02	15613	p3	(AAT)5	15	83953803	83953817	TCATTATAAACGTCATTGATCGAAA	TCAGTAAAATCAGCTACCTTAGTCCA	222
Ad02A15615	Aradu.A02	15615	p4	(AAGA)5	20	83956336	83956355	ATGGGATGAGAATGCCAGAG	CTTTTACATTGACCGCGTGA	247
Ad02A15616	Aradu.A02	15616	p3	(TTG)5	15	83957502	83957516	GGGTGAGTCATGATGCATTG	AGCTATTTCGGGGCATGTAA	193
Ad02A15617	Aradu.A02	15617	c	(ACT)6ctctttgt	94	83960441	83960534	ACCACCTCTGACAACCGAAC	ACCTCCCTCCACGTTCTTCT	191
Ad02A15618	Aradu.A02	15618	c	(T)10cctttttcac	108	83960744	83960851	GAATTGCAAATGGGGTTCTG	TCCGTTCTTCTTCGTCGTCT	211
Ad02A15619	Aradu.A02	15619	p3	(ATA)5	15	83965005	83965019	CTTGCACTATCTGCAAGGCA	GGGTTCTGAAGAAAATTTCGG	195
Ad02A15620	Aradu.A02	15620	p2	(AG)8	16	83966836	83966851	GTTAAATTTTTGTTGCAATTTTTGA	ATTGGCTCCTCACTCCTTTG	170

ID	Chromosome	SSR nr.	SSR type	SSR	Size	Start	End	FORWARD PRIMER (5'-3')	REVERSE PRIMER (5'-3')	Product (bp)
Ad02A15622	Aradu.A02	15622	c	(TA)9attaatctc	134	83975286	83975419	CAGCCAATACACCAAAGCAA	GACGACGATAATGAAAGAGAACAA	272
Ad02A15624	Aradu.A02	15624	p3	(GAA)5	15	83976427	83976441	GCAAAATTAATGAAAAGAGGAGGA	TATGCGTGCGTGAAATTAGG	230
Ad02A15625	Aradu.A02	15625	p2	(AG)9	18	83985757	83985774	AGTACCGCAATCACCGAAAC	CCGCTCTCCAAGTACCACAT	273
Ad02A15626	Aradu.A02	15626	p3	(ATA)6	18	83986145	83986162	TGCTAGTTCGATGTTGTCGG	AGGTCAGAGTTGGTGTTGGC	264
Ad02A15627	Aradu.A02	15627	p2	(TA)7	14	83986455	83986468	CCCAACAGGCCAACACTAAA	TCAAGTATTAGGCATATTCATCTCG	279
Ad02A15629	Aradu.A02	15629	c*	(TTA)5(ATTA	41	83989292	83989332	TTCCGTACGATTTTGATCCC	TGAAAAAGACGTGAACGACG	211
Ad02A15634	Aradu.A02	15634	c	(AT)8agtaaata	109	83995643	83995751	AACAGCAGCGTTGCTATCTTA	CACATTTGAAATCTTCCGCA	238
Ad02A15635	Aradu.A02	15635	p2	(TA)7	14	83996508	83996521	AAAAACAAAACATTACTCGGAAA	ATGTTGCTCGCAACCCTAAT	153
Ad02A15638	Aradu.A02	15638	p3	(ATT)5	15	84001028	84001042	GGCACATGCAAGAAAAGTCA	GGCTATAAGTTACCCGCACG	256
Ad02A15639	Aradu.A02	15639	p3	(ATT)19	57	84002744	84002800	TGGAATTCAAATTGATAACTCGT	GTCCAACGCTGTATCCCTGT	151
Ad02A15641	Aradu.A02	15641	p3	(CAT)17	51	84003804	84003854	GATCAAGTTGAGATTCAAGTGAAAA	TCGGTGACATTTATTAATACCAATTAT	269
Ad02A15648	Aradu.A02	15648	p3	(CTT)6	18	84010899	84010916	ATGGTCCCTGAGTCCCTTTT	TTTGGTCTGCTCCTTTTGCT	240
Ad02A15660	Aradu.A02	15660	p2	(AT)9	18	84054952	84054969	TGTGAAGAAGGAGACCTGGTT	CACATTTGGTGATAAATGGGAA	225
Ad02A15666	Aradu.A02	15666	p2	(CT)13	26	84078516	84078541	GCCCATGAGGGTGTAAGTTG	GTAGCCATGGTTGTGGCTTT	267
Ad02A15667	Aradu.A02	15667	p3	(ATG)5	15	84079866	84079880	CATGCCATTGTTGGACTCAC	TTCCTCAATTGAAGGCAACC	177
Ad02A15668	Aradu.A02	15668	p2	(GA)21	42	84083816	84083857	ATCGGAAGTTCCAAGTGGTG	AAACGCAAAATTCTTCCCCT	131
Ad02A15674	Aradu.A02	15674	p3	(ATA)8	24	84089312	84089335	TCATTGGGCTAAAGCTTGAGTTA	TGTGATTTGTCCATGCATCC	156
Ad02A15679	Aradu.A02	15679	p2	(AT)11	22	84108745	84108766	AGAAGCAGAACATAACGGCG	TTTTCCTCGTTTTTGTGACCTT	277
Ad02A15682	Aradu.A02	15682	c	(AAT)7ag(TA	47	84112570	84112616	TGATATCATGGCAAAGGCAA	TGTGATGGATCGAGTGGAGA	239
Ad02A15683	Aradu.A02	15683	p3	(ATA)15	45	84113330	84113374	TTGGACTTGGACCACCATTT	TTGATGGAATCACCAACCAA	178
Ad02A15693	Aradu.A02	15693	p3	(AAG)5	15	84141682	84141696	GCAAAGAAAGGGAACCACAG	GACTGACGAGGAACAACGGT	159
Ad02A15697	Aradu.A02	15697	p3	(GGT)5	15	84159107	84159121	GAGACAAACGTGGAGGGAAA	GTCCACTACCACCTCCCTCA	150
Ad02A15698	Aradu.A02	15698	p3	(CAT)6	18	84160883	84160900	GTGCGTGGTATGTCATCGTC	GTGCCTCGTCAGTTGTGCTA	277
Ad02A15700	Aradu.A02	15700	p3	(AAT)7	21	84162329	84162349	GCAATGCCAATTTGTGAGAA	TGCATGGAGAACATCAGATTC	277
Ad02A15703	Aradu.A02	15703	p4	(ATGT)19	76	84164516	84164591	CCTCGTACGGATGATCAGGT	TTACCCCTTGTCCTTCCCTT	180
Ad02A15711	Aradu.A02	15711	p4	(TTAA)5	20	84199808	84199827	GCATGCTGAAAGCACATGAT	GGTGTCATTGGACACATTGC	224
Ad02A15712	Aradu.A02	15712	c	(A)11gaaaaaga	132	84201442	84201573	AAAACCATTTATGCATCTCTTTTT	CCAATGCTTGAAGTATAATGATTGA	278
Ad02A15713	Aradu.A02	15713	p2	(GA)7	14	84203733	84203746	TCCCTTTTCCGATCCTTTCT	GGGATTGATGTGGATGTGGT	264
Ad02A15714	Aradu.A02	15714	p2	(TA)6	12	84204387	84204398	TGGCCTTCTAAGCTAGTGCTC	TCACAAATCCAACTTATCCACC	249
Ad02A15715	Aradu.A02	15715	p4	(TTAT)5	20	84205780	84205799	CCAATCCAAACTGCACAATG	AATCAACAATGACATAACAAGTCAA	240
Ad02A15716	Aradu.A02	15716	p3	(TTA)7	21	84208271	84208291	CACCACACCACACTTTGTGA	CCTGCTTCTCCATCTTGCTC	191
Ad02A15720	Aradu.A02	15720	p5	(TTATT)5	25	84213675	84213699	TGAACAACCCGAATTGTCTAAA	AAAAGGTGGTAGGGTTTGGG	232
Ad02A15723	Aradu.A02	15723	p3	(TAA)12	36	84216513	84216548	TGCTGTTAGTGGTGCCAGAG	CACTTTTCAAATTCCTCTTCTCA	237
Ad02A15726	Aradu.A02	15726	p3	(ATA)9	27	84228314	84228340	CCTTCTGTTCCCAACTGCTC	GACAAGAGCTCGACGAAAGG	266

ID	Chromosome	SSR nr.	SSR type	SSR	Size	Start	End	FORWARD PRIMER (5'-3')	REVERSE PRIMER (5'-3')	Product (bp)
Ad02A15728	Aradu.A02	15728	p3	(GAT)5	15	84237661	84237675	TAAAAGGGGGTGGTTCAGTG	CGGATTGCTTCCCAGTAGAG	150
Ad02A15733	Aradu.A02	15733	p2	(TG)7	14	84245451	84245464	GAAGCGGAGCTGGTGATAGA	CAAACTCAACCCAAAACCAAA	243
Ad02A15736	Aradu.A02	15736	c	(A)10tatatacata	58	84253894	84253951	AAGCGTGGAGAAATTTGGAA	GGGCCTTTGTCCCTTTTTAT	260
Ad02A15741	Aradu.A02	15741	p3	(TAT)6	18	84296071	84296088	CTCACGGGTCTAAATCCAGG	TGGATGGACGGTTTCAAAAT	181
Ad02A15743	Aradu.A02	15743	c	(T)13gttttt(G)1	34	84304813	84304846	TGCCTGTTCATAGGAAACCA	TTGCTTAGTCAGTGCCATCG	211
Ad02A15744	Aradu.A02	15744	c*	(T)13(TG)6*	24	84306013	84306036	ATAGTCAAGAGACCCGCGAA	TGACGAAGTGGAGGAGACAA	170
Ad02A15745	Aradu.A02	15745	c	(GT)11(GA)14	50	84308725	84308774	TGCATATCATTTTTCCTTGGC	TGAGTTTTTGTTTGCGAATCC	248
Ad02A15746	Aradu.A02	15746	p3	(AAC)6	18	84310123	84310140	TCAACAGATGAGCTCGCAAT	AGTTCATTGTGCTATGGGGG	197
Ad02A15752	Aradu.A02	15752	c	(T)10cgtttctcta	119	84320238	84320356	AAGAGATTATGCTTTATTGATCTTTCA	AAATTTGGAATTTGGCCACC	235
Ad02A15754	Aradu.A02	15754	p3	(CAC)8	24	84323440	84323463	CCAAAAACAAAAAGGCTCCA	GTCGATGGATTGGACAACCT	266
Ad02A15759	Aradu.A02	15759	c	(T)20aaaacatgt	130	84328410	84328539	TGTGCCTGCATTGTAATGGT	CAGAATGCCAAAACAAAACAAA	259
Ad02A15760	Aradu.A02	15760	c	(T)10agacac(A	26	84330436	84330461	GACCCTGTGGGATAAGGGAT	TGGTTGAATAATCCTCCTGGTT	198
Ad02A15766	Aradu.A02	15766	p3	(AAT)6	18	84396888	84396905	CTTTGCAAGAGTTGAAATGATAGA	CCTCATTGCCACCCCTAAT	227
Ad02A15767	Aradu.A02	15767	p2	(TA)6	12	84397862	84397873	TTTTGGTGAATATAAGGCTAACCA	ATGCGAAAGCAACATTTGAC	252
Ad02A15772	Aradu.A02	15772	p2	(TG)6	12	84413989	84414000	TACGTACTCCCATGTCGGGT	GCATCCAAAGACCCACACTT	160
Ad02A15773	Aradu.A02	15773	p3	(ATA)7	21	84415828	84415848	GGATTTTGGAATGAATTTCACC	TCATGCACGCATATTGCTTT	266
Ad02A15780	Aradu.A02	15780	p2	(AT)7	14	84422877	84422890	GTCCGAGCTAGGAAACATGG	ATACAATGGGAGGCCGTACA	233
Ad02A15781	Aradu.A02	15781	p4	(AAGA)5	20	84423076	84423095	TGTACGGCCTCCCATTGTAT	TTGCATTGAAGGAACAACCA	242
Ad02A15788	Aradu.A02	15788	c	(TC)7ttctcacc(	34	84446857	84446890	TTAGTGATTGGGGTTGAGGC	GAATGACGAGATAGGGCTGC	253
Ad02A15789	Aradu.A02	15789	p2	(TC)13	26	84446993	84447018	GCAGCCCTATCTCGTCATTC	CAACAGTTGGTTGTCATCGG	180
Ad02A15791	Aradu.A02	15791	p6	(TTTAAT)5	30	84452402	84452431	TGAGAATGTGCATAAGTTGGAA	TTTTTAGAGGGACTTCCATCAC	252
Ad02A15793	Aradu.A02	15793	p2	(TG)8	16	84453693	84453708	TCGGTAGGGCAGGAGATAGA	TGTTGGATTGCACATTGTCAT	187
Ad02A15794	Aradu.A02	15794	p3	(ATA)8	24	84457099	84457122	AACCGCCGCTAATTTCAATA	ATGCAATTGAAGAGGGGCTA	214
Ad02A15795	Aradu.A02	15795	p2	(CT)13	26	84460825	84460850	TGATGGTGACCTCCTCCTTC	GCTGCCACCACTAAGCTTTC	173
Ad02A15800	Aradu.A02	15800	p2	(TA)7	14	84473437	84473450	TGCAATGTTGAAGAAACCCA	GATACATTTTTAAGCAATGACATGA	181
Ad02A15803	Aradu.A02	15803	p3	(AAT)11	33	84486452	84486484	GGCCTCAACATACGACCAAT	TGTACCGGTTAAATGGCGTT	262
Ad02A15804	Aradu.A02	15804	p3	(GAA)5	15	84491996	84492010	TCCAAAAACAAAAGAAGGAAGG	TTGACGCTACTACCAATCATCTTC	160
Ad02A15806	Aradu.A02	15806	p2	(AT)10	20	84502239	84502258	TGAGCACGTATCATTCTCTGC	GATCCATTCCAAGTGTTGGC	180
Ad02A15818	Aradu.A02	15818	p2	(TA)6	12	84523230	84523241	TTTCACTGGTGAGCGATCTG	CTCTTCTCCCCAAGTTCACG	267
Ad02A15821	Aradu.A02	15821	c	(A)11gaaaaaac	56	84527170	84527225	CCTGTTGGATATTGAAAGACAATG	ACCTTCCATCTTGGTCCTCC	237
Ad02A15825	Aradu.A02	15825	p2	(AT)6	12	84549141	84549152	CTTATTTAATTTGCCGCCCC	CGTGCAAATAAATCCCACTTC	131
Ad02A15828	Aradu.A02	15828	p2	(TA)6	12	84554653	84554664	CCATAAGCAAACCCAAAGGA	ACCACCATGATCATCAAGCA	250
Ad02A15836	Aradu.A02	15836	p4	(ATTA)5	20	84563609	84563628	CACAATAATGGCCATGCAAC	AAAGACTGCGTTTTGTTTTGAA	274
Ad02A15837	Aradu.A02	15837	p3	(TTG)5	15	84564046	84564060	CAATGCCATGAAAATGTTGC	ATGTCCCATTTGTTTGGGAA	188

ID	Chromosome	SSR nr.	SSR type	SSR	Size	Start	End	FORWARD PRIMER (5'-3')	REVERSE PRIMER (5'-3')	Product (bp)
Ad02A15838	Aradu.A02	15838	c	(T)13ggatctgat	88	84564310	84564397	AGATGGGTTTGCCATTGAAG	GGATGTGCAAATGGTGATGA	271
Ad02A15848	Aradu.A02	15848	p2	(TC)8	16	84588287	84588302	CCACCCAATCGCTACTTGTT	TTTCCAGTAACCAAGGCCAC	256
Ad02A15850	Aradu.A02	15850	p2	(TA)6	12	84599352	84599363	CATCTCCTGGCGAGAAACTC	ACCAATCACGTACCAAACGC	269
Ad02A15851	Aradu.A02	15851	p4	(TTCT)5	20	84599636	84599655	AGGGGTTGTTGAGTTCGTTG	TGATTACGGGACACTTCGTT	136
Ad02A15863	Aradu.A02	15863	c	(A)12caaacaaa	112	84659644	84659755	TTGATTAAAAACAAAAACAAAAGCA	TGGTAACCAAACAAACAAAACTAAA	274
Ad02A15871	Aradu.A02	15871	c	(T)10ctttctttttc	84	84675348	84675431	TGGTGTCGTTAGCATCTCTTTC	TTTGATGGGGTTTCCCAATA	263
Ad02A15872	Aradu.A02	15872	p3	(CTT)5	15	84676997	84677011	GCTACTCGCCGTTCTATTCG	TTAGGGTTTTGATTCGGTGC	131
Ad02A15876	Aradu.A02	15876	p3	(AAT)13	39	84682036	84682074	ACCTTTCCATGCAACTGGAG	TCACCAAACAAAATGCAAAAA	280
Ad02A15881	Aradu.A02	15881	p3	(ACC)5	15	84687389	84687403	AGCTGCAGAGCAACAACAAC	TGTTGGTGGTGTTGGAAAAA	256
Ad02A15883	Aradu.A02	15883	p4	(ATTT)5	20	84689430	84689449	CATGGGAAAATAAAATGGGG	GAGAAACCCAAAATGTCCCA	249
Ad02A15884	Aradu.A02	15884	c	(T)12aaataagaa	75	84691694	84691768	CTTTACGGGACACAAGGACC	CCAACCGAGTACGTGCCTAT	138
Ad02A15886	Aradu.A02	15886	p3	(TTC)5	15	84692531	84692545	CCCAAATTTGTTGCAAATACA	CCCTCAATGTTAGGGACCAA	260
Ad02A15887	Aradu.A02	15887	p3	(GAG)5	15	84693502	84693516	AAGCCATCAACGATGAAACA	CAACACCACCTCCTTCACCT	274
Ad02A15889	Aradu.A02	15889	p2	(AT)6	12	84698066	84698077	CCAAACACAAAGGAACAAGC	GCTTTGATTGGGGGAAGATT	146
Ad02A15894	Aradu.A02	15894	p2	(AT)7	14	84707687	84707700	TCTGCATGGTTGATGGTGAT	TGGCCATATTAGGGTTTGGA	241
Ad02A15897	Aradu.A02	15897	c	(TA)6(TG)7	26	84711978	84712003	TAATGGGGCCTCCAACTATG	GCATTGTCATCAAATGGCAC	235
Ad02A15899	Aradu.A02	15899	p3	(TTA)18	54	84717242	84717295	TGAAAAGAAAATACGTTTCACTACATC	TAATTTATGTTGTTTGTTTGGAAAA	269
Ad02A15901	Aradu.A02	15901	p2	(TA)6	12	84717688	84717699	AATCCAAAGTTGGAGGCACA	TGCTGAATCATGTTGTTTTGG	217
Ad02A15902	Aradu.A02	15902	c	(AT)8cacatcatt	101	84718601	84718701	AAAAGGGGTTGTATGTTGGATG	AATGGTGAAATGAAATGTTTGAA	271
Ad02A15903	Aradu.A02	15903	p3	(CAT)9	27	84722670	84722696	TGGGAGAGAACCATGAGAGG	TGTGGCAATGGAGATGGTTA	265
Ad02A15905	Aradu.A02	15905	p3	(AAT)5	15	84725195	84725209	ATTGCTCCTTTCCCCAAAAC	ATTTAACTCTTGGCACGCCC	240
Ad02A15906	Aradu.A02	15906	p2	(TA)7	14	84725341	84725354	ATTGCTCCTTTCCCCAAAAC	ATTTAACTCTTGGCACGCCC	240
Ad02A15908	Aradu.A02	15908	p3	(TAT)5	15	84730650	84730664	TATGGACGGACAACTGGACA	TAGGAAAAGAAGGACGCACC	246
Ad02A15909	Aradu.A02	15909	p4	(ACAT)6	24	84730957	84730980	GGTGCGTCCTTCTTTTCCTA	TTCACCCCAAATATTAAAAACCA	214
Ad02A15911	Aradu.A02	15911	p3	(ATG)5	15	84736188	84736202	TGTTTTGGATTGGGAATTGTT	GTGGCAAGCCATGGTACTTT	231
Ad02A15912	Aradu.A02	15912	p2	(GA)6	12	84740707	84740718	ACCACTAGCGCAGGAGAGAA	TGACGTCTTGACTCAGCAGC	280
Ad02A15915	Aradu.A02	15915	p3	(TAT)17	51	84748550	84748600	TGGATACAAAAGGGAGGTGG	TCAATTGTGTTTTCTTCTATCAATTT	243
Ad02A15919	Aradu.A02	15919	p2	(TA)15	30	84753579	84753608	TGCAACATAGATCTCTTGATGAGT	AAAACAAAATGATGGGCAGC	180
Ad02A15925	Aradu.A02	15925	p2	(GA)6	12	84768971	84768982	TTTGGTCAAATGAAATGGCTT	CACCTCATTGCTCTTCTCCC	280
Ad02A15926	Aradu.A02	15926	p3	(AAG)5	15	84770082	84770096	GGTAAACCAAAAAGAAAAAGATGA	GGCTGACTTTGGTGGTTGAT	277
Ad02A15927	Aradu.A02	15927	p3	(TTC)5	15	84771588	84771602	CATTGTTGGATGATTTTAATGGTT	CCTTCTCAAGTTTCTTCTTGTTTT	248
Ad02A15931	Aradu.A02	15931	c	(A)10tttacgata	42	84781061	84781102	GGTTTGTAGAAGAGGAAAATTGGA	TGCAGGTTTCTATATTTTTACCGA	242
Ad02A15933	Aradu.A02	15933	p3	(AAT)5	15	84790352	84790366	TGCCAAGTTGAGCATTGAAG	ACATCATCCACTCGTGCAAA	231
Ad02A15934	Aradu.A02	15934	p3	(CAT)5	15	84792806	84792820	TCACCTCCTTCTCCTCCTCA	AAAAAGACAACGATGGCGAC	124

ID	Chromosome	SSR nr.	SSR type	SSR	Size	Start	End	FORWARD PRIMER (5'-3')	REVERSE PRIMER (5'-3')	Product (bp)
Ad02A15935	Aradu.A02	15935	p4	(AAAG)5	20	84793445	84793464	GTGCTATTTTTGTTTCTGACAAGA	GGAAAGAAGGAGGAGGAAGG	278
Ad02A15936	Aradu.A02	15936	p2	(TA)7	14	84799402	84799415	GCGAGTACCCACCCTAATCTC	TTTATCCGGTAAGGACGGGT	272
Ad02A15937	Aradu.A02	15937	p3	(GAC)5	15	84799549	84799563	GGTAGAACGAATACCCGCAA	AAACGTCAGGGACAAAAACG	210
Ad02A15938	Aradu.A02	15938	p3	(TCT)5	15	84800594	84800608	GGACGATTGAAACGTTAGGG	CCTGGTGATATTGCAGCGTA	275
Ad02A15939	Aradu.A02	15939	p2	(GA)6	12	84801761	84801772	GCCGTTATTGGAGGTCTGTG	GAGCACTGTGGGTACAGCAA	256
Ad02A15952	Aradu.A02	15952	p2	(GA)11	22	84832911	84832932	GTCGTTGGATTTGGAGTCGT	CTATTTAGCGGTCGCAAAGC	111
Ad02A15959	Aradu.A02	15959	p2	(AT)6	12	84850976	84850987	AGTTAAACGGTGTCGGTATGA	AAATTAATAGTAGTGGTTGAAGGCA	280
Ad02A15961	Aradu.A02	15961	c	(TA)6tgcccctct	77	84860725	84860801	GAGGGTGATGATGAGGGCTA	GGAAGAGAGGTAGAGAGCCGA	216
Ad02A15966	Aradu.A02	15966	p3	(CAT)5	15	84869196	84869210	CGTATCGAATGCATGGTGAC	AAAACCCCTGAGCATTTTCC	196
Ad02A15970	Aradu.A02	15970	p3	(AAT)14	42	84874127	84874168	GGTAAACCAAACTTTGAATTGACTAA	TCAATGAACTACATCTTGAACTGG	278
Ad02A15972	Aradu.A02	15972	p2	(TA)9	18	84874906	84874923	TTGTCCATAAGAAACTTTGCCA	TGCAATTAGGTTTGTGAGACAG	256
Ad02A15979	Aradu.A02	15979	p2	(AT)6	12	84897083	84897094	CACACTGTGTCCACTGCTCA	CAGGAACCTCCTGTCTTTGG	245
Ad02A15982	Aradu.A02	15982	p2	(AG)9	18	84902075	84902092	TCACCGGAGAAGGTTAATCG	AAACGGTACAAACACATGCAA	221
Ad02A15985	Aradu.A02	15985	p2	(CT)7	14	84903439	84903452	AACTGGTAGGTGGGCAGATG	AGCAACATCCATCCACAACA	102
Ad02A15996	Aradu.A02	15996	p3	(GAA)5	15	84929923	84929937	CAACAGCGACAAAAGAACGA	CCTTCTTATTCGCATTCCTCC	156
Ad02A16001	Aradu.A02	16001	p2	(TC)6	12	84948822	84948833	TCTTCCCTTTGCTGCTGAGT	GGAGACGTAGGGATGAACCA	193
Ad02A16002	Aradu.A02	16002	p2	(TC)6	12	84950613	84950624	TCCTGCCTATTATTCCTCCC	CTGCCTTCAAAGCCACTAGG	220
Ad02A16004	Aradu.A02	16004	p3	(AAT)5	15	84954067	84954081	TGATCGTAGTTTATTGCCACAGA	TGGAGCAATGCAAAATCAGA	152
Ad02A16008	Aradu.A02	16008	p2	(AT)6	12	84974979	84974990	CGCGATGAAGAAAGAATTGA	TGAGGGTGTACGTGGATGTG	274
Ad02A16013	Aradu.A02	16013	p3	(TAT)5	15	84982883	84982897	TCTATTGATGAATGCCGCAG	TCAGGTGGCAAAAATAACCA	211
Ad02A16016	Aradu.A02	16016	p2	(TA)6	12	84988914	84988925	GAATGTCCAAATAATTCGAAACG	ACCCCAGACCGAGTTTTTCT	246
Ad02A16019	Aradu.A02	16019	c*	(GT)8(T)10*	25	84996890	84996914	AACAAGTTCGAAGGTGCATGT	GGCGTTCGTTTGGAAAAATA	222
Ad02A16029	Aradu.A02	16029	p2	(AT)6	12	85026383	85026394	TGTTGCTTTTCAGATGCAGG	GAAGCAAAGGTCGGTCAGAG	246
Ad02A16030	Aradu.A02	16030	p2	(AT)7	14	85028361	85028374	TTTTTGACAAAGGGAAACCA	TTTATGGTTACGAGTACGTTGATG	280
Ad02A16034	Aradu.A02	16034	p2	(TG)6	12	85035548	85035559	ACCCTGGTTCTGAGAGGCTT	CGCAATTAATTAACACAAGCAAA	265
[truncated: 647,878 more chars]
